# *In silico* regulatory analysis for exploring human disease progression

**DOI:** 10.1186/1745-6150-3-24

**Published:** 2008-06-18

**Authors:** Dustin T Holloway, Mark Kon, Charles DeLisi

**Affiliations:** 1 Molecular Biology Cell Biology and Biochemistry Department, Boston University, 5 Cummington Street, Boston, USA; 2 Department of Mathematics and Statistics, Boston University, 111 Cummington Street, Boston, USA; 3 Bioinformatics and Systems Biology, Boston University, 44 Cummington Street, Boston, USA

## Abstract

**Background:**

An important goal in bioinformatics is to unravel the network of transcription factors (TFs) and their targets. This is important in the human genome, where many TFs are involved in disease progression. Here, classification methods are applied to identify new targets for 152 transcriptional regulators using publicly-available targets as training examples. Three types of sequence information are used: composition, conservation, and overrepresentation.

**Results:**

Starting with 8817 TF-target interactions we predict an additional 9333 targets for 152 TFs. Randomized classifiers make few predictions (~2/18660) indicating that our predictions for many TFs are significantly enriched for true targets. An enrichment score is calculated and used to filter new predictions.

Two case-studies for the TFs OCT4 and WT1 illustrate the usefulness of our predictions:

• Many predicted OCT4 targets fall into the Wnt-pathway. This is consistent with known biology as OCT4 is developmentally related and Wnt pathway plays a role in early development.

• Beginning with 15 known targets, 354 predictions are made for WT1. WT1 has a role in formation of Wilms' tumor. Chromosomal regions previously implicated in Wilms' tumor by cytological evidence are statistically enriched in predicted WT1 targets. These findings may shed light on Wilms' tumor progression, suggesting that the tumor progresses either by loss of WT1 or by loss of regions harbouring its targets.

• Targets of WT1 are statistically enriched for cancer related functions including metastasis and apoptosis. Among new targets are *BAX *and *PDE4B*, which may help mediate the established anti-apoptotic effects of WT1.

• Of the thirteen TFs found which co-regulate genes with WT1 (*p *≤ 0.02), 8 have been previously implicated in cancer. The regulatory-network for WT1 targets in genomic regions relevant to Wilms' tumor is provided.

**Conclusion:**

We have assembled a set of features for the targets of human TFs and used them to develop classifiers for the determination of new regulatory targets. Many predicted targets are consistent with the known biology of their regulators, and new targets for the Wilms' tumor regulator, WT1, are proposed. We speculate that Wilms' tumor development is mediated by chromosomal rearrangements in the location of WT1 targets.

**Reviewers:**

This article was reviewed by Trey Ideker, Vladimir A. Kuznetsov(nominated by Frank Eisenhaber), and Tzachi Pilpel.

## Background

The first step in regulatory control is the binding of transcription factors (TFs) to specific regulatory sites in DNA. In simple eukaryotes such as yeast, an estimated 99% of TF sites occur within 800 bases from the transcription start site [1]. In humans, on the other hand, TFs may exert regulatory control at a distance of many kilobases from the start site [2-4]. Complex genomes also show greater incidence of binding sites occurring within 5' UTRs, introns, 3' UTRs, and even far downstream of a gene.

A particular TF may bind many similar, non identical promoter sites, with an affinity that varies with base sequence. The set of sites is often described as a motif or preferred pattern of bases. A popular representation of the binding motif is the position specific scoring matrix (PSSM) [5-8], which gives the frequency of observed nucleotide bases at each position of a known motif. However, results produced by scanning DNA with basic PSSM models are often overwhelmed by a high rate of false positive predictions [9]. In an effort to improve target prediction, we have previously employed a more sophisticated supervised learning method in *Saccharomyces cerevisiae *which combines many types of genomic data to assist binding site classification [10-12]. We have also developed a method to rank specific genomic features (*e.g*., presence or conservation of a particular *k*-mer) to select those which are most important for identifying target promoters for a particular TF [12,13]. We now adapt and apply these methods, which are based on the support vector machine (SVM), to produce separate classifiers for 152 TFs in the human genome in an attempt to discover new regulatory interactions important to human disease and development.

The genomic datasets used include sequence information from promoters (2 kb upstream and 5' UTR, introns, and 3' UTRs all taken from the UCSC genome browser database [14,15], see Methods) and take account of 1) sequence composition, 2) sequence conservation in 8 vertebrate genomes, and 3) statistical over-representation. These datasets have high dimensionality (see Methods), often containing thousands of numerical features. During classifier construction SVM recursive feature elimination (SVM-RFE) [16] is used to reduce the feature set to a manageable size.

**Figure 1:**
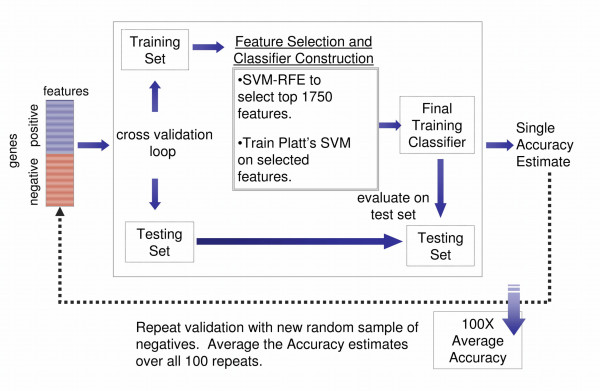
**SVM Framework**. This figure shows the data mining scheme for making TF classifiers. 100 classifiers are constructed for each TF, each using a different random sub-sample of the negative set. A classifier built on the training set is evaluated using cross-validation (center, gray box). This will usually be leave-one-out cross-validation, except for classifiers with large training sets where 5-fold cross-validation is used and repeated 10 times. For every cross-validation split, the top 1750 features are selected using SVM-RFE and the classifier is trained and finally used to classify the test set (left out sample). This process is repeated 100 times, and the accuracy for the procedure is the average of the 100 cross-validation accuracies.

Figure 1 provides a graphical scheme describing classifier construction. Feature ranking as well as feature set and classifier construction are described more completely in the Methods section. Each gene used in the analysis is described by a numerical or *feature *vector. Each component, or feature, represents one measurement taken in the genome, for example, the number of occurrences of a particular *k*-mer in the gene's promoter. SVMs efficiently handle high dimensional datasets and have proven effective in a wide range of biological systems [17-23].

SVMs require the input of positive (known target) genes and negative (non-target) genes to develop a decision rule which can be used to classify new genes as bound or not bound by a TF. Once a classifier is created an enrichment score is assigned to each predicted target using Platt's SVM [24]. Both the accuracy of the classifier and the enrichment score are dependent on the choice of positives and negatives used for training. An inaccurate training set will yield a noisy and less useful classifier. Furthermore, it is difficult to know the ratio of true positives to true negatives for any TF.

Positive examples are curated from several publicly available databases and also from the few ChIP-chip experiments which have been performed on human tissues (see Methods). The negative set is always chosen randomly from the genome. Clearly a random choice of negatives can introduce bias into the classifier since it could by chance contain unknown, but real, target genes even if the average number of expected targets is negligible. To resolve this difficulty, each TF classifier is constructed one hundred times, each with a new sampling of negatives. The performance of each classifier is evaluated by cross-validation and a final accuracy measurement is then the average accuracy from all one hundred trials (see Methods). In the cross-validation process, feature selection is done only on the basis of the training set, so that no information is used from the test set.

An enrichment score is assigned to each predicted target using the Platt SVM algorithm [24]. Platt's procedure was originally developed to estimate the likelihood (posterior probability) that any example is a positive (target) given the output of the SVM. In our case a true posterior probability is difficult to estimate, since the underlying class distributions are unknown, and Platt's estimate will be used simply to rank predicted targets so that only the best predictions are selected.

In our training and test sets we choose the negative and positive sets to be of equal size for each TF. Other studies have also employed balanced datasets [25,26]. This has several consequences for how cross-validation accuracy measurements and the posterior enrichment scores are interpreted. Since class priors are equal, a PPV measurement of 50% indicates a random classifier, only classifiers which achieve significantly better than 50% PPV will be useful for predicting new targets. By using a statistical test on the accuracy or PPV measurements it is possible to identify classifiers which perform better than chance. Similarly, the enrichment score will only be useful in ranking targets when the classifier is performing non-randomly. We note that these enrichment scores may actually be interpreted as confidence levels, but only on the balanced datasets used in SVM training and testing. A correction is required if one desires to use the enrichment scores as confidence levels in the full genome, wherein the number of negatives can outnumber the number of positives by a large factor. Although we use and report the uncorrected Platt scores here simply as a means to show enrichment for targets, we outline the calculations necessary to correct these scores to the genomic scale in the Methods section.

Our analysis produces informative classifiers for a number of human TFs, several of which are important to human development and disease. Although many factors are worth in-depth investigation we focus on the targets of two disease-relevant regulators: the targets of OCT4 and their relation to diabetes and, more extensively, the targets of WT1 and their relation to cancer development and progression. Alterations in transcription factors have been previously shown to be directly related to cancer progression [27].

Chromosomal regions known to be associated with progression of Wilms' tumor are significantly enriched for the predicted targets of WT1. This finding is significant since it provides a specific link between disease progression and disregulation or loss of WT1 targets in these regions. Motif discovery methods are also used to propose a new binding motif for WT1.

## Results and Discussion

For every TF here, all genes in the genome are given a score reflecting likelihood of being bound by the TF. The score, produced by Platt's procedure, ranges from 0 to 1, and will be denoted with a capital *P *(*e.g*., *P *= 0.5). Cross-validation performance measures (*e.g*., PPV or accuracy) are determined at the decision threshold of *P *= 0.5. This is the optimal discriminant threshold since, in a balanced test set (equal numbers of positives and negatives), it indicates that genes exceeding the threshold have better than a 50% chance of being a true target in the training set [24]. In practice the *P *= 0.5 threshold may be statistically significant because Platt scores in our method are *average scores *from 100 classifiers generated using different negative training sets. Genome-wide, fewer than half of all genes will exceed the 0.5 boundary. Nevertheless, *P *= 0.5 is not necessarily the best threshold for use in making new predictions. For the predictions we discuss below, we accept genes as targets only if they pass a threshold Platt score of 0.95 on average for 100 classifiers (one classifier for each negative training set) constructed for a particular TF. Starting with 8817 TF-target interactions curated from experimental datasets, 9333 new predictions can be made at this cutoff.

Not surprisingly, many classifiers show poor performance in cross validation (*P *= 0.5 threshold) although several do show high precision (33 have PPV > 0.6, see Methods). Poor performance may be partly due to the fact that our defined promoter region is large and in some cases may be thousands of base pairs long. This size may interfere with the ability of the SVM to identify important regions. The greater complexity in the human genome and likely presence of extensive combinatorial regulation may not always be captured well by individual classifiers trained for each TF. Finally and most importantly, human TFs generally have few known targets (small positive set), making it less likely that a classifier will find the correct decision rule. This is discussed in Additional File 1 where a hypothesis test is proposed to determine the significance of any classifier given the number of known targets.

Few supervised genome-scale strategies exist for predicting regulatory targets in mammalian genomes; however, several unsupervised approaches have been proposed. One successful unsupervised method [28] uses expression data and PWM models with a technique called MARS (multivariate adaptive regression splines) [29] to discover condition specific cis-regulatory networks. The advantages of MARS are that little prior information is necessary and the predictions represent a regulatory network specific to the expression conditions of interest. Our supervised method requires some known target genes and will predict condition *independent *binding. However, it is simple to integrate new types of data into SVM classifiers, whereas the method in [28] is restricted to sequence and expression data. In addition our system should function well in making condition-specific predictions if appropriate expression data are acquired.

**Figure 2:**
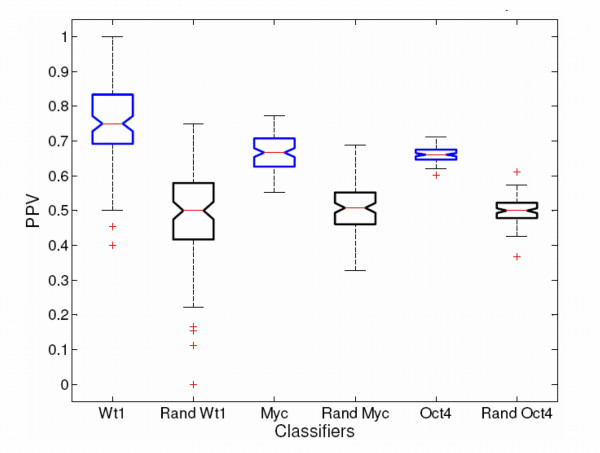
**Actual vs. Label-Shuffled Classifier Box-plots**. 100 classifiers represent each TF, meaning that cross-validation produces a population of PPV measurements to represent a TF classifier. These populations are used to compare the significance of the actual vs. the label-shuffled classifiers (denoted with the prefix "Rand"). Here the comparison is shown for WT1, Myc, and OCT4. Each box-and-whisker plot has a top line (the upper quartile value-not whisker line), a central red line (the median), and a bottom (the lower quartile value). If the notches on two different boxes do not overlap then one may conclude that the two population medians are significantly different (at the 5% level). Each box also has whiskers which look like standard error bars. The length of a whisker equals 1.5 times the interquartile range, which is the default value in Matlab [214]. Plus(+) signs represent potential outlier points existing beyond that default range.

Here, a control experiment was run to test the performance of SVM classifiers against randomized datasets. Three regulators were chosen according to the number of available targets (WT1–15 targets, MYC–67 targets, and OCT4–218 targets). For each regulator, the index of positives and negatives was shuffled during training (in all 100 classifiers representing the TF) to create randomized classifiers. These classifiers were then applied to the human genome and compared to the classifiers made with not-shuffled data. As expected, the shuffled classifiers make very few predictions in the genome which pass the 0.95 threshold. The shuffled WT1 classifier makes no predictions, while the shuffled OCT4 and MYC classifiers make 1 and 2 predictions respectively. In a genome of 18660 genes, this suggests that a random classifier will make fewer than 1 false positive per 10000 predictions when the threshold is set to 0.95 or greater. The performance of the randomized classifiers was tested using cross-validation (the classification threshold used in cross validation is 0.5). The real classifiers had performance measures which were significantly better than random in the cases which were tested (*p*-values for PPV and Accuracy less than 2.59e-28). This is shown in Figure 2 for PPV, where box-plots are used to compare the performance of actual and random classifiers.

Since cross-validation is performed at the 0.5 decision threshold, an immediate question that comes to mind is how to evaluate the significance of the performance accuracy of a given classifier (*e.g*., at P = 0.5, is 68% accuracy significantly better than random?) We have therefore constructed a hypothesis test to determine whether any measured accuracy is different than random. This test shows that the 68% accuracy measured for WT1 (averaged over 100 classifiers) is significant at *p *= 1.36e-4, making it unlikely that our results would have been obtained at random. Classifiers with larger numbers of known targets will show even stronger significance at the same accuracy. The full details of the hypothesis test as well as a brief discussion of its application to other TFs can be found in our Additional File 1.

**Figure 3:**
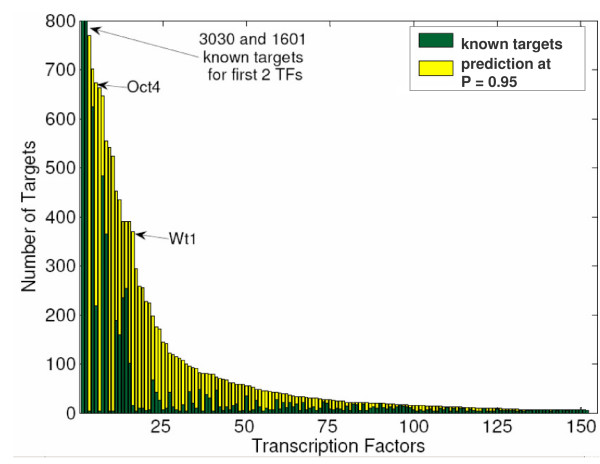
**Distribution of High Scoring Targets for TF Classifiers**. Each bar represents one TF classifier. The height of the bar indicates the number of genes known or predicted to be bound by the TF. The green portion of the bar indicates the number of previously known targets while the yellow portion indicates predictions made at greater than or equal to the Platt score 0.95 threshold.

Our method begins with 8817 known TF-gene interactions for 152 TFs. Many of these known interactions are confined to the few TFs for which ChIP-chip data is available (see Methods). The two largest, HNF4-α and CREB1, have 4627 known targets. In general, classifiers for TFs which include ChIP data do not necessarily perform better or worse than those without it. For example, OCT4 has ChIP data and performs about as well in cross-validation as WT1, which does not. *In fact, when large sets of known interactions exist, the classifiers make few or no new predictions*, perhaps suggesting that a significant subset of the targets for those factors have already been found (most strikingly, HNF4-α classifiers yield only 3 new predictions, and CREB1 yields only 1). Alternatively, since the positive sets for these two factors are very large, the possibility exists that the promoters of the positive sets have a large amount of variability. This variance, which could result from experimental noise or natural variability in target promoters, may prevent our classifiers from identifying features which distinguish potential new targets in the genome. Figure 3 displays the number of known targets for each TF along with the new predictions discovered at the average 0.95 threshold. The TFs OCT4 and WT1, which are discussed below, are indicated on this graph. In order to explore the best new predictions, for the remainder of this manuscript we discuss only targets predicted at the 0.95 Platt score threshold.

Results for all TFs are available in Additional File 2 and on our web server [30]. Additional File 2 also contains some brief notes on the naming conventions of TFs, and how the classifiers were constructed, as well as files listing the known targets used in classifier training (see Methods).

### SVM Classifiers Identify Biologically Relevant Targets for OCT4

Regulation by OCT4 is essential in early development, and expression of OCT4 is important for maintaining the pluripotency of embryonic stem cells [31,32]. ChIP-chip analysis of OCT4 and several other regulators revealed that OCT4 can act in concert with the TFs NANOG and SOX2 [32]. The SVM classifier for OCT4 has an accuracy of 67% and a PPV of 66% (at *P *= 0.5). This accuracy estimate is highly significant at a *p*-value << 0.01 (calculated with the method outlined in Additional File 1; again note that this is not the Platt score correction discussed elsewhere but a hypothesis test to determine the significance of the cross-validation accuracy).

It has been discovered that OCT4 targets are enriched for transcription factors, with many of these also being important for development [32]. In fact, the known targets in the training set for OCT4 are significantly enriched in the GO term "transcription regulatory activity" (50 genes, *p *= 2.1e-16), and new SVM predictions (at *P *≥ 0.95) also show enrichment in this category (111 genes, *p *= 6.7e-34). The known targets and new predictions share many statistically enriched functional terms, including "developmental protein", "homeobox", and "Wnt signalling pathway". Statistical enrichment of functional terms in gene groups throughout this chapter were calculated using the DAVID Bioinformatics Resource [33] (See Methods) For a complete list of enriched categories in OCT4 targets see Additional File 3.

**Figure 4:**
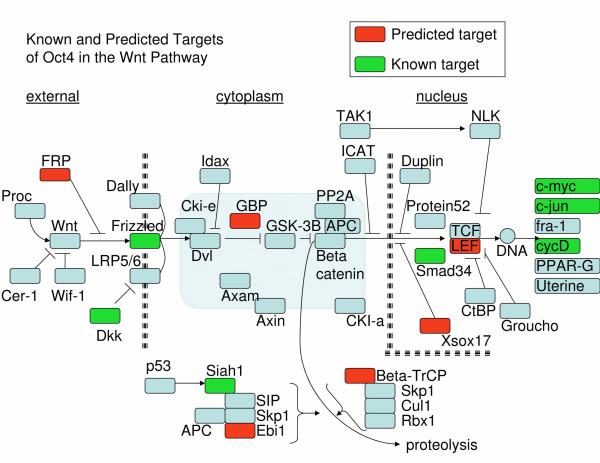
**Oct-4 Targets in the Wnt Signalling Pathway**. Known targets of Oct-4 are filled in green and new predictions are filled in red. Known targets and predicted targets are statistically enriched for genes falling in this pathway.

The authors in [32] noted that several targets of OCT4 fall into the Wnt signalling pathway. As mentioned above, both the known target set and the new predictions are enriched for genes in the Wnt pathway (*p *= 0.01, *p *= 0.0014 respectively), meaning that the predictions are consistent with the known biology of both Wnt and OCT4, implying a role in development. Figure 4 shows the Wnt pathway, highlighting SVM predictions alongside previous knowledge. Other research has shown that Wnt pathway activation is sufficient to preserve the self-renewal of human embryonic stem cells [34] and is important for maintaining pluripotency [35]. OCT4 itself is required to maintain the undifferentiated status of stem cells [36]. These results lend credibility to the SVM predictions since the predictions share a significant number of functional categories with the training set. They also impart specificity to the known role of OCT4 in Wnt pathway and maintenance of pluripotentcy.

### OCT4 regulates several genes involved in Diabetes

OCT4 is known to bind the promoters of several genes important for differentiation, and some of these are factors which can contribute to the onset of diabetes. The known targets of OCT4 are significantly enriched in genes falling into the KEGG pathway Maturity Onset Diabetes of the Young (MODY, *p *= 0.039). Particularly, OCT4 binds the gene *PDX1*, which causes Type IV MODY when mutated [37]. SVM predicts two new targets falling in this pathway. Most interesting is the transcription factor *NEUROD1*, which has been shown to cause Type VI MODY when mutated [38]. This evidence hints that OCT4 may play role in diabetes if its mode of regulation is disturbed. Others have hypothesized that disruption of normal transcriptional regulation is the ultimate cause of MODY TypeVI when *NEUROD1 *is lost [37]. This leaves open the possibility that the disruption of *NEUROD1 *targets could also be achieved by disruption or mutation of OCT4.

### Regulation by WT1

#### General findings

Wild type *WT1 *has a complex role in carcinogenesis, acting as both a tumor suppressor [39,40] and an oncogene [41] depending on context. To further complicate its role, the gene encodes four splice variants [42-48], each thought to have separate functions and slightly different DNA binding affinities. Regulation by WT1 is not well-defined, and its function may be modulated by post-translational modification [49,50] or by physical contact with other regulators, including possible dimerization with other proteins or with itself [51-58]. Two recent reviews of *WT1 *function and Wilms' tumor are available [59,60].

The classifier for WT1 has an average prediction accuracy of 68% and an average PPV of 75% (*P *= 0.5). Because the ratio of positives and negatives in the training sets are equal, these performance measures may not equal performance in the genome where the ratio of targets to non targets will be small. The number of expected false positives will be minimized in practice since any new targets must pass the 0.95 threshold (not 0.5) in its Platt score on average across 100 classifiers. Our randomized simulations and the hypothesis test in Additional File 1 show that the classifier for WT1 performs significantly better than would be expected at random.

Using the known set of 15 targets for WT1, the SVM method expands the set to include 354 new targets (at Platt score *P *≥ 0.95; see Methods The 15 training set target genes and original supporting references can be found in Additional File 2 in the spreadsheet "Wt1_known_targets_and_references.xls"). The new predictions show significant enrichment for several KEGG pathways in which there are previously annotated targets. These pathways are Map-kinase (*p *= 1.1e-3), adherens junction (*p *= 8.7e-3), and calcium signalling (*p *= 4.7e-2). Furthermore, one study has identified differentially expressed genes by comparing mutant and wild-type WT1 tissues [61]. Although the overlap with the set of predictions made here is small, the new predictions are significantly enriched for differentially expressed genes (*p *= 1.7e-4 by hypergeometric test). These data suggest that the classifier for this TF is revealing accurate biological hits.

Since the positive targets for all TFs are parsed from public databases, it is possible that other established targets have been confirmed in the literature but do not appear in our training sets. Such a set of identified targets can serve as an independent experimental validation set. A recent review [60] of *WT1 *compiles a list of 30 genes [62-96] which are possible targets of WT1 according to *in vitro *or *in vivo *studies (See spreadsheet in Additional File 2 entitled "Wt1_20_Targets.xls" for a list of these 20 genes, SVM predictions, and original references). Many of the *in vitro *studies showed transcriptional repression which has not yet been seen *in vivo*. This ambiguity makes it possible that, although experimental binding is observed in all cases, some of the binding sites are not biologically functional. 9 of the 30 genes in this list are already in our positive training set, and 1 gene [91] is an indirect target of WT1, leaving 20 genes which can be used as an independent validation set.

Of these 20 genes, 9 are predicted as targets by the WT1 classifier at the baseline 0.5 threshold. This is an encouraging result given the small size of the gene set and the possibility of experimental noise. Considering the number of genes which are predicted targets of WT1 at the 0.5 cutoff (3135 predictions out of 18660 total genes; recall that predictions are averaged over 100 test sets), correctly identifying 9 of these 20 genes is highly unlikely by chance (*p *= 6.34E-4, hypergeometric test); this is evidence that the positively classified genes are significantly enriched for binding targets of WT1. In addition to the discussion below, Additional File 1 contains an analysis of the role of WT1 in nervous tissue development and in cellular migration.

#### General Information Relating WT1 and Wilms' Tumor

To frame the new predictions for WT1 in a biological context, it is necessary to review some of what is known about regulation by this TF and the disease, Wilms' tumor, which is associated with it. Wilms' tumor is a renal malignancy accounting for 8% of childhood cancers [60]. The *Wilms tumor 1 *(*WT1*) gene codes for an essential transcription factor that plays a role in normal urogenital formation [97-101]. It is found to be overexpressed in an assortment of cancers including leukaemia [102], lung [103], colon [104], thyroid [105], breast [106], and several others [107-111]. The tumor may occur *sporadically *(no obviously heritable association) [112-115] or *syndromatically *(a genetic predisposition) [116-120]. The latter is relatively rare and often associated with a mutation in *WT1*, although mutation or loss of heterozygosity in other chromosomal regions (outside the cytological band that includes *WT1*) has been shown in some syndromatic cases. Overall, *WT1 *may also be mutated in 10–15% of sporadic cases [112,113,115,121,122]. Also in sporadic tumors, many chromosomal locations undergo loss of heterozygosity (LOH) or loss of imprinting (LOI) [123-128].

These changes are largely absent from syndromatic cases [123], suggesting that it is either loss of *WT1 *or loss of possible downstream targets which is the primary cause of Wilms' tumor. Recent evidence suggests that up to half of the sporadic tumors without a *WT1 *mutation may have some *WT1 *downregulation via epigenetic changes [129].

It is not completely understood how loss of *WT1 *precipitates cancer or how *WT1 *is linked to the other genomic changes observed in sporadic tumors. By combining known information with new predictions, a possible new model emerges which links past clinical and experimental observations of Wilms' tumor to the misregulation or loss of *WT1 *and/or the modification of its target genes.

#### WT1 May Regulate Apoptosis Through Factors Other than Bcl2

As a tumor suppressor, expression of *WT1 *has been shown to impede cell growth in some tumors [130,131]. This is consistent with Wilms' tumor resulting from the loss of *WT1*, either by mutation of the gene, or its downregulation.

On the other hand, ~90% of sporadic Wilms' tumors maintain a wildtype version of *WT1 *[61,132,133]. Indeed, in other cancers, WT1 is overexpressed [134,135]. This suggests that the presence or overexpression of WT1 may encourage malignancy in some conditions. Previous studies have shown that WT1 interacts with *P53 *[55,56,61], suppressing its apoptotic effects, and that it also directly activates the anti-apoptotic gene *BCL-2 *[41]. In addition, the results reported here include several new targets that are known to be anti-apoptotic or to otherwise regulate cell death (Additional File 4). One notable new prediction is that WT1 binds the promoter of *BAX*, a pro-apoptotic gene [136] whose protein product binds to *BCL-2 *and disrupts its repression of apoptosis [137]. A possible hypothesis is that the action of WT1 on the *BAX *promoter down regulates *BAX *gene expression, thereby allowing *BCL-2 *to repress apoptosis. Also interesting is the predicted target *PDE4B*, which can augment apoptosis when inactivated [138]. One possibility is that loss of WT1, and hence downregulation of *PDE4B*, may contribute to the sensitivity to apoptosis observed in *WT1 *mutant cells. Although the true expression relationships between WT1 and these genes awaits experimental validation, the SVM predictions provide insight into the possible targets of WT1 and can help in guiding further experimentation. For the results of additional analysis using the DAVID annotation system see Additional File 5 (genes related to cellular adhesion, cytoskeleton, or motility) and Additional File 6 (genes related to the nervous system).

#### Disease associated chromosomal loci are significantly enriched in predicted WT1 targets

In recent years it has become clear that there are distinct pathways of tumor formation in syndromatic versus sporadic tumors. As mentioned earlier, syndromatic tumors often contain a mutation in *WT1 *(Denys Drash and WAGR syndromes) or loss of the nearby region 11p15.5 (Beckwith-Wiedemann syndrome) [127,127]. The *WT1 *gene is located in 11p13, and naturally explains why disruption of this region contributes to tumor formation [139-142]. The syndromes resulting from these abnormalities and their associated chromosomal changes are listed in Table 1.

**Table 1:** Syndromes causing predisposition to Wilms' Tumor

**Syndrome**	**Occurrence of Wilms tumor**	**Chromosomal abnormality**	**Ref**.
WAGR	98% by age 6	Deletion at 11p13	OMIM: **#194072**
Beckwith- Wiedemann	96% by age 8	Duplication of paternal 11p15. May result in increased gene expression(IGF2) or inactivation(p57).	OMIM: **#130650**
Denys-Drash	96% by age 5	Missense mutation in WT1 (11p13 locus) causing dominant negative phenotype.	OMIM: **#194080**

This table highlights the syndromes causing predisposition to Wilms' Tumor development, and the genetic changes associated with the syndrome. The reference number for the syndrome in the Online Mendelian Inheritance in Man (OMIM) database [231] is given in the Ref. column. These include WAGR [139] Denys-Drash [232], and Beckwith-Wiedemann [140] syndromes.

**Figure 5:**
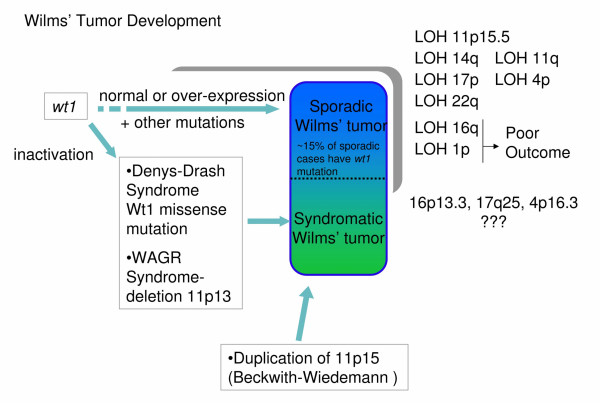
**Pathways to Wilms' Tumor**. Genetic changes leading to Wilms' Tumor. Cancer occurs through the sporadic or the syndromatic pathway. Loss of heterozygosity (LOH) and loss of imprinting (LOI) are generally associated with sporadic pathways, but are occasionally found in syndromatic tumors. The gray bar indicates that the LOH may occur anywhere along the development of the sporadic cancer. Most sporadic cases (but not all) have a wild-type overexpressed *WT1 *gene. It is possible that LOH, LOI, and other genetic changes in sporadic tumors compensate for the presence or over-expression of *WT1*. LOH at regions 16q and 1p correlate with poor prognosis. Other regions often showing LOH are listed. Regions 16p13.3, 17q25, and 4p16.3 are statistically enriched for predicted targets of WT1 but their involvement in tumor formation is unknown.

Only 10–15% of sporadic tumors have a *WT1 *mutation [112,113,115,121,122]; however, sporadic cases tend to have a variety of other genomic changes including loss of heterozygosity (LOH) and loss of imprinting (LOI). In sporadic tumors LOH occurs in 11p15 where the maternal copy of 11p15 is lost, often in conjunction with duplication of the paternal copy [128,143]. This causes the overexpression of some genes and the silencing of others, notably *IGF2 *[144-147] which is often upregulated, *H19 *[148-150] which is often silenced, and *p57 *[151-153]. Besides 11p15 [128], LOH in sporadic cases occurs in 1p, 4q, 7p, 11q, 14q,16q, and 17p [123]. LOI is an early stage event in sporadic tumors, and occurs in several regions including 11q, 16q, 4p, and 7p [123]. Figure 5 depicts some of the genetic changes which may lead to tumor formation by the syndromatic or sporadic pathways. These data suggest that regions shown to undergo LOH harbor genes regulated by WT1 or downstream effectors, yet these observations currently have no cohesive framework relating them. We show that by combining published data and the newly identified WT1 targets reported here, past observations on sporadic and syndromatic tumors can be tied together, relating them in molecular detail to misregulation or a loss of *WT1 *and/or modification of its targets.

Strikingly, examining the predicted targets of WT1 shows that these genes occur more frequently than expected by chance in several genomic regions including cytobands 11p15.5 (*p *= 6.3e-5, 8 new predictions), 1p36.3 (*p *= 6.3e-4, 3 new predictions), and 4p16.3 (*p *= 4.3e-3, 5 new predictions) (analysis in DAVID [33], see Methods). Three of the new targets for WT1 in 11p15.5 are possible tumor suppressors: RNH1 [154], IGF2AS [155], and CD151 [156,157]. *If in fact WT1 normally activates these genes it could explain why inactivation of WT1 or loss of genes in 11p15.5 contributes to cancer formation, since in both cases expression of these tumor suppressors would be abolished*. Also in these regions are 2 possible oncogenes (1 previously known–*IGF2*, 1–new *HRAS*), one gene expressed in the fetal kidney which may be involved in adhesion (*MUCDHL *[158]), and one known to contribute to cancer progression (*FGFR3 *[159-161]). Of particular interest in 11p15.5 is *MUCDHL*, the cadherin like protein. Loss of *MUCDHL *could conceivably contribute to loss of cell adhesion by disruption of adherens junctions, perhaps providing a relevant step toward metastasis. Subsequent to this analysis, one of our reviewers kindly pointed out that there had been previous evidence that MUCDHL and HRAS were linked to Wilms Tumor [158,162,163]. At the 4p16.3 locus the predicted target *FGFR3 *is associated with several types of cancer and may explain why sporadic tumors show disruption at this locus [164,165].

Although 16q and 22q, which correlate with poor prognosis [124,125], have no statistical enrichment, targets predicted at the 0.95 cutoff do lie in these regions. There are predicted target genes with known tumor suppressor activity in the regions 16q and 1p which could explain why loss of these regions correspond to poor clinical outcome (*CBFA2T3 *[166] in 16q, and *ENO1 *[167] in 1p). Also lying in 1p is the predicted target *PDE4B *which, as mentioned earlier, can augment apoptosis when inactivated [138].

Other chromosomal regions with strong enrichment include 16p13.3 (*p *= 4.3e-6, most significantly enriched location), 17q25 (*p *= 1.7e-5). These regions contain several new predictions which may be relevant to tumor formation. At 16p13.3 new targets include *TSC2*, which is thought to be tumor suppressor [168,169]. *TSC2 *has been shown to be mutated in renal disorders [170-173], suggesting that it has the potential to contribute to disease in some Wilms' tumor patients. At 17q25 lies the predicted target *FASN*. Inhibition of *FASN *can cause apoptosis [174] and also sensitizes cancer cells to treatment by chemotherapy [175]. Activation of *FASN *could provide another mechanism by which WT1 supports resistance to apoptosis. Regions 16p13.3 and 17q25 have never before been implicated in Wilms' tumor, and their strong enrichment in potential WT1 targets makes them excellent candidates for future experimental investigation. Since many chromosomal regions have been observed to undergo allele loss, duplication, or other mutation in Wilms' tumor, we have compiled a list of known targets and significant predictions which fall into several important chromosomal regions (Additional File 7).

Finally, WT1 is predicted to regulate the transcription factor POU6F2 (at 7p14-p13). This factor has been suggested to be a tumor suppressor, and mutations in *POU6F2 *confer a predisposition to Wilms' tumor [176]. Repression or activation of *POU6F2 *by WT1 could theoretically have an effect on carcinogenesis, and more studies will be necessary to uncover the expression relationship between these two factors. Since dysregulation of genes in Wilms' tumor is due to epigenetic changes as well as genetic mutations, it is difficult to predict the implications of regulation by WT1 without direct experimentation.

#### The Regulatory Network of Wilms Tumor Associated Loci

**Figure 6:**
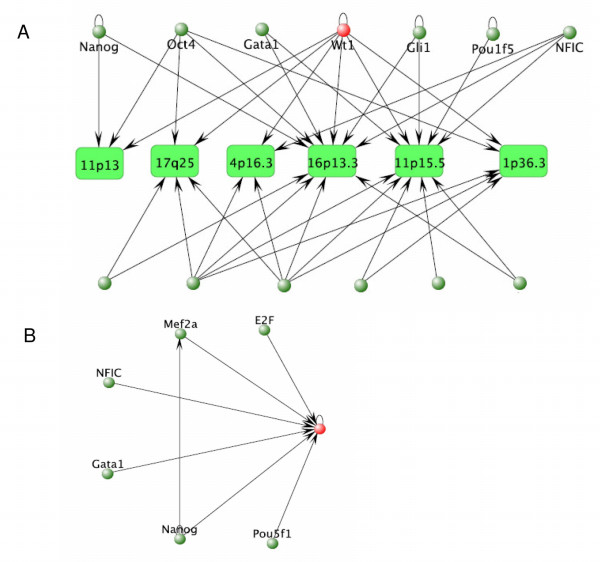
**Regulatory Network at Wilms' Tumor Associated Loci**. Figure 6A depicts the TFs, 13 in all, which target chromosomal loci thought to be involved in development of Wilms' tumor or which show significant enrichment of genes which are targets of WT1. Only TFs which have significant target overlap with WT1 are shown. Six of the TFs also regulate WT1 (based on our predictions). 6B shows only TFs that potentially regulate WT1, and the inferred interactions between them.

Since gene regulation is combinatorial, involving many TFs regulating common subsets of genes, it is of interest to determine which TFs also regulate the targets of WT1. Using the SVM predictions for only those TF classifiers which show high PPV (≥ 0.6), a statistical test (hypergeometric test) can be used to determine which regulators share more targets with WT1 than would be expected by chance. Thirteen regulators have been determined to significantly overlap the targets of WT1 (*p *≤ 0.02 see Table 2). This set includes several (8/13) TFs which have previously been implicated in cancer (NANOG [177], GLI1 [178], E2F1 [179], POU5F1/OCT4 [180], SPI-1 [181], YY-1 [182], GATA1 [183], and C/EBP-β [184]). Twelve of these factors bind to genes which are in chromosomal loci implicated in Wilms' tumor or which show enrichment of WT1 target genes. Figure 6A depicts a compact regulatory network of these factors generated in the VisAnt browser [185,186], showing which factors bind to genes in each chromosomal location.

**Table 2:** Transcription factors with significant target overlap to WT1

TF	Hypergeometric p-value	# of genes overlapped	Selected KEGG Pathways of targets shared with Wt1
GLI1	0	36	MapK Signalling, Tight Junction, Focal Adhesion
MEF2A	0	29	MapK Signalling, Regulation of Actin Cytoskeleton
NFIC	0	16	MapK Signalling, Regulation of Actin Cytoskeleton, Insulin Signalling
E2F	2.0e-12	52	Calcium Signaling, Notch Signalling, Regulation of Actin Cytoskeleton, WNT Signalling
SRF	1.3e-10	11	MapK Signalling
POU5F1	6.2e-10	38	Neuroactive Ligand Receptor Interaction, MapK Signalling
YY1	3.8e-6	6	MapK Signalling, Regulation of Actin Cytoskeleton
SPI1	1.1e-3	4	--
NANOG	6.7e-3	23	MapK Signalling, Regulation of Actin Cytoskeleton
POU1F1	7.7e-3	1	-
CEBPB	1.1e-2	6	Neuroactive Ligand Receptor Interaction
GATA1	1.5e-2	3	MapK Signalling, Regulation of Actin Cytoskeleton
T3R	1.9e-2	2	MapK Signalling

TFs that have been determined to have significant regulatory overlap to WT1 are given. The *p*-value for the significance of the overlap as calculated by hypergeometric test is given in column 2. Column 3 lists the number of genes regulated by WT1 and the TF listed in each row. Column 4 lists a selection of the most common pathways in which the targets fall.

Finally, six of the TFs are also predicted to directly regulate WT1. Figure 6B summarizes the regulatory relationships between these transcription factors (*WT1 *marked in red). Since several of the factors are known to be involved in cancer, it is possible that WT1 acts synergistically with several of these TFs to promote carcinogenesis. The fact that six of the TFs potentially bind the WT1 promoter suggests that WT1 normally acts downstream of these factors. Several of these TFs are master regulators acting in early development or in the embryonic stage (NANOG [187], GATA1 [188,189], SRF [190,191], MEF2A [192-194], and OCT4 [195,196]). These are logical coregulators for WT1 since WT1 is also known to be active in early development. Specifically, NANOG and OCT4 are critical for maintenance of the undifferentiated state in stem cells and may contribute to unbridled proliferation in some cancers [197-199]. Clearly, the regulation of WT1 and its targets is complex, possibly involving the combinatorial interactions of several TFs. The set of co-regulators determined here may serve as a basis for future investigation into the mechanisms of regulation by WT1.

### A New Binding Motif for WT1

**Figure 7:**
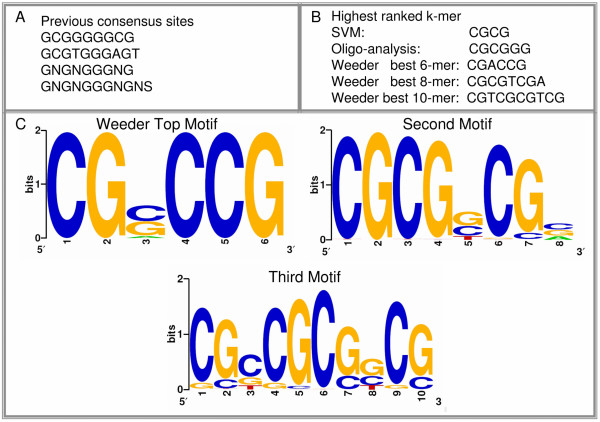
**Motif Discovery on WT1 Targets**. Figure 7A lists the proposed consensus binding sites for WT1 from the literature sources mentioned in the text. 7B shows the top ranked *k*-mer from each motif discovery method, including the best *k*-mer ranked by the SVM model. 7C shows the top 3 PSSMs created by the Weeder algorithm. Motif discovery was performed on all known and newly predicted targets of WT1.

Discovery of a binding site for WT1 has proven difficult since each isoform of the regulator may bind to slightly different sequences in DNA. Dimerization with other proteins and post-translational modifications may also alter the binding affinity in undetermined ways. Several binding sites for WT1 have nevertheless been proposed (GCGGGGGCG [45], GNGNGGGNG [200], GNGNGGGNGNS [74], and GCGTGGGAGT [201]). Unfortunately, showing that WT1 binds to a site *in vitro *has not always proven to be a good predictor of binding and regulatory action *in vivo *[60]. The four related consensus sites reported in the literature can be seen in Figure 7A. Our classification based approach has yielded a set of 354 high scoring targets to add to the set of 15 genes known to be bound. This provides a rich group from which to perform motif discovery.

A first approach (see Methods) comes from an SVM procedure which iteratively ranks each feature used by the classifier to determine those that are most useful in distinguishing the known targets (positives) form the non-targets (negatives). This method has been applied successfully to the *S. cerevisiae *genome to yield nucleotide strings which matched well with the known affinities of transcription factors. In this case it produces a ranking of *k*-mers based on information in the training set alone (*i.e*., new predictions do not contribute to the *k*-mer ranking). Two other methods have been applied to the entire set of predictions and known targets. The first of these is oligo-analysis [1,202], which scores each *k*-mer (up to *k *= 6) by its over-representation in promoters of the gene set (see Methods). The second is an algorithm called Weeder [203-205] which implements an efficient search to score and rank all possible *k*-mers of length 6, 8, and 10, while also allowing mismatches. Weeder was one of the best performing motif discovery algorithms in a recent comparison [206].

Figure 7B shows the top scoring *k*-mers from all methods. The results are uniform in that the discovered sites are GC-rich. The 4-mer ranked highest by SVM (CGCG) is also present in the result given by oligo-analysis and in the best 8 and 10-mers found by Weeder. The Weeder algorithm offers a further advantage since it automatically clusters the most similar of the significant *k*-mers (of any length), combines them into consensus site, and creates a position specific scoring matrix (PSSM) based on the occurrences of the consensus in the gene set. Figure 7C shows the top 3 PSSMs reported by Weeder. A scan of the known target promoters of WT1 with the best PSSM shows that all but 1 contains a perfect match to this matrix. Binding by WT1 is complex, and these motifs may describe only one possible binding mode of the regulator. Furthermore, since the identified sites are very GC-rich (as are many known WT1 target promoters) they may suffer from similar problems to the experimentally determined motifs, names that the sites may occur frequently in the genome at random. Although experimentation is required to validate any predictions, these motifs may aid investigators in future binding affinity studies with WT1 and serve as a useful comparison against experimentally determined sites. Additional File 8 contains the raw outputs from Weeder, oligo-analysis, and results of scanning previously proposed consensus sites against the promoters of predicted WT1 targets. All sites are within 2000 base pairs of the transcriptional start site.

## Conclusion

Prediction of transcription factor binding sites is a challenging problem in bioinformatics, especially in complex mammalian genomes. Here we have generated classifiers for each regulator developed methods to filter these using cross-validation performance. Comparison to randomized controls and a new hypothesis test show that many classifiers perform significantly better than would be expected by random target selection. Selecting the top new predictions by applying an enrichment threshold across 100 training sets reduces the effects of noise in the training data.

Functional enrichment analysis on the proposed targets of the TF OCT4 hints at its potential role in one type of diabetes. A similar analysis for WT1 confirms the role of WT1 in migration and Wnt signaling (Additional File 1) and suggests many new roles for WT1 in development, the nervous system, and in the progression of Wilms' tumor. Most strikingly, many of the newly proposed targets of WT1 are significantly enriched in chromosomal locations previously known to be associated with Wilms' tumor, indicating that the new targets could be relevant to this disease. Many of these new genes are tumor suppressors and oncogenes (including *ENO1, PDE4B, HRAS, MUCDHL, FGFR3, CBFA2T3, RNH1, IGF2AS*, and *CD151*), the loss or activation of which may now explain some of the clinical observations of Wilms' tumor patients. Two new chromosomal regions, 16p13.3 and 17q25, which were not previously connected to WT1 or Wilms' Tumor, are enriched for predicted WT1 targets. Two notable predictions in this region are *TSC2*, which is already known to be related to kidney disease, and *FASN*, which may be involved in apoptosis.

By applying statistical tests it has been discovered that the target sets of several other TFs significantly overlap the targets of WT1. This allows the construction of a potential transcriptional network for WT1, highlighting important genomic regions and the TFs known to bind genes in these locations. Important for their relation to Wilm's Tumor, several of these TFs have previously been implicated in cancer (NANOG[177], GLI1, E2F1, POU5F1/OCT4, SPI-1, YY-1, GATA1, and C/EBP-β). It is also seen that a large number of the identified regulators bind the *WT1 *promoter, suggesting that they are acting upstream of WT1 during development and/or carcinogenesis. Since the actual binding site of WT1 is ill-defined, three different motif discovery systems were applied to the known and newly identified targets of WT1 to produce PWM models which may assist in identifying specific WT1 binding sites. Finally, the underlying datasets as well as all predictions in the study are available for download from an online web-server.

The next step of this analysis has been to incorporate a more diverse set of data including expression studies which should allow the models to perform better for some regulators. Although sequence conservation has been used, our method finds sequences which are conserved *on average *in a promoter region. This ignores positional information and may allow elements which are not strongly conserved to go undetected if many similar but non-conserved sequences exist in a promoter. Work is ongoing to develop sequence conservation kernels for use with SVM that can 1) take into account the degree of conservation at every position in the promoter, and 2) handle missing data when good promoter alignments are unavailable. One possibility is a modification of the method described in [207]. Overall our approach shows that important biological insight can be gained about human disease and transcriptional networks using supervised machine learning methods. With future refinements these algorithms may be used to focus experiments, suggest new functional roles for human genes, and reveal the transcriptional circuitry underlying human development and disease.

## Methods

### SVM training and validation

SVM [208] is one of a number of binary decision processes for classifying objects based on their properties. In this paper the objects are genes which either are (positive set) or are not (negative set) targets of a particular regulator. Each gene is represented by a set of variables from which the SVM will learn a decision rule. We have previously applied machine learning to regulatory analysis [10,11,209], and results from application to both the yeast and human genomes are available on our website[30].

The SPIDER machine learning toolbox [210] in Matlab was used to select parameters and train the SVMs. The toolbox is an interface to several SVM optimizers written in other computer languages. Within this toolbox we have used the Andre [210] optimizer when training sets contained under 400 genes, and the "Libsvm [211] optimizer otherwise since it is faster on large training sets. Training an SVM involves setting a parameter *C*, which adjusts tolerance for misclassifications against the size "safety margin" about the separating hyperplane within which all classifications are considered to be in error. The classifier for the MYC transcription factor was used as the prototype for parameter selection. Five-fold cross validation was used to measure the performance of several values of *C*, and the value resulting in lowest classifier error was chosen for subsequent use in all classifiers. Tested values of *C *include: [2^-7^, 2^-5^, 2^-3^, 2^-1^, 1, 1.5, 2, 2^2^, 2^3^, 2^4^, 2^5^, 2^6^]. The value 2^-7 ^was selected [210] as having the best performance of all tested values. Initial experiments showed little change in the chosen value of *C *if other TFs were used to optimize the value. In principle, the choice of *C *and the type of SVM (linear vs. non-linear) could be specifically selected for each classifier, but this would become quite expensive computationally. The linear SVM was used in this study since previous studies in yeast have shown the linear version to be superior on the datasets used here. Preliminary results with human TFs (not shown) also indicated the linear SVM performs better than some common non-linear versions.

Choosing negatives for classifier construction is difficult since there is no defined set of genes known not to be targets. For every TF, a set of negatives is chosen randomly to be equal in size to the positive set. 100 classifiers are made in this way using different randomly selected negative sets, effectively smoothing out the negative background, from which the positive examples can stand out better. All 100 classifiers are tested using cross-validation, and the final performance measurements (accuracy, PPV, etc) are averaged over all trials. This is similar to the training set selection performed in [212]; however, their goal was not to predict new targets of transcription factors, but to filter existing target sets. Leave-one-out cross-validation (LOOCV) is the recommended procedure used for small sample classifiers and is applied for 141 of the 153 TFs in this study. For larger training sets LOOCV becomes computationally expensive and so a 5-Fold cross-validation (5CV) is used on all training sets with more than 100 genes (12 TFs fit this criteria). Because a single 5-Fold validation may not be as accurate as LOOCV, it is repeated 10 times for different random splits of the training set. For two TFs with very large training sets, HNF4-α and CREB1, SVM training would still be difficult. To make the training time more manageable, the training sets for these factors were under-sampled to a maximum size of 1000 genes. This is done independently for each of the 100 classifiers constructed for the TFs.

Accuracy and Positive Predictive Value (PPV) is used as the measure of classifier performance. As defined here, Accuracy is the ratio of correctly classified examples to all example points in the dataset:

$$\text{Accuracy}=\frac{\text{TP}+\text{TN}}{\text{TP}+\text{TN}+\text{FP}+\text{FN}}$$

Where TP = true positive, TN = true negative, FP = false positive, and FN = false negative. PPV is the number of correct positive predictions to all positive predictions:

$$\text{PPV}=\frac{\text{TP}}{\text{TP}+\text{FP}}$$

The entire analysis pipeline is described in Figure 1, and closely follows that reported in [213]. Below is an outline for the procedure, which is modified from our previous work [213]:

For a given TF :

1. Assemble positive set (denote size as *n*). Sample *n *genes randomly to construct the negative set.

2. Split the data for cross-validation.

3. Use SVM-RFE (SVM-Recursive Feature Elimination) to rank all features in the training set.

4. Construct SVM classifier on best 1750 features. Save full feature ranking.

5. Classify the left out genes.

6. Repeat steps 2–5 to complete cross-validation. Save all feature rankings.

7. Calculate all performance statistics (Accuracy, PPV, etc.)

8. Repeat steps 1–7 100 times.

9. Calculate final performance statistics for the TF (*i.e*., mean Accuracy, mean PPV, etc.).

Thus 100 classifiers represent any single TF. To classify a new example gene, the relevant feature data must be constructed and the 100 classifiers applied. Using the distance of the new gene from the hyperplane, Platt's method can be used to calculate an enrichment score (for each classifier) which can be used to rank the new prediction. Finally, the average is taken over all 100 Platt scores. Since the choice of negatives is random there will be fluctuations in the placement of the classifier in each training set. Using the WT1 classifier as an example, the genes lying between 0.45 and 0.55 (*i.e*., very near the classifier boundary of 0.5 have an average standard deviation of 0.21. Thus, these genes may find themselves on either side of the decision boundary depending on the training set used. By taking the average score over 100 classifiers, there is more confidence that a positively classified gene is actually a target according to the decision rule since a majority of training sets classify it as such.

We also noticed that genes lying deeper in the positive domain (i.e., farther on the positive side of the hyperplane) have less ambiguity in their classification. Those with an average Platt score of greater than 0.95 have a dispersion of only 0.1, meaning that they fall beyond the 0.5 boundary in most or all training sets.

Typically, if *P *> 0.5, a gene is classified as a positive, *only for cross-validation purposes*. In this paper we increase the Platt score cutoff to *P *≥ 0.95 for actual predictions, in order to select only the highest quality targets for each TF. Feature rankings on each training set are saved and used to calculate the final ranks of each feature (see below). All SVMs for classification and feature ranking were constructed in Matlab [214] using the SPIDER [210] machine learning toolbox.

### Classifying new targets and prediction significance

As described in [24] and applied in [213] the SVM can produce a probabilistic output. This is a class conditional probability of the form *P*(target is correct | SVM output), where "SVM output" refers to the distance from the gene to the hyperplane classifier. We refer to this output simply as the enrichment score and denote it using the upper-case *P *(e.g., *P *≥ 0.95), while other statistical tests which output *p*-values are denoted in lower-case (*e.g*., *p *≤ 0.01). The probability is calculated according to Platt's method by fitting a sigmoid function to the SVM output using 3-fold cross validation. Thus, genes lying at a greater distance from the hyperplane on the positive side will have higher scores (*i.e*., more likely to be positive). This form of output makes sense, as one would expect genes falling deep into the positive region to be more likely targets.

The true posterior probabilities are difficult to estimate since the underlying class distributions (number of true positives and true negatives for each TF) are unknown. Rather than guessing at the distributions, we employ balanced training sets (equal numbers of positives and negatives) and thus use the Platt estimates, not as hard probabilities as intended by Platt, but as a means to rank predicted targets. A new prediction must have an average Platt score of ≥ 0.95 across all 100 classifiers for a given TF. The Platt probability estimates, although accurate on the training set, will underestimate the number of false positives when the classifier is applied to the genome.

*This "balanced bias" is partly offset by the requirement that new targets achieve a Platt score of 0.95 on average across 100 classifiers for a TF*.

It may or course be of interest to future research to have the ability to correct the raw Platt scores to account for the large number of non-binders present in the whole genome (*e.g*., 90% or more of genes may be non-targets for any TF). We first make a conservative assumption about the proportion (p) of genes in the genome which are targets of a TF. For this example we choose 10% (*π *= 0.1) as the proportion of genes which are bound by a TF. The *π*-value for binding associated with any one gene, as corrected for genomic imbalance, will be given by

$$p\_full=\frac{p(1-\pi )}{p(1-2\pi )+\pi }$$

where *p *is the *p*-value (1-Platt score) and *p_full *is the *p*-value for the genome. As an example, if a gene is predicted to be a target of a TF with a Platt score of 0.99, the Platt conditional probability is equivalent to an uncorrected *p*-value of 0.01. The correction above is then used to transform the *p*-value of 0.01 to approximately a *p_full *of 0.1. Note that this is a very conservative correction since it does not take into account the fact that our Platt score is the *average* over 100 classifiers.

### Genomic feature selection and ranking

As demonstrated in the yeast genome [213], the SVM algorithm can be used to select and rank features. One main output of the SVM procedure is the vector **w**, which contains the learned weights of each data feature. The **w **vector is calculated directly as shown in [215]. Features with larger **w **components are more useful in distinguishing between the positives and negatives. The SVM recursive-feature-elimination (SVM-RFE) algorithm uses the **w **vector to iteratively select important features [16]. In this study, half of the features are removed during each iteration until there are 2050 left. They are then eliminated individually until 1750 are left. As indicated in the Discussion, the target of 1750 is determined by exploring the effect of feature selection on the prototype TF-classifier for MYC.

Since ranking is performed on each training set during a cross-validation, and because 100 classifiers are cross-validated for each TF, many feature rankings are accumulated for each TF. In contrast to the simple rankings by SVM-RFE, our method takes all rankings (on all cross-validation training sets for all classifiers representing a TF) into account when compiling a final feature rank for a particular regulator. To accomplish this, a count is taken of the number of times each feature appears in the top 40 of any ranking (40 chosen arbitrarily). The final rank is made by sorting the features according to the frequency of their appearance as a "top 40" feature. Genes high on this new list are consistently ranked highly over all cross validation trials and all choices of negative set, making them reliable in that they are robust to changes in the training set.

### Sequences and Transcription Factors

Several regulatory sequence regions were extracted for 18660 human genes from the UCSC genome browser database using the web based table retrieval tool [14,15]. These regions consist of: 1) 2 kb of sequence upstream of the transcription start site plus the 5'UTR, 2) all introns, 3) 3'UTR. All Refseq genes from the May 2004 human genome build in the UCSC database were selected. In some cases, UCSC reports that a Refseq mRNA matches more than one sequence region with greater than 95% similarity. We retain all sequence regions matched with 95% similarity and use them all as possible duplicate genes. These genes are indicated in our supplementary data by being suffixed with "_X_1", "_X_2" for copy 1, copy 2, etc.

Although we report results for 152 separate transcription factors, many regulators dimerize with others to form a protein complex (TF) which has its own specific regulatory action. For example, RARbeta/RXRalpha is a dimer of two proteins that has TF activity. Thus, an individual classifier is made for "RARbeta/RXRalpha". When one protein participates in more than one distinct TF complex, that protein may be represented more than once in our list of TFs. On a higher level, some groups of similar factors may share overlapping activity, and thus it might be possible to group them all together under one name, and thus make one classifier for the whole group which may be better than the smaller, individual classifiers when the individuals have small training sets. For example, the factors RARalpha, RARalpha/RXRalpha, RXR, RARbeta, and RARbeta/RXRalpha all have separate biological activity as transcription factors. Each has its own classifier in our study; however, we also create a "parent" classifier in which all their targets are grouped together, and we call this single, unified classifier "RetinoicAcidR". A more complete description of our naming conventions and classifier organization can be seen in Additional File 2 in the document entitled "notes_on_TF_names.doc".

### Feature Datasets

The sequence data described above was used to create three types of features vectors for use in the SVM:

1. *k*-mers–This feature is similar to that used in [213] on the yeast genome, and results in a feature set very similar to the spectrum kernel described in [216-218]. The frequency of *k*-mer counts in intergenic regions can discriminate between genes that are bound by a TF and those that are not. The appearances of all *k*-mers (length 4,5, and 6) are tallied in a gene's promoter region, 5'UTR, introns, and 3'UTR. The set of counts is assembled into the attribute vector for the gene. For each gene, the counts for 4-mers, 5-mers, and 6-mers are normalized separately to mean 0 and standard deviation 1. This is separate from the feature normalization which occurs prior to SVM training. *k*-mer counts are performed separately and summed for each regulatory region mentioned above. K-mer counting, which was used, in part, in datasets 1 and 3, was performed using code modified from a script that was kindly provided by Dr. William Stafford Noble of the University of Washington.

2. *k*-mer–Overrepresentation This method calculates the significance of occurrences of each *k*-mer in the a gene's regulatory regions. This method is the same as that reported in our previous work [213] and follows the equations set out by RSA tools [1,202]. Here, the background sequence set is all human gene promoters (2 kb upstream), 5'UTRs, introns, and 3'UTRs.

3. Conserved *k*-mer Counts–The feature vectors created here are made by using the output of the PhastCons algorithm [219,220] combined with *k*-mer counting and a customized weighting scheme. The procedure is as outlined in our work in the yeast genome [213]. Introns and 3'UTRs are included for the human genome. Essentially, *k*-mers are counted in gene regulatory regions as in data set 1, but each *k*-mer instance is weighted according to its level of conservation in a multiple alignment of sequences from human and seven other vertebrate genomes (chimp, dog, mouse, rat, chicken, zebrafish, fugu). Genomic alignments and PhastCons scores were downloaded from the UCSC genome browser website [14,15].

As in [213], the weighting metric we have chosen to use is:

$$\frac{1}{1-\beta P_{c}}$$

where *P*_*c *_is the PhastCons score which is averaged over the nucleotides of one *k*-mer instance. There is a parameter, *β*, which can be adjusted to control how heavily conservation is weighted in the *k*-mer count. When a *k*-mer is not conserved at all, it is given a baseline count of 1. *β *is chosen to equal 0.75, so that a *k*-mer with the highest possible conservation receives a weighted count of 4.

### Functional Analysis

Statistical enrichment of gene sets for particular gene functions was calculated using the Functional Annotation Tool in DAVID 2006 [33]. Enrichment for functions was calculated using default background sets provided in DAVID. DAVID uses the Fisher Exact test to measure functional enrichment in annotation categories from numerous public databases (e.g., KEGG pathways, GO terms, Spir keywords, etc). Enrichment for chromosomal locations was found using DAVID by searching only for enriched chromosomal cytobands. Genes were also clustered according to functional similarity using the Functional Annotation Clustering tool in DAVID. Many of the Additional Files showing gene annotation were modified from DAVID output.

### TF Coregulators with WT1

The set of potential TFs which may coregulate genes with WT1 was selected from the pool of factors whose classifiers had a measured PPV of 0.6 or greater. For each of the remaining TFs, the hypergeometric test was used to determine whether the number of overlapping targets was significant. Given 18660 genes in our study, 369 predicted targets for WT1 (known and new), and *x *targets predicted for a second TF, we ask what is the likelihood that *y *∈ *x *genes are shared targets of the TF and WT1. The test was implemented using the Matlab statistics toolbox [214].

### Positive Binding Targets

Known binding sites for human TFs were parsed from several public databases in January 2006. The databases used are Oregano [221], TRDD [222], Transfac [223], Ensembl [224], and the Eukaryotic Promoter Database [225]. Many binding sites were also manually curated from literature sources. Several large-scale experimental binding studies were also examined to identify binding sites [2,32,226-229]. In all cases, binding sites found outside of the sequence region studied (*i.e*., 2 kb upstream, 5' UTR, introns, and 3' UTR) were excluded. Lists of literature curated binding sites with Pub-med references and a spreadsheet of binding interactions parsed from the above databases can be downloaded in Additional File 2.

### Motif Discovery

Motif Discovery was performed on WT1 known targets and new predictions. Sequence data for each gene went to 1 kb upstream and 0.5 kb downstream of transcriptional start. The sequence data was downloaded from the human promoter extraction database at Cold Spring Harbor Laboratory [230]. Motif discovery was performed with Weeder [204] and Oligo-analysis [1] available at the RSA-tools website [202]. The full raw output from Weeder and Oligo-analysis along with the accompanying fasta files is available as Additional File 8. Matching of consensus strings to promoter regions was performed using RSAtools.

## Competing interests

The authors declare that they have no competing interests.

## Authors' contributions

DH coded the required software in Matlab and Perl, conceived of many of the design implementations, and wrote this article. All authors made contributions to this manuscript and the experimental design. CD initially conceived and motivated this work. All authors read and approved the final manuscript

## Reviewers Comments

### Reviewer's report 1

Trey Ideker, University of California San Diego

#### Reviewer Comments

I have now completed my review of your manuscript entitled "In Silico Regulatory Analysis for Exploring Human Disease Progression." As you know, predicting human transcription factor (TF) regulatory interactions is a timely endeavor and currently an area of active interest in genomics and computational biology. The topic is of course not novel, as many others have worked in this area, but continued efforts to predict protein-DNA interactions are highly significant, especially in human. Therefore the main questions are (1) whether your methods are sound and (2) represent a significant advance in the field over previous approaches.

In response to the first question, I did indeed find that your SVM-based classification system for TF targets was reasonable. In general, your method seems like an interesting way to predict TF targets and results in roughly doubling the number of protein-DNA interactions that are known for human at present.

As one major objection, I did not follow your logic as to why it is reasonable to use P > 0.50 as your cutoff for cross validation but P > 0.95 as your cutoff for final prediction of protein-DNA interactions. It would seem that you should consistently use P > 0.95 for both tasks. I suspect that using P > 0.95 for cross validation would result in performance estimates that appear much worse than the ones you give at present – perhaps this is why you avoided doing this. At P > 0.5 it seems that half of all genes (i.e. >10,000) would be chosen as targets for a given TF and this is clearly not reasonable. As it is, it appears that you may have chosen P to best suit your wish to make your approach look good in different circumstances (i.e. P = 0.5 gives good CV figures, P = 0.95 gives good GO enrichment). If I am being unfair here, please clarify the manuscript to make it clear why.

To get to the second question, that of how your method compares to previous attempts to predict transcription factor binding, this is not addressed by your manuscript as far as I could tell. In order to be publishable, I think it is reasonable to expect to see a comparison of the performance of your approach to at least one other leading method. You review these nicely in your paper, but never compare them to your method.

Finally, I have some recommendations regarding the organization of text. Sentence by sentence, I found the manuscript well written and easy to read. However, the larger scale structure of the manuscript is confusing. Much of the second half of the Introduction is really describing your Methods and some Results. Paragraphs 1–2 of the Results read like Discussion, and paragraph 3 (beginning "Few supervised genome-scale strategies exist...") reads like Introductory text. The second half of the Results goes into great detail on the biology of several of the TFs examined in your study, namely Oct4 and Wt1. At this point several pages of background are provided, reviewing what is known about Wt1 regulation. This text seems extraordinarily lengthy and at any rate inappropriate for the Results section of a paper that is really about a method for predicting large-scale TF binding interactions. I would recommend condensing the summary of Wt1 biology from several pages down into ~1 paragraph.

#### [Authors' Response]

We thank Dr. Ideker for his thoughtful criticisms. With respect to the choice of cutoff for validation versus prediction, we use the P > 0.5 for validation purposes so that each classifier can be compared on equal footing. In fact, the P = 0.5 threshold is similar to the maximum margin separator which is often the optimal separator when using SVMs. As we discussed in the manuscript, an Accuracy (or PPV) measurement of 50% is equivalent to random chance. This is due to the fact that balanced datasets are employed. If this is kept in mind, we see no reason for confusion when interpreting the results. Naively, it would seem that P = 0.5 is equivalent to random chance as well. This is not entirely true, however, because predicted targets at 0.5 must pass the 0.5 threshold across 100 classifiers repeated with slightly different negative sets. As such, far fewer than 50% of genes would be "hits" at P = 0.5. That being said, there are several TF classifiers which yield no new predictions at the stringent threshold of P = 0.95 (*i.e*, classifiers have a PPV = 0). In that case there is no difficulty since that TF will simply have no new hits by our method. Our use of random controls allows one to test whether a classifier is behaving "better than random" at the P = 0.5 cutoff. In this case, better than random means that the classifier shows a statistically significant improvement in the PPV as compared to the randomized control. Thus, if a classifier is showing statistical significance at P = 0.5, the targets identified at P = 0.95 should be the most relevant hits.

Regarding Dr. Ideker's comments on comparisons to other methods, we feel that showing statistical significance and biological interpretations is sufficient for a publication on this topic. As this method continues to develop, a more detailed comparison with other methods is desirable, and we hope to complete that in future work. We believe that the significance of this work lies not just in method, but in a combination of methodology and biology; for the latter, especially the insights on the important regulator, Wt1. Finally, regarding the writing and organization of the manuscript, we realize that there is content in the introduction which is methodological in nature and that there is also some background details given in the results. Given the nature of this topic and the fact that we are attempting to appeal to both computationally oriented and biologically oriented audiences, we felt the need to repeat certain methodological points which are important. We further felt that the biological meaning of the data presented in the Discussion section has greater depth given the detailed discussion of WT1. Moving this section to the Introduction may break the continuity of the story.

### Reviewer's report 2

Vladimir A. Kuznetsov, Division of Genome and Gene Expression Analysis Bioinformatics Institute, Singapore

Nominated by Frank Eisenhaber, Bioinformatics Institute (BII), Agency for Science, Technology and Research (A*STAR), Singapore

[Authors' Note]: Due to the extensive review provided by Dr. Kuznetsov, we respond section-by-section to his comments below. We are very grateful to Dr. Kuznetsov for his extensive comments and perspicacious analysis.

#### Reviewer Comments

The authors of this work have developed a pattern recognition method to identify new targets for a given transcriptional factor (TF). Based on Support Vector Machines (SVM) approach, they attempted to produce classifiers for that 152 TFs in order to predict new gene targets and to find new regulatory interactions and gene networks (genes that could control expression other TFs) which could be important in development of human disease (in particular, Wilm's cancer). The paper outlines the strategy behind this idea, the methods applied and the detailed findings of BSs for two important disease-relevant regulators: OCT4 and WT1. The authors used publicly available motif datasets and some other sequence information mapped on human genome as training set of SVM algorithm to predict new targets for selected TFs. Based on their counting of occurrence of several types of DNA fragments (e.g. motifs, preferred patterns of bases, evolutionarily conserved DNA fragments etc.) in RefSeq genes or their putative promoter regions the authors predicted 933 targets for 152 TFs, including 354 target genes for WT1 TF. An association of predicted OCT4 gene targets with Wnt pathway and some other biological and clinical correlations were considered. Main Comments Evaluation and control of accuracy, specificity, sensitivity of gene target predictions and consistency of the predictions with previous studies are my major focuses in consideration of the work. I concern regarding the predictive power of TFSVM methodology, performance of the method, biological significance of predicted targets, interpretation and extrapolation of the results, and independent validation.

1. At the beginning of the paragraph "Results and Discussion" the authors claim that cross-validation performance measures of their method are determined at the decision threshold of P = 0.5 and they further explain the reasons of their choice. That means that only genes with average scores higher than this threshold have a chance of being true targets. However, this P = 0.5 threshold might still be a small or a large number depending on the situation. Since P is an average score it is also sensitive to outliers. It may be better preferable to base selection on a criterion that also takes into account the variance of the 100 P scores for each gene or alternatively to use the additional information of how many times (percentage) is P exceeding a threshold over the 100 iterations.

#### [Authors' Response]

Our use of an average P = 0.5 threshold is due to the very observation that there may be variability between measurements. Since the choice of a negative set is random, and therefore potentially noisy, we felt that forcing an average cutoff is clearly better than any single classifier measurement. Nevertheless, Dr. Kuznetsov may be correct that using a criterion which accounts for the variance may improve prediction. For instance, perhaps we need not be as strict when the variance is very low (more strict when the variance is high)? This is something we intend to examine more closely in future work.

#### Reviewer Comments

2. How would different scores affect the number of false positives/negatives of the study?

3. At the same time, the authors mention that the best threshold for making predictions is set to 0.95. However, they do not explain the reasons behind this choice. Would it be the case that 0.95 may be too conservative in some situations, thus producing many false negatives and obscuring useful information of the study? How many new false positive predictions were made for lower cut-offs?

#### [Authors' Response]

Regarding the number of false positives/negatives, in the machine learning context, increasing the threshold value should have the effect of reducing false positives and false negatives. This is the primary reason why the 0.95 threshold was chosen to make new predictions, as it should yield a more enriched set of true positives. Given the noise in the negative sets and the higher threshold of 0.95, it is likely that there are many false negatives for some TFs, though this is preferable to a very high false positive rate.

#### Reviewer Comments

4. The authors mention that many classifiers show poor performance in cross-validation (at threshold P = 0.5) although several do show high precision (33 have PPV > 0.6). They claim that poor performance may be partly (i) due to the fact that the defined promoter region is large and in some cases maybe thousands of base pair long (the size interferes with the ability of the SVM to identify important regions); (ii) human TFs generally have few known targets making it less likely that a classifier would be able to find the correct decision rule. Although a test is developed to deal with the second problem, the first one is not discussed. How does the complexity of the genome region affect the findings? How is the number of false negatives affected by genome complexity? Is there a method to adjust for the P threshold depending on the complexity of the situation?

#### [Authors' Response]

As promoter regions get larger and encompass a greater variety of k-mers, it is more likely that important motifs will become lost in the background (i.e., less likely to stand out or discriminate between targets and non-targets). This is a problem for most TF motif detection algorithms as well as for our SVM classifiers. A related problem is that, since the negatives are chosen randomly, any given negative set may include unidentified positives, further hampering the discovery of an accurate decision rule. It was our intention that averaging the results of 100 classifiers would partially compensate for the noisy character of the negative sets in at least two ways: 1. negative sets which inadvertently contain positives will be in the minority, and their influence on the final predictions will be minimized, and 2. if the promoter regions are too complex or too variable, this will be detected as a low PPV since the classifiers will be unable to consistently identify true positives. It must be kept in mind that a PPV of 0.5 indicates a random classifier. A classifier which performs randomly at P = 0.5 could possibly show better results at a higher threshold of P = 0.95, although this is not necessarily expected and we did not examine this possibility. We would recommend focusing only on TFs where the PPV > 0.5.

#### Reviewer Comments

5. In order to identify new targets, genes are selected based on two decision rules (i) average P higher than 0.5 in cross-validation; (ii) average P higher than 0.95 in prediction. It is also mentioned that many classifiers show poor performance in cross-validation although several show high precision. Is it possible that genes just below the cross-validation threshold of 0.5 may have shown average P higher than 0.95 in the prediction phase? Should these genes be included in the analysis?

#### [Authors' Response]

A gene may have a low P score in any single classifier or cross-validation, due to variations in the negative sets. However, since we use the average P score (at P > 0.95) to make predictions, it is unlikely that genes with consistently low P scores in the cross-validation phase (across 100 classifiers) could make this cut-off.

#### Reviewer Comments

6. In the Regulation by WT1 paragraph, the authors mention that the new predictions show significant enrichment for several KEGG pathways in which there are previously annotated targets. However, the p-values of the pathways that are included in the analysis do not show significantly small p-values (especially for adherence function and calcium signaling). The p-values produced by this kind of analysis should be treated with caution since they depend on the number of the tests performed and the candidate genes for selection (genes with GO annotation, all genes in the chip set etc).

#### [Authors' Response]

The p-values obtained were calculated using the DAVID annotation system [33]. We understand that the p-values are the result of a modified Fisher Exact test (called the EASE score on the DAVID website) which takes into account the total size of the human genome as known in the DAVID system. This modified p-value is more conservative than a typical Fisher Exact test according to the description on their website. The p-values recited (adherens junction (*p *= 8.7e-3), and calcium signaling (*p *= 4.7e-2)) are both less than p = 0.05 which we believe to be reasonable threshold. Nevertheless, a discriminating reader may choose to apply a more stringent cutoff of 0.01, in which case calcium signaling would not be a significantly enriched pathway.

#### Reviewer Comments

7. In Materials and Methods, the authors outline their procedure consisting of 9 steps. Throughout the text some suggestions are given on the cross-validation scheme and SVM parameter selection. From the description of the procedure it seems that the cross-validation step might be the most time consuming one. To this extent, could the authors comment in more detail on the ability of their method to produce adequate results in a relatively short time?

#### [Authors' Response]

Cross-validation in itself does not take an extensive amount of time (<5 minutes on a 2 Ghz processor). Difficulties arise when performing the cross-validation 100 times for each TF, which can take significantly more computer resources. To complete all of the validations in our analysis we continuously ran the algorithm on ~25–30 nodes of a 200 node linux cluster for ~24 to 30 hours. This speed could be increased by changing the coding language from MATLAB, which is simpler to use, to a language like C++ or Java where there is greater control over memory usage.

#### Reviewer Comments

8. In the paragraph Classifying New Targets and Prediction Significance a measure is introduced that corrects Platt scores to account for the large number of non-binders present in the whole genome. The authors suggest estimating the average Platt score (p) and then calculating the p_full. Is this different from estimating p_full for each individual Platt score and then average the p_full scores? Which is should be preferred?

#### [Authors' Response]

Although the method for calculation of p_full is provided in the methods section, we noted in the manuscript that the p_full values were not used during validation or prediction in our experiments. The method for calculating p_full was added later, once we considered that it could be desirable to adjust for the uneven distribution of positives and negatives in the genome (whereas the classifiers are built using balanced datasets). We chose not to make the correction in our results since the true distribution of positives/negatives is unknown, and since the distribution may vary widely between transcription factors. In the absence of knowledge we felt that providing the results using balanced datasets was an objective approach so long as the reader understands the possibility for bias and has the ability to correct the P-score using the provided methods.

Moreover, the calculation of p_full does not take into account the fact that new predictions must meet the P-score cutoff *on average over 100 classifiers*. The average classifier criterion introduces a correction of its own which makes the results more conservative and partially offsets the need for higher stringency due to the uneven distribution of positives and negatives in the genome. If we apply the p_full correction to the average P-score obtained over the 100 classifiers, it would possibly likely produce an over-conservative assessment.

#### Reviewer Comments

9. Another important and open question is related to the small sample size which the authors used in training and relatively large number of genes in exam sets. How robust and representative is that set? What is the specificity and sensitivity of predictions in this case? What is the false discovery rate in such poorly-performed prediction studies? In particular using only 15 "known" WT1 TF gene targets, your algorithm predicted 354 new gene targets. I am not sure that such prediction is reliable and accurate. (See below). It must be validated in independent and direct detections of WT1 BSs for a specific cell type.

#### [Authors' Response]

If input data is of poor quality we expect that the 100 classifiers used to make predictions would not often agree and, thus, few or no new predictions would be made. However, there is always the possibility that, with small datasets especially, the input set may show a bias. This can happen, for example, when the small input set of promoters all happen to be AT rich. If there is a sequence bias in the positives, the classifiers may learn to detect this bias without identifying any true binding motifs. This can be very difficult to correct for (what if the true motif is AT-rich and exists in AT-rich promoters?) and is an obstacle to TF binding site prediction in general. This is part of the reason we attempted to provide additional evidence in the form of pathway analysis and chromosomal location to impart confidence that the results were biologically meaningful. Nevertheless, we agree that experimental validation is the ultimate "acid test" of new predictions, and we hope that the predicted targets are followed up in future studies.

#### Reviewer Comments

10. To illustrate my concern regarding correlation between limitation of training sets and reliability and predictive power of TFSVM, I provide the results of my own analysis of predictive power of training sets and the results of comparison of the prediction with published data. I collected experimentally-confirmed gene targets for well studied myc TFBSs. The 1-st report was from Li et al. (Li et al, 2003): 876 myc BSs associated with promoters in the Daudi Burkitt's lymphoma cell line were identified by ChIP-Seq method. 756 myc binding loci on Chr 21&Chr22 have been identified by tiling array in (Cawley et al., 2004). Fernandez et al, (2003) tested 6541 E-box BS regions for Myc binding by Chip-qPCR. About 3800 myc gene targets were found in ChIP-PET experiments and randomly validated with independent methods (Zeller et al, 2006). One of these 4 papers (Canwey et al, 2004, the paper was numerated twice by 2 and by 224, see References) has been cited by the authors and for some reason only very small set of gene targets (67 genes, P.5) have been used in their training set. I used author's software, TFSVM, to evaluate overlap between algorithm's prediction of myc gene targets and measurements of 688 direct gene targets observed in (Zeller et al, 2006). TFSVM program predicts 199 myc gene targets at score P = 0.95. Somebody expect that this subset will be strongly overlapped with experimentally defined myc targets. However, even for that "high reliable" cut-off value (P = 0.95), only 8% (16 of 199) of predicted genes were found in the list of 688 ChIP-PET direct myc target genes (2%). Additionally, I did not find any of 199 genes in the set of 15 genes (see Zeller et al, 2006) which is strongly confirmed by the four previous experimental studies. These analyses suggest that the small number of genes in the TFSVM training set and perhaps existence of significant fraction non- representative members in that set assure that the method has low predictive potential. Consequently, these are the two key issues that would need to be addressed in order to improve the predictive value of the method.

#### [Authors' Response]

We first wish to thank Dr. Kuznetsov for the detailed comparison he has provided with third party experimental results. Since our study included 152 transcription factors, it was not possible for us to conduct an exhaustive literature review on each TF to compile all known targets. We relied on public databases and a few large scale studies where possible. Including the results of large scale studies was difficult, especially when the results were derived from ChIP-chip or tiling arrays (e.g., Cawley et al. [2]). The tiling arrays will interrogate the whole genome and may include exon regions and very large intergenic regions. This is in contrast to our analysis which included regions of only several kb surrounding transcription start sites. Many of the positive hits identified by tiling array could not be included in our analysis simply because the identified binding site falls outside of the promoter regions we examined. Thus our site filtering by statistical significance and gene region is at least part of the reason why not all of the Cawley sites are included. Dr. Kuznetsov further points to the study by Zeller et al. {Zeller, 2006 #2124 and calculates that, of the 199 Myc targets identified in our study, only 16 overlap with the 688 targets identified by Zeller et al. using ChIP-PET. Although we would have hoped to see greater correspondence, this doesn't necessarily indicate that our method is finding poor targets. Given the size of the genome in our study (18660 genes), if we assume that the 688 targets identified by Zeller et al. are the gold standard set of true positives, we calculate that the p-value for identifying 16 correct targets is 0.0012 (by hypergeometric distribution), indicating that our target set is enriched for true targets in a statistical sense, and that the 199 gene set may represent an interesting group for further study (this calculation may be repeated using the MATLAB function "hygecdf" where "p-value = 1-hygecdf(16,18660,688,199)"). Therefore, while we acknowledge the limitations discussed by our reviewer and agree that these may be best addressed by follow-on experimental studies, we feel that many of the target sets we have identified merit further analysis.

#### Reviewer Comments

11. The authors used relatively large training set as well. For example, 4627 targets for CREB1 and HNF4-alpha [no references, V.K.]. They stressed that "*In fact, when large sets of known interactions exist, the classifiers make few or no new predictions, perhaps suggesting that a significant subset of the targets for those factors have already been found *(most strikingly, HNF4- classifiers yield only 3 new predictions, and CREB1 yields only 1)". This conclusion means that all specific gene targets for these TF are known. However, it contradicts to observation. For example, using CACO method, S. Impey et al (Cell, 2004) found 32700 potential CREB regulatory regions in the rat genome. These authors also found that ~60% CREB regulatory regions are located in 2 Kb 5' upstream promoter regions and in internal gene regions. This estimate assumes that at least 19634 genes could be considered as putative direct targets for CREB. For different TFs, Chip-seq method [Johnson et al, Science, 2007, Roberson et al, 2007, Nat Meth, 20007 ] (which sampling in 10–30 times deeper that was use before in ChIP-based sequencing/cloning experiments) identifies from 2000 to 42000 locations of TF binding sites in the human genome. These findings together with theoretical estimations of sensitivity of ChIP-PET data [Wei et al, Cell, 2006, Kuznetsov et al, Genome Informatics, 19, 2007] suggest that perhaps most of 152 TF training sets used in this work are represented by essentially incomplete, non-representative and bias variables.

#### [Authors Response]

We thank Dr. Kuznetsov this observation. Especially in the cases of CREB and HNF4-alpha, one alternative to the suggestion we provided in the manuscript is that, since the positive sets for these two factors are very large, the possibility exists that the promoters of the positive sets have a large amount of variability. This variance, which could result from experimental noise or natural variability in target promoters, may prevent our classifiers from identifying features which distinguish potential new targets in the genome. The problem may be compounded in situations where the TF binds to large numbers of sites in the genome (our reviewer recites a possible 32700 regions in the rat genome for CREB). In such cases it is increasingly likely that the randomly chosen negative sets may include target genes, interfering with the SVMs ability to find a sensible decision rule. However, we are glad to see that such variability in the promoter regions (or inability of the algorithm to find a good classifier) results in very few predictions for CREB and HNF4-alpha, indicating that our method of choosing targets (i.e., those which have P > 0.95 over 100 classifiers) has the desired effect of removing what might otherwise be false positives.

#### Reviewer Comments

12. The author's said that "OCT4 has ChIP data performs about as well as WT1, which does not" is in contrast with CACO, Chip-PET and ChIP-seq observations. Than they concluded: "Classifiers for TFs which include ChIP data do not necessarily perform better or worse than those without it". That conclusion did not consist with observation in [Johnson et al, Science, 2007, Roberson et al, 2007, Nath Meth,20007 ] and suggests that predictive power of the TFSVM is quite limited.

#### [Authors' Response]

We have no intention of refuting the articles cited by our reviewer, and believe that our remark in the manuscript may have been misunderstood. When we stated that "Classifiers for TFs which include ChIP data do not necessarily perform better or worse than those without it" we merely meant to indicate that the measured accuracy or PPV of our method did not seem to depend on the source of the input data. In all cases, positive sets came from experimental data; however, it did not seem that those TF classifiers which included large scale ChIP data yielded largely better or worse results than those which had other types of experimental data. Thus we draw no conclusions about the quality or sensitivity of ChIP datasets. Indeed, the recited methods of Chip-PET and ChIP-seq appear to have very high quality.

#### Reviewer Comments

13. The authors claimed that they found new potential suppressors and oncogenes in Wilms tumor cells including HRAS and MUCDHL. However, (i) I found in PubMed that HRAS and MUCDHL have been already considered as the genes which are strongly associated with WT1 functions and Wilms tumor phenotype. So the corresponding references should be presented and discussed). TFSVM predictions are not tissue-type and physiological condition specific. Are there these and other TFSVM predicted genes under-expressed or over-expressed in Wilms tumor versus original normal or benign cells? Predicted gene could be over-expressed or suppressed in many types of normal and pathological cells. Is it your case? I believe that gene expression and gene copy number (CGH) analyses should provide essential support and/or significantly improve author's work.

#### [Authors Response]

Our brief search of PubMed did not find the articles mentioned by our reviewer unless he was referring to Goldberg *et al*. [163], which discusses the biallelic expression of HRAS and MUCDHL in chromosomal region 11p15.5 (also perhaps'S Kumar, *et al*. [162], which we now cite in the manuscript). Another article by Goldberg *et al*. [158]further suggests a possible link between MUCDHL and Wilms' Tumor. Our initial searches did not uncover any direct experimental evidence of WT1 binding to the promoters of either HRAS or MUCDHL. If these two genes are indeed linked to or regulated by WT1, then SVM was successful in identifying them as potential targets, since they were not part of our original positive set. In retrospect, this result is not extremely surprising, since the genes lie in chromosomal regions which are affected in Wilms Tumor. Dr. Kuznetsov inquires about the expression of the target genes in various tissues, and we agree that this information would be valuable in validating the predictions. Thus far we have not analyzed gene expression or conducted any expression studies in our lab. This is clearly a high priority topic for future studies.

#### Reviewer Comments

14. There are too many references (229) in this manuscript. The significant proportion of the references could be omitted without leaving out any key information related to this work. On the other hand, the list of references does not include references to the papers (starting from spring of 2006) in which a several new ChIP-based sequencing methods have been used to detect many thousands TF targets on the genome scale. In particular, at least one thousand of high- and moderate- avidity gene regulatory regions for mouse OKT4 TF have been detected

[Loh YH et al, Nat Genet. 2006]. The data set is still incomplete; however it might be used for partial validation of TFSVM predictions.

#### [Author's Response]

The bulk of the coding and analysis on which this manuscript is based was undertaken in 2005 and 2006, which is the reason why many datasets published in mid-2006 onward were not included in our analysis. We intend to incorporate these newer datasets in future iterations of our algorithm.

#### Reviewer Comments

15. The title of the manuscript should be more concrete and reflect the major results.

#### [Author's Response]

Since this manuscript has already been cited in other work, we prefer not to alter the title so as not to confuse readers who cross-reference the article

#### Reviewer Comments

16. Finally, I agree with the authors that "prediction of transcription factor binding sites is a challenging problem in bioinformatics, especially in complex mammalian genomes". However, analysis of essentially incomplete, high-noisy and low-specific sequence data which poorly represent the full complexity of the genome demonstrate real limitations of machine learning approach for prediction and understanding of transcriptional machinery and networks. Using only pattern recognition approach for such TF binding information is not sufficient for providing reliable, specific and sensitive predictions of TF direct gene targets and considering TF controlled functional genes in different cell types at diverse physiological conditions.

#### [Author's Response]

Again, we thank Dr. Kuznetsov for his detailed response. We agree that no single approach will be entirely sufficient to unravel the complexities of predicting transcription factor binding sites. Along those lines we have begun developing related algorithms which are designed to learn the actual binding site motifs rather than simply make a prediction of "positive" or "negative" (Kon et al. 2007. ICMLA Proceedings of the Sixth International Conference on Machine Learning and Applications. P.573–580). It is hoped that alternative methods such as this, combined with experimental studies can provide better prediction methods for TF binding sites.

#### Reviewer Comments

I hope that the authors will find my comments constructive and useful.

Declaration of competing interests: I declare that I have no competing interests' in your report.

### Reviewer's report 3

Tzachi Pilpel, Weizmann Institute of Science Rehovot, Israel

#### Reviewer Comments

The authors study the problem of identification of targets for human transcription factors. They use state-of-the-art classifier to predict interactions based on training sets and identify thousands of new potential interactions. They focus on two particular cases of factors involved in cancer progression and among their targets on particular potential anti-apoptotic representatives.

Over-all this is a very well-written and interesting paper. The methodology is sound and robust. Various sources of information, including conservation and robust k-mer statistics are effectively utilized. The final output from the paper should be of wide interest, even beyond the cancer-related applications. As such the paper would add nicely to a growing body of data on human transcription factors and their targets. Two minor comments are:

1. I can t see how the current title of the paper "**In Silico Regulatory Analysis for Exploring Human Disease Progression" **reflects in main contribution. Particularly I think that delineating "disease progression" would amount to more than is shown here. On the other hand the title gives no clue about the actual content of the paper.

2. Despite an unusually high number of paper cited, I missed a mention of one of the best characterized cases of a mutation in human transcription factor with a clear implication in cancer – Cell. 2004 Nov 24;119(5):591–602

#### [Authors Response]

We thank Dr. Pipel for his thoughtful criticisms. Please see our response for Dr. Kuznetsov which addresses the comments about the manuscript title. We have updated our manuscript to recite the Cell reference kindly provided by Dr. Pipel.

## Supplementary Material

Additional file 1 Supplementary Notes. This is a word document describing and demonstrating the hypothesis test for classifier accuracy. Also described are the possible roles of WT1 in nervous tissue development and cellular migration. The hypothetical relationship between WT1 and the Wnt pathway are also discussed. Click here for file

Additional file 2 This file contains several sub-folders. The folder "Classifier Results" contains the SVM predictions for all TFs in this study as well as a list of classifiers and their associated performance measures. The new predictions for all TFs are also available for query and download on our website[30]. The folder "Literature_curated_targets" contains the known TF-target interactions taken from databases and the literature. Any interactions manually curated from primary literature are listed, and the Pubmed ID of the article used is given. All files are annotated so as to be self explainatory or have an accompanying Readme file. Click here for file

Additional file 3 This file contains two excel spreadsheets providing the functional annotations of known targets and predicted targets of OCT4 respectively. These are annotations as provided by the DAVID system at NIH and include the statistical significance of each functional category. Click here for file

Additional file 4 Using both known and newly predicted targets, this file contains a list of genes which relate to apoptosis as given by the DAVID functional analysis tools. The genes appear several times in various, similar annotation categories which are related to cell death pathways. Click here for file

Additional file 5 Using just the newly predicted targets, this file contains a list of genes which relate to cellular adhesion, cytoskeleton, or motility as given by the DAVID functional analysis tools. Click here for file

Additional file 6 Using both known and newly predicted targets, this file contains a list of genes which are annotated to terms by DAVID which are somehow related to the nervous system. Three main categories are present (represented by folders) which each contain several functional terms and the genes annotated to them. The three main categories are "Neuron related", "Sensory perception", and "Voltage gated channels and membrane receptors". Click here for file

Additional file 7 Using both known and newly predicted targets, this file contains a list of genes and the chromosomal cytobands to which they are mapped. *p*-values generated by DAVID are also given to show statistical enrichment. Click here for file

Additional file 8 This file contains the results of running the Weeder algorithm on 1) the set of known and newly predicted (Platt score *P *≥ 0.95) targets of WT1, and 2) the known targets of WT1. Sequence regions used are as defined in Methods. The file also contains the results of Oligo-analysis. Also included are the matching results after scanning the literature derived consensus sites for WT1 against the full set of WT1 targets (predicted and known). Click here for file
