# Supplementary material for: In silico regulatory analysis for exploring human disease progression
Source: Biol Direct. 2008 Jun 18;3:24. doi: 10.1186/1745-6150-3-24 (PMC2464594; doi:10.1186/1745-6150-3-24)
Supplement: Additional file 1 — Supplementary Notes. This is a word document describing and demonstrating the hypothesis test for classifier accuracy. Also described are the possible roles of WT1 in nervous tissue development and cellular migration. The hypothetical relationship between WT1 and the Wnt pathway are also discussed. [file 1745-6150-3-24-S1.pdf]

Hypothesis test for determining classifier significance given cross-validation accuracy.
A general calculation for Wt1 data (15 known target genes)

For any classifier we assume a "background" distribution of genomic feature data in a feature space F. For Wt1 we have a sample S of size 15 which has a possibly different distribution than background. Assume for the moment that both distributions are normal since all feature data are standardized to mean 0 and standard deviation of 1. There is then a fixed **w** vector which best differentiates the means of these distributions. The cross validation accuracy for Wt1 is 68% so our question is: How likely is it that the optimal separator of the target and the background distributions will have a 68% correct cross-validation rate? We will show here that the problem becomes approximately one dimensional.

To demonstrate that a 68% prediction rate under cross-validation is statistically significant even with such a small number of positive examples, we define a hypothesis test. We set the null hypothesis H_0 to be that we have picked 15 elements of the background distribution at random. Thus under H_0 in our feature space F, there is no linear information which differentiates binders from non-binders for Wt1. We test the likelihood under H_0 that a 68% positive cross-validation rate comes from the 15 positive genes. For the sample S of 15 positives, let $\bar{\mathbf{x}}$ denote their mean position in F. Then the direction of $\bar{\mathbf{x}}$ denotes an optimal choice of vector **w** which differentiates the distribution of S from the $N(0, 1)$ distribution of the background.

Comment: Assuming that under H_0, cross-validation yields an empirical choice of **w** in the SVM algorithm which is always close to the optimal **x**.

Assuming that the empirical distribution of S projected onto the direction of $\mathbf{w} = \bar{\mathbf{x}}$ has approximately the same unit variance (which would be true if it were normal), we now want the probability that the optimal separator for the distribution of S and $N(0, 1)$ (restricted now to the **w** direction) yields an empirical cross-validation rate of at least 68%. Note that by symmetry this optimal separator occurs at a distance $|\bar{\mathbf{x}}|/2$ from 0, and if the SVM can find this choice optimally, we would need the following (under H_0) for 68% discriminatory accuracy. We must require S have at least 68% of its distribution in the **w** direction at a distance of $|\bar{\mathbf{x}}|/2$ (location of separator) or more from 0. This would mean that the null distribution H_0 (now projected onto **w**) have at most 32% of its mass at a distance of $|\bar{\mathbf{x}}|$ or more from 0. This requires that the classification threshold occur at $z_{.32} = 0.47$.

Comment: We know that in order to have 32% of the mass of the null distribution to fall on the positive side of the hyperplane, the hyperplane must fall at Z=0.47. Because it is a maximal margin separator, the mean of x should be no closer than Z=0.94.

Note under our assumptions that the probability distribution of $\bar{\mathbf{x}}$ in the **w** direction is $N(0, 1/\sqrt{15}) = N(0, .2582)$. We now need to calculate under H_0 the probability we will have a classification threshold at such a location; namely the probability that $|\bar{\mathbf{x}}| \geq 0.94$ (so that the decision threshold is $b \geq 0.47$). Under the null hypothesis, projecting $\bar{\mathbf{x}}$ onto **w**, the probability $|\bar{\mathbf{x}}| \geq 0.94$ is

Comment: Of course the assumption here is that the data is normal and (by Central Limit Theorem) the standard deviation equals 1/sqrt(N).

$$P(Z \geq 0.94/.2582) = P(Z \geq 3.64) = 0.000136$$

Comment: mean of **x** divided by standard deviation gives the z-score of the mean of **x**, which is at 3.64.

or about 1/7,353. This makes it unlikely that these results are sampled from the background distribution, giving a p-value of .000136 that there is no difference in F between targets and non-targets. Multiplying by 152 to account for the number of TF's, we obtain a p-value of .0207 for such a result for *any* such TF. Certainly any factor with greater than 15 targets would have an even more significant score.

Role of WT1 in Nervous Tissue Development and Relation to Wilms' Tumor

The set of combined targets (known and newly predicted) for WT1 is significantly enriched in several annotation categories related to the nervous system and neuron growth: transmission of nerve impulse (p = 0.0069), synaptic transmission (p=0.013), and neurotransmitter receptor (p=0.058). Many genes are annotated to similar categories but do not show statistical significance (Additional File 6). These may still be important since they all relate to development or function of the nervous system. Observations have been made of neuronal differentiation markers in Wilms' Tumor [1], demonstrating that some mechanism in these tumors is activating nerve cell signature genes. WT1 has been shown to be required for normal development of the neurons in retinal [2] and olfactory [3] tissues. Furthermore, analysis of the developing mammalian embryo has shown presence of WT1 in brain, tongue, and retinal tissues [4]. Surprisingly, one highly significant predicted target for WT1 is the *TAS1R1* gene, which is a taste receptor responsible for detecting sweet compounds [5, 6]. This implies that, aside from its proven roles in eye and olfactory development, WT1 is also involved in taste sensation. Along these lines are also the potential new targets *EYA1* and *EYA4*, which are members of a gene family known to be involved in kidney, eye, and ear disease [7-11].

Another supporting target prediction is the *MTMR2* gene which, when mutated, can cause Charcot-Marie-Tooth Disease type 4B [12]. This is a demyelinating disease of the nervous system which causes sensory and motor defects. It is interesting that one of the chromosomal loci implicated in Charcot-Marie-Tooth Disease is 11p15 [13], a key Wilms' Tumor locus. Finally, 48 high confidence targets can be annotated as being either voltage gated ion channels, integral to the plasma membrane, or part of a neurotrophic ligand/receptor interaction (Additional File 6). Taken together, these predictions can provide new hypotheses about the role of WT1 in the nervous system, and point to several genes which may be examined further to elucidate WT1's function in nervous disease. These targets may be involved in producing many of the symptoms observed in Wilm's tumor patients. For example, patients with WAGR syndrome, which causes predisposition to Wilms' Tumor, show mental retardation and aniridia, a defect of the iris [14-18]. Also, there are reported cases of deafness and mental retardation accompanying Denys-Drash syndrome [19], which also predisposes patients to Wilms' Tumor.

Support for the Known Role of WT1 in Migration and Wnt Signalling

Recent evidence indicates that WT1 is involved in cellular migration [20], although few known targets of the TF have been previously reported to be directly involved in this process. *Functional grouping of our target predictions reveals a group of 67 genes which are annotated to cellular adhesion, cytoskeleton, or cell motility* (Additional File 5). This group includes many cadherin and contactin genes known to be involved in adhesion and migration. Notably, this set also contains *WASF1*, *IRSP53*, *AFADIN*, and *ARHGAP6*, which are all closely related to actin polymerization and associated with adherens junctions and cell migration [21-26]. Also of interest are *NECTIN* and *α-CATENIN*, core components of the adherens junction itself [24, 27-29]. Regulation of these genes by WT1 may play a role in the modulation of cellular adhesion and metastasis in cancer.

The complex behavior of WT1 suggests that different genetic changes must take place in wildtype-*WT1* vs. mutant-*WT1* tumors (whether they are sporadic or syndromatic cases). Tumors expressing (or overexpressing) wild type *WT1* have increased resistance to cell death[30, 31]. Tumors with *WT1* mutations may become sensitized to apoptosis [30] and thus may accumulate compensatory mutations which activate cellular growth and proliferation. In a study examining a group of *WT1*-mutant tumors, it was discovered that 75% also contained mutations in the *β-CATENIN* gene [32], a known oncogene and crucial component of the Wnt-signalling pathway. The Wnt pathway influences cell growth, development, migration, and adhesion. It is also a pathway often dysregulated in cancer, containing several oncogenes and tumor suppressors [33-35]. It makes sense that a tumor sensitized to apoptosis (*WT1*-mutation) may compensate for this sensitivity by maintaining a mutant copy of *β-CATENIN* which constitutively activates Wnt signalling.

Near the plasma membrane β-CATENIN links cadherins in adherens junctions to α-CATENIN [27, 29, 35-38]. As cancerous cells become metastatic, they progress through the Epithelial-Mesenchymal Transition (EMT), a hallmark of which is dissociation of the E-cadherin/β-CATENIN/α-CATENIN complex [38, 39]. This would result in loss of adherens junctions and increased cellular mobility. The disruption would release β-CATENIN, allowing it to translocate to the nucleus where it cooperates with the TCF/LEF complex to activate targets of the Wnt pathway [32, 39].

Although WT1 might conceivably act to repress Wnt signalling, it is more likely that WT1 is a Wnt activator. The case for repression of Wnt is supported by the fact that some (but not all) Wnt targets are upregulated in *WT1*-mutant as opposed to *WT1*-wildtype tumors [32]. However, the observed activation of Wnt in *WT1* mutants can, as indicated above, be attributed to gain of function mutations in *β-CATENIN*, not necessarily loss of repression by WT1. Also, several studies have directly shown that WT1 actually enhances Wnt signaling. This is supported by experiments showing that the expression of *WNT4* is reduced in *WT1* knockout cells and that induction of *WT1* causes an increase in *WNT4* expression [40]. A valid model, then, is that in sporadic tumors with wild-type *WT1*, the TF activates Wnt directly, possibly by downregulating DVL or CTBP (new predictions, $P > 0.95$), or by upregulating *WNT4*, *TCF*, *PP2A* (the latter 2 are new predictions). In syndromatic tumors with a mutant *WT1,* it is secondary lesions such as activating mutations in β-CATENIN that ensure the Wnt pathway remains active.

Finally, there is some evidence that WT1 may regulate both *α-CATENIN* and *β-CATENIN.* The prediction ($P \geq 0.95$) that WT1 regulates *α -CATENIN* is intriguing, since it suggests the possibility that WT1 could directly disrupt adherens junctions by repressing this gene. The disruption may activate Wnt signaling by freeing β-CATENIN from adherens junctions and allowing it to translocate to the nucleus. Figure A below summarizes the possible relationship between *WT1* and Wnt activation, showing the possible routes to Wnt activation when *WT1* is either active or inactive. Although less convincing, there is also some suggestive evidence that WT1 may bind the promoter of *β-CATENIN* itself, for which the SVM model assigns a score of 0.7. Closer inspection of the *β-CATENIN* promoter reveals 11 matches to the WT1 consensus site within 600bp of the *β-CATENIN* transcriptional start site (Figure B). The true relationship between WT1 and the Wnt pathway will have to be elucidated through further experiments, but there is strong evidence that WT1 may exert regulatory control on Wnt mediators and targets.

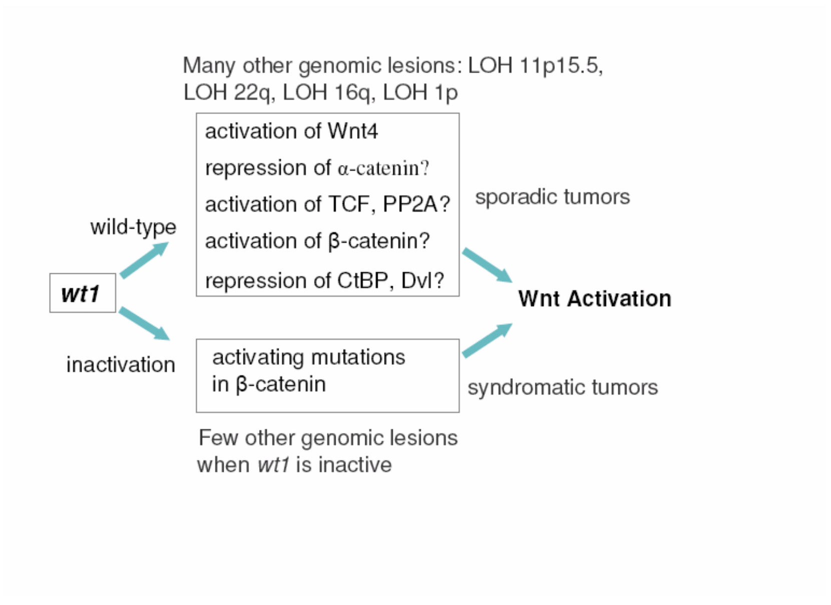

Figure A - Pathways to Wnt activation in Wilms' tumor

The path to possible Wnt activation is different depending on the state of *WT1*. If wild type WT1 is present, as in sporadic tumors, WT1 may activate Wnt directly by affecting key Wnt genes like wnt4, TCF, and β-catenin. If WT1 is inactivated, as in many syndromatic tumors, secondary mutations, such as activating mutations in β catenin cause Wnt activation.

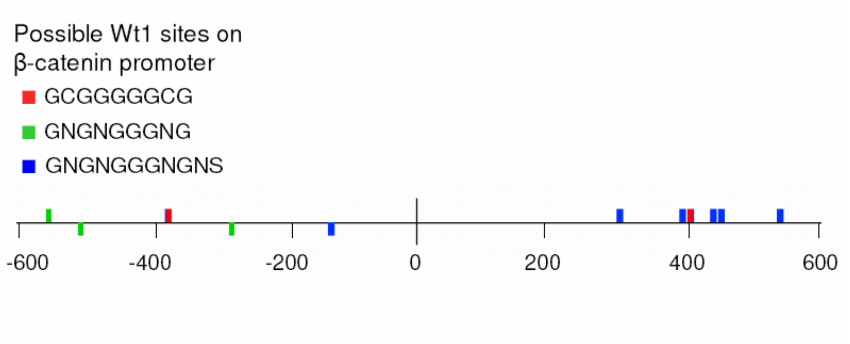

Figure B - Possible binding sites for WT1 near the *β-CATENIN* gene

This figure shows the region spanning 1200bp centered on the *β-CATENIN* transcriptional start site. Potential WT1 binding sites are highlighted as follows: red-GCGGGGGCG[41], green-GNGNGGGNG[42], blue-GNGNGGGNGNS[43]. The citations refer to the papers in which the binding sites have been established.

References

1. J Hussong, S Perkins, V Huff, M McDonald, T Pysher, B Beckwith, C Coffin: **Familial Wilms' Tumor with Neural Elements: Characterization by Histology, Immunohistochemistry, and Genetic Analysis**. *Pediatric and Developmental Pathology* 2000, **3**:561-567.
2. K-D Wagner, N Wagner, VP Vidal, G Schley, D Wilhelm, A Schedl, C Englert, H Scholz: **The Wilms' tumor gene Wt1 is required for normal development of the retina**. *EMBO* 2002, **21**:1398-1405.
3. N Wagner, K-D Wagner, A Hammes, KM Kirschner, VP Vidal, A Schedl, H Scholz: **A splice variant of the Wilms' tumour suppressor Wt1 is required for normal development of the olfactory system**. *Development* 2005, **132**:1327-1336.
4. J Armstrong, K Pritchard-Jones, W Bickmore, N Hastie, J Bard: **The expression of the Wilms' tumour gene, WT1, in the developing mammalian embryo**. *Mechanisms of Development* 1993, **40**:85-97.
5. M Sugita: **Taste perception and coding in the periphery**. *Cell Mol Life Sci* 2006, **63**:2000-15.
6. H Xu, L Staszewski, H Tang, E Adler, M Zoller, X Li: **Different functional roles of T1R subunits in the heteromeric taste receptors**. *Proc Natl Acad Sci U S A* 2004, **101**:14258-63.
7. J Schonberger, L Wang, JT Shin, SD Kim, FF Depreux, H Zhu, L Zon, A Pizard, JB Kim, CA Macrae, AJ Mungall, JG Seidman, CE Seidman: **Mutation in the transcriptional coactivator EYA4 causes dilated cardiomyopathy and sensorineural hearing loss**. *Nat Genet* 2005, **37**:418-22.
8. N Shimasaki, K Watanabe, M Hara, K Kosaki: **EYA1 mutation in a newborn female presenting with cardiofacial syndrome**. *Pediatr Cardiol* 2004, **25**:411-3.
9. Y Zhang, BM Knosp, M Maconochie, RA Friedman, RJ Smith: **A comparative study of Eya1 and Eya4 protein function and its implication in branchio-oto-renal syndrome and DFNA10**. *J Assoc Res Otolaryngol* 2004, **5**:295-304.
10. M Pfister, T Toth, H Thiele, B Haack, N Blin, HP Zenner, I Sziklai, P Nurnberg, S Kupka: **A 4-bp insertion in the eya-homologous region (eyaHR) of EYA4 causes hearing impairment in a Hungarian family linked to DFNA10**. *Mol Med* 2002, **8**:607-11.
11. S Fukuda, T Kuroda, E Chida, R Shimizu, S Usami, E Koda, S Abe, A Namba, K Kitamura, Y Inuyama: **A family affected by branchio-oto syndrome with EYA1 mutations**. *Auris Nasus Larynx* 2001, **28 Suppl**:S7-11.
12. A Bolino, M Muglia, FL Conforti, E LeGuern, MAM Salih, D-M Georgiou, K Christodoulou, I Hausmanowa-Petrusewicz, P Mandich, A Schenone, A Gambardella, F Bono, A Quattrone, M Devoto, AP Monaco: **Charcot-Marie-Tooth type 4B is caused by mutations in the gene encoding myotubularin-related protein-2**. 2000, **25**:17-19.
13. KB Othmane, E Johnson, M Menold, FL Graham, MB Hamida, O Hasegawa, AD Rogala, A Ohnishi, M Pericak-Vance, F Hentati, JM Vance: **Identification of a New Locus for Autosomal Recessive Charcot-Marie-Tooth Disease with Focally Folded Myelin on Chromosome 11p15**. *Genomics* 1999, **62**:344-349.

14. RD Schmickel: **Chromosomal deletions and enzyme deficiencies**. *J Pediatr* 1986, **108**:244-6.
15. VM Riccardi, E Sujansky, AC Smith, U Francke: **Chromosomal imbalance in the Aniridia-Wilms' tumor association: 11p interstitial deletion**. *Pediatrics* 1978, **61**:604-10.
16. C Termine, G Parigi, M Rossi, P Romano, U Balottin: **WAGR syndrome: is the 'R' always justified?** *Clin Dysmorphol* 2007, **16**:69-70.
17. P Mathur, HL Khamesera, AK Pendse, R Chittora, KK Porewal: **Wilm's tumour with WAGR complex**. *Indian J Cancer* 1996, **33**:136-8.
18. D Januszkiewicz, P Daszkiewicz: **[A case of Wilm's tumor with full symptomatic WAGR syndrome]**. *Pediatr Pol* 1995, **70**:255-7.
19. L Jadresic, J Leake, I Gordon, M Dillon, D Grant, J Pritchard, R Risdon, T Barrat: **Clinicopathologic review of twelve children with nephropathy, Wilms tumor, and genital abnormalities (Drash syndrome).** *Journal of Pediatrics* 1990, **117**:717-125.
20. T Jomgeow, Y Oji, N Tsuji, Y Ikeda, K Ito, A Tsuda, T Nakazawa, N Tatsumi, N Sakaguchi, S Takashima, T Shirakata, S Nishida, N Hosen, M Kawakami, A Tsuboi, Y Oka, K Itoh, H Sugiyama: **Wilms' tumor gene WT1 17AA(-)/KTS(-) isoform induces morphological changes and promotes cell migration and invasion in vitro**. *Cancer Science* 2006, **97**:259-270.
21. Y Funato, T Terabayashi, N Suenaga, M Seiki, T Takenawa, H Miki: **IRSp53/Eps8 complex is important for positive regulation of Rac and cancer cell motility/invasiveness**. *Cancer Res* 2004, **64**:5237-44.
22. S Kurisu, S Suetsugu, D Yamazaki, H Yamaguchi, T Takenawa: **Rac-WAVE2 signaling is involved in the invasive and metastatic phenotypes of murine melanoma cells**. *Oncogene* 2005, **24**:1309-19.
23. S Suetsugu, S Kurisu, T Oikawa, D Yamazaki, A Oda, T Takenawa: **Optimization of WAVE2 complex-induced actin polymerization by membrane-bound IRSp53, PIP(3), and Rac**. *J. Cell Biol.* 2006, **173**:571-85.
24. K Takahashi, H Nakanishi, M Miyahara, K Mandai, K Satoh, A Satoh, H Nishioka, J Aoki, A Nomoto, A Mizoguchi, Y Takai: **Nectin/PRR: an immunoglobulin-like cell adhesion molecule recruited to cadherin-based adherens junctions through interaction with Afadin, a PDZ domain-containing protein**. *J. Cell Biol.* 1999, **145**:539-49.
25. K Mandai, H Nakanishi, A Satoh, H Obaishi, M Wada, H Nishioka, M Itoh, A Mizoguchi, T Aoki, T Fujimoto, Y Matsuda, S Tsukita, Y Takai: **Afadin: A novel actin filament-binding protein with one PDZ domain localized at cadherin-based cell-to-cell adherens junction**. *J. Cell Biol.* 1997, **139**:517-28.
26. SK Prakash, R Paylor, S Jenna, N Lamarche-Vane, DL Armstrong, B Xu, MA Mancini, HY Zoghbi: **Functional analysis of ARHGAP6, a novel GTPase-activating protein for RhoA**. *Hum. Mol. Genet.* 2000, **9**:477-88.
27. M Perez-Moreno, E Fuchs: **Catenins: keeping cells from getting their signals crossed**. *Dev Cell* 2006, **11**:601-12.
28. WI Weis, WJ Nelson: **Re-solving the cadherin-catenin-actin conundrum**. *J Biol Chem* 2006, **281**:35593-7.

29. RM Mege, J Gavard, M Lambert: **Regulation of cell-cell junctions by the cytoskeleton**. *Curr Opin Cell Biol* 2006, **18**:541-8.
30. AJ Clark, DC Chan, MY Chen, H Fillmore, WG Dos Santos, TE Van Meter, MR Graf, WC Broaddus: **Down-regulation of Wilms' tumor 1 expression in glioblastoma cells increases radiosensitivity independently of p53**. *Journal of Neuro-oncology* 2007.
31. K Ito, Y Oji, N Tatsumi, S Shimizu, Y Kanai, T Nakazawa, M Asada, T Jomgeow, S Aoyagi, Y Nakano, H Tamaki, N Sakaguchi, T Shirakata, S Nishida, M Kawakami, A Tsuboi, Y Oka, Y Tsujimoto, H Sugiyama: **Antiapoptotic function of 17AA(+)WT1 (Wilms' tumor gene) isoforms on the intrinsic apoptosis pathway**. *Oncogene* 2006, **25**:4217-29.
32. C-M Li, CE Kim, AA Margolin, M Guo, J Zhu, JM Mason, TW Hensle, VVVS Murty, PE Grundy, ER Fearon, V D'Agati, JD Licht, B Tycko: **CTNNB1 Mutations and Overexpression of Wnt/{beta}-Catenin Target Genes in WT1-Mutant Wilms' Tumors**. *Am Journal of Pathology* 2004, **165**:1943-1953.
33. MF van Delft, DC Huang: **How the Bcl-2 family of proteins interact to regulate apoptosis**. *Cell Res* 2006, **16**:203-13.
34. P Polakis: **The many ways of Wnt in cancer**. *Curr Opin Genet Dev* 2007, **17**:45-51.
35. P Polakis: **Wnt signaling and cancer**. *Genes Dev* 2000, **14**:1837-51.
36. CY Logan, R Nusse: **THE WNT SIGNALING PATHWAY IN DEVELOPMENT AND DISEASE**. *Annual Review of Cell and Developmental Biology* 2004, **20**:781-810.
37. TJ Harris, M Peifer: **Decisions, decisions: beta-catenin chooses between adhesion and transcription**. *Trends Cell Biol* 2005, **15**:234-7.
38. M Bienz: **beta-Catenin: a pivot between cell adhesion and Wnt signalling**. *Current Biology* 2005, **15**:R64-7.
39. FH Brembeck, M Rosario, W Birchmeier: **Balancing cell adhesion and Wnt signaling, the key role of [beta]-catenin**. *Current Opinion in Genetics & Development Oncogenes and cell proliferation* 2006, **16**:51-59.
40. E Sim, A Smith, E Szilagi, F Rae, P Ioannou, M Lindsay, M Little: **Wnt-4 regulation by the Wilms' tumour suppressor gene, WT1**. *Oncogene* 2002, **21**:2948-2960.
41. F Rauscher, J Morris, O Tournay, D Cook, T Curran: **Binding of the Wilms' tumor locus zinc finger protein to the EGR-1 consensus sequence**. *Science* 1990, **250**:1259-1262.
42. G Fraizer, Y Wu, S Hewitt, T Maity, C Ton, V Huff, G Saunders: **Transcriptional regulation of the human Wilms' tumor gene (WT1). Cell type-specific enhancer and promiscuous promoter**. *J Biol Chem* 1994, **269**:8892-8900.
43. SM Hewitt, GC Fraizer, Y-J Wu, FJ Rauscher, III, GF Saunders: **Differential Function of Wilms' Tumor Gene WT1 Splice Isoforms in Transcriptional Regulation**. *J Biol Chem* 1996, **271**:8588-8592.
