# Supplementary material for: In silico regulatory analysis for exploring human disease progression
Source: Biol Direct. 2008 Jun 18;3:24. doi: 10.1186/1745-6150-3-24 (PMC2464594; doi:10.1186/1745-6150-3-24)
Supplement: Additional file 2 — This file contains several sub-folders. The folder "Classifier Results" contains the SVM predictions for all TFs in this study as well as a list of classifiers and their associated performance measures. The new predictions for all TFs are also available for query and download on our website[30]. The folder "Literature_curated_targets" contains the known TF-target interactions taken from databases and the literature. Any interactions manually curated from primary literature are listed, and the Pubmed ID of the article used is given. All files are annotated so as to be self explainatory or have an accompanying Readme file. [file 1745-6150-3-24-S2.zip › Literature_curated_targets/Excel and Text files giving known targets/notes_on_TF_names.doc]

Each header is a TF name used in our analysis and appearing in our files. The names listed underneath the header are names found in databases which are taken as synonomous with the header name. Sometimes dimers of different TF proteins interact, and so this new complex is given a new name. For example, RARbeta/RXRalpha is a dimer of two proteins that has a TF activity. Thus, an individual classifier is made for “RARbeta/RXRalpha.” On a higher level, some groups of similar factors may share overlapping activity, and thus it might be possible to group them all together under one name, and thus make one classifier for the whole group. For example, the factors RARalpha, RARalpha/RXRalpha, RXR, RARbeta, and RARbeta/RXRalpha all have separate biological activity as transcription factors. Each has their own classifier in our study, however, we also create a “parent” classifier in which all their targets are grouped together, and we call this single, unified classifier “RetinoicAcidR.”

Although not noted in this file there are many ambiguous cases in public databases where it is difficult to pin down a name. For example C/EBP-alpha and C/EBP-beta are separate factors with known binding affinities and thus separate classifiers in our analysis. In the TRDD databases there are entries simply stating “C/EBP”. Thus we created a separate classifier for these cases as they may represent either or both forms.

Note that the full name translations from database entries to the names used in our study can be seen in the excel file “Human Binding Data Parsed from Databases.xls”. The first column gives database names, the second provides the name we use in the analysis. Each unique name in the second column represents an individual classifier (as long as it has greater than 4 target genes).

Some further examples (not exhaustive) are:

AP1

- the name "AP1" is an umbrella term that encompasses all database interactions annotated to "JUN", "junD/c-fos", "junD/FRA1", "fos", and "c-jun/c-fos".

TFAP2A

- AP-2alpha, AP-2alphaA

STAT1

- stat1alpha, stat1beta (these are splice isoforms and do not have separate classifiers)

TCF4E

- TCF4E, TCF7L2

NFKB1

- NF-kappaB, NF-kappaB1, NFkappa, NF-kB. These include the Transfac factors (T00593 and T00590)

NFY

- NFY, NFYB, CP1, CP1A, NFYA/B/C, CBF

LXR/RXR

- LXR/RXR, LXR, and LXRalpha, and LXRalpha/RXRalpha

FXR/RXR

- any FXR/RXR heterodimer

SREBP

- SREBP,SREBP1, SREBP1a, SREBP1c
