# Supplementary material for: In silico regulatory analysis for exploring human disease progression
Source: Biol Direct. 2008 Jun 18;3:24. doi: 10.1186/1745-6150-3-24 (PMC2464594; doi:10.1186/1745-6150-3-24)
Supplement: Additional file 8 — This file contains the results of running the Weeder algorithm on 1) the set of known and newly predicted (Platt score P ≥ 0.95) targets of WT1, and 2) the known targets of WT1. Sequence regions used are as defined in Methods. The file also contains the results of Oligo-analysis. Also included are the matching results after scanning the literature derived consensus sites for WT1 against the full set of WT1 targets (predicted and known). [file 1745-6150-3-24-S8.zip › WeederOutout_known_and_predicted_targets_Wt1.zip!/Weeder_Output_known_and_predicted_targets.fasta.html]

Your Weeder Web Results  
  
NEW: Starting a medium job on file final.fasta  
  
Organism code: HS  
  
Processing **both** strands  
  
**weederlauncher.out final.fasta HS medium M S 15   
  
  
Searching for motifs of length 6 with 1 mutations.....  
  
(Actual commandline is :   
./weederTFBS.out -f final.fasta -R 50 -O HS -W 6 -e 1 -M -S -T 15 )  
1) CGACCG 0.61   
2) GTCGCG 0.59   
3) CCGACG 0.59   
4) CGCGTC 0.57   
5) TACGCG 0.57   
6) CGACGC 0.53   
7) ACGCCG 0.53   
8) CGCGTT 0.53   
9) TCGCGT 0.52   
10) CGTCCG 0.52   
11) CGCCGT 0.52   
12) GCGAGT 0.52   
13) GTCCGC 0.51   
14) TTCGCG 0.51   
15) CCGCGT 0.51   
  
Elapsed time : 0 min. 48 sec.  
  
  
Searching for motifs of length 8 with 2 mutations.....  
  
(Actual commandline is :   
./weederTFBS.out -f final.fasta -R 50 -O HS -W 8 -e 2 -M -S -T 15 )  
1) CGCGTCGA 0.66   
2) TCGCGTCG 0.56   
3) CGTCGACG 0.56   
4) TCGCGCGT 0.54   
5) GTACGCGG 0.51   
6) ACGCGCGT 0.51   
7) CGACGCGT 0.51   
8) TCGTCGCG 0.51   
9) CGCGTCGT 0.50   
10) CGACGCGC 0.50   
11) CGGTCGAC 0.49   
12) CCGACGAC 0.49   
13) CGCGTACG 0.49   
14) CCGCTCGA 0.49   
15) CGACGTCG 0.48   
  
Elapsed time : 23 min. 46 sec.  
  
  
Searching for motifs of length 10 with 3 mutations.....  
  
(Actual commandline is :   
./weederTFBS.out -f final.fasta -R 50 -O HS -W 10 -e 3 -M -S -T 15 )  
1) CGTCGCGTCG 0.63   
2) CGCGTACGCG 0.56   
3) CGGTCGAGCG 0.56   
4) CGTCGATCCG 0.53   
5) CGTCTACGCG 0.52   
6) CGCGAACGCG 0.52   
7) CGGGTACGCG 0.52   
8) CGTCCGAACG 0.51   
9) CGATGACCGC 0.51   
10) CGAACACGCG 0.51   
11) CGCGTCCGCG 0.51   
12) CGCCGTATCG 0.50   
13) CGACGCTTCG 0.49   
14) CGACGACCGC 0.49   
15) CGACGCGTCC 0.49   
  
Elapsed time : 777 min. 19 sec.  
  
  
Your sequences:  
  
Sequence 1 : >LL.374-Human-1 DBTSS:NM\_001657 chr4 +   
Sequence 2 : >LL.7015-Human-2 AF098956\_1 chr5 - 1347  
Sequence 3 : >LL.2297-Human-3 hchr5\_P021624 chr5 - 7  
Sequence 4 : >LL.4602-Human-4 HSU22376\_1 chr6 + 1355  
Sequence 5 : >LL.1956-Human-5 EP15043 chr7 + 5485956  
Sequence 6 : >LL.5243-Human-6 EP35012 chr7 - 8687435  
Sequence 7 : >LL.6736-Human-7 hchrY\_P90027 chrY - 26  
Sequence 8 : >LL.3481-Human-8 EP17072 chr11 - 211669  
Sequence 9 : >LL.51352-Human-9 HSWT1WIT\_1 chr11 + 32  
Sequence 10 : >LL.4192-Human-10 EP74560 chr11 + 46358  
Sequence 11 : >LL.596-Human-11 EP27006 chr18 - 591377  
Sequence 12 : >LL.3643-Human-12 HUMINSRB\_1 chr19 - 72  
Sequence 13 : >LL.898-Human-13 HSCYCEGEN\_1 chr19 + 34  
Sequence 14 : >LL.26155-Human-14 DBTSS:NM\_015658 chr1  
Sequence 15 : >LL.54991-Human-15 DBTSS:NM\_017891 chr1  
Sequence 16 : >LL.118424-Human-16 DBTSS:NM\_058167 chr  
Sequence 17 : >LL.1855-Human-17 hchr1\_P056916 chr1 -   
Sequence 18 : >LL.5590-Human-18 hchr1\_P001629 chr1 +   
Sequence 19 : >LL.79258-Human-19 hchr1\_P056017 chr1 -  
Sequence 20 : >LL.49856-Human-20 DBTSS:NM\_017818 chr1  
Sequence 21 : >LL.8514-Human-21 hchr1\_P003183 chr1 +   
Sequence 22 : >LL.80835-Human-22 hchr1\_P003562 chr1 +  
Sequence 23 : >LL.54897-Human-23 DBTSS:NM\_017766 chr1  
Sequence 24 : >LL.93611-Human-24 DBTSS:NM\_033182 chr1  
Sequence 25 : >LL.842-Human-25 AB019197S1\_1 chr1 - 15  
Sequence 26 : >LL.27245-Human-26 hchr1\_P049647 chr1 -  
Sequence 27 : >LL.399474-Human-27 hchr1\_P049365 chr1   
Sequence 28 : >LL.192669-Human-28 DBTSS:NM\_024852 chr  
Sequence 29 : >LL.114883-Human-29 hchr1\_P013395 chr1   
Sequence 30 : >LL.5142-Human-30 DBTSS:NM\_002600 chr1   
Sequence 31 : >LL.23266-Human-31 HSA244492\_1 chr1 + 8  
Sequence 32 : >LL.79739-Human-32 DBTSS:NM\_024686 chr1  
Sequence 33 : >LL.84146-Human-33 DBTSS:NM\_032186 chr1  
Sequence 34 : >LL.3749-Human-34 hchr1\_P016626 chr1 +   
Sequence 35 : >LL.5906-Human-35 hchr1\_P016813 chr1 +   
Sequence 36 : >LL.1942-Human-36 DBTSS:NM\_004428 chr1   
Sequence 37 : >LL.29922-Human-37 DBTSS:NM\_013330 chr1  
Sequence 38 : >LL.339479-Human-38 hchr1\_P034939 chr1   
Sequence 39 : >LL.54530-Human-39 DBTSS:NM\_144977 chr1  
Sequence 40 : >LL.4179-Human-40 EP73197 chr1 + 204312  
Sequence 41 : >LL.2104-Human-41 DBTSS:NM\_001438 chr1   
Sequence 42 : >LL.375-Human-42 DBTSS:NM\_001658 chr1 +  
Sequence 43 : >LL.80003-Human-43 hchr1\_P030219 chr1 -  
Sequence 44 : >LL.1131-Human-44 hchr1\_P028360 chr1 +   
Sequence 45 : >LL.1131-Human-45 AF331832S1\_2 chr1 + 2  
Sequence 46 : >LL.8648-Human-46 DBTSS:NM\_147233 chr2   
Sequence 47 : >LL.1788-Human-47 AB076659\_1 chr2 - 254  
Sequence 48 : >LL.53335-Human-48 hchr2\_P034273 chr2 -  
Sequence 49 : >LL.54812-Human-49 DBTSS:NM\_017657 chr2  
Sequence 50 : >LL.1496-Human-50 hchr2\_P90025 chr2 + 7  
Sequence 51 : >LL.151011-Human-51 DBTSS:NM\_144710 chr  
Sequence 52 : >LL.79712-Human-52 DBTSS:NM\_024659 chr2  
Sequence 53 : >LL.5000-Human-53 EP73754 chr2 - 148611  
Sequence 54 : >LL.10010-Human-54 hchr2\_P012128 chr2 +  
Sequence 55 : >LL.440925-Human-55 hchr2\_P025828 chr2   
Sequence 56 : >LL.7035-Human-56 DBTSS:NM\_006287 chr2   
Sequence 57 : >LL.116093-Human-57 AY039012S1\_2 chr2 -  
Sequence 58 : >LL.3329-Human-58 EP73740 chr2 - 198189  
Sequence 59 : >LL.117583-Human-59 hchr2\_P014673 chr2   
Sequence 60 : >LL.4133-Human-60 hchr2\_P014976 chr2 +   
Sequence 61 : >LL.9759-Human-61 DBTSS:NM\_006037 chr2   
Sequence 62 : >LL.51078-Human-62 DBTSS:NM\_015963 chr2  
Sequence 63 : >LL.23192-Human-63 DBTSS:NM\_013325 chr2  
Sequence 64 : >LL.27255-Human-64 hchr3\_P90001 chr3 +   
Sequence 65 : >LL.152330-Human-65 hchr3\_P000066 chr3   
Sequence 66 : >LL.5468-Human-66 AF012873\_1 chr3 + 123  
Sequence 67 : >LL.151648-Human-67 DBTSS:NM\_138484 chr  
Sequence 68 : >LL.7068-Human-68 DBTSS:NM\_000461 chr3   
Sequence 69 : >LL.6331-Human-69 AY313163\_2 chr3 - 386  
Sequence 70 : >LL.55152-Human-70 DBTSS:NM\_018114 chr3  
Sequence 71 : >LL.25915-Human-71 hchr3\_P003951 chr3 +  
Sequence 72 : >LL.27086-Human-72 DBTSS:NM\_032682 chr3  
Sequence 73 : >LL.11259-Human-73 DBTSS:NM\_014890 chr3  
Sequence 74 : >LL.56987-Human-74 DBTSS:NM\_020235 chr3  
Sequence 75 : >LL.26137-Human-75 DBTSS:NM\_015642 chr3  
Sequence 76 : >LL.60678-Human-76 hchr3\_P008399 chr3 +  
Sequence 77 : >LL.11342-Human-77 EP73895 chr3 + 15101  
Sequence 78 : >LL.64805-Human-78 hchr3\_P90121 chr3 -   
Sequence 79 : >LL.4154-Human-79 DBTSS:NM\_021038 chr3   
Sequence 80 : >LL.389170-Human-80 hchr3\_P010738 chr3   
Sequence 81 : >LL.84517-Human-81 hchr3\_P015928 chr3 -  
Sequence 82 : >LL.22871-Human-82 DBTSS:NM\_014932 chr3  
Sequence 83 : >LL.10242-Human-83 hchr3\_P90090 chr3 +   
Sequence 84 : >LL.10336-Human-84 DBTSS:NM\_006315 chr4  
Sequence 85 : >LL.10815-Human-85 DBTSS:NM\_006651 chr4  
Sequence 86 : >LL.53834-Human-86 hchr4\_P000721 chr4 +  
Sequence 87 : >LL.1487-Human-87 hchr4\_P020343 chr4 -   
Sequence 88 : >LL.2261-Human-88 HUMFGFRB\_1 chr4 + 176  
Sequence 89 : >LL.339983-Human-89 hchr4\_P001531 chr4   
Sequence 90 : >LL.2868-Human-90 HSGRKG01\_1 chr4 + 300  
Sequence 91 : >LL.170712-Human-91 hchr4\_P90111 chr4 -  
Sequence 92 : >LL.10886-Human-92 hchr4\_P004871 chr4 +  
Sequence 93 : >LL.3184-Human-93 hchr4\_P015060 chr4 -   
Sequence 94 : >LL.5602-Human-94 DBTSS:NM\_138980 chr4   
Sequence 95 : >LL.5783-Human-95 HSFAPI2\_1 chr4 + 8791  
Sequence 96 : >LL.401145-Human-96 hchr4\_P005838 chr4   
Sequence 97 : >LL.10611-Human-97 DBTSS:NM\_006457 chr4  
Sequence 98 : >LL.658-Human-98 hchr4\_P005927 chr4 + 9  
Sequence 99 : >LL.54790-Human-99 hchr4\_P006187 chr4 +  
Sequence 100 : >LL.27068-Human-100 EP73073 chr4 - 1067  
Sequence 101 : >LL.64579-Human-1 hchr4\_P90084 chr4 - 1  
Sequence 102 : >LL.55294-Human-2 DBTSS:NM\_018315 chr4   
Sequence 103 : >LL.27236-Human-3 DBTSS:NM\_014447 chr4   
Sequence 104 : >LL.56884-Human-4 hchr4\_P011808 chr4 -   
Sequence 105 : >LL.2823-Human-5 DBTSS:NM\_005277 chr4 -  
Sequence 106 : >LL.116966-Human-6 DBTSS:NM\_170710 chr4  
Sequence 107 : >LL.8470-Human-7 DBTSS:NM\_021069 chr4 -  
Sequence 108 : >LL.6531-Human-8 AF119117\_1 chr5 - 1498  
Sequence 109 : >LL.153571-Human-9 hchr5\_P001330 chr5 +  
Sequence 110 : >LL.1016-Human-10 hchr5\_P023904 chr5 -   
Sequence 111 : >LL.1010-Human-11 hchr5\_P023892 chr5 -   
Sequence 112 : >LL.1008-Human-12 DBTSS:NM\_006727 chr5   
Sequence 113 : >LL.1007-Human-13 DBTSS:NM\_016279 chr5   
Sequence 114 : >LL.25836-Human-14 hchr5\_P002465 chr5 +  
Sequence 115 : >LL.2668-Human-15 AF053749\_1 chr5 - 378  
Sequence 116 : >LL.153684-Human-16 hchr5\_P90010 chr5 +  
Sequence 117 : >LL.10571-Human-17 hchr6\_P024740 chr6 -  
Sequence 118 : >LL.153561-Human-18 hchr5\_P003594 chr5   
Sequence 119 : >LL.153561-Human-19 hchr5\_P021868 chr5   
Sequence 120 : >LL.153561-Human-20 hchr5\_P021885 chr5   
Sequence 121 : >LL.7518-Human-21 HSXRCC1\_2 chr5 + 8240  
Sequence 122 : >LL.4208-Human-22 DBTSS:NM\_002397 chr5   
Sequence 123 : >LL.6932-Human-23 DBTSS:NM\_003202 chr5   
Sequence 124 : >LL.4090-Human-24 DBTSS:NM\_005903 chr5   
Sequence 125 : >LL.5521-Human-25 DBTSS:NM\_004576 chr5   
Sequence 126 : >LL.9832-Human-26 DBTSS:NM\_014790 chr5   
Sequence 127 : >LL.64324-Human-27 hchr5\_P011324 chr5 +  
Sequence 128 : >LL.9260-Human-28 DBTSS:NM\_005451 chr5   
Sequence 129 : >LL.56897-Human-29 hchr6\_P000379 chr6 +  
Sequence 130 : >LL.7020-Human-30 DBTSS:NM\_003220 chr6   
Sequence 131 : >LL.3159-Human-31 HUMHMGIY\_1 chr6 + 343  
Sequence 132 : >LL.8464-Human-32 DBTSS:NM\_003599 chr6   
Sequence 133 : >LL.346007-Human-33 hchr6\_P90135 chr6 -  
Sequence 134 : >LL.8936-Human-34 DBTSS:NM\_003931 chr6   
Sequence 135 : >LL.5980-Human-35 DBTSS:NM\_002912 chr6   
Sequence 136 : >LL.51389-Human-36 DBTSS:NM\_015952 chr6  
Sequence 137 : >LL.5570-Human-37 DBTSS:NM\_032471 chr6   
Sequence 138 : >LL.154215-Human-38 DBTSS:NM\_153355 chr  
Sequence 139 : >LL.2070-Human-39 hchr6\_P010048 chr6 +   
Sequence 140 : >LL.23118-Human-40 hchr6\_P010724 chr6 +  
Sequence 141 : >LL.26575-Human-41 DBTSS:NM\_012419 chr6  
Sequence 142 : >LL.79683-Human-42 hchr6\_P011291 chr6 +  
Sequence 143 : >LL.79683-Human-43 hchr6\_P011317 chr6 +  
Sequence 144 : >LL.4301-Human-44 hchr6\_P012336 chr6 +   
Sequence 145 : >LL.79778-Human-45 DBTSS:NM\_024723 chr7  
Sequence 146 : >LL.202915-Human-46 DBTSS:NM\_152689 chr  
Sequence 147 : >LL.26100-Human-47 DBTSS:NM\_015610 chr7  
Sequence 148 : >LL.6119-Human-48 DBTSS:NM\_002947 chr7   
Sequence 149 : >LL.9734-Human-49 hchr7\_P90007 chr7 + 1  
Sequence 150 : >LL.3181-Human-50 DBTSS:NM\_031243 chr7   
Sequence 151 : >LL.11335-Human-51 EP73853 chr7 + 26013  
Sequence 152 : >LL.404744-Human-52 hchr7\_P90143 chr7 -  
Sequence 153 : >LL.11281-Human-53 hchr7\_P90019 chr7 +   
Sequence 154 : >LL.816-Human-54 hchr7\_P026002 chr7 - 4  
Sequence 155 : >LL.401337-Human-55 hchr7\_P90133 chr7 -  
Sequence 156 : >LL.948-Human-56 DBTSS:NM\_000072 chr7 +  
Sequence 157 : >LL.2913-Human-57 DBTSS:NM\_000840 chr7   
Sequence 158 : >LL.154661-Human-58 DBTSS:NM\_138290 chr  
Sequence 159 : >LL.889-Human-59 DBTSS:NM\_004912 chr7 -  
Sequence 160 : >LL.43-Human-60 HSU32675\_1 chr7 - 10013  
Sequence 161 : >LL.1523-Human-61 DBTSS:NM\_001913 chr7   
Sequence 162 : >LL.93986-Human-62 mealt\_P5-4 chr7 + 11  
Sequence 163 : >LL.22797-Human-63 hchr7\_P90102 chr7 -   
Sequence 164 : >LL.4232-Human-64 EP74533 chr7 + 129724  
Sequence 165 : >LL.4481-Human-65 HUMDMSR\_2 chr8 - 1609  
Sequence 166 : >LL.23362-Human-66 hchr8\_P022076 chr8 -  
Sequence 167 : >LL.649-Human-67 hchr8\_P002205 chr8 + 2  
Sequence 168 : >LL.64236-Human-68 DBTSS:NM\_021630 chr8  
Sequence 169 : >LL.148-Human-69 EP60012 chr8 - 2677861  
Sequence 170 : >LL.286-Human-70 DBTSS:NM\_020481 chr8 -  
Sequence 171 : >LL.9705-Human-71 DBTSS:NM\_014682 chr8   
Sequence 172 : >LL.79145-Human-72 DBTSS:NM\_024300 chr8  
Sequence 173 : >LL.6386-Human-73 EP74558 chr8 + 596273  
Sequence 174 : >LL.10499-Human-74 DBTSS:NM\_006540 chr8  
Sequence 175 : >LL.2138-Human-75 hchr8\_P018113 chr8 -   
Sequence 176 : >LL.5569-Human-76 EP73765 chr8 + 796650  
Sequence 177 : >LL.138046-Human-77 hchr8\_P006167 chr8   
Sequence 178 : >LL.157680-Human-78 hchr8\_P006992 chr8   
Sequence 179 : >LL.7227-Human-79 hchr8\_P016183 chr8 -   
Sequence 180 : >LL.11244-Human-80 DBTSS:NM\_007222 chr8  
Sequence 181 : >LL.51105-Human-81 DBTSS:NM\_032205 chr8  
Sequence 182 : >LL.1936-Human-82 EP74147 chr8 - 144750  
Sequence 183 : >LL.23513-Human-83 hchr8\_P013405 chr8 -  
Sequence 184 : >LL.5339-Human-84 DBTSS:NM\_000445 chr8   
Sequence 185 : >LL.83482-Human-85 hchr8\_P012566 chr8 -  
Sequence 186 : >LL.441381-Human-86 hchr8\_P012212 chr8   
Sequence 187 : >LL.414919-Human-87 hchr8\_P012185 chr8   
Sequence 188 : >LL.5991-Human-88 DBTSS:NM\_134428 chr9   
Sequence 189 : >LL.54875-Human-89 hchr9\_P000864 chr9 +  
Sequence 190 : >LL.158038-Human-90 hchr9\_P90127 chr9 -  
Sequence 191 : >LL.1271-Human-91 HUMCNFAR01\_1 chr9 - 3  
Sequence 192 : >LL.4969-Human-92 hchr9\_P90109 chr9 - 9  
Sequence 193 : >LL.8013-Human-93 D85243\_1 chr9 + 99662  
Sequence 194 : >LL.1902-Human-94 DBTSS:NM\_001401 chr9   
Sequence 195 : >LL.5524-Human-95 EP73218 chr9 + 128952  
Sequence 196 : >LL.84929-Human-96 DBTSS:NM\_032843 chr9  
Sequence 197 : >LL.6837-Human-97 HSA224639\_2 chr9 - 13  
Sequence 198 : >LL.29085-Human-98 DBTSS:NM\_014172 chr9  
Sequence 199 : >LL.20-Human-99 F327658S01\_1 chr9 - 137  
Sequence 200 : >LL.2902-Human-1 hchr9\_P012766 chr9 + 1  
Sequence 201 : >LL.54932-Human-2 hchr9\_P013871 chr9 -   
Sequence 202 : >LL.395-Human-3 hchrX\_P020102 chrX - 11  
Sequence 203 : >LL.11141-Human-4 hchrX\_P90011 chrX + 2  
Sequence 204 : >LL.257397-Human-5 DBTSS:NM\_152787 chrX  
Sequence 205 : >LL.1756-Human-6 AF213401\_1 chrX - 3298  
Sequence 206 : >LL.54880-Human-7 hchrX\_P018748 chrX -   
Sequence 207 : >LL.54830-Human-8 DBTSS:NM\_017681 chrX   
Sequence 208 : >LL.3358-Human-9 DBTSS:NM\_000868 chrX +  
Sequence 209 : >LL.2556-Human-10 DBTSS:NM\_000808 chrX   
Sequence 210 : >LL.1852-Human-11 hchrX\_P009789 chrX +   
Sequence 211 : >LL.54522-Human-12 hchr10\_P026705 chr10  
Sequence 212 : >LL.399717-Human-13 hchr10\_P026399 chr1  
Sequence 213 : >LL.1787-Human-14 hchr10\_P025839 chr10   
Sequence 214 : >LL.51322-Human-15 DBTSS:NM\_100264 chr1  
Sequence 215 : >LL.220929-Human-16 hchr10\_P024932 chr1  
Sequence 216 : >LL.6387-Human-17 DBTSS:NM\_000609 chr10  
Sequence 217 : >LL.220972-Human-18 DBTSS:NM\_145021 chr  
Sequence 218 : >LL.29974-Human-19 hchr10\_P90095 chr10   
Sequence 219 : >LL.65217-Human-20 DBTSS:NM\_033056 chr1  
Sequence 220 : >LL.9886-Human-21 hchr10\_P022865 chr10   
Sequence 221 : >LL.5076-Human-22 AF515729\_1 chr10 + 10  
Sequence 222 : >LL.2253-Human-23 AF079325\_1 chr10 - 10  
Sequence 223 : >LL.120-Human-24 DBTSS:NM\_019903 chr10   
Sequence 224 : >LL.2263-Human-25 AF233344\_1 chr10 - 12  
Sequence 225 : >LL.282973-Human-26 hchr10\_P012564 chr1  
Sequence 226 : >LL.84504-Human-27 hchr10\_P014662 chr10  
Sequence 227 : >LL.196743-Human-28 hchr10\_P013459 chr1  
Sequence 228 : >LL.6050-Human-29 DBTSS:NM\_002939 chr11  
Sequence 229 : >LL.3265-Human-30 EP16064 chr11 - 52512  
Sequence 230 : >LL.53841-Human-31 DBTSS:NM\_031265 chr1  
Sequence 231 : >LL.977-Human-32 DBTSS:NM\_004357 chr11   
Sequence 232 : >LL.7106-Human-33 DBTSS:NM\_003271 chr11  
Sequence 233 : >LL.4046-Human-34 DBTSS:NM\_002339 chr11  
Sequence 234 : >LL.51214-Human-35 hchr11\_P001671 chr11  
Sequence 235 : >LL.430-Human-36 meLONG\_P2-81 chr11 - 2  
Sequence 236 : >LL.65975-Human-37 hchr11\_P029984 chr11  
Sequence 237 : >LL.55553-Human-38 DBTSS:NM\_033326 chr1  
Sequence 238 : >LL.196296-Human-39 hchr11\_P028466 chr1  
Sequence 239 : >LL.5080-Human-40 HSU63833\_2 chr11 - 31  
Sequence 240 : >LL.7490-Human-41 EP58019 chr11 - 32413  
Sequence 241 : >LL.57689-Human-42 hchr11\_P028013 chr11  
Sequence 242 : >LL.219927-Human-43 hchr11\_P022842 chr1  
Sequence 243 : >LL.8898-Human-44 DBTSS:NM\_016156 chr11  
Sequence 244 : >LL.53942-Human-45 hchr11\_P013060 chr11  
Sequence 245 : >LL.838-Human-46 hchr11\_P90201 chr11 -   
Sequence 246 : >LL.5818-Human-47 DBTSS:NM\_002855 chr11  
Sequence 247 : >LL.27087-Human-48 hchr11\_P016734 chr11  
Sequence 248 : >LL.64581-Human-49 DBTSS:NM\_022570 chr1  
Sequence 249 : >LL.55729-Human-50 DBTSS:NM\_018179 chr1  
Sequence 250 : >LL.6660-Human-51 hchr12\_P023433 chr12   
Sequence 251 : >LL.11228-Human-52 DBTSS:NM\_007211 chr1  
Sequence 252 : >LL.283461-Human-53 hchr12\_P002496 chr1  
Sequence 253 : >LL.1272-Human-54 DBTSS:NM\_001843 chr12  
Sequence 254 : >LL.9194-Human-55 hchr12\_P90063 chr12 +  
Sequence 255 : >LL.338811-Human-56 hchr12\_P018900 chr1  
Sequence 256 : >LL.10818-Human-57 DBTSS:NM\_006654 chr1  
Sequence 257 : >LL.117177-Human-58 DBTSS:NM\_022456 chr  
Sequence 258 : >LL.84698-Human-59 DBTSS:NM\_032606 chr1  
Sequence 259 : >LL.114882-Human-60 DBTSS:NM\_020841 chr  
Sequence 260 : >LL.55117-Human-61 DBTSS:NM\_018057 chr1  
Sequence 261 : >LL.25834-Human-62 AB024906S1\_1 chr12 -  
Sequence 262 : >LL.9612-Human-63 hchr12\_P014185 chr12   
Sequence 263 : >LL.80324-Human-64 hchr12\_P011430 chr12  
Sequence 264 : >LL.7223-Human-65 DBTSS:NM\_016179 chr13  
Sequence 265 : >LL.55068-Human-66 hchr13\_P90040 chr13   
Sequence 266 : >LL.22862-Human-67 hchr13\_P002452 chr13  
Sequence 267 : >LL.27253-Human-68 DBTSS:NM\_014459 chr1  
Sequence 268 : >LL.81550-Human-69 DBTSS:NM\_030794 chr1  
Sequence 269 : >LL.11278-Human-70 AF312866S1\_1 chr13 -  
Sequence 270 : >LL.3621-Human-71 AB031270\_1 chr13 + 11  
Sequence 271 : >LL.404785-Human-72 hchr14\_P000022 chr1  
Sequence 272 : >LL.404785-Human-73 hchr14\_P018325 chr1  
Sequence 273 : >LL.57447-Human-74 DBTSS:NM\_016250 chr1  
Sequence 274 : >LL.145581-Human-75 DBTSS:NM\_152447 chr  
Sequence 275 : >LL.161357-Human-76 hchr14\_P016361 chr1  
Sequence 276 : >LL.23034-Human-77 DBTSS:NM\_015589 chr1  
Sequence 277 : >LL.5494-Human-78 DBTSS:NM\_021003 chr14  
Sequence 278 : >LL.2530-Human-79 DBTSS:NM\_004480 chr14  
Sequence 279 : >LL.56936-Human-80 hchr14\_P90087 chr14   
Sequence 280 : >LL.8788-Human-81 EP74027 chr14 + 10026  
Sequence 281 : >LL.122622-Human-82 hchr14\_P008226 chr1  
Sequence 282 : >LL.207-Human-83 F283818S01\_1 chr14 - 1  
Sequence 283 : >LL.283638-Human-84 hchr14\_P008383 chr1  
Sequence 284 : >LL.3714-Human-85 hchr14\_P009687 chr14   
Sequence 285 : >LL.2972-Human-86 DBTSS:NM\_145696 chr14  
Sequence 286 : >LL.54816-Human-87 DBTSS:NM\_017661 chr1  
Sequence 287 : >LL.7168-Human-88 EP73057 chr15 + 61121  
Sequence 288 : >LL.64799-Human-89 DBTSS:NM\_022784 chr1  
Sequence 289 : >LL.26035-Human-90 hchr15\_P004807 chr15  
Sequence 290 : >LL.11214-Human-91 hchr15\_P007617 chr15  
Sequence 291 : >LL.6650-Human-92 hchr16\_P000401 chr16   
Sequence 292 : >LL.146325-Human-93 hchr16\_P000596 chr1  
Sequence 293 : >LL.197335-Human-94 hchr16\_P000795 chr1  
Sequence 294 : >LL.124093-Human-95 hchr16\_P029203 chr1  
Sequence 295 : >LL.84264-Human-96 DBTSS:NM\_032304 chr1  
Sequence 296 : >LL.8912-Human-97 hchr16\_P001620 chr16   
Sequence 297 : >LL.65259-Human-98 DBTSS:NM\_023076 chr1  
Sequence 298 : >LL.197342-Human-99 hchr16\_P002420 chr1  
Sequence 299 : >LL.124056-Human-100 DBTSS:NM\_144603 ch  
Sequence 300 : >LL.7249-Human-1 AB014460\_1 chr16 + 203  
Sequence 301 : >LL.79412-Human-2 hchr16\_P003509 chr16   
Sequence 302 : >LL.11273-Human-3 hchr16\_P006607 chr16   
Sequence 303 : >LL.2294-Human-4 HS1FREAC1\_1 chr16 + 85  
Sequence 304 : >LL.4597-Human-5 DBTSS:NM\_002461 chr16   
Sequence 305 : >LL.863-Human-6 DBTSS:NM\_005187 chr16 -  
Sequence 306 : >LL.54849-Human-7 DBTSS:NM\_017702 chr16  
Sequence 307 : >LL.4641-Human-8 DBTSS:NM\_033375 chr17   
Sequence 308 : >LL.3090-Human-9 HSA404688\_1 chr17 + 19  
Sequence 309 : >LL.3090-Human-10 hchr17\_P000656 chr17   
Sequence 310 : >LL.23277-Human-11 DBTSS:NM\_015229 chr1  
Sequence 311 : >LL.84254-Human-12 hchr17\_P033822 chr17  
Sequence 312 : >LL.489-Human-13 HSSERCA1\_1 chr17 - 381  
Sequence 313 : >LL.50488-Human-14 hchr17\_P001384 chr17  
Sequence 314 : >LL.2027-Human-15 EP73659 chr17 + 47941  
Sequence 315 : >LL.90313-Human-16 AB110210S1\_1 chr17 +  
Sequence 316 : >LL.3975-Human-17 DBTSS:NM\_005568 chr17  
Sequence 317 : >LL.782-Human-18 DBTSS:NM\_000723 chr17   
Sequence 318 : >LL.6909-Human-19 hchr17\_P011804 chr17   
Sequence 319 : >LL.3773-Human-20 DBTSS:NM\_018658 chr17  
Sequence 320 : >LL.8877-Human-21 DBTSS:NM\_021972 chr17  
Sequence 321 : >LL.10458-Human-22 AB104726\_1 chr17 + 7  
Sequence 322 : >LL.71-Human-23 HUMACTGA\_1 chr17 - 7709  
Sequence 323 : >LL.2873-Human-24 hchr17\_P016517 chr17   
Sequence 324 : >LL.2194-Human-25 AF250144\_1 chr17 - 77  
Sequence 325 : >LL.9123-Human-26 DBTSS:NM\_004207 chr17  
Sequence 326 : >LL.3607-Human-27 hchr17\_P016865 chr17   
Sequence 327 : >LL.8715-Human-28 DBTSS:NM\_003787 chr18  
Sequence 328 : >LL.1837-Human-29 DBTSS:NM\_032981 chr18  
Sequence 329 : >LL.23556-Human-30 hchr18\_P007010 chr18  
Sequence 330 : >LL.28513-Human-31 DBTSS:NM\_021153 chr1  
Sequence 331 : >LL.81832-Human-32 hchr18\_P006734 chr18  
Sequence 332 : >LL.4772-Human-33 hchr18\_P004580 chr18   
Sequence 333 : >LL.22904-Human-34 hchr19\_P043688 chr19  
Sequence 334 : >LL.6915-Human-35 HSA308102\_1 chr19 - 3  
Sequence 335 : >LL.23030-Human-36 hchr19\_P004696 chr19  
Sequence 336 : >LL.7001-Human-37 EP73464 chr19 - 12773  
Sequence 337 : >LL.170463-Human-38 DBTSS:NM\_032627 chr  
Sequence 338 : >LL.7392-Human-39 hchr19\_P012522 chr19   
Sequence 339 : >LL.581-Human-40 hchr19\_P018042 chr19 +  
Sequence 340 : >LL.57106-Human-41 AB055058\_1 chr19 + 6  
Sequence 341 : >LL.11338-Human-42 DBTSS:NM\_007279 chr1  
Sequence 342 : >LL.126208-Human-43 hchr19\_P023336 chr1  
Sequence 343 : >LL.83959-Human-44 hchr20\_P018052 chr20  
Sequence 344 : >LL.57144-Human-45 DBTSS:NM\_020341 chr2  
Sequence 345 : >LL.23767-Human-46 hchr20\_P90091 chr20   
Sequence 346 : >LL.9935-Human-47 DBTSS:NM\_005461 chr20  
Sequence 347 : >LL.55544-Human-48 hchr20\_P006582 chr20  
Sequence 348 : >LL.5688-Human-49 EP73874 chr20 - 60151  
Sequence 349 : >LL.11047-Human-50 EP74602 chr20 + 6031  
Sequence 350 : >LL.3785-Human-51 hchr20\_P010123 chr20   
Sequence 351 : >LL.7165-Human-52 DBTSS:NM\_003288 chr20  
Sequence 352 : >LL.6493-Human-53 AP001726\_4 chr21 + 36  
Sequence 353 : >LL.51227-Human-54 EP74423 chr21 - 3736  
Sequence 354 : >LL.89766-Human-55 hchr21\_P001668 chr21  
Sequence 355 : >LL.56894-Human-56 DBTSS:NM\_020132 chr2  
Sequence 356 : >LL.23275-Human-57 DBTSS:NM\_015227 chr2  
Sequence 357 : >LL.6573-Human-58 DBTSS:NM\_003056 chr21  
Sequence 358 : >LL.6899-Human-59 hchr22\_P000828 chr22   
Sequence 359 : >LL.23598-Human-60 hchr22\_P013151 chr22  
Sequence 360 : >LL.4357-Human-61 DBTSS:NM\_021126 chr22  
Sequence 361 : >LL.400931-Human-62 hchr22\_P006415 chr2  
Sequence 362 : >LL.1890-Human-63 DBTSS:NM\_001953 chr22  
Sequence 363 : >LL.440836-Human-64 hchr22\_P008354 chr2  
Sequence 364 : >LL.8510-Human-65 hchr1\_P001510 chr1 +   
Sequence 365 : >LL.51078-Human-66 DBTSS:NM\_015963 chr2  
Sequence 366 : >LL.23192-Human-67 DBTSS:NM\_013325 chr2  
Sequence 367 : >LL.83482-Human-68 hchr8\_P012566 chr8 -  
  
  
**\*\*\*\* MY ADVICE \*\*\*\***  
  
  
 **\*\*\* Interesting motifs (highest-ranking) seem to be :**   
  
  
 **CGACCG**   
**CGGTCG**  
  
2 redundant motifs found:  
CGGTCGAC - CGTCCG -   
  
Best occurrences (match percentage):   
Seq St oligo pos match
  
 1 + **.CGGCCG.** 936 (95.29)   
1 + **.CGCCCG.** 1073 (95.73)   
1 + **[CGACCC]** 1391 (88.16)   
1 - **[CCACCG]** 1254 (88.59)   
1 - **[CGAGCG]** 1148 (88.31)   
1 - **.CGGCCG.** 936 (95.29)   
2 + **.CGCCCG.** 192 (95.73)   
2 + **[GGACCG]** 249 (87.21)   
2 + **[CGTCCG]** 479 (88.90)   
2 + **[CGTCCG]** 517 (88.90)   
2 + **[CGAGCG]** 698 (88.31)   
2 + **.CGCCCG.** 743 (95.73)   
2 + **[GGACCG]** 811 (87.21)   
2 + **[CGACCC]** 913 (88.16)   
2 + **.CGGCCG.** 1257 (95.29)   
2 + **[CGTCCG]** 1311 (88.90)   
2 + **.CGGCCG.** 1331 (95.29)   
2 + **[CGAGCG]** 1443 (88.31)   
2 - **.CGGCCG.** 1331 (95.29)   
2 - **[CGACCC]** 1304 (88.16)   
2 - **.CGGCCG.** 1257 (95.29)   
2 - **.CGCCCG.** 709 (95.73)   
2 - **[CGACCC]** 569 (88.16)   
2 - **[CCACCG]** 258 (88.59)   
2 - **.CGCCCG.** 106 (95.73)   
3 + **.CGCCCG.** 571 (95.73)   
3 + **[CGACCC]** 603 (88.16)   
3 + **.CGCCCG.** 1092 (95.73)   
3 + **.CGCCCG.** 1096 (95.73)   
3 + **.CGCCCG.** 1169 (95.73)   
3 + **[CGAGCG]** 1184 (88.31)   
3 + **.CGGCCG.** 1333 (95.29)   
3 + **.CGCCCG.** 1346 (95.73)   
3 - **.CGGCCG.** 1333 (95.29)   
3 - **.CGCCCG.** 1178 (95.73)   
4 + **[CCACCG]** 556 (88.59)   
4 + **.CGACCG.** 637 (100.00)   
4 + **[CGACCC]** 703 (88.16)   
4 + **[CGAGCG]** 832 (88.31)   
4 + **[CGACCC]** 852 (88.16)   
4 + **.CGACCG.** 894 (100.00)   
4 + **.CGCCCG.** 1201 (95.73)   
4 + **.CGGCCG.** 1258 (95.29)   
4 - **[CGTCCG]** 1440 (88.90)   
4 - **.CGGCCG.** 1258 (95.29)   
4 - **.CGCCCG.** 1254 (95.73)   
4 - **.CGCCCG.** 1250 (95.73)   
4 - **[GGACCG]** 1172 (87.21)   
4 - **.CGCCCG.** 877 (95.73)   
4 - **[CGACCC]** 688 (88.16)   
4 - **.CGCCCG.** 633 (95.73)   
4 - **[CCACCG]** 269 (88.59)   
4 - **.CGCCCG.** 199 (95.73)   
4 - **[CCACCG]** 180 (88.59)   
5 + **[GGACCG]** 1081 (87.21)   
5 + **[CCACCG]** 1199 (88.59)   
5 + **[CCACCG]** 1209 (88.59)   
5 + **.CGGCCG.** 1223 (95.29)   
5 + **[CGTCCG]** 1371 (88.90)   
5 + **.CGGCCG.** 1397 (95.29)   
5 + **.CGCCCG.** 1433 (95.73)   
5 + **.CGGCCG.** 1442 (95.29)   
5 + **.CGGCCG.** 1451 (95.29)   
5 + **.CGGCCG.** 1478 (95.29)   
5 - **[CGTCCG]** 1497 (88.90)   
5 - **.CGGCCG.** 1478 (95.29)   
5 - **.CGGCCG.** 1451 (95.29)   
5 - **.CGGCCG.** 1442 (95.29)   
5 - **.CGGCCG.** 1397 (95.29)   
5 - **.CGGCCG.** 1223 (95.29)   
5 - **[CGTCCG]** 793 (88.90)   
6 + **[CGTCCG]** 1436 (88.90)   
6 - **[CCACCG]** 1416 (88.59)   
6 - **.CGCCCG.** 1412 (95.73)   
7 + **[CGACCC]** 1347 (88.16)   
8 + **.CGGCCG.** 308 (95.29)   
8 + **.CGCCCG.** 501 (95.73)   
8 + **.CGCCCG.** 678 (95.73)   
8 + **.CGCCCG.** 1005 (95.73)   
8 + **.CGGCCG.** 1047 (95.29)   
8 + **.CGGCCG.** 1072 (95.29)   
8 + **.CGCCCG.** 1157 (95.73)   
8 + **[CGTCCG]** 1194 (88.90)   
8 + **.CGCCCG.** 1278 (95.73)   
8 + **[CCACCG]** 1433 (88.59)   
8 + **.CGCCCG.** 1506 (95.73)   
8 - **[GGACCG]** 1512 (87.21)   
8 - **[CGACCC]** 1202 (88.16)   
8 - **.CGCCCG.** 1187 (95.73)   
8 - **.CGGCCG.** 1072 (95.29)   
8 - **.CGGCCG.** 1047 (95.29)   
8 - **.CGCCCG.** 995 (95.73)   
8 - **.CGCCCG.** 931 (95.73)   
8 - **.CGCCCG.** 915 (95.73)   
8 - **[CGAGCG]** 424 (88.31)   
8 - **.CGGCCG.** 308 (95.29)   
9 + **.CGGCCG.** 751 (95.29)   
9 - **.CGCCCG.** 973 (95.73)   
9 - **.CGGCCG.** 751 (95.29)   
9 - **.CGCCCG.** 535 (95.73)   
9 - **[CCACCG]** 231 (88.59)   
9 - **[CGACCC]** 221 (88.16)   
10 + **.CGGCCG.** 33 (95.29)   
10 + **.CGACCG.** 412 (100.00)   
10 + **.CGACCG.** 438 (100.00)   
10 + **.CGGCCG.** 555 (95.29)   
10 + **[CCACCG]** 756 (88.59)   
10 + **.CGGCCG.** 1029 (95.29)   
10 + **[CGAGCG]** 1082 (88.31)   
10 + **.CGCCCG.** 1113 (95.73)   
10 + **.CGGCCG.** 1117 (95.29)   
10 + **.CGGCCG.** 1123 (95.29)   
10 + **[CGTCCG]** 1221 (88.90)   
10 + **[CCACCG]** 1258 (88.59)   
10 - **.CGACCG.** 1450 (100.00)   
10 - **[CCACCG]** 1340 (88.59)   
10 - **.CGGCCG.** 1123 (95.29)   
10 - **.CGGCCG.** 1117 (95.29)   
10 - **.CGGCCG.** 1029 (95.29)   
10 - **[CGACCC]** 903 (88.16)   
10 - **[CGTCCG]** 846 (88.90)   
10 - **[CGAGCG]** 760 (88.31)   
10 - **[CGACCC]** 745 (88.16)   
10 - **.CGCCCG.** 686 (95.73)   
10 - **.CGGCCG.** 555 (95.29)   
10 - **.CGCCCG.** 220 (95.73)   
10 - **[CGAGCG]** 39 (88.31)   
10 - **.CGGCCG.** 33 (95.29)   
11 + **.CGGCCG.** 213 (95.29)   
11 + **[CGAGCG]** 217 (88.31)   
11 + **.CGGCCG.** 495 (95.29)   
11 + **[CCACCG]** 602 (88.59)   
11 + **.CGGCCG.** 613 (95.29)   
11 + **.CGCCCG.** 635 (95.73)   
11 + **.CGGCCG.** 711 (95.29)   
11 + **.CGCCCG.** 959 (95.73)   
11 + **.CGCCCG.** 985 (95.73)   
11 + **.CGCCCG.** 989 (95.73)   
11 + **.CGCCCG.** 1004 (95.73)   
11 + **.CGGCCG.** 1090 (95.29)   
11 + **.CGGCCG.** 1138 (95.29)   
11 - **[CGAGCG]** 1358 (88.31)   
11 - **.CGGCCG.** 1138 (95.29)   
11 - **[CGAGCG]** 1100 (88.31)   
11 - **.CGGCCG.** 1090 (95.29)   
11 - **.CGACCG.** 812 (100.00)   
11 - **.CGGCCG.** 711 (95.29)   
11 - **[CGTCCG]** 696 (88.90)   
11 - **.CGCCCG.** 679 (95.73)   
11 - **.CGCCCG.** 665 (95.73)   
11 - **.CGGCCG.** 613 (95.29)   
11 - **.CGGCCG.** 495 (95.29)   
11 - **.CGGCCG.** 213 (95.29)   
11 - **[CCACCG]** 177 (88.59)   
11 - **[CGAGCG]** 89 (88.31)   
11 - **.CGCCCG.** 22 (95.73)   
12 + **.CGCCCG.** 779 (95.73)   
12 + **.CGCCCG.** 783 (95.73)   
12 + **.CGCCCG.** 811 (95.73)   
12 + **[CGACCC]** 872 (88.16)   
12 + **[CCACCG]** 990 (88.59)   
12 + **[CCACCG]** 1225 (88.59)   
12 + **.CGGCCG.** 1249 (95.29)   
12 + **.CGCCCG.** 1506 (95.73)   
12 - **[CCACCG]** 1437 (88.59)   
12 - **.CGCCCG.** 1337 (95.73)   
12 - **[CCACCG]** 1270 (88.59)   
12 - **.CGGCCG.** 1249 (95.29)   
12 - **.CGCCCG.** 1099 (95.73)   
12 - **[CGACCC]** 1093 (88.16)   
12 - **[CGACCC]** 978 (88.16)   
12 - **.CGCCCG.** 913 (95.73)   
12 - **[GGACCG]** 834 (87.21)   
12 - **.CGCCCG.** 666 (95.73)   
12 - **.CGCCCG.** 612 (95.73)   
12 - **.CGCCCG.** 592 (95.73)   
12 - **[GGACCG]** 534 (87.21)   
12 - **.CGCCCG.** 182 (95.73)   
13 + **[CCACCG]** 163 (88.59)   
13 + **.CGCCCG.** 169 (95.73)   
13 + **[CGACCC]** 331 (88.16)   
13 + **.CGCCCG.** 620 (95.73)   
13 + **.CGCCCG.** 699 (95.73)   
13 + **.CGCCCG.** 749 (95.73)   
13 + **.CGCCCG.** 789 (95.73)   
13 + **.CGGCCG.** 949 (95.29)   
13 + **.CGGCCG.** 1066 (95.29)   
13 + **[CCACCG]** 1105 (88.59)   
13 + **[CGTCCG]** 1192 (88.90)   
13 + **.CGCCCG.** 1331 (95.73)   
13 + **.CGCCCG.** 1512 (95.73)   
13 - **[CGACCC]** 1466 (88.16)   
13 - **[CGACCC]** 1449 (88.16)   
13 - **[CGACCC]** 1442 (88.16)   
13 - **.CGCCCG.** 1314 (95.73)   
13 - **[CGACCC]** 1300 (88.16)   
13 - **[CGAGCG]** 1116 (88.31)   
13 - **.CGGCCG.** 1066 (95.29)   
13 - **.CGCCCG.** 1044 (95.73)   
13 - **.CGGCCG.** 949 (95.29)   
13 - **[CGAGCG]** 680 (88.31)   
13 - **.CGCCCG.** 548 (95.73)   
14 + **[CGACCC]** 194 (88.16)   
14 + **.CGCCCG.** 275 (95.73)   
14 + **[CCACCG]** 424 (88.59)   
14 + **.CGCCCG.** 570 (95.73)   
14 + **[CGAGCG]** 816 (88.31)   
14 + **[CCACCG]** 839 (88.59)   
14 - **.CGCCCG.** 1335 (95.73)   
14 - **[CCACCG]** 1220 (88.59)   
14 - **[CCACCG]** 1102 (88.59)   
14 - **.CGACCG.** 903 (100.00)   
14 - **.CGCCCG.** 744 (95.73)   
14 - **.CGACCG.** 464 (100.00)   
15 + **.CGCCCG.** 249 (95.73)   
15 + **[CGTCCG]** 338 (88.90)   
15 + **.CGGCCG.** 430 (95.29)   
15 + **.CGGCCG.** 457 (95.29)   
15 + **.CGCCCG.** 541 (95.73)   
15 + **.CGGCCG.** 601 (95.29)   
15 + **.CGCCCG.** 677 (95.73)   
15 + **.CGGCCG.** 853 (95.29)   
15 + **[CCACCG]** 993 (88.59)   
15 + **.CGGCCG.** 1106 (95.29)   
15 + **.CGCCCG.** 1287 (95.73)   
15 - **[GGACCG]** 1268 (87.21)   
15 - **[CGACCC]** 1111 (88.16)   
15 - **.CGGCCG.** 1106 (95.29)   
15 - **.CGGCCG.** 853 (95.29)   
15 - **.CGACCG.** 673 (100.00)   
15 - **.CGGCCG.** 601 (95.29)   
15 - **[CGAGCG]** 499 (88.31)   
15 - **.CGGCCG.** 457 (95.29)   
15 - **.CGGCCG.** 430 (95.29)   
15 - **.CGCCCG.** 174 (95.73)   
15 - **[CGTCCG]** 119 (88.90)   
15 - **[CGTCCG]** 115 (88.90)   
16 + **[CCACCG]** 712 (88.59)   
16 + **.CGCCCG.** 825 (95.73)   
16 + **[GGACCG]** 962 (87.21)   
16 + **[CGAGCG]** 1005 (88.31)   
16 + **.CGCCCG.** 1302 (95.73)   
16 + **.CGCCCG.** 1309 (95.73)   
16 + **.CGGCCG.** 1413 (95.29)   
16 - **.CGGCCG.** 1413 (95.29)   
16 - **[CGAGCG]** 1223 (88.31)   
16 - **[CGAGCG]** 1191 (88.31)   
16 - **.CGCCCG.** 1091 (95.73)   
16 - **[CCACCG]** 1057 (88.59)   
16 - **[GGACCG]** 907 (87.21)   
16 - **[CGACCC]** 877 (88.16)   
16 - **[CGACCC]** 718 (88.16)   
16 - **[CGACCC]** 695 (88.16)   
16 - **[CGTCCG]** 496 (88.90)   
16 - **[CCACCG]** 304 (88.59)   
17 + **[GGACCG]** 801 (87.21)   
17 + **.CGACCG.** 1087 (100.00)   
17 + **.CGGCCG.** 1151 (95.29)   
17 - **[CCACCG]** 1358 (88.59)   
17 - **.CGGCCG.** 1151 (95.29)   
18 + **[CGTCCG]** 608 (88.90)   
18 + **.CGCCCG.** 893 (95.73)   
18 + **.CGCCCG.** 910 (95.73)   
18 + **.CGGCCG.** 934 (95.29)   
18 + **[CCACCG]** 1001 (88.59)   
18 + **.CGCCCG.** 1005 (95.73)   
18 + **[GGACCG]** 1016 (87.21)   
18 + **.CGCCCG.** 1020 (95.73)   
18 + **[GGACCG]** 1299 (87.21)   
18 + **.CGGCCG.** 1327 (95.29)   
18 + **[CGTCCG]** 1333 (88.90)   
18 + **.CGCCCG.** 1511 (95.73)   
18 - **[GGACCG]** 1504 (87.21)   
18 - **.CGGCCG.** 1327 (95.29)   
18 - **[GGACCG]** 1174 (87.21)   
18 - **[CGACCC]** 1161 (88.16)   
18 - **[GGACCG]** 1096 (87.21)   
18 - **[CGTCCG]** 1092 (88.90)   
18 - **[GGACCG]** 1055 (87.21)   
18 - **.CGGCCG.** 934 (95.29)   
18 - **[GGACCG]** 845 (87.21)   
18 - **[CGTCCG]** 598 (88.90)   
18 - **[CCACCG]** 371 (88.59)   
20 + **[GGACCG]** 128 (87.21)   
20 + **[GGACCG]** 188 (87.21)   
20 + **[CGAGCG]** 192 (88.31)   
20 + **.CGCCCG.** 196 (95.73)   
20 + **.CGCCCG.** 255 (95.73)   
20 + **.CGCCCG.** 1031 (95.73)   
20 + **.CGGCCG.** 1195 (95.29)   
20 - **.CGGCCG.** 1195 (95.29)   
20 - **[CGTCCG]** 1105 (88.90)   
20 - **.CGCCCG.** 1017 (95.73)   
20 - **[CGACCC]** 816 (88.16)   
20 - **[CGAGCG]** 313 (88.31)   
20 - **[CCACCG]** 295 (88.59)   
20 - **.CGCCCG.** 291 (95.73)   
21 + **[CGTCCG]** 1088 (88.90)   
21 - **[CCACCG]** 1311 (88.59)   
21 - **[CCACCG]** 1254 (88.59)   
21 - **.CGCCCG.** 1135 (95.73)   
22 + **.CGACCG.** 597 (100.00)   
22 + **.CGGCCG.** 620 (95.29)   
22 + **[CCACCG]** 841 (88.59)   
22 + **.CGACCG.** 857 (100.00)   
22 + **.CGGCCG.** 1203 (95.29)   
22 - **.CGGCCG.** 1203 (95.29)   
22 - **[GGACCG]** 1039 (87.21)   
22 - **[CGTCCG]** 816 (88.90)   
22 - **[CGACCC]** 743 (88.16)   
22 - **[CGACCC]** 718 (88.16)   
22 - **[CCACCG]** 663 (88.59)   
22 - **.CGGCCG.** 620 (95.29)   
22 - **[GGACCG]** 504 (87.21)   
22 - **.CGCCCG.** 178 (95.73)   
22 - **[CCACCG]** 47 (88.59)   
23 - **[CGTCCG]** 1126 (88.90)   
24 + **.CGCCCG.** 359 (95.73)   
24 + **[CGACCC]** 366 (88.16)   
24 + **[CGAGCG]** 393 (88.31)   
24 + **.CGACCG.** 542 (100.00)   
24 + **[CGACCC]** 571 (88.16)   
24 + **.CGCCCG.** 600 (95.73)   
24 + **.CGGCCG.** 604 (95.29)   
24 + **[GGACCG]** 662 (87.21)   
24 + **.CGGCCG.** 1045 (95.29)   
24 - **[CGACCC]** 1238 (88.16)   
24 - **[CGACCC]** 1141 (88.16)   
24 - **.CGGCCG.** 1045 (95.29)   
24 - **[CCACCG]** 895 (88.59)   
24 - **.CGGCCG.** 604 (95.29)   
24 - **[GGACCG]** 533 (87.21)   
24 - **.CGACCG.** 451 (100.00)   
24 - **[GGACCG]** 397 (87.21)   
24 - **[CGAGCG]** 355 (88.31)   
25 + **.CGCCCG.** 31 (95.73)   
25 + **[CGTCCG]** 909 (88.90)   
25 + **.CGGCCG.** 1002 (95.29)   
25 + **.CGGCCG.** 1225 (95.29)   
25 - **.CGGCCG.** 1225 (95.29)   
25 - **.CGGCCG.** 1002 (95.29)   
25 - **.CGCCCG.** 998 (95.73)   
25 - **[CCACCG]** 919 (88.59)   
25 - **.CGCCCG.** 631 (95.73)   
25 - **[CCACCG]** 491 (88.59)   
25 - **[CGACCC]** 378 (88.16)   
25 - **[CCACCG]** 356 (88.59)   
25 - **[CCACCG]** 293 (88.59)   
25 - **[CCACCG]** 261 (88.59)   
26 + **.CGGCCG.** 182 (95.29)   
26 + **.CGCCCG.** 735 (95.73)   
26 + **.CGGCCG.** 854 (95.29)   
26 + **[CGAGCG]** 1024 (88.31)   
26 - **[CGAGCG]** 1101 (88.31)   
26 - **.CGCCCG.** 1059 (95.73)   
26 - **.CGCCCG.** 1055 (95.73)   
26 - **.CGGCCG.** 854 (95.29)   
26 - **[CCACCG]** 683 (88.59)   
26 - **[CGAGCG]** 664 (88.31)   
26 - **[GGACCG]** 520 (87.21)   
26 - **.CGGCCG.** 182 (95.29)   
27 + **.CGACCG.** 746 (100.00)   
27 + **[GGACCG]** 846 (87.21)   
27 + **.CGCCCG.** 983 (95.73)   
27 + **[CGACCC]** 998 (88.16)   
27 + **[CGACCC]** 1075 (88.16)   
27 + **.CGGCCG.** 1266 (95.29)   
27 + **.CGACCG.** 1401 (100.00)   
27 + **.CGGCCG.** 1408 (95.29)   
27 - **[CGACCC]** 1497 (88.16)   
27 - **.CGGCCG.** 1408 (95.29)   
27 - **.CGACCG.** 1352 (100.00)   
27 - **.CGGCCG.** 1266 (95.29)   
27 - **[CCACCG]** 752 (88.59)   
28 + **.CGGCCG.** 662 (95.29)   
28 + **.CGGCCG.** 686 (95.29)   
28 + **.CGGCCG.** 745 (95.29)   
28 + **.CGGCCG.** 914 (95.29)   
28 + **[CGTCCG]** 1005 (88.90)   
28 + **.CGCCCG.** 1281 (95.73)   
28 - **[CGAGCG]** 1322 (88.31)   
28 - **.CGCCCG.** 1311 (95.73)   
28 - **[CGTCCG]** 936 (88.90)   
28 - **.CGCCCG.** 920 (95.73)   
28 - **.CGGCCG.** 914 (95.29)   
28 - **[CCACCG]** 899 (88.59)   
28 - **.CGGCCG.** 745 (95.29)   
28 - **.CGGCCG.** 686 (95.29)   
28 - **.CGGCCG.** 662 (95.29)   
29 + **[CCACCG]** 216 (88.59)   
29 + **[GGACCG]** 1243 (87.21)   
30 - **[CCACCG]** 1060 (88.59)   
33 + **.CGGCCG.** 1297 (95.29)   
33 + **.CGGCCG.** 1358 (95.29)   
33 + **.CGGCCG.** 1403 (95.29)   
33 - **.CGGCCG.** 1403 (95.29)   
33 - **.CGACCG.** 1367 (100.00)   
33 - **.CGGCCG.** 1358 (95.29)   
33 - **.CGGCCG.** 1297 (95.29)   
33 - **[CGAGCG]** 614 (88.31)   
34 + **.CGGCCG.** 855 (95.29)   
34 + **[CCACCG]** 905 (88.59)   
34 + **.CGCCCG.** 944 (95.73)   
34 + **.CGCCCG.** 984 (95.73)   
34 + **.CGGCCG.** 1118 (95.29)   
34 + **.CGCCCG.** 1255 (95.73)   
34 + **.CGCCCG.** 1356 (95.73)   
34 + **.CGCCCG.** 1360 (95.73)   
34 + **.CGGCCG.** 1364 (95.29)   
34 + **.CGCCCG.** 1402 (95.73)   
34 - **.CGGCCG.** 1364 (95.29)   
34 - **.CGCCCG.** 1311 (95.73)   
34 - **[CGTCCG]** 1282 (88.90)   
34 - **[GGACCG]** 1259 (87.21)   
34 - **.CGGCCG.** 1118 (95.29)   
34 - **[CGAGCG]** 1114 (88.31)   
34 - **.CGGCCG.** 855 (95.29)   
34 - **[CGAGCG]** 723 (88.31)   
34 - **[CGACCC]** 531 (88.16)   
34 - **.CGCCCG.** 510 (95.73)   
34 - **[GGACCG]** 363 (87.21)   
35 + **.CGGCCG.** 1024 (95.29)   
35 + **[GGACCG]** 1089 (87.21)   
35 + **.CGGCCG.** 1156 (95.29)   
35 + **.CGCCCG.** 1321 (95.73)   
35 + **[GGACCG]** 1328 (87.21)   
35 + **[CGACCC]** 1332 (88.16)   
35 + **.CGGCCG.** 1379 (95.29)   
35 - **.CGGCCG.** 1379 (95.29)   
35 - **.CGACCG.** 1337 (100.00)   
35 - **.CGGCCG.** 1156 (95.29)   
35 - **[CGACCC]** 1152 (88.16)   
35 - **.CGGCCG.** 1024 (95.29)   
36 + **.CGGCCG.** 509 (95.29)   
36 + **.CGCCCG.** 708 (95.73)   
36 + **[CCACCG]** 1392 (88.59)   
36 - **[CGAGCG]** 1222 (88.31)   
36 - **.CGCCCG.** 1099 (95.73)   
36 - **.CGCCCG.** 1071 (95.73)   
36 - **.CGCCCG.** 704 (95.73)   
36 - **.CGGCCG.** 509 (95.29)   
37 + **[CCACCG]** 389 (88.59)   
37 - **[GGACCG]** 1195 (87.21)   
37 - **.CGCCCG.** 1169 (95.73)   
38 + **[GGACCG]** 589 (87.21)   
38 + **.CGCCCG.** 843 (95.73)   
38 + **[CCACCG]** 862 (88.59)   
38 - **.CGACCG.** 910 (100.00)   
38 - **.CGCCCG.** 555 (95.73)   
39 + **.CGCCCG.** 1040 (95.73)   
39 + **[CCACCG]** 1270 (88.59)   
39 + **[CGACCC]** 1416 (88.16)   
39 + **.CGGCCG.** 1427 (95.29)   
39 - **[CGTCCG]** 1466 (88.90)   
39 - **.CGGCCG.** 1427 (95.29)   
39 - **.CGACCG.** 1201 (100.00)   
39 - **.CGCCCG.** 1191 (95.73)   
39 - **.CGCCCG.** 1044 (95.73)   
40 + **.CGGCCG.** 1125 (95.29)   
40 + **[CGTCCG]** 1237 (88.90)   
40 - **.CGGCCG.** 1125 (95.29)   
40 - **.CGCCCG.** 948 (95.73)   
41 - **.CGACCG.** 1175 (100.00)   
42 + **.CGGCCG.** 311 (95.29)   
42 + **.CGGCCG.** 452 (95.29)   
42 + **[CGACCC]** 1161 (88.16)   
42 + **.CGGCCG.** 1236 (95.29)   
42 + **.CGGCCG.** 1313 (95.29)   
42 + **.CGCCCG.** 1347 (95.73)   
42 + **.CGCCCG.** 1354 (95.73)   
42 + **.CGCCCG.** 1445 (95.73)   
42 + **.CGGCCG.** 1473 (95.29)   
42 - **[CGACCC]** 1492 (88.16)   
42 - **[CCACCG]** 1486 (88.59)   
42 - **.CGGCCG.** 1473 (95.29)   
42 - **.CGGCCG.** 1313 (95.29)   
42 - **[CGAGCG]** 1307 (88.31)   
42 - **[CGACCC]** 1265 (88.16)   
42 - **.CGCCCG.** 1260 (95.73)   
42 - **[CGTCCG]** 1256 (88.90)   
42 - **.CGGCCG.** 1236 (95.29)   
42 - **[CGTCCG]** 1202 (88.90)   
42 - **[CCACCG]** 921 (88.59)   
42 - **.CGCCCG.** 913 (95.73)   
42 - **.CGCCCG.** 749 (95.73)   
42 - **.CGGCCG.** 452 (95.29)   
42 - **.CGGCCG.** 311 (95.29)   
42 - **[GGACCG]** 208 (87.21)   
43 + **.CGGCCG.** 1078 (95.29)   
43 + **.CGGCCG.** 1244 (95.29)   
43 + **[CGACCC]** 1369 (88.16)   
43 - **[GGACCG]** 1472 (87.21)   
43 - **.CGGCCG.** 1244 (95.29)   
43 - **.CGCCCG.** 1118 (95.73)   
43 - **.CGGCCG.** 1078 (95.29)   
43 - **.CGCCCG.** 981 (95.73)   
44 + **[CCACCG]** 1337 (88.59)   
44 - **[CCACCG]** 139 (88.59)   
45 + **.CGACCG.** 731 (100.00)   
45 + **.CGGCCG.** 983 (95.29)   
45 + **.CGGCCG.** 1014 (95.29)   
45 + **.CGCCCG.** 1325 (95.73)   
45 - **[CGTCCG]** 1244 (88.90)   
45 - **.CGCCCG.** 1144 (95.73)   
45 - **.CGCCCG.** 1030 (95.73)   
45 - **.CGGCCG.** 1014 (95.29)   
45 - **.CGGCCG.** 983 (95.29)   
47 + **.CGGCCG.** 1138 (95.29)   
47 + **.CGCCCG.** 1152 (95.73)   
47 + **[GGACCG]** 1265 (87.21)   
47 + **.CGGCCG.** 1269 (95.29)   
47 + **.CGGCCG.** 1369 (95.29)   
47 + **[CGAGCG]** 1383 (88.31)   
47 + **.CGGCCG.** 1414 (95.29)   
47 + **[CGAGCG]** 1502 (88.31)   
47 - **.CGGCCG.** 1414 (95.29)   
47 - **.CGCCCG.** 1410 (95.73)   
47 - **.CGCCCG.** 1390 (95.73)   
47 - **.CGGCCG.** 1369 (95.29)   
47 - **.CGGCCG.** 1269 (95.29)   
47 - **.CGGCCG.** 1138 (95.29)   
47 - **[GGACCG]** 61 (87.21)   
48 + **[CCACCG]** 233 (88.59)   
48 + **[CGACCC]** 609 (88.16)   
48 + **.CGCCCG.** 868 (95.73)   
48 + **[CGAGCG]** 955 (88.31)   
48 + **[CGAGCG]** 1015 (88.31)   
48 + **.CGCCCG.** 1097 (95.73)   
48 + **[CCACCG]** 1279 (88.59)   
48 - **[GGACCG]** 747 (87.21)   
48 - **[GGACCG]** 685 (87.21)   
49 + **.CGCCCG.** 949 (95.73)   
49 + **.CGCCCG.** 975 (95.73)   
49 + **.CGCCCG.** 1508 (95.73)   
49 - **[GGACCG]** 1225 (87.21)   
49 - **[GGACCG]** 953 (87.21)   
49 - **.CGACCG.** 851 (100.00)   
50 - **[CGTCCG]** 1078 (88.90)   
51 + **.CGACCG.** 441 (100.00)   
51 + **.CGGCCG.** 504 (95.29)   
51 + **[CCACCG]** 583 (88.59)   
51 + **.CGCCCG.** 602 (95.73)   
51 + **[CGTCCG]** 916 (88.90)   
51 + **.CGCCCG.** 1005 (95.73)   
51 + **[CGACCC]** 1204 (88.16)   
51 + **[CCACCG]** 1208 (88.59)   
51 + **.CGCCCG.** 1261 (95.73)   
51 + **.CGGCCG.** 1339 (95.29)   
51 - **[CCACCG]** 1382 (88.59)   
51 - **.CGGCCG.** 1339 (95.29)   
51 - **[CGAGCG]** 1057 (88.31)   
51 - **.CGCCCG.** 606 (95.73)   
51 - **.CGGCCG.** 504 (95.29)   
51 - **[GGACCG]** 478 (87.21)   
51 - **.CGCCCG.** 464 (95.73)   
51 - **.CGCCCG.** 284 (95.73)   
51 - **.CGCCCG.** 278 (95.73)   
51 - **[CGTCCG]** 170 (88.90)   
53 + **[CGACCC]** 799 (88.16)   
53 - **[GGACCG]** 1402 (87.21)   
53 - **[CCACCG]** 1188 (88.59)   
53 - **[GGACCG]** 1024 (87.21)   
55 + **[CCACCG]** 42 (88.59)   
55 + **.CGCCCG.** 799 (95.73)   
55 + **[GGACCG]** 846 (87.21)   
55 + **.CGCCCG.** 856 (95.73)   
55 + **.CGCCCG.** 879 (95.73)   
55 + **.CGGCCG.** 932 (95.29)   
55 + **.CGCCCG.** 976 (95.73)   
55 + **[CGAGCG]** 987 (88.31)   
55 + **.CGACCG.** 991 (100.00)   
55 + **[CGACCC]** 1128 (88.16)   
55 + **.CGCCCG.** 1253 (95.73)   
55 - **[CGACCC]** 1482 (88.16)   
55 - **.CGACCG.** 1326 (100.00)   
55 - **.CGGCCG.** 932 (95.29)   
55 - **.CGCCCG.** 928 (95.73)   
55 - **[CCACCG]** 834 (88.59)   
55 - **[CGAGCG]** 212 (88.31)   
55 - **[CGAGCG]** 208 (88.31)   
58 + **.CGCCCG.** 198 (95.73)   
58 + **.CGGCCG.** 838 (95.29)   
58 + **[CGAGCG]** 1069 (88.31)   
58 + **[CGACCC]** 1137 (88.16)   
58 - **.CGCCCG.** 1183 (95.73)   
58 - **[GGACCG]** 1165 (87.21)   
58 - **.CGGCCG.** 838 (95.29)   
58 - **.CGACCG.** 594 (100.00)   
58 - **.CGACCG.** 552 (100.00)   
58 - **[CGAGCG]** 540 (88.31)   
58 - **.CGCCCG.** 305 (95.73)   
58 - **.CGCCCG.** 275 (95.73)   
58 - **[CGACCC]** 271 (88.16)   
59 + **[CGAGCG]** 474 (88.31)   
59 + **[GGACCG]** 689 (87.21)   
59 + **.CGGCCG.** 1014 (95.29)   
59 + **.CGCCCG.** 1165 (95.73)   
59 + **[CGACCC]** 1400 (88.16)   
59 - **[CCACCG]** 1405 (88.59)   
59 - **.CGCCCG.** 1139 (95.73)   
59 - **.CGGCCG.** 1014 (95.29)   
59 - **.CGCCCG.** 710 (95.73)   
60 + **.CGCCCG.** 632 (95.73)   
60 + **.CGGCCG.** 747 (95.29)   
60 + **.CGGCCG.** 829 (95.29)   
60 + **.CGACCG.** 1151 (100.00)   
60 - **[CGTCCG]** 1395 (88.90)   
60 - **[CCACCG]** 1139 (88.59)   
60 - **[CGAGCG]** 963 (88.31)   
60 - **.CGCCCG.** 959 (95.73)   
60 - **.CGGCCG.** 829 (95.29)   
60 - **.CGACCG.** 775 (100.00)   
60 - **.CGGCCG.** 747 (95.29)   
60 - **[GGACCG]** 733 (87.21)   
61 + **[CGAGCG]** 847 (88.31)   
61 + **[CGACCC]** 860 (88.16)   
61 + **.CGGCCG.** 912 (95.29)   
61 + **.CGACCG.** 1162 (100.00)   
61 + **.CGGCCG.** 1172 (95.29)   
61 + **.CGCCCG.** 1466 (95.73)   
61 - **.CGCCCG.** 1479 (95.73)   
61 - **.CGCCCG.** 1475 (95.73)   
61 - **[GGACCG]** 1208 (87.21)   
61 - **.CGGCCG.** 1172 (95.29)   
61 - **.CGGCCG.** 912 (95.29)   
61 - **.CGCCCG.** 748 (95.73)   
62 + **.CGGCCG.** 947 (95.29)   
62 - **.CGGCCG.** 947 (95.29)   
63 + **.CGGCCG.** 5 (95.29)   
63 + **.CGGCCG.** 39 (95.29)   
63 + **.CGCCCG.** 72 (95.73)   
63 + **.CGGCCG.** 91 (95.29)   
63 + **.CGCCCG.** 119 (95.73)   
63 + **.CGGCCG.** 138 (95.29)   
63 + **.CGGCCG.** 180 (95.29)   
63 + **.CGGCCG.** 307 (95.29)   
63 + **.CGCCCG.** 373 (95.73)   
63 + **.CGCCCG.** 377 (95.73)   
63 + **.CGCCCG.** 381 (95.73)   
63 + **[GGACCG]** 388 (87.21)   
63 + **[CGTCCG]** 450 (88.90)   
63 + **.CGCCCG.** 517 (95.73)   
63 + **[CGTCCG]** 587 (88.90)   
63 + **[CGTCCG]** 669 (88.90)   
63 + **[CGACCC]** 820 (88.16)   
63 + **.CGCCCG.** 831 (95.73)   
63 + **.CGGCCG.** 864 (95.29)   
63 + **.CGGCCG.** 1019 (95.29)   
63 + **[CGTCCG]** 1303 (88.90)   
63 + **.CGGCCG.** 1352 (95.29)   
63 + **.CGGCCG.** 1410 (95.29)   
63 + **[CGAGCG]** 1434 (88.31)   
63 - **.CGCCCG.** 1441 (95.73)   
63 - **[GGACCG]** 1416 (87.21)   
63 - **.CGGCCG.** 1410 (95.29)   
63 - **.CGCCCG.** 1379 (95.73)   
63 - **.CGGCCG.** 1352 (95.29)   
63 - **[GGACCG]** 1297 (87.21)   
63 - **[CCACCG]** 1219 (88.59)   
63 - **.CGCCCG.** 1176 (95.73)   
63 - **[CGACCC]** 1113 (88.16)   
63 - **[CGACCC]** 1061 (88.16)   
63 - **.CGGCCG.** 1019 (95.29)   
63 - **[CGAGCG]** 1003 (88.31)   
63 - **[CGAGCG]** 901 (88.31)   
63 - **.CGGCCG.** 864 (95.29)   
63 - **[CGTCCG]** 693 (88.90)   
63 - **.CGCCCG.** 609 (95.73)   
63 - **.CGCCCG.** 544 (95.73)   
63 - **.CGGCCG.** 307 (95.29)   
63 - **[CGACCC]** 212 (88.16)   
63 - **.CGGCCG.** 180 (95.29)   
63 - **.CGGCCG.** 138 (95.29)   
63 - **.CGGCCG.** 91 (95.29)   
63 - **.CGGCCG.** 39 (95.29)   
63 - **.CGGCCG.** 5 (95.29)   
65 + **.CGGCCG.** 782 (95.29)   
65 + **.CGGCCG.** 827 (95.29)   
65 + **.CGCCCG.** 894 (95.73)   
65 + **.CGCCCG.** 908 (95.73)   
65 + **[CGAGCG]** 930 (88.31)   
65 + **[CGAGCG]** 1005 (88.31)   
65 + **[CCACCG]** 1424 (88.59)   
65 + **[CGACCC]** 1441 (88.16)   
65 - **[CGACCC]** 1366 (88.16)   
65 - **.CGACCG.** 1228 (100.00)   
65 - **[CGAGCG]** 882 (88.31)   
65 - **.CGGCCG.** 827 (95.29)   
65 - **[CGAGCG]** 814 (88.31)   
65 - **.CGGCCG.** 782 (95.29)   
66 + **[CCACCG]** 487 (88.59)   
66 + **.CGGCCG.** 838 (95.29)   
66 + **.CGCCCG.** 939 (95.73)   
66 + **.CGCCCG.** 945 (95.73)   
66 + **[CGACCC]** 977 (88.16)   
66 + **.CGGCCG.** 1459 (95.29)   
66 - **.CGGCCG.** 1459 (95.29)   
66 - **[CGACCC]** 1402 (88.16)   
66 - **.CGCCCG.** 1344 (95.73)   
66 - **.CGCCCG.** 1340 (95.73)   
66 - **[CGACCC]** 1334 (88.16)   
66 - **[CGACCC]** 1059 (88.16)   
66 - **.CGGCCG.** 838 (95.29)   
66 - **.CGCCCG.** 831 (95.73)   
66 - **[CCACCG]** 799 (88.59)   
66 - **[CCACCG]** 732 (88.59)   
67 + **.CGACCG.** 833 (100.00)   
67 - **[CCACCG]** 1340 (88.59)   
67 - **[CGACCC]** 1191 (88.16)   
67 - **[GGACCG]** 1055 (87.21)   
67 - **[CCACCG]** 1046 (88.59)   
67 - **[CGTCCG]** 1042 (88.90)   
67 - **[CCACCG]** 936 (88.59)   
67 - **[CGACCC]** 819 (88.16)   
68 + **.CGGCCG.** 290 (95.29)   
68 + **.CGGCCG.** 346 (95.29)   
68 + **[CCACCG]** 584 (88.59)   
68 + **.CGCCCG.** 689 (95.73)   
68 + **.CGGCCG.** 826 (95.29)   
68 + **.CGGCCG.** 983 (95.29)   
68 + **[GGACCG]** 1146 (87.21)   
68 + **[CGAGCG]** 1265 (88.31)   
68 - **.CGCCCG.** 1275 (95.73)   
68 - **.CGCCCG.** 1252 (95.73)   
68 - **.CGGCCG.** 983 (95.29)   
68 - **.CGCCCG.** 979 (95.73)   
68 - **.CGCCCG.** 975 (95.73)   
68 - **.CGGCCG.** 826 (95.29)   
68 - **.CGCCCG.** 406 (95.73)   
68 - **[CGACCC]** 393 (88.16)   
68 - **[CGACCC]** 387 (88.16)   
68 - **[CGACCC]** 381 (88.16)   
68 - **.CGGCCG.** 346 (95.29)   
68 - **.CGGCCG.** 290 (95.29)   
68 - **[GGACCG]** 142 (87.21)   
68 - **.CGACCG.** 90 (100.00)   
69 + **[CGTCCG]** 692 (88.90)   
69 + **.CGGCCG.** 771 (95.29)   
69 + **[CGAGCG]** 859 (88.31)   
69 + **[CGACCC]** 962 (88.16)   
69 + **[CGACCC]** 975 (88.16)   
69 + **.CGCCCG.** 1057 (95.73)   
69 + **.CGGCCG.** 1170 (95.29)   
69 + **.CGCCCG.** 1205 (95.73)   
69 + **.CGGCCG.** 1224 (95.29)   
69 + **.CGCCCG.** 1381 (95.73)   
69 - **.CGGCCG.** 1224 (95.29)   
69 - **.CGGCCG.** 1170 (95.29)   
69 - **.CGCCCG.** 863 (95.73)   
69 - **.CGCCCG.** 836 (95.73)   
69 - **.CGGCCG.** 771 (95.29)   
69 - **[CGACCC]** 730 (88.16)   
70 + **.CGCCCG.** 354 (95.73)   
70 + **[CCACCG]** 921 (88.59)   
70 + **.CGGCCG.** 1209 (95.29)   
70 + **.CGCCCG.** 1248 (95.73)   
70 + **[CGACCC]** 1339 (88.16)   
70 + **[CGAGCG]** 1386 (88.31)   
70 + **.CGCCCG.** 1421 (95.73)   
70 - **[GGACCG]** 1371 (87.21)   
70 - **.CGGCCG.** 1209 (95.29)   
70 - **.CGCCCG.** 1195 (95.73)   
70 - **[GGACCG]** 1082 (87.21)   
70 - **[CGACCC]** 1035 (88.16)   
70 - **[GGACCG]** 949 (87.21)   
70 - **[CGACCC]** 730 (88.16)   
70 - **.CGCCCG.** 350 (95.73)   
70 - **.CGCCCG.** 338 (95.73)   
71 + **[GGACCG]** 747 (87.21)   
71 + **.CGCCCG.** 872 (95.73)   
71 + **.CGCCCG.** 962 (95.73)   
71 + **.CGACCG.** 1027 (100.00)   
71 + **.CGCCCG.** 1082 (95.73)   
71 - **.CGACCG.** 1492 (100.00)   
71 - **[CGAGCG]** 1450 (88.31)   
71 - **[CGAGCG]** 1078 (88.31)   
71 - **[CCACCG]** 933 (88.59)   
71 - **[CGAGCG]** 929 (88.31)   
71 - **[CCACCG]** 831 (88.59)   
73 + **[CGACCC]** 760 (88.16)   
73 + **[CCACCG]** 817 (88.59)   
73 + **.CGGCCG.** 874 (95.29)   
73 + **.CGGCCG.** 923 (95.29)   
73 + **.CGCCCG.** 1118 (95.73)   
73 - **[CGAGCG]** 1037 (88.31)   
73 - **.CGCCCG.** 1019 (95.73)   
73 - **.CGGCCG.** 923 (95.29)   
73 - **.CGGCCG.** 874 (95.29)   
73 - **[GGACCG]** 861 (87.21)   
74 + **.CGCCCG.** 1137 (95.73)   
74 + **[CGTCCG]** 1340 (88.90)   
74 + **.CGGCCG.** 1434 (95.29)   
74 + **[CGAGCG]** 1486 (88.31)   
74 - **.CGGCCG.** 1434 (95.29)   
74 - **[CGACCC]** 1425 (88.16)   
74 - **[CGACCC]** 1402 (88.16)   
76 + **[GGACCG]** 862 (87.21)   
76 + **[CCACCG]** 1025 (88.59)   
76 + **[CGAGCG]** 1060 (88.31)   
76 + **.CGCCCG.** 1153 (95.73)   
76 + **.CGACCG.** 1298 (100.00)   
76 - **[CGTCCG]** 1390 (88.90)   
76 - **[CGAGCG]** 1321 (88.31)   
76 - **.CGCCCG.** 1288 (95.73)   
76 - **.CGACCG.** 1124 (100.00)   
76 - **[CGAGCG]** 1074 (88.31)   
76 - **.CGCCCG.** 1003 (95.73)   
76 - **.CGCCCG.** 900 (95.73)   
76 - **[CGAGCG]** 764 (88.31)   
77 + **.CGCCCG.** 509 (95.73)   
77 + **.CGACCG.** 576 (100.00)   
77 + **[CGAGCG]** 1142 (88.31)   
77 + **[GGACCG]** 1287 (87.21)   
77 - **[CGACCC]** 998 (88.16)   
77 - **[GGACCG]** 594 (87.21)   
77 - **[CGAGCG]** 590 (88.31)   
77 - **.CGCCCG.** 419 (95.73)   
77 - **[GGACCG]** 373 (87.21)   
77 - **[CGAGCG]** 328 (88.31)   
79 + **[CGTCCG]** 635 (88.90)   
79 + **.CGCCCG.** 649 (95.73)   
79 + **.CGCCCG.** 721 (95.73)   
79 + **.CGACCG.** 767 (100.00)   
79 + **[CGACCC]** 813 (88.16)   
79 + **.CGGCCG.** 1275 (95.29)   
79 - **[CGTCCG]** 1410 (88.90)   
79 - **[CGAGCG]** 1306 (88.31)   
79 - **.CGGCCG.** 1275 (95.29)   
79 - **.CGCCCG.** 934 (95.73)   
79 - **[CCACCG]** 409 (88.59)   
80 + **[CGACCC]** 841 (88.16)   
80 + **.CGCCCG.** 950 (95.73)   
80 - **[GGACCG]** 1312 (87.21)   
80 - **[GGACCG]** 1141 (87.21)   
81 + **.CGCCCG.** 607 (95.73)   
81 + **[CGACCC]** 844 (88.16)   
81 + **[CGTCCG]** 1047 (88.90)   
81 + **[CCACCG]** 1411 (88.59)   
81 - **[CCACCG]** 1415 (88.59)   
81 - **[CCACCG]** 1036 (88.59)   
83 - **[CCACCG]** 1207 (88.59)   
84 + **.CGCCCG.** 53 (95.73)   
84 + **[CGACCC]** 616 (88.16)   
84 + **[CGACCC]** 635 (88.16)   
84 + **[CGAGCG]** 1019 (88.31)   
84 + **.CGCCCG.** 1179 (95.73)   
84 + **.CGCCCG.** 1239 (95.73)   
84 - **[CGTCCG]** 1087 (88.90)   
84 - **[CGAGCG]** 1042 (88.31)   
85 + **[CGACCC]** 72 (88.16)   
85 + **[CGTCCG]** 97 (88.90)   
85 + **.CGCCCG.** 412 (95.73)   
85 + **[CCACCG]** 430 (88.59)   
85 + **.CGCCCG.** 538 (95.73)   
85 + **.CGCCCG.** 869 (95.73)   
85 + **.CGCCCG.** 876 (95.73)   
85 + **[CGAGCG]** 1033 (88.31)   
85 + **.CGCCCG.** 1055 (95.73)   
85 + **[CGAGCG]** 1085 (88.31)   
85 + **.CGCCCG.** 1098 (95.73)   
85 + **[CGACCC]** 1335 (88.16)   
85 + **.CGCCCG.** 1452 (95.73)   
85 - **[GGACCG]** 1439 (87.21)   
85 - **.CGCCCG.** 1400 (95.73)   
85 - **.CGCCCG.** 1007 (95.73)   
85 - **.CGCCCG.** 932 (95.73)   
85 - **[CGTCCG]** 926 (88.90)   
85 - **[CGAGCG]** 922 (88.31)   
85 - **[CGACCC]** 528 (88.16)   
85 - **.CGCCCG.** 521 (95.73)   
85 - **[CGAGCG]** 388 (88.31)   
85 - **[CGACCC]** 307 (88.16)   
85 - **[CCACCG]** 137 (88.59)   
86 + **.CGGCCG.** 545 (95.29)   
86 + **.CGGCCG.** 660 (95.29)   
86 + **[CGACCC]** 799 (88.16)   
86 + **.CGCCCG.** 962 (95.73)   
86 + **.CGCCCG.** 966 (95.73)   
86 + **[CGTCCG]** 979 (88.90)   
86 + **[CGAGCG]** 1301 (88.31)   
86 + **.CGCCCG.** 1305 (95.73)   
86 + **[CGACCC]** 1322 (88.16)   
86 + **.CGCCCG.** 1337 (95.73)   
86 + **.CGCCCG.** 1406 (95.73)   
86 + **.CGCCCG.** 1463 (95.73)   
86 + **.CGCCCG.** 1467 (95.73)   
86 + **.CGGCCG.** 1508 (95.29)   
86 - **.CGGCCG.** 1508 (95.29)   
86 - **[GGACCG]** 1280 (87.21)   
86 - **[CGAGCG]** 1144 (88.31)   
86 - **[CGACCC]** 1122 (88.16)   
86 - **.CGCCCG.** 885 (95.73)   
86 - **[GGACCG]** 852 (87.21)   
86 - **.CGCCCG.** 815 (95.73)   
86 - **.CGCCCG.** 804 (95.73)   
86 - **[CGACCC]** 767 (88.16)   
86 - **.CGGCCG.** 660 (95.29)   
86 - **.CGGCCG.** 545 (95.29)   
87 + **[CGAGCG]** 784 (88.31)   
87 + **[CGACCC]** 973 (88.16)   
87 + **.CGGCCG.** 1106 (95.29)   
87 + **.CGCCCG.** 1126 (95.73)   
87 + **.CGCCCG.** 1148 (95.73)   
87 + **.CGCCCG.** 1186 (95.73)   
87 + **.CGGCCG.** 1369 (95.29)   
87 + **[GGACCG]** 1512 (87.21)   
87 - **.CGCCCG.** 1492 (95.73)   
87 - **.CGCCCG.** 1461 (95.73)   
87 - **.CGGCCG.** 1369 (95.29)   
87 - **[CGTCCG]** 1299 (88.90)   
87 - **.CGCCCG.** 1286 (95.73)   
87 - **.CGCCCG.** 1282 (95.73)   
87 - **.CGACCG.** 1219 (100.00)   
87 - **.CGCCCG.** 1110 (95.73)   
87 - **.CGGCCG.** 1106 (95.29)   
87 - **[CGTCCG]** 936 (88.90)   
87 - **[CGTCCG]** 681 (88.90)   
87 - **[GGACCG]** 590 (87.21)   
87 - **[CGACCC]** 538 (88.16)   
87 - **[CGACCC]** 419 (88.16)   
88 + **[CGACCC]** 112 (88.16)   
88 + **.CGCCCG.** 310 (95.73)   
88 + **[GGACCG]** 385 (87.21)   
88 + **[GGACCG]** 424 (87.21)   
88 + **.CGGCCG.** 552 (95.29)   
88 + **[GGACCG]** 845 (87.21)   
88 + **.CGGCCG.** 865 (95.29)   
88 + **[CCACCG]** 956 (88.59)   
88 + **[CGAGCG]** 1020 (88.31)   
88 + **.CGCCCG.** 1027 (95.73)   
88 + **[CGAGCG]** 1219 (88.31)   
88 + **.CGCCCG.** 1412 (95.73)   
88 + **[CGTCCG]** 1476 (88.90)   
88 - **[CGACCC]** 1304 (88.16)   
88 - **[CGACCC]** 935 (88.16)   
88 - **[CGACCC]** 926 (88.16)   
88 - **.CGCCCG.** 910 (95.73)   
88 - **.CGGCCG.** 865 (95.29)   
88 - **[CGACCC]** 698 (88.16)   
88 - **[CGAGCG]** 633 (88.31)   
88 - **.CGGCCG.** 552 (95.29)   
88 - **[CGTCCG]** 532 (88.90)   
88 - **.CGACCG.** 524 (100.00)   
88 - **[GGACCG]** 510 (87.21)   
88 - **[CCACCG]** 474 (88.59)   
88 - **.CGCCCG.** 389 (95.73)   
88 - **.CGCCCG.** 378 (95.73)   
88 - **.CGCCCG.** 300 (95.73)   
89 + **.CGGCCG.** 49 (95.29)   
89 + **.CGCCCG.** 96 (95.73)   
89 + **.CGCCCG.** 114 (95.73)   
89 + **.CGGCCG.** 141 (95.29)   
89 + **.CGGCCG.** 342 (95.29)   
89 + **.CGCCCG.** 346 (95.73)   
89 + **.CGGCCG.** 456 (95.29)   
89 + **.CGCCCG.** 472 (95.73)   
89 + **.CGGCCG.** 476 (95.29)   
89 + **.CGCCCG.** 500 (95.73)   
89 + **.CGGCCG.** 504 (95.29)   
89 + **.CGCCCG.** 650 (95.73)   
89 + **.CGGCCG.** 659 (95.29)   
89 + **.CGGCCG.** 771 (95.29)   
89 + **.CGCCCG.** 978 (95.73)   
89 + **[CGAGCG]** 1078 (88.31)   
89 + **[CCACCG]** 1146 (88.59)   
89 + **.CGCCCG.** 1207 (95.73)   
89 - **.CGACCG.** 1345 (100.00)   
89 - **[CGAGCG]** 1273 (88.31)   
89 - **.CGCCCG.** 1252 (95.73)   
89 - **.CGGCCG.** 771 (95.29)   
89 - **[CCACCG]** 692 (88.59)   
89 - **.CGGCCG.** 659 (95.29)   
89 - **.CGGCCG.** 504 (95.29)   
89 - **.CGGCCG.** 476 (95.29)   
89 - **.CGGCCG.** 456 (95.29)   
89 - **.CGGCCG.** 342 (95.29)   
89 - **.CGCCCG.** 264 (95.73)   
89 - **.CGGCCG.** 141 (95.29)   
89 - **[CGTCCG]** 70 (88.90)   
89 - **.CGGCCG.** 49 (95.29)   
89 - **.CGCCCG.** 24 (95.73)   
90 + **.CGCCCG.** 44 (95.73)   
90 + **.CGGCCG.** 250 (95.29)   
90 + **.CGCCCG.** 656 (95.73)   
90 + **.CGGCCG.** 773 (95.29)   
90 + **.CGCCCG.** 828 (95.73)   
90 + **.CGGCCG.** 846 (95.29)   
90 + **[CGACCC]** 1345 (88.16)   
90 - **.CGACCG.** 1023 (100.00)   
90 - **.CGCCCG.** 855 (95.73)   
90 - **.CGGCCG.** 846 (95.29)   
90 - **[CGACCC]** 839 (88.16)   
90 - **.CGGCCG.** 773 (95.29)   
90 - **.CGGCCG.** 250 (95.29)   
91 - **[GGACCG]** 333 (87.21)   
92 + **[CGACCC]** 1461 (88.16)   
92 - **[CGAGCG]** 1060 (88.31)   
93 + **[CGTCCG]** 489 (88.90)   
93 + **[GGACCG]** 912 (87.21)   
93 + **.CGGCCG.** 1074 (95.29)   
93 + **[GGACCG]** 1472 (87.21)   
93 - **.CGCCCG.** 1402 (95.73)   
93 - **.CGCCCG.** 1236 (95.73)   
93 - **.CGGCCG.** 1074 (95.29)   
93 - **[GGACCG]** 457 (87.21)   
93 - **.CGCCCG.** 411 (95.73)   
95 - **[CCACCG]** 1449 (88.59)   
96 + **.CGGCCG.** 1381 (95.29)   
96 - **.CGCCCG.** 1496 (95.73)   
96 - **[CGAGCG]** 1407 (88.31)   
96 - **[CGTCCG]** 1391 (88.90)   
96 - **.CGGCCG.** 1381 (95.29)   
96 - **[CGACCC]** 1348 (88.16)   
96 - **[CCACCG]** 1090 (88.59)   
96 - **[CGTCCG]** 776 (88.90)   
96 - **[CCACCG]** 679 (88.59)   
97 + **[CGAGCG]** 722 (88.31)   
97 + **[CCACCG]** 778 (88.59)   
97 + **[CGAGCG]** 840 (88.31)   
97 + **.CGCCCG.** 844 (95.73)   
97 + **.CGGCCG.** 1076 (95.29)   
97 + **.CGGCCG.** 1080 (95.29)   
97 + **.CGGCCG.** 1128 (95.29)   
97 + **.CGCCCG.** 1266 (95.73)   
97 + **.CGACCG.** 1339 (100.00)   
97 + **.CGCCCG.** 1380 (95.73)   
97 + **.CGCCCG.** 1446 (95.73)   
97 - **.CGGCCG.** 1128 (95.29)   
97 - **[CCACCG]** 1107 (88.59)   
97 - **.CGGCCG.** 1080 (95.29)   
97 - **.CGGCCG.** 1076 (95.29)   
97 - **[CCACCG]** 984 (88.59)   
97 - **.CGCCCG.** 980 (95.73)   
97 - **[CGACCC]** 934 (88.16)   
97 - **[CGTCCG]** 718 (88.90)   
97 - **.CGCCCG.** 357 (95.73)   
98 + **.CGGCCG.** 997 (95.29)   
98 + **[CGAGCG]** 1175 (88.31)   
98 + **.CGGCCG.** 1190 (95.29)   
98 + **.CGGCCG.** 1327 (95.29)   
98 - **.CGCCCG.** 1460 (95.73)   
98 - **.CGCCCG.** 1391 (95.73)   
98 - **[CCACCG]** 1366 (88.59)   
98 - **[CCACCG]** 1338 (88.59)   
98 - **.CGGCCG.** 1327 (95.29)   
98 - **.CGACCG.** 1266 (100.00)   
98 - **.CGGCCG.** 1190 (95.29)   
98 - **.CGCCCG.** 1029 (95.73)   
98 - **.CGGCCG.** 997 (95.29)   
98 - **[CGTCCG]** 700 (88.90)   
99 - **.CGACCG.** 823 (100.00)   
100 + **[CGAGCG]** 912 (88.31)   
100 + **[GGACCG]** 1004 (87.21)   
100 + **[GGACCG]** 1095 (87.21)   
100 + **[CGAGCG]** 1203 (88.31)   
100 - **[GGACCG]** 1311 (87.21)   
100 - **[CGACCC]** 1100 (88.16)   
100 - **[CGTCCG]** 939 (88.90)   
102 + **[GGACCG]** 775 (87.21)   
103 + **.CGCCCG.** 453 (95.73)   
103 + **[CCACCG]** 928 (88.59)   
103 + **[GGACCG]** 1511 (87.21)   
103 - **[CGACCC]** 1180 (88.16)   
103 - **[GGACCG]** 1069 (87.21)   
103 - **[CCACCG]** 544 (88.59)   
104 + **[CGAGCG]** 1144 (88.31)   
104 - **[CGTCCG]** 1259 (88.90)   
104 - **[GGACCG]** 686 (87.21)   
105 + **.CGCCCG.** 953 (95.73)   
105 - **[CGACCC]** 1366 (88.16)   
105 - **.CGCCCG.** 1140 (95.73)   
105 - **.CGCCCG.** 1136 (95.73)   
106 + **.CGGCCG.** 944 (95.29)   
106 + **.CGCCCG.** 1031 (95.73)   
106 - **[CCACCG]** 1098 (88.59)   
106 - **.CGCCCG.** 959 (95.73)   
106 - **.CGGCCG.** 944 (95.29)   
106 - **.CGCCCG.** 800 (95.73)   
108 + **[CGACCC]** 71 (88.16)   
108 + **.CGCCCG.** 199 (95.73)   
108 + **[CCACCG]** 546 (88.59)   
108 + **[CGTCCG]** 667 (88.90)   
108 + **.CGCCCG.** 753 (95.73)   
108 + **[CGTCCG]** 867 (88.90)   
108 + **[CGACCC]** 933 (88.16)   
108 + **[GGACCG]** 1179 (87.21)   
108 + **[CGAGCG]** 1254 (88.31)   
108 - **[CGAGCG]** 1110 (88.31)   
108 - **.CGCCCG.** 1086 (95.73)   
108 - **.CGCCCG.** 708 (95.73)   
108 - **[CGAGCG]** 622 (88.31)   
108 - **[CGACCC]** 581 (88.16)   
108 - **.CGACCG.** 384 (100.00)   
108 - **.CGCCCG.** 67 (95.73)   
109 + **.CGCCCG.** 457 (95.73)   
109 + **[CCACCG]** 584 (88.59)   
109 + **.CGCCCG.** 617 (95.73)   
109 + **.CGGCCG.** 621 (95.29)   
109 + **.CGGCCG.** 838 (95.29)   
109 + **.CGCCCG.** 1039 (95.73)   
109 + **.CGCCCG.** 1045 (95.73)   
109 + **.CGGCCG.** 1095 (95.29)   
109 + **[CGAGCG]** 1190 (88.31)   
109 + **.CGCCCG.** 1378 (95.73)   
109 + **.CGGCCG.** 1382 (95.29)   
109 + **.CGGCCG.** 1416 (95.29)   
109 - **[CCACCG]** 1513 (88.59)   
109 - **[CCACCG]** 1487 (88.59)   
109 - **.CGGCCG.** 1416 (95.29)   
109 - **[CGTCCG]** 1412 (88.90)   
109 - **.CGGCCG.** 1382 (95.29)   
109 - **.CGCCCG.** 1271 (95.73)   
109 - **[CCACCG]** 1125 (88.59)   
109 - **.CGGCCG.** 1095 (95.29)   
109 - **.CGGCCG.** 838 (95.29)   
109 - **.CGGCCG.** 621 (95.29)   
109 - **[CGAGCG]** 414 (88.31)   
109 - **.CGACCG.** 256 (100.00)   
110 - **[CGAGCG]** 953 (88.31)   
110 - **[CCACCG]** 817 (88.59)   
110 - **[CCACCG]** 546 (88.59)   
111 - **[CGAGCG]** 1254 (88.31)   
113 + **[CCACCG]** 18 (88.59)   
113 + **[GGACCG]** 1063 (87.21)   
113 - **[CGACCC]** 735 (88.16)   
114 + **.CGCCCG.** 261 (95.73)   
114 + **.CGCCCG.** 340 (95.73)   
114 + **.CGCCCG.** 693 (95.73)   
114 + **[CGTCCG]** 780 (88.90)   
114 + **[CCACCG]** 816 (88.59)   
114 + **.CGGCCG.** 1011 (95.29)   
114 - **.CGACCG.** 1140 (100.00)   
114 - **[CCACCG]** 1055 (88.59)   
114 - **.CGGCCG.** 1011 (95.29)   
114 - **.CGCCCG.** 954 (95.73)   
114 - **.CGCCCG.** 674 (95.73)   
114 - **.CGCCCG.** 572 (95.73)   
114 - **[CGAGCG]** 367 (88.31)   
114 - **.CGCCCG.** 354 (95.73)   
114 - **[CGAGCG]** 309 (88.31)   
114 - **[CCACCG]** 274 (88.59)   
114 - **[CGACCC]** 257 (88.16)   
114 - **[CCACCG]** 95 (88.59)   
115 + **.CGGCCG.** 95 (95.29)   
115 + **.CGGCCG.** 133 (95.29)   
115 + **[CGAGCG]** 199 (88.31)   
115 + **[CCACCG]** 540 (88.59)   
115 + **[CGACCC]** 568 (88.16)   
115 + **.CGCCCG.** 827 (95.73)   
115 + **[CGACCC]** 1043 (88.16)   
115 + **.CGGCCG.** 1250 (95.29)   
115 - **.CGGCCG.** 1250 (95.29)   
115 - **.CGCCCG.** 305 (95.73)   
115 - **.CGGCCG.** 133 (95.29)   
115 - **.CGGCCG.** 95 (95.29)   
116 + **[CCACCG]** 1221 (88.59)   
116 + **.CGACCG.** 1496 (100.00)   
117 + **.CGGCCG.** 1058 (95.29)   
117 - **[CCACCG]** 1100 (88.59)   
117 - **.CGGCCG.** 1058 (95.29)   
117 - **[GGACCG]** 987 (87.21)   
117 - **.CGCCCG.** 958 (95.73)   
117 - **[CGAGCG]** 796 (88.31)   
117 - **.CGACCG.** 687 (100.00)   
118 + **.CGCCCG.** 1041 (95.73)   
118 + **.CGGCCG.** 1047 (95.29)   
118 + **[GGACCG]** 1308 (87.21)   
118 - **[CCACCG]** 1090 (88.59)   
118 - **.CGGCCG.** 1047 (95.29)   
118 - **[GGACCG]** 977 (87.21)   
118 - **.CGCCCG.** 948 (95.73)   
118 - **[CGAGCG]** 772 (88.31)   
118 - **.CGACCG.** 665 (100.00)   
119 + **.CGCCCG.** 1032 (95.73)   
119 + **.CGGCCG.** 1038 (95.29)   
119 + **[GGACCG]** 1299 (87.21)   
119 - **[CCACCG]** 1081 (88.59)   
119 - **.CGGCCG.** 1038 (95.29)   
119 - **.CGCCCG.** 939 (95.73)   
119 - **[CGAGCG]** 770 (88.31)   
119 - **.CGACCG.** 663 (100.00)   
120 + **.CGCCCG.** 1499 (95.73)   
120 + **.CGGCCG.** 1505 (95.29)   
120 - **.CGGCCG.** 1505 (95.29)   
120 - **[GGACCG]** 1434 (87.21)   
120 - **.CGCCCG.** 1406 (95.73)   
120 - **[CGAGCG]** 1230 (88.31)   
120 - **.CGACCG.** 1122 (100.00)   
121 + **[CGTCCG]** 930 (88.90)   
121 + **[CCACCG]** 1000 (88.59)   
121 - **.CGCCCG.** 1092 (95.73)   
122 + **[CGAGCG]** 28 (88.31)   
122 + **[CGAGCG]** 61 (88.31)   
122 + **[CCACCG]** 180 (88.59)   
122 + **.CGACCG.** 804 (100.00)   
122 - **[CGACCC]** 1087 (88.16)   
122 - **[CGTCCG]** 587 (88.90)   
123 + **.CGGCCG.** 490 (95.29)   
123 + **[CGACCC]** 514 (88.16)   
123 + **.CGCCCG.** 597 (95.73)   
123 + **[CGAGCG]** 848 (88.31)   
123 + **.CGGCCG.** 902 (95.29)   
123 + **[CGACCC]** 1084 (88.16)   
123 + **[CCACCG]** 1098 (88.59)   
123 + **[GGACCG]** 1274 (87.21)   
123 + **[CCACCG]** 1301 (88.59)   
123 - **[CGACCC]** 1369 (88.16)   
123 - **.CGCCCG.** 1358 (95.73)   
123 - **[CCACCG]** 1254 (88.59)   
123 - **.CGCCCG.** 1140 (95.73)   
123 - **[GGACCG]** 1093 (87.21)   
123 - **.CGACCG.** 1000 (100.00)   
123 - **.CGGCCG.** 902 (95.29)   
123 - **[CGAGCG]** 877 (88.31)   
123 - **[GGACCG]** 842 (87.21)   
123 - **[CGTCCG]** 774 (88.90)   
123 - **.CGCCCG.** 747 (95.73)   
123 - **.CGCCCG.** 700 (95.73)   
123 - **.CGGCCG.** 490 (95.29)   
124 + **.CGCCCG.** 902 (95.73)   
124 + **[CGACCC]** 966 (88.16)   
124 + **[CGAGCG]** 1027 (88.31)   
124 + **.CGGCCG.** 1054 (95.29)   
124 + **.CGGCCG.** 1222 (95.29)   
124 + **[CGACCC]** 1243 (88.16)   
124 - **.CGCCCG.** 1258 (95.73)   
124 - **[CGTCCG]** 1228 (88.90)   
124 - **.CGGCCG.** 1222 (95.29)   
124 - **.CGCCCG.** 1157 (95.73)   
124 - **.CGGCCG.** 1054 (95.29)   
124 - **.CGCCCG.** 1031 (95.73)   
124 - **.CGCCCG.** 976 (95.73)   
124 - **[GGACCG]** 926 (87.21)   
127 + **.CGGCCG.** 257 (95.29)   
127 + **[CGTCCG]** 730 (88.90)   
127 + **.CGGCCG.** 799 (95.29)   
127 + **.CGCCCG.** 908 (95.73)   
127 + **.CGGCCG.** 1116 (95.29)   
127 + **[CGACCC]** 1149 (88.16)   
127 + **.CGGCCG.** 1484 (95.29)   
127 + **.CGGCCG.** 1507 (95.29)   
127 - **.CGGCCG.** 1507 (95.29)   
127 - **.CGGCCG.** 1484 (95.29)   
127 - **.CGCCCG.** 1338 (95.73)   
127 - **.CGCCCG.** 1120 (95.73)   
127 - **.CGGCCG.** 1116 (95.29)   
127 - **[CGACCC]** 1083 (88.16)   
127 - **[CGTCCG]** 1027 (88.90)   
127 - **.CGGCCG.** 799 (95.29)   
127 - **[CCACCG]** 685 (88.59)   
127 - **[GGACCG]** 572 (87.21)   
127 - **.CGCCCG.** 568 (95.73)   
127 - **.CGACCG.** 471 (100.00)   
127 - **.CGGCCG.** 257 (95.29)   
128 + **[CGTCCG]** 460 (88.90)   
128 + **[CGACCC]** 780 (88.16)   
128 + **.CGGCCG.** 1024 (95.29)   
128 + **.CGACCG.** 1096 (100.00)   
128 - **.CGCCCG.** 1321 (95.73)   
128 - **[CGACCC]** 1169 (88.16)   
128 - **.CGCCCG.** 1128 (95.73)   
128 - **.CGGCCG.** 1024 (95.29)   
128 - **[CGACCC]** 829 (88.16)   
128 - **.CGCCCG.** 735 (95.73)   
128 - **[CGACCC]** 430 (88.16)   
129 + **[CGACCC]** 583 (88.16)   
129 + **.CGGCCG.** 593 (95.29)   
129 + **[CGTCCG]** 740 (88.90)   
129 + **[CCACCG]** 746 (88.59)   
129 + **.CGGCCG.** 1036 (95.29)   
129 + **.CGGCCG.** 1040 (95.29)   
129 + **[CGAGCG]** 1107 (88.31)   
129 + **[CGAGCG]** 1127 (88.31)   
129 + **.CGGCCG.** 1198 (95.29)   
129 + **.CGGCCG.** 1227 (95.29)   
129 + **[CGACCC]** 1258 (88.16)   
129 + **[GGACCG]** 1343 (87.21)   
129 + **.CGGCCG.** 1388 (95.29)   
129 + **.CGCCCG.** 1433 (95.73)   
129 - **[CGACCC]** 1393 (88.16)   
129 - **.CGGCCG.** 1388 (95.29)   
129 - **.CGGCCG.** 1227 (95.29)   
129 - **.CGCCCG.** 1207 (95.73)   
129 - **.CGGCCG.** 1198 (95.29)   
129 - **[CGTCCG]** 1070 (88.90)   
129 - **[CGTCCG]** 1055 (88.90)   
129 - **.CGGCCG.** 1040 (95.29)   
129 - **.CGGCCG.** 1036 (95.29)   
129 - **[CGAGCG]** 775 (88.31)   
129 - **[CGAGCG]** 659 (88.31)   
129 - **.CGGCCG.** 593 (95.29)   
129 - **.CGCCCG.** 264 (95.73)   
130 + **[CGACCC]** 638 (88.16)   
130 + **.CGACCG.** 1056 (100.00)   
130 - **.CGCCCG.** 1323 (95.73)   
130 - **[CGACCC]** 700 (88.16)   
131 + **.CGCCCG.** 1 (95.73)   
131 + **[CCACCG]** 63 (88.59)   
131 + **[CGAGCG]** 585 (88.31)   
131 + **[CGTCCG]** 801 (88.90)   
131 + **.CGGCCG.** 918 (95.29)   
131 + **.CGGCCG.** 1119 (95.29)   
131 + **.CGCCCG.** 1512 (95.73)   
131 - **[GGACCG]** 1395 (87.21)   
131 - **[CCACCG]** 1128 (88.59)   
131 - **.CGGCCG.** 1119 (95.29)   
131 - **[CGACCC]** 1010 (88.16)   
131 - **.CGCCCG.** 984 (95.73)   
131 - **.CGGCCG.** 918 (95.29)   
131 - **.CGCCCG.** 914 (95.73)   
131 - **.CGACCG.** 825 (100.00)   
131 - **.CGCCCG.** 139 (95.73)   
131 - **.CGCCCG.** 132 (95.73)   
131 - **[CGAGCG]** 104 (88.31)   
132 + **[CGAGCG]** 774 (88.31)   
132 + **.CGCCCG.** 839 (95.73)   
132 + **[CGTCCG]** 863 (88.90)   
132 + **[CCACCG]** 904 (88.59)   
132 + **[CGAGCG]** 1351 (88.31)   
132 - **[CGACCC]** 1440 (88.16)   
132 - **.CGCCCG.** 1325 (95.73)   
132 - **.CGCCCG.** 1157 (95.73)   
132 - **[CGAGCG]** 794 (88.31)   
134 + **.CGCCCG.** 964 (95.73)   
134 + **[CGACCC]** 1142 (88.16)   
134 + **[GGACCG]** 1185 (87.21)   
134 + **.CGACCG.** 1192 (100.00)   
134 + **[CGAGCG]** 1417 (88.31)   
134 + **[CCACCG]** 1424 (88.59)   
134 + **[GGACCG]** 1497 (87.21)   
134 - **.CGCCCG.** 1471 (95.73)   
134 - **[GGACCG]** 933 (87.21)   
134 - **[GGACCG]** 509 (87.21)   
135 + **.CGCCCG.** 315 (95.73)   
135 + **[CGTCCG]** 393 (88.90)   
135 + **.CGCCCG.** 410 (95.73)   
135 + **.CGCCCG.** 422 (95.73)   
135 + **.CGCCCG.** 426 (95.73)   
135 + **.CGGCCG.** 767 (95.29)   
135 + **.CGGCCG.** 774 (95.29)   
135 + **.CGCCCG.** 845 (95.73)   
135 + **.CGCCCG.** 853 (95.73)   
135 + **[CGTCCG]** 956 (88.90)   
135 + **[GGACCG]** 999 (87.21)   
135 + **.CGCCCG.** 1141 (95.73)   
135 + **[CGACCC]** 1424 (88.16)   
135 - **.CGCCCG.** 1448 (95.73)   
135 - **.CGCCCG.** 1441 (95.73)   
135 - **[CCACCG]** 1403 (88.59)   
135 - **[CGACCC]** 1255 (88.16)   
135 - **[CCACCG]** 1026 (88.59)   
135 - **.CGCCCG.** 849 (95.73)   
135 - **.CGGCCG.** 774 (95.29)   
135 - **.CGGCCG.** 767 (95.29)   
135 - **.CGCCCG.** 757 (95.73)   
135 - **.CGCCCG.** 492 (95.73)   
136 + **[CGACCC]** 427 (88.16)   
136 + **[CGAGCG]** 734 (88.31)   
136 + **[CGAGCG]** 849 (88.31)   
136 + **.CGCCCG.** 1011 (95.73)   
136 + **.CGCCCG.** 1026 (95.73)   
136 + **.CGGCCG.** 1198 (95.29)   
136 - **.CGGCCG.** 1198 (95.29)   
136 - **[CGTCCG]** 651 (88.90)   
137 + **.CGGCCG.** 950 (95.29)   
137 + **[CCACCG]** 980 (88.59)   
137 + **.CGGCCG.** 1018 (95.29)   
137 + **[CGAGCG]** 1096 (88.31)   
137 + **[CGAGCG]** 1102 (88.31)   
137 + **[CGACCC]** 1327 (88.16)   
137 - **.CGCCCG.** 1163 (95.73)   
137 - **[CCACCG]** 1029 (88.59)   
137 - **.CGGCCG.** 1018 (95.29)   
137 - **.CGGCCG.** 950 (95.29)   
137 - **[CGAGCG]** 923 (88.31)   
137 - **[CCACCG]** 876 (88.59)   
137 - **.CGCCCG.** 741 (95.73)   
138 + **[CGAGCG]** 52 (88.31)   
138 + **.CGACCG.** 109 (100.00)   
138 + **.CGGCCG.** 227 (95.29)   
138 + **[GGACCG]** 420 (87.21)   
138 + **.CGCCCG.** 477 (95.73)   
138 + **[CGAGCG]** 584 (88.31)   
138 + **[CGAGCG]** 595 (88.31)   
138 + **.CGGCCG.** 613 (95.29)   
138 + **[CGAGCG]** 636 (88.31)   
138 + **[CGAGCG]** 735 (88.31)   
138 + **[CGAGCG]** 782 (88.31)   
138 - **.CGGCCG.** 613 (95.29)   
138 - **.CGCCCG.** 606 (95.73)   
138 - **[CGAGCG]** 443 (88.31)   
138 - **[GGACCG]** 252 (87.21)   
138 - **.CGGCCG.** 227 (95.29)   
138 - **[GGACCG]** 170 (87.21)   
139 + **.CGGCCG.** 975 (95.29)   
139 + **[GGACCG]** 1378 (87.21)   
139 + **[CGAGCG]** 1501 (88.31)   
139 - **.CGCCCG.** 1382 (95.73)   
139 - **[CGTCCG]** 1329 (88.90)   
139 - **.CGGCCG.** 975 (95.29)   
139 - **[CCACCG]** 904 (88.59)   
139 - **[GGACCG]** 755 (87.21)   
139 - **[CGAGCG]** 654 (88.31)   
139 - **.CGACCG.** 615 (100.00)   
140 + **[GGACCG]** 628 (87.21)   
140 + **[CGACCC]** 637 (88.16)   
140 + **.CGGCCG.** 680 (95.29)   
140 + **.CGGCCG.** 704 (95.29)   
140 + **.CGGCCG.** 741 (95.29)   
140 + **.CGCCCG.** 745 (95.73)   
140 + **.CGGCCG.** 1188 (95.29)   
140 + **[CGAGCG]** 1259 (88.31)   
140 + **[CGAGCG]** 1334 (88.31)   
140 + **[CGACCC]** 1415 (88.16)   
140 + **[CGACCC]** 1425 (88.16)   
140 + **.CGGCCG.** 1471 (95.29)   
140 + **[CGAGCG]** 1498 (88.31)   
140 - **.CGCCCG.** 1494 (95.73)   
140 - **.CGGCCG.** 1471 (95.29)   
140 - **.CGGCCG.** 1188 (95.29)   
140 - **.CGGCCG.** 741 (95.29)   
140 - **.CGGCCG.** 704 (95.29)   
140 - **.CGGCCG.** 680 (95.29)   
141 + **.CGGCCG.** 584 (95.29)   
141 + **[CGAGCG]** 622 (88.31)   
141 + **.CGCCCG.** 654 (95.73)   
141 + **.CGGCCG.** 686 (95.29)   
141 + **.CGGCCG.** 719 (95.29)   
141 + **[CGAGCG]** 772 (88.31)   
141 + **[CCACCG]** 797 (88.59)   
141 + **.CGCCCG.** 857 (95.73)   
141 + **[GGACCG]** 910 (87.21)   
141 + **.CGACCG.** 1101 (100.00)   
141 + **.CGGCCG.** 1346 (95.29)   
141 - **.CGGCCG.** 1346 (95.29)   
141 - **.CGCCCG.** 1267 (95.73)   
141 - **[CGAGCG]** 1237 (88.31)   
141 - **[GGACCG]** 1232 (87.21)   
141 - **.CGGCCG.** 719 (95.29)   
141 - **.CGGCCG.** 686 (95.29)   
141 - **.CGCCCG.** 636 (95.73)   
141 - **[CCACCG]** 602 (88.59)   
141 - **.CGGCCG.** 584 (95.29)   
141 - **.CGCCCG.** 558 (95.73)   
141 - **[CGTCCG]** 554 (88.90)   
141 - **.CGCCCG.** 493 (95.73)   
141 - **.CGCCCG.** 468 (95.73)   
141 - **.CGCCCG.** 191 (95.73)   
142 + **.CGGCCG.** 16 (95.29)   
142 + **.CGCCCG.** 33 (95.73)   
142 + **[CGACCC]** 101 (88.16)   
142 + **[CGTCCG]** 169 (88.90)   
142 + **.CGGCCG.** 314 (95.29)   
142 + **.CGCCCG.** 340 (95.73)   
142 + **.CGCCCG.** 680 (95.73)   
142 + **[CGACCC]** 684 (88.16)   
142 + **.CGCCCG.** 744 (95.73)   
142 + **.CGCCCG.** 748 (95.73)   
142 + **[CCACCG]** 851 (88.59)   
142 + **[CGAGCG]** 1110 (88.31)   
142 + **.CGGCCG.** 1202 (95.29)   
142 + **[CGAGCG]** 1404 (88.31)   
142 + **[CGACCC]** 1439 (88.16)   
142 + **.CGGCCG.** 1451 (95.29)   
142 - **.CGGCCG.** 1451 (95.29)   
142 - **[CGAGCG]** 1432 (88.31)   
142 - **.CGCCCG.** 1428 (95.73)   
142 - **[CGACCC]** 1351 (88.16)   
142 - **.CGGCCG.** 1202 (95.29)   
142 - **[CGACCC]** 1099 (88.16)   
142 - **.CGCCCG.** 799 (95.73)   
142 - **.CGCCCG.** 676 (95.73)   
142 - **[CCACCG]** 560 (88.59)   
142 - **.CGCCCG.** 442 (95.73)   
142 - **[GGACCG]** 351 (87.21)   
142 - **.CGGCCG.** 314 (95.29)   
142 - **.CGGCCG.** 16 (95.29)   
143 + **[CCACCG]** 940 (88.59)   
143 - **[GGACCG]** 1502 (87.21)   
144 + **[CGAGCG]** 8 (88.31)   
144 + **[CCACCG]** 46 (88.59)   
144 + **[CCACCG]** 83 (88.59)   
144 + **[CGTCCG]** 134 (88.90)   
144 + **[GGACCG]** 139 (87.21)   
144 + **.CGCCCG.** 155 (95.73)   
144 + **.CGCCCG.** 196 (95.73)   
144 + **.CGCCCG.** 216 (95.73)   
144 + **[CGTCCG]** 220 (88.90)   
144 + **.CGCCCG.** 317 (95.73)   
144 + **.CGCCCG.** 338 (95.73)   
144 + **.CGCCCG.** 382 (95.73)   
144 + **[GGACCG]** 412 (87.21)   
144 + **[CCACCG]** 505 (88.59)   
144 + **.CGCCCG.** 509 (95.73)   
144 + **.CGCCCG.** 529 (95.73)   
144 + **.CGCCCG.** 549 (95.73)   
144 + **.CGCCCG.** 592 (95.73)   
144 + **.CGCCCG.** 602 (95.73)   
144 + **[CGTCCG]** 606 (88.90)   
144 + **[GGACCG]** 611 (87.21)   
144 + **.CGCCCG.** 667 (95.73)   
144 + **[CGTCCG]** 671 (88.90)   
144 + **.CGCCCG.** 711 (95.73)   
144 + **.CGCCCG.** 752 (95.73)   
144 + **[CGTCCG]** 756 (88.90)   
144 + **[CGTCCG]** 776 (88.90)   
144 + **[CGTCCG]** 841 (88.90)   
144 + **[CCACCG]** 904 (88.59)   
144 + **.CGGCCG.** 1120 (95.29)   
144 + **.CGGCCG.** 1196 (95.29)   
144 + **.CGGCCG.** 1233 (95.29)   
144 + **.CGGCCG.** 1350 (95.29)   
144 + **.CGACCG.** 1437 (100.00)   
144 + **[CGACCC]** 1474 (88.16)   
144 + **.CGCCCG.** 1481 (95.73)   
144 - **.CGGCCG.** 1350 (95.29)   
144 - **.CGCCCG.** 1346 (95.73)   
144 - **.CGGCCG.** 1233 (95.29)   
144 - **.CGGCCG.** 1196 (95.29)   
144 - **.CGGCCG.** 1120 (95.29)   
144 - **.CGCCCG.** 1109 (95.73)   
144 - **.CGCCCG.** 1102 (95.73)   
144 - **.CGCCCG.** 1095 (95.73)   
144 - **.CGCCCG.** 1088 (95.73)   
144 - **.CGCCCG.** 1078 (95.73)   
144 - **.CGCCCG.** 1067 (95.73)   
144 - **.CGCCCG.** 1044 (95.73)   
144 - **[GGACCG]** 87 (87.21)   
145 + **.CGGCCG.** 1086 (95.29)   
145 + **[CCACCG]** 1288 (88.59)   
145 + **[CCACCG]** 1432 (88.59)   
145 - **.CGCCCG.** 1382 (95.73)   
145 - **.CGCCCG.** 1361 (95.73)   
145 - **.CGCCCG.** 1146 (95.73)   
145 - **.CGACCG.** 1094 (100.00)   
145 - **.CGCCCG.** 1090 (95.73)   
145 - **.CGGCCG.** 1086 (95.29)   
145 - **[GGACCG]** 1065 (87.21)   
145 - **.CGCCCG.** 675 (95.73)   
145 - **.CGCCCG.** 597 (95.73)   
145 - **[CCACCG]** 416 (88.59)   
145 - **[CGTCCG]** 268 (88.90)   
146 + **.CGCCCG.** 453 (95.73)   
146 + **.CGCCCG.** 1333 (95.73)   
147 + **.CGCCCG.** 702 (95.73)   
147 + **[CCACCG]** 740 (88.59)   
147 + **.CGGCCG.** 824 (95.29)   
147 + **.CGACCG.** 1073 (100.00)   
147 + **[CGAGCG]** 1085 (88.31)   
147 + **[CGACCC]** 1102 (88.16)   
147 + **.CGGCCG.** 1148 (95.29)   
147 - **[GGACCG]** 1460 (87.21)   
147 - **[CGACCC]** 1269 (88.16)   
147 - **[CGACCC]** 1262 (88.16)   
147 - **.CGGCCG.** 1148 (95.29)   
147 - **[CGAGCG]** 948 (88.31)   
147 - **[CGAGCG]** 831 (88.31)   
147 - **.CGGCCG.** 824 (95.29)   
148 + **[CGACCC]** 341 (88.16)   
148 + **[CGTCCG]** 1018 (88.90)   
148 - **[CGTCCG]** 1489 (88.90)   
150 + **.CGCCCG.** 96 (95.73)   
150 + **.CGCCCG.** 125 (95.73)   
150 + **.CGACCG.** 171 (100.00)   
150 + **.CGCCCG.** 201 (95.73)   
150 + **.CGACCG.** 716 (100.00)   
150 + **[CGTCCG]** 1116 (88.90)   
150 - **[CGTCCG]** 1439 (88.90)   
150 - **[CGAGCG]** 1141 (88.31)   
150 - **[CCACCG]** 269 (88.59)   
150 - **[CCACCG]** 205 (88.59)   
150 - **.CGCCCG.** 88 (95.73)   
150 - **.CGCCCG.** 62 (95.73)   
151 + **[CCACCG]** 737 (88.59)   
151 + **[CCACCG]** 800 (88.59)   
151 + **.CGCCCG.** 918 (95.73)   
151 + **.CGCCCG.** 943 (95.73)   
151 + **[CGACCC]** 1065 (88.16)   
151 + **[CGTCCG]** 1475 (88.90)   
151 + **.CGGCCG.** 1498 (95.29)   
151 - **.CGGCCG.** 1498 (95.29)   
151 - **[CGACCC]** 1432 (88.16)   
151 - **[CCACCG]** 1243 (88.59)   
151 - **.CGCCCG.** 1239 (95.73)   
151 - **[CGACCC]** 1235 (88.16)   
151 - **[CGAGCG]** 1121 (88.31)   
151 - **[CCACCG]** 1095 (88.59)   
151 - **.CGCCCG.** 910 (95.73)   
151 - **.CGCCCG.** 880 (95.73)   
151 - **.CGACCG.** 835 (100.00)   
151 - **.CGCCCG.** 804 (95.73)   
151 - **.CGACCG.** 289 (100.00)   
153 - **[CCACCG]** 388 (88.59)   
153 - **.CGCCCG.** 382 (95.73)   
154 + **[GGACCG]** 216 (87.21)   
154 + **.CGCCCG.** 1067 (95.73)   
154 + **.CGCCCG.** 1145 (95.73)   
154 + **.CGCCCG.** 1202 (95.73)   
154 + **.CGCCCG.** 1208 (95.73)   
154 + **[CGAGCG]** 1232 (88.31)   
154 + **.CGCCCG.** 1255 (95.73)   
154 + **[CGAGCG]** 1259 (88.31)   
154 + **[CGAGCG]** 1269 (88.31)   
154 + **[CGTCCG]** 1286 (88.90)   
154 - **[CGAGCG]** 1138 (88.31)   
154 - **.CGCCCG.** 1036 (95.73)   
154 - **.CGCCCG.** 1027 (95.73)   
154 - **.CGCCCG.** 863 (95.73)   
154 - **.CGCCCG.** 853 (95.73)   
154 - **.CGCCCG.** 784 (95.73)   
154 - **[CGACCC]** 747 (88.16)   
154 - **[CGACCC]** 99 (88.16)   
155 + **.CGGCCG.** 1007 (95.29)   
155 - **[CCACCG]** 1184 (88.59)   
155 - **.CGGCCG.** 1007 (95.29)   
155 - **.CGCCCG.** 642 (95.73)   
155 - **[CCACCG]** 511 (88.59)   
156 + **[CGTCCG]** 973 (88.90)   
157 + **[CCACCG]** 910 (88.59)   
157 + **[CCACCG]** 1124 (88.59)   
158 + **.CGCCCG.** 474 (95.73)   
158 + **[CCACCG]** 518 (88.59)   
158 + **[GGACCG]** 1169 (87.21)   
158 - **[CCACCG]** 1078 (88.59)   
160 + **.CGCCCG.** 104 (95.73)   
160 + **.CGACCG.** 176 (100.00)   
160 + **[CGAGCG]** 606 (88.31)   
160 + **[CGTCCG]** 638 (88.90)   
160 + **[CGTCCG]** 694 (88.90)   
160 + **.CGGCCG.** 1034 (95.29)   
160 + **.CGGCCG.** 1077 (95.29)   
160 + **.CGCCCG.** 1104 (95.73)   
160 + **[CGTCCG]** 1233 (88.90)   
160 + **.CGGCCG.** 1250 (95.29)   
160 + **.CGGCCG.** 1302 (95.29)   
160 - **.CGGCCG.** 1302 (95.29)   
160 - **.CGGCCG.** 1250 (95.29)   
160 - **.CGGCCG.** 1077 (95.29)   
160 - **.CGGCCG.** 1034 (95.29)   
160 - **.CGCCCG.** 941 (95.73)   
160 - **[CGACCC]** 778 (88.16)   
161 + **[CCACCG]** 450 (88.59)   
161 + **[CCACCG]** 625 (88.59)   
161 + **.CGACCG.** 756 (100.00)   
161 + **.CGGCCG.** 992 (95.29)   
161 + **.CGCCCG.** 1020 (95.73)   
161 + **.CGCCCG.** 1193 (95.73)   
161 - **.CGCCCG.** 1462 (95.73)   
161 - **.CGACCG.** 1217 (100.00)   
161 - **[CGAGCG]** 1181 (88.31)   
161 - **.CGGCCG.** 992 (95.29)   
161 - **[CCACCG]** 977 (88.59)   
161 - **[CCACCG]** 834 (88.59)   
161 - **.CGACCG.** 801 (100.00)   
161 - **[CGACCC]** 794 (88.16)   
161 - **.CGCCCG.** 707 (95.73)   
161 - **.CGCCCG.** 631 (95.73)   
161 - **[CGAGCG]** 618 (88.31)   
161 - **[CGACCC]** 505 (88.16)   
161 - **[GGACCG]** 411 (87.21)   
162 - **[CGTCCG]** 1145 (88.90)   
164 + **[GGACCG]** 203 (87.21)   
164 + **[CGAGCG]** 219 (88.31)   
164 + **.CGGCCG.** 548 (95.29)   
164 + **[CGAGCG]** 584 (88.31)   
164 + **.CGCCCG.** 1182 (95.73)   
164 + **[CGAGCG]** 1317 (88.31)   
164 + **.CGCCCG.** 1398 (95.73)   
164 - **[CCACCG]** 1267 (88.59)   
164 - **.CGCCCG.** 1207 (95.73)   
164 - **[CCACCG]** 869 (88.59)   
164 - **.CGGCCG.** 548 (95.29)   
164 - **[CCACCG]** 311 (88.59)   
165 + **[CGAGCG]** 616 (88.31)   
165 - **[CCACCG]** 573 (88.59)   
166 + **.CGGCCG.** 544 (95.29)   
166 + **[CCACCG]** 1010 (88.59)   
166 + **.CGCCCG.** 1207 (95.73)   
166 + **.CGGCCG.** 1231 (95.29)   
166 - **.CGGCCG.** 1231 (95.29)   
166 - **.CGCCCG.** 1063 (95.73)   
166 - **.CGCCCG.** 1059 (95.73)   
166 - **[CGAGCG]** 843 (88.31)   
166 - **[GGACCG]** 833 (87.21)   
166 - **[CGTCCG]** 829 (88.90)   
166 - **.CGCCCG.** 804 (95.73)   
166 - **[CGAGCG]** 775 (88.31)   
166 - **.CGGCCG.** 544 (95.29)   
166 - **[CCACCG]** 439 (88.59)   
167 + **[CGACCC]** 787 (88.16)   
167 + **.CGGCCG.** 861 (95.29)   
167 + **.CGCCCG.** 869 (95.73)   
167 + **[CGAGCG]** 1224 (88.31)   
167 + **[GGACCG]** 1239 (87.21)   
167 + **[CGAGCG]** 1243 (88.31)   
167 + **.CGGCCG.** 1470 (95.29)   
167 + **.CGGCCG.** 1474 (95.29)   
167 - **.CGGCCG.** 1474 (95.29)   
167 - **.CGGCCG.** 1470 (95.29)   
167 - **.CGCCCG.** 1348 (95.73)   
167 - **.CGACCG.** 1326 (100.00)   
167 - **.CGACCG.** 1320 (100.00)   
167 - **.CGCCCG.** 1090 (95.73)   
167 - **.CGCCCG.** 1000 (95.73)   
167 - **.CGCCCG.** 978 (95.73)   
167 - **.CGGCCG.** 861 (95.29)   
167 - **.CGCCCG.** 774 (95.73)   
167 - **[CGAGCG]** 119 (88.31)   
167 - **[CGAGCG]** 87 (88.31)   
168 + **[GGACCG]** 612 (87.21)   
168 + **.CGGCCG.** 677 (95.29)   
168 + **[GGACCG]** 749 (87.21)   
168 + **[CCACCG]** 814 (88.59)   
168 + **[CGTCCG]** 1201 (88.90)   
168 + **.CGGCCG.** 1267 (95.29)   
168 + **.CGCCCG.** 1381 (95.73)   
168 + **.CGGCCG.** 1433 (95.29)   
168 - **.CGGCCG.** 1433 (95.29)   
168 - **.CGGCCG.** 1267 (95.29)   
168 - **[CCACCG]** 1038 (88.59)   
168 - **.CGCCCG.** 997 (95.73)   
168 - **.CGACCG.** 826 (100.00)   
168 - **.CGCCCG.** 787 (95.73)   
168 - **.CGGCCG.** 677 (95.29)   
169 + **[CCACCG]** 510 (88.59)   
169 + **[CGAGCG]** 753 (88.31)   
169 + **[CCACCG]** 899 (88.59)   
169 + **[GGACCG]** 926 (87.21)   
169 - **[GGACCG]** 1228 (87.21)   
169 - **[CCACCG]** 1150 (88.59)   
169 - **.CGCCCG.** 909 (95.73)   
169 - **[CGAGCG]** 735 (88.31)   
169 - **[CGACCC]** 559 (88.16)   
169 - **[CGAGCG]** 435 (88.31)   
170 + **[GGACCG]** 1243 (87.21)   
171 + **[CCACCG]** 754 (88.59)   
171 + **[GGACCG]** 850 (87.21)   
171 - **[GGACCG]** 767 (87.21)   
172 + **[CCACCG]** 55 (88.59)   
172 + **[CGTCCG]** 366 (88.90)   
172 + **.CGCCCG.** 384 (95.73)   
172 + **[CCACCG]** 556 (88.59)   
172 + **[CCACCG]** 585 (88.59)   
172 + **.CGCCCG.** 623 (95.73)   
172 + **.CGCCCG.** 635 (95.73)   
172 + **.CGCCCG.** 658 (95.73)   
172 + **.CGACCG.** 871 (100.00)   
172 + **.CGGCCG.** 1093 (95.29)   
172 - **.CGGCCG.** 1093 (95.29)   
172 - **[CGACCC]** 797 (88.16)   
172 - **[CCACCG]** 772 (88.59)   
173 + **.CGACCG.** 666 (100.00)   
173 + **[CGACCC]** 834 (88.16)   
173 + **[CGACCC]** 894 (88.16)   
173 + **[CGAGCG]** 1033 (88.31)   
173 + **[CGACCC]** 1282 (88.16)   
173 + **[GGACCG]** 1414 (87.21)   
173 - **.CGCCCG.** 1099 (95.73)   
173 - **.CGCCCG.** 965 (95.73)   
173 - **[GGACCG]** 958 (87.21)   
173 - **.CGCCCG.** 784 (95.73)   
173 - **[CGACCC]** 513 (88.16)   
174 + **.CGCCCG.** 351 (95.73)   
174 + **.CGGCCG.** 386 (95.29)   
174 + **[CGACCC]** 418 (88.16)   
174 + **.CGGCCG.** 1038 (95.29)   
174 + **[CGACCC]** 1111 (88.16)   
174 + **.CGGCCG.** 1201 (95.29)   
174 + **[GGACCG]** 1275 (87.21)   
174 + **.CGGCCG.** 1509 (95.29)   
174 - **.CGGCCG.** 1509 (95.29)   
174 - **.CGCCCG.** 1484 (95.73)   
174 - **.CGCCCG.** 1459 (95.73)   
174 - **.CGGCCG.** 1201 (95.29)   
174 - **.CGCCCG.** 1166 (95.73)   
174 - **.CGCCCG.** 1155 (95.73)   
174 - **.CGGCCG.** 1038 (95.29)   
174 - **.CGCCCG.** 394 (95.73)   
174 - **[CGTCCG]** 390 (88.90)   
174 - **.CGGCCG.** 386 (95.29)   
175 - **[CCACCG]** 1248 (88.59)   
177 + **[CCACCG]** 224 (88.59)   
177 + **[CCACCG]** 1181 (88.59)   
177 + **.CGACCG.** 1470 (100.00)   
177 - **[CGAGCG]** 1238 (88.31)   
177 - **[GGACCG]** 1082 (87.21)   
177 - **[CGAGCG]** 838 (88.31)   
178 + **[CGACCC]** 188 (88.16)   
178 + **.CGGCCG.** 1271 (95.29)   
178 + **.CGGCCG.** 1448 (95.29)   
178 - **.CGGCCG.** 1448 (95.29)   
178 - **.CGGCCG.** 1271 (95.29)   
178 - **[GGACCG]** 976 (87.21)   
179 + **.CGGCCG.** 920 (95.29)   
179 - **.CGCCCG.** 966 (95.73)   
179 - **.CGCCCG.** 939 (95.73)   
179 - **[GGACCG]** 930 (87.21)   
179 - **.CGGCCG.** 920 (95.29)   
180 + **.CGGCCG.** 354 (95.29)   
180 + **.CGGCCG.** 869 (95.29)   
180 + **[CCACCG]** 931 (88.59)   
180 + **[CGAGCG]** 965 (88.31)   
180 + **.CGGCCG.** 1161 (95.29)   
180 + **[CGTCCG]** 1283 (88.90)   
180 + **[CCACCG]** 1384 (88.59)   
180 - **[CGAGCG]** 1243 (88.31)   
180 - **.CGGCCG.** 1161 (95.29)   
180 - **.CGGCCG.** 869 (95.29)   
180 - **[CGTCCG]** 865 (88.90)   
180 - **[CGACCC]** 733 (88.16)   
180 - **.CGACCG.** 571 (100.00)   
180 - **.CGCCCG.** 561 (95.73)   
180 - **[CGACCC]** 392 (88.16)   
180 - **.CGGCCG.** 354 (95.29)   
182 + **.CGCCCG.** 248 (95.73)   
182 + **.CGGCCG.** 459 (95.29)   
182 + **[CCACCG]** 730 (88.59)   
182 + **.CGCCCG.** 780 (95.73)   
182 + **[CGTCCG]** 1023 (88.90)   
182 + **.CGCCCG.** 1115 (95.73)   
182 + **[CGAGCG]** 1238 (88.31)   
182 + **[CGTCCG]** 1287 (88.90)   
182 - **[CGTCCG]** 1513 (88.90)   
182 - **.CGACCG.** 1334 (100.00)   
182 - **.CGCCCG.** 1136 (95.73)   
182 - **.CGCCCG.** 1100 (95.73)   
182 - **.CGCCCG.** 992 (95.73)   
182 - **[CGACCC]** 916 (88.16)   
182 - **.CGCCCG.** 626 (95.73)   
182 - **[CGAGCG]** 561 (88.31)   
182 - **[CGACCC]** 536 (88.16)   
182 - **.CGGCCG.** 459 (95.29)   
182 - **.CGCCCG.** 430 (95.73)   
182 - **.CGCCCG.** 384 (95.73)   
182 - **[CGAGCG]** 304 (88.31)   
182 - **[CGAGCG]** 286 (88.31)   
182 - **.CGACCG.** 125 (100.00)   
183 + **[CGTCCG]** 427 (88.90)   
183 + **[CGACCC]** 713 (88.16)   
183 + **.CGGCCG.** 761 (95.29)   
183 + **.CGGCCG.** 1066 (95.29)   
183 + **[CGAGCG]** 1135 (88.31)   
183 + **[CGAGCG]** 1258 (88.31)   
183 + **[CGAGCG]** 1267 (88.31)   
183 + **.CGGCCG.** 1290 (95.29)   
183 + **.CGGCCG.** 1297 (95.29)   
183 + **[CGAGCG]** 1404 (88.31)   
183 - **[CCACCG]** 1490 (88.59)   
183 - **.CGCCCG.** 1319 (95.73)   
183 - **.CGGCCG.** 1297 (95.29)   
183 - **.CGGCCG.** 1290 (95.29)   
183 - **.CGCCCG.** 1139 (95.73)   
183 - **.CGCCCG.** 1126 (95.73)   
183 - **.CGGCCG.** 1066 (95.29)   
183 - **.CGCCCG.** 1013 (95.73)   
183 - **.CGCCCG.** 778 (95.73)   
183 - **.CGCCCG.** 774 (95.73)   
183 - **.CGGCCG.** 761 (95.29)   
183 - **[GGACCG]** 445 (87.21)   
184 + **[CGACCC]** 225 (88.16)   
184 + **.CGGCCG.** 427 (95.29)   
184 + **[CGACCC]** 463 (88.16)   
184 + **.CGCCCG.** 506 (95.73)   
184 + **.CGCCCG.** 510 (95.73)   
184 + **.CGCCCG.** 530 (95.73)   
184 + **[CGAGCG]** 708 (88.31)   
184 + **.CGCCCG.** 821 (95.73)   
184 + **.CGCCCG.** 825 (95.73)   
184 + **.CGCCCG.** 882 (95.73)   
184 + **.CGGCCG.** 916 (95.29)   
184 - **.CGCCCG.** 1299 (95.73)   
184 - **.CGCCCG.** 1235 (95.73)   
184 - **[CGAGCG]** 1185 (88.31)   
184 - **[CGACCC]** 1115 (88.16)   
184 - **.CGCCCG.** 1098 (95.73)   
184 - **.CGGCCG.** 916 (95.29)   
184 - **[CGAGCG]** 906 (88.31)   
184 - **.CGCCCG.** 878 (95.73)   
184 - **.CGGCCG.** 427 (95.29)   
184 - **.CGCCCG.** 65 (95.73)   
185 + **.CGCCCG.** 11 (95.73)   
185 + **.CGGCCG.** 74 (95.29)   
185 + **[CGAGCG]** 309 (88.31)   
185 + **.CGCCCG.** 393 (95.73)   
185 + **[CGAGCG]** 427 (88.31)   
185 + **.CGCCCG.** 529 (95.73)   
185 + **[CGAGCG]** 790 (88.31)   
185 + **[GGACCG]** 1016 (87.21)   
185 + **.CGCCCG.** 1030 (95.73)   
185 + **[CCACCG]** 1123 (88.59)   
185 + **[CCACCG]** 1224 (88.59)   
185 - **.CGCCCG.** 1054 (95.73)   
185 - **[CGAGCG]** 933 (88.31)   
185 - **[CGACCC]** 178 (88.16)   
185 - **.CGGCCG.** 74 (95.29)   
185 - **.CGCCCG.** 70 (95.73)   
186 + **[GGACCG]** 172 (87.21)   
186 + **.CGCCCG.** 1242 (95.73)   
186 - **[CCACCG]** 1056 (88.59)   
187 + **[CCACCG]** 380 (88.59)   
187 + **[CGAGCG]** 597 (88.31)   
187 + **[CGAGCG]** 601 (88.31)   
187 + **[GGACCG]** 658 (87.21)   
187 + **.CGCCCG.** 846 (95.73)   
187 + **.CGGCCG.** 950 (95.29)   
187 + **[GGACCG]** 979 (87.21)   
187 - **[CCACCG]** 1504 (88.59)   
187 - **.CGACCG.** 1233 (100.00)   
187 - **.CGCCCG.** 1084 (95.73)   
187 - **[CGACCC]** 1072 (88.16)   
187 - **.CGCCCG.** 1025 (95.73)   
187 - **.CGGCCG.** 950 (95.29)   
187 - **[CGAGCG]** 859 (88.31)   
187 - **[GGACCG]** 840 (87.21)   
187 - **[CCACCG]** 231 (88.59)   
188 + **.CGCCCG.** 310 (95.73)   
188 + **.CGCCCG.** 1477 (95.73)   
188 + **.CGGCCG.** 1501 (95.29)   
188 - **.CGGCCG.** 1501 (95.29)   
188 - **[CGACCC]** 1452 (88.16)   
188 - **[CCACCG]** 855 (88.59)   
188 - **[CGAGCG]** 261 (88.31)   
189 + **[GGACCG]** 823 (87.21)   
189 - **[CGTCCG]** 1350 (88.90)   
189 - **[CGTCCG]** 1196 (88.90)   
191 + **.CGGCCG.** 571 (95.29)   
191 + **.CGCCCG.** 620 (95.73)   
191 + **[CGAGCG]** 746 (88.31)   
191 + **[GGACCG]** 751 (87.21)   
191 + **.CGGCCG.** 829 (95.29)   
191 + **.CGGCCG.** 885 (95.29)   
191 + **.CGCCCG.** 898 (95.73)   
191 + **[CGAGCG]** 976 (88.31)   
191 + **.CGGCCG.** 1193 (95.29)   
191 + **[CGTCCG]** 1360 (88.90)   
191 - **.CGCCCG.** 1364 (95.73)   
191 - **[CGTCCG]** 1279 (88.90)   
191 - **.CGGCCG.** 1193 (95.29)   
191 - **.CGACCG.** 1122 (100.00)   
191 - **[CGAGCG]** 1106 (88.31)   
191 - **.CGCCCG.** 1076 (95.73)   
191 - **[CCACCG]** 1063 (88.59)   
191 - **.CGCCCG.** 1001 (95.73)   
191 - **.CGCCCG.** 894 (95.73)   
191 - **.CGGCCG.** 885 (95.29)   
191 - **.CGGCCG.** 829 (95.29)   
191 - **[CGACCC]** 816 (88.16)   
191 - **[CGTCCG]** 664 (88.90)   
191 - **.CGGCCG.** 571 (95.29)   
193 + **[GGACCG]** 426 (87.21)   
193 + **[CGAGCG]** 754 (88.31)   
193 + **.CGGCCG.** 796 (95.29)   
193 + **.CGGCCG.** 824 (95.29)   
193 + **[CGAGCG]** 1034 (88.31)   
193 + **.CGCCCG.** 1371 (95.73)   
193 - **.CGGCCG.** 824 (95.29)   
193 - **.CGGCCG.** 796 (95.29)   
193 - **[GGACCG]** 696 (87.21)   
194 + **[CCACCG]** 187 (88.59)   
194 + **[CCACCG]** 367 (88.59)   
194 + **.CGCCCG.** 743 (95.73)   
194 + **.CGGCCG.** 747 (95.29)   
194 + **[CGAGCG]** 923 (88.31)   
194 + **[GGACCG]** 1195 (87.21)   
194 + **.CGCCCG.** 1228 (95.73)   
194 + **[CCACCG]** 1466 (88.59)   
194 - **[CGACCC]** 1316 (88.16)   
194 - **[CGTCCG]** 1044 (88.90)   
194 - **[CCACCG]** 841 (88.59)   
194 - **.CGCCCG.** 816 (95.73)   
194 - **.CGGCCG.** 747 (95.29)   
195 + **[CGTCCG]** 106 (88.90)   
195 + **[CGAGCG]** 269 (88.31)   
195 + **.CGCCCG.** 451 (95.73)   
195 + **.CGCCCG.** 857 (95.73)   
195 + **.CGGCCG.** 872 (95.29)   
195 + **.CGGCCG.** 899 (95.29)   
195 + **.CGCCCG.** 948 (95.73)   
195 + **[CGTCCG]** 962 (88.90)   
195 + **.CGGCCG.** 977 (95.29)   
195 + **.CGGCCG.** 1022 (95.29)   
195 + **.CGGCCG.** 1071 (95.29)   
195 - **[CGACCC]** 1260 (88.16)   
195 - **[CGACCC]** 1176 (88.16)   
195 - **.CGGCCG.** 1071 (95.29)   
195 - **.CGGCCG.** 1022 (95.29)   
195 - **.CGGCCG.** 977 (95.29)   
195 - **.CGGCCG.** 899 (95.29)   
195 - **[GGACCG]** 882 (87.21)   
195 - **.CGGCCG.** 872 (95.29)   
195 - **.CGCCCG.** 868 (95.73)   
195 - **.CGCCCG.** 505 (95.73)   
195 - **[CGACCC]** 462 (88.16)   
195 - **.CGCCCG.** 339 (95.73)   
195 - **[CCACCG]** 180 (88.59)   
195 - **[CGACCC]** 176 (88.16)   
195 - **[GGACCG]** 70 (87.21)   
195 - **[CGTCCG]** 2 (88.90)   
196 + **.CGGCCG.** 249 (95.29)   
196 + **.CGGCCG.** 528 (95.29)   
196 + **.CGGCCG.** 540 (95.29)   
196 + **[CGAGCG]** 544 (88.31)   
196 + **.CGCCCG.** 625 (95.73)   
196 + **.CGACCG.** 658 (100.00)   
196 + **[CCACCG]** 705 (88.59)   
196 + **[CCACCG]** 832 (88.59)   
196 + **.CGGCCG.** 939 (95.29)   
196 + **.CGCCCG.** 1072 (95.73)   
196 + **.CGGCCG.** 1096 (95.29)   
196 + **.CGGCCG.** 1100 (95.29)   
196 + **[CGAGCG]** 1363 (88.31)   
196 + **.CGCCCG.** 1413 (95.73)   
196 + **.CGCCCG.** 1445 (95.73)   
196 + **.CGCCCG.** 1449 (95.73)   
196 - **.CGCCCG.** 1370 (95.73)   
196 - **.CGGCCG.** 1100 (95.29)   
196 - **.CGGCCG.** 1096 (95.29)   
196 - **[GGACCG]** 995 (87.21)   
196 - **.CGGCCG.** 939 (95.29)   
196 - **[CGTCCG]** 686 (88.90)   
196 - **.CGCCCG.** 664 (95.73)   
196 - **.CGGCCG.** 540 (95.29)   
196 - **.CGGCCG.** 528 (95.29)   
196 - **.CGCCCG.** 520 (95.73)   
196 - **.CGCCCG.** 387 (95.73)   
196 - **.CGGCCG.** 249 (95.29)   
196 - **[CGTCCG]** 29 (88.90)   
197 + **.CGGCCG.** 656 (95.29)   
197 + **.CGCCCG.** 660 (95.73)   
197 + **[GGACCG]** 1043 (87.21)   
197 + **[CGACCC]** 1151 (88.16)   
197 - **.CGACCG.** 1370 (100.00)   
197 - **[CGAGCG]** 1366 (88.31)   
197 - **[CGACCC]** 1278 (88.16)   
197 - **.CGCCCG.** 1179 (95.73)   
197 - **[CGTCCG]** 1159 (88.90)   
197 - **[CGACCC]** 1115 (88.16)   
197 - **.CGGCCG.** 656 (95.29)   
198 + **.CGGCCG.** 314 (95.29)   
198 + **[GGACCG]** 332 (87.21)   
198 + **[CCACCG]** 744 (88.59)   
198 - **[CCACCG]** 1035 (88.59)   
198 - **[CGACCC]** 994 (88.16)   
198 - **[CCACCG]** 614 (88.59)   
198 - **[CCACCG]** 582 (88.59)   
198 - **.CGCCCG.** 578 (95.73)   
198 - **[CCACCG]** 548 (88.59)   
198 - **.CGCCCG.** 544 (95.73)   
198 - **[CCACCG]** 516 (88.59)   
198 - **.CGCCCG.** 512 (95.73)   
198 - **.CGCCCG.** 479 (95.73)   
198 - **[CCACCG]** 451 (88.59)   
198 - **.CGACCG.** 447 (100.00)   
198 - **.CGGCCG.** 314 (95.29)   
199 + **.CGCCCG.** 413 (95.73)   
199 + **[CGAGCG]** 488 (88.31)   
199 + **[CGTCCG]** 661 (88.90)   
199 + **.CGCCCG.** 763 (95.73)   
199 + **.CGCCCG.** 833 (95.73)   
199 + **.CGGCCG.** 1005 (95.29)   
199 + **.CGCCCG.** 1044 (95.73)   
199 + **.CGCCCG.** 1246 (95.73)   
199 + **.CGGCCG.** 1314 (95.29)   
199 + **[CGTCCG]** 1450 (88.90)   
199 - **[CGAGCG]** 1507 (88.31)   
199 - **[GGACCG]** 1442 (87.21)   
199 - **.CGACCG.** 1427 (100.00)   
199 - **.CGCCCG.** 1338 (95.73)   
199 - **.CGCCCG.** 1318 (95.73)   
199 - **.CGGCCG.** 1314 (95.29)   
199 - **[CGTCCG]** 1310 (88.90)   
199 - **.CGCCCG.** 1068 (95.73)   
199 - **[CGTCCG]** 1040 (88.90)   
199 - **.CGGCCG.** 1005 (95.29)   
199 - **.CGCCCG.** 973 (95.73)   
199 - **[CGTCCG]** 942 (88.90)   
199 - **.CGCCCG.** 936 (95.73)   
199 - **.CGCCCG.** 922 (95.73)   
199 - **.CGCCCG.** 915 (95.73)   
199 - **.CGCCCG.** 904 (95.73)   
199 - **[GGACCG]** 898 (87.21)   
199 - **[CGACCC]** 894 (88.16)   
199 - **[CCACCG]** 723 (88.59)   
199 - **[CGAGCG]** 665 (88.31)   
199 - **[GGACCG]** 461 (87.21)   
200 + **.CGACCG.** 604 (100.00)   
200 + **[CGACCC]** 661 (88.16)   
200 + **.CGCCCG.** 1230 (95.73)   
201 + **.CGGCCG.** 113 (95.29)   
201 + **[CGACCC]** 433 (88.16)   
201 + **.CGCCCG.** 495 (95.73)   
201 + **.CGCCCG.** 608 (95.73)   
201 + **.CGGCCG.** 708 (95.29)   
201 + **.CGCCCG.** 871 (95.73)   
201 + **.CGCCCG.** 998 (95.73)   
201 + **.CGCCCG.** 1016 (95.73)   
201 + **[CGACCC]** 1366 (88.16)   
201 + **.CGCCCG.** 1501 (95.73)   
201 - **[CCACCG]** 1485 (88.59)   
201 - **[CGTCCG]** 1344 (88.90)   
201 - **[GGACCG]** 1110 (87.21)   
201 - **.CGCCCG.** 1090 (95.73)   
201 - **.CGACCG.** 1068 (100.00)   
201 - **.CGCCCG.** 815 (95.73)   
201 - **[CGAGCG]** 721 (88.31)   
201 - **[CCACCG]** 712 (88.59)   
201 - **.CGGCCG.** 708 (95.29)   
201 - **.CGCCCG.** 593 (95.73)   
201 - **.CGACCG.** 444 (100.00)   
201 - **[CGACCC]** 415 (88.16)   
201 - **[GGACCG]** 253 (87.21)   
201 - **.CGGCCG.** 113 (95.29)   
202 + **[CCACCG]** 276 (88.59)   
202 + **.CGCCCG.** 369 (95.73)   
202 + **[CCACCG]** 1128 (88.59)   
202 + **.CGCCCG.** 1342 (95.73)   
202 - **[CCACCG]** 1388 (88.59)   
202 - **.CGCCCG.** 1298 (95.73)   
202 - **[CGAGCG]** 1170 (88.31)   
202 - **.CGCCCG.** 1159 (95.73)   
203 - **[CCACCG]** 605 (88.59)   
203 - **[CGACCC]** 315 (88.16)   
206 + **.CGCCCG.** 860 (95.73)   
207 + **[CGAGCG]** 853 (88.31)   
207 + **[CGACCC]** 904 (88.16)   
207 + **[CGAGCG]** 1060 (88.31)   
207 - **.CGCCCG.** 1333 (95.73)   
208 + **[CGTCCG]** 1175 (88.90)   
208 + **.CGACCG.** 1504 (100.00)   
208 - **[CGAGCG]** 1157 (88.31)   
208 - **[GGACCG]** 847 (87.21)   
210 + **[GGACCG]** 659 (87.21)   
210 + **.CGCCCG.** 1023 (95.73)   
210 + **.CGGCCG.** 1045 (95.29)   
210 + **.CGCCCG.** 1272 (95.73)   
210 + **[CCACCG]** 1422 (88.59)   
210 - **.CGGCCG.** 1045 (95.29)   
210 - **[CCACCG]** 988 (88.59)   
211 + **.CGACCG.** 691 (100.00)   
211 + **.CGGCCG.** 765 (95.29)   
211 + **.CGCCCG.** 779 (95.73)   
211 + **.CGGCCG.** 985 (95.29)   
211 + **.CGCCCG.** 1100 (95.73)   
211 - **.CGCCCG.** 1467 (95.73)   
211 - **.CGACCG.** 1329 (100.00)   
211 - **[CGAGCG]** 1255 (88.31)   
211 - **[CCACCG]** 1206 (88.59)   
211 - **[CGTCCG]** 1172 (88.90)   
211 - **.CGGCCG.** 985 (95.29)   
211 - **.CGGCCG.** 765 (95.29)   
211 - **[CGTCCG]** 761 (88.90)   
212 + **[GGACCG]** 69 (87.21)   
212 + **.CGGCCG.** 617 (95.29)   
212 + **[CGTCCG]** 1030 (88.90)   
212 + **.CGCCCG.** 1309 (95.73)   
212 + **[CGACCC]** 1382 (88.16)   
212 - **[CGACCC]** 738 (88.16)   
212 - **[CGTCCG]** 674 (88.90)   
212 - **.CGGCCG.** 617 (95.29)   
212 - **[GGACCG]** 61 (87.21)   
213 + **[GGACCG]** 1023 (87.21)   
213 + **[CCACCG]** 1484 (88.59)   
213 - **[GGACCG]** 1505 (87.21)   
213 - **[GGACCG]** 255 (87.21)   
214 + **[CGAGCG]** 115 (88.31)   
214 + **[CGAGCG]** 511 (88.31)   
214 + **.CGCCCG.** 683 (95.73)   
214 + **[CGTCCG]** 737 (88.90)   
214 + **[CGTCCG]** 866 (88.90)   
214 + **.CGCCCG.** 870 (95.73)   
214 + **.CGCCCG.** 906 (95.73)   
214 + **.CGGCCG.** 933 (95.29)   
214 + **.CGCCCG.** 946 (95.73)   
214 + **[CCACCG]** 1039 (88.59)   
214 + **.CGCCCG.** 1111 (95.73)   
214 + **[CGAGCG]** 1115 (88.31)   
214 + **[CGTCCG]** 1352 (88.90)   
214 - **[CCACCG]** 1145 (88.59)   
214 - **.CGGCCG.** 933 (95.29)   
214 - **[CCACCG]** 723 (88.59)   
215 - **[CCACCG]** 1285 (88.59)   
215 - **.CGCCCG.** 998 (95.73)   
215 - **.CGACCG.** 974 (100.00)   
215 - **.CGCCCG.** 18 (95.73)   
216 + **[CCACCG]** 547 (88.59)   
216 + **.CGCCCG.** 987 (95.73)   
216 + **[CCACCG]** 1004 (88.59)   
216 + **[CGACCC]** 1061 (88.16)   
216 + **[CGTCCG]** 1072 (88.90)   
216 + **.CGCCCG.** 1076 (95.73)   
216 + **.CGCCCG.** 1080 (95.73)   
216 + **.CGCCCG.** 1084 (95.73)   
216 + **.CGCCCG.** 1088 (95.73)   
216 + **.CGCCCG.** 1092 (95.73)   
216 + **.CGCCCG.** 1247 (95.73)   
216 + **.CGCCCG.** 1263 (95.73)   
216 + **.CGACCG.** 1370 (100.00)   
216 - **[CGAGCG]** 1171 (88.31)   
216 - **[CGAGCG]** 1068 (88.31)   
216 - **[CGAGCG]** 1042 (88.31)   
216 - **[CGACCC]** 97 (88.16)   
217 + **.CGCCCG.** 661 (95.73)   
217 + **.CGGCCG.** 898 (95.29)   
217 + **.CGGCCG.** 997 (95.29)   
217 + **[CGAGCG]** 1117 (88.31)   
217 + **.CGGCCG.** 1167 (95.29)   
217 - **.CGGCCG.** 1167 (95.29)   
217 - **.CGGCCG.** 997 (95.29)   
217 - **.CGGCCG.** 898 (95.29)   
220 + **[CCACCG]** 797 (88.59)   
220 + **[CGACCC]** 1064 (88.16)   
221 + **[CGTCCG]** 22 (88.90)   
221 + **[CCACCG]** 193 (88.59)   
221 + **.CGGCCG.** 1019 (95.29)   
221 + **.CGGCCG.** 1108 (95.29)   
221 + **[CCACCG]** 1303 (88.59)   
221 + **[CCACCG]** 1424 (88.59)   
221 - **.CGGCCG.** 1108 (95.29)   
221 - **[CGAGCG]** 1077 (88.31)   
221 - **.CGGCCG.** 1019 (95.29)   
221 - **[GGACCG]** 734 (87.21)   
221 - **[CCACCG]** 268 (88.59)   
221 - **.CGCCCG.** 5 (95.73)   
222 + **.CGGCCG.** 170 (95.29)   
222 + **.CGGCCG.** 256 (95.29)   
222 + **[CGTCCG]** 454 (88.90)   
222 + **[GGACCG]** 460 (87.21)   
222 + **.CGGCCG.** 514 (95.29)   
222 + **.CGGCCG.** 585 (95.29)   
222 + **.CGGCCG.** 599 (95.29)   
222 + **.CGCCCG.** 669 (95.73)   
222 + **.CGCCCG.** 693 (95.73)   
222 + **.CGCCCG.** 773 (95.73)   
222 + **[CCACCG]** 803 (88.59)   
222 + **.CGCCCG.** 1114 (95.73)   
222 + **.CGGCCG.** 1265 (95.29)   
222 - **.CGGCCG.** 1265 (95.29)   
222 - **.CGACCG.** 1238 (100.00)   
222 - **.CGCCCG.** 1202 (95.73)   
222 - **[CGAGCG]** 922 (88.31)   
222 - **.CGGCCG.** 599 (95.29)   
222 - **.CGGCCG.** 585 (95.29)   
222 - **.CGGCCG.** 514 (95.29)   
222 - **.CGACCG.** 381 (100.00)   
222 - **.CGCCCG.** 368 (95.73)   
222 - **.CGGCCG.** 256 (95.29)   
222 - **.CGGCCG.** 170 (95.29)   
223 + **.CGGCCG.** 311 (95.29)   
223 - **[CGACCC]** 501 (88.16)   
223 - **.CGCCCG.** 347 (95.73)   
223 - **.CGGCCG.** 311 (95.29)   
223 - **.CGACCG.** 108 (100.00)   
223 - **[CGAGCG]** 65 (88.31)   
223 - **[CGAGCG]** 54 (88.31)   
224 + **.CGCCCG.** 448 (95.73)   
224 + **.CGGCCG.** 523 (95.29)   
224 + **.CGGCCG.** 534 (95.29)   
224 + **[CGAGCG]** 757 (88.31)   
224 + **.CGGCCG.** 801 (95.29)   
224 + **[CGACCC]** 1171 (88.16)   
224 + **.CGGCCG.** 1413 (95.29)   
224 - **.CGCCCG.** 1508 (95.73)   
224 - **.CGGCCG.** 1413 (95.29)   
224 - **.CGCCCG.** 1317 (95.73)   
224 - **.CGCCCG.** 1030 (95.73)   
224 - **.CGCCCG.** 818 (95.73)   
224 - **.CGGCCG.** 801 (95.29)   
224 - **.CGCCCG.** 783 (95.73)   
224 - **.CGCCCG.** 765 (95.73)   
224 - **.CGCCCG.** 761 (95.73)   
224 - **[CCACCG]** 746 (88.59)   
224 - **.CGCCCG.** 722 (95.73)   
224 - **.CGCCCG.** 613 (95.73)   
224 - **.CGGCCG.** 534 (95.29)   
224 - **.CGGCCG.** 523 (95.29)   
224 - **[CGAGCG]** 444 (88.31)   
225 + **.CGCCCG.** 1249 (95.73)   
225 - **.CGACCG.** 1287 (100.00)   
225 - **[CCACCG]** 693 (88.59)   
225 - **[CCACCG]** 113 (88.59)   
226 + **.CGGCCG.** 127 (95.29)   
226 + **[CGAGCG]** 186 (88.31)   
226 + **.CGGCCG.** 339 (95.29)   
226 + **[CCACCG]** 507 (88.59)   
226 + **.CGGCCG.** 516 (95.29)   
226 + **.CGCCCG.** 559 (95.73)   
226 + **[CGTCCG]** 656 (88.90)   
226 + **[CGAGCG]** 749 (88.31)   
226 + **.CGGCCG.** 954 (95.29)   
226 + **[GGACCG]** 1002 (87.21)   
226 + **[CGTCCG]** 1067 (88.90)   
226 + **.CGACCG.** 1091 (100.00)   
226 + **.CGCCCG.** 1119 (95.73)   
226 + **.CGCCCG.** 1123 (95.73)   
226 + **.CGCCCG.** 1127 (95.73)   
226 + **.CGCCCG.** 1148 (95.73)   
226 + **.CGCCCG.** 1152 (95.73)   
226 + **.CGCCCG.** 1191 (95.73)   
226 + **.CGCCCG.** 1195 (95.73)   
226 + **.CGCCCG.** 1301 (95.73)   
226 + **.CGGCCG.** 1399 (95.29)   
226 + **.CGCCCG.** 1414 (95.73)   
226 - **.CGCCCG.** 1502 (95.73)   
226 - **[CGAGCG]** 1447 (88.31)   
226 - **.CGGCCG.** 1399 (95.29)   
226 - **.CGCCCG.** 1305 (95.73)   
226 - **.CGGCCG.** 954 (95.29)   
226 - **.CGCCCG.** 757 (95.73)   
226 - **.CGCCCG.** 687 (95.73)   
226 - **.CGGCCG.** 516 (95.29)   
226 - **.CGGCCG.** 339 (95.29)   
226 - **.CGGCCG.** 127 (95.29)   
227 + **[CGACCC]** 19 (88.16)   
227 + **[CCACCG]** 32 (88.59)   
227 + **[GGACCG]** 285 (87.21)   
227 + **.CGGCCG.** 372 (95.29)   
227 + **.CGCCCG.** 750 (95.73)   
227 + **.CGGCCG.** 754 (95.29)   
227 + **.CGGCCG.** 758 (95.29)   
227 + **.CGCCCG.** 796 (95.73)   
227 + **[GGACCG]** 1205 (87.21)   
227 + **.CGACCG.** 1223 (100.00)   
227 + **.CGCCCG.** 1442 (95.73)   
227 + **.CGGCCG.** 1491 (95.29)   
227 - **.CGGCCG.** 1491 (95.29)   
227 - **[CGAGCG]** 1380 (88.31)   
227 - **.CGCCCG.** 1247 (95.73)   
227 - **[CGAGCG]** 783 (88.31)   
227 - **.CGGCCG.** 758 (95.29)   
227 - **.CGGCCG.** 754 (95.29)   
227 - **[CGAGCG]** 708 (88.31)   
227 - **[CGTCCG]** 613 (88.90)   
227 - **.CGCCCG.** 435 (95.73)   
227 - **.CGGCCG.** 372 (95.29)   
227 - **[CGAGCG]** 15 (88.31)   
228 + **[CGACCC]** 48 (88.16)   
228 + **.CGGCCG.** 109 (95.29)   
228 + **[CGACCC]** 153 (88.16)   
228 + **.CGCCCG.** 277 (95.73)   
228 + **.CGCCCG.** 283 (95.73)   
228 + **.CGCCCG.** 418 (95.73)   
228 + **[CGACCC]** 437 (88.16)   
228 + **.CGCCCG.** 538 (95.73)   
228 + **.CGACCG.** 640 (100.00)   
228 + **[CCACCG]** 1249 (88.59)   
228 + **[GGACCG]** 1257 (87.21)   
228 - **[CGTCCG]** 1261 (88.90)   
228 - **.CGCCCG.** 1158 (95.73)   
228 - **[CGTCCG]** 877 (88.90)   
228 - **.CGCCCG.** 873 (95.73)   
228 - **[CGACCC]** 804 (88.16)   
228 - **.CGCCCG.** 773 (95.73)   
228 - **.CGGCCG.** 109 (95.29)   
229 + **[CCACCG]** 120 (88.59)   
229 + **.CGCCCG.** 126 (95.73)   
229 + **.CGCCCG.** 443 (95.73)   
229 + **.CGCCCG.** 450 (95.73)   
229 + **[CCACCG]** 461 (88.59)   
229 + **.CGGCCG.** 542 (95.29)   
229 + **.CGCCCG.** 933 (95.73)   
229 + **.CGACCG.** 937 (100.00)   
229 + **.CGCCCG.** 1063 (95.73)   
229 + **.CGCCCG.** 1078 (95.73)   
229 + **.CGCCCG.** 1091 (95.73)   
229 + **.CGGCCG.** 1239 (95.29)   
229 - **[GGACCG]** 1476 (87.21)   
229 - **.CGCCCG.** 1406 (95.73)   
229 - **.CGCCCG.** 1387 (95.73)   
229 - **.CGCCCG.** 1359 (95.73)   
229 - **.CGCCCG.** 1314 (95.73)   
229 - **.CGGCCG.** 1239 (95.29)   
229 - **[CGTCCG]** 1118 (88.90)   
229 - **.CGCCCG.** 1034 (95.73)   
229 - **[CGTCCG]** 1030 (88.90)   
229 - **[CCACCG]** 679 (88.59)   
229 - **.CGGCCG.** 542 (95.29)   
229 - **[CCACCG]** 496 (88.59)   
229 - **[CGACCC]** 361 (88.16)   
230 + **[CGACCC]** 294 (88.16)   
230 + **[CGTCCG]** 1135 (88.90)   
230 + **[CGACCC]** 1139 (88.16)   
230 - **.CGCCCG.** 1448 (95.73)   
230 - **.CGCCCG.** 1198 (95.73)   
230 - **.CGACCG.** 960 (100.00)   
231 + **[CGACCC]** 625 (88.16)   
231 + **.CGCCCG.** 830 (95.73)   
231 + **[CGACCC]** 854 (88.16)   
231 + **[CGAGCG]** 894 (88.31)   
231 + **.CGCCCG.** 898 (95.73)   
231 + **.CGGCCG.** 1086 (95.29)   
231 + **[CGAGCG]** 1090 (88.31)   
231 + **[CGAGCG]** 1099 (88.31)   
231 + **[CCACCG]** 1223 (88.59)   
231 + **[GGACCG]** 1343 (87.21)   
231 + **.CGCCCG.** 1437 (95.73)   
231 - **[CGACCC]** 1495 (88.16)   
231 - **.CGCCCG.** 1489 (95.73)   
231 - **.CGGCCG.** 1086 (95.29)   
231 - **[CGACCC]** 696 (88.16)   
232 + **.CGCCCG.** 151 (95.73)   
232 + **.CGCCCG.** 155 (95.73)   
232 + **[CGACCC]** 260 (88.16)   
232 + **.CGGCCG.** 662 (95.29)   
232 + **.CGGCCG.** 750 (95.29)   
232 + **[CCACCG]** 761 (88.59)   
232 + **[CCACCG]** 773 (88.59)   
232 + **.CGGCCG.** 865 (95.29)   
232 + **[CCACCG]** 899 (88.59)   
232 + **[CGAGCG]** 1017 (88.31)   
232 + **.CGACCG.** 1097 (100.00)   
232 + **[CGAGCG]** 1123 (88.31)   
232 - **[CGACCC]** 1451 (88.16)   
232 - **.CGCCCG.** 1203 (95.73)   
232 - **[CGTCCG]** 1117 (88.90)   
232 - **.CGCCCG.** 1008 (95.73)   
232 - **.CGGCCG.** 865 (95.29)   
232 - **.CGGCCG.** 750 (95.29)   
232 - **[CGAGCG]** 731 (88.31)   
232 - **.CGCCCG.** 697 (95.73)   
232 - **.CGGCCG.** 662 (95.29)   
233 + **.CGCCCG.** 1143 (95.73)   
234 + **[GGACCG]** 271 (87.21)   
234 + **[CGACCC]** 581 (88.16)   
234 + **.CGCCCG.** 596 (95.73)   
234 + **.CGGCCG.** 711 (95.29)   
234 + **.CGGCCG.** 736 (95.29)   
234 + **.CGCCCG.** 788 (95.73)   
234 + **.CGCCCG.** 852 (95.73)   
234 + **.CGCCCG.** 868 (95.73)   
234 + **[CGAGCG]** 1359 (88.31)   
234 + **.CGGCCG.** 1475 (95.29)   
234 - **.CGGCCG.** 1475 (95.29)   
234 - **.CGCCCG.** 1282 (95.73)   
234 - **.CGCCCG.** 1105 (95.73)   
234 - **.CGACCG.** 975 (100.00)   
234 - **.CGCCCG.** 778 (95.73)   
234 - **.CGGCCG.** 736 (95.29)   
234 - **.CGGCCG.** 711 (95.29)   
234 - **.CGCCCG.** 626 (95.73)   
234 - **[CGTCCG]** 589 (88.90)   
234 - **.CGCCCG.** 505 (95.73)   
234 - **[CCACCG]** 350 (88.59)   
234 - **.CGCCCG.** 277 (95.73)   
234 - **.CGCCCG.** 68 (95.73)   
235 + **[CGTCCG]** 1184 (88.90)   
235 + **.CGGCCG.** 1391 (95.29)   
235 + **[CGAGCG]** 1410 (88.31)   
235 - **.CGGCCG.** 1391 (95.29)   
235 - **.CGCCCG.** 787 (95.73)   
235 - **[CGAGCG]** 639 (88.31)   
235 - **.CGACCG.** 387 (100.00)   
235 - **[CCACCG]** 347 (88.59)   
236 + **.CGCCCG.** 223 (95.73)   
236 + **.CGGCCG.** 1152 (95.29)   
236 + **.CGGCCG.** 1289 (95.29)   
236 + **[GGACCG]** 1317 (87.21)   
236 + **[CCACCG]** 1431 (88.59)   
236 - **.CGGCCG.** 1289 (95.29)   
236 - **[CCACCG]** 1255 (88.59)   
236 - **.CGCCCG.** 1209 (95.73)   
236 - **.CGGCCG.** 1152 (95.29)   
238 + **[GGACCG]** 999 (87.21)   
239 + **.CGGCCG.** 539 (95.29)   
239 + **[CGACCC]** 711 (88.16)   
239 + **[CGAGCG]** 811 (88.31)   
239 + **[CCACCG]** 864 (88.59)   
239 + **[CGAGCG]** 1003 (88.31)   
239 - **.CGCCCG.** 1174 (95.73)   
239 - **[CCACCG]** 1159 (88.59)   
239 - **.CGCCCG.** 592 (95.73)   
239 - **.CGGCCG.** 539 (95.29)   
239 - **.CGCCCG.** 124 (95.73)   
240 + **.CGCCCG.** 142 (95.73)   
240 + **.CGGCCG.** 364 (95.29)   
240 + **.CGCCCG.** 580 (95.73)   
240 + **[CCACCG]** 883 (88.59)   
240 + **[CGACCC]** 894 (88.16)   
240 + **.CGGCCG.** 1152 (95.29)   
240 + **.CGACCG.** 1288 (100.00)   
240 + **.CGCCCG.** 1380 (95.73)   
240 - **.CGGCCG.** 1152 (95.29)   
240 - **.CGGCCG.** 364 (95.29)   
241 + **.CGACCG.** 191 (100.00)   
242 + **[GGACCG]** 766 (87.21)   
242 + **.CGACCG.** 770 (100.00)   
242 + **[GGACCG]** 959 (87.21)   
242 + **.CGGCCG.** 1020 (95.29)   
242 + **[CGTCCG]** 1078 (88.90)   
242 + **[CGAGCG]** 1165 (88.31)   
242 - **.CGGCCG.** 1020 (95.29)   
242 - **[CGACCC]** 694 (88.16)   
242 - **[CGTCCG]** 668 (88.90)   
242 - **[CGACCC]** 569 (88.16)   
242 - **[CGACCC]** 523 (88.16)   
243 + **[CCACCG]** 598 (88.59)   
243 + **.CGGCCG.** 922 (95.29)   
243 + **.CGGCCG.** 1220 (95.29)   
243 + **[CGTCCG]** 1278 (88.90)   
243 - **.CGGCCG.** 1220 (95.29)   
243 - **[GGACCG]** 1080 (87.21)   
243 - **.CGGCCG.** 922 (95.29)   
243 - **[GGACCG]** 694 (87.21)   
243 - **.CGACCG.** 59 (100.00)   
244 + **.CGGCCG.** 713 (95.29)   
244 - **[CGTCCG]** 783 (88.90)   
244 - **.CGGCCG.** 713 (95.29)   
244 - **.CGCCCG.** 709 (95.73)   
244 - **.CGCCCG.** 691 (95.73)   
244 - **[CGAGCG]** 519 (88.31)   
246 + **[CCACCG]** 232 (88.59)   
246 + **.CGGCCG.** 292 (95.29)   
246 + **.CGGCCG.** 374 (95.29)   
246 + **[CGAGCG]** 392 (88.31)   
246 + **.CGACCG.** 478 (100.00)   
246 + **[CGAGCG]** 557 (88.31)   
246 + **[CGAGCG]** 611 (88.31)   
246 + **[CGACCC]** 685 (88.16)   
246 + **.CGCCCG.** 701 (95.73)   
246 + **.CGGCCG.** 705 (95.29)   
246 + **.CGACCG.** 736 (100.00)   
246 + **.CGCCCG.** 752 (95.73)   
246 + **.CGCCCG.** 784 (95.73)   
246 + **.CGGCCG.** 816 (95.29)   
246 + **[CGAGCG]** 847 (88.31)   
246 + **[GGACCG]** 966 (87.21)   
246 + **.CGGCCG.** 1076 (95.29)   
246 + **.CGGCCG.** 1110 (95.29)   
246 + **[CGAGCG]** 1142 (88.31)   
246 + **.CGCCCG.** 1146 (95.73)   
246 + **.CGACCG.** 1153 (100.00)   
246 - **.CGCCCG.** 1268 (95.73)   
246 - **.CGGCCG.** 1110 (95.29)   
246 - **.CGGCCG.** 1076 (95.29)   
246 - **.CGCCCG.** 906 (95.73)   
246 - **.CGGCCG.** 816 (95.29)   
246 - **.CGGCCG.** 705 (95.29)   
246 - **.CGCCCG.** 553 (95.73)   
246 - **.CGCCCG.** 493 (95.73)   
246 - **[CGAGCG]** 467 (88.31)   
246 - **.CGACCG.** 449 (100.00)   
246 - **[CGAGCG]** 427 (88.31)   
246 - **.CGGCCG.** 374 (95.29)   
246 - **.CGGCCG.** 292 (95.29)   
246 - **[GGACCG]** 251 (87.21)   
246 - **.CGCCCG.** 102 (95.73)   
247 + **.CGCCCG.** 333 (95.73)   
247 + **[CGTCCG]** 337 (88.90)   
247 + **[CGTCCG]** 480 (88.90)   
247 + **[CCACCG]** 490 (88.59)   
247 + **[CCACCG]** 544 (88.59)   
247 + **.CGGCCG.** 609 (95.29)   
247 + **.CGGCCG.** 1090 (95.29)   
247 + **.CGCCCG.** 1124 (95.73)   
247 + **.CGGCCG.** 1220 (95.29)   
247 + **[GGACCG]** 1423 (87.21)   
247 - **.CGCCCG.** 1483 (95.73)   
247 - **.CGCCCG.** 1283 (95.73)   
247 - **.CGCCCG.** 1279 (95.73)   
247 - **.CGCCCG.** 1270 (95.73)   
247 - **.CGGCCG.** 1220 (95.29)   
247 - **.CGGCCG.** 1090 (95.29)   
247 - **.CGACCG.** 1075 (100.00)   
247 - **[CCACCG]** 830 (88.59)   
247 - **[CGAGCG]** 743 (88.31)   
247 - **.CGGCCG.** 609 (95.29)   
247 - **[GGACCG]** 570 (87.21)   
250 + **.CGACCG.** 610 (100.00)   
250 + **.CGGCCG.** 955 (95.29)   
250 + **[CGAGCG]** 1261 (88.31)   
250 + **[CGAGCG]** 1265 (88.31)   
250 + **[CGAGCG]** 1273 (88.31)   
250 - **.CGCCCG.** 1269 (95.73)   
250 - **.CGCCCG.** 1086 (95.73)   
250 - **.CGCCCG.** 959 (95.73)   
250 - **.CGGCCG.** 955 (95.29)   
252 + **.CGCCCG.** 542 (95.73)   
252 + **[GGACCG]** 964 (87.21)   
252 - **[CGACCC]** 1168 (88.16)   
252 - **[CCACCG]** 1020 (88.59)   
255 + **[GGACCG]** 990 (87.21)   
255 + **.CGCCCG.** 1067 (95.73)   
255 - **[CCACCG]** 1469 (88.59)   
255 - **[CCACCG]** 120 (88.59)   
256 + **[CCACCG]** 95 (88.59)   
256 + **.CGCCCG.** 101 (95.73)   
256 + **.CGCCCG.** 910 (95.73)   
256 + **.CGCCCG.** 1119 (95.73)   
256 + **[CGACCC]** 1126 (88.16)   
256 + **.CGGCCG.** 1193 (95.29)   
256 + **[CGTCCG]** 1276 (88.90)   
256 + **.CGACCG.** 1297 (100.00)   
256 + **[CGTCCG]** 1443 (88.90)   
256 + **[CGTCCG]** 1447 (88.90)   
256 + **[CGACCC]** 1451 (88.16)   
256 - **.CGGCCG.** 1193 (95.29)   
256 - **[CGTCCG]** 1160 (88.90)   
256 - **[CGACCC]** 1098 (88.16)   
257 + **.CGCCCG.** 812 (95.73)   
257 + **[GGACCG]** 966 (87.21)   
257 + **.CGGCCG.** 1164 (95.29)   
257 + **.CGGCCG.** 1192 (95.29)   
257 + **.CGCCCG.** 1241 (95.73)   
257 + **.CGCCCG.** 1249 (95.73)   
257 + **.CGGCCG.** 1280 (95.29)   
257 + **[CGACCC]** 1425 (88.16)   
257 + **.CGGCCG.** 1439 (95.29)   
257 - **.CGCCCG.** 1494 (95.73)   
257 - **.CGCCCG.** 1469 (95.73)   
257 - **.CGGCCG.** 1439 (95.29)   
257 - **.CGGCCG.** 1280 (95.29)   
257 - **.CGGCCG.** 1192 (95.29)   
257 - **[CCACCG]** 1170 (88.59)   
257 - **.CGGCCG.** 1164 (95.29)   
259 + **.CGCCCG.** 820 (95.73)   
259 + **[CCACCG]** 859 (88.59)   
259 + **.CGCCCG.** 1118 (95.73)   
259 + **.CGGCCG.** 1189 (95.29)   
259 + **.CGGCCG.** 1247 (95.29)   
259 - **.CGGCCG.** 1247 (95.29)   
259 - **.CGGCCG.** 1189 (95.29)   
259 - **[CGAGCG]** 1159 (88.31)   
259 - **.CGCCCG.** 925 (95.73)   
259 - **[CCACCG]** 723 (88.59)   
260 + **[CCACCG]** 441 (88.59)   
260 + **.CGGCCG.** 930 (95.29)   
260 + **[CGACCC]** 1120 (88.16)   
260 - **[CGTCCG]** 1248 (88.90)   
260 - **.CGCCCG.** 941 (95.73)   
260 - **.CGGCCG.** 930 (95.29)   
260 - **[CGAGCG]** 793 (88.31)   
260 - **.CGCCCG.** 759 (95.73)   
261 - **.CGCCCG.** 40 (95.73)   
261 - **.CGCCCG.** 28 (95.73)   
262 + **[CGAGCG]** 560 (88.31)   
262 + **.CGGCCG.** 1244 (95.29)   
262 + **.CGGCCG.** 1394 (95.29)   
262 - **[CGTCCG]** 1493 (88.90)   
262 - **.CGGCCG.** 1394 (95.29)   
262 - **.CGGCCG.** 1244 (95.29)   
262 - **[CCACCG]** 840 (88.59)   
263 + **[GGACCG]** 553 (87.21)   
263 + **.CGGCCG.** 852 (95.29)   
263 + **.CGGCCG.** 1009 (95.29)   
263 + **[CCACCG]** 1158 (88.59)   
263 - **[CGACCC]** 1497 (88.16)   
263 - **[CGACCC]** 1479 (88.16)   
263 - **[CGACCC]** 1441 (88.16)   
263 - **.CGCCCG.** 1162 (95.73)   
263 - **.CGACCG.** 1024 (100.00)   
263 - **.CGGCCG.** 1009 (95.29)   
263 - **[CGAGCG]** 1005 (88.31)   
263 - **.CGGCCG.** 852 (95.29)   
263 - **.CGCCCG.** 297 (95.73)   
263 - **.CGCCCG.** 163 (95.73)   
263 - **[CCACCG]** 34 (88.59)   
264 + **[CCACCG]** 576 (88.59)   
264 + **.CGACCG.** 1224 (100.00)   
264 - **.CGCCCG.** 1109 (95.73)   
264 - **.CGCCCG.** 417 (95.73)   
264 - **.CGACCG.** 413 (100.00)   
266 + **.CGGCCG.** 964 (95.29)   
266 + **.CGGCCG.** 1063 (95.29)   
266 + **.CGGCCG.** 1278 (95.29)   
266 - **.CGGCCG.** 1278 (95.29)   
266 - **.CGGCCG.** 1063 (95.29)   
266 - **[CCACCG]** 987 (88.59)   
266 - **.CGGCCG.** 964 (95.29)   
267 + **[GGACCG]** 188 (87.21)   
267 + **.CGGCCG.** 197 (95.29)   
267 + **[CCACCG]** 1488 (88.59)   
267 - **.CGCCCG.** 1441 (95.73)   
267 - **.CGCCCG.** 1304 (95.73)   
267 - **.CGGCCG.** 197 (95.29)   
268 + **[CGTCCG]** 31 (88.90)   
268 + **[CGTCCG]** 378 (88.90)   
268 + **.CGGCCG.** 797 (95.29)   
268 + **[CGAGCG]** 818 (88.31)   
268 + **.CGGCCG.** 844 (95.29)   
268 + **[CGTCCG]** 1146 (88.90)   
268 + **.CGGCCG.** 1352 (95.29)   
268 + **.CGACCG.** 1356 (100.00)   
268 + **[CGTCCG]** 1388 (88.90)   
268 + **.CGGCCG.** 1480 (95.29)   
268 - **.CGGCCG.** 1480 (95.29)   
268 - **.CGGCCG.** 1352 (95.29)   
268 - **.CGCCCG.** 1329 (95.73)   
268 - **.CGCCCG.** 1290 (95.73)   
268 - **.CGCCCG.** 1283 (95.73)   
268 - **.CGCCCG.** 1263 (95.73)   
268 - **.CGGCCG.** 844 (95.29)   
268 - **.CGGCCG.** 797 (95.29)   
268 - **[GGACCG]** 501 (87.21)   
268 - **.CGCCCG.** 382 (95.73)   
268 - **.CGCCCG.** 109 (95.73)   
268 - **[CGTCCG]** 9 (88.90)   
269 + **[CCACCG]** 452 (88.59)   
270 + **.CGGCCG.** 270 (95.29)   
270 + **[GGACCG]** 1363 (87.21)   
270 - **.CGCCCG.** 1297 (95.73)   
270 - **[CGAGCG]** 1065 (88.31)   
270 - **[CGACCC]** 1061 (88.16)   
270 - **[CGAGCG]** 452 (88.31)   
270 - **.CGGCCG.** 270 (95.29)   
270 - **[CGAGCG]** 256 (88.31)   
270 - **[GGACCG]** 163 (87.21)   
271 - **[CGACCC]** 1121 (88.16)   
271 - **[CGTCCG]** 740 (88.90)   
272 - **[CGACCC]** 1112 (88.16)   
273 + **.CGCCCG.** 820 (95.73)   
273 + **.CGCCCG.** 936 (95.73)   
273 + **.CGGCCG.** 1028 (95.29)   
273 + **.CGGCCG.** 1348 (95.29)   
273 + **[GGACCG]** 1433 (87.21)   
273 - **.CGGCCG.** 1348 (95.29)   
273 - **[CGTCCG]** 1299 (88.90)   
273 - **[CGAGCG]** 1172 (88.31)   
273 - **.CGCCCG.** 1168 (95.73)   
273 - **.CGCCCG.** 1103 (95.73)   
273 - **.CGGCCG.** 1028 (95.29)   
273 - **.CGCCCG.** 557 (95.73)   
273 - **[GGACCG]** 513 (87.21)   
274 + **.CGGCCG.** 11 (95.29)   
274 + **.CGGCCG.** 17 (95.29)   
274 + **[GGACCG]** 1192 (87.21)   
274 + **.CGCCCG.** 1271 (95.73)   
274 + **.CGGCCG.** 1324 (95.29)   
274 + **[CGAGCG]** 1498 (88.31)   
274 - **.CGCCCG.** 1350 (95.73)   
274 - **.CGGCCG.** 1324 (95.29)   
274 - **[CCACCG]** 1164 (88.59)   
274 - **[GGACCG]** 1159 (87.21)   
274 - **[GGACCG]** 318 (87.21)   
274 - **.CGCCCG.** 89 (95.73)   
274 - **.CGGCCG.** 17 (95.29)   
274 - **.CGGCCG.** 11 (95.29)   
275 + **.CGACCG.** 40 (100.00)   
275 + **.CGGCCG.** 212 (95.29)   
275 + **.CGCCCG.** 737 (95.73)   
275 + **[CGAGCG]** 1056 (88.31)   
275 + **[CGAGCG]** 1408 (88.31)   
275 + **[CGAGCG]** 1503 (88.31)   
275 - **.CGCCCG.** 1086 (95.73)   
275 - **[CGTCCG]** 1033 (88.90)   
275 - **[CGAGCG]** 1003 (88.31)   
275 - **.CGCCCG.** 561 (95.73)   
275 - **.CGCCCG.** 557 (95.73)   
275 - **[CCACCG]** 273 (88.59)   
275 - **[CCACCG]** 267 (88.59)   
275 - **.CGGCCG.** 212 (95.29)   
275 - **[CCACCG]** 105 (88.59)   
276 + **[GGACCG]** 924 (87.21)   
276 - **[GGACCG]** 39 (87.21)   
279 + **[CGAGCG]** 100 (88.31)   
279 + **.CGCCCG.** 586 (95.73)   
279 + **.CGACCG.** 659 (100.00)   
279 + **[GGACCG]** 698 (87.21)   
279 + **[CGACCC]** 955 (88.16)   
279 - **[CGAGCG]** 790 (88.31)   
279 - **.CGCCCG.** 525 (95.73)   
279 - **.CGCCCG.** 350 (95.73)   
279 - **[CGACCC]** 279 (88.16)   
279 - **[CGACCC]** 130 (88.16)   
279 - **.CGCCCG.** 104 (95.73)   
280 + **.CGGCCG.** 740 (95.29)   
280 + **[CGAGCG]** 829 (88.31)   
280 + **.CGCCCG.** 1068 (95.73)   
280 + **[CGTCCG]** 1086 (88.90)   
280 + **[GGACCG]** 1148 (87.21)   
280 + **[CGACCC]** 1154 (88.16)   
280 + **.CGGCCG.** 1160 (95.29)   
280 + **.CGACCG.** 1180 (100.00)   
280 + **.CGGCCG.** 1299 (95.29)   
280 + **.CGCCCG.** 1303 (95.73)   
280 + **.CGCCCG.** 1344 (95.73)   
280 - **[CGAGCG]** 1401 (88.31)   
280 - **.CGGCCG.** 1299 (95.29)   
280 - **.CGGCCG.** 1160 (95.29)   
280 - **[CCACCG]** 1013 (88.59)   
280 - **[CGAGCG]** 779 (88.31)   
280 - **.CGGCCG.** 740 (95.29)   
280 - **[CGACCC]** 308 (88.16)   
281 + **.CGCCCG.** 435 (95.73)   
281 + **.CGGCCG.** 533 (95.29)   
281 + **.CGCCCG.** 549 (95.73)   
281 + **.CGGCCG.** 928 (95.29)   
281 + **.CGCCCG.** 955 (95.73)   
281 + **.CGACCG.** 1111 (100.00)   
281 + **.CGACCG.** 1171 (100.00)   
281 + **[CCACCG]** 1311 (88.59)   
281 + **[CGACCC]** 1337 (88.16)   
281 + **[CGACCC]** 1467 (88.16)   
281 - **[CGTCCG]** 1253 (88.90)   
281 - **[CCACCG]** 1189 (88.59)   
281 - **[CGTCCG]** 1028 (88.90)   
281 - **.CGGCCG.** 928 (95.29)   
281 - **.CGGCCG.** 533 (95.29)   
281 - **.CGCCCG.** 439 (95.73)   
281 - **[CCACCG]** 287 (88.59)   
282 + **.CGCCCG.** 743 (95.73)   
283 + **.CGGCCG.** 583 (95.29)   
283 + **[GGACCG]** 611 (87.21)   
283 + **.CGGCCG.** 953 (95.29)   
283 + **[CGTCCG]** 1120 (88.90)   
283 + **.CGGCCG.** 1163 (95.29)   
283 + **[GGACCG]** 1173 (87.21)   
283 + **.CGCCCG.** 1284 (95.73)   
283 + **.CGGCCG.** 1385 (95.29)   
283 + **.CGCCCG.** 1389 (95.73)   
283 + **[CGACCC]** 1470 (88.16)   
283 + **[CCACCG]** 1475 (88.59)   
283 + **.CGCCCG.** 1479 (95.73)   
283 - **.CGGCCG.** 1385 (95.29)   
283 - **.CGGCCG.** 1163 (95.29)   
283 - **.CGGCCG.** 953 (95.29)   
283 - **.CGGCCG.** 583 (95.29)   
284 + **[GGACCG]** 188 (87.21)   
284 + **.CGCCCG.** 981 (95.73)   
284 + **[CCACCG]** 1037 (88.59)   
284 + **[CGAGCG]** 1144 (88.31)   
284 + **.CGGCCG.** 1285 (95.29)   
284 + **.CGCCCG.** 1323 (95.73)   
284 + **[CGACCC]** 1361 (88.16)   
284 + **.CGCCCG.** 1367 (95.73)   
284 - **[CGACCC]** 1503 (88.16)   
284 - **.CGCCCG.** 1498 (95.73)   
284 - **.CGCCCG.** 1494 (95.73)   
284 - **.CGCCCG.** 1475 (95.73)   
284 - **.CGCCCG.** 1469 (95.73)   
284 - **.CGCCCG.** 1434 (95.73)   
284 - **.CGCCCG.** 1430 (95.73)   
284 - **[CGAGCG]** 1419 (88.31)   
284 - **.CGCCCG.** 1415 (95.73)   
284 - **[CGAGCG]** 1357 (88.31)   
284 - **.CGGCCG.** 1285 (95.29)   
284 - **.CGCCCG.** 1196 (95.73)   
284 - **.CGCCCG.** 985 (95.73)   
284 - **[CCACCG]** 785 (88.59)   
285 + **.CGGCCG.** 550 (95.29)   
285 + **.CGGCCG.** 719 (95.29)   
285 + **[CGACCC]** 815 (88.16)   
285 + **.CGGCCG.** 856 (95.29)   
285 + **.CGCCCG.** 877 (95.73)   
285 + **.CGCCCG.** 881 (95.73)   
285 + **.CGCCCG.** 885 (95.73)   
285 + **.CGGCCG.** 1086 (95.29)   
285 + **.CGCCCG.** 1126 (95.73)   
285 - **[GGACCG]** 1314 (87.21)   
285 - **[CGTCCG]** 1281 (88.90)   
285 - **.CGGCCG.** 1086 (95.29)   
285 - **.CGGCCG.** 856 (95.29)   
285 - **.CGGCCG.** 719 (95.29)   
285 - **.CGCCCG.** 596 (95.73)   
285 - **.CGGCCG.** 550 (95.29)   
285 - **.CGCCCG.** 453 (95.73)   
285 - **.CGCCCG.** 447 (95.73)   
285 - **.CGCCCG.** 342 (95.73)   
285 - **[CGTCCG]** 168 (88.90)   
287 + **.CGCCCG.** 328 (95.73)   
287 + **.CGGCCG.** 758 (95.29)   
287 + **[CCACCG]** 801 (88.59)   
287 + **.CGGCCG.** 824 (95.29)   
287 + **[CGAGCG]** 842 (88.31)   
287 + **.CGCCCG.** 1027 (95.73)   
287 + **.CGACCG.** 1031 (100.00)   
287 + **.CGCCCG.** 1229 (95.73)   
287 - **[CGAGCG]** 1039 (88.31)   
287 - **[CGAGCG]** 1007 (88.31)   
287 - **.CGACCG.** 957 (100.00)   
287 - **.CGGCCG.** 824 (95.29)   
287 - **.CGGCCG.** 758 (95.29)   
288 + **.CGCCCG.** 683 (95.73)   
288 + **.CGGCCG.** 868 (95.29)   
288 + **[CGAGCG]** 1005 (88.31)   
288 + **[CGACCC]** 1103 (88.16)   
288 + **[CGAGCG]** 1181 (88.31)   
288 - **.CGGCCG.** 868 (95.29)   
288 - **[CGAGCG]** 817 (88.31)   
289 + **.CGCCCG.** 901 (95.73)   
289 - **.CGCCCG.** 1383 (95.73)   
289 - **.CGACCG.** 1230 (100.00)   
289 - **[CCACCG]** 1023 (88.59)   
290 + **[CGAGCG]** 1040 (88.31)   
290 + **.CGGCCG.** 1191 (95.29)   
290 - **.CGCCCG.** 1435 (95.73)   
290 - **[CGACCC]** 1428 (88.16)   
290 - **[CCACCG]** 1363 (88.59)   
290 - **[CGACCC]** 1323 (88.16)   
290 - **.CGGCCG.** 1191 (95.29)   
290 - **.CGACCG.** 1158 (100.00)   
290 - **[GGACCG]** 618 (87.21)   
290 - **[CCACCG]** 396 (88.59)   
291 + **.CGACCG.** 453 (100.00)   
291 + **[CCACCG]** 540 (88.59)   
291 + **[CGAGCG]** 674 (88.31)   
291 + **.CGGCCG.** 678 (95.29)   
291 + **.CGGCCG.** 905 (95.29)   
291 + **[CGTCCG]** 1229 (88.90)   
291 + **.CGGCCG.** 1242 (95.29)   
291 + **[CGAGCG]** 1308 (88.31)   
291 + **.CGGCCG.** 1391 (95.29)   
291 + **.CGGCCG.** 1401 (95.29)   
291 - **.CGCCCG.** 1498 (95.73)   
291 - **.CGCCCG.** 1426 (95.73)   
291 - **.CGCCCG.** 1405 (95.73)   
291 - **.CGGCCG.** 1401 (95.29)   
291 - **.CGGCCG.** 1391 (95.29)   
291 - **.CGGCCG.** 1242 (95.29)   
291 - **[CGACCC]** 1193 (88.16)   
291 - **[CGTCCG]** 1163 (88.90)   
291 - **.CGCCCG.** 1156 (95.73)   
291 - **.CGCCCG.** 1125 (95.73)   
291 - **.CGCCCG.** 1096 (95.73)   
291 - **.CGCCCG.** 1064 (95.73)   
291 - **.CGACCG.** 945 (100.00)   
291 - **.CGCCCG.** 941 (95.73)   
291 - **.CGGCCG.** 905 (95.29)   
291 - **[CGTCCG]** 703 (88.90)   
291 - **.CGGCCG.** 678 (95.29)   
291 - **.CGACCG.** 670 (100.00)   
291 - **[GGACCG]** 597 (87.21)   
291 - **[GGACCG]** 529 (87.21)   
292 + **.CGCCCG.** 110 (95.73)   
292 + **[CCACCG]** 348 (88.59)   
292 + **[CGTCCG]** 922 (88.90)   
292 + **.CGGCCG.** 944 (95.29)   
292 + **[CCACCG]** 952 (88.59)   
292 + **[CGAGCG]** 1086 (88.31)   
292 + **.CGCCCG.** 1142 (95.73)   
292 + **[CGAGCG]** 1173 (88.31)   
292 + **[GGACCG]** 1234 (87.21)   
292 + **[CGTCCG]** 1321 (88.90)   
292 + **.CGGCCG.** 1341 (95.29)   
292 + **.CGGCCG.** 1417 (95.29)   
292 - **.CGCCCG.** 1421 (95.73)   
292 - **.CGGCCG.** 1417 (95.29)   
292 - **.CGGCCG.** 1341 (95.29)   
292 - **[CGAGCG]** 1226 (88.31)   
292 - **.CGCCCG.** 1193 (95.73)   
292 - **.CGACCG.** 1092 (100.00)   
292 - **.CGCCCG.** 1032 (95.73)   
292 - **[GGACCG]** 978 (87.21)   
292 - **.CGGCCG.** 944 (95.29)   
292 - **.CGCCCG.** 875 (95.73)   
292 - **[GGACCG]** 561 (87.21)   
293 + **[GGACCG]** 423 (87.21)   
293 + **.CGGCCG.** 549 (95.29)   
293 + **.CGCCCG.** 625 (95.73)   
293 + **.CGGCCG.** 744 (95.29)   
293 + **[CGTCCG]** 748 (88.90)   
293 + **.CGGCCG.** 812 (95.29)   
293 + **.CGGCCG.** 816 (95.29)   
293 + **[CCACCG]** 983 (88.59)   
293 + **[CGAGCG]** 1123 (88.31)   
293 + **.CGCCCG.** 1328 (95.73)   
293 + **.CGCCCG.** 1465 (95.73)   
293 + **.CGCCCG.** 1472 (95.73)   
293 - **.CGCCCG.** 1324 (95.73)   
293 - **.CGCCCG.** 1284 (95.73)   
293 - **[CGACCC]** 1205 (88.16)   
293 - **.CGACCG.** 877 (100.00)   
293 - **.CGGCCG.** 816 (95.29)   
293 - **.CGGCCG.** 812 (95.29)   
293 - **.CGGCCG.** 744 (95.29)   
293 - **.CGCCCG.** 621 (95.73)   
293 - **.CGGCCG.** 549 (95.29)   
294 + **[GGACCG]** 137 (87.21)   
294 + **.CGGCCG.** 297 (95.29)   
294 + **[CCACCG]** 359 (88.59)   
294 + **[CCACCG]** 454 (88.59)   
294 + **.CGGCCG.** 656 (95.29)   
294 + **.CGCCCG.** 800 (95.73)   
294 + **.CGGCCG.** 864 (95.29)   
294 + **.CGCCCG.** 957 (95.73)   
294 + **[CGACCC]** 1041 (88.16)   
294 + **[CGTCCG]** 1290 (88.90)   
294 + **[CCACCG]** 1432 (88.59)   
294 - **.CGCCCG.** 1260 (95.73)   
294 - **[CCACCG]** 1017 (88.59)   
294 - **[CGTCCG]** 934 (88.90)   
294 - **.CGGCCG.** 864 (95.29)   
294 - **[GGACCG]** 660 (87.21)   
294 - **.CGGCCG.** 656 (95.29)   
294 - **.CGGCCG.** 297 (95.29)   
294 - **[CGACCC]** 272 (88.16)   
294 - **.CGCCCG.** 108 (95.73)   
294 - **[CCACCG]** 16 (88.59)   
294 - **[GGACCG]** 10 (87.21)   
295 + **.CGCCCG.** 96 (95.73)   
295 + **.CGCCCG.** 380 (95.73)   
295 + **[CCACCG]** 623 (88.59)   
295 + **[CGTCCG]** 706 (88.90)   
295 + **.CGGCCG.** 776 (95.29)   
295 + **[GGACCG]** 981 (87.21)   
295 + **.CGGCCG.** 985 (95.29)   
295 + **.CGGCCG.** 1343 (95.29)   
295 + **[CGACCC]** 1368 (88.16)   
295 - **[GGACCG]** 1503 (87.21)   
295 - **.CGGCCG.** 1343 (95.29)   
295 - **[CCACCG]** 1281 (88.59)   
295 - **[CCACCG]** 1186 (88.59)   
295 - **.CGGCCG.** 985 (95.29)   
295 - **.CGCCCG.** 840 (95.73)   
295 - **.CGGCCG.** 776 (95.29)   
295 - **.CGCCCG.** 683 (95.73)   
295 - **[CGACCC]** 599 (88.16)   
295 - **.CGCCCG.** 588 (95.73)   
295 - **[CGTCCG]** 350 (88.90)   
295 - **[CCACCG]** 208 (88.59)   
296 + **.CGCCCG.** 29 (95.73)   
296 + **.CGCCCG.** 122 (95.73)   
296 + **.CGCCCG.** 126 (95.73)   
296 + **[CGACCC]** 148 (88.16)   
296 + **.CGGCCG.** 266 (95.29)   
296 + **[CGAGCG]** 337 (88.31)   
296 + **.CGCCCG.** 360 (95.73)   
296 + **.CGCCCG.** 540 (95.73)   
296 + **[CGTCCG]** 736 (88.90)   
296 + **.CGGCCG.** 817 (95.29)   
296 + **[CGAGCG]** 926 (88.31)   
296 + **.CGCCCG.** 951 (95.73)   
296 + **.CGCCCG.** 1018 (95.73)   
296 + **.CGGCCG.** 1160 (95.29)   
296 + **.CGGCCG.** 1256 (95.29)   
296 + **.CGGCCG.** 1432 (95.29)   
296 + **[CGAGCG]** 1436 (88.31)   
296 - **.CGGCCG.** 1432 (95.29)   
296 - **.CGGCCG.** 1256 (95.29)   
296 - **.CGGCCG.** 1160 (95.29)   
296 - **.CGCCCG.** 1103 (95.73)   
296 - **.CGCCCG.** 955 (95.73)   
296 - **.CGCCCG.** 919 (95.73)   
296 - **.CGCCCG.** 828 (95.73)   
296 - **.CGGCCG.** 817 (95.29)   
296 - **.CGCCCG.** 788 (95.73)   
296 - **[GGACCG]** 685 (87.21)   
296 - **[CGAGCG]** 670 (88.31)   
296 - **[CGTCCG]** 653 (88.90)   
296 - **.CGACCG.** 587 (100.00)   
296 - **.CGCCCG.** 492 (95.73)   
296 - **[CGAGCG]** 488 (88.31)   
296 - **.CGCCCG.** 466 (95.73)   
296 - **.CGCCCG.** 423 (95.73)   
296 - **.CGGCCG.** 266 (95.29)   
296 - **[GGACCG]** 130 (87.21)   
297 + **[CGAGCG]** 652 (88.31)   
297 + **.CGACCG.** 670 (100.00)   
297 + **.CGCCCG.** 691 (95.73)   
297 + **[GGACCG]** 834 (87.21)   
297 + **.CGCCCG.** 844 (95.73)   
297 + **.CGGCCG.** 994 (95.29)   
297 + **[GGACCG]** 1001 (87.21)   
297 + **[CGACCC]** 1382 (88.16)   
297 - **[CGACCC]** 1288 (88.16)   
297 - **[CGAGCG]** 1235 (88.31)   
297 - **.CGCCCG.** 1231 (95.73)   
297 - **.CGGCCG.** 994 (95.29)   
297 - **[CGACCC]** 666 (88.16)   
297 - **[CGACCC]** 342 (88.16)   
298 + **.CGGCCG.** 717 (95.29)   
298 + **[GGACCG]** 770 (87.21)   
298 + **.CGGCCG.** 795 (95.29)   
298 + **[CCACCG]** 819 (88.59)   
298 + **.CGGCCG.** 953 (95.29)   
298 + **.CGGCCG.** 1006 (95.29)   
298 + **.CGGCCG.** 1242 (95.29)   
298 - **[GGACCG]** 1512 (87.21)   
298 - **[CGAGCG]** 1468 (88.31)   
298 - **[GGACCG]** 1441 (87.21)   
298 - **.CGCCCG.** 1304 (95.73)   
298 - **.CGGCCG.** 1242 (95.29)   
298 - **[GGACCG]** 1028 (87.21)   
298 - **.CGGCCG.** 1006 (95.29)   
298 - **.CGACCG.** 1000 (100.00)   
298 - **.CGGCCG.** 953 (95.29)   
298 - **[GGACCG]** 948 (87.21)   
298 - **[CGTCCG]** 905 (88.90)   
298 - **.CGGCCG.** 795 (95.29)   
298 - **.CGGCCG.** 717 (95.29)   
298 - **[CGTCCG]** 586 (88.90)   
298 - **.CGACCG.** 362 (100.00)   
299 + **.CGACCG.** 466 (100.00)   
299 + **[CGTCCG]** 735 (88.90)   
299 + **.CGGCCG.** 774 (95.29)   
299 + **.CGGCCG.** 849 (95.29)   
299 + **[CGTCCG]** 1285 (88.90)   
299 + **.CGGCCG.** 1348 (95.29)   
299 - **.CGGCCG.** 1348 (95.29)   
299 - **[CGACCC]** 1227 (88.16)   
299 - **.CGCCCG.** 974 (95.73)   
299 - **[CGAGCG]** 966 (88.31)   
299 - **.CGGCCG.** 849 (95.29)   
299 - **.CGGCCG.** 774 (95.29)   
299 - **[CCACCG]** 530 (88.59)   
300 - **.CGCCCG.** 776 (95.73)   
300 - **.CGCCCG.** 767 (95.73)   
300 - **[GGACCG]** 515 (87.21)   
300 - **[GGACCG]** 494 (87.21)   
300 - **[CCACCG]** 468 (88.59)   
300 - **[CGACCC]** 396 (88.16)   
301 + **.CGCCCG.** 174 (95.73)   
301 + **[CGAGCG]** 704 (88.31)   
301 + **[CGTCCG]** 818 (88.90)   
301 + **.CGCCCG.** 905 (95.73)   
301 + **[CGACCC]** 1362 (88.16)   
301 - **.CGCCCG.** 1391 (95.73)   
301 - **[CGTCCG]** 1303 (88.90)   
301 - **.CGCCCG.** 1110 (95.73)   
301 - **.CGCCCG.** 923 (95.73)   
301 - **[CGACCC]** 911 (88.16)   
301 - **.CGCCCG.** 263 (95.73)   
302 + **.CGGCCG.** 690 (95.29)   
302 + **.CGCCCG.** 752 (95.73)   
302 + **.CGCCCG.** 1087 (95.73)   
302 + **.CGGCCG.** 1371 (95.29)   
302 - **.CGGCCG.** 1371 (95.29)   
302 - **[CGTCCG]** 1198 (88.90)   
302 - **.CGGCCG.** 690 (95.29)   
302 - **[CGACCC]** 214 (88.16)   
303 + **.CGGCCG.** 37 (95.29)   
303 + **.CGGCCG.** 41 (95.29)   
303 + **[GGACCG]** 50 (87.21)   
303 + **.CGACCG.** 87 (100.00)   
303 + **.CGCCCG.** 175 (95.73)   
303 + **.CGGCCG.** 285 (95.29)   
303 + **[CGACCC]** 388 (88.16)   
303 + **[CGAGCG]** 407 (88.31)   
303 + **.CGCCCG.** 434 (95.73)   
303 + **[CGAGCG]** 452 (88.31)   
303 + **.CGGCCG.** 502 (95.29)   
303 + **.CGCCCG.** 572 (95.73)   
303 + **[CGTCCG]** 883 (88.90)   
303 + **.CGCCCG.** 947 (95.73)   
303 + **[CCACCG]** 964 (88.59)   
303 + **[CGTCCG]** 1026 (88.90)   
303 + **.CGCCCG.** 1071 (95.73)   
303 + **[CGACCC]** 1313 (88.16)   
303 + **.CGGCCG.** 1359 (95.29)   
303 + **.CGGCCG.** 1467 (95.29)   
303 - **.CGGCCG.** 1467 (95.29)   
303 - **.CGGCCG.** 1359 (95.29)   
303 - **[CGAGCG]** 757 (88.31)   
303 - **.CGCCCG.** 741 (95.73)   
303 - **.CGCCCG.** 722 (95.73)   
303 - **.CGGCCG.** 502 (95.29)   
303 - **.CGCCCG.** 461 (95.73)   
303 - **.CGGCCG.** 285 (95.29)   
303 - **.CGCCCG.** 222 (95.73)   
303 - **.CGCCCG.** 179 (95.73)   
303 - **.CGGCCG.** 41 (95.29)   
303 - **.CGGCCG.** 37 (95.29)   
303 - **[GGACCG]** 15 (87.21)   
304 + **[CGAGCG]** 797 (88.31)   
304 + **[CGTCCG]** 842 (88.90)   
304 + **.CGCCCG.** 879 (95.73)   
304 + **.CGCCCG.** 1335 (95.73)   
304 - **[CGACCC]** 1331 (88.16)   
304 - **.CGCCCG.** 1255 (95.73)   
304 - **[CCACCG]** 1248 (88.59)   
304 - **[CGACCC]** 1159 (88.16)   
304 - **[CCACCG]** 1019 (88.59)   
304 - **[CGTCCG]** 737 (88.90)   
305 + **[CCACCG]** 63 (88.59)   
305 + **.CGGCCG.** 202 (95.29)   
305 + **[CGACCC]** 309 (88.16)   
305 + **.CGGCCG.** 344 (95.29)   
305 - **.CGGCCG.** 344 (95.29)   
305 - **.CGGCCG.** 202 (95.29)   
306 + **.CGCCCG.** 621 (95.73)   
306 + **[CCACCG]** 627 (88.59)   
306 + **.CGCCCG.** 633 (95.73)   
306 + **.CGCCCG.** 719 (95.73)   
306 + **.CGCCCG.** 769 (95.73)   
306 + **[CGACCC]** 908 (88.16)   
306 + **[CGAGCG]** 950 (88.31)   
306 + **.CGGCCG.** 982 (95.29)   
306 + **.CGCCCG.** 1366 (95.73)   
306 + **.CGGCCG.** 1393 (95.29)   
306 - **.CGCCCG.** 1459 (95.73)   
306 - **.CGGCCG.** 1393 (95.29)   
306 - **[CGACCC]** 1267 (88.16)   
306 - **[CGACCC]** 1258 (88.16)   
306 - **[CGACCC]** 1222 (88.16)   
306 - **[CGACCC]** 1152 (88.16)   
306 - **[CGACCC]** 1115 (88.16)   
306 - **[CGACCC]** 1106 (88.16)   
306 - **[CGACCC]** 1055 (88.16)   
306 - **.CGCCCG.** 986 (95.73)   
306 - **.CGGCCG.** 982 (95.29)   
306 - **.CGCCCG.** 879 (95.73)   
306 - **[CCACCG]** 415 (88.59)   
307 + **.CGCCCG.** 537 (95.73)   
307 + **.CGGCCG.** 553 (95.29)   
307 + **.CGGCCG.** 735 (95.29)   
307 + **[CCACCG]** 988 (88.59)   
307 + **.CGGCCG.** 1042 (95.29)   
307 + **[GGACCG]** 1326 (87.21)   
307 + **.CGGCCG.** 1332 (95.29)   
307 - **.CGGCCG.** 1332 (95.29)   
307 - **[CGAGCG]** 1244 (88.31)   
307 - **.CGCCCG.** 1158 (95.73)   
307 - **.CGCCCG.** 1109 (95.73)   
307 - **.CGCCCG.** 1064 (95.73)   
307 - **.CGGCCG.** 1042 (95.29)   
307 - **.CGCCCG.** 1030 (95.73)   
307 - **.CGGCCG.** 735 (95.29)   
307 - **[CGACCC]** 716 (88.16)   
307 - **.CGGCCG.** 553 (95.29)   
307 - **.CGCCCG.** 544 (95.73)   
307 - **[CGAGCG]** 390 (88.31)   
308 + **.CGCCCG.** 788 (95.73)   
308 + **.CGCCCG.** 820 (95.73)   
308 + **.CGACCG.** 920 (100.00)   
308 + **[GGACCG]** 1030 (87.21)   
308 + **.CGGCCG.** 1077 (95.29)   
308 + **[CCACCG]** 1172 (88.59)   
308 + **[CCACCG]** 1363 (88.59)   
308 + **[GGACCG]** 1397 (87.21)   
308 - **.CGCCCG.** 1449 (95.73)   
308 - **[CGACCC]** 1445 (88.16)   
308 - **.CGCCCG.** 1284 (95.73)   
308 - **.CGGCCG.** 1077 (95.29)   
308 - **[GGACCG]** 1063 (87.21)   
308 - **.CGCCCG.** 935 (95.73)   
308 - **.CGCCCG.** 45 (95.73)   
309 + **[GGACCG]** 583 (87.21)   
309 + **.CGCCCG.** 652 (95.73)   
309 + **.CGCCCG.** 693 (95.73)   
309 + **.CGCCCG.** 707 (95.73)   
309 + **[CGAGCG]** 773 (88.31)   
309 - **.CGCCCG.** 1198 (95.73)   
309 - **[CCACCG]** 875 (88.59)   
309 - **[CGAGCG]** 670 (88.31)   
309 - **.CGCCCG.** 394 (95.73)   
310 + **[CCACCG]** 830 (88.59)   
310 + **.CGCCCG.** 836 (95.73)   
310 + **[CGACCC]** 1005 (88.16)   
310 + **[CGTCCG]** 1160 (88.90)   
310 + **[CGTCCG]** 1233 (88.90)   
310 + **.CGACCG.** 1482 (100.00)   
310 - **[CCACCG]** 1283 (88.59)   
310 - **[CCACCG]** 1250 (88.59)   
311 + **.CGCCCG.** 769 (95.73)   
311 + **.CGGCCG.** 877 (95.29)   
311 + **.CGGCCG.** 1061 (95.29)   
311 + **[CGAGCG]** 1089 (88.31)   
311 + **.CGCCCG.** 1111 (95.73)   
311 + **.CGCCCG.** 1203 (95.73)   
311 + **.CGCCCG.** 1228 (95.73)   
311 + **.CGGCCG.** 1451 (95.29)   
311 + **[GGACCG]** 1461 (87.21)   
311 - **.CGGCCG.** 1451 (95.29)   
311 - **.CGCCCG.** 1266 (95.73)   
311 - **[CCACCG]** 1152 (88.59)   
311 - **[CCACCG]** 1074 (88.59)   
311 - **.CGCCCG.** 1065 (95.73)   
311 - **.CGGCCG.** 1061 (95.29)   
311 - **[GGACCG]** 975 (87.21)   
311 - **.CGGCCG.** 877 (95.29)   
311 - **[GGACCG]** 841 (87.21)   
311 - **[CCACCG]** 150 (88.59)   
312 + **[GGACCG]** 816 (87.21)   
312 + **[GGACCG]** 822 (87.21)   
312 + **.CGGCCG.** 952 (95.29)   
312 + **.CGCCCG.** 1099 (95.73)   
312 + **.CGCCCG.** 1103 (95.73)   
312 + **.CGGCCG.** 1183 (95.29)   
312 + **.CGGCCG.** 1222 (95.29)   
312 - **.CGGCCG.** 1222 (95.29)   
312 - **.CGCCCG.** 1191 (95.73)   
312 - **.CGCCCG.** 1187 (95.73)   
312 - **.CGGCCG.** 1183 (95.29)   
312 - **[CGTCCG]** 1170 (88.90)   
312 - **.CGCCCG.** 1014 (95.73)   
312 - **.CGGCCG.** 952 (95.29)   
312 - **.CGCCCG.** 881 (95.73)   
313 + **.CGGCCG.** 828 (95.29)   
313 + **.CGGCCG.** 837 (95.29)   
313 + **[CGACCC]** 1245 (88.16)   
313 + **.CGCCCG.** 1257 (95.73)   
313 + **[CGACCC]** 1419 (88.16)   
313 - **.CGCCCG.** 1385 (95.73)   
313 - **[CCACCG]** 1127 (88.59)   
313 - **[GGACCG]** 1081 (87.21)   
313 - **[CGACCC]** 1077 (88.16)   
313 - **[CCACCG]** 1073 (88.59)   
313 - **.CGACCG.** 999 (100.00)   
313 - **[CGAGCG]** 960 (88.31)   
313 - **.CGACCG.** 872 (100.00)   
313 - **.CGGCCG.** 837 (95.29)   
313 - **.CGGCCG.** 828 (95.29)   
314 + **[GGACCG]** 963 (87.21)   
314 - **[CCACCG]** 1484 (88.59)   
314 - **.CGCCCG.** 386 (95.73)   
314 - **.CGCCCG.** 354 (95.73)   
314 - **[GGACCG]** 237 (87.21)   
314 - **.CGACCG.** 65 (100.00)   
315 + **[CGTCCG]** 134 (88.90)   
315 + **[CGACCC]** 231 (88.16)   
315 + **[CCACCG]** 391 (88.59)   
315 + **[GGACCG]** 1392 (87.21)   
315 - **.CGCCCG.** 1021 (95.73)   
315 - **.CGCCCG.** 1012 (95.73)   
315 - **[CGTCCG]** 1004 (88.90)   
315 - **[CGTCCG]** 813 (88.90)   
315 - **[GGACCG]** 700 (87.21)   
316 + **[GGACCG]** 29 (87.21)   
316 + **.CGCCCG.** 204 (95.73)   
316 + **[CGAGCG]** 464 (88.31)   
316 + **[CGAGCG]** 507 (88.31)   
316 + **.CGGCCG.** 534 (95.29)   
316 + **.CGGCCG.** 1415 (95.29)   
316 - **.CGGCCG.** 1415 (95.29)   
316 - **[CGACCC]** 1232 (88.16)   
316 - **.CGACCG.** 852 (100.00)   
316 - **[CGACCC]** 627 (88.16)   
316 - **.CGCCCG.** 615 (95.73)   
316 - **.CGGCCG.** 534 (95.29)   
316 - **[GGACCG]** 252 (87.21)   
316 - **.CGCCCG.** 236 (95.73)   
316 - **.CGCCCG.** 72 (95.73)   
317 + **[CCACCG]** 542 (88.59)   
317 + **[GGACCG]** 656 (87.21)   
317 + **[GGACCG]** 1114 (87.21)   
317 + **[GGACCG]** 1353 (87.21)   
317 - **.CGCCCG.** 1406 (95.73)   
317 - **[CGACCC]** 1340 (88.16)   
317 - **.CGCCCG.** 979 (95.73)   
317 - **.CGCCCG.** 975 (95.73)   
317 - **[CGAGCG]** 764 (88.31)   
317 - **[CCACCG]** 670 (88.59)   
318 + **.CGCCCG.** 99 (95.73)   
318 + **.CGGCCG.** 172 (95.29)   
318 + **.CGCCCG.** 303 (95.73)   
318 + **.CGGCCG.** 382 (95.29)   
318 + **.CGCCCG.** 386 (95.73)   
318 + **[CCACCG]** 412 (88.59)   
318 + **[CGACCC]** 478 (88.16)   
318 + **.CGGCCG.** 524 (95.29)   
318 + **.CGGCCG.** 862 (95.29)   
318 + **.CGCCCG.** 1182 (95.73)   
318 + **[CGTCCG]** 1198 (88.90)   
318 + **[CCACCG]** 1205 (88.59)   
318 + **.CGCCCG.** 1222 (95.73)   
318 + **.CGCCCG.** 1429 (95.73)   
318 + **.CGGCCG.** 1481 (95.29)   
318 + **.CGGCCG.** 1508 (95.29)   
318 - **.CGGCCG.** 1508 (95.29)   
318 - **.CGGCCG.** 1481 (95.29)   
318 - **.CGCCCG.** 1273 (95.73)   
318 - **[CGACCC]** 1194 (88.16)   
318 - **[CGTCCG]** 1152 (88.90)   
318 - **.CGGCCG.** 862 (95.29)   
318 - **[CCACCG]** 735 (88.59)   
318 - **.CGCCCG.** 731 (95.73)   
318 - **[CGACCC]** 574 (88.16)   
318 - **[CGAGCG]** 568 (88.31)   
318 - **.CGGCCG.** 524 (95.29)   
318 - **[CGACCC]** 491 (88.16)   
318 - **.CGGCCG.** 382 (95.29)   
318 - **.CGCCCG.** 248 (95.73)   
318 - **.CGGCCG.** 172 (95.29)   
318 - **[CGACCC]** 72 (88.16)   
319 + **[CCACCG]** 26 (88.59)   
320 + **.CGCCCG.** 602 (95.73)   
320 + **.CGGCCG.** 696 (95.29)   
320 + **[CGAGCG]** 787 (88.31)   
320 + **.CGGCCG.** 847 (95.29)   
320 + **[GGACCG]** 1165 (87.21)   
320 + **[CGACCC]** 1317 (88.16)   
320 + **[CGAGCG]** 1337 (88.31)   
320 + **[CCACCG]** 1394 (88.59)   
320 - **[CGAGCG]** 1203 (88.31)   
320 - **[CCACCG]** 1142 (88.59)   
320 - **.CGGCCG.** 847 (95.29)   
320 - **.CGCCCG.** 824 (95.73)   
320 - **.CGGCCG.** 696 (95.29)   
320 - **[CGAGCG]** 690 (88.31)   
320 - **.CGACCG.** 662 (100.00)   
320 - **[CGAGCG]** 609 (88.31)   
320 - **.CGCCCG.** 421 (95.73)   
321 + **.CGGCCG.** 373 (95.29)   
321 + **.CGCCCG.** 388 (95.73)   
321 + **.CGCCCG.** 392 (95.73)   
321 + **[CGTCCG]** 467 (88.90)   
321 + **[CGACCC]** 504 (88.16)   
321 + **.CGCCCG.** 517 (95.73)   
321 + **.CGCCCG.** 1219 (95.73)   
321 + **.CGCCCG.** 1227 (95.73)   
321 + **.CGGCCG.** 1347 (95.29)   
321 + **.CGCCCG.** 1371 (95.73)   
321 - **[CGAGCG]** 1474 (88.31)   
321 - **.CGGCCG.** 1347 (95.29)   
321 - **[CGACCC]** 1207 (88.16)   
321 - **[CGTCCG]** 987 (88.90)   
321 - **.CGCCCG.** 907 (95.73)   
321 - **.CGCCCG.** 874 (95.73)   
321 - **[CGACCC]** 422 (88.16)   
321 - **[CGACCC]** 381 (88.16)   
321 - **.CGGCCG.** 373 (95.29)   
321 - **[CGACCC]** 30 (88.16)   
322 + **.CGACCG.** 95 (100.00)   
322 + **.CGCCCG.** 99 (95.73)   
322 + **.CGCCCG.** 526 (95.73)   
322 + **[CGACCC]** 740 (88.16)   
322 + **.CGCCCG.** 770 (95.73)   
322 + **[CGAGCG]** 794 (88.31)   
322 + **.CGGCCG.** 1259 (95.29)   
322 + **.CGGCCG.** 1344 (95.29)   
322 + **[GGACCG]** 1411 (87.21)   
322 + **.CGCCCG.** 1428 (95.73)   
322 - **.CGGCCG.** 1344 (95.29)   
322 - **.CGGCCG.** 1259 (95.29)   
322 - **[CGTCCG]** 1218 (88.90)   
322 - **.CGACCG.** 1007 (100.00)   
322 - **.CGCCCG.** 965 (95.73)   
322 - **.CGCCCG.** 814 (95.73)   
322 - **[CGACCC]** 788 (88.16)   
322 - **.CGCCCG.** 406 (95.73)   
322 - **[CGACCC]** 307 (88.16)   
323 + **[CCACCG]** 244 (88.59)   
323 + **[CGACCC]** 707 (88.16)   
323 + **.CGGCCG.** 1085 (95.29)   
323 + **[CGAGCG]** 1109 (88.31)   
323 + **.CGCCCG.** 1498 (95.73)   
323 - **.CGACCG.** 1385 (100.00)   
323 - **.CGGCCG.** 1085 (95.29)   
323 - **.CGCCCG.** 983 (95.73)   
323 - **[CGACCC]** 794 (88.16)   
323 - **[CGACCC]** 625 (88.16)   
323 - **.CGCCCG.** 577 (95.73)   
323 - **.CGACCG.** 62 (100.00)   
324 + **.CGCCCG.** 877 (95.73)   
324 - **[GGACCG]** 1144 (87.21)   
324 - **[CGTCCG]** 1000 (88.90)   
324 - **[GGACCG]** 207 (87.21)   
324 - **[CGACCC]** 203 (88.16)   
325 + **[GGACCG]** 580 (87.21)   
325 - **[CGACCC]** 1465 (88.16)   
325 - **[CCACCG]** 276 (88.59)   
326 + **[CCACCG]** 450 (88.59)   
326 + **.CGCCCG.** 456 (95.73)   
326 + **.CGGCCG.** 710 (95.29)   
326 + **.CGCCCG.** 790 (95.73)   
326 + **.CGCCCG.** 818 (95.73)   
326 + **.CGCCCG.** 871 (95.73)   
326 + **.CGCCCG.** 943 (95.73)   
326 + **[CGTCCG]** 1071 (88.90)   
326 + **.CGGCCG.** 1115 (95.29)   
326 + **.CGGCCG.** 1160 (95.29)   
326 + **.CGGCCG.** 1207 (95.29)   
326 + **[CCACCG]** 1245 (88.59)   
326 + **.CGCCCG.** 1252 (95.73)   
326 + **.CGGCCG.** 1299 (95.29)   
326 + **.CGGCCG.** 1461 (95.29)   
326 - **[CCACCG]** 1484 (88.59)   
326 - **.CGGCCG.** 1461 (95.29)   
326 - **[CGAGCG]** 1446 (88.31)   
326 - **.CGCCCG.** 1387 (95.73)   
326 - **.CGCCCG.** 1338 (95.73)   
326 - **.CGCCCG.** 1317 (95.73)   
326 - **.CGGCCG.** 1299 (95.29)   
326 - **.CGGCCG.** 1207 (95.29)   
326 - **[CGAGCG]** 1174 (88.31)   
326 - **.CGGCCG.** 1160 (95.29)   
326 - **[CGAGCG]** 1153 (88.31)   
326 - **[CGAGCG]** 1149 (88.31)   
326 - **[CGAGCG]** 1145 (88.31)   
326 - **.CGGCCG.** 1115 (95.29)   
326 - **[CCACCG]** 1107 (88.59)   
326 - **[CGTCCG]** 978 (88.90)   
326 - **.CGCCCG.** 964 (95.73)   
326 - **.CGGCCG.** 710 (95.29)   
326 - **[CGAGCG]** 703 (88.31)   
326 - **[CGAGCG]** 407 (88.31)   
327 + **.CGGCCG.** 18 (95.29)   
327 + **[CGAGCG]** 22 (88.31)   
327 + **[CGAGCG]** 44 (88.31)   
327 + **[CCACCG]** 668 (88.59)   
327 + **[CCACCG]** 677 (88.59)   
327 + **.CGGCCG.** 707 (95.29)   
327 - **[CCACCG]** 1283 (88.59)   
327 - **[CCACCG]** 792 (88.59)   
327 - **.CGGCCG.** 707 (95.29)   
327 - **.CGCCCG.** 595 (95.73)   
327 - **[CGACCC]** 591 (88.16)   
327 - **[CGTCCG]** 237 (88.90)   
327 - **.CGGCCG.** 18 (95.29)   
328 - **[CCACCG]** 734 (88.59)   
329 + **.CGGCCG.** 982 (95.29)   
329 + **.CGCCCG.** 1229 (95.73)   
329 - **[CGTCCG]** 1235 (88.90)   
329 - **.CGGCCG.** 982 (95.29)   
330 + **[CGTCCG]** 712 (88.90)   
331 + **.CGGCCG.** 340 (95.29)   
331 + **[CGTCCG]** 574 (88.90)   
331 + **.CGGCCG.** 761 (95.29)   
331 + **.CGGCCG.** 914 (95.29)   
331 + **.CGCCCG.** 1193 (95.73)   
331 + **[CGACCC]** 1326 (88.16)   
331 + **.CGGCCG.** 1389 (95.29)   
331 + **.CGCCCG.** 1396 (95.73)   
331 + **[CGAGCG]** 1411 (88.31)   
331 + **.CGCCCG.** 1474 (95.73)   
331 - **[CCACCG]** 1420 (88.59)   
331 - **.CGGCCG.** 1389 (95.29)   
331 - **.CGGCCG.** 914 (95.29)   
331 - **.CGGCCG.** 761 (95.29)   
331 - **.CGGCCG.** 340 (95.29)   
332 + **.CGGCCG.** 54 (95.29)   
332 + **.CGCCCG.** 127 (95.73)   
332 + **.CGACCG.** 131 (100.00)   
332 + **.CGCCCG.** 379 (95.73)   
332 + **[CGAGCG]** 1120 (88.31)   
332 + **[CCACCG]** 1409 (88.59)   
332 + **.CGGCCG.** 1432 (95.29)   
332 - **.CGGCCG.** 1432 (95.29)   
332 - **[CGAGCG]** 1153 (88.31)   
332 - **.CGCCCG.** 1149 (95.73)   
332 - **.CGCCCG.** 1031 (95.73)   
332 - **.CGCCCG.** 1015 (95.73)   
332 - **[CGTCCG]** 88 (88.90)   
332 - **.CGGCCG.** 54 (95.29)   
332 - **[CCACCG]** 15 (88.59)   
333 + **[CCACCG]** 124 (88.59)   
333 + **.CGCCCG.** 656 (95.73)   
333 + **.CGCCCG.** 1069 (95.73)   
333 + **.CGCCCG.** 1136 (95.73)   
333 + **.CGGCCG.** 1140 (95.29)   
333 + **[CGACCC]** 1175 (88.16)   
333 + **[CCACCG]** 1192 (88.59)   
333 + **[CGACCC]** 1198 (88.16)   
333 + **.CGCCCG.** 1255 (95.73)   
333 + **[CGACCC]** 1325 (88.16)   
333 + **[CGACCC]** 1335 (88.16)   
333 + **[CCACCG]** 1382 (88.59)   
333 + **[CGACCC]** 1421 (88.16)   
333 - **.CGGCCG.** 1140 (95.29)   
333 - **[CCACCG]** 23 (88.59)   
334 + **[CGTCCG]** 40 (88.90)   
334 + **[CGACCC]** 428 (88.16)   
334 + **[CCACCG]** 622 (88.59)   
334 + **[CGACCC]** 875 (88.16)   
334 + **.CGCCCG.** 1039 (95.73)   
334 + **.CGCCCG.** 1073 (95.73)   
334 - **.CGCCCG.** 1085 (95.73)   
334 - **[CGAGCG]** 981 (88.31)   
334 - **[CCACCG]** 626 (88.59)   
334 - **[GGACCG]** 25 (87.21)   
335 + **.CGGCCG.** 502 (95.29)   
335 + **[CGAGCG]** 1009 (88.31)   
335 + **[CGAGCG]** 1042 (88.31)   
335 + **[CGAGCG]** 1059 (88.31)   
335 + **[GGACCG]** 1115 (87.21)   
335 + **.CGCCCG.** 1160 (95.73)   
335 + **.CGGCCG.** 1164 (95.29)   
335 + **[CGAGCG]** 1174 (88.31)   
335 + **.CGCCCG.** 1232 (95.73)   
335 + **.CGCCCG.** 1325 (95.73)   
335 + **.CGCCCG.** 1340 (95.73)   
335 + **.CGGCCG.** 1497 (95.29)   
335 - **.CGGCCG.** 1497 (95.29)   
335 - **.CGCCCG.** 1354 (95.73)   
335 - **.CGCCCG.** 1257 (95.73)   
335 - **.CGCCCG.** 1226 (95.73)   
335 - **.CGGCCG.** 1164 (95.29)   
335 - **.CGACCG.** 1028 (100.00)   
335 - **.CGCCCG.** 941 (95.73)   
335 - **[CGAGCG]** 937 (88.31)   
335 - **[CCACCG]** 863 (88.59)   
335 - **.CGCCCG.** 664 (95.73)   
335 - **.CGACCG.** 657 (100.00)   
335 - **.CGGCCG.** 502 (95.29)   
335 - **[CCACCG]** 341 (88.59)   
336 + **.CGGCCG.** 863 (95.29)   
336 + **.CGCCCG.** 892 (95.73)   
336 + **.CGGCCG.** 912 (95.29)   
336 + **.CGACCG.** 951 (100.00)   
336 + **[CGTCCG]** 964 (88.90)   
336 + **[CGTCCG]** 1074 (88.90)   
336 + **.CGCCCG.** 1267 (95.73)   
336 + **.CGGCCG.** 1343 (95.29)   
336 - **[CGAGCG]** 1352 (88.31)   
336 - **.CGGCCG.** 1343 (95.29)   
336 - **[CGAGCG]** 960 (88.31)   
336 - **.CGCCCG.** 936 (95.73)   
336 - **.CGGCCG.** 912 (95.29)   
336 - **.CGGCCG.** 863 (95.29)   
336 - **.CGCCCG.** 827 (95.73)   
336 - **[CGACCC]** 706 (88.16)   
337 + **[CGTCCG]** 795 (88.90)   
337 + **.CGCCCG.** 918 (95.73)   
337 + **.CGGCCG.** 1032 (95.29)   
337 + **.CGGCCG.** 1075 (95.29)   
337 + **.CGGCCG.** 1260 (95.29)   
337 + **[CGTCCG]** 1324 (88.90)   
337 + **[CGACCC]** 1444 (88.16)   
337 - **[CGACCC]** 1493 (88.16)   
337 - **[CGACCC]** 1419 (88.16)   
337 - **[CGAGCG]** 1411 (88.31)   
337 - **.CGCCCG.** 1383 (95.73)   
337 - **.CGCCCG.** 1352 (95.73)   
337 - **.CGGCCG.** 1260 (95.29)   
337 - **[CGTCCG]** 1098 (88.90)   
337 - **.CGGCCG.** 1075 (95.29)   
337 - **.CGGCCG.** 1032 (95.29)   
337 - **.CGCCCG.** 942 (95.73)   
337 - **.CGCCCG.** 924 (95.73)   
337 - **[CCACCG]** 529 (88.59)   
337 - **[CCACCG]** 441 (88.59)   
337 - **.CGCCCG.** 251 (95.73)   
338 + **[CGACCC]** 153 (88.16)   
338 + **[CCACCG]** 172 (88.59)   
338 + **.CGGCCG.** 640 (95.29)   
338 + **[GGACCG]** 658 (87.21)   
338 + **.CGCCCG.** 756 (95.73)   
338 + **.CGGCCG.** 768 (95.29)   
338 + **.CGCCCG.** 797 (95.73)   
338 + **[CGTCCG]** 880 (88.90)   
338 + **[CCACCG]** 935 (88.59)   
338 + **[CGACCC]** 988 (88.16)   
338 + **.CGCCCG.** 1094 (95.73)   
338 + **.CGCCCG.** 1098 (95.73)   
338 + **[CGTCCG]** 1156 (88.90)   
338 + **.CGGCCG.** 1303 (95.29)   
338 + **.CGGCCG.** 1452 (95.29)   
338 + **.CGCCCG.** 1482 (95.73)   
338 - **.CGCCCG.** 1499 (95.73)   
338 - **.CGCCCG.** 1495 (95.73)   
338 - **.CGGCCG.** 1452 (95.29)   
338 - **.CGCCCG.** 1425 (95.73)   
338 - **.CGGCCG.** 1303 (95.29)   
338 - **.CGGCCG.** 768 (95.29)   
338 - **.CGGCCG.** 640 (95.29)   
338 - **[GGACCG]** 433 (87.21)   
338 - **[CGACCC]** 264 (88.16)   
339 + **.CGGCCG.** 1037 (95.29)   
339 + **.CGCCCG.** 1041 (95.73)   
339 + **[CGTCCG]** 1171 (88.90)   
339 + **.CGCCCG.** 1392 (95.73)   
339 - **.CGCCCG.** 1242 (95.73)   
339 - **.CGCCCG.** 1138 (95.73)   
339 - **.CGGCCG.** 1037 (95.29)   
339 - **.CGCCCG.** 1026 (95.73)   
339 - **[CGTCCG]** 832 (88.90)   
340 + **[CGACCC]** 560 (88.16)   
340 + **.CGCCCG.** 691 (95.73)   
340 + **.CGCCCG.** 761 (95.73)   
340 + **[CCACCG]** 947 (88.59)   
340 + **.CGCCCG.** 975 (95.73)   
340 + **[CGACCC]** 981 (88.16)   
340 + **.CGACCG.** 1029 (100.00)   
340 + **[GGACCG]** 1105 (87.21)   
340 + **[CGAGCG]** 1119 (88.31)   
340 + **[CGTCCG]** 1203 (88.90)   
340 - **.CGCCCG.** 931 (95.73)   
340 - **.CGCCCG.** 611 (95.73)   
340 - **[CGACCC]** 351 (88.16)   
341 + **.CGGCCG.** 885 (95.29)   
341 + **.CGGCCG.** 1063 (95.29)   
341 + **.CGGCCG.** 1154 (95.29)   
341 + **[CGAGCG]** 1187 (88.31)   
341 + **.CGGCCG.** 1313 (95.29)   
341 + **[CGAGCG]** 1501 (88.31)   
341 - **.CGGCCG.** 1313 (95.29)   
341 - **[CGACCC]** 1229 (88.16)   
341 - **.CGGCCG.** 1154 (95.29)   
341 - **.CGCCCG.** 1113 (95.73)   
341 - **.CGGCCG.** 1063 (95.29)   
341 - **.CGCCCG.** 1000 (95.73)   
341 - **.CGCCCG.** 977 (95.73)   
341 - **.CGCCCG.** 973 (95.73)   
341 - **.CGGCCG.** 885 (95.29)   
341 - **[CCACCG]** 478 (88.59)   
341 - **.CGACCG.** 209 (100.00)   
342 + **[CGAGCG]** 660 (88.31)   
342 + **.CGGCCG.** 710 (95.29)   
342 + **.CGCCCG.** 907 (95.73)   
342 + **.CGGCCG.** 1039 (95.29)   
342 + **.CGCCCG.** 1068 (95.73)   
342 + **[CGAGCG]** 1277 (88.31)   
342 + **.CGGCCG.** 1337 (95.29)   
342 + **.CGCCCG.** 1402 (95.73)   
342 + **[CGAGCG]** 1424 (88.31)   
342 - **[CCACCG]** 1511 (88.59)   
342 - **.CGCCCG.** 1417 (95.73)   
342 - **.CGCCCG.** 1406 (95.73)   
342 - **.CGCCCG.** 1351 (95.73)   
342 - **.CGGCCG.** 1337 (95.29)   
342 - **.CGGCCG.** 1039 (95.29)   
342 - **.CGCCCG.** 1029 (95.73)   
342 - **[CCACCG]** 935 (88.59)   
342 - **.CGGCCG.** 710 (95.29)   
343 + **[CCACCG]** 165 (88.59)   
343 + **.CGGCCG.** 558 (95.29)   
343 + **.CGGCCG.** 651 (95.29)   
343 + **.CGGCCG.** 923 (95.29)   
343 + **.CGCCCG.** 959 (95.73)   
343 + **.CGCCCG.** 1037 (95.73)   
343 + **.CGGCCG.** 1093 (95.29)   
343 + **.CGCCCG.** 1196 (95.73)   
343 + **[CCACCG]** 1355 (88.59)   
343 + **[CGAGCG]** 1494 (88.31)   
343 - **[CGACCC]** 1434 (88.16)   
343 - **.CGCCCG.** 1338 (95.73)   
343 - **[CCACCG]** 1275 (88.59)   
343 - **[CCACCG]** 1159 (88.59)   
343 - **.CGGCCG.** 1093 (95.29)   
343 - **.CGCCCG.** 963 (95.73)   
343 - **.CGGCCG.** 923 (95.29)   
343 - **.CGCCCG.** 919 (95.73)   
343 - **.CGCCCG.** 858 (95.73)   
343 - **[CCACCG]** 714 (88.59)   
343 - **[CGTCCG]** 668 (88.90)   
343 - **.CGGCCG.** 651 (95.29)   
343 - **.CGACCG.** 626 (100.00)   
343 - **.CGCCCG.** 602 (95.73)   
343 - **.CGGCCG.** 558 (95.29)   
343 - **.CGCCCG.** 472 (95.73)   
343 - **[GGACCG]** 302 (87.21)   
343 - **[CGACCC]** 292 (88.16)   
343 - **.CGCCCG.** 287 (95.73)   
343 - **[CGACCC]** 89 (88.16)   
344 + **[CGACCC]** 752 (88.16)   
344 + **.CGGCCG.** 777 (95.29)   
344 + **.CGCCCG.** 1004 (95.73)   
344 + **[GGACCG]** 1299 (87.21)   
344 - **.CGGCCG.** 777 (95.29)   
344 - **[GGACCG]** 491 (87.21)   
345 - **.CGCCCG.** 384 (95.73)   
346 + **.CGGCCG.** 4 (95.29)   
346 + **[CGAGCG]** 733 (88.31)   
346 + **.CGCCCG.** 945 (95.73)   
346 + **[CGAGCG]** 1033 (88.31)   
346 + **[CGAGCG]** 1043 (88.31)   
346 + **[CCACCG]** 1117 (88.59)   
346 + **.CGCCCG.** 1148 (95.73)   
346 + **.CGGCCG.** 1349 (95.29)   
346 + **.CGGCCG.** 1353 (95.29)   
346 - **.CGCCCG.** 1369 (95.73)   
346 - **.CGGCCG.** 1353 (95.29)   
346 - **.CGGCCG.** 1349 (95.29)   
346 - **[GGACCG]** 1167 (87.21)   
346 - **[CGAGCG]** 834 (88.31)   
346 - **[CCACCG]** 305 (88.59)   
346 - **[CGACCC]** 53 (88.16)   
346 - **.CGACCG.** 19 (100.00)   
346 - **.CGCCCG.** 15 (95.73)   
346 - **.CGGCCG.** 4 (95.29)   
347 + **.CGCCCG.** 416 (95.73)   
347 + **.CGGCCG.** 802 (95.29)   
347 + **.CGCCCG.** 915 (95.73)   
347 + **[CGAGCG]** 1271 (88.31)   
347 + **.CGACCG.** 1444 (100.00)   
347 + **.CGCCCG.** 1496 (95.73)   
347 - **.CGCCCG.** 1151 (95.73)   
347 - **.CGCCCG.** 1117 (95.73)   
347 - **.CGACCG.** 1073 (100.00)   
347 - **[GGACCG]** 957 (87.21)   
347 - **.CGGCCG.** 802 (95.29)   
347 - **.CGACCG.** 572 (100.00)   
347 - **[CGACCC]** 463 (88.16)   
348 + **.CGCCCG.** 22 (95.73)   
348 + **[CCACCG]** 34 (88.59)   
348 + **.CGCCCG.** 101 (95.73)   
348 + **[CGTCCG]** 182 (88.90)   
348 + **.CGCCCG.** 186 (95.73)   
348 + **[CCACCG]** 196 (88.59)   
348 + **[GGACCG]** 262 (87.21)   
348 + **.CGGCCG.** 277 (95.29)   
348 + **.CGGCCG.** 346 (95.29)   
348 + **.CGCCCG.** 350 (95.73)   
348 + **.CGCCCG.** 384 (95.73)   
348 + **.CGCCCG.** 419 (95.73)   
348 + **.CGCCCG.** 423 (95.73)   
348 + **[CGTCCG]** 677 (88.90)   
348 + **[CGACCC]** 776 (88.16)   
348 + **.CGCCCG.** 794 (95.73)   
348 + **[CCACCG]** 833 (88.59)   
348 + **[CGTCCG]** 876 (88.90)   
348 + **.CGCCCG.** 880 (95.73)   
348 + **.CGCCCG.** 921 (95.73)   
348 + **.CGGCCG.** 1044 (95.29)   
348 + **.CGACCG.** 1071 (100.00)   
348 + **.CGCCCG.** 1094 (95.73)   
348 + **.CGACCG.** 1152 (100.00)   
348 + **.CGCCCG.** 1254 (95.73)   
348 + **[CGAGCG]** 1367 (88.31)   
348 + **.CGGCCG.** 1472 (95.29)   
348 - **[CGACCC]** 1485 (88.16)   
348 - **.CGCCCG.** 1476 (95.73)   
348 - **.CGGCCG.** 1472 (95.29)   
348 - **[CGAGCG]** 1371 (88.31)   
348 - **.CGCCCG.** 1283 (95.73)   
348 - **.CGCCCG.** 1260 (95.73)   
348 - **.CGCCCG.** 1236 (95.73)   
348 - **.CGCCCG.** 1194 (95.73)   
348 - **.CGGCCG.** 1044 (95.29)   
348 - **.CGCCCG.** 965 (95.73)   
348 - **[CCACCG]** 837 (88.59)   
348 - **[CCACCG]** 763 (88.59)   
348 - **.CGGCCG.** 346 (95.29)   
348 - **.CGGCCG.** 277 (95.29)   
349 + **[GGACCG]** 581 (87.21)   
349 + **.CGCCCG.** 642 (95.73)   
349 + **[GGACCG]** 848 (87.21)   
349 + **.CGGCCG.** 1082 (95.29)   
349 + **.CGACCG.** 1092 (100.00)   
349 - **.CGCCCG.** 1402 (95.73)   
349 - **.CGCCCG.** 1271 (95.73)   
349 - **.CGCCCG.** 1217 (95.73)   
349 - **.CGCCCG.** 1195 (95.73)   
349 - **.CGGCCG.** 1082 (95.29)   
349 - **[CGTCCG]** 1019 (88.90)   
349 - **.CGACCG.** 875 (100.00)   
349 - **.CGCCCG.** 651 (95.73)   
349 - **[CGTCCG]** 487 (88.90)   
349 - **.CGCCCG.** 85 (95.73)   
350 + **.CGGCCG.** 1161 (95.29)   
350 + **[CGACCC]** 1227 (88.16)   
350 + **.CGCCCG.** 1347 (95.73)   
350 - **.CGCCCG.** 1441 (95.73)   
350 - **.CGCCCG.** 1432 (95.73)   
350 - **.CGGCCG.** 1161 (95.29)   
350 - **.CGCCCG.** 1033 (95.73)   
351 + **.CGCCCG.** 36 (95.73)   
351 + **.CGCCCG.** 790 (95.73)   
351 + **.CGCCCG.** 997 (95.73)   
351 + **.CGCCCG.** 1017 (95.73)   
351 + **.CGCCCG.** 1041 (95.73)   
351 + **[GGACCG]** 1310 (87.21)   
351 + **.CGCCCG.** 1337 (95.73)   
351 - **[GGACCG]** 1437 (87.21)   
351 - **[CGACCC]** 1137 (88.16)   
351 - **[CGTCCG]** 1034 (88.90)   
351 - **[GGACCG]** 1029 (87.21)   
351 - **[CGAGCG]** 1001 (88.31)   
351 - **.CGCCCG.** 900 (95.73)   
351 - **[GGACCG]** 668 (87.21)   
351 - **[CGACCC]** 528 (88.16)   
351 - **[CGTCCG]** 291 (88.90)   
352 + **.CGGCCG.** 70 (95.29)   
352 + **.CGACCG.** 173 (100.00)   
352 + **.CGCCCG.** 410 (95.73)   
352 + **.CGGCCG.** 446 (95.29)   
352 + **.CGCCCG.** 549 (95.73)   
352 + **[CCACCG]** 626 (88.59)   
352 + **.CGGCCG.** 723 (95.29)   
352 + **[CGAGCG]** 1008 (88.31)   
352 + **.CGGCCG.** 1035 (95.29)   
352 + **.CGGCCG.** 1264 (95.29)   
352 - **.CGGCCG.** 1264 (95.29)   
352 - **.CGGCCG.** 1035 (95.29)   
352 - **.CGCCCG.** 854 (95.73)   
352 - **[GGACCG]** 817 (87.21)   
352 - **.CGGCCG.** 723 (95.29)   
352 - **[GGACCG]** 650 (87.21)   
352 - **.CGGCCG.** 446 (95.29)   
352 - **[GGACCG]** 357 (87.21)   
352 - **.CGGCCG.** 70 (95.29)   
353 + **[CGAGCG]** 308 (88.31)   
353 + **.CGGCCG.** 346 (95.29)   
353 + **.CGCCCG.** 693 (95.73)   
353 + **[CCACCG]** 854 (88.59)   
353 + **.CGCCCG.** 894 (95.73)   
353 - **[CGTCCG]** 1393 (88.90)   
353 - **[CGAGCG]** 1368 (88.31)   
353 - **[CGACCC]** 930 (88.16)   
353 - **[CGAGCG]** 906 (88.31)   
353 - **[CGTCCG]** 706 (88.90)   
353 - **[CGAGCG]** 421 (88.31)   
353 - **.CGGCCG.** 346 (95.29)   
353 - **[CGACCC]** 147 (88.16)   
354 + **.CGCCCG.** 503 (95.73)   
354 - **[CGTCCG]** 1186 (88.90)   
356 + **[CGACCC]** 479 (88.16)   
356 + **[CGACCC]** 516 (88.16)   
356 + **[CCACCG]** 678 (88.59)   
356 + **[CGACCC]** 690 (88.16)   
356 + **[CGACCC]** 706 (88.16)   
356 + **.CGCCCG.** 768 (95.73)   
356 + **.CGGCCG.** 1001 (95.29)   
356 + **.CGCCCG.** 1022 (95.73)   
356 + **.CGGCCG.** 1128 (95.29)   
356 + **[GGACCG]** 1203 (87.21)   
356 - **.CGGCCG.** 1128 (95.29)   
356 - **.CGGCCG.** 1001 (95.29)   
356 - **.CGACCG.** 110 (100.00)   
357 + **[CGACCC]** 539 (88.16)   
357 + **[GGACCG]** 641 (87.21)   
357 + **.CGCCCG.** 688 (95.73)   
357 + **[GGACCG]** 696 (87.21)   
357 + **.CGGCCG.** 867 (95.29)   
357 + **.CGCCCG.** 913 (95.73)   
357 + **.CGCCCG.** 986 (95.73)   
357 - **[CGAGCG]** 1509 (88.31)   
357 - **.CGCCCG.** 1467 (95.73)   
357 - **[CCACCG]** 1459 (88.59)   
357 - **[CGACCC]** 1452 (88.16)   
357 - **[CCACCG]** 1088 (88.59)   
357 - **.CGGCCG.** 867 (95.29)   
357 - **.CGACCG.** 762 (100.00)   
357 - **[GGACCG]** 456 (87.21)   
357 - **.CGCCCG.** 416 (95.73)   
357 - **[CCACCG]** 25 (88.59)   
358 + **[CGACCC]** 660 (88.16)   
358 + **.CGGCCG.** 742 (95.29)   
358 + **.CGGCCG.** 776 (95.29)   
358 + **[GGACCG]** 1427 (87.21)   
358 - **[CGACCC]** 1204 (88.16)   
358 - **[CCACCG]** 1194 (88.59)   
358 - **[CCACCG]** 1045 (88.59)   
358 - **[CGACCC]** 1009 (88.16)   
358 - **.CGCCCG.** 878 (95.73)   
358 - **.CGGCCG.** 776 (95.29)   
358 - **.CGGCCG.** 742 (95.29)   
358 - **.CGACCG.** 417 (100.00)   
359 + **.CGCCCG.** 357 (95.73)   
359 + **.CGCCCG.** 1034 (95.73)   
359 + **.CGCCCG.** 1038 (95.73)   
359 + **[CCACCG]** 1066 (88.59)   
359 + **[CCACCG]** 1100 (88.59)   
359 - **.CGCCCG.** 1421 (95.73)   
359 - **.CGCCCG.** 1417 (95.73)   
359 - **.CGCCCG.** 1325 (95.73)   
359 - **.CGCCCG.** 773 (95.73)   
359 - **[CGAGCG]** 715 (88.31)   
359 - **[CGTCCG]** 572 (88.90)   
359 - **.CGCCCG.** 547 (95.73)   
359 - **[CGAGCG]** 361 (88.31)   
359 - **.CGACCG.** 296 (100.00)   
359 - **.CGCCCG.** 182 (95.73)   
360 + **.CGCCCG.** 465 (95.73)   
360 + **.CGACCG.** 593 (100.00)   
360 + **.CGCCCG.** 609 (95.73)   
360 + **.CGGCCG.** 613 (95.29)   
360 + **.CGGCCG.** 671 (95.29)   
360 + **.CGGCCG.** 705 (95.29)   
360 + **[CCACCG]** 741 (88.59)   
360 + **.CGCCCG.** 766 (95.73)   
360 + **.CGGCCG.** 796 (95.29)   
360 - **[CGACCC]** 1302 (88.16)   
360 - **.CGCCCG.** 1047 (95.73)   
360 - **[CCACCG]** 978 (88.59)   
360 - **.CGCCCG.** 913 (95.73)   
360 - **.CGGCCG.** 796 (95.29)   
360 - **.CGGCCG.** 705 (95.29)   
360 - **.CGGCCG.** 671 (95.29)   
360 - **[CGAGCG]** 662 (88.31)   
360 - **.CGGCCG.** 613 (95.29)   
361 + **.CGGCCG.** 910 (95.29)   
361 - **.CGCCCG.** 1103 (95.73)   
361 - **.CGGCCG.** 910 (95.29)   
361 - **[CGAGCG]** 813 (88.31)   
361 - **.CGCCCG.** 634 (95.73)   
362 + **.CGGCCG.** 34 (95.29)   
362 + **[CGAGCG]** 701 (88.31)   
362 + **[CGTCCG]** 740 (88.90)   
362 + **[CCACCG]** 758 (88.59)   
362 + **.CGCCCG.** 796 (95.73)   
362 + **[GGACCG]** 819 (87.21)   
362 + **[GGACCG]** 1016 (87.21)   
362 + **[GGACCG]** 1194 (87.21)   
362 + **[CGACCC]** 1401 (88.16)   
362 - **[CGTCCG]** 1285 (88.90)   
362 - **[CGACCC]** 1231 (88.16)   
362 - **[CGTCCG]** 1171 (88.90)   
362 - **.CGCCCG.** 1126 (95.73)   
362 - **.CGCCCG.** 1098 (95.73)   
362 - **.CGCCCG.** 1059 (95.73)   
362 - **[GGACCG]** 1045 (87.21)   
362 - **.CGCCCG.** 813 (95.73)   
362 - **.CGCCCG.** 708 (95.73)   
362 - **[CGTCCG]** 96 (88.90)   
362 - **.CGGCCG.** 34 (95.29)   
363 + **[CCACCG]** 699 (88.59)   
363 + **.CGGCCG.** 1085 (95.29)   
363 + **.CGGCCG.** 1224 (95.29)   
363 - **.CGGCCG.** 1224 (95.29)   
363 - **[CGAGCG]** 1172 (88.31)   
363 - **.CGGCCG.** 1085 (95.29)   
363 - **[CGAGCG]** 942 (88.31)   
363 - **[CGTCCG]** 761 (88.90)   
363 - **[GGACCG]** 613 (87.21)   
364 + **.CGCCCG.** 896 (95.73)   
364 + **.CGGCCG.** 1181 (95.29)   
364 + **.CGCCCG.** 1188 (95.73)   
364 + **.CGCCCG.** 1362 (95.73)   
364 + **.CGCCCG.** 1393 (95.73)   
364 - **.CGGCCG.** 1181 (95.29)   
364 - **[GGACCG]** 1043 (87.21)   
364 - **.CGCCCG.** 301 (95.73)   
365 + **.CGGCCG.** 947 (95.29)   
365 - **.CGGCCG.** 947 (95.29)   
366 + **.CGGCCG.** 5 (95.29)   
366 + **.CGGCCG.** 39 (95.29)   
366 + **.CGCCCG.** 72 (95.73)   
366 + **.CGGCCG.** 91 (95.29)   
366 + **.CGCCCG.** 119 (95.73)   
366 + **.CGGCCG.** 138 (95.29)   
366 + **.CGGCCG.** 180 (95.29)   
366 + **.CGGCCG.** 307 (95.29)   
366 + **.CGCCCG.** 373 (95.73)   
366 + **.CGCCCG.** 377 (95.73)   
366 + **.CGCCCG.** 381 (95.73)   
366 + **[GGACCG]** 388 (87.21)   
366 + **[CGTCCG]** 450 (88.90)   
366 + **.CGCCCG.** 517 (95.73)   
366 + **[CGTCCG]** 587 (88.90)   
366 + **[CGTCCG]** 669 (88.90)   
366 + **[CGACCC]** 820 (88.16)   
366 + **.CGCCCG.** 831 (95.73)   
366 + **.CGGCCG.** 864 (95.29)   
366 + **.CGGCCG.** 1019 (95.29)   
366 + **[CGTCCG]** 1303 (88.90)   
366 + **.CGGCCG.** 1352 (95.29)   
366 + **.CGGCCG.** 1410 (95.29)   
366 + **[CGAGCG]** 1434 (88.31)   
366 - **.CGCCCG.** 1441 (95.73)   
366 - **[GGACCG]** 1416 (87.21)   
366 - **.CGGCCG.** 1410 (95.29)   
366 - **.CGCCCG.** 1379 (95.73)   
366 - **.CGGCCG.** 1352 (95.29)   
366 - **[GGACCG]** 1297 (87.21)   
366 - **[CCACCG]** 1219 (88.59)   
366 - **.CGCCCG.** 1176 (95.73)   
366 - **[CGACCC]** 1113 (88.16)   
366 - **[CGACCC]** 1061 (88.16)   
366 - **.CGGCCG.** 1019 (95.29)   
366 - **[CGAGCG]** 1003 (88.31)   
366 - **[CGAGCG]** 901 (88.31)   
366 - **.CGGCCG.** 864 (95.29)   
366 - **[CGTCCG]** 693 (88.90)   
366 - **.CGCCCG.** 609 (95.73)   
366 - **.CGCCCG.** 544 (95.73)   
366 - **.CGGCCG.** 307 (95.29)   
366 - **[CGACCC]** 212 (88.16)   
366 - **.CGGCCG.** 180 (95.29)   
366 - **.CGGCCG.** 138 (95.29)   
366 - **.CGGCCG.** 91 (95.29)   
366 - **.CGGCCG.** 39 (95.29)   
366 - **.CGGCCG.** 5 (95.29)   
367 + **.CGCCCG.** 11 (95.73)   
367 + **.CGGCCG.** 74 (95.29)   
367 + **[CGAGCG]** 309 (88.31)   
367 + **.CGCCCG.** 393 (95.73)   
367 + **[CGAGCG]** 427 (88.31)   
367 + **.CGCCCG.** 529 (95.73)   
367 + **[CGAGCG]** 790 (88.31)   
367 + **[GGACCG]** 1016 (87.21)   
367 + **.CGCCCG.** 1030 (95.73)   
367 + **[CCACCG]** 1123 (88.59)   
367 + **[CCACCG]** 1224 (88.59)   
367 - **.CGCCCG.** 1054 (95.73)   
367 - **[CGAGCG]** 933 (88.31)   
367 - **[CGACCC]** 178 (88.16)   
367 - **.CGGCCG.** 74 (95.29)   
367 - **.CGCCCG.** 70 (95.73)   
  

**Frequency Matrix**

| All Occs Best Occs|  |  |  |  |  |  |  |  |  |  |  |  |  |  |  |  |  |  |  |  |  |  |  |  |  |  |  |  |  |  |  |  |  |  |  |  |  |  |  |  |  |  |  |  |  |  |  |  |  |  |  |  |  |  |  |  |  |  |  |  |  |  |  |  |  |  |  |  |  |  |  |  |  |  |  |  |  |  |  |  |  |  |  |  | | --- | --- | --- | --- | --- | --- | --- | --- | --- | --- | --- | --- | --- | --- | --- | --- | --- | --- | --- | --- | --- | --- | --- | --- | --- | --- | --- | --- | --- | --- | --- | --- | --- | --- | --- | --- | --- | --- | --- | --- | --- | --- | --- | --- | --- | --- | --- | --- | --- | --- | --- | --- | --- | --- | --- | --- | --- | --- | --- | --- | --- | --- | --- | --- | --- | --- | --- | --- | --- | --- | --- | --- | --- | --- | --- | --- | --- | --- | --- | --- | --- | --- | --- | --- | | **A C G T A C G T | | | | | | | | | | | |**| **1** 156 4776 294 131 | 0 2368 0 0|  |  |  |  |  |  |  |  |  |  |  |  |  |  |  |  |  |  |  |  |  |  |  |  |  |  |  |  |  |  |  |  |  |  |  |  |  |  |  |  |  |  |  |  |  |  |  |  |  |  |  |  |  |  |  |  |  |  |  |  | | --- | --- | --- | --- | --- | --- | --- | --- | --- | --- | --- | --- | --- | --- | --- | --- | --- | --- | --- | --- | --- | --- | --- | --- | --- | --- | --- | --- | --- | --- | --- | --- | --- | --- | --- | --- | --- | --- | --- | --- | --- | --- | --- | --- | --- | --- | --- | --- | --- | --- | --- | --- | --- | --- | --- | --- | --- | --- | --- | --- | | **2** 108 395 4752 102 | 0 0 2368 0|  |  |  |  |  |  |  |  |  |  |  |  |  |  |  |  |  |  |  |  |  |  |  |  |  |  |  |  |  |  |  |  |  |  |  |  |  |  |  |  |  |  |  |  |  |  |  |  | | --- | --- | --- | --- | --- | --- | --- | --- | --- | --- | --- | --- | --- | --- | --- | --- | --- | --- | --- | --- | --- | --- | --- | --- | --- | --- | --- | --- | --- | --- | --- | --- | --- | --- | --- | --- | --- | --- | --- | --- | --- | --- | --- | --- | --- | --- | --- | --- | | **3** 2909 1147 1042 259 | 179 1147 1042 0|  |  |  |  |  |  |  |  |  |  |  |  |  |  |  |  |  |  |  |  |  |  |  |  |  |  |  |  |  |  |  |  |  |  |  |  | | --- | --- | --- | --- | --- | --- | --- | --- | --- | --- | --- | --- | --- | --- | --- | --- | --- | --- | --- | --- | --- | --- | --- | --- | --- | --- | --- | --- | --- | --- | --- | --- | --- | --- | --- | --- | | **4** 71 4858 380 48 | 0 2368 0 0|  |  |  |  |  |  |  |  |  |  |  |  |  |  |  |  |  |  |  |  |  |  |  |  | | --- | --- | --- | --- | --- | --- | --- | --- | --- | --- | --- | --- | --- | --- | --- | --- | --- | --- | --- | --- | --- | --- | --- | --- | | **5** 145 4909 164 139 | 0 2368 0 0|  |  |  |  |  |  |  |  |  |  |  |  | | --- | --- | --- | --- | --- | --- | --- | --- | --- | --- | --- | --- | | **6** 81 361 4760 155 | 0 0 2368 0 | | | | | | | | | | | | | | | | | | | | | | | | | | | | | | | | | | | | | | | | | | | | | | | | | | | | | | | | | | | | | | | | | | | | | | | | | | | | | | | | | | | | |

  
  

---

  
  
  
 **CGCGTCGA**   
**TCGACGCG**  
  
7 redundant motifs found:  
CGCGTCGT - CGACGCGC - CGACGCGT - TCGCGTCG - CGTCGACG - CGACGC - CGCGTC -   
  
Best occurrences (match percentage):   
Seq St oligo pos match
  
 1 + **.CGCGCCGC.** 1000 (92.40)   
1 + **[CGCCGCGC]** 1068 (85.13)   
1 + **[CGCCCCGA]** 1080 (88.75)   
1 - **.CGCGGCGC.** 1067 (93.00)   
1 - **[CGCCTCGG]** 1062 (87.36)   
1 - **[CGCGCGGA]** 882 (88.78)   
2 + **.CGCGGCCA.** 201 (90.02)   
2 + **[CGCGGGGA]** 364 (89.38)   
2 + **[CGCGTCTA]** 449 (87.79)   
2 + **[CGCGTCCG]** 515 (88.04)   
2 + **[CGCGGCCC]** 721 (85.80)   
2 + **[CGCGCGGA]** 892 (88.78)   
2 + **[CGCGGCCC]** 977 (85.80)   
2 + **.CGCGGCGC.** 996 (93.00)   
2 + **[CGCGGGGA]** 1190 (89.38)   
2 + **.CGCGGCGC.** 1447 (93.00)   
2 - **.CGCGCCGC.** 1448 (92.40)   
2 - **[CGCCGCGC]** 1446 (85.13)   
2 - **.CGCGCCGC.** 997 (92.40)   
2 - **[CGCCGCGA]** 995 (89.34)   
2 - **.CGCGCCGC.** 701 (92.40)   
2 - **[CGCGGGGC]** 511 (85.16)   
2 - **[GGCGTCGG]** 506 (85.99)   
2 - **[CGCGTAGA]** 453 (87.53)   
2 - **[GGCGGCGA]** 307 (87.97)   
3 + **.CGCGGCGG.** 1223 (92.45)   
3 + **[CGCCGCGC]** 1415 (85.13)   
3 - **[CACGTCGA]** 1486 (86.48)   
3 - **.CGCGGCGG.** 1414 (92.45)   
3 - **[CGCTGCGA]** 1306 (85.51)   
3 - **.CGCGCCGA.** 1247 (96.62)   
4 + **[CGGGTCGG]** 687 (85.44)   
4 + **[CGCCGCGA]** 698 (89.34)   
4 + **[CGCGGCCC]** 742 (85.80)   
4 + **.CGCGGCGC.** 802 (93.00)   
4 + **.CGCGGCGG.** 822 (92.45)   
4 + **[CGCCGCGC]** 1205 (85.13)   
4 + **[CGCGGGGC]** 1286 (85.16)   
4 - **[CGCGCCCC]** 1280 (85.20)   
4 - **[CGCGCCCC]** 1270 (85.20)   
4 - **.CGCGGCGG.** 1204 (92.45)   
4 - **[CGCGGGGC]** 1195 (85.16)   
4 - **[CGCGGGGA]** 1175 (89.38)   
4 - **[CGCGGGGC]** 871 (85.16)   
4 - **[CGGGTCGC]** 851 (85.99)   
4 - **.CGCGCCGC.** 826 (92.40)   
4 - **[CGCCGCGA]** 801 (89.34)   
4 - **[CGCGACGG]** 738 (85.91)   
4 - **[CGGGTCGC]** 702 (85.99)   
4 - **.CGCGGCGG.** 697 (92.45)   
4 - **[GGCGCCGA]** 691 (87.37)   
4 - **[CGCGCCCA]** 681 (89.42)   
5 + **[CGCGGGGA]** 1076 (89.38)   
5 + **[CGCCTCGG]** 1348 (87.36)   
5 + **[CGCGGCCG]** 1395 (85.25)   
5 + **[CGCGGCCG]** 1440 (85.25)   
5 - **[CGCCTCGG]** 1445 (87.36)   
5 - **.CGCGTCGG.** 1436 (95.23)   
5 - **[CGGGCCGA]** 1352 (86.82)   
5 - **[CGCGGGGA]** 1342 (89.38)   
6 + **.CGCGGCGA.** 1440 (97.21)   
8 + **[CGCGTCTA]** 149 (87.79)   
8 + **[CGCGTCCC]** 372 (88.59)   
8 + **[CGCGTGGC]** 454 (87.95)   
8 + **[CGCGCCCC]** 494 (85.20)   
8 + **[CGGGGCGA]** 899 (87.42)   
8 + **.CGCGTCGA.** 1146 (100.00)   
8 + **[CGCGACGG]** 1466 (85.91)   
8 - **.GGCGTCGA.** 1150 (90.75)   
8 - **[CGCGGGGC]** 1142 (85.16)   
8 - **.CGCGGCGC.** 450 (93.00)   
8 - **[CGCGCGGA]** 368 (88.78)   
8 - **[CGGGCCGA]** 347 (86.82)   
9 + **[CGCGCCCC]** 1062 (85.20)   
9 + **[CGCGACGG]** 1268 (85.91)   
9 - **[CCCGTCGC]** 1269 (85.16)   
10 + **[CGCGCCCC]** 513 (85.20)   
10 + **[CGCGGGGC]** 830 (85.16)   
10 + **.CGCGGCGG.** 1024 (92.45)   
10 + **.CGCGGCGG.** 1096 (92.45)   
10 + **[CGCGGCCG]** 1121 (85.25)   
10 + **[CGCGGCCC]** 1331 (85.80)   
10 - **.CGCCTCGA.** 1184 (92.13)   
10 - **[CGCGGCCG]** 1117 (85.25)   
10 - **[CGCCGCGC]** 1095 (85.13)   
10 - **[CGCGGCCC]** 1020 (85.80)   
10 - **[CGCGCCCC]** 869 (85.20)   
10 - **[CGCTGCGA]** 792 (85.51)   
10 - **[CGCGGCCC]** 509 (85.80)   
10 - **[CGCGGCCG]** 33 (85.25)   
11 + **[CCCGTCGC]** 526 (85.16)   
11 + **.CGCGCCGC.** 606 (92.40)   
11 + **[CGCGGCCG]** 611 (85.25)   
11 + **[CGCGGCCC]** 715 (85.80)   
11 + **.CGCGGCGC.** 769 (93.00)   
11 + **[CGCCGCGC]** 993 (85.13)   
11 + **[CGCCGCGC]** 1026 (85.13)   
11 + **.CGCGGCGG.** 1116 (92.45)   
11 + **[CGCCGCGC]** 1154 (85.13)   
11 - **.CGCGGCGG.** 1153 (92.45)   
11 - **[CGCCGCGC]** 1115 (85.13)   
11 - **.CGCGGCGG.** 1025 (92.45)   
11 - **.CGCGGCGG.** 992 (92.45)   
11 - **[CGCGTCCC]** 745 (88.59)   
11 - **[CGCGGGGC]** 719 (85.16)   
11 - **[CGCGGCCG]** 711 (85.25)   
11 - **[CGCGTCCG]** 696 (88.04)   
11 - **[CGCGGGGC]** 659 (85.16)   
11 - **[CGCGGCCG]** 613 (85.25)   
11 - **.CGCGGCGC.** 607 (93.00)   
11 - **[CGCGGGGC]** 589 (85.16)   
11 - **[CGCGACGG]** 527 (85.91)   
11 - **[CGCGGCCG]** 495 (85.25)   
11 - **[CGCCCCGA]** 92 (88.75)   
12 + **[CGCGCGGA]** 511 (88.78)   
12 + **[CGCGCCCA]** 543 (89.42)   
12 + **.CGCGCCGC.** 696 (92.40)   
12 + **[CGCGGCCC]** 711 (85.80)   
12 + **[CGCGTCCC]** 841 (88.59)   
12 + **[CGGGTCGC]** 1092 (85.99)   
12 + **[CGCGGCCC]** 1120 (85.80)   
12 + **[CGCCTCGG]** 1148 (87.36)   
12 + **[CGCCGCGC]** 1177 (85.13)   
12 + **.CGCGCCGC.** 1253 (92.40)   
12 + **[CGCCGCGA]** 1354 (89.34)   
12 - **.CGCGGCGC.** 1353 (93.00)   
12 - **[CGCGCCCC]** 1331 (85.20)   
12 - **.CGCGGCCA.** 1273 (90.02)   
12 - **[CGCGGCCG]** 1249 (85.25)   
12 - **.CGCGGCGC.** 1176 (93.00)   
12 - **[CGCGCGGA]** 837 (88.78)   
12 - **[CGCGGCCC]** 738 (85.80)   
12 - **.CGCGCCGG.** 539 (91.85)   
12 - **[CGCGCCCC]** 522 (85.20)   
12 - **[CGCGTGGG]** 203 (87.40)   
13 + **[CGCGCGGA]** 533 (88.78)   
13 + **.CGCGCCGC.** 615 (92.40)   
13 + **[CGCGGCCC]** 703 (85.80)   
13 + **[CGCCGCGC]** 720 (85.13)   
13 + **[CGCGTCCC]** 742 (88.59)   
13 + **[CGCGTCCC]** 766 (88.59)   
13 + **[CGCGCCCC]** 773 (85.20)   
13 + **[CGCGCCCC]** 780 (85.20)   
13 + **[CGCCTCGC]** 868 (87.91)   
13 + **[CGGGGCGA]** 909 (87.42)   
13 + **[CGCGGCCG]** 947 (85.25)   
13 + **[CGCGGCCG]** 1064 (85.25)   
13 + **[CGCCGCGC]** 1109 (85.13)   
13 + **[CCCGCCGA]** 1123 (86.00)   
13 + **[CGCGCCGT]** 1187 (87.13)   
13 + **.CGCGCCGG.** 1264 (91.85)   
13 + **[CGGGTCGC]** 1299 (85.99)   
13 + **[GGCGTCGC]** 1319 (86.54)   
13 + **[CGCGGGGC]** 1324 (85.16)   
13 + **.CGCGGCGG.** 1352 (92.45)   
13 + **[CGGGTCGG]** 1441 (85.44)   
13 + **[CGGGTCGC]** 1465 (85.99)   
13 - **[CCCGTCGC]** 1403 (85.16)   
13 - **[CGCCGCGC]** 1351 (85.13)   
13 - **[CGCGACGC]** 1320 (86.46)   
13 - **.CGCGGCGG.** 1195 (92.45)   
13 - **[GGCGGCGA]** 1159 (87.97)   
13 - **[CGGGCCGA]** 1119 (86.82)   
13 - **.CGCGGCGG.** 1108 (92.45)   
13 - **.CGCGCCGG.** 1060 (91.85)   
13 - **[CGCGCGGA]** 1008 (88.78)   
13 - **.CGCGCCGA.** 799 (96.62)   
13 - **[CGCGGGGC]** 783 (85.16)   
13 - **[CGCGGGGC]** 776 (85.16)   
13 - **[CGCGGGGC]** 762 (85.16)   
13 - **.CGCGGCGG.** 719 (92.45)   
13 - **[GGCGGCGA]** 716 (87.97)   
13 - **[CGCGTGGC]** 652 (87.95)   
14 + **[CGCGCCCC]** 292 (85.20)   
14 + **.CGCGGCGC.** 337 (93.00)   
14 + **.CGCGGCGG.** 748 (92.45)   
14 + **[GGCGCCGA]** 811 (87.37)   
14 + **.CGCGGCGG.** 940 (92.45)   
14 - **[CGCCGCGC]** 939 (85.13)   
14 - **[CGCCGCGC]** 747 (85.13)   
14 - **[CGCGGGGC]** 333 (85.16)   
14 - **[CGCGGGGC]** 231 (85.16)   
15 + **[CGCCGCGC]** 414 (85.13)   
15 + **.CGCGGCCA.** 545 (90.02)   
15 + **[CGCGCCCC]** 827 (85.20)   
15 + **.CGCGGCGC.** 986 (93.00)   
15 + **[CGGGTCGG]** 1110 (85.44)   
15 + **[CGCGTCCC]** 1183 (88.59)   
15 + **[CGCGCGGA]** 1202 (88.78)   
15 - **.CGCGGCCA.** 1262 (90.02)   
15 - **[GGCGGCGA]** 1242 (87.97)   
15 - **.CGCGCCGC.** 1198 (92.40)   
15 - **[CGCCGCGC]** 985 (85.13)   
15 - **[CGCGCCCC]** 982 (85.20)   
15 - **[CGCGGCCG]** 853 (85.25)   
15 - **.CGCGCCGG.** 731 (91.85)   
15 - **[CGCGGGGC]** 533 (85.16)   
15 - **[CGCCCCGA]** 502 (88.75)   
15 - **[CGCCTCGC]** 493 (87.91)   
15 - **[CGCGGCCG]** 457 (85.25)   
15 - **.CGCGGCGG.** 413 (92.45)   
15 - **[GGCGTCGC]** 267 (86.54)   
15 - **.CGCGGCGC.** 177 (93.00)   
15 - **[CGCGGCCC]** 21 (85.80)   
16 + **.CGCGCCGC.** 623 (92.40)   
16 + **[CGCCGCGC]** 625 (85.13)   
16 + **[CGCGCCCA]** 799 (89.42)   
16 + **.CGCGGCGG.** 1150 (92.45)   
16 + **[CGCGACGC]** 1282 (86.46)   
16 + **[CGCGGGGC]** 1313 (85.16)   
16 - **[CGCGTGGG]** 1161 (87.40)   
16 - **[CGCCGCGC]** 1149 (85.13)   
16 - **[CGCGGGGC]** 999 (85.16)   
16 - **[CGCGCCCA]** 901 (89.42)   
16 - **.CGCGGCGC.** 624 (93.00)   
16 - **[CGCGGCCC]** 619 (85.80)   
16 - **[CGCGTGGC]** 518 (87.95)   
16 - **.CGCGCCGC.** 490 (92.40)   
16 - **[CGCGGGGC]** 409 (85.16)   
17 + **[CGGGGCGA]** 983 (87.42)   
17 + **[CGCATCGA]** 1155 (86.64)   
17 + **[CCCGGCGA]** 1164 (86.59)   
17 - **.CGCGGCCA.** 1015 (90.02)   
17 - **[CGCCCCGA]** 982 (88.75)   
18 + **.CGCGCCGC.** 710 (92.40)   
18 + **.CGCGCCGC.** 834 (92.40)   
18 + **[CGCCGCGC]** 836 (85.13)   
18 + **[CGCCGCGC]** 865 (85.13)   
18 + **[CGCGCCCC]** 868 (85.20)   
18 + **[CGCCTCGC]** 916 (87.91)   
18 + **[CGCGGCCG]** 932 (85.25)   
18 + **[CGCCTCGC]** 975 (87.91)   
18 + **[CGCGCCCC]** 1109 (85.20)   
18 + **[CGGGTCGG]** 1160 (85.44)   
18 + **[CGCGGCCC]** 1204 (85.80)   
18 + **[CGCGTCCG]** 1331 (88.04)   
18 + **[GGCGGCGA]** 1460 (87.97)   
18 - **.CGCGCCGC.** 1384 (92.40)   
18 - **[CGCGGCCG]** 1327 (85.25)   
18 - **[CGCGGGGC]** 1227 (85.16)   
18 - **.CGCGGCGG.** 1200 (92.45)   
18 - **[GGCGCCGA]** 1164 (87.37)   
18 - **[CGCGGGGC]** 1125 (85.16)   
18 - **[CGCGGGGC]** 1103 (85.16)   
18 - **[CGCGGGGC]** 1025 (85.16)   
18 - **[CGCGGCCC]** 928 (85.80)   
18 - **.CGCGGCGC.** 864 (93.00)   
18 - **.CGCGGCGC.** 835 (93.00)   
20 + **[CGGGTCGG]** 815 (85.44)   
20 + **[CGCGCGGA]** 898 (88.78)   
20 + **[CGCGCCCA]** 992 (89.42)   
20 + **[CGCGTGGG]** 1199 (87.40)   
20 - **[CGCGGCCG]** 1195 (85.25)   
20 - **.CGCGTGGA.** 307 (92.16)   
20 - **.CGCGGCCA.** 298 (90.02)   
22 + **[CGCGGCCG]** 618 (85.25)   
22 + **[CGCCGCGC]** 771 (85.13)   
22 - **.CGCGGCGG.** 770 (92.45)   
22 - **.CGCCTCGA.** 673 (92.13)   
22 - **[CGCCTCGG]** 85 (87.36)   
24 + **[CCCGCCGA]** 361 (86.00)   
24 + **[GGCGGCGA]** 500 (87.97)   
24 + **[CCCGGCGA]** 537 (86.59)   
24 + **[CGCGGGGC]** 925 (85.16)   
24 + **[CGCGGGGC]** 937 (85.16)   
24 + **.CGCGGCGG.** 1040 (92.45)   
24 + **[CGGGTCGC]** 1140 (85.99)   
24 + **.CGCGCCGC.** 1145 (92.40)   
24 + **[CGCGGGGC]** 1355 (85.16)   
24 - **[CGCGCCCC]** 1096 (85.20)   
24 - **[CGCGCCCC]** 1073 (85.20)   
24 - **[CGCGCCCC]** 940 (85.20)   
24 - **[CGCGCCCC]** 933 (85.20)   
24 - **[CGCGGGGC]** 594 (85.16)   
24 - **[CGGGTCGG]** 570 (85.44)   
25 + **[CGGGGCGA]** 414 (87.42)   
25 + **[CGGGGCGA]** 445 (87.42)   
25 + **[CGGGGCGA]** 603 (87.42)   
25 + **[CGGGGCGA]** 824 (87.42)   
25 - **[CGCCTCGC]** 828 (87.91)   
25 - **.CGCGCCGC.** 790 (92.40)   
25 - **[CGCCTCGC]** 607 (87.91)   
25 - **[CGCCTCGC]** 570 (87.91)   
25 - **[CGCCTCGC]** 545 (87.91)   
25 - **[CGCCTCGC]** 386 (87.91)   
25 - **.CGCGCCGA.** 381 (96.62)   
26 + **[CGCGGCCC]** 826 (85.80)   
26 + **[CGCGCGGA]** 845 (88.78)   
26 + **[CGCGGGGC]** 1348 (85.16)   
26 + **.CGCGGCGG.** 1425 (92.45)   
26 - **.CGCGGCGC.** 1483 (93.00)   
26 - **[CGCCGCGC]** 1424 (85.13)   
26 - **[CGCGCCCC]** 1351 (85.20)   
26 - **[CGCTGCGA]** 1104 (85.51)   
26 - **[CGCCTCGC]** 1027 (87.91)   
26 - **[CGCGGGGA]** 839 (89.38)   
26 - **.CGCGCCGG.** 822 (91.85)   
26 - **.CGCGGCCA.** 801 (90.02)   
26 - **[GGCGGCGA]** 309 (87.97)   
26 - **[CGCGGCCG]** 182 (85.25)   
27 + **.CGCGTCGG.** 1146 (95.23)   
27 + **.CGCGGCGC.** 1204 (93.00)   
27 + **.CGCGCCGG.** 1249 (91.85)   
27 - **[GGCGGCGA]** 1355 (87.97)   
27 - **.CGCGCCGC.** 1205 (92.40)   
27 - **[CGCGGGGC]** 1142 (85.16)   
28 + **[CGCGTGGG]** 435 (87.40)   
28 + **.CGCGCCGG.** 740 (91.85)   
28 + **[CGCCTCGG]** 1072 (87.36)   
28 + **[CCCGTCGC]** 1095 (85.16)   
28 + **.CGCGTCGC.** 1100 (95.78)   
28 + **.CGCGCCGC.** 1105 (92.40)   
28 + **.CGCGTCGC.** 1110 (95.78)   
28 - **[CGCCTCGC]** 1412 (87.91)   
28 - **.CGCGGCGC.** 1106 (93.00)   
28 - **[CGCGACGC]** 1101 (86.46)   
28 - **[CGCGACGG]** 1096 (85.91)   
28 - **[GGCGGCGA]** 1062 (87.97)   
28 - **[CGCGGCCG]** 914 (85.25)   
28 - **[CGCCTCGC]** 793 (87.91)   
28 - **.CGCGCCGC.** 626 (92.40)   
28 - **.CGCGCCGC.** 431 (92.40)   
29 + **[CGCGCCCA]** 220 (89.42)   
29 - **[CGCGGCCC]** 1216 (85.80)   
33 + **[CGCGGCCC]** 904 (85.80)   
33 + **[CGCGGGGA]** 1031 (89.38)   
33 + **.CGCGGCGG.** 1362 (92.45)   
33 + **[CGCGGGGC]** 1413 (85.16)   
33 + **[GGCGTCGG]** 1461 (85.99)   
33 - **[CCCGCCGA]** 1426 (86.00)   
33 - **.CGCGGCGG.** 1409 (92.45)   
33 - **[CGCGGCCG]** 1358 (85.25)   
33 - **[CGGGGCGA]** 1058 (87.42)   
33 - **[CGCGGCGT]** 1038 (87.72)   
34 + **[CGCGGGGA]** 245 (89.38)   
34 + **[GGCGGCGA]** 669 (87.97)   
34 + **[CGCCGCGC]** 770 (85.13)   
34 + **[CGCGCCCC]** 773 (85.20)   
34 + **[GGCGCCGA]** 910 (87.37)   
34 + **[CGCGTAGA]** 1002 (87.53)   
34 + **[CGCGGCCC]** 1082 (85.80)   
34 + **[CGCCGCGC]** 1195 (85.13)   
34 + **[CGCGGCCC]** 1236 (85.80)   
34 + **[CGCCTCGC]** 1335 (87.91)   
34 - **.CGCGGCGC.** 1194 (93.00)   
34 - **[GGCGCCGA]** 1191 (87.37)   
34 - **[CGCGCGGA]** 1078 (88.78)   
34 - **[CGCCTCGG]** 914 (87.36)   
34 - **[CGCCTCGC]** 813 (87.91)   
34 - **[CGCGGGGC]** 790 (85.16)   
34 - **.CGCGGCGC.** 769 (93.00)   
34 - **[CGCGGGGC]** 759 (85.16)   
35 + **[CGCCGCGC]** 997 (85.13)   
35 + **[CGGGTCGG]** 1151 (85.44)   
35 - **[CGGGTCGG]** 1331 (85.44)   
35 - **[CGCCCCGA]** 1236 (88.75)   
35 - **[CGCGCCCC]** 1145 (85.20)   
35 - **.CGCGGCGA.** 996 (97.21)   
36 + **[CGCGCGGA]** 781 (88.78)   
36 + **.CGCGCCGG.** 1066 (91.85)   
36 + **[CGGGGCGA]** 1075 (87.42)   
36 - **[CGCGGGGC]** 1238 (85.16)   
36 - **.CGCGGCGC.** 1169 (93.00)   
36 - **[CGCGCGGA]** 777 (88.78)   
38 + **.CGCCTCGA.** 559 (92.13)   
39 + **[CGCGTCCC]** 1122 (88.59)   
39 + **[CCCGGCGA]** 1149 (86.59)   
39 + **[CGCGGCCG]** 1425 (85.25)   
39 - **[CGCGCCGT]** 1410 (87.13)   
39 - **[CGCGGGGA]** 1126 (89.38)   
39 - **[CGCGCCCA]** 1118 (89.42)   
40 + **[CGCGGCCC]** 895 (85.80)   
40 + **[CCCGCCGA]** 900 (86.00)   
40 + **.CGCGCCGC.** 1104 (92.40)   
40 + **[CGCCGCGC]** 1106 (85.13)   
40 + **[CGCGTCCG]** 1235 (88.04)   
40 + **.CGCGGCGA.** 1241 (97.21)   
40 + **[CGCGGGGC]** 1279 (85.16)   
40 + **.CGCGGCCA.** 1425 (90.02)   
40 - **.CGCGGCGC.** 1105 (93.00)   
40 - **[TGCGTCGA]** 931 (86.06)   
41 + **[CGCGGCCC]** 523 (85.80)   
41 + **[CCCGTCGC]** 913 (85.16)   
42 + **[GGCGCCGA]** 462 (87.37)   
42 + **[CCCGGCGA]** 597 (86.59)   
42 + **[CGCCTCGC]** 609 (87.91)   
42 + **[CGCGCCCC]** 813 (85.20)   
42 + **[CGCGGCCC]** 843 (85.80)   
42 + **[CACGTCGA]** 1156 (86.48)   
42 + **.CGCGGCGG.** 1168 (92.45)   
42 + **[GGCGGCGA]** 1177 (87.97)   
42 + **[CGGGTCGG]** 1264 (85.44)   
42 + **[CGCGGCCG]** 1311 (85.25)   
42 + **[CCCGTCGC]** 1349 (85.16)   
42 - **[CGCGGCCG]** 1473 (85.25)   
42 - **[CGCGCCCC]** 1068 (85.20)   
42 - **[CGCGGGGC]** 963 (85.16)   
42 - **[CGCGACGC]** 907 (86.46)   
42 - **[CGCGGGGC]** 521 (85.16)   
43 + **[GGCGGCGA]** 1180 (87.97)   
43 + **[CGCGGCCC]** 1197 (85.80)   
43 - **[CGCGGCCC]** 1341 (85.80)   
43 - **[CGCGCCCC]** 1249 (85.20)   
43 - **[CGCGCCCC]** 1193 (85.20)   
43 - **.CGCGGCGC.** 1099 (93.00)   
45 + **[CGCGGCCG]** 981 (85.25)   
45 + **[CGCGGCCG]** 1012 (85.25)   
45 + **[CGCGGGGC]** 1065 (85.16)   
45 - **.CGCGCCGA.** 1417 (96.62)   
45 - **.CGCGGCGC.** 395 (93.00)   
47 + **[CGCGGCCG]** 1136 (85.25)   
47 + **.CGCGCCGA.** 1249 (96.62)   
47 + **[CGCGCCCA]** 1275 (89.42)   
47 + **[CGCGGCCG]** 1367 (85.25)   
47 - **[CGCGGCCG]** 1369 (85.25)   
47 - **[CGCGGCCG]** 1269 (85.25)   
47 - **[CGCCTCGG]** 1141 (87.36)   
47 - **.CGCGCCGC.** 832 (92.40)   
47 - **[CGCGCCCC]** 348 (85.20)   
48 + **.CGCGGCGG.** 204 (92.45)   
48 + **.CGCGGCGC.** 433 (93.00)   
48 + **[CGCCTCGG]** 515 (87.36)   
48 + **[CGCGGGGA]** 1006 (89.38)   
48 + **.CGCGGCGG.** 1019 (92.45)   
48 + **.CGCGGCGC.** 1286 (93.00)   
48 + **[CGCGGCCC]** 1317 (85.80)   
48 - **[CGCGGCCC]** 1360 (85.80)   
48 - **[CGGGCCGA]** 1340 (86.82)   
48 - **.CGCGGCGG.** 1282 (92.45)   
48 - **[CGCCGCGC]** 1018 (85.13)   
48 - **[CGCCTCGG]** 216 (87.36)   
48 - **[CGCCGCGC]** 203 (85.13)   
49 + **[CGGGGCGA]** 577 (87.42)   
49 + **[CGCCCCGA]** 1283 (88.75)   
49 - **.CGCGGCGG.** 1405 (92.45)   
49 - **[CGCCCCGA]** 576 (88.75)   
51 + **[CGCGGCCC]** 36 (85.80)   
51 + **.CGCGTCGG.** 70 (95.23)   
51 + **[CGCCTCGG]** 92 (87.36)   
51 + **.CGCGGCGG.** 147 (92.45)   
51 + **[CGCCTCGG]** 386 (87.36)   
51 + **[CGCGCCCA]** 636 (89.42)   
51 + **[CGCGCCGT]** 782 (87.13)   
51 + **.GGCGTCGA.** 847 (90.75)   
51 + **[CCCGTCGC]** 974 (85.16)   
51 + **[CGCGCCCC]** 998 (85.20)   
51 + **[CCCGCCGA]** 1145 (86.00)   
51 + **[CGCGCCCC]** 1244 (85.20)   
51 + **.CGCGGCGG.** 1286 (92.45)   
51 + **[CGCGGCCG]** 1337 (85.25)   
51 + **[CGCGGGGC]** 1355 (85.16)   
51 + **.CGCGGCGC.** 1362 (93.00)   
51 - **[CGCCGCGC]** 1361 (85.13)   
51 - **[CGCGCCCC]** 1358 (85.20)   
51 - **[CGCGCCCA]** 1333 (89.42)   
51 - **[CGCGGGGC]** 1166 (85.16)   
51 - **[GGCGTCGG]** 1149 (85.99)   
51 - **[CCCGGCGA]** 1042 (86.59)   
51 - **[CGCGACGG]** 975 (85.91)   
51 - **[CGCGGGGC]** 632 (85.16)   
51 - **[CGCGGCCC]** 596 (85.80)   
51 - **[CCCGTCGC]** 489 (85.16)   
51 - **[GGCGGCGA]** 302 (87.97)   
51 - **.CGCGTCGA.** 229 (100.00)   
51 - **[CCCGCCGA]** 220 (86.00)   
51 - **[CGCCGCGA]** 146 (89.34)   
51 - **.CGCGGCGG.** 66 (92.45)   
53 + **.GGCGTCGA.** 783 (90.75)   
53 + **[CGCGGGGC]** 996 (85.16)   
53 + **.CGCGGCCA.** 1059 (90.02)   
53 + **[GGCGTCGG]** 1183 (85.99)   
53 - **[CGGGTCGC]** 798 (85.99)   
53 - **.CGCCTCGA.** 787 (92.13)   
54 - **[CGCGTCCC]** 1156 (88.59)   
54 - **[CGCGCGGA]** 959 (88.78)   
55 + **.CGCGGCCA.** 46 (90.02)   
55 + **[CGCCGCGC]** 198 (85.13)   
55 + **[CGCGCCCC]** 201 (85.20)   
55 + **[CGCGGCGT]** 943 (87.72)   
55 + **.CGCGCCGC.** 1082 (92.40)   
55 + **[CGCCGCGC]** 1084 (85.13)   
55 - **.CGCGGCGC.** 1083 (93.00)   
55 - **[CGCCGCGC]** 942 (85.13)   
55 - **.CGCGGCGG.** 882 (92.45)   
55 - **.CGCGGCGG.** 864 (92.45)   
55 - **[CGCGGGGC]** 217 (85.16)   
55 - **[CGGGGCGA]** 215 (87.42)   
55 - **.CGCGGCGA.** 197 (97.21)   
58 + **[CGCCCCGA]** 598 (88.75)   
58 + **[GGCGGCGA]** 1185 (87.97)   
58 - **[CGCGCCCA]** 1303 (89.42)   
58 - **.CGCGTCGC.** 1278 (95.78)   
58 - **[GGCGGCGA]** 1045 (87.97)   
58 - **.CGCGTCGG.** 602 (95.23)   
58 - **[CGGGGCGA]** 597 (87.42)   
58 - **[CGCGTGGC]** 416 (87.95)   
59 + **[CGCGGCCC]** 652 (85.80)   
59 + **[CGCGGCCG]** 1012 (85.25)   
59 + **.CGCGTCGG.** 1286 (95.23)   
59 + **.CGCGGCGC.** 1349 (93.00)   
59 - **[CGGGTCGC]** 1399 (85.99)   
59 - **[CGCGCCCC]** 1365 (85.20)   
59 - **.CGCGCCGC.** 1350 (92.40)   
59 - **[CGCCGCGC]** 1348 (85.13)   
59 - **[CCCGGCGA]** 1207 (86.59)   
59 - **[CGGGCCGA]** 1188 (86.82)   
59 - **[CCCGGCGA]** 683 (86.59)   
60 + **[CGCGGCCG]** 827 (85.25)   
60 + **[CGCGGGGC]** 866 (85.16)   
60 + **[CGCGGGGC]** 882 (85.16)   
60 - **[CGCCTCGG]** 903 (87.36)   
60 - **.CGCGGCGG.** 823 (92.45)   
61 + **[CGGGCCGA]** 685 (86.82)   
61 + **.CGCGGCGA.** 842 (97.21)   
61 + **[CGCGGGGC]** 927 (85.16)   
61 + **.CGCGGCGA.** 1039 (97.21)   
61 - **[CGCGGGGC]** 1378 (85.16)   
61 - **[CGCGGGGA]** 1060 (89.38)   
61 - **[CGCGGCGT]** 1035 (87.72)   
61 - **.CGCGGCGG.** 923 (92.45)   
61 - **[CGCGGCCG]** 912 (85.25)   
61 - **[CGCGTCCC]** 838 (88.59)   
61 - **.CGCGCCGG.** 499 (91.85)   
61 - **[CGCTTCGA]** 434 (88.30)   
63 + **[CGCGCCCC]** 131 (85.20)   
63 + **[CGCGGGGC]** 184 (85.16)   
63 + **[CGGGTCGG]** 211 (85.44)   
63 + **[CGCCCCGA]** 392 (88.75)   
63 + **.CGCGGCGG.** 410 (92.45)   
63 + **[GGCGGCGA]** 413 (87.97)   
63 + **[CGCGGCGT]** 445 (87.72)   
63 + **[CGCGCCGT]** 454 (87.13)   
63 + **.CGCGCCGA.** 658 (96.62)   
63 + **.CGCCTCGA.** 719 (92.13)   
63 + **.CGCGTCGG.** 854 (95.23)   
63 + **[CGCGCCGT]** 907 (87.13)   
63 + **[CGCGGCGT]** 954 (87.72)   
63 + **[CCCGTCGC]** 962 (85.16)   
63 + **[CGGGTCGG]** 1112 (85.44)   
63 + **[CGCGGGGC]** 1191 (85.16)   
63 + **.CGCGGCGG.** 1393 (92.45)   
63 + **[GGCGGCGA]** 1396 (87.97)   
63 - **[CGCGTCCC]** 1428 (88.59)   
63 - **[CGCGGCCG]** 1410 (85.25)   
63 - **[CGCCGCGC]** 1392 (85.13)   
63 - **.CGCGGCGC.** 1251 (93.00)   
63 - **[GGCGTCGC]** 996 (86.54)   
63 - **[GGCGGCGA]** 922 (87.97)   
63 - **[CGGGCCGA]** 858 (86.82)   
63 - **[CGCGCCCA]** 654 (89.42)   
63 - **[CGGGGCGA]** 621 (87.42)   
63 - **[CGCTGCGA]** 581 (85.51)   
63 - **[CGCCGCGC]** 409 (85.13)   
63 - **[CGCGCCGT]** 264 (87.13)   
63 - **[CGCCCCGA]** 215 (88.75)   
63 - **[CGCGGCCG]** 180 (85.25)   
63 - **[CGCCTCGG]** 66 (87.36)   
65 + **[CGCCTCGG]** 636 (87.36)   
65 + **[CGCGGGGC]** 788 (85.16)   
65 + **[CGCGGCTA]** 948 (85.00)   
65 + **[CGCCGCGA]** 1000 (89.34)   
65 + **[CGCCGCGC]** 1027 (85.13)   
65 + **[CGCGGGGC]** 1310 (85.16)   
65 + **[CGCTGCGA]** 1483 (85.51)   
65 - **[CGCGCGGA]** 1259 (88.78)   
65 - **[CGCGTCCC]** 1222 (88.59)   
65 - **[CGCGCCCA]** 1203 (89.42)   
65 - **[GGCGGCGA]** 1086 (87.97)   
65 - **.CGCGGCGC.** 1026 (93.00)   
65 - **.CGCGGCGG.** 999 (92.45)   
65 - **.CGCGCCGG.** 977 (91.85)   
65 - **[CGCGGGGC]** 944 (85.16)   
65 - **.CGCGCCGG.** 862 (91.85)   
65 - **[CGCGGCCG]** 827 (85.25)   
65 - **[CGCGGCCG]** 782 (85.25)   
66 + **[CGCGGGGC]** 770 (85.16)   
66 + **.CGCGCCGC.** 869 (92.40)   
66 + **.CGCGCCGG.** 955 (91.85)   
66 + **[CGGGTCGC]** 1333 (85.99)   
66 + **[CGCGTCTA]** 1406 (87.79)   
66 - **.CGCGTCCA.** 1478 (92.80)   
66 - **[CGCGCCCC]** 1177 (85.20)   
66 - **.CGCGGCGG.** 987 (92.45)   
66 - **[CGGGTCGG]** 976 (85.44)   
66 - **.CGCGTCCA.** 784 (92.80)   
67 + **[CGGGTCGC]** 818 (85.99)   
67 + **[CGCGGGGC]** 1075 (85.16)   
67 + **.CGCGTCCA.** 1198 (92.80)   
67 - **.CGCGGCGA.** 1194 (97.21)   
67 - **.CGCGTCCA.** 1171 (92.80)   
67 - **[CGCCTCGC]** 995 (87.91)   
68 + **.CGCGGCCA.** 245 (90.02)   
68 + **.CGCGTCGG.** 419 (95.23)   
68 + **[CGCCGCGC]** 588 (85.13)   
68 + **.CGCGGCGC.** 649 (93.00)   
68 + **[CGCGGCCC]** 812 (85.80)   
68 + **[CGCGACGC]** 871 (86.46)   
68 + **.CGCGGCGG.** 992 (92.45)   
68 + **[CGCGCGGA]** 1150 (88.78)   
68 + **[CGCGGGGC]** 1336 (85.16)   
68 + **.CGCGGCGG.** 1394 (92.45)   
68 - **[CGCTGCGA]** 1461 (85.51)   
68 - **[CGCCGCGC]** 1393 (85.13)   
68 - **[GGCGTCGC]** 872 (86.54)   
68 - **.CGCGCCGC.** 650 (92.40)   
68 - **.CGCGGCGG.** 587 (92.45)   
69 + **[CGCGTCCG]** 690 (88.04)   
69 + **[CGCGTGGG]** 696 (87.40)   
69 + **[CGCGGCCG]** 769 (85.25)   
69 + **.CGCGTCGC.** 894 (95.78)   
69 + **[CGCCCCGA]** 957 (88.75)   
69 + **.CGCGCCGC.** 998 (92.40)   
69 + **.CGCGCCGA.** 1121 (96.62)   
69 - **[CGCGGGGC]** 1117 (85.16)   
69 - **[CGCGGCCG]** 771 (85.25)   
70 + **[CGCGGCCC]** 1064 (85.80)   
70 + **[CGCTTCGA]** 1165 (88.30)   
70 + **.CGCGCCGA.** 1334 (96.62)   
70 + **[CGCCTCGC]** 1425 (87.91)   
70 - **[CGCGTTGA]** 1060 (87.22)   
70 - **[CGCCTCGG]** 247 (87.36)   
71 + **.CGCGTCCA.** 904 (92.80)   
71 - **[CGCGCGGA]** 1469 (88.78)   
71 - **.CGCGTGGA.** 1271 (92.16)   
71 - **[CGCGGGGC]** 1088 (85.16)   
73 + **[CGCGGCCG]** 921 (85.25)   
73 + **[CCCGCCGA]** 952 (86.00)   
73 - **[CGCTGCGA]** 1081 (85.51)   
73 - **.CGCGGCGG.** 917 (92.45)   
74 + **[CCCGGCGA]** 1139 (86.59)   
74 + **[CGCGTCCG]** 1338 (88.04)   
74 + **.CGCGGCGG.** 1429 (92.45)   
74 + **.CGCGCCGG.** 1438 (91.85)   
74 - **[CGCGGCCG]** 1434 (85.25)   
74 - **[CGCCGCGA]** 1428 (89.34)   
76 + **.CGCGCCGG.** 829 (91.85)   
76 + **.CGCGGCGA.** 866 (97.21)   
76 + **[CCCGGCGA]** 1173 (86.59)   
76 - **[CGCGTCCC]** 1282 (88.59)   
76 - **[CGCCTCGG]** 911 (87.36)   
77 + **.CGCGGCCA.** 781 (90.02)   
77 + **[CGCGTGGG]** 933 (87.40)   
79 + **[CGCCGCGA]** 653 (89.34)   
79 + **.CGCGCCGC.** 1202 (92.40)   
79 + **[CGCGTCCC]** 1325 (88.59)   
79 - **[CGCGGCCG]** 1275 (85.25)   
79 - **[CGCGGGGA]** 1198 (89.38)   
79 - **[CGCGACGG]** 1086 (85.91)   
79 - **[CGCGGCCC]** 946 (85.80)   
79 - **[CTCGTCGA]** 897 (86.25)   
79 - **.CGCGGCGG.** 876 (92.45)   
79 - **[GGCGGCGA]** 873 (87.97)   
79 - **[GGCGCCGA]** 753 (87.37)   
79 - **.CGCGGCGG.** 652 (92.45)   
80 + **[CGCCGCGC]** 1015 (85.13)   
80 + **[CGCGGGGC]** 1297 (85.16)   
80 - **.CGCGCCGC.** 1042 (92.40)   
80 - **.CGCGGCGA.** 1014 (97.21)   
80 - **.CGCGCCGG.** 953 (91.85)   
81 + **[CGCCCCGA]** 839 (88.75)   
81 - **[CGGGTCGG]** 843 (85.44)   
81 - **[CGCCTCGC]** 686 (87.91)   
81 - **[CGCGTAGA]** 681 (87.53)   
84 + **[CGCGGCCC]** 798 (85.80)   
84 + **[CGCGCCCC]** 864 (85.20)   
84 + **.CGCGGCGC.** 1023 (93.00)   
84 + **.CGCGGCGC.** 1037 (93.00)   
84 - **[CGGGCCGA]** 1262 (86.82)   
84 - **[GGCGCCGA]** 1235 (87.37)   
84 - **.CGCGCCGG.** 1151 (91.85)   
84 - **.CGCGTCGG.** 1033 (95.23)   
84 - **[CGCCGCGC]** 1022 (85.13)   
84 - **.CGCGTCGG.** 898 (95.23)   
84 - **[CGCGGGGC]** 867 (85.16)   
85 + **[CGCGTCCG]** 95 (88.04)   
85 + **[CGCGCGGA]** 440 (88.78)   
85 + **[CGCGGCCC]** 880 (85.80)   
85 + **[CGCGCCCC]** 1037 (85.20)   
85 + **[CGCGCCCC]** 1059 (85.20)   
85 + **[CGCGCCCC]** 1078 (85.20)   
85 + **[CGCCCCGA]** 1080 (88.75)   
85 + **[CGCGGCCC]** 1217 (85.80)   
85 - **[CGCGCGGA]** 1378 (88.78)   
85 - **[CGCGTCCG]** 926 (88.04)   
85 - **[CGCGCCCC]** 585 (85.20)   
85 - **[CGCGGGGC]** 436 (85.16)   
85 - **[CGCCCCGA]** 310 (88.75)   
86 + **[CGCGGCCG]** 543 (85.25)   
86 + **[CGCGGCCG]** 658 (85.25)   
86 + **.CGCGGCGG.** 836 (92.45)   
86 + **.CGCGGCGA.** 902 (97.21)   
86 + **[CGCGCGGA]** 1034 (88.78)   
86 + **[CGCGCGGA]** 1090 (88.78)   
86 + **[CGGGTCGG]** 1121 (85.44)   
86 + **[CGCCTCGC]** 1185 (87.91)   
86 + **.CGCGGCGC.** 1190 (93.00)   
86 + **.CGCGGCGC.** 1195 (93.00)   
86 + **[CGCGCCCA]** 1341 (89.42)   
86 + **[CGCCCCGA]** 1362 (88.75)   
86 + **[CGCGGGGC]** 1386 (85.16)   
86 + **[GGCGGCGA]** 1413 (87.97)   
86 + **[CCCGCCGA]** 1481 (86.00)   
86 + **[CGCGGCCG]** 1506 (85.25)   
86 - **[CGCGGCCG]** 1508 (85.25)   
86 - **[CGCGGCGT]** 1435 (87.72)   
86 - **[CGCGCCCC]** 1382 (85.20)   
86 - **[CGGGTCGG]** 1321 (85.44)   
86 - **[CGCGGGGC]** 1251 (85.16)   
86 - **[CGCCGCGC]** 1194 (85.13)   
86 - **.CGCGCCGC.** 1191 (92.40)   
86 - **[CGCCGCGA]** 1189 (89.34)   
86 - **[CGCCGCGC]** 901 (85.13)   
86 - **[CGCGCCCC]** 898 (85.20)   
86 - **[CGCCGCGC]** 835 (85.13)   
86 - **[CGGGTCGG]** 798 (85.44)   
86 - **.CGCGGCGA.** 539 (97.21)   
86 - **[CGCCTCGC]** 285 (87.91)   
87 + **.CGCGGCGC.** 310 (93.00)   
87 + **[CGGGTCGG]** 418 (85.44)   
87 + **[CGCGGGGC]** 770 (85.16)   
87 + **[CGGGGCGA]** 772 (87.42)   
87 + **[CGCGTCCC]** 1099 (88.59)   
87 + **.CGCGCCGC.** 1194 (92.40)   
87 + **.CGCGGCGG.** 1244 (92.45)   
87 + **[GGCGCCGA]** 1304 (87.37)   
87 + **.CGCGCCGG.** 1322 (91.85)   
87 + **.CGCGGCGG.** 1333 (92.45)   
87 + **.CGCGCCGG.** 1346 (91.85)   
87 + **[CGCGGCCG]** 1367 (85.25)   
87 - **[CGCGCCCC]** 1499 (85.20)   
87 - **[CGCCGCGA]** 1332 (89.34)   
87 - **[CGCGTGGG]** 1318 (87.40)   
87 - **[CGCCGCGC]** 1243 (85.13)   
87 - **.CGCGCCGC.** 1240 (92.40)   
87 - **[CGCCCCGA]** 930 (88.75)   
87 - **.CGCGCCGC.** 841 (92.40)   
87 - **.CGCGCCGC.** 311 (92.40)   
87 - **[CGCCGCGA]** 309 (89.34)   
87 - **[CGCGTGGC]** 264 (87.95)   
88 + **[CCCGCCGA]** 117 (86.00)   
88 + **[CGCCCCGA]** 343 (88.75)   
88 + **[CGCGGGGC]** 449 (85.16)   
88 + **[CGCCGCGC]** 503 (85.13)   
88 + **.CGCGGCGG.** 849 (92.45)   
88 + **[CGCGGCCC]** 1099 (85.80)   
88 + **.CGCGTCGT.** 1206 (90.51)   
88 + **[CGGGTCGG]** 1303 (85.44)   
88 + **.CGCGGCCA.** 1380 (90.02)   
88 + **.CGCGGCGA.** 1395 (97.21)   
88 - **[CGCCGCGC]** 1394 (85.13)   
88 - **[CGCGGCGT]** 1095 (87.72)   
88 - **[CGCGCCCC]** 753 (85.20)   
88 - **[CGCGCCCC]** 703 (85.20)   
88 - **[CGCCCCGA]** 701 (88.75)   
88 - **.CGCGGCGC.** 502 (93.00)   
88 - **.CGCGCCGC.** 443 (92.40)   
88 - **[CGCGCCCC]** 417 (85.20)   
89 + **[CGCGGCCG]** 47 (85.25)   
89 + **.CGCGCCGC.** 100 (92.40)   
89 + **[CCCGTCGC]** 148 (85.16)   
89 + **[CGCGCCCA]** 153 (89.42)   
89 + **[CCCGGCGA]** 170 (86.59)   
89 + **[CGCCGCGA]** 268 (89.34)   
89 + **[CGCGCCCC]** 335 (85.20)   
89 + **[CGCGCCCC]** 364 (85.20)   
89 + **[CGCCGCGC]** 434 (85.13)   
89 + **.CGCGCCGC.** 495 (92.40)   
89 + **[CGCCGCGC]** 663 (85.13)   
89 + **[CGCGCCCC]** 666 (85.20)   
89 + **[CGCGCCCC]** 675 (85.20)   
89 + **[CGCGGCGT]** 743 (87.72)   
89 + **.CGCGCCGC.** 789 (92.40)   
89 + **.CGCGCCGG.** 1023 (91.85)   
89 + **[CGCCGCGC]** 1125 (85.13)   
89 + **[CCCGGCGA]** 1226 (86.59)   
89 - **.CGCGGCGC.** 1308 (93.00)   
89 - **.CGCGGCGG.** 1124 (92.45)   
89 - **[CGCGGGGC]** 1031 (85.16)   
89 - **.CGCGTGGA.** 962 (92.16)   
89 - **.CGCGCCGG.** 935 (91.85)   
89 - **.CGCGCCGC.** 785 (92.40)   
89 - **[CGCCGCGC]** 742 (85.13)   
89 - **[CGCGCCCC]** 739 (85.20)   
89 - **[CGCGCCCC]** 719 (85.20)   
89 - **.CGCGGCGG.** 662 (92.45)   
89 - **[GGCGGCGA]** 610 (87.97)   
89 - **[CGCGGCCG]** 476 (85.25)   
89 - **.CGCGGCGC.** 433 (93.00)   
89 - **.CGCGGCGG.** 416 (92.45)   
89 - **[CGCGGGGC]** 360 (85.16)   
89 - **.CGCGGCGC.** 267 (93.00)   
89 - **[CGCGGCCC]** 258 (85.80)   
89 - **[CGCCTCGC]** 174 (87.91)   
89 - **[CGCGACGG]** 149 (85.91)   
90 + **[CGCGCCCC]** 514 (85.20)   
90 + **.CGCGCCGC.** 715 (92.40)   
90 + **[CGCGGCCC]** 720 (85.80)   
90 + **.CGCGGCGG.** 777 (92.45)   
90 + **[GGCGGCGA]** 780 (87.97)   
90 + **[CGGGTCGC]** 838 (85.99)   
90 + **.CGCGGCGG.** 850 (92.45)   
90 + **[CGGGGCGA]** 982 (87.42)   
90 + **[CGCCCCGA]** 1045 (88.75)   
90 + **[CCCGGCGA]** 1170 (86.59)   
90 + **[CGCGTCAA]** 1314 (86.67)   
90 - **[CGGGGCGA]** 1044 (87.42)   
90 - **.CGCGGCGG.** 1017 (92.45)   
90 - **[CGCCTCGC]** 950 (87.91)   
90 - **[CGCGGCCG]** 846 (85.25)   
90 - **[CGCGGCCG]** 773 (85.25)   
90 - **.CGCGGCGC.** 716 (93.00)   
90 - **[CGCGTGGC]** 711 (87.95)   
90 - **[CGCGGCCC]** 585 (85.80)   
90 - **[CCCGGCGA]** 433 (86.59)   
92 + **.CGCGGCGG.** 1228 (92.45)   
92 - **[CCCGGCGA]** 1403 (86.59)   
92 - **[CGCCGCGC]** 1227 (85.13)   
93 + **[GGCGGCGA]** 413 (87.97)   
93 + **[CGCGCCGT]** 1003 (87.13)   
93 + **.CGCGGCGG.** 1037 (92.45)   
93 + **[GGCGGCGA]** 1427 (87.97)   
93 - **[CCCGCCGA]** 1398 (86.00)   
93 - **[CCCGCCGA]** 1350 (86.00)   
93 - **[CGCGTGGC]** 1190 (87.95)   
93 - **[CGCGTCCC]** 999 (88.59)   
93 - **[CGCGTCCC]** 961 (88.59)   
93 - **[CGCGGGGA]** 802 (89.38)   
96 + **.CGCGTCGA.** 740 (100.00)   
96 + **[CGGGTCGC]** 1347 (85.99)   
96 + **.CGCGTCGC.** 1354 (95.78)   
96 + **.CGCGTCGC.** 1359 (95.78)   
96 + **[CGCGGGGA]** 1395 (89.38)   
96 + **[CGCGCCGT]** 1422 (87.13)   
96 - **[CGCGTCCG]** 1391 (88.04)   
96 - **[CGCGACGC]** 1355 (86.46)   
97 + **.CGCGGCGC.** 877 (93.00)   
97 + **[CGGGCCGA]** 1334 (86.82)   
97 + **[GGCGTCGC]** 1441 (86.54)   
97 - **[CGCGTCCC]** 1365 (88.59)   
97 - **[CGCGCCCA]** 1221 (89.42)   
97 - **[CGCGGGGC]** 1043 (85.16)   
97 - **.CGCGCCGC.** 878 (92.40)   
97 - **[CGCCGCGC]** 876 (85.13)   
98 + **.CGCGGCGG.** 1001 (92.45)   
98 + **[CGCGGGGC]** 1033 (85.16)   
98 + **.CGCGGCGC.** 1086 (93.00)   
98 + **.CGCGGCGG.** 1137 (92.45)   
98 + **[CGCGGCCG]** 1325 (85.25)   
98 - **.CGCGCCGC.** 1225 (92.40)   
98 - **[CGCCGCGC]** 1136 (85.13)   
98 - **[CGCGCCCC]** 1036 (85.20)   
98 - **[CGCGGCCG]** 997 (85.25)   
99 + **[CCCGCCGA]** 1509 (86.00)   
100 + **[CGGGTCGC]** 1099 (85.99)   
100 + **[CGCGCCGT]** 1104 (87.13)   
100 + **.CGCGGCCA.** 1140 (90.02)   
100 + **.CGCGGCGG.** 1500 (92.45)   
100 - **[CGCGGCTA]** 1303 (85.00)   
100 - **[CGCGGGGC]** 1259 (85.16)   
100 - **[CGCGCGGA]** 989 (88.78)   
103 + **[CGGGTCGC]** 1179 (85.99)   
103 - **[CGCGGGGC]** 778 (85.16)   
104 - **[CGCGGGGA]** 780 (89.38)   
105 - **[CGCCTCGC]** 1507 (87.91)   
105 - **[CGCGGGGC]** 1058 (85.16)   
106 + **.CGCGGCGC.** 870 (93.00)   
106 + **[CGCCTCGC]** 1018 (87.91)   
106 + **[CGCGGCGT]** 1160 (87.72)   
106 - **[CGCCGCGA]** 1159 (89.34)   
106 - **[CGCGCGGA]** 999 (88.78)   
106 - **[CGCCGCGA]** 869 (89.34)   
108 + **.CGCGGCCA.** 101 (90.02)   
108 + **.CGCCTCGA.** 388 (92.13)   
108 + **[CGCGGGGC]** 539 (85.16)   
108 + **.CGCGTGGA.** 742 (92.16)   
108 + **[CGCGTCCC]** 1203 (88.59)   
108 + **[CGCGCCGT]** 1218 (87.13)   
108 + **[CGCGTGGG]** 1387 (87.40)   
108 - **[CGCGTCCC]** 1383 (88.59)   
108 - **[CGCGGGGA]** 1199 (89.38)   
108 - **[CGCCCCGA]** 1121 (88.75)   
108 - **[CCCGCCGA]** 941 (86.00)   
108 - **[CGCGTCCC]** 816 (88.59)   
108 - **[CGCGGGGA]** 608 (89.38)   
108 - **[CGGGCCGA]** 584 (86.82)   
108 - **[CCCGCCGA]** 470 (86.00)   
108 - **[CGCCTCGC]** 310 (87.91)   
108 - **[CGCGCGGA]** 174 (88.78)   
108 - **[CGCGCCCA]** 97 (89.42)   
109 + **[CGCGGCCC]** 339 (85.80)   
109 + **[CGCCTCGG]** 519 (87.36)   
109 + **[CGCGCCCC]** 659 (85.20)   
109 + **[CGCGTCCC]** 1032 (88.59)   
109 + **[GGCGTCGG]** 1090 (85.99)   
109 + **[CGCGCCGT]** 1233 (87.13)   
109 + **[CGCGGGGC]** 1315 (85.16)   
109 + **[CGCGGGGC]** 1322 (85.16)   
109 + **.CGCGGCGC.** 1359 (93.00)   
109 + **.CGCGGCGC.** 1391 (93.00)   
109 + **.CGCGGCGG.** 1396 (92.45)   
109 - **[CGCCGCGC]** 1395 (85.13)   
109 - **.CGCGCCGC.** 1392 (92.40)   
109 - **[CGCCGCGC]** 1390 (85.13)   
109 - **[CGCGCCCC]** 1387 (85.20)   
109 - **[CGCCGCGC]** 1358 (85.13)   
109 - **[CGCGCCCC]** 1318 (85.20)   
109 - **[CGCGCCCA]** 1311 (89.42)   
109 - **.CGCGTCGG.** 1229 (95.23)   
109 - **[GGCGTCGG]** 1183 (85.99)   
109 - **[CGCGCCCC]** 823 (85.20)   
109 - **[CGCGTCCC]** 812 (88.59)   
109 - **[CGCGGGGC]** 596 (85.16)   
109 - **.CGCGGCGA.** 335 (97.21)   
114 + **[CGGGTCGC]** 256 (85.99)   
114 + **.CGCGGCGC.** 1004 (93.00)   
114 + **[CGCGGCCG]** 1009 (85.25)   
114 + **[CCCGCCGA]** 1277 (86.00)   
114 + **.CGCGGCGA.** 1308 (97.21)   
114 - **.CGCGCCGC.** 1005 (92.40)   
114 - **[CGCCGCGC]** 1003 (85.13)   
114 - **[CGCGGGGA]** 301 (89.38)   
115 + **[CGCGGCGT]** 413 (87.72)   
115 + **[CGCCTCGC]** 461 (87.91)   
115 + **.CGCGTCGC.** 466 (95.78)   
115 + **[CGCCTCGG]** 495 (87.36)   
115 + **.CGCGCCGG.** 689 (91.85)   
115 + **[CGCGACGC]** 867 (86.46)   
115 - **[CGCGACGC]** 1234 (86.46)   
115 - **[CGCGGGGC]** 1035 (85.16)   
115 - **.CGCGTCGC.** 868 (95.78)   
115 - **[CGCGGGGA]** 685 (89.38)   
115 - **[CGCGGGGC]** 586 (85.16)   
115 - **[GGCGTCGG]** 556 (85.99)   
115 - **[CGCGGGGC]** 409 (85.16)   
115 - **.CGCGGCGC.** 308 (93.00)   
115 - **.CGCGCCGG.** 299 (91.85)   
115 - **.CGCGCCGC.** 279 (92.40)   
115 - **[CGCTGCGA]** 56 (85.51)   
116 - **.CGCGTCGT.** 959 (90.51)   
117 + **[CGCGCCCC]** 669 (85.20)   
117 + **[CGCCGCGC]** 791 (85.13)   
117 + **[CGCGGGGC]** 831 (85.16)   
117 + **[CGCGGCCG]** 1056 (85.25)   
117 - **[CGCGGGGA]** 827 (89.38)   
117 - **.CGCGGCGC.** 790 (93.00)   
117 - **.CGCGCCGA.** 665 (96.62)   
117 - **[CGCGCCCA]** 654 (89.42)   
117 - **[GGCGCCGA]** 454 (87.37)   
117 - **[CGCCTCGG]** 195 (87.36)   
118 + **[CGCCGCGC]** 767 (85.13)   
118 + **[CGCGGCCG]** 1045 (85.25)   
118 - **[CGCGGGGA]** 802 (89.38)   
118 - **.CGCGGCGC.** 766 (93.00)   
118 - **[CGCGCCCA]** 630 (89.42)   
118 - **[CGCGCCCA]** 602 (89.42)   
118 - **.CGCGGCCA.** 431 (90.02)   
118 - **[CGCCTCGG]** 97 (87.36)   
119 + **[CGCCGCGC]** 765 (85.13)   
119 + **[CGCGGCCG]** 1036 (85.25)   
119 - **[CGCGGGGA]** 800 (89.38)   
119 - **.CGCGGCGC.** 764 (93.00)   
119 - **.CGCGCCGA.** 639 (96.62)   
119 - **[CGCGCCCA]** 628 (89.42)   
119 - **.CGCGCCGA.** 611 (96.62)   
119 - **[CGCGCCCA]** 600 (89.42)   
119 - **.CGCGGCCA.** 429 (90.02)   
119 - **[CGCCTCGG]** 95 (87.36)   
120 + **[CGCGCCCC]** 1103 (85.20)   
120 + **[CGCCGCGC]** 1225 (85.13)   
120 + **[CGCGGGGC]** 1264 (85.16)   
120 + **[CGCGGCCG]** 1503 (85.25)   
120 - **[CGCGGGGA]** 1260 (89.38)   
120 - **.CGCGGCGC.** 1224 (93.00)   
120 - **.CGCGCCGA.** 1099 (96.62)   
120 - **[CGCGCCCA]** 1088 (89.42)   
120 - **[CGCGCCCA]** 1060 (89.42)   
120 - **[GGCGCCGA]** 860 (87.37)   
120 - **[CGCCTCGG]** 554 (87.36)   
122 + **.CGCGCCGC.** 32 (92.40)   
122 + **[CGCGGCCC]** 916 (85.80)   
122 + **[CGCGCCCC]** 924 (85.20)   
122 - **[CGCGGGGC]** 920 (85.16)   
122 - **.CGCGCCGA.** 900 (96.62)   
122 - **[CGCTGCGA]** 5 (85.51)   
123 + **[CGCGTCCC]** 392 (88.59)   
123 + **[CGCCGCGA]** 601 (89.34)   
123 + **[CGCGCCCC]** 660 (85.20)   
123 + **[CGCGCCCC]** 690 (85.20)   
123 + **[GGCGGCGA]** 752 (87.97)   
123 + **.CGCGCCGG.** 769 (91.85)   
123 + **[CGCGGCCG]** 900 (85.25)   
123 + **.CGCGGCGA.** 952 (97.21)   
123 + **[CGCCGCGC]** 1004 (85.13)   
123 + **.CGCGCCGC.** 1007 (92.40)   
123 + **[CGCCGCGC]** 1009 (85.13)   
123 + **.CGCGCCGC.** 1012 (92.40)   
123 + **[CGCGGCCC]** 1030 (85.80)   
123 + **[GGCGTCGG]** 1073 (85.99)   
123 + **[CGCGGGGA]** 1278 (89.38)   
123 + **.CGGGTCGA.** 1368 (90.21)   
123 - **.CGCGACGA.** 1231 (90.68)   
123 - **.CGCGGCGC.** 1008 (93.00)   
123 - **.CGCGGCGA.** 1003 (97.21)   
123 - **.CGCGCCGA.** 765 (96.62)   
123 - **.CGCGCCGC.** 741 (92.40)   
123 - **[CGCGGGGC]** 686 (85.16)   
123 - **[GGCGGCGA]** 637 (87.97)   
123 - **.CGCGGCGG.** 600 (92.45)   
123 - **[CGCGGGGC]** 541 (85.16)   
123 - **[CGGGTCGG]** 513 (85.44)   
123 - **[CGCGCCCA]** 388 (89.42)   
123 - **.CGCGGCGC.** 13 (93.00)   
124 + **[GGCGTCGG]** 1099 (85.99)   
124 + **[CGGGCCGA]** 1201 (86.82)   
124 - **[CGCGGCCG]** 1222 (85.25)   
124 - **[CGCCTCGG]** 1205 (87.36)   
124 - **.CGCGCCGG.** 1147 (91.85)   
124 - **.CGCGCCGG.** 878 (91.85)   
124 - **.CGCGCCGG.** 866 (91.85)   
124 - **.CGCGTCGA.** 826 (100.00)   
127 + **[CGCGGCCC]** 746 (85.80)   
127 + **.GGCGTCGA.** 1340 (90.75)   
127 - **[CGCGGCCG]** 1507 (85.25)   
127 - **[CGGGTCGG]** 1148 (85.44)   
127 - **[CGCGTCCG]** 1027 (88.04)   
127 - **[CGGGGCGA]** 902 (87.42)   
127 - **.CGCGCCGG.** 774 (91.85)   
127 - **[CGCCTCGC]** 740 (87.91)   
127 - **[CGCCTCGC]** 626 (87.91)   
127 - **[CGCGTGGC]** 465 (87.95)   
128 + **[CGCGGGGC]** 340 (85.16)   
128 + **.CGCGGCGC.** 408 (93.00)   
128 + **[CGGGTCGG]** 429 (85.44)   
128 + **[CGGGTCGG]** 828 (85.44)   
128 + **.CGCGACGA.** 938 (90.68)   
128 + **[CGCGGGGC]** 1102 (85.16)   
128 - **[CGCGCGGA]** 973 (88.78)   
128 - **[CGGGTCGC]** 779 (85.99)   
128 - **[CGCCTCGC]** 623 (87.91)   
129 + **[CGCCTCGC]** 779 (87.91)   
129 + **.CGCGGCGC.** 1141 (93.00)   
129 + **[CGCGGGGC]** 1146 (85.16)   
129 + **.CGCGCCGG.** 1202 (91.85)   
129 + **[CGCCGCGC]** 1319 (85.13)   
129 + **[CGCGGCCG]** 1386 (85.25)   
129 + **[CGGGTCGC]** 1392 (85.99)   
129 + **.CGCGCCGG.** 1402 (91.85)   
129 - **[GGCGGCGA]** 1429 (87.97)   
129 - **.CGCGGCGG.** 1318 (92.45)   
129 - **[CGCGGCCG]** 1198 (85.25)   
129 - **.CGCGCCGC.** 1142 (92.40)   
129 - **[CGCCGCGA]** 1140 (89.34)   
129 - **[CGCGTCCG]** 1055 (88.04)   
129 - **[CGCGGCGT]** 1014 (87.72)   
129 - **[CGCCTCGC]** 962 (87.91)   
129 - **.CGCGGCCA.** 868 (90.02)   
129 - **[CGCTTCGA]** 361 (88.30)   
130 + **[CGCGGGGA]** 584 (89.38)   
130 + **[CGCGGCTA]** 867 (85.00)   
130 + **[CGCGGGGC]** 1368 (85.16)   
131 + **.CGCGCCGC.** 33 (92.40)   
131 + **.CGCGTGGA.** 614 (92.16)   
131 + **[CGCGGCCG]** 1117 (85.25)   
131 + **.CGCGGCGG.** 1306 (92.45)   
131 + **.CGCGGCGG.** 1379 (92.45)   
131 + **.CGCGGCGG.** 1488 (92.45)   
131 - **[CGAGTCGA]** 828 (86.95)   
132 + **[CGCGCGGA]** 357 (88.78)   
132 + **[CGCGTGGC]** 712 (87.95)   
132 + **[CCCGGCGA]** 769 (86.59)   
132 + **.CGCGCCGG.** 1224 (91.85)   
132 - **[CGGGGCGA]** 944 (87.42)   
132 - **.CGCGGCGG.** 708 (92.45)   
134 + **[CGCGGGGA]** 987 (89.38)   
134 + **.CGCGTCGA.** 1035 (100.00)   
134 - **[CGCGCCCA]** 1368 (89.42)   
135 + **[CGCGCGGA]** 355 (88.78)   
135 + **[CGCGGCGT]** 365 (87.72)   
135 + **[CGCCTCGC]** 470 (87.91)   
135 + **[CCCGCCGA]** 875 (86.00)   
135 + **[CGGGTCGC]** 1254 (85.99)   
135 + **[GGCGGCGA]** 1285 (87.97)   
135 + **[CGCCGCGC]** 1499 (85.13)   
135 - **.CGCGGCGG.** 1498 (92.45)   
135 - **[CCCGCCGA]** 1472 (86.00)   
135 - **.CGCGGCGA.** 786 (97.21)   
135 - **[CGCGTGGC]** 351 (87.95)   
136 + **[CGAGTCGA]** 900 (86.95)   
136 + **.CGCGCCGC.** 1065 (92.40)   
136 + **[CGCGGCCG]** 1196 (85.25)   
136 + **[CGCCCCGA]** 1217 (88.75)   
136 + **.CGCGGCCA.** 1302 (90.02)   
136 - **.CGCGCCGG.** 1037 (91.85)   
136 - **.CGCGCCGG.** 934 (91.85)   
137 + **.CGCGGCGG.** 945 (92.45)   
137 + **[CGCGGCCG]** 1016 (85.25)   
137 - **[CGGGTCGC]** 1326 (85.99)   
137 - **[CGCGTCCC]** 941 (88.59)   
137 - **[CGCCCCGA]** 507 (88.75)   
138 + **.CGCGCCGC.** 192 (92.40)   
138 + **.CGCGGCGC.** 367 (93.00)   
138 + **[CGCGCCCC]** 452 (85.20)   
138 + **.CGCGGCGA.** 579 (97.21)   
138 + **.CGCGTCGG.** 601 (95.23)   
138 + **.CGCGGCGG.** 691 (92.45)   
138 + **.CGCGGCGC.** 763 (93.00)   
138 + **[CGCGGCGT]** 768 (87.72)   
138 + **[CGCCGCGC]** 837 (85.13)   
138 - **[CGCGGGGA]** 934 (89.38)   
138 - **.CGCGGCGG.** 836 (92.45)   
138 - **[CGCCGCGC]** 767 (85.13)   
138 - **.CGCGCCGC.** 764 (92.40)   
138 - **[CGCCGCGC]** 690 (85.13)   
138 - **.CGCGCCGC.** 639 (92.40)   
138 - **[CGCGGCCG]** 613 (85.25)   
138 - **[CGCCGCGC]** 578 (85.13)   
138 - **.CGCGCCGG.** 573 (91.85)   
138 - **[CGGGCCGA]** 446 (86.82)   
138 - **.CGCGCCGC.** 368 (92.40)   
138 - **[CGCGTCCC]** 363 (88.59)   
139 + **.CGCGCCGC.** 832 (92.40)   
139 + **[CGCGTCCC]** 847 (88.59)   
139 + **.CGCGGCGC.** 960 (93.00)   
139 - **[CGCGCCCA]** 1089 (89.42)   
139 - **[CGCCGCGC]** 959 (85.13)   
139 - **[CGCGTGGG]** 760 (87.40)   
139 - **.CGCGCCGC.** 688 (92.40)   
139 - **[CGGGCCGA]** 662 (86.82)   
140 + **.CGCGGCGC.** 730 (93.00)   
140 + **[CGCCGCGC]** 1192 (85.13)   
140 + **.CGCGCCGC.** 1195 (92.40)   
140 + **.CGCGGCGA.** 1248 (97.21)   
140 + **[CGGGGCGA]** 1410 (87.42)   
140 - **[GGCGTCGC]** 1365 (86.54)   
140 - **.CGCGGCGG.** 1191 (92.45)   
140 - **.CGCGGCGG.** 655 (92.45)   
140 - **[CGGGTCGC]** 636 (85.99)   
141 + **[CGCCGCGC]** 609 (85.13)   
141 + **[CGCGCCCA]** 612 (89.42)   
141 + **[CGCGGCCG]** 684 (85.25)   
141 + **.CGCGGCGC.** 1158 (93.00)   
141 + **.CGCGCCGC.** 1185 (92.40)   
141 + **[CGCGGGGA]** 1383 (89.38)   
141 - **[CGCGTCCC]** 1387 (88.59)   
141 - **[CGCGCCCC]** 1366 (85.20)   
141 - **[GGCGGCGA]** 1240 (87.97)   
141 - **.CGCGTCCA.** 1226 (92.80)   
141 - **.CGCGGCGC.** 1186 (93.00)   
141 - **[CGCGGCCC]** 1181 (85.80)   
141 - **.CGCGGCCA.** 1154 (90.02)   
141 - **.CGCGCCGA.** 945 (96.62)   
141 - **[CGCCTCGC]** 752 (87.91)   
141 - **[CGCGGCCG]** 719 (85.25)   
141 - **[CGCGGCCG]** 686 (85.25)   
141 - **.CGCGGCGC.** 608 (93.00)   
142 + **[CGCGCCCA]** 457 (89.42)   
142 + **.CGCGCCGC.** 513 (92.40)   
142 + **.CGCGGCGG.** 663 (92.45)   
142 + **[CGCCGCGC]** 893 (85.13)   
142 + **.CGCGCCGG.** 902 (91.85)   
142 + **[CGCGGCCC]** 946 (85.80)   
142 + **.CGCGCCGG.** 958 (91.85)   
142 + **[CGCCGCGC]** 1005 (85.13)   
142 + **[CGCGGCCC]** 1010 (85.80)   
142 + **[CGGGTCGC]** 1098 (85.99)   
142 + **[CGCGGCCG]** 1200 (85.25)   
142 + **.CGCGCCGC.** 1274 (92.40)   
142 + **[CGCGCCCC]** 1468 (85.20)   
142 - **[CGCGTCCC]** 1476 (88.59)   
142 - **[CGCGGGGC]** 1464 (85.16)   
142 - **[CGGGTCGC]** 1438 (85.99)   
142 - **[CGCCTCGG]** 1368 (87.36)   
142 - **[CCCGCCGA]** 1354 (86.00)   
142 - **[CGCCCCGA]** 1333 (88.75)   
142 - **.CGCGGCCA.** 1270 (90.02)   
142 - **[CGCGGCCC]** 1059 (85.80)   
142 - **[CGGGCCGA]** 1035 (86.82)   
142 - **.CGCGGCGA.** 1004 (97.21)   
142 - **[CGCGGGGA]** 942 (89.38)   
142 - **.CGCGGCGC.** 892 (93.00)   
142 - **[CGCGGGGC]** 718 (85.16)   
142 - **[CGCGGCCC]** 670 (85.80)   
142 - **[CGCCGCGC]** 662 (85.13)   
142 - **[CGCCTCGG]** 586 (87.36)   
142 - **.CGCGGCGC.** 514 (93.00)   
142 - **.CGCGTCGC.** 445 (95.78)   
142 - **[CGGGCCGA]** 301 (86.82)   
142 - **[CGCGGCCC]** 27 (85.80)   
144 + **[CGCGGGGA]** 12 (89.38)   
144 + **[CGCGTCCG]** 774 (88.04)   
144 + **[CGCGTCCG]** 839 (88.04)   
144 + **[CGCGGGGC]** 936 (85.16)   
144 + **[CGCCGCGC]** 1017 (85.13)   
144 + **[CGCGGGGC]** 1022 (85.16)   
144 + **.CGCGGCGC.** 1142 (93.00)   
144 + **.CGCGGCGC.** 1147 (93.00)   
144 + **.CGCGGCGC.** 1189 (93.00)   
144 + **[CGCGGCCG]** 1194 (85.25)   
144 + **.CGCGGCCA.** 1326 (90.02)   
144 + **[CGCGGGGC]** 1461 (85.16)   
144 + **[CGCCGCGC]** 1490 (85.13)   
144 + **[CGCGCCCC]** 1493 (85.20)   
144 - **.CGCGGCGC.** 1489 (93.00)   
144 - **[CCCGCCGA]** 1342 (86.00)   
144 - **[CGCGGGGC]** 1306 (85.16)   
144 - **[CGGGCCGA]** 1284 (86.82)   
144 - **[CGCGGCCG]** 1196 (85.25)   
144 - **.CGCGCCGC.** 1190 (92.40)   
144 - **.CGCGCCGC.** 1148 (92.40)   
144 - **[CGCCGCGC]** 1146 (85.13)   
144 - **.CGCGCCGC.** 1143 (92.40)   
144 - **[CGCCGCGC]** 1141 (85.13)   
144 - **.CGCGCCGC.** 1138 (92.40)   
144 - **[CGCGCCCC]** 1072 (85.20)   
144 - **.CGCGCCGC.** 1031 (92.40)   
144 - **.CGCGGCGC.** 1016 (93.00)   
145 + **[GGCGGCGA]** 445 (87.97)   
145 + **[CGCGCCCC]** 510 (85.20)   
145 + **[CGCCGCGC]** 1136 (85.13)   
145 + **.CGCGGCGG.** 1141 (92.45)   
145 + **.CGCGCCGC.** 1150 (92.40)   
145 + **[CGCGACGG]** 1253 (85.91)   
145 - **[CGCCTCGG]** 1404 (87.36)   
145 - **[CGCCGCGC]** 1140 (85.13)   
145 - **.CGCGGCGG.** 1135 (92.45)   
147 + **[CGCGCCCC]** 854 (85.20)   
147 + **[CGCCCCGA]** 856 (88.75)   
147 + **[CGCGTCCC]** 864 (88.59)   
147 + **[GGCGGCGA]** 1068 (87.97)   
147 + **[GGCGGCGA]** 1080 (87.97)   
147 + **[GGCGCCGA]** 1097 (87.37)   
147 + **[CGCCGCGC]** 1162 (85.13)   
147 + **[CGCGCCCA]** 1167 (89.42)   
147 + **[GGCGTCGC]** 1338 (86.54)   
147 - **[CGCGGGGA]** 1356 (89.38)   
147 - **.CGCGGCGA.** 1161 (97.21)   
147 - **[CGCCTCGG]** 1076 (87.36)   
147 - **[CGCGGCCC]** 976 (85.80)   
147 - **[CGCGACGC]** 961 (86.46)   
147 - **.CGCGTCGG.** 860 (95.23)   
148 + **[CGCCGCGC]** 827 (85.13)   
148 + **[CGCGGGGC]** 997 (85.16)   
148 + **[CCCGTCGC]** 1394 (85.16)   
148 - **[CGCGGCGT]** 826 (87.72)   
148 - **[GGCGTCGG]** 437 (85.99)   
150 + **[CGCGGGGC]** 24 (85.16)   
150 + **.CGCGCCGC.** 118 (92.40)   
150 + **[CGCCGCGC]** 120 (85.13)   
150 + **[GGCGCCGA]** 163 (87.37)   
150 + **[CGCGGCGT]** 191 (87.72)   
150 + **[CGCGACGG]** 933 (85.91)   
150 - **[CGCGCCCC]** 1467 (85.20)   
150 - **[CGCCGCGA]** 190 (89.34)   
150 - **.CGCGGCGC.** 119 (93.00)   
150 - **[CGCGGCCC]** 112 (85.80)   
151 + **[CGCCGCGA]** 814 (89.34)   
151 + **[CGCGGCCC]** 892 (85.80)   
151 + **[CGCGTCCC]** 1151 (88.59)   
151 + **.CGCGCCGG.** 1206 (91.85)   
151 + **.CGCGGCCA.** 1268 (90.02)   
151 + **[CGCGGGGC]** 1423 (85.16)   
151 + **[CGCGTCCG]** 1473 (88.04)   
151 + **[CGCGGCCG]** 1496 (85.25)   
151 - **.CGCGCCGC.** 1490 (92.40)   
151 - **.CGCGCCGC.** 1459 (92.40)   
151 - **[CGCGTGGC]** 1419 (87.95)   
151 - **[CGGGTCGC]** 1064 (85.99)   
151 - **[CGCGGGGC]** 980 (85.16)   
151 - **[CGCCGCGC]** 883 (85.13)   
151 - **[GGCGCCGA]** 841 (87.37)   
151 - **[CGCGGCGT]** 813 (87.72)   
151 - **[CGGGGCGA]** 767 (87.42)   
151 - **[CGCGACGG]** 70 (85.91)   
154 + **[CGGGTCGC]** 98 (85.99)   
154 + **[CGCGGGGA]** 1000 (89.38)   
154 + **.CGCGGCGG.** 1040 (92.45)   
154 + **[CGCCGCGA]** 1178 (89.34)   
154 + **.CGCGCCGC.** 1432 (92.40)   
154 + **.CGCGCCGC.** 1466 (92.40)   
154 - **[CGCGGGGC]** 1506 (85.16)   
154 - **[GGCGGCGA]** 1248 (87.97)   
154 - **[GGCGGCGA]** 1217 (87.97)   
154 - **.CGCGGCGC.** 1177 (93.00)   
154 - **[GGCGGCGA]** 1141 (87.97)   
154 - **.CGCGCCGC.** 1094 (92.40)   
154 - **[CGCGGGGA]** 1077 (89.38)   
154 - **[CGCCGCGC]** 1039 (85.13)   
155 + **[GGCGTCGC]** 1254 (86.54)   
155 + **[CGCGTTGA]** 1259 (87.22)   
155 - **[CGCGACGC]** 1255 (86.46)   
155 - **.CGCGCCGG.** 505 (91.85)   
157 + **[CGCGTGGG]** 1238 (87.40)   
158 + **[CGCGCCCA]** 522 (89.42)   
158 + **.CGCGTCGA.** 1123 (100.00)   
158 + **.CGCGTCGT.** 1179 (90.51)   
158 - **.CGCGCCGG.** 1510 (91.85)   
158 - **[CGCGTCTA]** 1367 (87.79)   
160 + **[CGCGTGGC]** 135 (87.95)   
160 + **[CGCCTCGC]** 277 (87.91)   
160 + **[CGCGGCCC]** 1055 (85.80)   
160 + **.CGCGCCGC.** 1206 (92.40)   
160 + **[CGCGGGGA]** 1329 (89.38)   
160 - **[GGCGTCGG]** 1267 (85.99)   
160 - **[CGCGGGGC]** 1202 (85.16)   
160 - **.CGCGGCGG.** 264 (92.45)   
161 + **[CGCCGCGC]** 893 (85.13)   
161 + **.CGCGGCGG.** 1268 (92.45)   
161 + **[CGCGGGGC]** 1302 (85.16)   
161 + **.CGCGCCGC.** 1481 (92.40)   
161 - **[CGCGGGGC]** 1477 (85.16)   
161 - **.CGCGGCGG.** 892 (92.45)   
161 - **[CGCGGCCC]** 882 (85.80)   
161 - **[CGCGGCCC]** 863 (85.80)   
161 - **.CGCGCCGG.** 750 (91.85)   
161 - **[CGCCCCGA]** 456 (88.75)   
161 - **.CGCGTGGA.** 314 (92.16)   
161 - **.CGCGCCGC.** 152 (92.40)   
164 + **[CGCTTCGA]** 517 (88.30)   
164 + **[CGCCTCGC]** 1045 (87.91)   
164 + **.CGCGGCGC.** 1099 (93.00)   
164 + **.CGCGGCCA.** 1225 (90.02)   
164 - **[CGCCGCGC]** 1098 (85.13)   
164 - **.CGCGCCGG.** 1095 (91.85)   
164 - **[CGCGCCCC]** 954 (85.20)   
164 - **[CGCGGGGC]** 297 (85.16)   
166 + **[CCCGGCGA]** 638 (86.59)   
166 + **[CGCCTCGG]** 926 (87.36)   
166 + **[GGCGTCGG]** 961 (85.99)   
166 + **.CGCGGCGC.** 1159 (93.00)   
166 + **[CGCGGCCG]** 1229 (85.25)   
166 + **.CGCGGCGC.** 1312 (93.00)   
166 - **[CACGTCGA]** 1326 (86.48)   
166 - **[CGCGCCCC]** 1288 (85.20)   
166 - **.CGCGCCGA.** 1223 (96.62)   
166 - **[CGCCTCGG]** 957 (87.36)   
166 - **[CGCGGCCG]** 544 (85.25)   
166 - **[CCCGTCGA]** 517 (89.38)   
166 - **[CGCGGGGA]** 421 (89.38)   
167 + **.CGCGGCGG.** 843 (92.45)   
167 + **[CGCGGCCC]** 956 (85.80)   
167 + **[CGCGGCCC]** 963 (85.80)   
167 + **[CGCGGCCC]** 1039 (85.80)   
167 + **[CGCGTCCC]** 1463 (88.59)   
167 - **[GGCGGCGA]** 1394 (87.97)   
167 - **[CGCGGCCG]** 861 (85.25)   
167 - **.CGCGCCGC.** 816 (92.40)   
167 - **[CGCGCCCC]** 799 (85.20)   
167 - **[CGCCTCGG]** 791 (87.36)   
167 - **[CGGGTCGG]** 786 (85.44)   
167 - **[GGCGCCGA]** 655 (87.37)   
167 - **[CCCGCCGA]** 612 (86.00)   
168 + **[CGCGGCCG]** 675 (85.25)   
168 + **[CGCGGGGC]** 734 (85.16)   
168 + **[CGCCGCGC]** 908 (85.13)   
168 + **[CGCGCGGA]** 911 (88.78)   
168 + **[CGCGCCCA]** 1114 (89.42)   
168 + **[CGCGGCCC]** 1137 (85.80)   
168 + **[CGCGCCCC]** 1171 (85.20)   
168 + **[CGCGGCCC]** 1205 (85.80)   
168 - **[CGCGGCCG]** 1433 (85.25)   
168 - **[CGCCCCGA]** 1427 (88.75)   
168 - **.CGCGCCGA.** 1181 (96.62)   
168 - **.CGCGGCGA.** 907 (97.21)   
168 - **[CGCGCCCC]** 730 (85.20)   
169 + **[CGCCGCGC]** 110 (85.13)   
169 + **[AGCGTCGA]** 1243 (87.51)   
169 + **[CGCGCCCC]** 1499 (85.20)   
169 - **[CGCGGGGC]** 1493 (85.16)   
169 - **.CGCGTCCA.** 1043 (92.80)   
169 - **[CGCGCCCC]** 869 (85.20)   
169 - **[CGCGGCGT]** 109 (87.72)   
171 + **[CGCGGCGT]** 774 (87.72)   
171 - **[GGCGCCGA]** 824 (87.37)   
171 - **.CGCGTGGA.** 770 (92.16)   
172 + **[CGCCTCGC]** 300 (87.91)   
172 + **[CGCGGGGC]** 337 (85.16)   
172 + **.CGCGGCGG.** 431 (92.45)   
172 + **[CGCGCCCA]** 551 (89.42)   
172 + **[CGCGACGC]** 716 (86.46)   
172 + **[CGCCTCGG]** 829 (87.36)   
172 + **[CGGGGCGA]** 859 (87.42)   
172 + **.CGCGCCGA.** 924 (96.62)   
172 + **[CGCGCCGT]** 1419 (87.13)   
172 + **[GGCGGCGA]** 1462 (87.97)   
172 + **[CGCGCCCA]** 1505 (89.42)   
172 - **.CGCGCCGC.** 1158 (92.40)   
172 - **.CGCGCCGC.** 918 (92.40)   
172 - **[CGCGGGGC]** 893 (85.16)   
172 - **[CGCGCCCC]** 766 (85.20)   
172 - **[CGCGGCCC]** 753 (85.80)   
172 - **[GGCGTCGC]** 717 (86.54)   
172 - **[CGCGGCCC]** 712 (85.80)   
172 - **[CGGGCCGA]** 604 (86.82)   
172 - **.CGCGTCCA.** 427 (92.80)   
172 - **[GGCGGCGA]** 304 (87.97)   
172 - **[CGCGGGGC]** 130 (85.16)   
173 + **[CGCCTCGG]** 947 (87.36)   
173 + **[GGCGGCGA]** 1028 (87.97)   
173 + **[CGCGGGGA]** 1103 (89.38)   
173 - **[GGCGTCGC]** 1189 (86.54)   
173 - **[CGGGTCGG]** 893 (85.44)   
174 + **[GGCGGCGA]** 1064 (87.97)   
174 + **[CGCGTCCC]** 1187 (88.59)   
174 + **.CGCGGCGC.** 1477 (93.00)   
174 + **[CGCGGCCG]** 1507 (85.25)   
174 - **[CGCCTCGG]** 1501 (87.36)   
174 - **.CGCGCCGC.** 1478 (92.40)   
174 - **[CCCGTCGC]** 1369 (85.16)   
174 - **[CGCGGCCG]** 1201 (85.25)   
174 - **.CGCGGCGC.** 1183 (93.00)   
174 - **[CGCGGCCC]** 1143 (85.80)   
174 - **[CGGGTCGG]** 1110 (85.44)   
174 - **[CGCGCCCC]** 781 (85.20)   
175 - **.CGCGGCGC.** 1464 (93.00)   
175 - **[CGCGCCCC]** 925 (85.20)   
177 + **[CGCCTCGG]** 821 (87.36)   
177 + **[CGCCGCGA]** 951 (89.34)   
177 + **[CCCGTCGC]** 1015 (85.16)   
177 + **[CGCCTCGC]** 1032 (87.91)   
177 + **.CGCGGCGA.** 1465 (97.21)   
177 - **[GGCGGCGA]** 1019 (87.97)   
177 - **.CGCGGCGA.** 950 (97.21)   
177 - **[CGCCTCGC]** 746 (87.91)   
178 + **.CGCGGCGG.** 1114 (92.45)   
178 - **[CGCGTCCC]** 1110 (88.59)   
179 + **.CGCGCCGC.** 858 (92.40)   
179 + **[CGCGGCCG]** 918 (85.25)   
179 + **[CGCGGGGA]** 988 (89.38)   
179 + **[CGCCGCGC]** 1473 (85.13)   
179 - **.CGCGGCGG.** 1472 (92.45)   
179 - **[CGCGTCCC]** 992 (88.59)   
179 - **.CGCGCCGC.** 984 (92.40)   
179 - **.CGCGGCGA.** 914 (97.21)   
180 + **[CGCGCGGA]** 798 (88.78)   
180 + **[CGCGGGGC]** 924 (85.16)   
180 + **[CCCGCCGA]** 960 (86.00)   
180 + **.CGCGGCGG.** 1065 (92.45)   
180 + **[CGCGGCCG]** 1159 (85.25)   
180 + **[CGCGGGGC]** 1186 (85.16)   
180 + **.CGCGGCGG.** 1247 (92.45)   
180 - **[CGCCGCGA]** 1246 (89.34)   
180 - **[CGCCGCGC]** 1064 (85.13)   
180 - **[CGCGCCCC]** 877 (85.20)   
180 - **[CGCGCCCC]** 794 (85.20)   
180 - **[GGCGCCGA]** 776 (87.37)   
180 - **[CGAGTCGA]** 121 (86.95)   
182 + **[CGCGGGGC]** 369 (85.16)   
182 + **[CGCGGCCC]** 377 (85.80)   
182 + **.CGCGGCGC.** 463 (93.00)   
182 + **[CGGGTCGC]** 535 (85.99)   
182 + **.CGCGTCGA.** 876 (100.00)   
182 + **[CGCGTCCC]** 920 (88.59)   
182 + **[CGCGGCCC]** 985 (85.80)   
182 + **[CGCGTCCG]** 1021 (88.04)   
182 + **[CGCGGCCC]** 1078 (85.80)   
182 + **[CGCGCCGT]** 1163 (87.13)   
182 + **[CGGGGCGA]** 1252 (87.42)   
182 - **[CGCGCCGT]** 872 (87.13)   
182 - **[CGCGCCCC]** 764 (85.20)   
182 - **.CGCGGCCA.** 746 (90.02)   
182 - **[CGCGGGGC]** 664 (85.16)   
182 - **[CGCGGCCC]** 527 (85.80)   
182 - **.CGCGCCGC.** 464 (92.40)   
182 - **[CGCGGCCG]** 459 (85.25)   
182 - **[CGCGGCCC]** 373 (85.80)   
182 - **[CGCGTGGC]** 365 (87.95)   
182 - **[CGCGTCAA]** 298 (86.67)   
182 - **[GGCGGCGA]** 289 (87.97)   
182 - **[CGCGCCGT]** 119 (87.13)   
183 + **[CGCGGGGC]** 838 (85.16)   
183 + **[CGCGGGGC]** 918 (85.16)   
183 + **[CGCGGGGC]** 987 (85.16)   
183 + **[GGCGGCGA]** 1024 (87.97)   
183 + **.CGCGGCGG.** 1061 (92.45)   
183 + **[CGCGCCCC]** 1232 (85.20)   
183 + **[CGCGTCCC]** 1301 (88.59)   
183 + **.CGCGGCGC.** 1356 (93.00)   
183 + **[CCCGCCGA]** 1399 (86.00)   
183 + **.CGCGCCGC.** 1424 (92.40)   
183 - **[CGCCGCGC]** 1355 (85.13)   
183 - **[CGCGGCCG]** 1297 (85.25)   
183 - **[CGCGGCCC]** 1228 (85.80)   
183 - **[CGCGGGGA]** 1195 (89.38)   
183 - **[CCCGCCGA]** 1080 (86.00)   
183 - **[CGCCGCGC]** 1060 (85.13)   
183 - **.CGCGCCGC.** 981 (92.40)   
183 - **[CGGGTCGC]** 712 (85.99)   
184 + **[CGCGGCCC]** 633 (85.80)   
184 + **[GGCGTCGG]** 669 (85.99)   
184 + **[CGGGGCGA]** 703 (87.42)   
184 + **.CGCGGCGC.** 928 (93.00)   
184 + **.CGCGGCCA.** 1068 (90.02)   
184 + **.CGCGCCGC.** 1087 (92.40)   
184 + **[CGCCGCGC]** 1164 (85.13)   
184 + **[CGCGCCCC]** 1167 (85.20)   
184 + **.CGCGGCGG.** 1352 (92.45)   
184 - **[CGCCGCGC]** 1351 (85.13)   
184 - **[CGCGCCCC]** 1348 (85.20)   
184 - **.CGCGGCGC.** 1163 (93.00)   
184 - **.CGCGGCGC.** 1088 (93.00)   
184 - **[CGCCGCGC]** 927 (85.13)   
184 - **[CGCGGGGC]** 864 (85.16)   
184 - **[CGCGGGGC]** 207 (85.16)   
184 - **.CGCGGCGG.** 52 (92.45)   
185 + **[CGCGCGGA]** 130 (88.78)   
185 + **[CGCGCCCC]** 413 (85.20)   
185 + **[CCCGCCGA]** 979 (86.00)   
185 + **.CGCGCCGG.** 1034 (91.85)   
185 + **.CGCGGCGG.** 1192 (92.45)   
185 - **.CGCGGCGG.** 1188 (92.45)   
185 - **.CGCGGCCA.** 1088 (90.02)   
185 - **.CGCGGCCA.** 1061 (90.02)   
185 - **[CGGGCCGA]** 842 (86.82)   
185 - **[CGCGGCCC]** 553 (85.80)   
185 - **[GGCGTCGC]** 219 (86.54)   
185 - **[CGCTGCGA]** 214 (85.51)   
186 + **.CGCGCCGC.** 1012 (92.40)   
186 + **.CGCGTCGT.** 1082 (90.51)   
186 - **[CGCGCGGA]** 1338 (88.78)   
187 + **[CGCCCCGA]** 863 (88.75)   
187 + **[CGCGGCCG]** 948 (85.25)   
187 + **[CGGGTCGC]** 1071 (85.99)   
187 + **[CGCGGGGC]** 1106 (85.16)   
187 + **[CGCGGGGA]** 1237 (89.38)   
187 - **.CGCGCCGG.** 1174 (91.85)   
187 - **[CGCGCCCA]** 944 (89.42)   
187 - **[CGGGGCGA]** 862 (87.42)   
187 - **[CGGGCCGA]** 151 (86.82)   
188 + **.CGCGCCGC.** 1440 (92.40)   
188 + **[CGCCGCGC]** 1469 (85.13)   
188 + **.CGCGCCGC.** 1472 (92.40)   
188 - **.CGCGGCGC.** 1468 (93.00)   
188 - **.CGCGGCGC.** 1441 (93.00)   
189 - **[CCCGGCGA]** 980 (86.59)   
189 - **[CGCGCCGT]** 847 (87.13)   
191 + **[CGCGCCCA]** 491 (89.42)   
191 + **[CGGGCCGA]** 514 (86.82)   
191 + **[CGCGGCCG]** 569 (85.25)   
191 + **[CGCGGGGC]** 794 (85.16)   
191 + **[CGCGGCCG]** 827 (85.25)   
191 + **[CGCGGGGA]** 841 (89.38)   
191 + **[CGCGGCCG]** 883 (85.25)   
191 + **.CGCGGCGC.** 1045 (93.00)   
191 + **[CGCGGCCG]** 1191 (85.25)   
191 - **.CGCGCCGC.** 1135 (92.40)   
191 - **[CGCCGCGC]** 1044 (85.13)   
191 - **.CGCGCCGG.** 1041 (91.85)   
191 - **[CGCGGCCG]** 829 (85.25)   
191 - **[CGCGCCCC]** 788 (85.20)   
191 - **[GGCGTCGC]** 332 (86.54)   
191 - **[CGCGCCCC]** 95 (85.20)   
191 - **[CGCCCCGA]** 93 (88.75)   
193 + **.CGCGGCGC.** 1249 (93.00)   
193 - **[CGCCGCGC]** 1248 (85.13)   
194 + **[GGCGTCGG]** 555 (85.99)   
194 + **.CGCGGCGG.** 912 (92.45)   
194 + **[CGCGGCGT]** 945 (87.72)   
194 + **.CGCGGCGA.** 990 (97.21)   
194 + **.CGCGGCGC.** 1112 (93.00)   
194 - **.CGCGCCGC.** 1113 (92.40)   
194 - **[CGCGGCCC]** 1108 (85.80)   
194 - **[CGCGCCCC]** 1064 (85.20)   
194 - **.CGCGGCGG.** 1038 (92.45)   
194 - **[CGCGCCGT]** 1014 (87.13)   
194 - **[CGGGCCGA]** 1002 (86.82)   
194 - **[CGCCGCGC]** 944 (85.13)   
194 - **.CGCGCCGG.** 941 (91.85)   
194 - **[CGCCGCGC]** 911 (85.13)   
194 - **[CGCGTCCC]** 906 (88.59)   
194 - **[CGCGCCCA]** 865 (89.42)   
194 - **[CGAGTCGA]** 286 (86.95)   
194 - **[CGCGGGGC]** 110 (85.16)   
195 + **.CGCGCCGC.** 153 (92.40)   
195 + **[CGCGCCCA]** 251 (89.42)   
195 + **[GGCGTCGC]** 446 (86.54)   
195 + **[CGGGTCGC]** 461 (85.99)   
195 + **.CGCGGCGC.** 852 (93.00)   
195 + **[CGCGCCCC]** 924 (85.20)   
195 + **[CGCGTCCG]** 960 (88.04)   
195 + **[CGGGTCGG]** 1175 (85.44)   
195 - **[CGCGCGGA]** 956 (88.78)   
195 - **[CGCGGGGC]** 918 (85.16)   
195 - **[CGCCGCGA]** 851 (89.34)   
195 - **[CGCCTCGC]** 307 (87.91)   
195 - **[CGCGGGGC]** 247 (85.16)   
195 - **.CGCGGCGG.** 164 (92.45)   
196 + **[CGCGTAGA]** 33 (87.53)   
196 + **.CGCGGCGG.** 120 (92.45)   
196 + **[CGCGGCGT]** 284 (87.72)   
196 + **[GGCGTCGG]** 287 (85.99)   
196 + **[CGCCGCGC]** 341 (85.13)   
196 + **[CGCGGGGC]** 499 (85.16)   
196 + **.CGCGCCGG.** 506 (91.85)   
196 + **[CGCGGCCG]** 538 (85.25)   
196 + **.CGCGTCGG.** 721 (95.23)   
196 + **[CGCCGCGC]** 910 (85.13)   
196 + **.CGCGCCGG.** 1317 (91.85)   
196 + **[CGCGGGGC]** 1473 (85.16)   
196 + **[CGCGCCCC]** 1508 (85.20)   
196 - **[CGCGGGGC]** 1511 (85.16)   
196 - **.CGCGCCGG.** 1469 (91.85)   
196 - **.CGCGCCGC.** 1425 (92.40)   
196 - **[CGCGCCGT]** 1350 (87.13)   
196 - **[CGCGGCCG]** 1100 (85.25)   
196 - **.CGCGGCGC.** 909 (93.00)   
196 - **[CGCGTCCG]** 686 (88.04)   
196 - **[CGCGCCCC]** 502 (85.20)   
196 - **.CGCGGCGA.** 340 (97.21)   
196 - **[CGCCGCGC]** 283 (85.13)   
196 - **[CCCGCCGA]** 261 (86.00)   
196 - **[CGCCGCGC]** 119 (85.13)   
196 - **[CGCGTCCG]** 29 (88.04)   
197 + **.CGCGTCGC.** 431 (95.78)   
197 + **[CGCGGCCC]** 478 (85.80)   
197 + **[CGGGTCGC]** 1114 (85.99)   
197 + **[CGCGGCGT]** 1163 (87.72)   
197 + **.CGCGGCGC.** 1506 (93.00)   
197 - **.CGCGCCGC.** 1507 (92.40)   
197 - **[CGCCGCGC]** 1505 (85.13)   
197 - **[CGCGTCCG]** 1159 (88.04)   
197 - **[CGGGTCGG]** 1150 (85.44)   
197 - **.CGCCTCGA.** 1001 (92.13)   
198 + **[CGCGCCCA]** 35 (89.42)   
198 + **[CGCCGCGC]** 256 (85.13)   
198 + **[CGCGCCCC]** 259 (85.20)   
198 + **[CGCGCCCC]** 266 (85.20)   
198 + **[CGGGTCGG]** 993 (85.44)   
198 + **[CGCGGCTA]** 1165 (85.00)   
198 - **[CGCCTCGC]** 1233 (87.91)   
198 - **[CGCGGGGC]** 262 (85.16)   
198 - **.CGCGGCGG.** 255 (92.45)   
198 - **[CGCGGGGA]** 210 (89.38)   
199 + **[CGCCTCGG]** 456 (87.36)   
199 + **[CGCGCCCC]** 672 (85.20)   
199 + **.CGCGTCGC.** 821 (95.78)   
199 + **[CCCGGCGA]** 835 (86.59)   
199 + **[CGGGTCGG]** 893 (85.44)   
199 + **.CGCGGCGC.** 908 (93.00)   
199 + **.CGCGGCGC.** 951 (93.00)   
199 + **[CGCGGCCG]** 1003 (85.25)   
199 + **.CGCGGCGG.** 1085 (92.45)   
199 + **.CGCGGCCA.** 1276 (90.02)   
199 + **[CGCGTCCG]** 1448 (88.04)   
199 - **.CGCGCCGC.** 1350 (92.40)   
199 - **[CGCGTGGG]** 1240 (87.40)   
199 - **[CGCGGCCC]** 1125 (85.80)   
199 - **[CGCGCCCC]** 1108 (85.20)   
199 - **[CGCCGCGC]** 1084 (85.13)   
199 - **.CGCGCCGC.** 1081 (92.40)   
199 - **.CGCGGCGA.** 999 (97.21)   
199 - **[CGCGCCCC]** 978 (85.20)   
199 - **[CGCCGCGC]** 950 (85.13)   
199 - **.CGCGCCGC.** 909 (92.40)   
199 - **[CGCCGCGC]** 907 (85.13)   
199 - **.CGCGCCGC.** 868 (92.40)   
199 - **[CGCGACGC]** 822 (86.46)   
199 - **[CGCGCCCA]** 817 (89.42)   
199 - **.CGCGCCGA.** 668 (96.62)   
199 - **[CGCGTGGC]** 309 (87.95)   
200 - **[CCCGGCGA]** 1249 (86.59)   
200 - **[GGCGCCGA]** 964 (87.37)   
200 - **.CGCGTCCA.** 570 (92.80)   
201 + **[CGCCGCGC]** 336 (85.13)   
201 + **[CGCGCCCC]** 339 (85.20)   
201 + **[GGCGTCGC]** 490 (86.54)   
201 + **[CGCGTTGA]** 573 (87.22)   
201 + **[CGCGCCCA]** 661 (89.42)   
201 + **[CGCGGGGC]** 778 (85.16)   
201 + **.CGCGGCGC.** 849 (93.00)   
201 + **.CGCGCCGG.** 1085 (91.85)   
201 + **[CGCGGGGA]** 1351 (89.38)   
201 - **[CGCGTCCC]** 1489 (88.59)   
201 - **[CGCGGCGT]** 1347 (87.72)   
201 - **[CGCGGCCC]** 1140 (85.80)   
201 - **[CGGGGCGA]** 1010 (87.42)   
201 - **[CGCGGGGC]** 845 (85.16)   
201 - **[CGCCTCGG]** 796 (87.36)   
201 - **.CGCGCCGC.** 774 (92.40)   
201 - **.CGCGGCGG.** 335 (92.45)   
202 + **[CGCGGGGA]** 595 (89.38)   
202 - **.CGCGTCCA.** 1391 (92.80)   
202 - **[CGCGCCCC]** 591 (85.20)   
202 - **[CGCGTCCC]** 308 (88.59)   
202 - **[CGCCTCGG]** 291 (87.36)   
207 + **.CGCGTCCA.** 857 (92.80)   
207 + **[GGCGGCGA]** 1065 (87.97)   
207 - **[CGCGTGGG]** 1287 (87.40)   
208 + **.CGCGCCGA.** 1359 (96.62)   
208 + **.CGCGGCGA.** 1499 (97.21)   
208 - **.CGCGGCGG.** 1495 (92.45)   
208 - **.CGCGCCGC.** 1407 (92.40)   
208 - **[CGCCTCGG]** 1392 (87.36)   
208 - **[CGAGTCGA]** 1337 (86.95)   
208 - **.CGCGACGA.** 1139 (90.68)   
210 + **[CGCGCCCC]** 616 (85.20)   
210 + **.CGCGTCGG.** 677 (95.23)   
210 + **[CGCGGCCC]** 780 (85.80)   
210 + **[CGCCCCGA]** 1151 (88.75)   
210 + **[CGCGCCCC]** 1223 (85.20)   
210 - **.CGCGCCGG.** 1064 (91.85)   
210 - **[CGCGGCCG]** 1045 (85.25)   
210 - **[CGCGGGGA]** 800 (89.38)   
211 + **[CGCCGCGC]** 734 (85.13)   
211 + **.CGCGCCGC.** 977 (92.40)   
211 + **.CGCGGCCA.** 1216 (90.02)   
211 - **[CGCGGGGA]** 1312 (89.38)   
211 - **[CGCGTCCC]** 1143 (88.59)   
211 - **.CGCGGCGG.** 733 (92.45)   
211 - **[CGCGGGGA]** 610 (89.38)   
212 + **[CGCGACGC]** 422 (86.46)   
212 + **.CGCGCCGG.** 455 (91.85)   
212 + **[CGCGGGGC]** 659 (85.16)   
212 + **[CGGGTCGG]** 737 (85.44)   
212 + **[CGCGTCAA]** 1034 (86.67)   
212 - **[CGCCTCGG]** 1493 (87.36)   
212 - **[CGGGGCGA]** 1303 (87.42)   
212 - **.CGCGGCCA.** 711 (90.02)   
212 - **[CGCGGGGC]** 553 (85.16)   
213 + **[CGCGGGGA]** 1063 (89.38)   
213 + **.CGCGCCGG.** 1193 (91.85)   
213 + **[GGCGCCGA]** 1335 (87.37)   
213 - **.CGCGCCGC.** 995 (92.40)   
214 + **[CCCGGCGA]** 685 (86.59)   
214 + **[CGCGGCGT]** 732 (87.72)   
214 + **[CGCGGCCG]** 931 (85.25)   
214 + **[CGCGCCCC]** 1033 (85.20)   
214 - **[CGCGGCCC]** 927 (85.80)   
214 - **[CGCCGCGC]** 731 (85.13)   
214 - **[CGCGGGGC]** 709 (85.16)   
214 - **[CGCCCCGA]** 650 (88.75)   
215 + **.CGCGTCGA.** 654 (100.00)   
215 + **.CGCGGCGC.** 1092 (93.00)   
215 + **[CGGGGCGA]** 1105 (87.42)   
215 + **[CGCGCCCC]** 1140 (85.20)   
215 + **[CGCGCCCC]** 1415 (85.20)   
215 - **.CGCGGCGG.** 1088 (92.45)   
215 - **[GGCGGCGA]** 955 (87.97)   
215 - **[CGCCTCGG]** 12 (87.36)   
216 + **[CGCGGCGT]** 212 (87.72)   
216 + **[CGCGCCCC]** 650 (85.20)   
216 + **[CGCCTCGG]** 821 (87.36)   
216 + **[CGCGCCCC]** 947 (85.20)   
216 - **[CCCGCCGA]** 1174 (86.00)   
216 - **[CGGGTCGG]** 1060 (85.44)   
216 - **.CGCGCCGG.** 990 (91.85)   
216 - **[CGCCGCGA]** 211 (89.34)   
217 + **.CGCGGCGG.** 577 (92.45)   
217 + **[CGCCGCGC]** 859 (85.13)   
217 + **.CGCGTCGG.** 941 (95.23)   
217 + **[CGGGCCGA]** 1112 (86.82)   
217 - **[CGCGGCGT]** 858 (87.72)   
217 - **.CGCCTCGA.** 775 (92.13)   
217 - **[CCCGGCGA]** 618 (86.59)   
217 - **[CGCCGCGC]** 576 (85.13)   
221 + **[CGCGTCCG]** 20 (88.04)   
221 + **.CGCGTGGA.** 305 (92.16)   
221 + **[CGCGGCCC]** 519 (85.80)   
221 + **[CGCGGGGA]** 939 (89.38)   
221 + **.CGCGGCGG.** 1112 (92.45)   
221 - **[CGCGGGGC]** 1284 (85.16)   
221 - **[CGCGGCCG]** 1108 (85.25)   
221 - **.CGCGCCGG.** 935 (91.85)   
221 - **[CGCGGCCC]** 513 (85.80)   
222 + **[CGCGGCCG]** 168 (85.25)   
222 + **[CGCGTCCG]** 452 (88.04)   
222 + **.CGCGGCGA.** 843 (97.21)   
222 + **.CGCGTCGT.** 879 (90.51)   
222 + **.CGCGGCGG.** 950 (92.45)   
222 + **[CGCGCCCC]** 1081 (85.20)   
222 + **.CGCGCCGG.** 1206 (91.85)   
222 + **.CGCGGCCA.** 1484 (90.02)   
222 - **.CGCGCCGG.** 1268 (91.85)   
222 - **[CGCGGCCC]** 999 (85.80)   
222 - **[CGCCGCGC]** 842 (85.13)   
222 - **.CGCGCCGC.** 839 (92.40)   
222 - **[CGCGGGGC]** 674 (85.16)   
222 - **[CGCGGGGA]** 446 (89.38)   
223 + **[GGCGTCGG]** 187 (85.99)   
223 + **[CGCTGCGA]** 394 (85.51)   
223 + **[CGCGCCGT]** 424 (87.13)   
223 - **[CGCGTCCC]** 454 (88.59)   
224 + **[CGCGGGGC]** 473 (85.16)   
224 + **[CGCGGCCG]** 532 (85.25)   
224 + **[GGCGTCGG]** 580 (85.99)   
224 + **.CGCGGCGC.** 619 (93.00)   
224 + **[CGCCGCGA]** 689 (89.34)   
224 + **[CGCGGCCG]** 799 (85.25)   
224 + **[CGCGGGGC]** 825 (85.16)   
224 + **.CGCGGCGA.** 856 (97.21)   
224 + **[CGCGGGGC]** 1178 (85.16)   
224 + **[CGCGGGGA]** 1185 (89.38)   
224 + **[CGCCGCGC]** 1233 (85.13)   
224 + **[CGCCTCGG]** 1245 (87.36)   
224 - **[CGCGCCCC]** 1324 (85.20)   
224 - **.CGCGGCGA.** 1232 (97.21)   
224 - **[CGCGCCCC]** 1181 (85.20)   
224 - **[CGCGTGGG]** 1174 (87.40)   
224 - **[CGCCGCGC]** 855 (85.13)   
224 - **.CGCGCCGG.** 852 (91.85)   
224 - **.CGCGGCGC.** 821 (93.00)   
224 - **.CGCGCCGC.** 716 (92.40)   
224 - **.CGCGGCGG.** 688 (92.45)   
224 - **[CGCCGCGC]** 618 (85.13)   
224 - **[CGCCCCGA]** 602 (88.75)   
224 - **[CGCGCCCC]** 476 (85.20)   
226 + **[CGCGGCCG]** 125 (85.25)   
226 + **[CGCGGGGC]** 138 (85.16)   
226 + **[CGCGTCTA]** 235 (87.79)   
226 + **.CGCGCCGG.** 511 (91.85)   
226 + **[CGGGGCGA]** 894 (87.42)   
226 + **[CGCGGCCG]** 952 (85.25)   
226 + **.CGCGCCGC.** 1032 (92.40)   
226 + **[CGCCGCGC]** 1034 (85.13)   
226 + **[CGCGCCCC]** 1037 (85.20)   
226 + **[CGCGTCCG]** 1065 (88.04)   
226 + **.CGCGGCGC.** 1078 (93.00)   
226 + **.CGCGCCGG.** 1137 (91.85)   
226 + **[CGCGCCCC]** 1163 (85.20)   
226 + **[CGCGCCCC]** 1170 (85.20)   
226 + **.CGCGGCGG.** 1497 (92.45)   
226 - **[CGCCGCGC]** 1496 (85.13)   
226 - **[CGCGCCCA]** 1493 (89.42)   
226 - **[CGCGCCCA]** 1432 (89.42)   
226 - **.CGCGGCCA.** 1335 (90.02)   
226 - **[CGCGGCCC]** 1200 (85.80)   
226 - **[GGCGTCGG]** 1187 (85.99)   
226 - **[CGCGGGGC]** 1173 (85.16)   
226 - **[CGCGGGGC]** 1166 (85.16)   
226 - **[CGCCGCGC]** 1077 (85.13)   
226 - **.CGCGGCGC.** 1033 (93.00)   
226 - **[CGCGCCCC]** 963 (85.20)   
226 - **[CGCCCCGA]** 893 (88.75)   
226 - **[CGCGTCCC]** 710 (88.59)   
226 - **[CGCGGCCG]** 516 (85.25)   
227 + **[CGCGGCCC]** 268 (85.80)   
227 + **[CGCGGGGC]** 848 (85.16)   
227 + **[CGCGGGGC]** 1284 (85.16)   
227 + **.CGCGGCGC.** 1297 (93.00)   
227 + **.CGCGCCGG.** 1361 (91.85)   
227 - **[CGCGGGGA]** 1085 (89.38)   
227 - **[CGCGGGGA]** 1075 (89.38)   
227 - **[CGCGGGGA]** 1034 (89.38)   
227 - **[CGCGGGGA]** 1014 (89.38)   
227 - **[CGCGGGGA]** 994 (89.38)   
227 - **[CGCGGGGA]** 984 (89.38)   
227 - **[CGCGGGGA]** 974 (89.38)   
227 - **[CGCGGGGA]** 963 (89.38)   
227 - **[CGGGCCGA]** 919 (86.82)   
227 - **[CGCCTCGG]** 900 (87.36)   
227 - **[CGCGTGGG]** 844 (87.40)   
227 - **.CGCGTCGA.** 685 (100.00)   
227 - **[CGCGTCCG]** 613 (88.04)   
227 - **[CGCGTCCC]** 305 (88.59)   
228 + **[CGCGCCCC]** 129 (85.20)   
228 + **[CGCGCCCC]** 136 (85.20)   
228 + **[CGCCCCGA]** 432 (88.75)   
228 + **.CGCGCCGA.** 471 (96.62)   
228 + **.CGCGCCGC.** 542 (92.40)   
228 + **[CGCGGGGC]** 954 (85.16)   
228 - **[CGCGTGGG]** 1425 (87.40)   
228 - **[CGCGGCCC]** 1269 (85.80)   
228 - **[CGCGCCCC]** 950 (85.20)   
228 - **.CGCCTCGA.** 834 (92.13)   
228 - **[CGCGTCTA]** 736 (87.79)   
228 - **[CGGGTCGG]** 152 (85.44)   
228 - **[CGCGGGGC]** 132 (85.16)   
228 - **[CGCGGCCC]** 125 (85.80)   
228 - **[CGCCTCGG]** 103 (87.36)   
229 + **[CGCGGCCG]** 540 (85.25)   
229 + **.CGCGGCCA.** 587 (90.02)   
229 + **[CGCCGCGC]** 689 (85.13)   
229 + **[CGCGGGGC]** 1038 (85.16)   
229 + **[CGGGGCGA]** 1136 (87.42)   
229 + **[CGCGCCCC]** 1148 (85.20)   
229 + **.CGCGGCGC.** 1284 (93.00)   
229 + **[CGCGGGGC]** 1303 (85.16)   
229 + **[GGCGTCGG]** 1389 (85.99)   
229 - **[CGCGCCCC]** 1299 (85.20)   
229 - **.CGCGCCGC.** 1285 (92.40)   
229 - **[CGCCGCGC]** 1283 (85.13)   
229 - **[CGCGGCCC]** 1244 (85.80)   
229 - **.CGCGGCGG.** 1112 (92.45)   
229 - **.CGCGGCGC.** 688 (93.00)   
230 + **[CGCCCCGA]** 289 (88.75)   
230 - **[CGGGGCGA]** 288 (87.42)   
231 + **[CGGGTCGC]** 695 (85.99)   
231 + **.CGCGGCCA.** 1070 (90.02)   
231 + **[CGGGGCGA]** 1094 (87.42)   
231 + **[CGGGGCGA]** 1103 (87.42)   
231 + **[CGCGCCCC]** 1165 (85.20)   
231 + **.CGCGCCGG.** 1484 (91.85)   
231 - **.CGCGGCCA.** 1480 (90.02)   
231 - **[CGCGCCCA]** 1468 (89.42)   
231 - **[CGCGTCCC]** 1381 (88.59)   
231 - **[CGCGCCCC]** 1139 (85.20)   
231 - **[CGCGCCCC]** 1120 (85.20)   
231 - **[CGCGGCCC]** 978 (85.80)   
231 - **.CGCGGCGG.** 904 (92.45)   
231 - **[CGCGTGGG]** 872 (87.40)   
232 + **[CGCCTCGG]** 106 (87.36)   
232 + **[CGCCGCGC]** 146 (85.13)   
232 + **[CGCGTCCC]** 635 (88.59)   
232 + **.CGCGTCCA.** 701 (92.80)   
232 + **[CGCCGCGA]** 793 (89.34)   
232 + **[CGCGGCCG]** 863 (85.25)   
232 + **.CGCGGCGG.** 997 (92.45)   
232 + **.CGCGCCGA.** 1246 (96.62)   
232 + **[CGCCTCGG]** 1268 (87.36)   
232 + **[CGCGGCCC]** 1335 (85.80)   
232 - **.CGCGTCCA.** 1331 (92.80)   
232 - **.CGCGGCCA.** 1242 (90.02)   
232 - **.CGCGCCGG.** 1178 (91.85)   
232 - **[CGCGTCCG]** 1117 (88.04)   
232 - **.CGCGCCGC.** 1011 (92.40)   
232 - **[CGCCGCGC]** 996 (85.13)   
232 - **[CGCGGCCG]** 865 (85.25)   
232 - **.CGCGGCGG.** 792 (92.45)   
232 - **.CGCGCCGC.** 631 (92.40)   
232 - **.CGCGCCGG.** 442 (91.85)   
232 - **[CGGGTCGC]** 259 (85.99)   
232 - **.CGCGGCGG.** 145 (92.45)   
233 + **[CGCGTGGG]** 1380 (87.40)   
233 - **[GGCGTCGC]** 1139 (86.54)   
234 + **[CGCGGGGA]** 181 (89.38)   
234 + **[CACGTCGA]** 215 (86.48)   
234 + **.CGCGCCGG.** 411 (91.85)   
234 + **.GGCGTCGA.** 631 (90.75)   
234 + **[CGCGGGGC]** 639 (85.16)   
234 + **[CGCGGCCC]** 816 (85.80)   
234 + **.CGCGGCGC.** 1331 (93.00)   
234 + **[CGCGCGGA]** 1413 (88.78)   
234 + **[CGGGCCGA]** 1434 (86.82)   
234 - **[CGCGTCCC]** 1409 (88.59)   
234 - **[CGCGTGGC]** 1327 (87.95)   
234 - **[CGCGCCCC]** 1287 (85.20)   
234 - **[CGGGGCGA]** 882 (87.42)   
234 - **.CGCGTCGA.** 635 (100.00)   
234 - **[CGCGACGG]** 315 (85.91)   
234 - **[CGCGCCCC]** 177 (85.20)   
235 + **.CGCGTGGA.** 476 (92.16)   
235 + **[CGCGCCCA]** 670 (89.42)   
235 + **.CGCGGCGC.** 900 (93.00)   
235 + **[CGCCGCGC]** 1361 (85.13)   
235 - **.CGCGGCGC.** 1360 (93.00)   
235 - **.CGCGCCGC.** 1315 (92.40)   
235 - **[CGCCTCGC]** 1275 (87.91)   
235 - **[CGCGGCCC]** 959 (85.80)   
235 - **.CGCGCCGC.** 901 (92.40)   
235 - **[CGCCGCGC]** 899 (85.13)   
235 - **[CGCGTGGC]** 758 (87.95)   
235 - **[CGCGTGGC]** 751 (87.95)   
235 - **[CGCGCCCC]** 633 (85.20)   
236 + **[CGCGGCCC]** 1026 (85.80)   
236 + **[CGGGCCGA]** 1321 (86.82)   
236 - **[CGCGGCCG]** 1152 (85.25)   
236 - **[CGCGCCCA]** 908 (89.42)   
238 - **[CGCGGGGC]** 1224 (85.16)   
238 - **.CGCGTCGG.** 1104 (95.23)   
239 + **[CGCTGCGA]** 128 (85.51)   
239 + **.CGCGCCGA.** 770 (96.62)   
239 - **[CGCGGCCC]** 766 (85.80)   
239 - **[CGCGCCCC]** 705 (85.20)   
239 - **.CGCGGCGA.** 443 (97.21)   
239 - **[CGGGCCGA]** 412 (86.82)   
240 + **.CGCGCCGC.** 1182 (92.40)   
240 + **[CGCCGCGA]** 1402 (89.34)   
240 - **.CGCGGCGA.** 1401 (97.21)   
240 - **[CGCGGGGA]** 1178 (89.38)   
240 - **[CGCGCCCC]** 51 (85.20)   
241 - **.CGCGCCGC.** 1282 (92.40)   
242 + **[CGGGTCGG]** 568 (85.44)   
242 + **.CGCGTCGT.** 1003 (90.51)   
242 - **[GGCGGCGA]** 893 (87.97)   
243 + **[CGCGGCCG]** 920 (85.25)   
243 - **[CGCGGCTA]** 1118 (85.00)   
244 + **[CGCGGGGC]** 765 (85.16)   
244 + **[CGCGCGGA]** 779 (88.78)   
244 - **[CGCCCCGA]** 921 (88.75)   
244 - **.CGCGTCCA.** 685 (92.80)   
246 + **[CGCCGCGC]** 273 (85.13)   
246 + **[CGCCTCGG]** 278 (87.36)   
246 + **[CGCATCGA]** 499 (86.64)   
246 + **.CGCGGCGC.** 674 (93.00)   
246 + **.CGCGCCGG.** 910 (91.85)   
246 + **[CGCGCGGA]** 1080 (88.78)   
246 + **[CCCGCCGA]** 1148 (86.00)   
246 - **[GGCGGCGA]** 1216 (87.97)   
246 - **[CGCGGCCG]** 1076 (85.25)   
246 - **[CGCGGGGC]** 746 (85.16)   
246 - **[CGCGGCCG]** 705 (85.25)   
246 - **[CGGGTCGG]** 684 (85.44)   
246 - **[CGCCGCGC]** 673 (85.13)   
246 - **[CGCGTCCC]** 668 (88.59)   
246 - **[GGCGCCGA]** 282 (87.37)   
246 - **.CGCGGCGG.** 272 (92.45)   
246 - **[CGCGGGGC]** 1 (85.16)   
247 + **[CGCCGCGA]** 18 (89.34)   
247 + **[CCCGCCGA]** 262 (86.00)   
247 + **.CGCGGCGG.** 422 (92.45)   
247 + **.CGCGGCGG.** 1024 (92.45)   
247 + **[CGCGCCCC]** 1096 (85.20)   
247 + **[CGCCTCGC]** 1227 (87.91)   
247 + **[CGCGCGGA]** 1287 (88.78)   
247 + **[CGCGGCTA]** 1299 (85.00)   
247 + **[CGCGGCCC]** 1360 (85.80)   
247 + **[CGCGCCCC]** 1473 (85.20)   
247 + **[CGCCGCGC]** 1492 (85.13)   
247 - **.CGCGGCGG.** 1491 (92.45)   
247 - **[CGCGGGGA]** 1469 (89.38)   
247 - **[CGCGGCCG]** 1090 (85.25)   
247 - **[CGCCCCGA]** 959 (88.75)   
247 - **[CGCCGCGC]** 421 (85.13)   
247 - **[CGCGGGGA]** 327 (89.38)   
247 - **.CGCGGCGG.** 17 (92.45)   
248 - **[CGCCTCGG]** 1405 (87.36)   
250 + **[CGCGCCCA]** 139 (89.42)   
250 + **.CGCGGCGC.** 457 (93.00)   
250 + **[CGCGCCCA]** 528 (89.42)   
250 + **[GGCGGCGA]** 961 (87.97)   
250 - **[CGCGGCCC]** 1094 (85.80)   
250 - **.CGCGGCGA.** 868 (97.21)   
250 - **.CGCGCCGC.** 458 (92.40)   
250 - **[CGCCGCGC]** 456 (85.13)   
250 - **[CGCGTCCC]** 451 (88.59)   
250 - **.CGCGTGGA.** 135 (92.16)   
252 + **[CGCGTCCC]** 394 (88.59)   
255 - **[CCCGCCGA]** 1508 (86.00)   
256 + **[CGCCTCGG]** 55 (87.36)   
256 + **[CGCGGGGC]** 862 (85.16)   
256 + **[CCCGGCGA]** 912 (86.59)   
256 + **[CGGGTCGG]** 1097 (85.44)   
256 + **[CCCGCCGA]** 1121 (86.00)   
256 + **.CGCGTCGG.** 1205 (95.23)   
256 - **.CGCGGCGG.** 1300 (92.45)   
256 - **[CGCGGCCG]** 1193 (85.25)   
256 - **[CGGGTCGG]** 1125 (85.44)   
257 + **[CCCGGCGA]** 719 (86.59)   
257 + **[GGCGTCGG]** 1464 (85.99)   
257 - **[CGGGTCGC]** 1424 (85.99)   
257 - **[CGCGGCCG]** 1280 (85.25)   
257 - **[GGCGCCGA]** 1237 (87.37)   
257 - **[CGCGGCCG]** 1192 (85.25)   
257 - **[CGCGGCCG]** 1164 (85.25)   
257 - **[CGCGTCCC]** 1117 (88.59)   
257 - **[CGGGGCGA]** 916 (87.42)   
257 - **[CGCGCCCC]** 874 (85.20)   
257 - **.CGCGGCCA.** 605 (90.02)   
259 + **[GGCGGCGA]** 989 (87.97)   
259 + **.CGCGGCGG.** 997 (92.45)   
259 + **[CGCGACGC]** 1020 (86.46)   
259 + **[CGCGGCCG]** 1187 (85.25)   
259 + **[CGCGCCCC]** 1221 (85.20)   
259 + **.CGCGGCGA.** 1304 (97.21)   
259 + **[CCCGCCGA]** 1477 (86.00)   
259 - **.CGCGGCGC.** 1300 (93.00)   
259 - **.CGCGTCGC.** 1021 (95.78)   
259 - **.CGCGTCGC.** 993 (95.78)   
260 + **[CGCGCCCC]** 879 (85.20)   
260 + **[CGGGCCGA]** 945 (86.82)   
260 + **[GGCGGCGA]** 1115 (87.97)   
262 - **[CCCGGCGA]** 1195 (86.59)   
263 + **[CGCGTGGC]** 167 (87.95)   
263 + **[CGCGGCCG]** 850 (85.25)   
263 + **.CGCGTCGC.** 1078 (95.78)   
263 + **[CGCGGGGC]** 1284 (85.16)   
263 - **[CGCGCCCA]** 1280 (89.42)   
263 - **.CGCGTCGG.** 1115 (95.23)   
263 - **[CGCGACGC]** 1079 (86.46)   
263 - **[CGCGGCCG]** 852 (85.25)   
263 - **[CGCGCCCC]** 538 (85.20)   
263 - **[CGCGCCCA]** 28 (89.42)   
264 + **[CGCGCCGT]** 1138 (87.13)   
264 + **[CGCCTCGG]** 1206 (87.36)   
264 - **[CGCGTCCC]** 750 (88.59)   
266 + **[CGCGGCCG]** 962 (85.25)   
266 + **.CGCGCCGG.** 982 (91.85)   
266 + **[CGCGGGGA]** 996 (89.38)   
266 + **.CGCGGCGG.** 1030 (92.45)   
266 + **.CGCGCCGA.** 1106 (96.62)   
266 + **.CGCGCCGG.** 1270 (91.85)   
266 + **.CGCGGCGG.** 1282 (92.45)   
266 - **.CGCGGCGC.** 1473 (93.00)   
266 - **[CGCGGCCG]** 1278 (85.25)   
266 - **.CGCGCCGA.** 1102 (96.62)   
266 - **[CCCGCCGA]** 1078 (86.00)   
266 - **[CGCGGCCG]** 1063 (85.25)   
266 - **.CGCCTCGA.** 1040 (92.13)   
266 - **[CGCCGCGC]** 1029 (85.13)   
266 - **.CGCGCCGG.** 1026 (91.85)   
266 - **[CGCGGGGC]** 992 (85.16)   
266 - **[CGCGTGGC]** 978 (87.95)   
266 - **.CGCGTGGA.** 543 (92.16)   
267 + **[CGCGGCCG]** 195 (85.25)   
267 + **[CGCGGGGA]** 699 (89.38)   
267 - **.CGCGGCGG.** 191 (92.45)   
267 - **.CGCCTCGA.** 95 (92.13)   
268 + **[CGCGGGGA]** 338 (89.38)   
268 + **.CGCGGCGA.** 813 (97.21)   
268 + **[CGCGGCGT]** 1015 (87.72)   
268 + **[CGCGGCCG]** 1478 (85.25)   
268 - **[CGCGCGGA]** 1390 (88.78)   
268 - **[CGCGGCCC]** 1318 (85.80)   
268 - **[CGCGTGGC]** 1197 (87.95)   
268 - **[CGCGGCCC]** 1066 (85.80)   
268 - **[CGCCGCGC]** 1014 (85.13)   
268 - **.CGCGCCGG.** 1011 (91.85)   
268 - **.CGCGTCCA.** 507 (92.80)   
268 - **[CGCGGCCC]** 178 (85.80)   
268 - **[CCCGCCGA]** 122 (86.00)   
268 - **.CGCGCCGC.** 55 (92.40)   
270 + **[GGCGCCGA]** 97 (87.37)   
270 + **[CGGGTCGC]** 1060 (85.99)   
270 + **[CGGGGCGA]** 1204 (87.42)   
270 + **[CGCGGGGC]** 1382 (85.16)   
270 + **[CGCGTGGC]** 1442 (87.95)   
270 - **[CGCGGCCC]** 1436 (85.80)   
270 - **[CGCTGCGA]** 812 (85.51)   
270 - **[CGCGCCCC]** 460 (85.20)   
270 - **[GGCGTCGC]** 342 (86.54)   
270 - **[CGCGCCGT]** 54 (87.13)   
271 - **[CGCGTCTA]** 1286 (87.79)   
271 - **[CGCGTCCG]** 740 (88.04)   
272 - **[CGCGTCTA]** 1277 (87.79)   
273 + **[CGCGCCCC]** 201 (85.20)   
273 + **[GGCGTCGG]** 361 (85.99)   
273 + **.CGCGGCGG.** 1032 (92.45)   
273 + **[CGCGTCCC]** 1121 (88.59)   
273 - **[CGCGGCCG]** 1028 (85.25)   
273 - **[GGCGCCGA]** 457 (87.37)   
274 + **[CGCGGCCG]** 15 (85.25)   
274 + **.CGCGGCGC.** 108 (93.00)   
274 + **[CGCGGCCG]** 1322 (85.25)   
274 - **.CGCGCCGC.** 109 (92.40)   
274 - **[CGCGGCCG]** 11 (85.25)   
275 + **[CGCGTAGA]** 114 (87.53)   
275 + **.CGCGCCGC.** 755 (92.40)   
275 + **[CGCCTCGC]** 815 (87.91)   
275 + **[CGCGACGC]** 1265 (86.46)   
275 + **[CGCGCCGT]** 1330 (87.13)   
275 - **[GGCGGCGA]** 1006 (87.97)   
275 - **.CGCGCCGC.** 71 (92.40)   
276 + **[CACGTCGA]** 930 (86.48)   
276 - **.CGCGTCGA.** 753 (100.00)   
279 + **[CGCGCCCA]** 22 (89.42)   
279 + **[CGCGGCCC]** 33 (85.80)   
279 + **[CGGGCCGA]** 95 (86.82)   
279 + **[CCCGGCGA]** 260 (86.59)   
279 + **[CGCGACGC]** 376 (86.46)   
279 + **.CGCGGCGC.** 415 (93.00)   
279 + **[CGCGTGGG]** 420 (87.40)   
279 - **.CGCGCCGC.** 416 (92.40)   
279 - **[CGCCGCGC]** 414 (85.13)   
279 - **[CCCGCCGA]** 282 (86.00)   
280 + **[CGCGGCTA]** 169 (85.00)   
280 + **[GGCGTCGG]** 504 (85.99)   
280 + **.CGCGGCGG.** 735 (92.45)   
280 + **.CGCGGCGG.** 982 (92.45)   
280 + **[CGCGCCCC]** 1072 (85.20)   
280 + **[CGCGTCCG]** 1084 (88.04)   
280 + **[CGCGCCCC]** 1264 (85.20)   
280 - **[CGCGGGGC]** 1366 (85.16)   
280 - **[CGCCGCGC]** 981 (85.13)   
280 - **.CGCGCCGC.** 974 (92.40)   
280 - **[CGCGGCCC]** 942 (85.80)   
280 - **[CGCGGCCG]** 740 (85.25)   
280 - **[CGCCGCGC]** 734 (85.13)   
281 + **[CGCGGGGC]** 448 (85.16)   
281 + **[CGCGGCCG]** 531 (85.25)   
281 + **[CGCGCCCA]** 553 (89.42)   
281 + **[GGCGGCGA]** 1166 (87.97)   
281 - **[CGCGCCCA]** 1198 (89.42)   
281 - **[CGCGCCCC]** 543 (85.20)   
281 - **[CGCGTCCC]** 444 (88.59)   
283 + **[CGCGCCCC]** 134 (85.20)   
283 - **[CTCGTCGA]** 1069 (86.25)   
284 + **[CGCCTCGC]** 555 (87.91)   
284 + **[GGCGTCGG]** 579 (85.99)   
284 + **[CGCGTGGG]** 718 (87.40)   
284 + **.CGCGGCGG.** 780 (92.45)   
284 + **[CGCCTCGC]** 905 (87.91)   
284 + **[CGCGGCCC]** 943 (85.80)   
284 + **.CGCGCCGG.** 1003 (91.85)   
284 + **.CGCGGCGC.** 1089 (93.00)   
284 + **[CGCGGCCC]** 1094 (85.80)   
284 + **[CGCGGCCG]** 1283 (85.25)   
284 + **.CGCGCCGC.** 1342 (92.40)   
284 + **.CGCGGCGC.** 1347 (93.00)   
284 + **[CGGGTCGC]** 1502 (85.99)   
284 - **.CGCGCCGG.** 1448 (91.85)   
284 - **.CGCGGCGC.** 1343 (93.00)   
284 - **[GGCGGCGA]** 1247 (87.97)   
284 - **.CGCGCCGC.** 1090 (92.40)   
284 - **[GGCGTCGC]** 1027 (86.54)   
284 - **[CGCGCCCC]** 997 (85.20)   
284 - **[CGCCCCGA]** 823 (88.75)   
284 - **[CGCCGCGC]** 779 (85.13)   
284 - **[CGCGGCCC]** 704 (85.80)   
284 - **[CGCGCCCC]** 666 (85.20)   
284 - **.CGCGCCGC.** 629 (92.40)   
284 - **.GGCGTCGA.** 488 (90.75)   
285 + **[CGCGGGGC]** 355 (85.16)   
285 + **[CGCGGCCC]** 495 (85.80)   
285 + **[CGCGGCCG]** 548 (85.25)   
285 + **.CGCGGCGC.** 554 (93.00)   
285 + **[CGCCGCGC]** 633 (85.13)   
285 + **[CGCGCCCC]** 636 (85.20)   
285 + **.CGCGCCGC.** 652 (92.40)   
285 + **[CGCCGCGC]** 654 (85.13)   
285 + **.CGCGCCGG.** 657 (91.85)   
285 + **[CGCGGCCG]** 717 (85.25)   
285 + **[CGCGGGGC]** 774 (85.16)   
285 + **.CGCGGCGC.** 909 (93.00)   
285 + **[CGCGCCCC]** 941 (85.20)   
285 - **.CGCGCCGG.** 1024 (91.85)   
285 - **[CGCGGGGC]** 944 (85.16)   
285 - **[CGCGGCCC]** 937 (85.80)   
285 - **.CGCGGCGG.** 905 (92.45)   
285 - **[CGCGGCCG]** 856 (85.25)   
285 - **.CGCGTCGC.** 713 (95.78)   
285 - **.CGCGGCGC.** 653 (93.00)   
285 - **[CGCGGGGC]** 639 (85.16)   
285 - **[CGCGGCGT]** 632 (87.72)   
285 - **[GGCGCCGA]** 619 (87.37)   
285 - **[CGCGGCCG]** 550 (85.25)   
285 - **.CGCGCCGC.** 441 (92.40)   
285 - **[CGCGGCCC]** 351 (85.80)   
285 - **[CGCGCCCA]** 332 (89.42)   
287 + **.CGCGCCGG.** 537 (91.85)   
287 + **[CGCCTCGG]** 688 (87.36)   
287 + **[CGCCTCGG]** 765 (87.36)   
287 + **[CGCGCCCA]** 1233 (89.42)   
287 - **[GGCGGCGA]** 1078 (87.97)   
287 - **[CGGGGCGA]** 1042 (87.42)   
288 + **.CGCGCCGA.** 1195 (96.62)   
288 - **.CGCGCCGG.** 874 (91.85)   
289 + **[CGCGCGGA]** 1045 (88.78)   
289 + **.CGCGGCGC.** 1300 (93.00)   
289 - **[CGCGCCCA]** 1315 (89.42)   
289 - **[CGCCGCGC]** 1299 (85.13)   
289 - **[CGCGTCTA]** 538 (87.79)   
290 + **[CGCCTCGG]** 1153 (87.36)   
290 + **.CGCGGCCA.** 1339 (90.02)   
290 + **[CGGGTCGC]** 1427 (85.99)   
290 - **[CCCGGCGA]** 1431 (86.59)   
290 - **[CGCGCCCC]** 1328 (85.20)   
290 - **[CGCCCCGA]** 1326 (88.75)   
290 - **[CGGGCCGA]** 1241 (86.82)   
290 - **[GGCGGCGA]** 1161 (87.97)   
291 + **[CGCGTCCC]** 203 (88.59)   
291 + **[CGCGTCCC]** 407 (88.59)   
291 + **[CGCTGCGA]** 496 (85.51)   
291 + **.CGCGGCGC.** 871 (93.00)   
291 + **[CGCCCCGA]** 1010 (88.75)   
291 + **[CGCCGCGC]** 1034 (85.13)   
291 + **[CGCGCCGT]** 1100 (87.13)   
291 + **[CGCGGGGC]** 1111 (85.16)   
291 + **[CGGGTCGG]** 1192 (85.44)   
291 + **[CGCGGCCG]** 1240 (85.25)   
291 + **[GGCGGCGA]** 1247 (87.97)   
291 + **.CGCGCCGG.** 1502 (91.85)   
291 - **[CCCGCCGA]** 1470 (86.00)   
291 - **[CGCGGCCC]** 1341 (85.80)   
291 - **[CGCGGCCC]** 1327 (85.80)   
291 - **[CGCGGGGC]** 1203 (85.16)   
291 - **[CGCCCCGA]** 1196 (88.75)   
291 - **[CGCGTCCG]** 1163 (88.04)   
291 - **[CGCGGCTA]** 1107 (85.00)   
291 - **.CGCGGCGC.** 1081 (93.00)   
291 - **.CGCGGCGC.** 1033 (93.00)   
291 - **[GGCGCCGA]** 1030 (87.37)   
291 - **[CGCCGCGC]** 870 (85.13)   
291 - **[CGCGGCGT]** 765 (87.72)   
291 - **[CGCGTGGG]** 734 (87.40)   
291 - **[CGCGGGGC]** 690 (85.16)   
291 - **[CGCGACGC]** 582 (86.46)   
291 - **[CGGGGCGA]** 361 (87.42)   
292 + **[CGCGCCCC]** 767 (85.20)   
292 + **.CGCGCCGC.** 774 (92.40)   
292 + **.CGCGGCGG.** 797 (92.45)   
292 + **[CGCGCCCC]** 958 (85.20)   
292 + **[CGCCCCGA]** 960 (88.75)   
292 + **[CGCCGCGA]** 1081 (89.34)   
292 + **[GGCGTCGG]** 1178 (85.99)   
292 + **[CGCGTCCG]** 1319 (88.04)   
292 + **[CGCGGCCG]** 1339 (85.25)   
292 + **[CGCGGGGC]** 1348 (85.16)   
292 + **[GGCGTCGC]** 1466 (86.54)   
292 + **[CGCGGGGC]** 1471 (85.16)   
292 + **[CGCGGGGC]** 1487 (85.16)   
292 - **[CGCGCCCC]** 1490 (85.20)   
292 - **.CGCGTCCA.** 1483 (92.80)   
292 - **[CGCGACGC]** 1467 (86.46)   
292 - **.CGCGCCGG.** 1344 (91.85)   
292 - **[CGCGTCCC]** 1187 (88.59)   
292 - **[CGCTGCGA]** 1102 (85.51)   
292 - **.CGCGGCGG.** 1080 (92.45)   
292 - **[CGCGCCCC]** 1041 (85.20)   
292 - **[CGCGCCCC]** 1024 (85.20)   
292 - **[CGCGGCGT]** 793 (87.72)   
292 - **[CGCGGGGC]** 770 (85.16)   
293 + **[CGCCTCGC]** 781 (87.91)   
293 + **.CGCGGCGG.** 839 (92.45)   
293 + **[CGCGGGGC]** 912 (85.16)   
293 + **[GGCGTCGC]** 1070 (86.54)   
293 + **[CGCGGGGC]** 1075 (85.16)   
293 + **[CGCGCGGA]** 1098 (88.78)   
293 + **[GGCGTCGC]** 1362 (86.54)   
293 + **[CGCGTGGC]** 1444 (87.95)   
293 - **[CCCGGCGA]** 1366 (86.59)   
293 - **[CGCGCCCA]** 1094 (89.42)   
293 - **[CGCGACGC]** 1071 (86.46)   
293 - **.CGCGCCGG.** 858 (91.85)   
293 - **[CGCCGCGC]** 838 (85.13)   
293 - **[CGCGCCCA]** 835 (89.42)   
293 - **[CGCCTCGG]** 819 (87.36)   
294 + **[CGCCGCGC]** 45 (85.13)   
294 + **[CGCCTCGC]** 363 (87.91)   
294 + **.CGCGTGGA.** 613 (92.16)   
294 + **.CGCGGCGC.** 894 (93.00)   
294 + **[CGCGCCCC]** 938 (85.20)   
294 - **[GGCGTCGG]** 1087 (85.99)   
294 - **[CGCGTGGC]** 1026 (87.95)   
294 - **[CGCGTCCG]** 934 (88.04)   
294 - **[CGCCGCGC]** 893 (85.13)   
294 - **.CGCGGCGG.** 44 (92.45)   
295 + **[GGCGTCGG]** 551 (85.99)   
295 + **.CGCGGCGG.** 592 (92.45)   
295 + **[CGCGTGGC]** 612 (87.95)   
295 + **[CGCGTCCG]** 704 (88.04)   
295 + **[CGCCGCGC]** 746 (85.13)   
295 - **[CGCCTCGC]** 1275 (87.91)   
295 - **.CGCGTGGA.** 1025 (92.16)   
295 - **.CGCGGCGC.** 745 (93.00)   
295 - **[CGCGCCCC]** 700 (85.20)   
295 - **[CGCCGCGC]** 591 (85.13)   
296 + **[CGCGCCCC]** 39 (85.20)   
296 + **.CGCGGCGG.** 85 (92.45)   
296 + **[GGCGGCGA]** 468 (87.97)   
296 + **.CGCGCCGG.** 591 (91.85)   
296 + **[CGCGCGGA]** 649 (88.78)   
296 + **[CGCGCCCC]** 697 (85.20)   
296 + **[CGCGCCCC]** 704 (85.20)   
296 + **[CGCGCCCC]** 711 (85.20)   
296 + **[CGCGCCCC]** 718 (85.20)   
296 + **[CGCCGCGC]** 756 (85.13)   
296 + **.CGCGCCGA.** 759 (96.62)   
296 + **[CGCGGCCG]** 815 (85.25)   
296 + **[GGCGCCGA]** 921 (87.37)   
296 + **.CGCGCCGC.** 946 (92.40)   
296 + **.CGCGGCGA.** 1003 (97.21)   
296 + **.CGCGCCGC.** 1184 (92.40)   
296 + **[GGCGTCGC]** 1207 (86.54)   
296 + **.CGCGCCGC.** 1315 (92.40)   
296 + **.CGCGGCGC.** 1440 (93.00)   
296 + **[CGCGTCCC]** 1474 (88.59)   
296 - **.CGCGCCGC.** 1441 (92.40)   
296 - **[CGCCGCGC]** 1439 (85.13)   
296 - **[CGCCTCGC]** 1381 (87.91)   
296 - **[CGCGCCCA]** 1311 (89.42)   
296 - **.CGCGGCGC.** 1185 (93.00)   
296 - **[CGCGGCCG]** 1160 (85.25)   
296 - **[CGCGCCCC]** 1116 (85.20)   
296 - **[CGCGTCCC]** 839 (88.59)   
296 - **[CGCGGCGT]** 755 (87.72)   
296 - **[CGCGGGGC]** 714 (85.16)   
296 - **[CGCGGGGC]** 707 (85.16)   
296 - **[CGCGGGGC]** 700 (85.16)   
296 - **[CGCGCCCC]** 417 (85.20)   
296 - **[CGCGCCCC]** 246 (85.20)   
296 - **.CGCGCCGC.** 193 (92.40)   
296 - **[CGGGTCGG]** 147 (85.44)   
297 + **[CGCGCCCA]** 792 (89.42)   
297 + **[CGCGGGGA]** 829 (89.38)   
297 + **[CGCGGGGC]** 987 (85.16)   
297 + **[CGCGTGGC]** 1033 (87.95)   
297 + **[GGCGTCGC]** 1201 (86.54)   
297 + **[GGCGTCGG]** 1443 (85.99)   
297 - **[CGCCTCGC]** 1191 (87.91)   
297 - **[CGCGGCCG]** 994 (85.25)   
297 - **.CGCGCCGC.** 983 (92.40)   
297 - **[CGCGCCCC]** 952 (85.20)   
297 - **[CCCGTCGC]** 898 (85.16)   
298 + **[CGCCTCGC]** 295 (87.91)   
298 + **.CGCGGCGC.** 746 (93.00)   
298 + **[CGCGTCAA]** 799 (86.67)   
298 + **[CGCGTAGA]** 823 (87.53)   
298 + **.CGCGGCGG.** 876 (92.45)   
298 + **[CGCGGCCG]** 1004 (85.25)   
298 + **[CGCCGCGA]** 1177 (89.34)   
298 + **[CGCCTCGC]** 1263 (87.91)   
298 - **[CGCGCCCC]** 1367 (85.20)   
298 - **[CGCCTCGG]** 1222 (87.36)   
298 - **.CGCGGCGG.** 1176 (92.45)   
298 - **[CGCGGCCG]** 795 (85.25)   
298 - **[CGCCGCGA]** 745 (89.34)   
299 + **[CGCGCGGA]** 579 (88.78)   
299 + **[CGCGGGGC]** 690 (85.16)   
299 + **.GGCGTCGA.** 833 (90.75)   
299 + **[CGCGCCCC]** 868 (85.20)   
299 + **.CGCGTCGG.** 883 (95.23)   
299 + **[CGCGCCCC]** 920 (85.20)   
299 + **.CGCGGCCA.** 1059 (90.02)   
299 + **[CGCGCCGT]** 1082 (87.13)   
299 + **.CGCGGCGC.** 1180 (93.00)   
299 + **.GGCGTCGA.** 1252 (90.75)   
299 + **.CGCGGCGC.** 1262 (93.00)   
299 + **[CGCCTCGG]** 1343 (87.36)   
299 + **.CGCGTCCA.** 1475 (92.80)   
299 - **[CGCGTTGA]** 1506 (87.22)   
299 - **[CGCGCCCC]** 1471 (85.20)   
299 - **[CGCGGCCG]** 1348 (85.25)   
299 - **[CGCGGGGC]** 877 (85.16)   
299 - **[CGCGGGGC]** 864 (85.16)   
299 - **[CGCGGCCG]** 849 (85.25)   
299 - **[CGCCTCGG]** 843 (87.36)   
299 - **[GGCGCCGA]** 819 (87.37)   
299 - **[TGCGTCGA]** 757 (86.06)   
299 - **[CGCGTGGG]** 575 (87.40)   
300 + **.CGCGGCGC.** 113 (93.00)   
300 + **[CGCGGCCC]** 175 (85.80)   
300 + **[CGGGTCGC]** 395 (85.99)   
300 + **.CGCGTCGG.** 446 (95.23)   
300 + **.CGCGGCGC.** 474 (93.00)   
300 + **[CGCGCCCA]** 581 (89.42)   
300 + **.CGCGGCGG.** 736 (92.45)   
300 - **[CGCCGCGA]** 735 (89.34)   
300 - **.CGCGCCGC.** 475 (92.40)   
300 - **[CGCCGCGC]** 473 (85.13)   
300 - **[CGCCCCGA]** 450 (88.75)   
300 - **[CGCCGCGC]** 112 (85.13)   
300 - **[CGCGCCGT]** 109 (87.13)   
301 + **[CGCGTGGG]** 1483 (87.40)   
301 - **[CGCCTCGC]** 1034 (87.91)   
301 - **[CGCGCCCC]** 763 (85.20)   
301 - **[CGCGCCCA]** 577 (89.42)   
302 + **[CGCGACGC]** 593 (86.46)   
302 + **.CGCGCCGC.** 756 (92.40)   
302 + **.CGCGCCGG.** 841 (91.85)   
302 + **[CCCGGCGA]** 945 (86.59)   
302 + **[CGCCTCGC]** 1021 (87.91)   
302 + **.CGCGGCGC.** 1026 (93.00)   
302 + **.CGCGCCGC.** 1039 (92.40)   
302 + **[CGCGGGGC]** 1073 (85.16)   
302 - **.CGCGGCGC.** 1040 (93.00)   
302 - **[CGCCGCGA]** 1025 (89.34)   
302 - **.CGCGTCGC.** 949 (95.78)   
303 + **.CGCGTCGG.** 130 (95.23)   
303 + **[CGCCGCGC]** 167 (85.13)   
303 + **.CGCGCCGC.** 170 (92.40)   
303 + **[CGCGGGGC]** 289 (85.16)   
303 + **[GGCGGCGA]** 294 (87.97)   
303 + **.CGCGCCGC.** 745 (92.40)   
303 + **.CGCGGCCA.** 1006 (90.02)   
303 + **.CGCGTCGT.** 1021 (90.51)   
303 - **[CGCGGCCG]** 1359 (85.25)   
303 - **.CGGGTCGA.** 1312 (90.21)   
303 - **.CGCGCCGC.** 1002 (92.40)   
303 - **[GGCGCCGA]** 943 (87.37)   
303 - **[CGCGGGGC]** 800 (85.16)   
303 - **.CGCGGCGC.** 746 (93.00)   
303 - **.CGCGGCGC.** 455 (93.00)   
303 - **[CGGGTCGG]** 387 (85.44)   
303 - **[CGCGGCCG]** 285 (85.25)   
303 - **.CGCGGCGG.** 166 (92.45)   
303 - **[CGCGGCCG]** 41 (85.25)   
304 + **[CGCCGCGC]** 707 (85.13)   
304 + **[CGCGCCCA]** 710 (89.42)   
304 + **.CGCGGCGG.** 1243 (92.45)   
304 + **[CGCGACGG]** 1309 (85.91)   
304 - **[CGCGGCCC]** 1261 (85.80)   
304 - **[CGCCGCGC]** 1242 (85.13)   
304 - **[CGCGCCCC]** 1239 (85.20)   
304 - **.CGCGCCGC.** 1177 (92.40)   
304 - **.CGCGCCGA.** 902 (96.62)   
304 - **.CGCGTCGG.** 731 (95.23)   
304 - **.CGCGGCGA.** 706 (97.21)   
305 - **[CGCGGGGC]** 1076 (85.16)   
306 + **.CGCGCCGG.** 342 (91.85)   
306 + **[CGCGGGGC]** 378 (85.16)   
306 + **[CGCCGCGC]** 764 (85.13)   
306 + **.CGCGCCGG.** 930 (91.85)   
306 + **[CGCGGGGC]** 954 (85.16)   
306 + **[CGCGGGGC]** 1041 (85.16)   
306 + **[CGCGTGGC]** 1477 (87.95)   
306 - **[CCCGCCGA]** 1261 (86.00)   
306 - **[CCCGCCGA]** 1155 (86.00)   
306 - **[CCCGCCGA]** 1109 (86.00)   
306 - **.CGCGCCGC.** 1037 (92.40)   
306 - **[CGCCTCGC]** 1017 (87.91)   
306 - **[CGGGTCGC]** 907 (85.99)   
306 - **.CGCGGCGG.** 763 (92.45)   
306 - **.CGCGTGGA.** 142 (92.16)   
307 + **.CGCGCCGG.** 548 (91.85)   
307 + **[CGGGTCGG]** 715 (85.44)   
307 + **.CGCGGCGG.** 730 (92.45)   
307 + **[CGCCCCGA]** 837 (88.75)   
307 + **[GGCGTCGG]** 1066 (85.99)   
307 + **[CGCCTCGG]** 1137 (87.36)   
307 + **[CGCGGCCC]** 1162 (85.80)   
307 + **[CGCGGCCG]** 1330 (85.25)   
307 - **.CGCGCCGA.** 1353 (96.62)   
307 - **.CGCGCCGC.** 1233 (92.40)   
307 - **[CGCCTCGG]** 1219 (87.36)   
307 - **[CGCGGGGC]** 920 (85.16)   
307 - **[CGCGGCCG]** 735 (85.25)   
307 - **.CGCGGCGG.** 677 (92.45)   
307 - **.CGCGCCGG.** 495 (91.85)   
307 - **[CGCGCCCC]** 428 (85.20)   
307 - **[CGCGCGGA]** 384 (88.78)   
307 - **[CGCGTCCC]** 332 (88.59)   
308 + **.CGCGGCGG.** 863 (92.45)   
308 + **[CGCCTCGG]** 1081 (87.36)   
308 + **.CGCGCCGG.** 1189 (91.85)   
308 + **[CGCGGGGC]** 1415 (85.16)   
308 - **[CGCGGGGC]** 1421 (85.16)   
308 - **[CGCGGCCC]** 1411 (85.80)   
308 - **.CGCGGCGG.** 1366 (92.45)   
308 - **[CGCGGCCC]** 1177 (85.80)   
308 - **[CGCCGCGC]** 862 (85.13)   
308 - **[CGCGGGGA]** 707 (89.38)   
308 - **.CGCGTGGA.** 69 (92.16)   
309 + **[GGCGGCGA]** 768 (87.97)   
309 + **.CGCGGCGG.** 1029 (92.45)   
309 - **.CGCGTGGA.** 1223 (92.16)   
309 - **[CGCGCCCC]** 1146 (85.20)   
309 - **.CGCGCCGC.** 1076 (92.40)   
309 - **[CGCCGCGC]** 1028 (85.13)   
309 - **[CGCGGCCC]** 992 (85.80)   
309 - **[CGCGGCTA]** 687 (85.00)   
310 + **[CGCCTCGG]** 790 (87.36)   
310 + **[CGCGGCTA]** 1301 (85.00)   
310 - **[CGCGCCCA]** 977 (89.42)   
311 + **[CGCCTCGC]** 764 (87.91)   
311 + **[CGCGGCCG]** 875 (85.25)   
311 + **.CGCGCCGC.** 960 (92.40)   
311 + **.CGCGGCGG.** 1069 (92.45)   
311 + **.CGCGGCGC.** 1106 (93.00)   
311 + **[CGCGTCTA]** 1345 (87.79)   
311 - **.CGCGGCGA.** 1469 (97.21)   
311 - **[CGCGTCCC]** 1260 (88.59)   
311 - **[CCCGCCGA]** 1249 (86.00)   
311 - **[CGCGCCCC]** 1176 (85.20)   
311 - **[CGCCTCGC]** 1146 (87.91)   
311 - **[CGCCGCGC]** 1068 (85.13)   
311 - **[CGCGGCGT]** 871 (87.72)   
312 + **[CGGGGCGA]** 223 (87.42)   
312 + **[CGCGCCCC]** 843 (85.20)   
312 + **[CGCGCCCC]** 872 (85.20)   
312 + **.CGCGCCGC.** 985 (92.40)   
312 + **.CGCGGCGC.** 1094 (93.00)   
312 + **[CGCGCCCC]** 1122 (85.20)   
312 + **.CGCGCCGC.** 1361 (92.40)   
312 + **.CGCGGCCA.** 1473 (90.02)   
312 - **[CGCGGCCC]** 1353 (85.80)   
312 - **.CGCGCCGG.** 1288 (91.85)   
312 - **[CGCCGCGC]** 1093 (85.13)   
312 - **[CGCGGGGC]** 981 (85.16)   
312 - **[CGCGCCCC]** 939 (85.20)   
312 - **[CGCGGGGC]** 875 (85.16)   
312 - **[CGCGTGGC]** 839 (87.95)   
312 - **.CGCGTCGC.** 739 (95.78)   
313 + **[CGCGTGGG]** 583 (87.40)   
313 + **[CGCGGCCG]** 826 (85.25)   
313 + **[CGCCTCGG]** 832 (87.36)   
313 + **.CGCGCCGG.** 1088 (91.85)   
313 + **[CGCGCCGT]** 1303 (87.13)   
313 - **.CGCGGCGG.** 1444 (92.45)   
313 - **[CGGGTCGC]** 1418 (85.99)   
313 - **.CGCGCCGG.** 954 (91.85)   
313 - **[GGCGCCGA]** 931 (87.37)   
313 - **[CGCGCCCA]** 815 (89.42)   
313 - **.CGCGTGGA.** 254 (92.16)   
314 + **[GGCGCCGA]** 12 (87.37)   
314 + **[CGGGCCGA]** 38 (86.82)   
314 - **[CGCCTCGG]** 42 (87.36)   
315 + **.CGCGGCGA.** 357 (97.21)   
315 + **[CGGGGCGA]** 946 (87.42)   
315 + **.CGCGCCGA.** 1273 (96.62)   
315 + **.CGCGTCCA.** 1344 (92.80)   
315 - **.CGCGGCGA.** 1340 (97.21)   
315 - **[CGCGTCCC]** 1283 (88.59)   
315 - **[CGGGCCGA]** 1221 (86.82)   
315 - **[CGCGCCCC]** 1055 (85.20)   
315 - **.CGCGCCGC.** 1024 (92.40)   
315 - **.CGCGCCGC.** 1015 (92.40)   
315 - **[CGCGTCCG]** 1004 (88.04)   
315 - **[CGCGCCCC]** 979 (85.20)   
315 - **[CGCGTCTA]** 377 (87.79)   
315 - **[CGCCGCGA]** 356 (89.34)   
316 + **[GGCGGCGA]** 166 (87.97)   
316 + **[CGCGGCCC]** 229 (85.80)   
316 + **.CGCGCCGA.** 257 (96.62)   
316 + **.CGCGTCCA.** 898 (92.80)   
316 + **.CGCGGCGC.** 910 (93.00)   
316 - **[CGGGGCGA]** 1267 (87.42)   
316 - **.CGCGCCGC.** 911 (92.40)   
316 - **.CGCGCCGA.** 894 (96.62)   
316 - **[CGCGGCCG]** 534 (85.25)   
316 - **[CGCGTCCC]** 198 (88.59)   
316 - **[CCCGCCGA]** 68 (86.00)   
316 - **[CGCCTCGC]** 34 (87.91)   
317 + **[CGCGGGGA]** 1007 (89.38)   
317 + **[CGCGCCCC]** 1433 (85.20)   
317 - **[CGCGCCCC]** 1003 (85.20)   
318 + **[CGCGTCCC]** 290 (88.59)   
318 + **.CGCGCCGG.** 307 (91.85)   
318 + **[CGCGCCCC]** 1246 (85.20)   
318 + **[CCCGCCGA]** 1358 (86.00)   
318 + **.CGCGGCGG.** 1485 (92.45)   
318 - **[CGCCTCGG]** 1511 (87.36)   
318 - **[CGCGGCCG]** 1481 (85.25)   
318 - **.CGCGCCGG.** 1432 (91.85)   
318 - **.CGCGTGGA.** 1344 (92.16)   
318 - **[CGCGGGGC]** 1092 (85.16)   
318 - **[CCCGCCGA]** 669 (86.00)   
318 - **[CGCGCCCA]** 536 (89.42)   
318 - **[CGCGGGGA]** 265 (89.38)   
320 + **[CGCGTCCC]** 107 (88.59)   
320 + **[GGCGCCGA]** 522 (87.37)   
320 + **.GGCGTCGA.** 554 (90.75)   
320 + **[CGCGCGGA]** 629 (88.78)   
320 + **[CGCGCCCC]** 674 (85.20)   
320 + **[CGCGGCCG]** 694 (85.25)   
320 + **[CGCCTCGC]** 716 (87.91)   
320 + **.CGCGGCGC.** 755 (93.00)   
320 + **.CGCGCCGG.** 913 (91.85)   
320 + **[CGCGGGGC]** 943 (85.16)   
320 + **[GGCGCCGA]** 962 (87.37)   
320 + **.CGCGGCGC.** 992 (93.00)   
320 + **.CGCGCCGC.** 1198 (92.40)   
320 + **[CGCGGGGC]** 1232 (85.16)   
320 + **[CGCCGCGC]** 1341 (85.13)   
320 + **.CGCGTCGC.** 1348 (95.78)   
320 + **.CGCGCCGC.** 1381 (92.40)   
320 - **.CGCGGCGC.** 1382 (93.00)   
320 - **.CGCGGCGC.** 1340 (93.00)   
320 - **[CGGGTCGC]** 1316 (85.99)   
320 - **.CGCGCCGC.** 874 (92.40)   
320 - **[CGCGCCCA]** 853 (89.42)   
320 - **[CGCGCCCC]** 796 (85.20)   
320 - **[CGCCGCGA]** 754 (89.34)   
321 + **[CGCGGCGT]** 455 (87.72)   
321 + **[CGCCCCGA]** 521 (88.75)   
321 + **.CGCGCCGC.** 696 (92.40)   
321 + **[CGCCGCGC]** 698 (85.13)   
321 + **[CGCGGGGC]** 945 (85.16)   
321 + **[GGCGCCGA]** 1175 (87.37)   
321 + **.CGCGCCGG.** 1260 (91.85)   
321 + **.CGCGGCCA.** 1302 (90.02)   
321 + **.CGCGCCGC.** 1395 (92.40)   
321 + **[CGCGGCCC]** 1400 (85.80)   
321 + **[GGCGTCGG]** 1484 (85.99)   
321 + **.CGCGCCGG.** 1506 (91.85)   
321 - **[CCCGCCGA]** 1488 (86.00)   
321 - **.CGCGGCGC.** 1396 (93.00)   
321 - **.CGCGCCGG.** 1298 (91.85)   
321 - **[CGCGGCCC]** 1254 (85.80)   
321 - **.CGCGGCGC.** 697 (93.00)   
321 - **.CGCGTGGA.** 553 (92.16)   
321 - **[CGGGTCGC]** 503 (85.99)   
321 - **[GGCGGCGA]** 384 (87.97)   
322 + **[CGGGTCGG]** 306 (85.44)   
322 + **[CGGGGCGA]** 431 (87.42)   
322 + **[CGCGCCCA]** 463 (89.42)   
322 + **[CGCCGCGC]** 496 (85.13)   
322 + **[CGCGCCCC]** 499 (85.20)   
322 + **[CGCGGCCC]** 632 (85.80)   
322 + **[CCCGGCGA]** 735 (86.59)   
322 + **.CGCGCCGC.** 948 (92.40)   
322 + **[CGCCGCGC]** 1480 (85.13)   
322 - **.CGCGGCGA.** 1479 (97.21)   
322 - **.CGCGACGA.** 1170 (90.68)   
322 - **[CGCGACGG]** 1063 (85.91)   
322 - **[CGCGGCCC]** 957 (85.80)   
322 - **[CGCGGCCC]** 940 (85.80)   
322 - **[CGCGCCGT]** 684 (87.13)   
322 - **[CGCCCCGA]** 660 (88.75)   
322 - **.CGCGCCGG.** 628 (91.85)   
322 - **.CGCGGCGG.** 495 (92.45)   
323 + **[CGCGCGGA]** 717 (88.78)   
323 + **[CGCGGCCC]** 860 (85.80)   
323 + **[CGCGGGGC]** 951 (85.16)   
323 + **[CGCGGGGC]** 989 (85.16)   
323 + **.CGCGGCCA.** 1020 (90.02)   
323 + **.CGCGGCCA.** 1032 (90.02)   
323 + **[CGCTGCGA]** 1104 (85.51)   
323 + **[CGCGCCCC]** 1182 (85.20)   
323 + **.CGCGGCGG.** 1231 (92.45)   
323 + **[CGCGCCCC]** 1313 (85.20)   
323 + **[CGCGCCCC]** 1321 (85.20)   
323 + **[CGCGGGGC]** 1363 (85.16)   
323 + **.CGCGTCGG.** 1424 (95.23)   
323 + **[CGCTGCGA]** 1472 (85.51)   
323 - **[CGCGGCCC]** 1492 (85.80)   
323 - **[CGCGCCCC]** 1359 (85.20)   
323 - **[CGCGGCGT]** 1227 (87.72)   
323 - **[CGCGGGGC]** 1178 (85.16)   
323 - **[CGCGTGGG]** 1066 (87.40)   
323 - **[CGCGGCCC]** 1028 (85.80)   
323 - **[CGCGCCCC]** 943 (85.20)   
323 - **[CGCGGGGC]** 713 (85.16)   
323 - **[CGGGTCGG]** 706 (85.44)   
325 + **[CGGGTCGG]** 1464 (85.44)   
325 - **[CGCGCCCA]** 1304 (89.42)   
326 + **[CGCCTCGG]** 411 (87.36)   
326 + **[CGCGGCCC]** 660 (85.80)   
326 + **[CGCGGGGA]** 689 (89.38)   
326 + **[CGCCGCGC]** 822 (85.13)   
326 + **.CGCGGCGC.** 938 (93.00)   
326 + **[CGCGGCCG]** 1113 (85.25)   
326 + **.CGCGGCGG.** 1202 (92.45)   
326 + **[CGCGCGGA]** 1256 (88.78)   
326 + **.CGCGGCGG.** 1303 (92.45)   
326 - **[CGCGGCCC]** 1415 (85.80)   
326 - **[CGCGGCCG]** 1299 (85.25)   
326 - **[CGGGCCGA]** 1235 (86.82)   
326 - **[CGCCGCGC]** 1201 (85.13)   
326 - **[GGCGGCGA]** 1138 (87.97)   
326 - **[CGCGGCCG]** 1115 (85.25)   
326 - **.CGCGGCGG.** 821 (92.45)   
326 - **[CGCGTGGC]** 649 (87.95)   
327 + **[CGCGGGGA]** 26 (89.38)   
327 + **[CGAGTCGA]** 166 (86.95)   
327 + **[CGCCTCGG]** 549 (87.36)   
327 + **[CGGGTCGG]** 590 (85.44)   
327 + **[CGCCGCGA]** 1112 (89.34)   
327 + **[AGCGTCGA]** 1193 (87.51)   
327 - **.CGCGGCGC.** 1111 (93.00)   
329 - **[CGCGTCCG]** 1235 (88.04)   
329 - **[GGCGCCGA]** 1205 (87.37)   
329 - **[GGCGGCGA]** 494 (87.97)   
329 - **.CGCGGCCA.** 294 (90.02)   
329 - **[GGCGTCGG]** 272 (85.99)   
331 + **[CGCGCCCC]** 359 (85.20)   
331 + **.CGCGGCGA.** 397 (97.21)   
331 + **[CGGGCCGA]** 632 (86.82)   
331 + **[CCCGGCGA]** 1010 (86.59)   
331 + **.CGCGGCGC.** 1135 (93.00)   
331 + **.CGCGCCGC.** 1311 (92.40)   
331 + **.CGCGGCGG.** 1332 (92.45)   
331 + **[GGCGGCGA]** 1335 (87.97)   
331 + **.CGCGGCGC.** 1441 (93.00)   
331 - **[CGCCGCGC]** 1440 (85.13)   
331 - **.CGCGGCGC.** 1312 (93.00)   
331 - **[CGCCCCGA]** 1269 (88.75)   
331 - **.CGCGCCGC.** 1136 (92.40)   
331 - **[CGCGGCCG]** 761 (85.25)   
331 - **.CGCGGCGG.** 719 (92.45)   
331 - **[CGCGCGGA]** 690 (88.78)   
331 - **[CGCGCCCC]** 355 (85.20)   
332 + **[CGCGCCCA]** 460 (89.42)   
332 + **[CGCGTCCC]** 504 (88.59)   
332 + **.CGCGTGGA.** 815 (92.16)   
332 + **[CGCGCCCC]** 1088 (85.20)   
332 + **[CGCGGGGA]** 1137 (89.38)   
332 + **[CGCGGCCC]** 1298 (85.80)   
332 + **[CGCCGCGC]** 1350 (85.13)   
332 + **[CGCGGCCG]** 1430 (85.25)   
332 + **.CGCGGCGC.** 1487 (93.00)   
332 + **[CGGGGCGA]** 1501 (87.42)   
332 - **[CGCCGCGC]** 1486 (85.13)   
332 - **[CGCGCCCC]** 1481 (85.20)   
332 - **[CGCGTGGC]** 1444 (87.95)   
332 - **[CGCGGCCC]** 1426 (85.80)   
332 - **[CGCGCCCC]** 1387 (85.20)   
332 - **.CGCGGCGA.** 1349 (97.21)   
332 - **[CGCGCCCC]** 1223 (85.20)   
332 - **[CGCGGGGC]** 1175 (85.16)   
332 - **.CGCGGCCA.** 99 (90.02)   
332 - **[CGCCCCGA]** 48 (88.75)   
333 + **.CGCGGCGG.** 1299 (92.45)   
333 + **[CGCCGCGA]** 1320 (89.34)   
333 + **[CCCGGCGA]** 1416 (86.59)   
333 - **[GGCGGCGA]** 1347 (87.97)   
333 - **.CGCGGCGG.** 1319 (92.45)   
333 - **[CGCCGCGC]** 1298 (85.13)   
333 - **[CGCGGGGA]** 1239 (89.38)   
333 - **.CGCGGCGC.** 1120 (93.00)   
333 - **[CGCGGGGC]** 1026 (85.16)   
334 - **[CGGGGCGA]** 1142 (87.42)   
334 - **[GGCGCCGA]** 1035 (87.37)   
335 + **[CGCCTCGG]** 612 (87.36)   
335 + **.CGCGGCGC.** 854 (93.00)   
335 + **.CGCGCCGC.** 1100 (92.40)   
335 + **.CGCGTCGC.** 1205 (95.78)   
335 + **[CGCGCCCA]** 1389 (89.42)   
335 - **[CGCGCCCC]** 1428 (85.20)   
335 - **[CGCGCCCC]** 1385 (85.20)   
335 - **[CGCGACGC]** 1206 (86.46)   
335 - **[CGCGGCCC]** 1120 (85.80)   
335 - **[GGCGTCGC]** 1077 (86.54)   
335 - **.CGCGCCGC.** 855 (92.40)   
335 - **[CGCCGCGA]** 853 (89.34)   
336 + **[CCCGCCGA]** 528 (86.00)   
336 + **[CGCGCCGT]** 981 (87.13)   
336 + **[CGCGGCCC]** 1023 (85.80)   
336 - **.CGCGTGGA.** 1060 (92.16)   
336 - **[CGCGTGGG]** 838 (87.40)   
337 + **[CGCTGCGA]** 672 (85.51)   
337 + **[CGCGCCCC]** 827 (85.20)   
337 + **.CGCGGCGG.** 842 (92.45)   
337 + **.CGCGGCGG.** 956 (92.45)   
337 + **[CGCGCGGA]** 997 (88.78)   
337 + **[CGCGGCCG]** 1073 (85.25)   
337 + **[CGCGCGGA]** 1094 (88.78)   
337 + **[CGCGGCCC]** 1176 (85.80)   
337 + **.CGCGGCGC.** 1183 (93.00)   
337 + **[CGCGGGGC]** 1365 (85.16)   
337 - **[CGCGCCCC]** 1473 (85.20)   
337 - **[CGCGCCCC]** 1436 (85.20)   
337 - **.CGCGCCGG.** 1346 (91.85)   
337 - **[CGCGCCCC]** 1146 (85.20)   
337 - **.CGCGGCGG.** 1069 (92.45)   
337 - **[CGCCGCGC]** 955 (85.13)   
337 - **[CGCGCCCC]** 948 (85.20)   
337 - **[CGCGGCTA]** 823 (85.00)   
338 + **[CGCGTCCC]** 694 (88.59)   
338 + **.CGCGCCGC.** 760 (92.40)   
338 + **[CGCGCCCC]** 812 (85.20)   
338 + **.CGCGGCGA.** 1054 (97.21)   
338 + **[CGCGCCCC]** 1065 (85.20)   
338 + **[CGCGCCCC]** 1126 (85.20)   
338 + **[CGCGTCCG]** 1154 (88.04)   
338 + **.CGCGCCGC.** 1172 (92.40)   
338 + **[CGCCGCGC]** 1192 (85.13)   
338 + **[CGCGGCGT]** 1459 (87.72)   
338 - **[CGCGGCCC]** 1412 (85.80)   
338 - **[CGCGGCCG]** 1303 (85.25)   
338 - **.CGCGGCGG.** 1191 (92.45)   
338 - **[CGCGGGGC]** 1068 (85.16)   
338 - **[CGCGGGGC]** 782 (85.16)   
338 - **.CGCGGCGC.** 690 (93.00)   
338 - **[CGCGGCCG]** 640 (85.25)   
338 - **[CGCGGGGA]** 436 (89.38)   
339 + **[CGCGCGGA]** 1045 (88.78)   
339 + **[CCCGGCGA]** 1054 (86.59)   
340 + **[GGCGTCGC]** 683 (86.54)   
340 - **[GGCGGCGA]** 687 (87.97)   
340 - **.CGCGTGGA.** 673 (92.16)   
340 - **[CGGGGCGA]** 502 (87.42)   
341 + **[CGCGGCCG]** 1152 (85.25)   
341 + **[CGCGGGGC]** 1233 (85.16)   
341 + **.CGCGTCGC.** 1260 (95.78)   
341 + **.CGCGGCGC.** 1319 (93.00)   
341 + **[CGCCGCGC]** 1383 (85.13)   
341 - **[CGCGTCCC]** 1490 (88.59)   
341 - **[CGCGCCCC]** 1415 (85.20)   
341 - **.CGCGGCGG.** 1382 (92.45)   
341 - **[CGCCGCGC]** 1318 (85.13)   
341 - **[CGCGGCCG]** 1313 (85.25)   
341 - **[CTCGTCGA]** 1177 (86.25)   
341 - **[CGCCTCGC]** 1124 (87.91)   
341 - **[CGCCTCGG]** 925 (87.36)   
342 + **[CGGGCCGA]** 325 (86.82)   
342 + **[CGCGGGGC]** 838 (85.16)   
342 + **.CGCGGCGG.** 1155 (92.45)   
342 + **[CGCGGCCG]** 1335 (85.25)   
342 + **[GGCGGCGA]** 1419 (87.97)   
342 + **[CGCGTGGC]** 1483 (87.95)   
342 - **[CGCGTCCC]** 1394 (88.59)   
342 - **[CGCGCCCA]** 1329 (89.42)   
342 - **[CGCGCCCA]** 1219 (89.42)   
342 - **[CGCCGCGC]** 1154 (85.13)   
342 - **.CGCGCCGC.** 1151 (92.40)   
342 - **.CGCGCCGC.** 1023 (92.40)   
342 - **.CGCGCCGA.** 834 (96.62)   
342 - **[CGCGGCCG]** 710 (85.25)   
343 + **[CGCCCCGA]** 266 (88.75)   
343 + **[CGCGTCCC]** 279 (88.59)   
343 + **[CGGGTCGC]** 291 (85.99)   
343 + **[CGCGACGC]** 781 (86.46)   
343 + **[CGCGGGGC]** 912 (85.16)   
343 + **[CGCGGCGT]** 998 (87.72)   
343 + **.CGCGGCCA.** 1122 (90.02)   
343 + **.CGCGGCGG.** 1182 (92.45)   
343 + **[CGCGGGGA]** 1307 (89.38)   
343 + **.CGCGGCCA.** 1350 (90.02)   
343 + **.CGGGTCGA.** 1433 (90.21)   
343 + **[CGCCCCGA]** 1489 (88.75)   
343 - **[CGCCGCGC]** 1181 (85.13)   
343 - **[CGCGCCCC]** 1178 (85.20)   
343 - **.CGCGGCCA.** 1118 (90.02)   
343 - **.CGCGGCGC.** 1076 (93.00)   
343 - **[CGCCGCGC]** 997 (85.13)   
343 - **[CGCGTCCG]** 668 (88.04)   
343 - **[CGCGGCCG]** 651 (85.25)   
343 - **[CGCGCCCC]** 522 (85.20)   
343 - **[CGCCCCGA]** 520 (88.75)   
343 - **[CGCTGCGA]** 425 (85.51)   
344 + **.CGCGCCGC.** 1166 (92.40)   
346 + **.CGCGGCGC.** 23 (93.00)   
346 + **.CGCGGCGG.** 85 (92.45)   
346 + **[CCCGTCGC]** 278 (85.16)   
346 + **[CGCGCCCC]** 882 (85.20)   
346 + **.CGCGCCGC.** 1231 (92.40)   
346 + **[CGCGTCCC]** 1239 (88.59)   
346 + **[CGCCTCGC]** 1389 (87.91)   
346 - **[CACGTCGA]** 1490 (86.48)   
346 - **.CGCGGCCA.** 1405 (90.02)   
346 - **.CGCGCCGA.** 1338 (96.62)   
346 - **.CGCGGCGG.** 1235 (92.45)   
346 - **.CGCGCCGG.** 1227 (91.85)   
346 - **[CGCGGCCC]** 904 (85.80)   
346 - **[CGCGCCCC]** 715 (85.20)   
346 - **[CGCCGCGA]** 22 (89.34)   
347 + **[CGCGCCCA]** 553 (89.42)   
347 + **[CGCGCCCA]** 838 (89.42)   
347 + **[CGCCGCGC]** 908 (85.13)   
347 + **.CGCGGCGC.** 1088 (93.00)   
347 + **[CGCGGCCC]** 1207 (85.80)   
347 + **[CGCGCCGT]** 1257 (87.13)   
347 + **[CGCCCCGA]** 1266 (88.75)   
347 + **[CGCGGCCC]** 1286 (85.80)   
347 + **[CGCCTCGC]** 1384 (87.91)   
347 + **[CGCGGCTA]** 1467 (85.00)   
347 - **[GGCGTCGG]** 1380 (85.99)   
347 - **[CGCGGGGC]** 1201 (85.16)   
347 - **[CGCGGGGC]** 1166 (85.16)   
347 - **.CGCGCCGA.** 1099 (96.62)   
347 - **[CGCCGCGC]** 1087 (85.13)   
347 - **[CGCGGGGA]** 960 (89.38)   
347 - **.CGCGGCGA.** 907 (97.21)   
347 - **[CGCGGGGC]** 176 (85.16)   
348 + **.CGCGCCGC.** 132 (92.40)   
348 + **[CGCGCCCC]** 228 (85.20)   
348 + **[CGCGGGGA]** 310 (89.38)   
348 + **[CGCGGCCG]** 344 (85.25)   
348 + **[CGCGCCCC]** 375 (85.20)   
348 + **[CGCGGGGC]** 547 (85.16)   
348 + **.CGCGTCGT.** 664 (90.51)   
348 + **[CCCGTCGC]** 684 (85.16)   
348 + **[CGCCGCGC]** 695 (85.13)   
348 + **.CGCGGCGC.** 712 (93.00)   
348 + **[CCCGGCGA]** 771 (86.59)   
348 + **[CCCGTCGC]** 903 (85.16)   
348 + **[CCCGTCGC]** 923 (85.16)   
348 + **[GGCGCCGA]** 947 (87.37)   
348 + **.CGCGGCGG.** 1028 (92.45)   
348 + **[CGCGGGGC]** 1178 (85.16)   
348 + **.CGCGGCGC.** 1240 (93.00)   
348 + **[CGCGGGGC]** 1268 (85.16)   
348 + **[CGCCTCGC]** 1392 (87.91)   
348 + **[CGCGTGGG]** 1414 (87.40)   
348 + **[CGCGTGGG]** 1480 (87.40)   
348 + **.CGCGCCGC.** 1489 (92.40)   
348 + **[CCCGGCGA]** 1500 (86.59)   
348 - **.CGCGCCGC.** 1277 (92.40)   
348 - **[CGCCGCGC]** 1239 (85.13)   
348 - **[CGCGTCCC]** 1188 (88.59)   
348 - **[CGCCGCGA]** 1027 (89.34)   
348 - **[CGCGCCCC]** 959 (85.20)   
348 - **[GGCGGCGA]** 907 (87.97)   
348 - **[GGCGTCGG]** 883 (85.99)   
348 - **.CGCGCCGG.** 860 (91.85)   
348 - **.CGCGGCGA.** 694 (97.21)   
348 - **.CGCGGCGC.** 660 (93.00)   
348 - **[CGCCTCGG]** 602 (87.36)   
348 - **.CGCGCCGC.** 543 (92.40)   
348 - **[CGCGGGGA]** 409 (89.38)   
348 - **[CGCGGGGC]** 378 (85.16)   
348 - **[CGCGTCCC]** 306 (88.59)   
348 - **.CGCGCCGG.** 291 (91.85)   
348 - **.CGCGGCGC.** 133 (93.00)   
348 - **.CGCGGCGA.** 67 (97.21)   
349 + **.CGCGTGGA.** 491 (92.16)   
349 + **[GGCGGCGA]** 937 (87.97)   
349 + **[CGGGCCGA]** 960 (86.82)   
349 + **[GGCGCCGA]** 995 (87.37)   
349 + **.CGCGGCGG.** 1165 (92.45)   
349 + **.CGCGTCGA.** 1310 (100.00)   
349 - **[CGCGGGGC]** 1325 (85.16)   
349 - **[CCCGGCGA]** 1298 (86.59)   
349 - **[CGCGGGGC]** 1282 (85.16)   
349 - **[CCCGCCGA]** 1259 (86.00)   
349 - **[CGCCGCGC]** 1164 (85.13)   
349 - **.CGCGCCGG.** 1161 (91.85)   
349 - **[CCCGGCGA]** 1067 (86.59)   
349 - **[CGCGCCCA]** 1043 (89.42)   
349 - **.CGCGCCGG.** 835 (91.85)   
349 - **[GGCGTCGC]** 654 (86.54)   
349 - **[CGCGTCCG]** 487 (88.04)   
350 + **[GGCGTCGC]** 996 (86.54)   
350 + **[CGCGGGGC]** 1013 (85.16)   
350 + **[CGGGGCGA]** 1020 (87.42)   
350 + **[GGCGCCGA]** 1062 (87.37)   
350 + **[CGCGCGGA]** 1074 (88.78)   
350 + **[CGCGGCCC]** 1107 (85.80)   
350 + **[CGCCTCGG]** 1141 (87.36)   
350 + **.CGCGCCGC.** 1186 (92.40)   
350 - **.CGCGCCGC.** 1435 (92.40)   
350 - **.CGCGCCGG.** 1341 (91.85)   
350 - **[CGGGTCGG]** 1226 (85.44)   
350 - **[CGCGCGGA]** 1101 (88.78)   
350 - **.CGCGCCGG.** 1042 (91.85)   
350 - **[CGCCTCGC]** 1024 (87.91)   
350 - **[CGCGACGC]** 997 (86.46)   
351 + **[CCCGCCGA]** 760 (86.00)   
351 + **.CGCGGCGA.** 975 (97.21)   
351 + **[CGCGGCCC]** 1185 (85.80)   
351 + **[CGCGGCCC]** 1255 (85.80)   
351 + **[CGCGGGGC]** 1446 (85.16)   
351 - **[CGCGGCGT]** 935 (87.72)   
351 - **[CGCCTCGG]** 572 (87.36)   
351 - **[CGCCTCGG]** 197 (87.36)   
351 - **[CGCCTCGC]** 44 (87.91)   
351 - **[CGCCTCGG]** 39 (87.36)   
352 + **.CGCGCCGC.** 55 (92.40)   
352 + **.CGCGGCGC.** 198 (93.00)   
352 + **[CGCGCCGT]** 203 (87.13)   
352 + **.CGCGGCGG.** 312 (92.45)   
352 + **.CGCGGCCA.** 621 (90.02)   
352 + **.CGCGCCGG.** 718 (91.85)   
352 + **[CGCGGCCC]** 942 (85.80)   
352 + **[CGCGCCGT]** 1228 (87.13)   
352 - **[CGCCTCGG]** 1060 (87.36)   
352 - **.CGCGGCGG.** 351 (92.45)   
352 - **[GGCGGCGA]** 336 (87.97)   
352 - **[CGCGGCCC]** 308 (85.80)   
352 - **.CGCGTCGC.** 280 (95.78)   
352 - **.CGCGCCGC.** 199 (92.40)   
352 - **[CGCGGGGA]** 194 (89.38)   
352 - **[CGCGGCCG]** 70 (85.25)   
353 + **.CGCGGCCA.** 167 (90.02)   
353 + **.CGCGCCGG.** 663 (91.85)   
353 + **.CGCCTCGA.** 1123 (92.13)   
353 + **[CGCCGCGA]** 1178 (89.34)   
353 + **[CGCGTCTA]** 1278 (87.79)   
353 + **.CGCGGCGG.** 1420 (92.45)   
353 + **.CGCGGCGG.** 1438 (92.45)   
353 - **[CGCCGCGC]** 1419 (85.13)   
353 - **[CGCGCCCC]** 1380 (85.20)   
353 - **[CGCGCCCC]** 1338 (85.20)   
353 - **.CGCGGCGC.** 1177 (93.00)   
353 - **[CGCGACGG]** 964 (85.91)   
353 - **[CGCGACGG]** 872 (85.91)   
353 - **[CGCGTCCG]** 706 (88.04)   
353 - **[CGCGTCCC]** 537 (88.59)   
353 - **[CGCGTCCC]** 185 (88.59)   
353 - **[CGCGTGGC]** 171 (87.95)   
356 + **[CGCCGCGC]** 90 (85.13)   
356 + **[CGCGCCCC]** 93 (85.20)   
356 + **[CGCCTCGC]** 219 (87.91)   
356 + **[CGCCCCGA]** 474 (88.75)   
356 + **[GGCGCCGA]** 511 (87.37)   
356 + **[CGCCGCGA]** 783 (89.34)   
356 + **[CGCGGGGA]** 918 (89.38)   
356 + **[CGCGGCCG]** 999 (85.25)   
356 + **.CGCGTCCA.** 1173 (92.80)   
356 - **[CGCGCCCC]** 995 (85.20)   
356 - **.CGCGGCGG.** 782 (92.45)   
356 - **[CGCGGGGC]** 607 (85.16)   
356 - **[CCCGGCGA]** 376 (86.59)   
356 - **[CGCGGGGC]** 306 (85.16)   
356 - **.CGCGGCGC.** 89 (93.00)   
357 + **[GGCGCCGA]** 435 (87.37)   
357 + **[CGCGTGGG]** 529 (87.40)   
357 + **[CGCGTCCC]** 544 (88.59)   
357 + **[CTCGTCGA]** 615 (86.25)   
357 + **[CGCCGCGC]** 990 (85.13)   
357 + **.CGCGCCGC.** 993 (92.40)   
357 + **[CGCGGGGC]** 1033 (85.16)   
357 + **[CCCGCCGA]** 1069 (86.00)   
357 + **[CGGGCCGA]** 1165 (86.82)   
357 + **[CGCGGGGC]** 1221 (85.16)   
357 + **[CGCGGGGC]** 1282 (85.16)   
357 - **[CGCGCCCC]** 1159 (85.20)   
357 - **[CGCGGCCC]** 1108 (85.80)   
357 - **.CGCGGCGG.** 989 (92.45)   
357 - **[CCCGCCGA]** 951 (86.00)   
357 - **[CGCGTGGC]** 919 (87.95)   
357 - **[CGGGTCGG]** 538 (85.44)   
358 + **[CGCGGCCG]** 774 (85.25)   
358 + **.CGCGGCGG.** 978 (92.45)   
358 + **[CGCTTCGA]** 1088 (88.30)   
358 + **[CCCGTCGC]** 1248 (85.16)   
358 - **[CGCCGCGC]** 977 (85.13)   
358 - **[CGCGGCTA]** 667 (85.00)   
359 + **[CGCGCCCC]** 1114 (85.20)   
359 + **[CGCGCCCC]** 1206 (85.20)   
359 + **.CGCGGCGC.** 1234 (93.00)   
359 + **.CGCGGCGG.** 1255 (92.45)   
359 + **.CGCGCCGG.** 1307 (91.85)   
359 - **[CGCGGGGA]** 1277 (89.38)   
359 - **[CGCCGCGC]** 1254 (85.13)   
359 - **.CGCGCCGC.** 1235 (92.40)   
359 - **[CGCGGGGC]** 541 (85.16)   
360 + **[CGCCGCGC]** 651 (85.13)   
360 + **[CGCGGCCG]** 794 (85.25)   
360 + **[CGCGGGGC]** 832 (85.16)   
360 + **.CGCGGCGG.** 973 (92.45)   
360 + **[CGCCGCGC]** 1065 (85.13)   
360 + **.CGCGCCGC.** 1068 (92.40)   
360 + **[CGCGGGGC]** 1187 (85.16)   
360 + **.CGCGCCGC.** 1245 (92.40)   
360 - **.CGCGCCGA.** 1183 (96.62)   
360 - **.CGCGGCGC.** 1069 (93.00)   
360 - **.CGCGGCGC.** 1064 (93.00)   
360 - **[CGGGGCGA]** 665 (87.42)   
360 - **[CGCGGCGT]** 650 (87.72)   
360 - **[CGGGGCGA]** 640 (87.42)   
361 + **[CCCGCCGA]** 1360 (86.00)   
361 + **.CGCGGCCA.** 1418 (90.02)   
361 - **[CGCCTCGC]** 1461 (87.91)   
361 - **[CGCCTCGG]** 606 (87.36)   
362 + **[CGCGTCCG]** 738 (88.04)   
362 + **[CCCGCCGA]** 838 (86.00)   
362 + **[CGCGGCGT]** 911 (87.72)   
362 + **[GGCGGCGA]** 934 (87.97)   
362 - **[GGCGTCGG]** 1261 (85.99)   
362 - **[CGCCTCGC]** 1144 (87.91)   
362 - **[CGCCGCGC]** 910 (85.13)   
362 - **[CGCGCCCC]** 907 (85.20)   
362 - **.CGCGGCGC.** 734 (93.00)   
362 - **.CGCGTCGG.** 523 (95.23)   
362 - **[CGCGGGGC]** 466 (85.16)   
362 - **[CGCGGGGC]** 423 (85.16)   
363 + **.CGCGGCCA.** 694 (90.02)   
363 + **[CGCGGCCC]** 799 (85.80)   
363 + **.CGCGGCGC.** 811 (93.00)   
363 + **[CCCGTCGC]** 1078 (85.16)   
363 + **[CGCGGCCG]** 1083 (85.25)   
363 + **[CGCGCCCC]** 1091 (85.20)   
363 - **.CGCGCCGA.** 1110 (96.62)   
363 - **[CGCGGCCG]** 1085 (85.25)   
363 - **[CGCGACGG]** 1079 (85.91)   
363 - **[CGCGGGGC]** 795 (85.16)   
363 - **[CGCGGCCC]** 690 (85.80)   
363 - **[CGCGGGGC]** 631 (85.16)   
364 + **[CGCGGCCG]** 1179 (85.25)   
364 + **.CGCGGCGG.** 1257 (92.45)   
364 - **[CGCGCCCC]** 1175 (85.20)   
364 - **[CGCGGCCC]** 819 (85.80)   
366 + **[CGCGCCCC]** 131 (85.20)   
366 + **[CGCGGGGC]** 184 (85.16)   
366 + **[CGGGTCGG]** 211 (85.44)   
366 + **[CGCCCCGA]** 392 (88.75)   
366 + **.CGCGGCGG.** 410 (92.45)   
366 + **[GGCGGCGA]** 413 (87.97)   
366 + **[CGCGGCGT]** 445 (87.72)   
366 + **[CGCGCCGT]** 454 (87.13)   
366 + **.CGCGCCGA.** 658 (96.62)   
366 + **.CGCCTCGA.** 719 (92.13)   
366 + **.CGCGTCGG.** 854 (95.23)   
366 + **[CGCGCCGT]** 907 (87.13)   
366 + **[CGCGGCGT]** 954 (87.72)   
366 + **[CCCGTCGC]** 962 (85.16)   
366 + **[CGGGTCGG]** 1112 (85.44)   
366 + **[CGCGGGGC]** 1191 (85.16)   
366 + **.CGCGGCGG.** 1393 (92.45)   
366 + **[GGCGGCGA]** 1396 (87.97)   
366 - **[CGCGTCCC]** 1428 (88.59)   
366 - **[CGCGGCCG]** 1410 (85.25)   
366 - **[CGCCGCGC]** 1392 (85.13)   
366 - **.CGCGGCGC.** 1251 (93.00)   
366 - **[GGCGTCGC]** 996 (86.54)   
366 - **[GGCGGCGA]** 922 (87.97)   
366 - **[CGGGCCGA]** 858 (86.82)   
366 - **[CGCGCCCA]** 654 (89.42)   
366 - **[CGGGGCGA]** 621 (87.42)   
366 - **[CGCTGCGA]** 581 (85.51)   
366 - **[CGCCGCGC]** 409 (85.13)   
366 - **[CGCGCCGT]** 264 (87.13)   
366 - **[CGCCCCGA]** 215 (88.75)   
366 - **[CGCGGCCG]** 180 (85.25)   
366 - **[CGCCTCGG]** 66 (87.36)   
367 + **[CGCGCGGA]** 130 (88.78)   
367 + **[CGCGCCCC]** 413 (85.20)   
367 + **[CCCGCCGA]** 979 (86.00)   
367 + **.CGCGCCGG.** 1034 (91.85)   
367 + **.CGCGGCGG.** 1192 (92.45)   
367 - **.CGCGGCGG.** 1188 (92.45)   
367 - **.CGCGGCCA.** 1088 (90.02)   
367 - **.CGCGGCCA.** 1061 (90.02)   
367 - **[CGGGCCGA]** 842 (86.82)   
367 - **[CGCGGCCC]** 553 (85.80)   
367 - **[GGCGTCGC]** 219 (86.54)   
367 - **[CGCTGCGA]** 214 (85.51)   
  

**Frequency Matrix**

| All Occs Best Occs|  |  |  |  |  |  |  |  |  |  |  |  |  |  |  |  |  |  |  |  |  |  |  |  |  |  |  |  |  |  |  |  |  |  |  |  |  |  |  |  |  |  |  |  |  |  |  |  |  |  |  |  |  |  |  |  |  |  |  |  |  |  |  |  |  |  |  |  |  |  |  |  |  |  |  |  |  |  |  |  |  |  |  |  |  |  |  |  |  |  |  |  |  |  |  |  |  |  |  |  |  |  |  |  |  |  |  |  | | --- | --- | --- | --- | --- | --- | --- | --- | --- | --- | --- | --- | --- | --- | --- | --- | --- | --- | --- | --- | --- | --- | --- | --- | --- | --- | --- | --- | --- | --- | --- | --- | --- | --- | --- | --- | --- | --- | --- | --- | --- | --- | --- | --- | --- | --- | --- | --- | --- | --- | --- | --- | --- | --- | --- | --- | --- | --- | --- | --- | --- | --- | --- | --- | --- | --- | --- | --- | --- | --- | --- | --- | --- | --- | --- | --- | --- | --- | --- | --- | --- | --- | --- | --- | --- | --- | --- | --- | --- | --- | --- | --- | --- | --- | --- | --- | --- | --- | --- | --- | --- | --- | --- | --- | --- | --- | --- | --- | | **A C G T A C G T | | | | | | | | | | | |**| **1** 156 3428 348 109 | 0 912 9 0|  |  |  |  |  |  |  |  |  |  |  |  |  |  |  |  |  |  |  |  |  |  |  |  |  |  |  |  |  |  |  |  |  |  |  |  |  |  |  |  |  |  |  |  |  |  |  |  |  |  |  |  |  |  |  |  |  |  |  |  |  |  |  |  |  |  |  |  |  |  |  |  |  |  |  |  |  |  |  |  |  |  |  |  | | --- | --- | --- | --- | --- | --- | --- | --- | --- | --- | --- | --- | --- | --- | --- | --- | --- | --- | --- | --- | --- | --- | --- | --- | --- | --- | --- | --- | --- | --- | --- | --- | --- | --- | --- | --- | --- | --- | --- | --- | --- | --- | --- | --- | --- | --- | --- | --- | --- | --- | --- | --- | --- | --- | --- | --- | --- | --- | --- | --- | --- | --- | --- | --- | --- | --- | --- | --- | --- | --- | --- | --- | --- | --- | --- | --- | --- | --- | --- | --- | --- | --- | --- | --- | | **2** 125 256 3542 118 | 0 0 921 0|  |  |  |  |  |  |  |  |  |  |  |  |  |  |  |  |  |  |  |  |  |  |  |  |  |  |  |  |  |  |  |  |  |  |  |  |  |  |  |  |  |  |  |  |  |  |  |  |  |  |  |  |  |  |  |  |  |  |  |  |  |  |  |  |  |  |  |  |  |  |  |  | | --- | --- | --- | --- | --- | --- | --- | --- | --- | --- | --- | --- | --- | --- | --- | --- | --- | --- | --- | --- | --- | --- | --- | --- | --- | --- | --- | --- | --- | --- | --- | --- | --- | --- | --- | --- | --- | --- | --- | --- | --- | --- | --- | --- | --- | --- | --- | --- | --- | --- | --- | --- | --- | --- | --- | --- | --- | --- | --- | --- | --- | --- | --- | --- | --- | --- | --- | --- | --- | --- | --- | --- | | **3** 139 3506 311 85 | 0 918 3 0|  |  |  |  |  |  |  |  |  |  |  |  |  |  |  |  |  |  |  |  |  |  |  |  |  |  |  |  |  |  |  |  |  |  |  |  |  |  |  |  |  |  |  |  |  |  |  |  |  |  |  |  |  |  |  |  |  |  |  |  | | --- | --- | --- | --- | --- | --- | --- | --- | --- | --- | --- | --- | --- | --- | --- | --- | --- | --- | --- | --- | --- | --- | --- | --- | --- | --- | --- | --- | --- | --- | --- | --- | --- | --- | --- | --- | --- | --- | --- | --- | --- | --- | --- | --- | --- | --- | --- | --- | --- | --- | --- | --- | --- | --- | --- | --- | --- | --- | --- | --- | | **4** 120 467 3273 181 | 0 13 908 0|  |  |  |  |  |  |  |  |  |  |  |  |  |  |  |  |  |  |  |  |  |  |  |  |  |  |  |  |  |  |  |  |  |  |  |  |  |  |  |  |  |  |  |  |  |  |  |  | | --- | --- | --- | --- | --- | --- | --- | --- | --- | --- | --- | --- | --- | --- | --- | --- | --- | --- | --- | --- | --- | --- | --- | --- | --- | --- | --- | --- | --- | --- | --- | --- | --- | --- | --- | --- | --- | --- | --- | --- | --- | --- | --- | --- | --- | --- | --- | --- | | **5** 198 860 997 1986 | 4 322 461 134|  |  |  |  |  |  |  |  |  |  |  |  |  |  |  |  |  |  |  |  |  |  |  |  |  |  |  |  |  |  |  |  |  |  |  |  | | --- | --- | --- | --- | --- | --- | --- | --- | --- | --- | --- | --- | --- | --- | --- | --- | --- | --- | --- | --- | --- | --- | --- | --- | --- | --- | --- | --- | --- | --- | --- | --- | --- | --- | --- | --- | | **6** 150 3280 472 139 | 0 898 23 0|  |  |  |  |  |  |  |  |  |  |  |  |  |  |  |  |  |  |  |  |  |  |  |  | | --- | --- | --- | --- | --- | --- | --- | --- | --- | --- | --- | --- | --- | --- | --- | --- | --- | --- | --- | --- | --- | --- | --- | --- | | **7** 119 543 3222 157 | 0 77 844 0|  |  |  |  |  |  |  |  |  |  |  |  | | --- | --- | --- | --- | --- | --- | --- | --- | --- | --- | --- | --- | | **8** 2302 811 708 220 | 229 370 314 8 | | | | | | | | | | | | | | | | | | | | | | | | | | | | | | | | | | | | | | | | | | | | | | | | | | | | | | | | | | | | | | | | | | | | | | | | | | | | | | | | | | | | | | | | | | | | | | | | | | | | | | | | | | | | |

  
  

---

  
  
  
 **CGTCGCGTCG**   
**CGACGCGACG**  
  
5 redundant motifs found:  
CGACGCTTCG - CGACGCGTCC - CGCCGTATCG - CGTCGATCCG - TCGCGTCG -   
  
Best occurrences (match percentage):   
Seq St oligo pos match
  
 1 + **.CGCCGCGCCC.** 1068 (90.72)   
1 + **.CGCCGCCCCG.** 1077 (91.18)   
1 - **[CGGGGCGGCG]** 1077 (89.73)   
1 - **[GGGCGCGGCG]** 1068 (89.72)   
1 - **.CGGCGCCTCG.** 1063 (92.73)   
2 + **[CGACGCCCCG]** 507 (85.41)   
2 + **[CCCCGCGTCC]** 512 (87.66)   
2 + **[CGGCGCGCGG]** 702 (88.50)   
2 + **[CCCCGCCCCG]** 900 (85.75)   
2 + **[CGCCCCGTCC]** 903 (86.19)   
2 + **.CCTCGCGGCG.** 993 (92.97)   
2 + **[CGGCGCCTGG]** 1154 (85.88)   
2 + **[CGCCGCCCCC]** 1268 (85.73)   
2 - **[CGCCGGGTCC]** 1195 (85.44)   
2 - **[CCGCGCGCCG]** 702 (89.93)   
2 - **.CGGGGCGTCG.** 507 (91.33)   
2 - **[GGTCCCGTCG]** 244 (86.71)   
3 + **.CGCGGCGGCG.** 1223 (90.54)   
3 + **[CGTGGCGCCC]** 1321 (85.80)   
3 + **.CGCCGCCCCG.** 1401 (91.18)   
3 + **[CGCCCCGCCG]** 1404 (89.28)   
3 + **[CGCCGCCGCC]** 1409 (86.49)   
3 + **.CGCCGCCGCG.** 1412 (91.94)   
3 + **[CGCCGCGCCA]** 1415 (85.85)   
3 - **[TGGCGCGGCG]** 1415 (85.50)   
3 - **.CGCGGCGGCG.** 1412 (90.54)   
3 - **[CGGCGGGGCG]** 1404 (88.48)   
3 - **[CGGGGCGGCG]** 1401 (89.73)   
3 - **[GGTGGCGGCG]** 1263 (85.61)   
3 - **.CGCCGCCGCG.** 1223 (91.94)   
4 + **[CGCCCCCGCG]** 568 (85.05)   
4 + **[CGGCGCCGCC]** 692 (85.68)   
4 + **.CGCCGCCGCG.** 695 (91.94)   
4 + **[CGCCGCGACC]** 698 (85.19)   
4 + **.CGTCGCGGCC.** 739 (92.95)   
4 + **.CCTCGCGGCG.** 799 (92.97)   
4 + **[CACCGCGGCG]** 819 (86.91)   
4 + **.CGCGGCGGCG.** 822 (90.54)   
4 + **[CGCCGCTGCG]** 1107 (88.00)   
4 + **[CCCCGCGCCC]** 1196 (85.30)   
4 + **[CGCCGCGCCA]** 1205 (85.85)   
4 - **[CGCCCCCGCG]** 1276 (85.05)   
4 - **[TGGCGCGGCG]** 1205 (85.50)   
4 - **[CGCAGCGGCG]** 1107 (86.06)   
4 - **[CGGCGCTCCG]** 1102 (86.43)   
4 - **[CGCCGCCTCC]** 901 (88.09)   
4 - **[GCTCGCGCCG]** 827 (85.81)   
4 - **.CGCCGCCGCG.** 822 (91.94)   
4 - **[CGCCGCGGTG]** 819 (86.42)   
4 - **.GGTCGCGGCG.** 698 (92.00)   
4 - **.CGCGGCGGCG.** 695 (90.54)   
4 - **[CGCCGCCTCC]** 667 (88.09)   
4 - **[CCCCGCCCCG]** 203 (85.75)   
5 + **[CGCCGCCTGG]** 1310 (86.68)   
5 + **[CCGCGCCTCG]** 1345 (87.31)   
5 + **[CGGCGCAGCG]** 1387 (85.09)   
5 + **[CGACGCGGCC]** 1437 (85.71)   
5 + **[CGCCGCCGCC]** 1482 (86.49)   
5 - **[GGCCGCCTCG]** 1446 (87.14)   
5 - **.GGCCGCGTCG.** 1437 (92.13)   
5 - **[CGCTGCGCCG]** 1387 (85.22)   
6 + **[CGCCGGGGCG]** 954 (89.29)   
6 - **.CGCCCCGGCG.** 954 (90.04)   
8 + **[CGGCCCGGCG]** 348 (89.23)   
8 + **[CCGCGCGTCC]** 369 (86.85)   
8 + **[CGGCGCGGCA]** 406 (85.80)   
8 + **.CGCCGCGTGG.** 451 (91.67)   
8 + **[CGCCCCCGCG]** 782 (85.05)   
8 + **[CGCCGCCCCC]** 813 (85.73)   
8 + **[CCCCGCCCCG]** 855 (85.75)   
8 + **.CCCCGCGTCG.** 1143 (93.10)   
8 + **[CGCCGCCTCC]** 1234 (88.09)   
8 - **[GGCGGCGTCG]** 1151 (85.74)   
8 - **[CCTCGCGCCC]** 932 (86.77)   
8 - **[CCTCGCCCCG]** 899 (87.22)   
8 - **[CCACGCGGCG]** 451 (85.73)   
8 - **[TGCCGCGCCG]** 406 (85.55)   
8 - **[CGCCGGGCCG]** 348 (88.53)   
9 + **[CCCCGCGCCC]** 1059 (85.30)   
9 + **[CGGCGCAGCG]** 1245 (85.09)   
9 - **[CCTCCCGTCG]** 1270 (87.68)   
9 - **[CGTCGCGCCT]** 1265 (89.01)   
9 - **[CGCTGCGCCG]** 1245 (85.22)   
10 + **[CGTCGGGGCC]** 43 (85.31)   
10 + **[CGTCGCAGCG]** 790 (87.37)   
10 + **.GGCCGCGGCG.** 1021 (90.53)   
10 + **[CGCGGCGGCC]** 1024 (85.09)   
10 + **[CGCGGCGGCC]** 1096 (85.09)   
10 + **[GGCCGCGGCC]** 1118 (85.08)   
10 - **[GGTGGCGGCG]** 1262 (85.61)   
10 - **[CGGCCCCTCG]** 1236 (85.84)   
10 - **[GGCCGCGGCC]** 1118 (85.08)   
10 - **[GGCCGCCGCG]** 1096 (85.54)   
10 - **[GGCCGCCGCG]** 1024 (85.54)   
10 - **.CGCCGCGGCC.** 1021 (91.48)   
10 - **[CCCCGCGTCC]** 827 (87.66)   
10 - **[CCTCGCGTCT]** 819 (85.95)   
10 - **[CGCCGCCGCC]** 688 (86.49)   
11 + **[CGGGGCGTCC]** 93 (85.89)   
11 + **[CACCGCGCCG]** 603 (86.15)   
11 + **[CCGCGCCGCG]** 605 (85.70)   
11 + **.CGCCGCGGCC.** 608 (91.48)   
11 + **[CGCGGCCGCG]** 611 (85.55)   
11 + **[GGCCGCGGCC]** 712 (85.08)   
11 + **[TGCCGCGGCG]** 766 (86.31)   
11 + **[CGTGGCCCCG]** 1018 (86.26)   
11 + **[CCCCGCCGCG]** 1023 (86.51)   
11 + **[CGCCGCGCTG]** 1026 (85.67)   
11 + **[CGCCGCCGCC]** 1037 (86.49)   
11 + **[CTGCGCGGCG]** 1113 (87.85)   
11 + **.CGCCGCCGCG.** 1151 (91.94)   
11 + **[CGCCGCGCAG]** 1154 (85.94)   
11 - **[CTGCGCGGCG]** 1154 (87.85)   
11 - **.CGCGGCGGCG.** 1151 (90.54)   
11 - **[CGCCGCGCAG]** 1113 (85.94)   
11 - **[CAGCGCGGCG]** 1026 (86.10)   
11 - **[CGCCGCGGCA]** 766 (86.61)   
11 - **.CGTCCCGCCG.** 741 (90.75)   
11 - **[GGCCGCGGCC]** 712 (85.08)   
11 - **[GGGCGCGTCC]** 697 (85.88)   
11 - **[CCGCGCGGCC]** 614 (85.25)   
11 - **[CGCGGCCGCG]** 611 (85.55)   
11 - **.GGCCGCGGCG.** 608 (90.53)   
11 - **[CGGCGCGGTG]** 603 (85.62)   
12 + **[CGCTGCGTCG]** 445 (87.58)   
12 + **[GGTCCCGGCG]** 535 (85.11)   
12 + **[CGGCGCGCCC]** 540 (89.91)   
12 + **[CCGCGCGTCC]** 838 (86.85)   
12 + **[GGCCGCCTCG]** 1145 (87.14)   
12 + **[GGCCGCGCCG]** 1250 (89.77)   
12 - **[CATCGCGGCG]** 1354 (88.38)   
12 - **.CGGCGCGGCC.** 1250 (90.67)   
12 - **[CGCCGCCCCC]** 1241 (85.73)   
12 - **[CCGCGCGTCC]** 1115 (86.85)   
12 - **[CCCCGCCCCG]** 601 (85.75)   
12 - **[CCCCGCCCCG]** 596 (85.75)   
12 - **[GGGCGCGCCG]** 540 (88.96)   
13 + **.CGCCGCCGCG.** 717 (91.94)   
13 + **[CGTCCCGCCC]** 744 (85.30)   
13 + **.CGCCGCCCCG.** 753 (91.18)   
13 + **[CCCCGCCCCG]** 758 (85.75)   
13 + **[CCCCGCGTCC]** 763 (87.66)   
13 + **[CCCCGCGCCC]** 777 (85.30)   
13 + **[CCCCGCGCCC]** 784 (85.30)   
13 + **[CGGCGCGCAG]** 800 (85.13)   
13 + **[CCGCGCGGCC]** 944 (85.25)   
13 + **[CGGCGGGGCG]** 974 (88.48)   
13 + **.CGGCGCGGCC.** 1061 (90.67)   
13 + **[CGGCCCGCCG]** 1120 (88.47)   
13 + **[CGGCGCCGCC]** 1136 (85.68)   
13 + **[CGCCGCCGCC]** 1139 (86.49)   
13 + **[CGCCGCCGCC]** 1142 (86.49)   
13 + **[CGTCGCCGCC]** 1157 (87.96)   
13 + **[CGCCGCCGCC]** 1160 (86.49)   
13 + **[CGCCGCCGCC]** 1163 (86.49)   
13 + **[GGCGGCGTCG]** 1316 (85.74)   
13 + **.CGTCGCGGGG.** 1321 (91.54)   
13 + **[CCCCGGGTCG]** 1438 (85.46)   
13 - **[CGCCGCCCCC]** 1425 (85.73)   
13 - **.CGGCCCGTCG.** 1404 (90.83)   
13 - **[CGTCGCCTCC]** 1399 (89.56)   
13 - **[CCCCGCCGCG]** 1352 (86.51)   
13 - **[CCCCGCGACG]** 1321 (85.21)   
13 - **[CGGCGGGCCG]** 1120 (87.72)   
13 - **[GGCCGCGCCG]** 1061 (89.77)   
13 - **[CGCCCCGCCG]** 974 (89.28)   
13 - **[CGTCCCGCCC]** 923 (85.30)   
13 - **[CGCCCCGTCC]** 918 (86.19)   
13 - **[CCTCGCCCCG]** 909 (87.22)   
13 - **[CTGCGCGCCG]** 800 (87.09)   
13 - **[CGGGGCGGCG]** 753 (89.73)   
13 - **.CGCGGCGGCG.** 717 (90.54)   
14 + **.CCCCGCGGCG.** 334 (91.50)   
14 + **[GGGCGCGGCG]** 745 (89.72)   
14 + **[TGGCGCGGCG]** 937 (85.50)   
14 + **[CGGCGGGGCG]** 942 (88.48)   
14 - **[CCCCGCCCCG]** 945 (85.75)   
14 - **[CGCCCCGCCG]** 942 (89.28)   
14 - **[CCCCGCCGCG]** 940 (86.51)   
14 - **[CGCCGCGCCA]** 937 (85.85)   
14 - **.CGCCGCGCCC.** 745 (90.72)   
14 - **.CGCCGCGGGG.** 334 (90.07)   
15 + **[CGACGCCCCG]** 694 (85.41)   
15 + **[CCCCGCCCCG]** 699 (85.75)   
15 + **[CCCCGCCCCG]** 831 (85.75)   
15 + **.CGCCCCGGCG.** 834 (90.04)   
15 + **[CCTGGCGCCG]** 938 (85.82)   
15 + **[GGGCGCGGCG]** 983 (89.72)   
15 + **[CGGCGCGCGG]** 1199 (88.50)   
15 + **[CGTCGCCGCC]** 1240 (87.96)   
15 + **[CGCCGCCTCC]** 1243 (88.09)   
15 - **[CCGCGCGCCG]** 1199 (89.93)   
15 - **.CGCCGCGCCC.** 983 (90.72)   
15 - **[CGCCGGGGCG]** 834 (89.29)   
15 - **.CGGGGCGTCG.** 694 (91.33)   
15 - **[GGCGGCGTCG]** 268 (85.74)   
16 + **[GGCCGCGCCG]** 620 (89.77)   
16 + **[CCGCGCCGCG]** 622 (85.70)   
16 + **[CGCCGCGCCT]** 625 (87.54)   
16 + **.CGCCGCCCCG.** 843 (91.18)   
16 + **[CGGCGCTGCG]** 1039 (87.19)   
16 + **[CGCTGCGGCG]** 1042 (85.98)   
16 + **.CGCGGCGGCG.** 1150 (90.54)   
16 + **[CGCCGGGTCC]** 1205 (85.44)   
16 - **[CGTCGCGCAG]** 1279 (87.41)   
16 - **.CGCCGCCGCG.** 1150 (91.94)   
16 - **[CGCCGCAGCG]** 1042 (85.90)   
16 - **[CGCAGCGCCG]** 1039 (85.31)   
16 - **[CGCCGCCGCC]** 1034 (86.49)   
16 - **[CGCCGCCGCC]** 1031 (86.49)   
16 - **[CGCCGCCGCC]** 1028 (86.49)   
16 - **[CGGGGCGGCG]** 843 (89.73)   
16 - **[AGGCGCGGCG]** 625 (86.78)   
16 - **.CGGCGCGGCC.** 620 (90.67)   
17 + **[CCTCGGGGCG]** 980 (85.33)   
17 - **[CCTCGCCCCG]** 983 (87.22)   
18 + **[CGCCGCTCCG]** 785 (87.24)   
18 + **[AGCCGCGCCG]** 831 (86.83)   
18 + **[CCGCGCCGCG]** 833 (85.70)   
18 + **.CGCCGCGCCC.** 865 (90.72)   
18 + **[CCGCGCCTCG]** 913 (87.31)   
18 + **[GGCCGCGGCC]** 929 (85.08)   
18 + **.CGCCGCGGCC.** 1201 (91.48)   
18 + **[CCCCGCCCCG]** 1223 (85.75)   
18 + **[GGCCGCGTCC]** 1328 (86.69)   
18 - **.GGCCGCGGCG.** 1201 (90.53)   
18 - **[GGCCGCGGCC]** 929 (85.08)   
18 - **[GGGCGCGGCG]** 865 (89.72)   
18 - **[CGGCGCGGCT]** 831 (87.49)   
18 - **[CGGAGCGGCG]** 785 (85.26)   
20 + **[GGCCGCGTGG]** 1196 (85.28)   
22 + **.CGCCGCCGCG.** 768 (91.94)   
22 - **[CGGCGGCTCG]** 1084 (85.09)   
22 - **[CGCCGGCTCG]** 879 (85.90)   
22 - **.CGCGGCGGCG.** 768 (90.54)   
24 + **[CGCCGCAGCG]** 385 (85.90)   
24 + **[CGGGGCGGCG]** 497 (89.73)   
24 + **[GGTCCCGGCG]** 534 (85.11)   
24 + **[CCCCGCCCCG]** 578 (85.75)   
24 + **[CCCCGCGCCC]** 595 (85.30)   
24 + **[CGGGGCCTCG]** 973 (86.34)   
24 + **[TGCCGCGGCG]** 1037 (86.31)   
24 + **[CGCGGCGGCC]** 1040 (85.09)   
24 + **.GGTCGCGCCG.** 1142 (91.24)   
24 - **[CCCCGCGCCC]** 1074 (85.30)   
24 - **[GGCCGCCGCG]** 1040 (85.54)   
24 - **[CGCCGCGGCA]** 1037 (86.61)   
24 - **[CGCCCCCGCG]** 944 (85.05)   
24 - **[CCCCGCGCCC]** 941 (85.30)   
24 - **[CCCCGCGCCC]** 934 (85.30)   
24 - **[CCCCGCCCCG]** 927 (85.75)   
24 - **.CGCCGCCCCG.** 497 (91.18)   
24 - **[CGCTGCGGCG]** 385 (85.98)   
25 + **[CGGCGCGGTG]** 791 (85.62)   
25 - **[CCTCGCCCCG]** 824 (87.22)   
25 - **[CACCGCGCCG]** 791 (86.15)   
25 - **[CGTCCCGCCC]** 632 (85.30)   
25 - **[CCTCGCCCCG]** 603 (87.22)   
25 - **[CCGCGCCTCG]** 546 (87.31)   
25 - **.CCTCGCGCCG.** 382 (92.21)   
25 - **[CCCCGCCCCG]** 59 (85.75)   
26 + **.CGGCGCGGCC.** 823 (90.67)   
26 - **[CCTCCCGCCG]** 1427 (85.32)   
26 - **[CGCCGCCGCC]** 960 (86.49)   
26 - **[CGCCGCCGCC]** 957 (86.49)   
26 - **[CCCCGCCCCG]** 858 (85.75)   
26 - **[GGCCGCGCCG]** 823 (89.77)   
27 + **.CCCCGCGTCG.** 1143 (93.10)   
27 + **[CTGCGCGCCG]** 1246 (87.09)   
27 + **[CGCCGCCTCC]** 1356 (88.09)   
27 - **[CGGCGCGCAG]** 1246 (85.13)   
28 + **.CGGCGCGTGG.** 432 (90.87)   
28 + **[CGTGGCGGGG]** 458 (85.15)   
28 + **[CGGCCCGGCG]** 671 (89.23)   
28 + **[CTCCGCGCCG]** 737 (87.90)   
28 + **[CGTCGCTCCG]** 763 (88.71)   
28 + **[CGTCGCCGCC]** 1060 (87.96)   
28 + **[CGCCGCCCCC]** 1063 (85.73)   
28 + **[CCCCGCCTCG]** 1069 (88.11)   
28 + **.CGTCGCGTCG.** 1097 (100.00)   
28 + **.CGTCGCGCCG.** 1102 (97.64)   
28 + **.CGCCGCGTCG.** 1107 (98.53)   
28 + **[CGTCGCCCCC]** 1112 (87.20)   
28 - **[CCCCGCCTCG]** 1413 (88.11)   
28 - **.CGACGCGGCG.** 1107 (91.16)   
28 - **[CGGCGCGACG]** 1102 (89.83)   
28 - **[CGCCGCCGCC]** 869 (86.49)   
28 - **[CCTCGCCTCG]** 794 (89.58)   
28 - **[CGGCGCGGAG]** 737 (85.89)   
28 - **[CGCCGGGCCG]** 671 (88.53)   
28 - **[CGTCGCGGCT]** 586 (89.77)   
28 - **[CGCCGCCTCC]** 427 (88.09)   
33 + **[CCCCGCGGCC]** 901 (86.05)   
33 + **[CGCCGCGTGC]** 1039 (86.23)   
33 + **.CGTCGCCCCG.** 1056 (92.65)   
33 + **.GGCCGCGGCG.** 1359 (90.53)   
33 + **.CGCCGCCGCG.** 1407 (91.94)   
33 + **.CGCCGCGGGG.** 1410 (90.07)   
33 - **.CCCCGCGGCG.** 1410 (91.50)   
33 - **.CGCGGCGGCG.** 1407 (90.54)   
33 - **.CGCCGCGGCC.** 1359 (91.48)   
33 - **[CGCCGCCTCC]** 1350 (88.09)   
33 - **[CGCGGCGTCC]** 1036 (86.69)   
33 - **[CGTCCCCGCG]** 1031 (86.52)   
34 + **[CGCTGCGCCG]** 765 (85.22)   
34 + **.CGCCGCGCCC.** 770 (90.72)   
34 + **[CCCCGCCCCG]** 786 (85.75)   
34 + **[CGTGGCGGCC]** 850 (86.56)   
34 + **[CGCCGAGGCG]** 912 (86.08)   
34 + **[CCTCGCGCCC]** 939 (86.77)   
34 + **.CGGCGCCCCG.** 976 (90.37)   
34 + **[CCGCGCGGCC]** 1079 (85.25)   
34 + **[CGCCGCCCCC]** 1122 (85.73)   
34 + **.CGGCGCCGCG.** 1192 (91.13)   
34 + **[CGCCGCGCAG]** 1195 (85.94)   
34 + **[CGCCGCCGCC]** 1445 (86.49)   
34 + **[CGCCGCCGCC]** 1448 (86.49)   
34 - **[CGGGGCGTCC]** 1204 (85.89)   
34 - **[CTGCGCGGCG]** 1195 (87.85)   
34 - **[CGCGGCGCCG]** 1192 (89.78)   
34 - **[CGGGGCGCCG]** 976 (88.97)   
34 - **[GGGCGCCTCG]** 915 (86.33)   
34 - **[CGCCTCGGCG]** 912 (86.77)   
34 - **[CCCCGCCTCG]** 814 (88.11)   
34 - **[GGGCGCGGCG]** 770 (89.72)   
34 - **[CGGCGCAGCG]** 765 (85.09)   
34 - **[CCCCGCCCCG]** 334 (85.75)   
35 + **[CGCCGCCGCC]** 948 (86.49)   
35 + **[CGCCGCGCAG]** 997 (85.94)   
35 + **[CGGGGCGGCG]** 1237 (89.73)   
35 - **[CCTGGCGGCG]** 1297 (86.58)   
35 - **[CCTCCCGCCG]** 1262 (85.32)   
35 - **.CGCCGCCCCG.** 1237 (91.18)   
35 - **[CGGCGCCTCC]** 1080 (87.29)   
35 - **[CTGCGCGGCG]** 997 (87.85)   
35 - **[CGGCGCCTCC]** 943 (87.29)   
36 + **[CCGCGCGCCG]** 1063 (89.93)   
36 + **[CGCCGCGGAG]** 1170 (86.69)   
36 - **[CTCCGCGGCG]** 1170 (88.66)   
36 - **[CGGCGCCGCC]** 1101 (85.68)   
36 - **[CCCCGCCCCG]** 1086 (85.75)   
36 - **[CCTCGCCCCG]** 1075 (87.22)   
36 - **[CGGCGCGCGG]** 1063 (88.50)   
38 + **[GGGCGCCTCG]** 556 (86.33)   
39 + **[GGGCGCGTCC]** 1119 (85.88)   
39 + **[CGTCCCCGCG]** 1124 (86.52)   
39 + **[CCTCCCGGCG]** 1146 (86.08)   
39 + **[CGCCGCCACG]** 1350 (85.65)   
39 + **[CCTCGCGCGG]** 1420 (85.36)   
39 - **.GGTCGCGCCG.** 1411 (91.24)   
39 - **.CGTGGCGGCG.** 1350 (92.01)   
39 - **[CGCCGCCCCC]** 1254 (85.73)   
40 + **[CCCCGCGGCC]** 892 (86.05)   
40 + **[CGGCCCGCCG]** 897 (88.47)   
40 + **[CCGCGCCGCG]** 1103 (85.70)   
40 + **[GGTGGCGTCG]** 1411 (87.21)   
40 - **[CCCCGCCCCG]** 1281 (85.75)   
40 - **[CGGCGGGCCG]** 897 (87.72)   
41 + **[CCTCCCGTCG]** 910 (87.68)   
41 + **[CGTCGCCTCC]** 915 (89.56)   
42 + **[CGCCGGGGCG]** 456 (89.29)   
42 + **[CGGGGCGCCG]** 459 (88.97)   
42 + **[CGTGGCCCCG]** 517 (86.26)   
42 + **[GGGCGCCTCG]** 606 (86.33)   
42 + **[CGTCGCGGGC]** 908 (86.09)   
42 + **.CCCCGCGGCG.** 1165 (91.50)   
42 + **.CGCGGCGGCG.** 1168 (90.54)   
42 + **[CCGCCCGTCG]** 1346 (85.41)   
42 + **[CGTCGCCCGG]** 1351 (85.79)   
42 + **[CCGCGCGTGG]** 1448 (85.44)   
42 - **[CCCCGCGGCC]** 1474 (86.05)   
42 - **[CGCCGCCGCC]** 1174 (86.49)   
42 - **[CGCCGCCGCC]** 1171 (86.49)   
42 - **.CGCCGCCGCG.** 1168 (91.94)   
42 - **.CGCCGCGGGG.** 1165 (90.07)   
42 - **.CGGCGCCCCG.** 459 (90.37)   
42 - **.CGCCCCGGCG.** 456 (90.04)   
43 + **.CGGCGCCGCG.** 1097 (91.13)   
43 - **[CGTCGCCGCC]** 1180 (87.96)   
43 - **[CGCCGCCGCC]** 1177 (86.49)   
43 - **[CGTCGCCCCC]** 1168 (87.20)   
43 - **[CGCCGCCTCC]** 1159 (88.09)   
43 - **[CGCGGCGCCG]** 1097 (89.78)   
45 - **[CGCCCCCGCG]** 931 (85.05)   
45 - **[CCCCGCGCCC]** 928 (85.30)   
47 + **[AGTCGCGCCG]** 1246 (88.30)   
47 + **.CGGCGCCCCG.** 1298 (90.37)   
47 + **[CGCAGCGCCG]** 1344 (85.31)   
47 + **[CGCGGCCGCG]** 1367 (85.55)   
47 - **[CGCCGGGTCC]** 1464 (85.44)   
47 - **[CGCGGCCGCG]** 1367 (85.55)   
47 - **[CGGCGCTGCG]** 1344 (87.19)   
47 - **[CGGGGCGCCG]** 1298 (88.97)   
47 - **[CCCCGCCTCG]** 1142 (88.11)   
48 + **[CGGCGCCTGG]** 190 (85.88)   
48 + **[CGCGGCGCCG]** 433 (89.78)   
48 + **.CGCGGCGGCG.** 1019 (90.54)   
48 + **.CGCCGCGGCG.** 1283 (96.93)   
48 + **[CCCCGCCCCG]** 1387 (85.75)   
48 - **.CGCCGCGGCG.** 1283 (96.93)   
48 - **[CGCCGCCGCC]** 1022 (86.49)   
48 - **.CGCCGCCGCG.** 1019 (91.94)   
48 - **[CCGCGCGGCC]** 763 (85.25)   
48 - **.CGGCGCCGCG.** 433 (91.13)   
49 + **[CGCCGCTCCG]** 1309 (87.24)   
49 + **[CGCCGCGGAG]** 1406 (86.69)   
49 - **[CTCCGCGGCG]** 1406 (88.66)   
49 - **[CGGAGCGGCG]** 1309 (85.26)   
49 - **[CGCCGCCGCC]** 1078 (86.49)   
49 - **[CGCCGCCGCC]** 1075 (86.49)   
49 - **[CGCCGCCGCC]** 1072 (86.49)   
49 - **[CGCCGCCTCC]** 1069 (88.09)   
51 + **[GGCCGCCGCG]** 64 (85.54)   
51 + **.CGCCGCGTCG.** 67 (98.53)   
51 + **[CCCCGCCCCG]** 628 (85.75)   
51 + **[CCCCGCGCCC]** 633 (85.30)   
51 + **.CCTCGCGCCG.** 779 (92.21)   
51 + **[CGTCGCGGAG]** 976 (88.16)   
51 + **[CCTCGCGCCC]** 995 (86.77)   
51 + **[CGTCGCCGGG]** 1040 (86.55)   
51 + **[CGCGGCGGCC]** 1286 (85.09)   
51 + **[GGGCGCGGCG]** 1359 (89.72)   
51 - **.CGCCGCGCCC.** 1359 (90.72)   
51 - **[GGCCGCCGCG]** 1286 (85.54)   
51 - **[CCCGGCGTCG]** 1150 (86.71)   
51 - **[GGTCCCGGCG]** 1043 (85.11)   
51 - **.CGACGCGGCG.** 67 (91.16)   
51 - **[CGCGGCGGCC]** 64 (85.09)   
51 - **[CGCCCCCGCG]** 4 (85.05)   
53 + **[CGTCGAGGCG]** 785 (87.55)   
53 - **[GGCCGCGTCC]** 1056 (86.69)   
54 - **[CGGCGCCTGG]** 1023 (85.88)   
55 + **[CGCCGCCGCC]** 92 (86.49)   
55 + **[CCTCGCCGCG]** 195 (87.98)   
55 + **.CGCCGCGCCC.** 198 (90.72)   
55 + **[CGGCCCGCCG]** 860 (88.47)   
55 + **[CAGCGCGGCG]** 940 (86.10)   
55 + **[CCGCGCCGCG]** 1081 (85.70)   
55 - **[CGCCGCGCTG]** 940 (85.67)   
55 - **[CGGCGGGCCG]** 860 (87.72)   
55 - **[CGGCGCCTGG]** 713 (85.88)   
55 - **[CGCTGCGTCG]** 651 (87.58)   
55 - **[GGGCGCGGCG]** 198 (89.72)   
58 + **[GGTCGCCCCG]** 595 (86.25)   
58 + **.CGCCGCCCCG.** 1085 (91.18)   
58 - **[CGGGGCGGCG]** 1085 (89.73)   
58 - **.CGTCGGGGCG.** 598 (90.76)   
58 - **[CGTGGCCCCG]** 412 (86.26)   
59 + **.CGCCGCGGCC.** 649 (91.48)   
59 + **[CGTCGCCGGG]** 681 (86.55)   
59 + **[CGCCGCCGCC]** 771 (86.49)   
59 + **[CGCCGCCGCC]** 774 (86.49)   
59 + **[CGCCGCCGCC]** 777 (86.49)   
59 + **[CGCCGCCGCC]** 780 (86.49)   
59 + **.CGGCGCCCCG.** 1107 (90.37)   
59 + **[CGGCCCGGCG]** 1194 (89.23)   
59 + **[CTGCGCGTCG]** 1283 (89.45)   
59 + **[CGGCGCGGAG]** 1351 (85.89)   
59 - **[CTCCGCGCCG]** 1351 (87.90)   
59 - **[CCGCGCCGCG]** 1349 (85.70)   
59 - **[CGCCGGGCCG]** 1194 (88.53)   
59 - **[CGGGGCGCCG]** 1107 (88.97)   
59 - **[CCTGGCGGCG]** 826 (86.58)   
59 - **[GGTCCCGGCG]** 684 (85.11)   
59 - **.GGCCGCGGCG.** 649 (90.53)   
60 + **[CCCCGCCGCG]** 821 (86.51)   
60 + **.CGCCGCGGCC.** 824 (91.48)   
60 - **[CTCCGCCTCG]** 904 (85.27)   
60 - **.GGCCGCGGCG.** 824 (90.53)   
61 + **.CGCCGCGGGG.** 924 (90.07)   
61 + **.CGCCGCGGCG.** 1036 (96.93)   
61 - **[CCTCGCCGCG]** 1039 (87.98)   
61 - **.CGCCGCGGCG.** 1036 (96.93)   
61 - **[CGCGGCGTCC]** 1033 (86.69)   
61 - **.CCCCGCGGCG.** 924 (91.50)   
61 - **.CGCCGCGTCC.** 839 (93.08)   
63 + **[CCCCGCGCCC]** 128 (85.30)   
63 + **[CGTGGCGGCC]** 227 (86.56)   
63 + **.CGCGGCGGCG.** 410 (90.54)   
63 + **[GCCCGCGGCG]** 442 (85.10)   
63 + **[CGCGGCGTCC]** 445 (86.69)   
63 + **.CGCCCCGGCG.** 622 (90.04)   
63 + **[GGGCGCGCCG]** 655 (88.96)   
63 + **[GCTCGCGTCG]** 851 (88.18)   
63 + **[CGTCGGCCCG]** 856 (85.01)   
63 + **.CCCCGCGGCG.** 951 (91.50)   
63 + **[CGCGGCGTCC]** 954 (86.69)   
63 + **.CGTCCCGTCG.** 959 (93.11)   
63 + **[CGTCGCGTTG]** 973 (89.50)   
63 + **[CGGGGCGGCG]** 1168 (89.73)   
63 + **[AGGCGCGGCG]** 1390 (86.78)   
63 + **.CGCGGCGGCG.** 1393 (90.54)   
63 - **.CGCCGCCGCG.** 1393 (91.94)   
63 - **[CGCCGCGCCT]** 1390 (87.54)   
63 - **.CGCCGCCCCG.** 1168 (91.18)   
63 - **[CGTCGCTCCG]** 992 (88.71)   
63 - **.CGCCGCGGGG.** 951 (90.07)   
63 - **[CGGCGCGCCC]** 655 (89.91)   
63 - **[CGCCGGGGCG]** 622 (89.29)   
63 - **.CGCCGCCGCG.** 410 (91.94)   
63 - **[CCTCGGGGCG]** 392 (85.33)   
63 - **[CCCCGCCCCG]** 216 (85.75)   
63 - **[CCCCGCGGCC]** 181 (86.05)   
63 - **[GGGCGCCTCG]** 67 (86.33)   
65 + **[CGCCTCGGCG]** 636 (86.77)   
65 + **[CGGCGCGTCT]** 863 (89.10)   
65 + **[CCCCGCCCCG]** 940 (85.75)   
65 + **[CGGCGCGCCA]** 978 (85.05)   
65 + **[CGCCGCGCCA]** 1027 (85.85)   
65 + **[CGCCGCCGCC]** 1087 (86.49)   
65 + **.CGCCGCCCCG.** 1090 (91.18)   
65 + **.CGGCGCCCCG.** 1147 (90.37)   
65 - **[GGTGGCGTCG]** 1419 (87.21)   
65 - **[CGTCGGGGCC]** 1411 (85.31)   
65 - **[CGGGGCGCCG]** 1147 (88.97)   
65 - **[CGGGGCGGCG]** 1090 (89.73)   
65 - **[CCTGGCGTCG]** 1054 (88.18)   
65 - **[TGGCGCGGCG]** 1027 (85.50)   
65 - **[CCTCCCGGCG]** 1009 (86.08)   
65 - **[GCTCGCGGCG]** 1000 (86.57)   
65 - **[CCGCGCGGCC]** 783 (85.25)   
65 - **[CGCCGAGGCG]** 636 (86.08)   
66 + **[CGCCGCCGCC]** 871 (86.49)   
66 + **[CGCCGCCCCC]** 874 (85.73)   
66 + **[CGCCCCCGCG]** 949 (85.05)   
66 + **.CCCCGCGCCG.** 952 (90.74)   
66 + **[CGCCGCCTGG]** 1041 (86.68)   
66 + **[CGCCACGCCG]** 1088 (85.53)   
66 + **[GCCCGCGGCG]** 1124 (85.10)   
66 - **[CGTGGCGCCC]** 1085 (85.80)   
66 - **[GCCCGCGGCG]** 988 (85.10)   
66 - **[CGGCGCGGGG]** 952 (89.26)   
67 + **.CGGCGGGTCG.** 815 (90.08)   
67 + **[CGTCGCCTGG]** 904 (88.15)   
67 + **[GGTCGCCGCG]** 1192 (87.01)   
67 + **.CGCCGCGTCC.** 1195 (93.08)   
67 - **[CGTCGCCTGG]** 1031 (88.15)   
68 + **[CGCCGCCGCC]** 576 (86.49)   
68 + **[CGGCGCGGAG]** 651 (85.89)   
68 + **[CGCCGCCGGG]** 906 (85.08)   
68 + **[CGGAGCGTCG]** 1224 (86.86)   
68 + **.CCGCGCGGCG.** 1391 (90.69)   
68 - **[CGCCGCGCGG]** 1391 (89.31)   
68 - **[CCCCGCCGCG]** 992 (86.51)   
68 - **[CGCCGCGTCA]** 989 (88.21)   
68 - **[CCTCCCGGCG]** 909 (86.08)   
68 - **[CCCGGCGGCG]** 906 (85.11)   
68 - **[CTCCGCGCCG]** 651 (87.90)   
68 - **[CCGCGCCGCG]** 649 (85.70)   
68 - **[GGTGGCGGCG]** 579 (85.61)   
68 - **[CGCCGCCGCC]** 499 (86.49)   
68 - **[CGCCGCCGCC]** 496 (86.49)   
69 + **[CGCGGCCGCG]** 769 (85.55)   
69 + **.CGCCCCGGCG.** 828 (90.04)   
69 + **.CCCCGCGCCG.** 1118 (90.74)   
69 + **[CGCCGAGCCG]** 1123 (85.33)   
69 + **[CCCCGCGCCC]** 1200 (85.30)   
69 - **[CGGCTCGGCG]** 1123 (85.97)   
69 - **[CGGCGCGGGG]** 1118 (89.26)   
69 - **[CGCCGGGGCG]** 828 (89.29)   
69 - **[CGCGGCCGCG]** 769 (85.55)   
70 + **[CTGCGCGCCG]** 1331 (87.09)   
70 - **[CGGCGCGCAG]** 1331 (85.13)   
71 + **[CCGCGCGTCC]** 901 (86.85)   
72 + **[CGTCGGGGCC]** 27 (85.31)   
73 + **[CGCCGCCGCC]** 912 (86.49)   
73 + **.CGCCGCCGCG.** 915 (91.94)   
73 + **.CGCCGCGGCC.** 918 (91.48)   
73 - **.GGCCGCGGCG.** 918 (90.53)   
73 - **.CGCGGCGGCG.** 915 (90.54)   
74 + **[CCGCGCGTCC]** 1335 (86.85)   
74 + **.GGTCGCGGCG.** 1426 (92.00)   
74 + **[CGCGGCGGCC]** 1429 (85.09)   
74 + **[GGCCGCGCCG]** 1435 (89.77)   
74 - **.CGGCGCGGCC.** 1435 (90.67)   
74 - **[GGCCGCCGCG]** 1429 (85.54)   
74 - **[CGCCGCGACC]** 1426 (85.19)   
74 - **[CGCCGCCGCC]** 1285 (86.49)   
74 - **[CGCCGCCGCC]** 1282 (86.49)   
74 - **[CGCCGCCGCC]** 1279 (86.49)   
74 - **[CGCCGCCGCC]** 1276 (86.49)   
74 - **[CGCCGCCGCC]** 1273 (86.49)   
74 - **[CGCCGCCGCC]** 1270 (86.49)   
74 - **[CGGCGGCTCG]** 1225 (85.09)   
76 + **[CGTCCCCCCG]** 309 (85.76)   
76 + **[CGTCCCCCCG]** 338 (85.76)   
76 + **[CAGCGCGCCG]** 826 (85.34)   
76 - **[CGCCGCCGCC]** 918 (86.49)   
76 - **[CGCCGCCGCC]** 915 (86.49)   
76 - **.CGCCGCCTCG.** 912 (93.54)   
79 + **.CGCCGCCGCG.** 874 (91.94)   
79 + **[CGCCGCGTGC]** 877 (86.23)   
79 + **[CGTCGCGGGC]** 1087 (86.09)   
79 + **.CCCCGCGCCG.** 1199 (90.74)   
79 - **[CGGCGCGGGG]** 1199 (89.26)   
79 - **.CGCGGCGGCG.** 874 (90.54)   
79 - **.CCTCGCGGCG.** 653 (92.97)   
80 + **.CGTCGCCGCG.** 1012 (93.41)   
80 + **.CGCCGCGCCC.** 1015 (90.72)   
80 - **[CGGGGCCTCG]** 1231 (86.34)   
80 - **[GGGCGCGGCG]** 1015 (89.72)   
84 + **[CGTCGGGTCC]** 778 (86.91)   
84 + **[CCTCGCGCCC]** 861 (86.77)   
84 + **.CGACGCGGCG.** 1034 (91.16)   
84 - **.CGCCGCGTCG.** 1034 (98.53)   
84 - **[AGGCGCGTCG]** 899 (88.38)   
85 + **[CCTCCCGCCG]** 14 (85.32)   
85 + **[CGCCGCCTGG]** 19 (86.68)   
85 + **[CGGGGCGGCG]** 311 (89.73)   
85 + **[CCCCGCCCCG]** 637 (85.75)   
85 + **[CCCCGCCCCG]** 705 (85.75)   
85 + **[CCCCGCCCCG]** 730 (85.75)   
85 + **[CCCCGCCCCG]** 792 (85.75)   
85 + **[CGCCCCGCCG]** 1039 (89.28)   
85 - **[CGGCGGGGCG]** 1039 (88.48)   
85 - **.CGCCGCCCCG.** 311 (91.18)   
86 + **.CGCCGCGGCC.** 540 (91.48)   
86 + **[GGGCGCGGCG]** 833 (89.72)   
86 + **[GGGCGCGGCG]** 899 (89.72)   
86 + **[CGGCGGGGCG]** 1011 (88.48)   
86 + **[CGGCGGGGCG]** 1059 (88.48)   
86 + **.CCTCGCGGCG.** 1187 (92.97)   
86 + **.CGGCGCGGCG.** 1192 (96.12)   
86 + **.CGGCGCCCCG.** 1197 (90.37)   
86 + **.CGCCGCCCCG.** 1220 (91.18)   
86 + **.CGCCGCCCCG.** 1354 (91.18)   
86 + **[CCCCGCCCCG]** 1359 (85.75)   
86 + **[CGGGGCGGCG]** 1388 (89.73)   
86 + **[CGGGGCGGCG]** 1410 (89.73)   
86 + **[CGCGGCCGCG]** 1506 (85.55)   
86 - **[GGTCGCGGCC]** 1509 (86.55)   
86 - **[CGCGGCCGCG]** 1506 (85.55)   
86 - **[GCCCGCGGCG]** 1436 (85.10)   
86 - **.CGCCGCCCCG.** 1410 (91.18)   
86 - **.CGCCGCCCCG.** 1388 (91.18)   
86 - **[CCCCGCGCCC]** 1383 (85.30)   
86 - **[CGGGGCGGCG]** 1354 (89.73)   
86 - **[CGGGGCGGCG]** 1220 (89.73)   
86 - **[CGGGGCGCCG]** 1197 (88.97)   
86 - **.CGCCGCGCCG.** 1192 (96.17)   
86 - **[CCGCGCCGCG]** 1190 (85.70)   
86 - **[CCCCGCCCCG]** 1062 (85.75)   
86 - **[CGCCCCGCCG]** 1059 (89.28)   
86 - **[CCCCGCCCCG]** 1014 (85.75)   
86 - **[CGCCCCGCCG]** 1011 (89.28)   
86 - **[CCCCGCCCCG]** 924 (85.75)   
86 - **[CCTCGCCGCG]** 902 (87.98)   
86 - **.CGCCGCGCCC.** 899 (90.72)   
86 - **.CGCCGCGCCC.** 833 (90.72)   
86 - **.GGCCGCGGCG.** 540 (90.53)   
87 + **[CGGCGCGGAG]** 842 (85.89)   
87 + **[CCTCGGGGCG]** 928 (85.33)   
87 + **[CCCCGCCCCG]** 1080 (85.75)   
87 + **[CCCCGCCCCG]** 1085 (85.75)   
87 + **[CGTCCCGGCC]** 1101 (86.06)   
87 + **.CGGCGCGGCG.** 1241 (96.12)   
87 + **[CGGCACGGCG]** 1257 (85.48)   
87 + **[GCTCGCGGCG]** 1330 (86.57)   
87 + **.CGCGGCGGCG.** 1333 (90.54)   
87 + **[CGGCGCAGCG]** 1338 (85.09)   
87 + **[CAGCGCGCCG]** 1343 (85.34)   
87 - **[CGCCCCCGCG]** 1503 (85.05)   
87 - **[CCCCGCGCCC]** 1500 (85.30)   
87 - **[CCCCGCCCCG]** 1471 (85.75)   
87 - **[CGCTGCGCCG]** 1338 (85.22)   
87 - **.CGCCGCCGCG.** 1333 (91.94)   
87 - **.CGGCGCGTGG.** 1319 (90.87)   
87 - **.CGCCGCGCCG.** 1241 (96.17)   
87 - **[CGGGGCCTCG]** 1178 (86.34)   
87 - **[CTCCGCGCCG]** 842 (87.90)   
87 - **[CCCCGCGGCC]** 767 (86.05)   
87 - **.CCTCGCGCCG.** 312 (92.21)   
88 + **[CGCCGCCCCC]** 222 (85.73)   
88 + **[CGCCCCCGCG]** 365 (85.05)   
88 + **[CGGCGCGCGG]** 444 (88.50)   
88 + **[GGTGGCGGCG]** 475 (85.61)   
88 + **[CGTCGCGGGC]** 487 (86.09)   
88 + **[CGCCGCGCGG]** 503 (89.31)   
88 + **[CGGCGGGCCG]** 541 (87.72)   
88 + **[CGCCGCCGCC]** 785 (86.49)   
88 + **[CGCCGCCGCC]** 788 (86.49)   
88 + **[CCTCCCGCCG]** 1033 (85.32)   
88 + **.CGCCGCGGCC.** 1096 (91.48)   
88 + **[CGGCGCCTCC]** 1172 (87.29)   
88 + **[CAGCGCGTCG]** 1203 (87.70)   
88 + **[CCCCGGGTCG]** 1300 (85.46)   
88 - **.GGCCGCGGCG.** 1096 (90.53)   
88 - **[CGCGGCGTCC]** 1093 (86.69)   
88 - **[CGCCGCCGCC]** 902 (86.49)   
88 - **[CGCCGCCGCC]** 899 (86.49)   
88 - **[CGCCGCCGCC]** 896 (86.49)   
88 - **[CCTGGCGCCG]** 869 (85.82)   
88 - **[CCCCGCCGCG]** 849 (86.51)   
88 - **[CGGCCCGCCG]** 541 (88.47)   
88 - **.CCGCGCGGCG.** 503 (90.69)   
88 - **[CCGCGCGCCG]** 444 (89.93)   
88 - **[CCCCGCCCCG]** 411 (85.75)   
88 - **[CGCCCCCTCG]** 348 (86.65)   
88 - **[CCTCGGGGCG]** 343 (85.33)   
89 + **[CGCCGCCGCC]** 102 (86.49)   
89 + **[CGCCGCCCCC]** 105 (85.73)   
89 + **.CGCCCCGTCG.** 145 (91.64)   
89 + **.CGTCGCGCCC.** 150 (92.19)   
89 + **[CGGCGAGGCG]** 172 (85.28)   
89 + **[CGGCGCCGCC]** 282 (85.68)   
89 + **[CGCCGCCTCC]** 350 (88.09)   
89 + **[CCCCGCGCCC]** 361 (85.30)   
89 + **[CGCCGCCGCC]** 380 (86.49)   
89 + **[CGCCGCCGCC]** 383 (86.49)   
89 + **[CGCCGCCGCC]** 386 (86.49)   
89 + **[CGCCGCCGCC]** 408 (86.49)   
89 + **[CGCCGCCGCC]** 411 (86.49)   
89 + **.CGCCGCCGCG.** 414 (91.94)   
89 + **.CGCCGCGCCC.** 434 (90.72)   
89 + **[CGCCGCCGCC]** 466 (86.49)   
89 + **[TGCCGCGCCG]** 492 (85.55)   
89 + **[CGCCGCCGCC]** 611 (86.49)   
89 + **[GGCCGCCGCG]** 660 (85.54)   
89 + **.CGCCGCGCCC.** 663 (90.72)   
89 + **[CCCCGCGCCC]** 672 (85.30)   
89 + **[CGGCGGGGCG]** 708 (88.48)   
89 + **[GGGCGCGGCG]** 740 (89.72)   
89 + **.CGGCGCGCCG.** 786 (95.36)   
89 + **[CGGCGCGGAG]** 936 (85.89)   
89 + **[CCCCGCCCCG]** 949 (85.75)   
89 + **.CGCCCCGGCG.** 952 (90.04)   
89 + **[CCCCGCGCCC]** 973 (85.30)   
89 + **[TGCCGCGCCG]** 1020 (85.55)   
89 + **[CCCCGCCGCG]** 1122 (86.51)   
89 + **.CGGCGCCGCG.** 1306 (91.13)   
89 + **.CGCCGCGGGG.** 1309 (90.07)   
89 - **[AGTCGCGTCC]** 1464 (85.22)   
89 - **.CCCCGCGGCG.** 1309 (91.50)   
89 - **[CGCGGCGCCG]** 1306 (89.78)   
89 - **[CGGCGCGGCA]** 1020 (85.80)   
89 - **[CGCCGGGGCG]** 952 (89.29)   
89 - **[CTCCGCGCCG]** 936 (87.90)   
89 - **.CGGCGCGCCG.** 786 (95.36)   
89 - **[CGCCGCCTCC]** 781 (88.09)   
89 - **.CGCCGCGCCC.** 740 (90.72)   
89 - **[CCCCGCCCCG]** 711 (85.75)   
89 - **[CGCCCCGCCG]** 708 (89.28)   
89 - **[GGGCGCGGCG]** 663 (89.72)   
89 - **[CGCGGCGGCC]** 660 (85.09)   
89 - **[CGGCGCGGCA]** 492 (85.80)   
89 - **[GGGCGCGGCG]** 434 (89.72)   
89 - **.CGCGGCGGCG.** 414 (90.54)   
89 - **[AGTCGCGGCG]** 268 (89.06)   
89 - **[CGCCGCCCCC]** 181 (85.73)   
89 - **[CCCCGCCTCG]** 175 (88.11)   
89 - **[CGCCTCGCCG]** 172 (86.01)   
90 + **[CGTCGTGGCG]** 354 (86.71)   
90 + **.CGGCGCCCCG.** 458 (90.37)   
90 + **.CGCCGCGGCC.** 717 (91.48)   
90 + **.GGCCGCGGCG.** 774 (90.53)   
90 + **.CGCGGCGGCG.** 777 (90.54)   
90 + **.GGCCGCGGCG.** 847 (90.53)   
90 + **[CCTCGCCCCG]** 1042 (87.22)   
90 + **[GGTGGCGGCG]** 1113 (85.61)   
90 - **[CCTGGCGCCG]** 1258 (85.82)   
90 - **[CGCCGCCGCC]** 1253 (86.49)   
90 - **[CGCCGCCGCC]** 1250 (86.49)   
90 - **[CGCCGCCGCC]** 1190 (86.49)   
90 - **[CGCCGCCCCC]** 1187 (85.73)   
90 - **[CGCCGCCGCC]** 1089 (86.49)   
90 - **[CGCCGCCGCC]** 1086 (86.49)   
90 - **[CGCCGCCGCC]** 1083 (86.49)   
90 - **[CGCCGCCTCC]** 1080 (88.09)   
90 - **[CCTCGCCTCG]** 951 (89.58)   
90 - **.CGCCGCGGCC.** 847 (91.48)   
90 - **.CGCCGCCGCG.** 777 (91.94)   
90 - **.CGCCGCGGCC.** 774 (91.48)   
90 - **.GGCCGCGGCG.** 717 (90.53)   
90 - **.CGGCGCGTGG.** 712 (90.87)   
90 - **[CGGGGCGCCG]** 458 (88.97)   
90 - **[CGGTGCGTCG]** 226 (86.78)   
92 + **[CAGCGCGGCG]** 1225 (86.10)   
92 - **[CGCCGCGCTG]** 1225 (85.67)   
93 + **[CGCTGCGGCG]** 462 (85.98)   
93 + **[CGGCGCCTGG]** 467 (85.88)   
93 + **.CGCGGCGGCG.** 1037 (90.54)   
93 + **[CGGGGCGGCG]** 1360 (89.73)   
93 + **[CGGCTCGGCG]** 1394 (85.97)   
93 + **[GGTGGCGGCG]** 1424 (85.61)   
93 - **[CGCCGCCGCC]** 1448 (86.49)   
93 - **[CGCCGAGCCG]** 1394 (85.33)   
93 - **.CGCCGCCCCG.** 1360 (91.18)   
93 - **[CGCCCCGTCC]** 1357 (86.19)   
93 - **.CGCCGCCGCG.** 1037 (91.94)   
93 - **.CGGCGCGTCC.** 1000 (92.28)   
93 - **[CGCCGCAGCG]** 462 (85.90)   
93 - **[CGTCGCCGCC]** 413 (87.96)   
96 + **[GCGCGCGTCG]** 737 (85.90)   
96 + **.CGTCGCGTCG.** 1356 (100.00)   
96 + **[CGCCGTGTCG]** 1424 (86.84)   
96 - **[CCCCGCGTCC]** 1392 (87.66)   
97 + **[CGGCGCCGCC]** 848 (85.68)   
97 + **[CGCCGCCGCC]** 851 (86.49)   
97 + **[CTGCGCGGCG]** 874 (87.85)   
97 + **[CGGCGCGGCT]** 879 (87.49)   
97 + **[CGGAGCGGCG]** 1227 (85.26)   
97 + **[CGTCGCCCGG]** 1443 (85.79)   
97 - **[CGCCGCTCCG]** 1227 (87.24)   
97 - **[AGCCGCGCCG]** 879 (86.83)   
97 - **[CCGCGCCGCG]** 877 (85.70)   
97 - **[CGCCGCGCAG]** 874 (85.94)   
97 - **[GGTGGCGGCG]** 854 (85.61)   
98 + **.GGCCGCGGCG.** 998 (90.53)   
98 + **[CGCGGCGGCC]** 1001 (85.09)   
98 + **[GGTGGCGGCG]** 1339 (85.61)   
98 - **[CGCCGCCGCC]** 1342 (86.49)   
98 - **[CGCCGGCTCG]** 1194 (85.90)   
98 - **[CCCCGCCGCG]** 1137 (86.51)   
98 - **[CCCCGCGCCC]** 1030 (85.30)   
98 - **[GGCCGCCGCG]** 1001 (85.54)   
98 - **.CGCCGCGGCC.** 998 (91.48)   
98 - **[CGCCGCCGCC]** 984 (86.49)   
98 - **[CGCCGCCGCC]** 981 (86.49)   
98 - **[CGCCGCCGCC]** 978 (86.49)   
98 - **[CGCCGCCGCC]** 975 (86.49)   
98 - **[CGCCGCCGCC]** 792 (86.49)   
99 - **[CGCCCCCGCG]** 1336 (85.05)   
99 - **[CCCCGCCCCG]** 1028 (85.75)   
100 + **[CGCCGGGGCG]** 916 (89.29)   
100 + **.GGTCGCGCCG.** 1101 (91.24)   
100 + **.CCCCGCGGCG.** 1497 (91.50)   
100 + **[CGCGGCGGCC]** 1500 (85.09)   
100 - **[GGCCGCCGCG]** 1500 (85.54)   
100 - **.CGCCGCGGGG.** 1497 (90.07)   
100 - **[CGGGGCGTCC]** 1255 (85.89)   
100 - **.CGCCCCGGCG.** 916 (90.04)   
105 - **[CCTCGCGCCC]** 1021 (86.77)   
106 + **[GCTCGCGGCG]** 867 (86.57)   
106 + **[TGTCGCGGCG]** 1157 (87.78)   
108 + **[GGTCGCCTCG]** 385 (88.61)   
108 + **[CCCCGCGTCC]** 1200 (87.66)   
108 - **[CCTGGCGCCG]** 1054 (85.82)   
108 - **[CCCCGCCCCG]** 953 (85.75)   
108 - **[CCTCCCGCCG]** 942 (85.32)   
108 - **[CCTCGCGTCC]** 922 (89.13)   
108 - **[CGTCGGGCCG]** 585 (90.00)   
109 + **[CTTCGCCGCG]** 333 (85.14)   
109 + **.CGCCGCGGCC.** 336 (91.48)   
109 + **[GGGCGCCTCG]** 516 (86.33)   
109 + **[CCTCGCGCCC]** 656 (86.77)   
109 + **[GGTCCCGGCG]** 1011 (85.11)   
109 + **[CGTCCCGCCC]** 1034 (85.30)   
109 + **[GGCGGCGTCG]** 1087 (85.74)   
109 + **.CGACGCGCCG.** 1230 (90.40)   
109 + **[CGTCACGCCG]** 1333 (87.00)   
109 + **[GGGCGCGGCG]** 1388 (89.72)   
109 + **.CGGCGCGGCG.** 1393 (96.12)   
109 + **.CGCGGCGGCG.** 1396 (90.54)   
109 + **[CGGGGCGGCG]** 1468 (89.73)   
109 + **[GGTGGCGGCG]** 1488 (85.61)   
109 - **[CGCCGCCGCC]** 1471 (86.49)   
109 - **.CGCCGCCCCG.** 1468 (91.18)   
109 - **.CGCCGCCGCG.** 1396 (91.94)   
109 - **.CGCCGCGCCG.** 1393 (96.17)   
109 - **[CCGCGCCGCG]** 1391 (85.70)   
109 - **.CGCCGCGCCC.** 1388 (90.72)   
109 - **[CCCCGCGCCC]** 1319 (85.30)   
109 - **[CCCCGCGCCC]** 1312 (85.30)   
109 - **.CGGCGCGTCG.** 1230 (97.72)   
109 - **[CGCCGCCGCC]** 1084 (86.49)   
109 - **[CGCCGCCGCC]** 1081 (86.49)   
109 - **[CGCCGCCGCC]** 1078 (86.49)   
109 - **[CGCCGCCGCC]** 1075 (86.49)   
109 - **[CGCCGGCTCG]** 918 (85.90)   
109 - **[CCGCGCGTCC]** 813 (86.85)   
109 - **[CGGCTCGCCG]** 799 (85.21)   
109 - **.GGCCGCGGCG.** 336 (90.53)   
114 + **[CGCAGCGTCG]** 373 (87.67)   
114 + **.CCGCGCGGCG.** 1001 (90.69)   
114 + **.CGGCGCGGCC.** 1006 (90.67)   
114 + **[GCCCGCGGCG]** 1305 (85.10)   
114 - **[GGCCGCGCCG]** 1006 (89.77)   
114 - **[CCGCGCCGCG]** 1004 (85.70)   
114 - **[CGCCGCGCGG]** 1001 (89.31)   
115 + **[CGGCGCGCGG]** 280 (88.50)   
115 + **.CCCCGCGGCG.** 410 (91.50)   
115 + **[GGGCGCCTCG]** 458 (86.33)   
115 + **.CCTCGCGTCG.** 463 (94.57)   
115 + **[CCCCGCCTCG]** 492 (88.11)   
115 + **.CCCCGCGCCG.** 686 (90.74)   
115 + **[CGCCGCCTCC]** 1011 (88.09)   
115 - **[CGCCGCCGCC]** 990 (86.49)   
115 - **[CGCCGCCGCC]** 987 (86.49)   
115 - **[CGCCGCCGCC]** 984 (86.49)   
115 - **[GCGCGCGTCG]** 869 (85.90)   
115 - **[CGGCGCGGGG]** 686 (89.26)   
115 - **.CGCCGCGGGG.** 410 (90.07)   
115 - **[CCGCGCGCCG]** 280 (89.93)   
116 + **[CGGCGCCGCC]** 1001 (85.68)   
116 + **[CGGCTCGCCG]** 1262 (85.21)   
116 - **[CGTTGCGCCG]** 1160 (86.69)   
116 - **[CGGCGCCGCC]** 999 (85.68)   
117 + **[CGGCGCCTCC]** 455 (87.29)   
117 + **[CGGCGCGCCC]** 666 (89.91)   
117 + **.CGGCGCCGCG.** 788 (91.13)   
117 - **[CGTCCCCTCG]** 950 (88.12)   
117 - **[CGCGGCGCCG]** 788 (89.78)   
117 - **[GGGCGCGCCG]** 666 (88.96)   
117 - **.CGGCGCCTCG.** 196 (92.73)   
118 + **.CGGCGCCGCG.** 764 (91.13)   
118 - **[CGTCCCCTCG]** 940 (88.12)   
118 - **[CGCGGCGCCG]** 764 (89.78)   
118 - **.CGGCGCCTCG.** 98 (92.73)   
119 + **.CGGCGCCGCG.** 762 (91.13)   
119 - **[CGTCCCCTCG]** 931 (88.12)   
119 - **[CGCGGCGCCG]** 762 (89.78)   
119 - **[AGTCGCGCCG]** 612 (88.30)   
119 - **.CGGCGCCTCG.** 96 (92.73)   
120 + **[CGGCGCCTCC]** 861 (87.29)   
120 + **[CGGCGCGCCC]** 1100 (89.91)   
120 + **.CGGCGCCGCG.** 1222 (91.13)   
120 - **[CGTCCCCTCG]** 1398 (88.12)   
120 - **[CGCGGCGCCG]** 1222 (89.78)   
120 - **[GGGCGCGCCG]** 1100 (88.96)   
120 - **.CGGCGCCTCG.** 555 (92.73)   
122 + **[CGCCGCAGCG]** 34 (85.90)   
122 + **[CCCCGCGCCC]** 921 (85.30)   
122 - **[CGCTGCGGCG]** 34 (85.98)   
123 + **[CGCCGCGGAG]** 14 (86.69)   
123 + **[GGGCGCGTCC]** 389 (85.88)   
123 + **[CGTCCCGGCC]** 394 (86.06)   
123 + **[CCCCGCGCCC]** 542 (85.30)   
123 + **[CGCCGCCCCC]** 638 (85.73)   
123 + **[CCCCGCGCCC]** 687 (85.30)   
123 + **.CGCCCCGGCG.** 692 (90.04)   
123 + **.CGGCGCGCCG.** 766 (95.36)   
123 + **[GCCCGCGGCG]** 949 (85.10)   
123 + **[GGTCGCCGCG]** 1001 (87.01)   
123 + **.CGCCGCGCCG.** 1004 (96.17)   
123 + **[CCGCGCCGCG]** 1006 (85.70)   
123 + **.CGCCGCGCCG.** 1009 (96.17)   
123 + **[CCCGGCGTCG]** 1070 (86.71)   
123 + **[CGCCGCCGGG]** 1362 (85.08)   
123 + **.CGCCGGGTCG.** 1365 (90.89)   
123 - **[CCCGGCGGCG]** 1362 (85.11)   
123 - **.CGGCGCGGCG.** 1009 (96.12)   
123 - **.CGGCGCGGCG.** 1004 (96.12)   
123 - **[CCTCGCCGCG]** 952 (87.98)   
123 - **.CGGCGCGCCG.** 766 (95.36)   
123 - **[CGTCGCCGCC]** 752 (87.96)   
123 - **[CGCCGCCGCC]** 749 (86.49)   
123 - **[CGCCGCCCCC]** 737 (85.73)   
123 - **[CGCCGGGGCG]** 692 (89.29)   
123 - **[CTCCGCGGCG]** 14 (88.66)   
124 + **[CGGCGCGGAG]** 867 (85.89)   
124 + **[CGGCGCGGAG]** 879 (85.89)   
124 + **[CGGGGCGTCC]** 1350 (85.89)   
124 - **[CGTCGCTGCG]** 1090 (89.47)   
124 - **[CTCCGCGCCG]** 879 (87.90)   
124 - **[CTCCGCGCCG]** 867 (87.90)   
124 - **.CTTCGCGTCG.** 827 (91.73)   
126 + **[CGCCGCCGCC]** 1037 (86.49)   
126 + **[CGCCGCCGCC]** 1040 (86.49)   
127 + **[CGGCCCGTCC]** 748 (85.39)   
127 + **[CGTCCCGTCC]** 753 (87.67)   
127 + **[CGTCCCGGCC]** 758 (86.06)   
127 + **[CCTCGCCCCG]** 900 (87.22)   
127 - **[CCGCGCCTCG]** 741 (87.31)   
127 - **[CGTGGCGTCC]** 461 (88.16)   
127 - **[CGTCGCCCCC]** 355 (87.20)   
128 + **[GGTCGCGACG]** 935 (85.71)   
128 - **[CGTCGCGACC]** 935 (86.66)   
128 - **[CGCCGCCTGG]** 368 (86.68)   
129 + **[CGCCGCCGGG]** 975 (85.08)   
129 + **[CGGCGCGGGG]** 1143 (89.26)   
129 + **[GGCCGCGCCG]** 1199 (89.77)   
129 + **[CACCGCGCCG]** 1399 (86.15)   
129 - **[CGGCGCGGTG]** 1399 (85.62)   
129 - **.CGGCGCGGCC.** 1199 (90.67)   
129 - **.CCCCGCGCCG.** 1143 (90.74)   
129 - **[CCGCGCCGCG]** 1141 (85.70)   
129 - **[CGGCGTGTCG]** 1010 (86.04)   
129 - **[CCCGGCGGCG]** 975 (85.11)   
129 - **[CCTCGCGCGG]** 958 (85.36)   
129 - **[CCTCGCGCGG]** 896 (85.36)   
131 + **[GGGCGCGCCG]** 30 (88.96)   
131 + **[CGCCGCTCCG]** 35 (87.24)   
131 + **.CGCGGCGGCG.** 1306 (90.54)   
131 + **[GCCCGCGGCG]** 1485 (85.10)   
131 + **.CGCGGCGGCG.** 1488 (90.54)   
131 - **[CGCCGCCGCC]** 1494 (86.49)   
131 - **[CGCCGCCGCC]** 1491 (86.49)   
131 - **.CGCCGCCGCG.** 1488 (91.94)   
131 - **[CGCCGCCGCC]** 1309 (86.49)   
131 - **.CGCCGCCGCG.** 1306 (91.94)   
131 - **[CGCCGCGTGC]** 1303 (86.23)   
131 - **[CGCCGCCGCC]** 899 (86.49)   
131 - **[CGCCGCCCCC]** 552 (85.73)   
131 - **[CGGAGCGGCG]** 35 (85.26)   
131 - **[CGGCGCGCCC]** 30 (89.91)   
132 + **.CGCCGCCGCG.** 706 (91.94)   
132 + **.CGCCGCGTGG.** 709 (91.67)   
132 + **.CCCCGCGCCG.** 1221 (90.74)   
132 - **[CGGCGCGGGG]** 1221 (89.26)   
132 - **[CCACGCGGCG]** 709 (85.73)   
132 - **.CGCGGCGGCG.** 706 (90.54)   
134 + **[CCCCGCCCCG]** 1381 (85.75)   
134 - **[CGCCGCCTGG]** 1076 (86.68)   
134 - **[CGCCCCCTCG]** 580 (86.65)   
135 + **[AGCCGCGGCG]** 362 (87.59)   
135 + **[CCTCGCCGCG]** 784 (87.98)   
135 + **[CGCCGCGTGC]** 787 (86.23)   
135 + **[CGCCGCCGCC]** 1205 (86.49)   
135 + **[CGGCGGGGCG]** 1473 (88.48)   
135 + **[CGGGGCGCCG]** 1476 (88.97)   
135 + **[CCCCGCCGCG]** 1496 (86.51)   
135 - **.CGGCGCCCCG.** 1476 (90.37)   
135 - **[CGCCCCGCCG]** 1473 (89.28)   
135 - **[CGCCGCCGCC]** 1282 (86.49)   
135 - **[CGCCGCCGCC]** 1279 (86.49)   
135 - **[CGCCGCCTCC]** 1109 (88.09)   
135 - **[CGCCGCGGCT]** 362 (88.30)   
135 - **[CCGCGCGTGG]** 352 (85.44)   
136 + **[CGCCACGGCG]** 609 (86.28)   
136 + **[CGGCGCGGAG]** 935 (85.89)   
136 + **[CGGCGCGGCA]** 1038 (85.80)   
136 + **[CTGCGCGCCG]** 1062 (87.09)   
136 + **[CCGCGCGGCC]** 1299 (85.25)   
136 - **[CCGCGCGGCC]** 1297 (85.25)   
136 - **[CCTCGGGGCG]** 1217 (85.33)   
136 - **[CGGCGCGCAG]** 1062 (85.13)   
136 - **[TGCCGCGCCG]** 1038 (85.55)   
136 - **[CTCCGCGCCG]** 935 (87.90)   
136 - **[CGCCGTGGCG]** 609 (85.24)   
137 + **[CGCGGCGGCC]** 945 (85.09)   
137 - **[GGCCGCCGCG]** 945 (85.54)   
137 - **.CGCCGCGTCC.** 942 (93.08)   
137 - **[CGTCCCGCCC]** 937 (85.30)   
138 + **[CGCCGCCGCC]** 177 (86.49)   
138 + **[CGGCGCGCGG]** 574 (88.50)   
138 + **[GCGCGCGTCG]** 598 (85.90)   
138 + **[GGGCGCGGCG]** 688 (89.72)   
138 + **.CGCGGCGGCG.** 691 (90.54)   
138 + **[GCCCGCGGCG]** 760 (85.10)   
138 + **.CGGCGCGGCG.** 765 (96.12)   
138 + **[CGCCGCCGCC]** 825 (86.49)   
138 + **[CGCCGCCGCC]** 828 (86.49)   
138 + **[CGCCGCCGCC]** 831 (86.49)   
138 + **.CGCCGCCGCG.** 834 (91.94)   
138 + **[CGCCGCGCCA]** 837 (85.85)   
138 - **[TGGCGCGGCG]** 837 (85.50)   
138 - **.CGCGGCGGCG.** 834 (90.54)   
138 - **.CGCCGCGCCG.** 765 (96.17)   
138 - **[CCGCGCCGCG]** 763 (85.70)   
138 - **.CGCCGCCGCG.** 691 (91.94)   
138 - **.CGCCGCGCCC.** 688 (90.72)   
138 - **[CCGCGCGCCG]** 574 (89.93)   
138 - **[CCGCGCCGCG]** 367 (85.70)   
138 - **.CGCCGCGTCC.** 364 (93.08)   
139 + **[CGGCGCGGAG]** 689 (85.89)   
139 + **[CCGCGCGCCG]** 829 (89.93)   
139 + **[CGCCGCCGCC]** 834 (86.49)   
139 + **[CGCCGCCCCC]** 837 (85.73)   
139 + **[CCCCGCGTCC]** 844 (87.66)   
139 + **[CGTCGCCCCC]** 913 (87.20)   
139 + **[CAGCGCGGCG]** 957 (86.10)   
139 - **[CGTCGCCCGG]** 1381 (85.79)   
139 - **[CGCCGCGCTG]** 957 (85.67)   
139 - **[CGGCGCGCGG]** 829 (88.50)   
139 - **[CTCCGCGCCG]** 689 (87.90)   
139 - **[CGCCGCCCCC]** 684 (85.73)   
140 + **[CGCCGCAGCG]** 684 (85.90)   
140 + **[CGCGGCGCCG]** 730 (89.78)   
140 + **[CGGCGCCGCC]** 732 (85.68)   
140 + **[CGCCGCCGCC]** 1086 (86.49)   
140 + **[CGCCGCCGCC]** 1089 (86.49)   
140 + **[CGCCGCCGCC]** 1092 (86.49)   
140 + **[CGCCGCCGCC]** 1095 (86.49)   
140 + **[GGCCGCCGCG]** 1189 (85.54)   
140 + **.CGCCGCGCCG.** 1192 (96.17)   
140 + **[AGCCGCGGCG]** 1245 (87.59)   
140 + **[CGGCGGGGCG]** 1407 (88.48)   
140 - **[CGCCGCCGCC]** 1486 (86.49)   
140 - **[CGCCGCCGCC]** 1463 (86.49)   
140 - **[CGCCGCCGCC]** 1460 (86.49)   
140 - **[GGTCGCCCCG]** 1410 (86.25)   
140 - **[CGCCCCGCCG]** 1407 (89.28)   
140 - **[GGCGGCGTCG]** 1366 (85.74)   
140 - **[CGCCGCGGCT]** 1245 (88.30)   
140 - **.CGGCGCGGCG.** 1192 (96.12)   
140 - **[CGCGGCGGCC]** 1189 (85.09)   
140 - **.CGGCGCCGCG.** 730 (91.13)   
140 - **[CGCTGCGGCG]** 684 (85.98)   
140 - **[GCCCGCGGCG]** 656 (85.10)   
140 - **.CGCCGGGTCG.** 637 (90.89)   
141 + **.CGCCGCGCCC.** 609 (90.72)   
141 + **[CGCGGCCGCG]** 684 (85.55)   
141 + **.GGCCGCGGCG.** 1155 (90.53)   
141 + **[GGCCGCGCCG]** 1182 (89.77)   
141 + **[CCGCGCCGCG]** 1184 (85.70)   
141 - **[CCTCGCGTCC]** 1388 (89.13)   
141 - **[CGTCCCCGCG]** 1383 (86.52)   
141 - **.CGGCGCGGCC.** 1182 (90.67)   
141 - **.CGCCGCGGCC.** 1155 (91.48)   
141 - **[CAGCGCGCCG]** 946 (85.34)   
141 - **[CGCGGCCGCG]** 684 (85.55)   
141 - **[GGGCGCGGCG]** 609 (89.72)   
142 + **[CTCCGCGCCG]** 510 (87.90)   
142 + **[CCGCGCCGCG]** 512 (85.70)   
142 + **[CGCCGCGGCT]** 515 (88.30)   
142 + **[CTGCGCGGCG]** 660 (87.85)   
142 + **[CGCTGCGCCG]** 775 (85.22)   
142 + **[CCCCGCGGCC]** 943 (86.05)   
142 + **.CCTCGCGCCG.** 955 (92.21)   
142 + **[CCTCGCCGCG]** 1002 (87.98)   
142 + **[CGCCGCGCGG]** 1005 (89.31)   
142 + **[CCGCGCGGCC]** 1007 (85.25)   
142 + **[CGCCGCCGCC]** 1024 (86.49)   
142 + **.CGGCGCCCCG.** 1114 (90.37)   
142 + **[GGCCGCGCCG]** 1271 (89.77)   
142 + **.CGCCGCCTCG.** 1316 (93.54)   
142 + **[CCTCGGGGCG]** 1331 (85.33)   
142 + **[CCCCGCGCCC]** 1465 (85.30)   
142 - **[CGTCGCTTCC]** 1488 (85.62)   
142 - **[CCCCGGGTCG]** 1439 (85.46)   
142 - **[CGCCCCCTCG]** 1420 (86.65)   
142 - **[CCTCGCGCGG]** 1415 (85.36)   
142 - **.CGGCGCGGCC.** 1271 (90.67)   
142 - **[CGGGGCGCCG]** 1114 (88.97)   
142 - **[CGGCGGCTCG]** 1019 (85.09)   
142 - **.CCGCGCGGCG.** 1005 (90.69)   
142 - **[CGGCGCAGCG]** 775 (85.09)   
142 - **[CCCCGCCGCG]** 663 (86.51)   
142 - **[CGCCGCGCAG]** 660 (85.94)   
142 - **[AGCCGCGGCG]** 515 (87.59)   
142 - **[CGGCGCGGAG]** 510 (85.89)   
142 - **.GGTCGCGTCG.** 446 (93.60)   
142 - **[CGTCGCCCGG]** 441 (85.79)   
144 + **[CGGCGCCGCC]** 441 (85.68)   
144 + **[CCGCGCGTCC]** 771 (86.85)   
144 + **.CGGCGCCGCG.** 1014 (91.13)   
144 + **[CGCCGCGCGG]** 1017 (89.31)   
144 + **[CGGGGCGGCG]** 1024 (89.73)   
144 + **.CGGCGCGGCG.** 1139 (96.12)   
144 + **.CGGCGCGGCG.** 1144 (96.12)   
144 + **.CGGCGCGGCC.** 1191 (90.67)   
144 + **[CGCGGCCGCG]** 1194 (85.55)   
144 + **[CGGCCCGTCC]** 1285 (85.39)   
144 + **[CGCTGCGCCG]** 1485 (85.22)   
144 + **.CGCCGCGCCC.** 1490 (90.72)   
144 - **[GGGCGCGGCG]** 1490 (89.72)   
144 - **[CGGCGCAGCG]** 1485 (85.09)   
144 - **[CGCGGCCGCG]** 1194 (85.55)   
144 - **[GGCCGCGCCG]** 1191 (89.77)   
144 - **[CCGCGCCGCG]** 1189 (85.70)   
144 - **.CGCCGCGTCC.** 1186 (93.08)   
144 - **[CCGCGCCGCG]** 1147 (85.70)   
144 - **.CGCCGCGCCG.** 1144 (96.17)   
144 - **[CCGCGCCGCG]** 1142 (85.70)   
144 - **.CGCCGCGCCG.** 1139 (96.17)   
144 - **[CGCCGCCGCC]** 1027 (86.49)   
144 - **.CGCCGCCCCG.** 1024 (91.18)   
144 - **.CCGCGCGGCG.** 1017 (90.69)   
144 - **[CGCGGCGCCG]** 1014 (89.78)   
144 - **[CGGCGCCGCC]** 1012 (85.68)   
145 + **[CGGCGGGGCG]** 546 (88.48)   
145 + **[CGCCGCGCGG]** 1136 (89.31)   
145 + **.CCGCGCGGCG.** 1138 (90.69)   
145 + **[GGGCGCGCCG]** 1147 (88.96)   
145 - **[CGGCGCGCCC]** 1147 (89.91)   
145 - **[CGCCGCGCGG]** 1138 (89.31)   
145 - **.CCGCGCGGCG.** 1136 (90.69)   
145 - **[CGCCGGGTCC]** 814 (85.44)   
145 - **[CGCCGCCTGG]** 572 (86.68)   
145 - **[CCCCGCCCCG]** 549 (85.75)   
145 - **[CGCCCCGCCG]** 546 (89.28)   
147 + **[CGACGCGTCC]** 861 (87.31)   
147 + **.CGTCGCGGGG.** 962 (91.54)   
147 + **[GGCGGCGTCG]** 1335 (85.74)   
147 - **.CGCCGCCTCG.** 1077 (93.54)   
147 - **[CCCCGCGACG]** 962 (85.21)   
147 - **[GGACGCGTCG]** 861 (86.36)   
147 - **.CGTCGGGGCG.** 856 (90.76)   
148 + **[CGGCGCCTCC]** 218 (87.29)   
148 + **[CGGCGCCTCC]** 627 (87.29)   
148 + **[CGTCGCCTCC]** 1396 (89.56)   
148 - **[CGCGGCGTCC]** 824 (86.69)   
150 + **[CCGCGCGCCG]** 115 (89.93)   
150 + **.CGCCGCGCCC.** 120 (90.72)   
150 + **[CGCGGCGTGG]** 191 (85.28)   
150 + **[CGTGGCGCCC]** 196 (85.80)   
150 + **[CCTCCCGTCG]** 687 (87.68)   
150 + **[CGCCGGGTCC]** 1023 (85.44)   
150 + **[CCTCCCGGCG]** 1384 (86.08)   
150 - **[CGCCACGCCG]** 193 (85.53)   
150 - **[GGGCGCGGCG]** 120 (89.72)   
150 - **[CGGCGCGCGG]** 115 (88.50)   
151 + **[CCTCGCCCCG]** 765 (87.22)   
151 + **[CGGCGCCTCC]** 842 (87.29)   
151 + **[GGGCGCGGCG]** 881 (89.72)   
151 + **.CCCCGCGCCG.** 1203 (90.74)   
151 + **.CGGCGCGTGG.** 1460 (90.87)   
151 + **[CCTGGCGGCG]** 1486 (86.58)   
151 + **[CGGCGCGCGG]** 1491 (88.50)   
151 - **[CCGCGCGCCG]** 1491 (89.93)   
151 - **[CCCCGCGTGG]** 1420 (86.25)   
151 - **[CGGCGCGGGG]** 1203 (89.26)   
151 - **.CGCCGCGCCC.** 881 (90.72)   
151 - **[CGCGGCGTGG]** 811 (85.28)   
151 - **[CCTCCCGTCG]** 314 (87.68)   
154 + **[GGGCGCGGCG]** 1037 (89.72)   
154 + **.CGCGGCGGCG.** 1040 (90.54)   
154 + **[CCTGGCGCCG]** 1173 (85.82)   
154 + **[CGCCGCCGCC]** 1249 (86.49)   
154 + **[CTCCGCGCCG]** 1429 (87.90)   
154 + **[CCGCGCGCCG]** 1463 (89.93)   
154 + **[CCGCGCGGCC]** 1509 (85.25)   
154 - **[CGGCGCGCGG]** 1463 (88.50)   
154 - **[CGGCGCGGAG]** 1429 (85.89)   
154 - **[GCTCGCGGCG]** 1178 (86.57)   
154 - **[CGCCGCCGCC]** 1043 (86.49)   
154 - **.CGCCGCCGCG.** 1040 (91.94)   
154 - **.CGCCGCGCCC.** 1037 (90.72)   
154 - **[CGCCGCCCCC]** 1019 (85.73)   
154 - **[CCCCGCGCCC]** 997 (85.30)   
155 + **[CGGCGCGGTG]** 506 (85.62)   
155 + **[CGTCGCGTTG]** 1256 (89.50)   
155 - **[CACCGCGCCG]** 506 (86.15)   
158 + **.GGCCGCGTCG.** 1176 (92.13)   
158 - **[CGACGCGGCC]** 1176 (85.71)   
158 - **[CGCGGCCTCG]** 1173 (87.15)   
160 + **[CGCCGCGGAG]** 265 (86.69)   
160 + **.CCCCGCGCCG.** 1203 (90.74)   
160 - **[CGGCGCGGGG]** 1203 (89.26)   
160 - **[CGCCGCCTCC]** 988 (88.09)   
160 - **[CTCCGCGGCG]** 265 (88.66)   
160 - **[CCCCGCCCCG]** 52 (85.75)   
161 + **.CGTCGGGGCG.** 454 (90.76)   
161 + **.CGCCGCGCCC.** 893 (90.72)   
161 + **[GGTGGCGGCG]** 978 (85.61)   
161 + **[CCTCGCGCCC]** 1188 (86.77)   
161 + **[GGTCGCGGCC]** 1218 (86.55)   
161 + **[GCCCGCGGCG]** 1265 (85.10)   
161 + **.CGCGGCGGCG.** 1268 (90.54)   
161 + **[CGCCGCCGCC]** 1422 (86.49)   
161 + **.CCCCGCGCCG.** 1478 (90.74)   
161 + **[CGCCGCCGCC]** 1483 (86.49)   
161 + **[CGCCGCCGCC]** 1486 (86.49)   
161 + **[CGCCGCCGCC]** 1489 (86.49)   
161 + **[CGCCGCCGCC]** 1501 (86.49)   
161 + **[CGCCGCCGCC]** 1504 (86.49)   
161 - **[CGGCGCGGGG]** 1478 (89.26)   
161 - **[GGTGGCGGCG]** 1425 (85.61)   
161 - **.CGCCGCCGCG.** 1268 (91.94)   
161 - **[CGCCGCCGCC]** 984 (86.49)   
161 - **[CGCCGCCGCC]** 981 (86.49)   
161 - **[GGGCGCGGCG]** 893 (89.72)   
161 - **.GGTCGCGCCG.** 751 (91.24)   
164 + **[CGTCGCGCTG]** 272 (87.14)   
164 + **[CGCAGCGGCG]** 303 (86.06)   
164 + **.CGGCGCGGCG.** 1096 (96.12)   
164 + **[CGCGGCGCCG]** 1099 (89.78)   
164 + **[CGGCGCCGCC]** 1101 (85.68)   
164 + **[CGCTGCGGCG]** 1199 (85.98)   
164 - **[CGCCGCAGCG]** 1199 (85.90)   
164 - **.CGGCGCCGCG.** 1099 (91.13)   
164 - **.CGCCGCGCCG.** 1096 (96.17)   
164 - **[CGCCGCTGCG]** 303 (88.00)   
166 + **[CCCCGCCTCG]** 923 (88.11)   
166 + **[CGAGGCGTCG]** 958 (86.37)   
166 + **[CGGCGCGCGG]** 1224 (88.50)   
166 + **[CGCGGCGCCG]** 1312 (89.78)   
166 - **.CGGCGCCGCG.** 1312 (91.13)   
166 - **[CGCCGCGTGC]** 1309 (86.23)   
166 - **[CCGCGCGCCG]** 1224 (89.93)   
166 - **[CGCCGCGTGC]** 1156 (86.23)   
166 - **[CGACGCCTCG]** 958 (87.77)   
167 + **[CGGCGGGGCG]** 845 (88.48)   
167 + **[CCTCGCGGCC]** 953 (87.52)   
167 + **[CCCCGCCCCG]** 1022 (85.75)   
167 + **[CGCCGCCGCC]** 1395 (86.49)   
167 + **.CGCCGCCCCG.** 1398 (91.18)   
167 + **[CGTCCCGGCC]** 1465 (86.06)   
167 - **[CGGGGCGGCG]** 1398 (89.73)   
167 - **[CCCCGCCCCG]** 848 (85.75)   
167 - **[CGCCCCGCCG]** 845 (89.28)   
167 - **[CCCCGCCGCG]** 843 (86.51)   
167 - **.CCTCGCGCCG.** 817 (92.21)   
167 - **[CCCCGCGCCC]** 800 (85.30)   
167 - **[CCCCGCCTCG]** 792 (88.11)   
167 - **[CCTCGGGTCG]** 787 (86.93)   
168 + **[CGCCGCGCGG]** 908 (89.31)   
168 + **[CGCCCCCGCG]** 1243 (85.05)   
168 - **[CGCCGCCTCC]** 916 (88.09)   
168 - **.CCGCGCGGCG.** 908 (90.69)   
168 - **[CCCCGCGCCC]** 731 (85.30)   
169 + **[CGCCGCGCTG]** 110 (85.67)   
169 - **[CAGCGCGGCG]** 110 (86.10)   
171 + **[CCACGCGGCG]** 771 (85.73)   
171 + **[CGCGGCGTGG]** 774 (85.28)   
171 - **.CGCCGCGTGG.** 771 (91.67)   
172 + **[CGCCTCGCCG]** 300 (86.01)   
172 + **[CGCCGCCGCC]** 305 (86.49)   
172 + **.CGCCGCCCCG.** 308 (91.18)   
172 + **[CGGCCCGTCC]** 361 (85.39)   
172 + **[CGCCGCCGCC]** 521 (86.49)   
172 + **[CGCCGCCGCC]** 524 (86.49)   
172 + **[CGCCGCCGCC]** 527 (86.49)   
172 + **[CGCCGCCGCC]** 530 (86.49)   
172 + **[CGCCGCCGCC]** 533 (86.49)   
172 + **[CGCCGCCGCC]** 536 (86.49)   
172 + **.CGTCGCGCCC.** 548 (92.19)   
172 + **[CCTCCCGTCG]** 597 (87.68)   
172 + **[CGTCGGCCCG]** 602 (85.01)   
172 + **[CGCCGCTGCG]** 627 (88.00)   
172 + **[CGGCGCGGAG]** 1159 (85.89)   
172 + **[CGCCGCAGCG]** 1186 (85.90)   
172 - **[CGCTGCGGCG]** 1186 (85.98)   
172 - **[CTCCGCGCCG]** 1159 (87.90)   
172 - **[CGCCGCCGCC]** 914 (86.49)   
172 - **[CCTCGCCCCG]** 859 (87.22)   
172 - **[GGCGGCGTCG]** 718 (85.74)   
172 - **.CGTCGCGGCC.** 713 (92.95)   
172 - **[CGCAGCGGCG]** 627 (86.06)   
172 - **.CGCCGCGTCC.** 428 (93.08)   
172 - **[CGGGGCGGCG]** 308 (89.73)   
172 - **[CGGCGAGGCG]** 300 (85.28)   
173 - **[CCCCGCGCCC]** 1100 (85.30)   
173 - **[CGCCGCCGCC]** 1025 (86.49)   
173 - **[CGCCGCCGCC]** 1022 (86.49)   
173 - **[CGCCGCCTCC]** 1019 (88.09)   
173 - **.CGCCGGGTCG.** 894 (90.89)   
174 + **.CGCCGCGTCC.** 1184 (93.08)   
174 + **[GCCCGCGGCG]** 1474 (85.10)   
174 + **[CGCCGAGGCG]** 1499 (86.08)   
174 - **[CCGCGCCTCG]** 1502 (87.31)   
174 - **[CGCCTCGGCG]** 1499 (86.77)   
174 - **[CCGCGCCGCG]** 1477 (85.70)   
174 - **[CGTCGCTTCC]** 1365 (85.62)   
174 - **[CGTCGCTGCG]** 1322 (89.47)   
174 - **[CCTCGCGGCC]** 1144 (87.52)   
174 - **[CCTCGCGCCC]** 395 (86.77)   
175 + **[CGCCGCGGCT]** 1465 (88.30)   
175 - **[AGCCGCGGCG]** 1465 (87.59)   
177 + **[CCTCGCCGCG]** 948 (87.98)   
177 + **[CGTCGCCGCC]** 1017 (87.96)   
177 + **[CGCCGCCGCC]** 1020 (86.49)   
177 + **[CGCCGCCGCC]** 1023 (86.49)   
177 + **[CGCCGCCGCC]** 1026 (86.49)   
177 + **.CGCCGCCTCG.** 1029 (93.54)   
177 - **[GGTCGCCGCG]** 1465 (87.01)   
177 - **[GCTCGCGGCG]** 951 (86.57)   
177 - **[CGCCGCCGCC]** 750 (86.49)   
177 - **.CGCCGCCTCG.** 747 (93.54)   
178 + **[CGGCGCAGCG]** 760 (85.09)   
178 + **[CCTCGCGGGG]** 1236 (86.11)   
178 - **.CGCCGCGTCC.** 1111 (93.08)   
178 - **[CGCTGCGCCG]** 760 (85.22)   
179 + **[CACCGCGCCG]** 855 (86.15)   
179 + **[CGGCGCTCCG]** 875 (86.43)   
179 + **[CGGCTCGCCG]** 910 (85.21)   
179 + **.CGCCGCGGCC.** 915 (91.48)   
179 + **[GGTCCCGGCG]** 931 (85.11)   
179 + **[CGGCGCGGGG]** 985 (89.26)   
179 + **[CCTCGCGTCC]** 1201 (89.13)   
179 + **[GGCCGCCGCG]** 1470 (85.54)   
179 + **[CGCCGCGCCA]** 1473 (85.85)   
179 - **[TGGCGCGGCG]** 1473 (85.50)   
179 - **[CGCGGCGGCC]** 1470 (85.09)   
179 - **[CGTCCCCGCG]** 988 (86.52)   
179 - **.CCCCGCGCCG.** 985 (90.74)   
179 - **[CGCCGCCGCC]** 980 (86.49)   
179 - **[CGCCGCCGCC]** 977 (86.49)   
179 - **[CGCCGCCGCC]** 974 (86.49)   
179 - **[CGCCGCCGCC]** 944 (86.49)   
179 - **[CGCCGCCGCC]** 941 (86.49)   
179 - **.GGCCGCGGCG.** 915 (90.53)   
179 - **[CGGCGCGGTG]** 855 (85.62)   
180 + **[AGGCGCGGCG]** 1062 (86.78)   
180 + **[CGCGGCGGCC]** 1065 (85.09)   
180 + **[GCTCGCGGCG]** 1244 (86.57)   
180 + **.CGCGGCGGCG.** 1247 (90.54)   
180 - **[CGCCGGGTCC]** 1268 (85.44)   
180 - **.CGCCGCCGCG.** 1247 (91.94)   
180 - **[GGCCGCCGCG]** 1065 (85.54)   
180 - **[CGCCGCGCCT]** 1062 (87.54)   
180 - **[CGCCGCCGCC]** 1041 (86.49)   
180 - **[CCTCGCTTCG]** 285 (85.64)   
182 + **[GGCCGCGGCC]** 374 (85.08)   
182 + **.GGCCGCGGCG.** 460 (90.53)   
182 + **[CCCCGCCCCG]** 660 (85.75)   
182 + **[GGCCGCCTCG]** 682 (87.14)   
182 + **.CGGCGCGTCG.** 873 (97.72)   
182 + **[GGTCGCGTCC]** 917 (88.16)   
182 + **[GCTCGCGCCG]** 1160 (85.81)   
182 - **.CGTCGCCCCG.** 1252 (92.65)   
182 - **[CGGCCCCTCG]** 1119 (85.84)   
182 - **.CGACGCGCCG.** 873 (90.40)   
182 - **[CCGCGCGGCC]** 528 (85.25)   
182 - **[CCGCGCCGCG]** 463 (85.70)   
182 - **.CGCCGCGGCC.** 460 (91.48)   
182 - **[CCCCGCGCCC]** 431 (85.30)   
182 - **[CGCCGCCCCC]** 392 (85.73)   
182 - **[CCCCGCGCCC]** 385 (85.30)   
182 - **[GGCCGCGGCC]** 374 (85.08)   
182 - **[CCCCGCGTGG]** 366 (86.25)   
183 + **[GGTGGCGGCG]** 601 (85.61)   
183 + **[CGGCGCGCGG]** 982 (88.50)   
183 + **[CTGCGCGGCG]** 1058 (87.85)   
183 + **[CGCGGCGGCC]** 1061 (85.09)   
183 + **[CGCCGCCGCC]** 1180 (86.49)   
183 + **[CGCCGCCGCC]** 1183 (86.49)   
183 + **[CGCCGGGCCG]** 1224 (88.53)   
183 + **[GGCCGCGTCC]** 1298 (86.69)   
183 + **[CGCCGCCGCC]** 1408 (86.49)   
183 - **[CGGCCCGGCG]** 1224 (89.23)   
183 - **[CGTCCCGCCC]** 1140 (85.30)   
183 - **[GGCCGCCGCG]** 1061 (85.54)   
183 - **[CGCCGCGCAG]** 1058 (85.94)   
183 - **[CCGCGCGCCG]** 982 (89.93)   
183 - **[CGCCGCCGCC]** 977 (86.49)   
184 + **[GGCCGCCGCG]** 50 (85.54)   
184 + **.CCGCGCGGCG.** 925 (90.69)   
184 + **[CGCCGCCTCC]** 983 (88.09)   
184 + **[AGGCGCGCCG]** 1084 (86.02)   
184 + **[CGCCGCGTGC]** 1089 (86.23)   
184 + **.CGCCGCGCCC.** 1164 (90.72)   
184 + **[GGGCGCGGCG]** 1349 (89.72)   
184 + **[CGCGGCGGCC]** 1352 (85.09)   
184 - **[GGCCGCCGCG]** 1352 (85.54)   
184 - **.CGCCGCGCCC.** 1349 (90.72)   
184 - **[GGGCGCGGCG]** 1164 (89.72)   
184 - **[CGGCGCGCCT]** 1084 (86.73)   
184 - **[CCCCGCCCCG]** 952 (85.75)   
184 - **[CGCCGCGCGG]** 925 (89.31)   
184 - **[CCCCGCCCCG]** 681 (85.75)   
184 - **[CGCGGCGGCC]** 50 (85.09)   
185 + **[CCCCGCGCCC]** 6 (85.30)   
185 + **.CGGCGCCCCG.** 15 (90.37)   
185 + **[CGCCGCTGCG]** 223 (88.00)   
185 + **[CGCTGCGCCG]** 226 (85.22)   
185 + **[CGCCGCCGCC]** 894 (86.49)   
185 + **[CGCCGCCGCC]** 897 (86.49)   
185 + **[CGCCGCCGCC]** 900 (86.49)   
185 + **[CGCCGCCGCC]** 903 (86.49)   
185 + **[CGCCGCCGCC]** 906 (86.49)   
185 + **[CGCCGCCGCC]** 909 (86.49)   
185 + **[CGCCGCCGCC]** 912 (86.49)   
185 + **[CGGCGCCGCC]** 1143 (85.68)   
185 + **[CGCCGCCGCC]** 1146 (86.49)   
185 + **[CGCCGCCCCC]** 1149 (85.73)   
185 + **.CGCCGCGGCG.** 1189 (96.93)   
185 + **.CGCGGCGGCG.** 1192 (90.54)   
185 - **.CGCCGCCGCG.** 1192 (91.94)   
185 - **.CGCCGCGGCG.** 1189 (96.93)   
185 - **[CGGCGCAGCG]** 226 (85.09)   
185 - **[CGCAGCGGCG]** 223 (86.06)   
185 - **[CGTCGCTGCG]** 215 (89.47)   
185 - **[CGGGGCGCCG]** 15 (88.97)   
186 + **[CTCCGCGCCG]** 1009 (87.90)   
186 + **[CGCAGCGGCG]** 1398 (86.06)   
186 - **[CGCCGCTGCG]** 1398 (88.00)   
186 - **[CGGCGCGGAG]** 1009 (85.89)   
187 + **[GGTCGCGGGG]** 1234 (85.14)   
187 - **[CCCCGCCCCG]** 1403 (85.75)   
187 - **.CGTCGGGGCG.** 863 (90.76)   
188 + **[CGCTGCGGCG]** 212 (85.98)   
188 + **[CGCCCCGCCG]** 302 (89.28)   
188 + **[CCCCGCCCCG]** 531 (85.75)   
188 + **[CGCTGCGGCG]** 1382 (85.98)   
188 + **[CGGCGCCGCC]** 1387 (85.68)   
188 + **[CGCCGCCGCC]** 1390 (86.49)   
188 + **[CTGCGCGCCG]** 1437 (87.09)   
188 + **[CGCCGCGGCT]** 1442 (88.30)   
188 + **.CGCCGCGCCG.** 1469 (96.17)   
188 - **.CGGCGCGGCG.** 1469 (96.12)   
188 - **[AGCCGCGGCG]** 1442 (87.59)   
188 - **[CGGCGCGCAG]** 1437 (85.13)   
188 - **[CGCCGCAGCG]** 1382 (85.90)   
188 - **[CGGCGGGGCG]** 302 (88.48)   
188 - **[CGCCGCAGCG]** 212 (85.90)   
189 + **[CGGCGCGTGC]** 848 (85.42)   
189 + **[CGGCGCAGCG]** 1173 (85.09)   
189 - **[CGCTGCGCCG]** 1173 (85.22)   
191 + **[CCTCGGGGCG]** 91 (85.33)   
191 + **[CGCGGCCGCG]** 827 (85.55)   
191 + **.CGGCGCGGCG.** 1042 (96.12)   
191 - **[CTTCGCGCCG]** 1136 (89.37)   
191 - **.CGCCGCGCCG.** 1042 (96.17)   
191 - **[CGCCGCCTCC]** 1024 (88.09)   
191 - **[CGCCGCCGCC]** 1009 (86.49)   
191 - **[CGCCGCCGCC]** 1006 (86.49)   
191 - **[CGCCGCCGCC]** 1003 (86.49)   
191 - **[CGCGGCCGCG]** 827 (85.55)   
191 - **[CCTCGCGCGG]** 204 (85.36)   
191 - **[CCTCGCGCCC]** 96 (86.77)   
193 + **[CAGCGCGGCG]** 1246 (86.10)   
193 + **[CGCGGCGCCG]** 1249 (89.78)   
193 - **.CGGCGCCGCG.** 1249 (91.13)   
193 - **[CGCCGCGCTG]** 1246 (85.67)   
194 + **[CGGGGCGTCC]** 179 (85.89)   
194 + **.CGGCGCGGCG.** 942 (96.12)   
194 + **.GGCCGCGGCG.** 987 (90.53)   
194 + **.GGCCGCGGCG.** 1109 (90.53)   
194 - **[CCGCGCCGCG]** 1112 (85.70)   
194 - **.CGCCGCGGCC.** 1109 (91.48)   
194 - **[CAGCGCGCCG]** 1015 (85.34)   
194 - **.CGCCGCGGCC.** 987 (91.48)   
194 - **.CGCCGCGCCG.** 942 (96.17)   
194 - **[CCGCGCGTCC]** 907 (86.85)   
194 - **[CCCCGCCCCG]** 298 (85.75)   
195 + **[CGCCGCGGCT]** 165 (88.30)   
195 + **[CCCCGCGCCC]** 248 (85.30)   
195 + **.CGGGGCGTCG.** 443 (91.33)   
195 + **[CGTCGCCCGG]** 448 (85.79)   
195 + **[AGTCGCGGCG]** 849 (89.06)   
195 + **[CGTCGCCCGG]** 945 (85.79)   
195 + **[CCGCGCGTCC]** 957 (86.85)   
195 - **[CCTGGCGGCG]** 1223 (86.58)   
195 - **[CGACGCCCCG]** 443 (85.41)   
195 - **[CCCCGCCTCG]** 308 (88.11)   
195 - **[AGCCGCGGCG]** 165 (87.59)   
196 + **[CAGCGCGGCG]** 117 (86.10)   
196 + **[CGCGGCGGCC]** 120 (85.09)   
196 + **[CGGCGGGGCG]** 262 (88.48)   
196 + **[CGGGGCGGCG]** 265 (89.73)   
196 + **.CGCGGCGTCG.** 284 (92.14)   
196 + **[CGCCGCGCAG]** 341 (85.94)   
196 + **[CCTCCCGGCG]** 463 (86.08)   
196 + **[GGGCGCGCCG]** 503 (88.96)   
196 + **[CGCCGCGCCA]** 910 (85.85)   
196 + **[CGCCGCCGGG]** 1022 (85.08)   
196 + **[CGGCGCGCAG]** 1351 (85.13)   
196 + **.CGCCCCGGCG.** 1465 (90.04)   
196 + **[CGGCGCGGGG]** 1470 (89.26)   
196 - **[CGCCGGGTCC]** 1491 (85.44)   
196 - **.CCCCGCGCCG.** 1470 (90.74)   
196 - **[CGCCGGGGCG]** 1465 (89.29)   
196 - **[CTGCGCGCCG]** 1351 (87.09)   
196 - **[CGGCGCCTCC]** 1325 (87.29)   
196 - **[CCCGGCGGCG]** 1022 (85.11)   
196 - **[TGGCGCGGCG]** 910 (85.50)   
196 - **[CCTCGCGCCC]** 665 (86.77)   
196 - **[CGGCGCGCCC]** 503 (89.91)   
196 - **[CCCCGCGTGG]** 496 (86.25)   
196 - **[CTGCGCGGCG]** 341 (87.85)   
196 - **[CGACGCCGCG]** 284 (86.17)   
196 - **[CCTCCCGCCG]** 270 (85.32)   
196 - **.CGCCGCCCCG.** 265 (91.18)   
196 - **[CGCCCCGCCG]** 262 (89.28)   
196 - **[GGCCGCCGCG]** 120 (85.54)   
196 - **[CGCCGCGCTG]** 117 (85.67)   
197 + **[CGCCGCCTCC]** 210 (88.09)   
197 + **.CTTCGCGTCG.** 428 (91.73)   
197 + **[AGGCGCGGCG]** 1503 (86.78)   
197 - **[CCGCGCCGCG]** 1506 (85.70)   
197 - **[CGCCGCGCCT]** 1503 (87.54)   
197 - **.CGCCGCGTCC.** 1160 (93.08)   
197 - **.CGCCGGGTCG.** 1151 (90.89)   
197 - **[CCTCCCGCCG]** 1074 (85.32)   
197 - **[CCCCGCCTCG]** 1002 (88.11)   
197 - **[CCTCCCGCCG]** 874 (85.32)   
197 - **[CGCGGCCTCG]** 785 (87.15)   
198 + **[CGTCCCCGCG]** 208 (86.52)   
198 + **.CGCCGCCGCG.** 253 (91.94)   
198 + **.CGCCGCGCCC.** 256 (90.72)   
198 + **[CCCCGCGCCC]** 263 (85.30)   
198 + **[CCCCGGGTCG]** 990 (85.46)   
198 - **[CCCCGCCTCG]** 1234 (88.11)   
198 - **[GGGCGCGGCG]** 256 (89.72)   
198 - **.CGCGGCGGCG.** 253 (90.54)   
199 + **[GGCCGCCTCG]** 453 (87.14)   
199 + **[CGGCGCGCCC]** 669 (89.91)   
199 + **.GGGCGCGTCG.** 818 (91.32)   
199 + **[CCCGGCGGCG]** 861 (85.11)   
199 + **[GGGCGCGGCG]** 905 (89.72)   
199 + **.CGCCGCGGCC.** 1000 (91.48)   
199 + **[CGCCGGGGCG]** 1009 (89.29)   
199 + **[CGCCCCCGCG]** 1058 (85.05)   
199 + **.CGGCGCGGCG.** 1082 (96.12)   
199 + **[CGGCGGGGCG]** 1104 (88.48)   
199 + **[CGTGGCCCCG]** 1151 (86.26)   
199 + **[CGGCGCTGCG]** 1399 (87.19)   
199 - **[CGCAGCGCCG]** 1399 (85.31)   
199 - **[CGCCGCCCCC]** 1346 (85.73)   
199 - **[CGCGGCCTCG]** 1261 (87.15)   
199 - **[CGCCCCGCCG]** 1104 (89.28)   
199 - **.CGCCGCGCCG.** 1082 (96.17)   
199 - **.CGCCCCGGCG.** 1009 (90.04)   
199 - **.GGCCGCGGCG.** 1000 (90.53)   
199 - **[CCGCGCCGCG]** 908 (85.70)   
199 - **.CGCCGCGCCC.** 905 (90.72)   
199 - **[CGCCGCCGCC]** 864 (86.49)   
199 - **[CGCCGCCGGG]** 861 (85.08)   
199 - **[GGGCGCGCCG]** 669 (88.96)   
200 + **[CCCCGCCCCG]** 695 (85.75)   
200 + **[CGCCGAGCCG]** 1003 (85.33)   
200 + **.CGTGGCGGCG.** 1079 (92.01)   
200 - **[CGCCGCCACG]** 1079 (85.65)   
200 - **[CGGCTCGGCG]** 1003 (85.97)   
201 + **.CGCCGCGCCC.** 336 (90.72)   
201 + **[CGCGGCCTCG]** 351 (87.15)   
201 + **[CGCGGCCTCG]** 533 (87.15)   
201 + **[GGTGGCGGCG]** 713 (85.61)   
201 + **[CGGCGCGGGG]** 775 (89.26)   
201 + **.CCCCGCGGCG.** 846 (91.50)   
201 + **[CCCCGCCCCG]** 961 (85.75)   
201 + **[CCTCGCCCCG]** 1008 (87.22)   
201 + **.CGCCGCGGGG.** 1348 (90.07)   
201 - **.CCCCGCGGCG.** 1348 (91.50)   
201 - **[CGCGGCGTCC]** 1345 (86.69)   
201 - **[CCGCGCGGCC]** 1141 (85.25)   
201 - **.CGCCGCGGGG.** 846 (90.07)   
201 - **.CCCCGCGCCG.** 775 (90.74)   
201 - **[CGCCGCCGCC]** 770 (86.49)   
201 - **[CGCCGCCTGG]** 767 (86.68)   
201 - **.CGTCCCGGCG.** 600 (91.51)   
201 - **[CGGCGCCGCC]** 595 (85.68)   
201 - **[GGGCGCGGCG]** 336 (89.72)   
202 + **[CGCCGCCGGG]** 1109 (85.08)   
202 - **[GGGCGCGTCC]** 1392 (85.88)   
202 - **[CCCGGCGGCG]** 1109 (85.11)   
202 - **[CCCCGCGCCC]** 592 (85.30)   
208 + **[CGTCGTGGCG]** 1149 (86.71)   
208 + **[CGCCGAGGCG]** 1390 (86.08)   
208 + **[CGGCGCGCAG]** 1408 (85.13)   
208 + **.CGCCGCGGCG.** 1496 (96.93)   
208 - **[GGTCGCCGCG]** 1499 (87.01)   
208 - **.CGCCGCGGCG.** 1496 (96.93)   
208 - **[CTGCGCGCCG]** 1408 (87.09)   
208 - **[CGCCTCGGCG]** 1390 (86.77)   
208 - **[CGGCGAGTCG]** 1338 (86.88)   
208 - **[CCTCGCGCGG]** 952 (85.36)   
210 + **.GGGCGCGTCG.** 674 (91.32)   
210 + **[GGCCGCGGCC]** 777 (85.08)   
210 + **[CGCAGCGGCG]** 931 (86.06)   
210 + **[CGGCGCGGAG]** 1065 (85.89)   
210 + **.CGGCGCCCCG.** 1106 (90.37)   
210 + **[CCCCGCCCCG]** 1148 (85.75)   
210 - **.CGTCGGGGCG.** 1151 (90.76)   
210 - **[CGGGGCGCCG]** 1106 (88.97)   
210 - **[CTCCGCGCCG]** 1065 (87.90)   
210 - **[CGCCGCCGCC]** 937 (86.49)   
210 - **[CGCCGCTGCG]** 931 (88.00)   
210 - **[CGCCGCCTCC]** 869 (88.09)   
210 - **[GGCCGCGGCC]** 777 (85.08)   
211 + **[CGTCCCCGCG]** 608 (86.52)   
211 + **[CCCCGCCGCG]** 731 (86.51)   
211 + **.CGCCGCGCCC.** 734 (90.72)   
211 + **[CGCCGCCGCC]** 961 (86.49)   
211 + **[CGCCCCGCCG]** 1077 (89.28)   
211 - **[CGGCGGGGCG]** 1077 (88.48)   
211 - **[GGGCGCGGCG]** 734 (89.72)   
211 - **[CGTGGCCTCG]** 600 (88.62)   
212 + **[CCCCGCCCCG]** 549 (85.75)   
212 - **[GGGCGCCTCG]** 1494 (86.33)   
212 - **[CGTCGCGCCA]** 419 (87.32)   
212 - **[CCTGGCGCCG]** 384 (85.82)   
212 - **[CGCCGCCGCC]** 231 (86.49)   
212 - **[CGCCGCCTCC]** 228 (88.09)   
213 + **[CCTGGCGCCG]** 1332 (85.82)   
213 - **[CGGCCCCTCG]** 1351 (85.84)   
213 - **[CATCGCGCCG]** 996 (87.62)   
213 - **[CCTCGCGGCC]** 911 (87.52)   
214 + **[CGCGGCGTCC]** 732 (86.69)   
214 + **[GGCCGCGGCC]** 928 (85.08)   
214 + **[CCTCGCGCCC]** 1030 (86.77)   
214 - **[CGCCGCCTCC]** 1486 (88.09)   
214 - **[CGCCGCCGCC]** 1462 (86.49)   
214 - **[CGCCGCCTGG]** 1443 (86.68)   
214 - **[CCTCCCGCCG]** 1414 (85.32)   
214 - **[CGCCGCCGCC]** 1409 (86.49)   
214 - **[CGCCGCCGCC]** 1406 (86.49)   
214 - **[CGCCGCCGCC]** 1403 (86.49)   
214 - **[GGCCGCGGCC]** 928 (85.08)   
215 + **[AGCCGCGTCG]** 651 (89.19)   
215 + **[CGTCGAGCCG]** 656 (86.80)   
215 + **.CGCCGCGGCG.** 1089 (96.93)   
215 + **[CGCGGCGCCG]** 1092 (89.78)   
215 + **[CGCCGGGGCG]** 1097 (89.29)   
215 - **[CGCGGCCTCG]** 1409 (87.15)   
215 - **[CCTCGCCCCG]** 1105 (87.22)   
215 - **[CCCCGCCCCG]** 1100 (85.75)   
215 - **.CGCCCCGGCG.** 1097 (90.04)   
215 - **.CGGCGCCGCG.** 1092 (91.13)   
215 - **.CGCCGCGGCG.** 1089 (96.93)   
215 - **[CCTGGCGCCG]** 488 (85.82)   
216 + **[CGCGGCGTGG]** 212 (85.28)   
216 + **[CCCCGCCCCG]** 951 (85.75)   
216 + **[CCCCGCCCCG]** 956 (85.75)   
217 + **.CCGCGCGGCG.** 574 (90.69)   
217 + **[CAGCGCGTCG]** 938 (87.70)   
217 - **[CGCGGCGTGG]** 856 (85.28)   
217 - **[CGCCGCGCGG]** 574 (89.31)   
217 - **[CCGCGCGGCC]** 572 (85.25)   
221 + **[CCGCGCGGCC]** 516 (85.25)   
221 + **[CGGCGCGGGG]** 936 (89.26)   
221 + **.GGCCGCGGCG.** 1109 (90.53)   
221 + **.CGCGGCGGCG.** 1112 (90.54)   
221 + **[CCTCCCGGCG]** 1258 (86.08)   
221 + **[CCCCGCCCCG]** 1280 (85.75)   
221 - **.CGCCGCCGCG.** 1112 (91.94)   
221 - **.CGCCGCGGCC.** 1109 (91.48)   
221 - **.CCCCGCGCCG.** 936 (90.74)   
221 - **[CCGCGCGGCC]** 514 (85.25)   
222 + **[CCGCGCGTCC]** 449 (86.85)   
222 + **[CCCCGCCCCG]** 611 (85.75)   
222 + **[CCTCGCGCCC]** 688 (86.77)   
222 + **[CGGTGCGGCG]** 697 (85.17)   
222 + **[CGGTGCGGCG]** 835 (85.17)   
222 + **.CGGCGCGGCG.** 840 (96.12)   
222 + **[AGCCGCGTCG]** 876 (89.19)   
222 + **[CGCGGCGGCC]** 950 (85.09)   
222 + **[CCCCGCGCCC]** 1078 (85.30)   
222 + **[GGGCGCGCCG]** 1203 (88.96)   
222 - **[CGGCGCGCCC]** 1203 (89.91)   
222 - **[GGCCGCCGCG]** 950 (85.54)   
222 - **.CGCCGCGCCG.** 840 (96.17)   
222 - **[CGCCGCACCG]** 835 (85.14)   
222 - **[CGCCGCACCG]** 697 (85.14)   
223 + **[CGCGCCGTCG]** 424 (85.25)   
223 + **[CGGCGCCGCC]** 672 (85.68)   
223 + **[CGCCGCCGGG]** 675 (85.08)   
223 - **[CCCGGCGGCG]** 675 (85.11)   
223 - **[CGTCCCGGCC]** 450 (86.06)   
223 - **.CGGCGCGTCC.** 421 (92.28)   
224 + **.CGCCGCGGGG.** 822 (90.07)   
224 + **.CGGCGCGGCG.** 853 (96.12)   
224 - **[CGTGGCGCCC]** 1276 (85.80)   
224 - **[CCCCGCGCCC]** 1182 (85.30)   
224 - **[CCCCGCGTGG]** 1175 (86.25)   
224 - **.CGCCGCGCCG.** 853 (96.17)   
224 - **.CCCCGCGGCG.** 822 (91.50)   
224 - **[CGCCGCCGCC]** 712 (86.49)   
224 - **[CGCCGCCGCC]** 709 (86.49)   
224 - **[CGCCGCCGCC]** 706 (86.49)   
224 - **[CGCCGCCCCC]** 703 (85.73)   
224 - **.CCTCGCGGCG.** 689 (92.97)   
226 + **[CGCAGCGGCG]** 6 (86.06)   
226 + **[CCCCGCCCCG]** 476 (85.75)   
226 + **[CACCGCGCCG]** 508 (86.15)   
226 + **.CGCCGCGCCC.** 1034 (90.72)   
226 + **.CCGCGCGGCG.** 1075 (90.69)   
226 + **[CGCGGCGCCG]** 1078 (89.78)   
226 + **[CGCCGCTCCG]** 1083 (87.24)   
226 + **[CGCCGCCGCC]** 1107 (86.49)   
226 + **[CGCCGCCGCC]** 1110 (86.49)   
226 + **[CGCCGCCGCC]** 1113 (86.49)   
226 + **[CCCCGCGCCC]** 1160 (85.30)   
226 + **[CCCCGCGCCC]** 1167 (85.30)   
226 + **[GGGCGCGGCG]** 1494 (89.72)   
226 - **.CGCCGCGCCC.** 1494 (90.72)   
226 - **[CGCCGCCGCC]** 1275 (86.49)   
226 - **[CGCCGCCGCC]** 1272 (86.49)   
226 - **[CGCCGCCGCC]** 1239 (86.49)   
226 - **[CGCCGCCGCC]** 1236 (86.49)   
226 - **[CGGAGCGGCG]** 1083 (85.26)   
226 - **.CGGCGCCGCG.** 1078 (91.13)   
226 - **[CGCCGCGCGG]** 1075 (89.31)   
226 - **[GGGCGCGGCG]** 1034 (89.72)   
226 - **[CGGCGCGGTG]** 508 (85.62)   
226 - **[CGCCGCTGCG]** 6 (88.00)   
227 + **[CGGCCCCTCG]** 270 (85.84)   
227 - **[CCCCGCGTGG]** 845 (86.25)   
228 + **[CCCCGCGCCC]** 133 (85.30)   
228 + **[CGCCGCTGCG]** 383 (88.00)   
228 + **[CCCCGCCCCG]** 429 (85.75)   
228 + **[CGCCCCCGCG]** 465 (85.05)   
228 + **.CCCCGCGCCG.** 468 (90.74)   
228 + **[CGTGGCGGGG]** 792 (85.15)   
228 + **[CGCCGCCGGG]** 867 (85.08)   
228 + **[CGCCGGGCCG]** 1265 (88.53)   
228 - **[CGGCCCGGCG]** 1265 (89.23)   
228 - **[CCCCGCGCCC]** 951 (85.30)   
228 - **[CCCGGCGGCG]** 867 (85.11)   
228 - **[CCCCGCCTCG]** 835 (88.11)   
228 - **[CCCCGCCCCG]** 797 (85.75)   
228 - **[CGGCGCGGGG]** 468 (89.26)   
228 - **[CGCAGCGGCG]** 383 (86.06)   
228 - **[GGCCGCCTCG]** 104 (87.14)   
229 + **[CCCCGCGGCC]** 584 (86.05)   
229 + **[GGTCCCGGCG]** 628 (85.11)   
229 + **.CGGCGCCGCG.** 686 (91.13)   
229 + **[CGCCGCGCTG]** 689 (85.67)   
229 + **[CCCCGCCCCG]** 1159 (85.75)   
229 + **[CCCCGCCCCG]** 1164 (85.75)   
229 - **[CGCCGCCGCC]** 1337 (86.49)   
229 - **[CGCCGCCGCC]** 1334 (86.49)   
229 - **[CGCCGCCGCC]** 1331 (86.49)   
229 - **[CGCCGCCGCC]** 1328 (86.49)   
229 - **[CGCCGCCGCC]** 1325 (86.49)   
229 - **[CCCCGCGCCC]** 1300 (85.30)   
229 - **[CCGCGCCGCG]** 1284 (85.70)   
229 - **[CCCCGCCCCG]** 1040 (85.75)   
229 - **[CCCCGCGCCC]** 1035 (85.30)   
229 - **[CAGCGCGGCG]** 689 (86.10)   
229 - **[CGCGGCGCCG]** 686 (89.78)   
229 - **[CGGCGCCGCC]** 684 (85.68)   
230 + **[CCTCGCCCCG]** 286 (87.22)   
231 + **.CGCCGCCGCG.** 902 (91.94)   
231 + **[CGGGGCGTCC]** 953 (85.89)   
231 + **[CGCCGCCCCC]** 1005 (85.73)   
231 + **[CGCCGCCGCC]** 1026 (86.49)   
231 + **[GGCCGCGCCG]** 1481 (89.77)   
231 - **[CGCCGCCGCC]** 1500 (86.49)   
231 - **.CGGCGCGGCC.** 1481 (90.67)   
231 - **[CCTCCCGGCG]** 1196 (86.08)   
231 - **[CCTCGCGCCC]** 1140 (86.77)   
231 - **[CCTCGCGCCC]** 1121 (86.77)   
231 - **[CCTCGCCCCG]** 1103 (87.22)   
231 - **[CGTGGCCGCG]** 1070 (87.02)   
231 - **[CCTGGCGGCG]** 1029 (86.58)   
231 - **[CCCCGCCCCG]** 948 (85.75)   
231 - **[CCCCGCCCCG]** 943 (85.75)   
231 - **[CCCCGCCCCG]** 938 (85.75)   
231 - **[CCCCGCCCCG]** 927 (85.75)   
231 - **.CGCGGCGGCG.** 902 (90.54)   
232 + **.CGCCGCGCCC.** 146 (90.72)   
232 + **.CGCCCCGGCG.** 438 (90.04)   
232 + **.CGGCGCGTCC.** 632 (92.28)   
232 + **[CCCCGCCCCG]** 689 (85.75)   
232 + **[GGGCGCGTCC]** 698 (85.88)   
232 + **[CCGCGCGGCC]** 860 (85.25)   
232 + **[CGCGGCCGCG]** 863 (85.55)   
232 + **[CTGCGCGGCG]** 994 (87.85)   
232 + **[CGGCGCGGGG]** 1179 (89.26)   
232 + **[GGCCGCGCCG]** 1243 (89.77)   
232 + **[CTCCGCCTCG]** 1265 (85.27)   
232 + **[CCCCGCCCCG]** 1402 (85.75)   
232 - **[GGCCGCGTCC]** 1332 (86.69)   
232 - **.CGGCGCGGCC.** 1243 (90.67)   
232 - **.CCCCGCGCCG.** 1179 (90.74)   
232 - **[CGCCGCCGCC]** 1026 (86.49)   
232 - **[CGCCGCCTCC]** 1023 (88.09)   
232 - **[GCTCGCGCCG]** 1012 (85.81)   
232 - **[CGCCGCGCAG]** 994 (85.94)   
232 - **[CGTAGCGCCG]** 918 (86.78)   
232 - **[CGCGGCCGCG]** 863 (85.55)   
232 - **[CGCCGCCGCC]** 627 (86.49)   
232 - **[CGCCGGGGCG]** 438 (89.29)   
232 - **[GGGCGCGGCG]** 146 (89.72)   
233 - **[CCGGGCGTCG]** 1140 (85.91)   
233 - **[CGTCCCCCCG]** 366 (85.76)   
234 + **[CGCCACGTCG]** 212 (87.89)   
234 + **[CTGCGCGCCG]** 408 (87.09)   
234 + **[GGCGGCGTCG]** 628 (85.74)   
234 + **[CCTCGCGCCC]** 847 (86.77)   
234 + **[CCTCGCCCCG]** 880 (87.22)   
234 + **[CCACGCGGCG]** 1328 (85.73)   
234 + **[CGCCGGGCCG]** 1431 (88.53)   
234 - **[CGGCCCGGCG]** 1431 (89.23)   
234 - **[CCGCGCGTCC]** 1410 (86.85)   
234 - **.CGCCGCGTGG.** 1328 (91.67)   
234 - **[CGCCCCCGCG]** 997 (85.05)   
234 - **[CCCCGCCCCG]** 924 (85.75)   
234 - **.CCCCGCGTCG.** 636 (93.10)   
234 - **[CGCCGCCTCC]** 545 (88.09)   
234 - **[CGGCGCGCAG]** 408 (85.13)   
234 - **[CGGCCCCTCG]** 220 (85.84)   
234 - **[CGTGGCGCCC]** 209 (85.80)   
234 - **[CCCCGCGCCC]** 178 (85.30)   
235 + **[CTGCGCGGCG]** 897 (87.85)   
235 + **[CGGCGCGTGC]** 902 (85.42)   
235 + **[CGGCGCGGAG]** 1316 (85.89)   
235 + **[CGCCGCGCCT]** 1361 (87.54)   
235 + **[CCTGGCGGCG]** 1507 (86.58)   
235 - **[AGGCGCGGCG]** 1361 (86.78)   
235 - **[CTCCGCGCCG]** 1316 (87.90)   
235 - **[CGCCGCCGCC]** 1311 (86.49)   
235 - **[CTTCGCCTCG]** 1276 (86.74)   
235 - **[CGCCGCGCAG]** 897 (85.94)   
235 - **[CGCCCCCGCG]** 685 (85.05)   
236 + **[CCTCGCGCGG]** 1021 (85.36)   
238 - **[CGGAGCGTCG]** 1088 (86.86)   
239 + **[CTTCGCCGCG]** 441 (85.14)   
239 + **.CGCCGCGGCC.** 444 (91.48)   
239 + **[GGCCGCGCCG]** 767 (89.77)   
239 - **[CGGGGCCTCG]** 956 (86.34)   
239 - **.CGGCGCGGCC.** 767 (90.67)   
239 - **[GGTCGCGCCC]** 706 (85.79)   
239 - **.GGCCGCGGCG.** 444 (90.53)   
240 + **.CCCCGCGCCG.** 1179 (90.74)   
240 + **[CCTCGCCGCG]** 1399 (87.98)   
240 - **[CGGCGCGGGG]** 1179 (89.26)   
240 - **[CCCCGCGCCC]** 52 (85.30)   
241 + **[CGGCGCGGAG]** 1283 (85.89)   
241 - **[CTCCGCGCCG]** 1283 (87.90)   
242 + **[CGCCGCCGCC]** 894 (86.49)   
242 + **[CGCCGGGCCG]** 939 (88.53)   
242 + **[GTTCGCGTCG]** 1000 (85.33)   
242 - **[CGGCCCGGCG]** 939 (89.23)   
244 - **[CGGCGCCTCC]** 831 (87.29)   
246 + **[CGCCGCGCCT]** 273 (87.54)   
246 + **[CCGCGCCTCG]** 275 (87.31)   
246 + **[CGCCTCGGCG]** 278 (86.77)   
246 + **[CGGCGCCGCC]** 283 (85.68)   
246 + **[CGGCGCCTCC]** 676 (87.29)   
246 + **[CCTCGCGCCC]** 696 (86.77)   
246 + **[CCCCGCGCCC]** 747 (85.30)   
246 + **[CGCCGCCTGG]** 756 (86.68)   
246 + **[GGGCGCGCCG]** 907 (88.96)   
246 + **[CCCCGCCCCG]** 1102 (85.75)   
246 - **[CCGCGCGGCC]** 1077 (85.25)   
246 - **[CCTCCCGGCG]** 974 (86.08)   
246 - **[CGGCGCGCCC]** 907 (89.91)   
246 - **[CCGCGCGTCC]** 669 (86.85)   
246 - **[CGCCGAGGCG]** 278 (86.08)   
246 - **[AGGCGCGGCG]** 273 (86.78)   
247 + **[CCCCGCGCCC]** 328 (85.30)   
247 + **[CGGCGGCTCG]** 424 (85.09)   
247 + **[CGCCGCCCCC]** 581 (85.73)   
247 + **[AGCCGCGGCG]** 1021 (87.59)   
247 + **[CGCCCCCGCG]** 1116 (85.05)   
247 + **[CCCCGCGCCC]** 1119 (85.30)   
247 + **.CGCCGCCTCG.** 1224 (93.54)   
247 + **[CGGGGCGGCG]** 1256 (89.73)   
247 + **[CCCCGCGCCC]** 1470 (85.30)   
247 + **[CGCCCCCGCG]** 1475 (85.05)   
247 + **[CGCCCCGCCG]** 1487 (89.28)   
247 + **[CCCCGCCGCG]** 1489 (86.51)   
247 - **[CGGCGGGGCG]** 1487 (88.48)   
247 - **.CGCCGCCCCG.** 1256 (91.18)   
247 - **[CGCCGCGGCT]** 1021 (88.30)   
247 - **[CGGCGCCTCC]** 1007 (87.29)   
247 - **[CCCCGCCCCG]** 960 (85.75)   
250 + **[CGGCGCGGAG]** 459 (85.89)   
250 + **[CGCCCCCGCG]** 1043 (85.05)   
250 + **[CGCCGGGCCG]** 1090 (88.53)   
250 - **[CGGCCCGGCG]** 1090 (89.23)   
250 - **[CTCCGCGCCG]** 459 (87.90)   
250 - **[CCGCGCCGCG]** 457 (85.70)   
250 - **[CCGCGCGTCC]** 452 (86.85)   
250 - **[CGCCGCCGCC]** 441 (86.49)   
256 + **[CCCCGCCCCG]** 926 (85.75)   
256 + **[GCCCGCGTCG]** 1202 (86.70)   
257 - **[CGCCGCCCCC]** 1398 (85.73)   
259 + **.CGACGCGGCG.** 994 (91.16)   
259 + **[CGGGGCCTCG]** 1282 (86.34)   
259 + **.CGGCGCCGCG.** 1298 (91.13)   
259 + **.CGCCGCGGCG.** 1301 (96.93)   
259 - **[GGTCGCCGCG]** 1304 (87.01)   
259 - **.CGCCGCGGCG.** 1301 (96.93)   
259 - **[CGCGGCGCCG]** 1298 (89.78)   
259 - **[CGCCGCCTGG]** 1036 (86.68)   
259 - **.CGTCGCGGCC.** 1017 (92.95)   
259 - **.CGCCGCGTCG.** 994 (98.53)   
259 - **[CGTCGCCGCC]** 989 (87.96)   
260 + **[CGCCCCGCCG]** 881 (89.28)   
260 - **[CGGCGGGGCG]** 881 (88.48)   
263 + **[CGCGGCCGCG]** 850 (85.55)   
263 + **[AGCCGCGTCG]** 1075 (89.19)   
263 - **[CCCCGCGCCC]** 1281 (85.30)   
263 - **[CGCGGCCGCG]** 850 (85.55)   
263 - **[CGCCGCCGCC]** 827 (86.49)   
263 - **[CGCCGCCTCC]** 824 (88.09)   
266 + **.CGGCGCGGCG.** 1027 (96.12)   
266 + **.CGGCGCGCCG.** 1103 (95.36)   
266 + **[CAGCGCGCCG]** 1267 (85.34)   
266 + **.GGCCGCGGCG.** 1279 (90.53)   
266 + **[CGCGGCGGCC]** 1282 (85.09)   
266 - **[CGGCGCCTCC]** 1469 (87.29)   
266 - **[GGCCGCCGCG]** 1282 (85.54)   
266 - **.CGCCGCGGCC.** 1279 (91.48)   
266 - **[CGCCGCCGCC]** 1230 (86.49)   
266 - **[CGCCGCCGCC]** 1153 (86.49)   
266 - **[CGCCGCCGCC]** 1150 (86.49)   
266 - **.CGGCGCGCCG.** 1103 (95.36)   
266 - **[CCCCGCCTCG]** 1041 (88.11)   
266 - **.CGCCGCGCCG.** 1027 (96.17)   
266 - **.CGGCGCGTGG.** 979 (90.87)   
267 + **.CGCCGCGGCC.** 192 (91.48)   
267 + **[CGCCGCCGCC]** 1023 (86.49)   
267 - **.GGCCGCGGCG.** 192 (90.53)   
268 + **.CGGCGCGGCG.** 1012 (96.12)   
268 - **[CGGCGCTCCG]** 1360 (86.43)   
268 - **[CGCCGCCGCC]** 1243 (86.49)   
268 - **[CGCCGCCGCC]** 1240 (86.49)   
268 - **[CGCCGCCGCC]** 1237 (86.49)   
268 - **[CGCCGCCTCC]** 1234 (88.09)   
268 - **.CGTGGCGCCG.** 1193 (91.25)   
268 - **[CGCCGCCCCC]** 1106 (85.73)   
268 - **.CGCCGCGCCG.** 1012 (96.17)   
268 - **[CGGCACGGCG]** 35 (85.48)   
270 + **[CGTCGCCCCC]** 198 (87.20)   
270 + **[CCGCGCGTGG]** 1439 (85.44)   
270 - **[GGCGGCGTCG]** 343 (85.74)   
270 - **[CGTCTCGGCG]** 99 (88.24)   
270 - **[CGCCGCCCCC]** 8 (85.73)   
271 + **[CGTGGGGTCG]** 1117 (85.97)   
272 + **[CGTGGGGTCG]** 1108 (85.97)   
273 + **[CCCCGCGCCC]** 198 (85.30)   
273 + **.GGCCGCGGCG.** 1029 (90.53)   
273 - **[CGGCCCCTCG]** 1064 (85.84)   
273 - **.CGCCGCGGCC.** 1029 (91.48)   
273 - **[CGTCCCGACG]** 363 (85.22)   
274 + **[GGCCGCGGCC]** 12 (85.08)   
274 + **[CGGCGCGCAG]** 110 (85.13)   
274 - **[CTGCGCGCCG]** 110 (87.09)   
274 - **[GGCCGCGGCC]** 12 (85.08)   
275 + **[CAGCGCGCCG]** 752 (85.34)   
275 + **[CGCCGCCGCC]** 757 (86.49)   
275 + **[CGCCGCCGCC]** 760 (86.49)   
275 + **[CGCCGCCGCC]** 763 (86.49)   
275 + **[CTTCGCCTCG]** 812 (86.74)   
275 + **[CGCCGCCGCC]** 1007 (86.49)   
275 + **[CGCCGCCGCC]** 1010 (86.49)   
275 + **[CGCCGCCGGG]** 1013 (85.08)   
275 + **[CCTCCCGGCG]** 1347 (86.08)   
275 + **[CGGCGCCTGG]** 1444 (85.88)   
275 - **[CCCGGCGGCG]** 1013 (85.11)   
275 - **[CGCCGCCCCC]** 67 (85.73)   
279 + **[AGGCGCGGCG]** 412 (86.78)   
279 + **.CGGCGCGTGG.** 417 (90.87)   
279 - **[CGCCGCGCCT]** 412 (87.54)   
279 - **[CGTCGCGTGC]** 373 (87.70)   
280 + **[CGCGGCGGCC]** 735 (85.09)   
280 + **.CGCGGCGGCG.** 982 (90.54)   
280 - **[CCTGGCGCCG]** 1109 (85.82)   
280 - **[CGCCGCCGCC]** 985 (86.49)   
280 - **.CGCCGCCGCG.** 982 (91.94)   
280 - **[CCTCGCGGCC]** 943 (87.52)   
280 - **[GGCCGCCGCG]** 735 (85.54)   
281 - **[CGCCGCCGCC]** 1163 (86.49)   
281 - **[CGCCGCCTCC]** 1160 (88.09)   
281 - **[CGCCGCCGCC]** 1036 (86.49)   
281 - **[CGCCCCCTCG]** 909 (86.65)   
281 - **[CCCCGCGTCC]** 445 (87.66)   
281 - **[CGTCCCGCCC]** 440 (85.30)   
284 + **[CGGCGCGGAG]** 630 (85.89)   
284 + **[CCCCGCGTGG]** 715 (86.25)   
284 + **[CCTCGGGGCG]** 821 (85.33)   
284 + **[CGCCTCGCCG]** 905 (86.01)   
284 + **[CCCCGCGGCC]** 940 (86.05)   
284 + **[CGCCGGGCCG]** 1005 (88.53)   
284 + **.CCCCGCGGCG.** 1086 (91.50)   
284 + **.CGGCGCGGCC.** 1091 (90.67)   
284 + **[CGCCGCCGCC]** 1236 (86.49)   
284 + **[CGTCGCCGCC]** 1245 (87.96)   
284 + **[CGCCGCCGGG]** 1248 (85.08)   
284 + **[CCGCGCGCCG]** 1339 (89.93)   
284 + **.CGCCGCGGCG.** 1344 (96.93)   
284 + **[CGGCGCTGCG]** 1349 (87.19)   
284 + **[CGCCGCCGCC]** 1380 (86.49)   
284 + **[CGCCGCCGCC]** 1383 (86.49)   
284 + **[GGTCGCGGGG]** 1504 (85.14)   
284 - **[CGCCGCCGCC]** 1480 (86.49)   
284 - **[CGCCGCCGCC]** 1477 (86.49)   
284 - **[CGCAGCGCCG]** 1349 (85.31)   
284 - **.CGCCGCGGCG.** 1344 (96.93)   
284 - **[CGGCGCGCGG]** 1339 (88.50)   
284 - **[CCCGGCGGCG]** 1248 (85.11)   
284 - **[GGCCGCGCCG]** 1091 (89.77)   
284 - **[CCGCGCCGCG]** 1089 (85.70)   
284 - **.CGCCGCGGGG.** 1086 (90.07)   
284 - **[CGGCCCGGCG]** 1005 (89.23)   
284 - **[CGGGGCCTCG]** 961 (86.34)   
284 - **[CGGCGAGGCG]** 905 (85.28)   
284 - **[CTCCGCGCCG]** 630 (87.90)   
285 + **[CGCGGCCGCG]** 548 (85.55)   
285 + **.GGCCGCGGCG.** 551 (90.53)   
285 + **[CGGGGCGGCG]** 600 (89.73)   
285 + **.CGCCGCGCCC.** 633 (90.72)   
285 + **.CCTCGCGCCG.** 649 (92.21)   
285 + **.CGCCGCGCCG.** 654 (96.17)   
285 + **[CGGGGCCTCG]** 662 (86.34)   
285 + **[CGACGCGGCC]** 714 (85.71)   
285 + **[CGTCCCGCCC]** 872 (85.30)   
285 + **.CGCCGCGGCG.** 906 (96.93)   
285 + **[CGCGGCGCCG]** 909 (89.78)   
285 - **[GCTCGCGCCG]** 1025 (85.81)   
285 - **.CGTCGCGCGG.** 947 (90.78)   
285 - **.CGGCGCCGCG.** 909 (91.13)   
285 - **.CGCCGCGGCG.** 906 (96.93)   
285 - **[CCCCGCGGCC]** 857 (86.05)   
285 - **[CGCCGCCGCC]** 756 (86.49)   
285 - **[CGCCGCCGCC]** 753 (86.49)   
285 - **.GGCCGCGTCG.** 714 (92.13)   
285 - **.CGGCGCGGCG.** 654 (96.12)   
285 - **[GGGCGCGGCG]** 633 (89.72)   
285 - **[CGCGGCGTCC]** 630 (86.69)   
285 - **.CGCCGCCCCG.** 600 (91.18)   
285 - **.CGCCGCGGCC.** 551 (91.48)   
285 - **[CGCGGCCGCG]** 548 (85.55)   
285 - **[CGGCGCCGCC]** 518 (85.68)   
285 - **[CGCCCCCGCG]** 472 (85.05)   
285 - **[CGCCGCCGCC]** 458 (86.49)   
285 - **[CGCCGCCGCC]** 455 (86.49)   
285 - **[CCCCGCGGCC]** 352 (86.05)   
285 - **[CCCCGCGCCC]** 343 (85.30)   
287 + **.CGCCGCCTCG.** 762 (93.54)   
287 + **[CGCCTCGGCG]** 765 (86.77)   
287 + **[CGGCGGCTCG]** 770 (85.09)   
287 + **[CGCCCCGCCG]** 1043 (89.28)   
287 + **[CGCCGCCGCC]** 1079 (86.49)   
287 - **[GGTGGCGGCG]** 1082 (85.61)   
287 - **[CGGCGGGGCG]** 1043 (88.48)   
287 - **[CGCCGAGGCG]** 765 (86.08)   
287 - **[CGTCGCCCCC]** 557 (87.20)   
288 + **[CCGCGCGCCG]** 1192 (89.93)   
288 - **[CGGCGCGCGG]** 1192 (88.50)   
288 - **.CCTCGCGCCG.** 875 (92.21)   
289 + **[CTGCGCGGCG]** 1297 (87.85)   
289 - **[CCTCGCGCCC]** 1316 (86.77)   
289 - **[CGCCGCGCAG]** 1297 (85.94)   
290 - **[GGTCCCGGCG]** 1442 (85.11)   
290 - **[CGGCGCCGCC]** 1437 (85.68)   
291 + **[CGTCCCGCCC]** 205 (85.30)   
291 + **[CGCAGCGTCG]** 578 (87.67)   
291 + **[CGCCGCGGCT]** 766 (88.30)   
291 + **[CGGCGCCTCC]** 873 (87.29)   
291 + **.CGCCCCGTCG.** 1023 (91.64)   
291 + **.CGGCGCCGCG.** 1031 (91.13)   
291 + **[CGCCGCGCAG]** 1034 (85.94)   
291 + **[CGCCGCGTGC]** 1082 (86.23)   
291 + **[GGGCGCGCCG]** 1097 (88.96)   
291 + **[CGGCGCCGCC]** 1358 (85.68)   
291 + **[CGCCGCCGGG]** 1361 (85.08)   
291 + **[CGGCGGGGCG]** 1471 (88.48)   
291 + **[GGGCGCGCCG]** 1499 (88.96)   
291 - **[CGGCGCGCCC]** 1499 (89.91)   
291 - **[CGCCCCGCCG]** 1471 (89.28)   
291 - **[CCCGGCGGCG]** 1361 (85.11)   
291 - **[CGGCGCCGCC]** 1356 (85.68)   
291 - **.CGCCGCCTCG.** 1353 (93.54)   
291 - **[CCTCGCGGCC]** 1342 (87.52)   
291 - **[CGGCGCGCCC]** 1097 (89.91)   
291 - **[CTGCGCGGCG]** 1034 (87.85)   
291 - **[CGCGGCGCCG]** 1031 (89.78)   
291 - **[AGCCGCGGCG]** 766 (87.59)   
291 - **[CGCAGCGTCG]** 493 (87.67)   
292 + **[CGTGGCCTCG]** 417 (88.62)   
292 + **.CCCCGCGCCG.** 771 (90.74)   
292 + **.CGCCGCGGCG.** 794 (96.93)   
292 + **[GGTCCCGGCG]** 979 (85.11)   
292 + **[CCTCCCGCCG]** 1076 (85.32)   
292 + **[CGGCGCGGGG]** 1345 (89.26)   
292 + **[CGCGGCCTCG]** 1386 (87.15)   
292 + **.CGTCGCGGGG.** 1468 (91.54)   
292 - **[CCCCGCGCCC]** 1491 (85.30)   
292 - **[CCCCGCGTCC]** 1484 (87.66)   
292 - **[CCCCGCGACG]** 1468 (85.21)   
292 - **[CGCCCCCGCG]** 1447 (85.05)   
292 - **[CCCCGCCCCG]** 1350 (85.75)   
292 - **.CCCCGCGCCG.** 1345 (90.74)   
292 - **[CGCCGCCGCC]** 1195 (86.49)   
292 - **[CGTCCCCCCG]** 1183 (85.76)   
292 - **[GCTCGCGGCG]** 1081 (86.57)   
292 - **[CCCCGCGCCC]** 1042 (85.30)   
292 - **[CGCCGCCCCC]** 1013 (85.73)   
292 - **[CGGCGCCTCC]** 867 (87.29)   
292 - **[CCCCGCCGCG]** 797 (86.51)   
292 - **.CGCCGCGGCG.** 794 (96.93)   
292 - **[CGGCGCGGGG]** 771 (89.26)   
292 - **[CGGCCCCTCG]** 553 (85.84)   
293 + **[CCTCGCCTCG]** 778 (89.58)   
293 + **[GGGCGCGGCG]** 836 (89.72)   
293 + **[CCTGGCGTCG]** 1067 (88.18)   
293 + **.CGTCGCGGGG.** 1072 (91.54)   
293 + **[CGTCGCCGGG]** 1364 (86.55)   
293 - **[CCCCGCGACG]** 1072 (85.21)   
293 - **.CGCCGCGCCC.** 836 (90.72)   
294 + **[CGGGGCCTCG]** 856 (86.34)   
294 + **[CGACGCCCCG]** 1088 (85.41)   
294 - **.CGGGGCGTCG.** 1088 (91.33)   
294 - **[CGTGGCGCCC]** 1022 (85.80)   
294 - **[GGGCGCGTCC]** 935 (85.88)   
295 + **.CGGGGCGTCG.** 548 (91.33)   
295 + **[GGGCGCGGCG]** 589 (89.72)   
295 + **[CGTGGCGCCC]** 614 (85.80)   
295 + **[GGGCGCGTCC]** 701 (85.88)   
295 - **[CGGGGCCTCG]** 780 (86.34)   
295 - **[CCCCGCCGCG]** 592 (86.51)   
295 - **.CGCCGCGCCC.** 589 (90.72)   
295 - **[CGACGCCCCG]** 548 (85.41)   
296 + **[CGCCCCCGCG]** 33 (85.05)   
296 + **[CCCCGCGCCC]** 36 (85.30)   
296 + **[CGGCGGCTCG]** 258 (85.09)   
296 + **[CGGGGCGCCG]** 458 (88.97)   
296 + **[CCCCGCCCCG]** 579 (85.75)   
296 + **.GGTCGCGCCG.** 588 (91.24)   
296 + **[CCCCGCCCCG]** 599 (85.75)   
296 + **[CCCCGCCCCG]** 604 (85.75)   
296 + **[CCCCGCGCCC]** 701 (85.30)   
296 + **[CCCCGCGCCC]** 708 (85.30)   
296 + **[CCCCGCGCCC]** 715 (85.30)   
296 + **.CGCCGCGCCG.** 756 (96.17)   
296 + **[CGTCGCCTCC]** 776 (89.56)   
296 + **[CTCCGCGCCG]** 1181 (87.90)   
296 + **[CCGCGCCGCG]** 1183 (85.70)   
296 + **[GGGCGCGCCG]** 1312 (88.96)   
296 + **[CGCCGCCCCC]** 1317 (85.73)   
296 + **[CGGGGCGCCG]** 1366 (88.97)   
296 + **[CGGCGCGGAG]** 1442 (85.89)   
296 - **[CTCCGCGCCG]** 1442 (87.90)   
296 - **[CCGCGCCGCG]** 1440 (85.70)   
296 - **[CTCCGCCTCG]** 1382 (85.27)   
296 - **.CGGCGCCCCG.** 1366 (90.37)   
296 - **[CGGCGCGCCC]** 1312 (89.91)   
296 - **[CGGCGCGGAG]** 1181 (85.89)   
296 - **[CCTCGCCGCG]** 1003 (87.98)   
296 - **[CGCCGCGTCT]** 1000 (89.90)   
296 - **.CGGCGCGGCG.** 756 (96.12)   
296 - **[CGCGGCGTGG]** 753 (85.28)   
296 - **.CGGCGCCCCG.** 458 (90.37)   
296 - **[CGCCGCCGCC]** 189 (86.49)   
296 - **[CGCCGCCGCC]** 186 (86.49)   
296 - **[CGCCGCCCCC]** 183 (85.73)   
296 - **[CCCCGCCGCG]** 85 (86.51)   
297 + **.CGCCGCCCCG.** 636 (91.18)   
297 + **[CCCGGCGGCG]** 976 (85.11)   
297 + **[CGGCGCGGGG]** 984 (89.26)   
297 + **[CCTGGCGTCG]** 1440 (88.18)   
297 - **[CCCCGCCCCG]** 1365 (85.75)   
297 - **[CTTCGCCTCG]** 1192 (86.74)   
297 - **.CCCCGCGCCG.** 984 (90.74)   
297 - **[CGCCGCCGCC]** 979 (86.49)   
297 - **[CGCCGCCGGG]** 976 (85.08)   
297 - **[CCCCGCCCCG]** 926 (85.75)   
297 - **[CGGGGCGGCG]** 636 (89.73)   
298 + **[CCGCGCCTCG]** 292 (87.31)   
298 + **[CGGCTCGTCG]** 650 (87.57)   
298 + **[GGTCGCGGCC]** 1001 (86.55)   
298 + **[CGCCGGCTCG]** 1162 (85.90)   
298 + **[GGCCGCCGCG]** 1174 (85.54)   
298 - **[CGCCGCCTGG]** 1339 (86.68)   
298 - **[CGCGGCGGCC]** 1174 (85.09)   
298 - **[CGCCCCGTCC]** 1093 (86.19)   
298 - **[CGCCGCGTGC]** 873 (86.23)   
299 + **[CGGCGCCTGG]** 820 (85.88)   
299 + **[CCCCGCGCCC]** 865 (85.30)   
299 + **.CCGCGCGTCG.** 880 (92.30)   
299 + **.GGCCGCGGCG.** 1177 (90.53)   
299 + **[GGGCGCGTCC]** 1472 (85.88)   
299 - **[CGTCGCGGGC]** 1398 (86.09)   
299 - **.CGCCGCGGCC.** 1177 (91.48)   
299 - **[GGCCGCCTCG]** 844 (87.14)   
299 - **[CCGCGCGTGG]** 576 (85.44)   
300 + **.CGGCGCGGCG.** 110 (96.12)   
300 + **[CCCCGCGGCC]** 172 (86.05)   
300 + **.CTCCGCGTCG.** 443 (90.26)   
300 + **.CGTCGGGGCG.** 448 (90.76)   
300 + **[TGGCGCGGCG]** 471 (85.50)   
300 + **[CGGCGCGGGG]** 476 (89.26)   
300 + **.CTTCGCGGCG.** 733 (90.13)   
300 - **.CCCCGCGCCG.** 476 (90.74)   
300 - **[CCGCGCCGCG]** 474 (85.70)   
300 - **[CGCCGCGCCA]** 471 (85.85)   
300 - **.CGCCGCGCCG.** 110 (96.17)   
301 - **[CCCCGCGCCC]** 578 (85.30)   
302 + **.CCTCGCGGCG.** 1023 (92.97)   
302 + **.CCTCGCGCCG.** 1036 (92.21)   
302 - **[CGTCGCCGGG]** 945 (86.55)   
302 - **[CGTCGCTGCG]** 598 (89.47)   
302 - **[CGCTGCGTCG]** 595 (87.58)   
303 + **.CGCCGCGCCG.** 167 (96.17)   
303 + **[CGGGGCGGCG]** 291 (89.73)   
303 + **[GGGCGCGCCG]** 742 (88.96)   
303 + **[CGCCGCGGCT]** 747 (88.30)   
303 + **[CCCCCCGTCG]** 838 (86.21)   
303 + **[CGGCCCGTCC]** 878 (85.39)   
303 + **.CGGCGCGGCC.** 1003 (90.67)   
303 + **.CCCCGCGTCG.** 1018 (93.10)   
303 + **[CGGCCCGTCC]** 1030 (85.39)   
303 - **[CCGCGCGGCC]** 1360 (85.25)   
303 - **[GGCCGCGCCG]** 1003 (89.77)   
303 - **[CGCCGCCTCC]** 998 (88.09)   
303 - **[CGCCGCCGCC]** 986 (86.49)   
303 - **[CGCCGCCGCC]** 983 (86.49)   
303 - **[CGCCGCCGCC]** 980 (86.49)   
303 - **[CGCCGCCGCC]** 977 (86.49)   
303 - **[CGCCGCCGCC]** 974 (86.49)   
303 - **[CGCCGCCGCC]** 913 (86.49)   
303 - **[AGCCGCGGCG]** 747 (87.59)   
303 - **[CGGCGCGCCC]** 742 (89.91)   
303 - **[CGCCCCCGCG]** 733 (85.05)   
303 - **[GCCCGCGGCG]** 456 (85.10)   
303 - **.CGCCGCCCCG.** 291 (91.18)   
303 - **[CCCCGCGGCC]** 286 (86.05)   
303 - **.CGGCGCGGCG.** 167 (96.12)   
303 - **[CCTCGCGGCC]** 42 (87.52)   
304 + **[CCCCGCCCCG]** 111 (85.75)   
304 + **[CGTGGCGGGG]** 140 (85.15)   
304 + **[CCTCGCCGCG]** 704 (87.98)   
304 + **.CGCCGCGCCC.** 707 (90.72)   
304 + **[GGGCGCGGCG]** 1240 (89.72)   
304 + **[CCCCGCGACG]** 1306 (85.21)   
304 - **.CGTCGCGGGG.** 1306 (91.54)   
304 - **.CGCCGCGCCC.** 1240 (90.72)   
304 - **[GGGCGCGGCG]** 707 (89.72)   
306 + **[AGCCGCGCCG]** 339 (86.83)   
306 + **[CGCCGGGTCC]** 344 (85.44)   
306 + **.CGCCGCGCCC.** 764 (90.72)   
306 + **[CTCCGCGCCG]** 927 (87.90)   
306 + **[CGGCGCGGGG]** 1038 (89.26)   
306 - **.CCCCGCGCCG.** 1038 (90.74)   
306 - **.CGCCGCCTCG.** 1018 (93.54)   
306 - **[CGGCGCGGAG]** 927 (85.89)   
306 - **[GGGCGCGGCG]** 764 (89.72)   
306 - **[CGGCGCGGCT]** 339 (87.49)   
307 + **[GGGCGCGCCG]** 545 (88.96)   
307 + **[CGCCGCGGAG]** 678 (86.69)   
307 + **[CGCGGCGGCC]** 730 (85.09)   
307 + **[CCCCGCCCCG]** 834 (85.75)   
307 + **[CGCCGCCGCC]** 980 (86.49)   
307 + **[CCGGGCGTCG]** 1063 (85.91)   
307 - **[CGCCCCCTCG]** 1315 (86.65)   
307 - **[CCCCGCCTCG]** 1220 (88.11)   
307 - **[CGCCGCCCCC]** 1081 (85.73)   
307 - **[GGTGGCGGCG]** 983 (85.61)   
307 - **[CCGCGCGGCC]** 736 (85.25)   
307 - **[GGCCGCCGCG]** 730 (85.54)   
307 - **[CTCCGCGGCG]** 678 (88.66)   
307 - **[CGGCGCGCCC]** 545 (89.91)   
308 + **[GGCCGCCTCG]** 1078 (87.14)   
308 + **[CTCCGCGCCG]** 1186 (87.90)   
308 + **.CGCCGCGGGG.** 1367 (90.07)   
308 + **[CGGCCCGCCG]** 1387 (88.47)   
308 - **[CCCCGCGGCC]** 1412 (86.05)   
308 - **[GGTCCCGGCG]** 1392 (85.11)   
308 - **[CGGCGGGCCG]** 1387 (87.72)   
308 - **.CCCCGCGGCG.** 1367 (91.50)   
308 - **[CGGCGCGGAG]** 1186 (85.89)   
308 - **[CCCCGCCGCG]** 863 (86.51)   
309 - **[TGTCGCGCCG]** 1077 (87.02)   
309 - **[CCCCGCCGCG]** 1029 (86.51)   
309 - **[CGCCGCCCCC]** 765 (85.73)   
310 + **[CGTGGCGGCC]** 1193 (86.56)   
310 + **[CGTGGCGCCC]** 1205 (85.80)   
311 + **[CCCCGCCCCG]** 681 (85.75)   
311 + **[CCGCGCCTCG]** 761 (87.31)   
311 + **.CGCCGCGGCC.** 872 (91.48)   
311 + **[GGGCGCGGCG]** 1066 (89.72)   
311 + **[AGCCGCGGCG]** 1103 (87.59)   
311 + **[CGGCGGGGCG]** 1250 (88.48)   
311 - **[CCCCGCCCCG]** 1253 (85.75)   
311 - **[CGCCCCGCCG]** 1250 (89.28)   
311 - **[CCCCGCCCCG]** 1170 (85.75)   
311 - **[CCCCGCCCCG]** 1165 (85.75)   
311 - **[CCCCGCCCCG]** 1160 (85.75)   
311 - **[CGCCGCGGCT]** 1103 (88.30)   
311 - **.CGCCGCGCCC.** 1066 (90.72)   
311 - **.GGCCGCGGCG.** 872 (90.53)   
311 - **[CGCGGCGTGG]** 869 (85.28)   
312 + **.CCCCGCGCCG.** 982 (90.74)   
312 + **.CGCCGCCCCG.** 987 (91.18)   
312 + **[CCCCGCCCCG]** 992 (85.75)   
312 + **[CCTCGCGCGG]** 1089 (85.36)   
312 + **[CCCCGCGCCC]** 1119 (85.30)   
312 - **[CGCCGCCTCC]** 1201 (88.09)   
312 - **[CGGGGCGGCG]** 987 (89.73)   
312 - **[CGGCGCGGGG]** 982 (89.26)   
312 - **[CTGCGCGTCG]** 740 (89.45)   
312 - **[CGTCGCCTCC]** 735 (89.56)   
312 - **[CGGGGCGTCC]** 706 (85.89)   
313 + **[CCTCGCGTGG]** 580 (87.72)   
313 + **[GGCCGCCTCG]** 829 (87.14)   
313 + **.CCTCGCGCCG.** 1085 (92.21)   
313 + **[CGCCGCGGCT]** 1445 (88.30)   
313 - **[AGCCGCGGCG]** 1445 (87.59)   
314 - **.CGGCGCCTCG.** 43 (92.73)   
315 + **[CGGCGCGGGG]** 1025 (89.26)   
315 + **[CGGCGGGCCG]** 1092 (87.72)   
315 + **[CCTCGCCGCG]** 1338 (87.98)   
315 + **.CGCCGCGTCC.** 1341 (93.08)   
315 - **[CGGCGCGCCA]** 1270 (85.05)   
315 - **[CGGCCCGCCG]** 1092 (88.47)   
315 - **[CGCCCCCGCG]** 1028 (85.05)   
315 - **.CCCCGCGCCG.** 1025 (90.74)   
315 - **[CCGCGCGTCC]** 1005 (86.85)   
315 - **[GGTCGCCGCG]** 357 (87.01)   
316 + **[CGGGGCGGCG]** 163 (89.73)   
316 + **[CGCCGAGCCG]** 259 (85.33)   
316 + **.CGGCGCGTCC.** 895 (92.28)   
316 + **[CCTCGCCCCG]** 1265 (87.22)   
316 - **[CGGCGCCGCC]** 1283 (85.68)   
316 - **[CATCGCGCCG]** 912 (87.62)   
316 - **[CGGCTCGGCG]** 259 (85.97)   
316 - **[GGGCGCGTCC]** 199 (85.88)   
316 - **.CGCCGCCCCG.** 163 (91.18)   
317 + **[CGCCGAGCCG]** 939 (85.33)   
317 - **[CCTCCCGCCG]** 1199 (85.32)   
317 - **[CGCCGCCGCC]** 1035 (86.49)   
317 - **[CGCCGCCTCC]** 1032 (88.09)   
317 - **[CCCCGCGCCC]** 1004 (85.30)   
317 - **[CGCCGGCTCG]** 942 (85.90)   
317 - **[CGGCTCGGCG]** 939 (85.97)   
318 + **[GGCCGCGTCC]** 287 (86.69)   
318 + **[CGCCGCCCCC]** 1109 (85.73)   
318 + **[CGCCCCCGCG]** 1112 (85.05)   
318 + **[CGCCGCCGCC]** 1216 (86.49)   
318 + **[CGGCCCGCCG]** 1355 (88.47)   
318 + **.GGCCGCGGCG.** 1482 (90.53)   
318 + **.CGCGGCGGCG.** 1485 (90.54)   
318 - **[CGCCGCCGCC]** 1491 (86.49)   
318 - **[CGCCGCCGCC]** 1488 (86.49)   
318 - **.CGCCGCCGCG.** 1485 (91.94)   
318 - **.CGCCGCGGCC.** 1482 (91.48)   
318 - **[CGCCGCCCCC]** 1473 (85.73)   
318 - **[CAGCGCGCCG]** 1433 (85.34)   
318 - **[CGCCGCCGCC]** 1388 (86.49)   
318 - **[CGGCGGGCCG]** 1355 (87.72)   
318 - **.CGTGGCGCCG.** 470 (91.25)   
318 - **[CGCCGCCTGG]** 465 (86.68)   
320 + **.CGTGGCGTCG.** 551 (93.61)   
320 + **[CCGCGCCTCG]** 713 (87.31)   
320 + **[CCTCGCGGCC]** 718 (87.52)   
320 + **.GGTCGCGGCG.** 752 (92.00)   
320 + **[CGCGGCGCCG]** 755 (89.78)   
320 + **[CGGCGCCGCC]** 757 (85.68)   
320 + **[GGCCGCGCCG]** 910 (89.77)   
320 + **[CTCCGCGGCG]** 989 (88.66)   
320 + **[CGCGGCGCCG]** 992 (89.78)   
320 + **[CTTCGCGCCG]** 1195 (89.37)   
320 + **[CCTCGCGGGG]** 1229 (86.11)   
320 + **[GCGCGCGTCG]** 1345 (85.90)   
320 + **[CGCCGCGGCT]** 1383 (88.30)   
320 - **[AGCCGCGGCG]** 1383 (87.59)   
320 - **.CGGCGCCGCG.** 992 (91.13)   
320 - **[CGCCGCGGAG]** 989 (86.69)   
320 - **.CGGCGCGGCC.** 910 (90.67)   
320 - **[CGCGGCCTCG]** 907 (87.15)   
320 - **[CAGCGCGCCG]** 875 (85.34)   
320 - **[CGCCGCCTCC]** 870 (88.09)   
320 - **.CGGCGCCGCG.** 755 (91.13)   
320 - **[CGCCGCGACC]** 752 (85.19)   
320 - **[CGCCCCCGCG]** 537 (85.05)   
321 + **[GCCCGCGGCG]** 452 (85.10)   
321 + **[CGCGGCGTCC]** 455 (86.69)   
321 + **[CGGCGGGCCG]** 509 (87.72)   
321 + **[CCGCGCCGCG]** 695 (85.70)   
321 + **[CGCCGCGCCT]** 698 (87.54)   
321 + **[CCGCGCGCCG]** 1257 (89.93)   
321 + **.CGGCGCGGCC.** 1299 (90.67)   
321 + **.CGCCGCGGCC.** 1397 (91.48)   
321 + **[CTTGGCGTCG]** 1481 (85.34)   
321 + **[CCGCGCGCCG]** 1503 (89.93)   
321 - **[CGGCGCGCGG]** 1503 (88.50)   
321 - **.GGCCGCGGCG.** 1397 (90.53)   
321 - **[GGCCGCGCCG]** 1299 (89.77)   
321 - **[CGGCGCGCGG]** 1257 (88.50)   
321 - **[CCCCGCCCCG]** 947 (85.75)   
321 - **[CGTCCCGCCC]** 919 (85.30)   
321 - **[AGGCGCGGCG]** 698 (86.78)   
321 - **[CGGCCCGCCG]** 509 (88.47)   
321 - **.CGCCGGGTCG.** 504 (90.89)   
321 - **[GGTCGCGGCC]** 499 (86.55)   
321 - **[CGCCGCTTCG]** 412 (89.60)   
321 - **[CGGCCCCTCG]** 365 (85.84)   
322 + **[CCCCGCGCCC]** 460 (85.30)   
322 + **[CCCCGCCGCG]** 493 (86.51)   
322 + **.CGCCGCGCCC.** 496 (90.72)   
322 + **.CGGCGCGGCC.** 629 (90.67)   
322 + **[CCCCGCCCCG]** 637 (85.75)   
322 + **[CGGCGCGGCA]** 685 (85.80)   
322 + **[CCTCCCGGCG]** 732 (86.08)   
322 + **[CGCCGCCGGG]** 950 (85.08)   
322 + **[CGCCGGGCCG]** 953 (88.53)   
322 + **[CCCCGCCCCG]** 1089 (85.75)   
322 + **[CGTCGCGCCT]** 1171 (89.01)   
322 + **[CGCCGCGCTG]** 1480 (85.67)   
322 - **[CAGCGCGGCG]** 1480 (86.10)   
322 - **[CCGCGCGGCC]** 958 (85.25)   
322 - **[CGGCCCGGCG]** 953 (89.23)   
322 - **[CCCGGCGGCG]** 950 (85.11)   
322 - **[TGCCGCGCCG]** 685 (85.55)   
322 - **[GGCCGCGCCG]** 629 (89.77)   
322 - **[GGGCGCGGCG]** 496 (89.72)   
322 - **[CCTCGCCCCG]** 431 (87.22)   
323 + **[CGTCGGGGCC]** 471 (85.31)   
323 + **[GGCCGCGGCC]** 1029 (85.08)   
323 + **[CGGCGCTGCG]** 1101 (87.19)   
323 + **[CCCCGCCCCG]** 1169 (85.75)   
323 + **[CCCCGCCCCG]** 1174 (85.75)   
323 + **[CCCCGCGCCC]** 1179 (85.30)   
323 + **.CGCCGCGGCG.** 1228 (96.93)   
323 + **[CGCCCCCGCG]** 1315 (85.05)   
323 + **[CCCCGCGCCC]** 1318 (85.30)   
323 + **[GCGCGCGTCG]** 1421 (85.90)   
323 + **[CGCCGGGCCG]** 1488 (88.53)   
323 - **[CGGCCCGGCG]** 1488 (89.23)   
323 - **[CCCCGCGCCC]** 1360 (85.30)   
323 - **[CCCCGCCGCG]** 1231 (86.51)   
323 - **.CGCCGCGGCG.** 1228 (96.93)   
323 - **[CGTCGCTTCC]** 1190 (85.62)   
323 - **[CGCAGCGCCG]** 1101 (85.31)   
323 - **[GGTCCCGGCG]** 1089 (85.11)   
323 - **[GGCCGCGGCC]** 1029 (85.08)   
323 - **[CCCCGCCCCG]** 959 (85.75)   
323 - **[CGTCCCGGCC]** 933 (86.06)   
326 + **[CCTCGCGGGG]** 686 (86.11)   
326 + **[CGCCGCGCCT]** 822 (87.54)   
326 + **[CGCGGCCGCG]** 1113 (85.55)   
326 + **[CGCGGCGGCC]** 1202 (85.09)   
326 + **[CCGCGCGGCC]** 1219 (85.25)   
326 + **.GGCCGCGGCG.** 1300 (90.53)   
326 + **.CGCGGCGGCG.** 1303 (90.54)   
326 - **[CGCCGCCCCC]** 1367 (85.73)   
326 - **[CGCCGCCCCC]** 1352 (85.73)   
326 - **.CGCCGCCGCG.** 1303 (91.94)   
326 - **.CGCCGCGGCC.** 1300 (91.48)   
326 - **[GGCCGCCGCG]** 1202 (85.54)   
326 - **[CGCGGCCGCG]** 1113 (85.55)   
326 - **[AGGCGCGGCG]** 822 (86.78)   
327 + **[CCCCGCCTCG]** 546 (88.11)   
327 + **[CCCCGGGTCG]** 587 (85.46)   
327 - **.CCTCGCGGCG.** 1112 (92.97)   
327 - **[CGGCGCCTGG]** 1107 (85.88)   
327 - **[GGTGGCGGCG]** 672 (85.61)   
327 - **[CGCCGCCGCC]** 597 (86.49)   
327 - **[CGCCGCCGCC]** 258 (86.49)   
327 - **[CGCCGCCTCC]** 255 (88.09)   
329 - **.CGGCCCGTCG.** 544 (90.83)   
329 - **[GGTGGCGGCG]** 460 (85.61)   
331 + **.CGCCGCCGCG.** 717 (91.94)   
331 + **[CGGGGCGCCG]** 753 (88.97)   
331 + **[CGGCGCGTCT]** 1137 (89.10)   
331 + **[CGCCGCCGCC]** 1169 (86.49)   
331 + **[CGGCGCCGCC]** 1178 (85.68)   
331 + **[CGCCGCCGCC]** 1181 (86.49)   
331 + **[CGCCGCCGCC]** 1184 (86.49)   
331 + **[CGCCGCCGCC]** 1187 (86.49)   
331 + **[CGGGGCGTCC]** 1270 (85.89)   
331 + **[CGCCGCCTCC]** 1299 (88.09)   
331 + **[CCGCGCGCCG]** 1308 (89.93)   
331 + **.CGCCGCGTCC.** 1313 (93.08)   
331 + **.CCCCGCGGCG.** 1329 (91.50)   
331 + **.CGCGGCGGCG.** 1332 (90.54)   
331 + **[CTGCGCGGCG]** 1438 (87.85)   
331 - **[CGCCGCGCAG]** 1438 (85.94)   
331 - **[CGTCGCCGCC]** 1335 (87.96)   
331 - **.CGCCGCCGCG.** 1332 (91.94)   
331 - **.CGCCGCGGGG.** 1329 (90.07)   
331 - **[CGGCGCGCGG]** 1308 (88.50)   
331 - **[CGGCTCGCCG]** 1012 (85.21)   
331 - **.CGGCGCCCCG.** 753 (90.37)   
331 - **.CGCGGCGGCG.** 717 (90.54)   
332 + **[CGCCGGGCCG]** 1236 (88.53)   
332 + **[CGCCGCGCCA]** 1350 (85.85)   
332 + **[GGCCGCGGCC]** 1427 (85.08)   
332 - **[CCTCGCCCCG]** 1501 (87.22)   
332 - **[CGTGGCGGGG]** 1440 (85.15)   
332 - **[GGCCGCGGCC]** 1427 (85.08)   
332 - **[TGGCGCGGCG]** 1350 (85.50)   
332 - **[CGGCCCGGCG]** 1236 (89.23)   
333 + **[CCCCGCCCCG]** 1022 (85.75)   
333 + **[CCCCGCCCCG]** 1179 (85.75)   
333 + **[CGCCGCGACC]** 1320 (85.19)   
333 - **.GGTCGCGGCG.** 1320 (92.00)   
333 - **[GCCCGCGGCG]** 1121 (85.10)   
334 + **[CCTGGCGGCG]** 510 (86.58)   
334 + **[GGTCGCCCCG]** 1140 (86.25)   
334 - **[GGTCGCGGCC]** 870 (86.55)   
335 + **[CCGCGCCTCG]** 609 (87.31)   
335 + **[CGGCGCGCGG]** 856 (88.50)   
335 + **[CGCCCCCGCG]** 1199 (85.05)   
335 + **.CCCCGCGTCG.** 1202 (93.10)   
335 + **[CGGCGGGGCG]** 1301 (88.48)   
335 + **.CGGCGCCCCG.** 1460 (90.37)   
335 - **[CGGGGCGCCG]** 1460 (88.97)   
335 - **[CGCCGCCCCC]** 1359 (85.73)   
335 - **[CGCCCCGCCG]** 1301 (89.28)   
335 - **[CCGCGCGCCG]** 856 (89.93)   
336 + **.CCCCGCGCCG.** 978 (90.74)   
336 - **[CGGCGCGGGG]** 978 (89.26)   
337 + **.CCCCGCGGCG.** 839 (91.50)   
337 + **[CGCCGCCGCC]** 1064 (86.49)   
337 + **.CGCCGCCGCG.** 1067 (91.94)   
337 + **.CGCCGCGGCC.** 1070 (91.48)   
337 + **[GCCCGCGGCG]** 1180 (85.10)   
337 + **[CGCGGCGCCG]** 1183 (89.78)   
337 + **[CGGCGCCGCC]** 1185 (85.68)   
337 - **[CGCCGCCGCC]** 1216 (86.49)   
337 - **.CGGCGCCGCG.** 1183 (91.13)   
337 - **[CCCCGCGCCC]** 1147 (85.30)   
337 - **.GGCCGCGGCG.** 1070 (90.53)   
337 - **.CGCGGCGGCG.** 1067 (90.54)   
337 - **[CCTCCCGCCG]** 958 (85.32)   
337 - **.CGCCGCGGGG.** 839 (90.07)   
337 - **[CGTCCCGCCC]** 252 (85.30)   
338 + **[CGCCGCCCCC]** 326 (85.73)   
338 + **.CGCCGCGTCC.** 691 (93.08)   
338 + **[CCTCGCGCCC]** 1062 (86.77)   
338 + **[GCTCGCGCCG]** 1169 (85.81)   
338 + **[CGCCGCCGCC]** 1174 (86.49)   
338 + **[CGCCGCCGCC]** 1177 (86.49)   
338 + **[CGCCGCCGCC]** 1180 (86.49)   
338 + **[CGCCGCCGCC]** 1183 (86.49)   
338 + **[CGCCGCCGCC]** 1186 (86.49)   
338 + **.CGCCGCCGCG.** 1189 (91.94)   
338 + **[CGCGGCGTCC]** 1459 (86.69)   
338 - **[CGCCGCCGCC]** 1379 (86.49)   
338 - **[CGCCGGCTCG]** 1311 (85.90)   
338 - **.CGCGGCGGCG.** 1189 (90.54)   
338 - **[GGTCGCCGCG]** 1054 (87.01)   
338 - **[CCCCGCCCCG]** 649 (85.75)   
339 + **[CCCGGCGGCG]** 944 (85.11)   
339 + **[CGGCGCTGCG]** 949 (87.19)   
339 + **[CGGCCCGTCC]** 1166 (85.39)   
339 - **[CGCCGCCGCC]** 1064 (86.49)   
339 - **[CGCAGCGCCG]** 949 (85.31)   
339 - **[CGCCGCCGGG]** 944 (85.08)   
340 + **[GGTCGCCCCG]** 500 (86.25)   
340 + **[CGTCGCCGCC]** 685 (87.96)   
340 + **.CGCCGCCCCG.** 935 (91.18)   
340 - **[CGGGGCGGCG]** 935 (89.73)   
340 - **[CCCCGCCCCG]** 201 (85.75)   
341 + **[GGTCGCGGGG]** 1230 (85.14)   
341 + **.CCTCGCGTCG.** 1257 (94.57)   
341 + **.CCGCGCGGCG.** 1316 (90.69)   
341 + **[CGGCGGGGCG]** 1411 (88.48)   
341 - **[CGCCCCGCCG]** 1411 (89.28)   
341 - **[CGCCGCCCCC]** 1406 (85.73)   
341 - **[CGCCGCGCGG]** 1316 (89.31)   
341 - **[CCGCGCGGCC]** 1314 (85.25)   
341 - **[CCCCGCCCCG]** 1004 (85.75)   
341 - **[CGTCCCGGCC]** 618 (86.06)   
342 + **[CGGCGCGGGG]** 835 (89.26)   
342 + **.CGGCGCGGCG.** 1152 (96.12)   
342 + **[CGCGGCGGCC]** 1155 (85.09)   
342 + **[CGTGGCGGGG]** 1485 (85.15)   
342 - **[GGCCGCCGCG]** 1155 (85.54)   
342 - **.CGCCGCGCCG.** 1152 (96.17)   
342 - **.CCCCGCGCCG.** 835 (90.74)   
343 + **[AGGCGCGGCG]** 995 (86.78)   
343 + **[CGCGGCGTGG]** 998 (85.28)   
343 + **[GGCCGCGGCC]** 1119 (85.08)   
343 + **[GGGCGCGGCG]** 1179 (89.72)   
343 - **.CGCCGCGCCC.** 1179 (90.72)   
343 - **[GGCCGCGGCC]** 1119 (85.08)   
343 - **[CGGCGCTCCG]** 1072 (86.43)   
343 - **[CGCCGCGCCT]** 995 (87.54)   
343 - **[CCCCGCGCCC]** 909 (85.30)   
343 - **[CCCCGCGTCC]** 669 (87.66)   
344 + **[CCTCCCGCCG]** 796 (85.32)   
344 + **[CGCCGCTTCG]** 1069 (89.60)   
346 + **.GGTCGCGGCG.** 20 (92.00)   
346 + **[CTCCGCGGCG]** 82 (88.66)   
346 + **[CGCCGCTCCG]** 820 (87.24)   
346 + **[CGGCGCCGCC]** 994 (85.68)   
346 + **.CGGCGCGCCG.** 1228 (95.36)   
346 + **.CGCCGCCGCG.** 1233 (91.94)   
346 + **.CGCCGCGTCC.** 1236 (93.08)   
346 - **.CGCGGCGGCG.** 1233 (90.54)   
346 - **.CGGCGCGCCG.** 1228 (95.36)   
346 - **[CGGAGCGGCG]** 820 (85.26)   
346 - **[CGCCGCGGAG]** 82 (86.69)   
346 - **[CGCCGCGACC]** 20 (85.19)   
347 + **[CCTCGCGCCC]** 550 (86.77)   
347 + **[CGCAGCGCCG]** 1065 (85.31)   
347 + **[CAGCGCGGCG]** 1085 (86.10)   
347 + **[CGTCGGCGCG]** 1097 (85.77)   
347 + **[CGCCGCCCCC]** 1181 (85.73)   
347 + **[CCGCGCGGCC]** 1204 (85.25)   
347 + **[CGGCGGGGCG]** 1227 (88.48)   
347 + **[CGACGCCTCG]** 1381 (87.77)   
347 + **[CCCCGCGCCC]** 1491 (85.30)   
347 - **[CGAGGCGTCG]** 1381 (86.37)   
347 - **[CGCCCCGCCG]** 1227 (89.28)   
347 - **.CGTCCCGCCG.** 1109 (90.75)   
347 - **[CGCCGCGCTG]** 1085 (85.67)   
347 - **[CGGCGCTGCG]** 1065 (87.19)   
347 - **[CCCCGCCCCG]** 978 (85.75)   
347 - **[CCCCGCCCCG]** 973 (85.75)   
348 + **[CCTCGCCGCG]** 65 (87.98)   
348 + **[CGCCGCGGCT]** 68 (88.30)   
348 + **[CGGCGCGCAG]** 292 (85.13)   
348 + **[CCCCGCGCCC]** 379 (85.30)   
348 + **[CGTCCCCGCG]** 407 (86.52)   
348 + **[CGCCGCTCCG]** 448 (87.24)   
348 + **[GGTGGCGGCG]** 539 (85.61)   
348 + **[CGGCGCGGGG]** 544 (89.26)   
348 + **.CGCCGCGTCG.** 661 (98.53)   
348 + **.CGCCCCGTCG.** 681 (91.64)   
348 + **.CGTCGCCGCG.** 692 (93.41)   
348 + **[CGCCGCGCCT]** 695 (87.54)   
348 + **[GCCCGCGGCG]** 709 (85.10)   
348 + **[CGCGGCGCCG]** 712 (89.78)   
348 + **[CGGCGCCGCC]** 714 (85.68)   
348 + **[CCCCGCCCCG]** 853 (85.75)   
348 + **.CGCCCCGGCG.** 856 (90.04)   
348 + **[CGTCGCCGCC]** 905 (87.96)   
348 + **[CCGCCCGTCG]** 920 (85.41)   
348 + **[AGTCGCGGCG]** 1025 (89.06)   
348 + **[GGGCGCGGCG]** 1237 (89.72)   
348 + **[CCTCGCGTGG]** 1411 (87.72)   
348 + **.GGTCGCGCCG.** 1486 (91.24)   
348 + **[CGCCGCCGCC]** 1491 (86.49)   
348 + **.CGCCGCCCCG.** 1494 (91.18)   
348 + **.CGCCCCGGCG.** 1497 (90.04)   
348 - **[CGCCGGGGCG]** 1497 (89.29)   
348 - **[CGGGGCGGCG]** 1494 (89.73)   
348 - **.CGCCGCGCCC.** 1237 (90.72)   
348 - **[CGCCGGGGCG]** 856 (89.29)   
348 - **.CGGCGCCGCG.** 712 (91.13)   
348 - **[AGGCGCGGCG]** 695 (86.78)   
348 - **.CGACGCGGCG.** 661 (91.16)   
348 - **.CCCCGCGCCG.** 544 (90.74)   
348 - **[CGGAGCGGCG]** 448 (85.26)   
348 - **[CCCCGCGTCC]** 307 (87.66)   
348 - **[CTGCGCGCCG]** 292 (87.09)   
348 - **[AGCCGCGGCG]** 68 (87.59)   
349 + **[CGGCGCGGGG]** 836 (89.26)   
349 + **.CGGCGCGGCG.** 1162 (96.12)   
349 + **.CGCCGCCCCG.** 1221 (91.18)   
349 + **[TGGCGCGTCG]** 1307 (87.10)   
349 - **[CGGGGCGGCG]** 1221 (89.73)   
349 - **.CGCCGCGCCG.** 1162 (96.17)   
349 - **[CGCCCCCGCG]** 839 (85.05)   
349 - **.CCCCGCGCCG.** 836 (90.74)   
350 + **.CGTCGCGGGG.** 998 (91.54)   
350 + **.CGGCGCGGCC.** 1043 (90.67)   
350 + **.CGCCGCCTCG.** 1138 (93.54)   
350 + **[CGCCGCCCCC]** 1188 (85.73)   
350 + **[CGGCGCGCCC]** 1342 (89.91)   
350 + **[CCTCGCGCGG]** 1425 (85.36)   
350 - **[CGCCCCGTCC]** 1363 (86.19)   
350 - **[GGGCGCGCCG]** 1342 (88.96)   
350 - **[CCCCGGGTCG]** 1227 (85.46)   
350 - **[CGGCGCCTGG]** 1059 (85.88)   
350 - **[GGCCGCGCCG]** 1043 (89.77)   
350 - **.CGCCGCCTCG.** 1025 (93.54)   
350 - **[CCTCGCCCCG]** 1020 (87.22)   
350 - **[CCCCGCCCCG]** 1015 (85.75)   
350 - **[CCCCGCGACG]** 998 (85.21)   
351 + **[CGCCGAGGCG]** 570 (86.08)   
351 + **[CGTCGCATCG]** 914 (88.97)   
351 + **[CGCCGCGGAG]** 936 (86.69)   
351 + **[CGGCGCCGCC]** 991 (85.68)   
351 + **[CCCCGCGGCC]** 1182 (86.05)   
351 - **[CTCCGCGGCG]** 936 (88.66)   
351 - **[CGCCTCGGCG]** 570 (86.77)   
351 - **[CGTCCCCTCG]** 70 (88.12)   
351 - **[CCTCGCCTCG]** 40 (89.58)   
352 + **[AGTCGCGCCG]** 52 (88.30)   
352 + **[CGCCGCCTCC]** 57 (88.09)   
352 + **.CCCCGCGGCG.** 195 (91.50)   
352 + **.CGGCGCGCCG.** 200 (95.36)   
352 + **.GGCCGCGGCG.** 309 (90.53)   
352 + **[CGCCGCCCCC]** 337 (85.73)   
352 + **[CCCCGCCGCG]** 349 (86.51)   
352 + **[CGGCGCTGCG]** 499 (87.19)   
352 + **[CTCCGCGCCG]** 715 (87.90)   
352 + **[CGGCGCCGCC]** 858 (85.68)   
352 + **[CGCCGCCGGG]** 861 (85.08)   
352 - **[CCCGGCGGCG]** 861 (85.11)   
352 - **[CGGCGCCGCC]** 856 (85.68)   
352 - **[CGGCGCGGAG]** 715 (85.89)   
352 - **[CGCAGCGCCG]** 499 (85.31)   
352 - **[CCCCGCCGCG]** 312 (86.51)   
352 - **.CGCCGCGGCC.** 309 (91.48)   
352 - **.CTCCGCGTCG.** 281 (90.26)   
352 - **.CGGCGCGCCG.** 200 (95.36)   
352 - **.CGCCGCGGGG.** 195 (90.07)   
353 + **[CAGCGCGCCG]** 660 (85.34)   
353 + **[CGCCGCCGCC]** 733 (86.49)   
353 + **[CGCCGCCGCC]** 736 (86.49)   
353 + **[CGCCGCCGCC]** 739 (86.49)   
353 + **[CGCCGCCGCC]** 742 (86.49)   
353 + **[GGTGGCGGCG]** 792 (85.61)   
353 + **[CGCCCCCGCG]** 898 (85.05)   
353 + **[CCCCGCCTCG]** 1120 (88.11)   
353 + **[CGCCGCGACC]** 1178 (85.19)   
353 + **[CTGCGCGGCG]** 1417 (87.85)   
353 - **[CCCCGCCGCG]** 1420 (86.51)   
353 - **[CGCCGCGCAG]** 1417 (85.94)   
353 - **.GGTCGCGGCG.** 1178 (92.00)   
353 - **[CCTCCCGCCG]** 777 (85.32)   
353 - **[GGTGGCGGCG]** 745 (85.61)   
353 - **[CCTCGCGTCC]** 538 (89.13)   
353 - **[CGCCTCGTCG]** 293 (88.38)   
353 - **[CGTGGCCGCG]** 167 (87.02)   
356 + **[CGTGGCGTCC]** 56 (88.16)   
356 + **.CGCCGCGCCC.** 90 (90.72)   
356 + **[CCCCGCGCCC]** 307 (85.30)   
356 - **[CCCCGCCCCG]** 1236 (85.75)   
356 - **[CCCCGCCCCG]** 989 (85.75)   
356 - **[CGCCGCCGCC]** 172 (86.49)   
356 - **[GGGCGCGGCG]** 90 (89.72)   
357 + **.CGCCGCGCCG.** 990 (96.17)   
357 + **[CGCCCCGCCG]** 1066 (89.28)   
357 + **[CGCCGAGTCG]** 1071 (87.69)   
357 - **[CGTGGCCCCG]** 1098 (86.26)   
357 - **[CGGCGGGGCG]** 1066 (88.48)   
357 - **.CGGCGCGGCG.** 990 (96.12)   
357 - **.CGTCCCGCCG.** 952 (90.75)   
357 - **[CGGGGCCTCG]** 927 (86.34)   
358 + **[CGGCGGGGCG]** 980 (88.48)   
358 - **[CGCCCCGCCG]** 980 (89.28)   
358 - **[CCCCGCCGCG]** 978 (86.51)   
359 + **[CGGCGCTTCG]** 493 (88.79)   
359 + **[CGCCGCTCCG]** 976 (87.24)   
359 + **[CGCCGCCGCC]** 984 (86.49)   
359 + **[CGCCGCCGCC]** 1025 (86.49)   
359 + **[CGCCGCCGCC]** 1028 (86.49)   
359 + **[CGCCGCCGCC]** 1055 (86.49)   
359 + **[CGCCGCCGCC]** 1058 (86.49)   
359 + **[CGCCCCGCCG]** 1116 (89.28)   
359 + **[CGCCGCCGCC]** 1121 (86.49)   
359 + **[CGCCGCCGCC]** 1124 (86.49)   
359 + **[CCCCGCCCCG]** 1170 (85.75)   
359 + **[AGCCGCGGCG]** 1231 (87.59)   
359 + **[CTGCGCGGCG]** 1252 (87.85)   
359 + **[CTGCGCGCCG]** 1304 (87.09)   
359 + **[CGGCGCCGCC]** 1312 (85.68)   
359 + **[CGCCGCCTGG]** 1315 (86.68)   
359 - **[CGGCGCGCAG]** 1304 (85.13)   
359 - **[CGCCGCGCAG]** 1252 (85.94)   
359 - **[CGCCGCGGCT]** 1231 (88.30)   
359 - **[CGGCGGGGCG]** 1116 (88.48)   
359 - **[GGTGGCGGCG]** 1061 (85.61)   
359 - **[CGGAGCGGCG]** 976 (85.26)   
360 + **[CCCCGCCCCG]** 623 (85.75)   
360 + **[CGCCGCGCTG]** 651 (85.67)   
360 + **[CCGCGCGGCC]** 791 (85.25)   
360 + **.CGCCGCGCCG.** 1065 (96.17)   
360 + **[CCGCGCCGCG]** 1067 (85.70)   
360 + **.CGCCGCGGGG.** 1070 (90.07)   
360 + **[TGCCGCGGCG]** 1124 (86.31)   
360 + **[CGGCGCGGGG]** 1184 (89.26)   
360 + **[AGGCGCGCCG]** 1242 (86.02)   
360 - **[CGTAGCGGCG]** 1247 (87.54)   
360 - **[CGGCGCGCCT]** 1242 (86.73)   
360 - **.CCCCGCGCCG.** 1184 (90.74)   
360 - **[CGCCGCGGCA]** 1124 (86.61)   
360 - **.CCCCGCGGCG.** 1070 (91.50)   
360 - **.CGGCGCGGCG.** 1065 (96.12)   
360 - **[CGCCGCCTGG]** 718 (86.68)   
360 - **[CAGCGCGGCG]** 651 (86.10)   
361 - **[CCCCGCCTCG]** 1462 (88.11)   
362 + **[CGCTGCGCCG]** 730 (85.22)   
362 + **.CGCCGCGTCC.** 735 (93.08)   
362 + **[GGGCGCGGCG]** 908 (89.72)   
362 + **[CGCCGCCGCC]** 992 (86.49)   
362 + **.CGCCGCCCCG.** 995 (91.18)   
362 + **[CGCCCCGCCG]** 998 (89.28)   
362 - **[CCTCCCGGCG]** 1265 (86.08)   
362 - **[CCCGGCGTCG]** 1262 (86.71)   
362 - **[CCCCGCCTCG]** 1145 (88.11)   
362 - **[CGGCGGGGCG]** 998 (88.48)   
362 - **[CGGGGCGGCG]** 995 (89.73)   
362 - **[CGCCGCCCCC]** 931 (85.73)   
362 - **.CGCCGCGCCC.** 908 (90.72)   
362 - **[CGTCGGGGCC]** 854 (85.31)   
362 - **[CGGCGCAGCG]** 730 (85.09)   
362 - **[CCCCGCCCCG]** 689 (85.75)   
362 - **[CCTCGCGGGG]** 424 (86.11)   
363 + **[CCCCGCCCCG]** 622 (85.75)   
363 + **[CCCCGCCCCG]** 627 (85.75)   
363 + **[GGCCGCGGCC]** 691 (85.08)   
363 + **[CCCCGCGGCC]** 796 (86.05)   
363 + **[CGGCGCCTGG]** 900 (85.88)   
363 + **.CGTCGCGGCC.** 1080 (92.95)   
363 + **[CGCGGCCGCG]** 1083 (85.55)   
363 - **[CGCGGCCGCG]** 1083 (85.55)   
363 - **[GGCCGCGGCC]** 691 (85.08)   
364 + **[CGTCGCTGCG]** 1020 (89.47)   
364 - **[CGCCGCGTCT]** 1254 (89.90)   
366 + **[CCCCGCGCCC]** 128 (85.30)   
366 + **[CGTGGCGGCC]** 227 (86.56)   
366 + **.CGCGGCGGCG.** 410 (90.54)   
366 + **[GCCCGCGGCG]** 442 (85.10)   
366 + **[CGCGGCGTCC]** 445 (86.69)   
366 + **.CGCCCCGGCG.** 622 (90.04)   
366 + **[GGGCGCGCCG]** 655 (88.96)   
366 + **[GCTCGCGTCG]** 851 (88.18)   
366 + **[CGTCGGCCCG]** 856 (85.01)   
366 + **.CCCCGCGGCG.** 951 (91.50)   
366 + **[CGCGGCGTCC]** 954 (86.69)   
366 + **.CGTCCCGTCG.** 959 (93.11)   
366 + **[CGTCGCGTTG]** 973 (89.50)   
366 + **[CGGGGCGGCG]** 1168 (89.73)   
366 + **[AGGCGCGGCG]** 1390 (86.78)   
366 + **.CGCGGCGGCG.** 1393 (90.54)   
366 - **.CGCCGCCGCG.** 1393 (91.94)   
366 - **[CGCCGCGCCT]** 1390 (87.54)   
366 - **.CGCCGCCCCG.** 1168 (91.18)   
366 - **[CGTCGCTCCG]** 992 (88.71)   
366 - **.CGCCGCGGGG.** 951 (90.07)   
366 - **[CGGCGCGCCC]** 655 (89.91)   
366 - **[CGCCGGGGCG]** 622 (89.29)   
366 - **.CGCCGCCGCG.** 410 (91.94)   
366 - **[CCTCGGGGCG]** 392 (85.33)   
366 - **[CCCCGCCCCG]** 216 (85.75)   
366 - **[CCCCGCGGCC]** 181 (86.05)   
366 - **[GGGCGCCTCG]** 67 (86.33)   
367 + **[CCCCGCGCCC]** 6 (85.30)   
367 + **.CGGCGCCCCG.** 15 (90.37)   
367 + **[CGCCGCTGCG]** 223 (88.00)   
367 + **[CGCTGCGCCG]** 226 (85.22)   
367 + **[CGCCGCCGCC]** 894 (86.49)   
367 + **[CGCCGCCGCC]** 897 (86.49)   
367 + **[CGCCGCCGCC]** 900 (86.49)   
367 + **[CGCCGCCGCC]** 903 (86.49)   
367 + **[CGCCGCCGCC]** 906 (86.49)   
367 + **[CGCCGCCGCC]** 909 (86.49)   
367 + **[CGCCGCCGCC]** 912 (86.49)   
367 + **[CGGCGCCGCC]** 1143 (85.68)   
367 + **[CGCCGCCGCC]** 1146 (86.49)   
367 + **[CGCCGCCCCC]** 1149 (85.73)   
367 + **.CGCCGCGGCG.** 1189 (96.93)   
367 + **.CGCGGCGGCG.** 1192 (90.54)   
367 - **.CGCCGCCGCG.** 1192 (91.94)   
367 - **.CGCCGCGGCG.** 1189 (96.93)   
367 - **[CGGCGCAGCG]** 226 (85.09)   
367 - **[CGCAGCGGCG]** 223 (86.06)   
367 - **[CGTCGCTGCG]** 215 (89.47)   
367 - **[CGGGGCGCCG]** 15 (88.97)   
  

**Frequency Matrix**

| All Occs Best Occs|  |  |  |  |  |  |  |  |  |  |  |  |  |  |  |  |  |  |  |  |  |  |  |  |  |  |  |  |  |  |  |  |  |  |  |  |  |  |  |  |  |  |  |  |  |  |  |  |  |  |  |  |  |  |  |  |  |  |  |  |  |  |  |  |  |  |  |  |  |  |  |  |  |  |  |  |  |  |  |  |  |  |  |  |  |  |  |  |  |  |  |  |  |  |  |  |  |  |  |  |  |  |  |  |  |  |  |  |  |  |  |  |  |  |  |  |  |  |  |  |  |  |  |  |  |  |  |  |  |  |  |  | | --- | --- | --- | --- | --- | --- | --- | --- | --- | --- | --- | --- | --- | --- | --- | --- | --- | --- | --- | --- | --- | --- | --- | --- | --- | --- | --- | --- | --- | --- | --- | --- | --- | --- | --- | --- | --- | --- | --- | --- | --- | --- | --- | --- | --- | --- | --- | --- | --- | --- | --- | --- | --- | --- | --- | --- | --- | --- | --- | --- | --- | --- | --- | --- | --- | --- | --- | --- | --- | --- | --- | --- | --- | --- | --- | --- | --- | --- | --- | --- | --- | --- | --- | --- | --- | --- | --- | --- | --- | --- | --- | --- | --- | --- | --- | --- | --- | --- | --- | --- | --- | --- | --- | --- | --- | --- | --- | --- | --- | --- | --- | --- | --- | --- | --- | --- | --- | --- | --- | --- | --- | --- | --- | --- | --- | --- | --- | --- | --- | --- | --- | --- | | **A C G T A C G T | | | | | | | | | | | |**| **1** 255 3641 589 177 | 0 567 51 0|  |  |  |  |  |  |  |  |  |  |  |  |  |  |  |  |  |  |  |  |  |  |  |  |  |  |  |  |  |  |  |  |  |  |  |  |  |  |  |  |  |  |  |  |  |  |  |  |  |  |  |  |  |  |  |  |  |  |  |  |  |  |  |  |  |  |  |  |  |  |  |  |  |  |  |  |  |  |  |  |  |  |  |  |  |  |  |  |  |  |  |  |  |  |  |  |  |  |  |  |  |  |  |  |  |  |  |  | | --- | --- | --- | --- | --- | --- | --- | --- | --- | --- | --- | --- | --- | --- | --- | --- | --- | --- | --- | --- | --- | --- | --- | --- | --- | --- | --- | --- | --- | --- | --- | --- | --- | --- | --- | --- | --- | --- | --- | --- | --- | --- | --- | --- | --- | --- | --- | --- | --- | --- | --- | --- | --- | --- | --- | --- | --- | --- | --- | --- | --- | --- | --- | --- | --- | --- | --- | --- | --- | --- | --- | --- | --- | --- | --- | --- | --- | --- | --- | --- | --- | --- | --- | --- | --- | --- | --- | --- | --- | --- | --- | --- | --- | --- | --- | --- | --- | --- | --- | --- | --- | --- | --- | --- | --- | --- | --- | --- | | **2** 197 728 3413 324 | 0 82 531 5|  |  |  |  |  |  |  |  |  |  |  |  |  |  |  |  |  |  |  |  |  |  |  |  |  |  |  |  |  |  |  |  |  |  |  |  |  |  |  |  |  |  |  |  |  |  |  |  |  |  |  |  |  |  |  |  |  |  |  |  |  |  |  |  |  |  |  |  |  |  |  |  |  |  |  |  |  |  |  |  |  |  |  |  |  |  |  |  |  |  |  |  |  |  |  |  | | --- | --- | --- | --- | --- | --- | --- | --- | --- | --- | --- | --- | --- | --- | --- | --- | --- | --- | --- | --- | --- | --- | --- | --- | --- | --- | --- | --- | --- | --- | --- | --- | --- | --- | --- | --- | --- | --- | --- | --- | --- | --- | --- | --- | --- | --- | --- | --- | --- | --- | --- | --- | --- | --- | --- | --- | --- | --- | --- | --- | --- | --- | --- | --- | --- | --- | --- | --- | --- | --- | --- | --- | --- | --- | --- | --- | --- | --- | --- | --- | --- | --- | --- | --- | --- | --- | --- | --- | --- | --- | --- | --- | --- | --- | --- | --- | | **3** 257 1329 1056 2020 | 7 405 135 71|  |  |  |  |  |  |  |  |  |  |  |  |  |  |  |  |  |  |  |  |  |  |  |  |  |  |  |  |  |  |  |  |  |  |  |  |  |  |  |  |  |  |  |  |  |  |  |  |  |  |  |  |  |  |  |  |  |  |  |  |  |  |  |  |  |  |  |  |  |  |  |  |  |  |  |  |  |  |  |  |  |  |  |  | | --- | --- | --- | --- | --- | --- | --- | --- | --- | --- | --- | --- | --- | --- | --- | --- | --- | --- | --- | --- | --- | --- | --- | --- | --- | --- | --- | --- | --- | --- | --- | --- | --- | --- | --- | --- | --- | --- | --- | --- | --- | --- | --- | --- | --- | --- | --- | --- | --- | --- | --- | --- | --- | --- | --- | --- | --- | --- | --- | --- | --- | --- | --- | --- | --- | --- | --- | --- | --- | --- | --- | --- | --- | --- | --- | --- | --- | --- | --- | --- | --- | --- | --- | --- | | **4** 169 3724 604 165 | 0 564 54 0|  |  |  |  |  |  |  |  |  |  |  |  |  |  |  |  |  |  |  |  |  |  |  |  |  |  |  |  |  |  |  |  |  |  |  |  |  |  |  |  |  |  |  |  |  |  |  |  |  |  |  |  |  |  |  |  |  |  |  |  |  |  |  |  |  |  |  |  |  |  |  |  | | --- | --- | --- | --- | --- | --- | --- | --- | --- | --- | --- | --- | --- | --- | --- | --- | --- | --- | --- | --- | --- | --- | --- | --- | --- | --- | --- | --- | --- | --- | --- | --- | --- | --- | --- | --- | --- | --- | --- | --- | --- | --- | --- | --- | --- | --- | --- | --- | --- | --- | --- | --- | --- | --- | --- | --- | --- | --- | --- | --- | --- | --- | --- | --- | --- | --- | --- | --- | --- | --- | --- | --- | | **5** 181 527 3746 208 | 0 26 592 0|  |  |  |  |  |  |  |  |  |  |  |  |  |  |  |  |  |  |  |  |  |  |  |  |  |  |  |  |  |  |  |  |  |  |  |  |  |  |  |  |  |  |  |  |  |  |  |  |  |  |  |  |  |  |  |  |  |  |  |  | | --- | --- | --- | --- | --- | --- | --- | --- | --- | --- | --- | --- | --- | --- | --- | --- | --- | --- | --- | --- | --- | --- | --- | --- | --- | --- | --- | --- | --- | --- | --- | --- | --- | --- | --- | --- | --- | --- | --- | --- | --- | --- | --- | --- | --- | --- | --- | --- | --- | --- | --- | --- | --- | --- | --- | --- | --- | --- | --- | --- | | **6** 178 3901 443 140 | 0 606 12 0|  |  |  |  |  |  |  |  |  |  |  |  |  |  |  |  |  |  |  |  |  |  |  |  |  |  |  |  |  |  |  |  |  |  |  |  |  |  |  |  |  |  |  |  |  |  |  |  | | --- | --- | --- | --- | --- | --- | --- | --- | --- | --- | --- | --- | --- | --- | --- | --- | --- | --- | --- | --- | --- | --- | --- | --- | --- | --- | --- | --- | --- | --- | --- | --- | --- | --- | --- | --- | --- | --- | --- | --- | --- | --- | --- | --- | --- | --- | --- | --- | | **7** 148 826 3419 269 | 0 134 484 0|  |  |  |  |  |  |  |  |  |  |  |  |  |  |  |  |  |  |  |  |  |  |  |  |  |  |  |  |  |  |  |  |  |  |  |  | | --- | --- | --- | --- | --- | --- | --- | --- | --- | --- | --- | --- | --- | --- | --- | --- | --- | --- | --- | --- | --- | --- | --- | --- | --- | --- | --- | --- | --- | --- | --- | --- | --- | --- | --- | --- | | **8** 219 1058 1313 2072 | 0 181 345 92|  |  |  |  |  |  |  |  |  |  |  |  |  |  |  |  |  |  |  |  |  |  |  |  | | --- | --- | --- | --- | --- | --- | --- | --- | --- | --- | --- | --- | --- | --- | --- | --- | --- | --- | --- | --- | --- | --- | --- | --- | | **9** 203 3740 531 188 | 0 583 35 0|  |  |  |  |  |  |  |  |  |  |  |  | | --- | --- | --- | --- | --- | --- | --- | --- | --- | --- | --- | --- | | **10** 183 732 3451 296 | 0 118 500 0 | | | | | | | | | | | | | | | | | | | | | | | | | | | | | | | | | | | | | | | | | | | | | | | | | | | | | | | | | | | | | | | | | | | | | | | | | | | | | | | | | | | | | | | | | | | | | | | | | | | | | | | | | | | | | | | | | | | | | | | | | | | | | | | | | | | | |

  
  

---

  
  
 **\*\*\* Interesting motifs (not highest-ranking) can also be :**   
  
  
 **CCGACG**   
**CGTCGG**  
  
3 redundant motifs found:  
CCGACGAC - CGACGC - CGCGTC -   
  
Best occurrences (match percentage):   
Seq St oligo pos match
  
 1 + **[CCCACG]** 932 (88.40)   
1 + **[CAGACG]** 1012 (85.30)   
1 + **.CCGAGG.** 1062 (90.88)   
1 + **.CCGCCG.** 1076 (96.68)   
1 + **.CCGCCG.** 1206 (96.68)   
1 + **.CCGCCG.** 1244 (96.68)   
1 + **.CCGGCG.** 1247 (94.52)   
1 + **.CCGGCG.** 1299 (94.52)   
1 + **[CCCCCG]** 1387 (85.08)   
1 + **[CCGACC]** 1390 (88.08)   
1 - **.CCGGCG.** 1251 (94.52)   
1 - **.CCGGCG.** 1245 (94.52)   
2 + **.CCGAGG.** 54 (90.88)   
2 + **.CCGAGG.** 83 (90.88)   
2 + **[CCGGGG]** 152 (85.40)   
2 + **.CCGACG.** 506 (100.00)   
2 + **[CCCCCG]** 733 (85.08)   
2 + **[CAGACG]** 800 (85.30)   
2 + **[CCCACG]** 822 (88.40)   
2 + **[CCGACC]** 912 (88.08)   
2 + **[CCGCGG]** 976 (87.56)   
2 + **[CCCCCG]** 1061 (85.08)   
2 + **.CCGGCG.** 1199 (94.52)   
2 + **[CCCCCG]** 1264 (85.08)   
2 + **.CCGCCG.** 1267 (96.68)   
2 + **[CCGGGG]** 1301 (85.40)   
2 + **[CCGGGG]** 1334 (85.40)   
2 + **[CCCCCG]** 1387 (85.08)   
2 + **[CCGCGG]** 1498 (87.56)   
2 + **[CCCCCG]** 1509 (85.08)   
2 + **.CCGAGG.** 1512 (90.88)   
2 - **[CCCCCG]** 1501 (85.08)   
2 - **[CCGCGG]** 1498 (87.56)   
2 - **[CCGAAG]** 1471 (86.09)   
2 - **[CCCCCG]** 1335 (85.08)   
2 - **[CGGACG]** 1311 (86.87)   
2 - **[CCGACC]** 1305 (88.08)   
2 - **[CCGGGG]** 1299 (85.40)   
2 - **.CCGCCG.** 1200 (96.68)   
2 - **[CCCACG]** 1036 (88.40)   
2 - **[CCGCGG]** 976 (87.56)   
2 - **[CCGGGG]** 928 (85.40)   
2 - **[CCGGGG]** 734 (85.40)   
2 - **.CCGAGG.** 617 (90.88)   
2 - **[CGGACG]** 517 (86.87)   
2 - **[CGGACG]** 479 (86.87)   
2 - **.CCGAGG.** 430 (90.88)   
2 - **[CCGGGG]** 255 (85.40)   
2 - **[CCGTCG]** 244 (86.99)   
2 - **[CCGACT]** 30 (85.38)   
3 + **[CCGACC]** 602 (88.08)   
3 + **[CCCCCG]** 653 (85.08)   
3 + **.CCGAGG.** 987 (90.88)   
3 + **[CCGCGG]** 1222 (87.56)   
3 + **.CCGCCG.** 1262 (96.68)   
3 + **.CCGGCG.** 1281 (94.52)   
3 + **[CCCCCG]** 1329 (85.08)   
3 + **.CCGGCG.** 1387 (94.52)   
3 + **.CCGCCG.** 1408 (96.68)   
3 + **.CCGCCG.** 1411 (96.68)   
3 + **.CCGCCG.** 1414 (96.68)   
3 + **.CCGAGG.** 1471 (90.88)   
3 - **[CCGGGG]** 1330 (85.40)   
3 - **[CCGGGG]** 1279 (85.40)   
3 - **[CCGACT]** 1245 (85.38)   
3 - **.CCGCCG.** 1225 (96.68)   
3 - **[CCGCGG]** 1222 (87.56)   
3 - **.CCGAGG.** 932 (90.88)   
3 - **.CCGAGG.** 775 (90.88)   
3 - **[CCGGGG]** 654 (85.40)   
3 - **.CCGAGG.** 131 (90.88)   
4 + **[CCCCCG]** 570 (85.08)   
4 + **[CCGCGG]** 640 (87.56)   
4 + **.CCGCCG.** 697 (96.68)   
4 + **[CCGTCG]** 738 (86.99)   
4 + **[CCCCCG]** 747 (85.08)   
4 + **[CCGCGG]** 821 (87.56)   
4 + **[CCGCGG]** 874 (87.56)   
4 + **[CCGCGG]** 897 (87.56)   
4 + **[CCGGGG]** 1120 (85.40)   
4 + **[CCGCGG]** 1178 (87.56)   
4 + **.CCGCCG.** 1204 (96.68)   
4 + **[CCGAAG]** 1220 (86.09)   
4 + **.CCGAGG.** 1261 (90.88)   
4 + **.CCGAGG.** 1357 (90.88)   
4 + **[CGGACG]** 1440 (86.87)   
4 - **.CCGAGG.** 1378 (90.88)   
4 - **[CCCCCG]** 1278 (85.08)   
4 - **[CCCCCG]** 1268 (85.08)   
4 - **[CCGGGG]** 1227 (85.40)   
4 - **.CCGCCG.** 1188 (96.68)   
4 - **[CCGCGG]** 1178 (87.56)   
4 - **.CCGCCG.** 1142 (96.68)   
4 - **.CCGCCG.** 906 (96.68)   
4 - **[CCGCGG]** 897 (87.56)   
4 - **[CCGCGG]** 874 (87.56)   
4 - **.CCGCCG.** 824 (96.68)   
4 - **[CCGCGG]** 821 (87.56)   
4 - **[CCGGGG]** 768 (85.40)   
4 - **[CCGGGG]** 748 (85.40)   
4 - **[GCGACG]** 739 (85.28)   
4 - **[CCGACC]** 689 (88.08)   
4 - **.CCGCCG.** 672 (96.68)   
4 - **[CCGCGG]** 640 (87.56)   
4 - **[CCGTCG]** 266 (86.99)   
4 - **[CCCACG]** 39 (88.40)   
5 + **[CGGACG]** 793 (86.87)   
5 + **[CCGTCG]** 921 (86.99)   
5 + **.CCGACG.** 937 (100.00)   
5 + **[CCGCGG]** 1075 (87.56)   
5 + **.CCGCCG.** 1173 (96.68)   
5 + **[CCGCGG]** 1190 (87.56)   
5 + **.CCGGCG.** 1219 (94.52)   
5 + **.CCGCCG.** 1309 (96.68)   
5 + **[CCCCCG]** 1383 (85.08)   
5 + **.CCGGCG.** 1386 (94.52)   
5 + **[CCCCCG]** 1416 (85.08)   
5 + **.CCGACG.** 1436 (100.00)   
5 + **.CCGAGG.** 1445 (90.88)   
5 + **.CCGGCG.** 1474 (94.52)   
5 + **.CCGCCG.** 1481 (96.68)   
5 + **.CCGCCG.** 1484 (96.68)   
5 + **[CGGACG]** 1497 (86.87)   
5 - **.CCGCCG.** 1475 (96.68)   
5 - **[CCGGGG]** 1472 (85.40)   
5 - **[CCGGGG]** 1384 (85.40)   
5 - **[CGGACG]** 1371 (86.87)   
5 - **.CCGAGG.** 1350 (90.88)   
5 - **.CCGCCG.** 1220 (96.68)   
5 - **[CCCCCG]** 1193 (85.08)   
5 - **[CCGCGG]** 1190 (87.56)   
5 - **.CCGGCG.** 1174 (94.52)   
5 - **[CCGCGG]** 1075 (87.56)   
5 - **.CCGACG.** 922 (100.00)   
5 - **.CCGAGG.** 790 (90.88)   
5 - **.CCGAGG.** 770 (90.88)   
6 + **[CCGGGG]** 956 (85.40)   
6 + **[CCGCGG]** 1409 (87.56)   
6 + **[CCGCGG]** 1439 (87.56)   
6 - **.CCGCCG.** 1477 (96.68)   
6 - **[CCGCGG]** 1439 (87.56)   
6 - **[CGGACG]** 1436 (86.87)   
6 - **[CCGCGG]** 1409 (87.56)   
6 - **[CCGACT]** 1088 (85.38)   
6 - **[CCCACG]** 962 (88.40)   
6 - **.CCGGCG.** 954 (94.52)   
6 - **[CCGACC]** 640 (88.08)   
8 + **[CCCCCG]** 133 (85.08)   
8 + **.CCGGCG.** 352 (94.52)   
8 + **[CCGGGG]** 396 (85.40)   
8 + **[CCGGGG]** 416 (85.40)   
8 + **[CCCCCG]** 546 (85.08)   
8 + **[CCGACT]** 549 (85.38)   
8 + **[CAGACG]** 583 (85.30)   
8 + **[CCCCCG]** 784 (85.08)   
8 + **.CCGCCG.** 812 (96.68)   
8 + **[CCCCCG]** 823 (85.08)   
8 + **[CCGGGG]** 826 (85.40)   
8 + **[CCCCCG]** 854 (85.08)   
8 + **[CCGGGG]** 892 (85.40)   
8 + **[CCGCGG]** 912 (87.56)   
8 + **.CCGGCG.** 1026 (94.52)   
8 + **[CCCCCG]** 1078 (85.08)   
8 + **[CCCACG]** 1092 (88.40)   
8 + **[CCGCGG]** 1197 (87.56)   
8 + **.CCGCCG.** 1233 (96.68)   
8 + **.CCGAGG.** 1281 (90.88)   
8 + **[CCCCCG]** 1297 (85.08)   
8 + **.CCGCCG.** 1323 (96.68)   
8 + **.CCGGCG.** 1326 (94.52)   
8 + **[CCCCCG]** 1342 (85.08)   
8 + **[CCCCCG]** 1367 (85.08)   
8 + **.CCGGCG.** 1370 (94.52)   
8 + **.CCGAGG.** 1451 (90.88)   
8 + **[GCGACG]** 1467 (85.28)   
8 + **[CCGTCG]** 1483 (86.99)   
8 + **[CCGCGG]** 1509 (87.56)   
8 - **[CCGCGG]** 1509 (87.56)   
8 - **.CCGACG.** 1484 (100.00)   
8 - **[CCGTCG]** 1468 (86.99)   
8 - **[CCGGGG]** 1422 (85.40)   
8 - **.CCGAGG.** 1382 (90.88)   
8 - **[CCGGGG]** 1368 (85.40)   
8 - **.CCGGCG.** 1324 (94.52)   
8 - **[CCGCGG]** 1197 (87.56)   
8 - **[CGGACG]** 1194 (86.87)   
8 - **[CCGGGG]** 1055 (85.40)   
8 - **.CCGCCG.** 1027 (96.68)   
8 - **[CCCACG]** 999 (88.40)   
8 - **[CCGCGG]** 912 (87.56)   
8 - **[CCCCCG]** 893 (85.08)   
8 - **[CCCCCG]** 881 (85.08)   
8 - **[CCGGGG]** 824 (85.40)   
8 - **[CCCCCG]** 417 (85.08)   
8 - **[CCCCCG]** 388 (85.08)   
8 - **[CCCCCG]** 7 (85.08)   
8 - **.CCGCCG.** 4 (96.68)   
9 + **.CCGAGG.** 54 (90.88)   
9 + **.CCGCCG.** 849 (96.68)   
9 + **[CCCCCG]** 1058 (85.08)   
9 + **[CCGGGG]** 1174 (85.40)   
9 + **[GCGACG]** 1241 (85.28)   
9 + **[GCGACG]** 1269 (85.28)   
9 + **[CCGAAG]** 1485 (86.09)   
9 - **[CCGTCG]** 1270 (86.99)   
9 - **[CCCACG]** 1253 (88.40)   
9 - **[CCGTCG]** 1242 (86.99)   
9 - **[CCCCCG]** 1175 (85.08)   
9 - **[CCGACC]** 473 (88.08)   
10 + **.CCGGCG.** 29 (94.52)   
10 + **[CCGGGG]** 51 (85.40)   
10 + **[CCGCGG]** 88 (87.56)   
10 + **[CAGACG]** 301 (85.30)   
10 + **[CCGCGG]** 489 (87.56)   
10 + **[CCGCGG]** 578 (87.56)   
10 + **[CCGGGG]** 596 (85.40)   
10 + **[CCGCGG]** 604 (87.56)   
10 + **[CCGGGG]** 709 (85.40)   
10 + **[CCGGGG]** 742 (85.40)   
10 + **.CCGCCG.** 786 (96.68)   
10 + **[CCGTCG]** 789 (86.99)   
10 + **[CGGACG]** 846 (86.87)   
10 + **[CCGGGG]** 900 (85.40)   
10 + **[CCGCGG]** 1023 (87.56)   
10 + **[CCGCGG]** 1120 (87.56)   
10 + **[CCCCCG]** 1193 (85.08)   
10 + **[CCGGGG]** 1196 (85.40)   
10 + **.CCGCCG.** 1261 (96.68)   
10 + **[CCGACT]** 1280 (85.38)   
10 + **[CCCCCG]** 1336 (85.08)   
10 + **.CCGAGG.** 1400 (90.88)   
10 + **[CCGCGG]** 1447 (87.56)   
10 - **[CCGCGG]** 1447 (87.56)   
10 - **[CCGGGG]** 1337 (85.40)   
10 - **[CGGACG]** 1221 (86.87)   
10 - **[CCGGGG]** 1194 (85.40)   
10 - **[CCCCCG]** 1153 (85.08)   
10 - **[CCGCGG]** 1120 (87.56)   
10 - **.CCGCCG.** 1098 (96.68)   
10 - **.CCGCCG.** 1026 (96.68)   
10 - **[CCGCGG]** 1023 (87.56)   
10 - **[CCCCCG]** 976 (85.08)   
10 - **[CCCCCG]** 907 (85.08)   
10 - **[CCGACC]** 904 (88.08)   
10 - **.CCGGCG.** 898 (94.52)   
10 - **[CCCCCG]** 885 (85.08)   
10 - **[CCCCCG]** 866 (85.08)   
10 - **.CCGGCG.** 850 (94.52)   
10 - **.CCGCCG.** 811 (96.68)   
10 - **[GCGACG]** 790 (85.28)   
10 - **[CCGACC]** 746 (88.08)   
10 - **[CCCCCG]** 710 (85.08)   
10 - **.CCGCCG.** 690 (96.68)   
10 - **[CCGCGG]** 604 (87.56)   
10 - **.CCGGCG.** 594 (94.52)   
10 - **[CCGCGG]** 578 (87.56)   
10 - **[CCGCGG]** 489 (87.56)   
10 - **[CCCCCG]** 194 (85.08)   
10 - **.CCGAGG.** 191 (90.88)   
10 - **[CCCCCG]** 126 (85.08)   
10 - **[CCGCGG]** 88 (87.56)   
10 - **[CCCCCG]** 52 (85.08)   
10 - **.CCGACG.** 43 (100.00)   
10 - **.CCGCCG.** 30 (96.68)   
11 + **[CCCCCG]** 206 (85.08)   
11 + **.CCGGCG.** 209 (94.52)   
11 + **.CCGGCG.** 414 (94.52)   
11 + **[CCGTCG]** 477 (86.99)   
11 + **[CCGTCG]** 527 (86.99)   
11 + **[CCGCGG]** 610 (87.56)   
11 + **[CCGCGG]** 662 (87.56)   
11 + **[CGGACG]** 696 (86.87)   
11 + **[CCGCGG]** 714 (87.56)   
11 + **[CCCCCG]** 737 (85.08)   
11 + **.CCGGCG.** 740 (94.52)   
11 + **[CCGCGG]** 768 (87.56)   
11 + **.CCGCCG.** 992 (96.68)   
11 + **.CCGCCG.** 1007 (96.68)   
11 + **.CCGCCG.** 1025 (96.68)   
11 + **.CCGCCG.** 1036 (96.68)   
11 + **.CCGCCG.** 1039 (96.68)   
11 + **.CCGCCG.** 1042 (96.68)   
11 + **[CCGGGG]** 1064 (85.40)   
11 + **.CCGGCG.** 1086 (94.52)   
11 + **.CCGCCG.** 1153 (96.68)   
11 + **.CCGGCG.** 1367 (94.52)   
11 - **[CCGGGG]** 1468 (85.40)   
11 - **.CCGCCG.** 1368 (96.68)   
11 - **.CCGGCG.** 1207 (94.52)   
11 - **.CCGCCG.** 1118 (96.68)   
11 - **.CCGCCG.** 1087 (96.68)   
11 - **[CCGACC]** 813 (88.08)   
11 - **[CCGCGG]** 768 (87.56)   
11 - **.CCGCCG.** 741 (96.68)   
11 - **[CCGGGG]** 738 (85.40)   
11 - **[CCGCGG]** 714 (87.56)   
11 - **[CCGGGG]** 708 (85.40)   
11 - **[CCGCGG]** 662 (87.56)   
11 - **[CCGCGG]** 610 (87.56)   
11 - **[GCGACG]** 528 (85.28)   
11 - **[CCGGGG]** 510 (85.40)   
11 - **.CCGGCG.** 221 (94.52)   
11 - **.CCGCCG.** 210 (96.68)   
11 - **[CCGGGG]** 207 (85.40)   
12 + **.CCGGCG.** 17 (94.52)   
12 + **[CCGGGG]** 168 (85.40)   
12 + **[CCCACG]** 203 (88.40)   
12 + **[CCGGGG]** 471 (85.40)   
12 + **.CCGGCG.** 539 (94.52)   
12 + **[CCGCGG]** 710 (87.56)   
12 + **.CCGCCG.** 775 (96.68)   
12 + **[CCGTCG]** 830 (86.99)   
12 + **[CCGGGG]** 973 (85.40)   
12 + **[CCGGGG]** 1046 (85.40)   
12 + **[CCGCGG]** 1089 (87.56)   
12 + **[CCCCCG]** 1163 (85.08)   
12 + **[CCGCGG]** 1166 (87.56)   
12 + **.CCGAGG.** 1193 (90.88)   
12 + **[CCGGGG]** 1228 (85.40)   
12 + **.CCGGCG.** 1235 (94.52)   
12 + **[CCCCCG]** 1396 (85.08)   
12 - **[CCCCCG]** 1430 (85.08)   
12 - **[CCCACG]** 1341 (88.40)   
12 - **[CCGGGG]** 1311 (85.40)   
12 - **.CCGCCG.** 1246 (96.68)   
12 - **[CCCCCG]** 1239 (85.08)   
12 - **.CCGCCG.** 1236 (96.68)   
12 - **[CCCCCG]** 1229 (85.08)   
12 - **[CCGCGG]** 1166 (87.56)   
12 - **.CCGAGG.** 1150 (90.88)   
12 - **[CCGCGG]** 1089 (87.56)   
12 - **[CCGGGG]** 1068 (85.40)   
12 - **[CCGACC]** 979 (88.08)   
12 - **[CCCCCG]** 974 (85.08)   
12 - **.CCGACG.** 831 (100.00)   
12 - **[CCGCGG]** 710 (87.56)   
12 - **.CCGAGG.** 531 (90.88)   
12 - **[CCCCCG]** 472 (85.08)   
12 - **.CCGACG.** 450 (100.00)   
12 - **[CAGACG]** 56 (85.30)   
13 + **[CCGGGG]** 601 (85.40)   
13 + **.CCGCCG.** 623 (96.68)   
13 + **[CCCCCG]** 692 (85.08)   
13 + **.CCGGCG.** 695 (94.52)   
13 + **[CCGCGG]** 702 (87.56)   
13 + **.CCGCCG.** 719 (96.68)   
13 + **.CCGCCG.** 752 (96.68)   
13 + **.CCGCCG.** 836 (96.68)   
13 + **.CCGGCG.** 879 (94.52)   
13 + **[CCGGGG]** 902 (85.40)   
13 + **[CCGGGG]** 997 (85.40)   
13 + **.CCGGCG.** 1060 (94.52)   
13 + **.CCGCCG.** 1108 (96.68)   
13 + **.CCGCCG.** 1124 (96.68)   
13 + **[CCGACT]** 1127 (85.38)   
13 + **.CCGGCG.** 1135 (94.52)   
13 + **.CCGCCG.** 1141 (96.68)   
13 + **.CCGCCG.** 1144 (96.68)   
13 + **[CCGTCG]** 1156 (86.99)   
13 + **.CCGCCG.** 1162 (96.68)   
13 + **.CCGCCG.** 1165 (96.68)   
13 + **.CCGCCG.** 1195 (96.68)   
13 + **[CCGCGG]** 1198 (87.56)   
13 + **[CCGCGG]** 1228 (87.56)   
13 + **[CCGGGG]** 1268 (85.40)   
13 + **[GCGACG]** 1403 (85.28)   
13 - **[CCGACC]** 1450 (88.08)   
13 - **[CCCCCG]** 1446 (85.08)   
13 - **[CCGACC]** 1443 (88.08)   
13 - **[CCGGGG]** 1438 (85.40)   
13 - **.CCGCCG.** 1430 (96.68)   
13 - **[CCCCCG]** 1424 (85.08)   
13 - **.CCGCCG.** 1421 (96.68)   
13 - **[CCGTCG]** 1404 (86.99)   
13 - **[CCGGGG]** 1394 (85.40)   
13 - **.CCGCCG.** 1354 (96.68)   
13 - **[GCGACG]** 1321 (85.28)   
13 - **[CCGGGG]** 1311 (85.40)   
13 - **.CCGGCG.** 1266 (94.52)   
13 - **[CCGCGG]** 1228 (87.56)   
13 - **[CCGCGG]** 1198 (87.56)   
13 - **[CGGACG]** 1192 (86.87)   
13 - **[GCGACG]** 1157 (85.28)   
13 - **.CCGCCG.** 974 (96.68)   
13 - **[CCCCCG]** 903 (85.08)   
13 - **.CCGGCG.** 837 (94.52)   
13 - **.CCGAGG.** 797 (90.88)   
13 - **[CCGCGG]** 702 (87.56)   
13 - **[CCGGGG]** 693 (85.40)   
13 - **.CCGAGG.** 559 (90.88)   
13 - **.CCGCCG.** 545 (96.68)   
13 - **[CCCCCG]** 499 (85.08)   
13 - **[CCCCCG]** 262 (85.08)   
14 + **[CCGAAG]** 63 (86.09)   
14 + **.CCGAGG.** 136 (90.88)   
14 + **[CCCCCG]** 169 (85.08)   
14 + **[CCCCCG]** 190 (85.08)   
14 + **[CCGACC]** 193 (88.08)   
14 + **[CCGGGG]** 216 (85.40)   
14 + **[CCGGGG]** 226 (85.40)   
14 + **[CCGCGG]** 234 (87.56)   
14 + **.CCGAGG.** 263 (90.88)   
14 + **[CCGCGG]** 336 (87.56)   
14 + **[CCGGGG]** 373 (85.40)   
14 + **[CCGCGG]** 461 (87.56)   
14 + **[CCGCGG]** 532 (87.56)   
14 + **.CCGCCG.** 685 (96.68)   
14 + **[CCGGGG]** 771 (85.40)   
14 + **.CCGGCG.** 783 (94.52)   
14 + **[CCGGGG]** 842 (85.40)   
14 + **.CCGGCG.** 894 (94.52)   
14 + **[CCGCGG]** 1073 (87.56)   
14 + **.CCGAGG.** 1081 (90.88)   
14 + **[CCGACC]** 1461 (88.08)   
14 - **[CCGGGG]** 1405 (85.40)   
14 - **.CCGAGG.** 1347 (90.88)   
14 - **[CCGAAG]** 1238 (86.09)   
14 - **[CCGCGG]** 1073 (87.56)   
14 - **[CCGAAG]** 1026 (86.09)   
14 - **.CCGCCG.** 974 (96.68)   
14 - **.CCGCCG.** 942 (96.68)   
14 - **[CCGACC]** 904 (88.08)   
14 - **.CCGGCG.** 892 (94.52)   
14 - **[CCGGGG]** 769 (85.40)   
14 - **.CCGCCG.** 750 (96.68)   
14 - **.CCGGCG.** 730 (94.52)   
14 - **[CCGAAG]** 721 (86.09)   
14 - **.CCGAGG.** 561 (90.88)   
14 - **[CCGCGG]** 532 (87.56)   
14 - **[CCGACC]** 465 (88.08)   
14 - **[CCGCGG]** 461 (87.56)   
14 - **[CCGGGG]** 449 (85.40)   
14 - **.CCGGCG.** 413 (94.52)   
14 - **[CCCCCG]** 374 (85.08)   
14 - **[CCGCGG]** 336 (87.56)   
14 - **[CCGCGG]** 234 (87.56)   
14 - **[CCGGGG]** 224 (85.40)   
14 - **[CCGGGG]** 170 (85.40)   
14 - **[CCGGGG]** 24 (85.40)   
15 + **[CGGACG]** 115 (86.87)   
15 + **[CGGACG]** 119 (86.87)   
15 + **[CCGCGG]** 180 (87.56)   
15 + **.CCGAGG.** 208 (90.88)   
15 + **[GCGACG]** 267 (85.28)   
15 + **[CCGGGG]** 284 (85.40)   
15 + **.CCGCCG.** 290 (96.68)   
15 + **.CCGCCG.** 413 (96.68)   
15 + **.CCGCCG.** 433 (96.68)   
15 + **.CCGAGG.** 436 (90.88)   
15 + **[CCGCGG]** 460 (87.56)   
15 + **[CCGCGG]** 544 (87.56)   
15 + **[CCGAAG]** 589 (86.09)   
15 + **.CCGGCG.** 640 (94.52)   
15 + **.CCGACG.** 661 (100.00)   
15 + **.CCGACG.** 690 (100.00)   
15 + **.CCGGCG.** 731 (94.52)   
15 + **[CCGGGG]** 747 (85.40)   
15 + **.CCGGCG.** 838 (94.52)   
15 + **[CCCACG]** 849 (88.40)   
15 + **.CCGCCG.** 901 (96.68)   
15 + **[CCGGGG]** 945 (85.40)   
15 + **[CCGGGG]** 951 (85.40)   
15 + **[CCGGGG]** 1038 (85.40)   
15 + **.CCGAGG.** 1166 (90.88)   
15 + **[CCGCGG]** 1265 (87.56)   
15 + **[CCGGGG]** 1349 (85.40)   
15 + **[CCGGGG]** 1436 (85.40)   
15 - **[CCGGGG]** 1347 (85.40)   
15 - **[CAGACG]** 1326 (85.30)   
15 - **[CCGCGG]** 1265 (87.56)   
15 - **[GCGACG]** 1240 (85.28)   
15 - **.CCGCCG.** 1148 (96.68)   
15 - **[CCGACC]** 1145 (88.08)   
15 - **[CCGACC]** 1112 (88.08)   
15 - **[CAGACG]** 1098 (85.30)   
15 - **.CCGGCG.** 1036 (94.52)   
15 - **.CCGGCG.** 943 (94.52)   
15 - **.CCGGCG.** 902 (94.52)   
15 - **[CCGGGG]** 836 (85.40)   
15 - **[CCCCCG]** 748 (85.08)   
15 - **.CCGCCG.** 641 (96.68)   
15 - **[CCGGGG]** 598 (85.40)   
15 - **[CCGCGG]** 544 (87.56)   
15 - **[CCCACG]** 513 (88.40)   
15 - **[CCGCGG]** 460 (87.56)   
15 - **.CCGGCG.** 454 (94.52)   
15 - **[CCGGGG]** 354 (85.40)   
15 - **[CGGACG]** 338 (86.87)   
15 - **[CCCCCG]** 303 (85.08)   
15 - **[CCGGGG]** 282 (85.40)   
15 - **[CCGGGG]** 231 (85.40)   
15 - **[CCGCGG]** 180 (87.56)   
15 - **[CCGAAG]** 135 (86.09)   
16 + **[CCCACG]** 25 (88.40)   
16 + **[CCGGGG]** 454 (85.40)   
16 + **[CGGACG]** 496 (86.87)   
16 + **[CCGGGG]** 715 (85.40)   
16 + **[CCCCCG]** 854 (85.08)   
16 + **[CCGGGG]** 874 (85.40)   
16 + **[CCGGGG]** 1071 (85.40)   
16 + **[CCCACG]** 1161 (88.40)   
16 + **[CCGGGG]** 1261 (85.40)   
16 + **[GCGACG]** 1283 (85.28)   
16 + **.CCGCCG.** 1305 (96.68)   
16 + **[CCGCGG]** 1312 (87.56)   
16 + **.CCGAGG.** 1348 (90.88)   
16 - **[CCCCCG]** 1506 (85.08)   
16 - **.CCGCCG.** 1361 (96.68)   
16 - **[CCGACC]** 1352 (88.08)   
16 - **[CCGGGG]** 1320 (85.40)   
16 - **[CCGCGG]** 1312 (87.56)   
16 - **.CCGCCG.** 1254 (96.68)   
16 - **.CCGGCG.** 1205 (94.52)   
16 - **.CCGCCG.** 1155 (96.68)   
16 - **.CCGCCG.** 1152 (96.68)   
16 - **.CCGCCG.** 1047 (96.68)   
16 - **.CCGCCG.** 1036 (96.68)   
16 - **.CCGCCG.** 1033 (96.68)   
16 - **.CCGCCG.** 1030 (96.68)   
16 - **[CCGGGG]** 958 (85.40)   
16 - **[CCCACG]** 899 (88.40)   
16 - **[CCGTCG]** 881 (86.99)   
16 - **[CCGGGG]** 872 (85.40)   
16 - **[CCGAAG]** 780 (86.09)   
16 - **[CCGACC]** 696 (88.08)   
16 - **[CCCACG]** 656 (88.40)   
16 - **[CCCACG]** 500 (88.40)   
17 + **[CCCCCG]** 622 (85.08)   
17 + **.CCGGCG.** 771 (94.52)   
17 + **[CCGACT]** 925 (85.38)   
17 + **[CCGCGG]** 1018 (87.56)   
17 + **.CCGGCG.** 1165 (94.52)   
17 + **[CAGACG]** 1483 (85.30)   
17 - **[CCCCCG]** 1352 (85.08)   
17 - **[CCCCCG]** 1313 (85.08)   
17 - **[CCCACG]** 1232 (88.40)   
17 - **.CCGCCG.** 1099 (96.68)   
17 - **[CCCACG]** 1072 (88.40)   
17 - **[CCGCGG]** 1018 (87.56)   
17 - **.CCGAGG.** 980 (90.88)   
17 - **[CCGAAG]** 790 (86.09)   
17 - **.CCGCCG.** 772 (96.68)   
17 - **.CCGGCG.** 769 (94.52)   
18 + **.CCGAGG.** 211 (90.88)   
18 + **[CGGACG]** 598 (86.87)   
18 + **[CCGGGG]** 778 (85.40)   
18 + **[CCGGGG]** 804 (85.40)   
18 + **[CCGCGG]** 931 (87.56)   
18 + **[CCCCCG]** 945 (85.08)   
18 + **.CCGAGG.** 948 (90.88)   
18 + **[CCGCGG]** 1028 (87.56)   
18 + **[CCCCCG]** 1079 (85.08)   
18 + **[CCCCCG]** 1085 (85.08)   
18 + **.CCGCCG.** 1088 (96.68)   
18 + **[CGGACG]** 1092 (86.87)   
18 + **[CCCCCG]** 1114 (85.08)   
18 + **.CCGCCG.** 1136 (96.68)   
18 + **[CCGCGG]** 1147 (87.56)   
18 + **[CCGTCG]** 1170 (86.99)   
18 + **.CCGCCG.** 1200 (96.68)   
18 + **[CCGCGG]** 1203 (87.56)   
18 + **[CCCCCG]** 1222 (85.08)   
18 + **[CCGGGG]** 1250 (85.40)   
18 + **[CCGTCG]** 1422 (86.99)   
18 + **[CCGGGG]** 1481 (85.40)   
18 - **.CCGAGG.** 1501 (90.88)   
18 - **.CCGACG.** 1423 (100.00)   
18 - **.CCGCCG.** 1365 (96.68)   
18 - **[CCCCCG]** 1354 (85.08)   
18 - **[CGGACG]** 1333 (86.87)   
18 - **.CCGCCG.** 1324 (96.68)   
18 - **[CCCCCG]** 1273 (85.08)   
18 - **.CCGCCG.** 1270 (96.68)   
18 - **.CCGCCG.** 1267 (96.68)   
18 - **[CCGCGG]** 1203 (87.56)   
18 - **.CCGACG.** 1171 (100.00)   
18 - **[CCGACC]** 1162 (88.08)   
18 - **[CCGCGG]** 1147 (87.56)   
18 - **.CCGGCG.** 1137 (94.52)   
18 - **.CCGGCG.** 1089 (94.52)   
18 - **[CCGCGG]** 1028 (87.56)   
18 - **[CCGGGG]** 1011 (85.40)   
18 - **[CCGCGG]** 931 (87.56)   
18 - **[CCCCCG]** 779 (85.08)   
18 - **.CCGAGG.** 694 (90.88)   
18 - **.CCGCCG.** 685 (96.68)   
18 - **[CGGACG]** 608 (86.87)   
18 - **.CCGCCG.** 368 (96.68)   
19 + **[CCCCCG]** 558 (85.08)   
19 - **[CAGACG]** 1156 (85.30)   
20 + **.CCGGCG.** 54 (94.52)   
20 + **[CCGGGG]** 131 (85.40)   
20 + **.CCGGCG.** 908 (94.52)   
20 + **.CCGGCG.** 965 (94.52)   
20 + **[CCGCGG]** 1006 (87.56)   
20 + **[CCGCGG]** 1034 (87.56)   
20 + **.CCGAGG.** 1063 (90.88)   
20 + **[CGGACG]** 1105 (86.87)   
20 + **.CCGGCG.** 1175 (94.52)   
20 + **[CCGGGG]** 1188 (85.40)   
20 + **[CCGGGG]** 1271 (85.40)   
20 + **[CCGGGG]** 1350 (85.40)   
20 - **[CCCACG]** 1201 (88.40)   
20 - **[CCCCCG]** 1189 (85.08)   
20 - **.CCGCCG.** 1176 (96.68)   
20 - **[CCGGGG]** 1173 (85.40)   
20 - **.CCGAGG.** 1158 (90.88)   
20 - **.CCGCCG.** 1127 (96.68)   
20 - **[CCGGGG]** 1102 (85.40)   
20 - **.CCGCCG.** 1044 (96.68)   
20 - **[CCGCGG]** 1034 (87.56)   
20 - **[CCGCGG]** 1006 (87.56)   
20 - **.CCGAGG.** 956 (90.88)   
20 - **.CCGCCG.** 909 (96.68)   
20 - **[CCCACG]** 843 (88.40)   
20 - **[CCGACC]** 831 (88.08)   
20 - **[CCGACC]** 817 (88.08)   
20 - **[CCGACC]** 406 (88.08)   
20 - **.CCGCCG.** 55 (96.68)   
20 - **.CCGGCG.** 52 (94.52)   
21 + **[CAGACG]** 1095 (85.30)   
21 - **[CGGACG]** 1088 (86.87)   
21 - **[CAGACG]** 976 (85.30)   
21 - **[CCCACG]** 942 (88.40)   
21 - **[CCCCCG]** 451 (85.08)   
22 + **.CCGAGG.** 85 (90.88)   
22 + **[CCGACT]** 468 (85.38)   
22 + **[CCGGGG]** 493 (85.40)   
22 + **[CCGGGG]** 530 (85.40)   
22 + **.CCGGCG.** 574 (94.52)   
22 + **[CCGGGG]** 623 (85.40)   
22 + **[CCCACG]** 629 (88.40)   
22 + **[CCGGGG]** 715 (85.40)   
22 + **.CCGCCG.** 767 (96.68)   
22 + **.CCGCCG.** 770 (96.68)   
22 + **[CGGACG]** 816 (86.87)   
22 + **.CCGCCG.** 853 (96.68)   
22 + **[CCGACC]** 856 (88.08)   
22 + **.CCGGCG.** 883 (94.52)   
22 + **.CCGGCG.** 896 (94.52)   
22 + **[CCGTCG]** 1035 (86.99)   
22 + **.CCGCCG.** 1088 (96.68)   
22 + **.CCGGCG.** 1091 (94.52)   
22 - **[CCGAAG]** 1341 (86.09)   
22 - **.CCGGCG.** 1089 (94.52)   
22 - **.CCGACG.** 1036 (100.00)   
22 - **.CCGCCG.** 897 (96.68)   
22 - **.CCGCCG.** 884 (96.68)   
22 - **.CCGGCG.** 813 (94.52)   
22 - **[CCGACC]** 719 (88.08)   
22 - **.CCGAGG.** 558 (90.88)   
22 - **[CCCCCG]** 494 (85.08)   
23 + **[CCCCCG]** 900 (85.08)   
23 + **.CCGAGG.** 1026 (90.88)   
23 + **[CGGACG]** 1126 (86.87)   
23 + **[CCGACT]** 1265 (85.38)   
23 + **[CAGACG]** 1479 (85.30)   
23 - **.CCGCCG.** 1100 (96.68)   
23 - **[CCGGGG]** 852 (85.40)   
23 - **.CCGGCG.** 818 (94.52)   
24 + **[CCCACG]** 19 (88.40)   
24 + **[CAGACG]** 31 (85.30)   
24 + **[CAGACG]** 94 (85.30)   
24 + **[CCCCCG]** 109 (85.08)   
24 + **.CCGCCG.** 362 (96.68)   
24 + **[CCGACC]** 365 (88.08)   
24 + **.CCGCCG.** 384 (96.68)   
24 + **[CCGGGG]** 496 (85.40)   
24 + **.CCGGCG.** 511 (94.52)   
24 + **.CCGGCG.** 538 (94.52)   
24 + **[CCCCCG]** 561 (85.08)   
24 + **[CCGACC]** 570 (88.08)   
24 + **[CCGGGG]** 639 (85.40)   
24 + **.CCGAGG.** 665 (90.88)   
24 + **.CCGAGG.** 802 (90.88)   
24 + **[CCGGGG]** 966 (85.40)   
24 + **[CCGCGG]** 1039 (87.56)   
24 + **.CCGAGG.** 1158 (90.88)   
24 + **[CCGACC]** 1175 (88.08)   
24 + **.CCGCCG.** 1190 (96.68)   
24 + **[CCGCGG]** 1354 (87.56)   
24 + **[CCGCGG]** 1494 (87.56)   
24 - **[CCGCGG]** 1494 (87.56)   
24 - **[CCGCGG]** 1354 (87.56)   
24 - **[CCCACG]** 1299 (88.40)   
24 - **[CCCCCG]** 1229 (85.08)   
24 - **[CCCCCG]** 1110 (85.08)   
24 - **[CCCCCG]** 1079 (85.08)   
24 - **.CCGCCG.** 1042 (96.68)   
24 - **[CCGCGG]** 1039 (87.56)   
24 - **[CCCCCG]** 946 (85.08)   
24 - **[CCGACT]** 833 (85.38)   
24 - **[CAGACG]** 524 (85.30)   
24 - **.CCGACG.** 407 (100.00)   
25 + **[CCGGGG]** 470 (85.40)   
25 + **[CCGGGG]** 940 (85.40)   
25 + **.CCGAGG.** 1005 (90.88)   
25 + **[CCCCCG]** 1197 (85.08)   
25 + **[CCCACG]** 1235 (88.40)   
25 - **[CCGACT]** 1328 (85.38)   
25 - **[CCGGGG]** 1198 (85.40)   
25 - **.CCGCCG.** 1086 (96.68)   
25 - **.CCGCCG.** 1074 (96.68)   
25 - **[CCCCCG]** 973 (85.08)   
25 - **[CCCCCG]** 954 (85.08)   
25 - **[CGGACG]** 909 (86.87)   
25 - **[CCCACG]** 742 (88.40)   
25 - **[CCCCCG]** 649 (85.08)   
25 - **[CCGACC]** 379 (88.08)   
25 - **[CCCACG]** 331 (88.40)   
25 - **[CCCCCG]** 53 (85.08)   
26 + **[CCGGGG]** 59 (85.40)   
26 + **[CCCCCG]** 178 (85.08)   
26 + **[CCGCGG]** 185 (87.56)   
26 + **.CCGAGG.** 357 (90.88)   
26 + **[CCCCCG]** 417 (85.08)   
26 + **[CCCCCG]** 425 (85.08)   
26 + **[CCCCCG]** 679 (85.08)   
26 + **[CAGACG]** 766 (85.30)   
26 + **[CCGGGG]** 791 (85.40)   
26 + **.CCGGCG.** 822 (94.52)   
26 + **[CCGGGG]** 857 (85.40)   
26 + **.CCGGCG.** 878 (94.52)   
26 + **[CCGCGG]** 1486 (87.56)   
26 - **[CCCCCG]** 1495 (85.08)   
26 - **[CCGCGG]** 1486 (87.56)   
26 - **.CCGCCG.** 1427 (96.68)   
26 - **[CCCCCG]** 1395 (85.08)   
26 - **[CAGACG]** 1195 (85.30)   
26 - **.CCGCCG.** 965 (96.68)   
26 - **.CCGCCG.** 962 (96.68)   
26 - **.CCGCCG.** 959 (96.68)   
26 - **.CCGCCG.** 956 (96.68)   
26 - **.CCGACG.** 811 (100.00)   
26 - **[CCGGGG]** 789 (85.40)   
26 - **[CCGGGG]** 680 (85.40)   
26 - **[CCGACC]** 361 (88.08)   
26 - **[CCGCGG]** 185 (87.56)   
26 - **[CCGGGG]** 179 (85.40)   
26 - **[CCCCCG]** 60 (85.08)   
27 + **[CCGGGG]** 266 (85.40)   
27 + **[CCGCGG]** 749 (87.56)   
27 + **.CCGAGG.** 945 (90.88)   
27 + **[CCGACC]** 997 (88.08)   
27 + **[CCCCCG]** 1002 (85.08)   
27 + **.CCGAGG.** 1005 (90.88)   
27 + **[CCGGGG]** 1039 (85.40)   
27 + **.CCGCCG.** 1071 (96.68)   
27 + **[CCGACC]** 1074 (88.08)   
27 + **[CCGCGG]** 1203 (87.56)   
27 + **[CCCCCG]** 1239 (85.08)   
27 + **[CCGGGG]** 1253 (85.40)   
27 + **[CCGGGG]** 1269 (85.40)   
27 + **[CCGGGG]** 1317 (85.40)   
27 + **[CCCCCG]** 1364 (85.08)   
27 + **[CCCCCG]** 1378 (85.08)   
27 + **[CCGACC]** 1400 (88.08)   
27 + **.CCGCCG.** 1404 (96.68)   
27 + **[CCCCCG]** 1422 (85.08)   
27 + **.CCGGCG.** 1491 (94.52)   
27 - **.CCGCCG.** 1492 (96.68)   
27 - **.CCGGCG.** 1489 (94.52)   
27 - **[CCGAAG]** 1480 (86.09)   
27 - **.CCGGCG.** 1405 (94.52)   
27 - **[CCCCCG]** 1318 (85.08)   
27 - **[CCCCCG]** 1270 (85.08)   
27 - **[CCCCCG]** 1254 (85.08)   
27 - **.CCGGCG.** 1251 (94.52)   
27 - **[CCGGGG]** 1240 (85.40)   
27 - **[CCGCGG]** 1203 (87.56)   
27 - **.CCGAGG.** 1184 (90.88)   
27 - **[CCGGGG]** 1161 (85.40)   
27 - **[CCCCCG]** 1151 (85.08)   
27 - **.CCGACG.** 1148 (100.00)   
27 - **[CCCCCG]** 1040 (85.08)   
27 - **[CCGACT]** 869 (85.38)   
27 - **[CCCCCG]** 801 (85.08)   
27 - **[CCGCGG]** 749 (87.56)   
27 - **.CCGGCG.** 441 (94.52)   
28 + **[CCGCGG]** 517 (87.56)   
28 + **.CCGCCG.** 541 (96.68)   
28 + **.CCGCCG.** 578 (96.68)   
28 + **[GCGACG]** 590 (85.28)   
28 + **.CCGGCG.** 675 (94.52)   
28 + **.CCGCCG.** 695 (96.68)   
28 + **.CCGACG.** 759 (100.00)   
28 + **.CCGGCG.** 836 (94.52)   
28 + **[CCGGGG]** 847 (85.40)   
28 + **[CCGCGG]** 917 (87.56)   
28 + **[CGGACG]** 936 (86.87)   
28 + **[CCGTCG]** 1059 (86.99)   
28 + **[CCCCCG]** 1068 (85.08)   
28 + **[CCGTCG]** 1096 (86.99)   
28 + **[CCCCCG]** 1118 (85.08)   
28 + **[CCGGGG]** 1386 (85.40)   
28 - **[CCGACT]** 1399 (85.38)   
28 - **[CCCCCG]** 1387 (85.08)   
28 - **[CCGGGG]** 1384 (85.40)   
28 - **.CCGACG.** 1308 (100.00)   
28 - **[CCGGGG]** 1119 (85.40)   
28 - **[GCGACG]** 1112 (85.28)   
28 - **[GCGACG]** 1102 (85.28)   
28 - **[GCGACG]** 1097 (85.28)   
28 - **.CCGAGG.** 1074 (90.88)   
28 - **[GCGACG]** 1060 (85.28)   
28 - **[CGGACG]** 1005 (86.87)   
28 - **[CCGCGG]** 917 (87.56)   
28 - **.CCGCCG.** 911 (96.68)   
28 - **.CCGACG.** 908 (100.00)   
28 - **.CCGCCG.** 874 (96.68)   
28 - **.CCGCCG.** 871 (96.68)   
28 - **.CCGCCG.** 868 (96.68)   
28 - **[CCGACC]** 865 (88.08)   
28 - **[CCGGGG]** 856 (85.40)   
28 - **.CCGGCG.** 834 (94.52)   
28 - **[GCGACG]** 763 (85.28)   
28 - **.CCGGCG.** 742 (94.52)   
28 - **.CCGCCG.** 676 (96.68)   
28 - **[GCGACG]** 597 (85.28)   
28 - **.CCGGCG.** 542 (94.52)   
28 - **[CCGCGG]** 517 (87.56)   
28 - **[CCCCCG]** 463 (85.08)   
28 - **[CCCACG]** 437 (88.40)   
29 + **.CCGAGG.** 32 (90.88)   
29 + **[CCCCCG]** 999 (85.08)   
29 + **[CCGGGG]** 1246 (85.40)   
29 + **.CCGCCG.** 1290 (96.68)   
29 - **.CCGAGG.** 179 (90.88)   
30 - **[CCCCCG]** 1029 (85.08)   
30 - **[CCCCCG]** 912 (85.08)   
31 + **.CCGAGG.** 1438 (90.88)   
33 + **[CAGACG]** 852 (85.30)   
33 + **[CCCCCG]** 900 (85.08)   
33 + **[CCGCGG]** 903 (87.56)   
33 + **[CCGGGG]** 957 (85.40)   
33 + **[CCGGGG]** 1219 (85.40)   
33 + **.CCGAGG.** 1300 (90.88)   
33 + **[CCGCGG]** 1361 (87.56)   
33 + **.CCGCCG.** 1406 (96.68)   
33 + **.CCGCCG.** 1409 (96.68)   
33 + **[CCGCGG]** 1412 (87.56)   
33 - **.CCGACG.** 1463 (100.00)   
33 - **[CCCCCG]** 1430 (85.08)   
33 - **.CCGCCG.** 1427 (96.68)   
33 - **[CCGCGG]** 1412 (87.56)   
33 - **.CCGCCG.** 1400 (96.68)   
33 - **.CCGCCG.** 1364 (96.68)   
33 - **[CCGCGG]** 1361 (87.56)   
33 - **.CCGCCG.** 1355 (96.68)   
33 - **[CCGGGG]** 1338 (85.40)   
33 - **.CCGCCG.** 1290 (96.68)   
33 - **.CCGCCG.** 1162 (96.68)   
33 - **[CCGGGG]** 1061 (85.40)   
33 - **[GCGACG]** 1056 (85.28)   
33 - **[CCCACG]** 949 (88.40)   
33 - **[CCGCGG]** 903 (87.56)   
33 - **.CCGAGG.** 418 (90.88)   
34 + **.CCGAGG.** 318 (90.88)   
34 + **[CCGGGG]** 583 (85.40)   
34 + **.CCGGCG.** 627 (94.52)   
34 + **[CCGGGG]** 707 (85.40)   
34 + **.CCGGCG.** 908 (94.52)   
34 + **.CCGAGG.** 914 (90.88)   
34 + **.CCGGCG.** 975 (94.52)   
34 + **[CAGACG]** 998 (85.30)   
34 + **.CCGCCG.** 1121 (96.68)   
34 + **[CCCCCG]** 1127 (85.08)   
34 + **[CCCCCG]** 1224 (85.08)   
34 + **[CCCCCG]** 1241 (85.08)   
34 + **.CCGGCG.** 1263 (94.52)   
34 + **[CCGCGG]** 1279 (87.56)   
34 + **[CGGACG]** 1282 (86.87)   
34 + **[CCCCCG]** 1352 (85.08)   
34 + **.CCGCCG.** 1367 (96.68)   
34 + **.CCGGCG.** 1405 (94.52)   
34 + **.CCGCCG.** 1444 (96.68)   
34 + **.CCGCCG.** 1447 (96.68)   
34 + **.CCGCCG.** 1450 (96.68)   
34 + **[CCCACG]** 1484 (88.40)   
34 - **[CCGCGG]** 1279 (87.56)   
34 - **[CCGGGG]** 1128 (85.40)   
34 - **.CCGGCG.** 868 (94.52)   
34 - **[CCGGGG]** 824 (85.40)   
34 - **[CCGACT]** 749 (85.38)   
34 - **.CCGCCG.** 628 (96.68)   
34 - **[CCCCCG]** 584 (85.08)   
34 - **[CCGAAG]** 543 (86.09)   
35 + **[CCGAAG]** 858 (86.09)   
35 + **.CCGCCG.** 950 (96.68)   
35 + **.CCGCCG.** 953 (96.68)   
35 + **.CCGAGG.** 962 (90.88)   
35 + **.CCGCCG.** 975 (96.68)   
35 + **.CCGCCG.** 978 (96.68)   
35 + **[CCGGGG]** 1092 (85.40)   
35 + **[CAGACG]** 1122 (85.30)   
35 + **.CCGAGG.** 1164 (90.88)   
35 + **[CCGGGG]** 1249 (85.40)   
35 + **.CCGGCG.** 1261 (94.52)   
35 + **[CCGCGG]** 1324 (87.56)   
35 + **[CCGACC]** 1331 (88.08)   
35 + **[CCGTCG]** 1362 (86.99)   
35 + **.CCGAGG.** 1431 (90.88)   
35 - **.CCGACG.** 1363 (100.00)   
35 - **[CCGCGG]** 1324 (87.56)   
35 - **.CCGCCG.** 1262 (96.68)   
35 - **[CCCCCG]** 1250 (85.08)   
35 - **.CCGCCG.** 1242 (96.68)   
35 - **.CCGGCG.** 1186 (94.52)   
35 - **[CCGACC]** 1153 (88.08)   
35 - **[CCCCCG]** 1142 (85.08)   
35 - **[CCCCCG]** 1093 (85.08)   
35 - **.CCGGCG.** 1085 (94.52)   
35 - **[CCCACG]** 842 (88.40)   
35 - **.CCGAGG.** 146 (90.88)   
36 + **[CCGACT]** 42 (85.38)   
36 + **[CCCCCG]** 132 (85.08)   
36 + **[CCCCCG]** 811 (85.08)   
36 + **[CCGCGG]** 1172 (87.56)   
36 + **.CCGAGG.** 1444 (90.88)   
36 + **[CCGGGG]** 1513 (85.40)   
36 - **[CCGGGG]** 1511 (85.40)   
36 - **[CCGCGG]** 1172 (87.56)   
36 - **.CCGGCG.** 1106 (94.52)   
36 - **.CCGGCG.** 1068 (94.52)   
36 - **[CCCCCG]** 959 (85.08)   
36 - **[CCGGGG]** 506 (85.40)   
36 - **[CAGACG]** 197 (85.30)   
36 - **.CCGGCG.** 143 (94.52)   
36 - **[CCCCCG]** 10 (85.08)   
37 + **.CCGACG.** 43 (100.00)   
37 + **[CCGGGG]** 306 (85.40)   
37 + **[CCCACG]** 981 (88.40)   
37 + **[CCGACC]** 1203 (88.08)   
37 + **[CCCACG]** 1236 (88.40)   
37 - **[CCGAAG]** 1166 (86.09)   
37 - **[CCGAAG]** 511 (86.09)   
37 - **.CCGAGG.** 449 (90.88)   
38 + **[CCCCCG]** 787 (85.08)   
38 + **[CCGAAG]** 1114 (86.09)   
38 - **[CCGAAG]** 53 (86.09)   
39 + **[CCCACG]** 469 (88.40)   
39 + **[CCCCCG]** 1062 (85.08)   
39 + **[CCGACT]** 1083 (85.38)   
39 + **.CCGGCG.** 1150 (94.52)   
39 + **.CCGGCG.** 1243 (94.52)   
39 + **[CCGGGG]** 1326 (85.40)   
39 + **.CCGCCG.** 1349 (96.68)   
39 + **.CCGCCG.** 1392 (96.68)   
39 + **.CCGGCG.** 1395 (94.52)   
39 + **[CGGACG]** 1466 (86.87)   
39 - **[CCCCCG]** 1459 (85.08)   
39 - **[CCCACG]** 1399 (88.40)   
39 - **.CCGGCG.** 1393 (94.52)   
39 - **[CCCCCG]** 1327 (85.08)   
39 - **.CCGCCG.** 1259 (96.68)   
39 - **[CCCCCG]** 1253 (85.08)   
39 - **.CCGCCG.** 1244 (96.68)   
39 - **[CCGGGG]** 1198 (85.40)   
39 - **[CCCACG]** 993 (88.40)   
40 + **[CCCACG]** 586 (88.40)   
40 + **.CCGAGG.** 880 (90.88)   
40 + **[CCGCGG]** 894 (87.56)   
40 + **.CCGCCG.** 901 (96.68)   
40 + **.CCGCCG.** 1128 (96.68)   
40 + **[CCGGGG]** 1221 (85.40)   
40 + **[CCGCGG]** 1240 (87.56)   
40 + **[CCGGGG]** 1352 (85.40)   
40 + **.CCGAGG.** 1393 (90.88)   
40 - **[CCGACT]** 1498 (85.38)   
40 - **[CCCCCG]** 1353 (85.08)   
40 - **[CCGACT]** 1265 (85.38)   
40 - **[CCGCGG]** 1240 (87.56)   
40 - **[CGGACG]** 1237 (86.87)   
40 - **[CCGGGG]** 1219 (85.40)   
40 - **[CCGGGG]** 945 (85.40)   
40 - **[CCGCGG]** 894 (87.56)   
40 - **.CCGAGG.** 802 (90.88)   
41 + **[CCGCGG]** 522 (87.56)   
41 + **[CCCCCG]** 528 (85.08)   
41 + **[CCGTCG]** 914 (86.99)   
41 - **[CCCCCG]** 1285 (85.08)   
41 - **[CCCACG]** 1275 (88.40)   
41 - **[GCGACG]** 915 (85.28)   
41 - **[CCGAAG]** 857 (86.09)   
41 - **[CCGGGG]** 529 (85.40)   
41 - **[CCGCGG]** 522 (87.56)   
42 + **[CCCCCG]** 21 (85.08)   
42 + **[CCGACC]** 57 (88.08)   
42 + **[CCCACG]** 339 (88.40)   
42 + **[CCCCCG]** 346 (85.08)   
42 + **[CCGGGG]** 359 (85.40)   
42 + **.CCGGCG.** 409 (94.52)   
42 + **.CCGCCG.** 455 (96.68)   
42 + **[CCGGGG]** 458 (85.40)   
42 + **.CCGAGG.** 466 (90.88)   
42 + **.CCGCCG.** 513 (96.68)   
42 + **[CCGGGG]** 551 (85.40)   
42 + **.CCGGCG.** 598 (94.52)   
42 + **[CCCCCG]** 618 (85.08)   
42 + **.CCGAGG.** 638 (90.88)   
42 + **[CCCCCG]** 717 (85.08)   
42 + **[CAGACG]** 802 (85.30)   
42 + **[CCGCGG]** 842 (87.56)   
42 + **[CCGGGG]** 849 (85.40)   
42 + **.CCGGCG.** 936 (94.52)   
42 + **.CCGGCG.** 956 (94.52)   
42 + **.CCGCCG.** 991 (96.68)   
42 + **.CCGGCG.** 1105 (94.52)   
42 + **[CCCCCG]** 1164 (85.08)   
42 + **[CCGCGG]** 1167 (87.56)   
42 + **[CCGGGG]** 1195 (85.40)   
42 + **[CGGACG]** 1202 (86.87)   
42 + **[CAGACG]** 1208 (85.30)   
42 + **[CGGACG]** 1256 (86.87)   
42 + **[CCGAAG]** 1300 (86.09)   
42 + **[CCCCCG]** 1343 (85.08)   
42 + **[CCGTCG]** 1350 (86.99)   
42 + **.CCGAGG.** 1438 (90.88)   
42 + **[CCGCGG]** 1476 (87.56)   
42 - **[CCCACG]** 1496 (88.40)   
42 - **[CCCCCG]** 1479 (85.08)   
42 - **[CCGCGG]** 1476 (87.56)   
42 - **[GCGACG]** 1351 (85.28)   
42 - **[CCGACC]** 1266 (88.08)   
42 - **[CCGACT]** 1243 (85.38)   
42 - **[CCCCCG]** 1196 (85.08)   
42 - **.CCGCCG.** 1176 (96.68)   
42 - **.CCGCCG.** 1173 (96.68)   
42 - **.CCGCCG.** 1170 (96.68)   
42 - **[CCGCGG]** 1167 (87.56)   
42 - **.CCGAGG.** 1126 (90.88)   
42 - **[CCCCCG]** 1109 (85.08)   
42 - **.CCGCCG.** 1106 (96.68)   
42 - **[GCGACG]** 908 (85.28)   
42 - **[CCGGGG]** 863 (85.40)   
42 - **[CCGCGG]** 842 (87.56)   
42 - **[CCGGGG]** 718 (85.40)   
42 - **[CCGGGG]** 596 (85.40)   
42 - **[CCGAAG]** 502 (86.09)   
42 - **.CCGGCG.** 456 (94.52)   
42 - **[CAGACG]** 413 (85.30)   
42 - **[CCGGGG]** 407 (85.40)   
42 - **[CCGGGG]** 357 (85.40)   
42 - **.CCGAGG.** 29 (90.88)   
42 - **[CCGGGG]** 22 (85.40)   
43 + **.CCGGCG.** 1096 (94.52)   
43 + **[CCGCGG]** 1102 (87.56)   
43 + **[GCGACG]** 1172 (85.28)   
43 + **[GCGACG]** 1184 (85.28)   
43 + **[CCGCGG]** 1203 (87.56)   
43 + **[CCGGGG]** 1247 (85.40)   
43 + **[CCGGGG]** 1284 (85.40)   
43 + **[CCGGGG]** 1353 (85.40)   
43 + **[CCGGGG]** 1477 (85.40)   
43 - **[CCCCCG]** 1354 (85.08)   
43 - **[CCGGGG]** 1282 (85.40)   
43 - **[CCGCGG]** 1203 (87.56)   
43 - **[CCCCCG]** 1191 (85.08)   
43 - **.CCGCCG.** 1188 (96.68)   
43 - **[CCGTCG]** 1185 (86.99)   
43 - **.CCGCCG.** 1179 (96.68)   
43 - **.CCGCCG.** 1176 (96.68)   
43 - **[CCGTCG]** 1173 (86.99)   
43 - **[CCCCCG]** 1167 (85.08)   
43 - **.CCGCCG.** 1164 (96.68)   
43 - **[CCCACG]** 1147 (88.40)   
43 - **[CCGCGG]** 1102 (87.56)   
43 - **.CCGAGG.** 1054 (90.88)   
43 - **.CCGAGG.** 997 (90.88)   
43 - **[CAGACG]** 525 (85.30)   
44 + **.CCGAGG.** 227 (90.88)   
44 + **[CCCCCG]** 1260 (85.08)   
44 + **[CAGACG]** 1328 (85.30)   
44 - **[CAGACG]** 1365 (85.30)   
44 - **[CCGGGG]** 1261 (85.40)   
45 + **[CCGCGG]** 398 (87.56)   
45 + **[CCGGGG]** 664 (85.40)   
45 + **[CCGCGG]** 980 (87.56)   
45 + **[CCGCGG]** 1027 (87.56)   
45 + **[CCGCGG]** 1064 (87.56)   
45 + **.CCGCCG.** 1111 (96.68)   
45 + **[CCGGGG]** 1114 (85.40)   
45 + **[CGGACG]** 1244 (86.87)   
45 + **.CCGGCG.** 1278 (94.52)   
45 - **[CCCACG]** 1282 (88.40)   
45 - **.CCGGCG.** 1112 (94.52)   
45 - **[CCGCGG]** 1064 (87.56)   
45 - **[CCGCGG]** 1027 (87.56)   
45 - **[CCGCGG]** 980 (87.56)   
45 - **[CCCCCG]** 933 (85.08)   
45 - **[CCCACG]** 761 (88.40)   
45 - **.CCGGCG.** 576 (94.52)   
45 - **[CCGCGG]** 398 (87.56)   
46 - **[CCGGGG]** 1087 (85.40)   
47 + **[CCGAAG]** 21 (86.09)   
47 + **[CCCACG]** 527 (88.40)   
47 + **[CCGACC]** 770 (88.08)   
47 + **.CCGCCG.** 933 (96.68)   
47 + **[CCGGGG]** 936 (85.40)   
47 + **[CCGCGG]** 945 (87.56)   
47 + **[CCGGGG]** 980 (85.40)   
47 + **.CCGAGG.** 1141 (90.88)   
47 + **[CCGGGG]** 1155 (85.40)   
47 + **[CCCCCG]** 1166 (85.08)   
47 + **.CCGGCG.** 1222 (94.52)   
47 + **.CCGAGG.** 1253 (90.88)   
47 + **[CCGGGG]** 1305 (85.40)   
47 + **[CCGGGG]** 1351 (85.40)   
47 + **[CCGGGG]** 1357 (85.40)   
47 + **[CCGCGG]** 1407 (87.56)   
47 + **.CCGGCG.** 1468 (94.52)   
47 - **.CCGCCG.** 1469 (96.68)   
47 - **[CCGCGG]** 1407 (87.56)   
47 - **.CCGCCG.** 1387 (96.68)   
47 - **.CCGGCG.** 1349 (94.52)   
47 - **[CCGACT]** 1317 (85.38)   
47 - **[CCGGGG]** 1303 (85.40)   
47 - **.CCGCCG.** 1223 (96.68)   
47 - **[CCGGGG]** 1220 (85.40)   
47 - **[CCGGGG]** 1167 (85.40)   
47 - **[CCCACG]** 1069 (88.40)   
47 - **[CCCCCG]** 955 (85.08)   
47 - **[CCGCGG]** 945 (87.56)   
47 - **[CCCCCG]** 937 (85.08)   
47 - **.CCGGCG.** 934 (94.52)   
47 - **[CCGACC]** 908 (88.08)   
47 - **[CCCCCG]** 371 (85.08)   
47 - **[CAGACG]** 38 (85.30)   
48 + **[CCCACG]** 18 (88.40)   
48 + **.CCGGCG.** 186 (94.52)   
48 + **.CCGAGG.** 216 (90.88)   
48 + **[CCGCGG]** 432 (87.56)   
48 + **[CCCCCG]** 540 (85.08)   
48 + **.CCGGCG.** 543 (94.52)   
48 + **[CCGGGG]** 556 (85.40)   
48 + **[CCGGGG]** 622 (85.40)   
48 + **[CCGCGG]** 964 (87.56)   
48 + **[CCGGGG]** 1115 (85.40)   
48 + **.CCGCCG.** 1282 (96.68)   
48 + **[CCGCGG]** 1285 (87.56)   
48 + **[CCGCGG]** 1316 (87.56)   
48 + **[CCCCCG]** 1353 (85.08)   
48 + **[CCGGGG]** 1356 (85.40)   
48 + **[CCCCCG]** 1386 (85.08)   
48 - **[CAGACG]** 1366 (85.30)   
48 - **[CCCCCG]** 1357 (85.08)   
48 - **[CCGGGG]** 1354 (85.40)   
48 - **.CCGAGG.** 1338 (90.88)   
48 - **[CCGCGG]** 1316 (87.56)   
48 - **[CCGCGG]** 1285 (87.56)   
48 - **.CCGCCG.** 1027 (96.68)   
48 - **.CCGCCG.** 1024 (96.68)   
48 - **.CCGCCG.** 1021 (96.68)   
48 - **[CCGAAG]** 985 (86.09)   
48 - **[CCGCGG]** 964 (87.56)   
48 - **.CCGAGG.** 938 (90.88)   
48 - **[CCGAAG]** 909 (86.09)   
48 - **[CCGGGG]** 744 (85.40)   
48 - **[CCGGGG]** 719 (85.40)   
48 - **.CCGAGG.** 712 (90.88)   
48 - **.CCGAGG.** 591 (90.88)   
48 - **[CCCCCG]** 557 (85.08)   
48 - **[CCGGGG]** 541 (85.40)   
48 - **.CCGAGG.** 517 (90.88)   
48 - **.CCGAGG.** 492 (90.88)   
48 - **[CCGCGG]** 432 (87.56)   
48 - **.CCGCCG.** 206 (96.68)   
48 - **.CCGCCG.** 187 (96.68)   
48 - **[CAGACG]** 134 (85.30)   
49 + **[CAGACG]** 20 (85.30)   
49 + **.CCGGCG.** 53 (94.52)   
49 + **.CCGGCG.** 755 (94.52)   
49 + **[CCGACC]** 958 (88.08)   
49 + **[CCCCCG]** 1007 (85.08)   
49 + **[CCGCGG]** 1119 (87.56)   
49 + **[CCGGGG]** 1235 (85.40)   
49 + **[CCGAAG]** 1287 (86.09)   
49 + **.CCGCCG.** 1308 (96.68)   
49 + **[CCGGGG]** 1333 (85.40)   
49 + **.CCGCCG.** 1405 (96.68)   
49 + **[CCGCGG]** 1408 (87.56)   
49 + **[CCGAAG]** 1511 (86.09)   
49 - **[CCGCGG]** 1408 (87.56)   
49 - **.CCGGCG.** 1331 (94.52)   
49 - **[CCGGGG]** 1252 (85.40)   
49 - **[CCGGGG]** 1233 (85.40)   
49 - **[CCGCGG]** 1119 (87.56)   
49 - **.CCGCCG.** 1083 (96.68)   
49 - **.CCGCCG.** 1080 (96.68)   
49 - **.CCGCCG.** 1077 (96.68)   
49 - **.CCGCCG.** 1074 (96.68)   
49 - **[CCCCCG]** 1042 (85.08)   
49 - **.CCGCCG.** 993 (96.68)   
49 - **[CCGACC]** 852 (88.08)   
49 - **[CCCCCG]** 57 (85.08)   
49 - **.CCGCCG.** 54 (96.68)   
50 + **[CGGACG]** 1078 (86.87)   
50 - **[CCGAAG]** 1024 (86.09)   
51 + **[CCGGGG]** 60 (85.40)   
51 + **.CCGCCG.** 66 (96.68)   
51 + **[CCGTCG]** 111 (86.99)   
51 + **[CGGACG]** 170 (86.87)   
51 + **[CCGTCG]** 217 (86.99)   
51 + **[CCCCCG]** 357 (85.08)   
51 + **[CCCCCG]** 454 (85.08)   
51 + **[GCGACG]** 489 (85.28)   
51 + **.CCGACG.** 514 (100.00)   
51 + **[CCCCCG]** 619 (85.08)   
51 + **[CCCCCG]** 627 (85.08)   
51 + **[CCCACG]** 640 (88.40)   
51 + **[CCCCCG]** 933 (85.08)   
51 + **[CCGTCG]** 975 (86.99)   
51 + **.CCGCCG.** 1033 (96.68)   
51 + **.CCGCCG.** 1036 (96.68)   
51 + **[CCGTCG]** 1039 (86.99)   
51 + **.CCGAGG.** 1118 (90.88)   
51 + **.CCGCCG.** 1146 (96.68)   
51 + **.CCGACG.** 1149 (100.00)   
51 + **[CCGGGG]** 1155 (85.40)   
51 + **[CCCCCG]** 1182 (85.08)   
51 + **[CCGCGG]** 1185 (87.56)   
51 + **[CCGCGG]** 1285 (87.56)   
51 - **[CCGGGG]** 1347 (85.40)   
51 - **.CCGCCG.** 1288 (96.68)   
51 - **[CCGCGG]** 1285 (87.56)   
51 - **[CCGGGG]** 1276 (85.40)   
51 - **[CCGCGG]** 1185 (87.56)   
51 - **[CCGGGG]** 1176 (85.40)   
51 - **.CCGGCG.** 1153 (94.52)   
51 - **.CCGGCG.** 1043 (94.52)   
51 - **[GCGACG]** 1040 (85.28)   
51 - **[GCGACG]** 976 (85.28)   
51 - **[CCGGGG]** 934 (85.40)   
51 - **[CGGACG]** 916 (86.87)   
51 - **[CAGACG]** 560 (85.30)   
51 - **.CCGCCG.** 521 (96.68)   
51 - **.CCGCCG.** 518 (96.68)   
51 - **[CCGTCG]** 515 (86.99)   
51 - **[CCGTCG]** 490 (86.99)   
51 - **.CCGAGG.** 407 (90.88)   
51 - **.CCGAGG.** 388 (90.88)   
51 - **.CCGCCG.** 221 (96.68)   
51 - **.CCGACG.** 218 (100.00)   
51 - **.CCGCCG.** 149 (96.68)   
51 - **.CCGCCG.** 130 (96.68)   
51 - **.CCGAGG.** 121 (90.88)   
51 - **[CCCCCG]** 115 (85.08)   
51 - **.CCGACG.** 112 (100.00)   
51 - **.CCGAGG.** 94 (90.88)   
51 - **.CCGACG.** 72 (100.00)   
51 - **[CCCCCG]** 12 (85.08)   
51 - **[CCCCCG]** 6 (85.08)   
52 - **[CCGAAG]** 956 (86.09)   
53 + **.CCGAGG.** 1330 (90.88)   
53 - **.CCGACG.** 1185 (100.00)   
53 - **[CCCCCG]** 835 (85.08)   
54 + **.CCGGCG.** 1030 (94.52)   
54 + **.CCGAGG.** 1194 (90.88)   
54 + **[CCCCCG]** 1268 (85.08)   
54 + **[CCGACT]** 1445 (85.38)   
54 - **.CCGACG.** 1326 (100.00)   
54 - **[CCGGGG]** 1269 (85.40)   
54 - **.CCGACG.** 1126 (100.00)   
54 - **.CCGGCG.** 1028 (94.52)   
54 - **[CCGACT]** 618 (85.38)   
55 + **[CCGCGG]** 45 (87.56)   
55 + **.CCGCCG.** 94 (96.68)   
55 + **.CCGCCG.** 97 (96.68)   
55 + **.CCGAGG.** 100 (90.88)   
55 + **[CCGAAG]** 115 (86.09)   
55 + **[CCGGGG]** 172 (85.40)   
55 + **.CCGACG.** 650 (100.00)   
55 + **.CCGCCG.** 864 (96.68)   
55 + **[CCCCCG]** 875 (85.08)   
55 + **.CCGCCG.** 882 (96.68)   
55 + **[CCGCGG]** 885 (87.56)   
55 + **.CCGAGG.** 897 (90.88)   
55 + **[CCGGGG]** 994 (85.40)   
55 + **.CCGAGG.** 1146 (90.88)   
55 + **[CCGAAG]** 1386 (86.09)   
55 - **[CCGCGG]** 885 (87.56)   
55 - **[CCCACG]** 842 (88.40)   
55 - **[CCGGGG]** 831 (85.40)   
55 - **.CCGGCG.** 718 (94.52)   
55 - **[CCGAAG]** 704 (86.09)   
55 - **.CCGGCG.** 170 (94.52)   
55 - **[CCGCGG]** 45 (87.56)   
56 + **[CCGACT]** 997 (85.38)   
56 - **[CCCCCG]** 1325 (85.08)   
58 + **.CCGACG.** 602 (100.00)   
58 + **.CCGAGG.** 710 (90.88)   
58 + **.CCGCCG.** 1084 (96.68)   
58 + **[CCCCCG]** 1123 (85.08)   
58 + **[CCCCCG]** 1140 (85.08)   
58 + **[GCGACG]** 1278 (85.28)   
58 + **[CCGACT]** 1385 (85.38)   
58 + **[CCGGGG]** 1399 (85.40)   
58 + **[CCCCCG]** 1436 (85.08)   
58 - **[CCCACG]** 1506 (88.40)   
58 - **[CCCACG]** 1323 (88.40)   
58 - **[CCGGGG]** 1124 (85.40)   
58 - **.CCGAGG.** 409 (90.88)   
58 - **[CCGACC]** 272 (88.08)   
59 + **[CCGGGG]** 607 (85.40)   
59 + **[CCGCGG]** 651 (87.56)   
59 + **[CCGTCG]** 680 (86.99)   
59 + **.CCGGCG.** 735 (94.52)   
59 + **.CCGCCG.** 773 (96.68)   
59 + **.CCGCCG.** 776 (96.68)   
59 + **.CCGCCG.** 779 (96.68)   
59 + **.CCGCCG.** 782 (96.68)   
59 + **[CCCCCG]** 798 (85.08)   
59 + **[CCGCGG]** 958 (87.56)   
59 + **.CCGCCG.** 982 (96.68)   
59 + **[CCGCGG]** 1011 (87.56)   
59 + **[CCGGGG]** 1099 (85.40)   
59 + **[CCGCGG]** 1168 (87.56)   
59 + **.CCGGCG.** 1198 (94.52)   
59 - **.CCGACG.** 1288 (100.00)   
59 - **[CCGAAG]** 1240 (86.09)   
59 - **.CCGGCG.** 1208 (94.52)   
59 - **[CCGCGG]** 1168 (87.56)   
59 - **[CCGGGG]** 1136 (85.40)   
59 - **[CCGGGG]** 1112 (85.40)   
59 - **[CCGCGG]** 1011 (87.56)   
59 - **.CCGGCG.** 983 (94.52)   
59 - **[CCGCGG]** 958 (87.56)   
59 - **[CCGGGG]** 799 (85.40)   
59 - **.CCGGCG.** 684 (94.52)   
59 - **[GCGACG]** 681 (85.28)   
59 - **[CCGCGG]** 651 (87.56)   
59 - **.CCGCCG.** 632 (96.68)   
59 - **[GCGACG]** 618 (85.28)   
59 - **.CCGAGG.** 438 (90.88)   
59 - **.CCGAGG.** 283 (90.88)   
60 + **[CCCCCG]** 585 (85.08)   
60 + **.CCGCCG.** 628 (96.68)   
60 + **.CCGAGG.** 715 (90.88)   
60 + **.CCGGCG.** 750 (94.52)   
60 + **.CCGCCG.** 823 (96.68)   
60 + **[CCGCGG]** 826 (87.56)   
60 + **[CCCACG]** 835 (88.40)   
60 + **.CCGAGG.** 903 (90.88)   
60 + **[CCGCGG]** 1154 (87.56)   
60 + **[CGGACG]** 1395 (86.87)   
60 - **.CCGAGG.** 1234 (90.88)   
60 - **[CCGCGG]** 1154 (87.56)   
60 - **[CCGCGG]** 826 (87.56)   
60 - **.CCGCCG.** 802 (96.68)   
60 - **.CCGCCG.** 751 (96.68)   
60 - **[CAGACG]** 169 (85.30)   
61 + **[CCCACG]** 475 (88.40)   
61 + **[CCCACG]** 491 (88.40)   
61 + **.CCGGCG.** 499 (94.52)   
61 + **[CCGGGG]** 544 (85.40)   
61 + **[CCGGGG]** 674 (85.40)   
61 + **[CCGACC]** 689 (88.08)   
61 + **[CCCACG]** 856 (88.40)   
61 + **.CCGCCG.** 923 (96.68)   
61 + **[CCGCGG]** 926 (87.56)   
61 + **[CCCACG]** 944 (88.40)   
61 + **[CCGCGG]** 1038 (87.56)   
61 + **[CCGGGG]** 1175 (85.40)   
61 + **[CCCCCG]** 1204 (85.08)   
61 + **[CCGCGG]** 1381 (87.56)   
61 + **[CAGACG]** 1416 (85.30)   
61 + **[CCGGGG]** 1469 (85.40)   
61 - **[CCGCGG]** 1381 (87.56)   
61 - **[CCGGGG]** 1205 (85.40)   
61 - **[CCGCGG]** 1038 (87.56)   
61 - **[CCGCGG]** 926 (87.56)   
61 - **.CCGAGG.** 864 (90.88)   
62 + **[CCCACG]** 943 (88.40)   
62 + **[CCCCCG]** 1471 (85.08)   
62 - **[CAGACG]** 1290 (85.30)   
63 + **[CCGGGG]** 42 (85.40)   
63 + **.CCGAGG.** 66 (90.88)   
63 + **[CCCACG]** 115 (88.40)   
63 + **[CCCCCG]** 127 (85.08)   
63 + **.CCGCCG.** 141 (96.68)   
63 + **.CCGAGG.** 144 (90.88)   
63 + **.CCGGCG.** 151 (94.52)   
63 + **[CCCCCG]** 165 (85.08)   
63 + **[CCGCGG]** 183 (87.56)   
63 + **[CCGGGG]** 236 (85.40)   
63 + **[CCGCGG]** 384 (87.56)   
63 + **.CCGAGG.** 396 (90.88)   
63 + **[CCGCGG]** 444 (87.56)   
63 + **[CCCCCG]** 501 (85.08)   
63 + **[CCCCCG]** 513 (85.08)   
63 + **[CCGCGG]** 535 (87.56)   
63 + **[CCGTCG]** 590 (86.99)   
63 + **[CAGACG]** 605 (85.30)   
63 + **.CCGGCG.** 626 (94.52)   
63 + **[CGGACG]** 693 (86.87)   
63 + **[CCGTCG]** 783 (86.99)   
63 + **[CCGTCG]** 803 (86.99)   
63 + **[CCCACG]** 811 (88.40)   
63 + **[CCGGGG]** 846 (85.40)   
63 + **[CCCCCG]** 950 (85.08)   
63 + **[CCGCGG]** 953 (87.56)   
63 + **[CCGTCG]** 963 (86.99)   
63 + **[CCGTCG]** 988 (86.99)   
63 + **[GCGACG]** 996 (85.28)   
63 + **[CCGGGG]** 1057 (85.40)   
63 + **[CCGGGG]** 1167 (85.40)   
63 + **[CCGCGG]** 1254 (87.56)   
63 + **.CCGCCG.** 1263 (96.68)   
63 + **[CCGTCG]** 1266 (86.99)   
63 + **[CCGCGG]** 1413 (87.56)   
63 + **[CCGGGG]** 1425 (85.40)   
63 - **.CCGCCG.** 1438 (96.68)   
63 - **[CCGCGG]** 1413 (87.56)   
63 - **.CCGCCG.** 1395 (96.68)   
63 - **.CCGCCG.** 1365 (96.68)   
63 - **[CCGACC]** 1349 (88.08)   
63 - **[CGGACG]** 1303 (86.87)   
63 - **.CCGACG.** 1267 (100.00)   
63 - **[CCGCGG]** 1254 (87.56)   
63 - **.CCGCCG.** 1239 (96.68)   
63 - **.CCGCCG.** 1173 (96.68)   
63 - **.CCGGCG.** 1145 (94.52)   
63 - **.CCGAGG.** 1135 (90.88)   
63 - **[CCGACC]** 1114 (88.08)   
63 - **[CCGACC]** 1062 (88.08)   
63 - **[CCCCCG]** 1058 (85.08)   
63 - **.CCGCCG.** 1016 (96.68)   
63 - **[CCGACT]** 1013 (85.38)   
63 - **.CCGACG.** 989 (100.00)   
63 - **[GCGACG]** 973 (85.28)   
63 - **[GCGACG]** 964 (85.28)   
63 - **[CCGCGG]** 953 (87.56)   
63 - **.CCGGCG.** 932 (94.52)   
63 - **.CCGACG.** 856 (100.00)   
63 - **[CCGGGG]** 844 (85.40)   
63 - **.CCGACG.** 784 (100.00)   
63 - **[CGGACG]** 669 (86.87)   
63 - **.CCGCCG.** 627 (96.68)   
63 - **[CCGGGG]** 624 (85.40)   
63 - **[CCCCCG]** 613 (85.08)   
63 - **.CCGACG.** 591 (100.00)   
63 - **[CGGACG]** 587 (86.87)   
63 - **[CCGAAG]** 551 (86.09)   
63 - **[CCGCGG]** 535 (87.56)   
63 - **[CGGACG]** 450 (86.87)   
63 - **[CCGCGG]** 444 (87.56)   
63 - **[CCCACG]** 433 (88.40)   
63 - **.CCGCCG.** 412 (96.68)   
63 - **[CCGCGG]** 384 (87.56)   
63 - **[CCGGGG]** 360 (85.40)   
63 - **[CCCCCG]** 221 (85.08)   
63 - **[CCGACC]** 213 (88.08)   
63 - **[CCGCGG]** 183 (87.56)   
63 - **[CCGGGG]** 135 (85.40)   
63 - **.CCGCCG.** 88 (96.68)   
63 - **.CCGGCG.** 2 (94.52)   
65 + **.CCGAGG.** 98 (90.88)   
65 + **[CCCCCG]** 227 (85.08)   
65 + **[CCCCCG]** 296 (85.08)   
65 + **[CCCCCG]** 302 (85.08)   
65 + **[CAGACG]** 491 (85.30)   
65 + **[CCCCCG]** 536 (85.08)   
65 + **.CCGGCG.** 862 (94.52)   
65 + **[CCCCCG]** 939 (85.08)   
65 + **[CCGCGG]** 947 (87.56)   
65 + **.CCGGCG.** 977 (94.52)   
65 + **.CCGCCG.** 999 (96.68)   
65 + **[CAGACG]** 1035 (85.30)   
65 + **[CCGGGG]** 1045 (85.40)   
65 + **.CCGCCG.** 1089 (96.68)   
65 + **.CCGGCG.** 1116 (94.52)   
65 + **.CCGAGG.** 1128 (90.88)   
65 + **.CCGGCG.** 1146 (94.52)   
65 + **[CCGGGG]** 1219 (85.40)   
65 + **[CCGAAG]** 1273 (86.09)   
65 + **[CCGCGG]** 1309 (87.56)   
65 + **.CCGACG.** 1415 (100.00)   
65 - **[CCGACC]** 1367 (88.08)   
65 - **[CAGACG]** 1325 (85.30)   
65 - **[CCGCGG]** 1309 (87.56)   
65 - **[CCGGGG]** 1217 (85.40)   
65 - **[CCCACG]** 1201 (88.40)   
65 - **[CCGGGG]** 1152 (85.40)   
65 - **.CCGAGG.** 1104 (90.88)   
65 - **[CCGGGG]** 1095 (85.40)   
65 - **.CCGGCG.** 1009 (94.52)   
65 - **[CCGCGG]** 947 (87.56)   
65 - **.CCGAGG.** 638 (90.88)   
65 - **[CCGGGG]** 467 (85.40)   
66 + **[CCGGGG]** 664 (85.40)   
66 + **[CCGCGG]** 769 (87.56)   
66 + **.CCGCCG.** 873 (96.68)   
66 + **[CCCCCG]** 879 (85.08)   
66 + **[CCCCCG]** 885 (85.08)   
66 + **[CCCCCG]** 892 (85.08)   
66 + **[CCCCCG]** 898 (85.08)   
66 + **.CCGGCG.** 935 (94.52)   
66 + **[CCCCCG]** 951 (85.08)   
66 + **[CCGACC]** 976 (88.08)   
66 + **.CCGCCG.** 987 (96.68)   
66 + **[CCGCGG]** 990 (87.56)   
66 + **.CCGCCG.** 1040 (96.68)   
66 + **.CCGCCG.** 1095 (96.68)   
66 + **[CCGCGG]** 1126 (87.56)   
66 + **.CCGAGG.** 1196 (90.88)   
66 + **.CCGAGG.** 1218 (90.88)   
66 + **[CAGACG]** 1245 (85.30)   
66 + **[CCGGGG]** 1289 (85.40)   
66 + **[CCGACT]** 1468 (85.38)   
66 - **[CCGCGG]** 1126 (87.56)   
66 - **[CCGACC]** 1060 (88.08)   
66 - **[CCGCGG]** 990 (87.56)   
66 - **.CCGGCG.** 957 (94.52)   
66 - **.CCGCCG.** 835 (96.68)   
66 - **[CCCACG]** 813 (88.40)   
66 - **[CCGCGG]** 769 (87.56)   
66 - **.CCGAGG.** 695 (90.88)   
66 - **[CCGAAG]** 683 (86.09)   
66 - **[CCCCCG]** 665 (85.08)   
66 - **.CCGAGG.** 637 (90.88)   
66 - **.CCGAGG.** 346 (90.88)   
67 + **[CCGACT]** 371 (85.38)   
67 + **[CCCACG]** 790 (88.40)   
67 + **.CCGGCG.** 814 (94.52)   
67 + **[CCGACC]** 832 (88.08)   
67 + **[GCGACG]** 932 (85.28)   
67 + **[GCGACG]** 1035 (85.28)   
67 + **[CGGACG]** 1042 (86.87)   
67 + **[CCCCCG]** 1226 (85.08)   
67 - **[CCGAAG]** 1313 (86.09)   
67 - **[CCGGGG]** 1227 (85.40)   
67 - **.CCGCCG.** 1039 (96.68)   
67 - **[CCGTCG]** 1036 (86.99)   
67 - **.CCGCCG.** 1020 (96.68)   
67 - **[CCGTCG]** 933 (86.99)   
67 - **[GCGACG]** 904 (85.28)   
67 - **.CCGCCG.** 815 (96.68)   
67 - **[CCGACT]** 436 (85.38)   
68 + **[CCGGGG]** 354 (85.40)   
68 + **[CCGGGG]** 475 (85.40)   
68 + **.CCGCCG.** 575 (96.68)   
68 + **.CCGCCG.** 578 (96.68)   
68 + **.CCGCCG.** 587 (96.68)   
68 + **[CCGGGG]** 704 (85.40)   
68 + **[CCGGGG]** 797 (85.40)   
68 + **[GCGACG]** 872 (85.28)   
68 + **.CCGCCG.** 905 (96.68)   
68 + **.CCGCCG.** 908 (96.68)   
68 + **[CCGCGG]** 1054 (87.56)   
68 + **[CCGCGG]** 1335 (87.56)   
68 - **.CCGCCG.** 1396 (96.68)   
68 - **[CCGGGG]** 1351 (85.40)   
68 - **[CCGCGG]** 1335 (87.56)   
68 - **[GCGACG]** 1229 (85.28)   
68 - **[CCGGGG]** 1089 (85.40)   
68 - **[CCGCGG]** 1054 (87.56)   
68 - **.CCGCCG.** 994 (96.68)   
68 - **.CCGGCG.** 909 (94.52)   
68 - **[CCGGGG]** 895 (85.40)   
68 - **.CCGGCG.** 884 (94.52)   
68 - **[CCCCCG]** 723 (85.08)   
68 - **[CCGGGG]** 667 (85.40)   
68 - **[CCGACT]** 597 (85.38)   
68 - **.CCGCCG.** 504 (96.68)   
68 - **.CCGCCG.** 501 (96.68)   
68 - **.CCGCCG.** 498 (96.68)   
68 - **[CAGACG]** 454 (85.30)   
68 - **.CCGACG.** 421 (100.00)   
68 - **[CCGACC]** 394 (88.08)   
68 - **[CCGACC]** 388 (88.08)   
68 - **[CCGACC]** 382 (88.08)   
68 - **[CCGACC]** 91 (88.08)   
68 - **[CCGACC]** 82 (88.08)   
69 + **[CCCCCG]** 747 (85.08)   
69 + **[CCGCGG]** 768 (87.56)   
69 + **[CCCCCG]** 803 (85.08)   
69 + **.CCGGCG.** 832 (94.52)   
69 + **[CCGGGG]** 919 (85.40)   
69 + **[CCGACC]** 961 (88.08)   
69 + **[CCGACC]** 974 (88.08)   
69 + **[CCGCGG]** 1033 (87.56)   
69 + **[CCCCCG]** 1081 (85.08)   
69 + **[CCGGGG]** 1084 (85.40)   
69 + **[CCGGGG]** 1162 (85.40)   
69 + **[CCGGGG]** 1173 (85.40)   
69 + **[CCCCCG]** 1199 (85.08)   
69 + **.CCGCCG.** 1246 (96.68)   
69 + **.CCGAGG.** 1266 (90.88)   
69 + **[CCGAAG]** 1273 (86.09)   
69 + **[CCGGGG]** 1401 (85.40)   
69 + **[CCGGGG]** 1481 (85.40)   
69 - **[CCCCCG]** 1482 (85.08)   
69 - **[CCCCCG]** 1402 (85.08)   
69 - **[CCGGGG]** 1399 (85.40)   
69 - **[CCGGGG]** 1307 (85.40)   
69 - **[CCCCCG]** 1174 (85.08)   
69 - **[CCGGGG]** 1160 (85.40)   
69 - **[CCGGGG]** 1082 (85.40)   
69 - **.CCGCCG.** 1047 (96.68)   
69 - **[CCGCGG]** 1033 (87.56)   
69 - **[GCGACG]** 896 (85.28)   
69 - **.CCGCCG.** 833 (96.68)   
69 - **[CCGGGG]** 830 (85.40)   
69 - **[CCGGGG]** 804 (85.40)   
69 - **[CCGCGG]** 768 (87.56)   
69 - **[GCGACG]** 722 (85.28)   
69 - **[CCCACG]** 698 (88.40)   
69 - **[CGGACG]** 692 (86.87)   
69 - **[CCCACG]** 575 (88.40)   
70 + **.CCGAGG.** 247 (90.88)   
70 + **[CCGCGG]** 542 (87.56)   
70 + **[CCGGGG]** 815 (85.40)   
70 + **.CCGCCG.** 924 (96.68)   
70 + **[CCGACC]** 1338 (88.08)   
70 + **[CCCCCG]** 1342 (85.08)   
70 + **[CCGCGG]** 1345 (87.56)   
70 - **[CCGCGG]** 1345 (87.56)   
70 - **[CCGGGG]** 1318 (85.40)   
70 - **[CCGGGG]** 1307 (85.40)   
70 - **[CCGACC]** 1036 (88.08)   
70 - **[CCGACT]** 946 (85.38)   
70 - **[CCGCGG]** 542 (87.56)   
71 + **[CCCACG]** 351 (88.40)   
71 + **[CCCACG]** 800 (88.40)   
71 + **[CCCCCG]** 923 (85.08)   
71 + **[CCGAAG]** 981 (86.09)   
71 + **[CCGCGG]** 1002 (87.56)   
71 + **[CCCCCG]** 1010 (85.08)   
71 + **[CCGCGG]** 1091 (87.56)   
71 + **.CCGGCG.** 1138 (94.52)   
71 - **[CCCCCG]** 1223 (85.08)   
71 - **[CCCCCG]** 1216 (85.08)   
71 - **[CCCCCG]** 1167 (85.08)   
71 - **[CCGCGG]** 1091 (87.56)   
71 - **[CCGGGG]** 1011 (85.40)   
71 - **[CCGCGG]** 1002 (87.56)   
71 - **.CCGAGG.** 912 (90.88)   
71 - **[CCCACG]** 876 (88.40)   
71 - **[CCGAAG]** 846 (86.09)   
71 - **[CCCACG]** 774 (88.40)   
71 - **[CCCCCG]** 577 (85.08)   
71 - **[CCCCCG]** 570 (85.08)   
71 - **.CCGAGG.** 341 (90.88)   
72 - **.CCGACG.** 27 (100.00)   
73 + **.CCGGCG.** 803 (94.52)   
73 + **.CCGCCG.** 914 (96.68)   
73 + **.CCGCCG.** 917 (96.68)   
73 + **[CCGCGG]** 920 (87.56)   
73 + **.CCGAGG.** 926 (90.88)   
73 + **[CCGCGG]** 935 (87.56)   
73 + **.CCGGCG.** 944 (94.52)   
73 + **[CCCCCG]** 950 (85.08)   
73 + **.CCGCCG.** 953 (96.68)   
73 + **[CCCCCG]** 1114 (85.08)   
73 + **.CCGGCG.** 1127 (94.52)   
73 - **[CCGGGG]** 1125 (85.40)   
73 - **[CCGGGG]** 1103 (85.40)   
73 - **[CCGACT]** 1074 (85.38)   
73 - **[CCGCGG]** 935 (87.56)   
73 - **[CCGCGG]** 920 (87.56)   
73 - **.CCGGCG.** 801 (94.52)   
74 + **.CCGGCG.** 1140 (94.52)   
74 + **.CCGCCG.** 1229 (96.68)   
74 + **[CCGCGG]** 1254 (87.56)   
74 + **.CCGCCG.** 1349 (96.68)   
74 + **[CCGGGG]** 1452 (85.40)   
74 - **[CCCCCG]** 1461 (85.08)   
74 - **.CCGGCG.** 1440 (94.52)   
74 - **.CCGCCG.** 1431 (96.68)   
74 - **[CCCCCG]** 1416 (85.08)   
74 - **[CCGACC]** 1413 (88.08)   
74 - **[CCGACC]** 1403 (88.08)   
74 - **[CGGACG]** 1340 (86.87)   
74 - **.CCGCCG.** 1290 (96.68)   
74 - **.CCGCCG.** 1287 (96.68)   
74 - **.CCGCCG.** 1284 (96.68)   
74 - **.CCGCCG.** 1281 (96.68)   
74 - **.CCGCCG.** 1278 (96.68)   
74 - **.CCGCCG.** 1275 (96.68)   
74 - **.CCGCCG.** 1272 (96.68)   
74 - **.CCGCCG.** 1269 (96.68)   
74 - **[CCGCGG]** 1254 (87.56)   
74 - **.CCGGCG.** 1230 (94.52)   
76 + **[CCGCGG]** 128 (87.56)   
76 + **[CCCACG]** 305 (88.40)   
76 + **[CCCCCG]** 313 (85.08)   
76 + **[CCCCCG]** 331 (85.08)   
76 + **.CCGCCG.** 334 (96.68)   
76 + **[CCCCCG]** 342 (85.08)   
76 + **[CCCCCG]** 354 (85.08)   
76 + **[CCCCCG]** 360 (85.08)   
76 + **[CCCCCG]** 377 (85.08)   
76 + **[CCCCCG]** 383 (85.08)   
76 + **[CCCCCG]** 394 (85.08)   
76 + **[CCCCCG]** 413 (85.08)   
76 + **[CCGCGG]** 865 (87.56)   
76 + **.CCGAGG.** 877 (90.88)   
76 + **.CCGAGG.** 911 (90.88)   
76 + **.CCGCCG.** 1028 (96.68)   
76 + **.CCGAGG.** 1156 (90.88)   
76 + **.CCGGCG.** 1174 (94.52)   
76 + **[CCGGGG]** 1261 (85.40)   
76 + **[CGGACG]** 1390 (86.87)   
76 - **.CCGCCG.** 1245 (96.68)   
76 - **[CCCACG]** 956 (88.40)   
76 - **.CCGCCG.** 939 (96.68)   
76 - **.CCGCCG.** 923 (96.68)   
76 - **.CCGCCG.** 920 (96.68)   
76 - **.CCGCCG.** 917 (96.68)   
76 - **[CCGCGG]** 865 (87.56)   
76 - **[CCCCCG]** 840 (85.08)   
76 - **.CCGGCG.** 831 (94.52)   
76 - **[CCCCCG]** 266 (85.08)   
76 - **[CCGCGG]** 128 (87.56)   
77 + **[CCGGGG]** 512 (85.40)   
77 + **[CCGACC]** 575 (88.08)   
77 + **[CAGACG]** 734 (85.30)   
77 - **.CCGAGG.** 1445 (90.88)   
77 - **[CCCCCG]** 1283 (85.08)   
77 - **.CCGAGG.** 1280 (90.88)   
77 - **[CCGACT]** 1188 (85.38)   
77 - **[CCCCCG]** 1080 (85.08)   
77 - **[CCGACC]** 999 (88.08)   
77 - **[CCCACG]** 935 (88.40)   
77 - **[CCCCCG]** 513 (85.08)   
77 - **[CCGACC]** 454 (88.08)   
77 - **[CCGGGG]** 237 (85.40)   
78 - **[CCGACC]** 1176 (88.08)   
79 + **[CAGACG]** 70 (85.30)   
79 + **.CCGCCG.** 652 (96.68)   
79 + **.CCGAGG.** 705 (90.88)   
79 + **[CCGGGG]** 825 (85.40)   
79 + **.CCGGCG.** 846 (94.52)   
79 + **.CCGCCG.** 876 (96.68)   
79 + **[CCGTCG]** 1086 (86.99)   
79 + **.CCGGCG.** 1226 (94.52)   
79 + **[CCGCGG]** 1278 (87.56)   
79 + **[CCGGGG]** 1347 (85.40)   
79 + **[CCGCGG]** 1398 (87.56)   
79 + **[CGGACG]** 1410 (86.87)   
79 - **[CCGCGG]** 1398 (87.56)   
79 - **[CCCCCG]** 1348 (85.08)   
79 - **[CCGCGG]** 1278 (87.56)   
79 - **.CCGGCG.** 1272 (94.52)   
79 - **.CCGCCG.** 1227 (96.68)   
79 - **[GCGACG]** 1087 (85.28)   
79 - **.CCGAGG.** 975 (90.88)   
79 - **[CCCCCG]** 938 (85.08)   
79 - **.CCGCCG.** 847 (96.68)   
79 - **.CCGGCG.** 844 (94.52)   
79 - **[CGGACG]** 635 (86.87)   
79 - **[CCGACT]** 612 (85.38)   
80 + **[CCGACT]** 752 (85.38)   
80 + **[CCGACT]** 759 (85.38)   
80 + **[CCCACG]** 789 (88.40)   
80 + **[CCGACC]** 840 (88.08)   
80 + **.CCGGCG.** 953 (94.52)   
80 + **[CAGACG]** 996 (85.30)   
80 + **[CCGACT]** 1035 (85.38)   
80 + **[CCGGGG]** 1183 (85.40)   
80 + **[CCGCGG]** 1208 (87.56)   
80 + **[CCGGGG]** 1333 (85.40)   
80 + **[CCCCCG]** 1361 (85.08)   
80 - **[CCGGGG]** 1331 (85.40)   
80 - **[CCGGGG]** 1236 (85.40)   
80 - **[CCGCGG]** 1208 (87.56)   
80 - **[GCGACG]** 1012 (85.28)   
80 - **[CCCACG]** 1000 (88.40)   
81 + **[CCGACC]** 843 (88.08)   
81 + **.CCGGCG.** 1101 (94.52)   
81 + **[CCCCCG]** 1165 (85.08)   
81 + **[CCGAAG]** 1168 (86.09)   
81 + **[CCGGGG]** 1283 (85.40)   
81 + **.CCGAGG.** 1297 (90.88)   
81 + **[CCGCGG]** 1318 (87.56)   
81 - **.CCGAGG.** 1401 (90.88)   
81 - **[CCCCCG]** 1357 (85.08)   
81 - **[CCGCGG]** 1318 (87.56)   
81 - **[CAGACG]** 1269 (85.30)   
81 - **.CCGGCG.** 1105 (94.52)   
81 - **[CGGACG]** 1047 (86.87)   
82 + **[CCGGGG]** 1101 (85.40)   
84 + **[CCCACG]** 34 (88.40)   
84 + **[CCGACC]** 615 (88.08)   
84 + **[CCGACC]** 634 (88.08)   
84 + **[CCCCCG]** 710 (85.08)   
84 + **[CCGTCG]** 777 (86.99)   
84 + **[CCGCGG]** 870 (87.56)   
84 + **.CCGCCG.** 882 (96.68)   
84 + **.CCGACG.** 898 (100.00)   
84 + **[CCGCGG]** 981 (87.56)   
84 + **[CAGACG]** 997 (85.30)   
84 + **[CCCCCG]** 1030 (85.08)   
84 + **.CCGACG.** 1033 (100.00)   
84 + **[CGGACG]** 1087 (86.87)   
84 + **.CCGGCG.** 1151 (94.52)   
84 + **[CCGTCG]** 1309 (86.99)   
84 + **[CCGTCG]** 1318 (86.99)   
84 - **[CCGGGG]** 1452 (85.40)   
84 - **.CCGACG.** 1319 (100.00)   
84 - **.CCGAGG.** 1260 (90.88)   
84 - **.CCGAGG.** 1233 (90.88)   
84 - **[CCGCGG]** 981 (87.56)   
84 - **[CCGCGG]** 870 (87.56)   
84 - **.CCGACG.** 778 (100.00)   
84 - **[CCGGGG]** 711 (85.40)   
84 - **[CCCACG]** 105 (88.40)   
84 - **[CCGACC]** 89 (88.08)   
84 - **.CCGAGG.** 1 (90.88)   
85 + **.CCGCCG.** 18 (96.68)   
85 + **[CCGACC]** 60 (88.08)   
85 + **[CCGGGG]** 117 (85.40)   
85 + **.CCGAGG.** 415 (90.88)   
85 + **[CCGTCG]** 470 (86.99)   
85 + **[CCGGGG]** 499 (85.40)   
85 + **.CCGCCG.** 541 (96.68)   
85 + **[CCGGGG]** 544 (85.40)   
85 + **[CCCCCG]** 606 (85.08)   
85 + **[CCCCCG]** 612 (85.08)   
85 + **[CCCCCG]** 618 (85.08)   
85 + **[CCCCCG]** 630 (85.08)   
85 + **[CCCCCG]** 636 (85.08)   
85 + **[CCCCCG]** 655 (85.08)   
85 + **[CCCCCG]** 661 (85.08)   
85 + **[CCCCCG]** 667 (85.08)   
85 + **[CCCCCG]** 674 (85.08)   
85 + **[CCCCCG]** 680 (85.08)   
85 + **[CCCCCG]** 686 (85.08)   
85 + **[CCCCCG]** 698 (85.08)   
85 + **[CCCCCG]** 704 (85.08)   
85 + **[CCCCCG]** 722 (85.08)   
85 + **[CCCCCG]** 747 (85.08)   
85 + **[CCCCCG]** 753 (85.08)   
85 + **[CCCCCG]** 760 (85.08)   
85 + **[CCCCCG]** 766 (85.08)   
85 + **[CCCCCG]** 772 (85.08)   
85 + **[CCCCCG]** 785 (85.08)   
85 + **[CCCCCG]** 791 (85.08)   
85 + **[CCCCCG]** 817 (85.08)   
85 + **[CCCCCG]** 823 (85.08)   
85 + **[CCCCCG]** 841 (85.08)   
85 + **[CCCCCG]** 847 (85.08)   
85 + **[CCCCCG]** 859 (85.08)   
85 + **[CCCCCG]** 865 (85.08)   
85 + **.CCGGCG.** 872 (94.52)   
85 + **[CCGCGG]** 879 (87.56)   
85 + **[CGGACG]** 926 (86.87)   
85 + **.CCGCCG.** 990 (96.68)   
85 + **.CCGCCG.** 993 (96.68)   
85 + **.CCGCCG.** 1043 (96.68)   
85 + **.CCGGCG.** 1136 (94.52)   
85 + **[CCCCCG]** 1154 (85.08)   
85 + **.CCGCCG.** 1176 (96.68)   
85 + **[CCGCGG]** 1216 (87.56)   
85 + **[CCCCCG]** 1244 (85.08)   
85 + **[CCCCCG]** 1319 (85.08)   
85 + **[CCCCCG]** 1331 (85.08)   
85 + **[CCGACC]** 1334 (88.08)   
85 + **[CCCCCG]** 1357 (85.08)   
85 + **[CCGGGG]** 1394 (85.40)   
85 + **[CCGCGG]** 1416 (87.56)   
85 + **[CCCCCG]** 1433 (85.08)   
85 + **[CCGCGG]** 1436 (87.56)   
85 - **[CCGCGG]** 1436 (87.56)   
85 - **[CCGCGG]** 1416 (87.56)   
85 - **[CCGGGG]** 1338 (85.40)   
85 - **[CCGGGG]** 1274 (85.40)   
85 - **[CCGGGG]** 1259 (85.40)   
85 - **[CCGGGG]** 1245 (85.40)   
85 - **[CCGGGG]** 1222 (85.40)   
85 - **[CCGCGG]** 1216 (87.56)   
85 - **[CCGGGG]** 1183 (85.40)   
85 - **.CCGGCG.** 1177 (94.52)   
85 - **[CCGGGG]** 1148 (85.40)   
85 - **[CCGGGG]** 1113 (85.40)   
85 - **[CCGGGG]** 1063 (85.40)   
85 - **[CCCCCG]** 936 (85.08)   
85 - **.CCGAGG.** 893 (90.88)   
85 - **[CCGCGG]** 879 (87.56)   
85 - **[CCGGGG]** 848 (85.40)   
85 - **[CCGGGG]** 824 (85.40)   
85 - **[CCGGGG]** 797 (85.40)   
85 - **[CCGGGG]** 773 (85.40)   
85 - **[CCGGGG]** 735 (85.40)   
85 - **[CCGGGG]** 710 (85.40)   
85 - **[CCGGGG]** 687 (85.40)   
85 - **[CCGGGG]** 642 (85.40)   
85 - **[CCGGGG]** 619 (85.40)   
85 - **[CCCCCG]** 583 (85.08)   
85 - **[CCCCCG]** 545 (85.08)   
85 - **.CCGGCG.** 542 (94.52)   
85 - **[CCCCCG]** 525 (85.08)   
85 - **[CCCCCG]** 500 (85.08)   
85 - **.CCGACG.** 471 (100.00)   
85 - **.CCGCCG.** 316 (96.68)   
85 - **[CCGACC]** 308 (88.08)   
85 - **[CCGACC]** 294 (88.08)   
85 - **[CCCCCG]** 118 (85.08)   
85 - **[CGGACG]** 97 (86.87)   
86 + **[CCGCGG]** 137 (87.56)   
86 + **[CCGGGG]** 517 (85.40)   
86 + **[CCGCGG]** 542 (87.56)   
86 + **[CCGAAG]** 614 (86.09)   
86 + **[CCCCCG]** 622 (85.08)   
86 + **[CCGGGG]** 625 (85.40)   
86 + **[CCGCGG]** 657 (87.56)   
86 + **[CCGGGG]** 713 (85.40)   
86 + **[CCGCGG]** 739 (87.56)   
86 + **[CCGACC]** 798 (88.08)   
86 + **[CCGGGG]** 895 (85.40)   
86 + **[CCGGGG]** 952 (85.40)   
86 + **.CCGGCG.** 1010 (94.52)   
86 + **[CCGGGG]** 1114 (85.40)   
86 + **.CCGCCG.** 1219 (96.68)   
86 + **[CCCCCG]** 1245 (85.08)   
86 + **[CCGCGG]** 1254 (87.56)   
86 + **[CCCCCG]** 1265 (85.08)   
86 + **.CCGCCG.** 1268 (96.68)   
86 + **[CCGACC]** 1321 (88.08)   
86 + **[CCGGGG]** 1379 (85.40)   
86 + **[CCGGGG]** 1409 (85.40)   
86 + **[CCGCGG]** 1424 (87.56)   
86 + **[CCGCGG]** 1438 (87.56)   
86 + **.CCGCCG.** 1459 (96.68)   
86 + **[CCCCCG]** 1479 (85.08)   
86 + **.CCGCCG.** 1482 (96.68)   
86 + **.CCGAGG.** 1485 (90.88)   
86 + **[CCCCCG]** 1496 (85.08)   
86 - **[CCGCGG]** 1438 (87.56)   
86 - **[CCGCGG]** 1424 (87.56)   
86 - **.CCGCCG.** 1393 (96.68)   
86 - **[CCCCCG]** 1380 (85.08)   
86 - **[CCGCGG]** 1254 (87.56)   
86 - **[CCGGGG]** 1246 (85.40)   
86 - **[CCGGGG]** 1239 (85.40)   
86 - **[CCGGGG]** 1225 (85.40)   
86 - **[CCGGGG]** 1202 (85.40)   
86 - **[CCCCCG]** 1126 (85.08)   
86 - **[CCGACC]** 1123 (88.08)   
86 - **[CCCCCG]** 1115 (85.08)   
86 - **[CCCCCG]** 1074 (85.08)   
86 - **.CCGCCG.** 1059 (96.68)   
86 - **[CCCCCG]** 1019 (85.08)   
86 - **.CCGCCG.** 1011 (96.68)   
86 - **[CGGACG]** 979 (86.87)   
86 - **[CCCCCG]** 935 (85.08)   
86 - **[CCCCCG]** 929 (85.08)   
86 - **[CCCCCG]** 896 (85.08)   
86 - **.CCGCCG.** 838 (96.68)   
86 - **[CCGCGG]** 739 (87.56)   
86 - **[CCGCGG]** 657 (87.56)   
86 - **[CCGGGG]** 623 (85.40)   
86 - **[CCGCGG]** 542 (87.56)   
86 - **[CCGGGG]** 515 (85.40)   
86 - **[CCGCGG]** 137 (87.56)   
86 - **.CCGAGG.** 1 (90.88)   
87 + **[CCCCCG]** 613 (85.08)   
87 + **.CCGCCG.** 670 (96.68)   
87 + **[CGGACG]** 681 (86.87)   
87 + **[CCGCGG]** 721 (87.56)   
87 + **[CCGCGG]** 769 (87.56)   
87 + **[CGGACG]** 936 (86.87)   
87 + **[CCGACT]** 982 (85.38)   
87 + **[CCGGGG]** 1029 (85.40)   
87 + **.CCGCCG.** 1038 (96.68)   
87 + **.CCGCCG.** 1198 (96.68)   
87 + **[CGGACG]** 1299 (86.87)   
87 + **.CCGAGG.** 1308 (90.88)   
87 + **[CCCACG]** 1318 (88.40)   
87 + **[CCGCGG]** 1366 (87.56)   
87 - **[CCCCCG]** 1505 (85.08)   
87 - **[CCCCCG]** 1496 (85.08)   
87 - **[CCCCCG]** 1465 (85.08)   
87 - **[CCGCGG]** 1366 (87.56)   
87 - **[CCGACC]** 1357 (88.08)   
87 - **.CCGGCG.** 1348 (94.52)   
87 - **.CCGCCG.** 1335 (96.68)   
87 - **.CCGGCG.** 1324 (94.52)   
87 - **.CCGCCG.** 1265 (96.68)   
87 - **.CCGCCG.** 1262 (96.68)   
87 - **.CCGCCG.** 1246 (96.68)   
87 - **.CCGACG.** 1223 (100.00)   
87 - **.CCGGCG.** 1039 (94.52)   
87 - **.CCGAGG.** 989 (90.88)   
87 - **.CCGAGG.** 928 (90.88)   
87 - **[CCGCGG]** 769 (87.56)   
87 - **.CCGAGG.** 747 (90.88)   
87 - **[CCGCGG]** 721 (87.56)   
87 - **.CCGGCG.** 671 (94.52)   
87 - **[CCGACT]** 603 (85.38)   
87 - **[CCGACC]** 539 (88.08)   
87 - **[CCGACC]** 420 (88.08)   
87 - **.CCGAGG.** 337 (90.88)   
88 + **[CCGACC]** 111 (88.08)   
88 + **[CCCCCG]** 115 (85.08)   
88 + **.CCGCCG.** 118 (96.68)   
88 + **.CCGCCG.** 221 (96.68)   
88 + **[CCCACG]** 281 (88.40)   
88 + **.CCGCCG.** 296 (96.68)   
88 + **[CCCCCG]** 306 (85.08)   
88 + **.CCGAGG.** 347 (90.88)   
88 + **[CCCCCG]** 367 (85.08)   
88 + **[CCGGGG]** 410 (85.40)   
88 + **[CGGACG]** 532 (86.87)   
88 + **.CCGGCG.** 540 (94.52)   
88 + **.CCGCCG.** 548 (96.68)   
88 + **[CCGCGG]** 582 (87.56)   
88 + **.CCGGCG.** 629 (94.52)   
88 + **[CCGGGG]** 751 (85.40)   
88 + **.CCGCCG.** 784 (96.68)   
88 + **.CCGCCG.** 787 (96.68)   
88 + **.CCGCCG.** 790 (96.68)   
88 + **[CCGCGG]** 848 (87.56)   
88 + **.CCGGCG.** 868 (94.52)   
88 + **.CCGCCG.** 959 (96.68)   
88 + **.CCGCCG.** 1037 (96.68)   
88 + **[CCGCGG]** 1098 (87.56)   
88 + **[CCCCCG]** 1104 (85.08)   
88 + **[CCCCCG]** 1110 (85.08)   
88 + **.CCGGCG.** 1171 (94.52)   
88 + **.CCGCCG.** 1316 (96.68)   
88 + **[CCGGGG]** 1337 (85.40)   
88 - **[CGGACG]** 1476 (86.87)   
88 - **[CCGGGG]** 1335 (85.40)   
88 - **.CCGGCG.** 1317 (94.52)   
88 - **[CCGACC]** 1305 (88.08)   
88 - **[CCGGGG]** 1300 (85.40)   
88 - **.CCGAGG.** 1180 (90.88)   
88 - **[CCGCGG]** 1098 (87.56)   
88 - **[CCCACG]** 1059 (88.40)   
88 - **.CCGGCG.** 1038 (94.52)   
88 - **[CCGACC]** 936 (88.08)   
88 - **[CCGACC]** 927 (88.08)   
88 - **.CCGCCG.** 907 (96.68)   
88 - **.CCGCCG.** 904 (96.68)   
88 - **.CCGCCG.** 901 (96.68)   
88 - **.CCGCCG.** 898 (96.68)   
88 - **.CCGCCG.** 851 (96.68)   
88 - **[CCGCGG]** 848 (87.56)   
88 - **[CCGACC]** 699 (88.08)   
88 - **[CCGCGG]** 582 (87.56)   
88 - **.CCGGCG.** 549 (94.52)   
88 - **.CCGCCG.** 541 (96.68)   
88 - **[GCGACG]** 487 (85.28)   
88 - **.CCGCCG.** 480 (96.68)   
88 - **.CCGAGG.** 326 (90.88)   
88 - **.CCGGCG.** 297 (94.52)   
88 - **[CCGGGG]** 273 (85.40)   
88 - **.CCGAGG.** 203 (90.88)   
89 + **[CCGCGG]** 46 (87.56)   
89 + **.CCGCCG.** 66 (96.68)   
89 + **[CGGACG]** 70 (86.87)   
89 + **[CCCCCG]** 92 (85.08)   
89 + **.CCGCCG.** 104 (96.68)   
89 + **[CCCCCG]** 110 (85.08)   
89 + **[CCGTCG]** 149 (86.99)   
89 + **.CCGGCG.** 171 (94.52)   
89 + **[CCCCCG]** 200 (85.08)   
89 + **[CCCCCG]** 220 (85.08)   
89 + **[CCGCGG]** 261 (87.56)   
89 + **.CCGGCG.** 281 (94.52)   
89 + **.CCGCCG.** 287 (96.68)   
89 + **.CCGCCG.** 349 (96.68)   
89 + **.CCGCCG.** 379 (96.68)   
89 + **.CCGCCG.** 382 (96.68)   
89 + **.CCGCCG.** 385 (96.68)   
89 + **.CCGCCG.** 388 (96.68)   
89 + **.CCGCCG.** 391 (96.68)   
89 + **.CCGCCG.** 407 (96.68)   
89 + **.CCGCCG.** 410 (96.68)   
89 + **.CCGCCG.** 413 (96.68)   
89 + **.CCGCCG.** 416 (96.68)   
89 + **[CCGCGG]** 419 (87.56)   
89 + **.CCGAGG.** 427 (90.88)   
89 + **.CCGCCG.** 465 (96.68)   
89 + **.CCGCCG.** 468 (96.68)   
89 + **.CCGCCG.** 561 (96.68)   
89 + **[CCGGGG]** 585 (85.40)   
89 + **.CCGCCG.** 613 (96.68)   
89 + **.CCGCCG.** 616 (96.68)   
89 + **.CCGGCG.** 619 (94.52)   
89 + **[CCCCCG]** 633 (85.08)   
89 + **.CCGCCG.** 662 (96.68)   
89 + **[CCCCCG]** 671 (85.08)   
89 + **.CCGCCG.** 731 (96.68)   
89 + **[CCGGGG]** 734 (85.40)   
89 + **[CCGCGG]** 842 (87.56)   
89 + **[CCCCCG]** 910 (85.08)   
89 + **.CCGGCG.** 935 (94.52)   
89 + **[CCCCCG]** 948 (85.08)   
89 + **.CCGGCG.** 956 (94.52)   
89 + **[CCGGGG]** 988 (85.40)   
89 + **[CCCCCG]** 1009 (85.08)   
89 + **[CCGGGG]** 1092 (85.40)   
89 + **[CCGGGG]** 1103 (85.40)   
89 + **.CCGCCG.** 1124 (96.68)   
89 + **.CCGGCG.** 1227 (94.52)   
89 + **.CCGGCG.** 1305 (94.52)   
89 + **[CCGCGG]** 1311 (87.56)   
89 + **[CCGGGG]** 1339 (85.40)   
89 + **[CCGGGG]** 1405 (85.40)   
89 - **[CCCCCG]** 1314 (85.08)   
89 - **[CCGCGG]** 1311 (87.56)   
89 - **[CCCCCG]** 1256 (85.08)   
89 - **[CCGGGG]** 1225 (85.40)   
89 - **[CCCACG]** 1056 (88.40)   
89 - **.CCGGCG.** 1025 (94.52)   
89 - **[CCGGGG]** 954 (85.40)   
89 - **.CCGAGG.** 928 (90.88)   
89 - **[CCGGGG]** 911 (85.40)   
89 - **.CCGGCG.** 903 (94.52)   
89 - **[CCGCGG]** 842 (87.56)   
89 - **[CCCCCG]** 735 (85.08)   
89 - **.CCGGCG.** 732 (94.52)   
89 - **[CCCCCG]** 716 (85.08)   
89 - **.CCGCCG.** 708 (96.68)   
89 - **[CCGGGG]** 689 (85.40)   
89 - **.CCGGCG.** 617 (94.52)   
89 - **[CCCCCG]** 586 (85.08)   
89 - **[CCGCGG]** 419 (87.56)   
89 - **[CCGGGG]** 339 (85.40)   
89 - **[CCGCGG]** 261 (87.56)   
89 - **[CCGGGG]** 221 (85.40)   
89 - **[CCCCCG]** 180 (85.08)   
89 - **[GCGACG]** 150 (85.28)   
89 - **.CCGGCG.** 67 (94.52)   
89 - **[CCGCGG]** 46 (87.56)   
89 - **[CCCCCG]** 12 (85.08)   
89 - **[CCCACG]** 4 (88.40)   
90 + **[CCGAAG]** 47 (86.09)   
90 + **.CCGACG.** 225 (100.00)   
90 + **[CCCCCG]** 301 (85.08)   
90 + **[CCCACG]** 350 (88.40)   
90 + **.CCGCCG.** 454 (96.68)   
90 + **.CCGGCG.** 457 (94.52)   
90 + **[CCGACC]** 487 (88.08)   
90 + **[CCGAAG]** 544 (86.09)   
90 + **[CCGCGG]** 588 (87.56)   
90 + **[CCGACT]** 631 (85.38)   
90 + **[CCGCGG]** 719 (87.56)   
90 + **[CCGCGG]** 776 (87.56)   
90 + **[CCGCGG]** 849 (87.56)   
90 + **[CCGAAG]** 882 (86.09)   
90 + **[CCGGGG]** 916 (85.40)   
90 + **[CCGGGG]** 981 (85.40)   
90 + **.CCGCCG.** 1017 (96.68)   
90 + **[CCGCGG]** 1020 (87.56)   
90 + **.CCGGCG.** 1171 (94.52)   
90 + **.CCGCCG.** 1245 (96.68)   
90 + **.CCGGCG.** 1248 (94.52)   
90 + **[CCCCCG]** 1341 (85.08)   
90 + **[CCGACC]** 1344 (88.08)   
90 + **[CCGGGG]** 1487 (85.40)   
90 - **[CCGGGG]** 1410 (85.40)   
90 - **.CCGCCG.** 1255 (96.68)   
90 - **.CCGCCG.** 1252 (96.68)   
90 - **.CCGCCG.** 1249 (96.68)   
90 - **.CCGGCG.** 1246 (94.52)   
90 - **.CCGAGG.** 1228 (90.88)   
90 - **.CCGCCG.** 1192 (96.68)   
90 - **[CCCCCG]** 1186 (85.08)   
90 - **.CCGCCG.** 1118 (96.68)   
90 - **.CCGCCG.** 1091 (96.68)   
90 - **.CCGCCG.** 1088 (96.68)   
90 - **.CCGCCG.** 1085 (96.68)   
90 - **[CCGACC]** 1024 (88.08)   
90 - **[CCGCGG]** 1020 (87.56)   
90 - **[CCCCCG]** 917 (85.08)   
90 - **.CCGCCG.** 852 (96.68)   
90 - **[CCGCGG]** 849 (87.56)   
90 - **.CCGGCG.** 843 (94.52)   
90 - **.CCGCCG.** 779 (96.68)   
90 - **[CCGCGG]** 776 (87.56)   
90 - **[CCGGGG]** 770 (85.40)   
90 - **[CCGCGG]** 719 (87.56)   
90 - **[CCGCGG]** 588 (87.56)   
90 - **[CCGGGG]** 463 (85.40)   
90 - **.CCGGCG.** 455 (94.52)   
90 - **.CCGGCG.** 434 (94.52)   
90 - **[CCGGGG]** 339 (85.40)   
90 - **[CCCACG]** 263 (88.40)   
91 - **[CCGGGG]** 926 (85.40)   
92 + **[CCGCGG]** 1274 (87.56)   
92 + **[CAGACG]** 1284 (85.30)   
92 + **[CAGACG]** 1360 (85.30)   
92 + **.CCGCCG.** 1390 (96.68)   
92 + **[CCCCCG]** 1444 (85.08)   
92 - **.CCGGCG.** 1404 (94.52)   
92 - **.CCGACG.** 1288 (100.00)   
92 - **[CCCCCG]** 1277 (85.08)   
92 - **[CCGCGG]** 1274 (87.56)   
92 - **.CCGCCG.** 1230 (96.68)   
92 - **[CCGAAG]** 1123 (86.09)   
93 + **[GCGACG]** 417 (85.28)   
93 + **.CCGCCG.** 572 (96.68)   
93 + **.CCGCCG.** 680 (96.68)   
93 + **.CCGAGG.** 915 (90.88)   
93 + **[CCGTCG]** 1015 (86.99)   
93 + **[CCGCGG]** 1036 (87.56)   
93 + **.CCGCCG.** 1077 (96.68)   
93 + **[CCGGGG]** 1475 (85.40)   
93 + **[CCGAAG]** 1502 (86.09)   
93 - **[CAGACG]** 1490 (85.30)   
93 - **[CCCCCG]** 1476 (85.08)   
93 - **.CCGCCG.** 1450 (96.68)   
93 - **.CCGCCG.** 1447 (96.68)   
93 - **.CCGCCG.** 1399 (96.68)   
93 - **.CCGCCG.** 1384 (96.68)   
93 - **.CCGCCG.** 1381 (96.68)   
93 - **.CCGCCG.** 1365 (96.68)   
93 - **.CCGCCG.** 1351 (96.68)   
93 - **.CCGCCG.** 1315 (96.68)   
93 - **.CCGCCG.** 1306 (96.68)   
93 - **.CCGCCG.** 1290 (96.68)   
93 - **.CCGCCG.** 1250 (96.68)   
93 - **.CCGCCG.** 1039 (96.68)   
93 - **[CCGCGG]** 1036 (87.56)   
93 - **.CCGACG.** 1016 (100.00)   
93 - **[CCCCCG]** 738 (85.08)   
93 - **[CCGGGG]** 658 (85.40)   
93 - **[CGGACG]** 489 (86.87)   
93 - **[CCGTCG]** 418 (86.99)   
94 + **.CCGACG.** 802 (100.00)   
94 - **.CCGAGG.** 549 (90.88)   
96 + **.CCGGCG.** 772 (94.52)   
96 + **[CGGACG]** 776 (86.87)   
96 + **[CCGGGG]** 894 (85.40)   
96 + **[CCCACG]** 962 (88.40)   
96 + **[CCGACC]** 1024 (88.08)   
96 + **[CCCCCG]** 1244 (85.08)   
96 + **[CCGAAG]** 1254 (86.09)   
96 + **[CGGACG]** 1391 (86.87)   
96 + **.CCGGCG.** 1465 (94.52)   
96 - **[GCGACG]** 1361 (85.28)   
96 - **[GCGACG]** 1356 (85.28)   
96 - **[CCGGGG]** 876 (85.40)   
96 - **.CCGCCG.** 773 (96.68)   
97 + **[CCGGGG]** 193 (85.40)   
97 + **[CGGACG]** 718 (86.87)   
97 + **.CCGGCG.** 847 (94.52)   
97 + **.CCGCCG.** 853 (96.68)   
97 + **[CCGCGG]** 977 (87.56)   
97 + **.CCGAGG.** 1063 (90.88)   
97 + **.CCGGCG.** 1083 (94.52)   
97 + **[CCGACC]** 1338 (88.08)   
97 + **.CCGGCG.** 1439 (94.52)   
97 - **[GCGACG]** 1443 (85.28)   
97 - **.CCGCCG.** 1232 (96.68)   
97 - **.CCGAGG.** 1188 (90.88)   
97 - **.CCGGCG.** 1055 (94.52)   
97 - **[CCGCGG]** 977 (87.56)   
97 - **[CCCCCG]** 938 (85.08)   
97 - **[CCGACC]** 935 (88.08)   
97 - **.CCGAGG.** 354 (90.88)   
98 + **[CGGACG]** 700 (86.87)   
98 + **.CCGCCG.** 755 (96.68)   
98 + **.CCGAGG.** 923 (90.88)   
98 + **[CCGCGG]** 1000 (87.56)   
98 + **[CCGCGG]** 1085 (87.56)   
98 + **[CCGCGG]** 1120 (87.56)   
98 + **.CCGGCG.** 1198 (94.52)   
98 + **.CCGAGG.** 1411 (90.88)   
98 - **[CCGACT]** 1506 (85.38)   
98 - **[CCGAAG]** 1475 (86.09)   
98 - **.CCGCCG.** 1450 (96.68)   
98 - **[CCGACT]** 1439 (85.38)   
98 - **.CCGCCG.** 1347 (96.68)   
98 - **.CCGCCG.** 1344 (96.68)   
98 - **[CCCACG]** 1331 (88.40)   
98 - **.CCGCCG.** 1263 (96.68)   
98 - **.CCGCCG.** 1139 (96.68)   
98 - **[CCGCGG]** 1120 (87.56)   
98 - **[CCGCGG]** 1085 (87.56)   
98 - **.CCGGCG.** 1067 (94.52)   
98 - **[CCGACT]** 1045 (85.38)   
98 - **.CCGCCG.** 1003 (96.68)   
98 - **[CCGCGG]** 1000 (87.56)   
98 - **.CCGCCG.** 989 (96.68)   
98 - **.CCGCCG.** 986 (96.68)   
98 - **.CCGCCG.** 983 (96.68)   
98 - **.CCGCCG.** 980 (96.68)   
98 - **.CCGCCG.** 977 (96.68)   
98 - **.CCGCCG.** 794 (96.68)   
98 - **[CCGACT]** 768 (85.38)   
99 + **[CCCACG]** 997 (88.40)   
99 + **[CCGGGG]** 1027 (85.40)   
99 + **.CCGCCG.** 1510 (96.68)   
99 + **.CCGAGG.** 1513 (90.88)   
99 - **[CCCCCG]** 1338 (85.08)   
99 - **[CCCCCG]** 1283 (85.08)   
99 - **.CCGAGG.** 1114 (90.88)   
99 - **[CCCCCG]** 851 (85.08)   
99 - **[CCGACC]** 824 (88.08)   
100 + **[CCGGGG]** 918 (85.40)   
100 + **[CGGACG]** 939 (86.87)   
100 + **.CCGGCG.** 955 (94.52)   
100 + **[CCGCGG]** 1332 (87.56)   
100 + **.CCGAGG.** 1468 (90.88)   
100 + **[CCGCGG]** 1499 (87.56)   
100 - **.CCGCCG.** 1502 (96.68)   
100 - **[CCGCGG]** 1499 (87.56)   
100 - **[CCGCGG]** 1332 (87.56)   
100 - **.CCGGCG.** 916 (94.52)   
100 - **[CCGACT]** 561 (85.38)   
100 - **[CCGACC]** 220 (88.08)   
101 + **[CCGCGG]** 796 (87.56)   
101 + **[CCGACT]** 1017 (85.38)   
101 - **[CCGCGG]** 796 (87.56)   
102 - **[CCCCCG]** 1399 (85.08)   
103 + **[CCGGGG]** 391 (85.40)   
103 + **[CCCCCG]** 510 (85.08)   
103 + **.CCGCCG.** 827 (96.68)   
103 + **[CCGACT]** 1073 (85.38)   
103 + **.CCGAGG.** 1142 (90.88)   
103 + **[CCCCCG]** 1484 (85.08)   
103 - **[CCCCCG]** 1316 (85.08)   
103 - **.CCGCCG.** 1231 (96.68)   
103 - **[CCCACG]** 1150 (88.40)   
103 - **.CCGCCG.** 1084 (96.68)   
103 - **.CCGAGG.** 1081 (90.88)   
103 - **.CCGGCG.** 1066 (94.52)   
103 - **[CCCACG]** 909 (88.40)   
103 - **.CCGGCG.** 828 (94.52)   
103 - **[CCGGGG]** 511 (85.40)   
103 - **[CAGACG]** 464 (85.30)   
103 - **[CCCCCG]** 434 (85.08)   
103 - **[CCCCCG]** 392 (85.08)   
103 - **[CCCCCG]** 90 (85.08)   
104 + **.CCGAGG.** 517 (90.88)   
104 + **[CCCACG]** 867 (88.40)   
104 + **[CCCCCG]** 1009 (85.08)   
104 + **[CGGACG]** 1259 (86.87)   
104 - **[CCCACG]** 1263 (88.40)   
104 - **[CCGGGG]** 1010 (85.40)   
105 + **[CCCCCG]** 1244 (85.08)   
105 + **[CCCCCG]** 1260 (85.08)   
105 + **[CCCCCG]** 1267 (85.08)   
105 + **[CCGACT]** 1351 (85.38)   
105 - **.CCGGCG.** 1410 (94.52)   
105 - **[CCGACC]** 1367 (88.08)   
105 - **.CCGAGG.** 1207 (90.88)   
105 - **.CCGAGG.** 1170 (90.88)   
105 - **.CCGAGG.** 1113 (90.88)   
105 - **[CAGACG]** 1069 (85.30)   
105 - **[CCGACT]** 755 (85.38)   
106 + **[CCGCGG]** 935 (87.56)   
106 + **[CCCCCG]** 955 (85.08)   
106 + **.CCGCCG.** 1034 (96.68)   
106 + **.CCGCCG.** 1061 (96.68)   
106 + **.CCGCCG.** 1435 (96.68)   
106 - **.CCGGCG.** 1436 (94.52)   
106 - **.CCGGCG.** 1035 (94.52)   
106 - **[CCGGGG]** 956 (85.40)   
106 - **[CCGCGG]** 935 (87.56)   
106 - **.CCGAGG.** 926 (90.88)   
106 - **[CCCACG]** 835 (88.40)   
107 + **.CCGAGG.** 839 (90.88)   
108 + **[CCGGGG]** 59 (85.40)   
108 + **[GCGACG]** 83 (85.28)   
108 + **.CCGACG.** 589 (100.00)   
108 + **[CCGAAG]** 670 (86.09)   
108 + **[CCGGGG]** 687 (85.40)   
108 + **[GCGACG]** 768 (85.28)   
108 + **[CCCCCG]** 789 (85.08)   
108 + **.CCGCCG.** 843 (96.68)   
108 + **[CCGGGG]** 897 (85.40)   
108 + **[CCGTCG]** 938 (86.99)   
108 + **.CCGAGG.** 989 (90.88)   
108 + **.CCGAGG.** 995 (90.88)   
108 + **[CCGAAG]** 1150 (86.09)   
108 + **[CCGGGG]** 1175 (85.40)   
108 + **[CCCCCG]** 1261 (85.08)   
108 + **.CCGCCG.** 1497 (96.68)   
108 - **[CCCCCG]** 1513 (85.08)   
108 - **[CCCCCG]** 1412 (85.08)   
108 - **[CCCACG]** 1389 (88.40)   
108 - **[CCCCCG]** 1169 (85.08)   
108 - **[GCGACG]** 1107 (85.28)   
108 - **.CCGCCG.** 942 (96.68)   
108 - **.CCGACG.** 939 (100.00)   
108 - **[CCCCCG]** 884 (85.08)   
108 - **[CCCCCG]** 878 (85.08)   
108 - **[CGGACG]** 867 (86.87)   
108 - **[CGGACG]** 667 (86.87)   
108 - **[CCGTCG]** 590 (86.99)   
108 - **[CCGACC]** 582 (88.08)   
108 - **.CCGCCG.** 508 (96.68)   
108 - **.CCGCCG.** 471 (96.68)   
108 - **.CCGAGG.** 468 (90.88)   
108 - **.CCGGCG.** 448 (94.52)   
108 - **.CCGAGG.** 381 (90.88)   
108 - **.CCGAGG.** 223 (90.88)   
108 - **.CCGAGG.** 113 (90.88)   
108 - **.CCGGCG.** 57 (94.52)   
109 + **.CCGCCG.** 275 (96.68)   
109 + **.CCGAGG.** 278 (90.88)   
109 + **[CCGCGG]** 338 (87.56)   
109 + **[CCGTCG]** 624 (86.99)   
109 + **[CCCACG]** 664 (88.40)   
109 + **.CCGGCG.** 758 (94.52)   
109 + **.CCGGCG.** 798 (94.52)   
109 + **[CCGGGG]** 884 (85.40)   
109 + **.CCGGCG.** 922 (94.52)   
109 + **.CCGGCG.** 957 (94.52)   
109 + **[CCCCCG]** 964 (85.08)   
109 + **.CCGGCG.** 1015 (94.52)   
109 + **.CCGGCG.** 1073 (94.52)   
109 + **[CCGAAG]** 1118 (86.09)   
109 + **.CCGCCG.** 1150 (96.68)   
109 + **.CCGACG.** 1183 (100.00)   
109 + **.CCGACG.** 1229 (100.00)   
109 + **[CCGGGG]** 1385 (85.40)   
109 + **[CGGACG]** 1412 (86.87)   
109 + **.CCGGCG.** 1419 (94.52)   
109 + **[CCGCGG]** 1460 (87.56)   
109 + **[CCGGGG]** 1467 (85.40)   
109 - **.CCGCCG.** 1493 (96.68)   
109 - **.CCGCCG.** 1476 (96.68)   
109 - **.CCGCCG.** 1473 (96.68)   
109 - **[CCGCGG]** 1460 (87.56)   
109 - **.CCGCCG.** 1420 (96.68)   
109 - **.CCGCCG.** 1401 (96.68)   
109 - **.CCGCCG.** 1398 (96.68)   
109 - **.CCGACG.** 1092 (100.00)   
109 - **.CCGCCG.** 1086 (96.68)   
109 - **.CCGCCG.** 1083 (96.68)   
109 - **.CCGCCG.** 1080 (96.68)   
109 - **.CCGCCG.** 1077 (96.68)   
109 - **.CCGCCG.** 1074 (96.68)   
109 - **[CAGACG]** 1005 (85.30)   
109 - **.CCGCCG.** 958 (96.68)   
109 - **[CCCCCG]** 820 (85.08)   
109 - **.CCGCCG.** 759 (96.68)   
109 - **[CCGGGG]** 756 (85.40)   
109 - **[CCGGGG]** 750 (85.40)   
109 - **.CCGACG.** 625 (100.00)   
109 - **.CCGAGG.** 521 (90.88)   
109 - **.CCGGCG.** 396 (94.52)   
109 - **[CCGCGG]** 338 (87.56)   
110 + **[CCCCCG]** 605 (85.08)   
110 + **[CCGCGG]** 860 (87.56)   
110 + **.CCGAGG.** 1025 (90.88)   
110 - **.CCGAGG.** 978 (90.88)   
110 - **[CCGCGG]** 860 (87.56)   
111 - **.CCGAGG.** 1071 (90.88)   
111 - **[CCGAAG]** 976 (86.09)   
112 + **[CCCCCG]** 978 (85.08)   
112 - **[CCGAAG]** 988 (86.09)   
112 - **[CCCCCG]** 718 (85.08)   
114 + **[CCGCGG]** 253 (87.56)   
114 + **.CCGGCG.** 291 (94.52)   
114 + **[CCGCGG]** 351 (87.56)   
114 + **.CCGCCG.** 754 (96.68)   
114 + **.CCGCCG.** 757 (96.68)   
114 + **[CCGTCG]** 760 (86.99)   
114 + **[CCCACG]** 799 (88.40)   
114 + **.CCGAGG.** 1014 (90.88)   
114 + **.CCGGCG.** 1105 (94.52)   
114 + **[CCCCCG]** 1204 (85.08)   
114 + **[CCGTCG]** 1228 (86.99)   
114 + **[CCGACT]** 1237 (85.38)   
114 + **[CCCCCG]** 1275 (85.08)   
114 + **.CCGCCG.** 1278 (96.68)   
114 + **[CCGCGG]** 1307 (87.56)   
114 + **[CCCCCG]** 1320 (85.08)   
114 + **[CCCCCG]** 1427 (85.08)   
114 - **[CCGGGG]** 1428 (85.40)   
114 - **[CCGACC]** 1341 (88.08)   
114 - **[CCGGGG]** 1321 (85.40)   
114 - **[CCGCGG]** 1307 (87.56)   
114 - **.CCGACG.** 1229 (100.00)   
114 - **[CCGACC]** 1141 (88.08)   
114 - **.CCGCCG.** 1106 (96.68)   
114 - **.CCGGCG.** 1103 (94.52)   
114 - **.CCGCCG.** 1042 (96.68)   
114 - **[GCGACG]** 900 (85.28)   
114 - **[CGGACG]** 780 (86.87)   
114 - **.CCGACG.** 761 (100.00)   
114 - **[CCGACT]** 569 (85.38)   
114 - **[GCGACG]** 378 (85.28)   
114 - **[CCGCGG]** 351 (87.56)   
114 - **.CCGCCG.** 292 (96.68)   
114 - **[CCGACT]** 283 (85.38)   
114 - **[CCGCGG]** 253 (87.56)   
115 + **.CCGGCG.** 299 (94.52)   
115 + **[CCGCGG]** 311 (87.56)   
115 + **[CCGCGG]** 412 (87.56)   
115 + **[CCCCCG]** 491 (85.08)   
115 + **.CCGACG.** 556 (100.00)   
115 + **[CCGGGG]** 581 (85.40)   
115 + **[GCGACG]** 868 (85.28)   
115 + **.CCGGCG.** 948 (94.52)   
115 + **.CCGCCG.** 1010 (96.68)   
115 + **.CCGAGG.** 1184 (90.88)   
115 + **[CCGGGG]** 1206 (85.40)   
115 + **[CCGACT]** 1487 (85.38)   
115 - **[CCCACG]** 1416 (88.40)   
115 - **[CAGACG]** 1284 (85.30)   
115 - **[GCGACG]** 1235 (85.28)   
115 - **.CCGCCG.** 995 (96.68)   
115 - **.CCGCCG.** 992 (96.68)   
115 - **.CCGCCG.** 989 (96.68)   
115 - **.CCGCCG.** 986 (96.68)   
115 - **.CCGCCG.** 949 (96.68)   
115 - **[CCCCCG]** 941 (85.08)   
115 - **.CCGAGG.** 923 (90.88)   
115 - **.CCGCCG.** 908 (96.68)   
115 - **[CCGGGG]** 838 (85.40)   
115 - **.CCGGCG.** 691 (94.52)   
115 - **[CCGGGG]** 615 (85.40)   
115 - **[CCGGGG]** 608 (85.40)   
115 - **.CCGAGG.** 497 (90.88)   
115 - **[GCGACG]** 468 (85.28)   
115 - **[CCGCGG]** 412 (87.56)   
115 - **[CCGCGG]** 311 (87.56)   
115 - **[CCCCCG]** 287 (85.08)   
116 + **.CCGAGG.** 718 (90.88)   
116 + **.CCGGCG.** 997 (94.52)   
116 + **[CCCCCG]** 1117 (85.08)   
116 + **[CCGCGG]** 1200 (87.56)   
116 + **.CCGGCG.** 1308 (94.52)   
116 + **[CCGTCG]** 1370 (86.99)   
116 + **.CCGCCG.** 1444 (96.68)   
116 - **.CCGGCG.** 1484 (94.52)   
116 - **.CCGGCG.** 1445 (94.52)   
116 - **.CCGCCG.** 1309 (96.68)   
116 - **[CCGCGG]** 1200 (87.56)   
116 - **[CCCCCG]** 1168 (85.08)   
116 - **.CCGACG.** 1107 (100.00)   
116 - **.CCGCCG.** 998 (96.68)   
116 - **.CCGGCG.** 995 (94.52)   
116 - **[CCCACG]** 974 (88.40)   
116 - **.CCGGCG.** 428 (94.52)   
117 + **[CCCACG]** 137 (88.40)   
117 + **.CCGAGG.** 195 (90.88)   
117 + **.CCGGCG.** 571 (94.52)   
117 + **.CCGCCG.** 700 (96.68)   
117 + **.CCGCCG.** 784 (96.68)   
117 + **.CCGGCG.** 787 (94.52)   
117 + **[CCGCGG]** 830 (87.56)   
117 + **[CCCCCG]** 899 (85.08)   
117 + **[CCCCCG]** 913 (85.08)   
117 + **[CCGCGG]** 927 (87.56)   
117 + **.CCGAGG.** 949 (90.88)   
117 + **[CCGCGG]** 1055 (87.56)   
117 - **[CCGCGG]** 1055 (87.56)   
117 - **[CCGCGG]** 927 (87.56)   
117 - **[CCGCGG]** 830 (87.56)   
117 - **.CCGGCG.** 785 (94.52)   
117 - **.CCGGCG.** 701 (94.52)   
117 - **[CCGACC]** 688 (88.08)   
117 - **.CCGCCG.** 572 (96.68)   
117 - **[CCCACG]** 521 (88.40)   
117 - **.CCGAGG.** 463 (90.88)   
118 + **.CCGAGG.** 97 (90.88)   
118 + **.CCGCCG.** 678 (96.68)   
118 + **.CCGCCG.** 760 (96.68)   
118 + **.CCGGCG.** 763 (94.52)   
118 + **[CCCCCG]** 874 (85.08)   
118 + **[CCCCCG]** 896 (85.08)   
118 + **[CCCCCG]** 903 (85.08)   
118 + **.CCGAGG.** 939 (90.88)   
118 + **[CCGCGG]** 1044 (87.56)   
118 - **[CCGCGG]** 1044 (87.56)   
118 - **.CCGGCG.** 761 (94.52)   
118 - **.CCGGCG.** 679 (94.52)   
118 - **[CCGACC]** 666 (88.08)   
118 - **[CCCACG]** 469 (88.40)   
118 - **.CCGAGG.** 412 (90.88)   
119 + **.CCGAGG.** 95 (90.88)   
119 + **.CCGCCG.** 676 (96.68)   
119 + **.CCGCCG.** 758 (96.68)   
119 + **.CCGGCG.** 761 (94.52)   
119 + **[CCGCGG]** 803 (87.56)   
119 + **[CCCCCG]** 872 (85.08)   
119 + **[CCCCCG]** 894 (85.08)   
119 + **.CCGAGG.** 930 (90.88)   
119 + **[CCGCGG]** 1035 (87.56)   
119 - **[CCGCGG]** 1035 (87.56)   
119 - **[CCGCGG]** 803 (87.56)   
119 - **.CCGGCG.** 759 (94.52)   
119 - **.CCGGCG.** 677 (94.52)   
119 - **[CCGACC]** 664 (88.08)   
119 - **[CCCACG]** 467 (88.40)   
119 - **.CCGAGG.** 410 (90.88)   
120 + **.CCGAGG.** 554 (90.88)   
120 + **.CCGCCG.** 1136 (96.68)   
120 + **.CCGCCG.** 1218 (96.68)   
120 + **.CCGGCG.** 1221 (94.52)   
120 + **[CCGCGG]** 1263 (87.56)   
120 + **[CCCCCG]** 1332 (85.08)   
120 + **[CCCCCG]** 1346 (85.08)   
120 + **[CCCCCG]** 1353 (85.08)   
120 + **[CCCCCG]** 1360 (85.08)   
120 + **.CCGAGG.** 1397 (90.88)   
120 + **[CCGCGG]** 1502 (87.56)   
120 - **[CCGCGG]** 1502 (87.56)   
120 - **[CCGCGG]** 1263 (87.56)   
120 - **.CCGGCG.** 1219 (94.52)   
120 - **.CCGGCG.** 1137 (94.52)   
120 - **[CCGACC]** 1123 (88.08)   
120 - **[CCCACG]** 927 (88.40)   
120 - **.CCGAGG.** 869 (90.88)   
120 - **[CCGGGG]** 298 (85.40)   
121 + **[CCGACC]** 642 (88.08)   
121 + **.CCGGCG.** 844 (94.52)   
121 + **[CCGGGG]** 872 (85.40)   
121 + **.CCGGCG.** 1038 (94.52)   
121 - **.CCGCCG.** 1039 (96.68)   
121 - **[CGGACG]** 930 (86.87)   
121 - **[CAGACG]** 904 (85.30)   
121 - **[CCGGGG]** 744 (85.40)   
121 - **.CCGAGG.** 659 (90.88)   
122 + **[CGGACG]** 587 (86.87)   
122 + **[CCCCCG]** 637 (85.08)   
122 + **[CCCCCG]** 670 (85.08)   
122 + **[CCGACC]** 803 (88.08)   
122 - **[CCGAAG]** 1047 (86.09)   
122 - **.CCGAGG.** 898 (90.88)   
122 - **[CCGGGG]** 14 (85.40)   
123 + **[CCGCGG]** 16 (87.56)   
123 + **[CCCCCG]** 190 (85.08)   
123 + **.CCGACG.** 331 (100.00)   
123 + **[CCCCCG]** 444 (85.08)   
123 + **[CCCCCG]** 478 (85.08)   
123 + **.CCGGCG.** 493 (94.52)   
123 + **[CCGACC]** 513 (88.08)   
123 + **.CCGCCG.** 600 (96.68)   
123 + **.CCGGCG.** 674 (94.52)   
123 + **.CCGGCG.** 696 (94.52)   
123 + **.CCGGCG.** 732 (94.52)   
123 + **[GCGACG]** 756 (85.28)   
123 + **[CGGACG]** 774 (86.87)   
123 + **.CCGCCG.** 838 (96.68)   
123 + **.CCGAGG.** 893 (90.88)   
123 + **.CCGGCG.** 905 (94.52)   
123 + **[CCGGGG]** 922 (85.40)   
123 + **[CCGGGG]** 931 (85.40)   
123 + **.CCGGCG.** 938 (94.52)   
123 + **[CCGGGG]** 944 (85.40)   
123 + **[CCGCGG]** 951 (87.56)   
123 + **.CCGAGG.** 962 (90.88)   
123 + **[CCCCCG]** 973 (85.08)   
123 + **.CCGCCG.** 976 (96.68)   
123 + **.CCGGCG.** 979 (94.52)   
123 + **[CCCCCG]** 994 (85.08)   
123 + **[CCGCGG]** 997 (87.56)   
123 + **.CCGGCG.** 1071 (94.52)   
123 + **[CCGACC]** 1083 (88.08)   
123 + **[CCCCCG]** 1087 (85.08)   
123 + **[CCGCGG]** 1090 (87.56)   
123 + **[CCGCGG]** 1277 (87.56)   
123 + **.CCGCCG.** 1364 (96.68)   
123 + **[CCCCCG]** 1416 (85.08)   
123 + **.CCGCCG.** 1462 (96.68)   
123 - **[CCCCCG]** 1510 (85.08)   
123 - **.CCGGCG.** 1365 (94.52)   
123 - **[CCGCGG]** 1277 (87.56)   
123 - **.CCGAGG.** 1251 (90.88)   
123 - **[GCGACG]** 1232 (85.28)   
123 - **[CCGCGG]** 1090 (87.56)   
123 - **.CCGACG.** 1075 (100.00)   
123 - **[CCGGGG]** 1069 (85.40)   
123 - **.CCGAGG.** 1036 (90.88)   
123 - **[CCGCGG]** 997 (87.56)   
123 - **.CCGGCG.** 983 (94.52)   
123 - **.CCGGCG.** 977 (94.52)   
123 - **[CCGCGG]** 951 (87.56)   
123 - **.CCGGCG.** 942 (94.52)   
123 - **[CCCCCG]** 932 (85.08)   
123 - **[CCCCCG]** 923 (85.08)   
123 - **.CCGCCG.** 906 (96.68)   
123 - **[GCGACG]** 874 (85.28)   
123 - **.CCGGCG.** 839 (94.52)   
123 - **.CCGGCG.** 771 (94.52)   
123 - **.CCGAGG.** 763 (90.88)   
123 - **.CCGCCG.** 751 (96.68)   
123 - **[CCCCCG]** 736 (85.08)   
123 - **.CCGCCG.** 733 (96.68)   
123 - **.CCGCCG.** 697 (96.68)   
123 - **[CCGGGG]** 694 (85.40)   
123 - **.CCGCCG.** 494 (96.68)   
123 - **[CCGGGG]** 479 (85.40)   
123 - **[CCGGGG]** 445 (85.40)   
123 - **[CCGCGG]** 16 (87.56)   
124 + **[CCCACG]** 359 (88.40)   
124 + **.CCGGCG.** 866 (94.52)   
124 + **.CCGGCG.** 878 (94.52)   
124 + **[CCGACC]** 965 (88.08)   
124 + **[GCGACG]** 1094 (85.28)   
124 + **.CCGGCG.** 1147 (94.52)   
124 + **.CCGGCG.** 1191 (94.52)   
124 + **.CCGAGG.** 1205 (90.88)   
124 + **[CCGCGG]** 1225 (87.56)   
124 + **[CGGACG]** 1228 (86.87)   
124 + **[CCGGGG]** 1288 (85.40)   
124 + **[CCGGGG]** 1349 (85.40)   
124 + **[CCGGGG]** 1370 (85.40)   
124 - **[CAGACG]** 1426 (85.30)   
124 - **[CCGCGG]** 1225 (87.56)   
124 - **.CCGCCG.** 1192 (96.68)   
124 - **[CCGGGG]** 1189 (85.40)   
124 - **[CCGGGG]** 1145 (85.40)   
124 - **.CCGACG.** 1101 (100.00)   
124 - **[CCGTCG]** 1095 (86.99)   
124 - **.CCGAGG.** 923 (90.88)   
124 - **[CCGACT]** 854 (85.38)   
124 - **[CCCACG]** 514 (88.40)   
125 + **[CCGACT]** 561 (85.38)   
125 - **.CCGAGG.** 1090 (90.88)   
126 + **.CCGCCG.** 1036 (96.68)   
126 + **.CCGCCG.** 1039 (96.68)   
126 + **.CCGCCG.** 1042 (96.68)   
126 + **[CCGACT]** 1172 (85.38)   
126 - **[CCGAAG]** 120 (86.09)   
127 + **[GCGACG]** 359 (85.28)   
127 + **.CCGAGG.** 387 (90.88)   
127 + **.CCGAGG.** 532 (90.88)   
127 + **.CCGGCG.** 584 (94.52)   
127 + **[CCGGGG]** 642 (85.40)   
127 + **[CCGCGG]** 733 (87.56)   
127 + **.CCGGCG.** 774 (94.52)   
127 + **[CCCACG]** 792 (88.40)   
127 + **.CCGGCG.** 802 (94.52)   
127 + **[CCCCCG]** 822 (85.08)   
127 + **.CCGAGG.** 911 (90.88)   
127 + **.CCGAGG.** 1019 (90.88)   
127 + **[CGGACG]** 1027 (86.87)   
127 + **[CCGGGG]** 1062 (85.40)   
127 + **[CCCCCG]** 1101 (85.08)   
127 + **[CCGCGG]** 1104 (87.56)   
127 + **[CCGACC]** 1148 (88.08)   
127 + **[CCGCGG]** 1304 (87.56)   
127 - **[CAGACG]** 1513 (85.30)   
127 - **[CCGCGG]** 1304 (87.56)   
127 - **[CAGACG]** 1277 (85.30)   
127 - **[CCGGGG]** 1269 (85.40)   
127 - **[CCGCGG]** 1104 (87.56)   
127 - **[CCCCCG]** 1063 (85.08)   
127 - **[CCCCCG]** 998 (85.08)   
127 - **[CCCCCG]** 936 (85.08)   
127 - **.CCGGCG.** 796 (94.52)   
127 - **[CCGCGG]** 733 (87.56)   
127 - **[CGGACG]** 730 (86.87)   
127 - **.CCGCCG.** 682 (96.68)   
127 - **[CCGGGG]** 640 (85.40)   
127 - **[CCGTCG]** 360 (86.99)   
128 + **.CCGCCG.** 313 (96.68)   
128 + **.CCGACG.** 418 (100.00)   
128 + **.CCGGCG.** 456 (94.52)   
128 + **.CCGAGG.** 582 (90.88)   
128 + **[CCGACT]** 803 (85.38)   
128 + **.CCGACG.** 857 (100.00)   
128 + **[GCGACG]** 939 (85.28)   
128 + **[CCCCCG]** 966 (85.08)   
128 + **.CCGACG.** 1054 (100.00)   
128 + **[CCGGGG]** 1136 (85.40)   
128 + **[CCCCCG]** 1222 (85.08)   
128 + **[CCCCCG]** 1228 (85.08)   
128 - **.CCGACG.** 1351 (100.00)   
128 - **[CCCCCG]** 1325 (85.08)   
128 - **.CCGCCG.** 1257 (96.68)   
128 - **[CCGTCG]** 858 (86.99)   
128 - **[CCGACC]** 830 (88.08)   
128 - **[CCGGGG]** 790 (85.40)   
128 - **[CCGACC]** 691 (88.08)   
128 - **[CCGAAG]** 487 (86.09)   
128 - **[CGGACG]** 460 (86.87)   
128 - **[CCGACC]** 431 (88.08)   
128 - **[CCGTCG]** 419 (86.99)   
128 - **.CCGGCG.** 314 (94.52)   
128 - **.CCGCCG.** 148 (96.68)   
129 + **[CCCCCG]** 795 (85.08)   
129 + **[CCCCCG]** 813 (85.08)   
129 + **[CCGCGG]** 871 (87.56)   
129 + **.CCGCCG.** 974 (96.68)   
129 + **.CCGCCG.** 977 (96.68)   
129 + **[CCGGGG]** 980 (85.40)   
129 + **.CCGAGG.** 1043 (90.88)   
129 + **[CGGACG]** 1055 (86.87)   
129 + **[CGGACG]** 1070 (86.87)   
129 + **.CCGCCG.** 1318 (96.68)   
129 + **.CCGGCG.** 1365 (94.52)   
129 + **[CCGCGG]** 1385 (87.56)   
129 + **[CCGGGG]** 1406 (85.40)   
129 + **.CCGGCG.** 1436 (94.52)   
129 + **.CCGACG.** 1493 (100.00)   
129 - **[CCGTCG]** 1494 (86.99)   
129 - **.CCGCCG.** 1450 (96.68)   
129 - **.CCGGCG.** 1404 (94.52)   
129 - **[CCGCGG]** 1385 (87.56)   
129 - **.CCGCCG.** 1366 (96.68)   
129 - **.CCGCCG.** 1224 (96.68)   
129 - **.CCGGCG.** 1204 (94.52)   
129 - **.CCGGCG.** 978 (94.52)   
129 - **.CCGCCG.** 912 (96.68)   
129 - **[CCGCGG]** 871 (87.56)   
129 - **[CCGACT]** 835 (85.38)   
129 - **[CGGACG]** 740 (86.87)   
130 + **[CCGGGG]** 480 (85.40)   
130 + **[CCCCCG]** 634 (85.08)   
130 + **[CCGACC]** 637 (88.08)   
130 + **.CCGAGG.** 681 (90.88)   
130 + **[CCGCGG]** 866 (87.56)   
130 + **[CCGACC]** 1055 (88.08)   
130 + **.CCGGCG.** 1079 (94.52)   
130 + **[CCGCGG]** 1367 (87.56)   
130 + **[CCGGGG]** 1383 (85.40)   
130 + **.CCGGCG.** 1393 (94.52)   
130 - **.CCGGCG.** 1381 (94.52)   
130 - **[CCGCGG]** 1367 (87.56)   
130 - **[CCCCCG]** 1152 (85.08)   
130 - **.CCGCCG.** 1080 (96.68)   
130 - **.CCGACG.** 957 (100.00)   
130 - **[CCGCGG]** 866 (87.56)   
130 - **[CCGAAG]** 787 (86.09)   
130 - **[CCCACG]** 530 (88.40)   
130 - **[CCGGGG]** 478 (85.40)   
131 + **[CCGCGG]** 569 (87.56)   
131 + **[CCCCCG]** 622 (85.08)   
131 + **[CCCCCG]** 746 (85.08)   
131 + **[CCGGGG]** 1071 (85.40)   
131 + **[CCCCCG]** 1252 (85.08)   
131 + **[CCGCGG]** 1281 (87.56)   
131 + **[CCGCGG]** 1378 (87.56)   
131 + **.CCGCCG.** 1414 (96.68)   
131 + **.CCGGCG.** 1450 (94.52)   
131 + **[CCGGGG]** 1480 (85.40)   
131 + **[CCGCGG]** 1487 (87.56)   
131 - **.CCGCCG.** 1499 (96.68)   
131 - **.CCGCCG.** 1496 (96.68)   
131 - **.CCGCCG.** 1493 (96.68)   
131 - **.CCGCCG.** 1490 (96.68)   
131 - **[CCGCGG]** 1487 (87.56)   
131 - **[CCGGGG]** 1478 (85.40)   
131 - **.CCGCCG.** 1451 (96.68)   
131 - **.CCGCCG.** 1381 (96.68)   
131 - **[CCGCGG]** 1378 (87.56)   
131 - **.CCGCCG.** 1371 (96.68)   
131 - **.CCGCCG.** 1314 (96.68)   
131 - **.CCGCCG.** 1311 (96.68)   
131 - **.CCGCCG.** 1308 (96.68)   
131 - **[CCCCCG]** 1284 (85.08)   
131 - **[CCGCGG]** 1281 (87.56)   
131 - **[CCCCCG]** 1228 (85.08)   
131 - **[CCGAAG]** 1225 (86.09)   
131 - **[CCGGGG]** 935 (85.40)   
131 - **.CCGCCG.** 904 (96.68)   
131 - **.CCGCCG.** 901 (96.68)   
131 - **.CCGCCG.** 898 (96.68)   
131 - **[CGGACG]** 801 (86.87)   
131 - **[CCGCGG]** 569 (87.56)   
131 - **.CCGGCG.** 136 (94.52)   
131 - **[CCGGGG]** 13 (85.40)   
132 + **.CCGCCG.** 708 (96.68)   
132 + **[CCCCCG]** 767 (85.08)   
132 + **.CCGGCG.** 770 (94.52)   
132 + **[CCCCCG]** 896 (85.08)   
132 + **[CCCCCG]** 1220 (85.08)   
132 - **[CCCACG]** 1396 (88.40)   
132 - **.CCGGCG.** 1282 (94.52)   
132 - **[CCGGGG]** 1243 (85.40)   
132 - **.CCGGCG.** 1226 (94.52)   
132 - **[GCGACG]** 1209 (85.28)   
132 - **[CCCCCG]** 1161 (85.08)   
132 - **[CCCCCG]** 1137 (85.08)   
132 - **[CCGACT]** 912 (85.38)   
132 - **[CGGACG]** 863 (86.87)   
132 - **[CCCCCG]** 856 (85.08)   
132 - **[CCGGGG]** 768 (85.40)   
132 - **.CCGAGG.** 546 (90.88)   
133 - **[CCCACG]** 1202 (88.40)   
134 + **[CCGACT]** 144 (85.38)   
134 + **[CCCCCG]** 619 (85.08)   
134 + **.CCGGCG.** 1057 (94.52)   
134 + **[CCGACC]** 1141 (88.08)   
134 + **.CCGAGG.** 1178 (90.88)   
134 + **.CCGGCG.** 1188 (94.52)   
134 + **[CCGAAG]** 1201 (86.09)   
134 + **[CCGCGG]** 1405 (87.56)   
134 + **[CCGGGG]** 1505 (85.40)   
134 - **[CCGGGG]** 1503 (85.40)   
134 - **.CCGCCG.** 1468 (96.68)   
134 - **.CCGCCG.** 1465 (96.68)   
134 - **[CCGCGG]** 1405 (87.56)   
134 - **[CCGGGG]** 1386 (85.40)   
134 - **.CCGCCG.** 1061 (96.68)   
134 - **.CCGCCG.** 1058 (96.68)   
134 - **[CCCCCG]** 1000 (85.08)   
134 - **[CCCCCG]** 894 (85.08)   
134 - **.CCGAGG.** 802 (90.88)   
134 - **[CCGAAG]** 757 (86.09)   
134 - **[CCCCCG]** 593 (85.08)   
134 - **.CCGCCG.** 125 (96.68)   
134 - **[CCGACT]** 122 (85.38)   
135 + **.CCGAGG.** 59 (90.88)   
135 + **.CCGGCG.** 210 (94.52)   
135 + **[CCGCGG]** 248 (87.56)   
135 + **[CCGAAG]** 305 (86.09)   
135 + **[CCCCCG]** 344 (85.08)   
135 + **[CCGCGG]** 364 (87.56)   
135 + **.CCGCCG.** 418 (96.68)   
135 + **[CCCCCG]** 535 (85.08)   
135 + **.CCGCCG.** 690 (96.68)   
135 + **.CCGCCG.** 770 (96.68)   
135 + **[CCGGGG]** 797 (85.40)   
135 + **[CCCCCG]** 827 (85.08)   
135 + **.CCGCCG.** 876 (96.68)   
135 + **.CCGAGG.** 1002 (90.88)   
135 + **.CCGGCG.** 1022 (94.52)   
135 + **.CCGACG.** 1056 (100.00)   
135 + **.CCGCCG.** 1144 (96.68)   
135 + **.CCGCCG.** 1207 (96.68)   
135 + **.CCGCCG.** 1229 (96.68)   
135 + **.CCGCCG.** 1232 (96.68)   
135 + **[CCGGGG]** 1483 (85.40)   
135 + **.CCGCCG.** 1498 (96.68)   
135 - **.CCGGCG.** 1481 (94.52)   
135 - **.CCGCCG.** 1473 (96.68)   
135 - **[CCGACT]** 1470 (85.38)   
135 - **.CCGGCG.** 1445 (94.52)   
135 - **[CCGGGG]** 1427 (85.40)   
135 - **[CCGAAG]** 1416 (86.09)   
135 - **.CCGCCG.** 1284 (96.68)   
135 - **.CCGCCG.** 1281 (96.68)   
135 - **.CCGCCG.** 1114 (96.68)   
135 - **.CCGCCG.** 1023 (96.68)   
135 - **[CGGACG]** 956 (86.87)   
135 - **.CCGGCG.** 771 (94.52)   
135 - **.CCGAGG.** 661 (90.88)   
135 - **[CGGACG]** 393 (86.87)   
135 - **[CCGCGG]** 364 (87.56)   
135 - **[CCGGGG]** 345 (85.40)   
135 - **[CCGCGG]** 248 (87.56)   
136 + **[CCGACC]** 426 (88.08)   
136 + **[CGGACG]** 651 (86.87)   
136 + **.CCGGCG.** 934 (94.52)   
136 + **[CCGGGG]** 993 (85.40)   
136 + **.CCGCCG.** 1022 (96.68)   
136 + **.CCGGCG.** 1037 (94.52)   
136 + **[CCGCGG]** 1195 (87.56)   
136 + **.CCGAGG.** 1221 (90.88)   
136 - **[CCGACT]** 1438 (85.38)   
136 - **[CCGCGG]** 1195 (87.56)   
137 + **[CCGGGG]** 863 (85.40)   
137 + **[CCCCCG]** 965 (85.08)   
137 + **[CAGACG]** 1012 (85.30)   
137 - **[CCGAAG]** 1486 (86.09)   
137 - **[CCCACG]** 1403 (88.40)   
137 - **[CCCCCG]** 1219 (85.08)   
137 - **[CCGACT]** 1216 (85.38)   
137 - **.CCGCCG.** 947 (96.68)   
137 - **[CCGGGG]** 861 (85.40)   
138 + **[CCCCCG]** 48 (85.08)   
138 + **[CCGCGG]** 87 (87.56)   
138 + **[CCGACT]** 112 (85.38)   
138 + **[CCGCGG]** 131 (87.56)   
138 + **.CCGCCG.** 176 (96.68)   
138 + **.CCGCCG.** 179 (96.68)   
138 + **.CCGCCG.** 182 (96.68)   
138 + **.CCGCCG.** 217 (96.68)   
138 + **.CCGCCG.** 413 (96.68)   
138 + **[CCGGGG]** 416 (85.40)   
138 + **[CCGCGG]** 423 (87.56)   
138 + **[CCCCCG]** 439 (85.08)   
138 + **[CCCCCG]** 473 (85.08)   
138 + **.CCGCCG.** 480 (96.68)   
138 + **[CCGCGG]** 514 (87.56)   
138 + **.CCGGCG.** 573 (94.52)   
138 + **.CCGAGG.** 684 (90.88)   
138 + **[CCGCGG]** 762 (87.56)   
138 + **.CCGCCG.** 824 (96.68)   
138 + **.CCGCCG.** 827 (96.68)   
138 + **.CCGCCG.** 830 (96.68)   
138 + **.CCGCCG.** 833 (96.68)   
138 + **.CCGCCG.** 836 (96.68)   
138 + **[CCCCCG]** 903 (85.08)   
138 + **.CCGACG.** 976 (100.00)   
138 - **[CCCACG]** 980 (88.40)   
138 - **[CCGTCG]** 966 (86.99)   
138 - **.CCGAGG.** 912 (90.88)   
138 - **[CCGCGG]** 762 (87.56)   
138 - **.CCGCCG.** 693 (96.68)   
138 - **.CCGCCG.** 610 (96.68)   
138 - **.CCGACG.** 603 (100.00)   
138 - **[CCGCGG]** 514 (87.56)   
138 - **[CCGCGG]** 423 (87.56)   
138 - **.CCGGCG.** 414 (94.52)   
138 - **[CCGGGG]** 167 (85.40)   
138 - **[CCGCGG]** 131 (87.56)   
138 - **[CCGCGG]** 87 (87.56)   
139 + **[CCGGGG]** 549 (85.40)   
139 + **.CCGAGG.** 742 (90.88)   
139 + **[CCCACG]** 760 (88.40)   
139 + **.CCGAGG.** 798 (90.88)   
139 + **[CCCCCG]** 826 (85.08)   
139 + **.CCGCCG.** 836 (96.68)   
139 + **[CCCCCG]** 843 (85.08)   
139 + **[CCCCCG]** 864 (85.08)   
139 + **.CCGAGG.** 867 (90.88)   
139 + **[CCCCCG]** 873 (85.08)   
139 + **.CCGGCG.** 876 (94.52)   
139 + **[GCGACG]** 909 (85.28)   
139 + **[CCCCCG]** 918 (85.08)   
139 + **[CCGGGG]** 1065 (85.40)   
139 + **[CCCCCG]** 1207 (85.08)   
139 + **[CCGAAG]** 1242 (86.09)   
139 + **[CGGACG]** 1329 (86.87)   
139 + **[CCGCGG]** 1342 (87.56)   
139 + **.CCGAGG.** 1370 (90.88)   
139 + **[GCGACG]** 1385 (85.28)   
139 + **[CCCCCG]** 1414 (85.08)   
139 + **[CCGGGG]** 1431 (85.40)   
139 + **.CCGCCG.** 1488 (96.68)   
139 + **[CCGGGG]** 1491 (85.40)   
139 - **.CCGGCG.** 1489 (94.52)   
139 - **[CCCCCG]** 1432 (85.08)   
139 - **[CCGAAG]** 1360 (86.09)   
139 - **[CCGCGG]** 1342 (87.56)   
139 - **.CCGAGG.** 1173 (90.88)   
139 - **.CCGCCG.** 1082 (96.68)   
139 - **[GCGACG]** 913 (85.28)   
139 - **.CCGCCG.** 877 (96.68)   
139 - **[CCGGGG]** 874 (85.40)   
139 - **.CCGCCG.** 712 (96.68)   
139 - **[CCGACT]** 660 (85.38)   
139 - **[CCCCCG]** 550 (85.08)   
139 - **[CAGACG]** 247 (85.30)   
140 + **.CCGGCG.** 592 (94.52)   
140 + **.CCGGCG.** 641 (94.52)   
140 + **.CCGCCG.** 655 (96.68)   
140 + **[CCGCGG]** 658 (87.56)   
140 + **.CCGCCG.** 683 (96.68)   
140 + **[CCGGGG]** 696 (85.40)   
140 + **[CCCCCG]** 712 (85.08)   
140 + **.CCGCCG.** 737 (96.68)   
140 + **[CCCCCG]** 854 (85.08)   
140 + **.CCGCCG.** 1085 (96.68)   
140 + **.CCGCCG.** 1088 (96.68)   
140 + **.CCGCCG.** 1091 (96.68)   
140 + **.CCGCCG.** 1094 (96.68)   
140 + **.CCGCCG.** 1097 (96.68)   
140 + **[CCCCCG]** 1112 (85.08)   
140 + **.CCGCCG.** 1115 (96.68)   
140 + **.CCGCCG.** 1148 (96.68)   
140 + **[CCGCGG]** 1160 (87.56)   
140 + **.CCGCCG.** 1191 (96.68)   
140 + **[CCGCGG]** 1247 (87.56)   
140 + **[GCGACG]** 1365 (85.28)   
140 + **[CCGTCG]** 1380 (86.99)   
140 + **.CCGCCG.** 1403 (96.68)   
140 + **.CCGGCG.** 1406 (94.52)   
140 + **[CCGACC]** 1424 (88.08)   
140 - **.CCGCCG.** 1491 (96.68)   
140 - **.CCGCCG.** 1488 (96.68)   
140 - **.CCGCCG.** 1468 (96.68)   
140 - **.CCGCCG.** 1465 (96.68)   
140 - **.CCGCCG.** 1462 (96.68)   
140 - **.CCGCCG.** 1407 (96.68)   
140 - **.CCGGCG.** 1404 (94.52)   
140 - **[CCGCGG]** 1247 (87.56)   
140 - **[CCGCGG]** 1160 (87.56)   
140 - **[CCGGGG]** 1066 (85.40)   
140 - **[CCCCCG]** 1033 (85.08)   
140 - **.CCGCCG.** 976 (96.68)   
140 - **[CCGGGG]** 933 (85.40)   
140 - **.CCGGCG.** 738 (94.52)   
140 - **[CCGGGG]** 713 (85.40)   
140 - **[CCGGGG]** 694 (85.40)   
140 - **[CCGCGG]** 658 (87.56)   
140 - **.CCGCCG.** 593 (96.68)   
140 - **[CAGACG]** 265 (85.30)   
141 + **[CCCACG]** 354 (88.40)   
141 + **[CGGACG]** 554 (86.87)   
141 + **[CCGAAG]** 568 (86.09)   
141 + **[CCGGGG]** 657 (85.40)   
141 + **[CCCCCG]** 933 (85.08)   
141 + **.CCGGCG.** 1115 (94.52)   
141 + **[CCGCGG]** 1157 (87.56)   
141 + **[CCGCGG]** 1189 (87.56)   
141 + **.CCGCCG.** 1283 (96.68)   
141 + **[CCGGGG]** 1286 (85.40)   
141 + **[CCGCGG]** 1318 (87.56)   
141 + **[CCGCGG]** 1382 (87.56)   
141 - **[CCGCGG]** 1382 (87.56)   
141 - **[CCGCGG]** 1318 (87.56)   
141 - **.CCGGCG.** 1284 (94.52)   
141 - **[GCGACG]** 1216 (85.28)   
141 - **[CCGCGG]** 1189 (87.56)   
141 - **[CCCCCG]** 1178 (85.08)   
141 - **[CCGCGG]** 1157 (87.56)   
141 - **[CCCCCG]** 1119 (85.08)   
141 - **.CCGCCG.** 1116 (96.68)   
141 - **[CCGGGG]** 1113 (85.40)   
141 - **[CCGGGG]** 934 (85.40)   
141 - **.CCGGCG.** 743 (94.52)   
141 - **[CCGACC]** 633 (88.08)   
141 - **[CCGAAG]** 310 (86.09)   
142 + **.CCGAGG.** 115 (90.88)   
142 + **[CCGGGG]** 257 (85.40)   
142 + **[CCGAAG]** 356 (86.09)   
142 + **[GCGACG]** 445 (85.28)   
142 + **[CCGGGG]** 496 (85.40)   
142 + **[CCGCGG]** 517 (87.56)   
142 + **[CCGGGG]** 534 (85.40)   
142 + **.CCGAGG.** 586 (90.88)   
142 + **[CCGCGG]** 673 (87.56)   
142 + **[CCGACC]** 683 (88.08)   
142 + **.CCGAGG.** 751 (90.88)   
142 + **[CCGCGG]** 832 (87.56)   
142 + **[CCGCGG]** 945 (87.56)   
142 + **[CCGGGG]** 962 (85.40)   
142 + **.CCGCCG.** 1023 (96.68)   
142 + **.CCGCCG.** 1026 (96.68)   
142 + **[CCGCGG]** 1062 (87.56)   
142 + **.CCGGCG.** 1303 (94.52)   
142 + **[CCGGGG]** 1309 (85.40)   
142 + **.CCGCCG.** 1315 (96.68)   
142 + **.CCGAGG.** 1368 (90.88)   
142 + **[CCGGGG]** 1380 (85.40)   
142 + **[CCGGGG]** 1388 (85.40)   
142 + **[CCGGGG]** 1443 (85.40)   
142 + **[GCGACG]** 1492 (85.28)   
142 + **[CCGCGG]** 1509 (87.56)   
142 - **[CCGCGG]** 1509 (87.56)   
142 - **[CCGTCG]** 1493 (86.99)   
142 - **[CCGGGG]** 1472 (85.40)   
142 - **[CCCCCG]** 1444 (85.08)   
142 - **[CCCCCG]** 1389 (85.08)   
142 - **.CCGCCG.** 1355 (96.68)   
142 - **[CCGACC]** 1352 (88.08)   
142 - **[CCCCCG]** 1348 (85.08)   
142 - **.CCGAGG.** 1331 (90.88)   
142 - **.CCGGCG.** 1307 (94.52)   
142 - **[CCGGGG]** 1119 (85.40)   
142 - **[CCGCGG]** 1062 (87.56)   
142 - **[CCCCCG]** 1056 (85.08)   
142 - **.CCGAGG.** 1033 (90.88)   
142 - **.CCGGCG.** 960 (94.52)   
142 - **[CCGCGG]** 945 (87.56)   
142 - **.CCGGCG.** 904 (94.52)   
142 - **[CCGCGG]** 832 (87.56)   
142 - **.CCGGCG.** 780 (94.52)   
142 - **[CCGCGG]** 673 (87.56)   
142 - **.CCGCCG.** 665 (96.68)   
142 - **[CCGACT]** 642 (85.38)   
142 - **[CCCACG]** 568 (88.40)   
142 - **[CCGCGG]** 517 (87.56)   
142 - **[CCGGGG]** 439 (85.40)   
142 - **[CCCACG]** 173 (88.40)   
142 - **[CGGACG]** 169 (86.87)   
142 - **[CCCCCG]** 129 (85.08)   
143 + **.CCGCCG.** 943 (96.68)   
143 + **[CCGGGG]** 1329 (85.40)   
143 - **.CCGGCG.** 1327 (94.52)   
143 - **.CCGGCG.** 944 (94.52)   
143 - **.CCGAGG.** 505 (90.88)   
144 + **.CCGGCG.** 440 (94.52)   
144 + **[CAGACG]** 916 (85.30)   
144 + **[CCGCGG]** 974 (87.56)   
144 + **.CCGGCG.** 1123 (94.52)   
144 + **[CCGCGG]** 1199 (87.56)   
144 + **[CCGCGG]** 1218 (87.56)   
144 + **.CCGGCG.** 1236 (94.52)   
144 + **[CCCCCG]** 1264 (85.08)   
144 + **[CCGCGG]** 1267 (87.56)   
144 + **[CCGACC]** 1436 (88.08)   
144 + **.CCGAGG.** 1440 (90.88)   
144 + **.CCGCCG.** 1452 (96.68)   
144 + **[CCCCCG]** 1477 (85.08)   
144 + **[CCCCCG]** 1509 (85.08)   
144 + **.CCGCCG.** 1512 (96.68)   
144 - **.CCGCCG.** 1366 (96.68)   
144 - **.CCGCCG.** 1343 (96.68)   
144 - **[CCGGGG]** 1301 (85.40)   
144 - **.CCGAGG.** 1282 (90.88)   
144 - **[CCGCGG]** 1267 (87.56)   
144 - **.CCGGCG.** 1257 (94.52)   
144 - **[CCCCCG]** 1240 (85.08)   
144 - **.CCGCCG.** 1237 (96.68)   
144 - **[CCGCGG]** 1218 (87.56)   
144 - **[CCGCGG]** 1199 (87.56)   
144 - **[CCGACT]** 1179 (85.38)   
144 - **.CCGCCG.** 1124 (96.68)   
144 - **.CCGGCG.** 1106 (94.52)   
144 - **.CCGCCG.** 1099 (96.68)   
144 - **.CCGCCG.** 1092 (96.68)   
144 - **.CCGCCG.** 1064 (96.68)   
144 - **.CCGCCG.** 1061 (96.68)   
144 - **.CCGCCG.** 1041 (96.68)   
144 - **.CCGCCG.** 1029 (96.68)   
144 - **.CCGCCG.** 1011 (96.68)   
144 - **[CCGCGG]** 974 (87.56)   
144 - **[CCGACC]** 866 (88.08)   
144 - **[CGGACG]** 841 (86.87)   
144 - **[CGGACG]** 776 (86.87)   
144 - **[CGGACG]** 756 (86.87)   
144 - **[CGGACG]** 671 (86.87)   
144 - **[CGGACG]** 606 (86.87)   
144 - **[CAGACG]** 533 (85.30)   
144 - **[CAGACG]** 513 (85.30)   
144 - **[CGGACG]** 220 (86.87)   
144 - **[CGGACG]** 134 (86.87)   
144 - **[CCGAAG]** 30 (86.09)   
145 + **[CCCCCG]** 226 (85.08)   
145 + **[CGGACG]** 268 (86.87)   
145 + **.CCGAGG.** 329 (90.88)   
145 + **[CCGGGG]** 772 (85.40)   
145 + **.CCGGCG.** 818 (94.52)   
145 + **[CCGGGG]** 876 (85.40)   
145 + **.CCGGCG.** 958 (94.52)   
145 + **.CCGCCG.** 1082 (96.68)   
145 + **[CCGCGG]** 1108 (87.56)   
145 + **.CCGCCG.** 1135 (96.68)   
145 + **[GCGACG]** 1218 (85.28)   
145 + **[GCGACG]** 1254 (85.28)   
145 + **[CCGACC]** 1297 (88.08)   
145 + **.CCGAGG.** 1404 (90.88)   
145 + **[CCCCCG]** 1444 (85.08)   
145 + **.CCGAGG.** 1447 (90.88)   
145 - **[CCCACG]** 1410 (88.40)   
145 - **[CCGTCG]** 1255 (86.99)   
145 - **.CCGCCG.** 1143 (96.68)   
145 - **[CCGCGG]** 1108 (87.56)   
145 - **.CCGGCG.** 1083 (94.52)   
145 - **[CAGACG]** 1074 (85.30)   
145 - **[CCCACG]** 1042 (88.40)   
145 - **[GCGACG]** 1006 (85.28)   
145 - **[CCCCCG]** 962 (85.08)   
145 - **.CCGCCG.** 959 (96.68)   
145 - **.CCGCCG.** 819 (96.68)   
145 - **[CCCACG]** 786 (88.40)   
145 - **[CCGACT]** 741 (85.38)   
145 - **[CCCACG]** 682 (88.40)   
145 - **[CCCACG]** 614 (88.40)   
145 - **[CCCACG]** 580 (88.40)   
145 - **.CCGCCG.** 546 (96.68)   
145 - **[CCGGGG]** 310 (85.40)   
146 + **[CCCACG]** 716 (88.40)   
146 + **[CCGGGG]** 1310 (85.40)   
146 - **[CCCACG]** 846 (88.40)   
146 - **[CAGACG]** 751 (85.30)   
147 + **.CCGAGG.** 111 (90.88)   
147 + **[CCCCCG]** 176 (85.08)   
147 + **.CCGCCG.** 827 (96.68)   
147 + **.CCGACG.** 860 (100.00)   
147 + **[CCCACG]** 870 (88.40)   
147 + **.CCGAGG.** 1076 (90.88)   
147 + **.CCGGCG.** 1095 (94.52)   
147 + **[CCGACC]** 1101 (88.08)   
147 + **.CCGGCG.** 1206 (94.52)   
147 + **[CCGGGG]** 1259 (85.40)   
147 + **[CCGCGG]** 1359 (87.56)   
147 + **[CCGGGG]** 1400 (85.40)   
147 - **.CCGAGG.** 1436 (90.88)   
147 - **[CCGCGG]** 1359 (87.56)   
147 - **[GCGACG]** 1340 (85.28)   
147 - **.CCGCCG.** 1334 (96.68)   
147 - **[CCGACT]** 1276 (85.38)   
147 - **[CCGACC]** 1270 (88.08)   
147 - **[CCCCCG]** 1266 (85.08)   
147 - **[CCGACC]** 1263 (88.08)   
147 - **[CCGGGG]** 1145 (85.40)   
147 - **.CCGCCG.** 1041 (96.68)   
147 - **[CCGGGG]** 999 (85.40)   
147 - **[GCGACG]** 962 (85.28)   
148 + **.CCGGCG.** 217 (94.52)   
148 + **.CCGACG.** 437 (100.00)   
148 + **[CCCACG]** 479 (88.40)   
148 + **[CCGCGG]** 547 (87.56)   
148 + **[CCCCCG]** 672 (85.08)   
148 + **.CCGCCG.** 675 (96.68)   
148 + **[CCGGGG]** 710 (85.40)   
148 + **[CCGCGG]** 996 (87.56)   
148 + **[CCCCCG]** 1266 (85.08)   
148 + **[CCGACT]** 1269 (85.38)   
148 + **[CCGTCG]** 1395 (86.99)   
148 + **[CAGACG]** 1480 (85.30)   
148 + **[CGGACG]** 1489 (86.87)   
148 - **[GCGACG]** 1396 (85.28)   
148 - **[CCCCCG]** 1227 (85.08)   
148 - **.CCGGCG.** 1099 (94.52)   
148 - **[CCCACG]** 1022 (88.40)   
148 - **[CGGACG]** 1018 (86.87)   
148 - **[CCGCGG]** 996 (87.56)   
148 - **[CCGCGG]** 547 (87.56)   
149 + **[CCGACC]** 905 (88.08)   
149 + **[CCGAAG]** 1193 (86.09)   
149 - **[CCCACG]** 1145 (88.40)   
149 - **[CCCACG]** 510 (88.40)   
150 + **.CCGCCG.** 31 (96.68)   
150 + **[CCGGGG]** 34 (85.40)   
150 + **[CCCACG]** 55 (88.40)   
150 + **.CCGGCG.** 99 (94.52)   
150 + **.CCGACG.** 167 (100.00)   
150 + **[CCGCGG]** 174 (87.56)   
150 + **[CAGACG]** 364 (85.30)   
150 + **[CAGACG]** 463 (85.30)   
150 + **.CCGCCG.** 553 (96.68)   
150 + **[CCGTCG]** 691 (86.99)   
150 + **[GCGACG]** 934 (85.28)   
150 + **[CCGCGG]** 1119 (87.56)   
150 + **.CCGGCG.** 1388 (94.52)   
150 + **[CGGACG]** 1439 (86.87)   
150 - **[CCGAAG]** 1512 (86.09)   
150 - **[CCCACG]** 1272 (88.40)   
150 - **[CCGACT]** 1255 (85.38)   
150 - **[CCGCGG]** 1119 (87.56)   
150 - **[CGGACG]** 1116 (86.87)   
150 - **.CCGGCG.** 1023 (94.52)   
150 - **.CCGCCG.** 990 (96.68)   
150 - **[CCGTCG]** 935 (86.99)   
150 - **.CCGCCG.** 915 (96.68)   
150 - **.CCGACG.** 832 (100.00)   
150 - **.CCGACG.** 692 (100.00)   
150 - **[CCCCCG]** 608 (85.08)   
150 - **[CCGCGG]** 174 (87.56)   
150 - **[CCCCCG]** 103 (85.08)   
150 - **.CCGCCG.** 100 (96.68)   
150 - **.CCGGCG.** 59 (94.52)   
150 - **[CCCCCG]** 35 (85.08)   
150 - **.CCGGCG.** 32 (94.52)   
151 + **.CCGCCG.** 16 (96.68)   
151 + **[CCGTCG]** 70 (86.99)   
151 + **.CCGCCG.** 91 (96.68)   
151 + **.CCGACG.** 174 (100.00)   
151 + **.CCGACG.** 313 (100.00)   
151 + **[CCCCCG]** 397 (85.08)   
151 + **[CCGCGG]** 832 (87.56)   
151 + **[CCCCCG]** 903 (85.08)   
151 + **.CCGCCG.** 906 (96.68)   
151 + **.CCGGCG.** 946 (94.52)   
151 + **.CCGGCG.** 974 (94.52)   
151 + **[CCGGGG]** 1157 (85.40)   
151 + **[GCGACG]** 1331 (85.28)   
151 + **.CCGGCG.** 1392 (94.52)   
151 + **.CCGAGG.** 1510 (90.88)   
151 - **[CGGACG]** 1475 (86.87)   
151 - **.CCGCCG.** 1393 (96.68)   
151 - **[CCGTCG]** 1332 (86.99)   
151 - **.CCGGCG.** 1277 (94.52)   
151 - **[CCGACC]** 1236 (88.08)   
151 - **.CCGGCG.** 1208 (94.52)   
151 - **.CCGGCG.** 1196 (94.52)   
151 - **[CCCCCG]** 1158 (85.08)   
151 - **[CCGACT]** 1092 (85.38)   
151 - **[CCGAAG]** 1010 (86.09)   
151 - **.CCGCCG.** 975 (96.68)   
151 - **[CCCACG]** 950 (88.40)   
151 - **.CCGGCG.** 907 (94.52)   
151 - **.CCGACG.** 839 (100.00)   
151 - **[CCGCGG]** 832 (87.56)   
151 - **[CAGACG]** 641 (85.30)   
151 - **[CAGACG]** 542 (85.30)   
151 - **.CCGCCG.** 452 (96.68)   
151 - **[CCGTCG]** 314 (86.99)   
151 - **[GCGACG]** 71 (85.28)   
153 + **.CCGAGG.** 426 (90.88)   
154 + **.CCGGCG.** 1148 (94.52)   
154 + **[CCCCCG]** 1167 (85.08)   
154 + **.CCGAGG.** 1187 (90.88)   
154 + **.CCGCCG.** 1220 (96.68)   
154 + **.CCGCCG.** 1251 (96.68)   
154 + **.CCGCCG.** 1289 (96.68)   
154 + **.CCGACG.** 1332 (100.00)   
154 + **[CCGGGG]** 1395 (85.40)   
154 + **.CCGCCG.** 1436 (96.68)   
154 + **.CCGCCG.** 1470 (96.68)   
154 - **.CCGGCG.** 1471 (94.52)   
154 - **.CCGGCG.** 1437 (94.52)   
154 - **[CGGACG]** 1286 (86.87)   
154 - **.CCGCCG.** 1045 (96.68)   
154 - **.CCGCCG.** 1042 (96.68)   
154 - **.CCGCCG.** 1024 (96.68)   
154 - **.CCGGCG.** 954 (94.52)   
154 - **[CCCCCG]** 857 (85.08)   
154 - **[CCCCCG]** 847 (85.08)   
154 - **[CCGACC]** 748 (88.08)   
155 + **.CCGGCG.** 505 (94.52)   
155 - **[GCGACG]** 1256 (85.28)   
155 - **[CCGGGG]** 351 (85.40)   
156 + **[CCGAAG]** 976 (86.09)   
156 - **[CGGACG]** 973 (86.87)   
157 + **[CCGACC]** 450 (88.08)   
157 + **[GCGACG]** 689 (85.28)   
157 + **[CCGCGG]** 803 (87.56)   
157 + **.CCGCCG.** 1112 (96.68)   
157 + **.CCGCCG.** 1115 (96.68)   
157 + **[CCGCGG]** 1127 (87.56)   
157 + **[CCCACG]** 1369 (88.40)   
157 - **[CCCACG]** 1240 (88.40)   
157 - **[CCGCGG]** 1127 (87.56)   
157 - **[CCGCGG]** 803 (87.56)   
157 - **[CCGTCG]** 690 (86.99)   
157 - **[CCGACT]** 497 (85.38)   
157 - **[CAGACG]** 137 (85.30)   
158 + **.CCGAGG.** 1172 (90.88)   
158 + **.CCGGCG.** 1510 (94.52)   
158 - **[CCGACC]** 1495 (88.08)   
158 - **.CCGAGG.** 1406 (90.88)   
159 + **[CCGAAG]** 1249 (86.09)   
160 + **[CCGACC]** 175 (88.08)   
160 + **[CCGAAG]** 200 (86.09)   
160 + **[CCGCGG]** 236 (87.56)   
160 + **.CCGCCG.** 264 (96.68)   
160 + **[CCGCGG]** 267 (87.56)   
160 + **[CCCACG]** 454 (88.40)   
160 + **[CCGGGG]** 560 (85.40)   
160 + **.CCGCCG.** 760 (96.68)   
160 + **[CCGGGG]** 842 (85.40)   
160 + **[CCGGGG]** 1023 (85.40)   
160 + **.CCGAGG.** 1152 (90.88)   
160 + **.CCGACG.** 1267 (100.00)   
160 + **[CCCACG]** 1273 (88.40)   
160 + **.CCGCCG.** 1305 (96.68)   
160 + **[CCGCGG]** 1328 (87.56)   
160 + **[CCCCCG]** 1337 (85.08)   
160 + **[CCGGGG]** 1371 (85.40)   
160 + **.CCGGCG.** 1428 (94.52)   
160 + **.CCGAGG.** 1505 (90.88)   
160 - **.CCGGCG.** 1432 (94.52)   
160 - **[CCGCGG]** 1328 (87.56)   
160 - **[CCGGGG]** 1247 (85.40)   
160 - **[CGGACG]** 1233 (86.87)   
160 - **[CCGGGG]** 1192 (85.40)   
160 - **.CCGGCG.** 1021 (94.52)   
160 - **[CCGACT]** 1006 (85.38)   
160 - **.CCGCCG.** 993 (96.68)   
160 - **[CCCCCG]** 979 (85.08)   
160 - **[CCCCCG]** 945 (85.08)   
160 - **[CCCCCG]** 935 (85.08)   
160 - **[CCCCCG]** 916 (85.08)   
160 - **[CCGGGG]** 840 (85.40)   
160 - **[CAGACG]** 832 (85.30)   
160 - **[CAGACG]** 815 (85.30)   
160 - **.CCGACG.** 804 (100.00)   
160 - **[CAGACG]** 784 (85.30)   
160 - **[CCCACG]** 746 (88.40)   
160 - **[CAGACG]** 733 (85.30)   
160 - **[CAGACG]** 698 (85.30)   
160 - **[CGGACG]** 694 (86.87)   
160 - **[CAGACG]** 642 (85.30)   
160 - **[CGGACG]** 638 (86.87)   
160 - **[CAGACG]** 546 (85.30)   
160 - **[CCGCGG]** 267 (87.56)   
160 - **[CCGCGG]** 236 (87.56)   
160 - **.CCGAGG.** 85 (90.88)   
160 - **[CCCCCG]** 57 (85.08)   
161 + **.CCGCCG.** 255 (96.68)   
161 + **[GCGACG]** 278 (85.28)   
161 + **.CCGCCG.** 290 (96.68)   
161 + **[CCGTCG]** 453 (86.99)   
161 + **[CCCACG]** 467 (88.40)   
161 + **.CCGACG.** 582 (100.00)   
161 + **[CCGCGG]** 628 (87.56)   
161 + **[CCCCCG]** 667 (85.08)   
161 + **[CCGGGG]** 696 (85.40)   
161 + **.CCGGCG.** 750 (94.52)   
161 + **[CCGCGG]** 759 (87.56)   
161 + **.CCGAGG.** 783 (90.88)   
161 + **[CCGGGG]** 860 (85.40)   
161 + **[CCGCGG]** 885 (87.56)   
161 + **.CCGCCG.** 892 (96.68)   
161 + **[CCGGGG]** 1196 (85.40)   
161 + **.CCGCCG.** 1229 (96.68)   
161 + **[CCGGGG]** 1232 (85.40)   
161 + **.CCGCCG.** 1255 (96.68)   
161 + **[CCGGGG]** 1258 (85.40)   
161 + **[CCGCGG]** 1267 (87.56)   
161 + **[CCGCGG]** 1301 (87.56)   
161 + **.CCGCCG.** 1421 (96.68)   
161 + **.CCGCCG.** 1424 (96.68)   
161 + **[CCCCCG]** 1439 (85.08)   
161 + **[CCCCCG]** 1470 (85.08)   
161 + **.CCGCCG.** 1485 (96.68)   
161 + **.CCGCCG.** 1488 (96.68)   
161 + **.CCGCCG.** 1491 (96.68)   
161 + **.CCGCCG.** 1503 (96.68)   
161 + **.CCGCCG.** 1506 (96.68)   
161 + **.CCGCCG.** 1509 (96.68)   
161 - **[CCGGGG]** 1471 (85.40)   
161 - **[CCGGGG]** 1459 (85.40)   
161 - **[CCGGGG]** 1440 (85.40)   
161 - **.CCGGCG.** 1412 (94.52)   
161 - **[CCGGGG]** 1336 (85.40)   
161 - **[CCGACT]** 1312 (85.38)   
161 - **[CCGCGG]** 1301 (87.56)   
161 - **.CCGCCG.** 1273 (96.68)   
161 - **.CCGCCG.** 1270 (96.68)   
161 - **[CCGCGG]** 1267 (87.56)   
161 - **[CCCCCG]** 1259 (85.08)   
161 - **.CCGGCG.** 1256 (94.52)   
161 - **[CCGGGG]** 1239 (85.40)   
161 - **[CCCCCG]** 1233 (85.08)   
161 - **.CCGGCG.** 1230 (94.52)   
161 - **[CCCCCG]** 1197 (85.08)   
161 - **.CCGCCG.** 989 (96.68)   
161 - **.CCGCCG.** 986 (96.68)   
161 - **.CCGCCG.** 983 (96.68)   
161 - **.CCGAGG.** 974 (90.88)   
161 - **.CCGCCG.** 945 (96.68)   
161 - **[CCGCGG]** 885 (87.56)   
161 - **.CCGCCG.** 831 (96.68)   
161 - **[CCGACC]** 802 (88.08)   
161 - **.CCGGCG.** 798 (94.52)   
161 - **[CCCCCG]** 791 (85.08)   
161 - **[CCGCGG]** 759 (87.56)   
161 - **.CCGAGG.** 730 (90.88)   
161 - **[CCGGGG]** 704 (85.40)   
161 - **[CCCCCG]** 697 (85.08)   
161 - **[CCGCGG]** 628 (87.56)   
161 - **[CCGACC]** 506 (88.08)   
161 - **.CCGACG.** 454 (100.00)   
162 + **.CCGAGG.** 896 (90.88)   
162 + **[CGGACG]** 1145 (86.87)   
164 + **.CCGGCG.** 206 (94.52)   
164 + **[CCCACG]** 513 (88.40)   
164 + **[CCGCGG]** 1002 (87.56)   
164 + **.CCGGCG.** 1095 (94.52)   
164 + **[CCCCCG]** 1140 (85.08)   
164 - **[CCGGGG]** 1490 (85.40)   
164 - **.CCGCCG.** 1204 (96.68)   
164 - **.CCGGCG.** 1074 (94.52)   
164 - **[CCGGGG]** 1019 (85.40)   
164 - **[CCGCGG]** 1002 (87.56)   
164 - **[CCGACT]** 976 (85.38)   
164 - **.CCGCCG.** 308 (96.68)   
164 - **[GCGACG]** 272 (85.28)   
164 - **[CAGACG]** 254 (85.30)   
166 + **[CCGGGG]** 379 (85.40)   
166 + **.CCGAGG.** 453 (90.88)   
166 + **[CCGCGG]** 547 (87.56)   
166 + **[CCGGGG]** 560 (85.40)   
166 + **.CCGAGG.** 592 (90.88)   
166 + **[CCGAAG]** 616 (86.09)   
166 + **.CCGGCG.** 639 (94.52)   
166 + **[CGGACG]** 829 (86.87)   
166 + **[CCCCCG]** 922 (85.08)   
166 + **.CCGAGG.** 957 (90.88)   
166 + **[CCCCCG]** 997 (85.08)   
166 + **[CCGGGG]** 1000 (85.40)   
166 + **.CCGCCG.** 1013 (96.68)   
166 + **[CCGCGG]** 1073 (87.56)   
166 + **.CCGCCG.** 1118 (96.68)   
166 + **.CCGCCG.** 1170 (96.68)   
166 + **[CCGGGG]** 1280 (85.40)   
166 + **[CCGGGG]** 1286 (85.40)   
166 - **.CCGAGG.** 1512 (90.88)   
166 - **[CCCACG]** 1330 (88.40)   
166 - **[CCGGGG]** 1199 (85.40)   
166 - **.CCGGCG.** 1171 (94.52)   
166 - **[CCGCGG]** 1073 (87.56)   
166 - **.CCGGCG.** 1014 (94.52)   
166 - **[CCGGGG]** 998 (85.40)   
166 - **.CCGACG.** 963 (100.00)   
166 - **.CCGAGG.** 928 (90.88)   
166 - **[CCGGGG]** 637 (85.40)   
166 - **[CCCCCG]** 561 (85.08)   
166 - **[CCGCGG]** 547 (87.56)   
166 - **[CCGGGG]** 526 (85.40)   
166 - **[CCGTCG]** 518 (86.99)   
166 - **[CCCACG]** 63 (88.40)   
167 + **[CCGACC]** 786 (88.08)   
167 + **.CCGAGG.** 791 (90.88)   
167 + **[CCGCGG]** 842 (87.56)   
167 + **[CCGCGG]** 872 (87.56)   
167 + **.CCGCCG.** 884 (96.68)   
167 + **[CCGGGG]** 929 (85.40)   
167 + **[CCGCGG]** 962 (87.56)   
167 + **.CCGAGG.** 1073 (90.88)   
167 + **.CCGAGG.** 1217 (90.88)   
167 + **[CCGCGG]** 1235 (87.56)   
167 + **.CCGCCG.** 1397 (96.68)   
167 + **.CCGGCG.** 1417 (94.52)   
167 + **[CCGACT]** 1490 (85.38)   
167 - **[CCGACC]** 1298 (88.08)   
167 - **[CCCCCG]** 1247 (85.08)   
167 - **[CCGCGG]** 1235 (87.56)   
167 - **[CCGACT]** 1143 (85.38)   
167 - **[CCGGGG]** 1027 (85.40)   
167 - **[CCGGGG]** 997 (85.40)   
167 - **[CCGCGG]** 962 (87.56)   
167 - **[CCGGGG]** 947 (85.40)   
167 - **[CCGGGG]** 927 (85.40)   
167 - **[CCCCCG]** 875 (85.08)   
167 - **[CCGCGG]** 872 (87.56)   
167 - **[CCCCCG]** 853 (85.08)   
167 - **.CCGCCG.** 845 (96.68)   
167 - **[CCGCGG]** 842 (87.56)   
167 - **[CCCCCG]** 797 (85.08)   
167 - **[CCCCCG]** 616 (85.08)   
167 - **.CCGCCG.** 613 (96.68)   
167 - **.CCGAGG.** 610 (90.88)   
167 - **[CCCCCG]** 518 (85.08)   
167 - **[CCGGGG]** 291 (85.40)   
167 - **[CCCCCG]** 176 (85.08)   
168 + **[CCGGGG]** 228 (85.40)   
168 + **[CCCACG]** 352 (88.40)   
168 + **.CCGGCG.** 680 (94.52)   
168 + **[CCGGGG]** 795 (85.40)   
168 + **[CCCCCG]** 869 (85.08)   
168 + **.CCGGCG.** 872 (94.52)   
168 + **.CCGGCG.** 1152 (94.52)   
168 + **[CCGCGG]** 1204 (87.56)   
168 + **[CCCCCG]** 1245 (85.08)   
168 - **.CCGAGG.** 1447 (90.88)   
168 - **.CCGAGG.** 1315 (90.88)   
168 - **[CCGGGG]** 1264 (85.40)   
168 - **[CCCCCG]** 1237 (85.08)   
168 - **[CCGCGG]** 1204 (87.56)   
168 - **[CGGACG]** 1201 (86.87)   
168 - **.CCGGCG.** 1156 (94.52)   
168 - **[CCGGGG]** 1150 (85.40)   
168 - **[CCGACT]** 1015 (85.38)   
168 - **[CCGGGG]** 994 (85.40)   
168 - **[CCGGGG]** 958 (85.40)   
168 - **.CCGCCG.** 943 (96.68)   
168 - **.CCGCCG.** 921 (96.68)   
168 - **.CCGCCG.** 873 (96.68)   
168 - **[CCGGGG]** 870 (85.40)   
168 - **[CCCCCG]** 777 (85.08)   
168 - **[CCGACT]** 774 (85.38)   
168 - **[CAGACG]** 717 (85.30)   
168 - **[CCCACG]** 684 (88.40)   
168 - **[CCGGGG]** 180 (85.40)   
168 - **[CCCACG]** 13 (88.40)   
169 + **.CCGAGG.** 56 (90.88)   
169 + **[CCCCCG]** 315 (85.08)   
169 + **.CCGGCG.** 513 (94.52)   
169 + **[CCGGGG]** 699 (85.40)   
169 - **[CCGGGG]** 1485 (85.40)   
169 - **[CCGAAG]** 1425 (86.09)   
169 - **[CCGTCG]** 1248 (86.99)   
169 - **[CCGAAG]** 1147 (86.09)   
169 - **[CCGAAG]** 804 (86.09)   
169 - **[CCGGGG]** 652 (85.40)   
169 - **[CCGAAG]** 415 (86.09)   
169 - **.CCGAGG.** 352 (90.88)   
170 + **[CCGGGG]** 666 (85.40)   
170 + **[CCGGGG]** 1223 (85.40)   
170 + **.CCGGCG.** 1468 (94.52)   
170 - **.CCGCCG.** 1472 (96.68)   
170 - **.CCGCCG.** 1469 (96.68)   
171 + **[CCCACG]** 835 (88.40)   
171 + **[CCCACG]** 1187 (88.40)   
171 - **[CCCACG]** 251 (88.40)   
172 + **[CCGACC]** 58 (88.08)   
172 + **.CCGCCG.** 227 (96.68)   
172 + **.CCGCCG.** 230 (96.68)   
172 + **.CCGCCG.** 307 (96.68)   
172 + **[CCGCGG]** 336 (87.56)   
172 + **.CCGCCG.** 369 (96.68)   
172 + **[CCGCGG]** 387 (87.56)   
172 + **.CCGCCG.** 520 (96.68)   
172 + **.CCGCCG.** 523 (96.68)   
172 + **.CCGCCG.** 526 (96.68)   
172 + **.CCGCCG.** 529 (96.68)   
172 + **.CCGCCG.** 532 (96.68)   
172 + **.CCGCCG.** 535 (96.68)   
172 + **.CCGCCG.** 538 (96.68)   
172 + **.CCGCCG.** 541 (96.68)   
172 + **.CCGACG.** 544 (100.00)   
172 + **[CCCCCG]** 591 (85.08)   
172 + **[CCGTCG]** 601 (86.99)   
172 + **.CCGCCG.** 626 (96.68)   
172 + **[CCCCCG]** 642 (85.08)   
172 + **.CCGCCG.** 682 (96.68)   
172 + **[GCGACG]** 717 (85.28)   
172 + **[CCGAAG]** 736 (86.09)   
172 + **[CCGCGG]** 756 (87.56)   
172 + **[CCGACC]** 870 (88.08)   
172 + **.CCGCCG.** 944 (96.68)   
172 + **[CCGGGG]** 947 (85.40)   
172 + **[CCCCCG]** 1073 (85.08)   
172 + **[CCGAAG]** 1087 (86.09)   
172 + **[CCGGGG]** 1245 (85.40)   
172 - **.CCGAGG.** 1472 (90.88)   
172 - **[CCCCCG]** 1246 (85.08)   
172 - **[CCCCCG]** 955 (85.08)   
172 - **.CCGGCG.** 945 (94.52)   
172 - **.CCGCCG.** 916 (96.68)   
172 - **[CCCCCG]** 853 (85.08)   
172 - **.CCGAGG.** 831 (90.88)   
172 - **[CCCCCG]** 780 (85.08)   
172 - **[CCGCGG]** 756 (87.56)   
172 - **.CCGACG.** 602 (100.00)   
172 - **[GCGACG]** 548 (85.28)   
172 - **.CCGCCG.** 433 (96.68)   
172 - **[CCGCGG]** 387 (87.56)   
172 - **[CGGACG]** 366 (86.87)   
172 - **[CCGCGG]** 336 (87.56)   
172 - **[CCGGGG]** 313 (85.40)   
172 - **.CCGAGG.** 251 (90.88)   
172 - **[CCGAAG]** 113 (86.09)   
172 - **[CCGAAG]** 68 (86.09)   
173 + **[GCGACG]** 651 (85.28)   
173 + **[CCGCGG]** 713 (87.56)   
173 + **[CCGAAG]** 730 (86.09)   
173 + **[CCGACC]** 893 (88.08)   
173 + **.CCGGCG.** 898 (94.52)   
173 + **[GCGACG]** 1189 (85.28)   
173 + **[CCCCCG]** 1385 (85.08)   
173 - **.CCGGCG.** 1083 (94.52)   
173 - **.CCGCCG.** 1027 (96.68)   
173 - **.CCGCCG.** 1024 (96.68)   
173 - **.CCGAGG.** 962 (90.88)   
173 - **[CCCCCG]** 952 (85.08)   
173 - **.CCGAGG.** 949 (90.88)   
173 - **[CCCCCG]** 928 (85.08)   
173 - **[CCGCGG]** 713 (87.56)   
174 + **[CCGGGG]** 307 (85.40)   
174 + **.CCGAGG.** 354 (90.88)   
174 + **[CCGAAG]** 376 (86.09)   
174 + **[CGGACG]** 390 (86.87)   
174 + **.CCGACG.** 703 (100.00)   
174 + **[CCGGGG]** 847 (85.40)   
174 + **.CCGACG.** 1081 (100.00)   
174 + **[CCGACC]** 1110 (88.08)   
174 + **[CCCCCG]** 1137 (85.08)   
174 + **[CCGGGG]** 1140 (85.40)   
174 + **[CCCCCG]** 1197 (85.08)   
174 + **.CCGCCG.** 1278 (96.68)   
174 + **.CCGCCG.** 1281 (96.68)   
174 + **[GCGACG]** 1326 (85.28)   
174 + **[GCGACG]** 1369 (85.28)   
174 + **[CCGCGG]** 1476 (87.56)   
174 + **.CCGAGG.** 1501 (90.88)   
174 + **[CCGGGG]** 1512 (85.40)   
174 - **[CCCCCG]** 1513 (85.08)   
174 - **[CCGCGG]** 1476 (87.56)   
174 - **[CCGGGG]** 1390 (85.40)   
174 - **[CCGTCG]** 1370 (86.99)   
174 - **.CCGGCG.** 1282 (94.52)   
174 - **[CCGGGG]** 1198 (85.40)   
174 - **[CCCCCG]** 1177 (85.08)   
174 - **[CCCCCG]** 1159 (85.08)   
174 - **[CCGGGG]** 1138 (85.40)   
174 - **[CCGTCG]** 1082 (86.99)   
174 - **.CCGCCG.** 1063 (96.68)   
174 - **[CCGAAG]** 1060 (86.09)   
174 - **.CCGAGG.** 1047 (90.88)   
174 - **.CCGCCG.** 835 (96.68)   
174 - **[CCCCCG]** 779 (85.08)   
174 - **.CCGAGG.** 776 (90.88)   
174 - **[CCGGGG]** 743 (85.40)   
174 - **[CCGTCG]** 704 (86.99)   
174 - **[CCGGGG]** 675 (85.40)   
174 - **[CAGACG]** 470 (85.30)   
175 + **.CCGAGG.** 425 (90.88)   
175 + **.CCGAGG.** 1097 (90.88)   
175 + **[CCGACT]** 1306 (85.38)   
175 + **[CCGCGG]** 1467 (87.56)   
175 + **[CCCCCG]** 1494 (85.08)   
175 - **[CCGCGG]** 1467 (87.56)   
175 - **[CCCACG]** 922 (88.40)   
177 + **.CCGGCG.** 857 (94.52)   
177 + **[CCGTCG]** 1016 (86.99)   
177 + **.CCGCCG.** 1022 (96.68)   
177 + **.CCGCCG.** 1025 (96.68)   
177 + **.CCGCCG.** 1028 (96.68)   
177 + **[CCGCGG]** 1464 (87.56)   
177 - **[CCGCGG]** 1464 (87.56)   
177 - **.CCGAGG.** 1079 (90.88)   
177 - **[CCCACG]** 1058 (88.40)   
177 - **[GCGACG]** 1017 (85.28)   
177 - **[CAGACG]** 924 (85.30)   
177 - **.CCGGCG.** 855 (94.52)   
177 - **.CCGAGG.** 823 (90.88)   
177 - **.CCGCCG.** 755 (96.68)   
177 - **.CCGCCG.** 752 (96.68)   
178 + **[CCGACC]** 187 (88.08)   
178 + **[CCCACG]** 293 (88.40)   
178 + **[CCCCCG]** 350 (85.08)   
178 + **.CCGCCG.** 753 (96.68)   
178 + **.CCGCCG.** 756 (96.68)   
178 + **.CCGGCG.** 759 (94.52)   
178 + **.CCGGCG.** 1074 (94.52)   
178 + **.CCGAGG.** 1139 (90.88)   
178 + **[CCGAAG]** 1274 (86.09)   
178 - **.CCGCCG.** 1116 (96.68)   
178 - **[CCCCCG]** 1078 (85.08)   
178 - **.CCGCCG.** 1075 (96.68)   
178 - **[CCGGGG]** 795 (85.40)   
178 - **.CCGGCG.** 757 (94.52)   
179 + **[CAGACG]** 791 (85.30)   
179 + **[CCGACT]** 882 (85.38)   
179 + **[CCGCGG]** 917 (87.56)   
179 + **[CCGGGG]** 923 (85.40)   
179 + **.CCGGCG.** 935 (94.52)   
179 + **[CCCCCG]** 1413 (85.08)   
179 + **.CCGCCG.** 1472 (96.68)   
179 + **.CCGAGG.** 1498 (90.88)   
179 - **.CCGCCG.** 982 (96.68)   
179 - **.CCGCCG.** 979 (96.68)   
179 - **.CCGCCG.** 976 (96.68)   
179 - **.CCGCCG.** 949 (96.68)   
179 - **.CCGCCG.** 946 (96.68)   
179 - **.CCGCCG.** 943 (96.68)   
179 - **.CCGCCG.** 936 (96.68)   
179 - **[CCGCGG]** 917 (87.56)   
179 - **.CCGACG.** 666 (100.00)   
179 - **[CCCCCG]** 524 (85.08)   
179 - **[CCCACG]** 465 (88.40)   
180 + **.CCGACG.** 102 (100.00)   
180 + **.CCGGCG.** 208 (94.52)   
180 + **[CCGCGG]** 267 (87.56)   
180 + **[CCGAAG]** 284 (86.09)   
180 + **[CCCCCG]** 373 (85.08)   
180 + **.CCGAGG.** 376 (90.88)   
180 + **[CCGCGG]** 700 (87.56)   
180 + **[CCGGGG]** 730 (85.40)   
180 + **[CCCCCG]** 861 (85.08)   
180 + **[CGGACG]** 865 (86.87)   
180 + **[CCGCGG]** 923 (87.56)   
180 + **.CCGCCG.** 961 (96.68)   
180 + **[CCGCGG]** 1158 (87.56)   
180 + **.CCGGCG.** 1272 (94.52)   
180 + **.CCGAGG.** 1286 (90.88)   
180 + **.CCGCCG.** 1510 (96.68)   
180 - **.CCGGCG.** 1511 (94.52)   
180 - **[CCCCCG]** 1404 (85.08)   
180 - **[CGGACG]** 1283 (86.87)   
180 - **[CCCACG]** 1276 (88.40)   
180 - **.CCGCCG.** 1252 (96.68)   
180 - **.CCGCCG.** 1249 (96.68)   
180 - **[CCGCGG]** 1158 (87.56)   
180 - **.CCGCCG.** 1067 (96.68)   
180 - **.CCGCCG.** 1043 (96.68)   
180 - **[CCGCGG]** 923 (87.56)   
180 - **[CCGGGG]** 862 (85.40)   
180 - **[CCGACC]** 734 (88.08)   
180 - **[CCGGGG]** 715 (85.40)   
180 - **[CCGCGG]** 700 (87.56)   
180 - **[CCGCGG]** 267 (87.56)   
180 - **.CCGCCG.** 209 (96.68)   
180 - **[CCGTCG]** 103 (86.99)   
180 - **.CCGGCG.** 35 (94.52)   
182 + **[CCGAAG]** 259 (86.09)   
182 + **[CCGCGG]** 376 (87.56)   
182 + **[CCGCGG]** 462 (87.56)   
182 + **[CCGCGG]** 494 (87.56)   
182 + **.CCGGCG.** 733 (94.52)   
182 + **[CCGGGG]** 811 (85.40)   
182 + **[CCGCGG]** 828 (87.56)   
182 + **[CCCCCG]** 929 (85.08)   
182 + **[CCCCCG]** 937 (85.08)   
182 + **.CCGCCG.** 1026 (96.68)   
182 + **[CCGCGG]** 1077 (87.56)   
182 + **[CCCCCG]** 1083 (85.08)   
182 + **.CCGCCG.** 1086 (96.68)   
182 + **.CCGAGG.** 1118 (90.88)   
182 + **[GCGACG]** 1256 (85.28)   
182 + **[CCGCGG]** 1290 (87.56)   
182 + **[CGGACG]** 1513 (86.87)   
182 - **[CCCACG]** 1396 (88.40)   
182 - **.CCGAGG.** 1348 (90.88)   
182 - **.CCGGCG.** 1305 (94.52)   
182 - **[CCCCCG]** 1299 (85.08)   
182 - **[CCGCGG]** 1290 (87.56)   
182 - **[CGGACG]** 1287 (86.87)   
182 - **[CCGTCG]** 1257 (86.99)   
182 - **[CCGACT]** 1097 (85.38)   
182 - **.CCGGCG.** 1087 (94.52)   
182 - **[CCGCGG]** 1077 (87.56)   
182 - **[CGGACG]** 1023 (86.87)   
182 - **[CCGGGG]** 938 (85.40)   
182 - **[CCGGGG]** 930 (85.40)   
182 - **[CCGCGG]** 828 (87.56)   
182 - **[CCCCCG]** 580 (85.08)   
182 - **[CCCCCG]** 515 (85.08)   
182 - **.CCGCCG.** 512 (96.68)   
182 - **[CCGCGG]** 494 (87.56)   
182 - **[CCGCGG]** 462 (87.56)   
182 - **.CCGCCG.** 456 (96.68)   
182 - **[CCCCCG]** 436 (85.08)   
182 - **[CCCCCG]** 418 (85.08)   
182 - **.CCGCCG.** 397 (96.68)   
182 - **[CCCCCG]** 390 (85.08)   
182 - **[CCGCGG]** 376 (87.56)   
182 - **.CCGAGG.** 44 (90.88)   
183 + **[CCGGGG]** 355 (85.40)   
183 + **[CCGTCG]** 441 (86.99)   
183 + **[CCGCGG]** 596 (87.56)   
183 + **[CCGCGG]** 837 (87.56)   
183 + **.CCGGCG.** 1093 (94.52)   
183 + **.CCGCCG.** 1179 (96.68)   
183 + **.CCGCCG.** 1182 (96.68)   
183 + **.CCGCCG.** 1185 (96.68)   
183 + **.CCGCCG.** 1188 (96.68)   
183 + **.CCGCCG.** 1223 (96.68)   
183 + **[CCCCCG]** 1385 (85.08)   
183 + **.CCGCCG.** 1400 (96.68)   
183 + **.CCGCCG.** 1410 (96.68)   
183 + **.CCGCCG.** 1413 (96.68)   
183 - **[CCGACT]** 1487 (85.38)   
183 - **.CCGGCG.** 1414 (94.52)   
183 - **[CCGGGG]** 1386 (85.40)   
183 - **.CCGGCG.** 1287 (94.52)   
183 - **.CCGGCG.** 1224 (94.52)   
183 - **.CCGCCG.** 1104 (96.68)   
183 - **.CCGCCG.** 1094 (96.68)   
183 - **.CCGCCG.** 1081 (96.68)   
183 - **.CCGCCG.** 1063 (96.68)   
183 - **.CCGCCG.** 979 (96.68)   
183 - **[CCGCGG]** 837 (87.56)   
183 - **.CCGCCG.** 606 (96.68)   
183 - **[CCGCGG]** 596 (87.56)   
183 - **[CCGGGG]** 582 (85.40)   
183 - **[CCCACG]** 551 (88.40)   
183 - **.CCGACG.** 442 (100.00)   
183 - **[CGGACG]** 427 (86.87)   
183 - **[CCGGGG]** 353 (85.40)   
183 - **[CCGGGG]** 295 (85.40)   
184 + **.CCGCCG.** 52 (96.68)   
184 + **[CCGCGG]** 55 (87.56)   
184 + **[CCCCCG]** 218 (85.08)   
184 + **.CCGACG.** 221 (100.00)   
184 + **[CCGAAG]** 266 (86.09)   
184 + **[CCGGGG]** 362 (85.40)   
184 + **.CCGAGG.** 430 (90.88)   
184 + **[CCGACC]** 462 (88.08)   
184 + **.CCGCCG.** 513 (96.68)   
184 + **[CCGCGG]** 632 (87.56)   
184 + **.CCGAGG.** 662 (90.88)   
184 + **[CCGGGG]** 702 (85.40)   
184 + **.CCGGCG.** 739 (94.52)   
184 + **[CCGGGG]** 783 (85.40)   
184 + **.CCGCCG.** 841 (96.68)   
184 + **[CCGGGG]** 951 (85.40)   
184 + **.CCGCCG.** 991 (96.68)   
184 + **[CCGCGG]** 1067 (87.56)   
184 + **[CCGGGG]** 1245 (85.40)   
184 + **[CCGGGG]** 1316 (85.40)   
184 + **[CCCCCG]** 1433 (85.08)   
184 - **[CCCCCG]** 1503 (85.08)   
184 - **.CCGAGG.** 1366 (90.88)   
184 - **.CCGCCG.** 1354 (96.68)   
184 - **[CCCCCG]** 1303 (85.08)   
184 - **[CCCCCG]** 1119 (85.08)   
184 - **[CCGACC]** 1116 (88.08)   
184 - **[CCGCGG]** 1067 (87.56)   
184 - **.CCGCCG.** 1023 (96.68)   
184 - **.CCGCCG.** 859 (96.68)   
184 - **[CCCCCG]** 784 (85.08)   
184 - **.CCGGCG.** 781 (94.52)   
184 - **.CCGGCG.** 737 (94.52)   
184 - **.CCGACG.** 671 (100.00)   
184 - **[CCGCGG]** 632 (87.56)   
184 - **[CCGACT]** 536 (85.38)   
184 - **[CCGGGG]** 360 (85.40)   
184 - **[CCGCGG]** 55 (87.56)   
185 + **[CCCCCG]** 5 (85.08)   
185 + **.CCGGCG.** 14 (94.52)   
185 + **[GCGACG]** 219 (85.28)   
185 + **[CCGGGG]** 246 (85.40)   
185 + **[CCGGGG]** 299 (85.40)   
185 + **[CCCCCG]** 389 (85.08)   
185 + **[CCCCCG]** 399 (85.08)   
185 + **[CCGCGG]** 556 (87.56)   
185 + **[CCGGGG]** 639 (85.40)   
185 + **.CCGCCG.** 667 (96.68)   
185 + **[CCCCCG]** 878 (85.08)   
185 + **[CCCCCG]** 885 (85.08)   
185 + **.CCGCCG.** 893 (96.68)   
185 + **.CCGCCG.** 896 (96.68)   
185 + **.CCGCCG.** 899 (96.68)   
185 + **.CCGCCG.** 902 (96.68)   
185 + **.CCGCCG.** 905 (96.68)   
185 + **.CCGCCG.** 908 (96.68)   
185 + **.CCGCCG.** 911 (96.68)   
185 + **.CCGCCG.** 914 (96.68)   
185 + **.CCGCCG.** 917 (96.68)   
185 + **[CCGTCG]** 920 (86.99)   
185 + **[CCCCCG]** 977 (85.08)   
185 + **.CCGCCG.** 980 (96.68)   
185 + **.CCGAGG.** 983 (90.88)   
185 + **.CCGGCG.** 997 (94.52)   
185 + **[CCCCCG]** 1009 (85.08)   
185 + **[CCGCGG]** 1012 (87.56)   
185 + **[CCGCGG]** 1064 (87.56)   
185 + **.CCGGCG.** 1081 (94.52)   
185 + **[CCGCGG]** 1091 (87.56)   
185 + **.CCGGCG.** 1108 (94.52)   
185 + **.CCGGCG.** 1142 (94.52)   
185 + **.CCGCCG.** 1148 (96.68)   
185 + **[CCCCCG]** 1154 (85.08)   
185 + **[CCGGGG]** 1157 (85.40)   
185 + **.CCGCCG.** 1188 (96.68)   
185 + **[CCGCGG]** 1191 (87.56)   
185 + **[CCCCCG]** 1313 (85.08)   
185 - **[CCCCCG]** 1200 (85.08)   
185 - **.CCGCCG.** 1197 (96.68)   
185 - **.CCGCCG.** 1194 (96.68)   
185 - **[CCGCGG]** 1191 (87.56)   
185 - **[CCCCCG]** 1158 (85.08)   
185 - **[CCGGGG]** 1155 (85.40)   
185 - **[CCGCGG]** 1091 (87.56)   
185 - **[CCGCGG]** 1064 (87.56)   
185 - **.CCGGCG.** 1036 (94.52)   
185 - **[CCGCGG]** 1012 (87.56)   
185 - **.CCGCCG.** 998 (96.68)   
185 - **[GCGACG]** 921 (85.28)   
185 - **.CCGAGG.** 840 (90.88)   
185 - **.CCGGCG.** 668 (94.52)   
185 - **[CCGGGG]** 611 (85.40)   
185 - **[CCCCCG]** 577 (85.08)   
185 - **[CCGCGG]** 556 (87.56)   
185 - **[CCGAAG]** 374 (86.09)   
185 - **.CCGCCG.** 313 (96.68)   
185 - **[CCCCCG]** 261 (85.08)   
185 - **[CCCCCG]** 247 (85.08)   
185 - **[CCCACG]** 114 (88.40)   
186 + **[CCGCGG]** 921 (87.56)   
186 + **.CCGCCG.** 1016 (96.68)   
186 + **[CCCCCG]** 1172 (85.08)   
186 + **.CCGGCG.** 1329 (94.52)   
186 - **.CCGGCG.** 1285 (94.52)   
186 - **[CCGGGG]** 1202 (85.40)   
186 - **.CCGGCG.** 1017 (94.52)   
186 - **.CCGAGG.** 948 (90.88)   
186 - **[CCGCGG]** 921 (87.56)   
186 - **[CAGACG]** 907 (85.30)   
186 - **[CCGACT]** 561 (85.38)   
186 - **[CCGGGG]** 461 (85.40)   
187 + **[CCGACC]** 122 (88.08)   
187 + **[CCGGGG]** 212 (85.40)   
187 + **.CCGAGG.** 793 (90.88)   
187 + **[CCGGGG]** 828 (85.40)   
187 + **.CCGACG.** 867 (100.00)   
187 + **[CCGGGG]** 953 (85.40)   
187 + **[CCCCCG]** 1033 (85.08)   
187 + **.CCGAGG.** 1036 (90.88)   
187 + **.CCGAGG.** 1043 (90.88)   
187 + **.CCGCCG.** 1090 (96.68)   
187 + **[CCCCCG]** 1148 (85.08)   
187 + **.CCGGCG.** 1151 (94.52)   
187 + **.CCGGCG.** 1174 (94.52)   
187 + **[CCGCGG]** 1303 (87.56)   
187 - **[CCCCCG]** 1408 (85.08)   
187 - **[CCCCCG]** 1353 (85.08)   
187 - **[CCGCGG]** 1303 (87.56)   
187 - **[CCGGGG]** 1149 (85.40)   
187 - **.CCGGCG.** 1091 (94.52)   
187 - **.CCGGCG.** 1062 (94.52)   
187 - **[CCCCCG]** 954 (85.08)   
187 - **[CCGACT]** 816 (85.38)   
187 - **.CCGGCG.** 613 (94.52)   
187 - **.CCGAGG.** 149 (90.88)   
187 - **[CCGGGG]** 50 (85.40)   
188 + **[CCGTCG]** 46 (86.99)   
188 + **[CCGCGG]** 225 (87.56)   
188 + **[CCGACT]** 235 (85.38)   
188 + **[CCCACG]** 246 (88.40)   
188 + **[CCCACG]** 257 (88.40)   
188 + **.CCGCCG.** 306 (96.68)   
188 + **[CCGCGG]** 415 (87.56)   
188 + **.CCGCCG.** 476 (96.68)   
188 + **.CCGGCG.** 479 (94.52)   
188 + **.CCGGCG.** 950 (94.52)   
188 + **.CCGCCG.** 1392 (96.68)   
188 + **.CCGCCG.** 1395 (96.68)   
188 + **.CCGAGG.** 1398 (90.88)   
188 + **[CCGCGG]** 1444 (87.56)   
188 + **.CCGCCG.** 1480 (96.68)   
188 - **.CCGGCG.** 1498 (94.52)   
188 - **[CCGGGG]** 1487 (85.40)   
188 - **.CCGGCG.** 1481 (94.52)   
188 - **[CCGCGG]** 1444 (87.56)   
188 - **[CCGGGG]** 1413 (85.40)   
188 - **[CCCCCG]** 1371 (85.08)   
188 - **[CCGAAG]** 1253 (86.09)   
188 - **.CCGCCG.** 951 (96.68)   
188 - **[CCGAAG]** 842 (86.09)   
188 - **[CCGGGG]** 649 (85.40)   
188 - **[CCGGGG]** 536 (85.40)   
188 - **.CCGCCG.** 503 (96.68)   
188 - **.CCGGCG.** 477 (94.52)   
188 - **[CCGCGG]** 415 (87.56)   
188 - **[CCGCGG]** 225 (87.56)   
188 - **.CCGCCG.** 217 (96.68)   
189 + **.CCGGCG.** 818 (94.52)   
189 + **[CCGCGG]** 926 (87.56)   
189 + **[CCCACG]** 1112 (88.40)   
189 + **.CCGGCG.** 1172 (94.52)   
189 + **[CGGACG]** 1196 (86.87)   
189 + **.CCGGCG.** 1243 (94.52)   
189 + **[CAGACG]** 1307 (85.30)   
189 + **[CGGACG]** 1350 (86.87)   
189 + **[GCGACG]** 1513 (85.28)   
189 - **[CCCACG]** 1464 (88.40)   
189 - **.CCGAGG.** 1160 (90.88)   
189 - **.CCGGCG.** 981 (94.52)   
189 - **[CCGCGG]** 926 (87.56)   
189 - **.CCGCCG.** 819 (96.68)   
190 - **[CCGAAG]** 295 (86.09)   
191 + **[CCCCCG]** 75 (85.08)   
191 + **[CCCCCG]** 201 (85.08)   
191 + **[GCGACG]** 332 (85.28)   
191 + **[CCGGGG]** 364 (85.40)   
191 + **[CCGGGG]** 477 (85.40)   
191 + **[CCGCGG]** 568 (87.56)   
191 + **[CCGGGG]** 574 (85.40)   
191 + **[CGGACG]** 664 (86.87)   
191 + **[CCGCGG]** 832 (87.56)   
191 + **[CCGCGG]** 901 (87.56)   
191 + **.CCGGCG.** 1041 (94.52)   
191 + **.CCGGCG.** 1072 (94.52)   
191 + **[CCGGGG]** 1115 (85.40)   
191 + **.CCGAGG.** 1156 (90.88)   
191 + **.CCGGCG.** 1229 (94.52)   
191 + **[CCCACG]** 1275 (88.40)   
191 + **[CGGACG]** 1279 (86.87)   
191 + **[CCCACG]** 1356 (88.40)   
191 + **[CCGACT]** 1404 (85.38)   
191 + **.CCGGCG.** 1431 (94.52)   
191 - **.CCGCCG.** 1432 (96.68)   
191 - **[CGGACG]** 1360 (86.87)   
191 - **[CCCCCG]** 1333 (85.08)   
191 - **[CCCCCG]** 1327 (85.08)   
191 - **[CCCCCG]** 1283 (85.08)   
191 - **[CCCACG]** 1269 (88.40)   
191 - **.CCGCCG.** 1230 (96.68)   
191 - **.CCGCCG.** 1179 (96.68)   
191 - **[CCGACC]** 1123 (88.08)   
191 - **[CCCCCG]** 1116 (85.08)   
191 - **.CCGCCG.** 1073 (96.68)   
191 - **.CCGCCG.** 1060 (96.68)   
191 - **.CCGCCG.** 1029 (96.68)   
191 - **.CCGCCG.** 1014 (96.68)   
191 - **.CCGCCG.** 1011 (96.68)   
191 - **.CCGCCG.** 1008 (96.68)   
191 - **.CCGCCG.** 1005 (96.68)   
191 - **[CCGCGG]** 901 (87.56)   
191 - **[CCGCGG]** 832 (87.56)   
191 - **[CCCCCG]** 786 (85.08)   
191 - **[CCCCCG]** 680 (85.08)   
191 - **[CCGCGG]** 568 (87.56)   
191 - **[CCCCCG]** 478 (85.08)   
191 - **[CCGACT]** 380 (85.38)   
191 - **.CCGGCG.** 336 (94.52)   
191 - **[CCGGGG]** 179 (85.40)   
191 - **.CCGAGG.** 91 (90.88)   
193 + **.CCGAGG.** 4 (90.88)   
193 + **[CCCACG]** 311 (88.40)   
193 + **[CAGACG]** 508 (85.30)   
193 + **[CAGACG]** 738 (85.30)   
193 + **[CCGGGG]** 789 (85.40)   
193 + **.CCGAGG.** 839 (90.88)   
193 + **[CAGACG]** 1122 (85.30)   
193 - **[CCGACT]** 1406 (85.38)   
193 - **.CCGCCG.** 1332 (96.68)   
193 - **.CCGCCG.** 804 (96.68)   
193 - **[CCCACG]** 711 (88.40)   
193 - **.CCGAGG.** 635 (90.88)   
193 - **.CCGAGG.** 624 (90.88)   
193 - **[CCGAAG]** 494 (86.09)   
193 - **[CCGGGG]** 346 (85.40)   
194 + **[CCGGGG]** 96 (85.40)   
194 + **[CCGGGG]** 178 (85.40)   
194 + **.CCGGCG.** 209 (94.52)   
194 + **[CCGACT]** 632 (85.38)   
194 + **.CCGCCG.** 739 (96.68)   
194 + **.CCGGCG.** 764 (94.52)   
194 + **.CCGCCG.** 855 (96.68)   
194 + **.CCGGCG.** 941 (94.52)   
194 + **[CCGCGG]** 989 (87.56)   
194 + **.CCGCCG.** 1038 (96.68)   
194 + **[CCGCGG]** 1041 (87.56)   
194 + **[CGGACG]** 1044 (86.87)   
194 + **[CCGCGG]** 1111 (87.56)   
194 + **.CCGCCG.** 1236 (96.68)   
194 + **[CCGGGG]** 1273 (85.40)   
194 + **.CCGAGG.** 1397 (90.88)   
194 - **[CCGACC]** 1317 (88.08)   
194 - **[CCGGGG]** 1271 (85.40)   
194 - **.CCGGCG.** 1237 (94.52)   
194 - **.CCGCCG.** 1191 (96.68)   
194 - **[CCGCGG]** 1111 (87.56)   
194 - **[CCGCGG]** 1041 (87.56)   
194 - **[CCGCGG]** 989 (87.56)   
194 - **.CCGCCG.** 914 (96.68)   
194 - **.CCGGCG.** 856 (94.52)   
194 - **[CCCCCG]** 849 (85.08)   
194 - **.CCGCCG.** 765 (96.68)   
194 - **[CCGGGG]** 762 (85.40)   
194 - **.CCGACG.** 557 (100.00)   
194 - **[CCCACG]** 371 (88.40)   
194 - **[CCGGGG]** 353 (85.40)   
194 - **[CCCCCG]** 303 (85.08)   
194 - **[CCCCCG]** 292 (85.08)   
194 - **[CCCCCG]** 236 (85.08)   
195 + **[CGGACG]** 2 (86.87)   
195 + **.CCGCCG.** 55 (96.68)   
195 + **[CCGCGG]** 109 (87.56)   
195 + **.CCGCCG.** 164 (96.68)   
195 + **[CCGCGG]** 167 (87.56)   
195 + **[CCGGGG]** 291 (85.40)   
195 + **.CCGAGG.** 528 (90.88)   
195 + **[CCGGGG]** 707 (85.40)   
195 + **.CCGACG.** 860 (100.00)   
195 + **[CCGTCG]** 944 (86.99)   
195 + **.CCGCCG.** 965 (96.68)   
195 + **.CCGGCG.** 1074 (94.52)   
195 + **[CCGGGG]** 1118 (85.40)   
195 + **.CCGCCG.** 1222 (96.68)   
195 + **.CCGGCG.** 1238 (94.52)   
195 + **[CCGGGG]** 1257 (85.40)   
195 + **.CCGAGG.** 1356 (90.88)   
195 - **[CCGACC]** 1261 (88.08)   
195 - **.CCGCCG.** 1239 (96.68)   
195 - **[CCGACC]** 1177 (88.08)   
195 - **.CCGGCG.** 974 (94.52)   
195 - **[CGGACG]** 962 (86.87)   
195 - **[GCGACG]** 945 (85.28)   
195 - **[CAGACG]** 651 (85.30)   
195 - **.CCGCCG.** 502 (96.68)   
195 - **[CCCCCG]** 488 (85.08)   
195 - **[GCGACG]** 448 (85.28)   
195 - **[CCGGGG]** 336 (85.40)   
195 - **[CCGAAG]** 200 (86.09)   
195 - **[CCGACC]** 177 (88.08)   
195 - **[CCGCGG]** 167 (87.56)   
195 - **[CCGCGG]** 109 (87.56)   
195 - **[CGGACG]** 106 (86.87)   
196 + **[CGGACG]** 29 (86.87)   
196 + **.CCGGCG.** 252 (94.52)   
196 + **[CCGAAG]** 374 (86.09)   
196 + **.CCGGCG.** 467 (94.52)   
196 + **[CCGGGG]** 510 (85.40)   
196 + **.CCGGCG.** 580 (94.52)   
196 + **[CCCACG]** 616 (88.40)   
196 + **[CCGACC]** 657 (88.08)   
196 + **[CCGCGG]** 661 (87.56)   
196 + **[CGGACG]** 686 (86.87)   
196 + **.CCGGCG.** 733 (94.52)   
196 + **[CCGGGG]** 750 (85.40)   
196 + **[CCCCCG]** 844 (85.08)   
196 + **.CCGGCG.** 847 (94.52)   
196 + **.CCGGCG.** 942 (94.52)   
196 + **.CCGCCG.** 1024 (96.68)   
196 + **[CCGGGG]** 1027 (85.40)   
196 + **[CCGCGG]** 1075 (87.56)   
196 + **[CCGCGG]** 1175 (87.56)   
196 + **.CCGAGG.** 1221 (90.88)   
196 + **[CCGGGG]** 1321 (85.40)   
196 + **.CCGGCG.** 1469 (94.52)   
196 + **.CCGGCG.** 1495 (94.52)   
196 - **.CCGCCG.** 1496 (96.68)   
196 - **[CCGGGG]** 1467 (85.40)   
196 - **.CCGCCG.** 1367 (96.68)   
196 - **.CCGGCG.** 1330 (94.52)   
196 - **.CCGGCG.** 1319 (94.52)   
196 - **[CCGCGG]** 1175 (87.56)   
196 - **[CCGCGG]** 1075 (87.56)   
196 - **[CCCCCG]** 1028 (85.08)   
196 - **.CCGGCG.** 1025 (94.52)   
196 - **.CCGAGG.** 955 (90.88)   
196 - **[CCCCCG]** 946 (85.08)   
196 - **.CCGCCG.** 943 (96.68)   
196 - **[CCGGGG]** 922 (85.40)   
196 - **[CCGGGG]** 845 (85.40)   
196 - **[CCGGGG]** 785 (85.40)   
196 - **[CCCCCG]** 751 (85.08)   
196 - **[CCGGGG]** 748 (85.40)   
196 - **.CCGACG.** 723 (100.00)   
196 - **[CCGCGG]** 661 (87.56)   
196 - **.CCGCCG.** 581 (96.68)   
196 - **.CCGGCG.** 578 (94.52)   
196 - **.CCGGCG.** 508 (94.52)   
196 - **.CCGCCG.** 468 (96.68)   
196 - **.CCGACG.** 289 (100.00)   
196 - **.CCGCCG.** 270 (96.68)   
196 - **.CCGCCG.** 262 (96.68)   
196 - **.CCGCCG.** 253 (96.68)   
196 - **[CCCCCG]** 183 (85.08)   
196 - **[CCCCCG]** 170 (85.08)   
196 - **[CCGGGG]** 136 (85.40)   
196 - **.CCGCCG.** 122 (96.68)   
197 + **.CCGCCG.** 209 (96.68)   
197 + **[CCGGGG]** 663 (85.40)   
197 + **.CCGAGG.** 784 (90.88)   
197 + **[CCGCGG]** 1046 (87.56)   
197 + **[CCGACC]** 1150 (88.08)   
197 + **.CCGGCG.** 1155 (94.52)   
197 + **[CGGACG]** 1159 (86.87)   
197 + **.CCGAGG.** 1465 (90.88)   
197 - **[CCGGGG]** 1402 (85.40)   
197 - **[CCGACC]** 1279 (88.08)   
197 - **[CAGACG]** 1168 (85.30)   
197 - **.CCGCCG.** 1156 (96.68)   
197 - **[CCCACG]** 1091 (88.40)   
197 - **.CCGCCG.** 1074 (96.68)   
197 - **.CCGCCG.** 1055 (96.68)   
197 - **[CCGCGG]** 1046 (87.56)   
197 - **.CCGCCG.** 1023 (96.68)   
197 - **.CCGAGG.** 925 (90.88)   
197 - **.CCGCCG.** 874 (96.68)   
197 - **.CCGAGG.** 720 (90.88)   
197 - **[GCGACG]** 524 (85.28)   
197 - **[CCGGGG]** 453 (85.40)   
197 - **[GCGACG]** 433 (85.28)   
197 - **[CCGGGG]** 376 (85.40)   
197 - **[CCCCCG]** 231 (85.08)   
197 - **.CCGACG.** 228 (100.00)   
198 + **[CCCCCG]** 166 (85.08)   
198 + **.CCGCCG.** 252 (96.68)   
198 + **.CCGCCG.** 255 (96.68)   
198 + **.CCGACG.** 935 (100.00)   
198 + **[CCCCCG]** 989 (85.08)   
198 + **.CCGACG.** 1078 (100.00)   
198 - **[CCGGGG]** 1445 (85.40)   
198 - **[CCCCCG]** 1300 (85.08)   
198 - **[CCCCCG]** 1239 (85.08)   
198 - **[CCGTCG]** 1079 (86.99)   
198 - **[CCGACC]** 995 (88.08)   
198 - **[CCGGGG]** 990 (85.40)   
198 - **[GCGACG]** 949 (85.28)   
198 - **[CCGTCG]** 936 (86.99)   
198 - **.CCGAGG.** 883 (90.88)   
198 - **[CCGAAG]** 639 (86.09)   
198 - **[CCGAAG]** 575 (86.09)   
198 - **[CCGAAG]** 541 (86.09)   
198 - **[CCGAAG]** 509 (86.09)   
198 - **[CCGAAG]** 476 (86.09)   
198 - **[CCGACC]** 448 (88.08)   
198 - **[CCGAAG]** 444 (86.09)   
198 - **[CAGACG]** 218 (85.30)   
198 - **[CCGAAG]** 121 (86.09)   
199 + **[CCGGGG]** 336 (85.40)   
199 + **[CCGGGG]** 678 (85.40)   
199 + **.CCGAGG.** 766 (90.88)   
199 + **[CCCCCG]** 774 (85.08)   
199 + **[CCGGGG]** 796 (85.40)   
199 + **.CCGGCG.** 836 (94.52)   
199 + **.CCGGCG.** 862 (94.52)   
199 + **[CGGACG]** 942 (86.87)   
199 + **[CCGCGG]** 1002 (87.56)   
199 + **.CCGCCG.** 1008 (96.68)   
199 + **[CCGGGG]** 1011 (85.40)   
199 + **[CGGACG]** 1040 (86.87)   
199 + **[CCCCCG]** 1060 (85.08)   
199 + **.CCGGCG.** 1103 (94.52)   
199 + **[CCGCGG]** 1128 (87.56)   
199 + **[CCCACG]** 1240 (88.40)   
199 + **.CCGAGG.** 1260 (90.88)   
199 + **[CCGCGG]** 1275 (87.56)   
199 + **[CGGACG]** 1310 (86.87)   
199 + **.CCGGCG.** 1334 (94.52)   
199 + **.CCGGCG.** 1398 (94.52)   
199 + **[CCGGGG]** 1413 (85.40)   
199 - **[CGGACG]** 1450 (86.87)   
199 - **.CCGCCG.** 1335 (96.68)   
199 - **[CCGGGG]** 1332 (85.40)   
199 - **[CCGCGG]** 1275 (87.56)   
199 - **.CCGGCG.** 1218 (94.52)   
199 - **.CCGCCG.** 1143 (96.68)   
199 - **[CCGCGG]** 1128 (87.56)   
199 - **.CCGCCG.** 1104 (96.68)   
199 - **.CCGCCG.** 1087 (96.68)   
199 - **.CCGGCG.** 1009 (94.52)   
199 - **[CCGCGG]** 1002 (87.56)   
199 - **[CCCCCG]** 966 (85.08)   
199 - **.CCGCCG.** 919 (96.68)   
199 - **[CCGACC]** 895 (88.08)   
199 - **.CCGCCG.** 866 (96.68)   
199 - **.CCGCCG.** 863 (96.68)   
199 - **[GCGACG]** 823 (85.28)   
199 - **[CCGGGG]** 775 (85.40)   
199 - **[CCGGGG]** 676 (85.40)   
199 - **[CGGACG]** 661 (86.87)   
199 - **[CCCCCG]** 635 (85.08)   
199 - **.CCGAGG.** 458 (90.88)   
199 - **.CCGAGG.** 145 (90.88)   
200 + **[CCGAAG]** 351 (86.09)   
200 + **[CCGGGG]** 451 (85.40)   
200 + **[CAGACG]** 745 (85.30)   
200 + **.CCGAGG.** 753 (90.88)   
200 + **[CCCACG]** 941 (88.40)   
200 + **.CCGCCG.** 1002 (96.68)   
200 + **.CCGCCG.** 1107 (96.68)   
200 + **[CCGGGG]** 1252 (85.40)   
200 - **.CCGGCG.** 1250 (94.52)   
200 - **[CCCACG]** 1087 (88.40)   
200 - **.CCGAGG.** 962 (90.88)   
200 - **[CAGACG]** 739 (85.30)   
200 - **.CCGAGG.** 707 (90.88)   
200 - **[CCGGGG]** 700 (85.40)   
200 - **[CCGGGG]** 682 (85.40)   
200 - **[CCCCCG]** 452 (85.08)   
200 - **[CCGGGG]** 449 (85.40)   
200 - **.CCGAGG.** 385 (90.88)   
200 - **[CCCACG]** 359 (88.40)   
201 + **.CCGCCG.** 335 (96.68)   
201 + **[CCCACG]** 511 (88.40)   
201 + **[CCCACG]** 642 (88.40)   
201 + **[CCGCGG]** 744 (87.56)   
201 + **[CCGGGG]** 755 (85.40)   
201 + **[CCGGGG]** 761 (85.40)   
201 + **.CCGAGG.** 796 (90.88)   
201 + **[CCGCGG]** 848 (87.56)   
201 + **[CCCCCG]** 899 (85.08)   
201 + **[CCCCCG]** 960 (85.08)   
201 + **.CCGCCG.** 981 (96.68)   
201 + **[CCGGGG]** 1172 (85.40)   
201 + **[CCGGGG]** 1237 (85.40)   
201 + **.CCGCCG.** 1248 (96.68)   
201 + **[CCGGGG]** 1260 (85.40)   
201 + **.CCGCCG.** 1332 (96.68)   
201 + **[CGGACG]** 1344 (86.87)   
201 + **[CCGCGG]** 1350 (87.56)   
201 + **[CCGACC]** 1365 (88.08)   
201 + **.CCGGCG.** 1371 (94.52)   
201 + **.CCGCCG.** 1381 (96.68)   
201 + **.CCGAGG.** 1467 (90.88)   
201 - **[CCGGGG]** 1369 (85.40)   
201 - **[CCGCGG]** 1350 (87.56)   
201 - **.CCGGCG.** 1249 (94.52)   
201 - **[CCCCCG]** 1238 (85.08)   
201 - **[CCCCCG]** 1173 (85.08)   
201 - **.CCGGCG.** 1170 (94.52)   
201 - **[CCGGGG]** 1136 (85.40)   
201 - **.CCGGCG.** 1087 (94.52)   
201 - **[CCGACC]** 1069 (88.08)   
201 - **.CCGGCG.** 1036 (94.52)   
201 - **.CCGGCG.** 982 (94.52)   
201 - **[CCGGGG]** 966 (85.40)   
201 - **[CCGGGG]** 882 (85.40)   
201 - **[CCGCGG]** 848 (87.56)   
201 - **.CCGCCG.** 772 (96.68)   
201 - **[CCGCGG]** 744 (87.56)   
201 - **.CCGGCG.** 600 (94.52)   
201 - **[CCGGGG]** 547 (85.40)   
201 - **.CCGAGG.** 538 (90.88)   
201 - **[GCGACG]** 492 (85.28)   
201 - **[CCGACC]** 445 (88.08)   
201 - **[CCCCCG]** 419 (85.08)   
201 - **[CCGACC]** 416 (88.08)   
201 - **[CCGGGG]** 315 (85.40)   
201 - **.CCGAGG.** 308 (90.88)   
201 - **.CCGAGG.** 258 (90.88)   
201 - **.CCGAGG.** 110 (90.88)   
201 - **[CCGGGG]** 61 (85.40)   
201 - **[CCCCCG]** 16 (85.08)   
202 + **.CCGAGG.** 291 (90.88)   
202 + **.CCGGCG.** 372 (94.52)   
202 + **.CCGGCG.** 1040 (94.52)   
202 + **.CCGCCG.** 1111 (96.68)   
202 + **[CCGCGG]** 1206 (87.56)   
202 + **[CCGGGG]** 1304 (85.40)   
202 - **.CCGAGG.** 1446 (90.88)   
202 - **.CCGAGG.** 1434 (90.88)   
202 - **.CCGGCG.** 1302 (94.52)   
202 - **[CCGACT]** 1248 (85.38)   
202 - **[CCGCGG]** 1206 (87.56)   
202 - **.CCGGCG.** 1112 (94.52)   
202 - **.CCGCCG.** 1041 (96.68)   
202 - **[CAGACG]** 994 (85.30)   
202 - **[CCGGGG]** 913 (85.40)   
202 - **[CCCCCG]** 753 (85.08)   
203 + **[CCCCCG]** 231 (85.08)   
203 + **[CCCCCG]** 910 (85.08)   
203 + **[CCGACT]** 913 (85.38)   
203 + **[CCGTCG]** 1034 (86.99)   
203 - **.CCGACG.** 1035 (100.00)   
203 - **[CCGAAG]** 573 (86.09)   
205 + **[CCGGGG]** 995 (85.40)   
205 - **[CCGAAG]** 1100 (86.09)   
205 - **[CCCCCG]** 996 (85.08)   
206 + **[CAGACG]** 1079 (85.30)   
206 - **[CCGGGG]** 1262 (85.40)   
206 - **[CCCCCG]** 383 (85.08)   
206 - **[CCGACC]** 339 (88.08)   
207 + **[CCGGGG]** 1230 (85.40)   
207 + **[CCCACG]** 1287 (88.40)   
207 + **[CCGGGG]** 1418 (85.40)   
207 - **[CCCCCG]** 1419 (85.08)   
207 - **[CCGGGG]** 1416 (85.40)   
207 - **[CCCCCG]** 1231 (85.08)   
207 - **[CCCCCG]** 1202 (85.08)   
207 - **.CCGCCG.** 1064 (96.68)   
208 + **[CCGACT]** 219 (85.38)   
208 + **[CCGGGG]** 353 (85.40)   
208 + **[CCGGGG]** 861 (85.40)   
208 + **[CCGGGG]** 1308 (85.40)   
208 + **.CCGGCG.** 1345 (94.52)   
208 + **.CCGCCG.** 1389 (96.68)   
208 + **.CCGAGG.** 1392 (90.88)   
208 + **[CCGACT]** 1437 (85.38)   
208 + **[CCGCGG]** 1444 (87.56)   
208 + **.CCGCCG.** 1495 (96.68)   
208 + **[CCGCGG]** 1498 (87.56)   
208 - **[CCGCGG]** 1498 (87.56)   
208 - **[CCGCGG]** 1444 (87.56)   
208 - **.CCGGCG.** 1343 (94.52)   
208 - **[CCCCCG]** 1309 (85.08)   
208 - **[CGGACG]** 1175 (86.87)   
208 - **[GCGACG]** 1140 (85.28)   
208 - **[CCCCCG]** 862 (85.08)   
208 - **[CCGACT]** 522 (85.38)   
209 + **[CCCCCG]** 1238 (85.08)   
210 + **[CCCACG]** 20 (88.40)   
210 + **[CCGGGG]** 662 (85.40)   
210 + **[CCGCGG]** 779 (87.56)   
210 + **[CCGGGG]** 979 (85.40)   
210 + **.CCGCCG.** 1019 (96.68)   
210 + **.CCGGCG.** 1064 (94.52)   
210 + **[CCGGGG]** 1113 (85.40)   
210 + **[CCCCCG]** 1147 (85.08)   
210 + **.CCGACG.** 1155 (100.00)   
210 + **[CCCACG]** 1205 (88.40)   
210 + **[CCCCCG]** 1261 (85.08)   
210 - **[CCGGGG]** 1111 (85.40)   
210 - **[CCGAAG]** 1031 (86.09)   
210 - **.CCGAGG.** 953 (90.88)   
210 - **.CCGCCG.** 942 (96.68)   
210 - **.CCGCCG.** 939 (96.68)   
210 - **.CCGCCG.** 936 (96.68)   
210 - **.CCGCCG.** 902 (96.68)   
210 - **.CCGCCG.** 899 (96.68)   
210 - **.CCGCCG.** 874 (96.68)   
210 - **[CCGCGG]** 779 (87.56)   
210 - **.CCGGCG.** 768 (94.52)   
210 - **[CCCACG]** 749 (88.40)   
210 - **[CCCCCG]** 733 (85.08)   
210 - **.CCGCCG.** 730 (96.68)   
210 - **.CCGACG.** 679 (100.00)   
210 - **.CCGGCG.** 627 (94.52)   
211 + **[CAGACG]** 93 (85.30)   
211 + **.CCGAGG.** 599 (90.88)   
211 + **.CCGAGG.** 633 (90.88)   
211 + **[CCGACC]** 690 (88.08)   
211 + **[CCCCCG]** 719 (85.08)   
211 + **[CCCCCG]** 730 (85.08)   
211 + **.CCGCCG.** 733 (96.68)   
211 + **[CCCACG]** 752 (88.40)   
211 + **[CGGACG]** 761 (86.87)   
211 + **[CCCACG]** 775 (88.40)   
211 + **[CCGGGG]** 813 (85.40)   
211 + **[CCCCCG]** 819 (85.08)   
211 + **[CCCCCG]** 847 (85.08)   
211 + **.CCGCCG.** 960 (96.68)   
211 + **.CCGCCG.** 963 (96.68)   
211 + **.CCGCCG.** 966 (96.68)   
211 + **.CCGCCG.** 981 (96.68)   
211 + **[CCCCCG]** 1004 (85.08)   
211 + **.CCGCCG.** 1054 (96.68)   
211 + **[CCGACT]** 1057 (85.38)   
211 + **.CCGCCG.** 1081 (96.68)   
211 + **.CCGCCG.** 1103 (96.68)   
211 + **.CCGCCG.** 1106 (96.68)   
211 + **[CCCCCG]** 1127 (85.08)   
211 + **[CGGACG]** 1172 (86.87)   
211 + **[CCGGGG]** 1263 (85.40)   
211 + **[CCGCGG]** 1315 (87.56)   
211 - **.CCGGCG.** 1464 (94.52)   
211 - **.CCGGCG.** 1365 (94.52)   
211 - **[CCCCCG]** 1346 (85.08)   
211 - **.CCGCCG.** 1326 (96.68)   
211 - **[CCGCGG]** 1315 (87.56)   
211 - **.CCGGCG.** 1107 (94.52)   
211 - **[CCGGGG]** 1090 (85.40)   
211 - **[CCGGGG]** 1005 (85.40)   
211 - **.CCGGCG.** 982 (94.52)   
211 - **[CAGACG]** 913 (85.30)   
211 - **.CCGCCG.** 883 (96.68)   
211 - **[CCGGGG]** 860 (85.40)   
211 - **[CCGGGG]** 833 (85.40)   
211 - **[CCGGGG]** 811 (85.40)   
211 - **[CCCCCG]** 678 (85.08)   
211 - **[CCCCCG]** 666 (85.08)   
211 - **.CCGAGG.** 663 (90.88)   
211 - **[CCGAAG]** 237 (86.09)   
212 + **[GCGACG]** 423 (85.28)   
212 + **[CCGGGG]** 459 (85.40)   
212 + **[CCCCCG]** 548 (85.08)   
212 + **.CCGCCG.** 620 (96.68)   
212 + **.CCGGCG.** 623 (94.52)   
212 + **[CCGCGG]** 658 (87.56)   
212 + **[CGGACG]** 674 (86.87)   
212 + **[CCGCGG]** 714 (87.56)   
212 + **[CCGACT]** 975 (85.38)   
212 + **.CCGGCG.** 1336 (94.52)   
212 + **[CCGACC]** 1381 (88.08)   
212 + **[CCGGGG]** 1471 (85.40)   
212 + **.CCGAGG.** 1493 (90.88)   
212 - **[CCGGGG]** 1469 (85.40)   
212 - **[CCGGGG]** 1425 (85.40)   
212 - **[CCGACC]** 1402 (88.08)   
212 - **[CCGGGG]** 1391 (85.40)   
212 - **.CCGAGG.** 1368 (90.88)   
212 - **[CGGACG]** 1030 (86.87)   
212 - **[CCGACC]** 739 (88.08)   
212 - **[CCGCGG]** 714 (87.56)   
212 - **[CCGCGG]** 658 (87.56)   
212 - **.CCGCCG.** 635 (96.68)   
212 - **.CCGCCG.** 624 (96.68)   
212 - **.CCGGCG.** 621 (94.52)   
212 - **[CCGGGG]** 502 (85.40)   
212 - **[CCCCCG]** 460 (85.08)   
212 - **.CCGGCG.** 457 (94.52)   
212 - **.CCGCCG.** 233 (96.68)   
212 - **.CCGAGG.** 49 (90.88)   
213 + **[GCGACG]** 1018 (85.28)   
213 + **[CCGGGG]** 1041 (85.40)   
213 + **[CAGACG]** 1313 (85.30)   
213 - **[CCGACT]** 1391 (85.38)   
213 - **.CCGAGG.** 1263 (90.88)   
213 - **.CCGGCG.** 1195 (94.52)   
213 - **[CCCCCG]** 1173 (85.08)   
213 - **[CCCACG]** 1105 (88.40)   
213 - **[CCGTCG]** 1019 (86.99)   
213 - **[CCCCCG]** 955 (85.08)   
214 + **.CCGGCG.** 686 (94.52)   
214 + **[CCGCGG]** 712 (87.56)   
214 + **[CCGAAG]** 788 (86.09)   
214 + **[CCGCGG]** 833 (87.56)   
214 + **[CCGACC]** 896 (88.08)   
214 + **[CCGCGG]** 930 (87.56)   
214 + **[CCGACT]** 1151 (85.38)   
214 + **.CCGAGG.** 1241 (90.88)   
214 + **.CCGAGG.** 1251 (90.88)   
214 + **.CCGGCG.** 1316 (94.52)   
214 + **.CCGAGG.** 1329 (90.88)   
214 + **[CCGAAG]** 1339 (86.09)   
214 - **.CCGCCG.** 1491 (96.68)   
214 - **[CCCCCG]** 1470 (85.08)   
214 - **.CCGCCG.** 1467 (96.68)   
214 - **.CCGCCG.** 1464 (96.68)   
214 - **.CCGCCG.** 1461 (96.68)   
214 - **.CCGCCG.** 1448 (96.68)   
214 - **.CCGCCG.** 1414 (96.68)   
214 - **.CCGCCG.** 1411 (96.68)   
214 - **.CCGCCG.** 1408 (96.68)   
214 - **.CCGCCG.** 1405 (96.68)   
214 - **.CCGCCG.** 1402 (96.68)   
214 - **.CCGCCG.** 1390 (96.68)   
214 - **[CGGACG]** 1352 (86.87)   
214 - **.CCGCCG.** 1317 (96.68)   
214 - **.CCGCCG.** 1142 (96.68)   
214 - **[CCGCGG]** 930 (87.56)   
214 - **[CCCCCG]** 924 (85.08)   
214 - **[CGGACG]** 866 (86.87)   
214 - **[CCGAAG]** 856 (86.09)   
214 - **[CCGCGG]** 833 (87.56)   
214 - **.CCGGCG.** 795 (94.52)   
214 - **[CGGACG]** 737 (86.87)   
214 - **[CCGCGG]** 712 (87.56)   
215 + **.CCGAGG.** 12 (90.88)   
215 + **[CCGCGG]** 519 (87.56)   
215 + **[CCGGGG]** 663 (85.40)   
215 + **[CCGACT]** 918 (85.38)   
215 + **.CCGCCG.** 958 (96.68)   
215 + **.CCGCCG.** 1088 (96.68)   
215 + **[CCGCGG]** 1091 (87.56)   
215 + **[CCGGGG]** 1099 (85.40)   
215 + **[CCGACT]** 1162 (85.38)   
215 + **.CCGGCG.** 1281 (94.52)   
215 - **[CCCACG]** 1422 (88.40)   
215 - **.CCGCCG.** 1282 (96.68)   
215 - **.CCGGCG.** 1097 (94.52)   
215 - **[CCGCGG]** 1091 (87.56)   
215 - **[CCCCCG]** 1074 (85.08)   
215 - **[CAGACG]** 1011 (85.30)   
215 - **[CCGACC]** 975 (88.08)   
215 - **[CCCACG]** 586 (88.40)   
215 - **[CCGCGG]** 519 (87.56)   
216 + **[CAGACG]** 35 (85.30)   
216 + **[CCGCGG]** 253 (87.56)   
216 + **.CCGCCG.** 334 (96.68)   
216 + **.CCGGCG.** 337 (94.52)   
216 + **.CCGGCG.** 461 (94.52)   
216 + **[CCCACG]** 505 (88.40)   
216 + **.CCGCCG.** 876 (96.68)   
216 + **.CCGAGG.** 879 (90.88)   
216 + **.CCGGCG.** 990 (94.52)   
216 + **.CCGCCG.** 1007 (96.68)   
216 + **[CCCCCG]** 1057 (85.08)   
216 + **[CCGACC]** 1060 (88.08)   
216 + **[GCGACG]** 1158 (85.28)   
216 + **.CCGGCG.** 1218 (94.52)   
216 + **[CCGCGG]** 1229 (87.56)   
216 + **.CCGAGG.** 1345 (90.88)   
216 + **[CCGCGG]** 1482 (87.56)   
216 - **[CCGCGG]** 1482 (87.56)   
216 - **[CCGCGG]** 1229 (87.56)   
216 - **[CCGAAG]** 1204 (86.09)   
216 - **.CCGCCG.** 1175 (96.68)   
216 - **[CCGTCG]** 1159 (86.99)   
216 - **[CGGACG]** 1072 (86.87)   
216 - **[CCGGGG]** 903 (85.40)   
216 - **.CCGAGG.** 823 (90.88)   
216 - **[GCGACG]** 731 (85.28)   
216 - **.CCGAGG.** 528 (90.88)   
216 - **.CCGCCG.** 462 (96.68)   
216 - **[CCCACG]** 394 (88.40)   
216 - **.CCGGCG.** 335 (94.52)   
216 - **[CCGACC]** 294 (88.08)   
216 - **[CCGCGG]** 253 (87.56)   
216 - **[CCCACG]** 217 (88.40)   
216 - **[CCGACC]** 67 (88.08)   
216 - **[CCGACT]** 60 (85.38)   
216 - **[CCCACG]** 6 (88.40)   
217 + **[CCGGGG]** 621 (85.40)   
217 + **[CCGCGG]** 696 (87.56)   
217 + **[GCGACG]** 723 (85.28)   
217 + **[CCCACG]** 855 (88.40)   
217 + **.CCGCCG.** 871 (96.68)   
217 + **.CCGCCG.** 874 (96.68)   
217 + **[CCCCCG]** 906 (85.08)   
217 + **.CCGGCG.** 1030 (94.52)   
217 + **[CCGCGG]** 1042 (87.56)   
217 + **[CCGTCG]** 1231 (86.99)   
217 - **[CAGACG]** 1304 (85.30)   
217 - **[CCGGGG]** 1173 (85.40)   
217 - **[CAGACG]** 1096 (85.30)   
217 - **[CCGGGG]** 1084 (85.40)   
217 - **[CCGCGG]** 1042 (87.56)   
217 - **.CCGACG.** 943 (100.00)   
217 - **[CCGGGG]** 907 (85.40)   
217 - **[CCGCGG]** 696 (87.56)   
217 - **.CCGGCG.** 619 (94.52)   
217 - **.CCGCCG.** 579 (96.68)   
217 - **[CAGACG]** 142 (85.30)   
219 + **[CCCCCG]** 1331 (85.08)   
220 + **[CCGACC]** 1111 (88.08)   
220 + **.CCGAGG.** 1166 (90.88)   
221 + **[CCGGGG]** 48 (85.40)   
221 + **.CCGAGG.** 142 (90.88)   
221 + **[CCCCCG]** 164 (85.08)   
221 + **.CCGAGG.** 196 (90.88)   
221 + **.CCGAGG.** 469 (90.88)   
221 + **[CCGGGG]** 510 (85.40)   
221 + **.CCGAGG.** 603 (90.88)   
221 + **[CCCCCG]** 730 (85.08)   
221 + **[CCGAAG]** 856 (86.09)   
221 + **.CCGGCG.** 935 (94.52)   
221 + **.CCGAGG.** 1003 (90.88)   
221 + **[CCGCGG]** 1111 (87.56)   
221 + **[CCGGGG]** 1137 (85.40)   
221 + **.CCGGCG.** 1262 (94.52)   
221 + **[CCCCCG]** 1279 (85.08)   
221 + **[CCGACC]** 1299 (88.08)   
221 + **[CCCCCG]** 1352 (85.08)   
221 + **[CCGGGG]** 1432 (85.40)   
221 - **[CCGGGG]** 1430 (85.40)   
221 - **[CCGGGG]** 1412 (85.40)   
221 - **[CCGGGG]** 1193 (85.40)   
221 - **.CCGAGG.** 1183 (90.88)   
221 - **[CCCCCG]** 1144 (85.08)   
221 - **[CCCCCG]** 1138 (85.08)   
221 - **.CCGCCG.** 1114 (96.68)   
221 - **[CCGCGG]** 1111 (87.56)   
221 - **[CCGAAG]** 1105 (86.09)   
221 - **.CCGGCG.** 763 (94.52)   
221 - **[CCGGGG]** 731 (85.40)   
221 - **[CCCACG]** 373 (88.40)   
221 - **.CCGGCG.** 214 (94.52)   
221 - **[CCGGGG]** 165 (85.40)   
221 - **[CGGACG]** 22 (86.87)   
222 + **.CCGGCG.** 273 (94.52)   
222 + **.CCGGCG.** 315 (94.52)   
222 + **[CCGGGG]** 463 (85.40)   
222 + **[CCGGGG]** 488 (85.40)   
222 + **[CCCCCG]** 520 (85.08)   
222 + **[CCGCGG]** 532 (87.56)   
222 + **[CCGTCG]** 581 (86.99)   
222 + **[CCCCCG]** 610 (85.08)   
222 + **.CCGCCG.** 665 (96.68)   
222 + **[CCCCCG]** 708 (85.08)   
222 + **[CCGCGG]** 870 (87.56)   
222 + **.CCGCCG.** 893 (96.68)   
222 + **[CCGCGG]** 949 (87.56)   
222 + **[CCGCGG]** 959 (87.56)   
222 + **.CCGCCG.** 977 (96.68)   
222 + **[CCGGGG]** 996 (85.40)   
222 + **[CCCCCG]** 1019 (85.08)   
222 + **[CCCCCG]** 1077 (85.08)   
222 + **.CCGGCG.** 1268 (94.52)   
222 + **.CCGAGG.** 1318 (90.88)   
222 + **.CCGCCG.** 1464 (96.68)   
222 + **.CCGAGG.** 1467 (90.88)   
222 + **[CCGCGG]** 1483 (87.56)   
222 - **[CCGCGG]** 1483 (87.56)   
222 - **.CCGGCG.** 1434 (94.52)   
222 - **[CCCCCG]** 1242 (85.08)   
222 - **[CCGACC]** 1239 (88.08)   
222 - **.CCGGCG.** 1235 (94.52)   
222 - **.CCGGCG.** 1208 (94.52)   
222 - **[CCCCCG]** 1195 (85.08)   
222 - **[CCGGGG]** 994 (85.40)   
222 - **.CCGGCG.** 978 (94.52)   
222 - **[CCGCGG]** 959 (87.56)   
222 - **.CCGCCG.** 952 (96.68)   
222 - **[CCGCGG]** 949 (87.56)   
222 - **.CCGGCG.** 894 (94.52)   
222 - **[CCGCGG]** 870 (87.56)   
222 - **[CCCCCG]** 649 (85.08)   
222 - **[CCGGGG]** 616 (85.40)   
222 - **[CCGGGG]** 596 (85.40)   
222 - **.CCGACG.** 582 (100.00)   
222 - **[CCGCGG]** 532 (87.56)   
222 - **[CGGACG]** 454 (86.87)   
222 - **[CCGACC]** 382 (88.08)   
222 - **.CCGCCG.** 316 (96.68)   
222 - **.CCGCCG.** 274 (96.68)   
222 - **.CCGAGG.** 122 (90.88)   
223 + **.CCGGCG.** 329 (94.52)   
223 + **[CCGTCG]** 428 (86.99)   
223 + **[CCGGGG]** 588 (85.40)   
223 + **.CCGCCG.** 677 (96.68)   
223 + **.CCGACG.** 690 (100.00)   
223 + **[CCGGGG]** 772 (85.40)   
223 - **[CCGGGG]** 770 (85.40)   
223 - **[CAGACG]** 703 (85.30)   
223 - **.CCGGCG.** 678 (94.52)   
223 - **[CCCCCG]** 649 (85.08)   
223 - **[CCCCCG]** 636 (85.08)   
223 - **.CCGCCG.** 522 (96.68)   
223 - **[CCCCCG]** 509 (85.08)   
223 - **[CCCCCG]** 472 (85.08)   
223 - **.CCGCCG.** 330 (96.68)   
223 - **.CCGACG.** 189 (100.00)   
224 + **[CCGGGG]** 451 (85.40)   
224 + **.CCGCCG.** 688 (96.68)   
224 + **[CCGCGG]** 824 (87.56)   
224 + **.CCGGCG.** 852 (94.52)   
224 + **[CCGGGG]** 900 (85.40)   
224 + **.CCGAGG.** 909 (90.88)   
224 + **.CCGAGG.** 1016 (90.88)   
224 + **[CCGCGG]** 1027 (87.56)   
224 + **.CCGGCG.** 1093 (94.52)   
224 + **[CCGACC]** 1170 (88.08)   
224 + **[CCCACG]** 1174 (88.40)   
224 + **[CCGGGG]** 1266 (85.40)   
224 + **.CCGGCG.** 1416 (94.52)   
224 - **[CCCCCG]** 1321 (85.08)   
224 - **.CCGGCG.** 1298 (94.52)   
224 - **[CCCACG]** 1273 (88.40)   
224 - **.CCGAGG.** 1247 (90.88)   
224 - **[GCGACG]** 1154 (85.28)   
224 - **[CCGGGG]** 1145 (85.40)   
224 - **.CCGGCG.** 1091 (94.52)   
224 - **[CCGCGG]** 1027 (87.56)   
224 - **[CCCCCG]** 901 (85.08)   
224 - **[CCGGGG]** 898 (85.40)   
224 - **[CCCCCG]** 880 (85.08)   
224 - **[CCGCGG]** 824 (87.56)   
224 - **.CCGCCG.** 769 (96.68)   
224 - **.CCGCCG.** 714 (96.68)   
224 - **.CCGCCG.** 711 (96.68)   
224 - **.CCGCCG.** 708 (96.68)   
224 - **[CCCCCG]** 702 (85.08)   
224 - **.CCGACG.** 582 (100.00)   
224 - **[CCGAAG]** 371 (86.09)   
225 + **.CCGAGG.** 1331 (90.88)   
225 + **[CCGACT]** 1443 (85.38)   
225 + **[CCGAAG]** 1496 (86.09)   
226 + **[CCCACG]** 40 (88.40)   
226 + **[CAGACG]** 105 (85.30)   
226 + **.CCGCCG.** 130 (96.68)   
226 + **[CCCCCG]** 227 (85.08)   
226 + **[CCCACG]** 279 (88.40)   
226 + **[CCGGGG]** 342 (85.40)   
226 + **[CCGACC]** 625 (88.08)   
226 + **.CCGCCG.** 649 (96.68)   
226 + **.CCGGCG.** 652 (94.52)   
226 + **[CCGGGG]** 879 (85.40)   
226 + **.CCGGCG.** 957 (94.52)   
226 + **[CCGCGG]** 1005 (87.56)   
226 + **[CCGGGG]** 1043 (85.40)   
226 + **[CCGACC]** 1090 (88.08)   
226 + **.CCGCCG.** 1106 (96.68)   
226 + **.CCGCCG.** 1109 (96.68)   
226 + **.CCGCCG.** 1112 (96.68)   
226 + **.CCGCCG.** 1115 (96.68)   
226 + **[CCCCCG]** 1159 (85.08)   
226 + **.CCGACG.** 1187 (100.00)   
226 + **.CCGGCG.** 1224 (94.52)   
226 + **.CCGGCG.** 1270 (94.52)   
226 - **[CCCCCG]** 1506 (85.08)   
226 - **.CCGCCG.** 1499 (96.68)   
226 - **[CCCACG]** 1491 (88.40)   
226 - **[GCGACG]** 1368 (85.28)   
226 - **.CCGCCG.** 1277 (96.68)   
226 - **.CCGCCG.** 1274 (96.68)   
226 - **.CCGCCG.** 1271 (96.68)   
226 - **.CCGCCG.** 1244 (96.68)   
226 - **.CCGCCG.** 1241 (96.68)   
226 - **.CCGCCG.** 1238 (96.68)   
226 - **.CCGCCG.** 1225 (96.68)   
226 - **.CCGGCG.** 1139 (94.52)   
226 - **[CCGGGG]** 1098 (85.40)   
226 - **[CGGACG]** 1067 (86.87)   
226 - **[CCGGGG]** 1041 (85.40)   
226 - **[CCGCGG]** 1005 (87.56)   
226 - **.CCGGCG.** 987 (94.52)   
226 - **[CCCCCG]** 976 (85.08)   
226 - **[CCCCCG]** 961 (85.08)   
226 - **.CCGCCG.** 958 (96.68)   
226 - **[CCGGGG]** 877 (85.40)   
226 - **.CCGCCG.** 691 (96.68)   
226 - **[CGGACG]** 656 (86.87)   
226 - **.CCGGCG.** 650 (94.52)   
226 - **.CCGGCG.** 513 (94.52)   
226 - **[CCCACG]** 377 (88.40)   
226 - **[CCCACG]** 365 (88.40)   
226 - **[CCCCCG]** 211 (85.08)   
226 - **[CCCACG]** 203 (88.40)   
226 - **.CCGACG.** 109 (100.00)   
226 - **[CCCACG]** 49 (88.40)   
227 + **[CCGACT]** 52 (85.38)   
227 + **[CCCACG]** 220 (88.40)   
227 + **[CCGGGG]** 288 (85.40)   
227 + **[CCCCCG]** 342 (85.08)   
227 + **[CCGCGG]** 345 (87.56)   
227 + **[CCGGGG]** 384 (85.40)   
227 + **[CGGACG]** 613 (86.87)   
227 + **[CCCACG]** 844 (88.40)   
227 + **.CCGAGG.** 900 (90.88)   
227 + **[CCGGGG]** 1365 (85.40)   
227 + **[CCGACC]** 1481 (88.08)   
227 + **.CCGAGG.** 1511 (90.88)   
227 - **.CCGGCG.** 1452 (94.52)   
227 - **[CCGAAG]** 1394 (86.09)   
227 - **[CCCCCG]** 1366 (85.08)   
227 - **.CCGGCG.** 1363 (94.52)   
227 - **.CCGCCG.** 1275 (96.68)   
227 - **[CCGGGG]** 1255 (85.40)   
227 - **[CCGGGG]** 1244 (85.40)   
227 - **.CCGACG.** 1233 (100.00)   
227 - **.CCGAGG.** 1192 (90.88)   
227 - **.CCGAGG.** 461 (90.88)   
227 - **[CCGCGG]** 345 (87.56)   
227 - **.CCGAGG.** 275 (90.88)   
228 + **.CCGAGG.** 103 (90.88)   
228 + **[CCGGGG]** 121 (85.40)   
228 + **[CCGACC]** 152 (88.08)   
228 + **.CCGAGG.** 214 (90.88)   
228 + **[CCCCCG]** 299 (85.08)   
228 + **.CCGCCG.** 382 (96.68)   
228 + **.CCGCCG.** 421 (96.68)   
228 + **[CCGACC]** 436 (88.08)   
228 + **[CCCCCG]** 467 (85.08)   
228 + **[CCCACG]** 500 (88.40)   
228 + **[CCCACG]** 518 (88.40)   
228 + **.CCGAGG.** 660 (90.88)   
228 + **[CCCACG]** 707 (88.40)   
228 + **[CCGGGG]** 825 (85.40)   
228 + **.CCGAGG.** 845 (90.88)   
228 + **.CCGCCG.** 869 (96.68)   
228 + **[CGGACG]** 877 (86.87)   
228 + **[CCGGGG]** 948 (85.40)   
228 + **[CCGGGG]** 1252 (85.40)   
228 + **[CGGACG]** 1261 (86.87)   
228 + **[CCGGGG]** 1354 (85.40)   
228 + **[CCCACG]** 1425 (88.40)   
228 + **.CCGAGG.** 1469 (90.88)   
228 - **.CCGGCG.** 1265 (94.52)   
228 - **[CCCCCG]** 1253 (85.08)   
228 - **[CCGACC]** 1197 (88.08)   
228 - **.CCGGCG.** 870 (94.52)   
228 - **[CCGACC]** 721 (88.08)   
228 - **.CCGGCG.** 711 (94.52)   
228 - **.CCGGCG.** 422 (94.52)   
228 - **[CCGGGG]** 300 (85.40)   
228 - **[CCGGGG]** 199 (85.40)   
228 - **[CCCCCG]** 122 (85.08)   
229 + **[CCGAAG]** 93 (86.09)   
229 + **.CCGCCG.** 446 (96.68)   
229 + **[CCGGGG]** 464 (85.40)   
229 + **[CCGCGG]** 586 (87.56)   
229 + **[CCGGGG]** 596 (85.40)   
229 + **.CCGGCG.** 632 (94.52)   
229 + **[CCGCGG]** 676 (87.56)   
229 + **.CCGGCG.** 699 (94.52)   
229 + **[CCGGGG]** 774 (85.40)   
229 + **[CCGACC]** 936 (88.08)   
229 + **[CCGCGG]** 940 (87.56)   
229 + **[CAGACG]** 953 (85.30)   
229 + **[CCGGGG]** 974 (85.40)   
229 + **[CAGACG]** 1026 (85.30)   
229 + **[CGGACG]** 1030 (86.87)   
229 + **.CCGCCG.** 1074 (96.68)   
229 + **.CCGCCG.** 1112 (96.68)   
229 + **[CCGCGG]** 1115 (87.56)   
229 + **[CGGACG]** 1118 (86.87)   
229 + **[CCCCCG]** 1152 (85.08)   
229 + **[CCCCCG]** 1158 (85.08)   
229 + **[CCGGGG]** 1195 (85.40)   
229 + **.CCGGCG.** 1310 (94.52)   
229 + **[CCGCGG]** 1369 (87.56)   
229 + **[CCCACG]** 1481 (88.40)   
229 + **.CCGGCG.** 1487 (94.52)   
229 + **.CCGGCG.** 1511 (94.52)   
229 - **.CCGAGG.** 1503 (90.88)   
229 - **.CCGGCG.** 1485 (94.52)   
229 - **[CCCCCG]** 1410 (85.08)   
229 - **.CCGCCG.** 1403 (96.68)   
229 - **[CCCCCG]** 1394 (85.08)   
229 - **.CCGACG.** 1391 (100.00)   
229 - **[CCGCGG]** 1369 (87.56)   
229 - **.CCGCCG.** 1342 (96.68)   
229 - **.CCGCCG.** 1339 (96.68)   
229 - **.CCGCCG.** 1336 (96.68)   
229 - **.CCGCCG.** 1333 (96.68)   
229 - **.CCGCCG.** 1330 (96.68)   
229 - **.CCGCCG.** 1327 (96.68)   
229 - **.CCGCCG.** 1324 (96.68)   
229 - **.CCGCCG.** 1311 (96.68)   
229 - **[CCCCCG]** 1297 (85.08)   
229 - **[CCCCCG]** 1196 (85.08)   
229 - **[CCGGGG]** 1193 (85.40)   
229 - **[CCGGGG]** 1181 (85.40)   
229 - **.CCGAGG.** 1175 (90.88)   
229 - **[CCGGGG]** 1169 (85.40)   
229 - **[CCGCGG]** 1115 (87.56)   
229 - **[CCGCGG]** 940 (87.56)   
229 - **[CCGGGG]** 798 (85.40)   
229 - **.CCGGCG.** 751 (94.52)   
229 - **[CCGACT]** 738 (85.38)   
229 - **[CCGCGG]** 676 (87.56)   
229 - **[CCGGGG]** 665 (85.40)   
229 - **.CCGCCG.** 633 (96.68)   
229 - **[CCCCCG]** 597 (85.08)   
229 - **[CCGCGG]** 586 (87.56)   
229 - **.CCGAGG.** 480 (90.88)   
230 + **[CCGACC]** 293 (88.08)   
230 + **[CCGACT]** 487 (85.38)   
230 + **[CCCCCG]** 723 (85.08)   
230 + **[CCCACG]** 787 (88.40)   
230 + **[CCGACC]** 1138 (88.08)   
230 + **[CCCCCG]** 1142 (85.08)   
230 + **[CCGGGG]** 1145 (85.40)   
230 - **.CCGAGG.** 1354 (90.88)   
230 - **.CCGCCG.** 1202 (96.68)   
230 - **[CCGGGG]** 1143 (85.40)   
230 - **[CGGACG]** 1135 (86.87)   
230 - **[CAGACG]** 989 (85.30)   
230 - **.CCGAGG.** 832 (90.88)   
230 - **[CCGAAG]** 228 (86.09)   
230 - **.CCGAGG.** 100 (90.88)   
231 + **[CCCACG]** 596 (88.40)   
231 + **[CCGACC]** 624 (88.08)   
231 + **[CCCACG]** 872 (88.40)   
231 + **.CCGCCG.** 901 (96.68)   
231 + **.CCGCCG.** 904 (96.68)   
231 + **[CCGCGG]** 907 (87.56)   
231 + **[CCGGGG]** 926 (85.40)   
231 + **[CCGGGG]** 937 (85.40)   
231 + **.CCGCCG.** 1004 (96.68)   
231 + **[CCCCCG]** 1010 (85.08)   
231 + **.CCGCCG.** 1025 (96.68)   
231 + **.CCGCCG.** 1028 (96.68)   
231 + **[CCGAAG]** 1153 (86.09)   
231 + **[CCGCGG]** 1274 (87.56)   
231 + **[CCCCCG]** 1449 (85.08)   
231 - **.CCGCCG.** 1505 (96.68)   
231 - **.CCGCCG.** 1502 (96.68)   
231 - **.CCGCCG.** 1499 (96.68)   
231 - **[CCGACC]** 1496 (88.08)   
231 - **.CCGGCG.** 1486 (94.52)   
231 - **[CCGCGG]** 1274 (87.56)   
231 - **.CCGGCG.** 1196 (94.52)   
231 - **[CCGGGG]** 1175 (85.40)   
231 - **[CCGCGG]** 907 (87.56)   
231 - **[CCGACT]** 287 (85.38)   
231 - **.CCGAGG.** 210 (90.88)   
232 + **.CCGCCG.** 145 (96.68)   
232 + **.CCGGCG.** 442 (94.52)   
232 + **[CCCACG]** 490 (88.40)   
232 + **.CCGACG.** 583 (100.00)   
232 + **.CCGCCG.** 792 (96.68)   
232 + **[CCGGGG]** 821 (85.40)   
232 + **[CCGGGG]** 845 (85.40)   
232 + **[CCGCGG]** 868 (87.56)   
232 + **[CCCCCG]** 892 (85.08)   
232 + **.CCGGCG.** 917 (94.52)   
232 + **[CCGACC]** 1096 (88.08)   
232 + **[CGGACG]** 1117 (86.87)   
232 + **.CCGGCG.** 1178 (94.52)   
232 + **[CCGCGG]** 1283 (87.56)   
232 + **[CCCCCG]** 1387 (85.08)   
232 + **.CCGCCG.** 1390 (96.68)   
232 + **.CCGACG.** 1510 (100.00)   
232 - **[CCGACC]** 1452 (88.08)   
232 - **[CCGGGG]** 1407 (85.40)   
232 - **.CCGGCG.** 1391 (94.52)   
232 - **[CCCCCG]** 1317 (85.08)   
232 - **[CCGCGG]** 1283 (87.56)   
232 - **.CCGAGG.** 1270 (90.88)   
232 - **[CCCCCG]** 1207 (85.08)   
232 - **[CCCCCG]** 1184 (85.08)   
232 - **.CCGCCG.** 1028 (96.68)   
232 - **.CCGCCG.** 999 (96.68)   
232 - **.CCGCCG.** 956 (96.68)   
232 - **.CCGCCG.** 953 (96.68)   
232 - **[CAGACG]** 929 (85.30)   
232 - **[CCGCGG]** 868 (87.56)   
232 - **[CCGGGG]** 694 (85.40)   
232 - **.CCGCCG.** 629 (96.68)   
232 - **.CCGAGG.** 532 (90.88)   
232 - **[CCGGGG]** 513 (85.40)   
232 - **.CCGCCG.** 494 (96.68)   
232 - **[CCGGGG]** 476 (85.40)   
232 - **[CCGGGG]** 440 (85.40)   
232 - **.CCGAGG.** 108 (90.88)   
233 + **.CCGACG.** 433 (100.00)   
233 + **[CAGACG]** 636 (85.30)   
233 + **.CCGAGG.** 1100 (90.88)   
233 + **[GCGACG]** 1139 (85.28)   
233 + **.CCGGCG.** 1146 (94.52)   
233 - **[CCCACG]** 1382 (88.40)   
233 - **[CCGTCG]** 434 (86.99)   
233 - **[CCCCCG]** 366 (85.08)   
233 - **.CCGAGG.** 249 (90.88)   
234 + **[CAGACG]** 165 (85.30)   
234 + **[CCGGGG]** 227 (85.40)   
234 + **[CCGCGG]** 274 (87.56)   
234 + **.CCGACG.** 299 (100.00)   
234 + **[CCGTCG]** 315 (86.99)   
234 + **[CCGGGG]** 361 (85.40)   
234 + **[CCGGGG]** 415 (85.40)   
234 + **.CCGGCG.** 459 (94.52)   
234 + **.CCGGCG.** 570 (94.52)   
234 + **[CCGCGG]** 586 (87.56)   
234 + **[CGGACG]** 589 (86.87)   
234 + **.CCGCCG.** 756 (96.68)   
234 + **[CCCACG]** 784 (88.40)   
234 + **[CCGCGG]** 815 (87.56)   
234 + **[CCGCGG]** 871 (87.56)   
234 + **[CCCCCG]** 902 (85.08)   
234 + **[CCGGGG]** 959 (85.40)   
234 + **[CCCCCG]** 1366 (85.08)   
234 + **[CCCCCG]** 1395 (85.08)   
234 - **.CCGGCG.** 1431 (94.52)   
234 - **[CCGGGG]** 1387 (85.40)   
234 - **.CCGGCG.** 1379 (94.52)   
234 - **[CCGGGG]** 1367 (85.40)   
234 - **[CCCCCG]** 1237 (85.08)   
234 - **[CCGACT]** 1234 (85.38)   
234 - **[CAGACG]** 1200 (85.30)   
234 - **[CCCCCG]** 999 (85.08)   
234 - **[CCCCCG]** 960 (85.08)   
234 - **[CCGGGG]** 957 (85.40)   
234 - **[CCCCCG]** 929 (85.08)   
234 - **[CCGGGG]** 892 (85.40)   
234 - **[CCGCGG]** 871 (87.56)   
234 - **[CCGCGG]** 815 (87.56)   
234 - **.CCGGCG.** 757 (94.52)   
234 - **[CCCCCG]** 705 (85.08)   
234 - **[CCCACG]** 691 (88.40)   
234 - **[CCGCGG]** 586 (87.56)   
234 - **.CCGCCG.** 571 (96.68)   
234 - **.CCGCCG.** 550 (96.68)   
234 - **.CCGAGG.** 502 (90.88)   
234 - **[CCCCCG]** 487 (85.08)   
234 - **.CCGCCG.** 460 (96.68)   
234 - **.CCGGCG.** 457 (94.52)   
234 - **[CCCCCG]** 441 (85.08)   
234 - **[CCCCCG]** 416 (85.08)   
234 - **.CCGGCG.** 413 (94.52)   
234 - **.CCGAGG.** 332 (90.88)   
234 - **[GCGACG]** 316 (85.28)   
234 - **[CCGTCG]** 300 (86.99)   
234 - **[CCGCGG]** 274 (87.56)   
234 - **[CCCCCG]** 228 (85.08)   
234 - **[CCCCCG]** 151 (85.08)   
234 - **[CCGACT]** 89 (85.38)   
235 + **.CCGACG.** 271 (100.00)   
235 + **[CCGGGG]** 445 (85.40)   
235 + **[CCCACG]** 830 (88.40)   
235 + **[CCCCCG]** 853 (85.08)   
235 + **[CCCCCG]** 914 (85.08)   
235 + **[CCGGGG]** 917 (85.40)   
235 + **[CCGGGG]** 1187 (85.40)   
235 + **[GCGACG]** 1306 (85.28)   
235 + **[CCCACG]** 1417 (88.40)   
235 + **[CAGACG]** 1474 (85.30)   
235 - **[CCGGGG]** 1464 (85.40)   
235 - **.CCGCCG.** 1313 (96.68)   
235 - **.CCGCCG.** 1310 (96.68)   
235 - **[CCGTCG]** 1307 (86.99)   
235 - **[CCCCCG]** 1188 (85.08)   
235 - **[CGGACG]** 1184 (86.87)   
235 - **[CCGGGG]** 915 (85.40)   
235 - **[CCCACG]** 884 (88.40)   
235 - **.CCGAGG.** 862 (90.88)   
235 - **[CCCCCG]** 687 (85.08)   
235 - **[CCGGGG]** 519 (85.40)   
235 - **[CCCCCG]** 446 (85.08)   
235 - **[CCGACC]** 388 (88.08)   
235 - **[CCGTCG]** 272 (86.99)   
236 + **[CCCCCG]** 335 (85.08)   
236 + **[CCGAAG]** 338 (86.09)   
236 + **[CAGACG]** 867 (85.30)   
236 + **[CCGCGG]** 977 (87.56)   
236 + **.CCGCCG.** 1004 (96.68)   
236 + **.CCGCCG.** 1007 (96.68)   
236 + **[CCCCCG]** 1031 (85.08)   
236 + **.CCGCCG.** 1148 (96.68)   
236 + **[CCGCGG]** 1155 (87.56)   
236 + **[CCGCGG]** 1196 (87.56)   
236 + **[CCGGGG]** 1218 (85.40)   
236 + **[CCGCGG]** 1331 (87.56)   
236 + **[CCCCCG]** 1383 (85.08)   
236 - **[CCGGGG]** 1418 (85.40)   
236 - **[CCGGGG]** 1384 (85.40)   
236 - **[CCGCGG]** 1331 (87.56)   
236 - **[CCGCGG]** 1196 (87.56)   
236 - **[CCGCGG]** 1155 (87.56)   
236 - **.CCGGCG.** 1149 (94.52)   
236 - **[CCGCGG]** 977 (87.56)   
236 - **[CCCACG]** 937 (88.40)   
237 + **[CCCCCG]** 671 (85.08)   
237 - **[CCCACG]** 507 (88.40)   
237 - **[CCCACG]** 341 (88.40)   
237 - **[CCCACG]** 263 (88.40)   
237 - **[CCGACT]** 232 (85.38)   
238 + **[CCGGGG]** 831 (85.40)   
238 + **.CCGCCG.** 1015 (96.68)   
238 + **.CCGACG.** 1033 (100.00)   
238 + **[GCGACG]** 1087 (85.28)   
238 + **.CCGACG.** 1104 (100.00)   
238 - **[CCCCCG]** 1506 (85.08)   
238 - **[CCCCCG]** 1382 (85.08)   
238 - **.CCGGCG.** 1016 (94.52)   
238 - **[CCGACT]** 876 (85.38)   
239 + **[CCGCGG]** 298 (87.56)   
239 + **[CCGCGG]** 446 (87.56)   
239 + **.CCGCCG.** 542 (96.68)   
239 + **.CCGAGG.** 666 (90.88)   
239 + **[CCGGGG]** 844 (85.40)   
239 + **.CCGGCG.** 867 (94.52)   
239 + **.CCGGCG.** 963 (94.52)   
239 - **.CCGGCG.** 1178 (94.52)   
239 - **[CCGGGG]** 961 (85.40)   
239 - **.CCGCCG.** 868 (96.68)   
239 - **.CCGGCG.** 842 (94.52)   
239 - **[CCCCCG]** 831 (85.08)   
239 - **[CCCCCG]** 596 (85.08)   
239 - **[CCGGGG]** 569 (85.40)   
239 - **[CCGCGG]** 446 (87.56)   
239 - **[CCGCGG]** 298 (87.56)   
239 - **[CCCACG]** 167 (88.40)   
240 + **[CCGACC]** 641 (88.08)   
240 + **.CCGCCG.** 1186 (96.68)   
240 + **[CCGGGG]** 1269 (85.40)   
240 + **[CCGACC]** 1287 (88.08)   
240 + **.CCGCCG.** 1390 (96.68)   
240 + **.CCGGCG.** 1461 (94.52)   
240 - **[CCCCCG]** 1270 (85.08)   
240 - **.CCGGCG.** 1187 (94.52)   
240 - **.CCGAGG.** 1061 (90.88)   
240 - **.CCGCCG.** 266 (96.68)   
240 - **[CCCACG]** 146 (88.40)   
240 - **[CCCCCG]** 57 (85.08)   
241 + **[CAGACG]** 415 (85.30)   
241 - **[CCCACG]** 812 (88.40)   
242 + **.CCGCCG.** 390 (96.68)   
242 + **[CCGGGG]** 438 (85.40)   
242 + **[CGGACG]** 668 (86.87)   
242 + **[CCGACC]** 769 (88.08)   
242 + **.CCGGCG.** 789 (94.52)   
242 + **.CCGAGG.** 883 (90.88)   
242 + **.CCGCCG.** 896 (96.68)   
242 + **.CCGCCG.** 899 (96.68)   
242 + **.CCGACG.** 922 (100.00)   
242 + **.CCGCCG.** 973 (96.68)   
242 + **.CCGAGG.** 1023 (90.88)   
242 + **[CCGACC]** 1207 (88.08)   
242 - **[CCCCCG]** 1268 (85.08)   
242 - **[CCGACT]** 1265 (85.38)   
242 - **[CCGAAG]** 1108 (86.09)   
242 - **[CGGACG]** 1078 (86.87)   
242 - **.CCGGCG.** 974 (94.52)   
242 - **.CCGGCG.** 939 (94.52)   
242 - **.CCGGCG.** 900 (94.52)   
242 - **.CCGCCG.** 790 (96.68)   
242 - **.CCGGCG.** 787 (94.52)   
242 - **.CCGCCG.** 761 (96.68)   
242 - **.CCGAGG.** 738 (90.88)   
242 - **[CCGACC]** 695 (88.08)   
242 - **[CCGACC]** 570 (88.08)   
242 - **[CCGACC]** 524 (88.08)   
242 - **[CCCCCG]** 407 (85.08)   
243 + **.CCGGCG.** 954 (94.52)   
243 + **.CCGGCG.** 1056 (94.52)   
243 + **.CCGGCG.** 1207 (94.52)   
243 + **[CCGAAG]** 1281 (86.09)   
243 + **[CCGGGG]** 1302 (85.40)   
243 - **[CCGGGG]** 1371 (85.40)   
243 - **.CCGGCG.** 1357 (94.52)   
243 - **[CGGACG]** 1278 (86.87)   
243 - **.CCGCCG.** 1208 (96.68)   
243 - **[CCGGGG]** 1077 (85.40)   
243 - **[CAGACG]** 958 (85.30)   
244 + **[CCGACC]** 331 (88.08)   
244 + **[CCGGGG]** 381 (85.40)   
244 + **[CCGACT]** 449 (85.38)   
244 + **[CCGGGG]** 716 (85.40)   
244 + **[CGGACG]** 783 (86.87)   
244 + **.CCGAGG.** 1043 (90.88)   
244 - **[CAGACG]** 952 (85.30)   
244 - **[CCCACG]** 870 (88.40)   
244 - **[CCGGGG]** 738 (85.40)   
244 - **.CCGGCG.** 695 (94.52)   
246 + **[CCCACG]** 192 (88.40)   
246 + **.CCGCCG.** 235 (96.68)   
246 + **.CCGCCG.** 272 (96.68)   
246 + **.CCGCCG.** 288 (96.68)   
246 + **.CCGAGG.** 377 (90.88)   
246 + **[CCGACC]** 477 (88.08)   
246 + **.CCGGCG.** 489 (94.52)   
246 + **[CCGACC]** 684 (88.08)   
246 + **.CCGCCG.** 732 (96.68)   
246 + **[CCGACC]** 735 (88.08)   
246 + **.CCGCCG.** 739 (96.68)   
246 + **.CCGCCG.** 755 (96.68)   
246 + **[CCGGGG]** 869 (85.40)   
246 + **[CCGGGG]** 914 (85.40)   
246 + **[CCGGGG]** 1061 (85.40)   
246 + **.CCGACG.** 1113 (100.00)   
246 + **.CCGCCG.** 1149 (96.68)   
246 + **[CCGACC]** 1152 (88.08)   
246 + **[CCCCCG]** 1236 (85.08)   
246 + **[CCCCCG]** 1243 (85.08)   
246 - **.CCGCCG.** 1478 (96.68)   
246 - **[CCGGGG]** 1237 (85.40)   
246 - **.CCGGCG.** 1229 (94.52)   
246 - **[CCGTCG]** 1114 (86.99)   
246 - **[CCGGGG]** 1107 (85.40)   
246 - **.CCGCCG.** 1054 (96.68)   
246 - **.CCGCCG.** 1023 (96.68)   
246 - **.CCGGCG.** 974 (94.52)   
246 - **[CCCCCG]** 915 (85.08)   
246 - **.CCGGCG.** 912 (94.52)   
246 - **.CCGCCG.** 660 (96.68)   
246 - **.CCGCCG.** 657 (96.68)   
246 - **.CCGGCG.** 638 (94.52)   
246 - **.CCGCCG.** 490 (96.68)   
246 - **[CCGGGG]** 487 (85.40)   
246 - **[CCGACC]** 450 (88.08)   
246 - **[CCGAAG]** 446 (86.09)   
246 - **.CCGGCG.** 289 (94.52)   
246 - **.CCGAGG.** 280 (90.88)   
246 - **[CAGACG]** 135 (85.30)   
247 + **.CCGCCG.** 17 (96.68)   
247 + **[CCCCCG]** 260 (85.08)   
247 + **.CCGCCG.** 263 (96.68)   
247 + **[CCGACC]** 266 (88.08)   
247 + **[CCGCGG]** 278 (87.56)   
247 + **[CCCCCG]** 317 (85.08)   
247 + **[CCCACG]** 343 (88.40)   
247 + **[CCGGGG]** 508 (85.40)   
247 + **[CCCCCG]** 587 (85.08)   
247 + **.CCGCCG.** 771 (96.68)   
247 + **[CCGCGG]** 796 (87.56)   
247 + **[CCGGGG]** 812 (85.40)   
247 + **[CCGGGG]** 852 (85.40)   
247 + **[CCGACC]** 917 (88.08)   
247 + **.CCGCCG.** 939 (96.68)   
247 + **.CCGGCG.** 942 (94.52)   
247 + **[CCGCGG]** 1023 (87.56)   
247 + **.CCGGCG.** 1034 (94.52)   
247 + **.CCGCCG.** 1043 (96.68)   
247 + **[CCCCCG]** 1118 (85.08)   
247 + **[CCCCCG]** 1165 (85.08)   
247 + **[CCCCCG]** 1172 (85.08)   
247 + **.CCGCCG.** 1223 (96.68)   
247 + **[CCGGGG]** 1255 (85.40)   
247 + **[CCGCGG]** 1298 (87.56)   
247 + **[CCCCCG]** 1315 (85.08)   
247 + **[CCGGGG]** 1318 (85.40)   
247 + **[CCGGGG]** 1343 (85.40)   
247 + **[CCGGGG]** 1389 (85.40)   
247 + **[CCGGGG]** 1395 (85.40)   
247 + **[CCCCCG]** 1477 (85.08)   
247 + **[CCGCGG]** 1480 (87.56)   
247 + **.CCGCCG.** 1491 (96.68)   
247 - **[CCGCGG]** 1480 (87.56)   
247 - **[GCGACG]** 1449 (85.28)   
247 - **.CCGGCG.** 1387 (94.52)   
247 - **[CCGGGG]** 1341 (85.40)   
247 - **[CCCCCG]** 1319 (85.08)   
247 - **[CCGGGG]** 1316 (85.40)   
247 - **[CCGCGG]** 1298 (87.56)   
247 - **.CCGCCG.** 1261 (96.68)   
247 - **[CCGAAG]** 1217 (86.09)   
247 - **[CCGACC]** 1076 (88.08)   
247 - **.CCGGCG.** 1067 (94.52)   
247 - **.CCGCCG.** 1026 (96.68)   
247 - **[CCGCGG]** 1023 (87.56)   
247 - **.CCGGCG.** 1012 (94.52)   
247 - **[CCCCCG]** 977 (85.08)   
247 - **[CCCCCG]** 965 (85.08)   
247 - **.CCGGCG.** 940 (94.52)   
247 - **.CCGGCG.** 869 (94.52)   
247 - **[CCCCCG]** 853 (85.08)   
247 - **[CCCCCG]** 813 (85.08)   
247 - **[CCCCCG]** 799 (85.08)   
247 - **[CCGCGG]** 796 (87.56)   
247 - **[CCCCCG]** 699 (85.08)   
247 - **[CGGACG]** 480 (86.87)   
247 - **[CCGGGG]** 466 (85.40)   
247 - **.CCGCCG.** 424 (96.68)   
247 - **.CCGGCG.** 375 (94.52)   
247 - **[CGGACG]** 337 (86.87)   
247 - **[CCGGGG]** 318 (85.40)   
247 - **[CCGCGG]** 278 (87.56)   
248 + **.CCGAGG.** 1405 (90.88)   
248 - **[CCGACC]** 92 (88.08)   
250 + **.CCGACG.** 85 (100.00)   
250 + **.CCGAGG.** 414 (90.88)   
250 + **.CCGGCG.** 439 (94.52)   
250 + **[CCGCGG]** 541 (87.56)   
250 + **[CCCCCG]** 583 (85.08)   
250 + **[CCGACC]** 609 (88.08)   
250 + **[CAGACG]** 903 (85.30)   
250 + **[CCCCCG]** 1033 (85.08)   
250 + **[CCCCCG]** 1045 (85.08)   
250 + **[CCCCCG]** 1059 (85.08)   
250 + **[CCCCCG]** 1065 (85.08)   
250 + **.CCGCCG.** 1068 (96.68)   
250 + **[CCCCCG]** 1077 (85.08)   
250 + **[CCGGGG]** 1080 (85.40)   
250 + **[CCGCGG]** 1097 (87.56)   
250 + **.CCGCCG.** 1106 (96.68)   
250 + **[CCGGGG]** 1407 (85.40)   
250 + **[CCCACG]** 1444 (88.40)   
250 - **.CCGGCG.** 1448 (94.52)   
250 - **[CCCCCG]** 1408 (85.08)   
250 - **[CCCCCG]** 1284 (85.08)   
250 - **.CCGGCG.** 1107 (94.52)   
250 - **[CCGCGG]** 1097 (87.56)   
250 - **.CCGGCG.** 1090 (94.52)   
250 - **[CCGGGG]** 1078 (85.40)   
250 - **[CCGGGG]** 1034 (85.40)   
250 - **[CCCCCG]** 1007 (85.08)   
250 - **[CCGAAG]** 935 (86.09)   
250 - **.CCGGCG.** 685 (94.52)   
250 - **[CCGCGG]** 541 (87.56)   
250 - **.CCGCCG.** 446 (96.68)   
250 - **.CCGCCG.** 443 (96.68)   
250 - **.CCGCCG.** 440 (96.68)   
250 - **[CCGGGG]** 360 (85.40)   
250 - **[CCGACT]** 169 (85.38)   
250 - **[CCGTCG]** 86 (86.99)   
250 - **.CCGGCG.** 14 (94.52)   
251 + **[CCCCCG]** 843 (85.08)   
251 + **.CCGACG.** 1058 (100.00)   
252 - **[CCCCCG]** 1172 (85.08)   
252 - **[CCGACC]** 1169 (88.08)   
252 - **[CCGACC]** 923 (88.08)   
255 + **.CCGCCG.** 1338 (96.68)   
255 - **.CCGCCG.** 1509 (96.68)   
255 - **.CCGAGG.** 1466 (90.88)   
255 - **.CCGAGG.** 1361 (90.88)   
255 - **.CCGGCG.** 1339 (94.52)   
255 - **[CCCCCG]** 1151 (85.08)   
256 + **[CCGAAG]** 602 (86.09)   
256 + **[CCGGGG]** 845 (85.40)   
256 + **.CCGGCG.** 913 (94.52)   
256 + **[CCCCCG]** 995 (85.08)   
256 + **[CCCCCG]** 1003 (85.08)   
256 + **.CCGCCG.** 1122 (96.68)   
256 + **[CCGACC]** 1125 (88.08)   
256 + **[CGGACG]** 1160 (86.87)   
256 + **[CCGCGG]** 1196 (87.56)   
256 + **[CCCCCG]** 1230 (85.08)   
256 + **.CCGGCG.** 1272 (94.52)   
256 + **[CCGGGG]** 1279 (85.40)   
256 + **.CCGCCG.** 1300 (96.68)   
256 + **[CCGCGG]** 1303 (87.56)   
256 + **[CCGACC]** 1450 (88.08)   
256 - **[CGGACG]** 1447 (86.87)   
256 - **[CGGACG]** 1443 (86.87)   
256 - **[CCGCGG]** 1303 (87.56)   
256 - **[CGGACG]** 1276 (86.87)   
256 - **[CCGAAG]** 1243 (86.09)   
256 - **.CCGACG.** 1207 (100.00)   
256 - **[CCGCGG]** 1196 (87.56)   
256 - **[CCGACC]** 1099 (88.08)   
256 - **[CCGGGG]** 1004 (85.40)   
256 - **.CCGAGG.** 763 (90.88)   
256 - **[CCGACT]** 712 (85.38)   
256 - **.CCGAGG.** 57 (90.88)   
257 + **[CCGCGG]** 608 (87.56)   
257 + **.CCGGCG.** 720 (94.52)   
257 + **.CCGACG.** 742 (100.00)   
257 + **.CCGACG.** 763 (100.00)   
257 + **[CCGCGG]** 815 (87.56)   
257 + **[GCGACG]** 850 (85.28)   
257 + **[CCCCCG]** 991 (85.08)   
257 + **.CCGGCG.** 1006 (94.52)   
257 + **.CCGGCG.** 1043 (94.52)   
257 + **.CCGCCG.** 1058 (96.68)   
257 + **.CCGCCG.** 1061 (96.68)   
257 + **[CCGCGG]** 1167 (87.56)   
257 + **[CCGCGG]** 1195 (87.56)   
257 + **[CCGCGG]** 1283 (87.56)   
257 + **[CCCCCG]** 1484 (85.08)   
257 - **[CCGGGG]** 1485 (85.40)   
257 - **.CCGCCG.** 1473 (96.68)   
257 - **.CCGACG.** 1466 (100.00)   
257 - **[CCCCCG]** 1335 (85.08)   
257 - **[CCCCCG]** 1327 (85.08)   
257 - **[CCGCGG]** 1283 (87.56)   
257 - **[CCGACT]** 1264 (85.38)   
257 - **.CCGAGG.** 1235 (90.88)   
257 - **[CCGCGG]** 1195 (87.56)   
257 - **[CCGCGG]** 1167 (87.56)   
257 - **.CCGGCG.** 1155 (94.52)   
257 - **[CCGAAG]** 1113 (86.09)   
257 - **.CCGCCG.** 1034 (96.68)   
257 - **.CCGAGG.** 1031 (90.88)   
257 - **[CCGGGG]** 992 (85.40)   
257 - **.CCGAGG.** 826 (90.88)   
257 - **[CCGCGG]** 815 (87.56)   
257 - **[CCGCGG]** 608 (87.56)   
258 + **[CCGACT]** 1035 (85.38)   
258 + **[CCGACT]** 1206 (85.38)   
259 + **.CCGACG.** 719 (100.00)   
259 + **[CCGAAG]** 903 (86.09)   
259 + **[CCGGGG]** 917 (85.40)   
259 + **[GCGACG]** 993 (85.28)   
259 + **[GCGACG]** 1021 (85.28)   
259 + **[CCGACT]** 1095 (85.38)   
259 + **[CCGCGG]** 1186 (87.56)   
259 + **.CCGCCG.** 1234 (96.68)   
259 + **.CCGACG.** 1237 (100.00)   
259 + **.CCGGCG.** 1297 (94.52)   
259 + **[CCGCGG]** 1303 (87.56)   
259 + **[CCCCCG]** 1475 (85.08)   
259 + **.CCGCCG.** 1478 (96.68)   
259 + **.CCGAGG.** 1481 (90.88)   
259 - **.CCGAGG.** 1312 (90.88)   
259 - **[CCGCGG]** 1303 (87.56)   
259 - **.CCGAGG.** 1287 (90.88)   
259 - **[CCGCGG]** 1186 (87.56)   
259 - **[CCCCCG]** 1163 (85.08)   
259 - **.CCGAGG.** 1135 (90.88)   
259 - **.CCGCCG.** 1041 (96.68)   
259 - **.CCGCCG.** 999 (96.68)   
259 - **[CCCCCG]** 918 (85.08)   
259 - **[CCGTCG]** 720 (86.99)   
259 - **[CCGAAG]** 137 (86.09)   
260 + **.CCGCCG.** 885 (96.68)   
260 + **[CGGACG]** 1248 (86.87)   
260 - **[CCGAAG]** 1187 (86.09)   
260 - **[CCCACG]** 956 (88.40)   
260 - **[CCGGGG]** 772 (85.40)   
262 + **.CCGAGG.** 406 (90.88)   
262 + **.CCGACG.** 785 (100.00)   
262 + **[CCGGGG]** 1247 (85.40)   
262 + **.CCGAGG.** 1321 (90.88)   
262 + **[CGGACG]** 1493 (86.87)   
262 - **[CCCCCG]** 1248 (85.08)   
262 - **[CCGGGG]** 1241 (85.40)   
262 - **.CCGGCG.** 1196 (94.52)   
262 - **.CCGGCG.** 837 (94.52)   
262 - **[CCCCCG]** 390 (85.08)   
262 - **.CCGAGG.** 375 (90.88)   
263 + **.CCGAGG.** 205 (90.88)   
263 + **[CCCCCG]** 436 (85.08)   
263 + **[CCGCGG]** 855 (87.56)   
263 + **.CCGCCG.** 1012 (96.68)   
263 + **[CCCACG]** 1047 (88.40)   
263 + **.CCGACG.** 1115 (100.00)   
263 + **[CCGGGG]** 1247 (85.40)   
263 - **[CCGACC]** 1498 (88.08)   
263 - **[CCGACC]** 1480 (88.08)   
263 - **[CCGACC]** 1442 (88.08)   
263 - **[GCGACG]** 1080 (85.28)   
263 - **[CCGACT]** 1021 (85.38)   
263 - **[CCGCGG]** 855 (87.56)   
263 - **.CCGCCG.** 832 (96.68)   
263 - **.CCGCCG.** 829 (96.68)   
263 - **[CCCACG]** 775 (88.40)   
263 - **[CCCACG]** 736 (88.40)   
263 - **[CCGAAG]** 60 (86.09)   
264 + **[CCGACT]** 579 (85.38)   
264 + **[CCCCCG]** 966 (85.08)   
264 + **[CCGAAG]** 1172 (86.09)   
264 + **.CCGCCG.** 1445 (96.68)   
264 + **[CCCCCG]** 1490 (85.08)   
264 - **[CCGGGG]** 1491 (85.40)   
264 - **.CCGAGG.** 1208 (90.88)   
264 - **.CCGAGG.** 1150 (90.88)   
264 - **[CCCACG]** 1029 (88.40)   
264 - **[CCCCCG]** 908 (85.08)   
264 - **[CCCACG]** 867 (88.40)   
264 - **[CCCACG]** 747 (88.40)   
264 - **[CCGGGG]** 649 (85.40)   
264 - **.CCGCCG.** 421 (96.68)   
264 - **[CCGACC]** 414 (88.08)   
265 - **[CAGACG]** 552 (85.30)   
266 + **.CCGGCG.** 412 (94.52)   
266 + **[CCCACG]** 584 (88.40)   
266 + **[CCGCGG]** 995 (87.56)   
266 + **.CCGGCG.** 1026 (94.52)   
266 + **[CCGCGG]** 1066 (87.56)   
266 + **[CCGTCG]** 1075 (86.99)   
266 + **[CCGACT]** 1110 (85.38)   
266 + **.CCGGCG.** 1274 (94.52)   
266 + **[CCGCGG]** 1281 (87.56)   
266 + **[CCGCGG]** 1476 (87.56)   
266 + **[CCGGGG]** 1506 (85.40)   
266 - **[CCGCGG]** 1476 (87.56)   
266 - **[CCGGGG]** 1462 (85.40)   
266 - **[CCCCCG]** 1428 (85.08)   
266 - **.CCGCCG.** 1284 (96.68)   
266 - **[CCGCGG]** 1281 (87.56)   
266 - **.CCGCCG.** 1275 (96.68)   
266 - **.CCGGCG.** 1272 (94.52)   
266 - **.CCGCCG.** 1232 (96.68)   
266 - **.CCGAGG.** 1172 (90.88)   
266 - **.CCGCCG.** 1155 (96.68)   
266 - **.CCGCCG.** 1152 (96.68)   
266 - **[CCGAAG]** 1114 (86.09)   
266 - **.CCGCCG.** 1079 (96.68)   
266 - **.CCGACG.** 1076 (100.00)   
266 - **[CCGCGG]** 1066 (87.56)   
266 - **[CCCCCG]** 1046 (85.08)   
266 - **.CCGCCG.** 1032 (96.68)   
266 - **[CCGGGG]** 1024 (85.40)   
266 - **[CCGCGG]** 995 (87.56)   
266 - **.CCGGCG.** 984 (94.52)   
266 - **.CCGAGG.** 910 (90.88)   
266 - **.CCGCCG.** 413 (96.68)   
267 + **[CCGGGG]** 167 (85.40)   
267 + **.CCGCCG.** 191 (96.68)   
267 + **[CCGCGG]** 194 (87.56)   
267 + **.CCGAGG.** 875 (90.88)   
267 + **.CCGCCG.** 1022 (96.68)   
267 + **.CCGCCG.** 1025 (96.68)   
267 + **.CCGCCG.** 1028 (96.68)   
267 + **[CAGACG]** 1382 (85.30)   
267 + **[CCGAAG]** 1433 (86.09)   
267 - **[CCCCCG]** 859 (85.08)   
267 - **[CCGCGG]** 194 (87.56)   
267 - **[CCGGGG]** 165 (85.40)   
267 - **.CCGAGG.** 150 (90.88)   
268 + **[CGGACG]** 9 (86.87)   
268 + **.CCGCCG.** 34 (96.68)   
268 + **[GCGACG]** 49 (85.28)   
268 + **[CCGACT]** 313 (85.38)   
268 + **[CCGCGG]** 337 (87.56)   
268 + **[CCGACC]** 874 (88.08)   
268 + **[CCCCCG]** 977 (85.08)   
268 + **.CCGGCG.** 1011 (94.52)   
268 + **.CCGCCG.** 1054 (96.68)   
268 + **[CCGCGG]** 1069 (87.56)   
268 + **[CCGGGG]** 1082 (85.40)   
268 + **[CCCCCG]** 1152 (85.08)   
268 + **[CCCCCG]** 1189 (85.08)   
268 + **.CCGGCG.** 1192 (94.52)   
268 + **[CCGGGG]** 1314 (85.40)   
268 + **[CCGACC]** 1355 (88.08)   
268 + **[CCGGGG]** 1367 (85.40)   
268 + **[CCGGGG]** 1404 (85.40)   
268 + **[CCGCGG]** 1490 (87.56)   
268 - **[CCGCGG]** 1490 (87.56)   
268 - **.CCGCCG.** 1461 (96.68)   
268 - **[CGGACG]** 1388 (86.87)   
268 - **.CCGGCG.** 1365 (94.52)   
268 - **[CCGGGG]** 1349 (85.40)   
268 - **[CCCCCG]** 1315 (85.08)   
268 - **.CCGCCG.** 1287 (96.68)   
268 - **[CCGGGG]** 1280 (85.40)   
268 - **.CCGCCG.** 1267 (96.68)   
268 - **[CCGGGG]** 1260 (85.40)   
268 - **.CCGCCG.** 1248 (96.68)   
268 - **.CCGCCG.** 1245 (96.68)   
268 - **.CCGCCG.** 1242 (96.68)   
268 - **.CCGCCG.** 1239 (96.68)   
268 - **[CCGGGG]** 1190 (85.40)   
268 - **[CCGAAG]** 1173 (86.09)   
268 - **[CCGGGG]** 1164 (85.40)   
268 - **[CCGGGG]** 1153 (85.40)   
268 - **[CGGACG]** 1146 (86.87)   
268 - **.CCGCCG.** 1111 (96.68)   
268 - **[CCGCGG]** 1069 (87.56)   
268 - **.CCGGCG.** 1055 (94.52)   
268 - **[CCGGGG]** 978 (85.40)   
268 - **.CCGGCG.** 841 (94.52)   
268 - **[CCGAAG]** 681 (86.09)   
268 - **[CGGACG]** 378 (86.87)   
268 - **[CCGCGG]** 337 (87.56)   
268 - **.CCGCCG.** 123 (96.68)   
268 - **.CCGCCG.** 53 (96.68)   
268 - **[CCGTCG]** 50 (86.99)   
268 - **[CGGACG]** 31 (86.87)   
270 + **[CCGGGG]** 136 (85.40)   
270 + **[CCGCGG]** 145 (87.56)   
270 + **[CCCCCG]** 276 (85.08)   
270 + **[CCGACT]** 329 (85.38)   
270 + **[GCGACG]** 342 (85.28)   
270 + **.CCGCCG.** 348 (96.68)   
270 + **.CCGCCG.** 576 (96.68)   
270 + **[CCGGGG]** 1348 (85.40)   
270 + **[CCGCGG]** 1381 (87.56)   
270 + **.CCGAGG.** 1427 (90.88)   
270 - **.CCGAGG.** 1493 (90.88)   
270 - **[CCGCGG]** 1381 (87.56)   
270 - **.CCGCCG.** 1187 (96.68)   
270 - **[CCGAAG]** 1057 (86.09)   
270 - **.CCGGCG.** 577 (94.52)   
270 - **[CCGGGG]** 541 (85.40)   
270 - **[CCCCCG]** 456 (85.08)   
270 - **.CCGGCG.** 349 (94.52)   
270 - **[CCGACC]** 267 (88.08)   
270 - **[GCGACG]** 198 (85.28)   
270 - **[CCGCGG]** 145 (87.56)   
270 - **.CCGCCG.** 13 (96.68)   
270 - **[CCCCCG]** 1 (85.08)   
271 + **[CGGACG]** 740 (86.87)   
271 - **[CCCACG]** 1464 (88.40)   
271 - **[CCGACC]** 1218 (88.08)   
271 - **[CCCACG]** 1117 (88.40)   
271 - **[CCGACT]** 875 (85.38)   
272 - **[CCGACC]** 1208 (88.08)   
272 - **[CCCACG]** 1123 (88.40)   
272 - **[CCCACG]** 1108 (88.40)   
272 - **[CCGGGG]** 131 (85.40)   
273 + **[CCCCCG]** 124 (85.08)   
273 + **[CCCCCG]** 197 (85.08)   
273 + **[CCCCCG]** 255 (85.08)   
273 + **[CCCCCG]** 293 (85.08)   
273 + **[CCCCCG]** 442 (85.08)   
273 + **[CCCCCG]** 495 (85.08)   
273 + **.CCGAGG.** 705 (90.88)   
273 + **[CCCCCG]** 834 (85.08)   
273 + **[CCGGGG]** 960 (85.40)   
273 + **[CCGGGG]** 989 (85.40)   
273 + **[CCGCGG]** 1031 (87.56)   
273 + **.CCGGCG.** 1099 (94.52)   
273 + **[CCGGGG]** 1254 (85.40)   
273 + **[CGGACG]** 1299 (86.87)   
273 + **[CCGTCG]** 1404 (86.99)   
273 + **[GCGACG]** 1498 (85.28)   
273 - **[CCGTCG]** 1499 (86.99)   
273 - **[CCCACG]** 1462 (88.40)   
273 - **[CCCCCG]** 1408 (85.08)   
273 - **.CCGACG.** 1405 (100.00)   
273 - **[CCCCCG]** 1145 (85.08)   
273 - **.CCGCCG.** 1100 (96.68)   
273 - **.CCGCCG.** 1034 (96.68)   
273 - **[CCGCGG]** 1031 (87.56)   
273 - **[CCCCCG]** 961 (85.08)   
273 - **[CCCACG]** 940 (88.40)   
273 - **[CCGGGG]** 835 (85.40)   
273 - **[CCCACG]** 578 (88.40)   
273 - **[CCCCCG]** 561 (85.08)   
273 - **.CCGCCG.** 510 (96.68)   
273 - **[CCGACT]** 415 (85.38)   
273 - **.CCGACG.** 363 (100.00)   
273 - **[CCGGGG]** 186 (85.40)   
274 + **[CCGCGG]** 14 (87.56)   
274 + **[CCGCGG]** 107 (87.56)   
274 + **[CCGACT]** 1015 (85.38)   
274 + **[CCGCGG]** 1156 (87.56)   
274 + **[CCGCGG]** 1361 (87.56)   
274 - **.CCGCCG.** 1420 (96.68)   
274 - **[CCGCGG]** 1361 (87.56)   
274 - **[CCGGGG]** 1347 (85.40)   
274 - **[CCGCGG]** 1156 (87.56)   
274 - **[CCCCCG]** 355 (85.08)   
274 - **[CCGCGG]** 107 (87.56)   
274 - **.CCGAGG.** 33 (90.88)   
274 - **[CCGGGG]** 26 (85.40)   
274 - **[CCGCGG]** 14 (87.56)   
274 - **[CCGGGG]** 8 (85.40)   
275 + **[CCGACC]** 39 (88.08)   
275 + **[CAGACG]** 308 (85.30)   
275 + **[CCCCCG]** 381 (85.08)   
275 + **[CCGGGG]** 594 (85.40)   
275 + **[CCCCCG]** 718 (85.08)   
275 + **.CCGCCG.** 740 (96.68)   
275 + **.CCGCCG.** 743 (96.68)   
275 + **.CCGCCG.** 759 (96.68)   
275 + **.CCGCCG.** 762 (96.68)   
275 + **.CCGCCG.** 765 (96.68)   
275 + **.CCGCCG.** 768 (96.68)   
275 + **.CCGGCG.** 857 (94.52)   
275 + **.CCGCCG.** 1009 (96.68)   
275 + **.CCGCCG.** 1012 (96.68)   
275 + **.CCGCCG.** 1015 (96.68)   
275 + **[CCGGGG]** 1018 (85.40)   
275 + **[CAGACG]** 1029 (85.30)   
275 + **[CGGACG]** 1033 (86.87)   
275 + **.CCGCCG.** 1114 (96.68)   
275 + **[GCGACG]** 1266 (85.28)   
275 + **[CCGGGG]** 1339 (85.40)   
275 + **.CCGGCG.** 1351 (94.52)   
275 + **.CCGGCG.** 1443 (94.52)   
275 - **[CCGGGG]** 1441 (85.40)   
275 - **.CCGGCG.** 1115 (94.52)   
275 - **.CCGAGG.** 1073 (90.88)   
275 - **.CCGGCG.** 1016 (94.52)   
275 - **[CAGACG]** 861 (85.30)   
275 - **[CCGGGG]** 855 (85.40)   
275 - **.CCGGCG.** 744 (94.52)   
275 - **[CCGGGG]** 719 (85.40)   
275 - **.CCGAGG.** 682 (90.88)   
275 - **[CCGAAG]** 547 (86.09)   
275 - **[CCCCCG]** 66 (85.08)   
276 + **.CCGAGG.** 957 (90.88)   
276 - **[CCGGGG]** 1197 (85.40)   
276 - **[CCGAAG]** 36 (86.09)   
279 + **.CCGGCG.** 261 (94.52)   
279 + **[GCGACG]** 361 (85.28)   
279 + **[GCGACG]** 377 (85.28)   
279 + **.CCGAGG.** 390 (90.88)   
279 + **.CCGCCG.** 498 (96.68)   
279 + **.CCGGCG.** 501 (94.52)   
279 + **[CAGACG]** 850 (85.30)   
279 + **[CCGACC]** 954 (88.08)   
279 - **[CCCACG]** 778 (88.40)   
279 - **.CCGGCG.** 499 (94.52)   
279 - **[CCCACG]** 422 (88.40)   
279 - **[CCGTCG]** 362 (86.99)   
279 - **[CCCCCG]** 354 (85.08)   
279 - **.CCGCCG.** 283 (96.68)   
279 - **[CCGACC]** 280 (88.08)   
279 - **[CCGACC]** 131 (88.08)   
280 + **[CCGGGG]** 673 (85.40)   
280 + **.CCGCCG.** 848 (96.68)   
280 + **.CCGGCG.** 899 (94.52)   
280 + **[CCGAAG]** 1183 (86.09)   
280 + **.CCGGCG.** 1250 (94.52)   
280 - **.CCGCCG.** 1251 (96.68)   
280 - **[CCGGGG]** 1248 (85.40)   
280 - **[CCGGGG]** 1157 (85.40)   
280 - **[CGGACG]** 1086 (86.87)   
280 - **.CCGCCG.** 1010 (96.68)   
280 - **[CCGGGG]** 1001 (85.40)   
280 - **.CCGCCG.** 990 (96.68)   
280 - **.CCGCCG.** 987 (96.68)   
280 - **.CCGCCG.** 984 (96.68)   
280 - **[CCCCCG]** 939 (85.08)   
280 - **[CCCCCG]** 932 (85.08)   
280 - **[CCCCCG]** 926 (85.08)   
280 - **.CCGGCG.** 903 (94.52)   
280 - **[CCCCCG]** 858 (85.08)   
280 - **[CCGACC]** 855 (88.08)   
280 - **.CCGCCG.** 737 (96.68)   
280 - **.CCGACG.** 506 (100.00)   
280 - **[CAGACG]** 394 (85.30)   
280 - **[CCGACC]** 309 (88.08)   
280 - **[CAGACG]** 294 (85.30)   
280 - **[CCCCCG]** 203 (85.08)   
280 - **[CCGACT]** 146 (85.38)   
281 + **[CCCACG]** 283 (88.40)   
281 + **[CCGAAG]** 702 (86.09)   
281 + **.CCGACG.** 767 (100.00)   
281 + **[CCGGGG]** 941 (85.40)   
281 + **[CGGACG]** 1028 (86.87)   
281 + **.CCGGCG.** 1034 (94.52)   
281 + **[CCCCCG]** 1119 (85.08)   
281 + **.CCGGCG.** 1122 (94.52)   
281 + **[CGGACG]** 1253 (86.87)   
281 + **[CCCCCG]** 1333 (85.08)   
281 + **[CCGACC]** 1336 (88.08)   
281 + **[CAGACG]** 1342 (85.30)   
281 + **[CCCACG]** 1432 (88.40)   
281 + **[CCCCCG]** 1463 (85.08)   
281 + **[CCGACC]** 1466 (88.08)   
281 - **[CCGGGG]** 1512 (85.40)   
281 - **.CCGCCG.** 1165 (96.68)   
281 - **[CCCCCG]** 1143 (85.08)   
281 - **[CCGGGG]** 1120 (85.40)   
281 - **.CCGCCG.** 1041 (96.68)   
281 - **.CCGCCG.** 1038 (96.68)   
281 - **.CCGCCG.** 1035 (96.68)   
281 - **.CCGGCG.** 1032 (94.52)   
281 - **[CCGGGG]** 1002 (85.40)   
281 - **[CCGGGG]** 973 (85.40)   
281 - **[CCGTCG]** 768 (86.99)   
281 - **[CCCCCG]** 579 (85.08)   
281 - **.CCGAGG.** 502 (90.88)   
281 - **[CCCACG]** 462 (88.40)   
282 + **[CCGACT]** 624 (85.38)   
282 - **[CCCCCG]** 584 (85.08)   
282 - **.CCGAGG.** 230 (90.88)   
282 - **.CCGAGG.** 31 (90.88)   
283 + **.CCGGCG.** 167 (94.52)   
283 + **[CCCACG]** 195 (88.40)   
283 + **[CCGTCG]** 216 (86.99)   
283 + **[CCCCCG]** 226 (85.08)   
283 + **.CCGAGG.** 229 (90.88)   
283 + **[CCCACG]** 253 (88.40)   
283 + **[CCGACT]** 457 (85.38)   
283 + **[CCGGGG]** 1123 (85.40)   
283 + **[CCCCCG]** 1466 (85.08)   
283 + **[CCGACC]** 1469 (88.08)   
283 + **[CCCACG]** 1487 (88.40)   
283 - **[CCCACG]** 1459 (88.40)   
283 - **[CCGGGG]** 1382 (85.40)   
283 - **[CCCACG]** 1322 (88.40)   
283 - **[CCGAAG]** 1313 (86.09)   
283 - **.CCGGCG.** 1160 (94.52)   
283 - **[CGGACG]** 1120 (86.87)   
283 - **.CCGAGG.** 950 (90.88)   
283 - **.CCGCCG.** 395 (96.68)   
283 - **[GCGACG]** 217 (85.28)   
283 - **.CCGCCG.** 168 (96.68)   
284 + **[CCGACT]** 365 (85.38)   
284 + **.CCGGCG.** 609 (94.52)   
284 + **[CCCCCG]** 836 (85.08)   
284 + **.CCGGCG.** 897 (94.52)   
284 + **[CCGACT]** 912 (85.38)   
284 + **[CCCCCG]** 939 (85.08)   
284 + **[CCGCGG]** 942 (87.56)   
284 + **.CCGAGG.** 960 (90.88)   
284 + **[CCCCCG]** 974 (85.08)   
284 + **.CCGCCG.** 977 (96.68)   
284 + **[CCGGGG]** 1012 (85.40)   
284 + **[GCGACG]** 1027 (85.28)   
284 + **[CCCCCG]** 1062 (85.08)   
284 + **[CCCCCG]** 1074 (85.08)   
284 + **[CCGCGG]** 1088 (87.56)   
284 + **.CCGAGG.** 1209 (90.88)   
284 + **.CCGCCG.** 1235 (96.68)   
284 + **.CCGCCG.** 1238 (96.68)   
284 + **.CCGCCG.** 1241 (96.68)   
284 + **[CCGTCG]** 1244 (86.99)   
284 + **.CCGCCG.** 1250 (96.68)   
284 + **.CCGCCG.** 1309 (96.68)   
284 + **.CCGCCG.** 1326 (96.68)   
284 + **[CCGCGG]** 1346 (87.56)   
284 + **.CCGCCG.** 1379 (96.68)   
284 + **.CCGCCG.** 1382 (96.68)   
284 + **.CCGCCG.** 1385 (96.68)   
284 + **.CCGGCG.** 1448 (94.52)   
284 - **[CCCCCG]** 1509 (85.08)   
284 - **.CCGCCG.** 1485 (96.68)   
284 - **.CCGCCG.** 1482 (96.68)   
284 - **.CCGCCG.** 1479 (96.68)   
284 - **[CCGCGG]** 1346 (87.56)   
284 - **.CCGGCG.** 1251 (94.52)   
284 - **[GCGACG]** 1245 (85.28)   
284 - **[CCGCGG]** 1088 (87.56)   
284 - **[CCGGGG]** 1075 (85.40)   
284 - **[CCGGGG]** 1063 (85.40)   
284 - **.CCGGCG.** 1005 (94.52)   
284 - **[CCGCGG]** 942 (87.56)   
284 - **.CCGGCG.** 895 (94.52)   
284 - **[CCGGGG]** 837 (85.40)   
284 - **.CCGAGG.** 821 (90.88)   
284 - **.CCGCCG.** 782 (96.68)   
284 - **[CCCACG]** 720 (88.40)   
284 - **.CCGACG.** 581 (100.00)   
285 + **.CCGCCG.** 112 (96.68)   
285 + **[CGGACG]** 168 (86.87)   
285 + **.CCGCCG.** 195 (96.68)   
285 + **[CCCCCG]** 235 (85.08)   
285 + **[CCCACG]** 247 (88.40)   
285 + **[CCGCGG]** 354 (87.56)   
285 + **[CCGGGG]** 407 (85.40)   
285 + **[CCCACG]** 513 (88.40)   
285 + **.CCGGCG.** 525 (94.52)   
285 + **[CCGCGG]** 547 (87.56)   
285 + **[CCGCGG]** 553 (87.56)   
285 + **.CCGCCG.** 581 (96.68)   
285 + **[CCGGGG]** 661 (85.40)   
285 + **[GCGACG]** 713 (85.28)   
285 + **[CCGCGG]** 773 (87.56)   
285 + **[CCCCCG]** 802 (85.08)   
285 + **[CCGCGG]** 859 (87.56)   
285 + **.CCGCCG.** 905 (96.68)   
285 + **[CCGCGG]** 908 (87.56)   
285 + **[GCGACG]** 951 (85.28)   
285 + **.CCGGCG.** 1024 (94.52)   
285 + **.CCGGCG.** 1072 (94.52)   
285 + **[CGGACG]** 1281 (86.87)   
285 + **.CCGGCG.** 1424 (94.52)   
285 - **.CCGCCG.** 1425 (96.68)   
285 - **[GCGACG]** 955 (85.28)   
285 - **.CCGGCG.** 914 (94.52)   
285 - **[CCGCGG]** 908 (87.56)   
285 - **[CCCCCG]** 862 (85.08)   
285 - **[CCGCGG]** 859 (87.56)   
285 - **[CCGCGG]** 773 (87.56)   
285 - **.CCGCCG.** 758 (96.68)   
285 - **.CCGCCG.** 755 (96.68)   
285 - **.CCGAGG.** 667 (90.88)   
285 - **.CCGGCG.** 659 (94.52)   
285 - **.CCGAGG.** 617 (90.88)   
285 - **[CCCCCG]** 608 (85.08)   
285 - **.CCGCCG.** 605 (96.68)   
285 - **[CCGCGG]** 553 (87.56)   
285 - **[CCGCGG]** 547 (87.56)   
285 - **.CCGGCG.** 523 (94.52)   
285 - **.CCGCCG.** 517 (96.68)   
285 - **[CCCCCG]** 474 (85.08)   
285 - **.CCGCCG.** 460 (96.68)   
285 - **.CCGCCG.** 457 (96.68)   
285 - **[CCCACG]** 384 (88.40)   
285 - **[CCGCGG]** 354 (87.56)   
285 - **[CCCCCG]** 348 (85.08)   
285 - **[CCCACG]** 330 (88.40)   
285 - **.CCGACG.** 263 (100.00)   
285 - **[CCGGGG]** 236 (85.40)   
285 - **.CCGCCG.** 155 (96.68)   
285 - **[CAGACG]** 7 (85.30)   
287 + **[CCGCGG]** 43 (87.56)   
287 + **[CCCACG]** 368 (88.40)   
287 + **[CCGGGG]** 449 (85.40)   
287 + **[CCCCCG]** 517 (85.08)   
287 + **[CCGGGG]** 541 (85.40)   
287 + **[GCGACG]** 561 (85.28)   
287 + **.CCGCCG.** 761 (96.68)   
287 + **.CCGGCG.** 820 (94.52)   
287 + **[CCCCCG]** 849 (85.08)   
287 + **[CCGCGG]** 852 (87.56)   
287 + **[CCGACC]** 1030 (88.08)   
287 + **.CCGCCG.** 1047 (96.68)   
287 + **.CCGCCG.** 1081 (96.68)   
287 + **[CCCCCG]** 1265 (85.08)   
287 + **.CCGAGG.** 1285 (90.88)   
287 - **[CAGACG]** 1345 (85.30)   
287 - **[CCGGGG]** 1266 (85.40)   
287 - **.CCGGCG.** 1244 (94.52)   
287 - **[CCGGGG]** 1218 (85.40)   
287 - **.CCGCCG.** 1184 (96.68)   
287 - **[CCGCGG]** 852 (87.56)   
287 - **.CCGCCG.** 821 (96.68)   
287 - **.CCGCCG.** 770 (96.68)   
287 - **.CCGAGG.** 767 (90.88)   
287 - **[CCGGGG]** 696 (85.40)   
287 - **.CCGAGG.** 690 (90.88)   
287 - **[CCCCCG]** 542 (85.08)   
287 - **.CCGGCG.** 539 (94.52)   
287 - **[CCGGGG]** 518 (85.40)   
287 - **[CCGCGG]** 43 (87.56)   
287 - **.CCGAGG.** 2 (90.88)   
288 + **[CCGAAG]** 535 (86.09)   
288 + **[CCGTCG]** 679 (86.99)   
288 + **.CCGCCG.** 871 (96.68)   
288 + **.CCGGCG.** 874 (94.52)   
288 + **[CCCCCG]** 1147 (85.08)   
288 + **.CCGACG.** 1207 (100.00)   
288 - **.CCGACG.** 1470 (100.00)   
288 - **[CCGGGG]** 1148 (85.40)   
288 - **.CCGGCG.** 872 (94.52)   
288 - **[CCGACC]** 710 (88.08)   
288 - **[GCGACG]** 680 (85.28)   
289 + **[CCCCCG]** 960 (85.08)   
289 + **.CCGCCG.** 963 (96.68)   
289 + **.CCGACG.** 1181 (100.00)   
289 + **[CCGGGG]** 1345 (85.40)   
289 + **.CCGAGG.** 1420 (90.88)   
289 - **[CCCCCG]** 1346 (85.08)   
289 - **[CCCCCG]** 1278 (85.08)   
289 - **[CCGACC]** 1231 (88.08)   
289 - **.CCGCCG.** 1020 (96.68)   
289 - **.CCGCCG.** 687 (96.68)   
289 - **.CCGAGG.** 632 (90.88)   
289 - **[CCGAAG]** 599 (86.09)   
290 + **.CCGCCG.** 842 (96.68)   
290 + **[CCGCGG]** 1105 (87.56)   
290 + **.CCGCCG.** 1164 (96.68)   
290 + **[CCGGGG]** 1182 (85.40)   
290 + **[CCGCGG]** 1246 (87.56)   
290 + **[CAGACG]** 1423 (85.30)   
290 - **.CCGGCG.** 1442 (94.52)   
290 - **.CCGGCG.** 1432 (94.52)   
290 - **[CCCCCG]** 1408 (85.08)   
290 - **[CCCCCG]** 1399 (85.08)   
290 - **[CCCCCG]** 1390 (85.08)   
290 - **[CCGACC]** 1324 (88.08)   
290 - **[CCGGGG]** 1257 (85.40)   
290 - **[CCCCCG]** 1249 (85.08)   
290 - **[CCGCGG]** 1246 (87.56)   
290 - **[CCCCCG]** 1183 (85.08)   
290 - **.CCGAGG.** 1155 (90.88)   
290 - **[CCGCGG]** 1105 (87.56)   
290 - **.CCGCCG.** 856 (96.68)   
290 - **[CCCACG]** 784 (88.40)   
291 + **[CCGGGG]** 312 (85.40)   
291 + **.CCGGCG.** 423 (94.52)   
291 + **.CCGAGG.** 456 (90.88)   
291 + **[GCGACG]** 492 (85.28)   
291 + **.CCGCCG.** 543 (96.68)   
291 + **[CCGCGG]** 693 (87.56)   
291 + **[CGGACG]** 703 (86.87)   
291 + **[CCCACG]** 734 (88.40)   
291 + **[CAGACG]** 762 (85.30)   
291 + **[CCGCGG]** 768 (87.56)   
291 + **[CCGGGG]** 882 (85.40)   
291 + **[CCGTCG]** 1027 (86.99)   
291 + **[CCGCGG]** 1110 (87.56)   
291 + **[CGGACG]** 1163 (86.87)   
291 + **[CCGGGG]** 1179 (85.40)   
291 + **[CCGCGG]** 1206 (87.56)   
291 + **.CCGGCG.** 1245 (94.52)   
291 + **.CCGCCG.** 1263 (96.68)   
291 + **.CCGGCG.** 1298 (94.52)   
291 + **[CCGGGG]** 1317 (85.40)   
291 + **[CCGGGG]** 1324 (85.40)   
291 + **.CCGCCG.** 1363 (96.68)   
291 + **[CCGTCG]** 1387 (86.99)   
291 + **[CCGGGG]** 1416 (85.40)   
291 - **.CCGGCG.** 1504 (94.52)   
291 - **[CCCCCG]** 1491 (85.08)   
291 - **.CCGAGG.** 1488 (90.88)   
291 - **.CCGCCG.** 1471 (96.68)   
291 - **.CCGCCG.** 1452 (96.68)   
291 - **[CCGGGG]** 1423 (85.40)   
291 - **.CCGACG.** 1388 (100.00)   
291 - **.CCGGCG.** 1364 (94.52)   
291 - **[CCCCCG]** 1318 (85.08)   
291 - **[CCCCCG]** 1302 (85.08)   
291 - **.CCGCCG.** 1299 (96.68)   
291 - **.CCGCCG.** 1246 (96.68)   
291 - **[CGGACG]** 1229 (86.87)   
291 - **[CCGCGG]** 1206 (87.56)   
291 - **[CCGACC]** 1194 (88.08)   
291 - **.CCGGCG.** 1160 (94.52)   
291 - **[CCGCGG]** 1110 (87.56)   
291 - **[CCGGGG]** 1093 (85.40)   
291 - **.CCGACG.** 1028 (100.00)   
291 - **.CCGCCG.** 949 (96.68)   
291 - **[CCGACC]** 946 (88.08)   
291 - **.CCGAGG.** 896 (90.88)   
291 - **.CCGAGG.** 837 (90.88)   
291 - **[CCGCGG]** 768 (87.56)   
291 - **[CAGACG]** 740 (85.30)   
291 - **[CCGCGG]** 693 (87.56)   
291 - **.CCGAGG.** 667 (90.88)   
291 - **[CCCACG]** 660 (88.40)   
291 - **.CCGCCG.** 639 (96.68)   
291 - **[GCGACG]** 583 (85.28)   
291 - **[CCCACG]** 399 (88.40)   
291 - **[CCGGGG]** 364 (85.40)   
291 - **.CCGCCG.** 98 (96.68)   
292 + **[CCGCGG]** 658 (87.56)   
292 + **.CCGGCG.** 711 (94.52)   
292 + **[CCGCGG]** 796 (87.56)   
292 + **[CCCCCG]** 836 (85.08)   
292 + **[CCGCGG]** 839 (87.56)   
292 + **.CCGGCG.** 983 (94.52)   
292 + **[CCGGGG]** 1059 (85.40)   
292 + **.CCGCCG.** 1080 (96.68)   
292 + **[CCCCCG]** 1111 (85.08)   
292 + **[CCGCGG]** 1237 (87.56)   
292 + **.CCGGCG.** 1261 (94.52)   
292 + **[CCCACG]** 1283 (88.40)   
292 + **[CCGCGG]** 1324 (87.56)   
292 + **.CCGGCG.** 1344 (94.52)   
292 + **[CCGCGG]** 1446 (87.56)   
292 - **[CCCCCG]** 1496 (85.08)   
292 - **[GCGACG]** 1468 (85.28)   
292 - **[CCCCCG]** 1449 (85.08)   
292 - **[CCGCGG]** 1446 (87.56)   
292 - **.CCGCCG.** 1437 (96.68)   
292 - **[CCGCGG]** 1324 (87.56)   
292 - **[CGGACG]** 1321 (86.87)   
292 - **.CCGGCG.** 1265 (94.52)   
292 - **[CCGCGG]** 1237 (87.56)   
292 - **.CCGCCG.** 1200 (96.68)   
292 - **.CCGCCG.** 1197 (96.68)   
292 - **[CCCCCG]** 1183 (85.08)   
292 - **.CCGACG.** 1180 (100.00)   
292 - **[CCGAAG]** 1161 (86.09)   
292 - **[CCGGGG]** 1112 (85.40)   
292 - **[CCGACC]** 1093 (88.08)   
292 - **[CCCCCG]** 1060 (85.08)   
292 - **[CCCCCG]** 1039 (85.08)   
292 - **.CCGCCG.** 1036 (96.68)   
292 - **[CCCCCG]** 1021 (85.08)   
292 - **.CCGCCG.** 1018 (96.68)   
292 - **[CGGACG]** 922 (86.87)   
292 - **.CCGGCG.** 872 (94.52)   
292 - **[CCCCCG]** 859 (85.08)   
292 - **[CCGCGG]** 839 (87.56)   
292 - **.CCGCCG.** 799 (96.68)   
292 - **[CCGCGG]** 796 (87.56)   
292 - **.CCGCCG.** 712 (96.68)   
292 - **[CCGCGG]** 658 (87.56)   
292 - **[CCCACG]** 425 (88.40)   
292 - **[CCCACG]** 381 (88.40)   
293 + **[CCGAAG]** 426 (86.09)   
293 + **.CCGCCG.** 539 (96.68)   
293 + **[CCGCGG]** 618 (87.56)   
293 + **.CCGAGG.** 819 (90.88)   
293 + **.CCGGCG.** 858 (94.52)   
293 + **[CCGCGG]** 911 (87.56)   
293 + **[CCGGGG]** 986 (85.40)   
293 + **.CCGAGG.** 1143 (90.88)   
293 + **.CCGAGG.** 1171 (90.88)   
293 + **[CCGGGG]** 1187 (85.40)   
293 + **[CCGGGG]** 1195 (85.40)   
293 + **[CCCCCG]** 1231 (85.08)   
293 + **[CCGGGG]** 1369 (85.40)   
293 + **.CCGAGG.** 1409 (90.88)   
293 + **.CCGGCG.** 1468 (94.52)   
293 - **[CCGGGG]** 1436 (85.40)   
293 - **[CCGGGG]** 1389 (85.40)   
293 - **.CCGGCG.** 1367 (94.52)   
293 - **[GCGACG]** 1364 (85.28)   
293 - **.CCGAGG.** 1321 (90.88)   
293 - **[CAGACG]** 1277 (85.30)   
293 - **.CCGCCG.** 1209 (96.68)   
293 - **[CCGACC]** 1206 (88.08)   
293 - **[CCCCCG]** 1163 (85.08)   
293 - **.CCGCCG.** 1127 (96.68)   
293 - **[GCGACG]** 1072 (85.28)   
293 - **.CCGCCG.** 1020 (96.68)   
293 - **[CCGCGG]** 911 (87.56)   
293 - **[CCGACC]** 878 (88.08)   
293 - **.CCGCCG.** 841 (96.68)   
293 - **.CCGCCG.** 799 (96.68)   
293 - **[CGGACG]** 748 (86.87)   
293 - **[CCGCGG]** 618 (87.56)   
293 - **[CCCACG]** 601 (88.40)   
293 - **[CCCACG]** 578 (88.40)   
293 - **.CCGGCG.** 540 (94.52)   
293 - **[CCGGGG]** 463 (85.40)   
293 - **[CAGACG]** 85 (85.30)   
294 + **[CAGACG]** 6 (85.30)   
294 + **.CCGCCG.** 44 (96.68)   
294 + **[CCGGGG]** 89 (85.40)   
294 + **[CCGCGG]** 315 (87.56)   
294 + **[CCCACG]** 488 (88.40)   
294 + **[CCCCCG]** 747 (85.08)   
294 + **.CCGCCG.** 758 (96.68)   
294 + **[CCCCCG]** 793 (85.08)   
294 + **.CCGCCG.** 796 (96.68)   
294 + **[CCGAAG]** 834 (86.09)   
294 + **.CCGCCG.** 867 (96.68)   
294 + **[CCGCGG]** 931 (87.56)   
294 + **[CGGACG]** 934 (86.87)   
294 + **[CCCCCG]** 947 (85.08)   
294 + **[CCCCCG]** 953 (85.08)   
294 + **.CCGCCG.** 997 (96.68)   
294 + **.CCGGCG.** 1000 (94.52)   
294 + **[CCGACC]** 1040 (88.08)   
294 + **.CCGCCG.** 1046 (96.68)   
294 + **.CCGGCG.** 1056 (94.52)   
294 + **.CCGACG.** 1087 (100.00)   
294 + **[CCCCCG]** 1185 (85.08)   
294 + **[CCGGGG]** 1193 (85.40)   
294 - **[CGGACG]** 1290 (86.87)   
294 - **[CAGACG]** 1141 (85.30)   
294 - **[CCGGGG]** 1093 (85.40)   
294 - **.CCGCCG.** 1001 (96.68)   
294 - **.CCGGCG.** 998 (94.52)   
294 - **[CCGCGG]** 931 (87.56)   
294 - **.CCGGCG.** 868 (94.52)   
294 - **.CCGAGG.** 861 (90.88)   
294 - **.CCGAGG.** 740 (90.88)   
294 - **[CCGGGG]** 674 (85.40)   
294 - **[CCGGGG]** 603 (85.40)   
294 - **[CCGCGG]** 315 (87.56)   
294 - **[CCCCCG]** 288 (85.08)   
294 - **[CCGACC]** 273 (88.08)   
294 - **[CCCACG]** 248 (88.40)   
294 - **.CCGGCG.** 87 (94.52)   
295 + **[CGGACG]** 350 (86.87)   
295 + **[CAGACG]** 500 (85.30)   
295 + **[CCGGGG]** 547 (85.40)   
295 + **.CCGCCG.** 639 (96.68)   
295 + **.CCGGCG.** 642 (94.52)   
295 + **[CCGCGG]** 709 (87.56)   
295 + **.CCGGCG.** 772 (94.52)   
295 + **.CCGAGG.** 779 (90.88)   
295 + **.CCGAGG.** 901 (90.88)   
295 + **[CCGGGG]** 966 (85.40)   
295 + **[CCGGGG]** 1037 (85.40)   
295 + **[CCGCGG]** 1325 (87.56)   
295 + **[CCCCCG]** 1352 (85.08)   
295 + **[CCGACC]** 1367 (88.08)   
295 + **[CCCACG]** 1393 (88.40)   
295 - **[CCGCGG]** 1325 (87.56)   
295 - **[CCCACG]** 1153 (88.40)   
295 - **[CCCCCG]** 894 (85.08)   
295 - **.CCGCCG.** 882 (96.68)   
295 - **[CCCCCG]** 847 (85.08)   
295 - **.CCGCCG.** 844 (96.68)   
295 - **.CCGCCG.** 773 (96.68)   
295 - **[CCGCGG]** 709 (87.56)   
295 - **[CGGACG]** 706 (86.87)   
295 - **[CCCCCG]** 693 (85.08)   
295 - **[CCCCCG]** 687 (85.08)   
295 - **.CCGGCG.** 640 (94.52)   
295 - **[CCGACC]** 600 (88.08)   
295 - **.CCGCCG.** 594 (96.68)   
295 - **.CCGGCG.** 585 (94.52)   
295 - **.CCGACG.** 553 (100.00)   
295 - **[CCCCCG]** 455 (85.08)   
295 - **[CCGGGG]** 447 (85.40)   
296 + **[CCCCCG]** 25 (85.08)   
296 + **[CCCCCG]** 35 (85.08)   
296 + **.CCGCCG.** 144 (96.68)   
296 + **[CCGACC]** 147 (88.08)   
296 + **[CCCCCG]** 272 (85.08)   
296 + **[CCCACG]** 356 (88.40)   
296 + **[CCGGGG]** 447 (85.40)   
296 + **[CCGGGG]** 511 (85.40)   
296 + **[CCCCCG]** 536 (85.08)   
296 + **[CCCCCG]** 551 (85.08)   
296 + **[CCCCCG]** 615 (85.08)   
296 + **.CCGCCG.** 618 (96.68)   
296 + **[CCGAAG]** 630 (86.09)   
296 + **[CGGACG]** 653 (86.87)   
296 + **.CCGAGG.** 763 (90.88)   
296 + **.CCGCCG.** 769 (96.68)   
296 + **.CCGCCG.** 772 (96.68)   
296 + **[CCGTCG]** 775 (86.99)   
296 + **.CCGCCG.** 784 (96.68)   
296 + **[CCGCGG]** 814 (87.56)   
296 + **[CCGGGG]** 849 (85.40)   
296 + **[CCGGGG]** 871 (85.40)   
296 + **[CCGGGG]** 877 (85.40)   
296 + **[CCGGGG]** 883 (85.40)   
296 + **[CCGGGG]** 906 (85.40)   
296 + **[CCGGGG]** 912 (85.40)   
296 + **[CCGCGG]** 936 (87.56)   
296 + **[CCGCGG]** 965 (87.56)   
296 + **.CCGCCG.** 977 (96.68)   
296 + **.CCGGCG.** 1156 (94.52)   
296 + **[CCGCGG]** 1163 (87.56)   
296 + **[CCGCGG]** 1188 (87.56)   
296 + **.CCGAGG.** 1271 (90.88)   
296 + **.CCGCCG.** 1286 (96.68)   
296 + **.CCGACG.** 1289 (100.00)   
296 + **[CCGGGG]** 1365 (85.40)   
296 + **.CCGGCG.** 1428 (94.52)   
296 + **.CCGACG.** 1460 (100.00)   
296 - **.CCGCCG.** 1429 (96.68)   
296 - **[CCCCCG]** 1392 (85.08)   
296 - **.CCGGCG.** 1371 (94.52)   
296 - **[CCGGGG]** 1363 (85.40)   
296 - **[CCGGGG]** 1353 (85.40)   
296 - **[CCGGGG]** 1222 (85.40)   
296 - **[GCGACG]** 1209 (85.28)   
296 - **[CCCCCG]** 1203 (85.08)   
296 - **[CCGCGG]** 1188 (87.56)   
296 - **[CCGCGG]** 1163 (87.56)   
296 - **.CCGCCG.** 1157 (96.68)   
296 - **[CCCCCG]** 1113 (85.08)   
296 - **[CCCCCG]** 1107 (85.08)   
296 - **.CCGAGG.** 1063 (90.88)   
296 - **.CCGGCG.** 978 (94.52)   
296 - **[CCGCGG]** 965 (87.56)   
296 - **[CCGCGG]** 936 (87.56)   
296 - **[CCCCCG]** 884 (85.08)   
296 - **[CCCCCG]** 850 (85.08)   
296 - **[CCGCGG]** 814 (87.56)   
296 - **.CCGGCG.** 785 (94.52)   
296 - **[GCGACG]** 776 (85.28)   
296 - **[CGGACG]** 736 (86.87)   
296 - **[CCGGGG]** 722 (85.40)   
296 - **[CCGGGG]** 689 (85.40)   
296 - **.CCGGCG.** 593 (94.52)   
296 - **[CCGGGG]** 584 (85.40)   
296 - **[CCGGGG]** 552 (85.40)   
296 - **[CCCCCG]** 512 (85.08)   
296 - **.CCGGCG.** 463 (94.52)   
296 - **[CCCCCG]** 448 (85.08)   
296 - **.CCGCCG.** 258 (96.68)   
296 - **.CCGCCG.** 191 (96.68)   
296 - **.CCGCCG.** 188 (96.68)   
296 - **.CCGCCG.** 87 (96.68)   
297 + **[CCCCCG]** 781 (85.08)   
297 + **[CCGCGG]** 828 (87.56)   
297 + **[GCGACG]** 898 (85.28)   
297 + **.CCGGCG.** 977 (94.52)   
297 + **[CCGCGG]** 997 (87.56)   
297 + **[CCCCCG]** 1110 (85.08)   
297 + **[CCCCCG]** 1126 (85.08)   
297 + **[CCGCGG]** 1156 (87.56)   
297 + **.CCGAGG.** 1300 (90.88)   
297 + **[CCGACC]** 1381 (88.08)   
297 - **[CCGGGG]** 1513 (85.40)   
297 - **.CCGCCG.** 1448 (96.68)   
297 - **.CCGACG.** 1445 (100.00)   
297 - **[CCCCCG]** 1370 (85.08)   
297 - **[CCGACC]** 1289 (88.08)   
297 - **[GCGACG]** 1203 (85.28)   
297 - **[CCGCGG]** 1156 (87.56)   
297 - **.CCGCCG.** 1076 (96.68)   
297 - **.CCGCCG.** 1021 (96.68)   
297 - **[CCGCGG]** 997 (87.56)   
297 - **.CCGCCG.** 981 (96.68)   
297 - **.CCGCCG.** 978 (96.68)   
297 - **[CCGGGG]** 975 (85.40)   
297 - **[CCCCCG]** 902 (85.08)   
297 - **[CCGTCG]** 899 (86.99)   
297 - **[CCCCCG]** 880 (85.08)   
297 - **[CCCCCG]** 853 (85.08)   
297 - **[CCGACT]** 850 (85.38)   
297 - **[CCGCGG]** 828 (87.56)   
298 + **.CCGGCG.** 206 (94.52)   
298 + **.CCGGCG.** 212 (94.52)   
298 + **[CCGTCG]** 355 (86.99)   
298 + **[CCCCCG]** 495 (85.08)   
298 + **.CCGAGG.** 498 (90.88)   
298 + **[CGGACG]** 586 (86.87)   
298 + **[CCGAAG]** 702 (86.09)   
298 + **.CCGGCG.** 780 (94.52)   
298 + **[CCGCGG]** 902 (87.56)   
298 + **[CGGACG]** 905 (86.87)   
298 + **[CCGGGG]** 1083 (85.40)   
298 + **[CCGGGG]** 1089 (85.40)   
298 + **.CCGACG.** 1146 (100.00)   
298 + **.CCGCCG.** 1161 (96.68)   
298 + **.CCGCCG.** 1176 (96.68)   
298 + **.CCGAGG.** 1222 (90.88)   
298 + **[CCGACT]** 1357 (85.38)   
298 + **.CCGAGG.** 1380 (90.88)   
298 + **[CCCCCG]** 1508 (85.08)   
298 - **[CCGGGG]** 1509 (85.40)   
298 - **[CCGGGG]** 1438 (85.40)   
298 - **[CCCCCG]** 1413 (85.08)   
298 - **[CCGACT]** 1410 (85.38)   
298 - **.CCGGCG.** 1162 (94.52)   
298 - **[CCCCCG]** 1064 (85.08)   
298 - **[CCGACC]** 1025 (88.08)   
298 - **[CCGCGG]** 902 (87.56)   
298 - **.CCGCCG.** 878 (96.68)   
298 - **.CCGACG.** 755 (100.00)   
298 - **[CCGACT]** 615 (85.38)   
298 - **[CCGACC]** 363 (88.08)   
298 - **.CCGCCG.** 359 (96.68)   
298 - **.CCGACG.** 356 (100.00)   
298 - **[CAGACG]** 334 (85.30)   
298 - **.CCGGCG.** 210 (94.52)   
298 - **.CCGAGG.** 115 (90.88)   
298 - **[CCCCCG]** 24 (85.08)   
299 + **.CCGGCG.** 418 (94.52)   
299 + **[CCGACC]** 465 (88.08)   
299 + **[CCCACG]** 575 (88.40)   
299 + **[CCGGGG]** 611 (85.40)   
299 + **[CCGGGG]** 655 (85.40)   
299 + **.CCGAGG.** 843 (90.88)   
299 + **[CCCCCG]** 925 (85.08)   
299 + **.CCGCCG.** 948 (96.68)   
299 + **[CCGGGG]** 1112 (85.40)   
299 + **[CCGCGG]** 1179 (87.56)   
299 + **.CCGAGG.** 1197 (90.88)   
299 + **[CCGCGG]** 1261 (87.56)   
299 + **[GCGACG]** 1402 (85.28)   
299 - **.CCGAGG.** 1345 (90.88)   
299 - **[CGGACG]** 1285 (86.87)   
299 - **[CCGCGG]** 1261 (87.56)   
299 - **[CCGACC]** 1228 (88.08)   
299 - **[CCGCGG]** 1179 (87.56)   
299 - **[CCGACT]** 1128 (85.38)   
299 - **[CCCCCG]** 1095 (85.08)   
299 - **[CCGGGG]** 926 (85.40)   
299 - **.CCGACG.** 885 (100.00)   
299 - **[CCGGGG]** 872 (85.40)   
299 - **[CCGACC]** 817 (88.08)   
299 - **.CCGAGG.** 794 (90.88)   
299 - **[CGGACG]** 735 (86.87)   
299 - **[CCCCCG]** 669 (85.08)   
299 - **[CCCCCG]** 612 (85.08)   
299 - **.CCGCCG.** 419 (96.68)   
299 - **[CCGACC]** 351 (88.08)   
300 + **[CCCACG]** 95 (88.40)   
300 + **[CCCCCG]** 171 (85.08)   
300 + **[CCGCGG]** 174 (87.56)   
300 + **.CCGGCG.** 266 (94.52)   
300 + **.CCGGCG.** 408 (94.52)   
300 + **[CCGGGG]** 419 (85.40)   
300 + **[CCCACG]** 499 (88.40)   
300 + **[CCCACG]** 520 (88.40)   
300 + **[CCGACT]** 596 (85.38)   
300 + **.CCGAGG.** 1112 (90.88)   
300 + **[CAGACG]** 1146 (85.30)   
300 + **[CCGCGG]** 1165 (87.56)   
300 - **[CCGCGG]** 1165 (87.56)   
300 - **[CAGACG]** 786 (85.30)   
300 - **[CCGACT]** 764 (85.38)   
300 - **.CCGCCG.** 738 (96.68)   
300 - **.CCGACG.** 715 (100.00)   
300 - **.CCGACG.** 448 (100.00)   
300 - **.CCGCCG.** 409 (96.68)   
300 - **.CCGGCG.** 304 (94.52)   
300 - **.CCGCCG.** 267 (96.68)   
300 - **[CCGGGG]** 185 (85.40)   
300 - **[CCGCGG]** 174 (87.56)   
301 + **[CCGGGG]** 35 (85.40)   
301 + **[CCCCCG]** 197 (85.08)   
301 + **[CCCCCG]** 204 (85.08)   
301 + **[CCCACG]** 220 (88.40)   
301 + **[CCCACG]** 336 (88.40)   
301 + **[CCCCCG]** 369 (85.08)   
301 + **[CCGAAG]** 656 (86.09)   
301 + **[CCGACC]** 869 (88.08)   
301 + **[CCGGGG]** 908 (85.40)   
301 + **[CCCACG]** 1021 (88.40)   
301 + **[CCCCCG]** 1166 (85.08)   
301 + **[CCCCCG]** 1181 (85.08)   
301 + **[CAGACG]** 1278 (85.30)   
301 + **[CGGACG]** 1303 (86.87)   
301 + **[CCGAAG]** 1356 (86.09)   
301 + **[CCGGGG]** 1367 (85.40)   
301 - **.CCGAGG.** 1492 (90.88)   
301 - **[CCCACG]** 1485 (88.40)   
301 - **[CCCCCG]** 1368 (85.08)   
301 - **[CCGGGG]** 1365 (85.40)   
301 - **[CCGACT]** 1300 (85.38)   
301 - **[CCGGGG]** 1182 (85.40)   
301 - **[CCGGGG]** 1167 (85.40)   
301 - **[CAGACG]** 936 (85.30)   
301 - **[CCCACG]** 927 (88.40)   
301 - **[CCCCCG]** 915 (85.08)   
301 - **[CCGACC]** 912 (88.08)   
301 - **[CGGACG]** 818 (86.87)   
301 - **[CCCCCG]** 583 (85.08)   
301 - **[CCCACG]** 575 (88.40)   
301 - **[CCGACT]** 300 (85.38)   
301 - **[CCGGGG]** 198 (85.40)   
301 - **[CCGACC]** 74 (88.08)   
302 + **[CCCACG]** 524 (88.40)   
302 + **[CCGCGG]** 581 (87.56)   
302 + **[GCGACG]** 594 (85.28)   
302 + **[GCGACG]** 602 (85.28)   
302 + **.CCGCCG.** 693 (96.68)   
302 + **[CCGACT]** 801 (85.38)   
302 + **.CCGCCG.** 826 (96.68)   
302 + **[CCCCCG]** 904 (85.08)   
302 + **.CCGGCG.** 946 (94.52)   
302 + **[GCGACG]** 949 (85.28)   
302 + **[CCGCGG]** 1043 (87.56)   
302 + **[CCCCCG]** 1063 (85.08)   
302 + **[CCGCGG]** 1072 (87.56)   
302 + **[CCCCCG]** 1083 (85.08)   
302 + **[CCCACG]** 1093 (88.40)   
302 + **[CGGACG]** 1198 (86.87)   
302 + **[CCCACG]** 1264 (88.40)   
302 + **[CCGTCG]** 1284 (86.99)   
302 + **[CCGGGG]** 1328 (85.40)   
302 + **.CCGCCG.** 1421 (96.68)   
302 + **[CCGGGG]** 1424 (85.40)   
302 + **[CCGAAG]** 1432 (86.09)   
302 + **.CCGCCG.** 1459 (96.68)   
302 + **.CCGCCG.** 1462 (96.68)   
302 + **[CCGGGG]** 1486 (85.40)   
302 - **[CCCCCG]** 1487 (85.08)   
302 - **.CCGGCG.** 1422 (94.52)   
302 - **.CCGCCG.** 1318 (96.68)   
302 - **[CCCCCG]** 1297 (85.08)   
302 - **.CCGACG.** 1285 (100.00)   
302 - **[CCGGGG]** 1156 (85.40)   
302 - **[CCGGGG]** 1104 (85.40)   
302 - **.CCGCCG.** 1097 (96.68)   
302 - **[CCGCGG]** 1072 (87.56)   
302 - **[CCGCGG]** 1043 (87.56)   
302 - **[CCGGGG]** 944 (85.40)   
302 - **[CCGGGG]** 905 (85.40)   
302 - **.CCGGCG.** 843 (94.52)   
302 - **.CCGGCG.** 827 (94.52)   
302 - **.CCGGCG.** 694 (94.52)   
302 - **[CCGAAG]** 642 (86.09)   
302 - **[CCCACG]** 609 (88.40)   
302 - **[CCGTCG]** 603 (86.99)   
302 - **[CCGCGG]** 581 (87.56)   
302 - **[CCGACC]** 215 (88.08)   
303 + **.CCGCCG.** 98 (96.68)   
303 + **.CCGCCG.** 166 (96.68)   
303 + **[CCGGGG]** 208 (85.40)   
303 + **[CCGCGG]** 219 (87.56)   
303 + **[CCGCGG]** 288 (87.56)   
303 + **.CCGAGG.** 340 (90.88)   
303 + **[CCGACC]** 387 (88.08)   
303 + **[CCGCGG]** 458 (87.56)   
303 + **.CCGGCG.** 543 (94.52)   
303 + **.CCGCCG.** 689 (96.68)   
303 + **.CCGGCG.** 692 (94.52)   
303 + **[CCGCGG]** 749 (87.56)   
303 + **.CCGCCG.** 773 (96.68)   
303 + **[CCCCCG]** 839 (85.08)   
303 + **[CCGTCG]** 842 (86.99)   
303 + **.CCGGCG.** 1067 (94.52)   
303 + **[CCGGGG]** 1194 (85.40)   
303 + **.CCGGCG.** 1470 (94.52)   
303 - **.CCGCCG.** 1471 (96.68)   
303 - **.CCGCCG.** 1356 (96.68)   
303 - **.CCGCCG.** 1353 (96.68)   
303 - **.CCGGCG.** 1058 (94.52)   
303 - **[CGGACG]** 1026 (86.87)   
303 - **[CCCCCG]** 994 (85.08)   
303 - **.CCGCCG.** 991 (96.68)   
303 - **.CCGCCG.** 988 (96.68)   
303 - **.CCGCCG.** 985 (96.68)   
303 - **.CCGCCG.** 982 (96.68)   
303 - **.CCGCCG.** 979 (96.68)   
303 - **.CCGCCG.** 976 (96.68)   
303 - **.CCGCCG.** 973 (96.68)   
303 - **[CCGAAG]** 941 (86.09)   
303 - **.CCGCCG.** 918 (96.68)   
303 - **.CCGCCG.** 915 (96.68)   
303 - **.CCGCCG.** 912 (96.68)   
303 - **.CCGCCG.** 903 (96.68)   
303 - **[CGGACG]** 883 (86.87)   
303 - **[CCGCGG]** 749 (87.56)   
303 - **[CCCCCG]** 735 (85.08)   
303 - **.CCGAGG.** 710 (90.88)   
303 - **.CCGCCG.** 693 (96.68)   
303 - **.CCGGCG.** 690 (94.52)   
303 - **.CCGAGG.** 584 (90.88)   
303 - **[CCCCCG]** 547 (85.08)   
303 - **.CCGCCG.** 544 (96.68)   
303 - **.CCGCCG.** 465 (96.68)   
303 - **[CCGCGG]** 458 (87.56)   
303 - **[CCCCCG]** 333 (85.08)   
303 - **[CCGCGG]** 288 (87.56)   
303 - **[CCGCGG]** 219 (87.56)   
303 - **[CCGGGG]** 144 (85.40)   
303 - **.CCGACG.** 132 (100.00)   
304 + **[CCGGGG]** 118 (85.40)   
304 + **[CCCACG]** 356 (88.40)   
304 + **[CCCACG]** 392 (88.40)   
304 + **[CCCACG]** 414 (88.40)   
304 + **[CCCCCG]** 601 (85.08)   
304 + **.CCGACG.** 731 (100.00)   
304 + **[CGGACG]** 737 (86.87)   
304 + **[CCGACT]** 882 (85.38)   
304 + **[CCGCGG]** 1107 (87.56)   
304 + **[CCCCCG]** 1305 (85.08)   
304 + **[GCGACG]** 1310 (85.28)   
304 + **.CCGGCG.** 1338 (94.52)   
304 + **.CCGCCG.** 1411 (96.68)   
304 - **[CCGTCG]** 1311 (86.99)   
304 - **[CCGACC]** 1252 (88.08)   
304 - **.CCGCCG.** 1245 (96.68)   
304 - **.CCGCCG.** 1175 (96.68)   
304 - **[CCCCCG]** 1156 (85.08)   
304 - **[CCGCGG]** 1107 (87.56)   
304 - **.CCGGCG.** 1070 (94.52)   
304 - **.CCGAGG.** 1033 (90.88)   
304 - **[CCGACC]** 900 (88.08)   
304 - **[CGGACG]** 842 (86.87)   
304 - **[CCGGGG]** 720 (85.40)   
304 - **.CCGAGG.** 258 (90.88)   
304 - **[CCGAAG]** 205 (86.09)   
304 - **.CCGCCG.** 189 (96.68)   
304 - **[CCCCCG]** 145 (85.08)   
304 - **[CCGGGG]** 116 (85.40)   
305 + **[CCGAAG]** 86 (86.09)   
305 + **[CCCCCG]** 305 (85.08)   
305 + **[CCGACC]** 308 (88.08)   
305 + **[CCCCCG]** 312 (85.08)   
305 + **[CCCCCG]** 382 (85.08)   
305 + **.CCGAGG.** 914 (90.88)   
305 + **[CCGGGG]** 1432 (85.40)   
305 + **[CCGGGG]** 1513 (85.40)   
305 - **[CCGGGG]** 1430 (85.40)   
305 - **.CCGAGG.** 1346 (90.88)   
305 - **[CCGACC]** 1235 (88.08)   
305 - **.CCGAGG.** 1152 (90.88)   
305 - **[CCGGGG]** 992 (85.40)   
305 - **[CCCCCG]** 615 (85.08)   
305 - **[CCGGGG]** 516 (85.40)   
305 - **[CCGGGG]** 383 (85.40)   
305 - **[CCCACG]** 356 (88.40)   
306 + **[CCGAAG]** 258 (86.09)   
306 + **.CCGGCG.** 273 (94.52)   
306 + **[CCCCCG]** 374 (85.08)   
306 + **[CCGCGG]** 377 (87.56)   
306 + **[CCGAAG]** 736 (86.09)   
306 + **.CCGCCG.** 763 (96.68)   
306 + **[CCGCGG]** 867 (87.56)   
306 + **.CCGGCG.** 1064 (94.52)   
306 + **.CCGGCG.** 1079 (94.52)   
306 + **.CCGGCG.** 1094 (94.52)   
306 + **.CCGGCG.** 1140 (94.52)   
306 + **.CCGGCG.** 1185 (94.52)   
306 + **.CCGGCG.** 1200 (94.52)   
306 + **.CCGGCG.** 1216 (94.52)   
306 + **.CCGGCG.** 1246 (94.52)   
306 + **.CCGGCG.** 1307 (94.52)   
306 + **.CCGGCG.** 1322 (94.52)   
306 + **.CCGGCG.** 1337 (94.52)   
306 + **.CCGGCG.** 1369 (94.52)   
306 + **[CCGGGG]** 1406 (85.40)   
306 + **.CCGCCG.** 1415 (96.68)   
306 + **.CCGGCG.** 1426 (94.52)   
306 + **[CCCCCG]** 1489 (85.08)   
306 - **[CCGGGG]** 1404 (85.40)   
306 - **.CCGAGG.** 1390 (90.88)   
306 - **.CCGCCG.** 1338 (96.68)   
306 - **[CCGACC]** 1329 (88.08)   
306 - **.CCGCCG.** 1308 (96.68)   
306 - **[CCGACC]** 1268 (88.08)   
306 - **.CCGCCG.** 1262 (96.68)   
306 - **[CCGACC]** 1259 (88.08)   
306 - **.CCGCCG.** 1247 (96.68)   
306 - **[CCGACC]** 1223 (88.08)   
306 - **.CCGCCG.** 1217 (96.68)   
306 - **.CCGCCG.** 1201 (96.68)   
306 - **.CCGCCG.** 1186 (96.68)   
306 - **.CCGCCG.** 1156 (96.68)   
306 - **[CCGACC]** 1153 (88.08)   
306 - **.CCGCCG.** 1141 (96.68)   
306 - **[CCGACC]** 1116 (88.08)   
306 - **.CCGCCG.** 1110 (96.68)   
306 - **[CCGACC]** 1107 (88.08)   
306 - **.CCGCCG.** 1095 (96.68)   
306 - **.CCGCCG.** 1080 (96.68)   
306 - **.CCGCCG.** 1065 (96.68)   
306 - **[CCGACC]** 1056 (88.08)   
306 - **.CCGCCG.** 1023 (96.68)   
306 - **.CCGGCG.** 932 (94.52)   
306 - **[CCGCGG]** 867 (87.56)   
306 - **[CCGCGG]** 377 (87.56)   
306 - **.CCGGCG.** 344 (94.52)   
306 - **[CCCCCG]** 172 (85.08)   
306 - **[CCCCCG]** 125 (85.08)   
307 + **.CCGGCG.** 495 (94.52)   
307 + **.CCGCCG.** 540 (96.68)   
307 + **.CCGCCG.** 677 (96.68)   
307 + **[CCGCGG]** 680 (87.56)   
307 + **[CCCCCG]** 811 (85.08)   
307 + **[CCCCCG]** 817 (85.08)   
307 + **[CCGCGG]** 825 (87.56)   
307 + **[CCCCCG]** 833 (85.08)   
307 + **[CCGGGG]** 912 (85.40)   
307 + **.CCGCCG.** 982 (96.68)   
307 + **.CCGCCG.** 991 (96.68)   
307 + **.CCGCCG.** 994 (96.68)   
307 + **.CCGAGG.** 1219 (90.88)   
307 + **[CCGGGG]** 1253 (85.40)   
307 + **.CCGCCG.** 1271 (96.68)   
307 + **[CCGGGG]** 1274 (85.40)   
307 + **.CCGAGG.** 1308 (90.88)   
307 + **[CCGCGG]** 1329 (87.56)   
307 + **[CCCCCG]** 1404 (85.08)   
307 + **.CCGGCG.** 1444 (94.52)   
307 - **.CCGCCG.** 1445 (96.68)   
307 - **[CCGGGG]** 1405 (85.40)   
307 - **[CCGAAG]** 1351 (86.09)   
307 - **[CCGCGG]** 1329 (87.56)   
307 - **.CCGGCG.** 1272 (94.52)   
307 - **.CCGAGG.** 1139 (90.88)   
307 - **[CCCCCG]** 1099 (85.08)   
307 - **.CCGCCG.** 1086 (96.68)   
307 - **.CCGACG.** 1068 (100.00)   
307 - **[CCCCCG]** 954 (85.08)   
307 - **[CCCCCG]** 903 (85.08)   
307 - **[CCGCGG]** 825 (87.56)   
307 - **[CCGGGG]** 818 (85.40)   
307 - **.CCGCCG.** 732 (96.68)   
307 - **[CCGACC]** 717 (88.08)   
307 - **[CCGCGG]** 680 (87.56)   
307 - **.CCGAGG.** 651 (90.88)   
307 - **[CCCCCG]** 605 (85.08)   
307 - **.CCGGCG.** 550 (94.52)   
307 - **.CCGGCG.** 541 (94.52)   
307 - **[CCGGGG]** 493 (85.40)   
307 - **[CCCCCG]** 327 (85.08)   
308 + **[CCCCCG]** 136 (85.08)   
308 + **[CCGCGG]** 710 (87.56)   
308 + **.CCGCCG.** 823 (96.68)   
308 + **[CCGACC]** 919 (88.08)   
308 + **[CCGGGG]** 923 (85.40)   
308 + **.CCGGCG.** 931 (94.52)   
308 + **[CCGGGG]** 988 (85.40)   
308 + **[CCGGGG]** 1026 (85.40)   
308 + **[CCGCGG]** 1180 (87.56)   
308 + **[CCGGGG]** 1219 (85.40)   
308 + **.CCGCCG.** 1366 (96.68)   
308 + **[CCGCGG]** 1369 (87.56)   
308 + **.CCGCCG.** 1391 (96.68)   
308 + **[CCGCGG]** 1414 (87.56)   
308 + **[CCGCGG]** 1424 (87.56)   
308 + **.CCGCCG.** 1501 (96.68)   
308 - **.CCGGCG.** 1502 (94.52)   
308 - **[CCGACC]** 1446 (88.08)   
308 - **[CCGCGG]** 1424 (87.56)   
308 - **[CCGCGG]** 1414 (87.56)   
308 - **.CCGGCG.** 1392 (94.52)   
308 - **[CCGCGG]** 1369 (87.56)   
308 - **.CCGCCG.** 1335 (96.68)   
308 - **.CCGCCG.** 1202 (96.68)   
308 - **.CCGGCG.** 1191 (94.52)   
308 - **[CCGCGG]** 1180 (87.56)   
308 - **.CCGAGG.** 1083 (90.88)   
308 - **.CCGACG.** 1074 (100.00)   
308 - **[CCCCCG]** 1043 (85.08)   
308 - **.CCGCCG.** 932 (96.68)   
308 - **[CCCCCG]** 924 (85.08)   
308 - **.CCGCCG.** 865 (96.68)   
308 - **.CCGGCG.** 824 (94.52)   
308 - **[CCGCGG]** 710 (87.56)   
308 - **[CCGACC]** 649 (88.08)   
308 - **[CCCACG]** 184 (88.40)   
308 - **[CCGGGG]** 137 (85.40)   
308 - **[CCGAAG]** 42 (86.09)   
309 + **[CCGAAG]** 586 (86.09)   
309 + **[CCGAAG]** 605 (86.09)   
309 + **[CCGCGG]** 872 (87.56)   
309 + **[CCCCCG]** 932 (85.08)   
309 + **[CCGCGG]** 995 (87.56)   
309 + **.CCGGCG.** 1005 (94.52)   
309 + **[CCCCCG]** 1289 (85.08)   
309 - **[CCCACG]** 1337 (88.40)   
309 - **[CCGGGG]** 1290 (85.40)   
309 - **[CCGAAG]** 1195 (86.09)   
309 - **[CCCACG]** 1143 (88.40)   
309 - **.CCGCCG.** 1031 (96.68)   
309 - **[CCGCGG]** 995 (87.56)   
309 - **[CCCACG]** 989 (88.40)   
309 - **[CCCCCG]** 956 (85.08)   
309 - **.CCGCCG.** 905 (96.68)   
309 - **[CCGCGG]** 872 (87.56)   
309 - **.CCGAGG.** 800 (90.88)   
309 - **[CCCCCG]** 763 (85.08)   
309 - **[CCCCCG]** 674 (85.08)   
309 - **.CCGCCG.** 557 (96.68)   
309 - **.CCGAGG.** 391 (90.88)   
309 - **[CCCCCG]** 119 (85.08)   
309 - **[CCGACT]** 116 (85.38)   
310 + **[CCGACT]** 332 (85.38)   
310 + **[CCGCGG]** 930 (87.56)   
310 + **[CCCCCG]** 1008 (85.08)   
310 + **[CCGCGG]** 1034 (87.56)   
310 + **[CCGCGG]** 1300 (87.56)   
310 + **[CCGCGG]** 1413 (87.56)   
310 - **.CCGAGG.** 1511 (90.88)   
310 - **[CCGACT]** 1443 (85.38)   
310 - **[CCGCGG]** 1413 (87.56)   
310 - **[CCGAAG]** 1388 (86.09)   
310 - **[CCGGGG]** 1333 (85.40)   
310 - **[CCGCGG]** 1300 (87.56)   
310 - **[CGGACG]** 1233 (86.87)   
310 - **[CCCCCG]** 1184 (85.08)   
310 - **[CCGAAG]** 1181 (86.09)   
310 - **[CGGACG]** 1160 (86.87)   
310 - **[CCGCGG]** 1034 (87.56)   
310 - **[CCGCGG]** 930 (87.56)   
310 - **.CCGAGG.** 792 (90.88)   
311 + **[CCCCCG]** 680 (85.08)   
311 + **[CCCACG]** 868 (88.40)   
311 + **[CCGCGG]** 874 (87.56)   
311 + **[CCGGGG]** 910 (85.40)   
311 + **.CCGCCG.** 933 (96.68)   
311 + **[CCGACC]** 1045 (88.08)   
311 + **[CCGCGG]** 1105 (87.56)   
311 + **[CCGCGG]** 1114 (87.56)   
311 + **.CCGCCG.** 1206 (96.68)   
311 + **.CCGGCG.** 1231 (94.52)   
311 + **[CCGCGG]** 1472 (87.56)   
311 + **.CCGCCG.** 1483 (96.68)   
311 - **[CAGACG]** 1487 (85.30)   
311 - **[CCGCGG]** 1472 (87.56)   
311 - **.CCGGCG.** 1448 (94.52)   
311 - **[CCCCCG]** 1326 (85.08)   
311 - **.CCGCCG.** 1250 (96.68)   
311 - **.CCGGCG.** 1207 (94.52)   
311 - **[CCGCGG]** 1114 (87.56)   
311 - **[CCGCGG]** 1105 (87.56)   
311 - **.CCGCCG.** 1071 (96.68)   
311 - **.CCGGCG.** 1058 (94.52)   
311 - **[CCGACC]** 925 (88.08)   
311 - **[CCCCCG]** 911 (85.08)   
311 - **.CCGGCG.** 908 (94.52)   
311 - **[CCGCGG]** 874 (87.56)   
311 - **[CCGGGG]** 732 (85.40)   
311 - **.CCGGCG.** 622 (94.52)   
311 - **.CCGAGG.** 595 (90.88)   
312 + **[GCGACG]** 739 (85.28)   
312 + **[CCGCGG]** 812 (87.56)   
312 + **.CCGAGG.** 825 (90.88)   
312 + **[CCCCCG]** 832 (85.08)   
312 + **[CCCCCG]** 847 (85.08)   
312 + **[CCGCGG]** 878 (87.56)   
312 + **[CCGGGG]** 937 (85.40)   
312 + **[CCGGGG]** 999 (85.40)   
312 + **[CCGGGG]** 1037 (85.40)   
312 + **.CCGCCG.** 1043 (96.68)   
312 + **.CCGCCG.** 1046 (96.68)   
312 + **[CCGTCG]** 1059 (86.99)   
312 + **[CCCCCG]** 1118 (85.08)   
312 + **[CCCCCG]** 1141 (85.08)   
312 + **.CCGGCG.** 1144 (94.52)   
312 + **[CGGACG]** 1170 (86.87)   
312 + **[CAGACG]** 1179 (85.30)   
312 + **.CCGCCG.** 1225 (96.68)   
312 + **.CCGACG.** 1228 (100.00)   
312 + **.CCGAGG.** 1258 (90.88)   
312 + **.CCGGCG.** 1275 (94.52)   
312 + **.CCGGCG.** 1288 (94.52)   
312 + **[CCGGGG]** 1342 (85.40)   
312 + **.CCGGCG.** 1461 (94.52)   
312 + **.CCGGCG.** 1487 (94.52)   
312 + **[CCGGGG]** 1493 (85.40)   
312 - **.CCGGCG.** 1491 (94.52)   
312 - **.CCGGCG.** 1485 (94.52)   
312 - **.CCGCCG.** 1462 (96.68)   
312 - **[CCGAAG]** 1439 (86.09)   
312 - **[CCCCCG]** 1350 (85.08)   
312 - **[CCGTCG]** 1162 (86.99)   
312 - **.CCGCCG.** 1145 (96.68)   
312 - **[CCGGGG]** 1142 (85.40)   
312 - **.CCGACG.** 1060 (100.00)   
312 - **[CCGGGG]** 997 (85.40)   
312 - **[CCGGGG]** 935 (85.40)   
312 - **[CCCCCG]** 915 (85.08)   
312 - **[CCGCGG]** 878 (87.56)   
312 - **[CCGGGG]** 848 (85.40)   
312 - **[CCGGGG]** 833 (85.40)   
312 - **[CCGCGG]** 812 (87.56)   
312 - **[CCGGGG]** 711 (85.40)   
312 - **.CCGGCG.** 559 (94.52)   
313 + **[CCCACG]** 464 (88.40)   
313 + **[CCGCGG]** 825 (87.56)   
313 + **.CCGCCG.** 840 (96.68)   
313 + **.CCGGCG.** 954 (94.52)   
313 + **[CCGGGG]** 1115 (85.40)   
313 + **[CCCCCG]** 1140 (85.08)   
313 + **[CCGGGG]** 1143 (85.40)   
313 + **[GCGACG]** 1170 (85.28)   
313 + **[CCCACG]** 1228 (88.40)   
313 + **[CCCCCG]** 1253 (85.08)   
313 + **.CCGCCG.** 1338 (96.68)   
313 + **.CCGCCG.** 1444 (96.68)   
313 + **[CCGCGG]** 1447 (87.56)   
313 + **[CCCCCG]** 1459 (85.08)   
313 - **[CCGCGG]** 1447 (87.56)   
313 - **.CCGCCG.** 1174 (96.68)   
313 - **[CCGTCG]** 1171 (86.99)   
313 - **[CCGGGG]** 1141 (85.40)   
313 - **[CCCCCG]** 1116 (85.08)   
313 - **[CCGGGG]** 1106 (85.40)   
313 - **.CCGGCG.** 1090 (94.52)   
313 - **[CCGACC]** 1078 (88.08)   
313 - **[CCGACT]** 1070 (85.38)   
313 - **[CCGGGG]** 952 (85.40)   
313 - **.CCGAGG.** 834 (90.88)   
313 - **[CCGCGG]** 825 (87.56)   
313 - **[CCCACG]** 585 (88.40)   
313 - **[CAGACG]** 342 (85.30)   
314 + **.CCGGCG.** 10 (94.52)   
314 + **.CCGACG.** 16 (100.00)   
314 + **.CCGAGG.** 42 (90.88)   
314 + **[CCCCCG]** 58 (85.08)   
314 + **.CCGCCG.** 61 (96.68)   
314 + **.CCGAGG.** 144 (90.88)   
314 + **[CCCACG]** 347 (88.40)   
314 + **.CCGGCG.** 446 (94.52)   
314 + **[CCGAAG]** 1250 (86.09)   
314 - **[CCGACT]** 383 (85.38)   
314 - **.CCGGCG.** 351 (94.52)   
314 - **.CCGAGG.** 234 (90.88)   
314 - **.CCGGCG.** 187 (94.52)   
314 - **[CCGACC]** 66 (88.08)   
314 - **.CCGGCG.** 62 (94.52)   
314 - **[CCGACT]** 35 (85.38)   
314 - **[CCGTCG]** 17 (86.99)   
314 - **[CCGGGG]** 8 (85.40)   
315 + **[CCGACC]** 137 (88.08)   
315 + **[CCGGGG]** 164 (85.40)   
315 + **[CCCACG]** 248 (88.40)   
315 + **[CCGGGG]** 452 (85.40)   
315 + **.CCGAGG.** 497 (90.88)   
315 + **.CCGACG.** 548 (100.00)   
315 + **[CGGACG]** 813 (86.87)   
315 + **[CCCCCG]** 830 (85.08)   
315 + **[CCGGGG]** 956 (85.40)   
315 + **[CGGACG]** 1004 (86.87)   
315 + **[CCGGGG]** 1105 (85.40)   
315 + **.CCGAGG.** 1277 (90.88)   
315 + **.CCGGCG.** 1395 (94.52)   
315 - **.CCGCCG.** 1396 (96.68)   
315 - **.CCGAGG.** 1219 (90.88)   
315 - **.CCGCCG.** 1092 (96.68)   
315 - **[CCCCCG]** 1030 (85.08)   
315 - **[CCCCCG]** 957 (85.08)   
315 - **[CCGTCG]** 549 (86.99)   
315 - **.CCGCCG.** 406 (96.68)   
315 - **[CGGACG]** 134 (86.87)   
315 - **[CCGACT]** 89 (85.38)   
316 + **[CCGGGG]** 2 (85.40)   
316 + **.CCGCCG.** 137 (96.68)   
316 + **[CCGGGG]** 207 (85.40)   
316 + **[CCGCGG]** 228 (87.56)   
316 + **.CCGCCG.** 275 (96.68)   
316 + **[CCGACT]** 346 (85.38)   
316 + **[CCGAAG]** 604 (86.09)   
316 + **.CCGAGG.** 670 (90.88)   
316 + **[CCGCGG]** 849 (87.56)   
316 + **[CCGCGG]** 909 (87.56)   
316 + **.CCGCCG.** 1182 (96.68)   
316 + **.CCGCCG.** 1185 (96.68)   
316 + **[CCGGGG]** 1301 (85.40)   
316 - **[CCGGGG]** 1317 (85.40)   
316 - **[CCGGGG]** 1307 (85.40)   
316 - **[CCGGGG]** 1270 (85.40)   
316 - **[CCGGGG]** 1090 (85.40)   
316 - **[CCGCGG]** 909 (87.56)   
316 - **[CCGACC]** 853 (88.08)   
316 - **[CCGCGG]** 849 (87.56)   
316 - **[CCCACG]** 500 (88.40)   
316 - **.CCGGCG.** 276 (94.52)   
316 - **.CCGCCG.** 249 (96.68)   
316 - **[CCGCGG]** 228 (87.56)   
316 - **.CCGGCG.** 138 (94.52)   
316 - **.CCGCCG.** 69 (96.68)   
316 - **.CCGACG.** 66 (100.00)   
316 - **[CCCCCG]** 3 (85.08)   
317 + **[CCGGGG]** 149 (85.40)   
317 + **[CCCACG]** 856 (88.40)   
317 + **.CCGGCG.** 946 (94.52)   
317 + **[CCGAAG]** 1092 (86.09)   
317 + **[CCCACG]** 1151 (88.40)   
317 + **.CCGGCG.** 1195 (94.52)   
317 + **[CCGGGG]** 1247 (85.40)   
317 + **[CCGCGG]** 1349 (87.56)   
317 - **[CCGACC]** 1365 (88.08)   
317 - **[CCGCGG]** 1349 (87.56)   
317 - **.CCGCCG.** 1199 (96.68)   
317 - **.CCGCCG.** 1196 (96.68)   
317 - **.CCGCCG.** 1040 (96.68)   
317 - **.CCGCCG.** 1037 (96.68)   
317 - **[CCCCCG]** 1028 (85.08)   
317 - **.CCGCCG.** 947 (96.68)   
318 + **[CCGGGG]** 91 (85.40)   
318 + **.CCGAGG.** 153 (90.88)   
318 + **[CCGACT]** 398 (85.38)   
318 + **[CCGGGG]** 448 (85.40)   
318 + **.CCGGCG.** 602 (94.52)   
318 + **.CCGGCG.** 704 (94.52)   
318 + **[CCGGGG]** 744 (85.40)   
318 + **.CCGGCG.** 771 (94.52)   
318 + **[CCCCCG]** 897 (85.08)   
318 + **[CCCCCG]** 1105 (85.08)   
318 + **.CCGCCG.** 1108 (96.68)   
318 + **[CCCCCG]** 1114 (85.08)   
318 + **[CGGACG]** 1152 (86.87)   
318 + **.CCGAGG.** 1165 (90.88)   
318 + **.CCGCCG.** 1218 (96.68)   
318 + **[CCGGGG]** 1230 (85.40)   
318 + **[CCCCCG]** 1250 (85.08)   
318 + **[CCCCCG]** 1269 (85.08)   
318 + **.CCGGCG.** 1307 (94.52)   
318 + **.CCGCCG.** 1359 (96.68)   
318 + **[CCGACT]** 1362 (85.38)   
318 + **.CCGGCG.** 1432 (94.52)   
318 + **[CCGCGG]** 1484 (87.56)   
318 + **.CCGAGG.** 1511 (90.88)   
318 - **.CCGCCG.** 1496 (96.68)   
318 - **.CCGCCG.** 1493 (96.68)   
318 - **.CCGCCG.** 1490 (96.68)   
318 - **.CCGCCG.** 1487 (96.68)   
318 - **[CCGCGG]** 1484 (87.56)   
318 - **.CCGCCG.** 1478 (96.68)   
318 - **[CCCCCG]** 1472 (85.08)   
318 - **.CCGCCG.** 1390 (96.68)   
318 - **[CCGGGG]** 1270 (85.40)   
318 - **[CCGGGG]** 1263 (85.40)   
318 - **[CCGGGG]** 1257 (85.40)   
318 - **[CCGGGG]** 1251 (85.40)   
318 - **[CCCCCG]** 1231 (85.08)   
318 - **[CGGACG]** 1198 (86.87)   
318 - **.CCGGCG.** 1149 (94.52)   
318 - **.CCGGCG.** 1057 (94.52)   
318 - **[CCGGGG]** 898 (85.40)   
318 - **.CCGCCG.** 772 (96.68)   
318 - **[CCGACC]** 680 (88.08)   
318 - **.CCGCCG.** 670 (96.68)   
318 - **[CCGGGG]** 633 (85.40)   
318 - **[CCGACC]** 492 (88.08)   
318 - **.CCGAGG.** 379 (90.88)   
318 - **.CCGGCG.** 309 (94.52)   
320 + **.CCGCCG.** 15 (96.68)   
320 + **.CCGGCG.** 154 (94.52)   
320 + **.CCGACG.** 224 (100.00)   
320 + **[CCCCCG]** 280 (85.08)   
320 + **.CCGGCG.** 520 (94.52)   
320 + **[CCGCGG]** 536 (87.56)   
320 + **[CCCCCG]** 578 (85.08)   
320 + **.CCGGCG.** 605 (94.52)   
320 + **[CAGACG]** 774 (85.30)   
320 + **.CCGAGG.** 906 (90.88)   
320 + **[CCCCCG]** 933 (85.08)   
320 + **.CCGGCG.** 960 (94.52)   
320 + **.CCGAGG.** 966 (90.88)   
320 + **[CCGCGG]** 991 (87.56)   
320 + **[CCGGGG]** 1045 (85.40)   
320 + **[CAGACG]** 1082 (85.30)   
320 + **[CCGACT]** 1168 (85.38)   
320 + **[CCCACG]** 1333 (88.40)   
320 + **[CCGCGG]** 1385 (87.56)   
320 - **[CCGCGG]** 1385 (87.56)   
320 - **[GCGACG]** 1350 (85.28)   
320 - **[CCGGGG]** 1239 (85.40)   
320 - **.CCGCCG.** 1139 (96.68)   
320 - **[CCCACG]** 1086 (88.40)   
320 - **.CCGGCG.** 997 (94.52)   
320 - **[CCGCGG]** 991 (87.56)   
320 - **[CCGGGG]** 934 (85.40)   
320 - **.CCGGCG.** 915 (94.52)   
320 - **[CCCACG]** 851 (88.40)   
320 - **[CCCCCG]** 794 (85.08)   
320 - **.CCGCCG.** 791 (96.68)   
320 - **[CAGACG]** 649 (85.30)   
320 - **[CCGGGG]** 579 (85.40)   
320 - **[CCCCCG]** 539 (85.08)   
320 - **[CCGCGG]** 536 (87.56)   
320 - **[CCGGGG]** 450 (85.40)   
320 - **[CCGGGG]** 432 (85.40)   
320 - **.CCGCCG.** 418 (96.68)   
320 - **[CCCCCG]** 386 (85.08)   
320 - **[CCCACG]** 376 (88.40)   
320 - **.CCGACG.** 352 (100.00)   
320 - **[CCGGGG]** 281 (85.40)   
320 - **[CCCCCG]** 228 (85.08)   
320 - **[CCGTCG]** 225 (86.99)   
320 - **[CCCCCG]** 189 (85.08)   
321 + **[CCCCCG]** 262 (85.08)   
321 + **.CCGAGG.** 364 (90.88)   
321 + **[CCGGGG]** 376 (85.40)   
321 + **.CCGGCG.** 395 (94.52)   
321 + **[CCGCGG]** 454 (87.56)   
321 + **.CCGGCG.** 508 (94.52)   
321 + **[CCGAAG]** 525 (86.09)   
321 + **.CCGGCG.** 570 (94.52)   
321 + **[CCCCCG]** 577 (85.08)   
321 + **[CCCCCG]** 627 (85.08)   
321 + **.CCGCCG.** 734 (96.68)   
321 + **[CCGCGG]** 944 (87.56)   
321 + **.CCGCCG.** 980 (96.68)   
321 + **.CCGCCG.** 983 (96.68)   
321 + **[CGGACG]** 987 (86.87)   
321 + **.CCGCCG.** 1042 (96.68)   
321 + **.CCGCCG.** 1055 (96.68)   
321 + **[CCCCCG]** 1068 (85.08)   
321 + **[CCCCCG]** 1170 (85.08)   
321 + **.CCGGCG.** 1173 (94.52)   
321 + **[CCGACC]** 1236 (88.08)   
321 + **[CCGGGG]** 1251 (85.40)   
321 + **.CCGGCG.** 1298 (94.52)   
321 + **.CCGGCG.** 1340 (94.52)   
321 + **[CCCCCG]** 1367 (85.08)   
321 + **[CCGCGG]** 1399 (87.56)   
321 + **[CCGGGG]** 1429 (85.40)   
321 + **.CCGACG.** 1448 (100.00)   
321 + **[CCCCCG]** 1466 (85.08)   
321 - **.CCGGCG.** 1508 (94.52)   
321 - **.CCGCCG.** 1489 (96.68)   
321 - **.CCGACG.** 1486 (100.00)   
321 - **[CCGTCG]** 1449 (86.99)   
321 - **[CCGCGG]** 1399 (87.56)   
321 - **.CCGGCG.** 1344 (94.52)   
321 - **.CCGGCG.** 1262 (94.52)   
321 - **[CCGGGG]** 1249 (85.40)   
321 - **[CCGGGG]** 1171 (85.40)   
321 - **.CCGGCG.** 991 (94.52)   
321 - **.CCGGCG.** 984 (94.52)   
321 - **[CCGCGG]** 944 (87.56)   
321 - **[CCCCCG]** 916 (85.08)   
321 - **[CCCCCG]** 893 (85.08)   
321 - **[CCCCCG]** 878 (85.08)   
321 - **.CCGGCG.** 871 (94.52)   
321 - **[CCCCCG]** 835 (85.08)   
321 - **[CCGACT]** 759 (85.38)   
321 - **.CCGGCG.** 568 (94.52)   
321 - **.CCGCCG.** 509 (96.68)   
321 - **[CGGACG]** 467 (86.87)   
321 - **[CCGCGG]** 454 (87.56)   
321 - **[CCGACC]** 423 (88.08)   
321 - **.CCGCCG.** 417 (96.68)   
321 - **.CCGCCG.** 396 (96.68)   
321 - **[CCCCCG]** 377 (85.08)   
322 + **[CCCACG]** 34 (88.40)   
322 + **[CCCCCG]** 91 (85.08)   
322 + **[CCGACC]** 94 (88.08)   
322 + **[CCGAAG]** 102 (86.09)   
322 + **[CCGACC]** 116 (88.08)   
322 + **[CCGGGG]** 351 (85.40)   
322 + **[CCGGGG]** 430 (85.40)   
322 + **[CCCCCG]** 459 (85.08)   
322 + **[CCCCCG]** 492 (85.08)   
322 + **.CCGCCG.** 495 (96.68)   
322 + **.CCGAGG.** 547 (90.88)   
322 + **[CCGGGG]** 601 (85.40)   
322 + **.CCGGCG.** 628 (94.52)   
322 + **[CCCACG]** 681 (88.40)   
322 + **[CCGGGG]** 698 (85.40)   
322 + **.CCGGCG.** 736 (94.52)   
322 + **.CCGCCG.** 773 (96.68)   
322 + **[CCGGGG]** 859 (85.40)   
322 + **[CCCACG]** 866 (88.40)   
322 + **.CCGGCG.** 881 (94.52)   
322 + **.CCGCCG.** 952 (96.68)   
322 + **[CCGCGG]** 1004 (87.56)   
322 + **.CCGCCG.** 1060 (96.68)   
322 + **[CCGTCG]** 1063 (86.99)   
322 + **[CCGGGG]** 1096 (85.40)   
322 + **.CCGAGG.** 1162 (90.88)   
322 + **[CGGACG]** 1218 (86.87)   
322 + **[CCGGGG]** 1238 (85.40)   
322 + **.CCGAGG.** 1367 (90.88)   
322 + **[CCGCGG]** 1379 (87.56)   
322 + **[CAGACG]** 1422 (85.30)   
322 - **[CCGCGG]** 1379 (87.56)   
322 - **[CCGAAG]** 1310 (86.09)   
322 - **[CCCCCG]** 1239 (85.08)   
322 - **.CCGGCG.** 1236 (94.52)   
322 - **[CCGGGG]** 1182 (85.40)   
322 - **[GCGACG]** 1171 (85.28)   
322 - **[CCGGGG]** 1137 (85.40)   
322 - **[GCGACG]** 1113 (85.28)   
322 - **[CCGGGG]** 1094 (85.40)   
322 - **[GCGACG]** 1064 (85.28)   
322 - **[CCGACC]** 1008 (88.08)   
322 - **[CCGCGG]** 1004 (87.56)   
322 - **[CCCCCG]** 995 (85.08)   
322 - **.CCGCCG.** 992 (96.68)   
322 - **.CCGGCG.** 953 (94.52)   
322 - **.CCGCCG.** 882 (96.68)   
322 - **[CCCCCG]** 802 (85.08)   
322 - **.CCGGCG.** 774 (94.52)   
322 - **.CCGAGG.** 744 (90.88)   
322 - **[CCGGGG]** 642 (85.40)   
322 - **.CCGAGG.** 326 (90.88)   
322 - **[CCGACC]** 308 (88.08)   
323 + **.CCGCCG.** 36 (96.68)   
323 + **.CCGAGG.** 247 (90.88)   
323 + **[CCCACG]** 376 (88.40)   
323 + **[CCGCGG]** 574 (87.56)   
323 + **[CCGGGG]** 583 (85.40)   
323 + **[CCGTCG]** 634 (86.99)   
323 + **[CCGACC]** 706 (88.08)   
323 + **[CCGGGG]** 730 (85.40)   
323 + **[CCGTCG]** 788 (86.99)   
323 + **.CCGGCG.** 804 (94.52)   
323 + **[CCGGGG]** 958 (85.40)   
323 + **[CCGGGG]** 977 (85.40)   
323 + **[CCGCGG]** 1031 (87.56)   
323 + **[CCCACG]** 1066 (88.40)   
323 + **.CCGCCG.** 1088 (96.68)   
323 + **[CCCCCG]** 1097 (85.08)   
323 + **.CCGGCG.** 1100 (94.52)   
323 + **[GCGACG]** 1194 (85.28)   
323 + **[CCGCGG]** 1230 (87.56)   
323 + **[CCCCCG]** 1317 (85.08)   
323 + **[CCCCCG]** 1326 (85.08)   
323 + **[CCGCGG]** 1412 (87.56)   
323 + **.CCGAGG.** 1443 (90.88)   
323 + **.CCGCCG.** 1487 (96.68)   
323 - **.CCGGCG.** 1488 (94.52)   
323 - **[CCCCCG]** 1481 (85.08)   
323 - **[CCCCCG]** 1429 (85.08)   
323 - **.CCGACG.** 1426 (100.00)   
323 - **[CCGCGG]** 1412 (87.56)   
323 - **[CCGACC]** 1386 (88.08)   
323 - **[CCGACT]** 1382 (85.38)   
323 - **[CCGGGG]** 1302 (85.40)   
323 - **.CCGCCG.** 1233 (96.68)   
323 - **[CCGCGG]** 1230 (87.56)   
323 - **[CCGGGG]** 1209 (85.40)   
323 - **[CCGTCG]** 1195 (86.99)   
323 - **[CCGGGG]** 1186 (85.40)   
323 - **.CCGCCG.** 1143 (96.68)   
323 - **[CCGGGG]** 1098 (85.40)   
323 - **.CCGGCG.** 1089 (94.52)   
323 - **[CCGCGG]** 1031 (87.56)   
323 - **[CCCCCG]** 964 (85.08)   
323 - **[CCCCCG]** 941 (85.08)   
323 - **[CCGACC]** 795 (88.08)   
323 - **.CCGACG.** 789 (100.00)   
323 - **[CCCACG]** 777 (88.40)   
323 - **[CCCCCG]** 731 (85.08)   
323 - **[CAGACG]** 652 (85.30)   
323 - **.CCGACG.** 635 (100.00)   
323 - **.CCGGCG.** 581 (94.52)   
323 - **[CCGCGG]** 574 (87.56)   
323 - **[CCCCCG]** 560 (85.08)   
323 - **[CCGAAG]** 541 (86.09)   
323 - **.CCGAGG.** 490 (90.88)   
323 - **.CCGACG.** 471 (100.00)   
323 - **[CCGGGG]** 382 (85.40)   
324 + **.CCGGCG.** 6 (94.52)   
324 + **.CCGAGG.** 826 (90.88)   
324 + **[CGGACG]** 1000 (86.87)   
324 + **[CCGTCG]** 1010 (86.99)   
324 + **.CCGAGG.** 1307 (90.88)   
324 - **[CCCCCG]** 1406 (85.08)   
324 - **[GCGACG]** 1011 (85.28)   
324 - **.CCGCCG.** 980 (96.68)   
324 - **[CCGACT]** 942 (85.38)   
324 - **[CCCCCG]** 839 (85.08)   
324 - **[CCGGGG]** 380 (85.40)   
324 - **[CCGACC]** 204 (88.08)   
324 - **[CAGACG]** 96 (85.30)   
324 - **.CCGCCG.** 7 (96.68)   
325 + **[CCGGGG]** 1278 (85.40)   
325 - **[CCGACC]** 1466 (88.08)   
325 - **[CCGAAG]** 1097 (86.09)   
325 - **.CCGCCG.** 1073 (96.68)   
325 - **[CCCCCG]** 691 (85.08)   
325 - **[CAGACG]** 593 (85.30)   
325 - **[CCCACG]** 155 (88.40)   
325 - **[CCCACG]** 130 (88.40)   
325 - **.CCGAGG.** 59 (90.88)   
326 + **.CCGCCG.** 733 (96.68)   
326 + **.CCGGCG.** 751 (94.52)   
326 + **.CCGCCG.** 821 (96.68)   
326 + **[CCCCCG]** 857 (85.08)   
326 + **[CCGACT]** 915 (85.38)   
326 + **[CCGCGG]** 937 (87.56)   
326 + **.CCGCCG.** 974 (96.68)   
326 + **[CGGACG]** 978 (86.87)   
326 + **[GCGACG]** 999 (85.28)   
326 + **.CCGGCG.** 1061 (94.52)   
326 + **.CCGGCG.** 1093 (94.52)   
326 + **.CCGCCG.** 1141 (96.68)   
326 + **.CCGGCG.** 1163 (94.52)   
326 + **.CCGGCG.** 1248 (94.52)   
326 + **[CCGGGG]** 1269 (85.40)   
326 + **[CCGCGG]** 1302 (87.56)   
326 + **[CCGCGG]** 1335 (87.56)   
326 + **[CCGGGG]** 1350 (85.40)   
326 + **[CCGGGG]** 1365 (85.40)   
326 + **.CCGCCG.** 1383 (96.68)   
326 - **[GCGACG]** 1472 (85.28)   
326 - **.CCGGCG.** 1384 (94.52)   
326 - **[CCCCCG]** 1366 (85.08)   
326 - **.CCGCCG.** 1357 (96.68)   
326 - **[CCCCCG]** 1351 (85.08)   
326 - **.CCGCCG.** 1342 (96.68)   
326 - **[CCGCGG]** 1335 (87.56)   
326 - **.CCGCCG.** 1305 (96.68)   
326 - **[CCGCGG]** 1302 (87.56)   
326 - **[CCCCCG]** 1270 (85.08)   
326 - **.CCGAGG.** 1233 (90.88)   
326 - **.CCGCCG.** 1204 (96.68)   
326 - **.CCGCCG.** 1164 (96.68)   
326 - **.CCGGCG.** 1157 (94.52)   
326 - **[CGGACG]** 1071 (86.87)   
326 - **[CCGAAG]** 1023 (86.09)   
326 - **.CCGGCG.** 975 (94.52)   
326 - **.CCGACG.** 961 (100.00)   
326 - **[CCGACT]** 950 (85.38)   
326 - **[CCGCGG]** 937 (87.56)   
326 - **[CCGGGG]** 926 (85.40)   
326 - **.CCGGCG.** 761 (94.52)   
326 - **[CCGGGG]** 749 (85.40)   
326 - **.CCGCCG.** 707 (96.68)   
326 - **.CCGAGG.** 413 (90.88)   
327 + **[CGGACG]** 237 (86.87)   
327 + **[CCGCGG]** 248 (87.56)   
327 + **.CCGGCG.** 347 (94.52)   
327 + **.CCGCCG.** 397 (96.68)   
327 + **.CCGCCG.** 671 (96.68)   
327 - **.CCGAGG.** 615 (90.88)   
327 - **[CAGACG]** 605 (85.30)   
327 - **.CCGCCG.** 599 (96.68)   
327 - **[CCGACC]** 592 (88.08)   
327 - **[CCGGGG]** 587 (85.40)   
327 - **.CCGAGG.** 551 (90.88)   
327 - **.CCGCCG.** 348 (96.68)   
327 - **.CCGCCG.** 260 (96.68)   
327 - **[CCCCCG]** 251 (85.08)   
327 - **[CCGCGG]** 248 (87.56)   
329 + **[CCGACC]** 6 (88.08)   
329 + **.CCGGCG.** 62 (94.52)   
329 + **.CCGAGG.** 121 (90.88)   
329 + **[CCGGGG]** 232 (85.40)   
329 + **.CCGCCG.** 269 (96.68)   
329 + **.CCGACG.** 272 (100.00)   
329 + **.CCGGCG.** 371 (94.52)   
329 + **[CCGAAG]** 382 (86.09)   
329 + **.CCGCCG.** 394 (96.68)   
329 + **.CCGACG.** 543 (100.00)   
329 + **[CCGACT]** 626 (85.38)   
329 + **[CCCACG]** 893 (88.40)   
329 + **[CCGCGG]** 1232 (87.56)   
329 + **[CGGACG]** 1235 (86.87)   
329 - **[CCCCCG]** 1448 (85.08)   
329 - **[CCGGGG]** 1417 (85.40)   
329 - **[CCGACT]** 1332 (85.38)   
329 - **[CCGCGG]** 1232 (87.56)   
329 - **[CCGGGG]** 601 (85.40)   
329 - **[CCGTCG]** 544 (86.99)   
329 - **.CCGCCG.** 63 (96.68)   
329 - **[CCGGGG]** 60 (85.40)   
329 - **.CCGGCG.** 26 (94.52)   
330 - **[CAGACG]** 1018 (85.30)   
330 - **[CGGACG]** 712 (86.87)   
331 + **[CCCCCG]** 28 (85.08)   
331 + **.CCGGCG.** 50 (94.52)   
331 + **[CCGCGG]** 396 (87.56)   
331 + **[CCGCGG]** 641 (87.56)   
331 + **.CCGCCG.** 716 (96.68)   
331 + **.CCGCCG.** 719 (96.68)   
331 + **[CCGCGG]** 722 (87.56)   
331 + **[CCGGGG]** 752 (85.40)   
331 + **[CCCCCG]** 898 (85.08)   
331 + **[CCCCCG]** 910 (85.08)   
331 + **[CAGACG]** 955 (85.30)   
331 + **.CCGGCG.** 1011 (94.52)   
331 + **[CCGGGG]** 1109 (85.40)   
331 + **[CCGACC]** 1123 (88.08)   
331 + **.CCGCCG.** 1168 (96.68)   
331 + **.CCGCCG.** 1171 (96.68)   
331 + **.CCGCCG.** 1174 (96.68)   
331 + **.CCGGCG.** 1177 (94.52)   
331 + **.CCGCCG.** 1183 (96.68)   
331 + **.CCGCCG.** 1186 (96.68)   
331 + **.CCGCCG.** 1189 (96.68)   
331 + **[CCGTCG]** 1201 (86.99)   
331 + **[CCGGGG]** 1279 (85.40)   
331 + **.CCGCCG.** 1298 (96.68)   
331 + **[CCGACC]** 1325 (88.08)   
331 + **[CCGCGG]** 1331 (87.56)   
331 + **[GCGACG]** 1339 (85.28)   
331 + **.CCGGCG.** 1392 (94.52)   
331 + **[CCGGGG]** 1477 (85.40)   
331 - **.CCGCCG.** 1370 (96.68)   
331 - **.CCGCCG.** 1367 (96.68)   
331 - **.CCGCCG.** 1334 (96.68)   
331 - **[CCGCGG]** 1331 (87.56)   
331 - **[CCGGGG]** 1262 (85.40)   
331 - **[CCGGGG]** 1256 (85.40)   
331 - **[CCGGGG]** 1250 (85.40)   
331 - **[GCGACG]** 1202 (85.28)   
331 - **.CCGGCG.** 1175 (94.52)   
331 - **[CAGACG]** 1142 (85.30)   
331 - **[CCGGGG]** 911 (85.40)   
331 - **[CCGGGG]** 899 (85.40)   
331 - **.CCGGCG.** 758 (94.52)   
331 - **[CCGGGG]** 750 (85.40)   
331 - **[CCGCGG]** 722 (87.56)   
331 - **[CCGCGG]** 641 (87.56)   
331 - **[CGGACG]** 574 (86.87)   
331 - **[CCGCGG]** 396 (87.56)   
331 - **[CCCCCG]** 200 (85.08)   
331 - **[CCCCCG]** 192 (85.08)   
332 + **.CCGAGG.** 5 (90.88)   
332 + **[CCGGGG]** 26 (85.40)   
332 + **[CGGACG]** 88 (86.87)   
332 + **[CCGACC]** 130 (88.08)   
332 + **[CCCCCG]** 140 (85.08)   
332 + **[CCCACG]** 174 (88.40)   
332 + **[CCGAAG]** 382 (86.09)   
332 + **[CCGGGG]** 426 (85.40)   
332 + **[CCCCCG]** 438 (85.08)   
332 + **[CCCACG]** 520 (88.40)   
332 + **[CCCCCG]** 757 (85.08)   
332 + **[CCCCCG]** 764 (85.08)   
332 + **[CCCCCG]** 771 (85.08)   
332 + **[CCCCCG]** 849 (85.08)   
332 + **[CCCCCG]** 858 (85.08)   
332 + **[CCGGGG]** 1170 (85.40)   
332 + **.CCGCCG.** 1235 (96.68)   
332 + **.CCGGCG.** 1243 (94.52)   
332 + **.CCGGCG.** 1340 (94.52)   
332 + **[CCGGGG]** 1385 (85.40)   
332 + **[CCGCGG]** 1429 (87.56)   
332 + **[CCGGGG]** 1479 (85.40)   
332 + **[CCGGGG]** 1500 (85.40)   
332 - **[CCGCGG]** 1429 (87.56)   
332 - **.CCGCCG.** 1341 (96.68)   
332 - **.CCGGCG.** 1338 (94.52)   
332 - **[CCCCCG]** 1255 (85.08)   
332 - **.CCGCCG.** 1244 (96.68)   
332 - **.CCGGCG.** 1236 (94.52)   
332 - **[CCGGGG]** 1168 (85.40)   
332 - **[CCGGGG]** 1028 (85.40)   
332 - **[CCGGGG]** 1012 (85.40)   
332 - **[CAGACG]** 834 (85.30)   
332 - **[CCGGGG]** 758 (85.40)   
332 - **[CCGGGG]** 751 (85.40)   
332 - **[CCCCCG]** 690 (85.08)   
332 - **.CCGGCG.** 524 (94.52)   
332 - **[CCGGGG]** 509 (85.40)   
332 - **[CCCCCG]** 427 (85.08)   
332 - **[CCGGGG]** 141 (85.40)   
332 - **.CCGAGG.** 72 (90.88)   
332 - **[CCGACT]** 46 (85.38)   
333 + **[CCCACG]** 97 (88.40)   
333 + **[CCCCCG]** 149 (85.08)   
333 + **[CCGACC]** 590 (88.08)   
333 + **[CCCCCG]** 987 (85.08)   
333 + **[CCCCCG]** 1006 (85.08)   
333 + **[CCGCGG]** 1029 (87.56)   
333 + **[CCGCGG]** 1123 (87.56)   
333 + **.CCGAGG.** 1155 (90.88)   
333 + **[CCCCCG]** 1178 (85.08)   
333 + **.CCGGCG.** 1232 (94.52)   
333 + **[CCGCGG]** 1242 (87.56)   
333 + **[CCGGGG]** 1258 (85.40)   
333 + **[CCCCCG]** 1309 (85.08)   
333 + **.CCGCCG.** 1319 (96.68)   
333 + **[CCGACC]** 1334 (88.08)   
333 + **.CCGCCG.** 1385 (96.68)   
333 + **[CCCCCG]** 1407 (85.08)   
333 + **[CCCCCG]** 1414 (85.08)   
333 + **.CCGGCG.** 1417 (94.52)   
333 - **[CCGGGG]** 1415 (85.40)   
333 - **.CCGCCG.** 1301 (96.68)   
333 - **[CCCCCG]** 1288 (85.08)   
333 - **[CCCACG]** 1281 (88.40)   
333 - **[CCGCGG]** 1242 (87.56)   
333 - **[CCGGGG]** 1184 (85.40)   
333 - **[CCGCGG]** 1123 (87.56)   
333 - **.CCGCCG.** 1090 (96.68)   
333 - **[CCGCGG]** 1029 (87.56)   
334 + **[CCCCCG]** 431 (85.08)   
334 + **.CCGAGG.** 576 (90.88)   
334 + **.CCGGCG.** 681 (94.52)   
334 + **.CCGAGG.** 730 (90.88)   
334 + **[CCCCCG]** 852 (85.08)   
334 + **[CCGCGG]** 855 (87.56)   
334 + **[CAGACG]** 1066 (85.30)   
334 + **[CCCCCG]** 1081 (85.08)   
334 + **.CCGGCG.** 1204 (94.52)   
334 + **[CCGCGG]** 1245 (87.56)   
334 + **.CCGGCG.** 1287 (94.52)   
334 + **[CCGGGG]** 1368 (85.40)   
334 + **[CCGGGG]** 1439 (85.40)   
334 - **[CCCCCG]** 1369 (85.08)   
334 - **.CCGCCG.** 1288 (96.68)   
334 - **[CCGCGG]** 1245 (87.56)   
334 - **[CCGGGG]** 1082 (85.40)   
334 - **.CCGAGG.** 1033 (90.88)   
334 - **[CCGCGG]** 855 (87.56)   
334 - **[CCCCCG]** 689 (85.08)   
334 - **.CCGGCG.** 524 (94.52)   
334 - **.CCGCCG.** 515 (96.68)   
334 - **[CCGGGG]** 432 (85.40)   
334 - **[CAGACG]** 280 (85.30)   
334 - **[CGGACG]** 40 (86.87)   
335 + **[CCGAAG]** 286 (86.09)   
335 + **.CCGCCG.** 541 (96.68)   
335 + **[CCGTCG]** 707 (86.99)   
335 + **.CCGGCG.** 792 (94.52)   
335 + **.CCGCCG.** 803 (96.68)   
335 + **.CCGGCG.** 832 (94.52)   
335 + **.CCGGCG.** 894 (94.52)   
335 + **[CCCCCG]** 1003 (85.08)   
335 + **[GCGACG]** 1077 (85.28)   
335 + **.CCGAGG.** 1083 (90.88)   
335 + **[CCCACG]** 1147 (88.40)   
335 + **.CCGCCG.** 1186 (96.68)   
335 + **[CCCCCG]** 1201 (85.08)   
335 + **.CCGGCG.** 1300 (94.52)   
335 + **[CCGGGG]** 1333 (85.40)   
335 + **[CCGGGG]** 1425 (85.40)   
335 + **[CAGACG]** 1449 (85.30)   
335 + **.CCGGCG.** 1459 (94.52)   
335 - **[CCGGGG]** 1494 (85.40)   
335 - **[CCCACG]** 1485 (88.40)   
335 - **[CCCCCG]** 1436 (85.08)   
335 - **[CCCCCG]** 1426 (85.08)   
335 - **[CCCCCG]** 1407 (85.08)   
335 - **.CCGCCG.** 1364 (96.68)   
335 - **[CCCCCG]** 1358 (85.08)   
335 - **[CCCCCG]** 1334 (85.08)   
335 - **.CCGCCG.** 1301 (96.68)   
335 - **.CCGAGG.** 1254 (90.88)   
335 - **[GCGACG]** 1207 (85.28)   
335 - **[CCCCCG]** 1090 (85.08)   
335 - **.CCGCCG.** 895 (96.68)   
335 - **.CCGGCG.** 804 (94.52)   
335 - **.CCGGCG.** 790 (94.52)   
335 - **[CCCACG]** 780 (88.40)   
335 - **.CCGACG.** 708 (100.00)   
335 - **.CCGACG.** 661 (100.00)   
335 - **.CCGAGG.** 614 (90.88)   
336 + **[CCGACC]** 149 (88.08)   
336 + **.CCGCCG.** 529 (96.68)   
336 + **.CCGAGG.** 532 (90.88)   
336 + **.CCGCCG.** 601 (96.68)   
336 + **[CCCCCG]** 749 (85.08)   
336 + **[CCGGGG]** 752 (85.40)   
336 + **[CCCACG]** 838 (88.40)   
336 + **[CCGAAG]** 866 (86.09)   
336 + **[CCCCCG]** 884 (85.08)   
336 + **[CCCCCG]** 924 (85.08)   
336 + **[CCGACC]** 950 (88.08)   
336 + **[CCCCCG]** 977 (85.08)   
336 + **[CCCACG]** 1095 (88.40)   
336 + **.CCGAGG.** 1235 (90.88)   
336 + **[CCGACT]** 1270 (85.38)   
336 + **[CCGACT]** 1346 (85.38)   
336 + **.CCGCCG.** 1478 (96.68)   
336 - **[CCCACG]** 1356 (88.40)   
336 - **[CCCCCG]** 1226 (85.08)   
336 - **[CGGACG]** 1074 (86.87)   
336 - **[CGGACG]** 964 (86.87)   
336 - **.CCGCCG.** 824 (96.68)   
336 - **[CCGGGG]** 750 (85.40)   
337 + **.CCGAGG.** 100 (90.88)   
337 + **[CCCCCG]** 838 (85.08)   
337 + **[CCGCGG]** 841 (87.56)   
337 + **.CCGGCG.** 868 (94.52)   
337 + **[CCGCGG]** 921 (87.56)   
337 + **[CCGGGG]** 1035 (85.40)   
337 + **.CCGCCG.** 1054 (96.68)   
337 + **.CCGCCG.** 1063 (96.68)   
337 + **.CCGCCG.** 1066 (96.68)   
337 + **.CCGCCG.** 1069 (96.68)   
337 + **[CCGCGG]** 1072 (87.56)   
337 + **[CCCCCG]** 1090 (85.08)   
337 + **[CGGACG]** 1098 (86.87)   
337 + **[CCGGGG]** 1144 (85.40)   
337 + **[GCGACG]** 1165 (85.28)   
337 + **[CCGCGG]** 1182 (87.56)   
337 + **[CCGGGG]** 1307 (85.40)   
337 + **.CCGGCG.** 1346 (94.52)   
337 + **[CCCCCG]** 1447 (85.08)   
337 + **.CCGAGG.** 1450 (90.88)   
337 - **.CCGGCG.** 1497 (94.52)   
337 - **[CCCCCG]** 1470 (85.08)   
337 - **[CCCCCG]** 1434 (85.08)   
337 - **.CCGCCG.** 1387 (96.68)   
337 - **[CCCACG]** 1338 (88.40)   
337 - **[CGGACG]** 1324 (86.87)   
337 - **[CCCCCG]** 1308 (85.08)   
337 - **.CCGCCG.** 1218 (96.68)   
337 - **[CCGGGG]** 1208 (85.40)   
337 - **[CCGCGG]** 1182 (87.56)   
337 - **[CCGTCG]** 1166 (86.99)   
337 - **[CAGACG]** 1079 (85.30)   
337 - **[CCGCGG]** 1072 (87.56)   
337 - **.CCGCCG.** 958 (96.68)   
337 - **[CCCCCG]** 946 (85.08)   
337 - **[CCGCGG]** 921 (87.56)   
337 - **.CCGCCG.** 872 (96.68)   
337 - **.CCGCCG.** 869 (96.68)   
337 - **.CCGCCG.** 844 (96.68)   
337 - **[CCGCGG]** 841 (87.56)   
337 - **[CGGACG]** 795 (86.87)   
337 - **[CCCACG]** 358 (88.40)   
337 - **[CCGAAG]** 248 (86.09)   
338 + **[CCCACG]** 3 (88.40)   
338 + **[CCCCCG]** 39 (85.08)   
338 + **[CCGCGG]** 42 (87.56)   
338 + **[CCCACG]** 96 (88.40)   
338 + **[CCGACC]** 152 (88.08)   
338 + **[CCGGGG]** 210 (85.40)   
338 + **[CCCCCG]** 331 (85.08)   
338 + **[CCGACT]** 347 (85.38)   
338 + **[CCGCGG]** 439 (87.56)   
338 + **[CCGGGG]** 661 (85.40)   
338 + **[CCGGGG]** 700 (85.40)   
338 + **[CCGGGG]** 716 (85.40)   
338 + **.CCGCCG.** 764 (96.68)   
338 + **.CCGCCG.** 793 (96.68)   
338 + **[CCGACC]** 938 (88.08)   
338 + **[CCCCCG]** 981 (85.08)   
338 + **.CCGACG.** 984 (100.00)   
338 + **.CCGACG.** 1014 (100.00)   
338 + **[CCGCGG]** 1071 (87.56)   
338 + **[CCCCCG]** 1104 (85.08)   
338 + **[CCGGGG]** 1164 (85.40)   
338 + **.CCGCCG.** 1176 (96.68)   
338 + **.CCGCCG.** 1179 (96.68)   
338 + **.CCGCCG.** 1182 (96.68)   
338 + **.CCGCCG.** 1185 (96.68)   
338 + **.CCGCCG.** 1188 (96.68)   
338 + **.CCGCCG.** 1191 (96.68)   
338 + **.CCGCCG.** 1273 (96.68)   
338 + **.CCGCCG.** 1276 (96.68)   
338 + **.CCGGCG.** 1315 (94.52)   
338 + **.CCGGCG.** 1491 (94.52)   
338 - **.CCGCCG.** 1492 (96.68)   
338 - **.CCGAGG.** 1474 (90.88)   
338 - **.CCGCCG.** 1384 (96.68)   
338 - **.CCGCCG.** 1381 (96.68)   
338 - **.CCGCCG.** 1378 (96.68)   
338 - **.CCGCCG.** 1335 (96.68)   
338 - **.CCGCCG.** 1300 (96.68)   
338 - **.CCGGCG.** 1277 (94.52)   
338 - **.CCGCCG.** 1254 (96.68)   
338 - **.CCGCCG.** 1251 (96.68)   
338 - **[CCGGGG]** 1162 (85.40)   
338 - **[CGGACG]** 1156 (86.87)   
338 - **[CCGGGG]** 1105 (85.40)   
338 - **[CCGGGG]** 1079 (85.40)   
338 - **[CCGCGG]** 1071 (87.56)   
338 - **[CGGACG]** 880 (86.87)   
338 - **[CCGGGG]** 845 (85.40)   
338 - **[CCGGGG]** 816 (85.40)   
338 - **.CCGGCG.** 765 (94.52)   
338 - **[CCCCCG]** 701 (85.08)   
338 - **[CAGACG]** 680 (85.30)   
338 - **[CCCCCG]** 654 (85.08)   
338 - **[CAGACG]** 505 (85.30)   
338 - **[CCGACT]** 488 (85.38)   
338 - **[CCGCGG]** 439 (87.56)   
338 - **[CCGACC]** 265 (88.08)   
338 - **.CCGAGG.** 188 (90.88)   
338 - **[CCGCGG]** 42 (87.56)   
339 + **[CCGGGG]** 73 (85.40)   
339 + **[CGGACG]** 832 (86.87)   
339 + **.CCGAGG.** 913 (90.88)   
339 + **.CCGGCG.** 945 (94.52)   
339 + **.CCGGCG.** 1055 (94.52)   
339 + **[CCGGGG]** 1096 (85.40)   
339 + **[CCCCCG]** 1368 (85.08)   
339 + **[CCGGGG]** 1500 (85.40)   
339 - **[CCGGGG]** 1498 (85.40)   
339 - **[CCGGGG]** 1369 (85.40)   
339 - **.CCGAGG.** 1272 (90.88)   
339 - **[CGGACG]** 1171 (86.87)   
339 - **.CCGGCG.** 1163 (94.52)   
339 - **[CCCCCG]** 1115 (85.08)   
339 - **.CCGCCG.** 1078 (96.68)   
339 - **.CCGCCG.** 1069 (96.68)   
339 - **.CCGCCG.** 1066 (96.68)   
339 - **.CCGCCG.** 946 (96.68)   
339 - **[CCCACG]** 619 (88.40)   
340 + **[CCGGGG]** 10 (85.40)   
340 + **[CCGACT]** 185 (85.38)   
340 + **[CCGGGG]** 200 (85.40)   
340 + **[CCCCCG]** 311 (85.08)   
340 + **.CCGGCG.** 694 (94.52)   
340 + **.CCGCCG.** 757 (96.68)   
340 + **[CCGGGG]** 896 (85.40)   
340 + **[CCGGGG]** 1108 (85.40)   
340 + **.CCGAGG.** 1333 (90.88)   
340 + **[CCGGGG]** 1478 (85.40)   
340 - **[CCCCCG]** 1388 (85.08)   
340 - **.CCGAGG.** 1385 (90.88)   
340 - **[CCCCCG]** 1244 (85.08)   
340 - **[CGGACG]** 1203 (86.87)   
340 - **[GCGACG]** 685 (85.28)   
340 - **[CCGGGG]** 655 (85.40)   
340 - **[CCGACT]** 608 (85.38)   
340 - **[CCCACG]** 576 (88.40)   
340 - **[CCGGGG]** 8 (85.40)   
341 + **[CCGCGG]** 155 (87.56)   
341 + **.CCGAGG.** 421 (90.88)   
341 + **[CCCACG]** 429 (88.40)   
341 + **[CCGAAG]** 494 (86.09)   
341 + **.CCGAGG.** 580 (90.88)   
341 + **[CCGAAG]** 613 (86.09)   
341 + **.CCGGCG.** 667 (94.52)   
341 + **[CCGAAG]** 879 (86.09)   
341 + **.CCGAGG.** 925 (90.88)   
341 + **.CCGGCG.** 1056 (94.52)   
341 + **[CCGAAG]** 1076 (86.09)   
341 + **[CCGAAG]** 1098 (86.09)   
341 + **[CCGGGG]** 1226 (85.40)   
341 + **[CCCCCG]** 1276 (85.08)   
341 + **.CCGCCG.** 1382 (96.68)   
341 + **[CCCACG]** 1461 (88.40)   
341 - **[CCCCCG]** 1487 (85.08)   
341 - **.CCGCCG.** 1442 (96.68)   
341 - **.CCGCCG.** 1411 (96.68)   
341 - **[GCGACG]** 1262 (85.28)   
341 - **.CCGCCG.** 1086 (96.68)   
341 - **.CCGCCG.** 1060 (96.68)   
341 - **.CCGCCG.** 1057 (96.68)   
341 - **.CCGGCG.** 1054 (94.52)   
341 - **[CCCCCG]** 948 (85.08)   
341 - **[CCGAAG]** 933 (86.09)   
341 - **[CCGACT]** 840 (85.38)   
341 - **.CCGCCG.** 668 (96.68)   
341 - **[CCGGGG]** 452 (85.40)   
341 - **[CCGCGG]** 155 (87.56)   
342 + **.CCGAGG.** 329 (90.88)   
342 + **[CCGGGG]** 550 (85.40)   
342 + **[CCGGGG]** 649 (85.40)   
342 + **[CCGGGG]** 701 (85.40)   
342 + **[CCGAAG]** 824 (86.09)   
342 + **.CCGCCG.** 910 (96.68)   
342 + **[CCGGGG]** 991 (85.40)   
342 + **.CCGCCG.** 1106 (96.68)   
342 + **[CCGGGG]** 1340 (85.40)   
342 + **[CCGCGG]** 1414 (87.56)   
342 - **.CCGAGG.** 1508 (90.88)   
342 - **[CCCCCG]** 1502 (85.08)   
342 - **[CCCCCG]** 1490 (85.08)   
342 - **[CCGCGG]** 1414 (87.56)   
342 - **[CCCCCG]** 1355 (85.08)   
342 - **[CCGAAG]** 1229 (86.09)   
342 - **[CCCCCG]** 1193 (85.08)   
342 - **[CCGGGG]** 1163 (85.40)   
342 - **.CCGCCG.** 1157 (96.68)   
342 - **[CCCCCG]** 992 (85.08)   
342 - **.CCGGCG.** 911 (94.52)   
342 - **.CCGAGG.** 832 (90.88)   
342 - **[CCCCCG]** 742 (85.08)   
342 - **.CCGCCG.** 693 (96.68)   
342 - **[CCCCCG]** 230 (85.08)   
343 + **.CCGAGG.** 37 (90.88)   
343 + **.CCGAGG.** 385 (90.88)   
343 + **[CCGGGG]** 489 (85.40)   
343 + **[CGGACG]** 668 (86.87)   
343 + **[CCGGGG]** 688 (85.40)   
343 + **[GCGACG]** 782 (85.28)   
343 + **.CCGGCG.** 803 (94.52)   
343 + **[CCGCGG]** 899 (87.56)   
343 + **.CCGCCG.** 1025 (96.68)   
343 + **.CCGCCG.** 1028 (96.68)   
343 + **[CCGCGG]** 1121 (87.56)   
343 + **[CCGGGG]** 1175 (85.40)   
343 + **[CCGTCG]** 1221 (86.99)   
343 + **[CCGGGG]** 1233 (85.40)   
343 + **[CCGGGG]** 1268 (85.40)   
343 + **[CCGCGG]** 1306 (87.56)   
343 - **[CCGCGG]** 1306 (87.56)   
343 - **[CAGACG]** 1252 (85.30)   
343 - **[CCGGGG]** 1231 (85.40)   
343 - **.CCGACG.** 1222 (100.00)   
343 - **.CCGCCG.** 1184 (96.68)   
343 - **[CCCCCG]** 1176 (85.08)   
343 - **[CCGCGG]** 1121 (87.56)   
343 - **.CCGGCG.** 1069 (94.52)   
343 - **.CCGGCG.** 1029 (94.52)   
343 - **.CCGGCG.** 1016 (94.52)   
343 - **[CCCACG]** 1003 (88.40)   
343 - **[CAGACG]** 973 (85.30)   
343 - **.CCGAGG.** 938 (90.88)   
343 - **[CCGCGG]** 899 (87.56)   
343 - **.CCGCCG.** 862 (96.68)   
343 - **[CCGGGG]** 755 (85.40)   
343 - **[CCCCCG]** 674 (85.08)   
343 - **[CCGGGG]** 623 (85.40)   
343 - **[CCGACT]** 518 (85.38)   
343 - **[CCGGGG]** 284 (85.40)   
343 - **[CCGACC]** 90 (88.08)   
344 + **[CCGGGG]** 71 (85.40)   
344 + **[CCCCCG]** 653 (85.08)   
344 + **.CCGCCG.** 656 (96.68)   
344 + **[CCGGGG]** 659 (85.40)   
344 + **[CCCCCG]** 748 (85.08)   
344 + **[CCGACC]** 751 (88.08)   
344 + **[CCCCCG]** 773 (85.08)   
344 + **.CCGCCG.** 780 (96.68)   
344 + **.CCGCCG.** 800 (96.68)   
344 + **.CCGAGG.** 854 (90.88)   
344 + **[CCCCCG]** 955 (85.08)   
344 + **[CCGGGG]** 1007 (85.40)   
344 + **.CCGCCG.** 1068 (96.68)   
344 - **[CCGGGG]** 1403 (85.40)   
344 - **.CCGGCG.** 801 (94.52)   
344 - **[CCGGGG]** 774 (85.40)   
344 - **.CCGGCG.** 657 (94.52)   
345 - **[CCCCCG]** 1147 (85.08)   
345 - **[CCCACG]** 1108 (88.40)   
345 - **.CCGAGG.** 458 (90.88)   
346 + **[CCGCGG]** 84 (87.56)   
346 + **.CCGAGG.** 202 (90.88)   
346 + **[CCGGGG]** 210 (85.40)   
346 + **[CCGTCG]** 279 (86.99)   
346 + **.CCGGCG.** 601 (94.52)   
346 + **.CCGCCG.** 819 (96.68)   
346 + **[CCGGGG]** 827 (85.40)   
346 + **[CCGCGG]** 907 (87.56)   
346 + **[CCCCCG]** 953 (85.08)   
346 + **.CCGCCG.** 999 (96.68)   
346 + **.CCGCCG.** 1022 (96.68)   
346 + **[CCCACG]** 1098 (88.40)   
346 + **[CCGACT]** 1151 (85.38)   
346 + **.CCGGCG.** 1227 (94.52)   
346 + **.CCGCCG.** 1235 (96.68)   
346 + **.CCGGCG.** 1262 (94.52)   
346 + **[CCCCCG]** 1324 (85.08)   
346 + **[CCGCGG]** 1408 (87.56)   
346 - **[CCGCGG]** 1408 (87.56)   
346 - **.CCGCCG.** 1380 (96.68)   
346 - **[CCGGGG]** 1269 (85.40)   
346 - **.CCGCCG.** 1263 (96.68)   
346 - **.CCGGCG.** 1000 (94.52)   
346 - **[CCGCGG]** 907 (87.56)   
346 - **[CCCCCG]** 866 (85.08)   
346 - **[CCCCCG]** 859 (85.08)   
346 - **[CCCCCG]** 764 (85.08)   
346 - **[CCCACG]** 741 (88.40)   
346 - **[CCCCCG]** 560 (85.08)   
346 - **[GCGACG]** 546 (85.28)   
346 - **.CCGCCG.** 327 (96.68)   
346 - **[GCGACG]** 280 (85.28)   
346 - **[CCCCCG]** 211 (85.08)   
346 - **.CCGAGG.** 194 (90.88)   
346 - **.CCGGCG.** 98 (94.52)   
346 - **.CCGCCG.** 87 (96.68)   
346 - **[CCGCGG]** 84 (87.56)   
346 - **[CCCACG]** 8 (88.40)   
347 + **[CCGGGG]** 67 (85.40)   
347 + **[CCGGGG]** 114 (85.40)   
347 + **[CCGCGG]** 397 (87.56)   
347 + **[CCGGGG]** 460 (85.40)   
347 + **[CCGACT]** 667 (85.38)   
347 + **[CAGACG]** 733 (85.30)   
347 + **[CCGCGG]** 812 (87.56)   
347 + **.CCGGCG.** 953 (94.52)   
347 + **[CCGGGG]** 1035 (85.40)   
347 + **[CCGGGG]** 1047 (85.40)   
347 + **.CCGCCG.** 1180 (96.68)   
347 + **[CCGCGG]** 1285 (87.56)   
347 + **[CCCCCG]** 1301 (85.08)   
347 + **.CCGGCG.** 1304 (94.52)   
347 + **.CCGACG.** 1380 (100.00)   
347 + **[CCGACC]** 1443 (88.08)   
347 + **[CAGACG]** 1451 (85.30)   
347 + **[CCGCGG]** 1466 (87.56)   
347 + **[CCCCCG]** 1490 (85.08)   
347 + **.CCGCCG.** 1512 (96.68)   
347 - **[CCGCGG]** 1466 (87.56)   
347 - **[CCGAAG]** 1411 (86.09)   
347 - **[CCCACG]** 1353 (88.40)   
347 - **[CCGGGG]** 1302 (85.40)   
347 - **[CCGCGG]** 1285 (87.56)   
347 - **.CCGCCG.** 1227 (96.68)   
347 - **.CCGGCG.** 1121 (94.52)   
347 - **.CCGCCG.** 1109 (96.68)   
347 - **.CCGACG.** 1097 (100.00)   
347 - **[CCGACC]** 1074 (88.08)   
347 - **.CCGGCG.** 1070 (94.52)   
347 - **[CCCCCG]** 1036 (85.08)   
347 - **[CCCCCG]** 1013 (85.08)   
347 - **.CCGCCG.** 954 (96.68)   
347 - **[CCCCCG]** 815 (85.08)   
347 - **[CCGCGG]** 812 (87.56)   
347 - **[CCGACC]** 573 (88.08)   
347 - **[CCCACG]** 513 (88.40)   
347 - **[CCGCGG]** 397 (87.56)   
348 + **.CCGCCG.** 25 (96.68)   
348 + **[CCGTCG]** 28 (86.99)   
348 + **[CCGAAG]** 37 (86.09)   
348 + **[CCGCGG]** 70 (87.56)   
348 + **.CCGAGG.** 85 (90.88)   
348 + **[CCGCGG]** 136 (87.56)   
348 + **.CCGAGG.** 216 (90.88)   
348 + **[CCGGGG]** 258 (85.40)   
348 + **.CCGCCG.** 288 (96.68)   
348 + **.CCGGCG.** 291 (94.52)   
348 + **.CCGCCG.** 330 (96.68)   
348 + **[CCGTCG]** 333 (86.99)   
348 + **.CCGCCG.** 434 (96.68)   
348 + **.CCGCCG.** 447 (96.68)   
348 + **[CAGACG]** 517 (85.30)   
348 + **.CCGAGG.** 602 (90.88)   
348 + **[CCGTCG]** 685 (86.99)   
348 + **[CCGTCG]** 691 (86.99)   
348 + **[CCGCGG]** 711 (87.56)   
348 + **.CCGCCG.** 719 (96.68)   
348 + **[CCCCCG]** 769 (85.08)   
348 + **.CCGGCG.** 772 (94.52)   
348 + **[CCCCCG]** 826 (85.08)   
348 + **.CCGGCG.** 860 (94.52)   
348 + **.CCGACG.** 883 (100.00)   
348 + **[CCGTCG]** 904 (86.99)   
348 + **[CCGTCG]** 924 (86.99)   
348 + **.CCGAGG.** 951 (90.88)   
348 + **.CCGCCG.** 1047 (96.68)   
348 + **.CCGCCG.** 1055 (96.68)   
348 + **.CCGACG.** 1097 (100.00)   
348 + **[CCGCGG]** 1155 (87.56)   
348 + **[CCGGGG]** 1185 (85.40)   
348 + **[CCGGGG]** 1229 (85.40)   
348 + **[CCGCGG]** 1257 (87.56)   
348 + **.CCGCCG.** 1493 (96.68)   
348 + **.CCGGCG.** 1501 (94.52)   
348 - **[CCGGGG]** 1499 (85.40)   
348 - **[CCCACG]** 1482 (88.40)   
348 - **[CCGGGG]** 1469 (85.40)   
348 - **[CCCCCG]** 1432 (85.08)   
348 - **[CCGGGG]** 1423 (85.40)   
348 - **[CCCACG]** 1416 (88.40)   
348 - **[CCGCGG]** 1257 (87.56)   
348 - **.CCGCCG.** 1166 (96.68)   
348 - **[CCGCGG]** 1155 (87.56)   
348 - **[CCGTCG]** 1098 (86.99)   
348 - **.CCGGCG.** 1056 (94.52)   
348 - **.CCGCCG.** 1030 (96.68)   
348 - **.CCGGCG.** 978 (94.52)   
348 - **.CCGGCG.** 928 (94.52)   
348 - **[GCGACG]** 925 (85.28)   
348 - **[GCGACG]** 905 (85.28)   
348 - **[CGGACG]** 876 (86.87)   
348 - **[CCGGGG]** 858 (85.40)   
348 - **[CCGGGG]** 827 (85.40)   
348 - **[CCGGGG]** 770 (85.40)   
348 - **[CCGCGG]** 711 (87.56)   
348 - **[GCGACG]** 692 (85.28)   
348 - **[GCGACG]** 686 (85.28)   
348 - **[CGGACG]** 677 (86.87)   
348 - **[CCCACG]** 669 (88.40)   
348 - **[CCCCCG]** 654 (85.08)   
348 - **[CCGGGG]** 362 (85.40)   
348 - **.CCGACG.** 334 (100.00)   
348 - **[CCCACG]** 303 (88.40)   
348 - **.CCGGCG.** 289 (94.52)   
348 - **[CCGGGG]** 209 (85.40)   
348 - **[CGGACG]** 182 (86.87)   
348 - **[CCCCCG]** 145 (85.08)   
348 - **[CCGCGG]** 136 (87.56)   
348 - **[CCGCGG]** 70 (87.56)   
348 - **[GCGACG]** 29 (85.28)   
349 + **[CGGACG]** 487 (86.87)   
349 + **[GCGACG]** 654 (85.28)   
349 + **.CCGAGG.** 660 (90.88)   
349 + **.CCGCCG.** 746 (96.68)   
349 + **.CCGCCG.** 749 (96.68)   
349 + **.CCGCCG.** 818 (96.68)   
349 + **.CCGCCG.** 832 (96.68)   
349 + **.CCGGCG.** 835 (94.52)   
349 + **[CCGGGG]** 851 (85.40)   
349 + **.CCGGCG.** 979 (94.52)   
349 + **.CCGAGG.** 999 (90.88)   
349 + **[CGGACG]** 1019 (86.87)   
349 + **[CCGGGG]** 1085 (85.40)   
349 + **.CCGGCG.** 1161 (94.52)   
349 + **[CCGCGG]** 1285 (87.56)   
349 + **[CCGGGG]** 1301 (85.40)   
349 + **[CCGCGG]** 1328 (87.56)   
349 + **.CCGGCG.** 1447 (94.52)   
349 - **[CCGGGG]** 1382 (85.40)   
349 - **[CCGCGG]** 1328 (87.56)   
349 - **.CCGGCG.** 1299 (94.52)   
349 - **[CCGCGG]** 1285 (87.56)   
349 - **.CCGCCG.** 1260 (96.68)   
349 - **[CCCACG]** 1251 (88.40)   
349 - **[CCGGGG]** 1226 (85.40)   
349 - **[CCCCCG]** 1199 (85.08)   
349 - **[CCCCCG]** 1183 (85.08)   
349 - **.CCGCCG.** 1167 (96.68)   
349 - **[CCCCCG]** 1135 (85.08)   
349 - **[CCCCCG]** 1102 (85.08)   
349 - **[CCCCCG]** 1086 (85.08)   
349 - **.CCGGCG.** 1068 (94.52)   
349 - **.CCGCCG.** 980 (96.68)   
349 - **.CCGCCG.** 936 (96.68)   
349 - **[CCCCCG]** 841 (85.08)   
349 - **.CCGGCG.** 833 (94.52)   
349 - **.CCGGCG.** 819 (94.52)   
349 - **.CCGAGG.** 591 (90.88)   
349 - **[CAGACG]** 184 (85.30)   
350 + **.CCGAGG.** 54 (90.88)   
350 + **[CCGGGG]** 608 (85.40)   
350 + **.CCGGCG.** 1042 (94.52)   
350 + **.CCGAGG.** 1066 (90.88)   
350 + **.CCGCCG.** 1137 (96.68)   
350 + **[CCGGGG]** 1164 (85.40)   
350 + **[CCCCCG]** 1194 (85.08)   
350 + **[CCCCCG]** 1207 (85.08)   
350 + **[CCGACC]** 1226 (88.08)   
350 + **[CCGGGG]** 1231 (85.40)   
350 + **.CCGGCG.** 1341 (94.52)   
350 + **[CCGACT]** 1350 (85.38)   
350 + **.CCGAGG.** 1390 (90.88)   
350 - **.CCGGCG.** 1445 (94.52)   
350 - **.CCGGCG.** 1382 (94.52)   
350 - **[CCGGGG]** 1339 (85.40)   
350 - **[CCCACG]** 1327 (88.40)   
350 - **[CCGGGG]** 1287 (85.40)   
350 - **.CCGCCG.** 1275 (96.68)   
350 - **[CCCCCG]** 1165 (85.08)   
350 - **.CCGAGG.** 1143 (90.88)   
350 - **.CCGCCG.** 1030 (96.68)   
350 - **[CCCCCG]** 1003 (85.08)   
350 - **[GCGACG]** 998 (85.28)   
350 - **[CCCCCG]** 609 (85.08)   
351 + **.CCGAGG.** 39 (90.88)   
351 + **.CCGAGG.** 197 (90.88)   
351 + **[CCCCCG]** 259 (85.08)   
351 + **[CGGACG]** 291 (86.87)   
351 + **.CCGCCG.** 569 (96.68)   
351 + **.CCGAGG.** 572 (90.88)   
351 + **.CCGCCG.** 761 (96.68)   
351 + **[CCGTCG]** 913 (86.99)   
351 + **[CCGCGG]** 938 (87.56)   
351 + **.CCGGCG.** 990 (94.52)   
351 + **[CGGACG]** 1034 (86.87)   
351 + **.CCGCCG.** 1058 (96.68)   
351 + **[CCGCGG]** 1184 (87.56)   
351 + **[CCGGGG]** 1204 (85.40)   
351 + **[CCGCGG]** 1254 (87.56)   
351 + **.CCGAGG.** 1318 (90.88)   
351 + **.CCGCCG.** 1340 (96.68)   
351 + **[CCCCCG]** 1433 (85.08)   
351 + **.CCGAGG.** 1489 (90.88)   
351 - **[CCGGGG]** 1434 (85.40)   
351 - **[CCGCGG]** 1254 (87.56)   
351 - **[CCGCGG]** 1184 (87.56)   
351 - **[CAGACG]** 1143 (85.30)   
351 - **.CCGGCG.** 1059 (94.52)   
351 - **[CCGCGG]** 938 (87.56)   
351 - **[GCGACG]** 914 (85.28)   
351 - **[CCGACT]** 818 (85.38)   
351 - **[CCGGGG]** 614 (85.40)   
351 - **[CCGACC]** 529 (88.08)   
351 - **[CCCCCG]** 501 (85.08)   
352 + **.CCGGCG.** 169 (94.52)   
352 + **.CCGGCG.** 176 (94.52)   
352 + **[CCGCGG]** 197 (87.56)   
352 + **[GCGACG]** 280 (85.28)   
352 + **[CCGCGG]** 311 (87.56)   
352 + **[CCCCCG]** 342 (85.08)   
352 + **[CCCCCG]** 348 (85.08)   
352 + **.CCGCCG.** 351 (96.68)   
352 + **[CCGCGG]** 354 (87.56)   
352 + **[CCGAAG]** 413 (86.09)   
352 + **.CCGGCG.** 498 (94.52)   
352 + **[CCGCGG]** 620 (87.56)   
352 + **.CCGCCG.** 629 (96.68)   
352 + **.CCGAGG.** 749 (90.88)   
352 + **.CCGCCG.** 863 (96.68)   
352 + **[CCCCCG]** 928 (85.08)   
352 + **[CCGCGG]** 941 (87.56)   
352 + **[CCGCGG]** 953 (87.56)   
352 + **.CCGCCG.** 994 (96.68)   
352 + **.CCGCCG.** 997 (96.68)   
352 + **[CCGCGG]** 1019 (87.56)   
352 + **.CCGAGG.** 1060 (90.88)   
352 + **[CCGTCG]** 1158 (86.99)   
352 + **[CCGAAG]** 1241 (86.09)   
352 + **[CCGGGG]** 1267 (85.40)   
352 + **.CCGGCG.** 1285 (94.52)   
352 + **[CCGCGG]** 1339 (87.56)   
352 - **[CCGAAG]** 1397 (86.09)   
352 - **[CCGAAG]** 1391 (86.09)   
352 - **[CAGACG]** 1365 (85.30)   
352 - **[CCGCGG]** 1339 (87.56)   
352 - **.CCGCCG.** 1286 (96.68)   
352 - **.CCGACG.** 1159 (100.00)   
352 - **[CCGCGG]** 1019 (87.56)   
352 - **[CCGCGG]** 953 (87.56)   
352 - **[CCGCGG]** 941 (87.56)   
352 - **[CCGGGG]** 917 (85.40)   
352 - **.CCGGCG.** 864 (94.52)   
352 - **.CCGCCG.** 851 (96.68)   
352 - **.CCGGCG.** 720 (94.52)   
352 - **[CCGCGG]** 620 (87.56)   
352 - **[CCGCGG]** 354 (87.56)   
352 - **.CCGCCG.** 314 (96.68)   
352 - **[CCGCGG]** 311 (87.56)   
352 - **[CCGCGG]** 197 (87.56)   
352 - **.CCGAGG.** 114 (90.88)   
352 - **[CCGGGG]** 28 (85.40)   
352 - **.CCGAGG.** 9 (90.88)   
353 + **[CCCACG]** 250 (88.40)   
353 + **[GCGACG]** 292 (85.28)   
353 + **.CCGAGG.** 349 (90.88)   
353 + **[CCCACG]** 579 (88.40)   
353 + **[CCCCCG]** 608 (85.08)   
353 + **.CCGCCG.** 689 (96.68)   
353 + **[CGGACG]** 706 (86.87)   
353 + **.CCGCCG.** 732 (96.68)   
353 + **.CCGCCG.** 735 (96.68)   
353 + **.CCGCCG.** 738 (96.68)   
353 + **.CCGCCG.** 741 (96.68)   
353 + **.CCGCCG.** 744 (96.68)   
353 + **.CCGACG.** 773 (100.00)   
353 + **[CCGTCG]** 872 (86.99)   
353 + **[CCGCGG]** 882 (87.56)   
353 + **[CCCCCG]** 900 (85.08)   
353 + **[CCGGGG]** 923 (85.40)   
353 + **[CCGTCG]** 964 (86.99)   
353 + **.CCGAGG.** 1057 (90.88)   
353 + **.CCGAGG.** 1097 (90.88)   
353 + **[CCGGGG]** 1166 (85.40)   
353 + **[CGGACG]** 1393 (86.87)   
353 + **[CCGCGG]** 1437 (87.56)   
353 - **.CCGCCG.** 1440 (96.68)   
353 - **[CCGCGG]** 1437 (87.56)   
353 - **[CCCCCG]** 1425 (85.08)   
353 - **.CCGCCG.** 1422 (96.68)   
353 - **[CCCCCG]** 1378 (85.08)   
353 - **[CCGACT]** 1019 (85.38)   
353 - **[GCGACG]** 965 (85.28)   
353 - **[CCGCGG]** 882 (87.56)   
353 - **[GCGACG]** 873 (85.28)   
353 - **[CCCACG]** 821 (88.40)   
353 - **.CCGCCG.** 777 (96.68)   
353 - **[CCGTCG]** 774 (86.99)   
353 - **.CCGAGG.** 703 (90.88)   
353 - **.CCGGCG.** 665 (94.52)   
353 - **[CCGGGG]** 609 (85.40)   
353 - **[CCCCCG]** 364 (85.08)   
353 - **[CCCCCG]** 144 (85.08)   
354 + **[CGGACG]** 1186 (86.87)   
354 - **.CCGAGG.** 1202 (90.88)   
356 + **[CCGGGG]** 379 (85.40)   
356 + **[CCCCCG]** 437 (85.08)   
356 + **[CCGACT]** 440 (85.38)   
356 + **[CCGACC]** 478 (88.08)   
356 + **.CCGGCG.** 509 (94.52)   
356 + **[CCGACC]** 515 (88.08)   
356 + **[CCCCCG]** 549 (85.08)   
356 + **.CCGCCG.** 552 (96.68)   
356 + **[CCCACG]** 596 (88.40)   
356 + **[CCGACT]** 681 (85.38)   
356 + **[CCGACC]** 689 (88.08)   
356 + **[CCGACC]** 705 (88.08)   
356 + **[CCCCCG]** 709 (85.08)   
356 + **.CCGCCG.** 782 (96.68)   
356 + **[CCGGGG]** 988 (85.40)   
356 + **.CCGCCG.** 1004 (96.68)   
356 + **[CCGGGG]** 1025 (85.40)   
356 + **[CAGACG]** 1160 (85.30)   
356 + **[CCCACG]** 1283 (88.40)   
356 + **[CCGGGG]** 1304 (85.40)   
356 - **[CCCCCG]** 1144 (85.08)   
356 - **.CCGCCG.** 1125 (96.68)   
356 - **.CCGGCG.** 1005 (94.52)   
356 - **[CCCACG]** 751 (88.40)   
356 - **[CCGGGG]** 710 (85.40)   
356 - **[CCGGGG]** 693 (85.40)   
356 - **[CCGGGG]** 582 (85.40)   
356 - **[CCGTCG]** 428 (86.99)   
356 - **.CCGGCG.** 377 (94.52)   
356 - **[CCGGGG]** 269 (85.40)   
356 - **.CCGCCG.** 177 (96.68)   
356 - **.CCGCCG.** 174 (96.68)   
356 - **.CCGCCG.** 171 (96.68)   
356 - **.CCGAGG.** 130 (90.88)   
356 - **[CCGACC]** 111 (88.08)   
356 - **[CCGACC]** 107 (88.08)   
357 + **[CCCACG]** 225 (88.40)   
357 + **[CCCCCG]** 307 (85.08)   
357 + **[CCGGGG]** 430 (85.40)   
357 + **[CCCACG]** 496 (88.40)   
357 + **[CCGACC]** 538 (88.08)   
357 + **.CCGAGG.** 691 (90.88)   
357 + **.CCGGCG.** 737 (94.52)   
357 + **.CCGAGG.** 754 (90.88)   
357 + **.CCGGCG.** 822 (94.52)   
357 + **.CCGGCG.** 847 (94.52)   
357 + **[CCGGGG]** 853 (85.40)   
357 + **.CCGCCG.** 989 (96.68)   
357 + **.CCGCCG.** 1070 (96.68)   
357 + **.CCGAGG.** 1169 (90.88)   
357 + **[CCGCGG]** 1220 (87.56)   
357 + **[CCGGGG]** 1253 (85.40)   
357 + **[CCGCGG]** 1281 (87.56)   
357 + **[CCGGGG]** 1315 (85.40)   
357 + **[CCGGGG]** 1437 (85.40)   
357 + **[CCGCGG]** 1446 (87.56)   
357 + **.CCGCCG.** 1505 (96.68)   
357 - **[CCCCCG]** 1449 (85.08)   
357 - **[CCGCGG]** 1446 (87.56)   
357 - **[CCCACG]** 1431 (88.40)   
357 - **[CCCACG]** 1309 (88.40)   
357 - **[CCGCGG]** 1281 (87.56)   
357 - **[CCCACG]** 1247 (88.40)   
357 - **[CCGCGG]** 1220 (87.56)   
357 - **[CCCCCG]** 1119 (85.08)   
357 - **.CCGCCG.** 952 (96.68)   
357 - **[CCCACG]** 871 (88.40)   
357 - **.CCGGCG.** 851 (94.52)   
357 - **.CCGCCG.** 823 (96.68)   
357 - **[CCGGGG]** 820 (85.40)   
357 - **[CCGACC]** 763 (88.08)   
357 - **[CCCCCG]** 741 (85.08)   
357 - **.CCGCCG.** 738 (96.68)   
357 - **.CCGGCG.** 735 (94.52)   
357 - **[CCCCCG]** 663 (85.08)   
357 - **[CCCACG]** 531 (88.40)   
357 - **[CCCCCG]** 431 (85.08)   
357 - **.CCGCCG.** 420 (96.68)   
357 - **[CCGGGG]** 344 (85.40)   
357 - **[CCGGGG]** 308 (85.40)   
357 - **[CAGACG]** 283 (85.30)   
357 - **.CCGCCG.** 203 (96.68)   
357 - **.CCGAGG.** 96 (90.88)   
358 + **[CCGCGG]** 406 (87.56)   
358 + **[CCGCGG]** 773 (87.56)   
358 + **[CCGCGG]** 811 (87.56)   
358 + **.CCGACG.** 874 (100.00)   
358 + **.CCGAGG.** 1116 (90.88)   
358 + **[CCGGGG]** 1157 (85.40)   
358 + **[CCGTCG]** 1249 (86.99)   
358 - **[GCGACG]** 1250 (85.28)   
358 - **[CCGACC]** 1205 (88.08)   
358 - **[CCCCCG]** 1158 (85.08)   
358 - **.CCGCCG.** 1055 (96.68)   
358 - **[CCGACC]** 1010 (88.08)   
358 - **.CCGCCG.** 980 (96.68)   
358 - **[CCGTCG]** 911 (86.99)   
358 - **[CCGTCG]** 875 (86.99)   
358 - **[CCGCGG]** 811 (87.56)   
358 - **[CCGCGG]** 773 (87.56)   
358 - **[CCGAAG]** 499 (86.09)   
358 - **.CCGAGG.** 414 (90.88)   
358 - **[CCGCGG]** 406 (87.56)   
359 + **[CCGGGG]** 42 (85.40)   
359 + **[CCGGGG]** 110 (85.40)   
359 + **[CCGAAG]** 342 (86.09)   
359 + **[CCGGGG]** 514 (85.40)   
359 + **[CCCCCG]** 535 (85.08)   
359 + **[CCGCGG]** 544 (87.56)   
359 + **[CGGACG]** 572 (86.87)   
359 + **[CCGGGG]** 792 (85.40)   
359 + **[CCGGGG]** 801 (85.40)   
359 + **[CCGGGG]** 960 (85.40)   
359 + **.CCGCCG.** 975 (96.68)   
359 + **.CCGCCG.** 983 (96.68)   
359 + **.CCGCCG.** 986 (96.68)   
359 + **.CCGCCG.** 1024 (96.68)   
359 + **.CCGCCG.** 1027 (96.68)   
359 + **.CCGCCG.** 1030 (96.68)   
359 + **.CCGCCG.** 1054 (96.68)   
359 + **.CCGCCG.** 1057 (96.68)   
359 + **.CCGCCG.** 1060 (96.68)   
359 + **[CCCCCG]** 1106 (85.08)   
359 + **.CCGCCG.** 1120 (96.68)   
359 + **.CCGCCG.** 1123 (96.68)   
359 + **.CCGCCG.** 1126 (96.68)   
359 + **.CCGGCG.** 1163 (94.52)   
359 + **[CCCCCG]** 1169 (85.08)   
359 + **[CCGCGG]** 1233 (87.56)   
359 + **.CCGGCG.** 1311 (94.52)   
359 + **[CCCCCG]** 1388 (85.08)   
359 - **[CCCCCG]** 1461 (85.08)   
359 - **[CCGGGG]** 1389 (85.40)   
359 - **.CCGGCG.** 1309 (94.52)   
359 - **.CCGCCG.** 1257 (96.68)   
359 - **[CCGCGG]** 1233 (87.56)   
359 - **[CCGGGG]** 1175 (85.40)   
359 - **.CCGGCG.** 799 (94.52)   
359 - **[CCCCCG]** 653 (85.08)   
359 - **[CCCCCG]** 636 (85.08)   
359 - **[CCCCCG]** 576 (85.08)   
359 - **[CCGCGG]** 544 (87.56)   
359 - **[CCGGGG]** 536 (85.40)   
359 - **[CCCCCG]** 515 (85.08)   
359 - **[CCGGGG]** 262 (85.40)   
359 - **[CCGGGG]** 108 (85.40)   
359 - **[CCCCCG]** 43 (85.08)   
360 + **[CCCCCG]** 89 (85.08)   
360 + **[CCGGGG]** 193 (85.40)   
360 + **.CCGAGG.** 231 (90.88)   
360 + **[CCGGGG]** 536 (85.40)   
360 + **[CCGACC]** 592 (88.08)   
360 + **.CCGGCG.** 596 (94.52)   
360 + **[CCCCCG]** 622 (85.08)   
360 + **[CCCCCG]** 701 (85.08)   
360 + **.CCGCCG.** 769 (96.68)   
360 + **[CCCCCG]** 788 (85.08)   
360 + **[CCGCGG]** 849 (87.56)   
360 + **[CCCACG]** 923 (88.40)   
360 + **[CCGCGG]** 1072 (87.56)   
360 + **[CCGCGG]** 1126 (87.56)   
360 - **[CCCCCG]** 1369 (85.08)   
360 - **[CCGCGG]** 1126 (87.56)   
360 - **[CCCCCG]** 1075 (85.08)   
360 - **[CCGCGG]** 1072 (87.56)   
360 - **.CCGCCG.** 975 (96.68)   
360 - **[CCCCCG]** 881 (85.08)   
360 - **.CCGGCG.** 869 (94.52)   
360 - **[CCCCCG]** 852 (85.08)   
360 - **[CCGCGG]** 849 (87.56)   
360 - **[CCGGGG]** 702 (85.40)   
360 - **[CCGGGG]** 668 (85.40)   
360 - **.CCGCCG.** 597 (96.68)   
360 - **[CCGGGG]** 574 (85.40)   
360 - **[CCCCCG]** 194 (85.08)   
360 - **[CCGGGG]** 90 (85.40)   
360 - **[CCGACT]** 33 (85.38)   
361 + **[CCGCGG]** 471 (87.56)   
361 + **.CCGAGG.** 606 (90.88)   
361 + **[CCCCCG]** 866 (85.08)   
361 + **[CCGGGG]** 902 (85.40)   
361 + **.CCGCCG.** 913 (96.68)   
361 + **.CCGAGG.** 1128 (90.88)   
361 + **.CCGACG.** 1319 (100.00)   
361 + **.CCGCCG.** 1361 (96.68)   
361 + **.CCGAGG.** 1364 (90.88)   
361 + **[CCGCGG]** 1417 (87.56)   
361 + **[CCGGGG]** 1473 (85.40)   
361 - **[CCGACT]** 1441 (85.38)   
361 - **[CCGCGG]** 1417 (87.56)   
361 - **[CCGGGG]** 1166 (85.40)   
361 - **.CCGGCG.** 914 (94.52)   
361 - **[CCCCCG]** 903 (85.08)   
361 - **[CCGCGG]** 471 (87.56)   
361 - **.CCGAGG.** 225 (90.88)   
362 + **[CCGGGG]** 90 (85.40)   
362 + **[CGGACG]** 96 (86.87)   
362 + **[CCGCGG]** 117 (87.56)   
362 + **[CCGCGG]** 282 (87.56)   
362 + **[CCGGGG]** 385 (85.40)   
362 + **[CCGCGG]** 469 (87.56)   
362 + **.CCGACG.** 523 (100.00)   
362 + **.CCGAGG.** 761 (90.88)   
362 + **[CAGACG]** 792 (85.30)   
362 + **[CCCCCG]** 836 (85.08)   
362 + **.CCGCCG.** 839 (96.68)   
362 + **.CCGACG.** 842 (100.00)   
362 + **.CCGACG.** 858 (100.00)   
362 + **[CCGGGG]** 883 (85.40)   
362 + **[CCGGGG]** 893 (85.40)   
362 + **[CCGGGG]** 905 (85.40)   
362 + **[CCGGGG]** 929 (85.40)   
362 + **.CCGCCG.** 994 (96.68)   
362 + **.CCGCCG.** 1002 (96.68)   
362 + **.CCGCCG.** 1005 (96.68)   
362 + **[CCGCGG]** 1056 (87.56)   
362 + **[CGGACG]** 1171 (86.87)   
362 + **.CCGAGG.** 1197 (90.88)   
362 + **.CCGACG.** 1261 (100.00)   
362 + **[CGGACG]** 1285 (86.87)   
362 + **[CCGGGG]** 1345 (85.40)   
362 + **[CCGACC]** 1400 (88.08)   
362 - **[CCGGGG]** 1336 (85.40)   
362 - **[CCGGGG]** 1282 (85.40)   
362 - **.CCGGCG.** 1265 (94.52)   
362 - **[CCGACC]** 1232 (88.08)   
362 - **[CCGCGG]** 1056 (87.56)   
362 - **.CCGGCG.** 1006 (94.52)   
362 - **[CCCCCG]** 930 (85.08)   
362 - **[CCCCCG]** 894 (85.08)   
362 - **[CCCCCG]** 884 (85.08)   
362 - **[CCGTCG]** 859 (86.99)   
362 - **[CCGGGG]** 849 (85.40)   
362 - **[CCGTCG]** 843 (86.99)   
362 - **[CGGACG]** 740 (86.87)   
362 - **.CCGACG.** 705 (100.00)   
362 - **[CCGCGG]** 469 (87.56)   
362 - **[CCCCCG]** 386 (85.08)   
362 - **[CCGGGG]** 383 (85.40)   
362 - **[CCGACC]** 360 (88.08)   
362 - **[CCGCGG]** 282 (87.56)   
362 - **[CCGGGG]** 164 (85.40)   
362 - **[CCGCGG]** 117 (87.56)   
363 + **[CCCACG]** 325 (88.40)   
363 + **[CCCACG]** 377 (88.40)   
363 + **[CCGGGG]** 393 (85.40)   
363 + **[CCGCGG]** 693 (87.56)   
363 + **[CGGACG]** 761 (86.87)   
363 + **[CCGCGG]** 798 (87.56)   
363 + **[CCGGGG]** 989 (85.40)   
363 + **[CAGACG]** 1036 (85.30)   
363 + **[CCGTCG]** 1079 (86.99)   
363 + **[CCCCCG]** 1095 (85.08)   
363 + **[CCCACG]** 1124 (88.40)   
363 + **.CCGACG.** 1195 (100.00)   
363 + **[CCCCCG]** 1204 (85.08)   
363 + **[CCCACG]** 1230 (88.40)   
363 + **[CCCCCG]** 1275 (85.08)   
363 + **[CCCCCG]** 1334 (85.08)   
363 + **[CCGACC]** 1337 (88.08)   
363 + **[CCCACG]** 1426 (88.40)   
363 + **[CCCACG]** 1502 (88.40)   
363 - **.CCGAGG.** 1358 (90.88)   
363 - **[CCGGGG]** 1276 (85.40)   
363 - **[CCGTCG]** 1196 (86.99)   
363 - **[CCGGGG]** 1148 (85.40)   
363 - **[CCGAAG]** 1108 (86.09)   
363 - **[GCGACG]** 1080 (85.28)   
363 - **[CCCCCG]** 990 (85.08)   
363 - **[CCGGGG]** 878 (85.40)   
363 - **[CCGGGG]** 829 (85.40)   
363 - **[CCGCGG]** 798 (87.56)   
363 - **[CCGCGG]** 693 (87.56)   
363 - **.CCGAGG.** 359 (90.88)   
363 - **[GCGACG]** 310 (85.28)   
363 - **.CCGCCG.** 172 (96.68)   
364 + **[CAGACG]** 51 (85.30)   
364 + **[CCGACC]** 262 (88.08)   
364 + **[CCGGGG]** 307 (85.40)   
364 + **[CAGACG]** 579 (85.30)   
364 + **.CCGAGG.** 730 (90.88)   
364 + **[CCCCCG]** 924 (85.08)   
364 + **[CCGCGG]** 949 (87.56)   
364 + **[CCCACG]** 990 (88.40)   
364 + **[CCCCCG]** 1078 (85.08)   
364 + **[CAGACG]** 1084 (85.30)   
364 + **[CCGGGG]** 1166 (85.40)   
364 + **.CCGGCG.** 1184 (94.52)   
364 + **[CCGCGG]** 1229 (87.56)   
364 + **[CCGCGG]** 1247 (87.56)   
364 + **[CCCCCG]** 1327 (85.08)   
364 + **[CCCCCG]** 1368 (85.08)   
364 + **[CCCACG]** 1488 (88.40)   
364 + **[CCCACG]** 1504 (88.40)   
364 - **[CCCCCG]** 1492 (85.08)   
364 - **[CCGTCG]** 1481 (86.99)   
364 - **.CCGCCG.** 1259 (96.68)   
364 - **[CCGCGG]** 1247 (87.56)   
364 - **[CCGCGG]** 1229 (87.56)   
364 - **[CCCCCG]** 1167 (85.08)   
364 - **[CCGGGG]** 1164 (85.40)   
364 - **.CCGCCG.** 1040 (96.68)   
364 - **[GCGACG]** 1020 (85.28)   
364 - **[CCGCGG]** 949 (87.56)   
364 - **[CCGACC]** 734 (88.08)   
364 - **[CCGGGG]** 468 (85.40)   
364 - **.CCGGCG.** 305 (94.52)   
365 + **[CCCACG]** 943 (88.40)   
365 + **[CCCCCG]** 1471 (85.08)   
365 - **[CAGACG]** 1290 (85.30)   
366 + **[CCGGGG]** 42 (85.40)   
366 + **.CCGAGG.** 66 (90.88)   
366 + **[CCCACG]** 115 (88.40)   
366 + **[CCCCCG]** 127 (85.08)   
366 + **.CCGCCG.** 141 (96.68)   
366 + **.CCGAGG.** 144 (90.88)   
366 + **.CCGGCG.** 151 (94.52)   
366 + **[CCCCCG]** 165 (85.08)   
366 + **[CCGCGG]** 183 (87.56)   
366 + **[CCGGGG]** 236 (85.40)   
366 + **[CCGCGG]** 384 (87.56)   
366 + **.CCGAGG.** 396 (90.88)   
366 + **[CCGCGG]** 444 (87.56)   
366 + **[CCCCCG]** 501 (85.08)   
366 + **[CCCCCG]** 513 (85.08)   
366 + **[CCGCGG]** 535 (87.56)   
366 + **[CCGTCG]** 590 (86.99)   
366 + **[CAGACG]** 605 (85.30)   
366 + **.CCGGCG.** 626 (94.52)   
366 + **[CGGACG]** 693 (86.87)   
366 + **[CCGTCG]** 783 (86.99)   
366 + **[CCGTCG]** 803 (86.99)   
366 + **[CCCACG]** 811 (88.40)   
366 + **[CCGGGG]** 846 (85.40)   
366 + **[CCCCCG]** 950 (85.08)   
366 + **[CCGCGG]** 953 (87.56)   
366 + **[CCGTCG]** 963 (86.99)   
366 + **[CCGTCG]** 988 (86.99)   
366 + **[GCGACG]** 996 (85.28)   
366 + **[CCGGGG]** 1057 (85.40)   
366 + **[CCGGGG]** 1167 (85.40)   
366 + **[CCGCGG]** 1254 (87.56)   
366 + **.CCGCCG.** 1263 (96.68)   
366 + **[CCGTCG]** 1266 (86.99)   
366 + **[CCGCGG]** 1413 (87.56)   
366 + **[CCGGGG]** 1425 (85.40)   
366 - **.CCGCCG.** 1438 (96.68)   
366 - **[CCGCGG]** 1413 (87.56)   
366 - **.CCGCCG.** 1395 (96.68)   
366 - **.CCGCCG.** 1365 (96.68)   
366 - **[CCGACC]** 1349 (88.08)   
366 - **[CGGACG]** 1303 (86.87)   
366 - **.CCGACG.** 1267 (100.00)   
366 - **[CCGCGG]** 1254 (87.56)   
366 - **.CCGCCG.** 1239 (96.68)   
366 - **.CCGCCG.** 1173 (96.68)   
366 - **.CCGGCG.** 1145 (94.52)   
366 - **.CCGAGG.** 1135 (90.88)   
366 - **[CCGACC]** 1114 (88.08)   
366 - **[CCGACC]** 1062 (88.08)   
366 - **[CCCCCG]** 1058 (85.08)   
366 - **.CCGCCG.** 1016 (96.68)   
366 - **[CCGACT]** 1013 (85.38)   
366 - **.CCGACG.** 989 (100.00)   
366 - **[GCGACG]** 973 (85.28)   
366 - **[GCGACG]** 964 (85.28)   
366 - **[CCGCGG]** 953 (87.56)   
366 - **.CCGGCG.** 932 (94.52)   
366 - **.CCGACG.** 856 (100.00)   
366 - **[CCGGGG]** 844 (85.40)   
366 - **.CCGACG.** 784 (100.00)   
366 - **[CGGACG]** 669 (86.87)   
366 - **.CCGCCG.** 627 (96.68)   
366 - **[CCGGGG]** 624 (85.40)   
366 - **[CCCCCG]** 613 (85.08)   
366 - **.CCGACG.** 591 (100.00)   
366 - **[CGGACG]** 587 (86.87)   
366 - **[CCGAAG]** 551 (86.09)   
366 - **[CCGCGG]** 535 (87.56)   
366 - **[CGGACG]** 450 (86.87)   
366 - **[CCGCGG]** 444 (87.56)   
366 - **[CCCACG]** 433 (88.40)   
366 - **.CCGCCG.** 412 (96.68)   
366 - **[CCGCGG]** 384 (87.56)   
366 - **[CCGGGG]** 360 (85.40)   
366 - **[CCCCCG]** 221 (85.08)   
366 - **[CCGACC]** 213 (88.08)   
366 - **[CCGCGG]** 183 (87.56)   
366 - **[CCGGGG]** 135 (85.40)   
366 - **.CCGCCG.** 88 (96.68)   
366 - **.CCGGCG.** 2 (94.52)   
367 + **[CCCCCG]** 5 (85.08)   
367 + **.CCGGCG.** 14 (94.52)   
367 + **[GCGACG]** 219 (85.28)   
367 + **[CCGGGG]** 246 (85.40)   
367 + **[CCGGGG]** 299 (85.40)   
367 + **[CCCCCG]** 389 (85.08)   
367 + **[CCCCCG]** 399 (85.08)   
367 + **[CCGCGG]** 556 (87.56)   
367 + **[CCGGGG]** 639 (85.40)   
367 + **.CCGCCG.** 667 (96.68)   
367 + **[CCCCCG]** 878 (85.08)   
367 + **[CCCCCG]** 885 (85.08)   
367 + **.CCGCCG.** 893 (96.68)   
367 + **.CCGCCG.** 896 (96.68)   
367 + **.CCGCCG.** 899 (96.68)   
367 + **.CCGCCG.** 902 (96.68)   
367 + **.CCGCCG.** 905 (96.68)   
367 + **.CCGCCG.** 908 (96.68)   
367 + **.CCGCCG.** 911 (96.68)   
367 + **.CCGCCG.** 914 (96.68)   
367 + **.CCGCCG.** 917 (96.68)   
367 + **[CCGTCG]** 920 (86.99)   
367 + **[CCCCCG]** 977 (85.08)   
367 + **.CCGCCG.** 980 (96.68)   
367 + **.CCGAGG.** 983 (90.88)   
367 + **.CCGGCG.** 997 (94.52)   
367 + **[CCCCCG]** 1009 (85.08)   
367 + **[CCGCGG]** 1012 (87.56)   
367 + **[CCGCGG]** 1064 (87.56)   
367 + **.CCGGCG.** 1081 (94.52)   
367 + **[CCGCGG]** 1091 (87.56)   
367 + **.CCGGCG.** 1108 (94.52)   
367 + **.CCGGCG.** 1142 (94.52)   
367 + **.CCGCCG.** 1148 (96.68)   
367 + **[CCCCCG]** 1154 (85.08)   
367 + **[CCGGGG]** 1157 (85.40)   
367 + **.CCGCCG.** 1188 (96.68)   
367 + **[CCGCGG]** 1191 (87.56)   
367 + **[CCCCCG]** 1313 (85.08)   
367 - **[CCCCCG]** 1200 (85.08)   
367 - **.CCGCCG.** 1197 (96.68)   
367 - **.CCGCCG.** 1194 (96.68)   
367 - **[CCGCGG]** 1191 (87.56)   
367 - **[CCCCCG]** 1158 (85.08)   
367 - **[CCGGGG]** 1155 (85.40)   
367 - **[CCGCGG]** 1091 (87.56)   
367 - **[CCGCGG]** 1064 (87.56)   
367 - **.CCGGCG.** 1036 (94.52)   
367 - **[CCGCGG]** 1012 (87.56)   
367 - **.CCGCCG.** 998 (96.68)   
367 - **[GCGACG]** 921 (85.28)   
367 - **.CCGAGG.** 840 (90.88)   
367 - **.CCGGCG.** 668 (94.52)   
367 - **[CCGGGG]** 611 (85.40)   
367 - **[CCCCCG]** 577 (85.08)   
367 - **[CCGCGG]** 556 (87.56)   
367 - **[CCGAAG]** 374 (86.09)   
367 - **.CCGCCG.** 313 (96.68)   
367 - **[CCCCCG]** 261 (85.08)   
367 - **[CCCCCG]** 247 (85.08)   
367 - **[CCCACG]** 114 (88.40)   
  

**Frequency Matrix**

| All Occs Best Occs|  |  |  |  |  |  |  |  |  |  |  |  |  |  |  |  |  |  |  |  |  |  |  |  |  |  |  |  |  |  |  |  |  |  |  |  |  |  |  |  |  |  |  |  |  |  |  |  |  |  |  |  |  |  |  |  |  |  |  |  |  |  |  |  |  |  |  |  |  |  |  |  |  |  |  |  |  |  |  |  |  |  |  |  | | --- | --- | --- | --- | --- | --- | --- | --- | --- | --- | --- | --- | --- | --- | --- | --- | --- | --- | --- | --- | --- | --- | --- | --- | --- | --- | --- | --- | --- | --- | --- | --- | --- | --- | --- | --- | --- | --- | --- | --- | --- | --- | --- | --- | --- | --- | --- | --- | --- | --- | --- | --- | --- | --- | --- | --- | --- | --- | --- | --- | --- | --- | --- | --- | --- | --- | --- | --- | --- | --- | --- | --- | --- | --- | --- | --- | --- | --- | --- | --- | --- | --- | --- | --- | | **A C G T A C G T | | | | | | | | | | | |**| **1** 62 5462 190 53 | 0 3224 0 0|  |  |  |  |  |  |  |  |  |  |  |  |  |  |  |  |  |  |  |  |  |  |  |  |  |  |  |  |  |  |  |  |  |  |  |  |  |  |  |  |  |  |  |  |  |  |  |  |  |  |  |  |  |  |  |  |  |  |  |  | | --- | --- | --- | --- | --- | --- | --- | --- | --- | --- | --- | --- | --- | --- | --- | --- | --- | --- | --- | --- | --- | --- | --- | --- | --- | --- | --- | --- | --- | --- | --- | --- | --- | --- | --- | --- | --- | --- | --- | --- | --- | --- | --- | --- | --- | --- | --- | --- | --- | --- | --- | --- | --- | --- | --- | --- | --- | --- | --- | --- | | **2** 181 5177 259 150 | 0 3224 0 0|  |  |  |  |  |  |  |  |  |  |  |  |  |  |  |  |  |  |  |  |  |  |  |  |  |  |  |  |  |  |  |  |  |  |  |  |  |  |  |  |  |  |  |  |  |  |  |  | | --- | --- | --- | --- | --- | --- | --- | --- | --- | --- | --- | --- | --- | --- | --- | --- | --- | --- | --- | --- | --- | --- | --- | --- | --- | --- | --- | --- | --- | --- | --- | --- | --- | --- | --- | --- | --- | --- | --- | --- | --- | --- | --- | --- | --- | --- | --- | --- | | **3** 105 370 5218 74 | 0 0 3224 0|  |  |  |  |  |  |  |  |  |  |  |  |  |  |  |  |  |  |  |  |  |  |  |  |  |  |  |  |  |  |  |  |  |  |  |  | | --- | --- | --- | --- | --- | --- | --- | --- | --- | --- | --- | --- | --- | --- | --- | --- | --- | --- | --- | --- | --- | --- | --- | --- | --- | --- | --- | --- | --- | --- | --- | --- | --- | --- | --- | --- | | **4** 3193 1496 914 164 | 814 1496 914 0|  |  |  |  |  |  |  |  |  |  |  |  |  |  |  |  |  |  |  |  |  |  |  |  | | --- | --- | --- | --- | --- | --- | --- | --- | --- | --- | --- | --- | --- | --- | --- | --- | --- | --- | --- | --- | --- | --- | --- | --- | | **5** 204 4877 609 77 | 0 2615 609 0|  |  |  |  |  |  |  |  |  |  |  |  | | --- | --- | --- | --- | --- | --- | --- | --- | --- | --- | --- | --- | | **6** 135 337 5113 182 | 0 0 3224 0 | | | | | | | | | | | | | | | | | | | | | | | | | | | | | | | | | | | | | | | | | | | | | | | | | | | | | | | | | | | | | | | | | | | | | | | | | | | | | | | | | | | | |

  
  

---

  
  
  
 **GTCGCG**   
**CGCGAC**  
  
7 redundant motifs found:  
TCGTCGCG - TTCGCG - TTCGCG - TCGCGT - CGCGTT - CGCGTC - TACGCG -   
  
Best occurrences (match percentage):   
Seq St oligo pos match
  
 1 + **.GCCGCG.** 1069 (95.35)   
1 - **[GTCGGG]** 1389 (87.52)   
1 - **.GGCGCG.** 1071 (95.43)   
1 - **.GGCGCG.** 1000 (95.43)   
2 + **[GTCGGG]** 31 (87.52)   
2 + **.GTCGCG.** 362 (100.00)   
2 + **[GTCCCG]** 398 (88.98)   
2 + **.GCCGCG.** 447 (95.35)   
2 + **[GTCGCC]** 571 (86.61)   
2 + **.GGCGCG.** 703 (95.43)   
2 + **[GTCGGG]** 762 (87.52)   
2 + **[GTGGCG]** 827 (87.04)   
2 + **[GTCCCG]** 909 (88.98)   
2 + **[CTCGCG]** 994 (88.37)   
2 + **.GGCGCG.** 999 (95.43)   
2 + **.GCCGCG.** 1071 (95.35)   
2 + **.GGCGCG.** 1450 (95.43)   
2 - **.GCCGCG.** 1447 (95.35)   
2 - **[GTCGCC]** 1369 (86.61)   
2 - **[CTCGCG]** 1123 (88.37)   
2 - **[CTCGCG]** 1001 (88.37)   
2 - **.GCCGCG.** 996 (95.35)   
2 - **.GCCGCG.** 977 (95.35)   
2 - **[GTCGGG]** 911 (87.52)   
2 - **.GCCGCG.** 721 (95.35)   
2 - **[CTCGCG]** 696 (88.37)   
2 - **[GACGCG]** 515 (89.80)   
2 - **[GTCGGG]** 505 (87.52)   
2 - **.GGCGCG.** 457 (95.43)   
2 - **[GACGCG]** 449 (89.80)   
2 - **[GTCCCG]** 247 (88.98)   
2 - **.GCCGCG.** 201 (95.35)   
3 + **.GTCGCG.** 701 (100.00)   
3 + **.GCCGCG.** 789 (95.35)   
3 + **[GTCCCG]** 1057 (88.98)   
3 + **.GGCGCG.** 1180 (95.43)   
3 + **.GGCGCG.** 1249 (95.43)   
3 + **[GTGGCG]** 1322 (87.04)   
3 + **.GCCGCG.** 1416 (95.35)   
3 - **[GTGGCG]** 1420 (87.04)   
3 - **.GGCGCG.** 1418 (95.43)   
3 - **[GTCCCG]** 1350 (88.98)   
3 - **[GTGGCG]** 1266 (87.04)   
3 - **[CTCGCG]** 1253 (88.37)   
3 - **.GCCGCG.** 1223 (95.35)   
3 - **[CTCGCG]** 1182 (88.37)   
3 - **[GTGGCG]** 1107 (87.04)   
4 + **[GTGGCG]** 271 (87.04)   
4 + **[GTCCCG]** 606 (88.98)   
4 + **.GGCGCG.** 683 (95.43)   
4 + **.GCCGCG.** 699 (95.35)   
4 + **.GTCGCG.** 740 (100.00)   
4 + **[CTCGCG]** 800 (88.37)   
4 + **.GGCGCG.** 828 (95.43)   
4 + **.GGCGCG.** 916 (95.43)   
4 + **.GCCGCG.** 1206 (95.35)   
4 + **.GGCGCG.** 1272 (95.43)   
4 + **.GGCGCG.** 1282 (95.43)   
4 - **.GGCGCG.** 1208 (95.43)   
4 - **.GGCGCG.** 1199 (95.43)   
4 - **.GCCGCG.** 1179 (95.35)   
4 - **[GTCGCC]** 892 (86.61)   
4 - **[GTCGCC]** 850 (86.61)   
4 - **[CTCGCG]** 830 (88.37)   
4 - **.GCCGCG.** 822 (95.35)   
4 - **.GCCGCG.** 802 (95.35)   
4 - **.GGCGCG.** 786 (95.43)   
4 - **.GCCGCG.** 742 (95.35)   
4 - **.GTCGCG.** 701 (100.00)   
4 - **[GTCCCG]** 675 (88.98)   
4 - **[GTCGCC]** 635 (86.61)   
4 - **.GTCGCG.** 574 (100.00)   
5 + **.GCCGCG.** 812 (95.35)   
5 + **[GTCCCG]** 869 (88.98)   
5 + **[GTCCCG]** 1187 (88.98)   
5 + **[GACGCG]** 1438 (89.80)   
5 + **[GTCCCG]** 1460 (88.98)   
5 - **.GCCGCG.** 1440 (95.35)   
5 - **[GTCGGG]** 1435 (87.52)   
5 - **.GCCGCG.** 1395 (95.35)   
5 - **[CTCGCG]** 1360 (88.37)   
5 - **.GGCGCG.** 1346 (95.43)   
5 - **[GTCGCC]** 825 (86.61)   
5 - **[CTCGCG]** 508 (88.37)   
6 - **[GTCGCC]** 1443 (86.61)   
6 - **.GCCGCG.** 1440 (95.35)   
6 - **[CTCGCG]** 1141 (88.37)   
8 + **[CTCGCG]** 147 (88.37)   
8 + **[GTCCCG]** 375 (88.98)   
8 + **.GGCGCG.** 407 (95.43)   
8 + **.GCCGCG.** 452 (95.35)   
8 + **.GGCGCG.** 927 (95.43)   
8 + **.GGCGCG.** 933 (95.43)   
8 + **.GGCGCG.** 978 (95.43)   
8 + **[GTCCCG]** 1183 (88.98)   
8 + **.GTCGCG.** 1204 (100.00)   
8 + **[GTCCCG]** 1410 (88.98)   
8 + **[CTCGCG]** 1464 (88.37)   
8 - **.GTCGCG.** 1466 (100.00)   
8 - **[GTGGCG]** 1431 (87.04)   
8 - **[GACGCG]** 1146 (89.80)   
8 - **.GTCGCG.** 980 (100.00)   
8 - **[CTCGCG]** 935 (88.37)   
8 - **[GTCGGG]** 548 (87.52)   
8 - **.GGCGCG.** 494 (95.43)   
8 - **.GCCGCG.** 409 (95.35)   
8 - **[GACGCG]** 372 (89.80)   
8 - **[GACGCG]** 149 (89.80)   
9 + **[GTCGGG]** 474 (87.52)   
9 + **.GGCGCG.** 975 (95.43)   
9 + **.GGCGCG.** 1266 (95.43)   
9 + **[GTCGCC]** 1459 (86.61)   
9 - **.GTCGCG.** 1268 (100.00)   
9 - **.GGCGCG.** 1062 (95.43)   
9 - **[CTCGCG]** 977 (88.37)   
9 - **[GTCCCG]** 476 (88.98)   
10 + **.GCCGCG.** 35 (95.35)   
10 + **[GTCGGG]** 44 (87.52)   
10 + **.GCCGCG.** 511 (95.35)   
10 + **[GTCCCG]** 575 (88.98)   
10 + **[GTCGGG]** 747 (87.52)   
10 + **[GACGCG]** 820 (89.80)   
10 + **[GACGCG]** 828 (89.80)   
10 + **.GGCGCG.** 871 (95.43)   
10 + **[GTCGGG]** 905 (87.52)   
10 + **.GCCGCG.** 1022 (95.35)   
10 + **.GCCGCG.** 1119 (95.35)   
10 + **[GTCGCC]** 1203 (86.61)   
10 + **[GTCGCC]** 1452 (86.61)   
10 - **.GCCGCG.** 1331 (95.35)   
10 - **[GTCGGG]** 1279 (87.52)   
10 - **[GTGGCG]** 1265 (87.04)   
10 - **.GCCGCG.** 1121 (95.35)   
10 - **.GCCGCG.** 1096 (95.35)   
10 - **[GTCCCG]** 1038 (88.98)   
10 - **.GCCGCG.** 1024 (95.35)   
10 - **[CTCGCG]** 822 (88.37)   
10 - **[GTCCCG]** 731 (88.98)   
10 - **.GCCGCG.** 605 (95.35)   
10 - **.GGCGCG.** 513 (95.43)   
10 - **[GTCGCC]** 436 (86.61)   
10 - **[GTCGCC]** 410 (86.61)   
11 + **.GGCGCG.** 250 (95.43)   
11 + **.GCCGCG.** 497 (95.35)   
11 + **.GTCGCG.** 529 (100.00)   
11 + **.GCCGCG.** 609 (95.35)   
11 + **.GCCGCG.** 615 (95.35)   
11 + **[GACGCG]** 698 (89.80)   
11 + **.GCCGCG.** 713 (95.35)   
11 + **[GACGCG]** 747 (89.80)   
11 + **.GCCGCG.** 767 (95.35)   
11 + **[GTCGGG]** 814 (87.52)   
11 + **.GCCGCG.** 994 (95.35)   
11 + **.GCCGCG.** 1027 (95.35)   
11 + **.GCCGCG.** 1155 (95.35)   
11 + **.GGCGCG.** 1379 (95.43)   
11 - **[GACGCG]** 1381 (89.80)   
11 - **.GCCGCG.** 1116 (95.35)   
11 - **.GGCGCG.** 957 (95.43)   
11 - **.GCCGCG.** 769 (95.35)   
11 - **[GTGGCG]** 751 (87.04)   
11 - **.GGCGCG.** 749 (95.43)   
11 - **[GTCCCG]** 744 (88.98)   
11 - **.GCCGCG.** 715 (95.35)   
11 - **.GGCGCG.** 700 (95.43)   
11 - **[GTGGCG]** 641 (87.04)   
11 - **.GGCGCG.** 639 (95.43)   
11 - **.GCCGCG.** 611 (95.35)   
11 - **.GGCGCG.** 606 (95.43)   
11 - **.GCCGCG.** 560 (95.35)   
11 - **[GTCGCC]** 291 (86.61)   
12 + **[CTCGCG]** 178 (88.37)   
12 + **[GTCGGG]** 451 (87.52)   
12 + **.GGCGCG.** 509 (95.43)   
12 + **.GGCGCG.** 524 (95.43)   
12 + **[GTCCCG]** 536 (88.98)   
12 + **.GGCGCG.** 541 (95.43)   
12 + **.GCCGCG.** 740 (95.35)   
12 + **[GTCCCG]** 852 (88.98)   
12 + **[GTCCCG]** 909 (88.98)   
12 + **[GTCGGG]** 980 (87.52)   
12 + **.GCCGCG.** 1030 (95.35)   
12 + **.GTCGCG.** 1095 (100.00)   
12 + **[GACGCG]** 1116 (89.80)   
12 + **.GCCGCG.** 1178 (95.35)   
12 + **.GCCGCG.** 1251 (95.35)   
12 + **[GTGGCG]** 1266 (87.04)   
12 + **.GCCGCG.** 1275 (95.35)   
12 + **.GGCGCG.** 1333 (95.43)   
12 + **.GCCGCG.** 1355 (95.35)   
12 - **.GGCGCG.** 1253 (95.43)   
12 - **.GCCGCG.** 1120 (95.35)   
12 - **.GGCGCG.** 1036 (95.43)   
12 - **.GTCGCG.** 870 (100.00)   
12 - **[GACGCG]** 841 (89.80)   
12 - **[GTGGCG]** 787 (87.04)   
12 - **.GCCGCG.** 711 (95.35)   
12 - **.GGCGCG.** 696 (95.43)   
12 - **.GGCGCG.** 543 (95.43)   
12 - **.GGCGCG.** 526 (95.43)   
12 - **[GTCCCG]** 186 (88.98)   
13 + **.GGCGCG.** 440 (95.43)   
13 + **.GCCGCG.** 721 (95.35)   
13 + **[GTCCCG]** 745 (88.98)   
13 + **[GTCCCG]** 769 (88.98)   
13 + **.GGCGCG.** 801 (95.43)   
13 + **[CTCGCG]** 871 (88.37)   
13 + **[GTCCCG]** 941 (88.98)   
13 + **[GTCCCG]** 1030 (88.98)   
13 + **.GGCGCG.** 1062 (95.43)   
13 + **.GGCGCG.** 1092 (95.43)   
13 + **.GCCGCG.** 1110 (95.35)   
13 + **[GTCGCC]** 1158 (86.61)   
13 + **.GCCGCG.** 1197 (95.35)   
13 + **.GCCGCG.** 1227 (95.35)   
13 + **.GTCGCG.** 1302 (100.00)   
13 + **.GTCGCG.** 1322 (100.00)   
13 + **[GTCGGG]** 1444 (87.52)   
13 + **.GTCGCG.** 1468 (100.00)   
13 - **[GTCCCG]** 1433 (88.98)   
13 - **[GTCGCC]** 1402 (86.61)   
13 - **.GCCGCG.** 1352 (95.35)   
13 - **.GGCGCG.** 1264 (95.43)   
13 - **.GGCGCG.** 1187 (95.43)   
13 - **[GTCCCG]** 1176 (88.98)   
13 - **[GTGGCG]** 1148 (87.04)   
13 - **[GTGGCG]** 1103 (87.04)   
13 - **.GCCGCG.** 1064 (95.35)   
13 - **.GCCGCG.** 947 (95.35)   
13 - **[GTCCCG]** 926 (88.98)   
13 - **.GGCGCG.** 787 (95.43)   
13 - **.GGCGCG.** 780 (95.43)   
13 - **.GGCGCG.** 773 (95.43)   
13 - **[GACGCG]** 766 (89.80)   
13 - **[GACGCG]** 742 (89.80)   
13 - **.GCCGCG.** 703 (95.35)   
13 - **.GCCGCG.** 656 (95.35)   
13 - **.GGCGCG.** 615 (95.43)   
13 - **[GTCCCG]** 523 (88.98)   
13 - **.GGCGCG.** 167 (95.43)   
14 + **.GTCGCG.** 349 (100.00)   
14 + **[GTCCCG]** 435 (88.98)   
14 + **.GCCGCG.** 460 (95.35)   
14 + **[GTGGCG]** 514 (87.04)   
14 + **[GTCGCC]** 568 (86.61)   
14 + **[CTCGCG]** 618 (88.37)   
14 + **.GGCGCG.** 712 (95.43)   
14 + **.GGCGCG.** 746 (95.43)   
14 + **[GTGGCG]** 936 (87.04)   
14 + **.GGCGCG.** 938 (95.43)   
14 + **.GCCGCG.** 989 (95.35)   
14 + **.GCCGCG.** 1072 (95.35)   
14 - **[CTCGCG]** 1307 (88.37)   
14 - **.GCCGCG.** 993 (95.35)   
14 - **.GCCGCG.** 940 (95.35)   
14 - **.GGCGCG.** 870 (95.43)   
14 - **[GACGCG]** 793 (89.80)   
14 - **[GTCCCG]** 753 (88.98)   
14 - **.GCCGCG.** 748 (95.35)   
14 - **[GTCGCC]** 590 (86.61)   
14 - **.GCCGCG.** 337 (95.35)   
14 - **.GGCGCG.** 292 (95.43)   
14 - **.GCCGCG.** 235 (95.35)   
14 - **[GTCGGG]** 192 (87.52)   
15 + **.GCCGCG.** 23 (95.35)   
15 + **.GCCGCG.** 179 (95.35)   
15 + **[GTCCCG]** 386 (88.98)   
15 + **.GCCGCG.** 415 (95.35)   
15 + **.GCCGCG.** 459 (95.35)   
15 + **[CTCGCG]** 490 (88.37)   
15 + **[GTCGCC]** 675 (86.61)   
15 + **.GGCGCG.** 733 (95.43)   
15 + **.GCCGCG.** 855 (95.35)   
15 + **.GGCGCG.** 984 (95.43)   
15 + **.GGCGCG.** 1200 (95.43)   
15 + **[GTCGCC]** 1241 (86.61)   
15 + **.GCCGCG.** 1264 (95.35)   
15 - **[GTCCCG]** 1301 (88.98)   
15 - **[GACGCG]** 1232 (89.80)   
15 - **[GACGCG]** 1183 (89.80)   
15 - **[GTGGCG]** 991 (87.04)   
15 - **.GCCGCG.** 986 (95.35)   
15 - **.GGCGCG.** 827 (95.43)   
15 - **[CTCGCG]** 737 (88.37)   
15 - **[GTCGGG]** 689 (87.52)   
15 - **.GCCGCG.** 545 (95.35)   
15 - **.GGCGCG.** 539 (95.43)   
15 - **[CTCGCG]** 492 (88.37)   
15 - **.GGCGCG.** 247 (95.43)   
15 - **[GTCCCG]** 183 (88.98)   
15 - **.GTCGCG.** 25 (100.00)   
16 + **[GTGGCG]** 224 (87.04)   
16 + **.GGCGCG.** 492 (95.43)   
16 + **.GCCGCG.** 621 (95.35)   
16 + **.GCCGCG.** 626 (95.35)   
16 + **[GTCGGG]** 697 (87.52)   
16 + **[GTCGCC]** 720 (86.61)   
16 + **[CTCGCG]** 797 (88.37)   
16 + **.GGCGCG.** 903 (95.43)   
16 + **.GGCGCG.** 1093 (95.43)   
16 + **[GTCGGG]** 1395 (87.52)   
16 + **[GTCGGG]** 1504 (87.52)   
16 - **.GGCGCG.** 1300 (95.43)   
16 - **.GTCGCG.** 1282 (100.00)   
16 - **.GCCGCG.** 1150 (95.35)   
16 - **[CTCGCG]** 1003 (88.37)   
16 - **[GTCGCC]** 885 (86.61)   
16 - **.GGCGCG.** 799 (95.43)   
16 - **.GCCGCG.** 775 (95.35)   
16 - **.GGCGCG.** 628 (95.43)   
16 - **.GGCGCG.** 623 (95.43)   
16 - **[GTCCCG]** 602 (88.98)   
16 - **[GTGGCG]** 348 (87.04)   
16 - **.GGCGCG.** 314 (95.43)   
16 - **[GTCCCG]** 126 (88.98)   
17 + **[GTCGGG]** 856 (87.52)   
17 + **.GCCGCG.** 1017 (95.35)   
17 - **[GTCGCC]** 1167 (86.61)   
17 - **.GCCGCG.** 1019 (95.35)   
18 + **.GCCGCG.** 474 (95.35)   
18 + **[CTCGCG]** 758 (88.37)   
18 + **.GCCGCG.** 832 (95.35)   
18 + **.GCCGCG.** 837 (95.35)   
18 + **.GCCGCG.** 866 (95.35)   
18 + **.GCCGCG.** 930 (95.35)   
18 + **[GTGGCG]** 964 (87.04)   
18 + **[GTCCCG]** 1098 (88.98)   
18 + **.GCCGCG.** 1202 (95.35)   
18 + **.GCCGCG.** 1329 (95.35)   
18 + **.GGCGCG.** 1386 (95.43)   
18 + **[GTCGGG]** 1424 (87.52)   
18 + **.GTCGCG.** 1489 (100.00)   
18 - **[CTCGCG]** 1437 (88.37)   
18 - **[GACGCG]** 1331 (89.80)   
18 - **.GGCGCG.** 1282 (95.43)   
18 - **.GGCGCG.** 1231 (95.43)   
18 - **.GCCGCG.** 1204 (95.35)   
18 - **.GGCGCG.** 1109 (95.43)   
18 - **[GTCCCG]** 1014 (88.98)   
18 - **.GCCGCG.** 932 (95.35)   
18 - **.GGCGCG.** 914 (95.43)   
18 - **[GTCGGG]** 895 (87.52)   
18 - **.GGCGCG.** 868 (95.43)   
18 - **.GGCGCG.** 834 (95.43)   
18 - **.GGCGCG.** 710 (95.43)   
18 - **[GACGCG]** 476 (89.80)   
18 - **[GTCGCC]** 458 (86.61)   
19 - **[GTCCCG]** 892 (88.98)   
19 - **[GTCGCC]** 254 (86.61)   
20 + **.GGCGCG.** 66 (95.43)   
20 + **.GCCGCG.** 300 (95.35)   
20 + **[GTCCCG]** 671 (88.98)   
20 + **.GCCGCG.** 896 (95.35)   
20 + **[GTCCCG]** 1003 (88.98)   
20 + **[GTGGCG]** 1027 (87.04)   
20 + **.GCCGCG.** 1197 (95.35)   
20 - **[GTCCCG]** 1482 (88.98)   
20 - **[GTCCCG]** 1314 (88.98)   
20 - **.GCCGCG.** 1035 (95.35)   
20 - **.GGCGCG.** 992 (95.43)   
22 + **[GTCGGG]** 720 (87.52)   
22 + **.GTCGCG.** 745 (100.00)   
22 + **.GCCGCG.** 772 (95.35)   
22 + **[GTCCCG]** 977 (88.98)   
22 + **[CTCGCG]** 1024 (88.37)   
22 + **[CTCGCG]** 1229 (88.37)   
22 - **.GTCGCG.** 1231 (100.00)   
22 - **[CTCGCG]** 1026 (88.37)   
22 - **[CTCGCG]** 1015 (88.37)   
22 - **[GTGGCG]** 839 (87.04)   
22 - **.GTCGCG.** 778 (100.00)   
22 - **.GCCGCG.** 618 (95.35)   
22 - **.GGCGCG.** 192 (95.43)   
23 - **[GTCGGG]** 1264 (87.52)   
23 - **[CTCGCG]** 1004 (88.37)   
23 - **[GTCCCG]** 855 (88.98)   
23 - **[GTCCCG]** 130 (88.98)   
24 + **[GTCGCC]** 213 (86.61)   
24 + **.GCCGCG.** 304 (95.35)   
24 + **.GTCGCG.** 453 (100.00)   
24 + **[GTCCCG]** 493 (88.98)   
24 + **[GTCCCG]** 508 (88.98)   
24 + **[GTCCCG]** 535 (88.98)   
24 + **.GGCGCG.** 935 (95.43)   
24 + **.GGCGCG.** 942 (95.43)   
24 + **.GCCGCG.** 1038 (95.35)   
24 + **.GGCGCG.** 1075 (95.43)   
24 + **.GGCGCG.** 1098 (95.43)   
24 + **.GTCGCG.** 1143 (100.00)   
24 + **[GTCGCC]** 1240 (86.61)   
24 + **.GGCGCG.** 1319 (95.43)   
24 + **.GCCGCG.** 1353 (95.35)   
24 + **[GTCGCC]** 1408 (86.61)   
24 - **.GCCGCG.** 1217 (95.35)   
24 - **.GGCGCG.** 1145 (95.43)   
24 - **.GCCGCG.** 1100 (95.35)   
24 - **.GCCGCG.** 1040 (95.35)   
24 - **.GGCGCG.** 598 (95.43)   
24 - **[GTCGGG]** 569 (87.52)   
24 - **.GGCGCG.** 546 (95.43)   
24 - **[GTCGCC]** 540 (86.61)   
24 - **[GACGCG]** 522 (89.80)   
24 - **[CTCGCG]** 488 (88.37)   
24 - **[CTCGCG]** 455 (88.37)   
24 - **.GGCGCG.** 306 (95.43)   
25 + **.GTCGCG.** 87 (100.00)   
25 + **[GTGGCG]** 295 (87.04)   
25 + **[GTGGCG]** 358 (87.04)   
25 + **.GGCGCG.** 360 (95.43)   
25 + **.GGCGCG.** 383 (95.43)   
25 + **[GTCCCG]** 467 (88.98)   
25 + **[GACGCG]** 487 (89.80)   
25 + **.GGCGCG.** 549 (95.43)   
25 + **[GACGCG]** 770 (89.80)   
25 + **.GGCGCG.** 792 (95.43)   
25 + **[GACGCG]** 950 (89.80)   
25 + **.GCCGCG.** 1151 (95.35)   
25 + **[GTCGGG]** 1329 (87.52)   
25 - **[CTCGCG]** 1153 (88.37)   
25 - **[GTCCCG]** 635 (88.98)   
25 - **[CTCGCG]** 385 (88.37)   
26 + **.GCCGCG.** 184 (95.35)   
26 + **[GACGCG]** 753 (89.80)   
26 + **.GGCGCG.** 779 (95.43)   
26 + **.GCCGCG.** 803 (95.35)   
26 + **.GGCGCG.** 824 (95.43)   
26 + **[GTCCCG]** 875 (88.98)   
26 + **.GCCGCG.** 885 (95.35)   
26 + **.GGCGCG.** 1353 (95.43)   
26 + **.GCCGCG.** 1485 (95.35)   
26 - **.GCCGCG.** 1425 (95.35)   
26 - **.GCCGCG.** 826 (95.35)   
26 - **.GCCGCG.** 186 (95.35)   
27 + **[GTCCCG]** 1036 (88.98)   
27 + **[GTCGGG]** 1149 (87.52)   
27 + **.GGCGCG.** 1207 (95.43)   
27 + **[GTCGCC]** 1354 (86.61)   
27 - **.GGCGCG.** 1249 (95.43)   
27 - **.GCCGCG.** 1204 (95.35)   
27 - **[GACGCG]** 1146 (89.80)   
27 - **[GTCCCG]** 1127 (88.98)   
28 + **.GGCGCG.** 433 (95.43)   
28 + **[GTGGCG]** 459 (87.04)   
28 + **.GCCGCG.** 587 (95.35)   
28 + **.GGCGCG.** 628 (95.43)   
28 + **.GCCGCG.** 916 (95.35)   
28 + **[GTCGCC]** 1061 (86.61)   
28 + **.GTCGCG.** 1098 (100.00)   
28 + **.GTCGCG.** 1103 (100.00)   
28 + **.GCCGCG.** 1108 (95.35)   
28 + **[GTCGCC]** 1113 (86.61)   
28 + **[GTGGCG]** 1144 (87.04)   
28 + **[GTCGGG]** 1309 (87.52)   
28 - **.GTCGCG.** 1354 (100.00)   
28 - **[CTCGCG]** 1297 (88.37)   
28 - **[GACGCG]** 1110 (89.80)   
28 - **.GGCGCG.** 1105 (95.43)   
28 - **[GACGCG]** 1100 (89.80)   
28 - **.GTCGCG.** 1009 (100.00)   
28 - **.GGCGCG.** 957 (95.43)   
28 - **[GTCCCG]** 940 (88.98)   
28 - **[GTCGGG]** 758 (87.52)   
28 - **.GGCGCG.** 740 (95.43)   
28 - **[CTCGCG]** 630 (88.37)   
28 - **.GTCGCG.** 589 (100.00)   
28 - **.GCCGCG.** 518 (95.35)   
29 + **.GCCGCG.** 1218 (95.35)   
29 - **[CTCGCG]** 1299 (88.37)   
29 - **.GGCGCG.** 220 (95.43)   
33 + **[GACGCG]** 1029 (89.80)   
33 + **.GCCGCG.** 1040 (95.35)   
33 + **[GTCGCC]** 1057 (86.61)   
33 + **[GTGGCG]** 1176 (87.04)   
33 + **.GCCGCG.** 1360 (95.35)   
33 + **.GCCGCG.** 1411 (95.35)   
33 - **[GTCCCG]** 1420 (88.98)   
33 - **.GCCGCG.** 1362 (95.35)   
33 - **[GTCCCG]** 1165 (88.98)   
33 - **.GGCGCG.** 938 (95.43)   
33 - **.GCCGCG.** 904 (95.35)   
33 - **[GTCCCG]** 127 (88.98)   
34 + **[CTCGCG]** 395 (88.37)   
34 + **.GGCGCG.** 591 (95.43)   
34 + **.GGCGCG.** 649 (95.43)   
34 + **[GACGCG]** 683 (89.80)   
34 + **[GTCGCC]** 730 (86.61)   
34 + **.GCCGCG.** 771 (95.35)   
34 + **.GGCGCG.** 801 (95.43)   
34 + **[GTGGCG]** 851 (87.04)   
34 + **[CTCGCG]** 940 (88.37)   
34 + **.GCCGCG.** 956 (95.35)   
34 + **[GACGCG]** 1000 (89.80)   
34 + **.GCCGCG.** 1196 (95.35)   
34 + **.GCCGCG.** 1278 (95.35)   
34 + **.GGCGCG.** 1313 (95.43)   
34 - **.GCCGCG.** 1236 (95.35)   
34 - **.GCCGCG.** 1082 (95.35)   
34 - **[CTCGCG]** 962 (88.37)   
34 - **.GGCGCG.** 942 (95.43)   
34 - **[GTGGCG]** 839 (87.04)   
34 - **[GACGCG]** 803 (89.80)   
34 - **.GGCGCG.** 773 (95.43)   
34 - **.GTCGCG.** 397 (100.00)   
35 + **[GTCGCC]** 995 (86.61)   
35 + **.GCCGCG.** 998 (95.35)   
35 + **.GGCGCG.** 1147 (95.43)   
35 + **[GTCGCC]** 1184 (86.61)   
35 + **[GTCGCC]** 1195 (86.61)   
35 + **[GTCGGG]** 1235 (87.52)   
35 + **[GTCGGG]** 1364 (87.52)   
35 - **.GCCGCG.** 812 (95.35)   
36 + **[GTCCCG]** 448 (88.98)   
36 + **.GCCGCG.** 627 (95.35)   
36 + **[GTCCCG]** 802 (88.98)   
36 + **.GCCGCG.** 1062 (95.35)   
36 + **[GTCGGG]** 1097 (87.52)   
36 + **.GCCGCG.** 1171 (95.35)   
36 + **[GTCCCG]** 1406 (88.98)   
36 - **[GTGGCG]** 1306 (87.04)   
36 - **.GGCGCG.** 1066 (95.43)   
36 - **[GTCCCG]** 892 (88.98)   
36 - **.GTCGCG.** 859 (100.00)   
36 - **.GGCGCG.** 269 (95.43)   
36 - **.GCCGCG.** 230 (95.35)   
36 - **[GACGCG]** 151 (89.80)   
36 - **[GTCGGG]** 41 (87.52)   
37 + **[GTGGCG]** 974 (87.04)   
37 - **[GTCGGG]** 1202 (87.52)   
37 - **[GTCGGG]** 42 (87.52)   
39 + **.GGCGCG.** 1046 (95.43)   
39 + **.GGCGCG.** 1120 (95.43)   
39 + **[GTGGCG]** 1233 (87.04)   
39 + **.GGCGCG.** 1412 (95.43)   
39 + **[CTCGCG]** 1421 (88.37)   
39 - **.GCCGCG.** 1425 (95.35)   
39 - **.GTCGCG.** 1414 (100.00)   
39 - **[GTGGCG]** 1353 (87.04)   
39 - **[GTGGCG]** 1268 (87.04)   
39 - **[GTCGCC]** 1152 (86.61)   
39 - **[GACGCG]** 1122 (89.80)   
40 + **.GGCGCG.** 950 (95.43)   
40 + **.GCCGCG.** 1107 (95.35)   
40 + **[GTCGGG]** 1266 (87.52)   
40 + **.GTCGCG.** 1277 (100.00)   
40 + **[GTGGCG]** 1362 (87.04)   
40 + **[GTGGCG]** 1412 (87.04)   
40 - **.GCCGCG.** 1425 (95.35)   
40 - **.GCCGCG.** 1241 (95.35)   
40 - **[GACGCG]** 1235 (89.80)   
40 - **.GGCGCG.** 1104 (95.43)   
40 - **.GCCGCG.** 952 (95.35)   
40 - **.GCCGCG.** 895 (95.35)   
41 + **.GCCGCG.** 521 (95.35)   
41 + **[GTCGCC]** 916 (86.61)   
41 - **.GCCGCG.** 958 (95.35)   
41 - **.GCCGCG.** 523 (95.35)   
42 + **.GTCGCG.** 909 (100.00)   
42 + **.GGCGCG.** 1070 (95.43)   
42 + **[GTCGGG]** 1244 (87.52)   
42 + **[CTCGCG]** 1309 (88.37)   
42 + **[GTCGCC]** 1352 (86.61)   
42 + **.GCCGCG.** 1475 (95.35)   
42 - **.GCCGCG.** 1311 (95.35)   
42 - **[GTCGCC]** 1180 (86.61)   
42 - **.GCCGCG.** 1168 (95.35)   
42 - **.GGCGCG.** 900 (95.43)   
42 - **.GCCGCG.** 843 (95.35)   
42 - **[CTCGCG]** 830 (88.37)   
42 - **.GGCGCG.** 813 (95.43)   
42 - **[CTCGCG]** 525 (88.37)   
42 - **[GACGCG]** 442 (89.80)   
42 - **[GTCGGG]** 56 (87.52)   
43 + **.GCCGCG.** 1101 (95.35)   
43 + **.GGCGCG.** 1195 (95.43)   
43 + **.GGCGCG.** 1251 (95.43)   
43 + **.GCCGCG.** 1343 (95.35)   
43 + **[GTCCCG]** 1474 (88.98)   
43 - **.GCCGCG.** 1197 (95.35)   
43 - **[GTCGCC]** 1183 (86.61)   
43 - **[GTCGCC]** 1171 (86.61)   
45 + **.GCCGCG.** 397 (95.35)   
45 + **.GGCGCG.** 929 (95.43)   
45 + **.GCCGCG.** 979 (95.35)   
45 + **[GACGCG]** 1008 (89.80)   
45 + **.GGCGCG.** 1268 (95.43)   
45 + **[GTGGCG]** 1355 (87.04)   
45 + **.GGCGCG.** 1419 (95.43)   
45 - **[GTGGCG]** 1329 (87.04)   
45 - **[GTGGCG]** 1248 (87.04)   
45 - **.GCCGCG.** 1012 (95.35)   
45 - **.GCCGCG.** 981 (95.35)   
45 - **[GTGGCG]** 735 (87.04)   
45 - **[CTCGCG]** 690 (88.37)   
47 + **[GTGGCG]** 106 (87.04)   
47 + **.GGCGCG.** 350 (95.43)   
47 + **.GGCGCG.** 834 (95.43)   
47 + **[GTCGGG]** 909 (87.52)   
47 + **.GTCGCG.** 1247 (100.00)   
47 + **.GCCGCG.** 1271 (95.35)   
47 + **.GCCGCG.** 1371 (95.35)   
47 - **.GTCGCG.** 1494 (100.00)   
47 - **.GCCGCG.** 1367 (95.35)   
47 - **.GGCGCG.** 1275 (95.43)   
47 - **.GGCGCG.** 1249 (95.43)   
47 - **.GCCGCG.** 1136 (95.35)   
47 - **[GTCCCG]** 743 (88.98)   
48 + **.GCCGCG.** 764 (95.35)   
48 + **.GCCGCG.** 963 (95.35)   
48 + **.GCCGCG.** 1284 (95.35)   
48 + **.GCCGCG.** 1362 (95.35)   
48 - **[GACGCG]** 1364 (89.80)   
48 - **.GCCGCG.** 1317 (95.35)   
48 - **.GCCGCG.** 1286 (95.35)   
48 - **[GTGGCG]** 1277 (87.04)   
48 - **.GCCGCG.** 1019 (95.35)   
48 - **[GTCCCG]** 471 (88.98)   
48 - **.GCCGCG.** 433 (95.35)   
48 - **.GCCGCG.** 204 (95.35)   
49 + **[GTCCCG]** 955 (88.98)   
49 + **[GTCCCG]** 1116 (88.98)   
49 + **.GCCGCG.** 1407 (95.35)   
49 + **[GTGGCG]** 1504 (87.04)   
49 - **.GCCGCG.** 1120 (95.35)   
49 - **[GTCGGG]** 957 (87.52)   
51 + **[CTCGCG]** 34 (88.37)   
51 + **.GCCGCG.** 68 (95.35)   
51 + **[GTCGGG]** 73 (87.52)   
51 + **[GTCGGG]** 113 (87.52)   
51 + **[GACGCG]** 231 (89.80)   
51 + **.GGCGCG.** 280 (95.43)   
51 + **.GGCGCG.** 286 (95.43)   
51 + **.GCCGCG.** 598 (95.35)   
51 + **[CTCGCG]** 735 (88.37)   
51 + **[CTCGCG]** 780 (88.37)   
51 + **.GTCGCG.** 977 (100.00)   
51 + **[CTCGCG]** 996 (88.37)   
51 + **[GTCGCC]** 1041 (86.61)   
51 + **[GTGGCG]** 1123 (87.04)   
51 + **.GGCGCG.** 1125 (95.43)   
51 + **[GTCCCG]** 1143 (88.98)   
51 + **.GGCGCG.** 1335 (95.43)   
51 + **.GGCGCG.** 1360 (95.43)   
51 - **.GCCGCG.** 1362 (95.35)   
51 - **.GCCGCG.** 1337 (95.35)   
51 - **.GCCGCG.** 1286 (95.35)   
51 - **.GGCGCG.** 1244 (95.43)   
51 - **[GTCCCG]** 1188 (88.98)   
51 - **.GCCGCG.** 1127 (95.35)   
51 - **.GTCGCG.** 1101 (100.00)   
51 - **[GTCCCG]** 1046 (88.98)   
51 - **.GGCGCG.** 998 (95.43)   
51 - **.GGCGCG.** 782 (95.43)   
51 - **[CTCGCG]** 737 (88.37)   
51 - **.GGCGCG.** 636 (95.43)   
51 - **.GGCGCG.** 600 (95.43)   
51 - **[GACGCG]** 558 (89.80)   
51 - **[GTCGGG]** 513 (87.52)   
51 - **.GCCGCG.** 147 (95.35)   
51 - **.GGCGCG.** 90 (95.43)   
51 - **[GACGCG]** 70 (89.80)   
51 - **.GCCGCG.** 36 (95.35)   
53 + **[GACGCG]** 1057 (89.80)   
53 + **[GTGGCG]** 1190 (87.04)   
53 - **.GCCGCG.** 1059 (95.35)   
54 + **[GACGCG]** 1158 (89.80)   
54 + **[GTCCCG]** 1364 (88.98)   
54 - **[GTCGGG]** 1444 (87.52)   
54 - **[CTCGCG]** 1257 (88.37)   
54 - **[GTCGCC]** 1032 (86.61)   
55 + **.GCCGCG.** 199 (95.35)   
55 + **.GCCGCG.** 866 (95.35)   
55 + **.GCCGCG.** 884 (95.35)   
55 + **[GTGGCG]** 954 (87.04)   
55 + **.GGCGCG.** 956 (95.43)   
55 + **.GCCGCG.** 1085 (95.35)   
55 + **[GTCCCG]** 1167 (88.98)   
55 + **.GGCGCG.** 1322 (95.43)   
55 + **.GTCGCG.** 1484 (100.00)   
55 - **.GGCGCG.** 1276 (95.43)   
55 - **[GTCGCC]** 1126 (86.61)   
55 - **.GGCGCG.** 1082 (95.43)   
55 - **[GTCGCC]** 1021 (86.61)   
55 - **.GCCGCG.** 943 (95.35)   
55 - **[GTCCCG]** 466 (88.98)   
55 - **.GGCGCG.** 201 (95.43)   
55 - **[GTCCCG]** 87 (88.98)   
55 - **.GCCGCG.** 46 (95.35)   
58 + **[GTCGGG]** 273 (87.52)   
58 + **.GGCGCG.** 536 (95.43)   
58 + **[GTCGCC]** 554 (86.61)   
58 + **[GTCGCC]** 596 (86.61)   
58 + **[GACGCG]** 604 (89.80)   
58 + **[GACGCG]** 751 (89.80)   
58 + **[CTCGCG]** 895 (88.37)   
58 + **[CTCGCG]** 1249 (88.37)   
58 + **[GACGCG]** 1280 (89.80)   
58 + **.GGCGCG.** 1305 (95.43)   
58 + **[CTCGCG]** 1512 (88.37)   
58 - **.GGCGCG.** 1459 (95.43)   
58 - **[GTCGCC]** 1188 (86.61)   
58 - **.GCCGCG.** 1101 (95.35)   
58 - **.GCCGCG.** 781 (95.35)   
58 - **[GTCGGG]** 601 (87.52)   
58 - **[GTCGCC]** 505 (86.61)   
58 - **[GTCCCG]** 422 (88.98)   
59 + **.GGCGCG.** 188 (95.43)   
59 + **[GTCGCC]** 619 (86.61)   
59 + **.GCCGCG.** 650 (95.35)   
59 + **[GTCGCC]** 682 (86.61)   
59 + **.GGCGCG.** 893 (95.43)   
59 + **.GCCGCG.** 957 (95.35)   
59 + **[GTCCCG]** 974 (88.98)   
59 + **.GCCGCG.** 1071 (95.35)   
59 + **[GTCGCC]** 1206 (86.61)   
59 + **.GGCGCG.** 1352 (95.43)   
59 + **.GGCGCG.** 1367 (95.43)   
59 + **[GTGGCG]** 1407 (87.04)   
59 - **[GTCGCC]** 1409 (86.61)   
59 - **.GCCGCG.** 1369 (95.35)   
59 - **.GCCGCG.** 1349 (95.35)   
59 - **[GACGCG]** 1286 (89.80)   
59 - **[CTCGCG]** 1073 (88.37)   
59 - **[GTGGCG]** 1035 (87.04)   
59 - **.GCCGCG.** 1012 (95.35)   
59 - **[GTCCCG]** 687 (88.98)   
59 - **.GCCGCG.** 652 (95.35)   
60 + **[GTCCCG]** 625 (88.98)   
60 + **.GTCGCG.** 656 (100.00)   
60 + **.GCCGCG.** 825 (95.35)   
60 + **[CTCGCG]** 843 (88.37)   
60 + **.GTCGCG.** 1147 (100.00)   
60 - **.GTCGCG.** 1149 (100.00)   
60 - **[GTCCCG]** 873 (88.98)   
60 - **[GTCCCG]** 857 (88.98)   
60 - **.GCCGCG.** 827 (95.35)   
61 + **.GGCGCG.** 501 (95.43)   
61 + **[GACGCG]** 840 (89.80)   
61 + **.GCCGCG.** 914 (95.35)   
61 + **.GCCGCG.** 925 (95.35)   
61 + **.GCCGCG.** 1037 (95.35)   
61 + **[GACGCG]** 1320 (89.80)   
61 - **.GCCGCG.** 1039 (95.35)   
61 - **.GCCGCG.** 842 (95.35)   
61 - **[GTGGCG]** 766 (87.04)   
62 - **[GTGGCG]** 1319 (87.04)   
62 - **[GTGGCG]** 1067 (87.04)   
63 + **.GCCGCG.** 182 (95.35)   
63 + **[GTCGGG]** 214 (87.52)   
63 + **[GTGGCG]** 228 (87.04)   
63 + **.GGCGCG.** 266 (95.43)   
63 + **.GCCGCG.** 346 (95.35)   
63 + **.GCCGCG.** 534 (95.35)   
63 + **[GTCGCC]** 620 (86.61)   
63 + **.GGCGCG.** 656 (95.43)   
63 + **[GTCCCG]** 780 (88.98)   
63 + **[CTCGCG]** 852 (88.37)   
63 + **[CTCGCG]** 903 (88.37)   
63 + **[GTCCCG]** 960 (88.98)   
63 + **[GTCGCC]** 965 (86.61)   
63 + **.GTCGCG.** 974 (100.00)   
63 + **[GTCGGG]** 1115 (87.52)   
63 + **.GCCGCG.** 1123 (95.35)   
63 + **.GCCGCG.** 1253 (95.35)   
63 + **[GTCCCG]** 1299 (88.98)   
63 + **.GGCGCG.** 1391 (95.43)   
63 + **.GCCGCG.** 1412 (95.35)   
63 + **[GACGCG]** 1430 (89.80)   
63 - **[CTCGCG]** 1432 (88.37)   
63 - **.GCCGCG.** 1393 (95.35)   
63 - **.GTCGCG.** 1125 (100.00)   
63 - **.GCCGCG.** 954 (95.35)   
63 - **.GGCGCG.** 907 (95.43)   
63 - **[GACGCG]** 854 (89.80)   
63 - **[GTGGCG]** 760 (87.04)   
63 - **.GGCGCG.** 658 (95.43)   
63 - **[GACGCG]** 521 (89.80)   
63 - **.GGCGCG.** 454 (95.43)   
63 - **.GCCGCG.** 445 (95.35)   
63 - **[GTCCCG]** 428 (88.98)   
63 - **[GTCGCC]** 416 (86.61)   
63 - **.GCCGCG.** 410 (95.35)   
63 - **.GGCGCG.** 131 (95.43)   
64 - **[GTCGGG]** 745 (87.52)   
65 + **.GTCGCG.** 587 (100.00)   
65 + **.GCCGCG.** 784 (95.35)   
65 + **.GCCGCG.** 829 (95.35)   
65 + **.GGCGCG.** 864 (95.43)   
65 + **.GGCGCG.** 979 (95.43)   
65 + **.GCCGCG.** 1001 (95.35)   
65 + **.GCCGCG.** 1028 (95.35)   
65 + **.GGCGCG.** 1205 (95.43)   
65 + **[GACGCG]** 1224 (89.80)   
65 + **.GTCGCG.** 1230 (100.00)   
65 + **[GTCGGG]** 1368 (87.52)   
65 - **[GTGGCG]** 1422 (87.04)   
65 - **[GTCGGG]** 1414 (87.52)   
65 - **.GGCGCG.** 1030 (95.43)   
65 - **[CTCGCG]** 1003 (88.37)   
65 - **.GGCGCG.** 981 (95.43)   
65 - **.GCCGCG.** 948 (95.35)   
65 - **.GGCGCG.** 912 (95.43)   
65 - **[GACGCG]** 866 (89.80)   
65 - **.GGCGCG.** 589 (95.43)   
65 - **[GACGCG]** 547 (89.80)   
66 + **[GTGGCG]** 737 (87.04)   
66 + **.GCCGCG.** 768 (95.35)   
66 + **[GACGCG]** 786 (89.80)   
66 + **.GTCGCG.** 865 (100.00)   
66 + **.GCCGCG.** 989 (95.35)   
66 + **.GGCGCG.** 1179 (95.43)   
66 + **.GTCGCG.** 1336 (100.00)   
66 + **.GTCGCG.** 1404 (100.00)   
66 + **.GGCGCG.** 1438 (95.43)   
66 + **[GACGCG]** 1480 (89.80)   
66 + **[GTCCCG]** 1495 (88.98)   
66 - **[CTCGCG]** 1482 (88.37)   
66 - **[GACGCG]** 1406 (89.80)   
66 - **.GCCGCG.** 1127 (95.35)   
66 - **[GTGGCG]** 1088 (87.04)   
66 - **[GTCGGG]** 975 (87.52)   
66 - **.GGCGCG.** 955 (95.43)   
66 - **.GGCGCG.** 943 (95.43)   
66 - **.GGCGCG.** 869 (95.43)   
67 + **[GTCCCG]** 811 (88.98)   
67 + **[GTCGCC]** 821 (86.61)   
67 + **[GTCGCC]** 905 (86.61)   
67 + **[GTGGCG]** 1016 (87.04)   
67 + **[GACGCG]** 1173 (89.80)   
67 + **[GTCGCC]** 1193 (86.61)   
67 + **.GCCGCG.** 1196 (95.35)   
67 - **[GACGCG]** 1198 (89.80)   
67 - **.GGCGCG.** 1175 (95.43)   
67 - **[GTCGCC]** 1034 (86.61)   
67 - **[GTGGCG]** 958 (87.04)   
67 - **[GTCCCG]** 919 (88.98)   
67 - **[GTCGGG]** 831 (87.52)   
67 - **[GTCCCG]** 691 (88.98)   
68 + **[GTCGGG]** 83 (87.52)   
68 + **[GTCGGG]** 92 (87.52)   
68 + **[GTCCCG]** 144 (88.98)   
68 + **[GTCGGG]** 383 (87.52)   
68 + **[GTCGGG]** 389 (87.52)   
68 + **.GCCGCG.** 589 (95.35)   
68 + **.GGCGCG.** 652 (95.43)   
68 + **[CTCGCG]** 867 (88.37)   
68 + **[GTCGCC]** 882 (86.61)   
68 + **[GACGCG]** 990 (89.80)   
68 - **.GCCGCG.** 1394 (95.35)   
68 - **[GTCGCC]** 1182 (86.61)   
68 - **.GCCGCG.** 992 (95.35)   
68 - **[GTCCCG]** 898 (88.98)   
68 - **.GTCGCG.** 871 (100.00)   
68 - **.GCCGCG.** 812 (95.35)   
68 - **.GCCGCG.** 649 (95.35)   
68 - **[GTGGCG]** 582 (87.04)   
68 - **[GACGCG]** 419 (89.80)   
68 - **.GCCGCG.** 245 (95.35)   
68 - **[GTGGCG]** 37 (87.04)   
68 - **.GGCGCG.** 35 (95.43)   
68 - **[GTCGGG]** 28 (87.52)   
69 + **[GTCGCC]** 723 (86.61)   
69 + **[GTCGCC]** 732 (86.61)   
69 + **.GCCGCG.** 773 (95.35)   
69 + **.GGCGCG.** 892 (95.43)   
69 + **.GCCGCG.** 1032 (95.35)   
69 - **.GGCGCG.** 1203 (95.43)   
69 - **.GGCGCG.** 1138 (95.43)   
69 - **.GGCGCG.** 1121 (95.43)   
69 - **.GGCGCG.** 998 (95.43)   
69 - **[GTCGGG]** 973 (87.52)   
69 - **[GTCGGG]** 960 (87.52)   
69 - **[GACGCG]** 894 (89.80)   
69 - **.GCCGCG.** 769 (95.35)   
69 - **[GACGCG]** 690 (89.80)   
70 + **[GTGGCG]** 346 (87.04)   
70 + **.GTCGCG.** 732 (100.00)   
70 + **[GTCGGG]** 1037 (87.52)   
70 + **[GACGCG]** 1103 (89.80)   
70 + **.GGCGCG.** 1161 (95.43)   
70 - **[GACGCG]** 1450 (89.80)   
70 - **[GACGCG]** 1390 (89.80)   
70 - **.GGCGCG.** 1334 (95.43)   
70 - **[GTGGCG]** 1113 (87.04)   
70 - **.GCCGCG.** 1064 (95.35)   
70 - **[GTCGCC]** 1018 (86.61)   
70 - **[CTCGCG]** 734 (88.37)   
71 + **[GTCCCG]** 754 (88.98)   
71 + **.GCCGCG.** 1001 (95.35)   
71 + **[CTCGCG]** 1182 (88.37)   
71 + **[GACGCG]** 1237 (89.80)   
71 + **.GTCGCG.** 1494 (100.00)   
71 - **[CTCGCG]** 1239 (88.37)   
71 - **.GGCGCG.** 1184 (95.43)   
71 - **[GTCCCG]** 1005 (88.98)   
71 - **[GACGCG]** 904 (89.80)   
71 - **.GGCGCG.** 870 (95.43)   
72 + **[GTCGGG]** 28 (87.52)   
73 + **.GCCGCG.** 919 (95.35)   
73 + **[GTCCCG]** 932 (88.98)   
73 + **.GGCGCG.** 1021 (95.43)   
73 - **.GCCGCG.** 1087 (95.35)   
73 - **.GCCGCG.** 921 (95.35)   
73 - **[GTCGGG]** 788 (87.52)   
74 + **[GTCCCG]** 829 (88.98)   
74 + **[GTCGGG]** 1404 (87.52)   
74 + **[GTCGGG]** 1414 (87.52)   
74 + **.GTCGCG.** 1427 (100.00)   
74 + **.GCCGCG.** 1436 (95.35)   
74 - **.GGCGCG.** 1438 (95.43)   
74 - **.GCCGCG.** 1429 (95.35)   
74 - **[GACGCG]** 1338 (89.80)   
74 - **.GCCGCG.** 1255 (95.35)   
74 - **[CTCGCG]** 1150 (88.37)   
74 - **[CTCGCG]** 1063 (88.37)   
74 - **.GCCGCG.** 934 (95.35)   
76 + **.GCCGCG.** 127 (95.35)   
76 + **.GGCGCG.** 999 (95.43)   
76 + **.GCCGCG.** 1056 (95.35)   
76 + **[GACGCG]** 1284 (89.80)   
76 - **[GTCCCG]** 1336 (88.98)   
76 - **.GGCGCG.** 1116 (95.43)   
76 - **.GCCGCG.** 1064 (95.35)   
76 - **[CTCGCG]** 1058 (88.37)   
76 - **[GTCGCC]** 869 (86.61)   
76 - **.GCCGCG.** 866 (95.35)   
76 - **.GGCGCG.** 829 (95.43)   
77 + **[GTGGCG]** 35 (87.04)   
77 + **.GGCGCG.** 248 (95.43)   
77 + **[GTCGGG]** 455 (87.52)   
77 + **[GTCCCG]** 596 (88.98)   
77 + **.GCCGCG.** 716 (95.35)   
77 + **.GGCGCG.** 931 (95.43)   
77 - **[CTCGCG]** 1316 (88.37)   
77 - **.GCCGCG.** 1117 (95.35)   
77 - **.GCCGCG.** 1054 (95.35)   
77 - **[GTCCCG]** 942 (88.98)   
77 - **.GCCGCG.** 781 (95.35)   
77 - **.GGCGCG.** 600 (95.43)   
79 + **.GCCGCG.** 654 (95.35)   
79 + **.GCCGCG.** 878 (95.35)   
79 + **.GCCGCG.** 948 (95.35)   
79 + **[GTGGCG]** 1037 (87.04)   
79 + **.GTCGCG.** 1088 (100.00)   
79 + **.GCCGCG.** 1277 (95.35)   
79 + **[CTCGCG]** 1308 (88.37)   
79 + **.GCCGCG.** 1397 (95.35)   
79 - **[GACGCG]** 1325 (89.80)   
79 - **.GGCGCG.** 1202 (95.43)   
79 - **[GTCGCC]** 811 (86.61)   
79 - **[GTCGCC]** 765 (86.61)   
79 - **[CTCGCG]** 656 (88.37)   
79 - **[GTCGCC]** 166 (86.61)   
80 + **.GTCGCG.** 396 (100.00)   
80 + **.GGCGCG.** 955 (95.43)   
80 + **[GTCGCC]** 1013 (86.61)   
80 + **.GCCGCG.** 1016 (95.35)   
80 + **.GGCGCG.** 1044 (95.43)   
80 + **.GCCGCG.** 1207 (95.35)   
80 + **.GGCGCG.** 1308 (95.43)   
80 - **.GCCGCG.** 1343 (95.35)   
80 - **[GTGGCG]** 1252 (87.04)   
80 - **.GCCGCG.** 1209 (95.35)   
80 - **.GGCGCG.** 1018 (95.43)   
80 - **[GTCGGG]** 839 (87.52)   
80 - **[GTCGGG]** 758 (87.52)   
80 - **[GTCGGG]** 751 (87.52)   
81 + **[CTCGCG]** 642 (88.37)   
81 + **[GTGGCG]** 1038 (87.04)   
81 - **[CTCGCG]** 1242 (88.37)   
81 - **[GTCGGG]** 842 (87.52)   
81 - **[CTCGCG]** 685 (88.37)   
83 + **[GTGGCG]** 1352 (87.04)   
84 + **[GTCCCG]** 666 (88.98)   
84 + **[GTCGGG]** 779 (87.52)   
84 + **[CTCGCG]** 862 (88.37)   
84 + **[GACGCG]** 900 (89.80)   
84 + **.GCCGCG.** 980 (95.35)   
84 + **[GACGCG]** 1035 (89.80)   
84 + **.GGCGCG.** 1153 (95.43)   
84 - **.GCCGCG.** 1037 (95.35)   
84 - **[GTCGGG]** 1032 (87.52)   
84 - **.GCCGCG.** 1023 (95.35)   
84 - **.GGCGCG.** 902 (95.43)   
84 - **[GTCGGG]** 897 (87.52)   
84 - **.GCCGCG.** 871 (95.35)   
84 - **.GGCGCG.** 864 (95.43)   
84 - **.GCCGCG.** 798 (95.35)   
84 - **[GTCGGG]** 614 (87.52)   
85 + **.GCCGCG.** 93 (95.35)   
85 + **[GTCGGG]** 295 (87.52)   
85 + **[GTCGGG]** 309 (87.52)   
85 + **[GTCGGG]** 472 (87.52)   
85 + **.GGCGCG.** 587 (95.43)   
85 + **[GACGCG]** 928 (89.80)   
85 + **[GACGCG]** 961 (89.80)   
85 + **[CTCGCG]** 973 (88.37)   
85 + **.GCCGCG.** 1415 (95.35)   
85 + **[GTCCCG]** 1441 (88.98)   
85 - **.GGCGCG.** 1450 (95.43)   
85 - **[GTCGGG]** 1333 (87.52)   
85 - **.GCCGCG.** 1217 (95.35)   
85 - **.GGCGCG.** 1096 (95.43)   
85 - **.GGCGCG.** 1078 (95.43)   
85 - **.GGCGCG.** 1059 (95.43)   
85 - **.GGCGCG.** 1037 (95.43)   
85 - **[GTGGCG]** 1011 (87.04)   
85 - **.GGCGCG.** 963 (95.43)   
85 - **.GCCGCG.** 880 (95.35)   
85 - **[GACGCG]** 589 (89.80)   
85 - **[GACGCG]** 95 (89.80)   
85 - **[GTCGCC]** 70 (86.61)   
85 - **[GTCGGG]** 31 (87.52)   
86 + **.GCCGCG.** 305 (95.35)   
86 + **.GCCGCG.** 541 (95.35)   
86 + **.GCCGCG.** 738 (95.35)   
86 + **.GTCGCG.** 769 (100.00)   
86 + **.GGCGCG.** 834 (95.43)   
86 + **.GGCGCG.** 879 (95.43)   
86 + **.GGCGCG.** 900 (95.43)   
86 + **[GACGCG]** 1086 (89.80)   
86 + **[GTCGGG]** 1124 (87.52)   
86 + **[CTCGCG]** 1188 (88.37)   
86 + **.GGCGCG.** 1193 (95.43)   
86 + **.GGCGCG.** 1384 (95.43)   
86 + **.GCCGCG.** 1437 (95.35)   
86 + **.GCCGCG.** 1510 (95.35)   
86 - **.GTCGCG.** 1512 (100.00)   
86 - **.GCCGCG.** 1506 (95.35)   
86 - **.GGCGCG.** 1341 (95.43)   
86 - **[GTCGGG]** 1320 (87.52)   
86 - **[GTCCCG]** 1285 (88.98)   
86 - **.GCCGCG.** 1195 (95.35)   
86 - **.GCCGCG.** 1190 (95.35)   
86 - **[CTCGCG]** 988 (88.37)   
86 - **.GCCGCG.** 902 (95.35)   
86 - **.GCCGCG.** 836 (95.35)   
86 - **[GTCGGG]** 797 (87.52)   
86 - **.GCCGCG.** 658 (95.35)   
86 - **.GCCGCG.** 543 (95.35)   
87 + **[GACGCG]** 133 (89.80)   
87 + **[CTCGCG]** 174 (88.37)   
87 + **[CTCGCG]** 308 (88.37)   
87 + **.GGCGCG.** 313 (95.43)   
87 + **[GTCGGG]** 421 (87.52)   
87 + **[GTCGGG]** 540 (87.52)   
87 + **[GACGCG]** 586 (89.80)   
87 + **[GTCGGG]** 604 (87.52)   
87 + **.GGCGCG.** 639 (95.43)   
87 + **.GCCGCG.** 768 (95.35)   
87 + **.GGCGCG.** 843 (95.43)   
87 + **.GCCGCG.** 1097 (95.35)   
87 + **[GTCCCG]** 1102 (88.98)   
87 + **.GGCGCG.** 1242 (95.43)   
87 + **[CTCGCG]** 1331 (88.37)   
87 + **[GTCGGG]** 1358 (87.52)   
87 + **.GCCGCG.** 1434 (95.35)   
87 + **.GCCGCG.** 1486 (95.35)   
87 + **.GGCGCG.** 1501 (95.43)   
87 - **.GGCGCG.** 1378 (95.43)   
87 - **.GCCGCG.** 1367 (95.35)   
87 - **.GGCGCG.** 1346 (95.43)   
87 - **.GCCGCG.** 1333 (95.35)   
87 - **.GGCGCG.** 1322 (95.43)   
87 - **.GCCGCG.** 1244 (95.35)   
87 - **.GCCGCG.** 1231 (95.35)   
87 - **.GGCGCG.** 1194 (95.43)   
87 - **[CTCGCG]** 1176 (88.37)   
87 - **[GACGCG]** 1099 (89.80)   
87 - **[CTCGCG]** 1056 (88.37)   
87 - **[GTCGGG]** 981 (87.52)   
87 - **.GGCGCG.** 874 (95.43)   
87 - **.GCCGCG.** 722 (95.35)   
87 - **[CTCGCG]** 315 (88.37)   
87 - **.GCCGCG.** 310 (95.35)   
87 - **[GACGCG]** 135 (89.80)   
88 + **.GGCGCG.** 419 (95.43)   
88 + **.GGCGCG.** 445 (95.43)   
88 + **[GTGGCG]** 476 (87.04)   
88 + **.GTCGCG.** 488 (100.00)   
88 + **.GCCGCG.** 504 (95.35)   
88 + **.GCCGCG.** 607 (95.35)   
88 + **[CTCGCG]** 667 (88.37)   
88 + **[CTCGCG]** 689 (88.37)   
88 + **[GTCGGG]** 700 (87.52)   
88 + **.GGCGCG.** 705 (95.43)   
88 + **.GTCGCG.** 714 (100.00)   
88 + **.GGCGCG.** 755 (95.43)   
88 + **[GTCGGG]** 937 (87.52)   
88 + **.GCCGCG.** 1097 (95.35)   
88 + **[CTCGCG]** 1140 (88.37)   
88 - **[GTCGCC]** 1398 (86.61)   
88 - **.GCCGCG.** 1395 (95.35)   
88 - **.GCCGCG.** 1380 (95.35)   
88 - **[GACGCG]** 1206 (89.80)   
88 - **.GCCGCG.** 1099 (95.35)   
88 - **[GTCCCG]** 939 (88.98)   
88 - **.GCCGCG.** 849 (95.35)   
88 - **.GCCGCG.** 707 (95.35)   
88 - **[CTCGCG]** 371 (88.37)   
88 - **[GTCGGG]** 110 (87.52)   
89 + **[GTCCCG]** 56 (88.98)   
89 + **[CTCGCG]** 130 (88.37)   
89 + **.GTCGCG.** 151 (100.00)   
89 + **.GCCGCG.** 260 (95.35)   
89 + **.GCCGCG.** 269 (95.35)   
89 + **.GCCGCG.** 418 (95.35)   
89 + **.GCCGCG.** 435 (95.35)   
89 + **.GCCGCG.** 478 (95.35)   
89 + **.GCCGCG.** 493 (95.35)   
89 + **[GACGCG]** 600 (89.80)   
89 + **.GCCGCG.** 664 (95.35)   
89 + **.GGCGCG.** 721 (95.43)   
89 + **.GGCGCG.** 741 (95.43)   
89 + **.GGCGCG.** 787 (95.43)   
89 + **.GGCGCG.** 937 (95.43)   
89 + **.GCCGCG.** 1021 (95.35)   
89 + **.GCCGCG.** 1126 (95.35)   
89 + **.GCCGCG.** 1310 (95.35)   
89 + **.GCCGCG.** 1451 (95.35)   
89 + **[GACGCG]** 1465 (89.80)   
89 - **.GTCGCG.** 1467 (100.00)   
89 - **.GGCGCG.** 1325 (95.43)   
89 - **.GGCGCG.** 1023 (95.43)   
89 - **.GGCGCG.** 976 (95.43)   
89 - **.GCCGCG.** 843 (95.35)   
89 - **.GGCGCG.** 789 (95.43)   
89 - **[CTCGCG]** 758 (88.37)   
89 - **.GCCGCG.** 743 (95.35)   
89 - **.GGCGCG.** 675 (95.43)   
89 - **.GGCGCG.** 666 (95.43)   
89 - **[GTCGGG]** 530 (87.52)   
89 - **.GGCGCG.** 495 (95.43)   
89 - **[GACGCG]** 480 (89.80)   
89 - **.GGCGCG.** 437 (95.43)   
89 - **.GGCGCG.** 364 (95.43)   
89 - **.GGCGCG.** 335 (95.43)   
89 - **.GTCGCG.** 271 (100.00)   
89 - **.GGCGCG.** 153 (95.43)   
89 - **.GGCGCG.** 100 (95.43)   
89 - **.GCCGCG.** 47 (95.35)   
90 + **[GTCGCC]** 84 (86.61)   
90 + **[GTGGCG]** 358 (87.04)   
90 + **.GGCGCG.** 360 (95.43)   
90 + **[CTCGCG]** 445 (88.37)   
90 + **.GGCGCG.** 510 (95.43)   
90 + **.GGCGCG.** 557 (95.43)   
90 + **.GCCGCG.** 587 (95.35)   
90 + **[CTCGCG]** 608 (88.37)   
90 + **[CTCGCG]** 635 (88.37)   
90 + **.GCCGCG.** 718 (95.35)   
90 + **.GCCGCG.** 775 (95.35)   
90 + **[GTCGCC]** 841 (86.61)   
90 + **.GCCGCG.** 848 (95.35)   
90 + **.GCCGCG.** 1019 (95.35)   
90 + **[GTCGGG]** 1025 (87.52)   
90 + **[GTGGCG]** 1114 (87.04)   
90 - **[GTCGGG]** 1440 (87.52)   
90 - **[GTCGGG]** 1402 (87.52)   
90 - **[GTCGGG]** 1343 (87.52)   
90 - **.GCCGCG.** 1330 (95.35)   
90 - **[GACGCG]** 1314 (89.80)   
90 - **.GCCGCG.** 993 (95.35)   
90 - **[CTCGCG]** 949 (88.37)   
90 - **.GCCGCG.** 850 (95.35)   
90 - **.GCCGCG.** 777 (95.35)   
90 - **.GCCGCG.** 720 (95.35)   
90 - **.GGCGCG.** 715 (95.43)   
90 - **.GGCGCG.** 654 (95.43)   
90 - **[CTCGCG]** 637 (88.37)   
90 - **[CTCGCG]** 610 (88.37)   
90 - **.GGCGCG.** 514 (95.43)   
90 - **.GGCGCG.** 480 (95.43)   
90 - **[GACGCG]** 362 (89.80)   
90 - **[CTCGCG]** 330 (88.37)   
92 + **.GCCGCG.** 1056 (95.35)   
92 - **.GTCGCG.** 1459 (100.00)   
92 - **.GCCGCG.** 1228 (95.35)   
93 + **[GTCCCG]** 407 (88.98)   
93 + **[GACGCG]** 963 (89.80)   
93 + **.GGCGCG.** 975 (95.43)   
93 + **[CTCGCG]** 983 (88.37)   
93 + **[GACGCG]** 1001 (89.80)   
93 + **.GGCGCG.** 1238 (95.43)   
93 + **[GTGGCG]** 1425 (87.04)   
93 - **[GACGCG]** 1488 (89.80)   
93 - **[GTCCCG]** 1470 (88.98)   
93 - **[GTCGCC]** 1430 (86.61)   
93 - **.GCCGCG.** 1282 (95.35)   
93 - **.GCCGCG.** 1242 (95.35)   
93 - **[CTCGCG]** 1224 (88.37)   
93 - **.GGCGCG.** 1198 (95.43)   
93 - **[CTCGCG]** 1135 (88.37)   
93 - **.GCCGCG.** 1037 (95.35)   
93 - **.GGCGCG.** 1003 (95.43)   
93 - **[GTCCCG]** 998 (88.98)   
93 - **[GACGCG]** 985 (89.80)   
93 - **[GTCCCG]** 910 (88.98)   
93 - **[GTCGCC]** 416 (86.61)   
94 - **[GTCGCC]** 561 (86.61)   
96 + **.GCCGCG.** 848 (95.35)   
96 + **[CTCGCG]** 908 (88.37)   
96 + **[GTCGCC]** 1020 (86.61)   
96 + **.GTCGCG.** 1350 (100.00)   
96 + **.GTCGCG.** 1357 (100.00)   
96 + **[GTCGCC]** 1362 (86.61)   
96 + **[GACGCG]** 1393 (89.80)   
96 + **[GTCGGG]** 1430 (87.52)   
96 - **.GGCGCG.** 1422 (95.43)   
96 - **[GACGCG]** 1359 (89.80)   
96 - **[GACGCG]** 1354 (89.80)   
96 - **.GCCGCG.** 884 (95.35)   
96 - **[GACGCG]** 740 (89.80)   
97 + **.GGCGCG.** 880 (95.43)   
97 + **[GTCGGG]** 936 (87.52)   
97 + **[GTGGCG]** 1072 (87.04)   
97 + **.GGCGCG.** 1223 (95.43)   
97 + **[GACGCG]** 1367 (89.80)   
97 + **[GTGGCG]** 1389 (87.04)   
97 + **[GTCGCC]** 1444 (86.61)   
97 - **[GTCCCG]** 1343 (88.98)   
97 - **[CTCGCG]** 1147 (88.37)   
97 - **.GGCGCG.** 1047 (95.43)   
97 - **[CTCGCG]** 1005 (88.37)   
97 - **.GCCGCG.** 882 (95.35)   
97 - **.GCCGCG.** 877 (95.35)   
97 - **[GTGGCG]** 857 (87.04)   
98 + **[GTCCCG]** 964 (88.98)   
98 + **[GTGGCG]** 973 (87.04)   
98 + **.GCCGCG.** 999 (95.35)   
98 + **.GGCGCG.** 1031 (95.43)   
98 + **.GGCGCG.** 1038 (95.43)   
98 + **[CTCGCG]** 1058 (88.37)   
98 + **[GACGCG]** 1098 (89.80)   
98 + **.GCCGCG.** 1119 (95.35)   
98 + **.GGCGCG.** 1227 (95.43)   
98 + **[GTCGCC]** 1268 (86.61)   
98 + **[GTGGCG]** 1340 (87.04)   
98 + **[GTCGGG]** 1440 (87.52)   
98 - **[GTCCCG]** 1464 (88.98)   
98 - **[GTCCCG]** 1404 (88.98)   
98 - **.GCCGCG.** 1325 (95.35)   
98 - **[GTCCCG]** 1231 (88.98)   
98 - **.GCCGCG.** 1137 (95.35)   
98 - **.GCCGCG.** 1086 (95.35)   
98 - **[GTCCCG]** 1062 (88.98)   
98 - **.GCCGCG.** 1001 (95.35)   
99 + **[GTCGGG]** 1069 (87.52)   
99 - **[GTCCCG]** 1352 (88.98)   
99 - **[GTGGCG]** 19 (87.04)   
99 - **.GGCGCG.** 17 (95.43)   
100 + **[GTCGGG]** 221 (87.52)   
100 + **[GTCCCG]** 575 (88.98)   
100 + **.GTCGCG.** 1102 (100.00)   
100 + **.GCCGCG.** 1305 (95.35)   
100 - **.GCCGCG.** 1500 (95.35)   
100 - **.GCCGCG.** 1140 (95.35)   
100 - **.GGCGCG.** 1104 (95.43)   
102 + **[GTCCCG]** 1221 (88.98)   
103 + **[GTCCCG]** 835 (88.98)   
103 + **[GTCGCC]** 1064 (86.61)   
103 + **.GTCGCG.** 1182 (100.00)   
103 - **[GTCCCG]** 514 (88.98)   
103 - **[CTCGCG]** 443 (88.37)   
103 - **[GTGGCG]** 85 (87.04)   
104 + **[GACGCG]** 1140 (89.80)   
104 - **[CTCGCG]** 1142 (88.37)   
105 + **[GTCGCC]** 963 (86.61)   
105 + **.GGCGCG.** 1022 (95.43)   
105 + **.GGCGCG.** 1142 (95.43)   
105 + **[GTCGGG]** 1368 (87.52)   
105 - **[GTCGGG]** 1350 (87.52)   
105 - **.GGCGCG.** 1248 (95.43)   
105 - **.GCCGCG.** 1039 (95.35)   
105 - **[CTCGCG]** 1024 (88.37)   
106 + **[CTCGCG]** 868 (88.37)   
106 + **.GTCGCG.** 1158 (100.00)   
106 - **.GCCGCG.** 1160 (95.35)   
106 - **.GCCGCG.** 870 (95.35)   
107 - **[GTGGCG]** 66 (87.04)   
108 + **.GGCGCG.** 99 (95.43)   
108 + **[GTCCCG]** 281 (88.98)   
108 + **[GTCGCC]** 386 (86.61)   
108 + **.GTCGCG.** 397 (100.00)   
108 + **[GTCCCG]** 478 (88.98)   
108 + **[GACGCG]** 818 (89.80)   
108 + **[GACGCG]** 923 (89.80)   
108 + **[CTCGCG]** 1065 (88.37)   
108 + **[CTCGCG]** 1112 (88.37)   
108 + **[GTCCCG]** 1206 (88.98)   
108 + **[GTCGCC]** 1233 (86.61)   
108 + **.GTCGCG.** 1243 (100.00)   
108 + **.GGCGCG.** 1300 (95.43)   
108 + **[GACGCG]** 1385 (89.80)   
108 + **[CTCGCG]** 1509 (88.37)   
108 - **[CTCGCG]** 1302 (88.37)   
108 - **[GTGGCG]** 1271 (87.04)   
108 - **.GGCGCG.** 1218 (95.43)   
108 - **[GACGCG]** 1203 (89.80)   
108 - **.GCCGCG.** 1114 (95.35)   
108 - **[GTCGCC]** 931 (86.61)   
108 - **[CTCGCG]** 925 (88.37)   
108 - **[GTCCCG]** 815 (88.98)   
108 - **.GTCGCG.** 767 (100.00)   
108 - **[GTCGGG]** 588 (87.52)   
108 - **[GACGCG]** 429 (89.80)   
108 - **.GCCGCG.** 101 (95.35)   
108 - **[GTCGCC]** 82 (86.61)   
108 - **[GTCGCC]** 69 (86.61)   
109 + **.GTCGCG.** 291 (100.00)   
109 + **.GCCGCG.** 337 (95.35)   
109 + **[CTCGCG]** 376 (88.37)   
109 + **[GTCGCC]** 394 (86.61)   
109 + **[CTCGCG]** 657 (88.37)   
109 + **[GACGCG]** 814 (89.80)   
109 + **.GGCGCG.** 825 (95.43)   
109 + **[GTCCCG]** 834 (88.98)   
109 + **.GCCGCG.** 865 (95.35)   
109 + **[GTCGCC]** 936 (86.61)   
109 + **.GCCGCG.** 943 (95.35)   
109 + **[GTCCCG]** 1012 (88.98)   
109 + **[GTCCCG]** 1028 (88.98)   
109 + **[GTCCCG]** 1035 (88.98)   
109 + **[GACGCG]** 1231 (89.80)   
109 + **.GGCGCG.** 1313 (95.43)   
109 + **.GGCGCG.** 1320 (95.43)   
109 + **.GGCGCG.** 1355 (95.43)   
109 + **.GGCGCG.** 1389 (95.43)   
109 + **.GGCGCG.** 1394 (95.43)   
109 + **[GTGGCG]** 1483 (87.04)   
109 + **[GTGGCG]** 1489 (87.04)   
109 - **.GCCGCG.** 1396 (95.35)   
109 - **.GCCGCG.** 1391 (95.35)   
109 - **.GCCGCG.** 1359 (95.35)   
109 - **[GACGCG]** 1331 (89.80)   
109 - **.GGCGCG.** 1233 (95.43)   
109 - **.GGCGCG.** 1194 (95.43)   
109 - **[GTCGGG]** 1182 (87.52)   
109 - **.GGCGCG.** 1043 (95.43)   
109 - **[GACGCG]** 1032 (89.80)   
109 - **[GTGGCG]** 947 (87.04)   
109 - **.GGCGCG.** 945 (95.43)   
109 - **[GTCGCC]** 924 (86.61)   
109 - **.GGCGCG.** 659 (95.43)   
109 - **[CTCGCG]** 600 (88.37)   
109 - **.GGCGCG.** 455 (95.43)   
109 - **[CTCGCG]** 378 (88.37)   
109 - **.GCCGCG.** 339 (95.35)   
109 - **.GTCGCG.** 293 (100.00)   
110 + **.GCCGCG.** 859 (95.35)   
110 + **.GGCGCG.** 949 (95.43)   
112 + **[CTCGCG]** 349 (88.37)   
114 + **[GTCCCG]** 9 (88.98)   
114 + **[GTCGCC]** 259 (86.61)   
114 + **[GTCGGG]** 284 (87.52)   
114 + **.GCCGCG.** 350 (95.35)   
114 + **[CTCGCG]** 369 (88.37)   
114 + **[GTCGCC]** 379 (86.61)   
114 + **[GTCGGG]** 570 (87.52)   
114 + **[GTCCCG]** 582 (88.98)   
114 + **.GCCGCG.** 1000 (95.35)   
114 + **.GGCGCG.** 1007 (95.43)   
114 + **[GACGCG]** 1019 (89.80)   
114 + **[GTGGCG]** 1032 (87.04)   
114 + **[GTGGCG]** 1057 (87.04)   
114 + **[GTCGGG]** 1342 (87.52)   
114 - **[GTGGCG]** 1512 (87.04)   
114 - **.GCCGCG.** 1308 (95.35)   
114 - **[GTCGGG]** 1245 (87.52)   
114 - **[CTCGCG]** 1021 (88.37)   
114 - **.GCCGCG.** 1009 (95.35)   
114 - **.GCCGCG.** 1004 (95.35)   
114 - **[GTCGCC]** 765 (86.61)   
114 - **.GCCGCG.** 742 (95.35)   
115 + **.GGCGCG.** 224 (95.43)   
115 + **.GGCGCG.** 281 (95.43)   
115 + **.GGCGCG.** 301 (95.43)   
115 + **.GCCGCG.** 310 (95.35)   
115 + **[CTCGCG]** 464 (88.37)   
115 + **[GTCGCC]** 469 (86.61)   
115 + **[GACGCG]** 870 (89.80)   
115 + **.GTCGCG.** 1236 (100.00)   
115 - **[GTCGGG]** 1486 (87.52)   
115 - **.GTCGCG.** 1041 (100.00)   
115 - **.GGCGCG.** 1023 (95.43)   
115 - **.GTCGCG.** 867 (100.00)   
115 - **.GGCGCG.** 689 (95.43)   
115 - **[GTCCCG]** 679 (88.98)   
115 - **.GGCGCG.** 590 (95.43)   
115 - **[GACGCG]** 466 (89.80)   
115 - **.GCCGCG.** 413 (95.35)   
115 - **[GTGGCG]** 264 (87.04)   
115 - **[CTCGCG]** 226 (88.37)   
115 - **[GTGGCG]** 153 (87.04)   
116 + **[GACGCG]** 961 (89.80)   
116 + **[GTCGGG]** 1108 (87.52)   
116 - **[GTGGCG]** 1219 (87.04)   
116 - **.GCCGCG.** 1201 (95.35)   
116 - **[GTGGCG]** 1007 (87.04)   
117 + **.GGCGCG.** 595 (95.43)   
117 + **.GGCGCG.** 656 (95.43)   
117 + **.GGCGCG.** 667 (95.43)   
117 + **.GCCGCG.** 792 (95.35)   
117 + **.GCCGCG.** 926 (95.35)   
117 + **[GTCCCG]** 989 (88.98)   
117 - **.GCCGCG.** 1056 (95.35)   
117 - **.GGCGCG.** 1026 (95.43)   
117 - **.GCCGCG.** 978 (95.35)   
117 - **[GTGGCG]** 962 (87.04)   
117 - **[GTGGCG]** 942 (87.04)   
117 - **.GGCGCG.** 940 (95.43)   
117 - **[GTCCCG]** 853 (88.98)   
117 - **.GGCGCG.** 669 (95.43)   
117 - **.GTCGCG.** 640 (100.00)   
118 + **.GCCGCG.** 433 (95.35)   
118 + **.GGCGCG.** 543 (95.43)   
118 + **.GGCGCG.** 604 (95.43)   
118 + **.GGCGCG.** 632 (95.43)   
118 + **.GCCGCG.** 768 (95.35)   
118 + **[GTCCCG]** 979 (88.98)   
118 - **.GCCGCG.** 1045 (95.35)   
118 - **.GGCGCG.** 1016 (95.43)   
118 - **[GTGGCG]** 952 (87.04)   
118 - **[GTGGCG]** 932 (87.04)   
118 - **.GGCGCG.** 930 (95.43)   
118 - **[GTCCCG]** 829 (88.98)   
118 - **.GTCGCG.** 617 (100.00)   
118 - **.GTCGCG.** 589 (100.00)   
119 + **.GCCGCG.** 431 (95.35)   
119 + **.GGCGCG.** 541 (95.43)   
119 + **.GGCGCG.** 602 (95.43)   
119 + **.GGCGCG.** 613 (95.43)   
119 + **.GGCGCG.** 630 (95.43)   
119 + **.GGCGCG.** 641 (95.43)   
119 + **.GCCGCG.** 766 (95.35)   
119 - **.GCCGCG.** 1036 (95.35)   
119 - **.GGCGCG.** 1007 (95.43)   
119 - **.GCCGCG.** 958 (95.35)   
119 - **[GTGGCG]** 943 (87.04)   
119 - **[GTGGCG]** 923 (87.04)   
119 - **.GGCGCG.** 921 (95.43)   
119 - **[GTCCCG]** 827 (88.98)   
119 - **.GTCGCG.** 615 (100.00)   
119 - **.GTCGCG.** 587 (100.00)   
120 + **.GGCGCG.** 1001 (95.43)   
120 + **.GGCGCG.** 1062 (95.43)   
120 + **.GGCGCG.** 1090 (95.43)   
120 + **.GGCGCG.** 1101 (95.43)   
120 + **.GCCGCG.** 1226 (95.35)   
120 + **[GTCCCG]** 1436 (88.98)   
120 - **.GCCGCG.** 1503 (95.35)   
120 - **.GGCGCG.** 1474 (95.43)   
120 - **.GCCGCG.** 1425 (95.35)   
120 - **[GTGGCG]** 1410 (87.04)   
120 - **[GTGGCG]** 1390 (87.04)   
120 - **.GGCGCG.** 1388 (95.43)   
120 - **[GTCCCG]** 1286 (88.98)   
120 - **.GGCGCG.** 1103 (95.43)   
120 - **.GTCGCG.** 1075 (100.00)   
120 - **.GTCGCG.** 1046 (100.00)   
121 + **[GTCCCG]** 900 (88.98)   
121 + **[GTCCCG]** 1028 (88.98)   
121 - **.GTCGCG.** 1282 (100.00)   
122 + **.GCCGCG.** 82 (95.35)   
122 + **[GTCCCG]** 413 (88.98)   
122 + **[CTCGCG]** 457 (88.37)   
122 + **.GGCGCG.** 902 (95.43)   
122 + **[CTCGCG]** 936 (88.37)   
122 + **[CTCGCG]** 951 (88.37)   
122 + **[GTCGCC]** 1089 (86.61)   
122 - **.GGCGCG.** 924 (95.43)   
122 - **.GCCGCG.** 916 (95.35)   
122 - **[CTCGCG]** 459 (88.37)   
122 - **[CTCGCG]** 84 (88.37)   
122 - **.GGCGCG.** 32 (95.43)   
123 + **.GCCGCG.** 15 (95.35)   
123 + **.GGCGCG.** 390 (95.43)   
123 + **[GTCCCG]** 395 (88.98)   
123 + **[GTCCCG]** 438 (88.98)   
123 + **[CTCGCG]** 525 (88.37)   
123 + **.GCCGCG.** 602 (95.35)   
123 + **.GGCGCG.** 743 (95.43)   
123 + **.GGCGCG.** 767 (95.43)   
123 + **.GCCGCG.** 826 (95.35)   
123 + **[GTCCCG]** 844 (88.98)   
123 + **.GGCGCG.** 898 (95.43)   
123 + **[GTCCCG]** 928 (88.98)   
123 + **[GTCGCC]** 1002 (86.61)   
123 + **.GCCGCG.** 1005 (95.35)   
123 + **.GCCGCG.** 1010 (95.35)   
123 + **.GTCGCG.** 1233 (100.00)   
123 - **[GTCGGG]** 1082 (87.52)   
123 - **.GCCGCG.** 1030 (95.35)   
123 - **.GGCGCG.** 1012 (95.43)   
123 - **.GGCGCG.** 1007 (95.43)   
123 - **.GCCGCG.** 952 (95.35)   
123 - **.GCCGCG.** 900 (95.35)   
123 - **.GTCGCG.** 852 (100.00)   
123 - **.GTCGCG.** 828 (100.00)   
123 - **.GGCGCG.** 769 (95.43)   
123 - **[GTCGCC]** 755 (86.61)   
123 - **.GGCGCG.** 690 (95.43)   
123 - **.GGCGCG.** 660 (95.43)   
123 - **.GGCGCG.** 557 (95.43)   
123 - **.GGCGCG.** 545 (95.43)   
123 - **[GACGCG]** 392 (89.80)   
124 + **[GACGCG]** 828 (89.80)   
124 + **.GGCGCG.** 868 (95.43)   
124 + **.GGCGCG.** 880 (95.43)   
124 + **[GTCCCG]** 928 (88.98)   
124 + **.GGCGCG.** 953 (95.43)   
124 + **[GTCCCG]** 1047 (88.98)   
124 + **[GTCGGG]** 1102 (87.52)   
124 + **[CTCGCG]** 1125 (88.37)   
124 + **.GGCGCG.** 1149 (95.43)   
124 + **.GCCGCG.** 1224 (95.35)   
124 + **[GTCGGG]** 1256 (87.52)   
124 - **[CTCGCG]** 1127 (88.37)   
124 - **.GGCGCG.** 1112 (95.43)   
127 + **[GTCCCG]** 432 (88.98)   
127 + **[GTCCCG]** 555 (88.98)   
127 + **[GTCCCG]** 574 (88.98)   
127 + **[GTCCCG]** 581 (88.98)   
127 + **.GGCGCG.** 630 (95.43)   
127 + **.GGCGCG.** 744 (95.43)   
127 + **[GTCCCG]** 754 (88.98)   
127 + **[GTCCCG]** 759 (88.98)   
127 + **.GGCGCG.** 776 (95.43)   
127 + **.GGCGCG.** 850 (95.43)   
127 + **[GTCCCG]** 957 (88.98)   
127 + **[GACGCG]** 1029 (89.80)   
127 + **[GTCGCC]** 1085 (86.61)   
127 + **[CTCGCG]** 1334 (88.37)   
127 + **.GCCGCG.** 1509 (95.35)   
127 - **[GACGCG]** 1511 (89.80)   
127 - **[GTCCCG]** 1314 (88.98)   
127 - **.GCCGCG.** 1305 (95.35)   
127 - **[GTCGGG]** 1147 (87.52)   
127 - **.GCCGCG.** 746 (95.35)   
127 - **[GTGGCG]** 464 (87.04)   
127 - **[GTCGCC]** 358 (86.61)   
128 + **[GACGCG]** 31 (89.80)   
128 + **[GACGCG]** 406 (89.80)   
128 + **[CTCGCG]** 609 (88.37)   
128 + **.GTCGCG.** 936 (100.00)   
128 + **[GTCCCG]** 1507 (88.98)   
128 - **.GTCGCG.** 938 (100.00)   
128 - **[GTCCCG]** 861 (88.98)   
128 - **[GTCGGG]** 856 (87.52)   
128 - **[GTCGGG]** 802 (87.52)   
128 - **[GTCGCC]** 778 (86.61)   
128 - **.GCCGCG.** 611 (95.35)   
128 - **.GCCGCG.** 408 (95.35)   
128 - **[GTCCCG]** 348 (88.98)   
128 - **[GTCGGG]** 283 (87.52)   
129 + **[CTCGCG]** 477 (88.37)   
129 + **[CTCGCG]** 678 (88.37)   
129 + **[CTCGCG]** 706 (88.37)   
129 + **[CTCGCG]** 758 (88.37)   
129 + **.GCCGCG.** 870 (95.35)   
129 + **.GCCGCG.** 895 (95.35)   
129 + **.GCCGCG.** 957 (95.35)   
129 + **.GCCGCG.** 1016 (95.35)   
129 + **[GACGCG]** 1057 (89.80)   
129 + **[CTCGCG]** 1139 (88.37)   
129 + **.GGCGCG.** 1144 (95.43)   
129 + **.GCCGCG.** 1200 (95.35)   
129 + **.GCCGCG.** 1320 (95.35)   
129 - **[GTCGGG]** 1492 (87.52)   
129 - **.GGCGCG.** 1402 (95.43)   
129 - **.GCCGCG.** 1386 (95.35)   
129 - **.GGCGCG.** 1202 (95.43)   
129 - **.GCCGCG.** 1141 (95.35)   
129 - **[CTCGCG]** 1105 (88.37)   
129 - **[GTCGGG]** 1008 (87.52)   
129 - **[CTCGCG]** 961 (88.37)   
129 - **[CTCGCG]** 899 (88.37)   
129 - **[GTGGCG]** 744 (87.04)   
129 - **.GTCGCG.** 710 (100.00)   
129 - **[CTCGCG]** 341 (88.37)   
130 + **.GCCGCG.** 865 (95.35)   
130 + **[GTCGCC]** 1092 (86.61)   
130 + **.GCCGCG.** 1109 (95.35)   
130 + **.GGCGCG.** 1267 (95.43)   
130 - **.GGCGCG.** 1269 (95.43)   
130 - **[GTCGGG]** 1054 (87.52)   
130 - **.GCCGCG.** 867 (95.35)   
130 - **[GTCGGG]** 636 (87.52)   
130 - **[GTCGCC]** 555 (86.61)   
131 + **.GGCGCG.** 31 (95.43)   
131 + **[GTGGCG]** 153 (87.04)   
131 + **.GCCGCG.** 568 (95.35)   
131 + **.GGCGCG.** 980 (95.43)   
131 + **.GGCGCG.** 1091 (95.43)   
131 + **.GCCGCG.** 1183 (95.35)   
131 + **[CTCGCG]** 1463 (88.37)   
131 - **.GCCGCG.** 1488 (95.35)   
131 - **.GCCGCG.** 1379 (95.35)   
131 - **.GCCGCG.** 1306 (95.35)   
131 - **.GCCGCG.** 1117 (95.35)   
131 - **.GCCGCG.** 1093 (95.35)   
131 - **[GTGGCG]** 730 (87.04)   
131 - **[GTCGCC]** 203 (86.61)   
131 - **.GGCGCG.** 33 (95.43)   
132 + **.GGCGCG.** 355 (95.43)   
132 + **.GCCGCG.** 710 (95.35)   
132 + **.GCCGCG.** 749 (95.35)   
132 + **[GTGGCG]** 779 (87.04)   
132 + **[GTCGGG]** 913 (87.52)   
132 + **[GTCCCG]** 1027 (88.98)   
132 + **[CTCGCG]** 1078 (88.37)   
132 - **.GGCGCG.** 1224 (95.43)   
132 - **[GTGGCG]** 816 (87.04)   
132 - **.GGCGCG.** 814 (95.43)   
134 + **[CTCGCG]** 505 (88.37)   
134 + **[CTCGCG]** 929 (88.37)   
134 + **.GGCGCG.** 950 (95.43)   
134 + **[CTCGCG]** 983 (88.37)   
134 + **[CTCGCG]** 1015 (88.37)   
134 + **[CTCGCG]** 1031 (88.37)   
134 + **[GTCCCG]** 1168 (88.98)   
134 + **.GGCGCG.** 1370 (95.43)   
134 + **.GGCGCG.** 1473 (95.43)   
134 - **.GGCGCG.** 1475 (95.43)   
134 - **[CTCGCG]** 1253 (88.37)   
134 - **[GTCGCC]** 1190 (86.61)   
134 - **[CTCGCG]** 1172 (88.37)   
134 - **[GTCGGG]** 1140 (87.52)   
134 - **[GACGCG]** 1035 (89.80)   
134 - **[CTCGCG]** 545 (88.37)   
134 - **.GCCGCG.** 533 (95.35)   
135 + **[CTCGCG]** 254 (88.37)   
135 + **.GCCGCG.** 363 (95.35)   
135 + **.GCCGCG.** 788 (95.35)   
135 + **[GTCCCG]** 841 (88.98)   
135 + **[GTGGCG]** 1028 (87.04)   
135 + **[GTGGCG]** 1037 (87.04)   
135 + **[GTCGCC]** 1257 (86.61)   
135 + **[GTGGCG]** 1277 (87.04)   
135 + **.GGCGCG.** 1450 (95.43)   
135 + **.GCCGCG.** 1500 (95.35)   
135 - **[GTGGCG]** 917 (87.04)   
135 - **[CTCGCG]** 443 (88.37)   
135 - **.GCCGCG.** 365 (95.35)   
135 - **.GGCGCG.** 313 (95.43)   
135 - **.GCCGCG.** 258 (95.35)   
135 - **.GCCGCG.** 249 (95.35)   
135 - **[GTCCCG]** 40 (88.98)   
136 + **[GACGCG]** 699 (89.80)   
136 + **[GTCCCG]** 845 (88.98)   
136 + **.GGCGCG.** 936 (95.43)   
136 + **.GGCGCG.** 1039 (95.43)   
136 + **.GCCGCG.** 1298 (95.35)   
136 + **[GTCGGG]** 1439 (87.52)   
136 - **.GCCGCG.** 1302 (95.35)   
136 - **.GCCGCG.** 1196 (95.35)   
136 - **.GGCGCG.** 1065 (95.43)   
136 - **.GCCGCG.** 1041 (95.35)   
136 - **[GTGGCG]** 609 (87.04)   
137 + **.GGCGCG.** 610 (95.43)   
137 + **[GTGGCG]** 737 (87.04)   
137 + **[GACGCG]** 752 (89.80)   
137 + **.GCCGCG.** 919 (95.35)   
137 + **[GACGCG]** 943 (89.80)   
137 + **[GACGCG]** 1014 (89.80)   
137 + **.GGCGCG.** 1165 (95.43)   
137 + **[GTCGGG]** 1217 (87.52)   
137 + **[CTCGCG]** 1494 (88.37)   
137 - **.GGCGCG.** 1496 (95.43)   
137 - **[GTCGCC]** 1325 (86.61)   
137 - **.GCCGCG.** 1138 (95.35)   
137 - **[CTCGCG]** 1100 (88.37)   
137 - **.GCCGCG.** 1016 (95.35)   
137 - **.GCCGCG.** 945 (95.35)   
137 - **[GTCCCG]** 940 (88.98)   
137 - **[GTCCCG]** 901 (88.98)   
138 + **[GACGCG]** 365 (89.80)   
138 + **.GGCGCG.** 370 (95.43)   
138 + **.GCCGCG.** 513 (95.35)   
138 + **[CTCGCG]** 546 (88.37)   
138 + **.GGCGCG.** 575 (95.43)   
138 + **[GTCGGG]** 604 (87.52)   
138 + **.GCCGCG.** 615 (95.35)   
138 + **.GGCGCG.** 641 (95.43)   
138 + **.GGCGCG.** 689 (95.43)   
138 + **.GGCGCG.** 766 (95.43)   
138 + **[GTGGCG]** 801 (87.04)   
138 + **.GCCGCG.** 838 (95.35)   
138 - **.GGCGCG.** 840 (95.43)   
138 - **.GCCGCG.** 768 (95.35)   
138 - **.GCCGCG.** 763 (95.35)   
138 - **.GGCGCG.** 739 (95.43)   
138 - **.GCCGCG.** 691 (95.35)   
138 - **[GACGCG]** 601 (89.80)   
138 - **.GCCGCG.** 579 (95.35)   
138 - **.GGCGCG.** 452 (95.43)   
138 - **[GTCCCG]** 432 (88.98)   
138 - **.GCCGCG.** 367 (95.35)   
138 - **.GGCGCG.** 192 (95.43)   
139 + **.GTCGCG.** 134 (100.00)   
139 + **.GTCGCG.** 617 (100.00)   
139 + **.GGCGCG.** 690 (95.43)   
139 + **[GTGGCG]** 906 (87.04)   
139 + **[GTCGCC]** 914 (86.61)   
139 + **.GGCGCG.** 1091 (95.43)   
139 - **[CTCGCG]** 1479 (88.37)   
139 - **[GTCGCC]** 1384 (86.61)   
139 - **.GCCGCG.** 1343 (95.35)   
139 - **.GCCGCG.** 960 (95.35)   
139 - **[GTCGCC]** 908 (86.61)   
139 - **[GACGCG]** 847 (89.80)   
139 - **.GGCGCG.** 832 (95.43)   
139 - **[CTCGCG]** 764 (88.37)   
139 - **[GTGGCG]** 718 (87.04)   
140 + **[GTCCCG]** 623 (88.98)   
140 + **.GCCGCG.** 657 (95.35)   
140 + **[GTCCCG]** 755 (88.98)   
140 + **[GTGGCG]** 859 (87.04)   
140 + **[GTCGGG]** 1031 (87.52)   
140 + **.GCCGCG.** 1159 (95.35)   
140 + **[CTCGCG]** 1174 (88.37)   
140 + **.GCCGCG.** 1193 (95.35)   
140 + **.GCCGCG.** 1246 (95.35)   
140 - **[GTCGGG]** 1423 (87.52)   
140 - **[GTCGCC]** 1413 (86.61)   
140 - **[GTCGCC]** 1364 (86.61)   
140 - **[CTCGCG]** 1298 (88.37)   
140 - **.GCCGCG.** 1248 (95.35)   
140 - **.GGCGCG.** 1195 (95.43)   
140 - **.GCCGCG.** 1161 (95.35)   
140 - **[GACGCG]** 984 (89.80)   
140 - **[GTGGCG]** 759 (87.04)   
140 - **.GCCGCG.** 730 (95.35)   
141 + **[GTCCCG]** 580 (88.98)   
141 + **.GCCGCG.** 610 (95.35)   
141 + **[GTCGGG]** 634 (87.52)   
141 + **[CTCGCG]** 682 (88.37)   
141 + **.GCCGCG.** 688 (95.35)   
141 + **.GCCGCG.** 721 (95.35)   
141 + **.GGCGCG.** 947 (95.43)   
141 + **.GCCGCG.** 1156 (95.35)   
141 + **.GCCGCG.** 1183 (95.35)   
141 + **.GCCGCG.** 1188 (95.35)   
141 + **[GACGCG]** 1228 (89.80)   
141 + **.GGCGCG.** 1368 (95.43)   
141 + **[GACGCG]** 1389 (89.80)   
141 - **[CTCGCG]** 1391 (88.37)   
141 - **.GGCGCG.** 1185 (95.43)   
141 - **.GCCGCG.** 1158 (95.35)   
141 - **.GTCGCG.** 1099 (100.00)   
141 - **[CTCGCG]** 751 (88.37)   
141 - **.GGCGCG.** 723 (95.43)   
141 - **.GCCGCG.** 684 (95.35)   
141 - **[GTCCCG]** 675 (88.98)   
141 - **[CTCGCG]** 628 (88.37)   
141 - **.GGCGCG.** 612 (95.43)   
141 - **[GTCCCG]** 527 (88.98)   
141 - **[GTCCCG]** 376 (88.98)   
142 + **.GCCGCG.** 29 (95.35)   
142 + **[GTCGGG]** 55 (87.52)   
142 + **[GTCCCG]** 353 (88.98)   
142 + **[GACGCG]** 447 (89.80)   
142 + **.GCCGCG.** 516 (95.35)   
142 + **[GTCCCG]** 531 (88.98)   
142 + **[GTCGCC]** 541 (86.61)   
142 + **[GTCCCG]** 583 (88.98)   
142 + **.GCCGCG.** 625 (95.35)   
142 + **.GCCGCG.** 672 (95.35)   
142 + **.GCCGCG.** 831 (95.35)   
142 + **.GGCGCG.** 842 (95.43)   
142 + **.GCCGCG.** 894 (95.35)   
142 + **[CTCGCG]** 956 (88.37)   
142 + **.GCCGCG.** 1006 (95.35)   
142 + **.GCCGCG.** 1061 (95.35)   
142 + **.GTCGCG.** 1101 (100.00)   
142 + **.GCCGCG.** 1194 (95.35)   
142 + **.GCCGCG.** 1272 (95.35)   
142 + **.GCCGCG.** 1414 (95.35)   
142 + **[GACGCG]** 1478 (89.80)   
142 + **.GCCGCG.** 1508 (95.35)   
142 - **[GTCCCG]** 1475 (88.98)   
142 - **.GGCGCG.** 1468 (95.43)   
142 - **[GTCGCC]** 1437 (86.61)   
142 - **[CTCGCG]** 1418 (88.37)   
142 - **.GGCGCG.** 1274 (95.43)   
142 - **.GCCGCG.** 1200 (95.35)   
142 - **[CTCGCG]** 1017 (88.37)   
142 - **.GCCGCG.** 1010 (95.35)   
142 - **.GGCGCG.** 958 (95.43)   
142 - **.GCCGCG.** 946 (95.35)   
142 - **.GGCGCG.** 902 (95.43)   
142 - **[CTCGCG]** 844 (88.37)   
142 - **.GGCGCG.** 722 (95.43)   
142 - **[GTCGGG]** 682 (87.52)   
142 - **.GCCGCG.** 663 (95.35)   
142 - **[CTCGCG]** 627 (88.37)   
142 - **.GCCGCG.** 518 (95.35)   
142 - **.GGCGCG.** 513 (95.43)   
142 - **.GGCGCG.** 457 (95.43)   
142 - **.GTCGCG.** 449 (100.00)   
142 - **[GTCGCC]** 444 (86.61)   
142 - **.GTCGCG.** 99 (100.00)   
142 - **.GGCGCG.** 31 (95.43)   
143 + **.GGCGCG.** 527 (95.43)   
144 + **[GTCGGG]** 867 (87.52)   
144 + **.GCCGCG.** 973 (95.35)   
144 + **.GCCGCG.** 1018 (95.35)   
144 + **.GGCGCG.** 1033 (95.43)   
144 + **.GGCGCG.** 1074 (95.43)   
144 + **.GGCGCG.** 1140 (95.43)   
144 + **.GGCGCG.** 1145 (95.43)   
144 + **.GGCGCG.** 1150 (95.43)   
144 + **[GACGCG]** 1187 (89.80)   
144 + **.GGCGCG.** 1192 (95.43)   
144 + **.GCCGCG.** 1198 (95.35)   
144 + **.GCCGCG.** 1217 (95.35)   
144 + **[GTGGCG]** 1246 (87.04)   
144 + **.GGCGCG.** 1324 (95.43)   
144 + **.GGCGCG.** 1459 (95.43)   
144 + **.GCCGCG.** 1491 (95.35)   
144 - **.GGCGCG.** 1493 (95.43)   
144 - **.GTCGCG.** 1472 (100.00)   
144 - **.GCCGCG.** 1326 (95.35)   
144 - **.GCCGCG.** 1312 (95.35)   
144 - **.GCCGCG.** 1194 (95.35)   
144 - **.GCCGCG.** 1189 (95.35)   
144 - **.GCCGCG.** 1147 (95.35)   
144 - **.GCCGCG.** 1142 (95.35)   
144 - **.GCCGCG.** 975 (95.35)   
144 - **[GACGCG]** 839 (89.80)   
144 - **[GACGCG]** 774 (89.80)   
144 - **[GTGGCG]** 74 (87.04)   
145 + **[GTCGGG]** 595 (87.52)   
145 + **.GGCGCG.** 654 (95.43)   
145 + **[GTCGGG]** 742 (87.52)   
145 + **[GTCGGG]** 840 (87.52)   
145 + **[GTCGCC]** 1007 (86.61)   
145 + **[GTCCCG]** 1079 (88.98)   
145 + **.GTCGCG.** 1096 (100.00)   
145 + **.GCCGCG.** 1107 (95.35)   
145 + **.GCCGCG.** 1137 (95.35)   
145 + **.GGCGCG.** 1148 (95.43)   
145 - **[GTGGCG]** 1286 (87.04)   
145 - **.GTCGCG.** 1253 (100.00)   
145 - **.GTCGCG.** 1217 (100.00)   
145 - **.GGCGCG.** 1150 (95.43)   
145 - **.GCCGCG.** 1141 (95.35)   
145 - **.GCCGCG.** 1098 (95.35)   
145 - **[CTCGCG]** 656 (88.37)   
145 - **[GTCCCG]** 639 (88.98)   
145 - **[GTCCCG]** 622 (88.98)   
145 - **[GTCCCG]** 601 (88.98)   
145 - **.GGCGCG.** 510 (95.43)   
146 + **[GTGGCG]** 569 (87.04)   
147 + **[GACGCG]** 862 (89.80)   
147 + **.GTCGCG.** 963 (100.00)   
147 + **.GCCGCG.** 978 (95.35)   
147 + **[GTGGCG]** 1059 (87.04)   
147 + **[GTGGCG]** 1066 (87.04)   
147 + **[CTCGCG]** 1128 (88.37)   
147 + **.GCCGCG.** 1163 (95.35)   
147 + **[GTCGGG]** 1264 (87.52)   
147 + **[GTCGGG]** 1277 (87.52)   
147 + **.GGCGCG.** 1370 (95.43)   
147 + **[GTCCCG]** 1462 (88.98)   
147 - **.GGCGCG.** 1167 (95.43)   
147 - **.GGCGCG.** 1137 (95.43)   
147 - **[GTCGCC]** 1071 (86.61)   
147 - **[GACGCG]** 864 (89.80)   
147 - **[GTCGGG]** 859 (87.52)   
147 - **.GGCGCG.** 854 (95.43)   
148 + **[GTCCCG]** 780 (88.98)   
148 + **.GCCGCG.** 828 (95.35)   
148 + **.GTCGCG.** 1087 (100.00)   
148 + **[GTCGCC]** 1397 (86.61)   
148 - **[GTCGGG]** 1268 (87.52)   
148 - **[GACGCG]** 832 (89.80)   
148 - **[GTCCCG]** 822 (88.98)   
148 - **[CTCGCG]** 637 (88.37)   
148 - **.GCCGCG.** 548 (95.35)   
148 - **.GGCGCG.** 472 (95.43)   
148 - **[GTGGCG]** 441 (87.04)   
148 - **[GTCGGG]** 436 (87.52)   
150 + **.GGCGCG.** 90 (95.43)   
150 + **.GCCGCG.** 114 (95.35)   
150 + **.GCCGCG.** 121 (95.35)   
150 + **[GTGGCG]** 197 (87.04)   
150 + **[GACGCG]** 465 (89.80)   
150 + **[GTCGGG]** 833 (87.52)   
150 + **[GACGCG]** 956 (89.80)   
150 + **[GTCCCG]** 1029 (88.98)   
150 + **.GGCGCG.** 1469 (95.43)   
150 - **.GTCGCG.** 933 (100.00)   
150 - **[GTCCCG]** 754 (88.98)   
150 - **.GTCGCG.** 714 (100.00)   
150 - **.GCCGCG.** 191 (95.35)   
150 - **.GCCGCG.** 175 (95.35)   
150 - **.GGCGCG.** 123 (95.43)   
150 - **.GGCGCG.** 118 (95.43)   
150 - **.GGCGCG.** 94 (95.43)   
151 + **.GTCGCG.** 72 (100.00)   
151 + **[GTCCCG]** 252 (88.98)   
151 + **.GTCGCG.** 291 (100.00)   
151 + **.GCCGCG.** 815 (95.35)   
151 + **.GCCGCG.** 831 (95.35)   
151 + **.GGCGCG.** 882 (95.43)   
151 + **.GGCGCG.** 912 (95.43)   
151 + **.GGCGCG.** 1033 (95.43)   
151 + **[GTCCCG]** 1154 (88.98)   
151 + **[GTCGGG]** 1237 (87.52)   
151 + **[CTCGCG]** 1250 (88.37)   
151 + **.GGCGCG.** 1264 (95.43)   
151 + **[GTGGCG]** 1328 (87.04)   
151 + **.GGCGCG.** 1461 (95.43)   
151 + **[GTCCCG]** 1469 (88.98)   
151 + **.GGCGCG.** 1492 (95.43)   
151 + **[GTCCCG]** 1507 (88.98)   
151 - **.GCCGCG.** 1496 (95.35)   
151 - **[CTCGCG]** 1479 (88.37)   
151 - **[GACGCG]** 1473 (89.80)   
151 - **[GTGGCG]** 1418 (87.04)   
151 - **[GTCGCC]** 1330 (86.61)   
151 - **.GCCGCG.** 1268 (95.35)   
151 - **.GCCGCG.** 1252 (95.35)   
151 - **.GGCGCG.** 1206 (95.43)   
151 - **[GACGCG]** 1151 (89.80)   
151 - **.GTCGCG.** 1063 (100.00)   
151 - **.GGCGCG.** 916 (95.43)   
151 - **.GCCGCG.** 892 (95.35)   
151 - **.GCCGCG.** 884 (95.35)   
151 - **[GACGCG]** 540 (89.80)   
151 - **[GTCGGG]** 173 (87.52)   
151 - **[GACGCG]** 49 (89.80)   
153 + **.GGCGCG.** 384 (95.43)   
154 + **[GTCCCG]** 36 (88.98)   
154 + **[GTCGCC]** 101 (86.61)   
154 + **.GGCGCG.** 824 (95.43)   
154 + **.GGCGCG.** 865 (95.43)   
154 + **.GGCGCG.** 933 (95.43)   
154 + **.GGCGCG.** 998 (95.43)   
154 + **.GGCGCG.** 1009 (95.43)   
154 + **.GGCGCG.** 1038 (95.43)   
154 + **.GGCGCG.** 1096 (95.43)   
154 + **.GCCGCG.** 1179 (95.35)   
154 + **[GTCGCC]** 1216 (86.61)   
154 + **[GTCGCC]** 1247 (86.61)   
154 + **.GGCGCG.** 1378 (95.43)   
154 - **.GCCGCG.** 1512 (95.35)   
154 - **.GGCGCG.** 1466 (95.43)   
154 - **.GGCGCG.** 1432 (95.43)   
154 - **[GTCCCG]** 1422 (88.98)   
154 - **[GTCCCG]** 1413 (88.98)   
154 - **[CTCGCG]** 1230 (88.37)   
154 - **.GGCGCG.** 1206 (95.43)   
154 - **.GGCGCG.** 1200 (95.43)   
154 - **[CTCGCG]** 1181 (88.37)   
154 - **.GGCGCG.** 1071 (95.43)   
154 - **.GCCGCG.** 1040 (95.35)   
154 - **[CTCGCG]** 775 (88.37)   
155 + **.GGCGCG.** 507 (95.43)   
155 + **[GTGGCG]** 1217 (87.04)   
155 + **.GTCGCG.** 1257 (100.00)   
155 - **.GCCGCG.** 994 (95.35)   
157 + **.GTCGCG.** 209 (100.00)   
157 + **.GGCGCG.** 1219 (95.43)   
157 + **.GCCGCG.** 1236 (95.35)   
157 - **.GGCGCG.** 731 (95.43)   
157 - **[CTCGCG]** 211 (88.37)   
158 + **[GTCGCC]** 268 (86.61)   
158 + **.GGCGCG.** 375 (95.43)   
158 + **.GCCGCG.** 1177 (95.35)   
158 + **[GACGCG]** 1242 (89.80)   
158 + **[GACGCG]** 1369 (89.80)   
158 + **[GTCGGG]** 1496 (87.52)   
158 + **.GGCGCG.** 1512 (95.43)   
158 - **.GGCGCG.** 1371 (95.43)   
158 - **[GACGCG]** 1179 (89.80)   
158 - **[GACGCG]** 1123 (89.80)   
158 - **.GGCGCG.** 522 (95.43)   
160 + **.GCCGCG.** 133 (95.35)   
160 + **.GCCGCG.** 266 (95.35)   
160 + **.GTCGCG.** 780 (100.00)   
160 - **.GGCGCG.** 1206 (95.43)   
160 - **[GTCCCG]** 1175 (88.98)   
160 - **[GTCCCG]** 1108 (88.98)   
160 - **.GCCGCG.** 1055 (95.35)   
160 - **[GACGCG]** 782 (89.80)   
160 - **[GTCGGG]** 174 (87.52)   
161 + **.GGCGCG.** 154 (95.43)   
161 + **[GTCCCG]** 407 (88.98)   
161 + **[GTCGGG]** 455 (87.52)   
161 + **[GTCGGG]** 507 (87.52)   
161 + **[GTCCCG]** 521 (88.98)   
161 + **.GGCGCG.** 752 (95.43)   
161 + **[GTCCCG]** 780 (88.98)   
161 + **[GTCGCC]** 796 (86.61)   
161 + **.GCCGCG.** 865 (95.35)   
161 + **.GCCGCG.** 884 (95.35)   
161 + **.GCCGCG.** 894 (95.35)   
161 + **[GTGGCG]** 979 (87.04)   
161 + **[CTCGCG]** 1189 (88.37)   
161 + **.GTCGCG.** 1219 (100.00)   
161 + **[GTCGGG]** 1313 (87.52)   
161 - **.GGCGCG.** 1481 (95.43)   
161 - **[GTGGCG]** 1428 (87.04)   
161 - **.GCCGCG.** 1268 (95.35)   
161 - **.GCCGCG.** 1221 (95.35)   
161 - **.GGCGCG.** 1191 (95.43)   
161 - **.GGCGCG.** 896 (95.43)   
161 - **[CTCGCG]** 873 (88.37)   
161 - **.GTCGCG.** 754 (100.00)   
161 - **[GTCCCG]** 237 (88.98)   
164 + **.GTCGCG.** 273 (100.00)   
164 + **.GGCGCG.** 956 (95.43)   
164 + **.GCCGCG.** 1001 (95.35)   
164 + **.GGCGCG.** 1097 (95.43)   
164 - **[GTCCCG]** 1461 (88.98)   
164 - **.GGCGCG.** 1396 (95.43)   
164 - **.GCCGCG.** 1225 (95.35)   
164 - **.GCCGCG.** 1099 (95.35)   
164 - **.GCCGCG.** 1003 (95.35)   
164 - **[GTGGCG]** 543 (87.04)   
164 - **[GTGGCG]** 491 (87.04)   
165 + **[CTCGCG]** 579 (88.37)   
165 - **.GGCGCG.** 581 (95.43)   
166 + **[GTCCCG]** 450 (88.98)   
166 + **.GCCGCG.** 546 (95.35)   
166 + **[GTCCCG]** 613 (88.98)   
166 + **[CTCGCG]** 659 (88.37)   
166 + **[CTCGCG]** 777 (88.37)   
166 + **.GCCGCG.** 1072 (95.35)   
166 + **[GTCCCG]** 1135 (88.98)   
166 + **[CTCGCG]** 1179 (88.37)   
166 + **.GGCGCG.** 1225 (95.43)   
166 + **.GGCGCG.** 1290 (95.43)   
166 + **.GGCGCG.** 1297 (95.43)   
166 + **[GTCGCC]** 1350 (86.61)   
166 - **.GCCGCG.** 1312 (95.35)   
166 - **.GCCGCG.** 1229 (95.35)   
166 - **.GGCGCG.** 1181 (95.43)   
166 - **.GCCGCG.** 1159 (95.35)   
166 - **[GTGGCG]** 1008 (87.04)   
166 - **.GCCGCG.** 811 (95.35)   
166 - **.GCCGCG.** 661 (95.35)   
166 - **[GTCCCG]** 585 (88.98)   
166 - **[GTCCCG]** 529 (88.98)   
167 + **[GTGGCG]** 3 (87.04)   
167 + **[CTCGCG]** 121 (88.37)   
167 + **[GTGGCG]** 411 (87.04)   
167 + **[GACGCG]** 731 (89.80)   
167 + **[GACGCG]** 770 (89.80)   
167 + **.GGCGCG.** 801 (95.43)   
167 + **.GGCGCG.** 818 (95.43)   
167 + **[GTCCCG]** 839 (88.98)   
167 + **.GCCGCG.** 863 (95.35)   
167 + **[CTCGCG]** 954 (88.37)   
167 + **.GGCGCG.** 1002 (95.43)   
167 + **.GGCGCG.** 1035 (95.43)   
167 + **[GTCCCG]** 1064 (88.98)   
167 + **.GCCGCG.** 1234 (95.35)   
167 + **[GTCGGG]** 1299 (87.52)   
167 + **.GTCGCG.** 1322 (100.00)   
167 + **.GTCGCG.** 1328 (100.00)   
167 + **[GTCCCG]** 1466 (88.98)   
167 - **[GACGCG]** 1463 (89.80)   
167 - **.GCCGCG.** 1039 (95.35)   
167 - **.GCCGCG.** 1006 (95.35)   
167 - **.GCCGCG.** 963 (95.35)   
167 - **.GCCGCG.** 956 (95.35)   
167 - **.GGCGCG.** 867 (95.43)   
167 - **.GCCGCG.** 843 (95.35)   
167 - **[CTCGCG]** 820 (88.37)   
167 - **[CTCGCG]** 733 (88.37)   
168 + **.GGCGCG.** 732 (95.43)   
168 + **[GTCGGG]** 775 (87.52)   
168 + **.GCCGCG.** 909 (95.35)   
168 + **[GTCGGG]** 1016 (87.52)   
168 + **[GTGGCG]** 1040 (87.04)   
168 + **.GGCGCG.** 1183 (95.43)   
168 + **.GCCGCG.** 1435 (95.35)   
168 - **[CTCGCG]** 1437 (88.37)   
168 - **[CTCGCG]** 1249 (88.37)   
168 - **.GCCGCG.** 1205 (95.35)   
168 - **.GGCGCG.** 1171 (95.43)   
168 - **.GCCGCG.** 1137 (95.35)   
168 - **.GGCGCG.** 1114 (95.43)   
168 - **.GCCGCG.** 675 (95.35)   
169 + **.GCCGCG.** 111 (95.35)   
169 + **[GTCCCG]** 325 (88.98)   
169 + **[GTCGCC]** 690 (86.61)   
169 + **.GGCGCG.** 871 (95.43)   
169 + **[GTCGCC]** 892 (86.61)   
169 + **[GACGCG]** 1045 (89.80)   
169 + **[GTCCCG]** 1448 (88.98)   
169 - **.GGCGCG.** 1499 (95.43)   
169 - **[CTCGCG]** 873 (88.37)   
169 - **[GTCCCG]** 439 (88.98)   
171 + **[GTGGCG]** 382 (87.04)   
171 - **[GTCCCG]** 875 (88.98)   
171 - **.GCCGCG.** 774 (95.35)   
172 + **[GACGCG]** 429 (89.80)   
172 + **.GTCGCG.** 549 (100.00)   
172 + **.GCCGCG.** 714 (95.35)   
172 + **.GCCGCG.** 755 (95.35)   
172 + **.GGCGCG.** 768 (95.43)   
172 + **.GTCGCG.** 799 (100.00)   
172 + **.GGCGCG.** 920 (95.43)   
172 + **.GGCGCG.** 1160 (95.43)   
172 - **.GGCGCG.** 1505 (95.43)   
172 - **.GGCGCG.** 1419 (95.43)   
172 - **[GTGGCG]** 1224 (87.04)   
172 - **.GGCGCG.** 924 (95.43)   
172 - **.GCCGCG.** 899 (95.35)   
172 - **[GTCGGG]** 869 (87.52)   
172 - **.GCCGCG.** 757 (95.35)   
172 - **.GTCGCG.** 716 (100.00)   
172 - **.GGCGCG.** 551 (95.43)   
172 - **.GCCGCG.** 431 (95.35)   
173 + **[GACGCG]** 943 (89.80)   
173 + **[GTCCCG]** 1095 (88.98)   
173 + **.GGCGCG.** 1101 (95.43)   
173 + **.GCCGCG.** 1278 (95.35)   
173 - **.GTCGCG.** 1280 (100.00)   
173 - **[GTCGCC]** 1188 (86.61)   
173 - **[CTCGCG]** 1177 (88.37)   
173 - **.GGCGCG.** 945 (95.43)   
173 - **[GTCGGG]** 892 (87.52)   
173 - **[GTCGCC]** 650 (86.61)   
174 + **.GTCGCG.** 212 (100.00)   
174 + **.GGCGCG.** 396 (95.43)   
174 + **.GGCGCG.** 783 (95.43)   
174 + **[CTCGCG]** 897 (88.37)   
174 + **[CTCGCG]** 905 (88.37)   
174 + **[GACGCG]** 936 (89.80)   
174 + **.GCCGCG.** 1145 (95.35)   
174 + **.GCCGCG.** 1185 (95.35)   
174 + **.GCCGCG.** 1203 (95.35)   
174 + **[GACGCG]** 1380 (89.80)   
174 + **[GTCCCG]** 1442 (88.98)   
174 + **.GGCGCG.** 1480 (95.43)   
174 + **.GGCGCG.** 1505 (95.43)   
174 - **.GCCGCG.** 1507 (95.35)   
174 - **.GCCGCG.** 1477 (95.35)   
174 - **[CTCGCG]** 1451 (88.37)   
174 - **.GTCGCG.** 1205 (100.00)   
174 - **[GACGCG]** 1187 (89.80)   
174 - **[CTCGCG]** 1147 (88.37)   
174 - **.GGCGCG.** 1128 (95.43)   
174 - **[CTCGCG]** 899 (88.37)   
174 - **[CTCGCG]** 785 (88.37)   
174 - **[GTCGCC]** 416 (86.61)   
174 - **[CTCGCG]** 398 (88.37)   
174 - **.GGCGCG.** 214 (95.43)   
175 + **[GACGCG]** 859 (89.80)   
175 + **.GGCGCG.** 927 (95.43)   
175 + **[GTGGCG]** 1337 (87.04)   
175 + **.GCCGCG.** 1466 (95.35)   
175 - **.GCCGCG.** 1468 (95.35)   
177 + **.GCCGCG.** 952 (95.35)   
177 + **[GTCGCC]** 1018 (86.61)   
177 - **[GTCGCC]** 1468 (86.61)   
177 - **.GCCGCG.** 1465 (95.35)   
177 - **[GTGGCG]** 980 (87.04)   
177 - **[CTCGCG]** 954 (88.37)   
177 - **.GGCGCG.** 819 (95.43)   
177 - **.GCCGCG.** 786 (95.35)   
177 - **[GTCCCG]** 255 (88.98)   
178 + **[GACGCG]** 941 (89.80)   
178 + **[GACGCG]** 1112 (89.80)   
178 + **[CTCGCG]** 1237 (88.37)   
178 - **[GTCCCG]** 1168 (88.98)   
178 - **.GCCGCG.** 1114 (95.35)   
178 - **[GTGGCG]** 803 (87.04)   
179 + **[CTCGCG]** 900 (88.37)   
179 + **.GCCGCG.** 916 (95.35)   
179 + **[GTCCCG]** 932 (88.98)   
179 + **.GGCGCG.** 986 (95.43)   
179 + **[GACGCG]** 994 (89.80)   
179 + **[GTGGCG]** 1030 (87.04)   
179 + **[CTCGCG]** 1202 (88.37)   
179 + **.GCCGCG.** 1474 (95.35)   
179 - **[GTGGCG]** 1478 (87.04)   
179 - **.GGCGCG.** 1476 (95.43)   
179 - **[GACGCG]** 1204 (89.80)   
179 - **.GCCGCG.** 918 (95.35)   
179 - **.GGCGCG.** 858 (95.43)   
180 + **[GTGGCG]** 152 (87.04)   
180 + **[GTCGCC]** 399 (86.61)   
180 + **.GGCGCG.** 796 (95.43)   
180 + **.GGCGCG.** 879 (95.43)   
180 + **.GGCGCG.** 1063 (95.43)   
180 + **[GTGGCG]** 1085 (87.04)   
180 + **.GGCGCG.** 1087 (95.43)   
180 + **[CTCGCG]** 1245 (88.37)   
180 + **[GTGGCG]** 1258 (87.04)   
180 + **[CTCGCG]** 1311 (88.37)   
180 + **[GTCGCC]** 1325 (86.61)   
180 - **[GTGGCG]** 1382 (87.04)   
180 - **.GCCGCG.** 1313 (95.35)   
180 - **.GCCGCG.** 1247 (95.35)   
180 - **.GCCGCG.** 1159 (95.35)   
180 - **.GTCGCG.** 1102 (100.00)   
180 - **[CTCGCG]** 1089 (88.37)   
180 - **.GCCGCG.** 1065 (95.35)   
180 - **.GGCGCG.** 883 (95.43)   
180 - **[GTCGCC]** 326 (86.61)   
182 + **.GGCGCG.** 32 (95.43)   
182 + **.GGCGCG.** 121 (95.43)   
182 + **.GCCGCG.** 233 (95.35)   
182 + **[GTCCCG]** 244 (88.98)   
182 + **.GTCGCG.** 264 (100.00)   
182 + **[GACGCG]** 300 (89.80)   
182 + **.GCCGCG.** 375 (95.35)   
182 + **.GGCGCG.** 386 (95.43)   
182 + **.GGCGCG.** 432 (95.43)   
182 + **.GCCGCG.** 461 (95.35)   
182 + **.GGCGCG.** 466 (95.43)   
182 + **.GCCGCG.** 529 (95.35)   
182 + **.GTCGCG.** 538 (100.00)   
182 + **[GTCCCG]** 612 (88.98)   
182 + **.GCCGCG.** 748 (95.35)   
182 + **.GGCGCG.** 766 (95.43)   
182 + **[GTCGGG]** 864 (87.52)   
182 + **.GGCGCG.** 874 (95.43)   
182 + **.GTCGCG.** 918 (100.00)   
182 + **[GTCGCC]** 1046 (86.61)   
182 + **[GTCGGG]** 1098 (87.52)   
182 + **.GGCGCG.** 1102 (95.43)   
182 + **[CTCGCG]** 1161 (88.37)   
182 - **[GTCGCC]** 1255 (86.61)   
182 - **.GGCGCG.** 1163 (95.43)   
182 - **.GTCGCG.** 1104 (100.00)   
182 - **.GCCGCG.** 1078 (95.35)   
182 - **[GACGCG]** 1021 (89.80)   
182 - **.GCCGCG.** 985 (95.35)   
182 - **[GACGCG]** 920 (89.80)   
182 - **[GACGCG]** 876 (89.80)   
182 - **[GTCGCC]** 735 (86.61)   
182 - **.GGCGCG.** 668 (95.43)   
182 - **.GCCGCG.** 463 (95.35)   
182 - **.GCCGCG.** 377 (95.35)   
182 - **.GCCGCG.** 266 (95.35)   
183 + **.GGCGCG.** 364 (95.43)   
183 + **[GTCCCG]** 593 (88.98)   
183 + **[GTGGCG]** 602 (87.04)   
183 + **.GCCGCG.** 836 (95.35)   
183 + **.GGCGCG.** 983 (95.43)   
183 + **.GCCGCG.** 1230 (95.35)   
183 + **.GCCGCG.** 1299 (95.35)   
183 + **[GTGGCG]** 1464 (87.04)   
183 - **.GGCGCG.** 1424 (95.43)   
183 - **.GCCGCG.** 1356 (95.35)   
183 - **[GACGCG]** 1301 (89.80)   
183 - **.GGCGCG.** 1232 (95.43)   
183 - **[GTCCCG]** 1143 (88.98)   
183 - **.GCCGCG.** 1061 (95.35)   
183 - **.GTCGCG.** 711 (100.00)   
183 - **[GTCCCG]** 703 (88.98)   
183 - **[GACGCG]** 82 (89.80)   
184 + **.GCCGCG.** 54 (95.35)   
184 + **[GTCGGG]** 672 (87.52)   
184 + **.GGCGCG.** 1085 (95.43)   
184 + **.GCCGCG.** 1090 (95.35)   
184 + **[GTCGGG]** 1117 (87.52)   
184 + **.GCCGCG.** 1165 (95.35)   
184 + **.GGCGCG.** 1276 (95.43)   
184 + **.GGCGCG.** 1350 (95.43)   
184 - **.GGCGCG.** 1437 (95.43)   
184 - **.GCCGCG.** 1352 (95.35)   
184 - **.GCCGCG.** 1278 (95.35)   
184 - **.GGCGCG.** 1167 (95.43)   
184 - **.GGCGCG.** 1087 (95.43)   
184 - **.GCCGCG.** 1068 (95.35)   
184 - **.GCCGCG.** 928 (95.35)   
184 - **.GCCGCG.** 633 (95.35)   
184 - **[GTCGCC]** 498 (86.61)   
184 - **[GTCGGG]** 220 (87.52)   
184 - **[GTCGCC]** 35 (86.61)   
185 + **.GCCGCG.** 128 (95.35)   
185 + **.GGCGCG.** 337 (95.43)   
185 + **.GCCGCG.** 555 (95.35)   
185 + **.GCCGCG.** 571 (95.35)   
185 + **[CTCGCG]** 592 (88.37)   
185 + **[GTCGCC]** 922 (86.61)   
185 + **.GCCGCG.** 1063 (95.35)   
185 + **.GCCGCG.** 1090 (95.35)   
185 + **.GCCGCG.** 1190 (95.35)   
185 + **[CTCGCG]** 1511 (88.37)   
185 - **[GTCCCG]** 1352 (88.98)   
185 - **[GTCCCG]** 1269 (88.98)   
185 - **.GCCGCG.** 1192 (95.35)   
185 - **.GGCGCG.** 1047 (95.43)   
185 - **.GGCGCG.** 1034 (95.43)   
185 - **[GTGGCG]** 924 (87.04)   
185 - **[GTCCCG]** 614 (88.98)   
185 - **[GTCGGG]** 531 (87.52)   
185 - **[GTCCCG]** 440 (88.98)   
185 - **.GGCGCG.** 413 (95.43)   
185 - **.GGCGCG.** 9 (95.43)   
186 + **[GTGGCG]** 1065 (87.04)   
186 + **[GTCCCG]** 1088 (88.98)   
186 + **.GCCGCG.** 1348 (95.35)   
186 + **.GGCGCG.** 1461 (95.43)   
186 - **.GCCGCG.** 1463 (95.35)   
186 - **.GCCGCG.** 1389 (95.35)   
186 - **.GGCGCG.** 1350 (95.43)   
186 - **.GGCGCG.** 1316 (95.43)   
186 - **[GTGGCG]** 1268 (87.04)   
186 - **[GACGCG]** 1082 (89.80)   
186 - **.GGCGCG.** 1012 (95.43)   
186 - **[GACGCG]** 905 (89.80)   
187 + **[GTCGGG]** 817 (87.52)   
187 + **[GTCCCG]** 825 (88.98)   
187 + **[GTCCCG]** 842 (88.98)   
187 + **.GGCGCG.** 946 (95.43)   
187 + **.GTCGCG.** 1074 (100.00)   
187 + **.GGCGCG.** 1125 (95.43)   
187 + **.GGCGCG.** 1176 (95.43)   
187 + **.GGCGCG.** 1229 (95.43)   
187 + **.GTCGCG.** 1235 (100.00)   
187 + **.GCCGCG.** 1302 (95.35)   
187 - **[GTCCCG]** 1434 (88.98)   
187 - **[CTCGCG]** 1312 (88.37)   
187 - **[GTCCCG]** 1204 (88.98)   
187 - **[CTCGCG]** 1127 (88.37)   
187 - **.GCCGCG.** 948 (95.35)   
187 - **[GACGCG]** 925 (89.80)   
187 - **[GTCGGG]** 866 (87.52)   
187 - **[GTCCCG]** 622 (88.98)   
187 - **[GTCGGG]** 121 (87.52)   
187 - **.GGCGCG.** 21 (95.43)   
188 + **[GACGCG]** 387 (89.80)   
188 + **.GCCGCG.** 414 (95.35)   
188 + **.GCCGCG.** 1443 (95.35)   
188 + **.GCCGCG.** 1470 (95.35)   
188 - **.GGCGCG.** 1472 (95.43)   
188 - **.GCCGCG.** 1445 (95.35)   
188 - **.GGCGCG.** 1440 (95.43)   
188 - **[GTCGCC]** 1120 (86.61)   
188 - **[GTGGCG]** 896 (87.04)   
188 - **[GTCCCG]** 680 (88.98)   
188 - **[GTGGCG]** 636 (87.04)   
188 - **[GTGGCG]** 207 (87.04)   
188 - **.GGCGCG.** 205 (95.43)   
189 + **.GGCGCG.** 849 (95.43)   
189 + **[CTCGCG]** 869 (88.37)   
189 + **[CTCGCG]** 949 (88.37)   
189 + **.GTCGCG.** 1192 (100.00)   
189 + **.GGCGCG.** 1472 (95.43)   
189 - **.GGCGCG.** 1474 (95.43)   
189 - **.GTCGCG.** 1090 (100.00)   
189 - **[GTCCCG]** 919 (88.98)   
191 + **.GGCGCG.** 97 (95.43)   
191 + **[CTCGCG]** 314 (88.37)   
191 + **.GGCGCG.** 616 (95.43)   
191 + **.GGCGCG.** 790 (95.43)   
191 + **[GTGGCG]** 804 (87.04)   
191 + **[GTGGCG]** 821 (87.04)   
191 + **.GGCGCG.** 823 (95.43)   
191 + **.GCCGCG.** 831 (95.35)   
191 + **.GGCGCG.** 839 (95.43)   
191 + **.GGCGCG.** 877 (95.43)   
191 + **.GGCGCG.** 1043 (95.43)   
191 + **.GGCGCG.** 1137 (95.43)   
191 + **.GGCGCG.** 1161 (95.43)   
191 - **[GTCGCC]** 1287 (86.61)   
191 - **.GCCGCG.** 1191 (95.35)   
191 - **.GCCGCG.** 1045 (95.35)   
191 - **[CTCGCG]** 938 (88.37)   
191 - **.GCCGCG.** 883 (95.35)   
191 - **.GCCGCG.** 827 (95.35)   
191 - **[CTCGCG]** 638 (88.37)   
191 - **.GGCGCG.** 618 (95.43)   
191 - **.GCCGCG.** 569 (95.35)   
191 - **.GGCGCG.** 491 (95.43)   
191 - **[GTCGCC]** 331 (86.61)   
191 - **[CTCGCG]** 207 (88.37)   
191 - **[CTCGCG]** 99 (88.37)   
193 + **.GGCGCG.** 642 (95.43)   
193 + **[GTCCCG]** 698 (88.98)   
193 + **[GACGCG]** 1124 (89.80)   
193 + **[GTCGGG]** 1407 (87.52)   
193 - **[GTCCCG]** 1482 (88.98)   
193 - **.GCCGCG.** 1249 (95.35)   
194 + **[GTGGCG]** 658 (87.04)   
194 + **.GCCGCG.** 723 (95.35)   
194 + **.GGCGCG.** 867 (95.43)   
194 + **[GACGCG]** 908 (89.80)   
194 + **.GGCGCG.** 943 (95.43)   
194 + **.GGCGCG.** 973 (95.43)   
194 + **.GCCGCG.** 988 (95.35)   
194 + **.GGCGCG.** 1016 (95.43)   
194 + **[GTCCCG]** 1035 (88.98)   
194 + **.GCCGCG.** 1040 (95.35)   
194 + **.GGCGCG.** 1066 (95.43)   
194 + **.GCCGCG.** 1110 (95.35)   
194 + **.GGCGCG.** 1115 (95.43)   
194 + **[GTCCCG]** 1250 (88.98)   
194 + **[GTCGGG]** 1318 (87.52)   
194 - **.GGCGCG.** 1254 (95.43)   
194 - **.GCCGCG.** 1112 (95.35)   
194 - **[GTGGCG]** 1008 (87.04)   
194 - **[GTCGCC]** 993 (86.61)   
194 - **.GCCGCG.** 990 (95.35)   
194 - **.GCCGCG.** 945 (95.35)   
194 - **.GCCGCG.** 912 (95.35)   
194 - **.GCCGCG.** 871 (95.35)   
194 - **[GTCCCG]** 859 (88.98)   
194 - **[GTCGCC]** 799 (86.61)   
194 - **[GTCCCG]** 757 (88.98)   
194 - **[GTCGCC]** 660 (86.61)   
194 - **.GGCGCG.** 608 (95.43)   
194 - **[GTGGCG]** 430 (87.04)   
195 + **.GCCGCG.** 166 (95.35)   
195 + **[CTCGCG]** 304 (88.37)   
195 + **[GTCGCC]** 449 (86.61)   
195 + **[GTCCCG]** 791 (88.98)   
195 + **.GTCGCG.** 850 (100.00)   
195 + **.GGCGCG.** 907 (95.43)   
195 + **[GTCGCC]** 946 (86.61)   
195 + **[GTCGGG]** 1262 (87.52)   
195 - **[GACGCG]** 960 (89.80)   
195 - **.GGCGCG.** 924 (95.43)   
195 - **[GTCGGG]** 859 (87.52)   
195 - **.GCCGCG.** 852 (95.35)   
195 - **[GTCCCG]** 777 (88.98)   
195 - **[GTCCCG]** 494 (88.98)   
195 - **[CTCGCG]** 306 (88.37)   
195 - **.GGCGCG.** 251 (95.43)   
195 - **.GCCGCG.** 168 (95.35)   
195 - **.GGCGCG.** 153 (95.43)   
196 + **[GACGCG]** 31 (89.80)   
196 + **[GTCGGG]** 290 (87.52)   
196 + **.GCCGCG.** 342 (95.35)   
196 + **.GGCGCG.** 504 (95.43)   
196 + **.GGCGCG.** 666 (95.43)   
196 + **[GACGCG]** 688 (89.80)   
196 + **.GCCGCG.** 911 (95.35)   
196 + **.GCCGCG.** 1102 (95.35)   
196 + **[GTCGGG]** 1209 (87.52)   
196 + **.GGCGCG.** 1352 (95.43)   
196 + **.GGCGCG.** 1427 (95.43)   
196 + **.GGCGCG.** 1471 (95.43)   
196 - **.GGCGCG.** 1508 (95.43)   
196 - **[CTCGCG]** 1361 (88.37)   
196 - **.GGCGCG.** 1317 (95.43)   
196 - **.GCCGCG.** 1076 (95.35)   
196 - **[GTGGCG]** 915 (87.04)   
196 - **.GGCGCG.** 913 (95.43)   
196 - **[GTCCCG]** 788 (88.98)   
196 - **[GACGCG]** 721 (89.80)   
196 - **[CTCGCG]** 668 (88.37)   
196 - **.GCCGCG.** 538 (95.35)   
196 - **.GGCGCG.** 506 (95.43)   
196 - **.GCCGCG.** 284 (95.35)   
196 - **.GCCGCG.** 120 (95.35)   
196 - **[GTCGCC]** 66 (86.61)   
197 + **[GTCGGG]** 229 (87.52)   
197 + **[GTCGCC]** 434 (86.61)   
197 + **.GTCGCG.** 476 (100.00)   
197 + **.GCCGCG.** 789 (95.35)   
197 + **[GACGCG]** 1100 (89.80)   
197 + **[GACGCG]** 1161 (89.80)   
197 + **.GGCGCG.** 1504 (95.43)   
197 + **.GGCGCG.** 1509 (95.43)   
197 - **.GCCGCG.** 1506 (95.35)   
197 - **.GCCGCG.** 1163 (95.35)   
197 - **.GCCGCG.** 1047 (95.35)   
197 - **[CTCGCG]** 935 (88.37)   
197 - **.GGCGCG.** 791 (95.43)   
197 - **.GCCGCG.** 478 (95.35)   
197 - **[GACGCG]** 431 (89.80)   
198 + **[GTCCCG]** 236 (88.98)   
198 + **.GCCGCG.** 257 (95.35)   
198 + **[CTCGCG]** 708 (88.37)   
198 + **[GTCGGG]** 996 (87.52)   
198 + **[GTGGCG]** 1037 (87.04)   
198 - **.GGCGCG.** 1437 (95.43)   
198 - **[GTCCCG]** 1254 (88.98)   
198 - **[GTCCCG]** 1200 (88.98)   
198 - **.GCCGCG.** 1165 (95.35)   
198 - **[GTCGGG]** 934 (87.52)   
198 - **[GTCCCG]** 654 (88.98)   
198 - **.GGCGCG.** 266 (95.43)   
198 - **.GGCGCG.** 259 (95.43)   
198 - **[GACGCG]** 216 (89.80)   
198 - **.GGCGCG.** 35 (95.43)   
199 + **.GCCGCG.** 438 (95.35)   
199 + **.GGCGCG.** 670 (95.43)   
199 + **[GTCGCC]** 713 (86.61)   
199 + **.GGCGCG.** 819 (95.43)   
199 + **.GTCGCG.** 824 (100.00)   
199 + **.GGCGCG.** 870 (95.43)   
199 + **[GTCCCG]** 900 (88.98)   
199 + **.GGCGCG.** 906 (95.43)   
199 + **.GGCGCG.** 911 (95.43)   
199 + **.GGCGCG.** 938 (95.43)   
199 + **.GGCGCG.** 980 (95.43)   
199 + **[GTCGCC]** 998 (86.61)   
199 + **.GCCGCG.** 1001 (95.35)   
199 + **[GTCCCG]** 1036 (88.98)   
199 + **.GGCGCG.** 1083 (95.43)   
199 + **.GGCGCG.** 1110 (95.43)   
199 + **.GCCGCG.** 1127 (95.35)   
199 + **.GCCGCG.** 1265 (95.35)   
199 + **.GGCGCG.** 1286 (95.43)   
199 + **.GGCGCG.** 1320 (95.43)   
199 + **.GGCGCG.** 1352 (95.43)   
199 + **[GTCCCG]** 1444 (88.98)   
199 - **.GGCGCG.** 1491 (95.43)   
199 - **[GACGCG]** 1448 (89.80)   
199 - **.GCCGCG.** 1276 (95.35)   
199 - **.GGCGCG.** 1244 (95.43)   
199 - **.GCCGCG.** 1085 (95.35)   
199 - **.GCCGCG.** 1003 (95.35)   
199 - **.GCCGCG.** 951 (95.35)   
199 - **.GCCGCG.** 908 (95.35)   
199 - **[GACGCG]** 821 (89.80)   
199 - **.GGCGCG.** 672 (95.43)   
199 - **[GTCCCG]** 541 (88.98)   
200 + **[GACGCG]** 572 (89.80)   
200 + **[GTGGCG]** 1080 (87.04)   
200 + **.GGCGCG.** 1140 (95.43)   
200 - **.GCCGCG.** 1142 (95.35)   
200 - **[GTGGCG]** 936 (87.04)   
201 + **.GCCGCG.** 337 (95.35)   
201 + **[GTCGGG]** 417 (87.52)   
201 + **[GTCGGG]** 446 (87.52)   
201 + **[GTCGCC]** 493 (86.61)   
201 + **.GGCGCG.** 627 (95.43)   
201 + **.GGCGCG.** 676 (95.43)   
201 + **[GTGGCG]** 714 (87.04)   
201 + **.GGCGCG.** 776 (95.43)   
201 + **[GTCGGG]** 1070 (87.52)   
201 + **[GTCCCG]** 1112 (88.98)   
201 + **.GCCGCG.** 1142 (95.35)   
201 + **.GCCGCG.** 1349 (95.35)   
201 + **[GTCCCG]** 1391 (88.98)   
201 + **[GACGCG]** 1491 (89.80)   
201 - **.GGCGCG.** 1499 (95.43)   
201 - **.GGCGCG.** 1414 (95.43)   
201 - **[GACGCG]** 1201 (89.80)   
201 - **.GGCGCG.** 1085 (95.43)   
201 - **.GCCGCG.** 849 (95.35)   
201 - **.GGCGCG.** 661 (95.43)   
201 - **[GTCCCG]** 603 (88.98)   
201 - **.GCCGCG.** 533 (95.35)   
201 - **[GTCGCC]** 431 (86.61)   
201 - **.GCCGCG.** 351 (95.35)   
201 - **.GGCGCG.** 339 (95.43)   
201 - **[CTCGCG]** 69 (88.37)   
202 + **[GACGCG]** 310 (89.80)   
202 + **.GGCGCG.** 593 (95.43)   
202 + **.GGCGCG.** 722 (95.43)   
202 + **[GTGGCG]** 813 (87.04)   
202 + **[GTCCCG]** 1155 (88.98)   
202 + **.GGCGCG.** 1285 (95.43)   
202 + **[CTCGCG]** 1369 (88.37)   
202 + **[GACGCG]** 1393 (89.80)   
202 - **[GTCCCG]** 1437 (88.98)   
202 - **.GGCGCG.** 1395 (95.43)   
202 - **.GCCGCG.** 1371 (95.35)   
202 - **[GACGCG]** 992 (89.80)   
202 - **[GTCCCG]** 572 (88.98)   
202 - **[GTCCCG]** 307 (88.98)   
203 + **.GTCGCG.** 317 (100.00)   
203 - **[GTCGGG]** 912 (87.52)   
203 - **.GCCGCG.** 102 (95.35)   
206 - **[GTGGCG]** 892 (87.04)   
206 - **[GTGGCG]** 152 (87.04)   
207 + **[GTGGCG]** 715 (87.04)   
207 + **[GTCGCC]** 879 (86.61)   
207 + **[GTCCCG]** 1108 (88.98)   
207 + **.GTCGCG.** 1329 (100.00)   
207 - **.GTCGCG.** 1502 (100.00)   
207 - **[GTCGCC]** 1068 (86.61)   
207 - **[GACGCG]** 857 (89.80)   
208 + **.GTCGCG.** 1141 (100.00)   
208 + **[GTGGCG]** 1153 (87.04)   
208 + **[GACGCG]** 1226 (89.80)   
208 + **.GGCGCG.** 1396 (95.43)   
208 + **.GGCGCG.** 1409 (95.43)   
208 + **.GCCGCG.** 1443 (95.35)   
208 + **.GCCGCG.** 1497 (95.35)   
208 - **[GTCGCC]** 1502 (86.61)   
208 - **.GCCGCG.** 1499 (95.35)   
208 - **[CTCGCG]** 1398 (88.37)   
208 - **.GGCGCG.** 1359 (95.43)   
208 - **[CTCGCG]** 955 (88.37)   
209 + **[GTGGCG]** 395 (87.04)   
209 + **.GCCGCG.** 838 (95.35)   
210 + **.GGCGCG.** 675 (95.43)   
210 + **[GTCGGG]** 680 (87.52)   
210 + **.GCCGCG.** 778 (95.35)   
210 + **[GTGGCG]** 990 (87.04)   
210 + **[GTGGCG]** 1009 (87.04)   
210 + **.GCCGCG.** 1047 (95.35)   
210 + **.GGCGCG.** 1066 (95.43)   
210 + **[CTCGCG]** 1319 (88.37)   
210 - **.GGCGCG.** 1223 (95.43)   
210 - **[GTCGGG]** 1154 (87.52)   
210 - **[GTGGCG]** 973 (87.04)   
210 - **[GTCGCC]** 854 (86.61)   
210 - **[CTCGCG]** 804 (88.37)   
210 - **.GCCGCG.** 780 (95.35)   
210 - **[GACGCG]** 677 (89.80)   
210 - **[GTGGCG]** 650 (87.04)   
210 - **.GGCGCG.** 616 (95.43)   
210 - **[GTCCCG]** 258 (88.98)   
211 + **.GCCGCG.** 735 (95.35)   
211 + **[CTCGCG]** 803 (88.37)   
211 + **[GTGGCG]** 933 (87.04)   
211 + **[GACGCG]** 1145 (89.80)   
211 + **[GACGCG]** 1405 (89.80)   
211 - **.GGCGCG.** 1481 (95.43)   
211 - **.GCCGCG.** 1216 (95.35)   
211 - **[CTCGCG]** 1147 (88.37)   
211 - **[GTCCCG]** 1142 (88.98)   
211 - **[CTCGCG]** 1068 (88.37)   
211 - **[GTCCCG]** 1031 (88.98)   
211 - **.GGCGCG.** 977 (95.43)   
211 - **.GGCGCG.** 737 (95.43)   
211 - **[GTCGGG]** 689 (87.52)   
212 + **[GTCCCG]** 63 (88.98)   
212 + **[GTGGCG]** 418 (87.04)   
212 + **.GGCGCG.** 420 (95.43)   
212 + **.GCCGCG.** 713 (95.35)   
212 + **[GTCGGG]** 740 (87.52)   
212 + **[GTCCCG]** 757 (88.98)   
212 + **[GTCGGG]** 1403 (87.52)   
212 + **[CTCGCG]** 1431 (88.37)   
212 - **.GCCGCG.** 1486 (95.35)   
212 - **[GTCGCC]** 1416 (86.61)   
212 - **[GTCCCG]** 1405 (88.98)   
212 - **[GACGCG]** 1034 (89.80)   
212 - **.GCCGCG.** 715 (95.35)   
212 - **.GGCGCG.** 455 (95.43)   
212 - **.GTCGCG.** 422 (100.00)   
212 - **[GTCCCG]** 67 (88.98)   
213 + **.GCCGCG.** 912 (95.35)   
213 + **.GGCGCG.** 997 (95.43)   
213 + **.GGCGCG.** 1061 (95.43)   
213 + **[GTCGGG]** 1392 (87.52)   
213 - **.GGCGCG.** 1290 (95.43)   
213 - **.GGCGCG.** 1193 (95.43)   
213 - **[CTCGCG]** 914 (88.37)   
214 + **.GCCGCG.** 929 (95.35)   
214 + **[CTCGCG]** 1031 (88.37)   
214 + **[CTCGCG]** 1155 (88.37)   
214 + **.GTCGCG.** 1190 (100.00)   
214 - **[CTCGCG]** 1157 (88.37)   
214 - **.GGCGCG.** 1033 (95.43)   
214 - **.GCCGCG.** 931 (95.35)   
214 - **[GTCGGG]** 908 (87.52)   
214 - **.GCCGCG.** 834 (95.35)   
214 - **.GCCGCG.** 732 (95.35)   
214 - **[GTCCCG]** 623 (88.98)   
214 - **[CTCGCG]** 384 (88.37)   
214 - **[GACGCG]** 355 (89.80)   
215 + **[GTGGCG]** 110 (87.04)   
215 + **.GGCGCG.** 112 (95.43)   
215 + **.GCCGCG.** 652 (95.35)   
215 + **[GTCGCC]** 954 (86.61)   
215 + **.GCCGCG.** 1090 (95.35)   
215 + **[GACGCG]** 1195 (89.80)   
215 + **[GTCCCG]** 1278 (88.98)   
215 + **[GTCGCC]** 1324 (86.61)   
215 + **[GTGGCG]** 1334 (87.04)   
215 + **.GCCGCG.** 1413 (95.35)   
215 + **.GGCGCG.** 1459 (95.43)   
215 - **[GACGCG]** 1461 (89.80)   
215 - **.GGCGCG.** 1415 (95.43)   
215 - **.GGCGCG.** 1369 (95.43)   
215 - **[GTCGGG]** 1161 (87.52)   
215 - **.GGCGCG.** 1140 (95.43)   
215 - **.GCCGCG.** 1092 (95.35)   
215 - **[GTCGGG]** 917 (87.52)   
215 - **[GTCGCC]** 846 (86.61)   
215 - **[GACGCG]** 654 (89.80)   
215 - **[GTCCCG]** 557 (88.98)   
215 - **[GTGGCG]** 203 (87.04)   
215 - **.GGCGCG.** 201 (95.43)   
215 - **.GGCGCG.** 116 (95.43)   
216 + **[GTCGGG]** 68 (87.52)   
216 + **[GTGGCG]** 387 (87.04)   
216 + **[GTGGCG]** 489 (87.04)   
216 + **[GTCCCG]** 631 (88.98)   
216 + **.GGCGCG.** 992 (95.43)   
216 + **.GCCGCG.** 1228 (95.35)   
216 + **.GTCGCG.** 1319 (100.00)   
216 + **.GGCGCG.** 1465 (95.43)   
216 + **.GCCGCG.** 1481 (95.35)   
216 - **[CTCGCG]** 1467 (88.37)   
216 - **[GTCCCG]** 1447 (88.98)   
216 - **[GTCCCG]** 1411 (88.98)   
216 - **[GTCGGG]** 1265 (87.52)   
216 - **.GCCGCG.** 1230 (95.35)   
216 - **.GGCGCG.** 1096 (95.43)   
216 - **[GTCGGG]** 1059 (87.52)   
216 - **.GGCGCG.** 996 (95.43)   
216 - **.GGCGCG.** 947 (95.43)   
216 - **[GACGCG]** 892 (89.80)   
216 - **.GGCGCG.** 650 (95.43)   
216 - **[GTCCCG]** 571 (88.98)   
216 - **[GTGGCG]** 545 (87.04)   
216 - **.GCCGCG.** 212 (95.35)   
217 + **[GTCGCC]** 534 (86.61)   
217 + **.GCCGCG.** 573 (95.35)   
217 + **[GTCGCC]** 617 (86.61)   
217 + **.GCCGCG.** 860 (95.35)   
217 + **[CTCGCG]** 1092 (88.37)   
217 - **[GACGCG]** 1094 (89.80)   
217 - **.GTCGCG.** 1057 (100.00)   
217 - **[GACGCG]** 941 (89.80)   
217 - **[GTCGCC]** 722 (86.61)   
217 - **[GACGCG]** 665 (89.80)   
217 - **.GCCGCG.** 577 (95.35)   
220 - **[GTCCCG]** 1345 (88.98)   
221 + **.GCCGCG.** 18 (95.35)   
221 + **.GTCGCG.** 303 (100.00)   
221 + **.GCCGCG.** 515 (95.35)   
221 + **.GGCGCG.** 937 (95.43)   
221 + **.GCCGCG.** 1110 (95.35)   
221 + **[GTCGCC]** 1345 (86.61)   
221 - **[GTCGGG]** 1467 (87.52)   
221 - **[CTCGCG]** 1388 (88.37)   
221 - **[GTGGCG]** 1178 (87.04)   
221 - **.GCCGCG.** 1112 (95.35)   
221 - **.GCCGCG.** 519 (95.35)   
221 - **[GACGCG]** 20 (89.80)   
222 + **[GTCGGG]** 383 (87.52)   
222 + **[CTCGCG]** 689 (88.37)   
222 + **.GGCGCG.** 841 (95.43)   
222 + **.GCCGCG.** 877 (95.35)   
222 + **[CTCGCG]** 924 (88.37)   
222 + **.GCCGCG.** 1001 (95.35)   
222 + **.GGCGCG.** 1204 (95.43)   
222 + **[GTCGGG]** 1240 (87.52)   
222 + **.GGCGCG.** 1270 (95.43)   
222 + **.GCCGCG.** 1482 (95.35)   
222 - **.GCCGCG.** 1484 (95.35)   
222 - **[GTCCCG]** 1251 (88.98)   
222 - **.GGCGCG.** 1206 (95.43)   
222 - **.GGCGCG.** 1099 (95.43)   
222 - **.GGCGCG.** 1081 (95.43)   
222 - **[GTGGCG]** 1067 (87.04)   
222 - **.GGCGCG.** 1005 (95.43)   
222 - **[GTCCCG]** 962 (88.98)   
222 - **.GCCGCG.** 950 (95.35)   
222 - **[GACGCG]** 940 (89.80)   
222 - **.GGCGCG.** 926 (95.43)   
222 - **[GACGCG]** 879 (89.80)   
222 - **[GTCCCG]** 864 (88.98)   
222 - **.GCCGCG.** 843 (95.35)   
222 - **.GGCGCG.** 691 (95.43)   
222 - **[CTCGCG]** 473 (88.37)   
222 - **[GTCCCG]** 458 (88.98)   
222 - **[GACGCG]** 452 (89.80)   
222 - **[GTCCCG]** 277 (88.98)   
222 - **[CTCGCG]** 216 (88.37)   
222 - **.GCCGCG.** 168 (95.35)   
223 + **[GTCCCG]** 75 (88.98)   
223 + **[GTCGCC]** 110 (86.61)   
223 + **[GTCGCC]** 205 (86.61)   
223 + **[GACGCG]** 422 (89.80)   
223 + **[GTCCCG]** 433 (88.98)   
223 + **[GACGCG]** 456 (89.80)   
223 + **.GTCGCG.** 503 (100.00)   
223 + **[GTGGCG]** 654 (87.04)   
223 - **[GACGCG]** 701 (89.80)   
223 - **[GTCGGG]** 689 (87.52)   
223 - **[GTCCCG]** 453 (88.98)   
223 - **.GGCGCG.** 424 (95.43)   
223 - **[CTCGCG]** 88 (88.37)   
223 - **[GTCCCG]** 41 (88.98)   
224 + **[GTGGCG]** 469 (87.04)   
224 + **.GGCGCG.** 471 (95.43)   
224 + **.GGCGCG.** 478 (95.43)   
224 + **[GTCGGG]** 583 (87.52)   
224 + **.GCCGCG.** 590 (95.35)   
224 + **.GGCGCG.** 615 (95.43)   
224 + **.GCCGCG.** 690 (95.35)   
224 + **.GGCGCG.** 718 (95.43)   
224 + **.GCCGCG.** 823 (95.35)   
224 + **.GGCGCG.** 854 (95.43)   
224 + **[CTCGCG]** 949 (88.37)   
224 + **.GGCGCG.** 1183 (95.43)   
224 + **.GTCGCG.** 1201 (100.00)   
224 + **.GCCGCG.** 1234 (95.35)   
224 + **.GGCGCG.** 1326 (95.43)   
224 - **[GTGGCG]** 1279 (87.04)   
224 - **[GTCGGG]** 1169 (87.52)   
224 - **[CTCGCG]** 951 (88.37)   
224 - **[CTCGCG]** 892 (88.37)   
224 - **[GTCGCC]** 859 (86.61)   
224 - **.GCCGCG.** 856 (95.35)   
224 - **.GCCGCG.** 799 (95.35)   
224 - **[CTCGCG]** 692 (88.37)   
224 - **.GCCGCG.** 619 (95.35)   
224 - **[GTCCCG]** 596 (88.98)   
224 - **.GCCGCG.** 532 (95.35)   
226 + **[CTCGCG]** 123 (88.37)   
226 + **.GCCGCG.** 246 (95.35)   
226 + **.GCCGCG.** 448 (95.35)   
226 + **[GTCCCG]** 460 (88.98)   
226 + **.GCCGCG.** 518 (95.35)   
226 + **.GTCGCG.** 701 (100.00)   
226 + **[GACGCG]** 712 (89.80)   
226 + **.GCCGCG.** 924 (95.35)   
226 + **.GGCGCG.** 965 (95.43)   
226 + **.GCCGCG.** 1035 (95.35)   
226 + **[GTCCCG]** 1072 (88.98)   
226 + **.GCCGCG.** 1202 (95.35)   
226 + **.GGCGCG.** 1307 (95.43)   
226 + **.GCCGCG.** 1337 (95.35)   
226 + **.GGCGCG.** 1434 (95.43)   
226 + **.GGCGCG.** 1495 (95.43)   
226 - **.GCCGCG.** 1497 (95.35)   
226 - **[GTCCCG]** 1451 (88.98)   
226 - **.GGCGCG.** 1170 (95.43)   
226 - **.GGCGCG.** 1163 (95.43)   
226 - **.GGCGCG.** 1137 (95.43)   
226 - **.GCCGCG.** 1078 (95.35)   
226 - **[GACGCG]** 1065 (89.80)   
226 - **.GGCGCG.** 1037 (95.43)   
226 - **.GGCGCG.** 1032 (95.43)   
226 - **.GCCGCG.** 952 (95.35)   
226 - **[GTCCCG]** 721 (88.98)   
226 - **.GTCGCG.** 703 (100.00)   
226 - **[GTCGGG]** 624 (87.52)   
226 - **[GACGCG]** 601 (89.80)   
226 - **.GGCGCG.** 511 (95.43)   
226 - **[GTCCCG]** 464 (88.98)   
226 - **[CTCGCG]** 351 (88.37)   
226 - **[GACGCG]** 235 (89.80)   
226 - **.GCCGCG.** 125 (95.35)   
227 + **[CTCGCG]** 95 (88.37)   
227 + **[GACGCG]** 307 (89.80)   
227 + **.GGCGCG.** 550 (95.43)   
227 + **[GACGCG]** 615 (89.80)   
227 + **[GACGCG]** 687 (89.80)   
227 + **[CTCGCG]** 785 (88.37)   
227 + **[CTCGCG]** 832 (88.37)   
227 + **[GTCGGG]** 1234 (87.52)   
227 - **.GGCGCG.** 1361 (95.43)   
227 - **.GCCGCG.** 1297 (95.35)   
227 - **.GGCGCG.** 1089 (95.43)   
227 - **.GGCGCG.** 1079 (95.43)   
227 - **.GGCGCG.** 1038 (95.43)   
227 - **.GGCGCG.** 1018 (95.43)   
227 - **.GGCGCG.** 998 (95.43)   
227 - **.GGCGCG.** 988 (95.43)   
227 - **.GGCGCG.** 978 (95.43)   
227 - **.GGCGCG.** 309 (95.43)   
227 - **.GCCGCG.** 268 (95.35)   
227 - **[GTCGGG]** 51 (87.52)   
227 - **[GTGGCG]** 30 (87.04)   
228 + **[GTCGCC]** 34 (86.61)   
228 + **.GTCGCG.** 44 (100.00)   
228 + **[GTCCCG]** 100 (88.98)   
228 + **.GCCGCG.** 127 (95.35)   
228 + **[GTCCCG]** 657 (88.98)   
228 + **[GTCGGG]** 722 (87.52)   
228 + **[GACGCG]** 738 (89.80)   
228 + **[GTGGCG]** 780 (87.04)   
228 + **.GGCGCG.** 782 (95.43)   
228 + **[GTGGCG]** 793 (87.04)   
228 + **[GTGGCG]** 882 (87.04)   
228 + **.GGCGCG.** 884 (95.43)   
228 + **.GGCGCG.** 952 (95.43)   
228 + **[GTCGGG]** 1198 (87.52)   
228 + **.GCCGCG.** 1271 (95.35)   
228 - **.GGCGCG.** 1429 (95.43)   
228 - **[GTCCCG]** 1244 (88.98)   
228 - **.GTCGCG.** 638 (100.00)   
228 - **.GGCGCG.** 542 (95.43)   
228 - **.GGCGCG.** 471 (95.43)   
228 - **[GTCGGG]** 435 (87.52)   
228 - **.GGCGCG.** 281 (95.43)   
228 - **.GGCGCG.** 275 (95.43)   
228 - **[GTCGGG]** 151 (87.52)   
228 - **.GGCGCG.** 136 (95.43)   
228 - **.GGCGCG.** 129 (95.43)   
228 - **[GTCGGG]** 52 (87.52)   
228 - **.GTCGCG.** 46 (100.00)   
229 + **[GTCGCC]** 363 (86.61)   
229 + **[GTCCCG]** 629 (88.98)   
229 + **.GCCGCG.** 690 (95.35)   
229 + **[GTCGGG]** 739 (87.52)   
229 + **.GGCGCG.** 996 (95.43)   
229 + **.GCCGCG.** 1015 (95.35)   
229 + **.GGCGCG.** 1036 (95.43)   
229 + **.GCCGCG.** 1114 (95.35)   
229 + **.GTCGCG.** 1204 (100.00)   
229 + **.GCCGCG.** 1246 (95.35)   
229 + **.GGCGCG.** 1287 (95.43)   
229 + **.GGCGCG.** 1301 (95.43)   
229 + **[GTCGGG]** 1392 (87.52)   
229 + **[CTCGCG]** 1463 (88.37)   
229 - **.GCCGCG.** 1284 (95.35)   
229 - **.GGCGCG.** 1250 (95.43)   
229 - **.GGCGCG.** 1206 (95.43)   
229 - **.GGCGCG.** 1148 (95.43)   
229 - **.GGCGCG.** 1017 (95.43)   
229 - **.GCCGCG.** 941 (95.35)   
229 - **[GTCGGG]** 935 (87.52)   
229 - **[GTCCCG]** 668 (88.98)   
229 - **.GCCGCG.** 587 (95.35)   
229 - **.GCCGCG.** 540 (95.35)   
229 - **.GGCGCG.** 124 (95.43)   
230 + **[GTCGGG]** 389 (87.52)   
230 + **[GTCCCG]** 1331 (88.98)   
230 + **[GTCCCG]** 1465 (88.98)   
230 - **[GTCGGG]** 704 (87.52)   
230 - **[GTCGGG]** 292 (87.52)   
231 + **[GTGGCG]** 126 (87.04)   
231 + **[GTCGCC]** 698 (86.61)   
231 + **[GTCGCC]** 731 (86.61)   
231 + **.GCCGCG.** 906 (95.35)   
231 + **.GCCGCG.** 980 (95.35)   
231 + **.GGCGCG.** 1122 (95.43)   
231 + **.GGCGCG.** 1141 (95.43)   
231 + **[GACGCG]** 1383 (89.80)   
231 + **.GGCGCG.** 1470 (95.43)   
231 + **.GCCGCG.** 1482 (95.35)   
231 - **.GGCGCG.** 1484 (95.43)   
231 - **.GGCGCG.** 1472 (95.43)   
231 - **[GTCCCG]** 1380 (88.98)   
231 - **.GGCGCG.** 1165 (95.43)   
231 - **[CTCGCG]** 1143 (88.37)   
231 - **[CTCGCG]** 1124 (88.37)   
231 - **.GCCGCG.** 1070 (95.35)   
231 - **[GTGGCG]** 700 (87.04)   
232 + **.GCCGCG.** 147 (95.35)   
232 + **.GGCGCG.** 444 (95.43)   
232 + **.GGCGCG.** 633 (95.43)   
232 + **.GGCGCG.** 699 (95.43)   
232 + **.GCCGCG.** 794 (95.35)   
232 + **[GTCCCG]** 818 (88.98)   
232 + **[GTCCCG]** 842 (88.98)   
232 + **.GCCGCG.** 867 (95.35)   
232 + **.GGCGCG.** 1013 (95.43)   
232 + **[GTCCCG]** 1077 (88.98)   
232 + **[GACGCG]** 1119 (89.80)   
232 + **.GGCGCG.** 1180 (95.43)   
232 + **.GCCGCG.** 1244 (95.35)   
232 + **[GACGCG]** 1333 (89.80)   
232 + **[GTCCCG]** 1474 (88.98)   
232 + **[GTCCCG]** 1499 (88.98)   
232 - **[GTCGGG]** 1509 (87.52)   
232 - **.GCCGCG.** 1335 (95.35)   
232 - **.GGCGCG.** 1246 (95.43)   
232 - **[CTCGCG]** 1135 (88.37)   
232 - **[CTCGCG]** 1121 (88.37)   
232 - **[CTCGCG]** 1015 (88.37)   
232 - **.GCCGCG.** 997 (95.35)   
232 - **.GCCGCG.** 869 (95.35)   
232 - **.GCCGCG.** 863 (95.35)   
232 - **[GTGGCG]** 771 (87.04)   
232 - **[GACGCG]** 701 (89.80)   
232 - **[GACGCG]** 635 (89.80)   
232 - **.GGCGCG.** 521 (95.43)   
232 - **.GGCGCG.** 149 (95.43)   
233 + **.GGCGCG.** 1378 (95.43)   
233 - **[GTCGCC]** 1138 (86.61)   
233 - **[GTCGGG]** 432 (87.52)   
233 - **[GTCGGG]** 385 (87.52)   
234 + **.GGCGCG.** 179 (95.43)   
234 + **.GTCGCG.** 317 (100.00)   
234 + **[GTGGCG]** 352 (87.04)   
234 + **[GACGCG]** 637 (89.80)   
234 + **.GTCGCG.** 803 (100.00)   
234 + **[CTCGCG]** 848 (88.37)   
234 + **[GTCGGG]** 1235 (87.52)   
234 + **.GGCGCG.** 1289 (95.43)   
234 + **[GACGCG]** 1411 (89.80)   
234 - **[GTCCCG]** 1408 (88.98)   
234 - **.GCCGCG.** 1331 (95.35)   
234 - **[GTGGCG]** 1300 (87.04)   
234 - **.GGCGCG.** 856 (95.43)   
234 - **.GGCGCG.** 850 (95.43)   
234 - **.GCCGCG.** 816 (95.35)   
234 - **[GTCCCG]** 600 (88.98)   
234 - **.GTCGCG.** 579 (100.00)   
234 - **.GGCGCG.** 411 (95.43)   
234 - **[GTCCCG]** 373 (88.98)   
234 - **[GTGGCG]** 212 (87.04)   
235 + **[GTCGGG]** 389 (87.52)   
235 + **.GGCGCG.** 635 (95.43)   
235 + **.GTCGCG.** 683 (100.00)   
235 + **.GCCGCG.** 797 (95.35)   
235 + **.GGCGCG.** 903 (95.43)   
235 + **[GTCGCC]** 952 (86.61)   
235 + **.GCCGCG.** 961 (95.35)   
235 + **[GTGGCG]** 1264 (87.04)   
235 + **.GGCGCG.** 1317 (95.43)   
235 + **.GCCGCG.** 1362 (95.35)   
235 + **.GGCGCG.** 1429 (95.43)   
235 + **[GACGCG]** 1486 (89.80)   
235 - **[GTCCCG]** 1403 (88.98)   
235 - **.GGCGCG.** 1364 (95.43)   
235 - **[GTCGCC]** 1305 (86.61)   
235 - **[CTCGCG]** 1244 (88.37)   
235 - **.GGCGCG.** 963 (95.43)   
235 - **.GGCGCG.** 929 (95.43)   
235 - **.GCCGCG.** 900 (95.35)   
235 - **.GGCGCG.** 799 (95.43)   
235 - **[GTGGCG]** 757 (87.04)   
235 - **.GGCGCG.** 755 (95.43)   
235 - **.GGCGCG.** 670 (95.43)   
236 + **.GGCGCG.** 910 (95.43)   
236 + **[CTCGCG]** 1022 (88.37)   
236 + **.GCCGCG.** 1154 (95.35)   
236 - **.GCCGCG.** 1026 (95.35)   
236 - **.GCCGCG.** 978 (95.35)   
236 - **.GGCGCG.** 912 (95.43)   
237 + **[GACGCG]** 1057 (89.80)   
237 + **[GTGGCG]** 1389 (87.04)   
237 - **[GACGCG]** 473 (89.80)   
237 - **[GTCCCG]** 34 (88.98)   
238 + **[GACGCG]** 1106 (89.80)   
238 + **[GTGGCG]** 1146 (87.04)   
238 - **.GTCGCG.** 1228 (100.00)   
238 - **[GTCGGG]** 1103 (87.52)   
238 - **[GTCCCG]** 849 (88.98)   
239 + **.GCCGCG.** 445 (95.35)   
239 + **[GTCCCG]** 679 (88.98)   
239 + **.GGCGCG.** 707 (95.43)   
239 + **.GCCGCG.** 768 (95.35)   
239 + **.GGCGCG.** 952 (95.43)   
239 - **[CTCGCG]** 954 (88.37)   
239 - **[GTCCCG]** 858 (88.98)   
239 - **.GGCGCG.** 770 (95.43)   
239 - **.GCCGCG.** 752 (95.35)   
239 - **.GTCGCG.** 709 (100.00)   
239 - **.GCCGCG.** 447 (95.35)   
239 - **.GCCGCG.** 299 (95.35)   
240 + **.GGCGCG.** 53 (95.43)   
240 + **[CTCGCG]** 138 (88.37)   
240 + **[GTCCCG]** 638 (88.98)   
240 + **.GCCGCG.** 1403 (95.35)   
240 + **[GTCCCG]** 1451 (88.98)   
240 - **[GTCCCG]** 1362 (88.98)   
240 - **[GTCGGG]** 1286 (87.52)   
240 - **.GGCGCG.** 1182 (95.43)   
240 - **[GTCGGG]** 710 (87.52)   
240 - **[GTCGGG]** 640 (87.52)   
240 - **.GGCGCG.** 140 (95.43)   
241 + **[GACGCG]** 417 (89.80)   
241 + **.GCCGCG.** 1242 (95.35)   
241 + **.GGCGCG.** 1284 (95.43)   
241 - **[GACGCG]** 1312 (89.80)   
241 - **[GTCGCC]** 1036 (86.61)   
241 - **.GGCGCG.** 421 (95.43)   
242 + **[GTCGGG]** 525 (87.52)   
242 + **[GTCGGG]** 571 (87.52)   
242 + **[GTCCCG]** 927 (88.98)   
242 + **[GTCGGG]** 1266 (87.52)   
242 - **[GTGGCG]** 1187 (87.04)   
242 - **[GACGCG]** 1003 (89.80)   
242 - **[GACGCG]** 824 (89.80)   
242 - **[CTCGCG]** 774 (88.37)   
242 - **[GTCCCG]** 764 (88.98)   
242 - **[GTCCCG]** 754 (88.98)   
242 - **.GTCGCG.** 325 (100.00)   
243 + **.GTCGCG.** 916 (100.00)   
243 + **.GGCGCG.** 1005 (95.43)   
243 + **.GCCGCG.** 1120 (95.35)   
243 + **.GCCGCG.** 1160 (95.35)   
243 - **.GCCGCG.** 920 (95.35)   
244 + **[GACGCG]** 687 (89.80)   
244 + **.GGCGCG.** 763 (95.43)   
244 + **.GGCGCG.** 777 (95.43)   
244 + **[GTCCCG]** 948 (88.98)   
244 - **[CTCGCG]** 804 (88.37)   
244 - **[GACGCG]** 345 (89.80)   
246 + **[GTCCCG]** 98 (88.98)   
246 + **.GCCGCG.** 274 (95.35)   
246 + **[CTCGCG]** 429 (88.37)   
246 + **[GTCGGG]** 451 (87.52)   
246 + **.GGCGCG.** 459 (95.43)   
246 + **.GGCGCG.** 495 (95.43)   
246 + **[GACGCG]** 670 (89.80)   
246 + **[CTCGCG]** 697 (88.37)   
246 + **.GCCGCG.** 707 (95.35)   
246 + **[GTCCCG]** 795 (88.98)   
246 + **.GGCGCG.** 908 (95.43)   
246 + **.GCCGCG.** 1078 (95.35)   
246 - **[CTCGCG]** 1198 (88.37)   
246 - **.GGCGCG.** 910 (95.43)   
246 - **.GGCGCG.** 884 (95.43)   
246 - **[GTGGCG]** 799 (87.04)   
246 - **.GGCGCG.** 750 (95.43)   
246 - **.GGCGCG.** 699 (95.43)   
246 - **.GCCGCG.** 674 (95.35)   
246 - **.GCCGCG.** 615 (95.35)   
246 - **.GGCGCG.** 276 (95.43)   
247 + **[GTCCCG]** 14 (88.98)   
247 + **.GCCGCG.** 19 (95.35)   
247 + **[GACGCG]** 418 (89.80)   
247 + **[CTCGCG]** 883 (88.37)   
247 + **[GTCCCG]** 936 (88.98)   
247 + **.GGCGCG.** 950 (95.43)   
247 + **.GCCGCG.** 1022 (95.35)   
247 + **.GCCGCG.** 1092 (95.35)   
247 + **.GGCGCG.** 1285 (95.43)   
247 + **[GTCGCC]** 1450 (86.61)   
247 + **.GCCGCG.** 1493 (95.35)   
247 - **.GGCGCG.** 1473 (95.43)   
247 - **.GCCGCG.** 1360 (95.35)   
247 - **[GTCGGG]** 1332 (87.52)   
247 - **.GCCGCG.** 1299 (95.35)   
247 - **.GGCGCG.** 1188 (95.43)   
247 - **.GGCGCG.** 1122 (95.43)   
247 - **.GGCGCG.** 1096 (95.43)   
247 - **[GTGGCG]** 1047 (87.04)   
247 - **.GCCGCG.** 1024 (95.35)   
247 - **.GGCGCG.** 885 (95.43)   
247 - **[GTGGCG]** 758 (87.04)   
247 - **.GCCGCG.** 422 (95.35)   
247 - **.GGCGCG.** 331 (95.43)   
247 - **.GCCGCG.** 279 (95.35)   
247 - **.GGCGCG.** 236 (95.43)   
249 + **[GTGGCG]** 134 (87.04)   
249 - **[GTCGCC]** 248 (86.61)   
250 + **.GTCGCG.** 154 (100.00)   
250 + **[GACGCG]** 453 (89.80)   
250 + **.GGCGCG.** 460 (95.43)   
250 + **[CTCGCG]** 511 (88.37)   
250 + **.GCCGCG.** 870 (95.35)   
250 + **.GCCGCG.** 1096 (95.35)   
250 - **[CTCGCG]** 1259 (88.37)   
250 - **[GTCGCC]** 1011 (86.61)   
250 - **[GTCGGG]** 608 (87.52)   
250 - **.GGCGCG.** 591 (95.43)   
250 - **.GCCGCG.** 542 (95.35)   
250 - **.GGCGCG.** 528 (95.43)   
250 - **.GCCGCG.** 513 (95.35)   
250 - **.GCCGCG.** 457 (95.35)   
250 - **.GCCGCG.** 156 (95.35)   
250 - **.GGCGCG.** 139 (95.43)   
251 - **[GTCGGG]** 1057 (87.52)   
252 + **[GTCGCC]** 295 (86.61)   
252 + **.GGCGCG.** 392 (95.43)   
252 + **[GTCGGG]** 1170 (87.52)   
252 + **[GTCCCG]** 1178 (88.98)   
252 - **[GACGCG]** 394 (89.80)   
253 + **[GTCGCC]** 975 (86.61)   
255 + **[GACGCG]** 1297 (89.80)   
255 - **[GTCGCC]** 1422 (86.61)   
255 - **[GTCGCC]** 1239 (86.61)   
256 + **[GTCGGG]** 713 (87.52)   
256 + **.GGCGCG.** 860 (95.43)   
256 + **.GCCGCG.** 1195 (95.35)   
256 + **.GCCGCG.** 1302 (95.35)   
256 - **[GTCCCG]** 1324 (88.98)   
256 - **[GACGCG]** 1205 (89.80)   
256 - **.GGCGCG.** 1117 (95.43)   
256 - **.GGCGCG.** 99 (95.43)   
257 + **.GCCGCG.** 607 (95.35)   
257 + **[CTCGCG]** 788 (88.37)   
257 + **.GGCGCG.** 876 (95.43)   
257 + **[GTCCCG]** 1020 (88.98)   
257 + **[GACGCG]** 1119 (89.80)   
257 + **.GCCGCG.** 1166 (95.35)   
257 + **.GCCGCG.** 1194 (95.35)   
257 + **.GCCGCG.** 1282 (95.35)   
257 + **.GGCGCG.** 1316 (95.43)   
257 + **[GTCGGG]** 1467 (87.52)   
257 + **.GGCGCG.** 1496 (95.43)   
257 - **[GTCGCC]** 1423 (86.61)   
257 - **.GCCGCG.** 1318 (95.35)   
257 - **.GCCGCG.** 1284 (95.35)   
257 - **.GGCGCG.** 1247 (95.43)   
257 - **[GTCCCG]** 1116 (88.98)   
257 - **[GTCGGG]** 762 (87.52)   
257 - **[GTCGGG]** 741 (87.52)   
257 - **[GTGGCG]** 574 (87.04)   
258 - **[GTCGGG]** 1205 (87.52)   
258 - **[GTCGCC]** 305 (86.61)   
259 + **[GACGCG]** 995 (89.80)   
259 + **.GCCGCG.** 1018 (95.35)   
259 + **[GACGCG]** 1023 (89.80)   
259 + **[CTCGCG]** 1219 (88.37)   
259 + **.GCCGCG.** 1302 (95.35)   
259 + **[GTCCCG]** 1335 (88.98)   
259 - **[GTCGCC]** 1415 (86.61)   
259 - **[GTCGCC]** 1307 (86.61)   
259 - **.GCCGCG.** 1304 (95.35)   
259 - **.GGCGCG.** 1221 (95.43)   
259 - **.GCCGCG.** 1187 (95.35)   
259 - **.GTCGCG.** 1020 (100.00)   
259 - **.GCCGCG.** 997 (95.35)   
259 - **[GTCGCC]** 992 (86.61)   
260 - **.GTCGCG.** 1226 (100.00)   
260 - **[GTCGCC]** 1118 (86.61)   
260 - **.GGCGCG.** 1042 (95.43)   
260 - **.GGCGCG.** 879 (95.43)   
261 - **[GTCGCC]** 149 (86.61)   
261 - **[GTGGCG]** 129 (87.04)   
261 - **.GGCGCG.** 127 (95.43)   
262 + **[GTCGCC]** 835 (86.61)   
262 + **[CTCGCG]** 944 (88.37)   
262 + **[GTCGCC]** 1194 (86.61)   
262 - **[GTCGGG]** 784 (87.52)   
262 - **[GTCCCG]** 378 (88.98)   
263 + **.GGCGCG.** 30 (95.43)   
263 + **.GGCGCG.** 165 (95.43)   
263 + **[GTGGCG]** 305 (87.04)   
263 + **.GGCGCG.** 307 (95.43)   
263 + **.GGCGCG.** 540 (95.43)   
263 + **.GTCGCG.** 846 (100.00)   
263 + **.GCCGCG.** 854 (95.35)   
263 + **.GCCGCG.** 1076 (95.35)   
263 + **.GTCGCG.** 1081 (100.00)   
263 + **[GTCCCG]** 1112 (88.98)   
263 + **[GACGCG]** 1117 (89.80)   
263 + **[GTCCCG]** 1244 (88.98)   
263 + **.GGCGCG.** 1282 (95.43)   
263 + **[GTCGGG]** 1481 (87.52)   
263 + **[GTCGGG]** 1499 (87.52)   
263 - **[GTCGGG]** 1114 (87.52)   
263 - **[GACGCG]** 1078 (89.80)   
263 - **.GCCGCG.** 850 (95.35)   
263 - **.GGCGCG.** 542 (95.43)   
263 - **[GTGGCG]** 395 (87.04)   
263 - **.GGCGCG.** 393 (95.43)   
263 - **.GGCGCG.** 311 (95.43)   
264 + **[CTCGCG]** 141 (88.37)   
264 + **[GTCGCC]** 353 (86.61)   
264 + **[GTCGGG]** 415 (87.52)   
264 + **[GACGCG]** 752 (89.80)   
264 + **[GTCGCC]** 1204 (86.61)   
264 - **.GGCGCG.** 1138 (95.43)   
264 - **[GTGGCG]** 574 (87.04)   
264 - **[GACGCG]** 143 (89.80)   
266 + **[CTCGCG]** 1004 (88.37)   
266 + **.GGCGCG.** 1028 (95.43)   
266 + **[GTCCCG]** 1059 (88.98)   
266 + **.GCCGCG.** 1065 (95.35)   
266 + **.GGCGCG.** 1104 (95.43)   
266 + **.GCCGCG.** 1280 (95.35)   
266 + **.GCCGCG.** 1475 (95.35)   
266 - **.GCCGCG.** 1282 (95.35)   
266 - **.GGCGCG.** 1270 (95.43)   
266 - **.GGCGCG.** 1106 (95.43)   
266 - **.GCCGCG.** 1030 (95.35)   
266 - **.GGCGCG.** 982 (95.43)   
266 - **[GTGGCG]** 977 (87.04)   
266 - **.GCCGCG.** 962 (95.35)   
267 + **.GCCGCG.** 193 (95.35)   
267 + **[GTCGCC]** 230 (86.61)   
267 + **[CTCGCG]** 697 (88.37)   
267 - **[GTCCCG]** 922 (88.98)   
267 - **.GCCGCG.** 195 (95.35)   
268 + **.GGCGCG.** 57 (95.43)   
268 + **.GCCGCG.** 180 (95.35)   
268 + **[GACGCG]** 509 (89.80)   
268 + **[CTCGCG]** 623 (88.37)   
268 + **[GTCCCG]** 750 (88.98)   
268 + **.GGCGCG.** 1013 (95.43)   
268 + **[GTGGCG]** 1043 (87.04)   
268 + **.GCCGCG.** 1068 (95.35)   
268 + **[GTCGGG]** 1124 (87.52)   
268 + **.GCCGCG.** 1320 (95.35)   
268 + **[GTGGCG]** 1336 (87.04)   
268 - **.GCCGCG.** 1478 (95.35)   
268 - **.GCCGCG.** 1394 (95.35)   
268 - **[CTCGCG]** 1322 (88.37)   
268 - **[GTGGCG]** 1196 (87.04)   
268 - **.GCCGCG.** 1015 (95.35)   
268 - **.GCCGCG.** 813 (95.35)   
268 - **.GTCGCG.** 625 (100.00)   
268 - **[GTGGCG]** 594 (87.04)   
268 - **[GTCGCC]** 48 (86.61)   
270 + **.GCCGCG.** 25 (95.35)   
270 + **.GGCGCG.** 56 (95.43)   
270 + **[GTCCCG]** 165 (88.98)   
270 + **[GTCGCC]** 199 (86.61)   
270 + **[GTCCCG]** 214 (88.98)   
270 + **.GGCGCG.** 462 (95.43)   
270 + **[GTGGCG]** 560 (87.04)   
270 + **[GTCCCG]** 855 (88.98)   
270 + **[GTCGCC]** 1257 (86.61)   
270 + **.GGCGCG.** 1299 (95.43)   
270 + **.GCCGCG.** 1438 (95.35)   
270 - **[GTCGCC]** 341 (86.61)   
270 - **[CTCGCG]** 58 (88.37)   
270 - **[GACGCG]** 27 (89.80)   
271 + **[GACGCG]** 742 (89.80)   
271 + **[GTCGCC]** 1123 (86.61)   
271 + **[GTCGGG]** 1219 (87.52)   
271 + **[GACGCG]** 1288 (89.80)   
271 - **[GTGGCG]** 1422 (87.04)   
271 - **[GTCCCG]** 1221 (88.98)   
271 - **.GCCGCG.** 681 (95.35)   
272 + **.GCCGCG.** 712 (95.35)   
272 + **[GTCGCC]** 1114 (86.61)   
272 + **[GTCGGG]** 1209 (87.52)   
272 + **[GACGCG]** 1279 (89.80)   
272 - **[GTGGCG]** 1413 (87.04)   
272 - **.GCCGCG.** 669 (95.35)   
273 + **[GTCGGG]** 364 (87.52)   
273 + **[GTCCCG]** 515 (88.98)   
273 + **.GCCGCG.** 1030 (95.35)   
273 + **[GTCGGG]** 1406 (87.52)   
273 - **[CTCGCG]** 1234 (88.37)   
273 - **[GACGCG]** 1121 (89.80)   
273 - **.GCCGCG.** 1032 (95.35)   
273 - **[GTCCCG]** 366 (88.98)   
273 - **.GGCGCG.** 201 (95.43)   
274 + **.GCCGCG.** 13 (95.35)   
274 + **[CTCGCG]** 47 (88.37)   
274 + **.GGCGCG.** 111 (95.43)   
274 + **.GGCGCG.** 1352 (95.43)   
274 + **.GCCGCG.** 1360 (95.35)   
274 - **.GCCGCG.** 1322 (95.35)   
274 - **.GGCGCG.** 1269 (95.43)   
274 - **[GTCGGG]** 1014 (87.52)   
274 - **.GGCGCG.** 587 (95.43)   
274 - **.GCCGCG.** 108 (95.35)   
274 - **.GGCGCG.** 49 (95.43)   
274 - **.GCCGCG.** 15 (95.35)   
275 + **[GTCCCG]** 36 (88.98)   
275 + **.GGCGCG.** 73 (95.43)   
275 + **[GACGCG]** 91 (89.80)   
275 + **[GTGGCG]** 170 (87.04)   
275 + **[GTGGCG]** 269 (87.04)   
275 + **[GTGGCG]** 275 (87.04)   
275 + **[GTCGCC]** 575 (86.61)   
275 + **.GGCGCG.** 1088 (95.43)   
275 + **[CTCGCG]** 1328 (88.37)   
275 - **.GGCGCG.** 1330 (95.43)   
275 - **.GTCGCG.** 1265 (100.00)   
275 - **.GGCGCG.** 1101 (95.43)   
275 - **[CTCGCG]** 1090 (88.37)   
275 - **.GGCGCG.** 755 (95.43)   
275 - **.GGCGCG.** 735 (95.43)   
275 - **.GCCGCG.** 711 (95.35)   
275 - **[CTCGCG]** 605 (88.37)   
275 - **[GTCGGG]** 38 (87.52)   
276 + **[GACGCG]** 755 (89.80)   
279 + **.GGCGCG.** 106 (95.43)   
279 + **.GGCGCG.** 413 (95.43)   
279 + **.GGCGCG.** 418 (95.43)   
279 + **[GACGCG]** 555 (89.80)   
279 - **[CTCGCG]** 632 (88.37)   
279 - **.GGCGCG.** 459 (95.43)   
279 - **.GCCGCG.** 415 (95.35)   
279 - **.GTCGCG.** 376 (100.00)   
279 - **[GTGGCG]** 110 (87.04)   
279 - **.GGCGCG.** 108 (95.43)   
279 - **.GGCGCG.** 40 (95.43)   
279 - **.GCCGCG.** 33 (95.35)   
279 - **.GGCGCG.** 22 (95.43)   
280 + **[GTCGGG]** 507 (87.52)   
280 + **.GCCGCG.** 742 (95.35)   
280 + **[CTCGCG]** 781 (88.37)   
280 + **[GTCGGG]** 856 (87.52)   
280 + **.GCCGCG.** 944 (95.35)   
280 + **.GGCGCG.** 976 (95.43)   
280 + **[CTCGCG]** 1262 (88.37)   
280 + **[GTCCCG]** 1349 (88.98)   
280 + **[GTGGCG]** 1484 (87.04)   
280 - **.GGCGCG.** 1264 (95.43)   
280 - **[GACGCG]** 1197 (89.80)   
280 - **.GTCGCG.** 1178 (100.00)   
280 - **.GTCGCG.** 1152 (100.00)   
280 - **[GTCCCG]** 1146 (88.98)   
280 - **.GGCGCG.** 1128 (95.43)   
280 - **[GACGCG]** 1084 (89.80)   
280 - **.GGCGCG.** 1072 (95.43)   
280 - **.GGCGCG.** 1066 (95.43)   
280 - **.GCCGCG.** 982 (95.35)   
280 - **[CTCGCG]** 946 (88.37)   
280 - **[CTCGCG]** 827 (88.37)   
280 - **.GCCGCG.** 735 (95.35)   
280 - **[GTCCCG]** 620 (88.98)   
280 - **.GCCGCG.** 169 (95.35)   
281 + **[GTGGCG]** 289 (87.04)   
281 + **[GACGCG]** 446 (89.80)   
281 + **[GACGCG]** 512 (89.80)   
281 + **.GGCGCG.** 545 (95.43)   
281 + **.GTCGCG.** 743 (100.00)   
281 + **[GTGGCG]** 924 (87.04)   
281 + **.GGCGCG.** 1024 (95.43)   
281 + **[GTCGGG]** 1090 (87.52)   
281 + **.GGCGCG.** 1200 (95.43)   
281 - **[GTCGGG]** 1465 (87.52)   
281 - **[GTCGGG]** 1335 (87.52)   
281 - **[GTCGCC]** 1169 (86.61)   
281 - **[GTCGGG]** 766 (87.52)   
281 - **.GGCGCG.** 553 (95.43)   
281 - **.GGCGCG.** 547 (95.43)   
281 - **.GCCGCG.** 531 (95.35)   
281 - **.GCCGCG.** 521 (95.35)   
281 - **[GTCCCG]** 443 (88.98)   
281 - **.GGCGCG.** 433 (95.43)   
282 - **[GTCGGG]** 623 (87.52)   
282 - **.GCCGCG.** 13 (95.35)   
283 + **[GTCCCG]** 594 (88.98)   
283 - **[GTCGGG]** 1468 (87.52)   
283 - **[GTCCCG]** 1016 (88.98)   
283 - **[GTCCCG]** 598 (88.98)   
283 - **[GTGGCG]** 517 (87.04)   
283 - **.GGCGCG.** 134 (95.43)   
284 + **.GGCGCG.** 631 (95.43)   
284 + **.GGCGCG.** 668 (95.43)   
284 + **.GCCGCG.** 706 (95.35)   
284 + **[GTGGCG]** 787 (87.04)   
284 + **.GGCGCG.** 999 (95.43)   
284 + **.GGCGCG.** 1092 (95.43)   
284 + **.GGCGCG.** 1198 (95.43)   
284 + **[GTCGCC]** 1246 (86.61)   
284 + **.GCCGCG.** 1345 (95.35)   
284 + **.GGCGCG.** 1450 (95.43)   
284 + **.GGCGCG.** 1471 (95.43)   
284 + **.GTCGCG.** 1505 (100.00)   
284 - **.GCCGCG.** 1452 (95.35)   
284 - **.GGCGCG.** 1371 (95.43)   
284 - **.GCCGCG.** 1347 (95.35)   
284 - **.GGCGCG.** 1342 (95.43)   
284 - **.GCCGCG.** 1283 (95.35)   
284 - **[GTCCCG]** 1217 (88.98)   
284 - **.GCCGCG.** 1094 (95.35)   
284 - **.GCCGCG.** 1089 (95.35)   
284 - **[GTCGCC]** 1026 (86.61)   
284 - **.GGCGCG.** 1003 (95.43)   
284 - **.GCCGCG.** 943 (95.35)   
284 - **.GCCGCG.** 780 (95.35)   
284 - **[GTGGCG]** 710 (87.04)   
284 - **.GGCGCG.** 708 (95.43)   
284 - **.GGCGCG.** 553 (95.43)   
285 + **[GTCGCC]** 231 (86.61)   
285 + **.GGCGCG.** 334 (95.43)   
285 + **.GGCGCG.** 344 (95.43)   
285 + **.GCCGCG.** 353 (95.35)   
285 + **[CTCGCG]** 375 (88.37)   
285 + **.GGCGCG.** 443 (95.43)   
285 + **.GGCGCG.** 449 (95.43)   
285 + **.GGCGCG.** 470 (95.43)   
285 + **.GCCGCG.** 552 (95.35)   
285 + **.GCCGCG.** 634 (95.35)   
285 + **[CTCGCG]** 650 (88.37)   
285 + **.GCCGCG.** 655 (95.35)   
285 + **[GACGCG]** 715 (89.80)   
285 + **.GCCGCG.** 858 (95.35)   
285 + **[GTCCCG]** 873 (88.98)   
285 + **.GCCGCG.** 907 (95.35)   
285 + **.GCCGCG.** 939 (95.35)   
285 + **[CTCGCG]** 964 (88.37)   
285 + **.GGCGCG.** 1026 (95.43)   
285 + **.GCCGCG.** 1033 (95.35)   
285 + **.GTCGCG.** 1401 (100.00)   
285 + **.GGCGCG.** 1486 (95.43)   
285 - **.GGCGCG.** 1488 (95.43)   
285 - **.GCCGCG.** 1216 (95.35)   
285 - **[CTCGCG]** 1028 (88.37)   
285 - **[GTCGGG]** 985 (87.52)   
285 - **.GCCGCG.** 966 (95.35)   
285 - **.GTCGCG.** 950 (100.00)   
285 - **.GGCGCG.** 941 (95.43)   
285 - **.GCCGCG.** 909 (95.35)   
285 - **.GCCGCG.** 717 (95.35)   
285 - **.GGCGCG.** 657 (95.43)   
285 - **.GGCGCG.** 652 (95.43)   
285 - **.GGCGCG.** 636 (95.43)   
285 - **.GCCGCG.** 554 (95.35)   
285 - **.GCCGCG.** 548 (95.35)   
285 - **.GCCGCG.** 495 (95.35)   
287 + **.GCCGCG.** 42 (95.35)   
287 + **[GACGCG]** 490 (89.80)   
287 + **[GTCGCC]** 959 (86.61)   
287 + **.GGCGCG.** 1339 (95.43)   
287 - **[GACGCG]** 1343 (89.80)   
287 - **[GTCCCG]** 1247 (88.98)   
287 - **.GGCGCG.** 1233 (95.43)   
287 - **[GTGGCG]** 1085 (87.04)   
287 - **[GTCGGG]** 1029 (87.52)   
287 - **[GTCGCC]** 560 (86.61)   
287 - **.GGCGCG.** 537 (95.43)   
287 - **.GGCGCG.** 326 (95.43)   
287 - **[GTGGCG]** 210 (87.04)   
288 + **[GTCGCC]** 681 (86.61)   
288 + **[CTCGCG]** 819 (88.37)   
288 + **.GGCGCG.** 876 (95.43)   
288 + **.GTCGCG.** 980 (100.00)   
288 + **.GCCGCG.** 1168 (95.35)   
288 + **[GTCCCG]** 1189 (88.98)   
288 + **[CTCGCG]** 1252 (88.37)   
288 + **[GTCGGG]** 1471 (87.52)   
288 - **[CTCGCG]** 1254 (88.37)   
288 - **.GGCGCG.** 1195 (95.43)   
288 - **[GTGGCG]** 1172 (87.04)   
288 - **.GGCGCG.** 1170 (95.43)   
288 - **.GGCGCG.** 1042 (95.43)   
288 - **[CTCGCG]** 878 (88.37)   
288 - **[CTCGCG]** 821 (88.37)   
289 + **[GACGCG]** 540 (89.80)   
289 + **[CTCGCG]** 998 (88.37)   
289 + **[GTCGGG]** 1232 (87.52)   
289 + **.GGCGCG.** 1317 (95.43)   
289 + **.GGCGCG.** 1379 (95.43)   
289 + **[GTGGCG]** 1483 (87.04)   
289 - **[CTCGCG]** 1319 (88.37)   
289 - **.GCCGCG.** 1300 (95.35)   
290 + **.GCCGCG.** 1104 (95.35)   
290 + **[GTCGCC]** 1160 (86.61)   
290 + **[GTCGGG]** 1325 (87.52)   
290 + **.GGCGCG.** 1330 (95.43)   
290 + **[GTCGCC]** 1430 (86.61)   
290 - **[GTCCCG]** 1445 (88.98)   
290 - **.GCCGCG.** 1339 (95.35)   
291 + **[CTCGCG]** 201 (88.37)   
291 + **[GTCCCG]** 206 (88.98)   
291 + **[GTCGCC]** 360 (86.61)   
291 + **.GCCGCG.** 510 (95.35)   
291 + **.GTCGCG.** 584 (100.00)   
291 + **.GGCGCG.** 608 (95.43)   
291 + **[CTCGCG]** 629 (88.37)   
291 + **.GCCGCG.** 767 (95.35)   
291 + **[CTCGCG]** 997 (88.37)   
291 + **.GCCGCG.** 1035 (95.35)   
291 + **.GGCGCG.** 1066 (95.43)   
291 + **.GCCGCG.** 1083 (95.35)   
291 + **.GGCGCG.** 1098 (95.43)   
291 + **.GCCGCG.** 1109 (95.35)   
291 + **[GACGCG]** 1165 (89.80)   
291 + **[GTCGGG]** 1195 (87.52)   
291 + **.GCCGCG.** 1329 (95.35)   
291 + **.GCCGCG.** 1343 (95.35)   
291 + **[GTCCCG]** 1384 (88.98)   
291 + **.GGCGCG.** 1500 (95.43)   
291 - **.GGCGCG.** 1502 (95.43)   
291 - **[CTCGCG]** 1345 (88.37)   
291 - **.GCCGCG.** 1240 (95.35)   
291 - **.GCCGCG.** 1207 (95.35)   
291 - **.GGCGCG.** 1100 (95.43)   
291 - **[GTGGCG]** 965 (87.04)   
291 - **.GCCGCG.** 871 (95.35)   
291 - **.GCCGCG.** 769 (95.35)   
291 - **[GACGCG]** 738 (89.80)   
291 - **[GTCCCG]** 696 (88.98)   
291 - **[GACGCG]** 512 (89.80)   
291 - **.GTCGCG.** 451 (100.00)   
291 - **[GACGCG]** 407 (89.80)   
291 - **[GACGCG]** 203 (89.80)   
291 - **[GTGGCG]** 59 (87.04)   
292 + **[GTCGGG]** 128 (87.52)   
292 + **.GCCGCG.** 795 (95.35)   
292 + **[GTCCCG]** 980 (88.98)   
292 + **.GGCGCG.** 1026 (95.43)   
292 + **.GGCGCG.** 1043 (95.43)   
292 + **.GCCGCG.** 1082 (95.35)   
292 + **[GTCGGG]** 1181 (87.52)   
292 + **[GACGCG]** 1189 (89.80)   
292 + **.GTCGCG.** 1337 (100.00)   
292 + **.GGCGCG.** 1346 (95.43)   
292 + **.GCCGCG.** 1445 (95.35)   
292 + **.GTCGCG.** 1469 (100.00)   
292 + **[GACGCG]** 1485 (89.80)   
292 + **.GGCGCG.** 1492 (95.43)   
292 - **.GCCGCG.** 1386 (95.35)   
292 - **.GCCGCG.** 1339 (95.35)   
292 - **[GACGCG]** 1319 (89.80)   
292 - **.GGCGCG.** 1140 (95.43)   
292 - **[CTCGCG]** 1084 (88.37)   
292 - **.GGCGCG.** 1006 (95.43)   
292 - **.GGCGCG.** 958 (95.43)   
292 - **.GCCGCG.** 840 (95.35)   
292 - **.GCCGCG.** 797 (95.35)   
292 - **.GGCGCG.** 774 (95.43)   
292 - **.GGCGCG.** 767 (95.43)   
292 - **.GCCGCG.** 659 (95.35)   
293 + **.GGCGCG.** 837 (95.43)   
293 + **.GGCGCG.** 860 (95.43)   
293 + **[GTCGGG]** 879 (87.52)   
293 + **.GTCGCG.** 1073 (100.00)   
293 + **.GGCGCG.** 1096 (95.43)   
293 + **[GTCCCG]** 1168 (88.98)   
293 + **[GTCCCG]** 1273 (88.98)   
293 + **[GTCCCG]** 1339 (88.98)   
293 + **[GTCGCC]** 1365 (86.61)   
293 + **.GGCGCG.** 1378 (95.43)   
293 + **[CTCGCG]** 1442 (88.37)   
293 - **.GCCGCG.** 1154 (95.35)   
293 - **.GCCGCG.** 936 (95.35)   
293 - **.GCCGCG.** 839 (95.35)   
293 - **.GTCGCG.** 773 (100.00)   
294 + **[GTCCCG]** 12 (88.98)   
294 + **.GCCGCG.** 46 (95.35)   
294 + **[GTCGGG]** 274 (87.52)   
294 + **[CTCGCG]** 366 (88.37)   
294 + **[CTCGCG]** 611 (88.37)   
294 + **.GCCGCG.** 785 (95.35)   
294 + **[GACGCG]** 936 (89.80)   
294 + **[GTCCCG]** 1190 (88.98)   
294 - **[GTCGGG]** 1086 (87.52)   
294 - **[GTGGCG]** 1025 (87.04)   
294 - **.GGCGCG.** 938 (95.43)   
294 - **.GCCGCG.** 894 (95.35)   
294 - **[GTCCCG]** 778 (88.98)   
294 - **.GCCGCG.** 316 (95.35)   
295 + **[GTCGGG]** 554 (87.52)   
295 + **.GGCGCG.** 590 (95.43)   
295 + **[GTGGCG]** 615 (87.04)   
295 + **.GGCGCG.** 702 (95.43)   
295 + **.GCCGCG.** 747 (95.35)   
295 + **[GTCCCG]** 862 (88.98)   
295 + **.GCCGCG.** 1324 (95.35)   
295 - **[GTCGGG]** 1366 (87.52)   
295 - **[CTCGCG]** 1274 (88.37)   
295 - **[CTCGCG]** 1029 (88.37)   
295 - **.GCCGCG.** 855 (95.35)   
295 - **[GACGCG]** 704 (89.80)   
295 - **.GCCGCG.** 592 (95.35)   
295 - **[GTCCCG]** 450 (88.98)   
296 + **.GGCGCG.** 195 (95.43)   
296 + **.GGCGCG.** 248 (95.43)   
296 + **.GGCGCG.** 419 (95.43)   
296 + **.GGCGCG.** 494 (95.43)   
296 + **.GTCGCG.** 589 (100.00)   
296 + **.GCCGCG.** 757 (95.35)   
296 + **[GTCGCC]** 777 (86.61)   
296 + **.GCCGCG.** 813 (95.35)   
296 + **[GACGCG]** 841 (89.80)   
296 + **[GACGCG]** 1001 (89.80)   
296 + **[GACGCG]** 1012 (89.80)   
296 + **.GGCGCG.** 1118 (95.43)   
296 + **.GCCGCG.** 1162 (95.35)   
296 + **.GCCGCG.** 1187 (95.35)   
296 + **.GGCGCG.** 1313 (95.43)   
296 + **[GACGCG]** 1378 (89.80)   
296 + **.GGCGCG.** 1443 (95.43)   
296 + **[GTCCCG]** 1477 (88.98)   
296 - **[GACGCG]** 1474 (89.80)   
296 - **.GCCGCG.** 1440 (95.35)   
296 - **[CTCGCG]** 1380 (88.37)   
296 - **.GGCGCG.** 1315 (95.43)   
296 - **[GTGGCG]** 1260 (87.04)   
296 - **.GGCGCG.** 1184 (95.43)   
296 - **.GCCGCG.** 1164 (95.35)   
296 - **[GTCCCG]** 1022 (88.98)   
296 - **.GGCGCG.** 1016 (95.43)   
296 - **.GCCGCG.** 1003 (95.35)   
296 - **.GGCGCG.** 946 (95.43)   
296 - **.GCCGCG.** 815 (95.35)   
296 - **.GGCGCG.** 759 (95.43)   
296 - **.GGCGCG.** 718 (95.43)   
296 - **.GGCGCG.** 711 (95.43)   
296 - **.GGCGCG.** 704 (95.43)   
296 - **.GGCGCG.** 697 (95.43)   
296 - **.GGCGCG.** 591 (95.43)   
296 - **[GACGCG]** 500 (89.80)   
296 - **[GTCGCC]** 471 (86.61)   
296 - **[CTCGCG]** 335 (88.37)   
296 - **.GCCGCG.** 250 (95.35)   
296 - **.GCCGCG.** 201 (95.35)   
296 - **.GCCGCG.** 85 (95.35)   
296 - **[GTCCCG]** 73 (88.98)   
296 - **.GGCGCG.** 39 (95.43)   
297 + **.GCCGCG.** 827 (95.35)   
297 + **[GTCGGG]** 851 (87.52)   
297 + **.GGCGCG.** 954 (95.43)   
297 + **.GGCGCG.** 985 (95.43)   
297 + **.GCCGCG.** 996 (95.35)   
297 + **.GCCGCG.** 1155 (95.35)   
297 + **[GTCGCC]** 1204 (86.61)   
297 + **[CTCGCG]** 1498 (88.37)   
297 - **.GGCGCG.** 792 (95.43)   
297 - **[CTCGCG]** 650 (88.37)   
297 - **[GTCCCG]** 247 (88.98)   
298 + **[GTCGGG]** 616 (87.52)   
298 + **[CTCGCG]** 744 (88.37)   
298 + **.GCCGCG.** 797 (95.35)   
298 + **.GTCGCG.** 838 (100.00)   
298 + **.GGCGCG.** 868 (95.43)   
298 + **.GCCGCG.** 901 (95.35)   
298 + **.GTCGCG.** 1002 (100.00)   
298 + **.GGCGCG.** 1040 (95.43)   
298 + **.GCCGCG.** 1178 (95.35)   
298 + **[CTCGCG]** 1266 (88.37)   
298 + **.GGCGCG.** 1369 (95.43)   
298 + **[GTCGGG]** 1411 (87.52)   
298 + **[CTCGCG]** 1470 (88.37)   
298 - **[CTCGCG]** 1472 (88.37)   
298 - **[GTCCCG]** 1190 (88.98)   
298 - **[CTCGCG]** 1180 (88.37)   
298 - **[GTCGCC]** 1115 (86.61)   
298 - **.GCCGCG.** 1004 (95.35)   
298 - **[GACGCG]** 937 (89.80)   
298 - **.GGCGCG.** 921 (95.43)   
298 - **.GCCGCG.** 876 (95.35)   
298 - **.GCCGCG.** 842 (95.35)   
298 - **[GACGCG]** 799 (89.80)   
298 - **.GCCGCG.** 746 (95.35)   
298 - **.GGCGCG.** 293 (95.43)   
299 + **[GTCGGG]** 352 (87.52)   
299 + **[GTGGCG]** 394 (87.04)   
299 + **[CTCGCG]** 410 (88.37)   
299 + **[GTCCCG]** 446 (88.98)   
299 + **.GCCGCG.** 851 (95.35)   
299 + **.GGCGCG.** 1078 (95.43)   
299 + **[CTCGCG]** 1091 (88.37)   
299 + **.GCCGCG.** 1178 (95.35)   
299 + **[GTGGCG]** 1290 (87.04)   
299 + **[GTCCCG]** 1300 (88.98)   
299 + **[GTGGCG]** 1330 (87.04)   
299 + **.GCCGCG.** 1350 (95.35)   
299 + **.GGCGCG.** 1473 (95.43)   
299 - **[GACGCG]** 1475 (89.80)   
299 - **.GTCGCG.** 1401 (100.00)   
299 - **.GCCGCG.** 1262 (95.35)   
299 - **.GCCGCG.** 1180 (95.35)   
299 - **.GGCGCG.** 1082 (95.43)   
299 - **.GCCGCG.** 1059 (95.35)   
299 - **.GGCGCG.** 920 (95.43)   
299 - **[GACGCG]** 883 (89.80)   
299 - **.GGCGCG.** 868 (95.43)   
299 - **[GACGCG]** 853 (89.80)   
299 - **[GTCGCC]** 798 (86.61)   
299 - **[GTCGGG]** 56 (87.52)   
300 + **.GGCGCG.** 111 (95.43)   
300 + **[CTCGCG]** 220 (88.37)   
300 + **.GTCGCG.** 398 (100.00)   
300 + **[GTCCCG]** 416 (88.98)   
300 + **[GTCGGG]** 449 (87.52)   
300 + **[GTGGCG]** 470 (87.04)   
300 + **.GGCGCG.** 472 (95.43)   
300 + **.GGCGCG.** 477 (95.43)   
300 + **[GTGGCG]** 490 (87.04)   
300 + **[GTGGCG]** 511 (87.04)   
300 + **[GTCGGG]** 716 (87.52)   
300 + **[GTCGGG]** 765 (87.52)   
300 - **.GCCGCG.** 736 (95.35)   
300 - **[GTCGGG]** 595 (87.52)   
300 - **.GGCGCG.** 581 (95.43)   
300 - **.GCCGCG.** 474 (95.35)   
300 - **[GACGCG]** 446 (89.80)   
300 - **.GCCGCG.** 175 (95.35)   
300 - **.GCCGCG.** 113 (95.35)   
300 - **[GTCCCG]** 59 (88.98)   
300 - **.GCCGCG.** 2 (95.35)   
301 + **[GTGGCG]** 270 (87.04)   
301 + **[GTCGGG]** 301 (87.52)   
301 + **[GTCCCG]** 539 (88.98)   
301 + **.GGCGCG.** 579 (95.43)   
301 + **.GGCGCG.** 765 (95.43)   
301 + **[GTCGGG]** 913 (87.52)   
301 + **.GCCGCG.** 1481 (95.35)   
301 - **.GCCGCG.** 1289 (95.35)   
302 + **[GTCGGG]** 216 (87.52)   
302 + **[GTCCCG]** 491 (88.98)   
302 + **[GTGGCG]** 539 (87.04)   
302 + **.GGCGCG.** 740 (95.43)   
302 + **[CTCGCG]** 861 (88.37)   
302 + **[GACGCG]** 951 (89.80)   
302 + **[CTCGCG]** 975 (88.37)   
302 + **.GCCGCG.** 999 (95.35)   
302 + **[CTCGCG]** 1024 (88.37)   
302 + **[CTCGCG]** 1037 (88.37)   
302 + **.GCCGCG.** 1042 (95.35)   
302 - **[GTCGGG]** 1065 (87.52)   
302 - **.GGCGCG.** 1039 (95.43)   
302 - **.GCCGCG.** 1026 (95.35)   
302 - **[CTCGCG]** 1001 (88.37)   
302 - **.GGCGCG.** 977 (95.43)   
302 - **.GGCGCG.** 961 (95.43)   
302 - **[GTCGCC]** 948 (86.61)   
302 - **[GTGGCG]** 865 (87.04)   
302 - **.GGCGCG.** 863 (95.43)   
302 - **.GCCGCG.** 853 (95.35)   
302 - **.GGCGCG.** 841 (95.43)   
302 - **.GGCGCG.** 756 (95.43)   
302 - **.GTCGCG.** 593 (100.00)   
303 + **[CTCGCG]** 21 (88.37)   
303 + **.GCCGCG.** 43 (95.35)   
303 + **[GTCGGG]** 133 (87.52)   
303 + **.GCCGCG.** 168 (95.35)   
303 + **.GCCGCG.** 287 (95.35)   
303 + **.GCCGCG.** 457 (95.35)   
303 + **.GGCGCG.** 743 (95.43)   
303 + **.GCCGCG.** 748 (95.35)   
303 + **.GGCGCG.** 1004 (95.43)   
303 + **.GCCGCG.** 1361 (95.35)   
303 - **.GCCGCG.** 1365 (95.35)   
303 - **[GTGGCG]** 1229 (87.04)   
303 - **[GACGCG]** 1021 (89.80)   
303 - **.GCCGCG.** 1006 (95.35)   
303 - **.GCCGCG.** 750 (95.35)   
303 - **.GGCGCG.** 745 (95.43)   
303 - **[CTCGCG]** 393 (88.37)   
303 - **.GGCGCG.** 170 (95.43)   
303 - **[GACGCG]** 130 (89.80)   
303 - **[CTCGCG]** 45 (88.37)   
304 + **[GTGGCG]** 141 (87.04)   
304 + **.GGCGCG.** 169 (95.43)   
304 + **.GCCGCG.** 708 (95.35)   
304 + **[GACGCG]** 733 (89.80)   
304 + **.GGCGCG.** 904 (95.43)   
304 + **.GCCGCG.** 1106 (95.35)   
304 + **.GTCGCG.** 1161 (100.00)   
304 + **.GGCGCG.** 1179 (95.43)   
304 + **.GGCGCG.** 1241 (95.43)   
304 + **[GTCGGG]** 1253 (87.52)   
304 + **.GCCGCG.** 1263 (95.35)   
304 + **[GTCGCC]** 1333 (86.61)   
304 - **[GTGGCG]** 1512 (87.04)   
304 - **.GGCGCG.** 1510 (95.43)   
304 - **.GTCGCG.** 1309 (100.00)   
304 - **.GCCGCG.** 1243 (95.35)   
304 - **[CTCGCG]** 908 (88.37)   
304 - **[GTCGGG]** 881 (87.52)   
304 - **.GGCGCG.** 710 (95.43)   
304 - **[GTCCCG]** 192 (88.98)   
305 + **.GCCGCG.** 924 (95.35)   
305 + **[GTGGCG]** 964 (87.04)   
305 + **[GTCGGG]** 1236 (87.52)   
305 - **[GACGCG]** 1080 (89.80)   
305 - **[GTCCCG]** 740 (88.98)   
305 - **[GTCGGG]** 307 (87.52)   
306 + **[GTCCCG]** 263 (88.98)   
306 + **[GTGGCG]** 308 (87.04)   
306 + **.GGCGCG.** 310 (95.43)   
306 + **.GCCGCG.** 340 (95.35)   
306 + **[GTCGCC]** 512 (86.61)   
306 + **[CTCGCG]** 710 (88.37)   
306 + **.GCCGCG.** 765 (95.35)   
306 + **[GACGCG]** 845 (89.80)   
306 + **[CTCGCG]** 904 (88.37)   
306 + **.GGCGCG.** 1039 (95.43)   
306 + **[GTCGGG]** 1057 (87.52)   
306 + **[GTCGGG]** 1117 (87.52)   
306 + **[GTCGGG]** 1224 (87.52)   
306 + **[GTCGGG]** 1269 (87.52)   
306 + **[GTCGGG]** 1330 (87.52)   
306 - **.GGCGCG.** 930 (95.43)   
306 - **.GTCGCG.** 906 (100.00)   
306 - **.GGCGCG.** 767 (95.43)   
306 - **.GGCGCG.** 631 (95.43)   
306 - **[GTGGCG]** 625 (87.04)   
306 - **.GGCGCG.** 342 (95.43)   
306 - **[GACGCG]** 312 (89.80)   
306 - **[CTCGCG]** 146 (88.37)   
306 - **[CTCGCG]** 49 (88.37)   
307 + **[GTGGCG]** 85 (87.04)   
307 + **[GTCCCG]** 285 (88.98)   
307 + **[GACGCG]** 334 (89.80)   
307 + **.GGCGCG.** 430 (95.43)   
307 + **.GGCGCG.** 497 (95.43)   
307 + **[GTCCCG]** 517 (88.98)   
307 + **.GGCGCG.** 546 (95.43)   
307 + **.GCCGCG.** 679 (95.35)   
307 + **.GCCGCG.** 737 (95.35)   
307 + **[GTCCCG]** 1105 (88.98)   
307 + **[CTCGCG]** 1126 (88.37)   
307 + **[GTCGCC]** 1135 (86.61)   
307 + **.GGCGCG.** 1160 (95.43)   
307 + **[GTCCCG]** 1191 (88.98)   
307 + **.GGCGCG.** 1235 (95.43)   
307 + **.GGCGCG.** 1355 (95.43)   
307 - **.GCCGCG.** 1330 (95.35)   
307 - **[GTCCCG]** 1239 (88.98)   
307 - **.GCCGCG.** 1162 (95.35)   
307 - **.GCCGCG.** 1128 (95.35)   
307 - **[GTCCCG]** 1004 (88.98)   
307 - **[GTGGCG]** 986 (87.04)   
307 - **[GTCGGG]** 840 (87.52)   
307 - **.GCCGCG.** 730 (95.35)   
307 - **[GTCCCG]** 592 (88.98)   
307 - **.GGCGCG.** 548 (95.43)   
307 - **[CTCGCG]** 432 (88.37)   
307 - **[GTGGCG]** 178 (87.04)   
307 - **.GGCGCG.** 176 (95.43)   
308 + **[GTCGCC]** 166 (86.61)   
308 + **.GCCGCG.** 387 (95.35)   
308 + **[GTCCCG]** 537 (88.98)   
308 + **[CTCGCG]** 545 (88.37)   
308 + **[CTCGCG]** 635 (88.37)   
308 + **[GTCGGG]** 650 (87.52)   
308 + **.GCCGCG.** 770 (95.35)   
308 + **[GTGGCG]** 798 (87.04)   
308 + **[GACGCG]** 877 (89.80)   
308 + **.GGCGCG.** 1100 (95.43)   
308 + **.GCCGCG.** 1179 (95.35)   
308 + **.GCCGCG.** 1368 (95.35)   
308 + **.GCCGCG.** 1413 (95.35)   
308 + **[GTCGGG]** 1447 (87.52)   
308 - **[GTCCCG]** 1395 (88.98)   
308 - **.GGCGCG.** 1189 (95.43)   
308 - **.GCCGCG.** 1181 (95.35)   
308 - **.GCCGCG.** 1116 (95.35)   
308 - **.GTCGCG.** 879 (100.00)   
308 - **.GCCGCG.** 863 (95.35)   
308 - **[CTCGCG]** 792 (88.37)   
308 - **.GGCGCG.** 786 (95.43)   
309 + **[GTCGGG]** 117 (87.52)   
309 + **[GTCGCC]** 618 (86.61)   
309 + **.GCCGCG.** 689 (95.35)   
309 + **[CTCGCG]** 864 (88.37)   
309 + **.GCCGCG.** 994 (95.35)   
309 + **.GGCGCG.** 1078 (95.43)   
309 + **.GGCGCG.** 1148 (95.43)   
309 + **[GTCGCC]** 1319 (86.61)   
309 - **[GTCCCG]** 1152 (88.98)   
309 - **.GTCGCG.** 1080 (100.00)   
309 - **.GCCGCG.** 1029 (95.35)   
309 - **.GGCGCG.** 691 (95.43)   
310 + **[GTCGGG]** 662 (87.52)   
310 + **.GGCGCG.** 682 (95.43)   
310 + **.GGCGCG.** 979 (95.43)   
310 + **[GTGGCG]** 1194 (87.04)   
310 + **[GTGGCG]** 1206 (87.04)   
310 - **[GTCCCG]** 1349 (88.98)   
310 - **.GCCGCG.** 1301 (95.35)   
310 - **.GCCGCG.** 1035 (95.35)   
310 - **.GCCGCG.** 981 (95.35)   
310 - **.GGCGCG.** 834 (95.43)   
310 - **[GTGGCG]** 688 (87.04)   
310 - **.GGCGCG.** 686 (95.43)   
311 + **[GTGGCG]** 524 (87.04)   
311 + **[GTCGCC]** 620 (86.61)   
311 + **.GCCGCG.** 873 (95.35)   
311 + **[GTGGCG]** 918 (87.04)   
311 + **[GTCCCG]** 1003 (88.98)   
311 + **.GGCGCG.** 1067 (95.43)   
311 + **.GCCGCG.** 1104 (95.35)   
311 + **.GGCGCG.** 1178 (95.43)   
311 + **[GTCGCC]** 1226 (86.61)   
311 + **[GACGCG]** 1262 (89.80)   
311 + **.GCCGCG.** 1343 (95.35)   
311 + **[CTCGCG]** 1425 (88.37)   
311 + **.GCCGCG.** 1471 (95.35)   
311 - **[GTCCCG]** 1459 (88.98)   
311 - **[GACGCG]** 1345 (89.80)   
311 - **.GCCGCG.** 1106 (95.35)   
311 - **.GCCGCG.** 1069 (95.35)   
311 - **.GGCGCG.** 960 (95.43)   
311 - **.GCCGCG.** 875 (95.35)   
311 - **.GGCGCG.** 762 (95.43)   
312 + **[GACGCG]** 741 (89.80)   
312 + **.GGCGCG.** 941 (95.43)   
312 + **[GTCCCG]** 1010 (88.98)   
312 + **.GGCGCG.** 1029 (95.43)   
312 + **[GTCGGG]** 1061 (87.52)   
312 + **[CTCGCG]** 1090 (88.37)   
312 + **.GGCGCG.** 1290 (95.43)   
312 + **.GCCGCG.** 1355 (95.35)   
312 - **.GCCGCG.** 1473 (95.35)   
312 - **.GGCGCG.** 1361 (95.43)   
312 - **[GTGGCG]** 1239 (87.04)   
312 - **.GGCGCG.** 1122 (95.43)   
312 - **.GCCGCG.** 1094 (95.35)   
312 - **.GGCGCG.** 985 (95.43)   
312 - **.GGCGCG.** 872 (95.43)   
312 - **[GTCGGG]** 856 (87.52)   
312 - **.GGCGCG.** 843 (95.43)   
312 - **[GTCCCG]** 820 (88.98)   
312 - **[GTCGCC]** 738 (86.61)   
313 + **[CTCGCG]** 581 (88.37)   
313 + **.GGCGCG.** 817 (95.43)   
313 + **[CTCGCG]** 866 (88.37)   
313 + **[CTCGCG]** 885 (88.37)   
313 + **.GGCGCG.** 956 (95.43)   
313 + **[CTCGCG]** 962 (88.37)   
313 + **[CTCGCG]** 974 (88.37)   
313 + **[CTCGCG]** 1086 (88.37)   
313 + **.GGCGCG.** 1123 (95.43)   
313 + **.GCCGCG.** 1446 (95.35)   
313 - **.GCCGCG.** 1448 (95.35)   
313 - **[GTCGCC]** 1417 (86.61)   
313 - **.GGCGCG.** 1303 (95.43)   
313 - **[GTCGCC]** 1243 (86.61)   
313 - **[GTCGCC]** 1169 (86.61)   
313 - **.GGCGCG.** 1088 (95.43)   
313 - **.GGCGCG.** 1008 (95.43)   
313 - **[GACGCG]** 964 (89.80)   
313 - **.GGCGCG.** 892 (95.43)   
313 - **.GCCGCG.** 826 (95.35)   
313 - **.GTCGCG.** 260 (100.00)   
314 + **[GTCGGG]** 36 (87.52)   
314 + **[GTCGGG]** 384 (87.52)   
314 + **[GTCCCG]** 548 (88.98)   
314 - **[GTCGCC]** 356 (86.61)   
315 + **[GTGGCG]** 342 (87.04)   
315 + **[CTCGCG]** 355 (88.37)   
315 + **[GACGCG]** 379 (89.80)   
315 + **[GACGCG]** 903 (89.80)   
315 + **.GGCGCG.** 981 (95.43)   
315 + **[GACGCG]** 1006 (89.80)   
315 + **.GGCGCG.** 1017 (95.43)   
315 + **.GGCGCG.** 1026 (95.43)   
315 + **.GGCGCG.** 1057 (95.43)   
315 + **[CTCGCG]** 1166 (88.37)   
315 + **[GTGGCG]** 1269 (87.04)   
315 + **.GGCGCG.** 1271 (95.43)   
315 + **[GACGCG]** 1285 (89.80)   
315 + **.GCCGCG.** 1342 (95.35)   
315 + **[GTCCCG]** 1420 (88.98)   
315 + **[GTCCCG]** 1503 (88.98)   
315 - **[GACGCG]** 1344 (89.80)   
315 - **.GGCGCG.** 1273 (95.43)   
315 - **[CTCGCG]** 1227 (88.37)   
315 - **[CTCGCG]** 1168 (88.37)   
315 - **[CTCGCG]** 987 (88.37)   
315 - **.GGCGCG.** 383 (95.43)   
315 - **[GTCGCC]** 360 (86.61)   
315 - **.GCCGCG.** 357 (95.35)   
315 - **[CTCGCG]** 4 (88.37)   
316 + **[GACGCG]** 200 (89.80)   
316 + **.GCCGCG.** 227 (95.35)   
316 + **.GCCGCG.** 536 (95.35)   
316 + **.GGCGCG.** 573 (95.43)   
316 + **[GTCGCC]** 629 (86.61)   
316 + **.GGCGCG.** 896 (95.43)   
316 + **.GGCGCG.** 913 (95.43)   
316 + **[GTCGCC]** 1029 (86.61)   
316 - **[GTCCCG]** 1310 (88.98)   
316 - **[GTCGGG]** 1042 (87.52)   
316 - **.GCCGCG.** 910 (95.35)   
316 - **[GACGCG]** 898 (89.80)   
316 - **[CTCGCG]** 538 (88.37)   
316 - **[GTCGGG]** 345 (87.52)   
316 - **.GGCGCG.** 257 (95.43)   
316 - **.GCCGCG.** 229 (95.35)   
316 - **.GGCGCG.** 202 (95.43)   
316 - **[GTCGCC]** 169 (86.61)   
316 - **[CTCGCG]** 33 (88.37)   
317 + **[GTGGCG]** 292 (87.04)   
317 + **.GGCGCG.** 1005 (95.43)   
317 + **[GTCCCG]** 1244 (88.98)   
317 + **.GGCGCG.** 1379 (95.43)   
317 + **.GGCGCG.** 1408 (95.43)   
317 + **[GTGGCG]** 1487 (87.04)   
317 - **.GGCGCG.** 1433 (95.43)   
317 - **[GTCCCG]** 654 (88.98)   
318 + **.GTCGCG.** 74 (100.00)   
318 + **.GGCGCG.** 250 (95.43)   
318 + **.GCCGCG.** 288 (95.35)   
318 + **[GTCCCG]** 293 (88.98)   
318 + **.GGCGCG.** 538 (95.43)   
318 + **.GGCGCG.** 722 (95.43)   
318 + **.GTCGCG.** 1038 (100.00)   
318 + **.GCCGCG.** 1242 (95.35)   
318 + **[CTCGCG]** 1419 (88.37)   
318 + **.GGCGCG.** 1434 (95.43)   
318 + **.GCCGCG.** 1483 (95.35)   
318 - **.GCCGCG.** 1485 (95.35)   
318 - **[GTGGCG]** 1350 (87.04)   
318 - **.GGCGCG.** 1348 (95.43)   
318 - **.GGCGCG.** 1246 (95.43)   
318 - **[GTGGCG]** 1186 (87.04)   
318 - **[GTCCCG]** 636 (88.98)   
318 - **[GTGGCG]** 473 (87.04)   
318 - **[GTCGGG]** 397 (87.52)   
318 - **[GTGGCG]** 355 (87.04)   
318 - **.GGCGCG.** 307 (95.43)   
318 - **[GACGCG]** 290 (89.80)   
320 + **[CTCGCG]** 49 (88.37)   
320 + **.GCCGCG.** 72 (95.35)   
320 + **[GTCGCC]** 290 (86.61)   
320 + **[GTCGGG]** 353 (87.52)   
320 + **[GTGGCG]** 456 (87.04)   
320 + **[GTGGCG]** 552 (87.04)   
320 + **.GCCGCG.** 594 (95.35)   
320 + **[CTCGCG]** 692 (88.37)   
320 + **[CTCGCG]** 719 (88.37)   
320 + **.GTCGCG.** 753 (100.00)   
320 + **.GGCGCG.** 798 (95.43)   
320 + **.GGCGCG.** 855 (95.43)   
320 + **.GGCGCG.** 876 (95.43)   
320 + **.GCCGCG.** 911 (95.35)   
320 + **[GTGGCG]** 1144 (87.04)   
320 + **[CTCGCG]** 1230 (88.37)   
320 + **.GGCGCG.** 1313 (95.43)   
320 + **.GCCGCG.** 1342 (95.35)   
320 + **.GCCGCG.** 1384 (95.35)   
320 - **.GCCGCG.** 1386 (95.35)   
320 - **.GGCGCG.** 1381 (95.43)   
320 - **[GACGCG]** 1348 (89.80)   
320 - **.GTCGCG.** 1315 (100.00)   
320 - **.GGCGCG.** 1198 (95.43)   
320 - **.GCCGCG.** 992 (95.35)   
320 - **.GGCGCG.** 913 (95.43)   
320 - **.GCCGCG.** 755 (95.35)   
320 - **.GCCGCG.** 721 (95.35)   
320 - **.GGCGCG.** 714 (95.43)   
320 - **[GTCCCG]** 706 (88.98)   
320 - **.GCCGCG.** 694 (95.35)   
320 - **.GGCGCG.** 674 (95.43)   
320 - **.GGCGCG.** 640 (95.43)   
320 - **.GGCGCG.** 600 (95.43)   
320 - **[GTCCCG]** 305 (88.98)   
320 - **[GTCGGG]** 223 (87.52)   
320 - **[GACGCG]** 107 (89.80)   
321 + **[GTCGCC]** 32 (86.61)   
321 + **[GTCGCC]** 383 (86.61)   
321 + **[GTCGGG]** 424 (87.52)   
321 + **.GCCGCG.** 500 (95.35)   
321 + **.GCCGCG.** 699 (95.35)   
321 + **.GCCGCG.** 943 (95.35)   
321 + **.GTCGCG.** 1209 (100.00)   
321 + **.GCCGCG.** 1256 (95.35)   
321 + **.GGCGCG.** 1300 (95.43)   
321 + **.GCCGCG.** 1398 (95.35)   
321 + **.GCCGCG.** 1502 (95.35)   
321 - **.GGCGCG.** 1506 (95.43)   
321 - **.GCCGCG.** 1400 (95.35)   
321 - **.GGCGCG.** 1395 (95.43)   
321 - **[GTCCCG]** 1312 (88.98)   
321 - **.GCCGCG.** 1302 (95.35)   
321 - **.GGCGCG.** 1260 (95.43)   
321 - **[GTCGGG]** 1235 (87.52)   
321 - **.GGCGCG.** 1225 (95.43)   
321 - **[GTCCCG]** 1093 (88.98)   
321 - **[GTCCCG]** 922 (88.98)   
321 - **.GGCGCG.** 701 (95.43)   
321 - **.GGCGCG.** 696 (95.43)   
321 - **[GTGGCG]** 617 (87.04)   
321 - **.GTCGCG.** 502 (100.00)   
321 - **.GCCGCG.** 455 (95.35)   
322 + **[GTCGGG]** 304 (87.52)   
322 + **[GTCGGG]** 309 (87.52)   
322 + **.GCCGCG.** 497 (95.35)   
322 + **.GGCGCG.** 630 (95.43)   
322 + **.GGCGCG.** 686 (95.43)   
322 + **[GTCCCG]** 695 (88.98)   
322 + **.GTCGCG.** 790 (100.00)   
322 + **.GCCGCG.** 942 (95.35)   
322 + **.GCCGCG.** 959 (95.35)   
322 + **.GCCGCG.** 1003 (95.35)   
322 + **[GTCGCC]** 1019 (86.61)   
322 + **.GTCGCG.** 1065 (100.00)   
322 + **.GTCGCG.** 1172 (100.00)   
322 + **[GACGCG]** 1424 (89.80)   
322 + **.GCCGCG.** 1481 (95.35)   
322 - **.GGCGCG.** 1426 (95.43)   
322 - **.GGCGCG.** 1174 (95.43)   
322 - **.GGCGCG.** 948 (95.43)   
322 - **[CTCGCG]** 792 (88.37)   
322 - **[GTCGCC]** 738 (86.61)   
322 - **.GCCGCG.** 688 (95.35)   
322 - **.GCCGCG.** 632 (95.35)   
322 - **.GGCGCG.** 499 (95.43)   
322 - **.GGCGCG.** 463 (95.43)   
322 - **.GGCGCG.** 259 (95.43)   
322 - **[GTCGGG]** 93 (87.52)   
323 + **[GTCGCC]** 64 (86.61)   
323 + **.GCCGCG.** 453 (95.35)   
323 + **[GTCGGG]** 472 (87.52)   
323 + **[GTCGGG]** 790 (87.52)   
323 + **[GTCGGG]** 796 (87.52)   
323 + **.GGCGCG.** 858 (95.43)   
323 + **.GGCGCG.** 945 (95.43)   
323 + **.GGCGCG.** 985 (95.43)   
323 + **.GCCGCG.** 1030 (95.35)   
323 + **.GCCGCG.** 1229 (95.35)   
323 + **.GGCGCG.** 1361 (95.43)   
323 + **[GTCGGG]** 1387 (87.52)   
323 + **[GTCGGG]** 1427 (87.52)   
323 + **.GGCGCG.** 1448 (95.43)   
323 + **.GCCGCG.** 1494 (95.35)   
323 - **.GGCGCG.** 1496 (95.43)   
323 - **[GACGCG]** 1450 (89.80)   
323 - **[GACGCG]** 1424 (89.80)   
323 - **[GTGGCG]** 1330 (87.04)   
323 - **.GGCGCG.** 1321 (95.43)   
323 - **.GGCGCG.** 1313 (95.43)   
323 - **.GCCGCG.** 1231 (95.35)   
323 - **.GGCGCG.** 1182 (95.43)   
323 - **[GTCCCG]** 1092 (88.98)   
323 - **.GCCGCG.** 1074 (95.35)   
323 - **[CTCGCG]** 1041 (88.37)   
323 - **.GCCGCG.** 1032 (95.35)   
323 - **.GCCGCG.** 1020 (95.35)   
323 - **[GTCCCG]** 936 (88.98)   
323 - **.GCCGCG.** 860 (95.35)   
325 + **[GTCGGG]** 689 (87.52)   
325 + **.GGCGCG.** 1306 (95.43)   
325 + **.GGCGCG.** 1432 (95.43)   
325 - **[GTCCCG]** 1240 (88.98)   
326 + **[GACGCG]** 177 (89.80)   
326 + **[CTCGCG]** 687 (88.37)   
326 + **[GTCCCG]** 814 (88.98)   
326 + **.GCCGCG.** 823 (95.35)   
326 + **[GTCGGG]** 951 (87.52)   
326 + **[GTCGGG]** 962 (87.52)   
326 + **[GTGGCG]** 1109 (87.04)   
326 + **.GGCGCG.** 1111 (95.43)   
326 + **.GCCGCG.** 1117 (95.35)   
326 + **.GCCGCG.** 1301 (95.35)   
326 + **.GGCGCG.** 1319 (95.43)   
326 + **[GTGGCG]** 1401 (87.04)   
326 + **.GGCGCG.** 1403 (95.43)   
326 + **.GCCGCG.** 1417 (95.35)   
326 - **[CTCGCG]** 1419 (88.37)   
326 - **.GGCGCG.** 1405 (95.43)   
326 - **[GTGGCG]** 1329 (87.04)   
326 - **.GCCGCG.** 1303 (95.35)   
326 - **[GTGGCG]** 1243 (87.04)   
326 - **.GGCGCG.** 1241 (95.43)   
326 - **.GCCGCG.** 1222 (95.35)   
326 - **.GCCGCG.** 1202 (95.35)   
326 - **.GCCGCG.** 1113 (95.35)   
326 - **[GTCCCG]** 953 (88.98)   
326 - **.GCCGCG.** 938 (95.35)   
326 - **.GGCGCG.** 875 (95.43)   
326 - **.GGCGCG.** 869 (95.43)   
326 - **.GGCGCG.** 825 (95.43)   
326 - **.GCCGCG.** 660 (95.35)   
326 - **.GCCGCG.** 653 (95.35)   
326 - **.GGCGCG.** 454 (95.43)   
327 + **.GGCGCG.** 468 (95.43)   
327 + **[GTCGGG]** 593 (87.52)   
327 + **.GGCGCG.** 658 (95.43)   
327 + **.GCCGCG.** 900 (95.35)   
327 + **.GCCGCG.** 1113 (95.35)   
327 - **[CTCGCG]** 1115 (88.37)   
327 - **.GTCGCG.** 902 (100.00)   
327 - **.GGCGCG.** 681 (95.43)   
327 - **[GTGGCG]** 675 (87.04)   
327 - **.GTCGCG.** 48 (100.00)   
329 + **.GTCGCG.** 112 (100.00)   
329 + **.GCCGCG.** 296 (95.35)   
329 + **[GTGGCG]** 330 (87.04)   
329 + **[CTCGCG]** 746 (88.37)   
329 + **.GTCGCG.** 1225 (100.00)   
329 + **[GACGCG]** 1237 (89.80)   
329 - **.GGCGCG.** 1227 (95.43)   
329 - **[CTCGCG]** 748 (88.37)   
329 - **[GTCGGG]** 625 (87.52)   
329 - **[GTGGCG]** 463 (87.04)   
329 - **[GTCGCC]** 332 (86.61)   
329 - **[GTCGGG]** 5 (87.52)   
331 + **[CTCGCG]** 60 (88.37)   
331 + **.GCCGCG.** 163 (95.35)   
331 + **[GTGGCG]** 186 (87.04)   
331 + **.GGCGCG.** 188 (95.43)   
331 + **.GGCGCG.** 357 (95.43)   
331 + **.GCCGCG.** 640 (95.35)   
331 + **.GCCGCG.** 721 (95.35)   
331 + **.GCCGCG.** 738 (95.35)   
331 + **.GCCGCG.** 763 (95.35)   
331 + **[GTGGCG]** 999 (87.04)   
331 + **.GGCGCG.** 1001 (95.43)   
331 + **.GGCGCG.** 1138 (95.43)   
331 + **[GTCCCG]** 1276 (88.98)   
331 + **.GCCGCG.** 1314 (95.35)   
331 + **[GTGGCG]** 1385 (87.04)   
331 - **.GCCGCG.** 1441 (95.35)   
331 - **[GTCGCC]** 1338 (86.61)   
331 - **.GCCGCG.** 1332 (95.35)   
331 - **[GTCGGG]** 1324 (87.52)   
331 - **[GACGCG]** 1316 (89.80)   
331 - **.GGCGCG.** 1311 (95.43)   
331 - **[GACGCG]** 1140 (89.80)   
331 - **.GCCGCG.** 1135 (95.35)   
331 - **.GCCGCG.** 723 (95.35)   
331 - **.GCCGCG.** 694 (95.35)   
331 - **.GCCGCG.** 397 (95.35)   
331 - **.GGCGCG.** 359 (95.43)   
331 - **[GACGCG]** 269 (89.80)   
331 - **[CTCGCG]** 167 (88.37)   
332 + **[GTCGGG]** 47 (87.52)   
332 + **.GCCGCG.** 101 (95.35)   
332 + **.GTCGCG.** 450 (100.00)   
332 + **.GTCGCG.** 458 (100.00)   
332 + **[GTCCCG]** 594 (88.98)   
332 + **[GTCCCG]** 830 (88.98)   
332 + **.GGCGCG.** 1135 (95.43)   
332 + **.GGCGCG.** 1225 (95.43)   
332 + **[GTCGCC]** 1348 (86.61)   
332 + **.GCCGCG.** 1351 (95.35)   
332 + **.GGCGCG.** 1389 (95.43)   
332 + **.GCCGCG.** 1428 (95.35)   
332 + **.GGCGCG.** 1483 (95.43)   
332 - **.GCCGCG.** 1487 (95.35)   
332 - **[GTGGCG]** 1443 (87.04)   
332 - **.GCCGCG.** 1430 (95.35)   
332 - **.GGCGCG.** 1353 (95.43)   
332 - **.GCCGCG.** 1298 (95.35)   
332 - **[GTCGCC]** 1158 (86.61)   
332 - **.GGCGCG.** 1088 (95.43)   
332 - **[GTCCCG]** 598 (88.98)   
332 - **[GACGCG]** 504 (89.80)   
332 - **.GGCGCG.** 460 (95.43)   
332 - **[GTCGGG]** 129 (87.52)   
332 - **[GTCCCG]** 83 (88.98)   
332 - **[GTCCCG]** 75 (88.98)   
333 + **.GCCGCG.** 1122 (95.35)   
333 + **.GCCGCG.** 1321 (95.35)   
333 - **[GTCGCC]** 1419 (86.61)   
333 - **.GTCGCG.** 1323 (100.00)   
333 - **[GTCCCG]** 1304 (88.98)   
333 - **.GCCGCG.** 1299 (95.35)   
333 - **.GTCGCG.** 1196 (100.00)   
333 - **.GCCGCG.** 1030 (95.35)   
333 - **[GTCGGG]** 589 (87.52)   
334 + **.GCCGCG.** 381 (95.35)   
334 + **[GTCCCG]** 678 (88.98)   
334 + **.GCCGCG.** 871 (95.35)   
334 + **[GTCCCG]** 977 (88.98)   
334 + **.GGCGCG.** 1087 (95.43)   
334 + **[GTCGCC]** 1141 (86.61)   
334 - **[GTGGCG]** 897 (87.04)   
334 - **.GTCGCG.** 873 (100.00)   
334 - **[GTCGCC]** 426 (86.61)   
335 + **[GTGGCG]** 343 (87.04)   
335 + **[GTCGGG]** 662 (87.52)   
335 + **[GTCGGG]** 709 (87.52)   
335 + **.GTCGCG.** 718 (100.00)   
335 + **.GGCGCG.** 857 (95.43)   
335 + **.GGCGCG.** 983 (95.43)   
335 + **[GTCGCC]** 1030 (86.61)   
335 + **.GCCGCG.** 1122 (95.35)   
335 + **.GTCGCG.** 1208 (100.00)   
335 + **.GGCGCG.** 1228 (95.43)   
335 + **.GGCGCG.** 1259 (95.43)   
335 + **[CTCGCG]** 1282 (88.37)   
335 + **.GGCGCG.** 1387 (95.43)   
335 + **.GGCGCG.** 1430 (95.43)   
335 - **.GGCGCG.** 1389 (95.43)   
335 - **.GCCGCG.** 1261 (95.35)   
335 - **[GTCCCG]** 1236 (88.98)   
335 - **.GGCGCG.** 1230 (95.43)   
335 - **[GACGCG]** 1205 (89.80)   
335 - **.GGCGCG.** 1100 (95.43)   
335 - **[CTCGCG]** 1007 (88.37)   
335 - **.GCCGCG.** 987 (95.35)   
335 - **.GCCGCG.** 854 (95.35)   
335 - **.GCCGCG.** 720 (95.35)   
335 - **.GGCGCG.** 610 (95.43)   
336 + **[GTCGCC]** 708 (86.61)   
336 + **[GTCCCG]** 859 (88.98)   
336 + **[GTCGCC]** 1471 (86.61)   
336 - **[GTCGGG]** 1269 (87.52)   
336 - **.GCCGCG.** 1023 (95.35)   
336 - **.GGCGCG.** 981 (95.43)   
336 - **[GTCGGG]** 949 (87.52)   
336 - **[GACGCG]** 876 (89.80)   
336 - **.GGCGCG.** 760 (95.43)   
337 + **.GCCGCG.** 318 (95.35)   
337 + **.GCCGCG.** 765 (95.35)   
337 + **.GCCGCG.** 825 (95.35)   
337 + **.GGCGCG.** 950 (95.43)   
337 + **.GCCGCG.** 1071 (95.35)   
337 + **.GGCGCG.** 1148 (95.43)   
337 + **.GGCGCG.** 1348 (95.43)   
337 + **.GGCGCG.** 1379 (95.43)   
337 + **.GTCGCG.** 1421 (100.00)   
337 + **.GGCGCG.** 1438 (95.43)   
337 + **.GGCGCG.** 1475 (95.43)   
337 + **[GTCGCC]** 1495 (86.61)   
337 - **[CTCGCG]** 1479 (88.37)   
337 - **.GTCGCG.** 1442 (100.00)   
337 - **[CTCGCG]** 1423 (88.37)   
337 - **[CTCGCG]** 1397 (88.37)   
337 - **[CTCGCG]** 1286 (88.37)   
337 - **.GCCGCG.** 1183 (95.35)   
337 - **.GCCGCG.** 1176 (95.35)   
337 - **[GTCGCC]** 1164 (86.61)   
337 - **.GCCGCG.** 1073 (95.35)   
337 - **.GCCGCG.** 956 (95.35)   
337 - **[GTCCCG]** 875 (88.98)   
337 - **.GCCGCG.** 842 (95.35)   
337 - **.GGCGCG.** 827 (95.43)   
337 - **[CTCGCG]** 813 (88.37)   
337 - **.GGCGCG.** 411 (95.43)   
337 - **[GTCCCG]** 255 (88.98)   
338 + **[GTCCCG]** 360 (88.98)   
338 + **[GTCGGG]** 489 (87.52)   
338 + **.GCCGCG.** 642 (95.35)   
338 + **.GCCGCG.** 692 (95.35)   
338 + **[GTCCCG]** 697 (88.98)   
338 + **[CTCGCG]** 1063 (88.37)   
338 + **[CTCGCG]** 1086 (88.37)   
338 + **[CTCGCG]** 1170 (88.37)   
338 + **.GCCGCG.** 1193 (95.35)   
338 + **.GCCGCG.** 1305 (95.35)   
338 + **.GCCGCG.** 1414 (95.35)   
338 + **[GTCCCG]** 1465 (88.98)   
338 - **.GCCGCG.** 1459 (95.35)   
338 - **[CTCGCG]** 1309 (88.37)   
338 - **.GGCGCG.** 1172 (95.43)   
338 - **[GACGCG]** 1154 (89.80)   
338 - **.GGCGCG.** 1126 (95.43)   
338 - **.GGCGCG.** 1092 (95.43)   
338 - **.GGCGCG.** 1065 (95.43)   
338 - **[GTCGCC]** 1057 (86.61)   
338 - **.GCCGCG.** 1054 (95.35)   
338 - **[GTCGGG]** 1013 (87.52)   
338 - **[GTCGGG]** 983 (87.52)   
338 - **[GTCCCG]** 884 (88.98)   
338 - **.GGCGCG.** 812 (95.43)   
338 - **.GGCGCG.** 760 (95.43)   
338 - **.GGCGCG.** 754 (95.43)   
338 - **[GACGCG]** 694 (89.80)   
338 - **.GCCGCG.** 440 (95.35)   
338 - **[GTCCCG]** 191 (88.98)   
338 - **[GTCCCG]** 182 (88.98)   
338 - **[GTCCCG]** 106 (88.98)   
338 - **[GTCCCG]** 57 (88.98)   
339 + **.GGCGCG.** 529 (95.43)   
339 + **.GGCGCG.** 1028 (95.43)   
339 + **[GTCCCG]** 1304 (88.98)   
339 + **[GTCGCC]** 1390 (86.61)   
339 - **[GTCCCG]** 899 (88.98)   
339 - **[GTGGCG]** 850 (87.04)   
340 + **[GTCGCC]** 501 (86.61)   
340 + **[GTCGGG]** 609 (87.52)   
340 + **[GTCGCC]** 686 (86.61)   
340 + **[CTCGCG]** 739 (88.37)   
340 + **[GTGGCG]** 1064 (87.04)   
340 + **.GGCGCG.** 1066 (95.43)   
340 - **[GTCCCG]** 1070 (88.98)   
340 - **[GTCGCC]** 1027 (86.61)   
340 - **.GTCGCG.** 979 (100.00)   
340 - **[GTCGGG]** 184 (87.52)   
341 + **[CTCGCG]** 37 (88.37)   
341 + **.GCCGCG.** 187 (95.35)   
341 + **.GTCGCG.** 211 (100.00)   
341 + **[GTGGCG]** 480 (87.04)   
341 + **[CTCGCG]** 964 (88.37)   
341 + **[GTGGCG]** 1044 (87.04)   
341 + **.GTCGCG.** 1231 (100.00)   
341 + **[CTCGCG]** 1258 (88.37)   
341 + **.GCCGCG.** 1315 (95.35)   
341 + **[GTGGCG]** 1344 (87.04)   
341 + **.GGCGCG.** 1346 (95.43)   
341 + **.GCCGCG.** 1384 (95.35)   
341 + **.GGCGCG.** 1417 (95.43)   
341 + **[GTGGCG]** 1481 (87.04)   
341 + **.GGCGCG.** 1483 (95.43)   
341 + **[GACGCG]** 1492 (89.80)   
341 - **.GGCGCG.** 1505 (95.43)   
341 - **.GCCGCG.** 1494 (95.35)   
341 - **.GGCGCG.** 1350 (95.43)   
341 - **.GCCGCG.** 1319 (95.35)   
341 - **[GACGCG]** 1260 (89.80)   
341 - **.GCCGCG.** 1152 (95.35)   
341 - **[GTCCCG]** 621 (88.98)   
341 - **[CTCGCG]** 272 (88.37)   
341 - **.GCCGCG.** 156 (95.35)   
341 - **[GTGGCG]** 107 (87.04)   
341 - **.GGCGCG.** 105 (95.43)   
342 + **.GCCGCG.** 712 (95.35)   
342 + **.GGCGCG.** 720 (95.43)   
342 + **[GTCGGG]** 740 (87.52)   
342 + **.GGCGCG.** 836 (95.43)   
342 + **[GTGGCG]** 983 (87.04)   
342 + **.GGCGCG.** 1025 (95.43)   
342 + **[GTCGCC]** 1096 (86.61)   
342 + **.GGCGCG.** 1153 (95.43)   
342 + **.GGCGCG.** 1221 (95.43)   
342 + **.GGCGCG.** 1297 (95.43)   
342 + **.GGCGCG.** 1331 (95.43)   
342 + **[GACGCG]** 1347 (89.80)   
342 + **[GACGCG]** 1396 (89.80)   
342 + **.GGCGCG.** 1471 (95.43)   
342 + **[GTGGCG]** 1486 (87.04)   
342 - **.GGCGCG.** 1400 (95.43)   
342 - **.GCCGCG.** 1335 (95.35)   
342 - **.GTCGCG.** 1281 (100.00)   
342 - **[GTCCCG]** 1166 (88.98)   
342 - **.GCCGCG.** 1155 (95.35)   
342 - **[CTCGCG]** 1123 (88.37)   
342 - **[CTCGCG]** 722 (88.37)   
343 + **[GTCGGG]** 91 (87.52)   
343 + **.GGCGCG.** 277 (95.43)   
343 + **[GTCGGG]** 519 (87.52)   
343 + **.GGCGCG.** 524 (95.43)   
343 + **.GCCGCG.** 653 (95.35)   
343 + **[GACGCG]** 670 (89.80)   
343 + **[CTCGCG]** 779 (88.37)   
343 + **.GGCGCG.** 910 (95.43)   
343 + **.GGCGCG.** 965 (95.43)   
343 + **.GGCGCG.** 996 (95.43)   
343 + **[GTCGCC]** 1014 (86.61)   
343 + **[GTCGCC]** 1044 (86.61)   
343 + **.GCCGCG.** 1078 (95.35)   
343 + **.GCCGCG.** 1120 (95.35)   
343 + **.GGCGCG.** 1180 (95.43)   
343 + **[GTCGGG]** 1223 (87.52)   
343 + **.GCCGCG.** 1305 (95.35)   
343 + **.GGCGCG.** 1319 (95.43)   
343 + **.GGCGCG.** 1340 (95.43)   
343 + **[GTCGCC]** 1389 (86.61)   
343 - **.GCCGCG.** 1350 (95.35)   
343 - **.GCCGCG.** 1342 (95.35)   
343 - **[GTGGCG]** 1208 (87.04)   
343 - **.GCCGCG.** 1182 (95.35)   
343 - **.GCCGCG.** 1122 (95.35)   
343 - **[CTCGCG]** 1105 (88.37)   
343 - **.GCCGCG.** 998 (95.35)   
343 - **.GGCGCG.** 957 (95.43)   
343 - **.GTCGCG.** 781 (100.00)   
343 - **[GACGCG]** 279 (89.80)   
343 - **.GCCGCG.** 55 (95.35)   
344 + **[GTCGCC]** 693 (86.61)   
344 + **.GGCGCG.** 1104 (95.43)   
344 + **[GTCGGG]** 1113 (87.52)   
344 - **.GGCGCG.** 1166 (95.43)   
344 - **[GTCCCG]** 1115 (88.98)   
344 - **.GGCGCG.** 1106 (95.43)   
344 - **[GTCGGG]** 750 (87.52)   
346 + **.GTCGCG.** 21 (100.00)   
346 + **[GTCCCG]** 182 (88.98)   
346 + **[GTGGCG]** 234 (87.04)   
346 + **[GTCGCC]** 281 (86.61)   
346 + **.GCCGCG.** 527 (95.35)   
346 + **[GTCGCC]** 547 (86.61)   
346 + **[GTCCCG]** 598 (88.98)   
346 + **.GGCGCG.** 717 (95.43)   
346 + **[GACGCG]** 855 (89.80)   
346 + **.GGCGCG.** 880 (95.43)   
346 + **.GCCGCG.** 906 (95.35)   
346 + **.GGCGCG.** 1229 (95.43)   
346 + **.GCCGCG.** 1237 (95.35)   
346 + **.GGCGCG.** 1340 (95.43)   
346 + **.GCCGCG.** 1407 (95.35)   
346 - **[GACGCG]** 1239 (89.80)   
346 - **.GGCGCG.** 1231 (95.43)   
346 - **[GTCCCG]** 1218 (88.98)   
346 - **[GTCGGG]** 1150 (87.52)   
346 - **.GGCGCG.** 882 (95.43)   
346 - **[GTCCCG]** 748 (88.98)   
346 - **[CTCGCG]** 679 (88.37)   
346 - **[CTCGCG]** 529 (88.37)   
346 - **.GCCGCG.** 85 (95.35)   
346 - **.GCCGCG.** 23 (95.35)   
347 + **[GTCCCG]** 22 (88.98)   
347 + **[CTCGCG]** 551 (88.37)   
347 + **[GTCGCC]** 649 (86.61)   
347 + **.GGCGCG.** 836 (95.43)   
347 + **.GCCGCG.** 909 (95.35)   
347 + **[GTCGGG]** 1075 (87.52)   
347 + **.GGCGCG.** 1101 (95.43)   
347 + **[GTCCCG]** 1463 (88.98)   
347 - **.GGCGCG.** 1494 (95.43)   
347 - **.GCCGCG.** 1467 (95.35)   
347 - **[GTCGCC]** 1415 (86.61)   
347 - **.GCCGCG.** 1286 (95.35)   
347 - **.GGCGCG.** 1257 (95.43)   
347 - **.GCCGCG.** 1207 (95.35)   
347 - **[GTCCCG]** 1112 (88.98)   
347 - **.GCCGCG.** 1088 (95.35)   
347 - **.GGCGCG.** 966 (95.43)   
347 - **.GGCGCG.** 913 (95.43)   
347 - **.GGCGCG.** 838 (95.43)   
347 - **.GGCGCG.** 553 (95.43)   
347 - **.GCCGCG.** 436 (95.35)   
347 - **[GTCCCG]** 420 (88.98)   
348 + **[GTCGCC]** 30 (86.61)   
348 + **.GCCGCG.** 69 (95.35)   
348 + **.GCCGCG.** 135 (95.35)   
348 + **.GGCGCG.** 293 (95.43)   
348 + **[GACGCG]** 308 (89.80)   
348 + **[GTCGGG]** 335 (87.52)   
348 + **[CTCGCG]** 342 (88.37)   
348 + **[GACGCG]** 519 (89.80)   
348 + **[GTGGCG]** 540 (87.04)   
348 + **.GGCGCG.** 545 (95.43)   
348 + **.GTCGCG.** 623 (100.00)   
348 + **.GCCGCG.** 662 (95.35)   
348 + **[GTCGCC]** 687 (86.61)   
348 + **[GTCGCC]** 693 (86.61)   
348 + **.GCCGCG.** 696 (95.35)   
348 + **.GGCGCG.** 862 (95.43)   
348 + **[GTCGCC]** 906 (86.61)   
348 + **[GTCGCC]** 926 (86.61)   
348 + **.GGCGCG.** 961 (95.43)   
348 + **[GTGGCG]** 974 (87.04)   
348 + **.GTCGCG.** 986 (100.00)   
348 + **.GTCGCG.** 1026 (100.00)   
348 + **[GACGCG]** 1190 (89.80)   
348 + **.GGCGCG.** 1238 (95.43)   
348 + **.GGCGCG.** 1279 (95.43)   
348 + **.GCCGCG.** 1363 (95.35)   
348 + **[CTCGCG]** 1412 (88.37)   
348 + **.GGCGCG.** 1478 (95.43)   
348 + **.GTCGCG.** 1487 (100.00)   
348 - **.GGCGCG.** 1489 (95.43)   
348 - **[CTCGCG]** 1365 (88.37)   
348 - **.GCCGCG.** 1240 (95.35)   
348 - **[GTCGGG]** 1096 (87.52)   
348 - **.GGCGCG.** 1075 (95.43)   
348 - **.GCCGCG.** 1028 (95.35)   
348 - **[GTCGGG]** 882 (87.52)   
348 - **.GGCGCG.** 866 (95.43)   
348 - **[GTCGCC]** 774 (86.61)   
348 - **.GCCGCG.** 712 (95.35)   
348 - **.GGCGCG.** 698 (95.43)   
348 - **[GACGCG]** 664 (89.80)   
348 - **.GGCGCG.** 417 (95.43)   
348 - **.GGCGCG.** 399 (95.43)   
348 - **.GGCGCG.** 382 (95.43)   
348 - **.GGCGCG.** 375 (95.43)   
348 - **.GCCGCG.** 344 (95.35)   
348 - **.GGCGCG.** 228 (95.43)   
348 - **.GGCGCG.** 132 (95.43)   
348 - **.GCCGCG.** 71 (95.35)   
348 - **.GTCGCG.** 55 (100.00)   
348 - **[GTGGCG]** 32 (87.04)   
349 + **[GACGCG]** 489 (89.80)   
349 + **.GGCGCG.** 837 (95.43)   
349 + **[GTCGCC]** 877 (86.61)   
349 + **.GGCGCG.** 1045 (95.43)   
349 + **[GTCGCC]** 1066 (86.61)   
349 + **.GGCGCG.** 1163 (95.43)   
349 + **[GTCGCC]** 1297 (86.61)   
349 + **[GTGGCG]** 1306 (87.04)   
349 + **.GGCGCG.** 1308 (95.43)   
349 - **[GACGCG]** 1310 (89.80)   
349 - **.GCCGCG.** 1165 (95.35)   
349 - **[GTCGCC]** 1090 (86.61)   
349 - **[CTCGCG]** 1047 (88.37)   
349 - **.GCCGCG.** 916 (95.35)   
349 - **[GTCGCC]** 653 (86.61)   
349 - **[GTCCCG]** 411 (88.98)   
349 - **[GTCCCG]** 352 (88.98)   
349 - **[GTCCCG]** 251 (88.98)   
349 - **[GTCGCC]** 87 (86.61)   
349 - **[GTGGCG]** 15 (87.04)   
350 + **[GTCCCG]** 51 (88.98)   
350 + **[GTCCCG]** 642 (88.98)   
350 + **.GTCGCG.** 999 (100.00)   
350 + **.GGCGCG.** 1044 (95.43)   
350 + **.GTCGCG.** 1266 (100.00)   
350 + **.GGCGCG.** 1343 (95.43)   
350 + **[CTCGCG]** 1426 (88.37)   
350 + **.GGCGCG.** 1437 (95.43)   
350 - **.GCCGCG.** 1404 (95.35)   
350 - **[GTCCCG]** 1361 (88.98)   
350 - **[GTCGGG]** 1349 (87.52)   
350 - **.GGCGCG.** 1345 (95.43)   
350 - **[GTCGGG]** 1225 (87.52)   
350 - **.GGCGCG.** 1186 (95.43)   
350 - **.GCCGCG.** 1107 (95.35)   
350 - **.GCCGCG.** 1046 (95.35)   
350 - **[GTGGCG]** 789 (87.04)   
351 + **[GTGGCG]** 250 (87.04)   
351 + **.GGCGCG.** 301 (95.43)   
351 + **[GTCGGG]** 530 (87.52)   
351 + **.GGCGCG.** 576 (95.43)   
351 + **[GTCGGG]** 819 (87.52)   
351 + **.GCCGCG.** 937 (95.35)   
351 + **.GTCGCG.** 1139 (100.00)   
351 - **.GCCGCG.** 1255 (95.35)   
351 - **.GCCGCG.** 1185 (95.35)   
351 - **[GACGCG]** 1141 (89.80)   
351 - **.GTCGCG.** 1021 (100.00)   
351 - **.GCCGCG.** 975 (95.35)   
351 - **[CTCGCG]** 580 (88.37)   
351 - **.GGCGCG.** 390 (95.43)   
351 - **.GGCGCG.** 305 (95.43)   
352 + **.GTCGCG.** 53 (100.00)   
352 + **.GCCGCG.** 72 (95.35)   
352 + **.GGCGCG.** 201 (95.43)   
352 + **[GACGCG]** 282 (89.80)   
352 + **.GGCGCG.** 290 (95.43)   
352 + **.GCCGCG.** 310 (95.35)   
352 + **.GCCGCG.** 353 (95.35)   
352 + **[GTCGCC]** 640 (86.61)   
352 + **.GGCGCG.** 1064 (95.43)   
352 + **.GCCGCG.** 1338 (95.35)   
352 - **[GTCGCC]** 1442 (86.61)   
352 - **.GCCGCG.** 1340 (95.35)   
352 - **.GGCGCG.** 1228 (95.43)   
352 - **.GCCGCG.** 942 (95.35)   
352 - **.GGCGCG.** 718 (95.43)   
352 - **.GCCGCG.** 621 (95.35)   
352 - **.GGCGCG.** 547 (95.43)   
352 - **.GCCGCG.** 439 (95.35)   
352 - **[GTCCCG]** 386 (88.98)   
352 - **.GCCGCG.** 312 (95.35)   
352 - **.GGCGCG.** 292 (95.43)   
352 - **.GTCGCG.** 221 (100.00)   
352 - **.GGCGCG.** 203 (95.43)   
352 - **.GCCGCG.** 198 (95.35)   
352 - **[GTCGCC]** 171 (86.61)   
352 - **.GGCGCG.** 74 (95.43)   
352 - **.GGCGCG.** 55 (95.43)   
353 + **.GGCGCG.** 165 (95.43)   
353 + **[GACGCG]** 187 (89.80)   
353 + **[CTCGCG]** 423 (88.37)   
353 + **[GACGCG]** 539 (89.80)   
353 + **[GACGCG]** 549 (89.80)   
353 + **[GACGCG]** 708 (89.80)   
353 + **[GTGGCG]** 793 (87.04)   
353 + **.GTCGCG.** 874 (100.00)   
353 + **.GCCGCG.** 881 (95.35)   
353 + **.GTCGCG.** 966 (100.00)   
353 + **[GTCGGG]** 1064 (87.52)   
353 + **.GCCGCG.** 1179 (95.35)   
353 + **.GGCGCG.** 1340 (95.43)   
353 + **.GGCGCG.** 1382 (95.43)   
353 + **.GGCGCG.** 1401 (95.43)   
353 - **.GCCGCG.** 1438 (95.35)   
353 - **.GCCGCG.** 1420 (95.35)   
353 - **.GGCGCG.** 1344 (95.43)   
353 - **[GTGGCG]** 1320 (87.04)   
353 - **[GACGCG]** 1278 (89.80)   
353 - **[CTCGCG]** 1217 (88.37)   
353 - **.GTCGCG.** 1181 (100.00)   
353 - **[CTCGCG]** 876 (88.37)   
353 - **[GTGGCG]** 748 (87.04)   
353 - **[CTCGCG]** 697 (88.37)   
353 - **.GGCGCG.** 663 (95.43)   
353 - **[GTCCCG]** 629 (88.98)   
353 - **[CTCGCG]** 541 (88.37)   
353 - **[GTCCCG]** 497 (88.98)   
353 - **[CTCGCG]** 306 (88.37)   
353 - **[GTCGCC]** 299 (86.61)   
353 - **[GTCGCC]** 291 (86.61)   
353 - **.GCCGCG.** 167 (95.35)   
353 - **[CTCGCG]** 126 (88.37)   
354 - **[GTCGCC]** 1388 (86.61)   
355 + **[GTCGGG]** 1152 (87.52)   
356 + **[GTGGCG]** 57 (87.04)   
356 + **.GCCGCG.** 91 (95.35)   
356 + **.GCCGCG.** 784 (95.35)   
356 + **.GGCGCG.** 997 (95.43)   
356 + **[GTCGGG]** 1142 (87.52)   
356 + **[GTGGCG]** 1428 (87.04)   
356 - **[GACGCG]** 1173 (89.80)   
356 - **[GTCGCC]** 1035 (86.61)   
356 - **.GCCGCG.** 999 (95.35)   
356 - **.GTCGCG.** 772 (100.00)   
356 - **[GTCGGG]** 704 (87.52)   
356 - **[GTCCCG]** 696 (88.98)   
356 - **[GTCGGG]** 688 (87.52)   
356 - **[GTGGCG]** 613 (87.04)   
356 - **.GGCGCG.** 611 (95.43)   
356 - **[GTCGGG]** 477 (87.52)   
356 - **.GCCGCG.** 465 (95.35)   
356 - **[GTCGGG]** 439 (87.52)   
356 - **.GGCGCG.** 310 (95.43)   
356 - **.GGCGCG.** 93 (95.43)   
357 + **[GTCCCG]** 427 (88.98)   
357 + **.GGCGCG.** 527 (95.43)   
357 + **[GTCCCG]** 547 (88.98)   
357 + **[GTCGGG]** 764 (87.52)   
357 + **.GCCGCG.** 811 (95.35)   
357 + **[GACGCG]** 944 (89.80)   
357 + **.GCCGCG.** 980 (95.35)   
357 + **.GCCGCG.** 991 (95.35)   
357 + **[GTCCCG]** 1016 (88.98)   
357 + **.GCCGCG.** 1110 (95.35)   
357 + **[GTCGGG]** 1117 (87.52)   
357 + **.GGCGCG.** 1161 (95.43)   
357 - **.GGCGCG.** 993 (95.43)   
357 - **.GGCGCG.** 984 (95.43)   
357 - **[GTCCCG]** 955 (88.98)   
357 - **[CTCGCG]** 925 (88.37)   
357 - **[CTCGCG]** 709 (88.37)   
357 - **[GACGCG]** 544 (89.80)   
357 - **[GTCGGG]** 537 (87.52)   
358 + **.GCCGCG.** 669 (95.35)   
358 + **.GGCGCG.** 880 (95.43)   
358 + **[GTGGCG]** 922 (87.04)   
358 + **[GTGGCG]** 952 (87.04)   
358 + **[GTGGCG]** 960 (87.04)   
358 + **[CTCGCG]** 1084 (88.37)   
358 + **[GTCGCC]** 1251 (86.61)   
358 - **.GCCGCG.** 978 (95.35)   
358 - **.GCCGCG.** 774 (95.35)   
358 - **.GGCGCG.** 671 (95.43)   
358 - **.GCCGCG.** 70 (95.35)   
359 + **.GGCGCG.** 184 (95.43)   
359 + **.GGCGCG.** 775 (95.43)   
359 + **[CTCGCG]** 1181 (88.37)   
359 + **.GCCGCG.** 1232 (95.35)   
359 + **.GGCGCG.** 1237 (95.43)   
359 + **.GGCGCG.** 1346 (95.43)   
359 + **[CTCGCG]** 1378 (88.37)   
359 + **[GTGGCG]** 1438 (87.04)   
359 + **.GGCGCG.** 1440 (95.43)   
359 - **.GGCGCG.** 1307 (95.43)   
359 - **.GCCGCG.** 1255 (95.35)   
359 - **.GGCGCG.** 1247 (95.43)   
359 - **.GCCGCG.** 1234 (95.35)   
359 - **.GGCGCG.** 1206 (95.43)   
359 - **.GGCGCG.** 1114 (95.43)   
359 - **[GTGGCG]** 1098 (87.04)   
359 - **.GGCGCG.** 1096 (95.43)   
359 - **[GTCCCG]** 1084 (88.98)   
359 - **[GTGGCG]** 1064 (87.04)   
359 - **[GTGGCG]** 442 (87.04)   
359 - **[GTCGCC]** 304 (86.61)   
360 + **.GCCGCG.** 419 (95.35)   
360 + **.GCCGCG.** 652 (95.35)   
360 + **[CTCGCG]** 826 (88.37)   
360 + **[GTGGCG]** 875 (87.04)   
360 + **.GGCGCG.** 877 (95.43)   
360 + **.GGCGCG.** 944 (95.43)   
360 + **[GTGGCG]** 1024 (87.04)   
360 + **.GCCGCG.** 1066 (95.35)   
360 + **.GCCGCG.** 1071 (95.35)   
360 + **.GCCGCG.** 1125 (95.35)   
360 + **.GGCGCG.** 1185 (95.43)   
360 + **.GGCGCG.** 1243 (95.43)   
360 + **[GTGGCG]** 1307 (87.04)   
360 + **[GTGGCG]** 1314 (87.04)   
360 - **.GGCGCG.** 1245 (95.43)   
360 - **[GTCCCG]** 1202 (88.98)   
360 - **.GCCGCG.** 1127 (95.35)   
360 - **.GGCGCG.** 1068 (95.43)   
360 - **.GCCGCG.** 973 (95.35)   
360 - **[GTCCCG]** 843 (88.98)   
360 - **.GCCGCG.** 794 (95.35)   
360 - **.GGCGCG.** 781 (95.43)   
360 - **[GTGGCG]** 739 (87.04)   
360 - **.GGCGCG.** 737 (95.43)   
360 - **[GTCGGG]** 591 (87.52)   
360 - **[CTCGCG]** 414 (88.37)   
361 + **.GCCGCG.** 470 (95.35)   
361 + **.GCCGCG.** 1416 (95.35)   
361 - **.GCCGCG.** 1418 (95.35)   
361 - **[GTCGCC]** 1105 (86.61)   
362 + **[GTCCCG]** 210 (88.98)   
362 + **.GGCGCG.** 237 (95.43)   
362 + **[GTCCCG]** 399 (88.98)   
362 + **[GACGCG]** 525 (89.80)   
362 + **[GTCGGG]** 706 (87.52)   
362 + **.GCCGCG.** 736 (95.35)   
362 + **.GGCGCG.** 909 (95.43)   
362 + **[GTCCCG]** 1047 (88.98)   
362 + **.GTCGCG.** 1141 (100.00)   
362 + **[GTCGGG]** 1233 (87.52)   
362 - **[GTCGGG]** 1399 (87.52)   
362 - **.GGCGCG.** 1358 (95.43)   
362 - **[GTCCCG]** 1235 (88.98)   
362 - **[GTCCCG]** 1192 (88.98)   
362 - **[GTCGCC]** 1179 (86.61)   
362 - **[CTCGCG]** 1143 (88.37)   
362 - **[GTCGCC]** 937 (86.61)   
362 - **.GCCGCG.** 911 (95.35)   
362 - **[GTCGGG]** 857 (87.52)   
362 - **[GTCCCG]** 817 (88.98)   
362 - **[GACGCG]** 738 (89.80)   
362 - **[GTCCCG]** 718 (88.98)   
362 - **[GACGCG]** 624 (89.80)   
362 - **.GCCGCG.** 470 (95.35)   
362 - **[CTCGCG]** 427 (88.37)   
362 - **.GCCGCG.** 283 (95.35)   
362 - **.GCCGCG.** 118 (95.35)   
363 + **[CTCGCG]** 583 (88.37)   
363 + **.GCCGCG.** 692 (95.35)   
363 + **.GTCGCG.** 1081 (100.00)   
363 + **.GCCGCG.** 1087 (95.35)   
363 + **.GGCGCG.** 1112 (95.43)   
363 - **[GTCGGG]** 1336 (87.52)   
363 - **[GTCCCG]** 1259 (88.98)   
363 - **.GCCGCG.** 1188 (95.35)   
363 - **.GGCGCG.** 1091 (95.43)   
363 - **.GCCGCG.** 1083 (95.35)   
363 - **.GCCGCG.** 811 (95.35)   
363 - **.GCCGCG.** 799 (95.35)   
363 - **.GCCGCG.** 694 (95.35)   
363 - **[CTCGCG]** 489 (88.37)   
364 + **[GACGCG]** 297 (89.80)   
364 + **.GCCGCG.** 821 (95.35)   
364 + **[GTCCCG]** 1061 (88.98)   
364 + **.GGCGCG.** 1177 (95.43)   
364 + **[GACGCG]** 1255 (89.80)   
364 - **.GGCGCG.** 1391 (95.43)   
364 - **[CTCGCG]** 1317 (88.37)   
364 - **.GCCGCG.** 1257 (95.35)   
364 - **.GCCGCG.** 1179 (95.35)   
364 - **.GCCGCG.** 950 (95.35)   
365 - **[GTGGCG]** 1319 (87.04)   
365 - **[GTGGCG]** 1067 (87.04)   
366 + **.GCCGCG.** 182 (95.35)   
366 + **[GTCGGG]** 214 (87.52)   
366 + **[GTGGCG]** 228 (87.04)   
366 + **.GGCGCG.** 266 (95.43)   
366 + **.GCCGCG.** 346 (95.35)   
366 + **.GCCGCG.** 534 (95.35)   
366 + **[GTCGCC]** 620 (86.61)   
366 + **.GGCGCG.** 656 (95.43)   
366 + **[GTCCCG]** 780 (88.98)   
366 + **[CTCGCG]** 852 (88.37)   
366 + **[CTCGCG]** 903 (88.37)   
366 + **[GTCCCG]** 960 (88.98)   
366 + **[GTCGCC]** 965 (86.61)   
366 + **.GTCGCG.** 974 (100.00)   
366 + **[GTCGGG]** 1115 (87.52)   
366 + **.GCCGCG.** 1123 (95.35)   
366 + **.GCCGCG.** 1253 (95.35)   
366 + **[GTCCCG]** 1299 (88.98)   
366 + **.GGCGCG.** 1391 (95.43)   
366 + **.GCCGCG.** 1412 (95.35)   
366 + **[GACGCG]** 1430 (89.80)   
366 - **[CTCGCG]** 1432 (88.37)   
366 - **.GCCGCG.** 1393 (95.35)   
366 - **.GTCGCG.** 1125 (100.00)   
366 - **.GCCGCG.** 954 (95.35)   
366 - **.GGCGCG.** 907 (95.43)   
366 - **[GACGCG]** 854 (89.80)   
366 - **[GTGGCG]** 760 (87.04)   
366 - **.GGCGCG.** 658 (95.43)   
366 - **[GACGCG]** 521 (89.80)   
366 - **.GGCGCG.** 454 (95.43)   
366 - **.GCCGCG.** 445 (95.35)   
366 - **[GTCCCG]** 428 (88.98)   
366 - **[GTCGCC]** 416 (86.61)   
366 - **.GCCGCG.** 410 (95.35)   
366 - **.GGCGCG.** 131 (95.43)   
367 + **.GCCGCG.** 128 (95.35)   
367 + **.GGCGCG.** 337 (95.43)   
367 + **.GCCGCG.** 555 (95.35)   
367 + **.GCCGCG.** 571 (95.35)   
367 + **[CTCGCG]** 592 (88.37)   
367 + **[GTCGCC]** 922 (86.61)   
367 + **.GCCGCG.** 1063 (95.35)   
367 + **.GCCGCG.** 1090 (95.35)   
367 + **.GCCGCG.** 1190 (95.35)   
367 + **[CTCGCG]** 1511 (88.37)   
367 - **[GTCCCG]** 1352 (88.98)   
367 - **[GTCCCG]** 1269 (88.98)   
367 - **.GCCGCG.** 1192 (95.35)   
367 - **.GGCGCG.** 1047 (95.43)   
367 - **.GGCGCG.** 1034 (95.43)   
367 - **[GTGGCG]** 924 (87.04)   
367 - **[GTCCCG]** 614 (88.98)   
367 - **[GTCGGG]** 531 (87.52)   
367 - **[GTCCCG]** 440 (88.98)   
367 - **.GGCGCG.** 413 (95.43)   
367 - **.GGCGCG.** 9 (95.43)   
  

**Frequency Matrix**

| All Occs Best Occs|  |  |  |  |  |  |  |  |  |  |  |  |  |  |  |  |  |  |  |  |  |  |  |  |  |  |  |  |  |  |  |  |  |  |  |  |  |  |  |  |  |  |  |  |  |  |  |  |  |  |  |  |  |  |  |  |  |  |  |  |  |  |  |  |  |  |  |  |  |  |  |  |  |  |  |  |  |  |  |  |  |  |  |  | | --- | --- | --- | --- | --- | --- | --- | --- | --- | --- | --- | --- | --- | --- | --- | --- | --- | --- | --- | --- | --- | --- | --- | --- | --- | --- | --- | --- | --- | --- | --- | --- | --- | --- | --- | --- | --- | --- | --- | --- | --- | --- | --- | --- | --- | --- | --- | --- | --- | --- | --- | --- | --- | --- | --- | --- | --- | --- | --- | --- | --- | --- | --- | --- | --- | --- | --- | --- | --- | --- | --- | --- | --- | --- | --- | --- | --- | --- | --- | --- | --- | --- | --- | --- | | **A C G T A C G T | | | | | | | | | | | |**| **1** 107 431 5448 115 | 0 0 2667 0|  |  |  |  |  |  |  |  |  |  |  |  |  |  |  |  |  |  |  |  |  |  |  |  |  |  |  |  |  |  |  |  |  |  |  |  |  |  |  |  |  |  |  |  |  |  |  |  |  |  |  |  |  |  |  |  |  |  |  |  | | --- | --- | --- | --- | --- | --- | --- | --- | --- | --- | --- | --- | --- | --- | --- | --- | --- | --- | --- | --- | --- | --- | --- | --- | --- | --- | --- | --- | --- | --- | --- | --- | --- | --- | --- | --- | --- | --- | --- | --- | --- | --- | --- | --- | --- | --- | --- | --- | --- | --- | --- | --- | --- | --- | --- | --- | --- | --- | --- | --- | | **2** 358 1203 1224 3316 | 0 1203 1224 240|  |  |  |  |  |  |  |  |  |  |  |  |  |  |  |  |  |  |  |  |  |  |  |  |  |  |  |  |  |  |  |  |  |  |  |  |  |  |  |  |  |  |  |  |  |  |  |  | | --- | --- | --- | --- | --- | --- | --- | --- | --- | --- | --- | --- | --- | --- | --- | --- | --- | --- | --- | --- | --- | --- | --- | --- | --- | --- | --- | --- | --- | --- | --- | --- | --- | --- | --- | --- | --- | --- | --- | --- | --- | --- | --- | --- | --- | --- | --- | --- | | **3** 83 5583 330 105 | 0 2667 0 0|  |  |  |  |  |  |  |  |  |  |  |  |  |  |  |  |  |  |  |  |  |  |  |  |  |  |  |  |  |  |  |  |  |  |  |  | | --- | --- | --- | --- | --- | --- | --- | --- | --- | --- | --- | --- | --- | --- | --- | --- | --- | --- | --- | --- | --- | --- | --- | --- | --- | --- | --- | --- | --- | --- | --- | --- | --- | --- | --- | --- | | **4** 127 479 5309 186 | 0 0 2667 0|  |  |  |  |  |  |  |  |  |  |  |  |  |  |  |  |  |  |  |  |  |  |  |  | | --- | --- | --- | --- | --- | --- | --- | --- | --- | --- | --- | --- | --- | --- | --- | --- | --- | --- | --- | --- | --- | --- | --- | --- | | **5** 98 5540 364 99 | 0 2667 0 0|  |  |  |  |  |  |  |  |  |  |  |  | | --- | --- | --- | --- | --- | --- | --- | --- | --- | --- | --- | --- | | **6** 102 299 5549 151 | 0 0 2667 0 | | | | | | | | | | | | | | | | | | | | | | | | | | | | | | | | | | | | | | | | | | | | | | | | | | | | | | | | | | | | | | | | | | | | | | | | | | | | | | | | | | | | |

  
  

---

  
  
  
 **TCGCGTCG**   
**CGACGCGA**  
  
6 redundant motifs found:  
CGTCGCGTCG - CGCGTCGT - CGCGTCGA - TCGCGT - CGACGC - CGCGTC -   
  
Best occurrences (match percentage):   
Seq St oligo pos match
  
 1 + **.GCGCGCCG.** 999 (91.32)   
1 - **[TCGGGGCG]** 1080 (87.91)   
1 - **.GCGCGGCG.** 1068 (91.99)   
1 - **[GCGCCTCG]** 1063 (87.99)   
2 + **[TCGCGGGG]** 363 (87.42)   
2 + **[CCGCGTCT]** 448 (85.23)   
2 + **[CCGCGTCC]** 514 (88.23)   
2 + **[CCGCGGCC]** 976 (85.01)   
2 + **.TCGCGGCG.** 995 (96.78)   
2 + **.GCGCGGCG.** 1446 (91.99)   
2 - **.TCGCGCCG.** 1449 (96.11)   
2 - **[TCGCGGGG]** 1062 (87.42)   
2 - **.TCGCGCCG.** 998 (96.11)   
2 - **.GCGCGCCG.** 702 (91.32)   
2 - **[GGGCGTCG]** 507 (86.23)   
2 - **.TCCCGTCG.** 244 (91.22)   
3 + **.CCGCGGCG.** 1222 (92.56)   
3 + **[CCGCCGCG]** 1414 (85.35)   
3 - **.GCGCGGCG.** 1415 (91.99)   
3 - **[TGGCGGCG]** 1263 (87.81)   
3 - **.GCGCGCCG.** 1248 (91.32)   
3 - **[CCGCCGCG]** 1223 (85.35)   
3 - **[TCGCGCCC]** 1179 (88.56)   
4 + **[GCGGGTCG]** 686 (86.33)   
4 + **[CCGCCGCG]** 697 (85.35)   
4 + **[TCGCGGCC]** 741 (89.23)   
4 + **.TCGCGGCG.** 801 (96.78)   
4 + **.CCGCGGCG.** 821 (92.56)   
4 + **[CCGCCGCG]** 1204 (85.35)   
4 - **.GCGCGGCG.** 1205 (91.99)   
4 - **[CCGGGTCG]** 852 (86.90)   
4 - **.TCGCGCCG.** 827 (96.11)   
4 - **[CCGCCGCG]** 822 (85.35)   
4 - **[CCGCGACG]** 739 (85.28)   
4 - **[CCGGGTCG]** 703 (86.90)   
4 - **.TCGCGGCG.** 698 (96.78)   
4 - **[TCCCGCCG]** 672 (87.33)   
4 - **[TCGCGGGG]** 571 (87.42)   
5 + **[CCCCGTCG]** 919 (87.00)   
5 + **[TCCCGCCG]** 1171 (87.33)   
5 + **[GCGCCTCG]** 1347 (87.99)   
5 - **[CCGCCTCG]** 1446 (88.56)   
5 - **.CCGCGTCG.** 1437 (95.78)   
5 - **[TCGCGCGG]** 1357 (86.75)   
6 + **[CCGCTTCG]** 979 (85.17)   
6 + **.CCGCGGCG.** 1439 (92.56)   
6 + **[TGGCGGCG]** 1474 (87.81)   
6 - **[TCGCCGCG]** 1440 (89.57)   
8 + **[TCGCGTCT]** 148 (89.45)   
8 + **[GCGCGTCC]** 371 (87.65)   
8 + **[CCGCGTGG]** 453 (86.42)   
8 + **.CCGCGTCG.** 1145 (95.78)   
8 + **[CCGCTTCG]** 1272 (85.17)   
8 + **[TCGGGCCG]** 1384 (87.23)   
8 + **[TCGCGACG]** 1465 (89.50)   
8 - **[CGGCGTCG]** 1151 (86.80)   
8 - **[TCGCGCCC]** 932 (88.56)   
8 - **[TCGCCCCG]** 899 (88.90)   
8 - **[ACGCGGCG]** 451 (86.77)   
8 - **.CCGCGCCG.** 406 (91.89)   
9 - **.TCCCGTCG.** 1270 (91.22)   
9 - **[TCGCGCCT]** 1265 (85.56)   
9 - **[TCGCGCCC]** 974 (88.56)   
10 + **.CCGCGGCG.** 1023 (92.56)   
10 + **.GCGCGGCG.** 1095 (91.99)   
10 + **[CCGCGGCC]** 1120 (85.01)   
10 - **[TGGCGGCG]** 1262 (87.81)   
10 - **[CCGCGGCC]** 1118 (85.01)   
10 - **[CCGCCGCG]** 1096 (85.35)   
10 - **[CCGCCGCG]** 1024 (85.35)   
10 - **[CCGCGGCC]** 1021 (85.01)   
10 - **[CCGCGTCC]** 827 (88.23)   
10 - **[TCGCGTCT]** 819 (89.45)   
11 + **[TCGGGGCG]** 92 (87.91)   
11 + **[TCCCGGCG]** 412 (88.00)   
11 + **[CCCCGTCG]** 525 (87.00)   
11 + **.CCGCGCCG.** 605 (91.89)   
11 + **[CCGCGGCC]** 610 (85.01)   
11 + **[CCGCGGCC]** 714 (85.01)   
11 + **.CCGCGGCG.** 768 (92.56)   
11 + **[CCGCCGCG]** 992 (85.35)   
11 + **[CCGCCGCG]** 1025 (85.35)   
11 + **.GCGCGGCG.** 1115 (91.99)   
11 + **[CCGCCGCG]** 1153 (85.35)   
11 + **[GCGCGTCC]** 1380 (87.65)   
11 - **.GCGCGGCG.** 1154 (91.99)   
11 - **[TCCCGCCG]** 1118 (87.33)   
11 - **[CCGCCGCG]** 1116 (85.35)   
11 - **.GCGCGGCG.** 1026 (91.99)   
11 - **.GCGCGGCG.** 993 (91.99)   
11 - **[GCGCGTCC]** 746 (87.65)   
11 - **[TCCCGCCG]** 741 (87.33)   
11 - **[CCGCGGCC]** 712 (85.01)   
11 - **[GCGCGTCC]** 697 (87.65)   
11 - **.CCGCGGCG.** 608 (92.56)   
11 - **[CCGCGACG]** 528 (85.28)   
12 + **.TGGCGTCG.** 30 (91.02)   
12 + **[TCCCGGCG]** 537 (88.00)   
12 + **.CCGCGCCG.** 695 (91.89)   
12 + **[CCGCGGCC]** 710 (85.01)   
12 + **[TCCCGCCG]** 773 (87.33)   
12 + **[GCGCGTCC]** 840 (87.65)   
12 + **[GCGGGTCG]** 1091 (86.33)   
12 + **[CCGCCTCG]** 1147 (88.56)   
12 + **.CCGCGCCG.** 1252 (91.89)   
12 + **[TCGCGTAG]** 1464 (86.48)   
12 - **.TCGCGGCG.** 1354 (96.78)   
12 - **.GCGCGGCG.** 1177 (91.99)   
12 - **[GCGCGTCC]** 1115 (87.65)   
12 - **.GCGCGCCG.** 540 (91.32)   
12 - **[GCGCGTGG]** 204 (85.84)   
13 + **.CCGCGCCG.** 614 (91.89)   
13 + **[CCGCGGCC]** 702 (85.01)   
13 + **[CCGCCGCG]** 719 (85.35)   
13 + **[CCGCGTCC]** 741 (88.23)   
13 + **[CCGCGTCC]** 765 (88.23)   
13 + **[GCGCCTCG]** 867 (87.99)   
13 + **[CCGCCGCG]** 1108 (85.35)   
13 + **.GCGCGCCG.** 1186 (91.32)   
13 + **[CCGCCGCG]** 1195 (85.35)   
13 + **.CCGCGCCG.** 1263 (91.89)   
13 + **[CCGGGTCG]** 1298 (86.90)   
13 + **[CGGCGTCG]** 1318 (86.80)   
13 + **[TCGCGGGG]** 1323 (87.42)   
13 + **.GCGCGGCG.** 1351 (91.99)   
13 + **[CCGGGTCG]** 1440 (86.90)   
13 + **[CCGGGTCG]** 1464 (86.90)   
13 - **[TCCCGCCG]** 1430 (87.33)   
13 - **[GCCCGTCG]** 1404 (86.42)   
13 - **[TCGCCTCC]** 1399 (85.23)   
13 - **[CCGCCGCG]** 1352 (85.35)   
13 - **[CCGCGACG]** 1321 (85.28)   
13 - **.CCGCGGCG.** 1196 (92.56)   
13 - **[TGGCGGCG]** 1145 (87.81)   
13 - **.GCGCGGCG.** 1109 (91.99)   
13 - **.CCGCGCCG.** 1061 (91.89)   
13 - **[TCGCCCCG]** 909 (88.90)   
13 - **.GCGCGCCG.** 800 (91.32)   
13 - **.GCGCGGCG.** 720 (91.99)   
13 - **[CCGCGTGG]** 653 (86.42)   
14 + **.CCGCGGCG.** 336 (92.56)   
14 + **[TCGCGGGG]** 350 (87.42)   
14 + **.GCGCGGCG.** 747 (91.99)   
14 + **[CCGCGTCC]** 792 (88.23)   
14 + **.GCGCGGCG.** 939 (91.99)   
14 - **[CCGCCGCG]** 940 (85.35)   
14 - **[TCCCGCCG]** 750 (87.33)   
14 - **[CCGCCGCG]** 748 (85.35)   
14 - **[TCGCCTCC]** 541 (85.23)   
15 + **[TCCCGCCG]** 411 (87.33)   
15 + **[CCGCCGCG]** 413 (85.35)   
15 + **[TGGCGCCG]** 451 (87.13)   
15 + **[TCGGGGCG]** 502 (87.91)   
15 + **[CCGCGGCC]** 544 (85.01)   
15 + **[TCGCGCCC]** 826 (88.56)   
15 + **[TCGCGCGG]** 892 (86.75)   
15 + **[TGGCGCCG]** 940 (87.13)   
15 + **.GCGCGGCG.** 985 (91.99)   
15 + **[CCGGGTCG]** 1109 (86.90)   
15 + **[GCGCGTCC]** 1182 (87.65)   
15 + **[CCGCGTCC]** 1231 (88.23)   
15 - **[CCGCGGCC]** 1263 (85.01)   
15 - **.GCGCGCCG.** 1199 (91.32)   
15 - **[TCGGGCCG]** 1162 (87.23)   
15 - **[TGGCGCCG]** 988 (87.13)   
15 - **[TCCCGGCG]** 902 (88.00)   
15 - **.GCGCGCCG.** 732 (91.32)   
15 - **[GGGCGTCG]** 694 (86.23)   
15 - **[GCGCCTCG]** 494 (87.99)   
15 - **[CCGCGGCC]** 458 (85.01)   
15 - **.GCGCGGCG.** 414 (91.99)   
15 - **[CGGCGTCG]** 268 (86.80)   
15 - **[GCGCGTGG]** 244 (85.84)   
15 - **.CCGCGGCG.** 178 (92.56)   
15 - **[TCGCGGCC]** 22 (89.23)   
16 + **.CCGCGCCG.** 622 (91.89)   
16 + **[TCGCGCCC]** 798 (88.56)   
16 + **.GCGCGGCG.** 1149 (91.99)   
16 + **[TCGCGCCC]** 1299 (88.56)   
16 - **[TCGGGCCG]** 1323 (87.23)   
16 - **[TCGCTGCG]** 1167 (86.18)   
16 - **[GCGCGTGG]** 1162 (85.84)   
16 - **[CCGCCGCG]** 1150 (85.35)   
16 - **[TCGCGGGG]** 1000 (87.42)   
16 - **.GCGCGGCG.** 625 (91.99)   
16 - **.CCGCGCCG.** 491 (91.89)   
17 + **[TCGGGGCG]** 982 (87.91)   
17 - **[CCGCGGCC]** 1016 (85.01)   
17 - **[TCGCCCCG]** 983 (88.90)   
17 - **[TCGCCTCC]** 841 (85.23)   
18 + **[CCGCGTCC]** 475 (88.23)   
18 + **.GCGCGCCG.** 709 (91.32)   
18 + **.CCGCGCCG.** 833 (91.89)   
18 + **[GCGCCTCG]** 915 (87.99)   
18 + **[CCGCGGCC]** 931 (85.01)   
18 + **[CCGCCTCG]** 974 (88.56)   
18 + **[CCGGGTCG]** 1159 (86.90)   
18 + **[CCGCCGCG]** 1200 (85.35)   
18 + **[CCGCGGCC]** 1203 (85.01)   
18 + **[CCGCGTCC]** 1330 (88.23)   
18 + **[TCGCTGCG]** 1470 (86.18)   
18 - **.CCGCGCCG.** 1385 (91.89)   
18 - **.CCGCGGCG.** 1201 (92.56)   
18 - **[CCGCGGCC]** 929 (85.01)   
18 - **.GCGCGGCG.** 865 (91.99)   
18 - **.GCGCGGCG.** 836 (91.99)   
20 + **.TCGGGTCG.** 814 (91.12)   
20 + **[CCGCGTGG]** 1198 (86.42)   
20 - **[GCGCGTGG]** 308 (85.84)   
20 - **[TCGCGGCC]** 299 (89.23)   
22 + **[CCGCCGCG]** 770 (85.35)   
22 + **.TCCCGTCG.** 1033 (91.22)   
22 - **[TCGCGCGG]** 1012 (86.75)   
22 - **.GCGCGGCG.** 771 (91.99)   
22 - **[CCGCCTCG]** 674 (88.56)   
22 - **[CCGCCTCG]** 86 (88.56)   
23 - **[CCGCGTGG]** 1076 (86.42)   
23 - **[TCGCTGCG]** 904 (86.18)   
24 + **[TCGCTGCG]** 438 (86.18)   
24 + **[TCCCGGCG]** 509 (88.00)   
24 + **[CCGCGTCT]** 521 (85.23)   
24 + **[TCCCGGCG]** 536 (88.00)   
24 + **.CCGCGGCG.** 1039 (92.56)   
24 + **[CCGGGTCG]** 1139 (86.90)   
24 + **.TCGCGCCG.** 1144 (96.11)   
24 - **[CCGCCGCG]** 1040 (85.35)   
24 - **[GCGGGTCG]** 571 (86.33)   
24 - **[TCGCTGCG]** 388 (86.18)   
25 - **[TCGCGGCT]** 1150 (86.24)   
25 - **[CCGCTTCG]** 1064 (85.17)   
25 - **[GCGCCTCG]** 829 (87.99)   
25 - **[TCGCCCCG]** 824 (88.90)   
25 - **.CCGCGCCG.** 791 (91.89)   
25 - **[TCGCCCCG]** 603 (88.90)   
25 - **[CCGCCTCG]** 571 (88.56)   
25 - **[GCGCCTCG]** 546 (87.99)   
25 - **[TCGCCCCG]** 445 (88.90)   
25 - **[TCGCCCCG]** 414 (88.90)   
25 - **[GCGCCTCG]** 387 (87.99)   
25 - **.TCGCGCCG.** 382 (96.11)   
26 + **[TCCCGGCG]** 876 (88.00)   
26 + **.GCGCGGCG.** 1424 (91.99)   
26 - **.CCGCGGCG.** 1484 (92.56)   
26 - **[TCCCGCCG]** 1427 (87.33)   
26 - **[CCGCCGCG]** 1425 (85.35)   
26 - **[CCGCCTCG]** 1028 (88.56)   
26 - **.CCGCGCCG.** 823 (91.89)   
26 - **[GCGCGTCC]** 752 (87.65)   
26 - **[CCGCGGCC]** 183 (85.01)   
27 + **.CCGCGTCG.** 1145 (95.78)   
27 + **.CCGCGGCG.** 1203 (92.56)   
27 + **.GCGCGCCG.** 1248 (91.32)   
27 - **.GCGCGCCG.** 1206 (91.32)   
27 - **[CCGCTTCG]** 779 (85.17)   
28 + **[GCGCGTGG]** 434 (85.84)   
28 + **[CCGCGACG]** 588 (85.28)   
28 + **.CCGCGCCG.** 739 (91.89)   
28 + **[TCGCTCCG]** 765 (85.50)   
28 + **[CCGCCTCG]** 1071 (88.56)   
28 + **[GCCCGTCG]** 1094 (86.42)   
28 + **.TCGCGTCG.** 1099 (100.00)   
28 + **.TCGCGCCG.** 1104 (96.11)   
28 + **.CCGCGTCG.** 1109 (95.78)   
28 - **[CCGCCTCG]** 1413 (88.56)   
28 - **[ACGCGGCG]** 1107 (86.77)   
28 - **[CCGCGGCC]** 915 (85.01)   
28 - **.TCGCCTCG.** 794 (92.78)   
28 - **.TCGCGCCG.** 627 (96.11)   
28 - **[TCGCGGCT]** 586 (86.24)   
28 - **[TGGCGGCG]** 579 (87.81)   
28 - **[ACGCGCCG]** 432 (86.10)   
28 - **[GCGCCTCG]** 252 (87.99)   
29 + **[GCGCCTCG]** 1207 (87.99)   
33 + **[CCGCGGCC]** 903 (85.01)   
33 + **[TCGCCCCG]** 1058 (88.90)   
33 + **[TGGCGGCG]** 1287 (87.81)   
33 + **.CCGCGGCG.** 1361 (92.56)   
33 + **[CCGCCGCG]** 1409 (85.35)   
33 + **[CGGCGTCG]** 1460 (86.80)   
33 - **.CCGCGGCG.** 1410 (92.56)   
33 - **[CCGCCGCG]** 1362 (85.35)   
33 - **[CCGCGGCC]** 1359 (85.01)   
33 - **[TCCCGCCG]** 1162 (87.33)   
33 - **[ACGCGGCG]** 1039 (86.77)   
34 + **[TCGCGGGG]** 244 (87.42)   
34 + **[TGGCGGCG]** 668 (87.81)   
34 + **[TCGCGCCC]** 941 (88.56)   
34 + **[CCGCCTCG]** 1334 (88.56)   
34 - **.GCGCGGCG.** 1195 (91.99)   
34 - **[TGGCGGCG]** 1025 (87.81)   
34 - **[GCGCCTCG]** 915 (87.99)   
34 - **[CCGCCTCG]** 814 (88.56)   
34 - **[TCGCGGGG]** 791 (87.42)   
34 - **.GCGCGGCG.** 770 (91.99)   
35 + **[TCGCCGCG]** 996 (89.57)   
35 + **[GCGGGTCG]** 1150 (86.33)   
35 + **[TCGGGGCG]** 1236 (87.91)   
35 - **[CCGGGTCG]** 1332 (86.90)   
35 - **[TGGCGGCG]** 1297 (87.81)   
35 - **[TCCCGCCG]** 1262 (87.33)   
35 - **.GCGCGGCG.** 997 (91.99)   
36 + **[TCGCCTCC]** 1007 (85.23)   
36 + **.GCGCGCCG.** 1065 (91.32)   
36 - **.CCGCGGCG.** 1170 (92.56)   
36 - **[TCGCCCCG]** 1075 (88.90)   
38 + **[GCGCCTCG]** 558 (87.99)   
38 + **[CCGCCTCG]** 865 (88.56)   
39 + **[GCGCGTCC]** 1121 (87.65)   
39 + **[TCCCGGCG]** 1148 (88.00)   
39 + **[TCGCGCGG]** 1422 (86.75)   
39 - **.TCGCGCCG.** 1411 (96.11)   
39 - **[TGGCGGCG]** 1350 (87.81)   
40 + **[CCGCGGCC]** 894 (85.01)   
40 + **.CCGCGCCG.** 1103 (91.89)   
40 + **[GCGCGTCC]** 1234 (87.65)   
40 + **.CCGCGGCG.** 1240 (92.56)   
40 + **[TCGCGGGG]** 1278 (87.42)   
40 + **.TGGCGTCG.** 1413 (91.02)   
40 - **[TCGCCGCG]** 1241 (89.57)   
40 - **.GCGCGGCG.** 1106 (91.99)   
41 + **[CCGCGGCC]** 522 (85.01)   
41 + **[TCGCTCCG]** 897 (85.50)   
41 + **.TCCCGTCG.** 912 (91.22)   
41 + **[TCGCCTCC]** 917 (85.23)   
42 + **[GCGCCTCG]** 608 (87.99)   
42 + **[CCGCGGCC]** 842 (85.01)   
42 + **[TCCCGGCG]** 934 (88.00)   
42 + **.CCGCGGCG.** 1167 (92.56)   
42 + **[GCGGGTCG]** 1263 (86.33)   
42 + **[TCGCGGCC]** 1310 (89.23)   
42 + **[GCCCGTCG]** 1348 (86.42)   
42 + **[GCGCGTGG]** 1450 (85.84)   
42 - **[CCGCGGCC]** 1474 (85.01)   
42 - **[CCGCCGCG]** 1168 (85.35)   
42 - **[CCGCGACG]** 908 (85.28)   
42 - **[TCGCGGGG]** 522 (87.42)   
43 - **[CCGGGTCG]** 1369 (86.90)   
43 - **.CCGCGGCG.** 1100 (92.56)   
45 + **[CCGCGGCC]** 980 (85.01)   
45 - **.CCGCGCCG.** 1418 (91.89)   
45 - **.CCGCGGCG.** 396 (92.56)   
47 + **.TCGCGCCG.** 1248 (96.11)   
47 - **[CCGCCTCG]** 1142 (88.56)   
47 - **[TCGAGTCG]** 1106 (87.13)   
47 - **.CCGCGCCG.** 833 (91.89)   
48 + **.GCGCGGCG.** 203 (91.99)   
48 + **.CCGCGGCG.** 432 (92.56)   
48 + **.GCGCGGCG.** 1018 (91.99)   
48 + **[CCGCCGCG]** 1282 (85.35)   
48 + **.CCGCGGCG.** 1285 (92.56)   
48 + **[CCGCGGCC]** 1316 (85.01)   
48 + **[CCGCGTCT]** 1363 (85.23)   
48 - **.CCGCGGCG.** 1283 (92.56)   
48 - **[CCGCCGCG]** 1019 (85.35)   
48 - **[CCGCCTCG]** 217 (88.56)   
48 - **[CCGCCGCG]** 204 (85.35)   
49 + **[TCGGGGCG]** 576 (87.91)   
49 + **[CCGCCGCG]** 1405 (85.35)   
49 - **.CCGCGGCG.** 1406 (92.56)   
49 - **[TCGGGGCG]** 1283 (87.91)   
49 - **[TCGCCCCG]** 577 (88.90)   
51 + **[TCGCGGCC]** 35 (89.23)   
51 + **[CCGCCGCG]** 66 (85.35)   
51 + **.CCGCGTCG.** 69 (95.78)   
51 + **[GCGCCTCG]** 91 (87.99)   
51 + **.TCGCGGCG.** 146 (96.78)   
51 + **[TGGCGCCG]** 212 (87.13)   
51 + **[CCGCCTCG]** 385 (88.56)   
51 + **.TCGCGCCG.** 781 (96.11)   
51 + **[CCCCGTCG]** 973 (87.00)   
51 + **[TCGCGCCC]** 997 (88.56)   
51 + **[TCCCGCCG]** 1031 (87.33)   
51 + **[TCCCGCCG]** 1144 (87.33)   
51 + **.CCGCGGCG.** 1285 (92.56)   
51 + **[TCGCGGGG]** 1354 (87.42)   
51 + **.GCGCGGCG.** 1361 (91.99)   
51 - **[TGGCGCCG]** 1364 (87.13)   
51 - **[CCGCCGCG]** 1286 (85.35)   
51 - **[CGGCGTCG]** 1150 (86.80)   
51 - **[TCCCGGCG]** 1043 (88.00)   
51 - **[CCGCGACG]** 976 (85.28)   
51 - **[GCCCGTCG]** 490 (86.42)   
51 - **.GCGCGTCG.** 230 (95.20)   
51 - **[TCCCGCCG]** 221 (87.33)   
51 - **[CCGCCGCG]** 147 (85.35)   
51 - **[ACGCGGCG]** 67 (86.77)   
53 + **[GGGCGTCG]** 1182 (86.23)   
53 - **[CCGCGTCC]** 1056 (88.23)   
53 - **[CCGGGTCG]** 799 (86.90)   
53 - **[GCGCCTCG]** 788 (87.99)   
53 - **[TCGCCTCC]** 368 (85.23)   
53 - **[TCGCCTCC]** 139 (85.23)   
54 - **.TCGCGTGG.** 1254 (90.64)   
54 - **[GCGCGTCC]** 1157 (87.65)   
55 + **[CCGCGGCC]** 45 (85.01)   
55 + **[TCGCCGCG]** 197 (89.57)   
55 + **[TCGCCCCG]** 215 (88.90)   
55 + **[CCGCCGCG]** 864 (85.35)   
55 + **[CCGCCGCG]** 882 (85.35)   
55 + **.GCGCGGCG.** 942 (91.99)   
55 + **.CCGCGCCG.** 1081 (91.89)   
55 - **.GCGCGGCG.** 1084 (91.99)   
55 - **.CCGCGGCG.** 883 (92.56)   
55 - **[ACGCGGCG]** 865 (86.77)   
55 - **.GCGCGGCG.** 198 (91.99)   
58 + **[TCGCCCCG]** 597 (88.90)   
58 - **.CCGCGTCG.** 1279 (95.78)   
58 - **[TCGCGTCA]** 750 (87.59)   
58 - **.GCGCGTCG.** 603 (95.20)   
58 - **[TCGGGGCG]** 598 (87.91)   
58 - **[CCGCGTGG]** 417 (86.42)   
59 + **[CCGCGGCC]** 651 (85.01)   
59 + **[CCGCGGCC]** 1011 (85.01)   
59 + **.GCGCGTCG.** 1285 (95.20)   
59 + **.GCGCGGCG.** 1348 (91.99)   
59 - **[CCGGGTCG]** 1400 (86.90)   
59 - **.CCGCGCCG.** 1351 (91.89)   
59 - **[TGGCGGCG]** 826 (87.81)   
59 - **[TCCCGGCG]** 684 (88.00)   
59 - **.CCGCGGCG.** 649 (92.56)   
60 + **[TCCCGCCG]** 626 (87.33)   
60 + **[CCGCCGCG]** 823 (85.35)   
60 + **[CCGCGGCC]** 826 (85.01)   
60 - **[CCGCCTCG]** 904 (88.56)   
60 - **.CCGCGGCG.** 824 (92.56)   
60 - **[CCGCTTCG]** 770 (85.17)   
61 + **[ACGCGGCG]** 841 (86.77)   
61 + **[TCCCGCCG]** 921 (87.33)   
61 + **[CCGCCGCG]** 923 (85.35)   
61 + **.CCGCGGCG.** 1038 (92.56)   
61 - **[TCGCCGCG]** 1039 (89.57)   
61 - **.CCGCGGCG.** 1036 (92.56)   
61 - **.CCGCGGCG.** 924 (92.56)   
61 - **[TCGCCGCG]** 842 (89.57)   
61 - **[CCGCGTCC]** 839 (88.23)   
61 - **.GCGCGCCG.** 500 (91.32)   
61 - **[TCGCTTCG]** 435 (89.39)   
63 + **[TCGGGCCG]** 174 (87.23)   
63 + **[CCGGGTCG]** 210 (86.90)   
63 + **[TCGGGGCG]** 215 (87.91)   
63 + **.GCGCGGCG.** 409 (91.99)   
63 + **.CCGCGGCG.** 444 (92.56)   
63 + **.CCGCGCCG.** 453 (91.89)   
63 + **[CCGCGTCC]** 520 (88.23)   
63 + **[TCGCCCCG]** 621 (88.90)   
63 + **.GCGCGCCG.** 657 (91.32)   
63 + **[CCGCCTCG]** 718 (88.56)   
63 + **.TCCCGTCG.** 781 (91.22)   
63 + **.TCGCGTCG.** 853 (100.00)   
63 + **.GCGCGCCG.** 906 (91.32)   
63 + **.CCGCGGCG.** 953 (92.56)   
63 + **.TCCCGTCG.** 961 (91.22)   
63 + **[TCGCGTTG]** 975 (87.97)   
63 + **[GCCCGTCG]** 986 (86.42)   
63 + **.TCGGGTCG.** 1111 (91.12)   
63 + **.GCGCGGCG.** 1392 (91.99)   
63 - **.TCGCGTCC.** 1429 (92.45)   
63 - **[CCGCGGCC]** 1411 (85.01)   
63 - **[CCGCCGCG]** 1393 (85.35)   
63 - **.CCGCGGCG.** 1252 (92.56)   
63 - **[CGGCGTCG]** 997 (86.80)   
63 - **[TCGCTCCG]** 992 (85.50)   
63 - **[TCGTGTCG]** 815 (85.92)   
63 - **[CCGCCGCG]** 410 (85.35)   
63 - **[TCGGGGCG]** 392 (87.91)   
63 - **.GCGCGCCG.** 265 (91.32)   
63 - **[CCGCGGCC]** 181 (85.01)   
63 - **[GCGCCTCG]** 67 (87.99)   
65 + **[CCGCGTCT]** 546 (85.23)   
65 + **[TCGCGCCT]** 588 (85.56)   
65 + **.TCGCCTCG.** 635 (92.78)   
65 + **[TCGCCTCC]** 817 (85.23)   
65 + **[CCGCCGCG]** 999 (85.35)   
65 - **.TGGCGTCG.** 1419 (91.02)   
65 - **[CCGCGTCC]** 1223 (88.23)   
65 - **.TGGCGTCG.** 1054 (91.02)   
65 - **.GCGCGGCG.** 1027 (91.99)   
65 - **[TCCCGGCG]** 1009 (88.00)   
65 - **.TCGCGGCG.** 1000 (96.78)   
65 - **.GCGCGCCG.** 978 (91.32)   
65 - **[ACGCGCCG]** 863 (86.10)   
66 + **.GCGCGCCG.** 868 (91.32)   
66 + **.CCGCGCCG.** 954 (91.89)   
66 + **[CCGCCGCG]** 987 (85.35)   
66 + **.CCGCGGCG.** 1126 (92.56)   
66 + **[TCGCGTCT]** 1405 (89.45)   
66 - **.TCGCGTCC.** 1479 (92.45)   
66 - **[TCGAGTCG]** 1469 (87.13)   
66 - **.CCGCGGCG.** 988 (92.56)   
66 - **[CCGGGTCG]** 977 (86.90)   
66 - **[CCGCGTCC]** 785 (88.23)   
67 + **[TCCCGGCG]** 812 (88.00)   
67 + **[GCGGGTCG]** 817 (86.33)   
67 + **[TGGCGGCG]** 1017 (87.81)   
67 + **[TCGCCGCG]** 1194 (89.57)   
67 + **[CCGCGTCC]** 1197 (88.23)   
67 + **[TCGGGCCG]** 1354 (87.23)   
67 - **[ACGCGGCG]** 1195 (86.77)   
67 - **[GCGCGTCC]** 1172 (87.65)   
67 - **[CCGCCTCG]** 996 (88.56)   
68 + **[TCGGGCCG]** 84 (87.23)   
68 + **[TCGGGCCG]** 93 (87.23)   
68 + **.GCGCGTCG.** 418 (95.20)   
68 + **[TGGCGGCG]** 495 (87.81)   
68 + **[TCGCTGCG]** 534 (86.18)   
68 + **[CCGCCGCG]** 587 (85.35)   
68 + **[ACGCGGCG]** 991 (86.77)   
68 + **.GCGCGGCG.** 1393 (91.99)   
68 - **[CCGCCGCG]** 1394 (85.35)   
68 - **[CCGCCGCG]** 992 (85.35)   
68 - **[TCCCGGCG]** 909 (88.00)   
68 - **[GGGCGTCG]** 873 (86.23)   
68 - **.CCGCGCCG.** 651 (91.89)   
68 - **.GCGCGGCG.** 588 (91.99)   
68 - **[TGGCGGCG]** 579 (87.81)   
69 + **[CCGCGTCC]** 689 (88.23)   
69 + **[CCGCGTGG]** 695 (86.42)   
69 + **[CCGCGGCC]** 768 (85.01)   
69 + **.GCGCGTCG.** 893 (95.20)   
69 + **.CCGCGCCG.** 997 (91.89)   
69 + **.CCGCGCCG.** 1120 (91.89)   
69 - **[TCGGGGCG]** 957 (87.91)   
70 + **.GCGCGCCG.** 1333 (91.32)   
70 + **[GCGCGTCC]** 1389 (87.65)   
70 + **[CCGCCTCG]** 1424 (88.56)   
70 - **[CCGCGTGG]** 839 (86.42)   
70 - **[CCGCCTCG]** 248 (88.56)   
71 + **[GCGCGTCC]** 903 (87.65)   
71 + **[TCGCGCGG]** 1495 (86.75)   
71 - **[GCGCGTGG]** 1272 (85.84)   
71 - **.TCGCGTCC.** 1236 (92.45)   
73 + **[CCGCCGCG]** 917 (85.35)   
73 + **[CCGCGGCC]** 920 (85.01)   
73 - **.CCGCGGCG.** 918 (92.56)   
74 + **[GCGCGTCC]** 1337 (87.65)   
74 + **.TCGCGGCG.** 1428 (96.78)   
74 + **.CCGCGCCG.** 1437 (91.89)   
74 - **[CCGCCGCG]** 1429 (85.35)   
74 - **[TGGCGGCG]** 1350 (87.81)   
76 + **.GCGCGCCG.** 828 (91.32)   
76 + **.CCGCGGCG.** 865 (92.56)   
76 - **[CCGCGTCC]** 1283 (88.23)   
76 - **[TCGCGGCT]** 1055 (86.24)   
76 - **[CCGCCTCG]** 912 (88.56)   
76 - **[TCGCCGCG]** 866 (89.57)   
77 + **[GCGCGTGG]** 932 (85.84)   
77 + **[CCGCTTCG]** 950 (85.17)   
77 - **[TCGCTGCG]** 1045 (86.18)   
79 + **[CCGCCGCG]** 652 (85.35)   
79 + **[CCGCCGCG]** 876 (85.35)   
79 + **.CCGCGCCG.** 1201 (91.89)   
79 + **.TCGCGTCC.** 1324 (92.45)   
79 - **[CCGCGGCC]** 1276 (85.01)   
79 - **[CCGCGACG]** 1087 (85.28)   
79 - **[ACGCGGCG]** 877 (86.77)   
79 - **.TCGCGGCG.** 653 (96.78)   
80 + **[TCGCGTAG]** 397 (86.48)   
80 + **[TCGCCGCG]** 1014 (89.57)   
80 + **[TGGCGGCG]** 1040 (87.81)   
80 - **.CCGCGCCG.** 1043 (91.89)   
80 - **.GCGCGGCG.** 1015 (91.99)   
80 - **[ACGCGCCG]** 954 (86.10)   
81 - **[TCGGGGCG]** 839 (87.91)   
81 - **[CCGCCTCG]** 687 (88.56)   
81 - **[TCGCGTAG]** 682 (86.48)   
84 + **[TCGCGCCC]** 863 (88.56)   
84 + **.GCGCGGCG.** 1022 (91.99)   
84 + **[ACGCGGCG]** 1036 (86.77)   
84 + **[CCCCGTCG]** 1316 (87.00)   
84 - **.CCGCGCCG.** 1152 (91.89)   
84 - **.CCGCGTCG.** 1034 (95.78)   
84 - **.GCGCGTCG.** 899 (95.20)   
85 + **[TCCCGCCG]** 16 (87.33)   
85 + **[CCGCGTCC]** 94 (88.23)   
85 + **[TCGGGGCG]** 310 (87.91)   
85 + **[CCGCGGCC]** 879 (85.01)   
85 + **[TCCCGCCG]** 1174 (87.33)   
85 + **[CCGCGGCC]** 1216 (85.01)   
85 - **[TCGGGGCG]** 1080 (87.91)   
85 - **[TCGGGCCG]** 1028 (87.23)   
85 - **[CCGCGTCC]** 927 (88.23)   
86 + **[TCGCCGCG]** 539 (89.57)   
86 + **[CCGCGGCC]** 542 (85.01)   
86 + **[CCGCGGCC]** 657 (85.01)   
86 + **.GCGCGGCG.** 835 (91.99)   
86 + **.GCGCGGCG.** 901 (91.99)   
86 + **[TGGCGGCG]** 1056 (87.81)   
86 + **[GCGGGTCG]** 1120 (86.33)   
86 + **[TCGCTCCG]** 1147 (85.50)   
86 + **.TCGCGGCG.** 1189 (96.78)   
86 + **.GCGCGGCG.** 1194 (91.99)   
86 - **[TCGCGGCC]** 1509 (89.23)   
86 - **.CCGCGGCG.** 1436 (92.56)   
86 - **[TCCCGCCG]** 1393 (87.33)   
86 - **[TCGGGGCG]** 1362 (87.91)   
86 - **[TCGGGCCG]** 1327 (87.23)   
86 - **[CCGGGTCG]** 1322 (86.90)   
86 - **.CCGCGCCG.** 1192 (91.89)   
86 - **[TCGCCGCG]** 902 (89.57)   
86 - **[CCGCCGCG]** 836 (85.35)   
86 - **[CCGGGTCG]** 799 (86.90)   
86 - **.CCGCGGCG.** 540 (92.56)   
86 - **[TCGCGGCT]** 304 (86.24)   
87 + **.TCGCGGCG.** 309 (96.78)   
87 + **[CCGGGTCG]** 417 (86.90)   
87 + **[CCGCGGCC]** 721 (85.01)   
87 + **[TCGGGGCG]** 930 (87.91)   
87 + **[CCGCGTCC]** 1098 (88.23)   
87 + **.GCGCGCCG.** 1193 (91.32)   
87 + **.GCGCGGCG.** 1243 (91.99)   
87 + **[ACGCGCCG]** 1321 (86.10)   
87 + **.TCGCGGCG.** 1332 (96.78)   
87 + **.GCGCGCCG.** 1345 (91.32)   
87 + **[CCGCGGCC]** 1366 (85.01)   
87 - **[CCGCCGCG]** 1333 (85.35)   
87 - **[GCGCGTGG]** 1319 (85.84)   
87 - **[CCGCCGCG]** 1244 (85.35)   
87 - **.CCGCGCCG.** 1241 (91.89)   
87 - **.CCGCGCCG.** 842 (91.89)   
87 - **[TCGCCCCG]** 772 (88.90)   
87 - **[CCGCGGCC]** 767 (85.01)   
87 - **[CCGCGTCC]** 585 (88.23)   
87 - **.TCGCGCCG.** 312 (96.11)   
87 - **[GCGCGTGG]** 265 (85.84)   
88 + **[TGGCGGCG]** 477 (87.81)   
88 + **[TGGCGCCG]** 500 (87.13)   
88 + **[TCGGGGCG]** 701 (87.91)   
88 + **.CCGCGGCG.** 848 (92.56)   
88 + **[TCCCGCCG]** 1035 (87.33)   
88 + **[CCGCGGCC]** 1098 (85.01)   
88 + **.GCGCGTCG.** 1205 (95.20)   
88 + **[CCGGGTCG]** 1302 (86.90)   
88 + **.GCGCGGCG.** 1394 (91.99)   
88 - **[TCGCCGCG]** 1395 (89.57)   
88 - **.CCGCGGCG.** 1096 (92.56)   
88 - **[TGGCGCCG]** 869 (87.13)   
88 - **[CCGCCGCG]** 849 (85.35)   
88 - **.GCGCGGCG.** 503 (91.99)   
88 - **[CCGCGACG]** 487 (85.28)   
88 - **.GCGCGCCG.** 444 (91.32)   
88 - **[TCGCGGGG]** 368 (87.42)   
88 - **[TCGGGGCG]** 343 (87.91)   
89 + **[CCGCGGCC]** 46 (85.01)   
89 + **.CCGCGCCG.** 99 (91.89)   
89 + **[CCCCGTCG]** 147 (87.00)   
89 + **[TCGCGCCC]** 152 (88.56)   
89 + **[CCGCCGCG]** 416 (85.35)   
89 + **.CCGCGCCG.** 494 (91.89)   
89 + **[CCGCCGCG]** 662 (85.35)   
89 + **.GCGCGGCG.** 742 (91.99)   
89 + **.GCGCGCCG.** 788 (91.32)   
89 + **[CCGCGGCC]** 842 (85.01)   
89 + **[TCGGGCCG]** 930 (87.23)   
89 + **.CCGCGCCG.** 1022 (91.89)   
89 + **[CCGCCGCG]** 1124 (85.35)   
89 - **.TCGCGTCC.** 1464 (92.45)   
89 - **.CCGCGGCG.** 1309 (92.56)   
89 - **.GCGCGGCG.** 1125 (91.99)   
89 - **[CCGCGTGG]** 963 (86.42)   
89 - **.CCGCGCCG.** 936 (91.89)   
89 - **.GCGCGCCG.** 786 (91.32)   
89 - **.GCGCGGCG.** 663 (91.99)   
89 - **[TGGCGCCG]** 620 (87.13)   
89 - **[GCGCGTCC]** 599 (87.65)   
89 - **.GCGCGGCG.** 434 (91.99)   
89 - **.CCGCGGCG.** 417 (92.56)   
89 - **.TCGCGGCG.** 268 (96.78)   
89 - **[CCGCGGCC]** 259 (85.01)   
89 - **[TCGCGGGG]** 201 (87.42)   
89 - **[CCGCCTCG]** 175 (88.56)   
90 + **[TCCCGCCG]** 452 (87.33)   
90 + **[ACGCGCCG]** 714 (86.10)   
90 + **[CCGCGGCC]** 719 (85.01)   
90 + **.CCGCGGCG.** 776 (92.56)   
90 + **[GCGGGTCG]** 837 (86.33)   
90 + **.CCGCGGCG.** 849 (92.56)   
90 + **[CCGCCGCG]** 1017 (85.35)   
90 + **[TCGCCCCG]** 1044 (88.90)   
90 + **[TGGCGGCG]** 1115 (87.81)   
90 - **[TGGCGCCG]** 1258 (87.13)   
90 - **[TCGGGGCG]** 1045 (87.91)   
90 - **.CCGCGGCG.** 1018 (92.56)   
90 - **[TCGCCCCG]** 982 (88.90)   
90 - **.TCGCCTCG.** 951 (92.78)   
90 - **[CCGCCGCG]** 850 (85.35)   
90 - **[CCGCGGCC]** 847 (85.01)   
90 - **[CCGCCGCG]** 777 (85.35)   
90 - **[CCGCGGCC]** 774 (85.01)   
90 - **.CCGCGGCG.** 717 (92.56)   
90 - **[GCGCGTGG]** 712 (85.84)   
90 - **[CCGCGGCC]** 586 (85.01)   
92 + **.GCGCGGCG.** 1227 (91.99)   
92 - **[TCCCGGCG]** 1404 (88.00)   
92 - **[CCGCCGCG]** 1228 (85.35)   
93 + **[TCCCGCCG]** 678 (87.33)   
93 + **[TCGCGTCA]** 984 (87.59)   
93 + **[ACGCGCCG]** 1002 (86.10)   
93 + **.CCGCGGCG.** 1036 (92.56)   
93 + **[TGGCGGCG]** 1426 (87.81)   
93 + **[CCGCGTCT]** 1487 (85.23)   
93 - **[GCGCGTGG]** 1191 (85.84)   
93 - **[TCGCCTCC]** 1145 (85.23)   
93 - **[TCGCCTCC]** 1063 (85.23)   
93 - **[TGGCGCCG]** 1042 (87.13)   
93 - **[CCGCCGCG]** 1037 (85.35)   
93 - **[GCGCGTCC]** 1000 (87.65)   
93 - **[GCGCGTCC]** 962 (87.65)   
96 + **.GCGCGTCG.** 739 (95.20)   
96 + **[GCGGGTCG]** 1346 (86.33)   
96 + **.GCGCGTCG.** 1353 (95.20)   
96 + **.TCGCGTCG.** 1358 (100.00)   
96 + **.CCGCGCCG.** 1421 (91.89)   
96 - **[CCGCGTCC]** 1392 (88.23)   
97 + **.GCGCGGCG.** 876 (91.99)   
97 + **[CGGCGTCG]** 1440 (86.80)   
97 - **[CCGCGTCC]** 1366 (88.23)   
97 - **.CCGCGCCG.** 879 (91.89)   
97 - **[TGGCGGCG]** 854 (87.81)   
98 + **[TGGCGGCG]** 974 (87.81)   
98 + **.CCGCGGCG.** 1000 (92.56)   
98 + **.CCGCGGCG.** 1085 (92.56)   
98 + **.GCGCGGCG.** 1136 (91.99)   
98 + **[TGGCGGCG]** 1341 (87.81)   
98 - **.CCGCGCCG.** 1226 (91.89)   
98 - **[CCGCCGCG]** 1137 (85.35)   
98 - **[CCGCGTCC]** 1097 (88.23)   
98 - **[CCGCCGCG]** 1001 (85.35)   
98 - **[CCGCGGCC]** 998 (85.01)   
100 + **[CCGGGTCG]** 1098 (86.90)   
100 + **.TCGCGCCG.** 1103 (96.11)   
100 + **.CCGCGGCG.** 1499 (92.56)   
100 - **[CCGCCGCG]** 1500 (85.35)   
103 + **[CCGGGTCG]** 1178 (86.90)   
104 - **[TCGCGTCT]** 1139 (89.45)   
104 - **[TCGCGGGG]** 781 (87.42)   
104 - **[TCGCTGCG]** 748 (86.18)   
105 - **.TCGCCTCG.** 1508 (92.78)   
105 - **[TCGCCTCC]** 1333 (85.23)   
105 - **[TCGCGCCC]** 1021 (88.56)   
106 + **.TCGCGGCG.** 869 (96.78)   
106 + **.TCGCCTCG.** 1017 (92.78)   
106 + **.TCGCGGCG.** 1159 (96.78)   
108 + **.TCGCCTCG.** 387 (92.78)   
108 + **[CCGCGTCT]** 428 (85.23)   
108 + **[CCGCGTGG]** 741 (86.42)   
108 + **[CCGCGACG]** 766 (85.28)   
108 + **[CCCCGTCG]** 936 (87.00)   
108 + **[TCGGGGCG]** 1121 (87.91)   
108 + **[CCGCGTCC]** 1202 (88.23)   
108 + **.CCGCGCCG.** 1217 (91.89)   
108 + **[TCGCGGGG]** 1510 (87.42)   
108 - **[TCGCGCCT]** 1299 (85.56)   
108 - **[GCGCCTCG]** 1297 (87.99)   
108 - **[TGGCGCCG]** 1054 (87.13)   
108 - **[TCCCGCCG]** 942 (87.33)   
108 - **.TCGCGTCC.** 922 (92.45)   
108 - **[GCGCGTCC]** 817 (87.65)   
108 - **[CCCCGTCG]** 590 (87.00)   
108 - **[TCGGGCCG]** 585 (87.23)   
108 - **[TCCCGGCG]** 448 (88.00)   
108 - **[CCGCCTCG]** 311 (88.56)   
109 + **[TCGCCGCG]** 335 (89.57)   
109 + **[CCGCGGCC]** 338 (85.01)   
109 + **[GCGCCTCG]** 518 (87.99)   
109 + **[TCGGGCCG]** 523 (87.23)   
109 + **[TCGCGCCC]** 658 (88.56)   
109 + **[TCCCGGCG]** 955 (88.00)   
109 + **[TCCCGGCG]** 1013 (88.00)   
109 + **[CCGCGTCC]** 1031 (88.23)   
109 + **[CGGCGTCG]** 1089 (86.80)   
109 + **[TCGCTGCG]** 1206 (86.18)   
109 + **[ACGCGCCG]** 1232 (86.10)   
109 + **.GCGCGGCG.** 1358 (91.99)   
109 + **.GCGCGGCG.** 1390 (91.99)   
109 + **.GCGCGGCG.** 1395 (91.99)   
109 + **[TGGCGGCG]** 1490 (87.81)   
109 - **[CCGCCGCG]** 1396 (85.35)   
109 - **.CCGCGCCG.** 1393 (91.89)   
109 - **.GCGCGTCG.** 1230 (95.20)   
109 - **[CGGCGTCG]** 1184 (86.80)   
109 - **[GCGCGTCC]** 813 (87.65)   
109 - **[TGGCGCCG]** 740 (87.13)   
109 - **[TCGCGGGG]** 597 (87.42)   
109 - **.CCGCGGCG.** 336 (92.56)   
110 - **[TGGCGGCG]** 931 (87.81)   
114 + **[GCGGGTCG]** 255 (86.33)   
114 + **.GCGCGGCG.** 1003 (91.99)   
114 + **[TGGCGCCG]** 1100 (87.13)   
114 + **.CCGCGGCG.** 1307 (92.56)   
114 - **[TCGCCGCG]** 1308 (89.57)   
114 - **.TCGCGTCC.** 1018 (92.45)   
114 - **.CCGCGCCG.** 1006 (91.89)   
115 + **.CCGCGGCG.** 412 (92.56)   
115 + **[GCGCCTCG]** 460 (87.99)   
115 + **.TCGCGTCG.** 465 (100.00)   
115 + **[CCGCCTCG]** 494 (88.56)   
115 + **.CCGCGCCG.** 688 (91.89)   
115 + **[TGGCGGCG]** 983 (87.81)   
115 - **[CCGCGACG]** 1235 (85.28)   
115 - **[TCGCGCGG]** 1038 (86.75)   
115 - **[TCCCGCCG]** 995 (87.33)   
115 - **[TCCCGCCG]** 949 (87.33)   
115 - **.GCGCGTCG.** 869 (95.20)   
115 - **.CCGCGGCG.** 309 (92.56)   
115 - **.CCGCGCCG.** 300 (91.89)   
115 - **.GCGCGCCG.** 280 (91.32)   
115 - **[TCGCGCCT]** 223 (85.56)   
116 + **[CCGCGGCC]** 1200 (85.01)   
116 - **[TGGCGGCG]** 1004 (87.81)   
116 - **.GCGCGTCG.** 960 (95.20)   
117 + **[TGGCGCCG]** 695 (87.13)   
117 + **[CCGCGGCC]** 1055 (85.01)   
117 - **.GCGCGGCG.** 791 (91.99)   
117 - **.GCGCGCCG.** 666 (91.32)   
117 - **[GCGCCTCG]** 196 (87.99)   
118 + **[TGGCGCCG]** 673 (87.13)   
118 + **[CCGCGGCC]** 1044 (85.01)   
118 - **.GCGCGGCG.** 767 (91.99)   
118 - **[GCGCCTCG]** 98 (87.99)   
119 + **[TGGCGCCG]** 671 (87.13)   
119 + **[CCGCGGCC]** 1035 (85.01)   
119 - **.GCGCGGCG.** 765 (91.99)   
119 - **.GCGCGCCG.** 640 (91.32)   
119 - **.TCGCGCCG.** 612 (96.11)   
119 - **[GCGCCTCG]** 96 (87.99)   
120 + **[CCGCGGCC]** 1502 (85.01)   
120 - **.GCGCGGCG.** 1225 (91.99)   
120 - **.GCGCGCCG.** 1100 (91.32)   
120 - **[GCGCCTCG]** 555 (87.99)   
122 + **.GCGCGCCG.** 31 (91.32)   
122 - **.GCGCGCCG.** 901 (91.32)   
123 + **[GCGCGTCC]** 391 (87.65)   
123 + **[CCGCCGCG]** 600 (85.35)   
123 + **[TCCCGGCG]** 672 (88.00)   
123 + **.GCGCGCCG.** 768 (91.32)   
123 + **.CCGCGGCG.** 951 (92.56)   
123 + **[TCGCCGCG]** 1003 (89.57)   
123 + **.CCGCGCCG.** 1006 (91.89)   
123 + **.CCGCGCCG.** 1011 (91.89)   
123 + **[CGGCGTCG]** 1072 (86.80)   
123 + **[CCGGGTCG]** 1367 (86.90)   
123 - **.GCGCGGCG.** 1009 (91.99)   
123 - **.GCGCGGCG.** 1004 (91.99)   
123 - **[TCGCCGCG]** 952 (89.57)   
123 - **[TCGCGGCT]** 825 (86.24)   
123 - **.GCGCGCCG.** 766 (91.32)   
123 - **.CCGCGCCG.** 742 (91.89)   
123 - **.TCGCGGCG.** 601 (96.78)   
123 - **[GCGGGTCG]** 514 (86.33)   
123 - **.CCGCGGCG.** 14 (92.56)   
124 + **[CGGCGTCG]** 1098 (86.80)   
124 + **[TCGGGCCG]** 1200 (87.23)   
124 - **[CCGCGGCC]** 1223 (85.01)   
124 - **[CCGCCTCG]** 1206 (88.56)   
124 - **.GCGCGCCG.** 1148 (91.32)   
124 - **[TCGCTGCG]** 1090 (86.18)   
124 - **.CCGCGCCG.** 879 (91.89)   
124 - **.CCGCGCCG.** 867 (91.89)   
124 - **.TCGCGTCG.** 827 (100.00)   
127 + **[TCCCGGCG]** 582 (88.00)   
127 + **[TCGCCCCG]** 902 (88.90)   
127 + **[GGGCGTCG]** 1339 (86.23)   
127 + **[CCGCGTCT]** 1510 (85.23)   
127 - **[CCGGGTCG]** 1149 (86.90)   
127 - **[GCGCGTCC]** 1028 (87.65)   
127 - **.GCGCGCCG.** 775 (91.32)   
127 - **[GCGCCTCG]** 741 (87.99)   
127 - **[GCGCCTCG]** 627 (87.99)   
127 - **[CCGCGTGG]** 466 (86.42)   
128 + **[ACGCGGCG]** 407 (86.77)   
128 + **[GCGGGTCG]** 428 (86.33)   
128 + **[CCGGGTCG]** 827 (86.90)   
128 + **[TCGCGACG]** 937 (89.50)   
128 + **[TCACGTCG]** 1348 (87.82)   
128 - **.TCCCGTCG.** 858 (91.22)   
128 - **[GCGGGTCG]** 780 (86.33)   
128 - **[TCGCCTCC]** 775 (85.23)   
128 - **.TCGCCTCG.** 624 (92.78)   
128 - **[TCTCGTCG]** 133 (87.92)   
128 - **[TCGCGTCT]** 30 (89.45)   
129 + **.TCGCCTCG.** 778 (92.78)   
129 + **.TCGCGGCG.** 1140 (96.78)   
129 + **.CCGCGCCG.** 1201 (91.89)   
129 + **[CCGCCGCG]** 1318 (85.35)   
129 + **[CCGCGGCC]** 1385 (85.01)   
129 + **[CCGGGTCG]** 1391 (86.90)   
129 + **.CCGCGCCG.** 1401 (91.89)   
129 - **[TGGCGCCG]** 1437 (87.13)   
129 - **.GCGCGGCG.** 1319 (91.99)   
129 - **.CCGCGCCG.** 1143 (91.89)   
129 - **[CCGCGTCC]** 1056 (88.23)   
129 - **[ACGCGGCG]** 1015 (86.77)   
129 - **[GCGCCTCG]** 963 (87.99)   
129 - **[TCGCGCGG]** 958 (86.75)   
129 - **[TCGCGCGG]** 896 (86.75)   
129 - **[CCGCGGCC]** 869 (85.01)   
129 - **[GCGGGTCG]** 583 (86.33)   
130 + **[TCGCATCG]** 752 (86.74)   
130 - **[TCGCGGCT]** 1108 (86.24)   
131 + **.GCGCGCCG.** 32 (91.32)   
131 + **[CCGCGTGG]** 613 (86.42)   
131 + **[TCGCGGCC]** 1116 (89.23)   
131 + **[ACGCGGCG]** 1305 (86.77)   
131 + **.CCGCGGCG.** 1378 (92.56)   
131 + **.CCGCGGCG.** 1487 (92.56)   
131 - **[CCGCCGCG]** 1488 (85.35)   
131 - **[CCGCCGCG]** 1379 (85.35)   
131 - **[CCGCCGCG]** 1306 (85.35)   
131 - **[TCGAGTCG]** 829 (87.13)   
132 + **[CCGCCGCG]** 708 (85.35)   
132 + **[CCGCGTGG]** 711 (86.42)   
132 + **[TCGCCCCG]** 944 (88.90)   
132 + **.CCGCGCCG.** 1223 (91.89)   
132 + **[GCGGGTCG]** 1438 (86.33)   
132 - **[ACGCGGCG]** 709 (86.77)   
134 + **[TCGCGCGG]** 984 (86.75)   
134 + **.GCGCGTCG.** 1034 (95.20)   
135 + **[TCGCGCGG]** 255 (86.75)   
135 + **.CCGCGGCG.** 364 (92.56)   
135 + **[GCGCCTCG]** 469 (87.99)   
135 + **[TCGCCTCC]** 474 (85.23)   
135 + **[TCGCCGCG]** 786 (89.57)   
135 + **[TCCCGCCG]** 874 (87.33)   
135 + **[TCCCGGCG]** 1020 (88.00)   
135 + **[CCGGGTCG]** 1253 (86.90)   
135 + **[TGGCGGCG]** 1278 (87.81)   
135 + **[CCGCCGCG]** 1498 (85.35)   
135 - **.GCGCGGCG.** 1499 (91.99)   
135 - **[TCGCTCCG]** 1419 (85.50)   
135 - **[ACGCGGCG]** 787 (86.77)   
135 - **[GCGCGTGG]** 352 (85.84)   
136 + **[TCCCGGCG]** 1035 (88.00)   
136 + **.GCGCGCCG.** 1064 (91.32)   
136 + **[CCGCGGCC]** 1195 (85.01)   
136 - **[TCGGGGCG]** 1217 (87.91)   
136 - **.CCGCGCCG.** 1038 (91.89)   
136 - **.CCGCGCCG.** 935 (91.89)   
137 + **[TCGGGGCG]** 507 (87.91)   
137 + **[ACGCGGCG]** 944 (86.77)   
137 - **.TCGGGTCG.** 1327 (91.12)   
137 - **[CCGCGTCT]** 1013 (85.23)   
137 - **[CCGCCGCG]** 945 (85.35)   
137 - **[CCGCGTCC]** 942 (88.23)   
137 - **[CCGCGTCC]** 751 (88.23)   
137 - **[TCGCATCG]** 639 (86.74)   
138 + **.TCGCGCCG.** 191 (96.11)   
138 + **[ACGCGGCG]** 366 (86.77)   
138 + **.GCGCGGCG.** 578 (91.99)   
138 + **.GCGCGTCG.** 600 (95.20)   
138 + **.GCGCGGCG.** 690 (91.99)   
138 + **.CCGCGGCG.** 762 (92.56)   
138 + **.GCGCGGCG.** 767 (91.99)   
138 + **[CCGCCGCG]** 836 (85.35)   
138 - **[TCGCGGGG]** 935 (87.42)   
138 - **.GCGCGGCG.** 837 (91.99)   
138 - **.CCGCGCCG.** 765 (91.89)   
138 - **[CCGCCGCG]** 691 (85.35)   
138 - **.CCGCGCCG.** 640 (91.89)   
138 - **[TCGCCGCG]** 579 (89.57)   
138 - **.GCGCGCCG.** 574 (91.32)   
138 - **.CCGCGCCG.** 369 (91.89)   
138 - **[CCGCGTCC]** 364 (88.23)   
139 + **[TCGCGTAG]** 135 (86.48)   
139 + **[TCGAGTCG]** 657 (87.13)   
139 + **.GCGCGCCG.** 831 (91.32)   
139 + **[CCGCGTCC]** 846 (88.23)   
139 + **.GCGCGGCG.** 959 (91.99)   
139 - **.TCGCGTGG.** 761 (90.64)   
139 - **[TGGCGCCG]** 715 (87.13)   
139 - **.CCGCGCCG.** 689 (91.89)   
140 + **[CCGCCGCG]** 655 (85.35)   
140 + **[CCGCGTCC]** 983 (88.23)   
140 + **[CCGCCGCG]** 1191 (85.35)   
140 + **.CCGCGCCG.** 1194 (91.89)   
140 + **.CCGCGGCG.** 1247 (92.56)   
140 + **[TGGCGGCG]** 1459 (87.81)   
140 + **[TGGCGGCG]** 1485 (87.81)   
140 - **[TCGCCCCG]** 1410 (88.90)   
140 - **[CGGCGTCG]** 1366 (86.80)   
140 - **[TCGCCGCG]** 1248 (89.57)   
140 - **[TGGCGGCG]** 1197 (87.81)   
140 - **.GCGCGGCG.** 1192 (91.99)   
140 - **.CCGCGGCG.** 656 (92.56)   
140 - **[CCGGGTCG]** 637 (86.90)   
140 - **[TCGCTGCG]** 632 (86.18)   
141 + **[TCGCGGCC]** 683 (89.23)   
141 + **.CCGCGGCG.** 1157 (92.56)   
141 + **.CCGCGCCG.** 1184 (91.89)   
141 - **.TCGCGTCC.** 1388 (92.45)   
141 - **[CCGCGTCC]** 1227 (88.23)   
141 - **.CCGCGGCG.** 1187 (92.56)   
141 - **[CCGCGGCC]** 1155 (85.01)   
141 - **.GCGCGCCG.** 946 (91.32)   
141 - **[GCGCCTCG]** 753 (87.99)   
141 - **.GCGCGGCG.** 609 (91.99)   
142 + **.CCGCGCCG.** 512 (91.89)   
142 + **.GCGCGGCG.** 662 (91.99)   
142 + **[TCGCTGCG]** 774 (86.18)   
142 + **.GCGCGCCG.** 901 (91.32)   
142 + **[CCGCGGCC]** 945 (85.01)   
142 + **.TCGCGCCG.** 957 (96.11)   
142 + **[TCGCCGCG]** 1004 (89.57)   
142 + **[CCGGGTCG]** 1097 (86.90)   
142 + **.CCGCGCCG.** 1273 (91.89)   
142 + **[CCGCCTCG]** 1318 (88.56)   
142 + **[TCGGGGCG]** 1333 (87.91)   
142 - **[CCGCGTCC]** 1477 (88.23)   
142 - **[CCGGGTCG]** 1439 (86.90)   
142 - **[TCGCGCGG]** 1415 (86.75)   
142 - **[GCGCCTCG]** 1369 (87.99)   
142 - **[TCGCTGCG]** 1087 (86.18)   
142 - **[CCGCGGCC]** 1060 (85.01)   
142 - **.GCGCGGCG.** 1005 (91.99)   
142 - **.GCGCGGCG.** 893 (91.99)   
142 - **[TCGCGCCT]** 841 (85.56)   
142 - **[CCGCGGCC]** 671 (85.01)   
142 - **[CCGCCGCG]** 663 (85.35)   
142 - **[TCGCGGCT]** 624 (86.24)   
142 - **[CCGCCTCG]** 587 (88.56)   
142 - **.CCGCGGCG.** 515 (92.56)   
142 - **[GCGCGTGG]** 454 (85.84)   
142 - **.TCGCGTCG.** 446 (100.00)   
144 + **[GCGCGTCC]** 773 (87.65)   
144 + **[GCGCGTCC]** 838 (87.65)   
144 + **.GCGCGGCG.** 1141 (91.99)   
144 + **.GCGCGGCG.** 1146 (91.99)   
144 + **[ACGCGGCG]** 1188 (86.77)   
144 - **.GCGCGGCG.** 1490 (91.99)   
144 - **[CCGCGGCC]** 1197 (85.01)   
144 - **.CCGCGCCG.** 1191 (91.89)   
144 - **[CCGCGTCC]** 1186 (88.23)   
144 - **.CCGCGCCG.** 1149 (91.89)   
144 - **.CCGCGCCG.** 1144 (91.89)   
144 - **.CCGCGCCG.** 1139 (91.89)   
144 - **.GCGCGCCG.** 1032 (91.32)   
144 - **.GCGCGGCG.** 1017 (91.99)   
145 + **[TCCCGCCG]** 1080 (87.33)   
145 + **[CCGCCGCG]** 1135 (85.35)   
145 + **.GCGCGGCG.** 1140 (91.99)   
145 + **.GCGCGCCG.** 1149 (91.32)   
145 + **[CCGCGACG]** 1216 (85.28)   
145 + **[CCGCGACG]** 1252 (85.28)   
145 - **[TCACGTCG]** 1219 (87.82)   
145 - **[CCGCCGCG]** 1141 (85.35)   
145 - **.GCGCGGCG.** 1136 (91.99)   
145 - **[TCGCGCCC]** 653 (88.56)   
147 + **[TCGCGGGG]** 964 (87.42)   
147 + **[TGGCGGCG]** 1067 (87.81)   
147 + **[TCGCCGCG]** 1161 (89.57)   
147 + **[CGGCGTCG]** 1337 (86.80)   
147 - **.GCGCGGCG.** 1162 (91.99)   
147 - **[CCGCCTCG]** 1077 (88.56)   
147 - **[CCGCGACG]** 962 (85.28)   
147 - **[ACGCGTCG]** 861 (89.99)   
147 - **[TCGGGGCG]** 856 (87.91)   
148 + **[GCCCGTCG]** 1393 (86.42)   
148 + **[TCGCCTCC]** 1398 (85.23)   
148 - **.GCGCGGCG.** 827 (91.99)   
148 - **.TGGCGTCG.** 438 (91.02)   
150 + **.GCGCGCCG.** 117 (91.32)   
150 + **.TCGCGGCG.** 190 (96.78)   
150 + **.TCCCGTCG.** 689 (91.22)   
150 + **[TGGCGGCG]** 912 (87.81)   
150 + **[TCCCGGCG]** 1386 (88.00)   
150 - **[TGGCGCCG]** 1389 (87.13)   
150 - **[CCGCGTCT]** 464 (85.23)   
150 - **.GCGCGGCG.** 120 (91.99)   
151 + **[CCGCGTCT]** 539 (85.23)   
151 + **[TCGCCCCG]** 767 (88.90)   
151 + **.GCGCGGCG.** 883 (91.99)   
151 + **.CCGCGCCG.** 1205 (91.89)   
151 + **[TCGCGGCT]** 1251 (86.24)   
151 + **[GCGCGTGG]** 1462 (85.84)   
151 + **[CCGCGTCC]** 1472 (88.23)   
151 + **[TGGCGGCG]** 1488 (87.81)   
151 - **.GCGCGCCG.** 1491 (91.32)   
151 - **[ACGCGCCG]** 1460 (86.10)   
151 - **[CCGCGTGG]** 1420 (86.42)   
151 - **[CCGGGTCG]** 1065 (86.90)   
151 - **.TCGCGGCG.** 814 (96.78)   
151 - **.TCCCGTCG.** 314 (91.22)   
151 - **[TGGCGGCG]** 92 (87.81)   
154 + **[CCGGGTCG]** 97 (86.90)   
154 + **.GCGCGGCG.** 1039 (91.99)   
154 + **[TGGCGCCG]** 1175 (87.13)   
154 + **.CCGCGCCG.** 1431 (91.89)   
154 + **.GCGCGCCG.** 1465 (91.32)   
154 - **.TCGCGGCG.** 1178 (96.78)   
154 - **[TCGCTCCG]** 1115 (85.50)   
154 - **.GCGCGCCG.** 1095 (91.32)   
154 - **[CCGCCGCG]** 1040 (85.35)   
155 + **[GGGCGTCG]** 1253 (86.23)   
155 + **[TCGCGTTG]** 1258 (87.97)   
155 - **.CCGCGCCG.** 506 (91.89)   
157 + **[CCGCGTGG]** 1237 (86.42)   
158 + **.GCGCGTCG.** 1122 (95.20)   
158 + **.CCGCGTCG.** 1178 (95.78)   
158 - **.GCGCGCCG.** 1511 (91.32)   
158 - **.TCGCGTCC.** 1241 (92.45)   
160 + **[CCGCGTGG]** 134 (86.42)   
160 + **[CCGCCGCG]** 264 (85.35)   
160 + **[GCGCCTCG]** 276 (87.99)   
160 + **[TCGCGTCT]** 781 (89.45)   
160 + **.CCGCGCCG.** 1205 (91.89)   
160 + **[TCCCGGCG]** 1426 (88.00)   
160 - **[GGGCGTCG]** 1268 (86.23)   
160 - **.CCGCGGCG.** 265 (92.56)   
161 + **[TCGTGTCG]** 396 (85.92)   
161 + **[TCGGGGCG]** 456 (87.91)   
161 + **[CCGCCGCG]** 892 (85.35)   
161 + **[TGGCGGCG]** 980 (87.81)   
161 + **[TGGCGCCG]** 1014 (87.13)   
161 + **[TCGCGCCC]** 1190 (88.56)   
161 + **[TCGCGGCC]** 1220 (89.23)   
161 + **.CCGCGGCG.** 1267 (92.56)   
161 + **[TGGCGCCG]** 1409 (87.13)   
161 + **.CCGCGCCG.** 1480 (91.89)   
161 - **[TGGCGGCG]** 1492 (87.81)   
161 - **[TGGCGGCG]** 1425 (87.81)   
161 - **[TCCCGGCG]** 1412 (88.00)   
161 - **[CCGCCGCG]** 1268 (85.35)   
161 - **.GCGCGGCG.** 893 (91.99)   
161 - **[CCGCGGCC]** 883 (85.01)   
161 - **.TCGCGCCG.** 751 (96.11)   
161 - **[GCGCGTGG]** 315 (85.84)   
161 - **.CCGCGCCG.** 153 (91.89)   
164 + **[TCGCTCCG]** 989 (85.50)   
164 + **[GCGCCTCG]** 1044 (87.99)   
164 + **.GCGCGGCG.** 1098 (91.99)   
164 + **[TCGCCTCC]** 1246 (85.23)   
164 - **.CCGCGCCG.** 1096 (91.89)   
165 + **[TCGCGCCT]** 580 (85.56)   
166 + **[TCGCGGCT]** 660 (86.24)   
166 + **[TCGCCTCC]** 873 (85.23)   
166 + **[CCGCCTCG]** 925 (88.56)   
166 + **[ACGCGGCG]** 1158 (86.77)   
166 + **[TCGCGCCC]** 1180 (88.56)   
166 + **[ACGCGGCG]** 1311 (86.77)   
166 - **.GCGCGCCG.** 1224 (91.32)   
166 - **[CCGCGGCC]** 545 (85.01)   
167 + **.CCGCGGCG.** 842 (92.56)   
167 + **[TCGCGGCC]** 955 (89.23)   
167 + **[CCGCGGCC]** 962 (85.01)   
167 + **[CCGCGTCC]** 1462 (88.23)   
167 - **[TCGCCTCC]** 1271 (85.23)   
167 - **[TCGGGCCG]** 982 (87.23)   
167 - **[CCGCCGCG]** 843 (85.35)   
167 - **.TCGCGCCG.** 817 (96.11)   
167 - **[CCGCCTCG]** 792 (88.56)   
167 - **.TCGGGTCG.** 787 (91.12)   
167 - **[CCGCGTCT]** 769 (85.23)   
167 - **[TCGCGTCT]** 730 (89.45)   
168 + **[TCGCCGCG]** 907 (89.57)   
168 + **[CCGCGGCC]** 1204 (85.01)   
168 + **[TCGGGGCG]** 1427 (87.91)   
168 - **[TCGCGGCC]** 1434 (89.23)   
168 - **[TCGCTCCG]** 1318 (85.50)   
168 - **[TCGCGGGG]** 1246 (87.42)   
168 - **.CCGCGCCG.** 1182 (91.89)   
168 - **.GCGCGGCG.** 908 (91.99)   
169 + **[TCGCTTCG]** 801 (89.39)   
169 + **[TCGCCTCC]** 893 (85.23)   
169 - **[CCGCGTCC]** 1044 (88.23)   
169 - **[TCGCGCCC]** 870 (88.56)   
169 - **.GCGCGGCG.** 110 (91.99)   
170 - **[TCGCCTCC]** 1258 (85.23)   
171 + **[ACGCGGCG]** 773 (86.77)   
171 - **[TGGCGCCG]** 825 (87.13)   
171 - **[CCGCGTGG]** 771 (86.42)   
172 + **[ACGCGGCG]** 430 (86.77)   
172 + **[TCGCGCCC]** 550 (88.56)   
172 + **.TCCCGTCG.** 599 (91.22)   
172 + **[CCGCGACG]** 715 (85.28)   
172 + **[CCGCCTCG]** 828 (88.56)   
172 + **[TGGCGGCG]** 913 (87.81)   
172 + **.GCGCGCCG.** 923 (91.32)   
172 + **[TCGCTCCG]** 1240 (85.50)   
172 + **.GCGCGCCG.** 1418 (91.32)   
172 - **.CCGCGCCG.** 1159 (91.89)   
172 - **[CCGCTTCG]** 1088 (85.17)   
172 - **[CCGCTTCG]** 1045 (85.17)   
172 - **.GCGCGCCG.** 919 (91.32)   
172 - **[TCGCCCCG]** 859 (88.90)   
172 - **[CCGCGGCC]** 754 (85.01)   
172 - **[CGGCGTCG]** 718 (86.80)   
172 - **[TCGCGGCC]** 713 (89.23)   
172 - **[CCGCCGCG]** 431 (85.35)   
172 - **[CCGCGTCC]** 428 (88.23)   
173 + **[GCGCCTCG]** 946 (87.99)   
173 - **[GGGCGTCG]** 1190 (86.23)   
173 - **[CCGGGTCG]** 894 (86.90)   
173 - **[TCTCGTCG]** 652 (87.92)   
174 + **[TCGCGCCT]** 213 (85.56)   
174 + **[TGGCGGCG]** 832 (87.81)   
174 + **[CCGCGTCC]** 1186 (88.23)   
174 + **.CCGCGGCG.** 1476 (92.56)   
174 - **[GCGCCTCG]** 1502 (87.99)   
174 - **.CCGCGCCG.** 1479 (91.89)   
174 - **[CCGCGTCT]** 1379 (85.23)   
174 - **[TCGCTGCG]** 1322 (86.18)   
174 - **[TCGCGGCC]** 1202 (89.23)   
174 - **[ACGCGGCG]** 1184 (86.77)   
174 - **[TCGCGGCC]** 1144 (89.23)   
174 - **.TCGGGTCG.** 1111 (91.12)   
174 - **[CCGCGTCT]** 935 (85.23)   
174 - **[TCGCGCCC]** 782 (88.56)   
174 - **[TCGCGCCC]** 395 (88.56)   
175 - **.CCGCGGCG.** 1465 (92.56)   
177 + **[GCGCCTCG]** 820 (87.99)   
177 + **[TCGCCGCG]** 950 (89.57)   
177 + **[CCCCGTCG]** 1014 (87.00)   
177 + **[CCGCCTCG]** 1031 (88.56)   
177 + **.CCGCGGCG.** 1464 (92.56)   
177 - **[TCGCCGCG]** 1465 (89.57)   
177 - **.TCGCGGCG.** 951 (96.78)   
177 - **[CCGCCTCG]** 747 (88.56)   
178 + **[ACGCGGCG]** 1113 (86.77)   
178 + **[TCGCGGGG]** 1238 (87.42)   
178 - **[TCGCCTCC]** 1439 (85.23)   
178 - **[CCGCCGCG]** 1114 (85.35)   
178 - **[CCGCGTCC]** 1111 (88.23)   
179 + **.CCGCGCCG.** 857 (91.89)   
179 + **[TCGCCGCG]** 914 (89.57)   
179 + **[CCGCGGCC]** 917 (85.01)   
179 + **[TCCCGGCG]** 933 (88.00)   
179 + **.TCGCGTCC.** 1203 (92.45)   
179 + **[CCGCCGCG]** 1472 (85.35)   
179 - **.GCGCGGCG.** 1473 (91.99)   
179 - **[GCGCGTCC]** 993 (87.65)   
179 - **.CCGCGCCG.** 985 (91.89)   
179 - **.CCGCGGCG.** 915 (92.56)   
180 + **[TCGTGTCG]** 395 (85.92)   
180 + **[TGGCGGCG]** 1040 (87.81)   
180 + **.GCGCGGCG.** 1064 (91.99)   
180 + **[CCGCGGCC]** 1158 (85.01)   
180 + **.TCGCGGCG.** 1246 (96.78)   
180 + **[TCGCGGCT]** 1312 (86.24)   
180 - **[CCGCCGCG]** 1247 (85.35)   
180 - **[CCGCCGCG]** 1065 (85.35)   
180 - **[TCGCTTCG]** 285 (89.39)   
180 - **.TCCCGTCG.** 103 (91.22)   
182 + **[CCGCGGCC]** 376 (85.01)   
182 + **.CCGCGGCG.** 462 (92.56)   
182 + **[GCGGGTCG]** 534 (86.33)   
182 + **[CCGCCTCG]** 684 (88.56)   
182 + **.GCGCGTCG.** 875 (95.20)   
182 + **.TCGCGTCC.** 919 (92.45)   
182 + **[CCGCGTCC]** 1020 (88.23)   
182 + **[CCGCGGCC]** 1077 (85.01)   
182 + **.TCGCGCCG.** 1162 (96.11)   
182 - **[TCCCGGCG]** 1305 (88.00)   
182 - **[TCGCCCCG]** 1252 (88.90)   
182 - **[TCGCGCCC]** 1101 (88.56)   
182 - **[ACGCGCCG]** 873 (86.10)   
182 - **[TCGCGGCC]** 747 (89.23)   
182 - **.CCGCGCCG.** 465 (91.89)   
182 - **[CCGCGGCC]** 460 (85.01)   
182 - **[CCGCGGCC]** 374 (85.01)   
182 - **[CCGCGTGG]** 366 (86.42)   
182 - **.CCGCGCCG.** 120 (91.89)   
183 + **[TGGCGGCG]** 603 (87.81)   
183 + **.GCGCGGCG.** 1060 (91.99)   
183 + **[CCGCGTCC]** 1300 (88.23)   
183 + **.GCGCGGCG.** 1355 (91.99)   
183 + **.CCGCGCCG.** 1423 (91.89)   
183 - **[CCGCCGCG]** 1061 (85.35)   
183 - **.GCGCGCCG.** 982 (91.32)   
183 - **[GCGGGTCG]** 713 (86.33)   
184 + **[CCGCCGCG]** 52 (85.35)   
184 + **[CCGCGGCC]** 632 (85.01)   
184 + **.GCGCGGCG.** 927 (91.99)   
184 + **[CCGCGGCC]** 1067 (85.01)   
184 + **.GCGCGCCG.** 1086 (91.32)   
184 + **.GCGCGGCG.** 1351 (91.99)   
184 - **[CCGCCGCG]** 1352 (85.35)   
184 - **.GCGCGGCG.** 1164 (91.99)   
184 - **[ACGCGGCG]** 1089 (86.77)   
184 - **[TCGGGCCG]** 1003 (87.23)   
184 - **[TCGCCCCG]** 703 (88.90)   
184 - **[TCGCCTCC]** 495 (85.23)   
184 - **.CCGCGGCG.** 53 (92.56)   
185 + **[TCGCTCCG]** 936 (85.50)   
185 + **.CCGCGCCG.** 1033 (91.89)   
185 + **[CCGCCGCG]** 1188 (85.35)   
185 + **.CCGCGGCG.** 1191 (92.56)   
185 - **[CCGCCGCG]** 1192 (85.35)   
185 - **.CCGCGGCG.** 1189 (92.56)   
185 - **[CCGCGGCC]** 1089 (85.01)   
185 - **[CCGCGGCC]** 1062 (85.01)   
185 - **[CCGCGGCC]** 554 (85.01)   
185 - **[TCCCGCCG]** 313 (87.33)   
185 - **[CGGCGTCG]** 220 (86.80)   
185 - **[TCGCTGCG]** 215 (86.18)   
186 + **.CCGCGCCG.** 1011 (91.89)   
186 + **.GCGCGTCG.** 1081 (95.20)   
187 + **[TCGCCCCG]** 862 (88.90)   
187 + **[GCGCGTCC]** 924 (87.65)   
187 + **[CCGGGTCG]** 1070 (86.90)   
187 + **[TCGCGGGG]** 1236 (87.42)   
187 - **.CCGCGCCG.** 1175 (91.89)   
187 - **[TCGCGCCT]** 1124 (85.56)   
187 - **[TCGTGTCG]** 892 (85.92)   
187 - **[TCGGGGCG]** 863 (87.91)   
188 + **.GCGCGCCG.** 1439 (91.32)   
188 + **.CCGCGCCG.** 1471 (91.89)   
188 - **.GCGCGGCG.** 1469 (91.99)   
188 - **.CCGCGGCG.** 1442 (92.56)   
188 - **[CCGCGGCC]** 413 (85.01)   
189 + **[TCGCCTCC]** 1059 (85.23)   
189 - **[TCGCCTCC]** 1334 (85.23)   
189 - **[TCGCTGCG]** 1150 (86.18)   
189 - **[ACGCGCCG]** 848 (86.10)   
191 + **[TCGGGGCG]** 93 (87.91)   
191 + **[CCGCGGCC]** 568 (85.01)   
191 + **.GCGCGGCG.** 1044 (91.99)   
191 - **.TCGCGCCG.** 1136 (96.11)   
191 - **.CCGCGCCG.** 1042 (91.89)   
191 - **[CCGCGGCC]** 830 (85.01)   
191 - **[CGGCGTCG]** 333 (86.80)   
191 - **[TCGCGCGG]** 204 (86.75)   
191 - **[TCGCGCCC]** 96 (88.56)   
193 + **.GCGCGGCG.** 1248 (91.99)   
194 + **.TGGCGTCG.** 554 (91.02)   
194 + **.GCGCGGCG.** 911 (91.99)   
194 + **.GCGCGGCG.** 944 (91.99)   
194 + **.CCGCGGCG.** 989 (92.56)   
194 + **[TCCCGCCG]** 1036 (87.33)   
194 + **[CCGCCGCG]** 1038 (85.35)   
194 + **.CCGCGGCG.** 1111 (92.56)   
194 - **[TCGCCTCC]** 1158 (85.23)   
194 - **.CCGCGCCG.** 1114 (91.89)   
194 - **[CCGCGGCC]** 1109 (85.01)   
194 - **.CCGCGGCG.** 1039 (92.56)   
194 - **.GCGCGCCG.** 1015 (91.32)   
194 - **[TCGCCGCG]** 990 (89.57)   
194 - **[CCGCGGCC]** 987 (85.01)   
194 - **.CCGCGCCG.** 942 (91.89)   
194 - **[CCGCCGCG]** 912 (85.35)   
194 - **[GCGCGTCC]** 907 (87.65)   
194 - **[TCCCGGCG]** 856 (88.00)   
194 - **[TCGCGGGG]** 111 (87.42)   
195 + **.CCGCGCCG.** 152 (91.89)   
195 + **[CCGCCGCG]** 164 (85.35)   
195 + **[GGGCGTCG]** 445 (86.23)   
195 + **[GCGGGTCG]** 460 (86.33)   
195 + **.TCGCGGCG.** 851 (96.78)   
195 + **[GCGCGTCC]** 959 (87.65)   
195 + **[CCGGGTCG]** 1174 (86.90)   
195 - **[TGGCGGCG]** 1223 (87.81)   
195 - **[CCGCCTCG]** 308 (88.56)   
195 - **.CCGCGGCG.** 165 (92.56)   
196 + **.GCGCGGCG.** 119 (91.99)   
196 + **.GCGCGGCG.** 283 (91.99)   
196 + **[CGGCGTCG]** 286 (86.80)   
196 + **[TCGCCGCG]** 340 (89.57)   
196 + **[TCCCGGCG]** 465 (88.00)   
196 + **.GCGCGCCG.** 505 (91.32)   
196 + **[GGGCGTCG]** 638 (86.23)   
196 + **[ACGCGTCG]** 720 (89.99)   
196 + **.GCGCGCCG.** 1316 (91.32)   
196 - **.CCGCGCCG.** 1470 (91.89)   
196 - **.GCGCGCCG.** 1426 (91.32)   
196 - **.GCGCGCCG.** 1351 (91.32)   
196 - **.GCGCGGCG.** 910 (91.99)   
196 - **[CCGCGTCC]** 687 (88.23)   
196 - **[TCGCGCCC]** 665 (88.56)   
196 - **[CCGCGTGG]** 496 (86.42)   
196 - **.GCGCGGCG.** 341 (91.99)   
196 - **[TCCCGCCG]** 270 (87.33)   
196 - **[CCGCCGCG]** 120 (85.35)   
197 + **[TCGCTTCG]** 222 (89.39)   
197 + **.TCGCGTCG.** 430 (100.00)   
197 + **[TCGCGGCC]** 477 (89.23)   
197 + **[GCGGGTCG]** 1113 (86.33)   
197 + **[ACGCGGCG]** 1162 (86.77)   
197 + **.GCGCGGCG.** 1505 (91.99)   
197 - **.CCGCGCCG.** 1508 (91.89)   
197 - **[CCGCGTCC]** 1160 (88.23)   
197 - **[CCGGGTCG]** 1151 (86.90)   
197 - **[CCGCGTCT]** 1099 (85.23)   
197 - **[TCCCGCCG]** 1074 (87.33)   
197 - **[CCGCCTCG]** 1002 (88.56)   
197 - **[TCCCGCCG]** 874 (87.33)   
198 + **[CCGCCGCG]** 255 (85.35)   
198 + **[CCGGGTCG]** 992 (86.90)   
198 - **[CCGCCTCG]** 1234 (88.56)   
198 - **.TCCCGTCG.** 936 (91.22)   
198 - **.GCGCGGCG.** 256 (91.99)   
199 + **[CCGCCTCG]** 455 (88.56)   
199 + **.GCGCGTCG.** 820 (95.20)   
199 + **[TCCCGGCG]** 860 (88.00)   
199 + **[CCGGGTCG]** 892 (86.90)   
199 + **.GCGCGGCG.** 907 (91.99)   
199 + **.GCGCGGCG.** 950 (91.99)   
199 + **[TCGCCGCG]** 999 (89.57)   
199 + **[CCGCGGCC]** 1002 (85.01)   
199 + **.GCGCGGCG.** 1084 (91.99)   
199 + **[CCGCGGCC]** 1275 (85.01)   
199 + **[CCGCGTCC]** 1447 (88.23)   
199 - **.CCGCGCCG.** 1351 (91.89)   
199 - **[GCGCGTGG]** 1241 (85.84)   
199 - **[CCGCGGCC]** 1126 (85.01)   
199 - **[CCGCCGCG]** 1085 (85.35)   
199 - **.CCGCGCCG.** 1082 (91.89)   
199 - **.CCGCGGCG.** 1000 (92.56)   
199 - **.CCGCGCCG.** 910 (91.89)   
199 - **.GCGCGCCG.** 869 (91.32)   
199 - **.GCGCGCCG.** 669 (91.32)   
199 - **[CCGCGTGG]** 310 (86.42)   
200 + **[TGGCGGCG]** 1081 (87.81)   
200 - **[CCGCGTCC]** 571 (88.23)   
201 + **[CCGCCGCG]** 335 (85.35)   
201 + **[TGGCGGCG]** 715 (87.81)   
201 + **.CCGCGGCG.** 848 (92.56)   
201 + **[TCGCCCCG]** 1010 (88.90)   
201 + **.CCGCGCCG.** 1084 (91.89)   
201 + **[GCGCGTCC]** 1200 (87.65)   
201 - **[GCGCGTCC]** 1490 (87.65)   
201 - **.CCGCGGCG.** 1348 (92.56)   
201 - **[TCCCGGCG]** 982 (88.00)   
201 - **[CCGCCTCG]** 797 (88.56)   
201 - **.CCGCGCCG.** 775 (91.89)   
201 - **[TCCCGGCG]** 600 (88.00)   
201 - **.GCGCGGCG.** 336 (91.99)   
202 + **[CCGCGTCT]** 991 (85.23)   
202 + **[TCGGGCCG]** 1448 (87.23)   
202 - **[GCGCGTCC]** 1392 (87.65)   
202 - **[TCCCGGCG]** 1112 (88.00)   
202 - **[CCGCGTCC]** 309 (88.23)   
203 + **[CCCCGTCG]** 1032 (87.00)   
207 + **[GCGCGTCC]** 856 (87.65)   
207 - **.TCGCGTGG.** 1288 (90.64)   
208 + **.TCGCGCCG.** 1358 (96.11)   
208 + **[CCGCCGCG]** 1495 (85.35)   
208 + **.CCGCGGCG.** 1498 (92.56)   
208 - **[TCGCCGCG]** 1499 (89.57)   
208 - **.CCGCGGCG.** 1496 (92.56)   
208 - **.GCGCGCCG.** 1408 (91.32)   
208 - **[TCGCGCCT]** 1395 (85.56)   
208 - **[GCGCCTCG]** 1393 (87.99)   
208 - **[TCGCTCCG]** 1283 (85.50)   
208 - **[TCGCGTCT]** 1225 (89.45)   
208 - **[TCGCGACG]** 1140 (89.50)   
208 - **[TCGCGCGG]** 952 (86.75)   
209 - **[TCGCCTCC]** 1270 (85.23)   
210 + **.GCGCGTCG.** 676 (95.20)   
210 + **[CCGCGGCC]** 779 (85.01)   
210 + **[TCCCGCCG]** 1017 (87.33)   
210 - **[TCGGGGCG]** 1151 (87.91)   
210 - **.CCGCGCCG.** 1065 (91.89)   
210 - **[TCGCGGGG]** 801 (87.42)   
210 - **[CCGCGGCC]** 777 (85.01)   
211 + **[CCGCCGCG]** 733 (85.35)   
211 + **.CCGCGCCG.** 976 (91.89)   
211 + **[TCGGGCCG]** 1258 (87.23)   
211 - **.TCGCGTCC.** 1144 (92.45)   
211 - **.GCGCGGCG.** 734 (91.99)   
212 + **.CCGCGCCG.** 454 (91.89)   
212 + **.TCGGGTCG.** 736 (91.12)   
212 + **[TCGCCCCG]** 1303 (88.90)   
212 + **[TCCCGGCG]** 1334 (88.00)   
212 - **[GCGCCTCG]** 1494 (87.99)   
212 - **[CCGCGGCC]** 712 (85.01)   
212 - **[TTGCGTCG]** 424 (86.27)   
212 - **[TGGCGCCG]** 384 (87.13)   
213 + **.CCGCGCCG.** 1192 (91.89)   
213 + **[TGGCGCCG]** 1334 (87.13)   
213 - **[TCCCGGCG]** 1195 (88.00)   
213 - **[GCGCGTGG]** 1120 (85.84)   
213 - **.TCGCGCCG.** 996 (96.11)   
213 - **[TCGCGGCC]** 911 (89.23)   
214 + **[TCGCGTCA]** 354 (87.59)   
214 + **[TCGGGGCG]** 650 (87.91)   
214 + **.GCGCGGCG.** 731 (91.99)   
214 + **[CCGCGGCC]** 930 (85.01)   
214 + **[TCGCGCCC]** 1032 (88.56)   
214 - **[TCCCGCCG]** 1414 (87.33)   
214 - **[CCGCGGCC]** 928 (85.01)   
214 - **[TCGGGCCG]** 784 (87.23)   
215 + **.CCGCGTCG.** 653 (95.78)   
215 + **[TCCCGCCG]** 1086 (87.33)   
215 + **[CCGCCGCG]** 1088 (85.35)   
215 + **.CCGCGGCG.** 1091 (92.56)   
215 + **[TCCCGGCG]** 1279 (88.00)   
215 - **[TCGCCTCC]** 1404 (85.23)   
215 - **[TCGCCCCG]** 1105 (88.90)   
215 - **.CCGCGGCG.** 1089 (92.56)   
215 - **[TGGCGCCG]** 488 (87.13)   
215 - **[CCGCCTCG]** 13 (88.56)   
216 + **.TCGCGGCG.** 211 (96.78)   
216 + **[TGGCGCCG]** 388 (87.13)   
216 + **[GCGCCTCG]** 820 (87.99)   
216 - **[TCGCGCCT]** 1464 (85.56)   
216 - **[GCGGGTCG]** 1061 (86.33)   
216 - **.GCGCGCCG.** 991 (91.32)   
217 + **.GCGCGGCG.** 576 (91.99)   
217 + **.GCGCGTCG.** 940 (95.20)   
217 + **[TCGCGTCT]** 1093 (89.45)   
217 - **.GCGCGGCG.** 859 (91.99)   
217 - **[TGGCGGCG]** 797 (87.81)   
217 - **[CCGCCTCG]** 776 (88.56)   
217 - **[CCGCCGCG]** 577 (85.35)   
221 + **[CCGCGTCC]** 19 (88.23)   
221 + **.TCGCGTGG.** 304 (90.64)   
221 + **[TCCCGGCG]** 933 (88.00)   
221 + **.CCGCGGCG.** 1111 (92.56)   
221 + **[TCCCGGCG]** 1260 (88.00)   
221 + **[TCGCCTCC]** 1346 (85.23)   
221 - **[CCGCCGCG]** 1112 (85.35)   
221 - **[CCGCGGCC]** 1109 (85.01)   
221 - **.CCGCGCCG.** 936 (91.89)   
222 + **[GCGCGTCC]** 451 (87.65)   
222 + **[TCCCGCCG]** 663 (87.33)   
222 + **[TCGCGCCC]** 690 (88.56)   
222 + **.GCGCGGCG.** 842 (91.99)   
222 + **.CCGCGTCG.** 878 (95.78)   
222 + **[TCGCGCCC]** 925 (88.56)   
222 + **[GCGCGTCC]** 939 (87.65)   
222 + **.CCGCGGCG.** 949 (92.56)   
222 + **.GCGCGCCG.** 1205 (91.32)   
222 + **[CCGCGGCC]** 1483 (85.01)   
222 - **.CCGCGCCG.** 1269 (91.89)   
222 - **[CCGCCGCG]** 950 (85.35)   
222 - **[TCGCCGCG]** 843 (89.57)   
222 - **.CCGCGCCG.** 840 (91.89)   
222 - **[TCCCGCCG]** 274 (87.33)   
223 + **[ACGCGCCG]** 423 (86.10)   
223 + **[TCGCGCGG]** 504 (86.75)   
223 - **[CCGCGTCC]** 455 (88.23)   
223 - **[GCGCGTCC]** 421 (87.65)   
224 + **[TCGCGGCC]** 531 (89.23)   
224 + **[TCGCCTCC]** 545 (85.23)   
224 + **[TCGGGGCG]** 602 (87.91)   
224 + **.GCGCGGCG.** 618 (91.99)   
224 + **[CCGCCGCG]** 688 (85.35)   
224 + **.GCGCGGCG.** 855 (91.99)   
224 + **[TGGCGCCG]** 1088 (87.13)   
224 + **[TCGCCGCG]** 1232 (89.57)   
224 - **.GCGCGGCG.** 1233 (91.99)   
224 - **[CCGCGTGG]** 1175 (86.42)   
224 - **[TCGCCGCG]** 856 (89.57)   
224 - **.CCGCGCCG.** 853 (91.89)   
224 - **.CCGCGGCG.** 822 (92.56)   
224 - **.CCGCGCCG.** 717 (91.89)   
224 - **.TCGCGGCG.** 689 (96.78)   
226 + **[TCGCGGCC]** 124 (89.23)   
226 + **.CCGCGCCG.** 510 (91.89)   
226 + **[CCGCGTCC]** 600 (88.23)   
226 + **[TCGGGGCG]** 893 (87.91)   
226 + **.GCGCGCCG.** 1031 (91.32)   
226 + **[GCGCGTCC]** 1064 (87.65)   
226 + **.GCGCGGCG.** 1077 (91.99)   
226 + **.CCGCGCCG.** 1136 (91.89)   
226 + **.GCGCGGCG.** 1496 (91.99)   
226 - **[CCGCCGCG]** 1497 (85.35)   
226 - **[GGGCGTCG]** 1188 (86.23)   
226 - **.GCGCGGCG.** 1034 (91.99)   
226 - **[TCGCCCCG]** 894 (88.90)   
226 - **[CCGCGTCC]** 711 (88.23)   
227 + **[TCGCGGGG]** 96 (87.42)   
227 + **[TCGCTGCG]** 711 (86.18)   
227 + **[TCGCTCCG]** 1137 (85.50)   
227 + **[TCGCGGGG]** 1283 (87.42)   
227 + **.CCGCGCCG.** 1360 (91.89)   
227 - **[GCGCCTCG]** 901 (87.99)   
227 - **[CCGCGTGG]** 845 (86.42)   
227 - **.CCGCGTCG.** 686 (95.78)   
227 - **[GCGCGTCC]** 306 (87.65)   
228 + **.CCGCGCCG.** 470 (91.89)   
228 + **.CCGCGCCG.** 541 (91.89)   
228 + **[TCGCTCCG]** 1238 (85.50)   
228 - **[GCGCGTGG]** 1426 (85.84)   
228 - **[TCGCGGCC]** 1270 (89.23)   
228 - **[CCGCCTCG]** 835 (88.56)   
228 - **[CCGCGTCT]** 737 (85.23)   
228 - **[TCGGGGCG]** 432 (87.91)   
228 - **[GCGGGTCG]** 153 (86.33)   
228 - **[CCGCCTCG]** 104 (88.56)   
229 + **[CCGCGGCC]** 586 (85.01)   
229 + **[TCCCGGCG]** 630 (88.00)   
229 + **[CCGCCGCG]** 1112 (85.35)   
229 + **[TCGCGCCT]** 1205 (85.56)   
229 + **.GCGCGGCG.** 1283 (91.99)   
229 + **[GGGCGTCG]** 1388 (86.23)   
229 - **.CCGCGCCG.** 1286 (91.89)   
229 - **[TCGCCCCG]** 1136 (88.90)   
229 - **.CCGCGGCG.** 1113 (92.56)   
229 - **.GCGCGGCG.** 689 (91.99)   
230 + **[TCGCCCCG]** 288 (88.90)   
230 - **[TCGGGGCG]** 289 (87.91)   
231 + **[CCGGGTCG]** 694 (86.90)   
231 + **[CCGCCGCG]** 904 (85.35)   
231 + **.CCGCGCCG.** 1483 (91.89)   
231 - **[GCGCGTCC]** 1382 (87.65)   
231 - **[TCCCGGCG]** 1196 (88.00)   
231 - **[TCGCGCCC]** 1140 (88.56)   
231 - **[TCGCGCCC]** 1121 (88.56)   
231 - **[TCGCCCCG]** 1103 (88.90)   
231 - **[TCGCCCCG]** 1094 (88.90)   
231 - **[TGGCGGCG]** 1029 (87.81)   
231 - **.CCGCGGCG.** 905 (92.56)   
231 - **[CCGCGTGG]** 873 (86.42)   
232 + **.TCGCCTCG.** 105 (92.78)   
232 + **[CCGCCGCG]** 145 (85.35)   
232 + **[TGGCGGCG]** 626 (87.81)   
232 + **[GCGCGTCC]** 634 (87.65)   
232 + **[GCGCGTCC]** 700 (87.65)   
232 + **[CCGCCGCG]** 792 (85.35)   
232 + **[TCCCGGCG]** 915 (88.00)   
232 + **.GCGCGGCG.** 996 (91.99)   
232 + **.CCGCGCCG.** 1245 (91.89)   
232 + **[CCGCCTCG]** 1267 (88.56)   
232 - **[CCGCGTCC]** 1332 (88.23)   
232 - **.CCGCGCCG.** 1179 (91.89)   
232 - **.TCGCGTCC.** 1118 (92.45)   
232 - **.TCGCGCCG.** 1012 (96.11)   
232 - **[TCCCGCCG]** 999 (87.33)   
232 - **[CCGCCGCG]** 997 (85.35)   
232 - **[CCGCGGCC]** 866 (85.01)   
232 - **.TCGCGGCG.** 793 (96.78)   
232 - **[ACGCGCCG]** 632 (86.10)   
232 - **.GCGCGCCG.** 443 (91.32)   
232 - **[GCGGGTCG]** 260 (86.33)   
232 - **.GCGCGGCG.** 146 (91.99)   
233 + **[GCGCGTGG]** 1379 (85.84)   
233 - **[GGGCGTCG]** 1140 (86.23)   
233 - **[CCGCCTCG]** 374 (88.56)   
234 + **.GCGCGCCG.** 410 (91.32)   
234 + **[CGGCGTCG]** 630 (86.80)   
234 + **[CCGCGGCC]** 815 (85.01)   
234 + **[TCGCGCCC]** 849 (88.56)   
234 + **[TCGCCCCG]** 882 (88.90)   
234 + **[ACGCGGCG]** 1330 (86.77)   
234 - **[GCGCGTCC]** 1410 (87.65)   
234 - **[CCGCGTGG]** 1328 (86.42)   
234 - **.CCGCGTCG.** 636 (95.78)   
234 - **[CCGCTTCG]** 509 (85.17)   
234 - **[TCGGGCCG]** 397 (87.23)   
234 - **[TCGCGACG]** 316 (89.50)   
235 + **[GCGCGTGG]** 475 (85.84)   
235 + **[TCGCGGGG]** 684 (87.42)   
235 + **.GCGCGGCG.** 899 (91.99)   
235 + **[TGGCGGCG]** 1509 (87.81)   
235 - **.GCGCGGCG.** 1361 (91.99)   
235 - **.CCGCGCCG.** 1316 (91.89)   
235 - **.TCGCCTCG.** 1276 (92.78)   
235 - **[ACGCGCCG]** 902 (86.10)   
235 - **[GCGCGTGG]** 752 (85.84)   
235 - **.TCCCGTCG.** 272 (91.22)   
236 + **[TCGCGCGG]** 1023 (86.75)   
236 - **[CCGCGGCC]** 1153 (85.01)   
237 + **[CCGCGTCT]** 472 (85.23)   
237 - **[CCGCGTCT]** 1056 (85.23)   
238 - **[TCGCGGGG]** 1225 (87.42)   
238 - **.CCGCGTCG.** 1105 (95.78)   
239 + **[TCGCCGCG]** 443 (89.57)   
239 + **[CCGCGGCC]** 446 (85.01)   
239 + **.CCGCGCCG.** 769 (91.89)   
239 - **[TCGCCTCC]** 1163 (85.23)   
239 - **[TCGCGCCC]** 706 (88.56)   
239 - **.CCGCGGCG.** 444 (92.56)   
240 + **[TCGCGCCC]** 139 (88.56)   
240 + **.CCGCGCCG.** 1181 (91.89)   
240 + **[TCGCCGCG]** 1401 (89.57)   
240 - **.TCGCGGCG.** 1402 (96.78)   
241 - **.CCGCGCCG.** 1283 (91.89)   
242 + **[TCGCTGCG]** 505 (86.18)   
242 + **[CCGCGTCT]** 823 (85.23)   
242 + **.TCGCGTCG.** 1002 (100.00)   
242 - **[TCCCGCCG]** 761 (87.33)   
243 + **[TCGCGCGG]** 917 (86.75)   
243 - **[TCGCGCGG]** 1161 (86.75)   
243 - **[TCGCGGCT]** 1119 (86.24)   
244 + **[TCGGGGCG]** 921 (87.91)   
244 - **[CCGCGTCC]** 686 (88.23)   
246 + **[CCGCCGCG]** 272 (85.35)   
246 + **[GCGCCTCG]** 277 (87.99)   
246 + **.GCGCGGCG.** 673 (91.99)   
246 + **[TCGCGCCC]** 698 (88.56)   
246 + **.CCGCGCCG.** 883 (91.89)   
246 + **.GCGCGCCG.** 909 (91.32)   
246 - **[TCGCCTCC]** 992 (85.23)   
246 - **[TCCCGGCG]** 974 (88.00)   
246 - **[GCGCGTCC]** 669 (87.65)   
246 - **.GCGCGGCG.** 273 (91.99)   
247 + **[TCCCGCCG]** 15 (87.33)   
247 + **[CCGCCGCG]** 17 (85.35)   
247 + **.GCGCGGCG.** 421 (91.99)   
247 + **[TCCCGCCG]** 937 (87.33)   
247 + **[TCGGGGCG]** 959 (87.91)   
247 + **.CCGCGGCG.** 1023 (92.56)   
247 + **[CCGCCTCG]** 1226 (88.56)   
247 + **[CCGCCGCG]** 1491 (85.35)   
247 - **.GCGCGGCG.** 1492 (91.99)   
247 - **[TGGCGGCG]** 1044 (87.81)   
247 - **[CCGCCGCG]** 1024 (85.35)   
247 - **[CCGCCGCG]** 422 (85.35)   
247 - **.TCGCGGCG.** 18 (96.78)   
248 - **[CCGCCTCG]** 1406 (88.56)   
250 + **.GCGCGGCG.** 456 (91.99)   
250 + **[TCGCGGCT]** 512 (86.24)   
250 + **[TCGCCTCC]** 548 (85.23)   
250 + **[TCGCCGCG]** 868 (89.57)   
250 + **[TCGCTGCG]** 1490 (86.18)   
250 - **[CCGCGGCC]** 1095 (85.01)   
250 - **[ACGCGGCG]** 869 (86.77)   
250 - **[TCCCGGCG]** 685 (88.00)   
250 - **[TCGGGCCG]** 605 (87.23)   
250 - **.CCGCGCCG.** 459 (91.89)   
250 - **[GCGCGTCC]** 452 (87.65)   
250 - **[GCGCGTGG]** 136 (85.84)   
252 + **[GCGCGTCC]** 393 (87.65)   
256 + **[CCGCCTCG]** 54 (88.56)   
256 + **[TCGGGCCG]** 1027 (87.23)   
256 + **[GCGGGTCG]** 1096 (86.33)   
256 + **[TCGCGCCC]** 1116 (88.56)   
256 + **.CCGCGTCG.** 1204 (95.78)   
256 + **[CCGCTTCG]** 1240 (85.17)   
256 + **[CCGCCGCG]** 1300 (85.35)   
256 - **.CCGCGGCG.** 1301 (92.56)   
256 - **[CCGCGGCC]** 1194 (85.01)   
256 - **[GCGGGTCG]** 1126 (86.33)   
257 + **[TCGCCCCG]** 916 (88.90)   
257 + **[TGGCGCCG]** 1303 (87.13)   
257 + **[GGGCGTCG]** 1463 (86.23)   
257 - **[CCGGGTCG]** 1425 (86.90)   
257 - **[CCGCGGCC]** 1281 (85.01)   
257 - **[CCGCGGCC]** 1193 (85.01)   
257 - **[CCGCGGCC]** 1165 (85.01)   
257 - **[GCGCGTCC]** 1118 (87.65)   
257 - **[CCGCGGCC]** 606 (85.01)   
259 + **[ACGCGGCG]** 996 (86.77)   
259 + **[CCGCGACG]** 1019 (85.28)   
259 + **[CCGCGGCC]** 1186 (85.01)   
259 + **[TCGCGCCC]** 1220 (88.56)   
259 + **.CCGCGGCG.** 1303 (92.56)   
259 - **[TCGCCGCG]** 1304 (89.57)   
259 - **.CCGCGGCG.** 1301 (92.56)   
259 - **.GCGCGTCG.** 1022 (95.20)   
259 - **[TCGCGGCC]** 1017 (89.23)   
259 - **[CCGCCGCG]** 997 (85.35)   
259 - **.CCGCGTCG.** 994 (95.78)   
260 + **[TCGCTGCG]** 1415 (86.18)   
263 + **[GCGCGTGG]** 166 (85.84)   
263 + **[TCGCGCGG]** 847 (86.75)   
263 + **.CCGCGTCG.** 1077 (95.78)   
263 - **.CCGCGTCG.** 1116 (95.78)   
263 - **[TCGCGACG]** 1080 (89.50)   
263 - **[CCGCGGCC]** 853 (85.01)   
263 - **[TCGCTCCG]** 835 (85.50)   
264 + **[TCGCGTCT]** 142 (89.45)   
264 + **.CCGCGCCG.** 1137 (91.89)   
264 + **.TCGCCTCG.** 1205 (92.78)   
264 - **[GCGCGTCC]** 751 (87.65)   
266 + **[ACGCGCCG]** 981 (86.10)   
266 + **.GCGCGGCG.** 1029 (91.99)   
266 + **.GCGCGCCG.** 1105 (91.32)   
266 + **[TGGCGGCG]** 1149 (87.81)   
266 + **.GCGCGCCG.** 1269 (91.32)   
266 + **.CCGCGGCG.** 1281 (92.56)   
266 - **.CCGCGGCG.** 1474 (92.56)   
266 - **[CCGCCGCG]** 1282 (85.35)   
266 - **[CCGCGGCC]** 1279 (85.01)   
266 - **.GCGCGCCG.** 1103 (91.32)   
266 - **[CCGCGGCC]** 1064 (85.01)   
266 - **[CCGCCTCG]** 1041 (88.56)   
266 - **[CCGCCGCG]** 1030 (85.35)   
266 - **.CCGCGCCG.** 1027 (91.89)   
266 - **[GCGCGTGG]** 979 (85.84)   
266 - **.TCGCGTGG.** 544 (90.64)   
267 + **[CCGCCGCG]** 191 (85.35)   
267 + **[CCGCGGCC]** 194 (85.01)   
267 + **[TCGCGGGG]** 698 (87.42)   
267 - **[TCGCCTCC]** 1203 (85.23)   
267 - **.CCGCGGCG.** 192 (92.56)   
267 - **[GCGCCTCG]** 96 (87.99)   
268 + **[TCGCGCGG]** 20 (86.75)   
268 + **[ACGCGGCG]** 812 (86.77)   
268 + **.GCGCGGCG.** 1014 (91.99)   
268 + **[TCGGGCCG]** 1175 (87.23)   
268 - **[TCGCCTCC]** 1435 (85.23)   
268 - **[TCGCGGCC]** 1319 (89.23)   
268 - **[CCGCGTGG]** 1198 (86.42)   
268 - **[TGGCGCCG]** 1193 (87.13)   
268 - **[CCGCGGCC]** 1067 (85.01)   
268 - **.CCGCGCCG.** 1012 (91.89)   
268 - **[TCGCCGCG]** 813 (89.57)   
268 - **.GCGCGCCG.** 56 (91.32)   
270 + **[CCGCGTCC]** 26 (88.23)   
270 + **.TCGGGTCG.** 1059 (91.12)   
270 + **[GCGCGTGG]** 1441 (85.84)   
270 - **[TCGCCCCG]** 1204 (88.90)   
270 - **[TCGCTGCG]** 813 (86.18)   
270 - **[TCCCGGCG]** 577 (88.00)   
270 - **[CGGCGTCG]** 343 (86.80)   
270 - **.TCGCGCCG.** 55 (96.11)   
271 - **[TCGCGTCT]** 1287 (89.45)   
272 - **[TCGCGTCT]** 1278 (89.45)   
273 + **.CCGCGGCG.** 1031 (92.56)   
273 + **[CCGCGTCC]** 1120 (88.23)   
273 - **[CCGCCGCG]** 1032 (85.35)   
273 - **[CCGCGGCC]** 1029 (85.01)   
273 - **[TGGCGCCG]** 458 (87.13)   
274 + **[CCGCGGCC]** 14 (85.01)   
274 + **[TCGCGCCT]** 48 (85.56)   
274 + **.CCGCGGCG.** 107 (92.56)   
274 - **.GCGCGCCG.** 110 (91.32)   
274 - **[CCGCGGCC]** 12 (85.01)   
275 + **.GCGCGCCG.** 754 (91.32)   
275 + **.TCGCCTCG.** 814 (92.78)   
275 + **[TCGCGACG]** 1264 (89.50)   
275 + **.TCGCGCCG.** 1329 (96.11)   
275 + **[TCCCGGCG]** 1349 (88.00)   
275 - **[TCGCGCCC]** 1087 (88.56)   
275 - **[TCGCATCG]** 461 (86.74)   
275 - **[GCGCGTCC]** 90 (87.65)   
275 - **.CCGCGCCG.** 72 (91.89)   
276 - **.TCGCGTCG.** 754 (100.00)   
279 + **.GCGCGGCG.** 414 (91.99)   
279 + **[GCGCGTGG]** 419 (85.84)   
279 - **[TCGCTGCG]** 541 (86.18)   
279 - **[ACGCGCCG]** 417 (86.10)   
279 - **[GCGCCTCG]** 115 (87.99)   
280 + **[GGGCGTCG]** 503 (86.23)   
280 + **.GCGCGGCG.** 734 (91.99)   
280 + **.GCGCGGCG.** 981 (91.99)   
280 + **[TCGCTGCG]** 1060 (86.18)   
280 + **.TCGCGTCC.** 1083 (92.45)   
280 + **[GCGCGTCC]** 1196 (87.65)   
280 + **[TCGCGCCC]** 1263 (88.56)   
280 - **[TGGCGCCG]** 1109 (87.13)   
280 - **[CCGCCGCG]** 982 (85.35)   
280 - **.GCGCGCCG.** 975 (91.32)   
280 - **[TCGCGGCC]** 943 (89.23)   
280 - **[CCGCCGCG]** 735 (85.35)   
281 + **[TCGCCTCC]** 205 (85.23)   
281 + **[TCGCGGCC]** 530 (89.23)   
281 - **[GCCCGTCG]** 768 (86.42)   
281 - **[GCGCGTCC]** 511 (87.65)   
281 - **[CCGCGTCC]** 445 (88.23)   
283 - **[TCCCGCCG]** 168 (87.33)   
284 + **[GCGCCTCG]** 554 (87.99)   
284 + **[GGGCGTCG]** 578 (86.23)   
284 + **[CCGCGTGG]** 717 (86.42)   
284 + **.GCGCGGCG.** 779 (91.99)   
284 + **[TCGGGGCG]** 823 (87.91)   
284 + **[CCGCGGCC]** 942 (85.01)   
284 + **.GCGCGCCG.** 1002 (91.32)   
284 + **.CCGCGGCG.** 1088 (92.56)   
284 + **.GCGCGCCG.** 1341 (91.32)   
284 + **.CCGCGGCG.** 1346 (92.56)   
284 + **[GCGGGTCG]** 1501 (86.33)   
284 + **[TCGCGGGG]** 1506 (87.42)   
284 - **.CCGCGCCG.** 1449 (91.89)   
284 - **.CCGCGGCG.** 1344 (92.56)   
284 - **.CCGCGCCG.** 1091 (91.89)   
284 - **[GGGCGTCG]** 1028 (86.23)   
284 - **[CCGCCGCG]** 780 (85.35)   
284 - **.CCGCGCCG.** 630 (91.89)   
284 - **.TGGCGTCG.** 489 (91.02)   
285 + **[CCGCGGCC]** 547 (85.01)   
285 + **.CCGCGGCG.** 553 (92.56)   
285 + **.TCGCGCCG.** 651 (96.11)   
285 + **.CCGCGCCG.** 656 (91.89)   
285 + **[CCGCCGCG]** 905 (85.35)   
285 + **.CCGCGGCG.** 908 (92.56)   
285 + **[TCCCGGCG]** 1070 (88.00)   
285 - **.TCGCGCCG.** 1025 (96.11)   
285 - **[TCGCGCGG]** 947 (86.75)   
285 - **.CCGCGGCG.** 906 (92.56)   
285 - **[CCGCGGCC]** 857 (85.01)   
285 - **.CCGCGTCG.** 714 (95.78)   
285 - **.GCGCGGCG.** 654 (91.99)   
285 - **.GCGCGGCG.** 633 (91.99)   
285 - **[TGGCGGCG]** 582 (87.81)   
285 - **[CCGCGGCC]** 551 (85.01)   
285 - **.CCGCGCCG.** 442 (91.89)   
285 - **[CCGCGGCC]** 352 (85.01)   
287 + **.GCGCGCCG.** 536 (91.32)   
287 + **[GCGCCTCG]** 687 (87.99)   
287 + **[CCGCCTCG]** 764 (88.56)   
287 + **[TCGCCCCG]** 1042 (88.90)   
287 - **[TCCCGGCG]** 1244 (88.00)   
287 - **[TGGCGGCG]** 1082 (87.81)   
288 + **.GCGCGCCG.** 1194 (91.32)   
288 - **.TCGCGCCG.** 875 (96.11)   
289 + **.GCGCGGCG.** 1299 (91.99)   
289 - **[TCGCGCCC]** 1316 (88.56)   
289 - **[CCGCGTCT]** 539 (85.23)   
290 + **.TCGCCTCG.** 1152 (92.78)   
290 + **[TCGGGGCG]** 1326 (87.91)   
290 - **[TCCCGGCG]** 1442 (88.00)   
291 + **.TCGCGTCC.** 202 (92.45)   
291 + **[TCGCCCCG]** 361 (88.90)   
291 + **[CCGCGTCC]** 406 (88.23)   
291 + **[CCGCGTCT]** 511 (85.23)   
291 + **[TCGGGCCG]** 839 (87.23)   
291 + **.GCGCGGCG.** 870 (91.99)   
291 + **[CCCCGTCG]** 1025 (87.00)   
291 + **.GCGCGCCG.** 1099 (91.32)   
291 + **[CCGGGTCG]** 1191 (86.90)   
291 + **[TCGGGGCG]** 1196 (87.91)   
291 + **.TCCCGTCG.** 1385 (91.22)   
291 + **.GCGCGCCG.** 1501 (91.32)   
291 - **[CCGCCTCG]** 1353 (88.56)   
291 - **[TCGCGGCC]** 1342 (89.23)   
291 - **[CCGCGTCC]** 1164 (88.23)   
291 - **[ACGCGGCG]** 1082 (86.77)   
291 - **.GCGCGGCG.** 1034 (91.99)   
291 - **[TCGGGGCG]** 1010 (87.91)   
291 - **.CCGCGGCG.** 766 (92.56)   
291 - **[TCGCGCCT]** 607 (85.56)   
291 - **[TCGCGACG]** 583 (89.50)   
292 + **.CCGCGCCG.** 773 (91.89)   
292 + **.CCGCGGCG.** 796 (92.56)   
292 + **[TCCCGGCG]** 981 (88.00)   
292 + **[TCGGGCCG]** 1054 (87.23)   
292 + **[TCCCGCCG]** 1078 (87.33)   
292 + **[CCGCCGCG]** 1080 (85.35)   
292 + **[CGGCGTCG]** 1177 (86.80)   
292 + **[TCCCGGCG]** 1259 (88.00)   
292 + **[GCGCGTCC]** 1318 (87.65)   
292 + **[TCGCGGCC]** 1338 (89.23)   
292 + **[GGGCGTCG]** 1465 (86.23)   
292 + **[TCGCGGGG]** 1470 (87.42)   
292 - **[CCGCGTCC]** 1484 (88.23)   
292 - **[CCGCGACG]** 1468 (85.28)   
292 - **[TCCCGCCG]** 1437 (87.33)   
292 - **.CCGCGCCG.** 1345 (91.89)   
292 - **[CCGCGTCC]** 1188 (88.23)   
292 - **.TCGCGGCG.** 1081 (96.78)   
292 - **[TCGGGGCG]** 960 (87.91)   
292 - **[CCGCCGCG]** 797 (85.35)   
292 - **.CCGCGGCG.** 794 (92.56)   
293 + **.TCGCCTCG.** 780 (92.78)   
293 + **.GCGCGGCG.** 838 (91.99)   
293 + **[TCGCGGCT]** 935 (86.24)   
293 + **.TGGCGTCG.** 1069 (91.02)   
293 + **[TCGCGGGG]** 1074 (87.42)   
293 + **.TGGCGTCG.** 1361 (91.02)   
293 + **.TCGCGTGG.** 1443 (90.64)   
293 - **[CCGCGACG]** 1072 (85.28)   
293 - **[TGGCGGCG]** 1009 (87.81)   
293 - **.CCGCGCCG.** 859 (91.89)   
293 - **[CCGCCGCG]** 839 (85.35)   
293 - **[GCGCCTCG]** 820 (87.99)   
293 - **[TCCCGCCG]** 799 (87.33)   
294 + **[CCGCCGCG]** 44 (85.35)   
294 + **[CCGCGGCC]** 315 (85.01)   
294 + **[CCGCCTCG]** 362 (88.56)   
294 + **.TCGCGTGG.** 612 (90.64)   
294 + **.GCGCGGCG.** 893 (91.99)   
294 - **[GGGCGTCG]** 1088 (86.23)   
294 - **[GCGCGTGG]** 1027 (85.84)   
294 - **[GCGCGTCC]** 935 (87.65)   
294 - **[CCGCTTCG]** 543 (85.17)   
294 - **.GCGCGGCG.** 45 (91.99)   
295 + **[GGGCGTCG]** 550 (86.23)   
295 + **.GCGCGGCG.** 591 (91.99)   
295 + **[GCGCGTGG]** 611 (85.84)   
295 + **[GCGCGTCC]** 703 (87.65)   
295 + **[CCGCTTCG]** 1095 (85.17)   
295 - **[CCGCGGCC]** 1323 (85.01)   
295 - **[CCGCCTCG]** 1276 (88.56)   
295 - **.TCGCGTGG.** 1026 (90.64)   
295 - **.GCGCGGCG.** 746 (91.99)   
295 - **[CCGCCGCG]** 592 (85.35)   
296 + **[ACGCGGCG]** 84 (86.77)   
296 + **[TCGGGCCG]** 506 (87.23)   
296 + **.TCGCGCCG.** 590 (96.11)   
296 + **.CCGCGCCG.** 758 (91.89)   
296 + **[TCGCCTCC]** 778 (85.23)   
296 + **[CCGCGGCC]** 814 (85.01)   
296 + **.CCGCGCCG.** 945 (91.89)   
296 + **[ACGCGGCG]** 1002 (86.77)   
296 + **[TCCCGGCG]** 1154 (88.00)   
296 + **.CCGCGCCG.** 1183 (91.89)   
296 + **[GGGCGTCG]** 1206 (86.23)   
296 + **.GCGCGCCG.** 1314 (91.32)   
296 + **.GCGCGGCG.** 1439 (91.99)   
296 + **[GCGCGTCC]** 1473 (87.65)   
296 - **.CCGCGCCG.** 1442 (91.89)   
296 - **[CCGCCTCG]** 1382 (88.56)   
296 - **.CCGCGGCG.** 1186 (92.56)   
296 - **[CCGCGGCC]** 1161 (85.01)   
296 - **[CCGCTTCG]** 1043 (85.17)   
296 - **[GCGCGTCC]** 1011 (87.65)   
296 - **[TCGCCGCG]** 1003 (89.57)   
296 - **[CCGCGTCT]** 1000 (85.23)   
296 - **[CCGCGTCC]** 840 (88.23)   
296 - **.GCGCGGCG.** 756 (91.99)   
296 - **.GCGCGCCG.** 194 (91.32)   
296 - **[CCGGGTCG]** 148 (86.90)   
296 - **[CCGCCGCG]** 85 (85.35)   
297 + **[TGGCGGCG]** 1018 (87.81)   
297 + **[GCGCGTGG]** 1032 (85.84)   
297 + **[TCGGGCCG]** 1259 (87.23)   
297 + **[CCGCGTGG]** 1420 (86.42)   
297 + **.TGGCGTCG.** 1442 (91.02)   
297 - **.TCGCCTCG.** 1192 (92.78)   
297 - **[TCGCTCCG]** 1187 (85.50)   
297 - **[CCGCGGCC]** 995 (85.01)   
297 - **.CCGCGCCG.** 984 (91.89)   
297 - **[CCCCGTCG]** 899 (87.00)   
298 + **[GCGCCTCG]** 294 (87.99)   
298 + **[TCGGGCCG]** 617 (87.23)   
298 + **.TCGCGGCG.** 745 (96.78)   
298 + **[TCGCGCGG]** 839 (86.75)   
298 + **[ACGCGGCG]** 875 (86.77)   
298 + **[CCGCGTCC]** 936 (88.23)   
298 + **[TCGCGGCC]** 1003 (89.23)   
298 + **[CCGCCGCG]** 1176 (85.35)   
298 + **[GCGCCTCG]** 1262 (87.99)   
298 - **.TCGCGGCG.** 1177 (96.78)   
298 - **[CCGCCGCG]** 876 (85.35)   
299 + **.TGGCGTCG.** 832 (91.02)   
299 + **[CCGCGTCT]** 852 (85.23)   
299 + **.GCGCGTCG.** 882 (95.20)   
299 + **[TCCCGCCG]** 946 (87.33)   
299 + **.GCGCGCCG.** 1081 (91.32)   
299 + **[TCGCGGGG]** 1092 (87.42)   
299 + **.CCGCGGCG.** 1179 (92.56)   
299 + **.CCGCGGCG.** 1261 (92.56)   
299 + **[GCGCCTCG]** 1342 (87.99)   
299 + **[CCGCGACG]** 1400 (85.28)   
299 + **[TCGCTCCG]** 1414 (85.50)   
299 + **[GCGCGTCC]** 1474 (87.65)   
299 - **[CCGCGGCC]** 1177 (85.01)   
299 - **[TGGCGGCG]** 978 (87.81)   
299 - **[CCGCCTCG]** 844 (88.56)   
299 - **[GCGCGTGG]** 576 (85.84)   
300 + **[TCGCGGCT]** 1 (86.24)   
300 + **.GCGCGGCG.** 112 (91.99)   
300 + **[CCGCGGCC]** 174 (85.01)   
300 + **[TCCCGGCG]** 264 (88.00)   
300 + **[GCGGGTCG]** 394 (86.33)   
300 + **.CCGCGTCG.** 445 (95.78)   
300 + **[TCGGGGCG]** 450 (87.91)   
300 + **.GCGCGGCG.** 473 (91.99)   
300 + **.TCGCGGCG.** 735 (96.78)   
300 - **[CCGCCGCG]** 736 (85.35)   
300 - **.CCGCGCCG.** 476 (91.89)   
300 - **.CCGCGCCG.** 110 (91.89)   
301 + **[CCGCGTGG]** 1482 (86.42)   
301 - **[TCGCTTCG]** 1357 (89.39)   
301 - **[CCGCCTCG]** 1035 (88.56)   
302 + **[TCGCGACG]** 592 (89.50)   
302 + **.CCGCGCCG.** 755 (91.89)   
302 + **[TCCCGCCG]** 824 (87.33)   
302 + **.CCGCGCCG.** 840 (91.89)   
302 + **[TCGCGCCC]** 976 (88.56)   
302 + **[CCGCCTCG]** 1020 (88.56)   
302 + **.TCGCGGCG.** 1025 (96.78)   
302 + **.TCGCGCCG.** 1038 (96.11)   
302 + **[GCCCGTCG]** 1282 (86.42)   
302 + **[TGGCGCCG]** 1444 (87.13)   
302 - **.CCGCGGCG.** 1041 (92.56)   
302 - **[TCGCGGCT]** 998 (86.24)   
302 - **.GCGCGTCG.** 950 (95.20)   
302 - **[TCCCGGCG]** 827 (88.00)   
302 - **[TCGCTGCG]** 598 (86.18)   
303 + **.GCGCGTCG.** 129 (95.20)   
303 + **[TCCCGCCG]** 164 (87.33)   
303 + **[CCGCCGCG]** 166 (85.35)   
303 + **.CCGCGCCG.** 169 (91.89)   
303 + **.GCGCGCCG.** 744 (91.32)   
303 + **[CCCCGTCG]** 840 (87.00)   
303 + **.CCGCGTCG.** 1020 (95.78)   
303 - **[CCGGGTCG]** 1313 (86.90)   
303 - **.CCGCGCCG.** 1003 (91.89)   
303 - **.CCGCGGCG.** 747 (92.56)   
303 - **.CCGCGGCG.** 456 (92.56)   
303 - **[GCGGGTCG]** 388 (86.33)   
303 - **[CCGCGGCC]** 286 (85.01)   
303 - **.GCGCGGCG.** 167 (91.99)   
303 - **[TCGCGGCC]** 42 (89.23)   
304 + **[TCGCCGCG]** 706 (89.57)   
304 + **.GCGCGGCG.** 1242 (91.99)   
304 + **[CCGCGACG]** 1308 (85.28)   
304 - **[TCGCGGGG]** 1306 (87.42)   
304 - **[CCGCCGCG]** 1243 (85.35)   
304 - **.GCGCGCCG.** 1178 (91.32)   
304 - **.GCGCGCCG.** 903 (91.32)   
304 - **.CCGCGTCG.** 732 (95.78)   
304 - **.GCGCGGCG.** 707 (91.99)   
304 - **[TCCCGCCG]** 189 (87.33)   
306 + **[GCGCGTCC]** 311 (87.65)   
306 + **.CCGCGCCG.** 341 (91.89)   
306 + **[CCGCCGCG]** 763 (85.35)   
306 + **.CCGCGCCG.** 929 (91.89)   
306 + **[CCGCGTGG]** 1476 (86.42)   
306 - **.CCGCGCCG.** 1038 (91.89)   
306 - **[CCGCCTCG]** 1018 (88.56)   
306 - **[GCGGGTCG]** 908 (86.33)   
306 - **[GCGCGTCC]** 844 (87.65)   
306 - **.GCGCGGCG.** 764 (91.99)   
306 - **.TCGCGTGG.** 143 (90.64)   
307 + **.GCGCGCCG.** 547 (91.32)   
307 + **[CCGCCGCG]** 677 (85.35)   
307 + **[CCGGGTCG]** 714 (86.90)   
307 + **[GGGCGTCG]** 1065 (86.23)   
307 + **.TCGCCTCG.** 1136 (92.78)   
307 + **[CCGCGGCC]** 1329 (85.01)   
307 + **[CCGCTTCG]** 1348 (85.17)   
307 - **.CCGCGCCG.** 1354 (91.89)   
307 - **.CCGCGCCG.** 1234 (91.89)   
307 - **[CCGCCTCG]** 1220 (88.56)   
307 - **[TGGCGGCG]** 983 (87.81)   
307 - **[TCGGGGCG]** 837 (87.91)   
307 - **[CCGCCGCG]** 730 (85.35)   
307 - **.CCGCGGCG.** 678 (92.56)   
307 - **[ACGCGCCG]** 496 (86.10)   
307 - **[TCGCGCCC]** 429 (88.56)   
307 - **.TCGCGTCC.** 333 (92.45)   
308 + **.GCGCGGCG.** 862 (91.99)   
308 + **[CCGCCTCG]** 1080 (88.56)   
308 + **[TCGCGGCT]** 1115 (86.24)   
308 + **.CCGCGCCG.** 1188 (91.89)   
308 + **[CCGCCGCG]** 1366 (85.35)   
308 - **[CCGCGGCC]** 1412 (85.01)   
308 - **[TCCCGGCG]** 1392 (88.00)   
308 - **.CCGCGGCG.** 1367 (92.56)   
308 - **[CCGCGGCC]** 1178 (85.01)   
308 - **[TCGCGTCT]** 876 (89.45)   
308 - **[CCGCCGCG]** 863 (85.35)   
308 - **[CCGCGTGG]** 70 (86.42)   
309 + **.GCGCGGCG.** 1028 (91.99)   
309 - **[CCGCGTGG]** 1224 (86.42)   
309 - **.TCGCGCCG.** 1077 (96.11)   
309 - **[CCGCCGCG]** 1029 (85.35)   
309 - **[CCGCGGCC]** 993 (85.01)   
309 - **[TCCCGCCG]** 557 (87.33)   
310 + **[CCGCCTCG]** 789 (88.56)   
311 + **[GCGCCTCG]** 763 (87.99)   
311 + **[CCGCGGCC]** 874 (85.01)   
311 + **.CCGCGCCG.** 959 (91.89)   
311 + **[TCGCTCCG]** 1040 (85.50)   
311 + **.GCGCGGCG.** 1068 (91.99)   
311 + **.CCGCGGCG.** 1105 (92.56)   
311 + **[CCGCGTCT]** 1344 (85.23)   
311 + **[TCGCCGCG]** 1469 (89.57)   
311 - **.CCGCGGCG.** 1470 (92.56)   
311 - **[CCGCGTCC]** 1261 (88.23)   
311 - **[CCGCCTCG]** 1147 (88.56)   
311 - **[CCGCCGCG]** 1069 (85.35)   
311 - **.CCGCGGCG.** 872 (92.56)   
312 + **.CCGCGCCG.** 984 (91.89)   
312 + **[TCGCGCGG]** 1091 (86.75)   
312 + **.GCGCGGCG.** 1093 (91.99)   
312 + **.GCGCGCCG.** 1360 (91.32)   
312 - **[GCGCGTGG]** 840 (85.84)   
312 - **.GCGCGTCG.** 740 (95.20)   
312 - **[TCGCCTCC]** 735 (85.23)   
312 - **[TCGCCCCG]** 223 (88.90)   
313 + **.TCGCGTGG.** 582 (90.64)   
313 + **[CCGCGGCC]** 825 (85.01)   
313 + **[CCGCCTCG]** 831 (88.56)   
313 + **[TCGCGCGG]** 867 (86.75)   
313 + **[TCGCTCCG]** 875 (85.50)   
313 + **[TCGCGTCA]** 963 (87.59)   
313 + **[TCGCTCCG]** 1002 (85.50)   
313 + **.TCGCGCCG.** 1087 (96.11)   
313 + **.GCGCGCCG.** 1302 (91.32)   
313 + **[TCCCGCCG]** 1442 (87.33)   
313 + **[CCGCCGCG]** 1444 (85.35)   
313 - **.CCGCGGCG.** 1445 (92.56)   
313 - **.GCGCGCCG.** 955 (91.32)   
313 - **[GCGCGTGG]** 255 (85.84)   
314 + **[TCGGGCCG]** 37 (87.23)   
314 + **[TCCCGGCG]** 444 (88.00)   
314 - **[GCGCCTCG]** 43 (87.99)   
314 - **.TCCCGTCG.** 17 (91.22)   
315 + **.TCGCGGCG.** 356 (96.78)   
315 + **.GCGCGCCG.** 1272 (91.32)   
315 + **[TCGCCGCG]** 1340 (89.57)   
315 + **[CCGCGTCC]** 1343 (88.23)   
315 - **[ACGCGGCG]** 1341 (86.77)   
315 - **[CCGCGTCC]** 1284 (88.23)   
315 - **.CCGCGCCG.** 1025 (91.89)   
315 - **.CCGCGCCG.** 1016 (91.89)   
315 - **[GCGCGTCC]** 1005 (87.65)   
315 - **[TCGCCCCG]** 946 (88.90)   
315 - **[TCGCCGCG]** 357 (89.57)   
315 - **[TCGCGGGG]** 1 (87.42)   
316 + **[CCGCGGCC]** 228 (85.01)   
316 + **.CCGCGCCG.** 256 (91.89)   
316 + **[GCGCGTCC]** 897 (87.65)   
316 + **.CCGCGGCG.** 909 (92.56)   
316 + **[TCCCGCCG]** 1180 (87.33)   
316 + **[TCGCCCCG]** 1267 (88.90)   
316 - **.TCGCGCCG.** 912 (96.11)   
316 - **[ACGCGCCG]** 895 (86.10)   
316 - **[TCGCGGCC]** 535 (89.23)   
316 - **[GCGCGTCC]** 199 (87.65)   
316 - **[GCGCCTCG]** 35 (87.99)   
317 - **[TCCCGCCG]** 1199 (87.33)   
318 + **[CCGCGTCC]** 289 (88.23)   
318 + **.CCGCGCCG.** 306 (91.89)   
318 + **[GCGCGTGG]** 1041 (85.84)   
318 + **[CCGGGTCG]** 1192 (86.90)   
318 + **[TGGCGGCG]** 1387 (87.81)   
318 + **.CCGCGGCG.** 1484 (92.56)   
318 - **[CCGCCGCG]** 1485 (85.35)   
318 - **[CCGCGGCC]** 1482 (85.01)   
318 - **.GCGCGCCG.** 1433 (91.32)   
318 - **[GCGCGTGG]** 1345 (85.84)   
318 - **[CCGGGTCG]** 478 (86.90)   
318 - **[TGGCGCCG]** 470 (87.13)   
320 + **[TCCCGGCG]** 518 (88.00)   
320 + **.TGGCGTCG.** 553 (91.02)   
320 + **[TCGCTCCG]** 612 (85.50)   
320 + **[TCGCGCGG]** 628 (86.75)   
320 + **[TCGCGGCC]** 693 (89.23)   
320 + **[GCGCCTCG]** 715 (87.99)   
320 + **[TCGCGGCC]** 720 (89.23)   
320 + **.TCGCGGCG.** 754 (96.78)   
320 + **.CCGCGCCG.** 912 (91.89)   
320 + **.CCGCGGCG.** 991 (92.56)   
320 + **.TCGCGCCG.** 1197 (96.11)   
320 + **[TCGCGGGG]** 1231 (87.42)   
320 + **.GCGCGTCG.** 1347 (95.20)   
320 + **.GCGCGCCG.** 1380 (91.32)   
320 - **.CCGCGGCG.** 1383 (92.56)   
320 - **.GCGCGGCG.** 1341 (91.99)   
320 - **[CCGGGTCG]** 1317 (86.90)   
320 - **.GCGCGCCG.** 875 (91.32)   
320 - **[TCGTGTCG]** 618 (85.92)   
320 - **[CCCCGTCG]** 225 (87.00)   
321 + **.CCGCGGCG.** 454 (92.56)   
321 + **.CCGCGCCG.** 695 (91.89)   
321 + **.GCGCGCCG.** 1259 (91.32)   
321 + **.GCGCGCCG.** 1394 (91.32)   
321 + **[CCGCGGCC]** 1399 (85.01)   
321 + **.TGGCGTCG.** 1483 (91.02)   
321 + **.GCGCGCCG.** 1505 (91.32)   
321 - **[TCCCGCCG]** 1489 (87.33)   
321 - **.CCGCGGCG.** 1397 (92.56)   
321 - **.CCGCGCCG.** 1299 (91.89)   
321 - **.GCGCGGCG.** 698 (91.99)   
321 - **[GCGCGTGG]** 554 (85.84)   
321 - **[TCGGGGCG]** 521 (87.91)   
321 - **[CCGGGTCG]** 504 (86.90)   
321 - **[TCGCGGCC]** 499 (89.23)   
321 - **[CCGCTTCG]** 412 (85.17)   
322 + **.TCGGGTCG.** 305 (91.12)   
322 + **[CCGCCGCG]** 495 (85.35)   
322 + **[TCGGGGCG]** 660 (87.91)   
322 + **[TCCCGGCG]** 734 (88.00)   
322 + **.GCGCGCCG.** 947 (91.32)   
322 + **[TCGCGCCT]** 1173 (85.56)   
322 + **[TCGCCGCG]** 1479 (89.57)   
322 - **.GCGCGGCG.** 1480 (91.99)   
322 - **[CCGCGGCC]** 1002 (85.01)   
322 - **.CCGCGCCG.** 685 (91.89)   
322 - **.CCGCGCCG.** 629 (91.89)   
322 - **.GCGCGGCG.** 496 (91.99)   
322 - **[TCGCCCCG]** 431 (88.90)   
323 + **[CCCCGTCG]** 632 (87.00)   
323 + **[CCGCGGCC]** 1031 (85.01)   
323 + **.CCGCGGCG.** 1230 (92.56)   
323 + **.GCGCGTCG.** 1423 (95.20)   
323 - **[CCGCCGCG]** 1231 (85.35)   
323 - **.CCGCGGCG.** 1228 (92.56)   
323 - **[TCCCGGCG]** 1089 (88.00)   
323 - **[GCGCGTGG]** 1067 (85.84)   
323 - **[CCGCGGCC]** 1029 (85.01)   
323 - **[GCGGGTCG]** 707 (86.33)   
323 - **[TCGCTCCG]** 544 (85.50)   
325 - **[TCGCGCCC]** 1431 (88.56)   
326 + **.TCGCCTCG.** 410 (92.78)   
326 + **[TCGCGGGG]** 688 (87.42)   
326 + **[CCGCCGCG]** 821 (85.35)   
326 + **.CCGCGGCG.** 937 (92.56)   
326 + **.GCGCGGCG.** 1201 (91.99)   
326 + **.CCGCGGCG.** 1302 (92.56)   
326 - **[TCGCGGCC]** 1416 (89.23)   
326 - **[CCGCCGCG]** 1303 (85.35)   
326 - **[CCGCGGCC]** 1300 (85.01)   
326 - **[CCGCCGCG]** 1202 (85.35)   
326 - **.GCGCGGCG.** 822 (91.99)   
326 - **[CCGCGTGG]** 650 (86.42)   
326 - **[TCGCGTCA]** 176 (87.59)   
327 + **[CCGCCTCG]** 548 (88.56)   
327 + **[CCGGGTCG]** 589 (86.90)   
327 - **.TCGCGGCG.** 1112 (96.78)   
327 - **[TGGCGGCG]** 672 (87.81)   
327 - **[TGGCGCCG]** 263 (87.13)   
329 + **[TCGGGCCG]** 227 (87.23)   
329 + **[TCGCGCCC]** 1226 (88.56)   
329 - **[GCGCGTCC]** 1236 (87.65)   
329 - **[GCCCGTCG]** 544 (86.42)   
329 - **[TGGCGGCG]** 495 (87.81)   
329 - **[TGGCGGCG]** 460 (87.81)   
331 + **[TCCCGGCG]** 48 (88.00)   
331 + **[CCGCGTCT]** 268 (85.23)   
331 + **.CCGCGGCG.** 396 (92.56)   
331 + **[CCGCCGCG]** 719 (85.35)   
331 + **[TCGCTGCG]** 1236 (86.18)   
331 + **[TCGGGGCG]** 1269 (87.91)   
331 + **.GCGCGCCG.** 1310 (91.32)   
331 + **[CCGCGTCC]** 1315 (88.23)   
331 + **.CCGCGGCG.** 1331 (92.56)   
331 + **.GCGCGGCG.** 1440 (91.99)   
331 - **[CCGCCGCG]** 1332 (85.35)   
331 - **[ACGCGGCG]** 1313 (86.77)   
331 - **[ACGCGCCG]** 1137 (86.10)   
331 - **.CCGCGGCG.** 720 (92.56)   
331 - **[TCGCCGCG]** 397 (89.57)   
331 - **[TCGCGCGG]** 164 (86.75)   
332 + **[TCGGGGCG]** 48 (87.91)   
332 + **[TCGCGCCC]** 459 (88.56)   
332 + **[GCGCGTCC]** 503 (87.65)   
332 + **[CCGCGTGG]** 814 (86.42)   
332 + **[TCGCCGCG]** 1349 (89.57)   
332 + **[CCGCGGCC]** 1429 (85.01)   
332 + **.GCGCGGCG.** 1486 (91.99)   
332 - **[TCGCCCCG]** 1501 (88.90)   
332 - **[GCGCGTGG]** 1445 (85.84)   
332 - **[CCGCGGCC]** 1427 (85.01)   
332 - **.GCGCGGCG.** 1350 (91.99)   
333 + **.GCGCGGCG.** 1298 (91.99)   
333 + **[CCGCCGCG]** 1319 (85.35)   
333 - **[TGGCGGCG]** 1348 (87.81)   
333 - **.TCGCGGCG.** 1320 (96.78)   
333 - **[TCCCGCCG]** 1301 (87.33)   
333 - **[CCGCCGCG]** 1299 (85.35)   
333 - **.CCGCGGCG.** 1121 (92.56)   
334 + **[TGGCGGCG]** 512 (87.81)   
334 + **[TCCCGGCG]** 679 (88.00)   
334 + **[TCGCCCCG]** 1142 (88.90)   
334 - **[TCGCGGCC]** 870 (89.23)   
334 - **[TCCCGGCG]** 524 (88.00)   
334 - **[TCCCGCCG]** 515 (87.33)   
335 + **[GCGCCTCG]** 611 (87.99)   
335 + **.TCGCGGCG.** 853 (96.78)   
335 + **.GCGCGCCG.** 1099 (91.32)   
335 + **.CCGCGTCG.** 1204 (95.78)   
335 + **[TCGGGCCG]** 1271 (87.23)   
335 - **[CCGCGACG]** 1207 (85.28)   
335 - **[TGGCGGCG]** 1187 (87.81)   
335 - **[CGGCGTCG]** 1078 (86.80)   
335 - **[TCGGGCCG]** 1054 (87.23)   
335 - **[TCGCGGGG]** 1004 (87.42)   
335 - **.GCGCGCCG.** 856 (91.32)   
336 + **.CCGCGCCG.** 980 (91.89)   
336 + **[TCGGGCCG]** 1001 (87.23)   
336 + **[TCGCGGCC]** 1022 (89.23)   
336 + **[TCGCCTCC]** 1472 (85.23)   
336 - **[CCGCGTGG]** 839 (86.42)   
337 + **[TCGCTGCG]** 671 (86.18)   
337 + **.CCGCGGCG.** 841 (92.56)   
337 + **.GCGCGGCG.** 955 (91.99)   
337 + **[CCGCCGCG]** 1069 (85.35)   
337 + **[CCGCGGCC]** 1072 (85.01)   
337 + **.CCGCGGCG.** 1182 (92.56)   
337 - **.CCGCGCCG.** 1347 (91.89)   
337 - **.CCGCGGCG.** 1070 (92.56)   
337 - **[TCCCGCCG]** 958 (87.33)   
337 - **[CCGCCGCG]** 956 (85.35)   
337 - **[TCCCGCCG]** 872 (87.33)   
337 - **[CCGCCGCG]** 842 (85.35)   
337 - **[TCGCGGCT]** 764 (86.24)   
338 + **[CCGCGTCC]** 693 (88.23)   
338 + **.CCGCGCCG.** 759 (91.89)   
338 + **[TCGCGCCC]** 1064 (88.56)   
338 + **[GCGCGTCC]** 1153 (87.65)   
338 + **.TCGCGCCG.** 1171 (96.11)   
338 + **[CCGCCGCG]** 1191 (85.35)   
338 + **[TGGCGGCG]** 1332 (87.81)   
338 + **[TCGGGCCG]** 1476 (87.23)   
338 - **[TCGCGCGG]** 1306 (86.75)   
338 - **.GCGCGGCG.** 1192 (91.99)   
338 - **[TCGCCGCG]** 1054 (89.57)   
338 - **[ACGCGGCG]** 691 (86.77)   
339 - **[TCCCGCCG]** 1069 (87.33)   
339 - **[TGGCGCCG]** 847 (87.13)   
340 + **[TCGCCCCG]** 502 (88.90)   
340 + **.TGGCGTCG.** 682 (91.02)   
340 - **[GCGCGTGG]** 674 (85.84)   
341 + **[TCGCGGGG]** 1232 (87.42)   
341 + **.TCGCGTCG.** 1259 (100.00)   
341 + **.GCGCGGCG.** 1318 (91.99)   
341 + **[GCGCCTCG]** 1351 (87.99)   
341 + **[CCGCCGCG]** 1382 (85.35)   
341 - **[CCGCGTCC]** 1491 (88.23)   
341 - **.GCGCGGCG.** 1383 (91.99)   
341 - **.TCGCCTCG.** 1125 (92.78)   
341 - **[CCGCTTCG]** 1099 (85.17)   
341 - **[GCGCCTCG]** 926 (87.99)   
341 - **[CCGCTTCG]** 880 (85.17)   
341 - **[CCGCTTCG]** 473 (85.17)   
342 + **.GCGCGGCG.** 1154 (91.99)   
342 + **[GCGCGTGG]** 1482 (85.84)   
342 - **[GCGCGTCC]** 1395 (87.65)   
342 - **[CCGCCGCG]** 1155 (85.35)   
342 - **.CCGCGCCG.** 1152 (91.89)   
342 - **.CCGCGCCG.** 1024 (91.89)   
342 - **.CCGCGCCG.** 835 (91.89)   
342 - **[TCGCGCCT]** 719 (85.56)   
343 + **[TCGCATCG]** 209 (86.74)   
343 + **[GCGCGTCC]** 278 (87.65)   
343 + **[GCGGGTCG]** 290 (86.33)   
343 + **[TCGGGGCG]** 520 (87.91)   
343 + **[TCGCGACG]** 780 (89.50)   
343 + **.GCGCGGCG.** 997 (91.99)   
343 + **[TCCCGCCG]** 1023 (87.33)   
343 + **[CCGCGGCC]** 1121 (85.01)   
343 + **.GCGCGGCG.** 1181 (91.99)   
343 + **.TCCCGTCG.** 1219 (91.22)   
343 + **[TCGCGGCC]** 1349 (89.23)   
343 + **[GCGGGTCG]** 1432 (86.33)   
343 - **[TCGGGGCG]** 1489 (87.91)   
343 - **[TCCCGCCG]** 1184 (87.33)   
343 - **[CCGCCGCG]** 1182 (85.35)   
343 - **[CCGCGGCC]** 1119 (85.01)   
343 - **[ACGCGGCG]** 1077 (86.77)   
343 - **[CCGCGTCC]** 669 (88.23)   
343 - **[TCGCTGCG]** 426 (86.18)   
343 - **[TCGGGGCG]** 266 (87.91)   
344 + **[TCCCGCCG]** 798 (87.33)   
344 + **[CCGCTTCG]** 1071 (85.17)   
344 + **.GCGCGCCG.** 1165 (91.32)   
345 + **[TCGCCTCC]** 144 (85.23)   
346 + **.TCGCGGCG.** 22 (96.78)   
346 + **.CCGCGGCG.** 84 (92.56)   
346 + **.TCCCGTCG.** 277 (91.22)   
346 + **[TCCCGGCG]** 599 (88.00)   
346 + **[TCGGGCCG]** 837 (87.23)   
346 + **.GCGCGCCG.** 1230 (91.32)   
346 + **[CCGCCGCG]** 1235 (85.35)   
346 + **[CCGCGTCC]** 1238 (88.23)   
346 + **[GCGCCTCG]** 1388 (87.99)   
346 - **[TCACGTCG]** 1491 (87.82)   
346 - **[CCGCGGCC]** 1406 (85.01)   
346 - **.GCGCGCCG.** 1339 (91.32)   
346 - **[ACGCGGCG]** 1236 (86.77)   
346 - **.GCGCGCCG.** 1228 (91.32)   
346 - **[TCGCCTCC]** 976 (85.23)   
346 - **[CCGCGGCC]** 905 (85.01)   
346 - **[TCGCGGCT]** 526 (86.24)   
346 - **[TCCCGGCG]** 98 (88.00)   
346 - **[CCGCCGCG]** 85 (85.35)   
347 + **[TCGCGCCC]** 552 (88.56)   
347 + **[TCGCCGCG]** 907 (89.57)   
347 + **[TCCCGGCG]** 951 (88.00)   
347 + **.GCGCGGCG.** 1087 (91.99)   
347 + **.CCGCGCCG.** 1256 (91.89)   
347 + **[CCGCGGCC]** 1285 (85.01)   
347 - **[TGGCGCCG]** 1305 (87.13)   
347 - **[TCGGGGCG]** 1266 (87.91)   
347 - **[TCCCGCCG]** 1109 (87.33)   
347 - **.GCGCGCCG.** 1100 (91.32)   
347 - **.GCGCGGCG.** 908 (91.99)   
348 + **[TCGCCGCG]** 67 (89.57)   
348 + **.GCGCGCCG.** 131 (91.32)   
348 + **[TCGCGGCC]** 343 (89.23)   
348 + **[TCGCGCCC]** 374 (88.56)   
348 + **[TGGCGGCG]** 541 (87.81)   
348 + **.CCGCGTCG.** 663 (95.78)   
348 + **[CCCCGTCG]** 683 (87.00)   
348 + **[TCGCCGCG]** 694 (89.57)   
348 + **.CCGCGGCG.** 711 (92.56)   
348 + **[GCCCGTCG]** 902 (86.42)   
348 + **[GCCCGTCG]** 922 (86.42)   
348 + **[TGGCGCCG]** 975 (87.13)   
348 + **.TCGCGGCG.** 1027 (96.78)   
348 + **.GCGCGGCG.** 1239 (91.99)   
348 + **[GCGCCTCG]** 1391 (87.99)   
348 + **.TCGCGTGG.** 1413 (90.64)   
348 + **[GCGCGTGG]** 1479 (85.84)   
348 + **.TCGCGCCG.** 1488 (96.11)   
348 - **.TGGCGTCG.** 1444 (91.02)   
348 - **[TCGCGGCT]** 1362 (86.24)   
348 - **.CCGCGCCG.** 1278 (91.89)   
348 - **[CCGCGTCC]** 1189 (88.23)   
348 - **[CCGCCGCG]** 1028 (85.35)   
348 - **[TCCCGGCG]** 978 (88.00)   
348 - **.GCGCGCCG.** 861 (91.32)   
348 - **.GCGCGGCG.** 695 (91.99)   
348 - **[ACGCGGCG]** 661 (86.77)   
348 - **[GCGCCTCG]** 603 (87.99)   
348 - **[TCGGGCCG]** 598 (87.23)   
348 - **.CCGCGCCG.** 544 (91.89)   
348 - **[TCGCGTCT]** 518 (89.45)   
348 - **[CCGCGTCC]** 307 (88.23)   
348 - **.GCGCGCCG.** 292 (91.32)   
348 - **[TCGGGCCG]** 212 (87.23)   
348 - **.CCGCGGCG.** 134 (92.56)   
348 - **.CCGCGGCG.** 68 (92.56)   
349 + **.GCGCGGCG.** 1164 (91.99)   
349 + **.GCGCGTCG.** 1309 (95.20)   
349 - **[TCCCGCCG]** 1260 (87.33)   
349 - **[CCGCCGCG]** 1165 (85.35)   
349 - **.CCGCGCCG.** 1162 (91.89)   
349 - **[TCGCGCCC]** 1044 (88.56)   
349 - **.CCGCGCCG.** 836 (91.89)   
349 - **[CGGCGTCG]** 655 (86.80)   
350 + **.TGGCGTCG.** 995 (91.02)   
350 + **[TCGCGGGG]** 1000 (87.42)   
350 + **[CCGCCTCG]** 1140 (88.56)   
350 + **.CCGCGCCG.** 1185 (91.89)   
350 + **[TCGCGCGG]** 1427 (86.75)   
350 - **[TCCCGGCG]** 1445 (88.00)   
350 - **.CCGCGCCG.** 1436 (91.89)   
350 - **.GCGCGCCG.** 1342 (91.32)   
350 - **[CCGGGTCG]** 1227 (86.90)   
350 - **[TCGCTCCG]** 1078 (85.50)   
350 - **.CCGCGCCG.** 1043 (91.89)   
350 - **[CCGCCTCG]** 1025 (88.56)   
350 - **[TCGCCCCG]** 1020 (88.90)   
350 - **[CCGCGACG]** 998 (85.28)   
351 + **[TCCCGCCG]** 759 (87.33)   
351 + **[TCGCATCG]** 916 (86.74)   
351 + **[ACGCGGCG]** 974 (86.77)   
351 + **[TCCCGGCG]** 988 (88.00)   
351 + **[TCGCGTCT]** 1140 (89.45)   
351 + **[CCGCGGCC]** 1184 (85.01)   
351 + **[CCGCGGCC]** 1254 (85.01)   
351 - **[TCGCCGCG]** 975 (89.57)   
351 - **.CCGCGGCG.** 936 (92.56)   
351 - **[GCGCCTCG]** 573 (87.99)   
351 - **[CCGCCTCG]** 198 (88.56)   
351 - **[CCGCCTCG]** 45 (88.56)   
351 - **.TCGCCTCG.** 40 (92.78)   
352 + **.TCGCGCCG.** 54 (96.11)   
352 + **.CCGCGGCG.** 197 (92.56)   
352 + **.GCGCGCCG.** 202 (91.32)   
352 + **.CCGCGGCG.** 311 (92.56)   
352 + **[CCGCCGCG]** 351 (85.35)   
352 + **[CCGCGGCC]** 620 (85.01)   
352 + **.CCGCGCCG.** 717 (91.89)   
352 + **[CCGCGGCC]** 941 (85.01)   
352 + **.GCGCGCCG.** 1227 (91.32)   
352 + **[CCGCGGCC]** 1339 (85.01)   
352 - **[TCGCGCCT]** 1063 (85.56)   
352 - **[GCGCCTCG]** 1061 (87.99)   
352 - **.CCGCGGCG.** 352 (92.56)   
352 - **[CCGCCGCG]** 312 (85.35)   
352 - **[CCGCGGCC]** 309 (85.01)   
352 - **.CCGCGTCG.** 281 (95.78)   
352 - **.GCGCGCCG.** 200 (91.32)   
353 + **[TCGCGCGG]** 492 (86.75)   
353 + **.GCGCGCCG.** 662 (91.32)   
353 + **[TGGCGGCG]** 794 (87.81)   
353 + **[CCGCCTCG]** 1122 (88.56)   
353 + **.GCGCGGCG.** 1419 (91.99)   
353 + **.CCGCGGCG.** 1437 (92.56)   
353 - **[TCGCTCCG]** 1443 (85.50)   
353 - **[CCGCCGCG]** 1438 (85.35)   
353 - **[CCGCCGCG]** 1420 (85.35)   
353 - **.TCGCGGCG.** 1178 (96.78)   
353 - **[TCGCGACG]** 873 (89.50)   
353 - **[TCCCGCCG]** 777 (87.33)   
353 - **[TGGCGGCG]** 745 (87.81)   
353 - **[CCGCGTCC]** 707 (88.23)   
353 - **.TCGCGTCC.** 538 (92.45)   
353 - **.TCGCCTCG.** 296 (92.78)   
353 - **[GCGCGTCC]** 186 (87.65)   
353 - **[GCGCGTGG]** 172 (85.84)   
356 + **[CCGCCGCG]** 782 (85.35)   
356 + **[GCGCGTCC]** 1172 (87.65)   
356 - **.TCGCGGCG.** 783 (96.78)   
356 - **[TCGGGGCG]** 474 (87.91)   
356 - **.GCGCGGCG.** 90 (91.99)   
357 + **[GCGCGTGG]** 528 (85.84)   
357 + **[CCGCGTCC]** 543 (88.23)   
357 + **[CCGCCGCG]** 989 (85.35)   
357 + **.CCGCGCCG.** 992 (91.89)   
357 + **[TCCCGCCG]** 1503 (87.33)   
357 - **.GCGCGGCG.** 990 (91.99)   
357 - **[TCCCGCCG]** 952 (87.33)   
357 - **[GCGCGTGG]** 920 (85.84)   
357 - **[GCGGGTCG]** 539 (86.33)   
358 + **[TCGCTGCG]** 578 (86.18)   
358 + **[CCGCGGCC]** 773 (85.01)   
358 + **.GCGCGGCG.** 977 (91.99)   
358 + **.TCCCGTCG.** 1247 (91.22)   
358 - **[CCGCCGCG]** 978 (85.35)   
358 - **[GCCCGTCG]** 875 (86.42)   
359 + **.CCGCGGCG.** 1233 (92.56)   
359 + **.GCGCGGCG.** 1254 (91.99)   
359 + **.GCGCGCCG.** 1306 (91.32)   
359 - **[CCGCCGCG]** 1255 (85.35)   
359 - **.GCGCGCCG.** 1236 (91.32)   
359 - **[TGGCGGCG]** 1061 (87.81)   
359 - **[GGGCGTCG]** 354 (86.23)   
360 + **[TCGCCCCG]** 640 (88.90)   
360 + **[TCGCCCCG]** 665 (88.90)   
360 + **[CCGCTTCG]** 799 (85.17)   
360 + **.CCGCGCCG.** 1067 (91.89)   
360 + **.CCGCGGCG.** 1126 (92.56)   
360 + **.GCGCGCCG.** 1244 (91.32)   
360 - **.CCGCGCCG.** 1184 (91.89)   
360 - **.CCGCGGCG.** 1070 (92.56)   
360 - **.GCGCGGCG.** 1065 (91.99)   
360 - **[CCGCCGCG]** 973 (85.35)   
360 - **[TGGCGGCG]** 770 (87.81)   
360 - **.GCGCGGCG.** 651 (91.99)   
361 + **[TCGCCTCC]** 680 (85.23)   
361 + **[CCGCGGCC]** 1417 (85.01)   
361 - **[CCGCCTCG]** 1462 (88.56)   
361 - **.TCGCCTCG.** 607 (92.78)   
362 + **[CCGCGGCC]** 282 (85.01)   
362 + **[GCGCGTCC]** 623 (87.65)   
362 + **[CCGCGTCC]** 737 (88.23)   
362 + **.GCGCGGCG.** 910 (91.99)   
362 - **[CCGCTTCG]** 1471 (85.17)   
362 - **[TCGCTCCG]** 1313 (85.50)   
362 - **[TCCCGGCG]** 1265 (88.00)   
362 - **[CGGCGTCG]** 1262 (86.80)   
362 - **[CCGCCTCG]** 1145 (88.56)   
362 - **[ACGCGGCG]** 735 (86.77)   
362 - **.CCGCGTCG.** 524 (95.78)   
362 - **[TCGCGGGG]** 424 (87.42)   
363 + **[CCGCGGCC]** 693 (85.01)   
363 + **[CCGCGGCC]** 798 (85.01)   
363 + **[TCGCGGCC]** 1082 (89.23)   
363 - **.GCGCGCCG.** 1111 (91.32)   
363 - **[CCGCGACG]** 1080 (85.28)   
363 - **[CCGCGGCC]** 691 (85.01)   
364 + **[TCGCTGCG]** 1022 (86.18)   
364 + **[ACGCGGCG]** 1256 (86.77)   
364 - **[CCGCCGCG]** 1257 (85.35)   
364 - **[CCGCGTCT]** 1254 (85.23)   
364 - **[CCGCGTCT]** 296 (85.23)   
366 + **[TCGGGCCG]** 174 (87.23)   
366 + **[CCGGGTCG]** 210 (86.90)   
366 + **[TCGGGGCG]** 215 (87.91)   
366 + **.GCGCGGCG.** 409 (91.99)   
366 + **.CCGCGGCG.** 444 (92.56)   
366 + **.CCGCGCCG.** 453 (91.89)   
366 + **[CCGCGTCC]** 520 (88.23)   
366 + **[TCGCCCCG]** 621 (88.90)   
366 + **.GCGCGCCG.** 657 (91.32)   
366 + **[CCGCCTCG]** 718 (88.56)   
366 + **.TCCCGTCG.** 781 (91.22)   
366 + **.TCGCGTCG.** 853 (100.00)   
366 + **.GCGCGCCG.** 906 (91.32)   
366 + **.CCGCGGCG.** 953 (92.56)   
366 + **.TCCCGTCG.** 961 (91.22)   
366 + **[TCGCGTTG]** 975 (87.97)   
366 + **[GCCCGTCG]** 986 (86.42)   
366 + **.TCGGGTCG.** 1111 (91.12)   
366 + **.GCGCGGCG.** 1392 (91.99)   
366 - **.TCGCGTCC.** 1429 (92.45)   
366 - **[CCGCGGCC]** 1411 (85.01)   
366 - **[CCGCCGCG]** 1393 (85.35)   
366 - **.CCGCGGCG.** 1252 (92.56)   
366 - **[CGGCGTCG]** 997 (86.80)   
366 - **[TCGCTCCG]** 992 (85.50)   
366 - **[TCGTGTCG]** 815 (85.92)   
366 - **[CCGCCGCG]** 410 (85.35)   
366 - **[TCGGGGCG]** 392 (87.91)   
366 - **.GCGCGCCG.** 265 (91.32)   
366 - **[CCGCGGCC]** 181 (85.01)   
366 - **[GCGCCTCG]** 67 (87.99)   
367 + **[TCGCTCCG]** 936 (85.50)   
367 + **.CCGCGCCG.** 1033 (91.89)   
367 + **[CCGCCGCG]** 1188 (85.35)   
367 + **.CCGCGGCG.** 1191 (92.56)   
367 - **[CCGCCGCG]** 1192 (85.35)   
367 - **.CCGCGGCG.** 1189 (92.56)   
367 - **[CCGCGGCC]** 1089 (85.01)   
367 - **[CCGCGGCC]** 1062 (85.01)   
367 - **[CCGCGGCC]** 554 (85.01)   
367 - **[TCCCGCCG]** 313 (87.33)   
367 - **[CGGCGTCG]** 220 (86.80)   
367 - **[TCGCTGCG]** 215 (86.18)   
  

**Frequency Matrix**

| All Occs Best Occs|  |  |  |  |  |  |  |  |  |  |  |  |  |  |  |  |  |  |  |  |  |  |  |  |  |  |  |  |  |  |  |  |  |  |  |  |  |  |  |  |  |  |  |  |  |  |  |  |  |  |  |  |  |  |  |  |  |  |  |  |  |  |  |  |  |  |  |  |  |  |  |  |  |  |  |  |  |  |  |  |  |  |  |  |  |  |  |  |  |  |  |  |  |  |  |  |  |  |  |  |  |  |  |  |  |  |  |  | | --- | --- | --- | --- | --- | --- | --- | --- | --- | --- | --- | --- | --- | --- | --- | --- | --- | --- | --- | --- | --- | --- | --- | --- | --- | --- | --- | --- | --- | --- | --- | --- | --- | --- | --- | --- | --- | --- | --- | --- | --- | --- | --- | --- | --- | --- | --- | --- | --- | --- | --- | --- | --- | --- | --- | --- | --- | --- | --- | --- | --- | --- | --- | --- | --- | --- | --- | --- | --- | --- | --- | --- | --- | --- | --- | --- | --- | --- | --- | --- | --- | --- | --- | --- | --- | --- | --- | --- | --- | --- | --- | --- | --- | --- | --- | --- | --- | --- | --- | --- | --- | --- | --- | --- | --- | --- | --- | --- | | **A C G T A C G T | | | | | | | | | | | |**| **1** 191 818 708 2361 | 0 365 346 183|  |  |  |  |  |  |  |  |  |  |  |  |  |  |  |  |  |  |  |  |  |  |  |  |  |  |  |  |  |  |  |  |  |  |  |  |  |  |  |  |  |  |  |  |  |  |  |  |  |  |  |  |  |  |  |  |  |  |  |  |  |  |  |  |  |  |  |  |  |  |  |  |  |  |  |  |  |  |  |  |  |  |  |  | | --- | --- | --- | --- | --- | --- | --- | --- | --- | --- | --- | --- | --- | --- | --- | --- | --- | --- | --- | --- | --- | --- | --- | --- | --- | --- | --- | --- | --- | --- | --- | --- | --- | --- | --- | --- | --- | --- | --- | --- | --- | --- | --- | --- | --- | --- | --- | --- | --- | --- | --- | --- | --- | --- | --- | --- | --- | --- | --- | --- | --- | --- | --- | --- | --- | --- | --- | --- | --- | --- | --- | --- | --- | --- | --- | --- | --- | --- | --- | --- | --- | --- | --- | --- | | **2** 80 3517 369 112 | 0 878 16 0|  |  |  |  |  |  |  |  |  |  |  |  |  |  |  |  |  |  |  |  |  |  |  |  |  |  |  |  |  |  |  |  |  |  |  |  |  |  |  |  |  |  |  |  |  |  |  |  |  |  |  |  |  |  |  |  |  |  |  |  |  |  |  |  |  |  |  |  |  |  |  |  | | --- | --- | --- | --- | --- | --- | --- | --- | --- | --- | --- | --- | --- | --- | --- | --- | --- | --- | --- | --- | --- | --- | --- | --- | --- | --- | --- | --- | --- | --- | --- | --- | --- | --- | --- | --- | --- | --- | --- | --- | --- | --- | --- | --- | --- | --- | --- | --- | --- | --- | --- | --- | --- | --- | --- | --- | --- | --- | --- | --- | --- | --- | --- | --- | --- | --- | --- | --- | --- | --- | --- | --- | | **3** 159 373 3383 163 | 0 20 874 0|  |  |  |  |  |  |  |  |  |  |  |  |  |  |  |  |  |  |  |  |  |  |  |  |  |  |  |  |  |  |  |  |  |  |  |  |  |  |  |  |  |  |  |  |  |  |  |  |  |  |  |  |  |  |  |  |  |  |  |  | | --- | --- | --- | --- | --- | --- | --- | --- | --- | --- | --- | --- | --- | --- | --- | --- | --- | --- | --- | --- | --- | --- | --- | --- | --- | --- | --- | --- | --- | --- | --- | --- | --- | --- | --- | --- | --- | --- | --- | --- | --- | --- | --- | --- | --- | --- | --- | --- | --- | --- | --- | --- | --- | --- | --- | --- | --- | --- | --- | --- | | **4** 137 3467 373 101 | 0 885 9 0|  |  |  |  |  |  |  |  |  |  |  |  |  |  |  |  |  |  |  |  |  |  |  |  |  |  |  |  |  |  |  |  |  |  |  |  |  |  |  |  |  |  |  |  |  |  |  |  | | --- | --- | --- | --- | --- | --- | --- | --- | --- | --- | --- | --- | --- | --- | --- | --- | --- | --- | --- | --- | --- | --- | --- | --- | --- | --- | --- | --- | --- | --- | --- | --- | --- | --- | --- | --- | --- | --- | --- | --- | --- | --- | --- | --- | --- | --- | --- | --- | | **5** 115 525 3214 224 | 0 21 873 0|  |  |  |  |  |  |  |  |  |  |  |  |  |  |  |  |  |  |  |  |  |  |  |  |  |  |  |  |  |  |  |  |  |  |  |  | | --- | --- | --- | --- | --- | --- | --- | --- | --- | --- | --- | --- | --- | --- | --- | --- | --- | --- | --- | --- | --- | --- | --- | --- | --- | --- | --- | --- | --- | --- | --- | --- | --- | --- | --- | --- | | **6** 154 811 960 2153 | 0 339 396 159|  |  |  |  |  |  |  |  |  |  |  |  |  |  |  |  |  |  |  |  |  |  |  |  | | --- | --- | --- | --- | --- | --- | --- | --- | --- | --- | --- | --- | --- | --- | --- | --- | --- | --- | --- | --- | --- | --- | --- | --- | | **7** 116 3463 330 169 | 0 883 11 0|  |  |  |  |  |  |  |  |  |  |  |  | | --- | --- | --- | --- | --- | --- | --- | --- | --- | --- | --- | --- | | **8** 143 484 3223 228 | 0 18 876 0 | | | | | | | | | | | | | | | | | | | | | | | | | | | | | | | | | | | | | | | | | | | | | | | | | | | | | | | | | | | | | | | | | | | | | | | | | | | | | | | | | | | | | | | | | | | | | | | | | | | | | | | | | | | | |

  
  

---

  
  
  
 **CGCGTACGCG**   
**CGCGTACGCG**  
  
6 redundant motifs found:  
CGAACACGCG - CGCGTCCGCG - CGTCTACGCG - CGCGAACGCG - CGGGTACGCG - CGCGTACG -   
  
Best occurrences (match percentage):   
Seq St oligo pos match
  
 1 + **.CGCGCCCGCC.** 1071 (90.62)   
1 - **.GGCGGGCGCG.** 1071 (90.62)   
2 + **.CGCGCGGGCG.** 705 (90.54)   
2 + **[CGCGGGCGGG]** 707 (89.87)   
2 + **[CGGGTCCGCC]** 737 (85.07)   
2 + **[CGGGCACCCG]** 848 (85.05)   
2 + **[CGCGGACCCC]** 894 (85.07)   
2 + **[CGGGTCCCCG]** 923 (85.22)   
2 + **.CGCGGCCCCG.** 977 (90.94)   
2 + **[CGGGGACCCG]** 1192 (85.22)   
2 - **[CGGGTCCCCG]** 1192 (85.22)   
2 - **.CGGGGCCGCG.** 977 (90.94)   
2 - **[CGGGGACCCG]** 923 (85.22)   
2 - **[GGGGTCCGCG]** 894 (85.07)   
2 - **[CGGGTGCCCG]** 848 (85.05)   
2 - **[GGCGGACCCG]** 737 (85.07)   
2 - **[CCCGCCCGCG]** 707 (89.87)   
2 - **.CGCCCGCGCG.** 705 (90.54)   
3 + **.CGCGGCGGCG.** 1223 (90.88)   
3 + **.CGCCGCCGCG.** 1412 (90.88)   
3 - **.CGCGGCGGCG.** 1412 (90.88)   
3 - **.CGCCGCCGCG.** 1223 (90.88)   
4 + **.CGCCCCCGCG.** 568 (90.71)   
4 + **[CGCGGAGCCG]** 625 (85.16)   
4 + **[GGCGACCGCG]** 635 (85.74)   
4 + **[CGGGTCGGCG]** 687 (85.16)   
4 + **.CGCCGCCGCG.** 695 (90.88)   
4 + **[CGCCGTCGCG]** 736 (85.83)   
4 + **.CGCGGCGGCG.** 822 (90.88)   
4 + **[CGCGAGCGCC]** 830 (85.57)   
4 + **[GGCGACCGCG]** 892 (85.74)   
4 + **.CGCGGAGGCG.** 898 (91.99)   
4 + **[CGCGTGCGCA]** 918 (88.64)   
4 + **.CGCGCCCGCC.** 1199 (90.62)   
4 + **.CGGGGGCGCG.** 1268 (90.77)   
4 + **.CGCGGGGGCG.** 1276 (90.71)   
4 + **.CGGGGGCGCG.** 1278 (90.77)   
4 - **.CGCGCCCCCG.** 1278 (90.77)   
4 - **.CGCCCCCGCG.** 1276 (90.71)   
4 - **.CGCGCCCCCG.** 1268 (90.77)   
4 - **.GGCGGGCGCG.** 1199 (90.62)   
4 - **[TGCGCACGCG]** 918 (88.64)   
4 - **.CGCCTCCGCG.** 898 (91.99)   
4 - **[CGCGGTCGCC]** 892 (85.74)   
4 - **[GGCGCTCGCG]** 830 (85.57)   
4 - **.CGCCGCCGCG.** 822 (90.88)   
4 - **[CGCGACGGCG]** 736 (85.83)   
4 - **.CGCGGCGGCG.** 695 (90.88)   
4 - **[CGCCGACCCG]** 687 (85.16)   
4 - **[CGCGGTCGCC]** 635 (85.74)   
4 - **[CGGCTCCGCG]** 625 (85.16)   
4 - **.CGCGGGGGCG.** 568 (90.71)   
5 + **[CGCGGGCACG]** 816 (86.75)   
5 + **[CGCGGCCGCA]** 1395 (87.70)   
5 + **[CGCGGCCGAG]** 1440 (86.41)   
5 - **[CTCGGCCGCG]** 1440 (86.41)   
5 - **[TGCGGCCGCG]** 1395 (87.70)   
5 - **[CGTGCCCGCG]** 816 (86.75)   
8 + **.CGCCCCCGCG.** 782 (90.71)   
8 + **.CGCGGGCGCC.** 913 (90.62)   
8 + **.CGCGGGCGCG.** 929 (97.61)   
8 + **[CGGGGTCGCG]** 1200 (85.89)   
8 + **[CGGGCTCGCG]** 1460 (85.72)   
8 + **.GGCGCCCGCG.** 1504 (90.62)   
8 - **.CGCGGGCGCC.** 1504 (90.62)   
8 - **[CGCGAGCCCG]** 1460 (85.72)   
8 - **[CGCGACCCCG]** 1200 (85.89)   
8 - **.CGCGCCCGCG.** 929 (97.61)   
8 - **.GGCGCCCGCG.** 913 (90.62)   
8 - **.CGCGGGGGCG.** 782 (90.71)   
10 + **.GGCGGCCGCG.** 31 (90.79)   
10 + **[CGAGGACGCG]** 824 (88.96)   
10 + **[CGGGGACGCC]** 892 (85.07)   
10 + **.CCCGGCCGCG.** 1115 (90.04)   
10 + **[CGCGGTCGCC]** 1448 (85.74)   
10 - **[GGCGACCGCG]** 1448 (85.74)   
10 - **.CGCGGCCGGG.** 1115 (90.04)   
10 - **[GGCGTCCCCG]** 892 (85.07)   
10 - **[CGCGTCCTCG]** 824 (88.96)   
10 - **.CGCGGCCGCC.** 31 (90.79)   
11 + **[CGCGTGCGTG]** 34 (87.55)   
11 + **.CCCGGCCGCG.** 493 (90.04)   
11 + **[CGCGGACCCC]** 531 (85.07)   
11 + **.CGCGGCCGCG.** 611 (97.78)   
11 + **[CGCGGGCGCA]** 663 (87.53)   
11 + **.CCCGGACGCG.** 694 (91.15)   
11 + **.CCCGGCCGCG.** 709 (90.04)   
11 + **.CGCGGCCCCG.** 715 (90.94)   
11 + **.CGCGCCCGCC.** 957 (90.62)   
11 + **[CGCGCTCCCG]** 996 (85.72)   
11 + **.CGCCGCCGCG.** 1151 (90.88)   
11 - **.CGCGGCGGCG.** 1151 (90.88)   
11 - **[CGGGAGCGCG]** 996 (85.72)   
11 - **.GGCGGGCGCG.** 957 (90.62)   
11 - **.CGGGGCCGCG.** 715 (90.94)   
11 - **.CGCGGCCGGG.** 709 (90.04)   
11 - **.CGCGTCCGGG.** 694 (91.15)   
11 - **[TGCGCCCGCG]** 663 (87.53)   
11 - **.CGCGGCCGCG.** 611 (97.78)   
11 - **[GGGGTCCGCG]** 531 (85.07)   
11 - **.CGCGGCCGGG.** 493 (90.04)   
11 - **[CACGCACGCG]** 34 (87.55)   
12 + **[CGCGGGCACG]** 117 (86.75)   
12 + **[CGCGGGCGGG]** 180 (89.87)   
12 + **.CGCCCACGCG.** 201 (91.82)   
12 + **[CGCGCCCTCG]** 526 (87.68)   
12 + **[CGGGGAGGCG]** 584 (85.16)   
12 + **.CGCGCGCGCC.** 1032 (90.45)   
12 + **[CGCGGGCGTG]** 1097 (86.44)   
12 + **[CGCGCTCCCG]** 1205 (85.72)   
12 + **.GGCGGCCGCG.** 1247 (90.79)   
12 + **[CGCGGGCGTG]** 1335 (86.44)   
12 + **[CGCGTAGGGG]** 1465 (85.36)   
12 - **[CCCCTACGCG]** 1465 (85.36)   
12 - **[CACGCCCGCG]** 1335 (86.44)   
12 - **.CGCGGCCGCC.** 1247 (90.79)   
12 - **[CGGGAGCGCG]** 1205 (85.72)   
12 - **[CACGCCCGCG]** 1097 (86.44)   
12 - **.GGCGCGCGCG.** 1032 (90.45)   
12 - **[CGCCTCCCCG]** 584 (85.16)   
12 - **[CGAGGGCGCG]** 526 (87.68)   
12 - **.CGCGTGGGCG.** 201 (91.82)   
12 - **[CCCGCCCGCG]** 180 (89.87)   
12 - **[CGTGCCCGCG]** 117 (86.75)   
13 + **[AGCGCGCGCG]** 529 (86.91)   
13 + **.GGCGCCCGCG.** 697 (90.62)   
13 + **.CGCCGCCGCG.** 717 (90.88)   
13 + **[CGCGGCCGCT]** 947 (87.25)   
13 + **.CGCGGCCGCC.** 1064 (90.79)   
13 + **[CGCCAGCGCG]** 1070 (85.66)   
13 + **[CGCGCGCTCG]** 1112 (87.51)   
13 + **[CGGGTCCCCG]** 1306 (85.22)   
13 - **[CGGGGACCCG]** 1306 (85.22)   
13 - **[CGAGCGCGCG]** 1112 (87.51)   
13 - **[CGCGCTGGCG]** 1070 (85.66)   
13 - **.GGCGGCCGCG.** 1064 (90.79)   
13 - **[AGCGGCCGCG]** 947 (87.25)   
13 - **.CGCGGCGGCG.** 717 (90.88)   
13 - **.CGCGGGCGCC.** 697 (90.62)   
13 - **[CGCGCGCGCT]** 529 (86.91)   
14 + **[GGCGTCCCCG]** 444 (85.07)   
14 + **[CGCAGGCGCG]** 708 (86.48)   
14 + **[TGCGGGCGCG]** 742 (87.53)   
14 + **[CGAGCACGCG]** 858 (88.79)   
14 + **.CGCGAACGCG.** 864 (93.84)   
14 + **[CGCAGCCGCG]** 985 (86.65)   
14 - **[CGCGGCTGCG]** 985 (86.65)   
14 - **.CGCGTTCGCG.** 864 (93.84)   
14 - **[CGCGTGCTCG]** 858 (88.79)   
14 - **[CGCGCCCGCA]** 742 (87.53)   
14 - **[CGCGCCTGCG]** 708 (86.48)   
14 - **[CGGGGACGCC]** 444 (85.07)   
15 + **.CGCGCGCCCG.** 537 (90.60)   
15 + **.CGCGCCCGCG.** 539 (97.61)   
15 + **[CGCCTCCGCC]** 620 (85.01)   
15 + **[CGCCTCCGCC]** 626 (85.01)   
15 + **[CACGGCCGCG]** 851 (86.61)   
15 + **[CGGGTCGGCG]** 1110 (85.16)   
15 + **[CGCGTCCCCC]** 1183 (85.07)   
15 + **[CGCGTCCTCG]** 1232 (88.96)   
15 - **[CGAGGACGCG]** 1232 (88.96)   
15 - **[GGGGGACGCG]** 1183 (85.07)   
15 - **[CGCCGACCCG]** 1110 (85.16)   
15 - **[CGCGGCCGTG]** 851 (86.61)   
15 - **[GGCGGAGGCG]** 626 (85.01)   
15 - **[GGCGGAGGCG]** 620 (85.01)   
15 - **.CGCGGGCGCG.** 539 (97.61)   
15 - **.CGGGCGCGCG.** 537 (90.60)   
16 + **[CGCGGACGTG]** 494 (87.72)   
16 + **[CGTGGGCGCG]** 899 (86.75)   
16 + **.CGCGGCGGCG.** 1150 (90.88)   
16 + **.CGCGCCCGCC.** 1300 (90.62)   
16 - **.GGCGGGCGCG.** 1300 (90.62)   
16 - **.CGCCGCCGCG.** 1150 (90.88)   
16 - **[CGCGCCCACG]** 899 (86.75)   
16 - **[CACGTCCGCG]** 494 (87.72)   
18 + **[CCCGCCCGCG]** 908 (89.87)   
18 + **.CGCGGCCGGG.** 932 (90.04)   
18 + **[CGGGTCGGCG]** 1160 (85.16)   
18 + **.GGCGGCCGCG.** 1325 (90.79)   
18 + **.CGCGTCCGCC.** 1331 (91.90)   
18 - **.GGCGGACGCG.** 1331 (91.90)   
18 - **.CGCGGCCGCC.** 1325 (90.79)   
18 - **[CGCCGACCCG]** 1160 (85.16)   
18 - **.CCCGGCCGCG.** 932 (90.04)   
18 - **[CGCGGGCGGG]** 908 (89.87)   
20 + **[AGCGCCCGCG]** 194 (87.08)   
20 + **.GGCGCCCGCG.** 1029 (90.62)   
20 + **.GGCGGCCGCG.** 1193 (90.79)   
20 - **.CGCGGCCGCC.** 1193 (90.79)   
20 - **.CGCGGGCGCC.** 1029 (90.62)   
20 - **[CGCGGGCGCT]** 194 (87.08)   
22 + **.CGCGGCCGGG.** 618 (90.04)   
22 + **.CGCCGCCGCG.** 768 (90.88)   
22 - **.CGCGGCGGCG.** 768 (90.88)   
22 - **.CCCGGCCGCG.** 618 (90.04)   
24 + **[GGCGGTCGCG]** 449 (85.74)   
24 + **[GGCGACCGCG]** 540 (85.74)   
24 + **.CGCGGGGGCG.** 944 (90.71)   
24 + **[CGCGGCTGCG]** 1217 (86.65)   
24 + **[CGCGCTGGCG]** 1321 (85.66)   
24 - **[CGCCAGCGCG]** 1321 (85.66)   
24 - **[CGCAGCCGCG]** 1217 (86.65)   
24 - **.CGCCCCCGCG.** 944 (90.71)   
24 - **[CGCGGTCGCC]** 540 (85.74)   
24 - **[CGCGACCGCC]** 449 (85.74)   
25 + **.CGCGGAGGCG.** 551 (91.99)   
25 - **.CGCCTCCGCG.** 551 (91.99)   
26 + **.CCCGGCCGCG.** 180 (90.04)   
26 + **[CGAGGACGCG]** 749 (88.96)   
26 + **.CGCGGCCCCG.** 826 (90.94)   
26 + **[CCCGCGCGCG]** 841 (89.70)   
26 + **[CGCGGGAGCG]** 1487 (86.11)   
26 - **[CGCTCCCGCG]** 1487 (86.11)   
26 - **[CGCGCGCGGG]** 841 (89.70)   
26 - **.CGGGGCCGCG.** 826 (90.94)   
26 - **[CGCGTCCTCG]** 749 (88.96)   
26 - **.CGCGGCCGGG.** 180 (90.04)   
28 + **[GGCCTCCGCG]** 512 (85.01)   
28 + **.GGCGGCCGCG.** 912 (90.79)   
28 - **.CGCGGCCGCC.** 912 (90.79)   
28 - **[CGCGGAGGCC]** 512 (85.01)   
33 + **[CGCGCCTGCG]** 938 (86.48)   
33 + **[CGGGGACGCC]** 1033 (85.07)   
33 + **[CGGGGAGGCG]** 1347 (85.16)   
33 + **.GGCGGCCGCG.** 1356 (90.79)   
33 + **.CGCCGCCGCG.** 1407 (90.88)   
33 - **.CGCGGCGGCG.** 1407 (90.88)   
33 - **.CGCGGCCGCC.** 1356 (90.79)   
33 - **[CGCCTCCCCG]** 1347 (85.16)   
33 - **[GGCGTCCCCG]** 1033 (85.07)   
33 - **[CGCAGGCGCG]** 938 (86.48)   
34 + **[CGCCGAGGCG]** 912 (85.09)   
34 + **.CGCGCCCGCC.** 942 (90.62)   
34 + **[CGCGCGCGAG]** 958 (86.07)   
34 + **[CGCGGACGCA]** 1280 (88.81)   
34 + **[TGCGGGCGCG]** 1309 (87.53)   
34 - **[CGCGCCCGCA]** 1309 (87.53)   
34 - **[TGCGTCCGCG]** 1280 (88.81)   
34 - **[CTCGCGCGCG]** 958 (86.07)   
34 - **.GGCGGGCGCG.** 942 (90.62)   
34 - **[CGCCTCGGCG]** 912 (85.09)   
35 + **[TGCGTGCGCG]** 926 (88.64)   
35 + **[CACGCCCGCG]** 1319 (86.44)   
35 - **[CGCGGGCGTG]** 1319 (86.44)   
35 - **[CGCGCACGCA]** 926 (88.64)   
36 + **[CCGGTACGCG]** 145 (85.42)   
36 + **.CGCGGGCGCC.** 702 (90.62)   
36 + **.CCCGCACGCG.** 853 (90.98)   
36 - **.CGCGTGCGGG.** 853 (90.98)   
36 - **.GGCGCCCGCG.** 702 (90.62)   
36 - **[CGCGTACCGG]** 145 (85.42)   
39 + **[CCCGGGCGCG]** 1042 (89.87)   
39 + **.CGCGTCCCCG.** 1122 (92.05)   
39 + **.CGCGGCCGGG.** 1425 (90.04)   
39 - **.CCCGGCCGCG.** 1425 (90.04)   
39 - **.CGGGGACGCG.** 1122 (92.05)   
39 - **[CGCGCCCGGG]** 1042 (89.87)   
40 + **[CCCGGGCGCG]** 946 (89.87)   
40 + **[CGGGTTCGCG]** 1227 (87.00)   
40 + **.CGCGTCCGCG.** 1235 (98.89)   
40 + **[CGTGTGCGCG]** 1419 (87.86)   
40 - **[CGCGCACACG]** 1419 (87.86)   
40 - **.CGCGGACGCG.** 1235 (98.89)   
40 - **[CGCGAACCCG]** 1227 (87.00)   
40 - **[CGCGCCCGGG]** 946 (89.87)   
42 + **[CGCGCCTGCG]** 900 (86.48)   
42 + **[CGCGGGCGCA]** 911 (87.53)   
42 + **.CGCGGCGGCG.** 1168 (90.88)   
42 + **[CGCGGCCGCT]** 1311 (87.25)   
42 + **.CGCCCGCGCG.** 1445 (90.54)   
42 + **[AGCGGCCGCG]** 1471 (87.25)   
42 - **[CGCGGCCGCT]** 1471 (87.25)   
42 - **.CGCGCGGGCG.** 1445 (90.54)   
42 - **[AGCGGCCGCG]** 1311 (87.25)   
42 - **.CGCCGCCGCG.** 1168 (90.88)   
42 - **[TGCGCCCGCG]** 911 (87.53)   
42 - **[CGCAGGCGCG]** 900 (86.48)   
43 + **.CGGGGGCGCG.** 1191 (90.77)   
43 + **.CGCGGGCCCG.** 1204 (90.77)   
43 - **.CGGGCCCGCG.** 1204 (90.77)   
43 - **.CGCGCCCCCG.** 1191 (90.77)   
45 + **.CGCGCGCGCG.** 53 (97.44)   
45 + **.CGCGCGCGCG.** 55 (97.44)   
45 + **.CGCGCGCGCG.** 57 (97.44)   
45 + **.CGCGCGCGCG.** 59 (97.44)   
45 + **.CGCGCGCGCG.** 61 (97.44)   
45 + **[CGCGCGCGTG]** 63 (86.27)   
45 + **[CGCGTGCACG]** 67 (87.86)   
45 + **.CGGGCACGCG.** 551 (91.88)   
45 + **.CGCGGGGGCG.** 931 (90.71)   
45 + **[CGCGGCCGCT]** 981 (87.25)   
45 + **[CGCGGCCGCA]** 1012 (87.70)   
45 - **[TGCGGCCGCG]** 1012 (87.70)   
45 - **[AGCGGCCGCG]** 981 (87.25)   
45 - **.CGCCCCCGCG.** 931 (90.71)   
45 - **.CGCGTGCCCG.** 551 (91.88)   
45 - **[CGTGCACGCG]** 67 (87.86)   
45 - **[CACGCGCGCG]** 63 (86.27)   
45 - **.CGCGCGCGCG.** 61 (97.44)   
45 - **.CGCGCGCGCG.** 59 (97.44)   
45 - **.CGCGCGCGCG.** 57 (97.44)   
45 - **.CGCGCGCGCG.** 55 (97.44)   
45 - **.CGCGCGCGCG.** 53 (97.44)   
47 + **[CGCGGCCGAG]** 1136 (86.41)   
47 + **.CGCGGCCGCG.** 1367 (97.78)   
47 - **.CGCGGCCGCG.** 1367 (97.78)   
47 - **[CTCGGCCGCG]** 1136 (86.41)   
48 + **[CGCAGCCGCG]** 959 (86.65)   
48 + **.CGCGGCGGCG.** 1019 (90.88)   
48 - **.CGCCGCCGCG.** 1019 (90.88)   
48 - **[CGCGGCTGCG]** 959 (86.65)   
49 + **[CGCGGCAGCG]** 1120 (86.28)   
49 + **[CGCGGAGCCG]** 1409 (85.16)   
49 - **[CGGCTCCGCG]** 1409 (85.16)   
49 - **[CGCTGCCGCG]** 1120 (86.28)   
51 + **.CGCGGGGGCG.** 4 (90.71)   
51 + **[CGGGTCCCCG]** 152 (85.22)   
51 + **.CGCGCGGGCG.** 274 (90.54)   
51 + **.CGCGGGCGCG.** 276 (97.61)   
51 + **.CGCGGGCGCG.** 282 (97.61)   
51 + **[CGCGCCCGGG]** 600 (89.87)   
51 + **[CGCGCCCACG]** 636 (86.75)   
51 + **[CGCCGACGCC]** 1147 (85.01)   
51 - **[GGCGTCGGCG]** 1147 (85.01)   
51 - **[CGTGGGCGCG]** 636 (86.75)   
51 - **[CCCGGGCGCG]** 600 (89.87)   
51 - **.CGCGCCCGCG.** 282 (97.61)   
51 - **.CGCGCCCGCG.** 276 (97.61)   
51 - **.CGCCCGCGCG.** 274 (90.54)   
51 - **[CGGGGACCCG]** 152 (85.22)   
51 - **.CGCCCCCGCG.** 4 (90.71)   
53 + **[TGCGTGCGCG]** 990 (88.64)   
53 - **[CGCGCACGCA]** 990 (88.64)   
54 + **.CGGGCCCGCG.** 1301 (90.77)   
54 - **.CGCGGGCCCG.** 1301 (90.77)   
55 + **[CCCGCGCGCG]** 219 (89.70)   
55 + **[CCCGCCCGCG]** 797 (89.87)   
55 + **[CGCGCACGCT]** 958 (88.19)   
55 - **[AGCGTGCGCG]** 958 (88.19)   
55 - **[CGCGGGCGGG]** 797 (89.87)   
55 - **[CGCGCGCGGG]** 219 (89.70)   
58 + **.CGCGTGCGCC.** 192 (91.73)   
58 + **[CCCGTACGCC]** 375 (85.27)   
58 + **.CGCGCAGGCG.** 530 (91.82)   
58 + **[CGCAGGCGCG]** 532 (86.48)   
58 + **[CGCGCTCGCC]** 538 (85.57)   
58 - **[GGCGAGCGCG]** 538 (85.57)   
58 - **[CGCGCCTGCG]** 532 (86.48)   
58 - **.CGCCTGCGCG.** 530 (91.82)   
58 - **[GGCGTACGGG]** 375 (85.27)   
58 - **.GGCGCACGCG.** 192 (91.73)   
59 + **.CGCGGGCGCC.** 708 (90.62)   
59 + **.CGCGGCCGCC.** 1012 (90.79)   
59 + **[CGCTGCCGCG]** 1067 (86.28)   
59 + **[TGCGCCCGCG]** 1163 (87.53)   
59 + **.CGCGTCGGCG.** 1286 (91.99)   
59 - **.CGCCGACGCG.** 1286 (91.99)   
59 - **[CGCGGGCGCA]** 1163 (87.53)   
59 - **[CGCGGCAGCG]** 1067 (86.28)   
59 - **.GGCGGCCGCG.** 1012 (90.79)   
59 - **.GGCGCCCGCG.** 708 (90.62)   
60 + **.CGCGGCCGCC.** 827 (90.79)   
60 + **[CGGGCTCGCG]** 839 (85.72)   
60 + **.CGCGACCGCG.** 1149 (92.73)   
60 - **.CGCGGTCGCG.** 1149 (92.73)   
60 - **[CGCGAGCCCG]** 839 (85.72)   
60 - **.GGCGGCCGCG.** 827 (90.79)   
61 + **[CACGGCCGCG]** 910 (86.61)   
61 + **[CGCGTTCCCG]** 916 (87.00)   
61 + **.CGCGTGCGGG.** 1322 (90.98)   
61 + **[CGGGGAGGCG]** 1328 (85.16)   
61 - **[CGCCTCCCCG]** 1328 (85.16)   
61 - **.CCCGCACGCG.** 1322 (90.98)   
61 - **[CGGGAACGCG]** 916 (87.00)   
61 - **[CGCGGCCGTG]** 910 (86.61)   
63 + **[CGCGTTCGGG]** 169 (86.10)   
63 + **[CCCGCCCGCG]** 379 (89.87)   
63 + **.CGCGGCGGCG.** 410 (90.88)   
63 + **.GGCGTCCGCG.** 448 (91.90)   
63 + **[CCCGCCCGCG]** 515 (89.87)   
63 + **[CGCGTCCTCG]** 521 (88.96)   
63 + **[CGCGTCGGCC]** 854 (85.01)   
63 + **[CGCTCGCGCG]** 901 (85.94)   
63 + **[CGGGAGCGCG]** 1185 (85.72)   
63 + **.CGCGGCGGCG.** 1393 (90.88)   
63 + **.GGCGGCCGCG.** 1408 (90.79)   
63 + **.CGGGGACGCG.** 1426 (92.05)   
63 - **.CGCGTCCCCG.** 1426 (92.05)   
63 - **.CGCGGCCGCC.** 1408 (90.79)   
63 - **.CGCCGCCGCG.** 1393 (90.88)   
63 - **[CGCGCTCCCG]** 1185 (85.72)   
63 - **[CGCGCGAGCG]** 901 (85.94)   
63 - **[GGCCGACGCG]** 854 (85.01)   
63 - **[CGAGGACGCG]** 521 (88.96)   
63 - **[CGCGGGCGGG]** 515 (89.87)   
63 - **.CGCGGACGCC.** 448 (91.90)   
63 - **.CGCCGCCGCG.** 410 (90.88)   
63 - **[CGCGGGCGGG]** 379 (89.87)   
63 - **[CCCGAACGCG]** 169 (86.10)   
65 + **[CGCCTCGGCG]** 636 (85.09)   
65 + **[CTCGGCCGCG]** 780 (86.41)   
65 + **[CCCGCCCGCG]** 906 (89.87)   
65 + **[CGCGAGCGCC]** 1003 (85.57)   
65 + **[CGTGGGCGCG]** 1201 (86.75)   
65 + **.CGGGGACGCG.** 1220 (92.05)   
65 + **.CGCGGTCGCG.** 1226 (92.73)   
65 - **.CGCGACCGCG.** 1226 (92.73)   
65 - **.CGCGTCCCCG.** 1220 (92.05)   
65 - **[CGCGCCCACG]** 1201 (86.75)   
65 - **[GGCGCTCGCG]** 1003 (85.57)   
65 - **[CGCGGGCGGG]** 906 (89.87)   
65 - **[CGCGGCCGAG]** 780 (86.41)   
65 - **[CGCCGAGGCG]** 636 (85.09)   
66 + **.GGCGCCCGCG.** 937 (90.62)   
66 + **.CGCGCCCGCC.** 943 (90.62)   
66 + **.CGCCCCCGCG.** 949 (90.71)   
66 + **[CGGGTGCCCG]** 1119 (85.05)   
66 + **[CGCGGGCGGG]** 1338 (89.87)   
66 - **[CCCGCCCGCG]** 1338 (89.87)   
66 - **[CGGGCACCCG]** 1119 (85.05)   
66 - **.CGCGGGGGCG.** 949 (90.71)   
66 - **.GGCGGGCGCG.** 943 (90.62)   
66 - **.CGCGGGCGCC.** 937 (90.62)   
67 + **[CGCGCCTGCG]** 1175 (86.48)   
67 - **[CGCAGGCGCG]** 1175 (86.48)   
68 + **.CGGGCACGCG.** 715 (91.88)   
68 + **[CGCGGGCTCG]** 1055 (87.68)   
68 + **[CGAGCGCGCG]** 1265 (87.51)   
68 + **[AGCGCGCGCG]** 1267 (86.91)   
68 + **[CGCGCGCGGG]** 1269 (89.70)   
68 + **.CGCGCGGGCG.** 1271 (90.54)   
68 + **[CGCGGGCGGG]** 1273 (89.87)   
68 - **[CCCGCCCGCG]** 1273 (89.87)   
68 - **.CGCCCGCGCG.** 1271 (90.54)   
68 - **[CCCGCGCGCG]** 1269 (89.70)   
68 - **[CGCGCGCGCT]** 1267 (86.91)   
68 - **[CGCGCGCTCG]** 1265 (87.51)   
68 - **[CGAGCCCGCG]** 1055 (87.68)   
68 - **.CGCGTGCCCG.** 715 (91.88)   
69 + **.CGCGTCCGCG.** 690 (98.89)   
69 + **.CGCGGCCGCG.** 769 (97.78)   
69 + **[CGCGTGTGCG]** 775 (87.59)   
69 + **[CGAGCCCGCG]** 992 (87.68)   
69 + **[CGCGGAGCCG]** 1034 (85.16)   
69 + **[CGGGTCCGCC]** 1310 (85.07)   
69 - **[GGCGGACCCG]** 1310 (85.07)   
69 - **[CGGCTCCGCG]** 1034 (85.16)   
69 - **[CGCGGGCTCG]** 992 (87.68)   
69 - **[CGCACACGCG]** 775 (87.59)   
69 - **.CGCGGCCGCG.** 769 (97.78)   
69 - **.CGCGGACGCG.** 690 (98.89)   
70 + **.CGCGCACCCG.** 1105 (91.88)   
70 - **.CGGGTGCGCG.** 1105 (91.88)   
71 + **[CGCGCCCGTG]** 870 (86.44)   
71 - **[CACGGGCGCG]** 870 (86.44)   
73 + **.CGCCGCCGCG.** 915 (90.88)   
73 + **[CGCGGCCGAG]** 921 (86.41)   
73 - **[CTCGGCCGCG]** 921 (86.41)   
73 - **.CGCGGCGGCG.** 915 (90.88)   
74 + **[CGGCTCCGCG]** 1057 (85.16)   
74 + **[CGAGCCCGCG]** 1144 (87.68)   
74 + **.GGCGGCCGCG.** 1432 (90.79)   
74 - **.CGCGGCCGCC.** 1432 (90.79)   
74 - **[CGCGGGCTCG]** 1144 (87.68)   
74 - **[CGCGGAGCCG]** 1057 (85.16)   
76 + **[CGCTGGCGCG]** 995 (86.11)   
76 + **[CGCGGGCGCT]** 1001 (87.08)   
76 + **.CGCGAGCGCG.** 1058 (92.56)   
76 + **[CGCGCCTGCG]** 1116 (86.48)   
76 - **[CGCAGGCGCG]** 1116 (86.48)   
76 - **.CGCGCTCGCG.** 1058 (92.56)   
76 - **[AGCGCCCGCG]** 1001 (87.08)   
76 - **[CGCGCCAGCG]** 995 (86.11)   
79 + **.GGCGCCCGCG.** 719 (90.62)   
79 + **.CGCCGCCGCG.** 874 (90.88)   
79 - **.CGCGGCGGCG.** 874 (90.88)   
79 - **.CGCGGGCGCC.** 719 (90.62)   
84 + **[CGCGCCTGCG]** 902 (86.48)   
84 + **[CGCGGACCCC]** 1155 (85.07)   
84 + **.CGCGTGCGCC.** 1164 (91.73)   
84 + **.CGGGCCCGCG.** 1297 (90.77)   
84 - **.CGCGGGCCCG.** 1297 (90.77)   
84 - **.GGCGCACGCG.** 1164 (91.73)   
84 - **[GGGGTCCGCG]** 1155 (85.07)   
84 - **[CGCAGGCGCG]** 902 (86.48)   
85 + **[CGCGTCCGCT]** 95 (88.36)   
85 + **.CGGGGGCGCG.** 583 (90.77)   
85 + **[CGGGTCCGCC]** 738 (85.07)   
85 + **[CGGGTCCGCC]** 827 (85.07)   
85 + **[CGCCTCCGCC]** 833 (85.01)   
85 + **.GGCGCCCGCG.** 874 (90.62)   
85 + **.CGCGGCCCCG.** 880 (90.94)   
85 + **[CTCGGACGCG]** 924 (87.52)   
85 + **[CGCGGGCGGG]** 930 (89.87)   
85 + **.CGCGCGCCCG.** 1094 (90.60)   
85 + **.CGCGGCCCCG.** 1217 (90.94)   
85 + **.CGCGCGCGCG.** 1380 (97.44)   
85 + **[CGCGCGCGGG]** 1382 (89.70)   
85 + **[CGCGGTCCCG]** 1437 (85.89)   
85 - **[CGGGACCGCG]** 1437 (85.89)   
85 - **[CCCGCGCGCG]** 1382 (89.70)   
85 - **.CGCGCGCGCG.** 1380 (97.44)   
85 - **.CGGGGCCGCG.** 1217 (90.94)   
85 - **.CGGGCGCGCG.** 1094 (90.60)   
85 - **[CCCGCCCGCG]** 930 (89.87)   
85 - **[CGCGTCCGAG]** 924 (87.52)   
85 - **.CGGGGCCGCG.** 880 (90.94)   
85 - **.CGCGGGCGCC.** 874 (90.62)   
85 - **[GGCGGAGGCG]** 833 (85.01)   
85 - **[GGCGGACCCG]** 827 (85.07)   
85 - **[GGCGGACCCG]** 738 (85.07)   
85 - **.CGCGCCCCCG.** 583 (90.77)   
85 - **[AGCGGACGCG]** 95 (88.36)   
86 + **.CGCGCGGGCG.** 811 (90.54)   
86 + **[CGCGGGCGCA]** 813 (87.53)   
86 + **.CGCGCGGGCG.** 881 (90.54)   
86 + **.CGGGGGCGCG.** 896 (90.77)   
86 + **[TGCGCGCGCG]** 1030 (87.36)   
86 + **[CGCGGACTCG]** 1255 (88.96)   
86 + **.CGGGGGCGCG.** 1380 (90.77)   
86 + **.CGCGGGCCCG.** 1439 (90.77)   
86 + **[CCCGCGCGCG]** 1498 (89.70)   
86 + **.CGCGCGCGCG.** 1500 (97.44)   
86 + **.CGCGGCCGCG.** 1506 (97.78)   
86 - **.CGCGGCCGCG.** 1506 (97.78)   
86 - **.CGCGCGCGCG.** 1500 (97.44)   
86 - **[CGCGCGCGGG]** 1498 (89.70)   
86 - **.CGGGCCCGCG.** 1439 (90.77)   
86 - **.CGCGCCCCCG.** 1380 (90.77)   
86 - **[CGAGTCCGCG]** 1255 (88.96)   
86 - **[CGCGCGCGCA]** 1030 (87.36)   
86 - **.CGCGCCCCCG.** 896 (90.77)   
86 - **.CGCCCGCGCG.** 881 (90.54)   
86 - **[TGCGCCCGCG]** 813 (87.53)   
86 - **.CGCCCGCGCG.** 811 (90.54)   
87 + **.CGGGCCCGCG.** 868 (90.77)   
87 + **[CGCAGCCGCG]** 1093 (86.65)   
87 + **[CCCGCCCGCG]** 1184 (89.87)   
87 + **.CGCCCGCGCG.** 1186 (90.54)   
87 + **[CCCGCGCGCG]** 1188 (89.70)   
87 + **.CGCGCGCGCC.** 1190 (90.45)   
87 + **[CGGGCTCGCG]** 1327 (85.72)   
87 + **.CGCGGCGGCG.** 1333 (90.88)   
87 + **[CGCGGCCGCT]** 1367 (87.25)   
87 + **.CGCGCGCGCG.** 1436 (97.44)   
87 + **.CGCGCGCGCG.** 1438 (97.44)   
87 + **.CGCGCGCGCG.** 1440 (97.44)   
87 + **[CGCGGGCGGG]** 1459 (89.87)   
87 + **.CGCGCGGGCG.** 1488 (90.54)   
87 + **[CGCGGGCGGG]** 1490 (89.87)   
87 + **.CGCGGGGGCG.** 1503 (90.71)   
87 - **.CGCCCCCGCG.** 1503 (90.71)   
87 - **[CCCGCCCGCG]** 1490 (89.87)   
87 - **.CGCCCGCGCG.** 1488 (90.54)   
87 - **[CCCGCCCGCG]** 1459 (89.87)   
87 - **.CGCGCGCGCG.** 1440 (97.44)   
87 - **.CGCGCGCGCG.** 1438 (97.44)   
87 - **.CGCGCGCGCG.** 1436 (97.44)   
87 - **[AGCGGCCGCG]** 1367 (87.25)   
87 - **.CGCCGCCGCG.** 1333 (90.88)   
87 - **[CGCGAGCCCG]** 1327 (85.72)   
87 - **.GGCGCGCGCG.** 1190 (90.45)   
87 - **[CGCGCGCGGG]** 1188 (89.70)   
87 - **.CGCGCGGGCG.** 1186 (90.54)   
87 - **[CGCGGGCGGG]** 1184 (89.87)   
87 - **[CGCGGCTGCG]** 1093 (86.65)   
87 - **.CGCGGGCCCG.** 868 (90.77)   
88 + **.CGCCCCCGCG.** 365 (90.71)   
88 + **[CGCGGTGGCG]** 472 (85.83)   
88 + **[CCCGCGCGCG]** 1075 (89.70)   
88 + **[CGCGCGCGCT]** 1077 (86.91)   
88 + **[CGCCCTCGCG]** 1136 (85.66)   
88 - **[CGCGAGGGCG]** 1136 (85.66)   
88 - **[AGCGCGCGCG]** 1077 (86.91)   
88 - **[CGCGCGCGGG]** 1075 (89.70)   
88 - **[CGCCACCGCG]** 472 (85.83)   
88 - **.CGCGGGGGCG.** 365 (90.71)   
89 + **.CGCGGCCGGG.** 47 (90.04)   
89 + **[CCCGCCCGCG]** 94 (89.87)   
89 + **.CGCGGGCGCC.** 262 (90.62)   
89 + **[CGCCTCCGCC]** 353 (85.01)   
89 + **.CGCCGCCGCG.** 414 (90.88)   
89 + **.CCCGGCCGCG.** 474 (90.04)   
89 + **[CGCTGCCGCG]** 489 (86.28)   
89 + **[CCCGCGCGCG]** 860 (89.70)   
89 + **[CGCGCGCGCA]** 862 (87.36)   
89 - **[TGCGCGCGCG]** 862 (87.36)   
89 - **[CGCGCGCGGG]** 860 (89.70)   
89 - **[CGCGGCAGCG]** 489 (86.28)   
89 - **.CGCGGCCGGG.** 474 (90.04)   
89 - **.CGCGGCGGCG.** 414 (90.88)   
89 - **[GGCGGAGGCG]** 353 (85.01)   
89 - **.GGCGCCCGCG.** 262 (90.62)   
89 - **[CGCGGGCGGG]** 94 (89.87)   
89 - **.CCCGGCCGCG.** 47 (90.04)   
90 + **[CGCGCTCCCG]** 447 (85.72)   
90 + **[CGCGCCCGCA]** 654 (87.53)   
90 + **.CCCGGCCGCG.** 771 (90.04)   
90 + **.CGCGGCGGCG.** 777 (90.88)   
90 + **[TGCGCGCGCG]** 943 (87.36)   
90 + **[CGCGCGCGAG]** 945 (86.07)   
90 + **[CGCGGCAGCG]** 993 (86.28)   
90 - **[CGCTGCCGCG]** 993 (86.28)   
90 - **[CTCGCGCGCG]** 945 (86.07)   
90 - **[CGCGCGCGCA]** 943 (87.36)   
90 - **.CGCCGCCGCG.** 777 (90.88)   
90 - **.CGCGGCCGGG.** 771 (90.04)   
90 - **[TGCGGGCGCG]** 654 (87.53)   
90 - **[CGGGAGCGCG]** 447 (85.72)   
93 + **[TGCGCGCGCG]** 449 (87.36)   
93 + **[GGGGGACGCG]** 959 (85.07)   
93 + **.CGCGGCGGCG.** 1037 (90.88)   
93 + **[CACGCGCGCG]** 1192 (86.27)   
93 + **.CGCGCGCGCC.** 1194 (90.45)   
93 + **[AGCGGGCGCG]** 1234 (87.08)   
93 + **.CGGGCGCGCG.** 1236 (90.60)   
93 + **[CGCGGCAGCG]** 1242 (86.28)   
93 + **[CGCGGCAGCG]** 1282 (86.28)   
93 - **[CGCTGCCGCG]** 1282 (86.28)   
93 - **[CGCTGCCGCG]** 1242 (86.28)   
93 - **.CGCGCGCCCG.** 1236 (90.60)   
93 - **[CGCGCCCGCT]** 1234 (87.08)   
93 - **.GGCGCGCGCG.** 1194 (90.45)   
93 - **[CGCGCGCGTG]** 1192 (86.27)   
93 - **.CGCCGCCGCG.** 1037 (90.88)   
93 - **[CGCGTCCCCC]** 959 (85.07)   
93 - **[CGCGCGCGCA]** 449 (87.36)   
96 + **[CGCGTCGCCG]** 1359 (85.16)   
96 + **.GGCGGACGCG.** 1389 (91.90)   
96 - **.CGCGTCCGCC.** 1389 (91.90)   
96 - **[CGGCGACGCG]** 1359 (85.16)   
97 + **.CGGGGACGCG.** 1363 (92.05)   
97 - **.CGCGTCCCCG.** 1363 (92.05)   
98 + **[AGCGGCCGCG]** 995 (87.25)   
98 + **[CGCAGCCGCG]** 1115 (86.65)   
98 + **[CGAGCGCGCG]** 1175 (87.51)   
98 + **[CGCGGCCGTG]** 1325 (86.61)   
98 - **[CACGGCCGCG]** 1325 (86.61)   
98 - **[CGCGCGCTCG]** 1175 (87.51)   
98 - **[CGCGGCTGCG]** 1115 (86.65)   
98 - **[CGCGGCCGCT]** 995 (87.25)   
99 + **.CGCGGGGGCG.** 1336 (90.71)   
99 + **[CGTGTGCGCG]** 1344 (87.86)   
99 - **[CGCGCACACG]** 1344 (87.86)   
99 - **.CGCCCCCGCG.** 1336 (90.71)   
100 + **[CGCGGGAGCG]** 1333 (86.11)   
100 + **[CGGGAGCGCG]** 1335 (85.72)   
100 - **[CGCGCTCCCG]** 1335 (85.72)   
100 - **[CGCTCCCGCG]** 1333 (86.11)   
105 + **.GGCGGGCGCG.** 1138 (90.62)   
105 - **.CGCGCCCGCC.** 1138 (90.62)   
106 + **[CGGGCTCGCG]** 864 (85.72)   
106 + **.CGCGGGCCCG.** 936 (90.77)   
106 - **.CGGGCCCGCG.** 936 (90.77)   
106 - **[CGCGAGCCCG]** 864 (85.72)   
108 + **[CGGGTGCCCG]** 680 (85.05)   
108 + **[CGCGAGGGCG]** 925 (85.66)   
108 - **[CGCCCTCGCG]** 925 (85.66)   
108 - **[CGGGCACCCG]** 680 (85.05)   
109 + **[CGCGCCCGCA]** 455 (87.53)   
109 + **[CCCGCCCGCG]** 1037 (89.87)   
109 + **.CGCGGCGGCG.** 1396 (90.88)   
109 + **[CGGGCACCCG]** 1404 (85.05)   
109 + **[CGCGGAGCCG]** 1505 (85.16)   
109 - **[CGGCTCCGCG]** 1505 (85.16)   
109 - **[CGGGTGCCCG]** 1404 (85.05)   
109 - **.CGCCGCCGCG.** 1396 (90.88)   
109 - **[CGCGGGCGGG]** 1037 (89.87)   
109 - **[TGCGGGCGCG]** 455 (87.53)   
114 + **[CGCGCGCTCG]** 305 (87.51)   
114 + **[CGCGGGCGAG]** 352 (86.24)   
114 + **[CGCGGCCGAG]** 1009 (86.41)   
114 + **[CGAGGACGCG]** 1015 (88.96)   
114 + **.CGGGCCCGCG.** 1302 (90.77)   
114 - **.CGCGGGCCCG.** 1302 (90.77)   
114 - **[CGCGTCCTCG]** 1015 (88.96)   
114 - **[CTCGGCCGCG]** 1009 (86.41)   
114 - **[CTCGCCCGCG]** 352 (86.24)   
114 - **[CGAGCGCGCG]** 305 (87.51)   
115 + **.CGCGGGCGCC.** 303 (90.62)   
115 + **.CGGGCCCGCG.** 841 (90.77)   
115 - **.CGCGGGCCCG.** 841 (90.77)   
115 - **.GGCGCCCGCG.** 303 (90.62)   
117 + **[CGCGGGCTCG]** 658 (87.68)   
117 + **[CGGGAGCGCG]** 1020 (85.72)   
117 + **.CGCGGCCGGG.** 1056 (90.04)   
117 - **.CCCGGCCGCG.** 1056 (90.04)   
117 - **[CGCGCTCCCG]** 1020 (85.72)   
117 - **[CGAGCCCGCG]** 658 (87.68)   
118 + **[CGCGGGCTCG]** 634 (87.68)   
118 - **[CGAGCCCGCG]** 634 (87.68)   
119 + **[CGCGGGCTCG]** 604 (87.68)   
119 + **[CGCGGGCTCG]** 632 (87.68)   
119 + **.CGCGGCCGGG.** 1036 (90.04)   
119 - **.CCCGGCCGCG.** 1036 (90.04)   
119 - **[CGAGCCCGCG]** 632 (87.68)   
119 - **[CGAGCCCGCG]** 604 (87.68)   
120 + **[CGCGGGCTCG]** 1092 (87.68)   
120 + **.CGCGGCCGGG.** 1503 (90.04)   
120 - **.CCCGGCCGCG.** 1503 (90.04)   
120 - **[CGAGCCCGCG]** 1092 (87.68)   
122 + **.GGCGCGCGCG.** 902 (90.45)   
122 + **.CGCGGCCCCG.** 916 (90.94)   
122 + **[CTCGCGCGCG]** 936 (86.07)   
122 + **[CGCGCGCGCT]** 938 (86.91)   
122 + **[CTCGCGCGCG]** 951 (86.07)   
122 + **.CGCGCGCGCG.** 953 (97.44)   
122 + **[CGCGCGCGCA]** 955 (87.36)   
122 - **[TGCGCGCGCG]** 955 (87.36)   
122 - **.CGCGCGCGCG.** 953 (97.44)   
122 - **[CGCGCGCGAG]** 951 (86.07)   
122 - **[AGCGCGCGCG]** 938 (86.91)   
122 - **[CGCGCGCGAG]** 936 (86.07)   
122 - **.CGGGGCCGCG.** 916 (90.94)   
122 - **.CGCGCGCGCC.** 902 (90.45)   
123 + **[CGCGGAGCCG]** 527 (85.16)   
123 + **[CGGCTCCGCG]** 654 (85.16)   
123 + **[CGAGGGCGCG]** 894 (87.68)   
123 + **[CGCGGTCGCC]** 998 (85.74)   
123 + **[CGGGGACGCC]** 1262 (85.07)   
123 - **[GGCGTCCCCG]** 1262 (85.07)   
123 - **[GGCGACCGCG]** 998 (85.74)   
123 - **[CGCGCCCTCG]** 894 (87.68)   
123 - **[CGCGGAGCCG]** 654 (85.16)   
123 - **[CGGCTCCGCG]** 527 (85.16)   
124 + **[CGCGCATGCG]** 955 (87.59)   
124 + **.GGCGCGCGCG.** 1149 (90.45)   
124 + **[CGCGCGCGGG]** 1151 (89.70)   
124 + **.CGCGCGGGCG.** 1153 (90.54)   
124 + **[CGCGGGCGAG]** 1155 (86.24)   
124 - **[CTCGCCCGCG]** 1155 (86.24)   
124 - **.CGCCCGCGCG.** 1153 (90.54)   
124 - **[CCCGCGCGCG]** 1151 (89.70)   
124 - **.CGCGCGCGCC.** 1149 (90.45)   
124 - **[CGCATGCGCG]** 955 (87.59)   
127 + **[CGCGCACGCA]** 778 (88.64)   
127 + **[TGCGGACGCG]** 1025 (88.81)   
127 + **[CGCGGAGGCC]** 1105 (85.01)   
127 + **[TGCGGCCGCG]** 1505 (87.70)   
127 - **[CGCGGCCGCA]** 1505 (87.70)   
127 - **[GGCCTCCGCG]** 1105 (85.01)   
127 - **[CGCGTCCGCA]** 1025 (88.81)   
127 - **[TGCGTGCGCG]** 778 (88.64)   
128 + **[CGGGTCGGCG]** 828 (85.16)   
128 - **[CGCCGACCCG]** 828 (85.16)   
129 + **[CGCCACCGCG]** 744 (85.83)   
129 + **.CGCGCACGCG.** 760 (98.72)   
129 + **[GGCCTCCGCG]** 1172 (85.01)   
129 + **[CGCGTGCGCA]** 1178 (88.64)   
129 + **[TGCGGCCGCG]** 1196 (87.70)   
129 + **.CGCGGCCGGG.** 1386 (90.04)   
129 - **.CCCGGCCGCG.** 1386 (90.04)   
129 - **[CGCGGCCGCA]** 1196 (87.70)   
129 - **[TGCGCACGCG]** 1178 (88.64)   
129 - **[CGCGGAGGCC]** 1172 (85.01)   
129 - **.CGCGTGCGCG.** 760 (98.72)   
129 - **[CGCGGTGGCG]** 744 (85.83)   
130 + **[CGCGCATGCG]** 949 (87.59)   
130 - **[CGCATGCGCG]** 949 (87.59)   
131 + **[CGCGGGCGGG]** 982 (89.87)   
131 + **[CGCTGGCGCG]** 1087 (86.11)   
131 + **[CGCGGCCGCT]** 1117 (87.25)   
131 + **.CGCGCACGGG.** 1185 (90.98)   
131 + **[CGAGCACGCG]** 1300 (88.79)   
131 + **.CGCGGCGGCG.** 1306 (90.88)   
131 + **.CGCGGCGGCG.** 1488 (90.88)   
131 - **.CGCCGCCGCG.** 1488 (90.88)   
131 - **.CGCCGCCGCG.** 1306 (90.88)   
131 - **[CGCGTGCTCG]** 1300 (88.79)   
131 - **.CCCGTGCGCG.** 1185 (90.98)   
131 - **[AGCGGCCGCG]** 1117 (87.25)   
131 - **[CGCGCCAGCG]** 1087 (86.11)   
131 - **[CCCGCCCGCG]** 982 (89.87)   
132 + **[CGCGGAAGCG]** 359 (87.39)   
132 + **.CGCCGCCGCG.** 706 (90.88)   
132 + **[CGCGCATGCG]** 831 (87.59)   
132 - **[CGCATGCGCG]** 831 (87.59)   
132 - **.CGCGGCGGCG.** 706 (90.88)   
132 - **[CGCTTCCGCG]** 359 (87.39)   
134 + **.GGCGGGCGCG.** 1469 (90.62)   
134 - **.CGCGCCCGCC.** 1469 (90.62)   
135 + **.CGCGTGCCCG.** 32 (91.88)   
135 + **.CGCGCCCGCG.** 313 (97.61)   
135 + **[CGCGGAGCCG]** 357 (85.16)   
135 + **.CGCGCGGGCG.** 753 (90.54)   
135 + **[CGCGGGCGCT]** 755 (87.08)   
135 + **.CGCGTGCCCG.** 790 (91.88)   
135 + **[CGCGCACACG]** 1502 (87.86)   
135 + **[CGCACACGCG]** 1504 (87.59)   
135 - **[CGCGTGTGCG]** 1504 (87.59)   
135 - **[CGTGTGCGCG]** 1502 (87.86)   
135 - **.CGGGCACGCG.** 790 (91.88)   
135 - **[AGCGCCCGCG]** 755 (87.08)   
135 - **.CGCCCGCGCG.** 753 (90.54)   
135 - **[CGGCTCCGCG]** 357 (85.16)   
135 - **.CGCGGGCGCG.** 313 (97.61)   
135 - **.CGGGCACGCG.** 32 (91.88)   
136 + **[CGCGGCCGCA]** 1196 (87.70)   
136 - **[TGCGGCCGCG]** 1196 (87.70)   
137 + **[CGCGGCCGCA]** 1016 (87.70)   
137 + **[GGCGAGCGCG]** 1094 (85.57)   
137 + **[CGCGAGCGCC]** 1100 (85.57)   
137 + **[AGCGGGCGCG]** 1161 (87.08)   
137 - **[CGCGCCCGCT]** 1161 (87.08)   
137 - **[GGCGCTCGCG]** 1100 (85.57)   
137 - **[CGCGCTCGCC]** 1094 (85.57)   
137 - **[TGCGGCCGCG]** 1016 (87.70)   
138 + **[CGAGCGCGCG]** 52 (87.51)   
138 + **[AGCGCGCGCG]** 54 (86.91)   
138 + **[CGCGCGCGCA]** 56 (87.36)   
138 + **[CGCTCCCGCG]** 82 (86.11)   
138 + **.CGCGGACCCG.** 372 (92.05)   
138 + **[CGCGGAGCCG]** 424 (85.16)   
138 + **[GGCGAGCGCG]** 593 (85.57)   
138 + **[CGAGCGCGCG]** 595 (87.51)   
138 + **.GGCGGCCGCG.** 611 (90.79)   
138 + **[CGAGGGCGCG]** 685 (87.68)   
138 + **.CGCGGCGGCG.** 691 (90.88)   
138 + **.CGCCGCCGCG.** 834 (90.88)   
138 - **.CGCGGCGGCG.** 834 (90.88)   
138 - **.CGCCGCCGCG.** 691 (90.88)   
138 - **[CGCGCCCTCG]** 685 (87.68)   
138 - **.CGCGGCCGCC.** 611 (90.79)   
138 - **[CGCGCGCTCG]** 595 (87.51)   
138 - **[CGCGCTCGCC]** 593 (85.57)   
138 - **[CGGCTCCGCG]** 424 (85.16)   
138 - **.CGGGTCCGCG.** 372 (92.05)   
138 - **[CGCGGGAGCG]** 82 (86.11)   
138 - **[TGCGCGCGCG]** 56 (87.36)   
138 - **[CGCGCGCGCT]** 54 (86.91)   
138 - **[CGCGCGCTCG]** 52 (87.51)   
139 + **.CGCGGTCGCG.** 613 (92.73)   
139 + **[CGGGGACGCC]** 1492 (85.07)   
139 - **[GGCGTCCCCG]** 1492 (85.07)   
139 - **.CGCGACCGCG.** 613 (92.73)   
141 + **[GGCGAGCGCG]** 620 (85.57)   
141 + **[CGAGCGCGCG]** 622 (87.51)   
141 + **.CGCGGCCGCG.** 684 (97.78)   
141 + **[CGCATGCGCG]** 758 (87.59)   
141 + **[CGCGACCGCC]** 1099 (85.74)   
141 + **.CGGGGACGCG.** 1385 (92.05)   
141 - **.CGCGTCCCCG.** 1385 (92.05)   
141 - **[GGCGGTCGCG]** 1099 (85.74)   
141 - **[CGCGCATGCG]** 758 (87.59)   
141 - **.CGCGGCCGCG.** 684 (97.78)   
141 - **[CGCGCGCTCG]** 622 (87.51)   
141 - **[CGCGCTCGCC]** 620 (85.57)   
142 + **[CGCGCCCGCA]** 31 (87.53)   
142 + **.CGGGGCCGCG.** 668 (90.94)   
142 + **.CGCGGGCGCC.** 674 (90.62)   
142 + **[CGCGGGCGAG]** 797 (86.24)   
142 + **.CGCGCGCGCG.** 896 (97.44)   
142 + **.CGCGCGCGCC.** 898 (90.45)   
142 + **.CGCGGCCGCC.** 1200 (90.79)   
142 - **.GGCGGCCGCG.** 1200 (90.79)   
142 - **.GGCGCGCGCG.** 898 (90.45)   
142 - **.CGCGCGCGCG.** 896 (97.44)   
142 - **[CTCGCCCGCG]** 797 (86.24)   
142 - **.GGCGCCCGCG.** 674 (90.62)   
142 - **.CGCGGCCCCG.** 668 (90.94)   
142 - **[TGCGGGCGCG]** 31 (87.53)   
144 + **[CGCGGGCGGG]** 1076 (89.87)   
144 + **.CGCGGCCGCG.** 1194 (97.78)   
144 + **.CGCGGAGGCG.** 1200 (91.99)   
144 + **.CGCGGAGGCG.** 1219 (91.99)   
144 + **[GGCGGAGGCG]** 1225 (85.01)   
144 - **[CGCCTCCGCC]** 1225 (85.01)   
144 - **.CGCCTCCGCG.** 1219 (91.99)   
144 - **.CGCCTCCGCG.** 1200 (91.99)   
144 - **.CGCGGCCGCG.** 1194 (97.78)   
144 - **[CCCGCCCGCG]** 1076 (89.87)   
145 + **[CGCGAGCCCG]** 656 (85.72)   
145 + **[GGCGGTCGCG]** 1092 (85.74)   
145 + **.GGCGGGCGCG.** 1144 (90.62)   
145 - **.CGCGCCCGCC.** 1144 (90.62)   
145 - **[CGCGACCGCC]** 1092 (85.74)   
145 - **[CGGGCTCGCG]** 656 (85.72)   
147 + **.CGGGGCCGCG.** 974 (90.94)   
147 + **.CGCGCAGGCG.** 980 (91.82)   
147 + **[CGCCCTCGCG]** 1124 (85.66)   
147 - **[CGCGAGGGCG]** 1124 (85.66)   
147 - **.CGCCTGCGCG.** 980 (91.82)   
147 - **.CGCGGCCCCG.** 974 (90.94)   
150 + **[AGCGGGCGCG]** 86 (87.08)   
150 + **.CGGGCGCGCG.** 88 (90.60)   
150 + **.CGCGCGCCCG.** 92 (90.60)   
150 + **[CGCGCCCGCT]** 123 (87.08)   
150 + **.CGCGGAGGCG.** 1471 (91.99)   
150 - **.CGCCTCCGCG.** 1471 (91.99)   
150 - **[AGCGGGCGCG]** 123 (87.08)   
150 - **.CGGGCGCGCG.** 92 (90.60)   
150 - **.CGCGCGCCCG.** 88 (90.60)   
150 - **[CGCGCCCGCT]** 86 (87.08)   
151 + **[CGCCTCCGCC]** 845 (85.01)   
151 + **[AGCGGGCGCG]** 878 (87.08)   
151 + **.CGGGCGCGCG.** 910 (90.60)   
151 + **.CGCGCGCCCG.** 914 (90.60)   
151 + **[CGCGCCCGCT]** 916 (87.08)   
151 + **[GGCGGAGGCG]** 1394 (85.01)   
151 + **.CGCGTCCGCG.** 1473 (98.89)   
151 - **.CGCGGACGCG.** 1473 (98.89)   
151 - **[CGCCTCCGCC]** 1394 (85.01)   
151 - **[AGCGGGCGCG]** 916 (87.08)   
151 - **.CGGGCGCGCG.** 914 (90.60)   
151 - **.CGCGCGCCCG.** 910 (90.60)   
151 - **[CGCGCCCGCT]** 878 (87.08)   
151 - **[GGCGGAGGCG]** 845 (85.01)   
154 + **[TGCGTGCGCG]** 769 (88.64)   
154 + **.GGCGGGCGCG.** 861 (90.62)   
154 + **.CGGGCGCGCG.** 863 (90.60)   
154 + **.GGCGGGCGCG.** 1034 (90.62)   
154 + **.CGCGGCGGCG.** 1040 (90.88)   
154 + **.GGCGCCCGCG.** 1065 (90.62)   
154 + **[CGCCTCCCCG]** 1073 (85.16)   
154 + **.CGCGCCCGCG.** 1200 (97.61)   
154 + **[CGTGCCCGCG]** 1224 (86.75)   
154 + **.GGCGCGCGCG.** 1378 (90.45)   
154 + **[CGCGCGCGGG]** 1380 (89.70)   
154 - **[CCCGCGCGCG]** 1380 (89.70)   
154 - **.CGCGCGCGCC.** 1378 (90.45)   
154 - **[CGCGGGCACG]** 1224 (86.75)   
154 - **.CGCGGGCGCG.** 1200 (97.61)   
154 - **[CGGGGAGGCG]** 1073 (85.16)   
154 - **.CGCGGGCGCC.** 1065 (90.62)   
154 - **.CGCCGCCGCG.** 1040 (90.88)   
154 - **.CGCGCCCGCC.** 1034 (90.62)   
154 - **.CGCGCGCCCG.** 863 (90.60)   
154 - **.CGCGCCCGCC.** 861 (90.62)   
154 - **[CGCGCACGCA]** 769 (88.64)   
158 + **[CGAGGCCGCG]** 1173 (87.85)   
158 - **[CGCGGCCTCG]** 1173 (87.85)   
161 + **.CGCGACCGCG.** 754 (92.73)   
161 + **.CGGGGCCGCG.** 861 (90.94)   
161 + **.CGCGCGCGCG.** 867 (97.44)   
161 + **[CGCGCGCGAG]** 869 (86.07)   
161 + **[CGCGCCCGGG]** 1191 (89.87)   
161 + **.CGCGGCGGCG.** 1268 (90.88)   
161 - **.CGCCGCCGCG.** 1268 (90.88)   
161 - **[CCCGGGCGCG]** 1191 (89.87)   
161 - **[CTCGCGCGCG]** 869 (86.07)   
161 - **.CGCGCGCGCG.** 867 (97.44)   
161 - **.CGCGGCCCCG.** 861 (90.94)   
161 - **.CGCGGTCGCG.** 754 (92.73)   
164 + **[CGCTGCCGCG]** 997 (86.28)   
164 + **[CGCGCCCGCT]** 1396 (87.08)   
164 - **[AGCGGGCGCG]** 1396 (87.08)   
164 - **[CGCGGCAGCG]** 997 (86.28)   
166 + **[CGCGCACGCA]** 425 (88.64)   
166 + **[CGGGTCCCCG]** 521 (85.22)   
166 + **[CGCGGCTGCG]** 811 (86.65)   
166 + **[CGCGGGCTCG]** 1074 (87.68)   
166 + **.CGCGGCCGGG.** 1229 (90.04)   
166 + **[TGCGCACGCG]** 1306 (88.64)   
166 - **[CGCGTGCGCA]** 1306 (88.64)   
166 - **.CCCGGCCGCG.** 1229 (90.04)   
166 - **[CGAGCCCGCG]** 1074 (87.68)   
166 - **[CGCAGCCGCG]** 811 (86.65)   
166 - **[CGGGGACCCG]** 521 (85.22)   
166 - **[TGCGTGCGCG]** 425 (88.64)   
167 + **.CGGGGGCGCG.** 797 (90.77)   
167 + **[CTCGGCCGCG]** 859 (86.41)   
167 + **.CGCGCGCCCG.** 865 (90.60)   
167 + **.CGCGCCCGCG.** 867 (97.61)   
167 + **[CCCGGGCGCG]** 998 (89.87)   
167 + **.CGGGCGCGCG.** 1000 (90.60)   
167 + **[CGCGGAGCCG]** 1228 (85.16)   
167 + **.CGCGGTCGCG.** 1324 (92.73)   
167 - **.CGCGACCGCG.** 1324 (92.73)   
167 - **[CGGCTCCGCG]** 1228 (85.16)   
167 - **.CGCGCGCCCG.** 1000 (90.60)   
167 - **[CGCGCCCGGG]** 998 (89.87)   
167 - **.CGCGGGCGCG.** 867 (97.61)   
167 - **.CGGGCGCGCG.** 865 (90.60)   
167 - **[CGCGGCCGAG]** 859 (86.41)   
167 - **.CGCGCCCCCG.** 797 (90.77)   
168 + **.CGCGGAGGCG.** 913 (91.99)   
168 + **[CTCGTCCGCG]** 1199 (87.52)   
168 + **.CGCCCCCGCG.** 1243 (90.71)   
168 + **.GGCGGCCGCG.** 1431 (90.79)   
168 - **.CGCGGCCGCC.** 1431 (90.79)   
168 - **.CGCGGGGGCG.** 1243 (90.71)   
168 - **[CGCGGACGAG]** 1199 (87.52)   
168 - **.CGCCTCCGCG.** 913 (91.99)   
172 + **[CGGGGTCGCG]** 795 (85.89)   
172 + **.CGCGGAGGCG.** 1162 (91.99)   
172 - **.CGCCTCCGCG.** 1162 (91.99)   
172 - **[CGCGACCCCG]** 795 (85.89)   
173 + **[CGCGGGCACG]** 714 (86.75)   
173 + **[CCCGGGCGCG]** 1097 (89.87)   
173 + **[CGCGACCCCG]** 1280 (85.89)   
173 - **[CGGGGTCGCG]** 1280 (85.89)   
173 - **[CGCGCCCGGG]** 1097 (89.87)   
173 - **[CGTGCCCGCG]** 714 (86.75)   
174 + **.CGGGGGCGCG.** 779 (90.77)   
174 + **.CGGGGCCGCG.** 1141 (90.94)   
174 + **[CGCGAGGGCG]** 1147 (85.66)   
174 + **.CCCGGCCGCG.** 1199 (90.04)   
174 + **[CGCGGGCGAG]** 1482 (86.24)   
174 + **[CGCCGAGGCG]** 1499 (85.09)   
174 + **.CGCGGCCGGG.** 1507 (90.04)   
174 - **.CCCGGCCGCG.** 1507 (90.04)   
174 - **[CGCCTCGGCG]** 1499 (85.09)   
174 - **[CTCGCCCGCG]** 1482 (86.24)   
174 - **.CGCGGCCGGG.** 1199 (90.04)   
174 - **[CGCCCTCGCG]** 1147 (85.66)   
174 - **.CGCGGCCCCG.** 1141 (90.94)   
174 - **.CGCGCCCCCG.** 779 (90.77)   
179 + **.CGCGGCCGGG.** 918 (90.04)   
179 + **.CGGGGACGCG.** 990 (92.05)   
179 - **.CGCGTCCCCG.** 990 (92.05)   
179 - **.CCCGGCCGCG.** 918 (90.04)   
180 + **[CGCGCGCGGG]** 812 (89.70)   
180 + **[CGGCTCCGCG]** 1096 (85.16)   
180 + **.CGCGGCGGCG.** 1247 (90.88)   
180 - **.CGCCGCCGCG.** 1247 (90.88)   
180 - **[CGCGGAGCCG]** 1096 (85.16)   
180 - **[CCCGCGCGCG]** 812 (89.70)   
182 + **.CGGGGCCGCG.** 371 (90.94)   
182 + **[CCCGGGCGCG]** 382 (89.87)   
182 + **.GGCGGGCGCG.** 428 (90.62)   
182 + **.GGCGGCCGCG.** 457 (90.79)   
182 + **.GGCGCACGCG.** 628 (91.73)   
182 + **[CGCACGCGCG]** 630 (86.31)   
182 + **[CGCGCCTGCG]** 668 (86.48)   
182 + **.CGCCTGCGCG.** 670 (91.82)   
182 + **.GGCGCGCGCG.** 766 (90.45)   
182 + **[CGCGCGCGCT]** 768 (86.91)   
182 + **[GGCCTCCGCG]** 823 (85.01)   
182 + **[CGCGTCCCCC]** 920 (85.07)   
182 + **.CCCGTGCGCG.** 979 (90.98)   
182 + **.CGCGTCCGCC.** 1021 (91.90)   
182 + **[CGCCAGCGCG]** 1191 (85.66)   
182 + **.GGCGTCCGCG.** 1285 (91.90)   
182 - **.CGCGGACGCC.** 1285 (91.90)   
182 - **[CGCGCTGGCG]** 1191 (85.66)   
182 - **.GGCGGACGCG.** 1021 (91.90)   
182 - **.CGCGCACGGG.** 979 (90.98)   
182 - **[GGGGGACGCG]** 920 (85.07)   
182 - **[CGCGGAGGCC]** 823 (85.01)   
182 - **[AGCGCGCGCG]** 768 (86.91)   
182 - **.CGCGCGCGCC.** 766 (90.45)   
182 - **.CGCGCAGGCG.** 670 (91.82)   
182 - **[CGCAGGCGCG]** 668 (86.48)   
182 - **[CGCGCGTGCG]** 630 (86.31)   
182 - **.CGCGTGCGCC.** 628 (91.73)   
182 - **.CGCGGCCGCC.** 457 (90.79)   
182 - **.CGCGCCCGCC.** 428 (90.62)   
182 - **[CGCGCCCGGG]** 382 (89.87)   
182 - **.CGCGGCCCCG.** 371 (90.94)   
183 + **[CGCGGGCTCG]** 652 (87.68)   
183 + **[CCCGCGCGCG]** 912 (89.70)   
183 + **[CGCGCGCGGG]** 914 (89.70)   
183 + **.CGCGTCCCCG.** 1301 (92.05)   
183 - **.CGGGGACGCG.** 1301 (92.05)   
183 - **[CCCGCGCGCG]** 914 (89.70)   
183 - **[CGCGCGCGGG]** 912 (89.70)   
183 - **[CGAGCCCGCG]** 652 (87.68)   
184 + **[CGGGGACGCC]** 363 (85.07)   
184 + **.CGCGCACGCC.** 731 (91.73)   
184 + **.CGGGCCCGCG.** 920 (90.77)   
184 + **[CGCCTCCGCC]** 986 (85.01)   
184 + **.CGCGTGCGGG.** 1092 (90.98)   
184 - **.CCCGCACGCG.** 1092 (90.98)   
184 - **[GGCGGAGGCG]** 986 (85.01)   
184 - **.CGCGGGCCCG.** 920 (90.77)   
184 - **.GGCGTGCGCG.** 731 (91.73)   
184 - **[GGCGTCCCCG]** 363 (85.07)   
185 + **.CGCGGCGGCG.** 1192 (90.88)   
185 - **.CGCCGCCGCG.** 1192 (90.88)   
186 + **[CGCGTCTGCG]** 905 (87.76)   
186 + **[CGCAGCCGCG]** 1344 (86.65)   
186 - **[CGCGGCTGCG]** 1344 (86.65)   
186 - **[CGCAGACGCG]** 905 (87.76)   
187 + **.CGCGGCCGGG.** 948 (90.04)   
187 + **.CGCGGAGGCG.** 1076 (91.99)   
187 + **.CGCGGTCGCG.** 1231 (92.73)   
187 + **.CGCGGACCCG.** 1304 (92.05)   
187 - **.CGGGTCCGCG.** 1304 (92.05)   
187 - **.CGCGACCGCG.** 1231 (92.73)   
187 - **.CGCCTCCGCG.** 1076 (91.99)   
187 - **.CCCGGCCGCG.** 948 (90.04)   
188 + **[CGCCAGCGCG]** 767 (85.66)   
188 - **[CGCGCTGGCG]** 767 (85.66)   
189 + **[CGCGCATGCG]** 951 (87.59)   
189 + **[CGCATGCGCG]** 953 (87.59)   
189 + **[CGCGTTCGCC]** 1054 (86.85)   
189 - **[GGCGAACGCG]** 1054 (86.85)   
189 - **[CGCGCATGCG]** 953 (87.59)   
189 - **[CGCATGCGCG]** 951 (87.59)   
191 + **[TGCGCGCGCG]** 545 (87.36)   
191 + **.CGCGCGCGCG.** 547 (97.44)   
191 + **.CGCGGCCGGG.** 569 (90.04)   
191 + **[CGCGCCCGAG]** 618 (86.24)   
191 + **.CGGGGGCGCG.** 786 (90.77)   
191 + **.CGCGGCCGCG.** 827 (97.78)   
191 + **.CGCGGAGGCG.** 833 (91.99)   
191 + **[CGAGGGCGCG]** 873 (87.68)   
191 + **.GGCGCGCGCG.** 877 (90.45)   
191 + **.GGCGCCCGCG.** 896 (90.62)   
191 + **[CGAGGGCGCG]** 1157 (87.68)   
191 - **[CGCGCCCTCG]** 1157 (87.68)   
191 - **.CGCGGGCGCC.** 896 (90.62)   
191 - **.CGCGCGCGCC.** 877 (90.45)   
191 - **[CGCGCCCTCG]** 873 (87.68)   
191 - **.CGCCTCCGCG.** 833 (91.99)   
191 - **.CGCGGCCGCG.** 827 (97.78)   
191 - **.CGCGCCCCCG.** 786 (90.77)   
191 - **[CTCGGGCGCG]** 618 (86.24)   
191 - **.CCCGGCCGCG.** 569 (90.04)   
191 - **.CGCGCGCGCG.** 547 (97.44)   
191 - **[CGCGCGCGCA]** 545 (87.36)   
193 + **.CGCGCGCGCG.** 847 (97.44)   
193 + **.CGCGCGCGCG.** 849 (97.44)   
193 + **.CGCGCGCGCG.** 851 (97.44)   
193 + **.CGCGCGCGCG.** 853 (97.44)   
193 + **.CGCGCGCGCG.** 855 (97.44)   
193 + **[CGCGCGCGCA]** 857 (87.36)   
193 + **[CGCGCACACG]** 861 (87.86)   
193 - **[CGTGTGCGCG]** 861 (87.86)   
193 - **[TGCGCGCGCG]** 857 (87.36)   
193 - **.CGCGCGCGCG.** 855 (97.44)   
193 - **.CGCGCGCGCG.** 853 (97.44)   
193 - **.CGCGCGCGCG.** 851 (97.44)   
193 - **.CGCGCGCGCG.** 849 (97.44)   
193 - **.CGCGCGCGCG.** 847 (97.44)   
194 + **[GGCGTCGGCG]** 555 (85.01)   
194 + **.CGCGGACGGG.** 1042 (91.15)   
194 + **[CGCGCGTGCG]** 1068 (86.31)   
194 + **[CGCGTGCGAG]** 1070 (87.35)   
194 - **[CTCGCACGCG]** 1070 (87.35)   
194 - **[CGCACGCGCG]** 1068 (86.31)   
194 - **.CCCGTCCGCG.** 1042 (91.15)   
194 - **[CGCCGACGCC]** 555 (85.01)   
195 + **.CCCGTCCGCG.** 104 (91.15)   
195 + **[CGCGGACACG]** 110 (88.03)   
195 + **[CGCGCATGCG]** 909 (87.59)   
195 + **.CGCGTCCGCC.** 960 (91.90)   
195 - **.GGCGGACGCG.** 960 (91.90)   
195 - **[CGCATGCGCG]** 909 (87.59)   
195 - **[CGTGTCCGCG]** 110 (88.03)   
195 - **.CGCGGACGGG.** 104 (91.15)   
196 + **[CGGGAGCGCG]** 532 (85.72)   
196 + **[CGCGGCCGAG]** 538 (86.41)   
196 + **.CGCGGGCGCG.** 662 (97.61)   
196 + **[TGCGGACGCG]** 684 (88.81)   
196 + **[AGCGCGCGCG]** 1186 (86.91)   
196 + **[CGCGCGCGTG]** 1188 (86.27)   
196 + **[CGGGGAGGCG]** 1322 (85.16)   
196 + **[CGCGCTCCCG]** 1393 (85.72)   
196 + **[CGGGGACCCG]** 1488 (85.22)   
196 - **[CGGGTCCCCG]** 1488 (85.22)   
196 - **[CGGGAGCGCG]** 1393 (85.72)   
196 - **[CGCCTCCCCG]** 1322 (85.16)   
196 - **[CACGCGCGCG]** 1188 (86.27)   
196 - **[CGCGCGCGCT]** 1186 (86.91)   
196 - **[CGCGTCCGCA]** 684 (88.81)   
196 - **.CGCGCCCGCG.** 662 (97.61)   
196 - **[CTCGGCCGCG]** 538 (86.41)   
196 - **[CGCGCTCCCG]** 532 (85.72)   
197 + **[CGAGGCCGCG]** 785 (87.85)   
197 + **.GGCGGACGCG.** 1157 (91.90)   
197 - **.CGCGTCCGCC.** 1157 (91.90)   
197 - **[CGCGGCCTCG]** 785 (87.85)   
198 + **.CGCCGCCGCG.** 253 (90.88)   
198 - **.CGCGGCGGCG.** 253 (90.88)   
199 + **[CGCCAGCGCG]** 715 (85.66)   
199 + **[CCCGGGCGCG]** 902 (89.87)   
199 + **.GGCGGGCGCG.** 934 (90.62)   
199 + **.CGCGGCCGCC.** 1003 (90.79)   
199 + **.CGCCCCCGCG.** 1058 (90.71)   
199 + **.CGCGCGGGCG.** 1064 (90.54)   
199 + **.CGCGCCCGCC.** 1244 (90.62)   
199 + **[CGAGGCCGCG]** 1261 (87.85)   
199 + **[CGCGCGCACG]** 1298 (86.58)   
199 + **[CGCGCACGTG]** 1300 (87.55)   
199 + **.CGGGCGCGCG.** 1318 (90.60)   
199 + **.CGCGGGCCCG.** 1354 (90.77)   
199 + **[CGCGTCCGCT]** 1448 (88.36)   
199 - **[AGCGGACGCG]** 1448 (88.36)   
199 - **.CGGGCCCGCG.** 1354 (90.77)   
199 - **.CGCGCGCCCG.** 1318 (90.60)   
199 - **[CACGTGCGCG]** 1300 (87.55)   
199 - **[CGTGCGCGCG]** 1298 (86.58)   
199 - **[CGCGGCCTCG]** 1261 (87.85)   
199 - **.GGCGGGCGCG.** 1244 (90.62)   
199 - **.CGCCCGCGCG.** 1064 (90.54)   
199 - **.CGCGGGGGCG.** 1058 (90.71)   
199 - **.GGCGGCCGCG.** 1003 (90.79)   
199 - **.CGCGCCCGCC.** 934 (90.62)   
199 - **[CGCGCCCGGG]** 902 (89.87)   
199 - **[CGCGCTGGCG]** 715 (85.66)   
201 + **[CGCGGCCTCG]** 351 (87.85)   
201 + **[CGCGGCCTCG]** 533 (87.85)   
201 + **[CCCGCCCGCG]** 1014 (89.87)   
201 + **.CGGGTGCGCG.** 1195 (91.88)   
201 + **.CGCGCGCGCG.** 1493 (97.44)   
201 + **.CGCGCGCGCC.** 1495 (90.45)   
201 + **.CGCGCGCCCG.** 1497 (90.60)   
201 + **.CGCGCCCGCC.** 1499 (90.62)   
201 - **.GGCGGGCGCG.** 1499 (90.62)   
201 - **.CGGGCGCGCG.** 1497 (90.60)   
201 - **.GGCGCGCGCG.** 1495 (90.45)   
201 - **.CGCGCGCGCG.** 1493 (97.44)   
201 - **.CGCGCACCCG.** 1195 (91.88)   
201 - **[CGCGGGCGGG]** 1014 (89.87)   
201 - **[CGAGGCCGCG]** 533 (87.85)   
201 - **[CGAGGCCGCG]** 351 (87.85)   
202 + **[CGGGAGCGCG]** 1380 (85.72)   
202 - **[CGCGCTCCCG]** 1380 (85.72)   
208 + **[CGCCGAGGCG]** 1390 (85.09)   
208 - **[CGCCTCGGCG]** 1390 (85.09)   
210 + **[GGCGGAGGCG]** 903 (85.01)   
210 + **.CCCGGCCGCG.** 1043 (90.04)   
210 + **.GGCGCACGCG.** 1217 (91.73)   
210 - **.CGCGTGCGCC.** 1217 (91.73)   
210 - **.CGCGGCCGGG.** 1043 (90.04)   
210 - **[CGCCTCCGCC]** 903 (85.01)   
211 + **[CACGCCCGCG]** 777 (86.44)   
211 + **.CGCGTGCGGG.** 1407 (90.98)   
211 - **.CCCGCACGCG.** 1407 (90.98)   
211 - **[CGCGGGCGTG]** 777 (86.44)   
212 + **[AGCGTCCGCG]** 1028 (88.36)   
212 - **[CGCGGACGCT]** 1028 (88.36)   
214 + **.GGCGCACGCG.** 980 (91.73)   
214 - **.CGCGTGCGCC.** 980 (91.73)   
215 + **.CGCCTGCGCG.** 1017 (91.82)   
215 + **[CGCGCACGCT]** 1197 (88.19)   
215 + **[CGAGGCCGCG]** 1409 (87.85)   
215 - **[CGCGGCCTCG]** 1409 (87.85)   
215 - **[AGCGTGCGCG]** 1197 (88.19)   
215 - **.CGCGCAGGCG.** 1017 (91.82)   
216 + **[CCCGCCCGCG]** 1090 (89.87)   
216 + **[CGCGGACCCC]** 1483 (85.07)   
216 - **[GGGGTCCGCG]** 1483 (85.07)   
216 - **[CGCGGGCGGG]** 1090 (89.87)   
221 + **[CGCGTCCGCA]** 20 (88.81)   
221 + **.CGGGGCCGCG.** 511 (90.94)   
221 + **.CGCGGCGGCG.** 1112 (90.88)   
221 - **.CGCCGCCGCG.** 1112 (90.88)   
221 - **.CGCGGCCCCG.** 511 (90.94)   
221 - **[TGCGGACGCG]** 20 (88.81)   
222 + **.CGCGTCCGGG.** 452 (91.15)   
222 + **[CGCGGAGCCG]** 871 (85.16)   
222 + **.CGGGGCCGCG.** 997 (90.94)   
222 + **.CGCGGAGGCG.** 1187 (91.99)   
222 - **.CGCCTCCGCG.** 1187 (91.99)   
222 - **.CGCGGCCCCG.** 997 (90.94)   
222 - **[CGGCTCCGCG]** 871 (85.16)   
222 - **.CCCGGACGCG.** 452 (91.15)   
223 + **[CGGCTACGCG]** 386 (86.27)   
223 + **[CGGGCACCCG]** 460 (85.05)   
223 - **[CGGGTGCCCG]** 460 (85.05)   
223 - **[CGCGTAGCCG]** 386 (86.27)   
224 + **[CGCGGCCGAG]** 532 (86.41)   
224 + **[CCCGGGCGCG]** 611 (89.87)   
224 + **.CGGGCGCGCG.** 613 (90.60)   
224 + **[AGCGCGCGCG]** 793 (86.91)   
224 + **.CGCGGCCGCC.** 799 (90.79)   
224 + **[GGGGTACGCG]** 1062 (86.18)   
224 - **[CGCGTACCCC]** 1062 (86.18)   
224 - **.GGCGGCCGCG.** 799 (90.79)   
224 - **[CGCGCGCGCT]** 793 (86.91)   
224 - **.CGCGCGCCCG.** 613 (90.60)   
224 - **[CGCGCCCGGG]** 611 (89.87)   
224 - **[CTCGGCCGCG]** 532 (86.41)   
226 + **.CGCGGCCGCC.** 125 (90.79)   
226 + **[CCCGCGCGCG]** 229 (89.70)   
226 + **[CGCCTCCCCG]** 471 (85.16)   
226 + **[CGAGCGCGCG]** 749 (87.51)   
226 + **.CGCGCGGGCG.** 753 (90.54)   
226 + **[CGCGGGCGGG]** 755 (89.87)   
226 + **.CGGGGGCGCG.** 961 (90.77)   
226 + **[AGCGCGCGCG]** 1026 (86.91)   
226 + **.CGCGCGCGCC.** 1028 (90.45)   
226 + **[CCCGGGCGCG]** 1303 (89.87)   
226 + **[CCCCTACGCG]** 1379 (85.36)   
226 + **.GGCGCCCGCG.** 1412 (90.62)   
226 + **[CGTGGGCGCG]** 1491 (86.75)   
226 - **[CGCGCCCACG]** 1491 (86.75)   
226 - **.CGCGGGCGCC.** 1412 (90.62)   
226 - **[CGCGTAGGGG]** 1379 (85.36)   
226 - **[CGCGCCCGGG]** 1303 (89.87)   
226 - **.GGCGCGCGCG.** 1028 (90.45)   
226 - **[CGCGCGCGCT]** 1026 (86.91)   
226 - **.CGCGCCCCCG.** 961 (90.77)   
226 - **[CCCGCCCGCG]** 755 (89.87)   
226 - **.CGCCCGCGCG.** 753 (90.54)   
226 - **[CGCGCGCTCG]** 749 (87.51)   
226 - **[CGGGGAGGCG]** 471 (85.16)   
226 - **[CGCGCGCGGG]** 229 (89.70)   
226 - **.GGCGGCCGCG.** 125 (90.79)   
227 + **.CGGGGACGCG.** 303 (92.05)   
227 + **[CGCGCACACG]** 538 (87.86)   
227 + **[CGCAGGCGCG]** 546 (86.48)   
227 + **[CGCGCACACG]** 554 (87.86)   
227 + **[CGCACACGCG]** 556 (87.59)   
227 + **[CGCTCGCGCG]** 783 (85.94)   
227 + **[CGCGGAGGCC]** 892 (85.01)   
227 + **[CGCCTCCCCG]** 980 (85.16)   
227 + **[CGCCTCCCCG]** 990 (85.16)   
227 + **[CGCCTCCCCG]** 1000 (85.16)   
227 + **[CGCCTCCCCG]** 1020 (85.16)   
227 + **[CGCCTCCCCG]** 1040 (85.16)   
227 + **[CGCCTCCCCG]** 1081 (85.16)   
227 + **[CGCCTCCCCG]** 1091 (85.16)   
227 + **[CGCCTCCCCG]** 1121 (85.16)   
227 - **[CGGGGAGGCG]** 1121 (85.16)   
227 - **[CGGGGAGGCG]** 1091 (85.16)   
227 - **[CGGGGAGGCG]** 1081 (85.16)   
227 - **[CGGGGAGGCG]** 1040 (85.16)   
227 - **[CGGGGAGGCG]** 1020 (85.16)   
227 - **[CGGGGAGGCG]** 1000 (85.16)   
227 - **[CGGGGAGGCG]** 990 (85.16)   
227 - **[CGGGGAGGCG]** 980 (85.16)   
227 - **[GGCCTCCGCG]** 892 (85.01)   
227 - **[CGCGCGAGCG]** 783 (85.94)   
227 - **[CGCGTGTGCG]** 556 (87.59)   
227 - **[CGTGTGCGCG]** 554 (87.86)   
227 - **[CGCGCCTGCG]** 546 (86.48)   
227 - **[CGTGTGCGCG]** 538 (87.86)   
227 - **.CGCGTCCCCG.** 303 (92.05)   
228 + **[CGCGACCCCG]** 46 (85.89)   
228 + **[CGCACACGCG]** 269 (87.59)   
228 + **.CGCGCCCGCG.** 275 (97.61)   
228 + **.CGCGCCCGCC.** 281 (90.62)   
228 + **.CGCCCCCGCG.** 465 (90.71)   
228 + **[CACGCCCGCG]** 536 (86.44)   
228 + **[CGCGGGCGAG]** 771 (86.24)   
228 + **[CGCGCTCCCG]** 784 (85.72)   
228 - **[CGGGAGCGCG]** 784 (85.72)   
228 - **[CTCGCCCGCG]** 771 (86.24)   
228 - **[CGCGGGCGTG]** 536 (86.44)   
228 - **.CGCGGGGGCG.** 465 (90.71)   
228 - **.GGCGGGCGCG.** 281 (90.62)   
228 - **.CGCGGGCGCG.** 275 (97.61)   
228 - **[CGCGTGTGCG]** 269 (87.59)   
228 - **[CGGGGTCGCG]** 46 (85.89)   
229 + **[CGCGGCCGCT]** 540 (87.25)   
229 + **.CGCGCCCCCG.** 1148 (90.77)   
229 + **.CGGGGGCGCG.** 1297 (90.77)   
229 + **[GGCGTACCCG]** 1489 (86.18)   
229 - **[CGGGTACGCC]** 1489 (86.18)   
229 - **.CGCGCCCCCG.** 1297 (90.77)   
229 - **.CGGGGGCGCG.** 1148 (90.77)   
229 - **[AGCGGCCGCG]** 540 (87.25)   
231 + **.CGCCGCCGCG.** 902 (90.88)   
231 + **[CGCGGCCACG]** 1070 (86.92)   
231 - **[CGTGGCCGCG]** 1070 (86.92)   
231 - **.CGCGGCGGCG.** 902 (90.88)   
232 + **.CGCGCCCGCC.** 149 (90.62)   
232 + **[CCCGGGCGCG]** 695 (89.87)   
232 + **.CGGGCCCGCG.** 855 (90.77)   
232 + **.CGCGGCCGCG.** 863 (97.78)   
232 - **.CGCGGCCGCG.** 863 (97.78)   
232 - **.CGCGGGCCCG.** 855 (90.77)   
232 - **[CGCGCCCGGG]** 695 (89.87)   
232 - **.GGCGGGCGCG.** 149 (90.62)   
234 + **.CGCGGGCGCC.** 275 (90.62)   
234 + **[CGCGACCCCG]** 579 (85.89)   
234 + **.CGCGCCCGCG.** 850 (97.61)   
234 + **.GGCGCCCGCG.** 866 (90.62)   
234 + **.CGCGGGGGCG.** 997 (90.71)   
234 - **.CGCCCCCGCG.** 997 (90.71)   
234 - **.CGCGGGCGCC.** 866 (90.62)   
234 - **.CGCGGGCGCG.** 850 (97.61)   
234 - **[CGGGGTCGCG]** 579 (85.89)   
234 - **.GGCGCCCGCG.** 275 (90.62)   
235 + **.CGCGGGGGCG.** 685 (90.71)   
235 + **.CGCGTACGCG.** 762 (100.00)   
235 + **[CGCGCCTGCG]** 1364 (86.48)   
235 + **[CGCACGCGCG]** 1395 (86.31)   
235 + **[CGCGTACGGC]** 1431 (85.27)   
235 - **[GCCGTACGCG]** 1431 (85.27)   
235 - **[CGCGCGTGCG]** 1395 (86.31)   
235 - **[CGCAGGCGCG]** 1364 (86.48)   
235 - **.CGCGTACGCG.** 762 (100.00)   
235 - **.CGCCCCCGCG.** 685 (90.71)   
236 + **.CGGGCCCGCG.** 1191 (90.77)   
236 + **[CGAGCCCGCG]** 1326 (87.68)   
236 + **[CGCGGACCCC]** 1332 (85.07)   
236 - **[GGGGTCCGCG]** 1332 (85.07)   
236 - **[CGCGGGCTCG]** 1326 (87.68)   
236 - **.CGCGGGCCCG.** 1191 (90.77)   
237 + **[CCCGTAGGCG]** 101 (85.36)   
237 - **[CGCCTACGGG]** 101 (85.36)   
239 + **.GGCGTGCGCG.** 783 (91.73)   
239 - **.CGCGCACGCC.** 783 (91.73)   
240 + **[CGCGCCCGTG]** 140 (86.44)   
240 - **[CACGGGCGCG]** 140 (86.44)   
242 + **.GGCGTCCGCG.** 1076 (91.90)   
242 - **.CGCGGACGCC.** 1076 (91.90)   
244 + **.CGCGGGCGCC.** 689 (90.62)   
244 + **[CGCGGACGCA]** 781 (88.81)   
244 - **[TGCGTCCGCG]** 781 (88.81)   
244 - **.GGCGCCCGCG.** 689 (90.62)   
246 + **[CGCCTCGGCG]** 278 (85.09)   
246 + **.CGCGCTCGCG.** 425 (92.56)   
246 + **[CGCGTGCGCT]** 461 (88.19)   
246 + **.GGCGGGCGCG.** 491 (90.62)   
246 + **.CCCGGCCGCG.** 703 (90.04)   
246 + **.CGCGCCCGCC.** 750 (90.62)   
246 + **[TGCGGCCGCG]** 1074 (87.70)   
246 - **[CGCGGCCGCA]** 1074 (87.70)   
246 - **.GGCGGGCGCG.** 750 (90.62)   
246 - **.CGCGGCCGGG.** 703 (90.04)   
246 - **.CGCGCCCGCC.** 491 (90.62)   
246 - **[AGCGCACGCG]** 461 (88.19)   
246 - **.CGCGAGCGCG.** 425 (92.56)   
246 - **[CGCCGAGGCG]** 278 (85.09)   
247 + **.CGCCTCCGCG.** 791 (91.99)   
247 + **[CGCTGGCGCG]** 946 (86.11)   
247 + **[CGCGGGCTCG]** 952 (87.68)   
247 + **.CGCCCCCGCG.** 1116 (90.71)   
247 + **[CGCGCCCGCT]** 1122 (87.08)   
247 + **.GGCGGGCGCG.** 1281 (90.62)   
247 + **.CGGGCGCGCG.** 1283 (90.60)   
247 + **.CGCGCCCCCG.** 1473 (90.77)   
247 + **.CGCCCCCGCG.** 1475 (90.71)   
247 + **.CGCGGGCGCC.** 1481 (90.62)   
247 - **.GGCGCCCGCG.** 1481 (90.62)   
247 - **.CGCGGGGGCG.** 1475 (90.71)   
247 - **.CGGGGGCGCG.** 1473 (90.77)   
247 - **.CGCGCGCCCG.** 1283 (90.60)   
247 - **.CGCGCCCGCC.** 1281 (90.62)   
247 - **[AGCGGGCGCG]** 1122 (87.08)   
247 - **.CGCGGGGGCG.** 1116 (90.71)   
247 - **[CGAGCCCGCG]** 952 (87.68)   
247 - **[CGCGCCAGCG]** 946 (86.11)   
247 - **.CGCGGAGGCG.** 791 (91.99)   
250 + **.CGGGGACGCG.** 449 (92.05)   
250 + **[CCCGCGCGCG]** 585 (89.70)   
250 + **.CGCGCGCGCC.** 587 (90.45)   
250 + **.CGCCCCCGCG.** 1043 (90.71)   
250 + **[CACGCGCGCG]** 1303 (86.27)   
250 + **[CGCGCGCGCA]** 1305 (87.36)   
250 - **[TGCGCGCGCG]** 1305 (87.36)   
250 - **[CGCGCGCGTG]** 1303 (86.27)   
250 - **.CGCGGGGGCG.** 1043 (90.71)   
250 - **.GGCGCGCGCG.** 587 (90.45)   
250 - **[CGCGCGCGGG]** 585 (89.70)   
250 - **.CGCGTCCCCG.** 449 (92.05)   
256 + **.CGCGCCCGCC.** 1117 (90.62)   
256 + **[CGCCGACCCG]** 1123 (85.16)   
256 + **.CGCGGGCCCG.** 1197 (90.77)   
256 + **.CGGGCCCGCG.** 1199 (90.77)   
256 - **.CGCGGGCCCG.** 1199 (90.77)   
256 - **.CGGGCCCGCG.** 1197 (90.77)   
256 - **[CGGGTCGGCG]** 1123 (85.16)   
256 - **.GGCGGGCGCG.** 1117 (90.62)   
257 + **.CGCGGGCCCG.** 735 (90.77)   
257 + **.CGCGGGCCCG.** 756 (90.77)   
257 + **[CGCGGAAGCG]** 878 (87.39)   
257 + **[AGCGGCCGCG]** 1162 (87.25)   
257 + **[TGCGGCCGCG]** 1190 (87.70)   
257 + **.GGCGCCCGCG.** 1239 (90.62)   
257 + **.CGCCCGCGCG.** 1241 (90.54)   
257 + **.CGCGCGCCCG.** 1245 (90.60)   
257 + **.CGCGCCCGCC.** 1247 (90.62)   
257 + **.GGCGGCCGCG.** 1278 (90.79)   
257 + **[CACGGGCGCG]** 1492 (86.44)   
257 - **[CGCGCCCGTG]** 1492 (86.44)   
257 - **.CGCGGCCGCC.** 1278 (90.79)   
257 - **.GGCGGGCGCG.** 1247 (90.62)   
257 - **.CGGGCGCGCG.** 1245 (90.60)   
257 - **.CGCGCGGGCG.** 1241 (90.54)   
257 - **.CGCGGGCGCC.** 1239 (90.62)   
257 - **[CGCGGCCGCA]** 1190 (87.70)   
257 - **[CGCGGCCGCT]** 1162 (87.25)   
257 - **[CGCTTCCGCG]** 878 (87.39)   
257 - **.CGGGCCCGCG.** 756 (90.77)   
257 - **.CGGGCCCGCG.** 735 (90.77)   
259 + **.CGCCTCCGCG.** 863 (91.99)   
259 + **[CGGCGACGCG]** 991 (85.16)   
259 + **[CGCGGCCGCT]** 1187 (87.25)   
259 - **[AGCGGCCGCG]** 1187 (87.25)   
259 - **[CGCGTCGCCG]** 991 (85.16)   
259 - **.CGCGGAGGCG.** 863 (91.99)   
260 + **[CACGCGCGCG]** 992 (86.27)   
260 + **.CGCGCGCGCG.** 994 (97.44)   
260 + **[CGCGCGCGCA]** 996 (87.36)   
260 + **.CGCGCACCCG.** 1026 (91.88)   
260 + **[CGCACCCGCG]** 1034 (86.48)   
260 - **[CGCGGGTGCG]** 1034 (86.48)   
260 - **.CGGGTGCGCG.** 1026 (91.88)   
260 - **[TGCGCGCGCG]** 996 (87.36)   
260 - **.CGCGCGCGCG.** 994 (97.44)   
260 - **[CGCGCGCGTG]** 992 (86.27)   
263 + **[CGCGCCTGCG]** 542 (86.48)   
263 + **.CGCGGCCGCG.** 850 (97.78)   
263 - **.CGCGGCCGCG.** 850 (97.78)   
263 - **[CGCAGGCGCG]** 542 (86.48)   
266 + **[CGCGGCCGCT]** 962 (87.25)   
266 + **.CCCGGCCGCG.** 1061 (90.04)   
266 + **.CGCGCCGGCG.** 1270 (90.71)   
266 + **.GGCGGCCGCG.** 1276 (90.79)   
266 - **.CGCGGCCGCC.** 1276 (90.79)   
266 - **.CGCCGGCGCG.** 1270 (90.71)   
266 - **.CGCGGCCGGG.** 1061 (90.04)   
266 - **[AGCGGCCGCG]** 962 (87.25)   
267 + **[CGCGGCCGCA]** 195 (87.70)   
267 - **[TGCGGCCGCG]** 195 (87.70)   
268 + **.CCCGCACGCG.** 827 (90.98)   
268 + **[CGCGGAAGCG]** 833 (87.39)   
268 + **[CGCGGGTGCG]** 1070 (86.48)   
268 - **[CGCACCCGCG]** 1070 (86.48)   
268 - **[CGCTTCCGCG]** 833 (87.39)   
268 - **.CGCGTGCGGG.** 827 (90.98)   
270 + **.CGCGGACCCG.** 146 (92.05)   
270 - **.CGGGTCCGCG.** 146 (92.05)   
271 + **.CGCGCACGCC.** 574 (91.73)   
271 + **.CGCGCACGCC.** 587 (91.73)   
271 + **.CGCGCACGCC.** 600 (91.73)   
271 + **.CGCGCACGCC.** 613 (91.73)   
271 + **.CGCGCACGCC.** 626 (91.73)   
271 + **.CGCGCACGCC.** 653 (91.73)   
271 + **.CGCGCACGCC.** 666 (91.73)   
271 + **[CGCACGCGCG]** 675 (86.31)   
271 - **[CGCGCGTGCG]** 675 (86.31)   
271 - **.GGCGTGCGCG.** 666 (91.73)   
271 - **.GGCGTGCGCG.** 653 (91.73)   
271 - **.GGCGTGCGCG.** 626 (91.73)   
271 - **.GGCGTGCGCG.** 613 (91.73)   
271 - **.GGCGTGCGCG.** 600 (91.73)   
271 - **.GGCGTGCGCG.** 587 (91.73)   
271 - **.GGCGTGCGCG.** 574 (91.73)   
272 + **.CGCGCACGCC.** 588 (91.73)   
272 + **.CGCGCACGCC.** 601 (91.73)   
272 + **.CGCGCACGCC.** 614 (91.73)   
272 + **.CGCGCACGCC.** 627 (91.73)   
272 + **.CGCGCACGCC.** 654 (91.73)   
272 + **[CGCACGCGCG]** 663 (86.31)   
272 + **[CGCGCGCGCA]** 714 (87.36)   
272 + **[CGCGCGCACG]** 716 (86.58)   
272 + **.CGCGCACGCC.** 718 (91.73)   
272 - **.GGCGTGCGCG.** 718 (91.73)   
272 - **[CGTGCGCGCG]** 716 (86.58)   
272 - **[TGCGCGCGCG]** 714 (87.36)   
272 - **[CGCGCGTGCG]** 663 (86.31)   
272 - **.GGCGTGCGCG.** 654 (91.73)   
272 - **.GGCGTGCGCG.** 627 (91.73)   
272 - **.GGCGTGCGCG.** 614 (91.73)   
272 - **.GGCGTGCGCG.** 601 (91.73)   
272 - **.GGCGTGCGCG.** 588 (91.73)   
273 + **[AGCGGCCGCG]** 1026 (87.25)   
273 + **.CGCGGACGGG.** 1297 (91.15)   
273 - **.CCCGTCCGCG.** 1297 (91.15)   
273 - **[CGCGGCCGCT]** 1026 (87.25)   
274 + **.CCCGGCCGCG.** 9 (90.04)   
274 + **[CGCGGCCGCA]** 15 (87.70)   
274 + **[CGCGCGCGTG]** 569 (86.27)   
274 + **.CGCGCGCGCC.** 583 (90.45)   
274 + **[TGCGCGCGCG]** 1263 (87.36)   
274 + **.CGCGCGCGCC.** 1265 (90.45)   
274 + **.CGCGCGCCCG.** 1267 (90.60)   
274 + **[CGCGCCCGGG]** 1269 (89.87)   
274 + **[TGCGTGCGCG]** 1316 (88.64)   
274 + **.CGCGGCCGCC.** 1322 (90.79)   
274 + **[CCCGGGCGCG]** 1348 (89.87)   
274 + **[CGCGGAGCCG]** 1354 (85.16)   
274 - **[CGGCTCCGCG]** 1354 (85.16)   
274 - **[CGCGCCCGGG]** 1348 (89.87)   
274 - **.GGCGGCCGCG.** 1322 (90.79)   
274 - **[CGCGCACGCA]** 1316 (88.64)   
274 - **[CCCGGGCGCG]** 1269 (89.87)   
274 - **.CGGGCGCGCG.** 1267 (90.60)   
274 - **.GGCGCGCGCG.** 1265 (90.45)   
274 - **[CGCGCGCGCA]** 1263 (87.36)   
274 - **.GGCGCGCGCG.** 583 (90.45)   
274 - **[CACGCGCGCG]** 569 (86.27)   
274 - **[TGCGGCCGCG]** 15 (87.70)   
274 - **.CGCGGCCGGG.** 9 (90.04)   
275 + **.CGCGCCCGCC.** 735 (90.62)   
275 + **[CCCGGGCGCG]** 1084 (89.87)   
275 - **[CGCGCCCGGG]** 1084 (89.87)   
275 - **.GGCGGGCGCG.** 735 (90.62)   
279 + **[AGCGGGCGCG]** 102 (87.08)   
279 + **[CGCGGAGGCC]** 1201 (85.01)   
279 - **[GGCCTCCGCG]** 1201 (85.01)   
279 - **[CGCGCCCGCT]** 102 (87.08)   
280 + **.GGCGGCCGCG.** 738 (90.79)   
280 + **[CGTGTGCGCG]** 821 (87.86)   
280 + **.GGCGCGCGCG.** 976 (90.45)   
280 + **.CGCGGCGGCG.** 982 (90.88)   
280 + **.CGCGCCCGCG.** 1066 (97.61)   
280 + **[CGCGTCCGCA]** 1084 (88.81)   
280 + **[CGGGACCGCG]** 1146 (85.89)   
280 + **[CGCGACCCCG]** 1152 (85.89)   
280 - **[CGGGGTCGCG]** 1152 (85.89)   
280 - **[CGCGGTCCCG]** 1146 (85.89)   
280 - **[TGCGGACGCG]** 1084 (88.81)   
280 - **.CGCGGGCGCG.** 1066 (97.61)   
280 - **.CGCCGCCGCG.** 982 (90.88)   
280 - **.CGCGCGCGCC.** 976 (90.45)   
280 - **[CGCGCACACG]** 821 (87.86)   
280 - **.CGCGGCCGCC.** 738 (90.79)   
281 + **[CGCGCCCGGG]** 433 (89.87)   
281 + **.CGCGGCCGCC.** 531 (90.79)   
281 + **.CGCGCCCGCG.** 547 (97.61)   
281 + **.CGCGGACGCC.** 1026 (91.90)   
281 + **[CGGGGACCCG]** 1092 (85.22)   
281 - **[CGGGTCCCCG]** 1092 (85.22)   
281 - **.GGCGTCCGCG.** 1026 (91.90)   
281 - **.CGCGGGCGCG.** 547 (97.61)   
281 - **.GGCGGCCGCG.** 531 (90.79)   
281 - **[CCCGGGCGCG]** 433 (89.87)   
282 + **.GGCGTGCGCG.** 7 (91.73)   
282 - **.CGCGCACGCC.** 7 (91.73)   
284 + **.CGCGGCCCCG.** 1094 (90.94)   
284 + **[CGGGAGCGCG]** 1188 (85.72)   
284 + **.CGCGGGCGCG.** 1194 (97.61)   
284 + **.CGCGGCCGCC.** 1283 (90.79)   
284 + **[CCCGCCCGCG]** 1365 (89.87)   
284 - **[CGCGGGCGGG]** 1365 (89.87)   
284 - **.GGCGGCCGCG.** 1283 (90.79)   
284 - **.CGCGCCCGCG.** 1194 (97.61)   
284 - **[CGCGCTCCCG]** 1188 (85.72)   
284 - **.CGGGGCCGCG.** 1094 (90.94)   
285 + **[CGTGGGCGCG]** 330 (86.75)   
285 + **.GGCGCGCGCG.** 334 (90.45)   
285 + **[CGCGCGCGGG]** 336 (89.70)   
285 + **.CGCGCGGGCG.** 338 (90.54)   
285 + **.CGCGGGCGCG.** 340 (97.61)   
285 + **.CGCGGGCGCG.** 445 (97.61)   
285 + **[CGCTGGCGCG]** 466 (86.11)   
285 + **.CGCGGGGGCG.** 472 (90.71)   
285 + **.CGCGGCCGCG.** 548 (97.78)   
285 + **[CGCGGGCGGG]** 594 (89.87)   
285 + **[CGCGGCCGTG]** 717 (86.61)   
285 + **.CCCGGCCGCG.** 854 (90.04)   
285 + **[CCCGTACCCG]** 898 (85.42)   
285 - **[CGGGTACGGG]** 898 (85.42)   
285 - **.CGCGGCCGGG.** 854 (90.04)   
285 - **[CACGGCCGCG]** 717 (86.61)   
285 - **[CCCGCCCGCG]** 594 (89.87)   
285 - **.CGCGGCCGCG.** 548 (97.78)   
285 - **.CGCCCCCGCG.** 472 (90.71)   
285 - **[CGCGCCAGCG]** 466 (86.11)   
285 - **.CGCGCCCGCG.** 445 (97.61)   
285 - **.CGCGCCCGCG.** 340 (97.61)   
285 - **.CGCCCGCGCG.** 338 (90.54)   
285 - **[CCCGCGCGCG]** 336 (89.70)   
285 - **.CGCGCGCGCC.** 334 (90.45)   
285 - **[CGCGCCCACG]** 330 (86.75)   
287 + **[CGCGCCCGCA]** 326 (87.53)   
287 + **[CGGGGAGGCG]** 450 (85.16)   
287 + **[CGCCTCGGCG]** 765 (85.09)   
287 + **[CGGCTCCGCG]** 999 (85.16)   
287 + **[CGCGCGCTCG]** 1035 (87.51)   
287 + **[CGCGCTCGCC]** 1037 (85.57)   
287 + **[TGCGCCCGCG]** 1227 (87.53)   
287 - **[CGCGGGCGCA]** 1227 (87.53)   
287 - **[GGCGAGCGCG]** 1037 (85.57)   
287 - **[CGAGCGCGCG]** 1035 (87.51)   
287 - **[CGCGGAGCCG]** 999 (85.16)   
287 - **[CGCCGAGGCG]** 765 (85.09)   
287 - **[CGCCTCCCCG]** 450 (85.16)   
287 - **[TGCGGGCGCG]** 326 (87.53)   
288 + **.CGCCGGCGCG.** 872 (90.71)   
288 - **.CGCGCCGGCG.** 872 (90.71)   
289 + **.CGCGGGCGCC.** 1381 (90.62)   
289 - **.GGCGCCCGCG.** 1381 (90.62)   
290 + **[CGCATGCGCG]** 1055 (87.59)   
290 - **[CGCGCATGCG]** 1055 (87.59)   
291 + **.CGGGCGCGCG.** 1064 (90.60)   
291 + **.CGCGTGCGCC.** 1085 (91.73)   
291 + **[CCCGGGCGCG]** 1094 (89.87)   
291 + **.CGGGGCCGCG.** 1325 (90.94)   
291 + **.CGCGGGCCCG.** 1437 (90.77)   
291 + **[CGCGGGCTCG]** 1463 (87.68)   
291 - **[CGAGCCCGCG]** 1463 (87.68)   
291 - **.CGGGCCCGCG.** 1437 (90.77)   
291 - **.CGCGGCCCCG.** 1325 (90.94)   
291 - **[CGCGCCCGGG]** 1094 (89.87)   
291 - **.GGCGCACGCG.** 1085 (91.73)   
291 - **.CGCGCGCCCG.** 1064 (90.60)   
292 + **.CGCGCGGGCG.** 1028 (90.54)   
292 + **.CGGGGGCGCG.** 1039 (90.77)   
292 + **.CGCGAGCGCG.** 1084 (92.56)   
292 + **.CGCGCGCCCG.** 1138 (90.60)   
292 + **.CGCGCCCGCC.** 1140 (90.62)   
292 + **[GGGGGACGCG]** 1185 (85.07)   
292 + **.CGCGGACCCG.** 1238 (92.05)   
292 + **.CGCGTCCGCG.** 1319 (98.89)   
292 + **[CGCGGCCTCG]** 1386 (87.85)   
292 + **.CGCGGGGGCG.** 1447 (90.71)   
292 - **.CGCCCCCGCG.** 1447 (90.71)   
292 - **[CGAGGCCGCG]** 1386 (87.85)   
292 - **.CGCGGACGCG.** 1319 (98.89)   
292 - **.CGGGTCCGCG.** 1238 (92.05)   
292 - **[CGCGTCCCCC]** 1185 (85.07)   
292 - **.GGCGGGCGCG.** 1140 (90.62)   
292 - **.CGGGCGCGCG.** 1138 (90.60)   
292 - **.CGCGCTCGCG.** 1084 (92.56)   
292 - **.CGCGCCCCCG.** 1039 (90.77)   
292 - **.CGCCCGCGCG.** 1028 (90.54)   
293 + **.CGCGGGCGCC.** 619 (90.62)   
293 + **[CGCGGAGGCC]** 1100 (85.01)   
293 - **[GGCCTCCGCG]** 1100 (85.01)   
293 - **.GGCGCCCGCG.** 619 (90.62)   
294 + **.CGCGGACGCG.** 932 (98.89)   
294 + **.CGCGTGCCCG.** 1009 (91.88)   
294 + **[CGCGCACGCT]** 1030 (88.19)   
294 + **[CGCGTGCTCG]** 1282 (88.79)   
294 - **[CGAGCACGCG]** 1282 (88.79)   
294 - **[AGCGTGCGCG]** 1030 (88.19)   
294 - **.CGGGCACGCG.** 1009 (91.88)   
294 - **.CGCGTCCGCG.** 932 (98.89)   
295 + **[CGAGCACGCG]** 354 (88.79)   
295 + **[AGCGTGCGCG]** 606 (88.19)   
295 + **.CGGGCACGCG.** 627 (91.88)   
295 + **.CGCGTCCGCG.** 704 (98.89)   
295 - **.CGCGGACGCG.** 704 (98.89)   
295 - **.CGCGTGCCCG.** 627 (91.88)   
295 - **[CGCGCACGCT]** 606 (88.19)   
295 - **[CGCGTGCTCG]** 354 (88.79)   
296 + **.CGCCCCCGCG.** 33 (90.71)   
296 + **[CACGCGCGCG]** 53 (86.27)   
296 + **.CGCGCGCGCG.** 55 (97.44)   
296 + **[CGCGCGCGGG]** 57 (89.70)   
296 + **[CGCCGACCCG]** 145 (85.16)   
296 + **.GGCGCGCGCG.** 195 (90.45)   
296 + **[CGCGGCAGCG]** 201 (86.28)   
296 + **[CGCGAGCGCC]** 335 (85.57)   
296 + **[CGCGGGCGGG]** 421 (89.87)   
296 + **[CTCGGGCGCG]** 490 (86.24)   
296 + **.CGGGCGCGCG.** 492 (90.60)   
296 + **.GGCGCGCGCG.** 494 (90.45)   
296 + **.CGCGGACGGG.** 651 (91.15)   
296 + **[CGCCTCCGCC]** 779 (85.01)   
296 + **.CGCGGCCGGG.** 815 (90.04)   
296 + **[GGCGGAGGCG]** 854 (85.01)   
296 + **[GGGGTCCGCG]** 931 (85.07)   
296 + **[CGAGGACGCG]** 1008 (88.96)   
296 + **.CGCGCGCCCG.** 1014 (90.60)   
296 + **[CGCGCCCGGG]** 1016 (89.87)   
296 + **.GGCGGCCGCG.** 1158 (90.79)   
296 + **[GGCGTCCCCG]** 1348 (85.07)   
296 + **[CCCGTACCCG]** 1479 (85.42)   
296 - **[CGGGTACGGG]** 1479 (85.42)   
296 - **[CGGGGACGCC]** 1348 (85.07)   
296 - **.CGCGGCCGCC.** 1158 (90.79)   
296 - **[CCCGGGCGCG]** 1016 (89.87)   
296 - **.CGGGCGCGCG.** 1014 (90.60)   
296 - **[CGCGTCCTCG]** 1008 (88.96)   
296 - **[CGCGGACCCC]** 931 (85.07)   
296 - **[CGCCTCCGCC]** 854 (85.01)   
296 - **.CCCGGCCGCG.** 815 (90.04)   
296 - **[GGCGGAGGCG]** 779 (85.01)   
296 - **.CCCGTCCGCG.** 651 (91.15)   
296 - **.CGCGCGCGCC.** 494 (90.45)   
296 - **.CGCGCGCCCG.** 492 (90.60)   
296 - **[CGCGCCCGAG]** 490 (86.24)   
296 - **[CCCGCCCGCG]** 421 (89.87)   
296 - **[GGCGCTCGCG]** 335 (85.57)   
296 - **[CGCTGCCGCG]** 201 (86.28)   
296 - **.CGCGCGCGCC.** 195 (90.45)   
296 - **[CGGGTCGGCG]** 145 (85.16)   
296 - **[CCCGCGCGCG]** 57 (89.70)   
296 - **.CGCGCGCGCG.** 55 (97.44)   
296 - **[CGCGCGCGTG]** 53 (86.27)   
296 - **.CGCGGGGGCG.** 33 (90.71)   
297 + **[CGCGAGCGCC]** 650 (85.57)   
297 + **.GGCGGCCGCG.** 992 (90.79)   
297 + **[CGCGGGCTCG]** 1252 (87.68)   
297 + **[GGCGTCGGCG]** 1443 (85.01)   
297 - **[CGCCGACGCC]** 1443 (85.01)   
297 - **[CGAGCCCGCG]** 1252 (87.68)   
297 - **.CGCGGCCGCC.** 992 (90.79)   
297 - **[GGCGCTCGCG]** 650 (85.57)   
298 + **[CGGGCACCCG]** 380 (85.05)   
298 + **[CGCACCCGCG]** 623 (86.48)   
298 + **.CGCGCACGCG.** 870 (98.72)   
298 + **[CGCGGACGCA]** 903 (88.81)   
298 + **.CGGGTCCGCG.** 931 (92.05)   
298 + **[GGCGCTCGCG]** 1466 (85.57)   
298 - **[CGCGAGCGCC]** 1466 (85.57)   
298 - **.CGCGGACCCG.** 931 (92.05)   
298 - **[TGCGTCCGCG]** 903 (88.81)   
298 - **.CGCGTGCGCG.** 870 (98.72)   
298 - **[CGCGGGTGCG]** 623 (86.48)   
298 - **[CGGGTGCCCG]** 380 (85.05)   
299 + **.GGCGGCCGCG.** 847 (90.79)   
299 + **[CTCGGCCGCG]** 1346 (86.41)   
299 - **[CGCGGCCGAG]** 1346 (86.41)   
299 - **.CGCGGCCGCC.** 847 (90.79)   
300 + **[CGCGGTGGCG]** 466 (85.83)   
300 - **[CGCCACCGCG]** 466 (85.83)   
301 + **[CGCCTCCGCC]** 208 (85.01)   
301 + **[CGTGGGCGCG]** 575 (86.75)   
301 + **[CGCGTGAGCG]** 670 (87.22)   
301 + **[CGCGTGCGTG]** 708 (87.55)   
301 - **[CACGCACGCG]** 708 (87.55)   
301 - **[CGCTCACGCG]** 670 (87.22)   
301 - **[CGCGCCCACG]** 575 (86.75)   
301 - **[GGCGGAGGCG]** 208 (85.01)   
302 + **[CGCGTGAGCG]** 744 (87.22)   
302 + **[AGCGCCCGCG]** 750 (87.08)   
302 + **[CGGCGACGCG]** 947 (85.16)   
302 + **.CGCGCACGCG.** 953 (98.72)   
302 + **[CGCACGCGCG]** 955 (86.31)   
302 - **[CGCGCGTGCG]** 955 (86.31)   
302 - **.CGCGTGCGCG.** 953 (98.72)   
302 - **[CGCGTCGCCG]** 947 (85.16)   
302 - **[CGCGGGCGCT]** 750 (87.08)   
302 - **[CGCTCACGCG]** 744 (87.22)   
303 + **.GGCGGCCGCG.** 283 (90.79)   
303 + **.CGCGGGGGCG.** 733 (90.71)   
303 + **.GGCGGGCGCG.** 739 (90.62)   
303 + **.CCCGTCCGCG.** 881 (91.15)   
303 + **.GGCGGCCGCG.** 1357 (90.79)   
303 - **.CGCGGCCGCC.** 1357 (90.79)   
303 - **.CGCGGACGGG.** 881 (91.15)   
303 - **.CGCGCCCGCC.** 739 (90.62)   
303 - **.CGCCCCCGCG.** 733 (90.71)   
303 - **.CGCGGCCGCC.** 283 (90.79)   
304 + **.CGCGGACGCC.** 735 (91.90)   
304 + **.CGGGTACGCG.** 1148 (93.16)   
304 + **.CGGGGCCGCG.** 1259 (90.94)   
304 - **.CGCGGCCCCG.** 1259 (90.94)   
304 - **.CGCGTACCCG.** 1148 (93.16)   
304 - **.GGCGTCCGCG.** 735 (91.90)   
306 + **[CGCCACCGCG]** 625 (85.83)   
306 + **.CGCGCCCGCC.** 767 (90.62)   
306 + **[CGGGGACGCC]** 1407 (85.07)   
306 - **[GGCGTCCCCG]** 1407 (85.07)   
306 - **.GGCGGGCGCG.** 767 (90.62)   
306 - **[CGCGGTGGCG]** 625 (85.83)   
307 + **[GGGGGACGCG]** 330 (85.07)   
307 + **[CGCGCGCTCG]** 386 (87.51)   
307 + **.GGCGGCCGCG.** 733 (90.79)   
307 + **[CGCGGACCCC]** 826 (85.07)   
307 + **[AGCGGGCGCG]** 1156 (87.08)   
307 + **[CGGGGACGCC]** 1275 (85.07)   
307 - **[GGCGTCCCCG]** 1275 (85.07)   
307 - **[CGCGCCCGCT]** 1156 (87.08)   
307 - **[GGGGTCCGCG]** 826 (85.07)   
307 - **.CGCGGCCGCC.** 733 (90.79)   
307 - **[CGAGCGCGCG]** 386 (87.51)   
307 - **[CGCGTCCCCC]** 330 (85.07)   
308 + **.CGCGCCCGCG.** 786 (97.61)   
308 + **[CGGCTCCGCG]** 1183 (85.16)   
308 - **[CGCGGAGCCG]** 1183 (85.16)   
308 - **.CGCGGGCGCG.** 786 (97.61)   
309 + **[CGCGCCCGCT]** 691 (87.08)   
309 + **[GGCGAGCGCG]** 771 (85.57)   
309 + **[GGCGGAGGCG]** 976 (85.01)   
309 - **[CGCCTCCGCC]** 976 (85.01)   
309 - **[CGCGCTCGCC]** 771 (85.57)   
309 - **[AGCGGGCGCG]** 691 (87.08)   
311 + **.CGCGGAGGCG.** 502 (91.99)   
311 + **.CGCGGCCGGG.** 875 (90.04)   
311 + **.GGCGCCCGCG.** 1109 (90.62)   
311 + **.CGGGGACGCG.** 1258 (92.05)   
311 + **[CGCGGGCGAG]** 1264 (86.24)   
311 - **[CTCGCCCGCG]** 1264 (86.24)   
311 - **.CGCGTCCCCG.** 1258 (92.05)   
311 - **.CGCGGGCGCC.** 1109 (90.62)   
311 - **.CCCGGCCGCG.** 875 (90.04)   
311 - **.CGCCTCCGCG.** 502 (91.99)   
312 + **.CGCGCCCCCG.** 843 (90.77)   
312 + **.CGCGCGCGCC.** 1357 (90.45)   
312 - **.GGCGCGCGCG.** 1357 (90.45)   
312 - **.CGGGGGCGCG.** 843 (90.77)   
313 + **[CGCGTGTGCG]** 788 (87.59)   
313 + **[TGCGCACGCG]** 794 (88.64)   
313 + **.CGCGGCCGCC.** 826 (90.79)   
313 + **.CGCCTGCGCG.** 915 (91.82)   
313 + **.CGCGCTCGCG.** 958 (92.56)   
313 + **[CGCGCCTGCG]** 1008 (86.48)   
313 - **[CGCAGGCGCG]** 1008 (86.48)   
313 - **.CGCGAGCGCG.** 958 (92.56)   
313 - **.CGCGCAGGCG.** 915 (91.82)   
313 - **.GGCGGCCGCG.** 826 (90.79)   
313 - **[CGCGTGCGCA]** 794 (88.64)   
313 - **[CGCACACGCG]** 788 (87.59)   
315 + **.CGCGTGCGCG.** 905 (98.72)   
315 + **.GGCGCGCGCG.** 981 (90.45)   
315 + **[CGCGCGCGAG]** 983 (86.07)   
315 + **.CGCGCGGGCG.** 1008 (90.54)   
315 + **.CGCGGGGGCG.** 1028 (90.71)   
315 + **.GGCGCGCGCG.** 1057 (90.45)   
315 + **[CGCGCGCGCT]** 1059 (86.91)   
315 + **[GGGGGACGCG]** 1281 (85.07)   
315 - **[CGCGTCCCCC]** 1281 (85.07)   
315 - **[AGCGCGCGCG]** 1059 (86.91)   
315 - **.CGCGCGCGCC.** 1057 (90.45)   
315 - **.CGCCCCCGCG.** 1028 (90.71)   
315 - **.CGCCCGCGCG.** 1008 (90.54)   
315 - **[CTCGCGCGCG]** 983 (86.07)   
315 - **.CGCGCGCGCC.** 981 (90.45)   
315 - **.CGCGCACGCG.** 905 (98.72)   
316 + **[CGCGCCCGGG]** 202 (89.87)   
316 + **.GGCGGCCGCG.** 532 (90.79)   
316 - **.CGCGGCCGCC.** 532 (90.79)   
316 - **[CCCGGGCGCG]** 202 (89.87)   
317 + **[CGCTCCCGCG]** 1344 (86.11)   
317 - **[CGCGGGAGCG]** 1344 (86.11)   
318 + **.CGCCCGCGCG.** 99 (90.54)   
318 + **.GGCGGGCGCG.** 246 (90.62)   
318 + **[CCCGCCCGCG]** 301 (89.87)   
318 + **[CGCAGGCGCG]** 718 (86.48)   
318 + **.CGCCCCCGCG.** 1112 (90.71)   
318 + **.CGCGCCCCCG.** 1246 (90.77)   
318 + **.GGCGGCCGCG.** 1479 (90.79)   
318 + **.CGCGGCGGCG.** 1485 (90.88)   
318 - **.CGCCGCCGCG.** 1485 (90.88)   
318 - **.CGCGGCCGCC.** 1479 (90.79)   
318 - **.CGGGGGCGCG.** 1246 (90.77)   
318 - **.CGCGGGGGCG.** 1112 (90.71)   
318 - **[CGCGCCTGCG]** 718 (86.48)   
318 - **[CGCGGGCGGG]** 301 (89.87)   
318 - **.CGCGCCCGCC.** 246 (90.62)   
318 - **.CGCGCGGGCG.** 99 (90.54)   
320 + **[CGCGGACACG]** 51 (88.03)   
320 + **.CGCGGGGGCG.** 537 (90.71)   
320 + **.CGCGCGCGCC.** 596 (90.45)   
320 + **.CGCGCGCCCG.** 598 (90.60)   
320 + **.CGCGGCCGGG.** 694 (90.04)   
320 + **.CGGGGGCGCG.** 794 (90.77)   
320 + **[CGTGGGCGCG]** 851 (86.75)   
320 + **[CGAGGCCGCG]** 907 (87.85)   
320 + **[CGGGAGCGCG]** 937 (85.72)   
320 + **[CGCCCTCGCG]** 1226 (85.66)   
320 - **[CGCGAGGGCG]** 1226 (85.66)   
320 - **[CGCGCTCCCG]** 937 (85.72)   
320 - **[CGCGGCCTCG]** 907 (87.85)   
320 - **[CGCGCCCACG]** 851 (86.75)   
320 - **.CGCGCCCCCG.** 794 (90.77)   
320 - **.CCCGGCCGCG.** 694 (90.04)   
320 - **.CGGGCGCGCG.** 598 (90.60)   
320 - **.GGCGCGCGCG.** 596 (90.45)   
320 - **.CGCCCCCGCG.** 537 (90.71)   
320 - **[CGTGTCCGCG]** 51 (88.03)   
321 + **[CGCCTCCCCG]** 631 (85.16)   
321 + **.CGCCCGCGCG.** 1219 (90.54)   
321 + **.CGCGCGCCCG.** 1223 (90.60)   
321 + **[CGCGCCCGAG]** 1225 (86.24)   
321 + **.CGGGGCCGCG.** 1252 (90.94)   
321 + **[CGCGCGCTCG]** 1470 (87.51)   
321 + **[CGCGCTCGCC]** 1472 (85.57)   
321 + **[GGCGTCGGCG]** 1484 (85.01)   
321 - **[CGCCGACGCC]** 1484 (85.01)   
321 - **[GGCGAGCGCG]** 1472 (85.57)   
321 - **[CGAGCGCGCG]** 1470 (87.51)   
321 - **.CGCGGCCCCG.** 1252 (90.94)   
321 - **[CTCGGGCGCG]** 1225 (86.24)   
321 - **.CGGGCGCGCG.** 1223 (90.60)   
321 - **.CGCGCGGGCG.** 1219 (90.54)   
321 - **[CGGGGAGGCG]** 631 (85.16)   
322 + **.CGCGGCCCCG.** 632 (90.94)   
322 + **.CGCGAGCGCG.** 792 (92.56)   
322 + **[CGAGCGCGCG]** 794 (87.51)   
322 + **[CGCGGGCGGG]** 812 (89.87)   
322 + **.CGCGCGCGCC.** 944 (90.45)   
322 + **.CGCGCGGGCG.** 961 (90.54)   
322 + **[CGCCGTCGCG]** 1061 (85.83)   
322 + **.CGCGCCCGCC.** 1426 (90.62)   
322 - **.GGCGGGCGCG.** 1426 (90.62)   
322 - **[CGCGACGGCG]** 1061 (85.83)   
322 - **.CGCCCGCGCG.** 961 (90.54)   
322 - **.GGCGCGCGCG.** 944 (90.45)   
322 - **[CCCGCCCGCG]** 812 (89.87)   
322 - **[CGCGCGCTCG]** 794 (87.51)   
322 - **.CGCGCTCGCG.** 792 (92.56)   
322 - **.CGGGGCCGCG.** 632 (90.94)   
323 + **.CGCGGGCGCC.** 575 (90.62)   
323 + **[CGCAGGCGCG]** 854 (86.48)   
323 + **.CGGGGGCGCG.** 941 (90.77)   
323 + **.GGCGCGCGCG.** 945 (90.45)   
323 + **[CGCGCGCGGG]** 947 (89.70)   
323 + **.GGCGGGCGCG.** 981 (90.62)   
323 + **.CGGGCGCGCG.** 983 (90.60)   
323 + **[CACGCGCGCG]** 1068 (86.27)   
323 + **[GGCCTCCGCG]** 1307 (85.01)   
323 + **.CGCGCCCCCG.** 1313 (90.77)   
323 + **.CGCCCCCGCG.** 1315 (90.71)   
323 + **[GGCCTCCGCG]** 1407 (85.01)   
323 + **[CGAGGGCGCG]** 1444 (87.68)   
323 + **.CGCGCCCGCC.** 1496 (90.62)   
323 - **.GGCGGGCGCG.** 1496 (90.62)   
323 - **[CGCGCCCTCG]** 1444 (87.68)   
323 - **[CGCGGAGGCC]** 1407 (85.01)   
323 - **.CGCGGGGGCG.** 1315 (90.71)   
323 - **.CGGGGGCGCG.** 1313 (90.77)   
323 - **[CGCGGAGGCC]** 1307 (85.01)   
323 - **[CGCGCGCGTG]** 1068 (86.27)   
323 - **.CGCGCGCCCG.** 983 (90.60)   
323 - **.CGCGCCCGCC.** 981 (90.62)   
323 - **[CCCGCGCGCG]** 947 (89.70)   
323 - **.CGCGCGCGCC.** 945 (90.45)   
323 - **.CGCGCCCCCG.** 941 (90.77)   
323 - **[CGCGCCTGCG]** 854 (86.48)   
323 - **.GGCGCCCGCG.** 575 (90.62)   
326 + **.CGCGCCCGCG.** 869 (97.61)   
326 + **.CGCGGCCGCG.** 1113 (97.78)   
326 + **.GGCGCCCGCG.** 1250 (90.62)   
326 + **.CGCCCGCGCG.** 1252 (90.54)   
326 + **.GGCGGCCGCG.** 1297 (90.79)   
326 + **.CGCGGCGGCG.** 1303 (90.88)   
326 + **[CTCGGGCGCG]** 1315 (86.24)   
326 + **[CGCGGGCACG]** 1321 (86.75)   
326 - **[CGTGCCCGCG]** 1321 (86.75)   
326 - **[CGCGCCCGAG]** 1315 (86.24)   
326 - **.CGCCGCCGCG.** 1303 (90.88)   
326 - **.CGCGGCCGCC.** 1297 (90.79)   
326 - **.CGCGCGGGCG.** 1252 (90.54)   
326 - **.CGCGGGCGCC.** 1250 (90.62)   
326 - **.CGCGGCCGCG.** 1113 (97.78)   
326 - **.CGCGGGCGCG.** 869 (97.61)   
327 + **[CGCCACCGCG]** 675 (85.83)   
327 - **[CGCGGTGGCG]** 675 (85.83)   
329 + **[CGCCGACGCC]** 270 (85.01)   
329 + **.CGCGCCCGCG.** 1227 (97.61)   
329 + **.CGCGGACGCG.** 1233 (98.89)   
329 - **.CGCGTCCGCG.** 1233 (98.89)   
329 - **.CGCGGGCGCG.** 1227 (97.61)   
329 - **[GGCGTCGGCG]** 270 (85.01)   
331 + **.CGCCGCCGCG.** 717 (90.88)   
331 + **.CGCGGCGGCG.** 1332 (90.88)   
331 - **.CGCCGCCGCG.** 1332 (90.88)   
331 - **.CGCGGCGGCG.** 717 (90.88)   
332 + **.CGCGTCCCCG.** 504 (92.05)   
332 + **.CCCGTGCGCG.** 1082 (90.98)   
332 + **.CGCGGGCCCG.** 1181 (90.77)   
332 + **[CGGGGAGGCG]** 1247 (85.16)   
332 + **.CGCGGCCGGG.** 1430 (90.04)   
332 - **.CCCGGCCGCG.** 1430 (90.04)   
332 - **[CGCCTCCCCG]** 1247 (85.16)   
332 - **.CGGGCCCGCG.** 1181 (90.77)   
332 - **.CGCGCACGGG.** 1082 (90.98)   
332 - **.CGGGGACGCG.** 504 (92.05)   
333 + **[CGCGGACCCC]** 1243 (85.07)   
333 - **[GGGGTCCGCG]** 1243 (85.07)   
334 + **[CCCCTACGCG]** 596 (85.36)   
334 + **[CGCGGACCCC]** 856 (85.07)   
334 + **[CCCGGGCGCG]** 1083 (89.87)   
334 - **[CGCGCCCGGG]** 1083 (89.87)   
334 - **[GGGGTCCGCG]** 856 (85.07)   
334 - **[CGCGTAGGGG]** 596 (85.36)   
335 + **.CGCGCACGCC.** 1124 (91.73)   
335 + **[CGCGGACCCC]** 1178 (85.07)   
335 + **.CGCCCCCGCG.** 1199 (90.71)   
335 + **[TGCGGGCGCG]** 1224 (87.53)   
335 + **[CGCGCCCGGG]** 1230 (89.87)   
335 + **[CTCGGGCGCG]** 1255 (86.24)   
335 + **.CGGGGGCGCG.** 1426 (90.77)   
335 - **.CGCGCCCCCG.** 1426 (90.77)   
335 - **[CGCGCCCGAG]** 1255 (86.24)   
335 - **[CCCGGGCGCG]** 1230 (89.87)   
335 - **[CGCGCCCGCA]** 1224 (87.53)   
335 - **.CGCGGGGGCG.** 1199 (90.71)   
335 - **[GGGGTCCGCG]** 1178 (85.07)   
335 - **.GGCGTGCGCG.** 1124 (91.73)   
336 + **[GGGGTCCGCG]** 754 (85.07)   
336 + **[CGGGTACCCG]** 1440 (86.33)   
336 + **[CGCCTCCGCC]** 1473 (85.01)   
336 - **[GGCGGAGGCG]** 1473 (85.01)   
336 - **[CGGGTACCCG]** 1440 (86.33)   
336 - **[CGCGGACCCC]** 754 (85.07)   
337 + **[CGCGCCTGCG]** 411 (86.48)   
337 + **[CACGCGCGCG]** 749 (86.27)   
337 + **[CGCGCGCGTG]** 751 (86.27)   
337 + **.CGCGTGCGCG.** 856 (98.72)   
337 + **[TGCGCCCGCG]** 916 (87.53)   
337 + **.CGGGGGCGCG.** 946 (90.77)   
337 + **.GGCGCGCGCG.** 950 (90.45)   
337 + **.CGCCGCCGCG.** 1067 (90.88)   
337 + **.GGCGTCCGCG.** 1322 (91.90)   
337 + **[CGCGGGCGGG]** 1350 (89.87)   
337 + **.CGGGGGCGCG.** 1434 (90.77)   
337 - **.CGCGCCCCCG.** 1434 (90.77)   
337 - **[CCCGCCCGCG]** 1350 (89.87)   
337 - **.CGCGGACGCC.** 1322 (91.90)   
337 - **.CGCGGCGGCG.** 1067 (90.88)   
337 - **.CGCGCGCGCC.** 950 (90.45)   
337 - **.CGCGCCCCCG.** 946 (90.77)   
337 - **[CGCGGGCGCA]** 916 (87.53)   
337 - **.CGCGCACGCG.** 856 (98.72)   
337 - **[CACGCGCGCG]** 751 (86.27)   
337 - **[CGCGCGCGTG]** 749 (86.27)   
337 - **[CGCAGGCGCG]** 411 (86.48)   
338 + **[CGCGGAGCCG]** 43 (85.16)   
338 + **.CGCGCCCGCG.** 754 (97.61)   
338 + **[CGCCTTCGCG]** 856 (86.94)   
338 + **[CGCGCGCGCT]** 892 (86.91)   
338 + **[CGGGCTCGCG]** 1082 (85.72)   
338 + **[CTCGCGCGCG]** 1086 (86.07)   
338 + **.CGCGCGCGCC.** 1088 (90.45)   
338 + **.CGCGCGCCCG.** 1090 (90.60)   
338 + **.CGCGCCCGCC.** 1092 (90.62)   
338 + **[AGCGCGCGCG]** 1120 (86.91)   
338 + **.CGCGCGCGCC.** 1122 (90.45)   
338 + **.CGCGTCCGCC.** 1154 (91.90)   
338 + **.CGCCGCCGCG.** 1189 (90.88)   
338 + **.CGCGCGCGCG.** 1195 (97.44)   
338 + **[CGCGCGCGCA]** 1197 (87.36)   
338 + **.GGCGGCCGCG.** 1301 (90.79)   
338 + **[GGCGGAGGCG]** 1501 (85.01)   
338 + **[GGCGGAGGCG]** 1507 (85.01)   
338 - **[CGCCTCCGCC]** 1507 (85.01)   
338 - **[CGCCTCCGCC]** 1501 (85.01)   
338 - **.CGCGGCCGCC.** 1301 (90.79)   
338 - **[TGCGCGCGCG]** 1197 (87.36)   
338 - **.CGCGCGCGCG.** 1195 (97.44)   
338 - **.CGCGGCGGCG.** 1189 (90.88)   
338 - **.GGCGGACGCG.** 1154 (91.90)   
338 - **.GGCGCGCGCG.** 1122 (90.45)   
338 - **[CGCGCGCGCT]** 1120 (86.91)   
338 - **.GGCGGGCGCG.** 1092 (90.62)   
338 - **.CGGGCGCGCG.** 1090 (90.60)   
338 - **.GGCGCGCGCG.** 1088 (90.45)   
338 - **[CGCGCGCGAG]** 1086 (86.07)   
338 - **[CGCGAGCCCG]** 1082 (85.72)   
338 - **[AGCGCGCGCG]** 892 (86.91)   
338 - **[CGCGAAGGCG]** 856 (86.94)   
338 - **.CGCGGGCGCG.** 754 (97.61)   
338 - **[CGGCTCCGCG]** 43 (85.16)   
339 + **[CCCGGGCGCG]** 1024 (89.87)   
339 + **.CGCCCGCGCG.** 1041 (90.54)   
339 - **.CGCGCGGGCG.** 1041 (90.54)   
339 - **[CGCGCCCGGG]** 1024 (89.87)   
340 + **[CGCGCTGGCG]** 677 (85.66)   
340 - **[CGCCAGCGCG]** 677 (85.66)   
341 + **.CGCGCACGCG.** 39 (98.72)   
341 + **.CGCGGCCGCC.** 1152 (90.79)   
341 + **[CGGGGTCGCG]** 1227 (85.89)   
341 + **[CACGGCCGCG]** 1311 (86.61)   
341 + **.CGCGGGCCCG.** 1419 (90.77)   
341 + **[GGGGGACGCG]** 1488 (85.07)   
341 - **[CGCGTCCCCC]** 1488 (85.07)   
341 - **.CGGGCCCGCG.** 1419 (90.77)   
341 - **[CGCGGCCGTG]** 1311 (86.61)   
341 - **[CGCGACCCCG]** 1227 (85.89)   
341 - **.GGCGGCCGCG.** 1152 (90.79)   
341 - **.CGCGTGCGCG.** 39 (98.72)   
342 + **[CGGGGACCCG]** 702 (85.22)   
342 + **.CCCGGCCGCG.** 708 (90.04)   
342 + **.CGCGCAGGCG.** 714 (91.82)   
342 + **[CGCAGGCGCG]** 716 (86.48)   
342 + **[GGCGAGCGCG]** 1275 (85.57)   
342 + **[CGCGCGTGCG]** 1299 (86.31)   
342 + **.CGCGTGCGCG.** 1301 (98.72)   
342 + **.CGCGGCCGGG.** 1335 (90.04)   
342 + **[CGCGGGCGGG]** 1349 (89.87)   
342 + **.CGCGTGCGCG.** 1366 (98.72)   
342 + **.CGCGCGCCCG.** 1398 (90.60)   
342 + **[CGCGCCCGGG]** 1400 (89.87)   
342 + **.CGCGTGCGCG.** 1447 (98.72)   
342 + **.GGCGCGCGCG.** 1471 (90.45)   
342 + **[CGCGCGCGTG]** 1473 (86.27)   
342 + **[CGCGCGTGCG]** 1475 (86.31)   
342 + **.CGCGTGCGCG.** 1477 (98.72)   
342 - **.CGCGCACGCG.** 1477 (98.72)   
342 - **[CGCACGCGCG]** 1475 (86.31)   
342 - **[CACGCGCGCG]** 1473 (86.27)   
342 - **.CGCGCGCGCC.** 1471 (90.45)   
342 - **.CGCGCACGCG.** 1447 (98.72)   
342 - **[CCCGGGCGCG]** 1400 (89.87)   
342 - **.CGGGCGCGCG.** 1398 (90.60)   
342 - **.CGCGCACGCG.** 1366 (98.72)   
342 - **[CCCGCCCGCG]** 1349 (89.87)   
342 - **.CCCGGCCGCG.** 1335 (90.04)   
342 - **.CGCGCACGCG.** 1301 (98.72)   
342 - **[CGCACGCGCG]** 1299 (86.31)   
342 - **[CGCGCTCGCC]** 1275 (85.57)   
342 - **[CGCGCCTGCG]** 716 (86.48)   
342 - **.CGCCTGCGCG.** 714 (91.82)   
342 - **.CGCGGCCGGG.** 708 (90.04)   
342 - **[CGGGTCCCCG]** 702 (85.22)   
343 + **.CGCGTCCCCG.** 279 (92.05)   
343 + **[AGCGGACGCG]** 666 (88.36)   
343 + **[CGCGCCCGGG]** 957 (89.87)   
343 + **[CCCGGGCGCG]** 961 (89.87)   
343 + **.CGGGGGCGCG.** 1176 (90.77)   
343 + **[CGCGGGTGCG]** 1321 (86.48)   
343 + **[CCCGGGCGCG]** 1336 (89.87)   
343 - **[CGCGCCCGGG]** 1336 (89.87)   
343 - **[CGCACCCGCG]** 1321 (86.48)   
343 - **.CGCGCCCCCG.** 1176 (90.77)   
343 - **[CGCGCCCGGG]** 961 (89.87)   
343 - **[CCCGGGCGCG]** 957 (89.87)   
343 - **[CGCGTCCGCT]** 666 (88.36)   
343 - **.CGGGGACGCG.** 279 (92.05)   
346 + **[GGCGGTCGCG]** 17 (85.74)   
346 + **[CGCGGAGGCC]** 908 (85.01)   
346 + **.CGCCGCCGCG.** 1233 (90.88)   
346 - **.CGCGGCGGCG.** 1233 (90.88)   
346 - **[GGCCTCCGCG]** 908 (85.01)   
346 - **[CGCGACCGCC]** 17 (85.74)   
347 + **[CGCGTGCGCT]** 876 (88.19)   
347 + **.CGCGCGCCCG.** 911 (90.60)   
347 + **[CGCGCCCGGG]** 913 (89.87)   
347 + **[CGCGCCCGGG]** 1494 (89.87)   
347 - **[CCCGGGCGCG]** 1494 (89.87)   
347 - **[CCCGGGCGCG]** 913 (89.87)   
347 - **.CGGGCGCGCG.** 911 (90.60)   
347 - **[AGCGCACGCG]** 876 (88.19)   
348 + **[CGCGGACTCG]** 137 (88.96)   
348 + **[CGGGTGCCCG]** 281 (85.05)   
348 + **.CGCCGGCGCG.** 289 (90.71)   
348 + **.CGCGCAGGCG.** 295 (91.82)   
348 + **.CGCGGCCGCC.** 344 (90.79)   
348 + **.CGCGCCCGCC.** 382 (90.62)   
348 + **[CCCGCGCGCG]** 411 (89.70)   
348 + **.CGCGCGCGCC.** 413 (90.45)   
348 + **.CGCGCGCCCG.** 415 (90.60)   
348 + **.CGCGCCCGCC.** 417 (90.62)   
348 + **.CGGGCCCGCG.** 706 (90.77)   
348 + **[CGCGGGTGCG]** 988 (86.48)   
348 + **.CGGGTGCGCG.** 990 (91.88)   
348 + **.CGGGGACGCG.** 1186 (92.05)   
348 + **.GGCGCCCGCG.** 1252 (90.62)   
348 + **[CGCGGGCGTG]** 1258 (86.44)   
348 + **.GGCGTGCGCG.** 1262 (91.73)   
348 - **.CGCGCACGCC.** 1262 (91.73)   
348 - **[CACGCCCGCG]** 1258 (86.44)   
348 - **.CGCGGGCGCC.** 1252 (90.62)   
348 - **.CGCGTCCCCG.** 1186 (92.05)   
348 - **.CGCGCACCCG.** 990 (91.88)   
348 - **[CGCACCCGCG]** 988 (86.48)   
348 - **.CGCGGGCCCG.** 706 (90.77)   
348 - **.GGCGGGCGCG.** 417 (90.62)   
348 - **.CGGGCGCGCG.** 415 (90.60)   
348 - **.GGCGCGCGCG.** 413 (90.45)   
348 - **[CGCGCGCGGG]** 411 (89.70)   
348 - **.GGCGGGCGCG.** 382 (90.62)   
348 - **.GGCGGCCGCG.** 344 (90.79)   
348 - **.CGCCTGCGCG.** 295 (91.82)   
348 - **.CGCGCCGGCG.** 289 (90.71)   
348 - **[CGGGCACCCG]** 281 (85.05)   
348 - **[CGAGTCCGCG]** 137 (88.96)   
349 + **.CGCCGGCGCG.** 833 (90.71)   
349 + **.CGCGGGGGCG.** 839 (90.71)   
349 - **.CGCCCCCGCG.** 839 (90.71)   
349 - **.CGCGCCGGCG.** 833 (90.71)   
350 + **.CGCGCGGGCG.** 1428 (90.54)   
350 + **.CGCGGGCGCC.** 1439 (90.62)   
350 - **.GGCGCCCGCG.** 1439 (90.62)   
350 - **.CGCCCGCGCG.** 1428 (90.54)   
351 + **[CGCCGAGGCG]** 570 (85.09)   
351 + **[AGCGCCCGCG]** 1015 (87.08)   
351 + **[CGGGGTCGCG]** 1135 (85.89)   
351 + **[CGCGTCTGCG]** 1141 (87.76)   
351 - **[CGCAGACGCG]** 1141 (87.76)   
351 - **[CGCGACCCCG]** 1135 (85.89)   
351 - **[CGCGGGCGCT]** 1015 (87.08)   
351 - **[CGCCTCGGCG]** 570 (85.09)   
352 + **[CTCGGCCGCG]** 68 (86.41)   
352 + **[CGGGTCCCCG]** 190 (85.22)   
352 + **.CGCGGAGGCG.** 284 (91.99)   
352 + **[CGCGCCCGGG]** 547 (89.87)   
352 + **[CGCCTCCGCC]** 706 (85.01)   
352 + **.CGCCTCCGCG.** 712 (91.99)   
352 + **[CGGGGAGGCG]** 899 (85.16)   
352 - **[CGCCTCCCCG]** 899 (85.16)   
352 - **.CGCGGAGGCG.** 712 (91.99)   
352 - **[GGCGGAGGCG]** 706 (85.01)   
352 - **[CCCGGGCGCG]** 547 (89.87)   
352 - **.CGCCTCCGCG.** 284 (91.99)   
352 - **[CGGGGACCCG]** 190 (85.22)   
352 - **[CGCGGCCGAG]** 68 (86.41)   
353 + **[CGCGGCCACG]** 167 (86.92)   
353 + **[CGCGAGCGCC]** 306 (85.57)   
353 + **[CTCGGACGCG]** 704 (87.52)   
353 + **[CGCGCGTGCG]** 834 (86.31)   
353 + **[CGCGTGCGCA]** 836 (88.64)   
353 + **[CGCCGTCGCG]** 870 (85.83)   
353 + **.CGCCCCCGCG.** 898 (90.71)   
353 + **[CGCTTGCGCG]** 1025 (87.22)   
353 + **.CGGGGGCGCG.** 1378 (90.77)   
353 - **.CGCGCCCCCG.** 1378 (90.77)   
353 - **[CGCGCAAGCG]** 1025 (87.22)   
353 - **.CGCGGGGGCG.** 898 (90.71)   
353 - **[CGCGACGGCG]** 870 (85.83)   
353 - **[TGCGCACGCG]** 836 (88.64)   
353 - **[CGCACGCGCG]** 834 (86.31)   
353 - **[CGCGTCCGAG]** 704 (87.52)   
353 - **[GGCGCTCGCG]** 306 (85.57)   
353 - **[CGTGGCCGCG]** 167 (86.92)   
356 + **.GGCGCCCGCG.** 766 (90.62)   
356 + **[TGCGCACGCG]** 859 (88.64)   
356 + **[CGCATGCGCG]** 912 (87.59)   
356 + **.CGCGGCCGCC.** 999 (90.79)   
356 + **[CGCGTCCACG]** 1173 (88.03)   
356 - **[CGTGGACGCG]** 1173 (88.03)   
356 - **.GGCGGCCGCG.** 999 (90.79)   
356 - **[CGCGCATGCG]** 912 (87.59)   
356 - **[CGCGTGCGCA]** 859 (88.64)   
356 - **.CGCGGGCGCC.** 766 (90.62)   
357 + **[CGCAGCCGCG]** 976 (86.65)   
357 + **.CGCGCGCCCG.** 982 (90.60)   
357 + **.CGCGCCCGCC.** 984 (90.62)   
357 + **.CGCGTGCGCG.** 1027 (98.72)   
357 + **.CGGGGCCGCG.** 1106 (90.94)   
357 + **[GGGGTCCGCG]** 1276 (85.07)   
357 - **[CGCGGACCCC]** 1276 (85.07)   
357 - **.CGCGGCCCCG.** 1106 (90.94)   
357 - **.CGCGCACGCG.** 1027 (98.72)   
357 - **.GGCGGGCGCG.** 984 (90.62)   
357 - **.CGGGCGCGCG.** 982 (90.60)   
357 - **[CGCGGCTGCG]** 976 (86.65)   
358 + **.CGCGGCCGGG.** 774 (90.04)   
358 - **.CCCGGCCGCG.** 774 (90.04)   
359 + **[CGCGGGCGGG]** 545 (89.87)   
359 + **[CGCGCACGTG]** 777 (87.55)   
359 + **[CGGGGACGCC]** 793 (85.07)   
359 + **.CCCGTGCGCG.** 1108 (90.98)   
359 + **.GGCGCGCGCG.** 1237 (90.45)   
359 + **.CGCGCGCGCG.** 1239 (97.44)   
359 + **.CGCGCGCGCG.** 1241 (97.44)   
359 + **.CGCGCGCGCC.** 1243 (90.45)   
359 + **[CGCGCCTGCG]** 1247 (86.48)   
359 + **.CGCCTGCGCG.** 1249 (91.82)   
359 + **.CGCGTGCGCG.** 1281 (98.72)   
359 + **.CGCGCCGGCG.** 1307 (90.71)   
359 - **.CGCCGGCGCG.** 1307 (90.71)   
359 - **.CGCGCACGCG.** 1281 (98.72)   
359 - **.CGCGCAGGCG.** 1249 (91.82)   
359 - **[CGCAGGCGCG]** 1247 (86.48)   
359 - **.GGCGCGCGCG.** 1243 (90.45)   
359 - **.CGCGCGCGCG.** 1241 (97.44)   
359 - **.CGCGCGCGCG.** 1239 (97.44)   
359 - **.CGCGCGCGCC.** 1237 (90.45)   
359 - **.CGCGCACGGG.** 1108 (90.98)   
359 - **[GGCGTCCCCG]** 793 (85.07)   
359 - **[CACGTGCGCG]** 777 (87.55)   
359 - **[CCCGCCCGCG]** 545 (89.87)   
360 + **[CGCGCACACG]** 421 (87.86)   
360 + **[CGCGCTGGCG]** 654 (85.66)   
360 + **[CGCGGCCGCT]** 794 (87.25)   
360 + **[CTCGCGCGCG]** 826 (86.07)   
360 + **[CGCGCGCGGG]** 828 (89.70)   
360 - **[CCCGCGCGCG]** 828 (89.70)   
360 - **[CGCGCGCGAG]** 826 (86.07)   
360 - **[AGCGGCCGCG]** 794 (87.25)   
360 - **[CGCCAGCGCG]** 654 (85.66)   
360 - **[CGTGTGCGCG]** 421 (87.86)   
362 + **[CGCGGGCGAG]** 1057 (86.24)   
362 - **[CTCGCCCGCG]** 1057 (86.24)   
363 + **.CGCGGCCGCG.** 1083 (97.78)   
363 + **.CGCGCCCCCG.** 1091 (90.77)   
363 - **.CGGGGGCGCG.** 1091 (90.77)   
363 - **.CGCGGCCGCG.** 1083 (97.78)   
364 + **.CGCGGGCGCC.** 299 (90.62)   
364 - **.GGCGCCCGCG.** 299 (90.62)   
366 + **[CGCGTTCGGG]** 169 (86.10)   
366 + **[CCCGCCCGCG]** 379 (89.87)   
366 + **.CGCGGCGGCG.** 410 (90.88)   
366 + **.GGCGTCCGCG.** 448 (91.90)   
366 + **[CCCGCCCGCG]** 515 (89.87)   
366 + **[CGCGTCCTCG]** 521 (88.96)   
366 + **[CGCGTCGGCC]** 854 (85.01)   
366 + **[CGCTCGCGCG]** 901 (85.94)   
366 + **[CGGGAGCGCG]** 1185 (85.72)   
366 + **.CGCGGCGGCG.** 1393 (90.88)   
366 + **.GGCGGCCGCG.** 1408 (90.79)   
366 + **.CGGGGACGCG.** 1426 (92.05)   
366 - **.CGCGTCCCCG.** 1426 (92.05)   
366 - **.CGCGGCCGCC.** 1408 (90.79)   
366 - **.CGCCGCCGCG.** 1393 (90.88)   
366 - **[CGCGCTCCCG]** 1185 (85.72)   
366 - **[CGCGCGAGCG]** 901 (85.94)   
366 - **[GGCCGACGCG]** 854 (85.01)   
366 - **[CGAGGACGCG]** 521 (88.96)   
366 - **[CGCGGGCGGG]** 515 (89.87)   
366 - **.CGCGGACGCC.** 448 (91.90)   
366 - **.CGCCGCCGCG.** 410 (90.88)   
366 - **[CGCGGGCGGG]** 379 (89.87)   
366 - **[CCCGAACGCG]** 169 (86.10)   
367 + **.CGCGGCGGCG.** 1192 (90.88)   
367 - **.CGCCGCCGCG.** 1192 (90.88)   
  

**Frequency Matrix**

| All Occs Best Occs|  |  |  |  |  |  |  |  |  |  |  |  |  |  |  |  |  |  |  |  |  |  |  |  |  |  |  |  |  |  |  |  |  |  |  |  |  |  |  |  |  |  |  |  |  |  |  |  |  |  |  |  |  |  |  |  |  |  |  |  |  |  |  |  |  |  |  |  |  |  |  |  |  |  |  |  |  |  |  |  |  |  |  |  |  |  |  |  |  |  |  |  |  |  |  |  |  |  |  |  |  |  |  |  |  |  |  |  |  |  |  |  |  |  |  |  |  |  |  |  |  |  |  |  |  |  |  |  |  |  |  |  | | --- | --- | --- | --- | --- | --- | --- | --- | --- | --- | --- | --- | --- | --- | --- | --- | --- | --- | --- | --- | --- | --- | --- | --- | --- | --- | --- | --- | --- | --- | --- | --- | --- | --- | --- | --- | --- | --- | --- | --- | --- | --- | --- | --- | --- | --- | --- | --- | --- | --- | --- | --- | --- | --- | --- | --- | --- | --- | --- | --- | --- | --- | --- | --- | --- | --- | --- | --- | --- | --- | --- | --- | --- | --- | --- | --- | --- | --- | --- | --- | --- | --- | --- | --- | --- | --- | --- | --- | --- | --- | --- | --- | --- | --- | --- | --- | --- | --- | --- | --- | --- | --- | --- | --- | --- | --- | --- | --- | --- | --- | --- | --- | --- | --- | --- | --- | --- | --- | --- | --- | --- | --- | --- | --- | --- | --- | --- | --- | --- | --- | --- | --- | | **A C G T A C G T | | | | | | | | | | | |**| **1** 253 4363 660 286 | 0 934 170 0|  |  |  |  |  |  |  |  |  |  |  |  |  |  |  |  |  |  |  |  |  |  |  |  |  |  |  |  |  |  |  |  |  |  |  |  |  |  |  |  |  |  |  |  |  |  |  |  |  |  |  |  |  |  |  |  |  |  |  |  |  |  |  |  |  |  |  |  |  |  |  |  |  |  |  |  |  |  |  |  |  |  |  |  |  |  |  |  |  |  |  |  |  |  |  |  |  |  |  |  |  |  |  |  |  |  |  |  | | --- | --- | --- | --- | --- | --- | --- | --- | --- | --- | --- | --- | --- | --- | --- | --- | --- | --- | --- | --- | --- | --- | --- | --- | --- | --- | --- | --- | --- | --- | --- | --- | --- | --- | --- | --- | --- | --- | --- | --- | --- | --- | --- | --- | --- | --- | --- | --- | --- | --- | --- | --- | --- | --- | --- | --- | --- | --- | --- | --- | --- | --- | --- | --- | --- | --- | --- | --- | --- | --- | --- | --- | --- | --- | --- | --- | --- | --- | --- | --- | --- | --- | --- | --- | --- | --- | --- | --- | --- | --- | --- | --- | --- | --- | --- | --- | --- | --- | --- | --- | --- | --- | --- | --- | --- | --- | --- | --- | | **2** 223 563 4565 211 | 0 48 1056 0|  |  |  |  |  |  |  |  |  |  |  |  |  |  |  |  |  |  |  |  |  |  |  |  |  |  |  |  |  |  |  |  |  |  |  |  |  |  |  |  |  |  |  |  |  |  |  |  |  |  |  |  |  |  |  |  |  |  |  |  |  |  |  |  |  |  |  |  |  |  |  |  |  |  |  |  |  |  |  |  |  |  |  |  |  |  |  |  |  |  |  |  |  |  |  |  | | --- | --- | --- | --- | --- | --- | --- | --- | --- | --- | --- | --- | --- | --- | --- | --- | --- | --- | --- | --- | --- | --- | --- | --- | --- | --- | --- | --- | --- | --- | --- | --- | --- | --- | --- | --- | --- | --- | --- | --- | --- | --- | --- | --- | --- | --- | --- | --- | --- | --- | --- | --- | --- | --- | --- | --- | --- | --- | --- | --- | --- | --- | --- | --- | --- | --- | --- | --- | --- | --- | --- | --- | --- | --- | --- | --- | --- | --- | --- | --- | --- | --- | --- | --- | --- | --- | --- | --- | --- | --- | --- | --- | --- | --- | --- | --- | | **3** 297 4349 685 231 | 0 982 122 0|  |  |  |  |  |  |  |  |  |  |  |  |  |  |  |  |  |  |  |  |  |  |  |  |  |  |  |  |  |  |  |  |  |  |  |  |  |  |  |  |  |  |  |  |  |  |  |  |  |  |  |  |  |  |  |  |  |  |  |  |  |  |  |  |  |  |  |  |  |  |  |  |  |  |  |  |  |  |  |  |  |  |  |  | | --- | --- | --- | --- | --- | --- | --- | --- | --- | --- | --- | --- | --- | --- | --- | --- | --- | --- | --- | --- | --- | --- | --- | --- | --- | --- | --- | --- | --- | --- | --- | --- | --- | --- | --- | --- | --- | --- | --- | --- | --- | --- | --- | --- | --- | --- | --- | --- | --- | --- | --- | --- | --- | --- | --- | --- | --- | --- | --- | --- | --- | --- | --- | --- | --- | --- | --- | --- | --- | --- | --- | --- | --- | --- | --- | --- | --- | --- | --- | --- | --- | --- | --- | --- | | **4** 220 690 4453 199 | 0 124 980 0|  |  |  |  |  |  |  |  |  |  |  |  |  |  |  |  |  |  |  |  |  |  |  |  |  |  |  |  |  |  |  |  |  |  |  |  |  |  |  |  |  |  |  |  |  |  |  |  |  |  |  |  |  |  |  |  |  |  |  |  |  |  |  |  |  |  |  |  |  |  |  |  | | --- | --- | --- | --- | --- | --- | --- | --- | --- | --- | --- | --- | --- | --- | --- | --- | --- | --- | --- | --- | --- | --- | --- | --- | --- | --- | --- | --- | --- | --- | --- | --- | --- | --- | --- | --- | --- | --- | --- | --- | --- | --- | --- | --- | --- | --- | --- | --- | --- | --- | --- | --- | --- | --- | --- | --- | --- | --- | --- | --- | --- | --- | --- | --- | --- | --- | --- | --- | --- | --- | --- | --- | | **5** 399 1492 1562 2109 | 12 442 519 131|  |  |  |  |  |  |  |  |  |  |  |  |  |  |  |  |  |  |  |  |  |  |  |  |  |  |  |  |  |  |  |  |  |  |  |  |  |  |  |  |  |  |  |  |  |  |  |  |  |  |  |  |  |  |  |  |  |  |  |  | | --- | --- | --- | --- | --- | --- | --- | --- | --- | --- | --- | --- | --- | --- | --- | --- | --- | --- | --- | --- | --- | --- | --- | --- | --- | --- | --- | --- | --- | --- | --- | --- | --- | --- | --- | --- | --- | --- | --- | --- | --- | --- | --- | --- | --- | --- | --- | --- | --- | --- | --- | --- | --- | --- | --- | --- | --- | --- | --- | --- | | **6** 2109 1562 1492 399 | 131 519 442 12|  |  |  |  |  |  |  |  |  |  |  |  |  |  |  |  |  |  |  |  |  |  |  |  |  |  |  |  |  |  |  |  |  |  |  |  |  |  |  |  |  |  |  |  |  |  |  |  | | --- | --- | --- | --- | --- | --- | --- | --- | --- | --- | --- | --- | --- | --- | --- | --- | --- | --- | --- | --- | --- | --- | --- | --- | --- | --- | --- | --- | --- | --- | --- | --- | --- | --- | --- | --- | --- | --- | --- | --- | --- | --- | --- | --- | --- | --- | --- | --- | | **7** 199 4453 690 220 | 0 980 124 0|  |  |  |  |  |  |  |  |  |  |  |  |  |  |  |  |  |  |  |  |  |  |  |  |  |  |  |  |  |  |  |  |  |  |  |  | | --- | --- | --- | --- | --- | --- | --- | --- | --- | --- | --- | --- | --- | --- | --- | --- | --- | --- | --- | --- | --- | --- | --- | --- | --- | --- | --- | --- | --- | --- | --- | --- | --- | --- | --- | --- | | **8** 231 685 4349 297 | 0 122 982 0|  |  |  |  |  |  |  |  |  |  |  |  |  |  |  |  |  |  |  |  |  |  |  |  | | --- | --- | --- | --- | --- | --- | --- | --- | --- | --- | --- | --- | --- | --- | --- | --- | --- | --- | --- | --- | --- | --- | --- | --- | | **9** 211 4565 563 223 | 0 1056 48 0|  |  |  |  |  |  |  |  |  |  |  |  | | --- | --- | --- | --- | --- | --- | --- | --- | --- | --- | --- | --- | | **10** 286 660 4363 253 | 0 170 934 0 | | | | | | | | | | | | | | | | | | | | | | | | | | | | | | | | | | | | | | | | | | | | | | | | | | | | | | | | | | | | | | | | | | | | | | | | | | | | | | | | | | | | | | | | | | | | | | | | | | | | | | | | | | | | | | | | | | | | | | | | | | | | | | | | | | | | |

  
  

---

  
  
  
 **TACGCG**   
**CGCGTA**  
  
8 redundant motifs found:  
CGCGTACG - GTACGCGG - TTCGCG - TTCGCG - CCGCGT - TCGCGT - CGCGTT - GTCGCG -   
  
Best occurrences (match percentage):   
Seq St oligo pos match
  
 1 + **.TCCGCG.** 882 (94.31)   
1 + **[CGCGCG]** 884 (85.98)   
1 + **.TGCGCG.** 998 (94.26)   
1 + **[GCCGCG]** 1069 (86.55)   
1 - **[GGCGCG]** 1071 (86.50)   
1 - **[GGCGCG]** 1000 (86.50)   
1 - **[CGCGCG]** 884 (85.98)   
2 + **.TGCGCG.** 199 (94.26)   
2 + **[GCCGCG]** 447 (86.55)   
2 + **.TACGCG.** 455 (100.00)   
2 + **[CCCGCG]** 513 (86.03)   
2 + **[GGCGCG]** 703 (86.50)   
2 + **[CGCGCG]** 705 (85.98)   
2 + **[TTCGCG]** 790 (87.60)   
2 + **[CGCGCG]** 892 (85.98)   
2 + **.TCCGCG.** 975 (94.31)   
2 + **[GGCGCG]** 999 (86.50)   
2 + **[CCCGCG]** 1063 (86.03)   
2 + **[GCCGCG]** 1071 (86.55)   
2 + **[CGCGCG]** 1073 (85.98)   
2 + **.TCCGCG.** 1209 (94.31)   
2 + **[GGCGCG]** 1450 (86.50)   
2 + **[TTCGCG]** 1478 (87.60)   
2 + **[CCCGCG]** 1497 (86.03)   
2 - **[CCCGCG]** 1499 (86.03)   
2 - **[TTCGCG]** 1452 (87.60)   
2 - **[GCCGCG]** 1447 (86.55)   
2 - **[CCCGCG]** 1190 (86.03)   
2 - **[CGCGCG]** 1073 (85.98)   
2 - **[GCCGCG]** 996 (86.55)   
2 - **[GCCGCG]** 977 (86.55)   
2 - **.TCCGCG.** 894 (94.31)   
2 - **[CGCGCG]** 892 (85.98)   
2 - **[CCCGCG]** 792 (86.03)   
2 - **[GCCGCG]** 721 (86.55)   
2 - **[CCCGCG]** 707 (86.03)   
2 - **[CGCGCG]** 705 (85.98)   
2 - **.GACGCG.** 515 (92.24)   
2 - **[GGCGCG]** 457 (86.50)   
2 - **.GACGCG.** 449 (92.24)   
2 - **[CCCGCG]** 364 (86.03)   
2 - **[GCCGCG]** 201 (86.55)   
3 + **[GCCGCG]** 789 (86.55)   
3 + **[GGCGCG]** 1180 (86.50)   
3 + **.TCCGCG.** 1209 (94.31)   
3 + **[GGCGCG]** 1249 (86.50)   
3 + **[CGCGCG]** 1251 (85.98)   
3 + **[GCCGCG]** 1416 (86.55)   
3 - **[GGCGCG]** 1418 (86.50)   
3 - **[CGCGCG]** 1251 (85.98)   
3 - **[GCCGCG]** 1223 (86.55)   
3 - **.TACGCG.** 791 (100.00)   
3 - **[CCCGCG]** 703 (86.03)   
3 - **[TAGGCG]** 166 (85.06)   
4 + **.TGCGCG.** 144 (94.26)   
4 + **[CCCGCG]** 572 (86.03)   
4 + **.CACGCG.** 623 (91.73)   
4 + **[GGCGCG]** 683 (86.50)   
4 + **[GCCGCG]** 699 (86.55)   
4 + **[CCCGCG]** 782 (86.03)   
4 + **[CGCGCG]** 784 (85.98)   
4 + **[TAGGCG]** 813 (85.06)   
4 + **[GGCGCG]** 828 (86.50)   
4 + **[CCCGCG]** 873 (86.03)   
4 + **[GGCGCG]** 916 (86.50)   
4 + **[CCCGCG]** 1177 (86.03)   
4 + **[CCCGCG]** 1197 (86.03)   
4 + **[GCCGCG]** 1206 (86.55)   
4 + **[GGCGCG]** 1272 (86.50)   
4 + **[CGCGCG]** 1274 (85.98)   
4 + **[GGCGCG]** 1282 (86.50)   
4 + **[CGCGCG]** 1284 (85.98)   
4 - **[CCCGCG]** 1286 (86.03)   
4 - **[CGCGCG]** 1284 (85.98)   
4 - **[CCCGCG]** 1276 (86.03)   
4 - **[CGCGCG]** 1274 (85.98)   
4 - **[GGCGCG]** 1208 (86.50)   
4 - **[GGCGCG]** 1199 (86.50)   
4 - **[GCCGCG]** 1179 (86.55)   
4 - **.CACGCG.** 918 (91.73)   
4 - **.TCCGCG.** 898 (94.31)   
4 - **[CCCGCG]** 875 (86.03)   
4 - **[GCCGCG]** 822 (86.55)   
4 - **[GCCGCG]** 802 (86.55)   
4 - **[GGCGCG]** 786 (86.50)   
4 - **[CGCGCG]** 784 (85.98)   
4 - **[GCCGCG]** 742 (86.55)   
4 - **[CCCGCG]** 685 (86.03)   
4 - **.TCCGCG.** 625 (94.31)   
5 + **.TCCGCG.** 506 (94.31)   
5 + **[GCCGCG]** 812 (86.55)   
5 + **[CGCGCG]** 814 (85.98)   
5 + **.TCCGCG.** 1074 (94.31)   
5 + **[CCCGCG]** 1189 (86.03)   
5 + **[CCCGCG]** 1344 (86.03)   
5 + **[CCCGCG]** 1356 (86.03)   
5 + **[CGCGCG]** 1358 (85.98)   
5 + **.GACGCG.** 1438 (92.24)   
5 - **[GCCGCG]** 1440 (86.55)   
5 - **[GCCGCG]** 1395 (86.55)   
5 - **[CGCGCG]** 1358 (85.98)   
5 - **[GGCGCG]** 1346 (86.50)   
5 - **[CCCGCG]** 1191 (86.03)   
5 - **[CCCGCG]** 1076 (86.03)   
5 - **[CCCGCG]** 816 (86.03)   
5 - **[CGCGCG]** 814 (85.98)   
6 + **.TGCGCG.** 790 (94.26)   
6 + **[CCCGCG]** 1378 (86.03)   
6 + **[CGCGCG]** 1380 (85.98)   
6 + **[CCCGCG]** 1408 (86.03)   
6 + **.TCCGCG.** 1438 (94.31)   
6 + **[AACGCG]** 1494 (87.55)   
6 - **[GCCGCG]** 1440 (86.55)   
6 - **[CCCGCG]** 1410 (86.03)   
6 - **[CGCGCG]** 1380 (85.98)   
6 - **.TGCGCG.** 1368 (94.26)   
6 - **[AACGCG]** 792 (87.55)   
8 + **.TCCGCG.** 368 (94.31)   
8 + **[CGCGCG]** 370 (85.98)   
8 + **[GGCGCG]** 407 (86.50)   
8 + **[GCCGCG]** 452 (86.55)   
8 + **.TGCGCG.** 492 (94.26)   
8 + **[CCCGCG]** 786 (86.03)   
8 + **.TCCGCG.** 911 (94.31)   
8 + **[GGCGCG]** 927 (86.50)   
8 + **[GGCGCG]** 933 (86.50)   
8 + **[CCCGCG]** 951 (86.03)   
8 + **[CGCGCG]** 953 (85.98)   
8 + **[GGCGCG]** 978 (86.50)   
8 + **[CCCGCG]** 1102 (86.03)   
8 + **[CCCGCG]** 1144 (86.03)   
8 + **.TCCGCG.** 1196 (94.31)   
8 + **[CCCGCG]** 1344 (86.03)   
8 + **.TCCGCG.** 1400 (94.31)   
8 + **[CCCGCG]** 1508 (86.03)   
8 - **[CCCGCG]** 1198 (86.03)   
8 - **.GACGCG.** 1146 (92.24)   
8 - **[CCCGCG]** 955 (86.03)   
8 - **[CGCGCG]** 953 (85.98)   
8 - **[CCCGCG]** 929 (86.03)   
8 - **[CCCGCG]** 913 (86.03)   
8 - **.TGCGCG.** 788 (94.26)   
8 - **[GGCGCG]** 494 (86.50)   
8 - **.CACGCG.** 454 (91.73)   
8 - **[GCCGCG]** 409 (86.55)   
8 - **.GACGCG.** 372 (92.24)   
8 - **[CGCGCG]** 370 (85.98)   
8 - **.GACGCG.** 149 (92.24)   
9 + **[CGCGCG]** 6 (85.98)   
9 + **.CACGCG.** 99 (91.73)   
9 + **[GGCGCG]** 975 (86.50)   
9 + **[CCCGCG]** 1060 (86.03)   
9 + **[GGCGCG]** 1266 (86.50)   
9 - **[GGCGCG]** 1062 (86.50)   
9 - **[TAGGCG]** 603 (85.06)   
9 - **[CGCGCG]** 6 (85.98)   
10 + **[GCCGCG]** 35 (86.55)   
10 + **[CCCGCG]** 87 (86.03)   
10 + **[CCCGCG]** 488 (86.03)   
10 + **[GCCGCG]** 511 (86.55)   
10 + **[CCCGCG]** 577 (86.03)   
10 + **.TCCGCG.** 603 (94.31)   
10 + **.TGCGCG.** 722 (94.26)   
10 + **.GACGCG.** 820 (92.24)   
10 + **.GACGCG.** 828 (92.24)   
10 + **[GGCGCG]** 871 (86.50)   
10 + **[GCCGCG]** 1022 (86.55)   
10 + **[CGCGCG]** 1094 (85.98)   
10 + **[GCCGCG]** 1119 (86.55)   
10 + **.CACGCG.** 1306 (91.73)   
10 + **.CACGCG.** 1329 (91.73)   
10 + **.TCCGCG.** 1446 (94.31)   
10 - **[GCCGCG]** 1331 (86.55)   
10 - **.TCCGCG.** 1308 (94.31)   
10 - **[GCCGCG]** 1121 (86.55)   
10 - **[GCCGCG]** 1096 (86.55)   
10 - **[CGCGCG]** 1094 (85.98)   
10 - **[CCCGCG]** 1073 (86.03)   
10 - **[GCCGCG]** 1024 (86.55)   
10 - **[CCCGCG]** 873 (86.03)   
10 - **[CCCGCG]** 864 (86.03)   
10 - **[CCCGCG]** 830 (86.03)   
10 - **[GCCGCG]** 605 (86.55)   
10 - **[CCCGCG]** 579 (86.03)   
10 - **[GGCGCG]** 513 (86.50)   
10 - **.TCCGCG.** 490 (94.31)   
10 - **.TCCGCG.** 89 (94.31)   
11 + **.TGCGCG.** 32 (94.26)   
11 + **.TGCGCG.** 49 (94.26)   
11 + **[TAGGCG]** 248 (85.06)   
11 + **[GGCGCG]** 250 (86.50)   
11 + **[GCCGCG]** 497 (86.55)   
11 + **.CACGCG.** 558 (91.73)   
11 + **[CCCGCG]** 591 (86.03)   
11 + **[GCCGCG]** 609 (86.55)   
11 + **[GCCGCG]** 615 (86.55)   
11 + **[CGCGCG]** 617 (85.98)   
11 + **[CCCGCG]** 637 (86.03)   
11 + **[CCCGCG]** 661 (86.03)   
11 + **.GACGCG.** 698 (92.24)   
11 + **[GCCGCG]** 713 (86.55)   
11 + **[CCCGCG]** 721 (86.03)   
11 + **[CGCGCG]** 723 (85.98)   
11 + **.GACGCG.** 747 (92.24)   
11 + **[GCCGCG]** 767 (86.55)   
11 + **[CCCGCG]** 955 (86.03)   
11 + **[GCCGCG]** 994 (86.55)   
11 + **[GCCGCG]** 1027 (86.55)   
11 + **.TGCGCG.** 1114 (94.26)   
11 + **[GCCGCG]** 1155 (86.55)   
11 + **[GGCGCG]** 1379 (86.50)   
11 - **.GACGCG.** 1381 (92.24)   
11 - **[CCCGCG]** 1345 (86.03)   
11 - **.TGCGCG.** 1157 (94.26)   
11 - **[GCCGCG]** 1116 (86.55)   
11 - **[GGCGCG]** 957 (86.50)   
11 - **[GCCGCG]** 769 (86.55)   
11 - **[GGCGCG]** 749 (86.50)   
11 - **[CGCGCG]** 723 (85.98)   
11 - **[GCCGCG]** 715 (86.55)   
11 - **[GGCGCG]** 700 (86.50)   
11 - **[CCCGCG]** 663 (86.03)   
11 - **[GGCGCG]** 639 (86.50)   
11 - **[CCCGCG]** 619 (86.03)   
11 - **[CGCGCG]** 617 (85.98)   
11 - **[GCCGCG]** 611 (86.55)   
11 - **[GGCGCG]** 606 (86.50)   
11 - **.TGCGCG.** 593 (94.26)   
11 - **[GCCGCG]** 560 (86.55)   
11 - **.TCCGCG.** 531 (94.31)   
11 - **.CACGCG.** 499 (91.73)   
11 - **.CACGCG.** 252 (91.73)   
11 - **.TCCGCG.** 51 (94.31)   
11 - **.CACGCG.** 34 (91.73)   
12 + **.CACGCG.** 115 (91.73)   
12 + **.CACGCG.** 205 (91.73)   
12 + **[GGCGCG]** 509 (86.50)   
12 + **[CGCGCG]** 511 (85.98)   
12 + **[GGCGCG]** 524 (86.50)   
12 + **[GGCGCG]** 541 (86.50)   
12 + **[CCCGCG]** 557 (86.03)   
12 + **[CGCGCG]** 559 (85.98)   
12 + **.TCCGCG.** 694 (94.31)   
12 + **[CCCGCG]** 709 (86.03)   
12 + **[GCCGCG]** 740 (86.55)   
12 + **.TCCGCG.** 837 (94.31)   
12 + **[CGCGCG]** 839 (85.98)   
12 + **[CGCGCG]** 882 (85.98)   
12 + **[GCCGCG]** 1030 (86.55)   
12 + **[CGCGCG]** 1032 (85.98)   
12 + **[CGCGCG]** 1034 (85.98)   
12 + **[CCCGCG]** 1088 (86.03)   
12 + **.GACGCG.** 1116 (92.24)   
12 + **[CGCGCG]** 1118 (85.98)   
12 + **[CCCGCG]** 1165 (86.03)   
12 + **[GCCGCG]** 1178 (86.55)   
12 + **[CGCGCG]** 1180 (85.98)   
12 + **[CCCGCG]** 1203 (86.03)   
12 + **[GCCGCG]** 1251 (86.55)   
12 + **[GCCGCG]** 1275 (86.55)   
12 + **[GGCGCG]** 1333 (86.50)   
12 + **[GCCGCG]** 1355 (86.55)   
12 - **.TACGCG.** 1465 (100.00)   
12 - **[CCCGCG]** 1335 (86.03)   
12 - **[GGCGCG]** 1253 (86.50)   
12 - **[CGCGCG]** 1180 (85.98)   
12 - **[CCCGCG]** 1167 (86.03)   
12 - **[GCCGCG]** 1120 (86.55)   
12 - **[CGCGCG]** 1118 (85.98)   
12 - **[CCCGCG]** 1097 (86.03)   
12 - **[CCCGCG]** 1090 (86.03)   
12 - **[GGCGCG]** 1036 (86.50)   
12 - **[CGCGCG]** 1034 (85.98)   
12 - **[CGCGCG]** 1032 (85.98)   
12 - **.TCCGCG.** 922 (94.31)   
12 - **[CCCGCG]** 884 (86.03)   
12 - **[CGCGCG]** 882 (85.98)   
12 - **.GACGCG.** 841 (92.24)   
12 - **[CGCGCG]** 839 (85.98)   
12 - **[AACGCG]** 742 (87.55)   
12 - **[GCCGCG]** 711 (86.55)   
12 - **[GGCGCG]** 696 (86.50)   
12 - **[CCCGCG]** 561 (86.03)   
12 - **[CGCGCG]** 559 (85.98)   
12 - **[GGCGCG]** 543 (86.50)   
12 - **[GGCGCG]** 526 (86.50)   
12 - **.TCCGCG.** 513 (94.31)   
12 - **[CGCGCG]** 511 (85.98)   
12 - **[CCCGCG]** 180 (86.03)   
12 - **[CCCGCG]** 117 (86.03)   
13 + **[GGCGCG]** 440 (86.50)   
13 + **[CGCGCG]** 531 (85.98)   
13 + **[CGCGCG]** 533 (85.98)   
13 + **[CCCGCG]** 613 (86.03)   
13 + **[CCCGCG]** 642 (86.03)   
13 + **.CACGCG.** 654 (91.73)   
13 + **[CCCGCG]** 701 (86.03)   
13 + **[GCCGCG]** 721 (86.55)   
13 + **[CGCGCG]** 723 (85.98)   
13 + **[CCCGCG]** 740 (86.03)   
13 + **[CCCGCG]** 764 (86.03)   
13 + **[CCCGCG]** 771 (86.03)   
13 + **[CCCGCG]** 778 (86.03)   
13 + **[CCCGCG]** 785 (86.03)   
13 + **[GGCGCG]** 801 (86.50)   
13 + **[CCCGCG]** 943 (86.03)   
13 + **[CGCGCG]** 945 (85.98)   
13 + **.TCCGCG.** 1008 (94.31)   
13 + **[CGCGCG]** 1010 (85.98)   
13 + **[GGCGCG]** 1062 (86.50)   
13 + **[GGCGCG]** 1092 (86.50)   
13 + **[GCCGCG]** 1110 (86.55)   
13 + **[CGCGCG]** 1112 (85.98)   
13 + **[GCCGCG]** 1197 (86.55)   
13 + **[GCCGCG]** 1227 (86.55)   
13 - **[CCCGCG]** 1470 (86.03)   
13 - **[GCCGCG]** 1352 (86.55)   
13 - **[CCCGCG]** 1324 (86.03)   
13 - **[CCCGCG]** 1304 (86.03)   
13 - **[GGCGCG]** 1264 (86.50)   
13 - **[CCCGCG]** 1229 (86.03)   
13 - **.TCCGCG.** 1199 (94.31)   
13 - **[GGCGCG]** 1187 (86.50)   
13 - **[CGCGCG]** 1112 (85.98)   
13 - **.TCCGCG.** 1094 (94.31)   
13 - **[GCCGCG]** 1064 (86.55)   
13 - **.TGCGCG.** 1012 (94.26)   
13 - **[CGCGCG]** 1010 (85.98)   
13 - **[GCCGCG]** 947 (86.55)   
13 - **[CGCGCG]** 945 (85.98)   
13 - **[CCCGCG]** 873 (86.03)   
13 - **.TGCGCG.** 803 (94.26)   
13 - **[GGCGCG]** 787 (86.50)   
13 - **[GGCGCG]** 780 (86.50)   
13 - **[GGCGCG]** 773 (86.50)   
13 - **.GACGCG.** 766 (92.24)   
13 - **.GACGCG.** 742 (92.24)   
13 - **[CGCGCG]** 723 (85.98)   
13 - **[GCCGCG]** 703 (86.55)   
13 - **[GCCGCG]** 656 (86.55)   
13 - **[GGCGCG]** 615 (86.50)   
13 - **.TCCGCG.** 535 (94.31)   
13 - **[CGCGCG]** 533 (85.98)   
13 - **[CGCGCG]** 531 (85.98)   
13 - **[GGCGCG]** 167 (86.50)   
14 + **[CCCGCG]** 233 (86.03)   
14 + **.CACGCG.** 290 (91.73)   
14 + **[CCCGCG]** 335 (86.03)   
14 + **[GCCGCG]** 460 (86.55)   
14 + **.TCCGCG.** 531 (94.31)   
14 + **[GGCGCG]** 712 (86.50)   
14 + **[GGCGCG]** 746 (86.50)   
14 + **.CACGCG.** 862 (91.73)   
14 + **[AACGCG]** 868 (87.55)   
14 + **[GGCGCG]** 938 (86.50)   
14 + **[GCCGCG]** 989 (86.55)   
14 + **[CGCGCG]** 991 (85.98)   
14 + **[GCCGCG]** 1072 (86.55)   
14 + **.CACGCG.** 1305 (91.73)   
14 - **[CCCGCG]** 1074 (86.03)   
14 - **[GCCGCG]** 993 (86.55)   
14 - **[CGCGCG]** 991 (85.98)   
14 - **[GCCGCG]** 940 (86.55)   
14 - **[GGCGCG]** 870 (86.50)   
14 - **[TTCGCG]** 864 (87.60)   
14 - **.GACGCG.** 793 (92.24)   
14 - **[GCCGCG]** 748 (86.55)   
14 - **.CACGCG.** 714 (91.73)   
14 - **[AACGCG]** 620 (87.55)   
14 - **.TCCGCG.** 533 (94.31)   
14 - **[CCCGCG]** 351 (86.03)   
14 - **[GCCGCG]** 337 (86.55)   
14 - **[GGCGCG]** 292 (86.50)   
14 - **[GCCGCG]** 235 (86.55)   
15 + **[GCCGCG]** 23 (86.55)   
15 + **.TGCGCG.** 52 (94.26)   
15 + **[GCCGCG]** 179 (86.55)   
15 + **.CACGCG.** 245 (91.73)   
15 + **[GCCGCG]** 415 (86.55)   
15 + **[GCCGCG]** 459 (86.55)   
15 + **[CCCGCG]** 535 (86.03)   
15 + **[CGCGCG]** 537 (85.98)   
15 + **[CCCGCG]** 543 (86.03)   
15 + **[GGCGCG]** 733 (86.50)   
15 + **[CGCGCG]** 735 (85.98)   
15 + **[TTCGCG]** 825 (87.60)   
15 + **[GCCGCG]** 855 (86.55)   
15 + **[CGCGCG]** 857 (85.98)   
15 + **[CGCGCG]** 859 (85.98)   
15 + **[CGCGCG]** 893 (85.98)   
15 + **[GGCGCG]** 984 (86.50)   
15 + **.TGCGCG.** 1181 (94.26)   
15 + **[GGCGCG]** 1200 (86.50)   
15 + **[CGCGCG]** 1202 (85.98)   
15 + **[CCCGCG]** 1230 (86.03)   
15 + **[GCCGCG]** 1264 (86.55)   
15 - **.GACGCG.** 1232 (92.24)   
15 - **.TCCGCG.** 1204 (94.31)   
15 - **[CGCGCG]** 1202 (85.98)   
15 - **.GACGCG.** 1183 (92.24)   
15 - **[GCCGCG]** 986 (86.55)   
15 - **[CCCGCG]** 895 (86.03)   
15 - **[CGCGCG]** 893 (85.98)   
15 - **[CGCGCG]** 859 (85.98)   
15 - **[CGCGCG]** 857 (85.98)   
15 - **[GGCGCG]** 827 (86.50)   
15 - **[CGCGCG]** 735 (85.98)   
15 - **[TAGGCG]** 632 (85.06)   
15 - **[GCCGCG]** 545 (86.55)   
15 - **[GGCGCG]** 539 (86.50)   
15 - **[CGCGCG]** 537 (85.98)   
15 - **.TCCGCG.** 461 (94.31)   
15 - **[GGCGCG]** 247 (86.50)   
15 - **[CCCGCG]** 181 (86.03)   
16 + **.TGCGCG.** 312 (94.26)   
16 + **[CCCGCG]** 411 (86.03)   
16 + **[GGCGCG]** 492 (86.50)   
16 + **.CACGCG.** 520 (91.73)   
16 + **[GCCGCG]** 621 (86.55)   
16 + **[GCCGCG]** 626 (86.55)   
16 + **[CGCGCG]** 773 (85.98)   
16 + **[GGCGCG]** 903 (86.50)   
16 + **[CCCGCG]** 1001 (86.03)   
16 + **[GGCGCG]** 1093 (86.50)   
16 + **.CACGCG.** 1163 (91.73)   
16 + **.TGCGCG.** 1280 (94.26)   
16 + **[TTCGCG]** 1298 (87.60)   
16 + **[CCCGCG]** 1311 (86.03)   
16 + **.TGCGCG.** 1338 (94.26)   
16 - **.TCCGCG.** 1340 (94.31)   
16 - **[CCCGCG]** 1313 (86.03)   
16 - **[GGCGCG]** 1300 (86.50)   
16 - **.TGCGCG.** 1165 (94.26)   
16 - **[GCCGCG]** 1150 (86.55)   
16 - **[CCCGCG]** 1095 (86.03)   
16 - **.TGCGCG.** 966 (94.26)   
16 - **[GGCGCG]** 799 (86.50)   
16 - **[GCCGCG]** 775 (86.55)   
16 - **[CGCGCG]** 773 (85.98)   
16 - **[GGCGCG]** 628 (86.50)   
16 - **[GGCGCG]** 623 (86.50)   
16 - **.CACGCG.** 522 (91.73)   
16 - **.TCCGCG.** 494 (94.31)   
16 - **[GGCGCG]** 314 (86.50)   
17 + **[GCCGCG]** 1017 (86.55)   
17 - **[GCCGCG]** 1019 (86.55)   
18 + **[GCCGCG]** 474 (86.55)   
18 + **[CGCGCG]** 708 (85.98)   
18 + **[GCCGCG]** 832 (86.55)   
18 + **[GCCGCG]** 837 (86.55)   
18 + **[CGCGCG]** 839 (85.98)   
18 + **[CGCGCG]** 841 (85.98)   
18 + **[GCCGCG]** 866 (86.55)   
18 + **[CCCGCG]** 912 (86.03)   
18 + **[GCCGCG]** 930 (86.55)   
18 + **[CCCGCG]** 1027 (86.03)   
18 + **[CCCGCG]** 1105 (86.03)   
18 + **[CGCGCG]** 1107 (85.98)   
18 + **[CCCGCG]** 1127 (86.03)   
18 + **.TCCGCG.** 1146 (94.31)   
18 + **.TCCGCG.** 1192 (94.31)   
18 + **[GCCGCG]** 1202 (86.55)   
18 + **[CCCGCG]** 1229 (86.03)   
18 + **[GCCGCG]** 1329 (86.55)   
18 + **[GGCGCG]** 1386 (86.50)   
18 + **.TGCGCG.** 1435 (94.26)   
18 - **[CCCGCG]** 1491 (86.03)   
18 - **[CCCGCG]** 1388 (86.03)   
18 - **.GACGCG.** 1331 (92.24)   
18 - **[GGCGCG]** 1282 (86.50)   
18 - **[GGCGCG]** 1231 (86.50)   
18 - **[GCCGCG]** 1204 (86.55)   
18 - **[AACGCG]** 1194 (87.55)   
18 - **.TCCGCG.** 1148 (94.31)   
18 - **[GGCGCG]** 1109 (86.50)   
18 - **[CGCGCG]** 1107 (85.98)   
18 - **.TCCGCG.** 1029 (94.31)   
18 - **[GCCGCG]** 932 (86.55)   
18 - **[GGCGCG]** 914 (86.50)   
18 - **[GGCGCG]** 868 (86.50)   
18 - **[CGCGCG]** 841 (85.98)   
18 - **[CGCGCG]** 839 (85.98)   
18 - **[GGCGCG]** 834 (86.50)   
18 - **[AACGCG]** 760 (87.55)   
18 - **[GGCGCG]** 710 (86.50)   
18 - **[CGCGCG]** 708 (85.98)   
18 - **.GACGCG.** 476 (92.24)   
20 + **.TGCGCG.** 27 (94.26)   
20 + **[GGCGCG]** 66 (86.50)   
20 + **[CCCGCG]** 198 (86.03)   
20 + **.TGCGCG.** 274 (94.26)   
20 + **[CGCGCG]** 276 (85.98)   
20 + **[GCCGCG]** 300 (86.55)   
20 + **.CACGCG.** 309 (91.73)   
20 + **[GCCGCG]** 896 (86.55)   
20 + **[CGCGCG]** 898 (85.98)   
20 + **.TGCGCG.** 990 (94.26)   
20 + **[CCCGCG]** 1005 (86.03)   
20 + **[CCCGCG]** 1033 (86.03)   
20 + **[GCCGCG]** 1197 (86.55)   
20 - **.CACGCG.** 1199 (91.73)   
20 - **[GCCGCG]** 1035 (86.55)   
20 - **[CCCGCG]** 1007 (86.03)   
20 - **[GGCGCG]** 992 (86.50)   
20 - **.TCCGCG.** 900 (94.31)   
20 - **[CGCGCG]** 898 (85.98)   
20 - **[CCCGCG]** 278 (86.03)   
20 - **[CGCGCG]** 276 (85.98)   
20 - **.TGCGCG.** 200 (94.26)   
20 - **.TCCGCG.** 68 (94.31)   
22 + **.CACGCG.** 190 (91.73)   
22 + **[AACGCG]** 614 (87.55)   
22 + **[CGCGCG]** 616 (85.98)   
22 + **[GCCGCG]** 772 (86.55)   
22 + **[CGCGCG]** 774 (85.98)   
22 + **[CGCGCG]** 776 (85.98)   
22 + **[TAGGCG]** 847 (85.06)   
22 + **[CGCGCG]** 1013 (85.98)   
22 - **[CGCGCG]** 1013 (85.98)   
22 - **[CGCGCG]** 776 (85.98)   
22 - **[CGCGCG]** 774 (85.98)   
22 - **[GCCGCG]** 618 (86.55)   
22 - **[CGCGCG]** 616 (85.98)   
22 - **[GGCGCG]** 192 (86.50)   
23 + **[CCCGCG]** 1002 (86.03)   
23 + **.CACGCG.** 1077 (91.73)   
23 - **.TCCGCG.** 1079 (94.31)   
24 + **[GCCGCG]** 304 (86.55)   
24 + **.TCCGCG.** 520 (94.31)   
24 + **[CCCGCG]** 596 (86.03)   
24 + **.TGCGCG.** 923 (94.26)   
24 + **[GGCGCG]** 935 (86.50)   
24 + **[GGCGCG]** 942 (86.50)   
24 + **[GCCGCG]** 1038 (86.55)   
24 + **[GGCGCG]** 1075 (86.50)   
24 + **[GGCGCG]** 1098 (86.50)   
24 + **[GGCGCG]** 1319 (86.50)   
24 + **[GCCGCG]** 1353 (86.55)   
24 + **.TCCGCG.** 1493 (94.31)   
24 - **[CCCGCG]** 1495 (86.03)   
24 - **[CCCGCG]** 1355 (86.03)   
24 - **[GCCGCG]** 1217 (86.55)   
24 - **[GGCGCG]** 1145 (86.50)   
24 - **[GCCGCG]** 1100 (86.55)   
24 - **[CCCGCG]** 1077 (86.03)   
24 - **[GCCGCG]** 1040 (86.55)   
24 - **[CCCGCG]** 944 (86.03)   
24 - **[CCCGCG]** 937 (86.03)   
24 - **[CCCGCG]** 925 (86.03)   
24 - **[GGCGCG]** 598 (86.50)   
24 - **[GGCGCG]** 546 (86.50)   
24 - **.GACGCG.** 522 (92.24)   
24 - **[GGCGCG]** 306 (86.50)   
25 + **[GGCGCG]** 360 (86.50)   
25 + **[GGCGCG]** 383 (86.50)   
25 + **.GACGCG.** 487 (92.24)   
25 + **[GGCGCG]** 549 (86.50)   
25 + **.GACGCG.** 770 (92.24)   
25 + **[GGCGCG]** 792 (86.50)   
25 + **.GACGCG.** 950 (92.24)   
25 + **[GCCGCG]** 1151 (86.55)   
25 - **[CCCGCG]** 952 (86.03)   
25 - **.TCCGCG.** 772 (94.31)   
25 - **.TCCGCG.** 551 (94.31)   
25 - **.TCCGCG.** 362 (94.31)   
25 - **.TCCGCG.** 89 (94.31)   
26 + **[GCCGCG]** 184 (86.55)   
26 + **.GACGCG.** 753 (92.24)   
26 + **[GGCGCG]** 779 (86.50)   
26 + **[GCCGCG]** 803 (86.55)   
26 + **[GGCGCG]** 824 (86.50)   
26 + **[CCCGCG]** 841 (86.03)   
26 + **[CGCGCG]** 843 (85.98)   
26 + **[CGCGCG]** 845 (85.98)   
26 + **[GCCGCG]** 885 (86.55)   
26 + **[CGCGCG]** 892 (85.98)   
26 + **.TACGCG.** 1346 (100.00)   
26 + **[GGCGCG]** 1353 (86.50)   
26 + **.TGCGCG.** 1423 (94.26)   
26 + **[GCCGCG]** 1485 (86.55)   
26 - **[CCCGCG]** 1487 (86.03)   
26 - **[GCCGCG]** 1425 (86.55)   
26 - **[AACGCG]** 1355 (87.55)   
26 - **[CCCGCG]** 1348 (86.03)   
26 - **[CGCGCG]** 892 (85.98)   
26 - **.TCCGCG.** 847 (94.31)   
26 - **[CGCGCG]** 845 (85.98)   
26 - **[CGCGCG]** 843 (85.98)   
26 - **[GCCGCG]** 826 (86.55)   
26 - **[CCCGCG]** 781 (86.03)   
26 - **[GCCGCG]** 186 (86.55)   
27 + **[CCCGCG]** 1144 (86.03)   
27 + **.TCCGCG.** 1202 (94.31)   
27 + **[GGCGCG]** 1207 (86.50)   
27 + **.TGCGCG.** 1247 (94.26)   
27 - **[TTCGCG]** 1460 (87.60)   
27 - **[GGCGCG]** 1249 (86.50)   
27 - **.TGCGCG.** 1209 (94.26)   
27 - **[GCCGCG]** 1204 (86.55)   
27 - **.GACGCG.** 1146 (92.24)   
28 + **[GGCGCG]** 433 (86.50)   
28 + **.TCCGCG.** 516 (94.31)   
28 + **[GCCGCG]** 587 (86.55)   
28 + **[GGCGCG]** 628 (86.50)   
28 + **.TCCGCG.** 649 (94.31)   
28 + **.TCCGCG.** 738 (94.31)   
28 + **[GCCGCG]** 916 (86.55)   
28 + **.TCCGCG.** 955 (94.31)   
28 + **.TCCGCG.** 1007 (94.31)   
28 + **[GCCGCG]** 1108 (86.55)   
28 + **.TCCGCG.** 1352 (94.31)   
28 - **.GACGCG.** 1110 (92.24)   
28 - **[GGCGCG]** 1105 (86.50)   
28 - **.GACGCG.** 1100 (92.24)   
28 - **[TAGGCG]** 983 (85.06)   
28 - **[GGCGCG]** 957 (86.50)   
28 - **[CCCGCG]** 918 (86.03)   
28 - **[GGCGCG]** 740 (86.50)   
28 - **[AACGCG]** 651 (87.55)   
28 - **[GCCGCG]** 518 (86.55)   
28 - **.CACGCG.** 435 (91.73)   
29 + **[TAGGCG]** 900 (85.06)   
29 + **[TAGGCG]** 1021 (85.06)   
29 + **[GCCGCG]** 1218 (86.55)   
29 + **.TACGCG.** 1297 (100.00)   
29 - **[AACGCG]** 1220 (87.55)   
29 - **[GGCGCG]** 220 (86.50)   
33 + **[CCCGCG]** 902 (86.03)   
33 + **.GACGCG.** 1029 (92.24)   
33 + **[GCCGCG]** 1040 (86.55)   
33 + **[GCCGCG]** 1360 (86.55)   
33 + **[GCCGCG]** 1411 (86.55)   
33 - **[CCCGCG]** 1413 (86.03)   
33 - **[GCCGCG]** 1362 (86.55)   
33 - **.CACGCG.** 1042 (91.73)   
33 - **[CCCGCG]** 1031 (86.03)   
33 - **[GGCGCG]** 938 (86.50)   
33 - **[GCCGCG]** 904 (86.55)   
33 - **[TTCGCG]** 867 (87.60)   
34 + **[TTCGCG]** 153 (87.60)   
34 + **[GGCGCG]** 591 (86.50)   
34 + **[GGCGCG]** 649 (86.50)   
34 + **.GACGCG.** 683 (92.24)   
34 + **[CCCGCG]** 761 (86.03)   
34 + **[GCCGCG]** 771 (86.55)   
34 + **[CCCGCG]** 792 (86.03)   
34 + **[GGCGCG]** 801 (86.50)   
34 + **[GCCGCG]** 956 (86.55)   
34 + **[CGCGCG]** 958 (85.98)   
34 + **[CGCGCG]** 960 (85.98)   
34 + **.GACGCG.** 1000 (92.24)   
34 + **.TCCGCG.** 1078 (94.31)   
34 + **[CGCGCG]** 1080 (85.98)   
34 + **[GCCGCG]** 1196 (86.55)   
34 + **[GCCGCG]** 1278 (86.55)   
34 + **[GGCGCG]** 1313 (86.50)   
34 - **.TCCGCG.** 1280 (94.31)   
34 - **[GCCGCG]** 1236 (86.55)   
34 - **.TGCGCG.** 1198 (94.26)   
34 - **[GCCGCG]** 1082 (86.55)   
34 - **[CGCGCG]** 1080 (85.98)   
34 - **.TACGCG.** 1002 (100.00)   
34 - **[CGCGCG]** 960 (85.98)   
34 - **[CGCGCG]** 958 (85.98)   
34 - **[GGCGCG]** 942 (86.50)   
34 - **.GACGCG.** 803 (92.24)   
34 - **[TTCGCG]** 794 (87.60)   
34 - **[GGCGCG]** 773 (86.50)   
34 - **.TGCGCG.** 637 (94.26)   
34 - **.TCCGCG.** 593 (94.31)   
34 - **[CCCGCG]** 245 (86.03)   
35 + **[CCCGCG]** 366 (86.03)   
35 + **.TGCGCG.** 930 (94.26)   
35 + **[GCCGCG]** 998 (86.55)   
35 + **[GGCGCG]** 1147 (86.50)   
35 + **.TCCGCG.** 1219 (94.31)   
35 + **[CCCGCG]** 1323 (86.03)   
35 - **.TCCGCG.** 1325 (94.31)   
35 - **[AACGCG]** 1221 (87.55)   
35 - **[CCCGCG]** 1149 (86.03)   
35 - **.TGCGCG.** 1000 (94.26)   
35 - **[AACGCG]** 932 (87.55)   
35 - **[GCCGCG]** 812 (86.55)   
35 - **.CACGCG.** 368 (91.73)   
36 + **.TACGCG.** 149 (100.00)   
36 + **.CACGCG.** 228 (91.73)   
36 + **.TACGCG.** 364 (100.00)   
36 + **[CCCGCG]** 450 (86.03)   
36 + **[GCCGCG]** 627 (86.55)   
36 + **[AACGCG]** 700 (87.55)   
36 + **.TCCGCG.** 777 (94.31)   
36 + **[CGCGCG]** 779 (85.98)   
36 + **[CGCGCG]** 781 (85.98)   
36 + **[CCCGCG]** 813 (86.03)   
36 + **.CACGCG.** 857 (91.73)   
36 + **[GCCGCG]** 1062 (86.55)   
36 + **[CGCGCG]** 1064 (85.98)   
36 + **[GCCGCG]** 1171 (86.55)   
36 + **[CCCGCG]** 1240 (86.03)   
36 - **.TCCGCG.** 1173 (94.31)   
36 - **[GGCGCG]** 1066 (86.50)   
36 - **[CGCGCG]** 1064 (85.98)   
36 - **[AACGCG]** 815 (87.55)   
36 - **.TCCGCG.** 783 (94.31)   
36 - **[CGCGCG]** 781 (85.98)   
36 - **[CGCGCG]** 779 (85.98)   
36 - **[CCCGCG]** 702 (86.03)   
36 - **.CACGCG.** 629 (91.73)   
36 - **.CACGCG.** 452 (91.73)   
36 - **.CACGCG.** 366 (91.73)   
36 - **[GGCGCG]** 269 (86.50)   
36 - **[GCCGCG]** 230 (86.55)   
36 - **.GACGCG.** 151 (92.24)   
36 - **[CCCGCG]** 8 (86.03)   
37 + **[AACGCG]** 1434 (87.55)   
38 - **[TAGGCG]** 797 (85.06)   
39 + **[GGCGCG]** 1046 (86.50)   
39 + **[GGCGCG]** 1120 (86.50)   
39 + **[CCCGCG]** 1128 (86.03)   
39 + **[GGCGCG]** 1412 (86.50)   
39 + **[CGCGCG]** 1423 (85.98)   
39 - **[GCCGCG]** 1425 (86.55)   
39 - **[CGCGCG]** 1423 (85.98)   
39 - **.GACGCG.** 1122 (92.24)   
39 - **[TAGGCG]** 503 (85.06)   
40 + **[CCCGCG]** 893 (86.03)   
40 + **[GGCGCG]** 950 (86.50)   
40 + **.TCCGCG.** 1102 (94.31)   
40 + **[GCCGCG]** 1107 (86.55)   
40 + **[TTCGCG]** 1231 (87.60)   
40 + **[CGCGCG]** 1233 (85.98)   
40 + **.TCCGCG.** 1239 (94.31)   
40 + **.TGCGCG.** 1423 (94.26)   
40 - **.TCCGCG.** 1513 (94.31)   
40 - **[GCCGCG]** 1425 (86.55)   
40 - **[CCCGCG]** 1279 (86.03)   
40 - **[GCCGCG]** 1241 (86.55)   
40 - **.GACGCG.** 1235 (92.24)   
40 - **[CGCGCG]** 1233 (85.98)   
40 - **.TGCGCG.** 1109 (94.26)   
40 - **[GGCGCG]** 1104 (86.50)   
40 - **[GCCGCG]** 952 (86.55)   
40 - **[GCCGCG]** 895 (86.55)   
41 + **[GCCGCG]** 521 (86.55)   
41 + **.TGCGCG.** 956 (94.26)   
41 - **[GCCGCG]** 958 (86.55)   
41 - **[GCCGCG]** 523 (86.55)   
42 + **[CCCGCG]** 438 (86.03)   
42 + **[CGCGCG]** 440 (85.98)   
42 + **[CCCGCG]** 523 (86.03)   
42 + **[TTCGCG]** 775 (87.60)   
42 + **.TGCGCG.** 811 (94.26)   
42 + **.TGCGCG.** 826 (94.26)   
42 + **[CGCGCG]** 828 (85.98)   
42 + **[CCCGCG]** 965 (86.03)   
42 + **[GGCGCG]** 1070 (86.50)   
42 + **[TTCGCG]** 1145 (87.60)   
42 + **[CCCGCG]** 1166 (86.03)   
42 + **[CCCGCG]** 1447 (86.03)   
42 + **[CGCGCG]** 1449 (85.98)   
42 + **[GCCGCG]** 1475 (86.55)   
42 - **[CCCGCG]** 1477 (86.03)   
42 - **.CACGCG.** 1451 (91.73)   
42 - **[CGCGCG]** 1449 (85.98)   
42 - **[GCCGCG]** 1311 (86.55)   
42 - **[GCCGCG]** 1168 (86.55)   
42 - **[TTCGCG]** 1147 (87.60)   
42 - **[CCCGCG]** 1072 (86.03)   
42 - **[CCCGCG]** 911 (86.03)   
42 - **[GGCGCG]** 900 (86.50)   
42 - **[GCCGCG]** 843 (86.55)   
42 - **[CGCGCG]** 828 (85.98)   
42 - **[GGCGCG]** 813 (86.50)   
42 - **.TCCGCG.** 777 (94.31)   
42 - **.TGCGCG.** 568 (94.26)   
42 - **.GACGCG.** 442 (92.24)   
42 - **[CGCGCG]** 440 (85.98)   
43 + **[GCCGCG]** 1101 (86.55)   
43 + **[TTCGCG]** 1108 (87.60)   
43 + **[GGCGCG]** 1195 (86.50)   
43 + **[CCCGCG]** 1202 (86.03)   
43 + **[GGCGCG]** 1251 (86.50)   
43 + **[GCCGCG]** 1343 (86.55)   
43 - **.TGCGCG.** 1253 (94.26)   
43 - **[CCCGCG]** 1204 (86.03)   
43 - **[GCCGCG]** 1197 (86.55)   
43 - **[CCCGCG]** 1110 (86.03)   
45 + **.TACGCG.** 51 (100.00)   
45 + **[CGCGCG]** 53 (85.98)   
45 + **[CGCGCG]** 55 (85.98)   
45 + **[CGCGCG]** 57 (85.98)   
45 + **[CGCGCG]** 59 (85.98)   
45 + **[CGCGCG]** 61 (85.98)   
45 + **[CGCGCG]** 63 (85.98)   
45 + **[CGCGCG]** 65 (85.98)   
45 + **[GCCGCG]** 397 (86.55)   
45 + **[AACGCG]** 537 (87.55)   
45 + **.CACGCG.** 555 (91.73)   
45 + **[TAGGCG]** 572 (85.06)   
45 + **[AACGCG]** 688 (87.55)   
45 + **[GGCGCG]** 929 (86.50)   
45 + **[GCCGCG]** 979 (86.55)   
45 + **.GACGCG.** 1008 (92.24)   
45 + **[CGCGCG]** 1010 (85.98)   
45 + **[CCCGCG]** 1026 (86.03)   
45 + **[AACGCG]** 1208 (87.55)   
45 + **[CCCGCG]** 1253 (86.03)   
45 + **[GGCGCG]** 1268 (86.50)   
45 + **[GGCGCG]** 1419 (86.50)   
45 - **[CCCGCG]** 1421 (86.03)   
45 - **.TCCGCG.** 1270 (94.31)   
45 - **.TGCGCG.** 1255 (94.26)   
45 - **.TCCGCG.** 1085 (94.31)   
45 - **[CCCGCG]** 1065 (86.03)   
45 - **[CCCGCG]** 1028 (86.03)   
45 - **[GCCGCG]** 1012 (86.55)   
45 - **[CGCGCG]** 1010 (85.98)   
45 - **[GCCGCG]** 981 (86.55)   
45 - **[CCCGCG]** 931 (86.03)   
45 - **[CCCGCG]** 557 (86.03)   
45 - **.TCCGCG.** 539 (94.31)   
45 - **[CCCGCG]** 399 (86.03)   
45 - **.CACGCG.** 67 (91.73)   
45 - **[CGCGCG]** 65 (85.98)   
45 - **[CGCGCG]** 63 (85.98)   
45 - **[CGCGCG]** 61 (85.98)   
45 - **[CGCGCG]** 59 (85.98)   
45 - **[CGCGCG]** 57 (85.98)   
45 - **[CGCGCG]** 55 (85.98)   
45 - **[CGCGCG]** 53 (85.98)   
47 + **[GGCGCG]** 350 (86.50)   
47 + **[GGCGCG]** 834 (86.50)   
47 + **.TCCGCG.** 944 (94.31)   
47 + **[GCCGCG]** 1271 (86.55)   
47 + **[CGCGCG]** 1273 (85.98)   
47 + **[GCCGCG]** 1371 (86.55)   
47 + **[CCCGCG]** 1406 (86.03)   
47 - **[CCCGCG]** 1408 (86.03)   
47 - **[GCCGCG]** 1367 (86.55)   
47 - **[GGCGCG]** 1275 (86.50)   
47 - **[CGCGCG]** 1273 (85.98)   
47 - **[GGCGCG]** 1249 (86.50)   
47 - **[GCCGCG]** 1136 (86.55)   
47 - **[CCCGCG]** 946 (86.03)   
47 - **.TGCGCG.** 352 (94.26)   
48 + **.CACGCG.** 56 (91.73)   
48 + **[CGCGCG]** 58 (85.98)   
48 + **[CCCGCG]** 431 (86.03)   
48 + **[CCCGCG]** 479 (86.03)   
48 + **[GCCGCG]** 764 (86.55)   
48 + **[CGCGCG]** 766 (85.98)   
48 + **.TCCGCG.** 834 (94.31)   
48 + **[GCCGCG]** 963 (86.55)   
48 + **.CACGCG.** 1004 (91.73)   
48 + **[CCCGCG]** 1099 (86.03)   
48 + **.TCCGCG.** 1186 (94.31)   
48 + **[GCCGCG]** 1284 (86.55)   
48 + **[CCCGCG]** 1315 (86.03)   
48 + **[GCCGCG]** 1362 (86.55)   
48 - **.TCCGCG.** 1378 (94.31)   
48 - **.GACGCG.** 1364 (92.24)   
48 - **[GCCGCG]** 1317 (86.55)   
48 - **[GCCGCG]** 1286 (86.55)   
48 - **.CACGCG.** 1188 (91.73)   
48 - **[GCCGCG]** 1019 (86.55)   
48 - **[CCCGCG]** 1006 (86.03)   
48 - **[CCCGCG]** 965 (86.03)   
48 - **[AACGCG]** 836 (87.55)   
48 - **[CCCGCG]** 768 (86.03)   
48 - **[CGCGCG]** 766 (85.98)   
48 - **[GCCGCG]** 433 (86.55)   
48 - **[GCCGCG]** 204 (86.55)   
48 - **.CACGCG.** 60 (91.73)   
48 - **[CGCGCG]** 58 (85.98)   
49 + **[CCCGCG]** 1118 (86.03)   
49 + **.TCCGCG.** 1208 (94.31)   
49 + **[GCCGCG]** 1407 (86.55)   
49 - **.TCCGCG.** 1409 (94.31)   
49 - **[GCCGCG]** 1120 (86.55)   
49 - **.TCCGCG.** 1054 (94.31)   
51 + **.TGCGCG.** 2 (94.26)   
51 + **[GCCGCG]** 68 (86.55)   
51 + **[CGCGCG]** 88 (85.98)   
51 + **[TTCGCG]** 145 (87.60)   
51 + **.GACGCG.** 231 (92.24)   
51 + **.TGCGCG.** 272 (94.26)   
51 + **[CGCGCG]** 274 (85.98)   
51 + **[GGCGCG]** 280 (86.50)   
51 + **[GGCGCG]** 286 (86.50)   
51 + **[CCCGCG]** 359 (86.03)   
51 + **.TACGCG.** 556 (100.00)   
51 + **[GCCGCG]** 598 (86.55)   
51 + **[CCCGCG]** 634 (86.03)   
51 + **[CCCGCG]** 1099 (86.03)   
51 + **[GGCGCG]** 1125 (86.50)   
51 + **[CCCGCG]** 1168 (86.03)   
51 + **[CCCGCG]** 1184 (86.03)   
51 + **[CCCGCG]** 1242 (86.03)   
51 + **.TCCGCG.** 1284 (94.31)   
51 + **[GGCGCG]** 1335 (86.50)   
51 + **[TTCGCG]** 1353 (87.60)   
51 + **[GGCGCG]** 1360 (86.50)   
51 - **[GCCGCG]** 1362 (86.55)   
51 - **[CCCGCG]** 1355 (86.03)   
51 - **[GCCGCG]** 1337 (86.55)   
51 - **[GCCGCG]** 1286 (86.55)   
51 - **[GGCGCG]** 1244 (86.50)   
51 - **[CCCGCG]** 1186 (86.03)   
51 - **[GCCGCG]** 1127 (86.55)   
51 - **[GGCGCG]** 998 (86.50)   
51 - **.TCCGCG.** 979 (94.31)   
51 - **.TGCGCG.** 902 (94.26)   
51 - **[GGCGCG]** 782 (86.50)   
51 - **[GGCGCG]** 636 (86.50)   
51 - **[GGCGCG]** 600 (86.50)   
51 - **.GACGCG.** 558 (92.24)   
51 - **[TAGGCG]** 306 (85.06)   
51 - **[CCCGCG]** 282 (86.03)   
51 - **[CCCGCG]** 276 (86.03)   
51 - **[CGCGCG]** 274 (85.98)   
51 - **.TGCGCG.** 233 (94.26)   
51 - **[GCCGCG]** 147 (86.55)   
51 - **[GGCGCG]** 90 (86.50)   
51 - **[CGCGCG]** 88 (85.98)   
51 - **.GACGCG.** 70 (92.24)   
51 - **[GCCGCG]** 36 (86.55)   
51 - **[CCCGCG]** 4 (86.03)   
53 + **.TGCGCG.** 994 (94.26)   
53 + **.GACGCG.** 1057 (92.24)   
53 - **[GCCGCG]** 1059 (86.55)   
53 - **[CCCGCG]** 996 (86.03)   
54 + **[TAGGCG]** 859 (85.06)   
54 + **.TCCGCG.** 959 (94.31)   
54 + **[CGCGCG]** 961 (85.98)   
54 + **.GACGCG.** 1158 (92.24)   
54 + **.CACGCG.** 1255 (91.73)   
54 + **[CCCGCG]** 1305 (86.03)   
54 - **.TGCGCG.** 963 (94.26)   
54 - **[CGCGCG]** 961 (85.98)   
55 + **[GCCGCG]** 199 (86.55)   
55 + **[CCCGCG]** 219 (86.03)   
55 + **[CGCGCG]** 221 (85.98)   
55 + **[CGCGCG]** 223 (85.98)   
55 + **[CCCGCG]** 559 (86.03)   
55 + **[CCCGCG]** 801 (86.03)   
55 + **[GCCGCG]** 866 (86.55)   
55 + **[GCCGCG]** 884 (86.55)   
55 + **[GGCGCG]** 956 (86.50)   
55 + **[CCCGCG]** 1080 (86.03)   
55 + **[GCCGCG]** 1085 (86.55)   
55 + **[GGCGCG]** 1322 (86.50)   
55 - **[GGCGCG]** 1276 (86.50)   
55 - **.TGCGCG.** 1087 (94.26)   
55 - **[GGCGCG]** 1082 (86.50)   
55 - **.TGCGCG.** 958 (94.26)   
55 - **[GCCGCG]** 943 (86.55)   
55 - **.CACGCG.** 868 (91.73)   
55 - **.TGCGCG.** 803 (94.26)   
55 - **[CGCGCG]** 223 (85.98)   
55 - **[CGCGCG]** 221 (85.98)   
55 - **[GGCGCG]** 201 (86.50)   
55 - **[GCCGCG]** 46 (86.55)   
58 + **.TGCGCG.** 190 (94.26)   
58 + **.CACGCG.** 418 (91.73)   
58 + **[GGCGCG]** 536 (86.50)   
58 + **.GACGCG.** 604 (92.24)   
58 + **[TAGGCG]** 699 (85.06)   
58 + **.GACGCG.** 751 (92.24)   
58 + **.TGCGCG.** 871 (94.26)   
58 + **[CGCGCG]** 897 (85.98)   
58 + **.CACGCG.** 960 (91.73)   
58 + **.TACGCG.** 1099 (100.00)   
58 + **.GACGCG.** 1280 (92.24)   
58 + **[GGCGCG]** 1305 (86.50)   
58 - **[GGCGCG]** 1459 (86.50)   
58 - **[CCCGCG]** 1307 (86.03)   
58 - **.TCCGCG.** 1282 (94.31)   
58 - **[CCCGCG]** 1251 (86.03)   
58 - **[GCCGCG]** 1101 (86.55)   
58 - **.TGCGCG.** 899 (94.26)   
58 - **[CGCGCG]** 897 (85.98)   
58 - **[CCCGCG]** 873 (86.03)   
58 - **[GCCGCG]** 781 (86.55)   
58 - **[TTCGCG]** 650 (87.60)   
58 - **.TGCGCG.** 606 (94.26)   
58 - **.TGCGCG.** 530 (94.26)   
58 - **[CCCGCG]** 420 (86.03)   
58 - **.CACGCG.** 192 (91.73)   
59 + **.CACGCG.** 102 (91.73)   
59 + **[GGCGCG]** 188 (86.50)   
59 + **[GCCGCG]** 650 (86.55)   
59 + **[GGCGCG]** 893 (86.50)   
59 + **[GCCGCG]** 957 (86.55)   
59 + **.TCCGCG.** 1010 (94.31)   
59 + **[GCCGCG]** 1071 (86.55)   
59 + **[CCCGCG]** 1167 (86.03)   
59 + **.TGCGCG.** 1284 (94.26)   
59 + **[GGCGCG]** 1352 (86.50)   
59 + **[GGCGCG]** 1367 (86.50)   
59 - **[GCCGCG]** 1369 (86.55)   
59 - **.TCCGCG.** 1354 (94.31)   
59 - **[GCCGCG]** 1349 (86.55)   
59 - **.GACGCG.** 1286 (92.24)   
59 - **[CCCGCG]** 1169 (86.03)   
59 - **[GCCGCG]** 1012 (86.55)   
59 - **.TCCGCG.** 959 (94.31)   
59 - **.TCCGCG.** 895 (94.31)   
59 - **[CCCGCG]** 708 (86.03)   
59 - **[GCCGCG]** 652 (86.55)   
59 - **[AACGCG]** 190 (87.55)   
60 + **[GCCGCG]** 825 (86.55)   
60 + **.TGCGCG.** 864 (94.26)   
60 + **.TGCGCG.** 880 (94.26)   
60 + **.TGCGCG.** 973 (94.26)   
60 - **[CCCGCG]** 1155 (86.03)   
60 - **[CCCGCG]** 975 (86.03)   
60 - **[CCCGCG]** 882 (86.03)   
60 - **[CCCGCG]** 866 (86.03)   
60 - **.TCCGCG.** 845 (94.31)   
60 - **[GCCGCG]** 827 (86.55)   
60 - **[CCCGCG]** 658 (86.03)   
61 + **[AACGCG]** 442 (87.55)   
61 + **[GGCGCG]** 501 (86.50)   
61 + **[CGCGCG]** 503 (85.98)   
61 + **[AACGCG]** 533 (87.55)   
61 + **.GACGCG.** 840 (92.24)   
61 + **[GCCGCG]** 914 (86.55)   
61 + **[GCCGCG]** 925 (86.55)   
61 + **[GCCGCG]** 1037 (86.55)   
61 + **[CCCGCG]** 1062 (86.03)   
61 + **.GACGCG.** 1320 (92.24)   
61 + **[CCCGCG]** 1380 (86.03)   
61 - **.CACGCG.** 1322 (91.73)   
61 - **[TTCGCG]** 1230 (87.60)   
61 - **[TTCGCG]** 1099 (87.60)   
61 - **[AACGCG]** 1064 (87.55)   
61 - **[GCCGCG]** 1039 (86.55)   
61 - **[CCCGCG]** 927 (86.03)   
61 - **[AACGCG]** 916 (87.55)   
61 - **[GCCGCG]** 842 (86.55)   
61 - **[CCCGCG]** 535 (86.03)   
61 - **[CGCGCG]** 503 (85.98)   
61 - **.TGCGCG.** 444 (94.26)   
63 + **[CGCGCG]** 18 (85.98)   
63 + **[CCCGCG]** 129 (86.03)   
63 + **[CCCGCG]** 167 (86.03)   
63 + **[GCCGCG]** 182 (86.55)   
63 + **[GGCGCG]** 266 (86.50)   
63 + **[GCCGCG]** 346 (86.55)   
63 + **[CCCGCG]** 383 (86.03)   
63 + **[CCCGCG]** 443 (86.03)   
63 + **.TCCGCG.** 452 (94.31)   
63 + **[CCCGCG]** 519 (86.03)   
63 + **[GCCGCG]** 534 (86.55)   
63 + **[GGCGCG]** 656 (86.50)   
63 + **[CGCGCG]** 905 (85.98)   
63 + **[CCCGCG]** 952 (86.03)   
63 + **[GCCGCG]** 1123 (86.55)   
63 + **[GCCGCG]** 1253 (86.55)   
63 + **[GGCGCG]** 1391 (86.50)   
63 + **[GCCGCG]** 1412 (86.55)   
63 + **.GACGCG.** 1430 (92.24)   
63 - **[GCCGCG]** 1393 (86.55)   
63 - **.TCCGCG.** 1255 (94.31)   
63 - **[CCCGCG]** 1191 (86.03)   
63 - **[AACGCG]** 976 (87.55)   
63 - **[GCCGCG]** 954 (86.55)   
63 - **[GGCGCG]** 907 (86.50)   
63 - **[CGCGCG]** 905 (85.98)   
63 - **.GACGCG.** 854 (92.24)   
63 - **[GGCGCG]** 658 (86.50)   
63 - **[CCCGCG]** 536 (86.03)   
63 - **.GACGCG.** 521 (92.24)   
63 - **[GGCGCG]** 454 (86.50)   
63 - **[GCCGCG]** 445 (86.55)   
63 - **[GCCGCG]** 410 (86.55)   
63 - **.TCCGCG.** 385 (94.31)   
63 - **.TGCGCG.** 348 (94.26)   
63 - **[CCCGCG]** 330 (86.03)   
63 - **.TACGCG.** 245 (100.00)   
63 - **[CCCGCG]** 184 (86.03)   
63 - **[AACGCG]** 169 (87.55)   
63 - **[GGCGCG]** 131 (86.50)   
63 - **.TGCGCG.** 20 (94.26)   
63 - **[CGCGCG]** 18 (85.98)   
65 + **[CCCGCG]** 229 (86.03)   
65 + **.TCCGCG.** 545 (94.31)   
65 + **[TTCGCG]** 749 (87.60)   
65 + **[CGCGCG]** 751 (85.98)   
65 + **[GCCGCG]** 784 (86.55)   
65 + **[CGCGCG]** 786 (85.98)   
65 + **[GCCGCG]** 829 (86.55)   
65 + **.TGCGCG.** 844 (94.26)   
65 + **[GGCGCG]** 864 (86.50)   
65 + **[CCCGCG]** 910 (86.03)   
65 + **[CCCGCG]** 946 (86.03)   
65 + **[GGCGCG]** 979 (86.50)   
65 + **[GCCGCG]** 1001 (86.55)   
65 + **[GCCGCG]** 1028 (86.55)   
65 + **[GGCGCG]** 1205 (86.50)   
65 + **[CGCGCG]** 1207 (85.98)   
65 + **.GACGCG.** 1224 (92.24)   
65 + **.TCCGCG.** 1259 (94.31)   
65 + **[CGCGCG]** 1261 (85.98)   
65 + **[CCCGCG]** 1308 (86.03)   
65 - **.TCCGCG.** 1397 (94.31)   
65 - **[CCCGCG]** 1310 (86.03)   
65 - **[CCCGCG]** 1263 (86.03)   
65 - **[CGCGCG]** 1261 (85.98)   
65 - **[CCCGCG]** 1232 (86.03)   
65 - **[CGCGCG]** 1207 (85.98)   
65 - **[GGCGCG]** 1030 (86.50)   
65 - **[GGCGCG]** 981 (86.50)   
65 - **[GCCGCG]** 948 (86.55)   
65 - **[GGCGCG]** 912 (86.50)   
65 - **.GACGCG.** 866 (92.24)   
65 - **[CCCGCG]** 846 (86.03)   
65 - **.TGCGCG.** 831 (94.26)   
65 - **[CCCGCG]** 788 (86.03)   
65 - **[CGCGCG]** 786 (85.98)   
65 - **[CGCGCG]** 751 (85.98)   
65 - **[GGCGCG]** 589 (86.50)   
65 - **.GACGCG.** 547 (92.24)   
65 - **[TAGGCG]** 387 (85.06)   
65 - **.TGCGCG.** 231 (94.26)   
66 + **[AACGCG]** 201 (87.55)   
66 + **[GCCGCG]** 768 (86.55)   
66 + **.GACGCG.** 786 (92.24)   
66 + **.TGCGCG.** 827 (94.26)   
66 + **[CGCGCG]** 867 (85.98)   
66 + **[CCCGCG]** 941 (86.03)   
66 + **[CCCGCG]** 953 (86.03)   
66 + **[GCCGCG]** 989 (86.55)   
66 + **[CCCGCG]** 1125 (86.03)   
66 + **[GGCGCG]** 1179 (86.50)   
66 + **[GGCGCG]** 1438 (86.50)   
66 + **.GACGCG.** 1480 (92.24)   
66 - **[CCCGCG]** 1440 (86.03)   
66 - **.TGCGCG.** 1420 (94.26)   
66 - **.GACGCG.** 1406 (92.24)   
66 - **[CCCGCG]** 1338 (86.03)   
66 - **.TCCGCG.** 1181 (94.31)   
66 - **[GCCGCG]** 1127 (86.55)   
66 - **[CCCGCG]** 991 (86.03)   
66 - **[GGCGCG]** 955 (86.50)   
66 - **[GGCGCG]** 943 (86.50)   
66 - **[GGCGCG]** 869 (86.50)   
66 - **[CGCGCG]** 867 (85.98)   
66 - **[CCCGCG]** 829 (86.03)   
66 - **[CCCGCG]** 788 (86.03)   
66 - **[CCCGCG]** 770 (86.03)   
66 - **.TGCGCG.** 203 (94.26)   
67 + **.TGCGCG.** 1073 (94.26)   
67 + **.GACGCG.** 1173 (92.24)   
67 + **[GCCGCG]** 1196 (86.55)   
67 - **.GACGCG.** 1198 (92.24)   
67 - **[GGCGCG]** 1175 (86.50)   
67 - **[CCCGCG]** 1075 (86.03)   
68 + **.CACGCG.** 33 (91.73)   
68 + **.TGCGCG.** 216 (94.26)   
68 + **[GCCGCG]** 589 (86.55)   
68 + **[GGCGCG]** 652 (86.50)   
68 + **.CACGCG.** 719 (91.73)   
68 + **[CGCGCG]** 869 (85.98)   
68 + **.GACGCG.** 990 (92.24)   
68 + **[CGCGCG]** 1150 (85.98)   
68 + **[CGCGCG]** 1269 (85.98)   
68 + **[CGCGCG]** 1271 (85.98)   
68 + **.TCCGCG.** 1334 (94.31)   
68 + **[CGCGCG]** 1392 (85.98)   
68 - **[GCCGCG]** 1394 (86.55)   
68 - **[CGCGCG]** 1392 (85.98)   
68 - **[CCCGCG]** 1336 (86.03)   
68 - **[CCCGCG]** 1273 (86.03)   
68 - **[CGCGCG]** 1271 (85.98)   
68 - **[CGCGCG]** 1269 (85.98)   
68 - **.TCCGCG.** 1152 (94.31)   
68 - **[CGCGCG]** 1150 (85.98)   
68 - **[CCCGCG]** 1055 (86.03)   
68 - **[GCCGCG]** 992 (86.55)   
68 - **[CGCGCG]** 869 (85.98)   
68 - **[GCCGCG]** 812 (86.55)   
68 - **[TAGGCG]** 777 (85.06)   
68 - **[CCCGCG]** 721 (86.03)   
68 - **.TCCGCG.** 654 (94.31)   
68 - **[GCCGCG]** 649 (86.55)   
68 - **.TGCGCG.** 591 (94.26)   
68 - **.GACGCG.** 419 (92.24)   
68 - **[GCCGCG]** 245 (86.55)   
68 - **.TGCGCG.** 218 (94.26)   
68 - **[GGCGCG]** 35 (86.50)   
69 + **.TGCGCG.** 156 (94.26)   
69 + **[CGCGCG]** 163 (85.98)   
69 + **.TCCGCG.** 688 (94.31)   
69 + **.TCCGCG.** 694 (94.31)   
69 + **.TCCGCG.** 767 (94.31)   
69 + **[GCCGCG]** 773 (86.55)   
69 + **[GGCGCG]** 892 (86.50)   
69 + **[CCCGCG]** 996 (86.03)   
69 + **[GCCGCG]** 1032 (86.55)   
69 + **[CCCGCG]** 1119 (86.03)   
69 + **.TCCGCG.** 1136 (94.31)   
69 + **[CCCGCG]** 1201 (86.03)   
69 - **[GGCGCG]** 1203 (86.50)   
69 - **[GGCGCG]** 1138 (86.50)   
69 - **[GGCGCG]** 1121 (86.50)   
69 - **.TCCGCG.** 1034 (94.31)   
69 - **[GGCGCG]** 998 (86.50)   
69 - **.GACGCG.** 894 (92.24)   
69 - **.CACGCG.** 775 (91.73)   
69 - **[GCCGCG]** 769 (86.55)   
69 - **[TAGGCG]** 751 (85.06)   
69 - **.CACGCG.** 696 (91.73)   
69 - **.GACGCG.** 690 (92.24)   
69 - **.CACGCG.** 165 (91.73)   
69 - **[CGCGCG]** 163 (85.98)   
70 + **.CACGCG.** 840 (91.73)   
70 + **[AACGCG]** 1062 (87.55)   
70 + **.GACGCG.** 1103 (92.24)   
70 + **[CGCGCG]** 1150 (85.98)   
70 + **[GGCGCG]** 1161 (86.50)   
70 + **.TGCGCG.** 1332 (94.26)   
70 + **[CCCGCG]** 1344 (86.03)   
70 - **.GACGCG.** 1450 (92.24)   
70 - **[TAGGCG]** 1410 (85.06)   
70 - **.GACGCG.** 1390 (92.24)   
70 - **[CCCGCG]** 1346 (86.03)   
70 - **[GGCGCG]** 1334 (86.50)   
70 - **[CGCGCG]** 1150 (85.98)   
70 - **.TGCGCG.** 1105 (94.26)   
70 - **[GCCGCG]** 1064 (86.55)   
70 - **.TCCGCG.** 842 (94.31)   
70 - **.TCCGCG.** 543 (94.31)   
71 + **[CCCGCG]** 900 (86.03)   
71 + **[CGCGCG]** 902 (85.98)   
71 + **[CCCGCG]** 925 (86.03)   
71 + **[GCCGCG]** 1001 (86.55)   
71 + **[CCCGCG]** 1090 (86.03)   
71 + **[CCCGCG]** 1161 (86.03)   
71 + **[CGCGCG]** 1163 (85.98)   
71 + **.GACGCG.** 1237 (92.24)   
71 + **.CACGCG.** 1273 (91.73)   
71 + **.TCCGCG.** 1469 (94.31)   
71 + **[CGCGCG]** 1471 (85.98)   
71 + **[CGCGCG]** 1496 (85.98)   
71 - **[CCCGCG]** 1498 (86.03)   
71 - **[CGCGCG]** 1496 (85.98)   
71 - **[CGCGCG]** 1471 (85.98)   
71 - **[GGCGCG]** 1184 (86.50)   
71 - **[CCCGCG]** 1165 (86.03)   
71 - **[CGCGCG]** 1163 (85.98)   
71 - **[CCCGCG]** 1092 (86.03)   
71 - **[CCCGCG]** 1003 (86.03)   
71 - **.GACGCG.** 904 (92.24)   
71 - **[CGCGCG]** 902 (85.98)   
71 - **[GGCGCG]** 870 (86.50)   
73 + **.TCCGCG.** 885 (94.31)   
73 + **[GCCGCG]** 919 (86.55)   
73 + **[CCCGCG]** 934 (86.03)   
73 + **[GGCGCG]** 1021 (86.50)   
73 - **[GCCGCG]** 1087 (86.55)   
73 - **.TGCGCG.** 1067 (94.26)   
73 - **.TCCGCG.** 973 (94.31)   
73 - **[GCCGCG]** 921 (86.55)   
73 - **[TAGGCG]** 622 (85.06)   
74 + **.TGCGCG.** 932 (94.26)   
74 + **.TGCGCG.** 951 (94.26)   
74 + **.TCCGCG.** 1061 (94.31)   
74 + **[CCCGCG]** 1148 (86.03)   
74 + **[TAGGCG]** 1157 (85.06)   
74 + **.TCCGCG.** 1253 (94.31)   
74 + **[CCCGCG]** 1334 (86.03)   
74 + **[CGCGCG]** 1336 (85.98)   
74 + **[GCCGCG]** 1436 (86.55)   
74 - **[GGCGCG]** 1438 (86.50)   
74 - **[GCCGCG]** 1429 (86.55)   
74 - **.GACGCG.** 1338 (92.24)   
74 - **[CGCGCG]** 1336 (85.98)   
74 - **[GCCGCG]** 1255 (86.55)   
74 - **[CCCGCG]** 953 (86.03)   
74 - **[GCCGCG]** 934 (86.55)   
76 + **[GCCGCG]** 127 (86.55)   
76 + **[GGCGCG]** 999 (86.50)   
76 + **[GCCGCG]** 1056 (86.55)   
76 + **[CCCGCG]** 1112 (86.03)   
76 + **[CGCGCG]** 1114 (85.98)   
76 + **.GACGCG.** 1284 (92.24)   
76 - **[CCCGCG]** 1286 (86.03)   
76 - **[CCCGCG]** 1255 (86.03)   
76 - **[GGCGCG]** 1116 (86.50)   
76 - **[CGCGCG]** 1114 (85.98)   
76 - **[GCCGCG]** 1064 (86.55)   
76 - **[CCCGCG]** 1001 (86.03)   
76 - **[GCCGCG]** 866 (86.55)   
76 - **[GGCGCG]** 829 (86.50)   
76 - **.TCCGCG.** 129 (94.31)   
77 + **[GGCGCG]** 248 (86.50)   
77 + **[CCCGCG]** 598 (86.03)   
77 + **[GCCGCG]** 716 (86.55)   
77 + **.CACGCG.** 779 (91.73)   
77 + **.TCCGCG.** 848 (94.31)   
77 + **[GGCGCG]** 931 (86.50)   
77 + **[TTCGCG]** 1115 (87.60)   
77 + **.TCCGCG.** 1314 (94.31)   
77 - **[GCCGCG]** 1117 (86.55)   
77 - **[GCCGCG]** 1054 (86.55)   
77 - **[TAGGCG]** 956 (85.06)   
77 - **.CACGCG.** 933 (91.73)   
77 - **.CACGCG.** 850 (91.73)   
77 - **[GCCGCG]** 781 (86.55)   
77 - **[GGCGCG]** 600 (86.50)   
77 - **.CACGCG.** 250 (91.73)   
79 + **[GCCGCG]** 654 (86.55)   
79 + **[CCCGCG]** 723 (86.03)   
79 + **[GCCGCG]** 878 (86.55)   
79 + **[GCCGCG]** 948 (86.55)   
79 + **[CCCGCG]** 1200 (86.03)   
79 + **[GCCGCG]** 1277 (86.55)   
79 + **[TTCGCG]** 1323 (87.60)   
79 + **[GCCGCG]** 1397 (86.55)   
79 + **[CCCGCG]** 1430 (86.03)   
79 - **.TGCGCG.** 1432 (94.26)   
79 - **[CCCGCG]** 1399 (86.03)   
79 - **.GACGCG.** 1325 (92.24)   
79 - **[GGCGCG]** 1202 (86.50)   
79 - **[CCCGCG]** 1090 (86.03)   
79 - **.TGCGCG.** 950 (94.26)   
79 - **.CACGCG.** 880 (91.73)   
79 - **[CCCGCG]** 834 (86.03)   
80 + **.TCCGCG.** 897 (94.31)   
80 + **[GGCGCG]** 955 (86.50)   
80 + **[GCCGCG]** 1016 (86.55)   
80 + **[GGCGCG]** 1044 (86.50)   
80 + **[GCCGCG]** 1207 (86.55)   
80 + **[GGCGCG]** 1308 (86.50)   
80 + **[AACGCG]** 1339 (87.55)   
80 + **[CGCGCG]** 1341 (85.98)   
80 - **[GCCGCG]** 1343 (86.55)   
80 - **[CGCGCG]** 1341 (85.98)   
80 - **[CCCGCG]** 1297 (86.03)   
80 - **[GCCGCG]** 1209 (86.55)   
80 - **[GGCGCG]** 1018 (86.50)   
80 - **.CACGCG.** 957 (91.73)   
80 - **.TACGCG.** 899 (100.00)   
80 - **.TACGCG.** 398 (100.00)   
81 + **.TACGCG.** 683 (100.00)   
81 + **[CCCGCG]** 767 (86.03)   
81 - **[CCCGCG]** 1319 (86.03)   
81 - **[TTCGCG]** 769 (87.60)   
84 + **[CCCGCG]** 869 (86.03)   
84 + **.GACGCG.** 900 (92.24)   
84 + **[GCCGCG]** 980 (86.55)   
84 + **.GACGCG.** 1035 (92.24)   
84 + **[GGCGCG]** 1153 (86.50)   
84 + **[CCCGCG]** 1162 (86.03)   
84 + **[CCCGCG]** 1301 (86.03)   
84 - **.CACGCG.** 1164 (91.73)   
84 - **.TCCGCG.** 1155 (94.31)   
84 - **[GCCGCG]** 1037 (86.55)   
84 - **[GCCGCG]** 1023 (86.55)   
84 - **.TGCGCG.** 1008 (94.26)   
84 - **[CCCGCG]** 982 (86.03)   
84 - **[GGCGCG]** 902 (86.50)   
84 - **[GCCGCG]** 871 (86.55)   
84 - **[GGCGCG]** 864 (86.50)   
84 - **[GCCGCG]** 798 (86.55)   
85 + **[GCCGCG]** 93 (86.55)   
85 + **[CCCGCG]** 438 (86.03)   
85 + **[CGCGCG]** 440 (85.98)   
85 + **[GGCGCG]** 587 (86.50)   
85 + **[CCCGCG]** 878 (86.03)   
85 + **.GACGCG.** 928 (92.24)   
85 + **.GACGCG.** 961 (92.24)   
85 + **[CCCGCG]** 1057 (86.03)   
85 + **[CGCGCG]** 1094 (85.98)   
85 + **.TCCGCG.** 1378 (94.31)   
85 + **[CGCGCG]** 1380 (85.98)   
85 + **[CGCGCG]** 1382 (85.98)   
85 + **[CGCGCG]** 1384 (85.98)   
85 + **[GCCGCG]** 1415 (86.55)   
85 + **[CCCGCG]** 1435 (86.03)   
85 + **[CCCGCG]** 1448 (86.03)   
85 - **[GGCGCG]** 1450 (86.50)   
85 - **[CCCGCG]** 1386 (86.03)   
85 - **[CGCGCG]** 1384 (85.98)   
85 - **[CGCGCG]** 1382 (85.98)   
85 - **[CGCGCG]** 1380 (85.98)   
85 - **[GCCGCG]** 1217 (86.55)   
85 - **[GGCGCG]** 1096 (86.50)   
85 - **[CGCGCG]** 1094 (85.98)   
85 - **[GGCGCG]** 1078 (86.50)   
85 - **[GGCGCG]** 1059 (86.50)   
85 - **[GGCGCG]** 1037 (86.50)   
85 - **[GGCGCG]** 963 (86.50)   
85 - **[CCCGCG]** 930 (86.03)   
85 - **[GCCGCG]** 880 (86.55)   
85 - **.GACGCG.** 589 (92.24)   
85 - **.TCCGCG.** 442 (94.31)   
85 - **[CGCGCG]** 440 (85.98)   
85 - **.GACGCG.** 95 (92.24)   
85 - **[TTCGCG]** 46 (87.60)   
86 + **.TCCGCG.** 136 (94.31)   
86 + **[GCCGCG]** 305 (86.55)   
86 + **[GCCGCG]** 541 (86.55)   
86 + **[CCCGCG]** 656 (86.03)   
86 + **[GCCGCG]** 738 (86.55)   
86 + **[CGCGCG]** 811 (85.98)   
86 + **[GGCGCG]** 834 (86.50)   
86 + **[GGCGCG]** 879 (86.50)   
86 + **[CGCGCG]** 881 (85.98)   
86 + **[GGCGCG]** 900 (86.50)   
86 + **.TGCGCG.** 1030 (94.26)   
86 + **[CGCGCG]** 1032 (85.98)   
86 + **[CGCGCG]** 1034 (85.98)   
86 + **.GACGCG.** 1086 (92.24)   
86 + **[CGCGCG]** 1088 (85.98)   
86 + **[CGCGCG]** 1090 (85.98)   
86 + **[GGCGCG]** 1193 (86.50)   
86 + **[CCCGCG]** 1253 (86.03)   
86 + **[CCCGCG]** 1339 (86.03)   
86 + **[GGCGCG]** 1384 (86.50)   
86 + **[GCCGCG]** 1437 (86.55)   
86 + **[CCCGCG]** 1498 (86.03)   
86 + **[CGCGCG]** 1500 (85.98)   
86 + **[CGCGCG]** 1502 (85.98)   
86 + **[CGCGCG]** 1504 (85.98)   
86 + **[GCCGCG]** 1510 (86.55)   
86 - **[GCCGCG]** 1506 (86.55)   
86 - **[CGCGCG]** 1504 (85.98)   
86 - **[CGCGCG]** 1502 (85.98)   
86 - **[CGCGCG]** 1500 (85.98)   
86 - **[CCCGCG]** 1439 (86.03)   
86 - **.TCCGCG.** 1425 (94.31)   
86 - **[CCCGCG]** 1386 (86.03)   
86 - **[GGCGCG]** 1341 (86.50)   
86 - **.TCCGCG.** 1255 (94.31)   
86 - **[GCCGCG]** 1195 (86.55)   
86 - **[GCCGCG]** 1190 (86.55)   
86 - **.TCCGCG.** 1092 (94.31)   
86 - **[CGCGCG]** 1090 (85.98)   
86 - **[CGCGCG]** 1088 (85.98)   
86 - **.TCCGCG.** 1036 (94.31)   
86 - **[CGCGCG]** 1034 (85.98)   
86 - **[CGCGCG]** 1032 (85.98)   
86 - **[GCCGCG]** 902 (86.55)   
86 - **[CCCGCG]** 883 (86.03)   
86 - **[CGCGCG]** 881 (85.98)   
86 - **[GCCGCG]** 836 (86.55)   
86 - **[CCCGCG]** 813 (86.03)   
86 - **[CGCGCG]** 811 (85.98)   
86 - **.TCCGCG.** 771 (94.31)   
86 - **[CCCGCG]** 740 (86.03)   
86 - **[GCCGCG]** 658 (86.55)   
86 - **[GCCGCG]** 543 (86.55)   
86 - **[TTCGCG]** 307 (87.60)   
86 - **.TCCGCG.** 138 (94.31)   
87 + **.GACGCG.** 133 (92.24)   
87 + **.CACGCG.** 266 (91.73)   
87 + **[GGCGCG]** 313 (86.50)   
87 + **.GACGCG.** 586 (92.24)   
87 + **[GGCGCG]** 639 (86.50)   
87 + **[CCCGCG]** 720 (86.03)   
87 + **[GCCGCG]** 768 (86.55)   
87 + **[GGCGCG]** 843 (86.50)   
87 + **[CCCGCG]** 872 (86.03)   
87 + **[CCCGCG]** 1054 (86.03)   
87 + **[GCCGCG]** 1097 (86.55)   
87 + **[CCCGCG]** 1128 (86.03)   
87 + **[CCCGCG]** 1150 (86.03)   
87 + **[CCCGCG]** 1188 (86.03)   
87 + **[CGCGCG]** 1190 (85.98)   
87 + **[CGCGCG]** 1192 (85.98)   
87 + **[GGCGCG]** 1242 (86.50)   
87 + **.CACGCG.** 1320 (91.73)   
87 + **[CCCGCG]** 1365 (86.03)   
87 + **[GCCGCG]** 1434 (86.55)   
87 + **[CGCGCG]** 1436 (85.98)   
87 + **[CGCGCG]** 1438 (85.98)   
87 + **[CGCGCG]** 1440 (85.98)   
87 + **[CGCGCG]** 1442 (85.98)   
87 + **[CGCGCG]** 1444 (85.98)   
87 + **[GCCGCG]** 1486 (86.55)   
87 + **[CGCGCG]** 1488 (85.98)   
87 + **[GGCGCG]** 1501 (86.50)   
87 - **[CCCGCG]** 1503 (86.03)   
87 - **[CCCGCG]** 1490 (86.03)   
87 - **[CGCGCG]** 1488 (85.98)   
87 - **[CCCGCG]** 1459 (86.03)   
87 - **[AACGCG]** 1446 (87.55)   
87 - **[CGCGCG]** 1444 (85.98)   
87 - **[CGCGCG]** 1442 (85.98)   
87 - **[CGCGCG]** 1440 (85.98)   
87 - **[CGCGCG]** 1438 (85.98)   
87 - **[CGCGCG]** 1436 (85.98)   
87 - **[GGCGCG]** 1378 (86.50)   
87 - **[GCCGCG]** 1367 (86.55)   
87 - **[GGCGCG]** 1346 (86.50)   
87 - **[GCCGCG]** 1333 (86.55)   
87 - **[GGCGCG]** 1322 (86.50)   
87 - **[GCCGCG]** 1244 (86.55)   
87 - **[GCCGCG]** 1231 (86.55)   
87 - **[GGCGCG]** 1194 (86.50)   
87 - **[CGCGCG]** 1192 (85.98)   
87 - **[CGCGCG]** 1190 (85.98)   
87 - **.GACGCG.** 1099 (92.24)   
87 - **[GGCGCG]** 874 (86.50)   
87 - **.TCCGCG.** 845 (94.31)   
87 - **[CCCGCG]** 788 (86.03)   
87 - **[CCCGCG]** 770 (86.03)   
87 - **[GCCGCG]** 722 (86.55)   
87 - **[GCCGCG]** 310 (86.55)   
87 - **.TGCGCG.** 268 (94.26)   
87 - **.TCCGCG.** 176 (94.31)   
87 - **.GACGCG.** 135 (92.24)   
88 + **.TGCGCG.** 171 (94.26)   
88 + **[CCCGCG]** 369 (86.03)   
88 + **[GGCGCG]** 419 (86.50)   
88 + **[GGCGCG]** 445 (86.50)   
88 + **[CGCGCG]** 447 (85.98)   
88 + **.TGCGCG.** 470 (94.26)   
88 + **[GCCGCG]** 504 (86.55)   
88 + **[CGCGCG]** 506 (85.98)   
88 + **[CCCGCG]** 581 (86.03)   
88 + **[GCCGCG]** 607 (86.55)   
88 + **[CGCGCG]** 609 (85.98)   
88 + **[GGCGCG]** 705 (86.50)   
88 + **[GGCGCG]** 755 (86.50)   
88 + **[CCCGCG]** 1046 (86.03)   
88 + **[CCCGCG]** 1075 (86.03)   
88 + **[CGCGCG]** 1077 (85.98)   
88 + **[CGCGCG]** 1079 (85.98)   
88 + **[GCCGCG]** 1097 (86.55)   
88 - **[GCCGCG]** 1395 (86.55)   
88 - **[GCCGCG]** 1380 (86.55)   
88 - **.GACGCG.** 1206 (92.24)   
88 - **[GCCGCG]** 1099 (86.55)   
88 - **[CGCGCG]** 1079 (85.98)   
88 - **[CGCGCG]** 1077 (85.98)   
88 - **[GCCGCG]** 849 (86.55)   
88 - **[CCCGCG]** 757 (86.03)   
88 - **.TCCGCG.** 716 (94.31)   
88 - **[GCCGCG]** 707 (86.55)   
88 - **.TCCGCG.** 691 (94.31)   
88 - **.TCCGCG.** 669 (94.31)   
88 - **.TACGCG.** 611 (100.00)   
88 - **[CGCGCG]** 609 (85.98)   
88 - **.TCCGCG.** 583 (94.31)   
88 - **[CGCGCG]** 506 (85.98)   
88 - **[CCCGCG]** 490 (86.03)   
88 - **[CCCGCG]** 449 (86.03)   
88 - **[CGCGCG]** 447 (85.98)   
88 - **.TCCGCG.** 421 (94.31)   
89 + **.CACGCG.** 38 (91.73)   
89 + **.TCCGCG.** 45 (94.31)   
89 + **[CCCGCG]** 98 (86.03)   
89 + **[CCCGCG]** 202 (86.03)   
89 + **[GCCGCG]** 260 (86.55)   
89 + **[GCCGCG]** 269 (86.55)   
89 + **.TGCGCG.** 331 (94.26)   
89 + **[CGCGCG]** 333 (85.98)   
89 + **[CCCGCG]** 362 (86.03)   
89 + **[GCCGCG]** 418 (86.55)   
89 + **[GCCGCG]** 435 (86.55)   
89 + **[GCCGCG]** 478 (86.55)   
89 + **[GCCGCG]** 493 (86.55)   
89 + **.GACGCG.** 600 (92.24)   
89 + **[GCCGCG]** 664 (86.55)   
89 + **[CCCGCG]** 673 (86.03)   
89 + **[GGCGCG]** 721 (86.50)   
89 + **[GGCGCG]** 741 (86.50)   
89 + **.TCCGCG.** 756 (94.31)   
89 + **[GGCGCG]** 787 (86.50)   
89 + **.TCCGCG.** 841 (94.31)   
89 + **[CCCGCG]** 860 (86.03)   
89 + **[CGCGCG]** 862 (85.98)   
89 + **[CGCGCG]** 864 (85.98)   
89 + **[GGCGCG]** 937 (86.50)   
89 + **.CACGCG.** 964 (91.73)   
89 + **[CCCGCG]** 974 (86.03)   
89 + **[GCCGCG]** 1021 (86.55)   
89 + **[CCCGCG]** 1033 (86.03)   
89 + **[GCCGCG]** 1126 (86.55)   
89 + **[GCCGCG]** 1310 (86.55)   
89 + **.TGCGCG.** 1321 (94.26)   
89 + **[CGCGCG]** 1323 (85.98)   
89 + **[GCCGCG]** 1451 (86.55)   
89 + **.GACGCG.** 1465 (92.24)   
89 - **[GGCGCG]** 1325 (86.50)   
89 - **[CGCGCG]** 1323 (85.98)   
89 - **[CCCGCG]** 1312 (86.03)   
89 - **.TGCGCG.** 1128 (94.26)   
89 - **.TGCGCG.** 1035 (94.26)   
89 - **[GGCGCG]** 1023 (86.50)   
89 - **[GGCGCG]** 976 (86.50)   
89 - **.TCCGCG.** 966 (94.31)   
89 - **.TCCGCG.** 939 (94.31)   
89 - **.TGCGCG.** 866 (94.26)   
89 - **[CGCGCG]** 864 (85.98)   
89 - **[CGCGCG]** 862 (85.98)   
89 - **[GCCGCG]** 843 (86.55)   
89 - **[GGCGCG]** 789 (86.50)   
89 - **[GCCGCG]** 743 (86.55)   
89 - **[CCCGCG]** 723 (86.03)   
89 - **[GGCGCG]** 675 (86.50)   
89 - **[GGCGCG]** 666 (86.50)   
89 - **[GGCGCG]** 495 (86.50)   
89 - **.GACGCG.** 480 (92.24)   
89 - **[GGCGCG]** 437 (86.50)   
89 - **[CCCGCG]** 420 (86.03)   
89 - **[GGCGCG]** 364 (86.50)   
89 - **[GGCGCG]** 335 (86.50)   
89 - **[CGCGCG]** 333 (85.98)   
89 - **[CCCGCG]** 262 (86.03)   
89 - **[GGCGCG]** 153 (86.50)   
89 - **[GGCGCG]** 100 (86.50)   
89 - **[GCCGCG]** 47 (86.55)   
90 + **[AACGCG]** 6 (87.55)   
90 + **.TGCGCG.** 328 (94.26)   
90 + **[GGCGCG]** 360 (86.50)   
90 + **[TTCGCG]** 478 (87.60)   
90 + **[GGCGCG]** 510 (86.50)   
90 + **[CGCGCG]** 512 (85.98)   
90 + **[GGCGCG]** 557 (86.50)   
90 + **[CGCGCG]** 559 (85.98)   
90 + **[GCCGCG]** 587 (86.55)   
90 + **.CACGCG.** 713 (91.73)   
90 + **[GCCGCG]** 718 (86.55)   
90 + **[GCCGCG]** 775 (86.55)   
90 + **[GCCGCG]** 848 (86.55)   
90 + **.TGCGCG.** 943 (94.26)   
90 + **[CGCGCG]** 945 (85.98)   
90 + **[CGCGCG]** 947 (85.98)   
90 + **[GCCGCG]** 1019 (86.55)   
90 + **.TGCGCG.** 1328 (94.26)   
90 - **[GCCGCG]** 1330 (86.55)   
90 - **.GACGCG.** 1314 (92.24)   
90 - **[GCCGCG]** 993 (86.55)   
90 - **[CGCGCG]** 947 (85.98)   
90 - **[CGCGCG]** 945 (85.98)   
90 - **[GCCGCG]** 850 (86.55)   
90 - **[GCCGCG]** 777 (86.55)   
90 - **[GCCGCG]** 720 (86.55)   
90 - **[GGCGCG]** 715 (86.50)   
90 - **[GGCGCG]** 654 (86.50)   
90 - **.TCCGCG.** 589 (94.31)   
90 - **[CGCGCG]** 559 (85.98)   
90 - **[GGCGCG]** 514 (86.50)   
90 - **[CGCGCG]** 512 (85.98)   
90 - **[GGCGCG]** 480 (86.50)   
90 - **.GACGCG.** 362 (92.24)   
90 - **.TCCGCG.** 8 (94.31)   
92 + **[GCCGCG]** 1056 (86.55)   
92 + **.TCCGCG.** 1273 (94.31)   
92 - **[CCCGCG]** 1275 (86.03)   
92 - **[GCCGCG]** 1228 (86.55)   
93 + **.TGCGCG.** 449 (94.26)   
93 + **[CGCGCG]** 451 (85.98)   
93 + **[CGCGCG]** 453 (85.98)   
93 + **.TCCGCG.** 480 (94.31)   
93 + **[CCCGCG]** 804 (86.03)   
93 + **[AACGCG]** 867 (87.55)   
93 + **.GACGCG.** 963 (92.24)   
93 + **[CGCGCG]** 965 (85.98)   
93 + **[GGCGCG]** 975 (86.50)   
93 + **.GACGCG.** 1001 (92.24)   
93 + **.TCCGCG.** 1035 (94.31)   
93 + **.CACGCG.** 1192 (91.73)   
93 + **[CGCGCG]** 1194 (85.98)   
93 + **[CGCGCG]** 1196 (85.98)   
93 + **[TTCGCG]** 1222 (87.60)   
93 + **[GGCGCG]** 1238 (86.50)   
93 + **[CGCGCG]** 1240 (85.98)   
93 + **[TTCGCG]** 1280 (87.60)   
93 + **[TAGGCG]** 1390 (85.06)   
93 - **.GACGCG.** 1488 (92.24)   
93 - **[GCCGCG]** 1282 (86.55)   
93 - **[GCCGCG]** 1242 (86.55)   
93 - **[CGCGCG]** 1240 (85.98)   
93 - **[GGCGCG]** 1198 (86.50)   
93 - **[CGCGCG]** 1196 (85.98)   
93 - **[CGCGCG]** 1194 (85.98)   
93 - **[GCCGCG]** 1037 (86.55)   
93 - **[GGCGCG]** 1003 (86.50)   
93 - **.GACGCG.** 985 (92.24)   
93 - **[CGCGCG]** 965 (85.98)   
93 - **.TCCGCG.** 869 (94.31)   
93 - **[CGCGCG]** 453 (85.98)   
93 - **[CGCGCG]** 451 (85.98)   
93 - **[TAGGCG]** 4 (85.06)   
96 + **.TGCGCG.** 736 (94.26)   
96 + **[CGCGCG]** 738 (85.98)   
96 + **[GCCGCG]** 848 (86.55)   
96 + **.TCCGCG.** 932 (94.31)   
96 + **[CGCGCG]** 1352 (85.98)   
96 + **.GACGCG.** 1393 (92.24)   
96 + **[CGCGCG]** 1451 (85.98)   
96 - **[CGCGCG]** 1451 (85.98)   
96 - **[GGCGCG]** 1422 (86.50)   
96 - **[CCCGCG]** 1395 (86.03)   
96 - **.GACGCG.** 1359 (92.24)   
96 - **.GACGCG.** 1354 (92.24)   
96 - **[CGCGCG]** 1352 (85.98)   
96 - **.TGCGCG.** 934 (94.26)   
96 - **[GCCGCG]** 884 (86.55)   
96 - **.GACGCG.** 740 (92.24)   
96 - **[CGCGCG]** 738 (85.98)   
97 + **.TGCGCG.** 875 (94.26)   
97 + **[GGCGCG]** 880 (86.50)   
97 + **[CCCGCG]** 976 (86.03)   
97 + **[CCCGCG]** 1045 (86.03)   
97 + **[AACGCG]** 1145 (87.55)   
97 + **[GGCGCG]** 1223 (86.50)   
97 + **.GACGCG.** 1367 (92.24)   
97 - **[CCCGCG]** 1369 (86.03)   
97 - **.TCCGCG.** 1225 (94.31)   
97 - **[GGCGCG]** 1047 (86.50)   
97 - **[CCCGCG]** 978 (86.03)   
97 - **[GCCGCG]** 882 (86.55)   
97 - **[GCCGCG]** 877 (86.55)   
98 + **[CCCGCG]** 966 (86.03)   
98 + **[GCCGCG]** 999 (86.55)   
98 + **[GGCGCG]** 1031 (86.50)   
98 + **[GGCGCG]** 1038 (86.50)   
98 + **.GACGCG.** 1098 (92.24)   
98 + **[GCCGCG]** 1119 (86.55)   
98 + **[CGCGCG]** 1135 (85.98)   
98 + **[CGCGCG]** 1179 (85.98)   
98 + **[GGCGCG]** 1227 (86.50)   
98 + **[CGCGCG]** 1289 (85.98)   
98 + **.TGCGCG.** 1323 (94.26)   
98 + **.TGCGCG.** 1387 (94.26)   
98 - **[CCCGCG]** 1389 (86.03)   
98 - **[GCCGCG]** 1325 (86.55)   
98 - **[CGCGCG]** 1289 (85.98)   
98 - **[CCCGCG]** 1229 (86.03)   
98 - **[CCCGCG]** 1181 (86.03)   
98 - **[CGCGCG]** 1179 (85.98)   
98 - **[GCCGCG]** 1137 (86.55)   
98 - **[CGCGCG]** 1135 (85.98)   
98 - **[CCCGCG]** 1121 (86.03)   
98 - **[CCCGCG]** 1100 (86.03)   
98 - **[GCCGCG]** 1086 (86.55)   
98 - **[CCCGCG]** 1060 (86.03)   
98 - **.TCCGCG.** 1040 (94.31)   
98 - **[CCCGCG]** 1033 (86.03)   
98 - **[GCCGCG]** 1001 (86.55)   
99 + **.TGCGCG.** 1332 (94.26)   
99 + **[CGCGCG]** 1334 (85.98)   
99 + **.TGCGCG.** 1348 (94.26)   
99 + **[TTCGCG]** 1472 (87.60)   
99 - **[CCCGCG]** 1350 (86.03)   
99 - **[CCCGCG]** 1336 (86.03)   
99 - **[CGCGCG]** 1334 (85.98)   
99 - **[CCCGCG]** 1180 (86.03)   
99 - **[GGCGCG]** 17 (86.50)   
100 + **.TGCGCG.** 156 (94.26)   
100 + **[TAGGCG]** 289 (85.06)   
100 + **.TCCGCG.** 989 (94.31)   
100 + **[CGCGCG]** 991 (85.98)   
100 + **[CCCGCG]** 1261 (86.03)   
100 + **[CGCGCG]** 1263 (85.98)   
100 + **[GCCGCG]** 1305 (86.55)   
100 + **[CGCGCG]** 1307 (85.98)   
100 + **[CCCGCG]** 1331 (86.03)   
100 + **[CCCGCG]** 1498 (86.03)   
100 - **[GCCGCG]** 1500 (86.55)   
100 - **.TGCGCG.** 1341 (94.26)   
100 - **[CCCGCG]** 1333 (86.03)   
100 - **[CGCGCG]** 1307 (85.98)   
100 - **[CGCGCG]** 1263 (85.98)   
100 - **[GCCGCG]** 1140 (86.55)   
100 - **[GGCGCG]** 1104 (86.50)   
100 - **.TGCGCG.** 993 (94.26)   
100 - **[CGCGCG]** 991 (85.98)   
103 + **[CCCGCG]** 780 (86.03)   
103 + **[CGCGCG]** 782 (85.98)   
103 - **[AACGCG]** 1184 (87.55)   
103 - **.TGCGCG.** 784 (94.26)   
103 - **[CGCGCG]** 782 (85.98)   
104 + **[CCCGCG]** 782 (86.03)   
104 + **.GACGCG.** 1140 (92.24)   
104 - **[TTCGCG]** 784 (87.60)   
105 + **[GGCGCG]** 1022 (86.50)   
105 + **[CCCGCG]** 1060 (86.03)   
105 + **[GGCGCG]** 1142 (86.50)   
105 + **[CCCGCG]** 1246 (86.03)   
105 - **[GGCGCG]** 1248 (86.50)   
105 - **.TACGCG.** 1144 (100.00)   
105 - **.TGCGCG.** 1062 (94.26)   
105 - **[GCCGCG]** 1039 (86.55)   
106 + **.TCCGCG.** 892 (94.31)   
106 + **[CCCGCG]** 934 (86.03)   
106 + **.TCCGCG.** 999 (94.31)   
106 + **[CGCGCG]** 1001 (85.98)   
106 - **.TCCGCG.** 1199 (94.31)   
106 - **[GCCGCG]** 1160 (86.55)   
106 - **[AACGCG]** 1003 (87.55)   
106 - **[CGCGCG]** 1001 (85.98)   
106 - **[CCCGCG]** 936 (86.03)   
106 - **[GCCGCG]** 870 (86.55)   
108 + **[GGCGCG]** 99 (86.50)   
108 + **.TCCGCG.** 174 (94.31)   
108 + **[CGCGCG]** 176 (85.98)   
108 + **[TAGGCG]** 301 (85.06)   
108 + **[CCCGCG]** 427 (86.03)   
108 + **[CCCGCG]** 610 (86.03)   
108 + **[CGCGCG]** 612 (85.98)   
108 + **[CCCGCG]** 740 (86.03)   
108 + **.TCCGCG.** 765 (94.31)   
108 + **.GACGCG.** 818 (92.24)   
108 + **.GACGCG.** 923 (92.24)   
108 + **[TTCGCG]** 1038 (87.60)   
108 + **[CCCGCG]** 1201 (86.03)   
108 + **[GGCGCG]** 1300 (86.50)   
108 + **.GACGCG.** 1385 (92.24)   
108 - **[CCCGCG]** 1511 (86.03)   
108 - **.CACGCG.** 1387 (91.73)   
108 - **[CCCGCG]** 1245 (86.03)   
108 - **[GGCGCG]** 1218 (86.50)   
108 - **.GACGCG.** 1203 (92.24)   
108 - **[GCCGCG]** 1114 (86.55)   
108 - **.CACGCG.** 1067 (91.73)   
108 - **.TCCGCG.** 1040 (94.31)   
108 - **.TGCGCG.** 820 (94.26)   
108 - **.CACGCG.** 742 (91.73)   
108 - **.TGCGCG.** 614 (94.26)   
108 - **[CGCGCG]** 612 (85.98)   
108 - **[CCCGCG]** 539 (86.03)   
108 - **.GACGCG.** 429 (92.24)   
108 - **.TCCGCG.** 399 (94.31)   
108 - **[CGCGCG]** 176 (85.98)   
108 - **[GCCGCG]** 101 (86.55)   
109 + **[GCCGCG]** 337 (86.55)   
109 + **.CACGCG.** 453 (91.73)   
109 + **[CCCGCG]** 598 (86.03)   
109 + **.GACGCG.** 814 (92.24)   
109 + **[CGCGCG]** 816 (85.98)   
109 + **[GGCGCG]** 825 (86.50)   
109 + **[CGCGCG]** 827 (85.98)   
109 + **[GCCGCG]** 865 (86.55)   
109 + **[GCCGCG]** 943 (86.55)   
109 + **[CCCGCG]** 1030 (86.03)   
109 + **[CCCGCG]** 1041 (86.03)   
109 + **.GACGCG.** 1231 (92.24)   
109 + **[GGCGCG]** 1313 (86.50)   
109 + **[GGCGCG]** 1320 (86.50)   
109 + **[CCCGCG]** 1329 (86.03)   
109 + **[GGCGCG]** 1355 (86.50)   
109 + **[CGCGCG]** 1357 (85.98)   
109 + **[GGCGCG]** 1389 (86.50)   
109 + **[GGCGCG]** 1394 (86.50)   
109 + **[CCCGCG]** 1459 (86.03)   
109 - **.TCCGCG.** 1505 (94.31)   
109 - **[CCCGCG]** 1461 (86.03)   
109 - **[GCCGCG]** 1396 (86.55)   
109 - **[GCCGCG]** 1391 (86.55)   
109 - **[GCCGCG]** 1359 (86.55)   
109 - **[CGCGCG]** 1357 (85.98)   
109 - **.GACGCG.** 1331 (92.24)   
109 - **[CCCGCG]** 1322 (86.03)   
109 - **[CCCGCG]** 1315 (86.03)   
109 - **[GGCGCG]** 1233 (86.50)   
109 - **[GGCGCG]** 1194 (86.50)   
109 - **[GGCGCG]** 1043 (86.50)   
109 - **.GACGCG.** 1032 (92.24)   
109 - **[GGCGCG]** 945 (86.50)   
109 - **[CCCGCG]** 829 (86.03)   
109 - **[CGCGCG]** 827 (85.98)   
109 - **[CCCGCG]** 818 (86.03)   
109 - **[CGCGCG]** 816 (85.98)   
109 - **[GGCGCG]** 659 (86.50)   
109 - **.TCCGCG.** 649 (94.31)   
109 - **[GGCGCG]** 455 (86.50)   
109 - **[GCCGCG]** 339 (86.55)   
110 + **[GCCGCG]** 859 (86.55)   
110 + **[GGCGCG]** 949 (86.50)   
110 + **.TGCGCG.** 1000 (94.26)   
110 - **.TCCGCG.** 1002 (94.31)   
110 - **.TCCGCG.** 861 (94.31)   
111 + **.TCCGCG.** 1504 (94.31)   
111 - **.TGCGCG.** 1506 (94.26)   
111 - **[TAGGCG]** 1012 (85.06)   
112 - **.TCCGCG.** 351 (94.31)   
114 + **[CCCGCG]** 252 (86.03)   
114 + **[CCCGCG]** 303 (86.03)   
114 + **[CGCGCG]** 305 (85.98)   
114 + **[GCCGCG]** 350 (86.55)   
114 + **[GCCGCG]** 1000 (86.55)   
114 + **[CGCGCG]** 1002 (85.98)   
114 + **[GGCGCG]** 1007 (86.50)   
114 + **.GACGCG.** 1019 (92.24)   
114 + **[CCCGCG]** 1306 (86.03)   
114 - **[GCCGCG]** 1308 (86.55)   
114 - **[GCCGCG]** 1009 (86.55)   
114 - **[GCCGCG]** 1004 (86.55)   
114 - **[CGCGCG]** 1002 (85.98)   
114 - **[GCCGCG]** 742 (86.55)   
114 - **[CCCGCG]** 672 (86.03)   
114 - **.TACGCG.** 457 (100.00)   
114 - **[TAGGCG]** 381 (85.06)   
114 - **.TGCGCG.** 371 (94.26)   
114 - **[CCCGCG]** 352 (86.03)   
114 - **[CGCGCG]** 305 (85.98)   
114 - **[CCCGCG]** 254 (86.03)   
115 + **[GGCGCG]** 224 (86.50)   
115 + **[GGCGCG]** 281 (86.50)   
115 + **[CGCGCG]** 283 (85.98)   
115 + **[GGCGCG]** 301 (86.50)   
115 + **[GCCGCG]** 310 (86.55)   
115 + **[CCCGCG]** 346 (86.03)   
115 + **[CCCGCG]** 411 (86.03)   
115 + **.TCCGCG.** 422 (94.31)   
115 + **[CCCGCG]** 588 (86.03)   
115 + **[CCCGCG]** 687 (86.03)   
115 + **[CCCGCG]** 845 (86.03)   
115 + **.GACGCG.** 870 (92.24)   
115 + **[CGCGCG]** 872 (85.98)   
115 + **[CCCGCG]** 1037 (86.03)   
115 + **[CGCGCG]** 1039 (85.98)   
115 + **.TCCGCG.** 1090 (94.31)   
115 - **[CCCGCG]** 1238 (86.03)   
115 - **[CGCGCG]** 1039 (85.98)   
115 - **[GGCGCG]** 1023 (86.50)   
115 - **.TGCGCG.** 874 (94.26)   
115 - **[CGCGCG]** 872 (85.98)   
115 - **.TGCGCG.** 847 (94.26)   
115 - **[GGCGCG]** 689 (86.50)   
115 - **[GGCGCG]** 590 (86.50)   
115 - **.GACGCG.** 466 (92.24)   
115 - **[GCCGCG]** 413 (86.55)   
115 - **.TGCGCG.** 348 (94.26)   
115 - **[CCCGCG]** 303 (86.03)   
115 - **[CCCGCG]** 285 (86.03)   
115 - **[CGCGCG]** 283 (85.98)   
116 + **.GACGCG.** 961 (92.24)   
116 + **[CGCGCG]** 963 (85.98)   
116 + **.TCCGCG.** 1199 (94.31)   
116 + **[TTCGCG]** 1452 (87.60)   
116 - **[TTCGCG]** 1500 (87.60)   
116 - **[GCCGCG]** 1201 (86.55)   
116 - **[TTCGCG]** 965 (87.60)   
116 - **[CGCGCG]** 963 (85.98)   
116 - **[TTCGCG]** 925 (87.60)   
117 + **[GGCGCG]** 595 (86.50)   
117 + **[GGCGCG]** 656 (86.50)   
117 + **[GGCGCG]** 667 (86.50)   
117 + **[GCCGCG]** 792 (86.55)   
117 + **[CCCGCG]** 829 (86.03)   
117 + **[GCCGCG]** 926 (86.55)   
117 + **[CCCGCG]** 936 (86.03)   
117 + **[CGCGCG]** 938 (85.98)   
117 + **.TACGCG.** 976 (100.00)   
117 + **[CCCGCG]** 1054 (86.03)   
117 - **[GCCGCG]** 1056 (86.55)   
117 - **[GGCGCG]** 1026 (86.50)   
117 - **[GCCGCG]** 978 (86.55)   
117 - **[GGCGCG]** 940 (86.50)   
117 - **[CGCGCG]** 938 (85.98)   
117 - **[CCCGCG]** 928 (86.03)   
117 - **[CCCGCG]** 831 (86.03)   
117 - **[GGCGCG]** 669 (86.50)   
117 - **[CCCGCG]** 658 (86.03)   
117 - **[CCCGCG]** 597 (86.03)   
118 + **[GCCGCG]** 433 (86.55)   
118 + **[GGCGCG]** 543 (86.50)   
118 + **[GGCGCG]** 604 (86.50)   
118 + **[GGCGCG]** 632 (86.50)   
118 + **[GCCGCG]** 768 (86.55)   
118 + **[CCCGCG]** 804 (86.03)   
118 + **[CCCGCG]** 926 (86.03)   
118 + **[CGCGCG]** 928 (85.98)   
118 + **.TACGCG.** 965 (100.00)   
118 + **[CCCGCG]** 1043 (86.03)   
118 - **[GCCGCG]** 1045 (86.55)   
118 - **[GGCGCG]** 1016 (86.50)   
118 - **[GGCGCG]** 930 (86.50)   
118 - **[CGCGCG]** 928 (85.98)   
118 - **[CCCGCG]** 634 (86.03)   
118 - **[CCCGCG]** 606 (86.03)   
118 - **[CCCGCG]** 545 (86.03)   
119 + **[GCCGCG]** 431 (86.55)   
119 + **[GGCGCG]** 541 (86.50)   
119 + **[GGCGCG]** 602 (86.50)   
119 + **[GGCGCG]** 613 (86.50)   
119 + **[GGCGCG]** 630 (86.50)   
119 + **[GGCGCG]** 641 (86.50)   
119 + **[GCCGCG]** 766 (86.55)   
119 + **[CCCGCG]** 802 (86.03)   
119 + **[CCCGCG]** 917 (86.03)   
119 + **[CGCGCG]** 919 (85.98)   
119 + **.TACGCG.** 956 (100.00)   
119 + **[CCCGCG]** 1034 (86.03)   
119 - **[GCCGCG]** 1036 (86.55)   
119 - **[GGCGCG]** 1007 (86.50)   
119 - **[GCCGCG]** 958 (86.55)   
119 - **[GGCGCG]** 921 (86.50)   
119 - **[CGCGCG]** 919 (85.98)   
119 - **[CCCGCG]** 804 (86.03)   
119 - **[CCCGCG]** 632 (86.03)   
119 - **[CCCGCG]** 604 (86.03)   
119 - **[CCCGCG]** 543 (86.03)   
120 + **[GGCGCG]** 1001 (86.50)   
120 + **[GGCGCG]** 1062 (86.50)   
120 + **[GGCGCG]** 1090 (86.50)   
120 + **[GGCGCG]** 1101 (86.50)   
120 + **[GCCGCG]** 1226 (86.55)   
120 + **[CCCGCG]** 1262 (86.03)   
120 + **[CCCGCG]** 1384 (86.03)   
120 + **[CGCGCG]** 1386 (85.98)   
120 + **.TACGCG.** 1423 (100.00)   
120 + **[CCCGCG]** 1501 (86.03)   
120 - **[GCCGCG]** 1503 (86.55)   
120 - **[GGCGCG]** 1474 (86.50)   
120 - **[GCCGCG]** 1425 (86.55)   
120 - **[GGCGCG]** 1388 (86.50)   
120 - **[CGCGCG]** 1386 (85.98)   
120 - **[CCCGCG]** 1264 (86.03)   
120 - **[GGCGCG]** 1103 (86.50)   
120 - **[CCCGCG]** 1092 (86.03)   
120 - **[CCCGCG]** 1064 (86.03)   
120 - **[CCCGCG]** 1003 (86.03)   
121 + **[CCCGCG]** 1280 (86.03)   
121 - **[TAGGCG]** 955 (85.06)   
122 + **[GCCGCG]** 82 (86.55)   
122 + **.TACGCG.** 699 (100.00)   
122 + **[GGCGCG]** 902 (86.50)   
122 + **[CGCGCG]** 904 (85.98)   
122 + **[CGCGCG]** 906 (85.98)   
122 + **.TGCGCG.** 914 (94.26)   
122 + **[CCCGCG]** 922 (86.03)   
122 + **[CGCGCG]** 938 (85.98)   
122 + **[CGCGCG]** 940 (85.98)   
122 + **[CGCGCG]** 953 (85.98)   
122 + **[CGCGCG]** 955 (85.98)   
122 + **[CGCGCG]** 957 (85.98)   
122 + **.CACGCG.** 1028 (91.73)   
122 - **.TACGCG.** 1030 (100.00)   
122 - **.TGCGCG.** 959 (94.26)   
122 - **[CGCGCG]** 957 (85.98)   
122 - **[CGCGCG]** 955 (85.98)   
122 - **[CGCGCG]** 953 (85.98)   
122 - **[CGCGCG]** 940 (85.98)   
122 - **[CGCGCG]** 938 (85.98)   
122 - **[GGCGCG]** 924 (86.50)   
122 - **[GCCGCG]** 916 (86.55)   
122 - **[TTCGCG]** 908 (87.60)   
122 - **[CGCGCG]** 906 (85.98)   
122 - **[CGCGCG]** 904 (85.98)   
122 - **[TTCGCG]** 522 (87.60)   
122 - **[GGCGCG]** 32 (86.50)   
123 + **[GCCGCG]** 15 (86.55)   
123 + **[GGCGCG]** 390 (86.50)   
123 + **[CCCGCG]** 543 (86.03)   
123 + **[CCCGCG]** 555 (86.03)   
123 + **[CCCGCG]** 588 (86.03)   
123 + **[GCCGCG]** 602 (86.55)   
123 + **.TCCGCG.** 658 (94.31)   
123 + **[CCCGCG]** 688 (86.03)   
123 + **[GGCGCG]** 743 (86.50)   
123 + **[GGCGCG]** 767 (86.50)   
123 + **[GCCGCG]** 826 (86.55)   
123 + **[GGCGCG]** 898 (86.50)   
123 + **[CCCGCG]** 950 (86.03)   
123 + **[CCCGCG]** 996 (86.03)   
123 + **[GCCGCG]** 1005 (86.55)   
123 + **[GCCGCG]** 1010 (86.55)   
123 + **.TGCGCG.** 1026 (94.26)   
123 + **[CGCGCG]** 1028 (85.98)   
123 + **[CCCGCG]** 1089 (86.03)   
123 - **[CCCGCG]** 1278 (86.03)   
123 - **[GCCGCG]** 1030 (86.55)   
123 - **[CGCGCG]** 1028 (85.98)   
123 - **[GGCGCG]** 1012 (86.50)   
123 - **[GGCGCG]** 1007 (86.50)   
123 - **[GCCGCG]** 952 (86.55)   
123 - **[GCCGCG]** 900 (86.55)   
123 - **[GGCGCG]** 769 (86.50)   
123 - **[CCCGCG]** 745 (86.03)   
123 - **[GGCGCG]** 690 (86.50)   
123 - **[GGCGCG]** 660 (86.50)   
123 - **[GGCGCG]** 557 (86.50)   
123 - **[GGCGCG]** 545 (86.50)   
123 - **.TCCGCG.** 527 (94.31)   
123 - **.GACGCG.** 392 (92.24)   
123 - **.TCCGCG.** 17 (94.31)   
124 + **.GACGCG.** 828 (92.24)   
124 + **[GGCGCG]** 868 (86.50)   
124 + **[GGCGCG]** 880 (86.50)   
124 + **[GGCGCG]** 953 (86.50)   
124 + **.TGCGCG.** 1040 (94.26)   
124 + **[GGCGCG]** 1149 (86.50)   
124 + **[CGCGCG]** 1151 (85.98)   
124 + **[CGCGCG]** 1153 (85.98)   
124 + **[GCCGCG]** 1224 (86.55)   
124 + **[CCCGCG]** 1459 (86.03)   
124 - **.TCCGCG.** 1226 (94.31)   
124 - **[CCCGCG]** 1155 (86.03)   
124 - **[CGCGCG]** 1153 (85.98)   
124 - **[CGCGCG]** 1151 (85.98)   
124 - **[TAGGCG]** 1114 (85.06)   
124 - **[GGCGCG]** 1112 (86.50)   
124 - **.CACGCG.** 1042 (91.73)   
124 - **.TGCGCG.** 955 (94.26)   
124 - **.TCCGCG.** 882 (94.31)   
124 - **.TCCGCG.** 870 (94.31)   
124 - **[TTCGCG]** 830 (87.60)   
124 - **[TAGGCG]** 771 (85.06)   
126 - **[TAGGCG]** 51 (85.06)   
127 + **.CACGCG.** 467 (91.73)   
127 + **[GGCGCG]** 630 (86.50)   
127 + **.TCCGCG.** 732 (94.31)   
127 + **[GGCGCG]** 744 (86.50)   
127 + **[GGCGCG]** 776 (86.50)   
127 + **[TAGGCG]** 848 (85.06)   
127 + **[GGCGCG]** 850 (86.50)   
127 + **.GACGCG.** 1029 (92.24)   
127 + **[CCCGCG]** 1103 (86.03)   
127 + **[CCCGCG]** 1303 (86.03)   
127 + **[GCCGCG]** 1509 (86.55)   
127 - **.GACGCG.** 1511 (92.24)   
127 - **[CCCGCG]** 1336 (86.03)   
127 - **[GCCGCG]** 1305 (86.55)   
127 - **.TCCGCG.** 1105 (94.31)   
127 - **[CCCGCG]** 852 (86.03)   
127 - **.TGCGCG.** 778 (94.26)   
127 - **[GCCGCG]** 746 (86.55)   
127 - **.TGCGCG.** 632 (94.26)   
128 + **.GACGCG.** 31 (92.24)   
128 + **[AACGCG]** 338 (87.55)   
128 + **.GACGCG.** 406 (92.24)   
128 + **.TGCGCG.** 425 (94.26)   
128 + **.TCCGCG.** 973 (94.31)   
128 + **[CGCGCG]** 975 (85.98)   
128 + **[CGCGCG]** 1100 (85.98)   
128 - **[CCCGCG]** 1102 (86.03)   
128 - **[CGCGCG]** 1100 (85.98)   
128 - **[CGCGCG]** 975 (85.98)   
128 - **[GCCGCG]** 611 (86.55)   
128 - **[TAGGCG]** 592 (85.06)   
128 - **[CCCGCG]** 427 (86.03)   
128 - **[GCCGCG]** 408 (86.55)   
128 - **[CCCGCG]** 340 (86.03)   
129 + **[CCCGCG]** 339 (86.03)   
129 + **[CGCGCG]** 708 (85.98)   
129 + **.CACGCG.** 764 (91.73)   
129 + **[GCCGCG]** 870 (86.55)   
129 + **[GCCGCG]** 895 (86.55)   
129 + **[CGCGCG]** 897 (85.98)   
129 + **[GCCGCG]** 957 (86.55)   
129 + **[CGCGCG]** 959 (85.98)   
129 + **[GCCGCG]** 1016 (86.55)   
129 + **.GACGCG.** 1057 (92.24)   
129 + **[CCCGCG]** 1103 (86.03)   
129 + **[GGCGCG]** 1144 (86.50)   
129 + **.TCCGCG.** 1176 (94.31)   
129 + **[GCCGCG]** 1200 (86.55)   
129 + **[GCCGCG]** 1320 (86.55)   
129 + **[CCCGCG]** 1384 (86.03)   
129 - **[GGCGCG]** 1402 (86.50)   
129 - **[GCCGCG]** 1386 (86.55)   
129 - **.TGCGCG.** 1322 (94.26)   
129 - **[GGCGCG]** 1202 (86.50)   
129 - **.CACGCG.** 1178 (91.73)   
129 - **[CCCGCG]** 1146 (86.03)   
129 - **[GCCGCG]** 1141 (86.55)   
129 - **[CCCGCG]** 1059 (86.03)   
129 - **.CACGCG.** 1018 (91.73)   
129 - **[CGCGCG]** 959 (85.98)   
129 - **[CGCGCG]** 897 (85.98)   
129 - **.TCCGCG.** 872 (94.31)   
129 - **.TGCGCG.** 766 (94.26)   
129 - **.TGCGCG.** 760 (94.26)   
129 - **.TGCGCG.** 750 (94.26)   
129 - **[CGCGCG]** 708 (85.98)   
129 - **.TGCGCG.** 680 (94.26)   
129 - **.TACGCG.** 479 (100.00)   
130 + **.TGCGCG.** 582 (94.26)   
130 + **[GCCGCG]** 865 (86.55)   
130 + **[GCCGCG]** 1109 (86.55)   
130 + **[GGCGCG]** 1267 (86.50)   
130 + **[CCCGCG]** 1366 (86.03)   
130 - **[CCCGCG]** 1368 (86.03)   
130 - **.TGCGCG.** 1359 (94.26)   
130 - **[GGCGCG]** 1269 (86.50)   
130 - **.TGCGCG.** 949 (94.26)   
130 - **[GCCGCG]** 867 (86.55)   
130 - **[CCCGCG]** 584 (86.03)   
131 + **[GGCGCG]** 31 (86.50)   
131 + **.CACGCG.** 474 (91.73)   
131 + **[GCCGCG]** 568 (86.55)   
131 + **[CCCGCG]** 612 (86.03)   
131 + **.TGCGCG.** 776 (94.26)   
131 + **[CCCGCG]** 885 (86.03)   
131 + **[GGCGCG]** 980 (86.50)   
131 + **[GGCGCG]** 1091 (86.50)   
131 + **[TTCGCG]** 1115 (87.60)   
131 + **[GCCGCG]** 1183 (86.55)   
131 + **[CCCGCG]** 1280 (86.03)   
131 + **.CACGCG.** 1304 (91.73)   
131 + **[CCCGCG]** 1486 (86.03)   
131 - **[GCCGCG]** 1488 (86.55)   
131 - **.TGCGCG.** 1437 (94.26)   
131 - **[GCCGCG]** 1379 (86.55)   
131 - **[GCCGCG]** 1306 (86.55)   
131 - **[CCCGCG]** 1282 (86.03)   
131 - **.TGCGCG.** 1185 (94.26)   
131 - **[GCCGCG]** 1117 (86.55)   
131 - **[GCCGCG]** 1093 (86.55)   
131 - **[CCCGCG]** 982 (86.03)   
131 - **.CACGCG.** 778 (91.73)   
131 - **.CACGCG.** 614 (91.73)   
131 - **[AACGCG]** 589 (87.55)   
131 - **.TCCGCG.** 570 (94.31)   
131 - **[CCCGCG]** 476 (86.03)   
131 - **[GGCGCG]** 33 (86.50)   
132 + **[GGCGCG]** 355 (86.50)   
132 + **[CGCGCG]** 357 (85.98)   
132 + **[AACGCG]** 476 (87.55)   
132 + **[GCCGCG]** 710 (86.55)   
132 + **[GCCGCG]** 749 (86.55)   
132 + **[CGCGCG]** 812 (85.98)   
132 + **[CCCGCG]** 1222 (86.03)   
132 + **.TGCGCG.** 1435 (94.26)   
132 - **[CCCGCG]** 1437 (86.03)   
132 - **[GGCGCG]** 1224 (86.50)   
132 - **[CCCGCG]** 1080 (86.03)   
132 - **.TGCGCG.** 831 (94.26)   
132 - **[GGCGCG]** 814 (86.50)   
132 - **[CGCGCG]** 812 (85.98)   
132 - **.TGCGCG.** 751 (94.26)   
132 - **.CACGCG.** 712 (91.73)   
132 - **.TGCGCG.** 478 (94.26)   
132 - **.TCCGCG.** 359 (94.31)   
132 - **[CGCGCG]** 357 (85.98)   
134 + **[TAGGCG]** 915 (85.06)   
134 + **[GGCGCG]** 950 (86.50)   
134 + **[CGCGCG]** 985 (85.98)   
134 + **[CGCGCG]** 1033 (85.98)   
134 + **[TAGGCG]** 1160 (85.06)   
134 + **[CCCGCG]** 1170 (86.03)   
134 + **[GGCGCG]** 1370 (86.50)   
134 + **.TCCGCG.** 1404 (94.31)   
134 + **[GGCGCG]** 1473 (86.50)   
134 - **[GGCGCG]** 1475 (86.50)   
134 - **[CCCGCG]** 1406 (86.03)   
134 - **.GACGCG.** 1035 (92.24)   
134 - **[CGCGCG]** 1033 (85.98)   
134 - **[CCCGCG]** 987 (86.03)   
134 - **[CGCGCG]** 985 (85.98)   
134 - **[CCCGCG]** 952 (86.03)   
134 - **[GCCGCG]** 533 (86.55)   
135 + **.TGCGCG.** 30 (94.26)   
135 + **[CGCGCG]** 256 (85.98)   
135 + **[CCCGCG]** 311 (86.03)   
135 + **[CCCGCG]** 317 (86.03)   
135 + **.CACGCG.** 353 (91.73)   
135 + **[CGCGCG]** 355 (85.98)   
135 + **[GCCGCG]** 363 (86.55)   
135 + **[CCCGCG]** 441 (86.03)   
135 + **.CACGCG.** 488 (91.73)   
135 + **[CCCGCG]** 751 (86.03)   
135 + **[CGCGCG]** 753 (85.98)   
135 + **[GCCGCG]** 788 (86.55)   
135 + **[GGCGCG]** 1450 (86.50)   
135 + **[GCCGCG]** 1500 (86.55)   
135 + **.CACGCG.** 1508 (91.73)   
135 - **.TGCGCG.** 1510 (94.26)   
135 - **.TGCGCG.** 1502 (94.26)   
135 - **[CCCGCG]** 1452 (86.03)   
135 - **[TAGGCG]** 1126 (85.06)   
135 - **.CACGCG.** 790 (91.73)   
135 - **[CCCGCG]** 755 (86.03)   
135 - **[CGCGCG]** 753 (85.98)   
135 - **[CCCGCG]** 490 (86.03)   
135 - **[GCCGCG]** 365 (86.55)   
135 - **.TCCGCG.** 357 (94.31)   
135 - **[CGCGCG]** 355 (85.98)   
135 - **[GGCGCG]** 313 (86.50)   
135 - **[GCCGCG]** 258 (86.55)   
135 - **[CGCGCG]** 256 (85.98)   
135 - **[GCCGCG]** 249 (86.55)   
135 - **.CACGCG.** 32 (91.73)   
136 + **.GACGCG.** 699 (92.24)   
136 + **[CGCGCG]** 701 (85.98)   
136 + **[CGCGCG]** 825 (85.98)   
136 + **[GGCGCG]** 936 (86.50)   
136 + **[GGCGCG]** 1039 (86.50)   
136 + **.TGCGCG.** 1063 (94.26)   
136 + **[CCCGCG]** 1194 (86.03)   
136 + **[GCCGCG]** 1298 (86.55)   
136 + **[CGCGCG]** 1300 (85.98)   
136 - **[GCCGCG]** 1302 (86.55)   
136 - **[CGCGCG]** 1300 (85.98)   
136 - **[GCCGCG]** 1196 (86.55)   
136 - **[TAGGCG]** 1070 (85.06)   
136 - **[GGCGCG]** 1065 (86.50)   
136 - **[GCCGCG]** 1041 (86.55)   
136 - **.TCCGCG.** 938 (94.31)   
136 - **.TGCGCG.** 827 (94.26)   
136 - **[CGCGCG]** 825 (85.98)   
136 - **[CGCGCG]** 701 (85.98)   
137 + **[GGCGCG]** 610 (86.50)   
137 + **.GACGCG.** 752 (92.24)   
137 + **[GCCGCG]** 919 (86.55)   
137 + **.GACGCG.** 943 (92.24)   
137 + **.GACGCG.** 1014 (92.24)   
137 + **[CGCGCG]** 1136 (85.98)   
137 + **[GGCGCG]** 1165 (86.50)   
137 - **[GGCGCG]** 1496 (86.50)   
137 - **.CACGCG.** 1167 (91.73)   
137 - **[GCCGCG]** 1138 (86.55)   
137 - **[CGCGCG]** 1136 (85.98)   
137 - **[GCCGCG]** 1016 (86.55)   
137 - **[GCCGCG]** 945 (86.55)   
137 - **[CCCGCG]** 754 (86.03)   
137 - **.TACGCG.** 612 (100.00)   
138 + **[CGCGCG]** 56 (85.98)   
138 + **[CGCGCG]** 58 (85.98)   
138 + **[CCCGCG]** 86 (86.03)   
138 + **[TTCGCG]** 190 (87.60)   
138 + **.GACGCG.** 365 (92.24)   
138 + **[GGCGCG]** 370 (86.50)   
138 + **.TGCGCG.** 388 (94.26)   
138 + **[CCCGCG]** 450 (86.03)   
138 + **[GCCGCG]** 513 (86.55)   
138 + **[CGCGCG]** 548 (85.98)   
138 + **[GGCGCG]** 575 (86.50)   
138 + **[CGCGCG]** 577 (85.98)   
138 + **[CGCGCG]** 599 (85.98)   
138 + **[GCCGCG]** 615 (86.55)   
138 + **[GGCGCG]** 641 (86.50)   
138 + **[TTCGCG]** 668 (87.60)   
138 + **[GGCGCG]** 689 (86.50)   
138 + **[CCCGCG]** 761 (86.03)   
138 + **[GGCGCG]** 766 (86.50)   
138 + **[GCCGCG]** 838 (86.55)   
138 + **[CCCGCG]** 936 (86.03)   
138 - **[GGCGCG]** 840 (86.50)   
138 - **[GCCGCG]** 768 (86.55)   
138 - **[GCCGCG]** 763 (86.55)   
138 - **[GGCGCG]** 739 (86.50)   
138 - **[GCCGCG]** 691 (86.55)   
138 - **[CCCGCG]** 670 (86.03)   
138 - **.GACGCG.** 601 (92.24)   
138 - **[CGCGCG]** 599 (85.98)   
138 - **[GCCGCG]** 579 (86.55)   
138 - **[CGCGCG]** 577 (85.98)   
138 - **[CGCGCG]** 548 (85.98)   
138 - **[CCCGCG]** 515 (86.03)   
138 - **[GGCGCG]** 452 (86.50)   
138 - **.TCCGCG.** 424 (94.31)   
138 - **.TGCGCG.** 390 (94.26)   
138 - **.TCCGCG.** 372 (94.31)   
138 - **[GCCGCG]** 367 (86.55)   
138 - **[TAGGCG]** 207 (85.06)   
138 - **[GGCGCG]** 192 (86.50)   
138 - **.TCCGCG.** 88 (94.31)   
138 - **.TGCGCG.** 60 (94.26)   
138 - **[CGCGCG]** 58 (85.98)   
138 - **[CGCGCG]** 56 (85.98)   
139 + **[TAGGCG]** 437 (85.06)   
139 + **[GGCGCG]** 690 (86.50)   
139 + **.CACGCG.** 762 (91.73)   
139 + **[CCCGCG]** 828 (86.03)   
139 + **[CGCGCG]** 830 (85.98)   
139 + **[CCCGCG]** 845 (86.03)   
139 + **.TCCGCG.** 990 (94.31)   
139 + **[GGCGCG]** 1091 (86.50)   
139 + **.TGCGCG.** 1477 (94.26)   
139 - **[GCCGCG]** 1343 (86.55)   
139 - **[TTCGCG]** 1277 (87.60)   
139 - **.TGCGCG.** 992 (94.26)   
139 - **[GCCGCG]** 960 (86.55)   
139 - **.GACGCG.** 847 (92.24)   
139 - **[GGCGCG]** 832 (86.50)   
139 - **[CGCGCG]** 830 (85.98)   
139 - **.TCCGCG.** 692 (94.31)   
139 - **[CCCGCG]** 619 (86.03)   
139 - **.TACGCG.** 136 (100.00)   
140 + **[GCCGCG]** 657 (86.55)   
140 + **[CCCGCG]** 982 (86.03)   
140 + **[GCCGCG]** 1159 (86.55)   
140 + **[GCCGCG]** 1193 (86.55)   
140 + **[GCCGCG]** 1246 (86.55)   
140 - **[GCCGCG]** 1248 (86.55)   
140 - **[GGCGCG]** 1195 (86.50)   
140 - **[GCCGCG]** 1161 (86.55)   
140 - **.GACGCG.** 984 (92.24)   
140 - **[GCCGCG]** 730 (86.55)   
140 - **[CCCGCG]** 659 (86.03)   
141 + **[CCCGCG]** 435 (86.03)   
141 + **[CGCGCG]** 457 (85.98)   
141 + **[CCCGCG]** 542 (86.03)   
141 + **[GCCGCG]** 610 (86.55)   
141 + **[CGCGCG]** 626 (85.98)   
141 + **[GCCGCG]** 688 (86.55)   
141 + **[CGCGCG]** 690 (85.98)   
141 + **[GCCGCG]** 721 (86.55)   
141 + **[CCCGCG]** 749 (86.03)   
141 + **.TGCGCG.** 762 (94.26)   
141 + **[GGCGCG]** 947 (86.50)   
141 + **[GCCGCG]** 1156 (86.55)   
141 + **[GCCGCG]** 1183 (86.55)   
141 + **[GCCGCG]** 1188 (86.55)   
141 + **.GACGCG.** 1228 (92.24)   
141 + **[CCCGCG]** 1317 (86.03)   
141 + **[GGCGCG]** 1368 (86.50)   
141 + **[CCCGCG]** 1381 (86.03)   
141 + **.GACGCG.** 1389 (92.24)   
141 - **[CCCGCG]** 1383 (86.03)   
141 - **[CCCGCG]** 1370 (86.03)   
141 - **.TCCGCG.** 1319 (94.31)   
141 - **[CCCGCG]** 1190 (86.03)   
141 - **[GGCGCG]** 1185 (86.50)   
141 - **[GCCGCG]** 1158 (86.55)   
141 - **[GGCGCG]** 723 (86.50)   
141 - **.TGCGCG.** 692 (94.26)   
141 - **[CGCGCG]** 690 (85.98)   
141 - **[GCCGCG]** 684 (86.55)   
141 - **[CGCGCG]** 626 (85.98)   
141 - **[GGCGCG]** 612 (86.50)   
141 - **[TTCGCG]** 544 (87.60)   
141 - **[CCCGCG]** 459 (86.03)   
141 - **[CGCGCG]** 457 (85.98)   
141 - **.TGCGCG.** 437 (94.26)   
142 + **[GCCGCG]** 29 (86.55)   
142 + **.CACGCG.** 97 (91.73)   
142 + **.GACGCG.** 447 (92.24)   
142 + **.CACGCG.** 455 (91.73)   
142 + **.TCCGCG.** 511 (94.31)   
142 + **[GCCGCG]** 516 (86.55)   
142 + **.TGCGCG.** 602 (94.26)   
142 + **[GCCGCG]** 625 (86.55)   
142 + **.TGCGCG.** 661 (94.26)   
142 + **[GCCGCG]** 672 (86.55)   
142 + **[CCCGCG]** 720 (86.03)   
142 + **[TTCGCG]** 795 (87.60)   
142 + **[GCCGCG]** 831 (86.55)   
142 + **[GGCGCG]** 842 (86.50)   
142 + **[GCCGCG]** 894 (86.55)   
142 + **[CGCGCG]** 896 (85.98)   
142 + **[CGCGCG]** 898 (85.98)   
142 + **[CGCGCG]** 900 (85.98)   
142 + **[CCCGCG]** 944 (86.03)   
142 + **[GCCGCG]** 1006 (86.55)   
142 + **[CGCGCG]** 1008 (85.98)   
142 + **[CCCGCG]** 1015 (86.03)   
142 + **[CCCGCG]** 1039 (86.03)   
142 + **[GCCGCG]** 1061 (86.55)   
142 + **[GCCGCG]** 1194 (86.55)   
142 + **[CGCGCG]** 1196 (85.98)   
142 + **[CGCGCG]** 1198 (85.98)   
142 + **[GCCGCG]** 1272 (86.55)   
142 + **[GCCGCG]** 1414 (86.55)   
142 + **[CGCGCG]** 1416 (85.98)   
142 + **[CCCGCG]** 1466 (86.03)   
142 + **.GACGCG.** 1478 (92.24)   
142 + **[GCCGCG]** 1508 (86.55)   
142 - **[CCCGCG]** 1510 (86.03)   
142 - **[CCCGCG]** 1480 (86.03)   
142 - **[GGCGCG]** 1468 (86.50)   
142 - **[CGCGCG]** 1416 (85.98)   
142 - **[GGCGCG]** 1274 (86.50)   
142 - **[GCCGCG]** 1200 (86.55)   
142 - **[CGCGCG]** 1198 (85.98)   
142 - **[CGCGCG]** 1196 (85.98)   
142 - **.TCCGCG.** 1063 (94.31)   
142 - **[CCCGCG]** 1054 (86.03)   
142 - **.TGCGCG.** 1041 (94.26)   
142 - **[GCCGCG]** 1010 (86.55)   
142 - **[CGCGCG]** 1008 (85.98)   
142 - **[GGCGCG]** 958 (86.50)   
142 - **[GCCGCG]** 946 (86.55)   
142 - **[GGCGCG]** 902 (86.50)   
142 - **[CGCGCG]** 900 (85.98)   
142 - **[CGCGCG]** 898 (85.98)   
142 - **[CGCGCG]** 896 (85.98)   
142 - **[CCCGCG]** 833 (86.03)   
142 - **[CCCGCG]** 797 (86.03)   
142 - **[GGCGCG]** 722 (86.50)   
142 - **[CCCGCG]** 674 (86.03)   
142 - **[GCCGCG]** 663 (86.55)   
142 - **[CCCGCG]** 604 (86.03)   
142 - **[GCCGCG]** 518 (86.55)   
142 - **[GGCGCG]** 513 (86.50)   
142 - **[GGCGCG]** 457 (86.50)   
142 - **[GGCGCG]** 31 (86.50)   
143 + **[GGCGCG]** 527 (86.50)   
143 + **.TGCGCG.** 1498 (94.26)   
143 - **[AACGCG]** 529 (87.55)   
144 + **[CGCGCG]** 772 (85.98)   
144 + **[CGCGCG]** 837 (85.98)   
144 + **[CGCGCG]** 934 (85.98)   
144 + **[GCCGCG]** 973 (86.55)   
144 + **[GCCGCG]** 1018 (86.55)   
144 + **[CGCGCG]** 1020 (85.98)   
144 + **[GGCGCG]** 1033 (86.50)   
144 + **[GGCGCG]** 1074 (86.50)   
144 + **[GGCGCG]** 1140 (86.50)   
144 + **[GGCGCG]** 1145 (86.50)   
144 + **[GGCGCG]** 1150 (86.50)   
144 + **.GACGCG.** 1187 (92.24)   
144 + **[GGCGCG]** 1192 (86.50)   
144 + **[GCCGCG]** 1198 (86.55)   
144 + **[GCCGCG]** 1217 (86.55)   
144 + **[CCCGCG]** 1266 (86.03)   
144 + **[CCCGCG]** 1308 (86.03)   
144 + **[CGCGCG]** 1310 (85.98)   
144 + **[GGCGCG]** 1324 (86.50)   
144 + **[GGCGCG]** 1459 (86.50)   
144 + **.TGCGCG.** 1470 (94.26)   
144 + **[GCCGCG]** 1491 (86.55)   
144 - **[GGCGCG]** 1493 (86.50)   
144 - **[CCCGCG]** 1461 (86.03)   
144 - **[GCCGCG]** 1326 (86.55)   
144 - **[GCCGCG]** 1312 (86.55)   
144 - **[CGCGCG]** 1310 (85.98)   
144 - **.TCCGCG.** 1268 (94.31)   
144 - **.TCCGCG.** 1219 (94.31)   
144 - **.TCCGCG.** 1200 (94.31)   
144 - **[GCCGCG]** 1194 (86.55)   
144 - **[GCCGCG]** 1189 (86.55)   
144 - **.TCCGCG.** 1152 (94.31)   
144 - **[GCCGCG]** 1147 (86.55)   
144 - **[GCCGCG]** 1142 (86.55)   
144 - **[CCCGCG]** 1076 (86.03)   
144 - **.TGCGCG.** 1055 (94.26)   
144 - **.TGCGCG.** 1035 (94.26)   
144 - **[CCCGCG]** 1022 (86.03)   
144 - **[CGCGCG]** 1020 (85.98)   
144 - **[GCCGCG]** 975 (86.55)   
144 - **[CCCGCG]** 936 (86.03)   
144 - **[CGCGCG]** 934 (85.98)   
144 - **.GACGCG.** 839 (92.24)   
144 - **[CGCGCG]** 837 (85.98)   
144 - **.GACGCG.** 774 (92.24)   
144 - **[CGCGCG]** 772 (85.98)   
144 - **[CCCGCG]** 12 (86.03)   
145 + **[GGCGCG]** 654 (86.50)   
145 + **[GCCGCG]** 1107 (86.55)   
145 + **[GCCGCG]** 1137 (86.55)   
145 + **[CGCGCG]** 1139 (85.98)   
145 + **[GGCGCG]** 1148 (86.50)   
145 + **.TCCGCG.** 1251 (94.31)   
145 + **.TGCGCG.** 1349 (94.26)   
145 - **[GGCGCG]** 1150 (86.50)   
145 - **[GCCGCG]** 1141 (86.55)   
145 - **[CGCGCG]** 1139 (85.98)   
145 - **[CCCGCG]** 1109 (86.03)   
145 - **[GCCGCG]** 1098 (86.55)   
145 - **[GGCGCG]** 510 (86.50)   
147 + **.GACGCG.** 862 (92.24)   
147 + **[GCCGCG]** 978 (86.55)   
147 + **[CGCGCG]** 1135 (85.98)   
147 + **[GCCGCG]** 1163 (86.55)   
147 + **[CGCGCG]** 1165 (85.98)   
147 + **[CCCGCG]** 1358 (86.03)   
147 + **[GGCGCG]** 1370 (86.50)   
147 - **[CCCGCG]** 1360 (86.03)   
147 - **[GGCGCG]** 1167 (86.50)   
147 - **[CGCGCG]** 1165 (85.98)   
147 - **[GGCGCG]** 1137 (86.50)   
147 - **[CGCGCG]** 1135 (85.98)   
147 - **.TGCGCG.** 980 (94.26)   
147 - **[CCCGCG]** 965 (86.03)   
147 - **.GACGCG.** 864 (92.24)   
147 - **[GGCGCG]** 854 (86.50)   
148 + **[CCCGCG]** 470 (86.03)   
148 + **[TTCGCG]** 595 (87.60)   
148 + **[CCCGCG]** 635 (86.03)   
148 + **[GCCGCG]** 828 (86.55)   
148 + **[CGCGCG]** 830 (85.98)   
148 + **.TCCGCG.** 921 (94.31)   
148 + **[CCCGCG]** 995 (86.03)   
148 - **.TGCGCG.** 1089 (94.26)   
148 - **[CCCGCG]** 997 (86.03)   
148 - **.TGCGCG.** 923 (94.26)   
148 - **.GACGCG.** 832 (92.24)   
148 - **[CGCGCG]** 830 (85.98)   
148 - **[CCCGCG]** 597 (86.03)   
148 - **[TAGGCG]** 574 (85.06)   
148 - **[GCCGCG]** 548 (86.55)   
148 - **[GGCGCG]** 472 (86.50)   
148 - **[TAGGCG]** 21 (85.06)   
150 + **.TGCGCG.** 20 (94.26)   
150 + **[CGCGCG]** 22 (85.98)   
150 + **[GGCGCG]** 90 (86.50)   
150 + **[CGCGCG]** 92 (85.98)   
150 + **[GCCGCG]** 114 (86.55)   
150 + **[CGCGCG]** 116 (85.98)   
150 + **[GCCGCG]** 121 (86.55)   
150 + **[TTCGCG]** 189 (87.60)   
150 + **.GACGCG.** 465 (92.24)   
150 + **.TGCGCG.** 712 (94.26)   
150 + **.TACGCG.** 931 (100.00)   
150 + **.GACGCG.** 956 (92.24)   
150 + **.TCCGCG.** 1118 (94.31)   
150 + **.TCCGCG.** 1177 (94.31)   
150 + **[GGCGCG]** 1469 (86.50)   
150 - **.TCCGCG.** 1471 (94.31)   
150 - **.TCCGCG.** 1120 (94.31)   
150 - **[AACGCG]** 958 (87.55)   
150 - **[CCCGCG]** 467 (86.03)   
150 - **[GCCGCG]** 191 (86.55)   
150 - **[GCCGCG]** 175 (86.55)   
150 - **[GGCGCG]** 123 (86.50)   
150 - **[GGCGCG]** 118 (86.50)   
150 - **[CGCGCG]** 116 (85.98)   
150 - **[GGCGCG]** 94 (86.50)   
150 - **[CGCGCG]** 92 (85.98)   
150 - **[CCCGCG]** 24 (86.03)   
150 - **[CGCGCG]** 22 (85.98)   
151 + **[AACGCG]** 47 (87.55)   
151 + **[CCCGCG]** 538 (86.03)   
151 + **[GCCGCG]** 815 (86.55)   
151 + **[GCCGCG]** 831 (86.55)   
151 + **[GGCGCG]** 882 (86.50)   
151 + **[GGCGCG]** 912 (86.50)   
151 + **[CGCGCG]** 914 (85.98)   
151 + **[CCCGCG]** 982 (86.03)   
151 + **[CGCGCG]** 984 (85.98)   
151 + **[TAGGCG]** 1031 (85.06)   
151 + **[GGCGCG]** 1033 (86.50)   
151 + **[CCCGCG]** 1061 (86.03)   
151 + **.CACGCG.** 1149 (91.73)   
151 + **[CCCGCG]** 1204 (86.03)   
151 + **[GGCGCG]** 1264 (86.50)   
151 + **[CGCGCG]** 1266 (85.98)   
151 + **[CCCGCG]** 1384 (86.03)   
151 + **.CACGCG.** 1421 (91.73)   
151 + **[GGCGCG]** 1461 (86.50)   
151 + **[CCCGCG]** 1471 (86.03)   
151 + **.TCCGCG.** 1477 (94.31)   
151 + **[GGCGCG]** 1492 (86.50)   
151 + **[CGCGCG]** 1494 (85.98)   
151 - **[GCCGCG]** 1496 (86.55)   
151 - **[CGCGCG]** 1494 (85.98)   
151 - **.GACGCG.** 1473 (92.24)   
151 - **.CACGCG.** 1463 (91.73)   
151 - **[CCCGCG]** 1423 (86.03)   
151 - **[AACGCG]** 1386 (87.55)   
151 - **[GCCGCG]** 1268 (86.55)   
151 - **[CGCGCG]** 1266 (85.98)   
151 - **[GCCGCG]** 1252 (86.55)   
151 - **[GGCGCG]** 1206 (86.50)   
151 - **.GACGCG.** 1151 (92.24)   
151 - **.TCCGCG.** 1035 (94.31)   
151 - **.TGCGCG.** 986 (94.26)   
151 - **[CGCGCG]** 984 (85.98)   
151 - **[GGCGCG]** 916 (86.50)   
151 - **[CGCGCG]** 914 (85.98)   
151 - **[GCCGCG]** 892 (86.55)   
151 - **[GCCGCG]** 884 (86.55)   
151 - **[TTCGCG]** 817 (87.60)   
151 - **.GACGCG.** 540 (92.24)   
151 - **.TGCGCG.** 293 (94.26)   
151 - **.TACGCG.** 74 (100.00)   
151 - **.GACGCG.** 49 (92.24)   
153 + **[GGCGCG]** 384 (86.50)   
154 + **.TGCGCG.** 773 (94.26)   
154 + **[GGCGCG]** 824 (86.50)   
154 + **[GGCGCG]** 865 (86.50)   
154 + **[CGCGCG]** 867 (85.98)   
154 + **[GGCGCG]** 933 (86.50)   
154 + **[GGCGCG]** 998 (86.50)   
154 + **[GGCGCG]** 1009 (86.50)   
154 + **[GGCGCG]** 1038 (86.50)   
154 + **[CCCGCG]** 1069 (86.03)   
154 + **[CCCGCG]** 1079 (86.03)   
154 + **[CGCGCG]** 1081 (85.98)   
154 + **[GGCGCG]** 1096 (86.50)   
154 + **[GCCGCG]** 1179 (86.55)   
154 + **[CCCGCG]** 1198 (86.03)   
154 + **[CCCGCG]** 1204 (86.03)   
154 + **[CCCGCG]** 1228 (86.03)   
154 + **[GGCGCG]** 1378 (86.50)   
154 + **[CGCGCG]** 1380 (85.98)   
154 + **[CGCGCG]** 1382 (85.98)   
154 + **.TCCGCG.** 1430 (94.31)   
154 + **[CCCGCG]** 1462 (86.03)   
154 + **[CGCGCG]** 1464 (85.98)   
154 + **[CCCGCG]** 1508 (86.03)   
154 + **[CGCGCG]** 1510 (85.98)   
154 - **[GCCGCG]** 1512 (86.55)   
154 - **[CGCGCG]** 1510 (85.98)   
154 - **[GGCGCG]** 1466 (86.50)   
154 - **[CGCGCG]** 1464 (85.98)   
154 - **[GGCGCG]** 1432 (86.50)   
154 - **[CCCGCG]** 1384 (86.03)   
154 - **[CGCGCG]** 1382 (85.98)   
154 - **[CGCGCG]** 1380 (85.98)   
154 - **[GGCGCG]** 1206 (86.50)   
154 - **[GGCGCG]** 1200 (86.50)   
154 - **.TACGCG.** 1083 (100.00)   
154 - **[CGCGCG]** 1081 (85.98)   
154 - **[GGCGCG]** 1071 (86.50)   
154 - **[GCCGCG]** 1040 (86.55)   
154 - **.CACGCG.** 1011 (91.73)   
154 - **[CCCGCG]** 1000 (86.03)   
154 - **.CACGCG.** 935 (91.73)   
154 - **.CACGCG.** 869 (91.73)   
154 - **[CGCGCG]** 867 (85.98)   
154 - **[CCCGCG]** 826 (86.03)   
155 + **[GGCGCG]** 507 (86.50)   
155 - **[AACGCG]** 1259 (87.55)   
155 - **[GCCGCG]** 994 (86.55)   
157 + **.TCCGCG.** 802 (94.31)   
157 + **.TGCGCG.** 977 (94.26)   
157 + **[GGCGCG]** 1219 (86.50)   
157 + **[GCCGCG]** 1236 (86.55)   
157 - **.CACGCG.** 1238 (91.73)   
157 - **.TCCGCG.** 1221 (94.31)   
157 - **.TGCGCG.** 979 (94.26)   
157 - **.TCCGCG.** 804 (94.31)   
157 - **[TAGGCG]** 733 (85.06)   
157 - **[GGCGCG]** 731 (86.50)   
158 + **[GGCGCG]** 375 (86.50)   
158 + **.CACGCG.** 1119 (91.73)   
158 + **[CGCGCG]** 1121 (85.98)   
158 + **[GCCGCG]** 1177 (86.55)   
158 + **.GACGCG.** 1242 (92.24)   
158 + **[TAGGCG]** 1280 (85.06)   
158 + **.GACGCG.** 1369 (92.24)   
158 + **[GGCGCG]** 1512 (86.50)   
158 - **[GGCGCG]** 1371 (86.50)   
158 - **[TTCGCG]** 1244 (87.60)   
158 - **.GACGCG.** 1179 (92.24)   
158 - **.GACGCG.** 1123 (92.24)   
158 - **[CGCGCG]** 1121 (85.98)   
158 - **[GGCGCG]** 522 (86.50)   
158 - **.CACGCG.** 377 (91.73)   
160 + **.TGCGCG.** 69 (94.26)   
160 + **[GCCGCG]** 133 (86.55)   
160 + **[CCCGCG]** 235 (86.03)   
160 + **[GCCGCG]** 266 (86.55)   
160 + **.TGCGCG.** 295 (94.26)   
160 + **.TGCGCG.** 702 (94.26)   
160 + **[CCCGCG]** 1204 (86.03)   
160 + **[CCCGCG]** 1327 (86.03)   
160 - **[CCCGCG]** 1329 (86.03)   
160 - **[GGCGCG]** 1206 (86.50)   
160 - **[GCCGCG]** 1055 (86.55)   
160 - **.GACGCG.** 782 (92.24)   
160 - **.CACGCG.** 704 (91.73)   
160 - **.CACGCG.** 297 (91.73)   
160 - **.TCCGCG.** 268 (94.31)   
160 - **[CCCGCG]** 237 (86.03)   
160 - **.CACGCG.** 135 (91.73)   
160 - **[CCCGCG]** 71 (86.03)   
161 + **[GGCGCG]** 154 (86.50)   
161 + **.CACGCG.** 316 (91.73)   
161 + **[CGCGCG]** 318 (85.98)   
161 + **.TCCGCG.** 546 (94.31)   
161 + **.TGCGCG.** 687 (94.26)   
161 + **[GGCGCG]** 752 (86.50)   
161 + **[GCCGCG]** 865 (86.55)   
161 + **[CGCGCG]** 867 (85.98)   
161 + **[CGCGCG]** 869 (85.98)   
161 + **[CGCGCG]** 871 (85.98)   
161 + **[GCCGCG]** 884 (86.55)   
161 + **[GCCGCG]** 894 (86.55)   
161 + **.TCCGCG.** 966 (94.31)   
161 + **.TCCGCG.** 1177 (94.31)   
161 + **[CCCGCG]** 1266 (86.03)   
161 + **[CCCGCG]** 1479 (86.03)   
161 - **[GGCGCG]** 1481 (86.50)   
161 - **[CCCGCG]** 1302 (86.03)   
161 - **[GCCGCG]** 1268 (86.55)   
161 - **[GCCGCG]** 1221 (86.55)   
161 - **[GGCGCG]** 1191 (86.50)   
161 - **[GGCGCG]** 896 (86.50)   
161 - **[CGCGCG]** 871 (85.98)   
161 - **[CGCGCG]** 869 (85.98)   
161 - **[CGCGCG]** 867 (85.98)   
161 - **.TCCGCG.** 811 (94.31)   
161 - **.TCCGCG.** 760 (94.31)   
161 - **[CCCGCG]** 629 (86.03)   
161 - **[CGCGCG]** 318 (85.98)   
161 - **[CCCGCG]** 156 (86.03)   
164 + **[CCCGCG]** 299 (86.03)   
164 + **[GGCGCG]** 956 (86.50)   
164 + **.TCCGCG.** 993 (94.31)   
164 + **[GCCGCG]** 1001 (86.55)   
164 + **[GGCGCG]** 1097 (86.50)   
164 + **.TGCGCG.** 1112 (94.26)   
164 + **[AACGCG]** 1223 (87.55)   
164 + **.TCCGCG.** 1394 (94.31)   
164 - **[CCCGCG]** 1459 (86.03)   
164 - **[GGCGCG]** 1396 (86.50)   
164 - **[GCCGCG]** 1225 (86.55)   
164 - **[CCCGCG]** 1114 (86.03)   
164 - **[GCCGCG]** 1099 (86.55)   
164 - **[GCCGCG]** 1003 (86.55)   
164 - **[CCCGCG]** 958 (86.03)   
164 - **.TGCGCG.** 301 (94.26)   
164 - **.TCCGCG.** 200 (94.31)   
165 - **[GGCGCG]** 581 (86.50)   
166 + **[CCCGCG]** 423 (86.03)   
166 + **[GCCGCG]** 546 (86.55)   
166 + **[CCCGCG]** 622 (86.03)   
166 + **[GCCGCG]** 1072 (86.55)   
166 + **.TGCGCG.** 1108 (94.26)   
166 + **[CGCGCG]** 1110 (85.98)   
166 + **.CACGCG.** 1157 (91.73)   
166 + **[GGCGCG]** 1225 (86.50)   
166 + **[CGCGCG]** 1227 (85.98)   
166 + **[GGCGCG]** 1290 (86.50)   
166 + **[GGCGCG]** 1297 (86.50)   
166 + **.CACGCG.** 1310 (91.73)   
166 + **[TTCGCG]** 1429 (87.60)   
166 - **[GCCGCG]** 1312 (86.55)   
166 - **[CCCGCG]** 1299 (86.03)   
166 - **[GCCGCG]** 1229 (86.55)   
166 - **[CGCGCG]** 1227 (85.98)   
166 - **[GGCGCG]** 1181 (86.50)   
166 - **[GCCGCG]** 1159 (86.55)   
166 - **[CCCGCG]** 1112 (86.03)   
166 - **[CGCGCG]** 1110 (85.98)   
166 - **[CCCGCG]** 1074 (86.03)   
166 - **[GCCGCG]** 811 (86.55)   
166 - **[CCCGCG]** 779 (86.03)   
166 - **[GCCGCG]** 661 (86.55)   
166 - **.TACGCG.** 624 (100.00)   
166 - **[TAGGCG]** 600 (85.06)   
166 - **.TCCGCG.** 548 (94.31)   
166 - **.TGCGCG.** 425 (94.26)   
167 + **.GACGCG.** 731 (92.24)   
167 + **.GACGCG.** 770 (92.24)   
167 + **[GGCGCG]** 801 (86.50)   
167 + **[GGCGCG]** 818 (86.50)   
167 + **[CCCGCG]** 841 (86.03)   
167 + **[GCCGCG]** 863 (86.55)   
167 + **[CGCGCG]** 865 (85.98)   
167 + **[CCCGCG]** 871 (86.03)   
167 + **[CCCGCG]** 961 (86.03)   
167 + **[GGCGCG]** 1002 (86.50)   
167 + **[CGCGCG]** 1004 (85.98)   
167 + **[GGCGCG]** 1035 (86.50)   
167 + **[CGCGCG]** 1037 (85.98)   
167 + **[GCCGCG]** 1234 (86.55)   
167 + **[CCCGCG]** 1461 (86.03)   
167 - **.GACGCG.** 1463 (92.24)   
167 - **.TCCGCG.** 1236 (94.31)   
167 - **.TCCGCG.** 1228 (94.31)   
167 - **[GCCGCG]** 1039 (86.55)   
167 - **[CGCGCG]** 1037 (85.98)   
167 - **[GCCGCG]** 1006 (86.55)   
167 - **[CGCGCG]** 1004 (85.98)   
167 - **[GCCGCG]** 963 (86.55)   
167 - **[GCCGCG]** 956 (86.55)   
167 - **[CCCGCG]** 873 (86.03)   
167 - **[GGCGCG]** 867 (86.50)   
167 - **[CGCGCG]** 865 (85.98)   
167 - **[GCCGCG]** 843 (86.55)   
167 - **[CCCGCG]** 803 (86.03)   
167 - **[CCCGCG]** 772 (86.03)   
168 + **[GGCGCG]** 732 (86.50)   
168 + **[GCCGCG]** 909 (86.55)   
168 + **[CGCGCG]** 911 (85.98)   
168 + **[CGCGCG]** 1135 (85.98)   
168 + **.TCCGCG.** 1169 (94.31)   
168 + **[GGCGCG]** 1183 (86.50)   
168 + **.TCCGCG.** 1203 (94.31)   
168 + **[CCCGCG]** 1247 (86.03)   
168 + **[GCCGCG]** 1435 (86.55)   
168 - **.TCCGCG.** 1390 (94.31)   
168 - **[GCCGCG]** 1205 (86.55)   
168 - **.TCCGCG.** 1185 (94.31)   
168 - **[GGCGCG]** 1171 (86.50)   
168 - **[GCCGCG]** 1137 (86.55)   
168 - **[CGCGCG]** 1135 (85.98)   
168 - **[GGCGCG]** 1114 (86.50)   
168 - **.TCCGCG.** 913 (94.31)   
168 - **[CGCGCG]** 911 (85.98)   
168 - **[CCCGCG]** 734 (86.03)   
168 - **[GCCGCG]** 675 (86.55)   
169 + **[GCCGCG]** 111 (86.55)   
169 + **[GGCGCG]** 871 (86.50)   
169 + **.GACGCG.** 1045 (92.24)   
169 + **[CCCGCG]** 1495 (86.03)   
169 + **[CGCGCG]** 1497 (85.98)   
169 - **[GGCGCG]** 1499 (86.50)   
169 - **[CGCGCG]** 1497 (85.98)   
169 - **.TCCGCG.** 1354 (94.31)   
169 - **[TAGGCG]** 1002 (85.06)   
170 + **.TACGCG.** 357 (100.00)   
170 - **[AACGCG]** 359 (87.55)   
171 + **.TGCGCG.** 763 (94.26)   
171 + **.CACGCG.** 772 (91.73)   
171 - **[GCCGCG]** 774 (86.55)   
172 + **[CCCGCG]** 132 (86.03)   
172 + **[CCCGCG]** 386 (86.03)   
172 + **.GACGCG.** 429 (92.24)   
172 + **[CGCGCG]** 649 (85.98)   
172 + **[GCCGCG]** 714 (86.55)   
172 + **[GCCGCG]** 755 (86.55)   
172 + **[GGCGCG]** 768 (86.50)   
172 + **[CCCGCG]** 895 (86.03)   
172 + **[CGCGCG]** 897 (85.98)   
172 + **[GGCGCG]** 920 (86.50)   
172 + **[CGCGCG]** 922 (85.98)   
172 + **.TGCGCG.** 975 (94.26)   
172 + **[GGCGCG]** 1160 (86.50)   
172 + **.TGCGCG.** 1417 (94.26)   
172 + **.TGCGCG.** 1503 (94.26)   
172 - **[GGCGCG]** 1505 (86.50)   
172 - **[GGCGCG]** 1419 (86.50)   
172 - **.TCCGCG.** 1194 (94.31)   
172 - **.TCCGCG.** 1162 (94.31)   
172 - **[GGCGCG]** 924 (86.50)   
172 - **[CGCGCG]** 922 (85.98)   
172 - **[GCCGCG]** 899 (86.55)   
172 - **[CGCGCG]** 897 (85.98)   
172 - **[GCCGCG]** 757 (86.55)   
172 - **[CGCGCG]** 649 (85.98)   
172 - **[GGCGCG]** 551 (86.50)   
172 - **[GCCGCG]** 431 (86.55)   
172 - **.TCCGCG.** 388 (94.31)   
172 - **[CCCGCG]** 337 (86.03)   
172 - **.TACGCG.** 134 (100.00)   
173 + **[TAGGCG]** 436 (85.06)   
173 + **[CCCGCG]** 712 (86.03)   
173 + **.TGCGCG.** 913 (94.26)   
173 + **.GACGCG.** 943 (92.24)   
173 + **[GGCGCG]** 1101 (86.50)   
173 + **[TTCGCG]** 1175 (87.60)   
173 + **[GCCGCG]** 1278 (86.55)   
173 + **[CCCGCG]** 1286 (86.03)   
173 + **[CGCGCG]** 1288 (85.98)   
173 - **[AACGCG]** 1290 (87.55)   
173 - **[CGCGCG]** 1288 (85.98)   
173 - **[CCCGCG]** 1103 (86.03)   
173 - **[GGCGCG]** 945 (86.50)   
173 - **[CCCGCG]** 915 (86.03)   
173 - **[CCCGCG]** 714 (86.03)   
174 + **[GGCGCG]** 396 (86.50)   
174 + **[CCCGCG]** 723 (86.03)   
174 + **[GGCGCG]** 783 (86.50)   
174 + **.GACGCG.** 936 (92.24)   
174 + **[GCCGCG]** 1145 (86.55)   
174 + **[GCCGCG]** 1185 (86.55)   
174 + **[GCCGCG]** 1203 (86.55)   
174 + **.TGCGCG.** 1290 (94.26)   
174 + **.GACGCG.** 1380 (92.24)   
174 + **[CCCGCG]** 1475 (86.03)   
174 + **[GGCGCG]** 1480 (86.50)   
174 + **[GGCGCG]** 1505 (86.50)   
174 - **[GCCGCG]** 1507 (86.55)   
174 - **[CCCGCG]** 1482 (86.03)   
174 - **[GCCGCG]** 1477 (86.55)   
174 - **.TCCGCG.** 1382 (94.31)   
174 - **.GACGCG.** 1187 (92.24)   
174 - **[GGCGCG]** 1128 (86.50)   
174 - **.TCCGCG.** 938 (94.31)   
174 - **[GGCGCG]** 214 (86.50)   
175 + **.GACGCG.** 859 (92.24)   
175 + **[GGCGCG]** 927 (86.50)   
175 + **[GCCGCG]** 1466 (86.55)   
175 + **[CCCGCG]** 1496 (86.03)   
175 + **[CGCGCG]** 1498 (85.98)   
175 - **.TACGCG.** 1500 (100.00)   
175 - **[CGCGCG]** 1498 (85.98)   
175 - **[GCCGCG]** 1468 (86.55)   
175 - **[AACGCG]** 929 (87.55)   
175 - **.CACGCG.** 861 (91.73)   
175 - **[TAGGCG]** 58 (85.06)   
177 + **.TGCGCG.** 817 (94.26)   
177 + **[GCCGCG]** 952 (86.55)   
177 + **[AACGCG]** 1161 (87.55)   
177 + **.TCCGCG.** 1463 (94.31)   
177 - **[GCCGCG]** 1465 (86.55)   
177 - **[GGCGCG]** 819 (86.50)   
177 - **[GCCGCG]** 786 (86.55)   
178 + **.GACGCG.** 941 (92.24)   
178 + **.GACGCG.** 1112 (92.24)   
178 - **[CCCGCG]** 1239 (86.03)   
178 - **[GCCGCG]** 1114 (86.55)   
178 - **.TGCGCG.** 943 (94.26)   
179 + **[AACGCG]** 749 (87.55)   
179 + **[GCCGCG]** 916 (86.55)   
179 + **[GGCGCG]** 986 (86.50)   
179 + **.GACGCG.** 994 (92.24)   
179 + **[CGCGCG]** 996 (85.98)   
179 + **.TCCGCG.** 1042 (94.31)   
179 + **.TGCGCG.** 1096 (94.26)   
179 + **[GCCGCG]** 1474 (86.55)   
179 - **[GGCGCG]** 1476 (86.50)   
179 - **.GACGCG.** 1204 (92.24)   
179 - **.TCCGCG.** 1098 (94.31)   
179 - **[TTCGCG]** 1044 (87.60)   
179 - **[CGCGCG]** 996 (85.98)   
179 - **[CCCGCG]** 988 (86.03)   
179 - **[GCCGCG]** 918 (86.55)   
179 - **.TGCGCG.** 902 (94.26)   
179 - **[GGCGCG]** 858 (86.50)   
179 - **[AACGCG]** 751 (87.55)   
180 + **[CCCGCG]** 266 (86.03)   
180 + **[CCCGCG]** 699 (86.03)   
180 + **[GGCGCG]** 796 (86.50)   
180 + **[CGCGCG]** 798 (85.98)   
180 + **[CGCGCG]** 812 (85.98)   
180 + **[CGCGCG]** 814 (85.98)   
180 + **[GGCGCG]** 879 (86.50)   
180 + **[CGCGCG]** 881 (85.98)   
180 + **.TCCGCG.** 922 (94.31)   
180 + **[GGCGCG]** 1063 (86.50)   
180 + **[GGCGCG]** 1087 (86.50)   
180 + **.TCCGCG.** 1100 (94.31)   
180 + **.TCCGCG.** 1157 (94.31)   
180 - **[GCCGCG]** 1313 (86.55)   
180 - **[GCCGCG]** 1247 (86.55)   
180 - **[CCCGCG]** 1186 (86.03)   
180 - **[GCCGCG]** 1159 (86.55)   
180 - **[GCCGCG]** 1065 (86.55)   
180 - **[CCCGCG]** 924 (86.03)   
180 - **[GGCGCG]** 883 (86.50)   
180 - **[CGCGCG]** 881 (85.98)   
180 - **[CCCGCG]** 816 (86.03)   
180 - **[CGCGCG]** 814 (85.98)   
180 - **[CGCGCG]** 812 (85.98)   
180 - **.TCCGCG.** 800 (94.31)   
180 - **[CGCGCG]** 798 (85.98)   
180 - **.TCCGCG.** 268 (94.31)   
182 + **[GGCGCG]** 32 (86.50)   
182 + **[CGCGCG]** 34 (85.98)   
182 + **[GGCGCG]** 121 (86.50)   
182 + **[GCCGCG]** 233 (86.55)   
182 + **.GACGCG.** 300 (92.24)   
182 + **.CACGCG.** 367 (91.73)   
182 + **[GCCGCG]** 375 (86.55)   
182 + **[GGCGCG]** 386 (86.50)   
182 + **[GGCGCG]** 432 (86.50)   
182 + **[GCCGCG]** 461 (86.55)   
182 + **[GGCGCG]** 466 (86.50)   
182 + **[CCCGCG]** 493 (86.03)   
182 + **[GCCGCG]** 529 (86.55)   
182 + **[CGCGCG]** 531 (85.98)   
182 + **.CACGCG.** 632 (91.73)   
182 + **[CGCGCG]** 634 (85.98)   
182 + **[CCCGCG]** 666 (86.03)   
182 + **.TGCGCG.** 674 (94.26)   
182 + **[GCCGCG]** 748 (86.55)   
182 + **[GGCGCG]** 766 (86.50)   
182 + **[CGCGCG]** 768 (85.98)   
182 + **[CGCGCG]** 770 (85.98)   
182 + **.TCCGCG.** 827 (94.31)   
182 + **[GGCGCG]** 874 (86.50)   
182 + **.TGCGCG.** 983 (94.26)   
182 + **[CCCGCG]** 1019 (86.03)   
182 + **[GGCGCG]** 1102 (86.50)   
182 + **.TCCGCG.** 1289 (94.31)   
182 - **[CCCGCG]** 1197 (86.03)   
182 - **[GGCGCG]** 1163 (86.50)   
182 - **[GCCGCG]** 1078 (86.55)   
182 - **.GACGCG.** 1021 (92.24)   
182 - **[GCCGCG]** 985 (86.55)   
182 - **.GACGCG.** 920 (92.24)   
182 - **.GACGCG.** 876 (92.24)   
182 - **[CGCGCG]** 770 (85.98)   
182 - **[CGCGCG]** 768 (85.98)   
182 - **[TTCGCG]** 750 (87.60)   
182 - **[GGCGCG]** 668 (86.50)   
182 - **[CGCGCG]** 634 (85.98)   
182 - **[CCCGCG]** 540 (86.03)   
182 - **[CCCGCG]** 533 (86.03)   
182 - **[CGCGCG]** 531 (85.98)   
182 - **[CCCGCG]** 495 (86.03)   
182 - **[GCCGCG]** 463 (86.55)   
182 - **[CCCGCG]** 434 (86.03)   
182 - **[CCCGCG]** 388 (86.03)   
182 - **[GCCGCG]** 377 (86.55)   
182 - **[CCCGCG]** 369 (86.03)   
182 - **[GCCGCG]** 266 (86.55)   
182 - **.TGCGCG.** 235 (94.26)   
182 - **[TTCGCG]** 36 (87.60)   
182 - **[CGCGCG]** 34 (85.98)   
183 + **[GGCGCG]** 364 (86.50)   
183 + **[CCCGCG]** 595 (86.03)   
183 + **[CGCGCG]** 650 (85.98)   
183 + **[GCCGCG]** 836 (86.55)   
183 + **[CCCGCG]** 912 (86.03)   
183 + **[CGCGCG]** 914 (85.98)   
183 + **[CGCGCG]** 916 (85.98)   
183 + **.TGCGCG.** 949 (94.26)   
183 + **[GGCGCG]** 983 (86.50)   
183 + **[CGCGCG]** 985 (85.98)   
183 + **[TAGGCG]** 1022 (85.06)   
183 + **.TGCGCG.** 1059 (94.26)   
183 + **[CCCGCG]** 1197 (86.03)   
183 + **[GCCGCG]** 1230 (86.55)   
183 + **[GCCGCG]** 1299 (86.55)   
183 + **.TGCGCG.** 1354 (94.26)   
183 + **[CCCGCG]** 1422 (86.03)   
183 - **[GGCGCG]** 1424 (86.50)   
183 - **[GCCGCG]** 1356 (86.55)   
183 - **.GACGCG.** 1301 (92.24)   
183 - **[CCCGCG]** 1271 (86.03)   
183 - **[GGCGCG]** 1232 (86.50)   
183 - **[GCCGCG]** 1061 (86.55)   
183 - **[CCCGCG]** 987 (86.03)   
183 - **[CGCGCG]** 985 (85.98)   
183 - **[CCCGCG]** 918 (86.03)   
183 - **[CGCGCG]** 916 (85.98)   
183 - **[CGCGCG]** 914 (85.98)   
183 - **[CCCGCG]** 838 (86.03)   
183 - **[CCCGCG]** 652 (86.03)   
183 - **[CGCGCG]** 650 (85.98)   
183 - **[CCCGCG]** 597 (86.03)   
183 - **.GACGCG.** 82 (92.24)   
184 + **[GCCGCG]** 54 (86.55)   
184 + **[CCCGCG]** 209 (86.03)   
184 + **.TCCGCG.** 631 (94.31)   
184 + **[CCCGCG]** 866 (86.03)   
184 + **[CCCGCG]** 924 (86.03)   
184 + **[CGCGCG]** 926 (85.98)   
184 + **[GGCGCG]** 1085 (86.50)   
184 + **[GCCGCG]** 1090 (86.55)   
184 + **[GCCGCG]** 1165 (86.55)   
184 + **[GGCGCG]** 1276 (86.50)   
184 + **[GGCGCG]** 1350 (86.50)   
184 + **[CCCGCG]** 1435 (86.03)   
184 - **[GGCGCG]** 1437 (86.50)   
184 - **[GCCGCG]** 1352 (86.55)   
184 - **[GCCGCG]** 1278 (86.55)   
184 - **[GGCGCG]** 1167 (86.50)   
184 - **.CACGCG.** 1092 (91.73)   
184 - **[GGCGCG]** 1087 (86.50)   
184 - **[GCCGCG]** 1068 (86.55)   
184 - **[GCCGCG]** 928 (86.55)   
184 - **[CGCGCG]** 926 (85.98)   
184 - **.TGCGCG.** 868 (94.26)   
184 - **.TGCGCG.** 731 (94.26)   
184 - **[GCCGCG]** 633 (86.55)   
185 + **[CCCGCG]** 7 (86.03)   
185 + **[GCCGCG]** 128 (86.55)   
185 + **[CGCGCG]** 130 (85.98)   
185 + **[GGCGCG]** 337 (86.50)   
185 + **[CGCGCG]** 339 (85.98)   
185 + **[CCCGCG]** 411 (86.03)   
185 + **[GCCGCG]** 555 (86.55)   
185 + **[GCCGCG]** 571 (86.55)   
185 + **[CGCGCG]** 573 (85.98)   
185 + **[CCCGCG]** 1011 (86.03)   
185 + **[CCCGCG]** 1032 (86.03)   
185 + **[GCCGCG]** 1063 (86.55)   
185 + **[GCCGCG]** 1090 (86.55)   
185 + **[GCCGCG]** 1190 (86.55)   
185 + **[CGCGCG]** 1513 (85.98)   
185 - **[CGCGCG]** 1513 (85.98)   
185 - **[GCCGCG]** 1192 (86.55)   
185 - **[AACGCG]** 1127 (87.55)   
185 - **[GGCGCG]** 1047 (86.50)   
185 - **[GGCGCG]** 1034 (86.50)   
185 - **.TCCGCG.** 1013 (94.31)   
185 - **.TGCGCG.** 594 (94.26)   
185 - **[CCCGCG]** 575 (86.03)   
185 - **[CGCGCG]** 573 (85.98)   
185 - **[GGCGCG]** 413 (86.50)   
185 - **[CGCGCG]** 339 (85.98)   
185 - **.TCCGCG.** 132 (94.31)   
185 - **[CGCGCG]** 130 (85.98)   
185 - **[GGCGCG]** 9 (86.50)   
186 + **.CACGCG.** 903 (91.73)   
186 + **.TCCGCG.** 1010 (94.31)   
186 + **.TGCGCG.** 1080 (94.26)   
186 + **.TGCGCG.** 1314 (94.26)   
186 + **.TCCGCG.** 1338 (94.31)   
186 + **[CGCGCG]** 1340 (85.98)   
186 + **[GCCGCG]** 1348 (86.55)   
186 + **.TGCGCG.** 1387 (94.26)   
186 + **.TGCGCG.** 1429 (94.26)   
186 + **[GGCGCG]** 1461 (86.50)   
186 - **[GCCGCG]** 1463 (86.55)   
186 - **.CACGCG.** 1431 (91.73)   
186 - **[GCCGCG]** 1389 (86.55)   
186 - **[GGCGCG]** 1350 (86.50)   
186 - **.TGCGCG.** 1342 (94.26)   
186 - **[CGCGCG]** 1340 (85.98)   
186 - **[GGCGCG]** 1316 (86.50)   
186 - **[TAGGCG]** 1246 (85.06)   
186 - **.GACGCG.** 1082 (92.24)   
186 - **[GGCGCG]** 1012 (86.50)   
186 - **.TCCGCG.** 922 (94.31)   
186 - **.GACGCG.** 905 (92.24)   
187 + **.TGCGCG.** 19 (94.26)   
187 + **.TGCGCG.** 923 (94.26)   
187 + **[GGCGCG]** 946 (86.50)   
187 + **[AACGCG]** 1097 (87.55)   
187 + **[GGCGCG]** 1125 (86.50)   
187 + **[GGCGCG]** 1176 (86.50)   
187 + **[GGCGCG]** 1229 (86.50)   
187 + **[GCCGCG]** 1302 (86.55)   
187 + **[CCCGCG]** 1310 (86.03)   
187 + **.TGCGCG.** 1500 (94.26)   
187 - **.TCCGCG.** 1304 (94.31)   
187 - **[CCCGCG]** 1237 (86.03)   
187 - **[CCCGCG]** 1178 (86.03)   
187 - **.TGCGCG.** 1136 (94.26)   
187 - **[CCCGCG]** 1106 (86.03)   
187 - **[TTCGCG]** 1099 (87.60)   
187 - **.TCCGCG.** 1076 (94.31)   
187 - **[GCCGCG]** 948 (86.55)   
187 - **.GACGCG.** 925 (92.24)   
187 - **[GGCGCG]** 21 (86.50)   
188 + **.TCCGCG.** 203 (94.31)   
188 + **[CCCGCG]** 312 (86.03)   
188 + **.GACGCG.** 387 (92.24)   
188 + **[GCCGCG]** 414 (86.55)   
188 + **[TAGGCG]** 831 (85.06)   
188 + **.TGCGCG.** 1438 (94.26)   
188 + **[GCCGCG]** 1443 (86.55)   
188 + **[GCCGCG]** 1470 (86.55)   
188 - **[GGCGCG]** 1472 (86.50)   
188 - **[GCCGCG]** 1445 (86.55)   
188 - **[GGCGCG]** 1440 (86.50)   
188 - **.CACGCG.** 1298 (91.73)   
188 - **.CACGCG.** 773 (91.73)   
188 - **.TCCGCG.** 416 (94.31)   
188 - **.TCCGCG.** 389 (94.31)   
188 - **.TCCGCG.** 363 (94.31)   
188 - **[CCCGCG]** 226 (86.03)   
188 - **[GGCGCG]** 205 (86.50)   
189 + **[GGCGCG]** 849 (86.50)   
189 + **.TGCGCG.** 957 (94.26)   
189 + **[GGCGCG]** 1472 (86.50)   
189 - **[GGCGCG]** 1474 (86.50)   
189 - **.TCCGCG.** 1194 (94.31)   
189 - **[CCCGCG]** 1181 (86.03)   
189 - **[AACGCG]** 1054 (87.55)   
189 - **.TGCGCG.** 951 (94.26)   
189 - **[CCCGCG]** 927 (86.03)   
189 - **.CACGCG.** 851 (91.73)   
191 + **[GGCGCG]** 97 (86.50)   
191 + **[CCCGCG]** 203 (86.03)   
191 + **[CGCGCG]** 205 (85.98)   
191 + **.TGCGCG.** 489 (94.26)   
191 + **.TGCGCG.** 545 (94.26)   
191 + **[CGCGCG]** 547 (85.98)   
191 + **[CGCGCG]** 549 (85.98)   
191 + **[CGCGCG]** 551 (85.98)   
191 + **.TGCGCG.** 581 (94.26)   
191 + **[GGCGCG]** 616 (86.50)   
191 + **[AACGCG]** 636 (87.55)   
191 + **.CACGCG.** 650 (91.73)   
191 + **[CCCGCG]** 735 (86.03)   
191 + **[GGCGCG]** 790 (86.50)   
191 + **[CGCGCG]** 792 (85.98)   
191 + **[GGCGCG]** 823 (86.50)   
191 + **[CGCGCG]** 825 (85.98)   
191 + **[GCCGCG]** 831 (86.55)   
191 + **[GGCGCG]** 839 (86.50)   
191 + **[GGCGCG]** 877 (86.50)   
191 + **[CGCGCG]** 879 (85.98)   
191 + **[CGCGCG]** 881 (85.98)   
191 + **[CCCGCG]** 900 (86.03)   
191 + **[CGCGCG]** 936 (85.98)   
191 + **[GGCGCG]** 1043 (86.50)   
191 + **.TCCGCG.** 1090 (94.31)   
191 + **[GGCGCG]** 1137 (86.50)   
191 + **[GGCGCG]** 1161 (86.50)   
191 + **.TGCGCG.** 1187 (94.26)   
191 + **[CGCGCG]** 1189 (85.98)   
191 + **.CACGCG.** 1477 (91.73)   
191 + **[CGCGCG]** 1479 (85.98)   
191 - **[CCCGCG]** 1481 (86.03)   
191 - **[CGCGCG]** 1479 (85.98)   
191 - **[GCCGCG]** 1191 (86.55)   
191 - **[CGCGCG]** 1189 (85.98)   
191 - **[TTCGCG]** 1139 (87.60)   
191 - **[GCCGCG]** 1045 (86.55)   
191 - **[CGCGCG]** 936 (85.98)   
191 - **[GCCGCG]** 883 (86.55)   
191 - **[CGCGCG]** 881 (85.98)   
191 - **[CGCGCG]** 879 (85.98)   
191 - **[CCCGCG]** 841 (86.03)   
191 - **.TCCGCG.** 833 (94.31)   
191 - **[GCCGCG]** 827 (86.55)   
191 - **[CGCGCG]** 825 (85.98)   
191 - **[CCCGCG]** 794 (86.03)   
191 - **[CGCGCG]** 792 (85.98)   
191 - **.CACGCG.** 755 (91.73)   
191 - **.TGCGCG.** 737 (94.26)   
191 - **.CACGCG.** 693 (91.73)   
191 - **.CACGCG.** 652 (91.73)   
191 - **[GGCGCG]** 618 (86.50)   
191 - **.CACGCG.** 583 (91.73)   
191 - **[GCCGCG]** 569 (86.55)   
191 - **[AACGCG]** 553 (87.55)   
191 - **[CGCGCG]** 551 (85.98)   
191 - **[CGCGCG]** 549 (85.98)   
191 - **[CGCGCG]** 547 (85.98)   
191 - **[GGCGCG]** 491 (86.50)   
191 - **[AACGCG]** 316 (87.55)   
191 - **[CGCGCG]** 205 (85.98)   
193 + **[CGCGCG]** 253 (85.98)   
193 + **[GGCGCG]** 642 (86.50)   
193 + **[AACGCG]** 845 (87.55)   
193 + **[CGCGCG]** 847 (85.98)   
193 + **[CGCGCG]** 849 (85.98)   
193 + **[CGCGCG]** 851 (85.98)   
193 + **[CGCGCG]** 853 (85.98)   
193 + **[CGCGCG]** 855 (85.98)   
193 + **[CGCGCG]** 857 (85.98)   
193 + **[CGCGCG]** 859 (85.98)   
193 + **.CACGCG.** 894 (91.73)   
193 + **[AACGCG]** 1040 (87.55)   
193 + **.GACGCG.** 1124 (92.24)   
193 + **[CCCGCG]** 1203 (86.03)   
193 + **[CGCGCG]** 1205 (85.98)   
193 - **[GCCGCG]** 1249 (86.55)   
193 - **[CGCGCG]** 1205 (85.98)   
193 - **.TGCGCG.** 1169 (94.26)   
193 - **.TGCGCG.** 1042 (94.26)   
193 - **.TCCGCG.** 896 (94.31)   
193 - **.TGCGCG.** 861 (94.26)   
193 - **[CGCGCG]** 859 (85.98)   
193 - **[CGCGCG]** 857 (85.98)   
193 - **[CGCGCG]** 855 (85.98)   
193 - **[CGCGCG]** 853 (85.98)   
193 - **[CGCGCG]** 851 (85.98)   
193 - **[CGCGCG]** 849 (85.98)   
193 - **[CGCGCG]** 847 (85.98)   
193 - **.CACGCG.** 255 (91.73)   
193 - **[CGCGCG]** 253 (85.98)   
194 + **[CCCGCG]** 112 (86.03)   
194 + **[GCCGCG]** 723 (86.55)   
194 + **[GGCGCG]** 867 (86.50)   
194 + **[CGCGCG]** 869 (85.98)   
194 + **.GACGCG.** 908 (92.24)   
194 + **[CGCGCG]** 910 (85.98)   
194 + **[GGCGCG]** 943 (86.50)   
194 + **[GGCGCG]** 973 (86.50)   
194 + **[GCCGCG]** 988 (86.55)   
194 + **[GGCGCG]** 1016 (86.50)   
194 + **[GCCGCG]** 1040 (86.55)   
194 + **[GGCGCG]** 1066 (86.50)   
194 + **[CGCGCG]** 1068 (85.98)   
194 + **.TGCGCG.** 1098 (94.26)   
194 + **[GCCGCG]** 1110 (86.55)   
194 + **[GGCGCG]** 1115 (86.50)   
194 + **[CCCGCG]** 1252 (86.03)   
194 + **.TGCGCG.** 1329 (94.26)   
194 - **[TAGGCG]** 1505 (85.06)   
194 - **[CCCGCG]** 1331 (86.03)   
194 - **[GGCGCG]** 1254 (86.50)   
194 - **[CCCGCG]** 1117 (86.03)   
194 - **[GCCGCG]** 1112 (86.55)   
194 - **.CACGCG.** 1070 (91.73)   
194 - **[CGCGCG]** 1068 (85.98)   
194 - **.TCCGCG.** 1042 (94.31)   
194 - **[GCCGCG]** 990 (86.55)   
194 - **[TTCGCG]** 975 (87.60)   
194 - **[GCCGCG]** 945 (86.55)   
194 - **[GCCGCG]** 912 (86.55)   
194 - **[CGCGCG]** 910 (85.98)   
194 - **[GCCGCG]** 871 (86.55)   
194 - **[CGCGCG]** 869 (85.98)   
194 - **[CCCGCG]** 690 (86.03)   
194 - **[GGCGCG]** 608 (86.50)   
195 + **.TCCGCG.** 108 (94.31)   
195 + **.TCCGCG.** 151 (94.31)   
195 + **[GCCGCG]** 166 (86.55)   
195 + **[CCCGCG]** 249 (86.03)   
195 + **[GGCGCG]** 907 (86.50)   
195 + **[CCCGCG]** 920 (86.03)   
195 + **[CGCGCG]** 922 (85.98)   
195 + **.TCCGCG.** 956 (94.31)   
195 + **[CGCGCG]** 958 (85.98)   
195 - **.GACGCG.** 960 (92.24)   
195 - **[CGCGCG]** 958 (85.98)   
195 - **[GGCGCG]** 924 (86.50)   
195 - **[CGCGCG]** 922 (85.98)   
195 - **.TGCGCG.** 909 (94.26)   
195 - **[GCCGCG]** 852 (86.55)   
195 - **[GGCGCG]** 251 (86.50)   
195 - **[GCCGCG]** 168 (86.55)   
195 - **[GGCGCG]** 153 (86.50)   
195 - **.TCCGCG.** 110 (94.31)   
196 + **.GACGCG.** 31 (92.24)   
196 + **[GCCGCG]** 342 (86.55)   
196 + **.CACGCG.** 497 (91.73)   
196 + **[GGCGCG]** 504 (86.50)   
196 + **[GGCGCG]** 666 (86.50)   
196 + **.GACGCG.** 688 (92.24)   
196 + **.CACGCG.** 719 (91.73)   
196 + **[GCCGCG]** 911 (86.55)   
196 + **[CCCGCG]** 1074 (86.03)   
196 + **[GCCGCG]** 1102 (86.55)   
196 + **.TCCGCG.** 1174 (94.31)   
196 + **[CGCGCG]** 1188 (85.98)   
196 + **[CGCGCG]** 1190 (85.98)   
196 + **[GGCGCG]** 1352 (86.50)   
196 + **[TTCGCG]** 1391 (87.60)   
196 + **[GGCGCG]** 1427 (86.50)   
196 + **[GGCGCG]** 1471 (86.50)   
196 + **[CCCGCG]** 1513 (86.03)   
196 - **[GGCGCG]** 1508 (86.50)   
196 - **[CCCGCG]** 1473 (86.03)   
196 - **.TGCGCG.** 1354 (94.26)   
196 - **[GGCGCG]** 1317 (86.50)   
196 - **.CACGCG.** 1192 (91.73)   
196 - **[CGCGCG]** 1190 (85.98)   
196 - **[CGCGCG]** 1188 (85.98)   
196 - **[CCCGCG]** 1176 (86.03)   
196 - **[AACGCG]** 1104 (87.55)   
196 - **[GCCGCG]** 1076 (86.55)   
196 - **[GGCGCG]** 913 (86.50)   
196 - **.GACGCG.** 721 (92.24)   
196 - **.TCCGCG.** 690 (94.31)   
196 - **[CCCGCG]** 662 (86.03)   
196 - **.TCCGCG.** 548 (94.31)   
196 - **[GCCGCG]** 538 (86.55)   
196 - **[CCCGCG]** 518 (86.03)   
196 - **[GGCGCG]** 506 (86.50)   
196 - **[CCCGCG]** 499 (86.03)   
196 - **.TGCGCG.** 344 (94.26)   
196 - **[GCCGCG]** 284 (86.55)   
196 - **[GCCGCG]** 120 (86.55)   
196 - **.TACGCG.** 33 (100.00)   
197 + **[TTCGCG]** 429 (87.60)   
197 + **[GCCGCG]** 789 (86.55)   
197 + **.GACGCG.** 1100 (92.24)   
197 + **.GACGCG.** 1161 (92.24)   
197 + **[TAGGCG]** 1269 (85.06)   
197 + **[CGCGCG]** 1423 (85.98)   
197 + **[GGCGCG]** 1504 (86.50)   
197 + **[GGCGCG]** 1509 (86.50)   
197 - **.TCCGCG.** 1511 (94.31)   
197 - **[GCCGCG]** 1506 (86.55)   
197 - **.TGCGCG.** 1425 (94.26)   
197 - **[CGCGCG]** 1423 (85.98)   
197 - **[GCCGCG]** 1163 (86.55)   
197 - **[CCCGCG]** 1102 (86.03)   
197 - **[GCCGCG]** 1047 (86.55)   
197 - **[GGCGCG]** 791 (86.50)   
197 - **[GCCGCG]** 478 (86.55)   
197 - **.GACGCG.** 431 (92.24)   
198 + **.TCCGCG.** 33 (94.31)   
198 + **[CCCGCG]** 212 (86.03)   
198 + **[CGCGCG]** 214 (85.98)   
198 + **[GCCGCG]** 257 (86.55)   
198 + **[CCCGCG]** 264 (86.03)   
198 + **.TGCGCG.** 1163 (94.26)   
198 + **[CCCGCG]** 1435 (86.03)   
198 - **[GGCGCG]** 1437 (86.50)   
198 - **[GCCGCG]** 1165 (86.55)   
198 - **[TAGGCG]** 717 (85.06)   
198 - **[CCCGCG]** 710 (86.03)   
198 - **[GGCGCG]** 266 (86.50)   
198 - **[GGCGCG]** 259 (86.50)   
198 - **.GACGCG.** 216 (92.24)   
198 - **[CGCGCG]** 214 (85.98)   
198 - **[GGCGCG]** 35 (86.50)   
199 + **.CACGCG.** 311 (91.73)   
199 + **[GCCGCG]** 438 (86.55)   
199 + **[GGCGCG]** 670 (86.50)   
199 + **[GGCGCG]** 819 (86.50)   
199 + **[GGCGCG]** 870 (86.50)   
199 + **[CGCGCG]** 872 (85.98)   
199 + **[GGCGCG]** 906 (86.50)   
199 + **[GGCGCG]** 911 (86.50)   
199 + **[GGCGCG]** 938 (86.50)   
199 + **[GGCGCG]** 980 (86.50)   
199 + **[GCCGCG]** 1001 (86.55)   
199 + **[CCCGCG]** 1062 (86.03)   
199 + **[CGCGCG]** 1064 (85.98)   
199 + **[GGCGCG]** 1083 (86.50)   
199 + **[GGCGCG]** 1110 (86.50)   
199 + **[GCCGCG]** 1127 (86.55)   
199 + **.CACGCG.** 1242 (91.73)   
199 + **[GCCGCG]** 1265 (86.55)   
199 + **[CGCGCG]** 1267 (85.98)   
199 + **[GGCGCG]** 1286 (86.50)   
199 + **[CGCGCG]** 1298 (85.98)   
199 + **[GGCGCG]** 1320 (86.50)   
199 + **[CGCGCG]** 1322 (85.98)   
199 + **[GGCGCG]** 1352 (86.50)   
199 + **[CCCGCG]** 1446 (86.03)   
199 + **[CCCGCG]** 1489 (86.03)   
199 - **[GGCGCG]** 1491 (86.50)   
199 - **.GACGCG.** 1448 (92.24)   
199 - **[CCCGCG]** 1354 (86.03)   
199 - **.TGCGCG.** 1324 (94.26)   
199 - **[CGCGCG]** 1322 (85.98)   
199 - **.TGCGCG.** 1300 (94.26)   
199 - **[CGCGCG]** 1298 (85.98)   
199 - **[GCCGCG]** 1276 (86.55)   
199 - **.TGCGCG.** 1269 (94.26)   
199 - **[CGCGCG]** 1267 (85.98)   
199 - **[GGCGCG]** 1244 (86.50)   
199 - **.TGCGCG.** 1112 (94.26)   
199 - **[GCCGCG]** 1085 (86.55)   
199 - **[CCCGCG]** 1066 (86.03)   
199 - **[CGCGCG]** 1064 (85.98)   
199 - **.TCCGCG.** 1026 (94.31)   
199 - **[GCCGCG]** 1003 (86.55)   
199 - **[CCCGCG]** 982 (86.03)   
199 - **[GCCGCG]** 951 (86.55)   
199 - **.TCCGCG.** 940 (94.31)   
199 - **[CCCGCG]** 913 (86.03)   
199 - **[GCCGCG]** 908 (86.55)   
199 - **.TGCGCG.** 874 (94.26)   
199 - **[CGCGCG]** 872 (85.98)   
199 - **.CACGCG.** 826 (91.73)   
199 - **.GACGCG.** 821 (92.24)   
199 - **[GGCGCG]** 672 (86.50)   
199 - **[CCCGCG]** 539 (86.03)   
199 - **.CACGCG.** 440 (91.73)   
199 - **.TCCGCG.** 313 (94.31)   
200 + **.GACGCG.** 572 (92.24)   
200 + **[GGCGCG]** 1140 (86.50)   
200 - **.TGCGCG.** 1504 (94.26)   
200 - **[GCCGCG]** 1142 (86.55)   
200 - **.TCCGCG.** 903 (94.31)   
200 - **.TCCGCG.** 574 (94.31)   
201 + **[AACGCG]** 67 (87.55)   
201 + **[GCCGCG]** 337 (86.55)   
201 + **[CCCGCG]** 571 (86.03)   
201 + **[GGCGCG]** 627 (86.50)   
201 + **.TGCGCG.** 659 (94.26)   
201 + **[GGCGCG]** 676 (86.50)   
201 + **[CCCGCG]** 743 (86.03)   
201 + **[GGCGCG]** 776 (86.50)   
201 + **[CCCGCG]** 847 (86.03)   
201 + **[CCCGCG]** 1018 (86.03)   
201 + **.TCCGCG.** 1083 (94.31)   
201 + **[GCCGCG]** 1142 (86.55)   
201 + **[CGCGCG]** 1144 (85.98)   
201 + **.TGCGCG.** 1199 (94.26)   
201 + **[GCCGCG]** 1349 (86.55)   
201 + **.TCCGCG.** 1412 (94.31)   
201 + **.GACGCG.** 1491 (92.24)   
201 + **[CGCGCG]** 1493 (85.98)   
201 + **[CGCGCG]** 1495 (85.98)   
201 + **[CGCGCG]** 1497 (85.98)   
201 - **[GGCGCG]** 1499 (86.50)   
201 - **[CGCGCG]** 1497 (85.98)   
201 - **[CGCGCG]** 1495 (85.98)   
201 - **[CGCGCG]** 1493 (85.98)   
201 - **[GGCGCG]** 1414 (86.50)   
201 - **[CCCGCG]** 1351 (86.03)   
201 - **.GACGCG.** 1201 (92.24)   
201 - **[CCCGCG]** 1146 (86.03)   
201 - **[CGCGCG]** 1144 (85.98)   
201 - **[GGCGCG]** 1085 (86.50)   
201 - **[GCCGCG]** 849 (86.55)   
201 - **[CCCGCG]** 778 (86.03)   
201 - **.TCCGCG.** 745 (94.31)   
201 - **.TGCGCG.** 678 (94.26)   
201 - **[GGCGCG]** 661 (86.50)   
201 - **[CCCGCG]** 629 (86.03)   
201 - **[AACGCG]** 573 (87.55)   
201 - **[GCCGCG]** 533 (86.55)   
201 - **[GCCGCG]** 351 (86.55)   
201 - **[GGCGCG]** 339 (86.50)   
202 + **.GACGCG.** 310 (92.24)   
202 + **[GGCGCG]** 593 (86.50)   
202 + **[GGCGCG]** 722 (86.50)   
202 + **.TCCGCG.** 990 (94.31)   
202 + **[CCCGCG]** 1205 (86.03)   
202 + **[GGCGCG]** 1285 (86.50)   
202 + **.GACGCG.** 1393 (92.24)   
202 - **[GGCGCG]** 1395 (86.50)   
202 - **[GCCGCG]** 1371 (86.55)   
202 - **.TGCGCG.** 1287 (94.26)   
202 - **[CCCGCG]** 1207 (86.03)   
202 - **.GACGCG.** 992 (92.24)   
202 - **[CCCGCG]** 595 (86.03)   
202 - **.TCCGCG.** 312 (94.31)   
203 - **[GCCGCG]** 102 (86.55)   
207 + **[CCCGCG]** 1149 (86.03)   
207 + **[CGCGCG]** 1151 (85.98)   
207 + **.CACGCG.** 1289 (91.73)   
207 - **[CCCGCG]** 1331 (86.03)   
207 - **.CACGCG.** 1153 (91.73)   
207 - **[CGCGCG]** 1151 (85.98)   
207 - **.GACGCG.** 857 (92.24)   
208 + **[CCCGCG]** 951 (86.03)   
208 + **[CGCGCG]** 953 (85.98)   
208 + **[TAGGCG]** 962 (85.06)   
208 + **.GACGCG.** 1226 (92.24)   
208 + **[GGCGCG]** 1396 (86.50)   
208 + **[GGCGCG]** 1409 (86.50)   
208 + **[GCCGCG]** 1443 (86.55)   
208 + **[GCCGCG]** 1497 (86.55)   
208 - **[GCCGCG]** 1499 (86.55)   
208 - **[CCCGCG]** 1445 (86.03)   
208 - **.TGCGCG.** 1411 (94.26)   
208 - **[GGCGCG]** 1359 (86.50)   
208 - **[CGCGCG]** 953 (85.98)   
209 + **[GCCGCG]** 838 (86.55)   
209 + **[CGCGCG]** 1000 (85.98)   
209 - **.TGCGCG.** 1002 (94.26)   
209 - **[CGCGCG]** 1000 (85.98)   
209 - **.CACGCG.** 840 (91.73)   
210 + **.TGCGCG.** 555 (94.26)   
210 + **[CCCGCG]** 614 (86.03)   
210 + **[GGCGCG]** 675 (86.50)   
210 + **[GCCGCG]** 778 (86.55)   
210 + **[CCCGCG]** 802 (86.03)   
210 + **[GCCGCG]** 1047 (86.55)   
210 + **[GGCGCG]** 1066 (86.50)   
210 + **.CACGCG.** 1221 (91.73)   
210 + **[CGCGCG]** 1321 (85.98)   
210 - **.TGCGCG.** 1323 (94.26)   
210 - **[CGCGCG]** 1321 (85.98)   
210 - **[GGCGCG]** 1223 (86.50)   
210 - **.TCCGCG.** 1068 (94.31)   
210 - **[GCCGCG]** 780 (86.55)   
210 - **.GACGCG.** 677 (92.24)   
210 - **[GGCGCG]** 616 (86.50)   
210 - **.TACGCG.** 557 (100.00)   
211 + **[CCCGCG]** 612 (86.03)   
211 + **[CCCGCG]** 721 (86.03)   
211 + **[GCCGCG]** 735 (86.55)   
211 + **[CCCGCG]** 781 (86.03)   
211 + **[CCCGCG]** 849 (86.03)   
211 + **[CCCGCG]** 975 (86.03)   
211 + **.TCCGCG.** 1066 (94.31)   
211 + **.GACGCG.** 1145 (92.24)   
211 + **.TCCGCG.** 1245 (94.31)   
211 + **[CCCGCG]** 1314 (86.03)   
211 + **.GACGCG.** 1405 (92.24)   
211 + **.TGCGCG.** 1479 (94.26)   
211 - **[GGCGCG]** 1481 (86.50)   
211 - **.CACGCG.** 1407 (91.73)   
211 - **.TCCGCG.** 1316 (94.31)   
211 - **[GCCGCG]** 1216 (86.55)   
211 - **[GGCGCG]** 977 (86.50)   
211 - **.TGCGCG.** 851 (94.26)   
211 - **[GGCGCG]** 737 (86.50)   
211 - **.TGCGCG.** 723 (94.26)   
211 - **[CCCGCG]** 676 (86.03)   
212 + **[GGCGCG]** 420 (86.50)   
212 + **.TCCGCG.** 453 (94.31)   
212 + **[CCCGCG]** 555 (86.03)   
212 + **[CCCGCG]** 657 (86.03)   
212 + **[AACGCG]** 686 (87.55)   
212 + **[CGCGCG]** 688 (85.98)   
212 + **[GCCGCG]** 713 (86.55)   
212 + **.TGCGCG.** 907 (94.26)   
212 + **.TCCGCG.** 1032 (94.31)   
212 + **.CACGCG.** 1484 (91.73)   
212 - **[GCCGCG]** 1486 (86.55)   
212 - **[CCCGCG]** 1433 (86.03)   
212 - **.GACGCG.** 1034 (92.24)   
212 - **[AACGCG]** 909 (87.55)   
212 - **[GCCGCG]** 715 (86.55)   
212 - **[CGCGCG]** 688 (85.98)   
212 - **[CCCGCG]** 659 (86.03)   
212 - **.TGCGCG.** 557 (94.26)   
212 - **[GGCGCG]** 455 (86.50)   
213 + **[GCCGCG]** 912 (86.55)   
213 + **[GGCGCG]** 997 (86.50)   
213 + **[GGCGCG]** 1061 (86.50)   
213 + **.CACGCG.** 1121 (91.73)   
213 + **[CCCGCG]** 1191 (86.03)   
213 + **.TGCGCG.** 1288 (94.26)   
213 - **[GGCGCG]** 1290 (86.50)   
213 - **[GGCGCG]** 1193 (86.50)   
213 - **[CCCGCG]** 1063 (86.03)   
213 - **.TCCGCG.** 973 (94.31)   
214 + **.CACGCG.** 631 (91.73)   
214 + **[CCCGCG]** 711 (86.03)   
214 + **[CGCGCG]** 730 (85.98)   
214 + **.TCCGCG.** 832 (94.31)   
214 + **[GCCGCG]** 929 (86.55)   
214 + **.CACGCG.** 984 (91.73)   
214 + **.TCCGCG.** 1016 (94.31)   
214 + **[CCCGCG]** 1270 (86.03)   
214 + **[CGCGCG]** 1272 (85.98)   
214 - **[CGCGCG]** 1272 (85.98)   
214 - **[TTCGCG]** 1192 (87.60)   
214 - **[GGCGCG]** 1033 (86.50)   
214 - **.TGCGCG.** 986 (94.26)   
214 - **[GCCGCG]** 931 (86.55)   
214 - **[GCCGCG]** 834 (86.55)   
214 - **[GCCGCG]** 732 (86.55)   
214 - **[CGCGCG]** 730 (85.98)   
214 - **.TCCGCG.** 713 (94.31)   
214 - **.GACGCG.** 355 (92.24)   
215 + **[GGCGCG]** 112 (86.50)   
215 + **[CGCGCG]** 114 (85.98)   
215 + **[CCCGCG]** 518 (86.03)   
215 + **[GCCGCG]** 652 (86.55)   
215 + **.TGCGCG.** 1021 (94.26)   
215 + **[GCCGCG]** 1090 (86.55)   
215 + **[CCCGCG]** 1138 (86.03)   
215 + **.GACGCG.** 1195 (92.24)   
215 + **[GCCGCG]** 1413 (86.55)   
215 + **[GGCGCG]** 1459 (86.50)   
215 - **.GACGCG.** 1461 (92.24)   
215 - **[GGCGCG]** 1415 (86.50)   
215 - **[GGCGCG]** 1369 (86.50)   
215 - **.TGCGCG.** 1197 (94.26)   
215 - **[GGCGCG]** 1140 (86.50)   
215 - **[GCCGCG]** 1092 (86.55)   
215 - **[CCCGCG]** 1023 (86.03)   
215 - **.GACGCG.** 654 (92.24)   
215 - **[CCCGCG]** 520 (86.03)   
215 - **[GGCGCG]** 201 (86.50)   
215 - **[GGCGCG]** 116 (86.50)   
215 - **[CGCGCG]** 114 (85.98)   
216 + **[TTCGCG]** 210 (87.60)   
216 + **.TCCGCG.** 252 (94.31)   
216 + **[CGCGCG]** 945 (85.98)   
216 + **[GGCGCG]** 992 (86.50)   
216 + **[CGCGCG]** 994 (85.98)   
216 + **[CCCGCG]** 1064 (86.03)   
216 + **[CCCGCG]** 1094 (86.03)   
216 + **[GCCGCG]** 1228 (86.55)   
216 + **[GGCGCG]** 1465 (86.50)   
216 + **[GCCGCG]** 1481 (86.55)   
216 - **.TCCGCG.** 1483 (94.31)   
216 - **[AACGCG]** 1321 (87.55)   
216 - **[GCCGCG]** 1230 (86.55)   
216 - **[GGCGCG]** 1096 (86.50)   
216 - **[GGCGCG]** 996 (86.50)   
216 - **[CGCGCG]** 994 (85.98)   
216 - **[GGCGCG]** 947 (86.50)   
216 - **[CGCGCG]** 945 (85.98)   
216 - **.GACGCG.** 892 (92.24)   
216 - **[TAGGCG]** 664 (85.06)   
216 - **[GGCGCG]** 650 (86.50)   
216 - **[CCCGCG]** 254 (86.03)   
216 - **[GCCGCG]** 212 (86.55)   
217 + **[GCCGCG]** 573 (86.55)   
217 + **[CGCGCG]** 575 (85.98)   
217 + **[CCCGCG]** 663 (86.03)   
217 + **[CCCGCG]** 695 (86.03)   
217 + **[GCCGCG]** 860 (86.55)   
217 + **[CGCGCG]** 862 (85.98)   
217 + **[CCCGCG]** 1041 (86.03)   
217 - **.GACGCG.** 1094 (92.24)   
217 - **.TCCGCG.** 1043 (94.31)   
217 - **.GACGCG.** 941 (92.24)   
217 - **.TGCGCG.** 864 (94.26)   
217 - **[CGCGCG]** 862 (85.98)   
217 - **.TCCGCG.** 697 (94.31)   
217 - **.GACGCG.** 665 (92.24)   
217 - **[GCCGCG]** 577 (86.55)   
217 - **[CGCGCG]** 575 (85.98)   
220 - **[TAGGCG]** 1093 (85.06)   
221 + **[GCCGCG]** 18 (86.55)   
221 + **[GCCGCG]** 515 (86.55)   
221 + **[CGCGCG]** 517 (85.98)   
221 + **[GGCGCG]** 937 (86.50)   
221 + **[GCCGCG]** 1110 (86.55)   
221 + **[CCCGCG]** 1155 (86.03)   
221 + **[CGCGCG]** 1157 (85.98)   
221 + **[CCCGCG]** 1286 (86.03)   
221 + **[CGCGCG]** 1288 (85.98)   
221 - **[CGCGCG]** 1288 (85.98)   
221 - **[CGCGCG]** 1157 (85.98)   
221 - **[GCCGCG]** 1112 (86.55)   
221 - **[CCCGCG]** 939 (86.03)   
221 - **[GCCGCG]** 519 (86.55)   
221 - **[CGCGCG]** 517 (85.98)   
221 - **.CACGCG.** 305 (91.73)   
221 - **.GACGCG.** 20 (92.24)   
222 + **.CACGCG.** 166 (91.73)   
222 + **.TGCGCG.** 214 (94.26)   
222 + **[TAGGCG]** 434 (85.06)   
222 + **[CCCGCG]** 448 (86.03)   
222 + **[CGCGCG]** 450 (85.98)   
222 + **[CGCGCG]** 471 (85.98)   
222 + **[CCCGCG]** 531 (86.03)   
222 + **[CCCGCG]** 676 (86.03)   
222 + **[GGCGCG]** 841 (86.50)   
222 + **[CCCGCG]** 869 (86.03)   
222 + **[GCCGCG]** 877 (86.55)   
222 + **[CCCGCG]** 948 (86.03)   
222 + **[CCCGCG]** 958 (86.03)   
222 + **[GCCGCG]** 1001 (86.55)   
222 + **[CGCGCG]** 1003 (85.98)   
222 + **.TCCGCG.** 1026 (94.31)   
222 + **[CCCGCG]** 1079 (86.03)   
222 + **[CCCGCG]** 1097 (86.03)   
222 + **.TGCGCG.** 1185 (94.26)   
222 + **[GGCGCG]** 1204 (86.50)   
222 + **[GGCGCG]** 1270 (86.50)   
222 + **[GCCGCG]** 1482 (86.55)   
222 - **[GCCGCG]** 1484 (86.55)   
222 - **[CCCGCG]** 1272 (86.03)   
222 - **[GGCGCG]** 1206 (86.50)   
222 - **.TCCGCG.** 1187 (94.31)   
222 - **[GGCGCG]** 1099 (86.50)   
222 - **[GGCGCG]** 1081 (86.50)   
222 - **[GGCGCG]** 1005 (86.50)   
222 - **[CGCGCG]** 1003 (85.98)   
222 - **[CCCGCG]** 960 (86.03)   
222 - **[GCCGCG]** 950 (86.55)   
222 - **.GACGCG.** 940 (92.24)   
222 - **[GGCGCG]** 926 (86.50)   
222 - **.GACGCG.** 879 (92.24)   
222 - **.TCCGCG.** 871 (94.31)   
222 - **[GCCGCG]** 843 (86.55)   
222 - **[GGCGCG]** 691 (86.50)   
222 - **[CCCGCG]** 533 (86.03)   
222 - **[CGCGCG]** 471 (85.98)   
222 - **.GACGCG.** 452 (92.24)   
222 - **[CGCGCG]** 450 (85.98)   
222 - **[GCCGCG]** 168 (86.55)   
223 + **.TACGCG.** 390 (100.00)   
223 + **.GACGCG.** 422 (92.24)   
223 + **.GACGCG.** 456 (92.24)   
223 + **[CCCGCG]** 466 (86.03)   
223 + **[CGCGCG]** 468 (85.98)   
223 + **[CGCGCG]** 505 (85.98)   
223 + **[TTCGCG]** 697 (87.60)   
223 + **[CGCGCG]** 699 (85.98)   
223 - **.GACGCG.** 701 (92.24)   
223 - **[CGCGCG]** 699 (85.98)   
223 - **[CCCGCG]** 507 (86.03)   
223 - **[CGCGCG]** 505 (85.98)   
223 - **[CCCGCG]** 470 (86.03)   
223 - **[CGCGCG]** 468 (85.98)   
223 - **[CCCGCG]** 458 (86.03)   
223 - **[GGCGCG]** 424 (86.50)   
224 + **[GGCGCG]** 471 (86.50)   
224 + **[GGCGCG]** 478 (86.50)   
224 + **[GCCGCG]** 590 (86.55)   
224 + **[CGCGCG]** 592 (85.98)   
224 + **[GGCGCG]** 615 (86.50)   
224 + **[CGCGCG]** 617 (85.98)   
224 + **[GCCGCG]** 690 (86.55)   
224 + **[GGCGCG]** 718 (86.50)   
224 + **[CGCGCG]** 795 (85.98)   
224 + **[CGCGCG]** 797 (85.98)   
224 + **[GCCGCG]** 823 (86.55)   
224 + **[GGCGCG]** 854 (86.50)   
224 + **.TGCGCG.** 927 (94.26)   
224 + **[CCCGCG]** 1026 (86.03)   
224 + **[CCCGCG]** 1041 (86.03)   
224 + **.TACGCG.** 1066 (100.00)   
224 + **.CACGCG.** 1176 (91.73)   
224 + **[GGCGCG]** 1183 (86.50)   
224 + **[GCCGCG]** 1234 (86.55)   
224 + **[GGCGCG]** 1326 (86.50)   
224 - **.TCCGCG.** 1328 (94.31)   
224 - **.TGCGCG.** 1236 (94.26)   
224 - **.TCCGCG.** 1203 (94.31)   
224 - **[CCCGCG]** 1185 (86.03)   
224 - **[CCCGCG]** 1178 (86.03)   
224 - **.CACGCG.** 1068 (91.73)   
224 - **[CCCGCG]** 1028 (86.03)   
224 - **[GCCGCG]** 856 (86.55)   
224 - **[CCCGCG]** 825 (86.03)   
224 - **[GCCGCG]** 799 (86.55)   
224 - **[CGCGCG]** 797 (85.98)   
224 - **[CGCGCG]** 795 (85.98)   
224 - **[CCCGCG]** 720 (86.03)   
224 - **[GCCGCG]** 619 (86.55)   
224 - **[CGCGCG]** 617 (85.98)   
224 - **[CCCGCG]** 594 (86.03)   
224 - **[CGCGCG]** 592 (85.98)   
224 - **[GCCGCG]** 532 (86.55)   
224 - **[CCCGCG]** 480 (86.03)   
224 - **[CCCGCG]** 473 (86.03)   
226 + **.TGCGCG.** 136 (94.26)   
226 + **[CCCGCG]** 229 (86.03)   
226 + **[CGCGCG]** 231 (85.98)   
226 + **[CGCGCG]** 233 (85.98)   
226 + **[GCCGCG]** 246 (86.55)   
226 + **.TCCGCG.** 305 (94.31)   
226 + **[TTCGCG]** 349 (87.60)   
226 + **[GCCGCG]** 448 (86.55)   
226 + **[CGCGCG]** 450 (85.98)   
226 + **[GCCGCG]** 518 (86.55)   
226 + **[CCCGCG]** 599 (86.03)   
226 + **.GACGCG.** 712 (92.24)   
226 + **[CGCGCG]** 753 (85.98)   
226 + **[GCCGCG]** 924 (86.55)   
226 + **.TGCGCG.** 950 (94.26)   
226 + **[GGCGCG]** 965 (86.50)   
226 + **[CGCGCG]** 1028 (85.98)   
226 + **[CGCGCG]** 1030 (85.98)   
226 + **[GCCGCG]** 1035 (86.55)   
226 + **[CCCGCG]** 1074 (86.03)   
226 + **[CGCGCG]** 1076 (85.98)   
226 + **[CCCGCG]** 1135 (86.03)   
226 + **[CCCGCG]** 1161 (86.03)   
226 + **[CCCGCG]** 1168 (86.03)   
226 + **[CCCGCG]** 1175 (86.03)   
226 + **[GCCGCG]** 1202 (86.55)   
226 + **[CGCGCG]** 1204 (85.98)   
226 + **[GGCGCG]** 1307 (86.50)   
226 + **[GCCGCG]** 1337 (86.55)   
226 + **.TACGCG.** 1383 (100.00)   
226 + **[CCCGCG]** 1416 (86.03)   
226 + **[GGCGCG]** 1434 (86.50)   
226 + **[GGCGCG]** 1495 (86.50)   
226 - **[GCCGCG]** 1497 (86.55)   
226 - **.TGCGCG.** 1436 (94.26)   
226 - **[AACGCG]** 1309 (87.55)   
226 - **.TGCGCG.** 1206 (94.26)   
226 - **[CGCGCG]** 1204 (85.98)   
226 - **[GGCGCG]** 1170 (86.50)   
226 - **[GGCGCG]** 1163 (86.50)   
226 - **[GGCGCG]** 1137 (86.50)   
226 - **[GCCGCG]** 1078 (86.55)   
226 - **[CGCGCG]** 1076 (85.98)   
226 - **.GACGCG.** 1065 (92.24)   
226 - **[GGCGCG]** 1037 (86.50)   
226 - **[GGCGCG]** 1032 (86.50)   
226 - **[CGCGCG]** 1030 (85.98)   
226 - **[CGCGCG]** 1028 (85.98)   
226 - **[CCCGCG]** 1006 (86.03)   
226 - **[GCCGCG]** 952 (86.55)   
226 - **[CCCGCG]** 755 (86.03)   
226 - **[CGCGCG]** 753 (85.98)   
226 - **[CCCGCG]** 714 (86.03)   
226 - **[AACGCG]** 640 (87.55)   
226 - **.GACGCG.** 601 (92.24)   
226 - **.CACGCG.** 520 (91.73)   
226 - **[GGCGCG]** 511 (86.50)   
226 - **.CACGCG.** 452 (91.73)   
226 - **[CGCGCG]** 450 (85.98)   
226 - **[AACGCG]** 248 (87.55)   
226 - **.GACGCG.** 235 (92.24)   
226 - **[CGCGCG]** 233 (85.98)   
226 - **[CGCGCG]** 231 (85.98)   
226 - **[CCCGCG]** 138 (86.03)   
226 - **[GCCGCG]** 125 (86.55)   
227 + **[CGCGCG]** 266 (85.98)   
227 + **.GACGCG.** 307 (92.24)   
227 + **[CCCGCG]** 344 (86.03)   
227 + **.CACGCG.** 536 (91.73)   
227 + **[GGCGCG]** 550 (86.50)   
227 + **[CGCGCG]** 552 (85.98)   
227 + **.CACGCG.** 560 (91.73)   
227 + **.GACGCG.** 615 (92.24)   
227 + **.GACGCG.** 687 (92.24)   
227 + **[CGCGCG]** 787 (85.98)   
227 + **.CACGCG.** 846 (91.73)   
227 + **[CCCGCG]** 965 (86.03)   
227 + **[CCCGCG]** 976 (86.03)   
227 + **[CCCGCG]** 986 (86.03)   
227 + **[CCCGCG]** 996 (86.03)   
227 + **[CCCGCG]** 1016 (86.03)   
227 + **[CCCGCG]** 1036 (86.03)   
227 + **[CCCGCG]** 1077 (86.03)   
227 + **[CCCGCG]** 1087 (86.03)   
227 + **[CGCGCG]** 1209 (85.98)   
227 + **[CCCGCG]** 1359 (86.03)   
227 + **.TGCGCG.** 1472 (94.26)   
227 - **.TGCGCG.** 1474 (94.26)   
227 - **[GGCGCG]** 1361 (86.50)   
227 - **[GCCGCG]** 1297 (86.55)   
227 - **[CCCGCG]** 1284 (86.03)   
227 - **[CGCGCG]** 1209 (85.98)   
227 - **[GGCGCG]** 1089 (86.50)   
227 - **[GGCGCG]** 1079 (86.50)   
227 - **[GGCGCG]** 1038 (86.50)   
227 - **[GGCGCG]** 1018 (86.50)   
227 - **[GGCGCG]** 998 (86.50)   
227 - **[GGCGCG]** 988 (86.50)   
227 - **[GGCGCG]** 978 (86.50)   
227 - **.TCCGCG.** 892 (94.31)   
227 - **[CCCGCG]** 848 (86.03)   
227 - **[CCCGCG]** 834 (86.03)   
227 - **.TGCGCG.** 789 (94.26)   
227 - **[CGCGCG]** 787 (85.98)   
227 - **[CCCGCG]** 689 (86.03)   
227 - **[AACGCG]** 617 (87.55)   
227 - **.TGCGCG.** 554 (94.26)   
227 - **[CGCGCG]** 552 (85.98)   
227 - **.TGCGCG.** 538 (94.26)   
227 - **[CCCGCG]** 346 (86.03)   
227 - **[GGCGCG]** 309 (86.50)   
227 - **[GCCGCG]** 268 (86.55)   
227 - **[CGCGCG]** 266 (85.98)   
227 - **[CCCGCG]** 97 (86.03)   
228 + **[GCCGCG]** 127 (86.55)   
228 + **[CCCGCG]** 134 (86.03)   
228 + **[CCCGCG]** 156 (86.03)   
228 + **.CACGCG.** 273 (91.73)   
228 + **[CCCGCG]** 279 (86.03)   
228 + **[CCCGCG]** 469 (86.03)   
228 + **[CCCGCG]** 540 (86.03)   
228 + **[CCCGCG]** 636 (86.03)   
228 + **.GACGCG.** 738 (92.24)   
228 + **.TGCGCG.** 769 (94.26)   
228 + **[GGCGCG]** 782 (86.50)   
228 + **[GGCGCG]** 884 (86.50)   
228 + **[GGCGCG]** 952 (86.50)   
228 + **[GCCGCG]** 1271 (86.55)   
228 + **.CACGCG.** 1427 (91.73)   
228 - **[GGCGCG]** 1429 (86.50)   
228 - **[TTCGCG]** 1273 (87.60)   
228 - **[CCCGCG]** 954 (86.03)   
228 - **[CCCGCG]** 771 (86.03)   
228 - **[CCCGCG]** 740 (86.03)   
228 - **[GGCGCG]** 542 (86.50)   
228 - **[GGCGCG]** 471 (86.50)   
228 - **[TAGGCG]** 316 (85.06)   
228 - **[GGCGCG]** 281 (86.50)   
228 - **[GGCGCG]** 275 (86.50)   
228 - **[GGCGCG]** 136 (86.50)   
228 - **[GGCGCG]** 129 (86.50)   
228 - **[TAGGCG]** 36 (85.06)   
229 + **[TAGGCG]** 110 (85.06)   
229 + **.TGCGCG.** 538 (94.26)   
229 + **.TGCGCG.** 555 (94.26)   
229 + **[CCCGCG]** 585 (86.03)   
229 + **.TCCGCG.** 675 (94.31)   
229 + **[GCCGCG]** 690 (86.55)   
229 + **[CCCGCG]** 911 (86.03)   
229 + **[GGCGCG]** 996 (86.50)   
229 + **[GCCGCG]** 1015 (86.55)   
229 + **[GGCGCG]** 1036 (86.50)   
229 + **[GCCGCG]** 1114 (86.55)   
229 + **[CGCGCG]** 1146 (85.98)   
229 + **[GCCGCG]** 1246 (86.55)   
229 + **[CGCGCG]** 1248 (85.98)   
229 + **.TACGCG.** 1280 (100.00)   
229 + **[CGCGCG]** 1282 (85.98)   
229 + **[GGCGCG]** 1287 (86.50)   
229 + **[GGCGCG]** 1301 (86.50)   
229 + **[CCCGCG]** 1368 (86.03)   
229 - **[CCCGCG]** 1370 (86.03)   
229 - **[CCCGCG]** 1303 (86.03)   
229 - **[CCCGCG]** 1289 (86.03)   
229 - **[GCCGCG]** 1284 (86.55)   
229 - **[CGCGCG]** 1282 (85.98)   
229 - **[GGCGCG]** 1250 (86.50)   
229 - **[CGCGCG]** 1248 (85.98)   
229 - **[GGCGCG]** 1206 (86.50)   
229 - **[GGCGCG]** 1148 (86.50)   
229 - **[CGCGCG]** 1146 (85.98)   
229 - **.TCCGCG.** 1116 (94.31)   
229 - **[CCCGCG]** 1038 (86.03)   
229 - **[GGCGCG]** 1017 (86.50)   
229 - **.TGCGCG.** 998 (94.26)   
229 - **[GCCGCG]** 941 (86.55)   
229 - **[AACGCG]** 913 (87.55)   
229 - **[GCCGCG]** 587 (86.55)   
229 - **.TCCGCG.** 557 (94.31)   
229 - **[GCCGCG]** 540 (86.55)   
229 - **[GGCGCG]** 124 (86.50)   
231 + **.CACGCG.** 87 (91.73)   
231 + **.CACGCG.** 874 (91.73)   
231 + **[GCCGCG]** 906 (86.55)   
231 + **[GCCGCG]** 980 (86.55)   
231 + **.TGCGCG.** 1068 (94.26)   
231 + **[GGCGCG]** 1122 (86.50)   
231 + **[GGCGCG]** 1141 (86.50)   
231 + **.TGCGCG.** 1163 (94.26)   
231 + **.TCCGCG.** 1273 (94.31)   
231 + **.GACGCG.** 1383 (92.24)   
231 + **[GGCGCG]** 1470 (86.50)   
231 + **[GCCGCG]** 1482 (86.55)   
231 - **[GGCGCG]** 1484 (86.50)   
231 - **[GGCGCG]** 1472 (86.50)   
231 - **.TGCGCG.** 1385 (94.26)   
231 - **[CCCGCG]** 1275 (86.03)   
231 - **[GGCGCG]** 1165 (86.50)   
231 - **[GCCGCG]** 1070 (86.55)   
231 - **.TGCGCG.** 982 (94.26)   
231 - **[CCCGCG]** 908 (86.03)   
231 - **[CCCGCG]** 876 (86.03)   
231 - **[TAGGCG]** 864 (85.06)   
231 - **[CCCGCG]** 117 (86.03)   
231 - **.CACGCG.** 89 (91.73)   
232 + **[GCCGCG]** 147 (86.55)   
232 + **[GGCGCG]** 444 (86.50)   
232 + **[CCCGCG]** 519 (86.03)   
232 + **[GGCGCG]** 633 (86.50)   
232 + **.CACGCG.** 668 (91.73)   
232 + **[GGCGCG]** 699 (86.50)   
232 + **[GCCGCG]** 794 (86.55)   
232 + **[CCCGCG]** 859 (86.03)   
232 + **[CGCGCG]** 861 (85.98)   
232 + **[GCCGCG]** 867 (86.55)   
232 + **[TTCGCG]** 962 (87.60)   
232 + **.TGCGCG.** 995 (94.26)   
232 + **[GGCGCG]** 1013 (86.50)   
232 + **.GACGCG.** 1119 (92.24)   
232 + **[GGCGCG]** 1180 (86.50)   
232 + **[GCCGCG]** 1244 (86.55)   
232 + **[CCCGCG]** 1282 (86.03)   
232 + **.GACGCG.** 1333 (92.24)   
232 + **.TCCGCG.** 1420 (94.31)   
232 - **[AACGCG]** 1422 (87.55)   
232 - **[GCCGCG]** 1335 (86.55)   
232 - **[CCCGCG]** 1284 (86.03)   
232 - **[GGCGCG]** 1246 (86.50)   
232 - **[CCCGCG]** 1182 (86.03)   
232 - **[GCCGCG]** 997 (86.55)   
232 - **[AACGCG]** 964 (87.55)   
232 - **[GCCGCG]** 869 (86.55)   
232 - **[GCCGCG]** 863 (86.55)   
232 - **[CGCGCG]** 861 (85.98)   
232 - **.GACGCG.** 701 (92.24)   
232 - **[TTCGCG]** 670 (87.60)   
232 - **.GACGCG.** 635 (92.24)   
232 - **[GGCGCG]** 521 (86.50)   
232 - **.TGCGCG.** 446 (94.26)   
232 - **[GGCGCG]** 149 (86.50)   
233 + **[GGCGCG]** 1378 (86.50)   
233 - **.CACGCG.** 1380 (91.73)   
234 + **[GGCGCG]** 179 (86.50)   
234 + **.TGCGCG.** 409 (94.26)   
234 + **[CCCGCG]** 585 (86.03)   
234 + **.GACGCG.** 637 (92.24)   
234 + **[CCCGCG]** 814 (86.03)   
234 + **[CCCGCG]** 828 (86.03)   
234 + **[CGCGCG]** 830 (85.98)   
234 + **[CCCGCG]** 854 (86.03)   
234 + **[CCCGCG]** 870 (86.03)   
234 + **.TGCGCG.** 995 (94.26)   
234 + **[GGCGCG]** 1289 (86.50)   
234 + **.CACGCG.** 1329 (91.73)   
234 + **.GACGCG.** 1411 (92.24)   
234 + **[CGCGCG]** 1413 (85.98)   
234 - **.TCCGCG.** 1415 (94.31)   
234 - **[CGCGCG]** 1413 (85.98)   
234 - **[GCCGCG]** 1331 (86.55)   
234 - **[CCCGCG]** 997 (86.03)   
234 - **.TCCGCG.** 872 (94.31)   
234 - **[GGCGCG]** 856 (86.50)   
234 - **[GGCGCG]** 850 (86.50)   
234 - **[CCCGCG]** 832 (86.03)   
234 - **[CGCGCG]** 830 (85.98)   
234 - **[GCCGCG]** 816 (86.55)   
234 - **[CCCGCG]** 681 (86.03)   
234 - **[CCCGCG]** 639 (86.03)   
234 - **.TCCGCG.** 587 (94.31)   
234 - **[CCCGCG]** 439 (86.03)   
234 - **[GGCGCG]** 411 (86.50)   
234 - **.TCCGCG.** 383 (94.31)   
234 - **[CCCGCG]** 275 (86.03)   
234 - **[CCCGCG]** 181 (86.03)   
235 + **.TGCGCG.** 474 (94.26)   
235 + **[GGCGCG]** 635 (86.50)   
235 + **.CACGCG.** 753 (91.73)   
235 + **.CACGCG.** 760 (91.73)   
235 + **.TACGCG.** 766 (100.00)   
235 + **[GCCGCG]** 797 (86.55)   
235 + **.TGCGCG.** 898 (94.26)   
235 + **[GGCGCG]** 903 (86.50)   
235 + **.TGCGCG.** 927 (94.26)   
235 + **[GCCGCG]** 961 (86.55)   
235 + **[CGCGCG]** 983 (85.98)   
235 + **.TGCGCG.** 990 (94.26)   
235 + **.TGCGCG.** 1031 (94.26)   
235 + **[CGCGCG]** 1033 (85.98)   
235 + **[CGCGCG]** 1242 (85.98)   
235 + **[GGCGCG]** 1317 (86.50)   
235 + **[GCCGCG]** 1362 (86.55)   
235 + **.CACGCG.** 1397 (91.73)   
235 + **[CGCGCG]** 1399 (85.98)   
235 + **[GGCGCG]** 1429 (86.50)   
235 + **.GACGCG.** 1486 (92.24)   
235 - **.CACGCG.** 1488 (91.73)   
235 - **.TACGCG.** 1431 (100.00)   
235 - **[CCCGCG]** 1401 (86.03)   
235 - **[CGCGCG]** 1399 (85.98)   
235 - **[GGCGCG]** 1364 (86.50)   
235 - **.TCCGCG.** 1319 (94.31)   
235 - **[CGCGCG]** 1242 (85.98)   
235 - **[CCCGCG]** 1035 (86.03)   
235 - **[CGCGCG]** 1033 (85.98)   
235 - **.CACGCG.** 992 (91.73)   
235 - **[CGCGCG]** 983 (85.98)   
235 - **[GGCGCG]** 963 (86.50)   
235 - **[GGCGCG]** 929 (86.50)   
235 - **.CACGCG.** 905 (91.73)   
235 - **[GCCGCG]** 900 (86.55)   
235 - **[GGCGCG]** 799 (86.50)   
235 - **.TACGCG.** 762 (100.00)   
235 - **[GGCGCG]** 755 (86.50)   
235 - **[CCCGCG]** 685 (86.03)   
235 - **[GGCGCG]** 670 (86.50)   
235 - **.CACGCG.** 476 (91.73)   
235 - **[AACGCG]** 1 (87.55)   
236 + **[GGCGCG]** 910 (86.50)   
236 + **[CCCGCG]** 976 (86.03)   
236 + **[CGCGCG]** 1024 (85.98)   
236 + **[GCCGCG]** 1154 (86.55)   
236 + **[CCCGCG]** 1195 (86.03)   
236 + **[AACGCG]** 1251 (87.55)   
236 + **[CCCGCG]** 1330 (86.03)   
236 - **.TCCGCG.** 1332 (94.31)   
236 - **[CCCGCG]** 1197 (86.03)   
236 - **[GCCGCG]** 1026 (86.55)   
236 - **[CGCGCG]** 1024 (85.98)   
236 - **[GCCGCG]** 978 (86.55)   
236 - **[GGCGCG]** 912 (86.50)   
237 + **[TAGGCG]** 105 (85.06)   
237 + **.TCCGCG.** 471 (94.31)   
237 + **.GACGCG.** 1057 (92.24)   
237 - **.GACGCG.** 473 (92.24)   
238 + **.TCCGCG.** 915 (94.31)   
238 + **[TAGGCG]** 954 (85.06)   
238 + **[TTCGCG]** 1041 (87.60)   
238 + **.GACGCG.** 1106 (92.24)   
238 + **[CCCGCG]** 1226 (86.03)   
238 - **[AACGCG]** 1043 (87.55)   
239 + **[GCCGCG]** 445 (86.55)   
239 + **[GGCGCG]** 707 (86.50)   
239 + **[TTCGCG]** 750 (87.60)   
239 + **[GCCGCG]** 768 (86.55)   
239 + **.TGCGCG.** 787 (94.26)   
239 + **[GGCGCG]** 952 (86.50)   
239 - **.TCCGCG.** 789 (94.31)   
239 - **[GGCGCG]** 770 (86.50)   
239 - **[GCCGCG]** 752 (86.55)   
239 - **[GCCGCG]** 447 (86.55)   
239 - **[GCCGCG]** 299 (86.55)   
240 + **[GGCGCG]** 53 (86.50)   
240 + **[TAGGCG]** 512 (85.06)   
240 + **[CGCGCG]** 1109 (85.98)   
240 + **[CCCGCG]** 1180 (86.03)   
240 + **[GCCGCG]** 1403 (86.55)   
240 - **[GGCGCG]** 1182 (86.50)   
240 - **[CGCGCG]** 1109 (85.98)   
240 - **.CACGCG.** 1016 (91.73)   
240 - **[GGCGCG]** 140 (86.50)   
240 - **[CCCGCG]** 55 (86.03)   
241 + **.GACGCG.** 417 (92.24)   
241 + **[CGCGCG]** 419 (85.98)   
241 + **[GCCGCG]** 1242 (86.55)   
241 + **[GGCGCG]** 1284 (86.50)   
241 + **[AACGCG]** 1310 (87.55)   
241 - **.GACGCG.** 1312 (92.24)   
241 - **.TCCGCG.** 1286 (94.31)   
241 - **.TGCGCG.** 1244 (94.26)   
241 - **[GGCGCG]** 421 (86.50)   
241 - **[CGCGCG]** 419 (85.98)   
242 + **[AACGCG]** 610 (87.55)   
242 + **.TCCGCG.** 822 (94.31)   
242 + **.CACGCG.** 994 (91.73)   
242 + **[TTCGCG]** 1001 (87.60)   
242 + **.TCCGCG.** 1080 (94.31)   
242 - **.CACGCG.** 1082 (91.73)   
242 - **.GACGCG.** 1003 (92.24)   
242 - **.GACGCG.** 824 (92.24)   
243 + **[CGCGCG]** 918 (85.98)   
243 + **[GGCGCG]** 1005 (86.50)   
243 + **[GCCGCG]** 1120 (86.55)   
243 + **[GCCGCG]** 1160 (86.55)   
243 + **[CGCGCG]** 1162 (85.98)   
243 + **[TTCGCG]** 1330 (87.60)   
243 - **.TCCGCG.** 1332 (94.31)   
243 - **[CGCGCG]** 1162 (85.98)   
243 - **[TTCGCG]** 1122 (87.60)   
243 - **[GCCGCG]** 920 (86.55)   
243 - **[CGCGCG]** 918 (85.98)   
244 + **[AACGCG]** 245 (87.55)   
244 + **[AACGCG]** 343 (87.55)   
244 + **.GACGCG.** 687 (92.24)   
244 + **[GGCGCG]** 763 (86.50)   
244 + **[GGCGCG]** 777 (86.50)   
244 + **[CGCGCG]** 779 (85.98)   
244 + **.CACGCG.** 802 (91.73)   
244 - **.TCCGCG.** 781 (94.31)   
244 - **[CGCGCG]** 779 (85.98)   
244 - **[CCCGCG]** 765 (86.03)   
244 - **[CCCGCG]** 707 (86.03)   
244 - **[CCCGCG]** 689 (86.03)   
244 - **.GACGCG.** 345 (92.24)   
244 - **.TACGCG.** 247 (100.00)   
246 + **[CCCGCG]** 3 (86.03)   
246 + **[GCCGCG]** 274 (86.55)   
246 + **[CCCGCG]** 342 (86.03)   
246 + **[CCCGCG]** 423 (86.03)   
246 + **[GGCGCG]** 459 (86.50)   
246 + **[GGCGCG]** 495 (86.50)   
246 + **.GACGCG.** 670 (92.24)   
246 + **[CGCGCG]** 672 (85.98)   
246 + **[GCCGCG]** 707 (86.55)   
246 + **[CCCGCG]** 748 (86.03)   
246 + **[CCCGCG]** 882 (86.03)   
246 + **[GGCGCG]** 908 (86.50)   
246 + **[GCCGCG]** 1078 (86.55)   
246 + **[CGCGCG]** 1080 (85.98)   
246 + **.TGCGCG.** 1196 (94.26)   
246 - **.TCCGCG.** 1082 (94.31)   
246 - **[CGCGCG]** 1080 (85.98)   
246 - **[GGCGCG]** 910 (86.50)   
246 - **[GGCGCG]** 884 (86.50)   
246 - **[GGCGCG]** 750 (86.50)   
246 - **.TGCGCG.** 709 (94.26)   
246 - **[GGCGCG]** 699 (86.50)   
246 - **[GCCGCG]** 674 (86.55)   
246 - **[CGCGCG]** 672 (85.98)   
246 - **[GCCGCG]** 615 (86.55)   
246 - **.TGCGCG.** 497 (94.26)   
246 - **.CACGCG.** 461 (91.73)   
246 - **[GGCGCG]** 276 (86.50)   
246 - **.TGCGCG.** 5 (94.26)   
247 + **[GCCGCG]** 19 (86.55)   
247 + **.TGCGCG.** 234 (94.26)   
247 + **[CCCGCG]** 329 (86.03)   
247 + **.GACGCG.** 418 (92.24)   
247 + **[CGCGCG]** 420 (85.98)   
247 + **.TCCGCG.** 795 (94.31)   
247 + **[GGCGCG]** 950 (86.50)   
247 + **[GCCGCG]** 1022 (86.55)   
247 + **[GCCGCG]** 1092 (86.55)   
247 + **[CGCGCG]** 1094 (85.98)   
247 + **[CCCGCG]** 1120 (86.03)   
247 + **[CCCGCG]** 1186 (86.03)   
247 + **[CCCGCG]** 1200 (86.03)   
247 + **[CGCGCG]** 1202 (85.98)   
247 + **[GGCGCG]** 1285 (86.50)   
247 + **[CGCGCG]** 1287 (85.98)   
247 + **[CCCGCG]** 1297 (86.03)   
247 + **.CACGCG.** 1358 (91.73)   
247 + **[CCCGCG]** 1415 (86.03)   
247 + **[CCCGCG]** 1471 (86.03)   
247 + **[CCCGCG]** 1479 (86.03)   
247 + **[GCCGCG]** 1493 (86.55)   
247 - **.TGCGCG.** 1495 (94.26)   
247 - **[CCCGCG]** 1481 (86.03)   
247 - **[GGCGCG]** 1473 (86.50)   
247 - **[GCCGCG]** 1360 (86.55)   
247 - **[GCCGCG]** 1299 (86.55)   
247 - **.TCCGCG.** 1289 (94.31)   
247 - **[CGCGCG]** 1287 (85.98)   
247 - **[CGCGCG]** 1202 (85.98)   
247 - **[GGCGCG]** 1188 (86.50)   
247 - **[GGCGCG]** 1122 (86.50)   
247 - **[GGCGCG]** 1096 (86.50)   
247 - **[CGCGCG]** 1094 (85.98)   
247 - **[GCCGCG]** 1024 (86.55)   
247 - **[CCCGCG]** 952 (86.03)   
247 - **[TAGGCG]** 894 (85.06)   
247 - **[GGCGCG]** 885 (86.50)   
247 - **[CCCGCG]** 797 (86.03)   
247 - **[GCCGCG]** 422 (86.55)   
247 - **[CGCGCG]** 420 (85.98)   
247 - **[GGCGCG]** 331 (86.50)   
247 - **[GCCGCG]** 279 (86.55)   
247 - **[GGCGCG]** 236 (86.50)   
247 - **[TTCGCG]** 21 (87.60)   
250 + **.CACGCG.** 137 (91.73)   
250 + **.GACGCG.** 453 (92.24)   
250 + **[CGCGCG]** 455 (85.98)   
250 + **[GGCGCG]** 460 (86.50)   
250 + **.TCCGCG.** 526 (94.31)   
250 + **[CCCGCG]** 540 (86.03)   
250 + **[CCCGCG]** 585 (86.03)   
250 + **[CGCGCG]** 587 (85.98)   
250 + **[CGCGCG]** 589 (85.98)   
250 + **[GCCGCG]** 870 (86.55)   
250 + **[CCCGCG]** 1047 (86.03)   
250 + **[GCCGCG]** 1096 (86.55)   
250 + **.CACGCG.** 1303 (91.73)   
250 + **[CGCGCG]** 1305 (85.98)   
250 + **[CGCGCG]** 1307 (85.98)   
250 - **.TGCGCG.** 1309 (94.26)   
250 - **[CGCGCG]** 1307 (85.98)   
250 - **[CGCGCG]** 1305 (85.98)   
250 - **[AACGCG]** 872 (87.55)   
250 - **[GGCGCG]** 591 (86.50)   
250 - **[CGCGCG]** 589 (85.98)   
250 - **[CGCGCG]** 587 (85.98)   
250 - **[GCCGCG]** 542 (86.55)   
250 - **[GGCGCG]** 528 (86.50)   
250 - **[GCCGCG]** 513 (86.55)   
250 - **.TCCGCG.** 462 (94.31)   
250 - **[GCCGCG]** 457 (86.55)   
250 - **[CGCGCG]** 455 (85.98)   
250 - **[GCCGCG]** 156 (86.55)   
250 - **[GGCGCG]** 139 (86.50)   
252 + **[GGCGCG]** 392 (86.50)   
252 + **[TAGGCG]** 526 (85.06)   
252 - **.GACGCG.** 394 (92.24)   
255 + **.GACGCG.** 1297 (92.24)   
255 - **.TCCGCG.** 1299 (94.31)   
256 + **[GGCGCG]** 860 (86.50)   
256 + **[TTCGCG]** 1115 (87.60)   
256 + **[GCCGCG]** 1195 (86.55)   
256 + **[CCCGCG]** 1203 (86.03)   
256 + **[GCCGCG]** 1302 (86.55)   
256 - **.TCCGCG.** 1304 (94.31)   
256 - **.GACGCG.** 1205 (92.24)   
256 - **[CCCGCG]** 1197 (86.03)   
256 - **[GGCGCG]** 1117 (86.50)   
256 - **[CCCGCG]** 1095 (86.03)   
256 - **[CCCGCG]** 862 (86.03)   
256 - **[GGCGCG]** 99 (86.50)   
257 + **[GCCGCG]** 607 (86.55)   
257 + **[CCCGCG]** 814 (86.03)   
257 + **[GGCGCG]** 876 (86.50)   
257 + **[CCCGCG]** 1022 (86.03)   
257 + **.GACGCG.** 1119 (92.24)   
257 + **[GCCGCG]** 1166 (86.55)   
257 + **[GCCGCG]** 1194 (86.55)   
257 + **[CCCGCG]** 1243 (86.03)   
257 + **[CGCGCG]** 1245 (85.98)   
257 + **[GCCGCG]** 1282 (86.55)   
257 + **[GGCGCG]** 1316 (86.50)   
257 + **.CACGCG.** 1323 (91.73)   
257 + **[GGCGCG]** 1496 (86.50)   
257 - **[AACGCG]** 1498 (87.55)   
257 - **[CCCGCG]** 1325 (86.03)   
257 - **[GCCGCG]** 1318 (86.55)   
257 - **[GCCGCG]** 1284 (86.55)   
257 - **[GGCGCG]** 1247 (86.50)   
257 - **[CGCGCG]** 1245 (85.98)   
257 - **[CCCGCG]** 1196 (86.03)   
257 - **[AACGCG]** 1024 (87.55)   
257 - **.TCCGCG.** 878 (94.31)   
257 - **.TCCGCG.** 816 (94.31)   
257 - **[CCCGCG]** 756 (86.03)   
257 - **[CCCGCG]** 735 (86.03)   
257 - **.TCCGCG.** 609 (94.31)   
259 + **[TAGGCG]** 120 (85.06)   
259 + **.TCCGCG.** 867 (94.31)   
259 + **[TTCGCG]** 964 (87.60)   
259 + **.GACGCG.** 995 (92.24)   
259 + **[GCCGCG]** 1018 (86.55)   
259 + **.GACGCG.** 1023 (92.24)   
259 + **.TCCGCG.** 1185 (94.31)   
259 + **[GCCGCG]** 1302 (86.55)   
259 - **[GCCGCG]** 1304 (86.55)   
259 - **[GGCGCG]** 1221 (86.50)   
259 - **[GCCGCG]** 1187 (86.55)   
259 - **[GCCGCG]** 997 (86.55)   
259 - **.TGCGCG.** 869 (94.26)   
259 - **[TAGGCG]** 835 (85.06)   
260 + **.TCCGCG.** 877 (94.31)   
260 + **[TAGGCG]** 926 (85.06)   
260 + **.CACGCG.** 992 (91.73)   
260 + **[CGCGCG]** 994 (85.98)   
260 + **[CGCGCG]** 996 (85.98)   
260 + **[CGCGCG]** 998 (85.98)   
260 + **.CACGCG.** 1024 (91.73)   
260 + **[CCCGCG]** 1038 (86.03)   
260 + **[CGCGCG]** 1040 (85.98)   
260 + **[TTCGCG]** 1224 (87.60)   
260 - **[TAGGCG]** 1305 (85.06)   
260 - **[GGCGCG]** 1042 (86.50)   
260 - **[CGCGCG]** 1040 (85.98)   
260 - **.TGCGCG.** 1026 (94.26)   
260 - **.TGCGCG.** 1000 (94.26)   
260 - **[CGCGCG]** 998 (85.98)   
260 - **[CGCGCG]** 996 (85.98)   
260 - **[CGCGCG]** 994 (85.98)   
260 - **[GGCGCG]** 879 (86.50)   
261 - **[GGCGCG]** 127 (86.50)   
262 - **[CCCGCG]** 946 (86.03)   
263 + **[GGCGCG]** 30 (86.50)   
263 + **[GGCGCG]** 165 (86.50)   
263 + **[GGCGCG]** 307 (86.50)   
263 + **[CGCGCG]** 309 (85.98)   
263 + **[GGCGCG]** 540 (86.50)   
263 + **[CGCGCG]** 848 (85.98)   
263 + **[GCCGCG]** 854 (86.55)   
263 + **.TCCGCG.** 1001 (94.31)   
263 + **[GCCGCG]** 1076 (86.55)   
263 + **.GACGCG.** 1117 (92.24)   
263 + **[GGCGCG]** 1282 (86.50)   
263 - **[CCCGCG]** 1284 (86.03)   
263 - **[CCCGCG]** 1119 (86.03)   
263 - **[TTCGCG]** 1083 (87.60)   
263 - **.GACGCG.** 1078 (92.24)   
263 - **[GCCGCG]** 850 (86.55)   
263 - **[CGCGCG]** 848 (85.98)   
263 - **[GGCGCG]** 542 (86.50)   
263 - **[GGCGCG]** 393 (86.50)   
263 - **[GGCGCG]** 311 (86.50)   
263 - **[CGCGCG]** 309 (85.98)   
263 - **.CACGCG.** 167 (91.73)   
264 + **[CGCGCG]** 711 (85.98)   
264 + **.GACGCG.** 752 (92.24)   
264 + **[CCCGCG]** 1136 (86.03)   
264 - **[GGCGCG]** 1138 (86.50)   
264 - **.TGCGCG.** 754 (94.26)   
264 - **[CCCGCG]** 713 (86.03)   
264 - **[CGCGCG]** 711 (85.98)   
264 - **.GACGCG.** 143 (92.24)   
266 + **.CACGCG.** 545 (91.73)   
266 + **[TTCGCG]** 738 (87.60)   
266 + **[AACGCG]** 960 (87.55)   
266 + **.CACGCG.** 980 (91.73)   
266 + **[CCCGCG]** 994 (86.03)   
266 + **[GGCGCG]** 1028 (86.50)   
266 + **[GCCGCG]** 1065 (86.55)   
266 + **[CCCGCG]** 1087 (86.03)   
266 + **[GGCGCG]** 1104 (86.50)   
266 + **.TACGCG.** 1198 (100.00)   
266 + **[GCCGCG]** 1280 (86.55)   
266 + **.TCCGCG.** 1417 (94.31)   
266 + **[GCCGCG]** 1475 (86.55)   
266 - **.TCCGCG.** 1477 (94.31)   
266 - **[TTCGCG]** 1419 (87.60)   
266 - **[CCCGCG]** 1404 (86.03)   
266 - **[GCCGCG]** 1282 (86.55)   
266 - **[GGCGCG]** 1270 (86.50)   
266 - **.TCCGCG.** 1200 (94.31)   
266 - **[GGCGCG]** 1106 (86.50)   
266 - **.TGCGCG.** 1089 (94.26)   
266 - **[CCCGCG]** 1067 (86.03)   
266 - **[GCCGCG]** 1030 (86.55)   
266 - **[AACGCG]** 1006 (87.55)   
266 - **[CCCGCG]** 996 (86.03)   
266 - **[GGCGCG]** 982 (86.50)   
266 - **[GCCGCG]** 962 (86.55)   
266 - **.CACGCG.** 740 (91.73)   
267 + **[GCCGCG]** 193 (86.55)   
267 + **[CCCGCG]** 1102 (86.03)   
267 - **.TGCGCG.** 1104 (94.26)   
267 - **[CCCGCG]** 699 (86.03)   
267 - **[GCCGCG]** 195 (86.55)   
268 + **[CGCGCG]** 21 (85.98)   
268 + **[GGCGCG]** 57 (86.50)   
268 + **[GCCGCG]** 180 (86.55)   
268 + **[CCCGCG]** 336 (86.03)   
268 + **.GACGCG.** 509 (92.24)   
268 + **[AACGCG]** 811 (87.55)   
268 + **.CACGCG.** 831 (91.73)   
268 + **[GGCGCG]** 1013 (86.50)   
268 + **[GCCGCG]** 1068 (86.55)   
268 + **.CACGCG.** 1199 (91.73)   
268 + **[GCCGCG]** 1320 (86.55)   
268 + **.TCCGCG.** 1390 (94.31)   
268 + **[CGCGCG]** 1392 (85.98)   
268 + **[AACGCG]** 1476 (87.55)   
268 + **[CCCGCG]** 1489 (86.03)   
268 - **[CCCGCG]** 1491 (86.03)   
268 - **[GCCGCG]** 1478 (86.55)   
268 - **[GCCGCG]** 1394 (86.55)   
268 - **[CGCGCG]** 1392 (85.98)   
268 - **.TCCGCG.** 1201 (94.31)   
268 - **[CCCGCG]** 1070 (86.03)   
268 - **[GCCGCG]** 1015 (86.55)   
268 - **.TCCGCG.** 833 (94.31)   
268 - **[GCCGCG]** 813 (86.55)   
268 - **[AACGCG]** 511 (87.55)   
268 - **[CCCGCG]** 338 (86.03)   
268 - **[CCCGCG]** 107 (86.03)   
268 - **.TGCGCG.** 59 (94.26)   
268 - **[CGCGCG]** 21 (85.98)   
270 + **[GCCGCG]** 25 (86.55)   
270 + **[GGCGCG]** 56 (86.50)   
270 + **[CCCGCG]** 144 (86.03)   
270 + **[GGCGCG]** 462 (86.50)   
270 + **.TGCGCG.** 786 (94.26)   
270 + **[TAGGCG]** 1183 (85.06)   
270 + **[GGCGCG]** 1299 (86.50)   
270 + **[CCCGCG]** 1380 (86.03)   
270 + **[GCCGCG]** 1438 (86.55)   
270 + **[CGCGCG]** 1440 (85.98)   
270 - **.CACGCG.** 1442 (91.73)   
270 - **[CGCGCG]** 1440 (85.98)   
270 - **[CCCGCG]** 1382 (86.03)   
270 - **.TCCGCG.** 1301 (94.31)   
270 - **[AACGCG]** 788 (87.55)   
270 - **[CCCGCG]** 464 (86.03)   
270 - **.TCCGCG.** 146 (94.31)   
270 - **.GACGCG.** 27 (92.24)   
271 + **.CACGCG.** 558 (91.73)   
271 + **.CACGCG.** 572 (91.73)   
271 + **.CACGCG.** 585 (91.73)   
271 + **.CACGCG.** 598 (91.73)   
271 + **.CACGCG.** 611 (91.73)   
271 + **.CACGCG.** 624 (91.73)   
271 + **.CACGCG.** 637 (91.73)   
271 + **.CACGCG.** 651 (91.73)   
271 + **.CACGCG.** 664 (91.73)   
271 + **.CACGCG.** 677 (91.73)   
271 + **[CGCGCG]** 679 (85.98)   
271 + **.GACGCG.** 742 (92.24)   
271 + **.CACGCG.** 779 (91.73)   
271 + **.CACGCG.** 830 (91.73)   
271 + **.CACGCG.** 843 (91.73)   
271 + **[CGCGCG]** 845 (85.98)   
271 + **.GACGCG.** 1288 (92.24)   
271 - **[AACGCG]** 847 (87.55)   
271 - **[CGCGCG]** 845 (85.98)   
271 - **.TGCGCG.** 832 (94.26)   
271 - **.TGCGCG.** 781 (94.26)   
271 - **.TACGCG.** 744 (100.00)   
271 - **.TGCGCG.** 731 (94.26)   
271 - **[GCCGCG]** 681 (86.55)   
271 - **[CGCGCG]** 679 (85.98)   
271 - **.TGCGCG.** 666 (94.26)   
271 - **.TGCGCG.** 653 (94.26)   
271 - **.TGCGCG.** 639 (94.26)   
271 - **.TGCGCG.** 626 (94.26)   
271 - **.TGCGCG.** 613 (94.26)   
271 - **.TGCGCG.** 600 (94.26)   
271 - **.TGCGCG.** 587 (94.26)   
271 - **.TGCGCG.** 574 (94.26)   
271 - **.TGCGCG.** 560 (94.26)   
272 + **.CACGCG.** 558 (91.73)   
272 + **.CACGCG.** 586 (91.73)   
272 + **.CACGCG.** 599 (91.73)   
272 + **.CACGCG.** 612 (91.73)   
272 + **.CACGCG.** 625 (91.73)   
272 + **.CACGCG.** 638 (91.73)   
272 + **.CACGCG.** 652 (91.73)   
272 + **.CACGCG.** 665 (91.73)   
272 + **[CGCGCG]** 667 (85.98)   
272 + **[GCCGCG]** 712 (86.55)   
272 + **[CGCGCG]** 714 (85.98)   
272 + **[CGCGCG]** 716 (85.98)   
272 + **.CACGCG.** 730 (91.73)   
272 + **.CACGCG.** 767 (91.73)   
272 + **.CACGCG.** 818 (91.73)   
272 + **.CACGCG.** 831 (91.73)   
272 + **[CGCGCG]** 833 (85.98)   
272 + **.GACGCG.** 1279 (92.24)   
272 - **[AACGCG]** 835 (87.55)   
272 - **[CGCGCG]** 833 (85.98)   
272 - **.TGCGCG.** 820 (94.26)   
272 - **.TGCGCG.** 769 (94.26)   
272 - **.TACGCG.** 732 (100.00)   
272 - **.TGCGCG.** 718 (94.26)   
272 - **[CGCGCG]** 716 (85.98)   
272 - **[CGCGCG]** 714 (85.98)   
272 - **[GCCGCG]** 669 (86.55)   
272 - **[CGCGCG]** 667 (85.98)   
272 - **.TGCGCG.** 654 (94.26)   
272 - **.TGCGCG.** 640 (94.26)   
272 - **.TGCGCG.** 627 (94.26)   
272 - **.TGCGCG.** 614 (94.26)   
272 - **.TGCGCG.** 601 (94.26)   
272 - **.TGCGCG.** 588 (94.26)   
272 - **.TGCGCG.** 560 (94.26)   
273 + **[CCCGCG]** 199 (86.03)   
273 + **[CCCGCG]** 265 (86.03)   
273 + **[GCCGCG]** 1030 (86.55)   
273 + **.TCCGCG.** 1119 (94.31)   
273 + **.TGCGCG.** 1230 (94.26)   
273 + **[CGCGCG]** 1232 (85.98)   
273 + **[TTCGCG]** 1396 (87.60)   
273 - **[CCCGCG]** 1398 (86.03)   
273 - **.TCCGCG.** 1297 (94.31)   
273 - **[CGCGCG]** 1232 (85.98)   
273 - **.GACGCG.** 1121 (92.24)   
273 - **[GCCGCG]** 1032 (86.55)   
273 - **[GGCGCG]** 201 (86.50)   
274 + **[GCCGCG]** 13 (86.55)   
274 + **.TCCGCG.** 106 (94.31)   
274 + **[GGCGCG]** 111 (86.50)   
274 + **.TGCGCG.** 173 (94.26)   
274 + **[CGCGCG]** 569 (85.98)   
274 + **[CGCGCG]** 571 (85.98)   
274 + **.TACGCG.** 581 (100.00)   
274 + **[CGCGCG]** 583 (85.98)   
274 + **[CGCGCG]** 585 (85.98)   
274 + **[CCCGCG]** 1155 (86.03)   
274 + **.TGCGCG.** 1263 (94.26)   
274 + **[CGCGCG]** 1265 (85.98)   
274 + **[CGCGCG]** 1267 (85.98)   
274 + **.TGCGCG.** 1320 (94.26)   
274 + **[GGCGCG]** 1352 (86.50)   
274 + **[GCCGCG]** 1360 (86.55)   
274 + **[CCCGCG]** 1507 (86.03)   
274 - **.TACGCG.** 1509 (100.00)   
274 - **[CCCGCG]** 1362 (86.03)   
274 - **.TCCGCG.** 1354 (94.31)   
274 - **[GCCGCG]** 1322 (86.55)   
274 - **[GGCGCG]** 1269 (86.50)   
274 - **[CGCGCG]** 1267 (85.98)   
274 - **[CGCGCG]** 1265 (85.98)   
274 - **[TAGGCG]** 589 (85.06)   
274 - **[GGCGCG]** 587 (86.50)   
274 - **[CGCGCG]** 585 (85.98)   
274 - **[CGCGCG]** 583 (85.98)   
274 - **.CACGCG.** 573 (91.73)   
274 - **[CGCGCG]** 571 (85.98)   
274 - **[CGCGCG]** 569 (85.98)   
274 - **.CACGCG.** 175 (91.73)   
274 - **.TGCGCG.** 113 (94.26)   
274 - **[GCCGCG]** 108 (86.55)   
274 - **[GGCGCG]** 49 (86.50)   
274 - **[GCCGCG]** 15 (86.55)   
275 + **[GGCGCG]** 73 (86.50)   
275 + **.GACGCG.** 91 (92.24)   
275 + **[CGCGCG]** 93 (85.98)   
275 + **.CACGCG.** 112 (91.73)   
275 + **[TTCGCG]** 603 (87.60)   
275 + **[CGCGCG]** 709 (85.98)   
275 + **[CCCGCG]** 733 (86.03)   
275 + **[GGCGCG]** 1088 (86.50)   
275 - **[GGCGCG]** 1330 (86.50)   
275 - **[GGCGCG]** 1101 (86.50)   
275 - **[GGCGCG]** 755 (86.50)   
275 - **[GGCGCG]** 735 (86.50)   
275 - **[GCCGCG]** 711 (86.55)   
275 - **[CGCGCG]** 709 (85.98)   
275 - **.TACGCG.** 114 (100.00)   
275 - **.TGCGCG.** 95 (94.26)   
275 - **[CGCGCG]** 93 (85.98)   
275 - **.TCCGCG.** 75 (94.31)   
276 + **.GACGCG.** 755 (92.24)   
276 - **[TTCGCG]** 757 (87.60)   
279 + **[CGCGCG]** 31 (85.98)   
279 + **[CCCGCG]** 38 (86.03)   
279 + **[GGCGCG]** 106 (86.50)   
279 + **.CACGCG.** 374 (91.73)   
279 + **[GGCGCG]** 413 (86.50)   
279 + **[GGCGCG]** 418 (86.50)   
279 + **.GACGCG.** 555 (92.24)   
279 + **.TGCGCG.** 630 (94.26)   
279 - **.TCCGCG.** 1201 (94.31)   
279 - **[CCCGCG]** 557 (86.03)   
279 - **[GGCGCG]** 459 (86.50)   
279 - **.CACGCG.** 420 (91.73)   
279 - **[GCCGCG]** 415 (86.55)   
279 - **[GGCGCG]** 108 (86.50)   
279 - **[GGCGCG]** 40 (86.50)   
279 - **[GCCGCG]** 33 (86.55)   
279 - **[CGCGCG]** 31 (85.98)   
279 - **[GGCGCG]** 22 (86.50)   
280 + **.TGCGCG.** 733 (94.26)   
280 + **[GCCGCG]** 742 (86.55)   
280 + **.TGCGCG.** 825 (94.26)   
280 + **[GCCGCG]** 944 (86.55)   
280 + **[GGCGCG]** 976 (86.50)   
280 + **[CGCGCG]** 978 (85.98)   
280 + **[CGCGCG]** 980 (85.98)   
280 + **.TGCGCG.** 1064 (94.26)   
280 + **[CCCGCG]** 1070 (86.03)   
280 + **[TTCGCG]** 1082 (87.60)   
280 + **[CCCGCG]** 1126 (86.03)   
280 + **.TGCGCG.** 1195 (94.26)   
280 + **[CCCGCG]** 1368 (86.03)   
280 - **[TAGGCG]** 1353 (85.06)   
280 - **[GGCGCG]** 1264 (86.50)   
280 - **.GACGCG.** 1197 (92.24)   
280 - **[GGCGCG]** 1128 (86.50)   
280 - **.GACGCG.** 1084 (92.24)   
280 - **[GGCGCG]** 1072 (86.50)   
280 - **[GGCGCG]** 1066 (86.50)   
280 - **.TGCGCG.** 1028 (94.26)   
280 - **[GCCGCG]** 982 (86.55)   
280 - **[CGCGCG]** 980 (85.98)   
280 - **[CGCGCG]** 978 (85.98)   
280 - **[CCCGCG]** 783 (86.03)   
280 - **.CACGCG.** 744 (91.73)   
280 - **[GCCGCG]** 735 (86.55)   
280 - **[GCCGCG]** 169 (86.55)   
281 + **.GACGCG.** 446 (92.24)   
281 + **.GACGCG.** 512 (92.24)   
281 + **.TGCGCG.** 519 (94.26)   
281 + **[TTCGCG]** 529 (87.60)   
281 + **[GGCGCG]** 545 (86.50)   
281 + **[CCCGCG]** 551 (86.03)   
281 + **[GGCGCG]** 1024 (86.50)   
281 + **[CCCGCG]** 1180 (86.03)   
281 + **[GGCGCG]** 1200 (86.50)   
281 - **.TGCGCG.** 1202 (94.26)   
281 - **.CACGCG.** 1182 (91.73)   
281 - **.TCCGCG.** 1026 (94.31)   
281 - **[GGCGCG]** 553 (86.50)   
281 - **[GGCGCG]** 547 (86.50)   
281 - **[GCCGCG]** 531 (86.55)   
281 - **[GCCGCG]** 521 (86.55)   
281 - **[CCCGCG]** 448 (86.03)   
281 - **[GGCGCG]** 433 (86.50)   
282 + **.TGCGCG.** 11 (94.26)   
282 - **[GCCGCG]** 13 (86.55)   
283 + **.TCCGCG.** 132 (94.31)   
283 - **[GGCGCG]** 134 (86.50)   
284 + **[GGCGCG]** 631 (86.50)   
284 + **[GGCGCG]** 668 (86.50)   
284 + **[GCCGCG]** 706 (86.55)   
284 + **[CCCGCG]** 716 (86.03)   
284 + **.CACGCG.** 918 (91.73)   
284 + **[CCCGCG]** 941 (86.03)   
284 + **[CCCGCG]** 948 (86.03)   
284 + **[GGCGCG]** 999 (86.50)   
284 + **[CGCGCG]** 1001 (85.98)   
284 + **[CCCGCG]** 1087 (86.03)   
284 + **[GGCGCG]** 1092 (86.50)   
284 + **.TGCGCG.** 1124 (94.26)   
284 + **[GGCGCG]** 1198 (86.50)   
284 + **[CCCGCG]** 1338 (86.03)   
284 + **[CGCGCG]** 1340 (85.98)   
284 + **[GCCGCG]** 1345 (86.55)   
284 + **[CCCGCG]** 1369 (86.03)   
284 + **[GGCGCG]** 1450 (86.50)   
284 + **[GGCGCG]** 1471 (86.50)   
284 - **[CCCGCG]** 1507 (86.03)   
284 - **[CCCGCG]** 1473 (86.03)   
284 - **[GCCGCG]** 1452 (86.55)   
284 - **[GGCGCG]** 1371 (86.50)   
284 - **[GCCGCG]** 1347 (86.55)   
284 - **[GGCGCG]** 1342 (86.50)   
284 - **[CGCGCG]** 1340 (85.98)   
284 - **[GCCGCG]** 1283 (86.55)   
284 - **.TGCGCG.** 1200 (94.26)   
284 - **[CCCGCG]** 1194 (86.03)   
284 - **[CCCGCG]** 1126 (86.03)   
284 - **[GCCGCG]** 1094 (86.55)   
284 - **[GCCGCG]** 1089 (86.55)   
284 - **[GGCGCG]** 1003 (86.50)   
284 - **[CGCGCG]** 1001 (85.98)   
284 - **[GCCGCG]** 943 (86.55)   
284 - **.TCCGCG.** 920 (94.31)   
284 - **[GCCGCG]** 780 (86.55)   
284 - **.CACGCG.** 718 (91.73)   
284 - **[GGCGCG]** 708 (86.50)   
284 - **.TGCGCG.** 670 (94.26)   
284 - **.TCCGCG.** 633 (94.31)   
284 - **[GGCGCG]** 553 (86.50)   
285 + **.CACGCG.** 177 (91.73)   
285 + **[GGCGCG]** 334 (86.50)   
285 + **[CGCGCG]** 336 (85.98)   
285 + **[CGCGCG]** 338 (85.98)   
285 + **[GGCGCG]** 344 (86.50)   
285 + **[GCCGCG]** 353 (86.55)   
285 + **[GGCGCG]** 443 (86.50)   
285 + **[GGCGCG]** 449 (86.50)   
285 + **[GGCGCG]** 470 (86.50)   
285 + **.TGCGCG.** 493 (94.26)   
285 + **.TCCGCG.** 546 (94.31)   
285 + **[GCCGCG]** 552 (86.55)   
285 + **[TTCGCG]** 592 (87.60)   
285 + **[GCCGCG]** 634 (86.55)   
285 + **[CCCGCG]** 641 (86.03)   
285 + **[GCCGCG]** 655 (86.55)   
285 + **.GACGCG.** 715 (92.24)   
285 + **[CCCGCG]** 772 (86.03)   
285 + **[GCCGCG]** 858 (86.55)   
285 + **[GCCGCG]** 907 (86.55)   
285 + **[GCCGCG]** 939 (86.55)   
285 + **[CCCGCG]** 946 (86.03)   
285 + **[CGCGCG]** 948 (85.98)   
285 + **[GGCGCG]** 1026 (86.50)   
285 + **[GCCGCG]** 1033 (86.55)   
285 + **[CGCGCG]** 1035 (85.98)   
285 + **[TTCGCG]** 1262 (87.60)   
285 + **[CGCGCG]** 1403 (85.98)   
285 + **[GGCGCG]** 1486 (86.50)   
285 - **[GGCGCG]** 1488 (86.50)   
285 - **[CGCGCG]** 1403 (85.98)   
285 - **[GCCGCG]** 1216 (86.55)   
285 - **[CGCGCG]** 1035 (85.98)   
285 - **[GCCGCG]** 966 (86.55)   
285 - **[CGCGCG]** 948 (85.98)   
285 - **[GGCGCG]** 941 (86.50)   
285 - **[GCCGCG]** 909 (86.55)   
285 - **[CCCGCG]** 860 (86.03)   
285 - **[CCCGCG]** 774 (86.03)   
285 - **[GCCGCG]** 717 (86.55)   
285 - **[GGCGCG]** 657 (86.50)   
285 - **[GGCGCG]** 652 (86.50)   
285 - **[GGCGCG]** 636 (86.50)   
285 - **[CCCGCG]** 594 (86.03)   
285 - **[GCCGCG]** 554 (86.55)   
285 - **[GCCGCG]** 548 (86.55)   
285 - **[GCCGCG]** 495 (86.55)   
285 - **[CCCGCG]** 472 (86.03)   
285 - **[CCCGCG]** 451 (86.03)   
285 - **[CCCGCG]** 445 (86.03)   
285 - **.TGCGCG.** 377 (94.26)   
285 - **[CCCGCG]** 355 (86.03)   
285 - **[CCCGCG]** 346 (86.03)   
285 - **[CCCGCG]** 340 (86.03)   
285 - **[CGCGCG]** 338 (85.98)   
285 - **[CGCGCG]** 336 (85.98)   
285 - **[AACGCG]** 294 (87.55)   
285 - **[AACGCG]** 179 (87.55)   
287 + **[GCCGCG]** 42 (86.55)   
287 + **.GACGCG.** 490 (92.24)   
287 + **.TCCGCG.** 584 (94.31)   
287 + **.CACGCG.** 667 (91.73)   
287 + **[CCCGCG]** 851 (86.03)   
287 + **.TCCGCG.** 1003 (94.31)   
287 + **[CGCGCG]** 1035 (85.98)   
287 + **[CCCGCG]** 1231 (86.03)   
287 + **[GGCGCG]** 1339 (86.50)   
287 + **[CGCGCG]** 1341 (85.98)   
287 + **[CCCGCG]** 1439 (86.03)   
287 - **[TTCGCG]** 1441 (87.60)   
287 - **.GACGCG.** 1343 (92.24)   
287 - **[CGCGCG]** 1341 (85.98)   
287 - **[GGCGCG]** 1233 (86.50)   
287 - **[CGCGCG]** 1035 (85.98)   
287 - **[CCCGCG]** 853 (86.03)   
287 - **.TCCGCG.** 669 (94.31)   
287 - **[GGCGCG]** 537 (86.50)   
287 - **[CCCGCG]** 492 (86.03)   
287 - **.TGCGCG.** 416 (94.26)   
287 - **[GGCGCG]** 326 (86.50)   
287 - **[CCCGCG]** 44 (86.03)   
288 + **[GGCGCG]** 876 (86.50)   
288 + **[CGCGCG]** 982 (85.98)   
288 + **[CGCGCG]** 984 (85.98)   
288 + **[GCCGCG]** 1168 (86.55)   
288 + **[CCCGCG]** 1191 (86.03)   
288 + **[CGCGCG]** 1193 (85.98)   
288 - **[GGCGCG]** 1195 (86.50)   
288 - **[CGCGCG]** 1193 (85.98)   
288 - **[GGCGCG]** 1170 (86.50)   
288 - **[GGCGCG]** 1042 (86.50)   
288 - **[CGCGCG]** 984 (85.98)   
288 - **[CGCGCG]** 982 (85.98)   
288 - **[TAGGCG]** 722 (85.06)   
289 + **.GACGCG.** 540 (92.24)   
289 + **.TGCGCG.** 1043 (94.26)   
289 + **[CGCGCG]** 1045 (85.98)   
289 + **.TGCGCG.** 1298 (94.26)   
289 + **[GGCGCG]** 1317 (86.50)   
289 + **[GGCGCG]** 1379 (86.50)   
289 - **[CCCGCG]** 1381 (86.03)   
289 - **[GCCGCG]** 1300 (86.55)   
289 - **.TCCGCG.** 1047 (94.31)   
289 - **[CGCGCG]** 1045 (85.98)   
289 - **[CCCGCG]** 1000 (86.03)   
289 - **[TTCGCG]** 747 (87.60)   
289 - **[CCCGCG]** 542 (86.03)   
290 + **.TCCGCG.** 975 (94.31)   
290 + **[CCCGCG]** 1017 (86.03)   
290 + **.TGCGCG.** 1059 (94.26)   
290 + **[GCCGCG]** 1104 (86.55)   
290 + **[CCCGCG]** 1245 (86.03)   
290 + **[GGCGCG]** 1330 (86.50)   
290 - **[GCCGCG]** 1339 (86.55)   
290 - **.TCCGCG.** 1332 (94.31)   
290 - **[CCCGCG]** 1247 (86.03)   
290 - **[TAGGCG]** 1195 (85.06)   
290 - **.TCCGCG.** 1089 (94.31)   
290 - **.TCCGCG.** 1061 (94.31)   
290 - **.CACGCG.** 1019 (91.73)   
290 - **.CACGCG.** 977 (91.73)   
291 + **.TCCGCG.** 449 (94.31)   
291 + **[GCCGCG]** 510 (86.55)   
291 + **[GGCGCG]** 608 (86.50)   
291 + **[CCCGCG]** 692 (86.03)   
291 + **.CACGCG.** 736 (91.73)   
291 + **[GCCGCG]** 767 (86.55)   
291 + **[GCCGCG]** 1035 (86.55)   
291 + **[GGCGCG]** 1066 (86.50)   
291 + **[CGCGCG]** 1068 (85.98)   
291 + **[GCCGCG]** 1083 (86.55)   
291 + **[GGCGCG]** 1098 (86.50)   
291 + **[GCCGCG]** 1109 (86.55)   
291 + **.GACGCG.** 1165 (92.24)   
291 + **[CCCGCG]** 1205 (86.03)   
291 + **[GCCGCG]** 1329 (86.55)   
291 + **[GCCGCG]** 1343 (86.55)   
291 + **[GGCGCG]** 1500 (86.50)   
291 - **[GGCGCG]** 1502 (86.50)   
291 - **[CCCGCG]** 1463 (86.03)   
291 - **[CCCGCG]** 1437 (86.03)   
291 - **[GCCGCG]** 1240 (86.55)   
291 - **[GCCGCG]** 1207 (86.55)   
291 - **[CCCGCG]** 1167 (86.03)   
291 - **[CCCGCG]** 1111 (86.03)   
291 - **[GGCGCG]** 1100 (86.50)   
291 - **.CACGCG.** 1085 (91.73)   
291 - **[CGCGCG]** 1068 (85.98)   
291 - **.TGCGCG.** 1037 (94.26)   
291 - **.TGCGCG.** 999 (94.26)   
291 - **[GCCGCG]** 871 (86.55)   
291 - **[GCCGCG]** 769 (86.55)   
291 - **.GACGCG.** 738 (92.24)   
291 - **[CCCGCG]** 694 (86.03)   
291 - **[TTCGCG]** 631 (87.60)   
291 - **[TTCGCG]** 610 (87.60)   
291 - **[TTCGCG]** 586 (87.60)   
291 - **.GACGCG.** 512 (92.24)   
291 - **.GACGCG.** 407 (92.24)   
291 - **.GACGCG.** 203 (92.24)   
292 + **.TCCGCG.** 657 (94.31)   
292 + **[CCCGCG]** 765 (86.03)   
292 + **[CCCGCG]** 772 (86.03)   
292 + **[GCCGCG]** 795 (86.55)   
292 + **[CCCGCG]** 838 (86.03)   
292 + **[CGCGCG]** 956 (85.98)   
292 + **.TGCGCG.** 1004 (94.26)   
292 + **[GGCGCG]** 1026 (86.50)   
292 + **[CGCGCG]** 1028 (85.98)   
292 + **[GGCGCG]** 1043 (86.50)   
292 + **[GCCGCG]** 1082 (86.55)   
292 + **.CACGCG.** 1136 (91.73)   
292 + **[CGCGCG]** 1138 (85.98)   
292 + **.GACGCG.** 1189 (92.24)   
292 + **.TCCGCG.** 1323 (94.31)   
292 + **[GGCGCG]** 1346 (86.50)   
292 + **.CACGCG.** 1384 (91.73)   
292 + **[GCCGCG]** 1445 (86.55)   
292 + **.GACGCG.** 1485 (92.24)   
292 + **[GGCGCG]** 1492 (86.50)   
292 - **[CCCGCG]** 1494 (86.03)   
292 - **[CCCGCG]** 1487 (86.03)   
292 - **[CCCGCG]** 1471 (86.03)   
292 - **[CCCGCG]** 1447 (86.03)   
292 - **[GCCGCG]** 1386 (86.55)   
292 - **[CCCGCG]** 1348 (86.03)   
292 - **[GCCGCG]** 1339 (86.55)   
292 - **[CCCGCG]** 1325 (86.03)   
292 - **.GACGCG.** 1319 (92.24)   
292 - **.TCCGCG.** 1238 (94.31)   
292 - **[CCCGCG]** 1191 (86.03)   
292 - **[GGCGCG]** 1140 (86.50)   
292 - **[CGCGCG]** 1138 (85.98)   
292 - **[CCCGCG]** 1045 (86.03)   
292 - **[CCCGCG]** 1030 (86.03)   
292 - **[CGCGCG]** 1028 (85.98)   
292 - **[GGCGCG]** 1006 (86.50)   
292 - **[GGCGCG]** 958 (86.50)   
292 - **[CGCGCG]** 956 (85.98)   
292 - **[GCCGCG]** 840 (86.55)   
292 - **[GCCGCG]** 797 (86.55)   
292 - **[GGCGCG]** 774 (86.50)   
292 - **[GGCGCG]** 767 (86.50)   
292 - **[GCCGCG]** 659 (86.55)   
292 - **[TAGGCG]** 114 (85.06)   
293 + **.TCCGCG.** 617 (94.31)   
293 + **[GGCGCG]** 837 (86.50)   
293 + **[GGCGCG]** 860 (86.50)   
293 + **[CCCGCG]** 910 (86.03)   
293 + **[TTCGCG]** 934 (87.60)   
293 + **[GGCGCG]** 1096 (86.50)   
293 + **[CGCGCG]** 1098 (85.98)   
293 + **[TAGGCG]** 1110 (85.06)   
293 + **[CGCGCG]** 1152 (85.98)   
293 + **[GGCGCG]** 1378 (86.50)   
293 - **.CACGCG.** 1444 (91.73)   
293 - **.TCCGCG.** 1380 (94.31)   
293 - **[TAGGCG]** 1235 (85.06)   
293 - **[GCCGCG]** 1154 (86.55)   
293 - **[CGCGCG]** 1152 (85.98)   
293 - **.TCCGCG.** 1100 (94.31)   
293 - **[CGCGCG]** 1098 (85.98)   
293 - **[CCCGCG]** 1075 (86.03)   
293 - **[GCCGCG]** 936 (86.55)   
293 - **[CCCGCG]** 912 (86.03)   
293 - **[CCCGCG]** 862 (86.03)   
293 - **[GCCGCG]** 839 (86.55)   
293 - **[CCCGCG]** 619 (86.03)   
294 + **[GCCGCG]** 46 (86.55)   
294 + **[CCCGCG]** 314 (86.03)   
294 + **[CGCGCG]** 368 (85.98)   
294 + **[GCCGCG]** 785 (86.55)   
294 + **.TCCGCG.** 930 (94.31)   
294 + **.GACGCG.** 936 (92.24)   
294 + **.TGCGCG.** 974 (94.26)   
294 + **[CGCGCG]** 976 (85.98)   
294 + **.TGCGCG.** 1007 (94.26)   
294 + **.CACGCG.** 1028 (91.73)   
294 + **.TCCGCG.** 1280 (94.31)   
294 - **.CACGCG.** 1282 (91.73)   
294 - **.TGCGCG.** 1030 (94.26)   
294 - **.CACGCG.** 1009 (91.73)   
294 - **[CGCGCG]** 976 (85.98)   
294 - **[GGCGCG]** 938 (86.50)   
294 - **.TCCGCG.** 932 (94.31)   
294 - **[GCCGCG]** 894 (86.55)   
294 - **[AACGCG]** 787 (87.55)   
294 - **.CACGCG.** 613 (91.73)   
294 - **.CACGCG.** 370 (91.73)   
294 - **[CGCGCG]** 368 (85.98)   
294 - **[GCCGCG]** 316 (86.55)   
294 - **.CACGCG.** 128 (91.73)   
295 + **.CACGCG.** 358 (91.73)   
295 + **[GGCGCG]** 590 (86.50)   
295 + **.TGCGCG.** 610 (94.26)   
295 + **.CACGCG.** 631 (91.73)   
295 + **[CGCGCG]** 665 (85.98)   
295 + **[GGCGCG]** 702 (86.50)   
295 + **.TCCGCG.** 708 (94.31)   
295 + **[GCCGCG]** 747 (86.55)   
295 + **[AACGCG]** 853 (87.55)   
295 + **.CACGCG.** 1027 (91.73)   
295 + **.CACGCG.** 1270 (91.73)   
295 + **[CGCGCG]** 1272 (85.98)   
295 + **[GCCGCG]** 1324 (86.55)   
295 + **.CACGCG.** 1512 (91.73)   
295 - **[CCCGCG]** 1326 (86.03)   
295 - **[CGCGCG]** 1272 (85.98)   
295 - **[GCCGCG]** 855 (86.55)   
295 - **.TCCGCG.** 710 (94.31)   
295 - **.GACGCG.** 704 (92.24)   
295 - **.TGCGCG.** 667 (94.26)   
295 - **[CGCGCG]** 665 (85.98)   
295 - **.TGCGCG.** 633 (94.26)   
295 - **.CACGCG.** 612 (91.73)   
295 - **[GCCGCG]** 592 (86.55)   
295 - **.TCCGCG.** 360 (94.31)   
296 + **[CCCGCG]** 37 (86.03)   
296 + **.CACGCG.** 53 (91.73)   
296 + **[CGCGCG]** 55 (85.98)   
296 + **[CGCGCG]** 57 (85.98)   
296 + **[CGCGCG]** 59 (85.98)   
296 + **.CACGCG.** 83 (91.73)   
296 + **[GGCGCG]** 195 (86.50)   
296 + **[CGCGCG]** 197 (85.98)   
296 + **[CGCGCG]** 199 (85.98)   
296 + **[GGCGCG]** 248 (86.50)   
296 + **[CCCGCG]** 333 (86.03)   
296 + **[GGCGCG]** 419 (86.50)   
296 + **[GGCGCG]** 494 (86.50)   
296 + **[CGCGCG]** 496 (85.98)   
296 + **[CGCGCG]** 498 (85.98)   
296 + **[CGCGCG]** 649 (85.98)   
296 + **[CCCGCG]** 695 (86.03)   
296 + **[CCCGCG]** 702 (86.03)   
296 + **[CCCGCG]** 709 (86.03)   
296 + **[CCCGCG]** 716 (86.03)   
296 + **[GCCGCG]** 757 (86.55)   
296 + **[GCCGCG]** 813 (86.55)   
296 + **.GACGCG.** 841 (92.24)   
296 + **.TCCGCG.** 935 (94.31)   
296 + **[CCCGCG]** 964 (86.03)   
296 + **.GACGCG.** 1001 (92.24)   
296 + **.GACGCG.** 1012 (92.24)   
296 + **[CGCGCG]** 1014 (85.98)   
296 + **.TGCGCG.** 1039 (94.26)   
296 + **[GGCGCG]** 1118 (86.50)   
296 + **[GCCGCG]** 1162 (86.55)   
296 + **.TCCGCG.** 1182 (94.31)   
296 + **[GCCGCG]** 1187 (86.55)   
296 + **[GGCGCG]** 1313 (86.50)   
296 + **.GACGCG.** 1378 (92.24)   
296 + **[GGCGCG]** 1443 (86.50)   
296 - **.GACGCG.** 1474 (92.24)   
296 - **.TCCGCG.** 1445 (94.31)   
296 - **[GCCGCG]** 1440 (86.55)   
296 - **[GGCGCG]** 1315 (86.50)   
296 - **[CCCGCG]** 1189 (86.03)   
296 - **[GGCGCG]** 1184 (86.50)   
296 - **[GCCGCG]** 1164 (86.55)   
296 - **[AACGCG]** 1120 (87.55)   
296 - **[TTCGCG]** 1041 (87.60)   
296 - **[GGCGCG]** 1016 (86.50)   
296 - **[CGCGCG]** 1014 (85.98)   
296 - **[GCCGCG]** 1003 (86.55)   
296 - **[CCCGCG]** 966 (86.03)   
296 - **[GGCGCG]** 946 (86.50)   
296 - **[CCCGCG]** 843 (86.03)   
296 - **[GCCGCG]** 815 (86.55)   
296 - **[GGCGCG]** 759 (86.50)   
296 - **[GGCGCG]** 718 (86.50)   
296 - **[GGCGCG]** 711 (86.50)   
296 - **[GGCGCG]** 704 (86.50)   
296 - **[GGCGCG]** 697 (86.50)   
296 - **.TCCGCG.** 651 (94.31)   
296 - **[CGCGCG]** 649 (85.98)   
296 - **[GGCGCG]** 591 (86.50)   
296 - **.GACGCG.** 500 (92.24)   
296 - **[CGCGCG]** 498 (85.98)   
296 - **[CGCGCG]** 496 (85.98)   
296 - **[CCCGCG]** 421 (86.03)   
296 - **[GCCGCG]** 250 (86.55)   
296 - **[TTCGCG]** 209 (87.60)   
296 - **[GCCGCG]** 201 (86.55)   
296 - **[CGCGCG]** 199 (85.98)   
296 - **[CGCGCG]** 197 (85.98)   
296 - **[GCCGCG]** 85 (86.55)   
296 - **[CCCGCG]** 61 (86.03)   
296 - **[CGCGCG]** 59 (85.98)   
296 - **[CGCGCG]** 57 (85.98)   
296 - **[CGCGCG]** 55 (85.98)   
296 - **[GGCGCG]** 39 (86.50)   
297 + **[CCCGCG]** 554 (86.03)   
297 + **.CACGCG.** 581 (91.73)   
297 + **[CCCGCG]** 790 (86.03)   
297 + **[GCCGCG]** 827 (86.55)   
297 + **[GGCGCG]** 954 (86.50)   
297 + **[GGCGCG]** 985 (86.50)   
297 + **[GCCGCG]** 996 (86.55)   
297 + **[CCCGCG]** 1128 (86.03)   
297 + **[GCCGCG]** 1155 (86.55)   
297 + **[TAGGCG]** 1282 (85.06)   
297 + **[CCCGCG]** 1419 (86.03)   
297 - **.CACGCG.** 1500 (91.73)   
297 - **.CACGCG.** 1421 (91.73)   
297 - **[CCCGCG]** 1252 (86.03)   
297 - **[CCCGCG]** 1157 (86.03)   
297 - **.CACGCG.** 1033 (91.73)   
297 - **.TCCGCG.** 998 (94.31)   
297 - **[CCCGCG]** 987 (86.03)   
297 - **[CCCGCG]** 956 (86.03)   
297 - **.TCCGCG.** 940 (94.31)   
297 - **[CCCGCG]** 878 (86.03)   
297 - **[CCCGCG]** 829 (86.03)   
297 - **[GGCGCG]** 792 (86.50)   
297 - **.TCCGCG.** 583 (94.31)   
297 - **[AACGCG]** 556 (87.55)   
298 + **[AACGCG]** 220 (87.55)   
298 + **[CCCGCG]** 291 (86.03)   
298 + **[CCCGCG]** 627 (86.03)   
298 + **.TGCGCG.** 679 (94.26)   
298 + **[GCCGCG]** 797 (86.55)   
298 + **[CGCGCG]** 840 (85.98)   
298 + **[GGCGCG]** 868 (86.50)   
298 + **.CACGCG.** 874 (91.73)   
298 + **[GCCGCG]** 901 (86.55)   
298 + **.TCCGCG.** 919 (94.31)   
298 + **.TCCGCG.** 935 (94.31)   
298 + **[GGCGCG]** 1040 (86.50)   
298 + **[GCCGCG]** 1178 (86.55)   
298 + **[GGCGCG]** 1369 (86.50)   
298 + **[CGCGCG]** 1371 (85.98)   
298 - **[CGCGCG]** 1371 (85.98)   
298 - **[CCCGCG]** 1042 (86.03)   
298 - **[GCCGCG]** 1004 (86.55)   
298 - **.GACGCG.** 937 (92.24)   
298 - **[GGCGCG]** 921 (86.50)   
298 - **.TCCGCG.** 903 (94.31)   
298 - **[GCCGCG]** 876 (86.55)   
298 - **.TGCGCG.** 870 (94.26)   
298 - **[GCCGCG]** 842 (86.55)   
298 - **[CGCGCG]** 840 (85.98)   
298 - **.TACGCG.** 823 (100.00)   
298 - **.GACGCG.** 799 (92.24)   
298 - **[GCCGCG]** 746 (86.55)   
298 - **.CACGCG.** 681 (91.73)   
298 - **.CACGCG.** 629 (91.73)   
298 - **[GGCGCG]** 293 (86.50)   
299 + **[TAGGCG]** 362 (85.06)   
299 + **.CACGCG.** 577 (91.73)   
299 + **[CGCGCG]** 579 (85.98)   
299 + **.TGCGCG.** 688 (94.26)   
299 + **[GCCGCG]** 851 (86.55)   
299 + **[CCCGCG]** 866 (86.03)   
299 + **[CCCGCG]** 879 (86.03)   
299 + **[CGCGCG]** 881 (85.98)   
299 + **[CGCGCG]** 918 (85.98)   
299 + **[CCCGCG]** 991 (86.03)   
299 + **[CGCGCG]** 1057 (85.98)   
299 + **[GGCGCG]** 1078 (86.50)   
299 + **[CGCGCG]** 1080 (85.98)   
299 + **[GCCGCG]** 1178 (86.55)   
299 + **.TCCGCG.** 1260 (94.31)   
299 + **[GCCGCG]** 1350 (86.55)   
299 + **[CCCGCG]** 1399 (86.03)   
299 + **[GGCGCG]** 1473 (86.50)   
299 + **[AACGCG]** 1508 (87.55)   
299 - **[CCCGCG]** 1510 (86.03)   
299 - **.GACGCG.** 1475 (92.24)   
299 - **[GCCGCG]** 1262 (86.55)   
299 - **[GCCGCG]** 1180 (86.55)   
299 - **[CCCGCG]** 1093 (86.03)   
299 - **[GGCGCG]** 1082 (86.50)   
299 - **[CGCGCG]** 1080 (85.98)   
299 - **[GCCGCG]** 1059 (86.55)   
299 - **[CGCGCG]** 1057 (85.98)   
299 - **.TGCGCG.** 993 (94.26)   
299 - **[GGCGCG]** 920 (86.50)   
299 - **[CGCGCG]** 918 (85.98)   
299 - **.GACGCG.** 883 (92.24)   
299 - **[CGCGCG]** 881 (85.98)   
299 - **[GGCGCG]** 868 (86.50)   
299 - **.GACGCG.** 853 (92.24)   
299 - **[CCCGCG]** 690 (86.03)   
299 - **.TCCGCG.** 581 (94.31)   
299 - **[CGCGCG]** 579 (85.98)   
300 + **[CCCGCG]** 28 (86.03)   
300 + **[GGCGCG]** 111 (86.50)   
300 + **[CCCGCG]** 173 (86.03)   
300 + **.TCCGCG.** 369 (94.31)   
300 + **.TCCGCG.** 444 (94.31)   
300 + **[GGCGCG]** 472 (86.50)   
300 + **[GGCGCG]** 477 (86.50)   
300 + **[CCCGCG]** 579 (86.03)   
300 + **[TTCGCG]** 734 (87.60)   
300 - **.TCCGCG.** 1166 (94.31)   
300 - **[GCCGCG]** 736 (86.55)   
300 - **[GGCGCG]** 581 (86.50)   
300 - **[CCCGCG]** 479 (86.03)   
300 - **[GCCGCG]** 474 (86.55)   
300 - **.GACGCG.** 446 (92.24)   
300 - **.TGCGCG.** 371 (94.26)   
300 - **[GCCGCG]** 175 (86.55)   
300 - **[GCCGCG]** 113 (86.55)   
300 - **.TGCGCG.** 30 (94.26)   
300 - **[GCCGCG]** 2 (86.55)   
301 + **[GGCGCG]** 579 (86.50)   
301 + **.TGCGCG.** 668 (94.26)   
301 + **[GGCGCG]** 765 (86.50)   
301 + **[GCCGCG]** 1481 (86.55)   
301 - **.CACGCG.** 1483 (91.73)   
301 - **[GCCGCG]** 1289 (86.55)   
301 - **.CACGCG.** 767 (91.73)   
301 - **.CACGCG.** 708 (91.73)   
301 - **.CACGCG.** 670 (91.73)   
301 - **[CCCGCG]** 581 (86.03)   
302 + **[CCCGCG]** 580 (86.03)   
302 + **[GGCGCG]** 740 (86.50)   
302 + **[CGCGCG]** 742 (85.98)   
302 + **[CCCGCG]** 754 (86.03)   
302 + **.TGCGCG.** 851 (94.26)   
302 + **[CCCGCG]** 920 (86.03)   
302 + **[CGCGCG]** 922 (85.98)   
302 + **.GACGCG.** 951 (92.24)   
302 + **.CACGCG.** 957 (91.73)   
302 + **[CGCGCG]** 959 (85.98)   
302 + **[GCCGCG]** 999 (86.55)   
302 + **[GCCGCG]** 1042 (86.55)   
302 - **[CCCGCG]** 1073 (86.03)   
302 - **[GGCGCG]** 1039 (86.50)   
302 - **[GCCGCG]** 1026 (86.55)   
302 - **[GGCGCG]** 977 (86.50)   
302 - **[GGCGCG]** 961 (86.50)   
302 - **[CGCGCG]** 959 (85.98)   
302 - **.TGCGCG.** 953 (94.26)   
302 - **[CGCGCG]** 922 (85.98)   
302 - **[GGCGCG]** 863 (86.50)   
302 - **[GCCGCG]** 853 (86.55)   
302 - **[GGCGCG]** 841 (86.50)   
302 - **[GGCGCG]** 756 (86.50)   
302 - **.CACGCG.** 744 (91.73)   
302 - **[CGCGCG]** 742 (85.98)   
302 - **.TCCGCG.** 582 (94.31)   
303 + **[TAGGCG]** 11 (85.06)   
303 + **[GCCGCG]** 43 (86.55)   
303 + **.TGCGCG.** 128 (94.26)   
303 + **[GCCGCG]** 168 (86.55)   
303 + **[GCCGCG]** 287 (86.55)   
303 + **[AACGCG]** 329 (87.55)   
303 + **[CCCGCG]** 391 (86.03)   
303 + **[GCCGCG]** 457 (86.55)   
303 + **[GGCGCG]** 743 (86.50)   
303 + **[GCCGCG]** 748 (86.55)   
303 + **[CCCGCG]** 802 (86.03)   
303 + **[CGCGCG]** 804 (85.98)   
303 + **.TCCGCG.** 885 (94.31)   
303 + **[GGCGCG]** 1004 (86.50)   
303 + **[CCCGCG]** 1019 (86.03)   
303 + **[GCCGCG]** 1361 (86.55)   
303 + **[CGCGCG]** 1363 (85.98)   
303 - **[GCCGCG]** 1365 (86.55)   
303 - **[CGCGCG]** 1363 (85.98)   
303 - **.GACGCG.** 1021 (92.24)   
303 - **[GCCGCG]** 1006 (86.55)   
303 - **[CGCGCG]** 804 (85.98)   
303 - **[GCCGCG]** 750 (86.55)   
303 - **[GGCGCG]** 745 (86.50)   
303 - **[CCCGCG]** 733 (86.03)   
303 - **[CCCGCG]** 459 (86.03)   
303 - **[CCCGCG]** 331 (86.03)   
303 - **[CCCGCG]** 289 (86.03)   
303 - **[CCCGCG]** 220 (86.03)   
303 - **.TGCGCG.** 202 (94.26)   
303 - **[GGCGCG]** 170 (86.50)   
303 - **.GACGCG.** 130 (92.24)   
303 - **[CCCGCG]** 23 (86.03)   
304 + **[GGCGCG]** 169 (86.50)   
304 + **[GCCGCG]** 708 (86.55)   
304 + **.GACGCG.** 733 (92.24)   
304 + **[GGCGCG]** 904 (86.50)   
304 + **[CGCGCG]** 906 (85.98)   
304 + **.TGCGCG.** 1015 (94.26)   
304 + **[GCCGCG]** 1106 (86.55)   
304 + **.TACGCG.** 1152 (100.00)   
304 + **[CGCGCG]** 1163 (85.98)   
304 + **[GGCGCG]** 1179 (86.50)   
304 + **[GGCGCG]** 1241 (86.50)   
304 + **[GCCGCG]** 1263 (86.55)   
304 + **[CGCGCG]** 1265 (85.98)   
304 + **[TTCGCG]** 1275 (87.60)   
304 + **[CCCGCG]** 1307 (86.03)   
304 + **.TGCGCG.** 1423 (94.26)   
304 - **[GGCGCG]** 1510 (86.50)   
304 - **[AACGCG]** 1425 (87.55)   
304 - **.CACGCG.** 1277 (91.73)   
304 - **[CGCGCG]** 1265 (85.98)   
304 - **[GCCGCG]** 1243 (86.55)   
304 - **[CGCGCG]** 1163 (85.98)   
304 - **[CCCGCG]** 1154 (86.03)   
304 - **[CCCGCG]** 1108 (86.03)   
304 - **[CCCGCG]** 981 (86.03)   
304 - **.CACGCG.** 947 (91.73)   
304 - **[CGCGCG]** 906 (85.98)   
304 - **.TCCGCG.** 735 (94.31)   
304 - **[GGCGCG]** 710 (86.50)   
304 - **.TCCGCG.** 171 (94.31)   
305 + **[GCCGCG]** 924 (86.55)   
305 + **[CCCGCG]** 1078 (86.03)   
305 - **.GACGCG.** 1080 (92.24)   
305 - **.TGCGCG.** 926 (94.26)   
306 + **[CCCGCG]** 47 (86.03)   
306 + **.CACGCG.** 144 (91.73)   
306 + **[CCCGCG]** 237 (86.03)   
306 + **[GGCGCG]** 310 (86.50)   
306 + **[GCCGCG]** 340 (86.55)   
306 + **[CCCGCG]** 376 (86.03)   
306 + **[CCCGCG]** 599 (86.03)   
306 + **[GCCGCG]** 765 (86.55)   
306 + **.GACGCG.** 845 (92.24)   
306 + **[CCCGCG]** 866 (86.03)   
306 + **[CCCGCG]** 911 (86.03)   
306 + **.TCCGCG.** 928 (94.31)   
306 + **[GGCGCG]** 1039 (86.50)   
306 + **[CCCGCG]** 1475 (86.03)   
306 - **.CACGCG.** 1477 (91.73)   
306 - **[CCCGCG]** 1041 (86.03)   
306 - **[CCCGCG]** 954 (86.03)   
306 - **[GGCGCG]** 930 (86.50)   
306 - **.CACGCG.** 913 (91.73)   
306 - **.TCCGCG.** 868 (94.31)   
306 - **.TGCGCG.** 847 (94.26)   
306 - **[GGCGCG]** 767 (86.50)   
306 - **[GGCGCG]** 631 (86.50)   
306 - **.TACGCG.** 601 (100.00)   
306 - **[CCCGCG]** 378 (86.03)   
306 - **[GGCGCG]** 342 (86.50)   
306 - **.GACGCG.** 312 (92.24)   
307 + **.GACGCG.** 334 (92.24)   
307 + **.TCCGCG.** 384 (94.31)   
307 + **[CGCGCG]** 386 (85.98)   
307 + **[GGCGCG]** 430 (86.50)   
307 + **[GGCGCG]** 497 (86.50)   
307 + **[GGCGCG]** 546 (86.50)   
307 + **[GCCGCG]** 679 (86.55)   
307 + **[GCCGCG]** 737 (86.55)   
307 + **[CGCGCG]** 739 (85.98)   
307 + **[CCCGCG]** 824 (86.03)   
307 + **[CGCGCG]** 892 (85.98)   
307 + **[CCCGCG]** 922 (86.03)   
307 + **[GGCGCG]** 1160 (86.50)   
307 + **[GGCGCG]** 1235 (86.50)   
307 + **[AACGCG]** 1289 (87.55)   
307 + **[GGCGCG]** 1355 (86.50)   
307 - **[CCCGCG]** 1357 (86.03)   
307 - **[GCCGCG]** 1330 (86.55)   
307 - **[CCCGCG]** 1237 (86.03)   
307 - **[GCCGCG]** 1162 (86.55)   
307 - **[GCCGCG]** 1128 (86.55)   
307 - **[CCCGCG]** 1016 (86.03)   
307 - **[CCCGCG]** 894 (86.03)   
307 - **[CGCGCG]** 892 (85.98)   
307 - **.TCCGCG.** 826 (94.31)   
307 - **[CCCGCG]** 741 (86.03)   
307 - **[CGCGCG]** 739 (85.98)   
307 - **[GCCGCG]** 730 (86.55)   
307 - **.TCCGCG.** 681 (94.31)   
307 - **[GGCGCG]** 548 (86.50)   
307 - **.CACGCG.** 499 (91.73)   
307 - **[CGCGCG]** 386 (85.98)   
307 - **[TTCGCG]** 336 (87.60)   
307 - **[GGCGCG]** 176 (86.50)   
308 + **.CACGCG.** 71 (91.73)   
308 + **[GCCGCG]** 387 (86.55)   
308 + **[CCCGCG]** 709 (86.03)   
308 + **.TGCGCG.** 746 (94.26)   
308 + **[GCCGCG]** 770 (86.55)   
308 + **.TGCGCG.** 784 (94.26)   
308 + **[CCCGCG]** 790 (86.03)   
308 + **.GACGCG.** 877 (92.24)   
308 + **[GGCGCG]** 1100 (86.50)   
308 + **[GCCGCG]** 1179 (86.55)   
308 + **.TCCGCG.** 1187 (94.31)   
308 + **[GCCGCG]** 1368 (86.55)   
308 + **[GCCGCG]** 1413 (86.55)   
308 + **[CCCGCG]** 1423 (86.03)   
308 - **[CCCGCG]** 1425 (86.03)   
308 - **[CCCGCG]** 1415 (86.03)   
308 - **[CCCGCG]** 1370 (86.03)   
308 - **[GGCGCG]** 1189 (86.50)   
308 - **[GCCGCG]** 1181 (86.55)   
308 - **[GCCGCG]** 1116 (86.55)   
308 - **[CCCGCG]** 1102 (86.03)   
308 - **[GCCGCG]** 863 (86.55)   
308 - **[GGCGCG]** 786 (86.50)   
308 - **.TGCGCG.** 772 (94.26)   
308 - **[CCCGCG]** 748 (86.03)   
308 - **[CCCGCG]** 711 (86.03)   
308 - **.CACGCG.** 637 (91.73)   
308 - **[CCCGCG]** 547 (86.03)   
308 - **.TGCGCG.** 389 (94.26)   
308 - **[CCCGCG]** 73 (86.03)   
309 + **[GCCGCG]** 689 (86.55)   
309 + **.TCCGCG.** 871 (94.31)   
309 + **[GCCGCG]** 994 (86.55)   
309 + **.TGCGCG.** 1027 (94.26)   
309 + **[GGCGCG]** 1078 (86.50)   
309 + **[GGCGCG]** 1148 (86.50)   
309 + **.CACGCG.** 1225 (91.73)   
309 - **[CCCGCG]** 1227 (86.03)   
309 - **[CCCGCG]** 1150 (86.03)   
309 - **[GCCGCG]** 1029 (86.55)   
309 - **.TCCGCG.** 996 (94.31)   
309 - **[CCCGCG]** 761 (86.03)   
309 - **[GGCGCG]** 691 (86.50)   
310 + **[GGCGCG]** 682 (86.50)   
310 + **[CGCGCG]** 684 (85.98)   
310 + **.TCCGCG.** 929 (94.31)   
310 + **[GGCGCG]** 979 (86.50)   
310 + **[AACGCG]** 1246 (87.55)   
310 + **[CCCGCG]** 1412 (86.03)   
310 - **[GCCGCG]** 1301 (86.55)   
310 - **[GCCGCG]** 1035 (86.55)   
310 - **[GCCGCG]** 981 (86.55)   
310 - **[GGCGCG]** 834 (86.50)   
310 - **[GGCGCG]** 686 (86.50)   
310 - **[CGCGCG]** 684 (85.98)   
311 + **[TAGGCG]** 61 (85.06)   
311 + **.CACGCG.** 500 (91.73)   
311 + **[GCCGCG]** 873 (86.55)   
311 + **[GGCGCG]** 1067 (86.50)   
311 + **[GCCGCG]** 1104 (86.55)   
311 + **[CCCGCG]** 1113 (86.03)   
311 + **[GGCGCG]** 1178 (86.50)   
311 + **.GACGCG.** 1262 (92.24)   
311 + **[GCCGCG]** 1343 (86.55)   
311 + **[GCCGCG]** 1471 (86.55)   
311 - **[CCCGCG]** 1473 (86.03)   
311 - **.TCCGCG.** 1427 (94.31)   
311 - **.GACGCG.** 1345 (92.24)   
311 - **[CCCGCG]** 1264 (86.03)   
311 - **.TCCGCG.** 1216 (94.31)   
311 - **.TGCGCG.** 1180 (94.26)   
311 - **[CCCGCG]** 1115 (86.03)   
311 - **[GCCGCG]** 1106 (86.55)   
311 - **[GCCGCG]** 1069 (86.55)   
311 - **[GGCGCG]** 960 (86.50)   
311 - **[GCCGCG]** 875 (86.55)   
311 - **[GGCGCG]** 762 (86.50)   
311 - **.TCCGCG.** 502 (94.31)   
312 + **.GACGCG.** 741 (92.24)   
312 + **.TCCGCG.** 811 (94.31)   
312 + **.CACGCG.** 841 (91.73)   
312 + **.CACGCG.** 870 (91.73)   
312 + **[CCCGCG]** 877 (86.03)   
312 + **[GGCGCG]** 941 (86.50)   
312 + **[CCCGCG]** 983 (86.03)   
312 + **[GGCGCG]** 1029 (86.50)   
312 + **[CGCGCG]** 1092 (85.98)   
312 + **[CCCGCG]** 1120 (86.03)   
312 + **[GGCGCG]** 1290 (86.50)   
312 + **[CGCGCG]** 1324 (85.98)   
312 + **[GCCGCG]** 1355 (86.55)   
312 + **[CGCGCG]** 1357 (85.98)   
312 + **[CGCGCG]** 1359 (85.98)   
312 + **.TGCGCG.** 1471 (94.26)   
312 - **[GCCGCG]** 1473 (86.55)   
312 - **[GGCGCG]** 1361 (86.50)   
312 - **[CGCGCG]** 1359 (85.98)   
312 - **[CGCGCG]** 1357 (85.98)   
312 - **[CCCGCG]** 1326 (86.03)   
312 - **[CGCGCG]** 1324 (85.98)   
312 - **[GGCGCG]** 1122 (86.50)   
312 - **[GCCGCG]** 1094 (86.55)   
312 - **[CGCGCG]** 1092 (85.98)   
312 - **[CCCGCG]** 1031 (86.03)   
312 - **[GGCGCG]** 985 (86.50)   
312 - **.TCCGCG.** 943 (94.31)   
312 - **[CCCGCG]** 879 (86.03)   
312 - **[GGCGCG]** 872 (86.50)   
312 - **[GGCGCG]** 843 (86.50)   
312 - **.TCCGCG.** 813 (94.31)   
312 - **.TGCGCG.** 743 (94.26)   
313 + **[TAGGCG]** 155 (85.06)   
313 + **.CACGCG.** 256 (91.73)   
313 + **[CGCGCG]** 258 (85.98)   
313 + **[AACGCG]** 786 (87.55)   
313 + **.CACGCG.** 798 (91.73)   
313 + **[GGCGCG]** 817 (86.50)   
313 + **.TCCGCG.** 824 (94.31)   
313 + **[CGCGCG]** 868 (85.98)   
313 + **.TGCGCG.** 919 (94.26)   
313 + **[GGCGCG]** 956 (86.50)   
313 + **.TGCGCG.** 995 (94.26)   
313 + **.TCCGCG.** 1006 (94.31)   
313 + **[GGCGCG]** 1123 (86.50)   
313 + **[GCCGCG]** 1446 (86.55)   
313 - **[GCCGCG]** 1448 (86.55)   
313 - **[GGCGCG]** 1303 (86.50)   
313 - **[GGCGCG]** 1088 (86.50)   
313 - **[GGCGCG]** 1008 (86.50)   
313 - **.GACGCG.** 964 (92.24)   
313 - **[TAGGCG]** 935 (85.06)   
313 - **.TGCGCG.** 921 (94.26)   
313 - **[GGCGCG]** 892 (86.50)   
313 - **[CGCGCG]** 868 (85.98)   
313 - **[GCCGCG]** 826 (86.55)   
313 - **.TGCGCG.** 800 (94.26)   
313 - **.CACGCG.** 788 (91.73)   
313 - **.CACGCG.** 583 (91.73)   
313 - **[CGCGCG]** 258 (85.98)   
315 + **[CCCGCG]** 2 (86.03)   
315 + **.GACGCG.** 379 (92.24)   
315 + **[CGCGCG]** 381 (85.98)   
315 + **.TCCGCG.** 507 (94.31)   
315 + **.TGCGCG.** 853 (94.26)   
315 + **.GACGCG.** 903 (92.24)   
315 + **.TGCGCG.** 909 (94.26)   
315 + **[GGCGCG]** 981 (86.50)   
315 + **[CGCGCG]** 983 (85.98)   
315 + **[CGCGCG]** 985 (85.98)   
315 + **.GACGCG.** 1006 (92.24)   
315 + **[CGCGCG]** 1008 (85.98)   
315 + **[GGCGCG]** 1017 (86.50)   
315 + **[GGCGCG]** 1026 (86.50)   
315 + **[GGCGCG]** 1057 (86.50)   
315 + **[CGCGCG]** 1059 (85.98)   
315 + **[CGCGCG]** 1061 (85.98)   
315 + **[CCCGCG]** 1225 (86.03)   
315 + **[GGCGCG]** 1271 (86.50)   
315 + **.GACGCG.** 1285 (92.24)   
315 + **[GCCGCG]** 1342 (86.55)   
315 - **.GACGCG.** 1344 (92.24)   
315 - **.TCCGCG.** 1287 (94.31)   
315 - **[GGCGCG]** 1273 (86.50)   
315 - **[CGCGCG]** 1061 (85.98)   
315 - **[CGCGCG]** 1059 (85.98)   
315 - **[CCCGCG]** 1028 (86.03)   
315 - **[CCCGCG]** 1019 (86.03)   
315 - **[CCCGCG]** 1010 (86.03)   
315 - **[CGCGCG]** 1008 (85.98)   
315 - **[CGCGCG]** 985 (85.98)   
315 - **[CGCGCG]** 983 (85.98)   
315 - **.TGCGCG.** 911 (94.26)   
315 - **.CACGCG.** 905 (91.73)   
315 - **[AACGCG]** 509 (87.55)   
315 - **[GGCGCG]** 383 (86.50)   
315 - **[CGCGCG]** 381 (85.98)   
315 - **[GCCGCG]** 357 (86.55)   
316 + **.GACGCG.** 200 (92.24)   
316 + **[GCCGCG]** 227 (86.55)   
316 + **.TCCGCG.** 255 (94.31)   
316 + **[GCCGCG]** 536 (86.55)   
316 + **[GGCGCG]** 573 (86.50)   
316 + **[CCCGCG]** 848 (86.03)   
316 + **[GGCGCG]** 896 (86.50)   
316 + **[CCCGCG]** 908 (86.03)   
316 + **[GGCGCG]** 913 (86.50)   
316 - **[GCCGCG]** 910 (86.55)   
316 - **.GACGCG.** 898 (92.24)   
316 - **[CCCGCG]** 884 (86.03)   
316 - **[GGCGCG]** 257 (86.50)   
316 - **[GCCGCG]** 229 (86.55)   
316 - **[GGCGCG]** 202 (86.50)   
317 + **[GGCGCG]** 1005 (86.50)   
317 + **[CCCGCG]** 1348 (86.03)   
317 + **[GGCGCG]** 1379 (86.50)   
317 + **[GGCGCG]** 1408 (86.50)   
317 + **.TGCGCG.** 1431 (94.26)   
317 - **[GGCGCG]** 1433 (86.50)   
317 - **.TCCGCG.** 1410 (94.31)   
317 - **[CCCGCG]** 1381 (86.03)   
317 - **.TCCGCG.** 1350 (94.31)   
317 - **[CCCGCG]** 1007 (86.03)   
317 - **[TAGGCG]** 815 (85.06)   
318 + **[CCCGCG]** 101 (86.03)   
318 + **[CGCGCG]** 103 (85.98)   
318 + **[GGCGCG]** 250 (86.50)   
318 + **[CCCGCG]** 267 (86.03)   
318 + **[GCCGCG]** 288 (86.55)   
318 + **[CCCGCG]** 305 (86.03)   
318 + **[GGCGCG]** 538 (86.50)   
318 + **.TCCGCG.** 689 (94.31)   
318 + **[GGCGCG]** 722 (86.50)   
318 + **[CGCGCG]** 1040 (85.98)   
318 + **[CCCGCG]** 1094 (86.03)   
318 + **[CCCGCG]** 1116 (86.03)   
318 + **[CGCGCG]** 1209 (85.98)   
318 + **[GCCGCG]** 1242 (86.55)   
318 + **[CGCGCG]** 1244 (85.98)   
318 + **.CACGCG.** 1346 (91.73)   
318 + **[GGCGCG]** 1434 (86.50)   
318 + **[GCCGCG]** 1483 (86.55)   
318 - **[GCCGCG]** 1485 (86.55)   
318 - **[GGCGCG]** 1348 (86.50)   
318 - **[GGCGCG]** 1246 (86.50)   
318 - **[CGCGCG]** 1244 (85.98)   
318 - **[CGCGCG]** 1209 (85.98)   
318 - **.TGCGCG.** 1118 (94.26)   
318 - **.TGCGCG.** 1096 (94.26)   
318 - **.CACGCG.** 1042 (91.73)   
318 - **[CGCGCG]** 1040 (85.98)   
318 - **[CCCGCG]** 540 (86.03)   
318 - **[GGCGCG]** 307 (86.50)   
318 - **.GACGCG.** 290 (92.24)   
318 - **[AACGCG]** 269 (87.55)   
318 - **[CCCGCG]** 252 (86.03)   
318 - **[CGCGCG]** 103 (85.98)   
320 + **[GCCGCG]** 72 (86.55)   
320 + **[AACGCG]** 105 (87.55)   
320 + **[TAGGCG]** 318 (85.06)   
320 + **[CCCGCG]** 535 (86.03)   
320 + **[GCCGCG]** 594 (86.55)   
320 + **[CGCGCG]** 596 (85.98)   
320 + **[CGCGCG]** 598 (85.98)   
320 + **[TTCGCG]** 627 (87.60)   
320 + **[CGCGCG]** 629 (85.98)   
320 + **[CCCGCG]** 638 (86.03)   
320 + **.TGCGCG.** 658 (94.26)   
320 + **[TTCGCG]** 670 (87.60)   
320 + **[CGCGCG]** 672 (85.98)   
320 + **.TCCGCG.** 712 (94.31)   
320 + **[GGCGCG]** 798 (86.50)   
320 + **[GGCGCG]** 855 (86.50)   
320 + **[GGCGCG]** 876 (86.50)   
320 + **[GCCGCG]** 911 (86.55)   
320 + **.TCCGCG.** 990 (94.31)   
320 + **[TTCGCG]** 1196 (87.60)   
320 + **[GGCGCG]** 1313 (86.50)   
320 + **[GCCGCG]** 1342 (86.55)   
320 + **[CGCGCG]** 1344 (85.98)   
320 + **[CGCGCG]** 1346 (85.98)   
320 + **[CGCGCG]** 1379 (85.98)   
320 + **[GCCGCG]** 1384 (86.55)   
320 - **[GCCGCG]** 1386 (86.55)   
320 - **[GGCGCG]** 1381 (86.50)   
320 - **[CGCGCG]** 1379 (85.98)   
320 - **.GACGCG.** 1348 (92.24)   
320 - **[CGCGCG]** 1346 (85.98)   
320 - **[CGCGCG]** 1344 (85.98)   
320 - **[CCCGCG]** 1232 (86.03)   
320 - **[GGCGCG]** 1198 (86.50)   
320 - **[GCCGCG]** 992 (86.55)   
320 - **[CCCGCG]** 943 (86.03)   
320 - **[GGCGCG]** 913 (86.50)   
320 - **[CCCGCG]** 857 (86.03)   
320 - **[TAGGCG]** 778 (85.06)   
320 - **[GCCGCG]** 755 (86.55)   
320 - **[GCCGCG]** 721 (86.55)   
320 - **[GGCGCG]** 714 (86.50)   
320 - **[GCCGCG]** 694 (86.55)   
320 - **[GGCGCG]** 674 (86.50)   
320 - **[CGCGCG]** 672 (85.98)   
320 - **[GGCGCG]** 640 (86.50)   
320 - **.TCCGCG.** 631 (94.31)   
320 - **[CGCGCG]** 629 (85.98)   
320 - **[GGCGCG]** 600 (86.50)   
320 - **[CGCGCG]** 598 (85.98)   
320 - **[CGCGCG]** 596 (85.98)   
320 - **[CCCGCG]** 537 (86.03)   
320 - **.TGCGCG.** 498 (94.26)   
320 - **[CCCGCG]** 384 (86.03)   
320 - **[AACGCG]** 147 (87.55)   
320 - **.GACGCG.** 107 (92.24)   
320 - **.TCCGCG.** 51 (94.31)   
321 + **[CCCGCG]** 453 (86.03)   
321 + **[GCCGCG]** 500 (86.55)   
321 + **[TTCGCG]** 537 (87.60)   
321 + **.CACGCG.** 555 (91.73)   
321 + **[CGCGCG]** 557 (85.98)   
321 + **[CCCGCG]** 694 (86.03)   
321 + **[GCCGCG]** 699 (86.55)   
321 + **[GCCGCG]** 943 (86.55)   
321 + **[CCCGCG]** 1221 (86.03)   
321 + **[CGCGCG]** 1223 (85.98)   
321 + **[GCCGCG]** 1256 (86.55)   
321 + **[CGCGCG]** 1258 (85.98)   
321 + **[GGCGCG]** 1300 (86.50)   
321 + **.TGCGCG.** 1393 (94.26)   
321 + **[GCCGCG]** 1398 (86.55)   
321 + **[CCCGCG]** 1468 (86.03)   
321 + **[CGCGCG]** 1470 (85.98)   
321 + **[GCCGCG]** 1502 (86.55)   
321 + **[CGCGCG]** 1504 (85.98)   
321 - **[GGCGCG]** 1506 (86.50)   
321 - **[CGCGCG]** 1504 (85.98)   
321 - **[CGCGCG]** 1470 (85.98)   
321 - **[GCCGCG]** 1400 (86.55)   
321 - **[GGCGCG]** 1395 (86.50)   
321 - **[GCCGCG]** 1302 (86.55)   
321 - **[GGCGCG]** 1260 (86.50)   
321 - **[CGCGCG]** 1258 (85.98)   
321 - **[GGCGCG]** 1225 (86.50)   
321 - **[CGCGCG]** 1223 (85.98)   
321 - **[CCCGCG]** 945 (86.03)   
321 - **[GGCGCG]** 701 (86.50)   
321 - **[GGCGCG]** 696 (86.50)   
321 - **[CGCGCG]** 557 (85.98)   
321 - **[TTCGCG]** 539 (87.60)   
321 - **[GCCGCG]** 455 (86.55)   
322 + **[CGCGCG]** 257 (85.98)   
322 + **[CCCGCG]** 461 (86.03)   
322 + **[GCCGCG]** 497 (86.55)   
322 + **[GGCGCG]** 630 (86.50)   
322 + **[GGCGCG]** 686 (86.50)   
322 + **[CCCGCG]** 780 (86.03)   
322 + **[CGCGCG]** 798 (85.98)   
322 + **[GCCGCG]** 942 (86.55)   
322 + **[CGCGCG]** 944 (85.98)   
322 + **[CGCGCG]** 946 (85.98)   
322 + **[GCCGCG]** 959 (86.55)   
322 + **[CGCGCG]** 961 (85.98)   
322 + **[GCCGCG]** 1003 (86.55)   
322 + **[CCCGCG]** 1378 (86.03)   
322 + **.GACGCG.** 1424 (92.24)   
322 + **[GCCGCG]** 1481 (86.55)   
322 - **[GGCGCG]** 1426 (86.50)   
322 - **[CCCGCG]** 1380 (86.03)   
322 - **[GGCGCG]** 1174 (86.50)   
322 - **[AACGCG]** 1067 (87.55)   
322 - **[CCCGCG]** 963 (86.03)   
322 - **[CGCGCG]** 961 (85.98)   
322 - **[GGCGCG]** 948 (86.50)   
322 - **[CGCGCG]** 946 (85.98)   
322 - **[CGCGCG]** 944 (85.98)   
322 - **[CCCGCG]** 812 (86.03)   
322 - **[CCCGCG]** 800 (86.03)   
322 - **[CGCGCG]** 798 (85.98)   
322 - **.TGCGCG.** 782 (94.26)   
322 - **[GCCGCG]** 688 (86.55)   
322 - **[GCCGCG]** 632 (86.55)   
322 - **[GGCGCG]** 499 (86.50)   
322 - **[GGCGCG]** 463 (86.50)   
322 - **[GGCGCG]** 259 (86.50)   
322 - **[CGCGCG]** 257 (85.98)   
323 + **.CACGCG.** 222 (91.73)   
323 + **[GCCGCG]** 453 (86.55)   
323 + **[CCCGCG]** 573 (86.03)   
323 + **[CCCGCG]** 715 (86.03)   
323 + **[CGCGCG]** 717 (85.98)   
323 + **[GGCGCG]** 858 (86.50)   
323 + **[GGCGCG]** 945 (86.50)   
323 + **[CGCGCG]** 947 (85.98)   
323 + **[CGCGCG]** 949 (85.98)   
323 + **[GGCGCG]** 985 (86.50)   
323 + **[CGCGCG]** 987 (85.98)   
323 + **[GCCGCG]** 1030 (86.55)   
323 + **.CACGCG.** 1068 (91.73)   
323 + **[CGCGCG]** 1070 (85.98)   
323 + **[CGCGCG]** 1072 (85.98)   
323 + **[CCCGCG]** 1180 (86.03)   
323 + **[GCCGCG]** 1229 (86.55)   
323 + **.TCCGCG.** 1311 (94.31)   
323 + **[CCCGCG]** 1319 (86.03)   
323 + **[GGCGCG]** 1361 (86.50)   
323 + **.TCCGCG.** 1411 (94.31)   
323 + **[CGCGCG]** 1422 (85.98)   
323 + **[GGCGCG]** 1448 (86.50)   
323 + **.TGCGCG.** 1468 (94.26)   
323 + **[GCCGCG]** 1494 (86.55)   
323 - **[GGCGCG]** 1496 (86.50)   
323 - **.GACGCG.** 1450 (92.24)   
323 - **.GACGCG.** 1424 (92.24)   
323 - **[CGCGCG]** 1422 (85.98)   
323 - **[CCCGCG]** 1413 (86.03)   
323 - **[CCCGCG]** 1363 (86.03)   
323 - **[GGCGCG]** 1321 (86.50)   
323 - **[GGCGCG]** 1313 (86.50)   
323 - **[GCCGCG]** 1231 (86.55)   
323 - **[GGCGCG]** 1182 (86.50)   
323 - **[GCCGCG]** 1074 (86.55)   
323 - **[CGCGCG]** 1072 (85.98)   
323 - **[CGCGCG]** 1070 (85.98)   
323 - **[GCCGCG]** 1032 (86.55)   
323 - **[GCCGCG]** 1020 (86.55)   
323 - **[CCCGCG]** 989 (86.03)   
323 - **[CGCGCG]** 987 (85.98)   
323 - **[CCCGCG]** 951 (86.03)   
323 - **[CGCGCG]** 949 (85.98)   
323 - **[CGCGCG]** 947 (85.98)   
323 - **[GCCGCG]** 860 (86.55)   
323 - **.TCCGCG.** 719 (94.31)   
323 - **[CGCGCG]** 717 (85.98)   
323 - **[CCCGCG]** 575 (86.03)   
323 - **.CACGCG.** 455 (91.73)   
324 + **[CCCGCG]** 110 (86.03)   
324 - **.TGCGCG.** 112 (94.26)   
325 + **[GGCGCG]** 1306 (86.50)   
325 + **[GGCGCG]** 1432 (86.50)   
325 - **[TTCGCG]** 1434 (87.60)   
325 - **.CACGCG.** 1308 (91.73)   
326 + **.GACGCG.** 177 (92.24)   
326 + **.CACGCG.** 651 (91.73)   
326 + **.CACGCG.** 658 (91.73)   
326 + **.TCCGCG.** 669 (94.31)   
326 + **[GCCGCG]** 823 (86.55)   
326 + **[CCCGCG]** 867 (86.03)   
326 + **[CCCGCG]** 873 (86.03)   
326 + **[CCCGCG]** 884 (86.03)   
326 + **[CCCGCG]** 902 (86.03)   
326 + **[CGCGCG]** 904 (85.98)   
326 + **.TCCGCG.** 936 (94.31)   
326 + **[GGCGCG]** 1111 (86.50)   
326 + **[GCCGCG]** 1117 (86.55)   
326 + **[CGCGCG]** 1119 (85.98)   
326 + **.TGCGCG.** 1200 (94.26)   
326 + **[CCCGCG]** 1218 (86.03)   
326 + **[CGCGCG]** 1220 (85.98)   
326 + **[CCCGCG]** 1239 (86.03)   
326 + **[CCCGCG]** 1254 (86.03)   
326 + **[CGCGCG]** 1256 (85.98)   
326 + **[GCCGCG]** 1301 (86.55)   
326 + **[GGCGCG]** 1319 (86.50)   
326 + **[CCCGCG]** 1334 (86.03)   
326 + **[GGCGCG]** 1403 (86.50)   
326 + **[GCCGCG]** 1417 (86.55)   
326 - **[GGCGCG]** 1405 (86.50)   
326 - **[CCCGCG]** 1336 (86.03)   
326 - **[CCCGCG]** 1321 (86.03)   
326 - **[GCCGCG]** 1303 (86.55)   
326 - **.TCCGCG.** 1258 (94.31)   
326 - **[CGCGCG]** 1256 (85.98)   
326 - **[GGCGCG]** 1241 (86.50)   
326 - **[GCCGCG]** 1222 (86.55)   
326 - **[CGCGCG]** 1220 (85.98)   
326 - **[GCCGCG]** 1202 (86.55)   
326 - **.CACGCG.** 1121 (91.73)   
326 - **[CGCGCG]** 1119 (85.98)   
326 - **[GCCGCG]** 1113 (86.55)   
326 - **[GCCGCG]** 938 (86.55)   
326 - **[CCCGCG]** 906 (86.03)   
326 - **[CGCGCG]** 904 (85.98)   
326 - **[GGCGCG]** 875 (86.50)   
326 - **[GGCGCG]** 869 (86.50)   
326 - **[TAGGCG]** 827 (85.06)   
326 - **[GGCGCG]** 825 (86.50)   
326 - **[TAGGCG]** 737 (85.06)   
326 - **[CCCGCG]** 689 (86.03)   
326 - **[GCCGCG]** 660 (86.55)   
326 - **[GCCGCG]** 653 (86.55)   
326 - **[GGCGCG]** 454 (86.50)   
327 + **.TCCGCG.** 247 (94.31)   
327 + **[GGCGCG]** 468 (86.50)   
327 + **[GGCGCG]** 658 (86.50)   
327 + **[GCCGCG]** 900 (86.55)   
327 + **[TAGGCG]** 1030 (85.06)   
327 + **[GCCGCG]** 1113 (86.55)   
327 - **[TAGGCG]** 683 (85.06)   
327 - **[GGCGCG]** 681 (86.50)   
327 - **.CACGCG.** 660 (91.73)   
327 - **[AACGCG]** 470 (87.55)   
327 - **[CCCGCG]** 249 (86.03)   
327 - **[CCCGCG]** 26 (86.03)   
329 + **[GCCGCG]** 296 (86.55)   
329 + **[TTCGCG]** 1118 (87.60)   
329 + **.TGCGCG.** 1192 (94.26)   
329 + **[CCCGCG]** 1231 (86.03)   
329 + **.GACGCG.** 1237 (92.24)   
329 - **.TCCGCG.** 1233 (94.31)   
329 - **[GGCGCG]** 1227 (86.50)   
329 - **.TCCGCG.** 1194 (94.31)   
329 - **.TGCGCG.** 298 (94.26)   
329 - **.TGCGCG.** 190 (94.26)   
329 - **.TGCGCG.** 114 (94.26)   
331 + **[CCCGCG]** 30 (86.03)   
331 + **[CGCGCG]** 32 (85.98)   
331 + **[GCCGCG]** 163 (86.55)   
331 + **[CGCGCG]** 165 (85.98)   
331 + **[GGCGCG]** 188 (86.50)   
331 + **[CCCGCG]** 267 (86.03)   
331 + **[GGCGCG]** 357 (86.50)   
331 + **.TCCGCG.** 395 (94.31)   
331 + **.TACGCG.** 451 (100.00)   
331 + **[GCCGCG]** 640 (86.55)   
331 + **.TCCGCG.** 690 (94.31)   
331 + **[CGCGCG]** 692 (85.98)   
331 + **[GCCGCG]** 721 (86.55)   
331 + **[GCCGCG]** 738 (86.55)   
331 + **[GCCGCG]** 763 (86.55)   
331 + **[CGCGCG]** 765 (85.98)   
331 + **[GGCGCG]** 1001 (86.50)   
331 + **[GGCGCG]** 1138 (86.50)   
331 + **[CCCGCG]** 1307 (86.03)   
331 + **[CGCGCG]** 1309 (85.98)   
331 + **[GCCGCG]** 1314 (86.55)   
331 + **[CCCGCG]** 1330 (86.03)   
331 + **.TGCGCG.** 1439 (94.26)   
331 - **[GCCGCG]** 1441 (86.55)   
331 - **[GCCGCG]** 1332 (86.55)   
331 - **.GACGCG.** 1316 (92.24)   
331 - **[GGCGCG]** 1311 (86.50)   
331 - **[CGCGCG]** 1309 (85.98)   
331 - **.GACGCG.** 1140 (92.24)   
331 - **[GCCGCG]** 1135 (86.55)   
331 - **.TGCGCG.** 1003 (94.26)   
331 - **.TGCGCG.** 767 (94.26)   
331 - **[CGCGCG]** 765 (85.98)   
331 - **.TGCGCG.** 740 (94.26)   
331 - **[GCCGCG]** 723 (86.55)   
331 - **[GCCGCG]** 694 (86.55)   
331 - **[CGCGCG]** 692 (85.98)   
331 - **.TCCGCG.** 642 (94.31)   
331 - **.TCCGCG.** 453 (94.31)   
331 - **[GCCGCG]** 397 (86.55)   
331 - **.TCCGCG.** 388 (94.31)   
331 - **[GGCGCG]** 359 (86.50)   
331 - **[TTCGCG]** 327 (87.60)   
331 - **.GACGCG.** 269 (92.24)   
331 - **[CCCGCG]** 190 (86.03)   
331 - **[CGCGCG]** 165 (85.98)   
331 - **[CGCGCG]** 32 (85.98)   
332 + **[GCCGCG]** 101 (86.55)   
332 + **[TTCGCG]** 111 (87.60)   
332 + **[CCCGCG]** 813 (86.03)   
332 + **.TGCGCG.** 1086 (94.26)   
332 + **[GGCGCG]** 1135 (86.50)   
332 + **[CCCGCG]** 1177 (86.03)   
332 + **[CGCGCG]** 1179 (85.98)   
332 + **[GGCGCG]** 1225 (86.50)   
332 + **[GCCGCG]** 1351 (86.55)   
332 + **[GGCGCG]** 1389 (86.50)   
332 + **[GCCGCG]** 1428 (86.55)   
332 + **.CACGCG.** 1446 (91.73)   
332 + **[CGCGCG]** 1448 (85.98)   
332 + **[GGCGCG]** 1483 (86.50)   
332 + **[CGCGCG]** 1485 (85.98)   
332 - **[GCCGCG]** 1487 (86.55)   
332 - **[CGCGCG]** 1485 (85.98)   
332 - **.TGCGCG.** 1450 (94.26)   
332 - **[CGCGCG]** 1448 (85.98)   
332 - **[GCCGCG]** 1430 (86.55)   
332 - **.TGCGCG.** 1413 (94.26)   
332 - **[CCCGCG]** 1391 (86.03)   
332 - **[GGCGCG]** 1353 (86.50)   
332 - **[GCCGCG]** 1298 (86.55)   
332 - **[CCCGCG]** 1227 (86.03)   
332 - **[CCCGCG]** 1181 (86.03)   
332 - **[CGCGCG]** 1179 (85.98)   
332 - **[CCCGCG]** 1137 (86.03)   
332 - **[GGCGCG]** 1088 (86.50)   
332 - **.CACGCG.** 815 (91.73)   
332 - **.GACGCG.** 504 (92.24)   
332 - **[GGCGCG]** 460 (86.50)   
332 - **.TGCGCG.** 452 (94.26)   
332 - **[CCCGCG]** 113 (86.03)   
332 - **.TGCGCG.** 103 (94.26)   
333 + **[CCCGCG]** 1028 (86.03)   
333 + **.TGCGCG.** 1047 (94.26)   
333 + **[GCCGCG]** 1122 (86.55)   
333 + **[CCCGCG]** 1241 (86.03)   
333 + **[GCCGCG]** 1321 (86.55)   
333 + **[CCCGCG]** 1364 (86.03)   
333 - **.TGCGCG.** 1508 (94.26)   
333 - **[GCCGCG]** 1299 (86.55)   
333 - **.TCCGCG.** 1243 (94.31)   
333 - **[CCCGCG]** 1124 (86.03)   
333 - **[GCCGCG]** 1030 (86.55)   
334 + **[GCCGCG]** 381 (86.55)   
334 + **.TACGCG.** 600 (100.00)   
334 + **[CCCGCG]** 854 (86.03)   
334 + **[GCCGCG]** 871 (86.55)   
334 + **[GGCGCG]** 1087 (86.50)   
334 + **[CCCGCG]** 1244 (86.03)   
334 - **[CCCGCG]** 1089 (86.03)   
334 - **.TCCGCG.** 856 (94.31)   
334 - **[CCCGCG]** 602 (86.03)   
334 - **[AACGCG]** 383 (87.55)   
335 + **.TCCGCG.** 608 (94.31)   
335 + **[GGCGCG]** 857 (86.50)   
335 + **[CGCGCG]** 859 (85.98)   
335 + **[GGCGCG]** 983 (86.50)   
335 + **[CGCGCG]** 985 (85.98)   
335 + **[CCCGCG]** 1005 (86.03)   
335 + **[GCCGCG]** 1122 (86.55)   
335 + **[CCCGCG]** 1203 (86.03)   
335 + **[GGCGCG]** 1228 (86.50)   
335 + **[GGCGCG]** 1259 (86.50)   
335 + **[GGCGCG]** 1387 (86.50)   
335 + **[GGCGCG]** 1430 (86.50)   
335 + **[CGCGCG]** 1432 (85.98)   
335 - **[CCCGCG]** 1434 (86.03)   
335 - **[CGCGCG]** 1432 (85.98)   
335 - **[GGCGCG]** 1389 (86.50)   
335 - **[GCCGCG]** 1261 (86.55)   
335 - **[GGCGCG]** 1230 (86.50)   
335 - **.GACGCG.** 1205 (92.24)   
335 - **.TCCGCG.** 1178 (94.31)   
335 - **.TGCGCG.** 1124 (94.26)   
335 - **[GGCGCG]** 1100 (86.50)   
335 - **[GCCGCG]** 987 (86.55)   
335 - **[CGCGCG]** 985 (85.98)   
335 - **[CGCGCG]** 859 (85.98)   
335 - **[GCCGCG]** 854 (86.55)   
335 - **[GCCGCG]** 720 (86.55)   
335 - **[GGCGCG]** 610 (86.50)   
336 + **.TCCGCG.** 758 (94.31)   
336 + **[AACGCG]** 788 (87.55)   
336 + **.TGCGCG.** 811 (94.26)   
336 + **.CACGCG.** 840 (91.73)   
336 + **[AACGCG]** 874 (87.55)   
336 + **[CCCGCG]** 979 (86.03)   
336 + **[TTCGCG]** 1021 (87.60)   
336 + **.CACGCG.** 1062 (91.73)   
336 - **.CACGCG.** 1064 (91.73)   
336 - **[CCCGCG]** 1054 (86.03)   
336 - **[GCCGCG]** 1023 (86.55)   
336 - **[GGCGCG]** 981 (86.50)   
336 - **.GACGCG.** 876 (92.24)   
336 - **[CCCGCG]** 842 (86.03)   
336 - **.TCCGCG.** 813 (94.31)   
336 - **[GGCGCG]** 760 (86.50)   
337 + **[GCCGCG]** 318 (86.55)   
337 + **[CGCGCG]** 409 (85.98)   
337 + **.CACGCG.** 749 (91.73)   
337 + **[CGCGCG]** 751 (85.98)   
337 + **[CGCGCG]** 753 (85.98)   
337 + **[GCCGCG]** 765 (86.55)   
337 + **[CCCGCG]** 811 (86.03)   
337 + **[GCCGCG]** 825 (86.55)   
337 + **[CCCGCG]** 840 (86.03)   
337 + **.TGCGCG.** 854 (94.26)   
337 + **.TGCGCG.** 860 (94.26)   
337 + **[CCCGCG]** 884 (86.03)   
337 + **[CCCGCG]** 920 (86.03)   
337 + **[GGCGCG]** 950 (86.50)   
337 + **[CGCGCG]** 952 (85.98)   
337 + **[CGCGCG]** 954 (85.98)   
337 + **[CGCGCG]** 976 (85.98)   
337 + **[CCCGCG]** 995 (86.03)   
337 + **[CGCGCG]** 997 (85.98)   
337 + **[GCCGCG]** 1071 (86.55)   
337 + **[CCCGCG]** 1092 (86.03)   
337 + **[CGCGCG]** 1094 (85.98)   
337 + **[GGCGCG]** 1148 (86.50)   
337 + **[CCCGCG]** 1181 (86.03)   
337 + **[CCCGCG]** 1284 (86.03)   
337 + **.TCCGCG.** 1326 (94.31)   
337 + **[GGCGCG]** 1348 (86.50)   
337 + **[CGCGCG]** 1363 (85.98)   
337 + **[GGCGCG]** 1379 (86.50)   
337 + **[GGCGCG]** 1438 (86.50)   
337 + **[CGCGCG]** 1440 (85.98)   
337 + **[GGCGCG]** 1475 (86.50)   
337 + **[CGCGCG]** 1477 (85.98)   
337 - **[CGCGCG]** 1477 (85.98)   
337 - **[CGCGCG]** 1440 (85.98)   
337 - **[CCCGCG]** 1381 (86.03)   
337 - **[CCCGCG]** 1365 (86.03)   
337 - **[CGCGCG]** 1363 (85.98)   
337 - **[CCCGCG]** 1350 (86.03)   
337 - **[GCCGCG]** 1183 (86.55)   
337 - **[GCCGCG]** 1176 (86.55)   
337 - **[CCCGCG]** 1150 (86.03)   
337 - **.TCCGCG.** 1096 (94.31)   
337 - **[CGCGCG]** 1094 (85.98)   
337 - **[GCCGCG]** 1073 (86.55)   
337 - **.TCCGCG.** 999 (94.31)   
337 - **[CGCGCG]** 997 (85.98)   
337 - **[AACGCG]** 978 (87.55)   
337 - **[CGCGCG]** 976 (85.98)   
337 - **[GCCGCG]** 956 (86.55)   
337 - **[CGCGCG]** 954 (85.98)   
337 - **[CGCGCG]** 952 (85.98)   
337 - **[CCCGCG]** 922 (86.03)   
337 - **.CACGCG.** 856 (91.73)   
337 - **[GCCGCG]** 842 (86.55)   
337 - **[GGCGCG]** 827 (86.50)   
337 - **.CACGCG.** 755 (91.73)   
337 - **[CGCGCG]** 753 (85.98)   
337 - **[CGCGCG]** 751 (85.98)   
337 - **[GGCGCG]** 411 (86.50)   
337 - **[CGCGCG]** 409 (85.98)   
338 + **[CCCGCG]** 41 (86.03)   
338 + **[CCCGCG]** 438 (86.03)   
338 + **[GCCGCG]** 642 (86.55)   
338 + **[GCCGCG]** 692 (86.55)   
338 + **[CCCGCG]** 752 (86.03)   
338 + **[CCCGCG]** 758 (86.03)   
338 + **[CCCGCG]** 784 (86.03)   
338 + **[TTCGCG]** 860 (87.60)   
338 + **[CGCGCG]** 892 (85.98)   
338 + **[CGCGCG]** 894 (85.98)   
338 + **[CCCGCG]** 1031 (86.03)   
338 + **[CCCGCG]** 1070 (86.03)   
338 + **[CGCGCG]** 1088 (85.98)   
338 + **[CGCGCG]** 1090 (85.98)   
338 + **[CGCGCG]** 1122 (85.98)   
338 + **[CGCGCG]** 1124 (85.98)   
338 + **[CGCGCG]** 1152 (85.98)   
338 + **[GCCGCG]** 1193 (86.55)   
338 + **[CGCGCG]** 1195 (85.98)   
338 + **[CGCGCG]** 1197 (85.98)   
338 + **[CGCGCG]** 1199 (85.98)   
338 + **[GCCGCG]** 1305 (86.55)   
338 + **[CGCGCG]** 1307 (85.98)   
338 + **[GCCGCG]** 1414 (86.55)   
338 - **[GCCGCG]** 1459 (86.55)   
338 - **.CACGCG.** 1416 (91.73)   
338 - **[CGCGCG]** 1307 (85.98)   
338 - **.TGCGCG.** 1201 (94.26)   
338 - **[CGCGCG]** 1199 (85.98)   
338 - **[CGCGCG]** 1197 (85.98)   
338 - **[CGCGCG]** 1195 (85.98)   
338 - **[GGCGCG]** 1172 (86.50)   
338 - **.GACGCG.** 1154 (92.24)   
338 - **[CGCGCG]** 1152 (85.98)   
338 - **[GGCGCG]** 1126 (86.50)   
338 - **[CGCGCG]** 1124 (85.98)   
338 - **[CGCGCG]** 1122 (85.98)   
338 - **[GGCGCG]** 1092 (86.50)   
338 - **[CGCGCG]** 1090 (85.98)   
338 - **[CGCGCG]** 1088 (85.98)   
338 - **.TCCGCG.** 1072 (94.31)   
338 - **[GGCGCG]** 1065 (86.50)   
338 - **[GCCGCG]** 1054 (86.55)   
338 - **[AACGCG]** 1033 (87.55)   
338 - **[CGCGCG]** 894 (85.98)   
338 - **[CGCGCG]** 892 (85.98)   
338 - **.TGCGCG.** 862 (94.26)   
338 - **[GGCGCG]** 812 (86.50)   
338 - **.TGCGCG.** 786 (94.26)   
338 - **[GGCGCG]** 760 (86.50)   
338 - **[GGCGCG]** 754 (86.50)   
338 - **.GACGCG.** 694 (92.24)   
338 - **[GCCGCG]** 440 (86.55)   
338 - **.TCCGCG.** 43 (94.31)   
339 + **[GGCGCG]** 529 (86.50)   
339 + **[GGCGCG]** 1028 (86.50)   
339 + **[CCCGCG]** 1043 (86.03)   
339 + **[CGCGCG]** 1045 (85.98)   
339 - **.TCCGCG.** 1047 (94.31)   
339 - **[CGCGCG]** 1045 (85.98)   
340 + **.CACGCG.** 675 (91.73)   
340 + **[CGCGCG]** 951 (85.98)   
340 + **[CGCGCG]** 953 (85.98)   
340 + **[CCCGCG]** 977 (86.03)   
340 + **[GGCGCG]** 1066 (86.50)   
340 - **[CCCGCG]** 1068 (86.03)   
340 - **[CGCGCG]** 953 (85.98)   
340 - **[CGCGCG]** 951 (85.98)   
341 + **.CACGCG.** 43 (91.73)   
341 + **[CCCGCG]** 154 (86.03)   
341 + **[GCCGCG]** 187 (86.55)   
341 + **[CGCGCG]** 213 (85.98)   
341 + **.TCCGCG.** 270 (94.31)   
341 + **.TACGCG.** 1150 (100.00)   
341 + **[GCCGCG]** 1315 (86.55)   
341 + **[CGCGCG]** 1317 (85.98)   
341 + **[GGCGCG]** 1346 (86.50)   
341 + **[CGCGCG]** 1348 (85.98)   
341 + **[GCCGCG]** 1384 (86.55)   
341 + **[CGCGCG]** 1386 (85.98)   
341 + **[TAGGCG]** 1393 (85.06)   
341 + **[GGCGCG]** 1417 (86.50)   
341 + **[GGCGCG]** 1483 (86.50)   
341 + **.GACGCG.** 1492 (92.24)   
341 - **[GGCGCG]** 1505 (86.50)   
341 - **[GCCGCG]** 1494 (86.55)   
341 - **[CCCGCG]** 1485 (86.03)   
341 - **[CCCGCG]** 1419 (86.03)   
341 - **[CGCGCG]** 1386 (85.98)   
341 - **[GGCGCG]** 1350 (86.50)   
341 - **[CGCGCG]** 1348 (85.98)   
341 - **[GCCGCG]** 1319 (86.55)   
341 - **[CGCGCG]** 1317 (85.98)   
341 - **.GACGCG.** 1260 (92.24)   
341 - **[CCCGCG]** 1233 (86.03)   
341 - **[GCCGCG]** 1152 (86.55)   
341 - **[CCCGCG]** 966 (86.03)   
341 - **.TGCGCG.** 215 (94.26)   
341 - **[CGCGCG]** 213 (85.98)   
341 - **[GCCGCG]** 156 (86.55)   
341 - **[GGCGCG]** 105 (86.50)   
341 - **.TGCGCG.** 45 (94.26)   
341 - **.TGCGCG.** 39 (94.26)   
342 + **[AACGCG]** 226 (87.55)   
342 + **[GCCGCG]** 712 (86.55)   
342 + **[GGCGCG]** 720 (86.50)   
342 + **[GGCGCG]** 836 (86.50)   
342 + **[GGCGCG]** 1025 (86.50)   
342 + **.TCCGCG.** 1121 (94.31)   
342 + **[GGCGCG]** 1153 (86.50)   
342 + **[GGCGCG]** 1221 (86.50)   
342 + **[CGCGCG]** 1223 (85.98)   
342 + **[GGCGCG]** 1297 (86.50)   
342 + **[CGCGCG]** 1299 (85.98)   
342 + **.TGCGCG.** 1305 (94.26)   
342 + **[GGCGCG]** 1331 (86.50)   
342 + **[CGCGCG]** 1333 (85.98)   
342 + **.GACGCG.** 1347 (92.24)   
342 + **.TGCGCG.** 1364 (94.26)   
342 + **.TGCGCG.** 1370 (94.26)   
342 + **.GACGCG.** 1396 (92.24)   
342 + **[CGCGCG]** 1398 (85.98)   
342 + **[CCCGCG]** 1413 (86.03)   
342 + **.TGCGCG.** 1445 (94.26)   
342 + **.TGCGCG.** 1451 (94.26)   
342 + **[GGCGCG]** 1471 (86.50)   
342 + **[CGCGCG]** 1473 (85.98)   
342 + **[CGCGCG]** 1475 (85.98)   
342 + **.TGCGCG.** 1481 (94.26)   
342 - **.CACGCG.** 1483 (91.73)   
342 - **.CACGCG.** 1477 (91.73)   
342 - **[CGCGCG]** 1475 (85.98)   
342 - **[CGCGCG]** 1473 (85.98)   
342 - **.CACGCG.** 1447 (91.73)   
342 - **[CCCGCG]** 1415 (86.03)   
342 - **[GGCGCG]** 1400 (86.50)   
342 - **[CGCGCG]** 1398 (85.98)   
342 - **.CACGCG.** 1366 (91.73)   
342 - **[CCCGCG]** 1349 (86.03)   
342 - **[GCCGCG]** 1335 (86.55)   
342 - **[CGCGCG]** 1333 (85.98)   
342 - **.CACGCG.** 1301 (91.73)   
342 - **[CGCGCG]** 1299 (85.98)   
342 - **[CGCGCG]** 1223 (85.98)   
342 - **[GCCGCG]** 1155 (86.55)   
342 - **[CCCGCG]** 1027 (86.03)   
342 - **[CCCGCG]** 838 (86.03)   
342 - **[TAGGCG]** 776 (85.06)   
342 - **.TGCGCG.** 714 (94.26)   
342 - **[CCCGCG]** 228 (86.03)   
343 + **.CACGCG.** 220 (91.73)   
343 + **.CACGCG.** 232 (91.73)   
343 + **[GGCGCG]** 277 (86.50)   
343 + **[GGCGCG]** 524 (86.50)   
343 + **[GCCGCG]** 653 (86.55)   
343 + **.GACGCG.** 670 (92.24)   
343 + **[CCCGCG]** 898 (86.03)   
343 + **[GGCGCG]** 910 (86.50)   
343 + **[GGCGCG]** 965 (86.50)   
343 + **[GGCGCG]** 996 (86.50)   
343 + **[GCCGCG]** 1078 (86.55)   
343 + **.TGCGCG.** 1103 (94.26)   
343 + **[GCCGCG]** 1120 (86.55)   
343 + **[GGCGCG]** 1180 (86.50)   
343 + **[AACGCG]** 1241 (87.55)   
343 + **.TGCGCG.** 1260 (94.26)   
343 + **[GCCGCG]** 1305 (86.55)   
343 + **[GGCGCG]** 1319 (86.50)   
343 + **[GGCGCG]** 1340 (86.50)   
343 + **[TTCGCG]** 1348 (87.60)   
343 + **[CGCGCG]** 1359 (85.98)   
343 - **[CGCGCG]** 1359 (85.98)   
343 - **[GCCGCG]** 1350 (86.55)   
343 - **[GCCGCG]** 1342 (86.55)   
343 - **[CCCGCG]** 1321 (86.03)   
343 - **[CCCGCG]** 1307 (86.03)   
343 - **[CCCGCG]** 1262 (86.03)   
343 - **.TCCGCG.** 1243 (94.31)   
343 - **[GCCGCG]** 1182 (86.55)   
343 - **.CACGCG.** 1136 (91.73)   
343 - **[GCCGCG]** 1122 (86.55)   
343 - **.CACGCG.** 1080 (91.73)   
343 - **[GCCGCG]** 998 (86.55)   
343 - **[GGCGCG]** 957 (86.50)   
343 - **[CCCGCG]** 912 (86.03)   
343 - **[CCCGCG]** 900 (86.03)   
343 - **[CCCGCG]** 672 (86.03)   
343 - **.TACGCG.** 655 (100.00)   
343 - **.GACGCG.** 279 (92.24)   
343 - **.TGCGCG.** 234 (94.26)   
343 - **.TGCGCG.** 222 (94.26)   
343 - **[GCCGCG]** 55 (86.55)   
344 + **[GGCGCG]** 1104 (86.50)   
344 + **.TGCGCG.** 1164 (94.26)   
344 - **[GGCGCG]** 1166 (86.50)   
344 - **[GGCGCG]** 1106 (86.50)   
346 + **.TCCGCG.** 83 (94.31)   
346 + **[AACGCG]** 315 (87.55)   
346 + **.TACGCG.** 478 (100.00)   
346 + **[GCCGCG]** 527 (86.55)   
346 + **.TGCGCG.** 677 (94.26)   
346 + **[GGCGCG]** 717 (86.50)   
346 + **.GACGCG.** 855 (92.24)   
346 + **[GGCGCG]** 880 (86.50)   
346 + **[GCCGCG]** 906 (86.55)   
346 + **[CCCGCG]** 931 (86.03)   
346 + **[CGCGCG]** 1121 (85.98)   
346 + **.TGCGCG.** 1174 (94.26)   
346 + **[GGCGCG]** 1229 (86.50)   
346 + **[GCCGCG]** 1237 (86.55)   
346 + **[CCCGCG]** 1326 (86.03)   
346 + **[GGCGCG]** 1340 (86.50)   
346 + **[GCCGCG]** 1407 (86.55)   
346 - **.TCCGCG.** 1409 (94.31)   
346 - **.GACGCG.** 1239 (92.24)   
346 - **[GGCGCG]** 1231 (86.50)   
346 - **[CCCGCG]** 1176 (86.03)   
346 - **[CGCGCG]** 1121 (85.98)   
346 - **.TGCGCG.** 933 (94.26)   
346 - **.TCCGCG.** 908 (94.31)   
346 - **[GGCGCG]** 882 (86.50)   
346 - **[CCCGCG]** 857 (86.03)   
346 - **.TGCGCG.** 719 (94.26)   
346 - **.TGCGCG.** 480 (94.26)   
346 - **[CCCGCG]** 317 (86.03)   
346 - **[GCCGCG]** 85 (86.55)   
346 - **[GCCGCG]** 23 (86.55)   
347 + **[CCCGCG]** 178 (86.03)   
347 + **[CCCGCG]** 396 (86.03)   
347 + **[CCCGCG]** 811 (86.03)   
347 + **[GGCGCG]** 836 (86.50)   
347 + **[CCCGCG]** 874 (86.03)   
347 + **[GCCGCG]** 909 (86.55)   
347 + **[CGCGCG]** 911 (85.98)   
347 + **[CCCGCG]** 962 (86.03)   
347 + **[CGCGCG]** 964 (85.98)   
347 + **[GGCGCG]** 1101 (86.50)   
347 + **[CCCGCG]** 1168 (86.03)   
347 + **[CGCGCG]** 1170 (85.98)   
347 + **[CCCGCG]** 1203 (86.03)   
347 + **[CGCGCG]** 1205 (85.98)   
347 + **[TAGGCG]** 1223 (85.06)   
347 + **[CCCGCG]** 1255 (86.03)   
347 + **[CCCGCG]** 1284 (86.03)   
347 + **[CCCGCG]** 1465 (86.03)   
347 + **[CCCGCG]** 1492 (86.03)   
347 - **[GGCGCG]** 1494 (86.50)   
347 - **[GCCGCG]** 1467 (86.55)   
347 - **[GCCGCG]** 1286 (86.55)   
347 - **[CCCGCG]** 1275 (86.03)   
347 - **[GGCGCG]** 1257 (86.50)   
347 - **[GCCGCG]** 1207 (86.55)   
347 - **[CGCGCG]** 1205 (85.98)   
347 - **[CGCGCG]** 1170 (85.98)   
347 - **.TGCGCG.** 1103 (94.26)   
347 - **[GCCGCG]** 1088 (86.55)   
347 - **[GGCGCG]** 966 (86.50)   
347 - **[CGCGCG]** 964 (85.98)   
347 - **[GGCGCG]** 913 (86.50)   
347 - **[CGCGCG]** 911 (85.98)   
347 - **.CACGCG.** 876 (91.73)   
347 - **[GGCGCG]** 838 (86.50)   
347 - **[CCCGCG]** 813 (86.03)   
347 - **[GGCGCG]** 553 (86.50)   
347 - **[GCCGCG]** 436 (86.55)   
347 - **[CCCGCG]** 398 (86.03)   
347 - **[AACGCG]** 180 (87.55)   
348 + **[GCCGCG]** 69 (86.55)   
348 + **[CGCGCG]** 130 (85.98)   
348 + **[GCCGCG]** 135 (86.55)   
348 + **[CCCGCG]** 224 (86.03)   
348 + **[CGCGCG]** 226 (85.98)   
348 + **[GGCGCG]** 293 (86.50)   
348 + **.GACGCG.** 308 (92.24)   
348 + **[TTCGCG]** 373 (87.60)   
348 + **[CCCGCG]** 380 (86.03)   
348 + **[CCCGCG]** 397 (86.03)   
348 + **[CCCGCG]** 411 (86.03)   
348 + **[CGCGCG]** 413 (85.98)   
348 + **[CGCGCG]** 415 (85.98)   
348 + **.GACGCG.** 519 (92.24)   
348 + **[GGCGCG]** 545 (86.50)   
348 + **[GCCGCG]** 662 (86.55)   
348 + **[GCCGCG]** 696 (86.55)   
348 + **[CCCGCG]** 710 (86.03)   
348 + **[GGCGCG]** 862 (86.50)   
348 + **[CGCGCG]** 864 (85.98)   
348 + **[GGCGCG]** 961 (86.50)   
348 + **.TGCGCG.** 994 (94.26)   
348 + **[CGCGCG]** 996 (85.98)   
348 + **.TACGCG.** 1123 (100.00)   
348 + **.TGCGCG.** 1176 (94.26)   
348 + **.GACGCG.** 1190 (92.24)   
348 + **[GGCGCG]** 1238 (86.50)   
348 + **[CCCGCG]** 1256 (86.03)   
348 + **.TGCGCG.** 1266 (94.26)   
348 + **[GGCGCG]** 1279 (86.50)   
348 + **[GCCGCG]** 1363 (86.55)   
348 + **[GGCGCG]** 1478 (86.50)   
348 - **[GGCGCG]** 1489 (86.50)   
348 - **.CACGCG.** 1480 (91.73)   
348 - **.CACGCG.** 1414 (91.73)   
348 - **[CCCGCG]** 1308 (86.03)   
348 - **[CCCGCG]** 1281 (86.03)   
348 - **[CCCGCG]** 1268 (86.03)   
348 - **[CCCGCG]** 1258 (86.03)   
348 - **[GCCGCG]** 1240 (86.55)   
348 - **[CCCGCG]** 1192 (86.03)   
348 - **[CCCGCG]** 1178 (86.03)   
348 - **.TGCGCG.** 1125 (94.26)   
348 - **[GGCGCG]** 1075 (86.50)   
348 - **[GCCGCG]** 1028 (86.55)   
348 - **[CCCGCG]** 998 (86.03)   
348 - **[CGCGCG]** 996 (85.98)   
348 - **[CCCGCG]** 988 (86.03)   
348 - **[CCCGCG]** 963 (86.03)   
348 - **[GGCGCG]** 866 (86.50)   
348 - **[CGCGCG]** 864 (85.98)   
348 - **[GCCGCG]** 712 (86.55)   
348 - **[GGCGCG]** 698 (86.50)   
348 - **.GACGCG.** 664 (92.24)   
348 - **[CCCGCG]** 652 (86.03)   
348 - **.TCCGCG.** 625 (94.31)   
348 - **[CCCGCG]** 547 (86.03)   
348 - **[TTCGCG]** 521 (87.60)   
348 - **[GGCGCG]** 417 (86.50)   
348 - **[CGCGCG]** 415 (85.98)   
348 - **[CGCGCG]** 413 (85.98)   
348 - **[GGCGCG]** 399 (86.50)   
348 - **[GGCGCG]** 382 (86.50)   
348 - **[GGCGCG]** 375 (86.50)   
348 - **[GCCGCG]** 344 (86.55)   
348 - **[CCCGCG]** 310 (86.03)   
348 - **.TGCGCG.** 295 (94.26)   
348 - **[GGCGCG]** 228 (86.50)   
348 - **[CGCGCG]** 226 (85.98)   
348 - **.TCCGCG.** 137 (94.31)   
348 - **[GGCGCG]** 132 (86.50)   
348 - **[CGCGCG]** 130 (85.98)   
348 - **[GCCGCG]** 71 (86.55)   
349 + **.GACGCG.** 489 (92.24)   
349 + **[GGCGCG]** 837 (86.50)   
349 + **.TGCGCG.** 914 (94.26)   
349 + **[GGCGCG]** 1045 (86.50)   
349 + **[GGCGCG]** 1163 (86.50)   
349 + **[CCCGCG]** 1284 (86.03)   
349 + **[GGCGCG]** 1308 (86.50)   
349 + **[CCCGCG]** 1327 (86.03)   
349 + **[AACGCG]** 1431 (87.55)   
349 - **.TCCGCG.** 1433 (94.31)   
349 - **.TCCGCG.** 1329 (94.31)   
349 - **.GACGCG.** 1310 (92.24)   
349 - **.TCCGCG.** 1286 (94.31)   
349 - **[GCCGCG]** 1165 (86.55)   
349 - **[GCCGCG]** 916 (86.55)   
349 - **[CCCGCG]** 839 (86.03)   
349 - **[TAGGCG]** 601 (85.06)   
349 - **.CACGCG.** 491 (91.73)   
350 + **[AACGCG]** 1011 (87.55)   
350 + **[GGCGCG]** 1044 (86.50)   
350 + **.TGCGCG.** 1072 (94.26)   
350 + **[CGCGCG]** 1074 (85.98)   
350 + **.TCCGCG.** 1101 (94.31)   
350 + **[CGCGCG]** 1103 (85.98)   
350 + **[CGCGCG]** 1105 (85.98)   
350 + **[CCCGCG]** 1184 (86.03)   
350 + **[CCCGCG]** 1196 (86.03)   
350 + **[GGCGCG]** 1343 (86.50)   
350 + **[CGCGCG]** 1428 (85.98)   
350 + **[GGCGCG]** 1437 (86.50)   
350 - **[CCCGCG]** 1439 (86.03)   
350 - **[CCCGCG]** 1430 (86.03)   
350 - **[CGCGCG]** 1428 (85.98)   
350 - **[GCCGCG]** 1404 (86.55)   
350 - **[GGCGCG]** 1345 (86.50)   
350 - **.TGCGCG.** 1268 (94.26)   
350 - **[GGCGCG]** 1186 (86.50)   
350 - **[GCCGCG]** 1107 (86.55)   
350 - **[CGCGCG]** 1105 (85.98)   
350 - **[CGCGCG]** 1103 (85.98)   
350 - **.TCCGCG.** 1076 (94.31)   
350 - **[CGCGCG]** 1074 (85.98)   
350 - **[GCCGCG]** 1046 (86.55)   
350 - **[CCCGCG]** 1013 (86.03)   
350 - **[CCCGCG]** 1001 (86.03)   
351 + **.CACGCG.** 8 (91.73)   
351 + **[CCCGCG]** 104 (86.03)   
351 + **[GGCGCG]** 301 (86.50)   
351 + **[CGCGCG]** 303 (85.98)   
351 + **[GGCGCG]** 576 (86.50)   
351 + **[CGCGCG]** 578 (85.98)   
351 + **[GCCGCG]** 937 (86.55)   
351 + **.TACGCG.** 973 (100.00)   
351 + **[CCCGCG]** 1019 (86.03)   
351 + **[CCCGCG]** 1183 (86.03)   
351 + **[CCCGCG]** 1253 (86.03)   
351 + **.TGCGCG.** 1444 (94.26)   
351 - **[CCCGCG]** 1446 (86.03)   
351 - **[GCCGCG]** 1255 (86.55)   
351 - **[GCCGCG]** 1185 (86.55)   
351 - **.GACGCG.** 1141 (92.24)   
351 - **[GCCGCG]** 975 (86.55)   
351 - **.TCCGCG.** 939 (94.31)   
351 - **.TCCGCG.** 922 (94.31)   
351 - **[CGCGCG]** 578 (85.98)   
351 - **[GGCGCG]** 390 (86.50)   
351 - **[GGCGCG]** 305 (86.50)   
351 - **[CGCGCG]** 303 (85.98)   
351 - **.TACGCG.** 106 (100.00)   
351 - **.TGCGCG.** 10 (94.26)   
352 + **[GCCGCG]** 72 (86.55)   
352 + **[CCCGCG]** 196 (86.03)   
352 + **[GGCGCG]** 201 (86.50)   
352 + **[CGCGCG]** 245 (85.98)   
352 + **.GACGCG.** 282 (92.24)   
352 + **[GGCGCG]** 290 (86.50)   
352 + **[GCCGCG]** 310 (86.55)   
352 + **[GCCGCG]** 353 (86.55)   
352 + **[AACGCG]** 437 (87.55)   
352 + **.TCCGCG.** 619 (94.31)   
352 + **.TCCGCG.** 716 (94.31)   
352 + **[CCCGCG]** 940 (86.03)   
352 + **.TCCGCG.** 952 (94.31)   
352 + **.TCCGCG.** 1018 (94.31)   
352 + **[GGCGCG]** 1064 (86.50)   
352 + **.TGCGCG.** 1226 (94.26)   
352 + **[GCCGCG]** 1338 (86.55)   
352 - **[GCCGCG]** 1340 (86.55)   
352 - **[GGCGCG]** 1228 (86.50)   
352 - **[CCCGCG]** 1020 (86.03)   
352 - **.TCCGCG.** 954 (94.31)   
352 - **[GCCGCG]** 942 (86.55)   
352 - **[GGCGCG]** 718 (86.50)   
352 - **[GCCGCG]** 621 (86.55)   
352 - **[GGCGCG]** 547 (86.50)   
352 - **[CCCGCG]** 490 (86.03)   
352 - **[TAGGCG]** 474 (85.06)   
352 - **[GCCGCG]** 439 (86.55)   
352 - **[GCCGCG]** 312 (86.55)   
352 - **[GGCGCG]** 292 (86.50)   
352 - **.TCCGCG.** 284 (94.31)   
352 - **[CCCGCG]** 247 (86.03)   
352 - **[CGCGCG]** 245 (85.98)   
352 - **[GGCGCG]** 203 (86.50)   
352 - **[GCCGCG]** 198 (86.55)   
352 - **[GGCGCG]** 74 (86.50)   
352 - **[GGCGCG]** 55 (86.50)   
353 + **.TGCGCG.** 124 (94.26)   
353 + **[TAGGCG]** 140 (85.06)   
353 + **[GGCGCG]** 165 (86.50)   
353 + **.CACGCG.** 173 (91.73)   
353 + **[CGCGCG]** 175 (85.98)   
353 + **.GACGCG.** 187 (92.24)   
353 + **.CACGCG.** 304 (91.73)   
353 + **[CGCGCG]** 493 (85.98)   
353 + **.GACGCG.** 539 (92.24)   
353 + **.GACGCG.** 549 (92.24)   
353 + **[CCCGCG]** 695 (86.03)   
353 + **.GACGCG.** 708 (92.24)   
353 + **[CGCGCG]** 834 (85.98)   
353 + **[GCCGCG]** 881 (86.55)   
353 + **[CCCGCG]** 902 (86.03)   
353 + **.TGCGCG.** 1029 (94.26)   
353 + **[GCCGCG]** 1179 (86.55)   
353 + **[CGCGCG]** 1276 (85.98)   
353 + **[GGCGCG]** 1340 (86.50)   
353 + **[CGCGCG]** 1342 (85.98)   
353 + **[GGCGCG]** 1382 (86.50)   
353 + **[GGCGCG]** 1401 (86.50)   
353 + **.TGCGCG.** 1418 (94.26)   
353 + **[CCCGCG]** 1436 (86.03)   
353 - **[GCCGCG]** 1438 (86.55)   
353 - **[GCCGCG]** 1420 (86.55)   
353 - **[CCCGCG]** 1403 (86.03)   
353 - **[CCCGCG]** 1384 (86.03)   
353 - **[GGCGCG]** 1344 (86.50)   
353 - **[CGCGCG]** 1342 (85.98)   
353 - **.GACGCG.** 1278 (92.24)   
353 - **[CGCGCG]** 1276 (85.98)   
353 - **.TCCGCG.** 1031 (94.31)   
353 - **[CCCGCG]** 883 (86.03)   
353 - **.CACGCG.** 836 (91.73)   
353 - **[CGCGCG]** 834 (85.98)   
353 - **[CCCGCG]** 710 (86.03)   
353 - **[GGCGCG]** 663 (86.50)   
353 - **.TGCGCG.** 551 (94.26)   
353 - **[CCCGCG]** 495 (86.03)   
353 - **[CGCGCG]** 493 (85.98)   
353 - **[TTCGCG]** 425 (87.60)   
353 - **.TCCGCG.** 270 (94.31)   
353 - **.TGCGCG.** 189 (94.26)   
353 - **[CGCGCG]** 175 (85.98)   
353 - **[GCCGCG]** 167 (86.55)   
354 + **[TAGGCG]** 12 (85.06)   
355 + **[CCCGCG]** 389 (86.03)   
355 + **[CCCGCG]** 1046 (86.03)   
355 - **[AACGCG]** 391 (87.55)   
356 + **[GCCGCG]** 91 (86.55)   
356 + **[CCCGCG]** 308 (86.03)   
356 + **.TGCGCG.** 463 (94.26)   
356 + **[CCCGCG]** 609 (86.03)   
356 + **[CCCGCG]** 770 (86.03)   
356 + **[GCCGCG]** 784 (86.55)   
356 + **.CACGCG.** 863 (91.73)   
356 + **.TGCGCG.** 916 (94.26)   
356 + **[GGCGCG]** 997 (86.50)   
356 + **[AACGCG]** 1169 (87.55)   
356 + **[CGCGCG]** 1171 (85.98)   
356 - **.GACGCG.** 1173 (92.24)   
356 - **[CGCGCG]** 1171 (85.98)   
356 - **[GCCGCG]** 999 (86.55)   
356 - **[CCCGCG]** 918 (86.03)   
356 - **.TGCGCG.** 865 (94.26)   
356 - **[TTCGCG]** 786 (87.60)   
356 - **.TACGCG.** 649 (100.00)   
356 - **[GGCGCG]** 611 (86.50)   
356 - **[GCCGCG]** 465 (86.55)   
356 - **[GGCGCG]** 310 (86.50)   
356 - **[GGCGCG]** 93 (86.50)   
357 + **.TGCGCG.** 296 (94.26)   
357 + **[GGCGCG]** 527 (86.50)   
357 + **[CCCGCG]** 542 (86.03)   
357 + **[AACGCG]** 659 (87.55)   
357 + **.TCCGCG.** 707 (94.31)   
357 + **[GCCGCG]** 811 (86.55)   
357 + **.CACGCG.** 921 (91.73)   
357 + **[CGCGCG]** 923 (85.98)   
357 + **.GACGCG.** 944 (92.24)   
357 + **[GCCGCG]** 980 (86.55)   
357 + **[CGCGCG]** 982 (85.98)   
357 + **[GCCGCG]** 991 (86.55)   
357 + **.TCCGCG.** 1025 (94.31)   
357 + **.TGCGCG.** 1031 (94.26)   
357 + **[GCCGCG]** 1110 (86.55)   
357 + **[GGCGCG]** 1161 (86.50)   
357 + **.TCCGCG.** 1219 (94.31)   
357 + **.TCCGCG.** 1280 (94.31)   
357 + **.TCCGCG.** 1445 (94.31)   
357 - **[CCCGCG]** 1447 (86.03)   
357 - **[CCCGCG]** 1282 (86.03)   
357 - **[CCCGCG]** 1221 (86.03)   
357 - **[CCCGCG]** 1163 (86.03)   
357 - **.CACGCG.** 1112 (91.73)   
357 - **[CCCGCG]** 1033 (86.03)   
357 - **.CACGCG.** 1027 (91.73)   
357 - **[GGCGCG]** 993 (86.50)   
357 - **[GGCGCG]** 984 (86.50)   
357 - **[CGCGCG]** 982 (85.98)   
357 - **[AACGCG]** 946 (87.55)   
357 - **[CGCGCG]** 923 (85.98)   
357 - **.CACGCG.** 813 (91.73)   
357 - **[CCCGCG]** 661 (86.03)   
357 - **.GACGCG.** 544 (92.24)   
357 - **.CACGCG.** 529 (91.73)   
357 - **[CCCGCG]** 298 (86.03)   
358 + **.TGCGCG.** 68 (94.26)   
358 + **[GCCGCG]** 669 (86.55)   
358 + **[CGCGCG]** 723 (85.98)   
358 + **[TTCGCG]** 836 (87.60)   
358 + **[CCCGCG]** 849 (86.03)   
358 + **[GGCGCG]** 880 (86.50)   
358 + **[TAGGCG]** 996 (85.06)   
358 + **.TGCGCG.** 1032 (94.26)   
358 - **[GCCGCG]** 978 (86.55)   
358 - **.CACGCG.** 851 (91.73)   
358 - **.CACGCG.** 838 (91.73)   
358 - **[CCCGCG]** 812 (86.03)   
358 - **[GCCGCG]** 774 (86.55)   
358 - **[CGCGCG]** 723 (85.98)   
358 - **[GGCGCG]** 671 (86.50)   
358 - **[CCCGCG]** 407 (86.03)   
358 - **[GCCGCG]** 70 (86.55)   
359 + **[GGCGCG]** 184 (86.50)   
359 + **[CCCGCG]** 543 (86.03)   
359 + **[GGCGCG]** 775 (86.50)   
359 + **[CCCGCG]** 1094 (86.03)   
359 + **.TGCGCG.** 1112 (94.26)   
359 + **[CGCGCG]** 1204 (85.98)   
359 + **[GCCGCG]** 1232 (86.55)   
359 + **[GGCGCG]** 1237 (86.50)   
359 + **[CGCGCG]** 1239 (85.98)   
359 + **[CGCGCG]** 1241 (85.98)   
359 + **[CGCGCG]** 1243 (85.98)   
359 + **[CGCGCG]** 1245 (85.98)   
359 + **.TGCGCG.** 1253 (94.26)   
359 + **[CCCGCG]** 1279 (86.03)   
359 + **.TGCGCG.** 1285 (94.26)   
359 + **.TGCGCG.** 1305 (94.26)   
359 + **[GGCGCG]** 1346 (86.50)   
359 + **[GGCGCG]** 1440 (86.50)   
359 - **.TGCGCG.** 1442 (94.26)   
359 - **.TGCGCG.** 1380 (94.26)   
359 - **[AACGCG]** 1348 (87.55)   
359 - **[GGCGCG]** 1307 (86.50)   
359 - **.CACGCG.** 1287 (91.73)   
359 - **.CACGCG.** 1281 (91.73)   
359 - **[GCCGCG]** 1255 (86.55)   
359 - **[GGCGCG]** 1247 (86.50)   
359 - **[CGCGCG]** 1245 (85.98)   
359 - **[CGCGCG]** 1243 (85.98)   
359 - **[CGCGCG]** 1241 (85.98)   
359 - **[CGCGCG]** 1239 (85.98)   
359 - **[GCCGCG]** 1234 (86.55)   
359 - **[GGCGCG]** 1206 (86.50)   
359 - **[CGCGCG]** 1204 (85.98)   
359 - **[GGCGCG]** 1114 (86.50)   
359 - **[GGCGCG]** 1096 (86.50)   
359 - **.TGCGCG.** 777 (94.26)   
359 - **[CCCGCG]** 545 (86.03)   
359 - **.TGCGCG.** 186 (94.26)   
360 + **.TCCGCG.** 412 (94.31)   
360 + **[GCCGCG]** 419 (86.55)   
360 + **[GCCGCG]** 652 (86.55)   
360 + **[CCCGCG]** 779 (86.03)   
360 + **[CCCGCG]** 790 (86.03)   
360 + **[CGCGCG]** 792 (85.98)   
360 + **[TTCGCG]** 803 (87.60)   
360 + **[CGCGCG]** 828 (85.98)   
360 + **[CGCGCG]** 830 (85.98)   
360 + **[CCCGCG]** 848 (86.03)   
360 + **[GGCGCG]** 877 (86.50)   
360 + **[GGCGCG]** 944 (86.50)   
360 + **[GCCGCG]** 1066 (86.55)   
360 + **[GCCGCG]** 1071 (86.55)   
360 + **[GCCGCG]** 1125 (86.55)   
360 + **[GGCGCG]** 1185 (86.50)   
360 + **[CCCGCG]** 1196 (86.03)   
360 + **[CGCGCG]** 1198 (85.98)   
360 + **[GGCGCG]** 1243 (86.50)   
360 - **[GGCGCG]** 1245 (86.50)   
360 - **[CCCGCG]** 1200 (86.03)   
360 - **[CGCGCG]** 1198 (85.98)   
360 - **[CCCGCG]** 1187 (86.03)   
360 - **[GCCGCG]** 1127 (86.55)   
360 - **[CCCGCG]** 1073 (86.03)   
360 - **[GGCGCG]** 1068 (86.50)   
360 - **[GCCGCG]** 973 (86.55)   
360 - **[CCCGCG]** 946 (86.03)   
360 - **[CCCGCG]** 879 (86.03)   
360 - **[CCCGCG]** 850 (86.03)   
360 - **[CCCGCG]** 832 (86.03)   
360 - **[CGCGCG]** 830 (85.98)   
360 - **[CGCGCG]** 828 (85.98)   
360 - **[GCCGCG]** 794 (86.55)   
360 - **[CGCGCG]** 792 (85.98)   
360 - **[GGCGCG]** 781 (86.50)   
360 - **[GGCGCG]** 737 (86.50)   
360 - **.TGCGCG.** 421 (94.26)   
361 + **[GCCGCG]** 470 (86.55)   
361 + **[AACGCG]** 1079 (87.55)   
361 + **[GCCGCG]** 1416 (86.55)   
361 - **[GCCGCG]** 1418 (86.55)   
361 - **.TCCGCG.** 1081 (94.31)   
361 - **[CCCGCG]** 472 (86.03)   
362 + **.TCCGCG.** 116 (94.31)   
362 + **[GGCGCG]** 237 (86.50)   
362 + **[CCCGCG]** 281 (86.03)   
362 + **[CCCGCG]** 425 (86.03)   
362 + **[CCCGCG]** 468 (86.03)   
362 + **.GACGCG.** 525 (92.24)   
362 + **.TGCGCG.** 622 (94.26)   
362 + **[GCCGCG]** 736 (86.55)   
362 + **.TCCGCG.** 874 (94.31)   
362 + **[GGCGCG]** 909 (86.50)   
362 + **.TGCGCG.** 1025 (94.26)   
362 + **[CCCGCG]** 1055 (86.03)   
362 + **[CCCGCG]** 1356 (86.03)   
362 - **[GGCGCG]** 1358 (86.50)   
362 - **[CCCGCG]** 1057 (86.03)   
362 - **[TTCGCG]** 1027 (87.60)   
362 - **[GCCGCG]** 911 (86.55)   
362 - **.CACGCG.** 876 (91.73)   
362 - **.GACGCG.** 738 (92.24)   
362 - **.GACGCG.** 624 (92.24)   
362 - **.TCCGCG.** 527 (94.31)   
362 - **[GCCGCG]** 470 (86.55)   
362 - **.TCCGCG.** 407 (94.31)   
362 - **[GCCGCG]** 283 (86.55)   
362 - **[TAGGCG]** 177 (85.06)   
362 - **[GCCGCG]** 118 (86.55)   
363 + **[CCCGCG]** 487 (86.03)   
363 + **[CCCGCG]** 633 (86.03)   
363 + **[GCCGCG]** 692 (86.55)   
363 + **[CCCGCG]** 746 (86.03)   
363 + **[CCCGCG]** 797 (86.03)   
363 + **[GCCGCG]** 1087 (86.55)   
363 + **[CGCGCG]** 1089 (85.98)   
363 + **[GGCGCG]** 1112 (86.50)   
363 + **[CGCGCG]** 1114 (85.98)   
363 + **.TGCGCG.** 1186 (94.26)   
363 - **[TAGGCG]** 1208 (85.06)   
363 - **[GCCGCG]** 1188 (86.55)   
363 - **[CGCGCG]** 1114 (85.98)   
363 - **[GGCGCG]** 1091 (86.50)   
363 - **[CGCGCG]** 1089 (85.98)   
363 - **[GCCGCG]** 1083 (86.55)   
363 - **[GCCGCG]** 811 (86.55)   
363 - **[GCCGCG]** 799 (86.55)   
363 - **[GCCGCG]** 694 (86.55)   
363 - **[AACGCG]** 585 (87.55)   
364 + **.GACGCG.** 297 (92.24)   
364 + **[GCCGCG]** 821 (86.55)   
364 + **.TCCGCG.** 948 (94.31)   
364 + **[GGCGCG]** 1177 (86.50)   
364 + **[CCCGCG]** 1228 (86.03)   
364 + **[CCCGCG]** 1246 (86.03)   
364 + **.GACGCG.** 1255 (92.24)   
364 + **.TCCGCG.** 1315 (94.31)   
364 + **.TGCGCG.** 1389 (94.26)   
364 + **.TCCGCG.** 1461 (94.31)   
364 - **[GGCGCG]** 1391 (86.50)   
364 - **[GCCGCG]** 1257 (86.55)   
364 - **[CCCGCG]** 1248 (86.03)   
364 - **.TCCGCG.** 1230 (94.31)   
364 - **[GCCGCG]** 1179 (86.55)   
364 - **[GCCGCG]** 950 (86.55)   
364 - **[AACGCG]** 823 (87.55)   
364 - **[CCCGCG]** 299 (86.03)   
366 + **[CGCGCG]** 18 (85.98)   
366 + **[CCCGCG]** 129 (86.03)   
366 + **[CCCGCG]** 167 (86.03)   
366 + **[GCCGCG]** 182 (86.55)   
366 + **[GGCGCG]** 266 (86.50)   
366 + **[GCCGCG]** 346 (86.55)   
366 + **[CCCGCG]** 383 (86.03)   
366 + **[CCCGCG]** 443 (86.03)   
366 + **.TCCGCG.** 452 (94.31)   
366 + **[CCCGCG]** 519 (86.03)   
366 + **[GCCGCG]** 534 (86.55)   
366 + **[GGCGCG]** 656 (86.50)   
366 + **[CGCGCG]** 905 (85.98)   
366 + **[CCCGCG]** 952 (86.03)   
366 + **[GCCGCG]** 1123 (86.55)   
366 + **[GCCGCG]** 1253 (86.55)   
366 + **[GGCGCG]** 1391 (86.50)   
366 + **[GCCGCG]** 1412 (86.55)   
366 + **.GACGCG.** 1430 (92.24)   
366 - **[GCCGCG]** 1393 (86.55)   
366 - **.TCCGCG.** 1255 (94.31)   
366 - **[CCCGCG]** 1191 (86.03)   
366 - **[AACGCG]** 976 (87.55)   
366 - **[GCCGCG]** 954 (86.55)   
366 - **[GGCGCG]** 907 (86.50)   
366 - **[CGCGCG]** 905 (85.98)   
366 - **.GACGCG.** 854 (92.24)   
366 - **[GGCGCG]** 658 (86.50)   
366 - **[CCCGCG]** 536 (86.03)   
366 - **.GACGCG.** 521 (92.24)   
366 - **[GGCGCG]** 454 (86.50)   
366 - **[GCCGCG]** 445 (86.55)   
366 - **[GCCGCG]** 410 (86.55)   
366 - **.TCCGCG.** 385 (94.31)   
366 - **.TGCGCG.** 348 (94.26)   
366 - **[CCCGCG]** 330 (86.03)   
366 - **.TACGCG.** 245 (100.00)   
366 - **[CCCGCG]** 184 (86.03)   
366 - **[AACGCG]** 169 (87.55)   
366 - **[GGCGCG]** 131 (86.50)   
366 - **.TGCGCG.** 20 (94.26)   
366 - **[CGCGCG]** 18 (85.98)   
367 + **[CCCGCG]** 7 (86.03)   
367 + **[GCCGCG]** 128 (86.55)   
367 + **[CGCGCG]** 130 (85.98)   
367 + **[GGCGCG]** 337 (86.50)   
367 + **[CGCGCG]** 339 (85.98)   
367 + **[CCCGCG]** 411 (86.03)   
367 + **[GCCGCG]** 555 (86.55)   
367 + **[GCCGCG]** 571 (86.55)   
367 + **[CGCGCG]** 573 (85.98)   
367 + **[CCCGCG]** 1011 (86.03)   
367 + **[CCCGCG]** 1032 (86.03)   
367 + **[GCCGCG]** 1063 (86.55)   
367 + **[GCCGCG]** 1090 (86.55)   
367 + **[GCCGCG]** 1190 (86.55)   
367 + **[CGCGCG]** 1513 (85.98)   
367 - **[CGCGCG]** 1513 (85.98)   
367 - **[GCCGCG]** 1192 (86.55)   
367 - **[AACGCG]** 1127 (87.55)   
367 - **[GGCGCG]** 1047 (86.50)   
367 - **[GGCGCG]** 1034 (86.50)   
367 - **.TCCGCG.** 1013 (94.31)   
367 - **.TGCGCG.** 594 (94.26)   
367 - **[CCCGCG]** 575 (86.03)   
367 - **[CGCGCG]** 573 (85.98)   
367 - **[GGCGCG]** 413 (86.50)   
367 - **[CGCGCG]** 339 (85.98)   
367 - **.TCCGCG.** 132 (94.31)   
367 - **[CGCGCG]** 130 (85.98)   
367 - **[GGCGCG]** 9 (86.50)   
  

**Frequency Matrix**

| All Occs Best Occs|  |  |  |  |  |  |  |  |  |  |  |  |  |  |  |  |  |  |  |  |  |  |  |  |  |  |  |  |  |  |  |  |  |  |  |  |  |  |  |  |  |  |  |  |  |  |  |  |  |  |  |  |  |  |  |  |  |  |  |  |  |  |  |  |  |  |  |  |  |  |  |  |  |  |  |  |  |  |  |  |  |  |  |  | | --- | --- | --- | --- | --- | --- | --- | --- | --- | --- | --- | --- | --- | --- | --- | --- | --- | --- | --- | --- | --- | --- | --- | --- | --- | --- | --- | --- | --- | --- | --- | --- | --- | --- | --- | --- | --- | --- | --- | --- | --- | --- | --- | --- | --- | --- | --- | --- | --- | --- | --- | --- | --- | --- | --- | --- | --- | --- | --- | --- | --- | --- | --- | --- | --- | --- | --- | --- | --- | --- | --- | --- | --- | --- | --- | --- | --- | --- | --- | --- | --- | --- | --- | --- | | **A C G T A C G T | | | | | | | | | | | |**| **1** 132 321 358 1867 | 0 321 358 1016|  |  |  |  |  |  |  |  |  |  |  |  |  |  |  |  |  |  |  |  |  |  |  |  |  |  |  |  |  |  |  |  |  |  |  |  |  |  |  |  |  |  |  |  |  |  |  |  |  |  |  |  |  |  |  |  |  |  |  |  | | --- | --- | --- | --- | --- | --- | --- | --- | --- | --- | --- | --- | --- | --- | --- | --- | --- | --- | --- | --- | --- | --- | --- | --- | --- | --- | --- | --- | --- | --- | --- | --- | --- | --- | --- | --- | --- | --- | --- | --- | --- | --- | --- | --- | --- | --- | --- | --- | --- | --- | --- | --- | --- | --- | --- | --- | --- | --- | --- | --- | | **2** 1610 479 474 115 | 742 479 474 0|  |  |  |  |  |  |  |  |  |  |  |  |  |  |  |  |  |  |  |  |  |  |  |  |  |  |  |  |  |  |  |  |  |  |  |  |  |  |  |  |  |  |  |  |  |  |  |  | | --- | --- | --- | --- | --- | --- | --- | --- | --- | --- | --- | --- | --- | --- | --- | --- | --- | --- | --- | --- | --- | --- | --- | --- | --- | --- | --- | --- | --- | --- | --- | --- | --- | --- | --- | --- | --- | --- | --- | --- | --- | --- | --- | --- | --- | --- | --- | --- | | **3** 55 2480 103 40 | 0 1695 0 0|  |  |  |  |  |  |  |  |  |  |  |  |  |  |  |  |  |  |  |  |  |  |  |  |  |  |  |  |  |  |  |  |  |  |  |  | | --- | --- | --- | --- | --- | --- | --- | --- | --- | --- | --- | --- | --- | --- | --- | --- | --- | --- | --- | --- | --- | --- | --- | --- | --- | --- | --- | --- | --- | --- | --- | --- | --- | --- | --- | --- | | **4** 58 91 2463 66 | 0 0 1695 0|  |  |  |  |  |  |  |  |  |  |  |  |  |  |  |  |  |  |  |  |  |  |  |  | | --- | --- | --- | --- | --- | --- | --- | --- | --- | --- | --- | --- | --- | --- | --- | --- | --- | --- | --- | --- | --- | --- | --- | --- | | **5** 39 2525 74 40 | 0 1695 0 0|  |  |  |  |  |  |  |  |  |  |  |  | | --- | --- | --- | --- | --- | --- | --- | --- | --- | --- | --- | --- | | **6** 57 77 2508 36 | 0 0 1695 0 | | | | | | | | | | | | | | | | | | | | | | | | | | | | | | | | | | | | | | | | | | | | | | | | | | | | | | | | | | | | | | | | | | | | | | | | | | | | | | | | | | | | |

  
  

---

  
  
  
 **CGCGTC**   
**GACGCG**  
  
12 redundant motifs found:  
CGCGTCGT - CGACGCGC - CGACGCGT - TCGCGTCG - CGCGTCGA - CCGCGT - TCGCGT - CGTCCG - CGACGC - CGCGTT - GTCGCG - CCGACG -   
  
Best occurrences (match percentage):   
Seq St oligo pos match
  
 1 + **[CGCTTC]** 101 (86.32)   
1 + **[CGCCGC]** 917 (85.72)   
1 + **.CGCGCC.** 1000 (94.60)   
1 + **[CGCCGC]** 1002 (85.72)   
1 + **[CGCCCC]** 1038 (85.80)   
1 + **[CGCCGC]** 1068 (85.72)   
1 + **.CGCGCC.** 1071 (94.60)   
1 + **[CGCCGC]** 1077 (85.72)   
1 + **[CGCCCC]** 1080 (85.80)   
1 + **[CGCCGC]** 1204 (85.72)   
1 + **[CGCCGC]** 1207 (85.72)   
1 + **[CGCCGC]** 1399 (85.72)   
1 - **[GGCGTC]** 1202 (87.08)   
1 - **[CGCCGC]** 1107 (85.72)   
1 - **.CGCGGC.** 1069 (94.52)   
1 - **.CGCCTC.** 1064 (91.20)   
1 - **[CGCCCC]** 913 (85.80)   
1 - **[CGCCCC]** 869 (85.80)   
1 - **.CGCCTC.** 864 (91.20)   
2 + **.CGCGGC.** 201 (94.52)   
2 + **[CGCCCC]** 253 (85.80)   
2 + **[CGCCGC]** 308 (85.72)   
2 + **[CCCGTC]** 409 (87.14)   
2 + **.CGCGTC.** 449 (100.00)   
2 + **.CGCGCC.** 457 (94.60)   
2 + **.CGCCTC.** 459 (91.20)   
2 + **[CGCCCC]** 510 (85.80)   
2 + **.CGCGTC.** 515 (100.00)   
2 + **[CGCCGC]** 573 (85.72)   
2 + **[GGCGTC]** 675 (87.08)   
2 + **.CGCGGC.** 721 (94.52)   
2 + **[CGGGTC]** 737 (87.94)   
2 + **[CGCTTC]** 817 (86.32)   
2 + **[CCCGTC]** 854 (87.14)   
2 + **.CGCCTC.** 882 (91.20)   
2 + **[CGCCCC]** 903 (85.80)   
2 + **[CCCGTC]** 906 (87.14)   
2 + **[CGGGTC]** 923 (87.94)   
2 + **.CGCGGC.** 977 (94.52)   
2 + **.CGCGGC.** 996 (94.52)   
2 + **[CGCCCC]** 1261 (85.80)   
2 + **[CGCCGC]** 1268 (85.72)   
2 + **[CGCCCC]** 1271 (85.80)   
2 + **[GGCGTC]** 1309 (87.08)   
2 + **[CGCTTC]** 1382 (86.32)   
2 + **.CGCGGC.** 1447 (94.52)   
2 - **[CCCGTC]** 1490 (87.14)   
2 - **.CGCGCC.** 1450 (94.60)   
2 - **[CGCCGC]** 1448 (85.72)   
2 - **[CGGGTC]** 1196 (87.94)   
2 - **[CGCCGC]** 1153 (85.72)   
2 - **.CGCGGC.** 1071 (94.52)   
2 - **.CGCGCC.** 999 (94.60)   
2 - **[CGCCGC]** 997 (85.72)   
2 - **[CGGGTC]** 844 (87.94)   
2 - **[GGCGTC]** 802 (87.08)   
2 - **[CGCTTC]** 717 (86.32)   
2 - **.CGCGCC.** 703 (94.60)   
2 - **[CGCCGC]** 701 (85.72)   
2 - **[GGCGTC]** 508 (87.08)   
2 - **.CGCGGC.** 447 (94.52)   
2 - **[CGCGAC]** 362 (87.27)   
2 - **[CCCGTC]** 337 (87.14)   
2 - **[CCCGTC]** 245 (87.14)   
2 - **.CGCCTC.** 188 (91.20)   
3 + **[CGGGTC]** 110 (87.94)   
3 + **[CGCCGC]** 163 (85.72)   
3 + **[CCCGTC]** 1054 (87.14)   
3 + **.CGCGGC.** 1223 (94.52)   
3 + **[CGCCGC]** 1263 (85.72)   
3 + **[CGCCCC]** 1312 (85.80)   
3 + **[CGCCCC]** 1326 (85.80)   
3 + **[CGCCCC]** 1391 (85.80)   
3 + **[CGCCGC]** 1401 (85.72)   
3 + **[CGCCCC]** 1404 (85.80)   
3 + **[CGCCGC]** 1409 (85.72)   
3 + **[CGCCGC]** 1412 (85.72)   
3 + **[CGCCGC]** 1415 (85.72)   
3 + **.CGCGCC.** 1418 (94.60)   
3 - **.CGCGGC.** 1416 (94.52)   
3 - **[CGCCCC]** 1363 (85.80)   
3 - **[CGGGTC]** 1353 (87.94)   
3 - **.CGCGCC.** 1249 (94.60)   
3 - **[CGCCGC]** 1227 (85.72)   
3 - **[CGCCGC]** 1224 (85.72)   
3 - **.CGCGCC.** 1180 (94.60)   
3 - **[CCCGTC]** 1126 (87.14)   
3 - **.CGCGGC.** 789 (94.52)   
3 - **[CGCGAC]** 701 (87.27)   
3 - **[CCCGTC]** 197 (87.14)   
3 - **.CGCCTC.** 34 (91.20)   
4 + **[CCCGTC]** 490 (87.14)   
4 + **[CGCCCC]** 568 (85.80)   
4 + **[CGCGAC]** 574 (87.27)   
4 + **[CGGGTC]** 687 (87.94)   
4 + **[CGCCGC]** 695 (85.72)   
4 + **[CGCCGC]** 698 (85.72)   
4 + **[CGCGAC]** 701 (87.27)   
4 + **.CGCGGC.** 742 (94.52)   
4 + **.CGCGCC.** 786 (94.60)   
4 + **.CGCCTC.** 788 (91.20)   
4 + **.CGCGGC.** 802 (94.52)   
4 + **.CGCGGC.** 822 (94.52)   
4 + **[CGCGTG]** 918 (86.67)   
4 + **[CGCCCC]** 1089 (85.80)   
4 + **[CGCCGC]** 1107 (85.72)   
4 + **.CGCGGC.** 1179 (94.52)   
4 + **.CGCGCC.** 1199 (94.60)   
4 + **[CGCCGC]** 1205 (85.72)   
4 + **.CGCGCC.** 1208 (94.60)   
4 - **[CGCCCC]** 1289 (85.80)   
4 - **.CGCGCC.** 1282 (94.60)   
4 - **[CGCCCC]** 1280 (85.80)   
4 - **.CGCGCC.** 1272 (94.60)   
4 - **[CGCCCC]** 1270 (85.80)   
4 - **.CGCGGC.** 1206 (94.52)   
4 - **.CGCCTC.** 1155 (91.20)   
4 - **[CGCCGC]** 1141 (85.72)   
4 - **[CGCCCC]** 933 (85.80)   
4 - **.CGCGCC.** 916 (94.60)   
4 - **[CGCCGC]** 905 (85.72)   
4 - **.CGCCTC.** 902 (91.20)   
4 - **[CGGGTC]** 853 (87.94)   
4 - **[CGCCGC]** 848 (85.72)   
4 - **.CGCGCC.** 828 (94.60)   
4 - **[CGCCGC]** 826 (85.72)   
4 - **[CGCCGC]** 823 (85.72)   
4 - **[CGCCGC]** 803 (85.72)   
4 - **[CGCGAC]** 740 (87.27)   
4 - **[CGGGTC]** 704 (87.94)   
4 - **.CGCGGC.** 699 (94.52)   
4 - **.CGCGCC.** 683 (94.60)   
4 - **[CGCCGC]** 671 (85.72)   
4 - **.CGCCTC.** 668 (91.20)   
4 - **[CGCGTG]** 623 (86.67)   
4 - **[CGCTTC]** 359 (86.32)   
4 - **[CGCCCC]** 312 (85.80)   
4 - **[CGCCCC]** 204 (85.80)   
4 - **[CGCCCC]** 63 (85.80)   
5 + **[CCCGTC]** 920 (87.14)   
5 + **[CGGGTC]** 1085 (87.94)   
5 + **[CGCCCC]** 1111 (85.80)   
5 + **[CGCCGC]** 1160 (85.72)   
5 + **[CGCCCC]** 1163 (85.80)   
5 + **[CGCCGC]** 1310 (85.72)   
5 + **.CGCGCC.** 1346 (94.60)   
5 + **.CGCCTC.** 1348 (91.20)   
5 + **.CGCGGC.** 1395 (94.52)   
5 + **[CGCCCC]** 1413 (85.80)   
5 + **.CGCGGC.** 1440 (94.52)   
5 + **[CGCCGC]** 1482 (85.72)   
5 + **[CGCCGC]** 1485 (85.72)   
5 - **.CGCCTC.** 1447 (91.20)   
5 - **.CGCGTC.** 1438 (100.00)   
5 - **[CGGGTC]** 897 (87.94)   
5 - **.CGCGGC.** 812 (94.52)   
5 - **[GGCGTC]** 795 (87.08)   
6 + **[CGCTTC]** 980 (86.32)   
6 + **.CGCGGC.** 1440 (94.52)   
6 - **[CGCCGC]** 1476 (85.72)   
6 - **[CGCCGC]** 1441 (85.72)   
6 - **.CGCCTC.** 1245 (91.20)   
6 - **[CGCCCC]** 958 (85.80)   
7 - **.CGCCTC.** 1386 (91.20)   
7 - **[CGCTTC]** 1343 (86.32)   
8 + **.CGCGTC.** 149 (100.00)   
8 + **.CGCGTC.** 372 (100.00)   
8 + **.CGCGGC.** 409 (94.52)   
8 + **[CGCCGC]** 451 (85.72)   
8 + **[CGCGTG]** 454 (86.67)   
8 + **.CGCGCC.** 494 (94.60)   
8 + **[CGCCCC]** 496 (85.80)   
8 + **[CGCCCC]** 782 (85.80)   
8 + **[CGCCGC]** 813 (85.72)   
8 + **[CGCCCC]** 816 (85.80)   
8 + **[CGCCCC]** 858 (85.80)   
8 + **[CGCGAC]** 980 (87.27)   
8 + **[CGCCCC]** 1076 (85.80)   
8 + **.CGCCTC.** 1082 (91.20)   
8 + **.CGCCTC.** 1121 (91.20)   
8 + **.CGCGTC.** 1146 (100.00)   
8 + **[CGCCGC]** 1154 (85.72)   
8 + **[CCCGTC]** 1180 (87.14)   
8 + **[CGCCGC]** 1234 (85.72)   
8 + **.CGCCTC.** 1237 (91.20)   
8 + **[CGCTTC]** 1273 (86.32)   
8 + **[CGCGAC]** 1466 (87.27)   
8 - **.CGCCTC.** 1286 (91.20)   
8 - **[CGCGAC]** 1204 (87.27)   
8 - **[GGCGTC]** 1152 (87.08)   
8 - **.CGCGCC.** 978 (94.60)   
8 - **.CGCGCC.** 933 (94.60)   
8 - **.CGCGCC.** 927 (94.60)   
8 - **[CGCCCC]** 900 (85.80)   
8 - **[CGCCCC]** 895 (85.80)   
8 - **[CGCCCC]** 883 (85.80)   
8 - **[CGCCCC]** 617 (85.80)   
8 - **[CGCCCC]** 588 (85.80)   
8 - **[CCCGTC]** 585 (87.14)   
8 - **.CGCGGC.** 452 (94.52)   
8 - **[CGCCCC]** 420 (85.80)   
8 - **.CGCGCC.** 407 (94.60)   
8 - **[CCCGTC]** 178 (87.14)   
8 - **[CGCCGC]** 3 (85.72)   
9 + **[CGGGTC]** 49 (87.94)   
9 + **.CGCGCC.** 1062 (94.60)   
9 + **[CGCCCC]** 1064 (85.80)   
9 + **[CGCGAC]** 1268 (87.27)   
9 - **[CGGGTC]** 1482 (87.94)   
9 - **[CCCGTC]** 1271 (87.14)   
9 - **.CGCGCC.** 1266 (94.60)   
9 - **[CGCCCC]** 1222 (85.80)   
9 - **[CGCCCC]** 1083 (85.80)   
9 - **.CGCGCC.** 975 (94.60)   
9 - **.CGCCTC.** 913 (91.20)   
9 - **[CGCTTC]** 351 (86.32)   
9 - **[CGCGTG]** 99 (86.67)   
9 - **[CGCCCC]** 22 (85.80)   
10 + **[CGCTTC]** 82 (86.32)   
10 + **[CGGGTC]** 442 (87.94)   
10 + **[CGCTTC]** 473 (86.32)   
10 + **[CGCCCC]** 499 (85.80)   
10 + **.CGCGCC.** 513 (94.60)   
10 + **[CGCCCC]** 515 (85.80)   
10 + **[CGGGTC]** 572 (87.94)   
10 + **.CGCGGC.** 605 (94.52)   
10 + **[CGGGTC]** 942 (87.94)   
10 + **.CGCGGC.** 1024 (94.52)   
10 + **.CGCGGC.** 1096 (94.52)   
10 + **.CGCGGC.** 1121 (94.52)   
10 + **[GGCGTC]** 1188 (87.08)   
10 + **.CGCCTC.** 1205 (91.20)   
10 + **[CGCCCC]** 1244 (85.80)   
10 + **[CGCCGC]** 1262 (85.72)   
10 + **.CGCGGC.** 1331 (94.52)   
10 - **[CCCGTC]** 1499 (87.14)   
10 - **[CCCGTC]** 1353 (87.14)   
10 - **[CGCGTG]** 1329 (86.67)   
10 - **[CGCGTG]** 1306 (86.67)   
10 - **[CGCCGC]** 1232 (85.72)   
10 - **.CGCCTC.** 1186 (91.20)   
10 - **.CGCCTC.** 1149 (91.20)   
10 - **.CGCGGC.** 1119 (94.52)   
10 - **[CGCCGC]** 1097 (85.72)   
10 - **[CCCGTC]** 1041 (87.14)   
10 - **[CGCCGC]** 1025 (85.72)   
10 - **.CGCGGC.** 1022 (94.52)   
10 - **[CGCCCC]** 938 (85.80)   
10 - **[CGCCCC]** 933 (85.80)   
10 - **[CGCCCC]** 909 (85.80)   
10 - **[GGCGTC]** 896 (87.08)   
10 - **.CGCGCC.** 871 (94.60)   
10 - **[CGCCCC]** 869 (85.80)   
10 - **[CGCTTC]** 860 (86.32)   
10 - **[GGCGTC]** 848 (87.08)   
10 - **.CGCGTC.** 828 (100.00)   
10 - **.CGCGTC.** 820 (100.00)   
10 - **[CCCGTC]** 734 (87.14)   
10 - **[CGCCGC]** 692 (85.72)   
10 - **[CGCCGC]** 689 (85.72)   
10 - **.CGCGGC.** 511 (94.52)   
10 - **[CGCCCC]** 128 (85.80)   
10 - **[CGCCCC]** 122 (85.80)   
10 - **[CGCCCC]** 54 (85.80)   
10 - **.CGCGGC.** 35 (94.52)   
11 + **[CGCGTG]** 34 (86.67)   
11 + **[GGCGTC]** 96 (87.08)   
11 + **[CGCGTG]** 252 (86.67)   
11 + **[CGCCCC]** 461 (85.80)   
11 + **[CGCGTG]** 499 (86.67)   
11 + **[CCCGTC]** 526 (87.14)   
11 + **.CGCGGC.** 560 (94.52)   
11 + **.CGCGCC.** 606 (94.60)   
11 + **[CGCCGC]** 608 (85.72)   
11 + **.CGCGGC.** 611 (94.52)   
11 + **.CGCGCC.** 639 (94.60)   
11 + **.CGCGCC.** 700 (94.60)   
11 + **.CGCGGC.** 715 (94.52)   
11 + **.CGCGCC.** 749 (94.60)   
11 + **.CGCGGC.** 769 (94.52)   
11 + **[CGCCCC]** 943 (85.80)   
11 + **.CGCGCC.** 957 (94.60)   
11 + **[CGCCCC]** 963 (85.80)   
11 + **[CGCCGC]** 974 (85.72)   
11 + **[CGCCGC]** 993 (85.72)   
11 + **[CGCCGC]** 1008 (85.72)   
11 + **[CGCCGC]** 1026 (85.72)   
11 + **[CGCCGC]** 1037 (85.72)   
11 + **[CGCCGC]** 1040 (85.72)   
11 + **[CGCCGC]** 1043 (85.72)   
11 + **.CGCGGC.** 1116 (94.52)   
11 + **[CGCCGC]** 1151 (85.72)   
11 + **[CGCCGC]** 1154 (85.72)   
11 + **[CGCCGC]** 1328 (85.72)   
11 + **.CGCGTC.** 1381 (100.00)   
11 - **.CGCCTC.** 1512 (91.20)   
11 - **.CGCGCC.** 1379 (94.60)   
11 - **.CGCCTC.** 1196 (91.20)   
11 - **.CGCGGC.** 1155 (94.52)   
11 - **[CGCCGC]** 1117 (85.72)   
11 - **.CGCGGC.** 1027 (94.52)   
11 - **.CGCGGC.** 994 (94.52)   
11 - **[CGCCCC]** 798 (85.80)   
11 - **[CGCCGC]** 770 (85.72)   
11 - **.CGCGGC.** 767 (94.52)   
11 - **.CGCGTC.** 747 (100.00)   
11 - **.CGCGGC.** 713 (94.52)   
11 - **.CGCGTC.** 698 (100.00)   
11 - **.CGCGGC.** 615 (94.52)   
11 - **.CGCGGC.** 609 (94.52)   
11 - **[CGCGTG]** 558 (86.67)   
11 - **[CGCGAC]** 529 (87.27)   
11 - **.CGCGGC.** 497 (94.52)   
11 - **.CGCGCC.** 250 (94.60)   
11 - **[CGCCCC]** 183 (85.80)   
11 - **[CGCCCC]** 173 (85.80)   
11 - **[CGCCCC]** 94 (85.80)   
12 + **[GGCGTC]** 19 (87.08)   
12 + **[GGCGTC]** 31 (87.08)   
12 + **.CGCCTC.** 466 (91.20)   
12 + **.CGCGCC.** 526 (94.60)   
12 + **.CGCGCC.** 543 (94.60)   
12 + **.CGCGCC.** 696 (94.60)   
12 + **[CGCCGC]** 698 (85.72)   
12 + **.CGCGGC.** 711 (94.52)   
12 + **[CGCCGC]** 776 (85.72)   
12 + **.CGCGTC.** 841 (100.00)   
12 + **[CGCGAC]** 870 (87.27)   
12 + **.CGCGCC.** 1036 (94.60)   
12 + **[CGGGTC]** 1092 (87.94)   
12 + **.CGCGGC.** 1120 (94.52)   
12 + **.CGCCTC.** 1148 (91.20)   
12 + **[CGCCGC]** 1177 (85.72)   
12 + **.CGCGCC.** 1253 (94.60)   
12 + **[CGCCGC]** 1255 (85.72)   
12 + **[CGCCGC]** 1354 (85.72)   
12 - **[CGCCCC]** 1433 (85.80)   
12 - **[CGCCCC]** 1426 (85.80)   
12 - **.CGCGGC.** 1355 (94.52)   
12 - **.CGCGCC.** 1333 (94.60)   
12 - **[CGCCCC]** 1331 (85.80)   
12 - **.CGCGGC.** 1275 (94.52)   
12 - **.CGCGGC.** 1251 (94.52)   
12 - **[CGCCGC]** 1245 (85.72)   
12 - **[CGCCCC]** 1242 (85.80)   
12 - **.CGCGGC.** 1178 (94.52)   
12 - **.CGCGTC.** 1116 (100.00)   
12 - **[CGCGAC]** 1095 (87.27)   
12 - **.CGCGGC.** 1030 (94.52)   
12 - **[CCCGTC]** 950 (87.14)   
12 - **.CGCGGC.** 740 (94.52)   
12 - **[CCCGTC]** 735 (87.14)   
12 - **[CGCCCC]** 602 (85.80)   
12 - **[CGCCCC]** 597 (85.80)   
12 - **.CGCCTC.** 588 (91.20)   
12 - **.CGCCTC.** 580 (91.20)   
12 - **.CGCGCC.** 541 (94.60)   
12 - **.CGCGCC.** 524 (94.60)   
12 - **[CGCCCC]** 522 (85.80)   
12 - **.CGCGCC.** 509 (94.60)   
12 - **[CGCCCC]** 462 (85.80)   
12 - **[CGCGTG]** 205 (86.67)   
12 - **[CGCCCC]** 197 (85.80)   
12 - **[CGCCCC]** 170 (85.80)   
12 - **[CGCGTG]** 115 (86.67)   
12 - **[CGCTTC]** 52 (86.32)   
13 + **.CGCGCC.** 167 (94.60)   
13 + **[GGCGTC]** 492 (87.08)   
13 + **.CGCGCC.** 615 (94.60)   
13 + **[CGCCGC]** 617 (85.72)   
13 + **.CGCGGC.** 656 (94.52)   
13 + **[CGCCGC]** 677 (85.72)   
13 + **[CGCCCC]** 690 (85.80)   
13 + **.CGCGGC.** 703 (94.52)   
13 + **[CGCCCC]** 710 (85.80)   
13 + **[CGCCGC]** 717 (85.72)   
13 + **[CGCCGC]** 720 (85.72)   
13 + **.CGCGTC.** 742 (100.00)   
13 + **[CGCCGC]** 753 (85.72)   
13 + **[CGCCCC]** 756 (85.80)   
13 + **[CGCCCC]** 761 (85.80)   
13 + **.CGCGTC.** 766 (100.00)   
13 + **.CGCGCC.** 773 (94.60)   
13 + **[CGCCCC]** 775 (85.80)   
13 + **.CGCGCC.** 780 (94.60)   
13 + **[CGCCCC]** 782 (85.80)   
13 + **.CGCGCC.** 787 (94.60)   
13 + **[CGCCCC]** 846 (85.80)   
13 + **[CCCGTC]** 858 (87.14)   
13 + **.CGCCTC.** 868 (91.20)   
13 + **[CGGGTC]** 897 (87.94)   
13 + **.CGCGGC.** 947 (94.52)   
13 + **.CGCGGC.** 1064 (94.52)   
13 + **[CGCCGC]** 1109 (85.72)   
13 + **[CGCCGC]** 1139 (85.72)   
13 + **[CGCCGC]** 1142 (85.72)   
13 + **[CGCCGC]** 1145 (85.72)   
13 + **[CGCCGC]** 1160 (85.72)   
13 + **[CGCCGC]** 1163 (85.72)   
13 + **[CGCCGC]** 1166 (85.72)   
13 + **.CGCGCC.** 1187 (94.60)   
13 + **[CGCCGC]** 1196 (85.72)   
13 + **.CGCGCC.** 1264 (94.60)   
13 + **[CGGGTC]** 1299 (87.94)   
13 + **[CGGGTC]** 1306 (87.94)   
13 + **[GGCGTC]** 1319 (87.08)   
13 + **.CGCGGC.** 1352 (94.52)   
13 + **[CGGGTC]** 1441 (87.94)   
13 + **[CGGGTC]** 1465 (87.94)   
13 - **[CGCGAC]** 1468 (87.27)   
13 - **[CGCCGC]** 1429 (85.72)   
13 - **[CGCCCC]** 1426 (85.80)   
13 - **[CGCCGC]** 1420 (85.72)   
13 - **[CCCGTC]** 1405 (87.14)   
13 - **.CGCCTC.** 1400 (91.20)   
13 - **[CGCCGC]** 1353 (85.72)   
13 - **[CGCCCC]** 1327 (85.80)   
13 - **[CGCGAC]** 1322 (87.27)   
13 - **[CGCCGC]** 1317 (85.72)   
13 - **[CGCGAC]** 1302 (87.27)   
13 - **[CGCCCC]** 1283 (85.80)   
13 - **.CGCGGC.** 1227 (94.52)   
13 - **.CGCGGC.** 1197 (94.52)   
13 - **.CGCGGC.** 1110 (94.52)   
13 - **.CGCGCC.** 1092 (94.60)   
13 - **.CGCGCC.** 1062 (94.60)   
13 - **[CGCCCC]** 978 (85.80)   
13 - **[CGCCGC]** 973 (85.72)   
13 - **[CCCGTC]** 929 (87.14)   
13 - **[CGCCCC]** 922 (85.80)   
13 - **[CCCGTC]** 919 (87.14)   
13 - **[CGCCCC]** 910 (85.80)   
13 - **[CGCCCC]** 905 (85.80)   
13 - **[CGCCCC]** 893 (85.80)   
13 - **.CGCGCC.** 801 (94.60)   
13 - **.CGCGGC.** 721 (94.52)   
13 - **[CGCGTG]** 654 (86.67)   
13 - **[CGCCCC]** 501 (85.80)   
13 - **.CGCGCC.** 440 (94.60)   
13 - **[CGCCCC]** 284 (85.80)   
13 - **[CGCCCC]** 269 (85.80)   
14 + **.CGCGGC.** 235 (94.52)   
14 + **.CGCCTC.** 285 (91.20)   
14 + **.CGCGCC.** 292 (94.60)   
14 + **[CGCCCC]** 294 (85.80)   
14 + **[CGCCCC]** 332 (85.80)   
14 + **.CGCGGC.** 337 (94.52)   
14 + **[GGCGTC]** 444 (87.08)   
14 + **[CCCGTC]** 572 (87.14)   
14 + **[GGCGTC]** 700 (87.08)   
14 + **[CGCGTG]** 714 (86.67)   
14 + **.CGCGGC.** 748 (94.52)   
14 + **[GGCGTC]** 785 (87.08)   
14 + **.CGCGTC.** 793 (100.00)   
14 + **.CGCGCC.** 870 (94.60)   
14 + **.CGCGGC.** 940 (94.52)   
14 + **.CGCGGC.** 993 (94.52)   
14 + **[CGCTTC]** 1024 (86.32)   
14 + **[CGGGTC]** 1076 (87.94)   
14 - **[CGCGTG]** 1305 (86.67)   
14 - **[CGGGTC]** 1193 (87.94)   
14 - **[CGCCCC]** 1154 (85.80)   
14 - **.CGCGGC.** 1072 (94.52)   
14 - **.CGCGGC.** 989 (94.52)   
14 - **[CGCCGC]** 973 (85.72)   
14 - **[CGCCCC]** 958 (85.80)   
14 - **[CGCCCC]** 946 (85.80)   
14 - **[CGCCGC]** 941 (85.72)   
14 - **.CGCGCC.** 938 (94.60)   
14 - **[CGCGTG]** 862 (86.67)   
14 - **.CGCCTC.** 823 (91.20)   
14 - **[CGCCCC]** 773 (85.80)   
14 - **[CGCCGC]** 749 (85.72)   
14 - **.CGCGCC.** 746 (94.60)   
14 - **.CGCGCC.** 712 (94.60)   
14 - **.CGCCTC.** 542 (91.20)   
14 - **[CCCGTC]** 488 (87.14)   
14 - **.CGCGGC.** 460 (94.52)   
14 - **[CGCCGC]** 442 (85.72)   
14 - **[CGCGAC]** 349 (87.27)   
14 - **[CGCCGC]** 338 (85.72)   
14 - **[CGCGTG]** 290 (86.67)   
15 + **[CGCGAC]** 25 (87.27)   
15 + **[CGCCCC]** 39 (85.80)   
15 + **[CGCCGC]** 178 (85.72)   
15 + **.CGCGCC.** 247 (94.60)   
15 + **[CGCCGC]** 271 (85.72)   
15 + **[CCCGTC]** 336 (87.14)   
15 + **[CGCCGC]** 414 (85.72)   
15 + **.CGCGCC.** 539 (94.60)   
15 + **.CGCGGC.** 545 (94.52)   
15 + **[CGCCGC]** 569 (85.72)   
15 + **[CGCCCC]** 596 (85.80)   
15 + **[CGCCCC]** 605 (85.80)   
15 + **.CGCCTC.** 620 (91.20)   
15 + **.CGCCTC.** 626 (91.20)   
15 + **[CGCCCC]** 697 (85.80)   
15 + **[CGCCCC]** 702 (85.80)   
15 + **[CCCGTC]** 705 (87.14)   
15 + **[CGCTTC]** 822 (86.32)   
15 + **.CGCGCC.** 827 (94.60)   
15 + **[CGCCCC]** 829 (85.80)   
15 + **[CGCCCC]** 834 (85.80)   
15 + **[CGCCCC]** 842 (85.80)   
15 + **.CGCGGC.** 986 (94.52)   
15 + **[CGGGTC]** 1110 (87.94)   
15 + **.CGCGTC.** 1183 (100.00)   
15 + **.CGCGTC.** 1232 (100.00)   
15 + **[CGCCGC]** 1243 (85.72)   
15 + **.CGCCTC.** 1246 (91.20)   
15 + **[GGCGTC]** 1324 (87.08)   
15 - **[CGCCCC]** 1322 (85.80)   
15 - **[GGCGTC]** 1304 (87.08)   
15 - **.CGCGGC.** 1264 (94.52)   
15 - **.CGCGCC.** 1200 (94.60)   
15 - **[CGCCGC]** 1198 (85.72)   
15 - **[CGCCCC]** 1084 (85.80)   
15 - **[CGCCGC]** 987 (85.72)   
15 - **.CGCGCC.** 984 (94.60)   
15 - **[CGCCCC]** 982 (85.80)   
15 - **[CGCCCC]** 953 (85.80)   
15 - **.CGCGGC.** 855 (94.52)   
15 - **[CGGGTC]** 776 (87.94)   
15 - **.CGCGCC.** 733 (94.60)   
15 - **[GGCGTC]** 695 (87.08)   
15 - **[GGCGTC]** 657 (87.08)   
15 - **[CGCCCC]** 504 (85.80)   
15 - **.CGCCTC.** 495 (91.20)   
15 - **[CGCCCC]** 470 (85.80)   
15 - **.CGCGGC.** 459 (94.52)   
15 - **.CGCGGC.** 415 (94.52)   
15 - **.CGCCTC.** 345 (91.20)   
15 - **[CGGGTC]** 334 (87.94)   
15 - **[GGCGTC]** 269 (87.08)   
15 - **[CGCGTG]** 245 (86.67)   
15 - **.CGCGGC.** 179 (94.52)   
15 - **.CGCGGC.** 23 (94.52)   
16 + **.CGCGCC.** 314 (94.60)   
16 + **[CGCCCC]** 343 (85.80)   
16 + **[CGCCCC]** 408 (85.80)   
16 + **[CGCGTG]** 522 (86.67)   
16 + **[CGGGTC]** 535 (87.94)   
16 + **.CGCGCC.** 623 (94.60)   
16 + **[CGCCGC]** 625 (85.72)   
16 + **.CGCGCC.** 628 (94.60)   
16 + **.CGCCTC.** 630 (91.20)   
16 + **.CGCGGC.** 775 (94.52)   
16 + **.CGCGCC.** 799 (94.60)   
16 + **[CGCCGC]** 843 (85.72)   
16 + **[CGCCCC]** 846 (85.80)   
16 + **[CGCCCC]** 851 (85.80)   
16 + **[CGCCCC]** 858 (85.80)   
16 + **[CGCCCC]** 956 (85.80)   
16 + **[CGCCCC]** 998 (85.80)   
16 + **.CGCGGC.** 1150 (94.52)   
16 + **[CGGGTC]** 1208 (87.94)   
16 + **[CGCGAC]** 1282 (87.27)   
16 + **[CGCTTC]** 1287 (86.32)   
16 + **.CGCGCC.** 1300 (94.60)   
16 + **[CGCCGC]** 1306 (85.72)   
16 + **[CGGGTC]** 1381 (87.94)   
16 + **[CCCGTC]** 1424 (87.14)   
16 - **[CGCCGC]** 1253 (85.72)   
16 - **[CGCGTG]** 1163 (86.67)   
16 - **[CGCCGC]** 1154 (85.72)   
16 - **[CGCCGC]** 1151 (85.72)   
16 - **.CGCGCC.** 1093 (94.60)   
16 - **[CGCCCC]** 1073 (85.80)   
16 - **[CGCCGC]** 1046 (85.72)   
16 - **[CGCCGC]** 1038 (85.72)   
16 - **[CGCCGC]** 1035 (85.72)   
16 - **[CGCCGC]** 1032 (85.72)   
16 - **[CGCCGC]** 1029 (85.72)   
16 - **.CGCCTC.** 1026 (91.20)   
16 - **.CGCGCC.** 903 (94.60)   
16 - **[CGCTTC]** 769 (86.32)   
16 - **.CGCCTC.** 686 (91.20)   
16 - **[CGCCCC]** 652 (85.80)   
16 - **.CGCGGC.** 626 (94.52)   
16 - **.CGCGGC.** 621 (94.52)   
16 - **[CGCGTG]** 520 (86.67)   
16 - **.CGCGCC.** 492 (94.60)   
16 - **[CGCCGC]** 490 (85.72)   
16 - **.CGCCTC.** 300 (91.20)   
17 + **[CGCCCC]** 883 (85.80)   
17 + **.CGCGGC.** 1019 (94.52)   
17 - **[CGCCCC]** 1501 (85.80)   
17 - **[CCCGTC]** 1485 (87.14)   
17 - **[CGCCCC]** 1354 (85.80)   
17 - **.CGCGGC.** 1017 (94.52)   
17 - **[CGCCCC]** 984 (85.80)   
17 - **.CGCCTC.** 842 (91.20)   
17 - **[CGCCCC]** 571 (85.80)   
18 + **.CGCGTC.** 476 (100.00)   
18 + **[CCCGTC]** 606 (87.14)   
18 + **.CGCGCC.** 710 (94.60)   
18 + **[CGCCGC]** 712 (85.72)   
18 + **[CGCCGC]** 785 (85.72)   
18 + **.CGCGCC.** 834 (94.60)   
18 + **[CGCCGC]** 836 (85.72)   
18 + **[CGCCGC]** 865 (85.72)   
18 + **.CGCGCC.** 868 (94.60)   
18 + **[CGCCCC]** 870 (85.80)   
18 + **.CGCGCC.** 914 (94.60)   
18 + **.CGCCTC.** 916 (91.20)   
18 + **.CGCGGC.** 932 (94.52)   
18 + **.CGCCTC.** 975 (91.20)   
18 + **[CGCCCC]** 1009 (85.80)   
18 + **[CGCCCC]** 1024 (85.80)   
18 + **[CGCCCC]** 1076 (85.80)   
18 + **[CGCCCC]** 1083 (85.80)   
18 + **[CGCCCC]** 1102 (85.80)   
18 + **.CGCGCC.** 1109 (94.60)   
18 + **[CGCCCC]** 1111 (85.80)   
18 + **[CGCCCC]** 1124 (85.80)   
18 + **[CGGGTC]** 1160 (87.94)   
18 + **[CGCCGC]** 1201 (85.72)   
18 + **.CGCGGC.** 1204 (94.52)   
18 + **[CGCCCC]** 1226 (85.80)   
18 + **.CGCGCC.** 1231 (94.60)   
18 + **.CGCGCC.** 1282 (94.60)   
18 + **.CGCGTC.** 1331 (100.00)   
18 + **.CGCCTC.** 1337 (91.20)   
18 - **[CGCGAC]** 1489 (87.27)   
18 - **[CGCCGC]** 1461 (85.72)   
18 - **.CGCGCC.** 1386 (94.60)   
18 - **[CGCCGC]** 1384 (85.72)   
18 - **[CGCCGC]** 1364 (85.72)   
18 - **.CGCGGC.** 1329 (94.52)   
18 - **[CGCCGC]** 1323 (85.72)   
18 - **[CGCCGC]** 1269 (85.72)   
18 - **[CGCCCC]** 1252 (85.80)   
18 - **.CGCGGC.** 1202 (94.52)   
18 - **.CGCGGC.** 930 (94.52)   
18 - **.CGCGGC.** 866 (94.52)   
18 - **.CGCGGC.** 837 (94.52)   
18 - **.CGCGGC.** 832 (94.52)   
18 - **[CGCCCC]** 781 (85.80)   
18 - **[CGCCCC]** 559 (85.80)   
18 - **.CGCGGC.** 474 (94.52)   
18 - **[CGCCGC]** 367 (85.72)   
18 - **.CGCCTC.** 95 (91.20)   
19 + **[GGCGTC]** 1229 (87.08)   
19 + **[CCCGTC]** 1378 (87.14)   
20 + **[CGCCCC]** 164 (85.80)   
20 + **[CGGGTC]** 815 (87.94)   
20 + **.CGCGCC.** 992 (94.60)   
20 + **.CGCGGC.** 1035 (94.52)   
20 + **[CGCGTG]** 1199 (86.67)   
20 - **.CGCGGC.** 1197 (94.52)   
20 - **[CGCCCC]** 1191 (85.80)   
20 - **[CGCCGC]** 1126 (85.72)   
20 - **[CGCCGC]** 1043 (85.72)   
20 - **[CGCCGC]** 911 (85.72)   
20 - **.CGCGGC.** 896 (94.52)   
20 - **[CGCGTG]** 309 (86.67)   
20 - **.CGCGGC.** 300 (94.52)   
20 - **.CGCGCC.** 66 (94.60)   
20 - **.CGCCTC.** 48 (91.20)   
21 + **[CGGGTC]** 839 (87.94)   
21 + **[CCCGTC]** 1086 (87.14)   
21 - **[CGCCCC]** 1449 (85.80)   
21 - **.CGCCTC.** 1350 (91.20)   
21 - **[CGCCCC]** 1250 (85.80)   
22 + **.CGCGCC.** 192 (94.60)   
22 + **[CGCTTC]** 236 (86.32)   
22 + **[CGCCCC]** 578 (85.80)   
22 + **.CGCGGC.** 618 (94.52)   
22 + **[CGCCGC]** 768 (85.72)   
22 + **[CGCCGC]** 771 (85.72)   
22 + **[CGCGAC]** 778 (87.27)   
22 + **[CGCCGC]** 851 (85.72)   
22 + **[CCCGTC]** 1034 (87.14)   
22 + **[CGCCCC]** 1044 (85.80)   
22 + **[CGCGAC]** 1231 (87.27)   
22 - **[CGGGTC]** 932 (87.94)   
22 - **[GGCGTC]** 818 (87.08)   
22 - **.CGCGGC.** 772 (94.52)   
22 - **[CGCGAC]** 745 (87.27)   
22 - **.CGCCTC.** 675 (91.20)   
22 - **[CGCGTG]** 190 (86.67)   
22 - **.CGCCTC.** 87 (91.20)   
23 + **[CGCCCC]** 378 (85.80)   
23 + **.CGCCTC.** 650 (91.20)   
23 - **[CCCGTC]** 1461 (87.14)   
23 - **[CCCGTC]** 1341 (87.14)   
23 - **[CCCGTC]** 1128 (87.14)   
23 - **[CGCGTG]** 1077 (86.67)   
23 - **[CGCCCC]** 991 (85.80)   
23 - **[CCCGTC]** 923 (87.14)   
23 - **[GGCGTC]** 816 (87.08)   
23 - **[CGGGTC]** 706 (87.94)   
24 + **.CGCCTC.** 215 (91.20)   
24 + **.CGCCTC.** 273 (91.20)   
24 + **.CGCGCC.** 306 (94.60)   
24 + **[CGCCCC]** 340 (85.80)   
24 + **[CGCCGC]** 385 (85.72)   
24 + **.CGCGTC.** 522 (100.00)   
24 + **.CGCGCC.** 546 (94.60)   
24 + **.CGCCTC.** 548 (91.20)   
24 + **[CGCCCC]** 576 (85.80)   
24 + **[CGCCCC]** 581 (85.80)   
24 + **.CGCCTC.** 586 (91.20)   
24 + **.CGCGCC.** 598 (94.60)   
24 + **[CGCCCC]** 608 (85.80)   
24 + **[CGCCCC]** 857 (85.80)   
24 + **.CGCGGC.** 1040 (94.52)   
24 + **.CGCGGC.** 1100 (94.52)   
24 + **[CGGGTC]** 1140 (87.94)   
24 + **.CGCGCC.** 1145 (94.60)   
24 + **[CGCCGC]** 1147 (85.72)   
24 + **.CGCGGC.** 1217 (94.52)   
24 - **.CGCGGC.** 1353 (94.52)   
24 - **.CGCGCC.** 1319 (94.60)   
24 - **[CGCGAC]** 1143 (87.27)   
24 - **.CGCGCC.** 1098 (94.60)   
24 - **[CGCCCC]** 1096 (85.80)   
24 - **[CGCCCC]** 1082 (85.80)   
24 - **.CGCGCC.** 1075 (94.60)   
24 - **[CGCCCC]** 1073 (85.80)   
24 - **[CGCCGC]** 1041 (85.72)   
24 - **.CGCGGC.** 1038 (94.52)   
24 - **[CGCCCC]** 948 (85.80)   
24 - **.CGCGCC.** 942 (94.60)   
24 - **[CGCCCC]** 940 (85.80)   
24 - **.CGCGCC.** 935 (94.60)   
24 - **[CGCCCC]** 933 (85.80)   
24 - **[CGCCCC]** 928 (85.80)   
24 - **[CGCCCC]** 813 (85.80)   
24 - **.CGCCTC.** 765 (91.20)   
24 - **[CGGGTC]** 572 (87.94)   
24 - **[CGCCGC]** 501 (85.72)   
24 - **[CGCCCC]** 498 (85.80)   
24 - **[CGCGAC]** 453 (87.27)   
24 - **[GGCGTC]** 338 (87.08)   
24 - **.CGCGGC.** 304 (94.52)   
24 - **.CGCCTC.** 139 (91.20)   
25 + **[GGCGTC]** 557 (87.08)   
25 + **[CGGGTC]** 979 (87.94)   
25 + **[CGCCCC]** 1195 (85.80)   
25 + **[CGGGTC]** 1229 (87.94)   
25 + **[CGCCCC]** 1239 (85.80)   
25 - **.CGCCTC.** 1250 (91.20)   
25 - **.CGCGGC.** 1151 (94.52)   
25 - **[GGCGTC]** 1138 (87.08)   
25 - **[CGCCGC]** 1085 (85.72)   
25 - **[CGCTTC]** 1065 (86.32)   
25 - **.CGCCTC.** 1025 (91.20)   
25 - **[CGCTTC]** 1015 (86.32)   
25 - **[CGCCCC]** 994 (85.80)   
25 - **[CGCCCC]** 975 (85.80)   
25 - **.CGCGTC.** 950 (100.00)   
25 - **[CGGGTC]** 937 (87.94)   
25 - **.CGCCTC.** 915 (91.20)   
25 - **[CGCCCC]** 883 (85.80)   
25 - **[CGCCCC]** 856 (85.80)   
25 - **.CGCCTC.** 830 (91.20)   
25 - **[CGCCCC]** 825 (85.80)   
25 - **[CGCCCC]** 820 (85.80)   
25 - **.CGCGCC.** 792 (94.60)   
25 - **[CGCCGC]** 790 (85.72)   
25 - **[CGCCCC]** 787 (85.80)   
25 - **.CGCGTC.** 770 (100.00)   
25 - **[CGCCCC]** 762 (85.80)   
25 - **[CGCCCC]** 757 (85.80)   
25 - **[CGCCCC]** 745 (85.80)   
25 - **[CGCCCC]** 732 (85.80)   
25 - **[CGCCCC]** 700 (85.80)   
25 - **[CGCCCC]** 688 (85.80)   
25 - **[CGCCCC]** 656 (85.80)   
25 - **[CGCCCC]** 651 (85.80)   
25 - **.CGCCTC.** 609 (91.20)   
25 - **[CGCCCC]** 604 (85.80)   
25 - **[CGCCCC]** 599 (85.80)   
25 - **.CGCCTC.** 572 (91.20)   
25 - **.CGCCTC.** 555 (91.20)   
25 - **.CGCGCC.** 549 (94.60)   
25 - **.CGCCTC.** 547 (91.20)   
25 - **.CGCGTC.** 487 (100.00)   
25 - **[CGCCCC]** 446 (85.80)   
25 - **[CGCCCC]** 441 (85.80)   
25 - **[CGCCCC]** 415 (85.80)   
25 - **.CGCCTC.** 388 (91.20)   
25 - **.CGCGCC.** 383 (94.60)   
25 - **.CGCGCC.** 360 (94.60)   
25 - **[CGCCCC]** 352 (85.80)   
25 - **[CGCCCC]** 347 (85.80)   
25 - **[CGCCCC]** 327 (85.80)   
25 - **[CGCCCC]** 315 (85.80)   
25 - **[CGCCCC]** 289 (85.80)   
25 - **[CGCCCC]** 284 (85.80)   
25 - **[CGCCCC]** 257 (85.80)   
25 - **.CGCCTC.** 252 (91.20)   
25 - **[CGCCCC]** 163 (85.80)   
25 - **[CGCCCC]** 111 (85.80)   
25 - **[CGCCCC]** 106 (85.80)   
25 - **[CGCGAC]** 87 (87.27)   
25 - **[CGCCCC]** 74 (85.80)   
25 - **[CGCCCC]** 60 (85.80)   
25 - **[CGCCCC]** 55 (85.80)   
26 + **.CGCGGC.** 186 (94.52)   
26 + **[CGCCGC]** 310 (85.72)   
26 + **[CGCCCC]** 429 (85.80)   
26 + **[CGCCCC]** 636 (85.80)   
26 + **.CGCGGC.** 826 (94.52)   
26 + **.CGCGGC.** 1425 (94.52)   
26 + **[CGCCGC]** 1484 (85.72)   
26 - **.CGCGGC.** 1485 (94.52)   
26 - **[CGCCCC]** 1480 (85.80)   
26 - **[CGCCCC]** 1443 (85.80)   
26 - **[CGCCGC]** 1426 (85.72)   
26 - **[CGCCCC]** 1391 (85.80)   
26 - **.CGCGCC.** 1353 (94.60)   
26 - **[CGCCCC]** 1351 (85.80)   
26 - **.CGCCTC.** 1029 (91.20)   
26 - **[CGCCGC]** 964 (85.72)   
26 - **[CGCCGC]** 961 (85.72)   
26 - **[CGCCGC]** 958 (85.72)   
26 - **[CGCCGC]** 955 (85.72)   
26 - **[CGCCGC]** 924 (85.72)   
26 - **.CGCGGC.** 885 (94.52)   
26 - **[CGCCCC]** 859 (85.80)   
26 - **.CGCGCC.** 824 (94.60)   
26 - **.CGCGGC.** 803 (94.52)   
26 - **.CGCGCC.** 779 (94.60)   
26 - **.CGCGTC.** 753 (100.00)   
26 - **[GGCGTC]** 560 (87.08)   
26 - **.CGCGGC.** 184 (94.52)   
26 - **[CGCCCC]** 62 (85.80)   
27 + **[CGCCCC]** 1141 (85.80)   
27 + **.CGCGTC.** 1146 (100.00)   
27 + **[CGCCCC]** 1176 (85.80)   
27 + **[CGGGTC]** 1187 (87.94)   
27 + **.CGCGGC.** 1204 (94.52)   
27 + **[CGGGTC]** 1218 (87.94)   
27 + **[CGCCCC]** 1231 (85.80)   
27 + **.CGCGCC.** 1249 (94.60)   
27 + **[CGCCGC]** 1356 (85.72)   
27 + **.CGCCTC.** 1359 (91.20)   
27 + **[CGCCCC]** 1474 (85.80)   
27 - **[CGCCCC]** 1447 (85.80)   
27 - **[CGCCCC]** 1278 (85.80)   
27 - **.CGCGCC.** 1207 (94.60)   
27 - **[CGCCGC]** 1205 (85.72)   
27 - **[CGCCCC]** 797 (85.80)   
27 - **[CGCTTC]** 780 (86.32)   
27 - **.CGCCTC.** 734 (91.20)   
27 - **[GGCGTC]** 439 (87.08)   
28 + **[GGCGTC]** 3 (87.08)   
28 + **[CGCGTG]** 435 (86.67)   
28 + **.CGCGGC.** 518 (94.52)   
28 + **[CGCCGC]** 579 (85.72)   
28 + **[CGCGAC]** 589 (87.27)   
28 + **[CGCCCC]** 613 (85.80)   
28 + **[CGCCGC]** 696 (85.72)   
28 + **[GGCGTC]** 731 (87.08)   
28 + **.CGCGCC.** 740 (94.60)   
28 + **.CGCGCC.** 957 (94.60)   
28 + **.CGCCTC.** 959 (91.20)   
28 + **[CGCGAC]** 1009 (87.27)   
28 + **[CGCCGC]** 1063 (85.72)   
28 + **[CGCCCC]** 1066 (85.80)   
28 + **.CGCCTC.** 1072 (91.20)   
28 + **[CCCGTC]** 1095 (87.14)   
28 + **.CGCGTC.** 1100 (100.00)   
28 + **.CGCGCC.** 1105 (94.60)   
28 + **[CGCCGC]** 1107 (85.72)   
28 + **.CGCGTC.** 1110 (100.00)   
28 + **[CGCCCC]** 1115 (85.80)   
28 + **.CGCCTC.** 1127 (91.20)   
28 + **[CGCCCC]** 1334 (85.80)   
28 + **[CGCGAC]** 1354 (87.27)   
28 - **.CGCCTC.** 1414 (91.20)   
28 - **.CGCGGC.** 1108 (94.52)   
28 - **[CGCGAC]** 1103 (87.27)   
28 - **[CGCGAC]** 1098 (87.27)   
28 - **[CCCGTC]** 938 (87.14)   
28 - **.CGCGGC.** 916 (94.52)   
28 - **[CGCCGC]** 873 (85.72)   
28 - **[CGCCGC]** 870 (85.72)   
28 - **[CGCCCC]** 849 (85.80)   
28 - **[GGCGTC]** 832 (87.08)   
28 - **[GGCGTC]** 803 (87.08)   
28 - **.CGCCTC.** 795 (91.20)   
28 - **.CGCCTC.** 790 (91.20)   
28 - **.CGCGCC.** 628 (94.60)   
28 - **[CGCCGC]** 626 (85.72)   
28 - **.CGCGGC.** 587 (94.52)   
28 - **.CGCGCC.** 433 (94.60)   
28 - **[CGCCGC]** 431 (85.72)   
28 - **.CGCCTC.** 428 (91.20)   
28 - **.CGCCTC.** 253 (91.20)   
29 + **.CGCGCC.** 220 (94.60)   
29 + **[CGCCCC]** 976 (85.80)   
29 + **[CGCCCC]** 981 (85.80)   
29 + **[CGCCCC]** 996 (85.80)   
29 + **[GGCGTC]** 1070 (87.08)   
29 + **.CGCCTC.** 1208 (91.20)   
29 - **[CGCCCC]** 1363 (85.80)   
29 - **.CGCGGC.** 1218 (94.52)   
29 - **[CGGGTC]** 960 (87.94)   
30 + **[CGGGTC]** 538 (87.94)   
33 + **.CGCGGC.** 904 (94.52)   
33 + **.CGCGCC.** 938 (94.60)   
33 + **[CGCCGC]** 1039 (85.72)   
33 + **[CGCGTG]** 1042 (86.67)   
33 + **[CGCCCC]** 1059 (85.80)   
33 + **.CGCGGC.** 1362 (94.52)   
33 + **[CGCCGC]** 1407 (85.72)   
33 + **[CGCCGC]** 1410 (85.72)   
33 + **[GGCGTC]** 1461 (87.08)   
33 - **[CGCCGC]** 1459 (85.72)   
33 - **[CGCCCC]** 1416 (85.80)   
33 - **.CGCGGC.** 1411 (94.52)   
33 - **[CGCCGC]** 1399 (85.72)   
33 - **[CGCCGC]** 1363 (85.72)   
33 - **.CGCGGC.** 1360 (94.52)   
33 - **[CGCCGC]** 1354 (85.72)   
33 - **.CGCCTC.** 1351 (91.20)   
33 - **.CGCCTC.** 1305 (91.20)   
33 - **[CGCCGC]** 1289 (85.72)   
33 - **[CGGGTC]** 1195 (87.94)   
33 - **[CGCCGC]** 1161 (85.72)   
33 - **[CGGGTC]** 1125 (87.94)   
33 - **.CGCGGC.** 1040 (94.52)   
33 - **[GGCGTC]** 1037 (87.08)   
33 - **.CGCGTC.** 1029 (100.00)   
33 - **[CGCCGC]** 1020 (85.72)   
33 - **.CGCCTC.** 966 (91.20)   
33 - **.CGCCTC.** 923 (91.20)   
33 - **[CGCCCC]** 732 (85.80)   
34 + **[CGCTTC]** 206 (86.32)   
34 + **[CCCGTC]** 227 (87.14)   
34 + **[CGCGAC]** 397 (87.27)   
34 + **[CGCTTC]** 541 (86.32)   
34 + **[CGCCGC]** 770 (85.72)   
34 + **.CGCGCC.** 773 (94.60)   
34 + **[CGCCCC]** 775 (85.80)   
34 + **[CGCCCC]** 789 (85.80)   
34 + **.CGCGTC.** 803 (100.00)   
34 + **[CGCCCC]** 920 (85.80)   
34 + **.CGCGCC.** 942 (94.60)   
34 + **[CGCCCC]** 948 (85.80)   
34 + **[CGCCCC]** 979 (85.80)   
34 + **[CGCCGC]** 1025 (85.72)   
34 + **.CGCGGC.** 1082 (94.52)   
34 + **[CGCCGC]** 1122 (85.72)   
34 + **[CGCCCC]** 1125 (85.80)   
34 + **[CGCCCC]** 1179 (85.80)   
34 + **[CGCCGC]** 1195 (85.72)   
34 + **[CGCCCC]** 1207 (85.80)   
34 + **[CGCCCC]** 1228 (85.80)   
34 + **.CGCGGC.** 1236 (94.52)   
34 + **.CGCCTC.** 1335 (91.20)   
34 + **[CGCCGC]** 1368 (85.72)   
34 + **[CGCCGC]** 1445 (85.72)   
34 + **[CGCCGC]** 1448 (85.72)   
34 + **[CGCCGC]** 1451 (85.72)   
34 - **.CGCGCC.** 1313 (94.60)   
34 - **.CGCGGC.** 1278 (94.52)   
34 - **[GGCGTC]** 1205 (87.08)   
34 - **.CGCGGC.** 1196 (94.52)   
34 - **[CGCCCC]** 1054 (85.80)   
34 - **.CGCGTC.** 1000 (100.00)   
34 - **.CGCGGC.** 956 (94.52)   
34 - **.CGCCTC.** 916 (91.20)   
34 - **[CGCCCC]** 846 (85.80)   
34 - **.CGCCTC.** 815 (91.20)   
34 - **.CGCGCC.** 801 (94.60)   
34 - **.CGCGGC.** 771 (94.52)   
34 - **.CGCGTC.** 683 (100.00)   
34 - **[CGCCGC]** 670 (85.72)   
34 - **.CGCGCC.** 649 (94.60)   
34 - **[CGCTTC]** 633 (86.32)   
34 - **[CGCCCC]** 611 (85.80)   
34 - **.CGCGCC.** 591 (94.60)   
34 - **.CGCCTC.** 589 (91.20)   
34 - **[CGCCCC]** 335 (85.80)   
34 - **.CGCCTC.** 270 (91.20)   
34 - **[CGCTTC]** 202 (86.32)   
35 + **[CGCGTG]** 368 (86.67)   
35 + **[CGCCCC]** 769 (85.80)   
35 + **.CGCGGC.** 812 (94.52)   
35 + **[CGCCGC]** 948 (85.72)   
35 + **[CGCCGC]** 951 (85.72)   
35 + **[CGCCGC]** 954 (85.72)   
35 + **[CGCCGC]** 976 (85.72)   
35 + **[CGCCGC]** 979 (85.72)   
35 + **[CGCCGC]** 997 (85.72)   
35 + **[CGGGTC]** 1151 (87.94)   
35 + **[CGCCGC]** 1297 (85.72)   
35 - **[CGGGTC]** 1333 (87.94)   
35 - **[CGCCGC]** 1241 (85.72)   
35 - **[CGCCCC]** 1238 (85.80)   
35 - **.CGCCTC.** 1172 (91.20)   
35 - **.CGCGCC.** 1147 (94.60)   
35 - **[CGCCCC]** 1145 (85.80)   
35 - **[CGCCCC]** 1108 (85.80)   
35 - **.CGCCTC.** 1081 (91.20)   
35 - **.CGCGGC.** 998 (94.52)   
35 - **.CGCCTC.** 944 (91.20)   
35 - **[CGCCCC]** 904 (85.80)   
35 - **[CGCCCC]** 885 (85.80)   
36 + **[CGCCCC]** 112 (85.80)   
36 + **.CGCGTC.** 151 (100.00)   
36 + **[CGCTTC]** 218 (86.32)   
36 + **.CGCGGC.** 230 (94.52)   
36 + **.CGCGCC.** 269 (94.60)   
36 + **[CGCGTG]** 366 (86.67)   
36 + **[CGCGTG]** 452 (86.67)   
36 + **[GGCGTC]** 495 (87.08)   
36 + **[CGCGTG]** 629 (86.67)   
36 + **[CGCGAC]** 859 (87.27)   
36 + **[CGCTTC]** 976 (86.32)   
36 + **.CGCCTC.** 1008 (91.20)   
36 + **[CGCCCC]** 1021 (85.80)   
36 + **.CGCGCC.** 1066 (94.60)   
36 + **[CGCCGC]** 1170 (85.72)   
36 - **[CGGGTC]** 1209 (87.94)   
36 - **.CGCGGC.** 1171 (94.52)   
36 - **[CGCCGC]** 1102 (85.72)   
36 - **[CGCCCC]** 1087 (85.80)   
36 - **[CGCCCC]** 1076 (85.80)   
36 - **.CGCGGC.** 1062 (94.52)   
36 - **[CGCGTG]** 857 (86.67)   
36 - **.CGCGGC.** 627 (94.52)   
36 - **.CGCCTC.** 493 (91.20)   
36 - **[CGCGTG]** 228 (86.67)   
37 + **[CGCTTC]** 636 (86.32)   
37 + **[GGCGTC]** 870 (87.08)   
37 + **[CGGGTC]** 906 (87.94)   
37 + **[GGCGTC]** 976 (87.08)   
37 + **.CGCCTC.** 985 (91.20)   
37 - **[CGCCCC]** 902 (85.80)   
37 - **[CGCTTC]** 278 (86.32)   
37 - **[CGCTTC]** 216 (86.32)   
38 + **.CGCCTC.** 559 (91.20)   
38 + **[CCCGTC]** 753 (87.14)   
38 + **[CGCCCC]** 785 (85.80)   
38 + **.CGCCTC.** 866 (91.20)   
38 - **[CGCTTC]** 551 (86.32)   
38 - **[CGCCCC]** 153 (85.80)   
38 - **.CGCCTC.** 34 (91.20)   
39 + **.CGCCTC.** 1066 (91.20)   
39 + **.CGCGTC.** 1122 (100.00)   
39 + **[CGCCCC]** 1135 (85.80)   
39 + **[CGCCGC]** 1350 (85.72)   
39 + **[CGCGAC]** 1414 (87.27)   
39 + **.CGCGGC.** 1425 (94.52)   
39 - **.CGCGCC.** 1412 (94.60)   
39 - **[CGCCCC]** 1329 (85.80)   
39 - **.CGCCTC.** 1312 (91.20)   
39 - **[CGCCGC]** 1258 (85.72)   
39 - **[CGCCCC]** 1255 (85.80)   
39 - **[CCCGTC]** 1251 (87.14)   
39 - **.CGCGCC.** 1120 (94.60)   
39 - **.CGCGCC.** 1046 (94.60)   
40 + **.CGCCTC.** 750 (91.20)   
40 + **.CGCGGC.** 895 (94.52)   
40 + **[CGCCCC]** 943 (85.80)   
40 + **.CGCGGC.** 952 (94.52)   
40 + **.CGCGCC.** 1104 (94.60)   
40 + **[CGCCGC]** 1106 (85.72)   
40 + **.CGCGTC.** 1235 (100.00)   
40 + **.CGCGGC.** 1241 (94.52)   
40 + **[GGCGTC]** 1414 (87.08)   
40 + **.CGCGGC.** 1425 (94.52)   
40 - **[CGCCCC]** 1282 (85.80)   
40 - **[CGCGAC]** 1277 (87.27)   
40 - **[CGCCGC]** 1242 (85.72)   
40 - **[CGCCCC]** 1223 (85.80)   
40 - **.CGCGGC.** 1107 (94.52)   
40 - **.CGCGCC.** 950 (94.60)   
40 - **[GGCGTC]** 748 (87.08)   
41 + **.CGCGGC.** 523 (94.52)   
41 + **[CCCGTC]** 913 (87.14)   
41 + **.CGCCTC.** 918 (91.20)   
41 + **.CGCGGC.** 958 (94.52)   
41 - **.CGCGGC.** 521 (94.52)   
42 + **.CGCCTC.** 247 (91.20)   
42 + **.CGCCTC.** 350 (91.20)   
42 + **[GGCGTC]** 411 (87.08)   
42 + **.CGCGTC.** 442 (100.00)   
42 + **.CGCCTC.** 609 (91.20)   
42 + **[CCCGTC]** 620 (87.14)   
42 + **.CGCGCC.** 813 (94.60)   
42 + **[CGCCCC]** 815 (85.80)   
42 + **.CGCGGC.** 843 (94.52)   
42 + **[CGCCCC]** 872 (85.80)   
42 + **.CGCGCC.** 900 (94.60)   
42 + **[GGCGTC]** 938 (87.08)   
42 + **[CGCCCC]** 946 (85.80)   
42 + **.CGCGGC.** 1168 (94.52)   
42 + **[CGGGTC]** 1264 (87.94)   
42 + **.CGCGGC.** 1311 (94.52)   
42 + **[CCCGTC]** 1349 (87.14)   
42 + **.CGCCTC.** 1417 (91.20)   
42 + **[CGCGTG]** 1451 (86.67)   
42 - **.CGCGGC.** 1475 (94.52)   
42 - **[CCCGTC]** 1258 (87.14)   
42 - **[CGCCCC]** 1198 (85.80)   
42 - **[CGCCGC]** 1178 (85.72)   
42 - **[CGCCGC]** 1175 (85.72)   
42 - **[CGCCGC]** 1172 (85.72)   
42 - **[CGCCGC]** 1169 (85.72)   
42 - **.CGCGCC.** 1070 (94.60)   
42 - **[CGCCCC]** 1068 (85.80)   
42 - **.CGCCTC.** 976 (91.20)   
42 - **[CGCGAC]** 909 (87.27)   
42 - **[CCCGTC]** 804 (87.14)   
42 - **[CGCCCC]** 605 (85.80)   
42 - **[CGCCCC]** 460 (85.80)   
43 + **.CGCCTC.** 1021 (91.20)   
43 + **[CGCCGC]** 1100 (85.72)   
43 + **[CGGGTC]** 1112 (87.94)   
43 + **.CGCGGC.** 1197 (94.52)   
43 - **[CGGGTC]** 1370 (87.94)   
43 - **.CGCGGC.** 1343 (94.52)   
43 - **[CGCCCC]** 1286 (85.80)   
43 - **[CGCCCC]** 1268 (85.80)   
43 - **.CGCGCC.** 1251 (94.60)   
43 - **[CGCCCC]** 1249 (85.80)   
43 - **.CGCGCC.** 1195 (94.60)   
43 - **[CGCCCC]** 1193 (85.80)   
43 - **[CGCCGC]** 1181 (85.72)   
43 - **[CGCCGC]** 1178 (85.72)   
43 - **[CGCCCC]** 1169 (85.80)   
43 - **[CGCCGC]** 1163 (85.72)   
43 - **.CGCCTC.** 1160 (91.20)   
43 - **.CGCGGC.** 1101 (94.52)   
45 + **[CGCGTG]** 67 (86.67)   
45 + **[CGCCGC]** 396 (85.72)   
45 + **[CGCCCC]** 518 (85.80)   
45 + **.CGCGGC.** 981 (94.52)   
45 + **.CGCGGC.** 1012 (94.52)   
45 - **.CGCGCC.** 1419 (94.60)   
45 - **.CGCGCC.** 1268 (94.60)   
45 - **[GGCGTC]** 1246 (87.08)   
45 - **[CCCGTC]** 1142 (87.14)   
45 - **.CGCGTC.** 1008 (100.00)   
45 - **.CGCCTC.** 996 (91.20)   
45 - **.CGCGGC.** 979 (94.52)   
45 - **[CGCCCC]** 935 (85.80)   
45 - **.CGCGCC.** 929 (94.60)   
45 - **.CGCCTC.** 865 (91.20)   
45 - **[CGCGTG]** 555 (86.67)   
45 - **.CGCGGC.** 397 (94.52)   
45 - **[CGCTTC]** 392 (86.32)   
47 + **[CCCGTC]** 36 (87.14)   
47 + **[CGGGTC]** 224 (87.94)   
47 + **.CGCGGC.** 1136 (94.52)   
47 + **.CGCGCC.** 1249 (94.60)   
47 + **.CGCGCC.** 1275 (94.60)   
47 + **[CGCCCC]** 1301 (85.80)   
47 + **.CGCGGC.** 1367 (94.52)   
47 + **[CGCGAC]** 1494 (87.27)   
47 - **.CGCCTC.** 1477 (91.20)   
47 - **[CGGGTC]** 1465 (87.94)   
47 - **[CGCCGC]** 1386 (85.72)   
47 - **[CGCCCC]** 1379 (85.80)   
47 - **.CGCGGC.** 1371 (94.52)   
47 - **[CGCCGC]** 1297 (85.72)   
47 - **.CGCGGC.** 1271 (94.52)   
47 - **[CGCGAC]** 1247 (87.27)   
47 - **[CGCCGC]** 1205 (85.72)   
47 - **[CGCCCC]** 1148 (85.80)   
47 - **.CGCCTC.** 1143 (91.20)   
47 - **[CGCCGC]** 1082 (85.72)   
47 - **[CGCCCC]** 1058 (85.80)   
47 - **.CGCGCC.** 834 (94.60)   
47 - **[CGCCGC]** 832 (85.72)   
47 - **.CGCCTC.** 367 (91.20)   
47 - **.CGCGCC.** 350 (94.60)   
47 - **[CGCCCC]** 348 (85.80)   
47 - **[CGCCCC]** 117 (85.80)   
48 + **[CGCGTG]** 60 (86.67)   
48 + **.CGCGGC.** 204 (94.52)   
48 + **.CGCGGC.** 433 (94.52)   
48 + **.CGCCTC.** 515 (91.20)   
48 + **[CGCCGC]** 730 (85.72)   
48 + **[GGCGTC]** 996 (87.08)   
48 + **.CGCGGC.** 1019 (94.52)   
48 + **[CGCTTC]** 1103 (86.32)   
48 + **[CGCGTG]** 1188 (86.67)   
48 + **[CGCCGC]** 1283 (85.72)   
48 + **.CGCGGC.** 1286 (94.52)   
48 + **.CGCGGC.** 1317 (94.52)   
48 + **[CGCCCC]** 1328 (85.80)   
48 + **[CGCCCC]** 1346 (85.80)   
48 + **.CGCGTC.** 1364 (100.00)   
48 + **[GGCGTC]** 1371 (87.08)   
48 + **[CGCCCC]** 1390 (85.80)   
48 - **.CGCGGC.** 1362 (94.52)   
48 - **[CGCCGC]** 1287 (85.72)   
48 - **.CGCGGC.** 1284 (94.52)   
48 - **[CGCCGC]** 1026 (85.72)   
48 - **[CGCCGC]** 1023 (85.72)   
48 - **[CGCCGC]** 1020 (85.72)   
48 - **[CGCGTG]** 1004 (86.67)   
48 - **.CGCGGC.** 963 (94.52)   
48 - **[CGGGTC]** 803 (87.94)   
48 - **.CGCGGC.** 764 (94.52)   
48 - **[CGCCCC]** 705 (85.80)   
48 - **[CGCCCC]** 669 (85.80)   
48 - **[GGCGTC]** 513 (87.08)   
48 - **[CGCCGC]** 434 (85.72)   
48 - **[CGGGTC]** 413 (87.94)   
48 - **.CGCCTC.** 218 (91.20)   
48 - **[CGGGTC]** 213 (87.94)   
48 - **[CGCCGC]** 205 (85.72)   
48 - **[CGCCGC]** 189 (85.72)   
48 - **[CGCGTG]** 56 (86.67)   
49 + **.CGCGGC.** 1120 (94.52)   
49 + **[GGCGTC]** 1146 (87.08)   
49 + **[CGCCCC]** 1283 (85.80)   
49 + **[CGCCGC]** 1306 (85.72)   
49 + **[CGCCGC]** 1309 (85.72)   
49 + **[CGCCGC]** 1406 (85.72)   
49 + **[CGCCGC]** 1496 (85.72)   
49 - **[CGCTTC]** 1513 (86.32)   
49 - **[CGCCGC]** 1480 (85.72)   
49 - **.CGCGGC.** 1407 (94.52)   
49 - **[CGCCGC]** 1082 (85.72)   
49 - **[CGCCGC]** 1079 (85.72)   
49 - **[CGCCGC]** 1076 (85.72)   
49 - **[CGCCGC]** 1073 (85.72)   
49 - **.CGCCTC.** 1070 (91.20)   
49 - **[CGCCCC]** 578 (85.80)   
51 + **.CGCGGC.** 36 (94.52)   
51 + **[CGCCGC]** 67 (85.72)   
51 + **.CGCGTC.** 70 (100.00)   
51 + **.CGCGCC.** 90 (94.60)   
51 + **.CGCCTC.** 92 (91.20)   
51 + **.CGCGGC.** 147 (94.52)   
51 + **[CGGGTC]** 152 (87.94)   
51 + **[CGCCGC]** 303 (85.72)   
51 + **[CGCCCC]** 315 (85.80)   
51 + **.CGCCTC.** 386 (91.20)   
51 + **.CGCGTC.** 558 (100.00)   
51 + **.CGCCTC.** 587 (91.20)   
51 + **.CGCGCC.** 600 (94.60)   
51 + **[CGCCCC]** 616 (85.80)   
51 + **[CGCCCC]** 631 (85.80)   
51 + **.CGCGCC.** 636 (94.60)   
51 + **.CGCCTC.** 708 (91.20)   
51 + **.CGCGCC.** 782 (94.60)   
51 + **[CGCCCC]** 820 (85.80)   
51 + **[GGCGTC]** 847 (87.08)   
51 + **[GGCGTC]** 914 (87.08)   
51 + **[CGCCCC]** 920 (85.80)   
51 + **[CGCCCC]** 931 (85.80)   
51 + **[CGCCCC]** 958 (85.80)   
51 + **[CCCGTC]** 974 (87.14)   
51 + **.CGCGCC.** 998 (94.60)   
51 + **[CGCCCC]** 1000 (85.80)   
51 + **[CGCCGC]** 1034 (85.72)   
51 + **[CGCTTC]** 1081 (86.32)   
51 + **[CGCGAC]** 1101 (87.27)   
51 + **.CGCGGC.** 1127 (94.52)   
51 + **.CGCGCC.** 1244 (94.60)   
51 + **[CGCCCC]** 1246 (85.80)   
51 + **.CGCGGC.** 1286 (94.52)   
51 + **.CGCGGC.** 1337 (94.52)   
51 + **.CGCGGC.** 1362 (94.52)   
51 - **[CGCCGC]** 1363 (85.72)   
51 - **.CGCGCC.** 1360 (94.60)   
51 - **[CGCCCC]** 1358 (85.80)   
51 - **.CGCGCC.** 1335 (94.60)   
51 - **[CGCCCC]** 1305 (85.80)   
51 - **[CGCCGC]** 1287 (85.72)   
51 - **[CGGGTC]** 1240 (87.94)   
51 - **[GGCGTC]** 1151 (87.08)   
51 - **.CGCGCC.** 1125 (94.60)   
51 - **[CGCGAC]** 977 (87.27)   
51 - **.CGCGGC.** 598 (94.52)   
51 - **[CGCCGC]** 520 (85.72)   
51 - **[CCCGTC]** 491 (87.14)   
51 - **[CGCTTC]** 374 (86.32)   
51 - **.CGCGCC.** 286 (94.60)   
51 - **.CGCGCC.** 280 (94.60)   
51 - **.CGCGTC.** 231 (100.00)   
51 - **[CGCCGC]** 148 (85.72)   
51 - **.CGCGGC.** 68 (94.52)   
51 - **[CGCCCC]** 8 (85.80)   
53 + **[GGCGTC]** 783 (87.08)   
53 + **.CGCGGC.** 1059 (94.52)   
53 + **[GGCGTC]** 1183 (87.08)   
53 + **[CGCCCC]** 1315 (85.80)   
53 - **[CGCCCC]** 1181 (85.80)   
53 - **[CGCTTC]** 1078 (86.32)   
53 - **.CGCGTC.** 1057 (100.00)   
53 - **[CGCCCC]** 837 (85.80)   
53 - **[CGGGTC]** 800 (87.94)   
53 - **.CGCCTC.** 789 (91.20)   
53 - **[CCCGTC]** 716 (87.14)   
53 - **[CCCGTC]** 683 (87.14)   
53 - **.CGCCTC.** 369 (91.20)   
53 - **.CGCCTC.** 140 (91.20)   
54 + **[CGCCGC]** 1177 (85.72)   
54 - **[CGCGTG]** 1255 (86.67)   
54 - **[CGCCCC]** 1173 (85.80)   
54 - **.CGCGTC.** 1158 (100.00)   
54 - **[CGCCCC]** 874 (85.80)   
54 - **[CGCCCC]** 864 (85.80)   
54 - **.CGCCTC.** 820 (91.20)   
55 + **.CGCGGC.** 46 (94.52)   
55 + **[CGCCGC]** 92 (85.72)   
55 + **[CGCCGC]** 95 (85.72)   
55 + **[CGCCGC]** 198 (85.72)   
55 + **.CGCGCC.** 201 (94.60)   
55 + **[CGCCCC]** 203 (85.80)   
55 + **[CGCCCC]** 216 (85.80)   
55 + **[CGCTTC]** 236 (86.32)   
55 + **[CGCCCC]** 591 (85.80)   
55 + **[CGCCGC]** 784 (85.72)   
55 + **[CGCCGC]** 865 (85.72)   
55 + **[CGCGTG]** 868 (86.67)   
55 + **[CGCCGC]** 883 (85.72)   
55 + **.CGCGGC.** 943 (94.52)   
55 + **.CGCGCC.** 1082 (94.60)   
55 + **[CGCCGC]** 1084 (85.72)   
55 + **[CGCCCC]** 1171 (85.80)   
55 + **.CGCGCC.** 1276 (94.60)   
55 - **[CGCGAC]** 1484 (87.27)   
55 - **[CGCCCC]** 1460 (85.80)   
55 - **.CGCGCC.** 1322 (94.60)   
55 - **.CGCGGC.** 1085 (94.52)   
55 - **.CGCGCC.** 956 (94.60)   
55 - **[CGCCGC]** 944 (85.72)   
55 - **[CGCCCC]** 918 (85.80)   
55 - **.CGCGGC.** 884 (94.52)   
55 - **.CGCGGC.** 866 (94.52)   
55 - **[CGCCCC]** 838 (85.80)   
55 - **[CGGGTC]** 557 (87.94)   
55 - **[CGCTTC]** 360 (86.32)   
55 - **.CGCGGC.** 199 (94.52)   
55 - **[GGCGTC]** 90 (87.08)   
58 + **[CGCGTG]** 192 (86.67)   
58 + **[CGCCCC]** 210 (85.80)   
58 + **[GGCGTC]** 362 (87.08)   
58 + **.CGCCTC.** 381 (91.20)   
58 + **.CGCCTC.** 444 (91.20)   
58 + **[CGCCGC]** 475 (85.72)   
58 + **[CGCCCC]** 544 (85.80)   
58 + **[CGCCGC]** 556 (85.72)   
58 + **[CGCCCC]** 572 (85.80)   
58 + **[CGCCCC]** 598 (85.80)   
58 + **[CGCCCC]** 620 (85.80)   
58 + **.CGCGGC.** 781 (94.52)   
58 + **[CGCCGC]** 1046 (85.72)   
58 + **[CGCCGC]** 1085 (85.72)   
58 + **[CGCCCC]** 1088 (85.80)   
58 + **.CGCGGC.** 1101 (94.52)   
58 + **[CGGGTC]** 1238 (87.94)   
58 + **[CGGGTC]** 1253 (87.94)   
58 + **.CGCGCC.** 1459 (94.60)   
58 + **.CGCCTC.** 1461 (91.20)   
58 - **[CGCCCC]** 1484 (85.80)   
58 - **.CGCGCC.** 1305 (94.60)   
58 - **.CGCGTC.** 1280 (100.00)   
58 - **[CGCCGC]** 1186 (85.72)   
58 - **.CGCCTC.** 986 (91.20)   
58 - **[CGCCCC]** 974 (85.80)   
58 - **[CGCGTG]** 960 (86.67)   
58 - **[CCCGTC]** 932 (87.14)   
58 - **[CGCCCC]** 885 (85.80)   
58 - **.CGCGTC.** 751 (100.00)   
58 - **.CGCCTC.** 662 (91.20)   
58 - **.CGCGTC.** 604 (100.00)   
58 - **.CGCGCC.** 536 (94.60)   
58 - **[CGCGTG]** 418 (86.67)   
58 - **[CGCCCC]** 360 (85.80)   
59 + **[CGCTTC]** 208 (86.32)   
59 + **[CGCCGC]** 649 (85.72)   
59 + **.CGCGGC.** 652 (94.52)   
59 + **[CGCCGC]** 714 (85.72)   
59 + **[CGCCGC]** 771 (85.72)   
59 + **[CGCCGC]** 774 (85.72)   
59 + **[CGCCGC]** 777 (85.72)   
59 + **[CGCCGC]** 780 (85.72)   
59 + **[CGCCGC]** 783 (85.72)   
59 + **[CGCCGC]** 826 (85.72)   
59 + **.CGCGGC.** 1012 (94.52)   
59 + **[CGCCCC]** 1018 (85.80)   
59 + **[CGCCCC]** 1110 (85.80)   
59 + **[CGGGTC]** 1115 (87.94)   
59 + **[GGCGTC]** 1141 (87.08)   
59 + **.CGCGTC.** 1286 (100.00)   
59 + **.CGCGGC.** 1349 (94.52)   
59 + **.CGCGGC.** 1369 (94.52)   
59 - **[CGGGTC]** 1401 (87.94)   
59 - **.CGCGCC.** 1367 (94.60)   
59 - **[CGCCCC]** 1365 (85.80)   
59 - **.CGCGCC.** 1352 (94.60)   
59 - **[CGCCGC]** 1350 (85.72)   
59 - **[CGGGTC]** 1331 (87.94)   
59 - **[CCCGTC]** 1249 (87.14)   
59 - **[CGCCGC]** 1106 (85.72)   
59 - **.CGCGGC.** 1071 (94.52)   
59 - **[CGCCCC]** 1063 (85.80)   
59 - **.CGCGGC.** 957 (94.52)   
59 - **.CGCGCC.** 893 (94.60)   
59 - **.CGCGGC.** 650 (94.52)   
59 - **[CGCCGC]** 631 (85.72)   
59 - **.CGCGCC.** 188 (94.60)   
59 - **[CGCGTG]** 102 (86.67)   
60 + **[CGCCCC]** 417 (85.80)   
60 + **.CGCCTC.** 513 (91.20)   
60 + **[CGCCGC]** 629 (85.72)   
60 + **[CGCCGC]** 824 (85.72)   
60 + **.CGCGGC.** 827 (94.52)   
60 + **[CGCCGC]** 940 (85.72)   
60 + **[CGCTTC]** 1035 (86.32)   
60 + **[CGCGAC]** 1149 (87.27)   
60 + **.CGCCTC.** 1220 (91.20)   
60 - **[CGCGAC]** 1147 (87.27)   
60 - **[CGCCCC]** 955 (85.80)   
60 - **[CGCCCC]** 936 (85.80)   
60 - **.CGCCTC.** 905 (91.20)   
60 - **[CGCCCC]** 885 (85.80)   
60 - **[CGCCCC]** 869 (85.80)   
60 - **.CGCGGC.** 825 (94.52)   
60 - **[CGCCGC]** 801 (85.72)   
60 - **[CGCTTC]** 771 (86.32)   
60 - **[CGCGAC]** 656 (87.27)   
60 - **[GGCGTC]** 601 (87.08)   
60 - **.CGCCTC.** 466 (91.20)   
61 + **[CGCTTC]** 519 (86.32)   
61 + **[CGCTTC]** 669 (86.32)   
61 + **.CGCGGC.** 842 (94.52)   
61 + **[CGCCGC]** 924 (85.72)   
61 + **.CGCCTC.** 983 (91.20)   
61 + **[CGCCGC]** 1036 (85.72)   
61 + **.CGCGGC.** 1039 (94.52)   
61 + **.CGCCTC.** 1218 (91.20)   
61 + **[CGCCCC]** 1272 (85.80)   
61 + **[CGCGTG]** 1322 (86.67)   
61 - **.CGCCTC.** 1486 (91.20)   
61 - **[CGCCCC]** 1471 (85.80)   
61 - **[CCCGTC]** 1418 (87.14)   
61 - **.CGCCTC.** 1332 (91.20)   
61 - **.CGCGTC.** 1320 (100.00)   
61 - **[CGCCGC]** 1040 (85.72)   
61 - **.CGCGGC.** 1037 (94.52)   
61 - **[GGCGTC]** 1034 (87.08)   
61 - **.CGCGGC.** 925 (94.52)   
61 - **.CGCGGC.** 914 (94.52)   
61 - **[CGCCGC]** 843 (85.72)   
61 - **.CGCGTC.** 840 (100.00)   
61 - **[CGGGTC]** 787 (87.94)   
61 - **.CGCGCC.** 501 (94.60)   
61 - **[CGCTTC]** 436 (86.32)   
62 + **[CGGGTC]** 587 (87.94)   
62 + **[CGCCCC]** 1382 (85.80)   
62 - **[CGCCCC]** 1396 (85.80)   
62 - **[CGCCCC]** 1339 (85.80)   
62 - **[CGCTTC]** 1286 (86.32)   
63 + **.CGCGCC.** 131 (94.60)   
63 + **[CGCCCC]** 133 (85.80)   
63 + **[CGCCCC]** 155 (85.80)   
63 + **[CGGGTC]** 211 (87.94)   
63 + **[CGCTTC]** 270 (86.32)   
63 + **[CGCCCC]** 392 (85.80)   
63 + **.CGCGGC.** 410 (94.52)   
63 + **.CGCGGC.** 445 (94.52)   
63 + **[GGCGTC]** 448 (87.08)   
63 + **.CGCGCC.** 454 (94.60)   
63 + **[CGCCCC]** 499 (85.80)   
63 + **[CGCCCC]** 505 (85.80)   
63 + **.CGCGTC.** 521 (100.00)   
63 + **[GGCGTC]** 546 (87.08)   
63 + **[CGCCGC]** 568 (85.72)   
63 + **[CGCCCC]** 622 (85.80)   
63 + **.CGCGCC.** 658 (94.60)   
63 + **.CGCCTC.** 710 (91.20)   
63 + **.CGCCTC.** 719 (91.20)   
63 + **[CCCGTC]** 782 (87.14)   
63 + **[CGCCCC]** 842 (85.80)   
63 + **.CGCGTC.** 854 (100.00)   
63 + **.CGCGCC.** 907 (94.60)   
63 + **[CGCCGC]** 923 (85.72)   
63 + **.CGCGGC.** 954 (94.52)   
63 + **[GGCGTC]** 957 (87.08)   
63 + **[CCCGTC]** 962 (87.14)   
63 + **[CCCGTC]** 987 (87.14)   
63 + **[CGCCGC]** 1000 (85.72)   
63 + **[CGGGTC]** 1007 (87.94)   
63 + **[CGGGTC]** 1112 (87.94)   
63 + **[CGCGAC]** 1125 (87.27)   
63 + **[CGCCGC]** 1252 (85.72)   
63 + **[CCCGTC]** 1301 (87.14)   
63 + **.CGCGGC.** 1393 (94.52)   
63 - **[CGCCGC]** 1437 (85.72)   
63 - **.CGCGTC.** 1430 (100.00)   
63 - **.CGCGGC.** 1412 (94.52)   
63 - **[CGCCCC]** 1406 (85.80)   
63 - **[CGCCGC]** 1397 (85.72)   
63 - **[CGCCGC]** 1394 (85.72)   
63 - **.CGCGCC.** 1391 (94.60)   
63 - **.CGCCTC.** 1389 (91.20)   
63 - **[CGCCGC]** 1364 (85.72)   
63 - **.CGCGGC.** 1253 (94.52)   
63 - **[CGCCGC]** 1241 (85.72)   
63 - **[CGCCCC]** 1194 (85.80)   
63 - **[CGCCGC]** 1172 (85.72)   
63 - **[CGCCCC]** 1169 (85.80)   
63 - **.CGCGGC.** 1123 (94.52)   
63 - **[GGCGTC]** 998 (87.08)   
63 - **[CGCGAC]** 974 (87.27)   
63 - **[CGCCGC]** 955 (85.72)   
63 - **[CGCTTC]** 756 (86.32)   
63 - **[CCCGTC]** 695 (87.14)   
63 - **.CGCGCC.** 656 (94.60)   
63 - **[CCCGTC]** 607 (87.14)   
63 - **.CGCGGC.** 534 (94.52)   
63 - **.CGCCTC.** 473 (91.20)   
63 - **[CGCCGC]** 446 (85.72)   
63 - **[CGCCGC]** 414 (85.72)   
63 - **[CGCCGC]** 411 (85.72)   
63 - **.CGCGGC.** 346 (94.52)   
63 - **.CGCGCC.** 266 (94.60)   
63 - **[CGCCCC]** 223 (85.80)   
63 - **[CGCCCC]** 217 (85.80)   
63 - **.CGCGGC.** 182 (94.52)   
63 - **.CGCCTC.** 68 (91.20)   
63 - **[CGCCCC]** 50 (85.80)   
64 - **[CCCGTC]** 1506 (87.14)   
64 - **[CGGGTC]** 743 (87.94)   
65 + **[CGCCCC]** 300 (85.80)   
65 + **[CGCCCC]** 306 (85.80)   
65 + **.CGCGTC.** 547 (100.00)   
65 + **.CGCGCC.** 589 (94.60)   
65 + **.CGCCTC.** 591 (91.20)   
65 + **.CGCCTC.** 636 (91.20)   
65 + **.CGCCTC.** 775 (91.20)   
65 + **.CGCCTC.** 818 (91.20)   
65 + **.CGCGTC.** 866 (100.00)   
65 + **[CCCGTC]** 874 (87.14)   
65 + **.CGCGCC.** 912 (94.60)   
65 + **.CGCCTC.** 914 (91.20)   
65 + **[CGCCCC]** 943 (85.80)   
65 + **.CGCGGC.** 948 (94.52)   
65 + **.CGCGCC.** 981 (94.60)   
65 + **[CGCCGC]** 1000 (85.72)   
65 + **[CGCCGC]** 1027 (85.72)   
65 + **.CGCGCC.** 1030 (94.60)   
65 + **[CGCCGC]** 1087 (85.72)   
65 + **[CGCCGC]** 1090 (85.72)   
65 + **[CGCCCC]** 1093 (85.80)   
65 + **[CGCCGC]** 1120 (85.72)   
65 + **[CGCCCC]** 1150 (85.80)   
65 + **[CGGGTC]** 1186 (87.94)   
65 + **[CGCTTC]** 1287 (86.32)   
65 - **[GGCGTC]** 1420 (87.08)   
65 - **[CGCCCC]** 1283 (85.80)   
65 - **[CGCGAC]** 1230 (87.27)   
65 - **.CGCGTC.** 1224 (100.00)   
65 - **.CGCGCC.** 1205 (94.60)   
65 - **.CGCCTC.** 1171 (91.20)   
65 - **[GGCGTC]** 1055 (87.08)   
65 - **.CGCGGC.** 1028 (94.52)   
65 - **.CGCGGC.** 1001 (94.52)   
65 - **.CGCGCC.** 979 (94.60)   
65 - **.CGCGCC.** 864 (94.60)   
65 - **.CGCGGC.** 829 (94.52)   
65 - **.CGCGGC.** 784 (94.52)   
65 - **[CGCGAC]** 587 (87.27)   
65 - **[CGCCGC]** 113 (85.72)   
66 + **.CGCCTC.** 721 (91.20)   
66 + **.CGCGCC.** 869 (94.60)   
66 + **[CGCCGC]** 871 (85.72)   
66 + **[CGCCGC]** 874 (85.72)   
66 + **[CGCCCC]** 877 (85.80)   
66 + **[CGCCCC]** 883 (85.80)   
66 + **[CGCCCC]** 896 (85.80)   
66 + **[CGCCCC]** 902 (85.80)   
66 + **.CGCGCC.** 943 (94.60)   
66 + **[CGCCCC]** 949 (85.80)   
66 + **.CGCGCC.** 955 (94.60)   
66 + **[CGCCGC]** 988 (85.72)   
66 + **[CGCCGC]** 1041 (85.72)   
66 + **[CGCCGC]** 1093 (85.72)   
66 + **.CGCGGC.** 1127 (94.52)   
66 + **[CGGGTC]** 1333 (87.94)   
66 + **.CGCGTC.** 1406 (100.00)   
66 - **.CGCGTC.** 1480 (100.00)   
66 - **.CGCGCC.** 1438 (94.60)   
66 - **[CGCCCC]** 1429 (85.80)   
66 - **[CGCGAC]** 1404 (87.27)   
66 - **[CGCGAC]** 1336 (87.27)   
66 - **[CCCGTC]** 1331 (87.14)   
66 - **[CGGGTC]** 1321 (87.94)   
66 - **[CCCGTC]** 1235 (87.14)   
66 - **.CGCGCC.** 1179 (94.60)   
66 - **[CGCCCC]** 1177 (85.80)   
66 - **[CGCCGC]** 1128 (85.72)   
66 - **[CGCCCC]** 1084 (85.80)   
66 - **.CGCGGC.** 989 (94.52)   
66 - **[CGGGTC]** 978 (87.94)   
66 - **[CGCGAC]** 865 (87.27)   
66 - **[CGCCGC]** 834 (85.72)   
66 - **.CGCGTC.** 786 (100.00)   
66 - **.CGCGGC.** 768 (94.52)   
66 - **[CGGGTC]** 513 (87.94)   
67 + **[CGGGTC]** 818 (87.94)   
67 + **[CGCCCC]** 846 (85.80)   
67 + **.CGCGCC.** 1175 (94.60)   
67 + **[CGCCGC]** 1195 (85.72)   
67 + **.CGCGTC.** 1198 (100.00)   
67 - **.CGCGGC.** 1196 (94.52)   
67 - **.CGCGTC.** 1173 (100.00)   
67 - **[CGCCGC]** 1019 (85.72)   
67 - **.CGCCTC.** 997 (91.20)   
67 - **[CGCCCC]** 915 (85.80)   
67 - **[CGGGTC]** 829 (87.94)   
67 - **[CCCGTC]** 378 (87.14)   
68 + **.CGCGCC.** 35 (94.60)   
68 + **.CGCGGC.** 245 (94.52)   
68 + **.CGCGTC.** 419 (100.00)   
68 + **[GGCGTC]** 452 (87.08)   
68 + **[CGCCCC]** 540 (85.80)   
68 + **[CGCCGC]** 576 (85.72)   
68 + **[CGCCGC]** 579 (85.72)   
68 + **[CGCCGC]** 588 (85.72)   
68 + **.CGCGGC.** 649 (94.52)   
68 + **[CGCCCC]** 665 (85.80)   
68 + **.CGCGGC.** 812 (94.52)   
68 + **.CGCCTC.** 830 (91.20)   
68 + **[CGCGAC]** 871 (87.27)   
68 + **[CGCCGC]** 906 (85.72)   
68 + **.CGCGGC.** 992 (94.52)   
68 + **[CGCCCC]** 1349 (85.80)   
68 + **.CGCGGC.** 1394 (94.52)   
68 - **[CGCCGC]** 1395 (85.72)   
68 - **[CGCCCC]** 1180 (85.80)   
68 - **[CGCCCC]** 1098 (85.80)   
68 - **[CGCCGC]** 993 (85.72)   
68 - **.CGCGTC.** 990 (100.00)   
68 - **[CGCCCC]** 921 (85.80)   
68 - **[GGCGTC]** 874 (87.08)   
68 - **.CGCCTC.** 737 (91.20)   
68 - **[CGCGTG]** 719 (86.67)   
68 - **[CGCCCC]** 706 (85.80)   
68 - **[CGCTTC]** 696 (86.32)   
68 - **.CGCGCC.** 652 (94.60)   
68 - **[CGCCGC]** 650 (85.72)   
68 - **.CGCGGC.** 589 (94.52)   
68 - **[CGCCGC]** 503 (85.72)   
68 - **[CGCCGC]** 500 (85.72)   
68 - **[CGCCGC]** 497 (85.72)   
68 - **[CGCCCC]** 477 (85.80)   
68 - **[CGCCCC]** 450 (85.80)   
68 - **.CGCCTC.** 342 (91.20)   
68 - **[GGCGTC]** 196 (87.08)   
68 - **[CGCGTG]** 33 (86.67)   
69 + **[CGCGTG]** 165 (86.67)   
69 + **.CGCGTC.** 690 (100.00)   
69 + **[CGCGTG]** 696 (86.67)   
69 + **.CGCGGC.** 769 (94.52)   
69 + **[CGCGTG]** 775 (86.67)   
69 + **[CGCCCC]** 828 (85.80)   
69 + **.CGCGTC.** 894 (100.00)   
69 + **[CGCCCC]** 957 (85.80)   
69 + **[CGCCCC]** 981 (85.80)   
69 + **.CGCGCC.** 998 (94.60)   
69 + **[CGCCGC]** 1000 (85.72)   
69 + **.CGCGCC.** 1121 (94.60)   
69 + **.CGCGCC.** 1138 (94.60)   
69 + **.CGCGCC.** 1203 (94.60)   
69 + **[CGCCGC]** 1247 (85.72)   
69 + **[CGGGTC]** 1310 (87.94)   
69 + **[CGCCCC]** 1428 (85.80)   
69 - **[CGCCCC]** 1368 (85.80)   
69 - **.CGCGGC.** 1032 (94.52)   
69 - **[CGCCCC]** 905 (85.80)   
69 - **.CGCGCC.** 892 (94.60)   
69 - **.CGCGGC.** 773 (94.52)   
69 - **[CGCTTC]** 718 (86.32)   
70 + **[CCCGTC]** 307 (87.14)   
70 + **[CGCCGC]** 925 (85.72)   
70 + **[CGCCCC]** 936 (85.80)   
70 + **.CGCGGC.** 1064 (94.52)   
70 + **[CGCTTC]** 1165 (86.32)   
70 + **.CGCGCC.** 1334 (94.60)   
70 + **[CGGGTC]** 1348 (87.94)   
70 + **.CGCGTC.** 1390 (100.00)   
70 + **.CGCCTC.** 1425 (91.20)   
70 + **.CGCGTC.** 1450 (100.00)   
70 - **.CGCGCC.** 1161 (94.60)   
70 - **.CGCGTC.** 1103 (100.00)   
70 - **[CGCCCC]** 858 (85.80)   
70 - **[CGCCCC]** 849 (85.80)   
70 - **[CGCGTG]** 840 (86.67)   
70 - **[CGCGAC]** 732 (87.27)   
70 - **.CGCCTC.** 249 (91.20)   
71 + **[CGGGTC]** 751 (87.94)   
71 + **.CGCGCC.** 870 (94.60)   
71 + **.CGCGTC.** 904 (100.00)   
71 + **.CGCGCC.** 1184 (94.60)   
71 + **[CGCTTC]** 1277 (86.32)   
71 + **[CGCCCC]** 1419 (85.80)   
71 + **[CGCCCC]** 1461 (85.80)   
71 - **[CGCGAC]** 1494 (87.27)   
71 - **[CGCGTG]** 1273 (86.67)   
71 - **.CGCGTC.** 1237 (100.00)   
71 - **[CCCGTC]** 1221 (87.14)   
71 - **.CGCGGC.** 1001 (94.52)   
71 - **[CGCTTC]** 866 (86.32)   
71 - **[CGCCCC]** 838 (85.80)   
71 - **[CGGGTC]** 653 (87.94)   
71 - **[CGCCCC]** 573 (85.80)   
72 + **[CCCGTC]** 950 (87.14)   
72 + **[CGCTTC]** 1097 (86.32)   
72 + **[CGCTTC]** 1145 (86.32)   
73 + **[CGCCGC]** 912 (85.72)   
73 + **[CGCCGC]** 915 (85.72)   
73 + **[CGCCGC]** 918 (85.72)   
73 + **.CGCGGC.** 921 (94.52)   
73 + **[CGCCCC]** 948 (85.80)   
73 + **[CGCCGC]** 963 (85.72)   
73 + **[CGCCCC]** 1032 (85.80)   
73 + **.CGCGGC.** 1087 (94.52)   
73 - **.CGCGCC.** 1021 (94.60)   
73 - **.CGCGGC.** 919 (94.52)   
74 + **[CGGGTC]** 593 (87.94)   
74 + **.CGCGGC.** 934 (94.52)   
74 + **[CGCCCC]** 1161 (85.80)   
74 + **.CGCGGC.** 1255 (94.52)   
74 + **.CGCGTC.** 1338 (100.00)   
74 + **[CGCCGC]** 1350 (85.72)   
74 + **.CGCGGC.** 1429 (94.52)   
74 + **.CGCGCC.** 1438 (94.60)   
74 - **[CGCCCC]** 1464 (85.80)   
74 - **.CGCGGC.** 1436 (94.52)   
74 - **[CGCCGC]** 1430 (85.72)   
74 - **[CGCGAC]** 1427 (87.27)   
74 - **[CGCCCC]** 1418 (85.80)   
74 - **[CGCCGC]** 1289 (85.72)   
74 - **[CGCCGC]** 1286 (85.72)   
74 - **[CGCCGC]** 1283 (85.72)   
74 - **[CGCCGC]** 1280 (85.72)   
74 - **[CGCCGC]** 1277 (85.72)   
74 - **[CGCCGC]** 1274 (85.72)   
74 - **[CGCCGC]** 1271 (85.72)   
74 - **[CGCCGC]** 1268 (85.72)   
74 - **[CGCTTC]** 1041 (86.32)   
76 + **[CGCCCC]** 328 (85.80)   
76 + **[CGCCCC]** 358 (85.80)   
76 + **[CCCGTC]** 362 (87.14)   
76 + **[CGCCCC]** 372 (85.80)   
76 + **[CGCCCC]** 381 (85.80)   
76 + **[CCCGTC]** 385 (87.14)   
76 + **[CGCCCC]** 408 (85.80)   
76 + **[CGCCCC]** 417 (85.80)   
76 + **[CGCCCC]** 427 (85.80)   
76 + **.CGCGCC.** 829 (94.60)   
76 + **.CGCGGC.** 866 (94.52)   
76 + **[CGCCGC]** 1029 (85.72)   
76 + **.CGCGGC.** 1064 (94.52)   
76 + **.CGCGCC.** 1116 (94.60)   
76 + **.CGCCTC.** 1221 (91.20)   
76 + **[CGCCCC]** 1349 (85.80)   
76 + **[CGCCCC]** 1378 (85.80)   
76 - **.CGCCTC.** 1386 (91.20)   
76 - **[CCCGTC]** 1339 (87.14)   
76 - **.CGCGTC.** 1284 (100.00)   
76 - **[CGCCCC]** 1263 (85.80)   
76 - **.CGCGGC.** 1056 (94.52)   
76 - **.CGCGCC.** 999 (94.60)   
76 - **[CGCCGC]** 938 (85.72)   
76 - **[CGCCGC]** 922 (85.72)   
76 - **[CGCCGC]** 919 (85.72)   
76 - **[CGCCGC]** 916 (85.72)   
76 - **.CGCCTC.** 913 (91.20)   
76 - **[CGCCGC]** 867 (85.72)   
76 - **.CGCGGC.** 127 (94.52)   
77 + **[CGCGTG]** 250 (86.67)   
77 + **.CGCCTC.** 441 (91.20)   
77 + **.CGCGCC.** 600 (94.60)   
77 + **.CGCGGC.** 781 (94.52)   
77 + **[CGCGTG]** 850 (86.67)   
77 + **[CGCGTG]** 933 (86.67)   
77 + **[CGCTTC]** 951 (86.32)   
77 + **.CGCGGC.** 1054 (94.52)   
77 + **.CGCGGC.** 1117 (94.52)   
77 - **[CGCCCC]** 938 (85.80)   
77 - **.CGCGCC.** 931 (94.60)   
77 - **.CGCCTC.** 929 (91.20)   
77 - **[CGCGTG]** 779 (86.67)   
77 - **[GGCGTC]** 736 (87.08)   
77 - **.CGCGGC.** 716 (94.52)   
77 - **.CGCGCC.** 248 (94.60)   
79 + **[GGCGTC]** 633 (87.08)   
79 + **[CGCCGC]** 653 (85.72)   
79 + **[CGCCGC]** 757 (85.72)   
79 + **[CGCCGC]** 874 (85.72)   
79 + **[CGCCGC]** 877 (85.72)   
79 + **[CGCGTG]** 880 (86.67)   
79 + **.CGCCTC.** 1151 (91.20)   
79 + **.CGCGCC.** 1202 (94.60)   
79 + **[CGCCGC]** 1204 (85.72)   
79 + **[CGCTTC]** 1207 (86.32)   
79 + **[CGGGTC]** 1230 (87.94)   
79 + **.CGCGTC.** 1325 (100.00)   
79 - **[CGCCCC]** 1476 (85.80)   
79 - **.CGCGGC.** 1397 (94.52)   
79 - **[CGCCCC]** 1297 (85.80)   
79 - **.CGCGGC.** 1277 (94.52)   
79 - **[CGCGAC]** 1088 (87.27)   
79 - **.CGCGGC.** 948 (94.52)   
79 - **.CGCGGC.** 878 (94.52)   
79 - **[CGCTTC]** 830 (86.32)   
79 - **[CGGGTC]** 778 (87.94)   
79 - **.CGCGGC.** 654 (94.52)   
79 - **[CGCTTC]** 623 (86.32)   
80 + **[CGCGTG]** 957 (86.67)   
80 + **[CGCCGC]** 1015 (85.72)   
80 + **.CGCGCC.** 1018 (94.60)   
80 + **[CCCGTC]** 1083 (87.14)   
80 + **.CGCGGC.** 1209 (94.52)   
80 + **[CGGGTC]** 1239 (87.94)   
80 + **.CGCGGC.** 1343 (94.52)   
80 + **[CGCTTC]** 1498 (86.32)   
80 - **.CGCGCC.** 1308 (94.60)   
80 - **.CGCGGC.** 1207 (94.52)   
80 - **.CGCCTC.** 1168 (91.20)   
80 - **[CGGGTC]** 1137 (87.94)   
80 - **.CGCGCC.** 1044 (94.60)   
80 - **[CGCCGC]** 1042 (85.72)   
80 - **.CGCGGC.** 1016 (94.52)   
80 - **.CGCGCC.** 955 (94.60)   
80 - **[CGCGAC]** 396 (87.27)   
81 + **[CGCCCC]** 839 (85.80)   
81 + **[CGCCCC]** 1017 (85.80)   
81 + **[GGCGTC]** 1040 (87.08)   
81 + **[GGCGTC]** 1179 (87.08)   
81 - **[CGGGTC]** 845 (87.94)   
81 - **.CGCCTC.** 688 (91.20)   
83 + **[CGCTTC]** 254 (86.32)   
84 + **[CGCCCC]** 751 (85.80)   
84 + **[CCCGTC]** 776 (87.14)   
84 + **[CGGGTC]** 781 (87.94)   
84 + **.CGCGGC.** 798 (94.52)   
84 + **[CGCCCC]** 824 (85.80)   
84 + **[CGCCCC]** 851 (85.80)   
84 + **.CGCGCC.** 864 (94.60)   
84 + **[CGCCCC]** 866 (85.80)   
84 + **.CGCGGC.** 871 (94.52)   
84 + **.CGCGCC.** 902 (94.60)   
84 + **[CGCCCC]** 923 (85.80)   
84 + **[CGCCCC]** 933 (85.80)   
84 + **.CGCGGC.** 1023 (94.52)   
84 + **[CGCCCC]** 1028 (85.80)   
84 + **.CGCGGC.** 1037 (94.52)   
84 + **.CGCCTC.** 1067 (91.20)   
84 + **[CGCCCC]** 1136 (85.80)   
84 + **[CGCGTG]** 1164 (86.67)   
84 + **[CCCGTC]** 1317 (87.14)   
84 - **.CGCGCC.** 1153 (94.60)   
84 - **[CCCGTC]** 1089 (87.14)   
84 - **[CGCCGC]** 1038 (85.72)   
84 - **.CGCGTC.** 1035 (100.00)   
84 - **[CGCCGC]** 1024 (85.72)   
84 - **.CGCGGC.** 980 (94.52)   
84 - **.CGCGTC.** 900 (100.00)   
84 - **[CGGGTC]** 774 (87.94)   
84 - **[CGGGTC]** 720 (87.94)   
84 - **[CGGGTC]** 167 (87.94)   
85 + **[CGCCCC]** 5 (85.80)   
85 + **[CGCCGC]** 19 (85.72)   
85 + **.CGCGTC.** 95 (100.00)   
85 + **[CGGGTC]** 215 (87.94)   
85 + **.CGCGTC.** 589 (100.00)   
85 + **[CGGGTC]** 598 (87.94)   
85 + **[CGCCCC]** 610 (85.80)   
85 + **[CGCCCC]** 616 (85.80)   
85 + **[CGGGTC]** 622 (87.94)   
85 + **[CGCCCC]** 634 (85.80)   
85 + **[CGCCCC]** 640 (85.80)   
85 + **[CGCCCC]** 659 (85.80)   
85 + **[CGCCCC]** 665 (85.80)   
85 + **[CGCCCC]** 671 (85.80)   
85 + **[CGCCCC]** 678 (85.80)   
85 + **[CGCCCC]** 684 (85.80)   
85 + **[CGGGTC]** 690 (87.94)   
85 + **[CGCCCC]** 702 (85.80)   
85 + **[CGCCCC]** 708 (85.80)   
85 + **[CGGGTC]** 713 (87.94)   
85 + **[CGCCCC]** 733 (85.80)   
85 + **[CGGGTC]** 738 (87.94)   
85 + **[CGCCCC]** 744 (85.80)   
85 + **[CGCCCC]** 751 (85.80)   
85 + **[CGCCCC]** 757 (85.80)   
85 + **[CGCCCC]** 764 (85.80)   
85 + **[CGCCCC]** 770 (85.80)   
85 + **[CGGGTC]** 776 (87.94)   
85 + **[CGCCCC]** 789 (85.80)   
85 + **[CGCCCC]** 795 (85.80)   
85 + **[CGGGTC]** 800 (87.94)   
85 + **[CGCCCC]** 814 (85.80)   
85 + **[CGCCCC]** 821 (85.80)   
85 + **[CGGGTC]** 827 (87.94)   
85 + **.CGCCTC.** 833 (91.20)   
85 + **[CGCCCC]** 839 (85.80)   
85 + **[CGCCCC]** 845 (85.80)   
85 + **[CGGGTC]** 851 (87.94)   
85 + **[CGCCCC]** 863 (85.80)   
85 + **.CGCGGC.** 880 (94.52)   
85 + **.CGCGCC.** 963 (94.60)   
85 + **[CGCCGC]** 991 (85.72)   
85 + **[CGCCGC]** 994 (85.72)   
85 + **.CGCGCC.** 1037 (94.60)   
85 + **[CGCCCC]** 1039 (85.80)   
85 + **[CGCCGC]** 1044 (85.72)   
85 + **.CGCGCC.** 1059 (94.60)   
85 + **[CGCCCC]** 1061 (85.80)   
85 + **.CGCGCC.** 1078 (94.60)   
85 + **[CGCCCC]** 1080 (85.80)   
85 + **.CGCGCC.** 1096 (94.60)   
85 + **[CGGGTC]** 1116 (87.94)   
85 + **[GGCGTC]** 1138 (87.08)   
85 + **[CGCTTC]** 1158 (86.32)   
85 + **[CGCTTC]** 1168 (86.32)   
85 + **.CGCGGC.** 1217 (94.52)   
85 + **[CGGGTC]** 1262 (87.94)   
85 + **.CGCGCC.** 1450 (94.60)   
85 + **[CGCTTC]** 1469 (86.32)   
85 - **[CGCTTC]** 1426 (86.32)   
85 - **.CGCGGC.** 1415 (94.52)   
85 - **[CGCCCC]** 1396 (85.80)   
85 - **.CGCGTC.** 961 (100.00)   
85 - **[CGCCCC]** 939 (85.80)   
85 - **.CGCGTC.** 928 (100.00)   
85 - **.CGCGCC.** 587 (94.60)   
85 - **[CGCCCC]** 585 (85.80)   
85 - **.CGCCTC.** 579 (91.20)   
85 - **[CGCCCC]** 517 (85.80)   
85 - **[CGCCCC]** 502 (85.80)   
85 - **[CGCCCC]** 355 (85.80)   
85 - **[CGCCGC]** 315 (85.72)   
85 - **[CGCCCC]** 312 (85.80)   
85 - **.CGCGGC.** 93 (94.52)   
85 - **[CGCCGC]** 68 (85.72)   
86 + **.CGCCTC.** 336 (91.20)   
86 + **[CCCGTC]** 435 (87.14)   
86 + **[CGCCCC]** 513 (85.80)   
86 + **[CGCCGC]** 540 (85.72)   
86 + **.CGCGGC.** 543 (94.52)   
86 + **.CGCGGC.** 658 (94.52)   
86 + **[CGCCCC]** 673 (85.80)   
86 + **.CGCGGC.** 836 (94.52)   
86 + **.CGCGGC.** 902 (94.52)   
86 + **[CGGGTC]** 1121 (87.94)   
86 + **.CGCCTC.** 1185 (91.20)   
86 + **.CGCGGC.** 1190 (94.52)   
86 + **.CGCGGC.** 1195 (94.52)   
86 + **[CGCCCC]** 1200 (85.80)   
86 + **[CGCCGC]** 1220 (85.72)   
86 + **[CGCCCC]** 1223 (85.80)   
86 + **[CGCCCC]** 1263 (85.80)   
86 + **[CGCCGC]** 1269 (85.72)   
86 + **.CGCGCC.** 1341 (94.60)   
86 + **[CGCCGC]** 1354 (85.72)   
86 + **[CGCCCC]** 1357 (85.80)   
86 + **[CGCCCC]** 1362 (85.80)   
86 + **[CGCCGC]** 1436 (85.72)   
86 + **[CGCCGC]** 1460 (85.72)   
86 + **.CGCGGC.** 1506 (94.52)   
86 + **[CGCGAC]** 1512 (87.27)   
86 - **.CGCGGC.** 1510 (94.52)   
86 - **.CGCGGC.** 1437 (94.52)   
86 - **[CGCCGC]** 1414 (85.72)   
86 - **[CGCCCC]** 1411 (85.80)   
86 - **[CGCCGC]** 1402 (85.72)   
86 - **[CGCCGC]** 1392 (85.72)   
86 - **[CGCCCC]** 1389 (85.80)   
86 - **.CGCGCC.** 1384 (94.60)   
86 - **[CGCCCC]** 1382 (85.80)   
86 - **[CGGGTC]** 1323 (87.94)   
86 - **[CGCCGC]** 1196 (85.72)   
86 - **.CGCGCC.** 1193 (94.60)   
86 - **[CGCCGC]** 1191 (85.72)   
86 - **[GGCGTC]** 1183 (87.08)   
86 - **[CGCCCC]** 1117 (85.80)   
86 - **.CGCGTC.** 1086 (100.00)   
86 - **[CGCCCC]** 1063 (85.80)   
86 - **[CGCCGC]** 1058 (85.72)   
86 - **[CGCCCC]** 1021 (85.80)   
86 - **[CGCCCC]** 1015 (85.80)   
86 - **[CGGGTC]** 949 (87.94)   
86 - **[CGCCCC]** 937 (85.80)   
86 - **[CGCCCC]** 931 (85.80)   
86 - **[CGCCCC]** 925 (85.80)   
86 - **[CGCCCC]** 920 (85.80)   
86 - **[CGCCGC]** 903 (85.72)   
86 - **.CGCGCC.** 900 (94.60)   
86 - **[CGCCCC]** 898 (85.80)   
86 - **.CGCGCC.** 879 (94.60)   
86 - **[CGCCGC]** 837 (85.72)   
86 - **.CGCGCC.** 834 (94.60)   
86 - **[CGGGTC]** 800 (87.94)   
86 - **[CGGGTC]** 795 (87.94)   
86 - **[CGCGAC]** 769 (87.27)   
86 - **.CGCGGC.** 738 (94.52)   
86 - **[CGCCCC]** 627 (85.80)   
86 - **.CGCGGC.** 541 (94.52)   
86 - **.CGCGGC.** 305 (94.52)   
86 - **.CGCCTC.** 287 (91.20)   
86 - **[CGCCCC]** 224 (85.80)   
86 - **.CGCCTC.** 155 (91.20)   
87 + **.CGCGTC.** 135 (100.00)   
87 + **.CGCGGC.** 310 (94.52)   
87 + **[CGGGTC]** 418 (87.94)   
87 + **.CGCGGC.** 722 (94.52)   
87 + **.CGCGCC.** 874 (94.60)   
87 + **[CGCCCC]** 1078 (85.80)   
87 + **[CGCCCC]** 1083 (85.80)   
87 + **[CGCCCC]** 1088 (85.80)   
87 + **.CGCGTC.** 1099 (100.00)   
87 + **[CGCCGC]** 1145 (85.72)   
87 + **.CGCGCC.** 1194 (94.60)   
87 + **[CGCCGC]** 1196 (85.72)   
87 + **.CGCGGC.** 1231 (94.52)   
87 + **.CGCGGC.** 1244 (94.52)   
87 + **[CGCCCC]** 1315 (85.80)   
87 + **.CGCGCC.** 1322 (94.60)   
87 + **.CGCGGC.** 1333 (94.52)   
87 + **.CGCGCC.** 1346 (94.60)   
87 + **.CGCGGC.** 1367 (94.52)   
87 + **.CGCGCC.** 1378 (94.60)   
87 + **.CGCCTC.** 1380 (91.20)   
87 - **[CGCCCC]** 1507 (85.80)   
87 - **.CGCGCC.** 1501 (94.60)   
87 - **[CGCCCC]** 1499 (85.80)   
87 - **.CGCGGC.** 1486 (94.52)   
87 - **[CGCCCC]** 1477 (85.80)   
87 - **[CGCCCC]** 1472 (85.80)   
87 - **[CGCCCC]** 1467 (85.80)   
87 - **.CGCGGC.** 1434 (94.52)   
87 - **[CGCCGC]** 1337 (85.72)   
87 - **[CGCCGC]** 1334 (85.72)   
87 - **[CGCGTG]** 1320 (86.67)   
87 - **[CGCCGC]** 1264 (85.72)   
87 - **[CGCCGC]** 1245 (85.72)   
87 - **.CGCGCC.** 1242 (94.60)   
87 - **[CGCCGC]** 1240 (85.72)   
87 - **.CGCGGC.** 1097 (94.52)   
87 - **[CGCCGC]** 954 (85.72)   
87 - **[CGCCCC]** 932 (85.80)   
87 - **.CGCGCC.** 843 (94.60)   
87 - **[CGCCGC]** 841 (85.72)   
87 - **[CGCCCC]** 826 (85.80)   
87 - **[CGCTTC]** 780 (86.32)   
87 - **[CGCCCC]** 773 (85.80)   
87 - **.CGCGGC.** 768 (94.52)   
87 - **.CGCCTC.** 752 (91.20)   
87 - **[CGCCCC]** 706 (85.80)   
87 - **.CGCGCC.** 639 (94.60)   
87 - **.CGCGTC.** 586 (100.00)   
87 - **.CGCGCC.** 313 (94.60)   
87 - **[CGCCGC]** 311 (85.72)   
87 - **[CGCTTC]** 298 (86.32)   
87 - **[CGCGTG]** 266 (86.67)   
87 - **.CGCGTC.** 133 (100.00)   
88 + **[CGCCCC]** 211 (85.80)   
88 + **[CGCCGC]** 222 (85.72)   
88 + **[CGCCCC]** 225 (85.80)   
88 + **[CGCCCC]** 237 (85.80)   
88 + **[CGCCCC]** 304 (85.80)   
88 + **[CGCCCC]** 343 (85.80)   
88 + **[CGCCCC]** 365 (85.80)   
88 + **[CGCCGC]** 503 (85.72)   
88 + **.CGCGGC.** 707 (94.52)   
88 + **[CGCCGC]** 785 (85.72)   
88 + **[CGCCGC]** 788 (85.72)   
88 + **[CGCCGC]** 791 (85.72)   
88 + **.CGCGGC.** 849 (94.52)   
88 + **[CGCCGC]** 960 (85.72)   
88 + **[CGCCCC]** 980 (85.80)   
88 + **.CGCCTC.** 1031 (91.20)   
88 + **[CGCCGC]** 1096 (85.72)   
88 + **.CGCGGC.** 1099 (94.52)   
88 + **[CGCCCC]** 1108 (85.80)   
88 + **[CGCCCC]** 1123 (85.80)   
88 + **.CGCCTC.** 1175 (91.20)   
88 + **.CGCGTC.** 1206 (100.00)   
88 + **[CGGGTC]** 1303 (87.94)   
88 + **[CGCCCC]** 1333 (85.80)   
88 + **.CGCGGC.** 1380 (94.52)   
88 + **.CGCGGC.** 1395 (94.52)   
88 + **[GGCGTC]** 1474 (87.08)   
88 - **[CGCCGC]** 1396 (85.72)   
88 - **[GGCGTC]** 1331 (87.08)   
88 - **[CCCGTC]** 1287 (87.14)   
88 - **.CGCCTC.** 1276 (91.20)   
88 - **.CGCGGC.** 1097 (94.52)   
88 - **[GGCGTC]** 1094 (87.08)   
88 - **[CGCCGC]** 1023 (85.72)   
88 - **[CGCCCC]** 915 (85.80)   
88 - **[CGCCGC]** 906 (85.72)   
88 - **[CGCCGC]** 903 (85.72)   
88 - **[CGCCGC]** 900 (85.72)   
88 - **[CGCCGC]** 897 (85.72)   
88 - **.CGCCTC.** 861 (91.20)   
88 - **[CGCCGC]** 850 (85.72)   
88 - **.CGCGCC.** 755 (94.60)   
88 - **[CGCCCC]** 753 (85.80)   
88 - **[CGCGAC]** 714 (87.27)   
88 - **.CGCGCC.** 705 (94.60)   
88 - **[CGCCCC]** 703 (85.80)   
88 - **.CGCGGC.** 607 (94.52)   
88 - **.CGCGGC.** 504 (94.52)   
88 - **[CGCGAC]** 488 (87.27)   
88 - **[CGCCGC]** 479 (85.72)   
88 - **.CGCGCC.** 445 (94.60)   
88 - **[CGCCGC]** 443 (85.72)   
88 - **.CGCGCC.** 419 (94.60)   
88 - **[CGCCCC]** 417 (85.80)   
88 - **[CGCCCC]** 412 (85.80)   
88 - **[CGCCCC]** 352 (85.80)   
89 + **.CGCGGC.** 47 (94.52)   
89 + **[CGGGTC]** 53 (87.94)   
89 + **[CGCCCC]** 74 (85.80)   
89 + **.CGCGCC.** 100 (94.60)   
89 + **[CGCCGC]** 102 (85.72)   
89 + **[CGCCGC]** 105 (85.72)   
89 + **[CGCCCC]** 108 (85.80)   
89 + **[CGCCCC]** 145 (85.80)   
89 + **[CCCGTC]** 148 (87.14)   
89 + **.CGCGCC.** 153 (94.60)   
89 + **[GGCGTC]** 187 (87.08)   
89 + **[CGCCGC]** 268 (85.72)   
89 + **[CGCGAC]** 271 (87.27)   
89 + **[CGCCGC]** 285 (85.72)   
89 + **[CGCCGC]** 288 (85.72)   
89 + **.CGCGCC.** 335 (94.60)   
89 + **[CGCCCC]** 337 (85.80)   
89 + **[CGCCGC]** 350 (85.72)   
89 + **.CGCCTC.** 353 (91.20)   
89 + **[CGCCCC]** 359 (85.80)   
89 + **.CGCGCC.** 364 (94.60)   
89 + **[CGCCCC]** 366 (85.80)   
89 + **[CGCCGC]** 380 (85.72)   
89 + **[CGCCGC]** 383 (85.72)   
89 + **[CGCCGC]** 386 (85.72)   
89 + **[CGCCGC]** 389 (85.72)   
89 + **[CGCCGC]** 392 (85.72)   
89 + **[CGCCGC]** 408 (85.72)   
89 + **[CGCCGC]** 411 (85.72)   
89 + **[CGCCGC]** 414 (85.72)   
89 + **[CGCCGC]** 417 (85.72)   
89 + **[CGGGTC]** 422 (87.94)   
89 + **[CGCCGC]** 434 (85.72)   
89 + **.CGCGCC.** 437 (94.60)   
89 + **[CGCCGC]** 466 (85.72)   
89 + **[CGCCGC]** 469 (85.72)   
89 + **.CGCGTC.** 480 (100.00)   
89 + **.CGCGCC.** 495 (94.60)   
89 + **[CGCCGC]** 497 (85.72)   
89 + **[CGCCGC]** 611 (85.72)   
89 + **[CGCCGC]** 614 (85.72)   
89 + **[CGCCGC]** 663 (85.72)   
89 + **.CGCGCC.** 666 (94.60)   
89 + **[CGCCCC]** 668 (85.80)   
89 + **.CGCGCC.** 675 (94.60)   
89 + **[CGCCCC]** 677 (85.80)   
89 + **.CGCGGC.** 743 (94.52)   
89 + **.CGCGCC.** 789 (94.60)   
89 + **[CGCCGC]** 791 (85.72)   
89 + **.CGCGGC.** 843 (94.52)   
89 + **[CGGGTC]** 893 (87.94)   
89 + **[CGCCCC]** 952 (85.80)   
89 + **[GGCGTC]** 958 (87.08)   
89 + **.CGCGCC.** 976 (94.60)   
89 + **.CGCGCC.** 1023 (94.60)   
89 + **[CGCCGC]** 1125 (85.72)   
89 + **.CGCCTC.** 1150 (91.20)   
89 + **.CGCCTC.** 1266 (91.20)   
89 + **[CGCCGC]** 1309 (85.72)   
89 + **.CGCGCC.** 1325 (94.60)   
89 + **[CGCGAC]** 1467 (87.27)   
89 - **[CGCTTC]** 1506 (86.32)   
89 - **.CGCGTC.** 1465 (100.00)   
89 - **.CGCGGC.** 1451 (94.52)   
89 - **.CGCCTC.** 1439 (91.20)   
89 - **[CGCCCC]** 1341 (85.80)   
89 - **.CGCGGC.** 1310 (94.52)   
89 - **[CGCCCC]** 1184 (85.80)   
89 - **.CGCGGC.** 1126 (94.52)   
89 - **.CGCGGC.** 1021 (94.52)   
89 - **[CGCGTG]** 964 (86.67)   
89 - **.CGCGCC.** 937 (94.60)   
89 - **.CGCGCC.** 787 (94.60)   
89 - **[CGCCGC]** 785 (85.72)   
89 - **.CGCCTC.** 782 (91.20)   
89 - **[CGCCGC]** 744 (85.72)   
89 - **.CGCGCC.** 741 (94.60)   
89 - **[CGCCCC]** 739 (85.80)   
89 - **.CGCGCC.** 721 (94.60)   
89 - **[CGCCCC]** 719 (85.80)   
89 - **[CGCCCC]** 712 (85.80)   
89 - **.CGCGGC.** 664 (94.52)   
89 - **.CGCGTC.** 600 (100.00)   
89 - **.CGCCTC.** 576 (91.20)   
89 - **.CGCGGC.** 493 (94.52)   
89 - **.CGCGGC.** 478 (94.52)   
89 - **.CGCGGC.** 435 (94.52)   
89 - **.CGCGGC.** 418 (94.52)   
89 - **[CGCCGC]** 316 (85.72)   
89 - **.CGCGGC.** 269 (94.52)   
89 - **.CGCGGC.** 260 (94.52)   
89 - **[CGCCGC]** 185 (85.72)   
89 - **[CGCCCC]** 182 (85.80)   
89 - **.CGCCTC.** 176 (91.20)   
89 - **[CGCGAC]** 151 (87.27)   
89 - **[GGCGTC]** 72 (87.08)   
89 - **[CGCGTG]** 38 (86.67)   
89 - **[CGCCGC]** 27 (85.72)   
89 - **[CGCCCC]** 20 (85.80)   
89 - **[CGCCCC]** 15 (85.80)   
90 + **[CGCCGC]** 41 (85.72)   
90 + **[CGCCCC]** 298 (85.80)   
90 + **.CGCGTC.** 362 (100.00)   
90 + **[CGCCCC]** 461 (85.80)   
90 + **.CGCGCC.** 480 (94.60)   
90 + **.CGCGCC.** 514 (94.60)   
90 + **[CGCCCC]** 516 (85.80)   
90 + **[CGCTTC]** 597 (86.32)   
90 + **.CGCGCC.** 654 (94.60)   
90 + **.CGCGCC.** 715 (94.60)   
90 + **[CGCCGC]** 717 (85.72)   
90 + **.CGCGGC.** 720 (94.52)   
90 + **.CGCGGC.** 777 (94.52)   
90 + **[CGGGTC]** 838 (87.94)   
90 + **.CGCGGC.** 850 (94.52)   
90 + **[CGCCCC]** 859 (85.80)   
90 + **.CGCGGC.** 993 (94.52)   
90 + **[CGCCGC]** 1018 (85.72)   
90 + **[CGCCCC]** 1045 (85.80)   
90 + **[GGCGTC]** 1196 (87.08)   
90 + **.CGCGTC.** 1314 (100.00)   
90 + **.CGCGGC.** 1330 (94.52)   
90 + **[CGCCCC]** 1339 (85.80)   
90 - **[CGCCGC]** 1257 (85.72)   
90 - **[CGCCGC]** 1254 (85.72)   
90 - **[CGCCGC]** 1251 (85.72)   
90 - **[CGCCGC]** 1194 (85.72)   
90 - **[CGCCGC]** 1191 (85.72)   
90 - **[CGCCCC]** 1188 (85.80)   
90 - **[CGCCGC]** 1117 (85.72)   
90 - **[CGCCGC]** 1093 (85.72)   
90 - **[CGCCGC]** 1090 (85.72)   
90 - **[CGCCGC]** 1087 (85.72)   
90 - **[CGCCGC]** 1084 (85.72)   
90 - **.CGCCTC.** 1081 (91.20)   
90 - **.CGCGGC.** 1019 (94.52)   
90 - **[CGGGTC]** 1014 (87.94)   
90 - **[CGCCCC]** 983 (85.80)   
90 - **.CGCCTC.** 952 (91.20)   
90 - **[CGCCGC]** 851 (85.72)   
90 - **.CGCGGC.** 848 (94.52)   
90 - **[CGCCCC]** 788 (85.80)   
90 - **[CGCCGC]** 781 (85.72)   
90 - **[CGCCGC]** 778 (85.72)   
90 - **.CGCGGC.** 775 (94.52)   
90 - **.CGCGGC.** 718 (94.52)   
90 - **[CGCGTG]** 713 (86.67)   
90 - **.CGCGGC.** 587 (94.52)   
90 - **.CGCGCC.** 557 (94.60)   
90 - **[CGGGTC]** 541 (87.94)   
90 - **.CGCGCC.** 510 (94.60)   
90 - **.CGCGCC.** 360 (94.60)   
90 - **[CGGGTC]** 68 (87.94)   
92 + **.CGCCTC.** 173 (91.20)   
92 + **.CGCGGC.** 1228 (94.52)   
92 + **[CGCGAC]** 1459 (87.27)   
92 - **[CCCGTC]** 1362 (87.14)   
92 - **[CGCCGC]** 1229 (85.72)   
92 - **.CGCCTC.** 1188 (91.20)   
92 - **.CGCGGC.** 1056 (94.52)   
93 + **[GGCGTC]** 487 (87.08)   
93 + **[CGCCGC]** 573 (85.72)   
93 + **[CGCCGC]** 609 (85.72)   
93 + **[CGCCGC]** 681 (85.72)   
93 + **.CGCGTC.** 985 (100.00)   
93 + **.CGCGCC.** 1003 (94.60)   
93 + **.CGCGGC.** 1037 (94.52)   
93 + **[CGCCGC]** 1078 (85.72)   
93 + **.CGCGCC.** 1198 (94.60)   
93 + **.CGCGGC.** 1242 (94.52)   
93 + **.CGCGGC.** 1282 (94.52)   
93 + **.CGCGTC.** 1488 (100.00)   
93 - **[CGCCCC]** 1478 (85.80)   
93 - **[CGCTTC]** 1460 (86.32)   
93 - **[CGCCGC]** 1452 (85.72)   
93 - **[CGCCGC]** 1449 (85.72)   
93 - **[CGCCGC]** 1446 (85.72)   
93 - **[CGCCGC]** 1428 (85.72)   
93 - **[CGCCGC]** 1383 (85.72)   
93 - **[CGCCGC]** 1364 (85.72)   
93 - **[CGCCCC]** 1361 (85.80)   
93 - **[CCCGTC]** 1358 (87.14)   
93 - **[CGCCGC]** 1314 (85.72)   
93 - **[CGCCGC]** 1289 (85.72)   
93 - **[CGCCGC]** 1249 (85.72)   
93 - **.CGCGCC.** 1238 (94.60)   
93 - **[CGCGTG]** 1192 (86.67)   
93 - **.CGCCTC.** 1170 (91.20)   
93 - **.CGCCTC.** 1146 (91.20)   
93 - **.CGCCTC.** 1064 (91.20)   
93 - **[CGCCGC]** 1041 (85.72)   
93 - **[CGCCGC]** 1038 (85.72)   
93 - **.CGCGTC.** 1001 (100.00)   
93 - **.CGCGCC.** 975 (94.60)   
93 - **.CGCGTC.** 963 (100.00)   
93 - **[CGCCCC]** 740 (85.80)   
93 - **[CGCCGC]** 466 (85.72)   
93 - **[CGCCGC]** 414 (85.72)   
93 - **[CGCCGC]** 374 (85.72)   
94 + **[CGCTTC]** 879 (86.32)   
94 - **[CGCCCC]** 1491 (85.80)   
96 + **.CGCGTC.** 740 (100.00)   
96 + **.CGCGGC.** 884 (94.52)   
96 + **.CGCCTC.** 956 (91.20)   
96 + **.CGCCTC.** 1041 (91.20)   
96 + **[CGCCCC]** 1323 (85.80)   
96 + **[CGGGTC]** 1347 (87.94)   
96 + **.CGCGTC.** 1354 (100.00)   
96 + **.CGCGTC.** 1359 (100.00)   
96 + **[CGCCGC]** 1364 (85.72)   
96 + **.CGCGCC.** 1422 (94.60)   
96 - **.CGCCTC.** 1512 (91.20)   
96 - **.CGCGTC.** 1393 (100.00)   
96 - **[CGCGAC]** 1357 (87.27)   
96 - **[CGCGAC]** 1350 (87.27)   
96 - **[CGCTTC]** 1086 (86.32)   
96 - **[CGCTTC]** 952 (86.32)   
96 - **.CGCGGC.** 848 (94.52)   
96 - **[CGCCGC]** 458 (85.72)   
97 + **[CGCCGC]** 851 (85.72)   
97 + **[CGCCGC]** 854 (85.72)   
97 + **[CGCCCC]** 869 (85.80)   
97 + **.CGCGGC.** 877 (94.52)   
97 + **.CGCGGC.** 882 (94.52)   
97 + **.CGCGCC.** 1047 (94.60)   
97 + **.CGCCTC.** 1092 (91.20)   
97 + **[GGCGTC]** 1441 (87.08)   
97 - **[CGCTTC]** 1511 (86.32)   
97 - **[CCCGTC]** 1407 (87.14)   
97 - **.CGCGTC.** 1367 (100.00)   
97 - **[CCCGTC]** 1361 (87.14)   
97 - **[CGCCGC]** 1231 (85.72)   
97 - **.CGCGCC.** 1223 (94.60)   
97 - **[CGCCCC]** 1180 (85.80)   
97 - **[CGGGTC]** 1060 (87.94)   
97 - **.CGCCTC.** 1031 (91.20)   
97 - **.CGCCTC.** 963 (91.20)   
97 - **[CGCCCC]** 945 (85.80)   
97 - **[CGCCCC]** 922 (85.80)   
97 - **.CGCCTC.** 902 (91.20)   
97 - **.CGCGCC.** 880 (94.60)   
97 - **[CGCCGC]** 878 (85.72)   
98 + **[CGCCGC]** 756 (85.72)   
98 + **.CGCGGC.** 1001 (94.52)   
98 + **.CGCGGC.** 1086 (94.52)   
98 + **.CGCGGC.** 1137 (94.52)   
98 + **[CCCGTC]** 1148 (87.14)   
98 + **.CGCGGC.** 1325 (94.52)   
98 + **[CGCCCC]** 1491 (85.80)   
98 - **[GGCGTC]** 1489 (87.08)   
98 - **[CGCCGC]** 1452 (85.72)   
98 - **[CGCCGC]** 1449 (85.72)   
98 - **[CGCCGC]** 1346 (85.72)   
98 - **[CGCCGC]** 1343 (85.72)   
98 - **[CGCCCC]** 1334 (85.80)   
98 - **[CGCCGC]** 1262 (85.72)   
98 - **[CCCGTC]** 1234 (87.14)   
98 - **.CGCGCC.** 1227 (94.60)   
98 - **[CGCCGC]** 1225 (85.72)   
98 - **[CGCCCC]** 1222 (85.80)   
98 - **.CGCCTC.** 1186 (91.20)   
98 - **[CGCCGC]** 1138 (85.72)   
98 - **.CGCGGC.** 1119 (94.52)   
98 - **.CGCGTC.** 1098 (100.00)   
98 - **[CGCCGC]** 1087 (85.72)   
98 - **[GGCGTC]** 1065 (87.08)   
98 - **.CGCGCC.** 1038 (94.60)   
98 - **[CGCCCC]** 1036 (85.80)   
98 - **.CGCGCC.** 1031 (94.60)   
98 - **[CGCCGC]** 1002 (85.72)   
98 - **.CGCGGC.** 999 (94.52)   
98 - **[CGCCGC]** 988 (85.72)   
98 - **[CGCCGC]** 985 (85.72)   
98 - **[CGCCGC]** 982 (85.72)   
98 - **[CGCCGC]** 979 (85.72)   
98 - **[CGCCGC]** 976 (85.72)   
98 - **[CGCCGC]** 796 (85.72)   
98 - **[CGCCGC]** 793 (85.72)   
98 - **[CGCTTC]** 714 (86.32)   
98 - **[CCCGTC]** 702 (87.14)   
99 + **.CGCGCC.** 17 (94.60)   
99 + **[CGCCCC]** 980 (85.80)   
99 + **[CGGGTC]** 1071 (87.94)   
99 + **[CGCCCC]** 1461 (85.80)   
99 - **[CGGGTC]** 1507 (87.94)   
99 - **[CGCCCC]** 1340 (85.80)   
99 - **.CGCCTC.** 1279 (91.20)   
99 - **[CGCCCC]** 1029 (85.80)   
99 - **[GGCGTC]** 978 (87.08)   
100 + **[GGCGTC]** 291 (87.08)   
100 + **[CGGGTC]** 1099 (87.94)   
100 + **.CGCGCC.** 1104 (94.60)   
100 + **.CGCGGC.** 1140 (94.52)   
100 + **.CGCCTC.** 1167 (91.20)   
100 + **[CGCCCC]** 1258 (85.80)   
100 + **.CGCGGC.** 1500 (94.52)   
100 - **[CGCCGC]** 1501 (85.72)   
100 - **.CGCCTC.** 1407 (91.20)   
100 - **.CGCGGC.** 1305 (94.52)   
100 - **[GGCGTC]** 1256 (87.08)   
100 - **[CGCCGC]** 1206 (85.72)   
100 - **[CGCGAC]** 1102 (87.27)   
100 - **[GGCGTC]** 1015 (87.08)   
100 - **[CGCCCC]** 920 (85.80)   
100 - **[CCCGTC]** 739 (87.14)   
100 - **.CGCCTC.** 361 (91.20)   
102 - **[CGCTTC]** 1228 (86.32)   
102 - **[CGCCCC]** 934 (85.80)   
103 + **.CGCCTC.** 879 (91.20)   
103 + **[CGCCCC]** 1128 (85.80)   
103 + **[CGGGTC]** 1179 (87.94)   
103 + **.CGCCTC.** 1396 (91.20)   
103 - **[CGCCCC]** 1345 (85.80)   
103 - **[CGCCGC]** 1230 (85.72)   
103 - **[CGCGAC]** 1182 (87.27)   
103 - **[CGCCCC]** 868 (85.80)   
103 - **[CGCCCC]** 735 (85.80)   
103 - **.CGCCTC.** 689 (91.20)   
104 + **.CGCCTC.** 675 (91.20)   
104 + **[CGCCCC]** 719 (85.80)   
104 + **[CCCGTC]** 722 (87.14)   
104 - **[CGGGTC]** 1207 (87.94)   
104 - **.CGCGTC.** 1140 (100.00)   
104 - **[CGCCCC]** 998 (85.80)   
104 - **[CGCCCC]** 940 (85.80)   
105 + **.CGCGGC.** 1039 (94.52)   
105 + **.CGCGCC.** 1248 (94.60)   
105 + **[GGCGTC]** 1255 (87.08)   
105 + **[CGCCCC]** 1264 (85.80)   
105 - **.CGCCTC.** 1509 (91.20)   
105 - **.CGCCTC.** 1334 (91.20)   
105 - **.CGCCTC.** 1307 (91.20)   
105 - **.CGCGCC.** 1142 (94.60)   
105 - **.CGCGCC.** 1022 (94.60)   
105 - **[CGCCCC]** 999 (85.80)   
106 + **.CGCCTC.** 787 (91.20)   
106 + **.CGCGGC.** 870 (94.52)   
106 + **[CGCTTC]** 915 (86.32)   
106 + **[CGCCCC]** 922 (85.80)   
106 + **[CGCCCC]** 948 (85.80)   
106 + **[CGCCCC]** 953 (85.80)   
106 + **.CGCCTC.** 1018 (91.20)   
106 + **[CGCCGC]** 1062 (85.72)   
106 + **.CGCCTC.** 1074 (91.20)   
106 + **.CGCGGC.** 1160 (94.52)   
106 + **[GGCGTC]** 1163 (87.08)   
106 - **[CGCCGC]** 1161 (85.72)   
106 - **[CGCGAC]** 1158 (87.27)   
106 - **[CGCCGC]** 871 (85.72)   
108 + **.CGCGGC.** 101 (94.52)   
108 + **[CCCGTC]** 278 (87.14)   
108 + **.CGCCTC.** 388 (91.20)   
108 + **.CGCGTC.** 429 (100.00)   
108 + **[CCCGTC]** 636 (87.14)   
108 + **[GGCGTC]** 665 (87.08)   
108 + **[CGCGTG]** 742 (86.67)   
108 + **[CCCGTC]** 755 (87.14)   
108 + **[CGCGAC]** 767 (87.27)   
108 + **[CGCCGC]** 844 (85.72)   
108 + **[CCCGTC]** 937 (87.14)   
108 + **[CGCGTG]** 1067 (86.67)   
108 + **.CGCGGC.** 1114 (94.52)   
108 + **[CGCCCC]** 1127 (85.80)   
108 + **.CGCGTC.** 1203 (100.00)   
108 + **.CGCGCC.** 1218 (94.60)   
108 + **[CGCCCC]** 1258 (85.80)   
108 + **[CGCGTG]** 1387 (86.67)   
108 + **[CGCCGC]** 1498 (85.72)   
108 - **.CGCGTC.** 1385 (100.00)   
108 - **.CGCGCC.** 1300 (94.60)   
108 - **.CGCCTC.** 1298 (91.20)   
108 - **[CGCGAC]** 1243 (87.27)   
108 - **[CGCCCC]** 1165 (85.80)   
108 - **[CGCCCC]** 1157 (85.80)   
108 - **[CGCCCC]** 1123 (85.80)   
108 - **[CGCCCC]** 959 (85.80)   
108 - **[CGCCCC]** 954 (85.80)   
108 - **.CGCGTC.** 923 (100.00)   
108 - **[CGCCCC]** 880 (85.80)   
108 - **.CGCCTC.** 874 (91.20)   
108 - **.CGCGTC.** 818 (100.00)   
108 - **[CCCGTC]** 591 (87.14)   
108 - **[CGCCGC]** 507 (85.72)   
108 - **[CGCTTC]** 454 (86.32)   
108 - **[CGGGTC]** 425 (87.94)   
108 - **[CGCGAC]** 397 (87.27)   
108 - **.CGCCTC.** 312 (91.20)   
108 - **.CGCGCC.** 99 (94.60)   
108 - **[GGCGTC]** 6 (87.08)   
109 + **[CGCGAC]** 293 (87.27)   
109 + **[CGCCGC]** 336 (85.72)   
109 + **.CGCGGC.** 339 (94.52)   
109 + **[CGCCCC]** 385 (85.80)   
109 + **[CGGGTC]** 448 (87.94)   
109 + **.CGCGCC.** 455 (94.60)   
109 + **.CGCCTC.** 519 (91.20)   
109 + **.CGCGCC.** 659 (94.60)   
109 + **[CGCCCC]** 661 (85.80)   
109 + **.CGCCTC.** 702 (91.20)   
109 + **[CGGGTC]** 831 (87.94)   
109 + **.CGCGCC.** 945 (94.60)   
109 + **[CCCGTC]** 1003 (87.14)   
109 + **.CGCGTC.** 1032 (100.00)   
109 + **.CGCGCC.** 1043 (94.60)   
109 + **[GGCGTC]** 1090 (87.08)   
109 + **.CGCGCC.** 1194 (94.60)   
109 + **.CGCGCC.** 1233 (94.60)   
109 + **.CGCGTC.** 1331 (100.00)   
109 + **.CGCGGC.** 1359 (94.52)   
109 + **.CGCGGC.** 1391 (94.52)   
109 + **.CGCGGC.** 1396 (94.52)   
109 - **[CGCCGC]** 1492 (85.72)   
109 - **[CGCCGC]** 1475 (85.72)   
109 - **[CGCCGC]** 1472 (85.72)   
109 - **[CGCCCC]** 1469 (85.80)   
109 - **[CGCCGC]** 1400 (85.72)   
109 - **[CGCCGC]** 1397 (85.72)   
109 - **.CGCGCC.** 1394 (94.60)   
109 - **[CGCCGC]** 1392 (85.72)   
109 - **.CGCGCC.** 1389 (94.60)   
109 - **[CGCCCC]** 1387 (85.80)   
109 - **[CGCCGC]** 1360 (85.72)   
109 - **.CGCGCC.** 1355 (94.60)   
109 - **.CGCGCC.** 1320 (94.60)   
109 - **[CGCCCC]** 1318 (85.80)   
109 - **.CGCGCC.** 1313 (94.60)   
109 - **.CGCGTC.** 1231 (100.00)   
109 - **[GGCGTC]** 1185 (87.08)   
109 - **[CGCCGC]** 1088 (85.72)   
109 - **[CGCCGC]** 1085 (85.72)   
109 - **[CGCCGC]** 1082 (85.72)   
109 - **[CGCCGC]** 1079 (85.72)   
109 - **[CGCCGC]** 1076 (85.72)   
109 - **[CCCGTC]** 1064 (87.14)   
109 - **.CGCGGC.** 943 (94.52)   
109 - **[CGCCCC]** 906 (85.80)   
109 - **.CGCGGC.** 865 (94.52)   
109 - **.CGCGCC.** 825 (94.60)   
109 - **[CGCCCC]** 823 (85.80)   
109 - **.CGCGTC.** 814 (100.00)   
109 - **[CGCGTG]** 453 (86.67)   
109 - **.CGCGGC.** 337 (94.52)   
109 - **[CGCGAC]** 291 (87.27)   
110 + **[CGCCGC]** 931 (85.72)   
110 - **.CGCGCC.** 949 (94.60)   
110 - **.CGCCTC.** 947 (91.20)   
110 - **.CGCGGC.** 859 (94.52)   
111 + **[CGCCGC]** 1251 (85.72)   
111 + **[CGCCGC]** 1258 (85.72)   
112 + **[CGCCCC]** 976 (85.80)   
114 + **.CGCCTC.** 193 (91.20)   
114 + **[CGGGTC]** 256 (87.94)   
114 + **[GGCGTC]** 676 (87.08)   
114 + **.CGCGGC.** 742 (94.52)   
114 + **[CGCCGC]** 755 (85.72)   
114 + **[CCCGTC]** 778 (87.14)   
114 + **.CGCCTC.** 975 (91.20)   
114 + **.CGCGGC.** 1004 (94.52)   
114 + **.CGCGGC.** 1009 (94.52)   
114 + **[CGCCCC]** 1273 (85.80)   
114 + **.CGCGGC.** 1308 (94.52)   
114 - **[CGCCGC]** 1309 (85.72)   
114 - **[GGCGTC]** 1271 (87.08)   
114 - **[CGCCGC]** 1041 (85.72)   
114 - **.CGCGTC.** 1019 (100.00)   
114 - **.CGCGCC.** 1007 (94.60)   
114 - **[CGCCGC]** 1005 (85.72)   
114 - **.CGCGGC.** 1000 (94.52)   
114 - **[CCCGTC]** 952 (87.14)   
114 - **.CGCGGC.** 350 (94.52)   
114 - **[CGCCCC]** 336 (85.80)   
115 + **[CGCCGC]** 309 (85.72)   
115 + **[CGCCGC]** 357 (85.72)   
115 + **.CGCGGC.** 413 (94.52)   
115 + **[GGCGTC]** 416 (87.08)   
115 + **.CGCCTC.** 461 (91.20)   
115 + **.CGCGTC.** 466 (100.00)   
115 + **.CGCCTC.** 495 (91.20)   
115 + **.CGCCTC.** 560 (91.20)   
115 + **.CGCGCC.** 590 (94.60)   
115 + **.CGCCTC.** 592 (91.20)   
115 + **.CGCGCC.** 689 (94.60)   
115 + **[CGCCCC]** 836 (85.80)   
115 + **[CGCGAC]** 867 (87.27)   
115 + **[CGCCGC]** 1011 (85.72)   
115 + **.CGCCTC.** 1014 (91.20)   
115 + **.CGCGCC.** 1023 (94.60)   
115 + **[CGCGAC]** 1041 (87.27)   
115 - **[CGGGTC]** 1452 (87.94)   
115 - **[CGCGAC]** 1236 (87.27)   
115 - **[CGGGTC]** 1176 (87.94)   
115 - **[CGCCGC]** 994 (85.72)   
115 - **[CGCCGC]** 991 (85.72)   
115 - **[CGCCGC]** 988 (85.72)   
115 - **[CGCCGC]** 985 (85.72)   
115 - **[CGCCGC]** 907 (85.72)   
115 - **.CGCGTC.** 870 (100.00)   
115 - **.CGCCTC.** 795 (91.20)   
115 - **[GGCGTC]** 558 (87.08)   
115 - **[CGCCCC]** 457 (85.80)   
115 - **[CGCCGC]** 414 (85.72)   
115 - **.CGCGGC.** 310 (94.52)   
115 - **.CGCGCC.** 301 (94.60)   
115 - **.CGCGCC.** 281 (94.60)   
115 - **[CGCCGC]** 279 (85.72)   
115 - **.CGCGCC.** 224 (94.60)   
115 - **[CGCTTC]** 178 (86.32)   
116 + **[CGCCGC]** 1004 (85.72)   
116 + **.CGCGGC.** 1201 (94.52)   
116 - **[CGCCGC]** 1047 (85.72)   
116 - **[CGCCGC]** 1000 (85.72)   
116 - **[GGCGTC]** 993 (87.08)   
116 - **.CGCGTC.** 961 (100.00)   
117 + **[CGCTTC]** 223 (86.32)   
117 + **.CGCCTC.** 458 (91.20)   
117 + **[CGCTTC]** 489 (86.32)   
117 + **[CGCGAC]** 640 (87.27)   
117 + **.CGCGCC.** 669 (94.60)   
117 + **[CGCCCC]** 671 (85.80)   
117 + **[CGCCGC]** 698 (85.72)   
117 + **[CGCCGC]** 791 (85.72)   
117 + **[CGCCCC]** 897 (85.80)   
117 + **[CCCGTC]** 901 (87.14)   
117 + **[CGCCCC]** 917 (85.80)   
117 + **.CGCGCC.** 940 (94.60)   
117 + **.CGCGGC.** 978 (94.52)   
117 + **[CCCGTC]** 991 (87.14)   
117 + **.CGCGCC.** 1026 (94.60)   
117 + **.CGCGGC.** 1056 (94.52)   
117 - **[CCCGTC]** 956 (87.14)   
117 - **.CGCGGC.** 926 (94.52)   
117 - **.CGCGGC.** 792 (94.52)   
117 - **.CGCGCC.** 667 (94.60)   
117 - **.CGCGCC.** 656 (94.60)   
117 - **.CGCGCC.** 595 (94.60)   
117 - **.CGCCTC.** 593 (91.20)   
117 - **.CGCCTC.** 197 (91.20)   
118 + **[CGCTTC]** 125 (86.32)   
118 + **.CGCCTC.** 407 (91.20)   
118 + **[CGCGAC]** 589 (87.27)   
118 + **[CGCGAC]** 617 (87.27)   
118 + **[CGCCGC]** 676 (85.72)   
118 + **[CGCCGC]** 767 (85.72)   
118 + **[CGCCCC]** 872 (85.80)   
118 + **[CCCGTC]** 876 (87.14)   
118 + **[CGCCCC]** 893 (85.80)   
118 + **[CGCCCC]** 900 (85.80)   
118 + **[CGCCCC]** 907 (85.80)   
118 + **.CGCGCC.** 930 (94.60)   
118 + **[CCCGTC]** 981 (87.14)   
118 + **.CGCGCC.** 1016 (94.60)   
118 + **.CGCGGC.** 1045 (94.52)   
118 - **[CCCGTC]** 946 (87.14)   
118 - **.CGCGGC.** 768 (94.52)   
118 - **.CGCGCC.** 632 (94.60)   
118 - **.CGCGCC.** 604 (94.60)   
118 - **.CGCGCC.** 543 (94.60)   
118 - **.CGCCTC.** 541 (91.20)   
118 - **.CGCGGC.** 433 (94.52)   
118 - **.CGCCTC.** 99 (91.20)   
119 + **[CGCTTC]** 123 (86.32)   
119 + **[CGCGAC]** 587 (87.27)   
119 + **[CGCGAC]** 615 (87.27)   
119 + **[CGCCGC]** 674 (85.72)   
119 + **[CGCCGC]** 765 (85.72)   
119 + **[CGCCCC]** 870 (85.80)   
119 + **[CCCGTC]** 874 (87.14)   
119 + **[CGCCCC]** 898 (85.80)   
119 + **.CGCGCC.** 921 (94.60)   
119 + **.CGCGGC.** 958 (94.52)   
119 + **.CGCGCC.** 1007 (94.60)   
119 + **.CGCGGC.** 1036 (94.52)   
119 - **[CCCGTC]** 937 (87.14)   
119 - **.CGCGGC.** 766 (94.52)   
119 - **.CGCGCC.** 641 (94.60)   
119 - **.CGCGCC.** 630 (94.60)   
119 - **.CGCGCC.** 613 (94.60)   
119 - **.CGCGCC.** 602 (94.60)   
119 - **.CGCGCC.** 541 (94.60)   
119 - **.CGCCTC.** 539 (91.20)   
119 - **.CGCGGC.** 431 (94.52)   
119 - **.CGCCTC.** 97 (91.20)   
120 + **[CGCTTC]** 583 (86.32)   
120 + **.CGCCTC.** 864 (91.20)   
120 + **[CGCGAC]** 1046 (87.27)   
120 + **[CGCGAC]** 1075 (87.27)   
120 + **.CGCGCC.** 1103 (94.60)   
120 + **[CGCCCC]** 1105 (85.80)   
120 + **[CGCCGC]** 1225 (85.72)   
120 + **[CGCCCC]** 1330 (85.80)   
120 + **[CCCGTC]** 1334 (87.14)   
120 + **[CGCCCC]** 1350 (85.80)   
120 + **[CGCCCC]** 1357 (85.80)   
120 + **[CGCCCC]** 1364 (85.80)   
120 + **.CGCGCC.** 1388 (94.60)   
120 + **.CGCGGC.** 1425 (94.52)   
120 + **[CCCGTC]** 1438 (87.14)   
120 + **.CGCGCC.** 1474 (94.60)   
120 + **.CGCGGC.** 1503 (94.52)   
120 - **[CCCGTC]** 1404 (87.14)   
120 - **.CGCGGC.** 1226 (94.52)   
120 - **.CGCGCC.** 1101 (94.60)   
120 - **.CGCGCC.** 1090 (94.60)   
120 - **.CGCGCC.** 1062 (94.60)   
120 - **.CGCGCC.** 1001 (94.60)   
120 - **.CGCCTC.** 999 (91.20)   
120 - **.CGCCTC.** 556 (91.20)   
121 + **[CCCGTC]** 902 (87.14)   
121 + **[CGCGAC]** 1282 (87.27)   
121 - **[CCCGTC]** 965 (87.14)   
121 - **[CGCCCC]** 631 (85.80)   
121 - **[CGCTTC]** 514 (86.32)   
122 + **.CGCGCC.** 32 (94.60)   
122 + **[CGCCGC]** 34 (85.72)   
122 + **[CGCCCC]** 417 (85.80)   
122 + **.CGCCTC.** 641 (91.20)   
122 + **[CGCCCC]** 674 (85.80)   
122 + **.CGCGGC.** 916 (94.52)   
122 + **.CGCGCC.** 924 (94.60)   
122 + **[CGCCCC]** 926 (85.80)   
122 + **[CGGGTC]** 1225 (87.94)   
122 - **[CGCGTG]** 1028 (86.67)   
122 - **.CGCGCC.** 902 (94.60)   
122 - **.CGCGGC.** 82 (94.52)   
123 + **[CGCCGC]** 14 (85.72)   
123 + **.CGCGTC.** 392 (100.00)   
123 + **[CGCCCC]** 442 (85.80)   
123 + **[CGCCCC]** 458 (85.80)   
123 + **[CGCCCC]** 471 (85.80)   
123 + **[CGCCCC]** 476 (85.80)   
123 + **.CGCGCC.** 545 (94.60)   
123 + **.CGCGCC.** 557 (94.60)   
123 + **[CGCCGC]** 601 (85.72)   
123 + **[CGCCGC]** 638 (85.72)   
123 + **[CGCCCC]** 641 (85.80)   
123 + **.CGCGCC.** 660 (94.60)   
123 + **[CGCCCC]** 662 (85.80)   
123 + **[CGCCCC]** 685 (85.80)   
123 + **.CGCGCC.** 690 (94.60)   
123 + **[CGCCCC]** 692 (85.80)   
123 + **.CGCGCC.** 769 (94.60)   
123 + **[CGCGAC]** 828 (87.27)   
123 + **[CGCCGC]** 836 (85.72)   
123 + **[CGCGAC]** 852 (87.27)   
123 + **.CGCGGC.** 900 (94.52)   
123 + **.CGCGGC.** 952 (94.52)   
123 + **[CGCCGC]** 1004 (85.72)   
123 + **.CGCGCC.** 1007 (94.60)   
123 + **[CGCCGC]** 1009 (85.72)   
123 + **.CGCGCC.** 1012 (94.60)   
123 + **[CGCCGC]** 1014 (85.72)   
123 + **[CGCCCC]** 1017 (85.80)   
123 + **.CGCGGC.** 1030 (94.52)   
123 + **[GGCGTC]** 1073 (87.08)   
123 + **[CGCCCC]** 1102 (85.80)   
123 + **[CGCCGC]** 1362 (85.72)   
123 + **[CGGGTC]** 1368 (87.94)   
123 + **.CGCCTC.** 1429 (91.20)   
123 + **[CGCCGC]** 1463 (85.72)   
123 - **[CGCCCC]** 1345 (85.80)   
123 - **.CGCCTC.** 1315 (91.20)   
123 - **[GGCGTC]** 1266 (87.08)   
123 - **[CCCGTC]** 1260 (87.14)   
123 - **[CGCGAC]** 1233 (87.27)   
123 - **.CGCGGC.** 1010 (94.52)   
123 - **.CGCGGC.** 1005 (94.52)   
123 - **[CGCCGC]** 953 (85.72)   
123 - **[CGCCGC]** 908 (85.72)   
123 - **.CGCGCC.** 898 (94.60)   
123 - **.CGCGGC.** 826 (94.52)   
123 - **.CGCGCC.** 767 (94.60)   
123 - **[CGCCGC]** 753 (85.72)   
123 - **[CGCCGC]** 750 (85.72)   
123 - **.CGCGCC.** 743 (94.60)   
123 - **[CGCCGC]** 741 (85.72)   
123 - **[CGCCCC]** 738 (85.80)   
123 - **.CGCGGC.** 602 (94.52)   
123 - **[CGGGTC]** 515 (87.94)   
123 - **[CGCCGC]** 496 (85.72)   
123 - **[CGCCCC]** 425 (85.80)   
123 - **.CGCCTC.** 420 (91.20)   
123 - **.CGCGCC.** 390 (94.60)   
123 - **.CGCGGC.** 15 (94.52)   
124 + **.CGCCTC.** 946 (91.20)   
124 + **[GGCGTC]** 978 (87.08)   
124 + **[CGGGTC]** 1016 (87.94)   
124 + **[CGCGTG]** 1042 (86.67)   
124 + **[GGCGTC]** 1099 (87.08)   
124 + **.CGCGCC.** 1112 (94.60)   
124 + **[GGCGTC]** 1353 (87.08)   
124 + **[GGCGTC]** 1424 (87.08)   
124 - **[GGCGTC]** 1433 (87.08)   
124 - **.CGCCTC.** 1422 (91.20)   
124 - **.CGCCTC.** 1361 (91.20)   
124 - **[CGCCCC]** 1351 (85.80)   
124 - **.CGCGGC.** 1224 (94.52)   
124 - **.CGCCTC.** 1207 (91.20)   
124 - **.CGCGCC.** 1149 (94.60)   
124 - **[CGCTTC]** 1086 (86.32)   
124 - **.CGCGCC.** 953 (94.60)   
124 - **.CGCGCC.** 880 (94.60)   
124 - **.CGCGCC.** 868 (94.60)   
124 - **.CGCGTC.** 828 (100.00)   
126 + **[CGCCGC]** 1037 (85.72)   
126 + **[CGCCGC]** 1040 (85.72)   
126 + **[CGCCGC]** 1043 (85.72)   
126 + **.CGCCTC.** 1046 (91.20)   
126 + **[CGGGTC]** 1235 (87.94)   
127 + **.CGCGGC.** 746 (94.52)   
127 + **[CCCGTC]** 751 (87.14)   
127 + **[CCCGTC]** 756 (87.14)   
127 + **[CGCCCC]** 903 (85.80)   
127 + **[CCCGTC]** 1275 (87.14)   
127 + **.CGCGGC.** 1305 (94.52)   
127 + **[GGCGTC]** 1340 (87.08)   
127 + **.CGCGTC.** 1511 (100.00)   
127 - **.CGCGGC.** 1509 (94.52)   
127 - **[CGGGTC]** 1401 (87.94)   
127 - **[CGGGTC]** 1150 (87.94)   
127 - **[CGCCCC]** 1136 (85.80)   
127 - **.CGCGTC.** 1029 (100.00)   
127 - **.CGCCTC.** 857 (91.20)   
127 - **.CGCGCC.** 850 (94.60)   
127 - **.CGCGCC.** 776 (94.60)   
127 - **.CGCGCC.** 744 (94.60)   
127 - **.CGCCTC.** 742 (91.20)   
127 - **[CGCCGC]** 681 (85.72)   
127 - **.CGCGCC.** 630 (94.60)   
127 - **.CGCCTC.** 628 (91.20)   
127 - **[CGCCCC]** 623 (85.80)   
127 - **[CGCGTG]** 467 (86.67)   
127 - **[GGCGTC]** 462 (87.08)   
127 - **[CGCCCC]** 356 (85.80)   
128 + **[CGCCCC]** 268 (85.80)   
128 + **.CGCCTC.** 362 (91.20)   
128 + **.CGCGGC.** 408 (94.52)   
128 + **[CGCTTC]** 413 (86.32)   
128 + **[CGGGTC]** 429 (87.94)   
128 + **[GGCGTC]** 458 (87.08)   
128 + **[CGCCCC]** 521 (85.80)   
128 + **.CGCGGC.** 611 (94.52)   
128 + **[CGCTTC]** 764 (86.32)   
128 + **[CGCCCC]** 811 (85.80)   
128 + **[CGGGTC]** 828 (87.94)   
128 + **[CGCGAC]** 938 (87.27)   
128 + **[CGCCCC]** 964 (85.80)   
128 + **[GGCGTC]** 1107 (87.08)   
128 + **[CGCCCC]** 1220 (85.80)   
128 + **[CGCCCC]** 1226 (85.80)   
128 + **[CGCCGC]** 1279 (85.72)   
128 + **[CGCTTC]** 1282 (86.32)   
128 - **[CGCCGC]** 1256 (85.72)   
128 - **[CGCCCC]** 1105 (85.80)   
128 - **[CGCCCC]** 1003 (85.80)   
128 - **[CGCGAC]** 936 (87.27)   
128 - **[CCCGTC]** 859 (87.14)   
128 - **[CGGGTC]** 781 (87.94)   
128 - **.CGCCTC.** 776 (91.20)   
128 - **.CGCCTC.** 625 (91.20)   
128 - **[CGCCGC]** 409 (85.72)   
128 - **.CGCGTC.** 406 (100.00)   
128 - **[CGCCGC]** 372 (85.72)   
128 - **[GGCGTC]** 360 (87.08)   
128 - **[GGCGTC]** 266 (87.08)   
128 - **.CGCGTC.** 31 (100.00)   
129 + **.CGCCTC.** 268 (91.20)   
129 + **.CGCCTC.** 597 (91.20)   
129 + **[CGCCGC]** 663 (85.72)   
129 + **[CGCGAC]** 710 (87.27)   
129 + **.CGCCTC.** 779 (91.20)   
129 + **[CGCCCC]** 799 (85.80)   
129 + **[CGCCCC]** 811 (85.80)   
129 + **[CGCCGC]** 975 (85.72)   
129 + **[CGCCGC]** 1015 (85.72)   
129 + **[CGCGTG]** 1018 (86.67)   
129 + **.CGCGGC.** 1141 (94.52)   
129 + **[CGCGTG]** 1178 (86.67)   
129 + **.CGCGCC.** 1202 (94.60)   
129 + **[CGCCGC]** 1319 (85.72)   
129 + **.CGCGGC.** 1386 (94.52)   
129 + **[CGGGTC]** 1392 (87.94)   
129 + **.CGCGCC.** 1402 (94.60)   
129 + **[CGCCGC]** 1430 (85.72)   
129 - **[CGCCGC]** 1449 (85.72)   
129 - **.CGCCTC.** 1446 (91.20)   
129 - **.CGCGGC.** 1320 (94.52)   
129 - **[CGCCGC]** 1223 (85.72)   
129 - **.CGCGGC.** 1200 (94.52)   
129 - **.CGCGCC.** 1144 (94.60)   
129 - **[CGCCGC]** 1142 (85.72)   
129 - **[CGCCGC]** 1086 (85.72)   
129 - **.CGCGTC.** 1057 (100.00)   
129 - **.CGCCTC.** 1023 (91.20)   
129 - **.CGCGGC.** 1016 (94.52)   
129 - **.CGCCTC.** 964 (91.20)   
129 - **.CGCGGC.** 957 (94.52)   
129 - **.CGCGGC.** 895 (94.52)   
129 - **.CGCGGC.** 870 (94.52)   
129 - **[CGCCCC]** 860 (85.80)   
129 - **[CGCGTG]** 764 (86.67)   
129 - **[CGCCGC]** 655 (85.72)   
129 - **[CGGGTC]** 584 (87.94)   
129 - **[CGCCCC]** 417 (85.80)   
129 - **[CGCTTC]** 363 (86.32)   
130 + **[CGCCCC]** 174 (85.80)   
130 + **[CGCTTC]** 606 (86.32)   
130 + **.CGCGGC.** 867 (94.52)   
130 + **.CGCGCC.** 1269 (94.60)   
130 - **.CGCGCC.** 1267 (94.60)   
130 - **.CGCCTC.** 1265 (91.20)   
130 - **[CGCTTC]** 1230 (86.32)   
130 - **.CGCGGC.** 1109 (94.52)   
130 - **[CGCCGC]** 906 (85.72)   
130 - **.CGCGGC.** 865 (94.52)   
130 - **.CGCCTC.** 544 (91.20)   
131 + **.CGCGCC.** 33 (94.60)   
131 + **[CGCCGC]** 35 (85.72)   
131 + **[CGCTTC]** 195 (86.32)   
131 + **[CGCGTG]** 614 (86.67)   
131 + **[CCCGTC]** 624 (87.14)   
131 + **[CCCGTC]** 735 (87.14)   
131 + **[CGCGTG]** 778 (86.67)   
131 + **[CGCCCC]** 860 (85.80)   
131 + **[CGCCCC]** 958 (85.80)   
131 + **[CCCGTC]** 992 (87.14)   
131 + **.CGCGGC.** 1093 (94.52)   
131 + **.CGCGGC.** 1117 (94.52)   
131 + **.CGCGGC.** 1306 (94.52)   
131 + **.CGCCTC.** 1328 (91.20)   
131 + **.CGCGGC.** 1379 (94.52)   
131 + **[CGCCGC]** 1415 (85.72)   
131 + **.CGCGGC.** 1488 (94.52)   
131 - **[CGCCCC]** 1508 (85.80)   
131 - **[CGCCGC]** 1498 (85.72)   
131 - **[CGCCGC]** 1495 (85.72)   
131 - **[CGCCGC]** 1492 (85.72)   
131 - **[CGCCGC]** 1489 (85.72)   
131 - **[CGCCGC]** 1380 (85.72)   
131 - **[CGCCGC]** 1370 (85.72)   
131 - **[CGCCGC]** 1313 (85.72)   
131 - **[CGCCGC]** 1310 (85.72)   
131 - **[CGCCGC]** 1307 (85.72)   
131 - **[CGCGTG]** 1304 (86.67)   
131 - **.CGCGGC.** 1183 (94.52)   
131 - **.CGCGCC.** 1091 (94.60)   
131 - **.CGCGCC.** 980 (94.60)   
131 - **[CGCCGC]** 903 (85.72)   
131 - **[CGCCGC]** 900 (85.72)   
131 - **[CGCCGC]** 897 (85.72)   
131 - **.CGCGGC.** 568 (94.52)   
131 - **[CGCCGC]** 556 (85.72)   
131 - **[CGCCCC]** 553 (85.80)   
131 - **[CGCGTG]** 474 (86.67)   
131 - **.CGCGCC.** 31 (94.60)   
132 + **[CGCCGC]** 706 (85.72)   
132 + **[CGCCGC]** 709 (85.72)   
132 + **[CGCGTG]** 712 (86.67)   
132 + **[CGCCCC]** 760 (85.80)   
132 + **[CGCCCC]** 765 (85.80)   
132 + **.CGCGCC.** 814 (94.60)   
132 + **[GGCGTC]** 861 (87.08)   
132 + **[CGCCCC]** 900 (85.80)   
132 + **[CGCCCC]** 945 (85.80)   
132 + **[CCCGTC]** 948 (87.14)   
132 + **[CGCCCC]** 1010 (85.80)   
132 + **.CGCGCC.** 1224 (94.60)   
132 + **[CGGGTC]** 1439 (87.94)   
132 - **[CGCTTC]** 1426 (86.32)   
132 - **.CGCCTC.** 1314 (91.20)   
132 - **[CGCCCC]** 1140 (85.80)   
132 - **[CGCTTC]** 1085 (86.32)   
132 - **[CGCCCC]** 859 (85.80)   
132 - **.CGCGGC.** 749 (94.52)   
132 - **.CGCGGC.** 710 (94.52)   
132 - **[CGCCGC]** 366 (85.72)   
132 - **[CGCTTC]** 363 (86.32)   
132 - **.CGCGCC.** 355 (94.60)   
134 + **.CGCGGC.** 533 (94.52)   
134 + **[CGCCCC]** 617 (85.80)   
134 + **[CGGGTC]** 954 (87.94)   
134 + **.CGCGTC.** 1035 (100.00)   
134 + **[CGCCCC]** 1072 (85.80)   
134 + **[CGCCCC]** 1384 (85.80)   
134 + **.CGCGCC.** 1475 (94.60)   
134 + **[CGCCCC]** 1501 (85.80)   
134 - **.CGCGCC.** 1473 (94.60)   
134 - **[CGCCGC]** 1467 (85.72)   
134 - **.CGCGCC.** 1370 (94.60)   
134 - **[CCCGTC]** 1356 (87.14)   
134 - **[CGCCGC]** 1080 (85.72)   
134 - **[CGCCGC]** 1060 (85.72)   
134 - **[CGCCCC]** 996 (85.80)   
134 - **.CGCGCC.** 950 (94.60)   
134 - **[CGCCCC]** 595 (85.80)   
134 - **[CGCCCC]** 584 (85.80)   
134 - **[CGCTTC]** 541 (86.32)   
134 - **[CGCTTC]** 197 (86.32)   
135 + **[CGCGTG]** 32 (86.67)   
135 + **.CGCGGC.** 249 (94.52)   
135 + **.CGCGGC.** 258 (94.52)   
135 + **.CGCGCC.** 313 (94.60)   
135 + **[CGCCCC]** 342 (85.80)   
135 + **.CGCGGC.** 365 (94.52)   
135 + **[CGCCGC]** 419 (85.72)   
135 + **.CGCCTC.** 470 (91.20)   
135 + **.CGCCTC.** 475 (91.20)   
135 + **.CGCCTC.** 539 (91.20)   
135 + **[CGCCCC]** 641 (85.80)   
135 + **[CGCCGC]** 691 (85.72)   
135 + **[CGCCCC]** 694 (85.80)   
135 + **.CGCCTC.** 778 (91.20)   
135 + **[CGCCGC]** 787 (85.72)   
135 + **[CGCGTG]** 790 (86.67)   
135 + **[CGCCCC]** 1136 (85.80)   
135 + **[CGCCGC]** 1145 (85.72)   
135 + **[CGCCGC]** 1205 (85.72)   
135 + **[CGCCGC]** 1208 (85.72)   
135 + **[CGCCGC]** 1230 (85.72)   
135 + **[CGCCGC]** 1233 (85.72)   
135 + **[CGGGTC]** 1254 (87.94)   
135 + **[CGCCGC]** 1499 (85.72)   
135 - **[CGCGTG]** 1508 (86.67)   
135 - **.CGCGGC.** 1500 (94.52)   
135 - **[CGCCCC]** 1477 (85.80)   
135 - **.CGCGCC.** 1450 (94.60)   
135 - **[CGCCGC]** 1286 (85.72)   
135 - **[CGCCGC]** 1283 (85.72)   
135 - **[CGCCGC]** 1280 (85.72)   
135 - **[CGCCCC]** 1201 (85.80)   
135 - **[CGCCGC]** 1113 (85.72)   
135 - **.CGCCTC.** 1110 (91.20)   
135 - **.CGCGGC.** 788 (94.52)   
135 - **[CGCGTG]** 488 (86.67)   
135 - **[CGCCGC]** 366 (85.72)   
135 - **.CGCGGC.** 363 (94.52)   
135 - **[CGCGTG]** 353 (86.67)   
135 - **[CGCCCC]** 105 (85.80)   
136 + **[GGCGTC]** 615 (87.08)   
136 + **[CGCCCC]** 655 (85.80)   
136 + **[CGCCCC]** 662 (85.80)   
136 + **[CGGGTC]** 961 (87.94)   
136 + **[CGCCGC]** 1023 (85.72)   
136 + **.CGCGGC.** 1041 (94.52)   
136 + **.CGCGCC.** 1065 (94.60)   
136 + **[CGCCGC]** 1067 (85.72)   
136 + **.CGCGGC.** 1196 (94.52)   
136 + **[CGCCCC]** 1217 (85.80)   
136 + **.CGCGGC.** 1302 (94.52)   
136 - **.CGCGGC.** 1298 (94.52)   
136 - **[CCCGTC]** 1235 (87.14)   
136 - **.CGCGCC.** 1039 (94.60)   
136 - **.CGCGCC.** 936 (94.60)   
136 - **.CGCGTC.** 699 (100.00)   
136 - **[GGCGTC]** 653 (87.08)   
136 - **[GGCGTC]** 523 (87.08)   
137 + **[CGCCCC]** 820 (85.80)   
137 + **.CGCGGC.** 945 (94.52)   
137 + **[CGCCCC]** 963 (85.80)   
137 + **[CGCCCC]** 994 (85.80)   
137 + **.CGCGGC.** 1016 (94.52)   
137 + **.CGCGGC.** 1138 (94.52)   
137 + **[CGCGTG]** 1167 (86.67)   
137 + **.CGCGCC.** 1496 (94.60)   
137 - **[CGGGTC]** 1328 (87.94)   
137 - **.CGCGCC.** 1165 (94.60)   
137 - **.CGCCTC.** 1041 (91.20)   
137 - **.CGCGTC.** 1014 (100.00)   
137 - **[CGCCGC]** 946 (85.72)   
137 - **.CGCGTC.** 943 (100.00)   
137 - **[CGCCCC]** 936 (85.80)   
137 - **.CGCGGC.** 919 (94.52)   
137 - **.CGCGTC.** 752 (100.00)   
137 - **.CGCGCC.** 610 (94.60)   
137 - **[CGCCCC]** 509 (85.80)   
138 + **[CGCCCC]** 156 (85.80)   
138 + **[CGCCGC]** 177 (85.72)   
138 + **[CGCCGC]** 180 (85.72)   
138 + **[CGCCGC]** 183 (85.72)   
138 + **.CGCGCC.** 192 (94.60)   
138 + **[CGCCGC]** 194 (85.72)   
138 + **[CGCCGC]** 218 (85.72)   
138 + **[CGCCGC]** 353 (85.72)   
138 + **.CGCGGC.** 367 (94.52)   
138 + **[CGCCCC]** 437 (85.80)   
138 + **.CGCGCC.** 452 (94.60)   
138 + **[CGCCCC]** 454 (85.80)   
138 + **[CGCCCC]** 466 (85.80)   
138 + **[CGCTTC]** 552 (86.32)   
138 + **[CGCCCC]** 561 (85.80)   
138 + **.CGCGGC.** 579 (94.52)   
138 + **.CGCGTC.** 601 (100.00)   
138 + **.CGCGGC.** 691 (94.52)   
138 + **.CGCGCC.** 739 (94.60)   
138 + **.CGCCTC.** 741 (91.20)   
138 + **.CGCGGC.** 763 (94.52)   
138 + **.CGCGGC.** 768 (94.52)   
138 + **[CGCCGC]** 825 (85.72)   
138 + **[CGCCGC]** 828 (85.72)   
138 + **[CGCCGC]** 831 (85.72)   
138 + **[CGCCGC]** 834 (85.72)   
138 + **[CGCCGC]** 837 (85.72)   
138 + **.CGCGCC.** 840 (94.60)   
138 - **.CGCGGC.** 838 (94.52)   
138 - **[CGCCGC]** 769 (85.72)   
138 - **.CGCGCC.** 766 (94.60)   
138 - **[CGCCGC]** 764 (85.72)   
138 - **[CGCCGC]** 695 (85.72)   
138 - **[CGCCGC]** 692 (85.72)   
138 - **.CGCGCC.** 689 (94.60)   
138 - **[CGCCCC]** 658 (85.80)   
138 - **.CGCGCC.** 641 (94.60)   
138 - **[CGCCGC]** 639 (85.72)   
138 - **.CGCCTC.** 632 (91.20)   
138 - **[CGCCCC]** 625 (85.80)   
138 - **.CGCGGC.** 615 (94.52)   
138 - **[CGCCGC]** 609 (85.72)   
138 - **[CGCCGC]** 580 (85.72)   
138 - **.CGCGCC.** 575 (94.60)   
138 - **.CGCCTC.** 530 (91.20)   
138 - **.CGCGGC.** 513 (94.52)   
138 - **[GGCGTC]** 435 (87.08)   
138 - **[CGGGTC]** 376 (87.94)   
138 - **.CGCGCC.** 370 (94.60)   
138 - **[CGCCGC]** 368 (85.72)   
138 - **.CGCGTC.** 365 (100.00)   
138 - **[CGCCCC]** 279 (85.80)   
139 + **[CGCCGC]** 206 (85.72)   
139 + **[GGCGTC]** 245 (87.08)   
139 + **[CCCGTC]** 376 (87.14)   
139 + **[CGCCCC]** 390 (85.80)   
139 + **[CGGGTC]** 621 (87.94)   
139 + **.CGCGCC.** 832 (94.60)   
139 + **[CGCCGC]** 834 (85.72)   
139 + **[CGCCGC]** 837 (85.72)   
139 + **[CGCCCC]** 840 (85.80)   
139 + **.CGCGTC.** 847 (100.00)   
139 + **[CGCCCC]** 916 (85.80)   
139 + **[CGCCCC]** 922 (85.80)   
139 + **.CGCGGC.** 960 (94.52)   
139 + **[CGCCCC]** 1195 (85.80)   
139 + **[CGCTTC]** 1200 (86.32)   
139 + **.CGCGGC.** 1343 (94.52)   
139 + **[CGCCCC]** 1418 (85.80)   
139 - **[GGCGTC]** 1496 (87.08)   
139 - **.CGCGCC.** 1091 (94.60)   
139 - **[CGCCGC]** 1081 (85.72)   
139 - **[CGCCGC]** 961 (85.72)   
139 - **[CGCGTG]** 762 (86.67)   
139 - **[CGCCGC]** 714 (85.72)   
139 - **.CGCGCC.** 690 (94.60)   
139 - **[CGCCGC]** 688 (85.72)   
139 - **[CGCCCC]** 685 (85.80)   
139 - **[CGCCCC]** 674 (85.80)   
139 - **[CGCGAC]** 617 (87.27)   
139 - **[GGCGTC]** 204 (87.08)   
139 - **[CGCGAC]** 134 (87.27)   
140 + **[CGGGTC]** 575 (87.94)   
140 + **[CGCCGC]** 656 (85.72)   
140 + **[CGCCGC]** 684 (85.72)   
140 + **[CGCCCC]** 692 (85.80)   
140 + **.CGCGGC.** 730 (94.52)   
140 + **[CGCCGC]** 735 (85.72)   
140 + **.CGCGTC.** 984 (100.00)   
140 + **[CGCCGC]** 1086 (85.72)   
140 + **[CGCCGC]** 1089 (85.72)   
140 + **[CGCCGC]** 1092 (85.72)   
140 + **[CGCCGC]** 1095 (85.72)   
140 + **[CGCCGC]** 1098 (85.72)   
140 + **[CGCCGC]** 1149 (85.72)   
140 + **.CGCCTC.** 1152 (91.20)   
140 + **.CGCGGC.** 1161 (94.52)   
140 + **[CGCCGC]** 1192 (85.72)   
140 + **.CGCGCC.** 1195 (94.60)   
140 + **[CGCCGC]** 1197 (85.72)   
140 + **.CGCGGC.** 1248 (94.52)   
140 + **[CGCCGC]** 1369 (85.72)   
140 - **[CGCCGC]** 1490 (85.72)   
140 - **[CGCCGC]** 1487 (85.72)   
140 - **[CGCCGC]** 1467 (85.72)   
140 - **[CGCCGC]** 1464 (85.72)   
140 - **[CGCCGC]** 1461 (85.72)   
140 - **[CGCCCC]** 1411 (85.80)   
140 - **[GGCGTC]** 1367 (87.08)   
140 - **[CGCCGC]** 1362 (85.72)   
140 - **[CGCCGC]** 1249 (85.72)   
140 - **.CGCGGC.** 1246 (94.52)   
140 - **.CGCGGC.** 1193 (94.52)   
140 - **.CGCGGC.** 1159 (94.52)   
140 - **[CGCCGC]** 975 (85.72)   
140 - **[CGGGTC]** 774 (87.94)   
140 - **[CGCCGC]** 731 (85.72)   
140 - **.CGCGGC.** 657 (94.52)   
140 - **[CGGGTC]** 638 (87.94)   
140 - **[CGCTTC]** 221 (86.32)   
141 + **[CGCTTC]** 296 (86.32)   
141 + **[GGCGTC]** 560 (87.08)   
141 + **[CGCCGC]** 609 (85.72)   
141 + **.CGCGCC.** 612 (94.60)   
141 + **.CGCGGC.** 684 (94.52)   
141 + **.CGCGCC.** 723 (94.60)   
141 + **[CGCCGC]** 837 (85.72)   
141 + **[CGCGAC]** 1099 (87.27)   
141 + **.CGCGGC.** 1158 (94.52)   
141 + **.CGCGCC.** 1185 (94.60)   
141 + **[CGCCGC]** 1187 (85.72)   
141 + **[CGGGTC]** 1192 (87.94)   
141 + **[CGCCGC]** 1241 (85.72)   
141 - **.CGCGTC.** 1389 (100.00)   
141 - **.CGCGCC.** 1368 (94.60)   
141 - **[CGCCCC]** 1366 (85.80)   
141 - **[CGCCCC]** 1342 (85.80)   
141 - **[CGCCCC]** 1329 (85.80)   
141 - **.CGCGTC.** 1228 (100.00)   
141 - **.CGCGGC.** 1188 (94.52)   
141 - **.CGCGGC.** 1183 (94.52)   
141 - **[CGCCGC]** 1159 (85.72)   
141 - **.CGCGGC.** 1156 (94.52)   
141 - **[CGCCCC]** 1121 (85.80)   
141 - **[CGCCCC]** 1088 (85.80)   
141 - **.CGCGCC.** 947 (94.60)   
141 - **.CGCCTC.** 754 (91.20)   
141 - **.CGCGGC.** 721 (94.52)   
141 - **.CGCGGC.** 688 (94.52)   
141 - **[CGCCCC]** 665 (85.80)   
141 - **.CGCGGC.** 610 (94.52)   
141 - **[CCCGTC]** 556 (87.14)   
141 - **[CGGGTC]** 379 (87.94)   
142 + **.CGCGCC.** 31 (94.60)   
142 + **[CGCGAC]** 99 (87.27)   
142 + **[CGCTTC]** 280 (86.32)   
142 + **[CGCCCC]** 379 (85.80)   
142 + **[CGCGAC]** 449 (87.27)   
142 + **.CGCGCC.** 457 (94.60)   
142 + **.CGCGCC.** 513 (94.60)   
142 + **[CGCCGC]** 515 (85.72)   
142 + **.CGCGGC.** 518 (94.52)   
142 + **.CGCGGC.** 663 (94.52)   
142 + **.CGCGCC.** 722 (94.60)   
142 + **[CGCCGC]** 893 (85.72)   
142 + **.CGCGCC.** 902 (94.60)   
142 + **.CGCGGC.** 946 (94.52)   
142 + **.CGCGCC.** 958 (94.60)   
142 + **[CGCCGC]** 1005 (85.72)   
142 + **.CGCGGC.** 1010 (94.52)   
142 + **[CGCCGC]** 1024 (85.72)   
142 + **[CGCCGC]** 1027 (85.72)   
142 + **[CGGGTC]** 1098 (87.94)   
142 + **[CGCCCC]** 1117 (85.80)   
142 + **.CGCGGC.** 1200 (94.52)   
142 + **.CGCGCC.** 1274 (94.60)   
142 + **[CGCCGC]** 1276 (85.72)   
142 + **[CGCCGC]** 1316 (85.72)   
142 + **.CGCCTC.** 1319 (91.20)   
142 + **.CGCGCC.** 1468 (94.60)   
142 + **[CGCCCC]** 1470 (85.80)   
142 - **.CGCGGC.** 1508 (94.52)   
142 - **[CGCTTC]** 1489 (86.32)   
142 - **.CGCGTC.** 1478 (100.00)   
142 - **[CGGGTC]** 1440 (87.94)   
142 - **[CGCCCC]** 1424 (85.80)   
142 - **.CGCGGC.** 1414 (94.52)   
142 - **.CGCCTC.** 1370 (91.20)   
142 - **[CGCCCC]** 1335 (85.80)   
142 - **.CGCGGC.** 1272 (94.52)   
142 - **.CGCGGC.** 1194 (94.52)   
142 - **[CGCCGC]** 1113 (85.72)   
142 - **[CGCGAC]** 1101 (87.27)   
142 - **.CGCGGC.** 1061 (94.52)   
142 - **.CGCGGC.** 1006 (94.52)   
142 - **.CGCGGC.** 894 (94.52)   
142 - **.CGCCTC.** 873 (91.20)   
142 - **.CGCGCC.** 842 (94.60)   
142 - **.CGCCTC.** 840 (91.20)   
142 - **.CGCGGC.** 831 (94.52)   
142 - **.CGCGGC.** 672 (94.52)   
142 - **[CGCCGC]** 664 (85.72)   
142 - **.CGCGGC.** 625 (94.52)   
142 - **.CGCCTC.** 588 (91.20)   
142 - **.CGCGGC.** 516 (94.52)   
142 - **[CGCGTG]** 455 (86.67)   
142 - **.CGCGTC.** 447 (100.00)   
142 - **[CGCGTG]** 97 (86.67)   
142 - **.CGCCTC.** 72 (91.20)   
142 - **.CGCGGC.** 29 (94.52)   
143 - **[GGCGTC]** 1057 (87.08)   
143 - **.CGCGCC.** 527 (94.60)   
144 + **[CCCGTC]** 132 (87.14)   
144 + **[CCCGTC]** 198 (87.14)   
144 + **[CCCGTC]** 218 (87.14)   
144 + **[CGCCGC]** 253 (85.72)   
144 + **[CCCGTC]** 340 (87.14)   
144 + **[CCCGTC]** 384 (87.14)   
144 + **[CGCCGC]** 444 (85.72)   
144 + **[CCCGTC]** 511 (87.14)   
144 + **[CCCGTC]** 531 (87.14)   
144 + **[CCCGTC]** 551 (87.14)   
144 + **[CCCGTC]** 594 (87.14)   
144 + **[CCCGTC]** 604 (87.14)   
144 + **[CCCGTC]** 669 (87.14)   
144 + **[CCCGTC]** 713 (87.14)   
144 + **[CCCGTC]** 754 (87.14)   
144 + **.CGCGTC.** 774 (100.00)   
144 + **.CGCGTC.** 839 (100.00)   
144 + **.CGCGGC.** 975 (94.52)   
144 + **[CGCCGC]** 1017 (85.72)   
144 + **.CGCGGC.** 1142 (94.52)   
144 + **.CGCGGC.** 1147 (94.52)   
144 + **.CGCGGC.** 1189 (94.52)   
144 + **.CGCGGC.** 1194 (94.52)   
144 + **[CCCGTC]** 1288 (87.14)   
144 + **.CGCGGC.** 1312 (94.52)   
144 + **.CGCGGC.** 1326 (94.52)   
144 + **[CGCGAC]** 1472 (87.27)   
144 + **[CGCCGC]** 1490 (85.72)   
144 + **.CGCGCC.** 1493 (94.60)   
144 + **[CGCCCC]** 1495 (85.80)   
144 + **[CGCCCC]** 1500 (85.80)   
144 + **[CGCCGC]** 1513 (85.72)   
144 - **.CGCGGC.** 1491 (94.52)   
144 - **.CGCGCC.** 1459 (94.60)   
144 - **[CGCCGC]** 1365 (85.72)   
144 - **.CGCGCC.** 1324 (94.60)   
144 - **.CGCCTC.** 1322 (91.20)   
144 - **[CGCCCC]** 1253 (85.80)   
144 - **.CGCCTC.** 1229 (91.20)   
144 - **.CGCCTC.** 1223 (91.20)   
144 - **.CGCGGC.** 1217 (94.52)   
144 - **.CGCCTC.** 1204 (91.20)   
144 - **.CGCGGC.** 1198 (94.52)   
144 - **.CGCGCC.** 1192 (94.60)   
144 - **[CGCCGC]** 1190 (85.72)   
144 - **.CGCGTC.** 1187 (100.00)   
144 - **.CGCGCC.** 1150 (94.60)   
144 - **[CGCCGC]** 1148 (85.72)   
144 - **.CGCGCC.** 1145 (94.60)   
144 - **[CGCCGC]** 1143 (85.72)   
144 - **.CGCGCC.** 1140 (94.60)   
144 - **[CGCCGC]** 1138 (85.72)   
144 - **.CGCCTC.** 1135 (91.20)   
144 - **[CGCCGC]** 1098 (85.72)   
144 - **[CGCCGC]** 1091 (85.72)   
144 - **.CGCGCC.** 1074 (94.60)   
144 - **[CGCCCC]** 1072 (85.80)   
144 - **[CGCCGC]** 1063 (85.72)   
144 - **[CGCCGC]** 1047 (85.72)   
144 - **.CGCGCC.** 1033 (94.60)   
144 - **[CGCCGC]** 1031 (85.72)   
144 - **[CGCCGC]** 1028 (85.72)   
144 - **[CGCCCC]** 1025 (85.80)   
144 - **.CGCGGC.** 1018 (94.52)   
144 - **[CGCCGC]** 1013 (85.72)   
144 - **[CGCCGC]** 1010 (85.72)   
144 - **.CGCGGC.** 973 (94.52)   
144 - **[CGGGTC]** 398 (87.94)   
144 - **[CGGGTC]** 392 (87.94)   
145 + **.CGCCTC.** 299 (91.20)   
145 + **.CGCGCC.** 510 (94.60)   
145 + **[CGCCCC]** 512 (85.80)   
145 + **.CGCGGC.** 1098 (94.52)   
145 + **[CGCCGC]** 1136 (85.72)   
145 + **.CGCGGC.** 1141 (94.52)   
145 + **.CGCGCC.** 1150 (94.60)   
145 + **[CGCCGC]** 1152 (85.72)   
145 + **[CGCGAC]** 1217 (87.27)   
145 + **[CGCGAC]** 1253 (87.27)   
145 - **.CGCCTC.** 1406 (91.20)   
145 - **.CGCGCC.** 1148 (94.60)   
145 - **[CGCCGC]** 1142 (85.72)   
145 - **.CGCGGC.** 1137 (94.52)   
145 - **.CGCGGC.** 1107 (94.52)   
145 - **[CGCGAC]** 1096 (87.27)   
145 - **[CGGGTC]** 995 (87.94)   
145 - **[CGCCCC]** 964 (85.80)   
145 - **.CGCCTC.** 949 (91.20)   
145 - **[CGCCCC]** 932 (85.80)   
145 - **[CCCGTC]** 897 (87.14)   
145 - **[CGCCCC]** 828 (85.80)   
145 - **[CGGGTC]** 815 (87.94)   
145 - **.CGCCTC.** 782 (91.20)   
145 - **[CGCCCC]** 722 (85.80)   
145 - **[CGCCCC]** 685 (85.80)   
145 - **[CGCCGC]** 678 (85.72)   
145 - **.CGCGCC.** 654 (94.60)   
145 - **.CGCCTC.** 635 (91.20)   
145 - **[CGCCCC]** 618 (85.80)   
145 - **[CGCCCC]** 583 (85.80)   
145 - **[CGCCGC]** 576 (85.72)   
145 - **[CGCCCC]** 550 (85.80)   
145 - **[CGCCGC]** 545 (85.72)   
145 - **[CGCCGC]** 446 (85.72)   
145 - **[CGCCCC]** 443 (85.80)   
146 + **.CGCCTC.** 605 (91.20)   
146 + **[CGCCCC]** 921 (85.80)   
146 + **[CGCCCC]** 926 (85.80)   
146 - **[CCCGTC]** 866 (87.14)   
146 - **[CGCCCC]** 493 (85.80)   
147 + **[CGCCCC]** 180 (85.80)   
147 + **.CGCCTC.** 540 (91.20)   
147 + **[CGCCGC]** 828 (85.72)   
147 + **.CGCGCC.** 854 (94.60)   
147 + **[CGCCCC]** 856 (85.80)   
147 + **.CGCGTC.** 864 (100.00)   
147 + **[CGCCGC]** 945 (85.72)   
147 + **.CGCGCC.** 1137 (94.60)   
147 + **[CGCCGC]** 1162 (85.72)   
147 + **.CGCGCC.** 1167 (94.60)   
147 + **[GGCGTC]** 1338 (87.08)   
147 + **[GGCGTC]** 1381 (87.08)   
147 - **[CGCCCC]** 1416 (85.80)   
147 - **[CGCCCC]** 1402 (85.80)   
147 - **[CGCCCC]** 1391 (85.80)   
147 - **.CGCCTC.** 1379 (91.20)   
147 - **.CGCGCC.** 1370 (94.60)   
147 - **[CGCCGC]** 1336 (85.72)   
147 - **[CGGGTC]** 1299 (87.94)   
147 - **.CGCGGC.** 1163 (94.52)   
147 - **[CGGGTC]** 1120 (87.94)   
147 - **[CGCCGC]** 1081 (85.72)   
147 - **.CGCCTC.** 1078 (91.20)   
147 - **[CGCCGC]** 1069 (85.72)   
147 - **[CGCCGC]** 1040 (85.72)   
147 - **.CGCCTC.** 1037 (91.20)   
147 - **[CCCGTC]** 1029 (87.14)   
147 - **.CGCGGC.** 978 (94.52)   
147 - **[CGCGAC]** 963 (87.27)   
147 - **.CGCGTC.** 862 (100.00)   
148 + **.CGCCTC.** 55 (91.20)   
148 + **.CGCCTC.** 221 (91.20)   
148 + **.CGCGCC.** 472 (94.60)   
148 + **.CGCCTC.** 474 (91.20)   
148 + **.CGCGGC.** 548 (94.52)   
148 + **.CGCCTC.** 630 (91.20)   
148 + **[CGCCGC]** 676 (85.72)   
148 + **[CGCCCC]** 767 (85.80)   
148 + **.CGCCTC.** 784 (91.20)   
148 + **[CGCCGC]** 827 (85.72)   
148 + **.CGCGTC.** 832 (100.00)   
148 + **[CGCCCC]** 1264 (85.80)   
148 + **[CCCGTC]** 1394 (87.14)   
148 + **.CGCCTC.** 1399 (91.20)   
148 - **[CGCGAC]** 1087 (87.27)   
148 - **[CGCCCC]** 1000 (85.80)   
148 - **.CGCGGC.** 828 (94.52)   
148 - **[GGCGTC]** 825 (87.08)   
148 - **[CGCCGC]** 626 (85.72)   
148 - **[GGCGTC]** 439 (87.08)   
148 - **[CGCCCC]** 419 (85.80)   
149 - **[CGCTTC]** 703 (86.32)   
150 + **.CGCGCC.** 94 (94.60)   
150 + **.CGCGCC.** 118 (94.60)   
150 + **[CGCCGC]** 120 (85.72)   
150 + **.CGCGCC.** 123 (94.60)   
150 + **.CGCGGC.** 175 (94.52)   
150 + **.CGCGGC.** 191 (94.52)   
150 + **[CGCCCC]** 333 (85.80)   
150 + **[CCCGTC]** 690 (87.14)   
150 + **[CGCGAC]** 714 (87.27)   
150 + **[CGCGAC]** 933 (87.27)   
150 + **[CGGGTC]** 1026 (87.94)   
150 + **.CGCCTC.** 1243 (91.20)   
150 + **.CGCCTC.** 1266 (91.20)   
150 - **.CGCCTC.** 1475 (91.20)   
150 - **.CGCGCC.** 1469 (94.60)   
150 - **[CGCCCC]** 1467 (85.80)   
150 - **[CGCTTC]** 1165 (86.32)   
150 - **[CGCCGC]** 989 (85.72)   
150 - **.CGCGTC.** 956 (100.00)   
150 - **[CGCCGC]** 914 (85.72)   
150 - **[CGCCCC]** 750 (85.80)   
150 - **[CGCCGC]** 490 (85.72)   
150 - **.CGCGTC.** 465 (100.00)   
150 - **[CGCCCC]** 237 (85.80)   
150 - **.CGCCTC.** 232 (91.20)   
150 - **[CGCCGC]** 192 (85.72)   
150 - **.CGCGGC.** 121 (94.52)   
150 - **.CGCGGC.** 114 (94.52)   
150 - **.CGCGCC.** 90 (94.60)   
151 + **[CGCCGC]** 17 (85.72)   
151 + **.CGCGTC.** 49 (100.00)   
151 + **[CGCCGC]** 92 (85.72)   
151 + **[CGCCCC]** 117 (85.80)   
151 + **[CGCCCC]** 256 (85.80)   
151 + **[CGCCGC]** 516 (85.72)   
151 + **.CGCGTC.** 540 (100.00)   
151 + **[CGCCCC]** 768 (85.80)   
151 + **.CGCCTC.** 773 (91.20)   
151 + **[CGCCGC]** 814 (85.72)   
151 + **.CGCCTC.** 845 (91.20)   
151 + **.CGCGGC.** 884 (94.52)   
151 + **.CGCGGC.** 892 (94.52)   
151 + **.CGCGCC.** 916 (94.60)   
151 + **[CGCGAC]** 1063 (87.27)   
151 + **.CGCGTC.** 1151 (100.00)   
151 + **.CGCGCC.** 1206 (94.60)   
151 + **.CGCGGC.** 1252 (94.52)   
151 + **.CGCGGC.** 1268 (94.52)   
151 + **[CGCCCC]** 1402 (85.80)   
151 + **[CGCGTG]** 1463 (86.67)   
151 + **.CGCGTC.** 1473 (100.00)   
151 + **.CGCGGC.** 1496 (94.52)   
151 - **.CGCGCC.** 1492 (94.60)   
151 - **[CGCCGC]** 1490 (85.72)   
151 - **.CGCGCC.** 1461 (94.60)   
151 - **[CGCCGC]** 1459 (85.72)   
151 - **[CGCGTG]** 1421 (86.67)   
151 - **.CGCCTC.** 1398 (91.20)   
151 - **.CGCGCC.** 1264 (94.60)   
151 - **[CGCGTG]** 1149 (86.67)   
151 - **.CGCCTC.** 1082 (91.20)   
151 - **[CGGGTC]** 1066 (87.94)   
151 - **[CGGGTC]** 1059 (87.94)   
151 - **.CGCGCC.** 1033 (94.60)   
151 - **.CGCGCC.** 912 (94.60)   
151 - **[CGCCGC]** 885 (85.72)   
151 - **.CGCGCC.** 882 (94.60)   
151 - **.CGCGGC.** 831 (94.52)   
151 - **.CGCGGC.** 815 (94.52)   
151 - **[CGCCCC]** 673 (85.80)   
151 - **[CCCGTC]** 315 (87.14)   
151 - **[CGCGAC]** 291 (87.27)   
151 - **[CGCGAC]** 72 (87.27)   
153 - **.CGCCTC.** 591 (91.20)   
153 - **.CGCGCC.** 384 (94.60)   
154 + **[CGGGTC]** 98 (87.94)   
154 + **[GGCGTC]** 811 (87.08)   
154 + **[CGCGTG]** 869 (86.67)   
154 + **[CGCGTG]** 935 (86.67)   
154 + **[CGCGTG]** 1011 (86.67)   
154 + **.CGCGGC.** 1040 (94.52)   
154 + **.CGCGCC.** 1071 (94.60)   
154 + **.CGCCTC.** 1073 (91.20)   
154 + **[CGCCGC]** 1142 (85.72)   
154 + **[CGCCGC]** 1178 (85.72)   
154 + **.CGCGCC.** 1200 (94.60)   
154 + **.CGCGCC.** 1206 (94.60)   
154 + **[CGCCGC]** 1218 (85.72)   
154 + **[CGCCGC]** 1249 (85.72)   
154 + **[CGCCGC]** 1252 (85.72)   
154 + **[CCCGTC]** 1284 (87.14)   
154 + **[CGCCGC]** 1290 (85.72)   
154 + **[CGCTTC]** 1325 (86.32)   
154 + **.CGCGCC.** 1432 (94.60)   
154 + **[CGCCGC]** 1434 (85.72)   
154 + **.CGCGCC.** 1466 (94.60)   
154 + **[CGCCGC]** 1468 (85.72)   
154 + **.CGCGGC.** 1512 (94.52)   
154 - **.CGCGCC.** 1378 (94.60)   
154 - **.CGCGGC.** 1179 (94.52)   
154 - **.CGCGCC.** 1096 (94.60)   
154 - **[CGCCGC]** 1094 (85.72)   
154 - **[CGCCGC]** 1063 (85.72)   
154 - **[CGCCGC]** 1047 (85.72)   
154 - **[CGCCGC]** 1044 (85.72)   
154 - **[CGCCGC]** 1041 (85.72)   
154 - **.CGCGCC.** 1038 (94.60)   
154 - **[CGCCCC]** 1032 (85.80)   
154 - **[CGCCGC]** 1023 (85.72)   
154 - **[CGCCCC]** 1020 (85.80)   
154 - **.CGCGCC.** 1009 (94.60)   
154 - **.CGCGCC.** 998 (94.60)   
154 - **.CGCGCC.** 933 (94.60)   
154 - **.CGCGCC.** 865 (94.60)   
154 - **[CGCCCC]** 859 (85.80)   
154 - **[CGCCCC]** 849 (85.80)   
154 - **.CGCGCC.** 824 (94.60)   
155 + **.CGCGGC.** 994 (94.52)   
155 + **[GGCGTC]** 1254 (87.08)   
155 - **[CGCGAC]** 1257 (87.27)   
155 - **.CGCGCC.** 507 (94.60)   
157 + **[CGCCCC]** 416 (85.80)   
157 + **.CGCGCC.** 731 (94.60)   
157 + **[CGCCGC]** 1113 (85.72)   
157 + **[CGCCGC]** 1116 (85.72)   
157 + **[CGCGTG]** 1238 (86.67)   
157 - **.CGCCTC.** 1326 (91.20)   
157 - **.CGCGGC.** 1236 (94.52)   
157 - **.CGCGCC.** 1219 (94.60)   
157 - **[CGCGAC]** 209 (87.27)   
158 + **.CGCCTC.** 317 (91.20)   
158 + **[CGCGTG]** 377 (86.67)   
158 + **.CGCCTC.** 478 (91.20)   
158 + **[GGCGTC]** 510 (87.08)   
158 + **.CGCGCC.** 522 (94.60)   
158 + **[CGCTTC]** 1109 (86.32)   
158 + **.CGCGTC.** 1123 (100.00)   
158 + **.CGCGTC.** 1179 (100.00)   
158 + **.CGCGCC.** 1371 (94.60)   
158 + **[GGCGTC]** 1445 (87.08)   
158 - **.CGCGCC.** 1512 (94.60)   
158 - **.CGCGTC.** 1369 (100.00)   
158 - **.CGCGTC.** 1242 (100.00)   
158 - **[CGGGTC]** 1207 (87.94)   
158 - **.CGCGGC.** 1177 (94.52)   
158 - **[CGCGTG]** 1119 (86.67)   
158 - **[CGCCCC]** 1105 (85.80)   
158 - **.CGCGCC.** 375 (94.60)   
160 + **[CGGGTC]** 73 (87.94)   
160 + **[CGCGTG]** 135 (86.67)   
160 + **[CCCGTC]** 209 (87.14)   
160 + **[CGCCGC]** 265 (85.72)   
160 + **.CGCCTC.** 277 (91.20)   
160 + **[CGCGTG]** 297 (86.67)   
160 + **[CCCGTC]** 544 (87.14)   
160 + **[CGCGTG]** 704 (86.67)   
160 + **[CGCCGC]** 761 (85.72)   
160 + **.CGCGTC.** 782 (100.00)   
160 + **[GGCGTC]** 813 (87.08)   
160 + **[CGGGTC]** 852 (87.94)   
160 + **.CGCCTC.** 1038 (91.20)   
160 + **.CGCGGC.** 1055 (94.52)   
160 + **.CGCCTC.** 1081 (91.20)   
160 + **[CGGGTC]** 1127 (87.94)   
160 + **[CGCTTC]** 1187 (86.32)   
160 + **.CGCGCC.** 1206 (94.60)   
160 + **[CGCCGC]** 1208 (85.72)   
160 + **.CGCCTC.** 1254 (91.20)   
160 + **[CGCCGC]** 1306 (85.72)   
160 + **.CGCCTC.** 1309 (91.20)   
160 + **[CGCCCC]** 1473 (85.80)   
160 + **[CGCCCC]** 1486 (85.80)   
160 - **[CGCCCC]** 1401 (85.80)   
160 - **[GGCGTC]** 1269 (87.08)   
160 - **[CGCCGC]** 992 (85.72)   
160 - **.CGCCTC.** 989 (91.20)   
160 - **[CGCCCC]** 947 (85.80)   
160 - **[CGCCCC]** 937 (85.80)   
160 - **[CGCCCC]** 918 (85.80)   
160 - **[CGCCCC]** 912 (85.80)   
160 - **[CGCGAC]** 780 (87.27)   
160 - **.CGCGGC.** 266 (94.52)   
160 - **.CGCGGC.** 133 (94.52)   
160 - **[CGCCCC]** 100 (85.80)   
160 - **[CGCCCC]** 53 (85.80)   
161 + **[CGCCGC]** 291 (85.72)   
161 + **.CGCCTC.** 462 (91.20)   
161 + **[GGCGTC]** 540 (87.08)   
161 + **[CGGGTC]** 739 (87.94)   
161 + **[CGCGAC]** 754 (87.27)   
161 + **[CGCCGC]** 893 (85.72)   
161 + **.CGCGCC.** 896 (94.60)   
161 + **[CGCCGC]** 1017 (85.72)   
161 + **.CGCGCC.** 1191 (94.60)   
161 + **.CGCGGC.** 1221 (94.52)   
161 + **.CGCGGC.** 1268 (94.52)   
161 + **[CGCCGC]** 1422 (85.72)   
161 + **[CGCCGC]** 1425 (85.72)   
161 + **[CGCCCC]** 1466 (85.80)   
161 + **.CGCGCC.** 1481 (94.60)   
161 + **[CGCCGC]** 1483 (85.72)   
161 + **[CGCCGC]** 1486 (85.72)   
161 + **[CGCCGC]** 1489 (85.72)   
161 + **[CGCCGC]** 1492 (85.72)   
161 + **[CGCCGC]** 1501 (85.72)   
161 + **[CGCCGC]** 1504 (85.72)   
161 + **[CGCCGC]** 1507 (85.72)   
161 - **[CGCCGC]** 1272 (85.72)   
161 - **[CGCCGC]** 1269 (85.72)   
161 - **[CGCGAC]** 1219 (87.27)   
161 - **[CGCTTC]** 1139 (86.32)   
161 - **[CGCCGC]** 988 (85.72)   
161 - **[CGCCGC]** 985 (85.72)   
161 - **[CGCCGC]** 982 (85.72)   
161 - **[CGCCGC]** 944 (85.72)   
161 - **.CGCGGC.** 894 (94.52)   
161 - **.CGCGGC.** 884 (94.52)   
161 - **.CGCGGC.** 865 (94.52)   
161 - **[CGCCGC]** 830 (85.72)   
161 - **[CGCCCC]** 787 (85.80)   
161 - **.CGCGCC.** 752 (94.60)   
161 - **[CGCCCC]** 574 (85.80)   
161 - **[CGCCGC]** 538 (85.72)   
161 - **[CGCCCC]** 458 (85.80)   
161 - **[CGCGTG]** 316 (86.67)   
161 - **.CGCGCC.** 154 (94.60)   
161 - **[CGCCGC]** 152 (85.72)   
161 - **[CGCTTC]** 149 (86.32)   
162 - **[GGCGTC]** 1147 (87.08)   
164 + **[GGCGTC]** 252 (87.08)   
164 + **[CGCTTC]** 517 (86.32)   
164 + **.CGCGGC.** 1003 (94.52)   
164 + **.CGCCTC.** 1045 (91.20)   
164 + **.CGCGGC.** 1099 (94.52)   
164 + **[CGCCGC]** 1104 (85.72)   
164 + **[CGCCCC]** 1138 (85.80)   
164 + **.CGCGGC.** 1225 (94.52)   
164 + **.CGCCTC.** 1247 (91.20)   
164 + **.CGCGCC.** 1396 (94.60)   
164 - **[CGCCGC]** 1203 (85.72)   
164 - **[CGCCGC]** 1178 (85.72)   
164 - **[CGCCGC]** 1100 (85.72)   
164 - **.CGCGCC.** 1097 (94.60)   
164 - **.CGCGGC.** 1001 (94.52)   
164 - **.CGCGCC.** 956 (94.60)   
164 - **[CGCCCC]** 954 (85.80)   
164 - **[CGCCCC]** 949 (85.80)   
164 - **[CGCCGC]** 931 (85.72)   
164 - **[CGCCGC]** 898 (85.72)   
164 - **[CGCCGC]** 307 (85.72)   
164 - **[CGCGAC]** 273 (87.27)   
165 + **.CGCCTC.** 249 (91.20)   
165 + **.CGCGCC.** 581 (94.60)   
166 + **[GGCGTC]** 137 (87.08)   
166 + **.CGCCTC.** 234 (91.20)   
166 + **[CGGGTC]** 374 (87.94)   
166 + **.CGCCTC.** 487 (91.20)   
166 + **[CGGGTC]** 521 (87.94)   
166 + **.CGCGGC.** 661 (94.52)   
166 + **.CGCGGC.** 811 (94.52)   
166 + **.CGCCTC.** 874 (91.20)   
166 + **[CGCCCC]** 900 (85.80)   
166 + **[CGCCCC]** 920 (85.80)   
166 + **.CGCCTC.** 926 (91.20)   
166 + **[GGCGTC]** 961 (87.08)   
166 + **.CGCCTC.** 973 (91.20)   
166 + **[CGCCCC]** 995 (85.80)   
166 + **[CGCCGC]** 1028 (85.72)   
166 + **[CGCCGC]** 1119 (85.72)   
166 + **.CGCCTC.** 1122 (91.20)   
166 + **.CGCGGC.** 1159 (94.52)   
166 + **.CGCGCC.** 1181 (94.60)   
166 + **.CGCGGC.** 1229 (94.52)   
166 + **.CGCGGC.** 1312 (94.52)   
166 + **[CGCCGC]** 1317 (85.72)   
166 + **[CGCCGC]** 1352 (85.72)   
166 - **[CGCCGC]** 1313 (85.72)   
166 - **[CGCGTG]** 1310 (86.67)   
166 - **.CGCGCC.** 1297 (94.60)   
166 - **.CGCGCC.** 1290 (94.60)   
166 - **[CGCCCC]** 1288 (85.80)   
166 - **.CGCGCC.** 1225 (94.60)   
166 - **[CGCCGC]** 1160 (85.72)   
166 - **[CGCGTG]** 1157 (86.67)   
166 - **.CGCGGC.** 1072 (94.52)   
166 - **.CGCCTC.** 959 (91.20)   
166 - **[GGCGTC]** 598 (87.08)   
166 - **.CGCGGC.** 546 (94.52)   
166 - **[CCCGTC]** 519 (87.14)   
166 - **[CGCCCC]** 468 (85.80)   
166 - **[CGCCCC]** 135 (85.80)   
167 + **.CGCCTC.** 34 (91.20)   
167 + **[GGCGTC]** 142 (87.08)   
167 + **[CGCCCC]** 289 (85.80)   
167 + **[GGCGTC]** 670 (87.08)   
167 + **.CGCGGC.** 843 (94.52)   
167 + **.CGCGCC.** 867 (94.60)   
167 + **[CGCCGC]** 885 (85.72)   
167 + **.CGCGGC.** 956 (94.52)   
167 + **.CGCGGC.** 963 (94.52)   
167 + **.CGCGGC.** 1006 (94.52)   
167 + **[CGCCCC]** 1025 (85.80)   
167 + **.CGCGGC.** 1039 (94.52)   
167 + **[CGCCGC]** 1383 (85.72)   
167 + **[CGCTTC]** 1386 (86.32)   
167 + **[CGCCGC]** 1395 (85.72)   
167 + **[CGCCGC]** 1398 (85.72)   
167 + **[CGCCCC]** 1401 (85.80)   
167 + **.CGCGTC.** 1463 (100.00)   
167 - **[GGCGTC]** 1358 (87.08)   
167 - **[CGCGAC]** 1328 (87.27)   
167 - **[CGCGAC]** 1322 (87.27)   
167 - **.CGCCTC.** 1272 (91.20)   
167 - **[CGCCCC]** 1250 (85.80)   
167 - **.CGCGGC.** 1234 (94.52)   
167 - **.CGCGCC.** 1035 (94.60)   
167 - **.CGCGCC.** 1002 (94.60)   
167 - **.CGCGGC.** 863 (94.52)   
167 - **[CGCCCC]** 849 (85.80)   
167 - **[CGCCGC]** 844 (85.72)   
167 - **.CGCGCC.** 818 (94.60)   
167 - **[CGCCGC]** 816 (85.72)   
167 - **[CGCCCC]** 813 (85.80)   
167 - **.CGCGCC.** 801 (94.60)   
167 - **[CGCCCC]** 799 (85.80)   
167 - **.CGCCTC.** 793 (91.20)   
167 - **[CGGGTC]** 788 (87.94)   
167 - **.CGCGTC.** 770 (100.00)   
167 - **.CGCGTC.** 731 (100.00)   
167 - **[CGCCGC]** 668 (85.72)   
167 - **[CGCTTC]** 152 (86.32)   
167 - **[CGCCCC]** 140 (85.80)   
167 - **[GGCGTC]** 32 (87.08)   
168 + **[CGGGTC]** 442 (87.94)   
168 + **.CGCGGC.** 675 (94.52)   
168 + **[CGCCGC]** 908 (85.72)   
168 + **[GGCGTC]** 1042 (87.08)   
168 + **.CGCGCC.** 1114 (94.60)   
168 + **.CGCCTC.** 1126 (91.20)   
168 + **.CGCGGC.** 1137 (94.52)   
168 + **.CGCGCC.** 1171 (94.60)   
168 + **[CGCCCC]** 1173 (85.80)   
168 + **.CGCGGC.** 1205 (94.52)   
168 + **[CGCCCC]** 1243 (85.80)   
168 - **[CGCCCC]** 1465 (85.80)   
168 - **.CGCGGC.** 1435 (94.52)   
168 - **[CGCCCC]** 1429 (85.80)   
168 - **[CGCCCC]** 1239 (85.80)   
168 - **.CGCGCC.** 1183 (94.60)   
168 - **[CGCCCC]** 951 (85.80)   
168 - **[CGCCGC]** 942 (85.72)   
168 - **[CGCCCC]** 939 (85.80)   
168 - **[CGCCGC]** 920 (85.72)   
168 - **.CGCCTC.** 917 (91.20)   
168 - **.CGCGGC.** 909 (94.52)   
168 - **.CGCGCC.** 732 (94.60)   
168 - **[CGCCCC]** 730 (85.80)   
168 - **[CGCCCC]** 663 (85.80)   
168 - **[CGCTTC]** 312 (86.32)   
168 - **.CGCCTC.** 155 (91.20)   
168 - **[CGCCCC]** 9 (85.80)   
169 + **[CGCCGC]** 110 (85.72)   
169 + **[CCCGTC]** 310 (87.14)   
169 + **[GGCGTC]** 382 (87.08)   
169 + **[CGGGTC]** 418 (87.94)   
169 + **[CGCCCC]** 587 (85.80)   
169 + **[CGCCCC]** 692 (85.80)   
169 + **[CGCTTC]** 802 (86.32)   
169 + **.CGCCTC.** 894 (91.20)   
169 + **[CGGGTC]** 1445 (87.94)   
169 + **.CGCGCC.** 1499 (94.60)   
169 + **[CGCCCC]** 1501 (85.80)   
169 - **.CGCGTC.** 1045 (100.00)   
169 - **[CGCCCC]** 998 (85.80)   
169 - **.CGCGCC.** 871 (94.60)   
169 - **[CGCCCC]** 869 (85.80)   
169 - **.CGCGGC.** 111 (94.52)   
170 + **[CGGGTC]** 1475 (87.94)   
170 - **[CGCCGC]** 1471 (85.72)   
170 - **.CGCCTC.** 1259 (91.20)   
171 + **[CGCCCC]** 731 (85.80)   
171 + **.CGCGGC.** 774 (94.52)   
171 + **.CGCCTC.** 999 (91.20)   
171 - **[CGCCGC]** 775 (85.72)   
171 - **[CGCGTG]** 772 (86.67)   
172 + **[CGGGTC]** 71 (87.94)   
172 + **[CGCCCC]** 89 (85.80)   
172 + **[CGCCGC]** 228 (85.72)   
172 + **[CGCCGC]** 231 (85.72)   
172 + **.CGCCTC.** 300 (91.20)   
172 + **[CGCCGC]** 305 (85.72)   
172 + **[CGCCGC]** 308 (85.72)   
172 + **[CGCCCC]** 311 (85.80)   
172 + **[CCCGTC]** 364 (87.14)   
172 + **[CGCCGC]** 370 (85.72)   
172 + **.CGCCTC.** 373 (91.20)   
172 + **.CGCGGC.** 431 (94.52)   
172 + **.CGCCTC.** 459 (91.20)   
172 + **[CGCCGC]** 521 (85.72)   
172 + **[CGCCGC]** 524 (85.72)   
172 + **[CGCCGC]** 527 (85.72)   
172 + **[CGCCGC]** 530 (85.72)   
172 + **[CGCCGC]** 533 (85.72)   
172 + **[CGCCGC]** 536 (85.72)   
172 + **[CGCCGC]** 539 (85.72)   
172 + **.CGCGCC.** 551 (94.60)   
172 + **[CGCCCC]** 589 (85.80)   
172 + **.CGCCTC.** 595 (91.20)   
172 + **[CCCGTC]** 600 (87.14)   
172 + **[CGCCCC]** 610 (85.80)   
172 + **[CGCCGC]** 627 (85.72)   
172 + **[CGCCCC]** 639 (85.80)   
172 + **[CGCCGC]** 683 (85.72)   
172 + **[CGCGAC]** 716 (87.27)   
172 + **[CGCCGC]** 721 (85.72)   
172 + **.CGCGGC.** 757 (94.52)   
172 + **[CGCCCC]** 811 (85.80)   
172 + **.CGCCTC.** 829 (91.20)   
172 + **[CGCCCC]** 875 (85.80)   
172 + **.CGCGGC.** 899 (94.52)   
172 + **.CGCGCC.** 924 (94.60)   
172 + **[CGCCGC]** 1186 (85.72)   
172 + **[CGGGTC]** 1231 (87.94)   
172 + **[CGCCCC]** 1364 (85.80)   
172 + **.CGCGCC.** 1419 (94.60)   
172 + **.CGCGCC.** 1505 (94.60)   
172 - **[CGCCGC]** 1463 (85.72)   
172 - **[CCCGTC]** 1442 (87.14)   
172 - **[CGCTTC]** 1320 (86.32)   
172 - **[CGCCCC]** 1248 (85.80)   
172 - **.CGCCTC.** 1166 (91.20)   
172 - **.CGCGCC.** 1160 (94.60)   
172 - **[CGCCGC]** 1158 (85.72)   
172 - **[CGCTTC]** 1089 (86.32)   
172 - **[CGCTTC]** 1046 (86.32)   
172 - **.CGCGCC.** 920 (94.60)   
172 - **[CGCCGC]** 918 (85.72)   
172 - **[CGCCGC]** 915 (85.72)   
172 - **[CGCCCC]** 860 (85.80)   
172 - **[CGCCCC]** 855 (85.80)   
172 - **[CGCGAC]** 799 (87.27)   
172 - **[CGCCCC]** 791 (85.80)   
172 - **[CGCCCC]** 776 (85.80)   
172 - **.CGCGCC.** 768 (94.60)   
172 - **[CGCCCC]** 766 (85.80)   
172 - **.CGCGGC.** 755 (94.52)   
172 - **[CCCGTC]** 750 (87.14)   
172 - **[GGCGTC]** 719 (87.08)   
172 - **.CGCGGC.** 714 (94.52)   
172 - **[CGCGAC]** 549 (87.27)   
172 - **[CGCCGC]** 432 (85.72)   
172 - **.CGCGTC.** 429 (100.00)   
172 - **[CGCTTC]** 106 (86.32)   
173 + **[CGCCCC]** 454 (85.80)   
173 + **[CGCCCC]** 798 (85.80)   
173 + **[CGCCCC]** 864 (85.80)   
173 + **.CGCGCC.** 945 (94.60)   
173 + **.CGCCTC.** 947 (91.20)   
173 + **[CGCCCC]** 1217 (85.80)   
173 + **[CGCGAC]** 1280 (87.27)   
173 + **[CGCCCC]** 1389 (85.80)   
173 - **.CGCGGC.** 1278 (94.52)   
173 - **[GGCGTC]** 1191 (87.08)   
173 - **[CGCCGC]** 1186 (85.72)   
173 - **.CGCGCC.** 1101 (94.60)   
173 - **[GGCGTC]** 1081 (87.08)   
173 - **[CGCCGC]** 1029 (85.72)   
173 - **[CGCCGC]** 1026 (85.72)   
173 - **[CGCCGC]** 1023 (85.72)   
173 - **.CGCCTC.** 1020 (91.20)   
173 - **[CGCCCC]** 954 (85.80)   
173 - **.CGCGTC.** 943 (100.00)   
173 - **[CGCCCC]** 930 (85.80)   
173 - **[CGGGTC]** 895 (87.94)   
173 - **[CCCGTC]** 881 (87.14)   
174 + **.CGCGCC.** 214 (94.60)   
174 + **.CGCCTC.** 216 (91.20)   
174 + **.CGCCTC.** 302 (91.20)   
174 + **[CCCGTC]** 517 (87.14)   
174 + **[CGCCCC]** 802 (85.80)   
174 + **.CGCGCC.** 1128 (94.60)   
174 + **[CGCCGC]** 1184 (85.72)   
174 + **.CGCGTC.** 1187 (100.00)   
174 + **[CGCGAC]** 1205 (87.27)   
174 + **.CGCCTC.** 1246 (91.20)   
174 + **[CGCCGC]** 1279 (85.72)   
174 + **.CGCGGC.** 1477 (94.52)   
174 + **.CGCGGC.** 1507 (94.52)   
174 - **.CGCGCC.** 1505 (94.60)   
174 - **.CGCCTC.** 1503 (91.20)   
174 - **.CGCGCC.** 1480 (94.60)   
174 - **[CGCCGC]** 1478 (85.72)   
174 - **.CGCCTC.** 1405 (91.20)   
174 - **.CGCGTC.** 1380 (100.00)   
174 - **[CCCGTC]** 1371 (87.14)   
174 - **[CGCTTC]** 1366 (86.32)   
174 - **.CGCCTC.** 1318 (91.20)   
174 - **.CGCGGC.** 1203 (94.52)   
174 - **.CGCGGC.** 1185 (94.52)   
174 - **[CGCCCC]** 1180 (85.80)   
174 - **[CGCCCC]** 1162 (85.80)   
174 - **.CGCGGC.** 1145 (94.52)   
174 - **[CGGGTC]** 1112 (87.94)   
174 - **[CGCCGC]** 1065 (85.72)   
174 - **.CGCGTC.** 936 (100.00)   
174 - **[CGCCGC]** 834 (85.72)   
174 - **.CGCGCC.** 783 (94.60)   
174 - **[CGCCCC]** 781 (85.80)   
174 - **.CGCGCC.** 396 (94.60)   
174 - **[CCCGTC]** 392 (87.14)   
174 - **[CGCCCC]** 298 (85.80)   
174 - **[CGCGAC]** 212 (87.27)   
175 + **[CGCGTG]** 861 (86.67)   
175 + **[CGCCGC]** 1465 (85.72)   
175 + **.CGCGGC.** 1468 (94.52)   
175 - **.CGCGGC.** 1466 (94.52)   
175 - **[CGCTTC]** 1461 (86.32)   
175 - **.CGCCTC.** 1443 (91.20)   
175 - **[CGCCCC]** 1274 (85.80)   
175 - **.CGCGCC.** 927 (94.60)   
175 - **[CGCCCC]** 925 (85.80)   
175 - **.CGCGTC.** 859 (100.00)   
177 + **[CGCCCC]** 387 (85.80)   
177 + **.CGCGGC.** 786 (94.52)   
177 + **.CGCGCC.** 819 (94.60)   
177 + **.CGCCTC.** 821 (91.20)   
177 + **[CGCCGC]** 951 (85.72)   
177 + **[CCCGTC]** 1015 (87.14)   
177 + **[CGCCGC]** 1020 (85.72)   
177 + **[CGCCGC]** 1023 (85.72)   
177 + **[CGCCGC]** 1026 (85.72)   
177 + **[CGCCGC]** 1029 (85.72)   
177 + **.CGCCTC.** 1032 (91.20)   
177 + **[CGCTTC]** 1165 (86.32)   
177 + **.CGCGGC.** 1465 (94.52)   
177 - **[CGCCGC]** 1466 (85.72)   
177 - **.CGCGGC.** 952 (94.52)   
177 - **.CGCCTC.** 802 (91.20)   
177 - **[CGCCGC]** 754 (85.72)   
177 - **[CGCCGC]** 751 (85.72)   
177 - **.CGCCTC.** 748 (91.20)   
177 - **[CCCGTC]** 258 (87.14)   
178 + **[CGCCCC]** 354 (85.80)   
178 + **[CGCCGC]** 754 (85.72)   
178 + **.CGCGGC.** 1114 (94.52)   
178 + **.CGCCTC.** 1287 (91.20)   
178 - **.CGCCTC.** 1440 (91.20)   
178 - **[CGGGTC]** 1316 (87.94)   
178 - **[CGCTTC]** 1226 (86.32)   
178 - **[CCCGTC]** 1171 (87.14)   
178 - **[CGCCGC]** 1115 (85.72)   
178 - **.CGCGTC.** 1112 (100.00)   
178 - **[CGCCCC]** 1080 (85.80)   
178 - **.CGCGTC.** 941 (100.00)   
179 + **[CGGGTC]** 845 (87.94)   
179 + **.CGCGCC.** 858 (94.60)   
179 + **[CGCCGC]** 860 (85.72)   
179 + **[CGCCGC]** 915 (85.72)   
179 + **.CGCGGC.** 918 (94.52)   
179 + **.CGCGTC.** 1204 (100.00)   
179 + **[CGCCGC]** 1473 (85.72)   
179 + **.CGCGCC.** 1476 (94.60)   
179 - **.CGCGGC.** 1474 (94.52)   
179 - **[CGGGTC]** 1142 (87.94)   
179 - **.CGCGTC.** 994 (100.00)   
179 - **.CGCGCC.** 986 (94.60)   
179 - **[CGCCGC]** 984 (85.72)   
179 - **[CGCCGC]** 981 (85.72)   
179 - **[CGCCGC]** 978 (85.72)   
179 - **[CGCCGC]** 975 (85.72)   
179 - **[CGCCGC]** 948 (85.72)   
179 - **[CGCCGC]** 945 (85.72)   
179 - **[CGCCGC]** 942 (85.72)   
179 - **.CGCGGC.** 916 (94.52)   
179 - **[CGCCGC]** 874 (85.72)   
179 - **[CGCCCC]** 829 (85.80)   
179 - **.CGCCTC.** 824 (91.20)   
179 - **[CCCGTC]** 793 (87.14)   
179 - **[CGCCCC]** 532 (85.80)   
180 + **[GGCGTC]** 617 (87.08)   
180 + **.CGCCTC.** 686 (91.20)   
180 + **[CGCCGC]** 780 (85.72)   
180 + **[CGCCCC]** 859 (85.80)   
180 + **.CGCGCC.** 883 (94.60)   
180 + **.CGCCTC.** 885 (91.20)   
180 + **.CGCGGC.** 1065 (94.52)   
180 + **[CGCGAC]** 1102 (87.27)   
180 + **.CGCGGC.** 1159 (94.52)   
180 + **.CGCGGC.** 1247 (94.52)   
180 + **[GGCGTC]** 1281 (87.08)   
180 + **.CGCGGC.** 1313 (94.52)   
180 + **[CGCCGC]** 1327 (85.72)   
180 + **[CGCCGC]** 1508 (85.72)   
180 - **[CGCCCC]** 1400 (85.80)   
180 - **[CCCGTC]** 1299 (87.14)   
180 - **[CGCCCC]** 1279 (85.80)   
180 - **[CGGGTC]** 1269 (87.94)   
180 - **[CGCCGC]** 1251 (85.72)   
180 - **[CGCCGC]** 1248 (85.72)   
180 - **.CGCGCC.** 1087 (94.60)   
180 - **[CGCCGC]** 1066 (85.72)   
180 - **.CGCGCC.** 1063 (94.60)   
180 - **.CGCCTC.** 1061 (91.20)   
180 - **[CGCCGC]** 1045 (85.72)   
180 - **[CGCCGC]** 1042 (85.72)   
180 - **.CGCGCC.** 879 (94.60)   
180 - **[CGCCCC]** 877 (85.80)   
180 - **.CGCGCC.** 796 (94.60)   
180 - **[CGCCCC]** 794 (85.80)   
180 - **[CGCCCC]** 682 (85.80)   
180 - **[CGCCCC]** 677 (85.80)   
180 - **[CGCCCC]** 654 (85.80)   
180 - **[CGCCCC]** 632 (85.80)   
180 - **[CGCCCC]** 615 (85.80)   
180 - **[CGGGTC]** 529 (87.94)   
180 - **[CGCCCC]** 431 (85.80)   
180 - **[CGCCCC]** 380 (85.80)   
180 - **[CGCCCC]** 318 (85.80)   
180 - **[CGCTTC]** 286 (86.32)   
180 - **[CCCGTC]** 104 (87.14)   
180 - **[GGCGTC]** 33 (87.08)   
180 - **[CGCCCC]** 25 (85.80)   
182 + **.CGCGGC.** 266 (94.52)   
182 + **[CGCCGC]** 290 (85.72)   
182 + **.CGCGGC.** 377 (94.52)   
182 + **.CGCGGC.** 463 (94.52)   
182 + **[CGGGTC]** 535 (87.94)   
182 + **[CGGGTC]** 596 (87.94)   
182 + **[CGCCCC]** 663 (85.80)   
182 + **.CGCGCC.** 668 (94.60)   
182 + **.CGCCTC.** 685 (91.20)   
182 + **.CGCGTC.** 876 (100.00)   
182 + **.CGCGTC.** 920 (100.00)   
182 + **.CGCGGC.** 985 (94.52)   
182 + **.CGCGTC.** 1021 (100.00)   
182 + **.CGCGGC.** 1078 (94.52)   
182 + **[CGCGAC]** 1104 (87.27)   
182 + **.CGCGCC.** 1163 (94.60)   
182 + **[GGCGTC]** 1285 (87.08)   
182 - **[CGCCCC]** 1301 (85.80)   
182 - **[CCCGTC]** 1297 (87.14)   
182 - **[CGCCCC]** 1253 (85.80)   
182 - **.CGCCTC.** 1248 (91.20)   
182 - **[CGCCCC]** 1234 (85.80)   
182 - **[GGCGTC]** 1113 (87.08)   
182 - **.CGCGCC.** 1102 (94.60)   
182 - **[CGCGAC]** 918 (87.27)   
182 - **[CGCCCC]** 903 (85.80)   
182 - **.CGCGCC.** 874 (94.60)   
182 - **.CGCGCC.** 766 (94.60)   
182 - **[CGCCCC]** 764 (85.80)   
182 - **[CGCTTC]** 753 (86.32)   
182 - **.CGCGGC.** 748 (94.52)   
182 - **[CGGGTC]** 720 (87.94)   
182 - **[CGCGTG]** 632 (86.67)   
182 - **[CGCCCC]** 582 (85.80)   
182 - **[CGCGAC]** 538 (87.27)   
182 - **.CGCGGC.** 529 (94.52)   
182 - **[CGGGTC]** 522 (87.94)   
182 - **[CGCCGC]** 511 (85.72)   
182 - **.CGCGCC.** 466 (94.60)   
182 - **[CGCCGC]** 464 (85.72)   
182 - **.CGCGGC.** 461 (94.52)   
182 - **.CGCGCC.** 432 (94.60)   
182 - **[CGCCCC]** 426 (85.80)   
182 - **[CCCGTC]** 416 (87.14)   
182 - **[CGCCGC]** 396 (85.72)   
182 - **[CGCCCC]** 393 (85.80)   
182 - **.CGCGCC.** 386 (94.60)   
182 - **.CGCGGC.** 375 (94.52)   
182 - **[CGCGTG]** 367 (86.67)   
182 - **.CGCGTC.** 300 (100.00)   
182 - **[CGCGAC]** 264 (87.27)   
182 - **.CGCGGC.** 233 (94.52)   
182 - **.CGCGCC.** 121 (94.60)   
182 - **.CGCGCC.** 32 (94.60)   
183 + **.CGCGTC.** 82 (100.00)   
183 + **[CGCCCC]** 580 (85.80)   
183 + **[CCCGTC]** 590 (87.14)   
183 + **[CGCGAC]** 711 (87.27)   
183 + **[CGCTTC]** 792 (86.32)   
183 + **[CGCCGC]** 879 (85.72)   
183 + **.CGCCTC.** 882 (91.20)   
183 + **[CGGGTC]** 964 (87.94)   
183 + **.CGCGGC.** 1061 (94.52)   
183 + **[CGCCGC]** 1180 (85.72)   
183 + **[CGCCGC]** 1183 (85.72)   
183 + **[CGCCGC]** 1186 (85.72)   
183 + **[CGCCGC]** 1189 (85.72)   
183 + **[CGCTTC]** 1201 (86.32)   
183 + **.CGCGCC.** 1232 (94.60)   
183 + **[CGCCCC]** 1234 (85.80)   
183 + **.CGCGTC.** 1301 (100.00)   
183 + **[CCCGTC]** 1307 (87.14)   
183 + **[CGCCCC]** 1328 (85.80)   
183 + **[CGCCCC]** 1341 (85.80)   
183 + **.CGCGGC.** 1356 (94.52)   
183 + **[CGCCCC]** 1382 (85.80)   
183 + **[CGCCGC]** 1408 (85.72)   
183 + **[CGCCGC]** 1411 (85.72)   
183 + **.CGCGCC.** 1424 (94.60)   
183 + **[CGCCGC]** 1426 (85.72)   
183 - **[CGCCGC]** 1357 (85.72)   
183 - **.CGCGGC.** 1299 (94.52)   
183 - **.CGCGGC.** 1230 (94.52)   
183 - **[CGGGTC]** 1220 (87.94)   
183 - **[CGCCGC]** 1103 (85.72)   
183 - **[CGCCGC]** 1062 (85.72)   
183 - **[CGCCGC]** 1025 (85.72)   
183 - **.CGCGCC.** 983 (94.60)   
183 - **[CGCCGC]** 981 (85.72)   
183 - **[CGCCGC]** 978 (85.72)   
183 - **.CGCCTC.** 975 (91.20)   
183 - **[CGCCCC]** 921 (85.80)   
183 - **.CGCGGC.** 836 (94.52)   
183 - **[CGGGTC]** 714 (87.94)   
183 - **[CGCCCC]** 632 (85.80)   
183 - **[CGCCGC]** 605 (85.72)   
183 - **.CGCGCC.** 364 (94.60)   
184 + **[CGCCGC]** 53 (85.72)   
184 + **[CGCCCC]** 69 (85.80)   
184 + **[CGCTTC]** 213 (86.32)   
184 + **[CGCCCC]** 332 (85.80)   
184 + **[CGCCCC]** 369 (85.80)   
184 + **[CGCCGC]** 514 (85.72)   
184 + **.CGCGGC.** 633 (94.52)   
184 + **[GGCGTC]** 669 (87.08)   
184 + **[CGCCCC]** 772 (85.80)   
184 + **[CGCCGC]** 800 (85.72)   
184 + **[CGCCGC]** 842 (85.72)   
184 + **.CGCGGC.** 928 (94.52)   
184 + **[CGCCGC]** 983 (85.72)   
184 + **.CGCCTC.** 986 (91.20)   
184 + **[CGCCGC]** 992 (85.72)   
184 + **.CGCGGC.** 1068 (94.52)   
184 + **.CGCGCC.** 1087 (94.60)   
184 + **[CGCCGC]** 1089 (85.72)   
184 + **[CGCGTG]** 1092 (86.67)   
184 + **[CGCCGC]** 1164 (85.72)   
184 + **.CGCGCC.** 1167 (94.60)   
184 + **[CGCCCC]** 1169 (85.80)   
184 + **.CGCGGC.** 1278 (94.52)   
184 + **.CGCGGC.** 1352 (94.52)   
184 + **.CGCGCC.** 1437 (94.60)   
184 - **[CCCGTC]** 1501 (87.14)   
184 - **[CGCCGC]** 1353 (85.72)   
184 - **.CGCGCC.** 1350 (94.60)   
184 - **[CGCCCC]** 1348 (85.80)   
184 - **[CGCCCC]** 1343 (85.80)   
184 - **[CGCCCC]** 1318 (85.80)   
184 - **.CGCGCC.** 1276 (94.60)   
184 - **.CGCCTC.** 1274 (91.20)   
184 - **[CCCGTC]** 1233 (87.14)   
184 - **.CGCGGC.** 1165 (94.52)   
184 - **[CGCCCC]** 1122 (85.80)   
184 - **.CGCGGC.** 1090 (94.52)   
184 - **.CGCGCC.** 1085 (94.60)   
184 - **.CGCCTC.** 1083 (91.20)   
184 - **[CGCCGC]** 1022 (85.72)   
184 - **[CGCCCC]** 953 (85.80)   
184 - **[CGCCGC]** 929 (85.72)   
184 - **[CGCCCC]** 704 (85.80)   
184 - **[CGCCCC]** 682 (85.80)   
184 - **.CGCCTC.** 667 (91.20)   
184 - **[CGCCCC]** 642 (85.80)   
184 - **[CGGGTC]** 586 (87.94)   
184 - **.CGCCTC.** 496 (91.20)   
184 - **[GGCGTC]** 367 (87.08)   
184 - **.CGCGGC.** 54 (94.52)   
185 + **.CGCGCC.** 9 (94.60)   
185 + **[CGCCCC]** 18 (85.80)   
185 + **[CGCCGC]** 223 (85.72)   
185 + **[CGCCGC]** 231 (85.72)   
185 + **.CGCCTC.** 384 (91.20)   
185 + **[CGCCCC]** 397 (85.80)   
185 + **.CGCGCC.** 413 (94.60)   
185 + **[CGCCCC]** 415 (85.80)   
185 + **[CGCCCC]** 804 (85.80)   
185 + **[CGCCCC]** 882 (85.80)   
185 + **[CGCCGC]** 894 (85.72)   
185 + **[CGCCGC]** 897 (85.72)   
185 + **[CGCCGC]** 900 (85.72)   
185 + **[CGCCGC]** 903 (85.72)   
185 + **[CGCCGC]** 906 (85.72)   
185 + **[CGCCGC]** 909 (85.72)   
185 + **[CGCCGC]** 912 (85.72)   
185 + **[CGCCGC]** 915 (85.72)   
185 + **[CGCCCC]** 1020 (85.80)   
185 + **.CGCGCC.** 1034 (94.60)   
185 + **.CGCGCC.** 1047 (94.60)   
185 + **[CGCCGC]** 1146 (85.72)   
185 + **[CGCCGC]** 1149 (85.72)   
185 + **[CGCCCC]** 1152 (85.80)   
185 + **[CGCCGC]** 1189 (85.72)   
185 + **.CGCGGC.** 1192 (94.52)   
185 - **[CGCCCC]** 1327 (85.80)   
185 - **[CGCCGC]** 1196 (85.72)   
185 - **[CGCCGC]** 1193 (85.72)   
185 - **.CGCGGC.** 1190 (94.52)   
185 - **.CGCGGC.** 1090 (94.52)   
185 - **.CGCGGC.** 1063 (94.52)   
185 - **.CGCCTC.** 749 (91.20)   
185 - **.CGCGGC.** 571 (94.52)   
185 - **.CGCGGC.** 555 (94.52)   
185 - **[CGCCCC]** 488 (85.80)   
185 - **.CGCGCC.** 337 (94.60)   
185 - **[CGCCGC]** 312 (85.72)   
185 - **[CGCCCC]** 263 (85.80)   
185 - **.CGCCTC.** 257 (91.20)   
185 - **[GGCGTC]** 221 (87.08)   
185 - **.CGCGGC.** 128 (94.52)   
186 + **[GGCGTC]** 103 (87.08)   
186 + **.CGCGTC.** 905 (100.00)   
186 + **.CGCGCC.** 1012 (94.60)   
186 + **[CGCCGC]** 1014 (85.72)   
186 + **.CGCGTC.** 1082 (100.00)   
186 + **.CGCCTC.** 1190 (91.20)   
186 + **[CGCCGC]** 1275 (85.72)   
186 + **.CGCGCC.** 1316 (94.60)   
186 + **.CGCGCC.** 1350 (94.60)   
186 + **.CGCGGC.** 1389 (94.52)   
186 + **[CGCGTG]** 1431 (86.67)   
186 + **.CGCGGC.** 1463 (94.52)   
186 - **.CGCGCC.** 1461 (94.60)   
186 - **[CGCCGC]** 1402 (85.72)   
186 - **.CGCGGC.** 1348 (94.52)   
186 - **[CCCGTC]** 1145 (87.14)   
186 - **[CGCGTG]** 903 (86.67)   
187 + **.CGCGCC.** 21 (94.60)   
187 + **[CGCCCC]** 48 (85.80)   
187 + **.CGCCTC.** 134 (91.20)   
187 + **[CCCGTC]** 478 (87.14)   
187 + **[CGCCCC]** 863 (85.80)   
187 + **.CGCGTC.** 925 (100.00)   
187 + **.CGCGGC.** 948 (94.52)   
187 + **[GGCGTC]** 990 (87.08)   
187 + **.CGCCTC.** 1005 (91.20)   
187 + **[CGGGTC]** 1071 (87.94)   
187 + **[CGCCGC]** 1088 (85.72)   
187 + **[CGCCCC]** 1146 (85.80)   
187 + **[CGCTTC]** 1169 (86.32)   
187 - **[CGCCCC]** 1450 (85.80)   
187 - **[CGCCCC]** 1416 (85.80)   
187 - **[CGCCCC]** 1404 (85.80)   
187 - **[CGGGTC]** 1308 (87.94)   
187 - **.CGCGGC.** 1302 (94.52)   
187 - **[CGCCCC]** 1267 (85.80)   
187 - **[CGCGAC]** 1235 (87.27)   
187 - **.CGCGCC.** 1229 (94.60)   
187 - **.CGCGCC.** 1176 (94.60)   
187 - **.CGCGCC.** 1125 (94.60)   
187 - **[CGCTTC]** 1102 (86.32)   
187 - **.CGCCTC.** 1080 (91.20)   
187 - **[CGCGAC]** 1074 (87.27)   
187 - **[CGCCCC]** 988 (85.80)   
187 - **[CGCCCC]** 974 (85.80)   
187 - **[CGCCCC]** 956 (85.80)   
187 - **.CGCGCC.** 946 (94.60)   
187 - **.CGCCTC.** 884 (91.20)   
187 - **[CGGGTC]** 877 (87.94)   
187 - **[CGCCGC]** 804 (85.72)   
187 - **[CGCCCC]** 318 (85.80)   
188 + **[CGCCCC]** 30 (85.80)   
188 + **.CGCGCC.** 205 (94.60)   
188 + **[CGCCCC]** 302 (85.80)   
188 + **[CGCCGC]** 307 (85.72)   
188 + **[CGCCCC]** 529 (85.80)   
188 + **[CGCCCC]** 534 (85.80)   
188 + **[CGCGTG]** 773 (86.67)   
188 + **[CCCGTC]** 877 (87.14)   
188 + **[CGCGTG]** 1298 (86.67)   
188 + **[CGCCGC]** 1390 (85.72)   
188 + **[CGCCGC]** 1393 (85.72)   
188 + **.CGCGCC.** 1440 (94.60)   
188 + **[CGCCGC]** 1442 (85.72)   
188 + **.CGCGGC.** 1445 (94.52)   
188 + **[CGCCGC]** 1469 (85.72)   
188 + **.CGCGCC.** 1472 (94.60)   
188 + **[CGCCGC]** 1474 (85.72)   
188 - **.CGCGGC.** 1470 (94.52)   
188 - **.CGCGGC.** 1443 (94.52)   
188 - **[CGCCGC]** 1386 (85.72)   
188 - **[CGCCGC]** 502 (85.72)   
188 - **[CGCTTC]** 427 (86.32)   
188 - **.CGCGGC.** 414 (94.52)   
188 - **.CGCGTC.** 387 (100.00)   
188 - **.CGCCTC.** 343 (91.20)   
188 - **[CGCCGC]** 216 (85.72)   
189 + **[CGCGTG]** 851 (86.67)   
189 + **.CGCCTC.** 1060 (91.20)   
189 + **[CGCCCC]** 1081 (85.80)   
189 + **[CGCGAC]** 1090 (87.27)   
189 + **.CGCGCC.** 1474 (94.60)   
189 - **.CGCGCC.** 1472 (94.60)   
189 - **.CGCCTC.** 1470 (91.20)   
189 - **[CGGGTC]** 1460 (87.94)   
189 - **[CGGGTC]** 1346 (87.94)   
189 - **.CGCCTC.** 1335 (91.20)   
189 - **[CGCGAC]** 1192 (87.27)   
189 - **[CGGGTC]** 1025 (87.94)   
189 - **.CGCGCC.** 849 (94.60)   
191 + **.CGCGCC.** 491 (94.60)   
191 + **.CGCGGC.** 569 (94.52)   
191 + **[CGCGTG]** 583 (86.67)   
191 + **.CGCGCC.** 618 (94.60)   
191 + **[CGCGTG]** 652 (86.67)   
191 + **[CGCGTG]** 693 (86.67)   
191 + **[CGCGTG]** 755 (86.67)   
191 + **.CGCGGC.** 827 (94.52)   
191 + **.CGCGGC.** 883 (94.52)   
191 + **.CGCGGC.** 1045 (94.52)   
191 + **.CGCGGC.** 1191 (94.52)   
191 - **[CGCGTG]** 1477 (86.67)   
191 - **[CGCCCC]** 1335 (85.80)   
191 - **[CGCCCC]** 1329 (85.80)   
191 - **[CGCCCC]** 1285 (85.80)   
191 - **[CCCGTC]** 1281 (87.14)   
191 - **[CGCCGC]** 1178 (85.72)   
191 - **.CGCGCC.** 1161 (94.60)   
191 - **.CGCGCC.** 1137 (94.60)   
191 - **[CGCCGC]** 1135 (85.72)   
191 - **[CGCCCC]** 1118 (85.80)   
191 - **[CGCCGC]** 1046 (85.72)   
191 - **.CGCGCC.** 1043 (94.60)   
191 - **[CGCCGC]** 1028 (85.72)   
191 - **.CGCCTC.** 1025 (91.20)   
191 - **[CGCCGC]** 1013 (85.72)   
191 - **[CGCCGC]** 1010 (85.72)   
191 - **[CGCCGC]** 1007 (85.72)   
191 - **[CGCCGC]** 1004 (85.72)   
191 - **.CGCGCC.** 877 (94.60)   
191 - **.CGCGCC.** 839 (94.60)   
191 - **.CGCCTC.** 837 (91.20)   
191 - **.CGCGGC.** 831 (94.52)   
191 - **.CGCGCC.** 823 (94.60)   
191 - **.CGCGCC.** 790 (94.60)   
191 - **[CGCCCC]** 788 (85.80)   
191 - **[CGCGTG]** 650 (86.67)   
191 - **.CGCGCC.** 616 (94.60)   
191 - **[GGCGTC]** 334 (87.08)   
191 - **.CGCCTC.** 262 (91.20)   
191 - **.CGCGCC.** 97 (94.60)   
191 - **[CGCCCC]** 95 (85.80)   
193 + **[CGCGTG]** 255 (86.67)   
193 + **[GGCGTC]** 489 (87.08)   
193 + **[GGCGTC]** 949 (87.08)   
193 + **[CGCCCC]** 1076 (85.80)   
193 + **.CGCGGC.** 1249 (94.52)   
193 + **[CGCCGC]** 1254 (85.72)   
193 + **[CGCCCC]** 1267 (85.80)   
193 + **.CGCCTC.** 1441 (91.20)   
193 - **[GGCGTC]** 1369 (87.08)   
193 - **[CGCCGC]** 1331 (85.72)   
193 - **[GGCGTC]** 1265 (87.08)   
193 - **[CGCCGC]** 1250 (85.72)   
193 - **.CGCGTC.** 1124 (100.00)   
193 - **[CGCGTG]** 894 (86.67)   
193 - **[CGCCGC]** 803 (85.72)   
193 - **[CGGGTC]** 707 (87.94)   
193 - **.CGCGCC.** 642 (94.60)   
193 - **.CGCCTC.** 640 (91.20)   
193 - **[GGCGTC]** 510 (87.08)   
193 - **[CGGGTC]** 190 (87.94)   
193 - **[CGGGTC]** 150 (87.94)   
194 + **[GGCGTC]** 182 (87.08)   
194 + **[CGGGTC]** 264 (87.94)   
194 + **[GGCGTC]** 555 (87.08)   
194 + **[CGCCCC]** 589 (85.80)   
194 + **.CGCGCC.** 608 (94.60)   
194 + **[CGCCGC]** 740 (85.72)   
194 + **.CGCGGC.** 871 (94.52)   
194 + **.CGCGGC.** 912 (94.52)   
194 + **.CGCGGC.** 945 (94.52)   
194 + **[CGCCCC]** 963 (85.80)   
194 + **.CGCGGC.** 990 (94.52)   
194 + **[CGCCGC]** 1039 (85.72)   
194 + **[CGCGTG]** 1070 (86.67)   
194 + **.CGCGGC.** 1112 (94.52)   
194 + **.CGCGCC.** 1254 (94.60)   
194 + **[CGCCCC]** 1269 (85.80)   
194 + **[CGCCCC]** 1411 (85.80)   
194 + **[CGCTTC]** 1434 (86.32)   
194 - **[CGCCGC]** 1190 (85.72)   
194 - **.CGCCTC.** 1159 (91.20)   
194 - **.CGCGCC.** 1115 (94.60)   
194 - **[CGCCGC]** 1113 (85.72)   
194 - **.CGCGGC.** 1110 (94.52)   
194 - **.CGCGCC.** 1066 (94.60)   
194 - **[CGCCCC]** 1064 (85.80)   
194 - **[CCCGTC]** 1046 (87.14)   
194 - **.CGCGGC.** 1040 (94.52)   
194 - **.CGCGCC.** 1016 (94.60)   
194 - **[CGCCGC]** 991 (85.72)   
194 - **.CGCGGC.** 988 (94.52)   
194 - **.CGCGCC.** 973 (94.60)   
194 - **[CGCCGC]** 946 (85.72)   
194 - **.CGCGCC.** 943 (94.60)   
194 - **[CGCCGC]** 913 (85.72)   
194 - **.CGCGTC.** 908 (100.00)   
194 - **[CGCCCC]** 892 (85.80)   
194 - **.CGCGCC.** 867 (94.60)   
194 - **[CGCCCC]** 845 (85.80)   
194 - **[CGCCCC]** 812 (85.80)   
194 - **[CGCCCC]** 782 (85.80)   
194 - **.CGCGGC.** 723 (94.52)   
194 - **[CGCCCC]** 704 (85.80)   
194 - **[CGCCCC]** 426 (85.80)   
194 - **[CGCCCC]** 417 (85.80)   
194 - **[CGCCCC]** 299 (85.80)   
194 - **[CGCCCC]** 294 (85.80)   
194 - **[CGGGTC]** 246 (87.94)   
194 - **[CGCCCC]** 232 (85.80)   
194 - **[CGCCCC]** 180 (85.80)   
194 - **[CGCCGC]** 25 (85.72)   
195 + **[CGCCGC]** 56 (85.72)   
195 + **[CCCGTC]** 104 (87.14)   
195 + **[CGCCCC]** 130 (85.80)   
195 + **.CGCGCC.** 153 (94.60)   
195 + **[CGCCGC]** 155 (85.72)   
195 + **[CGCCGC]** 165 (85.72)   
195 + **.CGCGGC.** 168 (94.52)   
195 + **.CGCGCC.** 251 (94.60)   
195 + **[GGCGTC]** 446 (87.08)   
195 + **[CGGGTC]** 461 (87.94)   
195 + **[CGCCCC]** 830 (85.80)   
195 + **.CGCGGC.** 852 (94.52)   
195 + **[CGCCCC]** 917 (85.80)   
195 + **.CGCGCC.** 924 (94.60)   
195 + **[CGCCCC]** 926 (85.80)   
195 + **.CGCGTC.** 960 (100.00)   
195 + **[CGCCGC]** 966 (85.72)   
195 + **[CGCCCC]** 1004 (85.80)   
195 + **[CGGGTC]** 1175 (87.94)   
195 + **[CGCCGC]** 1223 (85.72)   
195 + **[CGCCGC]** 1407 (85.72)   
195 - **[CGCCCC]** 1450 (85.80)   
195 - **[CGCCCC]** 1403 (85.80)   
195 - **[CGCCCC]** 1275 (85.80)   
195 - **.CGCGCC.** 907 (94.60)   
195 - **[CGCCGC]** 853 (85.72)   
195 - **[CGCGAC]** 850 (87.27)   
195 - **[GGCGTC]** 828 (87.08)   
195 - **[CGCCCC]** 490 (85.80)   
195 - **[CGCCCC]** 444 (85.80)   
195 - **[CGCCCC]** 439 (85.80)   
195 - **[CGCCCC]** 425 (85.80)   
195 - **[CGCCCC]** 394 (85.80)   
195 - **[CCCGTC]** 380 (87.14)   
195 - **[CGCCCC]** 372 (85.80)   
195 - **.CGCCTC.** 309 (91.20)   
195 - **.CGCGGC.** 166 (94.52)   
196 + **.CGCGGC.** 120 (94.52)   
196 + **[CGCCCC]** 134 (85.80)   
196 + **.CGCGGC.** 284 (94.52)   
196 + **[GGCGTC]** 287 (87.08)   
196 + **.CGCCTC.** 305 (91.20)   
196 + **[CGCCGC]** 341 (85.72)   
196 + **.CGCGCC.** 506 (94.60)   
196 + **.CGCGGC.** 538 (94.52)   
196 + **[GGCGTC]** 639 (87.08)   
196 + **[CGGGTC]** 678 (87.94)   
196 + **.CGCGTC.** 721 (100.00)   
196 + **[CGCCGC]** 910 (85.72)   
196 + **.CGCGCC.** 913 (94.60)   
196 + **[CGCCCC]** 965 (85.80)   
196 + **[CGCCGC]** 1022 (85.72)   
196 + **.CGCGGC.** 1076 (94.52)   
196 + **[CGCGTG]** 1192 (86.67)   
196 + **.CGCGCC.** 1317 (94.60)   
196 + **[CGCCCC]** 1465 (85.80)   
196 + **.CGCGCC.** 1508 (94.60)   
196 + **[CGCCCC]** 1510 (85.80)   
196 - **[CGGGTC]** 1492 (87.94)   
196 - **.CGCCTC.** 1484 (91.20)   
196 - **.CGCGCC.** 1471 (94.60)   
196 - **.CGCGCC.** 1427 (94.60)   
196 - **[CGCCGC]** 1425 (85.72)   
196 - **[CGCCGC]** 1366 (85.72)   
196 - **.CGCGCC.** 1352 (94.60)   
196 - **.CGCCTC.** 1326 (91.20)   
196 - **.CGCCTC.** 1152 (91.20)   
196 - **.CGCGGC.** 1102 (94.52)   
196 - **.CGCCTC.** 1018 (91.20)   
196 - **[CGCCCC]** 948 (85.80)   
196 - **[GGCGTC]** 929 (87.08)   
196 - **.CGCGGC.** 911 (94.52)   
196 - **[CGCCCC]** 866 (85.80)   
196 - **[CGCCCC]** 822 (85.80)   
196 - **[CGCGTG]** 719 (86.67)   
196 - **[GGCGTC]** 700 (87.08)   
196 - **.CGCGTC.** 688 (100.00)   
196 - **.CGCGCC.** 666 (94.60)   
196 - **[CGCCCC]** 637 (85.80)   
196 - **.CGCGCC.** 504 (94.60)   
196 - **[CGCCCC]** 502 (85.80)   
196 - **[CGCGTG]** 497 (86.67)   
196 - **.CGCGGC.** 342 (94.52)   
196 - **[CGCCGC]** 285 (85.72)   
196 - **[CGCCGC]** 269 (85.72)   
196 - **[CGCCCC]** 266 (85.80)   
196 - **[CGCCCC]** 245 (85.80)   
196 - **[CGCCCC]** 233 (85.80)   
196 - **[CGCCCC]** 172 (85.80)   
196 - **[CGCTTC]** 166 (86.32)   
196 - **[CGCCGC]** 121 (85.72)   
196 - **[CGCCCC]** 64 (85.80)   
196 - **.CGCGTC.** 31 (100.00)   
197 + **[CGCCGC]** 210 (85.72)   
197 + **.CGCCTC.** 213 (91.20)   
197 + **[CGCTTC]** 223 (86.32)   
197 + **.CGCGTC.** 431 (100.00)   
197 + **.CGCGGC.** 478 (94.52)   
197 + **.CGCGCC.** 791 (94.60)   
197 + **.CGCGGC.** 1047 (94.52)   
197 + **[CGGGTC]** 1114 (87.94)   
197 + **.CGCGGC.** 1163 (94.52)   
197 + **[GGCGTC]** 1166 (87.08)   
197 + **.CGCGGC.** 1506 (94.52)   
197 - **.CGCGCC.** 1509 (94.60)   
197 - **[CGCCGC]** 1507 (85.72)   
197 - **.CGCGCC.** 1504 (94.60)   
197 - **.CGCCTC.** 1502 (91.20)   
197 - **[CGCCCC]** 1187 (85.80)   
197 - **[CGCCGC]** 1164 (85.72)   
197 - **.CGCGTC.** 1161 (100.00)   
197 - **[CGGGTC]** 1152 (87.94)   
197 - **.CGCGTC.** 1100 (100.00)   
197 - **[CGCCGC]** 1073 (85.72)   
197 - **[CGCCGC]** 1054 (85.72)   
197 - **[CGCCGC]** 1022 (85.72)   
197 - **.CGCCTC.** 1003 (91.20)   
197 - **[CGCCGC]** 873 (85.72)   
197 - **.CGCGGC.** 789 (94.52)   
197 - **[CGCCCC]** 507 (85.80)   
197 - **[CGCGAC]** 476 (87.27)   
198 + **.CGCGCC.** 35 (94.60)   
198 + **.CGCCTC.** 170 (91.20)   
198 + **.CGCGTC.** 216 (100.00)   
198 + **[GGCGTC]** 233 (87.08)   
198 + **[CGCCGC]** 253 (85.72)   
198 + **[CGCCGC]** 256 (85.72)   
198 + **.CGCGCC.** 259 (94.60)   
198 + **[CGCCCC]** 261 (85.80)   
198 + **.CGCGCC.** 266 (94.60)   
198 + **[CGCCCC]** 268 (85.80)   
198 + **[CGCCCC]** 325 (85.80)   
198 + **[CGGGTC]** 993 (87.94)   
198 + **[GGCGTC]** 1083 (87.08)   
198 + **.CGCGGC.** 1165 (94.52)   
198 + **.CGCGCC.** 1437 (94.60)   
198 - **[CGCCCC]** 1302 (85.80)   
198 - **[CGCCCC]** 1281 (85.80)   
198 - **[CCCGTC]** 1263 (87.14)   
198 - **[CGCCCC]** 1241 (85.80)   
198 - **.CGCCTC.** 1235 (91.20)   
198 - **[CCCGTC]** 937 (87.14)   
198 - **.CGCCTC.** 908 (91.20)   
198 - **[CCCGTC]** 489 (87.14)   
198 - **.CGCGGC.** 257 (94.52)   
199 + **[CGCCCC]** 342 (85.80)   
199 + **[CGCGTG]** 440 (86.67)   
199 + **.CGCCTC.** 456 (91.20)   
199 + **.CGCGCC.** 672 (94.60)   
199 + **[CGCCCC]** 674 (85.80)   
199 + **.CGCCTC.** 701 (91.20)   
199 + **.CGCGTC.** 821 (100.00)   
199 + **[CGCGTG]** 826 (86.67)   
199 + **[CGGGTC]** 893 (87.94)   
199 + **.CGCGGC.** 908 (94.52)   
199 + **.CGCCTC.** 926 (91.20)   
199 + **.CGCGGC.** 951 (94.52)   
199 + **[CGCCGC]** 1000 (85.72)   
199 + **.CGCGGC.** 1003 (94.52)   
199 + **[CGGGTC]** 1033 (87.94)   
199 + **[CGCCCC]** 1058 (85.80)   
199 + **.CGCGGC.** 1085 (94.52)   
199 + **.CGCGCC.** 1244 (94.60)   
199 + **.CGCGGC.** 1276 (94.52)   
199 + **.CGCGTC.** 1448 (100.00)   
199 + **.CGCGCC.** 1491 (94.60)   
199 - **.CGCGCC.** 1352 (94.60)   
199 - **[CGCCGC]** 1350 (85.72)   
199 - **[CGCCCC]** 1347 (85.80)   
199 - **.CGCGCC.** 1320 (94.60)   
199 - **.CGCGCC.** 1286 (94.60)   
199 - **.CGCCTC.** 1284 (91.20)   
199 - **.CGCGGC.** 1265 (94.52)   
199 - **[CGCGTG]** 1242 (86.67)   
199 - **[CGCCGC]** 1142 (85.72)   
199 - **.CGCCTC.** 1139 (91.20)   
199 - **.CGCGGC.** 1127 (94.52)   
199 - **.CGCGCC.** 1110 (94.60)   
199 - **[CGCCCC]** 1108 (85.80)   
199 - **[CGCCGC]** 1086 (85.72)   
199 - **.CGCGCC.** 1083 (94.60)   
199 - **[CGCCGC]** 1081 (85.72)   
199 - **[GGCGTC]** 1042 (87.08)   
199 - **[CGCCCC]** 1013 (85.80)   
199 - **.CGCGGC.** 1001 (94.52)   
199 - **.CGCGCC.** 980 (94.60)   
199 - **[CGCCCC]** 978 (85.80)   
199 - **[CGCCGC]** 952 (85.72)   
199 - **.CGCGCC.** 938 (94.60)   
199 - **[CGCCGC]** 918 (85.72)   
199 - **.CGCGCC.** 911 (94.60)   
199 - **[CGCCGC]** 909 (85.72)   
199 - **.CGCGCC.** 906 (94.60)   
199 - **.CGCGCC.** 870 (94.60)   
199 - **[CGCCGC]** 868 (85.72)   
199 - **[CGCCGC]** 865 (85.72)   
199 - **[CGCCCC]** 843 (85.80)   
199 - **[GGCGTC]** 831 (87.08)   
199 - **[CGCGAC]** 824 (87.27)   
199 - **.CGCGCC.** 819 (94.60)   
199 - **[CGCCCC]** 697 (85.80)   
199 - **.CGCGCC.** 670 (94.60)   
199 - **.CGCGGC.** 438 (94.52)   
199 - **[CGCCCC]** 338 (85.80)   
199 - **[CGCGTG]** 311 (86.67)   
199 - **[CGCTTC]** 254 (86.32)   
200 + **[CGCCCC]** 653 (85.80)   
200 + **[CGCCCC]** 698 (85.80)   
200 + **[CCCGTC]** 737 (87.14)   
200 + **.CGCGGC.** 1142 (94.52)   
200 + **[CCCGTC]** 1371 (87.14)   
200 + **.CGCCTC.** 1385 (91.20)   
200 - **.CGCGCC.** 1140 (94.60)   
200 - **[CGCCGC]** 1083 (85.72)   
200 - **.CGCGTC.** 572 (100.00)   
200 - **[CGCCCC]** 539 (85.80)   
201 + **[CGCCGC]** 336 (85.72)   
201 + **.CGCGCC.** 339 (94.60)   
201 + **[CGCCCC]** 341 (85.80)   
201 + **.CGCGGC.** 351 (94.52)   
201 + **[GGCGTC]** 490 (87.08)   
201 + **[CGCCCC]** 515 (85.80)   
201 + **.CGCGGC.** 533 (94.52)   
201 + **.CGCGCC.** 661 (94.60)   
201 + **.CGCGGC.** 849 (94.52)   
201 + **[CGCCCC]** 854 (85.80)   
201 + **[CGCCCC]** 875 (85.80)   
201 + **[CGCCCC]** 897 (85.80)   
201 + **[CGCCCC]** 964 (85.80)   
201 + **[CGCCCC]** 1011 (85.80)   
201 + **.CGCGCC.** 1085 (94.60)   
201 + **.CGCGTC.** 1201 (100.00)   
201 + **[CGCCGC]** 1348 (85.72)   
201 + **[CGCCGC]** 1382 (85.72)   
201 + **.CGCGCC.** 1414 (94.60)   
201 + **.CGCGCC.** 1499 (94.60)   
201 - **.CGCGTC.** 1491 (100.00)   
201 - **.CGCCTC.** 1475 (91.20)   
201 - **[CGGGTC]** 1441 (87.94)   
201 - **.CGCGGC.** 1349 (94.52)   
201 - **[GGCGTC]** 1346 (87.08)   
201 - **[CGCCCC]** 1287 (85.80)   
201 - **[CGCCCC]** 1240 (85.80)   
201 - **.CGCGGC.** 1142 (94.52)   
201 - **[CGCCCC]** 1106 (85.80)   
201 - **[CGCCGC]** 850 (85.72)   
201 - **.CGCCTC.** 798 (91.20)   
201 - **.CGCGCC.** 776 (94.60)   
201 - **[CGCCGC]** 774 (85.72)   
201 - **[CGCCGC]** 771 (85.72)   
201 - **[CGCCGC]** 717 (85.72)   
201 - **.CGCGCC.** 676 (94.60)   
201 - **.CGCGCC.** 627 (94.60)   
201 - **[GGCGTC]** 606 (87.08)   
201 - **[CGCCGC]** 596 (85.72)   
201 - **.CGCCTC.** 488 (91.20)   
201 - **[CGCCCC]** 454 (85.80)   
201 - **[CGCCCC]** 421 (85.80)   
201 - **.CGCGGC.** 337 (94.52)   
201 - **[CGGGTC]** 198 (87.94)   
202 + **[GGCGTC]** 295 (87.08)   
202 + **[CGCCGC]** 835 (85.72)   
202 + **[CGCCCC]** 899 (85.80)   
202 + **[CGCCCC]** 946 (85.80)   
202 + **.CGCGTC.** 992 (100.00)   
202 + **[CGCCGC]** 1109 (85.72)   
202 + **.CGCGGC.** 1371 (94.52)   
202 + **.CGCGCC.** 1395 (94.60)   
202 - **.CGCGTC.** 1393 (100.00)   
202 - **.CGCCTC.** 1327 (91.20)   
202 - **.CGCGCC.** 1285 (94.60)   
202 - **[CGGGTC]** 800 (87.94)   
202 - **[CCCGTC]** 751 (87.14)   
202 - **.CGCGCC.** 722 (94.60)   
202 - **.CGCGCC.** 593 (94.60)   
202 - **[CGCCCC]** 591 (85.80)   
202 - **.CGCGTC.** 310 (100.00)   
202 - **.CGCCTC.** 293 (91.20)   
202 - **[CGGGTC]** 288 (87.94)   
203 + **.CGCGGC.** 102 (94.52)   
203 + **[CCCGTC]** 1033 (87.14)   
203 - **[CGCGAC]** 317 (87.27)   
203 - **[CGCCCC]** 283 (85.80)   
204 + **[CCCGTC]** 445 (87.14)   
205 + **[CGGGTC]** 953 (87.94)   
206 + **[CGCTTC]** 864 (86.32)   
206 + **[CCCGTC]** 874 (87.14)   
206 + **[CCCGTC]** 898 (87.14)   
207 + **[CGGGTC]** 516 (87.94)   
207 + **[CCCGTC]** 634 (87.14)   
207 + **[CCCGTC]** 675 (87.14)   
207 + **[GGCGTC]** 717 (87.08)   
207 + **[CGCCCC]** 759 (85.80)   
207 + **.CGCGTC.** 857 (100.00)   
207 + **[CGCCCC]** 1001 (85.80)   
207 + **[GGCGTC]** 1010 (87.08)   
207 + **[CGCGTG]** 1153 (86.67)   
207 + **[CGCCCC]** 1264 (85.80)   
207 + **[CGCGAC]** 1502 (87.27)   
207 - **[CGCGAC]** 1329 (87.27)   
207 - **[CGCGTG]** 1289 (86.67)   
207 - **[CCCGTC]** 1200 (87.14)   
207 - **[CGCCGC]** 1066 (85.72)   
207 - **[CGCCGC]** 1063 (85.72)   
207 - **[CGGGTC]** 997 (87.94)   
208 + **[CGCTTC]** 988 (86.32)   
208 + **[CGCTTC]** 1349 (86.32)   
208 + **.CGCGCC.** 1359 (94.60)   
208 + **[CGCCGC]** 1496 (85.72)   
208 + **.CGCGGC.** 1499 (94.52)   
208 - **[CGCCGC]** 1500 (85.72)   
208 - **.CGCGGC.** 1497 (94.52)   
208 - **.CGCGGC.** 1443 (94.52)   
208 - **.CGCGCC.** 1409 (94.60)   
208 - **[CGCCGC]** 1407 (85.72)   
208 - **.CGCGCC.** 1396 (94.60)   
208 - **.CGCCTC.** 1394 (91.20)   
208 - **[CGCCCC]** 1312 (85.80)   
208 - **.CGCGTC.** 1226 (100.00)   
208 - **[CGCGAC]** 1141 (87.27)   
209 + **[CGCGTG]** 840 (86.67)   
209 - **.CGCCTC.** 1338 (91.20)   
209 - **.CGCCTC.** 1271 (91.20)   
209 - **.CGCGGC.** 838 (94.52)   
210 + **[CGCTTC]** 289 (86.32)   
210 + **.CGCGCC.** 616 (94.60)   
210 + **[CGCCCC]** 618 (85.80)   
210 + **.CGCGTC.** 677 (100.00)   
210 + **.CGCGGC.** 780 (94.52)   
210 + **[CGCTTC]** 1013 (86.32)   
210 + **[CGCCGC]** 1020 (85.72)   
210 + **[CGCCCC]** 1109 (85.80)   
210 + **[CGCCCC]** 1151 (85.80)   
210 + **.CGCGCC.** 1223 (94.60)   
210 + **[CGCCCC]** 1225 (85.80)   
210 + **[CGCCGC]** 1269 (85.72)   
210 + **[CGCCCC]** 1439 (85.80)   
210 + **[CGCTTC]** 1470 (86.32)   
210 - **[CGGGTC]** 1347 (87.94)   
210 - **[CGCGTG]** 1221 (86.67)   
210 - **[CGCCGC]** 1105 (85.72)   
210 - **.CGCGCC.** 1066 (94.60)   
210 - **.CGCGGC.** 1047 (94.52)   
210 - **[CGCCGC]** 941 (85.72)   
210 - **[CGCCGC]** 938 (85.72)   
210 - **[CGCCGC]** 935 (85.72)   
210 - **.CGCCTC.** 907 (91.20)   
210 - **[CGCCGC]** 901 (85.72)   
210 - **[CGCCGC]** 898 (85.72)   
210 - **.CGCCTC.** 882 (91.20)   
210 - **[CGCCGC]** 873 (85.72)   
210 - **.CGCCTC.** 870 (91.20)   
210 - **[CGCCCC]** 840 (85.80)   
210 - **.CGCGGC.** 778 (94.52)   
210 - **[GGCGTC]** 766 (87.08)   
210 - **[CGCCCC]** 745 (85.80)   
210 - **.CGCGCC.** 675 (94.60)   
211 + **[CGCCGC]** 734 (85.72)   
211 + **.CGCGCC.** 737 (94.60)   
211 + **[CGCCGC]** 961 (85.72)   
211 + **[CGCCGC]** 964 (85.72)   
211 + **.CGCGCC.** 977 (94.60)   
211 + **[CGCCGC]** 979 (85.72)   
211 + **[CGCCCC]** 1077 (85.80)   
211 + **[CGCCGC]** 1082 (85.72)   
211 + **[CGCCGC]** 1104 (85.72)   
211 + **.CGCGGC.** 1216 (94.52)   
211 + **[CGCCCC]** 1238 (85.80)   
211 + **[CGCTTC]** 1249 (86.32)   
211 + **[CGCGTG]** 1407 (86.67)   
211 + **.CGCGCC.** 1481 (94.60)   
211 - **.CGCGTC.** 1405 (100.00)   
211 - **[CGCCCC]** 1391 (85.80)   
211 - **[CGCCCC]** 1348 (85.80)   
211 - **[CCCGTC]** 1344 (87.14)   
211 - **[CGCCCC]** 1336 (85.80)   
211 - **[CGCCGC]** 1325 (85.72)   
211 - **[CGCCCC]** 1265 (85.80)   
211 - **[CGCCCC]** 1160 (85.80)   
211 - **.CGCGTC.** 1145 (100.00)   
211 - **[CGCTTC]** 1096 (86.32)   
211 - **[CGGGTC]** 944 (87.94)   
211 - **.CGCGGC.** 735 (94.52)   
211 - **[CCCGTC]** 573 (87.14)   
212 + **[CGCGAC]** 422 (87.27)   
212 + **.CGCGCC.** 455 (94.60)   
212 + **[CGCCCC]** 552 (85.80)   
212 + **[CGCCCC]** 568 (85.80)   
212 + **[CGCCCC]** 580 (85.80)   
212 + **[CGCCGC]** 605 (85.72)   
212 + **.CGCGGC.** 715 (94.52)   
212 + **[CGGGTC]** 737 (87.94)   
212 + **.CGCGTC.** 1034 (100.00)   
212 + **[CGCTTC]** 1105 (86.32)   
212 + **[CGCCCC]** 1304 (85.80)   
212 + **[CGGGTC]** 1435 (87.94)   
212 + **.CGCGGC.** 1486 (94.52)   
212 + **[CGCCCC]** 1499 (85.80)   
212 - **[CGCCCC]** 1512 (85.80)   
212 - **.CGCCTC.** 1495 (91.20)   
212 - **[CGCGTG]** 1484 (86.67)   
212 - **[CGCCCC]** 1473 (85.80)   
212 - **.CGCGGC.** 713 (94.52)   
212 - **[CCCGTC]** 676 (87.14)   
212 - **[CGCCCC]** 670 (85.80)   
212 - **[CGCCGC]** 634 (85.72)   
212 - **[CGCCCC]** 631 (85.80)   
212 - **[CGCCCC]** 601 (85.80)   
212 - **.CGCGCC.** 420 (94.60)   
212 - **[CGCCGC]** 235 (85.72)   
212 - **[CGCCGC]** 232 (85.72)   
212 - **.CGCCTC.** 229 (91.20)   
213 + **.CGCGCC.** 1193 (94.60)   
213 + **.CGCGCC.** 1290 (94.60)   
213 + **.CGCCTC.** 1368 (91.20)   
213 - **[CGCCCC]** 1436 (85.80)   
213 - **[CGCCCC]** 1255 (85.80)   
213 - **[CGCCCC]** 1241 (85.80)   
213 - **[CGCCCC]** 1227 (85.80)   
213 - **[CGCCCC]** 1204 (85.80)   
213 - **[CGCGTG]** 1121 (86.67)   
213 - **[CGCCGC]** 1101 (85.72)   
213 - **.CGCGCC.** 1061 (94.60)   
213 - **.CGCCTC.** 1059 (91.20)   
213 - **.CGCGCC.** 997 (94.60)   
213 - **[CGCCGC]** 995 (85.72)   
213 - **[CCCGTC]** 953 (87.14)   
213 - **.CGCGGC.** 912 (94.52)   
214 + **[CGCTTC]** 174 (86.32)   
214 + **.CGCGTC.** 355 (100.00)   
214 + **[CGCCGC]** 490 (85.72)   
214 + **.CGCGGC.** 732 (94.52)   
214 + **[GGCGTC]** 735 (87.08)   
214 + **.CGCGGC.** 834 (94.52)   
214 + **.CGCGGC.** 931 (94.52)   
214 + **.CGCGCC.** 1033 (94.60)   
214 + **[CGCCCC]** 1035 (85.80)   
214 - **[CGCCGC]** 1490 (85.72)   
214 - **.CGCCTC.** 1487 (91.20)   
214 - **[CGCCGC]** 1466 (85.72)   
214 - **[CGCCGC]** 1463 (85.72)   
214 - **[CGCCGC]** 1460 (85.72)   
214 - **[CGCCGC]** 1447 (85.72)   
214 - **[CGCCGC]** 1413 (85.72)   
214 - **[CGCCGC]** 1410 (85.72)   
214 - **[CGCCGC]** 1407 (85.72)   
214 - **[CGCCGC]** 1404 (85.72)   
214 - **[CGCCGC]** 1401 (85.72)   
214 - **[CGCCGC]** 1389 (85.72)   
214 - **[CGGGTC]** 1268 (87.94)   
214 - **[CGCGAC]** 1190 (87.27)   
214 - **[CGCGTG]** 984 (86.67)   
214 - **.CGCGGC.** 929 (94.52)   
214 - **[CGCCCC]** 920 (85.80)   
214 - **[CGCTTC]** 862 (86.32)   
214 - **[CGCCGC]** 733 (85.72)   
214 - **[CGCCCC]** 652 (85.80)   
214 - **[CGCGTG]** 631 (86.67)   
214 - **[CGCCGC]** 514 (85.72)   
214 - **[CGGGTC]** 312 (87.94)   
215 + **.CGCGCC.** 116 (94.60)   
215 + **.CGCGCC.** 201 (94.60)   
215 + **.CGCGTC.** 654 (100.00)   
215 + **[GGCGTC]** 824 (87.08)   
215 + **[CGCCGC]** 956 (85.72)   
215 + **[CGCCGC]** 959 (85.72)   
215 + **[CGCCGC]** 1089 (85.72)   
215 + **.CGCGGC.** 1092 (94.52)   
215 + **.CGCGCC.** 1140 (94.60)   
215 + **[CGCCCC]** 1142 (85.80)   
215 + **[GGCGTC]** 1305 (87.08)   
215 + **.CGCGCC.** 1369 (94.60)   
215 + **.CGCGCC.** 1415 (94.60)   
215 + **[CGCCCC]** 1417 (85.80)   
215 + **.CGCGTC.** 1461 (100.00)   
215 - **[CGCCCC]** 1494 (85.80)   
215 - **.CGCGCC.** 1459 (94.60)   
215 - **[CGCCCC]** 1451 (85.80)   
215 - **.CGCGGC.** 1413 (94.52)   
215 - **.CGCCTC.** 1405 (91.20)   
215 - **[CGCCCC]** 1303 (85.80)   
215 - **.CGCGTC.** 1195 (100.00)   
215 - **[CGCCCC]** 1106 (85.80)   
215 - **[CGCCCC]** 1101 (85.80)   
215 - **[CGCCGC]** 1093 (85.72)   
215 - **.CGCGGC.** 1090 (94.52)   
215 - **[CGCCCC]** 1076 (85.80)   
215 - **[CGGGTC]** 711 (87.94)   
215 - **.CGCGGC.** 652 (94.52)   
215 - **[CGCCCC]** 538 (85.80)   
215 - **[CGCCGC]** 487 (85.72)   
215 - **.CGCGCC.** 112 (94.60)   
215 - **.CGCCTC.** 14 (91.20)   
216 + **.CGCGGC.** 212 (94.52)   
216 + **[CGGGTC]** 224 (87.94)   
216 + **[CGGGTC]** 432 (87.94)   
216 + **[CGCCGC]** 471 (85.72)   
216 + **[CCCGTC]** 628 (87.14)   
216 + **.CGCGCC.** 650 (94.60)   
216 + **[CGCCCC]** 652 (85.80)   
216 + **.CGCCTC.** 821 (91.20)   
216 + **.CGCGTC.** 892 (100.00)   
216 + **[CGCCCC]** 935 (85.80)   
216 + **.CGCGCC.** 947 (94.60)   
216 + **[CGCCCC]** 949 (85.80)   
216 + **[CGCCCC]** 954 (85.80)   
216 + **[CGCCCC]** 959 (85.80)   
216 + **.CGCGCC.** 996 (94.60)   
216 + **.CGCCTC.** 998 (91.20)   
216 + **[CGCCGC]** 1008 (85.72)   
216 + **[GGCGTC]** 1047 (87.08)   
216 + **.CGCGCC.** 1096 (94.60)   
216 + **.CGCGGC.** 1230 (94.52)   
216 + **[CGCCGC]** 1260 (85.72)   
216 + **[CGCCCC]** 1430 (85.80)   
216 - **.CGCGGC.** 1481 (94.52)   
216 - **.CGCGCC.** 1465 (94.60)   
216 - **[CGCGAC]** 1319 (87.27)   
216 - **.CGCGGC.** 1228 (94.52)   
216 - **[CGGGTC]** 1062 (87.94)   
216 - **.CGCGCC.** 992 (94.60)   
216 - **[CGCCCC]** 850 (85.80)   
216 - **[CCCGTC]** 430 (87.14)   
216 - **[CGCCCC]** 220 (85.80)   
216 - **[CGCCGC]** 213 (85.72)   
216 - **[GGCGTC]** 37 (87.08)   
217 + **.CGCGGC.** 577 (94.52)   
217 + **[CGCCCC]** 599 (85.80)   
217 + **.CGCCTC.** 630 (91.20)   
217 + **.CGCGTC.** 665 (100.00)   
217 + **[CGCCGC]** 797 (85.72)   
217 + **[CGCCGC]** 859 (85.72)   
217 + **[CGCCGC]** 872 (85.72)   
217 + **[CGCCGC]** 875 (85.72)   
217 + **.CGCGTC.** 941 (100.00)   
217 + **[CGCGAC]** 1057 (87.27)   
217 + **.CGCGTC.** 1094 (100.00)   
217 + **[CGCCCC]** 1171 (85.80)   
217 + **[CCCGTC]** 1223 (87.14)   
217 - **[CGCCCC]** 993 (85.80)   
217 - **.CGCGGC.** 860 (94.52)   
217 - **.CGCCTC.** 777 (91.20)   
217 - **[CGCCCC]** 703 (85.80)   
217 - **[GGCGTC]** 659 (87.08)   
217 - **[CGCCGC]** 578 (85.72)   
217 - **.CGCGGC.** 573 (94.52)   
220 - **[CGCCCC]** 1045 (85.80)   
221 + **.CGCGTC.** 20 (100.00)   
221 + **[CGCGTG]** 305 (86.67)   
221 + **.CGCGGC.** 519 (94.52)   
221 + **[CGCCGC]** 1074 (85.72)   
221 + **.CGCGGC.** 1112 (94.52)   
221 + **[CGCCCC]** 1277 (85.80)   
221 + **[CGCCCC]** 1283 (85.80)   
221 + **.CGCCTC.** 1307 (91.20)   
221 + **.CGCCTC.** 1347 (91.20)   
221 + **[CGCCCC]** 1399 (85.80)   
221 + **[CGCCCC]** 1428 (85.80)   
221 - **[CGCCCC]** 1140 (85.80)   
221 - **[CGCCGC]** 1116 (85.72)   
221 - **[CGCCGC]** 1113 (85.72)   
221 - **.CGCGGC.** 1110 (94.52)   
221 - **.CGCGCC.** 937 (94.60)   
221 - **.CGCGGC.** 515 (94.52)   
221 - **[CGCGAC]** 303 (87.27)   
221 - **[CGGGTC]** 264 (87.94)   
221 - **.CGCGGC.** 18 (94.52)   
221 - **[CGCCGC]** 8 (85.72)   
222 + **.CGCGGC.** 168 (94.52)   
222 + **[CGCCCC]** 438 (85.80)   
222 + **.CGCGTC.** 452 (100.00)   
222 + **[CGCCCC]** 518 (85.80)   
222 + **[CGCCCC]** 524 (85.80)   
222 + **[CGGGTC]** 535 (87.94)   
222 + **[CCCGTC]** 580 (87.14)   
222 + **[CGCCCC]** 589 (85.80)   
222 + **[CGCCCC]** 603 (85.80)   
222 + **[CGCCCC]** 608 (85.80)   
222 + **[CGCCCC]** 614 (85.80)   
222 + **[CGCCGC]** 666 (85.72)   
222 + **[CGCCCC]** 673 (85.80)   
222 + **.CGCGCC.** 691 (94.60)   
222 + **[CGCCCC]** 705 (85.80)   
222 + **[CGCCCC]** 712 (85.80)   
222 + **.CGCGGC.** 843 (94.52)   
222 + **.CGCGTC.** 879 (100.00)   
222 + **.CGCGCC.** 926 (94.60)   
222 + **.CGCGTC.** 940 (100.00)   
222 + **.CGCGGC.** 950 (94.52)   
222 + **.CGCGCC.** 1005 (94.60)   
222 + **[CGCCGC]** 1054 (85.72)   
222 + **[CGCCCC]** 1074 (85.80)   
222 + **.CGCGCC.** 1081 (94.60)   
222 + **[CGCCCC]** 1083 (85.80)   
222 + **.CGCCTC.** 1090 (91.20)   
222 + **.CGCGCC.** 1099 (94.60)   
222 + **.CGCCTC.** 1101 (91.20)   
222 + **.CGCGCC.** 1206 (94.60)   
222 + **.CGCGGC.** 1484 (94.52)   
222 - **.CGCGGC.** 1482 (94.52)   
222 - **.CGCGCC.** 1270 (94.60)   
222 - **[CGGGTC]** 1220 (87.94)   
222 - **.CGCGCC.** 1204 (94.60)   
222 - **.CGCCTC.** 1191 (91.20)   
222 - **.CGCGGC.** 1001 (94.52)   
222 - **[CGGGTC]** 974 (87.94)   
222 - **[CGCCGC]** 951 (85.72)   
222 - **.CGCGGC.** 877 (94.52)   
222 - **[CGGGTC]** 867 (87.94)   
222 - **[CGCCGC]** 844 (85.72)   
222 - **.CGCGCC.** 841 (94.60)   
222 - **[CGCCGC]** 839 (85.72)   
222 - **[CGCCGC]** 819 (85.72)   
222 - **[CGCCGC]** 701 (85.72)   
222 - **.CGCCTC.** 501 (91.20)   
222 - **[CGGGTC]** 355 (87.94)   
222 - **.CGCCTC.** 341 (91.20)   
222 - **[CGCTTC]** 325 (86.32)   
222 - **[CGCCGC]** 318 (85.72)   
222 - **[CGCGTG]** 166 (86.67)   
223 + **[GGCGTC]** 187 (87.08)   
223 + **[CGCCGC]** 207 (85.72)   
223 + **[CGCTTC]** 210 (86.32)   
223 + **.CGCGCC.** 424 (94.60)   
223 + **.CGCCTC.** 437 (91.20)   
223 + **[CGCCGC]** 675 (85.72)   
223 + **[CGCTTC]** 694 (86.32)   
223 + **.CGCGTC.** 701 (100.00)   
223 + **[CGCTTC]** 943 (86.32)   
223 + **[CGCCCC]** 983 (85.80)   
223 - **[CGCCGC]** 671 (85.72)   
223 - **[CGCCCC]** 590 (85.80)   
223 - **[CGCCGC]** 521 (85.72)   
223 - **[CGCGAC]** 503 (87.27)   
223 - **.CGCGTC.** 456 (100.00)   
223 - **.CGCGTC.** 422 (100.00)   
223 - **.CGCCTC.** 307 (91.20)   
223 - **[CGCCGC]** 296 (85.72)   
223 - **.CGCCTC.** 185 (91.20)   
223 - **[CGCCCC]** 123 (85.80)   
223 - **[CGCCCC]** 61 (85.80)   
223 - **[CGCCCC]** 37 (85.80)   
223 - **[CGCCCC]** 27 (85.80)   
223 - **[CGCCCC]** 22 (85.80)   
223 - **[CGCCCC]** 4 (85.80)   
224 + **.CGCGGC.** 532 (94.52)   
224 + **.CGCCTC.** 546 (91.20)   
224 + **[GGCGTC]** 580 (87.08)   
224 + **.CGCGGC.** 619 (94.52)   
224 + **[CGCCGC]** 689 (85.72)   
224 + **.CGCGGC.** 799 (94.52)   
224 + **[CGCCGC]** 822 (85.72)   
224 + **.CGCGGC.** 856 (94.52)   
224 + **[GGCGTC]** 1032 (87.08)   
224 + **[CGCGTG]** 1068 (86.67)   
224 + **[CGCCGC]** 1233 (85.72)   
224 + **.CGCCTC.** 1245 (91.20)   
224 + **[CGCCCC]** 1483 (85.80)   
224 - **[GGCGTC]** 1481 (87.08)   
224 - **.CGCGCC.** 1326 (94.60)   
224 - **[CGCCCC]** 1324 (85.80)   
224 - **[GGCGTC]** 1243 (87.08)   
224 - **.CGCGGC.** 1234 (94.52)   
224 - **[CGCGAC]** 1201 (87.27)   
224 - **.CGCGCC.** 1183 (94.60)   
224 - **[CGCCCC]** 1181 (85.80)   
224 - **[CGCGTG]** 1176 (86.67)   
224 - **[CGCCGC]** 857 (85.72)   
224 - **.CGCGCC.** 854 (94.60)   
224 - **[CGCCGC]** 838 (85.72)   
224 - **.CGCGGC.** 823 (94.52)   
224 - **[CGCCGC]** 768 (85.72)   
224 - **.CGCGCC.** 718 (94.60)   
224 - **[CGCCGC]** 716 (85.72)   
224 - **[CGCCGC]** 713 (85.72)   
224 - **[CGCCGC]** 710 (85.72)   
224 - **[CGCCGC]** 707 (85.72)   
224 - **[CGCCCC]** 704 (85.80)   
224 - **.CGCCTC.** 698 (91.20)   
224 - **.CGCGGC.** 690 (94.52)   
224 - **[CGGGTC]** 685 (87.94)   
224 - **[CGCCCC]** 678 (85.80)   
224 - **[CGCCGC]** 620 (85.72)   
224 - **.CGCGCC.** 615 (94.60)   
224 - **[CGCCCC]** 604 (85.80)   
224 - **.CGCGGC.** 590 (94.52)   
224 - **.CGCCTC.** 578 (91.20)   
224 - **.CGCGCC.** 478 (94.60)   
224 - **[CGCCCC]** 476 (85.80)   
224 - **.CGCGCC.** 471 (94.60)   
225 - **[CGCCCC]** 560 (85.80)   
225 - **[CGCCCC]** 271 (85.80)   
226 + **[CGCCCC]** 68 (85.80)   
226 + **.CGCGGC.** 125 (94.52)   
226 + **.CGCGTC.** 235 (100.00)   
226 + **[CGCCCC]** 264 (85.80)   
226 + **[CGCGTG]** 452 (86.67)   
226 + **.CGCCTC.** 471 (91.20)   
226 + **[CGCCCC]** 479 (85.80)   
226 + **.CGCGCC.** 511 (94.60)   
226 + **[CGCGTG]** 520 (86.67)   
226 + **.CGCGTC.** 601 (100.00)   
226 + **[GGCGTC]** 654 (87.08)   
226 + **[CGCGAC]** 703 (87.27)   
226 + **[CGGGTC]** 761 (87.94)   
226 + **.CGCGGC.** 952 (94.52)   
226 + **.CGCGCC.** 1032 (94.60)   
226 + **[CGCCGC]** 1034 (85.72)   
226 + **.CGCGCC.** 1037 (94.60)   
226 + **[CGCCCC]** 1039 (85.80)   
226 + **.CGCGTC.** 1065 (100.00)   
226 + **.CGCGGC.** 1078 (94.52)   
226 + **[CGCCGC]** 1083 (85.72)   
226 + **[CGCCGC]** 1107 (85.72)   
226 + **[CGCCGC]** 1110 (85.72)   
226 + **[CGCCGC]** 1113 (85.72)   
226 + **[CGCCGC]** 1116 (85.72)   
226 + **.CGCGCC.** 1137 (94.60)   
226 + **[CGCCCC]** 1156 (85.80)   
226 + **.CGCGCC.** 1163 (94.60)   
226 + **[CGCCCC]** 1165 (85.80)   
226 + **.CGCGCC.** 1170 (94.60)   
226 + **[CGCCCC]** 1172 (85.80)   
226 + **.CGCGGC.** 1497 (94.52)   
226 - **[CGCCGC]** 1498 (85.72)   
226 - **.CGCGCC.** 1495 (94.60)   
226 - **.CGCGCC.** 1434 (94.60)   
226 - **.CGCGGC.** 1337 (94.52)   
226 - **.CGCGCC.** 1307 (94.60)   
226 - **[CGCCGC]** 1279 (85.72)   
226 - **[CGCCGC]** 1276 (85.72)   
226 - **[CGCCGC]** 1273 (85.72)   
226 - **[CGCCGC]** 1243 (85.72)   
226 - **[CGCCGC]** 1240 (85.72)   
226 - **[CGCCGC]** 1237 (85.72)   
226 - **[CGCCCC]** 1234 (85.80)   
226 - **.CGCGGC.** 1202 (94.52)   
226 - **[GGCGTC]** 1189 (87.08)   
226 - **[CGCCGC]** 1079 (85.72)   
226 - **.CGCGGC.** 1035 (94.52)   
226 - **[CGCCCC]** 983 (85.80)   
226 - **.CGCGCC.** 965 (94.60)   
226 - **[CGCCCC]** 963 (85.80)   
226 - **.CGCGGC.** 924 (94.52)   
226 - **[CGCCCC]** 895 (85.80)   
226 - **[CGCCCC]** 740 (85.80)   
226 - **.CGCGTC.** 712 (100.00)   
226 - **[CGCGAC]** 701 (87.27)   
226 - **[CGCCGC]** 690 (85.72)   
226 - **.CGCGGC.** 518 (94.52)   
226 - **[CGGGTC]** 467 (87.94)   
226 - **.CGCGGC.** 448 (94.52)   
226 - **[CCCGTC]** 432 (87.14)   
226 - **.CGCCTC.** 356 (91.20)   
226 - **.CGCGGC.** 246 (94.52)   
226 - **[CGCCCC]** 207 (85.80)   
226 - **[CGCCGC]** 10 (85.72)   
227 + **[CGCCCC]** 68 (85.80)   
227 + **.CGCGGC.** 268 (94.52)   
227 + **.CGCGCC.** 309 (94.60)   
227 + **[CGGGTC]** 691 (87.94)   
227 + **[CGCTTC]** 855 (86.32)   
227 + **[CGCCCC]** 937 (85.80)   
227 + **[CGCCCC]** 947 (85.80)   
227 + **[CGCCCC]** 957 (85.80)   
227 + **.CGCGCC.** 978 (94.60)   
227 + **.CGCCTC.** 980 (91.20)   
227 + **.CGCGCC.** 988 (94.60)   
227 + **.CGCCTC.** 990 (91.20)   
227 + **.CGCGCC.** 998 (94.60)   
227 + **.CGCCTC.** 1000 (91.20)   
227 + **[CGCCCC]** 1008 (85.80)   
227 + **.CGCGCC.** 1018 (94.60)   
227 + **.CGCCTC.** 1020 (91.20)   
227 + **[CGCCCC]** 1028 (85.80)   
227 + **.CGCGCC.** 1038 (94.60)   
227 + **.CGCCTC.** 1040 (91.20)   
227 + **[CGCCCC]** 1059 (85.80)   
227 + **[CGCCCC]** 1069 (85.80)   
227 + **.CGCGCC.** 1079 (94.60)   
227 + **.CGCCTC.** 1081 (91.20)   
227 + **.CGCGCC.** 1089 (94.60)   
227 + **.CGCCTC.** 1091 (91.20)   
227 + **[CGCCCC]** 1099 (85.80)   
227 + **[CGCCCC]** 1109 (85.80)   
227 + **.CGCCTC.** 1121 (91.20)   
227 + **.CGCCTC.** 1143 (91.20)   
227 + **.CGCGGC.** 1297 (94.52)   
227 + **[CGGGTC]** 1340 (87.94)   
227 + **.CGCGCC.** 1361 (94.60)   
227 + **[CGCCCC]** 1404 (85.80)   
227 + **.CGCCTC.** 1495 (91.20)   
227 - **[CGCCGC]** 1298 (85.72)   
227 - **[CGCCGC]** 1274 (85.72)   
227 - **.CGCCTC.** 902 (91.20)   
227 - **[CGGGTC]** 868 (87.94)   
227 - **[CGCCCC]** 851 (85.80)   
227 - **[CGCGTG]** 846 (86.67)   
227 - **[GGCGTC]** 822 (87.08)   
227 - **[CGCTTC]** 704 (86.32)   
227 - **.CGCGTC.** 687 (100.00)   
227 - **.CGCGTC.** 615 (100.00)   
227 - **[CGCGTG]** 560 (86.67)   
227 - **.CGCGCC.** 550 (94.60)   
227 - **[CGCGTG]** 536 (86.67)   
227 - **.CGCGTC.** 307 (100.00)   
227 - **.CGCCTC.** 299 (91.20)   
228 + **[CGCGAC]** 46 (87.27)   
228 + **[CGCCCC]** 67 (85.80)   
228 + **[CGGGTC]** 88 (87.94)   
228 + **.CGCGCC.** 129 (94.60)   
228 + **[CGCCCC]** 131 (85.80)   
228 + **.CGCGCC.** 136 (94.60)   
228 + **[CGCCCC]** 138 (85.80)   
228 + **[CGCCCC]** 173 (85.80)   
228 + **.CGCGCC.** 275 (94.60)   
228 + **.CGCGCC.** 281 (94.60)   
228 + **[CGCCCC]** 297 (85.80)   
228 + **[CGCCCC]** 336 (85.80)   
228 + **[CGCCCC]** 368 (85.80)   
228 + **[CGCCGC]** 383 (85.72)   
228 + **[CGCCCC]** 432 (85.80)   
228 + **[CGCCCC]** 465 (85.80)   
228 + **.CGCGCC.** 471 (94.60)   
228 + **.CGCGCC.** 542 (94.60)   
228 + **[CGCCGC]** 544 (85.72)   
228 + **[CGCGAC]** 638 (87.27)   
228 + **[CGCCGC]** 867 (85.72)   
228 + **.CGCGCC.** 1429 (94.60)   
228 - **[CGCGTG]** 1427 (86.67)   
228 - **.CGCGGC.** 1271 (94.52)   
228 - **[GGCGTC]** 1263 (87.08)   
228 - **[CGCTTC]** 1099 (86.32)   
228 - **.CGCGCC.** 952 (94.60)   
228 - **[CGCCCC]** 950 (85.80)   
228 - **.CGCGCC.** 884 (94.60)   
228 - **.CGCCTC.** 836 (91.20)   
228 - **[CGCCCC]** 798 (85.80)   
228 - **.CGCGCC.** 782 (94.60)   
228 - **.CGCGTC.** 738 (100.00)   
228 - **[CGCGTG]** 273 (86.67)   
228 - **[CGGGTC]** 154 (87.94)   
228 - **.CGCGGC.** 127 (94.52)   
228 - **.CGCCTC.** 105 (91.20)   
228 - **[CGCGAC]** 44 (87.27)   
229 + **.CGCGCC.** 124 (94.60)   
229 + **[CGCCGC]** 447 (85.72)   
229 + **.CGCGGC.** 540 (94.52)   
229 + **.CGCGGC.** 587 (94.52)   
229 + **[CGCCGC]** 689 (85.72)   
229 + **[CGCCCC]** 796 (85.80)   
229 + **.CGCCTC.** 906 (91.20)   
229 + **.CGCGGC.** 941 (94.52)   
229 + **.CGCGCC.** 1017 (94.60)   
229 + **[CGCCGC]** 1075 (85.72)   
229 + **[CGCCGC]** 1113 (85.72)   
229 + **.CGCGCC.** 1148 (94.60)   
229 + **[CGCCCC]** 1150 (85.80)   
229 + **[CGCCCC]** 1156 (85.80)   
229 + **[CGCCCC]** 1162 (85.80)   
229 + **[CGCCCC]** 1167 (85.80)   
229 + **.CGCGCC.** 1206 (94.60)   
229 + **.CGCGCC.** 1250 (94.60)   
229 + **[CGCCCC]** 1261 (85.80)   
229 + **.CGCGGC.** 1284 (94.52)   
229 + **[GGCGTC]** 1389 (87.08)   
229 - **[CGCCGC]** 1402 (85.72)   
229 - **[CGCCGC]** 1341 (85.72)   
229 - **[CGCCGC]** 1338 (85.72)   
229 - **[CGCCGC]** 1335 (85.72)   
229 - **[CGCCGC]** 1332 (85.72)   
229 - **[CGCCGC]** 1329 (85.72)   
229 - **[CGCCGC]** 1326 (85.72)   
229 - **[CGCCGC]** 1323 (85.72)   
229 - **.CGCGCC.** 1301 (94.60)   
229 - **[CGCCCC]** 1299 (85.80)   
229 - **.CGCGCC.** 1287 (94.60)   
229 - **[CGCCGC]** 1285 (85.72)   
229 - **.CGCGGC.** 1246 (94.52)   
229 - **[CGCGAC]** 1204 (87.27)   
229 - **[CGCCCC]** 1137 (85.80)   
229 - **.CGCGGC.** 1114 (94.52)   
229 - **[CGCCCC]** 1046 (85.80)   
229 - **[CGCCCC]** 1041 (85.80)   
229 - **.CGCGCC.** 1036 (94.60)   
229 - **[CCCGTC]** 1032 (87.14)   
229 - **.CGCGGC.** 1015 (94.52)   
229 - **.CGCGCC.** 996 (94.60)   
229 - **[CGCCCC]** 981 (85.80)   
229 - **[CCCGTC]** 955 (87.14)   
229 - **.CGCGGC.** 690 (94.52)   
229 - **[CGCCGC]** 685 (85.72)   
229 - **[CGCCCC]** 406 (85.80)   
230 + **[CGCCCC]** 289 (85.80)   
230 + **[GGCGTC]** 987 (87.08)   
230 - **[CGCCGC]** 1201 (85.72)   
230 - **[CGGGTC]** 1044 (87.94)   
230 - **[GGCGTC]** 1037 (87.08)   
230 - **[CGCCCC]** 998 (85.80)   
230 - **[CGCCGC]** 985 (85.72)   
231 + **[CGCGTG]** 89 (86.67)   
231 + **[GGCGTC]** 382 (87.08)   
231 + **[CGGGTC]** 524 (87.94)   
231 + **[CGCCCC]** 600 (85.80)   
231 + **[CGGGTC]** 695 (87.94)   
231 + **.CGCCTC.** 753 (91.20)   
231 + **[CGCCCC]** 811 (85.80)   
231 + **[CGCCGC]** 902 (85.72)   
231 + **[CGCCGC]** 905 (85.72)   
231 + **[GGCGTC]** 956 (87.08)   
231 + **[CGCCGC]** 1005 (85.72)   
231 + **[CGCCCC]** 1008 (85.80)   
231 + **[CGCCGC]** 1026 (85.72)   
231 + **[CGCCGC]** 1029 (85.72)   
231 + **.CGCGGC.** 1070 (94.52)   
231 + **.CGCGCC.** 1165 (94.60)   
231 + **[CGCCCC]** 1167 (85.80)   
231 + **.CGCCTC.** 1327 (91.20)   
231 + **.CGCGCC.** 1472 (94.60)   
231 + **.CGCGCC.** 1484 (94.60)   
231 - **[CGCCGC]** 1504 (85.72)   
231 - **[CGCCGC]** 1501 (85.72)   
231 - **.CGCGGC.** 1482 (94.52)   
231 - **.CGCGCC.** 1470 (94.60)   
231 - **.CGCGTC.** 1383 (100.00)   
231 - **.CGCCTC.** 1323 (91.20)   
231 - **.CGCCTC.** 1305 (91.20)   
231 - **.CGCCTC.** 1280 (91.20)   
231 - **.CGCGCC.** 1141 (94.60)   
231 - **[CGCCCC]** 1139 (85.80)   
231 - **.CGCGCC.** 1122 (94.60)   
231 - **[CGCCCC]** 1120 (85.80)   
231 - **[CGCCCC]** 1113 (85.80)   
231 - **[CGCCCC]** 1104 (85.80)   
231 - **[CGCCCC]** 1095 (85.80)   
231 - **.CGCGGC.** 980 (94.52)   
231 - **[CGCCCC]** 954 (85.80)   
231 - **[CGCCCC]** 949 (85.80)   
231 - **[CGCCCC]** 944 (85.80)   
231 - **[CGCCCC]** 939 (85.80)   
231 - **[CGCCCC]** 928 (85.80)   
231 - **.CGCGGC.** 906 (94.52)   
231 - **[CGCGTG]** 874 (86.67)   
231 - **[CCCGTC]** 781 (87.14)   
231 - **[CGCGTG]** 87 (86.67)   
232 + **.CGCCTC.** 106 (91.20)   
232 + **[CGCCGC]** 146 (85.72)   
232 + **.CGCGCC.** 149 (94.60)   
232 + **.CGCCTC.** 431 (91.20)   
232 + **[CGCCCC]** 438 (85.80)   
232 + **[CGCCCC]** 487 (85.80)   
232 + **.CGCGCC.** 521 (94.60)   
232 + **.CGCCTC.** 523 (91.20)   
232 + **.CGCGTC.** 635 (100.00)   
232 + **[CGCCCC]** 692 (85.80)   
232 + **.CGCGTC.** 701 (100.00)   
232 + **[CGCCGC]** 793 (85.72)   
232 + **.CGCGGC.** 863 (94.52)   
232 + **.CGCGGC.** 869 (94.52)   
232 + **[GGCGTC]** 927 (87.08)   
232 + **.CGCGGC.** 997 (94.52)   
232 + **[CGGGTC]** 1220 (87.94)   
232 + **[CGCCCC]** 1233 (85.80)   
232 + **.CGCGCC.** 1246 (94.60)   
232 + **.CGCCTC.** 1268 (91.20)   
232 + **.CGCGGC.** 1335 (94.52)   
232 + **[CGCCGC]** 1355 (85.72)   
232 + **[CGCCCC]** 1405 (85.80)   
232 + **[CGGGTC]** 1471 (87.94)   
232 - **[CGCCCC]** 1351 (85.80)   
232 - **.CGCGTC.** 1333 (100.00)   
232 - **.CGCGGC.** 1244 (94.52)   
232 - **[CGCCCC]** 1216 (85.80)   
232 - **.CGCGCC.** 1180 (94.60)   
232 - **[CGCCCC]** 1128 (85.80)   
232 - **.CGCGTC.** 1119 (100.00)   
232 - **[CGCCCC]** 1061 (85.80)   
232 - **[CGCCGC]** 1030 (85.72)   
232 - **[CGCCGC]** 1027 (85.72)   
232 - **.CGCCTC.** 1024 (91.20)   
232 - **.CGCGCC.** 1013 (94.60)   
232 - **[CGCCGC]** 1011 (85.72)   
232 - **[CGCCGC]** 998 (85.72)   
232 - **[CGCCGC]** 955 (85.72)   
232 - **[CGCCGC]** 952 (85.72)   
232 - **.CGCGGC.** 867 (94.52)   
232 - **.CGCGGC.** 794 (94.52)   
232 - **.CGCGCC.** 699 (94.60)   
232 - **[CGCGTG]** 668 (86.67)   
232 - **.CGCGCC.** 633 (94.60)   
232 - **[CGCCGC]** 631 (85.72)   
232 - **[CGCCGC]** 628 (85.72)   
232 - **.CGCGCC.** 444 (94.60)   
232 - **[CGCCCC]** 406 (85.80)   
232 - **[CGGGTC]** 261 (87.94)   
232 - **[GGCGTC]** 252 (87.08)   
232 - **.CGCGGC.** 147 (94.52)   
233 + **[CCCGTC]** 784 (87.14)   
233 + **[CGCTTC]** 823 (86.32)   
233 + **[CGCCCC]** 987 (85.80)   
233 + **[CGCGTG]** 1380 (86.67)   
233 - **[CGCTTC]** 1412 (86.32)   
233 - **.CGCGCC.** 1378 (94.60)   
233 - **[GGCGTC]** 1141 (87.08)   
233 - **[CGCCGC]** 1136 (85.72)   
233 - **[CGGGTC]** 1090 (87.94)   
233 - **[CCCGTC]** 435 (87.14)   
233 - **.CGCCTC.** 375 (91.20)   
234 + **.CGCGCC.** 411 (94.60)   
234 + **.CGCCTC.** 497 (91.20)   
234 + **[CGCGAC]** 579 (87.27)   
234 + **[GGCGTC]** 631 (87.08)   
234 + **.CGCGGC.** 816 (94.52)   
234 + **.CGCGCC.** 850 (94.60)   
234 + **.CGCGCC.** 856 (94.60)   
234 + **[CGCCCC]** 883 (85.80)   
234 + **[CGCCCC]** 900 (85.80)   
234 + **[CGCCCC]** 1166 (85.80)   
234 + **[CGCCCC]** 1195 (85.80)   
234 + **[CCCGTC]** 1198 (87.14)   
234 + **.CGCGGC.** 1331 (94.52)   
234 + **[CGCCCC]** 1363 (85.80)   
234 - **.CGCGTC.** 1411 (100.00)   
234 - **[CGCCGC]** 1332 (85.72)   
234 - **[CGCGTG]** 1329 (86.67)   
234 - **.CGCGCC.** 1289 (94.60)   
234 - **[CGCCCC]** 1287 (85.80)   
234 - **[CGCCCC]** 1001 (85.80)   
234 - **[CGCCCC]** 925 (85.80)   
234 - **[CGCGAC]** 803 (87.27)   
234 - **[CGCCCC]** 707 (85.80)   
234 - **.CGCCTC.** 701 (91.20)   
234 - **.CGCCTC.** 662 (91.20)   
234 - **.CGCGTC.** 637 (100.00)   
234 - **[CGCCGC]** 629 (85.72)   
234 - **[CCCGTC]** 603 (87.14)   
234 - **[CGCCGC]** 549 (85.72)   
234 - **.CGCCTC.** 546 (91.20)   
234 - **[CGCTTC]** 510 (86.32)   
234 - **[CGCGAC]** 317 (87.27)   
234 - **[CGCCCC]** 248 (85.80)   
234 - **.CGCCTC.** 233 (91.20)   
234 - **[CGCTTC]** 196 (86.32)   
234 - **.CGCGCC.** 179 (94.60)   
234 - **[CGCCCC]** 177 (85.80)   
234 - **[GGCGTC]** 167 (87.08)   
234 - **[CCCGTC]** 113 (87.14)   
235 + **[CGCGTG]** 476 (86.67)   
235 + **[CGCCCC]** 517 (85.80)   
235 + **.CGCCTC.** 544 (91.20)   
235 + **[CGCCCC]** 579 (85.80)   
235 + **.CGCGCC.** 670 (94.60)   
235 + **.CGCGCC.** 755 (94.60)   
235 + **[CGCTTC]** 770 (86.32)   
235 + **.CGCGCC.** 799 (94.60)   
235 + **.CGCCTC.** 857 (91.20)   
235 + **.CGCGGC.** 900 (94.52)   
235 + **[CGCGTG]** 905 (86.67)   
235 + **.CGCGCC.** 929 (94.60)   
235 + **.CGCCTC.** 931 (91.20)   
235 + **.CGCGCC.** 963 (94.60)   
235 + **[CGCGTG]** 992 (86.67)   
235 + **[CGCCGC]** 1361 (85.72)   
235 + **.CGCGCC.** 1364 (94.60)   
235 + **[CGCGTG]** 1488 (86.67)   
235 - **[CGCCGC]** 1511 (85.72)   
235 - **.CGCGTC.** 1486 (100.00)   
235 - **.CGCGCC.** 1429 (94.60)   
235 - **[CGCGTG]** 1397 (86.67)   
235 - **.CGCGGC.** 1362 (94.52)   
235 - **.CGCGCC.** 1317 (94.60)   
235 - **[CGCCGC]** 1315 (85.72)   
235 - **[CGCCGC]** 1312 (85.72)   
235 - **.CGCCTC.** 1277 (91.20)   
235 - **[CGCTTC]** 1259 (86.32)   
235 - **[CGCCCC]** 1124 (85.80)   
235 - **[CGCCCC]** 1095 (85.80)   
235 - **.CGCGGC.** 961 (94.52)   
235 - **.CGCGCC.** 903 (94.60)   
235 - **[CGCCGC]** 901 (85.72)   
235 - **[CGCCCC]** 880 (85.80)   
235 - **.CGCGGC.** 797 (94.52)   
235 - **[CGCGTG]** 760 (86.67)   
235 - **[CGCGTG]** 753 (86.67)   
235 - **[CGCCCC]** 689 (85.80)   
235 - **[CGCGAC]** 683 (87.27)   
235 - **.CGCGCC.** 635 (94.60)   
235 - **[CGCCCC]** 633 (85.80)   
235 - **[CGCCCC]** 499 (85.80)   
235 - **[CCCGTC]** 487 (87.14)   
235 - **[CGCCCC]** 448 (85.80)   
235 - **[CCCGTC]** 305 (87.14)   
235 - **[CGCTTC]** 278 (86.32)   
235 - **[CCCGTC]** 273 (87.14)   
236 + **[CCCGTC]** 678 (87.14)   
236 + **.CGCGCC.** 912 (94.60)   
236 + **.CGCGGC.** 978 (94.52)   
236 + **[CGCCGC]** 1005 (85.72)   
236 + **[CGCCGC]** 1008 (85.72)   
236 + **.CGCGGC.** 1026 (94.52)   
236 + **[CCCGTC]** 1033 (87.14)   
236 + **[CGGGTC]** 1387 (87.94)   
236 - **[CGCTTC]** 1484 (86.32)   
236 - **[CGCCCC]** 1220 (85.80)   
236 - **[CGCCCC]** 1205 (85.80)   
236 - **.CGCGGC.** 1154 (94.52)   
236 - **[CGGGTC]** 1137 (87.94)   
236 - **.CGCGCC.** 910 (94.60)   
236 - **[CCCGTC]** 869 (87.14)   
237 + **.CGCGTC.** 473 (100.00)   
237 + **[CGCCCC]** 667 (85.80)   
237 + **.CGCCTC.** 675 (91.20)   
237 - **[CGCTTC]** 1260 (86.32)   
237 - **.CGCGTC.** 1057 (100.00)   
237 - **[CGCTTC]** 372 (86.32)   
238 + **[CGCGAC]** 1228 (87.27)   
238 + **[CGCTTC]** 1237 (86.32)   
238 - **.CGCGTC.** 1106 (100.00)   
238 - **[CGCTTC]** 1084 (86.32)   
238 - **.CGCCTC.** 686 (91.20)   
239 + **[CGCCCC]** 61 (85.80)   
239 + **.CGCGGC.** 299 (94.52)   
239 + **[CGCCGC]** 444 (85.72)   
239 + **.CGCGGC.** 447 (94.52)   
239 + **[CGCCCC]** 496 (85.80)   
239 + **[CGCCGC]** 543 (85.72)   
239 + **[CGCGAC]** 709 (87.27)   
239 + **.CGCGGC.** 752 (94.52)   
239 + **.CGCGCC.** 770 (94.60)   
239 - **.CGCCTC.** 1164 (91.20)   
239 - **.CGCGCC.** 952 (94.60)   
239 - **.CGCGGC.** 768 (94.52)   
239 - **.CGCGCC.** 707 (94.60)   
239 - **[CGCCCC]** 705 (85.80)   
239 - **[CGCCCC]** 603 (85.80)   
239 - **[CGCCCC]** 598 (85.80)   
239 - **[CGCCCC]** 588 (85.80)   
239 - **.CGCGGC.** 445 (94.52)   
240 + **[CGCCCC]** 32 (85.80)   
240 + **.CGCGCC.** 140 (94.60)   
240 + **.CGCCTC.** 202 (91.20)   
240 + **[CGCTTC]** 764 (86.32)   
240 + **[CGCGTG]** 1016 (86.67)   
240 + **[CGCCCC]** 1093 (85.80)   
240 + **.CGCGCC.** 1182 (94.60)   
240 + **[CGCCGC]** 1184 (85.72)   
240 + **[CGCCGC]** 1402 (85.72)   
240 + **[GGCGTC]** 1463 (87.08)   
240 - **[CGGGTC]** 1433 (87.94)   
240 - **.CGCGGC.** 1403 (94.52)   
240 - **[CGGGTC]** 1066 (87.94)   
240 - **.CGCGCC.** 53 (94.60)   
240 - **[CGCCCC]** 51 (85.80)   
241 + **.CGCGCC.** 421 (94.60)   
241 + **.CGCGTC.** 1312 (100.00)   
241 - **.CGCGCC.** 1284 (94.60)   
241 - **[CGCCGC]** 1282 (85.72)   
241 - **.CGCGGC.** 1242 (94.52)   
241 - **[CCCGTC]** 461 (87.14)   
241 - **.CGCGTC.** 417 (100.00)   
242 + **[CGCGAC]** 325 (87.27)   
242 + **[CGCCGC]** 391 (85.72)   
242 + **[CGGGTC]** 568 (87.94)   
242 + **[CGCCCC]** 713 (85.80)   
242 + **.CGCGTC.** 824 (100.00)   
242 + **[CGCCGC]** 894 (85.72)   
242 + **[CGCCGC]** 897 (85.72)   
242 + **[CGCTTC]** 931 (86.32)   
242 + **.CGCGTC.** 1003 (100.00)   
242 + **[GGCGTC]** 1076 (87.08)   
242 + **[CGCGTG]** 1082 (86.67)   
242 - **[CGCGTG]** 994 (86.67)   
242 - **[CGCCGC]** 783 (85.72)   
243 + **[CGCTTC]** 857 (86.32)   
243 + **.CGCGGC.** 920 (94.52)   
243 + **[GGCGTC]** 956 (87.08)   
243 + **[CGCTTC]** 1033 (86.32)   
243 + **[CGCTTC]** 1102 (86.32)   
243 - **[CGCCCC]** 1408 (85.80)   
243 - **[GGCGTC]** 1257 (87.08)   
243 - **.CGCGGC.** 1160 (94.52)   
243 - **.CGCGGC.** 1120 (94.52)   
243 - **.CGCGCC.** 1005 (94.60)   
243 - **[CGCGAC]** 916 (87.27)   
244 + **.CGCGTC.** 345 (100.00)   
244 + **[CGCCCC]** 736 (85.80)   
244 + **[CGCCGC]** 836 (85.72)   
244 + **[CCCGTC]** 950 (87.14)   
244 + **.CGCCTC.** 1017 (91.20)   
244 - **[CGGGTC]** 1040 (87.94)   
244 - **[CGCCCC]** 923 (85.80)   
244 - **.CGCCTC.** 832 (91.20)   
244 - **[CGCGTG]** 802 (86.67)   
244 - **.CGCGCC.** 777 (94.60)   
244 - **[CGCCCC]** 768 (85.80)   
244 - **.CGCGCC.** 763 (94.60)   
244 - **.CGCCTC.** 761 (91.20)   
244 - **[CGCCCC]** 723 (85.80)   
244 - **.CGCGTC.** 687 (100.00)   
244 - **.CGCCTC.** 678 (91.20)   
244 - **.CGCCTC.** 616 (91.20)   
246 + **[CGCCGC]** 236 (85.72)   
246 + **[CGCCGC]** 273 (85.72)   
246 + **.CGCGCC.** 276 (94.60)   
246 + **.CGCCTC.** 278 (91.20)   
246 + **[CGCCGC]** 286 (85.72)   
246 + **.CGCCTC.** 438 (91.20)   
246 + **[CGCGTG]** 461 (86.67)   
246 + **.CGCGGC.** 615 (94.52)   
246 + **.CGCGGC.** 674 (94.52)   
246 + **.CGCCTC.** 679 (91.20)   
246 + **.CGCGCC.** 699 (94.60)   
246 + **.CGCGCC.** 750 (94.60)   
246 + **[CGCCGC]** 756 (85.72)   
246 + **[CGCCGC]** 781 (85.72)   
246 + **.CGCGCC.** 884 (94.60)   
246 + **.CGCGCC.** 910 (94.60)   
246 + **[CGCCCC]** 1105 (85.80)   
246 + **[CGCCGC]** 1217 (85.72)   
246 + **[CGCCCC]** 1220 (85.80)   
246 + **[CGCCGC]** 1272 (85.72)   
246 - **.CGCGGC.** 1078 (94.52)   
246 - **[CGCCGC]** 1022 (85.72)   
246 - **.CGCCTC.** 993 (91.20)   
246 - **.CGCGCC.** 908 (94.60)   
246 - **[GGCGTC]** 828 (87.08)   
246 - **.CGCGGC.** 707 (94.52)   
246 - **[CGGGTC]** 686 (87.94)   
246 - **[CGCCGC]** 675 (85.72)   
246 - **.CGCGTC.** 670 (100.00)   
246 - **[CGCCGC]** 659 (85.72)   
246 - **[CGCCGC]** 656 (85.72)   
246 - **.CGCGCC.** 495 (94.60)   
246 - **.CGCGCC.** 459 (94.60)   
246 - **.CGCGGC.** 274 (94.52)   
247 + **[CGCCGC]** 18 (85.72)   
247 + **.CGCGCC.** 236 (94.60)   
247 + **.CGCGGC.** 279 (94.52)   
247 + **.CGCGCC.** 331 (94.60)   
247 + **[CCCGTC]** 335 (87.14)   
247 + **.CGCGGC.** 422 (94.52)   
247 + **[CGCCGC]** 581 (85.72)   
247 + **[CGCCCC]** 584 (85.80)   
247 + **[CGCCGC]** 772 (85.72)   
247 + **.CGCCTC.** 791 (91.20)   
247 + **.CGCGCC.** 885 (94.60)   
247 + **[CGGGTC]** 933 (87.94)   
247 + **.CGCGGC.** 1024 (94.52)   
247 + **[CGCCGC]** 1044 (85.72)   
247 + **.CGCGCC.** 1096 (94.60)   
247 + **[CGCCCC]** 1098 (85.80)   
247 + **[CGCCCC]** 1107 (85.80)   
247 + **[CGCCCC]** 1116 (85.80)   
247 + **.CGCGCC.** 1122 (94.60)   
247 + **[CGCCCC]** 1162 (85.80)   
247 + **[CGCCCC]** 1169 (85.80)   
247 + **.CGCCTC.** 1176 (91.20)   
247 + **.CGCGCC.** 1188 (94.60)   
247 + **[CGCCGC]** 1224 (85.72)   
247 + **.CGCCTC.** 1227 (91.20)   
247 + **.CGCGGC.** 1299 (94.52)   
247 + **.CGCGGC.** 1360 (94.52)   
247 + **[CGCCGC]** 1452 (85.72)   
247 + **.CGCGCC.** 1473 (94.60)   
247 + **[CGCCCC]** 1475 (85.80)   
247 + **[CGCCCC]** 1487 (85.80)   
247 + **[CGCCGC]** 1492 (85.72)   
247 - **.CGCGGC.** 1493 (94.52)   
247 - **[CGCCCC]** 1397 (85.80)   
247 - **[CGCCCC]** 1383 (85.80)   
247 - **[CGCGTG]** 1358 (86.67)   
247 - **.CGCGCC.** 1285 (94.60)   
247 - **[CGCCCC]** 1275 (85.80)   
247 - **[CGCCGC]** 1260 (85.72)   
247 - **[CGCCCC]** 1257 (85.80)   
247 - **.CGCGGC.** 1092 (94.52)   
247 - **[CGCCGC]** 1025 (85.72)   
247 - **.CGCGGC.** 1022 (94.52)   
247 - **.CGCCTC.** 1008 (91.20)   
247 - **[CGCCCC]** 1001 (85.80)   
247 - **[CGCCCC]** 996 (85.80)   
247 - **[CGCCCC]** 979 (85.80)   
247 - **[CGCCCC]** 973 (85.80)   
247 - **[CGCCCC]** 961 (85.80)   
247 - **.CGCGCC.** 950 (94.60)   
247 - **[CGCCCC]** 826 (85.80)   
247 - **[CGCTTC]** 780 (86.32)   
247 - **.CGCCTC.** 673 (91.20)   
247 - **[CGCCGC]** 423 (85.72)   
247 - **.CGCGTC.** 418 (100.00)   
247 - **.CGCGGC.** 19 (94.52)   
248 - **.CGCCTC.** 1407 (91.20)   
249 + **[CGCCCC]** 225 (85.80)   
249 - **.CGCCTC.** 198 (91.20)   
250 + **.CGCGCC.** 139 (94.60)   
250 + **.CGCGGC.** 156 (94.52)   
250 + **.CGCCTC.** 381 (91.20)   
250 + **.CGCGGC.** 457 (94.52)   
250 + **[CGCTTC]** 492 (86.32)   
250 + **.CGCGGC.** 513 (94.52)   
250 + **.CGCGCC.** 528 (94.60)   
250 + **.CGCGGC.** 542 (94.52)   
250 + **.CGCCTC.** 549 (91.20)   
250 + **.CGCGCC.** 591 (94.60)   
250 + **[CGCCGC]** 869 (85.72)   
250 + **[CGCCCC]** 1043 (85.80)   
250 + **[CGCCCC]** 1063 (85.80)   
250 + **[CGCCGC]** 1069 (85.72)   
250 + **.CGCCTC.** 1128 (91.20)   
250 + **.CGCCTC.** 1204 (91.20)   
250 + **.CGCCTC.** 1423 (91.20)   
250 - **[CGCGTG]** 1303 (86.67)   
250 - **[CCCGTC]** 1282 (87.14)   
250 - **.CGCGGC.** 1096 (94.52)   
250 - **[CGCCCC]** 1082 (85.80)   
250 - **[CGCCCC]** 1009 (85.80)   
250 - **[CGCCGC]** 962 (85.72)   
250 - **[CCCGTC]** 905 (87.14)   
250 - **.CGCGGC.** 870 (94.52)   
250 - **.CGCGCC.** 460 (94.60)   
250 - **[CGCCGC]** 458 (85.72)   
250 - **.CGCGTC.** 453 (100.00)   
250 - **[CGCCGC]** 445 (85.72)   
250 - **[CGCCGC]** 442 (85.72)   
250 - **[CGCGAC]** 154 (87.27)   
250 - **[CGCGTG]** 137 (86.67)   
252 + **.CGCGTC.** 394 (100.00)   
252 - **.CGCGCC.** 392 (94.60)   
253 + **.CGCCTC.** 977 (91.20)   
253 - **.CGCCTC.** 1255 (91.20)   
254 + **[CGCTTC]** 873 (86.32)   
255 + **[CGCCGC]** 1064 (85.72)   
255 - **.CGCGTC.** 1297 (100.00)   
256 + **.CGCCTC.** 55 (91.20)   
256 + **.CGCGCC.** 99 (94.60)   
256 + **.CGCCTC.** 365 (91.20)   
256 + **[CGCCCC]** 929 (85.80)   
256 + **[CGGGTC]** 1097 (87.94)   
256 + **.CGCGCC.** 1117 (94.60)   
256 + **.CGCGTC.** 1205 (100.00)   
256 + **[CGCTTC]** 1241 (86.32)   
256 + **[GGCGTC]** 1274 (87.08)   
256 + **[CGCCGC]** 1301 (85.72)   
256 + **[CGCCGC]** 1483 (85.72)   
256 - **[CGCCCC]** 1363 (85.80)   
256 - **[CCCGTC]** 1327 (87.14)   
256 - **.CGCGGC.** 1302 (94.52)   
256 - **.CGCGGC.** 1195 (94.52)   
256 - **[CGGGTC]** 1127 (87.94)   
256 - **[CGCCCC]** 865 (85.80)   
256 - **.CGCGCC.** 860 (94.60)   
256 - **.CGCCTC.** 858 (91.20)   
257 + **.CGCCTC.** 708 (91.20)   
257 + **[CGCCCC]** 893 (85.80)   
257 + **[CGCCCC]** 917 (85.80)   
257 + **[CGCCCC]** 922 (85.80)   
257 + **[CGCTTC]** 1001 (86.32)   
257 + **[CGCCGC]** 1059 (85.72)   
257 + **[CGCCCC]** 1145 (85.80)   
257 + **[CGCCCC]** 1229 (85.80)   
257 + **.CGCGCC.** 1247 (94.60)   
257 + **.CGCCTC.** 1253 (91.20)   
257 + **.CGCGGC.** 1284 (94.52)   
257 + **.CGCGGC.** 1318 (94.52)   
257 + **[CGGGTC]** 1430 (87.94)   
257 + **[CGCCCC]** 1443 (85.80)   
257 + **[GGCGTC]** 1464 (87.08)   
257 - **.CGCGCC.** 1496 (94.60)   
257 - **[CGCCGC]** 1472 (85.72)   
257 - **[CGGGTC]** 1426 (87.94)   
257 - **[CGCCGC]** 1402 (85.72)   
257 - **[CGCCCC]** 1399 (85.80)   
257 - **[CGCGTG]** 1323 (86.67)   
257 - **.CGCGCC.** 1316 (94.60)   
257 - **.CGCGGC.** 1282 (94.52)   
257 - **[CGCCCC]** 1276 (85.80)   
257 - **.CGCGGC.** 1194 (94.52)   
257 - **.CGCGGC.** 1166 (94.52)   
257 - **.CGCGTC.** 1119 (100.00)   
257 - **[CGCTTC]** 882 (86.32)   
257 - **.CGCGCC.** 876 (94.60)   
257 - **[CGCCCC]** 874 (85.80)   
257 - **.CGCGGC.** 607 (94.52)   
259 + **[CGCTTC]** 736 (86.32)   
259 + **.CGCCTC.** 863 (91.20)   
259 + **.CGCGGC.** 997 (94.52)   
259 + **[CGCGAC]** 1020 (87.27)   
259 + **[CGCCGC]** 1067 (85.72)   
259 + **[CGCCCC]** 1154 (85.80)   
259 + **.CGCGGC.** 1187 (94.52)   
259 + **.CGCGCC.** 1221 (94.60)   
259 + **[CGCCCC]** 1223 (85.80)   
259 + **[CGCCGC]** 1301 (85.72)   
259 + **.CGCGGC.** 1304 (94.52)   
259 + **.CGCCTC.** 1339 (91.20)   
259 - **[CGCCGC]** 1305 (85.72)   
259 - **.CGCGGC.** 1302 (94.52)   
259 - **[GGCGTC]** 1065 (87.08)   
259 - **[CGCCGC]** 1040 (85.72)   
259 - **.CGCGTC.** 1023 (100.00)   
259 - **.CGCGGC.** 1018 (94.52)   
259 - **[CGCCGC]** 998 (85.72)   
259 - **.CGCGTC.** 995 (100.00)   
259 - **[CGCCGC]** 990 (85.72)   
259 - **[CGCCCC]** 921 (85.80)   
259 - **[CCCGTC]** 884 (87.14)   
260 + **[CGCCGC]** 790 (85.72)   
260 + **.CGCGCC.** 879 (94.60)   
260 + **[CGCCCC]** 881 (85.80)   
260 + **.CGCCTC.** 962 (91.20)   
260 + **.CGCGCC.** 1042 (94.60)   
260 + **[CGGGTC]** 1141 (87.94)   
260 + **[CGCGAC]** 1226 (87.27)   
260 - **.CGCCTC.** 1266 (91.20)   
260 - **[CGCCGC]** 1116 (85.72)   
260 - **[CGCGTG]** 1024 (86.67)   
260 - **[CGCGTG]** 992 (86.67)   
260 - **[GGCGTC]** 788 (87.08)   
261 + **.CGCGCC.** 127 (94.60)   
261 - **.CGCCTC.** 100 (91.20)   
262 + **.CGCCTC.** 11 (91.20)   
262 + **[CGCCCC]** 976 (85.80)   
262 - **[CGCCCC]** 1304 (85.80)   
262 - **.CGCCTC.** 760 (91.20)   
262 - **[CGCTTC]** 527 (86.32)   
262 - **[CGCTTC]** 508 (86.32)   
262 - **[CCCGTC]** 388 (87.14)   
263 + **[CGCGTG]** 167 (86.67)   
263 + **.CGCGCC.** 311 (94.60)   
263 + **.CGCGCC.** 393 (94.60)   
263 + **[CGCCCC]** 440 (85.80)   
263 + **.CGCGCC.** 542 (94.60)   
263 + **[CGCCCC]** 631 (85.80)   
263 + **[CGCCCC]** 639 (85.80)   
263 + **[CGCTTC]** 786 (86.32)   
263 + **.CGCGGC.** 850 (94.52)   
263 + **[CGCCCC]** 910 (85.80)   
263 + **[CGCCCC]** 940 (85.80)   
263 + **.CGCGTC.** 1078 (100.00)   
263 + **[GGCGTC]** 1327 (87.08)   
263 - **.CGCCTC.** 1450 (91.20)   
263 - **[CGCCCC]** 1370 (85.80)   
263 - **[CGCCCC]** 1325 (85.80)   
263 - **.CGCGCC.** 1282 (94.60)   
263 - **[CCCGTC]** 1271 (87.14)   
263 - **[CGCCCC]** 1249 (85.80)   
263 - **[CGCCCC]** 1167 (85.80)   
263 - **.CGCGTC.** 1117 (100.00)   
263 - **[CGCGAC]** 1081 (87.27)   
263 - **.CGCGGC.** 1076 (94.52)   
263 - **.CGCGGC.** 854 (94.52)   
263 - **[CGCGAC]** 846 (87.27)   
263 - **[CGCCGC]** 831 (85.72)   
263 - **[CGCCGC]** 828 (85.72)   
263 - **.CGCCTC.** 825 (91.20)   
263 - **[CGGGTC]** 746 (87.94)   
263 - **[CCCGTC]** 663 (87.14)   
263 - **.CGCCTC.** 570 (91.20)   
263 - **.CGCGCC.** 540 (94.60)   
263 - **[CGCCCC]** 538 (85.80)   
263 - **[CCCGTC]** 345 (87.14)   
263 - **.CGCGCC.** 307 (94.60)   
263 - **.CGCGCC.** 165 (94.60)   
263 - **.CGCCTC.** 71 (91.20)   
263 - **.CGCGCC.** 30 (94.60)   
264 + **.CGCGTC.** 143 (100.00)   
264 + **[CGGGTC]** 715 (87.94)   
264 + **[CGCCCC]** 959 (85.80)   
264 + **.CGCGCC.** 1138 (94.60)   
264 + **.CGCCTC.** 1206 (91.20)   
264 + **[CGCCGC]** 1446 (85.72)   
264 - **.CGCCTC.** 1305 (91.20)   
264 - **.CGCGTC.** 752 (100.00)   
264 - **[CGCCGC]** 420 (85.72)   
264 - **[CGCTTC]** 363 (86.32)   
266 + **[CGCGTG]** 740 (86.67)   
266 + **.CGCGGC.** 962 (94.52)   
266 + **.CGCGCC.** 982 (94.60)   
266 + **.CGCGGC.** 1030 (94.52)   
266 + **.CGCGCC.** 1106 (94.60)   
266 + **[GGCGTC]** 1236 (87.08)   
266 + **.CGCGCC.** 1270 (94.60)   
266 + **.CGCGGC.** 1282 (94.52)   
266 + **[CCCGTC]** 1452 (87.14)   
266 + **[CGCCCC]** 1460 (85.80)   
266 + **[CGCCGC]** 1474 (85.72)   
266 - **.CGCGGC.** 1475 (94.52)   
266 - **.CGCCTC.** 1470 (91.20)   
266 - **.CGCCTC.** 1348 (91.20)   
266 - **[CGCCGC]** 1283 (85.72)   
266 - **.CGCGGC.** 1280 (94.52)   
266 - **[CGCCGC]** 1234 (85.72)   
266 - **[CGCCGC]** 1231 (85.72)   
266 - **.CGCCTC.** 1228 (91.20)   
266 - **[CGCCGC]** 1157 (85.72)   
266 - **[CGCCGC]** 1154 (85.72)   
266 - **[CGCCGC]** 1151 (85.72)   
266 - **.CGCGCC.** 1104 (94.60)   
266 - **.CGCGGC.** 1065 (94.52)   
266 - **.CGCCTC.** 1042 (91.20)   
266 - **[CGCCGC]** 1031 (85.72)   
266 - **.CGCGCC.** 1028 (94.60)   
266 - **[CGCGTG]** 980 (86.67)   
266 - **[CGCGTG]** 545 (86.67)   
267 + **.CGCCTC.** 101 (91.20)   
267 + **[CGCCGC]** 192 (85.72)   
267 + **.CGCGGC.** 195 (94.52)   
267 + **[CGCCGC]** 1023 (85.72)   
267 + **[CGCCGC]** 1026 (85.72)   
267 + **[CGCCGC]** 1029 (85.72)   
267 - **.CGCCTC.** 1204 (91.20)   
267 - **.CGCCTC.** 775 (91.20)   
267 - **.CGCGGC.** 193 (94.52)   
267 - **.CGCCTC.** 97 (91.20)   
267 - **.CGCCTC.** 4 (91.20)   
268 + **[CGCCCC]** 89 (85.80)   
268 + **[CCCGTC]** 530 (87.14)   
268 + **[CGCGAC]** 625 (87.27)   
268 + **[CGCCCC]** 658 (85.80)   
268 + **.CGCGGC.** 813 (94.52)   
268 + **.CGCGGC.** 1015 (94.52)   
268 + **[CGCCCC]** 1150 (85.80)   
268 + **[CGCCCC]** 1187 (85.80)   
268 + **.CGCGGC.** 1394 (94.52)   
268 + **.CGCGGC.** 1478 (94.52)   
268 - **[CGCCGC]** 1460 (85.72)   
268 - **.CGCCTC.** 1436 (91.20)   
268 - **.CGCGGC.** 1320 (94.52)   
268 - **[CGCCGC]** 1286 (85.72)   
268 - **[CGCCGC]** 1266 (85.72)   
268 - **[CGCCGC]** 1247 (85.72)   
268 - **[CGCCGC]** 1244 (85.72)   
268 - **[CGCCGC]** 1241 (85.72)   
268 - **[CGCCGC]** 1238 (85.72)   
268 - **.CGCCTC.** 1235 (91.20)   
268 - **[CGCGTG]** 1199 (86.67)   
268 - **[CGCCGC]** 1110 (85.72)   
268 - **[CGCCCC]** 1107 (85.80)   
268 - **.CGCGGC.** 1068 (94.52)   
268 - **[CGCCGC]** 1016 (85.72)   
268 - **.CGCGCC.** 1013 (94.60)   
268 - **[CGCTTC]** 837 (86.32)   
268 - **[CGCGTG]** 831 (86.67)   
268 - **[CGCCGC]** 814 (85.72)   
268 - **.CGCGTC.** 509 (100.00)   
268 - **.CGCGGC.** 180 (94.52)   
268 - **[CGCTTC]** 130 (86.32)   
268 - **.CGCGCC.** 57 (94.60)   
268 - **[CGCCGC]** 55 (85.72)   
268 - **[GGCGTC]** 11 (87.08)   
270 + **[CGGGTC]** 16 (87.94)   
270 + **.CGCGTC.** 27 (100.00)   
270 + **[CGCCCC]** 201 (85.80)   
270 + **[CGCCCC]** 274 (85.80)   
270 + **[CGCCGC]** 346 (85.72)   
270 + **[CGCCCC]** 539 (85.80)   
270 + **.CGCCTC.** 763 (91.20)   
270 + **[CGCCCC]** 780 (85.80)   
270 + **.CGCCTC.** 859 (91.20)   
270 + **[CGGGTC]** 1060 (87.94)   
270 + **[CGCCCC]** 1259 (85.80)   
270 + **.CGCCTC.** 1367 (91.20)   
270 + **[CGCGTG]** 1442 (86.67)   
270 - **.CGCGGC.** 1438 (94.52)   
270 - **[CGGGTC]** 1424 (87.94)   
270 - **[CGGGTC]** 1378 (87.94)   
270 - **.CGCGCC.** 1299 (94.60)   
270 - **[CGCCCC]** 1205 (85.80)   
270 - **[CGCCGC]** 1186 (85.72)   
270 - **[CGCCCC]** 1104 (85.80)   
270 - **.CGCCTC.** 939 (91.20)   
270 - **[GGCGTC]** 761 (87.08)   
270 - **.CGCGCC.** 462 (94.60)   
270 - **[CGCCCC]** 460 (85.80)   
270 - **.CGCCTC.** 424 (91.20)   
270 - **[GGCGTC]** 344 (87.08)   
270 - **[CGCCCC]** 339 (85.80)   
270 - **[CGGGTC]** 150 (87.94)   
270 - **[CCCGTC]** 105 (87.14)   
270 - **.CGCGCC.** 56 (94.60)   
270 - **.CGCGGC.** 25 (94.52)   
270 - **[CGCCGC]** 12 (85.72)   
270 - **[CGCCCC]** 9 (85.80)   
271 + **[CGCCGC]** 546 (85.72)   
271 + **[CGCCGC]** 553 (85.72)   
271 + **[CGCCGC]** 580 (85.72)   
271 + **[CGCCGC]** 593 (85.72)   
271 + **[CGCCGC]** 606 (85.72)   
271 + **[CGCCGC]** 619 (85.72)   
271 + **[CGCCGC]** 632 (85.72)   
271 + **[CGCCGC]** 659 (85.72)   
271 + **[CGCCGC]** 672 (85.72)   
271 + **.CGCGGC.** 681 (94.52)   
271 - **.CGCGTC.** 1288 (100.00)   
271 - **[CGCGTG]** 843 (86.67)   
271 - **[CGCGTG]** 830 (86.67)   
271 - **[CGCGTG]** 779 (86.67)   
271 - **.CGCGTC.** 742 (100.00)   
271 - **[CGCGTG]** 677 (86.67)   
271 - **[CGCGTG]** 664 (86.67)   
271 - **[CGCGTG]** 651 (86.67)   
271 - **[CGCGTG]** 637 (86.67)   
271 - **[CGCGTG]** 624 (86.67)   
271 - **[CGCGTG]** 611 (86.67)   
271 - **[CGCGTG]** 598 (86.67)   
271 - **[CGCGTG]** 585 (86.67)   
271 - **[CGCGTG]** 572 (86.67)   
271 - **[CGCGTG]** 558 (86.67)   
272 + **[CGCCGC]** 546 (85.72)   
272 + **[CGCCGC]** 553 (85.72)   
272 + **[CGCCGC]** 574 (85.72)   
272 + **[CGCCGC]** 581 (85.72)   
272 + **[CGCCGC]** 594 (85.72)   
272 + **[CGCCGC]** 607 (85.72)   
272 + **[CGCCGC]** 620 (85.72)   
272 + **[CGCCGC]** 633 (85.72)   
272 + **[CGCCGC]** 660 (85.72)   
272 + **.CGCGGC.** 669 (94.52)   
272 - **.CGCGTC.** 1279 (100.00)   
272 - **[CGCGTG]** 831 (86.67)   
272 - **[CGCGTG]** 818 (86.67)   
272 - **[CGCGTG]** 767 (86.67)   
272 - **[CGCGTG]** 730 (86.67)   
272 - **.CGCGGC.** 712 (94.52)   
272 - **[CGCGTG]** 665 (86.67)   
272 - **[CGCGTG]** 652 (86.67)   
272 - **[CGCGTG]** 638 (86.67)   
272 - **[CGCGTG]** 625 (86.67)   
272 - **[CGCGTG]** 612 (86.67)   
272 - **[CGCGTG]** 599 (86.67)   
272 - **[CGCGTG]** 586 (86.67)   
272 - **[CGCGTG]** 558 (86.67)   
273 + **[CGCCCC]** 142 (85.80)   
273 + **.CGCGCC.** 201 (94.60)   
273 + **[CGCCCC]** 203 (85.80)   
273 + **[CGCCCC]** 306 (85.80)   
273 + **[GGCGTC]** 361 (87.08)   
273 + **.CGCCTC.** 499 (91.20)   
273 + **[CGCCCC]** 752 (85.80)   
273 + **[CGCCCC]** 824 (85.80)   
273 + **[CGCCCC]** 831 (85.80)   
273 + **[CGGGTC]** 916 (87.94)   
273 + **.CGCGGC.** 1032 (94.52)   
273 + **.CGCGTC.** 1121 (100.00)   
273 - **[CGCTTC]** 1495 (86.32)   
273 - **[CCCGTC]** 1326 (87.14)   
273 - **[CCCGTC]** 1301 (87.14)   
273 - **[CGCCCC]** 1256 (85.80)   
273 - **[CGCCGC]** 1033 (85.72)   
273 - **.CGCGGC.** 1030 (94.52)   
273 - **[CGCTTC]** 1024 (86.32)   
273 - **[CGCCCC]** 991 (85.80)   
273 - **[CGCCCC]** 963 (85.80)   
273 - **[CGCCCC]** 952 (85.80)   
273 - **[CGCCCC]** 912 (85.80)   
273 - **[CGCCCC]** 686 (85.80)   
273 - **[CGCCCC]** 637 (85.80)   
273 - **[CGCCGC]** 509 (85.72)   
273 - **[CGCCGC]** 55 (85.72)   
274 + **[CGCCCC]** 6 (85.80)   
274 + **.CGCGGC.** 15 (94.52)   
274 + **.CGCGCC.** 49 (94.60)   
274 + **[CGCCCC]** 68 (85.80)   
274 + **.CGCGGC.** 108 (94.52)   
274 + **[CGCGTG]** 175 (86.67)   
274 + **[CGCGTG]** 573 (86.67)   
274 + **.CGCGCC.** 587 (94.60)   
274 + **.CGCGCC.** 1269 (94.60)   
274 + **.CGCGGC.** 1322 (94.52)   
274 - **[CGGGTC]** 1505 (87.94)   
274 - **[CGCCGC]** 1419 (85.72)   
274 - **.CGCGGC.** 1360 (94.52)   
274 - **.CGCGCC.** 1352 (94.60)   
274 - **[GGCGTC]** 692 (87.08)   
274 - **[CCCGTC]** 353 (87.14)   
274 - **.CGCCTC.** 121 (91.20)   
274 - **.CGCGCC.** 111 (94.60)   
274 - **[CGCCGC]** 109 (85.72)   
274 - **[CGCCCC]** 94 (85.80)   
274 - **.CGCGGC.** 13 (94.52)   
275 + **[CGCCGC]** 577 (85.72)   
275 + **[CGCCCC]** 587 (85.80)   
275 + **.CGCGGC.** 711 (94.52)   
275 + **.CGCGCC.** 735 (94.60)   
275 + **[CGCCGC]** 741 (85.72)   
275 + **.CGCGCC.** 755 (94.60)   
275 + **[CGCCGC]** 757 (85.72)   
275 + **[CGCCGC]** 760 (85.72)   
275 + **[CGCCGC]** 763 (85.72)   
275 + **[CGCCGC]** 766 (85.72)   
275 + **[CGCCGC]** 769 (85.72)   
275 + **[CGCTTC]** 794 (86.32)   
275 + **.CGCCTC.** 815 (91.20)   
275 + **[CGCCCC]** 841 (85.80)   
275 + **[GGCGTC]** 859 (87.08)   
275 + **[CGCCGC]** 1007 (85.72)   
275 + **[CGCCGC]** 1010 (85.72)   
275 + **[CGCCGC]** 1013 (85.72)   
275 + **[CGCTTC]** 1040 (86.32)   
275 + **.CGCGCC.** 1101 (94.60)   
275 + **[CGCGAC]** 1265 (87.27)   
275 + **.CGCGCC.** 1330 (94.60)   
275 + **[CGCTTC]** 1436 (86.32)   
275 - **.CGCGCC.** 1088 (94.60)   
275 - **[CGCCGC]** 895 (85.72)   
275 - **[GGCGTC]** 585 (87.08)   
275 - **[CGCGTG]** 112 (86.67)   
275 - **.CGCGTC.** 91 (100.00)   
275 - **.CGCGCC.** 73 (94.60)   
275 - **[CGCCGC]** 71 (85.72)   
275 - **[CGCCCC]** 68 (85.80)   
276 - **.CGCGTC.** 755 (100.00)   
278 - **.CGCCTC.** 1194 (91.20)   
279 + **.CGCGCC.** 22 (94.60)   
279 + **.CGCGGC.** 33 (94.52)   
279 + **.CGCGCC.** 40 (94.60)   
279 + **.CGCGCC.** 108 (94.60)   
279 + **[CGCGAC]** 376 (87.27)   
279 + **.CGCGGC.** 415 (94.52)   
279 + **[CGCGTG]** 420 (86.67)   
279 + **.CGCGCC.** 459 (94.60)   
279 - **.CGCGTC.** 555 (100.00)   
279 - **.CGCGCC.** 418 (94.60)   
279 - **[CGCCGC]** 416 (85.72)   
279 - **.CGCGCC.** 413 (94.60)   
279 - **[CGCGTG]** 374 (86.67)   
279 - **.CGCCTC.** 338 (91.20)   
279 - **.CGCCTC.** 295 (91.20)   
279 - **[CGGGTC]** 136 (87.94)   
279 - **.CGCCTC.** 116 (91.20)   
279 - **.CGCGCC.** 106 (94.60)   
280 + **.CGCGGC.** 169 (94.52)   
280 + **[GGCGTC]** 392 (87.08)   
280 + **[GGCGTC]** 504 (87.08)   
280 + **.CGCGGC.** 735 (94.52)   
280 + **[CGCGTG]** 744 (86.67)   
280 + **[CCCGTC]** 798 (87.14)   
280 + **[CGCCGC]** 846 (85.72)   
280 + **.CGCGGC.** 982 (94.52)   
280 + **.CGCGCC.** 1066 (94.60)   
280 + **.CGCGCC.** 1072 (94.60)   
280 + **[CGCCCC]** 1074 (85.80)   
280 + **.CGCGTC.** 1084 (100.00)   
280 + **.CGCGCC.** 1128 (94.60)   
280 + **[CGCGAC]** 1152 (87.27)   
280 + **[CGCGAC]** 1178 (87.27)   
280 + **.CGCGTC.** 1197 (100.00)   
280 + **.CGCGCC.** 1264 (94.60)   
280 + **[CGCCCC]** 1266 (85.80)   
280 + **[CGCCCC]** 1319 (85.80)   
280 + **[CCCGTC]** 1322 (87.14)   
280 + **[CCCGTC]** 1346 (87.14)   
280 + **[CCCGTC]** 1417 (87.14)   
280 - **[CGGGTC]** 1124 (87.94)   
280 - **[CGCCGC]** 1108 (85.72)   
280 - **[CGCCGC]** 1009 (85.72)   
280 - **[CGCCGC]** 989 (85.72)   
280 - **[CGCCGC]** 986 (85.72)   
280 - **[CGCCGC]** 983 (85.72)   
280 - **.CGCGCC.** 976 (94.60)   
280 - **[CGCCGC]** 974 (85.72)   
280 - **.CGCCTC.** 956 (91.20)   
280 - **.CGCGGC.** 944 (94.52)   
280 - **[CGCCCC]** 935 (85.80)   
280 - **[CGCCCC]** 928 (85.80)   
280 - **.CGCCTC.** 922 (91.20)   
280 - **[CGCCCC]** 860 (85.80)   
280 - **[GGCGTC]** 844 (87.08)   
280 - **.CGCGGC.** 742 (94.52)   
280 - **[CGCCGC]** 736 (85.72)   
280 - **[CGCCCC]** 502 (85.80)   
281 + **.CGCCTC.** 31 (91.20)   
281 + **.CGCCTC.** 206 (91.20)   
281 + **.CGCGCC.** 433 (94.60)   
281 + **.CGCGGC.** 521 (94.52)   
281 + **.CGCGGC.** 531 (94.52)   
281 + **.CGCGCC.** 547 (94.60)   
281 + **.CGCGCC.** 553 (94.60)   
281 + **[GGCGTC]** 945 (87.08)   
281 + **[CGCCCC]** 981 (85.80)   
281 + **[CGCCCC]** 993 (85.80)   
281 + **[CGCGTG]** 1182 (86.67)   
281 + **[CGGGTC]** 1299 (87.94)   
281 - **[GGCGTC]** 1255 (87.08)   
281 - **.CGCGCC.** 1200 (94.60)   
281 - **[CGCCGC]** 1167 (85.72)   
281 - **[CGCCGC]** 1164 (85.72)   
281 - **.CGCCTC.** 1161 (91.20)   
281 - **[CGCCCC]** 1145 (85.80)   
281 - **[CGGGTC]** 1096 (87.94)   
281 - **[CGCCGC]** 1040 (85.72)   
281 - **[CGCCGC]** 1037 (85.72)   
281 - **[GGCGTC]** 1030 (87.08)   
281 - **.CGCGCC.** 1024 (94.60)   
281 - **[CGCCCC]** 943 (85.80)   
281 - **[CGCCCC]** 913 (85.80)   
281 - **[CGCCGC]** 905 (85.72)   
281 - **[CCCGTC]** 769 (87.14)   
281 - **[CGCGAC]** 743 (87.27)   
281 - **.CGCGCC.** 545 (94.60)   
281 - **[CGCCCC]** 543 (85.80)   
281 - **.CGCGTC.** 512 (100.00)   
281 - **.CGCGTC.** 446 (100.00)   
281 - **[CGCCCC]** 147 (85.80)   
282 + **.CGCGGC.** 13 (94.52)   
282 - **[CGCCCC]** 790 (85.80)   
282 - **[GGCGTC]** 741 (87.08)   
282 - **.CGCCTC.** 5 (91.20)   
283 + **[CGCCCC]** 89 (85.80)   
283 + **.CGCGCC.** 134 (94.60)   
283 + **[CGCCCC]** 136 (85.80)   
283 + **.CGCCTC.** 309 (91.20)   
283 + **[CCCGTC]** 1118 (87.14)   
283 + **[CGCCCC]** 1177 (85.80)   
283 + **.CGCCTC.** 1288 (91.20)   
283 + **[CGCCCC]** 1308 (85.80)   
283 + **[CGCCGC]** 1329 (85.72)   
283 + **[CGCCCC]** 1483 (85.80)   
283 - **[CGCCCC]** 1325 (85.80)   
283 - **[CGCTTC]** 1318 (86.32)   
283 - **[CGCTTC]** 1091 (86.32)   
283 - **[CGGGTC]** 477 (87.94)   
283 - **[CGCCGC]** 394 (85.72)   
283 - **[CGCCCC]** 278 (85.80)   
283 - **[CCCGTC]** 113 (87.14)   
284 + **.CGCCTC.** 387 (91.20)   
284 + **[CGCCCC]** 394 (85.80)   
284 + **.CGCGCC.** 553 (94.60)   
284 + **.CGCCTC.** 555 (91.20)   
284 + **[GGCGTC]** 579 (87.08)   
284 + **[CGCCCC]** 597 (85.80)   
284 + **.CGCGCC.** 708 (94.60)   
284 + **[CGCGTG]** 718 (86.67)   
284 + **.CGCGGC.** 780 (94.52)   
284 + **[CGCCCC]** 829 (85.80)   
284 + **.CGCCTC.** 905 (91.20)   
284 + **.CGCGGC.** 943 (94.52)   
284 + **[CGCCGC]** 978 (85.72)   
284 + **.CGCGCC.** 1003 (94.60)   
284 + **[CGCCCC]** 1060 (85.80)   
284 + **.CGCGGC.** 1089 (94.52)   
284 + **.CGCGGC.** 1094 (94.52)   
284 + **[CGCCCC]** 1102 (85.80)   
284 + **[CGCCGC]** 1236 (85.72)   
284 + **[CGCCGC]** 1239 (85.72)   
284 + **[CGCCGC]** 1248 (85.72)   
284 + **.CGCGGC.** 1283 (94.52)   
284 + **[CGCCGC]** 1310 (85.72)   
284 + **[CGCCCC]** 1313 (85.80)   
284 + **[CGCCGC]** 1327 (85.72)   
284 + **.CGCGCC.** 1342 (94.60)   
284 + **[CGCCGC]** 1344 (85.72)   
284 + **.CGCGGC.** 1347 (94.52)   
284 + **.CGCGCC.** 1371 (94.60)   
284 + **[CGCCGC]** 1380 (85.72)   
284 + **[CGCCGC]** 1383 (85.72)   
284 + **[CGCCGC]** 1386 (85.72)   
284 + **.CGCCTC.** 1389 (91.20)   
284 + **.CGCGGC.** 1452 (94.52)   
284 + **[CGGGTC]** 1502 (87.94)   
284 - **[CGCGAC]** 1505 (87.27)   
284 - **[CGCCGC]** 1484 (85.72)   
284 - **[CGCCGC]** 1481 (85.72)   
284 - **[CGCCGC]** 1478 (85.72)   
284 - **.CGCGCC.** 1471 (94.60)   
284 - **.CGCGCC.** 1450 (94.60)   
284 - **[CGCCGC]** 1348 (85.72)   
284 - **.CGCGGC.** 1345 (94.52)   
284 - **[CGGGTC]** 1220 (87.94)   
284 - **.CGCGCC.** 1198 (94.60)   
284 - **.CGCGCC.** 1092 (94.60)   
284 - **[CGCCGC]** 1090 (85.72)   
284 - **[CGCCCC]** 1056 (85.80)   
284 - **[GGCGTC]** 1029 (87.08)   
284 - **[CGCCCC]** 1014 (85.80)   
284 - **.CGCGCC.** 999 (94.60)   
284 - **[CGCCCC]** 997 (85.80)   
284 - **[CGCGTG]** 918 (86.67)   
284 - **[CGCCCC]** 825 (85.80)   
284 - **[CGCCGC]** 781 (85.72)   
284 - **.CGCCTC.** 749 (91.20)   
284 - **.CGCGGC.** 706 (94.52)   
284 - **.CGCGCC.** 668 (94.60)   
284 - **[CGCCCC]** 666 (85.80)   
284 - **.CGCGCC.** 631 (94.60)   
284 - **[CGCCGC]** 629 (85.72)   
284 - **.CGCCTC.** 617 (91.20)   
284 - **[CGCCCC]** 588 (85.80)   
284 - **[CGCCCC]** 577 (85.80)   
284 - **[GGCGTC]** 490 (87.08)   
284 - **.CGCCTC.** 430 (91.20)   
284 - **[CGCCCC]** 423 (85.80)   
285 + **[CGCCGC]** 110 (85.72)   
285 + **[CGCCGC]** 196 (85.72)   
285 + **[CGCCCC]** 233 (85.80)   
285 + **.CGCGGC.** 495 (94.52)   
285 + **.CGCGGC.** 548 (94.52)   
285 + **.CGCGGC.** 554 (94.52)   
285 + **[CGCCGC]** 582 (85.72)   
285 + **[CGCCGC]** 633 (85.72)   
285 + **.CGCGCC.** 636 (94.60)   
285 + **[CGCCCC]** 638 (85.80)   
285 + **.CGCGCC.** 652 (94.60)   
285 + **[CGCCGC]** 654 (85.72)   
285 + **.CGCGCC.** 657 (94.60)   
285 + **.CGCGGC.** 717 (94.52)   
285 + **[CGCCGC]** 906 (85.72)   
285 + **.CGCGGC.** 909 (94.52)   
285 + **.CGCGCC.** 941 (94.60)   
285 + **[CGCCCC]** 943 (85.80)   
285 + **[CGCGAC]** 950 (87.27)   
285 + **.CGCGGC.** 966 (94.52)   
285 + **[CGCCCC]** 975 (85.80)   
285 + **[CGCCCC]** 1090 (85.80)   
285 + **.CGCGGC.** 1216 (94.52)   
285 + **.CGCGCC.** 1488 (94.60)   
285 - **.CGCGCC.** 1486 (94.60)   
285 - **[CGCGAC]** 1401 (87.27)   
285 - **[GGCGTC]** 1283 (87.08)   
285 - **[CGGGTC]** 1098 (87.94)   
285 - **.CGCGGC.** 1033 (94.52)   
285 - **.CGCGCC.** 1026 (94.60)   
285 - **.CGCGGC.** 939 (94.52)   
285 - **[CGCCGC]** 910 (85.72)   
285 - **.CGCGGC.** 907 (94.52)   
285 - **[CGCCCC]** 865 (85.80)   
285 - **.CGCGGC.** 858 (94.52)   
285 - **[CGGGTC]** 770 (87.94)   
285 - **[CGCCGC]** 760 (85.72)   
285 - **[CGCCGC]** 757 (85.72)   
285 - **[CGCCGC]** 754 (85.72)   
285 - **.CGCGTC.** 715 (100.00)   
285 - **.CGCGGC.** 655 (94.52)   
285 - **.CGCGGC.** 634 (94.52)   
285 - **[GGCGTC]** 631 (87.08)   
285 - **[CGCCGC]** 604 (85.72)   
285 - **[CGCCCC]** 601 (85.80)   
285 - **[CGCCGC]** 555 (85.72)   
285 - **.CGCGGC.** 552 (94.52)   
285 - **[CGCCGC]** 519 (85.72)   
285 - **[CGCCCC]** 476 (85.80)   
285 - **.CGCGCC.** 470 (94.60)   
285 - **[CGCCGC]** 462 (85.72)   
285 - **[CGCCGC]** 459 (85.72)   
285 - **[CGCCGC]** 456 (85.72)   
285 - **.CGCGCC.** 449 (94.60)   
285 - **.CGCGCC.** 443 (94.60)   
285 - **[CGCCGC]** 441 (85.72)   
285 - **[CGCCCC]** 387 (85.80)   
285 - **.CGCGGC.** 353 (94.52)   
285 - **.CGCGCC.** 344 (94.60)   
285 - **.CGCGCC.** 334 (94.60)   
285 - **[CGCCCC]** 326 (85.80)   
285 - **[CGCGTG]** 177 (86.67)   
285 - **[CCCGTC]** 170 (87.14)   
285 - **[CGCCCC]** 164 (85.80)   
287 + **[CGCCCC]** 305 (85.80)   
287 + **.CGCGCC.** 326 (94.60)   
287 + **[CGCCCC]** 353 (85.80)   
287 + **.CGCGCC.** 537 (94.60)   
287 + **.CGCCTC.** 688 (91.20)   
287 + **[CGCCGC]** 762 (85.72)   
287 + **.CGCCTC.** 765 (91.20)   
287 + **[CGGGTC]** 778 (87.94)   
287 + **[CGCCCC]** 961 (85.80)   
287 + **[CGCCCC]** 1043 (85.80)   
287 + **[CGCCGC]** 1079 (85.72)   
287 + **[CGCCGC]** 1082 (85.72)   
287 + **.CGCGCC.** 1233 (94.60)   
287 + **.CGCGTC.** 1343 (100.00)   
287 - **.CGCCTC.** 1459 (91.20)   
287 - **.CGCGCC.** 1339 (94.60)   
287 - **[CGCCGC]** 1183 (85.72)   
287 - **[GGCGTC]** 1095 (87.08)   
287 - **[CGCCCC]** 878 (85.80)   
287 - **[CGCGTG]** 667 (86.67)   
287 - **[CGCCCC]** 558 (85.80)   
287 - **.CGCGTC.** 490 (100.00)   
287 - **.CGCCTC.** 454 (91.20)   
287 - **.CGCGGC.** 42 (94.52)   
288 + **.CGCCTC.** 605 (91.20)   
288 + **[CGCTTC]** 845 (86.32)   
288 + **[CGCCCC]** 937 (85.80)   
288 + **.CGCGCC.** 1042 (94.60)   
288 + **.CGCCTC.** 1044 (91.20)   
288 + **.CGCGCC.** 1170 (94.60)   
288 + **.CGCGCC.** 1195 (94.60)   
288 + **[CGCCGC]** 1229 (85.72)   
288 + **[CGCCCC]** 1232 (85.80)   
288 + **[CGCCCC]** 1334 (85.80)   
288 - **.CGCGGC.** 1168 (94.52)   
288 - **[CGCGAC]** 980 (87.27)   
288 - **.CGCGCC.** 876 (94.60)   
289 + **[CCCGTC]** 903 (87.14)   
289 + **[CGCCCC]** 957 (85.80)   
289 + **[CGCCCC]** 976 (85.80)   
289 + **[CGCTTC]** 1078 (86.32)   
289 + **.CGCGGC.** 1300 (94.52)   
289 - **.CGCGCC.** 1379 (94.60)   
289 - **.CGCCTC.** 1357 (91.20)   
289 - **[CGCCCC]** 1349 (85.80)   
289 - **.CGCGCC.** 1317 (94.60)   
289 - **[CGCCGC]** 1301 (85.72)   
289 - **[CGCCCC]** 1280 (85.80)   
289 - **[CCCGTC]** 1276 (87.14)   
289 - **[CGCCCC]** 840 (85.80)   
289 - **[CGCCCC]** 829 (85.80)   
289 - **.CGCGTC.** 540 (100.00)   
290 + **[CCCGTC]** 895 (87.14)   
290 + **[CGCGTG]** 977 (86.67)   
290 + **[CGCGTG]** 1019 (86.67)   
290 + **.CGCCTC.** 1153 (91.20)   
290 + **[CGCCGC]** 1162 (85.72)   
290 + **[CGCCGC]** 1165 (85.72)   
290 + **.CGCCTC.** 1177 (91.20)   
290 + **.CGCGGC.** 1339 (94.52)   
290 + **[CGGGTC]** 1427 (87.94)   
290 - **[CGCCGC]** 1438 (85.72)   
290 - **[CCCGTC]** 1425 (87.14)   
290 - **[CGCCCC]** 1410 (85.80)   
290 - **[CGCCCC]** 1386 (85.80)   
290 - **[CGCCCC]** 1367 (85.80)   
290 - **.CGCGCC.** 1330 (94.60)   
290 - **[CGCCCC]** 1328 (85.80)   
290 - **[CGCTTC]** 1112 (86.32)   
290 - **.CGCGGC.** 1104 (94.52)   
290 - **[CGCCGC]** 855 (85.72)   
291 + **.CGCGTC.** 203 (100.00)   
291 + **[CGCCCC]** 362 (85.80)   
291 + **.CGCGTC.** 407 (100.00)   
291 + **[GGCGTC]** 441 (87.08)   
291 + **[CGCGAC]** 451 (87.27)   
291 + **.CGCGTC.** 512 (100.00)   
291 + **[CGCCGC]** 544 (85.72)   
291 + **[CGGGTC]** 624 (87.94)   
291 + **.CGCGTC.** 738 (100.00)   
291 + **[CGCCGC]** 749 (85.72)   
291 + **[CGCCGC]** 766 (85.72)   
291 + **.CGCGGC.** 769 (94.52)   
291 + **[CGCCCC]** 784 (85.80)   
291 + **[CGCCCC]** 845 (85.80)   
291 + **.CGCGGC.** 871 (94.52)   
291 + **.CGCCTC.** 876 (91.20)   
291 + **[CGCCCC]** 1010 (85.80)   
291 + **[CGCCCC]** 1023 (85.80)   
291 + **[CCCGTC]** 1026 (87.14)   
291 + **[CGCCGC]** 1034 (85.72)   
291 + **[CGCCGC]** 1082 (85.72)   
291 + **[CGCGTG]** 1085 (86.67)   
291 + **[CGCCCC]** 1091 (85.80)   
291 + **.CGCGCC.** 1100 (94.60)   
291 + **[CGGGTC]** 1192 (87.94)   
291 + **[CGCCCC]** 1202 (85.80)   
291 + **.CGCGGC.** 1207 (94.52)   
291 + **.CGCGGC.** 1240 (94.52)   
291 + **[CGCCGC]** 1264 (85.72)   
291 + **[CGCCGC]** 1361 (85.72)   
291 + **[CCCGTC]** 1386 (87.14)   
291 + **.CGCGCC.** 1502 (94.60)   
291 - **.CGCGCC.** 1500 (94.60)   
291 - **[CGCCCC]** 1475 (85.80)   
291 - **[CGCCGC]** 1451 (85.72)   
291 - **[CGCCGC]** 1357 (85.72)   
291 - **.CGCCTC.** 1354 (91.20)   
291 - **.CGCGGC.** 1343 (94.52)   
291 - **.CGCGGC.** 1329 (94.52)   
291 - **[CGCCCC]** 1304 (85.80)   
291 - **[CGCCGC]** 1248 (85.72)   
291 - **[CGCCCC]** 1198 (85.80)   
291 - **.CGCGTC.** 1165 (100.00)   
291 - **.CGCGGC.** 1109 (94.52)   
291 - **.CGCGCC.** 1098 (94.60)   
291 - **.CGCGGC.** 1083 (94.52)   
291 - **.CGCGCC.** 1066 (94.60)   
291 - **.CGCGGC.** 1035 (94.52)   
291 - **[CGCCGC]** 872 (85.72)   
291 - **.CGCGGC.** 767 (94.52)   
291 - **[GGCGTC]** 764 (87.08)   
291 - **[GGCGTC]** 747 (87.08)   
291 - **[CGCGTG]** 736 (86.67)   
291 - **[CGCCCC]** 650 (85.80)   
291 - **.CGCGCC.** 608 (94.60)   
291 - **[CGCCCC]** 593 (85.80)   
291 - **[CGCGAC]** 584 (87.27)   
291 - **.CGCGGC.** 510 (94.52)   
291 - **[GGCGTC]** 232 (87.08)   
292 + **[CGCCCC]** 352 (85.80)   
292 + **.CGCGGC.** 659 (94.52)   
292 + **.CGCGCC.** 767 (94.60)   
292 + **[CGCCCC]** 769 (85.80)   
292 + **.CGCGCC.** 774 (94.60)   
292 + **[CGCCGC]** 776 (85.72)   
292 + **[CGCCGC]** 794 (85.72)   
292 + **.CGCGGC.** 797 (94.52)   
292 + **.CGCGGC.** 840 (94.52)   
292 + **[CGCCCC]** 879 (85.80)   
292 + **[CGCCGC]** 905 (85.72)   
292 + **.CGCGCC.** 958 (94.60)   
292 + **[CGCCCC]** 960 (85.80)   
292 + **.CGCGCC.** 1006 (94.60)   
292 + **[CGCCGC]** 1081 (85.72)   
292 + **[CGCCCC]** 1108 (85.80)   
292 + **.CGCGCC.** 1140 (94.60)   
292 + **.CGCCTC.** 1146 (91.20)   
292 + **[GGCGTC]** 1178 (87.08)   
292 + **.CGCGTC.** 1319 (100.00)   
292 + **.CGCGGC.** 1339 (94.52)   
292 + **.CGCGGC.** 1386 (94.52)   
292 + **[GGCGTC]** 1466 (87.08)   
292 - **.CGCGCC.** 1492 (94.60)   
292 - **[CGCCCC]** 1490 (85.80)   
292 - **.CGCGTC.** 1485 (100.00)   
292 - **[CGCGAC]** 1469 (87.27)   
292 - **[CGCCCC]** 1451 (85.80)   
292 - **.CGCGGC.** 1445 (94.52)   
292 - **[CGCCGC]** 1436 (85.72)   
292 - **[CGCGTG]** 1384 (86.67)   
292 - **[CGCCCC]** 1361 (85.80)   
292 - **[CGCCCC]** 1351 (85.80)   
292 - **.CGCGCC.** 1346 (94.60)   
292 - **[CGCGAC]** 1337 (87.27)   
292 - **[CGGGTC]** 1242 (87.94)   
292 - **[CGCCGC]** 1199 (85.72)   
292 - **[CGCCGC]** 1196 (85.72)   
292 - **.CGCGTC.** 1189 (100.00)   
292 - **[CGCCGC]** 1176 (85.72)   
292 - **[CGCGTG]** 1136 (86.67)   
292 - **.CGCGGC.** 1082 (94.52)   
292 - **.CGCGCC.** 1043 (94.60)   
292 - **[CGCCCC]** 1041 (85.80)   
292 - **[CGCCGC]** 1035 (85.72)   
292 - **.CGCGCC.** 1026 (94.60)   
292 - **[CGCCCC]** 1024 (85.80)   
292 - **[CGCCGC]** 1017 (85.72)   
292 - **[CGCCCC]** 1014 (85.80)   
292 - **.CGCCTC.** 868 (91.20)   
292 - **[CGCCCC]** 861 (85.80)   
292 - **[CGCCGC]** 798 (85.72)   
292 - **.CGCGGC.** 795 (94.52)   
292 - **.CGCCTC.** 526 (91.20)   
292 - **[GGCGTC]** 108 (87.08)   
293 + **[CCCGTC]** 83 (87.14)   
293 + **[CGCGAC]** 773 (87.27)   
293 + **.CGCCTC.** 781 (91.20)   
293 + **.CGCGGC.** 839 (94.52)   
293 + **.CGCGGC.** 936 (94.52)   
293 + **[CGCCGC]** 1009 (85.72)   
293 + **[GGCGTC]** 1070 (87.08)   
293 + **.CGCGGC.** 1154 (94.52)   
293 + **[CCCGTC]** 1275 (87.14)   
293 + **[GGCGTC]** 1362 (87.08)   
293 + **[CGCGTG]** 1444 (86.67)   
293 - **[GGCGTC]** 1463 (87.08)   
293 - **.CGCGCC.** 1378 (94.60)   
293 - **[CGCCGC]** 1126 (85.72)   
293 - **.CGCGCC.** 1096 (94.60)   
293 - **[CGCCCC]** 1078 (85.80)   
293 - **[CGCGAC]** 1073 (87.27)   
293 - **.CGCGCC.** 860 (94.60)   
293 - **[CGCCGC]** 840 (85.72)   
293 - **.CGCGCC.** 837 (94.60)   
293 - **.CGCCTC.** 821 (91.20)   
293 - **[CGCCGC]** 798 (85.72)   
293 - **[CCCGTC]** 649 (87.14)   
293 - **[GGCGTC]** 255 (87.08)   
294 + **[CGCCGC]** 45 (85.72)   
294 + **[CGCGTG]** 128 (86.67)   
294 + **[CGGGTC]** 301 (87.94)   
294 + **.CGCGGC.** 316 (94.52)   
294 + **.CGCCTC.** 363 (91.20)   
294 + **[CGCGTG]** 370 (86.67)   
294 + **[CGCGTG]** 613 (86.67)   
294 + **[CGCCCC]** 683 (85.80)   
294 + **[CGCCCC]** 736 (85.80)   
294 + **[CGCCGC]** 759 (85.72)   
294 + **[CGCCGC]** 797 (85.72)   
294 + **.CGCCTC.** 804 (91.20)   
294 + **.CGCGGC.** 894 (94.52)   
294 + **[CGCCCC]** 922 (85.80)   
294 + **.CGCGCC.** 938 (94.60)   
294 + **[CGCCCC]** 940 (85.80)   
294 + **[CGCCCC]** 945 (85.80)   
294 + **[CGCCCC]** 951 (85.80)   
294 + **[CGCGTG]** 1009 (86.67)   
294 + **[CGCCGC]** 1047 (85.72)   
294 + **[CGCCCC]** 1091 (85.80)   
294 + **[CGCCCC]** 1147 (85.80)   
294 + **[CCCGTC]** 1187 (87.14)   
294 + **[CGCGTG]** 1282 (86.67)   
294 - **[CGCCCC]** 1355 (85.80)   
294 - **[GGCGTC]** 1089 (87.08)   
294 - **[CGCGTG]** 1028 (86.67)   
294 - **[CGCCCC]** 1021 (85.80)   
294 - **.CGCGTC.** 936 (100.00)   
294 - **[CGCCGC]** 895 (85.72)   
294 - **.CGCCTC.** 852 (91.20)   
294 - **[CGGGTC]** 831 (87.94)   
294 - **.CGCGGC.** 785 (94.52)   
294 - **[CGGGTC]** 774 (87.94)   
294 - **[CGCTTC]** 544 (86.32)   
294 - **.CGCGGC.** 46 (94.52)   
295 + **[CGCCCC]** 285 (85.80)   
295 + **[GGCGTC]** 551 (87.08)   
295 + **.CGCGGC.** 592 (94.52)   
295 + **[CGCGTG]** 612 (86.67)   
295 + **[CGCCCC]** 619 (85.80)   
295 + **.CGCGTC.** 704 (100.00)   
295 + **[CGCCGC]** 746 (85.72)   
295 + **.CGCCTC.** 788 (91.20)   
295 + **.CGCGGC.** 855 (94.52)   
295 + **[CGGGTC]** 866 (87.94)   
295 + **[CGCTTC]** 1096 (86.32)   
295 - **[CGCGTG]** 1512 (86.67)   
295 - **[CGGGTC]** 1339 (87.94)   
295 - **.CGCGGC.** 1324 (94.52)   
295 - **.CGCCTC.** 1277 (91.20)   
295 - **[CGCGTG]** 1270 (86.67)   
295 - **[CGCGTG]** 1027 (86.67)   
295 - **[CGCCCC]** 957 (85.80)   
295 - **[CGCCCC]** 905 (85.80)   
295 - **[CGCCGC]** 881 (85.72)   
295 - **[CGCCGC]** 843 (85.72)   
295 - **.CGCCTC.** 836 (91.20)   
295 - **.CGCGGC.** 747 (94.52)   
295 - **[CGCCCC]** 718 (85.80)   
295 - **.CGCGCC.** 702 (94.60)   
295 - **[CGCCCC]** 700 (85.80)   
295 - **[CGCCCC]** 695 (85.80)   
295 - **[CGCCCC]** 689 (85.80)   
295 - **[CGCGTG]** 631 (86.67)   
295 - **[CGCCGC]** 593 (85.72)   
295 - **.CGCGCC.** 590 (94.60)   
295 - **[CGCCCC]** 549 (85.80)   
295 - **[CGCCCC]** 494 (85.80)   
295 - **[CCCGTC]** 453 (87.14)   
295 - **[CGCGTG]** 358 (86.67)   
296 + **[CGCCCC]** 33 (85.80)   
296 + **.CGCGCC.** 39 (94.60)   
296 + **[CGCCCC]** 41 (85.80)   
296 + **.CGCGGC.** 85 (94.52)   
296 + **[CGCCCC]** 137 (85.80)   
296 + **.CGCGGC.** 201 (94.52)   
296 + **.CGCGGC.** 250 (94.52)   
296 + **[CGCCCC]** 270 (85.80)   
296 + **[CGCCCC]** 341 (85.80)   
296 + **.CGCGTC.** 500 (100.00)   
296 + **[CGCCCC]** 544 (85.80)   
296 + **[CGCCCC]** 549 (85.80)   
296 + **[CGCCCC]** 561 (85.80)   
296 + **[CGCCCC]** 582 (85.80)   
296 + **.CGCGCC.** 591 (94.60)   
296 + **[CGCCCC]** 602 (85.80)   
296 + **[CGCCCC]** 607 (85.80)   
296 + **[CGCCCC]** 612 (85.80)   
296 + **[CGCCGC]** 619 (85.72)   
296 + **.CGCGCC.** 697 (94.60)   
296 + **[CGCCCC]** 699 (85.80)   
296 + **.CGCGCC.** 704 (94.60)   
296 + **[CGCCCC]** 706 (85.80)   
296 + **.CGCGCC.** 711 (94.60)   
296 + **[CGCCCC]** 713 (85.80)   
296 + **.CGCGCC.** 718 (94.60)   
296 + **[CGCCCC]** 720 (85.80)   
296 + **[CCCGTC]** 734 (87.14)   
296 + **[CGCCGC]** 756 (85.72)   
296 + **.CGCGCC.** 759 (94.60)   
296 + **[CGCCGC]** 770 (85.72)   
296 + **.CGCCTC.** 779 (91.20)   
296 + **.CGCGGC.** 815 (94.52)   
296 + **.CGCGCC.** 946 (94.60)   
296 + **[CGCCGC]** 948 (85.72)   
296 + **[CGCCGC]** 975 (85.72)   
296 + **.CGCGGC.** 1003 (94.52)   
296 + **.CGCGCC.** 1016 (94.60)   
296 + **.CGCGGC.** 1164 (94.52)   
296 + **.CGCGCC.** 1184 (94.60)   
296 + **[CGCCGC]** 1186 (85.72)   
296 + **[GGCGTC]** 1207 (87.08)   
296 + **[CGGGTC]** 1225 (87.94)   
296 + **.CGCGCC.** 1315 (94.60)   
296 + **[CGCCGC]** 1317 (85.72)   
296 + **[CGCCCC]** 1320 (85.80)   
296 + **[GGCGTC]** 1348 (87.08)   
296 + **.CGCGGC.** 1440 (94.52)   
296 + **.CGCGTC.** 1474 (100.00)   
296 - **.CGCGCC.** 1443 (94.60)   
296 - **[CGCCGC]** 1441 (85.72)   
296 - **.CGCCTC.** 1383 (91.20)   
296 - **.CGCGTC.** 1378 (100.00)   
296 - **[CGCCCC]** 1367 (85.80)   
296 - **[CGCCCC]** 1346 (85.80)   
296 - **[CGCCGC]** 1334 (85.72)   
296 - **.CGCGCC.** 1313 (94.60)   
296 - **[CGCCCC]** 1205 (85.80)   
296 - **.CGCGGC.** 1187 (94.52)   
296 - **.CGCGGC.** 1162 (94.52)   
296 - **.CGCGCC.** 1118 (94.60)   
296 - **[CGCCCC]** 1116 (85.80)   
296 - **[CGCCCC]** 1109 (85.80)   
296 - **[CGCCCC]** 1099 (85.80)   
296 - **[CGCTTC]** 1044 (86.32)   
296 - **.CGCGTC.** 1012 (100.00)   
296 - **[CGCCGC]** 1004 (85.72)   
296 - **.CGCGTC.** 1001 (100.00)   
296 - **[GGCGTC]** 973 (87.08)   
296 - **.CGCCTC.** 893 (91.20)   
296 - **.CGCCTC.** 858 (91.20)   
296 - **[CGCCCC]** 852 (85.80)   
296 - **.CGCGTC.** 841 (100.00)   
296 - **.CGCGGC.** 813 (94.52)   
296 - **.CGCGGC.** 757 (94.52)   
296 - **[CCCGTC]** 655 (87.14)   
296 - **[CGCGAC]** 589 (87.27)   
296 - **[CGCCCC]** 514 (85.80)   
296 - **.CGCGCC.** 494 (94.60)   
296 - **[CGCCGC]** 469 (85.72)   
296 - **[CGCCCC]** 459 (85.80)   
296 - **[CGCCCC]** 454 (85.80)   
296 - **.CGCGCC.** 419 (94.60)   
296 - **[CGCCCC]** 417 (85.80)   
296 - **[CGCCCC]** 406 (85.80)   
296 - **.CGCGCC.** 248 (94.60)   
296 - **[CGCCCC]** 246 (85.80)   
296 - **.CGCGCC.** 195 (94.60)   
296 - **[CGCCGC]** 193 (85.72)   
296 - **[CGCCGC]** 190 (85.72)   
296 - **[CGCCGC]** 187 (85.72)   
296 - **[CGCCCC]** 184 (85.80)   
296 - **[CGGGTC]** 149 (87.94)   
296 - **[CGCCGC]** 86 (85.72)   
296 - **[CGCGTG]** 83 (86.67)   
296 - **[CGCGTG]** 53 (86.67)   
297 + **[CGCCCC]** 211 (85.80)   
297 + **[CGCCGC]** 636 (85.72)   
297 + **[CGCCCC]** 639 (85.80)   
297 + **[CGCCCC]** 656 (85.80)   
297 + **.CGCGCC.** 792 (94.60)   
297 + **[CGCGTG]** 1033 (86.67)   
297 + **[CGGGTC]** 1062 (87.94)   
297 + **[CGCCCC]** 1107 (85.80)   
297 + **[GGCGTC]** 1201 (87.08)   
297 + **[CGCCGC]** 1239 (85.72)   
297 + **[CGCTTC]** 1242 (86.32)   
297 + **[CGCGTG]** 1421 (86.67)   
297 + **[GGCGTC]** 1443 (87.08)   
297 + **[CGCGTG]** 1500 (86.67)   
297 - **[CGCCCC]** 1366 (85.80)   
297 - **.CGCCTC.** 1193 (91.20)   
297 - **.CGCGGC.** 1155 (94.52)   
297 - **[CGCCGC]** 1020 (85.72)   
297 - **.CGCGGC.** 996 (94.52)   
297 - **[CGCCCC]** 990 (85.80)   
297 - **.CGCGCC.** 985 (94.60)   
297 - **[CGCCGC]** 983 (85.72)   
297 - **[CGCCGC]** 980 (85.72)   
297 - **.CGCGCC.** 954 (94.60)   
297 - **[CGCCCC]** 952 (85.80)   
297 - **[CGCCCC]** 947 (85.80)   
297 - **[CGCCCC]** 927 (85.80)   
297 - **[CGCCCC]** 904 (85.80)   
297 - **[CCCGTC]** 900 (87.14)   
297 - **[CGCCCC]** 883 (85.80)   
297 - **.CGCGGC.** 827 (94.52)   
297 - **[CGCGTG]** 581 (86.67)   
298 + **.CGCGCC.** 293 (94.60)   
298 + **.CGCCTC.** 295 (91.20)   
298 + **[GGCGTC]** 332 (87.08)   
298 + **[CGCGTG]** 629 (86.67)   
298 + **[CGCGTG]** 681 (86.67)   
298 + **.CGCGGC.** 746 (94.52)   
298 + **[GGCGTC]** 782 (87.08)   
298 + **.CGCGTC.** 799 (100.00)   
298 + **.CGCGGC.** 842 (94.52)   
298 + **.CGCGGC.** 876 (94.52)   
298 + **.CGCGCC.** 921 (94.60)   
298 + **[CGGGTC]** 931 (87.94)   
298 + **.CGCGTC.** 937 (100.00)   
298 + **.CGCGGC.** 1004 (94.52)   
298 + **[CGCCGC]** 1177 (85.72)   
298 + **.CGCCTC.** 1263 (91.20)   
298 + **.CGCCTC.** 1402 (91.20)   
298 - **.CGCGCC.** 1369 (94.60)   
298 - **[CGCCCC]** 1367 (85.80)   
298 - **[CGCCGC]** 1343 (85.72)   
298 - **[CGCCGC]** 1290 (85.72)   
298 - **[CGCTTC]** 1259 (86.32)   
298 - **.CGCCTC.** 1224 (91.20)   
298 - **.CGCGGC.** 1178 (94.52)   
298 - **[CGCCGC]** 1113 (85.72)   
298 - **[CGCCCC]** 1097 (85.80)   
298 - **[CCCGTC]** 1094 (87.14)   
298 - **.CGCGCC.** 1040 (94.60)   
298 - **[CGCTTC]** 1012 (86.32)   
298 - **[CGCGAC]** 1002 (87.27)   
298 - **.CGCGGC.** 901 (94.52)   
298 - **[CGCCGC]** 877 (85.72)   
298 - **[CGCGTG]** 874 (86.67)   
298 - **.CGCGCC.** 868 (94.60)   
298 - **[CGCGAC]** 838 (87.27)   
298 - **.CGCGGC.** 797 (94.52)   
298 - **[CGCCGC]** 747 (85.72)   
298 - **.CGCCTC.** 735 (91.20)   
298 - **.CGCCTC.** 713 (91.20)   
298 - **.CGCCTC.** 634 (91.20)   
298 - **[CCCGTC]** 588 (87.14)   
298 - **[CGCCCC]** 575 (85.80)   
298 - **.CGCCTC.** 549 (91.20)   
298 - **[CGCTTC]** 465 (86.32)   
298 - **.CGCCTC.** 330 (91.20)   
298 - **.CGCCTC.** 173 (91.20)   
298 - **[CCCGTC]** 22 (87.14)   
299 + **.CGCCTC.** 778 (91.20)   
299 + **[GGCGTC]** 789 (87.08)   
299 + **[GGCGTC]** 833 (87.08)   
299 + **.CGCGTC.** 853 (100.00)   
299 + **[CGCCCC]** 863 (85.80)   
299 + **.CGCGCC.** 868 (94.60)   
299 + **[CGCCCC]** 870 (85.80)   
299 + **.CGCGTC.** 883 (100.00)   
299 + **.CGCGCC.** 920 (94.60)   
299 + **[CGCCCC]** 922 (85.80)   
299 + **[CGCCGC]** 978 (85.72)   
299 + **[CGGGTC]** 1007 (87.94)   
299 + **.CGCGGC.** 1059 (94.52)   
299 + **.CGCGCC.** 1082 (94.60)   
299 + **.CGCGGC.** 1180 (94.52)   
299 + **[GGCGTC]** 1252 (87.08)   
299 + **.CGCGGC.** 1262 (94.52)   
299 + **.CGCCTC.** 1343 (91.20)   
299 + **[CGCGAC]** 1401 (87.27)   
299 + **.CGCGTC.** 1475 (100.00)   
299 - **.CGCGCC.** 1473 (94.60)   
299 - **[CGCCCC]** 1471 (85.80)   
299 - **[CGGGTC]** 1361 (87.94)   
299 - **.CGCGGC.** 1350 (94.52)   
299 - **[CGCCGC]** 1263 (85.72)   
299 - **[CGCCCC]** 1206 (85.80)   
299 - **[CGCCGC]** 1181 (85.72)   
299 - **.CGCGGC.** 1178 (94.52)   
299 - **.CGCCTC.** 1163 (91.20)   
299 - **.CGCGCC.** 1078 (94.60)   
299 - **.CGCCTC.** 1076 (91.20)   
299 - **[CGCTTC]** 956 (86.32)   
299 - **.CGCGGC.** 851 (94.52)   
299 - **.CGCCTC.** 845 (91.20)   
299 - **.CGCCTC.** 787 (91.20)   
299 - **[CGCCCC]** 671 (85.80)   
299 - **[CGCGTG]** 577 (86.67)   
300 + **.CGCGGC.** 2 (94.52)   
300 + **.CGCCTC.** 87 (91.20)   
300 + **.CGCGGC.** 113 (94.52)   
300 + **.CGCGGC.** 175 (94.52)   
300 + **[CGGGTC]** 188 (87.94)   
300 + **[CGGGTC]** 208 (87.94)   
300 + **[CCCGTC]** 283 (87.14)   
300 + **[CGGGTC]** 395 (87.94)   
300 + **[GGCGTC]** 413 (87.08)   
300 + **.CGCGTC.** 446 (100.00)   
300 + **.CGCGGC.** 474 (94.52)   
300 + **[CGGGTC]** 561 (87.94)   
300 + **.CGCGCC.** 581 (94.60)   
300 + **[CGGGTC]** 649 (87.94)   
300 + **.CGCGGC.** 736 (94.52)   
300 - **[CCCGTC]** 1266 (87.14)   
300 - **[CGCCCC]** 772 (85.80)   
300 - **[CGCCGC]** 737 (85.72)   
300 - **.CGCCTC.** 541 (91.20)   
300 - **.CGCGCC.** 477 (94.60)   
300 - **[CGCCGC]** 475 (85.72)   
300 - **.CGCGCC.** 472 (94.60)   
300 - **[CGCCCC]** 452 (85.80)   
300 - **[CGCCGC]** 411 (85.72)   
300 - **[CGCGAC]** 398 (87.27)   
300 - **[CGCCGC]** 114 (85.72)   
300 - **.CGCGCC.** 111 (94.60)   
300 - **.CGCCTC.** 50 (91.20)   
301 + **.CGCCTC.** 208 (91.20)   
301 + **[CGCGTG]** 670 (86.67)   
301 + **[CGCGTG]** 708 (86.67)   
301 + **[CGCGTG]** 767 (86.67)   
301 + **[GGCGTC]** 934 (87.08)   
301 + **[CGCCCC]** 1164 (85.80)   
301 + **.CGCCTC.** 1220 (91.20)   
301 + **.CGCGGC.** 1289 (94.52)   
301 + **[CGCCCC]** 1331 (85.80)   
301 + **[CGCGTG]** 1483 (86.67)   
301 - **.CGCGGC.** 1481 (94.52)   
301 - **[CGCTTC]** 1358 (86.32)   
301 - **[GGCGTC]** 1218 (87.08)   
301 - **[CGGGTC]** 1191 (87.94)   
301 - **[CGCCCC]** 1106 (85.80)   
301 - **[GGCGTC]** 1079 (87.08)   
301 - **.CGCCTC.** 1036 (91.20)   
301 - **.CGCCTC.** 932 (91.20)   
301 - **[GGCGTC]** 903 (87.08)   
301 - **[CGCTTC]** 894 (86.32)   
301 - **.CGCGCC.** 765 (94.60)   
301 - **[CGCCCC]** 763 (85.80)   
301 - **[CGCCCC]** 642 (85.80)   
301 - **[CGCCCC]** 633 (85.80)   
301 - **.CGCGCC.** 579 (94.60)   
301 - **[CGCCCC]** 495 (85.80)   
301 - **[CGCCCC]** 318 (85.80)   
301 - **[CGCCCC]** 154 (85.80)   
302 + **.CGCCTC.** 569 (91.20)   
302 + **[CGCGAC]** 593 (87.27)   
302 + **.CGCCTC.** 661 (91.20)   
302 + **[CGCGTG]** 744 (86.67)   
302 + **.CGCGCC.** 756 (94.60)   
302 + **[CGCCGC]** 758 (85.72)   
302 + **.CGCGCC.** 841 (94.60)   
302 + **.CGCGGC.** 853 (94.52)   
302 + **.CGCGCC.** 863 (94.60)   
302 + **.CGCGCC.** 961 (94.60)   
302 + **.CGCGCC.** 977 (94.60)   
302 + **.CGCCTC.** 1021 (91.20)   
302 + **.CGCGGC.** 1026 (94.52)   
302 + **[CGCTTC]** 1031 (86.32)   
302 + **.CGCGCC.** 1039 (94.60)   
302 + **[CGCCGC]** 1041 (85.72)   
302 + **[CGCTTC]** 1125 (86.32)   
302 + **[CGCCCC]** 1259 (85.80)   
302 + **[CCCGTC]** 1283 (87.14)   
302 + **[CGCCGC]** 1447 (85.72)   
302 + **[CGCCGC]** 1460 (85.72)   
302 + **[CGCCGC]** 1463 (85.72)   
302 - **.CGCGGC.** 1042 (94.52)   
302 - **[CGCCGC]** 1027 (85.72)   
302 - **.CGCGGC.** 999 (94.52)   
302 - **[CGCGTG]** 957 (86.67)   
302 - **.CGCGTC.** 951 (100.00)   
302 - **.CGCGCC.** 740 (94.60)   
302 - **[CGCTTC]** 680 (86.32)   
302 - **.CGCCTC.** 345 (91.20)   
303 + **[CGCCGC]** 99 (85.72)   
303 + **.CGCGTC.** 130 (100.00)   
303 + **[CGCCGC]** 167 (85.72)   
303 + **.CGCGCC.** 170 (94.60)   
303 + **[CGCCGC]** 172 (85.72)   
303 + **[CGCCGC]** 456 (85.72)   
303 + **.CGCGCC.** 745 (94.60)   
303 + **[CGCCGC]** 747 (85.72)   
303 + **.CGCGGC.** 750 (94.52)   
303 + **[CCCGTC]** 841 (87.14)   
303 + **[CCCGTC]** 881 (87.14)   
303 + **.CGCGGC.** 1006 (94.52)   
303 + **.CGCGTC.** 1021 (100.00)   
303 + **[CCCGTC]** 1033 (87.14)   
303 + **[CGCTTC]** 1180 (86.32)   
303 + **.CGCGGC.** 1365 (94.52)   
303 - **.CGCGGC.** 1361 (94.52)   
303 - **[CGCCGC]** 1355 (85.72)   
303 - **[CGGGTC]** 1314 (87.94)   
303 - **.CGCGCC.** 1004 (94.60)   
303 - **[CGCCGC]** 1002 (85.72)   
303 - **.CGCCTC.** 999 (91.20)   
303 - **[CGCCGC]** 990 (85.72)   
303 - **[CGCCGC]** 987 (85.72)   
303 - **[CGCCGC]** 984 (85.72)   
303 - **[CGCCGC]** 981 (85.72)   
303 - **[CGCCGC]** 978 (85.72)   
303 - **[CGCCGC]** 975 (85.72)   
303 - **[CGCCGC]** 917 (85.72)   
303 - **[CGCCGC]** 914 (85.72)   
303 - **[CGCCGC]** 911 (85.72)   
303 - **[CGCCGC]** 902 (85.72)   
303 - **.CGCGGC.** 748 (94.52)   
303 - **.CGCGCC.** 743 (94.60)   
303 - **[CGCCCC]** 737 (85.80)   
303 - **.CGCCTC.** 653 (91.20)   
303 - **[CGCCCC]** 494 (85.80)   
303 - **[CGCCGC]** 464 (85.72)   
303 - **.CGCGGC.** 457 (94.52)   
303 - **[CGGGTC]** 389 (87.94)   
303 - **[CGCCGC]** 295 (85.72)   
303 - **[CGCCCC]** 292 (85.80)   
303 - **.CGCGGC.** 287 (94.52)   
303 - **[CGCCCC]** 268 (85.80)   
303 - **.CGCCTC.** 263 (91.20)   
303 - **.CGCGGC.** 168 (94.52)   
303 - **.CGCGGC.** 43 (94.52)   
304 + **[CGCCGC]** 7 (85.72)   
304 + **[CGCCCC]** 114 (85.80)   
304 + **[CGCCCC]** 389 (85.80)   
304 + **[CGCCCC]** 411 (85.80)   
304 + **.CGCCTC.** 443 (91.20)   
304 + **.CGCCTC.** 464 (91.20)   
304 + **[CGCCCC]** 509 (85.80)   
304 + **.CGCCTC.** 542 (91.20)   
304 + **.CGCCTC.** 576 (91.20)   
304 + **[CGCCGC]** 707 (85.72)   
304 + **.CGCGCC.** 710 (94.60)   
304 + **[CGCCCC]** 783 (85.80)   
304 + **[CCCGTC]** 850 (87.14)   
304 + **[CGCGTG]** 947 (86.67)   
304 + **.CGCGGC.** 1243 (94.52)   
304 + **[CGCGTG]** 1277 (86.67)   
304 + **[CGCGAC]** 1309 (87.27)   
304 + **[CGCCCC]** 1321 (85.80)   
304 + **.CGCGCC.** 1510 (94.60)   
304 - **.CGCGGC.** 1263 (94.52)   
304 - **[CGCCGC]** 1244 (85.72)   
304 - **.CGCGCC.** 1241 (94.60)   
304 - **[CGCCCC]** 1239 (85.80)   
304 - **.CGCGCC.** 1179 (94.60)   
304 - **[CGCCGC]** 1177 (85.72)   
304 - **[CGCGAC]** 1161 (87.27)   
304 - **[CCCGTC]** 1123 (87.14)   
304 - **.CGCGGC.** 1106 (94.52)   
304 - **.CGCGCC.** 904 (94.60)   
304 - **[CGCCCC]** 816 (85.80)   
304 - **[GGCGTC]** 739 (87.08)   
304 - **.CGCGTC.** 733 (100.00)   
304 - **.CGCGGC.** 708 (94.52)   
304 - **[CGGGTC]** 325 (87.94)   
304 - **[CGCCGC]** 188 (85.72)   
304 - **.CGCGCC.** 169 (94.60)   
304 - **[GGCGTC]** 62 (87.08)   
305 + **[CGCCCC]** 1075 (85.80)   
305 + **.CGCGTC.** 1080 (100.00)   
305 + **[CGCCCC]** 1188 (85.80)   
305 - **[GGCGTC]** 1186 (87.08)   
305 - **.CGCGGC.** 924 (94.52)   
305 - **[CGCCCC]** 352 (85.80)   
305 - **.CGCCTC.** 340 (91.20)   
306 + **.CGCGTC.** 312 (100.00)   
306 + **.CGCGCC.** 342 (94.60)   
306 + **[CGGGTC]** 347 (87.94)   
306 + **.CGCCTC.** 561 (91.20)   
306 + **.CGCCTC.** 588 (91.20)   
306 + **.CGCGCC.** 631 (94.60)   
306 + **.CGCCTC.** 723 (91.20)   
306 + **[CGCCGC]** 764 (85.72)   
306 + **.CGCGCC.** 767 (94.60)   
306 + **[CGCGAC]** 906 (87.27)   
306 + **[CGCGTG]** 913 (86.67)   
306 + **.CGCGCC.** 930 (94.60)   
306 + **[CGCCCC]** 1397 (85.80)   
306 + **[CGCCGC]** 1413 (85.72)   
306 + **[CGCCGC]** 1416 (85.72)   
306 + **[GGCGTC]** 1428 (87.08)   
306 + **[CGCGTG]** 1477 (86.67)   
306 - **[CGGGTC]** 1473 (87.94)   
306 - **[GGCGTC]** 1411 (87.08)   
306 - **[CCCGTC]** 1345 (87.14)   
306 - **[CCCGTC]** 1315 (87.14)   
306 - **[CCCGTC]** 1300 (87.14)   
306 - **[CCCGTC]** 1284 (87.14)   
306 - **[CCCGTC]** 1254 (87.14)   
306 - **[CCCGTC]** 1239 (87.14)   
306 - **[CCCGTC]** 1208 (87.14)   
306 - **[CCCGTC]** 1193 (87.14)   
306 - **[CCCGTC]** 1178 (87.14)   
306 - **[CCCGTC]** 1163 (87.14)   
306 - **[CCCGTC]** 1148 (87.14)   
306 - **[CCCGTC]** 1102 (87.14)   
306 - **[CCCGTC]** 1087 (87.14)   
306 - **[CCCGTC]** 1072 (87.14)   
306 - **.CGCGCC.** 1039 (94.60)   
306 - **[CGCCGC]** 1037 (85.72)   
306 - **[CGCCGC]** 1022 (85.72)   
306 - **.CGCCTC.** 1019 (91.20)   
306 - **[CGCCCC]** 978 (85.80)   
306 - **.CGCCTC.** 937 (91.20)   
306 - **[CGGGTC]** 909 (87.94)   
306 - **.CGCGTC.** 845 (100.00)   
306 - **.CGCGGC.** 765 (94.52)   
306 - **[CGCTTC]** 738 (86.32)   
306 - **[CCCGTC]** 665 (87.14)   
306 - **.CGCGGC.** 340 (94.52)   
306 - **.CGCGCC.** 310 (94.60)   
306 - **[CGCCCC]** 174 (85.80)   
306 - **[CGCGTG]** 144 (86.67)   
307 + **[CCCGTC]** 46 (87.14)   
307 + **.CGCGCC.** 176 (94.60)   
307 + **[CCCGTC]** 214 (87.14)   
307 + **[CGCGTG]** 499 (86.67)   
307 + **[GGCGTC]** 514 (87.08)   
307 + **.CGCGCC.** 548 (94.60)   
307 + **[CGCTTC]** 628 (86.32)   
307 + **[CGCCGC]** 678 (85.72)   
307 + **[CGGGTC]** 715 (87.94)   
307 + **.CGCGGC.** 730 (94.52)   
307 + **[GGCGTC]** 772 (87.08)   
307 + **[CGCCCC]** 815 (85.80)   
307 + **[CGCCCC]** 837 (85.80)   
307 + **[CGCTTC]** 926 (86.32)   
307 + **[CGCCGC]** 980 (85.72)   
307 + **[CGCCGC]** 983 (85.72)   
307 + **[CGCCGC]** 992 (85.72)   
307 + **[GGCGTC]** 1066 (87.08)   
307 + **[GGCGTC]** 1111 (87.08)   
307 + **.CGCGGC.** 1128 (94.52)   
307 + **.CGCCTC.** 1137 (91.20)   
307 + **.CGCGGC.** 1162 (94.52)   
307 + **.CGCGGC.** 1330 (94.52)   
307 + **[CGCTTC]** 1349 (86.32)   
307 - **[CGCCCC]** 1371 (85.80)   
307 - **.CGCGCC.** 1355 (94.60)   
307 - **[CGCCCC]** 1319 (85.80)   
307 - **[CGGGTC]** 1305 (87.94)   
307 - **[GGCGTC]** 1279 (87.08)   
307 - **[CGGGTC]** 1250 (87.94)   
307 - **.CGCGCC.** 1235 (94.60)   
307 - **[CGCCGC]** 1233 (85.72)   
307 - **.CGCCTC.** 1221 (91.20)   
307 - **.CGCGCC.** 1160 (94.60)   
307 - **[CGCTTC]** 1154 (86.32)   
307 - **[CGCCGC]** 1085 (85.72)   
307 - **[CGCCCC]** 1082 (85.80)   
307 - **[CGGGTC]** 1007 (87.94)   
307 - **[GGCGTC]** 978 (87.08)   
307 - **[CGCCCC]** 957 (85.80)   
307 - **[CGCCCC]** 945 (85.80)   
307 - **.CGCCTC.** 899 (91.20)   
307 - **.CGCGGC.** 737 (94.52)   
307 - **[CGCCGC]** 731 (85.72)   
307 - **.CGCGGC.** 679 (94.52)   
307 - **[CGGGTC]** 640 (87.94)   
307 - **.CGCGCC.** 546 (94.60)   
307 - **.CGCGCC.** 497 (94.60)   
307 - **.CGCGCC.** 430 (94.60)   
307 - **[CGCCCC]** 428 (85.80)   
307 - **.CGCGTC.** 334 (100.00)   
307 - **.CGCCTC.** 271 (91.20)   
308 + **[CGCCCC]** 27 (85.80)   
308 + **[CGCGTG]** 637 (86.67)   
308 + **.CGCCTC.** 731 (91.20)   
308 + **.CGCGCC.** 786 (94.60)   
308 + **.CGCGGC.** 863 (94.52)   
308 + **[CGCGAC]** 879 (87.27)   
308 + **.CGCCTC.** 1081 (91.20)   
308 + **.CGCGGC.** 1116 (94.52)   
308 + **.CGCGGC.** 1181 (94.52)   
308 + **.CGCGCC.** 1189 (94.60)   
308 + **[CGCCGC]** 1367 (85.72)   
308 - **.CGCGGC.** 1413 (94.52)   
308 - **.CGCGGC.** 1368 (94.52)   
308 - **[CGCCGC]** 1334 (85.72)   
308 - **[CGCCCC]** 1300 (85.80)   
308 - **.CGCCTC.** 1251 (91.20)   
308 - **.CGCGGC.** 1179 (94.52)   
308 - **.CGCGCC.** 1100 (94.60)   
308 - **[CCCGTC]** 1041 (87.14)   
308 - **.CGCGTC.** 877 (100.00)   
308 - **[CGCCGC]** 864 (85.72)   
308 - **.CGCGGC.** 770 (94.52)   
308 - **.CGCGGC.** 387 (94.52)   
308 - **[CGCCCC]** 309 (85.80)   
308 - **[CGGGTC]** 201 (87.94)   
308 - **.CGCCTC.** 142 (91.20)   
308 - **[CGCGTG]** 71 (86.67)   
309 + **.CGCGCC.** 691 (94.60)   
309 + **[CGCCCC]** 853 (85.80)   
309 + **.CGCGGC.** 1029 (94.52)   
309 + **[CGCGAC]** 1080 (87.27)   
309 + **[CGCCCC]** 1180 (85.80)   
309 - **[CGCCCC]** 1463 (85.80)   
309 - **[CGGGTC]** 1354 (87.94)   
309 - **[CGCGTG]** 1225 (86.67)   
309 - **.CGCGCC.** 1148 (94.60)   
309 - **[CGCCCC]** 1146 (85.80)   
309 - **.CGCGCC.** 1078 (94.60)   
309 - **[CGCCGC]** 1076 (85.72)   
309 - **[CGCCGC]** 1030 (85.72)   
309 - **.CGCGGC.** 994 (94.52)   
309 - **.CGCCTC.** 980 (91.20)   
309 - **[CGCCCC]** 974 (85.80)   
309 - **[CGCCGC]** 904 (85.72)   
309 - **[CGCTTC]** 901 (86.32)   
309 - **.CGCCTC.** 884 (91.20)   
309 - **[CGCCGC]** 769 (85.72)   
309 - **[CGCCCC]** 766 (85.80)   
309 - **.CGCGGC.** 689 (94.52)   
309 - **[CGCCGC]** 556 (85.72)   
309 - **[CGCCCC]** 540 (85.80)   
309 - **[CGCCCC]** 95 (85.80)   
310 + **.CGCGCC.** 686 (94.60)   
310 + **.CGCCTC.** 790 (91.20)   
310 + **.CGCGCC.** 834 (94.60)   
310 + **[CGGGTC]** 894 (87.94)   
310 + **.CGCGGC.** 981 (94.52)   
310 + **.CGCGGC.** 1035 (94.52)   
310 + **[GGCGTC]** 1231 (87.08)   
310 + **.CGCGGC.** 1301 (94.52)   
310 + **[CGGGTC]** 1310 (87.94)   
310 - **.CGCGCC.** 979 (94.60)   
310 - **.CGCGCC.** 682 (94.60)   
311 + **[CGCCCC]** 684 (85.80)   
311 + **[CGCCCC]** 742 (85.80)   
311 + **.CGCGCC.** 762 (94.60)   
311 + **.CGCCTC.** 764 (91.20)   
311 + **[CGCCGC]** 872 (85.72)   
311 + **.CGCGGC.** 875 (94.52)   
311 + **[CGCCGC]** 934 (85.72)   
311 + **.CGCGCC.** 960 (94.60)   
311 + **[CGCCGC]** 962 (85.72)   
311 + **.CGCGGC.** 1069 (94.52)   
311 + **.CGCGGC.** 1106 (94.52)   
311 + **.CGCGTC.** 1345 (100.00)   
311 + **[CGGGTC]** 1409 (87.94)   
311 + **[CGGGTC]** 1440 (87.94)   
311 + **[CGCCGC]** 1470 (85.72)   
311 + **[CGCTTC]** 1503 (86.32)   
311 - **.CGCGGC.** 1471 (94.52)   
311 - **.CGCGGC.** 1343 (94.52)   
311 - **.CGCCTC.** 1322 (91.20)   
311 - **.CGCGTC.** 1262 (100.00)   
311 - **[CGCCCC]** 1254 (85.80)   
311 - **.CGCGCC.** 1178 (94.60)   
311 - **[CGCCCC]** 1176 (85.80)   
311 - **[CGCCCC]** 1171 (85.80)   
311 - **[CGCCCC]** 1166 (85.80)   
311 - **[CGCCCC]** 1161 (85.80)   
311 - **[CGCCCC]** 1156 (85.80)   
311 - **.CGCCTC.** 1148 (91.20)   
311 - **[CGCCGC]** 1107 (85.72)   
311 - **.CGCGGC.** 1104 (94.52)   
311 - **[CGCCGC]** 1070 (85.72)   
311 - **.CGCGCC.** 1067 (94.60)   
311 - **[CGCCGC]** 1021 (85.72)   
311 - **[CGCCCC]** 994 (85.80)   
311 - **[CGCCCC]** 913 (85.80)   
311 - **[GGCGTC]** 906 (87.08)   
311 - **.CGCGGC.** 873 (94.52)   
311 - **[CGCCCC]** 813 (85.80)   
311 - **[CGCCCC]** 782 (85.80)   
311 - **.CGCCTC.** 506 (91.20)   
311 - **[CGCGTG]** 500 (86.67)   
312 + **[CGCCCC]** 709 (85.80)   
312 + **[CGCCCC]** 774 (85.80)   
312 + **.CGCGCC.** 843 (94.60)   
312 + **[CGCCCC]** 845 (85.80)   
312 + **.CGCGCC.** 872 (94.60)   
312 + **[CGCCCC]** 874 (85.80)   
312 + **[CGCCCC]** 933 (85.80)   
312 + **.CGCGCC.** 985 (94.60)   
312 + **[CGCCGC]** 987 (85.72)   
312 + **[CGCCCC]** 990 (85.80)   
312 + **[CGCCCC]** 995 (85.80)   
312 + **[CGCCGC]** 1044 (85.72)   
312 + **[CGCCGC]** 1047 (85.72)   
312 + **.CGCGGC.** 1094 (94.52)   
312 + **.CGCGCC.** 1122 (94.60)   
312 + **[CGCCCC]** 1124 (85.80)   
312 + **.CGCGCC.** 1361 (94.60)   
312 + **[CGCCGC]** 1363 (85.72)   
312 + **.CGCGGC.** 1473 (94.52)   
312 - **[CGCTTC]** 1417 (86.32)   
312 - **.CGCGGC.** 1355 (94.52)   
312 - **.CGCGCC.** 1290 (94.60)   
312 - **[CGCCGC]** 1205 (85.72)   
312 - **.CGCCTC.** 1202 (91.20)   
312 - **.CGCCTC.** 1152 (91.20)   
312 - **[CGCCGC]** 1095 (85.72)   
312 - **.CGCGCC.** 1029 (94.60)   
312 - **[CGCCCC]** 974 (85.80)   
312 - **.CGCGCC.** 941 (94.60)   
312 - **[CGCCCC]** 939 (85.80)   
312 - **[CGCGTG]** 870 (86.67)   
312 - **[CGCGTG]** 841 (86.67)   
312 - **.CGCGTC.** 741 (100.00)   
312 - **.CGCCTC.** 736 (91.20)   
312 - **[GGCGTC]** 707 (87.08)   
312 - **[CGGGTC]** 638 (87.94)   
312 - **.CGCCTC.** 318 (91.20)   
312 - **[CGCCCC]** 293 (85.80)   
312 - **[CGGGTC]** 264 (87.94)   
312 - **[CGCCCC]** 224 (85.80)   
313 + **[CGCGAC]** 260 (87.27)   
313 + **[CCCGTC]** 553 (87.14)   
313 + **[CGCGTG]** 583 (86.67)   
313 + **[CGCCCC]** 634 (85.80)   
313 + **[GGCGTC]** 717 (87.08)   
313 + **[CGCGTG]** 788 (86.67)   
313 + **.CGCGGC.** 826 (94.52)   
313 + **.CGCCTC.** 832 (91.20)   
313 + **[CGCCGC]** 841 (85.72)   
313 + **.CGCGCC.** 892 (94.60)   
313 + **.CGCGTC.** 964 (100.00)   
313 + **[CGCTTC]** 978 (86.32)   
313 + **.CGCGCC.** 1008 (94.60)   
313 + **.CGCGCC.** 1088 (94.60)   
313 + **[CGCCCC]** 1138 (85.80)   
313 + **.CGCGCC.** 1303 (94.60)   
313 + **[CGCCGC]** 1339 (85.72)   
313 + **.CGCCTC.** 1369 (91.20)   
313 + **[CGCCCC]** 1389 (85.80)   
313 + **[CGCCGC]** 1445 (85.72)   
313 + **.CGCGGC.** 1448 (94.52)   
313 - **.CGCGGC.** 1446 (94.52)   
313 - **[CGGGTC]** 1420 (87.94)   
313 - **[CGCCGC]** 1167 (85.72)   
313 - **[CGCTTC]** 1164 (86.32)   
313 - **.CGCGCC.** 1123 (94.60)   
313 - **.CGCCTC.** 1121 (91.20)   
313 - **.CGCGCC.** 956 (94.60)   
313 - **.CGCGCC.** 817 (94.60)   
313 - **[CGCGTG]** 798 (86.67)   
313 - **[CGCGTG]** 256 (86.67)   
314 + **[CCCGTC]** 229 (87.14)   
314 + **[CCCGTC]** 550 (87.14)   
314 - **[CGCCCC]** 1208 (85.80)   
314 - **.CGCCTC.** 946 (91.20)   
314 - **[CGCCGC]** 480 (85.72)   
314 - **[CGCCCC]** 477 (85.80)   
314 - **[CGCCCC]** 183 (85.80)   
314 - **.CGCCTC.** 44 (91.20)   
314 - **[CCCGTC]** 18 (87.14)   
315 + **[CCCGTC]** 132 (87.14)   
315 + **.CGCCTC.** 152 (91.20)   
315 + **[CGCCCC]** 291 (85.80)   
315 + **[GGCGTC]** 344 (87.08)   
315 + **.CGCGGC.** 357 (94.52)   
315 + **.CGCGCC.** 383 (94.60)   
315 + **[CCCGTC]** 676 (87.14)   
315 + **[CGCGTG]** 905 (86.67)   
315 + **.CGCCTC.** 1124 (91.20)   
315 + **[CGCCCC]** 1140 (85.80)   
315 + **.CGCGCC.** 1273 (94.60)   
315 + **[CGCCGC]** 1341 (85.72)   
315 + **.CGCGTC.** 1344 (100.00)   
315 + **[CGCCCC]** 1353 (85.80)   
315 - **.CGCCTC.** 1404 (91.20)   
315 - **.CGCGGC.** 1342 (94.52)   
315 - **.CGCGTC.** 1285 (100.00)   
315 - **.CGCGCC.** 1271 (94.60)   
315 - **[CGGGTC]** 1200 (87.94)   
315 - **[CGCCGC]** 1091 (85.72)   
315 - **.CGCGCC.** 1057 (94.60)   
315 - **[CGCCCC]** 1055 (85.80)   
315 - **[CGCCCC]** 1032 (85.80)   
315 - **.CGCGCC.** 1026 (94.60)   
315 - **[CGCCGC]** 1024 (85.72)   
315 - **.CGCGCC.** 1017 (94.60)   
315 - **[CGCCGC]** 1015 (85.72)   
315 - **.CGCGTC.** 1006 (100.00)   
315 - **.CGCGCC.** 981 (94.60)   
315 - **[CGCCCC]** 979 (85.80)   
315 - **[CGCCCC]** 947 (85.80)   
315 - **[CGCCCC]** 942 (85.80)   
315 - **.CGCGTC.** 903 (100.00)   
315 - **[CGGGTC]** 861 (87.94)   
315 - **[CGCTTC]** 651 (86.32)   
315 - **.CGCGTC.** 379 (100.00)   
315 - **[CGCCGC]** 358 (85.72)   
315 - **[CGCCCC]** 302 (85.80)   
316 + **.CGCGCC.** 202 (94.60)   
316 + **.CGCGGC.** 229 (94.52)   
316 + **.CGCGCC.** 257 (94.60)   
316 + **[CGCCCC]** 863 (85.80)   
316 + **.CGCGTC.** 898 (100.00)   
316 + **.CGCGGC.** 910 (94.52)   
316 + **[CGCCGC]** 1183 (85.72)   
316 + **[CGCCCC]** 1268 (85.80)   
316 + **[CGCCGC]** 1288 (85.72)   
316 + **[CGCCCC]** 1341 (85.80)   
316 - **[GGCGTC]** 1339 (87.08)   
316 - **[CGCCGC]** 1284 (85.72)   
316 - **[CGCCGC]** 1104 (85.72)   
316 - **.CGCGCC.** 913 (94.60)   
316 - **[CGCCGC]** 911 (85.72)   
316 - **[CGGGTC]** 906 (87.94)   
316 - **.CGCGCC.** 896 (94.60)   
316 - **[CGCCCC]** 686 (85.80)   
316 - **.CGCGCC.** 573 (94.60)   
316 - **.CGCGGC.** 536 (94.52)   
316 - **[CGCCGC]** 248 (85.72)   
316 - **.CGCGGC.** 227 (94.52)   
316 - **.CGCGTC.** 200 (100.00)   
316 - **[CGCCGC]** 167 (85.72)   
316 - **[CGCCCC]** 164 (85.80)   
316 - **.CGCCTC.** 36 (91.20)   
317 + **[CGCCCC]** 786 (85.80)   
317 + **[CGCCCC]** 834 (85.80)   
317 + **.CGCGCC.** 1433 (94.60)   
317 + **[CGCCCC]** 1435 (85.80)   
317 - **[CGCCCC]** 1511 (85.80)   
317 - **.CGCGCC.** 1408 (94.60)   
317 - **.CGCGCC.** 1379 (94.60)   
317 - **.CGCCTC.** 1311 (91.20)   
317 - **[CGCCGC]** 1198 (85.72)   
317 - **[CGCCGC]** 1039 (85.72)   
317 - **[CGCCGC]** 1036 (85.72)   
317 - **.CGCCTC.** 1033 (91.20)   
317 - **.CGCGCC.** 1005 (94.60)   
317 - **[CGCCCC]** 1003 (85.80)   
317 - **[CGCCCC]** 964 (85.80)   
317 - **[CGGGTC]** 699 (87.94)   
317 - **[CGCCCC]** 439 (85.80)   
318 + **.CGCGTC.** 290 (100.00)   
318 + **.CGCGCC.** 307 (94.60)   
318 + **[CGCCCC]** 556 (85.80)   
318 + **[CGGGTC]** 616 (87.94)   
318 + **.CGCCTC.** 655 (91.20)   
318 + **[CGGGTC]** 673 (87.94)   
318 + **[CCCGTC]** 778 (87.14)   
318 + **[CGCCCC]** 895 (85.80)   
318 + **[CGCGTG]** 1042 (86.67)   
318 + **[CGCCGC]** 1109 (85.72)   
318 + **[CGCCCC]** 1112 (85.80)   
318 + **[CGGGTC]** 1193 (87.94)   
318 + **[CGCCGC]** 1216 (85.72)   
318 + **[CGCCGC]** 1219 (85.72)   
318 + **.CGCGCC.** 1246 (94.60)   
318 + **[CGCCCC]** 1248 (85.80)   
318 + **.CGCGCC.** 1348 (94.60)   
318 + **.CGCGGC.** 1485 (94.52)   
318 - **.CGCCTC.** 1513 (91.20)   
318 - **[CGCCGC]** 1495 (85.72)   
318 - **[CGCCGC]** 1492 (85.72)   
318 - **[CGCCGC]** 1489 (85.72)   
318 - **[CGCCGC]** 1486 (85.72)   
318 - **.CGCGGC.** 1483 (94.52)   
318 - **[CGCCGC]** 1477 (85.72)   
318 - **[CGCCCC]** 1474 (85.80)   
318 - **[CGGGTC]** 1459 (87.94)   
318 - **.CGCGCC.** 1434 (94.60)   
318 - **[CGCCGC]** 1392 (85.72)   
318 - **[CGCCGC]** 1389 (85.72)   
318 - **[CGCGTG]** 1346 (86.67)   
318 - **.CGCGGC.** 1242 (94.52)   
318 - **[CCCGTC]** 1154 (87.14)   
318 - **[CGCGAC]** 1038 (87.27)   
318 - **.CGCGCC.** 722 (94.60)   
318 - **[CGGGTC]** 639 (87.94)   
318 - **.CGCGCC.** 538 (94.60)   
318 - **[CGGGTC]** 479 (87.94)   
318 - **[CGCCGC]** 469 (85.72)   
318 - **[CGCCCC]** 450 (85.80)   
318 - **.CGCGGC.** 288 (94.52)   
318 - **.CGCGCC.** 250 (94.60)   
318 - **[CGCGAC]** 74 (87.27)   
320 + **.CGCGTC.** 107 (100.00)   
320 + **[CGCCCC]** 430 (85.80)   
320 + **[CGCCCC]** 448 (85.80)   
320 + **[GGCGTC]** 554 (87.08)   
320 + **.CGCGCC.** 600 (94.60)   
320 + **.CGCGCC.** 640 (94.60)   
320 + **.CGCCTC.** 642 (91.20)   
320 + **.CGCGCC.** 674 (94.60)   
320 + **[CGCCCC]** 676 (85.80)   
320 + **.CGCGGC.** 694 (94.52)   
320 + **.CGCGCC.** 714 (94.60)   
320 + **.CGCCTC.** 716 (91.20)   
320 + **.CGCGGC.** 721 (94.52)   
320 + **.CGCCTC.** 732 (91.20)   
320 + **.CGCGGC.** 755 (94.52)   
320 + **[CGCCGC]** 760 (85.72)   
320 + **.CGCGCC.** 913 (94.60)   
320 + **.CGCGGC.** 992 (94.52)   
320 + **[CGCCCC]** 1116 (85.80)   
320 + **.CGCCTC.** 1148 (91.20)   
320 + **.CGCGCC.** 1198 (94.60)   
320 + **[CGCCGC]** 1200 (85.72)   
320 + **[CGCGAC]** 1315 (87.27)   
320 + **[CGCCGC]** 1341 (85.72)   
320 + **.CGCGTC.** 1348 (100.00)   
320 + **.CGCGCC.** 1381 (94.60)   
320 + **[CGCCGC]** 1383 (85.72)   
320 + **.CGCGGC.** 1386 (94.52)   
320 - **.CGCGGC.** 1384 (94.52)   
320 - **.CGCGGC.** 1342 (94.52)   
320 - **[CGGGTC]** 1318 (87.94)   
320 - **.CGCGCC.** 1313 (94.60)   
320 - **[CGCCCC]** 1065 (85.80)   
320 - **[CGCCGC]** 993 (85.72)   
320 - **[CGCCCC]** 946 (85.80)   
320 - **.CGCGGC.** 911 (94.52)   
320 - **.CGCCTC.** 892 (91.20)   
320 - **.CGCGCC.** 876 (94.60)   
320 - **[CGCCGC]** 874 (85.72)   
320 - **.CGCCTC.** 871 (91.20)   
320 - **.CGCGCC.** 855 (94.60)   
320 - **[CGGGTC]** 820 (87.94)   
320 - **.CGCGCC.** 798 (94.60)   
320 - **[CGCCCC]** 796 (85.80)   
320 - **[CGCCGC]** 790 (85.72)   
320 - **[GGCGTC]** 776 (87.08)   
320 - **[CGCCGC]** 756 (85.72)   
320 - **[CGCGAC]** 753 (87.27)   
320 - **.CGCGGC.** 594 (94.52)   
320 - **[CGCCCC]** 541 (85.80)   
320 - **[CGCCCC]** 507 (85.80)   
320 - **.CGCCTC.** 444 (91.20)   
320 - **[GGCGTC]** 428 (87.08)   
320 - **[CGCCGC]** 417 (85.72)   
320 - **[CCCGTC]** 226 (87.14)   
320 - **[CGCCCC]** 185 (85.80)   
320 - **.CGCGGC.** 72 (94.52)   
321 + **[CCCGTC]** 19 (87.14)   
321 + **[CGCCGC]** 385 (85.72)   
321 + **.CGCGGC.** 455 (94.52)   
321 + **[GGCGTC]** 458 (87.08)   
321 + **[CGCGAC]** 502 (87.27)   
321 + **[CGCCCC]** 521 (85.80)   
321 + **.CGCCTC.** 549 (91.20)   
321 + **[CGCCCC]** 574 (85.80)   
321 + **[CGCCCC]** 625 (85.80)   
321 + **.CGCCTC.** 631 (91.20)   
321 + **[CGCCCC]** 639 (85.80)   
321 + **.CGCGCC.** 696 (94.60)   
321 + **[CGCCGC]** 698 (85.72)   
321 + **.CGCGCC.** 701 (94.60)   
321 + **.CGCCTC.** 703 (91.20)   
321 + **[CGCCGC]** 732 (85.72)   
321 + **[CGCCGC]** 735 (85.72)   
321 + **[CGCCCC]** 788 (85.80)   
321 + **[CGCCGC]** 981 (85.72)   
321 + **[CGGGTC]** 1008 (87.94)   
321 + **[CGCCGC]** 1043 (85.72)   
321 + **[CGCCGC]** 1056 (85.72)   
321 + **[CGCCCC]** 1167 (85.80)   
321 + **[CGCCGC]** 1216 (85.72)   
321 + **.CGCGCC.** 1225 (94.60)   
321 + **.CGCGCC.** 1260 (94.60)   
321 + **.CGCGGC.** 1302 (94.52)   
321 + **.CGCGCC.** 1395 (94.60)   
321 + **[CGCCGC]** 1397 (85.72)   
321 + **.CGCGGC.** 1400 (94.52)   
321 + **[GGCGTC]** 1484 (87.08)   
321 + **.CGCGCC.** 1506 (94.60)   
321 - **.CGCGGC.** 1502 (94.52)   
321 - **.CGCGGC.** 1398 (94.52)   
321 - **[CGGGTC]** 1315 (87.94)   
321 - **.CGCGCC.** 1300 (94.60)   
321 - **.CGCGGC.** 1256 (94.52)   
321 - **[CGCGAC]** 1209 (87.27)   
321 - **[GGCGTC]** 989 (87.08)   
321 - **.CGCCTC.** 960 (91.20)   
321 - **[CGCCCC]** 948 (85.80)   
321 - **.CGCGGC.** 943 (94.52)   
321 - **[CGCCCC]** 931 (85.80)   
321 - **[CGCCCC]** 918 (85.80)   
321 - **[CGCCCC]** 895 (85.80)   
321 - **[CGCCCC]** 880 (85.80)   
321 - **[CGCCCC]** 837 (85.80)   
321 - **[GGCGTC]** 730 (87.08)   
321 - **.CGCGGC.** 699 (94.52)   
321 - **[CGCGTG]** 555 (86.67)   
321 - **[CGGGTC]** 505 (87.94)   
321 - **.CGCGGC.** 500 (94.52)   
321 - **[CGCCGC]** 456 (85.72)   
321 - **[CGCCGC]** 416 (85.72)   
321 - **[CGCTTC]** 413 (86.32)   
322 + **.CGCGCC.** 259 (94.60)   
322 + **[CGGGTC]** 306 (87.94)   
322 + **[CGCCCC]** 362 (85.80)   
322 + **.CGCGCC.** 463 (94.60)   
322 + **[CGCCGC]** 496 (85.72)   
322 + **.CGCGCC.** 499 (94.60)   
322 + **[CGCCCC]** 501 (85.80)   
322 + **.CGCGGC.** 632 (94.52)   
322 + **[CGCCCC]** 640 (85.80)   
322 + **.CGCGGC.** 688 (94.52)   
322 + **[CCCGTC]** 714 (87.14)   
322 + **[CGCCCC]** 754 (85.80)   
322 + **.CGCGCC.** 948 (94.60)   
322 + **[CGCCGC]** 950 (85.72)   
322 + **[CGCTTC]** 975 (86.32)   
322 + **[CGCCGC]** 1021 (85.72)   
322 + **[CGCCCC]** 1087 (85.80)   
322 + **[CGCCCC]** 1092 (85.80)   
322 + **[CGCCCC]** 1126 (85.80)   
322 + **[CGCTTC]** 1150 (86.32)   
322 + **.CGCGCC.** 1174 (94.60)   
322 + **[CGGGTC]** 1208 (87.94)   
322 + **.CGCGCC.** 1426 (94.60)   
322 + **[CGCCGC]** 1480 (85.72)   
322 - **.CGCGGC.** 1481 (94.52)   
322 - **.CGCGTC.** 1424 (100.00)   
322 - **[CGCCCC]** 1232 (85.80)   
322 - **[CCCGTC]** 1220 (87.14)   
322 - **[CGCGAC]** 1172 (87.27)   
322 - **[CGCGAC]** 1065 (87.27)   
322 - **.CGCGGC.** 1003 (94.52)   
322 - **.CGCGGC.** 959 (94.52)   
322 - **.CGCGGC.** 942 (94.52)   
322 - **[CGCCCC]** 851 (85.80)   
322 - **[CGCCCC]** 846 (85.80)   
322 - **.CGCCTC.** 830 (91.20)   
322 - **[CGCGAC]** 790 (87.27)   
322 - **[CGGGTC]** 766 (87.94)   
322 - **.CGCGCC.** 686 (94.60)   
322 - **[CGCCCC]** 662 (85.80)   
322 - **.CGCGCC.** 630 (94.60)   
322 - **.CGCGGC.** 497 (94.52)   
322 - **[CGCCCC]** 432 (85.80)   
322 - **[CGCCCC]** 231 (85.80)   
323 + **[CGCTTC]** 349 (86.32)   
323 + **[CGCCCC]** 380 (85.80)   
323 + **[CGCGTG]** 455 (86.67)   
323 + **[CCCGTC]** 633 (87.14)   
323 + **[CGCCCC]** 712 (85.80)   
323 + **.CGCGGC.** 860 (94.52)   
323 + **.CGCGGC.** 1020 (94.52)   
323 + **.CGCGGC.** 1032 (94.52)   
323 + **[CGCCCC]** 1063 (85.80)   
323 + **.CGCGGC.** 1074 (94.52)   
323 + **[CGCCCC]** 1172 (85.80)   
323 + **[CGCCCC]** 1177 (85.80)   
323 + **.CGCGCC.** 1182 (94.60)   
323 + **[CGCCCC]** 1184 (85.80)   
323 + **[CGCCGC]** 1228 (85.72)   
323 + **.CGCGGC.** 1231 (94.52)   
323 + **[CGCCCC]** 1300 (85.80)   
323 + **.CGCGCC.** 1313 (94.60)   
323 + **[CGCCCC]** 1315 (85.80)   
323 + **.CGCGCC.** 1321 (94.60)   
323 + **[CGCCCC]** 1323 (85.80)   
323 + **.CGCGTC.** 1424 (100.00)   
323 + **.CGCGTC.** 1450 (100.00)   
323 + **.CGCGCC.** 1496 (94.60)   
323 + **[CGCCCC]** 1502 (85.80)   
323 + **.CGCCTC.** 1507 (91.20)   
323 - **.CGCGGC.** 1494 (94.52)   
323 - **.CGCGCC.** 1448 (94.60)   
323 - **.CGCGCC.** 1361 (94.60)   
323 - **[CGCCCC]** 1359 (85.80)   
323 - **[CGCCGC]** 1232 (85.72)   
323 - **.CGCGGC.** 1229 (94.52)   
323 - **[CGCTTC]** 1191 (86.32)   
323 - **[CGCCGC]** 1142 (85.72)   
323 - **[CGCGTG]** 1068 (86.67)   
323 - **.CGCGGC.** 1030 (94.52)   
323 - **.CGCGCC.** 985 (94.60)   
323 - **[CGCCCC]** 979 (85.80)   
323 - **[CGCCCC]** 960 (85.80)   
323 - **.CGCGCC.** 945 (94.60)   
323 - **[CGCCCC]** 943 (85.80)   
323 - **[CCCGTC]** 939 (87.14)   
323 - **.CGCGCC.** 858 (94.60)   
323 - **[CGCCCC]** 765 (85.80)   
323 - **.CGCCTC.** 760 (91.20)   
323 - **[CGGGTC]** 708 (87.94)   
323 - **[CGCCCC]** 684 (85.80)   
323 - **.CGCGGC.** 453 (94.52)   
323 - **[CGCTTC]** 388 (86.32)   
323 - **[CGCGTG]** 222 (86.67)   
323 - **[CCCGTC]** 82 (87.14)   
324 + **[CGGGTC]** 28 (87.94)   
324 - **[CCCGTC]** 1371 (87.14)   
324 - **[CCCGTC]** 837 (87.14)   
324 - **.CGCCTC.** 668 (91.20)   
325 + **.CGCCTC.** 421 (91.20)   
325 + **[CGGGTC]** 636 (87.94)   
325 + **[GGCGTC]** 1056 (87.08)   
325 + **[CGCGTG]** 1308 (86.67)   
325 + **[CGGGTC]** 1464 (87.94)   
325 - **.CGCGCC.** 1432 (94.60)   
325 - **.CGCGCC.** 1306 (94.60)   
325 - **.CGCCTC.** 998 (91.20)   
325 - **[CGCCCC]** 560 (85.80)   
326 + **.CGCCTC.** 236 (91.20)   
326 + **.CGCCTC.** 411 (91.20)   
326 + **.CGCGCC.** 454 (94.60)   
326 + **[CGGGTC]** 629 (87.94)   
326 + **.CGCGGC.** 653 (94.52)   
326 + **.CGCGGC.** 660 (94.52)   
326 + **[CGCCGC]** 734 (85.72)   
326 + **[GGCGTC]** 753 (87.08)   
326 + **[CGCCGC]** 822 (85.72)   
326 + **.CGCGCC.** 825 (94.60)   
326 + **.CGCGCC.** 869 (94.60)   
326 + **.CGCGCC.** 875 (94.60)   
326 + **[CGCCCC]** 924 (85.80)   
326 + **.CGCGGC.** 938 (94.52)   
326 + **[GGCGTC]** 966 (87.08)   
326 + **[CGCCGC]** 1045 (85.72)   
326 + **.CGCGGC.** 1113 (94.52)   
326 + **[CGCGTG]** 1121 (86.67)   
326 + **[CGCCGC]** 1139 (85.72)   
326 + **[CGCCGC]** 1142 (85.72)   
326 + **.CGCGGC.** 1202 (94.52)   
326 + **.CGCGGC.** 1222 (94.52)   
326 + **.CGCGCC.** 1241 (94.60)   
326 + **.CGCGGC.** 1303 (94.52)   
326 + **.CGCGCC.** 1405 (94.60)   
326 - **[CGCTTC]** 1442 (86.32)   
326 - **.CGCGGC.** 1417 (94.52)   
326 - **.CGCGCC.** 1403 (94.60)   
326 - **[CGCCGC]** 1371 (85.72)   
326 - **[CGCCCC]** 1368 (85.80)   
326 - **[CGCCGC]** 1356 (85.72)   
326 - **[CGCCCC]** 1353 (85.80)   
326 - **[CGCCGC]** 1341 (85.72)   
326 - **.CGCGCC.** 1319 (94.60)   
326 - **[CGCCGC]** 1307 (85.72)   
326 - **[CGCCGC]** 1304 (85.72)   
326 - **.CGCGGC.** 1301 (94.52)   
326 - **[CGGGTC]** 1216 (87.94)   
326 - **[CGCCGC]** 1203 (85.72)   
326 - **.CGCGGC.** 1117 (94.52)   
326 - **.CGCGCC.** 1111 (94.60)   
326 - **[CGCCCC]** 1084 (85.80)   
326 - **[CGCCGC]** 939 (85.72)   
326 - **.CGCGGC.** 823 (94.52)   
326 - **[CGCGTG]** 658 (86.67)   
326 - **[CGCGTG]** 651 (86.67)   
326 - **.CGCGTC.** 177 (100.00)   
327 + **[CGCGAC]** 48 (87.27)   
327 + **.CGCCTC.** 549 (91.20)   
327 + **[CGGGTC]** 590 (87.94)   
327 + **[GGCGTC]** 603 (87.08)   
327 + **[GGCGTC]** 610 (87.08)   
327 + **[CGCGTG]** 660 (86.67)   
327 + **[CGCCGC]** 672 (85.72)   
327 + **.CGCGCC.** 681 (94.60)   
327 + **[CGCGAC]** 902 (87.27)   
327 + **[CGCCCC]** 948 (85.80)   
327 + **[CGCCGC]** 1112 (85.72)   
327 - **.CGCGGC.** 1113 (94.52)   
327 - **.CGCGGC.** 900 (94.52)   
327 - **.CGCGCC.** 658 (94.60)   
327 - **[CGCCGC]** 601 (85.72)   
327 - **[CGCCGC]** 598 (85.72)   
327 - **.CGCGCC.** 468 (94.60)   
327 - **.CGCCTC.** 370 (91.20)   
327 - **[CGCCGC]** 262 (85.72)   
327 - **[CGCCGC]** 259 (85.72)   
327 - **.CGCCTC.** 256 (91.20)   
327 - **[CGGGTC]** 120 (87.94)   
329 + **[CGCCCC]** 17 (85.80)   
329 + **[CGCCCC]** 51 (85.80)   
329 + **.CGCCTC.** 276 (91.20)   
329 + **[CGCCGC]** 460 (85.72)   
329 + **[CGCCGC]** 495 (85.72)   
329 + **.CGCCTC.** 526 (91.20)   
329 + **[CGCCCC]** 793 (85.80)   
329 + **[CGCCCC]** 804 (85.80)   
329 + **[CGCTTC]** 1060 (86.32)   
329 + **.CGCGCC.** 1227 (94.60)   
329 - **[CCCGTC]** 1446 (87.14)   
329 - **.CGCGTC.** 1237 (100.00)   
329 - **[CGCGAC]** 1225 (87.27)   
329 - **[CGCCCC]** 999 (85.80)   
329 - **[CCCGTC]** 942 (87.14)   
329 - **[CCCGTC]** 545 (87.14)   
329 - **.CGCGGC.** 296 (94.52)   
329 - **[GGCGTC]** 274 (87.08)   
329 - **[CGCGAC]** 112 (87.27)   
331 + **.CGCGTC.** 269 (100.00)   
331 + **.CGCGCC.** 359 (94.60)   
331 + **[CGCCCC]** 361 (85.80)   
331 + **.CGCGGC.** 397 (94.52)   
331 + **[CGCCCC]** 412 (85.80)   
331 + **[CGCTTC]** 558 (86.32)   
331 + **[CGGGTC]** 656 (87.94)   
331 + **[CGCCCC]** 675 (85.80)   
331 + **.CGCGGC.** 694 (94.52)   
331 + **[CGCCGC]** 717 (85.72)   
331 + **[CGCCGC]** 720 (85.72)   
331 + **.CGCGGC.** 723 (94.52)   
331 + **[CGCCCC]** 895 (85.80)   
331 + **[CGCCCC]** 918 (85.80)   
331 + **.CGCCTC.** 948 (91.20)   
331 + **.CGCGGC.** 1135 (94.52)   
331 + **.CGCGTC.** 1140 (100.00)   
331 + **[CGCCGC]** 1169 (85.72)   
331 + **[CGCCGC]** 1172 (85.72)   
331 + **[CGCCGC]** 1181 (85.72)   
331 + **[CGCCGC]** 1184 (85.72)   
331 + **[CGCCGC]** 1187 (85.72)   
331 + **[CGCCGC]** 1190 (85.72)   
331 + **[GGCGTC]** 1273 (87.08)   
331 + **[CGCCGC]** 1299 (85.72)   
331 + **.CGCCTC.** 1302 (91.20)   
331 + **.CGCGCC.** 1311 (94.60)   
331 + **[CGCCGC]** 1313 (85.72)   
331 + **.CGCGTC.** 1316 (100.00)   
331 + **.CGCGGC.** 1332 (94.52)   
331 + **.CGCGGC.** 1441 (94.52)   
331 - **[CGCCCC]** 1479 (85.80)   
331 - **[CGCCGC]** 1442 (85.72)   
331 - **[CGCCGC]** 1369 (85.72)   
331 - **[CGCCGC]** 1336 (85.72)   
331 - **[CGCCGC]** 1333 (85.72)   
331 - **.CGCGGC.** 1314 (94.52)   
331 - **[CGCCCC]** 1271 (85.80)   
331 - **.CGCGCC.** 1138 (94.60)   
331 - **[CGCCGC]** 1136 (85.72)   
331 - **.CGCGCC.** 1001 (94.60)   
331 - **[GGCGTC]** 957 (87.08)   
331 - **.CGCGGC.** 763 (94.52)   
331 - **[CGCCCC]** 754 (85.80)   
331 - **.CGCGGC.** 738 (94.52)   
331 - **.CGCGGC.** 721 (94.52)   
331 - **.CGCGGC.** 640 (94.52)   
331 - **[CGCCCC]** 434 (85.80)   
331 - **[CGCCGC]** 398 (85.72)   
331 - **.CGCGCC.** 357 (94.60)   
331 - **[CGCCCC]** 355 (85.80)   
331 - **.CGCGCC.** 188 (94.60)   
331 - **.CGCGGC.** 163 (94.52)   
331 - **[CGCCGC]** 1 (85.72)   
332 + **.CGCCTC.** 135 (91.20)   
332 + **[CCCGTC]** 440 (87.14)   
332 + **.CGCGCC.** 460 (94.60)   
332 + **[CGCCCC]** 473 (85.80)   
332 + **.CGCGTC.** 504 (100.00)   
332 + **[CGCCCC]** 768 (85.80)   
332 + **[CGCCCC]** 775 (85.80)   
332 + **[CGCGTG]** 815 (86.67)   
332 + **[GGCGTC]** 827 (87.08)   
332 + **[CCCGTC]** 832 (87.14)   
332 + **[CGCCCC]** 847 (85.80)   
332 + **[CGCCCC]** 874 (85.80)   
332 + **[CGCCCC]** 909 (85.80)   
332 + **[CGCCCC]** 917 (85.80)   
332 + **[CGCCCC]** 938 (85.80)   
332 + **[CGCCCC]** 952 (85.80)   
332 + **[CGCCCC]** 986 (85.80)   
332 + **[CGCCCC]** 1002 (85.80)   
332 + **[CGCCCC]** 1019 (85.80)   
332 + **[CGCCCC]** 1035 (85.80)   
332 + **[CGCCCC]** 1071 (85.80)   
332 + **.CGCGCC.** 1088 (94.60)   
332 + **[CGCCCC]** 1090 (85.80)   
332 + **.CGCCTC.** 1095 (91.20)   
332 + **[CGCCCC]** 1104 (85.80)   
332 + **.CGCGGC.** 1298 (94.52)   
332 + **[CGCCGC]** 1350 (85.72)   
332 + **.CGCGCC.** 1353 (94.60)   
332 + **.CGCGGC.** 1430 (94.52)   
332 + **[CGCCCC]** 1459 (85.80)   
332 + **.CGCGGC.** 1487 (94.52)   
332 - **[CGCCCC]** 1502 (85.80)   
332 - **[CGCCGC]** 1488 (85.72)   
332 - **.CGCGCC.** 1483 (94.60)   
332 - **[CGCCCC]** 1481 (85.80)   
332 - **[CGCGTG]** 1446 (86.67)   
332 - **.CGCGGC.** 1428 (94.52)   
332 - **.CGCGCC.** 1389 (94.60)   
332 - **[CGCCCC]** 1387 (85.80)   
332 - **.CGCGGC.** 1351 (94.52)   
332 - **.CGCCTC.** 1251 (91.20)   
332 - **.CGCGCC.** 1225 (94.60)   
332 - **[CGCCCC]** 1223 (85.80)   
332 - **[CGCCCC]** 1198 (85.80)   
332 - **[CGCCCC]** 1145 (85.80)   
332 - **.CGCGCC.** 1135 (94.60)   
332 - **[CGCTTC]** 665 (86.32)   
332 - **[CGCGAC]** 458 (87.27)   
332 - **[CGCGAC]** 450 (87.27)   
332 - **.CGCCTC.** 154 (91.20)   
332 - **.CGCGGC.** 101 (94.52)   
332 - **[CCCGTC]** 90 (87.14)   
332 - **[CGCCCC]** 50 (85.80)   
333 + **.CGCCTC.** 610 (91.20)   
333 + **[CGCCCC]** 985 (85.80)   
333 + **[CGCCCC]** 1004 (85.80)   
333 + **[CGCCCC]** 1025 (85.80)   
333 + **.CGCGGC.** 1030 (94.52)   
333 + **[CGCCGC]** 1121 (85.72)   
333 + **[CGCCCC]** 1182 (85.80)   
333 + **[CGCGAC]** 1196 (87.27)   
333 + **.CGCGGC.** 1299 (94.52)   
333 + **[CGCCGC]** 1320 (85.72)   
333 + **[CGCGAC]** 1323 (87.27)   
333 + **[CGCCGC]** 1348 (85.72)   
333 + **[CGCCCC]** 1411 (85.80)   
333 - **.CGCGGC.** 1321 (94.52)   
333 - **[CGCCGC]** 1300 (85.72)   
333 - **[CGCCCC]** 1290 (85.80)   
333 - **[CGCCCC]** 1284 (85.80)   
333 - **[CGGGTC]** 1146 (87.94)   
333 - **.CGCGGC.** 1122 (94.52)   
333 - **[CGCCGC]** 1089 (85.72)   
333 - **[CCCGTC]** 549 (87.14)   
333 - **[CGCTTC]** 237 (86.32)   
333 - **.CGCCTC.** 19 (91.20)   
334 + **[CGCCCC]** 462 (85.80)   
334 + **[CGCCCC]** 585 (85.80)   
334 + **[CGCCGC]** 650 (85.72)   
334 + **[CCCGTC]** 832 (87.14)   
334 + **[CGCGAC]** 873 (87.27)   
334 + **[GGCGTC]** 884 (87.08)   
334 + **[CGCCCC]** 935 (85.80)   
334 + **[CCCGTC]** 989 (87.14)   
334 + **[CGCCCC]** 1012 (85.80)   
334 + **[CGCCCC]** 1025 (85.80)   
334 + **[CGCCCC]** 1143 (85.80)   
334 - **.CGCGCC.** 1087 (94.60)   
334 - **.CGCGGC.** 871 (94.52)   
334 - **[CGCCCC]** 734 (85.80)   
334 - **[CGCTTC]** 581 (86.32)   
334 - **[CGCCGC]** 514 (85.72)   
334 - **.CGCGGC.** 381 (94.52)   
335 + **[CGCCGC]** 542 (85.72)   
335 + **.CGCGCC.** 610 (94.60)   
335 + **.CGCCTC.** 612 (91.20)   
335 + **.CGCGGC.** 720 (94.52)   
335 + **[CGCCCC]** 743 (85.80)   
335 + **.CGCGGC.** 854 (94.52)   
335 + **.CGCGGC.** 987 (94.52)   
335 + **.CGCGCC.** 1100 (94.60)   
335 + **[CGCCGC]** 1102 (85.72)   
335 + **[CGCCGC]** 1187 (85.72)   
335 + **[CGCCCC]** 1199 (85.80)   
335 + **.CGCGTC.** 1205 (100.00)   
335 + **.CGCGCC.** 1230 (94.60)   
335 + **.CGCGGC.** 1261 (94.52)   
335 + **.CGCGCC.** 1389 (94.60)   
335 + **[CGCCCC]** 1463 (85.80)   
335 - **.CGCGCC.** 1430 (94.60)   
335 - **[CGCCCC]** 1428 (85.80)   
335 - **[CGCCCC]** 1397 (85.80)   
335 - **.CGCGCC.** 1387 (94.60)   
335 - **[CGCCCC]** 1385 (85.80)   
335 - **[CGCCGC]** 1363 (85.72)   
335 - **[CGCCCC]** 1360 (85.80)   
335 - **[CGCCCC]** 1336 (85.80)   
335 - **[CGCCCC]** 1314 (85.80)   
335 - **[CGCCCC]** 1305 (85.80)   
335 - **.CGCGCC.** 1259 (94.60)   
335 - **.CGCGCC.** 1228 (94.60)   
335 - **[CGCGAC]** 1208 (87.27)   
335 - **.CGCGGC.** 1122 (94.52)   
335 - **[GGCGTC]** 1079 (87.08)   
335 - **.CGCGCC.** 983 (94.60)   
335 - **[CGCCCC]** 907 (85.80)   
335 - **.CGCGCC.** 857 (94.60)   
335 - **[CGCCGC]** 855 (85.72)   
335 - **.CGCCTC.** 823 (91.20)   
335 - **[CGCGAC]** 718 (87.27)   
335 - **[CCCGTC]** 96 (87.14)   
335 - **.CGCCTC.** 86 (91.20)   
336 + **[CGGGTC]** 278 (87.94)   
336 + **.CGCCTC.** 494 (91.20)   
336 + **[CGCCGC]** 602 (85.72)   
336 + **[CGCCCC]** 747 (85.80)   
336 + **.CGCGCC.** 760 (94.60)   
336 + **.CGCGTC.** 876 (100.00)   
336 + **.CGCGCC.** 981 (94.60)   
336 + **.CGCGGC.** 1023 (94.52)   
336 + **[CGGGTC]** 1056 (87.94)   
336 + **[CGCGTG]** 1064 (86.67)   
336 + **[CGCCCC]** 1279 (85.80)   
336 + **[CGGGTC]** 1408 (87.94)   
336 + **.CGCCTC.** 1473 (91.20)   
336 - **[CGCGTG]** 1062 (86.67)   
336 - **[CGGGTC]** 947 (87.94)   
336 - **[CGGGTC]** 908 (87.94)   
336 - **[CGCGTG]** 840 (86.67)   
336 - **[CGCCGC]** 823 (85.72)   
336 - **.CGCCTC.** 731 (91.20)   
336 - **[GGCGTC]** 492 (87.08)   
337 + **[CGCCCC]** 331 (85.80)   
337 + **.CGCGCC.** 411 (94.60)   
337 + **[CGCGTG]** 755 (86.67)   
337 + **.CGCGCC.** 827 (94.60)   
337 + **[CGCCCC]** 829 (85.80)   
337 + **.CGCGGC.** 842 (94.52)   
337 + **[CGCGTG]** 856 (86.67)   
337 + **[CGCCCC]** 901 (85.80)   
337 + **.CGCGGC.** 956 (94.52)   
337 + **[CGCCGC]** 1055 (85.72)   
337 + **[CGCCGC]** 1064 (85.72)   
337 + **[CGCCGC]** 1067 (85.72)   
337 + **[CGCCGC]** 1070 (85.72)   
337 + **.CGCGGC.** 1073 (94.52)   
337 + **.CGCGGC.** 1176 (94.52)   
337 + **.CGCGGC.** 1183 (94.52)   
337 + **[CGCCGC]** 1188 (85.72)   
337 + **[GGCGTC]** 1322 (87.08)   
337 + **[CGCGAC]** 1442 (87.27)   
337 - **.CGCGCC.** 1475 (94.60)   
337 - **[CGCCCC]** 1473 (85.80)   
337 - **.CGCGCC.** 1438 (94.60)   
337 - **[CGCCCC]** 1436 (85.80)   
337 - **[CGCGAC]** 1421 (87.27)   
337 - **[CGCCGC]** 1386 (85.72)   
337 - **.CGCGCC.** 1379 (94.60)   
337 - **.CGCGCC.** 1348 (94.60)   
337 - **[CGCCCC]** 1310 (85.80)   
337 - **[CGCCGC]** 1220 (85.72)   
337 - **[CGCCGC]** 1217 (85.72)   
337 - **[CGCCGC]** 1184 (85.72)   
337 - **.CGCGCC.** 1148 (94.60)   
337 - **[CGCCCC]** 1146 (85.80)   
337 - **.CGCGGC.** 1071 (94.52)   
337 - **[CGCCGC]** 957 (85.72)   
337 - **.CGCGCC.** 950 (94.60)   
337 - **[CGCCCC]** 948 (85.80)   
337 - **[CGCCGC]** 927 (85.72)   
337 - **[CGCCGC]** 871 (85.72)   
337 - **[CGCCGC]** 843 (85.72)   
337 - **.CGCGGC.** 825 (94.52)   
337 - **.CGCGGC.** 765 (94.52)   
337 - **[CGCGTG]** 749 (86.67)   
337 - **.CGCGGC.** 318 (94.52)   
338 + **[CGCCGC]** 326 (85.72)   
338 + **[CGCCCC]** 329 (85.80)   
338 + **[GGCGTC]** 357 (87.08)   
338 + **.CGCGGC.** 440 (94.52)   
338 + **[CGCCGC]** 691 (85.72)   
338 + **.CGCGTC.** 694 (100.00)   
338 + **.CGCGCC.** 754 (94.60)   
338 + **.CGCGCC.** 760 (94.60)   
338 + **[CGCCGC]** 762 (85.72)   
338 + **[CGCCGC]** 794 (85.72)   
338 + **.CGCGCC.** 812 (94.60)   
338 + **[CGCCCC]** 814 (85.80)   
338 + **[CGCCCC]** 843 (85.80)   
338 + **.CGCGGC.** 1054 (94.52)   
338 + **.CGCGCC.** 1065 (94.60)   
338 + **[CGCCCC]** 1067 (85.80)   
338 + **.CGCGCC.** 1092 (94.60)   
338 + **[CGCCCC]** 1102 (85.80)   
338 + **.CGCGCC.** 1126 (94.60)   
338 + **[CGCCCC]** 1128 (85.80)   
338 + **.CGCCTC.** 1139 (91.20)   
338 + **.CGCGTC.** 1154 (100.00)   
338 + **[CGCCCC]** 1160 (85.80)   
338 + **.CGCGCC.** 1172 (94.60)   
338 + **[CGCCGC]** 1174 (85.72)   
338 + **[CGCCGC]** 1177 (85.72)   
338 + **[CGCCGC]** 1180 (85.72)   
338 + **[CGCCGC]** 1183 (85.72)   
338 + **[CGCCGC]** 1186 (85.72)   
338 + **[CGCCGC]** 1189 (85.72)   
338 + **[CGCCGC]** 1192 (85.72)   
338 + **[CGCCGC]** 1274 (85.72)   
338 + **[CGCGTG]** 1416 (86.67)   
338 + **.CGCGGC.** 1459 (94.52)   
338 + **[GGCGTC]** 1462 (87.08)   
338 - **.CGCCTC.** 1511 (91.20)   
338 - **.CGCCTC.** 1505 (91.20)   
338 - **[CGCCGC]** 1460 (85.72)   
338 - **[CGCCCC]** 1448 (85.80)   
338 - **.CGCGGC.** 1414 (94.52)   
338 - **[CGCCGC]** 1383 (85.72)   
338 - **[CGCCGC]** 1380 (85.72)   
338 - **[CGCCGC]** 1334 (85.72)   
338 - **.CGCGGC.** 1305 (94.52)   
338 - **[CGCCGC]** 1299 (85.72)   
338 - **[CGCCGC]** 1253 (85.72)   
338 - **[CGCCGC]** 1250 (85.72)   
338 - **.CGCGGC.** 1193 (94.52)   
338 - **[CGCCGC]** 1055 (85.72)   
338 - **[CGCCGC]** 1047 (85.72)   
338 - **[CGGGTC]** 1029 (87.94)   
338 - **.CGCGGC.** 692 (94.52)   
338 - **[CGCCCC]** 650 (85.80)   
338 - **.CGCGGC.** 642 (94.52)   
339 + **[CGGGTC]** 1091 (87.94)   
339 + **[CCCGTC]** 1169 (87.14)   
339 + **[CGGGTC]** 1432 (87.94)   
339 + **.CGCCTC.** 1439 (91.20)   
339 - **[CGCCCC]** 1428 (85.80)   
339 - **[CCCGTC]** 1136 (87.14)   
339 - **.CGCCTC.** 1122 (91.20)   
339 - **.CGCCTC.** 1111 (91.20)   
339 - **[CCCGTC]** 1089 (87.14)   
339 - **[CGCCGC]** 1077 (85.72)   
339 - **[CGCCGC]** 1068 (85.72)   
339 - **[CGCCGC]** 1065 (85.72)   
339 - **.CGCCTC.** 1062 (91.20)   
339 - **.CGCGCC.** 1028 (94.60)   
339 - **[CGGGTC]** 1022 (87.94)   
339 - **[CGCCCC]** 978 (85.80)   
339 - **[CGCCGC]** 948 (85.72)   
339 - **[CGGGTC]** 942 (87.94)   
339 - **[CCCGTC]** 897 (87.14)   
339 - **[CCCGTC]** 575 (87.14)   
339 - **.CGCGCC.** 529 (94.60)   
339 - **[CGCCCC]** 347 (85.80)   
340 + **[CGCCCC]** 168 (85.80)   
340 + **[CGCCCC]** 503 (85.80)   
340 + **[CGCCCC]** 571 (85.80)   
340 + **[GGCGTC]** 683 (87.08)   
340 + **[CGCCGC]** 688 (85.72)   
340 + **[CGCCGC]** 758 (85.72)   
340 + **[CCCGTC]** 763 (87.14)   
340 + **[CGCTTC]** 844 (86.32)   
340 + **[CGCCGC]** 935 (85.72)   
340 + **[CGCCCC]** 938 (85.80)   
340 + **[CGCCCC]** 943 (85.80)   
340 + **[CGCGAC]** 979 (87.27)   
340 + **[GGCGTC]** 1201 (87.08)   
340 - **[CGCCCC]** 1095 (85.80)   
340 - **[CCCGTC]** 1073 (87.14)   
340 - **.CGCGCC.** 1066 (94.60)   
340 - **[CGGGTC]** 994 (87.94)   
340 - **[CGCGTG]** 675 (86.67)   
340 - **[CGCCCC]** 202 (85.80)   
341 + **.CGCGCC.** 105 (94.60)   
341 + **.CGCGGC.** 156 (94.52)   
341 + **[CGCTTC]** 931 (86.32)   
341 + **.CGCGGC.** 1152 (94.52)   
341 + **.CGCCTC.** 1158 (91.20)   
341 + **.CGCGTC.** 1260 (100.00)   
341 + **.CGCGGC.** 1319 (94.52)   
341 + **[CGCCCC]** 1324 (85.80)   
341 + **.CGCGCC.** 1350 (94.60)   
341 + **.CGCCTC.** 1352 (91.20)   
341 + **[CGCCGC]** 1383 (85.72)   
341 + **.CGCGGC.** 1494 (94.52)   
341 + **.CGCGCC.** 1505 (94.60)   
341 - **.CGCGTC.** 1492 (100.00)   
341 - **.CGCGCC.** 1483 (94.60)   
341 - **.CGCGCC.** 1417 (94.60)   
341 - **[CGCCCC]** 1415 (85.80)   
341 - **[CGCCGC]** 1410 (85.72)   
341 - **[CGCCCC]** 1407 (85.80)   
341 - **.CGCGGC.** 1384 (94.52)   
341 - **.CGCGCC.** 1346 (94.60)   
341 - **[CGCCGC]** 1320 (85.72)   
341 - **.CGCGGC.** 1315 (94.52)   
341 - **[CGCCCC]** 1236 (85.80)   
341 - **[CGCGAC]** 1231 (87.27)   
341 - **.CGCCTC.** 1126 (91.20)   
341 - **[CGCTTC]** 1100 (86.32)   
341 - **[CGCCGC]** 1085 (85.72)   
341 - **[CGCCGC]** 1059 (85.72)   
341 - **[CGCCCC]** 1005 (85.80)   
341 - **[CGCCCC]** 989 (85.80)   
341 - **.CGCCTC.** 927 (91.20)   
341 - **[CGCTTC]** 881 (86.32)   
341 - **.CGCCTC.** 511 (91.20)   
341 - **[CGCTTC]** 474 (86.32)   
341 - **[CGCGAC]** 211 (87.27)   
341 - **.CGCGGC.** 187 (94.52)   
341 - **[CGCGTG]** 43 (86.67)   
342 + **[CCCGTC]** 1070 (87.14)   
342 + **[CGCCGC]** 1098 (85.72)   
342 + **[CGCCGC]** 1107 (85.72)   
342 + **.CGCGGC.** 1155 (94.52)   
342 + **[CGCTTC]** 1227 (86.32)   
342 + **[CGCGAC]** 1281 (87.27)   
342 + **[CGCGTG]** 1301 (86.67)   
342 + **.CGCGGC.** 1335 (94.52)   
342 + **[CGCGTG]** 1366 (86.67)   
342 + **.CGCGCC.** 1400 (94.60)   
342 + **[CGCGTG]** 1447 (86.67)   
342 + **[CGGGTC]** 1462 (87.94)   
342 + **[CGCGTG]** 1477 (86.67)   
342 + **[CGCGTG]** 1483 (86.67)   
342 - **.CGCGCC.** 1471 (94.60)   
342 - **[CGCCGC]** 1420 (85.72)   
342 - **.CGCGTC.** 1396 (100.00)   
342 - **.CGCGTC.** 1347 (100.00)   
342 - **.CGCGCC.** 1331 (94.60)   
342 - **[CGCCCC]** 1319 (85.80)   
342 - **.CGCGCC.** 1297 (94.60)   
342 - **[CGCCGC]** 1290 (85.72)   
342 - **.CGCGCC.** 1221 (94.60)   
342 - **[CGCCCC]** 1189 (85.80)   
342 - **[CGCCGC]** 1156 (85.72)   
342 - **.CGCGCC.** 1153 (94.60)   
342 - **[CGCCGC]** 1151 (85.72)   
342 - **[CGCTTC]** 1035 (86.32)   
342 - **.CGCGCC.** 1025 (94.60)   
342 - **[CGCCGC]** 1023 (85.72)   
342 - **[CGCCCC]** 931 (85.80)   
342 - **[CGCCCC]** 841 (85.80)   
342 - **.CGCGCC.** 836 (94.60)   
342 - **[CGCCCC]** 815 (85.80)   
342 - **.CGCGCC.** 720 (94.60)   
342 - **.CGCGGC.** 712 (94.52)   
342 - **[CGGGTC]** 706 (87.94)   
342 - **[CGCTTC]** 515 (86.32)   
342 - **.CGCCTC.** 456 (91.20)   
343 + **.CGCGGC.** 55 (94.52)   
343 + **[CGCCCC]** 266 (85.80)   
343 + **.CGCGTC.** 279 (100.00)   
343 + **[CGGGTC]** 291 (87.94)   
343 + **[CGCTTC]** 528 (86.32)   
343 + **[CCCGTC]** 762 (87.14)   
343 + **[CGCGAC]** 781 (87.27)   
343 + **[CGGGTC]** 829 (87.94)   
343 + **[CGGGTC]** 927 (87.94)   
343 + **.CGCGCC.** 957 (94.60)   
343 + **.CGCGGC.** 998 (94.52)   
343 + **[CGCCGC]** 1026 (85.72)   
343 + **[CGCCGC]** 1077 (85.72)   
343 + **[CGCGTG]** 1080 (86.67)   
343 + **.CGCGGC.** 1122 (94.52)   
343 + **[CGCGTG]** 1136 (86.67)   
343 + **.CGCGGC.** 1182 (94.52)   
343 + **[CGGGTC]** 1200 (87.94)   
343 + **[CCCGTC]** 1220 (87.14)   
343 + **.CGCGGC.** 1342 (94.52)   
343 + **.CGCGGC.** 1350 (94.52)   
343 + **[CGGGTC]** 1433 (87.94)   
343 + **[CGCCCC]** 1489 (85.80)   
343 - **.CGCGCC.** 1340 (94.60)   
343 - **.CGCGCC.** 1319 (94.60)   
343 - **.CGCGGC.** 1305 (94.52)   
343 - **[CGCCGC]** 1183 (85.72)   
343 - **.CGCGCC.** 1180 (94.60)   
343 - **[CGCCCC]** 1178 (85.80)   
343 - **.CGCGGC.** 1120 (94.52)   
343 - **.CGCGGC.** 1078 (94.52)   
343 - **[CGCTTC]** 1065 (86.32)   
343 - **[CGCCGC]** 999 (85.72)   
343 - **.CGCGCC.** 996 (94.60)   
343 - **.CGCGCC.** 965 (94.60)   
343 - **[CGCCCC]** 915 (85.80)   
343 - **.CGCGCC.** 910 (94.60)   
343 - **[CGCCGC]** 861 (85.72)   
343 - **.CGCGTC.** 670 (100.00)   
343 - **.CGCGGC.** 653 (94.52)   
343 - **.CGCCTC.** 586 (91.20)   
343 - **.CGCGCC.** 524 (94.60)   
343 - **[CGCCCC]** 522 (85.80)   
343 - **[CGCCCC]** 353 (85.80)   
343 - **.CGCGCC.** 277 (94.60)   
343 - **[CGCGTG]** 232 (86.67)   
343 - **[CGCGTG]** 220 (86.67)   
344 + **[CGCCCC]** 745 (85.80)   
344 + **[CGCCGC]** 781 (85.72)   
344 + **[CGCCCC]** 959 (85.80)   
344 + **[CGCCGC]** 1069 (85.72)   
344 + **[CGCTTC]** 1072 (86.32)   
344 + **.CGCGCC.** 1106 (94.60)   
344 + **.CGCGCC.** 1166 (94.60)   
344 + **[CGCCGC]** 1168 (85.72)   
344 - **.CGCCTC.** 1148 (91.20)   
344 - **.CGCGCC.** 1104 (94.60)   
344 - **[CGCCCC]** 858 (85.80)   
345 + **.CGCCTC.** 145 (91.20)   
345 + **[CGCCCC]** 869 (85.80)   
345 - **[CCCGTC]** 1145 (87.14)   
346 + **.CGCGGC.** 23 (94.52)   
346 + **.CGCGGC.** 85 (94.52)   
346 + **[CCCGTC]** 278 (87.14)   
346 + **[CGCCGC]** 820 (85.72)   
346 + **.CGCGCC.** 882 (94.60)   
346 + **[CGCCCC]** 884 (85.80)   
346 + **[CGCCGC]** 897 (85.72)   
346 + **[CGCCGC]** 997 (85.72)   
346 + **[CGCCCC]** 1095 (85.80)   
346 + **.CGCCTC.** 1102 (91.20)   
346 + **.CGCGCC.** 1231 (94.60)   
346 + **[CGCCGC]** 1233 (85.72)   
346 + **[CGCCGC]** 1236 (85.72)   
346 + **.CGCGTC.** 1239 (100.00)   
346 + **.CGCCTC.** 1389 (91.20)   
346 + **[CGCTTC]** 1394 (86.32)   
346 - **[CGCCCC]** 1512 (85.80)   
346 - **.CGCGGC.** 1407 (94.52)   
346 - **[CGCCGC]** 1379 (85.72)   
346 - **.CGCGCC.** 1340 (94.60)   
346 - **.CGCGGC.** 1237 (94.52)   
346 - **.CGCGCC.** 1229 (94.60)   
346 - **[CGCCCC]** 1198 (85.80)   
346 - **[CGCCGC]** 993 (85.72)   
346 - **.CGCCTC.** 977 (91.20)   
346 - **.CGCGGC.** 906 (94.52)   
346 - **.CGCGCC.** 880 (94.60)   
346 - **[CGCCCC]** 862 (85.80)   
346 - **.CGCGTC.** 855 (100.00)   
346 - **.CGCGCC.** 717 (94.60)   
346 - **[CGCCCC]** 715 (85.80)   
346 - **.CGCCTC.** 590 (91.20)   
346 - **.CGCGGC.** 527 (94.52)   
346 - **[CGCCGC]** 326 (85.72)   
346 - **[GGCGTC]** 96 (87.08)   
346 - **[CGCCGC]** 86 (85.72)   
346 - **[CGCCGC]** 24 (85.72)   
346 - **[CGCGAC]** 21 (87.27)   
347 + **[CGCCGC]** 260 (85.72)   
347 + **[CGGGTC]** 297 (87.94)   
347 + **[CGCCCC]** 313 (85.80)   
347 + **[CGCTTC]** 427 (86.32)   
347 + **.CGCGGC.** 436 (94.52)   
347 + **.CGCGCC.** 553 (94.60)   
347 + **.CGCGCC.** 838 (94.60)   
347 + **[CGCCCC]** 847 (85.80)   
347 + **[CGCGTG]** 876 (86.67)   
347 + **[CGCCGC]** 908 (85.72)   
347 + **.CGCGCC.** 913 (94.60)   
347 + **[CGCTTC]** 947 (86.32)   
347 + **.CGCGCC.** 966 (94.60)   
347 + **.CGCGGC.** 1088 (94.52)   
347 + **[CGCCCC]** 1155 (85.80)   
347 + **[CGCCGC]** 1181 (85.72)   
347 + **[CGCCCC]** 1184 (85.80)   
347 + **.CGCGGC.** 1207 (94.52)   
347 + **[CGCCCC]** 1235 (85.80)   
347 + **.CGCGCC.** 1257 (94.60)   
347 + **[CGCCCC]** 1266 (85.80)   
347 + **.CGCGGC.** 1286 (94.52)   
347 + **[CGCCCC]** 1299 (85.80)   
347 + **.CGCCTC.** 1384 (91.20)   
347 + **.CGCGGC.** 1467 (94.52)   
347 + **.CGCGCC.** 1494 (94.60)   
347 + **[CGCCGC]** 1513 (85.72)   
347 - **[GGCGTC]** 1382 (87.08)   
347 - **[CGCCCC]** 1231 (85.80)   
347 - **[CGCCGC]** 1226 (85.72)   
347 - **[CCCGTC]** 1149 (87.14)   
347 - **[CCCGTC]** 1115 (87.14)   
347 - **.CGCGCC.** 1101 (94.60)   
347 - **[CGCCGC]** 1089 (85.72)   
347 - **[CGCCCC]** 1038 (85.80)   
347 - **[CGCCCC]** 1015 (85.80)   
347 - **[CGCCCC]** 995 (85.80)   
347 - **[CGCCCC]** 979 (85.80)   
347 - **[CGCCCC]** 974 (85.80)   
347 - **.CGCGGC.** 909 (94.52)   
347 - **.CGCGCC.** 836 (94.60)   
347 - **[CGGGTC]** 423 (87.94)   
347 - **[CGGGTC]** 293 (87.94)   
347 - **[CGGGTC]** 48 (87.94)   
347 - **.CGCCTC.** 1 (91.20)   
348 + **[CGCGAC]** 55 (87.27)   
348 + **[CGCCGC]** 68 (85.72)   
348 + **.CGCGGC.** 71 (94.52)   
348 + **.CGCGCC.** 132 (94.60)   
348 + **[CGCCGC]** 134 (85.72)   
348 + **.CGCGCC.** 228 (94.60)   
348 + **[CGCCCC]** 230 (85.80)   
348 + **[CGCCCC]** 266 (85.80)   
348 + **.CGCGGC.** 344 (94.52)   
348 + **[CGCCCC]** 360 (85.80)   
348 + **.CGCGCC.** 375 (94.60)   
348 + **[CGCCCC]** 377 (85.80)   
348 + **.CGCGCC.** 382 (94.60)   
348 + **.CGCGCC.** 399 (94.60)   
348 + **.CGCGCC.** 417 (94.60)   
348 + **[CGCCGC]** 435 (85.72)   
348 + **[CGCCGC]** 445 (85.72)   
348 + **[CGCCGC]** 448 (85.72)   
348 + **[CGCCGC]** 661 (85.72)   
348 + **.CGCGTC.** 664 (100.00)   
348 + **[CGCCCC]** 681 (85.80)   
348 + **[CCCGTC]** 684 (87.14)   
348 + **[CGCCGC]** 695 (85.72)   
348 + **.CGCGCC.** 698 (94.60)   
348 + **.CGCCTC.** 700 (91.20)   
348 + **.CGCGGC.** 712 (94.52)   
348 + **[CGCCGC]** 717 (85.72)   
348 + **[CGCCGC]** 720 (85.72)   
348 + **.CGCCTC.** 817 (91.20)   
348 + **[CGCCCC]** 856 (85.80)   
348 + **.CGCGCC.** 866 (94.60)   
348 + **[CCCGTC]** 903 (87.14)   
348 + **[CGCCGC]** 908 (85.72)   
348 + **[CGCCGC]** 918 (85.72)   
348 + **[CCCGTC]** 923 (87.14)   
348 + **.CGCGGC.** 1028 (94.52)   
348 + **.CGCGCC.** 1075 (94.60)   
348 + **.CGCGGC.** 1240 (94.52)   
348 + **.CGCCTC.** 1392 (91.20)   
348 + **[CGCGTG]** 1414 (86.67)   
348 + **[CGCGTG]** 1480 (86.67)   
348 + **.CGCGCC.** 1489 (94.60)   
348 + **[CGCCGC]** 1491 (85.72)   
348 + **[CGCCGC]** 1494 (85.72)   
348 + **[CGCCCC]** 1497 (85.80)   
348 - **[CGCGAC]** 1487 (87.27)   
348 - **.CGCGCC.** 1478 (94.60)   
348 - **[GGCGTC]** 1445 (87.08)   
348 - **.CGCGGC.** 1363 (94.52)   
348 - **[CGCCGC]** 1347 (85.72)   
348 - **.CGCCTC.** 1344 (91.20)   
348 - **.CGCGCC.** 1279 (94.60)   
348 - **[CGCCGC]** 1277 (85.72)   
348 - **[CGCCGC]** 1241 (85.72)   
348 - **.CGCGCC.** 1238 (94.60)   
348 - **.CGCGTC.** 1190 (100.00)   
348 - **[CGCCGC]** 1165 (85.72)   
348 - **.CGCCTC.** 1162 (91.20)   
348 - **[CGCCGC]** 1029 (85.72)   
348 - **[CGCGAC]** 1026 (87.27)   
348 - **[CGCGAC]** 986 (87.27)   
348 - **.CGCGCC.** 961 (94.60)   
348 - **[CGCCCC]** 959 (85.80)   
348 - **[GGCGTC]** 916 (87.08)   
348 - **[GGCGTC]** 885 (87.08)   
348 - **.CGCGCC.** 862 (94.60)   
348 - **[CGCCGC]** 713 (85.72)   
348 - **.CGCGGC.** 696 (94.52)   
348 - **.CGCGGC.** 662 (94.52)   
348 - **[CGCCCC]** 657 (85.80)   
348 - **[CGCCCC]** 641 (85.80)   
348 - **[CGCGAC]** 623 (87.27)   
348 - **.CGCCTC.** 604 (91.20)   
348 - **.CGCGCC.** 545 (94.60)   
348 - **[CGCCGC]** 543 (85.72)   
348 - **.CGCGTC.** 519 (100.00)   
348 - **[CGGGTC]** 431 (87.94)   
348 - **.CGCGTC.** 308 (100.00)   
348 - **.CGCGCC.** 293 (94.60)   
348 - **[CGGGTC]** 222 (87.94)   
348 - **.CGCGGC.** 135 (94.52)   
348 - **.CGCGGC.** 69 (94.52)   
349 + **[GGCGTC]** 127 (87.08)   
349 + **[CCCGTC]** 182 (87.14)   
349 + **[CGCGTG]** 491 (86.67)   
349 + **[CGCCCC]** 699 (85.80)   
349 + **[CGCCGC]** 731 (85.72)   
349 + **.CGCCTC.** 734 (91.20)   
349 + **[CGCCGC]** 747 (85.72)   
349 + **[CGCCGC]** 750 (85.72)   
349 + **[CGCTTC]** 813 (86.32)   
349 + **.CGCGGC.** 916 (94.52)   
349 + **.CGCCTC.** 1026 (91.20)   
349 + **.CGCGGC.** 1165 (94.52)   
349 + **[CGCCGC]** 1221 (85.72)   
349 + **[CGCCCC]** 1224 (85.80)   
349 + **[CGGGTC]** 1275 (87.94)   
349 + **.CGCGTC.** 1310 (100.00)   
349 + **[GGCGTC]** 1338 (87.08)   
349 - **[CGCCCC]** 1512 (85.80)   
349 - **.CGCGCC.** 1308 (94.60)   
349 - **[CGCCGC]** 1166 (85.72)   
349 - **.CGCGCC.** 1163 (94.60)   
349 - **.CGCCTC.** 1098 (91.20)   
349 - **[CGCCCC]** 1088 (85.80)   
349 - **[CGCCCC]** 1078 (85.80)   
349 - **.CGCGCC.** 1045 (94.60)   
349 - **[CGCCCC]** 956 (85.80)   
349 - **[CGCCGC]** 938 (85.72)   
349 - **[CGCCCC]** 843 (85.80)   
349 - **.CGCGCC.** 837 (94.60)   
349 - **[GGCGTC]** 656 (87.08)   
349 - **.CGCGTC.** 489 (100.00)   
349 - **[CGCCCC]** 11 (85.80)   
350 + **[GGCGTC]** 451 (87.08)   
350 + **[CCCGTC]** 781 (87.14)   
350 + **[CCCGTC]** 854 (87.14)   
350 + **[CCCGTC]** 935 (87.14)   
350 + **[GGCGTC]** 996 (87.08)   
350 + **.CGCGGC.** 1046 (94.52)   
350 + **.CGCGGC.** 1107 (94.52)   
350 + **[CGCCGC]** 1138 (85.72)   
350 + **.CGCCTC.** 1141 (91.20)   
350 + **.CGCGCC.** 1186 (94.60)   
350 + **[CGCCGC]** 1188 (85.72)   
350 + **[CGCCCC]** 1191 (85.80)   
350 + **[CGCCCC]** 1205 (85.80)   
350 + **.CGCCTC.** 1237 (91.20)   
350 + **.CGCGCC.** 1345 (94.60)   
350 + **.CGCGGC.** 1404 (94.52)   
350 - **.CGCGCC.** 1437 (94.60)   
350 - **[CGCCGC]** 1435 (85.72)   
350 - **[CGCCCC]** 1367 (85.80)   
350 - **[CCCGTC]** 1364 (87.14)   
350 - **.CGCGCC.** 1343 (94.60)   
350 - **[CGCCGC]** 1277 (85.72)   
350 - **[CGCGAC]** 1266 (87.27)   
350 - **[CGCCCC]** 1233 (85.80)   
350 - **[CGGGTC]** 1228 (87.94)   
350 - **.CGCGCC.** 1044 (94.60)   
350 - **[CGCCGC]** 1029 (85.72)   
350 - **.CGCCTC.** 1026 (91.20)   
350 - **[CGCCCC]** 1021 (85.80)   
350 - **[CGCCCC]** 1016 (85.80)   
350 - **[CGCGAC]** 999 (87.27)   
350 - **.CGCCTC.** 449 (91.20)   
351 + **[CCCGTC]** 261 (87.14)   
351 + **.CGCGCC.** 305 (94.60)   
351 + **.CGCGCC.** 390 (94.60)   
351 + **[CGCCCC]** 612 (85.80)   
351 + **.CGCCTC.** 772 (91.20)   
351 + **[CGCCGC]** 936 (85.72)   
351 + **.CGCGGC.** 975 (94.52)   
351 + **[CGCCGC]** 994 (85.72)   
351 + **[CGCGAC]** 1021 (87.27)   
351 + **[CGCCGC]** 1038 (85.72)   
351 + **[CGCCGC]** 1093 (85.72)   
351 + **.CGCGTC.** 1141 (100.00)   
351 + **.CGCGGC.** 1185 (94.52)   
351 + **.CGCGGC.** 1255 (94.52)   
351 + **[CGCTTC]** 1267 (86.32)   
351 - **[CGCGAC]** 1139 (87.27)   
351 - **[GGCGTC]** 1036 (87.08)   
351 - **[CGCCGC]** 976 (85.72)   
351 - **.CGCGGC.** 937 (94.52)   
351 - **[CGCCCC]** 871 (85.80)   
351 - **[CCCGTC]** 833 (87.14)   
351 - **.CGCGCC.** 576 (94.60)   
351 - **.CGCCTC.** 574 (91.20)   
351 - **.CGCGCC.** 301 (94.60)   
351 - **.CGCCTC.** 199 (91.20)   
351 - **[CGCCCC]** 155 (85.80)   
351 - **[CGCCGC]** 55 (85.72)   
351 - **.CGCCTC.** 46 (91.20)   
351 - **.CGCCTC.** 41 (91.20)   
351 - **[CGCGTG]** 8 (86.67)   
352 + **.CGCGCC.** 55 (94.60)   
352 + **[CGCCGC]** 57 (85.72)   
352 + **.CGCCTC.** 60 (91.20)   
352 + **.CGCGCC.** 74 (94.60)   
352 + **[CGGGTC]** 190 (87.94)   
352 + **.CGCGGC.** 198 (94.52)   
352 + **.CGCGCC.** 203 (94.60)   
352 + **[CGCGAC]** 221 (87.27)   
352 + **[CGGGTC]** 249 (87.94)   
352 + **.CGCGCC.** 292 (94.60)   
352 + **.CGCGGC.** 312 (94.52)   
352 + **[CGCCGC]** 337 (85.72)   
352 + **[CGCCCC]** 340 (85.80)   
352 + **[CGCCCC]** 346 (85.80)   
352 + **[CGCCGC]** 352 (85.72)   
352 + **[CGGGTC]** 420 (87.94)   
352 + **.CGCGGC.** 439 (94.52)   
352 + **.CGCGCC.** 547 (94.60)   
352 + **.CGCGGC.** 621 (94.52)   
352 + **[CGCCGC]** 630 (85.72)   
352 + **[CGCCCC]** 633 (85.80)   
352 + **.CGCCTC.** 706 (91.20)   
352 + **.CGCCTC.** 712 (91.20)   
352 + **.CGCGCC.** 718 (94.60)   
352 + **[CGCCGC]** 861 (85.72)   
352 + **[GGCGTC]** 905 (87.08)   
352 + **.CGCGGC.** 942 (94.52)   
352 + **[CGCCGC]** 995 (85.72)   
352 + **[CGCCGC]** 998 (85.72)   
352 + **[CGGGTC]** 1039 (87.94)   
352 + **.CGCCTC.** 1200 (91.20)   
352 + **.CGCGCC.** 1228 (94.60)   
352 + **.CGCGGC.** 1340 (94.52)   
352 + **[GGCGTC]** 1348 (87.08)   
352 + **[GGCGTC]** 1363 (87.08)   
352 - **[CGCCCC]** 1412 (85.80)   
352 - **[CGCCCC]** 1361 (85.80)   
352 - **.CGCGGC.** 1338 (94.52)   
352 - **[CGCTTC]** 1308 (86.32)   
352 - **.CGCGCC.** 1064 (94.60)   
352 - **.CGCCTC.** 1062 (91.20)   
352 - **.CGCCTC.** 903 (91.20)   
352 - **[CGCCGC]** 857 (85.72)   
352 - **[CGCCGC]** 850 (85.72)   
352 - **.CGCGGC.** 353 (94.52)   
352 - **[CGCCGC]** 313 (85.72)   
352 - **.CGCGGC.** 310 (94.52)   
352 - **.CGCGCC.** 290 (94.60)   
352 - **.CGCCTC.** 288 (91.20)   
352 - **.CGCGTC.** 282 (100.00)   
352 - **.CGCGCC.** 201 (94.60)   
352 - **[CGCCGC]** 199 (85.72)   
352 - **[CGCCCC]** 107 (85.80)   
352 - **.CGCGGC.** 72 (94.52)   
352 - **[CGCGAC]** 53 (87.27)   
353 + **.CGCGGC.** 167 (94.52)   
353 + **.CGCCTC.** 312 (91.20)   
353 + **.CGCGCC.** 663 (94.60)   
353 + **[CGCCGC]** 690 (85.72)   
353 + **[CGCCGC]** 733 (85.72)   
353 + **[CGCCGC]** 736 (85.72)   
353 + **[CGCCGC]** 739 (85.72)   
353 + **[CGCCGC]** 742 (85.72)   
353 + **[CGCCGC]** 745 (85.72)   
353 + **[CGCGTG]** 836 (86.67)   
353 + **[CGCCCC]** 858 (85.80)   
353 + **[CGCCCC]** 898 (85.80)   
353 + **.CGCCTC.** 1123 (91.20)   
353 + **[CGCCGC]** 1178 (85.72)   
353 + **[CGCGAC]** 1181 (87.27)   
353 + **.CGCGTC.** 1278 (100.00)   
353 + **[CGCCCC]** 1287 (85.80)   
353 + **.CGCGCC.** 1344 (94.60)   
353 + **.CGCGGC.** 1420 (94.52)   
353 + **.CGCGGC.** 1438 (94.52)   
353 - **.CGCCTC.** 1452 (91.20)   
353 - **[CGCCGC]** 1439 (85.72)   
353 - **[CGCCGC]** 1421 (85.72)   
353 - **.CGCCTC.** 1408 (91.20)   
353 - **.CGCGCC.** 1401 (94.60)   
353 - **.CGCCTC.** 1399 (91.20)   
353 - **.CGCGCC.** 1382 (94.60)   
353 - **[CGCCCC]** 1380 (85.80)   
353 - **.CGCGCC.** 1340 (94.60)   
353 - **[CGCCCC]** 1338 (85.80)   
353 - **.CGCGGC.** 1179 (94.52)   
353 - **[CGGGTC]** 1094 (87.94)   
353 - **[CGCGAC]** 966 (87.27)   
353 - **.CGCGGC.** 881 (94.52)   
353 - **[CGCGAC]** 874 (87.27)   
353 - **[CGCTTC]** 804 (86.32)   
353 - **[CGCCGC]** 796 (85.72)   
353 - **.CGCGTC.** 708 (100.00)   
353 - **.CGCGTC.** 549 (100.00)   
353 - **.CGCGTC.** 539 (100.00)   
353 - **[CGCGTG]** 304 (86.67)   
353 - **.CGCCTC.** 297 (91.20)   
353 - **[CGCCCC]** 289 (85.80)   
353 - **.CGCGTC.** 187 (100.00)   
353 - **[CGCGTG]** 173 (86.67)   
353 - **.CGCGCC.** 165 (94.60)   
354 + **.CGCCTC.** 1371 (91.20)   
354 - **.CGCCTC.** 1386 (91.20)   
354 - **[GGCGTC]** 1369 (87.08)   
355 + **[CGGGTC]** 211 (87.94)   
355 + **.CGCCTC.** 228 (91.20)   
355 - **[CCCGTC]** 331 (87.14)   
356 + **[GGCGTC]** 59 (87.08)   
356 + **[CGCCGC]** 90 (85.72)   
356 + **.CGCGCC.** 93 (94.60)   
356 + **[CGCCCC]** 95 (85.80)   
356 + **.CGCCTC.** 219 (91.20)   
356 + **.CGCGCC.** 310 (94.60)   
356 + **[CGCCCC]** 434 (85.80)   
356 + **.CGCGGC.** 465 (94.52)   
356 + **[CGCCCC]** 474 (85.80)   
356 + **[CCCGTC]** 520 (87.14)   
356 + **[CGCCCC]** 542 (85.80)   
356 + **[CGCCCC]** 547 (85.80)   
356 + **[CGCCCC]** 570 (85.80)   
356 + **[CGCCCC]** 606 (85.80)   
356 + **.CGCGCC.** 611 (94.60)   
356 + **[CGCGAC]** 772 (87.27)   
356 + **[CGCCGC]** 783 (85.72)   
356 + **[CGCCCC]** 882 (85.80)   
356 + **.CGCGGC.** 999 (94.52)   
356 + **.CGCGTC.** 1173 (100.00)   
356 - **[CGCCCC]** 1495 (85.80)   
356 - **[CGCCCC]** 1237 (85.80)   
356 - **[CGCCCC]** 1232 (85.80)   
356 - **[CGCCCC]** 1146 (85.80)   
356 - **.CGCGCC.** 997 (94.60)   
356 - **[CGCCCC]** 995 (85.80)   
356 - **[CGCCCC]** 990 (85.80)   
356 - **[CGCCCC]** 977 (85.80)   
356 - **[CGCGTG]** 863 (86.67)   
356 - **.CGCGGC.** 784 (94.52)   
356 - **[CGGGTC]** 602 (87.94)   
356 - **[CGCCCC]** 496 (85.80)   
356 - **[CGCCCC]** 259 (85.80)   
356 - **[CGCCCC]** 231 (85.80)   
356 - **[CGCCGC]** 176 (85.72)   
356 - **[CGCCGC]** 173 (85.72)   
356 - **[CGCCGC]** 170 (85.72)   
356 - **.CGCGGC.** 91 (94.52)   
357 + **[CGGGTC]** 300 (87.94)   
357 + **[CGGGTC]** 392 (87.94)   
357 + **[CGCGTG]** 529 (86.67)   
357 + **.CGCGTC.** 544 (100.00)   
357 + **[CGCGTG]** 813 (86.67)   
357 + **.CGCGCC.** 984 (94.60)   
357 + **[CGCCGC]** 990 (85.72)   
357 + **.CGCGCC.** 993 (94.60)   
357 + **[CGCCGC]** 995 (85.72)   
357 + **[CGCGTG]** 1027 (86.67)   
357 + **[CGGGTC]** 1041 (87.94)   
357 + **[CGCCCC]** 1066 (85.80)   
357 + **[CGCGTG]** 1112 (86.67)   
357 + **[CGCCGC]** 1506 (85.72)   
357 - **[CGCCCC]** 1427 (85.80)   
357 - **[CGCCCC]** 1366 (85.80)   
357 - **[CGCCCC]** 1305 (85.80)   
357 - **[CGCCCC]** 1243 (85.80)   
357 - **.CGCGCC.** 1161 (94.60)   
357 - **[CGCCCC]** 1159 (85.80)   
357 - **.CGCGGC.** 1110 (94.52)   
357 - **.CGCGGC.** 991 (94.52)   
357 - **.CGCGGC.** 980 (94.52)   
357 - **[CGGGTC]** 962 (87.94)   
357 - **.CGCGTC.** 944 (100.00)   
357 - **[CGCGTG]** 921 (86.67)   
357 - **.CGCGGC.** 811 (94.52)   
357 - **[CGCCCC]** 775 (85.80)   
357 - **[CGCCCC]** 758 (85.80)   
357 - **[GGCGTC]** 733 (87.08)   
357 - **[CGGGTC]** 540 (87.94)   
357 - **.CGCGCC.** 527 (94.60)   
357 - **[CGCCCC]** 433 (85.80)   
357 - **[CGCCGC]** 419 (85.72)   
358 + **.CGCGGC.** 70 (94.52)   
358 + **[CGGGTC]** 409 (87.94)   
358 + **[CGCCCC]** 542 (85.80)   
358 + **.CGCGCC.** 671 (94.60)   
358 + **.CGCCTC.** 673 (91.20)   
358 + **.CGCGGC.** 774 (94.52)   
358 + **[CGCGTG]** 838 (86.67)   
358 + **[CGCGTG]** 851 (86.67)   
358 + **.CGCGGC.** 978 (94.52)   
358 + **[CGCTTC]** 1088 (86.32)   
358 + **[CCCGTC]** 1248 (87.14)   
358 + **[CGCCCC]** 1319 (85.80)   
358 - **[CGCCGC]** 1054 (85.72)   
358 - **.CGCCTC.** 1041 (91.20)   
358 - **[CGCCCC]** 984 (85.80)   
358 - **[CGCCGC]** 979 (85.72)   
358 - **[CGCCCC]** 965 (85.80)   
358 - **[CCCGTC]** 912 (87.14)   
358 - **.CGCGCC.** 880 (94.60)   
358 - **[CCCGTC]** 876 (87.14)   
358 - **.CGCCTC.** 825 (91.20)   
358 - **.CGCGGC.** 669 (94.52)   
358 - **[CGCCCC]** 337 (85.80)   
358 - **.CGCCTC.** 214 (91.20)   
359 + **[CCCGTC]** 56 (87.14)   
359 + **[CGCTTC]** 378 (86.32)   
359 + **[CGCTTC]** 496 (86.32)   
359 + **[CGCCCC]** 871 (85.80)   
359 + **[CGCCGC]** 976 (85.72)   
359 + **[CGCCGC]** 984 (85.72)   
359 + **[CGCCGC]** 987 (85.72)   
359 + **[CGCCGC]** 1025 (85.72)   
359 + **[CGCCGC]** 1028 (85.72)   
359 + **[CGCCGC]** 1031 (85.72)   
359 + **[CGCCGC]** 1055 (85.72)   
359 + **[CGCCGC]** 1058 (85.72)   
359 + **[CGCCGC]** 1061 (85.72)   
359 + **.CGCGCC.** 1096 (94.60)   
359 + **[CGCCCC]** 1104 (85.80)   
359 + **.CGCGCC.** 1114 (94.60)   
359 + **[CGCCCC]** 1116 (85.80)   
359 + **[CGCCGC]** 1121 (85.72)   
359 + **[CGCCGC]** 1124 (85.72)   
359 + **[CGCCGC]** 1127 (85.72)   
359 + **[CGCCCC]** 1167 (85.80)   
359 + **[CGCCCC]** 1173 (85.80)   
359 + **.CGCGCC.** 1206 (94.60)   
359 + **[CGCCCC]** 1208 (85.80)   
359 + **.CGCGGC.** 1234 (94.52)   
359 + **.CGCGCC.** 1247 (94.60)   
359 + **.CGCGGC.** 1255 (94.52)   
359 + **[CGCGTG]** 1281 (86.67)   
359 + **[CGCGTG]** 1287 (86.67)   
359 + **.CGCGCC.** 1307 (94.60)   
359 + **[CGCCGC]** 1315 (85.72)   
359 - **.CGCGCC.** 1440 (94.60)   
359 - **.CGCCTC.** 1413 (91.20)   
359 - **[CGCCCC]** 1361 (85.80)   
359 - **.CGCGCC.** 1346 (94.60)   
359 - **[CGCCGC]** 1256 (85.72)   
359 - **.CGCGCC.** 1237 (94.60)   
359 - **[CGCCGC]** 1235 (85.72)   
359 - **.CGCGGC.** 1232 (94.52)   
359 - **[CGGGTC]** 1087 (87.94)   
359 - **[GGCGTC]** 797 (87.08)   
359 - **.CGCGCC.** 775 (94.60)   
359 - **[CGCCCC]** 711 (85.80)   
359 - **[CGCTTC]** 632 (86.32)   
359 - **[CCCGTC]** 574 (87.14)   
359 - **.CGCCTC.** 560 (91.20)   
359 - **[GGCGTC]** 355 (87.08)   
359 - **.CGCCTC.** 292 (91.20)   
359 - **.CGCGCC.** 184 (94.60)   
360 + **[CGCCCC]** 176 (85.80)   
360 + **[CGCTTC]** 252 (86.32)   
360 + **.CGCCTC.** 469 (91.20)   
360 + **[CCCGTC]** 545 (87.14)   
360 + **[CGCCCC]** 626 (85.80)   
360 + **[CGCCCC]** 641 (85.80)   
360 + **[CGCCGC]** 651 (85.72)   
360 + **[CGCCCC]** 666 (85.80)   
360 + **.CGCGCC.** 737 (94.60)   
360 + **[CGCCGC]** 763 (85.72)   
360 + **[CGCCGC]** 770 (85.72)   
360 + **.CGCGCC.** 781 (94.60)   
360 + **.CGCCTC.** 783 (91.20)   
360 + **.CGCGGC.** 794 (94.52)   
360 + **[CGCTTC]** 800 (86.32)   
360 + **.CGCGGC.** 973 (94.52)   
360 + **[CGCCGC]** 1065 (85.72)   
360 + **.CGCGCC.** 1068 (94.60)   
360 + **[CGCCGC]** 1070 (85.72)   
360 + **.CGCGGC.** 1127 (94.52)   
360 + **.CGCGCC.** 1245 (94.60)   
360 + **[CGCCGC]** 1247 (85.72)   
360 - **[CGCCCC]** 1283 (85.80)   
360 - **.CGCGCC.** 1243 (94.60)   
360 - **.CGCGCC.** 1185 (94.60)   
360 - **[CGCCGC]** 1128 (85.72)   
360 - **.CGCGGC.** 1125 (94.52)   
360 - **[CGGGTC]** 1112 (87.94)   
360 - **.CGCGGC.** 1071 (94.52)   
360 - **.CGCGGC.** 1066 (94.52)   
360 - **[CGCCCC]** 1061 (85.80)   
360 - **[CGCCGC]** 974 (85.72)   
360 - **.CGCGCC.** 944 (94.60)   
360 - **.CGCGCC.** 877 (94.60)   
360 - **[GGCGTC]** 867 (87.08)   
360 - **[CGGGTC]** 846 (87.94)   
360 - **[CGCCGC]** 722 (85.72)   
360 - **.CGCGGC.** 652 (94.52)   
360 - **[GGCGTC]** 649 (87.08)   
360 - **.CGCGGC.** 419 (94.52)   
360 - **[CGCCCC]** 338 (85.80)   
360 - **[CGCCCC]** 260 (85.80)   
360 - **[CGCCCC]** 248 (85.80)   
360 - **[CGCTTC]** 209 (86.32)   
360 - **[CGCCCC]** 126 (85.80)   
361 + **.CGCCTC.** 681 (91.20)   
361 + **.CGCCTC.** 722 (91.20)   
361 + **.CGCCTC.** 828 (91.20)   
361 + **[CGCCGC]** 1047 (85.72)   
361 + **.CGCGGC.** 1418 (94.52)   
361 - **.CGCCTC.** 1463 (91.20)   
361 - **.CGCGGC.** 1416 (94.52)   
361 - **[CGGGTC]** 1125 (87.94)   
361 - **[CCCGTC]** 1095 (87.14)   
361 - **[CCCGTC]** 848 (87.14)   
361 - **.CGCCTC.** 608 (91.20)   
361 - **.CGCGGC.** 470 (94.52)   
362 + **[CGCCCC]** 13 (85.80)   
362 + **.CGCGGC.** 118 (94.52)   
362 + **.CGCGGC.** 283 (94.52)   
362 + **.CGCGGC.** 470 (94.52)   
362 + **.CGCCTC.** 602 (91.20)   
362 + **.CGCGTC.** 624 (100.00)   
362 + **[CGCCGC]** 735 (85.72)   
362 + **.CGCGTC.** 738 (100.00)   
362 + **[CGCGTG]** 876 (86.67)   
362 + **.CGCGGC.** 911 (94.52)   
362 + **[CGCCGC]** 992 (85.72)   
362 + **[CGCCGC]** 995 (85.72)   
362 + **[CGCCCC]** 998 (85.80)   
362 + **[CGCCGC]** 1003 (85.72)   
362 + **.CGCGCC.** 1358 (94.60)   
362 - **[CGCTTC]** 1472 (86.32)   
362 - **[CGCTTC]** 1446 (86.32)   
362 - **[GGCGTC]** 1263 (87.08)   
362 - **[CGCCCC]** 1201 (85.80)   
362 - **[CGCTTC]** 1154 (86.32)   
362 - **.CGCCTC.** 1146 (91.20)   
362 - **[CGCGAC]** 1141 (87.27)   
362 - **[CGCCCC]** 988 (85.80)   
362 - **[CGCCGC]** 935 (85.72)   
362 - **[CGCCCC]** 932 (85.80)   
362 - **[CGCCGC]** 912 (85.72)   
362 - **.CGCGCC.** 909 (94.60)   
362 - **[CGCCCC]** 907 (85.80)   
362 - **[GGCGTC]** 794 (87.08)   
362 - **.CGCGGC.** 736 (94.52)   
362 - **[CGCCCC]** 690 (85.80)   
362 - **[CGGGTC]** 538 (87.94)   
362 - **.CGCGTC.** 525 (100.00)   
362 - **.CGCGCC.** 237 (94.60)   
362 - **.CGCCTC.** 128 (91.20)   
362 - **[CGCCCC]** 92 (85.80)   
363 + **[CGGGTC]** 370 (87.94)   
363 + **[CGCCCC]** 625 (85.80)   
363 + **[CGCCCC]** 630 (85.80)   
363 + **.CGCGGC.** 694 (94.52)   
363 + **[CGCCCC]** 737 (85.80)   
363 + **.CGCGGC.** 799 (94.52)   
363 + **.CGCGGC.** 811 (94.52)   
363 + **[CCCGTC]** 1078 (87.14)   
363 + **.CGCGGC.** 1083 (94.52)   
363 + **.CGCGCC.** 1091 (94.60)   
363 + **[CGCCCC]** 1093 (85.80)   
363 + **[CGCTTC]** 1118 (86.32)   
363 + **.CGCGGC.** 1188 (94.52)   
363 + **[CGCCCC]** 1202 (85.80)   
363 + **[CGCCGC]** 1234 (85.72)   
363 - **.CGCGCC.** 1112 (94.60)   
363 - **.CGCGGC.** 1087 (94.52)   
363 - **[CGCGAC]** 1081 (87.27)   
363 - **[CGGGTC]** 1076 (87.94)   
363 - **[CGCCGC]** 812 (85.72)   
363 - **[GGCGTC]** 763 (87.08)   
363 - **.CGCGGC.** 692 (94.52)   
363 - **[CGCCCC]** 285 (85.80)   
363 - **[CGCTTC]** 246 (86.32)   
363 - **.CGCCTC.** 199 (91.20)   
363 - **[CGCCGC]** 171 (85.72)   
364 + **[CGCCCC]** 749 (85.80)   
364 + **.CGCGGC.** 950 (94.52)   
364 + **[CGGGTC]** 1058 (87.94)   
364 + **[CGCCGC]** 1088 (85.72)   
364 + **.CGCGGC.** 1179 (94.52)   
364 + **.CGCGGC.** 1257 (94.52)   
364 + **[CGCCCC]** 1366 (85.80)   
364 + **.CGCGCC.** 1391 (94.60)   
364 - **[CGCCGC]** 1258 (85.72)   
364 - **.CGCGTC.** 1255 (100.00)   
364 - **.CGCGCC.** 1177 (94.60)   
364 - **[CGCCCC]** 1175 (85.80)   
364 - **[GGCGTC]** 1086 (87.08)   
364 - **[CGGGTC]** 863 (87.94)   
364 - **.CGCGGC.** 821 (94.52)   
364 - **[GGCGTC]** 747 (87.08)   
364 - **.CGCGTC.** 297 (100.00)   
365 + **[CGGGTC]** 587 (87.94)   
365 + **[CGCCCC]** 1382 (85.80)   
365 - **[CGCCCC]** 1396 (85.80)   
365 - **[CGCCCC]** 1339 (85.80)   
365 - **[CGCTTC]** 1286 (86.32)   
366 + **.CGCGCC.** 131 (94.60)   
366 + **[CGCCCC]** 133 (85.80)   
366 + **[CGCCCC]** 155 (85.80)   
366 + **[CGGGTC]** 211 (87.94)   
366 + **[CGCTTC]** 270 (86.32)   
366 + **[CGCCCC]** 392 (85.80)   
366 + **.CGCGGC.** 410 (94.52)   
366 + **.CGCGGC.** 445 (94.52)   
366 + **[GGCGTC]** 448 (87.08)   
366 + **.CGCGCC.** 454 (94.60)   
366 + **[CGCCCC]** 499 (85.80)   
366 + **[CGCCCC]** 505 (85.80)   
366 + **.CGCGTC.** 521 (100.00)   
366 + **[GGCGTC]** 546 (87.08)   
366 + **[CGCCGC]** 568 (85.72)   
366 + **[CGCCCC]** 622 (85.80)   
366 + **.CGCGCC.** 658 (94.60)   
366 + **.CGCCTC.** 710 (91.20)   
366 + **.CGCCTC.** 719 (91.20)   
366 + **[CCCGTC]** 782 (87.14)   
366 + **[CGCCCC]** 842 (85.80)   
366 + **.CGCGTC.** 854 (100.00)   
366 + **.CGCGCC.** 907 (94.60)   
366 + **[CGCCGC]** 923 (85.72)   
366 + **.CGCGGC.** 954 (94.52)   
366 + **[GGCGTC]** 957 (87.08)   
366 + **[CCCGTC]** 962 (87.14)   
366 + **[CCCGTC]** 987 (87.14)   
366 + **[CGCCGC]** 1000 (85.72)   
366 + **[CGGGTC]** 1007 (87.94)   
366 + **[CGGGTC]** 1112 (87.94)   
366 + **[CGCGAC]** 1125 (87.27)   
366 + **[CGCCGC]** 1252 (85.72)   
366 + **[CCCGTC]** 1301 (87.14)   
366 + **.CGCGGC.** 1393 (94.52)   
366 - **[CGCCGC]** 1437 (85.72)   
366 - **.CGCGTC.** 1430 (100.00)   
366 - **.CGCGGC.** 1412 (94.52)   
366 - **[CGCCCC]** 1406 (85.80)   
366 - **[CGCCGC]** 1397 (85.72)   
366 - **[CGCCGC]** 1394 (85.72)   
366 - **.CGCGCC.** 1391 (94.60)   
366 - **.CGCCTC.** 1389 (91.20)   
366 - **[CGCCGC]** 1364 (85.72)   
366 - **.CGCGGC.** 1253 (94.52)   
366 - **[CGCCGC]** 1241 (85.72)   
366 - **[CGCCCC]** 1194 (85.80)   
366 - **[CGCCGC]** 1172 (85.72)   
366 - **[CGCCCC]** 1169 (85.80)   
366 - **.CGCGGC.** 1123 (94.52)   
366 - **[GGCGTC]** 998 (87.08)   
366 - **[CGCGAC]** 974 (87.27)   
366 - **[CGCCGC]** 955 (85.72)   
366 - **[CGCTTC]** 756 (86.32)   
366 - **[CCCGTC]** 695 (87.14)   
366 - **.CGCGCC.** 656 (94.60)   
366 - **[CCCGTC]** 607 (87.14)   
366 - **.CGCGGC.** 534 (94.52)   
366 - **.CGCCTC.** 473 (91.20)   
366 - **[CGCCGC]** 446 (85.72)   
366 - **[CGCCGC]** 414 (85.72)   
366 - **[CGCCGC]** 411 (85.72)   
366 - **.CGCGGC.** 346 (94.52)   
366 - **.CGCGCC.** 266 (94.60)   
366 - **[CGCCCC]** 223 (85.80)   
366 - **[CGCCCC]** 217 (85.80)   
366 - **.CGCGGC.** 182 (94.52)   
366 - **.CGCCTC.** 68 (91.20)   
366 - **[CGCCCC]** 50 (85.80)   
367 + **.CGCGCC.** 9 (94.60)   
367 + **[CGCCCC]** 18 (85.80)   
367 + **[CGCCGC]** 223 (85.72)   
367 + **[CGCCGC]** 231 (85.72)   
367 + **.CGCCTC.** 384 (91.20)   
367 + **[CGCCCC]** 397 (85.80)   
367 + **.CGCGCC.** 413 (94.60)   
367 + **[CGCCCC]** 415 (85.80)   
367 + **[CGCCCC]** 804 (85.80)   
367 + **[CGCCCC]** 882 (85.80)   
367 + **[CGCCGC]** 894 (85.72)   
367 + **[CGCCGC]** 897 (85.72)   
367 + **[CGCCGC]** 900 (85.72)   
367 + **[CGCCGC]** 903 (85.72)   
367 + **[CGCCGC]** 906 (85.72)   
367 + **[CGCCGC]** 909 (85.72)   
367 + **[CGCCGC]** 912 (85.72)   
367 + **[CGCCGC]** 915 (85.72)   
367 + **[CGCCCC]** 1020 (85.80)   
367 + **.CGCGCC.** 1034 (94.60)   
367 + **.CGCGCC.** 1047 (94.60)   
367 + **[CGCCGC]** 1146 (85.72)   
367 + **[CGCCGC]** 1149 (85.72)   
367 + **[CGCCCC]** 1152 (85.80)   
367 + **[CGCCGC]** 1189 (85.72)   
367 + **.CGCGGC.** 1192 (94.52)   
367 - **[CGCCCC]** 1327 (85.80)   
367 - **[CGCCGC]** 1196 (85.72)   
367 - **[CGCCGC]** 1193 (85.72)   
367 - **.CGCGGC.** 1190 (94.52)   
367 - **.CGCGGC.** 1090 (94.52)   
367 - **.CGCGGC.** 1063 (94.52)   
367 - **.CGCCTC.** 749 (91.20)   
367 - **.CGCGGC.** 571 (94.52)   
367 - **.CGCGGC.** 555 (94.52)   
367 - **[CGCCCC]** 488 (85.80)   
367 - **.CGCGCC.** 337 (94.60)   
367 - **[CGCCGC]** 312 (85.72)   
367 - **[CGCCCC]** 263 (85.80)   
367 - **.CGCCTC.** 257 (91.20)   
367 - **[GGCGTC]** 221 (87.08)   
367 - **.CGCGGC.** 128 (94.52)   
  

**Frequency Matrix**

| All Occs Best Occs|  |  |  |  |  |  |  |  |  |  |  |  |  |  |  |  |  |  |  |  |  |  |  |  |  |  |  |  |  |  |  |  |  |  |  |  |  |  |  |  |  |  |  |  |  |  |  |  |  |  |  |  |  |  |  |  |  |  |  |  |  |  |  |  |  |  |  |  |  |  |  |  |  |  |  |  |  |  |  |  |  |  |  |  | | --- | --- | --- | --- | --- | --- | --- | --- | --- | --- | --- | --- | --- | --- | --- | --- | --- | --- | --- | --- | --- | --- | --- | --- | --- | --- | --- | --- | --- | --- | --- | --- | --- | --- | --- | --- | --- | --- | --- | --- | --- | --- | --- | --- | --- | --- | --- | --- | --- | --- | --- | --- | --- | --- | --- | --- | --- | --- | --- | --- | --- | --- | --- | --- | --- | --- | --- | --- | --- | --- | --- | --- | --- | --- | --- | --- | --- | --- | --- | --- | --- | --- | --- | --- | | **A C G T A C G T | | | | | | | | | | | |**| **1** 167 6068 343 156 | 0 3572 0 0|  |  |  |  |  |  |  |  |  |  |  |  |  |  |  |  |  |  |  |  |  |  |  |  |  |  |  |  |  |  |  |  |  |  |  |  |  |  |  |  |  |  |  |  |  |  |  |  |  |  |  |  |  |  |  |  |  |  |  |  | | --- | --- | --- | --- | --- | --- | --- | --- | --- | --- | --- | --- | --- | --- | --- | --- | --- | --- | --- | --- | --- | --- | --- | --- | --- | --- | --- | --- | --- | --- | --- | --- | --- | --- | --- | --- | --- | --- | --- | --- | --- | --- | --- | --- | --- | --- | --- | --- | --- | --- | --- | --- | --- | --- | --- | --- | --- | --- | --- | --- | | **2** 141 351 6122 120 | 0 0 3572 0|  |  |  |  |  |  |  |  |  |  |  |  |  |  |  |  |  |  |  |  |  |  |  |  |  |  |  |  |  |  |  |  |  |  |  |  |  |  |  |  |  |  |  |  |  |  |  |  | | --- | --- | --- | --- | --- | --- | --- | --- | --- | --- | --- | --- | --- | --- | --- | --- | --- | --- | --- | --- | --- | --- | --- | --- | --- | --- | --- | --- | --- | --- | --- | --- | --- | --- | --- | --- | --- | --- | --- | --- | --- | --- | --- | --- | --- | --- | --- | --- | | **3** 182 5980 409 163 | 0 3572 0 0|  |  |  |  |  |  |  |  |  |  |  |  |  |  |  |  |  |  |  |  |  |  |  |  |  |  |  |  |  |  |  |  |  |  |  |  | | --- | --- | --- | --- | --- | --- | --- | --- | --- | --- | --- | --- | --- | --- | --- | --- | --- | --- | --- | --- | --- | --- | --- | --- | --- | --- | --- | --- | --- | --- | --- | --- | --- | --- | --- | --- | | **4** 108 787 5573 266 | 0 787 2785 0|  |  |  |  |  |  |  |  |  |  |  |  |  |  |  |  |  |  |  |  |  |  |  |  | | --- | --- | --- | --- | --- | --- | --- | --- | --- | --- | --- | --- | --- | --- | --- | --- | --- | --- | --- | --- | --- | --- | --- | --- | | **5** 240 1224 1203 4067 | 0 1224 1203 1145|  |  |  |  |  |  |  |  |  |  |  |  | | --- | --- | --- | --- | --- | --- | --- | --- | --- | --- | --- | --- | | **6** 63 6218 321 132 | 0 3572 0 0 | | | | | | | | | | | | | | | | | | | | | | | | | | | | | | | | | | | | | | | | | | | | | | | | | | | | | | | | | | | | | | | | | | | | | | | | | | | | | | | | | | | | |

  
  

---

  
  
  
 **CGACGCGT**   
**ACGCGTCG**  
  
6 redundant motifs found:  
CGACGCGTCC - CGCGTCGT - CGACGCGC - CGCGTCGA - CGACGC - CGCGTC -   
  
Best occurrences (match percentage):   
Seq St oligo pos match
  
 1 + **[CGGGGCGT]** 868 (88.38)   
1 + **[CCGCGCGT]** 883 (86.82)   
1 + **[CGCCGCCT]** 917 (89.62)   
1 + **[CGCCGCCC]** 1002 (85.13)   
1 + **[CGAGGCGC]** 1063 (87.66)   
1 + **.CGCCGCGC.** 1068 (92.48)   
1 + **[CGCCGCCC]** 1077 (85.13)   
1 + **[CGCCGCCG]** 1204 (85.71)   
1 - **[CGGCGCCT]** 1065 (88.87)   
1 - **.CGGCGCGC.** 999 (91.73)   
2 + **[CGCCGCCT]** 308 (89.62)   
2 + **[AGCCGCGT]** 446 (86.16)   
2 + **[CGACGCCC]** 507 (88.15)   
2 + **[CCCCGCGT]** 512 (87.58)   
2 + **.CGGCGCGC.** 702 (91.73)   
2 + **[CGCCCCGT]** 903 (87.14)   
2 + **[CGGCGCGA]** 998 (87.47)   
2 + **[CGGCGCCT]** 1154 (88.87)   
2 + **[CGCCGCCC]** 1268 (85.13)   
2 + **[CGGCGCGA]** 1449 (87.47)   
2 - **.CGCCGCGC.** 1446 (92.48)   
2 - **[CGCCGGGT]** 1197 (85.88)   
2 - **[CGCCGCGA]** 995 (88.22)   
2 - **[GGCCGCGG]** 976 (85.06)   
2 - **[GGACGCGG]** 514 (88.08)   
2 - **[CGGGGCGT]** 509 (88.38)   
2 - **[AGGCGCGT]** 456 (85.41)   
2 - **[AGACGCGG]** 448 (85.28)   
3 + **[CGCCGCCT]** 163 (89.62)   
3 + **[AGCCGCGT]** 788 (86.16)   
3 + **[CGCGGCGG]** 1223 (85.22)   
3 + **.CGGCGCGC.** 1248 (91.73)   
3 + **[CGCCGCCC]** 1401 (85.13)   
3 + **[CGCCGCCG]** 1409 (85.71)   
3 + **[CGCCGCCG]** 1412 (85.71)   
3 + **.CGCCGCGC.** 1415 (92.48)   
3 - **[CGCGGCGG]** 1414 (85.22)   
3 - **[CGCCGCCG]** 1225 (85.71)   
3 - **.CGCCGCGG.** 1222 (93.07)   
4 + **[CGCCGCCG]** 695 (85.71)   
4 + **[CGCCGCGA]** 698 (88.22)   
4 + **[CGACCCGG]** 703 (86.25)   
4 + **[CGTCGCGG]** 739 (86.02)   
4 + **[CGCGGCGG]** 822 (85.22)   
4 + **[CGGCGCGA]** 827 (87.47)   
4 + **[CGACCCGG]** 852 (86.25)   
4 + **[GGGCGCGT]** 915 (88.21)   
4 + **.CGCCGCGC.** 1205 (92.48)   
4 - **[CGCGGCGG]** 1204 (85.22)   
4 - **[CGCCGCCT]** 1139 (89.62)   
4 - **[CGCCGCCT]** 903 (89.62)   
4 - **[CGCCGCCG]** 824 (85.71)   
4 - **.CGCCGCGG.** 821 (93.07)   
4 - **[CGCCGCGA]** 801 (88.22)   
4 - **[CGGCGCCT]** 733 (88.87)   
4 - **[CGCGGCGG]** 697 (85.22)   
4 - **[CGACCCGC]** 686 (85.67)   
4 - **[CGCCGCCT]** 669 (89.62)   
5 + **[CGCCGCCC]** 1160 (85.13)   
5 + **[CGCCGCCT]** 1310 (89.62)   
5 + **.CGACGCGG.** 1437 (96.09)   
5 + **[CGAGGCGG]** 1446 (88.25)   
5 + **[CGCCGCCG]** 1482 (85.71)   
5 + **[CGCCGCCC]** 1485 (85.13)   
5 - **[GGCCGCGT]** 1439 (88.97)   
5 - **[CGAGGCGC]** 1347 (87.66)   
6 + **[CGGGGCGT]** 957 (88.38)   
6 - **.CGCCGCGG.** 1439 (93.07)   
8 + **[CCGCGCGT]** 369 (86.82)   
8 + **.CGGCGCGG.** 406 (92.31)   
8 + **.CGCCGCGT.** 451 (96.98)   
8 + **[CGCCGCCC]** 813 (85.13)   
8 + **[CCCCGCGT]** 1143 (87.58)   
8 + **[CGACGCCG]** 1151 (88.74)   
8 + **[CGCCGCCC]** 1154 (85.13)   
8 + **[CGCCGCCT]** 1234 (89.62)   
8 - **.CGACGCGG.** 1145 (96.09)   
8 - **[CGCCCCGT]** 586 (87.14)   
8 - **[CCACGCGG]** 453 (86.69)   
8 - **[GGACGCGC]** 371 (87.50)   
8 - **[CGCCGCCT]** 1 (89.62)   
9 - **[CGTCGCGC]** 1267 (85.44)   
10 + **[GGACGCGG]** 827 (88.08)   
10 + **[GGCCGCGG]** 1021 (85.06)   
10 + **[CGCGGCGG]** 1024 (85.22)   
10 + **[CGCGGCGG]** 1096 (85.22)   
10 + **[GGCCGCGG]** 1118 (85.06)   
10 + **.CGAGGCGT.** 1185 (92.16)   
10 - **[GGCCGCGT]** 1330 (88.97)   
10 - **[GGCCGCGG]** 1120 (85.06)   
10 - **.CGCCGCGC.** 1095 (92.48)   
10 - **.CGCCGCGG.** 1023 (93.07)   
10 - **[CCCCGCGT]** 829 (87.58)   
10 - **[CGCCGCCG]** 690 (85.71)   
10 - **[CGCCGCCC]** 687 (85.13)   
10 - **[CGGCGCCT]** 591 (88.87)   
11 + **[CGGGGCGT]** 93 (88.38)   
11 + **[AGGCGCGT]** 249 (85.41)   
11 + **[GGCCGCGT]** 496 (88.97)   
11 + **[CGTCGCGG]** 528 (86.02)   
11 + **.CGCCGCGG.** 608 (93.07)   
11 + **[GGACGCGC]** 697 (87.50)   
11 + **[GGCCGCGG]** 712 (85.06)   
11 + **[GGACGCGC]** 746 (87.50)   
11 + **[CGGCGCCT]** 771 (88.87)   
11 + **[CGCCGCCT]** 974 (89.62)   
11 + **.CGCCGCGC.** 993 (92.48)   
11 + **.CGCCGCGC.** 1026 (92.48)   
11 + **[CGCCGCCG]** 1037 (85.71)   
11 + **[CGCCGCCG]** 1040 (85.71)   
11 + **[CGCGGCGG]** 1116 (85.22)   
11 + **[CGCCGCCG]** 1151 (85.71)   
11 + **.CGCCGCGC.** 1154 (92.48)   
11 + **[AGGCGCGT]** 1378 (85.41)   
11 - **[GGACGCGC]** 1380 (87.50)   
11 - **[CGCGGCGG]** 1153 (85.22)   
11 - **.CGCCGCGC.** 1115 (92.48)   
11 - **[CGCGGCGG]** 1025 (85.22)   
11 - **[CGCGGCGG]** 992 (85.22)   
11 - **.CGCCGCGG.** 768 (93.07)   
11 - **[GGCCGCGG]** 714 (85.06)   
11 - **[GGGCGCGT]** 699 (88.21)   
11 - **[GGCCGCGG]** 610 (85.06)   
11 - **.CGGCGCGG.** 605 (92.31)   
11 - **[GGCCGCGT]** 559 (88.97)   
12 + **[CCACGCGC]** 204 (86.11)   
12 + **.CGGCGCGC.** 540 (91.73)   
12 + **[CGCCGCCT]** 698 (89.62)   
12 + **[GGCCGCGT]** 739 (88.97)   
12 + **[CGCCGCCC]** 776 (85.13)   
12 + **[CCGCGCGT]** 838 (86.82)   
12 + **[GGACGCGC]** 1115 (87.50)   
12 + **.CGCCGCGC.** 1177 (92.48)   
12 + **[CGCCGCGA]** 1354 (88.22)   
12 - **.CGGCGCGG.** 1252 (92.31)   
12 - **[CGCCGCCC]** 1243 (85.13)   
12 - **[CGAGGCGG]** 1147 (88.25)   
12 - **[CCGCGCGT]** 1117 (86.82)   
12 - **[CGACCCGC]** 1091 (85.67)   
12 - **[GGACGCGC]** 840 (87.50)   
12 - **[CGGCCCGT]** 736 (86.38)   
12 - **[GGCCGCGG]** 710 (85.06)   
12 - **.CGGCGCGG.** 695 (92.31)   
13 + **[CGCCGCCC]** 617 (85.13)   
13 + **[CCACGCGG]** 653 (86.69)   
13 + **[CGCCGCCG]** 717 (85.71)   
13 + **.CGCCGCGC.** 720 (92.48)   
13 + **[CGCCGCCC]** 753 (85.13)   
13 + **[CCCCGCGT]** 763 (87.58)   
13 + **.CGGCGCGC.** 800 (91.73)   
13 + **.CGGCGCGG.** 1061 (92.31)   
13 + **.CGCCGCGC.** 1109 (92.48)   
13 + **[CGCCGCCG]** 1139 (85.71)   
13 + **[CGCCGCCG]** 1142 (85.71)   
13 + **[CGCCGCCG]** 1160 (85.71)   
13 + **[CGCCGCCG]** 1163 (85.71)   
13 + **[CGCCGCCT]** 1166 (89.62)   
13 + **.CGCCGCGG.** 1196 (93.07)   
13 + **[CGTCGCGG]** 1321 (86.02)   
13 + **[CGCGGCGG]** 1352 (85.22)   
13 - **[CGACCCGG]** 1464 (86.25)   
13 - **[CGACCCGG]** 1440 (86.25)   
13 - **[CGCCGCCC]** 1427 (85.13)   
13 - **[CGCCGCCC]** 1418 (85.13)   
13 - **[CGGCCCGT]** 1406 (86.38)   
13 - **.CGCCGCGC.** 1351 (92.48)   
13 - **[CGACGCCG]** 1318 (88.74)   
13 - **[CGCCGCCC]** 1315 (85.13)   
13 - **[CGACCCGG]** 1298 (86.25)   
13 - **.CGGCGCGG.** 1263 (92.31)   
13 - **[CGCGGCGG]** 1195 (85.22)   
13 - **.CGGCGCGC.** 1186 (91.73)   
13 - **[CGCGGCGG]** 1108 (85.22)   
13 - **[CGCCCCGT]** 920 (87.14)   
13 - **[CGAGGCGC]** 867 (87.66)   
13 - **[GGACGCGG]** 765 (88.08)   
13 - **[GGACGCGG]** 741 (88.08)   
13 - **[CGCGGCGG]** 719 (85.22)   
13 - **[GGCCGCGG]** 702 (85.06)   
13 - **[AGCCGCGT]** 655 (86.16)   
13 - **.CGGCGCGG.** 614 (92.31)   
14 + **[AGGCGCGT]** 711 (85.41)   
14 + **[CGCCGGGT]** 730 (85.88)   
14 + **[CGCGGCGG]** 748 (85.22)   
14 + **[CGCGGCGG]** 940 (85.22)   
14 - **[CGAAGCGT]** 1023 (88.01)   
14 - **.CGCCGCGC.** 939 (92.48)   
14 - **[GGGCGCGT]** 869 (88.21)   
14 - **[GGACGCGG]** 792 (88.08)   
14 - **.CGCCGCGC.** 747 (92.48)   
14 - **[CGGCGCCT]** 410 (88.87)   
14 - **.CGCCGCGG.** 336 (93.07)   
14 - **[GGGCGCGT]** 291 (88.21)   
15 + **.CGCCGCGG.** 178 (93.07)   
15 + **[CCACGCGC]** 244 (86.11)   
15 + **[CGACGCCG]** 268 (88.74)   
15 + **[CGCCGCCC]** 271 (85.13)   
15 + **.CGCCGCGC.** 414 (92.48)   
15 + **[GGCCGCGG]** 458 (85.06)   
15 + **[CGAGGCGC]** 494 (87.66)   
15 + **[CGCCGCCT]** 569 (89.62)   
15 + **[CGACGCTT]** 662 (88.71)   
15 + **[CGACGCCC]** 694 (88.15)   
15 + **[CGCCCCGT]** 702 (87.14)   
15 + **.CGGCGCGC.** 732 (91.73)   
15 + **.CGGCGCGC.** 1199 (91.73)   
15 + **[CGCCGCCT]** 1243 (89.62)   
15 + **[GGCCGCGG]** 1263 (85.06)   
15 - **[GGACGCGG]** 1231 (88.08)   
15 - **[GGACGCGC]** 1182 (87.50)   
15 - **[CGACCCGG]** 1109 (86.25)   
15 - **.CGCCGCGC.** 985 (92.48)   
15 - **[CGGGGCGT]** 696 (88.38)   
15 - **[GGCCGCGG]** 544 (85.06)   
15 - **[CGCGGCGG]** 413 (85.22)   
15 - **[GGGCGCGT]** 246 (88.21)   
16 + **.CGGCGCGG.** 491 (92.31)   
16 + **.CCACGCGT.** 519 (90.60)   
16 + **.CGCCGCGC.** 625 (92.48)   
16 + **[CGCCGCCC]** 843 (85.13)   
16 + **[CGCGGCGG]** 1150 (85.22)   
16 + **[CCACGCGC]** 1162 (86.11)   
16 + **[CGCCGGGT]** 1205 (85.88)   
16 + **[CGACGCTT]** 1284 (88.71)   
16 + **[CGCCGCCC]** 1306 (85.13)   
16 - **[CGTCGCGC]** 1281 (85.44)   
16 - **[CGCCGCCG]** 1152 (85.71)   
16 - **.CGCCGCGC.** 1149 (92.48)   
16 - **[CGCCGCCG]** 1036 (85.71)   
16 - **[CGCCGCCG]** 1033 (85.71)   
16 - **[CGCCGCCG]** 1030 (85.71)   
16 - **[CGCCGCCT]** 1027 (89.62)   
16 - **.CGGCGCGG.** 622 (92.31)   
17 + **[GGCCGCGG]** 1016 (85.06)   
18 + **.CGCCGCGC.** 836 (92.48)   
18 + **.CGCCGCGC.** 865 (92.48)   
18 + **[GGCCGCGG]** 929 (85.06)   
18 + **.CGCCGCGG.** 1201 (93.07)   
18 + **[GGCCGCGT]** 1328 (88.97)   
18 + **.CGGCGCGG.** 1385 (92.31)   
18 - **[CGCCGCCC]** 1459 (85.13)   
18 - **[GGACGCGG]** 1330 (88.08)   
18 - **[CGCCGCCG]** 1267 (85.71)   
18 - **[GGCCGCGG]** 1203 (85.06)   
18 - **[CGCGGCGG]** 1200 (85.22)   
18 - **[CGACCCGG]** 1159 (86.25)   
18 - **[CGAGGCGG]** 974 (88.25)   
18 - **[GGCCGCGG]** 931 (85.06)   
18 - **[CGAGGCGC]** 915 (87.66)   
18 - **.CGGCGCGG.** 833 (92.31)   
18 - **.CGGCGCGC.** 709 (91.73)   
18 - **[GGACGCGG]** 475 (88.08)   
18 - **[CGCCGCCC]** 365 (85.13)   
20 + **[CCACGCGC]** 308 (86.11)   
20 + **[GGCCGCGT]** 1196 (88.97)   
20 - **[CCACGCGG]** 1198 (86.69)   
20 - **[CGCCGCCT]** 1041 (89.62)   
20 - **[CGCCGCCG]** 909 (85.71)   
20 - **[CGGCGCCT]** 49 (88.87)   
22 + **[CGAGGCGG]** 86 (88.25)   
22 + **[CGAGGCGG]** 674 (88.25)   
22 + **[CGCCGCCG]** 768 (85.71)   
22 + **.CGCCGCGC.** 771 (92.48)   
22 + **[CGCCGCCG]** 851 (85.71)   
22 - **[CGGCGCCT]** 848 (88.87)   
22 - **[CGCGGCGG]** 770 (85.22)   
22 - **[CGGCCCGT]** 632 (86.38)   
22 - **[CCGCGCGT]** 615 (86.82)   
22 - **[AGGCGCGT]** 191 (85.41)   
23 + **[CCACGCGG]** 1076 (86.69)   
24 + **[CGACCCGC]** 571 (85.67)   
24 + **[CGCGGCGG]** 1040 (85.22)   
24 - **[CGGCGCGA]** 1144 (87.47)   
24 - **[CGACCCGG]** 1139 (86.25)   
24 - **.CGCCGCGG.** 1039 (93.07)   
24 - **[AGACGCGG]** 521 (85.28)   
24 - **[CGCCGCCC]** 499 (85.13)   
25 + **[CGGCGCGA]** 382 (87.47)   
25 + **[CGAGGCGC]** 387 (87.66)   
25 + **[CGAGGCGC]** 546 (87.66)   
25 + **[CGAGGCGG]** 571 (88.25)   
25 + **.CGAGGCGT.** 608 (92.16)   
25 + **.CGGCGCGG.** 791 (92.31)   
25 + **[CGAGGCGC]** 829 (87.66)   
25 - **[CCCCGCGT]** 951 (87.58)   
25 - **[CGCCGCCC]** 788 (85.13)   
25 - **[CGCCCCGT]** 686 (87.14)   
26 + **[GGCCGCGG]** 183 (85.06)   
26 + **[CGCCGCCC]** 310 (85.13)   
26 + **[GGACGCGC]** 752 (87.50)   
26 + **.CGGCGCGG.** 823 (92.31)   
26 + **[CGAGGCGG]** 1028 (88.25)   
26 + **[GGGCGCGT]** 1352 (88.21)   
26 + **[CGCGGCGG]** 1425 (85.22)   
26 + **.CGCCGCGG.** 1484 (93.07)   
26 - **.CGCCGCGC.** 1424 (92.48)   
26 - **[CCCCGCGT]** 1347 (87.58)   
26 - **[CGCCGCCG]** 962 (85.71)   
26 - **[CGCCGCCG]** 959 (85.71)   
26 - **[CGCCGCCG]** 956 (85.71)   
27 + **[CCCCGCGT]** 1143 (87.58)   
27 + **.CGGCGCGC.** 1206 (91.73)   
27 + **[CGCCGCCT]** 1356 (89.62)   
27 - **.CGGCGCGC.** 1248 (91.73)   
27 - **.CGCCGCGG.** 1203 (93.07)   
27 - **.CGACGCGG.** 1145 (96.09)   
28 + **[CGAGGCGC]** 252 (87.66)   
28 + **.CGGCGCGT.** 432 (96.22)   
28 + **[CGGCGCGA]** 627 (87.47)   
28 + **[GGCCGCGG]** 915 (85.06)   
28 + **[CGCCGCCC]** 1063 (85.13)   
28 + **[CGTCGCGT]** 1097 (89.93)   
28 + **[CGTCGCGC]** 1102 (85.44)   
28 + **.CGCCGCGT.** 1107 (96.98)   
28 + **[CGAGGCGG]** 1413 (88.25)   
28 - **.CGACGCGG.** 1109 (96.09)   
28 - **[CGGCGCGA]** 1104 (87.47)   
28 - **.CGACGCGA.** 1099 (91.25)   
28 - **[CGAGGCGG]** 1071 (88.25)   
28 - **[CGCCGCCG]** 871 (85.71)   
28 - **[CGCCGCCG]** 868 (85.71)   
28 - **.CGGCGCGG.** 739 (92.31)   
28 - **[CGTCGCGG]** 588 (86.02)   
28 - **[CGGCGGGT]** 539 (85.13)   
28 - **[CCACGCGC]** 434 (86.11)   
28 - **[CGCCGCCT]** 429 (89.62)   
29 + **[GGCCGCGT]** 1217 (88.97)   
29 - **[CGAGGCGC]** 1207 (87.66)   
33 + **.CGCCGCGT.** 1039 (96.98)   
33 + **[GGCCGCGG]** 1359 (85.06)   
33 + **[CGCGGCGG]** 1362 (85.22)   
33 + **[CGCCGCCG]** 1407 (85.71)   
33 + **.CGCCGCGG.** 1410 (93.07)   
33 - **[CGACGCCG]** 1460 (88.74)   
33 - **[CGCGGCGG]** 1409 (85.22)   
33 - **.CGCCGCGG.** 1361 (93.07)   
33 - **[CGCCGCCT]** 1352 (89.62)   
33 - **[CGCGGCGT]** 1038 (89.13)   
33 - **[CCCCGCGT]** 1030 (87.58)   
33 - **[GGCCGCGG]** 903 (85.06)   
34 + **.CGCCGCGC.** 770 (92.48)   
34 + **[GGGCGCGT]** 800 (88.21)   
34 + **[CGAGGCGG]** 814 (88.25)   
34 + **[CGAGGCGC]** 915 (87.66)   
34 + **[AGACGCGT]** 999 (89.19)   
34 + **[CGCCGCCC]** 1122 (85.13)   
34 + **.CGCCGCGC.** 1195 (92.48)   
34 + **[CGCCCCGT]** 1207 (87.14)   
34 + **[CGCCGCCG]** 1445 (85.71)   
34 + **[CGCCGCCG]** 1448 (85.71)   
34 - **[CGAGGCGG]** 1334 (88.25)   
34 - **[CGGGGCGT]** 1206 (88.38)   
34 - **[CTACGCGT]** 1001 (86.64)   
35 + **[CGCCGCCG]** 948 (85.71)   
35 + **[CGCCGCCG]** 951 (85.71)   
35 + **[CGCCGCCG]** 976 (85.71)   
35 + **.CGCCGCGC.** 997 (92.48)   
35 + **[CGACCCGG]** 1332 (86.25)   
35 - **[CGCCGCCC]** 1239 (85.13)   
35 - **[CGACCCGC]** 1150 (85.67)   
35 - **[CGGCGCCT]** 1082 (88.87)   
35 - **[CGGCGCCT]** 945 (88.87)   
36 + **[AGCCGCGT]** 626 (86.16)   
36 + **[CCCCGCGT]** 812 (87.58)   
36 + **.CGCCGCGG.** 1170 (93.07)   
36 - **[CGCCGCCC]** 1100 (85.13)   
36 - **.CGGCGCGC.** 1065 (91.73)   
36 - **[AGCCGCGT]** 229 (86.16)   
36 - **[AGACGCGT]** 150 (89.19)   
37 - **.CGAGGCGT.** 984 (92.16)   
38 - **[CGAGGCGG]** 865 (88.25)   
38 - **[CGAGGCGC]** 558 (87.66)   
39 + **[GGGCGCGT]** 1119 (88.21)   
39 + **[CGGCGCGA]** 1411 (87.47)   
39 - **[CGCCGCCC]** 1256 (85.13)   
39 - **[GGACGCGC]** 1121 (87.50)   
40 + **.CGCCGCGC.** 1106 (92.48)   
40 - **.CGCCGCGG.** 1240 (93.07)   
40 - **[GGACGCGC]** 1234 (87.50)   
40 - **.CGGCGCGG.** 1103 (92.31)   
40 - **[GGCCGCGG]** 894 (85.06)   
41 - **[CGACGAGT]** 1269 (86.53)   
41 - **[GGCCGCGG]** 522 (85.06)   
42 + **[CCGCGCGT]** 439 (86.82)   
42 + **[CGTCGCGG]** 908 (86.02)   
42 + **[CGCGGCGG]** 1168 (85.22)   
42 + **[CCGCGCGT]** 1448 (86.82)   
42 + **[GGCCGCGG]** 1474 (85.06)   
42 - **[CCACGCGC]** 1450 (86.11)   
42 - **[CGACCCGC]** 1263 (85.67)   
42 - **[CGCCGCCG]** 1176 (85.71)   
42 - **[CGCCGCCG]** 1173 (85.71)   
42 - **[CGCCGCCG]** 1170 (85.71)   
42 - **.CGCCGCGG.** 1167 (93.07)   
42 - **[CGCCGGGT]** 954 (85.88)   
42 - **[GGCCGCGG]** 842 (85.06)   
42 - **[CGAGGCGC]** 608 (87.66)   
43 + **.CGCCGCGG.** 1100 (93.07)   
43 + **[CGACCCGG]** 1369 (86.25)   
43 - **[CGCCGCCG]** 1179 (85.71)   
43 - **[CGCCGCCG]** 1176 (85.71)   
43 - **[CGCCGCCT]** 1161 (89.62)   
45 + **.CGCCGCGG.** 396 (93.07)   
45 + **.CGGCGCGG.** 1418 (92.31)   
45 - **[CCGCGCGT]** 1009 (86.82)   
45 - **[GGCCGCGG]** 980 (85.06)   
45 - **[CGGCGCCT]** 573 (88.87)   
47 + **.CGGCGCGG.** 833 (92.31)   
47 + **[CGAGGCGG]** 1142 (88.25)   
47 - **[CGCCGGGT]** 1466 (85.88)   
47 - **[CGGCGCGA]** 1248 (87.47)   
47 - **[CGCCGCTT]** 1203 (85.69)   
48 + **[CGGCGCCT]** 190 (88.87)   
48 + **[CGCGGCGG]** 204 (85.22)   
48 + **[CGAGGCGG]** 217 (88.25)   
48 + **[CGCCGCCT]** 730 (89.62)   
48 + **[CGCGGCGG]** 1019 (85.22)   
48 + **.CGCCGCGG.** 1283 (93.07)   
48 + **[GGCCGCGT]** 1361 (88.97)   
48 - **[AGACGCGG]** 1363 (85.28)   
48 - **[GGCCGCGG]** 1316 (85.06)   
48 - **.CGCCGCGG.** 1285 (93.07)   
48 - **[CGCGGCGG]** 1282 (85.22)   
48 - **[CGCCGCCG]** 1024 (85.71)   
48 - **[CGCCGCCG]** 1021 (85.71)   
48 - **.CGCCGCGC.** 1018 (92.48)   
48 - **[CCCCGCGT]** 1005 (87.58)   
48 - **.CGAGGCGT.** 514 (92.16)   
48 - **.CGCCGCGG.** 432 (93.07)   
48 - **.CGCCGCGC.** 203 (92.48)   
48 - **[CGCCGCCG]** 187 (85.71)   
49 + **[CGGCGCCT]** 756 (88.87)   
49 + **[CGCCGCCG]** 1306 (85.71)   
49 + **.CGCCGCGG.** 1406 (93.07)   
49 - **[CGCGGCGG]** 1405 (85.22)   
49 - **[CGCCGCCG]** 1080 (85.71)   
49 - **[CGCCGCCG]** 1077 (85.71)   
49 - **[CGCCGCCG]** 1074 (85.71)   
49 - **[CGCCGCCT]** 1071 (89.62)   
49 - **[CGACACGT]** 987 (87.00)   
51 + **.CGCCGCGT.** 67 (96.98)   
51 + **[CGCGGCGG]** 147 (85.22)   
51 + **[CGGCGGGT]** 149 (85.13)   
51 + **.CGACGCGC.** 230 (95.51)   
51 + **[CGCCGCCT]** 303 (89.62)   
51 + **[CTACGCGT]** 555 (86.64)   
51 + **[CGTCGCGG]** 976 (86.02)   
51 + **[CGCCGCCG]** 1034 (85.71)   
51 + **[CGACGCCG]** 1150 (88.74)   
51 + **[CGCGGCGG]** 1286 (85.22)   
51 - **.CGCCGCGC.** 1361 (92.48)   
51 - **.CGCCGCGG.** 1285 (93.07)   
51 - **.CGACGCCT.** 846 (92.65)   
51 - **[CGGCGCGA]** 781 (87.47)   
51 - **[AGACGCGT]** 557 (89.19)   
51 - **[CGCCGCCG]** 518 (85.71)   
51 - **[CGAGGCGG]** 385 (88.25)   
51 - **[CGCCGCGA]** 146 (88.22)   
51 - **[CGAGGCGC]** 91 (87.66)   
51 - **.CGACGCGG.** 69 (96.09)   
51 - **[CGCGGCGG]** 66 (85.22)   
53 + **[CGAGGCGC]** 788 (87.66)   
53 + **[CGACCCGG]** 799 (86.25)   
53 + **[GGACGCGG]** 1056 (88.08)   
53 + **[CGCCCCGT]** 1315 (87.14)   
53 - **[CGACGCCC]** 1182 (88.15)   
53 - **[GGCCGCGT]** 1058 (88.97)   
53 - **.CGACGCCT.** 782 (92.65)   
54 + **[GGACGCGC]** 1157 (87.50)   
54 - **[CGGCGCCT]** 1025 (88.87)   
55 + **[CGCCGCCG]** 92 (85.71)   
55 + **[CGCCGCCG]** 95 (85.71)   
55 + **.CGCCGCGC.** 198 (92.48)   
55 + **.CGCCGCGT.** 865 (96.98)   
55 + **.CGCCGCGG.** 883 (93.07)   
55 + **[CGCGGCGT]** 943 (89.13)   
55 + **.CGCCGCGC.** 1084 (92.48)   
55 + **[CGGGGCGT]** 1459 (88.38)   
55 - **.CGGCGCGG.** 1081 (92.31)   
55 - **.CGCCGCGC.** 942 (92.48)   
55 - **[CGCGGCGG]** 882 (85.22)   
55 - **[CGCGGCGG]** 864 (85.22)   
55 - **[CGGCGCCT]** 715 (88.87)   
55 - **[GGCCGCGG]** 45 (85.06)   
58 + **[CCACGCGG]** 417 (86.69)   
58 + **[CGACACGT]** 507 (87.00)   
58 + **.CGACGCGC.** 603 (95.51)   
58 + **[CGCCGCCC]** 1085 (85.13)   
58 + **.CGACGCGG.** 1279 (96.09)   
58 - **[CGCCGCCC]** 1184 (85.13)   
58 - **[GGCCGCGT]** 1100 (88.97)   
59 + **.CGCCGCGG.** 649 (93.07)   
59 + **[CGCCGCCG]** 771 (85.71)   
59 + **[CGCCGCCG]** 774 (85.71)   
59 + **[CGCCGCCG]** 777 (85.71)   
59 + **[CGCCGCCG]** 780 (85.71)   
59 + **[CGCCGCCC]** 783 (85.13)   
59 + **.CGGCGCGG.** 1351 (92.31)   
59 + **[CGACCCGG]** 1400 (86.25)   
59 - **.CGCCGCGC.** 1348 (92.48)   
59 - **.CGACGCGC.** 1285 (95.51)   
59 - **[GGCCGCGG]** 1011 (85.06)   
59 - **[GGCCGCGG]** 651 (85.06)   
60 + **[CGCCGCCC]** 629 (85.13)   
60 + **.CGCCGCGG.** 824 (93.07)   
60 + **[CGAGGCGG]** 904 (88.25)   
60 - **[GGCCGCGG]** 826 (85.06)   
60 - **[CGCGGCGG]** 823 (85.22)   
61 + **.CGGCGCGC.** 500 (91.73)   
61 + **[GGACGCGG]** 839 (88.08)   
61 + **[GGCCGCGT]** 913 (88.97)   
61 + **.CGCCGCGG.** 924 (93.07)   
61 + **[CGGCGCCT]** 948 (88.87)   
61 + **.CGCCGCGG.** 1036 (93.07)   
61 + **[CCCCGCGT]** 1061 (87.58)   
61 + **.GGACGCGT.** 1319 (91.99)   
61 - **.CGCCGCGG.** 1038 (93.07)   
61 - **[CGCGGCGT]** 1035 (89.13)   
61 - **[CGCGGCGG]** 923 (85.22)   
61 - **.CGCCGCGT.** 841 (96.98)   
61 - **[CGGCCCGT]** 684 (86.38)   
63 + **[CGAGGCGC]** 67 (87.66)   
63 + **[CCCCGCGT]** 166 (87.58)   
63 + **[GGCCGCGG]** 181 (85.06)   
63 + **.CGGCGCGC.** 265 (91.73)   
63 + **[CGCGGCGG]** 410 (85.22)   
63 + **[CGCGGCGT]** 445 (89.13)   
63 + **[CGCCGCCT]** 923 (89.62)   
63 + **[CGCGGCGT]** 954 (89.13)   
63 + **[CGTCGCGT]** 973 (89.93)   
63 + **[CGACGCCG]** 997 (88.74)   
63 + **.CGCCGCGG.** 1252 (93.07)   
63 + **[CGCGGCGG]** 1393 (85.22)   
63 + **[GGCCGCGG]** 1411 (85.06)   
63 - **[CGCCCCGT]** 1404 (87.14)   
63 - **[CGCCGCCG]** 1395 (85.71)   
63 - **.CGCCGCGC.** 1392 (92.48)   
63 - **[CGCCGCCC]** 1362 (85.13)   
63 - **[CGGCGGGT]** 1261 (85.13)   
63 - **[CGCCGCCG]** 1239 (85.71)   
63 - **[CGCCGCCC]** 1170 (85.13)   
63 - **.CGCCGCGG.** 953 (93.07)   
63 - **.CGGCGCGC.** 906 (91.73)   
63 - **.CGACGCGA.** 853 (91.25)   
63 - **[CGAGGCGG]** 718 (88.25)   
63 - **.CGGCGCGC.** 657 (91.73)   
63 - **[GGACGCGG]** 520 (88.08)   
63 - **.CGGCGCGG.** 453 (92.31)   
63 - **.CGCCGCGG.** 444 (93.07)   
63 - **[CGCCGCCG]** 412 (85.71)   
63 - **.CGCCGCGC.** 409 (92.48)   
63 - **[CGACCCGG]** 210 (86.25)   
65 + **.CGGCGCGT.** 863 (96.22)   
65 + **.CGGCGCGC.** 978 (91.73)   
65 + **[CGCCGCGA]** 1000 (88.22)   
65 + **.CGCCGCGC.** 1027 (92.48)   
65 + **[CGCCGCCG]** 1087 (85.71)   
65 + **[CGCCGCCC]** 1090 (85.13)   
65 + **[GGACGCGG]** 1223 (88.08)   
65 - **[CGCGGCGG]** 999 (85.22)   
65 - **[AGACGCGG]** 546 (85.28)   
66 + **[GGACGCGG]** 785 (88.08)   
66 + **[CGCCGCCG]** 871 (85.71)   
66 + **[CGCCGCCC]** 874 (85.13)   
66 + **[CGACCCGG]** 977 (86.25)   
66 + **.CGCCGCGG.** 988 (93.07)   
66 + **[CGCCGCCT]** 1041 (89.62)   
66 + **[CGCCGCCG]** 1093 (85.71)   
66 - **.CGACCCGT.** 1332 (90.16)   
66 - **.CGCCGCGG.** 1126 (93.07)   
66 - **[CGCGGCGG]** 987 (85.22)   
66 - **.CGGCGCGG.** 954 (92.31)   
66 - **.CGGCGCGC.** 868 (91.73)   
66 - **[CGCCGCCC]** 832 (85.13)   
67 + **[CGGCGGGT]** 815 (85.13)   
67 + **[CGAGGCGG]** 996 (88.25)   
67 + **[GGACGCGC]** 1172 (87.50)   
67 + **.CGCCGCGT.** 1195 (96.98)   
67 - **[GGACGCGG]** 1197 (88.08)   
67 - **[AGGCGCGT]** 1174 (85.41)   
67 - **[CGACCCGC]** 817 (85.67)   
68 + **[CGCCGCCG]** 576 (85.71)   
68 + **.CGCCGCGC.** 588 (92.48)   
68 + **.CGGCGCGG.** 651 (92.31)   
68 + **[CGACGCCC]** 873 (88.15)   
68 + **[CGCCGCCG]** 906 (85.71)   
68 + **[CGCGGCGG]** 992 (85.22)   
68 + **[CGCGGCGG]** 1394 (85.22)   
68 - **.CGCCGCGC.** 1393 (92.48)   
68 - **.CGCCGCGT.** 991 (96.98)   
68 - **[CGTCGCGC]** 870 (85.44)   
68 - **[CCCCGCGT]** 720 (87.58)   
68 - **[CGCGGCGG]** 587 (85.22)   
68 - **[CGCCGCCG]** 501 (85.71)   
68 - **[CGCCGCCG]** 498 (85.71)   
68 - **.CGACGCGC.** 418 (95.51)   
69 + **[GGCCGCGT]** 772 (88.97)   
69 + **[CGCCGCCT]** 1000 (89.62)   
69 + **[CGCCGCCC]** 1247 (85.13)   
69 - **.CGGCGCGG.** 1120 (92.31)   
69 - **.CGGCGCGG.** 997 (92.31)   
69 - **.CGACGCGC.** 893 (95.51)   
69 - **[GGCCGCGG]** 768 (85.06)   
69 - **[CGACGCTT]** 719 (88.71)   
69 - **[CCACGCGG]** 695 (86.69)   
69 - **[GGACGCGG]** 689 (88.08)   
70 + **[CGAGGCGG]** 248 (88.25)   
70 + **[CCACGCGG]** 839 (86.69)   
70 - **[CGAGGCGG]** 1424 (88.25)   
70 - **[GGACGCGC]** 1389 (87.50)   
70 - **.CGGCGCGC.** 1333 (91.73)   
70 - **[GGCCGCGT]** 1063 (88.97)   
71 + **[CCGCGCGT]** 901 (86.82)   
71 + **[CCACGCGC]** 1272 (86.11)   
71 - **[GGACGCGC]** 903 (87.50)   
73 + **[CGCCGCCG]** 912 (85.71)   
73 + **[CGCCGCCG]** 915 (85.71)   
73 + **.CGCCGCGG.** 918 (93.07)   
73 - **[GGCCGCGG]** 920 (85.06)   
73 - **[CGCGGCGG]** 917 (85.22)   
74 + **[CCGCGCGT]** 1335 (86.82)   
74 + **[CGCGGCGG]** 1429 (85.22)   
74 - **.CGGCGCGG.** 1437 (92.31)   
74 - **[CGCCGCGA]** 1428 (88.22)   
74 - **[GGACGCGC]** 1337 (87.50)   
74 - **[CGCCGCCG]** 1287 (85.71)   
74 - **[CGCCGCCG]** 1284 (85.71)   
74 - **[CGCCGCCG]** 1281 (85.71)   
74 - **[CGCCGCCG]** 1278 (85.71)   
74 - **[CGCCGCCG]** 1275 (85.71)   
74 - **[CGCCGCCG]** 1272 (85.71)   
74 - **[CGCCGCCG]** 1269 (85.71)   
76 + **[CGAGGCGG]** 912 (88.25)   
76 + **[CGGCGGGT]** 939 (85.13)   
76 + **[CGCCGCCT]** 1029 (89.62)   
76 + **[GGACGCGG]** 1283 (88.08)   
76 - **[CGCCGCCC]** 936 (85.13)   
76 - **[CGCCGCCG]** 920 (85.71)   
76 - **[CGCCGCCG]** 917 (85.71)   
76 - **[CGCCGCCT]** 914 (89.62)   
76 - **.CGCCGCGG.** 865 (93.07)   
76 - **[CGGCCCGT]** 855 (86.38)   
76 - **.CGGCGCGC.** 828 (91.73)   
77 + **[AGGCGCGT]** 930 (85.41)   
77 - **[CCACGCGC]** 932 (86.11)   
77 - **[GGCCGCGT]** 780 (88.97)   
79 + **[CGCCGCGA]** 653 (88.22)   
79 + **[CGCCGCCG]** 874 (85.71)   
79 + **.CGCCGCGT.** 877 (96.98)   
79 + **[CGACGAGT]** 898 (86.53)   
79 + **[CGTCGCGG]** 1087 (86.02)   
79 + **[CGCCGCTT]** 1204 (85.69)   
79 + **[CGGCGGGT]** 1227 (85.13)   
79 + **[GGCCGCGG]** 1276 (85.06)   
79 - **.CGGCGCGG.** 1201 (92.31)   
79 - **[CGCGGCGG]** 876 (85.22)   
79 - **[CGCGGCGG]** 652 (85.22)   
80 + **.CGGCGCGT.** 954 (96.22)   
80 + **.CGCCGCGC.** 1015 (92.48)   
80 + **.CGGCGCGG.** 1043 (92.31)   
80 - **[CCGCGCGT]** 1340 (86.82)   
81 + **[CGAGGCGG]** 687 (88.25)   
81 + **.CGACCCGT.** 844 (90.16)   
84 + **.CGACGCGC.** 899 (95.51)   
84 + **.CGACGCGG.** 1034 (96.09)   
84 + **.CGGCGCGG.** 1152 (92.31)   
84 + **[CCCCGCGT]** 1161 (87.58)   
84 + **[CGGCCCGT]** 1263 (86.38)   
84 - **.CGCCGCGT.** 1036 (96.98)   
84 - **.CGCCGCGC.** 1022 (92.48)   
84 - **[AGGCGCGT]** 901 (85.41)   
84 - **[CGACGGGT]** 775 (88.90)   
85 + **[CGCCGCCT]** 19 (89.62)   
85 + **[GGGCGCGT]** 586 (88.21)   
85 + **[GGACGCGG]** 927 (88.08)   
85 + **[CGCCGCCG]** 991 (85.71)   
85 - **[GGCCGCGG]** 1216 (85.06)   
85 - **[GGGCGCGT]** 962 (88.21)   
85 - **[GGCCGCGG]** 879 (85.06)   
85 - **[CGCCGCCC]** 313 (85.13)   
85 - **[GGACGCGG]** 94 (88.08)   
86 + **.CGAGGCGT.** 286 (92.16)   
86 + **.CGCCGCGG.** 540 (93.07)   
86 + **[CGACCCGG]** 799 (86.25)   
86 + **[CGCGGCGG]** 836 (85.22)   
86 + **.CGGCGCGG.** 1192 (92.31)   
86 + **[CGCCGCCC]** 1220 (85.13)   
86 + **[CGCCGCCT]** 1269 (89.62)   
86 + **[CGACCCGG]** 1322 (86.25)   
86 + **[CGCCGCCC]** 1354 (85.13)   
86 + **.CGCCGCGG.** 1436 (93.07)   
86 + **[CGCCGCCC]** 1460 (85.13)   
86 - **[CGCGGCGT]** 1435 (89.13)   
86 - **[CGCCGCCC]** 1412 (85.13)   
86 - **[CGCCGCCC]** 1390 (85.13)   
86 - **.CGCCGCGC.** 1194 (92.48)   
86 - **[CGCCGCGA]** 1189 (88.22)   
86 - **.CGAGGCGT.** 1184 (92.16)   
86 - **[CGACCCGC]** 1120 (85.67)   
86 - **.CGCCGCGC.** 901 (92.48)   
86 - **.CGCCGCGC.** 835 (92.48)   
86 - **[GGCCGCGG]** 657 (85.06)   
86 - **[GGCCGCGG]** 542 (85.06)   
86 - **[CGGGGCGT]** 512 (88.38)   
87 + **.GGACGCGT.** 132 (91.99)   
87 + **[CCACGCGC]** 265 (86.11)   
87 + **[CGGCGCGA]** 312 (87.47)   
87 + **[GGACGCGG]** 585 (88.08)   
87 + **[GGCCGCGG]** 767 (85.06)   
87 + **.CGGCGCGG.** 842 (92.31)   
87 + **[AGCCGCGT]** 1096 (86.16)   
87 + **[CGCCGCCC]** 1145 (85.13)   
87 + **[CGCCGCCG]** 1196 (85.71)   
87 + **.CGGCGCGG.** 1241 (92.31)   
87 + **[CGCGGCGG]** 1244 (85.22)   
87 + **[CCACGCGC]** 1319 (86.11)   
87 + **[CGCGGCGG]** 1333 (85.22)   
87 - **[GGCCGCGG]** 1366 (85.06)   
87 - **.CGGCGCGC.** 1345 (91.73)   
87 - **[CGCCGCCG]** 1335 (85.71)   
87 - **[CGCCGCGA]** 1332 (88.22)   
87 - **.CGGCGCGT.** 1321 (96.22)   
87 - **[CGCCGCCG]** 1262 (85.71)   
87 - **.CGCCGCGC.** 1243 (92.48)   
87 - **.CGGCGCGC.** 1193 (91.73)   
87 - **[GGACGCGG]** 1098 (88.08)   
87 - **[GGCCGCGG]** 721 (85.06)   
87 - **[CGACCCGG]** 417 (86.25)   
87 - **[CGCCGCGA]** 309 (88.22)   
87 - **.GGACGCGT.** 134 (91.99)   
88 + **[CGCCCCGT]** 211 (87.14)   
88 + **[CGCCGCCC]** 222 (85.13)   
88 + **.CGGCGCGC.** 444 (91.73)   
88 + **[CGTCGCGG]** 487 (86.02)   
88 + **.CGCCGCGC.** 503 (92.48)   
88 + **[CCGCGCGT]** 608 (86.82)   
88 + **[CGCCGCCG]** 785 (85.71)   
88 + **[CGCCGCCG]** 788 (85.71)   
88 + **[CGCCGCCC]** 791 (85.13)   
88 + **[CGCGGCGG]** 849 (85.22)   
88 + **[CGCCGCTT]** 960 (85.69)   
88 + **.CGCCGCGG.** 1096 (93.07)   
88 + **[CGGCGCCT]** 1172 (88.87)   
88 + **[CGGCCCGT]** 1261 (86.38)   
88 + **[CGCCGGGT]** 1317 (85.88)   
88 - **.CGCCGCGC.** 1394 (92.48)   
88 - **[CGGGGCGT]** 1332 (88.38)   
88 - **[CGACCCGG]** 1302 (86.25)   
88 - **.CGACGCGC.** 1205 (95.51)   
88 - **[GGCCGCGG]** 1098 (85.06)   
88 - **[CGCGGCGT]** 1095 (89.13)   
88 - **[CGCCGCCG]** 904 (85.71)   
88 - **[CGCCGCCG]** 901 (85.71)   
88 - **[CGCCGCCG]** 898 (85.71)   
88 - **[CGCCGCCC]** 895 (85.13)   
88 - **.CGCCGCGG.** 848 (93.07)   
89 + **[CGCCGCCG]** 102 (85.71)   
89 + **[CGCCGCCC]** 105 (85.13)   
89 + **[CGCCCCGT]** 145 (87.14)   
89 + **[CGTCGCGC]** 150 (85.44)   
89 + **[CGAGGCGG]** 175 (88.25)   
89 + **[GGCCGCGG]** 259 (85.06)   
89 + **[CGCCGCGA]** 268 (88.22)   
89 + **[CGCCGCCG]** 285 (85.71)   
89 + **[CGCCGCCT]** 350 (89.62)   
89 + **[CGCCGCCG]** 380 (85.71)   
89 + **[CGCCGCCG]** 383 (85.71)   
89 + **[CGCCGCCG]** 386 (85.71)   
89 + **[CGCCGCCG]** 389 (85.71)   
89 + **[CGCCGCCG]** 408 (85.71)   
89 + **[CGCCGCCG]** 411 (85.71)   
89 + **[CGCCGCCG]** 414 (85.71)   
89 + **.CGCCGCGG.** 417 (93.07)   
89 + **.CGCCGCGC.** 434 (92.48)   
89 + **[CGCCGCCG]** 466 (85.71)   
89 + **[CGCCGCCC]** 469 (85.13)   
89 + **[GGCCGCGT]** 477 (88.97)   
89 + **[CGCCGCCC]** 497 (85.13)   
89 + **[GGACGCGC]** 599 (87.50)   
89 + **[CGCCGCCG]** 611 (85.71)   
89 + **[CGCCGCCG]** 614 (85.71)   
89 + **.CGCCGCGC.** 663 (92.48)   
89 + **[CGCGGCGT]** 743 (89.13)   
89 + **.CGGCGCGC.** 786 (91.73)   
89 + **.CGGCGCGG.** 936 (92.31)   
89 + **[CCACGCGG]** 963 (86.69)   
89 + **.CGCCGCGC.** 1125 (92.48)   
89 + **.CGCCGCGG.** 1309 (93.07)   
89 + **[GGCCGCGT]** 1450 (88.97)   
89 - **[CGCGGCGG]** 1124 (85.22)   
89 - **.CGGCGCGG.** 1022 (92.31)   
89 - **[GGCCGCGG]** 842 (85.06)   
89 - **.CGGCGCGC.** 788 (91.73)   
89 - **[CGCCGCCT]** 783 (89.62)   
89 - **.CGCCGCGC.** 742 (92.48)   
89 - **[CGCGGCGG]** 662 (85.22)   
89 - **.CGGCGCGG.** 494 (92.31)   
89 - **[CGCGGCGG]** 416 (85.22)   
89 - **[CGCCGCCC]** 183 (85.13)   
89 - **[CGCCGGGT]** 169 (85.88)   
89 - **.CGGCGCGG.** 99 (92.31)   
89 - **[GGCCGCGG]** 46 (85.06)   
89 - **[CGCCGCCC]** 25 (85.13)   
90 + **[CGCCGCCC]** 41 (85.13)   
90 + **[GGCCGCGG]** 586 (85.06)   
90 + **[CCACGCGC]** 712 (86.11)   
90 + **.CGCCGCGG.** 717 (93.07)   
90 + **[GGCCGCGG]** 774 (85.06)   
90 + **[CGCGGCGG]** 777 (85.22)   
90 + **[GGCCGCGG]** 847 (85.06)   
90 + **[CGCGGCGG]** 850 (85.22)   
90 + **.CGCCGCGG.** 1018 (93.07)   
90 - **[CGCCGCCG]** 1255 (85.71)   
90 - **[CGCCGCCG]** 1252 (85.71)   
90 - **[CGCCGCCG]** 1249 (85.71)   
90 - **[CGCCGCCG]** 1192 (85.71)   
90 - **[CGCCGCCC]** 1189 (85.13)   
90 - **[CGCCGCCG]** 1091 (85.71)   
90 - **[CGCCGCCG]** 1088 (85.71)   
90 - **[CGCCGCCG]** 1085 (85.71)   
90 - **[CGCCGCCT]** 1082 (89.62)   
90 - **[CGCGGCGG]** 1017 (85.22)   
90 - **[CGGCGGGT]** 1015 (85.13)   
90 - **.CGCCGCGG.** 849 (93.07)   
90 - **[CGACCCGC]** 837 (85.67)   
90 - **[CGCCGCCG]** 779 (85.71)   
90 - **.CGCCGCGG.** 776 (93.07)   
90 - **[GGCCGCGG]** 719 (85.06)   
90 - **.CGGCGCGT.** 714 (96.22)   
92 + **[CGCGGCGG]** 1228 (85.22)   
92 - **.CGCCGCGC.** 1227 (92.48)   
93 + **[CGGCGCCT]** 467 (88.87)   
93 + **[GGACGCGC]** 962 (87.50)   
93 + **[GGACGCGC]** 1000 (87.50)   
93 + **[CGCGGCGG]** 1037 (85.22)   
93 + **[CCACGCGC]** 1191 (86.11)   
93 - **[AGACGCGG]** 1487 (85.28)   
93 - **[CGCCGCCG]** 1450 (85.71)   
93 - **[CGCCGCCG]** 1447 (85.71)   
93 - **[CGCCGCCG]** 1381 (85.71)   
93 - **[CGCCGCCC]** 1362 (85.13)   
93 - **[CGCCCCGT]** 1359 (87.14)   
93 - **[CGCCGCCG]** 1039 (85.71)   
93 - **.CGCCGCGG.** 1036 (93.07)   
93 - **.CGGCGCGT.** 1002 (96.22)   
93 - **[CGCCGCCC]** 412 (85.13)   
96 + **[CGTCGCGT]** 1356 (89.93)   
96 + **[CGCCGCTT]** 1364 (85.69)   
96 + **[GGACGCGG]** 1392 (88.08)   
96 - **.CGGCGCGG.** 1421 (92.31)   
96 - **[CCCCGCGT]** 1394 (87.58)   
96 - **.CGACGCGA.** 1358 (91.25)   
96 - **.CGACGCGC.** 1353 (95.51)   
96 - **[CGACCCGC]** 1346 (85.67)   
96 - **.CGACGCGC.** 739 (95.51)   
96 - **[CGCCGCTT]** 456 (85.69)   
97 + **[CGCCGCCG]** 851 (85.71)   
97 + **.CGGCGCGG.** 879 (92.31)   
97 + **[GGACGCGG]** 1366 (88.08)   
97 - **[CGACGCCG]** 1440 (88.74)   
97 - **.CGCCGCGC.** 876 (92.48)   
98 + **[GGCCGCGG]** 998 (85.06)   
98 + **[CGCGGCGG]** 1001 (85.22)   
98 + **[GGACGCGG]** 1097 (88.08)   
98 + **[CGCGGCGG]** 1137 (85.22)   
98 + **.CGGCGCGG.** 1226 (92.31)   
98 - **[CGCCGCCG]** 1450 (85.71)   
98 - **[CGCCGCCG]** 1344 (85.71)   
98 - **[CGCCGCCC]** 1260 (85.13)   
98 - **[CGCCGCCC]** 1223 (85.13)   
98 - **.CGCCGCGC.** 1136 (92.48)   
98 - **.CGCCGCGG.** 1085 (93.07)   
98 - **.CGCCGCGG.** 1000 (93.07)   
98 - **[CGCCGCCG]** 986 (85.71)   
98 - **[CGCCGCCG]** 983 (85.71)   
98 - **[CGCCGCCG]** 980 (85.71)   
98 - **[CGCCGCCG]** 977 (85.71)   
98 - **[CGCCGCCG]** 794 (85.71)   
98 - **[CGCCGCCT]** 791 (89.62)   
99 - **[CGGCGGGT]** 1508 (85.13)   
100 + **[CGCGGCGG]** 1500 (85.22)   
100 - **.CGCCGCGG.** 1499 (93.07)   
100 - **[CGGGGCGT]** 1257 (88.38)   
100 - **[CGGCGCGA]** 1103 (87.47)   
100 - **[CGACCCGG]** 1098 (86.25)   
100 - **.CGACGCCT.** 290 (92.65)   
103 - **[CGACCCGG]** 1178 (86.25)   
104 + **[CGCCCCGT]** 719 (87.14)   
105 + **[GGGCGCGT]** 1141 (88.21)   
106 + **[CGGCGCCT]** 872 (88.87)   
106 + **[CCGCGCGT]** 1000 (86.82)   
106 + **[CGCGGCGT]** 1160 (89.13)   
106 - **[CGCCGCGA]** 1159 (88.22)   
106 - **[CGCCGCGA]** 869 (88.22)   
108 + **[CGAGGCGG]** 311 (88.25)   
108 + **[GGACGCGC]** 817 (87.50)   
108 + **[CGCCGCTT]** 844 (85.69)   
108 + **[CCCCGCGT]** 1200 (87.58)   
108 + **[CGAGGCGC]** 1297 (87.66)   
108 + **.GGACGCGT.** 1384 (91.99)   
108 - **.CCACGCGT.** 1386 (90.60)   
108 - **.CGGCGCGG.** 1217 (92.31)   
108 - **[GGACGCGG]** 1202 (88.08)   
108 - **[CGTCGCGG]** 766 (86.02)   
108 - **[CCACGCGG]** 741 (86.69)   
108 - **[AGACGCGG]** 428 (85.28)   
109 + **.CGCCGCGG.** 336 (93.07)   
109 + **[GGACGCGC]** 813 (87.50)   
109 + **[CGACGCCG]** 1184 (88.74)   
109 + **.CGACGCGC.** 1230 (95.51)   
109 + **.CGGCGCGG.** 1393 (92.31)   
109 + **[CGCGGCGG]** 1396 (85.22)   
109 - **[CGCCGCCG]** 1473 (85.71)   
109 - **[CGCCGCCC]** 1470 (85.13)   
109 - **[CGCCGCCG]** 1398 (85.71)   
109 - **.CGCCGCGC.** 1395 (92.48)   
109 - **.CGCCGCGC.** 1390 (92.48)   
109 - **.CGCCGCGC.** 1358 (92.48)   
109 - **.CGGCGCGT.** 1232 (96.22)   
109 - **[CGACGCCG]** 1089 (88.74)   
109 - **[CGCCGCCG]** 1086 (85.71)   
109 - **[CGCCGCCG]** 1083 (85.71)   
109 - **[CGCCGCCG]** 1080 (85.71)   
109 - **[CGCCGCCG]** 1077 (85.71)   
109 - **[CGCCGCCG]** 1074 (85.71)   
109 - **[GGACGCGG]** 1031 (88.08)   
109 - **[CCGCGCGT]** 815 (86.82)   
109 - **[CGAGGCGC]** 518 (87.66)   
109 - **[GGGCGCGT]** 454 (88.21)   
109 - **[GGCCGCGG]** 338 (85.06)   
110 - **[CGGCGCCT]** 928 (88.87)   
114 + **[CGCCGCCG]** 755 (85.71)   
114 + **.CGGCGCGG.** 1006 (92.31)   
114 - **.CGCCGCGG.** 1307 (93.07)   
114 - **.CGCCGCGC.** 1003 (92.48)   
114 - **[CGACCCGC]** 255 (85.67)   
115 + **.CGGCGCGC.** 280 (91.73)   
115 + **.CGGCGCGG.** 300 (92.31)   
115 + **.CGCCGCGG.** 309 (93.07)   
115 + **[CGCGGCGT]** 413 (89.13)   
115 + **.CGACGCCT.** 557 (92.65)   
115 + **.CGACGCGC.** 869 (95.51)   
115 + **[CGCCGCCT]** 1011 (89.62)   
115 + **[CGTCGCGG]** 1235 (86.02)   
115 - **[CGCCGCCG]** 992 (85.71)   
115 - **[CGCCGCCG]** 989 (85.71)   
115 - **[CGCCGCCG]** 986 (85.71)   
115 - **[CGTCGCGC]** 866 (85.44)   
115 - **.CGGCGCGG.** 688 (92.31)   
115 - **[CGAGGCGG]** 494 (88.25)   
115 - **.CGACGCGA.** 465 (91.25)   
115 - **[CGAGGCGC]** 460 (87.66)   
115 - **.CGCCGCGG.** 412 (93.07)   
116 + **.CGACGCGC.** 960 (95.51)   
116 + **[CGCCGGGT]** 1484 (85.88)   
116 - **[GGCCGCGG]** 1200 (85.06)   
116 - **[CGCCGCCG]** 998 (85.71)   
117 + **[CGAGGCGC]** 196 (87.66)   
117 + **[CGGCGCCT]** 455 (88.87)   
117 + **.CGGCGCGC.** 666 (91.73)   
117 + **[CGCCGCCG]** 698 (85.71)   
117 + **.CGCCGCGC.** 791 (92.48)   
117 - **[GGCCGCGG]** 1055 (85.06)   
117 - **[CGGCGCCT]** 198 (88.87)   
118 + **[CGAGGCGC]** 98 (87.66)   
118 + **[CGCCGCCG]** 676 (85.71)   
118 + **.CGCCGCGC.** 767 (92.48)   
118 - **[GGCCGCGG]** 1044 (85.06)   
118 - **[CGGCGCCT]** 100 (88.87)   
119 + **[CGAGGCGC]** 96 (87.66)   
119 + **[CGGCGCGA]** 612 (87.47)   
119 + **.CGGCGCGC.** 640 (91.73)   
119 + **[CGCCGCCG]** 674 (85.71)   
119 + **.CGCCGCGC.** 765 (92.48)   
119 - **[GGCCGCGG]** 1035 (85.06)   
119 - **[CGGCGCCT]** 98 (88.87)   
120 + **[CGAGGCGC]** 555 (87.66)   
120 + **[CGGCGCCT]** 861 (88.87)   
120 + **.CGGCGCGC.** 1100 (91.73)   
120 + **.CGCCGCGC.** 1225 (92.48)   
120 - **[GGCCGCGG]** 1502 (85.06)   
120 - **[CGGCGCCT]** 557 (88.87)   
122 + **.CGGCGCGC.** 901 (91.73)   
122 - **.CGGCGCGC.** 31 (91.73)   
123 + **.CGCCGCGG.** 14 (93.07)   
123 + **[GGGCGCGT]** 389 (88.21)   
123 + **[CGGCGCCT]** 497 (88.87)   
123 + **[CGACCCGC]** 514 (85.67)   
123 + **[CGCCGCGA]** 601 (88.22)   
123 + **[CGCCGCCC]** 638 (85.13)   
123 + **.CGGCGCGG.** 742 (92.31)   
123 + **.CGGCGCGC.** 766 (91.73)   
123 + **[CGCCGCCG]** 836 (85.71)   
123 + **.CGCCGCGC.** 1004 (92.48)   
123 + **.CGCCGCGC.** 1009 (92.48)   
123 + **[CGCCGCCC]** 1014 (85.13)   
123 + **[CGTCGCGC]** 1232 (85.44)   
123 + **[CGCCGCCG]** 1362 (85.71)   
123 + **[CGCCGGGT]** 1365 (85.88)   
123 + **[CGCCGCCT]** 1463 (89.62)   
123 - **[CGACCCGG]** 1367 (86.25)   
123 - **[CGACGCCG]** 1072 (88.74)   
123 - **.CGGCGCGG.** 1011 (92.31)   
123 - **.CGGCGCGG.** 1006 (92.31)   
123 - **.CGCCGCGG.** 951 (93.07)   
123 - **[CGCCGCCG]** 906 (85.71)   
123 - **.CGGCGCGC.** 768 (91.73)   
123 - **[CGCCGCCG]** 751 (85.71)   
123 - **[CGCCGCCC]** 748 (85.13)   
123 - **[CGCCGCCC]** 739 (85.13)   
123 - **[CGCGGCGG]** 600 (85.22)   
123 - **[CGCCGCCG]** 494 (85.71)   
123 - **[GGACGCGC]** 391 (87.50)   
124 + **.CGACGCGA.** 827 (91.25)   
124 + **.CGGCGCGG.** 867 (92.31)   
124 + **.CGGCGCGG.** 879 (92.31)   
124 + **.CGGCGCGC.** 1148 (91.73)   
124 + **[CGAGGCGG]** 1206 (88.25)   
124 + **[GGCCGCGG]** 1223 (85.06)   
124 + **[CGGGGCGT]** 1350 (88.38)   
124 - **[CGACGCCG]** 1098 (88.74)   
126 + **[CGCCGCCG]** 1037 (85.71)   
126 + **[CGCCGCCG]** 1040 (85.71)   
126 + **[CGCCGCCT]** 1043 (89.62)   
127 + **[CCACGCGG]** 466 (86.69)   
127 + **[CGAGGCGC]** 627 (87.66)   
127 + **[CGAGGCGC]** 741 (87.66)   
127 + **[CGGCCCGT]** 748 (86.38)   
127 + **.CGGCGCGC.** 775 (91.73)   
127 + **[GGACGCGC]** 1028 (87.50)   
127 + **[CGACCCGG]** 1149 (86.25)   
127 + **[CGGCCCGT]** 1272 (86.38)   
127 + **[GGCCGCGT]** 1508 (88.97)   
127 - **[AGACGCGG]** 1510 (85.28)   
127 - **[CGACGCCC]** 1339 (88.15)   
127 - **[CGCCGCCC]** 679 (85.13)   
128 + **[CGACCCGC]** 780 (85.67)   
128 + **[CGCCCCGT]** 811 (87.14)   
128 + **[CGGGGCGT]** 1104 (88.38)   
128 + **[CGCCGCTT]** 1279 (85.69)   
128 - **[CGACCCGG]** 827 (86.25)   
128 - **[CGACCCGC]** 428 (85.67)   
128 - **.CGCCGCGT.** 407 (96.98)   
128 - **[CGCCGCCT]** 370 (89.62)   
128 - **[CCCCGCGT]** 339 (87.58)   
129 + **[CGACCCGC]** 583 (85.67)   
129 + **[GGCCGCGG]** 869 (85.06)   
129 + **[CGAGGCGC]** 963 (87.66)   
129 + **[CGCCGCCG]** 975 (85.71)   
129 + **.CGCCGCGT.** 1015 (96.98)   
129 + **[GGACGCGG]** 1056 (88.08)   
129 + **.CGGCGCGG.** 1143 (92.31)   
129 + **.CGCCGCGC.** 1319 (92.48)   
129 + **[CGCCGCCC]** 1430 (85.13)   
129 - **[CGCCGCCT]** 1447 (89.62)   
129 - **.CGGCGCGG.** 1401 (92.31)   
129 - **[CGACCCGG]** 1391 (86.25)   
129 - **[GGCCGCGG]** 1385 (85.06)   
129 - **[CGCCGGGT]** 1363 (85.88)   
129 - **[CGCGGCGG]** 1318 (85.22)   
129 - **[CGCCGCCC]** 1221 (85.13)   
129 - **.CGGCGCGG.** 1201 (92.31)   
129 - **[CGCCGCGA]** 1140 (88.22)   
129 - **[CGCGGCGT]** 1014 (89.13)   
129 - **[CGCCGCTT]** 653 (85.69)   
131 + **[CGCGGCGG]** 1306 (85.22)   
131 + **[CGCGGCGG]** 1379 (85.22)   
131 + **[CGCGGCGG]** 1488 (85.22)   
131 - **[CGCCGCCG]** 1496 (85.71)   
131 - **[CGCCGCCG]** 1493 (85.71)   
131 - **[CGCCGCCG]** 1490 (85.71)   
131 - **.CGCCGCGG.** 1487 (93.07)   
131 - **.CGCCGCGG.** 1378 (93.07)   
131 - **[CGCCGCCG]** 1311 (85.71)   
131 - **[CGCCGCCG]** 1308 (85.71)   
131 - **.CGCCGCGT.** 1305 (96.98)   
131 - **[CGGCCCGT]** 1190 (86.38)   
131 - **[CGCCGCCG]** 901 (85.71)   
131 - **[CGCCGCCG]** 898 (85.71)   
131 - **[CCACGCGG]** 613 (86.69)   
131 - **[CGCCGCCC]** 554 (85.13)   
131 - **.CGGCGCGC.** 32 (91.73)   
132 + **[CGCCGCCG]** 706 (85.71)   
132 + **.CGCCGCGT.** 709 (96.98)   
132 + **[CGCCCCGT]** 945 (87.14)   
132 - **[CGACCCGC]** 1438 (85.67)   
132 - **.CGGCGCGG.** 1223 (92.31)   
132 - **[CGGGGCGT]** 759 (88.38)   
132 - **[CCACGCGG]** 711 (86.69)   
132 - **[CGCGGCGG]** 708 (85.22)   
132 - **[CGCCGCTT]** 364 (85.69)   
134 + **[CGGCGGGT]** 1061 (85.13)   
134 - **[CGCCGCCG]** 1465 (85.71)   
134 - **[CGCCGCCT]** 1078 (89.62)   
134 - **[CGCCGCCG]** 1058 (85.71)   
134 - **.CGACGCGC.** 1034 (95.51)   
135 + **[CCACGCGC]** 352 (86.11)   
135 + **[CGCGGCGT]** 365 (89.13)   
135 + **[CGCCGCCC]** 419 (85.13)   
135 + **[CGCCGCCC]** 691 (85.13)   
135 + **.CGCCGCGT.** 787 (96.98)   
135 + **[CGCCGCCG]** 1205 (85.71)   
135 + **[CGCCGCCG]** 1230 (85.71)   
135 + **.CGCCGCGC.** 1499 (92.48)   
135 - **[CGCGGCGG]** 1498 (85.22)   
135 - **[CGCCGCCG]** 1284 (85.71)   
135 - **[CGCCGCCG]** 1281 (85.71)   
135 - **[CGACCCGG]** 1253 (86.25)   
135 - **[CGCCGCCT]** 1111 (89.62)   
135 - **[CGAGGCGC]** 469 (87.66)   
135 - **.CGCCGCGG.** 364 (93.07)   
135 - **[CCGCGCGT]** 354 (86.82)   
136 + **.CGGCGCGG.** 935 (92.31)   
136 + **[CGCCGCCC]** 1023 (85.13)   
136 + **.CGGCGCGG.** 1038 (92.31)   
136 + **[CGCCGCCT]** 1067 (89.62)   
136 - **[GGCCGCGG]** 1195 (85.06)   
136 - **.CGGCGCGC.** 1064 (91.73)   
136 - **[CGACTCGT]** 899 (86.94)   
137 + **[CGGGGCGT]** 508 (88.38)   
137 + **[GGGCGCGT]** 609 (88.21)   
137 + **[GGACGCGG]** 751 (88.08)   
137 + **[GGACGCGG]** 942 (88.08)   
137 + **[CGCGGCGG]** 945 (85.22)   
137 + **[AGACGCGG]** 1013 (85.28)   
137 + **[GGGCGCGT]** 1164 (88.21)   
137 - **[GGCCGCGT]** 1015 (88.97)   
137 - **.CGCCGCGT.** 944 (96.98)   
138 + **[CGCCGCCG]** 177 (85.71)   
138 + **[CGCCGCCG]** 180 (85.71)   
138 + **[CGCCGCTT]** 183 (85.69)   
138 + **[CGCCGCCT]** 218 (89.62)   
138 + **[GGACGCGG]** 364 (88.08)   
138 + **.CGGCGCGG.** 369 (92.31)   
138 + **.CGGCGCGC.** 574 (91.73)   
138 + **.CGGCGCGG.** 640 (92.31)   
138 + **[CGCGGCGG]** 691 (85.22)   
138 + **.CGGCGCGG.** 765 (92.31)   
138 + **[CGCGGCGT]** 768 (89.13)   
138 + **[CGCCGCCG]** 825 (85.71)   
138 + **[CGCCGCCG]** 828 (85.71)   
138 + **[CGCCGCCG]** 831 (85.71)   
138 + **[CGCCGCCG]** 834 (85.71)   
138 + **.CGCCGCGC.** 837 (92.48)   
138 - **[CGCGGCGG]** 836 (85.22)   
138 - **.CGCCGCGC.** 767 (92.48)   
138 - **.CGCCGCGG.** 762 (93.07)   
138 - **[CGCCGCCG]** 693 (85.71)   
138 - **.CGCCGCGC.** 690 (92.48)   
138 - **[CGCCGCCC]** 607 (85.13)   
138 - **.CGACGCGC.** 600 (95.51)   
138 - **.CGCCGCGC.** 578 (92.48)   
138 - **.CGCCGCGT.** 366 (96.98)   
138 - **[CGGCGCGA]** 191 (87.47)   
139 + **[CGCCGCCT]** 206 (89.62)   
139 + **.CGGCGCGG.** 689 (92.31)   
139 + **[CGCCGCCG]** 834 (85.71)   
139 + **[CGCCGCCC]** 837 (85.13)   
139 + **[CCCCGCGT]** 844 (87.58)   
139 - **.CGCCGCGC.** 959 (92.48)   
139 - **[GGACGCGG]** 846 (88.08)   
139 - **.CGGCGCGC.** 831 (91.73)   
139 - **[CGCCGCCG]** 712 (85.71)   
139 - **[CGCCGCCC]** 686 (85.13)   
140 + **[CGACCCGG]** 637 (86.25)   
140 + **.CGCCGCGG.** 656 (93.07)   
140 + **[CGCCGCCG]** 735 (85.71)   
140 + **[CGCCGCCG]** 1086 (85.71)   
140 + **[CGCCGCCG]** 1089 (85.71)   
140 + **[CGCCGCCG]** 1092 (85.71)   
140 + **[CGCCGCCG]** 1095 (85.71)   
140 + **[CGCCGCCT]** 1098 (89.62)   
140 + **[CGCCGCCT]** 1149 (89.62)   
140 + **.CGCCGCGC.** 1192 (92.48)   
140 + **[CGACGCCG]** 1366 (88.74)   
140 + **[CGCCGCCC]** 1369 (85.13)   
140 - **[CGCCGCCG]** 1488 (85.71)   
140 - **[CGCCGCCG]** 1465 (85.71)   
140 - **[CGCCGCCG]** 1462 (85.71)   
140 - **.CGCCGCGG.** 1247 (93.07)   
140 - **.CGGCGCGG.** 1194 (92.31)   
140 - **[CGCGGCGG]** 1191 (85.22)   
140 - **[GGACGCGG]** 983 (88.08)   
140 - **[CGCGGCGG]** 655 (85.22)   
140 - **[CGCCGGGT]** 639 (85.88)   
141 + **.CGCCGCGC.** 609 (92.48)   
141 + **[CGAGGCGC]** 753 (87.66)   
141 + **.CGGCGCGC.** 946 (91.73)   
141 + **[GGCCGCGG]** 1155 (85.06)   
141 + **.CGCCGCGG.** 1187 (93.07)   
141 + **[GGACGCGG]** 1227 (88.08)   
141 + **[CGCCGCCC]** 1241 (85.13)   
141 - **.CGGCGCGG.** 1184 (92.31)   
141 - **.CGCCGCGG.** 1157 (93.07)   
142 + **.CGACGCGA.** 446 (91.25)   
142 + **[CCACGCGC]** 454 (86.11)   
142 + **.CGCCGCGG.** 515 (93.07)   
142 + **[CGAGGCGG]** 587 (88.25)   
142 + **[CGCGGCGG]** 663 (85.22)   
142 + **[GGCCGCGG]** 671 (85.06)   
142 + **.CGCCGCGC.** 893 (92.48)   
142 + **.CGCCGCGC.** 1005 (92.48)   
142 + **[CGCCGCCG]** 1024 (85.71)   
142 + **[CGCCGCCC]** 1027 (85.13)   
142 + **[GGCCGCGG]** 1060 (85.06)   
142 + **[CGCCGCCT]** 1316 (89.62)   
142 + **[CGAGGCGC]** 1369 (87.66)   
142 + **[CGACCCGG]** 1439 (86.25)   
142 + **[GGACGCGG]** 1477 (88.08)   
142 - **[CGAGGCGG]** 1318 (88.25)   
142 - **.CGGCGCGG.** 1273 (92.31)   
142 - **[CGACCCGG]** 1097 (86.25)   
142 - **[CGGCGCGA]** 957 (87.47)   
142 - **[GGCCGCGG]** 945 (85.06)   
142 - **.CGGCGCGC.** 901 (91.73)   
142 - **.CGCCGCGC.** 662 (92.48)   
142 - **.CGGCGCGG.** 512 (92.31)   
142 - **[GGGCGCGT]** 456 (88.21)   
143 + **[AGGCGCGT]** 526 (85.41)   
144 + **[CGCCGCCC]** 253 (85.13)   
144 + **[CGCCGCCC]** 444 (85.13)   
144 + **[CCGCGCGT]** 771 (86.82)   
144 + **.CGCCGCGC.** 1017 (92.48)   
144 + **.CGGCGCGC.** 1032 (91.73)   
144 + **.CGGCGCGG.** 1139 (92.31)   
144 + **.CGGCGCGG.** 1144 (92.31)   
144 + **.CGGCGCGG.** 1149 (92.31)   
144 + **[GGACGCGG]** 1186 (88.08)   
144 + **.CGGCGCGG.** 1191 (92.31)   
144 + **[GGCCGCGG]** 1197 (85.06)   
144 + **[CGGCCCGT]** 1285 (86.38)   
144 + **.CGCCGCGC.** 1490 (92.48)   
144 - **.CGCCGCGT.** 1188 (96.98)   
144 - **.CGCCGCGC.** 1146 (92.48)   
144 - **.CGCCGCGC.** 1141 (92.48)   
144 - **[CGCCGCCT]** 1136 (89.62)   
144 - **[CGCCGCCC]** 1096 (85.13)   
144 - **[CGCCGCCC]** 1089 (85.13)   
144 - **[CGCCGCCG]** 1061 (85.71)   
144 - **[CGCCGCCC]** 1045 (85.13)   
144 - **[CGCCGCCG]** 1029 (85.71)   
144 - **[CGCCGCCC]** 1026 (85.13)   
144 - **[CGCCGCCG]** 1011 (85.71)   
144 - **[GGACGCGC]** 838 (87.50)   
144 - **[GGACGCGC]** 773 (87.50)   
145 + **.CGCCGCGC.** 1136 (92.48)   
145 + **[CGCGGCGG]** 1141 (85.22)   
145 + **[CGCCGCCC]** 1152 (85.13)   
145 + **.CGAGGCGT.** 1405 (92.16)   
145 - **[CGTCGCGG]** 1252 (86.02)   
145 - **[CGTCGCGG]** 1216 (86.02)   
145 - **.CGGCGCGC.** 1149 (91.73)   
145 - **.CGCCGCGC.** 1140 (92.48)   
145 - **[CGCGGCGG]** 1135 (85.22)   
145 - **[CGACGAGT]** 1003 (86.53)   
145 - **[CGCCGGGT]** 816 (85.88)   
145 - **[CGCCGCCC]** 676 (85.13)   
145 - **[CGCCGCCT]** 574 (89.62)   
145 - **[CGCCGCCC]** 444 (85.13)   
147 + **.CGACGCGT.** 861 (100.00)   
147 + **[CGTCGCGG]** 962 (86.02)   
147 + **[CGAGGCGG]** 1077 (88.25)   
147 + **.CGCCGCGC.** 1162 (92.48)   
147 + **[CGGGGCGT]** 1401 (88.38)   
147 - **[CGACGCCG]** 1337 (88.74)   
147 - **[CGCCGCCG]** 1334 (85.71)   
147 - **[CGCCGCCT]** 1079 (89.62)   
147 - **[CGCCGCCT]** 1038 (89.62)   
147 - **.GGACGCGT.** 863 (91.99)   
148 + **[CGGCGCCT]** 218 (88.87)   
148 + **[CGGCGCCT]** 627 (88.87)   
148 + **.CGCCGCGC.** 827 (92.48)   
148 + **[CCGCGCGT]** 829 (86.82)   
148 - **[CGCGGCGT]** 826 (89.13)   
150 + **.CGCCGCGC.** 120 (92.48)   
150 + **[CGCGGCGT]** 191 (89.13)   
150 + **[AGACGCGG]** 464 (85.28)   
150 + **[AGACGCGT]** 955 (89.19)   
150 + **[CGCCGGGT]** 1023 (85.88)   
150 - **[CGCCGCTT]** 987 (85.69)   
150 - **[CGTCGCGT]** 932 (89.93)   
150 - **[CGCCGCGA]** 190 (88.22)   
150 - **.CGGCGCGC.** 117 (91.73)   
151 + **[CGCCGCTT]** 17 (85.69)   
151 + **[CGTCGCGT]** 71 (89.93)   
151 + **[CGCCGCGA]** 814 (88.22)   
151 + **[CGGCGCCT]** 842 (88.87)   
151 + **[CGACCCGG]** 1065 (86.25)   
151 + **[CCACGCGG]** 1420 (86.69)   
151 + **.CGGCGCGT.** 1460 (96.22)   
151 + **.CGGCGCGC.** 1491 (91.73)   
151 - **[GGACGCGG]** 1472 (88.08)   
151 - **[CCACGCGC]** 1462 (86.11)   
151 - **[CCCCGCGT]** 1422 (87.58)   
151 - **.CGGCGCGG.** 1205 (92.31)   
151 - **.GGACGCGT.** 1150 (91.99)   
151 - **.CGCCGCGC.** 883 (92.48)   
151 - **[CGCGGCGT]** 813 (89.13)   
151 - **[AGACGCGG]** 539 (85.28)   
151 - **[AGACGCGT]** 48 (89.19)   
154 + **[GGGCGCGT]** 932 (88.21)   
154 + **[AGGCGCGT]** 1008 (85.41)   
154 + **[CGCGGCGG]** 1040 (85.22)   
154 + **[CCGCGCGT]** 1080 (86.82)   
154 + **.CGGCGCGC.** 1095 (91.73)   
154 + **[CGCCGCCC]** 1142 (85.13)   
154 + **[CGCCGCGA]** 1178 (88.22)   
154 + **[CGCCGCCG]** 1218 (85.71)   
154 + **[CGCCGCCG]** 1249 (85.71)   
154 + **[CGCCGCCC]** 1252 (85.13)   
154 + **[CGACGAGT]** 1333 (86.53)   
154 + **[CGCCGCCG]** 1434 (85.71)   
154 + **[CGCCGCCG]** 1468 (85.71)   
154 - **.CGGCGCGC.** 1465 (91.73)   
154 - **.CGGCGCGG.** 1431 (92.31)   
154 - **[CGCCGCCG]** 1045 (85.71)   
154 - **[CGCCGCCG]** 1042 (85.71)   
154 - **.CGCCGCGC.** 1039 (92.48)   
154 - **[CGCCGCCC]** 1021 (85.13)   
154 - **[CGACCCGG]** 97 (86.25)   
155 + **.CGGCGCGG.** 506 (92.31)   
155 + **[CGTCGCGT]** 1256 (89.93)   
155 - **[CGACGCCC]** 1253 (88.15)   
157 + **[CGCCGCCG]** 1113 (85.71)   
157 - **[CCACGCGG]** 1237 (86.69)   
158 + **[AGGCGCGT]** 374 (85.41)   
158 + **[GGCCGCGT]** 1176 (88.97)   
158 + **.CGGCGCGC.** 1511 (91.73)   
158 - **.CGACGCGG.** 1178 (96.09)   
158 - **.CGACGCGC.** 1122 (95.51)   
160 + **[AGCCGCGT]** 132 (86.16)   
160 + **.CGCCGCGG.** 265 (93.07)   
160 + **[CGACGCCC]** 1268 (88.15)   
160 + **[CGCCGCCT]** 1306 (89.62)   
160 - **.CGGCGCGG.** 1205 (92.31)   
160 - **[CGCCGCCT]** 990 (89.62)   
160 - **[CGAGGCGC]** 276 (87.66)   
160 - **[CGCGGCGG]** 264 (85.22)   
160 - **[CCACGCGG]** 134 (86.69)   
161 + **.CGGCGCGG.** 153 (92.31)   
161 + **[CCACGCGC]** 315 (86.11)   
161 + **[CGGCGCGA]** 751 (87.47)   
161 + **[GGCCGCGG]** 883 (85.06)   
161 + **.CGCCGCGC.** 893 (92.48)   
161 + **[CGCCGCCC]** 1017 (85.13)   
161 + **[CGCGGCGG]** 1268 (85.22)   
161 + **[CGCCGCCG]** 1422 (85.71)   
161 + **[CGCCGCCG]** 1483 (85.71)   
161 + **[CGCCGCCG]** 1486 (85.71)   
161 + **[CGCCGCCG]** 1489 (85.71)   
161 + **[CGCCGCCG]** 1501 (85.71)   
161 + **[CGCCGCCG]** 1504 (85.71)   
161 + **[CGCCGCCG]** 1507 (85.71)   
161 - **.CGGCGCGG.** 1480 (92.31)   
161 - **[CGCCGCCG]** 1270 (85.71)   
161 - **.CGCCGCGG.** 1267 (93.07)   
161 - **[CGCCGCCG]** 986 (85.71)   
161 - **[CGCCGCCG]** 983 (85.71)   
161 - **[CGCGGCGG]** 892 (85.22)   
161 - **[CGCCGCCC]** 828 (85.13)   
161 - **[CGCCGCTT]** 150 (85.69)   
164 + **[CGTCGCGC]** 272 (85.44)   
164 + **.CGGCGCGG.** 1096 (92.31)   
164 + **[CGCCGCCC]** 1104 (85.13)   
164 - **[GGCCGCGT]** 1224 (88.97)   
164 - **.CGCCGCGC.** 1098 (92.48)   
164 - **[CGAGGCGC]** 1044 (87.66)   
164 - **[CGAAGCGT]** 516 (88.01)   
166 + **[CGACGGGT]** 518 (88.90)   
166 + **[GGCCGCGG]** 545 (85.06)   
166 + **.CGAGGCGT.** 958 (92.16)   
166 + **[CGCCGCCT]** 1028 (89.62)   
166 + **[CGCCGCCT]** 1119 (89.62)   
166 + **.CGGCGCGC.** 1224 (91.73)   
166 - **.CGCCGCGT.** 1311 (96.98)   
166 - **.CGCCGCGT.** 1158 (96.98)   
166 - **.CGACGCCT.** 960 (92.65)   
166 - **[CGAGGCGG]** 925 (88.25)   
167 + **[AGACGCGG]** 769 (85.28)   
167 + **[CGAGGCGG]** 792 (88.25)   
167 + **[CGGCGCGA]** 817 (87.47)   
167 + **[CGCGGCGG]** 843 (85.22)   
167 + **[CGCCGCTT]** 1383 (85.69)   
167 + **[CGCCGCCG]** 1395 (85.71)   
167 + **[CGCCGCCC]** 1398 (85.13)   
167 - **[GGACGCGG]** 1462 (88.08)   
167 - **[GGCCGCGG]** 962 (85.06)   
167 - **.CGCCGCGG.** 842 (93.07)   
167 - **[CGCCGCCC]** 814 (85.13)   
167 - **[CGGGGCGT]** 288 (88.38)   
168 + **.CGCCGCGC.** 908 (92.48)   
168 + **.CGGCGCGG.** 1182 (92.31)   
168 - **[GGCCGCGG]** 1204 (85.06)   
168 - **[CGCCGCCC]** 940 (85.13)   
168 - **[CGCCGCCT]** 918 (89.62)   
169 + **.CGCCGCGC.** 110 (92.48)   
169 + **[GGACGCGG]** 1044 (88.08)   
169 - **.CGACGCCT.** 381 (92.65)   
169 - **[CGCGGCGT]** 109 (89.13)   
170 + **[CGGCGGGT]** 1472 (85.13)   
170 - **[CGCCGCCG]** 1469 (85.71)   
170 - **[CAACGCGT]** 358 (86.87)   
171 + **[CCACGCGG]** 771 (86.69)   
171 + **[CGCGGCGT]** 774 (89.13)   
171 - **.CGCCGCGT.** 773 (96.98)   
172 + **[CCCCGCGT]** 131 (87.58)   
172 + **[CGCCGCCG]** 228 (85.71)   
172 + **[CGCCGCCG]** 305 (85.71)   
172 + **[CGCCGCCC]** 308 (85.13)   
172 + **[CGGCCCGT]** 361 (86.38)   
172 + **[CGCCGCCT]** 370 (89.62)   
172 + **[GGACGCGG]** 428 (88.08)   
172 + **[CGCGGCGG]** 431 (85.22)   
172 + **[CGCCGCCG]** 521 (85.71)   
172 + **[CGCCGCCG]** 524 (85.71)   
172 + **[CGCCGCCG]** 527 (85.71)   
172 + **[CGCCGCCG]** 530 (85.71)   
172 + **[CGCCGCCG]** 533 (85.71)   
172 + **[CGCCGCCG]** 536 (85.71)   
172 + **[CGCCGCCG]** 539 (85.71)   
172 + **[CGTCGCGC]** 548 (85.44)   
172 + **[CGACGCCG]** 718 (88.74)   
172 + **[CGCCGCCC]** 721 (85.13)   
172 + **[GGCCGCGG]** 754 (85.06)   
172 + **.CGGCGCGC.** 919 (91.73)   
172 + **.CGGCGCGG.** 1159 (92.31)   
172 - **[CGCCGCCT]** 1461 (89.62)   
172 - **.CGGCGCGC.** 1418 (91.73)   
172 - **.CGGCGCGC.** 923 (91.73)   
172 - **[CGCCGCCG]** 916 (85.71)   
172 - **[CGAGGCGG]** 828 (88.25)   
172 - **[CGGCCCGT]** 751 (86.38)   
172 - **[CGTCGCGG]** 715 (86.02)   
172 - **.CGCCGCGT.** 430 (96.98)   
172 - **.CGAGGCGT.** 299 (92.16)   
173 + **[CGCCCCGT]** 864 (87.14)   
173 + **[CGACCCGG]** 894 (86.25)   
173 + **[CGACGCCC]** 1190 (88.15)   
173 + **[CCGCGCGT]** 1287 (86.82)   
173 - **[CGCCGCCG]** 1027 (85.71)   
173 - **[CGCCGCCG]** 1024 (85.71)   
173 - **[CGCCGCCT]** 1021 (89.62)   
173 - **[CGAGGCGC]** 946 (87.66)   
173 - **[AGGCGCGT]** 944 (85.41)   
173 - **[CGCCGGGT]** 896 (85.88)   
174 + **[CGCCCCGT]** 802 (87.14)   
174 + **[AGACGCGG]** 935 (85.28)   
174 + **.CGCCGCGT.** 1184 (96.98)   
174 + **[CGCCGCCG]** 1279 (85.71)   
174 + **[AGACGCGG]** 1379 (85.28)   
174 + **.CGGCGCGG.** 1479 (92.31)   
174 + **[CGAGGCGC]** 1502 (87.66)   
174 - **.CGCCGCGG.** 1476 (93.07)   
174 - **[GGACGCGG]** 1186 (88.08)   
174 - **[CGCCGCCG]** 1063 (85.71)   
175 + **.GGACGCGT.** 858 (91.99)   
175 + **[GGGCGCGT]** 926 (88.21)   
175 + **.CGCCGCGG.** 1465 (93.07)   
175 + **[CCGCGCGT]** 1497 (86.82)   
177 + **[CGAGGCGG]** 747 (88.25)   
177 + **[CGCCGCGA]** 951 (88.22)   
177 + **[CGCCGCCG]** 1020 (85.71)   
177 + **[CGCCGCCG]** 1023 (85.71)   
177 + **[CGCCGCCG]** 1026 (85.71)   
177 + **[CGCCGCCT]** 1029 (89.62)   
177 - **.CGCCGCGG.** 1464 (93.07)   
177 - **[CGAGGCGG]** 1031 (88.25)   
177 - **[CGAGGCGC]** 820 (87.66)   
177 - **[CGCCGCCG]** 752 (85.71)   
177 - **[CGCCGCCT]** 749 (89.62)   
178 + **[CGCCGCCG]** 754 (85.71)   
178 + **[GGACGCGG]** 1111 (88.08)   
178 + **[CGCGGCGG]** 1114 (85.22)   
178 - **.CGCCGCGT.** 1113 (96.98)   
179 + **.CGCCGCGG.** 915 (93.07)   
179 + **[CGGCGGGT]** 949 (85.13)   
179 + **.CGGCGCGG.** 985 (92.31)   
179 + **[GGACGCGC]** 993 (87.50)   
179 + **.CGCCGCGC.** 1473 (92.48)   
179 - **[CGCGGCGG]** 1472 (85.22)   
179 - **[CGCCGCCG]** 982 (85.71)   
179 - **[CGCCGCCG]** 979 (85.71)   
179 - **[CGCCGCCG]** 976 (85.71)   
179 - **[CGCCGCCC]** 973 (85.13)   
179 - **[CGCCGCCG]** 946 (85.71)   
179 - **[CGCCGCCG]** 943 (85.71)   
179 - **[CGCCGCCC]** 940 (85.13)   
179 - **[GGCCGCGG]** 917 (85.06)   
179 - **.CGGCGCGG.** 857 (92.31)   
180 + **[CGGGGCGT]** 614 (88.38)   
180 + **[CGCGGCGG]** 1065 (85.22)   
180 + **[CGCGGCGG]** 1247 (85.22)   
180 + **[CGGCGGGT]** 1252 (85.13)   
180 + **[CGCCGCCG]** 1508 (85.71)   
180 - **[CGCCGGGT]** 1270 (85.88)   
180 - **[CGCCGCCG]** 1249 (85.71)   
180 - **[CGCCGCGA]** 1246 (88.22)   
180 - **[GGCCGCGG]** 1158 (85.06)   
180 - **.CGCCGCGC.** 1064 (92.48)   
180 - **[CGCCGCCG]** 1043 (85.71)   
182 + **.CGGCGCGG.** 120 (92.31)   
182 + **[CGCCGCCC]** 290 (85.13)   
182 + **[CCACGCGG]** 366 (86.69)   
182 + **[GGCCGCGG]** 374 (85.06)   
182 + **[GGCCGCGG]** 460 (85.06)   
182 + **.CGGCGCGG.** 465 (92.31)   
182 + **.CGGCGCGT.** 873 (96.22)   
182 + **[CGGGGCGT]** 902 (88.38)   
182 + **[CGGCCCGT]** 941 (86.38)   
182 - **[CGGCGCGA]** 1162 (87.47)   
182 - **[GGCCGCGG]** 1077 (85.06)   
182 - **[GGACGCGG]** 1020 (88.08)   
182 - **.CGACGCGC.** 875 (95.51)   
182 - **[CGAGGCGG]** 684 (88.25)   
182 - **[CGACCCGC]** 534 (85.67)   
182 - **.CGCCGCGG.** 462 (93.07)   
182 - **[CGCCGCCC]** 394 (85.13)   
182 - **[GGCCGCGG]** 376 (85.06)   
182 - **[CCCCGCGT]** 368 (87.58)   
183 + **[CGACCCGC]** 713 (85.67)   
183 + **[CGCCGCCT]** 879 (89.62)   
183 + **.CGGCGCGC.** 982 (91.73)   
183 + **[CGCGGCGG]** 1061 (85.22)   
183 + **[CGCCGCCG]** 1180 (85.71)   
183 + **[CGCCGCCG]** 1183 (85.71)   
183 + **[CGCCGCCG]** 1186 (85.71)   
183 + **[GGCCGCGT]** 1298 (88.97)   
183 + **[CGCCGCCG]** 1408 (85.71)   
183 + **[CGCCGCCG]** 1411 (85.71)   
183 + **[CGCCGCCC]** 1426 (85.13)   
183 - **.CGGCGCGG.** 1423 (92.31)   
183 - **.CGCCGCGC.** 1355 (92.48)   
183 - **[GGACGCGG]** 1300 (88.08)   
183 - **[CGGCGGGT]** 1221 (85.13)   
183 - **.CGCCGCGC.** 1060 (92.48)   
183 - **[CGCCGCCT]** 1023 (89.62)   
183 - **[CGCCGCCG]** 979 (85.71)   
183 - **[CGCCGCCT]** 976 (89.62)   
184 + **.CGCCGCGG.** 53 (93.07)   
184 + **[CGCCCCGT]** 772 (87.14)   
184 + **[CGCCGCCT]** 983 (89.62)   
184 + **.CGCCGCGT.** 1089 (96.98)   
184 + **.CGCCGCGC.** 1164 (92.48)   
184 + **[CGGCCCGT]** 1322 (86.38)   
184 + **[CGCGGCGG]** 1352 (85.22)   
184 - **.CGCCGCGC.** 1351 (92.48)   
184 - **.CGGCGCGC.** 1086 (91.73)   
184 - **[GGCCGCGG]** 1067 (85.06)   
184 - **.CGCCGCGC.** 927 (92.48)   
184 - **.CGACGCCT.** 668 (92.65)   
184 - **[GGCCGCGG]** 632 (85.06)   
184 - **[CGCGGCGG]** 52 (85.22)   
185 + **[CGACGCCG]** 220 (88.74)   
185 + **[GGCCGCGG]** 554 (85.06)   
185 + **[CGCCGCCG]** 894 (85.71)   
185 + **[CGCCGCCG]** 897 (85.71)   
185 + **[CGCCGCCG]** 900 (85.71)   
185 + **[CGCCGCCG]** 903 (85.71)   
185 + **[CGCCGCCG]** 906 (85.71)   
185 + **[CGCCGCCG]** 909 (85.71)   
185 + **[CGCCGCCG]** 912 (85.71)   
185 + **[CGCCGCCG]** 915 (85.71)   
185 + **[GGCCGCGG]** 1062 (85.06)   
185 + **[GGCCGCGG]** 1089 (85.06)   
185 + **[CGCCGCCG]** 1146 (85.71)   
185 + **[CGCCGCCC]** 1149 (85.13)   
185 + **.CGCCGCGG.** 1189 (93.07)   
185 + **[CGCGGCGG]** 1192 (85.22)   
185 - **[CGCCGCCG]** 1194 (85.71)   
185 - **.CGCCGCGG.** 1191 (93.07)   
185 - **[CGCGGCGG]** 1188 (85.22)   
185 - **.CGGCGCGG.** 1033 (92.31)   
186 + **[CGCCGCCG]** 1014 (85.71)   
186 + **[CGGCGCCT]** 1330 (88.87)   
186 + **[CGGCGCCT]** 1403 (88.87)   
186 - **.CGACGCGC.** 1081 (95.51)   
186 - **.CGGCGCGG.** 1011 (92.31)   
186 - **[AGACGCGT]** 904 (89.19)   
187 + **[CGGCCCGT]** 152 (86.38)   
187 + **[CGCCGCCG]** 1088 (85.71)   
187 + **.CGGCGCGG.** 1175 (92.31)   
187 - **[CGACCCGG]** 1070 (86.25)   
187 - **[GGACGCGC]** 924 (87.50)   
187 - **[CGCCGCCC]** 802 (85.13)   
187 - **[CGGCGCCT]** 610 (88.87)   
188 + **[CGCCGCCC]** 307 (85.13)   
188 + **[GGCCGCGG]** 413 (85.06)   
188 + **[CGCCGCCG]** 1390 (85.71)   
188 + **[CGCCGCCG]** 1393 (85.71)   
188 + **.CGCCGCGG.** 1442 (93.07)   
188 + **.CGCCGCGC.** 1469 (92.48)   
188 + **[CGCCGCCC]** 1474 (85.13)   
188 - **.CGGCGCGG.** 1471 (92.31)   
188 - **.CGGCGCGC.** 1439 (91.73)   
188 - **[CGCCGCTT]** 500 (85.69)   
188 - **[CGGGGCGT]** 301 (88.38)   
189 + **.CGGCGCGT.** 848 (96.22)   
189 + **[CGCCGGGT]** 981 (85.88)   
191 + **[CGACGCCG]** 333 (88.74)   
191 + **.CCACGCGT.** 649 (90.60)   
191 + **[GGCCGCGG]** 830 (85.06)   
191 + **.CGGCGCGG.** 1042 (92.31)   
191 + **[CGGCGCGA]** 1136 (87.47)   
191 - **[CCGCGCGT]** 1478 (86.82)   
191 - **.CGCCGCGC.** 1044 (92.48)   
191 - **[CGCCGCCT]** 1026 (89.62)   
191 - **[CGCCGCCG]** 1011 (85.71)   
191 - **[CGCCGCCG]** 1008 (85.71)   
191 - **[CGCCGCCG]** 1005 (85.71)   
191 - **[CGCCGCCC]** 1002 (85.13)   
191 - **[GGCCGCGG]** 568 (85.06)   
193 - **.CGCCGCGC.** 1248 (92.48)   
194 + **[CGGGGCGT]** 179 (88.38)   
194 + **[CGCCGCCC]** 740 (85.13)   
194 + **[GGACGCGC]** 907 (87.50)   
194 + **[CGCGGCGG]** 912 (85.22)   
194 + **.CGGCGCGG.** 942 (92.31)   
194 + **[CGCGGCGT]** 945 (89.13)   
194 + **[GGCCGCGG]** 987 (85.06)   
194 + **.CGGCGCGC.** 1015 (91.73)   
194 + **.CGCCGCGG.** 1039 (93.07)   
194 + **[GGCCGCGG]** 1109 (85.06)   
194 + **.CGGCGCGG.** 1114 (92.31)   
194 - **.CGCCGCGG.** 1111 (93.07)   
194 - **[CGCGGCGG]** 1038 (85.22)   
194 - **.CGCCGCGG.** 989 (93.07)   
194 - **.CGCCGCGC.** 944 (92.48)   
194 - **.CGCCGCGC.** 911 (92.48)   
194 - **[CCGCGCGT]** 909 (86.82)   
194 - **[CGCCGCCC]** 23 (85.13)   
195 + **.CGCCGCGG.** 165 (93.07)   
195 + **[CGAGGCGG]** 308 (88.25)   
195 + **[CGGGGCGT]** 443 (88.38)   
195 + **[CCGCGCGT]** 957 (86.82)   
195 - **[CGACCCGG]** 1174 (86.25)   
195 - **[GGACGCGC]** 959 (87.50)   
195 - **[CGCCGCGA]** 851 (88.22)   
195 - **[CGGGGCGT]** 829 (88.38)   
195 - **[CGACCCGC]** 460 (85.67)   
195 - **[CGACGCCC]** 445 (88.15)   
195 - **[CGCGGCGG]** 164 (85.22)   
195 - **.CGGCGCGG.** 152 (92.31)   
196 + **.GGACGCGT.** 30 (91.99)   
196 + **[CGCGGCGG]** 120 (85.22)   
196 + **[CGCGGCGT]** 284 (89.13)   
196 + **.CGCCGCGC.** 341 (92.48)   
196 + **[CCACGCGG]** 496 (86.69)   
196 + **[GGACGCGG]** 687 (88.08)   
196 + **.CGCCGCGC.** 910 (92.48)   
196 + **[CGCCGCCG]** 1022 (85.71)   
196 + **[GGCCGCGT]** 1101 (88.97)   
196 + **.CGGCGCGC.** 1351 (91.73)   
196 + **.CGGCGCGC.** 1426 (91.73)   
196 + **.CGGCGCGG.** 1470 (92.31)   
196 - **[CGCCGGGT]** 1493 (85.88)   
196 - **[CGGCGCCT]** 1327 (88.87)   
196 - **.CGGCGCGC.** 1316 (91.73)   
196 - **[CGGCGCCT]** 1019 (88.87)   
196 - **.CGACGCGT.** 720 (100.00)   
196 - **[CGACGCCC]** 638 (88.15)   
196 - **[CGGCCCGT]** 619 (86.38)   
196 - **.CGGCGCGC.** 505 (91.73)   
196 - **[CCCCGCGT]** 498 (87.58)   
196 - **[CGACGCCG]** 286 (88.74)   
196 - **.CGCCGCGC.** 283 (92.48)   
196 - **[CGCCGCCC]** 267 (85.13)   
196 - **.CGCCGCGC.** 119 (92.48)   
196 - **[CTACGCGT]** 32 (86.64)   
197 + **[CGCCGCCT]** 210 (89.62)   
197 + **[CGAGGCGG]** 1002 (88.25)   
197 + **[AGACGCGG]** 1099 (85.28)   
197 + **[CGACCCGG]** 1151 (86.25)   
197 + **[GGACGCGG]** 1160 (88.08)   
197 + **[CGCGGCGT]** 1163 (89.13)   
197 + **.CGGCGCGG.** 1508 (92.31)   
197 - **.CGCCGCGC.** 1505 (92.48)   
197 - **.CGCCGCGT.** 1162 (96.98)   
197 - **[CGCCGGGT]** 1153 (85.88)   
197 - **[CGACCCGC]** 1113 (85.67)   
197 - **[CGCCGCCC]** 871 (85.13)   
197 - **[CGACGAGT]** 521 (86.53)   
197 - **.CGACGCGA.** 430 (91.25)   
198 + **[CCGCGCGT]** 213 (86.82)   
198 + **[CGCCGCCG]** 253 (85.71)   
198 + **.CGCCGCGC.** 256 (92.48)   
198 + **[CGAGGCGG]** 1234 (88.25)   
198 - **[CGACCCGG]** 992 (86.25)   
198 - **[CGCGGCGG]** 255 (85.22)   
198 - **[CGGCGGGT]** 250 (85.13)   
199 + **[CCACGCGG]** 310 (86.69)   
199 + **[AGCCGCGT]** 437 (86.16)   
199 + **.CGGCGCGC.** 669 (91.73)   
199 + **[GGGCGCGT]** 818 (88.21)   
199 + **[CGTCGCGT]** 823 (89.93)   
199 + **.CGGCGCGC.** 869 (91.73)   
199 + **.CGGCGCGG.** 910 (92.31)   
199 + **[CGGCGCCT]** 953 (88.87)   
199 + **.CGCCGCGG.** 1000 (93.07)   
199 + **.CGGCGCGG.** 1082 (92.31)   
199 + **[CGCGGCGG]** 1085 (85.22)   
199 + **[GGCCGCGG]** 1126 (85.06)   
199 + **[CCACGCGC]** 1241 (86.11)   
199 + **.CGGCGCGG.** 1351 (92.31)   
199 - **[GGACGCGG]** 1447 (88.08)   
199 - **[CGCCGCCC]** 1348 (85.13)   
199 - **[GGCCGCGG]** 1275 (85.06)   
199 - **[GGGCGCGT]** 1243 (88.21)   
199 - **[CGCCGCCT]** 1140 (89.62)   
199 - **.CGCCGCGC.** 1084 (92.48)   
199 - **[GGCCGCGG]** 1002 (85.06)   
199 - **.CGCCGCGC.** 950 (92.48)   
199 - **[CGCCGCCC]** 916 (85.13)   
199 - **.CGCCGCGC.** 907 (92.48)   
199 - **[CGACCCGG]** 892 (86.25)   
199 - **[CGCCGCCG]** 866 (85.71)   
199 - **[CGCCGCCG]** 863 (85.71)   
199 - **.CGACGCGC.** 820 (95.51)   
199 - **[CGAGGCGG]** 455 (88.25)   
200 + **[GGACGCGG]** 571 (88.08)   
200 - **[CGGCGGGT]** 1105 (85.13)   
201 + **.CGCCGCGC.** 336 (92.48)   
201 + **.CGGCGCGG.** 775 (92.31)   
201 + **[CGAGGCGG]** 797 (88.25)   
201 + **.CGCCGCGG.** 1348 (93.07)   
201 + **[CGCCGCCC]** 1382 (85.13)   
201 + **[GGACGCGC]** 1490 (87.50)   
201 - **[CGCGGCGT]** 1347 (89.13)   
201 - **[GGACGCGC]** 1200 (87.50)   
201 - **.CGGCGCGG.** 1084 (92.31)   
201 - **.CGCCGCGG.** 848 (93.07)   
201 - **[CGCCGCCG]** 772 (85.71)   
201 - **[CGCCGCCT]** 769 (89.62)   
201 - **[CGCCGCCC]** 594 (85.13)   
201 - **[CGGGGCGT]** 514 (88.38)   
201 - **.CGACGCCT.** 489 (92.65)   
201 - **[CGCGGCGG]** 335 (85.22)   
202 + **.CGAGGCGT.** 292 (92.16)   
202 + **[GGACGCGG]** 309 (88.08)   
202 + **[CGGCGCCT]** 373 (88.87)   
202 + **[CGCCGCTT]** 835 (85.69)   
202 + **[CGCCGCCG]** 1109 (85.71)   
202 + **[GGACGCGC]** 1392 (87.50)   
202 - **[GGGCGCGT]** 1394 (88.21)   
202 - **[AGACGCGG]** 991 (85.28)   
207 + **[CCGCGCGT]** 1150 (86.82)   
207 - **[CGACACGT]** 1475 (87.00)   
207 - **[CGCCGCCG]** 1064 (85.71)   
207 - **[GGACGCGC]** 856 (87.50)   
208 + **[CGAGGCGC]** 1393 (87.66)   
208 + **.CGGCGCGC.** 1408 (91.73)   
208 + **.CGCCGCGG.** 1496 (93.07)   
208 - **.CGCCGCGG.** 1498 (93.07)   
208 - **[CGCGGCGG]** 1495 (85.22)   
208 - **[CGGCGCGA]** 1358 (87.47)   
209 + **[AGCCGCGT]** 837 (86.16)   
210 + **[GGGCGCGT]** 674 (88.21)   
210 + **[GGCCGCGG]** 777 (85.06)   
210 + **[CGCCGCCC]** 1020 (85.13)   
210 + **.CGGCGCGG.** 1065 (92.31)   
210 + **[CGCCGCCC]** 1269 (85.13)   
210 - **[GGGCGCGT]** 1222 (88.21)   
210 - **[CGCCGCTT]** 1103 (85.69)   
210 - **[CGCCGCCG]** 939 (85.71)   
210 - **[CGCCGCCG]** 936 (85.71)   
210 - **[CGCCGCCG]** 899 (85.71)   
210 - **[CGCCGCCT]** 871 (89.62)   
210 - **[GGCCGCGG]** 779 (85.06)   
210 - **.CGACGCGC.** 676 (95.51)   
211 + **.CGCCGCGC.** 734 (92.48)   
211 + **[CGCCGCCG]** 961 (85.71)   
211 + **[CGCCGCCG]** 964 (85.71)   
211 + **[CGCCGCCG]** 979 (85.71)   
211 + **[CGCCGCCG]** 1104 (85.71)   
211 + **.GGACGCGT.** 1404 (91.99)   
211 - **.CGGCGCGG.** 976 (92.31)   
211 - **[CGCGGCGG]** 733 (85.22)   
212 + **[GGCCGCGG]** 712 (85.06)   
212 + **[CGAGGCGC]** 1494 (87.66)   
212 - **[AGCCGCGT]** 1485 (86.16)   
212 - **[CGCCGCCC]** 632 (85.13)   
212 - **.CGGCGCGG.** 454 (92.31)   
212 - **[CGTCGCGC]** 421 (85.44)   
212 - **[CGCCGCCG]** 233 (85.71)   
212 - **[CGCCGCCT]** 230 (89.62)   
213 + **[CGGCGCGA]** 996 (87.47)   
213 + **[CCACGCGC]** 1120 (86.11)   
213 - **.CGGCGCGG.** 1192 (92.31)   
214 + **[CGCCGCTT]** 490 (85.69)   
214 + **[CGCGGCGT]** 732 (89.13)   
214 + **[GGCCGCGG]** 928 (85.06)   
214 - **[CGCCGCCT]** 1488 (89.62)   
214 - **[CGCCGCCG]** 1464 (85.71)   
214 - **[CGCCGCCG]** 1461 (85.71)   
214 - **[CGCCGCCT]** 1445 (89.62)   
214 - **[CGCCGCCG]** 1411 (85.71)   
214 - **[CGCCGCCG]** 1408 (85.71)   
214 - **[CGCCGCCG]** 1405 (85.71)   
214 - **[CGCCGCCG]** 1402 (85.71)   
214 - **[GGCCGCGG]** 930 (85.06)   
214 - **[CGGCGCCT]** 792 (88.87)   
214 - **.CGCCGCGC.** 731 (92.48)   
215 + **[CGAGGCGG]** 13 (88.25)   
215 + **[AGCCGCGT]** 651 (86.16)   
215 + **[CGCCGCCG]** 956 (85.71)   
215 + **.CGCCGCGG.** 1089 (93.07)   
215 + **[CGCCCCGT]** 1142 (87.14)   
215 + **[CGCCCCGT]** 1417 (87.14)   
215 - **.CGCCGCGG.** 1091 (93.07)   
215 - **[CGCGGCGG]** 1088 (85.22)   
215 - **.CGACGCGG.** 653 (96.09)   
216 + **[CGCGGCGT]** 212 (89.13)   
216 + **[CGGCGCCT]** 338 (88.87)   
216 + **.CGGCGCGC.** 991 (91.73)   
216 + **[CGACCCGC]** 1061 (85.67)   
216 + **[CGCCGCCC]** 1260 (85.13)   
216 - **[CGAGGCGC]** 820 (87.66)   
216 - **[CGCCGCGA]** 211 (88.22)   
217 + **[CGCGGCGG]** 577 (85.22)   
217 + **[CGAGGCGG]** 776 (88.25)   
217 + **.CGCCGCGC.** 859 (92.48)   
217 + **[CGCCGCCG]** 872 (85.71)   
217 - **.CGACGCGC.** 940 (95.51)   
217 - **[CGCGGCGT]** 858 (89.13)   
217 - **.CGCCGCGC.** 576 (92.48)   
221 + **[AGCCGCGT]** 17 (86.16)   
221 + **.CGGCGCGG.** 936 (92.31)   
221 + **[GGCCGCGG]** 1109 (85.06)   
221 + **[CGCGGCGG]** 1112 (85.22)   
221 - **[CGCCGCCG]** 1114 (85.71)   
221 - **.CGCCGCGG.** 1111 (93.07)   
221 - **[GGACGCGG]** 19 (88.08)   
221 - **[CGCCGCCC]** 6 (85.13)   
222 + **[CCGCGCGT]** 449 (86.82)   
222 + **[CGCCGCCC]** 666 (85.13)   
222 + **.CGGCGCGG.** 840 (92.31)   
222 + **[AGCCGCGT]** 876 (86.16)   
222 + **[CGCGGCGG]** 950 (85.22)   
222 + **[CGCCGCCC]** 1054 (85.13)   
222 + **.CGGCGCGG.** 1269 (92.31)   
222 - **[GGCCGCGG]** 1483 (85.06)   
222 - **.CGGCGCGC.** 1205 (91.73)   
222 - **[CGGCGGGT]** 975 (85.13)   
222 - **.CGCCGCGG.** 949 (93.07)   
222 - **[GGACGCGC]** 939 (87.50)   
222 - **.CGACGCGG.** 878 (96.09)   
222 - **.CGCCGCGC.** 842 (92.48)   
222 - **[CGACGGGT]** 579 (88.90)   
222 - **[GGACGCGC]** 451 (87.50)   
222 - **[CGCCGCCG]** 316 (85.71)   
222 - **[GGCCGCGT]** 167 (88.97)   
223 + **[CGGGGCGT]** 26 (88.38)   
223 + **[CGCCGCTT]** 207 (85.69)   
223 + **[GGACGCGC]** 421 (87.50)   
223 + **[GGACGCGG]** 455 (88.08)   
223 + **[CGCCGCCG]** 675 (85.71)   
223 + **[CGCCGGGT]** 678 (85.88)   
223 + **[CGACGCTT]** 691 (88.71)   
223 - **[CGAAGCGT]** 693 (88.01)   
223 - **.CGGCGCGT.** 423 (96.22)   
223 - **.CGACGCCT.** 186 (92.65)   
224 + **[CGCCGCGA]** 689 (88.22)   
224 + **.CGGCGCGG.** 717 (92.31)   
224 + **[CGGCGGGT]** 769 (85.13)   
224 + **.CGCCGCGG.** 822 (93.07)   
224 + **.CGGCGCGG.** 853 (92.31)   
224 + **[CCACGCGG]** 1175 (86.69)   
224 + **.CGCCGCGC.** 1233 (92.48)   
224 + **[CGCCGGGT]** 1298 (85.88)   
224 - **.CGAGGCGT.** 1244 (92.16)   
224 - **[CCCCGCGT]** 1177 (87.58)   
224 - **.CGCCGCGC.** 855 (92.48)   
224 - **[CGCCGCCC]** 766 (85.13)   
224 - **[CGCCGCCG]** 714 (85.71)   
224 - **[CGCCGCCG]** 711 (85.71)   
224 - **[CGCCGCCG]** 708 (85.71)   
224 - **[CGCCGCCC]** 705 (85.13)   
224 - **[CGCGGCGG]** 688 (85.22)   
224 - **[CGGCGGGT]** 686 (85.13)   
224 - **.CGCCGCGC.** 618 (92.48)   
224 - **.CGACGCCT.** 579 (92.65)   
226 + **[CGCCCCGT]** 68 (87.14)   
226 + **[GGCCGCGT]** 245 (88.97)   
226 + **[CGGCCCGT]** 360 (86.38)   
226 + **[CCGCGCGT]** 449 (86.82)   
226 + **[GGCCGCGT]** 517 (88.97)   
226 + **[GGACGCGG]** 711 (88.08)   
226 + **.CGCCGCGC.** 1034 (92.48)   
226 + **[CGCCGCCG]** 1107 (85.71)   
226 + **[CGCCGCCG]** 1110 (85.71)   
226 + **[CGCCGCCG]** 1113 (85.71)   
226 + **[CGCCGCCC]** 1116 (85.13)   
226 + **[CGACGCCC]** 1188 (88.15)   
226 + **[GGGCGCGT]** 1306 (88.21)   
226 + **[CGGCCCGT]** 1486 (86.38)   
226 + **[CGCGGCGG]** 1497 (85.22)   
226 - **.CGCCGCGC.** 1496 (92.48)   
226 - **[CGCCGCCG]** 1277 (85.71)   
226 - **[CGCCGCCG]** 1274 (85.71)   
226 - **[CGCCGCCG]** 1271 (85.71)   
226 - **[CGCCGCCG]** 1241 (85.71)   
226 - **[CGCCGCCG]** 1238 (85.71)   
226 - **[CGCCGCCC]** 1235 (85.13)   
226 - **.CGGCGCGG.** 1136 (92.31)   
226 - **.CGCCGCGC.** 1077 (92.48)   
226 - **[GGACGCGC]** 1064 (87.50)   
226 - **.CGGCGCGC.** 1031 (91.73)   
226 - **[CGCCGCCC]** 688 (85.13)   
226 - **[GGACGCGG]** 600 (88.08)   
226 - **.CGGCGCGG.** 510 (92.31)   
227 + **[GGACGCGC]** 306 (87.50)   
227 + **.GGACGCGT.** 614 (91.99)   
227 + **.CGACGCGG.** 686 (96.09)   
227 + **[CCACGCGG]** 845 (86.69)   
227 + **[CGAGGCGC]** 901 (87.66)   
227 - **.CGGCGCGG.** 1360 (92.31)   
227 - **[CGCCGCCC]** 1272 (85.13)   
227 - **[CCCCGCGT]** 847 (87.58)   
227 - **[AGGCGCGT]** 308 (85.41)   
228 + **[CGAGGCGG]** 104 (88.25)   
228 + **[CGACCCGC]** 153 (85.67)   
228 + **[CGACACGT]** 571 (87.00)   
228 + **[AGACGCGG]** 737 (85.28)   
228 + **[CGAGGCGG]** 835 (88.25)   
228 + **[CGCCGCCG]** 867 (85.71)   
228 + **[CCACGCGC]** 1426 (86.11)   
228 - **[AGGCGCGT]** 1428 (85.41)   
228 - **.CGGCGCGG.** 541 (92.31)   
228 - **.CGGCGCGG.** 470 (92.31)   
228 - **[GGGCGCGT]** 274 (88.21)   
229 + **[CGCCGCCC]** 447 (85.13)   
229 + **.CGCCGCGC.** 689 (92.48)   
229 + **[CGGGGCGT]** 1045 (88.38)   
229 + **[CGCCGCCC]** 1075 (85.13)   
229 + **.CGCCGCGG.** 1113 (93.07)   
229 + **.CGGCGCGG.** 1286 (92.31)   
229 + **[CGGCGGGT]** 1342 (85.13)   
229 - **[CGACGCCC]** 1388 (88.15)   
229 - **[CGCCGCCG]** 1339 (85.71)   
229 - **[CGCCGCCG]** 1336 (85.71)   
229 - **[CGCCGCCG]** 1333 (85.71)   
229 - **[CGCCGCCG]** 1330 (85.71)   
229 - **[CGCCGCCG]** 1327 (85.71)   
229 - **[CGCCGCCG]** 1324 (85.71)   
229 - **[CGCCGCTT]** 1321 (85.69)   
229 - **.CGCCGCGC.** 1283 (92.48)   
229 - **[CCGCGCGT]** 1281 (86.82)   
229 - **[CGCGGCGG]** 1112 (85.22)   
229 - **[CGGCGGGT]** 1110 (85.13)   
229 - **[CGGCCCGT]** 956 (86.38)   
229 - **[CGCCGCCC]** 683 (85.13)   
229 - **[GGCCGCGG]** 586 (85.06)   
230 - **[CGCCGCCC]** 1199 (85.13)   
230 - **[CGCCGCCC]** 983 (85.13)   
231 + **[CCACGCGG]** 873 (86.69)   
231 + **[CGCCGCCG]** 902 (85.71)   
231 + **.CGCCGCGG.** 905 (93.07)   
231 + **[CGGGGCGT]** 953 (88.38)   
231 + **[CGCCGCCC]** 1005 (85.13)   
231 + **[CGCCGCCG]** 1026 (85.71)   
231 + **[GGACGCGC]** 1382 (87.50)   
231 - **[CGCCGCCG]** 1502 (85.71)   
231 - **[CGCCGCCG]** 1499 (85.71)   
231 - **.CGGCGCGG.** 1483 (92.31)   
231 - **[CGGCGCCT]** 1193 (88.87)   
231 - **[CGCGGCGG]** 904 (85.22)   
231 - **[CGACCCGG]** 694 (86.25)   
232 + **.CGCCGCGC.** 146 (92.48)   
232 + **[CGACCCGC]** 260 (85.67)   
232 + **.CGGCGCGC.** 443 (91.73)   
232 + **.CGGCGCGT.** 632 (96.22)   
232 + **[GGGCGCGT]** 698 (88.21)   
232 + **[CGCCGCGA]** 793 (88.22)   
232 + **[GGCCGCGG]** 866 (85.06)   
232 + **[CGCGGCGG]** 997 (85.22)   
232 + **[CGGCGCGA]** 1012 (87.47)   
232 + **.CGGCGCGG.** 1179 (92.31)   
232 + **[GGACGCGG]** 1332 (88.08)   
232 + **[CGCCGCCC]** 1355 (85.13)   
232 - **[GGCCGCGT]** 1334 (88.97)   
232 - **[CGAGGCGG]** 1267 (88.25)   
232 - **.CGGCGCGG.** 1245 (92.31)   
232 - **[CGCCGCCG]** 1028 (85.71)   
232 - **[CGCCGCCT]** 1025 (89.62)   
232 - **[CGCCGCCC]** 1009 (85.13)   
232 - **.CGCCGCGC.** 996 (92.48)   
232 - **[CGCCGCCG]** 953 (85.71)   
232 - **[CGCGGCGG]** 792 (85.22)   
232 - **[GGACGCGC]** 700 (87.50)   
232 - **[GGACGCGC]** 634 (87.50)   
232 - **[CGCCGCCG]** 629 (85.71)   
232 - **[CGCGGCGG]** 145 (85.22)   
233 + **[CGAGGCGG]** 374 (88.25)   
233 + **[CGACGGGT]** 434 (88.90)   
233 + **[CGACGCCC]** 1140 (88.15)   
233 - **[CCACGCGC]** 1379 (86.11)   
234 + **.CGACGCGG.** 636 (96.09)   
234 + **[CGCCCCGT]** 1195 (87.14)   
234 + **[CCACGCGG]** 1328 (86.69)   
234 + **[GGACGCGC]** 1410 (87.50)   
234 - **[CCGCGCGT]** 1412 (86.82)   
234 - **.CGCCGCGT.** 1330 (96.98)   
234 - **[GGCCGCGG]** 815 (85.06)   
234 - **[CCCCGCGT]** 638 (87.58)   
234 - **[CGACGCCG]** 630 (88.74)   
234 - **[CGCCGCCC]** 627 (85.13)   
234 - **[CGCCGCCT]** 547 (89.62)   
234 - **.CGGCGCGC.** 410 (91.73)   
235 + **[CCACGCGC]** 752 (86.11)   
235 + **.CCACGCGT.** 759 (90.60)   
235 + **.CGGCGCGT.** 902 (96.22)   
235 + **[CGAAGCGT]** 1258 (88.01)   
235 + **.CGGCGCGG.** 1316 (92.31)   
235 + **.CGCCGCGC.** 1361 (92.48)   
235 + **.GGACGCGT.** 1485 (91.99)   
235 - **[CCGCGCGT]** 1398 (86.82)   
235 - **[CGCCGCCG]** 1313 (85.71)   
235 - **[CGCCGCCG]** 1310 (85.71)   
235 - **.CGCCGCGC.** 899 (92.48)   
235 - **[CCACGCGC]** 475 (86.11)   
236 + **[CGCCGCCG]** 1005 (85.71)   
236 + **[GGCCGCGG]** 1153 (85.06)   
236 - **[CGGCGGGT]** 1002 (85.13)   
237 + **[AGACGCGG]** 1056 (85.28)   
237 - **[AGACGCGG]** 472 (85.28)   
238 + **[CGACGTGT]** 1034 (87.11)   
238 + **.CGACGCGG.** 1105 (96.09)   
239 + **.CGCCGCGG.** 444 (93.07)   
239 - **.CGGCGCGG.** 769 (92.31)   
239 - **[GGCCGCGG]** 446 (85.06)   
240 + **[CGCCGCCG]** 1184 (85.71)   
240 + **[CGCCGCGA]** 1402 (88.22)   
240 - **.CGGCGCGG.** 1181 (92.31)   
241 + **.CGGCGCGG.** 1283 (92.31)   
241 - **[AGACGCGT]** 1311 (89.19)   
241 - **[CGCCGCCC]** 1280 (85.13)   
242 + **[CGCCGCCG]** 894 (85.71)   
242 + **[CGCCGCCG]** 897 (85.71)   
242 - **.CGACGCGA.** 1002 (91.25)   
242 - **[AGACGCGG]** 823 (85.28)   
244 + **[GGACGCGG]** 686 (88.08)   
244 - **[CGGCGCCT]** 833 (88.87)   
244 - **[TGACGCGT]** 344 (87.02)   
246 + **.CGCCGCGC.** 273 (92.48)   
246 + **[CGCCGCCG]** 286 (85.71)   
246 + **[AGGCGCGT]** 458 (85.41)   
246 + **[GGACGCGC]** 669 (87.50)   
246 + **[CGGCGCCT]** 676 (88.87)   
246 + **.CGACCCGT.** 685 (90.16)   
246 + **[CGCCGCCT]** 756 (89.62)   
246 + **[CGCCGCCC]** 781 (85.13)   
246 + **[CGCCGCCC]** 1217 (85.13)   
246 - **.CGGCGCGC.** 909 (91.73)   
246 - **.CGGCGCGG.** 883 (92.31)   
246 - **.CGCCGCGC.** 673 (92.48)   
246 - **[CCGCGCGT]** 671 (86.82)   
246 - **[CGCCGCCG]** 657 (85.71)   
246 - **[CGAGGCGC]** 277 (87.66)   
246 - **[CGCGGCGG]** 272 (85.22)   
246 - **[CGGCGGGT]** 270 (85.13)   
247 + **[CGCCGCGA]** 18 (88.22)   
247 + **[CGCCGGGT]** 375 (85.88)   
247 + **[CGCGGCGG]** 422 (85.22)   
247 + **[CGCCGCCC]** 581 (85.13)   
247 + **[CGCCGCCT]** 772 (89.62)   
247 + **[CGCGGCGG]** 1024 (85.22)   
247 + **[CGCCGCCT]** 1224 (89.62)   
247 + **[CGGGGCGT]** 1396 (88.38)   
247 + **.CGCCGCGC.** 1492 (92.48)   
247 - **[CGCGGCGG]** 1491 (85.22)   
247 - **[CGACGAGT]** 1446 (86.53)   
247 - **[GGCCGCGT]** 1359 (88.97)   
247 - **[CGCCGCCC]** 1258 (85.13)   
247 - **[CGAGGCGG]** 1226 (88.25)   
247 - **.CGCCGCGG.** 1023 (93.07)   
247 - **[CGGCGCCT]** 1009 (88.87)   
247 - **.CGCCGCGC.** 421 (92.48)   
247 - **[CCGCGCGT]** 419 (86.82)   
247 - **[CGCGGCGG]** 17 (85.22)   
248 + **[CGAGGCGG]** 1406 (88.25)   
250 + **[CCACGCGC]** 136 (86.11)   
250 + **[GGACGCGC]** 452 (87.50)   
250 + **.CGGCGCGG.** 459 (92.31)   
250 + **.CGCCGCGT.** 869 (96.98)   
250 + **[GGCCGCGG]** 1095 (85.06)   
250 - **[CGCCGCCC]** 960 (85.13)   
250 - **[CGGCGCCT]** 682 (88.87)   
250 - **.CGCCGCGC.** 456 (92.48)   
250 - **[CCGCGCGT]** 454 (86.82)   
250 - **[CGCCGCCG]** 443 (85.71)   
250 - **[CGCCGCCG]** 440 (85.71)   
250 - **[GGGCGCGT]** 138 (88.21)   
252 + **[AGGCGCGT]** 391 (85.41)   
252 - **[GGACGCGC]** 393 (87.50)   
255 + **[CGCCGCCC]** 1064 (85.13)   
255 + **[CGGCGGGT]** 1509 (85.13)   
256 + **[CGACCCGC]** 1126 (85.67)   
256 + **[GGCCGCGG]** 1194 (85.06)   
256 + **.CGCCGCGG.** 1301 (93.07)   
256 - **[CGCGGCGG]** 1300 (85.22)   
256 - **.CGACGCGG.** 1204 (96.09)   
256 - **[CGACCCGC]** 1096 (85.67)   
256 - **[CGAGGCGG]** 54 (88.25)   
257 + **[GGCCGCGG]** 606 (85.06)   
257 + **[CGCCGCCG]** 1059 (85.71)   
257 + **[GGACGCGC]** 1118 (87.50)   
257 + **[GGCCGCGG]** 1165 (85.06)   
257 + **[GGCCGCGG]** 1193 (85.06)   
257 + **[GGCCGCGG]** 1281 (85.06)   
257 + **[CGACCCGG]** 1425 (86.25)   
257 + **[GGGCGCGT]** 1495 (88.21)   
257 - **[CGCCGCCC]** 1470 (85.13)   
257 - **[CGACGCCC]** 1463 (88.15)   
257 - **[CGCCGCCC]** 1400 (85.13)   
257 - **[CCCCGCGT]** 1324 (87.58)   
257 - **[CGACGTGT]** 586 (87.11)   
259 + **[CGATGCGT]** 548 (85.99)   
259 + **.CGACGCGG.** 994 (96.09)   
259 + **[CGCGGCGG]** 997 (85.22)   
259 + **.CGACGCGC.** 1022 (95.51)   
259 + **.CGCCGCGG.** 1301 (93.07)   
259 - **.CGCCGCGG.** 1303 (93.07)   
259 - **[GGCCGCGG]** 1186 (85.06)   
259 - **[CGCCGCCT]** 1038 (89.62)   
259 - **[CGTCGCGG]** 1019 (86.02)   
259 - **.CGCCGCGT.** 996 (96.98)   
259 - **[CGCCGCCT]** 988 (89.62)   
260 - **[CGCCGCCC]** 1114 (85.13)   
263 + **[GGGCGCGT]** 164 (88.21)   
263 + **[GGCCGCGG]** 853 (85.06)   
263 + **[AGCCGCGT]** 1075 (86.16)   
263 + **.CGACGCGG.** 1116 (96.09)   
263 - **.CGACGCGG.** 1077 (96.09)   
263 - **[CGCCGCCG]** 829 (85.71)   
263 - **[CGCCGCCT]** 826 (89.62)   
263 - **[CCACGCGC]** 166 (86.11)   
264 + **[GGACGCGC]** 751 (87.50)   
264 - **.CGGCGCGG.** 1137 (92.31)   
264 - **[CGCCGCCC]** 418 (85.13)   
266 + **[CCACGCGC]** 979 (86.11)   
266 + **.CGGCGCGG.** 1027 (92.31)   
266 + **[CGCGGCGG]** 1030 (85.22)   
266 + **[CGAGGCGG]** 1041 (88.25)   
266 + **[GGCCGCGG]** 1064 (85.06)   
266 + **.CGGCGCGC.** 1103 (91.73)   
266 + **[GGCCGCGG]** 1279 (85.06)   
266 + **[CGCGGCGG]** 1282 (85.22)   
266 + **.CGCCGCGG.** 1474 (93.07)   
266 - **[CGGCGCCT]** 1471 (88.87)   
266 - **.CGCCGCGG.** 1281 (93.07)   
266 - **.CGGCGCGC.** 1269 (91.73)   
266 - **[CGCCGCCG]** 1232 (85.71)   
266 - **[CGCCGCCT]** 1229 (89.62)   
266 - **[CGCCGCCG]** 1155 (85.71)   
266 - **[CGCCGCCG]** 1152 (85.71)   
266 - **.CGGCGCGC.** 1105 (91.73)   
266 - **.CGCCGCGC.** 1029 (92.48)   
266 - **.CGGCGCGT.** 981 (96.22)   
266 - **[GGCCGCGT]** 961 (88.97)   
267 + **[CGAGGCGC]** 96 (87.66)   
267 + **.CGCCGCGG.** 192 (93.07)   
267 + **[CGCCGCCG]** 1023 (85.71)   
267 + **[CGCCGCCG]** 1026 (85.71)   
267 - **[GGCCGCGG]** 194 (85.06)   
267 - **[CGCGGCGG]** 191 (85.22)   
268 + **.CGGCGCGC.** 56 (91.73)   
268 + **.GGACGCGT.** 508 (91.99)   
268 + **.CGGCGCGG.** 1012 (92.31)   
268 + **[CGCGGCGT]** 1015 (89.13)   
268 + **[GGCCGCGG]** 1067 (85.06)   
268 + **[CCACGCGG]** 1198 (86.69)   
268 - **[GGCCGCGT]** 1477 (88.97)   
268 - **[CGCCGCCC]** 1284 (85.13)   
268 - **[CGCCGCCC]** 1264 (85.13)   
268 - **[CGCCGCCG]** 1245 (85.71)   
268 - **[CGCCGCCG]** 1242 (85.71)   
268 - **[CGCCGCCG]** 1239 (85.71)   
268 - **[CGCCGCCT]** 1236 (89.62)   
268 - **[CGCCGCCC]** 1108 (85.13)   
268 - **.CGCCGCGC.** 1014 (92.48)   
268 - **.CGCCGCGT.** 812 (96.98)   
268 - **[CGCCGCCG]** 53 (85.71)   
270 + **[CGGCGGGT]** 13 (85.13)   
270 + **[AGCCGCGT]** 24 (86.16)   
270 + **[CGGCGCGA]** 55 (87.47)   
270 + **[CGACGCCG]** 343 (88.74)   
270 + **[CGCCGCCG]** 346 (85.71)   
270 + **[CCGCGCGT]** 1439 (86.82)   
270 - **[CCACGCGC]** 1441 (86.11)   
270 - **[CGCCGCCT]** 1184 (89.62)   
270 - **[GGACGCGG]** 26 (88.08)   
270 - **[CGCCGCCC]** 10 (85.13)   
271 + **.GGACGCGT.** 741 (91.99)   
271 - **[CCGCGCGT]** 678 (86.82)   
271 - **[CGATGCGT]** 494 (85.99)   
272 - **[CCGCGCGT]** 666 (86.82)   
272 - **[CGATGCGT]** 494 (85.99)   
273 + **[GGCCGCGG]** 1029 (85.06)   
273 + **[CGCGGCGG]** 1032 (85.22)   
273 - **[GGACGCGG]** 1120 (88.08)   
273 - **.CGCCGCGG.** 1031 (93.07)   
273 - **.CGACGCCT.** 360 (92.65)   
274 + **[GGCCGCGG]** 12 (85.06)   
274 + **.CGGCGCGC.** 110 (91.73)   
274 - **.CGCCGCGG.** 107 (93.07)   
274 - **[GGCCGCGG]** 14 (85.06)   
275 + **.CGGCGCGG.** 72 (92.31)   
275 + **[GGACGCGC]** 90 (87.50)   
275 + **[CGCCGCTT]** 577 (85.69)   
275 + **[CGCCGCCG]** 741 (85.71)   
275 + **[CGCCGCCG]** 757 (85.71)   
275 + **[CGCCGCCG]** 760 (85.71)   
275 + **[CGCCGCCG]** 763 (85.71)   
275 + **[CGCCGCCG]** 766 (85.71)   
275 + **[CGCCGCCG]** 1007 (85.71)   
275 + **[CGCCGCCG]** 1010 (85.71)   
275 + **[CGCCGCCG]** 1013 (85.71)   
275 + **[CGGCGCCT]** 1444 (88.87)   
275 - **[CGGCGCGA]** 1329 (87.47)   
275 - **.CGGCGCGC.** 754 (91.73)   
275 - **[CTACGCGT]** 113 (86.64)   
275 - **[CGCCGCCC]** 69 (85.13)   
276 + **.CGACGCGA.** 754 (91.25)   
279 + **[CGAGGCGC]** 115 (87.66)   
279 + **.CGGCGCGT.** 417 (96.22)   
279 - **[CCACGCGC]** 419 (86.11)   
279 - **.CGCCGCGC.** 414 (92.48)   
279 - **[CGTCGCGT]** 375 (89.93)   
280 + **[CGCGGCGG]** 735 (85.22)   
280 + **[GGCCGCGT]** 741 (88.97)   
280 + **[CGCCGCCG]** 846 (85.71)   
280 + **.CGGCGCGC.** 975 (91.73)   
280 + **[CGCGGCGG]** 982 (85.22)   
280 + **[CGCCCCGT]** 1319 (87.14)   
280 - **[CGGGGCGT]** 1318 (88.38)   
280 - **[GGACGCGC]** 1196 (87.50)   
280 - **[CGCCGCCG]** 987 (85.71)   
280 - **[CGCCGCCG]** 984 (85.71)   
280 - **.CGCCGCGC.** 981 (92.48)   
280 - **.CGCCGCGC.** 734 (92.48)   
280 - **[CGACGCCC]** 503 (88.15)   
281 + **[GGACGCGG]** 445 (88.08)   
281 + **[GGACGCGC]** 511 (87.50)   
281 + **[CGGGGCGT]** 942 (88.38)   
281 - **[CGCCGCCG]** 1165 (85.71)   
281 - **[CGCCGCCT]** 1162 (89.62)   
281 - **[CGCCGCCG]** 1038 (85.71)   
281 - **[CGCCGCCG]** 1035 (85.71)   
281 - **[CCCCGCGT]** 447 (87.58)   
284 + **.CGGCGCGG.** 630 (92.31)   
284 + **[CCCCGCGT]** 715 (87.58)   
284 + **[CGCGGCGG]** 780 (85.22)   
284 + **[CGCCGCCC]** 978 (85.13)   
284 + **[CGACGCCC]** 1028 (88.15)   
284 + **.CGGCGCGG.** 1091 (92.31)   
284 + **[CGCCGCCG]** 1236 (85.71)   
284 + **[CGCCGCCG]** 1239 (85.71)   
284 + **[CGCCGCCG]** 1248 (85.71)   
284 + **[CGCCGGGT]** 1251 (85.88)   
284 + **[CGCCGCCC]** 1310 (85.13)   
284 + **[CGCCGCCC]** 1327 (85.13)   
284 + **.CGCCGCGG.** 1344 (93.07)   
284 + **[CGCCGCCG]** 1380 (85.71)   
284 + **[CGCCGCCG]** 1383 (85.71)   
284 + **[CGCCGCCT]** 1386 (89.62)   
284 + **.CGGCGCGG.** 1449 (92.31)   
284 - **[CGACCCGC]** 1501 (85.67)   
284 - **[CGCCGCCG]** 1482 (85.71)   
284 - **[CGCCGCCG]** 1479 (85.71)   
284 - **[CGCCGCCC]** 1476 (85.13)   
284 - **.CGCCGCGG.** 1346 (93.07)   
284 - **.CGGCGCGC.** 1341 (91.73)   
284 - **.CGCCGCGG.** 1088 (93.07)   
284 - **.CGGCGCGC.** 1002 (91.73)   
284 - **[GGCCGCGG]** 942 (85.06)   
284 - **.CGAGGCGT.** 904 (92.16)   
284 - **.CGCCGCGC.** 779 (92.48)   
284 - **[CCACGCGG]** 717 (86.69)   
284 - **[CGACGCCC]** 578 (88.15)   
284 - **[CGAGGCGC]** 554 (87.66)   
285 + **[CGCCGCCG]** 110 (85.71)   
285 + **[GGCCGCGG]** 352 (85.06)   
285 + **.CGGCGCGG.** 442 (92.31)   
285 + **[GGCCGCGG]** 551 (85.06)   
285 + **[CGGCGCCT]** 620 (88.87)   
285 + **.CGCCGCGC.** 633 (92.48)   
285 + **.CGCCGCGC.** 654 (92.48)   
285 + **.CGACGCGG.** 714 (96.09)   
285 + **[GGCCGCGG]** 857 (85.06)   
285 + **.CGCCGCGG.** 906 (93.07)   
285 + **[CGGCGCGA]** 1025 (87.47)   
285 + **[CGGCGGGT]** 1425 (85.13)   
285 - **[CGTCGCGC]** 949 (85.44)   
285 - **.CGCCGCGG.** 908 (93.07)   
285 - **[CGCGGCGG]** 905 (85.22)   
285 - **[CGGCGGGT]** 903 (85.13)   
285 - **[CGCCGCCG]** 758 (85.71)   
285 - **[CGCCGCCG]** 755 (85.71)   
285 - **[CGCCGCCC]** 752 (85.13)   
285 - **[GGCCGCGT]** 716 (88.97)   
285 - **.CGGCGCGG.** 656 (92.31)   
285 - **[CGGCGCGA]** 651 (87.47)   
285 - **[CGCGGCGT]** 632 (89.13)   
285 - **[CGCCGCCC]** 602 (85.13)   
285 - **.CGCCGCGG.** 553 (93.07)   
285 - **[GGCCGCGG]** 547 (85.06)   
285 - **[CGCCGCCG]** 517 (85.71)   
285 - **[CGCCGCCG]** 460 (85.71)   
285 - **[CGCCGCCG]** 457 (85.71)   
285 - **[CGCCGCCC]** 454 (85.13)   
285 - **[CGCCGCCC]** 439 (85.13)   
287 + **[CGCCGCCT]** 762 (89.62)   
287 + **[CGCCGCCG]** 1079 (85.71)   
287 - **[CGCCGCCT]** 1181 (89.62)   
287 - **[CGCCCCGT]** 876 (87.14)   
287 - **[CGAGGCGG]** 764 (88.25)   
287 - **[CGAGGCGC]** 687 (87.66)   
287 - **.CGGCGCGC.** 536 (91.73)   
287 - **[CCCCGCGT]** 491 (87.58)   
288 + **[CGGCGCGA]** 875 (87.47)   
288 + **[CGCCGCCC]** 1229 (85.13)   
288 - **.CGGCGCGC.** 1194 (91.73)   
289 + **[AGACGCGG]** 539 (85.28)   
289 - **.CGCCGCGC.** 1299 (92.48)   
290 + **[CGCCGCCG]** 1162 (85.71)   
290 + **[CGCCGCTT]** 1165 (85.69)   
290 - **[CGCCGCCC]** 1436 (85.13)   
290 - **.CGACCCGT.** 1426 (90.16)   
291 + **[GGCCGCGT]** 509 (88.97)   
291 + **.CCACGCGT.** 735 (90.60)   
291 + **.CGCCGCGG.** 766 (93.07)   
291 + **[CGGCGCCT]** 873 (88.87)   
291 + **[CGCCCCGT]** 1023 (87.14)   
291 + **.CGCCGCGC.** 1034 (92.48)   
291 + **.CGCCGCGT.** 1082 (96.98)   
291 + **[GGACGCGG]** 1164 (88.08)   
291 + **[CGAGGCGG]** 1353 (88.25)   
291 + **[CGCCGCCG]** 1361 (85.71)   
291 - **.CGGCGCGC.** 1501 (91.73)   
291 - **[CGCCGCCC]** 1449 (85.13)   
291 - **[CGCCGCCT]** 1355 (89.62)   
291 - **[CGCCGCCG]** 1246 (85.71)   
291 - **[CGACCCGG]** 1191 (86.25)   
291 - **.CGGCGCGC.** 1099 (91.73)   
291 - **.CGCCGCGC.** 870 (92.48)   
291 - **[CGCGGCGT]** 765 (89.13)   
291 - **[AGACGCGT]** 737 (89.19)   
291 - **[AGACGCGG]** 511 (85.28)   
291 - **[GGACGCGG]** 406 (88.08)   
292 + **.CGCCGCGG.** 794 (93.07)   
292 + **[CGCGGCGG]** 797 (85.22)   
292 + **[CGCCGCGA]** 1081 (88.22)   
292 + **[GGACGCGG]** 1188 (88.08)   
292 + **.CGGCGCGG.** 1345 (92.31)   
292 + **[CGTCGCGG]** 1468 (86.02)   
292 + **[GGACGCGG]** 1484 (88.08)   
292 - **[CCCCGCGT]** 1486 (87.58)   
292 - **[CGACGCCC]** 1465 (88.15)   
292 - **[GGCCGCGT]** 1385 (88.97)   
292 - **[GGACGCGC]** 1318 (87.50)   
292 - **[CGCCGCCG]** 1197 (85.71)   
292 - **[CGCCGCCC]** 1194 (85.13)   
292 - **[CGACGCCG]** 1177 (88.74)   
292 - **[CGCGGCGG]** 1080 (85.22)   
292 - **[CGCCGCCC]** 1033 (85.13)   
292 - **[CGCCGCCC]** 1015 (85.13)   
292 - **[CGGCGCCT]** 869 (88.87)   
292 - **.CGCCGCGG.** 796 (93.07)   
292 - **[CGCGGCGT]** 793 (89.13)   
292 - **.CGGCGCGG.** 773 (92.31)   
293 + **[CGAGGCGC]** 820 (87.66)   
293 + **[CGCGGCGG]** 839 (85.22)   
293 + **.CGGCGCGG.** 859 (92.31)   
293 + **[CGTCGCGG]** 1072 (86.02)   
293 + **[CGGGGCGT]** 1077 (88.38)   
293 - **.CGCCGCGC.** 838 (92.48)   
294 + **.CGCCGCGC.** 45 (92.48)   
294 + **[AGCCGCGT]** 784 (86.16)   
294 + **[CGCCGCCC]** 797 (85.13)   
294 + **[GGACGCGC]** 935 (87.50)   
294 + **[CCACGCGC]** 1027 (86.11)   
294 + **[CGACGCCC]** 1088 (88.15)   
294 - **[CGGGGCGT]** 1090 (88.38)   
294 - **[GGGCGCGT]** 937 (88.21)   
294 - **.CGCCGCGC.** 893 (92.48)   
294 - **[CGAGGCGG]** 362 (88.25)   
294 - **[GGCCGCGG]** 315 (85.06)   
294 - **[CGCGGCGG]** 44 (85.22)   
295 + **[CGGGGCGT]** 548 (88.38)   
295 + **[CGCGGCGG]** 592 (85.22)   
295 + **[GGGCGCGT]** 701 (88.21)   
295 + **.CGCCGCGC.** 746 (92.48)   
295 + **[CGAGGCGG]** 1276 (88.25)   
295 + **[GGCCGCGG]** 1323 (85.06)   
295 - **[AGCCGCGT]** 854 (86.16)   
295 - **[CGCCGCCC]** 841 (85.13)   
295 - **[GGACGCGC]** 703 (87.50)   
295 - **[CCACGCGC]** 611 (86.11)   
295 - **.CGCCGCGC.** 591 (92.48)   
295 - **[CGACGCCC]** 550 (88.15)   
296 + **[CGCGGCGG]** 85 (85.22)   
296 + **[CGACCCGG]** 148 (86.25)   
296 + **.CGGCGCGC.** 194 (91.73)   
296 + **.CGCCGCGC.** 756 (92.48)   
296 + **[CGCCGCCG]** 770 (85.71)   
296 + **[GGACGCGG]** 840 (88.08)   
296 + **[CGCCGCCC]** 948 (85.13)   
296 + **[CGCCGCCG]** 975 (85.71)   
296 + **[AGACGCGG]** 1000 (85.28)   
296 + **[GGACGCGC]** 1011 (87.50)   
296 + **[GGGCGCGT]** 1117 (88.21)   
296 + **[GGCCGCGG]** 1161 (85.06)   
296 + **.CGCCGCGG.** 1186 (93.07)   
296 + **[CGCCGCCC]** 1317 (85.13)   
296 + **[CGAGGCGG]** 1382 (88.25)   
296 + **.CGGCGCGG.** 1442 (92.31)   
296 - **[GGACGCGC]** 1473 (87.50)   
296 - **.CGCCGCGC.** 1439 (92.48)   
296 - **.CGGCGCGC.** 1314 (91.73)   
296 - **[CGGCCCGT]** 1281 (86.38)   
296 - **[CGACGCCC]** 1206 (88.15)   
296 - **.CGGCGCGG.** 1183 (92.31)   
296 - **.CGCCGCGT.** 1002 (96.98)   
296 - **.CGGCGCGG.** 945 (92.31)   
296 - **[GGCCGCGG]** 814 (85.06)   
296 - **.CGGCGCGG.** 758 (92.31)   
296 - **[CGCGGCGT]** 755 (89.13)   
296 - **[CGGCGCGA]** 590 (87.47)   
296 - **[CGCCGCCC]** 467 (85.13)   
296 - **[CGCCGCCG]** 191 (85.71)   
296 - **[CGCCGCCG]** 188 (85.71)   
296 - **[CGCCGCCC]** 185 (85.13)   
296 - **.CGCCGCGT.** 84 (96.98)   
297 + **[CCCCGCGT]** 553 (87.58)   
297 + **[CGCCGCCC]** 636 (85.13)   
297 + **.CGGCGCGG.** 984 (92.31)   
297 + **[GGCCGCGG]** 995 (85.06)   
297 + **[CGCCGCTT]** 1239 (85.69)   
297 - **[CCACGCGG]** 1420 (86.69)   
297 - **[CGCCCCGT]** 1364 (87.14)   
297 - **.CGACGCCT.** 1200 (92.65)   
297 - **[CCACGCGC]** 1032 (86.11)   
297 - **[CGCCGCCG]** 981 (85.71)   
297 - **[CGCCGCCG]** 978 (85.71)   
298 + **[GGCCGCGT]** 796 (88.97)   
298 + **[CGCGGCGG]** 876 (85.22)   
298 + **[CGCCGCGA]** 1177 (88.22)   
298 + **.CGAGGCGT.** 1223 (92.16)   
298 - **[CGCCGCCT]** 1341 (89.62)   
298 - **[CGAGGCGC]** 1262 (87.66)   
298 - **[CGCGGCGG]** 1176 (85.22)   
298 - **[CGCCCCGT]** 1095 (87.14)   
298 - **[GGACGCGG]** 936 (88.08)   
298 - **.CGCCGCGT.** 875 (96.98)   
298 - **[CGCCGCGA]** 745 (88.22)   
298 - **[CGAGGCGC]** 294 (87.66)   
299 + **[CCACGCGC]** 576 (86.11)   
299 + **[CGGCGCCT]** 820 (88.87)   
299 + **[CGAGGCGG]** 844 (88.25)   
299 + **[GGCCGCGT]** 850 (88.97)   
299 + **[CCGCGCGT]** 880 (86.82)   
299 + **[GGCCGCGG]** 1177 (85.06)   
299 + **[GGGCGCGT]** 1472 (88.21)   
299 - **[GGACGCGC]** 1474 (87.50)   
299 - **[CGTCGCGG]** 1400 (86.02)   
299 - **[CGAGGCGC]** 1342 (87.66)   
299 - **.CGCCGCGG.** 1261 (93.07)   
299 - **.CGACGCCT.** 1251 (92.65)   
299 - **.CGCCGCGG.** 1179 (93.07)   
299 - **.CGGCGCGC.** 1081 (91.73)   
299 - **.CGACGCGC.** 882 (95.51)   
299 - **[AGACGCGG]** 852 (85.28)   
299 - **[CCGCGCGT]** 578 (86.82)   
300 + **.CGGCGCGG.** 110 (92.31)   
300 + **.CGGCGCGG.** 476 (92.31)   
300 + **[CGCGGCGG]** 736 (85.22)   
300 - **[CGCCGCGA]** 735 (88.22)   
300 - **.CGCCGCGC.** 473 (92.48)   
300 - **.CGACGCGG.** 445 (96.09)   
300 - **[CGCCGCCG]** 409 (85.71)   
300 - **[CGACCCGC]** 394 (85.67)   
300 - **[GGCCGCGG]** 174 (85.06)   
300 - **.CGCCGCGC.** 112 (92.48)   
301 + **[GGGCGCGT]** 764 (88.21)   
301 + **[CGAGGCGG]** 1035 (88.25)   
301 + **[AGCCGCGT]** 1480 (86.16)   
301 - **[CCACGCGG]** 1482 (86.69)   
302 + **.CGAGGCGT.** 344 (92.16)   
302 + **.CGACGCGC.** 950 (95.51)   
302 + **.CGCCGCGG.** 1041 (93.07)   
302 + **[CGCCGCCG]** 1460 (85.71)   
302 - **[CGGCGCGA]** 1038 (87.47)   
302 - **[CGCCGCGA]** 1025 (88.22)   
302 - **[CGAGGCGG]** 1020 (88.25)   
302 - **.CGGCGCGG.** 840 (92.31)   
302 - **.CGGCGCGG.** 755 (92.31)   
302 - **[CGGCCCGT]** 527 (86.38)   
303 + **.CGCCGCGC.** 167 (92.48)   
303 + **[CGCCGCCC]** 172 (85.13)   
303 + **[GGCCGCGG]** 286 (85.06)   
303 + **[CGACCCGC]** 388 (85.67)   
303 + **.CGCCGCGG.** 456 (93.07)   
303 + **.CGCCGCGG.** 747 (93.07)   
303 + **[CGGCCCGT]** 878 (86.38)   
303 + **.CGGCGCGG.** 1003 (92.31)   
303 + **[CCCCGCGT]** 1018 (87.58)   
303 + **[CGGCCCGT]** 1030 (86.38)   
303 + **[CGACCCGG]** 1313 (86.25)   
303 - **[CGCCGCCG]** 1353 (85.71)   
303 - **.CGACGCGG.** 1020 (96.09)   
303 - **[CGCCGCCT]** 1000 (89.62)   
303 - **[CGCCGCCG]** 988 (85.71)   
303 - **[CGCCGCCG]** 985 (85.71)   
303 - **[CGCCGCCG]** 982 (85.71)   
303 - **[CGCCGCCG]** 979 (85.71)   
303 - **[CGCCGCCG]** 976 (85.71)   
303 - **[CGCCGCCG]** 973 (85.71)   
303 - **[CGCCGCCG]** 915 (85.71)   
303 - **[CGCCGCCG]** 912 (85.71)   
303 - **.CGGCGCGC.** 744 (91.73)   
303 - **[CGCCGCCC]** 462 (85.13)   
303 - **[CCCCGCGT]** 330 (87.58)   
303 - **[CGCCGCCC]** 293 (85.13)   
303 - **.CGGCGCGG.** 169 (92.31)   
303 - **[CGCGGCGG]** 166 (85.22)   
303 - **.CGACGCGC.** 129 (95.51)   
304 + **.CGCCGCGC.** 707 (92.48)   
304 + **.CGACGCGG.** 732 (96.09)   
304 + **[CGGCGCCT]** 760 (88.87)   
304 + **.CGGCGCGC.** 903 (91.73)   
304 + **.CGGCGCGC.** 1178 (91.73)   
304 + **[CGCGGCGG]** 1243 (85.22)   
304 + **[CGGCGCCT]** 1339 (88.87)   
304 - **[CGTCGCGG]** 1308 (86.02)   
304 - **.CGCCGCGC.** 1242 (92.48)   
304 - **[CGCCGCCG]** 1175 (85.71)   
304 - **[CCCCGCGT]** 1153 (87.58)   
304 - **[CGCCGCCC]** 186 (85.13)   
305 + **[CCCCGCGT]** 1077 (87.58)   
306 + **[CGGCGCCT]** 274 (88.87)   
306 + **[CGCCGGGT]** 344 (85.88)   
306 + **.CGCCGCGC.** 764 (92.48)   
306 + **[GGACGCGC]** 844 (87.50)   
306 + **[CGACCCGC]** 908 (85.67)   
306 + **[CGAGGCGG]** 1018 (88.25)   
306 + **.CGGCGCGG.** 1038 (92.31)   
306 + **[CGCCGCCG]** 1413 (85.71)   
306 - **[CCACGCGG]** 1476 (86.69)   
306 - **[CGCCGCCT]** 1020 (89.62)   
306 - **.CGGCGCGG.** 929 (92.31)   
306 - **[CGCGGCGG]** 763 (85.22)   
306 - **.CGGCGCGG.** 341 (92.31)   
306 - **[GGACGCGC]** 311 (87.50)   
307 + **.CGGCGCGT.** 496 (96.22)   
307 + **.CGCCGCGG.** 678 (93.07)   
307 + **[CGCGGCGG]** 730 (85.22)   
307 + **[CGCCGCCG]** 980 (85.71)   
307 + **[CGCCGCCG]** 992 (85.71)   
307 + **[CGAGGCGG]** 1220 (88.25)   
307 + **.CGGCGCGG.** 1234 (92.31)   
307 + **.CGGCGCGG.** 1354 (92.31)   
307 - **[GGCCGCGG]** 1329 (85.06)   
307 - **[CGCCGCCC]** 1231 (85.13)   
307 - **[CGCCGCCC]** 1083 (85.13)   
307 - **[CGACGCCC]** 1065 (88.15)   
307 - **[CGACCCGG]** 714 (86.25)   
307 - **[CGCGGCGG]** 677 (85.22)   
307 - **.CGGCGCGC.** 547 (91.73)   
308 + **[CCACGCGG]** 70 (86.69)   
308 + **[CGCGGCGG]** 863 (85.22)   
308 + **[GGCCGCGG]** 1178 (85.06)   
308 + **[CGCCGGGT]** 1191 (85.88)   
308 + **[CGGCGGGT]** 1335 (85.13)   
308 + **.CGCCGCGG.** 1367 (93.07)   
308 + **[GGCCGCGG]** 1412 (85.06)   
308 - **[CGGCCCGT]** 1409 (86.38)   
308 - **[CGCGGCGG]** 1366 (85.22)   
308 - **.CGGCGCGG.** 1188 (92.31)   
308 - **[CGAGGCGG]** 1080 (88.25)   
308 - **.CGCCGCGC.** 862 (92.48)   
308 - **[CGACGAGT]** 178 (86.53)   
309 + **[GGCCGCGG]** 993 (85.06)   
309 + **[CGCGGCGG]** 1029 (85.22)   
309 + **[CGGCGCGA]** 1077 (87.47)   
309 + **[CCACGCGG]** 1224 (86.69)   
309 - **[CGACGAGT]** 1331 (86.53)   
309 - **.CGCCGCGC.** 1028 (92.48)   
309 - **[CGCCGCTT]** 902 (85.69)   
309 - **[CGCCGCCC]** 767 (85.13)   
310 - **[CGACTCGT]** 1440 (86.94)   
310 - **[CGAGGCGG]** 789 (88.25)   
311 + **.CGCCGCGG.** 872 (93.07)   
311 + **[CGCCGCCC]** 962 (85.13)   
311 + **[CGCGGCGG]** 1069 (85.22)   
311 + **[CGAGGCGG]** 1147 (88.25)   
311 + **[GGACGCGG]** 1261 (88.08)   
311 + **[GGCCGCGT]** 1342 (88.97)   
311 + **.CGCCGCGG.** 1470 (93.07)   
311 - **[AGACGCGG]** 1344 (85.28)   
311 - **.CGCCGCGG.** 1105 (93.07)   
311 - **.CGCCGCGC.** 1068 (92.48)   
311 - **[CGCCGCCC]** 1019 (85.13)   
311 - **.CGGCGCGG.** 959 (92.31)   
311 - **[GGCCGCGG]** 874 (85.06)   
311 - **[CGCGGCGT]** 871 (89.13)   
311 - **[CGAGGCGC]** 763 (87.66)   
312 + **.CGACGCGC.** 740 (95.51)   
312 + **[CCACGCGC]** 840 (86.11)   
312 + **[CGCCGCCC]** 987 (85.13)   
312 + **[CGCCGCCG]** 1044 (85.71)   
312 - **[CGCCGGGT]** 1459 (85.88)   
312 - **.CGGCGCGC.** 1360 (91.73)   
312 - **[CGCCGCCT]** 1203 (89.62)   
312 - **.CGCCGCGC.** 1093 (92.48)   
312 - **.CGGCGCGG.** 984 (92.31)   
312 - **[GGGCGCGT]** 871 (88.21)   
312 - **[GGGCGCGT]** 842 (88.21)   
312 - **[CGGGGCGT]** 708 (88.38)   
313 + **[CCACGCGC]** 255 (86.11)   
313 + **[CGCCGCCC]** 841 (85.13)   
313 + **[CGGCGCCT]** 932 (88.87)   
313 + **.CGGCGCGC.** 955 (91.73)   
313 + **.CGACCCGT.** 1419 (90.16)   
313 + **.CGCCGCGG.** 1445 (93.07)   
313 - **[CGCGGCGG]** 1444 (85.22)   
313 - **.CGGCGCGC.** 1302 (91.73)   
313 - **[CGCCGCTT]** 1165 (85.69)   
313 - **[CGGCGCGA]** 1087 (87.47)   
313 - **[CGAGGCGG]** 831 (88.25)   
313 - **[GGCCGCGG]** 825 (85.06)   
314 + **[CGAGGCGC]** 43 (87.66)   
314 - **[CGCCGCCC]** 478 (85.13)   
314 - **[CGGCGCCT]** 45 (88.87)   
315 + **[AGACGCGT]** 902 (89.19)   
315 + **[GGACGCGC]** 1005 (87.50)   
315 + **.CGGCGCGG.** 1016 (92.31)   
315 + **.CGGCGCGG.** 1025 (92.31)   
315 + **[GGACGCGG]** 1284 (88.08)   
315 + **.CGCCGCGT.** 1341 (96.98)   
315 - **[GGACGCGG]** 1343 (88.08)   
315 - **.CGGCGCGC.** 1272 (91.73)   
315 - **[CGCCGCCC]** 1089 (85.13)   
315 - **[CGCCGCCC]** 1022 (85.13)   
315 - **[CGCCGCCC]** 1013 (85.13)   
315 - **[CCGCGCGT]** 1007 (86.82)   
315 - **[CGCCGCGA]** 356 (88.22)   
315 - **.CGAGGCGT.** 151 (92.16)   
316 + **[CGAGGCGC]** 35 (87.66)   
316 + **[GGACGCGC]** 199 (87.50)   
316 + **.CGGCGCGT.** 895 (96.22)   
316 + **[CGGCGCGA]** 912 (87.47)   
316 + **[CGCCGCCG]** 1183 (85.71)   
316 - **[CGCCGCCT]** 1282 (89.62)   
316 - **.CGCCGCGG.** 909 (93.07)   
316 - **[GGACGCGC]** 897 (87.50)   
316 - **.CGGCGCGG.** 256 (92.31)   
316 - **[GGCCGCGG]** 228 (85.06)   
316 - **[GGGCGCGT]** 201 (88.21)   
316 - **[CGCCGCCC]** 165 (85.13)   
317 - **[CGCCGCCG]** 1196 (85.71)   
317 - **[CGCCGCCG]** 1037 (85.71)   
317 - **[CGCCGCCT]** 1034 (89.62)   
318 + **[CCCCGCGT]** 266 (87.58)   
318 + **[GGCCGCGT]** 287 (88.97)   
318 + **[CGACCCGG]** 478 (86.25)   
318 + **[CGGCGGGT]** 670 (85.13)   
318 + **[CGGCCCGT]** 775 (86.38)   
318 + **[CGCCGCCC]** 1109 (85.13)   
318 + **[CGCCGCCG]** 1216 (85.71)   
318 + **[CGCCGCCC]** 1219 (85.13)   
318 + **[CCACGCGC]** 1345 (86.11)   
318 + **.CGGCGCGC.** 1433 (91.73)   
318 + **[GGCCGCGG]** 1482 (85.06)   
318 + **[CGCGGCGG]** 1485 (85.22)   
318 - **[CGCCGCCG]** 1493 (85.71)   
318 - **[CGCCGCCG]** 1490 (85.71)   
318 - **[CGCCGCCG]** 1487 (85.71)   
318 - **.CGCCGCGG.** 1484 (93.07)   
318 - **[CGCCGCCC]** 1475 (85.13)   
318 - **[CGCCGCCG]** 1390 (85.71)   
318 - **[CGACCCGG]** 1192 (86.25)   
318 - **[CCACGCGC]** 1041 (86.11)   
318 - **[CGCCGCCT]** 467 (89.62)   
318 - **.CGGCGCGG.** 306 (92.31)   
318 - **[GGACGCGG]** 289 (88.08)   
320 + **[CGCCGCCT]** 760 (89.62)   
320 + **.CGGCGCGC.** 875 (91.73)   
320 + **[CGACCCGG]** 1317 (86.25)   
320 + **.CGCCGCGC.** 1341 (92.48)   
320 + **.CGCCGCGG.** 1383 (93.07)   
320 - **.CGGCGCGC.** 1380 (91.73)   
320 - **.CGACGCGC.** 1347 (95.51)   
320 - **[CGGCGCGA]** 1197 (87.47)   
320 - **.CGCCGCGG.** 991 (93.07)   
320 - **.CGGCGCGG.** 912 (92.31)   
320 - **[CGCCGCCT]** 872 (89.62)   
320 - **[CGCCGCGA]** 754 (88.22)   
320 - **[CGAGGCGC]** 715 (87.66)   
320 - **[CGGGGCGT]** 429 (88.38)   
320 - **[CGCCGCCC]** 415 (85.13)   
320 - **.GGACGCGT.** 106 (91.99)   
321 + **[CGCCGCCC]** 385 (85.13)   
321 + **[CGCGGCGT]** 455 (89.13)   
321 + **[CGACCCGG]** 504 (86.25)   
321 + **[CCACGCGC]** 554 (86.11)   
321 + **.CGCCGCGC.** 698 (92.48)   
321 + **[CGCCGCCG]** 732 (85.71)   
321 + **[CGCCGCCG]** 981 (85.71)   
321 + **[CGCCGCTT]** 1056 (85.69)   
321 + **[CGCCGCCC]** 1216 (85.13)   
321 + **.CGGCGCGG.** 1299 (92.31)   
321 + **.CGCCGCGG.** 1397 (93.07)   
321 - **.CGGCGCGC.** 1505 (91.73)   
321 - **[GGCCGCGG]** 1399 (85.06)   
321 - **.CGGCGCGC.** 1394 (91.73)   
321 - **.CGGCGCGC.** 1259 (91.73)   
321 - **.CGGCGCGG.** 695 (92.31)   
321 - **[CGCCGGGT]** 506 (85.88)   
321 - **.CGCCGCGG.** 454 (93.07)   
321 - **[CGCCGCTT]** 414 (85.69)   
322 + **[CGCCCCGT]** 362 (87.14)   
322 + **.CGCCGCGC.** 496 (92.48)   
322 + **.CGGCGCGG.** 629 (92.31)   
322 + **.CGGCGCGG.** 685 (92.31)   
322 + **[CGCCGCCG]** 950 (85.71)   
322 + **[GGCCGCGG]** 1002 (85.06)   
322 + **[CGTCGCGT]** 1064 (89.93)   
322 + **[CGTCGCGC]** 1171 (85.44)   
322 + **.CGCCGCGC.** 1480 (92.48)   
322 - **[GGGCGCGT]** 1425 (88.21)   
322 - **.CGGCGCGC.** 947 (91.73)   
322 - **[CGCGGCGG]** 495 (85.22)   
323 + **[GGCCGCGT]** 452 (88.97)   
323 + **[CGACCCGC]** 707 (85.67)   
323 + **[CGGCCCGT]** 862 (86.38)   
323 + **[GGCCGCGG]** 1029 (85.06)   
323 + **[CCACGCGC]** 1067 (86.11)   
323 + **.CGCCGCGG.** 1228 (93.07)   
323 + **[CGCGGCGG]** 1231 (85.22)   
323 + **[GGGCGCGT]** 1447 (88.21)   
323 - **.CGACGCGC.** 1423 (95.51)   
323 - **.CGCCGCGG.** 1230 (93.07)   
323 - **[CGCGGCGT]** 1227 (89.13)   
323 - **[CGCCGCCC]** 1140 (85.13)   
323 - **[GGCCGCGG]** 1031 (85.06)   
323 - **[CGGGGCGT]** 379 (88.38)   
325 + **[GGGCGCGT]** 1305 (88.21)   
325 - **.CGACCCGT.** 1463 (90.16)   
326 + **[CCACGCGG]** 650 (86.69)   
326 + **[CGCCGCCT]** 734 (89.62)   
326 + **[CGCCGGGT]** 761 (85.88)   
326 + **.CGCCGCGC.** 822 (92.48)   
326 + **[CGACGTGT]** 1000 (87.11)   
326 + **[CGCCGCCC]** 1045 (85.13)   
326 + **[CCGCGCGT]** 1118 (86.82)   
326 + **[CGCCGCCG]** 1139 (85.71)   
326 + **[CGCGGCGG]** 1202 (85.22)   
326 + **[GGCCGCGG]** 1300 (85.06)   
326 + **[CGCGGCGG]** 1303 (85.22)   
326 - **[CGACGAGT]** 1469 (86.53)   
326 - **[CGCCGCCC]** 1369 (85.13)   
326 - **[CGCCGCCC]** 1354 (85.13)   
326 - **[CGCCGCCC]** 1339 (85.13)   
326 - **[CGCCGCCG]** 1305 (85.71)   
326 - **.CGCCGCGG.** 1302 (93.07)   
326 - **.CGCCGCGC.** 1201 (92.48)   
326 - **.CGCCGCGG.** 937 (93.07)   
326 - **[CGCGGCGG]** 821 (85.22)   
326 - **[GGCCGCGT]** 659 (88.97)   
327 + **[AGGCGCGT]** 467 (85.41)   
327 + **[CGCCGCGA]** 1112 (88.22)   
327 - **[CGGCGCCT]** 1109 (88.87)   
327 - **[CGCCGCCG]** 599 (85.71)   
327 - **[CGCCGCCC]** 596 (85.13)   
327 - **[CGACCCGG]** 589 (86.25)   
327 - **[CGAGGCGG]** 548 (88.25)   
327 - **[CGCCGCCG]** 260 (85.71)   
327 - **[CGCCGCCT]** 257 (89.62)   
329 + **.CGACGCCT.** 273 (92.65)   
329 + **[GGACGCGC]** 1236 (87.50)   
329 - **[CGGCCCGT]** 546 (86.38)   
331 + **[CGCCGCCG]** 717 (85.71)   
331 + **.CGCCGCGG.** 720 (93.07)   
331 + **.CGGCGCGT.** 1137 (96.22)   
331 + **[CGCCGCCG]** 1169 (85.71)   
331 + **[CGCCGCCG]** 1172 (85.71)   
331 + **[CGCCGCCG]** 1181 (85.71)   
331 + **[CGCCGCCG]** 1184 (85.71)   
331 + **[CGCCGCCG]** 1187 (85.71)   
331 + **[CGCCGCCC]** 1190 (85.13)   
331 + **[CGGGGCGT]** 1270 (88.38)   
331 + **[CGCCGCCT]** 1299 (89.62)   
331 + **.CGCCGCGT.** 1313 (96.98)   
331 + **[CGCGGCGG]** 1332 (85.22)   
331 - **.CGCCGCGC.** 1440 (92.48)   
331 - **[CGCCGCCG]** 1367 (85.71)   
331 - **[CGCCGCCG]** 1334 (85.71)   
331 - **.CGCCGCGG.** 1331 (93.07)   
331 - **[GGACGCGG]** 1315 (88.08)   
331 - **.CGGCGCGC.** 1310 (91.73)   
331 - **[CGCGGCGG]** 719 (85.22)   
331 - **.CGCCGCGG.** 396 (93.07)   
331 - **[AGACGCGG]** 268 (85.28)   
332 + **[CGACTCGT]** 1160 (86.94)   
332 + **.CGCCGCGC.** 1350 (92.48)   
332 + **[GGCCGCGG]** 1427 (85.06)   
332 + **[CCACGCGC]** 1445 (86.11)   
332 - **.CGCCGCGC.** 1486 (92.48)   
332 - **[GGCCGCGG]** 1429 (85.06)   
332 - **[CCACGCGG]** 814 (86.69)   
332 - **[GGACGCGC]** 503 (87.50)   
333 + **.CGCCGCGG.** 1121 (93.07)   
333 + **[CGCGGCGG]** 1299 (85.22)   
333 + **[CGCCGCGA]** 1320 (88.22)   
333 - **[CGCGGCGG]** 1319 (85.22)   
333 - **.CGCCGCGC.** 1298 (92.48)   
333 - **[CGGCGCCT]** 1118 (88.87)   
334 + **[CGCCGCCT]** 650 (89.62)   
334 + **[CGGCGGGT]** 1288 (85.13)   
335 + **[CGCCGCCT]** 542 (89.62)   
335 + **.CGGCGCGC.** 856 (91.73)   
335 + **[CGACGCCG]** 1078 (88.74)   
335 + **[CCCCGCGT]** 1202 (87.58)   
335 + **[CGTCGCGG]** 1207 (86.02)   
335 - **[CGCCGCCC]** 1361 (85.13)   
335 - **.CGACGCGG.** 1204 (96.09)   
335 - **.CGGCGCGC.** 1099 (91.73)   
335 - **[CGCCGCGA]** 853 (88.22)   
335 - **[CGAGGCGC]** 611 (87.66)   
336 + **[CGCCGCCT]** 602 (89.62)   
336 + **[CCACGCGG]** 839 (86.69)   
336 + **[CAACGCGT]** 873 (86.87)   
336 + **.CCACGCGT.** 1061 (90.60)   
336 - **.CGGCGCGG.** 980 (92.31)   
336 - **[AGACGCGT]** 875 (89.19)   
337 + **[CGCGGCGG]** 842 (85.22)   
337 + **[CGCGGCGG]** 956 (85.22)   
337 + **[CGCCGCCG]** 1064 (85.71)   
337 + **[CGCCGCCG]** 1067 (85.71)   
337 + **.CGCCGCGG.** 1070 (93.07)   
337 + **[CGCCGCCT]** 1188 (89.62)   
337 + **.CGGCGCGG.** 1347 (92.31)   
337 - **[CGCCGCCC]** 1384 (85.13)   
337 - **[CGCCGCCG]** 1218 (85.71)   
337 - **.CGCCGCGG.** 1182 (93.07)   
337 - **[GGCCGCGG]** 1072 (85.06)   
337 - **[CGCGGCGG]** 1069 (85.22)   
337 - **.CGCCGCGC.** 955 (92.48)   
337 - **[CGCCGCCC]** 925 (85.13)   
337 - **[CGCCGCCG]** 869 (85.71)   
337 - **.CGCCGCGG.** 841 (93.07)   
338 + **[CGCCGCCC]** 326 (85.13)   
338 + **.CGCCGCGT.** 691 (96.98)   
338 + **[CGCCGCCG]** 762 (85.71)   
338 + **[CGCCGCCC]** 794 (85.13)   
338 + **[CGCCGCCG]** 1174 (85.71)   
338 + **[CGCCGCCG]** 1177 (85.71)   
338 + **[CGCCGCCG]** 1180 (85.71)   
338 + **[CGCCGCCG]** 1183 (85.71)   
338 + **[CGCCGCCG]** 1186 (85.71)   
338 + **[CGCCGCCG]** 1189 (85.71)   
338 + **.CGCCGCGC.** 1192 (92.48)   
338 + **[CGCCGCCG]** 1274 (85.71)   
338 + **[GGCCGCGT]** 1413 (88.97)   
338 + **[CGCGGCGT]** 1459 (89.13)   
338 - **[CGCCGCCG]** 1381 (85.71)   
338 - **[CGCCGCCG]** 1378 (85.71)   
338 - **[CGCCGCCC]** 1297 (85.13)   
338 - **[CGCCGCCG]** 1251 (85.71)   
338 - **[CGCCGCTT]** 1248 (85.69)   
338 - **[CGCGGCGG]** 1191 (85.22)   
338 - **[CGGCGCGA]** 1171 (87.47)   
338 - **[GGACGCGC]** 1153 (87.50)   
338 - **[CGCCGCCC]** 1045 (85.13)   
338 - **.CGGCGCGG.** 759 (92.31)   
338 - **[GGACGCGG]** 693 (88.08)   
339 + **[CGGCCCGT]** 1166 (86.38)   
339 - **[CGCCGCCG]** 1066 (85.71)   
339 - **[CGCCGCCT]** 1063 (89.62)   
339 - **[CGCCGCCG]** 946 (85.71)   
339 - **[CGCCGGGT]** 943 (85.88)   
340 + **[CGCCCCGT]** 571 (87.14)   
340 + **[CCACGCGC]** 674 (86.11)   
340 + **[CGCCGCCC]** 688 (85.13)   
340 + **[CGCCGCCC]** 758 (85.13)   
340 + **[CGCCGCCC]** 935 (85.13)   
341 + **[CGAGGCGC]** 926 (87.66)   
341 + **[CGACGAGT]** 1178 (86.53)   
341 + **.CGCCGCGC.** 1383 (92.48)   
341 + **[GGACGCGG]** 1491 (88.08)   
341 - **[CGCCGCCC]** 1408 (85.13)   
341 - **[CGCGGCGG]** 1382 (85.22)   
341 - **[CGAGGCGC]** 1351 (87.66)   
341 - **.CGCCGCGC.** 1318 (92.48)   
341 - **.CGACGCGA.** 1259 (91.25)   
341 - **[GGCCGCGT]** 1151 (88.97)   
341 - **[CGCCGCCG]** 1057 (85.71)   
342 + **.CGGCGCGG.** 835 (92.31)   
342 + **.CGGCGCGG.** 1024 (92.31)   
342 + **.CGGCGCGG.** 1152 (92.31)   
342 + **[CGCGGCGG]** 1155 (85.22)   
342 + **[GGACGCGC]** 1395 (87.50)   
342 - **[CCACGCGC]** 1482 (86.11)   
342 - **[CGCCGCCC]** 1418 (85.13)   
342 - **[CGCCGCCC]** 1288 (85.13)   
342 - **.CGCCGCGC.** 1154 (92.48)   
342 - **[CCCCGCGT]** 227 (87.58)   
343 + **[AGGCGCGT]** 276 (85.41)   
343 + **[GGCCGCGT]** 652 (88.97)   
343 + **[GGACGCGG]** 669 (88.08)   
343 + **[CGCGGCGT]** 998 (89.13)   
343 + **[CGCCGCCG]** 1026 (85.71)   
343 + **.CGCCGCGT.** 1077 (96.98)   
343 + **[GGCCGCGG]** 1119 (85.06)   
343 + **[CGCGGCGG]** 1182 (85.22)   
343 - **[CGACCCGC]** 1432 (85.67)   
343 - **.CGCCGCGC.** 1181 (92.48)   
343 - **[GGCCGCGG]** 1121 (85.06)   
343 - **.CGCCGCGC.** 997 (92.48)   
343 - **[CGCCGCCC]** 859 (85.13)   
343 - **[CCCCGCGT]** 671 (87.58)   
343 - **[CGACCCGC]** 290 (85.67)   
343 - **[GGACGCGC]** 278 (87.50)   
344 + **[CGCCGCTT]** 1069 (85.69)   
344 - **.CGGCGCGC.** 1165 (91.73)   
346 + **[CGGCGCCT]** 25 (88.87)   
346 + **[CGCGGCGG]** 85 (85.22)   
346 + **[GGCCGCGG]** 905 (85.06)   
346 + **[CGCCGCCG]** 997 (85.71)   
346 + **.CGGCGCGC.** 1228 (91.73)   
346 + **[CGCCGCCG]** 1233 (85.71)   
346 + **.CGCCGCGT.** 1236 (96.98)   
346 + **.CGGCGCGC.** 1339 (91.73)   
346 + **[GGCCGCGG]** 1406 (85.06)   
346 - **[CGAGGCGC]** 1388 (87.66)   
346 - **[GGACGCGG]** 1238 (88.08)   
346 - **[CGCGGCGG]** 1235 (85.22)   
346 - **.CGGCGCGC.** 1230 (91.73)   
346 - **[CCCCGCGT]** 856 (87.58)   
346 - **[CCCCGCGT]** 316 (87.58)   
346 - **.CGCCGCGG.** 84 (93.07)   
346 - **[CGCCGCGA]** 22 (88.22)   
347 + **[CCCCGCGT]** 177 (87.58)   
347 + **[CGCCGCCT]** 260 (89.62)   
347 + **.CGCCGCGC.** 908 (92.48)   
347 + **.CGGCGCGC.** 1100 (91.73)   
347 + **[CGCCGCCC]** 1181 (85.13)   
347 + **.CGACGCCT.** 1381 (92.65)   
347 - **.CGAGGCGT.** 1383 (92.16)   
347 - **[GGCCGCGG]** 1285 (85.06)   
347 - **.CGGCGCGG.** 1256 (92.31)   
347 - **[CGCCGCCT]** 1224 (89.62)   
347 - **.CGCCGCGC.** 1087 (92.48)   
348 + **.CGCCGCGG.** 68 (93.07)   
348 + **.CGCCGCGG.** 134 (93.07)   
348 + **.CGGCGCGC.** 292 (91.73)   
348 + **[GGACGCGG]** 307 (88.08)   
348 + **[CGCCGCCC]** 435 (85.13)   
348 + **[CGCCGCCG]** 445 (85.71)   
348 + **.CGGCGCGG.** 544 (92.31)   
348 + **[CGAGGCGC]** 603 (87.66)   
348 + **.CGCCGCGT.** 661 (96.98)   
348 + **[CGCCCCGT]** 681 (87.14)   
348 + **.CGCCGCGC.** 695 (92.48)   
348 + **[CGCCGCCG]** 717 (85.71)   
348 + **.CGGCGCGC.** 861 (91.73)   
348 + **[CGCCGCCT]** 908 (89.62)   
348 + **[CGCCGCCC]** 918 (85.13)   
348 + **[CGCGGCGG]** 1028 (85.22)   
348 + **[GGACGCGG]** 1189 (88.08)   
348 + **.CGGCGCGG.** 1278 (92.31)   
348 + **[GGGCGCGT]** 1477 (88.21)   
348 + **[CGCCGCCG]** 1491 (85.71)   
348 + **[CGCCGCCC]** 1494 (85.13)   
348 - **[CGGCGCGA]** 1488 (87.47)   
348 - **[CCACGCGC]** 1479 (86.11)   
348 - **[CGAGGCGC]** 1391 (87.66)   
348 - **[CGCCGCCT]** 1345 (89.62)   
348 - **.CGCCGCGC.** 1239 (92.48)   
348 - **[CGCCGCCT]** 1163 (89.62)   
348 - **[CGCCGCGA]** 1027 (88.22)   
348 - **.CGCCGCGG.** 711 (93.07)   
348 - **.CGACGCGG.** 663 (96.09)   
348 - **[CGGCGGGT]** 432 (85.13)   
348 - **[CCCCGCGT]** 309 (87.58)   
348 - **.CGGCGCGC.** 131 (91.73)   
349 + **.GGACGCGT.** 488 (91.99)   
349 + **[CGACGCCG]** 655 (88.74)   
349 + **[CGCCGCCT]** 731 (89.62)   
349 + **[CGCCGCCG]** 747 (85.71)   
349 + **.CGGCGCGG.** 836 (92.31)   
349 + **[CGGCGCCT]** 1023 (88.87)   
349 + **.CGGCGCGG.** 1162 (92.31)   
349 + **[CGCGGCGG]** 1165 (85.22)   
349 + **[CGGCGGGT]** 1167 (85.13)   
349 + **[CGCCGCCC]** 1221 (85.13)   
349 - **.CGACGCGC.** 1309 (95.51)   
349 - **.CGCCGCGC.** 1164 (92.48)   
349 - **[CGGCGCCT]** 994 (88.87)   
349 - **[CGCCGCCG]** 936 (85.71)   
349 - **.CCACGCGT.** 490 (90.60)   
350 + **[CGTCGCGG]** 998 (86.02)   
350 + **[CGAGGCGG]** 1025 (88.25)   
350 + **.CGGCGCGG.** 1043 (92.31)   
350 + **[CGCCGCCT]** 1138 (89.62)   
350 + **[CGCCGCCC]** 1188 (85.13)   
350 + **[CGACCCGG]** 1227 (86.25)   
350 + **.CGGCGCGC.** 1342 (91.73)   
350 + **.CGGCGCGG.** 1436 (92.31)   
350 - **[CGCCGCCC]** 1433 (85.13)   
350 - **[CGCCCCGT]** 1365 (87.14)   
350 - **[CGCCGCCG]** 1275 (85.71)   
350 - **.CGGCGCGG.** 1185 (92.31)   
350 - **[CGAGGCGG]** 1140 (88.25)   
350 - **[CGGCGCCT]** 1061 (88.87)   
350 - **[CGCCGCCT]** 1027 (89.62)   
350 - **[CCCCGCGT]** 1012 (87.58)   
351 + **[CGAGGCGG]** 45 (88.25)   
351 + **[CGGCGCCT]** 56 (88.87)   
351 + **[CGAGGCGG]** 198 (88.25)   
351 + **[CGAGGCGC]** 573 (87.66)   
351 + **.CGCCGCGG.** 936 (93.07)   
351 + **[CGCCGCCC]** 994 (85.13)   
351 + **[CGCCGCCC]** 1038 (85.13)   
351 - **[GGCCGCGG]** 1254 (85.06)   
351 - **[GGCCGCGG]** 1184 (85.06)   
351 - **.CGCCGCGT.** 974 (96.98)   
351 - **[CGCGGCGT]** 935 (89.13)   
352 + **[CGCCGCCT]** 57 (89.62)   
352 + **.CGGCGCGC.** 200 (91.73)   
352 + **.CGACGCGG.** 281 (96.09)   
352 + **[GGCCGCGG]** 309 (85.06)   
352 + **[CGCGGCGG]** 312 (85.22)   
352 + **[CGCCGCCC]** 337 (85.13)   
352 + **.CGCCGCGG.** 352 (93.07)   
352 + **[CGCCGCCC]** 630 (85.13)   
352 + **[CGCCGCCG]** 861 (85.71)   
352 + **[CGCCGGGT]** 864 (85.88)   
352 + **[CGCCGCCG]** 995 (85.71)   
352 + **[CGAGGCGC]** 1061 (87.66)   
352 - **[GGCCGCGG]** 1339 (85.06)   
352 - **.CGGCGCGC.** 1227 (91.73)   
352 - **[GGCCGCGG]** 941 (85.06)   
352 - **[CGCCGCCC]** 855 (85.13)   
352 - **.CGGCGCGG.** 717 (92.31)   
352 - **[GGCCGCGG]** 620 (85.06)   
352 - **[AGCCGCGT]** 438 (86.16)   
352 - **[CGCGGCGG]** 351 (85.22)   
352 - **.CGCCGCGG.** 311 (93.07)   
352 - **.CGGCGCGC.** 202 (91.73)   
352 - **.CGCCGCGG.** 197 (93.07)   
352 - **[CGGCGCGA]** 54 (87.47)   
352 - **[CGACTCGT]** 49 (86.94)   
353 + **[CCACGCGC]** 172 (86.11)   
353 + **[GGACGCGC]** 186 (87.50)   
353 + **[CGCCGGGT]** 665 (85.88)   
353 + **[CGCCGCCC]** 690 (85.13)   
353 + **[GGACGCGG]** 707 (88.08)   
353 + **[CGCCGCCG]** 733 (85.71)   
353 + **[CGCCGCCG]** 736 (85.71)   
353 + **[CGCCGCCG]** 739 (85.71)   
353 + **[CGCCGCCG]** 742 (85.71)   
353 + **[CGCCGCGA]** 1178 (88.22)   
353 + **[CGCGGCGG]** 1420 (85.22)   
353 + **[CGCGGCGG]** 1438 (85.22)   
353 - **.CGCCGCGG.** 1437 (93.07)   
353 - **.CGCCGCGC.** 1419 (92.48)   
353 - **[CGAGGCGG]** 1122 (88.25)   
353 - **.CGGCGCGC.** 662 (91.73)   
356 + **.CGCCGCGC.** 90 (92.48)   
356 + **[CGCCGCGA]** 783 (88.22)   
356 + **[CGGGGCGT]** 976 (88.38)   
356 - **[GGACGCGC]** 1172 (87.50)   
356 - **[CGCGGCGG]** 782 (85.22)   
356 - **[CGCCGGGT]** 507 (85.88)   
356 - **.CGAGGCGT.** 218 (92.16)   
356 - **[CGCCGCCG]** 174 (85.71)   
356 - **[CGCCGCCG]** 171 (85.71)   
357 + **[GGGCGCGT]** 526 (88.21)   
357 + **[CGACCCGC]** 539 (85.67)   
357 + **[CCACGCGC]** 920 (86.11)   
357 + **.GGACGCGT.** 943 (91.99)   
357 + **.CGCCGCGC.** 990 (92.48)   
357 + **[CGCCGCCT]** 995 (89.62)   
357 + **[GGCCGCGT]** 1109 (88.97)   
357 + **[CGGGGCGT]** 1242 (88.38)   
357 + **[CGGGGCGT]** 1304 (88.38)   
357 + **[CGGGGCGT]** 1365 (88.38)   
357 + **[CGGGGCGT]** 1426 (88.38)   
357 - **.CGGCGCGG.** 992 (92.31)   
357 - **[CGCGGCGG]** 989 (85.22)   
357 - **[CCCCGCGT]** 660 (87.58)   
357 - **[GGACGCGG]** 543 (88.08)   
357 - **[CCACGCGC]** 528 (86.11)   
357 - **[CGCCGCCC]** 417 (85.13)   
358 + **[CGACGGGT]** 911 (88.90)   
358 + **[CGCGGCGG]** 978 (85.22)   
358 - **.CGCCGCGC.** 977 (92.48)   
358 - **[GGCCGCGG]** 773 (85.06)   
359 + **[CGACGCCC]** 354 (88.15)   
359 + **[CGCCGCCG]** 984 (85.71)   
359 + **[CGCCGCCT]** 987 (89.62)   
359 + **[CGCCGCCG]** 1025 (85.71)   
359 + **[CGCCGCCG]** 1028 (85.71)   
359 + **[CGCCGCCC]** 1031 (85.13)   
359 + **[CGCCGCCG]** 1055 (85.71)   
359 + **[CGCCGCCG]** 1058 (85.71)   
359 + **[CGCCGCCG]** 1121 (85.71)   
359 + **[CGCCGCCG]** 1124 (85.71)   
359 + **.CGGCGCGC.** 1236 (91.73)   
359 + **[CGCGGCGG]** 1255 (85.22)   
359 + **[CCCCGCGT]** 1278 (87.58)   
359 + **[CGCCGCCT]** 1315 (89.62)   
359 + **[AGGCGCGT]** 1345 (85.41)   
359 - **.CGGCGCGC.** 1306 (91.73)   
359 - **.CGCCGCGC.** 1254 (92.48)   
359 - **.CGCCGCGG.** 1233 (93.07)   
360 + **[CGGCCCGT]** 491 (86.38)   
360 + **.CGCCGCGC.** 651 (92.48)   
360 + **[CGCCGCCC]** 763 (85.13)   
360 + **[CGCCGGGT]** 869 (85.88)   
360 + **[CGCGGCGG]** 973 (85.22)   
360 + **.CGCCGCGC.** 1065 (92.48)   
360 + **.CGCCGCGG.** 1070 (93.07)   
360 + **.CGGCGCGG.** 1184 (92.31)   
360 - **.CGGCGCGC.** 1244 (91.73)   
360 - **.CGCCGCGG.** 1126 (93.07)   
360 - **.CGGCGCGG.** 1067 (92.31)   
360 - **[CGCCGCCT]** 720 (89.62)   
360 - **[CGCGGCGT]** 650 (89.13)   
361 + **[CGAGGCGG]** 1462 (88.25)   
361 - **[GGCCGCGG]** 1417 (85.06)   
362 + **.CGACGCGG.** 524 (96.09)   
362 + **.CGCCGCGT.** 735 (96.98)   
362 + **[CGCGGCGT]** 911 (89.13)   
362 + **[CGCCGCCG]** 992 (85.71)   
362 + **[CGCCGCCC]** 995 (85.13)   
362 + **[CGCCGCCG]** 1003 (85.71)   
362 + **[CGAGGCGG]** 1145 (88.25)   
362 + **[CGACGCCG]** 1262 (88.74)   
362 - **[CGCCGCCC]** 933 (85.13)   
362 - **.CGCCGCGC.** 910 (92.48)   
362 - **[GGACGCGG]** 737 (88.08)   
362 - **[GGACGCGC]** 623 (87.50)   
362 - **[GGCCGCGG]** 282 (85.06)   
363 + **[GGCCGCGG]** 691 (85.06)   
363 + **[CGGCGCCT]** 900 (88.87)   
363 + **[CGTCGCGG]** 1080 (86.02)   
363 + **.CGGCGCGC.** 1111 (91.73)   
363 - **[CGACGGGT]** 1077 (88.90)   
363 - **[GGCCGCGG]** 798 (85.06)   
363 - **[GGCCGCGG]** 693 (85.06)   
364 + **[AGACGCGG]** 296 (85.28)   
364 + **[GGCCGCGT]** 820 (88.97)   
364 + **[AGACGCGG]** 1254 (85.28)   
364 + **[CGCGGCGG]** 1257 (85.22)   
364 - **.CGCCGCGT.** 1256 (96.98)   
366 + **[CGAGGCGC]** 67 (87.66)   
366 + **[CCCCGCGT]** 166 (87.58)   
366 + **[GGCCGCGG]** 181 (85.06)   
366 + **.CGGCGCGC.** 265 (91.73)   
366 + **[CGCGGCGG]** 410 (85.22)   
366 + **[CGCGGCGT]** 445 (89.13)   
366 + **[CGCCGCCT]** 923 (89.62)   
366 + **[CGCGGCGT]** 954 (89.13)   
366 + **[CGTCGCGT]** 973 (89.93)   
366 + **[CGACGCCG]** 997 (88.74)   
366 + **.CGCCGCGG.** 1252 (93.07)   
366 + **[CGCGGCGG]** 1393 (85.22)   
366 + **[GGCCGCGG]** 1411 (85.06)   
366 - **[CGCCCCGT]** 1404 (87.14)   
366 - **[CGCCGCCG]** 1395 (85.71)   
366 - **.CGCCGCGC.** 1392 (92.48)   
366 - **[CGCCGCCC]** 1362 (85.13)   
366 - **[CGGCGGGT]** 1261 (85.13)   
366 - **[CGCCGCCG]** 1239 (85.71)   
366 - **[CGCCGCCC]** 1170 (85.13)   
366 - **.CGCCGCGG.** 953 (93.07)   
366 - **.CGGCGCGC.** 906 (91.73)   
366 - **.CGACGCGA.** 853 (91.25)   
366 - **[CGAGGCGG]** 718 (88.25)   
366 - **.CGGCGCGC.** 657 (91.73)   
366 - **[GGACGCGG]** 520 (88.08)   
366 - **.CGGCGCGG.** 453 (92.31)   
366 - **.CGCCGCGG.** 444 (93.07)   
366 - **[CGCCGCCG]** 412 (85.71)   
366 - **.CGCCGCGC.** 409 (92.48)   
366 - **[CGACCCGG]** 210 (86.25)   
367 + **[CGACGCCG]** 220 (88.74)   
367 + **[GGCCGCGG]** 554 (85.06)   
367 + **[CGCCGCCG]** 894 (85.71)   
367 + **[CGCCGCCG]** 897 (85.71)   
367 + **[CGCCGCCG]** 900 (85.71)   
367 + **[CGCCGCCG]** 903 (85.71)   
367 + **[CGCCGCCG]** 906 (85.71)   
367 + **[CGCCGCCG]** 909 (85.71)   
367 + **[CGCCGCCG]** 912 (85.71)   
367 + **[CGCCGCCG]** 915 (85.71)   
367 + **[GGCCGCGG]** 1062 (85.06)   
367 + **[GGCCGCGG]** 1089 (85.06)   
367 + **[CGCCGCCG]** 1146 (85.71)   
367 + **[CGCCGCCC]** 1149 (85.13)   
367 + **.CGCCGCGG.** 1189 (93.07)   
367 + **[CGCGGCGG]** 1192 (85.22)   
367 - **[CGCCGCCG]** 1194 (85.71)   
367 - **.CGCCGCGG.** 1191 (93.07)   
367 - **[CGCGGCGG]** 1188 (85.22)   
367 - **.CGGCGCGG.** 1033 (92.31)   
  

**Frequency Matrix**

| All Occs Best Occs|  |  |  |  |  |  |  |  |  |  |  |  |  |  |  |  |  |  |  |  |  |  |  |  |  |  |  |  |  |  |  |  |  |  |  |  |  |  |  |  |  |  |  |  |  |  |  |  |  |  |  |  |  |  |  |  |  |  |  |  |  |  |  |  |  |  |  |  |  |  |  |  |  |  |  |  |  |  |  |  |  |  |  |  |  |  |  |  |  |  |  |  |  |  |  |  |  |  |  |  |  |  |  |  |  |  |  |  | | --- | --- | --- | --- | --- | --- | --- | --- | --- | --- | --- | --- | --- | --- | --- | --- | --- | --- | --- | --- | --- | --- | --- | --- | --- | --- | --- | --- | --- | --- | --- | --- | --- | --- | --- | --- | --- | --- | --- | --- | --- | --- | --- | --- | --- | --- | --- | --- | --- | --- | --- | --- | --- | --- | --- | --- | --- | --- | --- | --- | --- | --- | --- | --- | --- | --- | --- | --- | --- | --- | --- | --- | --- | --- | --- | --- | --- | --- | --- | --- | --- | --- | --- | --- | --- | --- | --- | --- | --- | --- | --- | --- | --- | --- | --- | --- | --- | --- | --- | --- | --- | --- | --- | --- | --- | --- | --- | --- | | **A C G T A C G T | | | | | | | | | | | |**| **1** 217 3146 434 127 | 0 830 16 0|  |  |  |  |  |  |  |  |  |  |  |  |  |  |  |  |  |  |  |  |  |  |  |  |  |  |  |  |  |  |  |  |  |  |  |  |  |  |  |  |  |  |  |  |  |  |  |  |  |  |  |  |  |  |  |  |  |  |  |  |  |  |  |  |  |  |  |  |  |  |  |  |  |  |  |  |  |  |  |  |  |  |  |  | | --- | --- | --- | --- | --- | --- | --- | --- | --- | --- | --- | --- | --- | --- | --- | --- | --- | --- | --- | --- | --- | --- | --- | --- | --- | --- | --- | --- | --- | --- | --- | --- | --- | --- | --- | --- | --- | --- | --- | --- | --- | --- | --- | --- | --- | --- | --- | --- | --- | --- | --- | --- | --- | --- | --- | --- | --- | --- | --- | --- | --- | --- | --- | --- | --- | --- | --- | --- | --- | --- | --- | --- | --- | --- | --- | --- | --- | --- | --- | --- | --- | --- | --- | --- | | **2** 130 327 3344 123 | 0 7 839 0|  |  |  |  |  |  |  |  |  |  |  |  |  |  |  |  |  |  |  |  |  |  |  |  |  |  |  |  |  |  |  |  |  |  |  |  |  |  |  |  |  |  |  |  |  |  |  |  |  |  |  |  |  |  |  |  |  |  |  |  |  |  |  |  |  |  |  |  |  |  |  |  | | --- | --- | --- | --- | --- | --- | --- | --- | --- | --- | --- | --- | --- | --- | --- | --- | --- | --- | --- | --- | --- | --- | --- | --- | --- | --- | --- | --- | --- | --- | --- | --- | --- | --- | --- | --- | --- | --- | --- | --- | --- | --- | --- | --- | --- | --- | --- | --- | --- | --- | --- | --- | --- | --- | --- | --- | --- | --- | --- | --- | --- | --- | --- | --- | --- | --- | --- | --- | --- | --- | --- | --- | | **3** 2013 953 791 167 | 127 398 321 0|  |  |  |  |  |  |  |  |  |  |  |  |  |  |  |  |  |  |  |  |  |  |  |  |  |  |  |  |  |  |  |  |  |  |  |  |  |  |  |  |  |  |  |  |  |  |  |  |  |  |  |  |  |  |  |  |  |  |  |  | | --- | --- | --- | --- | --- | --- | --- | --- | --- | --- | --- | --- | --- | --- | --- | --- | --- | --- | --- | --- | --- | --- | --- | --- | --- | --- | --- | --- | --- | --- | --- | --- | --- | --- | --- | --- | --- | --- | --- | --- | --- | --- | --- | --- | --- | --- | --- | --- | --- | --- | --- | --- | --- | --- | --- | --- | --- | --- | --- | --- | | **4** 165 3199 460 100 | 0 829 17 0|  |  |  |  |  |  |  |  |  |  |  |  |  |  |  |  |  |  |  |  |  |  |  |  |  |  |  |  |  |  |  |  |  |  |  |  |  |  |  |  |  |  |  |  |  |  |  |  | | --- | --- | --- | --- | --- | --- | --- | --- | --- | --- | --- | --- | --- | --- | --- | --- | --- | --- | --- | --- | --- | --- | --- | --- | --- | --- | --- | --- | --- | --- | --- | --- | --- | --- | --- | --- | --- | --- | --- | --- | --- | --- | --- | --- | --- | --- | --- | --- | | **5** 135 295 3361 133 | 0 6 840 0|  |  |  |  |  |  |  |  |  |  |  |  |  |  |  |  |  |  |  |  |  |  |  |  |  |  |  |  |  |  |  |  |  |  |  |  | | --- | --- | --- | --- | --- | --- | --- | --- | --- | --- | --- | --- | --- | --- | --- | --- | --- | --- | --- | --- | --- | --- | --- | --- | --- | --- | --- | --- | --- | --- | --- | --- | --- | --- | --- | --- | | **6** 123 3438 221 142 | 0 846 0 0|  |  |  |  |  |  |  |  |  |  |  |  |  |  |  |  |  |  |  |  |  |  |  |  | | --- | --- | --- | --- | --- | --- | --- | --- | --- | --- | --- | --- | --- | --- | --- | --- | --- | --- | --- | --- | --- | --- | --- | --- | | **7** 71 511 3149 193 | 0 15 831 0|  |  |  |  |  |  |  |  |  |  |  |  | | --- | --- | --- | --- | --- | --- | --- | --- | --- | --- | --- | --- | | **8** 247 708 818 2151 | 11 346 365 124 | | | | | | | | | | | | | | | | | | | | | | | | | | | | | | | | | | | | | | | | | | | | | | | | | | | | | | | | | | | | | | | | | | | | | | | | | | | | | | | | | | | | | | | | | | | | | | | | | | | | | | | | | | | | |

  
  

---**
