# Supplementary material for: In silico regulatory analysis for exploring human disease progression
Source: Biol Direct. 2008 Jun 18;3:24. doi: 10.1186/1745-6150-3-24 (PMC2464594; doi:10.1186/1745-6150-3-24)
Supplement: Additional file 8 — This file contains the results of running the Weeder algorithm on 1) the set of known and newly predicted (Platt score P ≥ 0.95) targets of WT1, and 2) the known targets of WT1. Sequence regions used are as defined in Methods. The file also contains the results of Oligo-analysis. Also included are the matching results after scanning the literature derived consensus sites for WT1 against the full set of WT1 targets (predicted and known). [file 1745-6150-3-24-S8.zip › WeederOutput_known_targets_Wt1.zip!/known.fasta.html]

Your Weeder Web Results  
  
NEW: Starting a medium job on file known.fasta  
  
Organism code: HS  
  
Processing **both** strands  
  
**weederlauncher.out known.fasta HS medium M S 15   
  
  
Searching for motifs of length 6 with 1 mutations.....  
  
(Actual commandline is :   
./weederTFBS.out -f known.fasta -R 50 -O HS -W 6 -e 1 -M -S -T 15 )  
1) CGACGG 1.32   
2) AGCGCG 1.20   
3) TACGCG 1.09   
4) CGACCC 1.09   
5) CGCGCG 1.08   
6) TCCGCG 1.07   
7) GTCGCG 1.07   
8) CCGCGT 1.07   
9) CGCGGG 1.05   
10) CCGACG 1.04   
11) GCGACC 1.03   
12) CGCACA 1.02   
13) GAGCGC 1.02   
14) GCGCGA 1.01   
15) CGGGCC 1.01   
  
Elapsed time : 0 min. 2 sec.  
  
  
Searching for motifs of length 8 with 2 mutations.....  
  
(Actual commandline is :   
./weederTFBS.out -f known.fasta -R 50 -O HS -W 8 -e 2 -M -S -T 15 )  
1) GCAGCGCG 1.66   
2) CCGTCGCG 1.62   
3) CGCGAGTT 1.59   
4) CCCGCGGG 1.56   
5) GTCCGCGG 1.52   
6) CCGCGCGG 1.50   
7) CGCGCTCT 1.49   
8) CTGCGCCG 1.47   
9) TCCGCGCG 1.45   
10) CAGCGCGG 1.38   
11) GCGGCGCA 1.38   
12) CGCGGGCA 1.38   
13) CGTCCCGC 1.36   
14) TGGGGCGC 1.34   
15) CGGCCACC 1.32   
  
Elapsed time : 1 min. 23 sec.  
  
  
Searching for motifs of length 10 with 3 mutations.....  
  
(Actual commandline is :   
./weederTFBS.out -f known.fasta -R 50 -O HS -W 10 -e 3 -M -S -T 15 )  
1) CGCTGCGTCG 1.96   
2) CCGGCGCAGC 1.86   
3) ACCGGGGGCC 1.83   
4) GACCCCCCGA 1.80   
5) CGTCGCAGCG 1.79   
6) CCATGCGCCG 1.73   
7) TTCCGCGCGT 1.71   
8) CCGGCCACCC 1.71   
9) CCCCGGCGCA 1.70   
10) CGTCCCGCAC 1.70   
11) CCGTCGGTGC 1.68   
12) CGAACTCGCG 1.65   
13) GTCGCAGCGC 1.64   
14) CGGCCCCCGG 1.63   
15) CCGCGGGCGC 1.63   
  
Elapsed time : 37 min. 39 sec.  
  
  
Your sequences:  
  
Sequence 1 : >LL.374-Human-1 DBTSS:NM\_001657 chr4 + GGCCGGTGAGGGCGGCGGGGGCGGCGGGACCCCGGGTCGGGGGTC�  
Sequence 2 : >LL.7015-Human-2 AF098956\_1 chr5 - 1347  
Sequence 3 : >LL.2297-Human-3 hchr5\_P021624 chr5 - 7  
Sequence 4 : >LL.4602-Human-4 HSU22376\_1 chr6 + 1355  
Sequence 5 : >LL.1956-Human-5 EP15043 chr7 + 5485956  
Sequence 6 : >LL.5243-Human-6 EP35012 chr7 - 8687435  
Sequence 7 : >LL.6736-Human-7 hchrY\_P90027 chrY - 26  
Sequence 8 : >LL.3481-Human-8 EP17072 chr11 - 211669  
Sequence 9 : >LL.51352-Human-9 HSWT1WIT\_1 chr11 + 32  
Sequence 10 : >LL.4192-Human-10 EP74560 chr11 + 46358  
Sequence 11 : >LL.596-Human-11 EP27006 chr18 - 591377  
Sequence 12 : >LL.3643-Human-12 HUMINSRB\_1 chr19 - 72  
Sequence 13 : >LL.898-Human-13 HSCYCEGEN\_1 chr19 + 34  
  
  
**\*\*\*\* MY ADVICE \*\*\*\***  
  
  
 **\*\*\* Interesting motifs (highest-ranking) seem to be :**   
  
  
 **CGACGG**   
**CCGTCG**  
  
2 redundant motifs found:  
CCGTCGCG - CCGACG -   
  
Best occurrences (match percentage):   
Seq St oligo pos match
  
 1 + **.CGAGGG.** 859 (90.65)   
1 + **[CCACGG]** 933 (85.35)   
1 + **[GGCCGG]** 937 (86.27)   
1 + **[CGAAGG]** 1216 (86.34)   
1 + **.CGCCGG.** 1245 (95.42)   
1 + **.CGCCGG.** 1251 (95.42)   
1 - **[CGGGGG]** 1387 (88.57)   
1 - **.CGCCGG.** 1299 (95.42)   
1 - **.CGCCGG.** 1247 (95.42)   
1 - **.CGGCGG.** 1244 (97.92)   
1 - **.CGGCGG.** 1206 (97.92)   
1 - **[CGAAGG]** 1098 (86.34)   
1 - **[GGGCGG]** 1079 (88.77)   
1 - **.CGGCGG.** 1076 (97.92)   
1 - **[GGGCGG]** 1004 (88.77)   
2 + **[GGGCGG]** 107 (88.77)   
2 + **.CGACGG.** 244 (100.00)   
2 + **[CGCGGG]** 364 (86.07)   
2 + **[CGACGC]** 507 (86.49)   
2 + **[CGCGGG]** 707 (86.07)   
2 + **[GGGCGG]** 710 (88.77)   
2 + **[GGCCGG]** 772 (86.27)   
2 + **[CGCGGG]** 792 (86.07)   
2 + **[CGCGGG]** 1190 (86.07)   
2 + **.CGGCGG.** 1200 (97.92)   
2 + **[GGGCGG]** 1328 (88.77)   
2 + **[GGCCGG]** 1332 (86.27)   
2 + **[CGGGGG]** 1335 (88.57)   
2 + **.GGACGG.** 1489 (90.85)   
2 + **[CGCGGG]** 1499 (86.07)   
2 + **[CGGGGG]** 1501 (88.57)   
2 - **[CGGGGG]** 1509 (88.57)   
2 - **[CGCGGG]** 1497 (86.07)   
2 - **[CGAAGG]** 1470 (86.34)   
2 - **[CGGGGG]** 1387 (88.57)   
2 - **[GGGCGG]** 1270 (88.77)   
2 - **.CGGCGG.** 1267 (97.92)   
2 - **[CGGGGG]** 1264 (88.57)   
2 - **[GGGCGG]** 1260 (88.77)   
2 - **.CGCCGG.** 1199 (95.42)   
2 - **[CGCGGG]** 1063 (86.07)   
2 - **[CGGGGG]** 1061 (88.57)   
2 - **[GGCCGG]** 1054 (86.27)   
2 - **[GGGCGG]** 984 (88.77)   
2 - **[GGCCGG]** 930 (86.27)   
2 - **.GGACGG.** 907 (90.85)   
2 - **[GGGCGG]** 902 (88.77)   
2 - **.GGACGG.** 855 (90.85)   
2 - **[GGGCGG]** 742 (88.77)   
2 - **[CGGGGG]** 733 (88.57)   
2 - **.CGAGGG.** 667 (90.65)   
2 - **.CGAGGG.** 616 (90.65)   
2 - **[CGCGGG]** 513 (86.07)   
2 - **[CGTCGG]** 506 (89.13)   
2 - **.GGACGG.** 464 (90.85)   
2 - **.GGACGG.** 410 (90.85)   
2 - **.CGAGGG.** 304 (90.65)   
2 - **[GGGCGG]** 252 (88.77)   
3 + **.CGAGGG.** 682 (90.65)   
3 + **[CGCGGG]** 703 (86.07)   
3 + **[GGCCGG]** 1175 (86.27)   
3 + **.CGGCGG.** 1225 (97.92)   
3 + **[GGCCGG]** 1334 (86.27)   
3 - **.CGGCGG.** 1414 (97.92)   
3 - **.CGGCGG.** 1411 (97.92)   
3 - **.CGGCGG.** 1408 (97.92)   
3 - **[GGGCGG]** 1403 (88.77)   
3 - **.CGCCGG.** 1387 (95.42)   
3 - **[GGGCGG]** 1345 (88.77)   
3 - **[GGCCGG]** 1332 (86.27)   
3 - **[CGGGGG]** 1329 (88.57)   
3 - **.CGCCGG.** 1281 (95.42)   
3 - **.CGGCGG.** 1262 (97.92)   
3 - **[GGGCGG]** 1168 (88.77)   
3 - **[GGGCGG]** 1095 (88.77)   
3 - **.GGACGG.** 1055 (90.85)   
3 - **[GGGCGG]** 998 (88.77)   
3 - **.CGAGGG.** 931 (90.65)   
3 - **.CGAGGG.** 774 (90.65)   
3 - **[CGGGGG]** 653 (88.57)   
4 + **[GGGCGG]** 64 (88.77)   
4 + **[GGCCGG]** 196 (86.27)   
4 + **[GGGCGG]** 200 (88.77)   
4 + **[GGGCGG]** 205 (88.77)   
4 + **.CGACGG.** 266 (100.00)   
4 + **[CGACCG]** 637 (85.35)   
4 + **.CGGCGG.** 672 (97.92)   
4 + **[CGCGGG]** 685 (86.07)   
4 + **.CGGCGG.** 824 (97.92)   
4 + **[CGCGGG]** 875 (86.07)   
4 + **[GGGCGG]** 878 (88.77)   
4 + **[CGACCG]** 894 (85.35)   
4 + **.CGGCGG.** 906 (97.92)   
4 + **.CGGCGG.** 1142 (97.92)   
4 + **.CGGCGG.** 1188 (97.92)   
4 + **[GGGCGG]** 1251 (88.77)   
4 + **[GGGCGG]** 1255 (88.77)   
4 + **.CGAGGG.** 1262 (90.65)   
4 + **[GGGCGG]** 1265 (88.77)   
4 + **[CGGGGG]** 1268 (88.57)   
4 + **[CGCGGG]** 1276 (86.07)   
4 + **[CGGGGG]** 1278 (88.57)   
4 + **[CGCGGG]** 1286 (86.07)   
4 - **.CGGCGG.** 1204 (97.92)   
4 - **[CGCGGG]** 1197 (86.07)   
4 - **[CGCGGG]** 1177 (86.07)   
4 - **[CGCGGG]** 873 (86.07)   
4 - **[GGCCGG]** 856 (86.27)   
4 - **[CGCGGG]** 782 (86.07)   
4 - **[GGCCGG]** 750 (86.27)   
4 - **[CGGGGG]** 747 (88.57)   
4 - **.CGACGG.** 738 (100.00)   
4 - **.CGGCGG.** 697 (97.92)   
4 - **[CGCGGG]** 572 (86.07)   
4 - **[CGGGGG]** 570 (88.57)   
4 - **[GGGCGG]** 344 (88.77)   
5 + **[CCACGG]** 582 (85.35)   
5 + **.CGAGGG.** 742 (90.65)   
5 + **[CGCGGG]** 816 (86.07)   
5 + **[CGTCGG]** 922 (89.13)   
5 + **[CGACGC]** 938 (86.49)   
5 + **[CGCGGG]** 1076 (86.07)   
5 + **.CGCCGG.** 1174 (95.42)   
5 + **[CGCGGG]** 1191 (86.07)   
5 + **[CGGGGG]** 1193 (88.57)   
5 + **.CGGCGG.** 1220 (97.92)   
5 + **[CGACGC]** 1437 (86.49)   
5 + **[GGCCGG]** 1452 (86.27)   
5 + **.CGGCGG.** 1475 (97.92)   
5 - **[GGGCGG]** 1487 (88.77)   
5 - **.CGGCGG.** 1484 (97.92)   
5 - **.CGGCGG.** 1481 (97.92)   
5 - **.CGCCGG.** 1474 (95.42)   
5 - **[CGTCGG]** 1436 (89.13)   
5 - **[CGGGGG]** 1416 (88.57)   
5 - **[GGGCGG]** 1412 (88.77)   
5 - **.CGCCGG.** 1386 (95.42)   
5 - **[CGGGGG]** 1383 (88.57)   
5 - **[CGCGGG]** 1356 (86.07)   
5 - **[CGCGGG]** 1344 (86.07)   
5 - **[GGGCGG]** 1332 (88.77)   
5 - **.CGGCGG.** 1309 (97.92)   
5 - **.CGCCGG.** 1219 (95.42)   
5 - **[CGCGGG]** 1189 (86.07)   
5 - **.CGGCGG.** 1173 (97.92)   
5 - **[GGGCGG]** 1162 (88.77)   
5 - **[CGTCGG]** 937 (89.13)   
5 - **.CGACGG.** 921 (100.00)   
5 - **.CGAGGG.** 769 (90.65)   
6 + **[GGCCGG]** 905 (86.27)   
6 + **.CGCCGG.** 954 (95.42)   
6 + **[CGCGGG]** 1410 (86.07)   
6 + **[GGGCGG]** 1413 (88.77)   
6 + **.CGGCGG.** 1477 (97.92)   
6 - **[CGCGGG]** 1408 (86.07)   
6 - **[CGCGGG]** 1378 (86.07)   
7 + **[GGGCGG]** 1005 (88.77)   
7 + **[GGGCGG]** 1028 (88.77)   
8 + **.CGGCGG.** 4 (97.92)   
8 + **[CGGGGG]** 7 (88.57)   
8 + **.GGACGG.** 177 (90.85)   
8 + **[GGCCGG]** 309 (86.27)   
8 + **[CGGGGG]** 388 (88.57)   
8 + **[CGGGGG]** 417 (88.57)   
8 + **[GGCCGG]** 459 (86.27)   
8 + **[CGGGGG]** 881 (88.57)   
8 + **[GGGCGG]** 884 (88.77)   
8 + **[CGGGGG]** 893 (88.57)   
8 + **[GGGCGG]** 896 (88.77)   
8 + **.CGAGGG.** 904 (90.65)   
8 + **[CGCGGG]** 913 (86.07)   
8 + **[CGCGGG]** 929 (86.07)   
8 + **[CGCGGG]** 955 (86.07)   
8 + **.CGGCGG.** 1027 (97.92)   
8 + **[CGACGC]** 1151 (86.49)   
8 + **[CGCGGG]** 1198 (86.07)   
8 + **.CGCCGG.** 1324 (95.42)   
8 + **.CGACGG.** 1468 (100.00)   
8 + **[CGTCGG]** 1484 (89.13)   
8 - **[CGCGGG]** 1508 (86.07)   
8 - **.CGACGG.** 1483 (100.00)   
8 - **.CGCCGG.** 1370 (95.42)   
8 - **[CGGGGG]** 1367 (88.57)   
8 - **[CGCGGG]** 1344 (86.07)   
8 - **[CGGGGG]** 1342 (88.57)   
8 - **.CGCCGG.** 1326 (95.42)   
8 - **.CGGCGG.** 1323 (97.92)   
8 - **[CGGGGG]** 1297 (88.57)   
8 - **.CGGCGG.** 1233 (97.92)   
8 - **.GGACGG.** 1193 (90.85)   
8 - **.GGACGG.** 1181 (90.85)   
8 - **[GGGCGG]** 1156 (88.77)   
8 - **[CGACGC]** 1147 (86.49)   
8 - **[CGCGGG]** 1144 (86.07)   
8 - **[CGCGGG]** 1102 (86.07)   
8 - **[CGGGGG]** 1078 (88.57)   
8 - **[GGGCGG]** 1075 (88.77)   
8 - **[GGCCGG]** 1071 (86.27)   
8 - **[GGCCGG]** 1057 (86.27)   
8 - **[GGCCGG]** 1046 (86.27)   
8 - **.CGCCGG.** 1026 (95.42)   
8 - **[CGCGGG]** 951 (86.07)   
8 - **[GGGCGG]** 857 (88.77)   
8 - **[CGGGGG]** 854 (88.57)   
8 - **[CGGGGG]** 823 (88.57)   
8 - **[GGGCGG]** 815 (88.77)   
8 - **.CGGCGG.** 812 (97.92)   
8 - **[CGCGGG]** 786 (86.07)   
8 - **[CGGGGG]** 784 (88.57)   
8 - **[GGGCGG]** 677 (88.77)   
8 - **[CGGGGG]** 546 (88.57)   
8 - **[GGGCGG]** 500 (88.77)   
8 - **[GGCCGG]** 361 (86.27)   
8 - **.CGCCGG.** 352 (95.42)   
8 - **[GGGCGG]** 284 (88.77)   
8 - **[CGGGGG]** 133 (88.57)   
9 + **[GGGCGG]** 46 (88.77)   
9 + **.CGAGGG.** 55 (90.65)   
9 + **[GGCCGG]** 114 (86.27)   
9 + **[GGGCGG]** 302 (88.77)   
9 + **[GGCCGG]** 509 (86.27)   
9 + **[GGCCGG]** 752 (86.27)   
9 + **[GGGCGG]** 1084 (88.77)   
9 + **[CGGGGG]** 1175 (88.57)   
9 + **[GGCCGG]** 1179 (86.27)   
9 + **.CGACGG.** 1242 (100.00)   
9 + **.GGACGG.** 1257 (90.85)   
9 + **.CGACGG.** 1270 (100.00)   
9 - **[GGCCGG]** 1299 (86.27)   
9 - **[CGCGGG]** 1060 (86.07)   
9 - **[CGGGGG]** 1058 (88.57)   
9 - **[CGAAGG]** 1032 (86.34)   
9 - **.CGGCGG.** 849 (97.92)   
9 - **[GGCCGG]** 750 (86.27)   
9 - **[CGAAGG]** 155 (86.34)   
9 - **.CGAGGG.** 28 (90.65)   
10 + **.CGGCGG.** 30 (97.92)   
10 + **[CGTCGG]** 43 (89.13)   
10 + **[GGCCGG]** 49 (86.27)   
10 + **[CGGGGG]** 52 (88.57)   
10 + **[GGGCGG]** 55 (88.77)   
10 + **[GGGCGG]** 123 (88.77)   
10 + **[CGGGGG]** 126 (88.57)   
10 + **[CGGGGG]** 194 (88.57)   
10 + **[CGACCG]** 412 (85.35)   
10 + **[CGACCG]** 438 (85.35)   
10 + **[CGCGGG]** 579 (86.07)   
10 + **.CGCCGG.** 594 (95.42)   
10 + **[GGGCGG]** 687 (88.77)   
10 + **.CGGCGG.** 690 (97.92)   
10 + **[GGCCGG]** 707 (86.27)   
10 + **[CGGGGG]** 710 (88.57)   
10 + **[GGCCGG]** 714 (86.27)   
10 + **.GGACGG.** 733 (90.85)   
10 + **[GGCCGG]** 774 (86.27)   
10 + **.CGGCGG.** 811 (97.92)   
10 + **[CGCGGG]** 830 (86.07)   
10 + **[GGCCGG]** 835 (86.27)   
10 + **.CGCCGG.** 850 (95.42)   
10 + **[CGCGGG]** 864 (86.07)   
10 + **[CGGGGG]** 866 (88.57)   
10 + **[CGCGGG]** 873 (86.07)   
10 + **[CGGGGG]** 885 (88.57)   
10 + **.CGCCGG.** 898 (95.42)   
10 + **[CGGGGG]** 907 (88.57)   
10 + **[GGGCGG]** 910 (88.77)   
10 + **[GGGCGG]** 934 (88.77)   
10 + **[GGGCGG]** 939 (88.77)   
10 + **[GGGCGG]** 973 (88.77)   
10 + **[CGGGGG]** 976 (88.57)   
10 + **.CGGCGG.** 1026 (97.92)   
10 + **[GGCCGG]** 1030 (86.27)   
10 + **.GGACGG.** 1040 (90.85)   
10 + **[CGCGGG]** 1073 (86.07)   
10 + **.CGGCGG.** 1098 (97.92)   
10 + **[CGGGGG]** 1153 (88.57)   
10 + **.CGAGGG.** 1236 (90.65)   
10 + **.GGACGG.** 1352 (90.85)   
10 + **[CGAAGG]** 1484 (86.34)   
10 + **.GGACGG.** 1498 (90.85)   
10 - **[CGACCG]** 1450 (85.35)   
10 - **[CGGGGG]** 1336 (88.57)   
10 - **.CGGCGG.** 1261 (97.92)   
10 - **[GGGCGG]** 1243 (88.77)   
10 - **[CGGGGG]** 1193 (88.57)   
10 - **[GGCCGG]** 1116 (86.27)   
10 - **[GGGCGG]** 1112 (88.77)   
10 - **.CGACGG.** 789 (100.00)   
10 - **.CGGCGG.** 786 (97.92)   
10 - **[CGCGGG]** 577 (86.07)   
10 - **[CCACGG]** 558 (85.35)   
10 - **[GGCCGG]** 554 (86.27)   
10 - **[GGGCGG]** 498 (88.77)   
10 - **[CGCGGG]** 488 (86.07)   
10 - **[CCACGG]** 421 (85.35)   
10 - **[CGCGGG]** 87 (86.07)   
10 - **.CGCCGG.** 29 (95.42)   
11 + **[GGGCGG]** 23 (88.77)   
11 + **[GGGCGG]** 174 (88.77)   
11 + **[GGGCGG]** 184 (88.77)   
11 + **.CGGCGG.** 210 (97.92)   
11 + **.CGCCGG.** 221 (95.42)   
11 + **[CGCGGG]** 619 (86.07)   
11 + **[GGCCGG]** 653 (86.27)   
11 + **[CGCGGG]** 663 (86.07)   
11 + **[GGGCGG]** 680 (88.77)   
11 + **[GGCCGG]** 688 (86.27)   
11 + **.CGGCGG.** 741 (97.92)   
11 + **[GGGCGG]** 799 (88.77)   
11 + **[CGAAGG]** 1056 (86.34)   
11 + **.CGGCGG.** 1087 (97.92)   
11 + **.CGGCGG.** 1118 (97.92)   
11 + **.CGCCGG.** 1207 (95.42)   
11 + **[CGCGGG]** 1345 (86.07)   
11 + **.CGGCGG.** 1368 (97.92)   
11 - **.GGACGG.** 1464 (90.85)   
11 - **.CGCCGG.** 1367 (95.42)   
11 - **.CGAGGG.** 1324 (90.65)   
11 - **.CGGCGG.** 1153 (97.92)   
11 - **.CGCCGG.** 1086 (95.42)   
11 - **.CGGCGG.** 1042 (97.92)   
11 - **.CGGCGG.** 1039 (97.92)   
11 - **.CGGCGG.** 1036 (97.92)   
11 - **.CGGCGG.** 1025 (97.92)   
11 - **[CCACGG]** 1017 (85.35)   
11 - **.CGGCGG.** 1007 (97.92)   
11 - **[GGGCGG]** 1003 (88.77)   
11 - **.CGGCGG.** 992 (97.92)   
11 - **[GGGCGG]** 988 (88.77)   
11 - **[GGGCGG]** 984 (88.77)   
11 - **[GGGCGG]** 962 (88.77)   
11 - **[CGCGGG]** 955 (86.07)   
11 - **[GGGCGG]** 942 (88.77)   
11 - **[CGACCG]** 812 (85.35)   
11 - **.CGCCGG.** 740 (95.42)   
11 - **[CGGGGG]** 737 (88.57)   
11 - **[CGCGGG]** 721 (86.07)   
11 - **[GGCCGG]** 710 (86.27)   
11 - **[GGCCGG]** 690 (86.27)   
11 - **[CGCGGG]** 661 (86.07)   
11 - **[CGCGGG]** 637 (86.07)   
11 - **[GGGCGG]** 634 (88.77)   
11 - **[CGCGGG]** 591 (86.07)   
11 - **.CGACGG.** 527 (100.00)   
11 - **[GGCCGG]** 494 (86.27)   
11 - **[GGCCGG]** 489 (86.27)   
11 - **.CGACGG.** 477 (100.00)   
11 - **[GGGCGG]** 460 (88.77)   
11 - **.CGCCGG.** 414 (95.42)   
11 - **.CGCCGG.** 209 (95.42)   
11 - **[CGGGGG]** 206 (88.57)   
12 + **[CGCGGG]** 117 (86.07)   
12 + **[GGCCGG]** 166 (86.27)   
12 + **[CGCGGG]** 180 (86.07)   
12 + **[GGGCGG]** 183 (88.77)   
12 + **[CGTCGG]** 450 (89.13)   
12 + **[CGGGGG]** 472 (88.57)   
12 + **[CGCGGG]** 561 (86.07)   
12 + **[GGGCGG]** 593 (88.77)   
12 + **[GGGCGG]** 598 (88.77)   
12 + **[GGGCGG]** 603 (88.77)   
12 + **[GGCCGG]** 609 (86.27)   
12 + **[GGGCGG]** 613 (88.77)   
12 + **[CGTCGG]** 831 (89.13)   
12 + **[CGCGGG]** 884 (86.07)   
12 + **.GGACGG.** 949 (90.85)   
12 + **[CGGGGG]** 974 (88.57)   
12 + **[CGCGGG]** 1090 (86.07)   
12 + **[CGCGGG]** 1097 (86.07)   
12 + **[CGCGGG]** 1167 (86.07)   
12 + **[CGGGGG]** 1229 (88.57)   
12 + **[GGCCGG]** 1233 (86.27)   
12 + **.CGGCGG.** 1236 (97.92)   
12 + **[CGGGGG]** 1239 (88.57)   
12 + **[GGGCGG]** 1243 (88.77)   
12 + **.CGGCGG.** 1246 (97.92)   
12 + **[CGCGGG]** 1335 (86.07)   
12 + **[GGGCGG]** 1344 (88.77)   
12 + **[GGGCGG]** 1427 (88.77)   
12 + **[CGGGGG]** 1430 (88.57)   
12 + **[GGGCGG]** 1434 (88.77)   
12 + **.GGACGG.** 1446 (90.85)   
12 - **[GGGCGG]** 1505 (88.77)   
12 - **[CGGGGG]** 1396 (88.57)   
12 - **.CGCCGG.** 1235 (95.42)   
12 - **[CGCGGG]** 1203 (86.07)   
12 - **[CGCGGG]** 1165 (86.07)   
12 - **[CGGGGG]** 1163 (88.57)   
12 - **[CGCGGG]** 1088 (86.07)   
12 - **.CGACGG.** 830 (100.00)   
12 - **[GGGCGG]** 782 (88.77)   
12 - **[GGGCGG]** 778 (88.77)   
12 - **.CGGCGG.** 775 (97.92)   
12 - **[CGCGGG]** 709 (86.07)   
12 - **[CGCGGG]** 557 (86.07)   
12 - **.CGCCGG.** 539 (95.42)   
12 - **.CGAGGG.** 530 (90.65)   
12 - **[CGACGC]** 449 (86.49)   
12 - **[CGACGC]** 32 (86.49)   
12 - **.CGCCGG.** 17 (95.42)   
13 + **[CGGGGG]** 262 (88.57)   
13 + **[CGGGGG]** 499 (88.57)   
13 + **[GGGCGG]** 502 (88.77)   
13 + **.CGGCGG.** 545 (97.92)   
13 + **[GGGCGG]** 549 (88.77)   
13 + **.CGCCGG.** 837 (95.42)   
13 + **[CGCGGG]** 873 (86.07)   
13 + **[GGCCGG]** 877 (86.27)   
13 + **[GGGCGG]** 894 (88.77)   
13 + **[CGGGGG]** 903 (88.57)   
13 + **[GGGCGG]** 906 (88.77)   
13 + **.CGAGGG.** 914 (90.65)   
13 + **.GGACGG.** 918 (90.85)   
13 + **[GGGCGG]** 923 (88.77)   
13 + **.GGACGG.** 928 (90.85)   
13 + **.CGGCGG.** 974 (97.92)   
13 + **[GGGCGG]** 979 (88.77)   
13 + **.CGAGGG.** 987 (90.65)   
13 + **[GGGCGG]** 990 (88.77)   
13 + **[GGCCGG]** 995 (86.27)   
13 + **[GGCCGG]** 1001 (86.27)   
13 + **[CGCGGG]** 1229 (86.07)   
13 + **.CGCCGG.** 1266 (95.42)   
13 + **[GGGCGG]** 1284 (88.77)   
13 + **[CGCGGG]** 1304 (86.07)   
13 + **[GGGCGG]** 1315 (88.77)   
13 + **[CGCGGG]** 1324 (86.07)   
13 + **.CGGCGG.** 1354 (97.92)   
13 + **.CGACGG.** 1404 (100.00)   
13 + **[GGCCGG]** 1409 (86.27)   
13 + **[GGGCGG]** 1418 (88.77)   
13 + **.CGGCGG.** 1421 (97.92)   
13 + **[CGGGGG]** 1424 (88.57)   
13 + **[GGGCGG]** 1427 (88.77)   
13 + **.CGGCGG.** 1430 (97.92)   
13 + **[CGGGGG]** 1446 (88.57)   
13 + **[CGCGGG]** 1470 (86.07)   
13 - **[CGACGC]** 1320 (86.49)   
13 - **.CGGCGG.** 1195 (97.92)   
13 - **.GGACGG.** 1191 (90.85)   
13 - **.CGGCGG.** 1165 (97.92)   
13 - **.CGGCGG.** 1162 (97.92)   
13 - **.CGACGG.** 1156 (100.00)   
13 - **.CGGCGG.** 1144 (97.92)   
13 - **.CGGCGG.** 1141 (97.92)   
13 - **.CGCCGG.** 1135 (95.42)   
13 - **.CGGCGG.** 1124 (97.92)   
13 - **.CGGCGG.** 1108 (97.92)   
13 - **.CGCCGG.** 1060 (95.42)   
13 - **[CGCGGG]** 943 (86.07)   
13 - **[CCACGG]** 885 (85.35)   
13 - **.CGCCGG.** 879 (95.42)   
13 - **.GGACGG.** 859 (90.85)   
13 - **[GGGCGG]** 845 (88.77)   
13 - **[GGCCGG]** 839 (86.27)   
13 - **.CGGCGG.** 836 (97.92)   
13 - **[GGGCGG]** 827 (88.77)   
13 - **.CGAGGG.** 796 (90.65)   
13 - **[GGCCGG]** 792 (86.27)   
13 - **[CGCGGG]** 785 (86.07)   
13 - **[CGCGGG]** 778 (86.07)   
13 - **[CGCGGG]** 771 (86.07)   
13 - **[CGCGGG]** 764 (86.07)   
13 - **[GGGCGG]** 760 (88.77)   
13 - **[GGGCGG]** 755 (88.77)   
13 - **.CGGCGG.** 752 (97.92)   
13 - **[GGGCGG]** 748 (88.77)   
13 - **[CGCGGG]** 740 (86.07)   
13 - **.CGGCGG.** 719 (97.92)   
13 - **.CGAGGG.** 713 (90.65)   
13 - **[GGGCGG]** 709 (88.77)   
13 - **[CGCGGG]** 701 (86.07)   
13 - **.CGCCGG.** 695 (95.42)   
13 - **[CGGGGG]** 692 (88.57)   
13 - **[GGGCGG]** 689 (88.77)   
13 - **[CGCGGG]** 642 (86.07)   
13 - **.CGGCGG.** 623 (97.92)   
13 - **[GGGCGG]** 619 (88.77)   
13 - **[CGCGGG]** 613 (86.07)   
13 - **[GGGCGG]** 464 (88.77)   
13 - **[GGCCGG]** 379 (86.27)   
13 - **[GGCCGG]** 172 (86.27)   
  

**Frequency Matrix**

| All Occs Best Occs|  |  |  |  |  |  |  |  |  |  |  |  |  |  |  |  |  |  |  |  |  |  |  |  |  |  |  |  |  |  |  |  |  |  |  |  |  |  |  |  |  |  |  |  |  |  |  |  |  |  |  |  |  |  |  |  |  |  |  |  |  |  |  |  |  |  |  |  |  |  |  |  |  |  |  |  |  |  |  |  |  |  |  |  | | --- | --- | --- | --- | --- | --- | --- | --- | --- | --- | --- | --- | --- | --- | --- | --- | --- | --- | --- | --- | --- | --- | --- | --- | --- | --- | --- | --- | --- | --- | --- | --- | --- | --- | --- | --- | --- | --- | --- | --- | --- | --- | --- | --- | --- | --- | --- | --- | --- | --- | --- | --- | --- | --- | --- | --- | --- | --- | --- | --- | --- | --- | --- | --- | --- | --- | --- | --- | --- | --- | --- | --- | --- | --- | --- | --- | --- | --- | --- | --- | --- | --- | --- | --- | | **A C G T A C G T | | | | | | | | | | | |**| **1** 5 195 21 5 | 0 139 21 0|  |  |  |  |  |  |  |  |  |  |  |  |  |  |  |  |  |  |  |  |  |  |  |  |  |  |  |  |  |  |  |  |  |  |  |  |  |  |  |  |  |  |  |  |  |  |  |  |  |  |  |  |  |  |  |  |  |  |  |  | | --- | --- | --- | --- | --- | --- | --- | --- | --- | --- | --- | --- | --- | --- | --- | --- | --- | --- | --- | --- | --- | --- | --- | --- | --- | --- | --- | --- | --- | --- | --- | --- | --- | --- | --- | --- | --- | --- | --- | --- | --- | --- | --- | --- | --- | --- | --- | --- | --- | --- | --- | --- | --- | --- | --- | --- | --- | --- | --- | --- | | **2** 2 6 213 5 | 0 0 160 0|  |  |  |  |  |  |  |  |  |  |  |  |  |  |  |  |  |  |  |  |  |  |  |  |  |  |  |  |  |  |  |  |  |  |  |  |  |  |  |  |  |  |  |  |  |  |  |  | | --- | --- | --- | --- | --- | --- | --- | --- | --- | --- | --- | --- | --- | --- | --- | --- | --- | --- | --- | --- | --- | --- | --- | --- | --- | --- | --- | --- | --- | --- | --- | --- | --- | --- | --- | --- | --- | --- | --- | --- | --- | --- | --- | --- | --- | --- | --- | --- | | **3** 113 37 68 8 | 55 37 68 0|  |  |  |  |  |  |  |  |  |  |  |  |  |  |  |  |  |  |  |  |  |  |  |  |  |  |  |  |  |  |  |  |  |  |  |  | | --- | --- | --- | --- | --- | --- | --- | --- | --- | --- | --- | --- | --- | --- | --- | --- | --- | --- | --- | --- | --- | --- | --- | --- | --- | --- | --- | --- | --- | --- | --- | --- | --- | --- | --- | --- | | **4** 7 195 20 4 | 0 140 20 0|  |  |  |  |  |  |  |  |  |  |  |  |  |  |  |  |  |  |  |  |  |  |  |  | | --- | --- | --- | --- | --- | --- | --- | --- | --- | --- | --- | --- | --- | --- | --- | --- | --- | --- | --- | --- | --- | --- | --- | --- | | **5** 5 6 213 2 | 0 0 160 0|  |  |  |  |  |  |  |  |  |  |  |  | | --- | --- | --- | --- | --- | --- | --- | --- | --- | --- | --- | --- | | **6** 2 8 215 1 | 0 0 160 0 | | | | | | | | | | | | | | | | | | | | | | | | | | | | | | | | | | | | | | | | | | | | | | | | | | | | | | | | | | | | | | | | | | | | | | | | | | | | | | | | | | | | |

  
  

---

  
  
  
 **GCAGCGCG**   
**CGCGCTGC**  
  
4 redundant motifs found:  
CAGCGCGG - GCGGCGCA - TCCGCGCG - AGCGCG -   
  
Best occurrences (match percentage):   
Seq St oligo pos match
  
 1 + **[GCGGGGCG]** 867 (89.21)   
1 + **[GGTGCGCG]** 996 (85.78)   
1 + **[GCGCCGCG]** 1067 (88.20)   
1 + **[GCCGCGCC]** 1069 (89.05)   
1 + **[GCGGCGCA]** 1107 (86.34)   
1 + **[GCAGCTCG]** 1162 (87.54)   
1 - **[GCGGCGCA]** 1397 (86.34)   
1 - **.GCAGCGGG.** 1234 (92.78)   
1 - **[GCGGCGGG]** 1075 (89.60)   
1 - **[GCGGCGCC]** 1066 (88.85)   
1 - **.GCGGCGCG.** 1000 (96.82)   
1 - **[GCGGCGCC]** 915 (88.85)   
2 + **[GGTGCGCG]** 197 (85.78)   
2 + **[GGAGCGCC]** 212 (85.37)   
2 + **[GGAGCCCG]** 501 (86.20)   
2 + **[GCCCCGCG]** 511 (88.39)   
2 + **.GGAGCGCG.** 692 (93.34)   
2 + **.GCGGCGCG.** 701 (96.82)   
2 + **.GGCGCGCG.** 703 (90.36)   
2 + **[GAAGCGCG]** 717 (87.75)   
2 + **.GCGGCGCG.** 997 (96.82)   
2 + **.GCAGCGCT.** 1013 (90.03)   
2 + **.GCCGCGCG.** 1071 (97.02)   
2 + **[GCCGTGCG]** 1093 (86.21)   
2 + **[GCGGCGCC]** 1153 (88.85)   
2 + **.GCAGCGCG.** 1186 (100.00)   
2 + **[GCAGCGCA]** 1361 (89.52)   
2 + **.GCGGCGCG.** 1448 (96.82)   
2 - **.GCAGCGCG.** 1480 (100.00)   
2 - **[GCGCCGCG]** 1447 (88.20)   
2 - **[GCCGCGCT]** 1445 (87.04)   
2 - **[GCGGCGGG]** 1266 (89.60)   
2 - **[GCCGTGCG]** 1253 (86.21)   
2 - **.GCAGCGGG.** 1082 (92.78)   
2 - **.GGAGCGCG.** 1075 (93.34)   
2 - **.GCAGCGCT.** 1015 (90.03)   
2 - **[GCGCCGCG]** 996 (88.20)   
2 - **[GCCGCGAG]** 994 (86.45)   
2 - **[GAAGCGCG]** 815 (87.75)   
2 - **[GGAGCCCG]** 775 (86.20)   
2 - **[GCCGCGCT]** 719 (87.04)   
2 - **[CCCGCGCG]** 705 (88.25)   
2 - **[GCGGGGCG]** 510 (89.21)   
2 - **[GCGGCGAG]** 306 (86.26)   
2 - **[GCCGCGCA]** 199 (86.54)   
3 + **.GCAGCGCT.** 937 (90.03)   
3 + **[GCCGGGCG]** 1176 (89.41)   
3 + **[GCGGCGCA]** 1227 (86.34)   
3 + **.GGCGCGCG.** 1249 (90.36)   
3 + **.GCAGCGCC.** 1308 (92.03)   
3 + **[GCCGCCCG]** 1344 (89.88)   
3 + **[GGAGCCCG]** 1382 (86.20)   
3 + **[GCCGCGCC]** 1416 (89.05)   
3 + **[GGAGGGCG]** 1499 (85.73)   
3 - **[GCGGCGGG]** 1407 (89.60)   
3 - **[GCGGGGCG]** 1404 (89.21)   
3 - **[GCGGCGCA]** 1399 (86.34)   
4 + **[GGTGCGCG]** 142 (85.78)   
4 + **[GCCGGGCG]** 197 (89.41)   
4 + **[GCGGGGCG]** 202 (89.21)   
4 + **[GCTGCCCG]** 611 (85.31)   
4 + **[GCCGGGCG]** 631 (89.41)   
4 + **[GCGGCGGG]** 671 (89.60)   
4 + **[GGAGCGGG]** 710 (86.12)   
4 + **[CCCGCGCG]** 782 (88.25)   
4 + **.GCACCGCG.** 818 (91.38)   
4 + **.GCGGCGCG.** 826 (96.82)   
4 + **[GCCCCGCG]** 871 (88.39)   
4 + **[GCGGCGGG]** 905 (89.60)   
4 + **.GCAGGGCG.** 912 (92.39)   
4 + **[GCGGTGCG]** 1096 (86.01)   
4 + **[GCGGAGCG]** 1101 (85.55)   
4 + **[GGAGCGCC]** 1103 (85.37)   
4 + **[GCAGCCGG]** 1116 (85.64)   
4 + **[GCGGCGGG]** 1141 (89.60)   
4 + **.GCAGCGGG.** 1148 (92.78)   
4 + **.GCAGCCCG.** 1161 (92.86)   
4 + **[GCCCCGCG]** 1195 (88.39)   
4 + **[GCCGCGCC]** 1206 (89.05)   
4 + **[GCCGGGCG]** 1248 (89.41)   
4 + **.GGGGCGCG.** 1270 (90.16)   
4 + **.GGCGCGCG.** 1272 (90.36)   
4 + **.GGGGCGCG.** 1280 (90.16)   
4 + **.GGCGCGCG.** 1282 (90.36)   
4 + **[GCGGGGCG]** 1287 (89.21)   
4 - **[GCCCCGCG]** 1286 (88.39)   
4 - **[CCCGCGCG]** 1284 (88.25)   
4 - **[CCCGCGCG]** 1274 (88.25)   
4 - **[GCCGCCCG]** 1254 (89.88)   
4 - **[GCGGCGGG]** 1203 (89.60)   
4 - **[GCCGCGGG]** 1177 (89.80)   
4 - **[GCTGCCCG]** 1145 (85.31)   
4 - **[GCGGCGCT]** 1105 (86.85)   
4 - **[GCAGTGCG]** 924 (89.19)   
4 - **[GCCGCCCG]** 877 (89.88)   
4 - **[GCGGTGCG]** 817 (86.01)   
4 - **[GCGCCGCG]** 802 (88.20)   
4 - **[GCCGCGAG]** 800 (86.45)   
4 - **.GGCGCGCG.** 784 (90.36)   
4 - **[GGAGCCCG]** 771 (86.20)   
4 - **[GCGGCGCC]** 693 (88.85)   
4 - **.GCAGCGGG.** 608 (92.78)   
4 - **[GGAGCGCC]** 314 (85.37)   
5 + **.GCCGCGCG.** 812 (97.02)   
5 + **[CCCGCGCG]** 1356 (88.25)   
5 + **.GCAGCGCG.** 1391 (100.00)   
5 - **[GCCGCGCT]** 1393 (87.04)   
5 - **.GCAGGGCG.** 1333 (92.39)   
5 - **.GCAGCGGG.** 1242 (92.78)   
5 - **[GCGGCGCA]** 1158 (86.34)   
5 - **[GGAGCGGG]** 1143 (86.12)   
5 - **[CCCGCGCG]** 814 (88.25)   
6 + **.GGAGCGCG.** 1137 (93.34)   
6 + **.GCAGCGCC.** 1325 (92.03)   
6 + **.GGAGCGCG.** 1364 (93.34)   
6 + **[CCCGCGCG]** 1378 (88.25)   
6 + **[GCGGTGCG]** 1383 (86.01)   
6 + **[GCGGCGGG]** 1476 (89.60)   
6 - **.GCAGCGCG.** 1496 (100.00)   
6 - **.GCACCGCG.** 1382 (91.38)   
8 + **[GCGGCGGG]** 3 (89.60)   
8 + **[GCAGCCGG]** 357 (85.64)   
8 + **[GCGCCGCG]** 450 (88.20)   
8 + **.GCTGCGCG.** 490 (92.44)   
8 + **[GGAGCCCG]** 626 (86.20)   
8 + **[GCCGCCCG]** 676 (89.88)   
8 + **[GCGGGGCG]** 898 (89.21)   
8 + **[CCCGCGCG]** 951 (88.25)   
8 + **[GCCGGGCG]** 993 (89.41)   
8 + **[GCCCCGCG]** 1142 (88.39)   
8 + **[GCCGCCCG]** 1155 (89.88)   
8 + **[GCAGGCCG]** 1318 (85.25)   
8 - **.GCAGCGCG.** 1402 (100.00)   
8 - **[GCGGCCCG]** 1385 (89.68)   
8 - **.GGAGCGCG.** 1346 (93.34)   
8 - **[GCGGCGGG]** 1232 (89.60)   
8 - **.GGAGCGCG.** 1206 (93.34)   
8 - **[GCAGCGAG]** 1167 (89.44)   
8 - **.GCAGCGGG.** 1159 (92.78)   
8 - **[GCAGAGCG]** 1106 (88.73)   
8 - **[CCCGCGCG]** 953 (88.25)   
8 - **[GCGGGGCG]** 858 (89.21)   
8 - **[GGTGCGCG]** 788 (85.78)   
8 - **[GCAGAGCG]** 750 (88.73)   
8 - **[GCGGGGCG]** 496 (89.21)   
8 - **.GGGGCGCG.** 494 (90.16)   
8 - **[GCGGCGCT]** 449 (86.85)   
8 - **[GCCGCGCC]** 407 (89.05)   
9 + **.GCAGCGCG.** 2 (100.00)   
9 + **[GCCGGGCG]** 533 (89.41)   
9 + **[GGAGCGGG]** 824 (86.12)   
9 + **[GCGGAGCG]** 1186 (85.55)   
9 + **[GCAGCGTG]** 1249 (85.64)   
9 - **.GGGGCGCG.** 1062 (90.16)   
9 - **.GCAGCGCG.** 8 (100.00)   
10 + **[GCCGCGCT]** 35 (87.04)   
10 + **[GCAGAGCG]** 467 (88.73)   
10 + **[GCCGCGCC]** 511 (89.05)   
10 + **.GCAGGGCG.** 535 (92.39)   
10 + **[GGAGCGGG]** 568 (86.12)   
10 + **.GCAGCGGG.** 682 (92.78)   
10 + **[GCGGCGCA]** 692 (86.34)   
10 + **[GCAGCGCA]** 794 (89.52)   
10 + **[GAAGCGCG]** 860 (87.75)   
10 + **.GGGGCGCG.** 869 (90.16)   
10 + **[GGAGCGGG]** 881 (86.12)   
10 + **[GCGGTGCG]** 912 (86.01)   
10 + **[GCGGGGCG]** 936 (89.21)   
10 + **[GGAGCGGG]** 1034 (86.12)   
10 + **.GCAGCGCG.** 1069 (100.00)   
10 + **[GCAGCGAG]** 1078 (89.44)   
10 + **[GCGGCGAG]** 1232 (86.26)   
10 + **.GCAGCCCG.** 1275 (92.86)   
10 - **.GCAGGGCG.** 1427 (92.39)   
10 - **[GCCGGGCG]** 1113 (89.41)   
10 - **.GCCGCGCG.** 1094 (97.02)   
10 - **[GCTGCCCG]** 1075 (85.31)   
10 - **[GCCCCGCG]** 830 (88.39)   
10 - **[GCCGCCCG]** 686 (89.88)   
10 - **.GGGGCGCG.** 513 (90.16)   
10 - **[GGAGCCCG]** 346 (86.20)   
11 + **.GCAGCGCT.** 149 (90.03)   
11 + **[GCCGAGCG]** 215 (85.75)   
11 + **[GCCCCGCG]** 589 (88.39)   
11 + **[GCGCCGCG]** 607 (88.20)   
11 + **.GCCGCGCG.** 615 (97.02)   
11 + **[GCCGCCCG]** 633 (89.88)   
11 + **[GCCCCGCG]** 659 (88.39)   
11 + **[GCGGAGCG]** 673 (85.55)   
11 + **[GGAGCGGG]** 675 (86.12)   
11 + **[GCCCCGCG]** 719 (88.39)   
11 + **[CCCGCGCG]** 721 (88.25)   
11 + **[GCGGCGCC]** 770 (88.85)   
11 + **[GGAGCGGG]** 793 (86.12)   
11 + **[GCGGGGCG]** 796 (89.21)   
11 + **[GCCGCGCT]** 994 (87.04)   
11 + **[GCCGCGCT]** 1027 (87.04)   
11 + **[GGAGCGGG]** 1105 (86.12)   
11 + **.GCTGCGCG.** 1112 (92.44)   
11 + **[GCGGCGGG]** 1117 (89.60)   
11 + **[GCCGCGCA]** 1155 (86.54)   
11 + **.GCAGCCCG.** 1188 (92.86)   
11 + **[ACAGCGCG]** 1341 (87.80)   
11 - **[GCCGAGCG]** 1358 (85.75)   
11 - **[GCGGCGAG]** 1326 (86.26)   
11 - **[GCGGCGCT]** 1149 (86.85)   
11 - **[GCCGCGCA]** 1114 (86.54)   
11 - **.GCAGCCCG.** 1109 (92.86)   
11 - **.GCAGCGCG.** 1029 (100.00)   
11 - **[GCGGCGGG]** 1024 (89.60)   
11 - **[GCGGCGGG]** 1006 (89.60)   
11 - **.GGAGCGCG.** 996 (93.34)   
11 - **[GCGGCGGG]** 991 (89.60)   
11 - **[GGAGCGGG]** 935 (86.12)   
11 - **[GCGCCGCG]** 769 (88.20)   
11 - **[GGAGGGCG]** 702 (85.73)   
11 - **[CCCGCGCG]** 617 (88.25)   
11 - **.GCGGCGCG.** 606 (96.82)   
12 + **.GGCGCGCG.** 509 (90.36)   
12 + **.GGGGCGCG.** 522 (90.16)   
12 + **[CCGGCGCG]** 539 (88.05)   
12 + **[CCCGCGCG]** 557 (88.25)   
12 + **[GCGGGGCG]** 595 (89.21)   
12 + **[GCGGGGCG]** 600 (89.21)   
12 + **[GCCGGGCG]** 610 (89.41)   
12 + **[GCGGCCCG]** 712 (89.68)   
12 + **[GCCGCCCG]** 777 (89.88)   
12 + **[GGAGCCCG]** 857 (86.20)   
12 + **.GCAGCGCG.** 918 (100.00)   
12 + **.GCCGCGCG.** 1030 (97.02)   
12 + **[GCAGGGGG]** 1073 (85.17)   
12 + **[GCGGCCCG]** 1084 (89.68)   
12 + **[GCGCCGCG]** 1176 (88.20)   
12 + **.GCCGCGCG.** 1178 (97.02)   
12 + **[GCCGCGCC]** 1251 (89.05)   
12 + **[GCCGCGCT]** 1275 (87.04)   
12 + **.GGGGCGCG.** 1331 (90.16)   
12 + **[GGAGCGCC]** 1350 (85.37)   
12 + **[GCGCCGCG]** 1353 (88.20)   
12 + **[GGAGCGGG]** 1474 (86.12)   
12 - **[GCGGCGCT]** 1352 (86.85)   
12 - **.GCAGCGCG.** 1277 (100.00)   
12 - **.GCGGCGCG.** 1253 (96.82)   
12 - **.GGAGCGCG.** 1205 (93.34)   
12 - **[GCGGCGCT]** 1175 (86.85)   
12 - **.GCCGCGCG.** 1118 (97.02)   
12 - **.GGCGCGCG.** 1034 (90.36)   
12 - **[GCGGCCCG]** 959 (89.68)   
12 - **[CCCGCGCG]** 882 (88.25)   
12 - **[GCGGCGGG]** 774 (89.60)   
12 - **[GCGGCCCG]** 737 (89.68)   
12 - **[GCCGCGGG]** 709 (89.80)   
12 - **.GCGGCGCG.** 696 (96.82)   
12 - **[GCCGCCCG]** 612 (89.88)   
12 - **[CCCGCGCG]** 559 (88.25)   
13 + **[GCAGCCGG]** 375 (85.64)   
13 + **[GGAGGGCG]** 508 (85.73)   
13 + **[ACAGCGCG]** 527 (87.80)   
13 + **[GCCGCCCG]** 618 (89.88)   
13 + **[GCGGCCCG]** 704 (89.68)   
13 + **.GCCGCGCG.** 721 (97.02)   
13 + **[GCCCCGCG]** 762 (88.39)   
13 + **[GCCCCGCG]** 776 (88.39)   
13 + **[GCCCCGCG]** 783 (88.39)   
13 + **[GCGGGGCG]** 908 (89.21)   
13 + **[CCCGCGCG]** 943 (88.25)   
13 + **[GCGGCGGG]** 973 (89.60)   
13 + **[GCGGGGCG]** 976 (89.21)   
13 + **[GCGGTGCG]** 981 (86.01)   
13 + **[GCCGGGCG]** 1042 (89.41)   
13 + **[GCAGCCGG]** 1056 (85.64)   
13 + **[CCGGCGCG]** 1060 (88.05)   
13 + **.CCAGCGCG.** 1072 (91.23)   
13 + **.GCCGCGCG.** 1110 (97.02)   
13 + **.GGAGCGCG.** 1183 (93.34)   
13 + **[GCCGCGGG]** 1227 (89.80)   
13 + **[GCGGGGCG]** 1325 (89.21)   
13 + **[GCGGCGGG]** 1353 (89.60)   
13 + **.GCAGCCCG.** 1384 (92.86)   
13 + **[GCGGCGGG]** 1420 (89.60)   
13 + **[GCGGCGGG]** 1429 (89.60)   
13 - **[GCCGCGCT]** 1350 (87.04)   
13 - **[GCCCCGCG]** 1324 (88.39)   
13 - **[GCCGCCCG]** 1314 (89.88)   
13 - **[CCGGCGCG]** 1264 (88.05)   
13 - **.GCAGCCCG.** 1231 (92.86)   
13 - **[GCGGCGCC]** 1137 (88.85)   
13 - **[GCCGAGCG]** 1116 (85.75)   
13 - **[GCCGCGCC]** 1062 (89.05)   
13 - **.GCCGCGCG.** 945 (97.02)   
13 - **[GCCGGGCG]** 789 (89.41)   
13 - **[GCGGGGCG]** 782 (89.21)   
13 - **.GGGGCGCG.** 780 (90.16)   
13 - **[GCGGGGCG]** 775 (89.21)   
13 - **.GGGGCGCG.** 773 (90.16)   
13 - **[GCGGGGCG]** 761 (89.21)   
13 - **[GCGGGGCG]** 756 (89.21)   
13 - **[GCGGCGGG]** 751 (89.60)   
13 - **[GCGGCGAG]** 715 (86.26)   
13 - **[GCCGCGGG]** 701 (89.80)   
13 - **[GCCGAGCG]** 680 (85.75)   
13 - **[GCGGCGCT]** 675 (86.85)   
13 - **.GCGGCGCG.** 615 (96.82)   
13 - **[GCCGGGCG]** 169 (89.41)   
  

**Frequency Matrix**

| All Occs Best Occs|  |  |  |  |  |  |  |  |  |  |  |  |  |  |  |  |  |  |  |  |  |  |  |  |  |  |  |  |  |  |  |  |  |  |  |  |  |  |  |  |  |  |  |  |  |  |  |  |  |  |  |  |  |  |  |  |  |  |  |  |  |  |  |  |  |  |  |  |  |  |  |  |  |  |  |  |  |  |  |  |  |  |  |  |  |  |  |  |  |  |  |  |  |  |  |  |  |  |  |  |  |  |  |  |  |  |  |  | | --- | --- | --- | --- | --- | --- | --- | --- | --- | --- | --- | --- | --- | --- | --- | --- | --- | --- | --- | --- | --- | --- | --- | --- | --- | --- | --- | --- | --- | --- | --- | --- | --- | --- | --- | --- | --- | --- | --- | --- | --- | --- | --- | --- | --- | --- | --- | --- | --- | --- | --- | --- | --- | --- | --- | --- | --- | --- | --- | --- | --- | --- | --- | --- | --- | --- | --- | --- | --- | --- | --- | --- | --- | --- | --- | --- | --- | --- | --- | --- | --- | --- | --- | --- | --- | --- | --- | --- | --- | --- | --- | --- | --- | --- | --- | --- | --- | --- | --- | --- | --- | --- | --- | --- | --- | --- | --- | --- | | **A C G T A C G T | | | | | | | | | | | |**| **1** 20 48 449 20 | 0 1 83 0|  |  |  |  |  |  |  |  |  |  |  |  |  |  |  |  |  |  |  |  |  |  |  |  |  |  |  |  |  |  |  |  |  |  |  |  |  |  |  |  |  |  |  |  |  |  |  |  |  |  |  |  |  |  |  |  |  |  |  |  |  |  |  |  |  |  |  |  |  |  |  |  |  |  |  |  |  |  |  |  |  |  |  |  | | --- | --- | --- | --- | --- | --- | --- | --- | --- | --- | --- | --- | --- | --- | --- | --- | --- | --- | --- | --- | --- | --- | --- | --- | --- | --- | --- | --- | --- | --- | --- | --- | --- | --- | --- | --- | --- | --- | --- | --- | --- | --- | --- | --- | --- | --- | --- | --- | --- | --- | --- | --- | --- | --- | --- | --- | --- | --- | --- | --- | --- | --- | --- | --- | --- | --- | --- | --- | --- | --- | --- | --- | --- | --- | --- | --- | --- | --- | --- | --- | --- | --- | --- | --- | | **2** 19 432 79 7 | 0 58 26 0|  |  |  |  |  |  |  |  |  |  |  |  |  |  |  |  |  |  |  |  |  |  |  |  |  |  |  |  |  |  |  |  |  |  |  |  |  |  |  |  |  |  |  |  |  |  |  |  |  |  |  |  |  |  |  |  |  |  |  |  |  |  |  |  |  |  |  |  |  |  |  |  | | --- | --- | --- | --- | --- | --- | --- | --- | --- | --- | --- | --- | --- | --- | --- | --- | --- | --- | --- | --- | --- | --- | --- | --- | --- | --- | --- | --- | --- | --- | --- | --- | --- | --- | --- | --- | --- | --- | --- | --- | --- | --- | --- | --- | --- | --- | --- | --- | --- | --- | --- | --- | --- | --- | --- | --- | --- | --- | --- | --- | --- | --- | --- | --- | --- | --- | --- | --- | --- | --- | --- | --- | | **3** 261 122 116 38 | 46 17 19 2|  |  |  |  |  |  |  |  |  |  |  |  |  |  |  |  |  |  |  |  |  |  |  |  |  |  |  |  |  |  |  |  |  |  |  |  |  |  |  |  |  |  |  |  |  |  |  |  |  |  |  |  |  |  |  |  |  |  |  |  | | --- | --- | --- | --- | --- | --- | --- | --- | --- | --- | --- | --- | --- | --- | --- | --- | --- | --- | --- | --- | --- | --- | --- | --- | --- | --- | --- | --- | --- | --- | --- | --- | --- | --- | --- | --- | --- | --- | --- | --- | --- | --- | --- | --- | --- | --- | --- | --- | --- | --- | --- | --- | --- | --- | --- | --- | --- | --- | --- | --- | | **4** 7 52 469 9 | 0 2 82 0|  |  |  |  |  |  |  |  |  |  |  |  |  |  |  |  |  |  |  |  |  |  |  |  |  |  |  |  |  |  |  |  |  |  |  |  |  |  |  |  |  |  |  |  |  |  |  |  | | --- | --- | --- | --- | --- | --- | --- | --- | --- | --- | --- | --- | --- | --- | --- | --- | --- | --- | --- | --- | --- | --- | --- | --- | --- | --- | --- | --- | --- | --- | --- | --- | --- | --- | --- | --- | --- | --- | --- | --- | --- | --- | --- | --- | --- | --- | --- | --- | | **5** 24 425 61 27 | 0 80 4 0|  |  |  |  |  |  |  |  |  |  |  |  |  |  |  |  |  |  |  |  |  |  |  |  |  |  |  |  |  |  |  |  |  |  |  |  | | --- | --- | --- | --- | --- | --- | --- | --- | --- | --- | --- | --- | --- | --- | --- | --- | --- | --- | --- | --- | --- | --- | --- | --- | --- | --- | --- | --- | --- | --- | --- | --- | --- | --- | --- | --- | | **6** 17 70 432 18 | 0 6 78 0|  |  |  |  |  |  |  |  |  |  |  |  |  |  |  |  |  |  |  |  |  |  |  |  | | --- | --- | --- | --- | --- | --- | --- | --- | --- | --- | --- | --- | --- | --- | --- | --- | --- | --- | --- | --- | --- | --- | --- | --- | | **7** 29 429 68 11 | 0 77 7 0|  |  |  |  |  |  |  |  |  |  |  |  | | --- | --- | --- | --- | --- | --- | --- | --- | --- | --- | --- | --- | | **8** 29 55 420 33 | 0 2 78 4 | | | | | | | | | | | | | | | | | | | | | | | | | | | | | | | | | | | | | | | | | | | | | | | | | | | | | | | | | | | | | | | | | | | | | | | | | | | | | | | | | | | | | | | | | | | | | | | | | | | | | | | | | | | | |

  
  

---

  
  
  
 **CGCTGCGTCG**   
**CGACGCAGCG**  
  
1 redundant motifs found:  
CCATGCGCCG -   
  
Best occurrences (match percentage):   
Seq St oligo pos match
  
 1 + **[GGCTGCGGCC]** 957 (89.57)   
1 + **[CGCGCCGCCC]** 1000 (86.26)   
1 + **[CGAGGCGCCG]** 1063 (85.53)   
1 + **.CGCCGCGCCC.** 1068 (92.63)   
1 + **.CGCCGCCCCG.** 1077 (92.34)   
1 + **[GCCGGCGCCG]** 1246 (85.85)   
1 + **.CCCTGCGCCG.** 1394 (92.91)   
1 - **[CGCAGGGTCG]** 1391 (88.69)   
1 - **[GGCGCCGGCG]** 1245 (85.37)   
1 - **[AGCGGCGGCG]** 1204 (88.74)   
1 - **[GGCGGCGTCT]** 1201 (85.38)   
1 - **[CGGGGCGGCG]** 1077 (89.44)   
1 - **[CGCGGCGCCT]** 1065 (89.75)   
1 - **[CGGCGCCTCG]** 1063 (85.40)   
1 - **[GGCGGCGCCC]** 914 (87.80)   
2 + **[CGGTGCGCGG]** 196 (85.00)   
2 + **[CCCCGCGTCC]** 512 (87.02)   
2 + **[CGCTGGGGCC]** 659 (88.99)   
2 + **[CCCCGCCCCG]** 900 (85.99)   
2 + **[CGCCCCGTCC]** 903 (86.74)   
2 + **.CGCGGCCCCG.** 977 (92.60)   
2 + **.CGCTGCGTCC.** 1017 (95.60)   
2 + **[CCCTGCTGCG]** 1103 (85.26)   
2 + **[CGCCGCCCCC]** 1268 (87.94)   
2 + **[CCCGGGGTCG]** 1300 (86.01)   
2 + **[CGCGGGGGCC]** 1499 (87.03)   
2 - **[GGCGGCGGGG]** 1265 (85.43)   
2 - **[CGCCGGGTCC]** 1195 (87.69)   
2 - **[CGCGGCATCG]** 1067 (87.33)   
2 - **[CGCCGCGAGG]** 993 (85.81)   
2 - **[GGCGGGGCCG]** 979 (86.51)   
2 - **[GGCTGGGCCG]** 931 (88.47)   
2 - **[GGCTGCCTCC]** 528 (85.80)   
2 - **.CGGGGCGTCG.** 507 (90.36)   
3 + **.CGCGGCGGCG.** 1223 (97.11)   
3 + **[CGCAGCTCCG]** 1285 (86.18)   
3 + **[CGCAGCGCCC]** 1307 (89.24)   
3 + **[GGCTGGGGCG]** 1359 (88.29)   
3 + **[CCCGGCGCCC]** 1386 (86.55)   
3 + **.CGCCGCCCCG.** 1401 (92.34)   
3 + **.CGCCCCGCCG.** 1404 (90.40)   
3 + **[CGCCGCCGCC]** 1409 (87.75)   
3 + **.CGCCGCCGCG.** 1412 (92.15)   
3 + **[CGCCGCGCCA]** 1415 (86.76)   
3 - **.CGCGGCGGCG.** 1412 (97.11)   
3 - **.GGCGGCGGCG.** 1409 (92.01)   
3 - **[GGCGGCGGGG]** 1406 (85.43)   
3 - **[CGGGGCGGCG]** 1401 (89.44)   
3 - **[CTCTGCGCCC]** 1364 (85.88)   
3 - **[CGCTGCGACT]** 1304 (87.06)   
3 - **.AGCTGCGCCG.** 1282 (90.89)   
3 - **.CGCCGCCGCG.** 1223 (92.15)   
4 + **.CGCTGGGGCG.** 308 (93.39)   
4 + **[CGCCCCCGCG]** 568 (85.51)   
4 + **[CGCTGCCCGG]** 610 (87.98)   
4 + **[CGCGGAGCCG]** 625 (87.59)   
4 + **[GGCGCCGCCG]** 693 (85.56)   
4 + **.CGCCGCCGCG.** 695 (92.15)   
4 + **[CGCCGCGACC]** 698 (87.99)   
4 + **[GGCGCCGTCG]** 734 (86.30)   
4 + **[CGCGGCCCCC]** 742 (88.21)   
4 + **[CACCGCGGCG]** 819 (87.15)   
4 + **.CGCGGCGGCG.** 822 (97.11)   
4 + **[CGCGGAGGCG]** 898 (87.40)   
4 + **[CGGAGCGCCG]** 1102 (85.96)   
4 + **[CGCCGCTGCG]** 1107 (89.39)   
4 + **[CGCTGCGCAG]** 1110 (89.52)   
4 + **[CCCCGCGCCC]** 1196 (86.28)   
4 + **[CGCCGCGCCA]** 1205 (86.76)   
4 + **[GGCGGGGGCG]** 1266 (86.33)   
4 + **.CGCGGGGGCG.** 1276 (91.43)   
4 + **[CCCTGCGGCT]** 1459 (85.17)   
4 - **[CGCGCCCCCG]** 1278 (85.97)   
4 - **[CGCCCCCGCG]** 1276 (85.51)   
4 - **[CGCGCCCCCG]** 1268 (85.97)   
4 - **[CGCGGCGGGC]** 1202 (86.13)   
4 - **[GGCTCCGCCG]** 1188 (87.52)   
4 - **.CGCAGCGGCG.** 1107 (93.45)   
4 - **.CGCTGGGGCG.** 1089 (93.39)   
4 - **[CGCCGCCTCC]** 901 (88.68)   
4 - **[GGCCGGGTCG]** 852 (86.98)   
4 - **[CGCCGCTCCC]** 844 (85.17)   
4 - **.CGCGCCGCCG.** 824 (90.66)   
4 - **.CGCCGCCGCG.** 822 (92.15)   
4 - **[CGCCGCGGTG]** 819 (87.60)   
4 - **[CGCCGCGAGG]** 799 (85.81)   
4 - **[GGCCGCGACG]** 739 (87.28)   
4 - **.CGCGGCGGCG.** 695 (97.11)   
4 - **.GGCGGCGCCG.** 692 (92.19)   
4 - **[CGCCGCCTCC]** 667 (88.68)   
4 - **.CGCGGGGGCG.** 568 (91.43)   
4 - **[CCCCGCCCCG]** 203 (85.99)   
4 - **[CGCCCCGCCC]** 200 (86.00)   
5 + **[CGCGGGGGCC]** 1191 (87.03)   
5 + **[CGCCGCCTGG]** 1310 (86.49)   
5 + **[CGCAGCGCGG]** 1390 (87.05)   
5 + **[CCCGGCGGCC]** 1473 (86.37)   
5 + **[CGCCGCCGCC]** 1482 (87.75)   
5 - **.GGCGGCGGCG.** 1482 (92.01)   
5 - **[GGCGGCGGCC]** 1479 (87.61)   
5 - **[GGCCGCCTCG]** 1446 (87.97)   
5 - **.GGCCGCGTCG.** 1437 (92.67)   
5 - **[GGCTGCTGCG]** 1401 (86.51)   
5 - **[TGCTGCGGCC]** 1398 (85.18)   
5 - **.CGCTGCGCCG.** 1387 (99.26)   
6 + **.CGCCGGGGCG.** 954 (91.16)   
6 + **[CGCGGCGACT]** 1440 (85.10)   
6 - **.CGCCCCGGCG.** 954 (90.21)   
8 + **[GGCGGCGGGG]** 2 (85.43)   
8 + **[CGCGGCACCG]** 409 (86.59)   
8 + **.CGCCGCGTGG.** 451 (91.19)   
8 + **[CGCCCCGCCC]** 496 (86.00)   
8 + **[CGCCCCCGCG]** 782 (85.51)   
8 + **[CGCCGCCCCC]** 813 (87.94)   
8 + **[CCCCGCCCCG]** 855 (85.99)   
8 + **[CGCGGGCGCG]** 929 (86.74)   
8 + **[CCCTGGGCCG]** 961 (87.23)   
8 + **[GGCGGCCCCG]** 1138 (87.50)   
8 + **.CCCCGCGTCG.** 1143 (91.42)   
8 + **.CGCTGCCTCG.** 1161 (95.30)   
8 + **.CGCGGGGTCG.** 1198 (92.36)   
8 + **[CGCCGCCTCC]** 1234 (88.68)   
8 + **.CGCTGCGTCC.** 1404 (95.60)   
8 + **[GGCTCCGTCG]** 1479 (88.26)   
8 - **[CGCAGCGCGG]** 1401 (87.05)   
8 - **[GGCGGCGGGG]** 1231 (85.43)   
8 - **.GGCGGCGTCG.** 1151 (92.93)   
8 - **.CGCGGGGCCG.** 1140 (91.62)   
8 - **[GGCGGGGGCG]** 1076 (86.33)   
8 - **[CGCAGCTCCG]** 1030 (86.18)   
8 - **[CGCGCCCGCG]** 929 (85.78)   
8 - **[CGCCCCGCCC]** 896 (86.00)   
8 - **.CGCGGGGGCG.** 782 (91.43)   
8 - **[GGCTGAGTCG]** 550 (85.18)   
8 - **[CGGTGCCGCG]** 409 (86.71)   
8 - **[TGCCGCGCCG]** 406 (87.54)   
8 - **.GGCTGCGCCG.** 353 (94.16)   
8 - **.CGCCGGGCCG.** 348 (91.35)   
9 + **[CCCCGCGCCC]** 1059 (86.28)   
9 + **[CGCAGCGTGG]** 1248 (87.79)   
9 - **.CGCTGCGCCG.** 1245 (99.26)   
9 - **[CGCTGCTGCC]** 1234 (87.21)   
9 - **[CGCTCCGGCC]** 1179 (88.04)   
9 - **[CGCTCCCTCG]** 55 (88.67)   
10 + **[CCCGGCGGCC]** 28 (86.37)   
10 + **[GGCGGCCGCG]** 31 (87.31)   
10 + **[GGCGGGGCCG]** 56 (86.51)   
10 + **[GGCGGGGGCG]** 124 (86.33)   
10 + **[GGCCGCGCCC]** 510 (87.53)   
10 + **.GGCGGCGGCG.** 688 (92.01)   
10 + **[CCCGGGGTCG]** 741 (86.01)   
10 + **.CGCGGGGCCG.** 830 (91.62)   
10 + **.GGCCGCGGCG.** 1021 (91.74)   
10 + **.CGCGGCGGCC.** 1024 (92.72)   
10 + **.CGCGGCGGCC.** 1096 (92.72)   
10 + **[CCCGGCCGCG]** 1115 (86.07)   
10 + **[GGCCGCGGCC]** 1118 (87.34)   
10 + **[AGCTGCGTCC]** 1169 (87.23)   
10 + **[CGGTGCGTCC]** 1216 (87.93)   
10 + **[CGCGGCCCCC]** 1331 (88.21)   
10 - **[GGCCGCGGCC]** 1118 (87.34)   
10 - **[CGCGGCCGGG]** 1115 (85.84)   
10 - **[GGCCGCCGCG]** 1096 (87.05)   
10 - **[CGCCGCGCGC]** 1093 (86.05)   
10 - **.CGCTGCTTCG.** 1065 (92.54)   
10 - **[CGCTCCGGCC]** 1030 (88.04)   
10 - **[GGCCGCCGCG]** 1024 (87.05)   
10 - **.CGCCGCGGCC.** 1021 (92.45)   
10 - **[CGCGGCCCCT]** 1018 (85.05)   
10 - **[CGCCCCGCCC]** 934 (86.00)   
10 - **[CCCGGCGTCC]** 895 (87.29)   
10 - **[CCCGGCCCCG]** 832 (86.26)   
10 - **[CCCCGCGTCC]** 827 (87.02)   
10 - **.CGCTGCGACG.** 790 (94.62)   
10 - **.CCCTGCGCCG.** 693 (92.91)   
10 - **[CGCCGCCGCC]** 688 (87.75)   
10 - **.CGCTGCCCCC.** 678 (90.17)   
10 - **[CCCGGCCCCG]** 46 (86.26)   
10 - **[CGCGGCCGCC]** 31 (88.02)   
11 + **[CGGGGCGTCC]** 93 (85.97)   
11 + **[CCCGGCGGCC]** 208 (86.37)   
11 + **[CCCGGCCGCG]** 493 (86.07)   
11 + **[CACCGCGCCG]** 603 (87.33)   
11 + **.CGCCGCGGCC.** 608 (92.45)   
11 + **.CGCGGCCGCG.** 611 (92.42)   
11 + **[GGCCGCGCGG]** 614 (85.34)   
11 + **[CCCGGCCGCG]** 709 (86.07)   
11 + **[GGCCGCGGCC]** 712 (87.34)   
11 + **.CGCGGCCCCG.** 715 (92.60)   
11 + **[TGCCGCGGCG]** 766 (87.35)   
11 + **[CGCGGCGCCT]** 769 (89.75)   
11 + **[GGCCCCGCCG]** 1021 (85.29)   
11 + **[CCCCGCCGCG]** 1023 (85.80)   
11 + **[CGCCGCGCTG]** 1026 (87.79)   
11 + **[CGCTGCCGCC]** 1031 (89.98)   
11 + **[CGCCGCCGCC]** 1037 (87.75)   
11 + **[GGCTGCGCGG]** 1111 (87.57)   
11 + **.CGCCGCCGCG.** 1151 (92.15)   
11 + **[CGCCGCGCAG]** 1154 (87.29)   
11 + **[GGGTGCGCCG]** 1202 (86.48)   
11 - **.CGCGGCGGCG.** 1151 (97.11)   
11 - **[CGCCGCGCAG]** 1113 (87.29)   
11 - **[GGCAGCGGCG]** 1043 (88.35)   
11 - **[AGCGGCGGCG]** 1040 (88.74)   
11 - **.GGCGGCGGCG.** 1037 (92.01)   
11 - **.CGCGGCGGGG.** 1023 (90.53)   
11 - **[CGCGGCGGGC]** 990 (86.13)   
11 - **[CGCCGCGGCA]** 766 (86.57)   
11 - **.CGCGGGGCCG.** 717 (91.62)   
11 - **[GGCCGCGGCC]** 712 (87.34)   
11 - **[CGCGGCCGGG]** 709 (85.84)   
11 - **.CGCTGCGCCC.** 666 (94.86)   
11 - **[CGCGGGGCCC]** 657 (87.22)   
11 - **.CGCGGCCGCG.** 611 (92.42)   
11 - **.GGCCGCGGCG.** 608 (91.74)   
11 - **.CGCGGCGCGG.** 605 (90.72)   
11 - **[CGCGGCCGGG]** 493 (85.84)   
11 - **[GGCCGGGCCG]** 490 (86.24)   
11 - **[CGCTCGGCCG]** 213 (86.94)   
11 - **[CGCTGCCCTG]** 145 (85.32)   
12 + **[GGCCGGGGCG]** 166 (86.06)   
12 + **.CGCTGCGTCG.** 445 (100.00)   
12 + **[CGCGCCCTCG]** 526 (86.71)   
12 + **[GGCGGGGCCG]** 604 (86.51)   
12 + **[CGCAGCGCGG]** 917 (87.05)   
12 + **[GGCTGCGGCC]** 1080 (89.57)   
12 + **[CGCGGCCCCC]** 1120 (88.21)   
12 + **[GGCCGCCTCG]** 1145 (87.97)   
12 + **[CGCCGCGCGC]** 1177 (86.05)   
12 + **[GGCGGCGGCC]** 1244 (87.61)   
12 + **[GGCGGCCGCG]** 1247 (87.31)   
12 + **.GGCCGCGCCG.** 1250 (91.93)   
12 - **[AGCAGCGGCG]** 1255 (85.08)   
12 - **[CGCGGCCGCC]** 1247 (88.02)   
12 - **[CGCCGCCCCC]** 1241 (87.94)   
12 - **[CGCGGCGCTG]** 1174 (88.06)   
12 - **[CGCTGGCCCG]** 1169 (88.88)   
12 - **.CGCGGCTTCG.** 1026 (90.58)   
12 - **.CGCTGCGGCC.** 961 (94.68)   
12 - **.CGCTGCGCCC.** 914 (94.86)   
12 - **[CTCTGGGTCG]** 872 (85.34)   
12 - **[GGCGGCGCGG]** 695 (85.61)   
12 - **[CTCTGCGCCC]** 667 (85.88)   
12 - **[CCCGGCCCCG]** 606 (86.26)   
12 - **[CCCCGCCCCG]** 601 (85.99)   
12 - **[CGCCCCGCCC]** 598 (86.00)   
12 - **[CCCCGCCCCG]** 596 (85.99)   
12 - **[CGCCCCGCCC]** 593 (86.00)   
12 - **[CGCCCCGGCC]** 166 (85.81)   
13 + **[CGCGCCGCCC]** 615 (86.26)   
13 + **[CCCGGCGCCC]** 694 (86.55)   
13 + **.CGCCGCCGCG.** 717 (92.15)   
13 + **.CGCCGCCCCG.** 753 (92.34)   
13 + **[CGCCCCGCCC]** 756 (86.00)   
13 + **[CCCCGCCCCG]** 758 (85.99)   
13 + **[CCCCGCGTCC]** 763 (87.02)   
13 + **[CCCCGCGCCC]** 777 (86.28)   
13 + **[CCCCGCGCCC]** 784 (86.28)   
13 + **[CCCTGCGCCT]** 863 (85.36)   
13 + **[GCCGGCGCCG]** 878 (85.85)   
13 + **[CGCGGCCGCC]** 1064 (88.02)   
13 + **[CGCCGCGCGC]** 1109 (86.05)   
13 + **[GGCGCCGCCG]** 1137 (85.56)   
13 + **[CGCCGCCGCC]** 1139 (87.75)   
13 + **[CGCCGCCGCC]** 1142 (87.75)   
13 + **[CGCCGCCGCC]** 1160 (87.75)   
13 + **[CGCCGCCGCC]** 1163 (87.75)   
13 + **[CGCGCCGTCC]** 1187 (87.00)   
13 + **.GGCGGCGTCG.** 1316 (92.93)   
13 + **[CGGGGCGCCC]** 1326 (85.23)   
13 + **.CGCGGCGGGG.** 1352 (90.53)   
13 + **[GGCGGCGGGG]** 1419 (85.43)   
13 + **[GGCGGGGGCG]** 1422 (86.33)   
13 + **[CCCCGGGTCG]** 1438 (85.74)   
13 - **[CGCCGCCCCC]** 1425 (87.94)   
13 - **[CCCCGCCGCG]** 1352 (85.80)   
13 - **[CCCCGCGACG]** 1321 (86.04)   
13 - **[CGCCGCCCGG]** 1313 (85.75)   
13 - **.GGCGGCGGCG.** 1163 (92.01)   
13 - **.GGCGGCGGCG.** 1160 (92.01)   
13 - **[GGCGGCGACG]** 1157 (87.55)   
13 - **.GGCGGCGGCG.** 1142 (92.01)   
13 - **.GGCGGCGGCG.** 1139 (92.01)   
13 - **.GGCGGCGCCG.** 1136 (92.19)   
13 - **[CGCGGCGGTG]** 1106 (87.87)   
13 - **[GGCGGCCGCG]** 1064 (87.31)   
13 - **.GGCCGCGCCG.** 1061 (91.93)   
13 - **.CGCCCCGCCG.** 974 (90.40)   
13 - **[GGCCGCGCGG]** 944 (85.34)   
13 - **[CGCCCCGTCC]** 918 (86.74)   
13 - **[CGCCCCGCCC]** 906 (86.00)   
13 - **[CACGGCGCCG]** 880 (87.60)   
13 - **[CGCCGGCCCG]** 875 (86.65)   
13 - **[GGCGGGGCCG]** 840 (86.51)   
13 - **[CGCGGGGCGG]** 760 (85.03)   
13 - **[CGGGGCGGCG]** 753 (89.44)   
13 - **.CGCGGCGGCG.** 717 (97.11)   
13 - **[GGCGGGGCCG]** 684 (86.51)   
13 - **[GGCGGCGCGG]** 614 (85.61)   
  

**Frequency Matrix**

| All Occs Best Occs|  |  |  |  |  |  |  |  |  |  |  |  |  |  |  |  |  |  |  |  |  |  |  |  |  |  |  |  |  |  |  |  |  |  |  |  |  |  |  |  |  |  |  |  |  |  |  |  |  |  |  |  |  |  |  |  |  |  |  |  |  |  |  |  |  |  |  |  |  |  |  |  |  |  |  |  |  |  |  |  |  |  |  |  |  |  |  |  |  |  |  |  |  |  |  |  |  |  |  |  |  |  |  |  |  |  |  |  |  |  |  |  |  |  |  |  |  |  |  |  |  |  |  |  |  |  |  |  |  |  |  |  | | --- | --- | --- | --- | --- | --- | --- | --- | --- | --- | --- | --- | --- | --- | --- | --- | --- | --- | --- | --- | --- | --- | --- | --- | --- | --- | --- | --- | --- | --- | --- | --- | --- | --- | --- | --- | --- | --- | --- | --- | --- | --- | --- | --- | --- | --- | --- | --- | --- | --- | --- | --- | --- | --- | --- | --- | --- | --- | --- | --- | --- | --- | --- | --- | --- | --- | --- | --- | --- | --- | --- | --- | --- | --- | --- | --- | --- | --- | --- | --- | --- | --- | --- | --- | --- | --- | --- | --- | --- | --- | --- | --- | --- | --- | --- | --- | --- | --- | --- | --- | --- | --- | --- | --- | --- | --- | --- | --- | --- | --- | --- | --- | --- | --- | --- | --- | --- | --- | --- | --- | --- | --- | --- | --- | --- | --- | --- | --- | --- | --- | --- | --- | | **A C G T A C G T | | | | | | | | | | | |**| **1** 17 230 47 12 | 1 61 18 0|  |  |  |  |  |  |  |  |  |  |  |  |  |  |  |  |  |  |  |  |  |  |  |  |  |  |  |  |  |  |  |  |  |  |  |  |  |  |  |  |  |  |  |  |  |  |  |  |  |  |  |  |  |  |  |  |  |  |  |  |  |  |  |  |  |  |  |  |  |  |  |  |  |  |  |  |  |  |  |  |  |  |  |  |  |  |  |  |  |  |  |  |  |  |  |  |  |  |  |  |  |  |  |  |  |  |  |  | | --- | --- | --- | --- | --- | --- | --- | --- | --- | --- | --- | --- | --- | --- | --- | --- | --- | --- | --- | --- | --- | --- | --- | --- | --- | --- | --- | --- | --- | --- | --- | --- | --- | --- | --- | --- | --- | --- | --- | --- | --- | --- | --- | --- | --- | --- | --- | --- | --- | --- | --- | --- | --- | --- | --- | --- | --- | --- | --- | --- | --- | --- | --- | --- | --- | --- | --- | --- | --- | --- | --- | --- | --- | --- | --- | --- | --- | --- | --- | --- | --- | --- | --- | --- | --- | --- | --- | --- | --- | --- | --- | --- | --- | --- | --- | --- | --- | --- | --- | --- | --- | --- | --- | --- | --- | --- | --- | --- | | **2** 12 34 245 15 | 0 3 77 0|  |  |  |  |  |  |  |  |  |  |  |  |  |  |  |  |  |  |  |  |  |  |  |  |  |  |  |  |  |  |  |  |  |  |  |  |  |  |  |  |  |  |  |  |  |  |  |  |  |  |  |  |  |  |  |  |  |  |  |  |  |  |  |  |  |  |  |  |  |  |  |  |  |  |  |  |  |  |  |  |  |  |  |  |  |  |  |  |  |  |  |  |  |  |  |  | | --- | --- | --- | --- | --- | --- | --- | --- | --- | --- | --- | --- | --- | --- | --- | --- | --- | --- | --- | --- | --- | --- | --- | --- | --- | --- | --- | --- | --- | --- | --- | --- | --- | --- | --- | --- | --- | --- | --- | --- | --- | --- | --- | --- | --- | --- | --- | --- | --- | --- | --- | --- | --- | --- | --- | --- | --- | --- | --- | --- | --- | --- | --- | --- | --- | --- | --- | --- | --- | --- | --- | --- | --- | --- | --- | --- | --- | --- | --- | --- | --- | --- | --- | --- | --- | --- | --- | --- | --- | --- | --- | --- | --- | --- | --- | --- | | **3** 7 272 25 2 | 0 79 1 0|  |  |  |  |  |  |  |  |  |  |  |  |  |  |  |  |  |  |  |  |  |  |  |  |  |  |  |  |  |  |  |  |  |  |  |  |  |  |  |  |  |  |  |  |  |  |  |  |  |  |  |  |  |  |  |  |  |  |  |  |  |  |  |  |  |  |  |  |  |  |  |  |  |  |  |  |  |  |  |  |  |  |  |  | | --- | --- | --- | --- | --- | --- | --- | --- | --- | --- | --- | --- | --- | --- | --- | --- | --- | --- | --- | --- | --- | --- | --- | --- | --- | --- | --- | --- | --- | --- | --- | --- | --- | --- | --- | --- | --- | --- | --- | --- | --- | --- | --- | --- | --- | --- | --- | --- | --- | --- | --- | --- | --- | --- | --- | --- | --- | --- | --- | --- | --- | --- | --- | --- | --- | --- | --- | --- | --- | --- | --- | --- | --- | --- | --- | --- | --- | --- | --- | --- | --- | --- | --- | --- | | **4** 24 69 75 138 | 1 24 37 18|  |  |  |  |  |  |  |  |  |  |  |  |  |  |  |  |  |  |  |  |  |  |  |  |  |  |  |  |  |  |  |  |  |  |  |  |  |  |  |  |  |  |  |  |  |  |  |  |  |  |  |  |  |  |  |  |  |  |  |  |  |  |  |  |  |  |  |  |  |  |  |  | | --- | --- | --- | --- | --- | --- | --- | --- | --- | --- | --- | --- | --- | --- | --- | --- | --- | --- | --- | --- | --- | --- | --- | --- | --- | --- | --- | --- | --- | --- | --- | --- | --- | --- | --- | --- | --- | --- | --- | --- | --- | --- | --- | --- | --- | --- | --- | --- | --- | --- | --- | --- | --- | --- | --- | --- | --- | --- | --- | --- | --- | --- | --- | --- | --- | --- | --- | --- | --- | --- | --- | --- | | **5** 5 33 260 8 | 0 4 76 0|  |  |  |  |  |  |  |  |  |  |  |  |  |  |  |  |  |  |  |  |  |  |  |  |  |  |  |  |  |  |  |  |  |  |  |  |  |  |  |  |  |  |  |  |  |  |  |  |  |  |  |  |  |  |  |  |  |  |  |  | | --- | --- | --- | --- | --- | --- | --- | --- | --- | --- | --- | --- | --- | --- | --- | --- | --- | --- | --- | --- | --- | --- | --- | --- | --- | --- | --- | --- | --- | --- | --- | --- | --- | --- | --- | --- | --- | --- | --- | --- | --- | --- | --- | --- | --- | --- | --- | --- | --- | --- | --- | --- | --- | --- | --- | --- | --- | --- | --- | --- | | **6** 12 246 42 6 | 0 69 11 0|  |  |  |  |  |  |  |  |  |  |  |  |  |  |  |  |  |  |  |  |  |  |  |  |  |  |  |  |  |  |  |  |  |  |  |  |  |  |  |  |  |  |  |  |  |  |  |  | | --- | --- | --- | --- | --- | --- | --- | --- | --- | --- | --- | --- | --- | --- | --- | --- | --- | --- | --- | --- | --- | --- | --- | --- | --- | --- | --- | --- | --- | --- | --- | --- | --- | --- | --- | --- | --- | --- | --- | --- | --- | --- | --- | --- | --- | --- | --- | --- | | **7** 8 52 224 22 | 0 15 63 2|  |  |  |  |  |  |  |  |  |  |  |  |  |  |  |  |  |  |  |  |  |  |  |  |  |  |  |  |  |  |  |  |  |  |  |  | | --- | --- | --- | --- | --- | --- | --- | --- | --- | --- | --- | --- | --- | --- | --- | --- | --- | --- | --- | --- | --- | --- | --- | --- | --- | --- | --- | --- | --- | --- | --- | --- | --- | --- | --- | --- | | **8** 21 89 84 112 | 1 27 39 13|  |  |  |  |  |  |  |  |  |  |  |  |  |  |  |  |  |  |  |  |  |  |  |  | | --- | --- | --- | --- | --- | --- | --- | --- | --- | --- | --- | --- | --- | --- | --- | --- | --- | --- | --- | --- | --- | --- | --- | --- | | **9** 12 248 32 14 | 0 76 4 0|  |  |  |  |  |  |  |  |  |  |  |  | | --- | --- | --- | --- | --- | --- | --- | --- | --- | --- | --- | --- | | **10** 9 56 220 21 | 0 11 69 0 | | | | | | | | | | | | | | | | | | | | | | | | | | | | | | | | | | | | | | | | | | | | | | | | | | | | | | | | | | | | | | | | | | | | | | | | | | | | | | | | | | | | | | | | | | | | | | | | | | | | | | | | | | | | | | | | | | | | | | | | | | | | | | | | | | | | |

  
  

---

  
  
 **\*\*\* Interesting motifs (not highest-ranking) can also be :**   
  
  
 **AGCGCG**   
**CGCGCT**  
  
11 redundant motifs found:  
CAGCGCGG - CGCGCTCT - GCAGCGCG - GCGCGA - GAGCGC - CGCGGG - TCCGCG - GTCGCG - CGCGCG - CGCGCG - TACGCG -   
  
Best occurrences (match percentage):   
Seq St oligo pos match
  
 1 + **.CGCGCG.** 884 (94.31)   
1 + **[TGCGCG]** 998 (88.18)   
1 + **[AGCGCC]** 1036 (88.80)   
1 + **[AGCCCG]** 1059 (89.62)   
1 + **[AGAGCG]** 1104 (87.91)   
1 - **[AGCGCC]** 1301 (88.80)   
1 - **[AGAGCG]** 1266 (87.91)   
1 - **[AGCGGG]** 1234 (88.67)   
1 - **[AGCGGG]** 1146 (88.67)   
1 - **.GGCGCG.** 1071 (93.80)   
1 - **[AGGGCG]** 1005 (89.10)   
1 - **.GGCGCG.** 1000 (93.80)   
1 - **.CGCGCG.** 884 (94.31)   
1 - **[AGGGCG]** 793 (89.10)   
2 + **[TGCGCG]** 199 (88.18)   
2 + **[AGCGCC]** 214 (88.80)   
2 + **[AGCCCG]** 503 (89.62)   
2 + **.AGCGCG.** 694 (100.00)   
2 + **.GGCGCG.** 703 (93.80)   
2 + **.CGCGCG.** 705 (94.31)   
2 + **.AGCGCG.** 719 (100.00)   
2 + **[ACCGCG]** 813 (86.51)   
2 + **.CGCGCG.** 892 (94.31)   
2 + **.GGCGCG.** 999 (93.80)   
2 + **[AGCGCT]** 1015 (85.80)   
2 + **.CGCGCG.** 1073 (94.31)   
2 + **[ACCGCG]** 1121 (86.51)   
2 + **.AGCGCG.** 1188 (100.00)   
2 + **[AGGGCG]** 1327 (89.10)   
2 + **[AGGGCG]** 1378 (89.10)   
2 + **.AGCGCG.** 1445 (100.00)   
2 + **.GGCGCG.** 1450 (93.80)   
2 - **.AGCGCG.** 1480 (100.00)   
2 - **[AGCGCC]** 1380 (88.80)   
2 - **[AGCGGG]** 1082 (88.67)   
2 - **.AGCGCG.** 1075 (100.00)   
2 - **.CGCGCG.** 1073 (94.31)   
2 - **[AGCGCT]** 1015 (85.80)   
2 - **[AGGGCG]** 985 (89.10)   
2 - **.CGCGCG.** 892 (94.31)   
2 - **.AGCGCG.** 815 (100.00)   
2 - **[AGCCCG]** 775 (89.62)   
2 - **.CGCGCG.** 705 (94.31)   
2 - **.GGCGCG.** 457 (93.80)   
3 + **[AGAGCG]** 610 (87.91)   
3 + **[AGCGCT]** 939 (85.80)   
3 + **.GGCGCG.** 1180 (93.80)   
3 + **[ACCGCG]** 1221 (86.51)   
3 + **.GGCGCG.** 1249 (93.80)   
3 + **.CGCGCG.** 1251 (94.31)   
3 + **[AGCGCC]** 1310 (88.80)   
3 + **[AGCCCG]** 1384 (89.62)   
3 + **[AGGGCG]** 1501 (89.10)   
3 - **.GGCGCG.** 1418 (93.80)   
3 - **[AGCCCG]** 1357 (89.62)   
3 - **.CGCGCG.** 1251 (94.31)   
3 - **[AGGGCG]** 999 (89.10)   
3 - **[AGAGCG]** 941 (87.91)   
3 - **[AGCGCT]** 939 (85.80)   
3 - **[AGAGCG]** 46 (87.91)   
4 + **[TGCGCG]** 144 (88.18)   
4 + **[ACCGCG]** 639 (86.51)   
4 + **.GGCGCG.** 683 (93.80)   
4 + **[AGCGGG]** 712 (88.67)   
4 + **.CGCGCG.** 784 (94.31)   
4 + **[ACCGCG]** 820 (86.51)   
4 + **.GGCGCG.** 828 (93.80)   
4 + **[AGCGCC]** 834 (88.80)   
4 + **[AGCCCG]** 862 (89.62)   
4 + **[ACCGCG]** 896 (86.51)   
4 + **[AGGGCG]** 914 (89.10)   
4 + **.GGCGCG.** 916 (93.80)   
4 + **[AGCGCC]** 1105 (88.80)   
4 + **[AGCGGG]** 1150 (88.67)   
4 + **[AGCCCG]** 1163 (89.62)   
4 + **[AGGGCG]** 1264 (89.10)   
4 + **.GGCGCG.** 1272 (93.80)   
4 + **.CGCGCG.** 1274 (94.31)   
4 + **.GGCGCG.** 1282 (93.80)   
4 + **.CGCGCG.** 1284 (94.31)   
4 - **.CGCGCG.** 1284 (94.31)   
4 - **.CGCGCG.** 1274 (94.31)   
4 - **.GGCGCG.** 1208 (93.80)   
4 - **.GGCGCG.** 1199 (93.80)   
4 - **.GGCGCG.** 786 (93.80)   
4 - **.CGCGCG.** 784 (94.31)   
4 - **[AGCCCG]** 771 (89.62)   
4 - **[ACCGCG]** 641 (86.51)   
4 - **[AGCGGG]** 608 (88.67)   
4 - **[AGCGCC]** 314 (88.80)   
4 - **[AGAGCG]** 148 (87.91)   
4 - **.AGCGCG.** 146 (100.00)   
4 - **[AGCCCG]** 67 (89.62)   
5 + **.CGCGCG.** 814 (94.31)   
5 + **.CGCGCG.** 1358 (94.31)   
5 + **.AGCGCG.** 1393 (100.00)   
5 + **[AGCGCC]** 1431 (88.80)   
5 - **.CGCGCG.** 1358 (94.31)   
5 - **.GGCGCG.** 1346 (93.80)   
5 - **[AGGGCG]** 1333 (89.10)   
5 - **[AGCGGG]** 1242 (88.67)   
5 - **[AGCGGG]** 1143 (88.67)   
5 - **.CGCGCG.** 814 (94.31)   
6 + **[TGCGCG]** 790 (88.18)   
6 + **[AGCGCC]** 952 (88.80)   
6 + **.AGCGCG.** 1139 (100.00)   
6 + **[AGCGCC]** 1327 (88.80)   
6 + **.AGCGCG.** 1366 (100.00)   
6 + **.CGCGCG.** 1380 (94.31)   
6 + **[AGCCCG]** 1406 (89.62)   
6 - **.AGCGCG.** 1496 (100.00)   
6 - **[ACCGCG]** 1382 (86.51)   
6 - **.CGCGCG.** 1380 (94.31)   
6 - **[TGCGCG]** 1368 (88.18)   
6 - **[AGAGCG]** 985 (87.91)   
7 + **[AGGGCG]** 1027 (89.10)   
7 - **[AGAGCG]** 1240 (87.91)   
7 - **[AGCGGG]** 1238 (88.67)   
8 + **.CGCGCG.** 370 (94.31)   
8 + **.GGCGCG.** 407 (93.80)   
8 + **[AGCGCC]** 449 (88.80)   
8 + **[TGCGCG]** 492 (88.18)   
8 + **[AGCCCG]** 628 (89.62)   
8 + **[AGCCCG]** 845 (89.62)   
8 + **.GGCGCG.** 927 (93.80)   
8 + **.GGCGCG.** 933 (93.80)   
8 + **.CGCGCG.** 953 (94.31)   
8 + **.GGCGCG.** 978 (93.80)   
8 + **[AGCGCC]** 1119 (88.80)   
8 - **[ACCGCG]** 1510 (86.51)   
8 - **[AGCCCG]** 1460 (89.62)   
8 - **.AGCGCG.** 1402 (100.00)   
8 - **.AGCGCG.** 1346 (100.00)   
8 - **[AGCGGG]** 1271 (88.67)   
8 - **.AGCGCG.** 1206 (100.00)   
8 - **[AGCGGG]** 1159 (88.67)   
8 - **[AGAGCG]** 1106 (87.91)   
8 - **.AGCGCG.** 1104 (100.00)   
8 - **.CGCGCG.** 953 (94.31)   
8 - **[AGAGCG]** 863 (87.91)   
8 - **[AGCGGG]** 861 (88.67)   
8 - **[TGCGCG]** 788 (88.18)   
8 - **[AGAGCG]** 750 (87.91)   
8 - **[AGCGCC]** 748 (88.80)   
8 - **[AGCGCC]** 619 (88.80)   
8 - **.GGCGCG.** 494 (93.80)   
8 - **[AGCGCC]** 422 (88.80)   
8 - **.CGCGCG.** 370 (94.31)   
8 - **[AGGGCG]** 285 (89.10)   
9 + **.AGCGCG.** 4 (100.00)   
9 + **.CGCGCG.** 6 (94.31)   
9 + **[AGGGCG]** 45 (89.10)   
9 + **[AGGGCG]** 301 (89.10)   
9 + **[AGCCCG]** 814 (89.62)   
9 + **[AGCGGG]** 826 (88.67)   
9 + **.GGCGCG.** 975 (93.80)   
9 + **.GGCGCG.** 1266 (93.80)   
9 - **[AGCCCG]** 1323 (89.62)   
9 - **.GGCGCG.** 1062 (93.80)   
9 - **[AGAGCG]** 103 (87.91)   
9 - **.AGCGCG.** 101 (100.00)   
9 - **[AGGGCG]** 26 (89.10)   
9 - **.AGCGCG.** 8 (100.00)   
9 - **.CGCGCG.** 6 (94.31)   
10 + **[AGCCCG]** 134 (89.62)   
10 + **[AGCGCC]** 316 (88.80)   
10 + **[AGAGCG]** 469 (87.91)   
10 + **[AGCGCT]** 471 (85.80)   
10 + **[AGGGCG]** 537 (89.10)   
10 + **[AGCGGG]** 570 (88.67)   
10 + **[AGCGGG]** 684 (88.67)   
10 + **[TGCGCG]** 722 (88.18)   
10 + **.AGCGCG.** 862 (100.00)   
10 + **.GGCGCG.** 871 (93.80)   
10 + **[AGCGGG]** 883 (88.67)   
10 + **[AGCGGG]** 1036 (88.67)   
10 + **.AGCGCG.** 1071 (100.00)   
10 + **.AGCGCG.** 1092 (100.00)   
10 + **.CGCGCG.** 1094 (94.31)   
10 + **[AGCCCG]** 1277 (89.62)   
10 - **[ACCGCG]** 1448 (86.51)   
10 - **[AGCGCC]** 1436 (88.80)   
10 - **[AGGGCG]** 1427 (89.10)   
10 - **[AGCCCG]** 1225 (89.62)   
10 - **.CGCGCG.** 1094 (94.31)   
10 - **[AGCCCG]** 875 (89.62)   
10 - **.GGCGCG.** 513 (93.80)   
10 - **[AGCGCT]** 471 (85.80)   
10 - **[AGCCCG]** 346 (89.62)   
10 - **.AGCGCG.** 37 (100.00)   
11 + **[TGCGCG]** 32 (88.18)   
11 + **[TGCGCG]** 49 (88.18)   
11 + **[AGCGCT]** 151 (85.80)   
11 + **[AGCGCC]** 219 (88.80)   
11 + **.GGCGCG.** 250 (93.80)   
11 + **[ACCGCG]** 604 (86.51)   
11 + **.CGCGCG.** 617 (94.31)   
11 + **[AGCGGG]** 677 (88.67)   
11 + **.CGCGCG.** 723 (94.31)   
11 + **[AGCGGG]** 795 (88.67)   
11 + **[AGCGCT]** 1098 (85.80)   
11 + **[AGCGGG]** 1107 (88.67)   
11 + **[TGCGCG]** 1114 (88.18)   
11 + **[AGCGCC]** 1149 (88.80)   
11 + **[AGCCCG]** 1190 (89.62)   
11 + **.AGCGCG.** 1343 (100.00)   
11 + **[AGCGCT]** 1356 (85.80)   
11 + **.GGCGCG.** 1379 (93.80)   
11 - **[AGCGCT]** 1356 (85.80)   
11 - **[AGCCCG]** 1347 (89.62)   
11 - **[TGCGCG]** 1157 (88.18)   
11 - **[AGCCCG]** 1109 (89.62)   
11 - **[AGCGCT]** 1098 (85.80)   
11 - **.AGCGCG.** 1029 (100.00)   
11 - **[AGAGCG]** 1011 (87.91)   
11 - **.AGCGCG.** 996 (100.00)   
11 - **.GGCGCG.** 957 (93.80)   
11 - **[AGCGGG]** 935 (88.67)   
11 - **[AGCCCG]** 893 (89.62)   
11 - **.GGCGCG.** 749 (93.80)   
11 - **.CGCGCG.** 723 (94.31)   
11 - **[AGGGCG]** 702 (89.10)   
11 - **.GGCGCG.** 700 (93.80)   
11 - **.GGCGCG.** 639 (93.80)   
11 - **.CGCGCG.** 617 (94.31)   
11 - **.GGCGCG.** 606 (93.80)   
11 - **[TGCGCG]** 593 (88.18)   
11 - **[AGCGCC]** 416 (88.80)   
11 - **[AGCGCT]** 151 (85.80)   
12 + **[AGGGCG]** 507 (89.10)   
12 + **.GGCGCG.** 509 (93.80)   
12 + **.CGCGCG.** 511 (94.31)   
12 + **.GGCGCG.** 524 (93.80)   
12 + **.GGCGCG.** 541 (93.80)   
12 + **.CGCGCG.** 559 (94.31)   
12 + **.CGCGCG.** 839 (94.31)   
12 + **[AGCCCG]** 859 (89.62)   
12 + **[AGAGCG]** 878 (87.91)   
12 + **.AGCGCG.** 880 (100.00)   
12 + **.CGCGCG.** 882 (94.31)   
12 + **.AGCGCG.** 920 (100.00)   
12 + **.CGCGCG.** 1032 (94.31)   
12 + **.CGCGCG.** 1034 (94.31)   
12 + **.CGCGCG.** 1118 (94.31)   
12 + **[AGCGCC]** 1175 (88.80)   
12 + **.CGCGCG.** 1180 (94.31)   
12 + **.GGCGCG.** 1333 (93.80)   
12 + **[AGCGCC]** 1352 (88.80)   
12 + **[AGCGGG]** 1476 (88.67)   
12 - **[AGCCCG]** 1510 (89.62)   
12 - **[AGCGGG]** 1398 (88.67)   
12 - **.AGCGCG.** 1277 (100.00)   
12 - **.GGCGCG.** 1253 (93.80)   
12 - **.AGCGCG.** 1205 (100.00)   
12 - **[AGAGCG]** 1184 (87.91)   
12 - **.AGCGCG.** 1182 (100.00)   
12 - **.CGCGCG.** 1180 (94.31)   
12 - **.CGCGCG.** 1118 (94.31)   
12 - **[AGGGCG]** 1038 (89.10)   
12 - **.GGCGCG.** 1036 (93.80)   
12 - **.CGCGCG.** 1034 (94.31)   
12 - **.CGCGCG.** 1032 (94.31)   
12 - **.CGCGCG.** 882 (94.31)   
12 - **.CGCGCG.** 839 (94.31)   
12 - **.GGCGCG.** 696 (93.80)   
12 - **.CGCGCG.** 559 (94.31)   
12 - **.GGCGCG.** 543 (93.80)   
12 - **[AGGGCG]** 528 (89.10)   
12 - **.GGCGCG.** 526 (93.80)   
12 - **.CGCGCG.** 511 (94.31)   
12 - **[AGAGCG]** 209 (87.91)   
12 - **.AGCGCG.** 207 (100.00)   
13 + **[ACCGCG]** 165 (86.51)   
13 + **.GGCGCG.** 440 (93.80)   
13 + **[AGGGCG]** 510 (89.10)   
13 + **.AGCGCG.** 529 (100.00)   
13 + **.CGCGCG.** 531 (94.31)   
13 + **.CGCGCG.** 533 (94.31)   
13 + **[AGCGCC]** 675 (88.80)   
13 + **.CGCGCG.** 723 (94.31)   
13 + **.GGCGCG.** 801 (93.80)   
13 + **.CGCGCG.** 945 (94.31)   
13 + **[AGGGCG]** 989 (89.10)   
13 + **.CGCGCG.** 1010 (94.31)   
13 + **.GGCGCG.** 1062 (93.80)   
13 + **.AGCGCG.** 1074 (100.00)   
13 + **.GGCGCG.** 1092 (93.80)   
13 + **.CGCGCG.** 1112 (94.31)   
13 + **.AGCGCG.** 1185 (100.00)   
13 + **[ACCGCG]** 1262 (86.51)   
13 + **[AGAGCG]** 1348 (87.91)   
13 + **.AGCGCG.** 1350 (100.00)   
13 + **[AGCCCG]** 1386 (89.62)   
13 + **[AGGGCG]** 1417 (89.10)   
13 - **.GGCGCG.** 1264 (93.80)   
13 - **[AGCCCG]** 1231 (89.62)   
13 - **.GGCGCG.** 1187 (93.80)   
13 - **.AGCGCG.** 1114 (100.00)   
13 - **.CGCGCG.** 1112 (94.31)   
13 - **[ACCGCG]** 1076 (86.51)   
13 - **[AGAGCG]** 1034 (87.91)   
13 - **[AGCGGG]** 1032 (88.67)   
13 - **[TGCGCG]** 1012 (88.18)   
13 - **.CGCGCG.** 1010 (94.31)   
13 - **.CGCGCG.** 945 (94.31)   
13 - **[AGCCCG]** 931 (89.62)   
13 - **[AGGGCG]** 828 (89.10)   
13 - **[TGCGCG]** 803 (88.18)   
13 - **.GGCGCG.** 787 (93.80)   
13 - **.GGCGCG.** 780 (93.80)   
13 - **.GGCGCG.** 773 (93.80)   
13 - **.CGCGCG.** 723 (94.31)   
13 - **.GGCGCG.** 615 (93.80)   
13 - **.CGCGCG.** 533 (94.31)   
13 - **.CGCGCG.** 531 (94.31)   
13 - **[ACCGCG]** 442 (86.51)   
13 - **.GGCGCG.** 167 (93.80)   
  

**Frequency Matrix**

| All Occs Best Occs|  |  |  |  |  |  |  |  |  |  |  |  |  |  |  |  |  |  |  |  |  |  |  |  |  |  |  |  |  |  |  |  |  |  |  |  |  |  |  |  |  |  |  |  |  |  |  |  |  |  |  |  |  |  |  |  |  |  |  |  |  |  |  |  |  |  |  |  |  |  |  |  |  |  |  |  |  |  |  |  |  |  |  |  | | --- | --- | --- | --- | --- | --- | --- | --- | --- | --- | --- | --- | --- | --- | --- | --- | --- | --- | --- | --- | --- | --- | --- | --- | --- | --- | --- | --- | --- | --- | --- | --- | --- | --- | --- | --- | --- | --- | --- | --- | --- | --- | --- | --- | --- | --- | --- | --- | --- | --- | --- | --- | --- | --- | --- | --- | --- | --- | --- | --- | --- | --- | --- | --- | --- | --- | --- | --- | --- | --- | --- | --- | --- | --- | --- | --- | --- | --- | --- | --- | --- | --- | --- | --- | | **A C G T A C G T | | | | | | | | | | | |**| **1** 231 62 55 15 | 37 62 55 0|  |  |  |  |  |  |  |  |  |  |  |  |  |  |  |  |  |  |  |  |  |  |  |  |  |  |  |  |  |  |  |  |  |  |  |  |  |  |  |  |  |  |  |  |  |  |  |  |  |  |  |  |  |  |  |  |  |  |  |  | | --- | --- | --- | --- | --- | --- | --- | --- | --- | --- | --- | --- | --- | --- | --- | --- | --- | --- | --- | --- | --- | --- | --- | --- | --- | --- | --- | --- | --- | --- | --- | --- | --- | --- | --- | --- | --- | --- | --- | --- | --- | --- | --- | --- | --- | --- | --- | --- | --- | --- | --- | --- | --- | --- | --- | --- | --- | --- | --- | --- | | **2** 3 15 340 5 | 0 0 154 0|  |  |  |  |  |  |  |  |  |  |  |  |  |  |  |  |  |  |  |  |  |  |  |  |  |  |  |  |  |  |  |  |  |  |  |  |  |  |  |  |  |  |  |  |  |  |  |  | | --- | --- | --- | --- | --- | --- | --- | --- | --- | --- | --- | --- | --- | --- | --- | --- | --- | --- | --- | --- | --- | --- | --- | --- | --- | --- | --- | --- | --- | --- | --- | --- | --- | --- | --- | --- | --- | --- | --- | --- | --- | --- | --- | --- | --- | --- | --- | --- | | **3** 19 311 25 8 | 0 154 0 0|  |  |  |  |  |  |  |  |  |  |  |  |  |  |  |  |  |  |  |  |  |  |  |  |  |  |  |  |  |  |  |  |  |  |  |  | | --- | --- | --- | --- | --- | --- | --- | --- | --- | --- | --- | --- | --- | --- | --- | --- | --- | --- | --- | --- | --- | --- | --- | --- | --- | --- | --- | --- | --- | --- | --- | --- | --- | --- | --- | --- | | **4** 5 29 320 9 | 0 0 154 0|  |  |  |  |  |  |  |  |  |  |  |  |  |  |  |  |  |  |  |  |  |  |  |  | | --- | --- | --- | --- | --- | --- | --- | --- | --- | --- | --- | --- | --- | --- | --- | --- | --- | --- | --- | --- | --- | --- | --- | --- | | **5** 5 330 24 4 | 0 154 0 0|  |  |  |  |  |  |  |  |  |  |  |  | | --- | --- | --- | --- | --- | --- | --- | --- | --- | --- | --- | --- | | **6** 7 24 320 12 | 0 0 154 0 | | | | | | | | | | | | | | | | | | | | | | | | | | | | | | | | | | | | | | | | | | | | | | | | | | | | | | | | | | | | | | | | | | | | | | | | | | | | | | | | | | | | |

  
  

---

  
  
  
 **CCGGCGCAGC**   
**GCTGCGCCGG**  
  
2 redundant motifs found:  
CCGTCGGTGC - CTGCGCCG -   
  
Best occurrences (match percentage):   
Seq St oligo pos match
  
 1 + **[GGGGCGCCGC]** 913 (88.17)   
1 + **[GAGGCGCCGC]** 1064 (85.25)   
1 + **[CCGCCGCCCC]** 1076 (87.88)   
1 + **[CCGCCGGCGC]** 1244 (87.76)   
1 + **.CCGGCGCCGG.** 1247 (91.95)   
1 + **[CCGGCGCTGA]** 1299 (86.77)   
1 + **.CCTGCGCCGC.** 1395 (90.59)   
1 - **[GCGGCGCAGG]** 1395 (87.91)   
1 - **.CCGGCGCCGG.** 1247 (91.95)   
1 - **.CGGGCGCGGC.** 1069 (91.84)   
1 - **[GCGGCGCCCC]** 913 (87.36)   
2 + **.CCGGAGCAGC.** 746 (91.28)   
2 + **.CCGGCCCAGC.** 930 (94.19)   
2 + **[GCGGCCCCGC]** 978 (88.27)   
2 + **[GCTGCGCAGC]** 1107 (86.54)   
2 + **.CCCCCGCCGC.** 1264 (90.16)   
2 + **[CCGCCGCCCC]** 1267 (87.88)   
2 - **[CCTGCGCTGC]** 1361 (87.24)   
2 - **.CCCGCGCTGC.** 1186 (91.27)   
2 - **.CAGGCGCCGC.** 1153 (90.22)   
2 - **[GCTGCGCAGC]** 1107 (86.54)   
2 - **[CGTGCGCAGC]** 1028 (85.61)   
2 - **[CTCGCGCCGC]** 997 (85.13)   
2 - **[GCGGGGCCGC]** 978 (87.92)   
2 - **[CCCGCGCGCC]** 703 (86.58)   
2 - **[CGCGCGCCGC]** 701 (88.70)   
2 - **[CCAGCCCAGC]** 19 (86.26)   
3 + **[CGGGCCCCGC]** 1100 (87.34)   
3 + **[CCGCGGCGGC]** 1222 (87.13)   
3 + **[GCGGCGGCGC]** 1224 (87.23)   
3 + **[GCGGCGCAGG]** 1227 (87.91)   
3 + **.CCGGCGCAGC.** 1281 (100.00)   
3 + **[CCGGCCGGGC]** 1332 (85.09)   
3 + **.CCGGCGCCCC.** 1387 (92.34)   
3 + **[GCCCCGCCGC]** 1405 (85.18)   
3 + **.CCGCCGCCGC.** 1408 (94.61)   
3 + **.CCGCCGCCGC.** 1411 (94.61)   
3 + **[CCGCCGCGCC]** 1414 (86.57)   
3 - **[GCGGCGGGGC]** 1405 (85.93)   
3 - **[GCGGGGCGGC]** 1402 (86.61)   
3 - **[CCCGGGCGGC]** 1344 (87.14)   
3 - **[CCGGAGCTGC]** 1286 (87.00)   
3 - **[CCGGGGCTGC]** 1275 (89.55)   
3 - **.CCTGCGCCGC.** 1227 (90.59)   
3 - **[GCGCCGCCGC]** 1224 (89.63)   
4 + **[TCGGCGCCGC]** 691 (89.45)   
4 + **[GCGCCGCCGC]** 694 (89.63)   
4 + **[CCGTCGCGGC]** 738 (87.79)   
4 + **[CCCGCGCGCC]** 782 (86.58)   
4 + **[CCGCGGCGGC]** 821 (87.13)   
4 + **[GCGGCGGCGC]** 823 (87.23)   
4 + **[GCGCCGCTGC]** 1106 (86.28)   
4 + **[GCTGCGCAGC]** 1111 (86.54)   
4 + **[CCGCCGCGCC]** 1204 (86.57)   
4 - **[CCCGCGCGCC]** 1282 (86.58)   
4 - **[CCCGCGCGCC]** 1272 (86.58)   
4 - **[CCCGCCCGGC]** 1248 (87.50)   
4 - **[GCTGCGCAGC]** 1111 (86.54)   
4 - **[CCCGCGGGGC]** 871 (86.46)   
4 - **[CTCGCGCCGC]** 826 (85.13)   
4 - **[GCGCCGCCGC]** 823 (89.63)   
4 - **[CGGGGGCCGC]** 743 (86.98)   
4 - **[GCGGCGGCGC]** 694 (87.23)   
4 - **[GCGGCGCCGA]** 691 (85.14)   
4 - **[CCGGCTCCGC]** 626 (89.14)   
4 - **[CCCGGGCAGC]** 611 (89.39)   
4 - **[CCAGCGGAGC]** 304 (85.22)   
4 - **[CCCGCCCCGC]** 202 (88.80)   
4 - **[CCCGCCCGGC]** 197 (87.50)   
4 - **[CCGGCCCAGG]** 192 (87.07)   
5 + **[CCTCCGCCGC]** 1306 (86.13)   
5 + **[CCCGCCCTGC]** 1331 (85.46)   
5 + **.CCGGCGCAGC.** 1386 (100.00)   
5 + **[CGGCCGCAGC]** 1397 (89.63)   
5 + **[CCGCAGCAGC]** 1400 (86.83)   
5 + **[CCGACGCGGC]** 1436 (87.79)   
5 + **[CGGCCGCCGC]** 1478 (88.69)   
5 + **.CCGCCGCCGC.** 1481 (94.61)   
5 - **[CCGCCGGGGC]** 1471 (86.45)   
5 - **[GCCGCGCTGC]** 1391 (86.29)   
5 - **[CAGGCCCAGC]** 1060 (85.35)   
5 - **.CCCGCGCGGC.** 812 (93.31)   
6 - **[CCGGCGCTGT]** 950 (85.08)   
6 - **[CCTGCCCAGC]** 939 (85.71)   
8 + **.CCGGCGCAGC.** 352 (100.00)   
8 + **[CCCGCGCACC]** 786 (88.83)   
8 + **[CCGCCGCCCC]** 812 (87.88)   
8 + **[CCGGGGCCCC]** 826 (86.17)   
8 + **[CCCGCCCCGC]** 856 (88.80)   
8 + **[CCCGCGCGGG]** 951 (86.19)   
8 + **.CCGGCGGAGC.** 1026 (93.15)   
8 + **[CCGGCCCCCC]** 1057 (86.53)   
8 + **[CCGCCCCCGC]** 1075 (88.80)   
8 + **[GCGGCCCCGC]** 1139 (88.27)   
8 + **[CCGGAGCGGC]** 1413 (89.04)   
8 + **[CCGGTGCCGC]** 1424 (88.56)   
8 - **.CCGGGGCCGC.** 1418 (92.90)   
8 - **[GCGGGGCCGC]** 1139 (87.92)   
8 - **[CGGGGGCGGC]** 1074 (85.67)   
8 - **[CCCGCGCGGG]** 951 (86.19)   
8 - **[CCCGCGGAGC]** 909 (88.70)   
8 - **[CCGCCCCCGC]** 880 (88.80)   
8 - **[CCCGGGCGGC]** 676 (87.14)   
8 - **[CCGGTGCCGC]** 410 (88.56)   
8 - **.CCCCCGCCGC.** 3 (90.16)   
9 + **[CGCGCGCTGC]** 6 (85.35)   
9 + **[CCCTCGCAGC]** 28 (85.59)   
9 + **[CCCGCGCCCC]** 1060 (87.89)   
9 + **[ACGGCGCAGC]** 1244 (86.95)   
9 - **[CCGGGCCAGC]** 1316 (88.02)   
9 - **[CCGTCGCTGC]** 1238 (85.75)   
9 - **[CGCGCGCTGC]** 2 (85.35)   
10 + **[GCGGCGGCGC]** 689 (87.23)   
10 + **[GCGGCGCAGG]** 692 (87.91)   
10 + **.CCGTCGCAGC.** 789 (90.04)   
10 + **[CCGCGGCGGC]** 1023 (87.13)   
10 + **[CGGGCCCGGC]** 1043 (86.03)   
10 + **[CGGCCGCGGC]** 1117 (87.38)   
10 + **[CCACCGCCGC]** 1258 (86.68)   
10 - **[CGGGGGCCGC]** 1332 (86.98)   
10 - **[CGGCCGCGGC]** 1119 (87.38)   
10 - **.CCCGCGCTGC.** 1069 (91.27)   
10 - **[CGGCCGCCGC]** 1025 (88.69)   
10 - **.CCGCCGCGGC.** 1022 (93.30)   
10 - **[CCCGCCCCGC]** 936 (88.80)   
10 - **[CCCGCGCCCC]** 869 (87.89)   
10 - **.CCGGCCCCGC.** 831 (93.25)   
10 - **.CCTGCGCCGC.** 692 (90.59)   
10 - **[GCGCCGCCGC]** 689 (89.63)   
10 - **.CCGGCTCAGC.** 548 (90.08)   
10 - **[GGGGCGCGGC]** 511 (86.86)   
11 + **.CCGGCCCGGC.** 489 (91.94)   
11 + **[GCGCCGCGGC]** 607 (88.32)   
11 + **[CCGCGGCCGC]** 610 (88.44)   
11 + **[CGGGCCCCGC]** 656 (87.34)   
11 + **.CGGGCGCAGC.** 665 (94.08)   
11 + **[CGGCCGCGGC]** 711 (87.38)   
11 + **[GCGGCCCCGC]** 716 (88.27)   
11 + **[GCCCCGCCGC]** 1022 (85.18)   
11 + **[GCCGCGCTGC]** 1027 (86.29)   
11 + **[CTGCCGCCGC]** 1033 (85.12)   
11 + **.CCGCCGCCGC.** 1036 (94.61)   
11 + **.CCGCCGCCGC.** 1039 (94.61)   
11 + **.CCGCCGCTGC.** 1042 (91.26)   
11 + **.CCAGCGCCGC.** 1147 (91.13)   
11 + **[GCGCCGCCGC]** 1150 (89.63)   
11 - **.CCGGCGCACC.** 1203 (93.28)   
11 - **[CCTGCGCGGC]** 1155 (89.28)   
11 - **[GCGGCGGCGC]** 1150 (87.23)   
11 - **.GCCGCGCAGC.** 1112 (90.57)   
11 - **[GCGGCGGGGC]** 1022 (85.93)   
11 - **[GCGCCGCGGC]** 767 (88.32)   
11 - **[GCGGGGCCGC]** 716 (87.92)   
11 - **[CCGGGCCGGC]** 689 (85.78)   
11 - **[CCCGCGGGGC]** 659 (86.46)   
11 - **.CCCGCGCGGC.** 615 (93.31)   
11 - **[CGGCCGCGGC]** 609 (87.38)   
12 + **.CCGGCGCGCC.** 539 (91.03)   
12 + **[CCCGCGCGGG]** 557 (86.19)   
12 + **[CGGGCGCAGA]** 666 (85.14)   
12 + **[TCCGCGCCGC]** 694 (85.00)   
12 + **[CCCGAGCAGC]** 757 (86.83)   
12 + **.CGGGCGCAGC.** 913 (94.08)   
12 + **[CCCGGGCCGC]** 957 (88.45)   
12 + **.CCAGCGCCGC.** 1173 (91.13)   
12 + **[GCCGCGCCGC]** 1251 (89.64)   
12 + **[GCGCCGCTGC]** 1254 (86.28)   
12 + **[GCCGCGCTGC]** 1275 (86.29)   
12 - **[CCCGCGCCCC]** 1331 (87.89)   
12 - **.GCGGCGCGGC.** 1251 (92.78)   
12 - **[CGGCCGCCGC]** 1245 (88.69)   
12 - **[CCGCCGCCCC]** 1242 (87.88)   
12 - **[CGCGCGCGGC]** 1030 (87.39)   
12 - **.CCGGCCCCGC.** 605 (93.25)   
12 - **[CCCGCCCCGC]** 600 (88.80)   
12 - **[CCCGCCCCGC]** 595 (88.80)   
12 - **[CCCGCGCGGG]** 557 (86.19)   
12 - **.CCGACGCAGC.** 446 (90.04)   
13 + **.CCCGCGCCGC.** 613 (94.62)   
13 + **.CCAGCGCCGC.** 673 (91.13)   
13 + **[CCGGCGCCCG]** 695 (85.22)   
13 + **[CCGCCGCCCC]** 752 (87.88)   
13 + **[CCCGCCCCGC]** 759 (88.80)   
13 + **[CCCGCGCCCC]** 771 (87.89)   
13 + **[CCCGCGCCCC]** 778 (87.89)   
13 + **.CCGGCCCCGC.** 839 (93.25)   
13 + **[CCGGCGCCGT]** 879 (88.43)   
13 + **.CCCGCGCGGC.** 943 (93.31)   
13 + **[GCGGCGGGGC]** 973 (85.93)   
13 + **[CCGGGGCCGG]** 997 (85.78)   
13 + **.CCGGCGCGGC.** 1060 (97.75)   
13 + **[CCACCGCCGC]** 1105 (86.68)   
13 + **.CCGGCGCCGC.** 1135 (99.06)   
13 + **[GCGCCGCCGC]** 1138 (89.63)   
13 + **.CCGCCGCCGC.** 1141 (94.61)   
13 + **[CCGTCGCCGC]** 1156 (89.10)   
13 + **.CCGCCGCCGC.** 1162 (94.61)   
13 + **[CCCGGGCGGC]** 1312 (87.14)   
13 + **[CGGGGGCGGC]** 1424 (85.67)   
13 - **[CCGCCGCCCC]** 1426 (87.88)   
13 - **[CCGCCCCCGC]** 1423 (88.80)   
13 - **.CCCCCGCCGC.** 1420 (90.16)   
13 - **[CCCGGGCTGC]** 1384 (85.10)   
13 - **[CGGGCGCCCC]** 1327 (86.42)   
13 - **[CCGGCGCGGT]** 1262 (87.12)   
13 - **[GCGGCGGCGC]** 1138 (87.23)   
13 - **[GCGGCGCCGG]** 1135 (86.97)   
13 - **[CCGGCCCCGG]** 997 (86.14)   
13 - **[CCGGCCCGCC]** 991 (85.22)   
13 - **[GCCCCGCCGC]** 973 (85.18)   
13 - **[GCGGGGCGGC]** 754 (86.61)   
13 - **[CGGGCGCCGG]** 695 (86.03)   
13 - **[CGGGCGGCGC]** 616 (86.30)   
13 - **[GCGGCGCGGG]** 613 (85.66)   
  

**Frequency Matrix**

| All Occs Best Occs|  |  |  |  |  |  |  |  |  |  |  |  |  |  |  |  |  |  |  |  |  |  |  |  |  |  |  |  |  |  |  |  |  |  |  |  |  |  |  |  |  |  |  |  |  |  |  |  |  |  |  |  |  |  |  |  |  |  |  |  |  |  |  |  |  |  |  |  |  |  |  |  |  |  |  |  |  |  |  |  |  |  |  |  |  |  |  |  |  |  |  |  |  |  |  |  |  |  |  |  |  |  |  |  |  |  |  |  |  |  |  |  |  |  |  |  |  |  |  |  |  |  |  |  |  |  |  |  |  |  |  |  | | --- | --- | --- | --- | --- | --- | --- | --- | --- | --- | --- | --- | --- | --- | --- | --- | --- | --- | --- | --- | --- | --- | --- | --- | --- | --- | --- | --- | --- | --- | --- | --- | --- | --- | --- | --- | --- | --- | --- | --- | --- | --- | --- | --- | --- | --- | --- | --- | --- | --- | --- | --- | --- | --- | --- | --- | --- | --- | --- | --- | --- | --- | --- | --- | --- | --- | --- | --- | --- | --- | --- | --- | --- | --- | --- | --- | --- | --- | --- | --- | --- | --- | --- | --- | --- | --- | --- | --- | --- | --- | --- | --- | --- | --- | --- | --- | --- | --- | --- | --- | --- | --- | --- | --- | --- | --- | --- | --- | --- | --- | --- | --- | --- | --- | --- | --- | --- | --- | --- | --- | --- | --- | --- | --- | --- | --- | --- | --- | --- | --- | --- | --- | | **A C G T A C G T | | | | | | | | | | | |**| **1** 11 435 107 29 | 0 49 2 0|  |  |  |  |  |  |  |  |  |  |  |  |  |  |  |  |  |  |  |  |  |  |  |  |  |  |  |  |  |  |  |  |  |  |  |  |  |  |  |  |  |  |  |  |  |  |  |  |  |  |  |  |  |  |  |  |  |  |  |  |  |  |  |  |  |  |  |  |  |  |  |  |  |  |  |  |  |  |  |  |  |  |  |  |  |  |  |  |  |  |  |  |  |  |  |  |  |  |  |  |  |  |  |  |  |  |  |  | | --- | --- | --- | --- | --- | --- | --- | --- | --- | --- | --- | --- | --- | --- | --- | --- | --- | --- | --- | --- | --- | --- | --- | --- | --- | --- | --- | --- | --- | --- | --- | --- | --- | --- | --- | --- | --- | --- | --- | --- | --- | --- | --- | --- | --- | --- | --- | --- | --- | --- | --- | --- | --- | --- | --- | --- | --- | --- | --- | --- | --- | --- | --- | --- | --- | --- | --- | --- | --- | --- | --- | --- | --- | --- | --- | --- | --- | --- | --- | --- | --- | --- | --- | --- | --- | --- | --- | --- | --- | --- | --- | --- | --- | --- | --- | --- | --- | --- | --- | --- | --- | --- | --- | --- | --- | --- | --- | --- | | **2** 36 434 82 30 | 1 47 3 0|  |  |  |  |  |  |  |  |  |  |  |  |  |  |  |  |  |  |  |  |  |  |  |  |  |  |  |  |  |  |  |  |  |  |  |  |  |  |  |  |  |  |  |  |  |  |  |  |  |  |  |  |  |  |  |  |  |  |  |  |  |  |  |  |  |  |  |  |  |  |  |  |  |  |  |  |  |  |  |  |  |  |  |  |  |  |  |  |  |  |  |  |  |  |  |  | | --- | --- | --- | --- | --- | --- | --- | --- | --- | --- | --- | --- | --- | --- | --- | --- | --- | --- | --- | --- | --- | --- | --- | --- | --- | --- | --- | --- | --- | --- | --- | --- | --- | --- | --- | --- | --- | --- | --- | --- | --- | --- | --- | --- | --- | --- | --- | --- | --- | --- | --- | --- | --- | --- | --- | --- | --- | --- | --- | --- | --- | --- | --- | --- | --- | --- | --- | --- | --- | --- | --- | --- | --- | --- | --- | --- | --- | --- | --- | --- | --- | --- | --- | --- | --- | --- | --- | --- | --- | --- | --- | --- | --- | --- | --- | --- | | **3** 42 112 392 36 | 3 10 35 3|  |  |  |  |  |  |  |  |  |  |  |  |  |  |  |  |  |  |  |  |  |  |  |  |  |  |  |  |  |  |  |  |  |  |  |  |  |  |  |  |  |  |  |  |  |  |  |  |  |  |  |  |  |  |  |  |  |  |  |  |  |  |  |  |  |  |  |  |  |  |  |  |  |  |  |  |  |  |  |  |  |  |  |  | | --- | --- | --- | --- | --- | --- | --- | --- | --- | --- | --- | --- | --- | --- | --- | --- | --- | --- | --- | --- | --- | --- | --- | --- | --- | --- | --- | --- | --- | --- | --- | --- | --- | --- | --- | --- | --- | --- | --- | --- | --- | --- | --- | --- | --- | --- | --- | --- | --- | --- | --- | --- | --- | --- | --- | --- | --- | --- | --- | --- | --- | --- | --- | --- | --- | --- | --- | --- | --- | --- | --- | --- | --- | --- | --- | --- | --- | --- | --- | --- | --- | --- | --- | --- | | **4** 25 118 414 25 | 1 12 37 1|  |  |  |  |  |  |  |  |  |  |  |  |  |  |  |  |  |  |  |  |  |  |  |  |  |  |  |  |  |  |  |  |  |  |  |  |  |  |  |  |  |  |  |  |  |  |  |  |  |  |  |  |  |  |  |  |  |  |  |  |  |  |  |  |  |  |  |  |  |  |  |  | | --- | --- | --- | --- | --- | --- | --- | --- | --- | --- | --- | --- | --- | --- | --- | --- | --- | --- | --- | --- | --- | --- | --- | --- | --- | --- | --- | --- | --- | --- | --- | --- | --- | --- | --- | --- | --- | --- | --- | --- | --- | --- | --- | --- | --- | --- | --- | --- | --- | --- | --- | --- | --- | --- | --- | --- | --- | --- | --- | --- | --- | --- | --- | --- | --- | --- | --- | --- | --- | --- | --- | --- | | **5** 38 443 78 23 | 1 49 1 0|  |  |  |  |  |  |  |  |  |  |  |  |  |  |  |  |  |  |  |  |  |  |  |  |  |  |  |  |  |  |  |  |  |  |  |  |  |  |  |  |  |  |  |  |  |  |  |  |  |  |  |  |  |  |  |  |  |  |  |  | | --- | --- | --- | --- | --- | --- | --- | --- | --- | --- | --- | --- | --- | --- | --- | --- | --- | --- | --- | --- | --- | --- | --- | --- | --- | --- | --- | --- | --- | --- | --- | --- | --- | --- | --- | --- | --- | --- | --- | --- | --- | --- | --- | --- | --- | --- | --- | --- | --- | --- | --- | --- | --- | --- | --- | --- | --- | --- | --- | --- | | **6** 27 86 442 27 | 0 5 45 1|  |  |  |  |  |  |  |  |  |  |  |  |  |  |  |  |  |  |  |  |  |  |  |  |  |  |  |  |  |  |  |  |  |  |  |  |  |  |  |  |  |  |  |  |  |  |  |  | | --- | --- | --- | --- | --- | --- | --- | --- | --- | --- | --- | --- | --- | --- | --- | --- | --- | --- | --- | --- | --- | --- | --- | --- | --- | --- | --- | --- | --- | --- | --- | --- | --- | --- | --- | --- | --- | --- | --- | --- | --- | --- | --- | --- | --- | --- | --- | --- | | **7** 17 482 70 13 | 0 50 1 0|  |  |  |  |  |  |  |  |  |  |  |  |  |  |  |  |  |  |  |  |  |  |  |  |  |  |  |  |  |  |  |  |  |  |  |  | | --- | --- | --- | --- | --- | --- | --- | --- | --- | --- | --- | --- | --- | --- | --- | --- | --- | --- | --- | --- | --- | --- | --- | --- | --- | --- | --- | --- | --- | --- | --- | --- | --- | --- | --- | --- | | **8** 224 172 119 67 | 13 26 9 3|  |  |  |  |  |  |  |  |  |  |  |  |  |  |  |  |  |  |  |  |  |  |  |  | | --- | --- | --- | --- | --- | --- | --- | --- | --- | --- | --- | --- | --- | --- | --- | --- | --- | --- | --- | --- | --- | --- | --- | --- | | **9** 17 71 472 22 | 0 3 48 0|  |  |  |  |  |  |  |  |  |  |  |  | | --- | --- | --- | --- | --- | --- | --- | --- | --- | --- | --- | --- | | **10** 37 460 62 23 | 0 49 2 0 | | | | | | | | | | | | | | | | | | | | | | | | | | | | | | | | | | | | | | | | | | | | | | | | | | | | | | | | | | | | | | | | | | | | | | | | | | | | | | | | | | | | | | | | | | | | | | | | | | | | | | | | | | | | | | | | | | | | | | | | | | | | | | | | | | | | |

  
  

---

  
  
  
 **CCCGCGGG**   
**CCCGCGGG**  
  
3 redundant motifs found:  
CGCGGGCA - TCCGCGCG - CGCGGG -   
  
Best occurrences (match percentage):   
Seq St oligo pos match
  
 1 + **[CCCGCCGC]** 1075 (86.41)   
1 - **[GCGGCGGG]** 1075 (86.41)   
2 + **[CGGGCGGG]** 106 (85.67)   
2 + **[CCCTCGGG]** 616 (88.81)   
2 + **.CGCGCGGG.** 705 (93.31)   
2 + **[CGGGCGGG]** 709 (85.67)   
2 + **.CCCCCGGG.** 733 (93.17)   
2 + **[CCCCCGCG]** 1061 (86.48)   
2 + **[GCCGCGCG]** 1071 (87.35)   
2 + **[CCCGGCGG]** 1198 (85.53)   
2 + **[CCGGCGGC]** 1199 (86.41)   
2 + **[CCCGCCGC]** 1266 (86.41)   
2 + **[CCCCGGGG]** 1299 (86.34)   
2 + **[GCCGGGGG]** 1333 (87.21)   
2 + **[CCGGGGGG]** 1334 (85.53)   
2 + **[CCCGCAGG]** 1389 (88.88)   
2 + **.CCCGCGGG.** 1497 (100.00)   
2 + **[CGCGGGGG]** 1499 (86.48)   
2 - **[CCCCCGCG]** 1499 (86.48)   
2 - **.CCCGCGGG.** 1497 (100.00)   
2 - **[CCTGCGGG]** 1389 (88.88)   
2 - **[CCCCCCGG]** 1334 (85.53)   
2 - **[CCCCCGGC]** 1333 (87.21)   
2 - **[CCCCGGGG]** 1299 (86.34)   
2 - **[GCGGCGGG]** 1266 (86.41)   
2 - **[GCCGCCGG]** 1199 (86.41)   
2 - **[CCGCCGGG]** 1198 (85.53)   
2 - **[CGCGCGGC]** 1071 (87.35)   
2 - **[CGCGGGGG]** 1061 (86.48)   
2 - **.CCCGGGGG.** 733 (93.17)   
2 - **[CCCGCCCG]** 709 (85.67)   
2 - **.CCCGCGCG.** 705 (93.31)   
2 - **[CCCGAGGG]** 616 (88.81)   
2 - **[CCCGCCCG]** 106 (85.67)   
3 + **[CCCCCGGC]** 653 (87.21)   
3 + **[CCCGCCCG]** 1094 (85.67)   
3 + **[CGCCCGGG]** 1096 (86.48)   
3 + **[CGCCCGGG]** 1169 (86.48)   
3 + **[CCCCCCGG]** 1328 (85.53)   
3 + **[CCCCCGGC]** 1329 (87.21)   
3 + **[CGCCCGGG]** 1346 (86.48)   
3 + **[CCCGCCGC]** 1407 (86.41)   
3 - **[GCGGCGGG]** 1407 (86.41)   
3 - **[CCCGGGCG]** 1346 (86.48)   
3 - **[GCCGGGGG]** 1329 (87.21)   
3 - **[CCGGGGGG]** 1328 (85.53)   
3 - **[CCCGGGCG]** 1169 (86.48)   
3 - **[CCCGGGCG]** 1096 (86.48)   
3 - **[CGGGCGGG]** 1094 (85.67)   
3 - **[GCCGGGGG]** 653 (87.21)   
4 + **[CGGGCGGG]** 199 (85.67)   
4 + **[CCCCCGCG]** 570 (86.48)   
4 + **[GCGGCGGG]** 671 (86.41)   
4 + **[GGCGCGGG]** 683 (87.35)   
4 + **[CCCCCGGC]** 747 (87.21)   
4 + **[GCCCCGGG]** 767 (87.21)   
4 + **.CCCGCGCG.** 782 (93.31)   
4 + **.CCCGCGGG.** 873 (100.00)   
4 + **[GCGGCGGG]** 905 (86.41)   
4 + **[GCGGCGGG]** 1141 (86.41)   
4 + **.CCCGCGGC.** 1177 (94.04)   
4 + **[CCCGCGCC]** 1197 (87.35)   
4 + **[CCCGCCGC]** 1203 (86.41)   
4 + **[CGGGCGGG]** 1250 (85.67)   
4 + **.CGCGCGGG.** 1274 (93.31)   
4 + **[CGCGGGGG]** 1276 (86.48)   
4 + **.CGCGCGGG.** 1284 (93.31)   
4 - **.CCCGCGCG.** 1284 (93.31)   
4 - **[CCCCCGCG]** 1276 (86.48)   
4 - **.CCCGCGCG.** 1274 (93.31)   
4 - **[CCCGCCCG]** 1250 (85.67)   
4 - **[GCGGCGGG]** 1203 (86.41)   
4 - **[GGCGCGGG]** 1197 (87.35)   
4 - **.GCCGCGGG.** 1177 (94.04)   
4 - **[CCCGCCGC]** 1141 (86.41)   
4 - **[CCCGCCGC]** 905 (86.41)   
4 - **.CCCGCGGG.** 873 (100.00)   
4 - **.CGCGCGGG.** 782 (93.31)   
4 - **[CCCGGGGC]** 767 (87.21)   
4 - **[GCCGGGGG]** 747 (87.21)   
4 - **[CCCGCGCC]** 683 (87.35)   
4 - **[CCCGCCGC]** 671 (86.41)   
4 - **[CGCGGGGG]** 570 (86.48)   
4 - **[CCCGCCCG]** 199 (85.67)   
5 + **[CCTGCGGG]** 530 (88.88)   
5 + **[GCCGCGCG]** 812 (87.35)   
5 + **.CGCGCGGG.** 814 (93.31)   
5 + **[TCCGCGGG]** 1074 (89.58)   
5 + **.CCCGCCGG.** 1172 (92.36)   
5 + **.CCCGCGGG.** 1189 (100.00)   
5 + **[CGCGGGGG]** 1191 (86.48)   
5 + **[CCGGCGGC]** 1219 (86.41)   
5 + **[CCCGCGCC]** 1344 (87.35)   
5 + **.CCCGCGCG.** 1356 (93.31)   
5 + **[CCCCCGGC]** 1383 (87.21)   
5 + **[CCCGGCGG]** 1473 (85.53)   
5 + **[CCGGCGGC]** 1474 (86.41)   
5 - **[GCCGCCGG]** 1474 (86.41)   
5 - **[CCGCCGGG]** 1473 (85.53)   
5 - **[GCCGGGGG]** 1383 (87.21)   
5 - **.CGCGCGGG.** 1356 (93.31)   
5 - **[GGCGCGGG]** 1344 (87.35)   
5 - **[GCCGCCGG]** 1219 (86.41)   
5 - **[CCCCCGCG]** 1191 (86.48)   
5 - **.CCCGCGGG.** 1189 (100.00)   
5 - **.CCGGCGGG.** 1172 (92.36)   
5 - **[CCCGCGGA]** 1074 (89.58)   
5 - **.CCCGCGCG.** 814 (93.31)   
5 - **[CGCGCGGC]** 812 (87.35)   
5 - **[CCCGCAGG]** 530 (88.88)   
6 + **.CCCGCGCG.** 1378 (93.31)   
6 + **.CCCGCGGG.** 1408 (100.00)   
6 + **[GCGGCGGG]** 1476 (86.41)   
6 - **[CCCGCCGC]** 1476 (86.41)   
6 - **.CCCGCGGG.** 1408 (100.00)   
6 - **.CGCGCGGG.** 1378 (93.31)   
8 + **[GCGGCGGG]** 3 (86.41)   
8 + **[CCGGGGGG]** 416 (85.53)   
8 + **[CCCGCCCG]** 499 (85.67)   
8 + **[CGCCCGGG]** 678 (86.48)   
8 + **[CCCCCGCG]** 784 (86.48)   
8 + **[CCCCCCGG]** 822 (85.53)   
8 + **.CCCCCGGG.** 823 (93.17)   
8 + **[CCCCGGGG]** 824 (86.34)   
8 + **[CCCGGGGC]** 825 (87.21)   
8 + **[TCCGCGGG]** 911 (89.58)   
8 + **[GGCGCGGG]** 927 (87.35)   
8 + **.CCCGCGCG.** 951 (93.31)   
8 + **.CGCGCGGG.** 953 (93.31)   
8 + **[CCCGGGCG]** 1185 (86.48)   
8 + **[TCCGCGGG]** 1196 (89.58)   
8 + **[CCCGCCGC]** 1232 (86.41)   
8 + **[CCCGCAGG]** 1299 (88.88)   
8 + **[GCCGCCGG]** 1322 (86.41)   
8 + **[CCCCCGCG]** 1342 (86.48)   
8 + **[CCCCCCGG]** 1366 (85.53)   
8 + **[CCCCCGGC]** 1367 (87.21)   
8 + **[CCCGCGGT]** 1508 (88.61)   
8 - **[ACCGCGGG]** 1508 (88.61)   
8 - **[GCCGGGGG]** 1367 (87.21)   
8 - **[CCGGGGGG]** 1366 (85.53)   
8 - **[CGCGGGGG]** 1342 (86.48)   
8 - **[CCGGCGGC]** 1322 (86.41)   
8 - **[CCTGCGGG]** 1299 (88.88)   
8 - **[GCGGCGGG]** 1232 (86.41)   
8 - **[CCCGCGGA]** 1196 (89.58)   
8 - **[CGCCCGGG]** 1185 (86.48)   
8 - **.CCCGCGCG.** 953 (93.31)   
8 - **.CGCGCGGG.** 951 (93.31)   
8 - **[CCCGCGCC]** 927 (87.35)   
8 - **[CCCGCGGA]** 911 (89.58)   
8 - **[GCCCCGGG]** 825 (87.21)   
8 - **[CCCCGGGG]** 824 (86.34)   
8 - **.CCCGGGGG.** 823 (93.17)   
8 - **[CCGGGGGG]** 822 (85.53)   
8 - **[CGCGGGGG]** 784 (86.48)   
8 - **[CCCGGGCG]** 678 (86.48)   
8 - **[CGGGCGGG]** 499 (85.67)   
8 - **[CCCCCCGG]** 416 (85.53)   
8 - **[CCCGCCGC]** 3 (86.41)   
9 + **[CCCCCGCG]** 1058 (86.48)   
9 + **[CCCGCGCC]** 1060 (87.35)   
9 - **[GGCGCGGG]** 1060 (87.35)   
9 - **[CGCGGGGG]** 1058 (86.48)   
10 + **[CCCGGCGG]** 28 (85.53)   
10 + **[CCGGCGGC]** 29 (86.41)   
10 + **[GCCGGGGG]** 50 (87.21)   
10 + **[CCCGCGGA]** 87 (89.58)   
10 + **[CCTGCGGG]** 342 (88.88)   
10 + **[CCCGCGGA]** 488 (89.58)   
10 + **.CCCGCGGG.** 577 (100.00)   
10 + **[GCCGGGGG]** 708 (87.21)   
10 + **[CGCGGGGG]** 864 (86.48)   
10 + **[GGCGCGGG]** 871 (87.35)   
10 + **[GCCGCGGC]** 1022 (88.09)   
10 + **[CGCGCGGC]** 1094 (87.35)   
10 + **[GCCGCGGC]** 1119 (88.09)   
10 + **.CCCCCGGG.** 1193 (93.17)   
10 + **[CCCCGGGG]** 1194 (86.34)   
10 - **[CCCCGGGG]** 1194 (86.34)   
10 - **.CCCGGGGG.** 1193 (93.17)   
10 - **[GCCGCGGC]** 1119 (88.09)   
10 - **[GCCGCGCG]** 1094 (87.35)   
10 - **[GCCGCGGC]** 1022 (88.09)   
10 - **[CCCGCGCC]** 871 (87.35)   
10 - **[CCCCCGCG]** 864 (86.48)   
10 - **[CCCCCGGC]** 708 (87.21)   
10 - **.CCCGCGGG.** 577 (100.00)   
10 - **[TCCGCGGG]** 488 (89.58)   
10 - **[CCCGCAGG]** 342 (88.88)   
10 - **[TCCGCGGG]** 87 (89.58)   
10 - **[CCCCCGGC]** 50 (87.21)   
10 - **[GCCGCCGG]** 29 (86.41)   
10 - **[CCGCCGGG]** 28 (85.53)   
11 + **[CGGGCGGG]** 22 (85.67)   
11 + **[CCCCCGGC]** 206 (87.21)   
11 + **[CCCGGCGG]** 208 (85.53)   
11 + **[CCGGCGGC]** 209 (86.41)   
11 + **[GCCGCGGC]** 609 (88.09)   
11 + **[GCCGCGCG]** 615 (87.35)   
11 + **.CGCGCGGG.** 617 (93.31)   
11 + **[CCCGCGCC]** 637 (87.35)   
11 + **.CCCGCGGG.** 661 (100.00)   
11 + **[CGGGCGGG]** 679 (85.67)   
11 + **[GCCGCGGC]** 713 (88.09)   
11 + **.CCCGCGCG.** 721 (93.31)   
11 + **[CCCCCGGC]** 737 (87.21)   
11 + **[CCCGGCGG]** 739 (85.53)   
11 + **.CCGGCGGG.** 740 (92.36)   
11 + **[GCCGCGGC]** 767 (88.09)   
11 + **[CCCGCGCC]** 955 (87.35)   
11 + **[CCCGCCCG]** 983 (85.67)   
11 + **[CCCGCCCG]** 987 (85.67)   
11 + **[CCCGCCGC]** 991 (86.41)   
11 + **[CCCGCCCG]** 1002 (85.67)   
11 + **[CCCGCCGC]** 1006 (86.41)   
11 + **[CCCGCCGC]** 1024 (86.41)   
11 + **[CCGGCGGC]** 1086 (86.41)   
11 + **[GCGGCGGG]** 1117 (86.41)   
11 + **.CCGGCGGG.** 1367 (92.36)   
11 - **.CCCGCCGG.** 1367 (92.36)   
11 - **[CCCGCCGC]** 1117 (86.41)   
11 - **[GCCGCCGG]** 1086 (86.41)   
11 - **[GCGGCGGG]** 1024 (86.41)   
11 - **[GCGGCGGG]** 1006 (86.41)   
11 - **[CGGGCGGG]** 1002 (85.67)   
11 - **[GCGGCGGG]** 991 (86.41)   
11 - **[CGGGCGGG]** 987 (85.67)   
11 - **[CGGGCGGG]** 983 (85.67)   
11 - **[GGCGCGGG]** 955 (87.35)   
11 - **[GCCGCGGC]** 767 (88.09)   
11 - **.CCCGCCGG.** 740 (92.36)   
11 - **[CCGCCGGG]** 739 (85.53)   
11 - **[GCCGGGGG]** 737 (87.21)   
11 - **.CGCGCGGG.** 721 (93.31)   
11 - **[GCCGCGGC]** 713 (88.09)   
11 - **[CCCGCCCG]** 679 (85.67)   
11 - **.CCCGCGGG.** 661 (100.00)   
11 - **[GGCGCGGG]** 637 (87.35)   
11 - **.CCCGCGCG.** 617 (93.31)   
11 - **[CGCGCGGC]** 615 (87.35)   
11 - **[GCCGCGGC]** 609 (88.09)   
11 - **[GCCGCCGG]** 209 (86.41)   
11 - **[CCGCCGGG]** 208 (85.53)   
11 - **[GCCGGGGG]** 206 (87.21)   
11 - **[CCCGCCCG]** 22 (85.67)   
12 + **[CACGCGGG]** 115 (86.59)   
12 + **[CTCGCGGG]** 178 (89.48)   
12 + **[CGGGCGGG]** 182 (85.67)   
12 + **[CCGGCGCG]** 539 (85.67)   
12 + **.CCCGCGCG.** 557 (93.31)   
12 + **.CGCGCGGG.** 559 (93.31)   
12 + **[CGGGCGGG]** 592 (85.67)   
12 + **[CCCGGGCG]** 664 (86.48)   
12 + **.CCCGCGGC.** 709 (94.04)   
12 + **[CCCGCCGC]** 774 (86.41)   
12 + **[CCCGCCCG]** 781 (85.67)   
12 + **.CGCGCGGG.** 882 (93.31)   
12 + **[CCCGGGCG]** 911 (86.48)   
12 + **[CCGGGGGG]** 973 (85.53)   
12 + **[GCCGCGCG]** 1030 (87.35)   
12 + **[CGCGCGCG]** 1032 (86.62)   
12 + **[CCCGGGGC]** 1045 (87.21)   
12 + **.CCCGCGGG.** 1088 (100.00)   
12 + **[CGCGCGGC]** 1118 (87.35)   
12 + **[CCCCCGCG]** 1163 (86.48)   
12 + **.CCCGCGGG.** 1165 (100.00)   
12 + **[GCCGCGCG]** 1178 (87.35)   
12 + **.CCGGCGGG.** 1235 (92.36)   
12 + **[GGCGCGGG]** 1333 (87.35)   
12 + **[CCCGCCCG]** 1504 (85.67)   
12 + **[CGCCCGGG]** 1506 (86.48)   
12 - **[CCCGGGCG]** 1506 (86.48)   
12 - **[CGGGCGGG]** 1504 (85.67)   
12 - **[CCCGCGCC]** 1333 (87.35)   
12 - **.CCCGCCGG.** 1235 (92.36)   
12 - **[CGCGCGGC]** 1178 (87.35)   
12 - **.CCCGCGGG.** 1165 (100.00)   
12 - **[CGCGGGGG]** 1163 (86.48)   
12 - **[GCCGCGCG]** 1118 (87.35)   
12 - **.CCCGCGGG.** 1088 (100.00)   
12 - **[GCCCCGGG]** 1045 (87.21)   
12 - **[CGCGCGCG]** 1032 (86.62)   
12 - **[CGCGCGGC]** 1030 (87.35)   
12 - **[CCCCCCGG]** 973 (85.53)   
12 - **[CGCCCGGG]** 911 (86.48)   
12 - **.CCCGCGCG.** 882 (93.31)   
12 - **[CGGGCGGG]** 781 (85.67)   
12 - **[GCGGCGGG]** 774 (86.41)   
12 - **.GCCGCGGG.** 709 (94.04)   
12 - **[CGCCCGGG]** 664 (86.48)   
12 - **[CCCGCCCG]** 592 (85.67)   
12 - **.CCCGCGCG.** 559 (93.31)   
12 - **.CGCGCGGG.** 557 (93.31)   
12 - **[CGCGCCGG]** 539 (85.67)   
12 - **[CCCGCCCG]** 182 (85.67)   
12 - **[CCCGCGAG]** 178 (89.48)   
12 - **[CCCGCGTG]** 115 (86.59)   
13 + **[CGCGCGCG]** 531 (86.62)   
13 + **[CCCGCGCC]** 613 (87.35)   
13 + **[CCCCCGGC]** 692 (87.21)   
13 + **.CCCGCGGC.** 701 (94.04)   
13 + **[GCCGCGCG]** 721 (87.35)   
13 + **[CCCGCCCG]** 747 (85.67)   
13 + **[CCCGCCGC]** 751 (86.41)   
13 + **[CCCGCGCC]** 771 (87.35)   
13 + **[CCCGCGCC]** 778 (87.35)   
13 + **[CCCGCGCC]** 785 (87.35)   
13 + **[CTCGCGGG]** 871 (89.48)   
13 + **.CCCGCGCG.** 943 (93.31)   
13 + **[CGCGCGGC]** 945 (87.35)   
13 + **[GCGGCGGG]** 973 (86.41)   
13 + **[CCGGCGCG]** 1060 (85.67)   
13 + **[GCCGCGCG]** 1110 (87.35)   
13 + **.GCCGCGGG.** 1227 (94.04)   
13 + **[CGCGCCGG]** 1264 (85.67)   
13 + **[CCCGGGCG]** 1312 (86.48)   
13 + **[CGCCCGGG]** 1331 (86.48)   
13 + **[GCGGCGGG]** 1353 (86.41)   
13 + **[GCCCCGGG]** 1393 (87.21)   
13 + **[GCGGCGGG]** 1420 (86.41)   
13 + **[GCGGCGGG]** 1429 (86.41)   
13 - **[CCCGCCGC]** 1429 (86.41)   
13 - **[CCCGCCGC]** 1420 (86.41)   
13 - **[CCCGGGGC]** 1393 (87.21)   
13 - **[CCCGCCGC]** 1353 (86.41)   
13 - **[CCCGGGCG]** 1331 (86.48)   
13 - **[CGCCCGGG]** 1312 (86.48)   
13 - **[CCGGCGCG]** 1264 (85.67)   
13 - **.CCCGCGGC.** 1227 (94.04)   
13 - **[CGCGCGGC]** 1110 (87.35)   
13 - **[CGCGCCGG]** 1060 (85.67)   
13 - **[CCCGCCGC]** 973 (86.41)   
13 - **[GCCGCGCG]** 945 (87.35)   
13 - **.CGCGCGGG.** 943 (93.31)   
13 - **[CCCGCGAG]** 871 (89.48)   
13 - **[GGCGCGGG]** 785 (87.35)   
13 - **[GGCGCGGG]** 778 (87.35)   
13 - **[GGCGCGGG]** 771 (87.35)   
13 - **[GCGGCGGG]** 751 (86.41)   
13 - **[CGGGCGGG]** 747 (85.67)   
13 - **[CGCGCGGC]** 721 (87.35)   
13 - **.GCCGCGGG.** 701 (94.04)   
13 - **[GCCGGGGG]** 692 (87.21)   
13 - **[GGCGCGGG]** 613 (87.35)   
13 - **[CGCGCGCG]** 531 (86.62)   
  

**Frequency Matrix**

| All Occs Best Occs|  |  |  |  |  |  |  |  |  |  |  |  |  |  |  |  |  |  |  |  |  |  |  |  |  |  |  |  |  |  |  |  |  |  |  |  |  |  |  |  |  |  |  |  |  |  |  |  |  |  |  |  |  |  |  |  |  |  |  |  |  |  |  |  |  |  |  |  |  |  |  |  |  |  |  |  |  |  |  |  |  |  |  |  |  |  |  |  |  |  |  |  |  |  |  |  |  |  |  |  |  |  |  |  |  |  |  |  | | --- | --- | --- | --- | --- | --- | --- | --- | --- | --- | --- | --- | --- | --- | --- | --- | --- | --- | --- | --- | --- | --- | --- | --- | --- | --- | --- | --- | --- | --- | --- | --- | --- | --- | --- | --- | --- | --- | --- | --- | --- | --- | --- | --- | --- | --- | --- | --- | --- | --- | --- | --- | --- | --- | --- | --- | --- | --- | --- | --- | --- | --- | --- | --- | --- | --- | --- | --- | --- | --- | --- | --- | --- | --- | --- | --- | --- | --- | --- | --- | --- | --- | --- | --- | --- | --- | --- | --- | --- | --- | --- | --- | --- | --- | --- | --- | --- | --- | --- | --- | --- | --- | --- | --- | --- | --- | --- | --- | | **A C G T A C G T | | | | | | | | | | | |**| **1** 33 556 127 42 | 0 64 4 0|  |  |  |  |  |  |  |  |  |  |  |  |  |  |  |  |  |  |  |  |  |  |  |  |  |  |  |  |  |  |  |  |  |  |  |  |  |  |  |  |  |  |  |  |  |  |  |  |  |  |  |  |  |  |  |  |  |  |  |  |  |  |  |  |  |  |  |  |  |  |  |  |  |  |  |  |  |  |  |  |  |  |  |  | | --- | --- | --- | --- | --- | --- | --- | --- | --- | --- | --- | --- | --- | --- | --- | --- | --- | --- | --- | --- | --- | --- | --- | --- | --- | --- | --- | --- | --- | --- | --- | --- | --- | --- | --- | --- | --- | --- | --- | --- | --- | --- | --- | --- | --- | --- | --- | --- | --- | --- | --- | --- | --- | --- | --- | --- | --- | --- | --- | --- | --- | --- | --- | --- | --- | --- | --- | --- | --- | --- | --- | --- | --- | --- | --- | --- | --- | --- | --- | --- | --- | --- | --- | --- | | **2** 21 583 111 43 | 0 53 15 0|  |  |  |  |  |  |  |  |  |  |  |  |  |  |  |  |  |  |  |  |  |  |  |  |  |  |  |  |  |  |  |  |  |  |  |  |  |  |  |  |  |  |  |  |  |  |  |  |  |  |  |  |  |  |  |  |  |  |  |  |  |  |  |  |  |  |  |  |  |  |  |  | | --- | --- | --- | --- | --- | --- | --- | --- | --- | --- | --- | --- | --- | --- | --- | --- | --- | --- | --- | --- | --- | --- | --- | --- | --- | --- | --- | --- | --- | --- | --- | --- | --- | --- | --- | --- | --- | --- | --- | --- | --- | --- | --- | --- | --- | --- | --- | --- | --- | --- | --- | --- | --- | --- | --- | --- | --- | --- | --- | --- | --- | --- | --- | --- | --- | --- | --- | --- | --- | --- | --- | --- | | **3** 32 598 90 38 | 0 64 4 0|  |  |  |  |  |  |  |  |  |  |  |  |  |  |  |  |  |  |  |  |  |  |  |  |  |  |  |  |  |  |  |  |  |  |  |  |  |  |  |  |  |  |  |  |  |  |  |  |  |  |  |  |  |  |  |  |  |  |  |  | | --- | --- | --- | --- | --- | --- | --- | --- | --- | --- | --- | --- | --- | --- | --- | --- | --- | --- | --- | --- | --- | --- | --- | --- | --- | --- | --- | --- | --- | --- | --- | --- | --- | --- | --- | --- | --- | --- | --- | --- | --- | --- | --- | --- | --- | --- | --- | --- | --- | --- | --- | --- | --- | --- | --- | --- | --- | --- | --- | --- | | **4** 19 109 593 37 | 0 3 65 0|  |  |  |  |  |  |  |  |  |  |  |  |  |  |  |  |  |  |  |  |  |  |  |  |  |  |  |  |  |  |  |  |  |  |  |  |  |  |  |  |  |  |  |  |  |  |  |  | | --- | --- | --- | --- | --- | --- | --- | --- | --- | --- | --- | --- | --- | --- | --- | --- | --- | --- | --- | --- | --- | --- | --- | --- | --- | --- | --- | --- | --- | --- | --- | --- | --- | --- | --- | --- | --- | --- | --- | --- | --- | --- | --- | --- | --- | --- | --- | --- | | **5** 37 593 109 19 | 0 65 3 0|  |  |  |  |  |  |  |  |  |  |  |  |  |  |  |  |  |  |  |  |  |  |  |  |  |  |  |  |  |  |  |  |  |  |  |  | | --- | --- | --- | --- | --- | --- | --- | --- | --- | --- | --- | --- | --- | --- | --- | --- | --- | --- | --- | --- | --- | --- | --- | --- | --- | --- | --- | --- | --- | --- | --- | --- | --- | --- | --- | --- | | **6** 38 90 598 32 | 0 4 64 0|  |  |  |  |  |  |  |  |  |  |  |  |  |  |  |  |  |  |  |  |  |  |  |  | | --- | --- | --- | --- | --- | --- | --- | --- | --- | --- | --- | --- | --- | --- | --- | --- | --- | --- | --- | --- | --- | --- | --- | --- | | **7** 43 111 583 21 | 0 15 53 0|  |  |  |  |  |  |  |  |  |  |  |  | | --- | --- | --- | --- | --- | --- | --- | --- | --- | --- | --- | --- | | **8** 42 127 556 33 | 0 4 64 0 | | | | | | | | | | | | | | | | | | | | | | | | | | | | | | | | | | | | | | | | | | | | | | | | | | | | | | | | | | | | | | | | | | | | | | | | | | | | | | | | | | | | | | | | | | | | | | | | | | | | | | | | | | | | |

  
  

---

  
  
  
 **CGCGCG**   
**CGCGCG**  
  
10 redundant motifs found:  
TCCGCGCG - CCGCGCGG - GCGCGA - CGCGGG - CGCGGG - TCCGCG - GTCGCG - TACGCG - AGCGCG - AGCGCG -   
  
Best occurrences (match percentage):   
Seq St oligo pos match
  
 1 + **.CGCGCG.** 884 (100.00)   
1 + **[CGCGCC]** 1000 (89.43)   
1 + **[CGCGCC]** 1071 (89.43)   
1 + **[CGCCCG]** 1073 (89.91)   
1 - **[CGGGCG]** 1073 (89.91)   
1 - **[GGCGCG]** 1071 (89.43)   
1 - **[GGCGCG]** 1000 (89.43)   
1 - **.CGCGCG.** 884 (100.00)   
2 + **[CGGGCG]** 106 (89.91)   
2 + **[CGCCCG]** 192 (89.91)   
2 + **.CGCGGG.** 364 (91.21)   
2 + **[CGCGCC]** 457 (89.43)   
2 + **.CCCGCG.** 513 (91.21)   
2 + **[AGCGCG]** 694 (87.64)   
2 + **[GGCGCG]** 703 (89.43)   
2 + **.CGCGCG.** 705 (100.00)   
2 + **.CGCGGG.** 707 (91.21)   
2 + **[CGGGCG]** 709 (89.91)   
2 + **[AGCGCG]** 719 (87.64)   
2 + **[CGCCCG]** 743 (89.91)   
2 + **.CGCGGG.** 792 (91.21)   
2 + **[CGCGCT]** 815 (87.64)   
2 + **.CGCGCG.** 892 (100.00)   
2 + **[GGCGCG]** 999 (89.43)   
2 + **.CCCGCG.** 1063 (91.21)   
2 + **.CGCGCG.** 1073 (100.00)   
2 + **[CGCGCT]** 1075 (87.64)   
2 + **[AGCGCG]** 1188 (87.64)   
2 + **.CGCGGG.** 1190 (91.21)   
2 + **[AGCGCG]** 1445 (87.64)   
2 + **[GGCGCG]** 1450 (89.43)   
2 + **[CGCGCT]** 1480 (87.64)   
2 + **.CCCGCG.** 1497 (91.21)   
2 + **.CGCGGG.** 1499 (91.21)   
2 - **.CCCGCG.** 1499 (91.21)   
2 - **.CGCGGG.** 1497 (91.21)   
2 - **[AGCGCG]** 1480 (87.64)   
2 - **[CGCGCC]** 1450 (89.43)   
2 - **[CGCGCT]** 1445 (87.64)   
2 - **.CCCGCG.** 1190 (91.21)   
2 - **[CGCGCT]** 1188 (87.64)   
2 - **[AGCGCG]** 1075 (87.64)   
2 - **.CGCGCG.** 1073 (100.00)   
2 - **.CGCGGG.** 1063 (91.21)   
2 - **[CGCGCC]** 999 (89.43)   
2 - **.CGCGCG.** 892 (100.00)   
2 - **[AGCGCG]** 815 (87.64)   
2 - **.CCCGCG.** 792 (91.21)   
2 - **[CGGGCG]** 743 (89.91)   
2 - **[CGCGCT]** 719 (87.64)   
2 - **[CGCCCG]** 709 (89.91)   
2 - **.CCCGCG.** 707 (91.21)   
2 - **.CGCGCG.** 705 (100.00)   
2 - **[CGCGCC]** 703 (89.43)   
2 - **[CGCGCT]** 694 (87.64)   
2 - **.CGCGGG.** 513 (91.21)   
2 - **[GGCGCG]** 457 (89.43)   
2 - **.CCCGCG.** 364 (91.21)   
2 - **[CGGGCG]** 192 (89.91)   
2 - **[CGCCCG]** 106 (89.91)   
3 + **[CGCCCG]** 571 (89.91)   
3 + **.CGCGGG.** 703 (91.21)   
3 + **[CGCCCG]** 1092 (89.91)   
3 + **[CGCCCG]** 1096 (89.91)   
3 + **[CGCCCG]** 1169 (89.91)   
3 + **[CGGGCG]** 1178 (89.91)   
3 + **[GGCGCG]** 1180 (89.43)   
3 + **[GGCGCG]** 1249 (89.43)   
3 + **.CGCGCG.** 1251 (100.00)   
3 + **[CGCCCG]** 1346 (89.91)   
3 + **[CGCGCC]** 1418 (89.43)   
3 - **[GGCGCG]** 1418 (89.43)   
3 - **[CGGGCG]** 1346 (89.91)   
3 - **.CGCGCG.** 1251 (100.00)   
3 - **[CGCGCC]** 1249 (89.43)   
3 - **[CGCGCC]** 1180 (89.43)   
3 - **[CGCCCG]** 1178 (89.91)   
3 - **[CGGGCG]** 1169 (89.91)   
3 - **[CGGGCG]** 1096 (89.91)   
3 - **[CGGGCG]** 1092 (89.91)   
3 - **.CCCGCG.** 703 (91.21)   
3 - **[CGGGCG]** 571 (89.91)   
4 + **[CGCGCT]** 146 (87.64)   
4 + **[CGGGCG]** 199 (89.91)   
4 + **.CCCGCG.** 572 (91.21)   
4 + **[CGGGCG]** 633 (89.91)   
4 + **[GGCGCG]** 683 (89.43)   
4 + **.CGCGGG.** 685 (91.21)   
4 + **.CCCGCG.** 782 (91.21)   
4 + **.CGCGCG.** 784 (100.00)   
4 + **[CGCGCC]** 786 (89.43)   
4 + **[GGCGCG]** 828 (89.43)   
4 + **.CCCGCG.** 873 (91.21)   
4 + **.CGCGGG.** 875 (91.21)   
4 + **[CGGGCG]** 877 (89.91)   
4 + **[GGCGCG]** 916 (89.43)   
4 + **.CCCGCG.** 1177 (91.21)   
4 + **.CCCGCG.** 1197 (91.21)   
4 + **[CGCGCC]** 1199 (89.43)   
4 + **[CGCCCG]** 1201 (89.91)   
4 + **[CGCGCC]** 1208 (89.43)   
4 + **[CGGGCG]** 1250 (89.91)   
4 + **[CGGGCG]** 1254 (89.91)   
4 + **[GGCGCG]** 1272 (89.43)   
4 + **.CGCGCG.** 1274 (100.00)   
4 + **.CGCGGG.** 1276 (91.21)   
4 + **[GGCGCG]** 1282 (89.43)   
4 + **.CGCGCG.** 1284 (100.00)   
4 + **.CGCGGG.** 1286 (91.21)   
4 - **.CCCGCG.** 1286 (91.21)   
4 - **.CGCGCG.** 1284 (100.00)   
4 - **[CGCGCC]** 1282 (89.43)   
4 - **.CCCGCG.** 1276 (91.21)   
4 - **.CGCGCG.** 1274 (100.00)   
4 - **[CGCGCC]** 1272 (89.43)   
4 - **[CGCCCG]** 1254 (89.91)   
4 - **[CGCCCG]** 1250 (89.91)   
4 - **[GGCGCG]** 1208 (89.43)   
4 - **[CGGGCG]** 1201 (89.91)   
4 - **[GGCGCG]** 1199 (89.43)   
4 - **.CGCGGG.** 1197 (91.21)   
4 - **.CGCGGG.** 1177 (91.21)   
4 - **[CGCGCC]** 916 (89.43)   
4 - **[CGCCCG]** 877 (89.91)   
4 - **.CCCGCG.** 875 (91.21)   
4 - **.CGCGGG.** 873 (91.21)   
4 - **[CGCGCC]** 828 (89.43)   
4 - **[GGCGCG]** 786 (89.43)   
4 - **.CGCGCG.** 784 (100.00)   
4 - **.CGCGGG.** 782 (91.21)   
4 - **.CCCGCG.** 685 (91.21)   
4 - **[CGCGCC]** 683 (89.43)   
4 - **[CGCCCG]** 633 (89.91)   
4 - **.CGCGGG.** 572 (91.21)   
4 - **[CGCCCG]** 199 (89.91)   
4 - **[AGCGCG]** 146 (87.64)   
5 + **.CGCGCG.** 814 (100.00)   
5 + **.CGCGGG.** 816 (91.21)   
5 + **.CGCGGG.** 1076 (91.21)   
5 + **.CCCGCG.** 1189 (91.21)   
5 + **.CGCGGG.** 1191 (91.21)   
5 + **.CCCGCG.** 1344 (91.21)   
5 + **[CGCGCC]** 1346 (89.43)   
5 + **.CCCGCG.** 1356 (91.21)   
5 + **.CGCGCG.** 1358 (100.00)   
5 + **[AGCGCG]** 1393 (87.64)   
5 + **[CGCCCG]** 1433 (89.91)   
5 - **[CGGGCG]** 1433 (89.91)   
5 - **[CGCGCT]** 1393 (87.64)   
5 - **.CGCGCG.** 1358 (100.00)   
5 - **.CGCGGG.** 1356 (91.21)   
5 - **[GGCGCG]** 1346 (89.43)   
5 - **.CGCGGG.** 1344 (91.21)   
5 - **.CCCGCG.** 1191 (91.21)   
5 - **.CGCGGG.** 1189 (91.21)   
5 - **.CCCGCG.** 1076 (91.21)   
5 - **.CCCGCG.** 816 (91.21)   
5 - **.CGCGCG.** 814 (100.00)   
6 + **[AGCGCG]** 1139 (87.64)   
6 + **[AGCGCG]** 1366 (87.64)   
6 + **.CCCGCG.** 1378 (91.21)   
6 + **.CGCGCG.** 1380 (100.00)   
6 + **.CCCGCG.** 1408 (91.21)   
6 + **.CGCGGG.** 1410 (91.21)   
6 + **[CGGGCG]** 1412 (89.91)   
6 + **[CGCGCT]** 1496 (87.64)   
6 - **[AGCGCG]** 1496 (87.64)   
6 - **[CGCCCG]** 1412 (89.91)   
6 - **.CCCGCG.** 1410 (91.21)   
6 - **.CGCGGG.** 1408 (91.21)   
6 - **.CGCGCG.** 1380 (100.00)   
6 - **.CGCGGG.** 1378 (91.21)   
6 - **[CGCGCT]** 1366 (87.64)   
6 - **[CGCGCT]** 1139 (87.64)   
8 + **.CGCGCG.** 370 (100.00)   
8 + **[GGCGCG]** 407 (89.43)   
8 + **[CGCGCC]** 494 (89.43)   
8 + **[CGCCCG]** 501 (89.91)   
8 + **[CGCCCG]** 678 (89.91)   
8 + **.CCCGCG.** 786 (91.21)   
8 + **.CGCGGG.** 913 (91.21)   
8 + **[CGGGCG]** 915 (89.91)   
8 + **[GGCGCG]** 927 (89.43)   
8 + **.CGCGGG.** 929 (91.21)   
8 + **[CGGGCG]** 931 (89.91)   
8 + **[GGCGCG]** 933 (89.43)   
8 + **.CCCGCG.** 951 (91.21)   
8 + **.CGCGCG.** 953 (100.00)   
8 + **.CGCGGG.** 955 (91.21)   
8 + **[GGCGCG]** 978 (89.43)   
8 + **[CGGGCG]** 995 (89.91)   
8 + **[CGCCCG]** 1005 (89.91)   
8 + **.CCCGCG.** 1102 (91.21)   
8 + **[CGCGCT]** 1104 (87.64)   
8 + **.CCCGCG.** 1144 (91.21)   
8 + **[CGCCCG]** 1157 (89.91)   
8 + **[CGGGCG]** 1187 (89.91)   
8 + **.CGCGGG.** 1198 (91.21)   
8 + **[CGCGCT]** 1206 (87.64)   
8 + **[CGCCCG]** 1278 (89.91)   
8 + **.CCCGCG.** 1344 (91.21)   
8 + **[CGCGCT]** 1346 (87.64)   
8 + **[CGCGCT]** 1402 (87.64)   
8 + **[CGCCCG]** 1506 (89.91)   
8 + **.CCCGCG.** 1508 (91.21)   
8 - **.CGCGGG.** 1508 (91.21)   
8 - **[CGGGCG]** 1506 (89.91)   
8 - **[AGCGCG]** 1402 (87.64)   
8 - **[AGCGCG]** 1346 (87.64)   
8 - **.CGCGGG.** 1344 (91.21)   
8 - **[CGGGCG]** 1278 (89.91)   
8 - **[AGCGCG]** 1206 (87.64)   
8 - **.CCCGCG.** 1198 (91.21)   
8 - **[CGCCCG]** 1187 (89.91)   
8 - **[CGGGCG]** 1157 (89.91)   
8 - **.CGCGGG.** 1144 (91.21)   
8 - **[AGCGCG]** 1104 (87.64)   
8 - **.CGCGGG.** 1102 (91.21)   
8 - **[CGGGCG]** 1005 (89.91)   
8 - **[CGCCCG]** 995 (89.91)   
8 - **[CGCGCC]** 978 (89.43)   
8 - **.CCCGCG.** 955 (91.21)   
8 - **.CGCGCG.** 953 (100.00)   
8 - **.CGCGGG.** 951 (91.21)   
8 - **[CGCGCC]** 933 (89.43)   
8 - **[CGCCCG]** 931 (89.91)   
8 - **.CCCGCG.** 929 (91.21)   
8 - **[CGCGCC]** 927 (89.43)   
8 - **[CGCCCG]** 915 (89.91)   
8 - **.CCCGCG.** 913 (91.21)   
8 - **.CGCGGG.** 786 (91.21)   
8 - **[CGGGCG]** 678 (89.91)   
8 - **[CGGGCG]** 501 (89.91)   
8 - **[GGCGCG]** 494 (89.43)   
8 - **[CGCGCC]** 407 (89.43)   
8 - **.CGCGCG.** 370 (100.00)   
9 + **[AGCGCG]** 4 (87.64)   
9 + **.CGCGCG.** 6 (100.00)   
9 + **[CGCGCT]** 8 (87.64)   
9 + **[CGCGCT]** 101 (87.64)   
9 + **[CGGGCG]** 535 (89.91)   
9 + **[CGGGCG]** 973 (89.91)   
9 + **[GGCGCG]** 975 (89.43)   
9 + **.CCCGCG.** 1060 (91.21)   
9 + **[CGCGCC]** 1062 (89.43)   
9 + **[GGCGCG]** 1266 (89.43)   
9 - **[CGCGCC]** 1266 (89.43)   
9 - **[GGCGCG]** 1062 (89.43)   
9 - **.CGCGGG.** 1060 (91.21)   
9 - **[CGCGCC]** 975 (89.43)   
9 - **[CGCCCG]** 973 (89.91)   
9 - **[CGCCCG]** 535 (89.91)   
9 - **[AGCGCG]** 101 (87.64)   
9 - **[AGCGCG]** 8 (87.64)   
9 - **.CGCGCG.** 6 (100.00)   
9 - **[CGCGCT]** 4 (87.64)   
10 + **[CGCGCT]** 37 (87.64)   
10 + **.CCCGCG.** 87 (91.21)   
10 + **[CGGGCG]** 220 (89.91)   
10 + **.CCCGCG.** 488 (91.21)   
10 + **[CGCGCC]** 513 (89.43)   
10 + **.CCCGCG.** 577 (91.21)   
10 + **.CGCGGG.** 579 (91.21)   
10 + **[CGGGCG]** 686 (89.91)   
10 + **.CGCGGG.** 830 (91.21)   
10 + **[AGCGCG]** 862 (87.64)   
10 + **.CGCGGG.** 864 (91.21)   
10 + **[GGCGCG]** 871 (89.43)   
10 + **.CGCGGG.** 873 (91.21)   
10 + **[AGCGCG]** 1071 (87.64)   
10 + **.CGCGGG.** 1073 (91.21)   
10 + **[AGCGCG]** 1092 (87.64)   
10 + **.CGCGCG.** 1094 (100.00)   
10 + **[CGCCCG]** 1113 (89.91)   
10 - **[CGGGCG]** 1113 (89.91)   
10 - **.CGCGCG.** 1094 (100.00)   
10 - **[CGCGCT]** 1092 (87.64)   
10 - **.CCCGCG.** 1073 (91.21)   
10 - **[CGCGCT]** 1071 (87.64)   
10 - **.CCCGCG.** 873 (91.21)   
10 - **[CGCGCC]** 871 (89.43)   
10 - **.CCCGCG.** 864 (91.21)   
10 - **[CGCGCT]** 862 (87.64)   
10 - **.CCCGCG.** 830 (91.21)   
10 - **[CGCCCG]** 686 (89.91)   
10 - **.CCCGCG.** 579 (91.21)   
10 - **.CGCGGG.** 577 (91.21)   
10 - **[GGCGCG]** 513 (89.43)   
10 - **.CGCGGG.** 488 (91.21)   
10 - **[CGCCCG]** 220 (89.91)   
10 - **.CGCGGG.** 87 (91.21)   
10 - **[AGCGCG]** 37 (87.64)   
11 + **[CGGGCG]** 22 (89.91)   
11 + **[GGCGCG]** 250 (89.43)   
11 + **.CCCGCG.** 591 (91.21)   
11 + **[CGCGCC]** 606 (89.43)   
11 + **.CGCGCG.** 617 (100.00)   
11 + **.CGCGGG.** 619 (91.21)   
11 + **[CGCCCG]** 635 (89.91)   
11 + **.CCCGCG.** 637 (91.21)   
11 + **[CGCGCC]** 639 (89.43)   
11 + **.CCCGCG.** 661 (91.21)   
11 + **.CGCGGG.** 663 (91.21)   
11 + **[CGGGCG]** 665 (89.91)   
11 + **[CGGGCG]** 679 (89.91)   
11 + **[CGCGCC]** 700 (89.43)   
11 + **.CCCGCG.** 721 (91.21)   
11 + **.CGCGCG.** 723 (100.00)   
11 + **[CGCGCC]** 749 (89.43)   
11 + **.CCCGCG.** 955 (91.21)   
11 + **[CGCGCC]** 957 (89.43)   
11 + **[CGCCCG]** 959 (89.91)   
11 + **[CGCCCG]** 985 (89.91)   
11 + **[CGCCCG]** 989 (89.91)   
11 + **[CGCGCT]** 996 (87.64)   
11 + **[CGCCCG]** 1004 (89.91)   
11 + **[CGCGCT]** 1029 (87.64)   
11 + **[AGCGCG]** 1343 (87.64)   
11 + **.CGCGGG.** 1345 (91.21)   
11 + **[GGCGCG]** 1379 (89.43)   
11 - **[CGCGCC]** 1379 (89.43)   
11 - **.CCCGCG.** 1345 (91.21)   
11 - **[CGCGCT]** 1343 (87.64)   
11 - **[AGCGCG]** 1029 (87.64)   
11 - **[CGGGCG]** 1004 (89.91)   
11 - **[AGCGCG]** 996 (87.64)   
11 - **[CGGGCG]** 989 (89.91)   
11 - **[CGGGCG]** 985 (89.91)   
11 - **[CGGGCG]** 959 (89.91)   
11 - **[GGCGCG]** 957 (89.43)   
11 - **.CGCGGG.** 955 (91.21)   
11 - **[GGCGCG]** 749 (89.43)   
11 - **.CGCGCG.** 723 (100.00)   
11 - **.CGCGGG.** 721 (91.21)   
11 - **[GGCGCG]** 700 (89.43)   
11 - **[CGCCCG]** 679 (89.91)   
11 - **[CGCCCG]** 665 (89.91)   
11 - **.CCCGCG.** 663 (91.21)   
11 - **.CGCGGG.** 661 (91.21)   
11 - **[GGCGCG]** 639 (89.43)   
11 - **.CGCGGG.** 637 (91.21)   
11 - **[CGGGCG]** 635 (89.91)   
11 - **.CCCGCG.** 619 (91.21)   
11 - **.CGCGCG.** 617 (100.00)   
11 - **[GGCGCG]** 606 (89.43)   
11 - **.CGCGGG.** 591 (91.21)   
11 - **[CGCGCC]** 250 (89.43)   
11 - **[CGCCCG]** 22 (89.91)   
12 + **.CGCGGG.** 117 (91.21)   
12 + **.CGCGGG.** 180 (91.21)   
12 + **[CGGGCG]** 182 (89.91)   
12 + **[CGCGCT]** 207 (87.64)   
12 + **[GGCGCG]** 509 (89.43)   
12 + **.CGCGCG.** 511 (100.00)   
12 + **[GGCGCG]** 524 (89.43)   
12 + **[CGCGCC]** 526 (89.43)   
12 + **[GGCGCG]** 541 (89.43)   
12 + **[CGCGCC]** 543 (89.43)   
12 + **.CCCGCG.** 557 (91.21)   
12 + **.CGCGCG.** 559 (100.00)   
12 + **.CGCGGG.** 561 (91.21)   
12 + **[CGGGCG]** 592 (89.91)   
12 + **[CGGGCG]** 612 (89.91)   
12 + **[CGGGCG]** 666 (89.91)   
12 + **[CGCGCC]** 696 (89.43)   
12 + **.CCCGCG.** 709 (91.21)   
12 + **[CGCCCG]** 779 (89.91)   
12 + **[CGCCCG]** 783 (89.91)   
12 + **[CGCCCG]** 811 (89.91)   
12 + **.CGCGCG.** 839 (100.00)   
12 + **[AGCGCG]** 880 (87.64)   
12 + **.CGCGCG.** 882 (100.00)   
12 + **.CGCGGG.** 884 (91.21)   
12 + **[CGGGCG]** 913 (89.91)   
12 + **[AGCGCG]** 920 (87.64)   
12 + **.CGCGCG.** 1032 (100.00)   
12 + **.CGCGCG.** 1034 (100.00)   
12 + **[CGCGCC]** 1036 (89.43)   
12 + **.CCCGCG.** 1088 (91.21)   
12 + **.CGCGGG.** 1090 (91.21)   
12 + **.CGCGGG.** 1097 (91.21)   
12 + **[CGGGCG]** 1099 (89.91)   
12 + **.CGCGCG.** 1118 (100.00)   
12 + **.CCCGCG.** 1165 (91.21)   
12 + **.CGCGGG.** 1167 (91.21)   
12 + **.CGCGCG.** 1180 (100.00)   
12 + **[CGCGCT]** 1182 (87.64)   
12 + **.CCCGCG.** 1203 (91.21)   
12 + **[CGCGCT]** 1205 (87.64)   
12 + **[CGCGCC]** 1253 (89.43)   
12 + **[CGCGCT]** 1277 (87.64)   
12 + **[GGCGCG]** 1333 (89.43)   
12 + **.CGCGGG.** 1335 (91.21)   
12 + **[CGGGCG]** 1337 (89.91)   
12 + **[CGCCCG]** 1506 (89.91)   
12 - **[CGGGCG]** 1506 (89.91)   
12 - **[CGCCCG]** 1337 (89.91)   
12 - **.CCCGCG.** 1335 (91.21)   
12 - **[CGCGCC]** 1333 (89.43)   
12 - **[AGCGCG]** 1277 (87.64)   
12 - **[GGCGCG]** 1253 (89.43)   
12 - **[AGCGCG]** 1205 (87.64)   
12 - **.CGCGGG.** 1203 (91.21)   
12 - **[AGCGCG]** 1182 (87.64)   
12 - **.CGCGCG.** 1180 (100.00)   
12 - **.CCCGCG.** 1167 (91.21)   
12 - **.CGCGGG.** 1165 (91.21)   
12 - **.CGCGCG.** 1118 (100.00)   
12 - **[CGCCCG]** 1099 (89.91)   
12 - **.CCCGCG.** 1097 (91.21)   
12 - **.CCCGCG.** 1090 (91.21)   
12 - **.CGCGGG.** 1088 (91.21)   
12 - **[GGCGCG]** 1036 (89.43)   
12 - **.CGCGCG.** 1034 (100.00)   
12 - **.CGCGCG.** 1032 (100.00)   
12 - **[CGCGCT]** 920 (87.64)   
12 - **[CGCCCG]** 913 (89.91)   
12 - **.CCCGCG.** 884 (91.21)   
12 - **.CGCGCG.** 882 (100.00)   
12 - **[CGCGCT]** 880 (87.64)   
12 - **.CGCGCG.** 839 (100.00)   
12 - **[CGGGCG]** 811 (89.91)   
12 - **[CGGGCG]** 783 (89.91)   
12 - **[CGGGCG]** 779 (89.91)   
12 - **.CGCGGG.** 709 (91.21)   
12 - **[GGCGCG]** 696 (89.43)   
12 - **[CGCCCG]** 666 (89.91)   
12 - **[CGCCCG]** 612 (89.91)   
12 - **[CGCCCG]** 592 (89.91)   
12 - **.CCCGCG.** 561 (91.21)   
12 - **.CGCGCG.** 559 (100.00)   
12 - **.CGCGGG.** 557 (91.21)   
12 - **[GGCGCG]** 543 (89.43)   
12 - **[CGCGCC]** 541 (89.43)   
12 - **[GGCGCG]** 526 (89.43)   
12 - **[CGCGCC]** 524 (89.43)   
12 - **.CGCGCG.** 511 (100.00)   
12 - **[CGCGCC]** 509 (89.43)   
12 - **[AGCGCG]** 207 (87.64)   
12 - **[CGCCCG]** 182 (89.91)   
12 - **.CCCGCG.** 180 (91.21)   
12 - **.CCCGCG.** 117 (91.21)   
13 + **[CGCGCC]** 167 (89.43)   
13 + **[CGCCCG]** 169 (89.91)   
13 + **[GGCGCG]** 440 (89.43)   
13 + **[AGCGCG]** 529 (87.64)   
13 + **.CGCGCG.** 531 (100.00)   
13 + **.CGCGCG.** 533 (100.00)   
13 + **[CGGGCG]** 548 (89.91)   
13 + **.CCCGCG.** 613 (91.21)   
13 + **[CGCGCC]** 615 (89.43)   
13 + **[CGCCCG]** 620 (89.91)   
13 + **.CCCGCG.** 642 (91.21)   
13 + **[CGCCCG]** 699 (89.91)   
13 + **.CCCGCG.** 701 (91.21)   
13 + **.CGCGCG.** 723 (100.00)   
13 + **.CCCGCG.** 740 (91.21)   
13 + **[CGCCCG]** 749 (89.91)   
13 + **.CCCGCG.** 764 (91.21)   
13 + **.CCCGCG.** 771 (91.21)   
13 + **[CGCGCC]** 773 (89.43)   
13 + **.CCCGCG.** 778 (91.21)   
13 + **[CGCGCC]** 780 (89.43)   
13 + **.CCCGCG.** 785 (91.21)   
13 + **[CGCGCC]** 787 (89.43)   
13 + **[CGCCCG]** 789 (89.91)   
13 + **[GGCGCG]** 801 (89.43)   
13 + **.CGCGGG.** 873 (91.21)   
13 + **.CCCGCG.** 943 (91.21)   
13 + **.CGCGCG.** 945 (100.00)   
13 + **.CGCGCG.** 1010 (100.00)   
13 + **[CGGGCG]** 1044 (89.91)   
13 + **[GGCGCG]** 1062 (89.43)   
13 + **[AGCGCG]** 1074 (87.64)   
13 + **[GGCGCG]** 1092 (89.43)   
13 + **.CGCGCG.** 1112 (100.00)   
13 + **[CGCGCT]** 1114 (87.64)   
13 + **[AGCGCG]** 1185 (87.64)   
13 + **[CGCGCC]** 1187 (89.43)   
13 + **.CGCGGG.** 1229 (91.21)   
13 + **[CGCGCC]** 1264 (89.43)   
13 + **.CGCGGG.** 1304 (91.21)   
13 + **[CGGGCG]** 1314 (89.91)   
13 + **.CGCGGG.** 1324 (91.21)   
13 + **[CGCCCG]** 1331 (89.91)   
13 + **[AGCGCG]** 1350 (87.64)   
13 + **.CGCGGG.** 1470 (91.21)   
13 + **[CGCCCG]** 1512 (89.91)   
13 - **[CGGGCG]** 1512 (89.91)   
13 - **.CCCGCG.** 1470 (91.21)   
13 - **[CGCGCT]** 1350 (87.64)   
13 - **[CGGGCG]** 1331 (89.91)   
13 - **.CCCGCG.** 1324 (91.21)   
13 - **[CGCCCG]** 1314 (89.91)   
13 - **.CCCGCG.** 1304 (91.21)   
13 - **[GGCGCG]** 1264 (89.43)   
13 - **.CCCGCG.** 1229 (91.21)   
13 - **[GGCGCG]** 1187 (89.43)   
13 - **[CGCGCT]** 1185 (87.64)   
13 - **[AGCGCG]** 1114 (87.64)   
13 - **.CGCGCG.** 1112 (100.00)   
13 - **[CGCGCC]** 1092 (89.43)   
13 - **[CGCGCT]** 1074 (87.64)   
13 - **[CGCGCC]** 1062 (89.43)   
13 - **[CGCCCG]** 1044 (89.91)   
13 - **.CGCGCG.** 1010 (100.00)   
13 - **.CGCGCG.** 945 (100.00)   
13 - **.CGCGGG.** 943 (91.21)   
13 - **.CCCGCG.** 873 (91.21)   
13 - **[CGCGCC]** 801 (89.43)   
13 - **[CGGGCG]** 789 (89.91)   
13 - **[GGCGCG]** 787 (89.43)   
13 - **.CGCGGG.** 785 (91.21)   
13 - **[GGCGCG]** 780 (89.43)   
13 - **.CGCGGG.** 778 (91.21)   
13 - **[GGCGCG]** 773 (89.43)   
13 - **.CGCGGG.** 771 (91.21)   
13 - **.CGCGGG.** 764 (91.21)   
13 - **[CGGGCG]** 749 (89.91)   
13 - **.CGCGGG.** 740 (91.21)   
13 - **.CGCGCG.** 723 (100.00)   
13 - **.CGCGGG.** 701 (91.21)   
13 - **[CGGGCG]** 699 (89.91)   
13 - **.CGCGGG.** 642 (91.21)   
13 - **[CGGGCG]** 620 (89.91)   
13 - **[GGCGCG]** 615 (89.43)   
13 - **.CGCGGG.** 613 (91.21)   
13 - **[CGCCCG]** 548 (89.91)   
13 - **.CGCGCG.** 533 (100.00)   
13 - **.CGCGCG.** 531 (100.00)   
13 - **[CGCGCT]** 529 (87.64)   
13 - **[CGCGCC]** 440 (89.43)   
13 - **[CGGGCG]** 169 (89.91)   
13 - **[GGCGCG]** 167 (89.43)   
  

**Frequency Matrix**

| All Occs Best Occs|  |  |  |  |  |  |  |  |  |  |  |  |  |  |  |  |  |  |  |  |  |  |  |  |  |  |  |  |  |  |  |  |  |  |  |  |  |  |  |  |  |  |  |  |  |  |  |  |  |  |  |  |  |  |  |  |  |  |  |  |  |  |  |  |  |  |  |  |  |  |  |  |  |  |  |  |  |  |  |  |  |  |  |  | | --- | --- | --- | --- | --- | --- | --- | --- | --- | --- | --- | --- | --- | --- | --- | --- | --- | --- | --- | --- | --- | --- | --- | --- | --- | --- | --- | --- | --- | --- | --- | --- | --- | --- | --- | --- | --- | --- | --- | --- | --- | --- | --- | --- | --- | --- | --- | --- | --- | --- | --- | --- | --- | --- | --- | --- | --- | --- | --- | --- | --- | --- | --- | --- | --- | --- | --- | --- | --- | --- | --- | --- | --- | --- | --- | --- | --- | --- | --- | --- | --- | --- | --- | --- | | **A C G T A C G T | | | | | | | | | | | |**| **1** 37 575 55 15 | 0 224 0 0|  |  |  |  |  |  |  |  |  |  |  |  |  |  |  |  |  |  |  |  |  |  |  |  |  |  |  |  |  |  |  |  |  |  |  |  |  |  |  |  |  |  |  |  |  |  |  |  |  |  |  |  |  |  |  |  |  |  |  |  | | --- | --- | --- | --- | --- | --- | --- | --- | --- | --- | --- | --- | --- | --- | --- | --- | --- | --- | --- | --- | --- | --- | --- | --- | --- | --- | --- | --- | --- | --- | --- | --- | --- | --- | --- | --- | --- | --- | --- | --- | --- | --- | --- | --- | --- | --- | --- | --- | --- | --- | --- | --- | --- | --- | --- | --- | --- | --- | --- | --- | | **2** 13 81 570 18 | 0 81 143 0|  |  |  |  |  |  |  |  |  |  |  |  |  |  |  |  |  |  |  |  |  |  |  |  |  |  |  |  |  |  |  |  |  |  |  |  |  |  |  |  |  |  |  |  |  |  |  |  | | --- | --- | --- | --- | --- | --- | --- | --- | --- | --- | --- | --- | --- | --- | --- | --- | --- | --- | --- | --- | --- | --- | --- | --- | --- | --- | --- | --- | --- | --- | --- | --- | --- | --- | --- | --- | --- | --- | --- | --- | --- | --- | --- | --- | --- | --- | --- | --- | | **3** 15 591 63 13 | 0 224 0 0|  |  |  |  |  |  |  |  |  |  |  |  |  |  |  |  |  |  |  |  |  |  |  |  |  |  |  |  |  |  |  |  |  |  |  |  | | --- | --- | --- | --- | --- | --- | --- | --- | --- | --- | --- | --- | --- | --- | --- | --- | --- | --- | --- | --- | --- | --- | --- | --- | --- | --- | --- | --- | --- | --- | --- | --- | --- | --- | --- | --- | | **4** 13 63 591 15 | 0 0 224 0|  |  |  |  |  |  |  |  |  |  |  |  |  |  |  |  |  |  |  |  |  |  |  |  | | --- | --- | --- | --- | --- | --- | --- | --- | --- | --- | --- | --- | --- | --- | --- | --- | --- | --- | --- | --- | --- | --- | --- | --- | | **5** 18 570 81 13 | 0 143 81 0|  |  |  |  |  |  |  |  |  |  |  |  | | --- | --- | --- | --- | --- | --- | --- | --- | --- | --- | --- | --- | | **6** 15 55 575 37 | 0 0 224 0 | | | | | | | | | | | | | | | | | | | | | | | | | | | | | | | | | | | | | | | | | | | | | | | | | | | | | | | | | | | | | | | | | | | | | | | | | | | | | | | | | | | | |

  
  

---

  
  
  
 **CCGCGCGG**   
**CCGCGCGG**  
  
4 redundant motifs found:  
CAGCGCGG - TCCGCGCG - CTGCGCCG - CGCGCG -   
  
Best occurrences (match percentage):   
Seq St oligo pos match
  
 1 + **[CCGCGCGT]** 883 (87.89)   
1 + **[GCGCGCCG]** 999 (87.56)   
1 + **[CCGCGCCC]** 1070 (87.56)   
1 + **[CCGCCCCG]** 1079 (86.75)   
1 + **[CGGCGCCG]** 1248 (87.28)   
1 - **[CGGCGCCG]** 1248 (87.28)   
1 - **[CGGGGCGG]** 1079 (86.75)   
1 - **[GGGCGCGG]** 1070 (87.56)   
1 - **[CGGCGCGC]** 999 (87.56)   
1 - **[ACGCGCGG]** 883 (87.89)   
2 + **[GCGGGCGG]** 105 (87.03)   
2 + **[GCGCCCGG]** 191 (87.03)   
2 + **[CCGCCCCG]** 252 (86.75)   
2 + **[CGGCGCGC]** 702 (87.56)   
2 + **.GCGCGCGG.** 704 (93.92)   
2 + **[GCGGGCGG]** 708 (87.03)   
2 + **.CCGCCCGG.** 742 (93.11)   
2 + **[CCGCCCCG]** 902 (86.75)   
2 + **[CCTCGCGG]** 993 (89.17)   
2 + **.CCGCGCGC.** 1072 (93.92)   
2 + **[CCGCGAGG]** 1122 (89.17)   
2 + **.CAGCGCGG.** 1187 (91.12)   
2 + **[GCGCGGGG]** 1189 (86.73)   
2 + **[CCCGGCGG]** 1198 (85.92)   
2 + **[CCCCGCCG]** 1265 (86.45)   
2 + **[CCCCGGGG]** 1299 (85.62)   
2 + **[CCGGGGGG]** 1334 (85.92)   
2 + **[GAGCGCGG]** 1444 (85.04)   
2 + **[GCCCGCGG]** 1496 (86.73)   
2 + **.CCGCGGGG.** 1498 (92.81)   
2 - **.CCCCGCGG.** 1498 (92.81)   
2 - **[CCGCGGGC]** 1496 (86.73)   
2 - **[CCGCGCTC]** 1444 (85.04)   
2 - **[CCCCCCGG]** 1334 (85.92)   
2 - **[CCCCGGGG]** 1299 (85.62)   
2 - **[CGGCGGGG]** 1265 (86.45)   
2 - **[CCGCCGGG]** 1198 (85.92)   
2 - **[CCCCGCGC]** 1189 (86.73)   
2 - **.CCGCGCTG.** 1187 (91.12)   
2 - **[CCTCGCGG]** 1122 (89.17)   
2 - **.GCGCGCGG.** 1072 (93.92)   
2 - **[CCGCGAGG]** 993 (89.17)   
2 - **[CGGGGCGG]** 902 (86.75)   
2 - **.CCGGGCGG.** 742 (93.11)   
2 - **[CCGCCCGC]** 708 (87.03)   
2 - **.CCGCGCGC.** 704 (93.92)   
2 - **[GCGCGCCG]** 702 (87.56)   
2 - **[CGGGGCGG]** 252 (86.75)   
2 - **[CCGGGCGC]** 191 (87.03)   
2 - **[CCGCCCGC]** 105 (87.03)   
3 + **.CCGCCCGG.** 1095 (93.11)   
3 + **.CCGCCCGG.** 1168 (93.11)   
3 + **[CCGGGCCG]** 1172 (86.75)   
3 + **[CCGGGCGC]** 1177 (87.03)   
3 + **[CCGCGGCG]** 1222 (86.45)   
3 + **[CGGCGCGC]** 1248 (87.56)   
3 + **[CCCCCCGG]** 1328 (85.92)   
3 + **[CCGGCCGG]** 1332 (86.22)   
3 + **.CCGCCCGG.** 1345 (93.11)   
3 + **[CCGCCCCG]** 1403 (86.75)   
3 + **[CCCCGCCG]** 1406 (86.45)   
3 - **[CGGCGGGG]** 1406 (86.45)   
3 - **[CGGGGCGG]** 1403 (86.75)   
3 - **.CCGGGCGG.** 1345 (93.11)   
3 - **[CCGGCCGG]** 1332 (86.22)   
3 - **[CCGGGGGG]** 1328 (85.92)   
3 - **[GCGCGCCG]** 1248 (87.56)   
3 - **[CGCCGCGG]** 1222 (86.45)   
3 - **[GCGCCCGG]** 1177 (87.03)   
3 - **[CGGCCCGG]** 1172 (86.75)   
3 - **.CCGGGCGG.** 1168 (93.11)   
3 - **.CCGGGCGG.** 1095 (93.11)   
4 + **.CCGGGCGG.** 198 (93.11)   
4 + **[CGGGGCGG]** 203 (86.75)   
4 + **[GGGCGCGG]** 682 (87.56)   
4 + **[CGGCGCCG]** 692 (87.28)   
4 + **.CCGCGCGC.** 783 (93.92)   
4 + **[CCTCGCGG]** 799 (89.17)   
4 + **[CCGCGGCG]** 821 (86.45)   
4 + **.CCCCGCGG.** 872 (92.81)   
4 + **[CCGCGGGC]** 874 (86.73)   
4 + **[GCGGGCGG]** 876 (87.03)   
4 + **.CCCCGCGG.** 1176 (92.81)   
4 + **[CCCCGCGC]** 1196 (86.73)   
4 + **[CCGCGCCC]** 1198 (87.56)   
4 + **.CCGGGCGG.** 1249 (93.11)   
4 + **[GCGGGCGG]** 1253 (87.03)   
4 + **.GCGCGCGG.** 1273 (93.92)   
4 + **[GCGCGGGG]** 1275 (86.73)   
4 + **.GCGCGCGG.** 1283 (93.92)   
4 + **[GCGCGGGG]** 1285 (86.73)   
4 - **[CCCCGCGC]** 1285 (86.73)   
4 - **.CCGCGCGC.** 1283 (93.92)   
4 - **[CCCCGCGC]** 1275 (86.73)   
4 - **.CCGCGCGC.** 1273 (93.92)   
4 - **[CCGCCCGC]** 1253 (87.03)   
4 - **.CCGCCCGG.** 1249 (93.11)   
4 - **[GGGCGCGG]** 1198 (87.56)   
4 - **[GCGCGGGG]** 1196 (86.73)   
4 - **.CCGCGGGG.** 1176 (92.81)   
4 - **[CCGCCCGC]** 876 (87.03)   
4 - **[GCCCGCGG]** 874 (86.73)   
4 - **.CCGCGGGG.** 872 (92.81)   
4 - **[CGCCGCGG]** 821 (86.45)   
4 - **[CCGCGAGG]** 799 (89.17)   
4 - **.GCGCGCGG.** 783 (93.92)   
4 - **[CGGCGCCG]** 692 (87.28)   
4 - **[CCGCGCCC]** 682 (87.56)   
4 - **[CCGCCCCG]** 203 (86.75)   
4 - **.CCGCCCGG.** 198 (93.11)   
5 + **.CCGCGCGG.** 813 (100.00)   
5 + **.CCGCGGGG.** 1075 (92.81)   
5 + **.CCGCGGGG.** 1190 (92.81)   
5 + **[CCCCGCGC]** 1343 (86.73)   
5 + **[CCGCGCGA]** 1357 (87.12)   
5 + **.CAGCGCGG.** 1392 (91.12)   
5 + **[CCGCACGG]** 1419 (86.00)   
5 + **[CCCGGCGG]** 1473 (85.92)   
5 - **[CCGCCGGG]** 1473 (85.92)   
5 - **[CCGTGCGG]** 1419 (86.00)   
5 - **.CCGCGCTG.** 1392 (91.12)   
5 - **[TCGCGCGG]** 1357 (87.12)   
5 - **[GCGCGGGG]** 1343 (86.73)   
5 - **.CCCCGCGG.** 1190 (92.81)   
5 - **.CCCCGCGG.** 1075 (92.81)   
5 - **.CCGCGCGG.** 813 (100.00)   
6 + **[CCGGGCCG]** 902 (86.75)   
6 + **.CCGCGCGG.** 1379 (100.00)   
6 + **[GCCCGCGG]** 1407 (86.73)   
6 + **[CCGCGGGC]** 1409 (86.73)   
6 + **[GCGGGCGG]** 1411 (87.03)   
6 + **[CCGCGGCG]** 1439 (86.45)   
6 - **[CGCCGCGG]** 1439 (86.45)   
6 - **[CCGCCCGC]** 1411 (87.03)   
6 - **[GCCCGCGG]** 1409 (86.73)   
6 - **[CCGCGGGC]** 1407 (86.73)   
6 - **.CCGCGCGG.** 1379 (100.00)   
6 - **[CGGCCCGG]** 902 (86.75)   
8 + **[CGGCGGGG]** 4 (86.45)   
8 + **[CGGCCCGG]** 348 (86.75)   
8 + **[CCGCGCGT]** 369 (87.89)   
8 + **.CGGCGCGG.** 406 (93.64)   
8 + **[CCGGGGGG]** 416 (85.92)   
8 + **[CCGCGTGG]** 453 (86.64)   
8 + **.CCGCCCGG.** 677 (93.11)   
8 + **[CCCCGCGC]** 785 (86.73)   
8 + **[CCCCCCGG]** 822 (85.92)   
8 + **[CCCCGGGG]** 824 (85.62)   
8 + **[CCGCCCCG]** 857 (86.75)   
8 + **[CCGCGGGC]** 912 (86.73)   
8 + **.CCGCGCGG.** 952 (100.00)   
8 + **[CCGCGCTC]** 1103 (85.04)   
8 + **[CCGCCCGC]** 1156 (87.03)   
8 + **[CCGGGCGC]** 1186 (87.03)   
8 + **.CCGCGGGG.** 1197 (92.81)   
8 + **[CCCCGCCG]** 1231 (86.45)   
8 + **[CCCCGCGC]** 1343 (86.73)   
8 + **[CCGCGCTC]** 1345 (85.04)   
8 + **[CCCCCCGG]** 1366 (85.92)   
8 + **.CCGCGCTG.** 1401 (91.12)   
8 + **[GCCCGCGG]** 1507 (86.73)   
8 - **[CCGCGGGC]** 1507 (86.73)   
8 - **.CAGCGCGG.** 1401 (91.12)   
8 - **[CCGGGGGG]** 1366 (85.92)   
8 - **[GAGCGCGG]** 1345 (85.04)   
8 - **[GCGCGGGG]** 1343 (86.73)   
8 - **[CGGCGGGG]** 1231 (86.45)   
8 - **.CCCCGCGG.** 1197 (92.81)   
8 - **[GCGCCCGG]** 1186 (87.03)   
8 - **[GCGGGCGG]** 1156 (87.03)   
8 - **[GAGCGCGG]** 1103 (85.04)   
8 - **.CCGCGCGG.** 952 (100.00)   
8 - **[GCCCGCGG]** 912 (86.73)   
8 - **[CGGGGCGG]** 857 (86.75)   
8 - **[CCCCGGGG]** 824 (85.62)   
8 - **[CCGGGGGG]** 822 (85.92)   
8 - **[GCGCGGGG]** 785 (86.73)   
8 - **.CCGGGCGG.** 677 (93.11)   
8 - **[CCACGCGG]** 453 (86.64)   
8 - **[CCCCCCGG]** 416 (85.92)   
8 - **.CCGCGCCG.** 406 (93.64)   
8 - **[ACGCGCGG]** 369 (87.89)   
8 - **[CCGGGCCG]** 348 (86.75)   
8 - **[CCCCGCCG]** 4 (86.45)   
9 + **[CAGCGCGC]** 3 (85.04)   
9 + **[GCGCGCGC]** 5 (87.84)   
9 + **[GCGCGCTG]** 7 (85.04)   
9 + **[CCGGCCGG]** 750 (86.22)   
9 + **[CCCCGCGC]** 1059 (86.73)   
9 + **[CCGCGCCC]** 1061 (87.56)   
9 - **[GGGCGCGG]** 1061 (87.56)   
9 - **[GCGCGGGG]** 1059 (86.73)   
9 - **[CCGGCCGG]** 750 (86.22)   
9 - **[CAGCGCGC]** 7 (85.04)   
9 - **[GCGCGCGC]** 5 (87.84)   
9 - **[GCGCGCTG]** 3 (85.04)   
10 + **[CCCGGCGG]** 28 (85.92)   
10 + **[CCGCGCTC]** 36 (85.04)   
10 + **[CCGCGCCC]** 512 (87.56)   
10 + **[CGGCCCGG]** 637 (86.75)   
10 + **[GCGGGCGG]** 685 (87.03)   
10 + **[CCGCTCGG]** 759 (87.74)   
10 + **[CGGCGGGG]** 811 (86.45)   
10 + **[GCGCGGGG]** 863 (86.73)   
10 + **[GGGCGCGG]** 870 (87.56)   
10 + **[CGGGGCGG]** 937 (86.75)   
10 + **[CCGCGGCG]** 1023 (86.45)   
10 + **.CAGCGCGG.** 1070 (91.12)   
10 + **.GCGCGCGG.** 1093 (93.92)   
10 + **.CCGCCCGG.** 1112 (93.11)   
10 + **[CCCCGGGG]** 1194 (85.62)   
10 - **[CCCCGGGG]** 1194 (85.62)   
10 - **.CCGGGCGG.** 1112 (93.11)   
10 - **.CCGCGCGC.** 1093 (93.92)   
10 - **.CCGCGCTG.** 1070 (91.12)   
10 - **[CGCCGCGG]** 1023 (86.45)   
10 - **[CCGCCCCG]** 937 (86.75)   
10 - **[CCGCGCCC]** 870 (87.56)   
10 - **[CCCCGCGC]** 863 (86.73)   
10 - **[CCCCGCCG]** 811 (86.45)   
10 - **[CCGAGCGG]** 759 (87.74)   
10 - **[CCGCCCGC]** 685 (87.03)   
10 - **[CCGGGCCG]** 637 (86.75)   
10 - **[GGGCGCGG]** 512 (87.56)   
10 - **[GAGCGCGG]** 36 (85.04)   
10 - **[CCGCCGGG]** 28 (85.92)   
11 + **[CCCGGCGG]** 208 (85.92)   
11 + **[CGGCCCGG]** 490 (86.75)   
11 + **[CCCCGCGC]** 590 (86.73)   
11 + **.CCGCGCCG.** 605 (93.64)   
11 + **[CGCCGCGG]** 608 (86.45)   
11 + **.CCGCGCGG.** 616 (100.00)   
11 + **[CCGCCCGC]** 634 (87.03)   
11 + **.CCCCGCGG.** 660 (92.81)   
11 + **[CCGCGGGC]** 662 (86.73)   
11 + **[GCGGGCGG]** 678 (87.03)   
11 + **[CGGCCCGG]** 691 (86.75)   
11 + **[CCCCGCGC]** 720 (86.73)   
11 + **[CCCGGCGG]** 739 (85.92)   
11 + **[CCGCGGCG]** 768 (86.45)   
11 + **[CGGGGCGG]** 797 (86.75)   
11 + **[CCGCGCCC]** 956 (87.56)   
11 + **[CCGCCCGC]** 984 (87.03)   
11 + **[CCGCCCGC]** 988 (87.03)   
11 + **[CCGCGCTC]** 995 (85.04)   
11 + **[CCGCCCGC]** 1003 (87.03)   
11 + **[CCCCGCCG]** 1023 (86.45)   
11 + **.CCGCGCTG.** 1028 (91.12)   
11 + **[CTGCGCGG]** 1113 (89.72)   
11 + **[CCGCGCAG]** 1156 (89.72)   
11 + **.CAGCGCGG.** 1342 (91.12)   
11 - **.CCGCGCTG.** 1342 (91.12)   
11 - **[CTGCGCGG]** 1156 (89.72)   
11 - **[CCGCGCAG]** 1113 (89.72)   
11 - **.CAGCGCGG.** 1028 (91.12)   
11 - **[CGGCGGGG]** 1023 (86.45)   
11 - **[GCGGGCGG]** 1003 (87.03)   
11 - **[GAGCGCGG]** 995 (85.04)   
11 - **[GCGGGCGG]** 988 (87.03)   
11 - **[GCGGGCGG]** 984 (87.03)   
11 - **[GGGCGCGG]** 956 (87.56)   
11 - **[CCGCCCCG]** 797 (86.75)   
11 - **[CGCCGCGG]** 768 (86.45)   
11 - **[CCGCCGGG]** 739 (85.92)   
11 - **[GCGCGGGG]** 720 (86.73)   
11 - **[CCGGGCCG]** 691 (86.75)   
11 - **[CCGCCCGC]** 678 (87.03)   
11 - **[GCCCGCGG]** 662 (86.73)   
11 - **.CCGCGGGG.** 660 (92.81)   
11 - **[GCGGGCGG]** 634 (87.03)   
11 - **.CCGCGCGG.** 616 (100.00)   
11 - **[CCGCGGCG]** 608 (86.45)   
11 - **.CGGCGCGG.** 605 (93.64)   
11 - **[GCGCGGGG]** 590 (86.73)   
11 - **[CCGGGCCG]** 490 (86.75)   
11 - **[CCGCCGGG]** 208 (85.92)   
12 + **[GCGGGCGG]** 181 (87.03)   
12 + **.GCGCGCGG.** 510 (93.92)   
12 + **[CGGCGCGC]** 540 (87.56)   
12 + **.CCGCGCGG.** 558 (100.00)   
12 + **[GCGGGCGG]** 591 (87.03)   
12 + **[CGGGGCGG]** 596 (86.75)   
12 + **[CGGGGCGG]** 601 (86.75)   
12 + **.CCGGGCGG.** 611 (93.11)   
12 + **[CCGGGCGC]** 665 (87.03)   
12 + **.CCGCGCCG.** 695 (93.64)   
12 + **[CCGCACGG]** 717 (86.00)   
12 + **[CCGCCCGC]** 778 (87.03)   
12 + **[CCGCCCGC]** 782 (87.03)   
12 + **[CCGCGCGT]** 838 (87.89)   
12 + **.GCGCGCGG.** 881 (93.92)   
12 + **[GCGCGGGG]** 883 (86.73)   
12 + **[CCGGGCGC]** 912 (87.03)   
12 + **.CAGCGCGG.** 919 (91.12)   
12 + **[CCGGGCCG]** 958 (86.75)   
12 + **[CCGGGGGG]** 973 (85.92)   
12 + **.CCGCGCGC.** 1031 (93.92)   
12 + **[GCGCGCGC]** 1033 (87.84)   
12 + **[GCCCGCGG]** 1087 (86.73)   
12 + **[ACGCGCGG]** 1117 (87.89)   
12 + **.CCCCGCGG.** 1164 (92.81)   
12 + **[CCGCGGGC]** 1166 (86.73)   
12 + **.CCGCGCGC.** 1179 (93.92)   
12 + **[CCCCGCGC]** 1202 (86.73)   
12 + **[CCGCGCTC]** 1204 (85.04)   
12 + **[CGGCGGGG]** 1236 (86.45)   
12 + **.CCGCGCCG.** 1252 (93.64)   
12 + **.CCGCGCTG.** 1276 (91.12)   
12 + **[GGGCGCGG]** 1332 (87.56)   
12 + **.CCGCCCGG.** 1505 (93.11)   
12 - **.CCGGGCGG.** 1505 (93.11)   
12 - **[CCGCGCCC]** 1332 (87.56)   
12 - **.CAGCGCGG.** 1276 (91.12)   
12 - **.CGGCGCGG.** 1252 (93.64)   
12 - **[CCCCGCCG]** 1236 (86.45)   
12 - **[GAGCGCGG]** 1204 (85.04)   
12 - **[GCGCGGGG]** 1202 (86.73)   
12 - **.GCGCGCGG.** 1179 (93.92)   
12 - **[GCCCGCGG]** 1166 (86.73)   
12 - **.CCGCGGGG.** 1164 (92.81)   
12 - **[CCGCGCGT]** 1117 (87.89)   
12 - **[CCGCGGGC]** 1087 (86.73)   
12 - **[GCGCGCGC]** 1033 (87.84)   
12 - **.GCGCGCGG.** 1031 (93.92)   
12 - **[CCCCCCGG]** 973 (85.92)   
12 - **[CGGCCCGG]** 958 (86.75)   
12 - **.CCGCGCTG.** 919 (91.12)   
12 - **[GCGCCCGG]** 912 (87.03)   
12 - **[CCCCGCGC]** 883 (86.73)   
12 - **.CCGCGCGC.** 881 (93.92)   
12 - **[ACGCGCGG]** 838 (87.89)   
12 - **[GCGGGCGG]** 782 (87.03)   
12 - **[GCGGGCGG]** 778 (87.03)   
12 - **[CCGTGCGG]** 717 (86.00)   
12 - **.CGGCGCGG.** 695 (93.64)   
12 - **[GCGCCCGG]** 665 (87.03)   
12 - **.CCGCCCGG.** 611 (93.11)   
12 - **[CCGCCCCG]** 601 (86.75)   
12 - **[CCGCCCCG]** 596 (86.75)   
12 - **[CCGCCCGC]** 591 (87.03)   
12 - **.CCGCGCGG.** 558 (100.00)   
12 - **[GCGCGCCG]** 540 (87.56)   
12 - **.CCGCGCGC.** 510 (93.92)   
12 - **[CCGCCCGC]** 181 (87.03)   
13 + **[CCGCGCCC]** 166 (87.56)   
13 + **[GCGCCCGG]** 168 (87.03)   
13 + **[CAGCGCGC]** 528 (85.04)   
13 + **[GCGCGCGC]** 530 (87.84)   
13 + **.GCGCGCGG.** 532 (93.92)   
13 + **[GCGGGCGG]** 547 (87.03)   
13 + **.CCGCGCCG.** 614 (93.64)   
13 + **[CCGCCCGC]** 619 (87.03)   
13 + **[CCACGCGG]** 653 (86.64)   
13 + **[CCGCTCGG]** 679 (87.74)   
13 + **[GCCCGCGG]** 700 (86.73)   
13 + **[CCGCCCGC]** 748 (87.03)   
13 + **[CCGCCCCG]** 755 (86.75)   
13 + **[CCGCCCCG]** 760 (86.75)   
13 + **[CCGCGCCC]** 772 (87.56)   
13 + **[CCCCGCGC]** 777 (86.73)   
13 + **[CCGCGCCC]** 779 (87.56)   
13 + **[CCCCGCGC]** 784 (86.73)   
13 + **[CCGCGCCC]** 786 (87.56)   
13 + **[GCGCCCGG]** 788 (87.03)   
13 + **[CGGCGCGC]** 800 (87.56)   
13 + **[CCTCGCGG]** 870 (89.17)   
13 + **[CGGCGCCG]** 880 (87.28)   
13 + **[CGGGGCGG]** 921 (86.75)   
13 + **.CCGCGCGG.** 944 (100.00)   
13 + **[CGGCGGGG]** 974 (86.45)   
13 + **[CGGGGCGG]** 977 (86.75)   
13 + **.CCGCGCGC.** 1009 (93.92)   
13 + **[CCGGGCGC]** 1043 (87.03)   
13 + **.CGGCGCGG.** 1061 (93.64)   
13 + **.CAGCGCGG.** 1073 (91.12)   
13 + **.CCGCGCGC.** 1111 (93.92)   
13 + **[CGGCGCCG]** 1136 (87.28)   
13 + **[GCGCGCCG]** 1186 (87.56)   
13 + **[CGCCGCGG]** 1196 (86.45)   
13 + **[CCGCGGGC]** 1228 (86.73)   
13 + **.CCGCGCCG.** 1263 (93.64)   
13 + **.CCGGGCGG.** 1313 (93.11)   
13 + **[GCGCCCGG]** 1330 (87.03)   
13 + **[GAGCGCGG]** 1349 (85.04)   
13 + **[CGGCGGGG]** 1354 (86.45)   
13 + **[CGGCGGGG]** 1421 (86.45)   
13 - **[CCCCGCCG]** 1421 (86.45)   
13 - **[CCCCGCCG]** 1354 (86.45)   
13 - **[CCGCGCTC]** 1349 (85.04)   
13 - **[CCGGGCGC]** 1330 (87.03)   
13 - **.CCGCCCGG.** 1313 (93.11)   
13 - **.CGGCGCGG.** 1263 (93.64)   
13 - **[GCCCGCGG]** 1228 (86.73)   
13 - **[CCGCGGCG]** 1196 (86.45)   
13 - **[CGGCGCGC]** 1186 (87.56)   
13 - **[CGGCGCCG]** 1136 (87.28)   
13 - **.GCGCGCGG.** 1111 (93.92)   
13 - **.CCGCGCTG.** 1073 (91.12)   
13 - **.CCGCGCCG.** 1061 (93.64)   
13 - **[GCGCCCGG]** 1043 (87.03)   
13 - **.GCGCGCGG.** 1009 (93.92)   
13 - **[CCGCCCCG]** 977 (86.75)   
13 - **[CCCCGCCG]** 974 (86.45)   
13 - **.CCGCGCGG.** 944 (100.00)   
13 - **[CCGCCCCG]** 921 (86.75)   
13 - **[CGGCGCCG]** 880 (87.28)   
13 - **[CCGCGAGG]** 870 (89.17)   
13 - **[GCGCGCCG]** 800 (87.56)   
13 - **[CCGGGCGC]** 788 (87.03)   
13 - **[GGGCGCGG]** 786 (87.56)   
13 - **[GCGCGGGG]** 784 (86.73)   
13 - **[GGGCGCGG]** 779 (87.56)   
13 - **[GCGCGGGG]** 777 (86.73)   
13 - **[GGGCGCGG]** 772 (87.56)   
13 - **[CGGGGCGG]** 760 (86.75)   
13 - **[CGGGGCGG]** 755 (86.75)   
13 - **[GCGGGCGG]** 748 (87.03)   
13 - **[CCGCGGGC]** 700 (86.73)   
13 - **[CCGAGCGG]** 679 (87.74)   
13 - **[CCGCGTGG]** 653 (86.64)   
13 - **[GCGGGCGG]** 619 (87.03)   
13 - **.CGGCGCGG.** 614 (93.64)   
13 - **[CCGCCCGC]** 547 (87.03)   
13 - **.CCGCGCGC.** 532 (93.92)   
13 - **[GCGCGCGC]** 530 (87.84)   
13 - **[GCGCGCTG]** 528 (85.04)   
13 - **[CCGGGCGC]** 168 (87.03)   
13 - **[GGGCGCGG]** 166 (87.56)   
  

**Frequency Matrix**

| All Occs Best Occs|  |  |  |  |  |  |  |  |  |  |  |  |  |  |  |  |  |  |  |  |  |  |  |  |  |  |  |  |  |  |  |  |  |  |  |  |  |  |  |  |  |  |  |  |  |  |  |  |  |  |  |  |  |  |  |  |  |  |  |  |  |  |  |  |  |  |  |  |  |  |  |  |  |  |  |  |  |  |  |  |  |  |  |  |  |  |  |  |  |  |  |  |  |  |  |  |  |  |  |  |  |  |  |  |  |  |  |  | | --- | --- | --- | --- | --- | --- | --- | --- | --- | --- | --- | --- | --- | --- | --- | --- | --- | --- | --- | --- | --- | --- | --- | --- | --- | --- | --- | --- | --- | --- | --- | --- | --- | --- | --- | --- | --- | --- | --- | --- | --- | --- | --- | --- | --- | --- | --- | --- | --- | --- | --- | --- | --- | --- | --- | --- | --- | --- | --- | --- | --- | --- | --- | --- | --- | --- | --- | --- | --- | --- | --- | --- | --- | --- | --- | --- | --- | --- | --- | --- | --- | --- | --- | --- | --- | --- | --- | --- | --- | --- | --- | --- | --- | --- | --- | --- | --- | --- | --- | --- | --- | --- | --- | --- | --- | --- | --- | --- | | **A C G T A C G T | | | | | | | | | | | |**| **1** 28 611 130 23 | 0 95 13 0|  |  |  |  |  |  |  |  |  |  |  |  |  |  |  |  |  |  |  |  |  |  |  |  |  |  |  |  |  |  |  |  |  |  |  |  |  |  |  |  |  |  |  |  |  |  |  |  |  |  |  |  |  |  |  |  |  |  |  |  |  |  |  |  |  |  |  |  |  |  |  |  |  |  |  |  |  |  |  |  |  |  |  |  | | --- | --- | --- | --- | --- | --- | --- | --- | --- | --- | --- | --- | --- | --- | --- | --- | --- | --- | --- | --- | --- | --- | --- | --- | --- | --- | --- | --- | --- | --- | --- | --- | --- | --- | --- | --- | --- | --- | --- | --- | --- | --- | --- | --- | --- | --- | --- | --- | --- | --- | --- | --- | --- | --- | --- | --- | --- | --- | --- | --- | --- | --- | --- | --- | --- | --- | --- | --- | --- | --- | --- | --- | --- | --- | --- | --- | --- | --- | --- | --- | --- | --- | --- | --- | | **2** 60 576 114 42 | 9 92 7 0|  |  |  |  |  |  |  |  |  |  |  |  |  |  |  |  |  |  |  |  |  |  |  |  |  |  |  |  |  |  |  |  |  |  |  |  |  |  |  |  |  |  |  |  |  |  |  |  |  |  |  |  |  |  |  |  |  |  |  |  |  |  |  |  |  |  |  |  |  |  |  |  | | --- | --- | --- | --- | --- | --- | --- | --- | --- | --- | --- | --- | --- | --- | --- | --- | --- | --- | --- | --- | --- | --- | --- | --- | --- | --- | --- | --- | --- | --- | --- | --- | --- | --- | --- | --- | --- | --- | --- | --- | --- | --- | --- | --- | --- | --- | --- | --- | --- | --- | --- | --- | --- | --- | --- | --- | --- | --- | --- | --- | --- | --- | --- | --- | --- | --- | --- | --- | --- | --- | --- | --- | | **3** 21 101 630 40 | 0 8 100 0|  |  |  |  |  |  |  |  |  |  |  |  |  |  |  |  |  |  |  |  |  |  |  |  |  |  |  |  |  |  |  |  |  |  |  |  |  |  |  |  |  |  |  |  |  |  |  |  |  |  |  |  |  |  |  |  |  |  |  |  | | --- | --- | --- | --- | --- | --- | --- | --- | --- | --- | --- | --- | --- | --- | --- | --- | --- | --- | --- | --- | --- | --- | --- | --- | --- | --- | --- | --- | --- | --- | --- | --- | --- | --- | --- | --- | --- | --- | --- | --- | --- | --- | --- | --- | --- | --- | --- | --- | --- | --- | --- | --- | --- | --- | --- | --- | --- | --- | --- | --- | | **4** 28 636 110 18 | 0 97 11 0|  |  |  |  |  |  |  |  |  |  |  |  |  |  |  |  |  |  |  |  |  |  |  |  |  |  |  |  |  |  |  |  |  |  |  |  |  |  |  |  |  |  |  |  |  |  |  |  | | --- | --- | --- | --- | --- | --- | --- | --- | --- | --- | --- | --- | --- | --- | --- | --- | --- | --- | --- | --- | --- | --- | --- | --- | --- | --- | --- | --- | --- | --- | --- | --- | --- | --- | --- | --- | --- | --- | --- | --- | --- | --- | --- | --- | --- | --- | --- | --- | | **5** 18 110 636 28 | 0 11 97 0|  |  |  |  |  |  |  |  |  |  |  |  |  |  |  |  |  |  |  |  |  |  |  |  |  |  |  |  |  |  |  |  |  |  |  |  | | --- | --- | --- | --- | --- | --- | --- | --- | --- | --- | --- | --- | --- | --- | --- | --- | --- | --- | --- | --- | --- | --- | --- | --- | --- | --- | --- | --- | --- | --- | --- | --- | --- | --- | --- | --- | | **6** 40 630 101 21 | 0 100 8 0|  |  |  |  |  |  |  |  |  |  |  |  |  |  |  |  |  |  |  |  |  |  |  |  | | --- | --- | --- | --- | --- | --- | --- | --- | --- | --- | --- | --- | --- | --- | --- | --- | --- | --- | --- | --- | --- | --- | --- | --- | | **7** 42 114 576 60 | 0 7 92 9|  |  |  |  |  |  |  |  |  |  |  |  | | --- | --- | --- | --- | --- | --- | --- | --- | --- | --- | --- | --- | | **8** 23 130 611 28 | 0 13 95 0 | | | | | | | | | | | | | | | | | | | | | | | | | | | | | | | | | | | | | | | | | | | | | | | | | | | | | | | | | | | | | | | | | | | | | | | | | | | | | | | | | | | | | | | | | | | | | | | | | | | | | | | | | | | | |

  
  

---

  
  
  
 **GTCGCG**   
**CGCGAC**  
  
8 redundant motifs found:  
TCCGCG - CCGTCGCG - GCGCGA - GCGACC - CGCGGG - CGCGCG - TACGCG - AGCGCG -   
  
Best occurrences (match percentage):   
Seq St oligo pos match
  
 1 + **[GGCGGG]** 866 (86.87)   
1 + **[GGGGCG]** 869 (86.37)   
1 + **[CGCGCG]** 884 (87.39)   
1 + **[GGGGCG]** 913 (86.37)   
1 + **[GGCGCC]** 915 (86.71)   
1 + **[GCCGCC]** 918 (87.14)   
1 + **[GCCGGG]** 938 (87.29)   
1 + **[GCCGCC]** 1003 (87.14)   
1 + **[GGCGCC]** 1066 (86.71)   
1 + **.GCCGCG.** 1069 (97.09)   
1 + **[GCCGCC]** 1078 (87.14)   
1 + **[GCCCCG]** 1081 (89.83)   
1 + **[GCGGCG]** 1107 (86.79)   
1 + **[GCCGCC]** 1205 (87.14)   
1 + **[GCCCCG]** 1232 (89.83)   
1 + **[GGCGCC]** 1249 (86.71)   
1 + **[GTCGCT]** 1264 (86.80)   
1 - **[GCGGCG]** 1399 (86.79)   
1 - **.GTCGGG.** 1389 (90.21)   
1 - **[GGCGCC]** 1249 (86.71)   
1 - **[GCGGCG]** 1207 (86.79)   
1 - **[GCGGCG]** 1204 (86.79)   
1 - **[GTCTCG]** 1199 (88.47)   
1 - **[GCCGCC]** 1158 (87.14)   
1 - **[GGGGCG]** 1080 (86.37)   
1 - **[GCGGCG]** 1077 (86.79)   
1 - **[GGCGGG]** 1075 (86.87)   
1 - **.GGCGCG.** 1071 (96.66)   
1 - **[GCGGCG]** 1068 (86.79)   
1 - **[GGCGCC]** 1066 (86.71)   
1 - **[GCCTCG]** 1063 (85.56)   
1 - **[GGGGCG]** 1038 (86.37)   
1 - **[GCGGCG]** 1002 (86.79)   
1 - **.GGCGCG.** 1000 (96.66)   
1 - **[GGCCCG]** 940 (89.40)   
1 - **[GCGGCG]** 917 (86.79)   
1 - **[GGCGCC]** 915 (86.71)   
1 - **[CGCGCG]** 884 (87.39)   
1 - **[GCCCCG]** 868 (89.83)   
2 + **.GTCGGG.** 31 (90.21)   
2 + **[GGCGGG]** 108 (86.87)   
2 + **[GCCGGG]** 151 (87.29)   
2 + **[GGCGCC]** 190 (86.71)   
2 + **[GCCCCG]** 254 (89.83)   
2 + **[GCCGCC]** 309 (87.14)   
2 + **.GTCGCG.** 362 (100.00)   
2 + **.GTCCCG.** 398 (92.74)   
2 + **.GCCGCG.** 447 (97.09)   
2 + **[GCCCCG]** 511 (89.83)   
2 + **[CCCGCG]** 513 (87.81)   
2 + **.GTCGCC.** 571 (90.05)   
2 + **[GCGGCG]** 701 (86.79)   
2 + **.GGCGCG.** 703 (96.66)   
2 + **[CGCGCG]** 705 (87.39)   
2 + **[GGCGGG]** 711 (86.87)   
2 + **.GTCGGG.** 762 (90.21)   
2 + **[GCCGGG]** 773 (87.29)   
2 + **[GTGGCG]** 827 (89.70)   
2 + **[CGCGCG]** 892 (87.39)   
2 + **[GCCCCG]** 904 (89.83)   
2 + **.GTCCCG.** 909 (92.74)   
2 + **[GCCCCG]** 981 (89.83)   
2 + **.CTCGCG.** 994 (90.72)   
2 + **[GCGGCG]** 997 (86.79)   
2 + **.GGCGCG.** 999 (96.66)   
2 + **[CCCGCG]** 1063 (87.81)   
2 + **.GCCGCG.** 1071 (97.09)   
2 + **[CGCGCG]** 1073 (87.39)   
2 + **[GCGGCG]** 1153 (86.79)   
2 + **[GGCGCC]** 1155 (86.71)   
2 + **[GCCGCC]** 1259 (87.14)   
2 + **[GCCGCC]** 1269 (87.14)   
2 + **[GCCGGG]** 1333 (87.29)   
2 + **[GGCCCG]** 1419 (89.40)   
2 + **[GCGGCG]** 1448 (86.79)   
2 + **.GGCGCG.** 1450 (96.66)   
2 + **[GGCCCG]** 1495 (89.40)   
2 + **[CCCGCG]** 1497 (87.81)   
2 - **[GCCTCG]** 1513 (85.56)   
2 - **[CCCGCG]** 1499 (87.81)   
2 - **[GCCCCG]** 1492 (89.83)   
2 - **.GCCGCG.** 1447 (97.09)   
2 - **.GTCGCC.** 1369 (90.05)   
2 - **[GTCGCT]** 1346 (86.80)   
2 - **[GCCGCC]** 1329 (87.14)   
2 - **[GGGGCG]** 1271 (86.37)   
2 - **[GCGGCG]** 1268 (86.79)   
2 - **[GGCGGG]** 1266 (86.87)   
2 - **[GGGGCG]** 1261 (86.37)   
2 - **[GCCGCC]** 1201 (87.14)   
2 - **[GCCGGG]** 1198 (87.29)   
2 - **[CCCGCG]** 1190 (87.81)   
2 - **[GCCGCC]** 1176 (87.14)   
2 - **[GGCGCC]** 1155 (86.71)   
2 - **.CTCGCG.** 1123 (90.72)   
2 - **[GGCTCG]** 1090 (85.14)   
2 - **[CGCGCG]** 1073 (87.39)   
2 - **[ATCGCG]** 1065 (86.18)   
2 - **.CTCGCG.** 1001 (90.72)   
2 - **.GCCGCG.** 996 (97.09)   
2 - **[GGCGGG]** 983 (86.87)   
2 - **.GCCGCG.** 977 (97.09)   
2 - **[GGCCCG]** 946 (89.40)   
2 - **[GCCGGG]** 929 (87.29)   
2 - **.GTCGGG.** 911 (90.21)   
2 - **[GGGGCG]** 903 (86.37)   
2 - **[GGCGGG]** 901 (86.87)   
2 - **[CGCGCG]** 892 (87.39)   
2 - **[CCCGCG]** 792 (87.81)   
2 - **[GCCCCG]** 764 (89.83)   
2 - **.GCCGCG.** 721 (97.09)   
2 - **[CCCGCG]** 707 (87.81)   
2 - **[CGCGCG]** 705 (87.39)   
2 - **.CTCGCG.** 696 (90.72)   
2 - **[GCGGCG]** 573 (86.79)   
2 - **.GACGCG.** 515 (92.06)   
2 - **[GGGGCG]** 510 (86.37)   
2 - **.GTCGGG.** 505 (90.21)   
2 - **.GGCGCG.** 457 (96.66)   
2 - **.GACGCG.** 449 (92.06)   
2 - **[CCCGCG]** 364 (87.81)   
2 - **[GGCCCG]** 339 (89.40)   
2 - **[GCGGCG]** 308 (86.79)   
2 - **[GGGGCG]** 253 (86.37)   
2 - **.GTCCCG.** 247 (92.74)   
2 - **.GCCGCG.** 201 (97.09)   
2 - **[GGCGCC]** 190 (86.71)   
2 - **[GCCCCG]** 153 (89.83)   
2 - **[GCCTCG]** 55 (85.56)   
2 - **[GGCCCG]** 33 (89.40)   
3 + **[GCCTCG]** 130 (85.56)   
3 + **[GCCGCC]** 164 (87.14)   
3 + **.GTCGCG.** 701 (100.00)   
3 + **.GCCGCG.** 789 (97.09)   
3 + **[GCCTCG]** 1023 (85.56)   
3 + **.GTCCCG.** 1057 (92.74)   
3 + **[GGCGCC]** 1073 (86.71)   
3 + **[GCCCCG]** 1103 (89.83)   
3 + **[GCCGGG]** 1176 (87.29)   
3 + **.GGCGCG.** 1180 (96.66)   
3 + **[GCGGCG]** 1224 (86.79)   
3 + **[GCGGCG]** 1227 (86.79)   
3 + **.GGCGCG.** 1249 (96.66)   
3 + **[CGCGCG]** 1251 (87.39)   
3 + **[GCCGCC]** 1264 (87.14)   
3 + **[GCCCCG]** 1278 (89.83)   
3 + **[GTGGCG]** 1322 (89.70)   
3 + **[GGCGCC]** 1324 (86.71)   
3 + **[GCCGGG]** 1335 (87.29)   
3 + **[GCCGCC]** 1344 (87.14)   
3 + **[GGGGCG]** 1363 (86.37)   
3 + **[GGCGCC]** 1389 (86.71)   
3 + **[GCCGCC]** 1402 (87.14)   
3 + **[GCCCCG]** 1405 (89.83)   
3 + **[GCCGCC]** 1410 (87.14)   
3 + **[GCCGCC]** 1413 (87.14)   
3 + **.GCCGCG.** 1416 (97.09)   
3 + **[GCCTCG]** 1465 (85.56)   
3 - **[GTGGCG]** 1420 (89.70)   
3 - **.GGCGCG.** 1418 (96.66)   
3 - **[GCGGCG]** 1415 (86.79)   
3 - **[GCGGCG]** 1412 (86.79)   
3 - **[GCGGCG]** 1409 (86.79)   
3 - **[GGCGGG]** 1407 (86.87)   
3 - **[GGGGCG]** 1404 (86.37)   
3 - **[GCGGCG]** 1401 (86.79)   
3 - **[GGGGCG]** 1391 (86.37)   
3 - **[GGCGCC]** 1389 (86.71)   
3 - **[GCCGGG]** 1386 (87.29)   
3 - **.GTCCCG.** 1350 (92.74)   
3 - **[GGCCCG]** 1337 (89.40)   
3 - **[GCCGGG]** 1331 (87.29)   
3 - **[GGGGCG]** 1326 (86.37)   
3 - **[GGCGCC]** 1324 (86.71)   
3 - **[GGGGCG]** 1312 (86.37)   
3 - **[GCCGGG]** 1280 (87.29)   
3 - **[GCCGGG]** 1271 (87.29)   
3 - **[GTGGCG]** 1266 (89.70)   
3 - **[GCGGCG]** 1263 (86.79)   
3 - **[GTCTCG]** 1255 (88.47)   
3 - **.CTCGCG.** 1253 (90.72)   
3 - **[CGCGCG]** 1251 (87.39)   
3 - **[GCCGCC]** 1226 (87.14)   
3 - **.GCCGCG.** 1223 (97.09)   
3 - **.CTCGCG.** 1182 (90.72)   
3 - **[GGCCCG]** 1173 (89.40)   
3 - **[GCCCCG]** 1128 (89.83)   
3 - **[GTGGCG]** 1107 (89.70)   
3 - **[GGCGGG]** 1105 (86.87)   
3 - **[GGCCCG]** 1100 (89.40)   
3 - **[GGCGGG]** 1094 (86.87)   
3 - **[GGCGCC]** 1073 (86.71)   
3 - **[CCCGCG]** 703 (87.81)   
3 - **[GCCGGG]** 655 (87.29)   
3 - **[GCGGCG]** 163 (86.79)   
4 + **[GGGGCG]** 63 (86.37)   
4 + **[GGCGGG]** 65 (86.87)   
4 + **[GCCGGG]** 197 (87.29)   
4 + **[GGCGGG]** 201 (86.87)   
4 + **[GGGGCG]** 204 (86.37)   
4 + **[GGCGGG]** 206 (86.87)   
4 + **[GGCCCG]** 220 (89.40)   
4 + **[GTGGCG]** 271 (89.70)   
4 + **[GGGGCG]** 312 (86.37)   
4 + **[GCCGCC]** 343 (87.14)   
4 + **[CCCGCG]** 572 (87.81)   
4 + **.GTCCCG.** 606 (92.74)   
4 + **[GCCGGG]** 631 (87.29)   
4 + **[GCGGCG]** 671 (86.79)   
4 + **[GGCGGG]** 673 (86.87)   
4 + **.GGCGCG.** 683 (96.66)   
4 + **[GGCGCC]** 693 (86.71)   
4 + **[GCCGCC]** 696 (87.14)   
4 + **.GCCGCG.** 699 (97.09)   
4 + **[GGCGCC]** 734 (86.71)   
4 + **.GTCGCG.** 740 (100.00)   
4 + **[GCCCCG]** 767 (89.83)   
4 + **[CCCGCG]** 782 (87.81)   
4 + **[CGCGCG]** 784 (87.39)   
4 + **[GCCTCG]** 798 (85.56)   
4 + **.CTCGCG.** 800 (90.72)   
4 + **[GCGGCG]** 803 (86.79)   
4 + **[GCGGCG]** 823 (86.79)   
4 + **[GCGGCG]** 826 (86.79)   
4 + **.GGCGCG.** 828 (96.66)   
4 + **[GCGGCG]** 848 (86.79)   
4 + **[GCCCCG]** 871 (89.83)   
4 + **[CCCGCG]** 873 (87.81)   
4 + **[GCGGCG]** 905 (86.79)   
4 + **[GGCGGG]** 907 (86.87)   
4 + **.GGCGCG.** 916 (96.66)   
4 + **[GGGGCG]** 933 (86.37)   
4 + **[GGCGCC]** 935 (86.71)   
4 + **[GGCGGG]** 947 (86.87)   
4 + **[GCCGGG]** 1119 (87.29)   
4 + **[GCGGCG]** 1141 (86.79)   
4 + **[GGCGGG]** 1143 (86.87)   
4 + **[CCCGCG]** 1177 (87.81)   
4 + **[GCCCCG]** 1195 (89.83)   
4 + **[CCCGCG]** 1197 (87.81)   
4 + **.GCCGCG.** 1206 (97.09)   
4 + **[GGCCCG]** 1217 (89.40)   
4 + **[GCCGGG]** 1248 (87.29)   
4 + **[GGCGGG]** 1252 (86.87)   
4 + **[GGCGGG]** 1266 (86.87)   
4 + **[GGGGCG]** 1270 (86.37)   
4 + **.GGCGCG.** 1272 (96.66)   
4 + **[CGCGCG]** 1274 (87.39)   
4 + **[GGGGCG]** 1280 (86.37)   
4 + **.GGCGCG.** 1282 (96.66)   
4 + **[CGCGCG]** 1284 (87.39)   
4 + **[GGGGCG]** 1289 (86.37)   
4 - **[GCCCCG]** 1288 (89.83)   
4 - **[CCCGCG]** 1286 (87.81)   
4 - **[CGCGCG]** 1284 (87.39)   
4 - **[CCCGCG]** 1276 (87.81)   
4 - **[CGCGCG]** 1274 (87.39)   
4 - **[GCCGCC]** 1256 (87.14)   
4 - **[GCCGGG]** 1228 (87.29)   
4 - **.GGCGCG.** 1208 (96.66)   
4 - **[GCGGCG]** 1205 (86.79)   
4 - **[GGCGGG]** 1203 (86.87)   
4 - **.GGCGCG.** 1199 (96.66)   
4 - **.GCCGCG.** 1179 (97.09)   
4 - **[GCCGCC]** 1157 (87.14)   
4 - **[GCCGCC]** 1140 (87.14)   
4 - **[GCGGCG]** 1107 (86.79)   
4 - **[GGGGCG]** 1089 (86.37)   
4 - **[GGCGCC]** 935 (86.71)   
4 - **[GCCGCC]** 904 (87.14)   
4 - **.GTCGCC.** 892 (90.05)   
4 - **[GCCGCC]** 879 (87.14)   
4 - **[CCCGCG]** 875 (87.81)   
4 - **[GCCGGG]** 864 (87.29)   
4 - **[GCCGGG]** 855 (87.29)   
4 - **.GTCGCC.** 850 (90.05)   
4 - **.CTCGCG.** 830 (90.72)   
4 - **[GCCGCC]** 825 (87.14)   
4 - **.GCCGCG.** 822 (97.09)   
4 - **.GCCGCG.** 802 (97.09)   
4 - **[GGCCCG]** 795 (89.40)   
4 - **.GGCGCG.** 786 (96.66)   
4 - **[CGCGCG]** 784 (87.39)   
4 - **[GCCGGG]** 749 (87.29)   
4 - **.GCCGCG.** 742 (97.09)   
4 - **[GGCGCC]** 734 (86.71)   
4 - **.GTCGCG.** 701 (100.00)   
4 - **[GCGGCG]** 698 (86.79)   
4 - **[GCGGCG]** 695 (86.79)   
4 - **[GGCGCC]** 693 (86.71)   
4 - **[CCCGCG]** 685 (87.81)   
4 - **.GTCCCG.** 675 (92.74)   
4 - **[GCCGCC]** 670 (87.14)   
4 - **.GTCGCC.** 635 (90.05)   
4 - **.GTCGCG.** 574 (100.00)   
4 - **[GGGGCG]** 568 (86.37)   
4 - **[GCCGCC]** 273 (87.14)   
4 - **[GCCCCG]** 203 (89.83)   
5 + **.GCCGCG.** 812 (97.09)   
5 + **[CGCGCG]** 814 (87.39)   
5 + **.GTCCCG.** 869 (92.74)   
5 + **[GGCTCG]** 953 (85.14)   
5 + **[GGCTCG]** 1041 (85.14)   
5 + **[GCCGCC]** 1161 (87.14)   
5 + **.GTCCCG.** 1187 (92.74)   
5 + **[CCCGCG]** 1189 (87.81)   
5 + **[GCCGCC]** 1311 (87.14)   
5 + **[CCCGCG]** 1344 (87.81)   
5 + **[GCCTCG]** 1349 (85.56)   
5 + **[GGCCCG]** 1354 (89.40)   
5 + **[CCCGCG]** 1356 (87.81)   
5 + **[CGCGCG]** 1358 (87.39)   
5 + **.GACGCG.** 1438 (92.06)   
5 + **.GTCCCG.** 1460 (92.74)   
5 + **[GCCCCG]** 1471 (89.83)   
5 + **[GCCGCC]** 1480 (87.14)   
5 + **[GCCGCC]** 1483 (87.14)   
5 + **[GCCGCC]** 1486 (87.14)   
5 - **[GCGGCG]** 1485 (86.79)   
5 - **[GCGGCG]** 1482 (86.79)   
5 - **[GCCGCC]** 1476 (87.14)   
5 - **[GCCGGG]** 1473 (87.29)   
5 - **[GCCGCC]** 1449 (87.14)   
5 - **[GCCTCG]** 1446 (85.56)   
5 - **.GCCGCG.** 1440 (97.09)   
5 - **.GTCGGG.** 1435 (90.21)   
5 - **[GGGGCG]** 1413 (86.37)   
5 - **.GCCGCG.** 1395 (97.09)   
5 - **[GCCGGG]** 1385 (87.29)   
5 - **.CTCGCG.** 1360 (90.72)   
5 - **[CGCGCG]** 1358 (87.39)   
5 - **.GGCGCG.** 1346 (96.66)   
5 - **[GGCGGG]** 1331 (86.87)   
5 - **[GCGGCG]** 1310 (86.79)   
5 - **[GCCGCC]** 1221 (87.14)   
5 - **[CCCGCG]** 1191 (87.81)   
5 - **[GGCGGG]** 1172 (86.87)   
5 - **[GGGGCG]** 1163 (86.37)   
5 - **[GCGGCG]** 1160 (86.79)   
5 - **[GGGGCG]** 1111 (86.37)   
5 - **[CCCGCG]** 1076 (87.81)   
5 - **.GTCGCC.** 825 (90.05)   
5 - **[CCCGCG]** 816 (87.81)   
5 - **[CGCGCG]** 814 (87.39)   
5 - **.CTCGCG.** 508 (90.72)   
5 - **[GTCTCG]** 182 (88.47)   
6 + **[GCCGGG]** 906 (87.29)   
6 + **[GCCGGG]** 955 (87.29)   
6 + **[GGGGCG]** 958 (86.37)   
6 + **[CCCGCG]** 1378 (87.81)   
6 + **[CGCGCG]** 1380 (87.39)   
6 + **[CCCGCG]** 1408 (87.81)   
6 + **[GCGGCG]** 1441 (86.79)   
6 + **[GCGGCG]** 1476 (86.79)   
6 + **[GGCGGG]** 1478 (86.87)   
6 - **[GCCGCC]** 1475 (87.14)   
6 - **.GTCGCC.** 1443 (90.05)   
6 - **.GCCGCG.** 1440 (97.09)   
6 - **[CCCGCG]** 1410 (87.81)   
6 - **[CGCGCG]** 1380 (87.39)   
6 - **.CTCGCG.** 1141 (90.72)   
6 - **[GCCCCG]** 957 (89.83)   
6 - **[GGCCCG]** 903 (89.40)   
7 + **[GGCGGG]** 1006 (86.87)   
7 - **[ATCGCG]** 1378 (86.18)   
7 - **[GTCGCT]** 1345 (86.80)   
8 + **[GCGGCG]** 3 (86.79)   
8 + **[GGCGGG]** 5 (86.87)   
8 + **[GGCGGG]** 16 (86.87)   
8 + **.CTCGCG.** 147 (90.72)   
8 + **[GGCCCG]** 349 (89.40)   
8 + **[CGCGCG]** 370 (87.39)   
8 + **.GTCCCG.** 375 (92.74)   
8 + **[GGCGGG]** 386 (86.87)   
8 + **.GGCGCG.** 407 (96.66)   
8 + **[GGGGCG]** 420 (86.37)   
8 + **.GCCGCG.** 452 (97.09)   
8 + **[GCCCCG]** 497 (89.83)   
8 + **[GGGGCG]** 588 (86.37)   
8 + **[GGGGCG]** 617 (86.37)   
8 + **[GCCGCC]** 676 (87.14)   
8 + **[GCCTCG]** 778 (85.56)   
8 + **[CCCGCG]** 786 (87.81)   
8 + **[GCCGCC]** 814 (87.14)   
8 + **[GCCCCG]** 859 (89.83)   
8 + **[GGCTCG]** 870 (85.14)   
8 + **[GGGGCG]** 883 (86.37)   
8 + **[GGCGGG]** 885 (86.87)   
8 + **[GGGGCG]** 895 (86.37)   
8 + **[GGCGGG]** 897 (86.87)   
8 + **[GGGGCG]** 900 (86.37)   
8 + **[GGCGCC]** 917 (86.71)   
8 + **.GGCGCG.** 927 (96.66)   
8 + **.GGCGCG.** 933 (96.66)   
8 + **[GGCCCG]** 949 (89.40)   
8 + **[CCCGCG]** 951 (87.81)   
8 + **[CGCGCG]** 953 (87.39)   
8 + **.GGCGCG.** 978 (96.66)   
8 + **[GCCGGG]** 993 (87.29)   
8 + **[GGCGCC]** 1003 (86.71)   
8 + **[GGCCCG]** 1043 (89.40)   
8 + **[GCCCCG]** 1054 (89.83)   
8 + **[GCCGCC]** 1074 (87.14)   
8 + **[CCCGCG]** 1102 (87.81)   
8 + **[GCCCCG]** 1142 (89.83)   
8 + **[CCCGCG]** 1144 (87.81)   
8 + **[GCCGCC]** 1155 (87.14)   
8 + **[GCCTCG]** 1165 (85.56)   
8 + **.GTCCCG.** 1183 (92.74)   
8 + **[GGCGCC]** 1189 (86.71)   
8 + **.GTCGCG.** 1204 (100.00)   
8 + **[GCCGCC]** 1235 (87.14)   
8 + **[GGCTCG]** 1313 (85.14)   
8 + **[GCCGCC]** 1322 (87.14)   
8 + **[CCCGCG]** 1344 (87.81)   
8 + **[GCCTCG]** 1381 (85.56)   
8 + **.GTCCCG.** 1410 (92.74)   
8 + **[GCCCCG]** 1421 (89.83)   
8 + **[GCCGCC]** 1429 (87.14)   
8 + **[GGCTCG]** 1462 (85.14)   
8 + **.CTCGCG.** 1464 (90.72)   
8 + **[GGCGCC]** 1504 (86.71)   
8 + **[CCCGCG]** 1508 (87.81)   
8 - **[GGCGCC]** 1504 (86.71)   
8 - **.GTCGCG.** 1466 (100.00)   
8 - **[GCCTCG]** 1452 (85.56)   
8 - **[GTGGCG]** 1431 (89.70)   
8 - **[GGCCCG]** 1385 (89.40)   
8 - **[GCCGGG]** 1369 (87.29)   
8 - **[GCGGCG]** 1234 (86.79)   
8 - **[GGCGGG]** 1232 (86.87)   
8 - **[GGCCCG]** 1217 (89.40)   
8 - **[CCCGCG]** 1198 (87.81)   
8 - **[GGCGCC]** 1189 (86.71)   
8 - **[GCGGCG]** 1154 (86.79)   
8 - **.GACGCG.** 1146 (92.06)   
8 - **[GCCGCC]** 1138 (87.14)   
8 - **[GGCGGG]** 1080 (86.87)   
8 - **[GGGGCG]** 1076 (86.37)   
8 - **[GCCGGG]** 1056 (87.29)   
8 - **[GCCGGG]** 1045 (87.29)   
8 - **[GGCGCC]** 1003 (86.71)   
8 - **.GTCGCG.** 980 (100.00)   
8 - **[GGCCCG]** 957 (89.40)   
8 - **[CCCGCG]** 955 (87.81)   
8 - **[CGCGCG]** 953 (87.39)   
8 - **[GCCTCG]** 946 (85.56)   
8 - **[GCCTCG]** 937 (85.56)   
8 - **.CTCGCG.** 935 (90.72)   
8 - **[CCCGCG]** 929 (87.81)   
8 - **[GGCGCC]** 917 (86.71)   
8 - **[CCCGCG]** 913 (87.81)   
8 - **[GCCCCG]** 899 (89.83)   
8 - **[GGGGCG]** 858 (86.37)   
8 - **[GGCGGG]** 856 (86.87)   
8 - **[GCCCCG]** 827 (89.83)   
8 - **[GGGGCG]** 816 (86.37)   
8 - **[GCGGCG]** 813 (86.79)   
8 - **[GGGGCG]** 782 (86.37)   
8 - **[GGCGGG]** 630 (86.87)   
8 - **[GCCCCG]** 587 (89.83)   
8 - **.GTCGGG.** 548 (90.21)   
8 - **[GGCTCG]** 505 (85.14)   
8 - **[GGCGGG]** 499 (86.87)   
8 - **[GGGGCG]** 496 (86.37)   
8 - **.GGCGCG.** 494 (96.66)   
8 - **[GCGGCG]** 451 (86.79)   
8 - **.GCCGCG.** 409 (97.09)   
8 - **[GCCCCG]** 397 (89.83)   
8 - **.GACGCG.** 372 (92.06)   
8 - **[CGCGCG]** 370 (87.39)   
8 - **[GCCGGG]** 351 (87.29)   
8 - **[GCCGCC]** 306 (87.14)   
8 - **[GGCGGG]** 283 (86.87)   
8 - **.GACGCG.** 149 (92.06)   
8 - **[GTCTCG]** 137 (88.47)   
8 - **[GCCGCC]** 2 (87.14)   
9 + **[CGCGCG]** 6 (87.39)   
9 + **[GGGGCG]** 22 (86.37)   
9 + **[GGCGCC]** 24 (86.71)   
9 + **[GGCGGG]** 47 (86.87)   
9 + **[GCCTCG]** 173 (85.56)   
9 + **[GGCGGG]** 303 (86.87)   
9 + **.GTCGGG.** 474 (90.21)   
9 + **[GCCGGG]** 510 (87.29)   
9 + **[GCCGGG]** 533 (87.29)   
9 + **[GCCGCC]** 601 (87.14)   
9 + **[GCCGGG]** 679 (87.29)   
9 + **[GGCGCC]** 766 (86.71)   
9 + **.GGCGCG.** 975 (96.66)   
9 + **[CCCGCG]** 1060 (87.81)   
9 + **[GGGGCG]** 1083 (86.37)   
9 + **[GGCGGG]** 1085 (86.87)   
9 + **[GGGGCG]** 1222 (86.37)   
9 + **[GGCGCC]** 1224 (86.71)   
9 + **.GGCGCG.** 1266 (96.66)   
9 + **[GGCCCG]** 1319 (89.40)   
9 + **.GTCGCC.** 1459 (90.05)   
9 - **[GGCCCG]** 1497 (89.40)   
9 - **.GTCGCG.** 1268 (100.00)   
9 - **[GTCGCT]** 1240 (86.80)   
9 - **[GGCGCC]** 1224 (86.71)   
9 - **[GGGGCG]** 1064 (86.37)   
9 - **.GGCGCG.** 1062 (96.66)   
9 - **.CTCGCG.** 977 (90.72)   
9 - **[GCCGGG]** 816 (87.29)   
9 - **[GGCGCC]** 766 (86.71)   
9 - **.GTCCCG.** 476 (92.74)   
9 - **[GGCGCC]** 24 (86.71)   
9 - **[CGCGCG]** 6 (87.39)   
10 + **.GCCGCG.** 35 (97.09)   
10 + **.GTCGGG.** 44 (90.21)   
10 + **[GCCGGG]** 50 (87.29)   
10 + **[GGGGCG]** 54 (86.37)   
10 + **[GGCGGG]** 56 (86.87)   
10 + **[CCCGCG]** 87 (87.81)   
10 + **[GGGGCG]** 122 (86.37)   
10 + **[GGCGGG]** 124 (86.87)   
10 + **[GGGGCG]** 128 (86.37)   
10 + **[GCCTCG]** 190 (85.56)   
10 + **[CCCGCG]** 488 (87.81)   
10 + **.GCCGCG.** 511 (97.09)   
10 + **[GGCGCC]** 539 (86.71)   
10 + **.GTCCCG.** 575 (92.74)   
10 + **[CCCGCG]** 577 (87.81)   
10 + **[GGCGCC]** 592 (86.71)   
10 + **[GCCGGG]** 595 (87.29)   
10 + **[GGCCCG]** 638 (89.40)   
10 + **[GCGGCG]** 689 (86.79)   
10 + **[GCGGCG]** 692 (86.79)   
10 + **[GCCGGG]** 708 (87.29)   
10 + **[GGCCCG]** 739 (89.40)   
10 + **.GTCGGG.** 747 (90.21)   
10 + **[GCCGGG]** 775 (87.29)   
10 + **[GCCGCC]** 785 (87.14)   
10 + **[GGCGGG]** 812 (86.87)   
10 + **.GACGCG.** 820 (92.06)   
10 + **.GACGCG.** 828 (92.06)   
10 + **[GCCGGG]** 836 (87.29)   
10 + **[GGGGCG]** 869 (86.37)   
10 + **.GGCGCG.** 871 (96.66)   
10 + **[GCCGGG]** 899 (87.29)   
10 + **.GTCGGG.** 905 (90.21)   
10 + **[GGGGCG]** 909 (86.37)   
10 + **[GGGGCG]** 933 (86.37)   
10 + **[GGCGGG]** 935 (86.87)   
10 + **[GGGGCG]** 938 (86.37)   
10 + **[GGCGGG]** 940 (86.87)   
10 + **[GGCGGG]** 974 (86.87)   
10 + **.GCCGCG.** 1022 (97.09)   
10 + **[GCGGCG]** 1025 (86.79)   
10 + **[GGCCCG]** 1045 (89.40)   
10 + **[CGCGCG]** 1094 (87.39)   
10 + **[GCGGCG]** 1097 (86.79)   
10 + **.GCCGCG.** 1119 (97.09)   
10 + **[GGCGGG]** 1151 (86.87)   
10 + **[GGCTCG]** 1181 (85.14)   
10 + **.GTCGCC.** 1203 (90.05)   
10 + **[GCGGCG]** 1232 (86.79)   
10 + **[GCCGCC]** 1242 (87.14)   
10 + **[GCCGCC]** 1263 (87.14)   
10 + **.GTCGCC.** 1452 (90.05)   
10 - **[GCCTCG]** 1509 (85.56)   
10 - **[GCCTCG]** 1401 (85.56)   
10 - **[GGCCCG]** 1355 (89.40)   
10 - **.GCCGCG.** 1331 (97.09)   
10 - **.GTCGGG.** 1279 (90.21)   
10 - **[GTGGCG]** 1265 (89.70)   
10 - **[GCGGCG]** 1262 (86.79)   
10 - **[GGGGCG]** 1244 (86.37)   
10 - **[GCCTCG]** 1185 (85.56)   
10 - **.GCCGCG.** 1121 (97.09)   
10 - **[GCCGGG]** 1115 (87.29)   
10 - **[GCCGCC]** 1099 (87.14)   
10 - **.GCCGCG.** 1096 (97.09)   
10 - **[CGCGCG]** 1094 (87.39)   
10 - **[CCCGCG]** 1073 (87.81)   
10 - **[GCCGGG]** 1047 (87.29)   
10 - **[GGCCCG]** 1043 (89.40)   
10 - **.GTCCCG.** 1038 (92.74)   
10 - **[GCCGCC]** 1027 (87.14)   
10 - **.GCCGCG.** 1024 (97.09)   
10 - **[GCCCCG]** 937 (89.83)   
10 - **[CCCGCG]** 873 (87.81)   
10 - **[CCCGCG]** 864 (87.81)   
10 - **[GCCCCG]** 832 (89.83)   
10 - **[CCCGCG]** 830 (87.81)   
10 - **.CTCGCG.** 822 (90.72)   
10 - **[GCCCCG]** 736 (89.83)   
10 - **.GTCCCG.** 731 (92.74)   
10 - **[GCCGCC]** 691 (87.14)   
10 - **[GCCGCC]** 688 (87.14)   
10 - **[GGCCCG]** 642 (89.40)   
10 - **.GCCGCG.** 605 (97.09)   
10 - **[GCCCCG]** 597 (89.83)   
10 - **[GGCGCC]** 592 (86.71)   
10 - **[CCCGCG]** 579 (87.81)   
10 - **[GGCGCC]** 539 (86.71)   
10 - **[GGGGCG]** 515 (86.37)   
10 - **.GGCGCG.** 513 (96.66)   
10 - **[GGGGCG]** 499 (86.37)   
10 - **.GTCGCC.** 436 (90.05)   
10 - **.GTCGCC.** 410 (90.05)   
10 - **[GCCTCG]** 224 (85.56)   
10 - **[GGCTCG]** 132 (85.14)   
10 - **[GCCCCG]** 58 (89.83)   
10 - **[GCCCCG]** 46 (89.83)   
10 - **[GCCGCC]** 31 (87.14)   
10 - **[GCCGGG]** 28 (87.29)   
11 + **[GGCGGG]** 24 (86.87)   
11 + **[GGGGCG]** 94 (86.37)   
11 + **[GGGGCG]** 173 (86.37)   
11 + **[GGGGCG]** 183 (86.37)   
11 + **.GGCGCG.** 250 (96.66)   
11 + **[GCCGCC]** 459 (87.14)   
11 + **[GGCCCG]** 491 (89.40)   
11 + **.GCCGCG.** 497 (97.09)   
11 + **.GTCGCG.** 529 (100.00)   
11 + **[GCCCCG]** 589 (89.83)   
11 + **[CCCGCG]** 591 (87.81)   
11 + **.GCCGCG.** 609 (97.09)   
11 + **.GCCGCG.** 615 (97.09)   
11 + **[CGCGCG]** 617 (87.39)   
11 + **[GCCGCC]** 633 (87.14)   
11 + **[CCCGCG]** 637 (87.81)   
11 + **[GCCGGG]** 654 (87.29)   
11 + **[GCCCCG]** 659 (89.83)   
11 + **[CCCGCG]** 661 (87.81)   
11 + **[GGCGGG]** 681 (86.87)   
11 + **[GGCCCG]** 692 (89.40)   
11 + **.GACGCG.** 698 (92.06)   
11 + **.GCCGCG.** 713 (97.09)   
11 + **[GCCCCG]** 719 (89.83)   
11 + **[CCCGCG]** 721 (87.81)   
11 + **[CGCGCG]** 723 (87.39)   
11 + **[GGCGGG]** 742 (86.87)   
11 + **.GACGCG.** 747 (92.06)   
11 + **.GCCGCG.** 767 (97.09)   
11 + **[GCGGCG]** 770 (86.79)   
11 + **[GGCGCC]** 772 (86.71)   
11 + **[GGGGCG]** 798 (86.37)   
11 + **.GTCGGG.** 814 (90.21)   
11 + **[GCCCCG]** 933 (89.83)   
11 + **[CCCGCG]** 955 (87.81)   
11 + **[GCCGCC]** 975 (87.14)   
11 + **.GCCGCG.** 994 (97.09)   
11 + **[GCCCCG]** 1022 (89.83)   
11 + **.GCCGCG.** 1027 (97.09)   
11 + **[GCCGCC]** 1035 (87.14)   
11 + **[GCCGCC]** 1038 (87.14)   
11 + **[GCCGCC]** 1041 (87.14)   
11 + **[GCCGGG]** 1063 (87.29)   
11 + **[GCGGCG]** 1117 (86.79)   
11 + **[GGCGGG]** 1119 (86.87)   
11 + **[GCCGCC]** 1152 (87.14)   
11 + **.GCCGCG.** 1155 (97.09)   
11 + **[GGCGGG]** 1198 (86.87)   
11 + **[GGCGGG]** 1369 (86.87)   
11 + **.GGCGCG.** 1379 (96.66)   
11 - **[GCCGGG]** 1469 (87.29)   
11 - **.GACGCG.** 1381 (92.06)   
11 - **[GGCCCG]** 1371 (89.40)   
11 - **[CCCGCG]** 1345 (87.81)   
11 - **[GCGGCG]** 1328 (86.79)   
11 - **[GCGGCG]** 1154 (86.79)   
11 - **[GCGGCG]** 1151 (86.79)   
11 - **[GCCGCC]** 1136 (87.14)   
11 - **.GCCGCG.** 1116 (97.09)   
11 - **[GCCGCC]** 1088 (87.14)   
11 - **[GGCCCG]** 1073 (89.40)   
11 - **[GCCCCG]** 1065 (89.83)   
11 - **[GCGGCG]** 1043 (86.79)   
11 - **[GCGGCG]** 1040 (86.79)   
11 - **[GCGGCG]** 1037 (86.79)   
11 - **[GCGGCG]** 1026 (86.79)   
11 - **[GGCGGG]** 1024 (86.87)   
11 - **[GCGGCG]** 1008 (86.79)   
11 - **[GGCGGG]** 1006 (86.87)   
11 - **[GGCGGG]** 1002 (86.87)   
11 - **[GCGGCG]** 993 (86.79)   
11 - **[GGCGGG]** 991 (86.87)   
11 - **[GGCGGG]** 987 (86.87)   
11 - **[GGCGGG]** 983 (86.87)   
11 - **[GCGGCG]** 974 (86.79)   
11 - **[GGGGCG]** 963 (86.37)   
11 - **[GGCGGG]** 961 (86.87)   
11 - **.GGCGCG.** 957 (96.66)   
11 - **[GGGGCG]** 943 (86.37)   
11 - **[GGCGGG]** 941 (86.87)   
11 - **[GCCCCG]** 797 (89.83)   
11 - **[GGCCCG]** 785 (89.40)   
11 - **[GGCGCC]** 772 (86.71)   
11 - **.GCCGCG.** 769 (97.09)   
11 - **[GGCCCG]** 760 (89.40)   
11 - **[GTGGCG]** 751 (89.70)   
11 - **.GGCGCG.** 749 (96.66)   
11 - **.GTCCCG.** 744 (92.74)   
11 - **[GCCGGG]** 739 (87.29)   
11 - **[CGCGCG]** 723 (87.39)   
11 - **.GCCGCG.** 715 (97.09)   
11 - **[GCCGGG]** 709 (87.29)   
11 - **.GGCGCG.** 700 (96.66)   
11 - **[CCCGCG]** 663 (87.81)   
11 - **[GGCCCG]** 656 (89.40)   
11 - **[GTGGCG]** 641 (89.70)   
11 - **.GGCGCG.** 639 (96.66)   
11 - **[CCCGCG]** 619 (87.81)   
11 - **[CGCGCG]** 617 (87.39)   
11 - **.GCCGCG.** 611 (97.09)   
11 - **[GCGGCG]** 608 (86.79)   
11 - **.GGCGCG.** 606 (96.66)   
11 - **.GCCGCG.** 560 (97.09)   
11 - **[GTCGCT]** 541 (86.80)   
11 - **[GCCGGG]** 493 (87.29)   
11 - **[GCCGGG]** 488 (87.29)   
11 - **[GGGGCG]** 461 (86.37)   
11 - **[GGCGGG]** 452 (86.87)   
11 - **[GCCGGG]** 413 (87.29)   
11 - **[GTCTCG]** 336 (88.47)   
11 - **.GTCGCC.** 291 (90.05)   
11 - **[GCCGCC]** 211 (87.14)   
11 - **[GCCGGG]** 208 (87.29)   
11 - **[GCCGCC]** 185 (87.14)   
11 - **[GCCCCG]** 93 (89.83)   
12 + **[GTCTCG]** 153 (88.47)   
12 + **[GCCGGG]** 167 (87.29)   
12 + **[GGGGCG]** 170 (86.37)   
12 + **.CTCGCG.** 178 (90.72)   
12 + **[GGCGGG]** 184 (86.87)   
12 + **[GGGGCG]** 197 (86.37)   
12 + **[GGCGCC]** 199 (86.71)   
12 + **[GTCGCT]** 224 (86.80)   
12 + **[GGCCCG]** 235 (89.40)   
12 + **.GTCGGG.** 451 (90.21)   
12 + **[GGGGCG]** 462 (86.37)   
12 + **[GGCGCC]** 464 (86.71)   
12 + **.GGCGCG.** 509 (96.66)   
12 + **[CGCGCG]** 511 (87.39)   
12 + **[GGGGCG]** 522 (86.37)   
12 + **.GGCGCG.** 524 (96.66)   
12 + **.GTCCCG.** 536 (92.74)   
12 + **.GGCGCG.** 541 (96.66)   
12 + **[CCCGCG]** 557 (87.81)   
12 + **[CGCGCG]** 559 (87.39)   
12 + **[GGCGGG]** 582 (86.87)   
12 + **[GGCGGG]** 590 (86.87)   
12 + **[GGCGGG]** 594 (86.87)   
12 + **[GGGGCG]** 597 (86.37)   
12 + **[GGCGGG]** 599 (86.87)   
12 + **[GGGGCG]** 602 (86.37)   
12 + **[GGCGGG]** 604 (86.87)   
12 + **[GCCGGG]** 610 (87.29)   
12 + **[GCCGCC]** 699 (87.14)   
12 + **[CCCGCG]** 709 (87.81)   
12 + **[GGCCCG]** 714 (89.40)   
12 + **.GCCGCG.** 740 (97.09)   
12 + **[GGCCCG]** 755 (89.40)   
12 + **[GCCGCC]** 777 (87.14)   
12 + **[CGCGCG]** 839 (87.39)   
12 + **.GTCCCG.** 852 (92.74)   
12 + **[ATCGCG]** 868 (86.18)   
12 + **[CGCGCG]** 882 (87.39)   
12 + **.GTCCCG.** 909 (92.74)   
12 + **[GCCCCG]** 935 (89.83)   
12 + **[GGCCCG]** 955 (89.40)   
12 + **.GTCGGG.** 980 (90.21)   
12 + **.GCCGCG.** 1030 (97.09)   
12 + **[CGCGCG]** 1032 (87.39)   
12 + **[CGCGCG]** 1034 (87.39)   
12 + **[GGCCCG]** 1086 (89.40)   
12 + **[CCCGCG]** 1088 (87.81)   
12 + **.GTCGCG.** 1095 (100.00)   
12 + **.GACGCG.** 1116 (92.06)   
12 + **[CGCGCG]** 1118 (87.39)   
12 + **[GCCGCC]** 1146 (87.14)   
12 + **[GCCTCG]** 1149 (85.56)   
12 + **[CCCGCG]** 1165 (87.81)   
12 + **.GCCGCG.** 1178 (97.09)   
12 + **[CGCGCG]** 1180 (87.39)   
12 + **[CCCGCG]** 1203 (87.81)   
12 + **[GGCGGG]** 1237 (86.87)   
12 + **[GGGGCG]** 1242 (86.37)   
12 + **[GCGGCG]** 1245 (86.79)   
12 + **.GCCGCG.** 1251 (97.09)   
12 + **[GTGGCG]** 1266 (89.70)   
12 + **.GCCGCG.** 1275 (97.09)   
12 + **[GGCGCC]** 1290 (86.71)   
12 + **[GGGGCG]** 1331 (86.37)   
12 + **.GGCGCG.** 1333 (96.66)   
12 + **[GGCGGG]** 1345 (86.87)   
12 + **.GCCGCG.** 1355 (97.09)   
12 + **[GGGGCG]** 1426 (86.37)   
12 + **[GGCGGG]** 1428 (86.87)   
12 + **[GGGGCG]** 1433 (86.37)   
12 + **[ATCGCG]** 1463 (86.18)   
12 + **[GGCTCG]** 1512 (85.14)   
12 - **[GGCGGG]** 1504 (86.87)   
12 - **[GGCTCG]** 1392 (85.14)   
12 - **[ATCGCG]** 1357 (86.18)   
12 - **[GCGGCG]** 1354 (86.79)   
12 - **[CCCGCG]** 1335 (87.81)   
12 - **[GGCCCG]** 1298 (89.40)   
12 - **[GGCGCC]** 1290 (86.71)   
12 - **[GCGGCG]** 1255 (86.79)   
12 - **.GGCGCG.** 1253 (96.66)   
12 - **[GCCGCC]** 1247 (87.14)   
12 - **[GCCGCC]** 1244 (87.14)   
12 - **[CGCGCG]** 1180 (87.39)   
12 - **[GCGGCG]** 1177 (86.79)   
12 - **[GGCCCG]** 1169 (89.40)   
12 - **[CCCGCG]** 1167 (87.81)   
12 - **.GCCGCG.** 1120 (97.09)   
12 - **[CGCGCG]** 1118 (87.39)   
12 - **[CCCGCG]** 1097 (87.81)   
12 - **[CCCGCG]** 1090 (87.81)   
12 - **[GCCGGG]** 1069 (87.29)   
12 - **[GCCCCG]** 1047 (89.83)   
12 - **.GGCGCG.** 1036 (96.66)   
12 - **[CGCGCG]** 1034 (87.39)   
12 - **[CGCGCG]** 1032 (87.39)   
12 - **[GCCCCG]** 982 (89.83)   
12 - **[GGCCCG]** 959 (89.40)   
12 - **[GCCCCG]** 952 (89.83)   
12 - **[GGCCCG]** 932 (89.40)   
12 - **[CCCGCG]** 884 (87.81)   
12 - **[CGCGCG]** 882 (87.39)   
12 - **.GTCGCG.** 870 (100.00)   
12 - **.GACGCG.** 841 (92.06)   
12 - **[CGCGCG]** 839 (87.39)   
12 - **[GGCGGG]** 813 (86.87)   
12 - **[GGCGGG]** 792 (86.87)   
12 - **[GTGGCG]** 787 (89.70)   
12 - **[GGCGGG]** 785 (86.87)   
12 - **[GGCGGG]** 781 (86.87)   
12 - **[GCGGCG]** 776 (86.79)   
12 - **[GGCGGG]** 774 (86.87)   
12 - **[GGCCCG]** 753 (89.40)   
12 - **[GGCCCG]** 737 (89.40)   
12 - **[GCCCCG]** 722 (89.83)   
12 - **.GCCGCG.** 711 (97.09)   
12 - **[GCGGCG]** 698 (86.79)   
12 - **.GGCGCG.** 696 (96.66)   
12 - **[GCCGCC]** 614 (87.14)   
12 - **[GCCCCG]** 606 (89.83)   
12 - **[GCCCCG]** 601 (89.83)   
12 - **[GCCCCG]** 596 (89.83)   
12 - **[GCCGGG]** 569 (87.29)   
12 - **[CCCGCG]** 561 (87.81)   
12 - **[CGCGCG]** 559 (87.39)   
12 - **.GGCGCG.** 543 (96.66)   
12 - **[GCCGGG]** 538 (87.29)   
12 - **.GGCGCG.** 526 (96.66)   
12 - **[CGCGCG]** 511 (87.39)   
12 - **[GCCCCG]** 498 (89.83)   
12 - **[GGCGCC]** 464 (86.71)   
12 - **[GGCCCG]** 453 (89.40)   
12 - **[GGCGCC]** 199 (86.71)   
12 - **.GTCCCG.** 186 (92.74)   
12 - **[CCCGCG]** 180 (87.81)   
12 - **[GCCCCG]** 169 (89.83)   
12 - **[CCCGCG]** 117 (87.81)   
13 + **[GTCTCG]** 98 (88.47)   
13 + **[GTCTCG]** 124 (88.47)   
13 + **[GGGGCG]** 269 (86.37)   
13 + **[GGGGCG]** 284 (86.37)   
13 + **.GGCGCG.** 440 (96.66)   
13 + **[GGCCCG]** 479 (89.40)   
13 + **[GTCTCG]** 495 (88.47)   
13 + **[GGGGCG]** 501 (86.37)   
13 + **[GGCGGG]** 503 (86.87)   
13 + **[GGCGGG]** 521 (86.87)   
13 + **[CGCGCG]** 531 (87.39)   
13 + **[CGCGCG]** 533 (87.39)   
13 + **[GGCGGG]** 546 (86.87)   
13 + **[GCCGGG]** 600 (87.29)   
13 + **[GGCCCG]** 605 (89.40)   
13 + **[CCCGCG]** 613 (87.81)   
13 + **[GCCGCC]** 618 (87.14)   
13 + **[CCCGCG]** 642 (87.81)   
13 + **[GCCCCG]** 686 (89.83)   
13 + **[GGCGCC]** 697 (86.71)   
13 + **[CCCGCG]** 701 (87.81)   
13 + **[GGCCCG]** 706 (89.40)   
13 + **[GCCGCC]** 718 (87.14)   
13 + **.GCCGCG.** 721 (97.09)   
13 + **[CGCGCG]** 723 (87.39)   
13 + **[CCCGCG]** 740 (87.81)   
13 + **.GTCCCG.** 745 (92.74)   
13 + **[GCCGCC]** 754 (87.14)   
13 + **[GCCCCG]** 757 (89.83)   
13 + **[GCCCCG]** 762 (89.83)   
13 + **[CCCGCG]** 764 (87.81)   
13 + **.GTCCCG.** 769 (92.74)   
13 + **[CCCGCG]** 771 (87.81)   
13 + **[GCCCCG]** 776 (89.83)   
13 + **[CCCGCG]** 778 (87.81)   
13 + **[GCCCCG]** 783 (89.83)   
13 + **[CCCGCG]** 785 (87.81)   
13 + **.GGCGCG.** 801 (96.66)   
13 + **[GCCCCG]** 824 (89.83)   
13 + **[GCCCCG]** 842 (89.83)   
13 + **[GCCTCG]** 869 (85.56)   
13 + **.CTCGCG.** 871 (90.72)   
13 + **[GGCGCC]** 881 (86.71)   
13 + **[GGGGCG]** 893 (86.37)   
13 + **[GGCGGG]** 895 (86.87)   
13 + **[GGGGCG]** 905 (86.37)   
13 + **[GGCGGG]** 907 (86.87)   
13 + **[GGGGCG]** 910 (86.37)   
13 + **[GGGGCG]** 922 (86.37)   
13 + **[GGCGGG]** 924 (86.87)   
13 + **.GTCCCG.** 941 (92.74)   
13 + **[CCCGCG]** 943 (87.81)   
13 + **[CGCGCG]** 945 (87.39)   
13 + **[GCGGCG]** 973 (86.79)   
13 + **[GGCGGG]** 975 (86.87)   
13 + **[GGGGCG]** 978 (86.37)   
13 + **[GGCGGG]** 991 (86.87)   
13 + **[GCCGGG]** 996 (87.29)   
13 + **[CGCGCG]** 1010 (87.39)   
13 + **.GTCCCG.** 1030 (92.74)   
13 + **[GCCGGG]** 1042 (87.29)   
13 + **.GGCGCG.** 1062 (96.66)   
13 + **[GCCGCC]** 1068 (87.14)   
13 + **.GGCGCG.** 1092 (96.66)   
13 + **.GCCGCG.** 1110 (97.09)   
13 + **[CGCGCG]** 1112 (87.39)   
13 + **[GGCCCG]** 1121 (89.40)   
13 + **[GGCGCC]** 1137 (86.71)   
13 + **[GCCGCC]** 1140 (87.14)   
13 + **[GCCGCC]** 1143 (87.14)   
13 + **[GCCGCC]** 1146 (87.14)   
13 + **.GTCGCC.** 1158 (90.05)   
13 + **[GCCGCC]** 1161 (87.14)   
13 + **[GCCGCC]** 1164 (87.14)   
13 + **[GCCGCC]** 1167 (87.14)   
13 + **[GCCGGG]** 1174 (87.29)   
13 + **.GCCGCG.** 1197 (97.09)   
13 + **.GCCGCG.** 1227 (97.09)   
13 + **[GCCGGG]** 1267 (87.29)   
13 + **[GGGGCG]** 1283 (86.37)   
13 + **[GGCGGG]** 1285 (86.87)   
13 + **[GCCGGG]** 1297 (87.29)   
13 + **.GTCGCG.** 1302 (100.00)   
13 + **[GCGGCG]** 1317 (86.79)   
13 + **.GTCGCG.** 1322 (100.00)   
13 + **[GGGGCG]** 1327 (86.37)   
13 + **[GGCGCC]** 1329 (86.71)   
13 + **[GCGGCG]** 1353 (86.79)   
13 + **[GGCGGG]** 1355 (86.87)   
13 + **[GCCCCG]** 1393 (89.83)   
13 + **[GCGGCG]** 1420 (86.79)   
13 + **[GGCGGG]** 1422 (86.87)   
13 + **[GGGGCG]** 1426 (86.37)   
13 + **[GCGGCG]** 1429 (86.79)   
13 + **[GGCGGG]** 1431 (86.87)   
13 + **.GTCGGG.** 1444 (90.21)   
13 + **.GTCGCG.** 1468 (100.00)   
13 + **[GGCTCG]** 1508 (85.14)   
13 - **[GGCGGG]** 1487 (86.87)   
13 - **[CCCGCG]** 1470 (87.81)   
13 - **.GTCCCG.** 1433 (92.74)   
13 - **[GCCGCC]** 1428 (87.14)   
13 - **[GCCGCC]** 1419 (87.14)   
13 - **[GGCCCG]** 1407 (89.40)   
13 - **.GTCGCC.** 1402 (90.05)   
13 - **[GGCCCG]** 1390 (89.40)   
13 - **.GCCGCG.** 1352 (97.09)   
13 - **[GGCGCC]** 1329 (86.71)   
13 - **[GCCCCG]** 1326 (89.83)   
13 - **[CCCGCG]** 1324 (87.81)   
13 - **[GCCGCC]** 1316 (87.14)   
13 - **[CCCGCG]** 1304 (87.81)   
13 - **.GGCGCG.** 1264 (96.66)   
13 - **[CCCGCG]** 1229 (87.81)   
13 - **[GCGGCG]** 1196 (86.79)   
13 - **.GGCGCG.** 1187 (96.66)   
13 - **.GTCCCG.** 1176 (92.74)   
13 - **[GCGGCG]** 1166 (86.79)   
13 - **[GCGGCG]** 1163 (86.79)   
13 - **[GCGGCG]** 1160 (86.79)   
13 - **[GTGGCG]** 1148 (89.70)   
13 - **[GCGGCG]** 1145 (86.79)   
13 - **[GCGGCG]** 1142 (86.79)   
13 - **[GCGGCG]** 1139 (86.79)   
13 - **[GGCGCC]** 1137 (86.71)   
13 - **[GGCGGG]** 1123 (86.87)   
13 - **[CGCGCG]** 1112 (87.39)   
13 - **[GCGGCG]** 1109 (86.79)   
13 - **[GTGGCG]** 1103 (89.70)   
13 - **[GGCGGG]** 1101 (86.87)   
13 - **.GCCGCG.** 1064 (97.09)   
13 - **[CGCGCG]** 1010 (87.39)   
13 - **[GCCCCG]** 998 (89.83)   
13 - **[GGCCCG]** 993 (89.40)   
13 - **[GCCCCG]** 977 (89.83)   
13 - **.GCCGCG.** 947 (97.09)   
13 - **[CGCGCG]** 945 (87.39)   
13 - **.GTCCCG.** 926 (92.74)   
13 - **[GCCCCG]** 921 (89.83)   
13 - **[GCCCCG]** 909 (89.83)   
13 - **[GGCGCC]** 881 (86.71)   
13 - **[GGCCCG]** 875 (89.40)   
13 - **[CCCGCG]** 873 (87.81)   
13 - **[GGGGCG]** 846 (86.37)   
13 - **[GGCGGG]** 844 (86.87)   
13 - **[GGCGGG]** 826 (86.87)   
13 - **[GCCGGG]** 791 (87.29)   
13 - **.GGCGCG.** 787 (96.66)   
13 - **[GGGGCG]** 782 (86.37)   
13 - **.GGCGCG.** 780 (96.66)   
13 - **[GGGGCG]** 775 (86.37)   
13 - **.GGCGCG.** 773 (96.66)   
13 - **.GACGCG.** 766 (92.06)   
13 - **[GGGGCG]** 761 (86.37)   
13 - **[GGCGGG]** 759 (86.87)   
13 - **[GGGGCG]** 756 (86.37)   
13 - **[GCGGCG]** 753 (86.79)   
13 - **[GGCGGG]** 751 (86.87)   
13 - **[GGCGGG]** 747 (86.87)   
13 - **.GACGCG.** 742 (92.06)   
13 - **[CGCGCG]** 723 (87.39)   
13 - **[GCGGCG]** 720 (86.79)   
13 - **[GCGGCG]** 717 (86.79)   
13 - **[GGGGCG]** 710 (86.37)   
13 - **[GGCGGG]** 708 (86.87)   
13 - **.GCCGCG.** 703 (97.09)   
13 - **[GGCGCC]** 697 (86.71)   
13 - **[GCCGGG]** 694 (87.29)   
13 - **[GGGGCG]** 690 (86.37)   
13 - **[GGCGGG]** 688 (86.87)   
13 - **[GCGGCG]** 677 (86.79)   
13 - **.GCCGCG.** 656 (97.09)   
13 - **[GGCGGG]** 622 (86.87)   
13 - **[GCGGCG]** 617 (86.79)   
13 - **.GGCGCG.** 615 (96.66)   
13 - **[GCCCCG]** 602 (89.83)   
13 - **[CGCGCG]** 533 (87.39)   
13 - **[CGCGCG]** 531 (87.39)   
13 - **.GTCCCG.** 523 (92.74)   
13 - **[GGCGGG]** 463 (86.87)   
13 - **[GCCGCC]** 361 (87.14)   
13 - **[GCCGGG]** 171 (87.29)   
13 - **.GGCGCG.** 167 (96.66)   
  

**Frequency Matrix**

| All Occs Best Occs|  |  |  |  |  |  |  |  |  |  |  |  |  |  |  |  |  |  |  |  |  |  |  |  |  |  |  |  |  |  |  |  |  |  |  |  |  |  |  |  |  |  |  |  |  |  |  |  |  |  |  |  |  |  |  |  |  |  |  |  |  |  |  |  |  |  |  |  |  |  |  |  |  |  |  |  |  |  |  |  |  |  |  |  | | --- | --- | --- | --- | --- | --- | --- | --- | --- | --- | --- | --- | --- | --- | --- | --- | --- | --- | --- | --- | --- | --- | --- | --- | --- | --- | --- | --- | --- | --- | --- | --- | --- | --- | --- | --- | --- | --- | --- | --- | --- | --- | --- | --- | --- | --- | --- | --- | --- | --- | --- | --- | --- | --- | --- | --- | --- | --- | --- | --- | --- | --- | --- | --- | --- | --- | --- | --- | --- | --- | --- | --- | --- | --- | --- | --- | --- | --- | --- | --- | --- | --- | --- | --- | | **A C G T A C G T | | | | | | | | | | | |**| **1** 5 18 247 3 | 0 18 208 0|  |  |  |  |  |  |  |  |  |  |  |  |  |  |  |  |  |  |  |  |  |  |  |  |  |  |  |  |  |  |  |  |  |  |  |  |  |  |  |  |  |  |  |  |  |  |  |  |  |  |  |  |  |  |  |  |  |  |  |  | | --- | --- | --- | --- | --- | --- | --- | --- | --- | --- | --- | --- | --- | --- | --- | --- | --- | --- | --- | --- | --- | --- | --- | --- | --- | --- | --- | --- | --- | --- | --- | --- | --- | --- | --- | --- | --- | --- | --- | --- | --- | --- | --- | --- | --- | --- | --- | --- | --- | --- | --- | --- | --- | --- | --- | --- | --- | --- | --- | --- | | **2** 15 62 55 141 | 15 62 55 94|  |  |  |  |  |  |  |  |  |  |  |  |  |  |  |  |  |  |  |  |  |  |  |  |  |  |  |  |  |  |  |  |  |  |  |  |  |  |  |  |  |  |  |  |  |  |  |  | | --- | --- | --- | --- | --- | --- | --- | --- | --- | --- | --- | --- | --- | --- | --- | --- | --- | --- | --- | --- | --- | --- | --- | --- | --- | --- | --- | --- | --- | --- | --- | --- | --- | --- | --- | --- | --- | --- | --- | --- | --- | --- | --- | --- | --- | --- | --- | --- | | **3** 1 256 14 2 | 0 226 0 0|  |  |  |  |  |  |  |  |  |  |  |  |  |  |  |  |  |  |  |  |  |  |  |  |  |  |  |  |  |  |  |  |  |  |  |  | | --- | --- | --- | --- | --- | --- | --- | --- | --- | --- | --- | --- | --- | --- | --- | --- | --- | --- | --- | --- | --- | --- | --- | --- | --- | --- | --- | --- | --- | --- | --- | --- | --- | --- | --- | --- | | **4** 1 30 233 9 | 0 30 196 0|  |  |  |  |  |  |  |  |  |  |  |  |  |  |  |  |  |  |  |  |  |  |  |  | | --- | --- | --- | --- | --- | --- | --- | --- | --- | --- | --- | --- | --- | --- | --- | --- | --- | --- | --- | --- | --- | --- | --- | --- | | **5** 3 254 16 0 | 0 210 16 0|  |  |  |  |  |  |  |  |  |  |  |  | | --- | --- | --- | --- | --- | --- | --- | --- | --- | --- | --- | --- | | **6** 3 15 249 6 | 0 15 211 0 | | | | | | | | | | | | | | | | | | | | | | | | | | | | | | | | | | | | | | | | | | | | | | | | | | | | | | | | | | | | | | | | | | | | | | | | | | | | | | | | | | | | |

  
  

---**
